# Supplementary material for: Kinetics of mRNA nuclear export regulate innate immune response gene expression
Source: Nat Commun. 2022 Nov 23;13:7197. doi: 10.1038/s41467-022-34635-5 (PMC9691726; doi:10.1038/s41467-022-34635-5)
Supplement: Supplementary file 1 — Supplementary Information [file 41467_2022_34635_MOESM1_ESM.pdf]

Supplementary Information

Supplementary Figure 1-7

Supplementary Data 1 (Manual Curation).xlsx
List of genes selected for downstream analysis, and genes removed, with reason indicated.

Supplementary Data 2 (Fitted Parameters).xlsx
Table of the optimized parameter sets for all replicates per genes with corresponding confidence intervals.

Source Data
Source Data File 1 (uncropped immunoblots).pdf
Uncropped immunoblots shown in Supplementary Figure 1A.
Source Data File 2 (all_genes_optim_naive).pdf
Fits and parameters of all the genes for all replicates produced from naïve macrophages.
Source Data File 3 (all_genes_optim_lpa).pdf
Fits and parameters of all the genes for all replicates produced from tolerized macrophages.
Source Data File 4 (Profile_dMod_merged_naive_with_optim_points).pdf
Profile analysis for all genes and replicates produced from naïve macrophages, with table of best fit parameters and confidence intervals.
Source Data File 5 (Profile_dMod_merged_lpa_with_optim_points).pdf
Profile analysis for all genes and replicates produced from tolerized macrophages, with table of best fit parameters and confidence intervals.

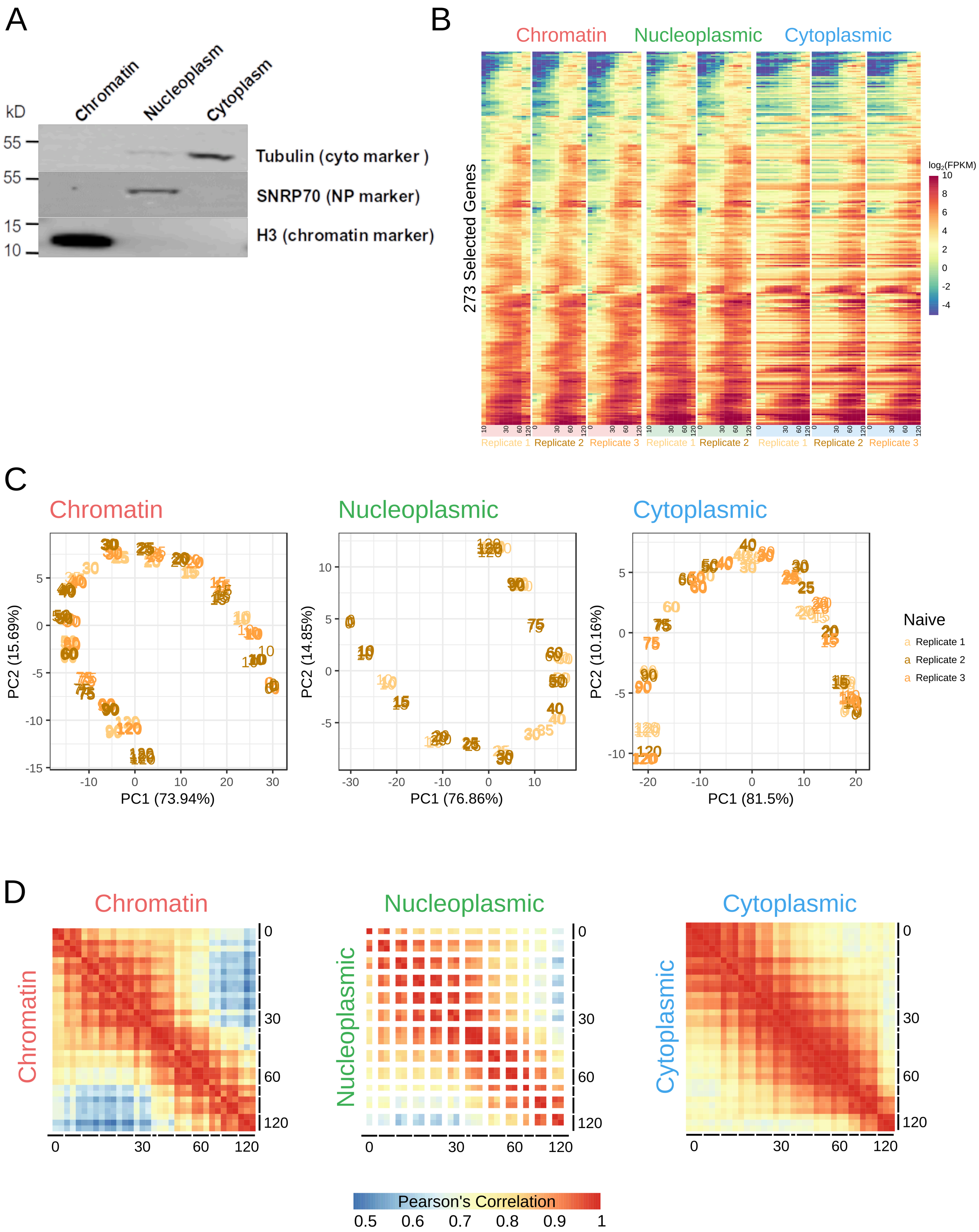
A
kD
Chromatin
Nucleoplasm
Cytoplasm
55
55
15
10
Tubulin (cyto marker)
SNRP70 (NP marker)
H3 (chromatin marker)
B
Chromatin
Nucleoplasmic
Cytoplasmic
273 Selected Genes
log2(FPKM)
10
8
6
4
2
0
-2
-4
Replicate 1
Replicate 2
Replicate 3
Replicate 1
Replicate 2
Replicate 1
Replicate 2
Replicate 3
C
Chromatin
PC1 (73.94%)
PC2 (15.69%)
Nucleoplasmic
PC1 (76.86%)
PC2 (14.85%)
Cytoplasmic
PC1 (81.5%)
PC2 (10.16%)
Naive
Replicate 1
Replicate 2
Replicate 3
D
Chromatin
Nucleoplasmic
Cytoplasmic
0
30
60
120
Pearson's Correlation
0.5
0.6
0.7
0.8
0.9
1

Supplementary Figure S1: Reproducibility of datasets produced from LPA-stimulated macrophages

A. Western blot of chromatin-associated, nucleoplasmic, and cytoplasmic proteins to document the clean separation of chromatin, nucleoplasmic and cytoplasmic fractions. This blot is representative of blots produced for each of the three biological replicates.
B. Heatmap of gene expression for the induced genes for the different replicates, showing high reproducibility between three replicates.
C. Principal component analysis showing each replicate. Technical sequencing replicates (same color) and biological replicates (different color) show high reproducibility. Numbers indicate the timpoint of the sample taken.
D. Correlation between timepoints of the replicates in each fraction. A good correlation between the different replicates and neighboring timepoints for each fraction is observed.

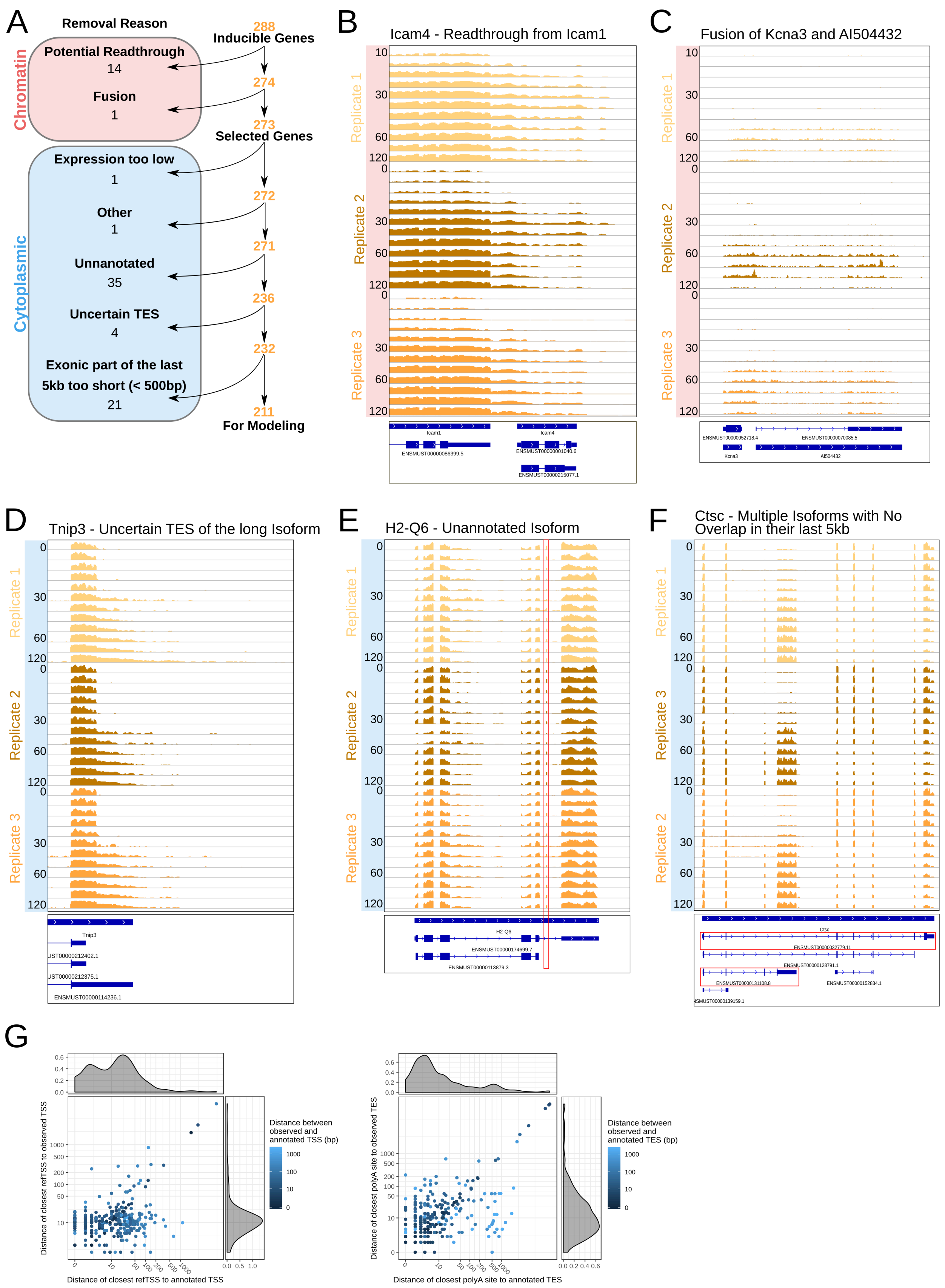
A
Removal Reason
Chromatin
Potential Readthrough
14
Fusion
1
Cytoplasmic
Expression too low
1
Other
1
Unnanotated
35
Uncertain TES
4
Exonic part of the last
5kb too short (< 500bp)
21
288
Inducible Genes
274
273
Selected Genes
272
271
236
232
211
For Modeling
B
Icam4 - Readthrough from Icam1
Replicate 1
Replicate 2
Replicate 3
C
Fusion of Kcna3 and AI504432
D
Tnip3 - Uncertain TES of the long Isoform
E
H2-Q6 - Unannotated Isoform
F
Ctsc - Multiple Isoforms with No Overlap in their last 5kb
G
Distance of closest refTSS to observed TSS
Distance of closest refTSS to annotated TSS
Distance between observed and annotated TSS (bp)
Distance of closest polyA site to observed TES
Distance of closest polyA site to annotated TES
Distance between observed and annotated TES (bp)

Supplementary Figure S2: Gene filtering based on visual inspection of genome browser tracks

A. Gene filtering steps (with corresponding numbers) to select high quality genes for the modeling.
B. *Icam4* chromatin-associated RNA tracks is an example of the genes that were removed from further analysis because of apparent read-through from another inducible gene (in this case *Icam1*).
C. Example of a gene that was removed from further analysis because of apparent gene fusion, where two genes locate to overlapping genomic regions.
D. *Tnip3* as an example of gene removed from further analysis due to uncertain TES given the presence of a shorter and longer isoform apparent in the cytoplasmic mRNA tracks.
E. *H2-Q6* as an example of gene removed from further analysis due to the presence of an unannotated isoform apparent in the cytoplasmic mRNA tracks).
F. *Ctsc* as an example of a gene removed from further analysis due to having two isoforms, one basal and one induced with very different TESs.
G. Comparison of observed and annotated TSS (left) and TES (right) to external database for 3′-UTR seq and CAGE peaks. The manual annotation of TSS is often closer to the external database. Though some observed TESs are slightly further from the external database polyA sites, a good number of genes for which the reference annotation was around 500-1000bp from the external database are brought to within 50bp with the observed TESs. Almost all observed TESs are within 100bp of the external database.

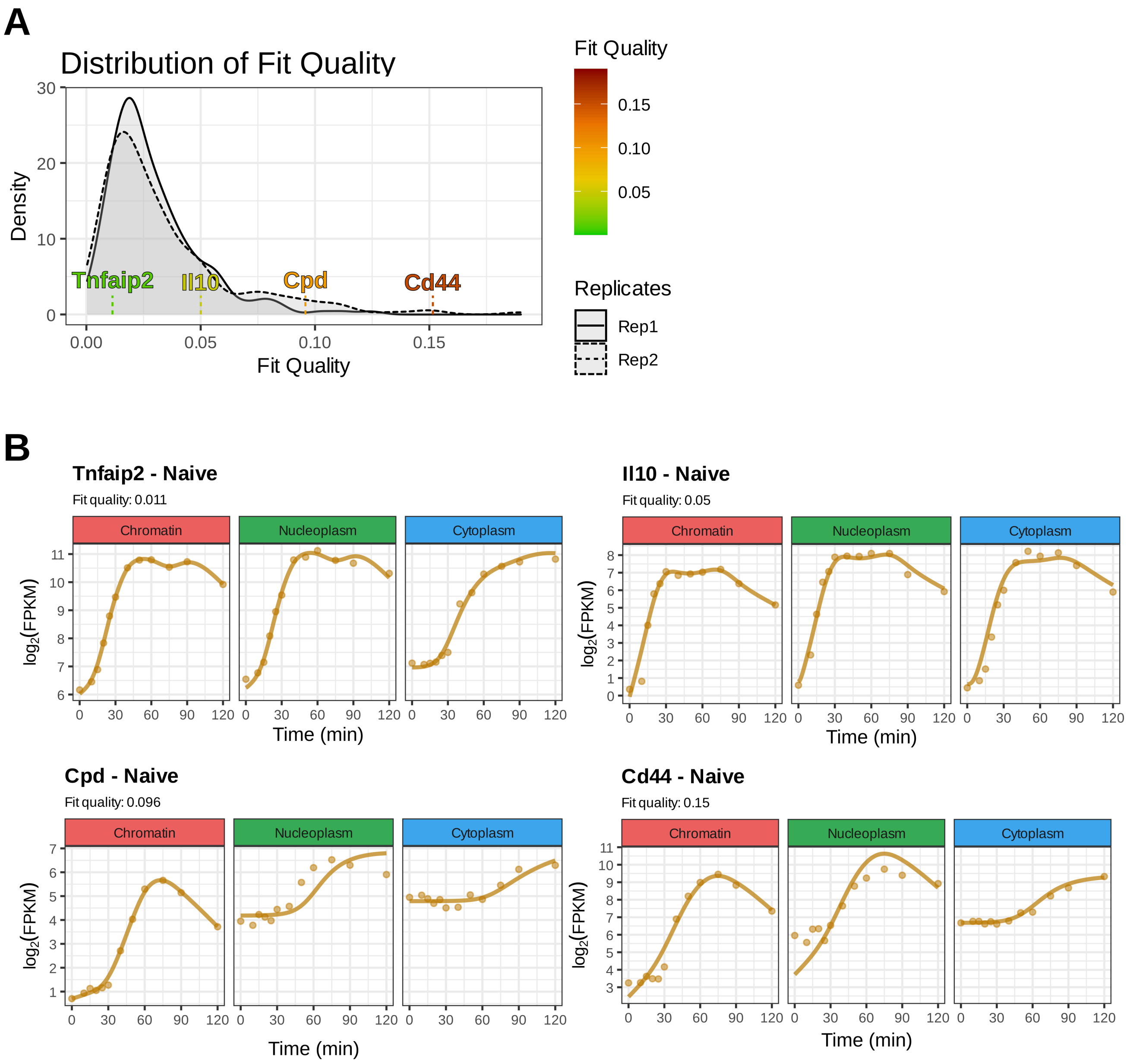

Supplementary Figure S3: Details of model fit quality and example genes.

A. Density of the fit quality for both replicates. Most genes exhibit a fit quality of <0.06 for both replicates.

B. Example genes across the fit quality spectrum. *Tnfaip2* is well fitted with a fit quality of 0.011 and barely show any difference with the data; *Il10* has a fit quality of 0.05 and shows slight differences between the model simulation and the data in the cytoplasmic fraction; *Cpd* and *Cd44* have a fit quality of 0.096 and 0.15 show discrepancies, primarily in the nucleoplasmic fraction.

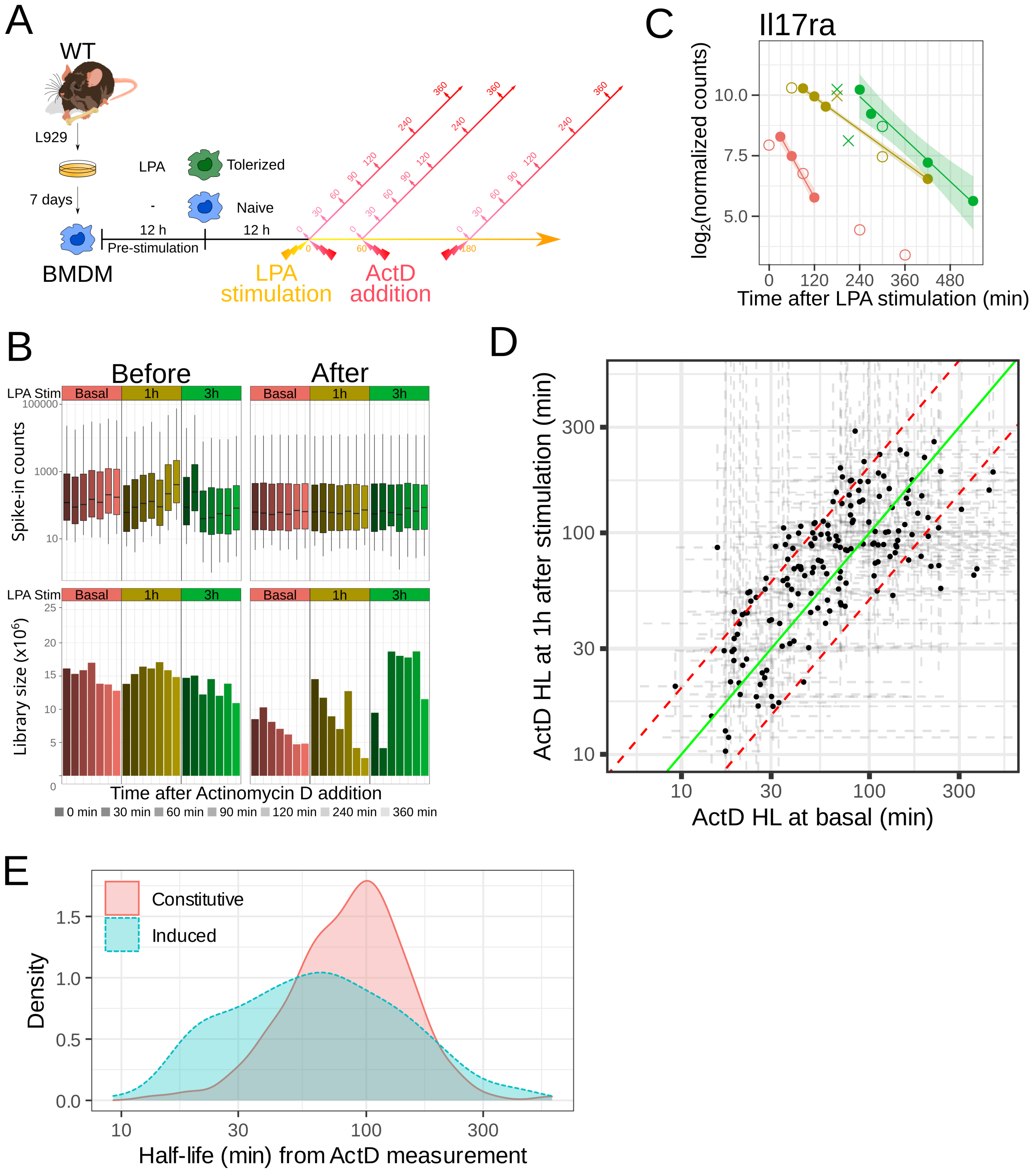
A
WT
L929
7 days
BMDM
LPA
Tolerized
-
Naive
12 h
Pre-stimulation
12 h
LPA
stimulation
ActD
addition
B
Before
After
LPA Stim
Basal
1h
3h
Spike-in counts
Library size (x10^6)
Time after Actinomycin D addition
0 min
30 min
60 min
90 min
120 min
240 min
360 min
C
Il17ra
log2(normalized counts)
Time after LPA stimulation (min)
D
ActD HL at 1h after stimulation (min)
ActD HL at basal (min)
E
Constitutive
Induced
Density
Half-life (min) from ActD measurement

Supplementary Figure S4: Deriving half-life from ActinomycinD mRNA sequencing data

A. Schematic of the experimental design for ActinomycinD mRNA half-life determination. BMDMs were either LPA-tolerized or used naïvely, and then stimulated with LPA for 0, 1, 2, or 3hr, at which time ActinomycinD was added for a timecourse. RNA was prepared at indicated time points for polyA+ RNA-seq.
B. RNA-seq data normalization using ERCC spike-ins. Before normalization, spike-in counts increased with time of Actinomycin D treatment. After normalization they remain constant, and the effective library size generally decreased with time of ActinomycinD treatment, as expected. This can be clearly seen for the basal and 1h LPA stimulation samples, but less at 3h, which suggests lower reliability in half-life determination. Box plots show the distribution of counts for all spike-ins (middle line: median, box:1st and 3rd quartiles, whiskers: smallest/largest value no further than 1.5x interquartile range from the box.
C. Regression to determine the mRNA half-life. The data for *Il17ra* is shown as an example. Circles indicate data that passes a quality control metrics, while X's indicate data that fail the quality control metrics. Filled circles indicate data selected by the model-aided algorithm to be used for half-life determination. The error band represents the 95% confidence interval of the regression lines.
D. Scatterplot to compare the measured half-life at basal and 1h after LPA stimulation for the selected induced genes. Gray dashed horizontal and vertical lines represent the 95% confidence interval of the regression. The measured half-life shows little change (remains within 2-fold, diagonal red dashed lines) within the first hour of LPA stimulation for the selected genes.
E. Distributions of mRNA half-lives associated with 273 induced genes identified in Figure 1D and 363 constitutively expressed genes (most highly expressed genes whose cytoplasmic abundance changes < $\log_2(1.5)$ at any timepoint of the LPA timecourse. The distributions are similarly wide, but short mRNA half-lives are rare among constitutively expressed genes.

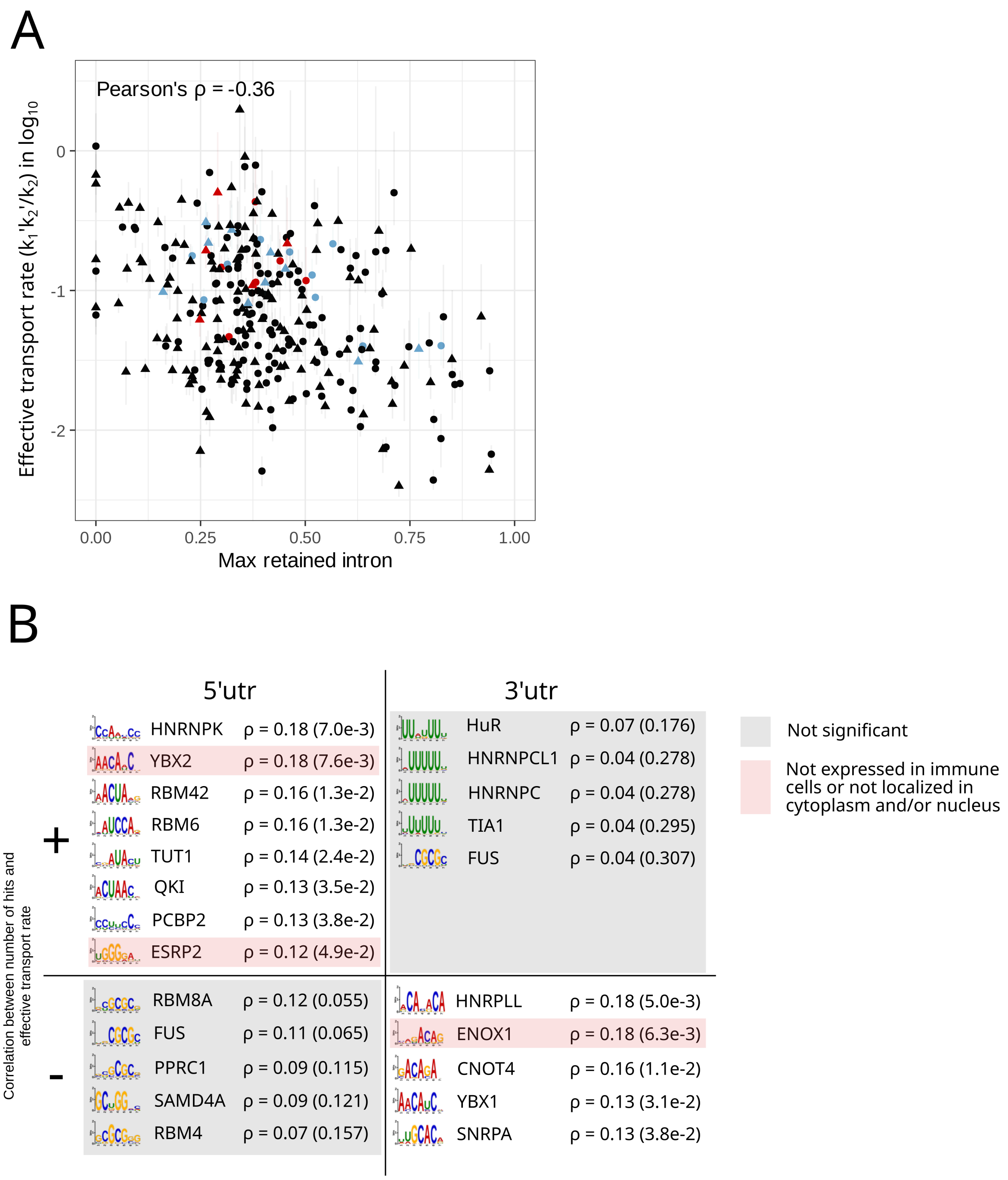

Supplementary Figure S5: RNA binding protein motifs analysis

A. Effective transport rates show a positive correlation with the splicing probability of the most highly retained intron. However, this correlation coefficient is lower than when the all introns are considered (Figure 5D).
B. Results of the RBP motifs analysis from AME tool. Motifs shaded in gray are not significant. Motifs shaded in pink are unlikely to be relevant though they exhibit some correlation. Significant motifs were found enriched in the 5′-UTR of genes having higher effective transport, and in the 3′-UTR of genes having lower effective transport.

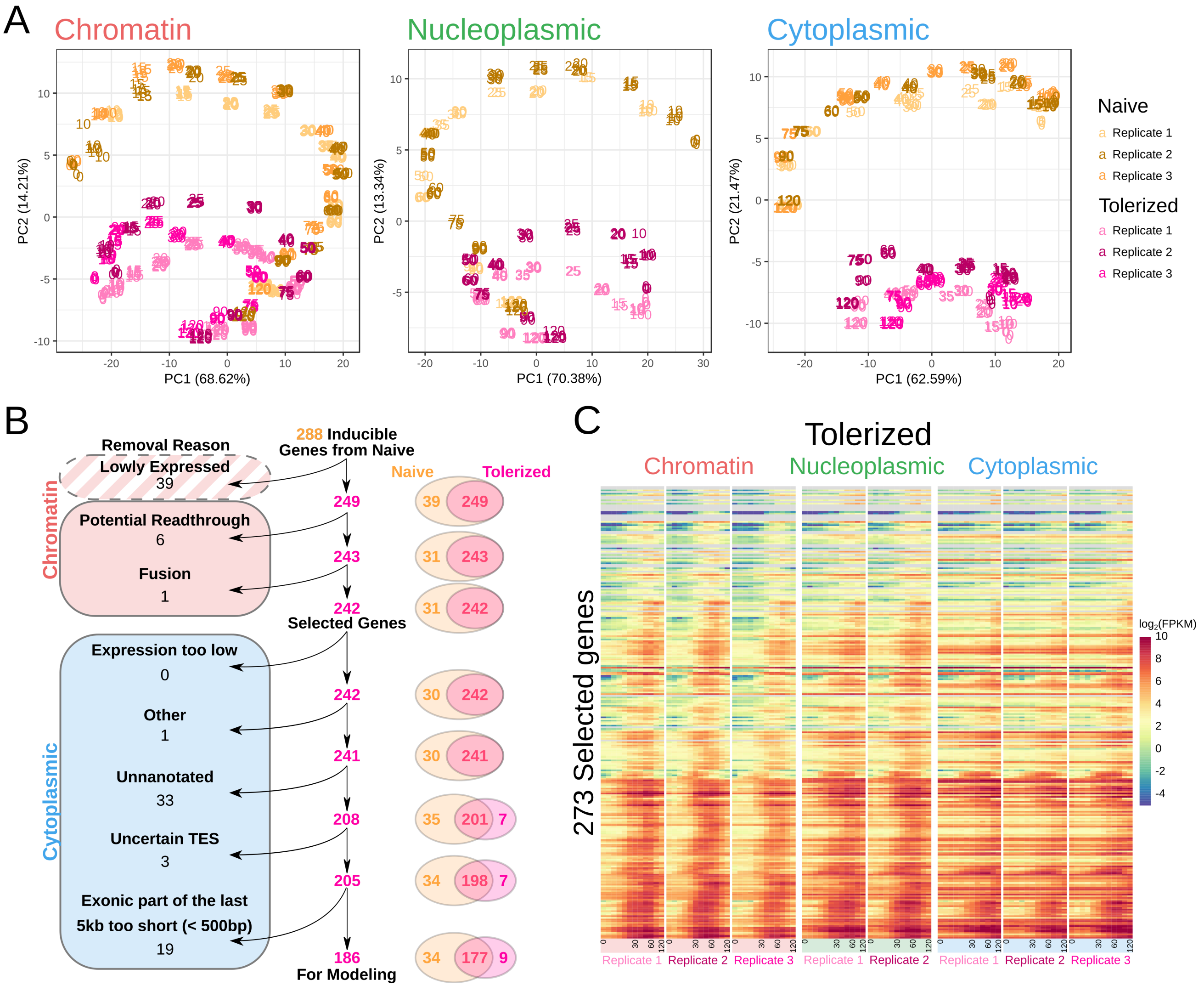

Supplementary Figure S6: Reproducibility and filtering of the datasets from LPA-tolerized macrophages.

A. Principal component analysis of the different replicates. Technical replicates (same color, i.e. biological replicate and number, i.e. timepoint) show high reproducibility, and the biological replicates also cluster together. Profound differences in the transcriptomic responses of tolerized and the naïve macrophages are apparent.
B. Gene filtering steps for the tolerized macrophage datasets (with corresponding numbers and Venn diagram) to select genes with high quality data for the model fitting.
C. Heatmap of gene expression of genes induced in the tolerized condition. These is good reproducibility between replicates.

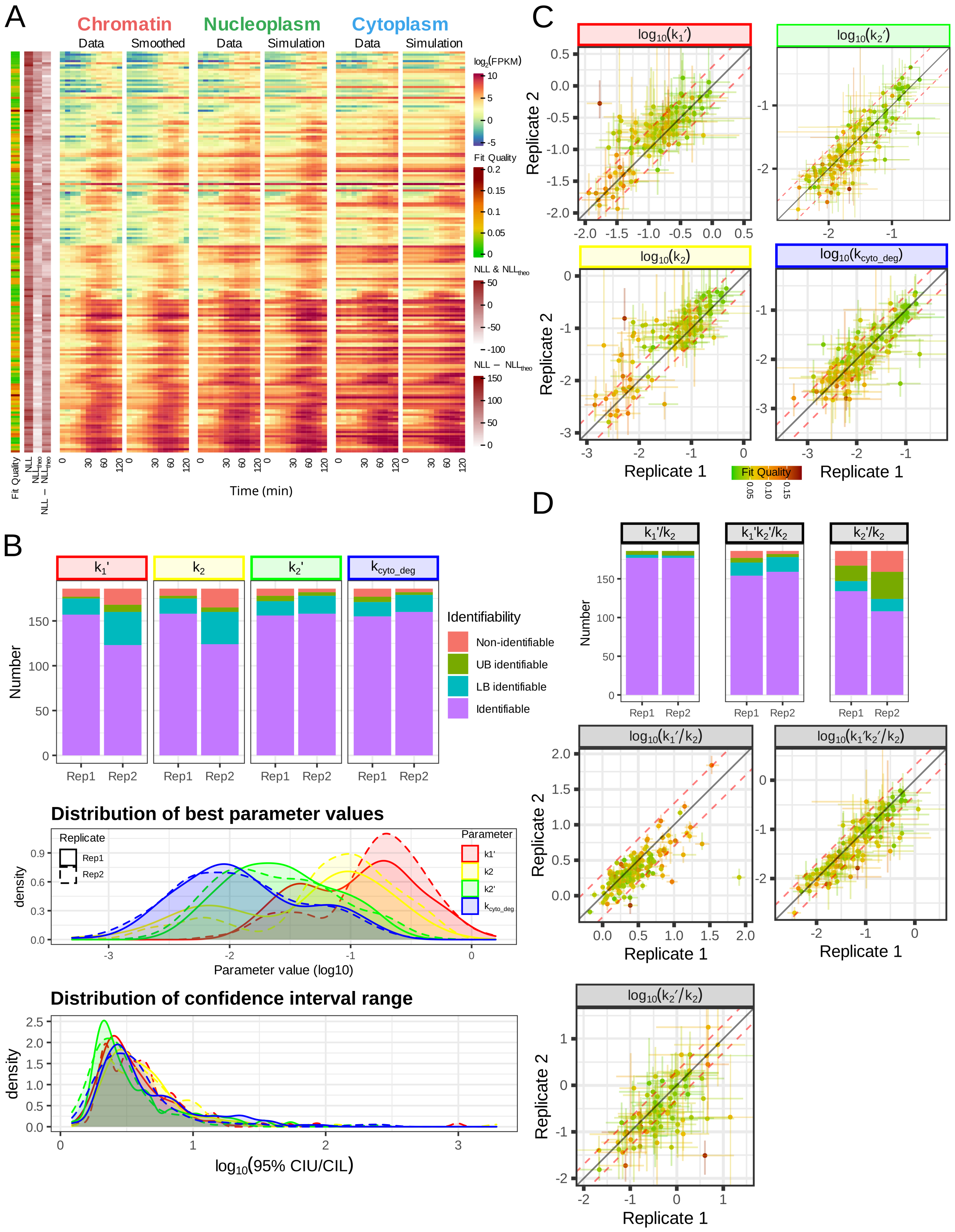
A
Chromatin
Nucleoplasm
Cytoplasm
Data
Smoothed
Data
Simulation
Data
Simulation
log2(FPKM)
Fit Quality
NLL & NLLtheo
NLL − NLLtheo
Time (min)
B
k1'
k2
k2'
kcyto_deg
Number
Identifiability
Non-identifiable
UB identifiable
LB identifiable
Identifiable
Rep1
Rep2
Distribution of best parameter values
Replicate
Parameter
density
Parameter value (log10)
Distribution of confidence interval range
log10(95% CIU/CIL)
C
log10(k1′)
log10(k2′)
log10(k2)
log10(kcyto_deg)
Replicate 2
Replicate 1
Fit Quality
D
k1'/k2
k1'k2'/k2
k2'/k2
log10(k1′/k2)
log10(k1′k2′/k2)
log10(k2′/k2)

Supplementary Figure S7: Parameter inference and reproducibility for the tolerized condition

A. Heatmap of fitted model simulations alongside the experimental data for the tolerized condition. Most genes fit the data well, though overall fits were not as good as the model fits for the naïve condition. The negative log likelihood used as cost function is shown on the left side (NLL), along with the best expected negative log likelihood that accounts for error arising from random sampling for time points with lower expression (NLLtheo), and the difference between them. An additional fit quality metric is shown that includes information on the autocorrelation of the residual (see, methods), which provides for a more reliable comparison between genes.
B. Identifiability and reproducibility of parameters. Top: bar graphs showing for how many genes the parameters are identifiable, defined by a single confidence interval of 95%. Middle: Distribution of the parameter values for the different replicates for the tolerized condition. The distributions are similar for the different biological replicates. Bottom: Distribution of the confidence intervals for the identifiable parameters.
C. Reproducibility of each parameter for genes for which the parameter is identifiable. The color of the point corresponds to the fit quality metric for the worst replicate, the line corresponds to 95% confidence interval, with the color corresponding to the fit quality of that replicate. The estimated parameters are quite reproducible and relatively well defined, though not as well as for the data from the naïve condition.
D. Identifiability and reproducibility of composite parameters k_1'/k_2, $k_2'k_2$ and $k_1'k_2'/k_2$. Top: bar graphs showing for how many genes the parameters are identifiable, defined by a single confidence interval of 95%. Bottom: Reproducibility of each parameter for genes for which the parameter is identifiable, as in (C). The effective transport rate parameter ($k_1'k_2'/k_2$) is relatively reproducible and well defined, more so than the transport efficiency (k_2'/k_2).
