# Supplementary material for: Kinetics of mRNA nuclear export regulate innate immune response gene expression
Source: Nat Commun. 2022 Nov 23;13:7197. doi: 10.1038/s41467-022-34635-5 (PMC9691726; doi:10.1038/s41467-022-34635-5)
Supplement: Supplementary file 3 — Description of Additional Supplementary Files [file 41467_2022_34635_MOESM3_ESM.pdf]

Description of Additional Supplementary Files

Supplementary Data 1: (Manual Curation).xlsx - List of genes selected for downstream analysis, and genes removed, with reason indicated.

Supplementary Data 2: (Fitted Parameters).xlsx - Table of the optimized parameter sets for all replicates per genes with corresponding confidence intervals.
