# Supplementary material for: Kinetics of mRNA nuclear export regulate innate immune response gene expression
Source: Nat Commun. 2022 Nov 23;13:7197. doi: 10.1038/s41467-022-34635-5 (PMC9691726; doi:10.1038/s41467-022-34635-5)
Supplement: Supplementary file 6 — Reporting Summary [file 41467_2022_34635_MOESM6_ESM.pdf]

nature portfolio

Corresponding author(s): Hoffmann, A

Last updated by author(s): Sept 15, 2022

Reporting Summary

Nature Portfolio wishes to improve the reproducibility of the work that we publish. This form provides structure for consistency and transparency in reporting. For further information on Nature Portfolio policies, see our Editorial Policies and the Editorial Policy Checklist.

Statistics

For all statistical analyses, confirm that the following items are present in the figure legend, table legend, main text, or Methods section.

n/a	Confirmed	
☐	☒	The exact sample size (n) for each experimental group/condition, given as a discrete number and unit of measurement
☐	☒	A statement on whether measurements were taken from distinct samples or whether the same sample was measured repeatedly
☐	☒	The statistical test(s) used AND whether they are one- or two-sided<br>*Only common tests should be described solely by name; describe more complex techniques in the Methods section.*
☐	☒	A description of all covariates tested
☐	☒	A description of any assumptions or corrections, such as tests of normality and adjustment for multiple comparisons
☐	☒	A full description of the statistical parameters including central tendency (e.g. means) or other basic estimates (e.g. regression coefficient) AND variation (e.g. standard deviation) or associated estimates of uncertainty (e.g. confidence intervals)
☐	☒	For null hypothesis testing, the test statistic (e.g. F, t, r) with confidence intervals, effect sizes, degrees of freedom and P value noted<br>*Give P values as exact values whenever suitable.*
☒	☐	For Bayesian analysis, information on the choice of priors and Markov chain Monte Carlo settings
☒	☐	For hierarchical and complex designs, identification of the appropriate level for tests and full reporting of outcomes
☐	☒	Estimates of effect sizes (e.g. Cohen's d, Pearson's r), indicating how they were calculated

Our web collection on statistics for biologists contains articles on many of the points above.

Software and code

Policy information about availability of computer code

Data collection	No software was used for data collection.
Data analysis	Python 3.7, cutadapt 1.12, STAR 2.52b, samtools 1.3.1, featuresCount 1.5.1, RSeQC, Homer 4.11, Meme suite,<br>R 3.5.1 and R packages edgeR: 3.22.5, deSolve: 1.21, shiny 1.1.0, ggplot2: 3.0.0, caret 6.0-8.0, RseQC: 2.6.4, IGV: 2.5.2, Meme suite: 5.0.3

For manuscripts utilizing custom algorithms or software that are central to the research but not yet described in published literature, software must be made available to editors and reviewers. We strongly encourage code deposition in a community repository (e.g. GitHub). See the Nature Portfolio guidelines for submitting code & software for further information.

Data

Policy information about availability of data

All manuscripts must include a data availability statement. This statement should provide the following information, where applicable:

- Accession codes, unique identifiers, or web links for publicly available datasets
- A description of any restrictions on data availability
- For clinical datasets or third party data, please ensure that the statement adheres to our policy

RNAseq Fractioned (fastq and bam files): https://www.encodeproject.org/awards/U01HG007912/
ChIPseq (fastq files): https://www.encodeproject.org/awards/U01HG007912/
ActinomycinD RNAseq (reviewer link): https://dataview.ncbi.nlm.nih.gov/object/PRJNA641336?reviewer=2gl9sjgsar9a52h4ii2upki7ps

Field-specific reporting

Please select the one below that is the best fit for your research. If you are not sure, read the appropriate sections before making your selection.

☒ Life sciences ☐ Behavioural & social sciences ☐ Ecological, evolutionary & environmental sciences

For a reference copy of the document with all sections, see nature.com/documents/nr-reporting-summary-flat.pdf

Life sciences study design

All studies must disclose on these points even when the disclosure is negative.

Sample size	The experimental design is a fine timecourse with over a dozen timepoints following a perturbation. Timepoint spacing was chosen such that several timepoints would describe the baseline, rise, peak, and decline of the molecular species. Thereby adjacent timepoints function to confirm measurements.
Data exclusions	No data was excluded.
Replication	Timecourses were undertaken in triplicate.
Randomization	N/A
Blinding	N/A

Reporting for specific materials, systems and methods

We require information from authors about some types of materials, experimental systems and methods used in many studies. Here, indicate whether each material, system or method listed is relevant to your study. If you are not sure if a list item applies to your research, read the appropriate section before selecting a response.

Materials & experimental systems

n/a	Involved in the study	
☐	☒	Antibodies
☒	☐	Eukaryotic cell lines
☒	☐	Palaeontology and archaeology
☐	☒	Animals and other organisms
☒	☐	Human research participants
☒	☐	Clinical data
☒	☐	Dual use research of concern

Methods

n/a	Involved in the study	
☐	☒	ChIP-seq
☒	☐	Flow cytometry
☒	☐	MRI-based neuroimaging

Antibodies

Antibodies used	Antibodies used for ChIP-seq studies of histone modification: H3K4me3 (05-745R, Millipore), H3K36me3 (ab9050, Abcam), H3K27ac (39133, Active-Motif), and H3K79me2 (ab3594, Abcam) antibodies.
Validation	Antibodies used in this study were recommended reagents by ENCODE. The recommended protocol was used. No other validation was undertaken.

Animals and other organisms

Policy information about studies involving animals; ARRIVE guidelines recommended for reporting animal research

Laboratory animals	Mus Musculus C57 Bl/6 were used to produce bone-marrow-derived macrophages.
Wild animals	N/A
Field-collected samples	N/A
Ethics oversight	University of California, Los Angeles Division of Laboratory Animal Medicine, accredited by Association for Assessment and Accreditation of Laboratory Animal Care International (AAALAC)

Note that full information on the approval of the study protocol must also be provided in the manuscript.

ChIP-seq

Data deposition

☒ Confirm that both raw and final processed data have been deposited in a public database such as GEO.

☒ Confirm that you have deposited or provided access to graph files (e.g. BED files) for the called peaks.

Data access links
May remain private before publication.

https://www.encodeproject.org/awards/U01HG007912/

Files in database submission

◦ H3K27ac: https://www.encodeproject.org/experiments/ENCSR933GNW/
▪ Replicate 1: ENCFF937IMG.fastq.gz and ENCFF412ZXF.fastq.gz
▪ Replicate 2: ENCFF065DFV.fastq.gz and ENCFF554XRW.fastq.gz
◦ H3K4me3: https://www.encodeproject.org/experiments/ENCSR916IRY/
▪ Replicate 1: ENCFF916ATM.fastq.gz and ENCFF588NZB.fastq.gz
▪ Replicate 2: ENCFF871YMY.fastq.gz and ENCFF934EMT.fastq.gz
◦ Controls for H3K27ac and H3K4me3: https://www.encodeproject.org/experiments/ENCSR601NNP/
▪ Replicate 1: ENCFF542ROM.fastq.gz and ENCFF451SQE.fastq.gz
▪ Replicate 2: ENCFF495GLD.fastq.gz and ENCFF666XOZ.fastq.gz
◦ H3K79me2: https://www.encodeproject.org/experiments/ENCSR570YMM/
▪ Replicate 1: ENCFF501QHM.fastq.gz
▪ Replicate 2: ENCFF850KGO.fastq.gz and ENCFF284JUF.fastq.gz
◦ H3K36me3: https://www.encodeproject.org/experiments/ENCSR280NNQ/
▪ Replicate 1: ENCFF902OXY.fastq.gz
▪ Replicate 2: ENCFF530USD.fastq.gz and ENCFF413ZVL.fastq.gz
◦ Controls for H3K79me2 and H3K36me3: https://www.encodeproject.org/experiments/ENCSR698GIN/
▪ Replicate 1: ENCFF016MGA.fastq.gz
▪ Replicate 2: ENCFF972NOO.fastq.gz and ENCFF503OGW.fastq.gz

Genome browser session
(e.g. UCSC)

no longer applicable

Methodology

Replicates

Two

Sequencing depth

Target File File Total number of reads Number of uniquely mapped read (deduplicated) Sequencing type
H3K27ac Replicate 1 ENCFF937IMG 29459940 42843707 50nt single-end
ENCFF412ZXF 28542463 50nt single-end
Replicate 2 ENCFF065DFV 28265698 42220623 50nt single-end
ENCFF554XRW 30843722 50nt single-end
H3K4me3 Replicate 1 ENCFF916ATM 25810990 36173982 50nt single-end
ENCFF588NZB 25577213 50nt single-end
Replicate 2 ENCFF871YMY 36963365 53009521 50nt single-end
ENCFF934EMT 36454161 50nt single-end
Control for H3K27ac and H3K4me3 Replicate 1 ENCFF542ROM 36760125 53141661 50nt single-end
ENCFF451SQE 36142515 50nt single-end
Replicate 2 ENCFF495GLD 37853102 55762075 50nt single-end
ENCFF666XOZ 37819924 50nt single-end
H3K79me2
Replicate 1 ENCFF501QHM 55619653 42482010 50nt single-end
Replicate 2 ENCFF850KGO 30780473 49074483 50nt single-end
ENCFF284JUF 33058178 50nt single-end
H3K36me3 Replicate 1 ENCFF902OXY 55619653 50753003 50nt single-end
Replicate 2 ENCFF530USD 30780473 43408409 50nt single-end
ENCFF413ZVL 33058178 50nt single-end
Control for H3K79me2 and H3K36me3 Replicate 1 ENCFF016MGA 63506632 46637714 50nt single-end
Replicate 2 ENCFF972NOO 28257365 42842683 50nt single-end
ENCFF503OGW 30228211 50nt single-end

Antibodies

Antibodies used for ChIP-seq studies of histone modification: H3K4me3 (05-745R, Millipore), H3K36me3 (ab9050, Abcam), H3K27ac (39133, Active-Motif), and H3K79me2 (ab3594, Abcam) antibodies.

Peak calling parameters

ChIP-seq was analysed using Encode chip-seq pipeline (https://github.com/ENCODE-DCC/chip-seq-pipeline2) v1.5.1:
conda activate encode-chip-seq-pipeline
caper run chip-seq-pipeline2 -i options.json

the options.json file lists the options to use for the pipeline:
- Path of files (replicates and inputs)
- "chip.pipeline_type": "histone"
- "chip.aligner": "bwa"
- "chip.align_only": false
- "chip.true_rep_only": false
- "chip.genome_tsv": mm10.tsv
- "chip.paired_end": false
- "chip.ctl_paired_end": false
- "chip.always_use_pooled_ctl": false

Options not listed here were kept to their default values.
The mm10.tsv index file was downloaded from Encode: https://storage.googleapis.com/encode-pipeline-genome-data/genome_tsv/v3/mm10.tsv and uses Encode ENCFF018NEO file as the bwa index (https://www.encodeproject.org/files/ENCFF018NEO/)
The peak calling is done by the pipeline using macs2 software with these default options:
- chip.cap_num_peak: 500000
- chip.pval_thresh: 0.01 (corresponds to macs2 callpeaks -p option)
- chip.idr_thresh: 0.05

Data quality

Quality was checked by ensuring library complexity as measured by a non redundant fraction (NRF) >= 0.8, a PCR bottlenecking coefficent 1 (PBC1) >= 0.8, a PCR bottlenecking coefficent 2 (PBC1) >= 3 as well as a irreproducible discovery rate (IDR) rescue ratio and self-constistency ratio <= 2

Target Nb of peaks at p-value < 0.01 and fold enrichement >= 1.2 Nb of peaks at fdr < 0.05 and fold enrichement >= 5
H3K27ac 34792 20338
H3K4me3 19552 15975
H3K79me2 27168 11404
H3K36me3 96240 1499

Software

ChIP-seq was analyzed using Encode chip-seq pipeline (https://github.com/ENCODE-DCC/chip-seq-pipeline2) v1.5.1
