# Supplementary material for: Kinetics of mRNA nuclear export regulate innate immune response gene expression
Source: Nat Commun. 2022 Nov 23;13:7197. doi: 10.1038/s41467-022-34635-5 (PMC9691726; doi:10.1038/s41467-022-34635-5)
Supplement: Supplementary file 7 — Source Data [file 41467_2022_34635_MOESM7_ESM.zip › Source Data File 3 all_genes_optim_lpa.pdf]

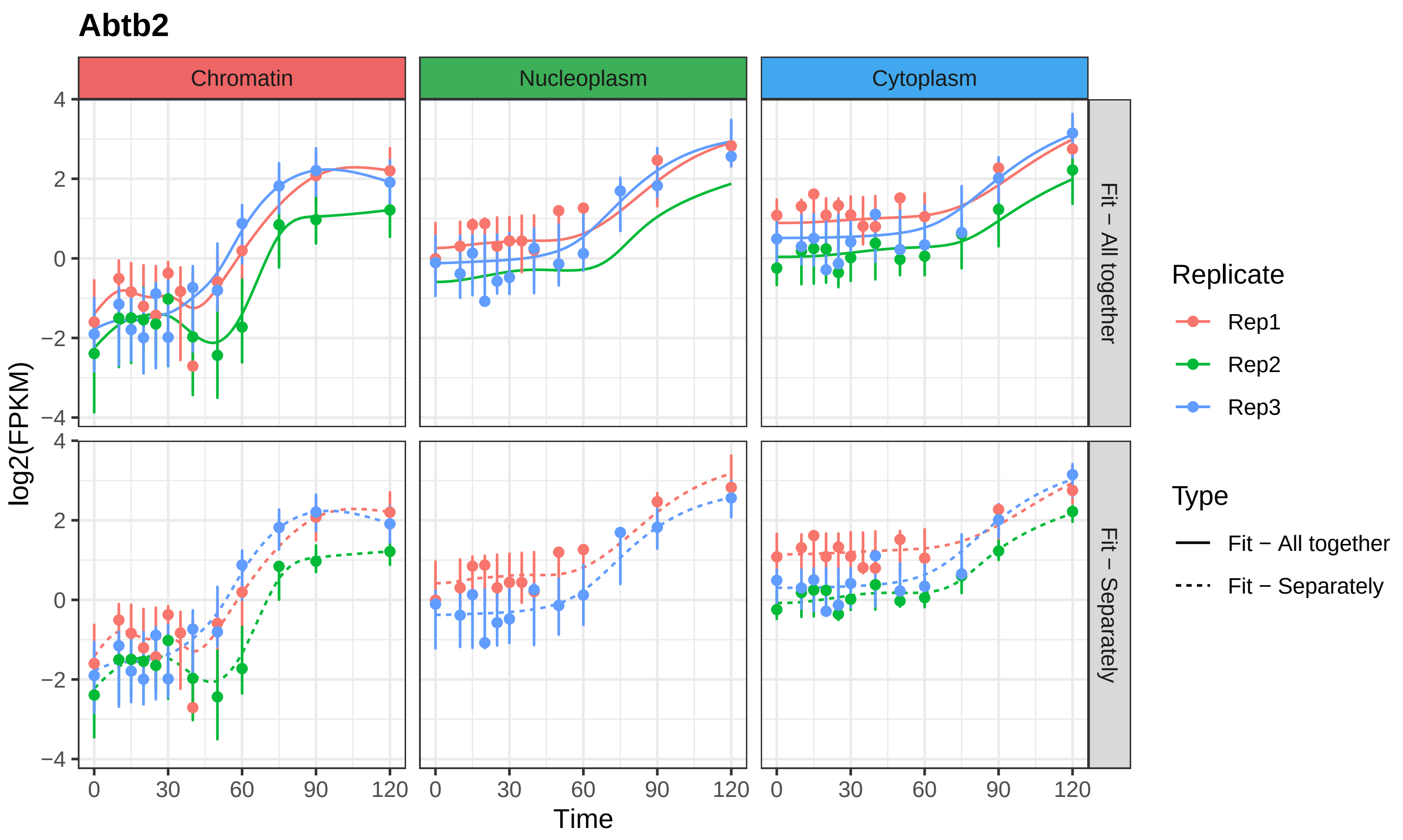

	Together	b1	b2	b3
**−NLL b1 ca**	5.663	4.958		
**−NLL b1 np**	6.602	4.744		
**−NLL b1 cyto**	4.856	2.12		
**−NLL b2 ca**	2.958		3.043	
**−NLL b2 np**				
**−NLL b2 cyto**	0.6252		−1.812	
**−NLL b3 ca**	3.763			4.064
**−NLL b3 np**	7.222			2.378
**−NLL b3 cyto**	8.093			5.247
Total	39.78	11.82	1.232	11.69
**Total with regul**	39.51	11.32	−0.979	10.36

	Together	b1	b2	b3
spar	4.092e−01	0.3955000	0.4268	4.289e−01
σ_b	1.854e−01	0.1564000	2.049e−06	1.338e−01
σ_t	3.261e−05	0.0001462	8.526e−06	4.593e−07
ca_{0,b_1}				
$\log_{10}(k_1')$	−1.373e+00	−1.2750000		−1.507e+00
$\log_{10}(k_2)$	−1.873e+00	−1.8280000	−0.4614 or −1.949	−1.926e+00
$\log_{10}(k_2')$	−1.172e+00	−1.4370000		−8.789e−01
$\log_{10}(k_{deg})$	−1.362e+00	−1.6560000	−1.949 or −0.4614	−1.083e+00
$\log_{10}(k_1'k_2')$	−2.545e+00	−2.7120000	−1.762	−2.385e+00
$\log_{10}(k_1'/k_2)$	4.997e−01	0.5530000		4.198e−01
$transport = \log_{10}(k_1'k_2'/k_2)$	−6.721e−01	−0.8839000	−1.301 or 0.1864	−4.591e−01

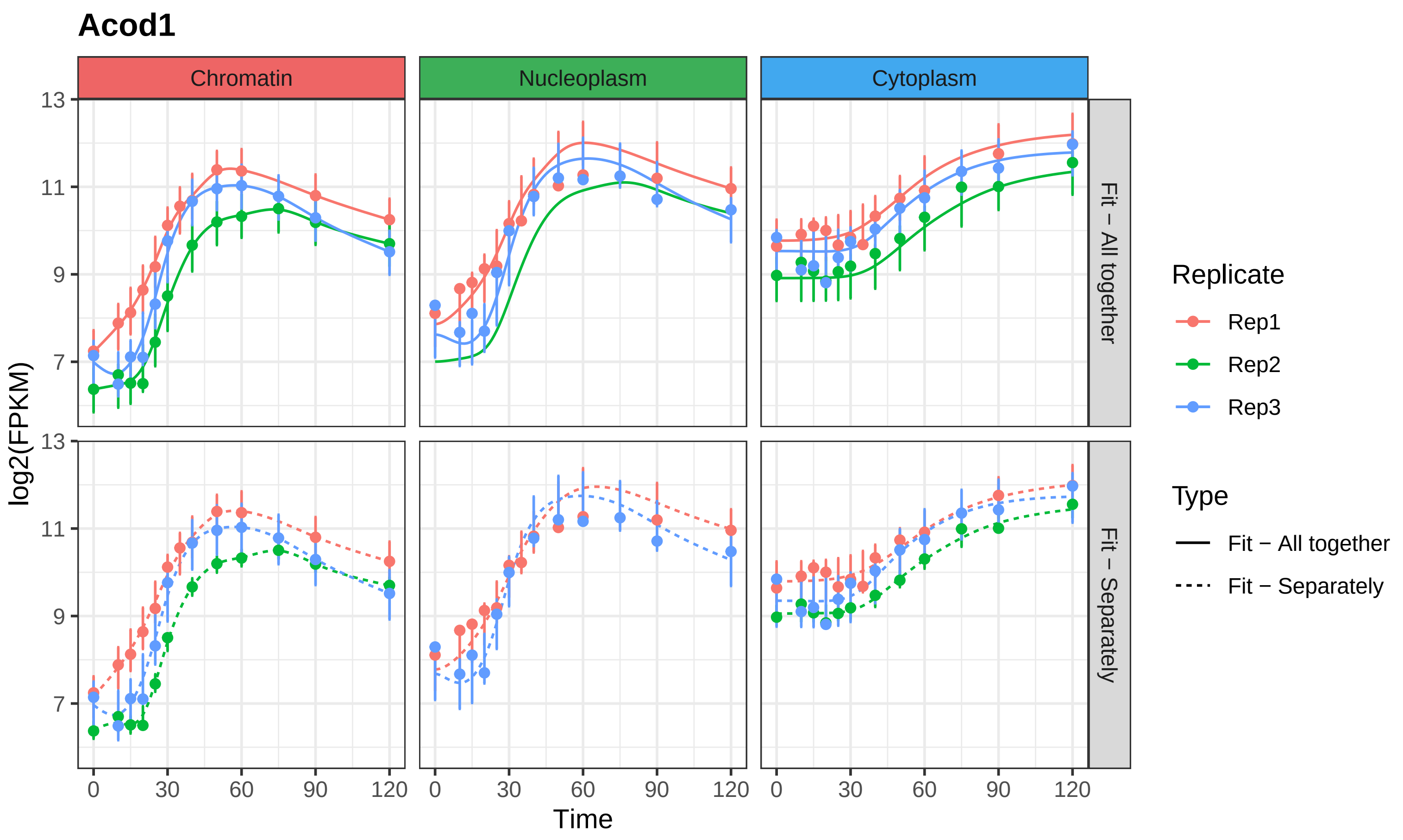

	Together	b1	b2	b3
**−NLL b1 ca**	−4.375	−4.98		
**−NLL b1 np**	9.08	8.938		
**−NLL b1 cyto**	−0.7884	−2.772		
**−NLL b2 ca**	−3.296		−10.67	
**−NLL b2 np**				
**−NLL b2 cyto**	−1.434		−8.217	
**−NLL b3 ca**	−2.212			−0.8222
**−NLL b3 np**	10.52			7.151
**−NLL b3 cyto**	2.384			1.395
Total	9.874	1.186	−18.88	7.723
**Total with regul**	11.42	0.4717	−14.71	9.155

	Together	b1	b2	b3
spar	0.3420	0.4147000	0.2731	0.354300
σ_b	0.1738	0.1646000	0.0692	0.195800
σ_t	1.0480	0.0006723	0.001894	0.000923
ca_{0,b_1}				
$\log_{10}(k_1')$	−0.4627	−0.6541000		−0.124900
$\log_{10}(k_2)$	−0.6533	−0.8388000	−0.2582 or −2.315	−0.339900
$\log_{10}(k_2')$	−1.7210	−1.8140000		−1.745000
$\log_{10}(k_{deg})$	−2.2960	−2.4200000	−2.315 or −0.2582	−2.248000
$\log_{10}(k_1'k_2')$	−2.1840	−2.4680000	−1.773	−1.869000
$\log_{10}(k_1'/k_2)$	0.1906	0.1847000		0.215000
$\text{transport} = \log_{10}(k_1'k_2'/k_2)$	−1.5310	−1.6290000	−1.515 or 0.5418	−1.530000

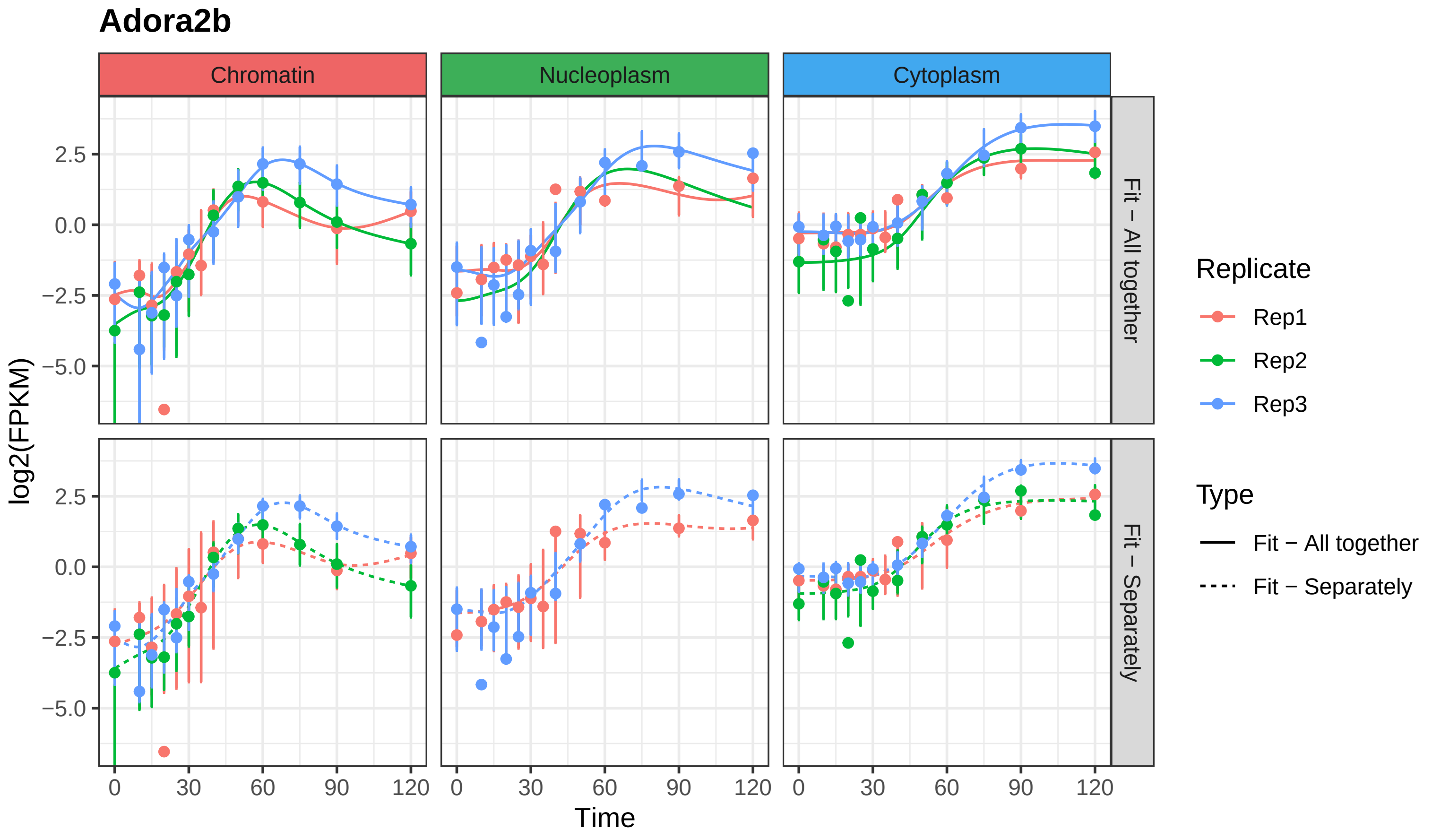

	Together	b1	b2	b3
**−NLL b1 ca**	11.23	13.34		
**−NLL b1 np**	12.57	8.533		
**−NLL b1 cyto**	6.699	3.258		
**−NLL b2 ca**	5.189		6.295	
**−NLL b2 np**				
**−NLL b2 cyto**	15.06		12.2	
**−NLL b3 ca**	6.88			6.67
**−NLL b3 np**	15.64			15.31
**−NLL b3 cyto**	−0.0648			−3.293
Total	73.21	25.13	18.5	18.69
**Total with regul**	73.7	25.39	18.08	17.07

	Together	b1	b2	b3
spar	0.3796	5.290e−01	0.4292	0.4069000
σ_b	0.1826	5.423e−06	0.1907	0.0807500
σ_t	2.7200	8.133e+00	5.703e−05	0.0005173
ca_{0,b_1}				
$\log_{10}(k_1')$	−0.9231	−1.070e+00		−0.9809000
$\log_{10}(k_2)$	−1.1740	−1.411e+00	0.8312 or −2.057	−1.2780000
$\log_{10}(k_2')$	−1.1370	−1.133e+00		−1.0080000
$\log_{10}(k_{deg})$	−1.5450	−1.479e+00	−2.057 or 0.8312	−1.3660000
$\log_{10}(k_1' k_2')$	−2.0610	−2.203e+00	−0.4292	−1.9890000
$\log_{10}(k_1'/k_2)$	0.2513	3.413e−01		0.2969000
$\text{transport} = \log_{10}(k_1' k_2'/k_2)$	−0.8861	−7.920e−01	−1.26 or 1.628	−0.7115000

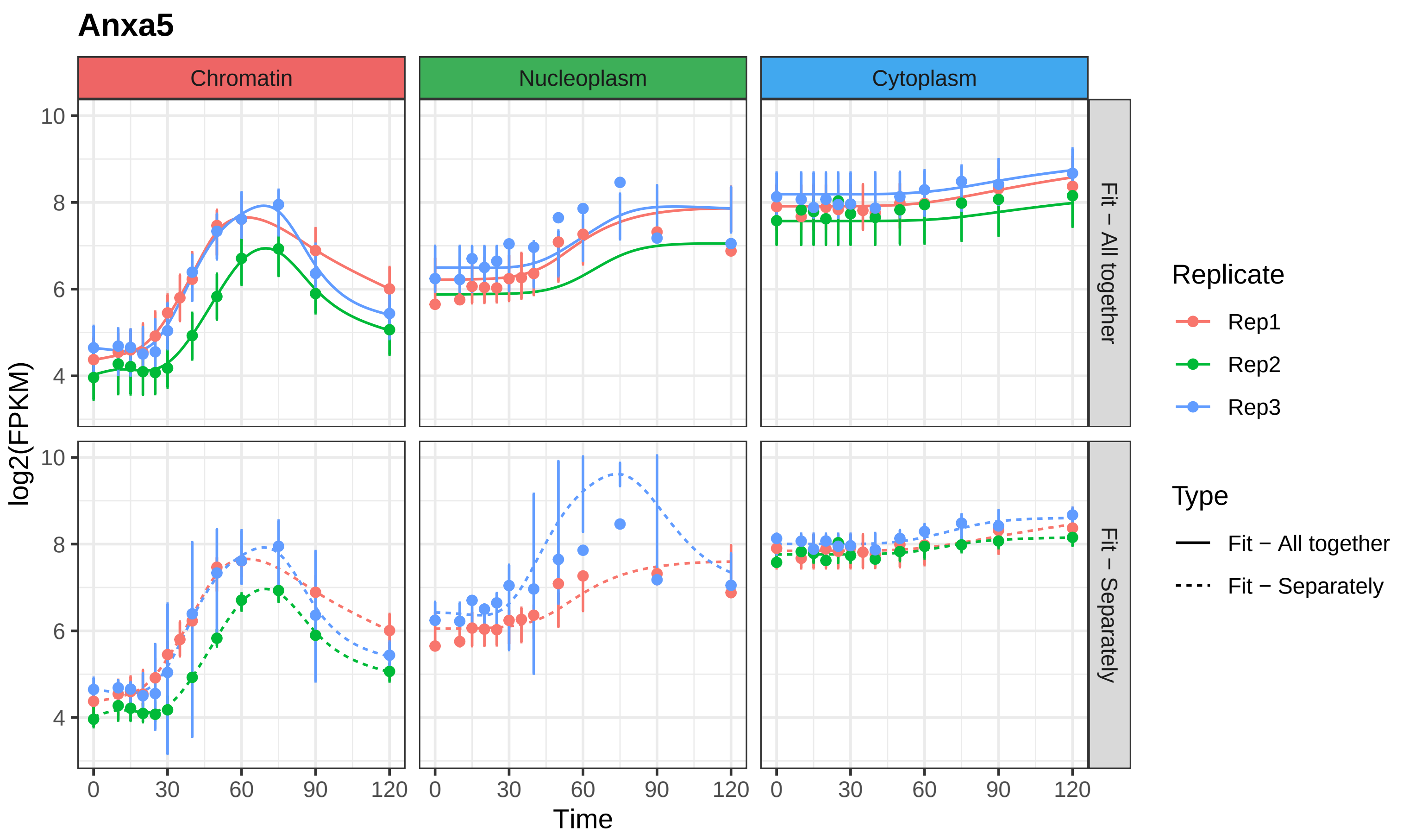

	Together	b1	b2	b3
**−NLL b1 ca**	−4.089	−6.976		
**−NLL b1 np**	8.142	8.301		
**−NLL b1 cyto**	−3.593	−7.244		
**−NLL b2 ca**	−4.144		−11.89	
**−NLL b2 np**				
**−NLL b2 cyto**	0.07443		−7.174	
**−NLL b3 ca**	−3.277			−0.5479
**−NLL b3 np**	20.1			12.07
**−NLL b3 cyto**	−1.873			−9.421
Total	11.34	−5.919	−19.07	2.102
**Total with regul**	11.11	−7.051	−20.21	1.839

	Together	b1	b2	b3
spar	4.027e−01	4.133e−01	0.3806	0.40440
σ_b	1.812e−01	1.354e−01	0.06532	0.08438
σ_t	2.455e−07	7.148e−06	0.0003866	8.01200
ca_{0,b_1}				
$\log_{10}(k_1')$	−1.660e+00	−1.772e+00		−0.27600
$\log_{10}(k_2)$	−2.218e+00	−2.280e+00	5.408 or −2.922	−0.81130
$\log_{10}(k_2')$	−1.710e+00	−1.674e+00		−2.32000
$\log_{10}(k_{deg})$	−2.220e+00	−2.215e+00	−2.922 or 5.408	−2.79600
$\log_{10}(k_1' k_2')$	−3.371e+00	−3.446e+00	3.613	−2.59600
$\log_{10}(k_1'/k_2)$	5.574e−01	5.079e−01		0.53530
$\text{transport} = \log_{10}(k_1' k_2'/k_2)$	−1.153e+00	−1.166e+00	−1.795 or 6.535	−1.78500

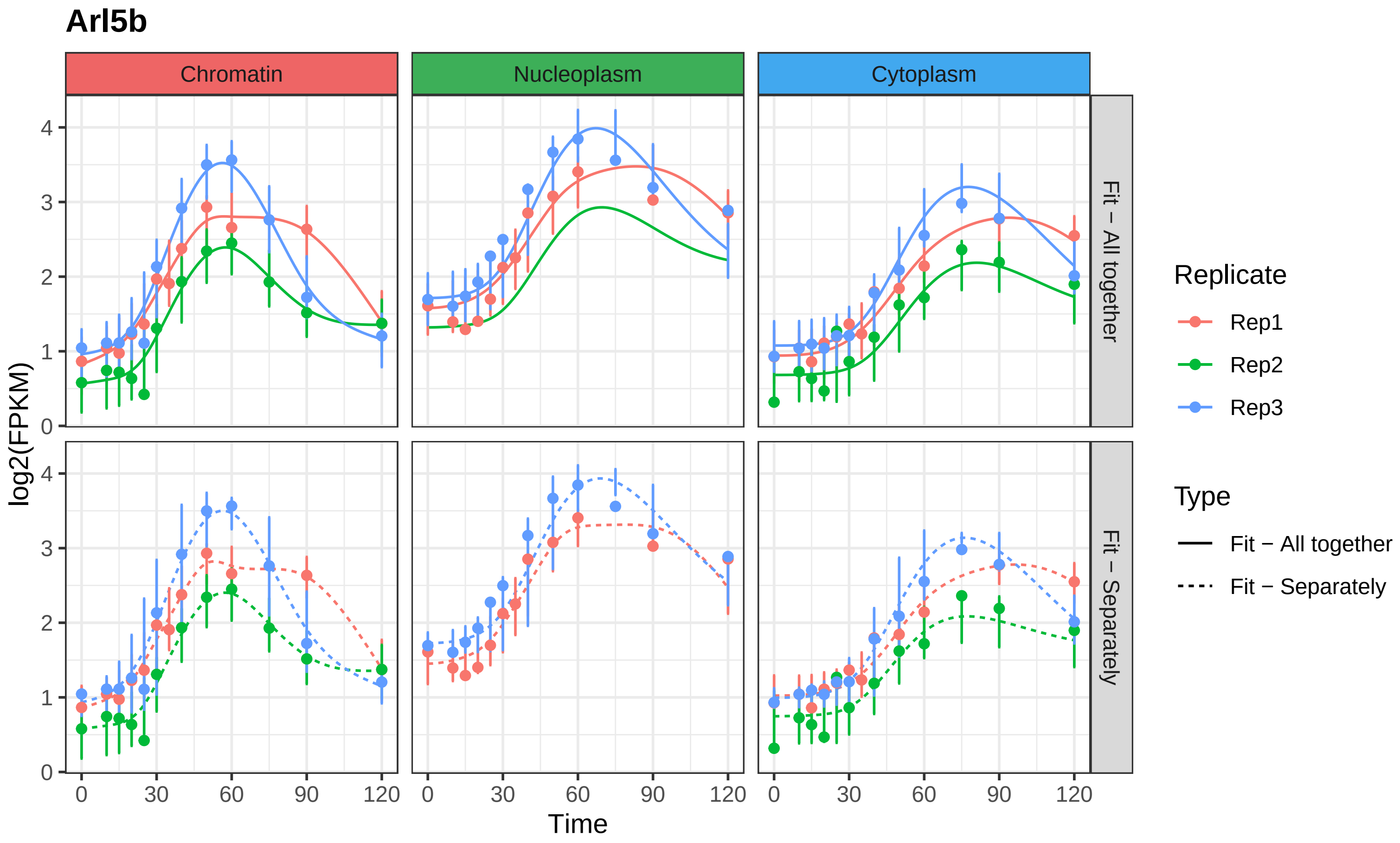

	Together	b1	b2	b3
**−NLL b1 ca**	−6.254	−8.056		
**−NLL b1 np**	1.099	−0.3334		
**−NLL b1 cyto**	−6.089	−7.436		
**−NLL b2 ca**	−5.48		−4.536	
**−NLL b2 np**				
**−NLL b2 cyto**	0.3696		0.04829	
**−NLL b3 ca**	−5.148			−4.527
**−NLL b3 np**	3.344			−0.3166
**−NLL b3 cyto**	−5.043			−8.254
Total	−23.2	−15.83	−4.487	−13.1
**Total with regul**	−24.82	−17.18	−6.126	−14.81

	Together	b1	b2	b3
spar	0.4510	0.39910	0.4383	4.668e−01
σ_b	0.1092	0.07733	0.1143	3.277e−06
σ_t	2.2810	2.69500	0.001688	5.245e+00
ca_{0,b_1}				
$\log_{10}(k_1')$	−0.9561	−0.80070		−1.035e+00
$\log_{10}(k_2)$	−1.1830	−0.98050	4.036 or −1.518	−1.274e+00
$\log_{10}(k_2')$	−1.2600	−1.43900		−1.083e+00
$\log_{10}(k_{deg})$	−1.0680	−1.31100	−1.518 or 4.036	−8.634e−01
$\log_{10}(k_1' k_2')$	−2.2160	−2.24000	2.569	−2.119e+00
$\log_{10}(k_1'/k_2)$	0.2269	0.17970		2.386e−01
$\text{transport} = \log_{10}(k_1' k_2'/k_2)$	−1.0330	−1.26000	−1.467 or 4.088	−8.448e−01

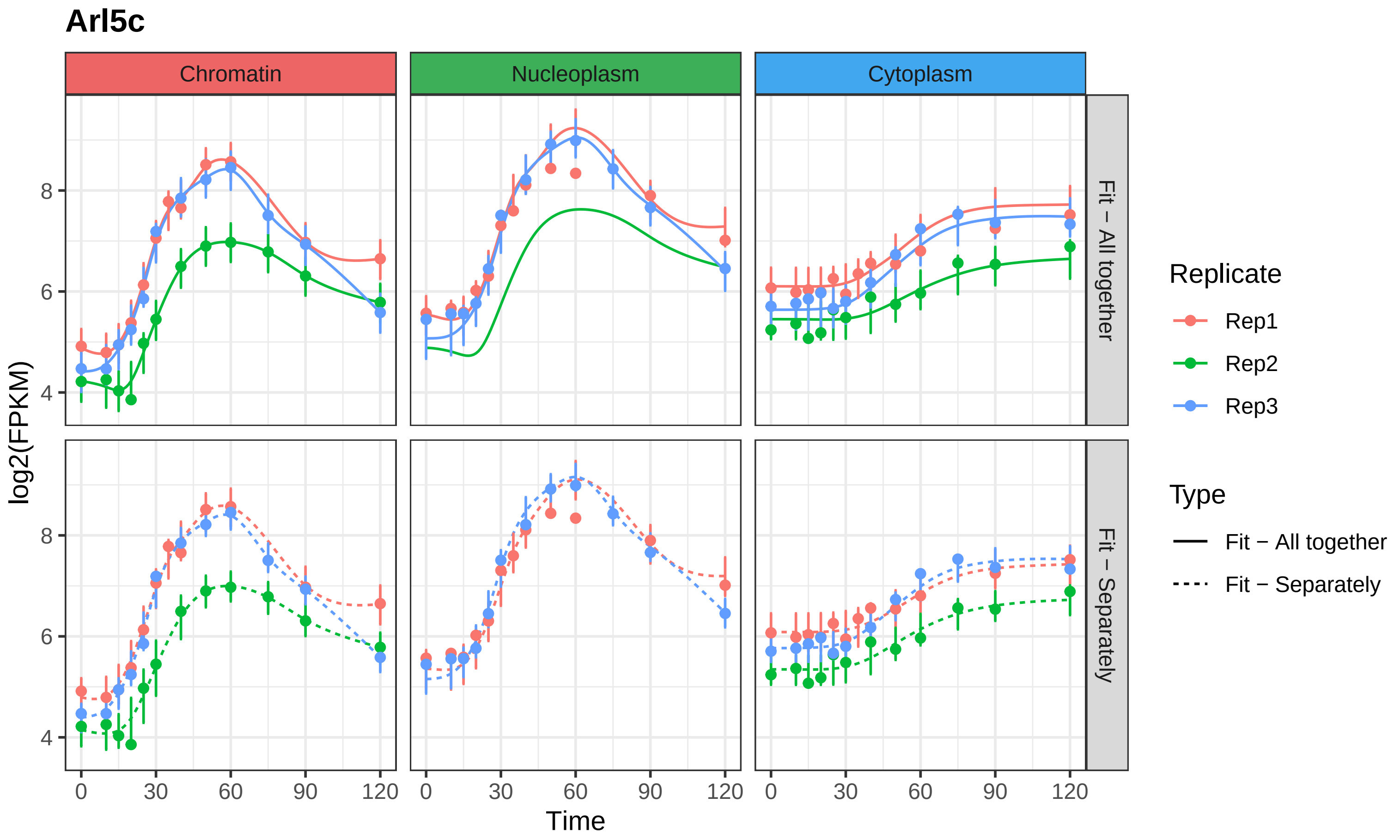

	Together	b1	b2	b3
**−NLL b1 ca**	−7.555	−5.973		
**−NLL b1 np**	8.538	4.858		
**−NLL b1 cyto**	−1.69	−5.938		
**−NLL b2 ca**	−6.184		−4.538	
**−NLL b2 np**				
**−NLL b2 cyto**	−1.825		−2.531	
**−NLL b3 ca**	−6.706			−7.634
**−NLL b3 np**	−1.664			−2.506
**−NLL b3 cyto**	−3.337			−6.07
Total	−20.42	−7.053	−7.069	−16.21
**Total with regul**	−17.96	−7.64	−8.249	−15.38

	Together	b1	b2	b3
spar	0.31000	3.845e−01	0.398	0.33360
σ_b	0.13170	1.322e−01	0.1017	0.09205
σ_t	0.00251	2.979e−05	2.008	0.76950
ca_{0,b_1}				
$\log_{10}(k_1')$	−0.31730	−4.747e−01		−0.15480
$\log_{10}(k_2)$	−0.51490	−6.451e−01	3.925 or −2.25	−0.38440
$\log_{10}(k_2')$	−2.15600	−2.310e+00		−2.19600
$\log_{10}(k_{deg})$	−2.32700	−2.529e+00	−2.25 or 3.925	−2.38100
$\log_{10}(k_1'k_2')$	−2.47300	−2.785e+00	2.035	−2.35100
$\log_{10}(k_1'/k_2)$	0.19770	1.704e−01		0.22960
$\text{transport} = \log_{10}(k_1'k_2'/k_2)$	−1.95800	−2.140e+00	−1.889 or 4.285	−1.96700

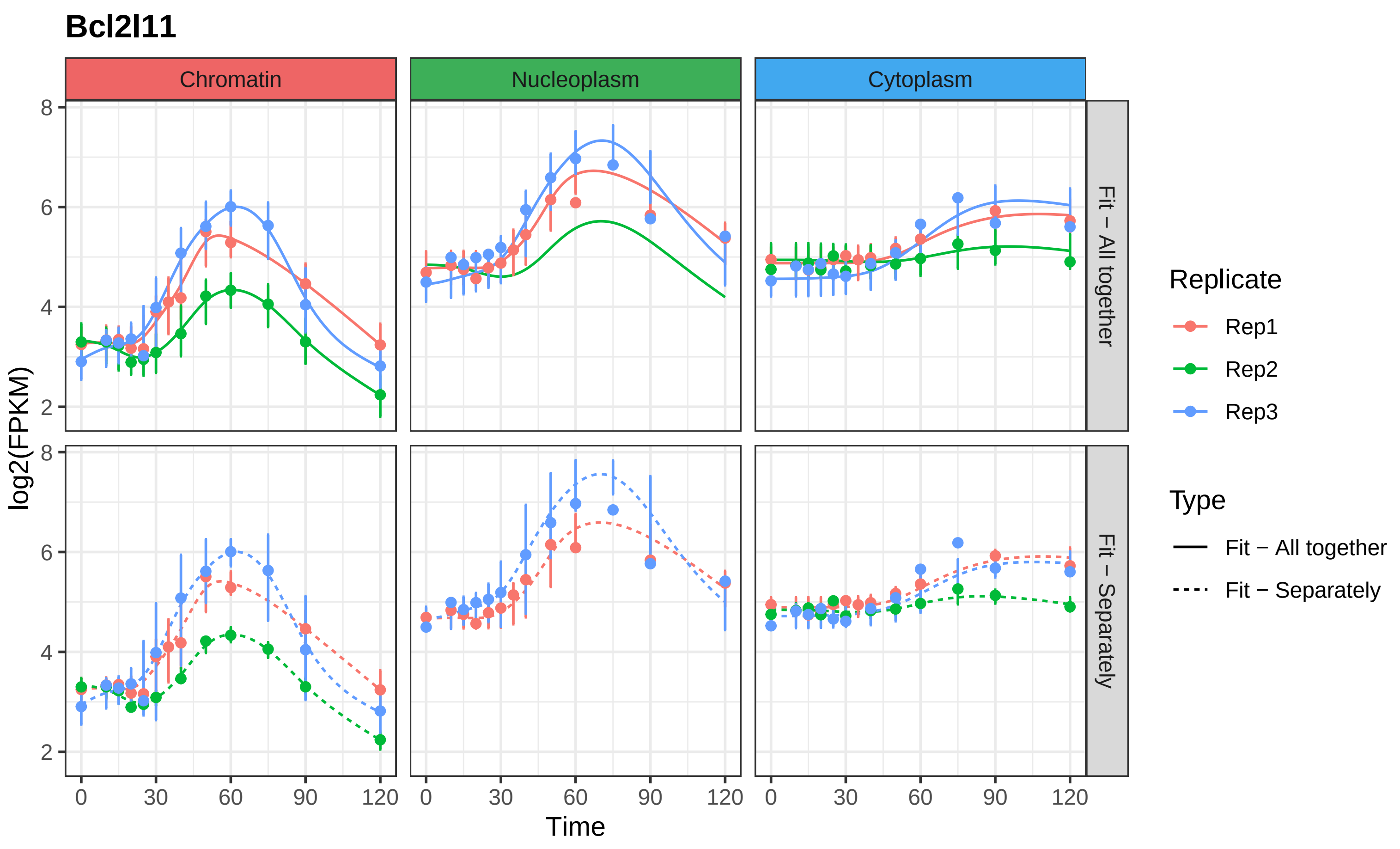

	Together	b1	b2	b3
**−NLL b1 ca**	−5.199	−6.462		
**−NLL b1 np**	0.2537	−1.409		
**−NLL b1 cyto**	−7.94	−10.39		
**−NLL b2 ca**	−7.553		−13.88	
**−NLL b2 np**				
**−NLL b2 cyto**	−6.103		−11.67	
**−NLL b3 ca**	−3.915			−2.837
**−NLL b3 np**	10.35			2.241
**−NLL b3 cyto**	2.553			−3.855
Total	−17.56	−18.26	−25.55	−4.451
**Total with regul**	−18.13	−19.64	−26.44	−5

	Together	b1	b2	b3
spar	0.3851	0.40680	0.3688	0.38650
σ_b	0.1190	0.06936	0.04676	0.08252
σ_t	3.0880	4.02900	0.001656	5.58500
ca_{0,b_1}				
$\log_{10}(k_1')$	−0.5899	−0.69230		−0.48150
$\log_{10}(k_2)$	−1.0460	−1.11800	3.098 or −2.056	−0.99820
$\log_{10}(k_2')$	−1.9760	−1.90800		−2.30800
$\log_{10}(k_{deg})$	−2.0050	−1.97400	−2.056 or 3.098	−2.32400
$\log_{10}(k_1'k_2')$	−2.5660	−2.60000	1.498	−2.79000
$\log_{10}(k_1'/k_2)$	0.4560	0.42550		0.51670
$transport = \log_{10}(k_1'k_2'/k_2)$	−1.5200	−1.48200	−1.6 or 3.555	−1.79100

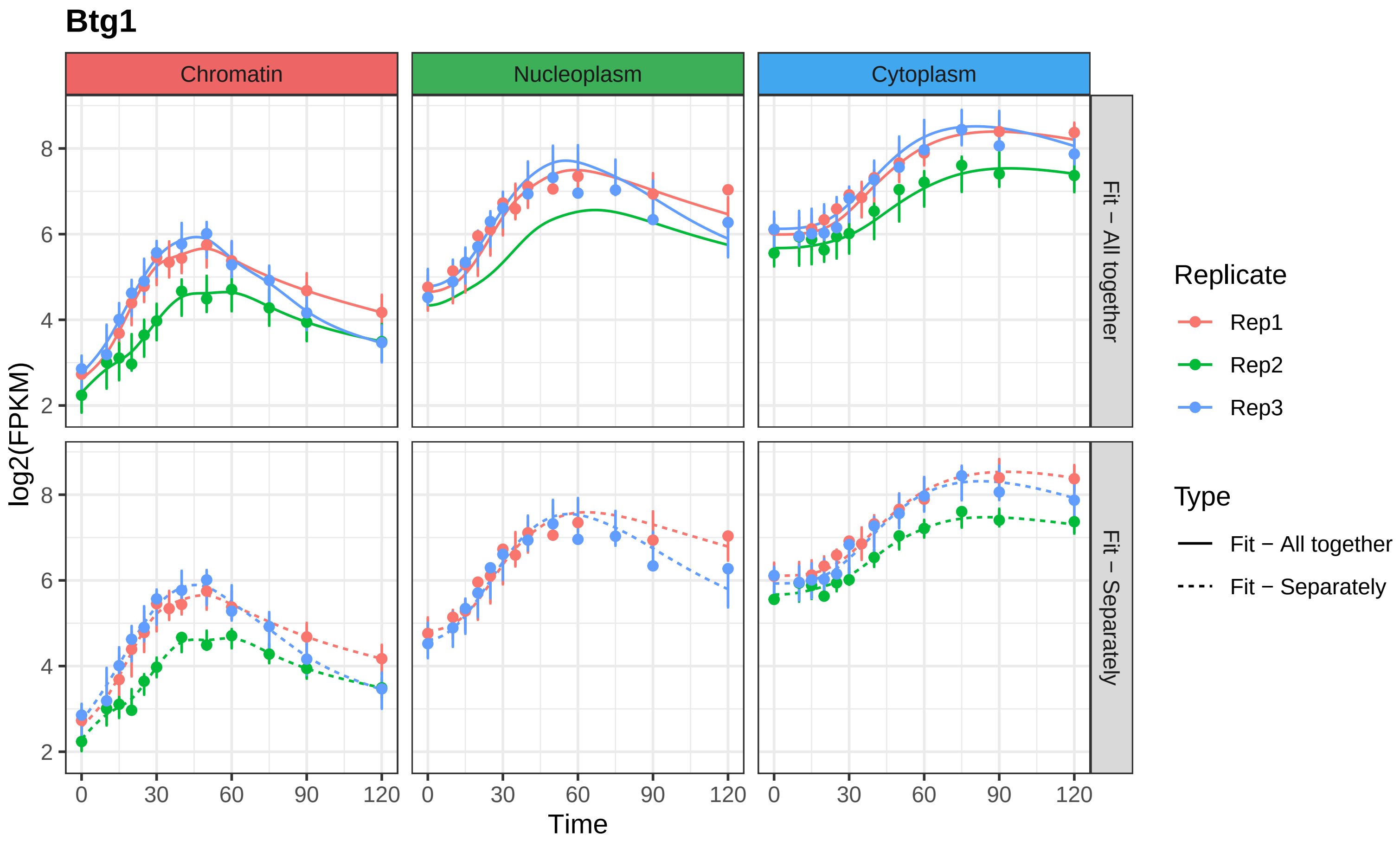

	Together	b1	b2	b3
**−NLL b1 ca**	−5.881	−6.015		
**−NLL b1 np**	4.709	2.3		
**−NLL b1 cyto**	−3.521	−5.915		
**−NLL b2 ca**	−5.653		−8.564	
**−NLL b2 np**				
**−NLL b2 cyto**	−3.882		−7.806	
**−NLL b3 ca**	−5.455			−3.822
**−NLL b3 np**	7.261			3.859
**−NLL b3 cyto**	0.09696			−4.174
Total	−12.32	−9.63	−16.37	−4.136
**Total with regul**	−12.48	−10.97	−16.5	−5.468

	Together	b1	b2	b3
spar	3.744e−01	0.4095	0.3517	4.448e−01
σ_b	1.430e−01	0.1072	0.07094	1.401e−01
σ_t	5.218e−06	1.9240	0.0003381	1.259e−05
ca_{0,b_1}				
$\log_{10}(k_1')$	−5.313e−01	−0.6238		−5.835e−01
$\log_{10}(k_2)$	−1.144e+00	−1.3000	−0.3672 or −1.667	−1.156e+00
$\log_{10}(k_2')$	−1.140e+00	−1.1100		−1.178e+00
$\log_{10}(k_{deg})$	−1.543e+00	−1.4960	−1.667 or −0.3672	−1.574e+00
$\log_{10}(k_1'k_2')$	−1.671e+00	−1.7340	−1.018	−1.762e+00
$\log_{10}(k_1'/k_2)$	6.125e−01	0.6757		5.729e−01
$\text{transport} = \log_{10}(k_1'k_2'/k_2)$	−5.271e−01	−0.4346	−0.6513 or 0.6487	−6.054e−01

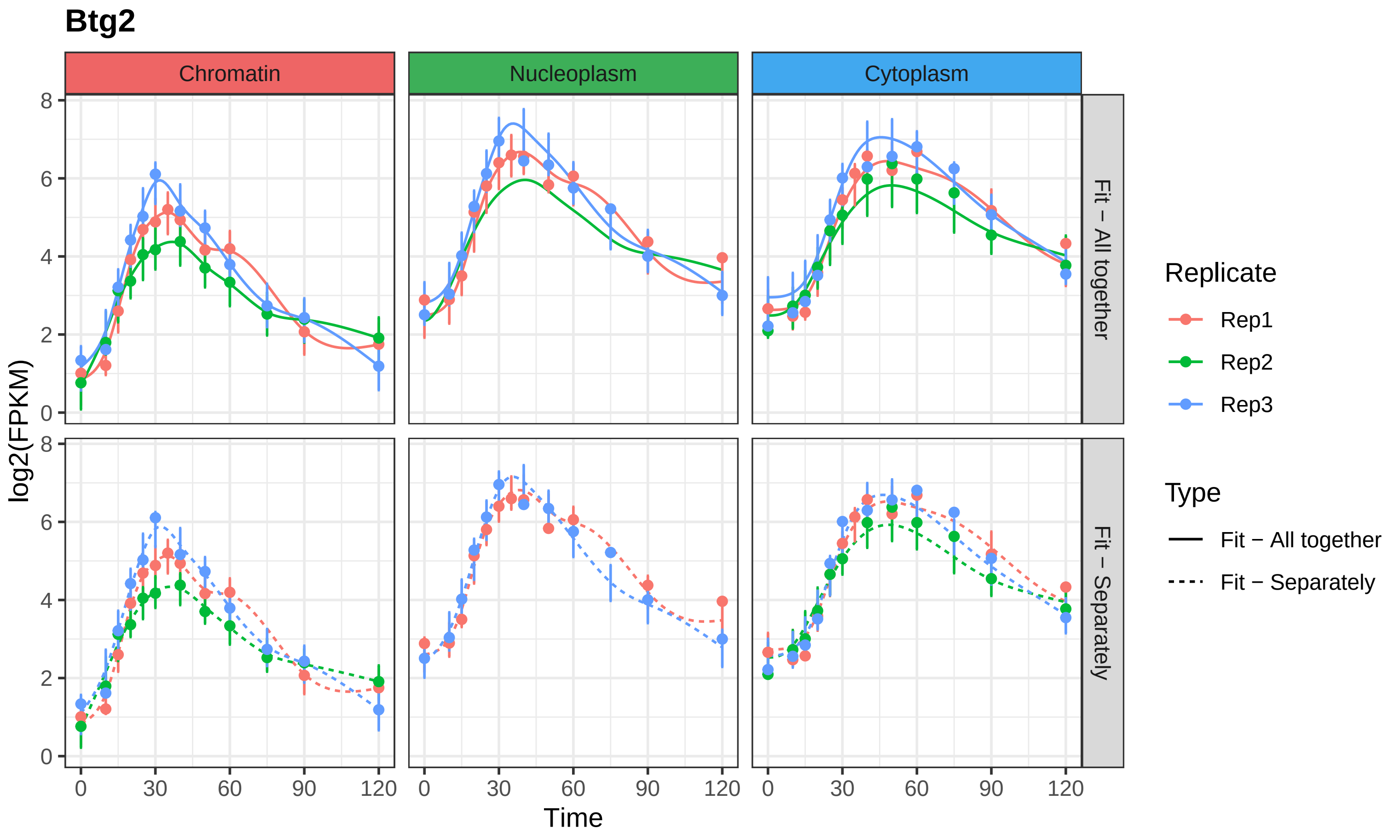

	Together	b1	b2	b3
**−NLL b1 ca**	−3.011	−4.688		
**−NLL b1 np**	2.189	1.179		
**−NLL b1 cyto**	1.942	1.482		
**−NLL b2 ca**	−3.259		−4.077	
**−NLL b2 np**				
**−NLL b2 cyto**	3.303		1.299	
**−NLL b3 ca**	−1.801			−0.3227
**−NLL b3 np**	3.81			−1.116
**−NLL b3 cyto**	9.88			2.683
Total	13.05	−2.027	−2.778	1.245
**Total with regul**	17	0.9804	−3.293	2.354

	Together	b1	b2	b3
spar	0.298400	3.044e−01	0.3851	0.3440
σ_b	0.182800	1.474e−01	0.1386	0.1532
σ_t	0.000246	7.925e−05	0.01278	0.6565
ca_{0,b_1}				
$\log_{10}(k_1')$	−0.167500	−8.408e−02		−0.1561
$\log_{10}(k_2)$	−0.664600	−6.185e−01	−0.8218 or −0.8581	−0.5792
$\log_{10}(k_2')$	−1.055000	−1.097e+00		−1.1330
$\log_{10}(k_{deg})$	−1.095000	−1.132e+00	−0.8581 or −0.8218	−1.1550
$\log_{10}(k_1'k_2')$	−1.222000	−1.181e+00	−1.136	−1.2890
$\log_{10}(k_1'/k_2)$	0.497100	5.344e−01		0.4231
transport $= \log_{10}(k_1'k_2'/k_2)$	−0.557800	−5.624e−01	−0.3138 or −0.2775	−0.7102

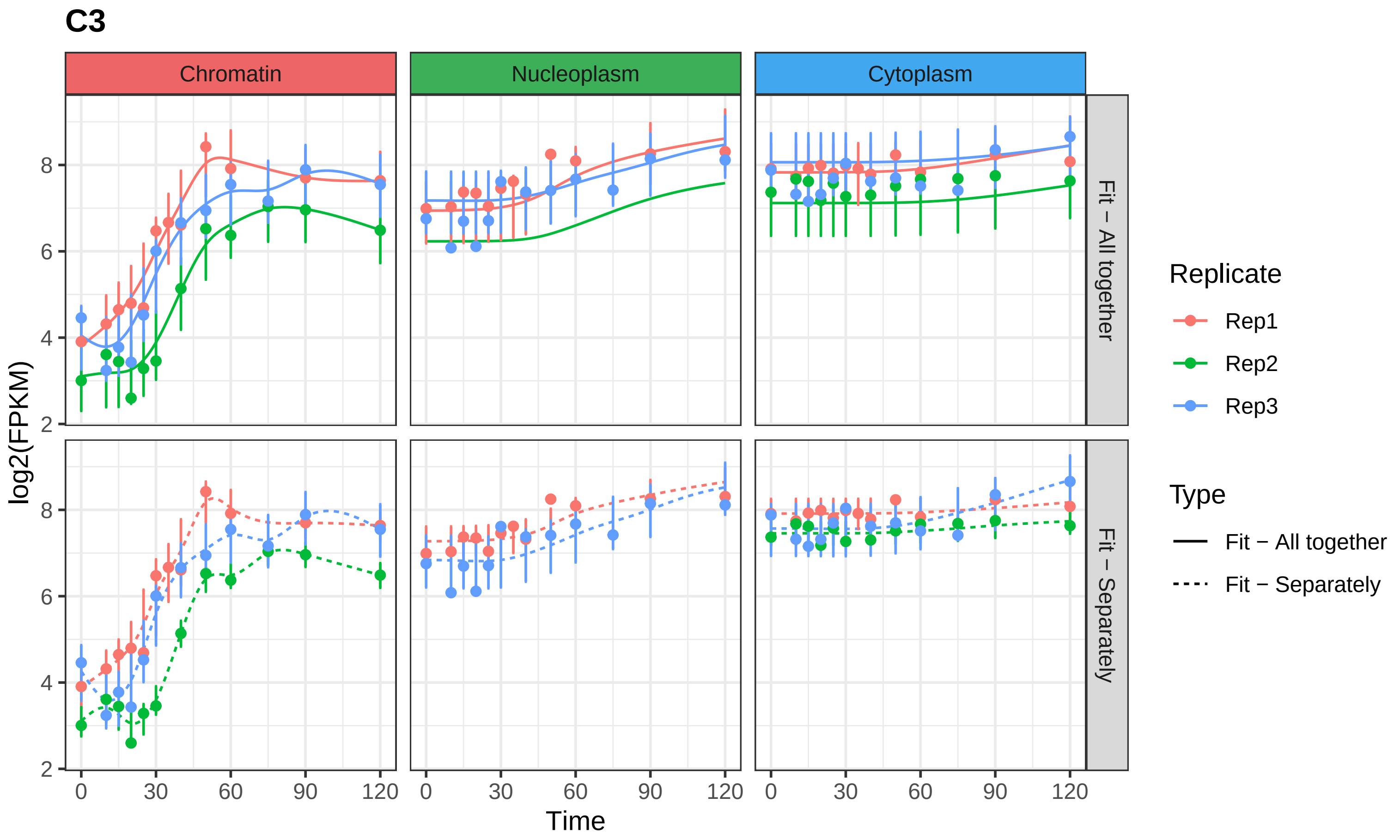

	Together	b1	b2	b3
**−NLL b1 ca**	3.344	−1.524		
**−NLL b1 np**	5.341	2.82		
**−NLL b1 cyto**	0.1362	−6.089		
**−NLL b2 ca**	3.274		−4.448	
**−NLL b2 np**				
**−NLL b2 cyto**	5.011		−4.413	
**−NLL b3 ca**	4.889			1.32
**−NLL b3 np**	10.93			8.457
**−NLL b3 cyto**	11.44			0.9584
Total	44.37	−4.794	−8.861	10.74
**Total with regul**	45.09	−4.899	−7.347	11.68

	Together	b1	b2	b3
spar	0.4472	0.3696	0.3205	0.3760
σ_b	0.2443	0.1215	0.09792	0.2074
σ_t	1.6030	3.0090	0.0002181	1.0540
ca_{0,b_1}				
$\log_{10}(k_1')$	−1.7960	−1.8300		−1.6900
$\log_{10}(k_2)$	−2.7380	−2.8480	3.84 or −3.617	−2.4650
$\log_{10}(k_2')$	−1.9270	−2.3330		−1.4070
$\log_{10}(k_{deg})$	−2.1930	−2.5260	−3.617 or 3.84	−1.6260
$\log_{10}(k_1' k_2')$	−3.7230	−4.1630	1.532	−3.0970
$\log_{10}(k_1'/k_2)$	0.9420	1.0180		0.7757
transport $= \log_{10}(k_1' k_2'/k_2)$	−0.9847	−1.3150	−2.307 or 5.149	−0.6312

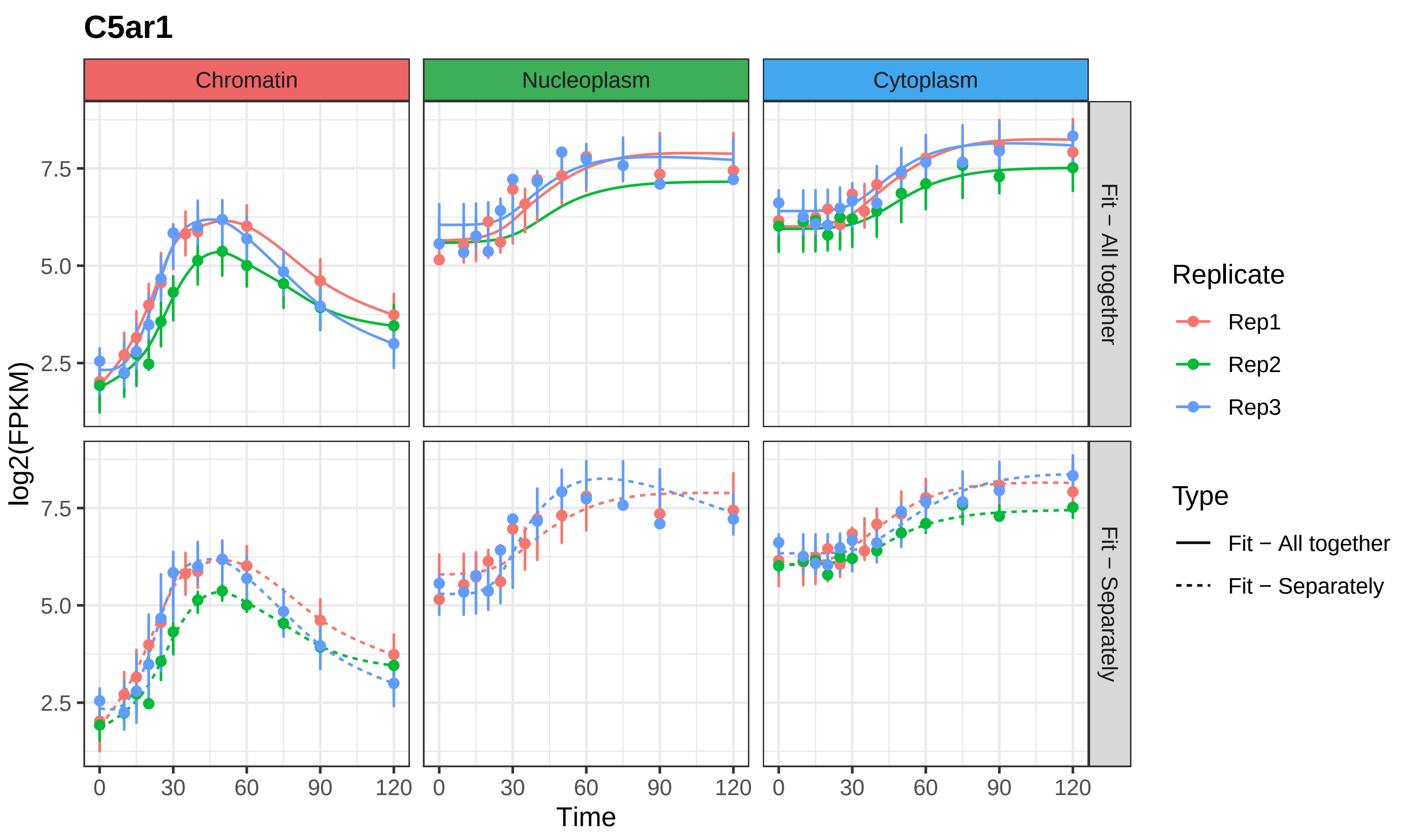

	Together	b1	b2	b3
**−NLL b1 ca**	−2.556	−2.496		
**−NLL b1 np**	8.707	9.834		
**−NLL b1 cyto**	0.1299	−1.496		
**−NLL b2 ca**	−2.34		−6.385	
**−NLL b2 np**				
**−NLL b2 cyto**	−2.43		−8.161	
**−NLL b3 ca**	−1.87			−0.2124
**−NLL b3 np**	16.77			9.626
**−NLL b3 cyto**	1.165			−0.7839
Total	17.57	5.842	−14.55	8.63
**Total with regul**	18.53	5.705	−15.9	9.847

	Together	b1	b2	b3
spar	3.657e−01	0.40070	0.3908	0.3538
σ_b	1.927e−01	0.18410	0.0665	0.1758
σ_t	3.030e−06	0.01241	1.504	2.6500
ca_{0,b_1}				
$\log_{10}(k_1')$	−1.174e+00	−1.21100		−0.7335
$\log_{10}(k_2)$	−2.294e+00	−2.38900	0.4006 or −2.41	−1.6180
$\log_{10}(k_2')$	−5.757e−01	0.57050		−1.6560
$\log_{10}(k_{deg})$	−6.825e−01	0.49150	−2.41 or 0.4006	−1.9690
$\log_{10}(k_1' k_2')$	−1.750e+00	−0.64040	−0.7446	−2.3890
$\log_{10}(k_1'/k_2)$	1.120e+00	1.17800		0.8844
$\text{transport} = \log_{10}(k_1' k_2'/k_2)$	5.444e−01	1.74800	−1.145 or 1.666	−0.7715

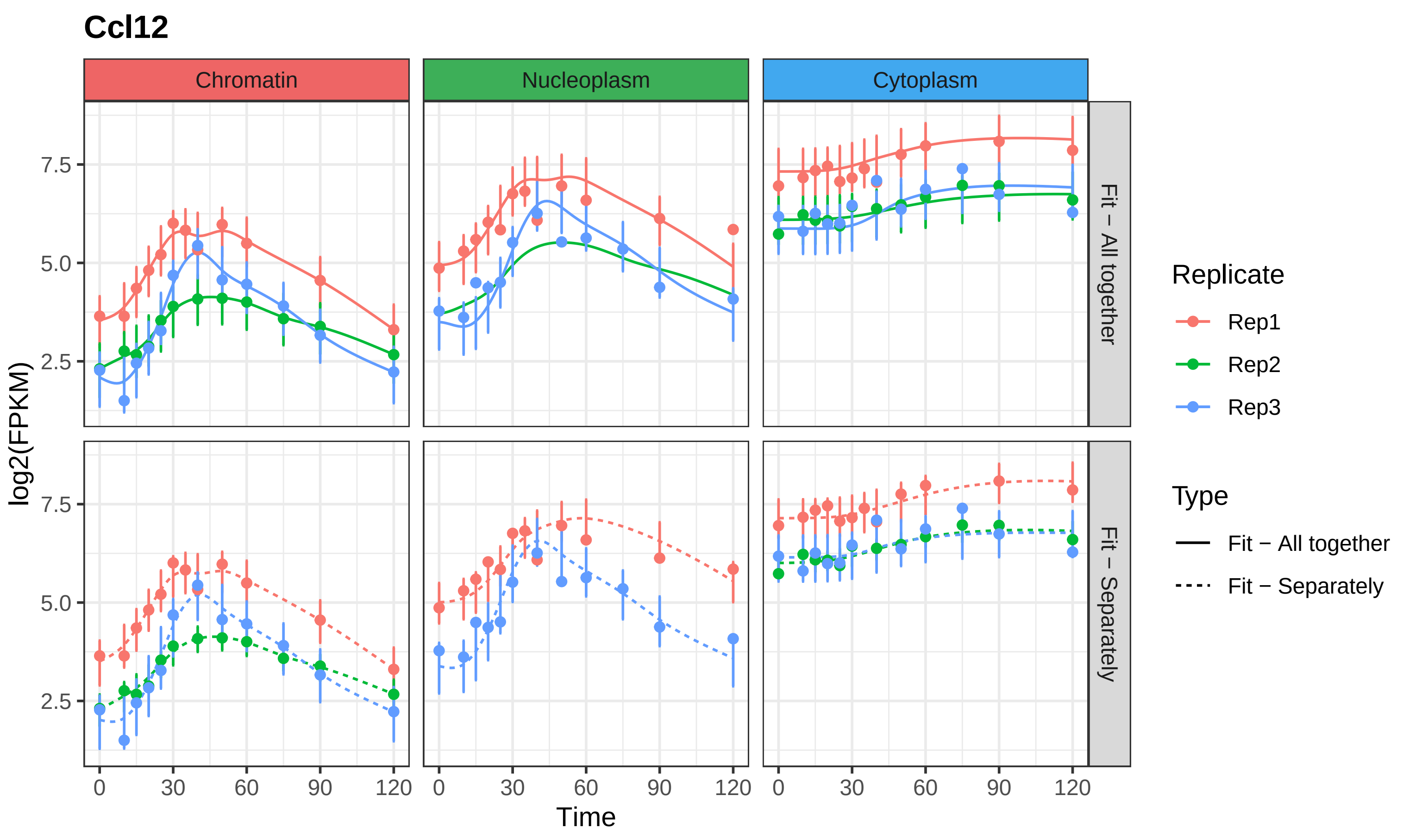

	Together	b1	b2	b3
**−NLL b1 ca**	−0.9887	−1.741		
**−NLL b1 np**	11.16	7.56		
**−NLL b1 cyto**	1.729	−2.057		
**−NLL b2 ca**	−1.834		−5.873	
**−NLL b2 np**				
**−NLL b2 cyto**	−0.7605		−4.318	
**−NLL b3 ca**	0.3186			1.746
**−NLL b3 np**	10.11			7.063
**−NLL b3 cyto**	7.886			3.2
Total	27.62	3.762	−10.19	12.01
**Total with regul**	28.88	3.446	−11.96	12.25

	Together	b1	b2	b3
spar	0.3659000	3.987e−01	0.4216	0.4001
σ_b	0.2082000	1.719e−01	0.08773	0.2009
σ_t	0.0003466	9.965e−07	0.003432	1.4270
ca_{0,b_1}				
$\log_{10}(k_1')$	−0.1912000	−7.259e−01		8.5520
$\log_{10}(k_2)$	−0.6136000	−1.178e+00	7.72 or −2.137	8.1410
$\log_{10}(k_2')$	−1.5930000	−1.587e+00		−1.8790
$\log_{10}(k_{deg})$	−2.3090000	−2.229e+00	−2.137 or 7.72	−2.7110
$\log_{10}(k_1'k_2')$	−1.7840000	−2.313e+00	6.704	6.6730
$\log_{10}(k_1'/k_2)$	0.4225000	4.524e−01		0.4113
$transport = \log_{10}(k_1'k_2'/k_2)$	−1.1700000	−1.134e+00	−1.016 or 8.84	−1.4670

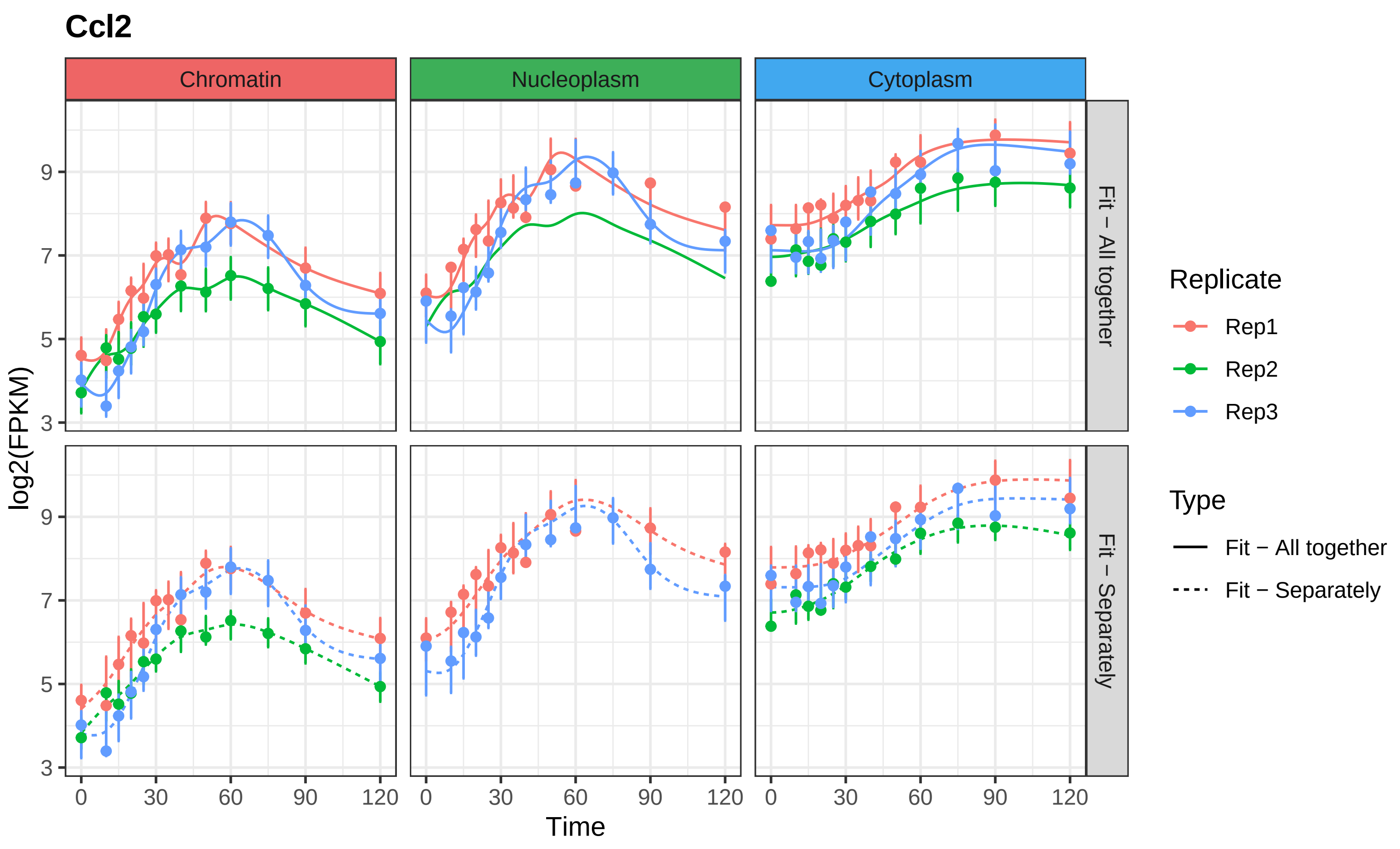

	Together	b1	b2	b3
**−NLL b1 ca**	−2.69	1.764		
**−NLL b1 np**	8.777	5.779		
**−NLL b1 cyto**	−0.2206	0.7075		
**−NLL b2 ca**	−4.131		−3.895	
**−NLL b2 np**				
**−NLL b2 cyto**	0.2329		−2.517	
**−NLL b3 ca**	−3.796			−1.312
**−NLL b3 np**	5.052			4.786
**−NLL b3 cyto**	4.242			2.947
Total	7.467	8.251	−6.412	6.422
**Total with regul**	12.44	7.658	−7.723	7.129

	Together	b1	b2	b3
spar	0.280100	0.4401	0.4092	0.370400
σ_b	0.174400	0.1767	0.1162	0.187500
σ_t	0.009238	2.6840	0.006273	0.007971
ca_{0,b_1}				
$\log_{10}(k_1')$	8.489000	−0.2697		4.808000
$\log_{10}(k_2)$	8.032000	−0.7736	4.841 or −1.65	4.359000
$\log_{10}(k_2')$	−1.435000	−1.4890		−1.588000
$\log_{10}(k_{deg})$	−1.938000	−2.0040	−1.65 or 4.841	−2.192000
$\log_{10}(k_1'k_2')$	7.053000	−1.7580	4.059	3.220000
$\log_{10}(k_1'/k_2)$	0.456500	0.5039		0.449300
transport = $\log_{10}(k_1'k_2'/k_2)$	−0.978800	−0.9848	−0.7814 or 5.709	−1.138000

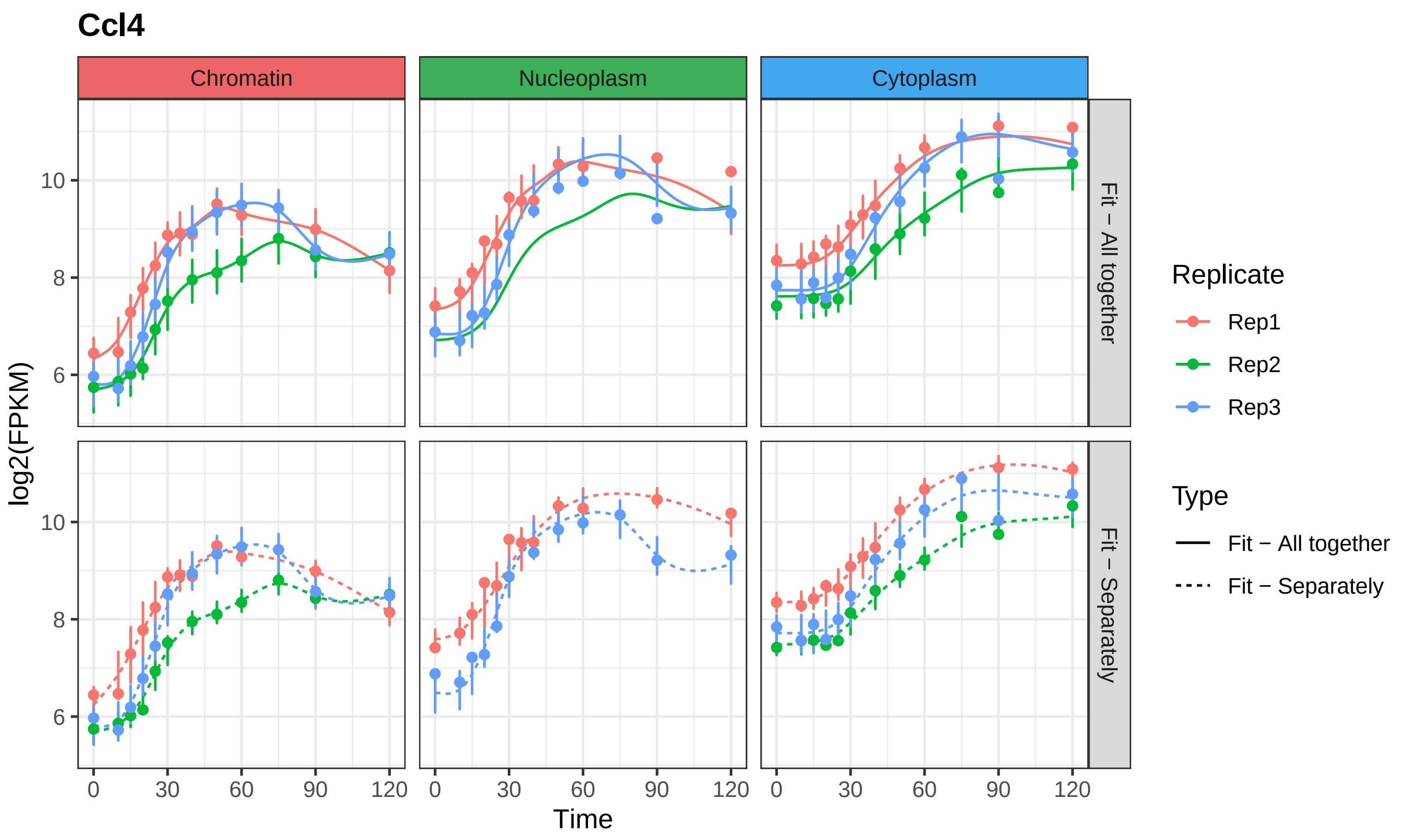

	Together	b1	b2	b3
**−NLL b1 ca**	−5.42	−5.017		
**−NLL b1 np**	7.858	0.0908		
**−NLL b1 cyto**	−3.879	−10.72		
**−NLL b2 ca**	−6.098		−10.18	
**−NLL b2 np**				
**−NLL b2 cyto**	−2.257		−5.349	
**−NLL b3 ca**	−5.3			−5.753
**−NLL b3 np**	4.966			−0.2208
**−NLL b3 cyto**	3.081			−0.1419
Total	−7.049	−15.65	−15.53	−6.115
**Total with regul**	−6.47	−17.58	−16.42	−5.678

	Together	b1	b2	b3
spar	0.3566000	0.44340	0.3746	0.3533000
σ_b	0.1517000	0.06601	0.07295	0.1331000
σ_t	0.0001945	2.85700	1.174	0.0005357
ca_{0,b_1}				
$\log_{10}(k_1')$	−0.4299000	−0.79290		0.3622000
$\log_{10}(k_2)$	−0.7378000	−1.20000	4.245 or −1.607	0.1620000
$\log_{10}(k_2')$	−1.2460000	−1.10000		−1.3570000
$\log_{10}(k_{deg})$	−1.5160000	−1.32700	−1.607 or 4.245	−1.7260000
$\log_{10}(k_1'k_2')$	−1.6760000	−1.89300	3.182	−0.9952000
$\log_{10}(k_1'/k_2)$	0.3079000	0.40680		0.2003000
$\text{transport} = \log_{10}(k_1'k_2'/k_2)$	−0.9379000	−0.69280	−1.063 or 4.789	−1.1570000

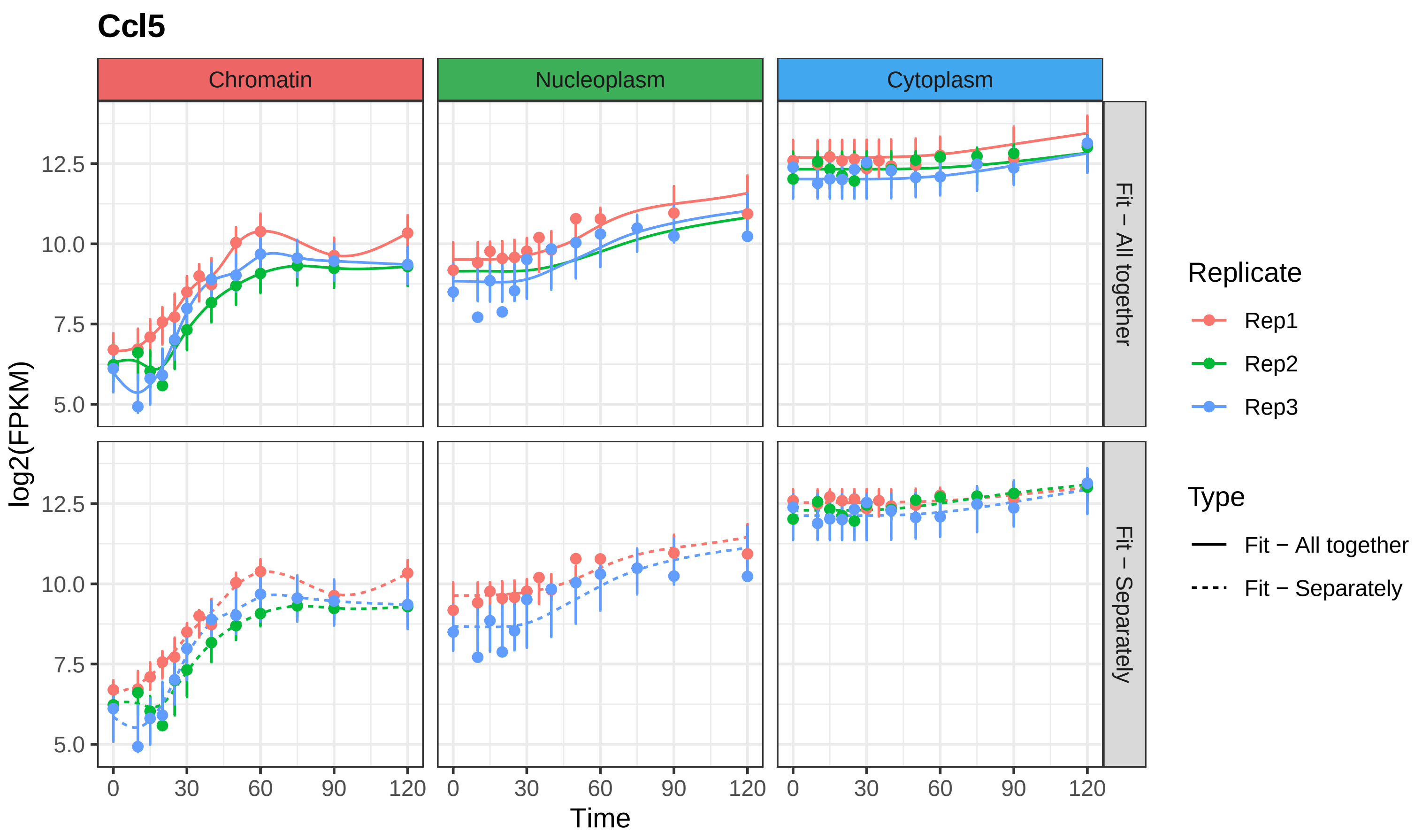

	Together	b1	b2	b3
**−NLL b1 ca**	−2.77	−4.08		
**−NLL b1 np**	4.282	6.416		
**−NLL b1 cyto**	0.7535	−5.787		
**−NLL b2 ca**	−0.8069		−0.826	
**−NLL b2 np**				
**−NLL b2 cyto**	0.1456		−2.572	
**−NLL b3 ca**	−1.762			1.402
**−NLL b3 np**	20.05			14.27
**−NLL b3 cyto**	0.3662			0.7165
Total	20.26	−3.452	−3.398	16.39
**Total with regul**	23.76	−4.444	−2.713	18.03

	Together	b1	b2	b3
spar	3.112e−01	4.139e−01	0.347	3.800e−01
σ_b	1.983e−01	1.460e−01	0.1179	2.439e−01
σ_t	2.838e−06	9.041e−05	3.049	7.266e−06
ca_{0,b_1}				
$\log_{10}(k_1')$	−1.426e+00	−1.526e+00		−1.365e+00
$\log_{10}(k_2)$	−2.283e+00	−2.446e+00	4.909 or −2.815	−2.214e+00
$\log_{10}(k_2')$	−1.311e+00	−1.563e+00		−1.343e+00
$\log_{10}(k_{deg})$	−2.269e+00	−2.435e+00	−2.815 or 4.909	−2.383e+00
$\log_{10}(k_1'k_2')$	−2.737e+00	−3.089e+00	3.9	−2.709e+00
$\log_{10}(k_1'/k_2)$	8.578e−01	9.199e−01		8.490e−01
$transport = \log_{10}(k_1'k_2'/k_2)$	−4.533e−01	−6.433e−01	−1.009 or 6.715	−4.941e−01

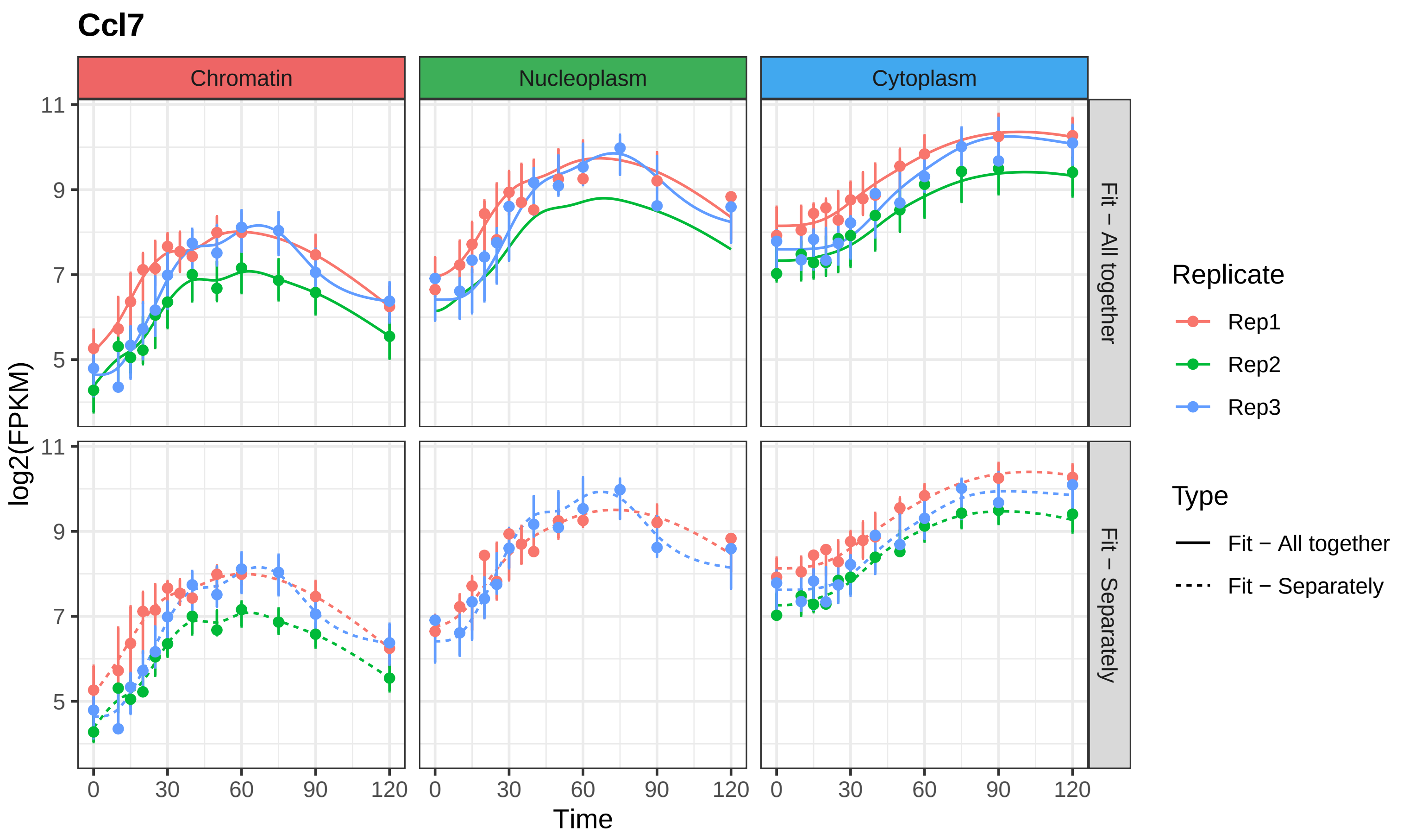

	Together	b1	b2	b3
**−NLL b1 ca**	−3.847	−4.021		
**−NLL b1 np**	9.852	2.673		
**−NLL b1 cyto**	−3.036	−4.174		
**−NLL b2 ca**	−3.03		−5.246	
**−NLL b2 np**				
**−NLL b2 cyto**	−1.817		−4.562	
**−NLL b3 ca**	−2.062			−2.588
**−NLL b3 np**	9.143			3.694
**−NLL b3 cyto**	2.327			0.4676
Total	7.531	−5.521	−9.808	1.574
**Total with regul**	8.369	−7.041	−9.502	2.313

	Together	b1	b2	b3
spar	0.3563	0.4343	0.347	0.3578000
σ_b	0.1622	0.0897	0.1002	0.1647000
σ_t	2.0710	4.1610	0.0008822	0.0001075
ca_{0,b_1}				
$\log_{10}(k_1')$	−0.3049	−0.6366		0.9680000
$\log_{10}(k_2)$	−0.8377	−1.1200	−0.4243 or −1.629	0.4334000
$\log_{10}(k_2')$	−1.3740	−1.2590		−1.5830000
$\log_{10}(k_{deg})$	−1.7310	−1.6660	−1.629 or −0.4243	−1.9470000
$\log_{10}(k_1' k_2')$	−1.6790	−1.8960	−1.184	−0.6146000
$\log_{10}(k_1'/k_2)$	0.5328	0.4833		0.5346000
transport = $\log_{10}(k_1' k_2'/k_2)$	−0.8408	−0.7758	−0.7592 or 0.4454	−1.0480000

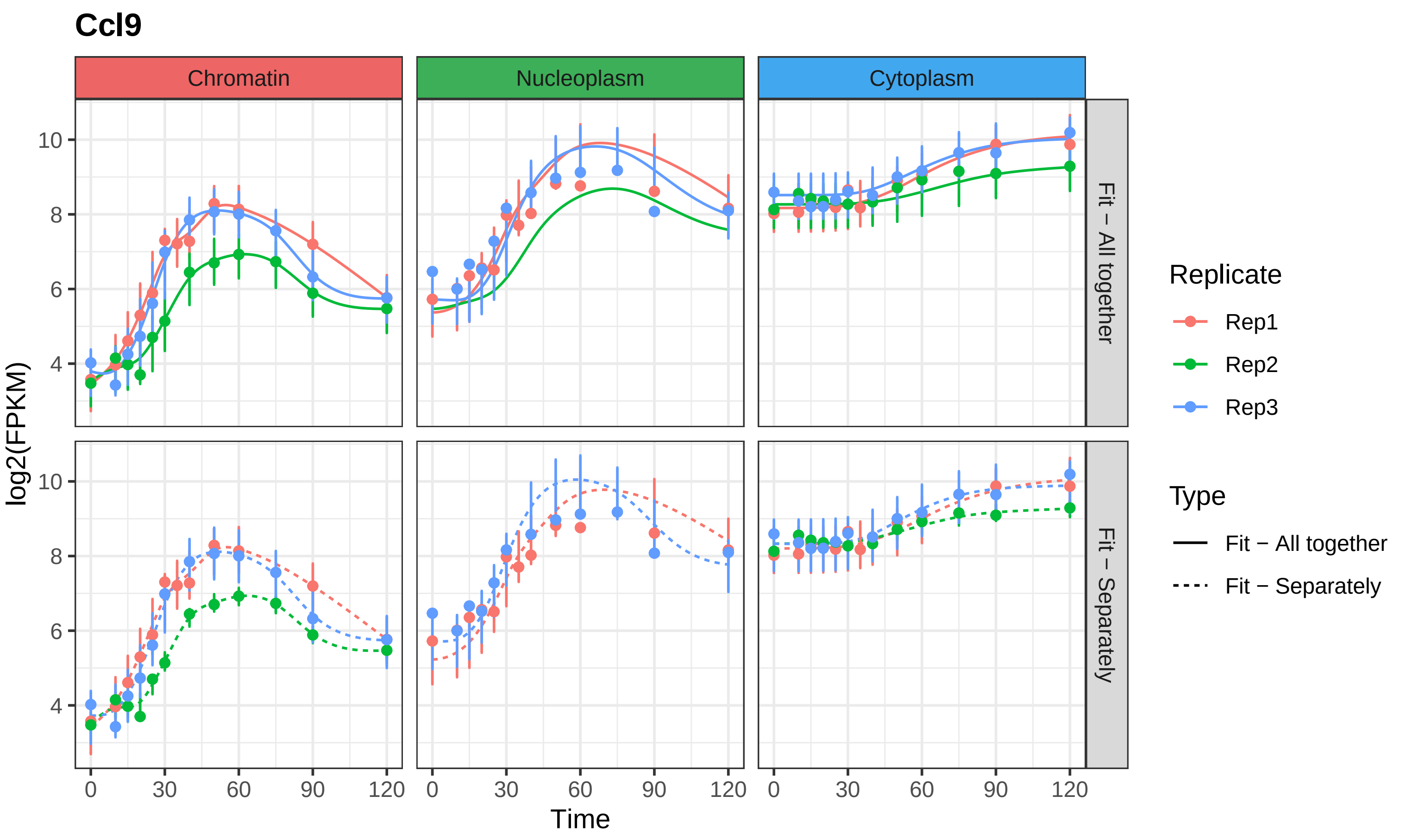

	Together	b1	b2	b3
**−NLL b1 ca**	−0.06145	−0.1694		
**−NLL b1 np**	15.75	14.59		
**−NLL b1 cyto**	−1.329	−1.131		
**−NLL b2 ca**	−0.7992		−6.217	
**−NLL b2 np**				
**−NLL b2 cyto**	−1.374		−7.996	
**−NLL b3 ca**	0.1377			0.754
**−NLL b3 np**	20.79			14.77
**−NLL b3 cyto**	−0.9828			−0.3328
Total	32.13	13.29	−14.21	15.19
**Total with regul**	33.59	14.24	−12.9	16.46

	Together	b1	b2	b3
spar	0.3621	0.3810	0.321	3.868e−01
σ_b	0.2078	0.2137	0.07815	2.363e−01
σ_t	1.9260	1.2160	0.000936	9.637e−05
ca_{0,b_1}				
$\log_{10}(k_1')$	−0.5535	−0.6268		−1.523e−01
$\log_{10}(k_2)$	−1.1330	−1.1730	3.861 or −2.724	−7.502e−01
$\log_{10}(k_2')$	−1.8080	−1.8000		−1.872e+00
$\log_{10}(k_{deg})$	−2.6500	−2.6950	−2.724 or 3.861	−2.659e+00
$\log_{10}(k_1' k_2')$	−2.3610	−2.4270	2.578	−2.025e+00
$\log_{10}(k_1'/k_2)$	0.5794	0.5459		5.978e−01
transport $= \log_{10}(k_1' k_2'/k_2)$	−1.2290	−1.2540	−1.283 or 5.302	−1.274e+00

Ccrl2

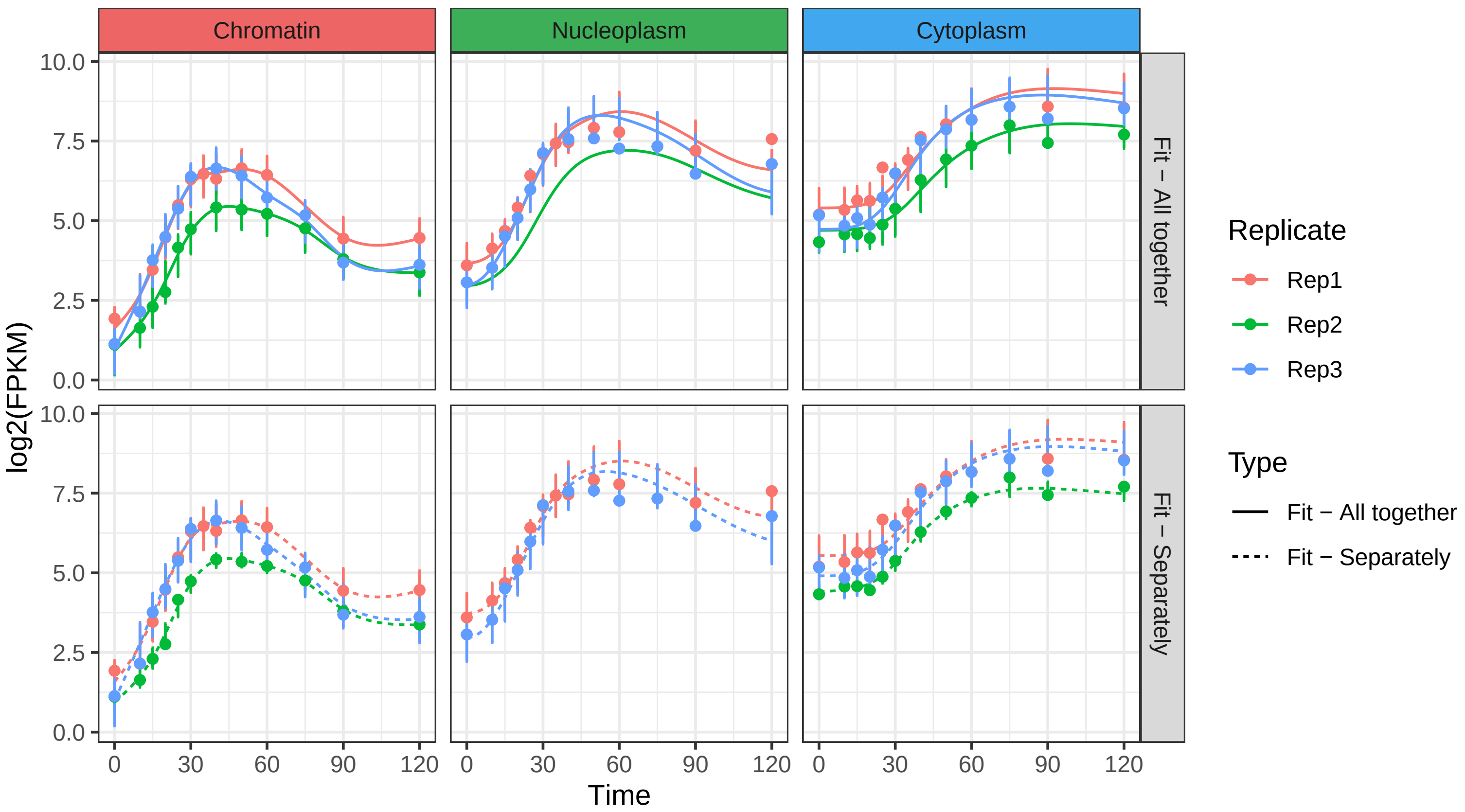

	Together	b1	b2	b3
**−NLL b1 ca**	−0.1633	0.4293		
**−NLL b1 np**	8.35	7.334		
**−NLL b1 cyto**	6.818	6.783		
**−NLL b2 ca**	−0.9327		−7.428	
**−NLL b2 np**				
**−NLL b2 cyto**	1.998		−6.696	
**−NLL b3 ca**	−0.2037			1.404
**−NLL b3 np**	11.85			8.705
**−NLL b3 cyto**	5.435			4.745
Total	33.15	14.55	−14.12	14.85
**Total with regul**	34.38	15.34	−14.69	15.26

	Together	b1	b2	b3
spar	0.377700	3.977e−01	0.3638	4.495e−01
σ_b	0.223700	2.267e−01	0.07199	2.334e−01
σ_t	0.000954	3.186e−05	0.678	4.832e−06
ca_{0,b_1}				
$\log_{10}(k_1')$	−0.571700	−5.672e−01		−6.404e−01
$\log_{10}(k_2)$	−1.187000	−1.215e+00	−0.2606 or −1.916	−1.239e+00
$\log_{10}(k_2')$	−1.294000	−1.342e+00		−1.283e+00
$\log_{10}(k_{deg})$	−1.817000	−1.886e+00	−1.916 or −0.2606	−1.865e+00
$\log_{10}(k_1'k_2')$	−1.866000	−1.909e+00	−1.129	−1.923e+00
$\log_{10}(k_1'/k_2)$	0.615700	6.483e−01		5.990e−01
$\text{transport} = \log_{10}(k_1'k_2'/k_2)$	−0.678800	−6.932e−01	−0.8688 or 0.7869	−6.837e−01

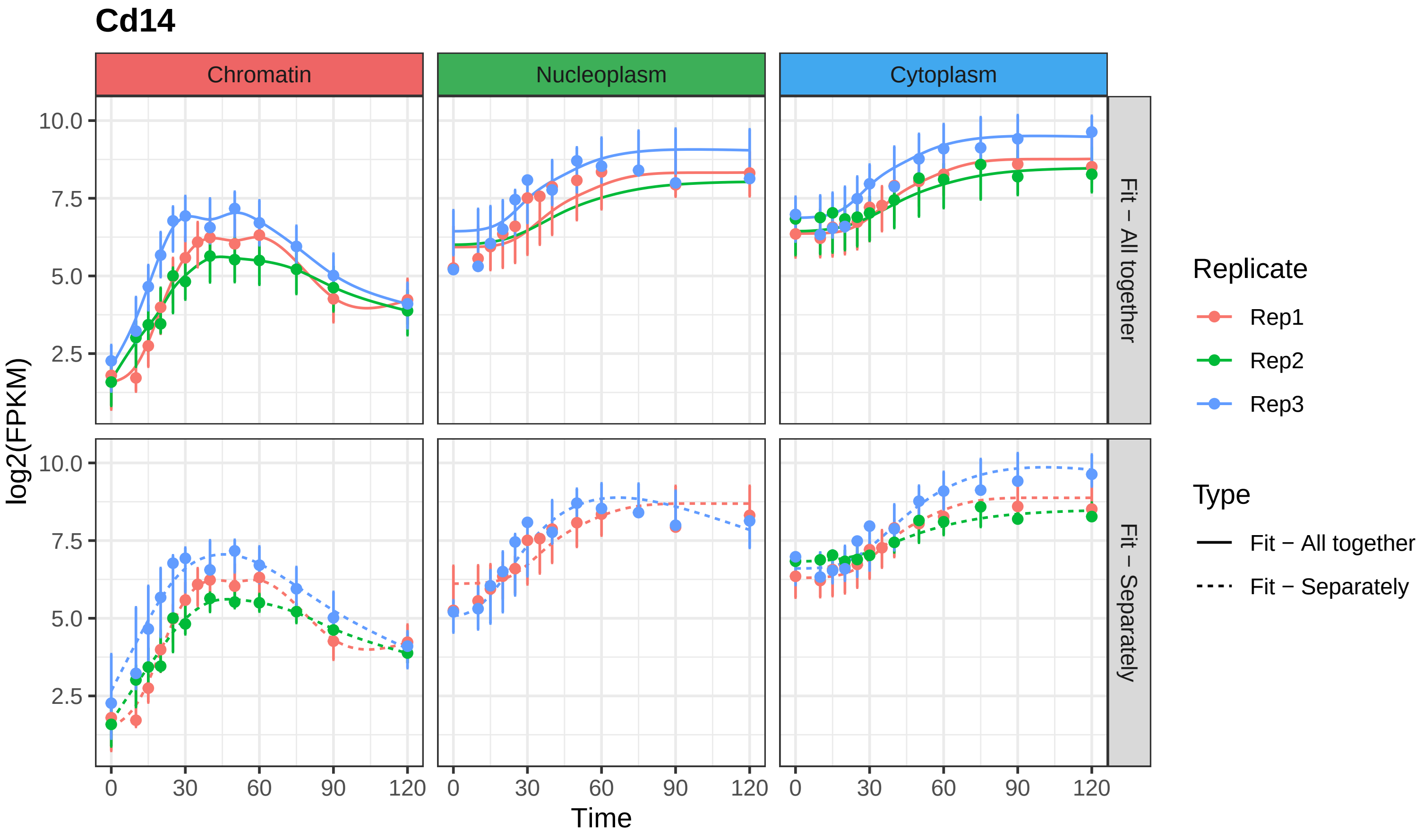

	Together	b1	b2	b3
**−NLL b1 ca**	0.08684	−0.8723		
**−NLL b1 np**	14.25	12.16		
**−NLL b1 cyto**	0.5199	−0.2421		
**−NLL b2 ca**	0.8955		−2.749	
**−NLL b2 np**				
**−NLL b2 cyto**	2.707		−3.488	
**−NLL b3 ca**	0.3345			4.835
**−NLL b3 np**	19.94			5.162
**−NLL b3 cyto**	4.699			3.674
Total	43.43	11.05	−6.237	13.67
**Total with regul**	46.75	12.15	−7.79	14.73

	Together	b1	b2	b3
spar	0.336100	3.707e−01	0.4172	0.5378
σ_b	0.246800	2.079e−01	0.09112	0.1773
σ_t	0.003175	8.323e−05	2.6	3.6470
ca_{0,b_1}				
$\log_{10}(k_1')$	−1.085000	−9.524e−01		−0.7832
$\log_{10}(k_2)$	−2.396000	−2.344e+00	4.77 or −2.589	−1.5120
$\log_{10}(k_2')$	6.069000	4.310e+00		−1.1980
$\log_{10}(k_{deg})$	5.938000	4.254e+00	−2.589 or 4.77	−1.6560
$\log_{10}(k_1' k_2')$	4.984000	3.358e+00	3.735	−1.9820
$\log_{10}(k_1'/k_2)$	1.311000	1.391e+00		0.7293
$\text{transport} = \log_{10}(k_1' k_2'/k_2)$	7.380000	5.701e+00	−1.035 or 6.324	−0.4691

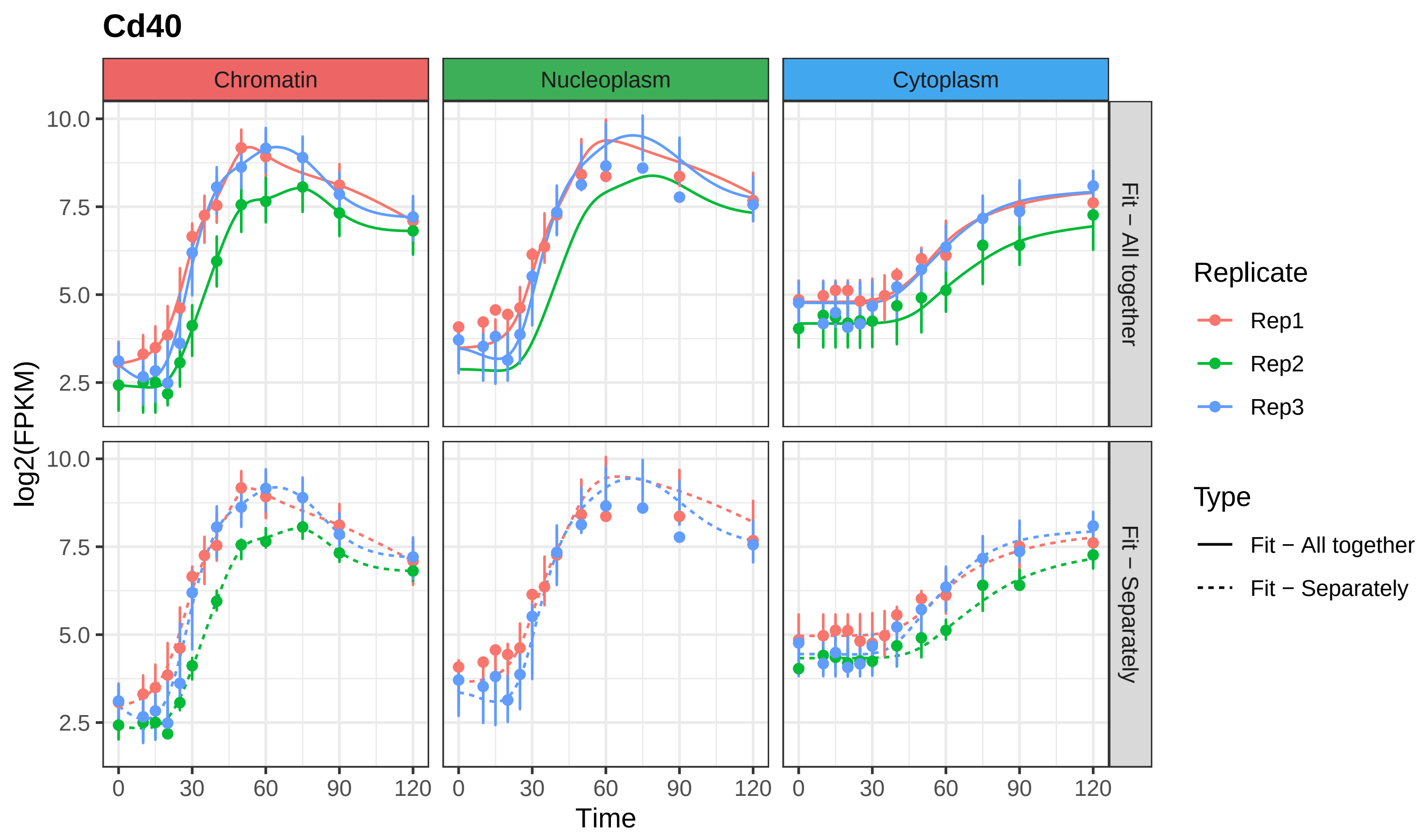

	Together	b1	b2	b3
**−NLL b1 ca**	−0.4585	0.346		
**−NLL b1 np**	14.63	13.52		
**−NLL b1 cyto**	0.9458	−0.02163		
**−NLL b2 ca**	−1.107		−5.578	
**−NLL b2 np**				
**−NLL b2 cyto**	0.4385		−3.389	
**−NLL b3 ca**	1.788			1.863
**−NLL b3 np**	12.9			10.27
**−NLL b3 cyto**	4.178			1.222
Total	33.32	13.84	−8.967	13.36
**Total with regul**	37.2	15.86	−8.686	16.12

	Together	b1	b2	b3
spar	0.3115	0.3514	0.3462	0.3280
σ_b	0.2171	0.2183	0.09429	0.2029
σ_t	0.7910	0.6916	6.127e−05	1.5450
ca_{0,b_1}				
$\log_{10}(k_1')$	−0.7597	−0.8403		−0.7858
$\log_{10}(k_2)$	−0.8960	−1.0440	−1.238 or −2.508	−0.8985
$\log_{10}(k_2')$	−2.1770	−2.3060		−2.1230
$\log_{10}(k_{deg})$	−2.5690	−2.7020	−2.508 or −1.238	−2.4550
$\log_{10}(k_1'k_2')$	−2.9370	−3.1470	−3.162	−2.9090
$\log_{10}(k_1'/k_2)$	0.1364	0.2037		0.1127
transport = $\log_{10}(k_1'k_2'/k_2)$	−2.0410	−2.1030	−1.924 or −0.6531	−2.0100

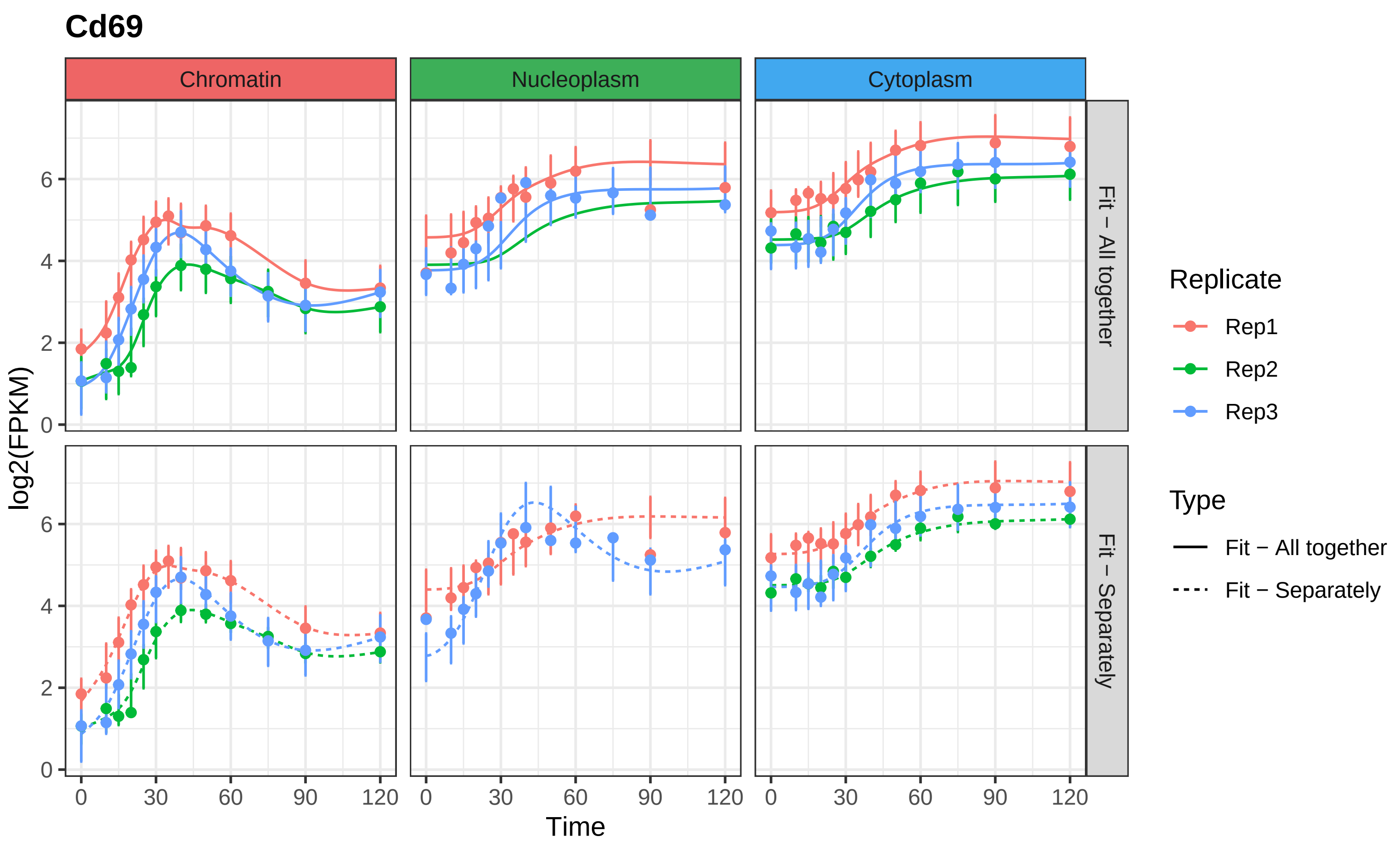

	Together	b1	b2	b3
**−NLL b1 ca**	−3.075	−3.213		
**−NLL b1 np**	11.72	10.33		
**−NLL b1 cyto**	−1.711	−3.292		
**−NLL b2 ca**	−2.1		−5.106	
**−NLL b2 np**				
**−NLL b2 cyto**	−2.989		−8.195	
**−NLL b3 ca**	−2.986			−2.073
**−NLL b3 np**	20.46			9.979
**−NLL b3 cyto**	−1.652			−0.5724
Total	17.66	3.827	−13.3	7.333
**Total with regul**	19.93	3.643	−14.44	7.804

	Together	b1	b2	b3
spar	0.33230	3.935e−01	0.3813	0.379300
σ_b	0.19070	1.730e−01	0.06006	0.189200
σ_t	0.00231	1.045e−08	1.725	0.000232
ca_{0,b_1}				
$\log_{10}(k_1')$	−1.21600	−1.321e+00		0.026810
$\log_{10}(k_2)$	−2.06900	−2.142e+00	4.111 or −2.059	−0.549900
$\log_{10}(k_2')$	0.61900	−2.781e−01		−1.582000
$\log_{10}(k_{deg})$	0.43360	−5.393e−01	−2.059 or 4.111	−2.087000
$\log_{10}(k_1'k_2')$	−0.59700	−1.599e+00	3.101	−1.555000
$\log_{10}(k_1'/k_2)$	0.85320	8.204e−01		0.576700
transport = $\log_{10}(k_1'k_2'/k_2)$	1.47200	5.422e−01	−1.01 or 5.16	−1.005000

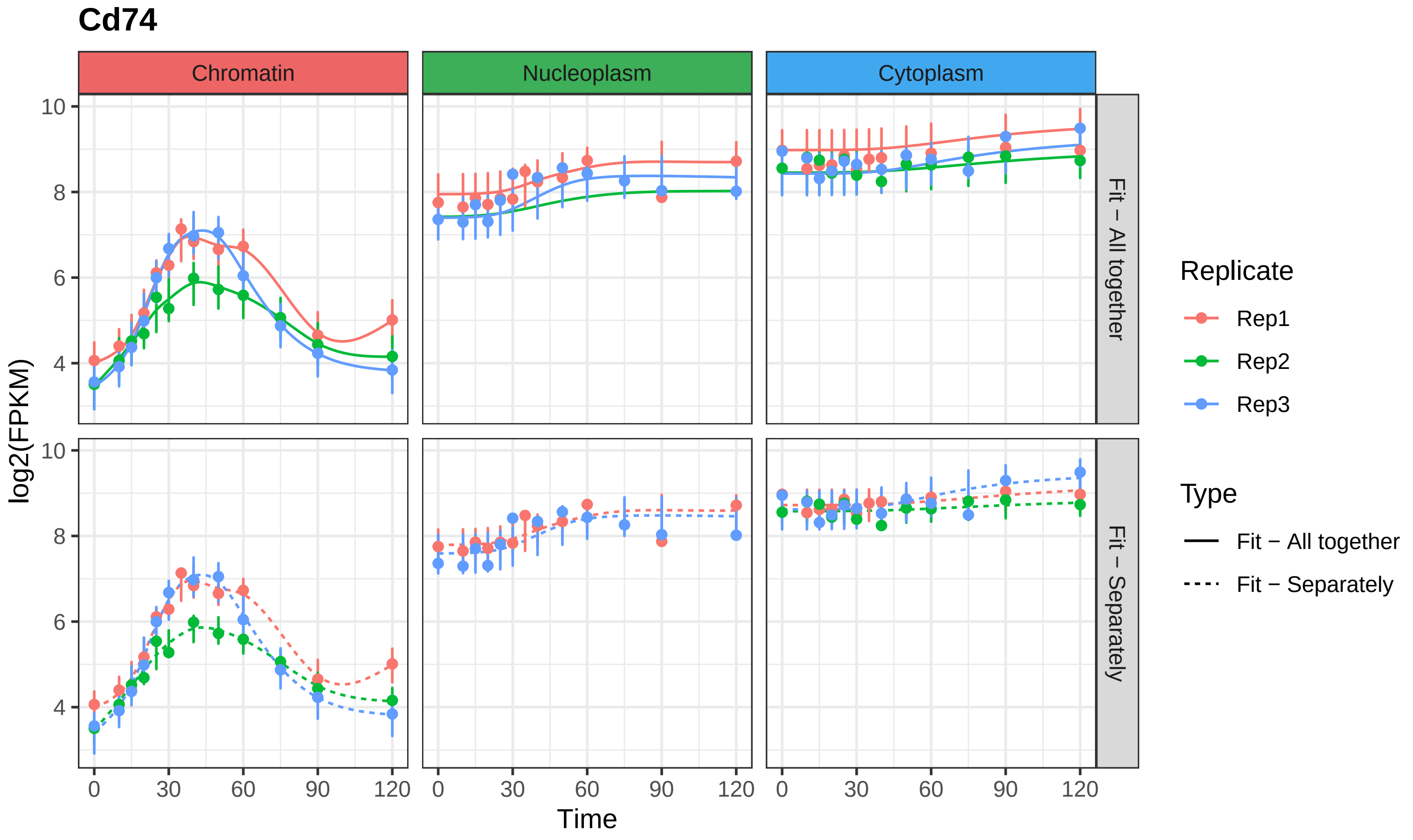

	Together	b1	b2	b3
**−NLL b1 ca**	−3.543	−4.739		
**−NLL b1 np**	3.087	2.769		
**−NLL b1 cyto**	4.522	−6.148		
**−NLL b2 ca**	−4.016		−5.305	
**−NLL b2 np**				
**−NLL b2 cyto**	−2.259		−3.882	
**−NLL b3 ca**	−4.713			−4.277
**−NLL b3 np**	7.471			6.477
**−NLL b3 cyto**	2.564			−2.862
Total	3.113	−8.118	−9.187	−0.662
**Total with regul**	3.915	−8.553	−10.84	−0.9976

	Together	b1	b2	b3
spar	3.574e−01	3.772e−01	0.425	3.879e−01
σ_b	1.677e−01	1.284e−01	0.1031	1.556e−01
σ_t	7.122e−07	1.548e−06	0.6383	3.678e−10
ca_{0,b_1}				
$\log_{10}(k_1')$	−1.391e+00	−1.400e+00		−1.400e+00
$\log_{10}(k_2)$	−2.578e+00	−2.547e+00	−1.758 or −3	−2.659e+00
$\log_{10}(k_2')$	−1.586e+00	−1.929e+00		−1.371e+00
$\log_{10}(k_{deg})$	−1.896e+00	−2.209e+00	−3 or −1.758	−1.681e+00
$\log_{10}(k_1'k_2')$	−2.977e+00	−3.329e+00	−3.219	−2.771e+00
$\log_{10}(k_1'/k_2)$	1.187e+00	1.147e+00		1.259e+00
transport = $\log_{10}(k_1'k_2'/k_2)$	−3.993e−01	−7.823e−01	−1.461 or −0.2188	−1.124e−01

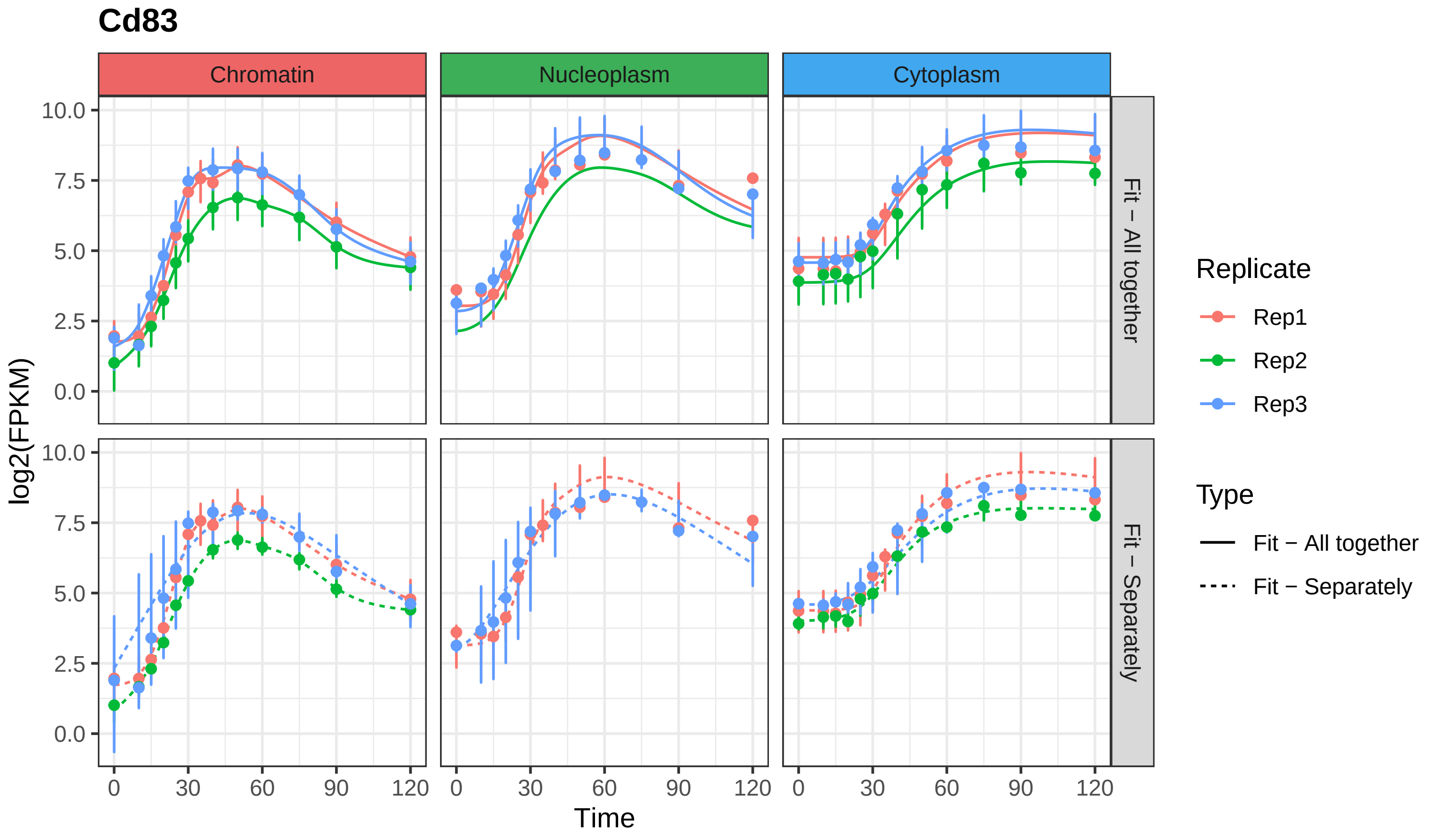

	Together	b1	b2	b3
**−NLL b1 ca**	−0.08698	0.1595		
**−NLL b1 np**	13.61	11.03		
**−NLL b1 cyto**	6.904	7.036		
**−NLL b2 ca**	−0.527		−7.922	
**−NLL b2 np**				
**−NLL b2 cyto**	7.206		−1.566	
**−NLL b3 ca**	1.702			8.762
**−NLL b3 np**	11.79			6.345
**−NLL b3 cyto**	3.773			−3.499
Total	44.37	18.23	−9.488	11.61
**Total with regul**	49.44	22.36	−8.707	14.87

	Together	b1	b2	b3
spar	0.3034000	3.197e−01	0.336	0.6047000
σ_b	0.2486000	2.474e−01	0.09993	0.0000255
σ_t	0.0003555	6.531e−06	0.003341	6.2960000
ca_{0,b_1}				
$\log_{10}(k_1')$	−0.5813000	−6.899e−01		−0.7293000
$\log_{10}(k_2)$	−0.9615000	−1.115e+00	6.432 or −2.198	−0.9683000
$\log_{10}(k_2')$	−1.5080000	−1.416e+00		−1.4910000
$\log_{10}(k_{deg})$	−2.0270000	−1.789e+00	−2.198 or 6.432	−1.9350000
$\log_{10}(k_1' k_2')$	−2.0890000	−2.106e+00	5.197	−2.2200000
$\log_{10}(k_1'/k_2)$	0.3802000	4.256e−01		0.2390000
$\text{transport} = \log_{10}(k_1' k_2'/k_2)$	−1.1270000	−9.904e−01	−1.235 or 7.395	−1.2520000

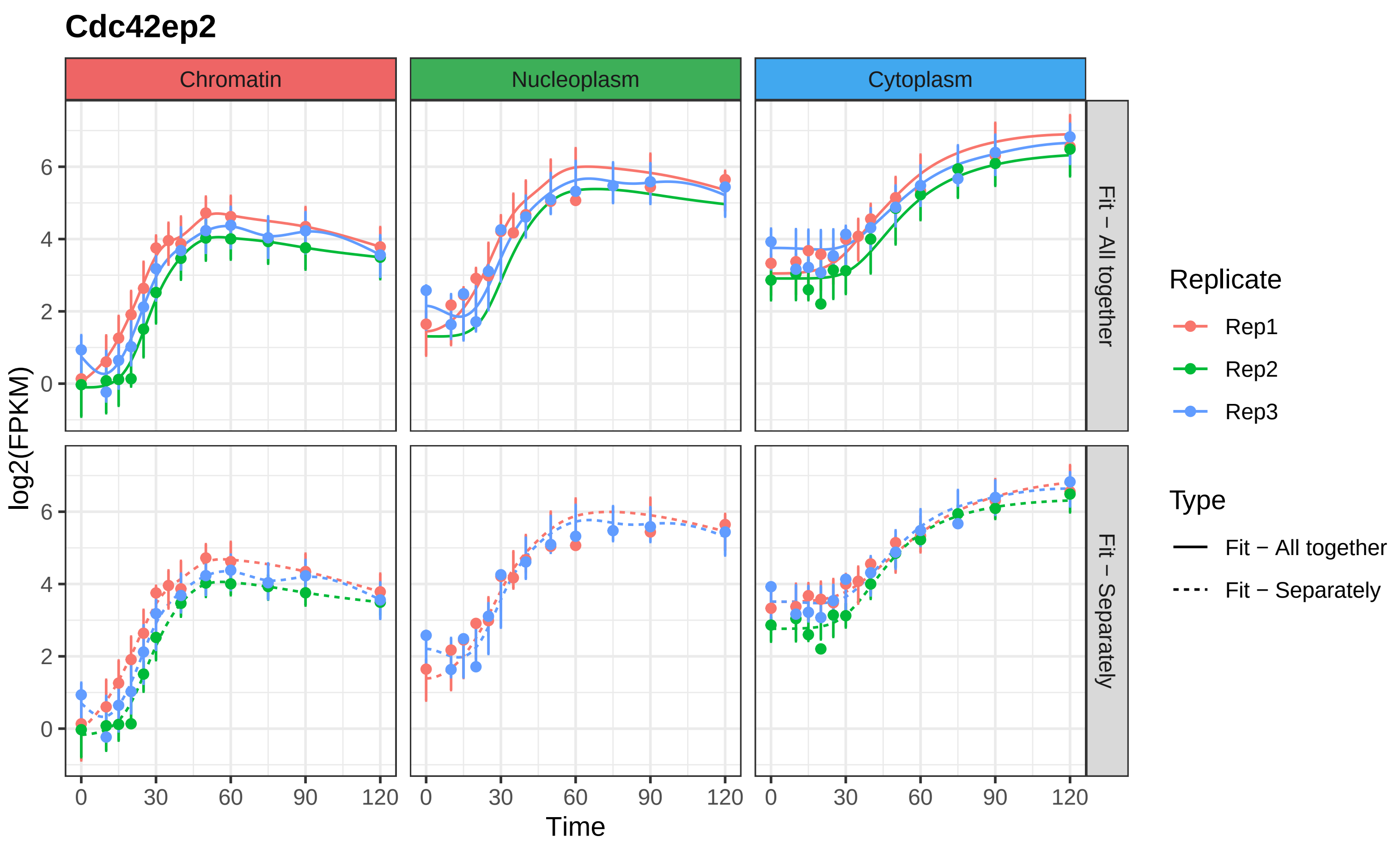

	Together	b1	b2	b3
**−NLL b1 ca**	−2.055	−2.219		
**−NLL b1 np**	10.16	9.475		
**−NLL b1 cyto**	4.043	−2.047		
**−NLL b2 ca**	−1.498		−3.262	
**−NLL b2 np**				
**−NLL b2 cyto**	1.762		−0.211	
**−NLL b3 ca**	−1.214			−0.4557
**−NLL b3 np**	6.617			5.905
**−NLL b3 cyto**	3.928			1.488
Total	21.75	5.209	−3.473	6.937
**Total with regul**	23.78	4.936	−4.124	7.685

	Together	b1	b2	b3
spar	0.3383	0.3990000	0.3773	0.3590
σ_b	0.1920	0.1747000	0.1106	0.1645
σ_t	0.6490	0.0004091	0.0001334	1.6920
ca_{0,b_1}				
$\log_{10}(k_1')$	−0.4693	−0.6421000		−0.4342
$\log_{10}(k_2)$	−0.8920	−1.0740000	−0.2605 or −1.8	−0.8867
$\log_{10}(k_2')$	−1.3430	−1.5190000		−1.2940
$\log_{10}(k_{deg})$	−1.8250	−2.1570000	−1.8 or −0.2605	−1.6880
$\log_{10}(k_1'k_2')$	−1.8120	−2.1610000	−1.176	−1.7280
$\log_{10}(k_1'/k_2)$	0.4227	0.4314000		0.4525
$transport = \log_{10}(k_1'k_2'/k_2)$	−0.9204	−1.0870000	−0.9156 or 0.6235	−0.8412

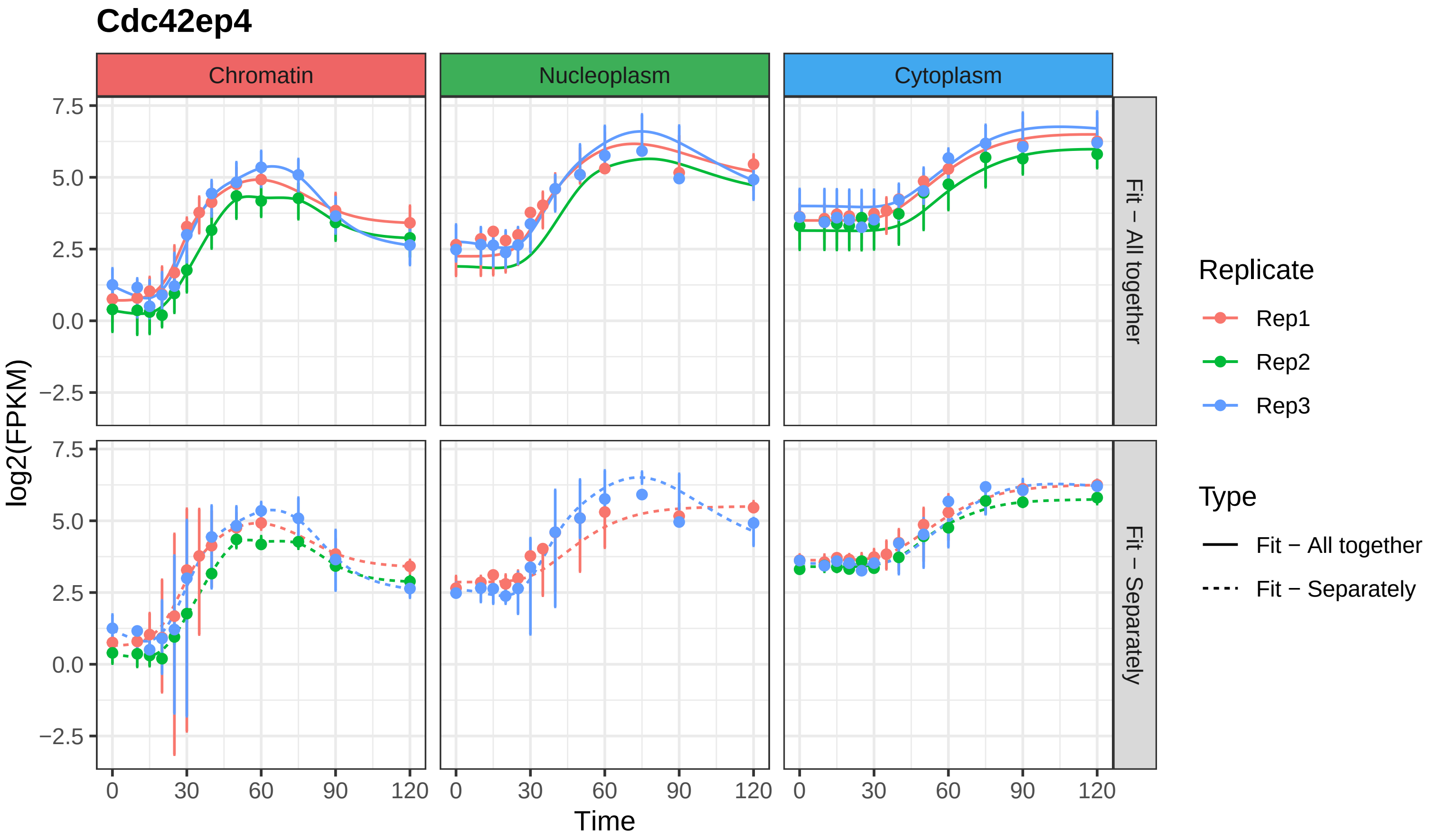

	Together	b1	b2	b3
**−NLL b1 ca**	−0.6982	1.405		
**−NLL b1 np**	12.13	8.468		
**−NLL b1 cyto**	−0.8987	−9.574		
**−NLL b2 ca**	−1.549		−8.536	
**−NLL b2 np**				
**−NLL b2 cyto**	3.114		−10.15	
**−NLL b3 ca**	0.3815			2.815
**−NLL b3 np**	9.061			5.643
**−NLL b3 cyto**	8.509			−3.51
Total	30.05	0.298	−18.69	4.948
**Total with regul**	32.01	−0.09543	−18.86	5.115

	Together	b1	b2	b3
spar	0.3512000	0.39250	0.3499	0.36270
σ_b	0.2156000	0.06278	0.05434	0.06788
σ_t	0.0001787	7.30600	0.001109	6.04400
ca_{0,b_1}				
$\log_{10}(k_1')$	−0.7474000	−1.45700		−0.72790
$\log_{10}(k_2)$	−1.2090000	−2.12200	4.373 or −2.162	−1.14900
$\log_{10}(k_2')$	−1.4650000	−0.68090		−1.58400
$\log_{10}(k_{deg})$	−1.8400000	−0.91040	−2.162 or 4.373	−1.86000
$\log_{10}(k_1'k_2')$	−2.2120000	−2.13800	3.126	−2.31200
$\log_{10}(k_1'/k_2)$	0.4618000	0.66520		0.42070
$\text{transport} = \log_{10}(k_1'k_2'/k_2)$	−1.0030000	−0.01567	−1.246 or 5.288	−1.16400

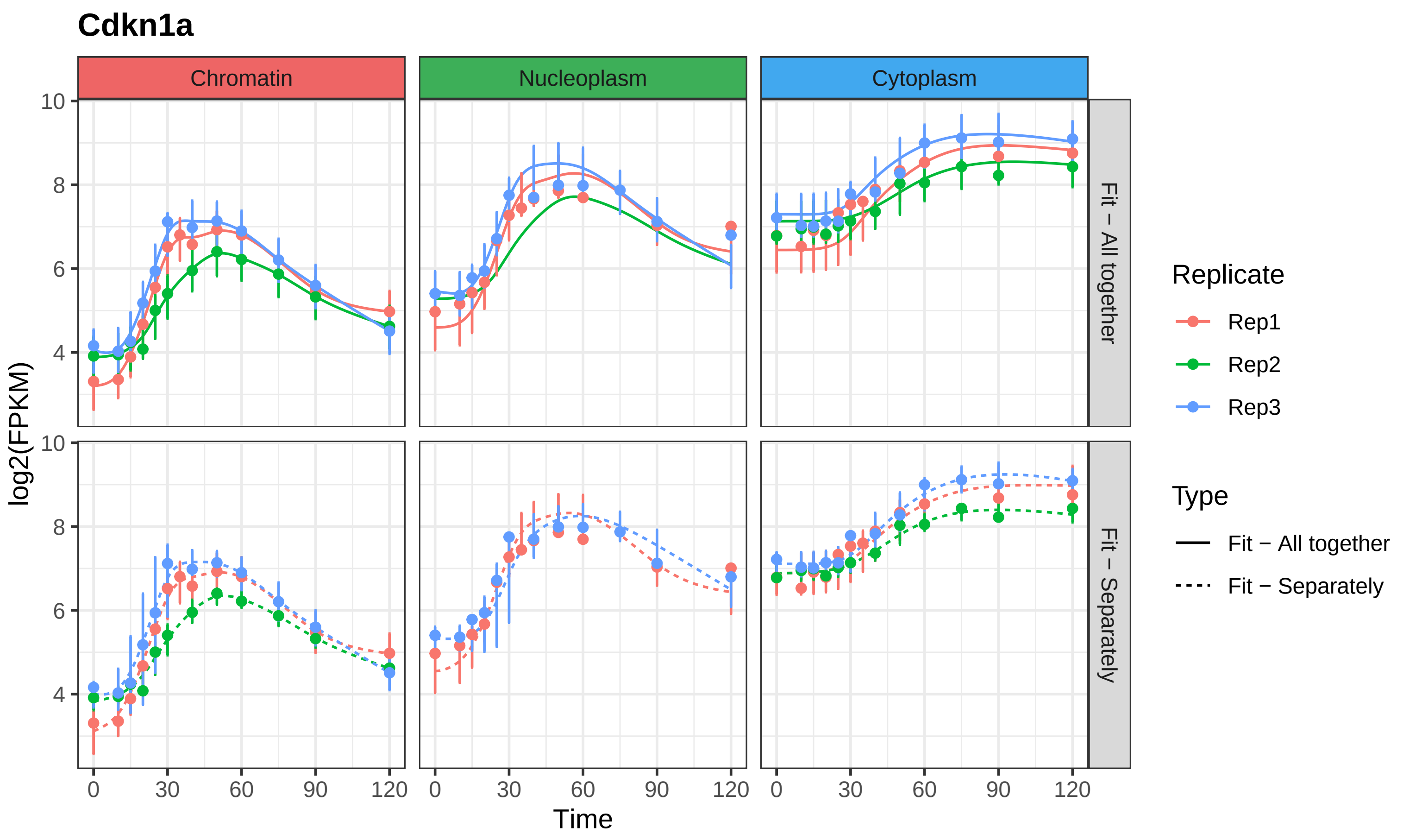

	Together	b1	b2	b3
**−NLL b1 ca**	−4.295	−3.772		
**−NLL b1 np**	7.278	7.489		
**−NLL b1 cyto**	8.255	−0.8461		
**−NLL b2 ca**	−4.073		−8.672	
**−NLL b2 np**				
**−NLL b2 cyto**	−1.176		−7.857	
**−NLL b3 ca**	−3.698			−2.157
**−NLL b3 np**	6.846			3.534
**−NLL b3 cyto**	−0.4722			−3.867
Total	8.664	2.871	−16.53	−2.49
**Total with regul**	10.59	2.978	−18.03	−2.597

	Together	b1	b2	b3
spar	3.343e−01	3.805e−01	0.3976	0.3655
σ_b	1.771e−01	1.709e−01	0.0629	0.1006
σ_t	7.081e−05	6.765e−05	1.912	3.7370
ca_{0,b_1}				
$\log_{10}(k_1')$	−2.408e−01	−1.394e−01		−0.7782
$\log_{10}(k_2)$	−6.610e−01	−5.707e−01	4.858 or −1.906	−1.1770
$\log_{10}(k_2')$	−1.379e+00	−1.492e+00		−1.2390
$\log_{10}(k_{deg})$	−1.936e+00	−2.197e+00	−1.906 or 4.858	−1.7770
$\log_{10}(k_1' k_2')$	−1.620e+00	−1.632e+00	3.872	−2.0170
$\log_{10}(k_1'/k_2)$	4.202e−01	4.313e−01		0.3990
transport = $\log_{10}(k_1' k_2'/k_2)$	−9.588e−01	−1.061e+00	−0.986 or 5.778	−0.8399

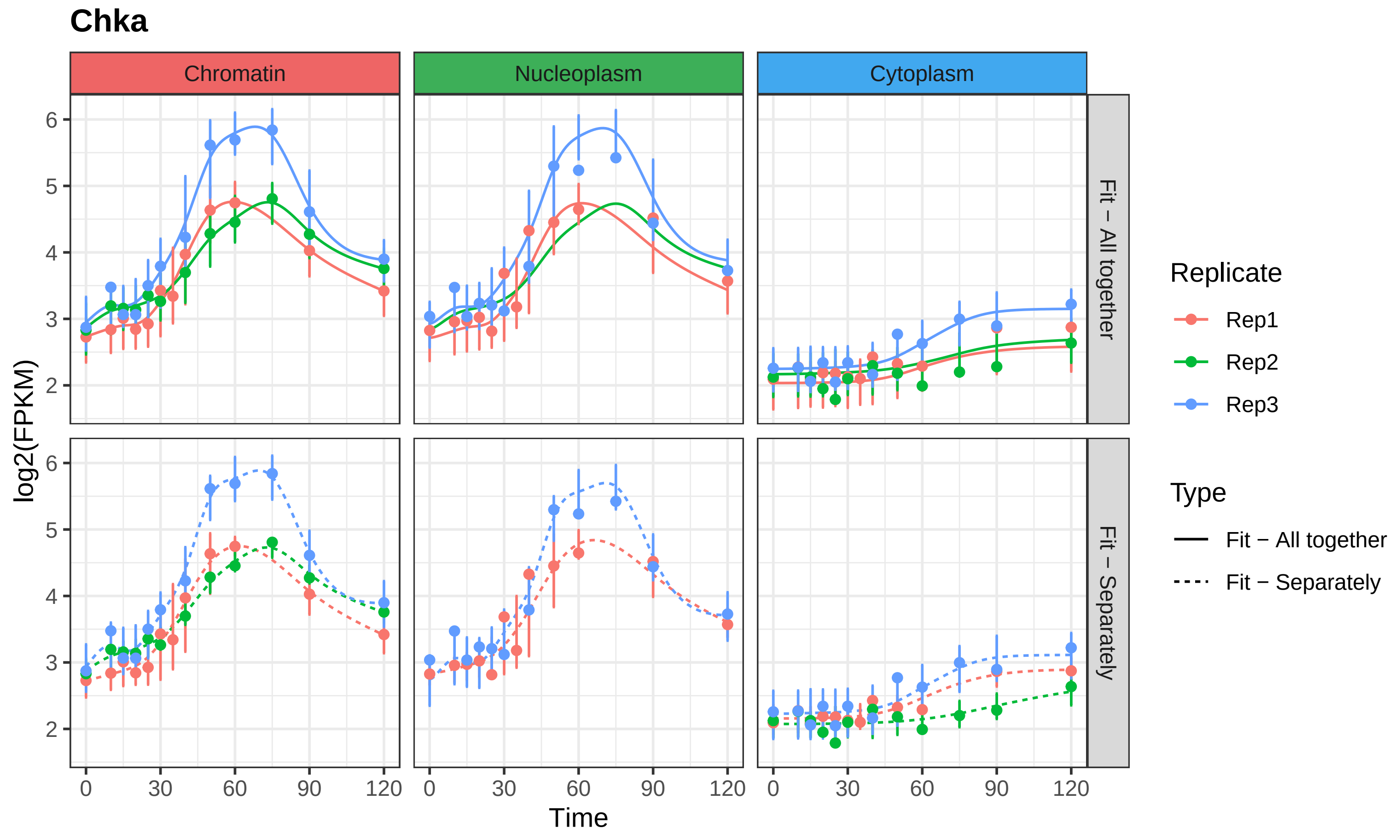

	Together	b1	b2	b3
**−NLL b1 ca**	−6.932	−8.466		
**−NLL b1 np**	1.041	−3.772		
**−NLL b1 cyto**	−2.294	−11.22		
**−NLL b2 ca**	−8.916		−12.75	
**−NLL b2 np**				
**−NLL b2 cyto**	−1.316		−4.797	
**−NLL b3 ca**	−4.169			−6.332
**−NLL b3 np**	5.385			1.543
**−NLL b3 cyto**	−5.342			−4.052
Total	−22.54	−23.46	−17.55	−8.841
**Total with regul**	−22.56	−25.29	−19.38	−6.783

	Together	b1	b2	b3
spar	0.361000	4.546e−01	0.4034	0.313100
σ_b	0.097560	5.327e−07	0.03027	0.112300
σ_t	3.472000	4.909e+00	0.004092	0.001326
ca_{0,b_1}				
$\log_{10}(k_1')$	−0.278000	−5.881e−01		−0.219600
$\log_{10}(k_2)$	−0.273500	−6.231e−01	−1.989 or −2	−0.164300
$\log_{10}(k_2')$	−2.674000	−2.484e+00		−2.643000
$\log_{10}(k_{deg})$	−2.469000	−2.278e+00	−2 or −1.989	−2.487000
$\log_{10}(k_1'k_2')$	−2.952000	−3.072e+00	−4.231	−2.863000
$\log_{10}(k_1'/k_2)$	−0.004506	3.497e−02		−0.055250
transport $= \log_{10}(k_1'k_2'/k_2)$	−2.679000	−2.449e+00	−2.241 or −2.231	−2.698000

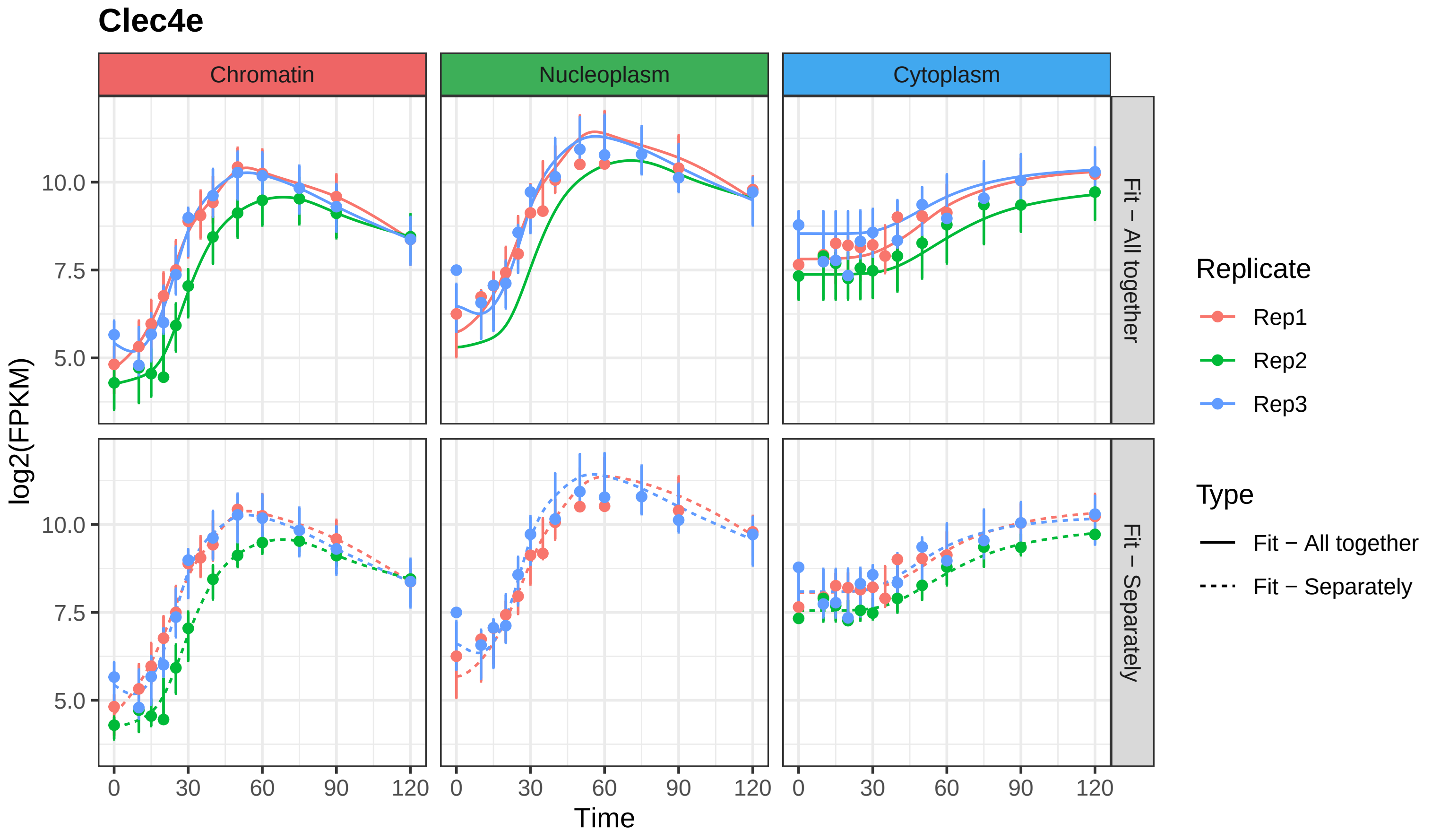

	Together	b1	b2	b3
**−NLL b1 ca**	−0.9069	−1.816		
**−NLL b1 np**	10.2	9.654		
**−NLL b1 cyto**	2.684	1.159		
**−NLL b2 ca**	0.2503		−2.588	
**−NLL b2 np**				
**−NLL b2 cyto**	1.556		−3.033	
**−NLL b3 ca**	0.7791			0.8857
**−NLL b3 np**	10.04			8.234
**−NLL b3 cyto**	12.26			5.997
Total	36.87	8.997	−5.621	15.12
**Total with regul**	39.61	9.418	−5.706	18.21

	Together	b1	b2	b3
spar	3.414e−01	3.888e−01	0.3599	3.357e−01
σ_b	2.325e−01	1.995e−01	0.1051	2.364e−01
σ_t	6.069e−05	5.660e−06	1.687	1.272e−05
ca_{0,b_1}				
$\log_{10}(k_1')$	−3.172e−02	−4.145e−01		2.756e−01
$\log_{10}(k_2)$	−3.469e−01	−7.354e−01	4.631 or −2.806	−7.345e−02
$\log_{10}(k_2')$	−2.136e+00	−2.158e+00		−2.186e+00
$\log_{10}(k_{deg})$	−2.759e+00	−2.877e+00	−2.806 or 4.631	−2.637e+00
$\log_{10}(k_1'k_2')$	−2.168e+00	−2.573e+00	2.827	−1.911e+00
$\log_{10}(k_1'/k_2)$	3.152e−01	3.209e−01		3.491e−01
transport $= \log_{10}(k_1'k_2'/k_2)$	−1.821e+00	−1.837e+00	−1.804 or 5.632	−1.837e+00

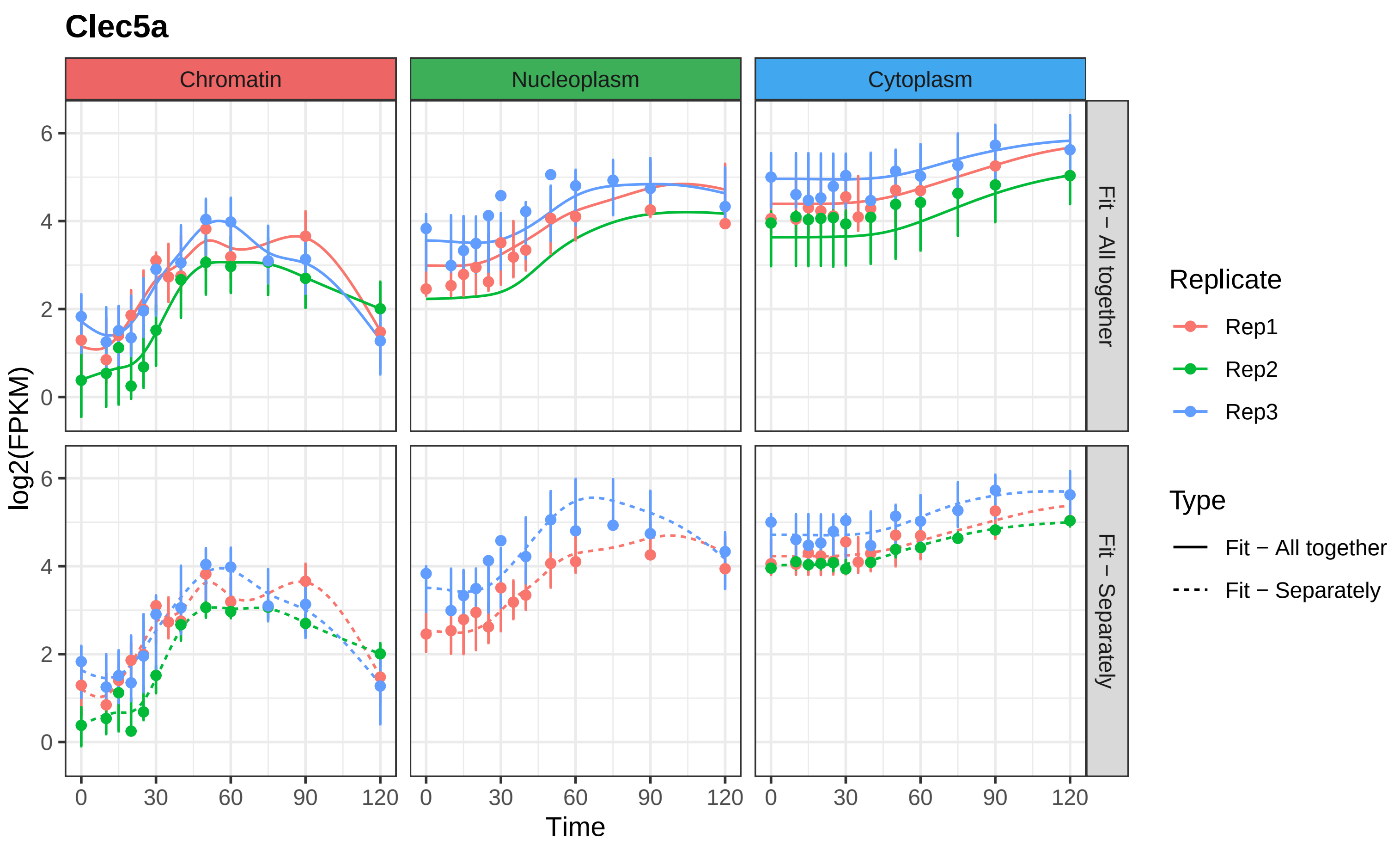

	Together	b1	b2	b3
**−NLL b1 ca**	1.027	−1.999		
**−NLL b1 np**	6.395	2.014		
**−NLL b1 cyto**	2.14	−2.373		
**−NLL b2 ca**	0.6638		−3.231	
**−NLL b2 np**				
**−NLL b2 cyto**	5.4		−15.7	
**−NLL b3 ca**	−0.216			0.4957
**−NLL b3 np**	12.51			8.867
**−NLL b3 cyto**	2.109			−1.325
Total	30.03	−2.358	−18.93	8.037
**Total with regul**	30.6	−1.407	−19.53	7.473

	Together	b1	b2	b3
spar	3.922e−01	0.3420	0.3573	0.4406
σ_b	2.109e−01	0.1365	0.01097	0.1689
σ_t	2.035e−05	0.4022	0.0004292	3.9330
ca_{0,b_1}				
$\log_{10}(k_1')$	−1.267e+00	−1.0430		−0.5987
$\log_{10}(k_2)$	−1.821e+00	−1.4410	0.07125 or −2.512	−1.1620
$\log_{10}(k_2')$	−1.425e+00	−1.6380		−1.7330
$\log_{10}(k_{deg})$	−1.847e+00	−2.1510	−2.512 or 0.07125	−2.0960
$\log_{10}(k_1' k_2')$	−2.693e+00	−2.6810	−1.349	−2.3320
$\log_{10}(k_1'/k_2)$	5.538e−01	0.3979		0.5633
$transport = \log_{10}(k_1' k_2'/k_2)$	−8.715e−01	−1.2400	−1.42 or 1.163	−1.1700

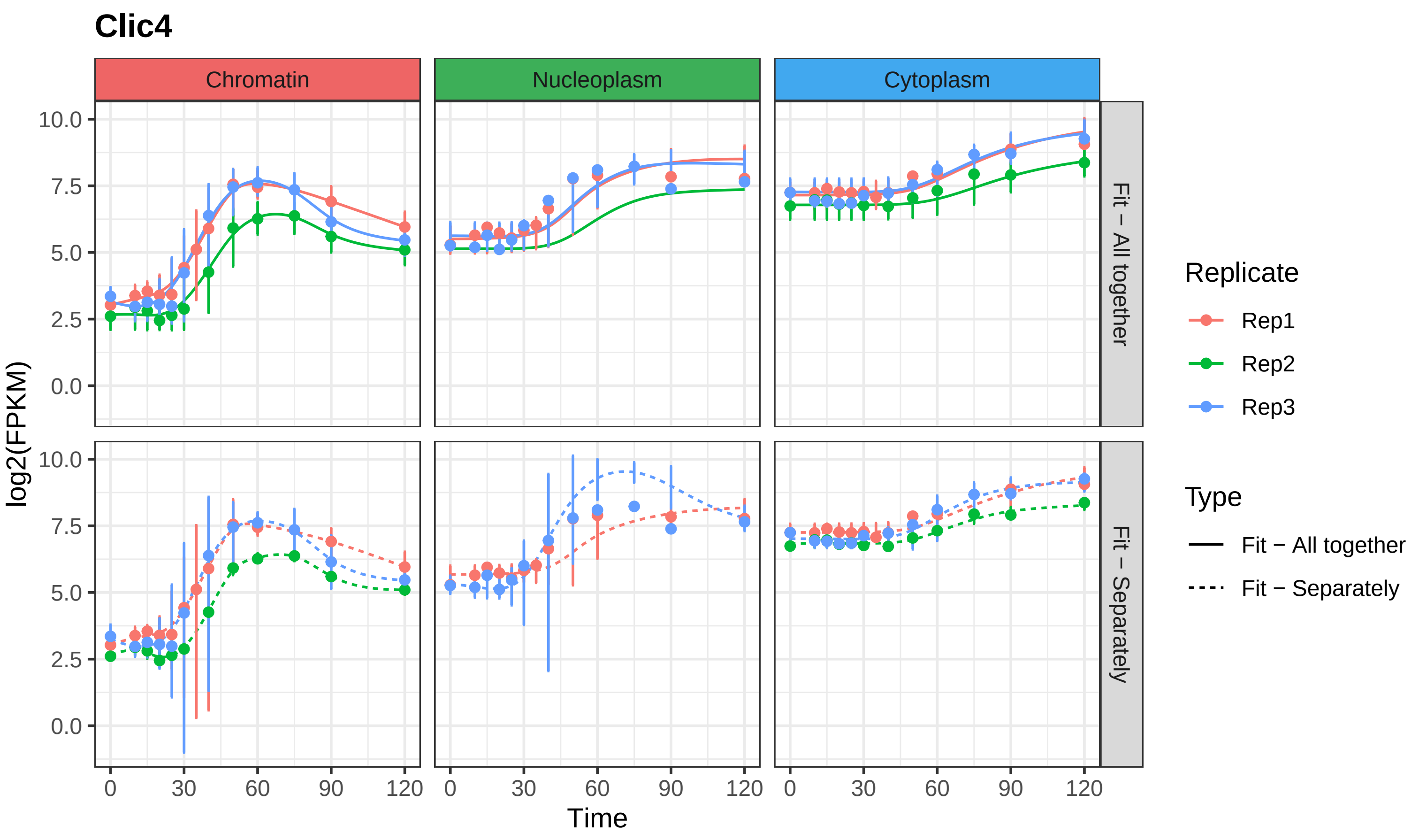

	Together	b1	b2	b3
**−NLL b1 ca**	1.484	2.788		
**−NLL b1 np**	9.487	7.613		
**−NLL b1 cyto**	−0.6364	−4.557		
**−NLL b2 ca**	0.01766		−12.39	
**−NLL b2 np**				
**−NLL b2 cyto**	−1.823		−9.79	
**−NLL b3 ca**	1.571			2.078
**−NLL b3 np**	16.72			14.5
**−NLL b3 cyto**	1.669			−3.663
Total	28.49	5.843	−22.18	12.92
**Total with regul**	28.31	5.956	−20.68	12.92

	Together	b1	b2	b3
spar	0.4269	0.3912	0.3147	0.3943
σ_b	0.1817	0.1169	0.05655	0.1157
σ_t	4.3870	7.2650	2.258e−05	7.1940
ca_{0,b_1}				
$\log_{10}(k_1')$	−1.3440	−1.5290		−0.4388
$\log_{10}(k_2)$	−2.0890	−2.3180	−0.6487 or −2.504	−1.0600
$\log_{10}(k_2')$	−1.2550	−1.1210		−1.8220
$\log_{10}(k_{deg})$	−1.7490	−1.5950	−2.504 or −0.6487	−2.3380
$\log_{10}(k_1' k_2')$	−2.5990	−2.6500	−1.895	−2.2600
$\log_{10}(k_1'/k_2)$	0.7448	0.7893		0.6208
$\text{transport} = \log_{10}(k_1' k_2'/k_2)$	−0.5097	−0.3320	−1.246 or 0.6088	−1.2010

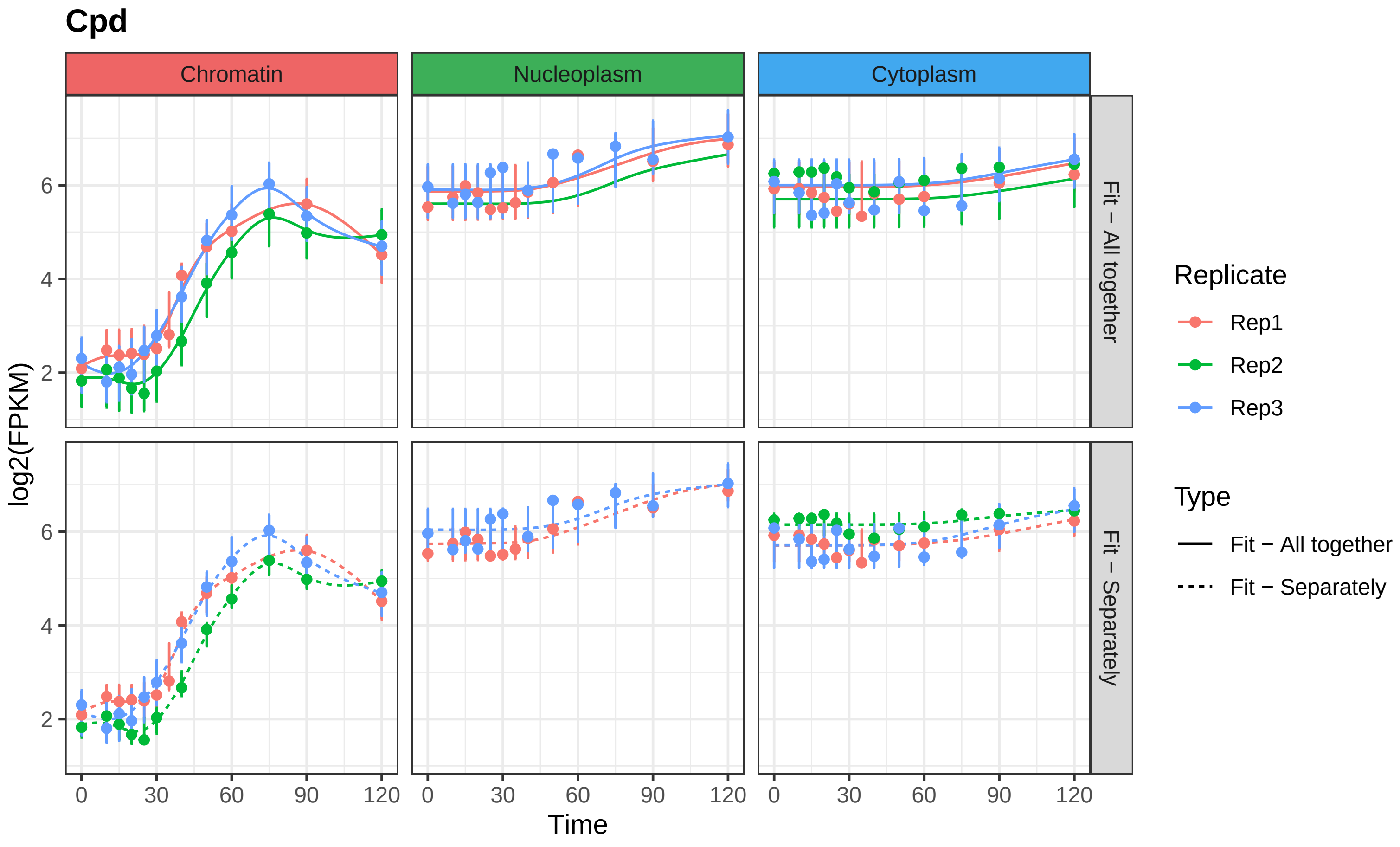

	Together	b1	b2	b3
**−NLL b1 ca**	−1.974	−6.263		
**−NLL b1 np**	1.136	0.974		
**−NLL b1 cyto**	2.954	−4.23		
**−NLL b2 ca**	−2.692		−8.802	
**−NLL b2 np**				
**−NLL b2 cyto**	12.44		−5.138	
**−NLL b3 ca**	−2.846			−4.129
**−NLL b3 np**	3.652			3.676
**−NLL b3 cyto**	7.183			1.118
Total	19.86	−9.519	−13.94	0.6649
**Total with regul**	19.91	−10.22	−14.89	−0.2648

	Together	b1	b2	b3
spar	4.042e−01	0.3844	0.3782	4.286e−01
σ_b	1.974e−01	0.1200	0.08224	1.611e−01
σ_t	2.208e−06	1.3990	4.126e−08	1.075e−07
ca_{0,b_1}				
$\log_{10}(k_1')$	−1.541e+00	−1.5000		−1.623e+00
$\log_{10}(k_2)$	−2.660e+00	−2.5840	−0.8569 or −3.289	−2.793e+00
$\log_{10}(k_2')$	−1.901e+00	−1.9780		−1.517e+00
$\log_{10}(k_{deg})$	−1.930e+00	−1.9680	−3.289 or −0.8569	−1.417e+00
$\log_{10}(k_1'k_2')$	−3.442e+00	−3.4780	−2.862	−3.140e+00
$\log_{10}(k_1'/k_2)$	1.118e+00	1.0840		1.169e+00
$transport = \log_{10}(k_1'k_2'/k_2)$	−7.827e−01	−0.8945	−2.005 or 0.4264	−3.472e−01

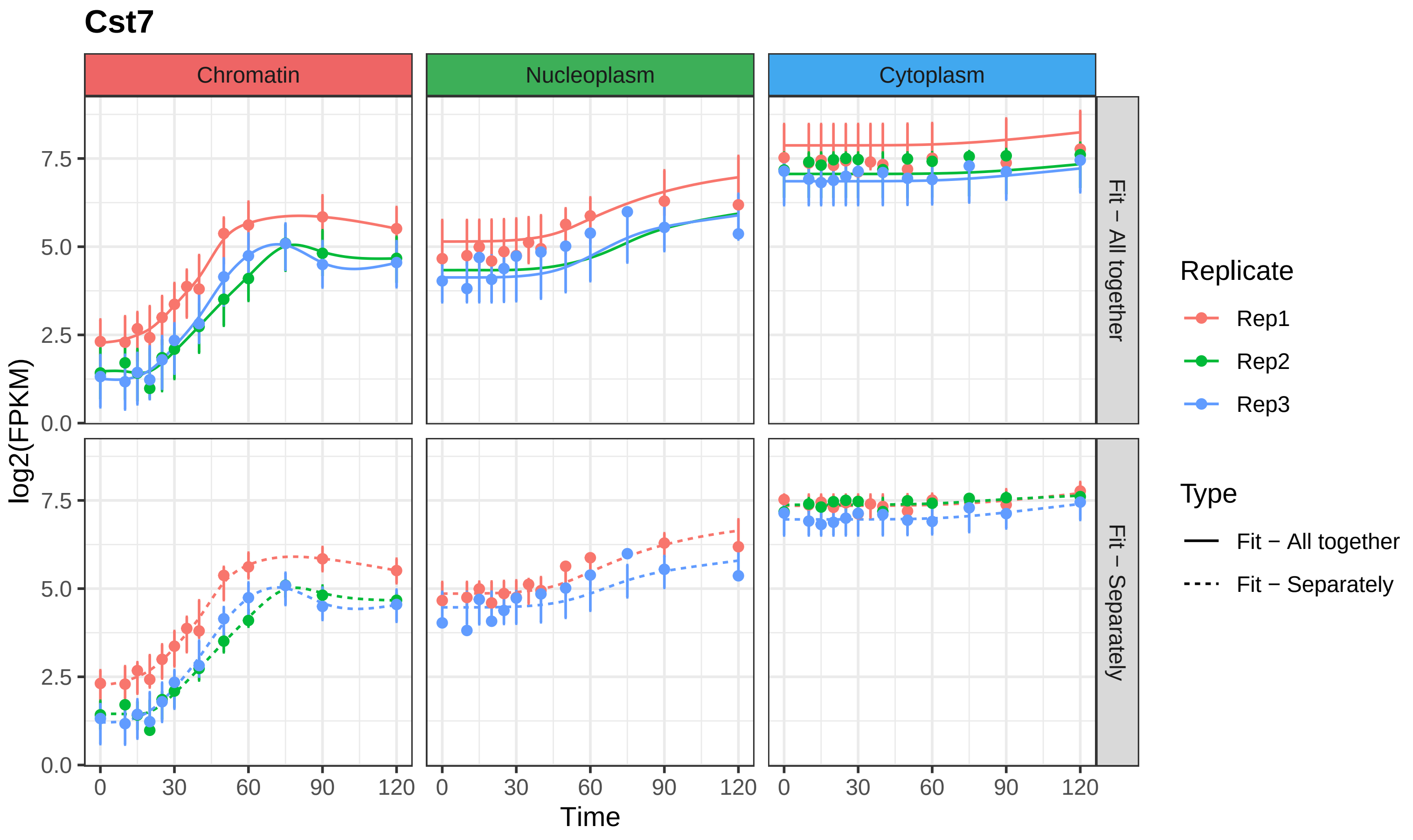

	Together	b1	b2	b3
**−NLL b1 ca**	−0.6254	−4.412		
**−NLL b1 np**	6.513	2.887		
**−NLL b1 cyto**	13.83	−7.533		
**−NLL b2 ca**	−0.3007		−5.006	
**−NLL b2 np**				
**−NLL b2 cyto**	3.817		−8.838	
**−NLL b3 ca**	−0.7636			−2.649
**−NLL b3 np**	10.96			8.486
**−NLL b3 cyto**	−0.8001			−5.182
Total	32.63	−9.058	−13.84	0.6545
**Total with regul**	33.69	−10.48	−15.71	−0.4376

	Together	b1	b2	b3
spar	0.3812000	0.4192	0.4151	4.310e−01
σ_b	0.2203000	0.1131	0.06507	1.520e−01
σ_t	0.0004268	1.8980	9.299e−05	3.079e−07
ca_{0,b_1}				
$\log_{10}(k_1')$	−1.5540000	−1.6640		−1.685e+00
$\log_{10}(k_2)$	−2.4180000	−2.4470	3.348 or −3.495	−2.667e+00
$\log_{10}(k_2')$	−1.6950000	−1.7700		−1.418e+00
$\log_{10}(k_{deg})$	−2.5150000	−2.5200	−3.495 or 3.348	−2.169e+00
$\log_{10}(k_1'k_2')$	−3.2490000	−3.4340	1.638	−3.104e+00
$\log_{10}(k_1'/k_2)$	0.8637000	0.7833		9.815e−01
transport $= \log_{10}(k_1'k_2'/k_2)$	−0.8312000	−0.9870	−1.71 or 5.133	−4.367e−01

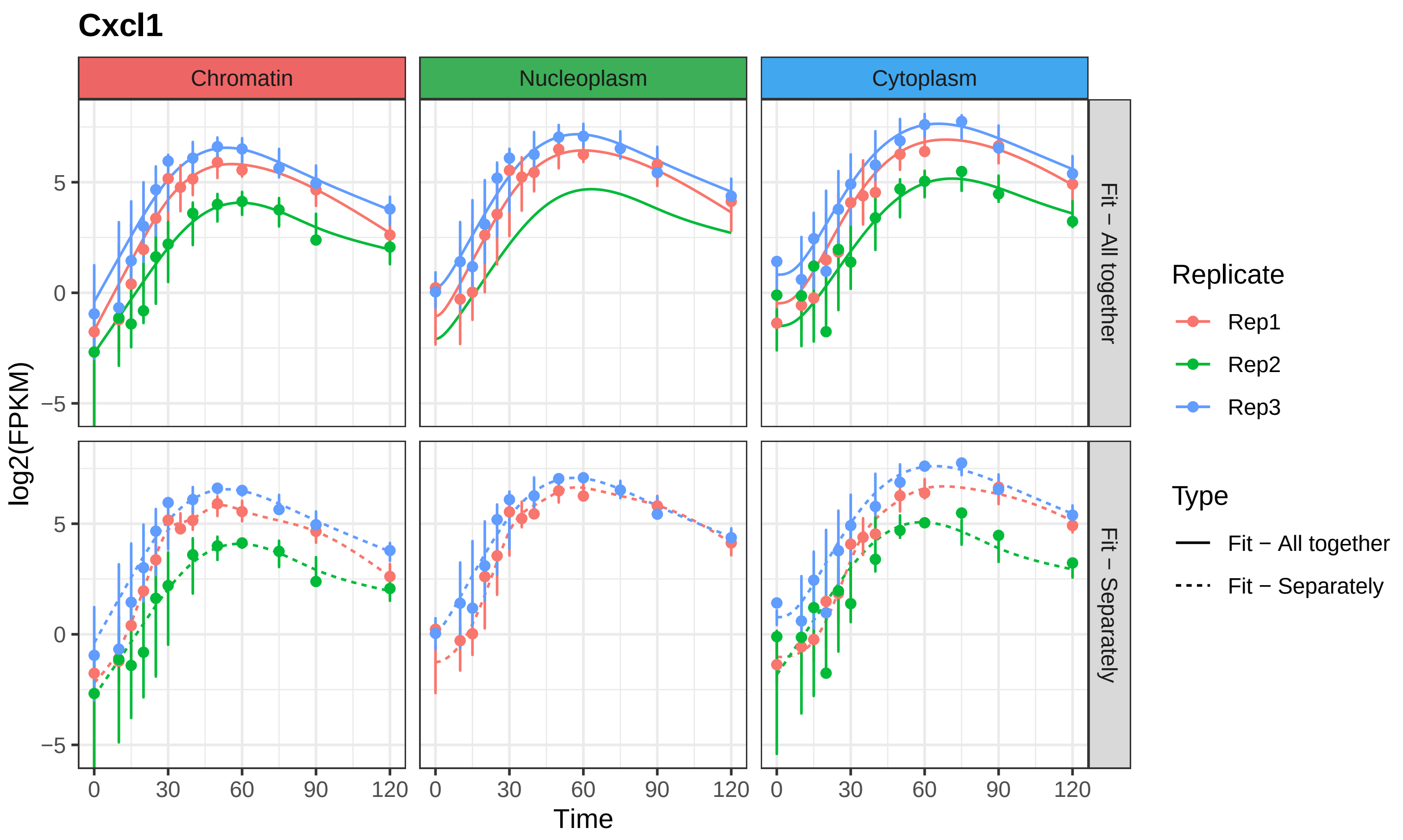

	Together	b1	b2	b3
**−NLL b1 ca**	8.486	1.901		
**−NLL b1 np**	13.35	12.61		
**−NLL b1 cyto**	11.68	5.855		
**−NLL b2 ca**	7.376		7.759	
**−NLL b2 np**				
**−NLL b2 cyto**	20.9		12.84	
**−NLL b3 ca**	8.139			5.543
**−NLL b3 np**	7.175			3.491
**−NLL b3 cyto**	10.26			11.62
Total	87.37	20.37	20.6	20.66
**Total with regul**	89.27	22.05	20.98	21.17

	Together	b1	b2	b3
spar	0.5608	0.3299	0.5493	5.617e−01
σ_b	0.1696	0.1448	9.287e−07	2.067e−07
σ_t	4.0090	1.8780	6.048	4.130e+00
ca_{0,b_1}				
$\log_{10}(k_1')$	−0.3901	−0.5192		−2.636e−01
$\log_{10}(k_2)$	−0.5831	−0.8022	4.522 or 3.486	−4.234e−01
$\log_{10}(k_2')$	−0.7412	−0.9431		−7.068e−01
$\log_{10}(k_{deg})$	−0.9167	−1.0130	3.486 or 4.522	−8.979e−01
$\log_{10}(k_1' k_2')$	−1.1310	−1.4620	8.302	−9.704e−01
$\log_{10}(k_1'/k_2)$	0.1930	0.2829		1.598e−01
transport $= \log_{10}(k_1' k_2'/k_2)$	−0.5482	−0.6602	3.78 or 4.816	−5.470e−01

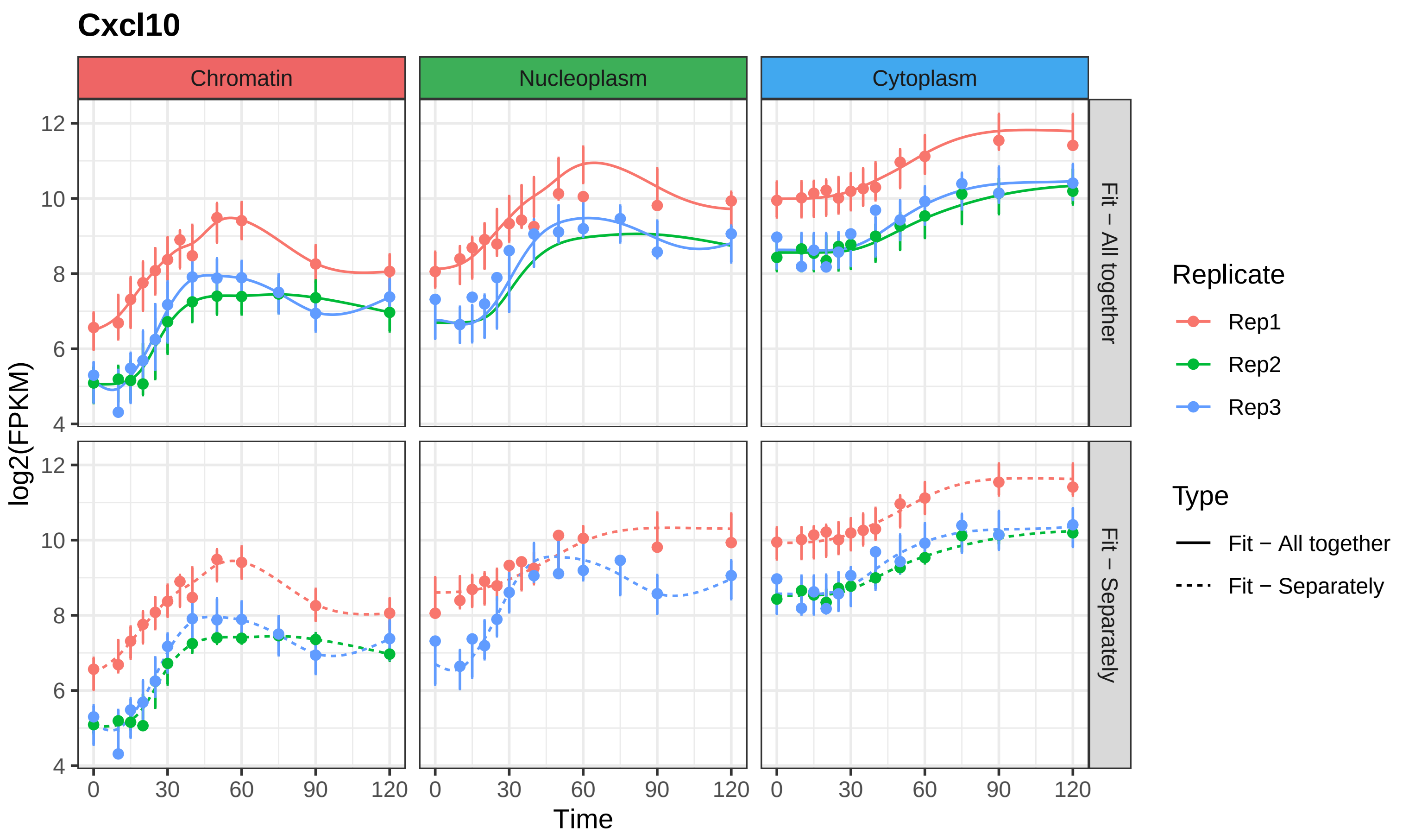

	Together	b1	b2	b3
**−NLL b1 ca**	−2.778	−4.204		
**−NLL b1 np**	10.66	6.385		
**−NLL b1 cyto**	−2.883	−5.238		
**−NLL b2 ca**	−3.5		−9.382	
**−NLL b2 np**				
**−NLL b2 cyto**	−3.759		−9.291	
**−NLL b3 ca**	−0.362			−0.697
**−NLL b3 np**	9.416			3.622
**−NLL b3 cyto**	4.076			1.574
Total	10.87	−3.057	−18.67	4.499
**Total with regul**	11.95	−3.896	−19.49	5.052

	Together	b1	b2	b3
spar	0.3527	4.058e−01	0.3706	0.370200
σ_b	0.1653	1.476e−01	0.05545	0.178800
σ_t	2.6200	4.745e−05	2.057	0.001016
ca_{0,b_1}				
$\log_{10}(k_1')$	−0.5107	−1.368e+00		7.124000
$\log_{10}(k_2)$	−1.0030	−2.015e+00	8.351 or −1.994	6.643000
$\log_{10}(k_2')$	−1.3400	−2.795e−01		−1.421000
$\log_{10}(k_{deg})$	−1.9020	−6.773e−01	−1.994 or 8.351	−1.985000
$\log_{10}(k_1' k_2')$	−1.8510	−1.647e+00	7.407	5.703000
$\log_{10}(k_1'/k_2)$	0.4919	6.474e−01		0.480900
$\text{transport} = \log_{10}(k_1' k_2'/k_2)$	−0.8480	3.679e−01	−0.9445 or 9.401	−0.940100

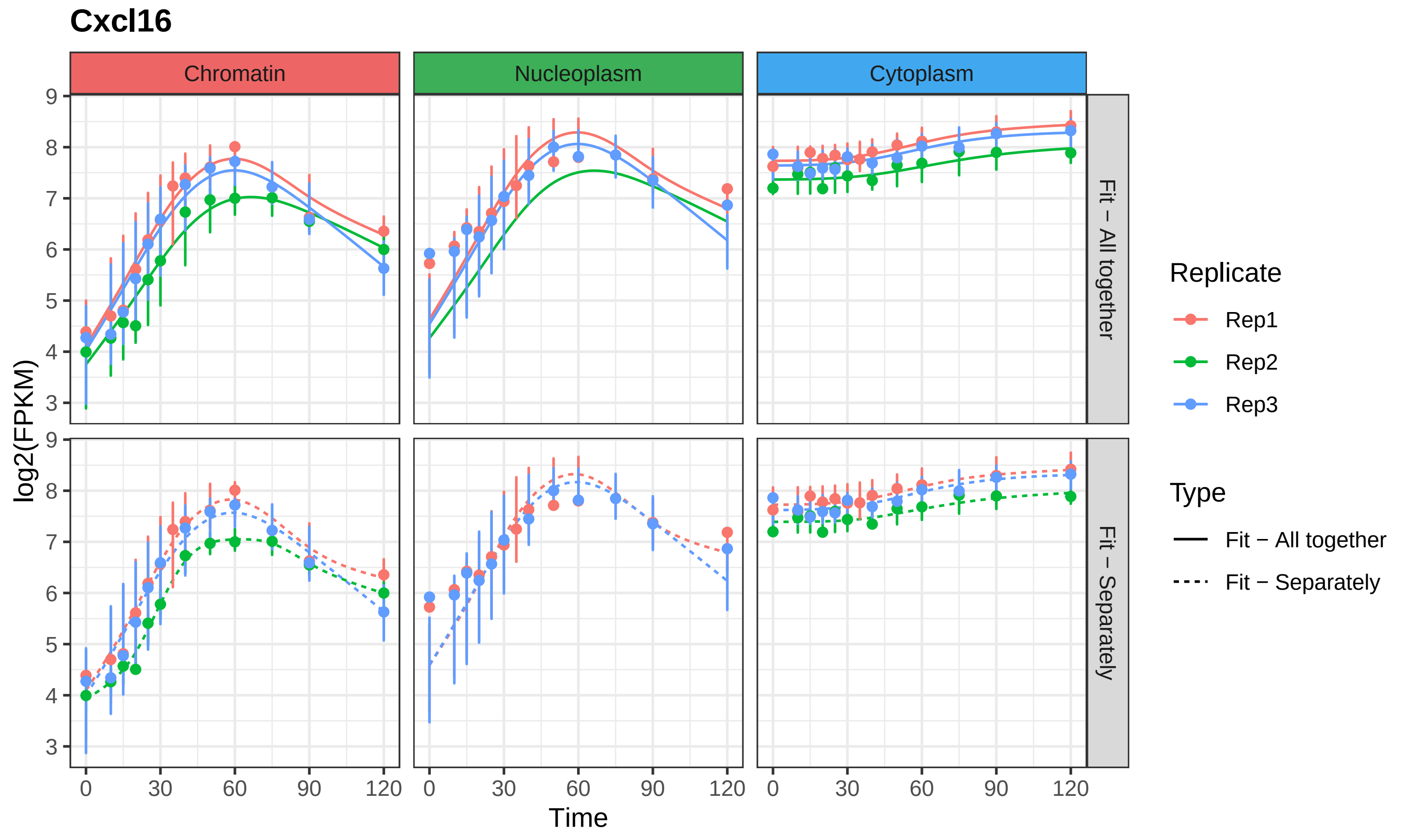

	Together	b1	b2	b3
**−NLL b1 ca**	2.386	0.7127		
**−NLL b1 np**	13.33	13.87		
**−NLL b1 cyto**	−10.37	−8.459		
**−NLL b2 ca**	−1.313		−8.904	
**−NLL b2 np**				
**−NLL b2 cyto**	−7.358		−7.05	
**−NLL b3 ca**	0.7833			1.257
**−NLL b3 np**	9.798			10.48
**−NLL b3 cyto**	−8.803			−8.229
Total	−1.553	6.127	−15.95	3.51
**Total with regul**	1.163	6.727	−17.34	5.49

	Together	b1	b2	b3
spar	0.59100	0.5417	0.3927	0.57580
σ_b	0.09502	0.1203	0.0709	0.09357
σ_t	5.51000	5.0720	1.003	5.90800
ca_{0,b_1}				
$\log_{10}(k_1')$	8.58700	7.1710		6.60000
$\log_{10}(k_2)$	8.43200	7.0210	5.306 or −3.025	6.42100
$\log_{10}(k_2')$	−2.11900	−2.1300		−2.11500
$\log_{10}(k_{deg})$	−3.05200	−3.0730	−3.025 or 5.306	−3.02800
$\log_{10}(k_1' k_2')$	6.46900	5.0410	3.323	4.48500
$\log_{10}(k_1'/k_2)$	0.15540	0.1498		0.17980
$\text{transport} = \log_{10}(k_1' k_2'/k_2)$	−1.96300	−1.9800	−1.983 or 6.348	−1.93600

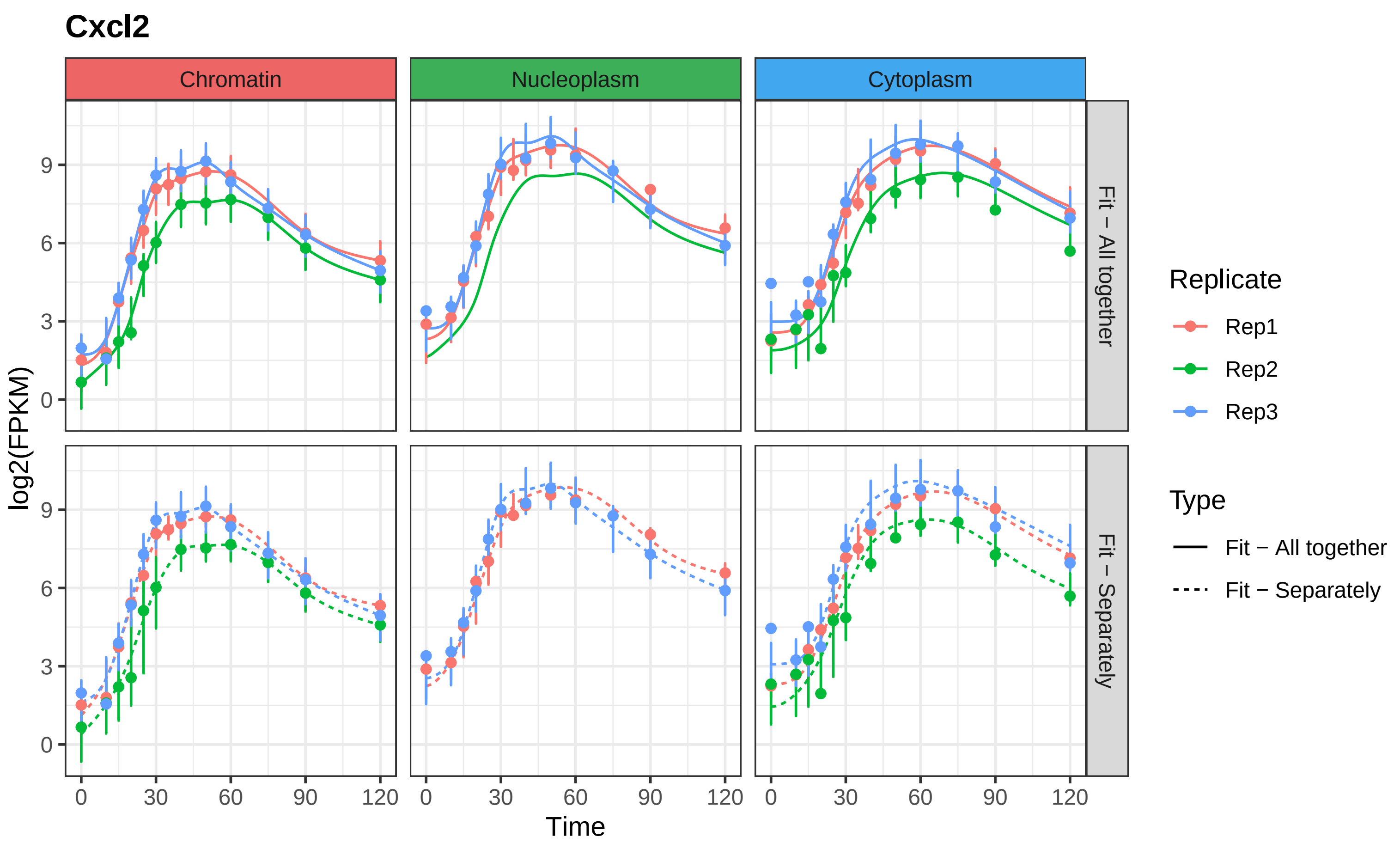

	Together	b1	b2	b3
**−NLL b1 ca**	1.188	−0.7254		
**−NLL b1 np**	4.312	7.407		
**−NLL b1 cyto**	4.209	0.7199		
**−NLL b2 ca**	1.612		2.683	
**−NLL b2 np**				
**−NLL b2 cyto**	15.67		14.46	
**−NLL b3 ca**	2.172			3.699
**−NLL b3 np**	4.48			4.628
**−NLL b3 cyto**	19.13			16.64
Total	52.78	7.401	17.14	24.96
**Total with regul**	62.7	9.754	19.36	32.97

	Together	b1	b2	b3
spar	0.2415000	0.3154	0.3397	0.2772000
σ_b	0.2664000	0.1422	0.1982	0.2953000
σ_t	0.0002808	1.3380	2.649	0.0003774
ca_{0,b_1}				
$\log_{10}(k_1')$	0.2092000	−0.2113		0.1888000
$\log_{10}(k_2)$	−0.0941300	−0.5547	4.094 or −0.9283	−0.0880200
$\log_{10}(k_2')$	−1.1030000	−1.0930		−1.0750000
$\log_{10}(k_{deg})$	−1.1790000	−1.1080	−0.9283 or 4.094	−1.2360000
$\log_{10}(k_1'k_2')$	−0.8943000	−1.3050	3.482	−0.8858000
$\log_{10}(k_1'/k_2)$	0.3033000	0.3435		0.2768000
$transport = \log_{10}(k_1'k_2'/k_2)$	−0.8001000	−0.7498	−0.6112 or 4.411	−0.7978000

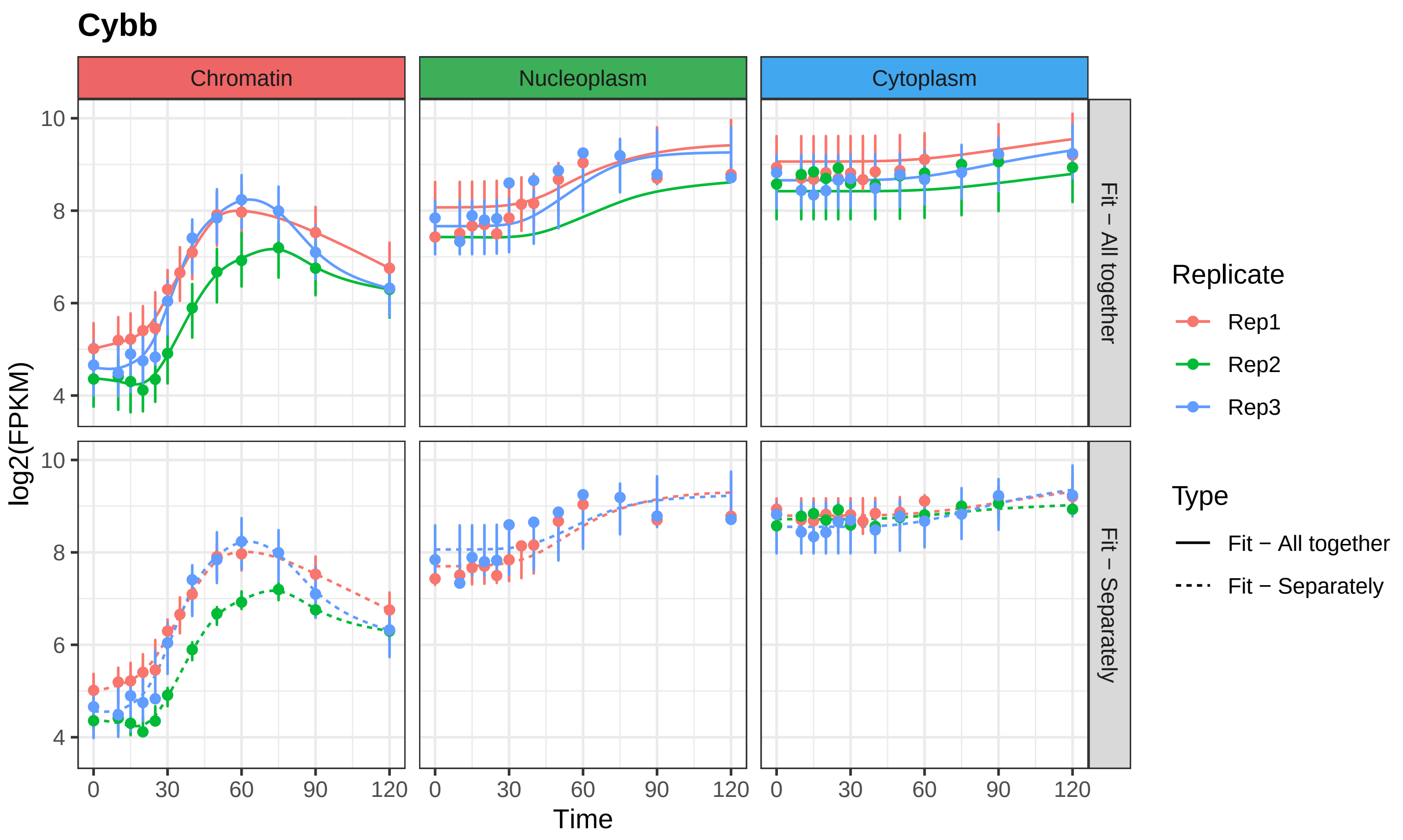

	Together	b1	b2	b3
**−NLL b1 ca**	−3.206	−7.135		
**−NLL b1 np**	9.505	6.859		
**−NLL b1 cyto**	2.388	−6.45		
**−NLL b2 ca**	−3.275		−12.42	
**−NLL b2 np**				
**−NLL b2 cyto**	4.028		−7.602	
**−NLL b3 ca**	−1.9			−1.328
**−NLL b3 np**	14.8			10.18
**−NLL b3 cyto**	−1.688			−2.357
Total	20.66	−6.727	−20.02	6.491
**Total with regul**	21.94	−7.863	−20.34	6.418

	Together	b1	b2	b3
spar	3.603e−01	4.125e−01	0.3554	4.026e−01
σ_b	1.995e−01	1.342e−01	0.0651	1.895e−01
σ_t	1.908e−06	4.847e−09	0.0002308	2.366e−05
ca_{0,b_1}				
$\log_{10}(k_1')$	−1.435e+00	−1.399e+00		−1.575e+00
$\log_{10}(k_2)$	−2.355e+00	−2.214e+00	0.2723 or −3.154	−2.629e+00
$\log_{10}(k_2')$	−1.928e+00	−2.010e+00		−1.591e+00
$\log_{10}(k_{deg})$	−2.227e+00	−2.339e+00	−3.154 or 0.2723	−1.739e+00
$\log_{10}(k_1'k_2')$	−3.363e+00	−3.409e+00	−1.574	−3.166e+00
$\log_{10}(k_1'/k_2)$	9.201e−01	8.149e−01		1.054e+00
$transport = \log_{10}(k_1'k_2'/k_2)$	−1.008e+00	−1.195e+00	−1.847 or 1.58	−5.371e−01

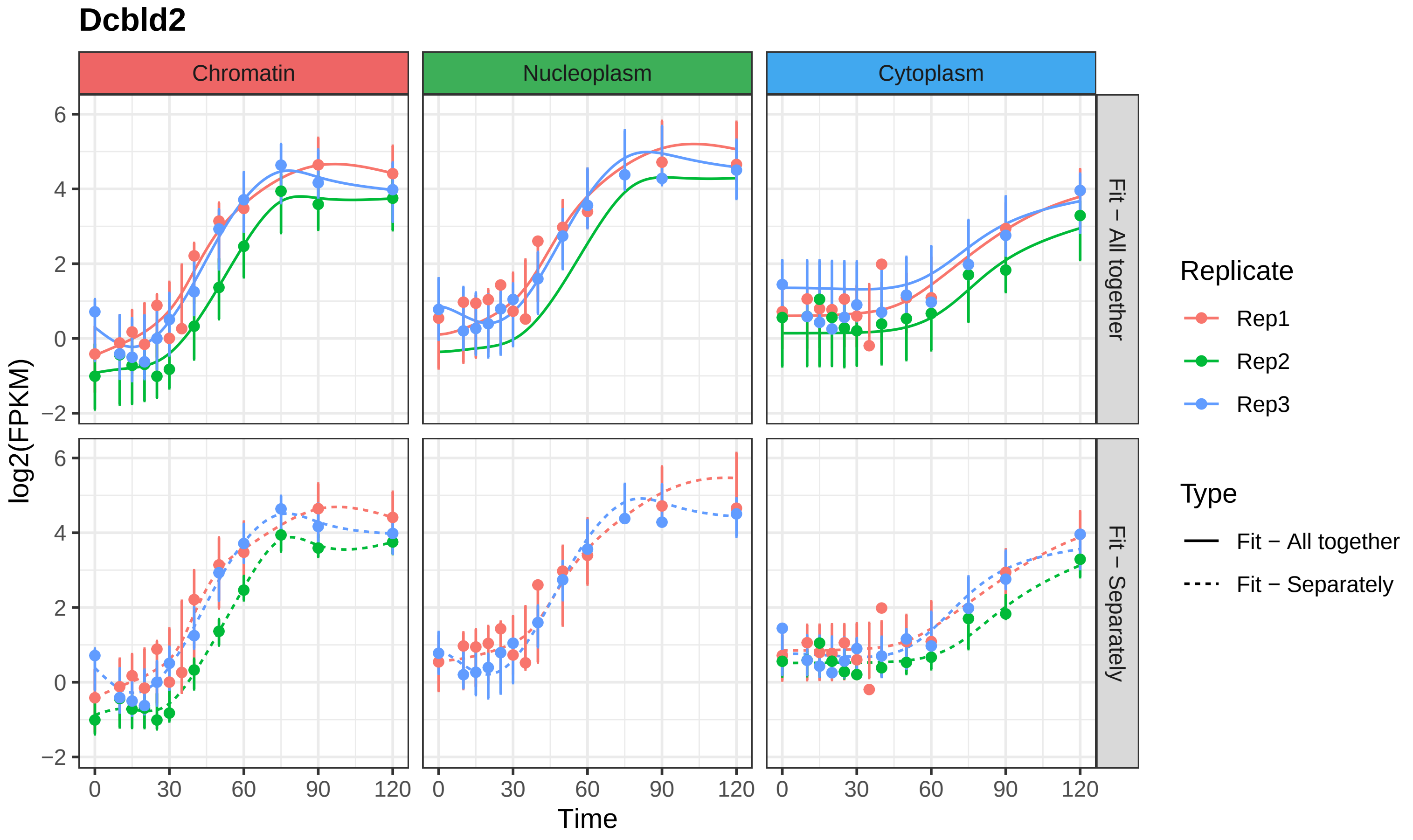

	Together	b1	b2	b3
**−NLL b1 ca**	6.02	4.53		
**−NLL b1 np**	10.8	8.761		
**−NLL b1 cyto**	9.62	9.033		
**−NLL b2 ca**	2.38		−4.358	
**−NLL b2 np**				
**−NLL b2 cyto**	5.514		0.09013	
**−NLL b3 ca**	2.66			−0.5988
**−NLL b3 np**	4.011			1.942
**−NLL b3 cyto**	14.25			5.12
Total	55.25	22.32	−4.268	6.462
**Total with regul**	56.82	23.4	−5.711	5.735

	Together	b1	b2	b3
spar	4.883e−01	0.4203	0.4088	4.654e−01
σ_b	2.680e−01	0.2445	0.1035	1.757e−01
σ_t	3.599e−05	3.4150	0.05937	4.627e−05
ca_{0,b_1}				
$\log_{10}(k_1')$	−5.893e−01	−1.0050		−4.731e−01
$\log_{10}(k_2)$	−7.579e−01	−1.3000	−1.826 or −1.84	−6.057e−01
$\log_{10}(k_2')$	−2.121e+00	−2.1000		−2.038e+00
$\log_{10}(k_{deg})$	−2.271e+00	−2.1840	−1.84 or −1.826	−2.020e+00
$\log_{10}(k_1'k_2')$	−2.710e+00	−3.1050	−3.248	−2.511e+00
$\log_{10}(k_1'/k_2)$	1.686e−01	0.2954		1.326e−01
transport $= \log_{10}(k_1'k_2'/k_2)$	−1.952e+00	−1.8050	−1.422 or −1.408	−1.905e+00

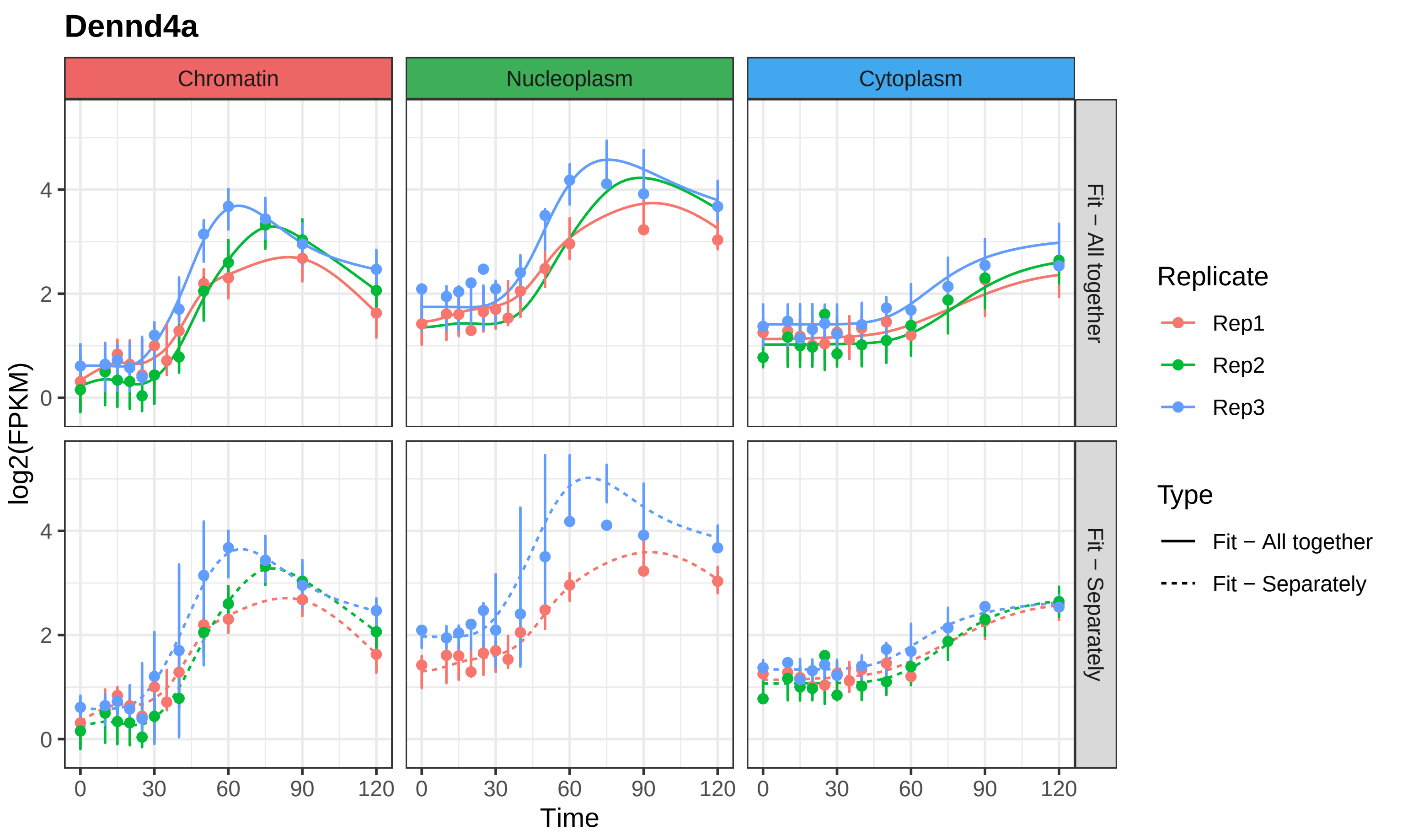

	Together	b1	b2	b3
**−NLL b1 ca**	−4.471	−6.13		
**−NLL b1 np**	−0.6476	−3.598		
**−NLL b1 cyto**	−4.176	−7.093		
**−NLL b2 ca**	−5.804		−5.711	
**−NLL b2 np**				
**−NLL b2 cyto**	−1.738		−0.7718	
**−NLL b3 ca**	−5.26			−2.516
**−NLL b3 np**	11.18			4.326
**−NLL b3 cyto**	−2.737			−7.934
Total	−13.65	−16.82	−6.483	−6.124
**Total with regul**	−14.35	−18.45	−8.05	−7.348

	Together	b1	b2	b3
spar	3.868e−01	4.060e−01	0.4091	0.44080
σ_b	1.278e−01	7.742e−02	0.09129	0.03490
σ_t	5.717e−09	1.997e−06	9.778e−06	7.13200
ca_{0,b_1}				
$\log_{10}(k_1')$	−7.604e−01	−7.752e−01		0.04797
$\log_{10}(k_2)$	−1.100e+00	−1.066e+00	−0.7199 or −2.145	−0.36890
$\log_{10}(k_2')$	−2.209e+00	−2.024e+00		−2.52700
$\log_{10}(k_{deg})$	−2.109e+00	−1.974e+00	−2.145 or −0.7199	−2.33700
$\log_{10}(k_1'k_2')$	−2.970e+00	−2.799e+00	−2.613	−2.47900
$\log_{10}(k_1'/k_2)$	3.401e−01	2.904e−01		0.41680
transport $= \log_{10}(k_1'k_2'/k_2)$	−1.869e+00	−1.733e+00	−1.893 or −0.4687	−2.11000

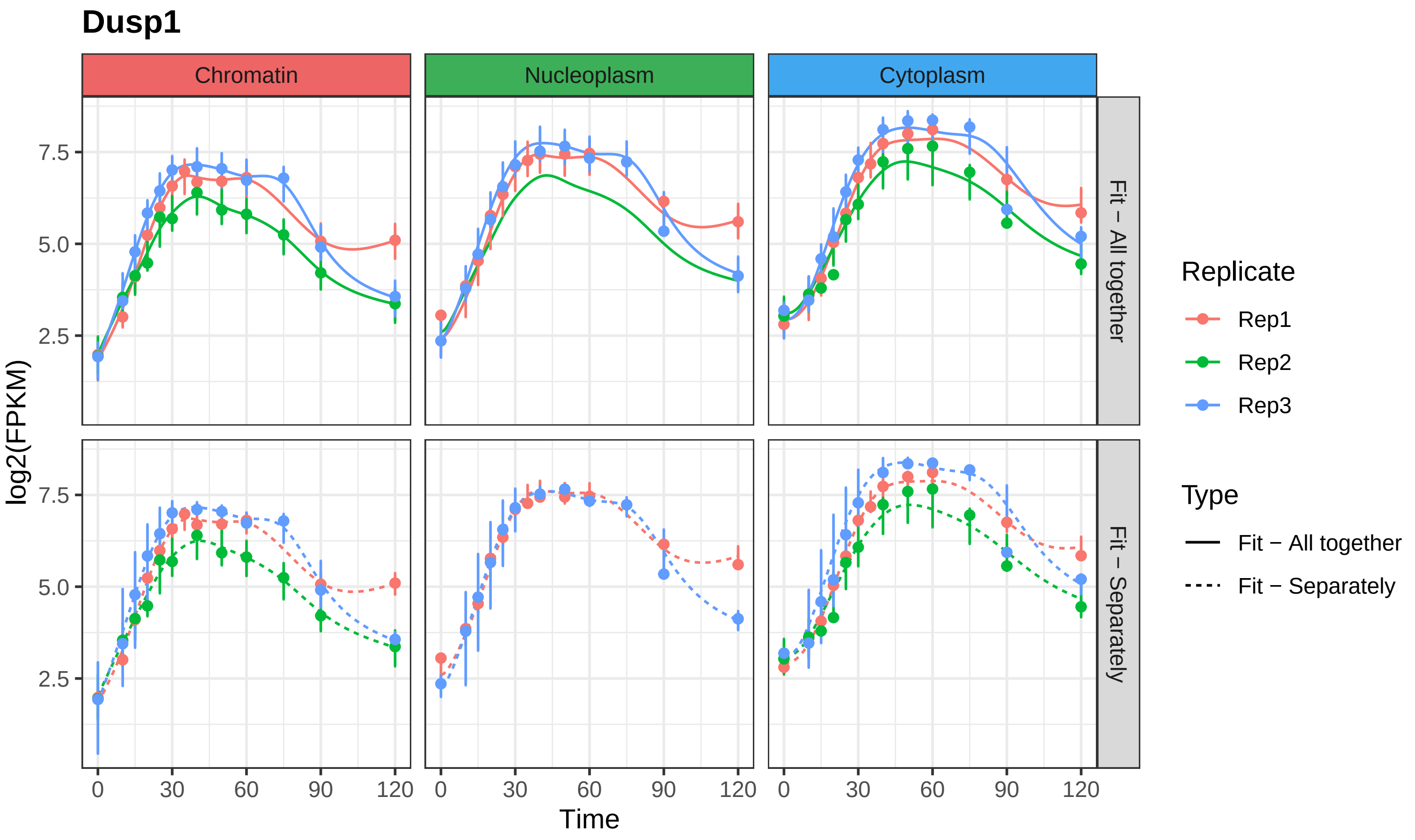

	Together	b1	b2	b3
**−NLL b1 ca**	−5.003	−8.606		
**−NLL b1 np**	1.337	−1.576		
**−NLL b1 cyto**	−4.183	−7.096		
**−NLL b2 ca**	−3.9		−2.233	
**−NLL b2 np**				
**−NLL b2 cyto**	7.455		5.847	
**−NLL b3 ca**	−4.649			−2.677
**−NLL b3 np**	0.2893			−2.867
**−NLL b3 cyto**	8.662			6.447
Total	0.008169	−17.28	3.615	0.9035
**Total with regul**	1.281	−17.59	2.991	0.2098

	Together	b1	b2	b3
spar	0.3430000	3.621e−01	0.4081	0.36820
σ_b	0.1614000	9.593e−02	0.1621	0.04585
σ_t	0.0003421	2.925e−06	1.207	3.00000
ca_{0,b_1}				
$\log_{10}(k_1')$	−0.1962000	−1.584e−01		−0.35310
$\log_{10}(k_2)$	−0.3762000	−3.947e−01	−0.6316 or −0.6809	−0.49180
$\log_{10}(k_2')$	−0.6801000	−7.047e−01		−0.39770
$\log_{10}(k_{deg})$	−0.8323000	−8.042e−01	−0.6809 or −0.6316	−0.64300
$\log_{10}(k_1'k_2')$	−0.8763000	−8.631e−01	−0.9853	−0.75080
$\log_{10}(k_1'/k_2)$	0.1799000	2.363e−01		0.13870
$transport = \log_{10}(k_1'k_2'/k_2)$	−0.5001000	−4.684e−01	−0.3537 or −0.3045	−0.25900

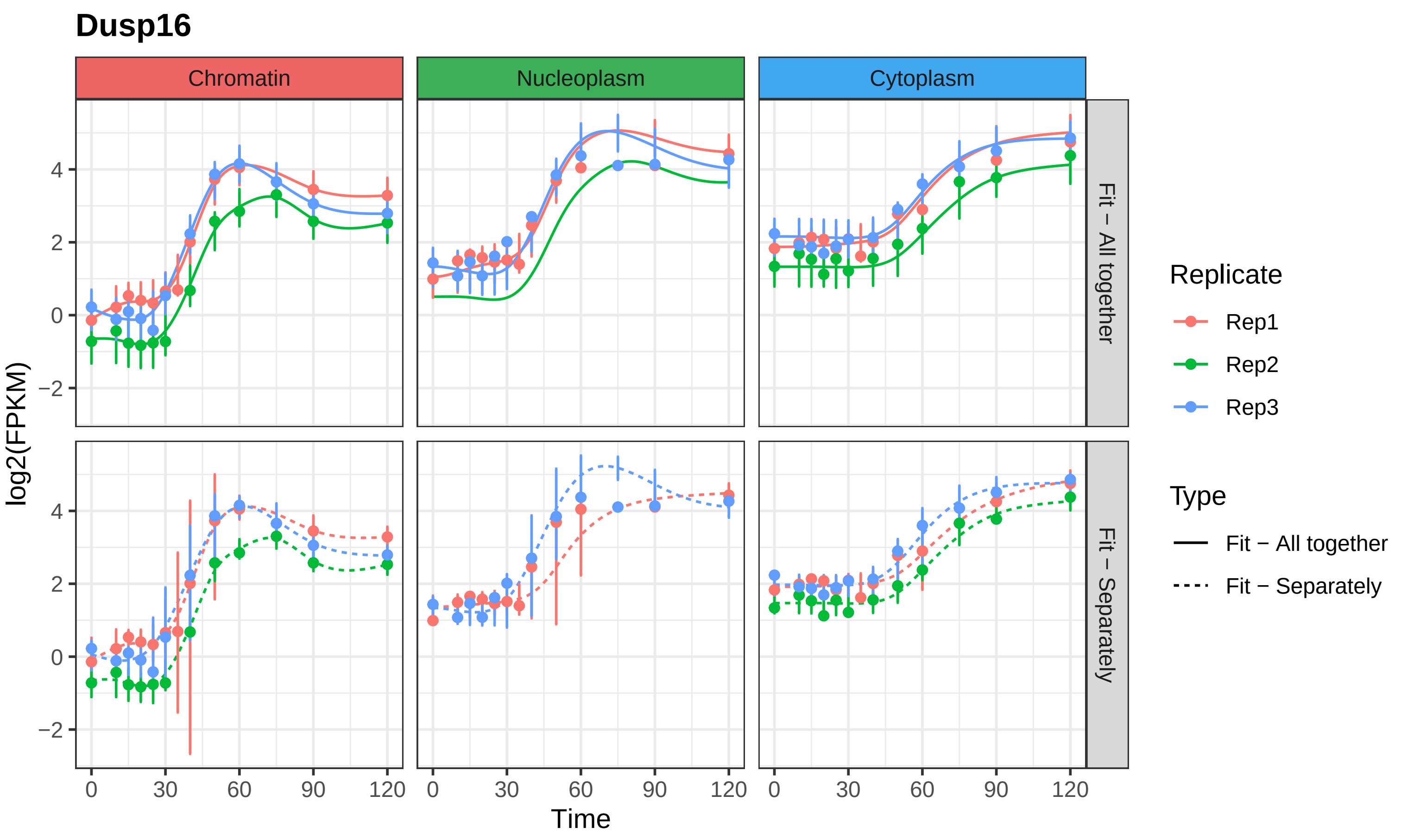

	Together	b1	b2	b3
**−NLL b1 ca**	−2.632	−0.7745		
**−NLL b1 np**	5.138	3.634		
**−NLL b1 cyto**	0.6325	−2.515		
**−NLL b2 ca**	−2.405		−4.865	
**−NLL b2 np**				
**−NLL b2 cyto**	0.2137		−3.844	
**−NLL b3 ca**	−2.46			−0.8037
**−NLL b3 np**	11.63			4.956
**−NLL b3 cyto**	−0.5787			−4.093
Total	9.543	0.345	−8.708	0.05855
**Total with regul**	9.591	0.07488	−9.285	−1.436

	Together	b1	b2	b3
spar	3.833e−01	0.38750	0.3664	0.44980
σ_b	1.718e−01	0.09131	0.08295	0.09165
σ_t	2.773e−05	6.51900	0.0002063	4.49400
ca_{0,b_1}				
$\log_{10}(k_1')$	−7.122e−01	−1.43400		−0.54990
$\log_{10}(k_2)$	−1.060e+00	−1.87700	−1.057 or −1.829	−0.93420
$\log_{10}(k_2')$	−1.579e+00	−1.22000		−1.65100
$\log_{10}(k_{deg})$	−1.826e+00	−1.38500	−1.829 or −1.057	−1.84200
$\log_{10}(k_1'k_2')$	−2.291e+00	−2.65300	−2.247	−2.20100
$\log_{10}(k_1'/k_2)$	3.481e−01	0.44290		0.38430
$transport = \log_{10}(k_1'k_2'/k_2)$	−1.231e+00	−0.77690	−1.19 or −0.4183	−1.26600

Dusp2

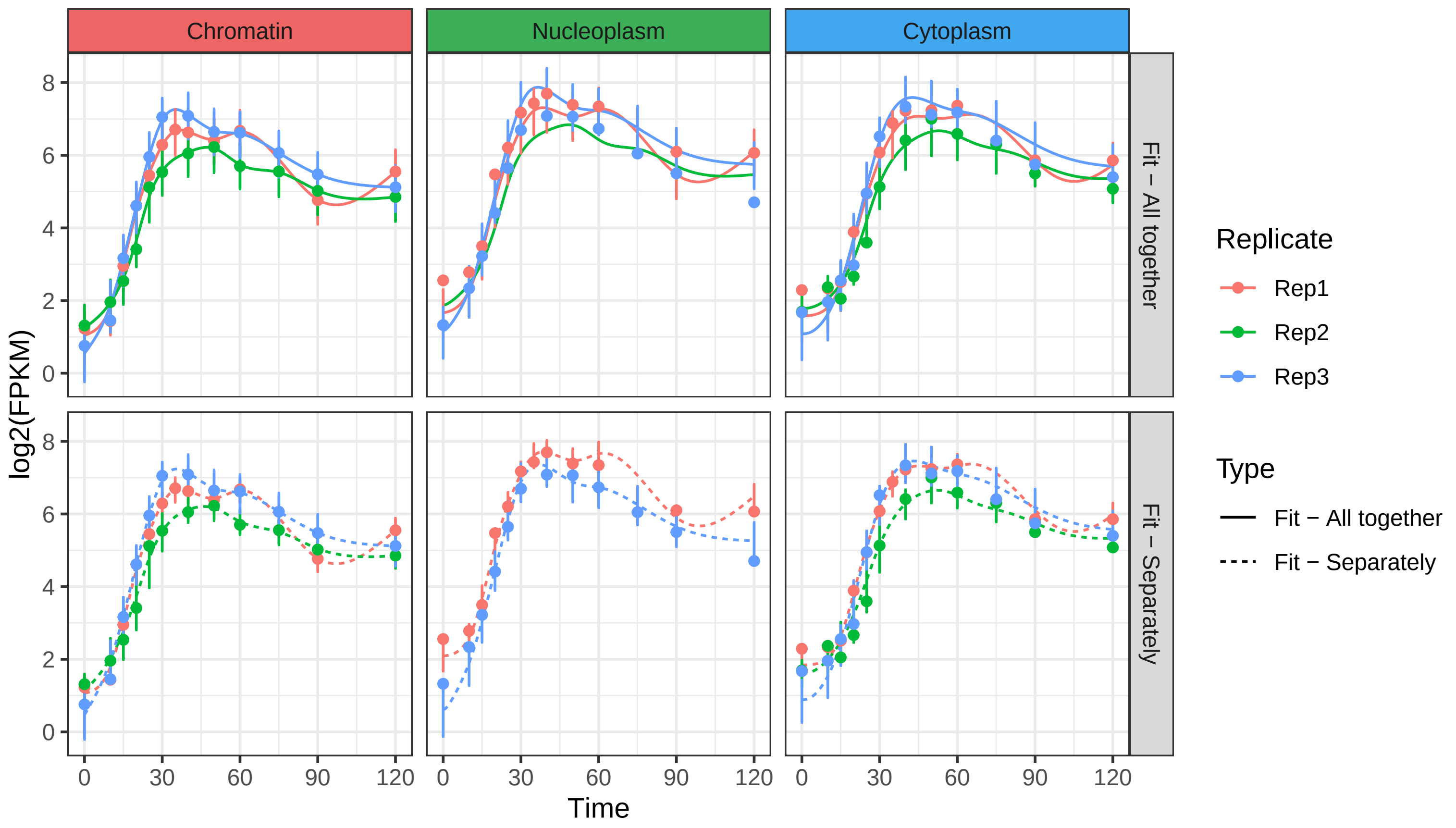

	Together	b1	b2	b3
**−NLL b1 ca**	−1.173	−7.258		
**−NLL b1 np**	9.485	2.286		
**−NLL b1 cyto**	2.621	−3.818		
**−NLL b2 ca**	−1.624		−3.713	
**−NLL b2 np**				
**−NLL b2 cyto**	3.236		4.002	
**−NLL b3 ca**	−1.111			−1.947
**−NLL b3 np**	15.9			3.614
**−NLL b3 cyto**	6.217			6.156
Total	33.55	−8.789	0.2883	7.823
**Total with regul**	39.81	−3.395	0.972	11.29

	Together	b1	b2	b3
spar	0.27360	0.2624	0.3427	0.306500
σ_b	0.21690	0.1163	0.1129	0.182300
σ_t	0.49100	0.1408	1.788	0.001179
ca_{0,b_1}				
$\log_{10}(k_1')$	0.07577	0.1092		−0.028410
$\log_{10}(k_2)$	−0.11300	−0.2015	−0.4909 or −0.5393	−0.069480
$\log_{10}(k_2')$	−0.79050	−0.8234		−0.692900
$\log_{10}(k_{deg})$	−0.76370	−0.7483	−0.5393 or −0.4909	−0.777500
$\log_{10}(k_1'k_2')$	−0.71470	−0.7143	−0.8806	−0.721300
$\log_{10}(k_1'/k_2)$	0.18880	0.3106		0.041080
transport $= \log_{10}(k_1'k_2'/k_2)$	−0.60180	−0.5128	−0.3897 or −0.3413	−0.651800

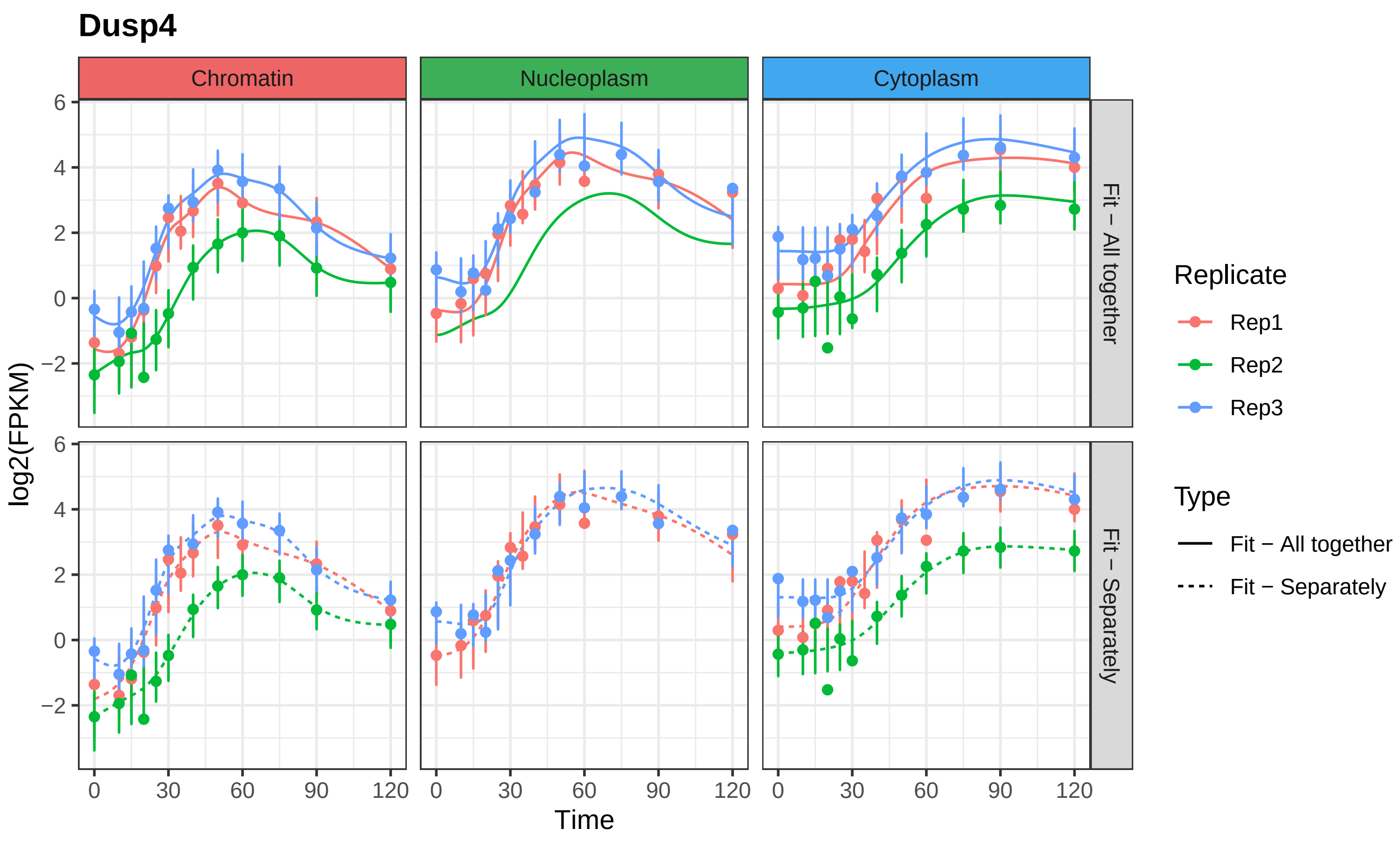

	Together	b1	b2	b3
**−NLL b1 ca**	2.406	4.379		
**−NLL b1 np**	9.384	7.307		
**−NLL b1 cyto**	12.91	10.57		
**−NLL b2 ca**	3.658		3.114	
**−NLL b2 np**				
**−NLL b2 cyto**	8.336		8.652	
**−NLL b3 ca**	2.699			1.83
**−NLL b3 np**	10.65			7.457
**−NLL b3 cyto**	4.813			4.26
Total	54.85	22.25	11.77	13.55
**Total with regul**	58.31	23.22	11.91	15.14

	Together	b1	b2	b3
spar	0.3446000	0.4199	0.4101	0.3531
σ_b	0.2667000	0.2468	0.207	0.1970
σ_t	0.0006047	1.7510	0.0006516	2.1000
ca_{0,b_1}				
$\log_{10}(k_1')$	−0.3473000	−0.3972		−0.8117
$\log_{10}(k_2)$	−0.7038000	−0.7990	4.815 or −1.821	−1.1550
$\log_{10}(k_2')$	−1.4430000	−1.3110		−1.2950
$\log_{10}(k_{deg})$	−1.6860000	−1.5760	−1.821 or 4.815	−1.5180
$\log_{10}(k_1'k_2')$	−1.7910000	−1.7090	3.58	−2.1060
$\log_{10}(k_1'/k_2)$	0.3565000	0.4018		0.3431
$transport = \log_{10}(k_1'k_2'/k_2)$	−1.0870000	−0.9095	−1.235 or 5.401	−0.9517

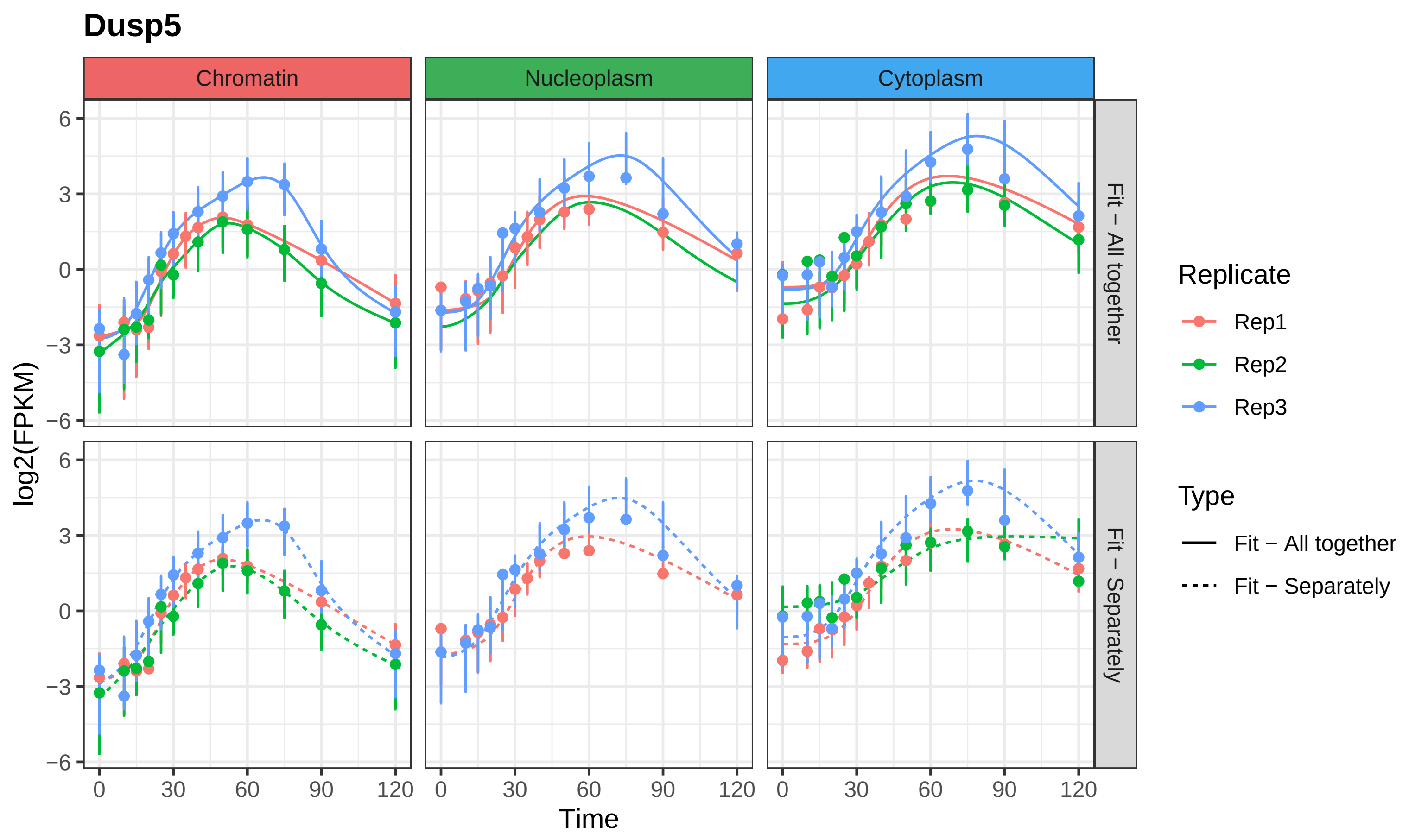

	Together	b1	b2	b3
**−NLL b1 ca**	6.247	3.394		
**−NLL b1 np**	7.44	8.102		
**−NLL b1 cyto**	12.46	5.319		
**−NLL b2 ca**	6.553		6.434	
**−NLL b2 np**				
**−NLL b2 cyto**	22.22		12.47	
**−NLL b3 ca**	5.825			6.073
**−NLL b3 np**	11.89			10.19
**−NLL b3 cyto**	12.41			12.94
Total	85.05	16.82	18.9	29.2
**Total with regul**	90.29	16.52	20.53	31.84

	Together	b1	b2	b3
spar	0.3497000	0.412900	0.4327	0.3902
σ_b	0.3332000	0.186900	0.2787	0.2901
σ_t	0.0004245	0.000485	0.006846	1.1750
ca_{0,b_1}				
$\log_{10}(k_1')$	−0.6528000	−0.659900		−0.6455
$\log_{10}(k_2)$	−0.9696000	−0.995000	4.075 or −2.329	−0.9601
$\log_{10}(k_2')$	−0.6494000	−0.825600		−0.6429
$\log_{10}(k_{deg})$	−0.9254000	−0.948200	−2.329 or 4.075	−0.8833
$\log_{10}(k_1'k_2')$	−1.3020000	−1.485000	2.838	−1.2880
$\log_{10}(k_1'/k_2)$	0.3168000	0.335200		0.3146
$transport = \log_{10}(k_1'k_2'/k_2)$	−0.3326000	−0.490400	−1.237 or 5.168	−0.3283

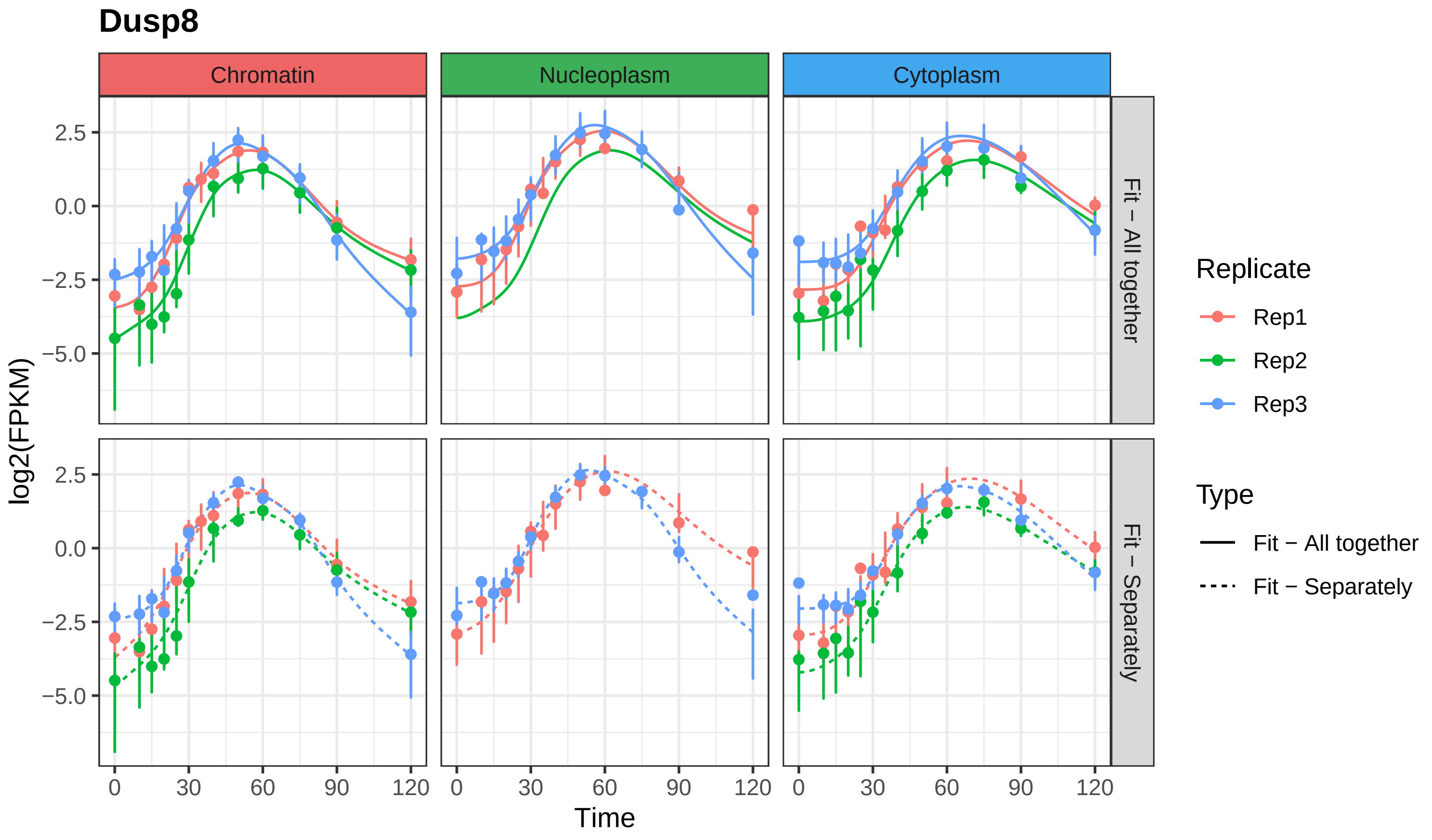

	Together	b1	b2	b3
**−NLL b1 ca**	2.12	4.251		
**−NLL b1 np**	9.347	6.769		
**−NLL b1 cyto**	12.3	8.624		
**−NLL b2 ca**	3.782		3.919	
**−NLL b2 np**				
**−NLL b2 cyto**	5.978		4.151	
**−NLL b3 ca**	2.115			0.1707
**−NLL b3 np**	4.301			5.606
**−NLL b3 cyto**	4.968			1.565
Total	44.91	19.64	8.07	7.342
**Total with regul**	44.75	19.2	5.886	6.792

	Together	b1	b2	b3
spar	0.4039	0.4723	0.4452	0.3628000
σ_b	0.1851	0.1842	0.0001165	0.0701700
σ_t	1.1720	1.9660	2.546	0.0002919
ca_{0,b_1}				
$\log_{10}(k_1')$	−0.4861	−0.6388		−0.3254000
$\log_{10}(k_2)$	−0.7032	−0.9002	4.802 or −1.138	−0.4911000
$\log_{10}(k_2')$	−1.0910	−0.9932		−1.1990000
$\log_{10}(k_{deg})$	−1.0570	−0.9635	−1.138 or 4.802	−1.1440000
$\log_{10}(k_1'k_2')$	−1.5770	−1.6320	3.806	−1.5240000
$\log_{10}(k_1'/k_2)$	0.2172	0.2614		0.1657000
$\text{transport} = \log_{10}(k_1'k_2'/k_2)$	−0.8740	−0.7318	−0.9956 or 4.944	−1.0330000

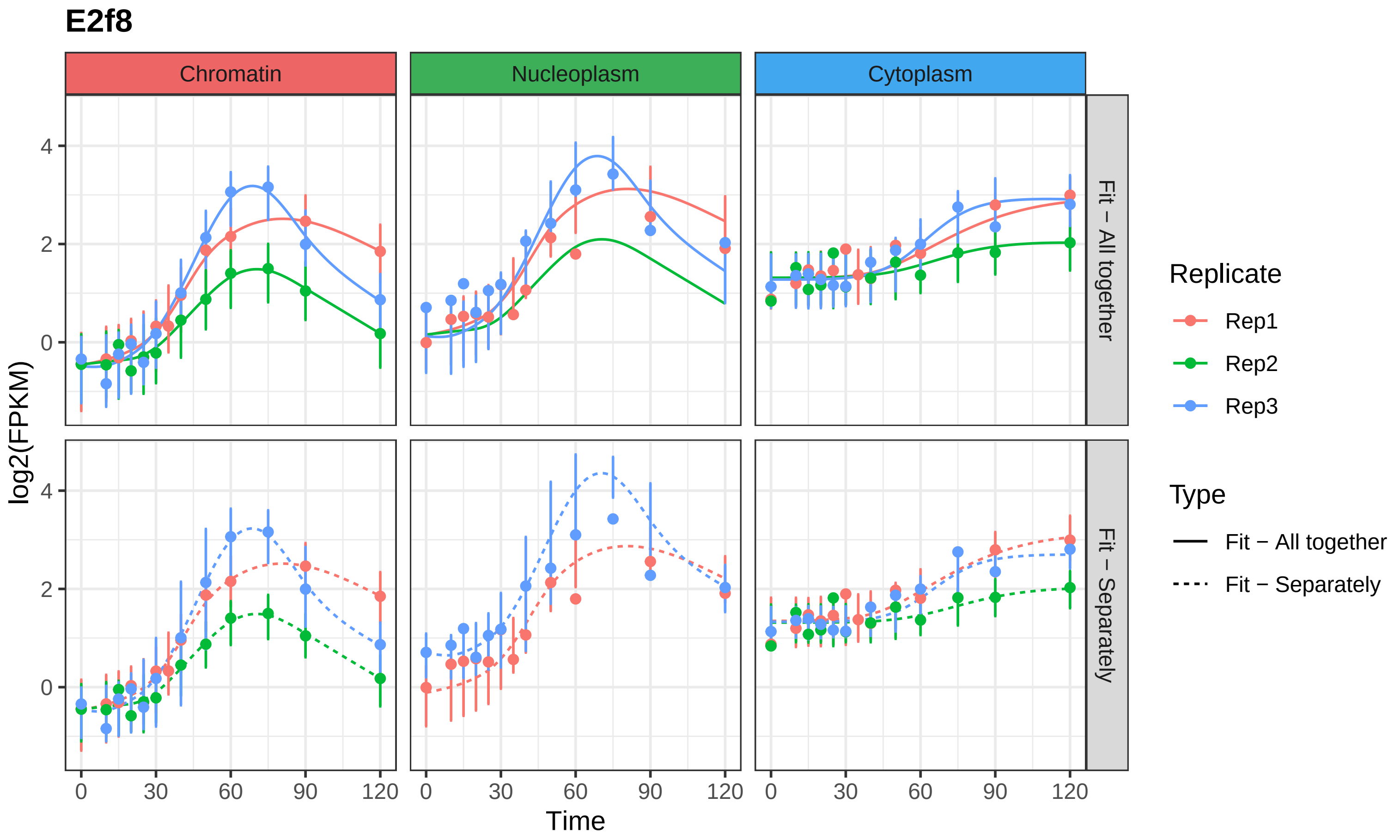

	Together	b1	b2	b3
**−NLL b1 ca**	−1.204	−2.087		
**−NLL b1 np**	10.47	7.844		
**−NLL b1 cyto**	0.7012	−0.4335		
**−NLL b2 ca**	−1.609		−1.936	
**−NLL b2 np**				
**−NLL b2 cyto**	0.3834		1.264	
**−NLL b3 ca**	−0.9904			1.279
**−NLL b3 np**	15.02			6.905
**−NLL b3 cyto**	−1.224			−2.553
Total	21.55	5.324	−0.672	5.63
**Total with regul**	20.78	4.153	−2.304	4.51

	Together	b1	b2	b3
spar	0.4459	0.452100	0.4419	0.41370
σ_b	0.1752	0.151400	0.1161	0.08673
σ_t	0.6121	0.006687	0.0001381	5.28400
ca_{0,b_1}				
$\log_{10}(k_1')$	4.4460	7.147000		0.05751
$\log_{10}(k_2)$	4.2630	7.040000	−1.021 or −2.273	−0.28520
$\log_{10}(k_2')$	−1.9190	−1.748000		−2.21500
$\log_{10}(k_{deg})$	−2.2670	−2.188000	−2.273 or −1.021	−2.41000
$\log_{10}(k_1'k_2')$	2.5270	5.399000	−2.762	−2.15700
$\log_{10}(k_1'/k_2)$	0.1832	0.107000		0.34270
transport = $\log_{10}(k_1'k_2'/k_2)$	−1.7360	−1.641000	−1.741 or −0.4897	−1.87200

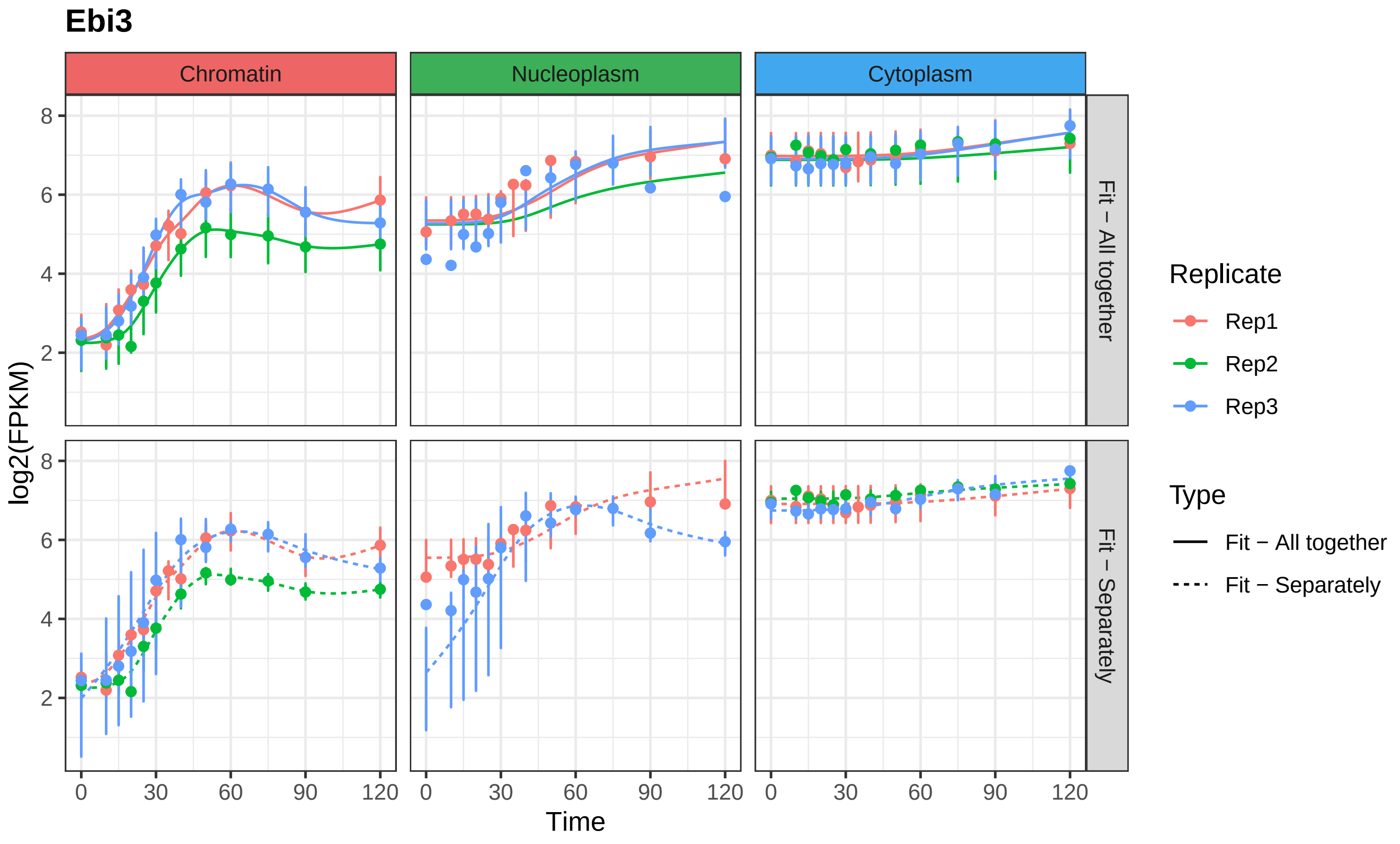

	Together	b1	b2	b3
**−NLL b1 ca**	−0.4206	−1.927		
**−NLL b1 np**	7.597	7.868		
**−NLL b1 cyto**	−1.161	−4.921		
**−NLL b2 ca**	−1.143		−6.531	
**−NLL b2 np**				
**−NLL b2 cyto**	−0.1376		−10.08	
**−NLL b3 ca**	−1.429			3.483
**−NLL b3 np**	25.77			9.265
**−NLL b3 cyto**	−1.504			−7.01
Total	27.57	1.02	−16.61	5.739
**Total with regul**	28.15	0.392	−17.83	6.248

	Together	b1	b2	b3
spar	3.921e−01	4.051e−01	0.3845	0.53270
σ_b	2.111e−01	1.604e−01	0.05796	0.06909
σ_t	3.057e−05	7.239e−06	1.842	7.81500
ca_{0,b_1}				
$\log_{10}(k_1')$	−1.499e+00	−1.437e+00		7.10200
$\log_{10}(k_2)$	−2.402e+00	−2.407e+00	3.95 or −3.165	6.90500
$\log_{10}(k_2')$	−1.922e+00	−2.250e+00		−1.96100
$\log_{10}(k_{deg})$	−2.414e+00	−2.660e+00	−3.165 or 3.95	−3.19700
$\log_{10}(k_1'k_2')$	−3.421e+00	−3.687e+00	2.226	5.14100
$\log_{10}(k_1'/k_2)$	9.038e−01	9.704e−01		0.19700
$\text{transport} = \log_{10}(k_1'k_2'/k_2)$	−1.019e+00	−1.280e+00	−1.724 or 5.391	−1.76400

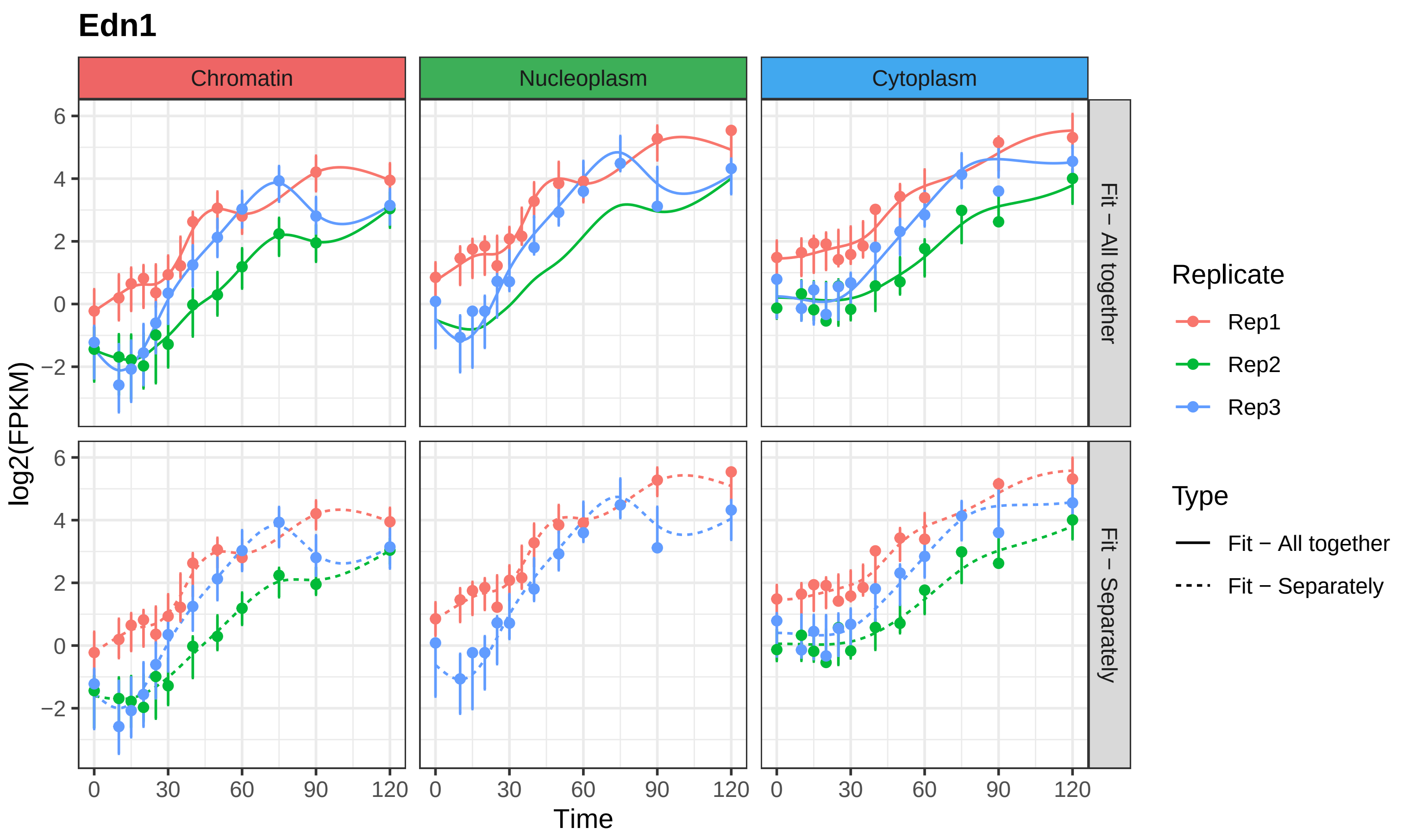

	Together	b1	b2	b3
**−NLL b1 ca**	−0.8876	−1.279		
**−NLL b1 np**	1.834	1.082		
**−NLL b1 cyto**	3.206	4.145		
**−NLL b2 ca**	0.3522		1.024	
**−NLL b2 np**				
**−NLL b2 cyto**	5.207		4.065	
**−NLL b3 ca**	−0.2433			1.948
**−NLL b3 np**	8.369			7.328
**−NLL b3 cyto**	8.941			7.109
Total	26.78	3.949	5.089	16.39
**Total with regul**	28.49	3.58	3.704	17.26

	Together	b1	b2	b3
spar	0.3446000	0.3872	0.4571	0.382500
σ_b	0.1900000	0.1476	0.1359	0.213600
σ_t	0.0007228	1.8950	0.0002526	0.002358
ca_{0,b_1}				
$\log_{10}(k_1')$	5.5780000	0.2289		6.242000
$\log_{10}(k_2)$	5.2870000	−0.1017	5.691 or −1.563	5.967000
$\log_{10}(k_2')$	−1.3290000	−1.3170		−1.453000
$\log_{10}(k_{deg})$	−1.5430000	−1.4890	−1.563 or 5.691	−1.761000
$\log_{10}(k_1'k_2')$	4.2490000	−1.0880	4.629	4.788000
$\log_{10}(k_1'/k_2)$	0.2911000	0.3306		0.274800
$\text{transport} = \log_{10}(k_1'k_2'/k_2)$	−1.0380000	−0.9861	−1.062 or 6.192	−1.179000

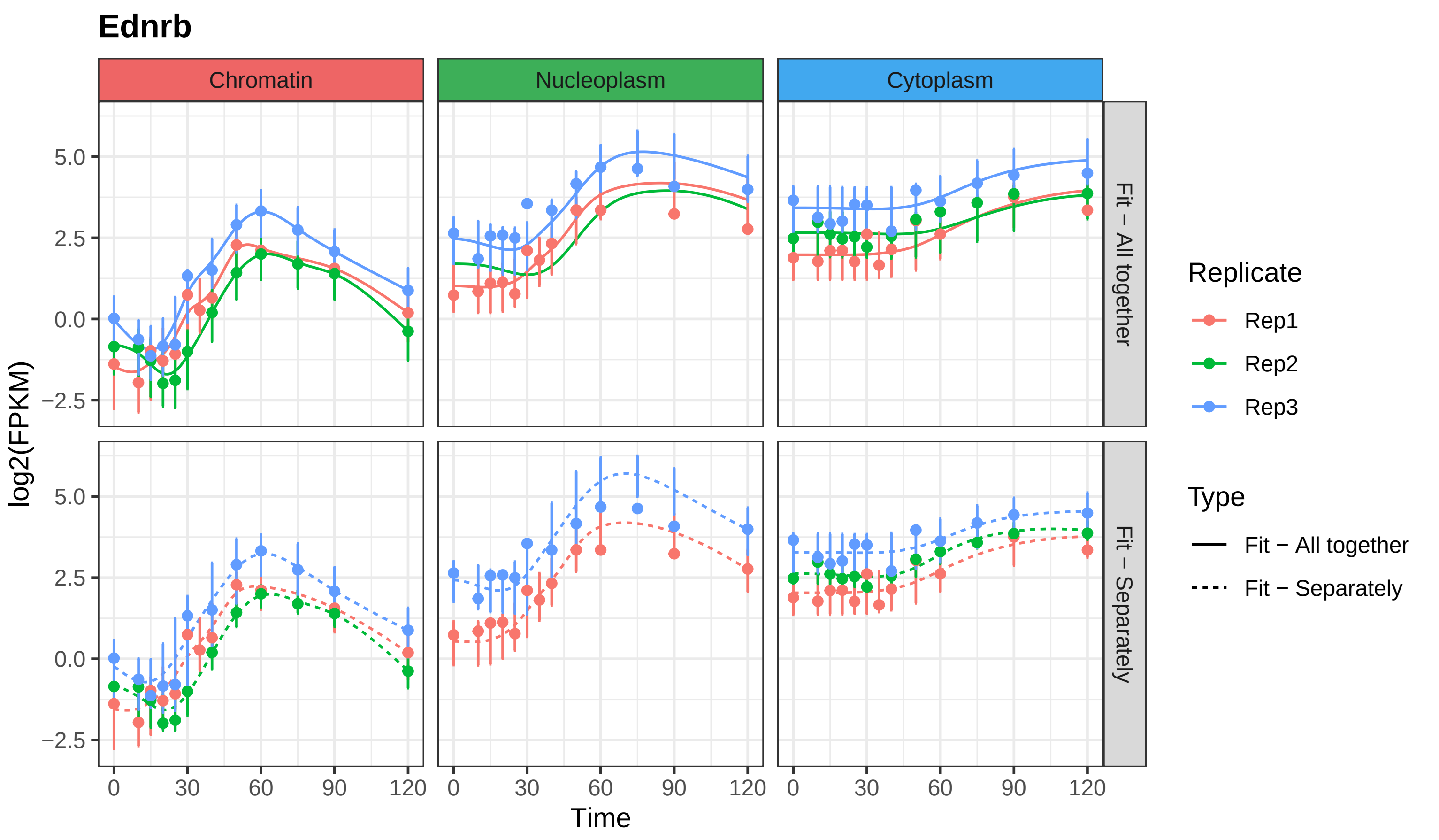

	Together	b1	b2	b3
**−NLL b1 ca**	3.766	4.45		
**−NLL b1 np**	8.934	6.882		
**−NLL b1 cyto**	5.575	4.324		
**−NLL b2 ca**	1.089		−1.712	
**−NLL b2 np**				
**−NLL b2 cyto**	3.098		−2.969	
**−NLL b3 ca**	2.581			4.608
**−NLL b3 np**	14.4			11.3
**−NLL b3 cyto**	4.5			3.09
Total	43.95	15.66	−4.681	19
**Total with regul**	47.17	15.97	−5.945	19.34

	Together	b1	b2	b3
spar	0.3340	0.4067	0.3958	0.4346
σ_b	0.2382	0.2091	0.09668	0.2079
σ_t	0.7812	1.8110	0.0005189	4.7690
ca_{0,b_1}				
$\log_{10}(k_1')$	−0.6729	−0.3154		−0.1878
$\log_{10}(k_2)$	−1.4220	−0.9437	5.542 or −2.079	−0.9876
$\log_{10}(k_2')$	−1.8300	−1.9340		−2.1550
$\log_{10}(k_{deg})$	−2.1180	−2.3840	−2.079 or 5.542	−2.4130
$\log_{10}(k_1' k_2')$	−2.5030	−2.2490	4.492	−2.3430
$\log_{10}(k_1'/k_2)$	0.7492	0.6283		0.7998
$\text{transport} = \log_{10}(k_1' k_2'/k_2)$	−1.0810	−1.3050	−1.05 or 6.571	−1.3550

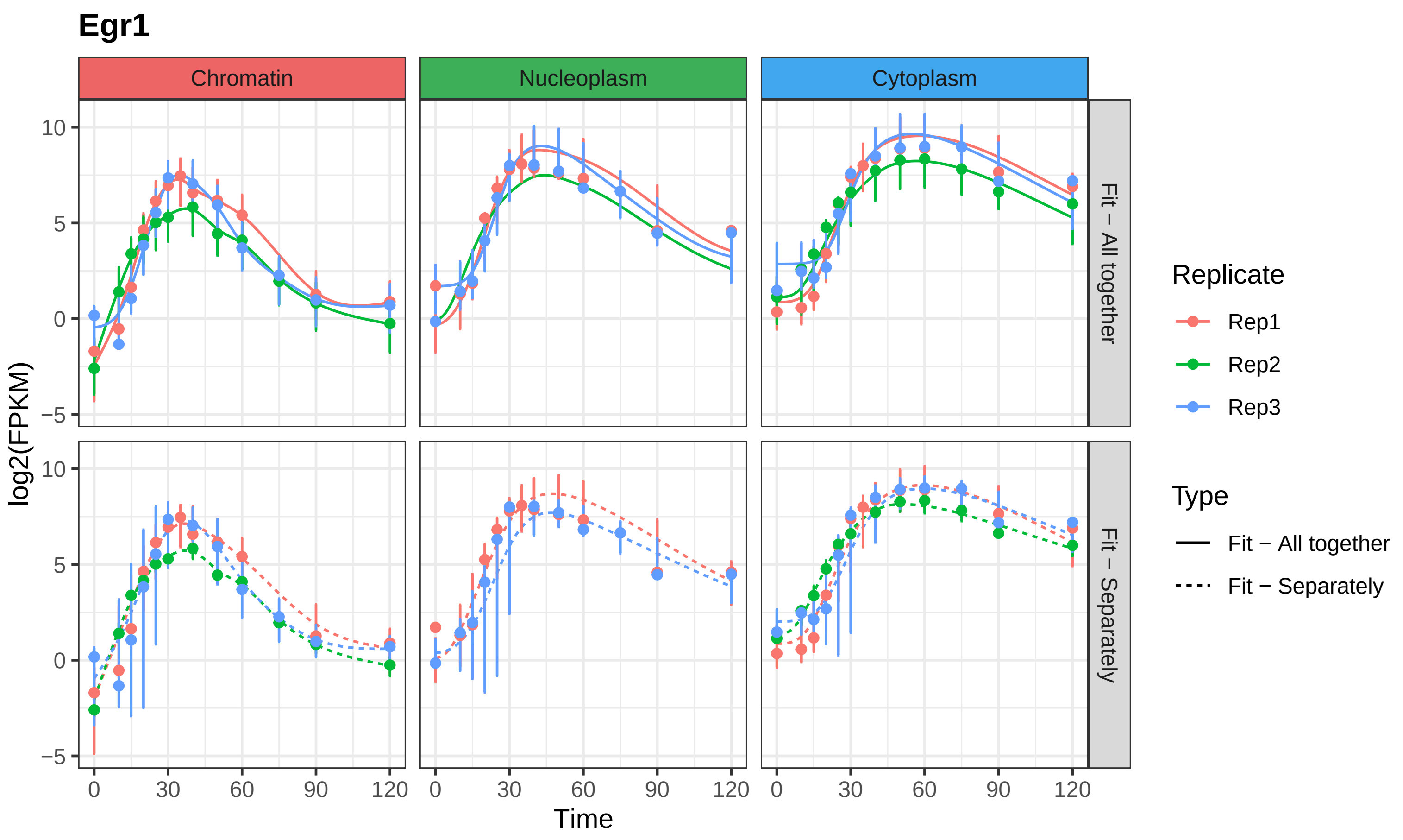

	Together	b1	b2	b3
**−NLL b1 ca**	7.689	10.79		
**−NLL b1 np**	22.38	21.09		
**−NLL b1 cyto**	10.66	11.28		
**−NLL b2 ca**	6.142		−2.237	
**−NLL b2 np**				
**−NLL b2 cyto**	9.247		−0.7024	
**−NLL b3 ca**	9.393			12.21
**−NLL b3 np**	17.87			14.28
**−NLL b3 cyto**	16.75			11.09
Total	100.1	43.17	−2.94	37.58
**Total with regul**	109.2	47.47	−1.898	38.4

	Together	b1	b2	b3
spar	0.3189000	0.4667	0.3374	0.4220
σ_b	0.3999000	0.3604	0.1283	0.2308
σ_t	0.0006616	1.8200	0.0003824	4.0280
ca_{0,b_1}				
$\log_{10}(k_1')$	−0.4266000	−0.5293		−0.9161
$\log_{10}(k_2)$	−1.0750000	−1.1680	0.002619 or −1.479	−1.3220
$\log_{10}(k_2')$	−0.8399000	−0.8832		−0.6619
$\log_{10}(k_{deg})$	−1.1890000	−1.1070	−1.479 or 0.002619	−1.1520
$\log_{10}(k_1' k_2')$	−1.2660000	−1.4130	−0.4482	−1.5780
$\log_{10}(k_1'/k_2)$	0.6488000	0.6386		0.4055
$\text{transport} = \log_{10}(k_1' k_2'/k_2)$	−0.1910000	−0.2447	−0.4508 or 1.031	−0.2564

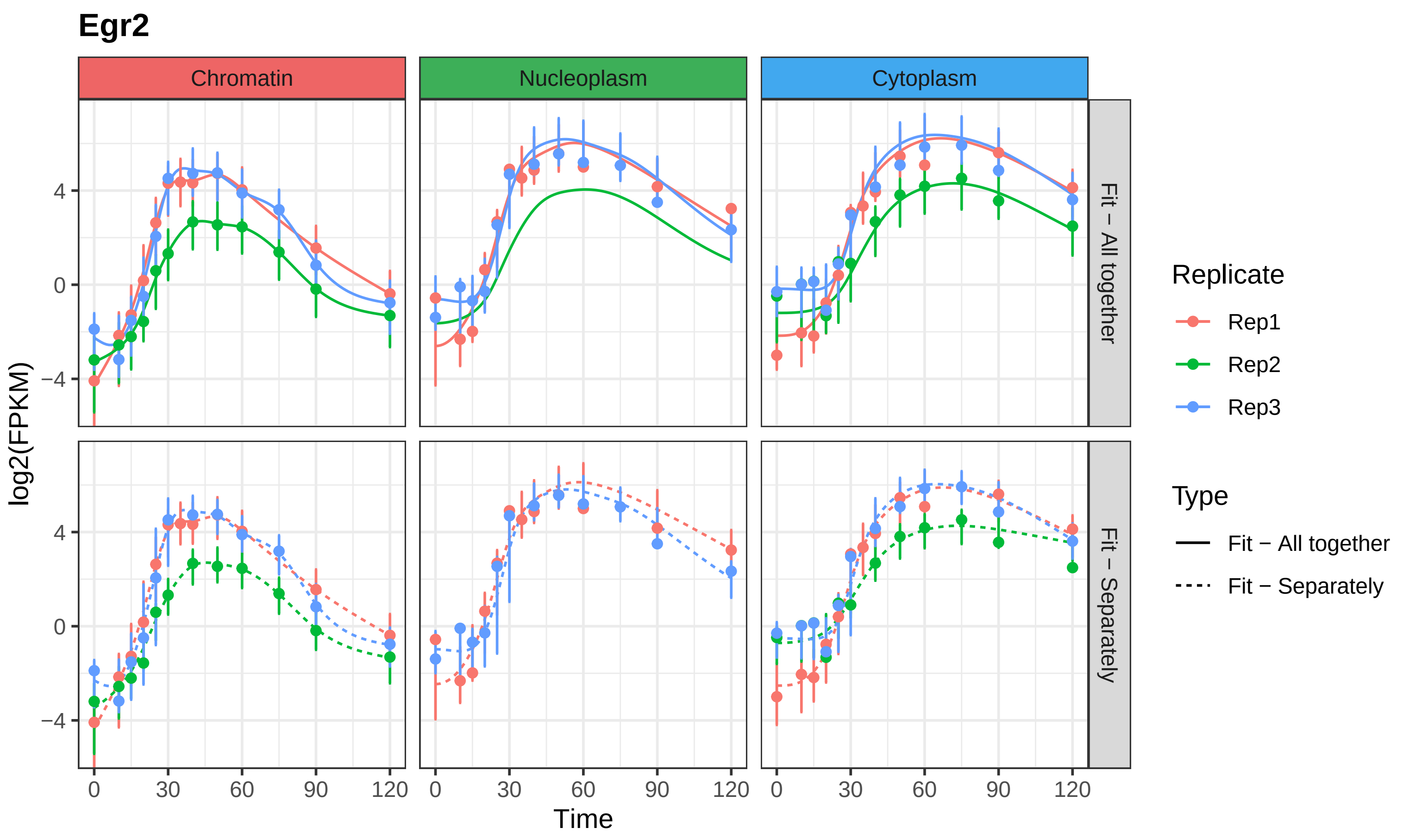

	Together	b1	b2	b3
**−NLL b1 ca**	5.09	4.849		
**−NLL b1 np**	21.15	22.53		
**−NLL b1 cyto**	10.16	7.572		
**−NLL b2 ca**	4.739		3.271	
**−NLL b2 np**				
**−NLL b2 cyto**	14.84		11.76	
**−NLL b3 ca**	5.899			5.794
**−NLL b3 np**	13.31			11.67
**−NLL b3 cyto**	11.32			9.308
Total	86.52	34.95	15.03	26.77
**Total with regul**	94.91	40.79	17.32	31.81

	Together	b1	b2	b3
spar	0.2896	0.3130	0.3617	0.3000
σ_b	0.3332	0.2956	0.2473	0.2355
σ_t	0.7761	1.2630	2.764e−05	2.0290
ca_{0,b_1}				
$\log_{10}(k_1')$	−0.6176	−0.6777		−0.7609
$\log_{10}(k_2)$	−1.1120	−1.2450	8.603 or −1.753	−1.1590
$\log_{10}(k_2')$	−0.9598	−1.0220		−0.9438
$\log_{10}(k_{deg})$	−1.0920	−1.0020	−1.753 or 8.603	−1.0830
$\log_{10}(k_1'k_2')$	−1.5770	−1.6990	7.683	−1.7050
$\log_{10}(k_1'/k_2)$	0.4946	0.5673		0.3985
transport = $\log_{10}(k_1'k_2'/k_2)$	−0.4653	−0.4544	−0.9201 or 9.436	−0.5453

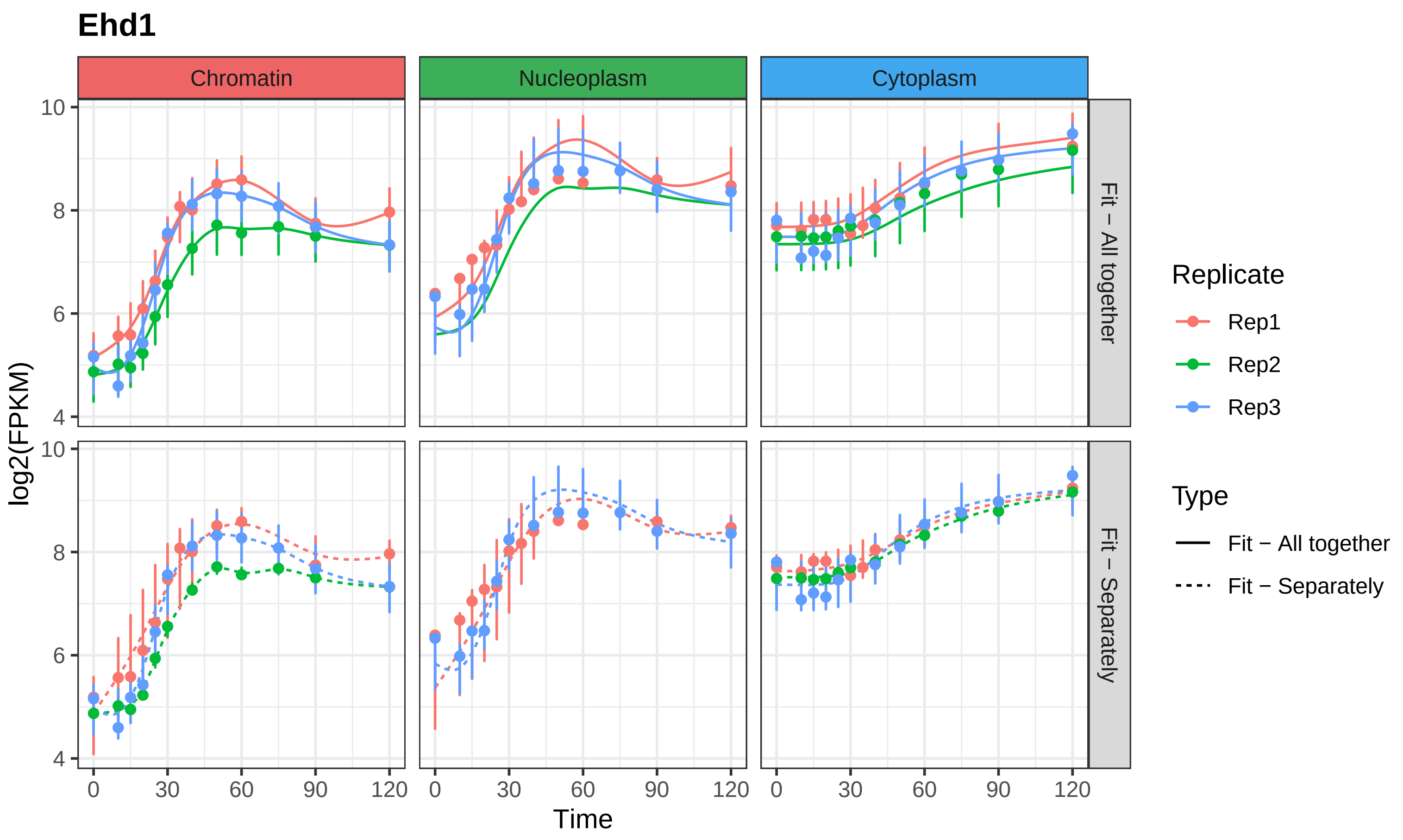

	Together	b1	b2	b3
**−NLL b1 ca**	−4.767	−0.5435		
**−NLL b1 np**	15.05	9.909		
**−NLL b1 cyto**	−1.952	−7.121		
**−NLL b2 ca**	−4.947		−16.28	
**−NLL b2 np**				
**−NLL b2 cyto**	−1.429		−17.45	
**−NLL b3 ca**	−3.036			−2.966
**−NLL b3 np**	4.221			4.284
**−NLL b3 cyto**	0.1353			−0.2447
Total	3.278	2.244	−33.73	1.074
**Total with regul**	3.407	2.851	−31.87	1.349

	Together	b1	b2	b3
spar	0.3778000	0.5472	0.3063	3.704e−01
σ_b	0.1677000	0.1084	0.02874	1.628e−01
σ_t	0.0009087	4.7290	0.323	4.345e−06
ca_{0,b_1}				
$\log_{10}(k_1')$	5.0080000	7.9070		5.511e+00
$\log_{10}(k_2)$	4.7720000	7.7610	4.667 or −2.262	5.250e+00
$\log_{10}(k_2')$	−1.8200000	−1.8910		−1.813e+00
$\log_{10}(k_{deg})$	−2.3470000	−2.5710	−2.262 or 4.667	−2.274e+00
$\log_{10}(k_1'k_2')$	3.1880000	6.0150	3.202	3.698e+00
$\log_{10}(k_1'/k_2)$	0.2358000	0.1460		2.607e−01
transport $= \log_{10}(k_1'k_2'/k_2)$	−1.5840000	−1.7450	−1.465 or 5.463	−1.552e+00

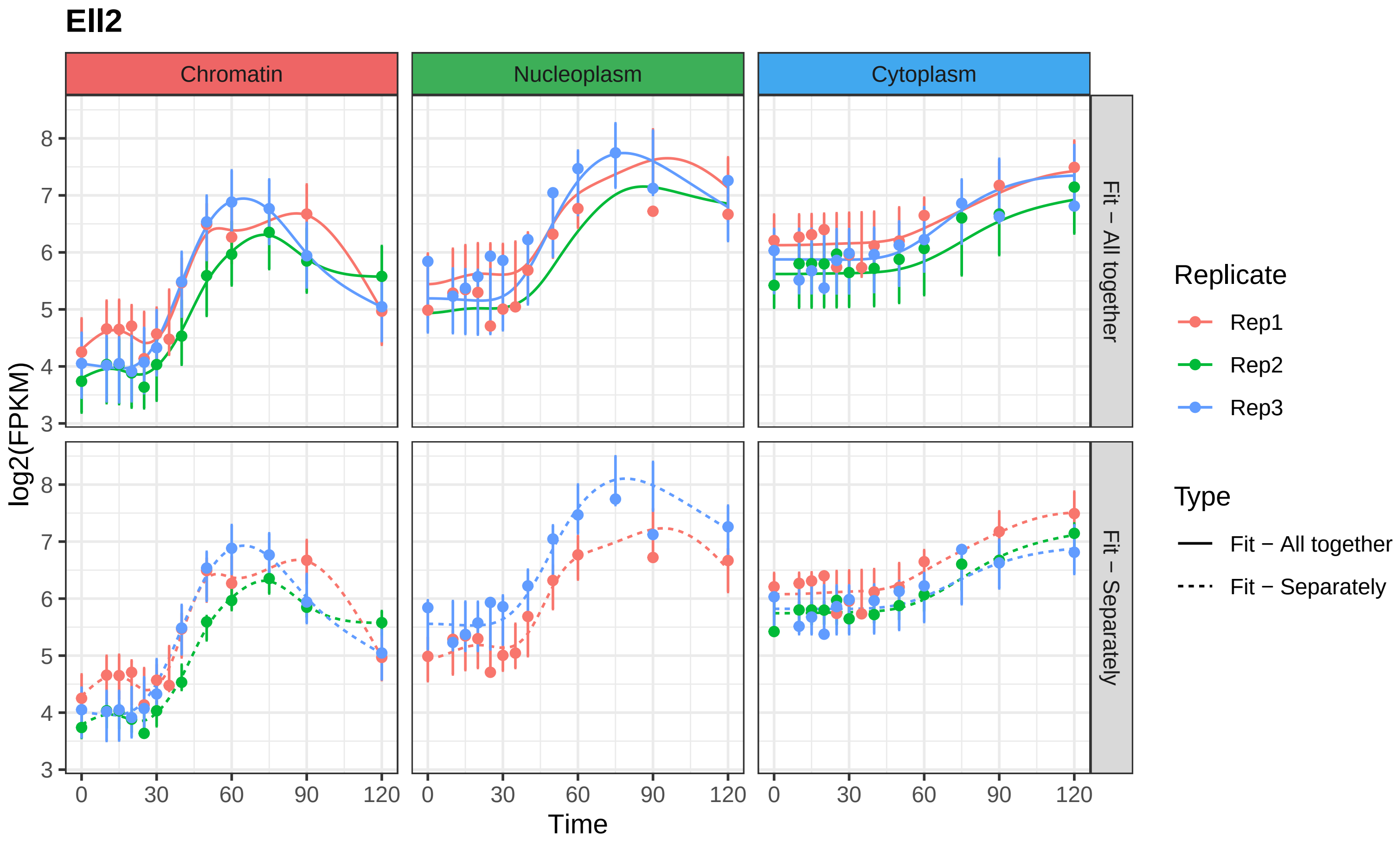

	Together	b1	b2	b3
**−NLL b1 ca**	−2.179	−5.277		
**−NLL b1 np**	14.03	0.3017		
**−NLL b1 cyto**	−0.4621	−1.606		
**−NLL b2 ca**	−3.251		−10.38	
**−NLL b2 np**				
**−NLL b2 cyto**	−0.933		−6.66	
**−NLL b3 ca**	−3.61			−5.383
**−NLL b3 np**	13.06			4.857
**−NLL b3 cyto**	2.094			−1.647
Total	18.75	−6.582	−17.04	−2.173
**Total with regul**	19.48	−6.434	−17.75	−3.283

	Together	b1	b2	b3
spar	3.745e−01	3.611e−01	0.3676	0.4277000
σ_b	1.949e−01	1.331e−01	0.07131	0.1489000
σ_t	5.969e−05	4.496e−05	6.999e−05	0.0001256
ca_{0,b_1}				
$\log_{10}(k_1')$	−9.283e−01	−9.182e−01		−0.8254000
$\log_{10}(k_2)$	−1.273e+00	−1.118e+00	−1.291 or −1.96	−1.2880000
$\log_{10}(k_2')$	−1.808e+00	−1.621e+00		−2.2300000
$\log_{10}(k_{deg})$	−2.014e+00	−1.960e+00	−1.96 or −1.291	−2.3100000
$\log_{10}(k_1' k_2')$	−2.737e+00	−2.539e+00	−2.663	−3.0550000
$\log_{10}(k_1'/k_2)$	3.444e−01	1.996e−01		0.4628000
transport = $\log_{10}(k_1' k_2'/k_2)$	−1.464e+00	−1.422e+00	−1.372 or −0.7026	−1.7670000

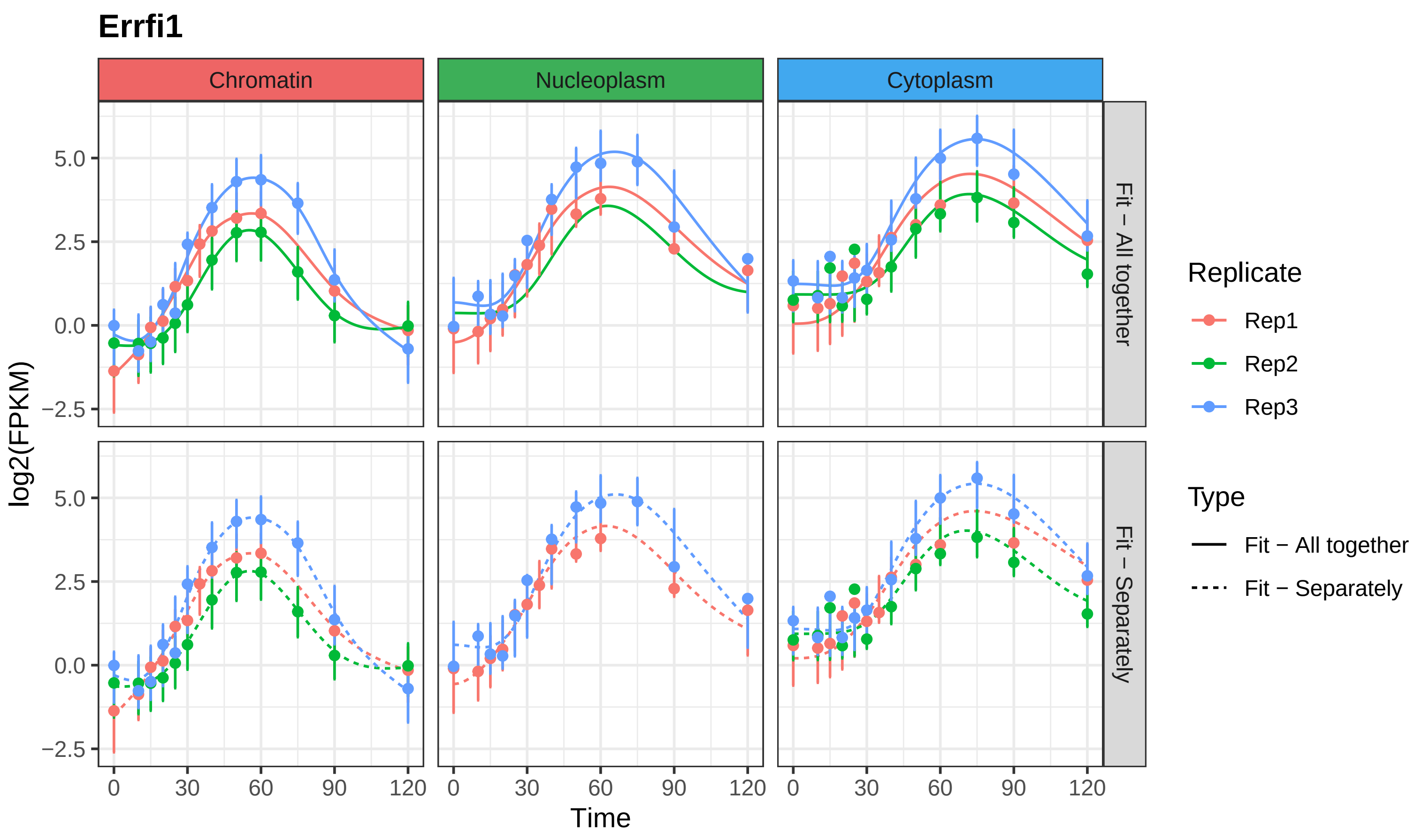

	Together	b1	b2	b3
**−NLL b1 ca**	1.185	0.5816		
**−NLL b1 np**	4.829	4.694		
**−NLL b1 cyto**	11.69	11.93		
**−NLL b2 ca**	0.2587		0.4533	
**−NLL b2 np**				
**−NLL b2 cyto**	11.33		12.16	
**−NLL b3 ca**	3.37			3.443
**−NLL b3 np**	9.349			8.855
**−NLL b3 cyto**	7.493			7.276
Total	49.51	17.21	12.61	19.57
**Total with regul**	51.37	17.91	13.44	20.84

	Together	b1	b2	b3
spar	0.38050	0.4126000	0.4171	0.3880
σ_b	0.25330	0.2344000	0.2423	0.2345
σ_t	0.00138	0.0001183	1.596e−05	1.8480
ca_{0,b_1}				
$\log_{10}(k_1')$	−0.70680	−0.6130000		−0.7666
$\log_{10}(k_2)$	−0.99520	−0.8941000	−0.9479 or −0.9792	−1.0380
$\log_{10}(k_2')$	−0.85960	−0.9946000		−0.8481
$\log_{10}(k_{deg})$	−1.02600	−1.2260000	−0.9792 or −0.9479	−0.9911
$\log_{10}(k_1'k_2')$	−1.56600	−1.6080000	−1.452	−1.6150
$\log_{10}(k_1'/k_2)$	0.28830	0.2811000		0.2718
transport $= \log_{10}(k_1'k_2'/k_2)$	−0.57130	−0.7135000	−0.504 or −0.4726	−0.5763

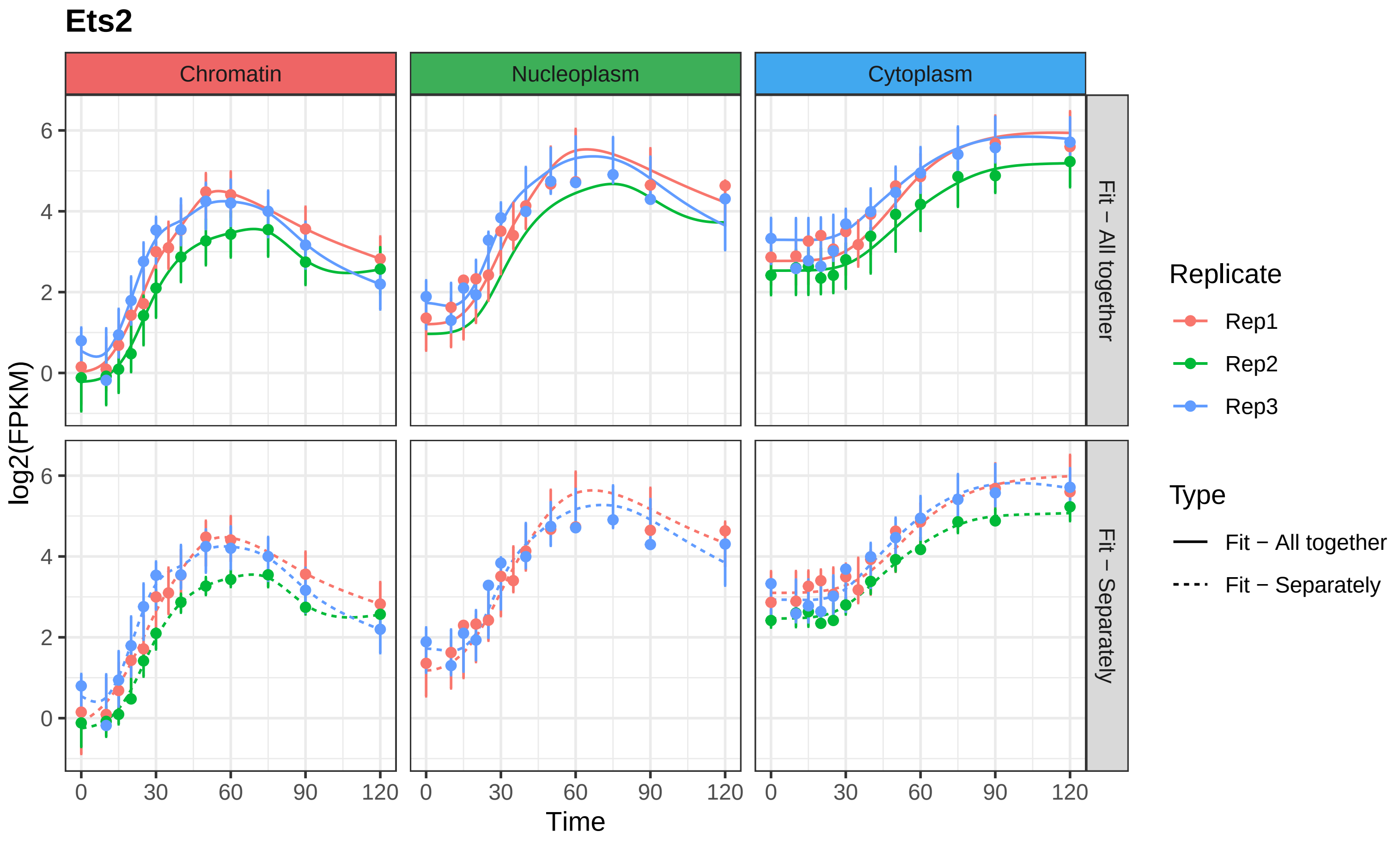

	Together	b1	b2	b3
**−NLL b1 ca**	−1.713	−1.04		
**−NLL b1 np**	9.87	8.917		
**−NLL b1 cyto**	4.201	0.5358		
**−NLL b2 ca**	−2.762		−8.253	
**−NLL b2 np**				
**−NLL b2 cyto**	−1.68		−6.539	
**−NLL b3 ca**	−0.3432			0.228
**−NLL b3 np**	8.722			6.986
**−NLL b3 cyto**	4.69			0.6786
Total	20.98	8.413	−14.79	7.893
**Total with regul**	22.47	8.041	−15.73	9.126

	Together	b1	b2	b3
spar	0.3523000	4.260e−01	0.3736	0.3527
σ_b	0.1945000	1.914e−01	0.06526	0.1800
σ_t	0.0001537	6.016e−05	0.0001017	1.3720
ca_{0,b_1}				
$\log_{10}(k_1')$	−0.5705000	−5.589e−01		−0.7642
$\log_{10}(k_2)$	−0.9270000	−9.457e−01	1.248 or −1.994	−1.1180
$\log_{10}(k_2')$	−1.4580000	−1.580e+00		−1.3290
$\log_{10}(k_{deg})$	−1.9300000	−2.158e+00	−1.994 or 1.248	−1.6940
$\log_{10}(k_1'k_2')$	−2.0290000	−2.139e+00	0.07252	−2.0930
$\log_{10}(k_1'/k_2)$	0.3565000	3.868e−01		0.3534
$\text{transport} = \log_{10}(k_1'k_2'/k_2)$	−1.1020000	−1.193e+00	−1.176 or 2.066	−0.9757

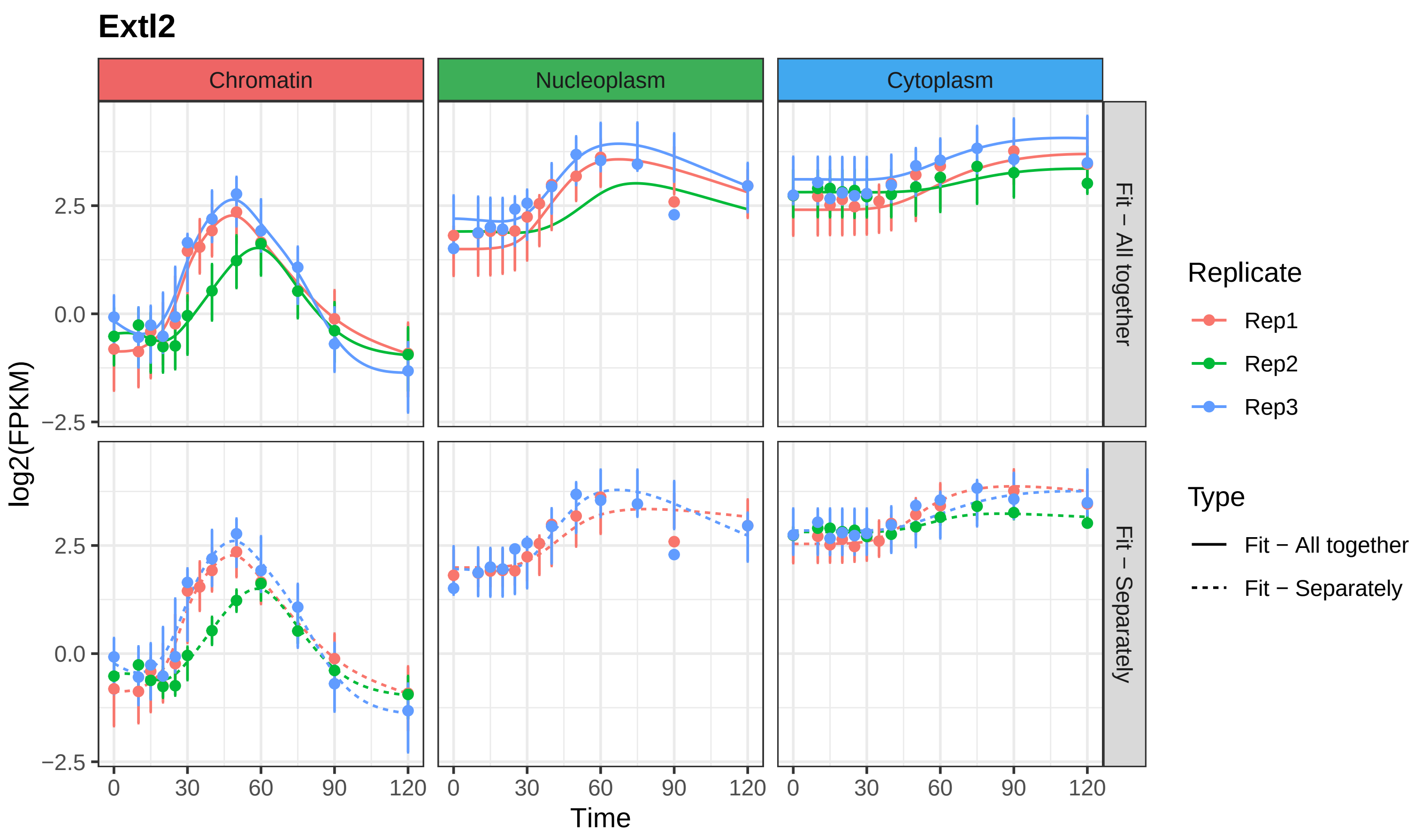

	Together	b1	b2	b3
**−NLL b1 ca**	1.032	−0.09324		
**−NLL b1 np**	5.274	5.008		
**−NLL b1 cyto**	2.489	−4.732		
**−NLL b2 ca**	−1.328		−4.028	
**−NLL b2 np**				
**−NLL b2 cyto**	−2.51		−12.75	
**−NLL b3 ca**	1.081			2.084
**−NLL b3 np**	11.08			8.111
**−NLL b3 cyto**	3.832			−1.115
Total	20.95	0.1833	−16.78	9.08
**Total with regul**	21.53	−0.1155	−18.47	8.859

	Together	b1	b2	b3
spar	0.3750	0.3790	0.3957	0.4082
σ_b	0.1874	0.1384	0.02746	0.1822
σ_t	0.9119	1.6280	0.003134	2.1150
ca_{0,b_1}				
$\log_{10}(k_1')$	−0.9674	−1.2640		−0.9891
$\log_{10}(k_2)$	−1.6790	−2.1260	4.857 or −2.352	−1.6450
$\log_{10}(k_2')$	−1.6780	−0.7617		−1.7880
$\log_{10}(k_{deg})$	−1.9520	−0.9262	−2.352 or 4.857	−2.0560
$\log_{10}(k_1'k_2')$	−2.6460	−2.0250	3.491	−2.7770
$\log_{10}(k_1'/k_2)$	0.7120	0.8620		0.6563
$\text{transport} = \log_{10}(k_1'k_2'/k_2)$	−0.9662	0.1003	−1.366 or 5.843	−1.1320

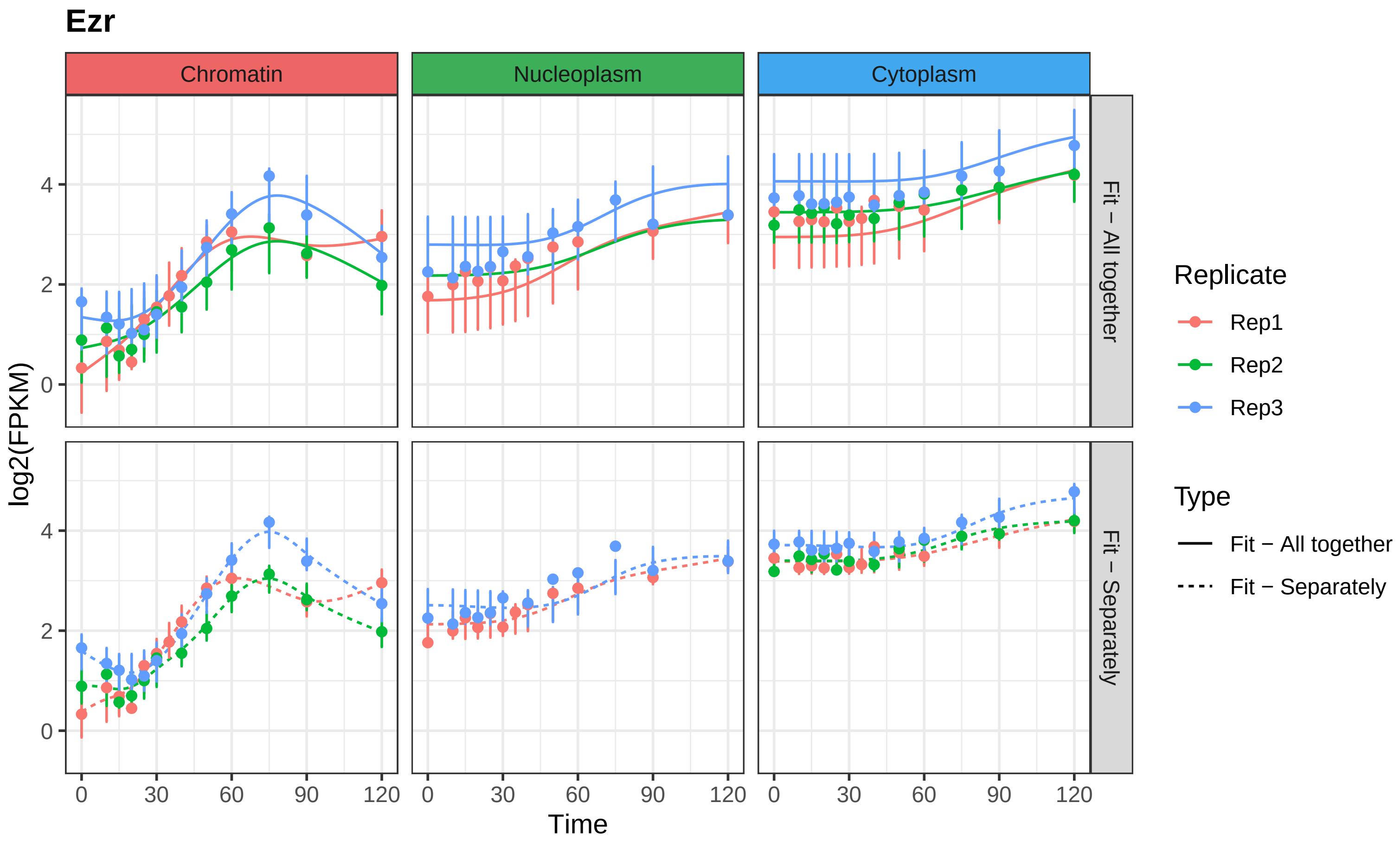

	Together	b1	b2	b3
**−NLL b1 ca**	−0.1641	−6.555		
**−NLL b1 np**	5.077	−3.528		
**−NLL b1 cyto**	6.114	−8.149		
**−NLL b2 ca**	−0.3095		−5.357	
**−NLL b2 np**				
**−NLL b2 cyto**	−2.159		−6.45	
**−NLL b3 ca**	0.3479			−6.962
**−NLL b3 np**	9.127			5.089
**−NLL b3 cyto**	5.063			−9.079
Total	23.1	−18.23	−11.81	−10.95
**Total with regul**	25.39	−20.07	−13.69	−12.77

	Together	b1	b2	b3
spar	5.660e−01	4.178e−01	0.421	4.574e−01
σ_b	1.961e−01	7.453e−02	0.07301	9.924e−02
σ_t	6.393e−06	3.224e−05	0.0001611	1.966e−05
ca_{0,b_1}				
$\log_{10}(k_1')$	−1.688e+00	−1.776e+00		−1.880e+00
$\log_{10}(k_2)$	−2.124e+00	−2.302e+00	5.439 or −2.284	−2.157e+00
$\log_{10}(k_2')$	−1.207e+00	−1.360e+00		−7.338e−01
$\log_{10}(k_{deg})$	−1.588e+00	−1.737e+00	−2.284 or 5.439	−1.096e+00
$\log_{10}(k_1'k_2')$	−2.895e+00	−3.136e+00	3.904	−2.614e+00
$\log_{10}(k_1'/k_2)$	4.362e−01	5.262e−01		2.764e−01
transport = $\log_{10}(k_1'k_2'/k_2)$	−7.709e−01	−8.335e−01	−1.535 or 6.188	−4.574e−01

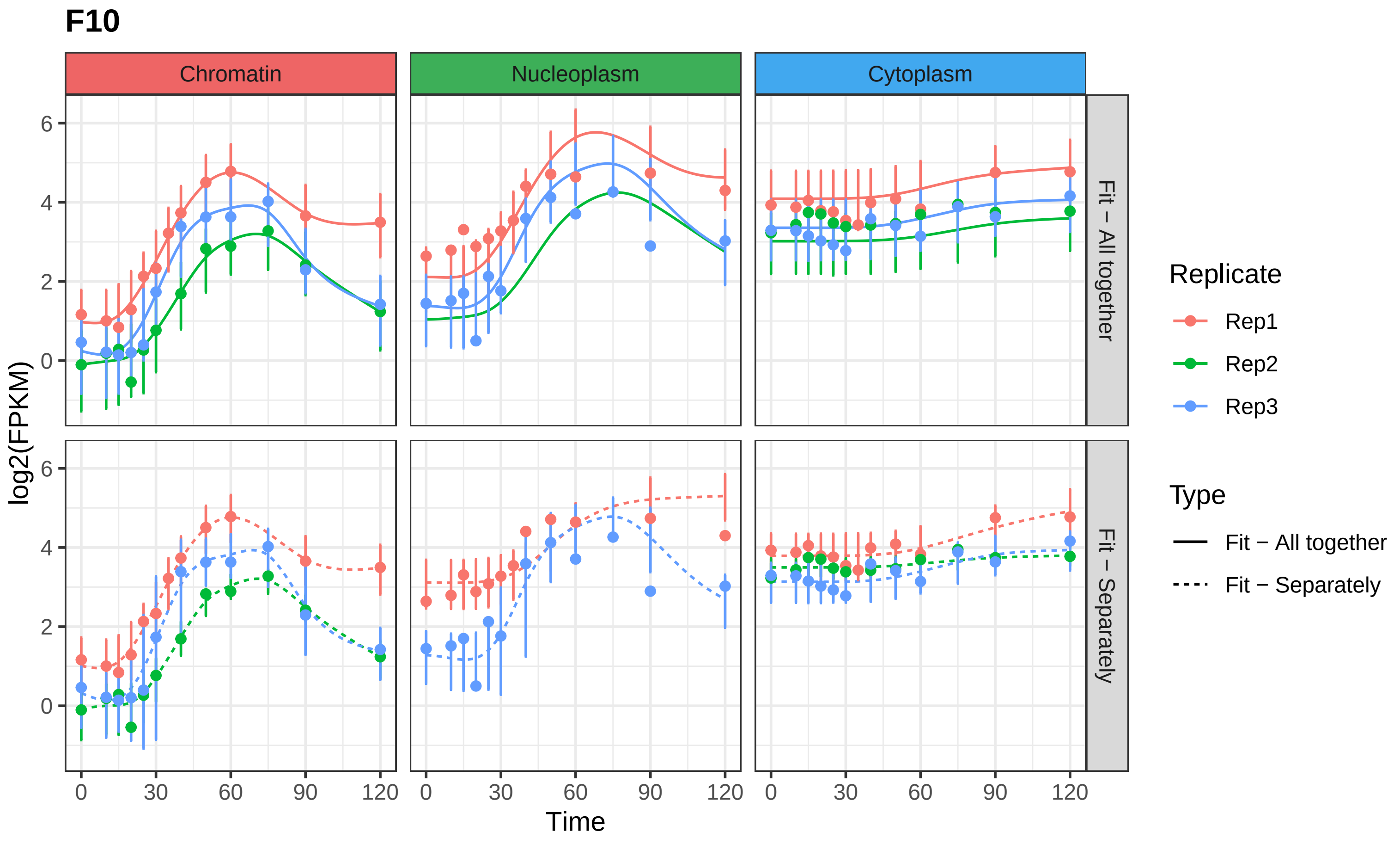

	Together	b1	b2	b3
−NLL b1 ca	1.275	−1.038		
−NLL b1 np	13.99	12.68		
−NLL b1 cyto	4.635	−0.8159		
−NLL b2 ca	2.811		−0.4471	
−NLL b2 np				
−NLL b2 cyto	8.105		−5.893	
−NLL b3 ca	3.513			3.424
−NLL b3 np	14.46			13.48
−NLL b3 cyto	3.512			−0.3793
Total	52.3	10.82	−6.34	16.53
Total with regul	53.25	10.61	−8.201	16.42

	Together	b1	b2	b3
spar	4.394e−01	4.233e−01	0.4146	0.4107
σ_b	2.548e−01	1.981e−01	0.06442	0.1654
σ_t	6.561e−07	8.845e−07	0.001666	5.1470
ca_{0,b_1}				
$\log_{10}(k_1')$	−5.779e−01	−1.466e+00		−0.7146
$\log_{10}(k_2)$	−9.205e−01	−2.099e+00	2.766 or −3.275	−1.0030
$\log_{10}(k_2')$	−2.325e+00	−1.941e+00		−2.2520
$\log_{10}(k_{deg})$	−2.920e+00	−2.145e+00	−3.275 or 2.766	−2.8110
$\log_{10}(k_1'k_2')$	−2.903e+00	−3.408e+00	0.5648	−2.9670
$\log_{10}(k_1'/k_2)$	3.426e−01	6.329e−01		0.2880
$\text{transport} = \log_{10}(k_1'k_2'/k_2)$	−1.983e+00	−1.309e+00	−2.201 or 3.84	−1.9640

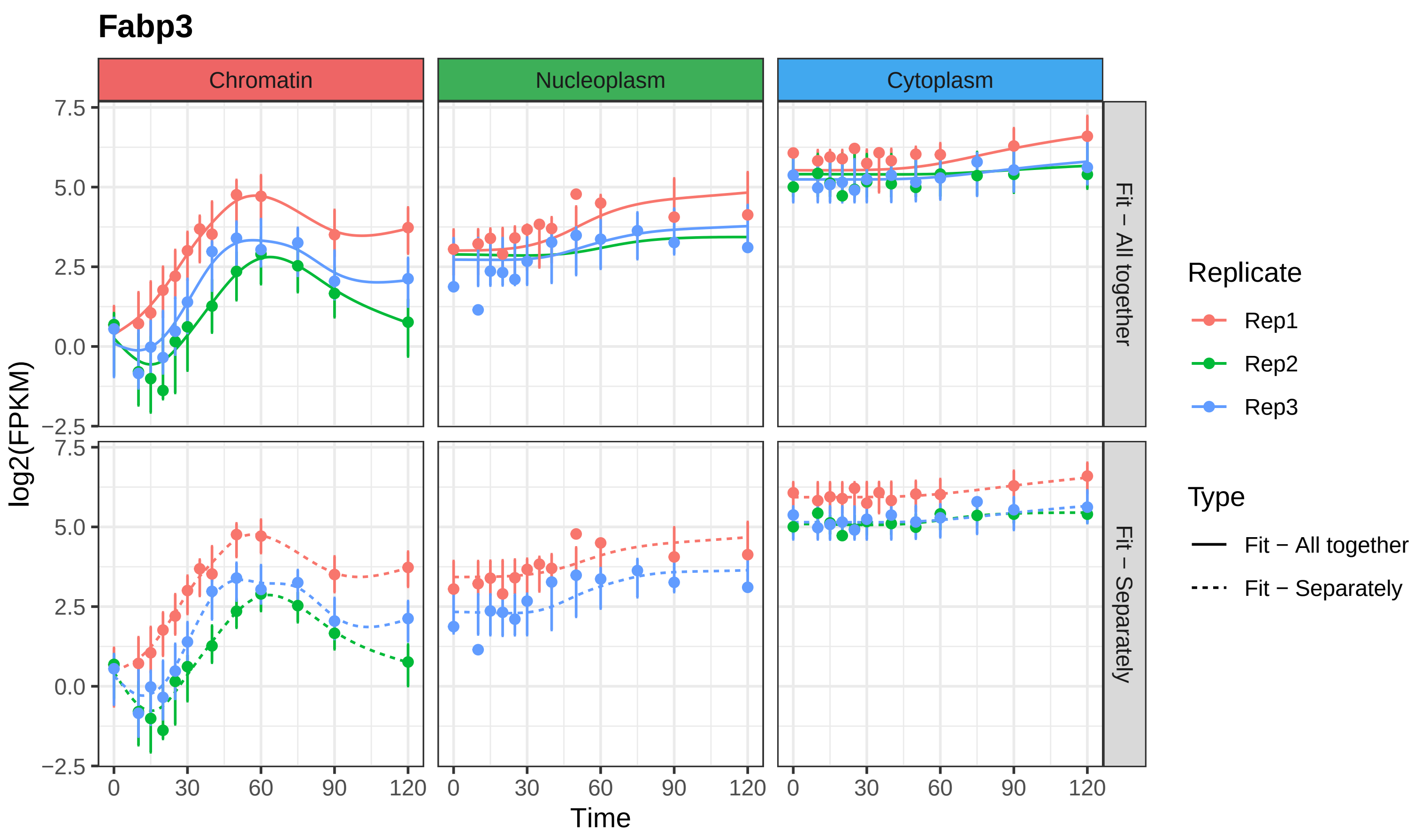

	Together	b1	b2	b3
**−NLL b1 ca**	1.312	−1.142		
**−NLL b1 np**	11.26	11.2		
**−NLL b1 cyto**	5.312	−4.222		
**−NLL b2 ca**	4.585		1.796	
**−NLL b2 np**				
**−NLL b2 cyto**	4.346		−3.379	
**−NLL b3 ca**	4.542			1.71
**−NLL b3 np**	14.06			13.05
**−NLL b3 cyto**	0.1411			−3.036
Total	45.56	5.836	−1.583	11.72
**Total with regul**	45.92	5.109	−3.086	11.61

	Together	b1	b2	b3
spar	4.668e−01	4.194e−01	0.4106	3.928e−01
σ_b	2.293e−01	1.676e−01	0.1006	1.764e−01
σ_t	3.929e−08	4.582e−06	0.000953	1.306e−05
ca_{0,b_1}				
$\log_{10}(k_1')$	−1.785e+00	−1.943e+00		−1.686e+00
$\log_{10}(k_2)$	−2.577e+00	−2.832e+00	7.927 or −2.718	−2.289e+00
$\log_{10}(k_2')$	−1.186e+00	−1.255e+00		−1.378e+00
$\log_{10}(k_{deg})$	−1.944e+00	−2.009e+00	−2.718 or 7.927	−2.226e+00
$\log_{10}(k_1'k_2')$	−2.971e+00	−3.198e+00	6.608	−3.064e+00
$\log_{10}(k_1'/k_2)$	7.915e−01	8.889e−01		6.028e−01
$transport = \log_{10}(k_1'k_2'/k_2)$	−3.942e−01	−3.663e−01	−1.319 or 9.326	−7.751e−01

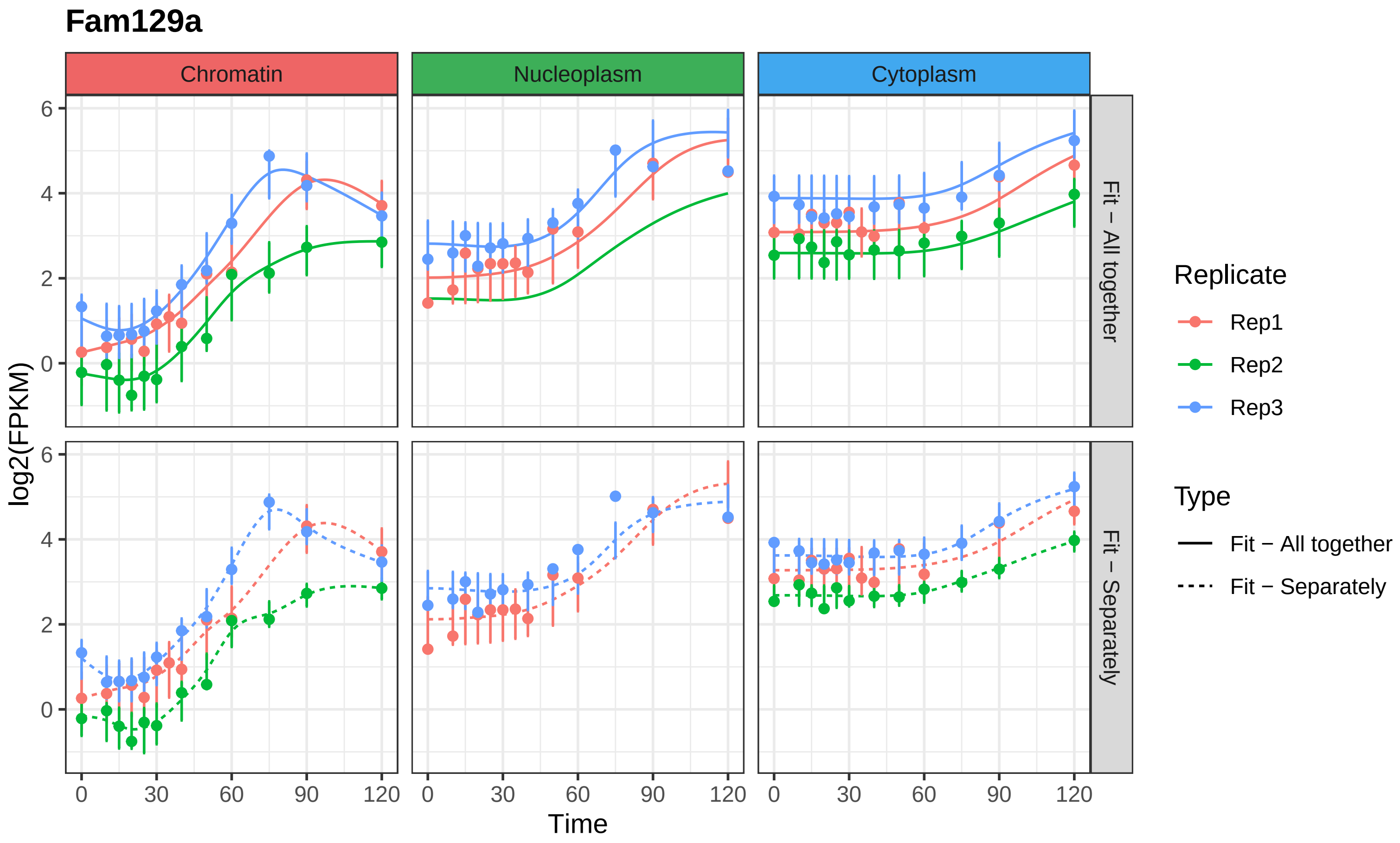

	Together	b1	b2	b3
**−NLL b1 ca**	−0.4156	−0.8762		
**−NLL b1 np**	7.988	7.836		
**−NLL b1 cyto**	3.534	1.483		
**−NLL b2 ca**	0.7897		−2.637	
**−NLL b2 np**				
**−NLL b2 cyto**	−1.876		−5.667	
**−NLL b3 ca**	−0.1088			−3.877
**−NLL b3 np**	7.28			5.903
**−NLL b3 cyto**	3.003			−4.554
Total	20.19	8.443	−8.304	−2.528
**Total with regul**	20.39	7.913	−9.773	−3.824

	Together	b1	b2	b3
spar	0.508700	4.433e−01	0.3974	4.312e−01
σ_b	0.190900	1.887e−01	0.07342	1.380e−01
σ_t	0.000135	2.294e−05	1.115	2.917e−06
ca_{0,b_1}				
$\log_{10}(k_1')$	−1.194000	−1.197e+00		−1.497e+00
$\log_{10}(k_2)$	−1.724000	−1.755e+00	−0.8427 or −2.302	−1.992e+00
$\log_{10}(k_2')$	−1.637000	−1.656e+00		−1.337e+00
$\log_{10}(k_{deg})$	−1.959000	−2.004e+00	−2.302 or −0.8427	−1.569e+00
$\log_{10}(k_1' k_2')$	−2.831000	−2.853e+00	−2.29	−2.833e+00
$\log_{10}(k_1'/k_2)$	0.530000	5.575e−01		4.957e−01
transport $= \log_{10}(k_1' k_2'/k_2)$	−1.107000	−1.098e+00	−1.447 or 0.0117	−8.411e−01

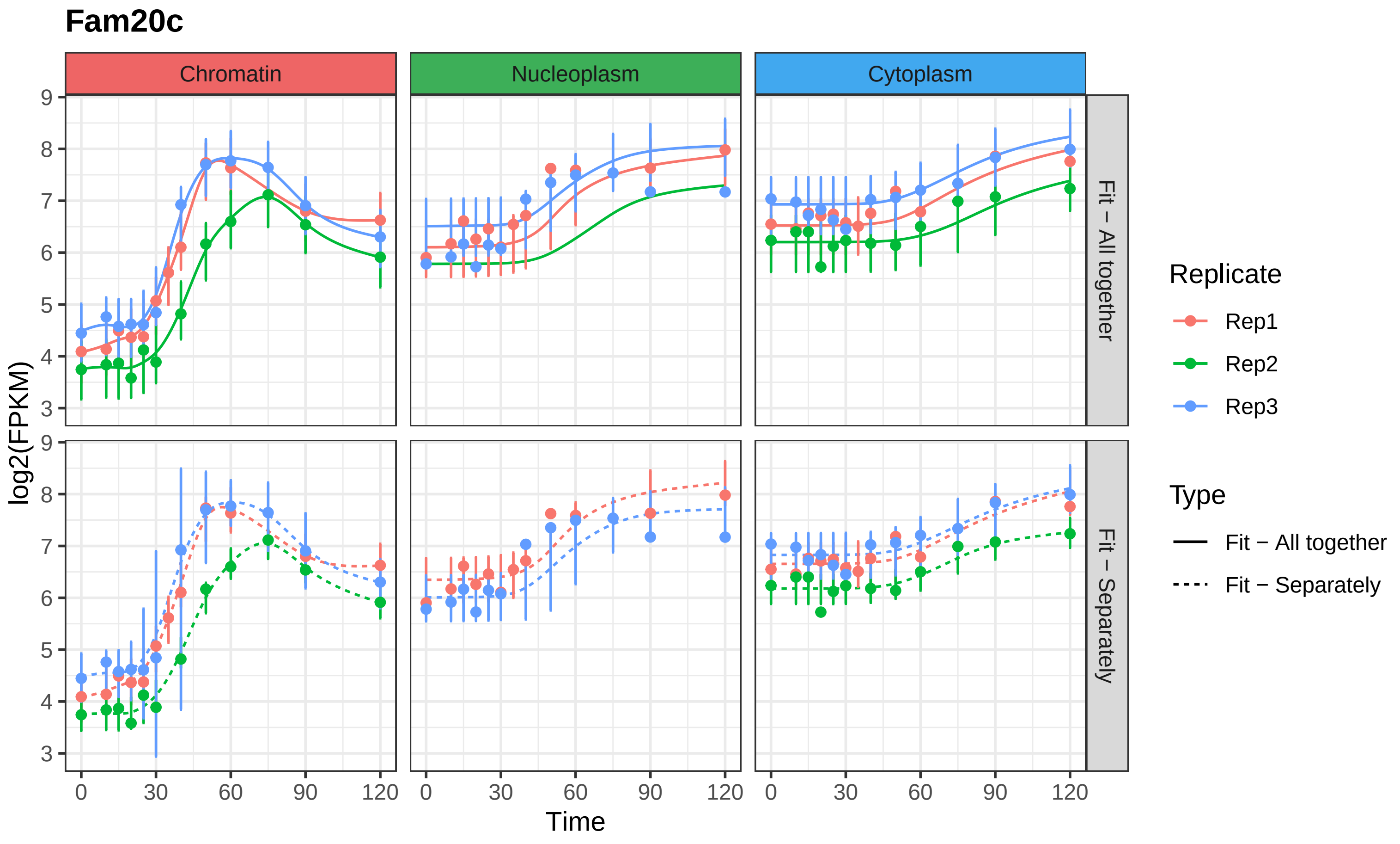

	Together	b1	b2	b3
**−NLL b1 ca**	−3.325	−5.104		
**−NLL b1 np**	9.366	5.32		
**−NLL b1 cyto**	−0.4879	−2.241		
**−NLL b2 ca**	−3.088		−6.147	
**−NLL b2 np**				
**−NLL b2 cyto**	−0.7845		−3.508	
**−NLL b3 ca**	−2.963			0.04997
**−NLL b3 np**	17.93			9.195
**−NLL b3 cyto**	−0.8063			−2.601
Total	15.84	−2.025	−9.655	6.644
**Total with regul**	17.28	−2.516	−10.86	7.003

	Together	b1	b2	b3
spar	3.510e−01	3.912e−01	0.3947	0.3967
σ_b	1.897e−01	1.507e−01	0.1003	0.1524
σ_t	2.653e−06	9.371e−06	0.0007542	6.8860
ca_{0,b_1}				
$\log_{10}(k_1')$	−1.688e+00	−1.564e+00		−1.7500
$\log_{10}(k_2)$	−2.298e+00	−2.248e+00	−0.3153 or −2.552	−2.2100
$\log_{10}(k_2')$	−1.406e+00	−1.605e+00		−1.4630
$\log_{10}(k_{deg})$	−1.532e+00	−1.697e+00	−2.552 or −0.3153	−1.7100
$\log_{10}(k_1'k_2')$	−3.095e+00	−3.169e+00	−2.138	−3.2140
$\log_{10}(k_1'/k_2)$	6.093e−01	6.840e−01		0.4596
transport $= \log_{10}(k_1'k_2'/k_2)$	−7.969e−01	−9.211e−01	−1.823 or 0.4139	−1.0040

Fnbp1l

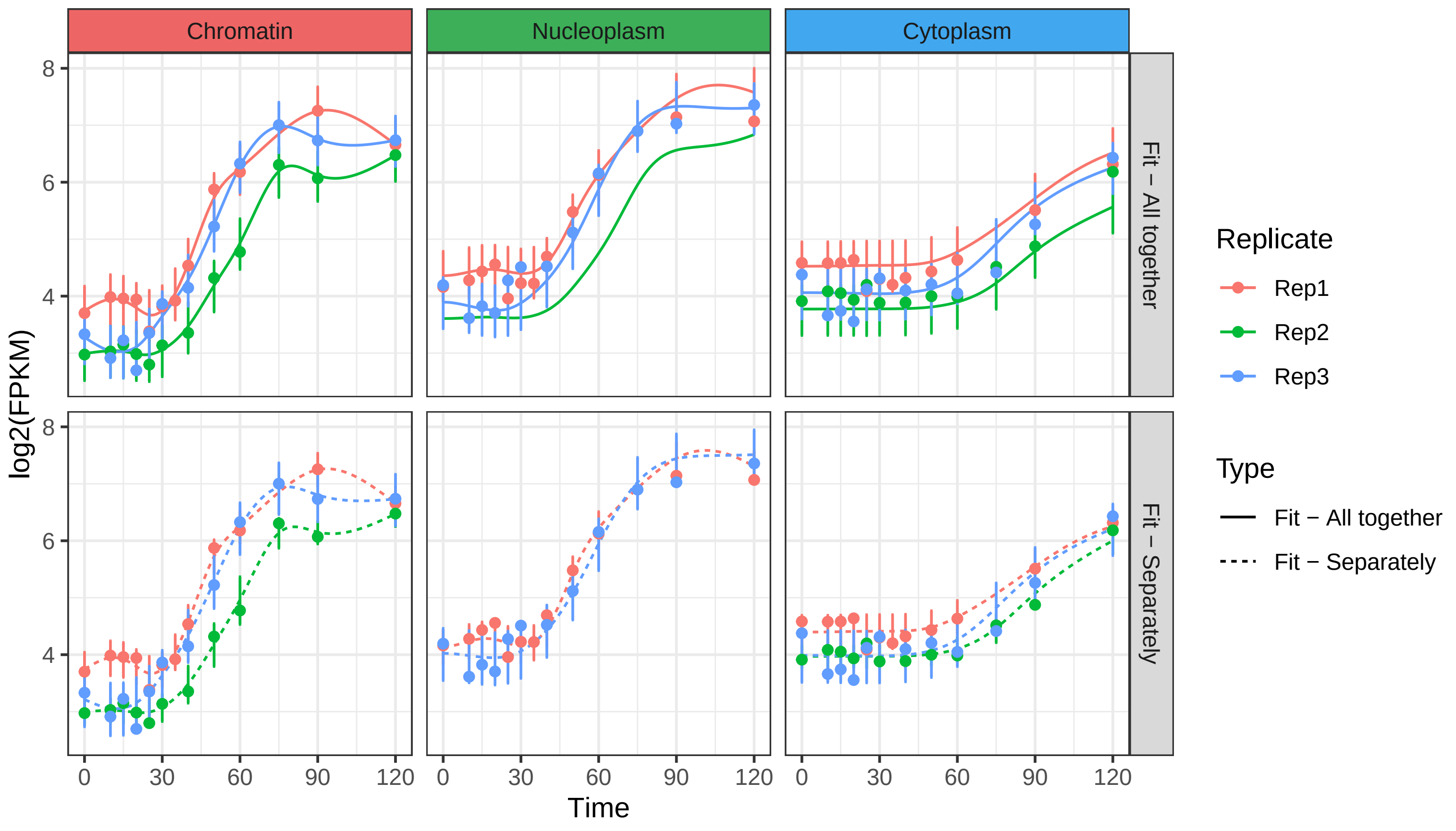

	Together	b1	b2	b3
**−NLL b1 ca**	−5.212	−8.132		
**−NLL b1 np**	0.6068	−3.207		
**−NLL b1 cyto**	−0.9696	−4.534		
**−NLL b2 ca**	−5.519		−8.643	
**−NLL b2 np**				
**−NLL b2 cyto**	2.292		−7.007	
**−NLL b3 ca**	−3.955			−2.53
**−NLL b3 np**	3.885			1.323
**−NLL b3 cyto**	4.045			1.299
Total	−4.828	−15.87	−15.65	0.09257
**Total with regul**	−4.741	−16.49	−17.34	−0.9601

	Together	b1	b2	b3
spar	3.719e−01	0.373500	0.4112	4.384e−01
σ_b	1.539e−01	0.103300	0.06888	1.574e−01
σ_t	1.535e−05	0.001354	2.111	8.887e−09
ca_{0,b_1}				
$\log_{10}(k_1')$	−1.005e+00	−0.815700		−1.024e+00
$\log_{10}(k_2)$	−1.191e+00	−0.934600	−1.633 or −1.661	−1.268e+00
$\log_{10}(k_2')$	−2.018e+00	−2.120000		−2.070e+00
$\log_{10}(k_{deg})$	−2.068e+00	−2.198000	−1.661 or −1.633	−2.059e+00
$\log_{10}(k_1'k_2')$	−3.023e+00	−2.936000	−3.001	−3.094e+00
$\log_{10}(k_1'/k_2)$	1.856e−01	0.118900		2.439e−01
$\text{transport} = \log_{10}(k_1'k_2'/k_2)$	−1.832e+00	−2.001000	−1.367 or −1.34	−1.826e+00

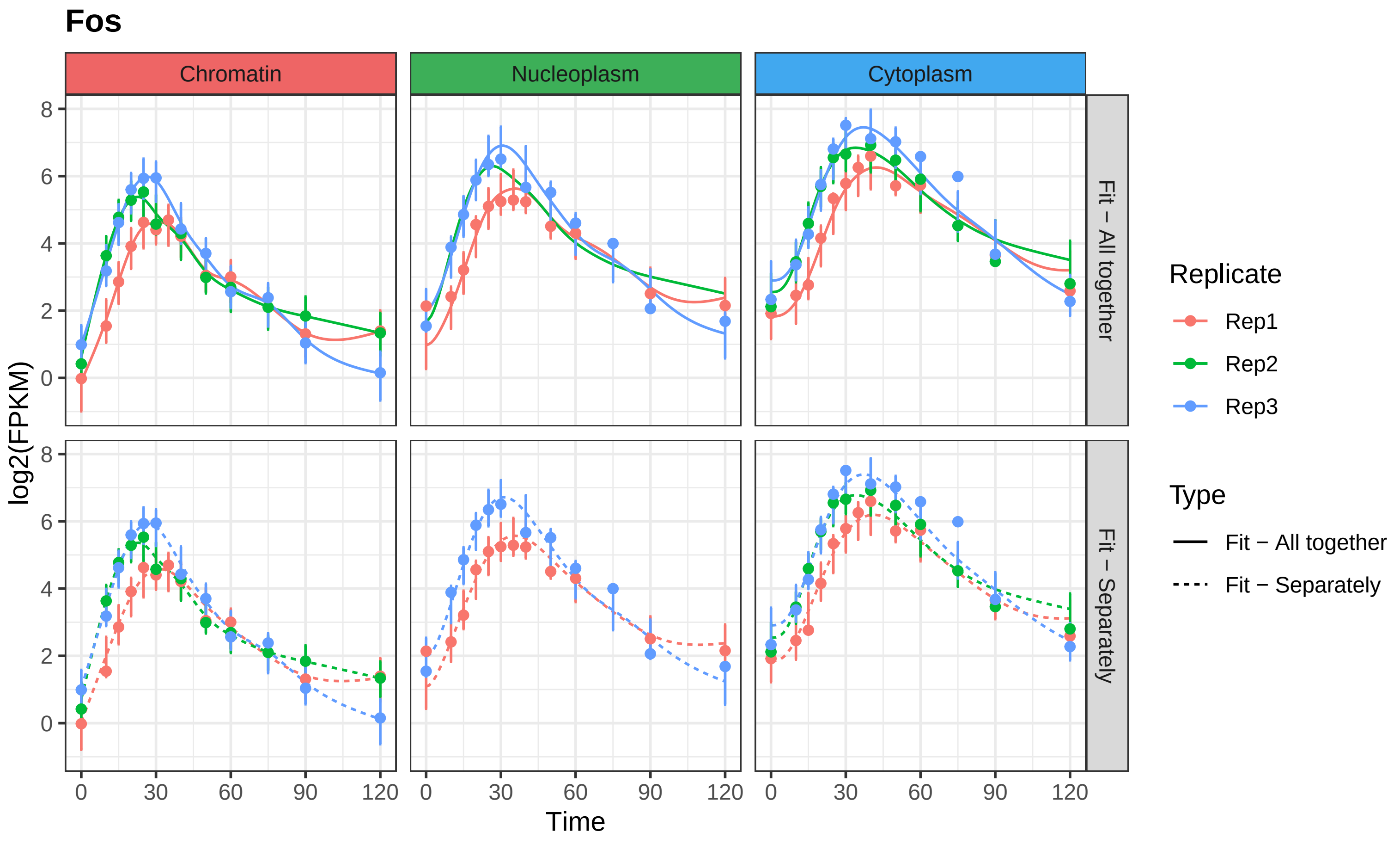

	Together	b1	b2	b3
**−NLL b1 ca**	−1.754	0.1857		
**−NLL b1 np**	8.453	6.691		
**−NLL b1 cyto**	3.173	2.563		
**−NLL b2 ca**	−1.62		−2.327	
**−NLL b2 np**				
**−NLL b2 cyto**	3.763		4.375	
**−NLL b3 ca**	−1.835			−1.417
**−NLL b3 np**	6.212			4.221
**−NLL b3 cyto**	9.969			4.261
Total	26.36	9.44	2.048	7.066
**Total with regul**	29.85	8.982	3.545	7.683

	Together	b1	b2	b3
spar	0.3140000	4.513e−01	0.3387	0.3727000
σ_b	0.2055000	1.929e−01	0.1635	0.1866000
σ_t	0.0001444	3.442e−05	0.0002329	0.0001982
ca_{0,b_1}				
$\log_{10}(k_1')$	−0.3555000	−2.963e−01		−0.4291000
$\log_{10}(k_2)$	−0.6832000	−6.172e−01	−0.7782 or −0.8168	−0.7189000
$\log_{10}(k_2')$	−0.6674000	−5.627e−01		−0.5940000
$\log_{10}(k_{deg})$	−0.9224000	−7.902e−01	−0.8168 or −0.7782	−0.8693000
$\log_{10}(k_1' k_2')$	−1.0230000	−8.590e−01	−1.042	−1.0230000
$\log_{10}(k_1'/k_2)$	0.3277000	3.209e−01		0.2898000
transport = $\log_{10}(k_1' k_2'/k_2)$	−0.3397000	−2.418e−01	−0.2637 or −0.2251	−0.3042000

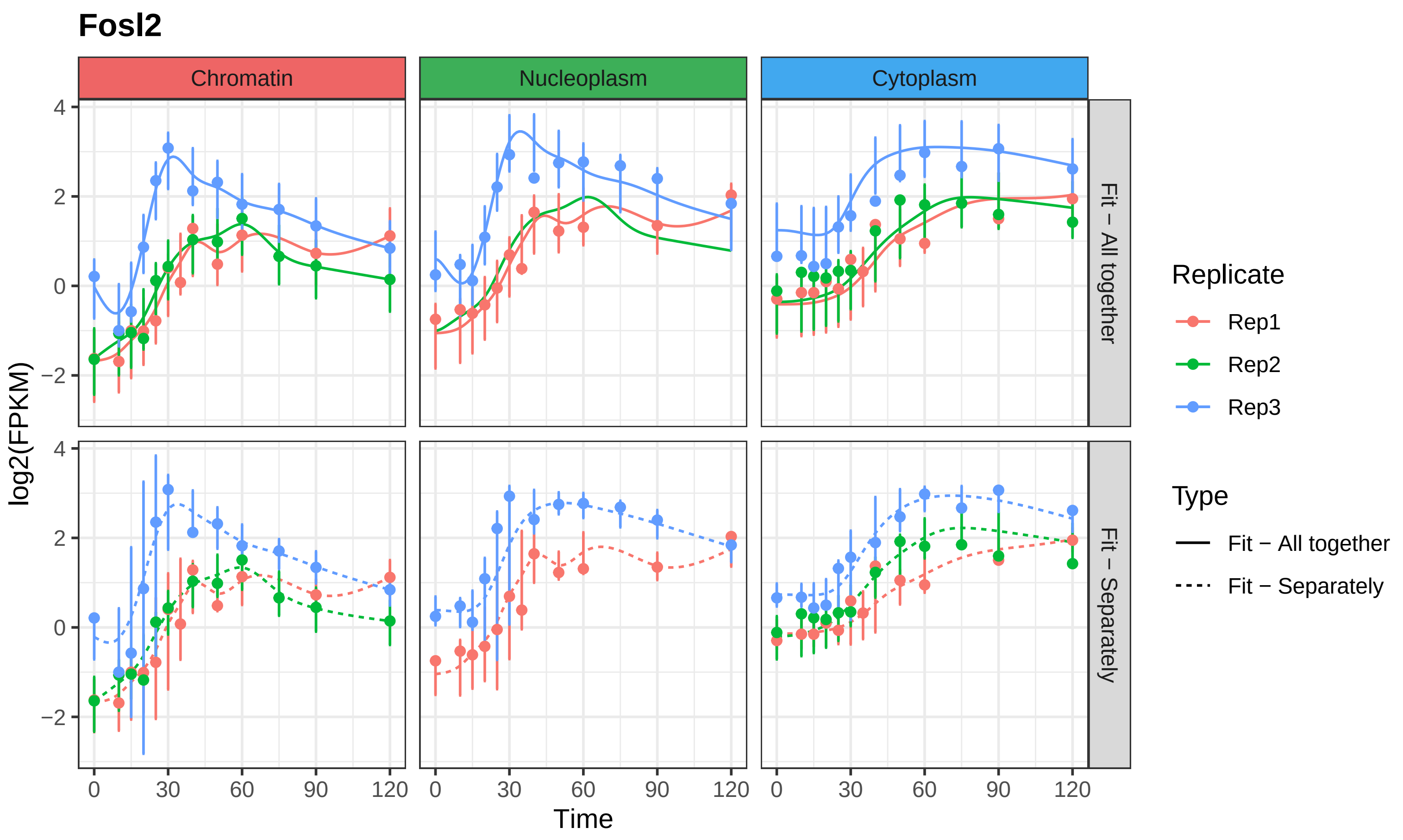

	Together	b1	b2	b3
**−NLL b1 ca**	1.541	0.7587		
**−NLL b1 np**	1.993	2.549		
**−NLL b1 cyto**	6.197	0.9752		
**−NLL b2 ca**	−0.3737		−1.322	
**−NLL b2 np**				
**−NLL b2 cyto**	6.209		3.128	
**−NLL b3 ca**	0.8971			5.742
**−NLL b3 np**	4.68			−1.368
**−NLL b3 cyto**	10.87			−0.1155
Total	32.01	4.283	1.806	4.258
**Total with regul**	34.25	5.131	1.426	3.25

	Together	b1	b2	b3
spar	0.3434000	0.34310	0.3874	0.40600
σ_b	0.2138000	0.07566	0.1519	0.07507
σ_t	0.0001768	5.45000	0.002893	5.66600
ca_{0,b_1}				
$\log_{10}(k_1')$	−0.1622000	6.23100		−1.10700
$\log_{10}(k_2)$	−0.3497000	6.04000	8.413 or −1.533	−1.28700
$\log_{10}(k_2')$	−1.3870000	−1.65300		−1.09900
$\log_{10}(k_{deg})$	−1.5810000	−1.92500	−1.533 or 8.413	−1.20400
$\log_{10}(k_1'k_2')$	−1.5500000	4.57800	7.316	−2.20600
$\log_{10}(k_1'/k_2)$	0.1875000	0.19110		0.18080
$transport = \log_{10}(k_1'k_2'/k_2)$	−1.2000000	−1.46200	−1.096 or 8.849	−0.91870

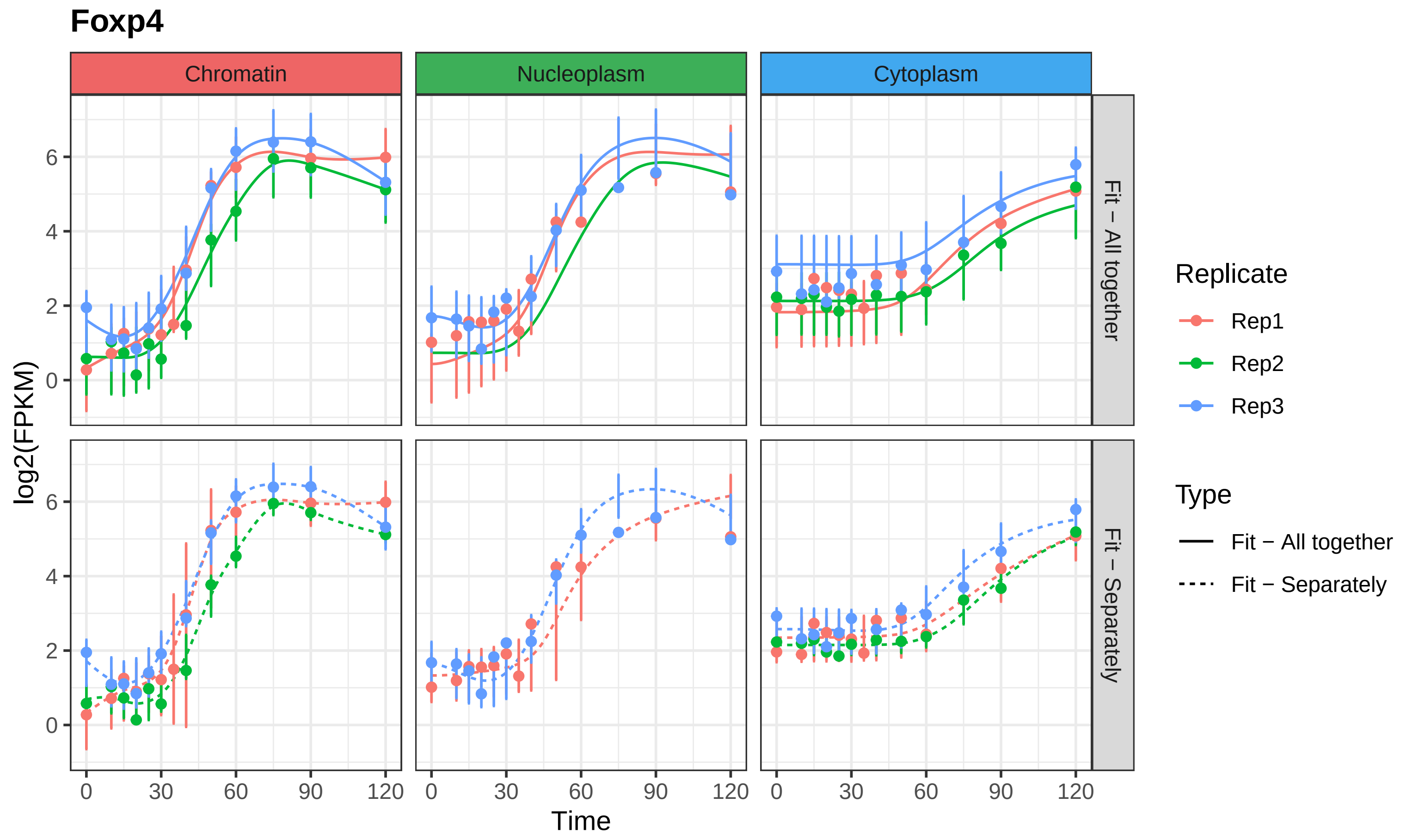

	Together	b1	b2	b3
**−NLL b1 ca**	4.053	3.106		
**−NLL b1 np**	14.9	11.45		
**−NLL b1 cyto**	9.898	3.209		
**−NLL b2 ca**	4.208		−0.8471	
**−NLL b2 np**				
**−NLL b2 cyto**	2.057		−5.248	
**−NLL b3 ca**	2.836			−0.3205
**−NLL b3 np**	10.96			9.18
**−NLL b3 cyto**	10.66			1.465
Total	59.57	17.76	−6.095	10.32
**Total with regul**	61.17	18.14	−7.282	9.991

	Together	b1	b2	b3
spar	0.4652000	0.4093	0.3846	0.436100
σ_b	0.2781000	0.1986	0.06657	0.197300
σ_t	0.0001116	4.4260	1.667	0.000202
ca_{0,b_1}				
$\log_{10}(k_1')$	−0.9821000	−1.6310		−0.970900
$\log_{10}(k_2)$	−1.0150000	−1.9320	−1.84 or −1.88	−0.948000
$\log_{10}(k_2')$	−2.0650000	−1.9200		−1.937000
$\log_{10}(k_{deg})$	−2.4840000	−2.2260	−1.88 or −1.84	−2.219000
$\log_{10}(k_1'k_2')$	−3.0470000	−3.5510	−3.281	−2.908000
$\log_{10}(k_1'/k_2)$	0.0330900	0.3012		−0.022840
transport $= \log_{10}(k_1'k_2'/k_2)$	−2.0320000	−1.6180	−1.441 or −1.401	−1.960000

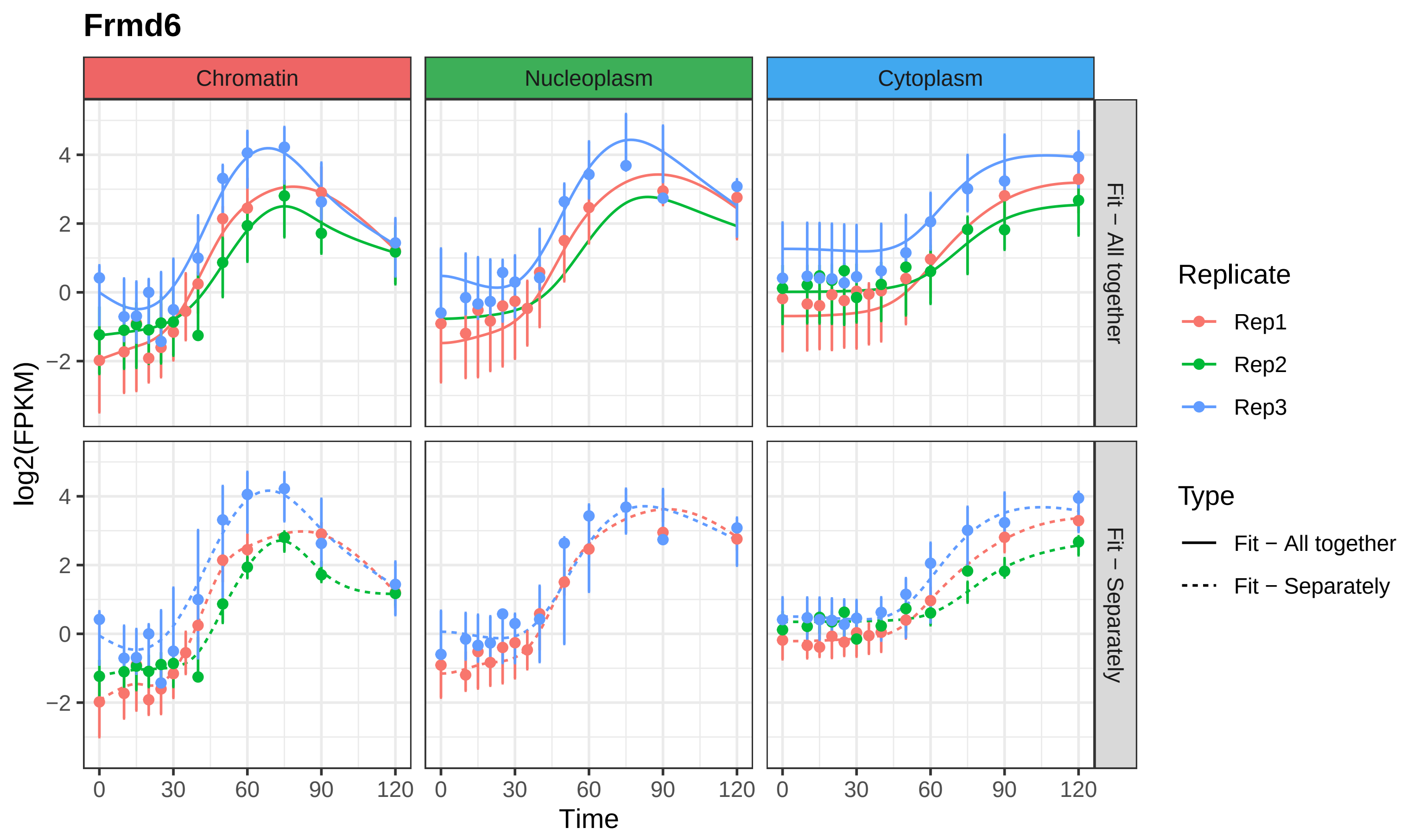

	Together	b1	b2	b3
**−NLL b1 ca**	5.052	−0.5437		
**−NLL b1 np**	6.94	6.505		
**−NLL b1 cyto**	5.369	−5.962		
**−NLL b2 ca**	4.38		−1.771	
**−NLL b2 np**				
**−NLL b2 cyto**	3.817		−0.9609	
**−NLL b3 ca**	7.917			8.282
**−NLL b3 np**	14.19			11.5
**−NLL b3 cyto**	14.68			0.254
**Total**	62.35	−0.0006126	−2.732	20.03
**Total with regul**	64.24	−0.9588	−4.097	20.72

	Together	b1	b2	b3
spar	4.944e−01	0.3929000	0.3962	0.5042
σ_b	2.764e−01	0.1190000	0.08646	0.1946
σ_t	2.869e−05	0.0000454	0.02682	5.1220
ca_{0,b_1}				
$\log_{10}(k_1')$	−8.835e−01	−0.8599000		−1.3240
$\log_{10}(k_2)$	−1.028e+00	−1.0930000	−1.667 or −1.721	−1.3580
$\log_{10}(k_2')$	−1.616e+00	−1.6870000		−1.3160
$\log_{10}(k_{deg})$	−1.853e+00	−1.9710000	−1.721 or −1.667	−1.4480
$\log_{10}(k_1'k_2')$	−2.500e+00	−2.5470000	−2.916	−2.6390
$\log_{10}(k_1'/k_2)$	1.449e−01	0.2330000		0.0346
transport = $\log_{10}(k_1'k_2'/k_2)$	−1.472e+00	−1.4540000	−1.249 or −1.195	−1.2810

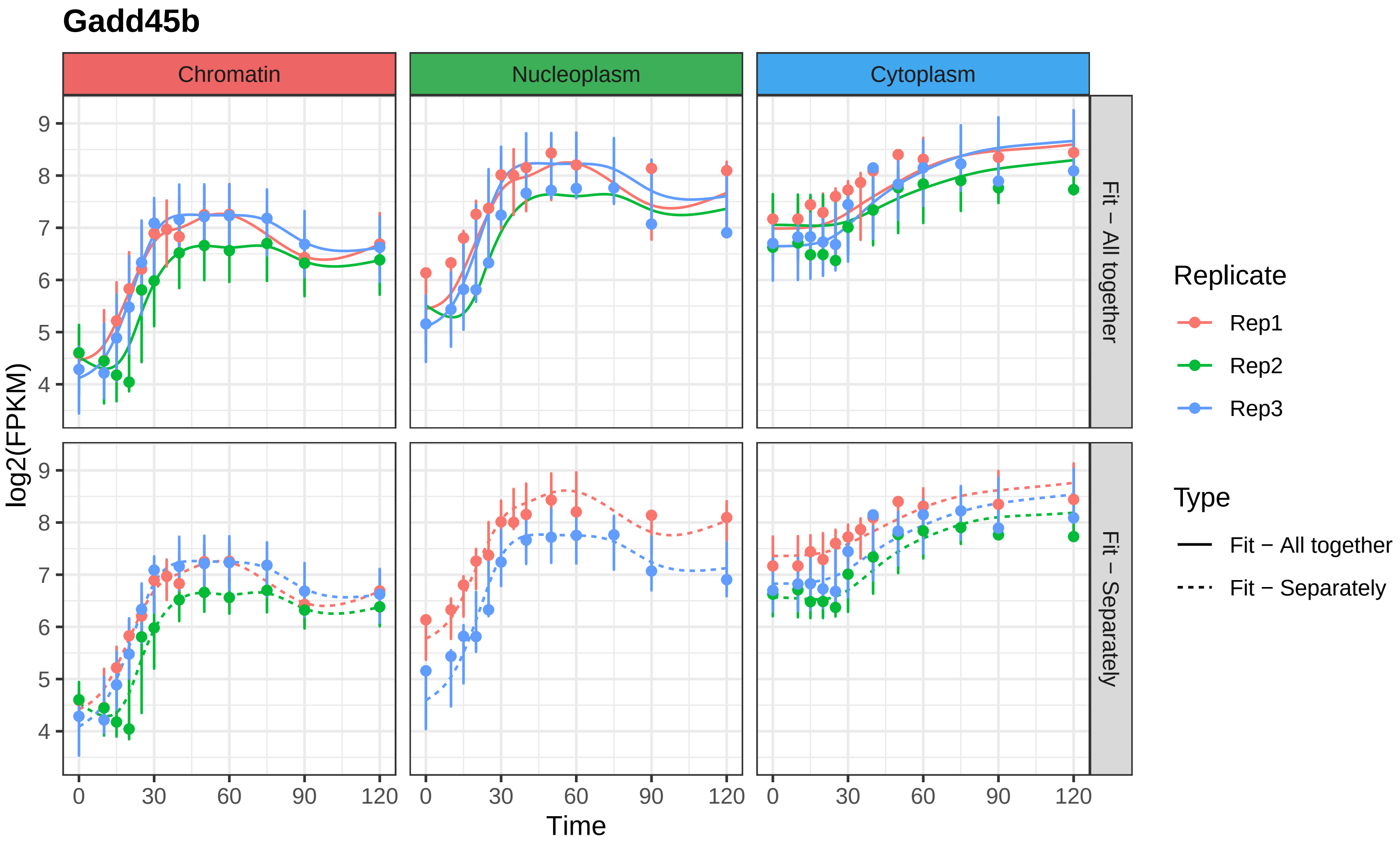

	Together	b1	b2	b3
**−NLL b1 ca**	−0.9253	−5.329		
**−NLL b1 np**	8.035	0.4415		
**−NLL b1 cyto**	4.032	−1.896		
**−NLL b2 ca**	0.3247		−3.201	
**−NLL b2 np**				
**−NLL b2 cyto**	6.898		1.049	
**−NLL b3 ca**	−0.6238			−2.543
**−NLL b3 np**	12.52			2.599
**−NLL b3 cyto**	4.318			4.908
Total	34.57	−6.783	−2.151	4.964
**Total with regul**	35.96	−7.747	−1.954	4.983

	Together	b1	b2	b3
spar	0.3692	0.401700	0.3612	0.3887
σ_b	0.2140	0.133100	0.1209	0.1771
σ_t	2.3230	0.001668	3.223	0.9205
ca_{0,b_1}				
$\log_{10}(k_1')$	5.6540	7.926000		6.3130
$\log_{10}(k_2)$	5.3580	7.518000	4.716 or −1.805	6.1600
$\log_{10}(k_2')$	−1.6790	−1.828000		−1.6680
$\log_{10}(k_{deg})$	−2.1440	−2.306000	−1.805 or 4.716	−2.3410
$\log_{10}(k_1'k_2')$	3.9750	6.098000	3.521	4.6450
$\log_{10}(k_1'/k_2)$	0.2966	0.408100		0.1529
$\text{transport} = \log_{10}(k_1'k_2'/k_2)$	−1.3830	−1.420000	−1.195 or 5.326	−1.5150

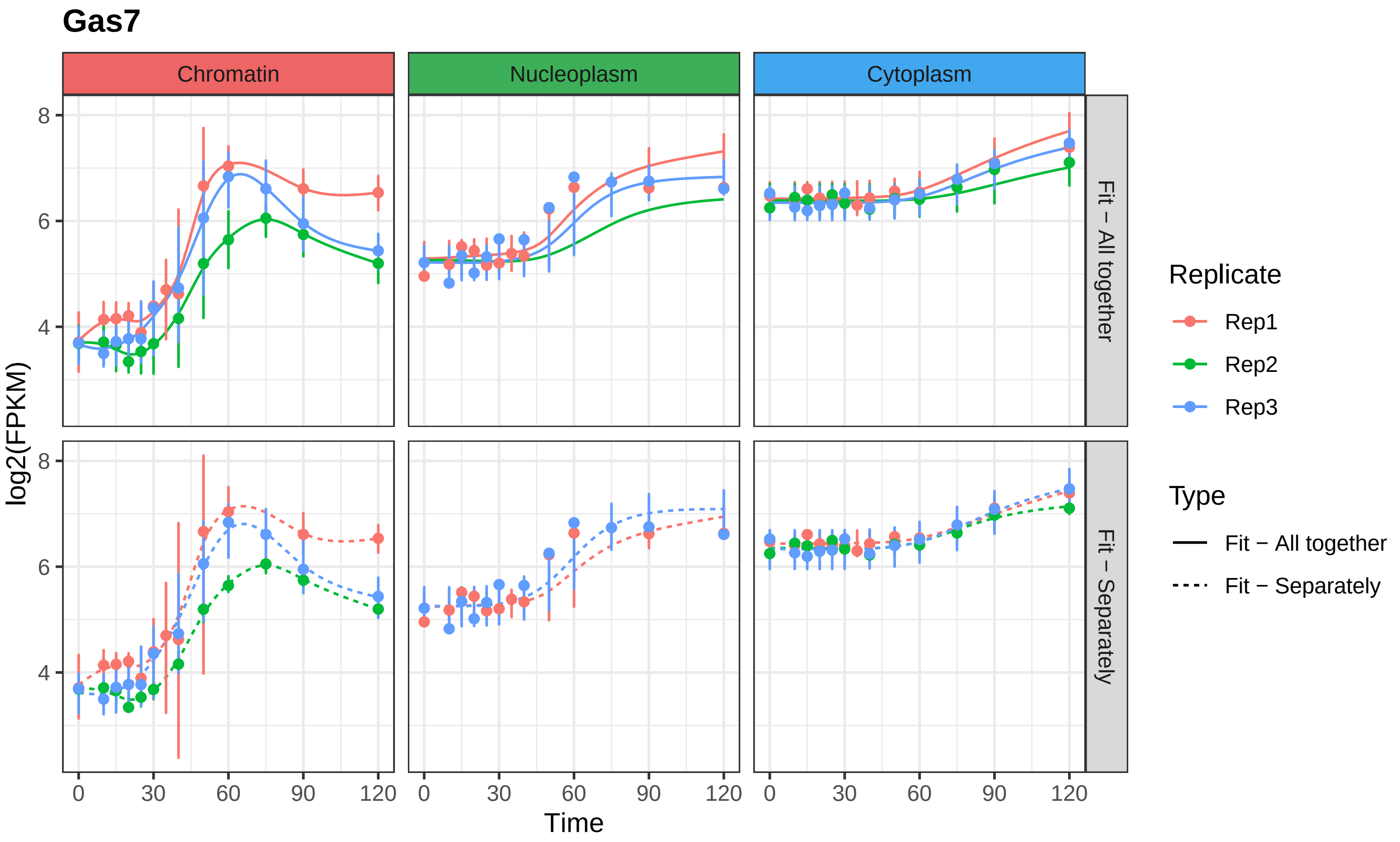

	Together	b1	b2	b3
**−NLL b1 ca**	−3.976	−2.581		
**−NLL b1 np**	8.103	3.829		
**−NLL b1 cyto**	−7.562	−10.97		
**−NLL b2 ca**	−6.276		−13.57	
**−NLL b2 np**				
**−NLL b2 cyto**	−7.549		−11.92	
**−NLL b3 ca**	−4.225			−3.495
**−NLL b3 np**	11.2			7.487
**−NLL b3 cyto**	−7.952			−6.584
Total	−18.24	−9.727	−25.49	−2.592
**Total with regul**	−17.1	−9.898	−26.2	−3.618

	Together	b1	b2	b3
spar	0.3429	0.39260	0.3638	0.4295
σ_b	0.1112	0.08459	0.0492	0.1277
σ_t	5.1870	7.51600	0.1641	4.4290
ca_{0,b_1}				
$\log_{10}(k_1')$	−1.6520	−1.84500		−1.5090
$\log_{10}(k_2)$	−2.1200	−2.29000	−0.6685 or −2.385	−2.0030
$\log_{10}(k_2')$	−1.5000	−1.49800		−1.5880
$\log_{10}(k_{deg})$	−1.8390	−1.85800	−2.385 or −0.6685	−1.9130
$\log_{10}(k_1' k_2')$	−3.1520	−3.34300	−2.258	−3.0970
$\log_{10}(k_1'/k_2)$	0.4673	0.44510		0.4937
transport = $\log_{10}(k_1' k_2'/k_2)$	−1.0320	−1.05300	−1.589 or 0.1276	−1.0950

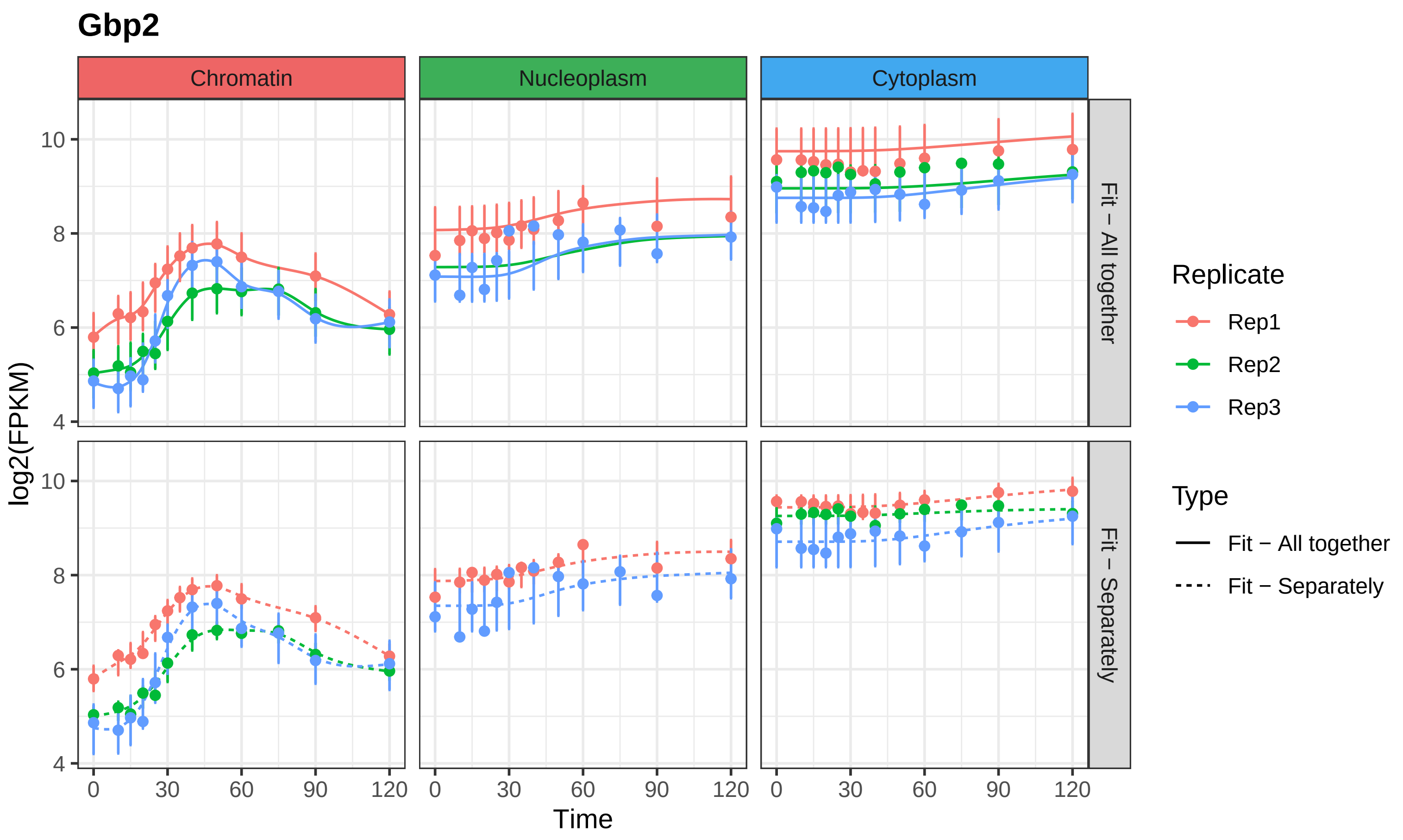

	Together	b1	b2	b3
**−NLL b1 ca**	−4.948	−10.91		
**−NLL b1 np**	2.583	−0.5043		
**−NLL b1 cyto**	3.732	−10.4		
**−NLL b2 ca**	−4.557		−9.933	
**−NLL b2 np**				
**−NLL b2 cyto**	3.726		−8.711	
**−NLL b3 ca**	−4.213			−2.46
**−NLL b3 np**	13.66			9.441
**−NLL b3 cyto**	−2.843			−2.324
Total	7.142	−21.81	−18.64	4.657
**Total with regul**	9.558	−23.26	−20.03	4.54

	Together	b1	b2	b3
spar	3.233e−01	4.013e−01	0.392	3.960e−01
σ_b	1.746e−01	8.920e−02	0.06371	1.799e−01
σ_t	5.876e−08	3.292e−08	1.646	7.492e−07
ca_{0,b_1}				
$\log_{10}(k_1')$	−1.691e+00	−1.794e+00		−1.795e+00
$\log_{10}(k_2)$	−2.369e+00	−2.415e+00	−0.3233 or −3.205	−2.577e+00
$\log_{10}(k_2')$	−1.560e+00	−1.406e+00		−1.315e+00
$\log_{10}(k_{deg})$	−2.064e+00	−1.877e+00	−3.205 or −0.3233	−1.724e+00
$\log_{10}(k_1' k_2')$	−3.251e+00	−3.200e+00	−2.247	−3.109e+00
$\log_{10}(k_1'/k_2)$	6.782e−01	6.210e−01		7.823e−01
transport = $\log_{10}(k_1' k_2'/k_2)$	−8.818e−01	−7.852e−01	−1.924 or 0.9575	−5.322e−01

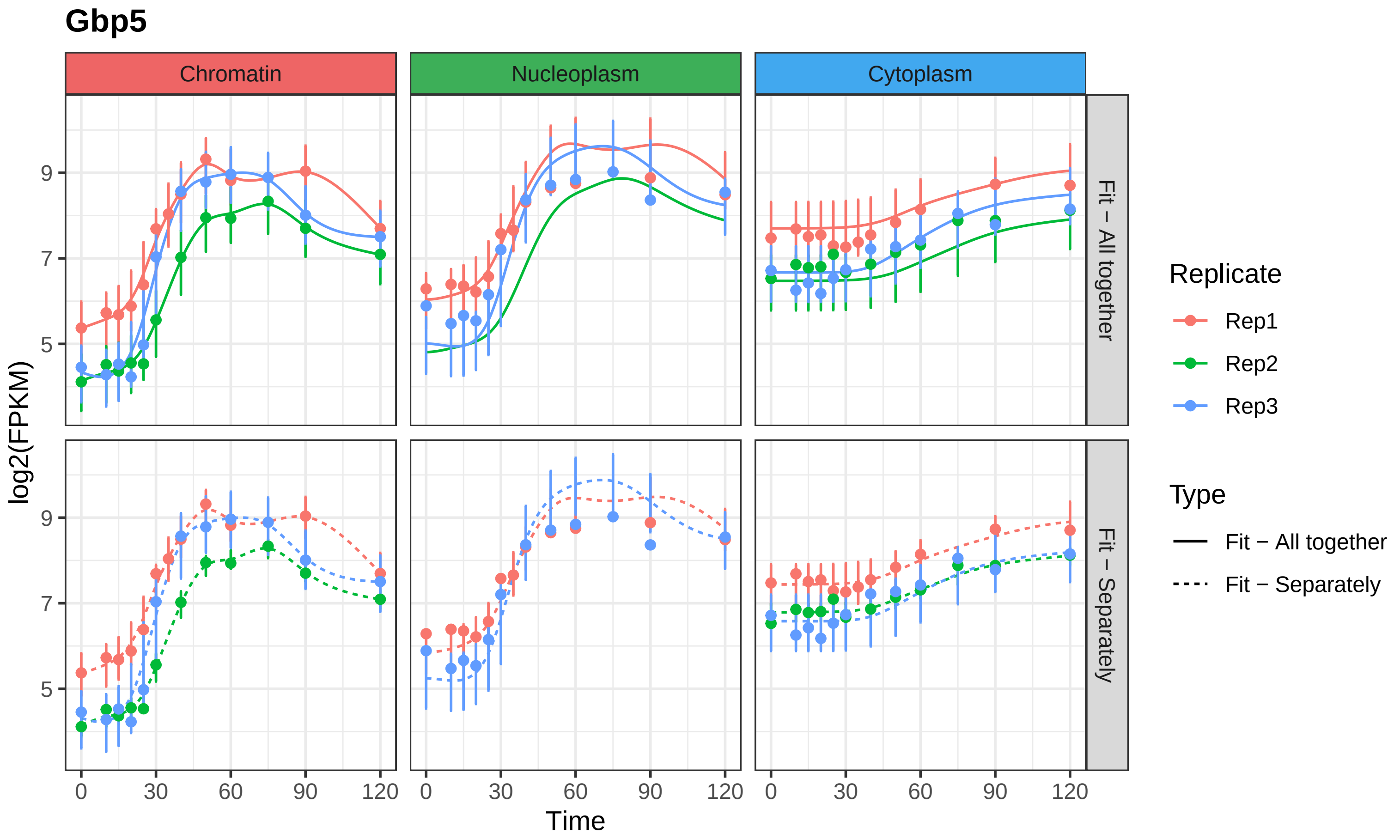

	Together	b1	b2	b3
**−NLL b1 ca**	−0.6362	−3.436		
**−NLL b1 np**	9.32	9.895		
**−NLL b1 cyto**	2.278	−3.701		
**−NLL b2 ca**	−0.7998		−7.94	
**−NLL b2 np**				
**−NLL b2 cyto**	5.081		−6.644	
**−NLL b3 ca**	1.362			1.82
**−NLL b3 np**	17.28			12.38
**−NLL b3 cyto**	2.68			1.569
Total	36.56	2.758	−14.58	15.77
**Total with regul**	38.31	2.914	−14.13	17.32

	Together	b1	b2	b3
spar	0.3627	0.3782	0.3389	0.3706
σ_b	0.2237	0.1696	0.07666	0.2260
σ_t	1.4420	0.4758	0.813	1.7860
ca_{0,b_1}				
$\log_{10}(k_1')$	−0.6891	−0.8100		−0.6048
$\log_{10}(k_2)$	−0.8908	−0.9596	3.836 or −2.773	−0.8840
$\log_{10}(k_2')$	−2.1900	−2.1520		−2.3940
$\log_{10}(k_{deg})$	−2.6910	−2.6300	−2.773 or 3.836	−2.7960
$\log_{10}(k_1'k_2')$	−2.8800	−2.9620	1.858	−2.9990
$\log_{10}(k_1'/k_2)$	0.2017	0.1496		0.2793
transport $= \log_{10}(k_1'k_2'/k_2)$	−1.9890	−2.0020	−1.978 or 4.631	−2.1150

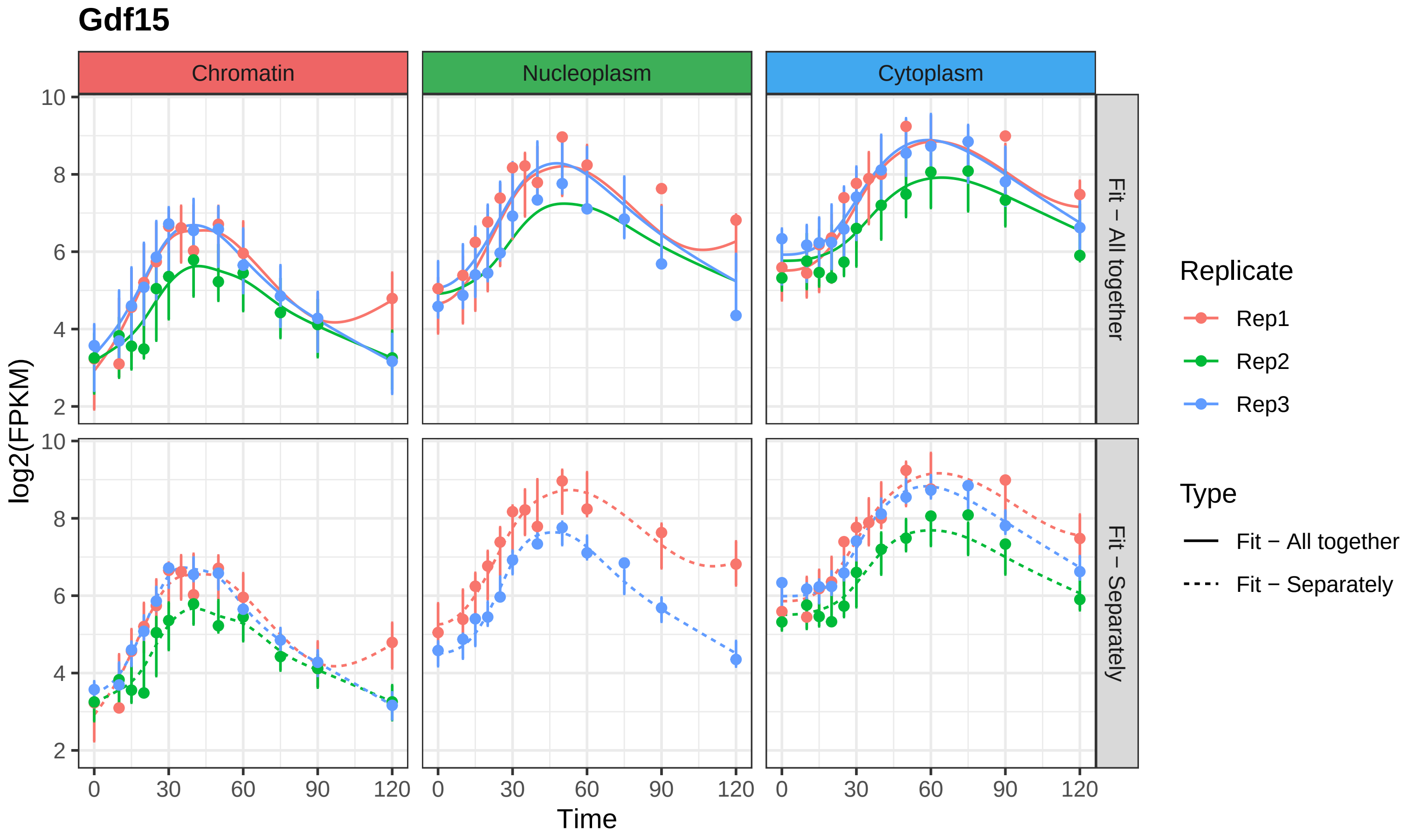

	Together	b1	b2	b3
**−NLL b1 ca**	4.083	2.711		
**−NLL b1 np**	14.07	3.037		
**−NLL b1 cyto**	7.415	4.17		
**−NLL b2 ca**	2.747		−0.5687	
**−NLL b2 np**				
**−NLL b2 cyto**	5.422		1.174	
**−NLL b3 ca**	2.41			−6.203
**−NLL b3 np**	16.95			−2.909
**−NLL b3 cyto**	1.925			−4.346
Total	55.02	9.918	0.6057	−13.46
**Total with regul**	55.93	9.56	−0.06949	−12.44

	Together	b1	b2	b3
spar	0.4377	0.4399	0.3966	0.3320000
σ_b	0.2461	0.1958	0.1358	0.1048000
σ_t	2.8950	1.0920	2.741	0.0008455
ca_{0,b_1}				
$\log_{10}(k_1')$	−0.3930	−0.3650		−0.5515000
$\log_{10}(k_2)$	−0.9177	−1.0700	−0.9275 or −0.9345	−0.8650000
$\log_{10}(k_2')$	−0.8736	−0.8976		−0.7344000
$\log_{10}(k_{deg})$	−1.1290	−1.0780	−0.9345 or −0.9275	−1.1790000
$\log_{10}(k_1'k_2')$	−1.2670	−1.2630	−1.177	−1.2860000
$\log_{10}(k_1'/k_2)$	0.5248	0.7046		0.3134000
$\text{transport} = \log_{10}(k_1'k_2'/k_2)$	−0.3488	−0.1930	−0.2494 or −0.2424	−0.4210000

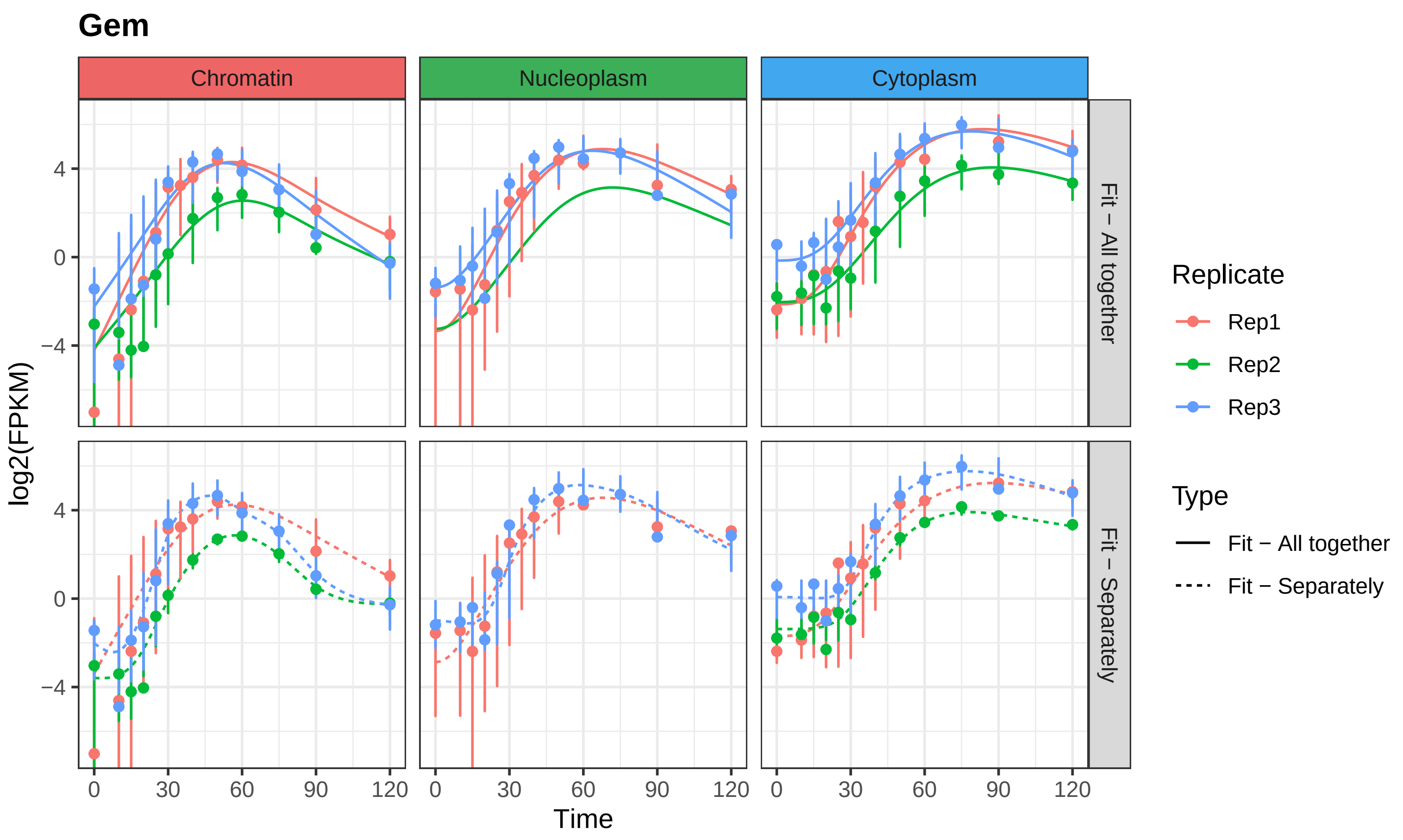

	Together	b1	b2	b3
**−NLL b1 ca**	14.58	15.02		
**−NLL b1 np**	18.12	17.64		
**−NLL b1 cyto**	11.17	7.355		
**−NLL b2 ca**	14.35		3.346	
**−NLL b2 np**				
**−NLL b2 cyto**	8.171		1.113	
**−NLL b3 ca**	18.66			9.838
**−NLL b3 np**	14.73			14.45
**−NLL b3 cyto**	10.99			8.665
Total	110.8	40.02	4.459	32.95
**Total with regul**	115.5	43.88	2.73	36.22

	Together	b1	b2	b3
spar	0.5867	6.149e−01	0.396	0.3482
σ_b	0.2353	2.586e−06	6.347e−05	0.2601
σ_t	4.9860	6.113e+00	0.5577	2.5140
ca_{0,b_1}				
$\log_{10}(k_1')$	−0.8892	−9.153e−01		−0.9237
$\log_{10}(k_2)$	−1.1510	−1.071e+00	7.586 or −1.695	−1.2340
$\log_{10}(k_2')$	−0.9068	−1.101e+00		−1.0050
$\log_{10}(k_{deg})$	−1.2720	−1.454e+00	−1.695 or 7.586	−1.3230
$\log_{10}(k_1'k_2')$	−1.7960	−2.016e+00	6.558	−1.9280
$\log_{10}(k_1'/k_2)$	0.2616	1.553e−01		0.3107
$transport = \log_{10}(k_1'k_2'/k_2)$	−0.6452	−9.456e−01	−1.028 or 8.253	−0.6940

Ggct

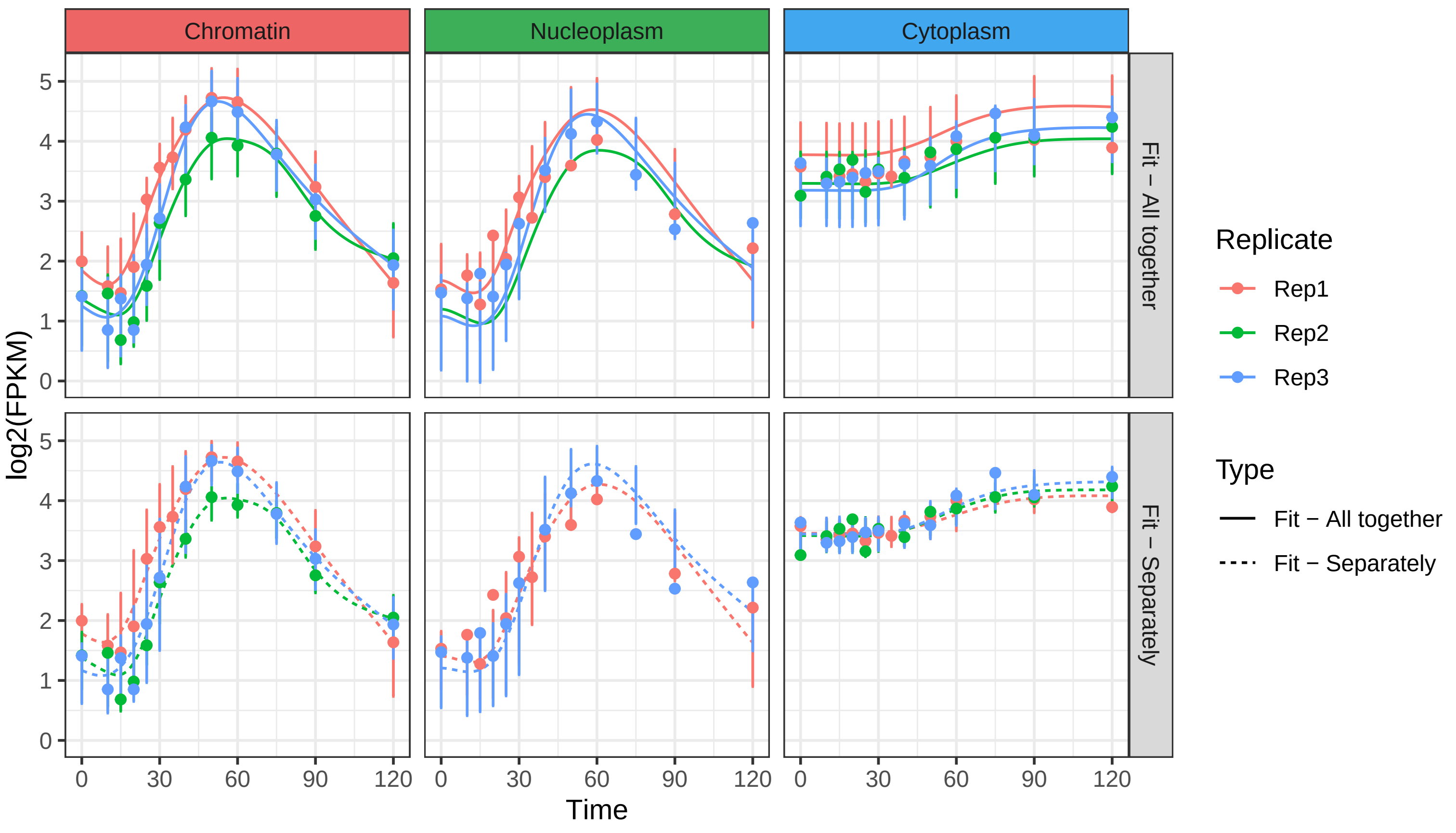

	Together	b1	b2	b3
**−NLL b1 ca**	−1.22	−0.867		
**−NLL b1 np**	11.17	9.811		
**−NLL b1 cyto**	8.055	−8.263		
**−NLL b2 ca**	−0.2303		−2.375	
**−NLL b2 np**				
**−NLL b2 cyto**	−0.8563		−3.901	
**−NLL b3 ca**	−0.5907			0.02634
**−NLL b3 np**	9.047			9.645
**−NLL b3 cyto**	0.5937			−7.115
Total	25.96	0.681	−6.275	2.557
**Total with regul**	25.86	−0.98	−7.649	0.8579

	Together	b1	b2	b3
spar	0.398500	0.42730	0.3907	0.43370
σ_b	0.183200	0.05666	0.06965	0.07951
σ_t	0.001119	4.43700	0.0003468	3.53500
ca_{0,b_1}				
$\log_{10}(k_1')$	−0.625300	−0.90920		−0.68850
$\log_{10}(k_2)$	−0.574100	−0.79490	4.207 or −2.491	−0.70050
$\log_{10}(k_2')$	−1.881000	−2.08600		−2.00100
$\log_{10}(k_{deg})$	−2.513000	−2.70400	−2.491 or 4.207	−2.67400
$\log_{10}(k_1' k_2')$	−2.507000	−2.99500	2.329	−2.69000
$\log_{10}(k_1'/k_2)$	−0.051250	−0.11430		0.01195
$\text{transport} = \log_{10}(k_1' k_2'/k_2)$	−1.933000	−2.20000	−1.878 or 4.82	−1.98900

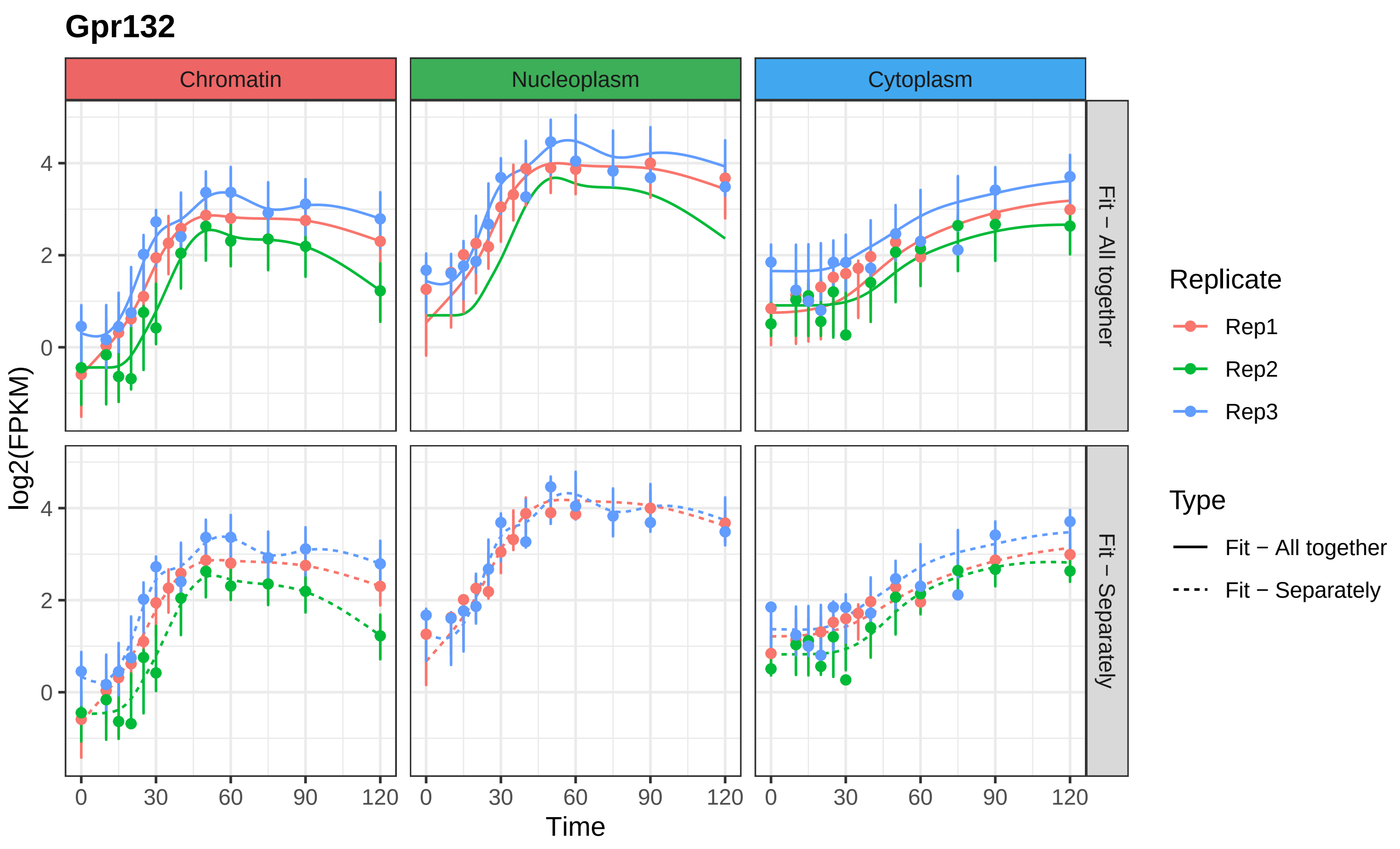

	Together	b1	b2	b3
**−NLL b1 ca**	−1.953	−5.509		
**−NLL b1 np**	4.08	2.603		
**−NLL b1 cyto**	4.814	−1.268		
**−NLL b2 ca**	1.154		0.5204	
**−NLL b2 np**				
**−NLL b2 cyto**	3.353		2.663	
**−NLL b3 ca**	−0.2066			−1.279
**−NLL b3 np**	5.199			2.858
**−NLL b3 cyto**	11.44			4.042
Total	27.88	−4.174	3.184	5.622
**Total with regul**	28.95	−5.691	2.414	6.846

	Together	b1	b2	b3
spar	0.3693000	0.4272	0.3971	0.3495000
σ_b	0.2038000	0.1134	0.1324	0.1744000
σ_t	0.0007624	1.6530	2.367	0.0001574
ca_{0,b_1}				
$\log_{10}(k_1')$	5.0670000	10.2600		6.2500000
$\log_{10}(k_2)$	4.7260000	9.8650	4.923 or −1.974	5.9650000
$\log_{10}(k_2')$	−2.0530000	−2.2380		−1.9830000
$\log_{10}(k_{deg})$	−2.1190000	−2.4010	−1.974 or 4.923	−2.0090000
$\log_{10}(k_1'k_2')$	3.0130000	8.0210	3.339	4.2670000
$\log_{10}(k_1'/k_2)$	0.3411000	0.3939		0.2845000
$transport = \log_{10}(k_1'k_2'/k_2)$	−1.7120000	−1.8440	−1.584 or 5.313	−1.6990000

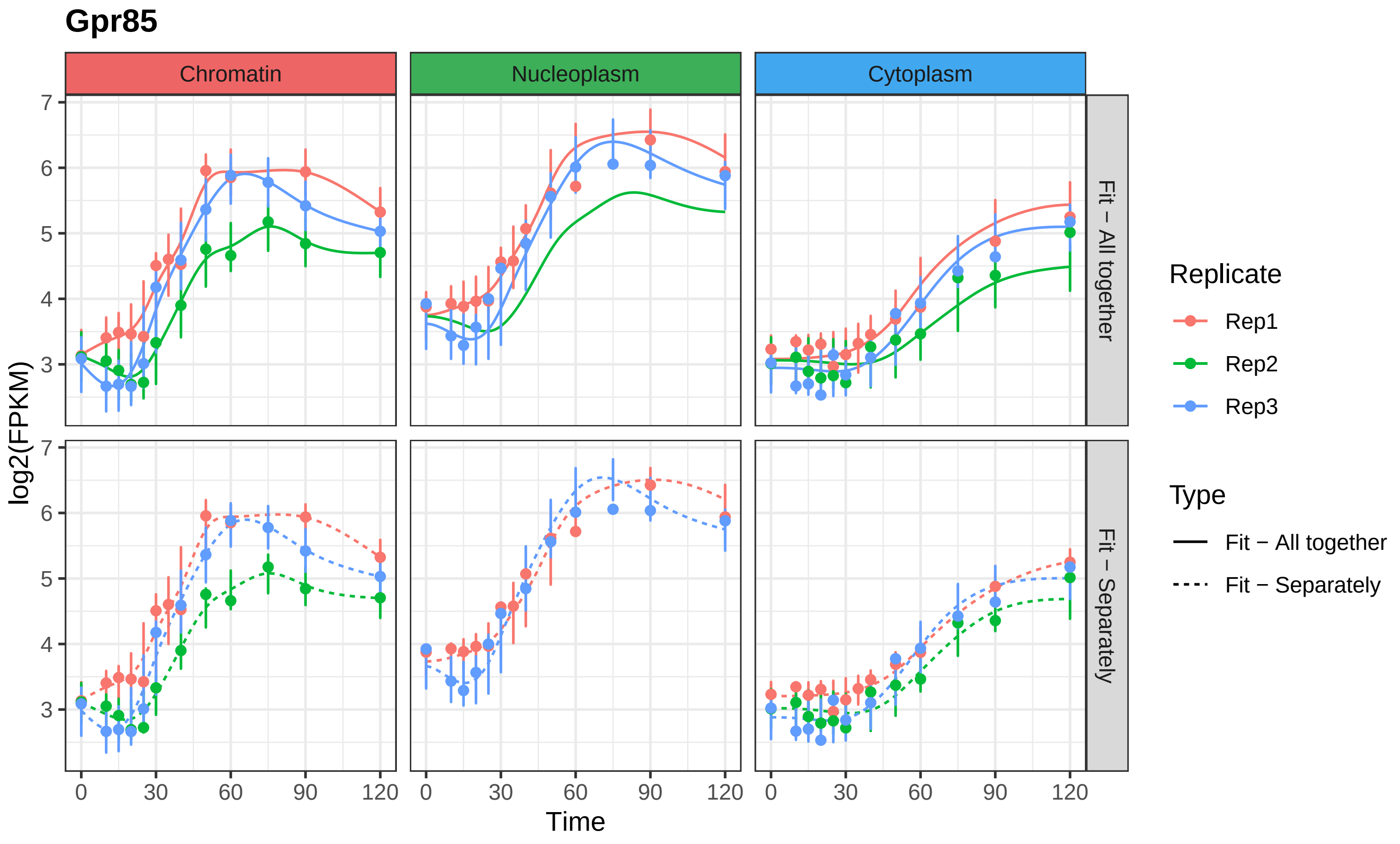

	Together	b1	b2	b3
**−NLL b1 ca**	−4.336	−6.593		
**−NLL b1 np**	−1.269	−2.077		
**−NLL b1 cyto**	−3.284	−9.39		
**−NLL b2 ca**	−6.599		−6.357	
**−NLL b2 np**				
**−NLL b2 cyto**	2.474		−2.432	
**−NLL b3 ca**	−5.454			−4.983
**−NLL b3 np**	2.217			−1.351
**−NLL b3 cyto**	−0.8629			−1.295
Total	−17.11	−18.06	−8.789	−7.629
**Total with regul**	−17.45	−19.12	−10.46	−8.74

	Together	b1	b2	b3
spar	0.3745	0.38670	0.4219	0.3977
σ_b	0.1208	0.06208	0.099	0.1056
σ_t	2.3900	3.62200	0.1615	2.2660
ca_{0,b_1}				
$\log_{10}(k_1')$	−0.7788	−0.96190		−0.4396
$\log_{10}(k_2)$	−0.9611	−1.13900	−1.272 or −1.277	−0.6431
$\log_{10}(k_2')$	−1.9040	−2.06200		−1.9990
$\log_{10}(k_{deg})$	−1.7020	−1.90200	−1.277 or −1.272	−1.7650
$\log_{10}(k_1'k_2')$	−2.6830	−3.02300	−2.574	−2.4380
$\log_{10}(k_1'/k_2)$	0.1823	0.17680		0.2036
transport $= \log_{10}(k_1'k_2'/k_2)$	−1.7220	−1.88500	−1.303 or −1.298	−1.7950

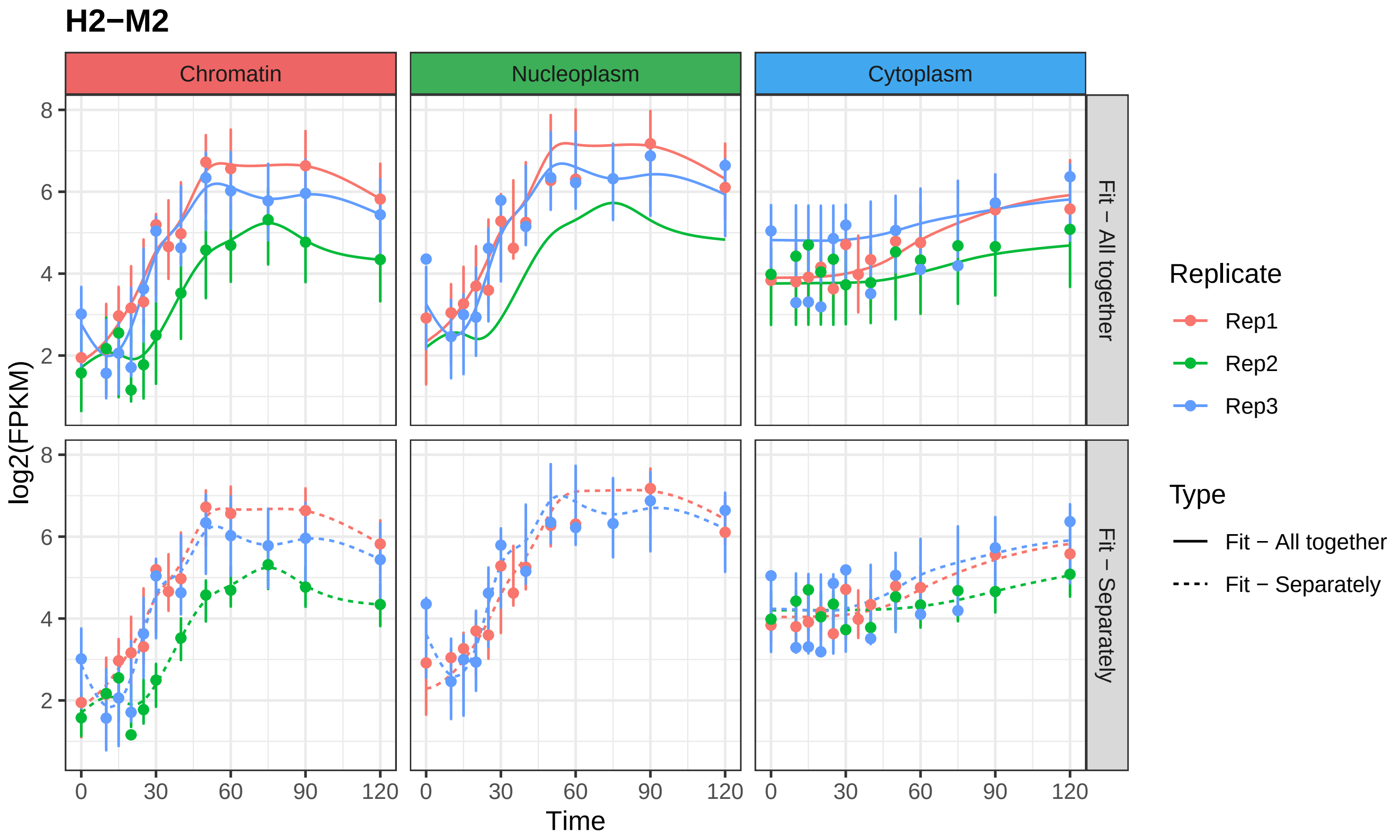

	Together	b1	b2	b3
**−NLL b1 ca**	4.842	2.571		
**−NLL b1 np**	9.354	9.129		
**−NLL b1 cyto**	4.205	2.243		
**−NLL b2 ca**	4.426		1.513	
**−NLL b2 np**				
**−NLL b2 cyto**	7.391		1.984	
**−NLL b3 ca**	6.367			5.125
**−NLL b3 np**	10.28			7.554
**−NLL b3 cyto**	25.05			15.9
Total	71.91	13.94	3.497	28.58
**Total with regul**	75.71	14.51	4.134	34.34

	Together	b1	b2	b3
spar	0.3722	0.3864	0.3632	3.298e−01
σ_b	0.3118	0.1976	0.1701	3.224e−01
σ_t	1.5010	2.5360	0.0001389	1.371e−06
ca_{0,b_1}				
$\log_{10}(k_1')$	4.7710	−0.4429		1.738e+00
$\log_{10}(k_2)$	4.6230	−0.5822	−1.717 or −2.541	1.513e+00
$\log_{10}(k_2')$	−2.3140	−2.3550		−2.166e+00
$\log_{10}(k_{deg})$	−2.7850	−2.8800	−2.541 or −1.717	−2.352e+00
$\log_{10}(k_1'k_2')$	2.4570	−2.7980	−3.504	−4.272e−01
$\log_{10}(k_1'/k_2)$	0.1479	0.1393		2.251e−01
transport $= \log_{10}(k_1'k_2'/k_2)$	−2.1660	−2.2160	−1.788 or −0.9635	−1.941e+00

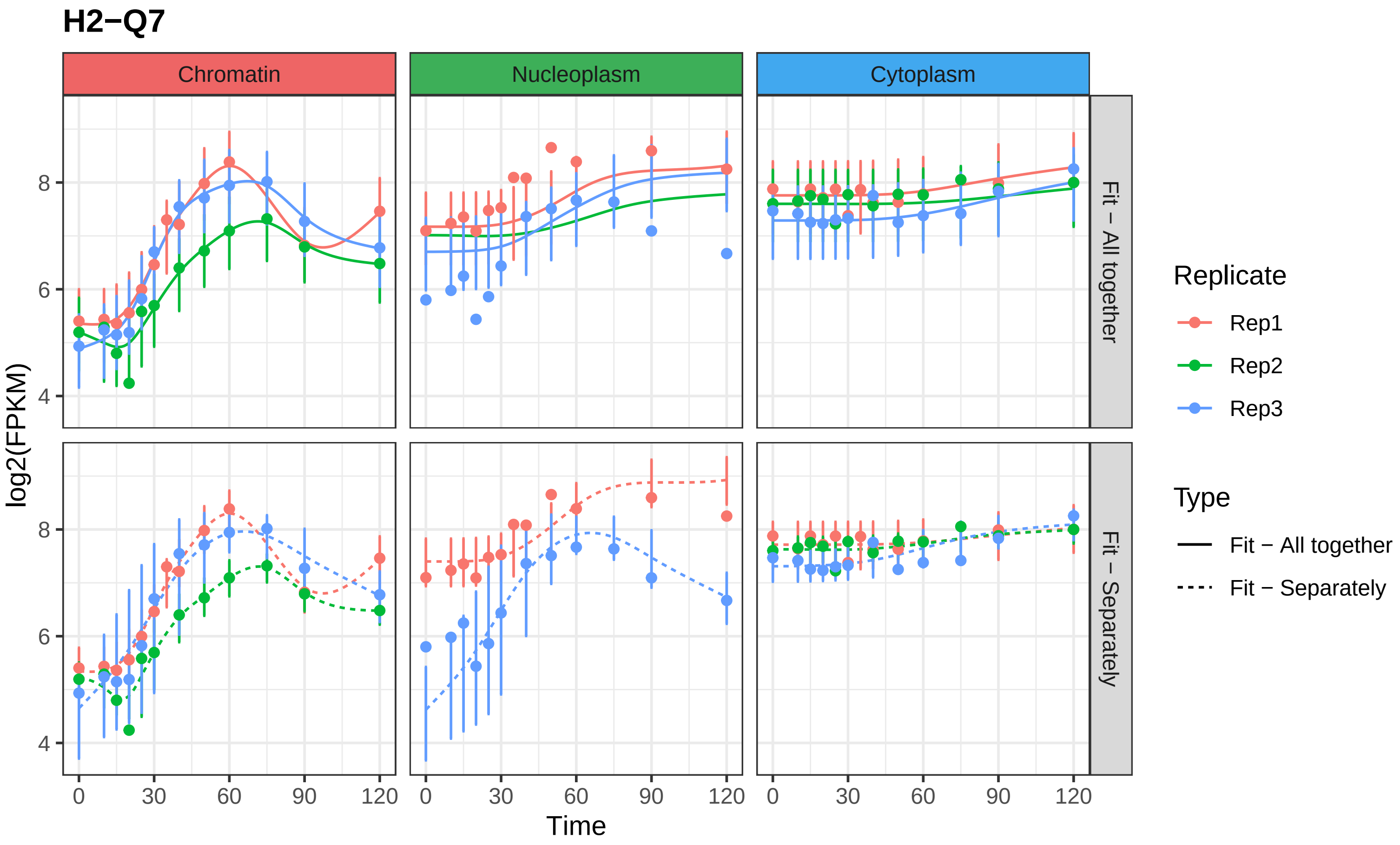

	Together	b1	b2	b3
**−NLL b1 ca**	−0.9847	−4.859		
**−NLL b1 np**	12.48	8.192		
**−NLL b1 cyto**	−0.1093	−4.475		
**−NLL b2 ca**	1.258		−2.294	
**−NLL b2 np**				
**−NLL b2 cyto**	−0.2664		−6.114	
**−NLL b3 ca**	−0.7008			2.152
**−NLL b3 np**	25.73			10.24
**−NLL b3 cyto**	−0.4399			−3.701
Total	36.96	−1.143	−8.408	8.689
**Total with regul**	37.75	−2.063	−7.901	9.912

	Together	b1	b2	b3
spar	4.046e−01	4.177e−01	0.3453	0.543200
σ_b	2.319e−01	1.542e−01	0.085	0.098380
σ_t	5.496e−05	1.955e−07	3.69	8.130000
ca_{0,b_1}				
$\log_{10}(k_1')$	−1.779e+00	−1.437e+00		4.832000
$\log_{10}(k_2)$	−2.326e+00	−2.056e+00	6.211 or −2.752	4.841000
$\log_{10}(k_2')$	−1.904e+00	−2.551e+00		−2.074000
$\log_{10}(k_{deg})$	−2.081e+00	−2.646e+00	−2.752 or 6.211	−2.884000
$\log_{10}(k_1'k_2')$	−3.683e+00	−3.988e+00	4.179	2.758000
$\log_{10}(k_1'/k_2)$	5.464e−01	6.192e−01		−0.008352
$\text{transport} = \log_{10}(k_1'k_2'/k_2)$	−1.358e+00	−1.932e+00	−2.032 or 6.931	−2.083000

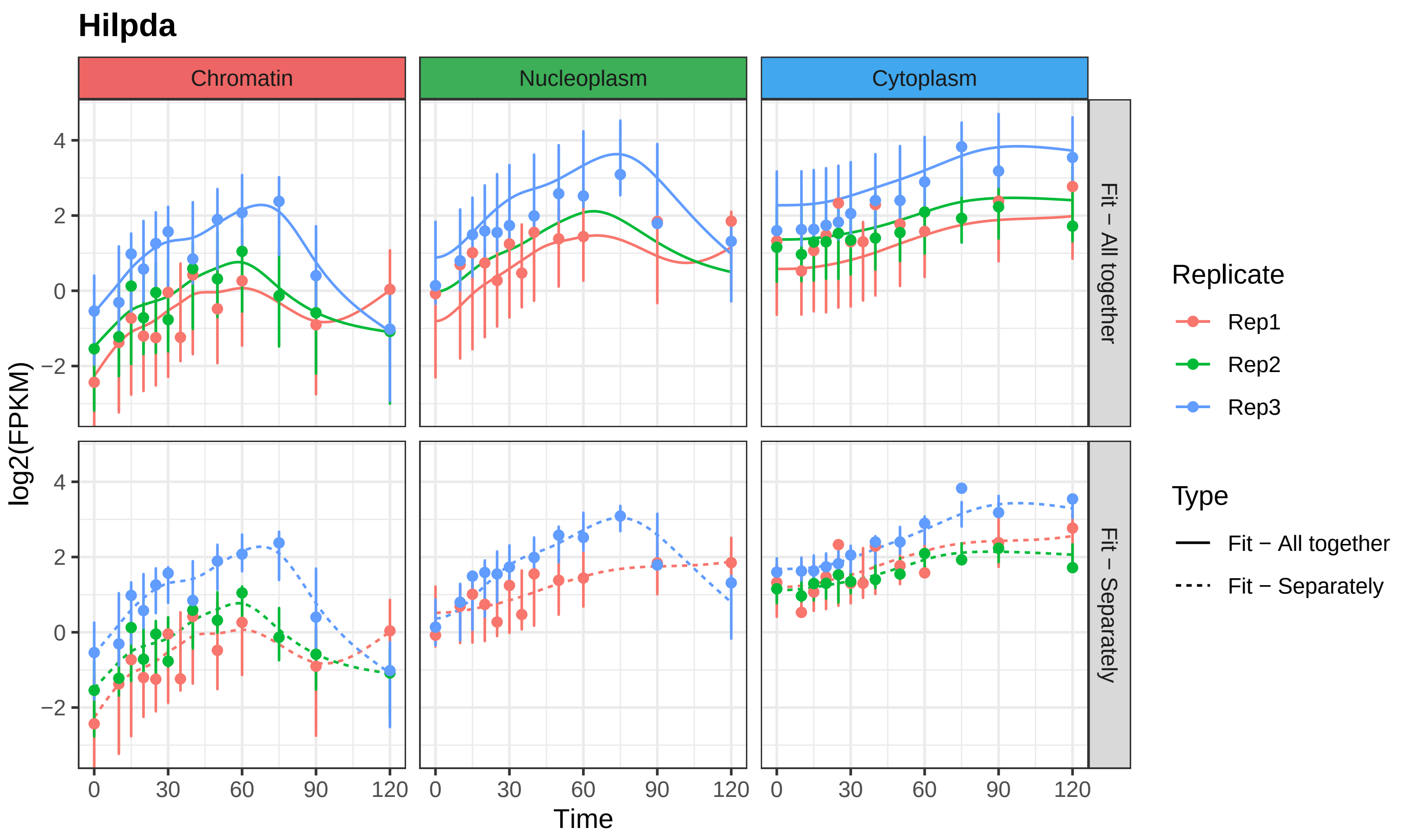

	Together	b1	b2	b3
**−NLL b1 ca**	8.937	7.784		
**−NLL b1 np**	12.58	3.9		
**−NLL b1 cyto**	18.3	8.658		
**−NLL b2 ca**	7.06		5.34	
**−NLL b2 np**				
**−NLL b2 cyto**	5.889		−3.708	
**−NLL b3 ca**	5.812			3.884
**−NLL b3 np**	12.91			3.947
**−NLL b3 cyto**	10.28			−4.608
Total	81.77	20.34	1.632	3.224
**Total with regul**	84.73	20.22	−0.409	1.612

	Together	b1	b2	b3
spar	0.4265000	0.4288000	0.42	0.43000
σ_b	0.3216000	0.2054000	0.04415	0.06256
σ_t	0.0001273	0.0004092	0.01815	4.63900
ca_{0,b_1}				
$\log_{10}(k_1')$	−0.5068000	−1.2600000		−0.80760
$\log_{10}(k_2)$	−0.9470000	−2.0990000	4.454 or −2.107	−1.09100
$\log_{10}(k_2')$	−1.6020000	4.5600000		−1.48100
$\log_{10}(k_{deg})$	−2.0200000	4.3560000	−2.107 or 4.454	−1.87700
$\log_{10}(k_1'k_2')$	−2.1090000	3.2990000	3.134	−2.28900
$\log_{10}(k_1'/k_2)$	0.4402000	0.8386000		0.28350
transport $= \log_{10}(k_1'k_2'/k_2)$	−1.1620000	5.3980000	−1.32 or 5.241	−1.19800

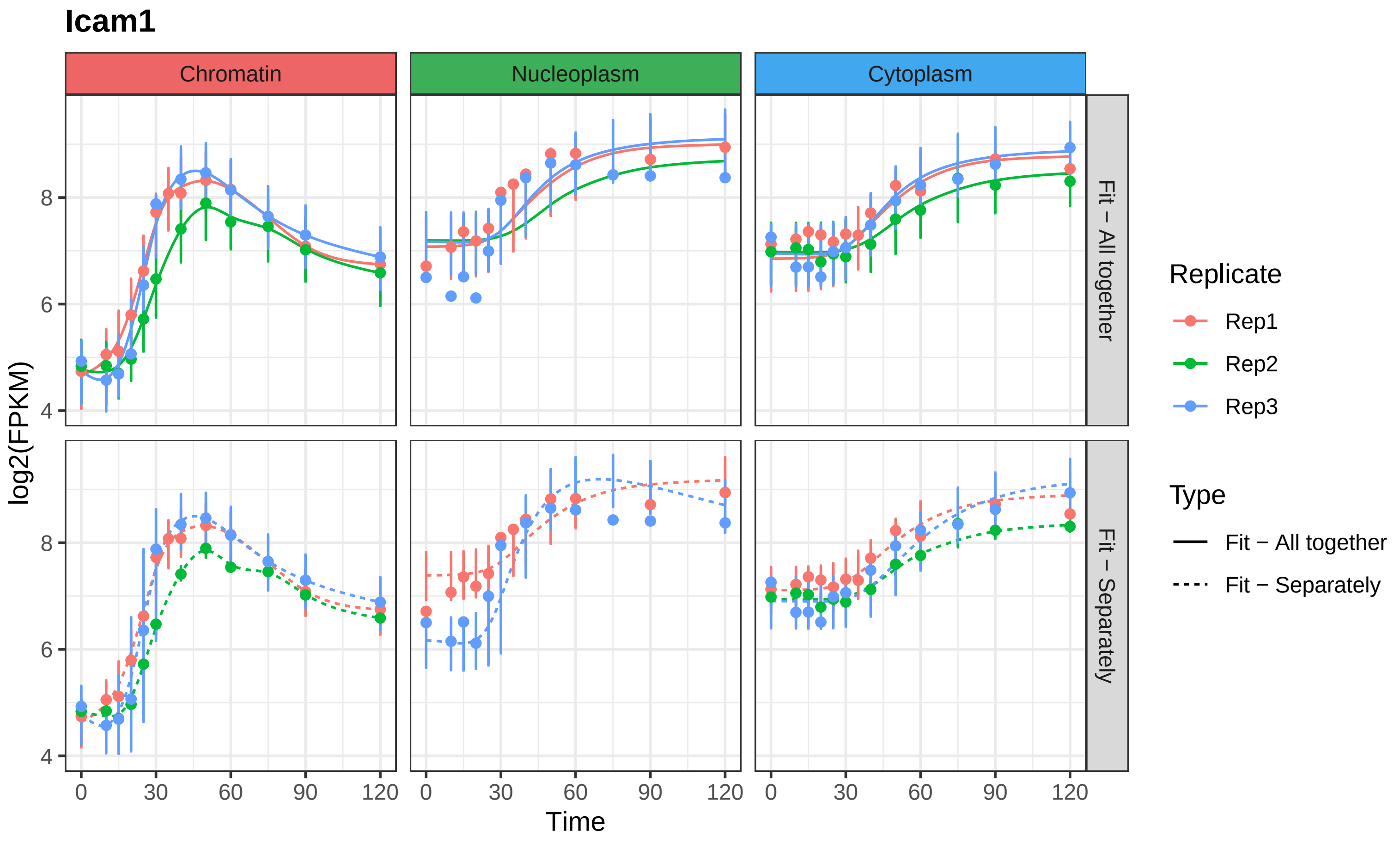

	Together	b1	b2	b3
**−NLL b1 ca**	−2.685	−5.039		
**−NLL b1 np**	8.065	7.634		
**−NLL b1 cyto**	1.299	−3.555		
**−NLL b2 ca**	−2.879		−15.16	
**−NLL b2 np**				
**−NLL b2 cyto**	−2.533		−12.2	
**−NLL b3 ca**	−0.789			−0.6452
**−NLL b3 np**	21.61			8.581
**−NLL b3 cyto**	−0.1746			0.2772
Total	21.92	−0.9603	−27.36	8.213
**Total with regul**	23.01	−1.058	−24.82	9.543

	Together	b1	b2	b3
spar	3.670e−01	3.790e−01	0.2958	0.3495
σ_b	2.016e−01	1.556e−01	0.04522	0.1698
σ_t	1.041e−07	1.174e−06	0.0004215	3.0670
ca_{0,b_1}				
$\log_{10}(k_1')$	−1.519e+00	−1.506e+00		−1.0890
$\log_{10}(k_2)$	−2.249e+00	−2.335e+00	−0.2569 or −2.277	−1.5040
$\log_{10}(k_2')$	−4.824e−01	−7.161e−01		−1.7350
$\log_{10}(k_{deg})$	−4.148e−01	−6.325e−01	−2.277 or −0.2569	−1.9580
$\log_{10}(k_1'k_2')$	−2.002e+00	−2.222e+00	−1.898	−2.8240
$\log_{10}(k_1'/k_2)$	7.294e−01	8.285e−01		0.4147
$\text{transport} = \log_{10}(k_1'k_2'/k_2)$	2.470e−01	1.124e−01	−1.641 or 0.3788	−1.3200

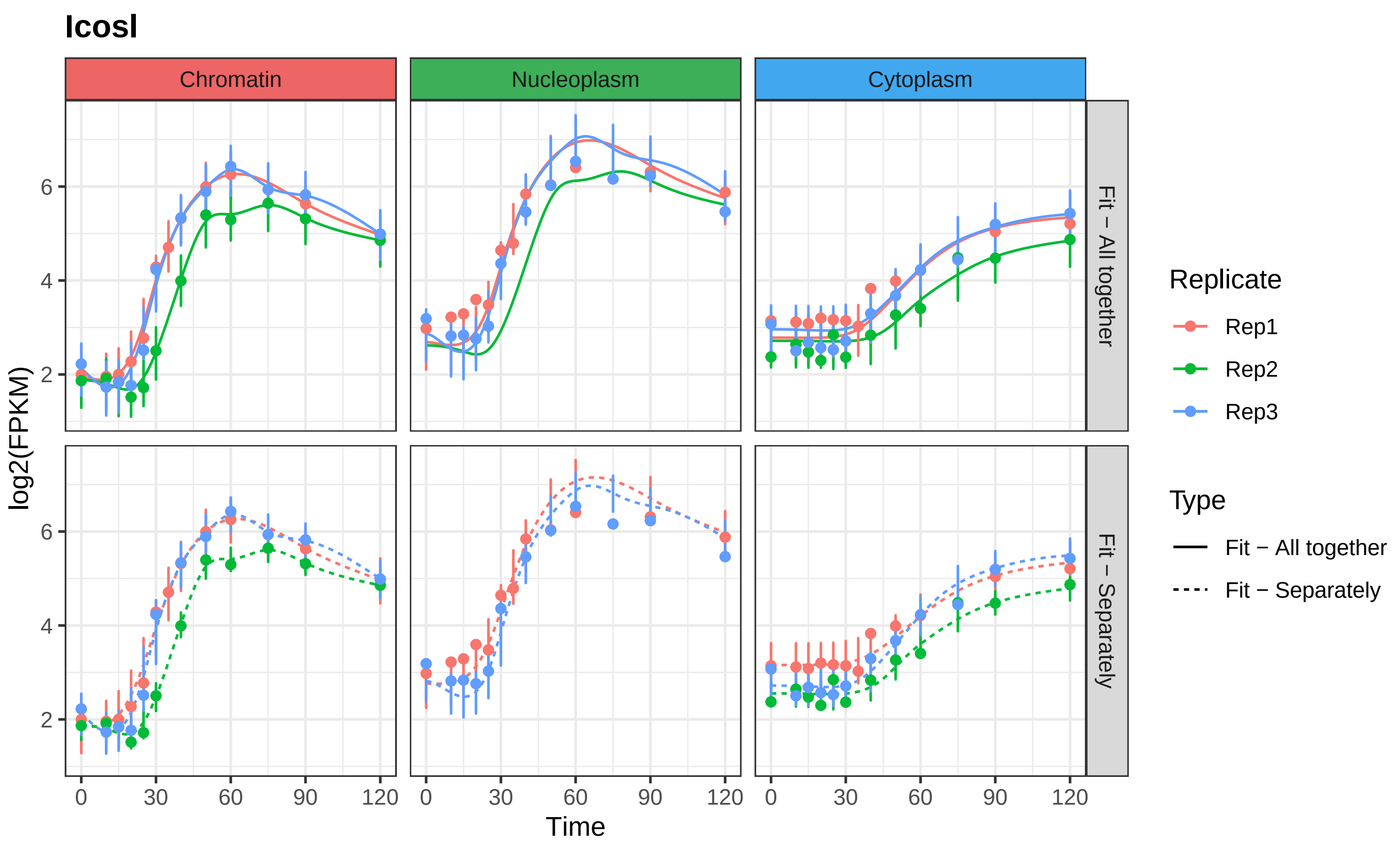

	Together	b1	b2	b3
**−NLL b1 ca**	−3.108	−2.825		
**−NLL b1 np**	10.02	8.899		
**−NLL b1 cyto**	4.066	−2.927		
**−NLL b2 ca**	−3.483		−7.644	
**−NLL b2 np**				
**−NLL b2 cyto**	0.0611		−3.278	
**−NLL b3 ca**	−1.718			−3.289
**−NLL b3 np**	6.393			5.005
**−NLL b3 cyto**	1.179			−3.815
Total	13.41	3.147	−10.92	−2.099
**Total with regul**	15.14	2.469	−10.72	−1.285

	Together	b1	b2	b3
spar	0.340500	0.4105	0.3455	0.3438
σ_b	0.181400	0.1617	0.08255	0.1291
σ_t	0.000737	1.0750	0.001387	1.4450
ca_{0,b_1}				
$\log_{10}(k_1')$	−0.232600	−0.3959		−0.5116
$\log_{10}(k_2)$	−0.451800	−0.6717	4.237 or −2.203	−0.7168
$\log_{10}(k_2')$	−2.216000	−2.3440		−2.1130
$\log_{10}(k_{deg})$	−2.245000	−2.4630	−2.203 or 4.237	−2.0850
$\log_{10}(k_1'k_2')$	−2.448000	−2.7400	2.234	−2.6240
$\log_{10}(k_1'/k_2)$	0.219100	0.2757		0.2052
$\text{transport} = \log_{10}(k_1'k_2'/k_2)$	−1.997000	−2.0680	−2.003 or 4.437	−1.9070

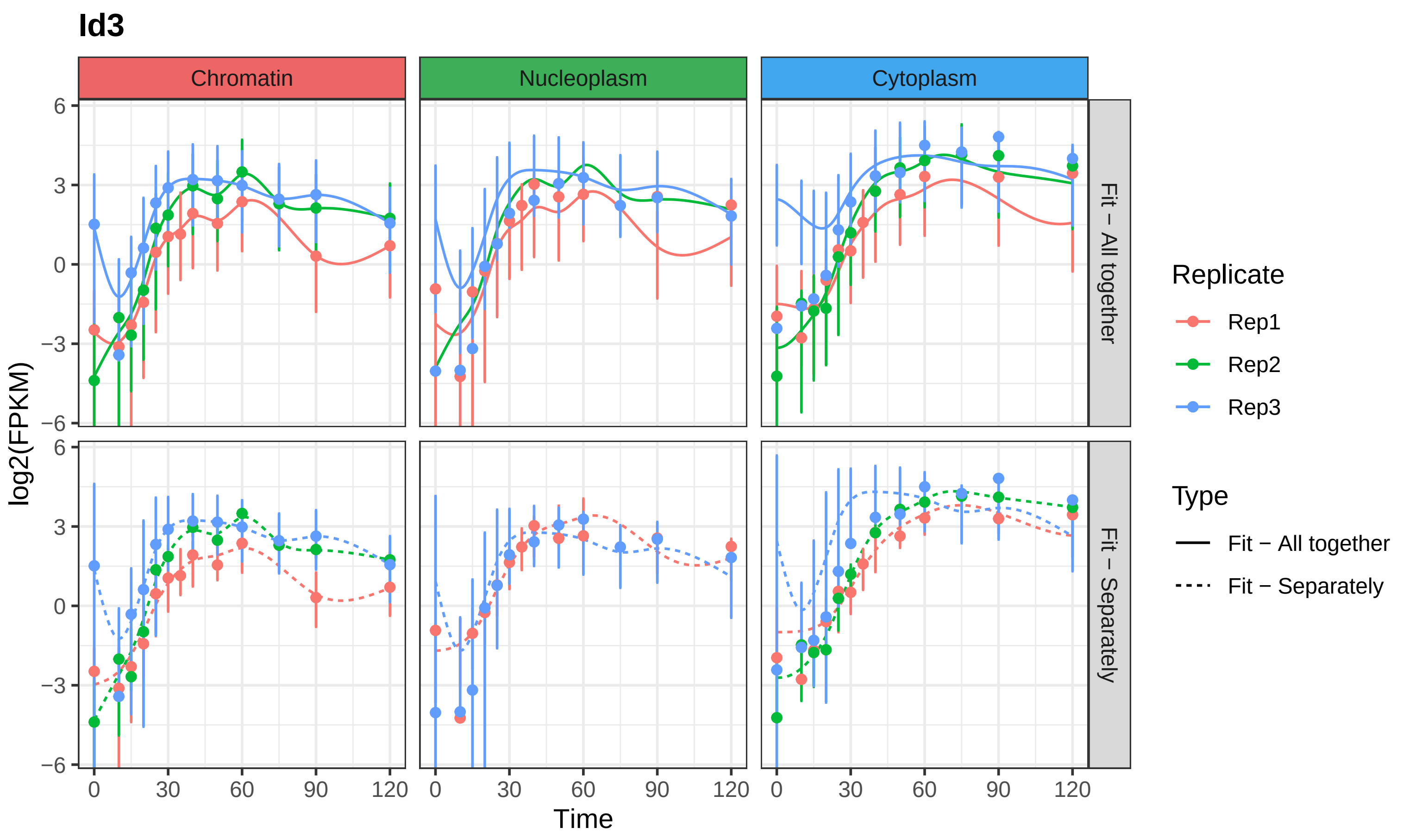

	Together	b1	b2	b3
**−NLL b1 ca**	10.27	5.994		
**−NLL b1 np**	17.93	11.82		
**−NLL b1 cyto**	15.58	13.71		
**−NLL b2 ca**	10.62		2.928	
**−NLL b2 np**				
**−NLL b2 cyto**	11.49		−1.288	
**−NLL b3 ca**	11.85			11.46
**−NLL b3 np**	26.82			17.13
**−NLL b3 cyto**	32.75			25.75
Total	137.3	31.52	1.64	54.35
**Total with regul**	152.6	32.43	2.956	65.28

	Together	b1	b2	b3
spar	0.2691	0.4132000	0.3154	0.2680
σ_b	0.4698	0.2432000	2.712e−05	0.3561
σ_t	2.0260	0.0003481	0.7221	3.8000
ca_{0,b_1}				
$\log_{10}(k_1')$	4.3440	−0.2824000		7.8950
$\log_{10}(k_2)$	4.2440	−0.6691000	0.09554 or −1.393	8.0310
$\log_{10}(k_2')$	−0.8859	−1.0390000		11.3900
$\log_{10}(k_{deg})$	−1.1110	−1.2500000	−1.393 or 0.09554	10.9300
$\log_{10}(k_1'k_2')$	3.4580	−1.3220000	−0.8159	19.2800
$\log_{10}(k_1'/k_2)$	0.1001	0.3867000		−0.1366
$\text{transport} = \log_{10}(k_1'k_2'/k_2)$	−0.7858	−0.6527000	−0.9114 or 0.577	11.2500

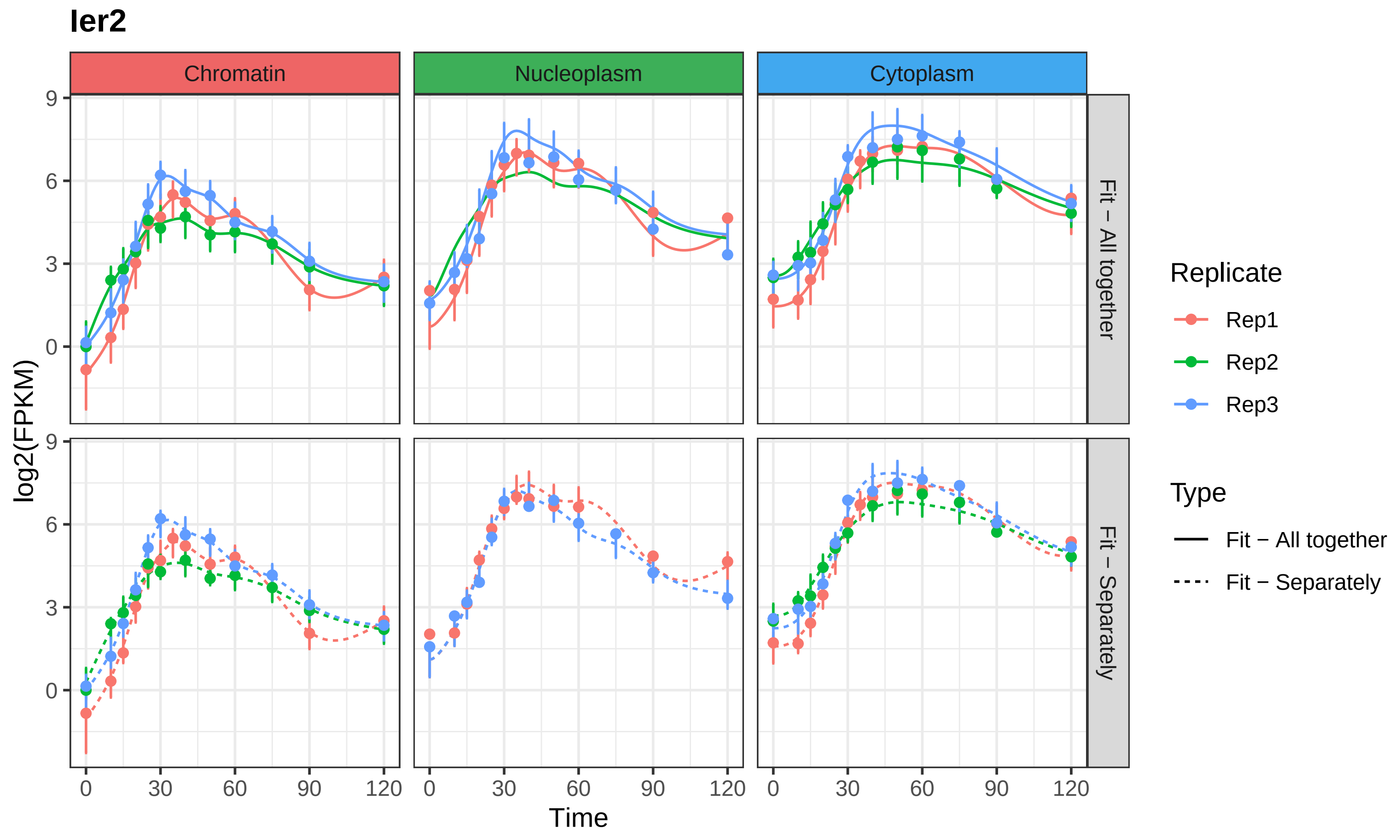

	Together	b1	b2	b3
**−NLL b1 ca**	−0.1364	−2.328		
**−NLL b1 np**	14.39	6.193		
**−NLL b1 cyto**	2.954	1.712		
**−NLL b2 ca**	−0.6685		−1.489	
**−NLL b2 np**				
**−NLL b2 cyto**	2.494		0.2965	
**−NLL b3 ca**	−0.7729			−3.369
**−NLL b3 np**	16.19			3.097
**−NLL b3 cyto**	4.003			2.749
**Total**	38.45	5.578	−1.193	2.477
**Total with regul**	43.99	8.698	−1.574	5.913

	Together	b1	b2	b3
spar	0.2852	3.098e−01	0.3849	0.3013000
σ_b	0.2196	1.735e−01	0.1477	0.1632000
σ_t	0.8171	1.769e−06	0.1358	0.0009297
ca_{0,b_1}				
$\log_{10}(k_1')$	0.1564	1.989e−01		−0.0316600
$\log_{10}(k_2)$	−0.3548	−4.431e−01	−0.8308 or −0.8868	−0.3675000
$\log_{10}(k_2')$	−0.8912	−9.102e−01		−0.7373000
$\log_{10}(k_{deg})$	−1.1130	−1.054e+00	−0.8868 or −0.8308	−1.0800000
$\log_{10}(k_1'k_2')$	−0.7348	−7.113e−01	−0.9899	−0.7689000
$\log_{10}(k_1'/k_2)$	0.5112	6.420e−01		0.3358000
$\text{transport} = \log_{10}(k_1'k_2'/k_2)$	−0.3800	−2.682e−01	−0.1591 or −0.1032	−0.4014000

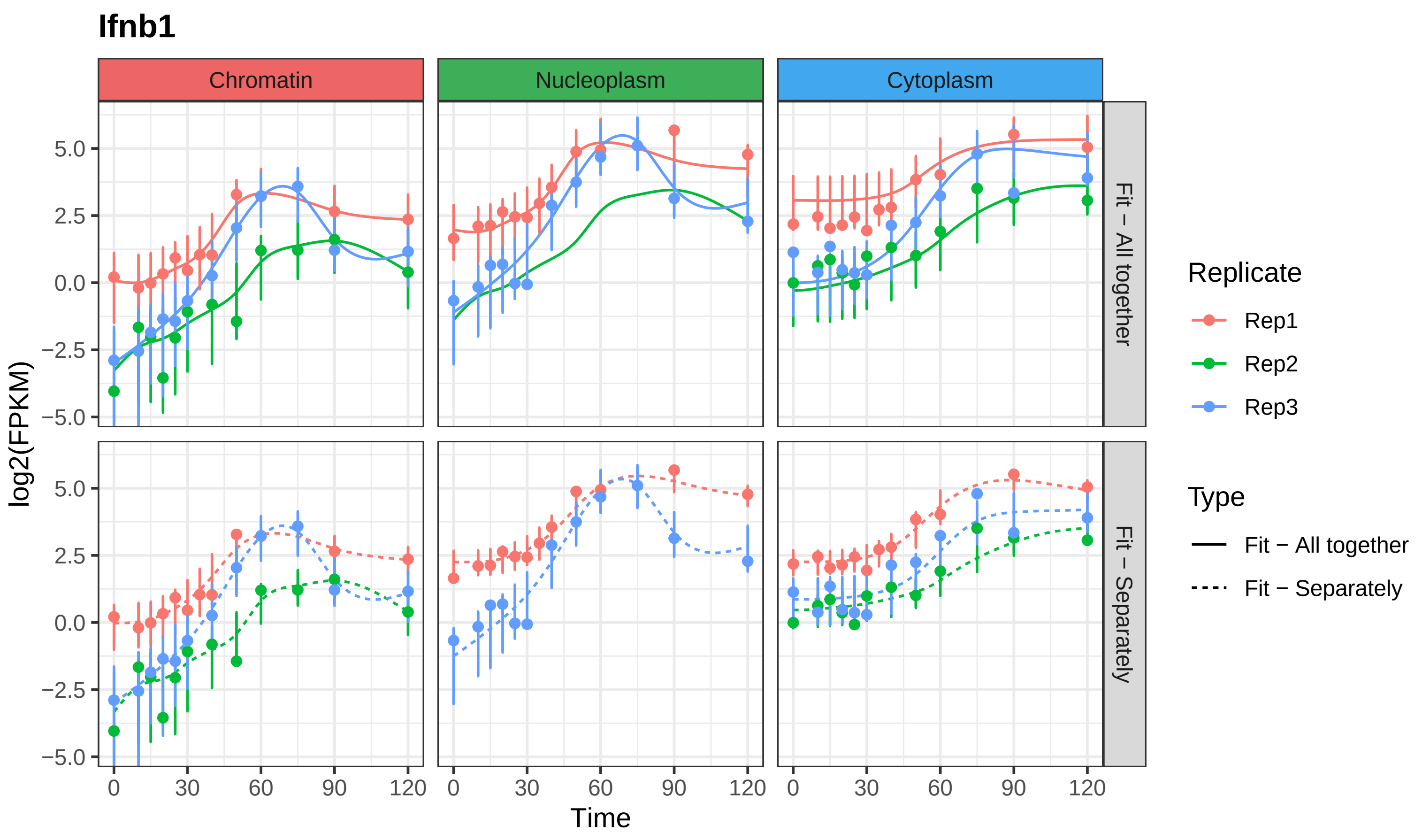

	Together	b1	b2	b3
**−NLL b1 ca**	5.477	2.55		
**−NLL b1 np**	7.12	3.471		
**−NLL b1 cyto**	14.3	−0.861		
**−NLL b2 ca**	11.15		10.19	
**−NLL b2 np**				
**−NLL b2 cyto**	11.19		4.794	
**−NLL b3 ca**	6.454			6.328
**−NLL b3 np**	10.32			10.06
**−NLL b3 cyto**	16.72			10.78
Total	82.74	5.159	14.99	27.17
**Total with regul**	85.65	4.268	14.13	28.45

	Together	b1	b2	b3
spar	0.429400	0.5006	0.4079	0.424800
σ_b	0.320500	0.1184	0.149	0.263800
σ_t	0.003936	3.2700	0.001254	0.003563
ca_{0,b_1}				
$\log_{10}(k_1')$	5.840000	−0.3117		7.494000
$\log_{10}(k_2)$	5.271000	−0.9965	8.139 or −2.259	6.971000
$\log_{10}(k_2')$	−1.493000	−1.1110		−1.857000
$\log_{10}(k_{deg})$	−1.822000	−1.1130	−2.259 or 8.139	−2.493000
$\log_{10}(k_1' k_2')$	4.347000	−1.4220	7.023	5.637000
$\log_{10}(k_1'/k_2)$	0.569100	0.6848		0.522700
$\text{transport} = \log_{10}(k_1' k_2'/k_2)$	−0.923600	−0.4257	−1.117 or 9.282	−1.334000

Ifrd1

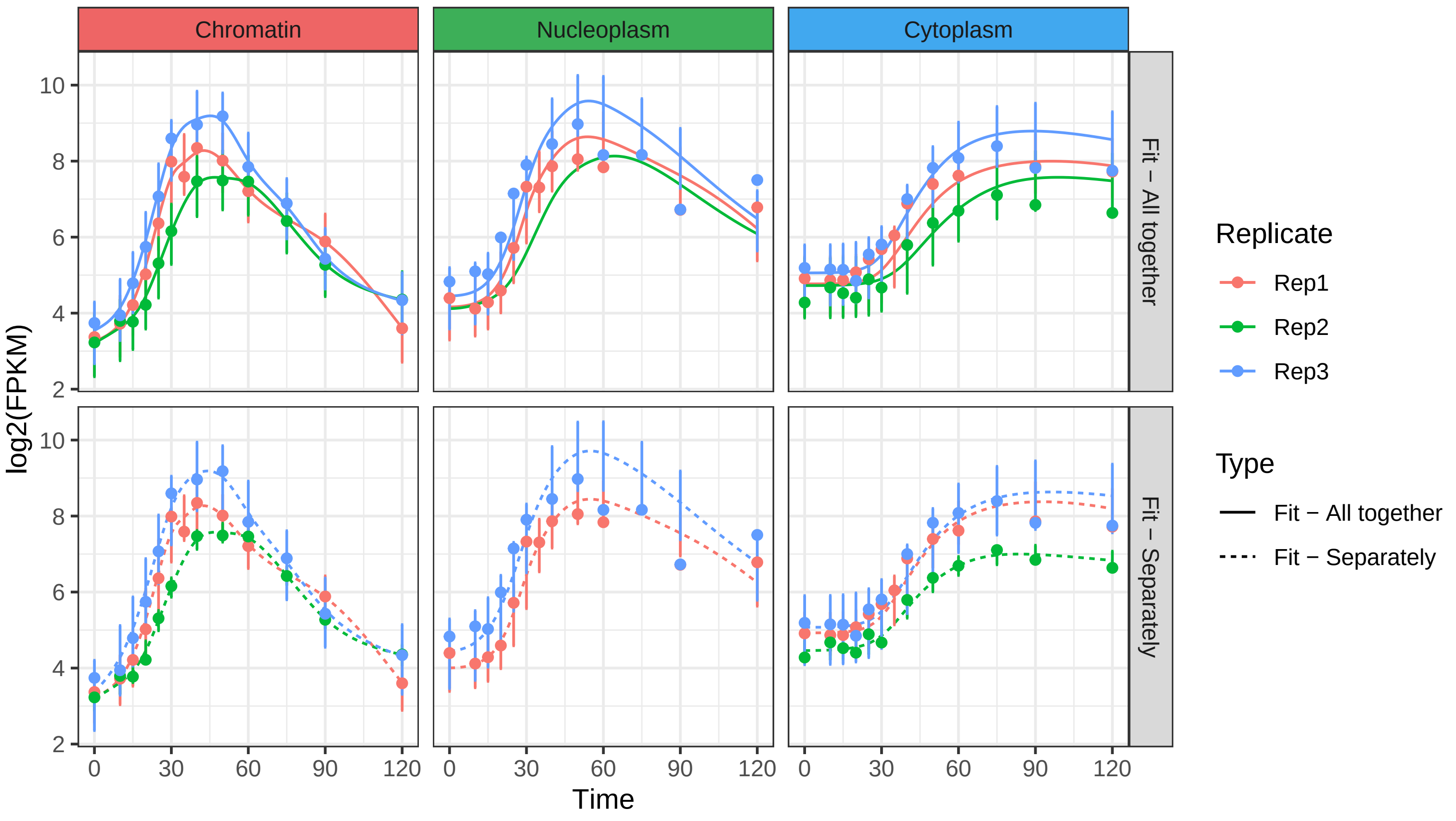

	Together	b1	b2	b3
**−NLL b1 ca**	1.58	0.2683		
**−NLL b1 np**	8.293	8.822		
**−NLL b1 cyto**	6.191	2.598		
**−NLL b2 ca**	0.5603		−8.226	
**−NLL b2 np**				
**−NLL b2 cyto**	6.185		−3.574	
**−NLL b3 ca**	1.058			3.424
**−NLL b3 np**	22.56			16.19
**−NLL b3 cyto**	6.332			6.226
Total	52.76	11.69	−11.8	25.84
**Total with regul**	57.14	13.66	−11.1	28.99

	Together	b1	b2	b3
spar	0.3259000	0.3415	0.3348	0.385600
σ_b	0.2688000	0.1944	0.08539	0.304500
σ_t	0.0008143	1.4850	1.113e−05	0.001491
ca_{0,b_1}				
$\log_{10}(k_1')$	−0.9739000	−1.0630		−0.938900
$\log_{10}(k_2)$	−1.2470000	−1.2980	4.977 or −2.117	−1.267000
$\log_{10}(k_2')$	−1.7140000	−1.4940		−1.868000
$\log_{10}(k_{deg})$	−1.8970000	−1.7720	−2.117 or 4.977	−2.055000
$\log_{10}(k_1' k_2')$	−2.6880000	−2.5570	3.239	−2.807000
$\log_{10}(k_1'/k_2)$	0.2734000	0.2354		0.328200
$transport = \log_{10}(k_1' k_2'/k_2)$	−1.4410000	−1.2590	−1.738 or 5.356	−1.540000

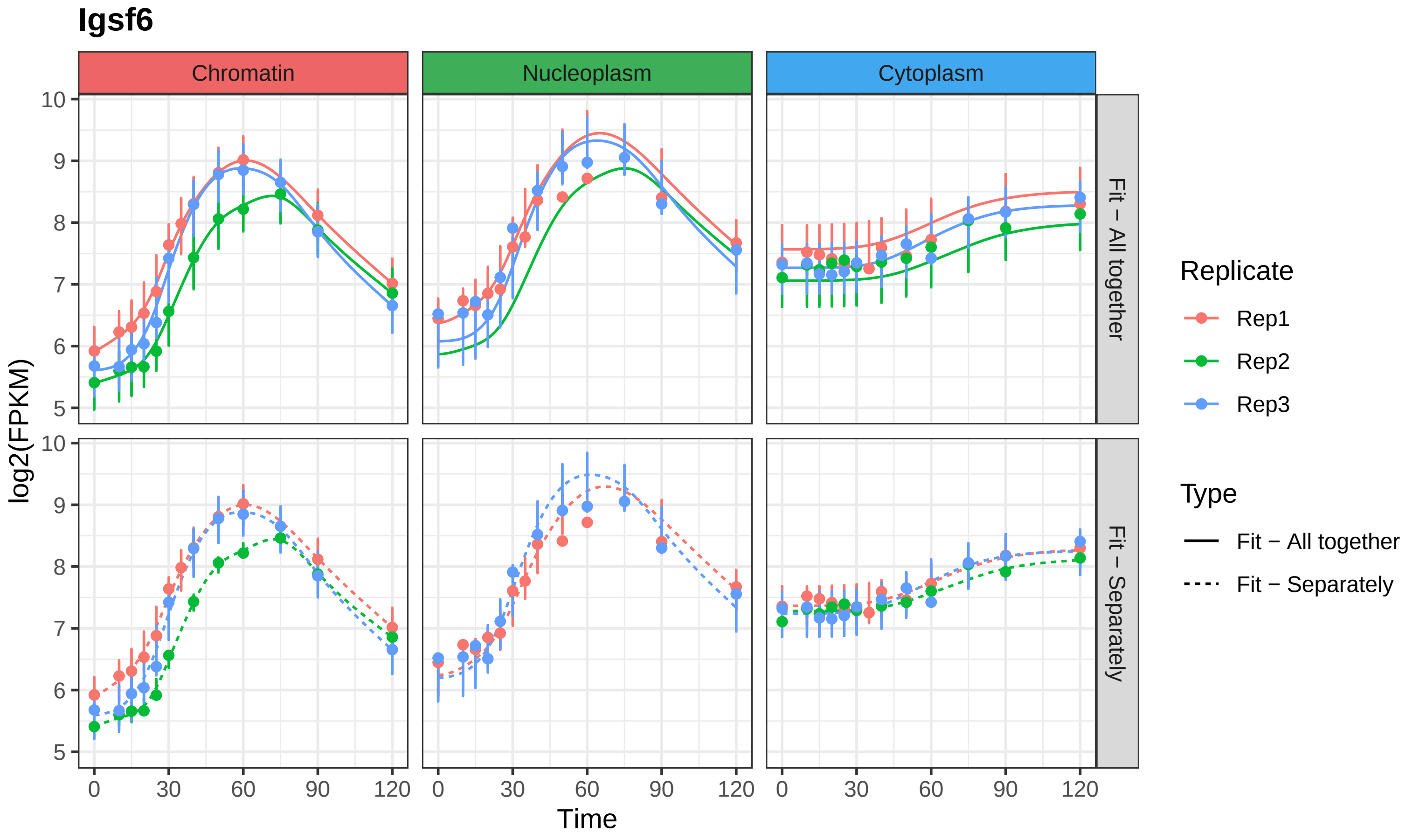

	Together	b1	b2	b3
**−NLL b1 ca**	−6.771	−9.456		
**−NLL b1 np**	6.48	4.999		
**−NLL b1 cyto**	0.3139	−8.512		
**−NLL b2 ca**	−6.776		−16.02	
**−NLL b2 np**				
**−NLL b2 cyto**	−0.2886		−13.68	
**−NLL b3 ca**	−5.935			−6.017
**−NLL b3 np**	7.888			4.721
**−NLL b3 cyto**	−5.801			−5.627
Total	−10.89	−12.97	−29.7	−6.923
**Total with regul**	−11.13	−14.26	−29.34	−7.748

	Together	b1	b2	b3
spar	0.3773	4.064e−01	0.336	0.3924
σ_b	0.1409	1.136e−01	0.03956	0.1279
σ_t	1.2710	1.989e−07	0.284	0.7834
ca_{0,b_1}				
$\log_{10}(k_1')$	−0.4889	−7.146e−01		−0.1247
$\log_{10}(k_2)$	−0.6303	−8.211e−01	−0.494 or −2.673	−0.3080
$\log_{10}(k_2')$	−2.2490	−2.293e+00		−2.3040
$\log_{10}(k_{deg})$	−2.6070	−2.630e+00	−2.673 or −0.494	−2.6170
$\log_{10}(k_1'k_2')$	−2.7380	−3.007e+00	−2.605	−2.4290
$\log_{10}(k_1'/k_2)$	0.1414	1.065e−01		0.1833
transport = $\log_{10}(k_1'k_2'/k_2)$	−2.1080	−2.186e+00	−2.111 or 0.06821	−2.1210

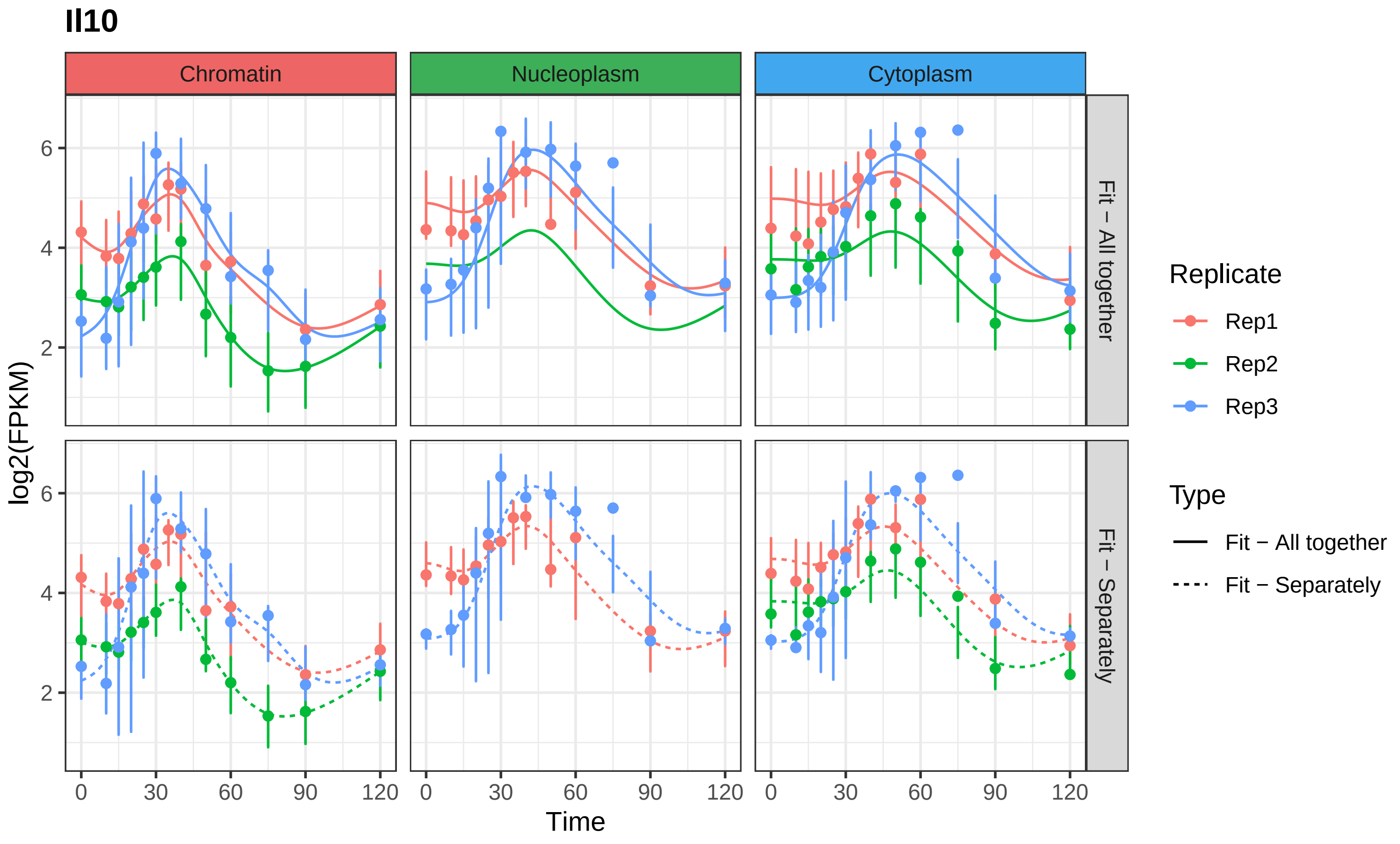

	Together	b1	b2	b3
**−NLL b1 ca**	1.723	0.893		
**−NLL b1 np**	5.328	0.8439		
**−NLL b1 cyto**	9.035	8.322		
**−NLL b2 ca**	0.8221		−2.237	
**−NLL b2 np**				
**−NLL b2 cyto**	5.34		5.47	
**−NLL b3 ca**	5.641			5.114
**−NLL b3 np**	11.46			2.67
**−NLL b3 cyto**	11.97			2.855
Total	51.32	10.06	3.234	10.64
**Total with regul**	52.32	9.697	3.022	9.452

	Together	b1	b2	b3
spar	0.4074	0.4347	0.3926	4.006e−01
σ_b	0.2283	0.1470	0.1702	2.868e−05
σ_t	4.1550	6.4370	0.04961	5.672e+00
ca_{0,b_1}				
$\log_{10}(k_1')$	−0.7739	−0.6865		−7.184e−01
$\log_{10}(k_2)$	−0.9808	−0.8138	−0.8451 or −0.8908	−9.732e−01
$\log_{10}(k_2')$	−0.9069	−0.7925		−7.113e−01
$\log_{10}(k_{deg})$	−0.9331	−0.8183	−0.8908 or −0.8451	−6.927e−01
$\log_{10}(k_1' k_2')$	−1.6810	−1.4790	−1.487	−1.430e+00
$\log_{10}(k_1'/k_2)$	0.2068	0.1272		2.548e−01
$\text{transport} = \log_{10}(k_1' k_2'/k_2)$	−0.7000	−0.6653	−0.6417 or −0.5961	−4.566e−01

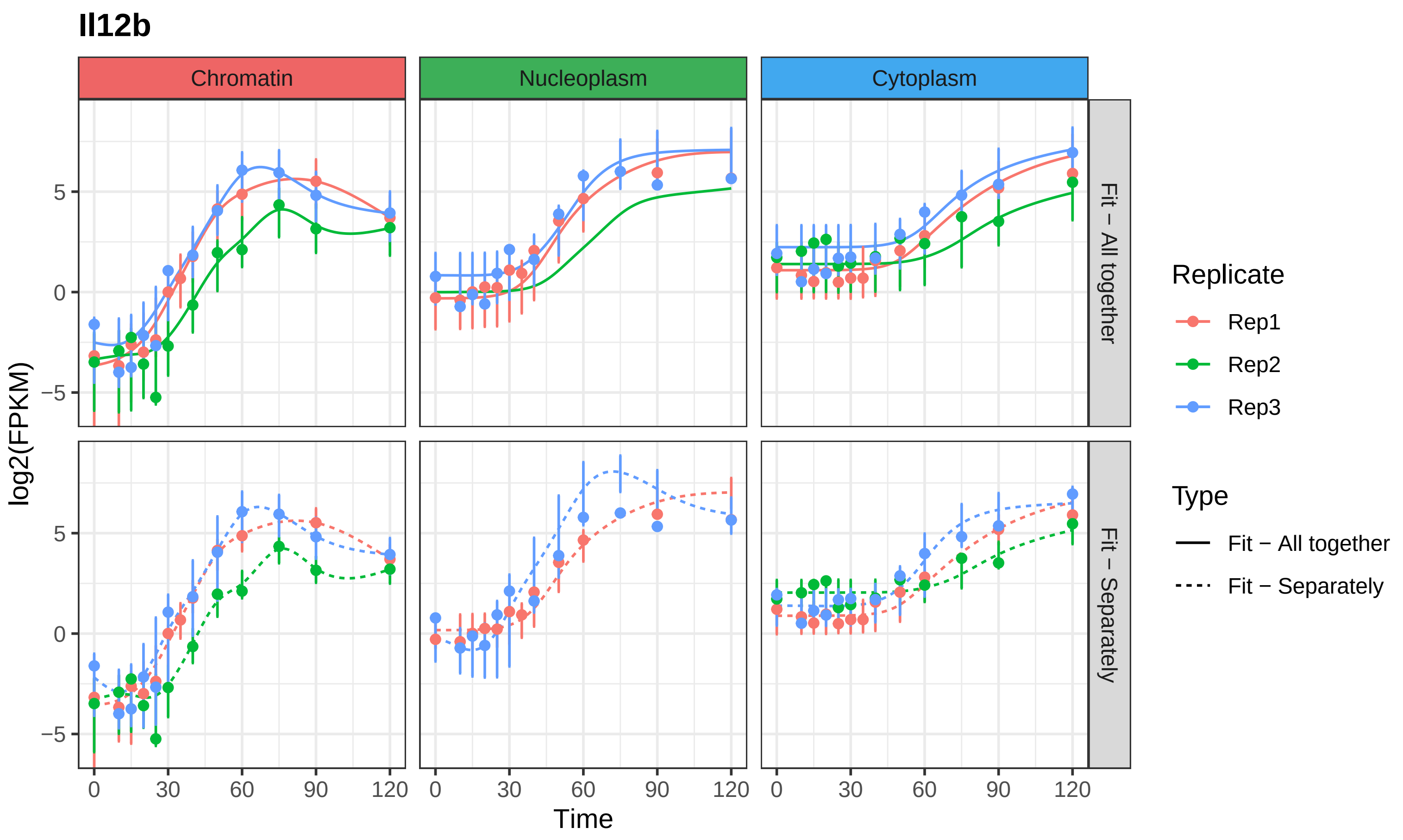

	Together	b1	b2	b3
**−NLL b1 ca**	8.771	5.647		
**−NLL b1 np**	11.82	13.18		
**−NLL b1 cyto**	8.814	5.717		
**−NLL b2 ca**	12.01		8.03	
**−NLL b2 np**				
**−NLL b2 cyto**	14.3		7.81	
**−NLL b3 ca**	14.61			11.1
**−NLL b3 np**	20.07			19.13
**−NLL b3 cyto**	15.24			8.172
Total	105.6	24.55	15.84	38.4
**Total with regul**	111.4	26.1	17.55	42.36

	Together	b1	b2	b3
spar	4.198e−01	0.4033000	0.3632	0.3616
σ_b	3.978e−01	0.2620000	0.2246	0.2986
σ_t	2.557e−06	0.0005962	0.06602	3.5350
ca_{0,b_1}				
$\log_{10}(k_1')$	−1.206e+00	−1.2130000		−0.1659
$\log_{10}(k_2)$	−2.213e+00	−2.3410000	−2.167 or −2.206	−0.7429
$\log_{10}(k_2')$	−1.589e+00	−1.6290000		−2.0190
$\log_{10}(k_{deg})$	−2.010e+00	−1.8440000	−2.206 or −2.167	−2.5180
$\log_{10}(k_1'k_2')$	−2.794e+00	−2.8410000	−2.769	−2.1850
$\log_{10}(k_1'/k_2)$	1.008e+00	1.1290000		0.5769
transport $= \log_{10}(k_1'k_2'/k_2)$	−5.810e−01	−0.5001000	−0.6021 or −0.5627	−1.4430

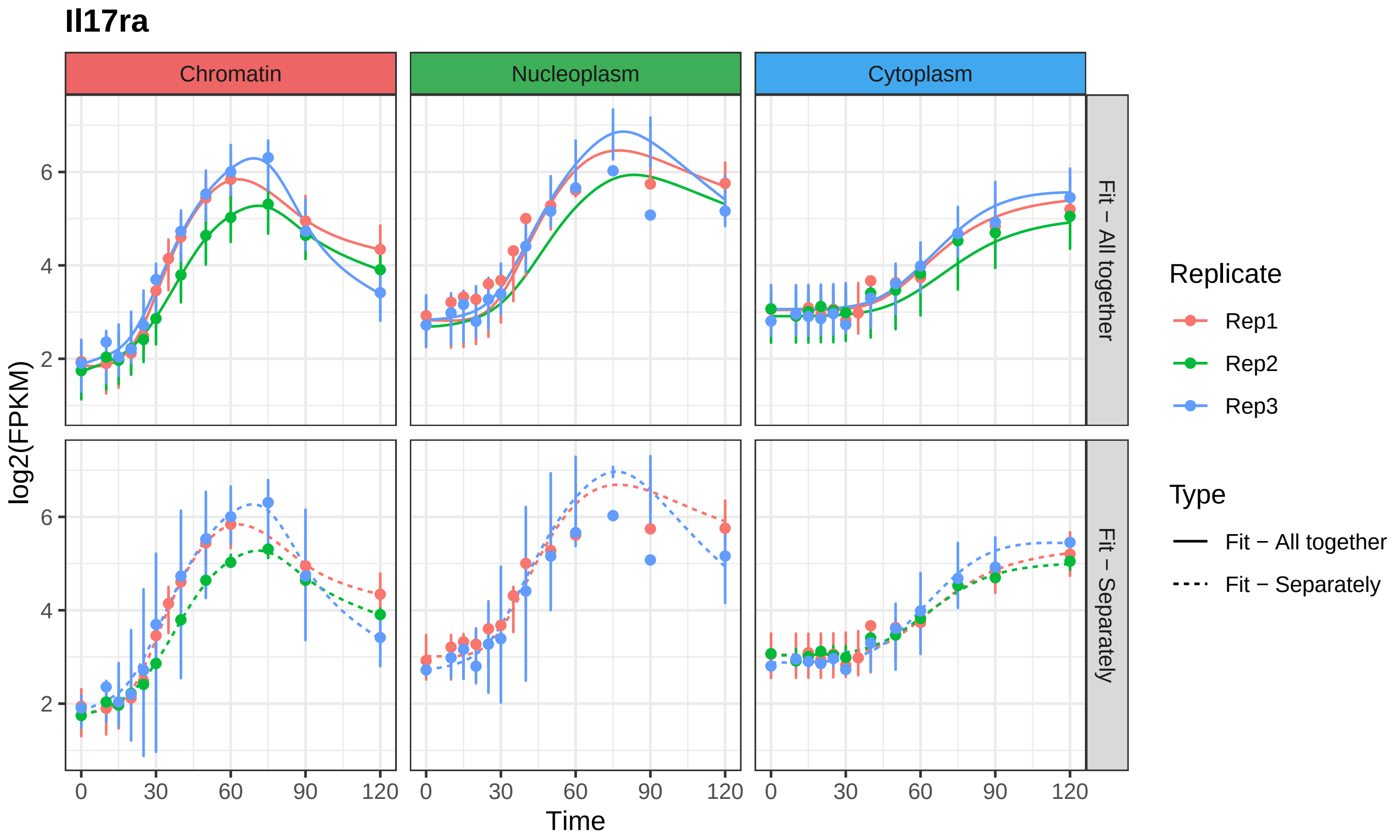

	Together	b1	b2	b3
**−NLL b1 ca**	−3.637	−4.742		
**−NLL b1 np**	10.31	7.95		
**−NLL b1 cyto**	−1.028	−2.269		
**−NLL b2 ca**	−4.11		−14.2	
**−NLL b2 np**				
**−NLL b2 cyto**	−0.2327		−11.25	
**−NLL b3 ca**	−2.243			3.29
**−NLL b3 np**	15.68			8.183
**−NLL b3 cyto**	−1.154			−7.19
Total	13.59	0.9394	−25.45	4.283
**Total with regul**	13.4	0.09838	−27.27	3.229

	Together	b1	b2	b3
spar	0.4034000	0.4190	0.4037	4.256e−01
σ_b	0.1843000	0.1599	0.03602	3.672e−06
σ_t	0.0006231	0.5680	5.338e−05	7.567e+00
ca_{0,b_1}				
$\log_{10}(k_1')$	−0.9731000	−0.8948		−7.781e−01
$\log_{10}(k_2)$	−1.2620000	−1.2500	−0.6584 or −2.176	−1.050e+00
$\log_{10}(k_2')$	−2.0210000	−2.1780		−2.060e+00
$\log_{10}(k_{deg})$	−2.0890000	−2.1880	−2.176 or −0.6584	−2.100e+00
$\log_{10}(k_1'k_2')$	−2.9940000	−3.0730	−2.441	−2.838e+00
$\log_{10}(k_1'/k_2)$	0.2891000	0.3547		2.716e−01
$transport = \log_{10}(k_1'k_2'/k_2)$	−1.7320000	−1.8230	−1.782 or −0.265	−1.788e+00

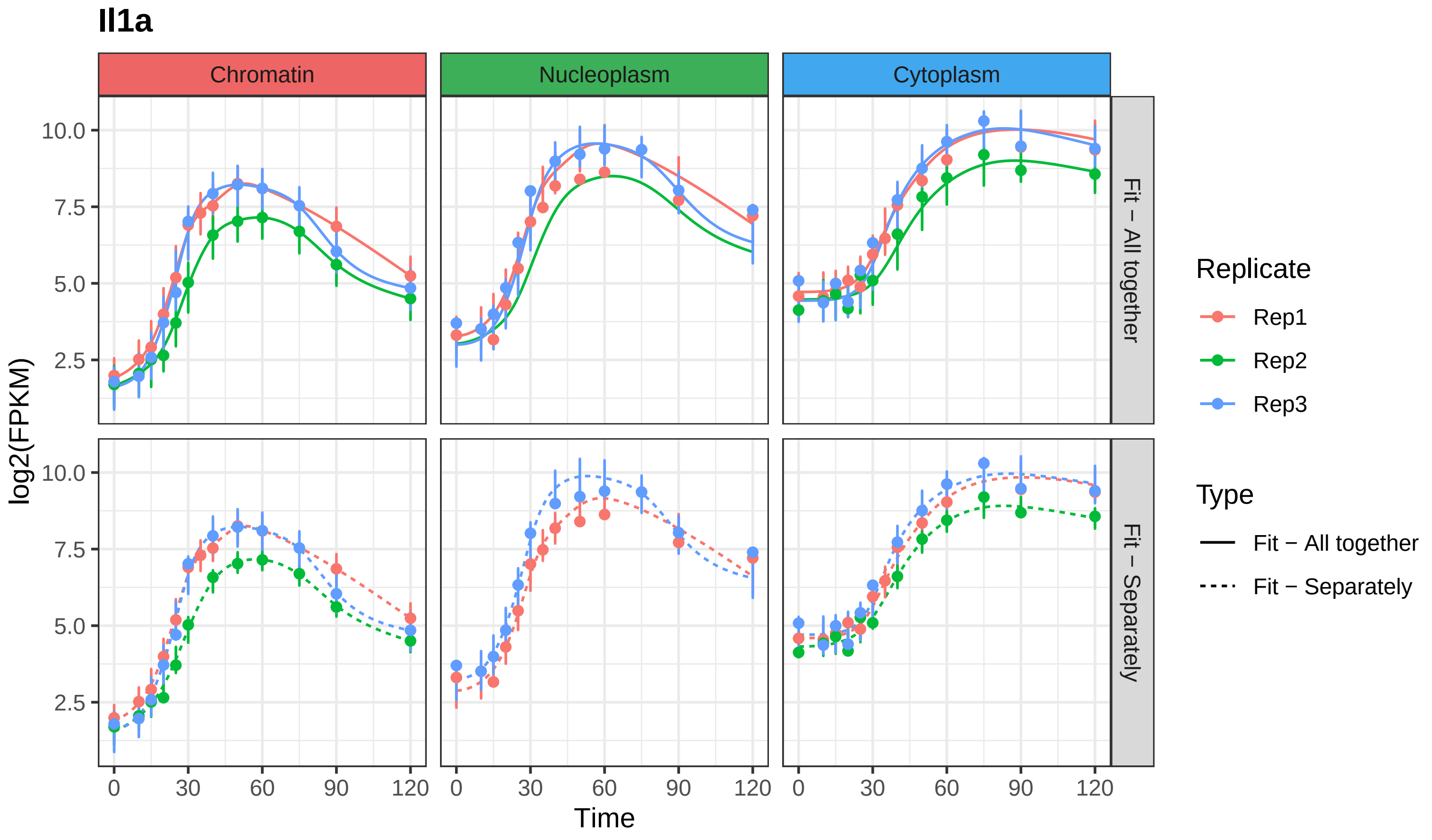

	Together	b1	b2	b3
**−NLL b1 ca**	−0.6166	−3.819		
**−NLL b1 np**	15.11	8.078		
**−NLL b1 cyto**	2.893	−0.89		
**−NLL b2 ca**	−1.022		−4.88	
**−NLL b2 np**				
**−NLL b2 cyto**	2.76		−0.4033	
**−NLL b3 ca**	0.08175			−0.2698
**−NLL b3 np**	12.97			7.502
**−NLL b3 cyto**	6.265			3.255
Total	38.44	3.369	−5.283	10.49
**Total with regul**	43.04	6.22	−5.632	14.12

	Together	b1	b2	b3
spar	0.3012	0.3141000	0.3691	3.129e−01
σ_b	0.2218	0.1718000	0.1143	2.092e−01
σ_t	1.0740	0.0003398	0.6229	3.229e−05
ca_{0,b_1}				
$\log_{10}(k_1')$	−0.2802	−0.4631000		1.254e−01
$\log_{10}(k_2)$	−0.6976	−0.7665000	5.125 or −1.739	−3.767e−01
$\log_{10}(k_2')$	−1.2710	−1.2310000		−1.466e+00
$\log_{10}(k_{deg})$	−1.7030	−1.7460000	−1.739 or 5.125	−1.905e+00
$\log_{10}(k_1'k_2')$	−1.5510	−1.6940000	4.224	−1.341e+00
$\log_{10}(k_1'/k_2)$	0.4174	0.3034000		5.020e−01
$transport = \log_{10}(k_1'k_2'/k_2)$	−0.8538	−0.9271000	−0.9012 or 5.964	−9.642e−01

Il1b

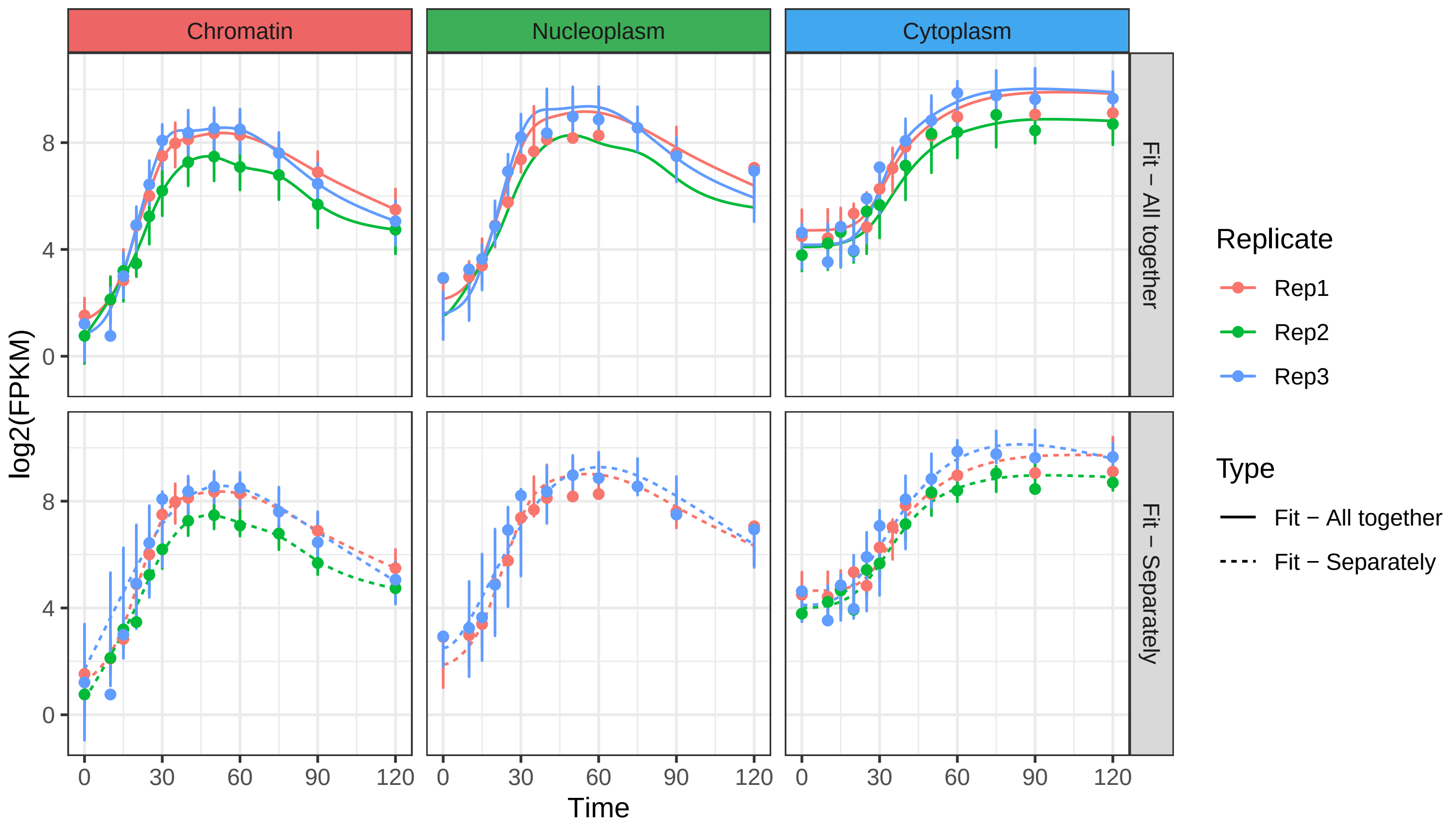

	Together	b1	b2	b3
**−NLL b1 ca**	1.51	0.9846		
**−NLL b1 np**	14.27	12.73		
**−NLL b1 cyto**	6.744	5.875		
**−NLL b2 ca**	1.457		−1.471	
**−NLL b2 np**				
**−NLL b2 cyto**	5.71		4.605	
**−NLL b3 ca**	3.613			10.75
**−NLL b3 np**	16			10.09
**−NLL b3 cyto**	9.359			8.578
Total	58.67	19.59	3.134	29.42
**Total with regul**	66.19	23.63	2.868	32.56

	Together	b1	b2	b3
spar	0.2781000	3.240e−01	0.3914	0.5737
σ_b	0.2806000	2.524e−01	0.1645	0.2017
σ_t	0.0002619	2.991e−05	0.7707	4.2670
ca_{0,b_1}				
$\log_{10}(k_1')$	−0.0668500	−3.646e−01		−0.6478
$\log_{10}(k_2)$	−0.3094000	−5.667e−01	6.42 or −2.135	−0.8920
$\log_{10}(k_2')$	−1.3760000	−1.410e+00		−1.1150
$\log_{10}(k_{deg})$	−2.1470000	−2.241e+00	−2.135 or 6.42	−1.5980
$\log_{10}(k_1'k_2')$	−1.4430000	−1.775e+00	5.32	−1.7620
$\log_{10}(k_1'/k_2)$	0.2426000	2.021e−01		0.2442
transport $= \log_{10}(k_1'k_2'/k_2)$	−1.1330000	−1.208e+00	−1.1 or 7.455	−0.8704

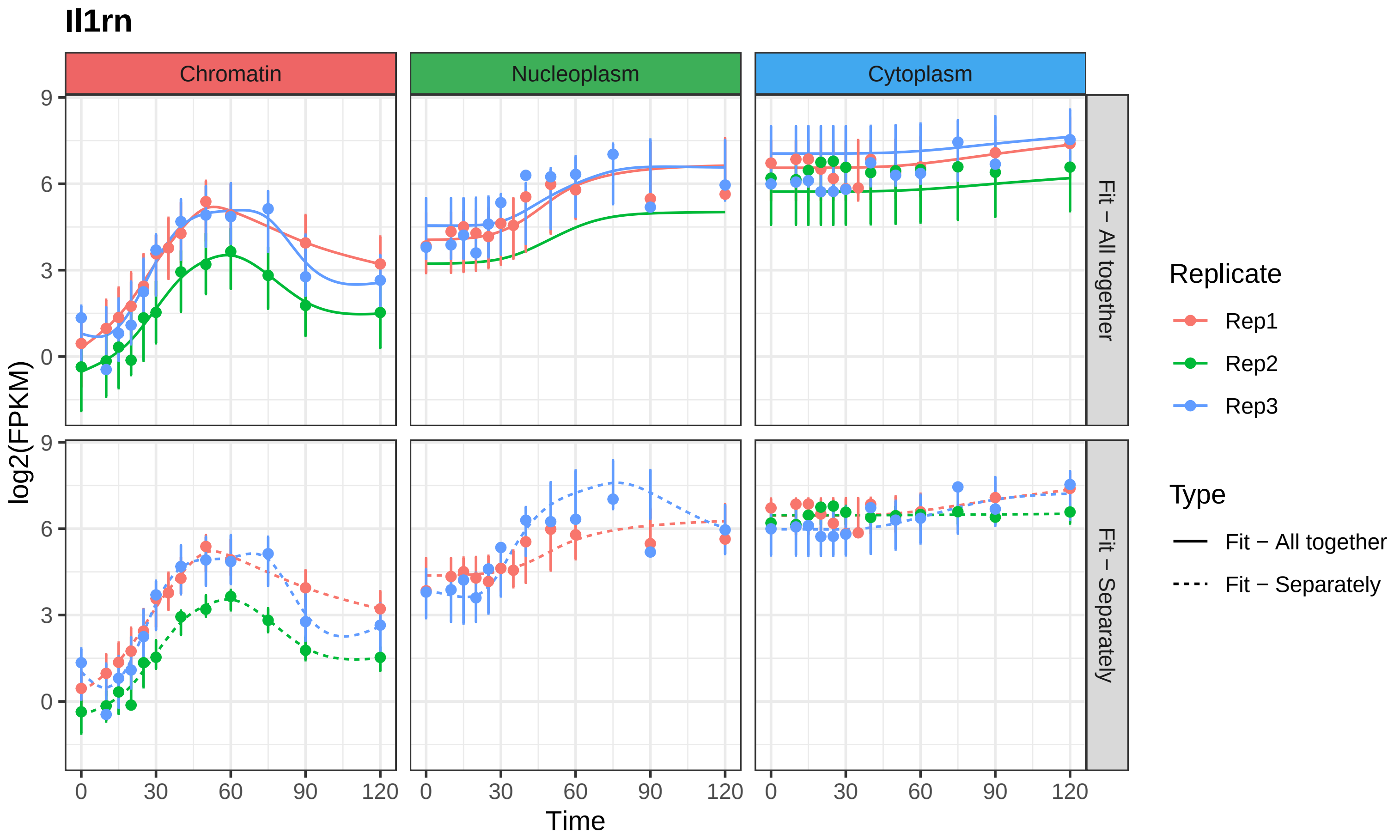

	Together	b1	b2	b3
**−NLL b1 ca**	4.203	−0.8159		
**−NLL b1 np**	8.847	9.491		
**−NLL b1 cyto**	6.186	3.936		
**−NLL b2 ca**	4.948		−0.8293	
**−NLL b2 np**				
**−NLL b2 cyto**	13.62		−2.604	
**−NLL b3 ca**	8.009			4.157
**−NLL b3 np**	16.14			16.17
**−NLL b3 cyto**	20.23			3.25
Total	82.18	12.61	−3.433	23.58
**Total with regul**	85.97	12.89	−4.746	26.67

	Together	b1	b2	b3
spar	4.308e−01	4.135e−01	0.4092	0.3681000
σ_b	3.473e−01	2.161e−01	0.1134	0.2852000
σ_t	1.758e−05	2.713e−06	1.216	0.0002982
ca_{0,b_1}				
$\log_{10}(k_1')$	−1.245e+00	−1.459e+00		−0.4885000
$\log_{10}(k_2)$	−2.375e+00	−2.676e+00	−3.006 or −3.062	−1.3270000
$\log_{10}(k_2')$	−1.807e+00	−1.526e+00		−2.0110000
$\log_{10}(k_{deg})$	−2.560e+00	−2.151e+00	−3.062 or −3.006	−2.6630000
$\log_{10}(k_1' k_2')$	−3.052e+00	−2.985e+00	−3.974	−2.4990000
$\log_{10}(k_1'/k_2)$	1.130e+00	1.217e+00		0.8388000
transport $= \log_{10}(k_1' k_2'/k_2)$	−6.769e−01	−3.091e−01	−0.9679 or −0.9124	−1.1720000

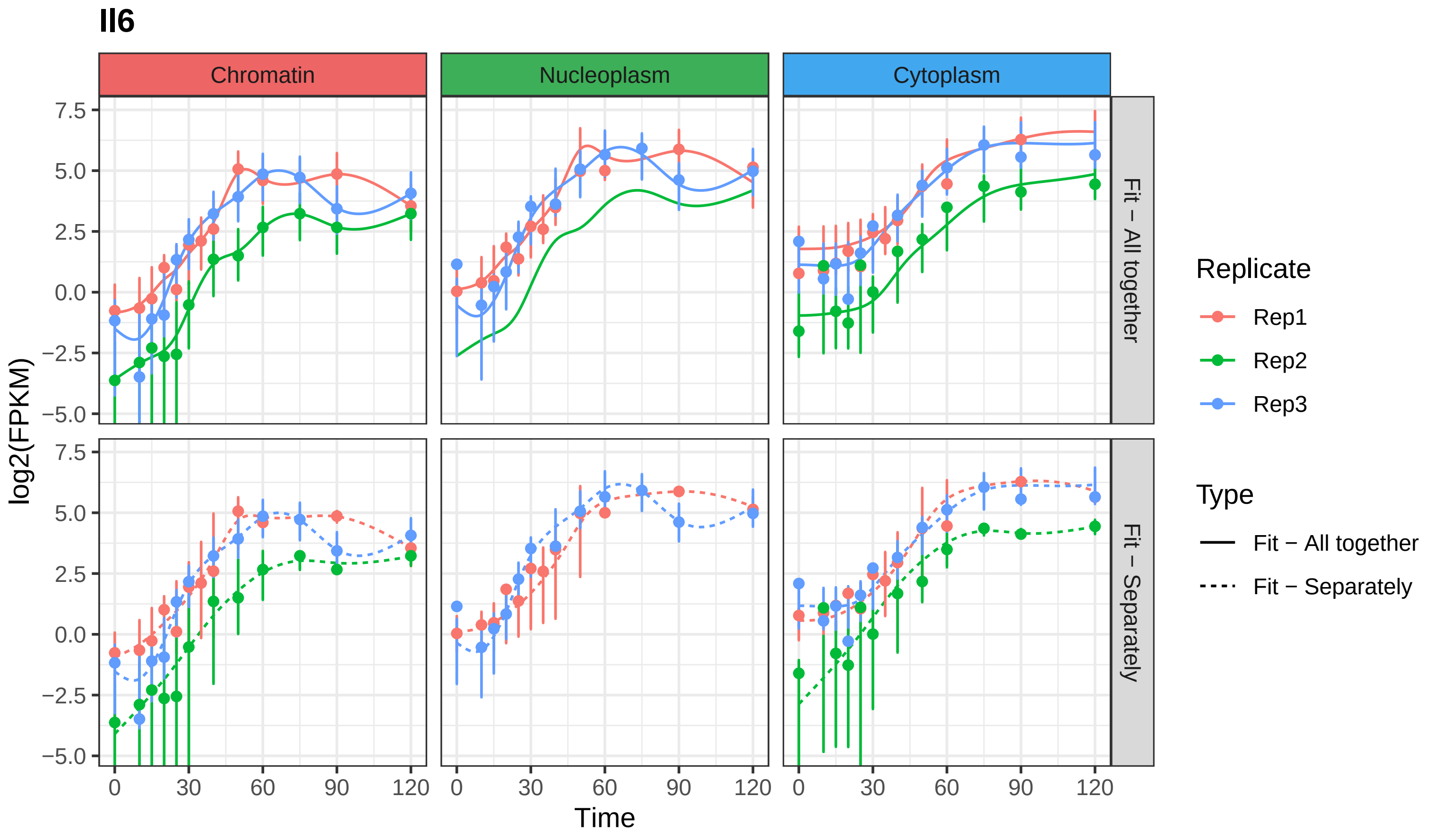

	Together	b1	b2	b3
**−NLL b1 ca**	5.93	6.266		
**−NLL b1 np**	8.769	6.026		
**−NLL b1 cyto**	13.57	6.871		
**−NLL b2 ca**	7.32		11.3	
**−NLL b2 np**				
**−NLL b2 cyto**	23.2		15.14	
**−NLL b3 ca**	6.511			5.501
**−NLL b3 np**	9.986			9.829
**−NLL b3 cyto**	11.27			12.22
**Total**	86.56	19.16	26.43	27.55
**Total with regul**	92.89	17.72	26.52	31.63

	Together	b1	b2	b3
spar	0.312500	4.120e−01	0.5295	0.3234000
σ_b	0.312100	1.159e−05	1.343e−05	0.2528000
σ_t	0.001108	5.418e+00	7.563	0.0005948
ca_{0,b_1}				
$\log_{10}(k_1')$	5.551000	−7.729e−01		4.5470000
$\log_{10}(k_2)$	5.261000	−1.092e+00	3.703 or 3.627	4.1920000
$\log_{10}(k_2')$	−1.357000	−5.832e−01		−1.4360000
$\log_{10}(k_{deg})$	−1.858000	−7.190e−01	3.627 or 3.703	−1.8950000
$\log_{10}(k_1'k_2')$	4.194000	−1.356e+00	7.7	3.1110000
$\log_{10}(k_1'/k_2)$	0.289700	3.195e−01		0.3554000
$\text{transport} = \log_{10}(k_1'k_2'/k_2)$	−1.067000	−2.637e−01	3.996 or 4.073	−1.0810000

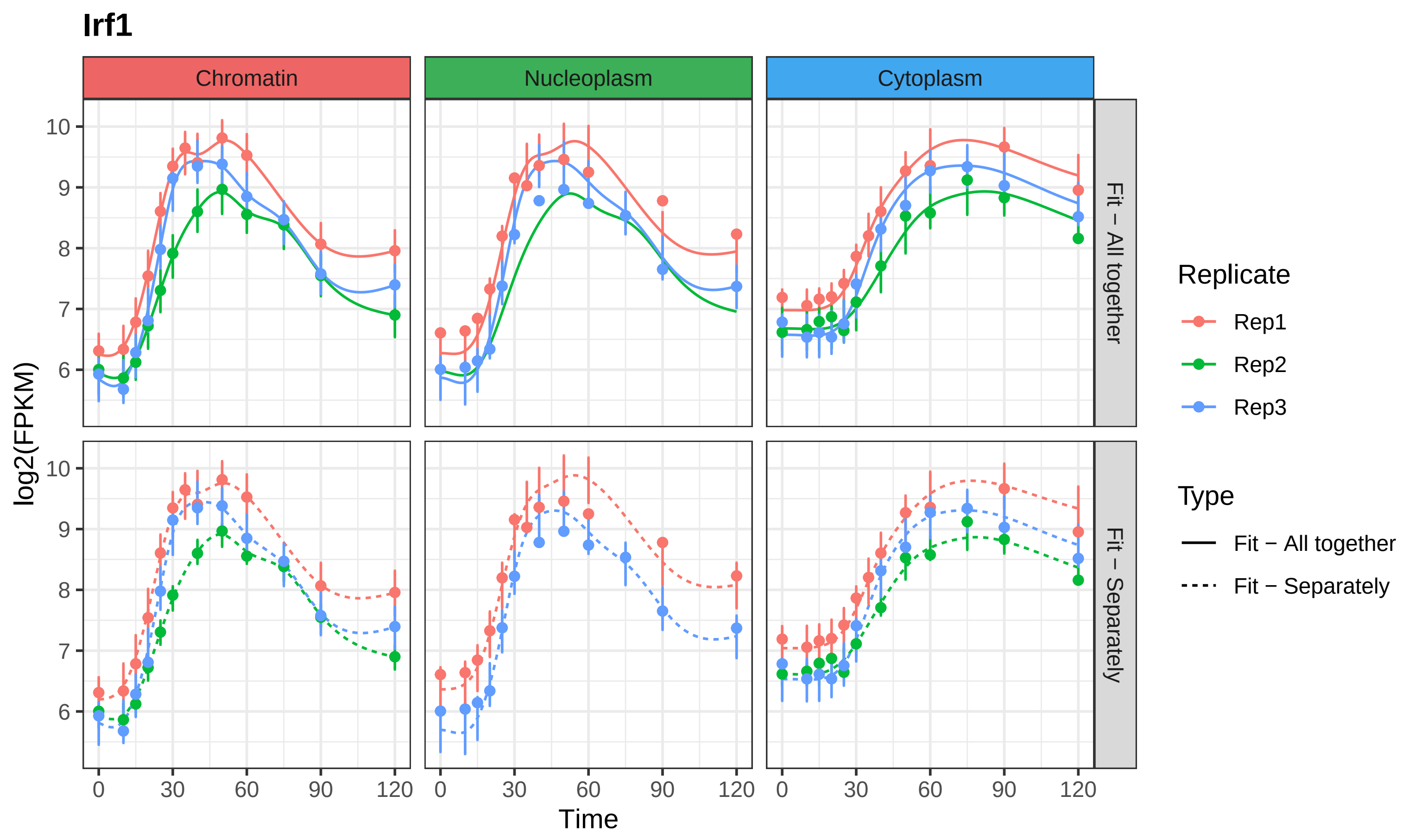

	Together	b1	b2	b3
**−NLL b1 ca**	−9.037	−7.429		
**−NLL b1 np**	9.435	4.741		
**−NLL b1 cyto**	−5.801	−5.231		
**−NLL b2 ca**	−9.417		−13.39	
**−NLL b2 np**				
**−NLL b2 cyto**	−4.962		−5.65	
**−NLL b3 ca**	−8.017			−6.463
**−NLL b3 np**	4.098			1.849
**−NLL b3 cyto**	−5.626			−4.891
Total	−29.33	−7.919	−19.04	−9.506
**Total with regul**	−26.23	−7.002	−17.94	−7.754

	Together	b1	b2	b3
spar	2.970e−01	0.3407000	0.3238	0.3208000
σ_b	1.212e−01	0.1300000	0.06855	0.1211000
σ_t	5.057e−05	0.0003805	0.0001888	0.0000595
ca_{0,b_1}				
$\log_{10}(k_1')$	−5.699e−01	−0.6155000		−0.5970000
$\log_{10}(k_2)$	−5.767e−01	−0.6653000	1.286 or −1.611	−0.5618000
$\log_{10}(k_2')$	−1.371e+00	−1.4440000		−1.3600000
$\log_{10}(k_{deg})$	−1.584e+00	−1.6480000	−1.611 or 1.286	−1.6110000
$\log_{10}(k_1'k_2')$	−1.941e+00	−2.0600000	−0.1178	−1.9570000
$\log_{10}(k_1'/k_2)$	6.884e−03	0.0498800		−0.0352200
$\text{transport} = \log_{10}(k_1'k_2'/k_2)$	−1.364e+00	−1.3940000	−1.403 or 1.493	−1.3950000

Irs2

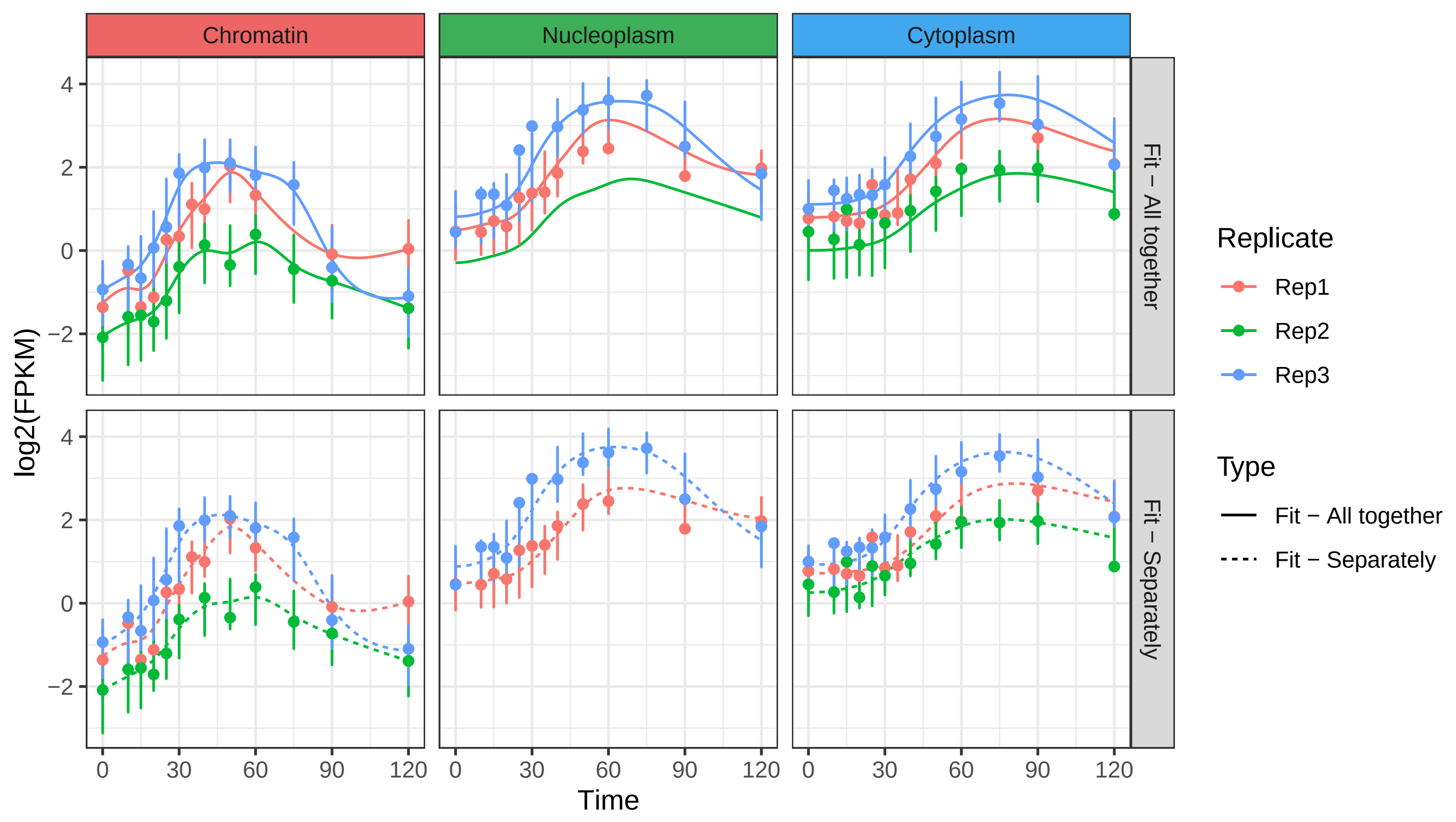

	Together	b1	b2	b3
**−NLL b1 ca**	2.666	2.296		
**−NLL b1 np**	4.443	2.061		
**−NLL b1 cyto**	6.355	4.312		
**−NLL b2 ca**	1.216		1.673	
**−NLL b2 np**				
**−NLL b2 cyto**	9.46		4.221	
**−NLL b3 ca**	0.9678			1.292
**−NLL b3 np**	7.743			5.901
**−NLL b3 cyto**	2.632			2.303
Total	35.48	8.67	5.895	9.496
**Total with regul**	37.18	8.641	5.025	9.063

	Together	b1	b2	b3
spar	0.3525	0.3885000	0.419	0.3994
σ_b	0.2004	0.1751000	0.1585	0.1517
σ_t	2.3160	0.0001939	0.009417	3.3300
ca_{0,b_1}				
$\log_{10}(k_1')$	−0.7091	−0.9935000		−0.6295
$\log_{10}(k_2)$	−1.2380	−1.5180000	4.315 or −1.538	−1.1960
$\log_{10}(k_2')$	−1.1080	−1.0310000		−1.1770
$\log_{10}(k_{deg})$	−1.1980	−1.1040000	−1.538 or 4.315	−1.1910
$\log_{10}(k_1' k_2')$	−1.8170	−2.0250000	3.479	−1.8070
$\log_{10}(k_1'/k_2)$	0.5289	0.5246000		0.5668
transport $= \log_{10}(k_1' k_2'/k_2)$	−0.5793	−0.5066000	−0.8355 or 5.017	−0.6103

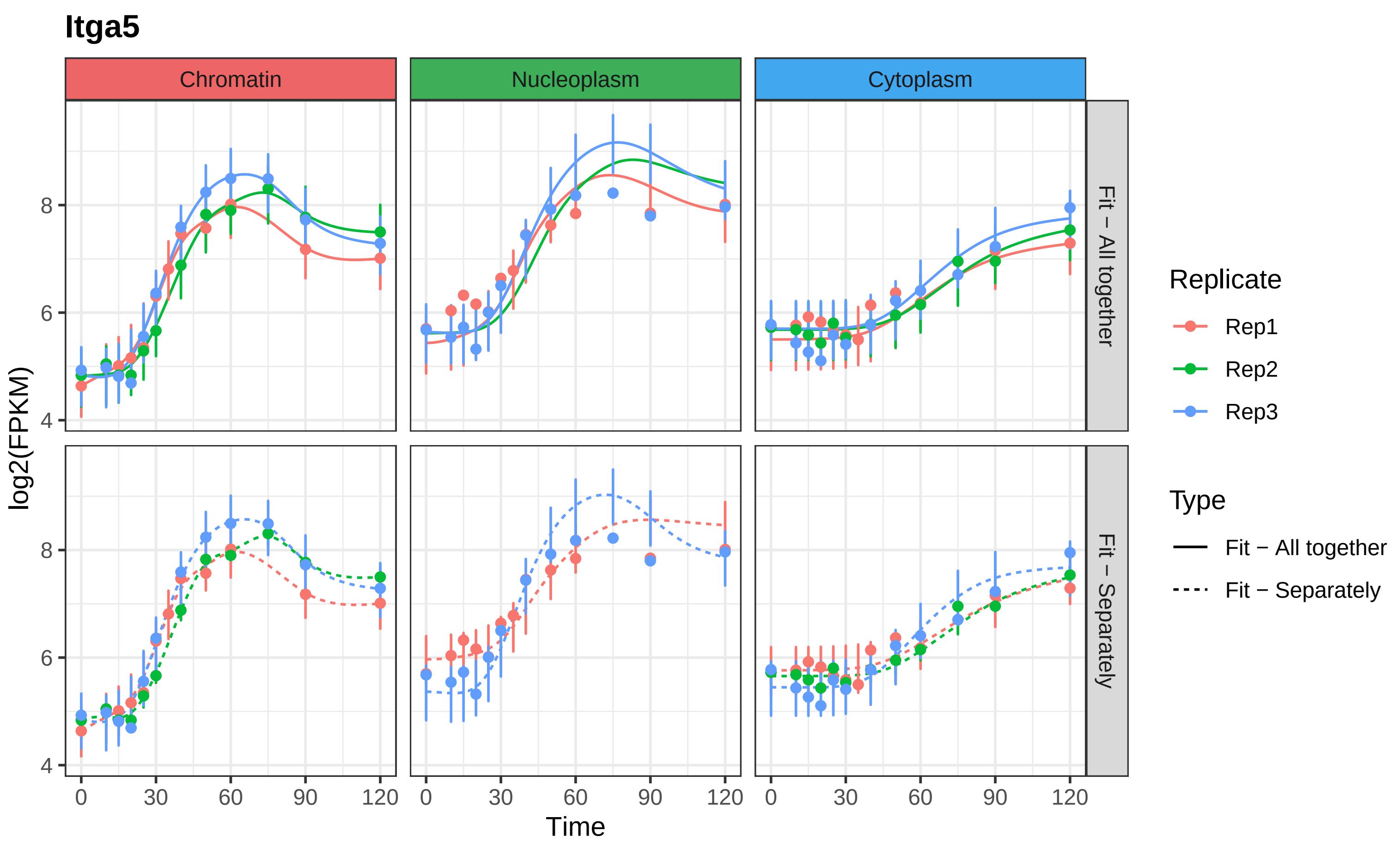

	Together	b1	b2	b3
**−NLL b1 ca**	−3.186	−4.876		
**−NLL b1 np**	9.085	6.288		
**−NLL b1 cyto**	1.135	−2.466		
**−NLL b2 ca**	−3.556		−12.71	
**−NLL b2 np**				
**−NLL b2 cyto**	−3.063		−8.985	
**−NLL b3 ca**	−2.564			−2.694
**−NLL b3 np**	13.99			7.353
**−NLL b3 cyto**	1.888			−1.22
Total	13.73	−1.055	−21.7	3.44
**Total with regul**	13.55	−1.721	−21.43	3.091

	Together	b1	b2	b3
spar	4.046e−01	0.4023000	0.3399	4.022e−01
σ_b	1.859e−01	0.1548000	0.05745	1.740e−01
σ_t	2.927e−07	0.0002059	2.316e−05	6.068e−06
ca_{0,b_1}				
$\log_{10}(k_1')$	−9.162e−01	−1.2680000		−6.917e−01
$\log_{10}(k_2)$	−1.156e+00	−1.6670000	−1.276 or −2.143	−8.482e−01
$\log_{10}(k_2')$	−2.161e+00	−2.0910000		−2.083e+00
$\log_{10}(k_{deg})$	−2.180e+00	−2.0300000	−2.143 or −1.276	−2.108e+00
$\log_{10}(k_1' k_2')$	−3.077e+00	−3.3590000	−3.178	−2.775e+00
$\log_{10}(k_1'/k_2)$	2.395e−01	0.3991000		1.566e−01
$\text{transport} = \log_{10}(k_1' k_2'/k_2)$	−1.922e+00	−1.6920000	−1.903 or −1.035	−1.927e+00

Itgav

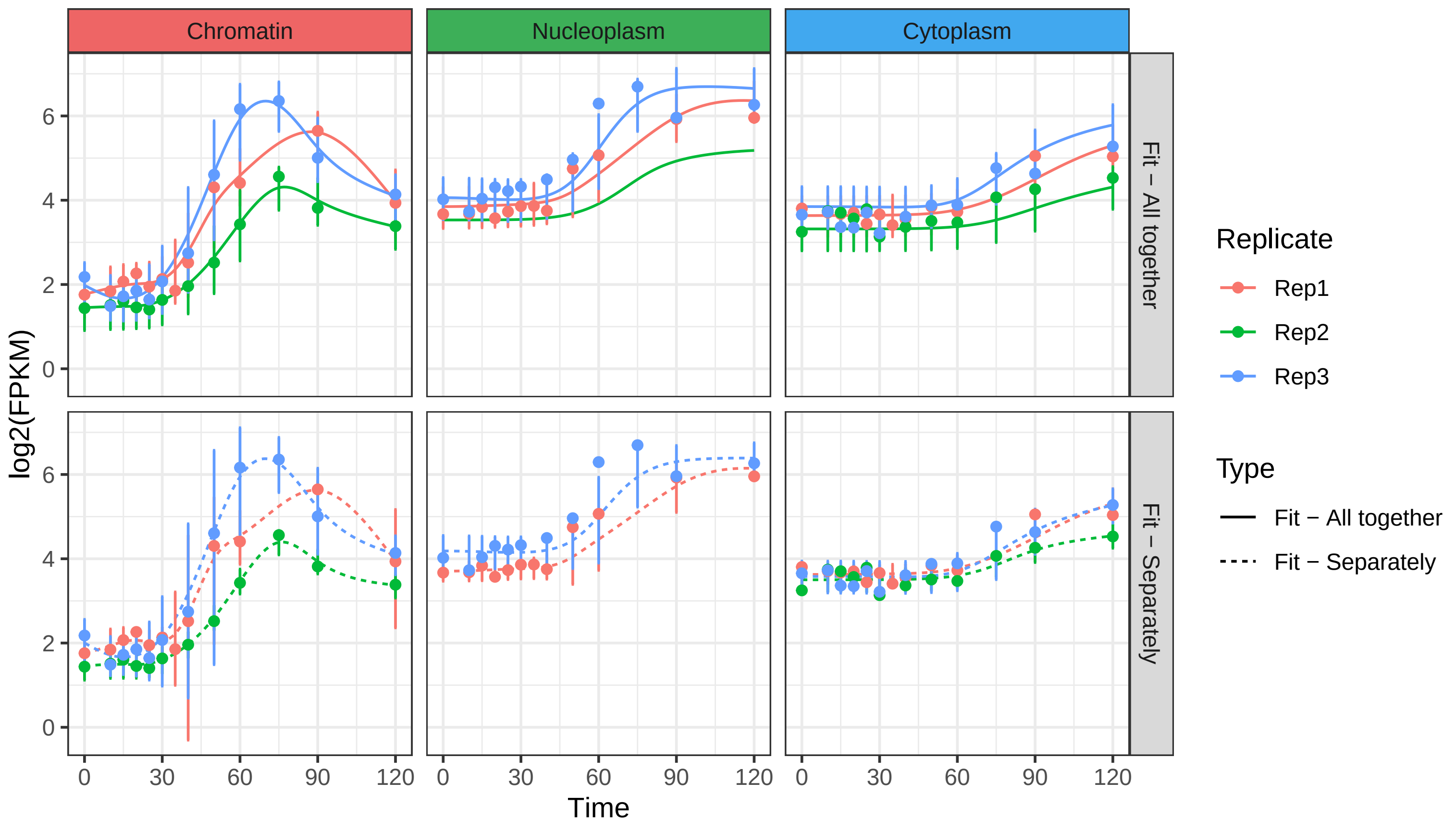

	Together	b1	b2	b3
**−NLL b1 ca**	−0.2694	−1.672		
**−NLL b1 np**	1.256	−3.391		
**−NLL b1 cyto**	−1.424	−3.693		
**−NLL b2 ca**	−2.598		−8.59	
**−NLL b2 np**				
**−NLL b2 cyto**	3.91		−1.331	
**−NLL b3 ca**	0.5251			1.935
**−NLL b3 np**	8.853			5.218
**−NLL b3 cyto**	6.389			−2.466
Total	16.64	−8.756	−9.921	4.686
**Total with regul**	16.13	−9.006	−11.51	4.039

	Together	b1	b2	b3
spar	0.4591	0.39670	0.4145	0.4500
σ_b	0.1711	0.07786	0.09824	0.1309
σ_t	4.0150	7.73600	3.398e−06	6.3820
ca_{0,b_1}				
$\log_{10}(k_1')$	−1.3630	−1.44400		−1.5390
$\log_{10}(k_2)$	−1.9890	−2.03300	−0.9409 or −2.342	−2.1960
$\log_{10}(k_2')$	−1.8680	−1.76100		−1.8820
$\log_{10}(k_{deg})$	−1.8040	−1.73800	−2.342 or −0.9409	−1.7000
$\log_{10}(k_1'k_2')$	−3.2310	−3.20600	−2.667	−3.4210
$\log_{10}(k_1'/k_2)$	0.6261	0.58870		0.6569
transport $= \log_{10}(k_1'k_2'/k_2)$	−1.2420	−1.17300	−1.726 or −0.3253	−1.2250

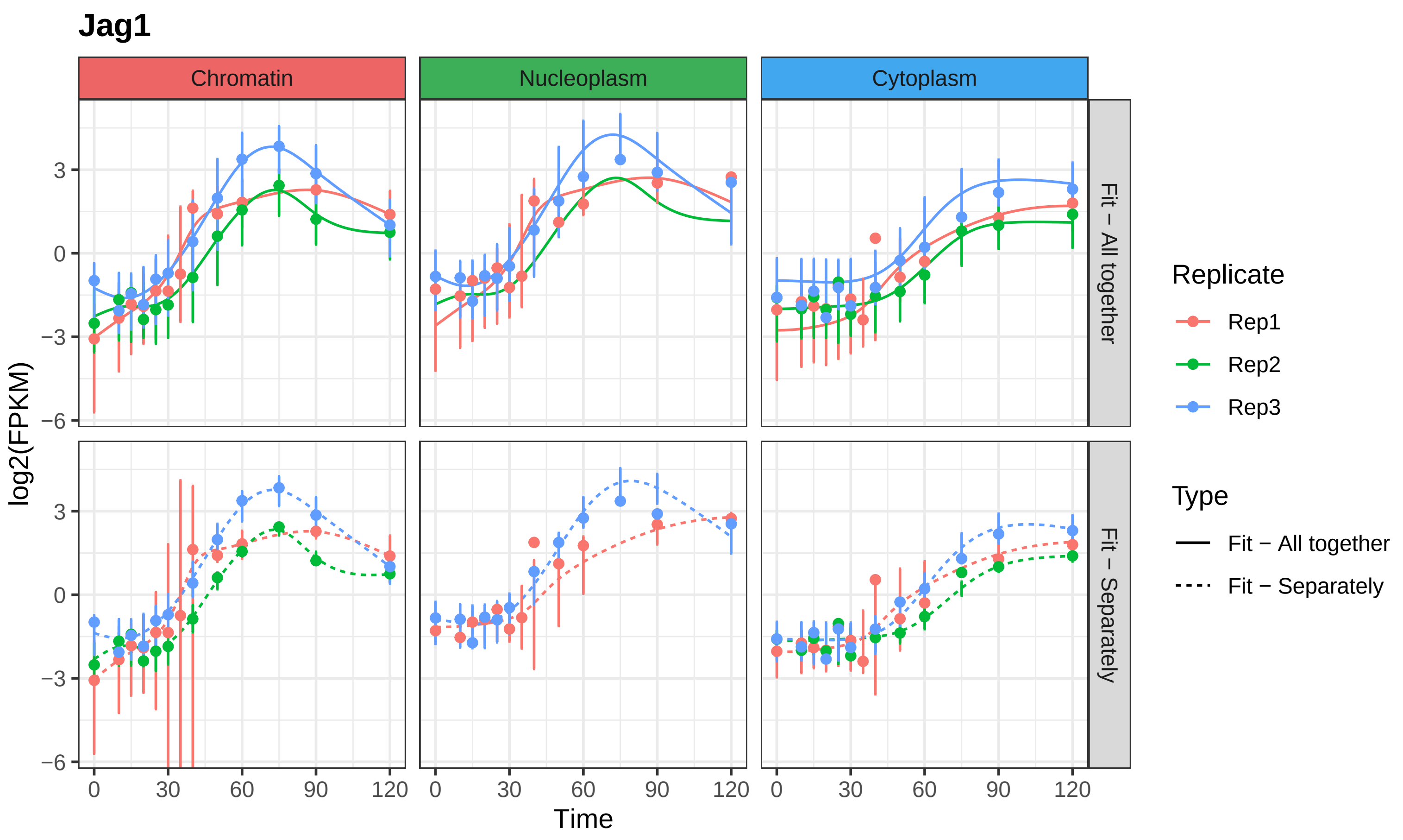

	Together	b1	b2	b3
**−NLL b1 ca**	6.733	7.314		
**−NLL b1 np**	15.33	6.44		
**−NLL b1 cyto**	20.04	8.029		
**−NLL b2 ca**	5.265		−1.419	
**−NLL b2 np**				
**−NLL b2 cyto**	5.059		0.4197	
**−NLL b3 ca**	5.334			0.9117
**−NLL b3 np**	12.31			7.498
**−NLL b3 cyto**	14.05			3.13
Total	84.12	21.78	−0.9995	11.54
**Total with regul**	86	21.79	−2.55	10.92

	Together	b1	b2	b3
spar	0.4297	3.913e−01	0.388	4.700e−01
σ_b	0.2752	2.577e−05	8.084e−08	1.801e−01
σ_t	4.0740	9.048e+00	6.255e−07	1.951e−05
ca_{0,b_1}				
$\log_{10}(k_1')$	6.1420	−1.554e+00		−7.450e−01
$\log_{10}(k_2)$	6.0110	−2.114e+00	−1.109 or −1.892	−8.853e−01
$\log_{10}(k_2')$	−1.9050	5.172e+00		−1.828e+00
$\log_{10}(k_{deg})$	−1.8550	5.440e+00	−1.892 or −1.109	−1.622e+00
$\log_{10}(k_1'k_2')$	4.2370	3.618e+00	−2.805	−2.573e+00
$\log_{10}(k_1'/k_2)$	0.1311	5.605e−01		1.403e−01
$\text{transport} = \log_{10}(k_1'k_2'/k_2)$	−1.7740	5.732e+00	−1.696 or −0.9132	−1.687e+00

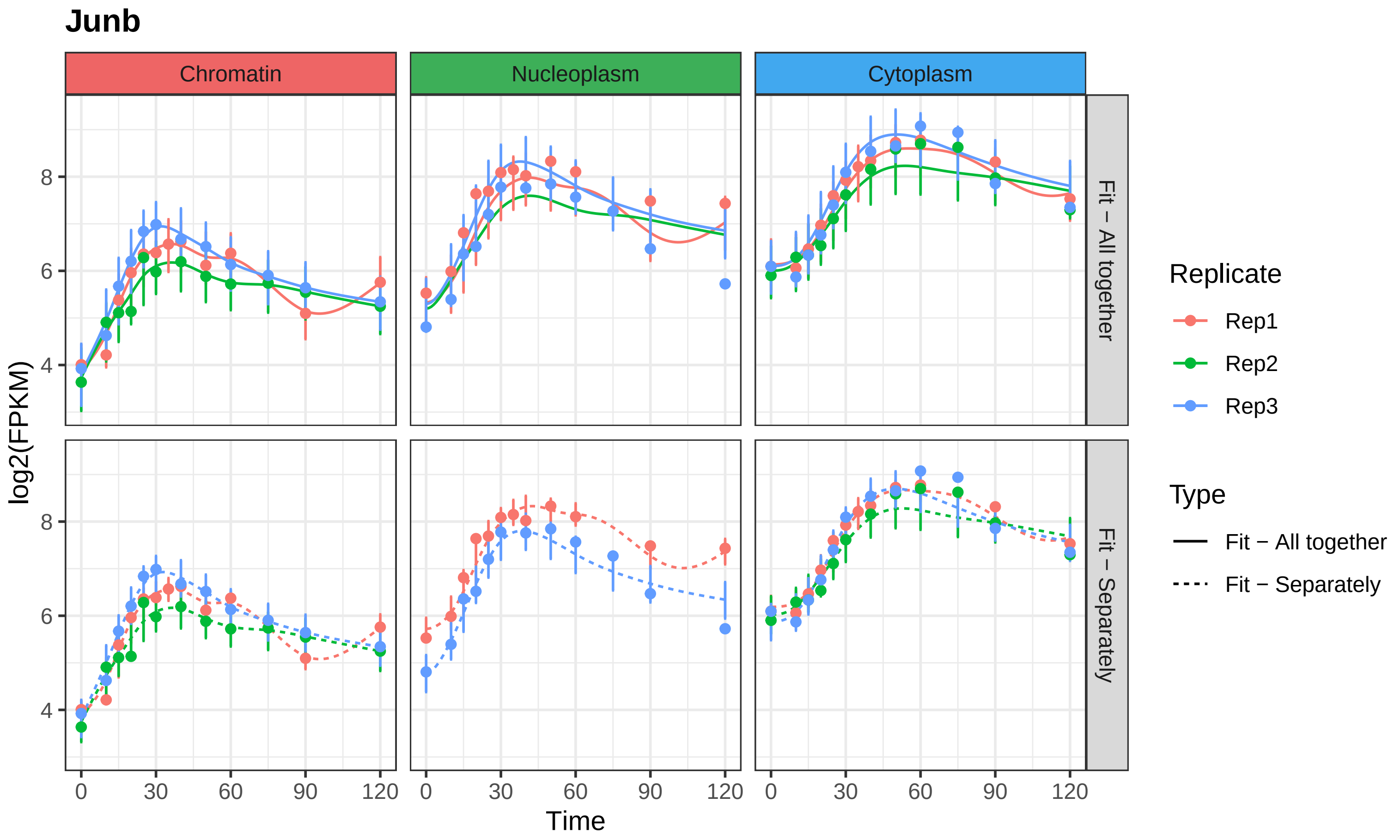

	Together	b1	b2	b3
**−NLL b1 ca**	−1.893	−7.423		
**−NLL b1 np**	9.009	−2.027		
**−NLL b1 cyto**	−2.138	−6.498		
**−NLL b2 ca**	−1.63		−2.737	
**−NLL b2 np**				
**−NLL b2 cyto**	2.266		0.1453	
**−NLL b3 ca**	−2.455			−5.203
**−NLL b3 np**	16.18			0.9727
**−NLL b3 cyto**	3.038			−1.761
Total	22.37	−15.95	−2.592	−5.991
**Total with regul**	23.03	−16.34	−3.491	−7.059

	Together	b1	b2	b3
spar	0.3769	0.36280	0.4028	4.077e−01
σ_b	0.1925	0.08184	0.1395	1.336e−01
σ_t	1.5720	1.87000	0.02796	9.198e−06
ca_{0,b_1}				
$\log_{10}(k_1')$	−0.2804	−0.28490		−4.290e−01
$\log_{10}(k_2)$	−0.7221	−0.84170	−0.8771 or −0.9006	−7.160e−01
$\log_{10}(k_2')$	−0.8452	−0.83800		−7.193e−01
$\log_{10}(k_{deg})$	−1.0880	−0.97840	−0.9006 or −0.8771	−1.048e+00
$\log_{10}(k_1'k_2')$	−1.1260	−1.12300	−1.092	−1.148e+00
$\log_{10}(k_1'/k_2)$	0.4416	0.55680		2.870e−01
$\text{transport} = \log_{10}(k_1'k_2'/k_2)$	−0.4036	−0.28120	−0.2148 or −0.1913	−4.323e−01

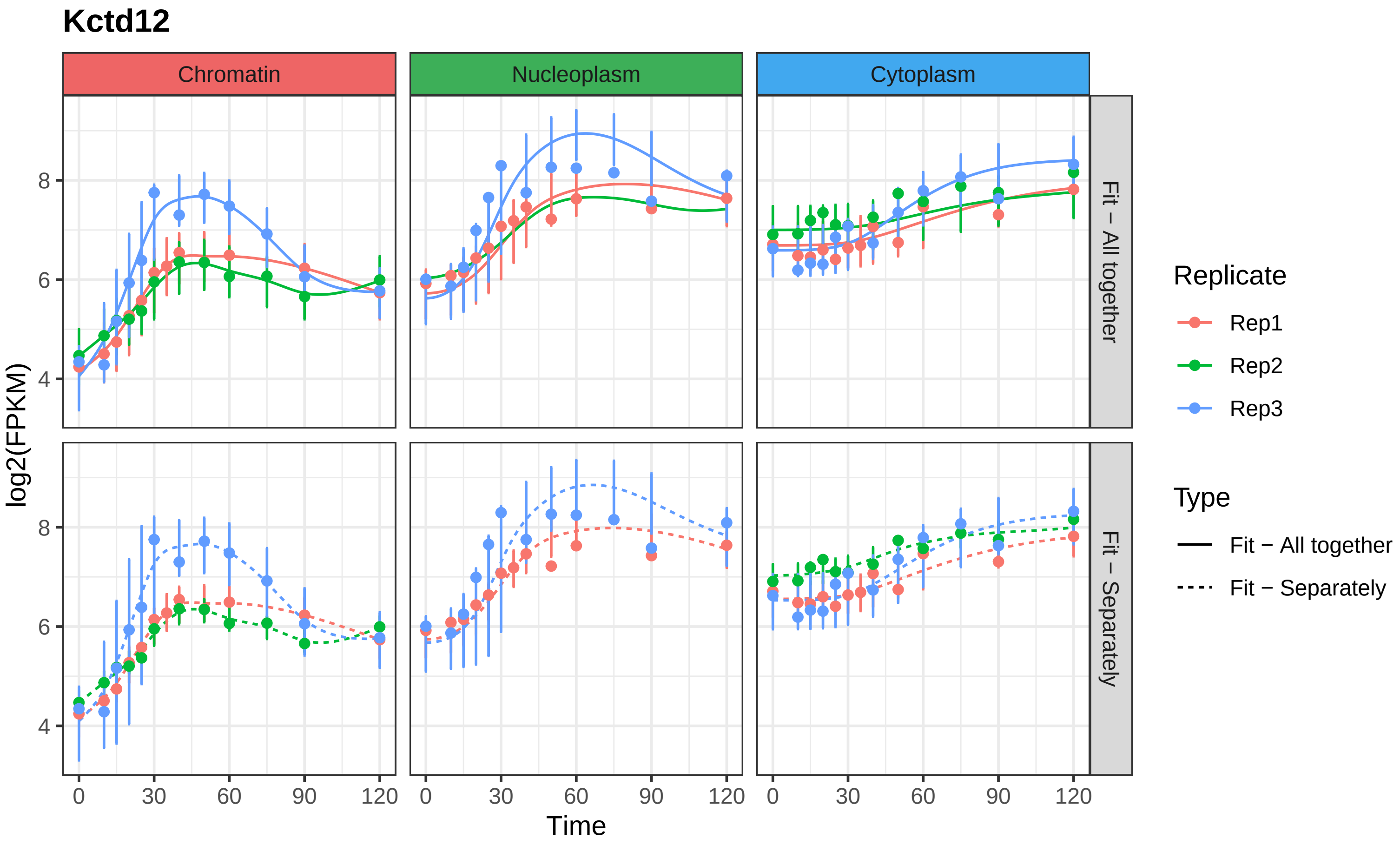

	Together	b1	b2	b3
**−NLL b1 ca**	−3.203	−8.1		
**−NLL b1 np**	1.59	2.804		
**−NLL b1 cyto**	−1.083	−3.308		
**−NLL b2 ca**	−3.721		−10.52	
**−NLL b2 np**				
**−NLL b2 cyto**	0.9672		−4.938	
**−NLL b3 ca**	1.481			2.934
**−NLL b3 np**	18.14			10.98
**−NLL b3 cyto**	2.026			1.563
Total	16.2	−8.604	−15.46	15.48
**Total with regul**	15.71	−9.893	−16.91	15.95

	Together	b1	b2	b3
spar	0.4250	4.174e−01	0.398	0.4004
σ_b	0.1731	1.278e−01	0.08092	0.1923
σ_t	3.1660	2.382e−06	0.001903	4.7400
ca_{0,b_1}				
$\log_{10}(k_1')$	−0.8198	−6.856e−01		−0.9232
$\log_{10}(k_2)$	−1.2930	−1.162e+00	3.679 or −2.132	−1.4000
$\log_{10}(k_2')$	−1.9620	−1.972e+00		−2.0100
$\log_{10}(k_{deg})$	−2.2520	−2.218e+00	−2.132 or 3.679	−2.2670
$\log_{10}(k_1' k_2')$	−2.7820	−2.657e+00	2.318	−2.9340
$\log_{10}(k_1'/k_2)$	0.4734	4.763e−01		0.4764
transport $= \log_{10}(k_1' k_2'/k_2)$	−1.4880	−1.495e+00	−1.362 or 4.45	−1.5340

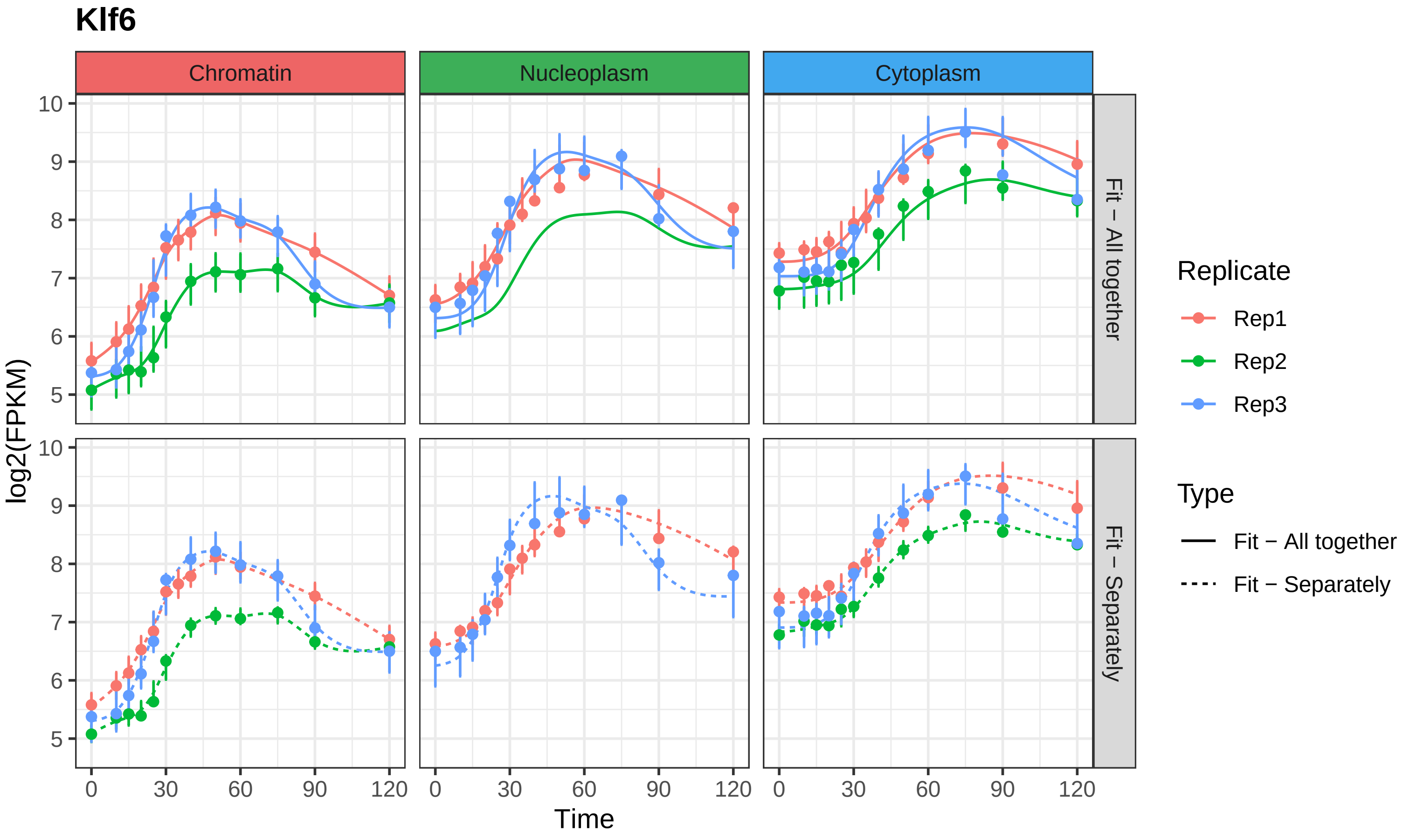

	Together	b1	b2	b3
**−NLL b1 ca**	−9.147	−12.87		
**−NLL b1 np**	0.2148	−5.519		
**−NLL b1 cyto**	−5.411	−6.939		
**−NLL b2 ca**	−9.067		−14.49	
**−NLL b2 np**				
**−NLL b2 cyto**	−4.666		−11.69	
**−NLL b3 ca**	−8.017			−7.105
**−NLL b3 np**	2.88			−1.104
**−NLL b3 cyto**	3.092			−1.7
Total	−30.12	−25.33	−26.18	−9.91
**Total with regul**	−29.55	−26.03	−25.91	−9.59

	Together	b1	b2	b3
spar	0.3450	3.698e−01	0.3389	0.3522000
σ_b	0.1138	8.164e−02	0.04515	0.1203000
σ_t	1.2930	5.527e−05	0.9227	0.0001342
ca_{0,b_1}				
$\log_{10}(k_1')$	−0.4367	−7.587e−01		1.0440000
$\log_{10}(k_2)$	−0.7389	−1.072e+00	4.78 or −1.332	0.7577000
$\log_{10}(k_2')$	−1.1020	−1.085e+00		−1.2480000
$\log_{10}(k_{deg})$	−1.3180	−1.310e+00	−1.332 or 4.78	−1.4450000
$\log_{10}(k_1'k_2')$	−1.5380	−1.844e+00	3.973	−0.2039000
$\log_{10}(k_1'/k_2)$	0.3022	3.133e−01		0.2860000
$\text{transport} = \log_{10}(k_1'k_2'/k_2)$	−0.7996	−7.720e−01	−0.8069 or 5.305	−0.9616000

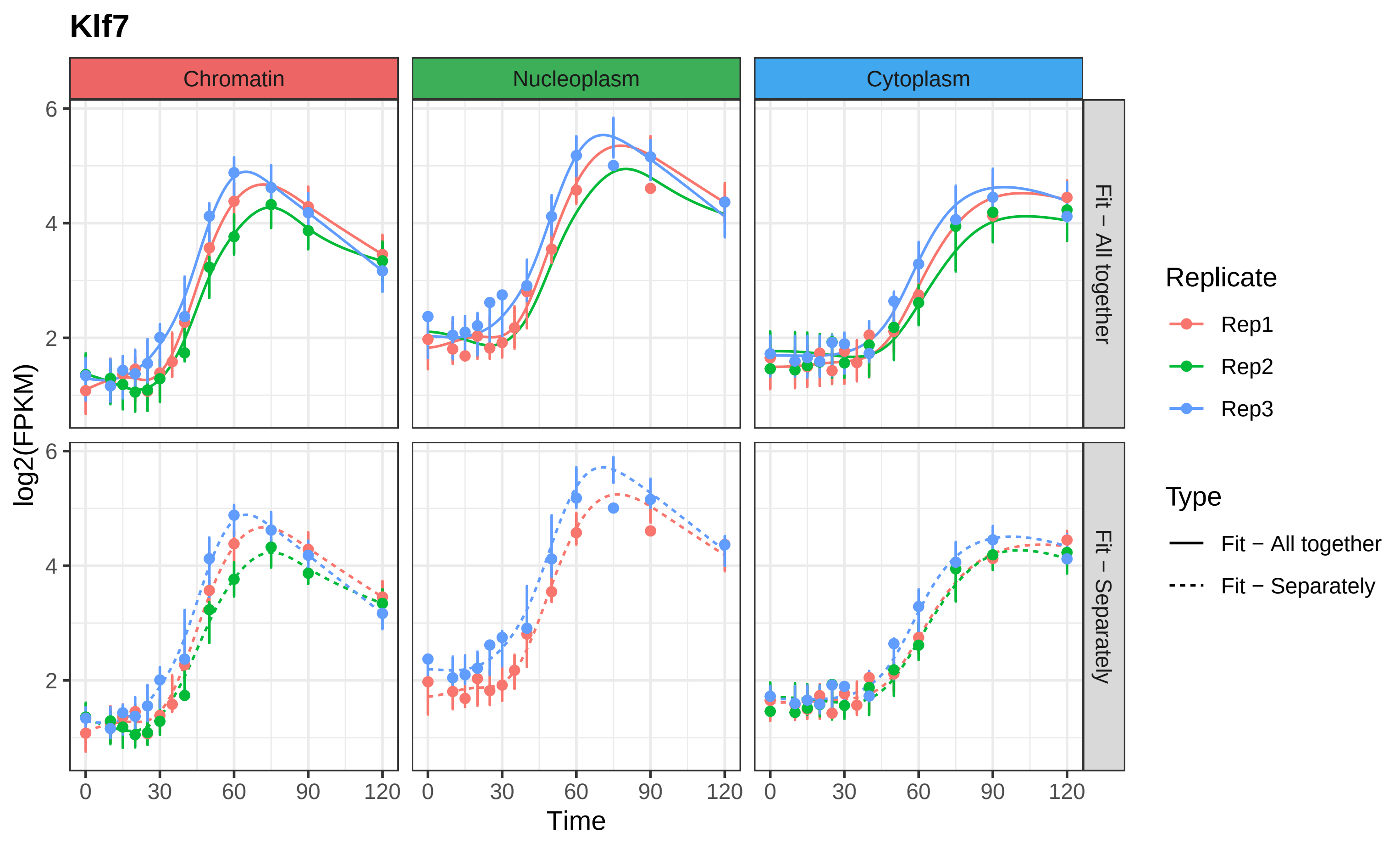

	Together	b1	b2	b3
**−NLL b1 ca**	−7.684	−8.425		
**−NLL b1 np**	−0.223	−1.787		
**−NLL b1 cyto**	−3.733	−7.14		
**−NLL b2 ca**	−7.808		−7.017	
**−NLL b2 np**				
**−NLL b2 cyto**	0.6615		−3.082	
**−NLL b3 ca**	−6.609			−7.52
**−NLL b3 np**	2.474			−3.419
**−NLL b3 cyto**	−4.25			−5.017
**Total**	−27.17	−17.35	−10.1	−15.96
**Total with regul**	−27.9	−19.16	−12.01	−17.33

	Together	b1	b2	b3
spar	3.837e−01	0.431400	0.4538	0.39570
σ_b	1.194e−01	0.093650	0.0859	0.07443
σ_t	8.757e−06	0.002362	1.315	1.98300
ca_{0,b_1}				
$\log_{10}(k_1')$	−5.460e−01	−0.447300		−0.45020
$\log_{10}(k_2)$	−7.674e−01	−0.630300	−1.003 or −1.204	−0.72260
$\log_{10}(k_2')$	−1.576e+00	−1.723000		−1.72200
$\log_{10}(k_{deg})$	−1.474e+00	−1.691000	−1.204 or −1.003	−1.56600
$\log_{10}(k_1'k_2')$	−2.121e+00	−2.170000	−2.096	−2.17200
$\log_{10}(k_1'/k_2)$	2.214e−01	0.183000		0.27240
$transport = \log_{10}(k_1'k_2'/k_2)$	−1.354e+00	−1.540000	−1.093 or −0.8914	−1.45000

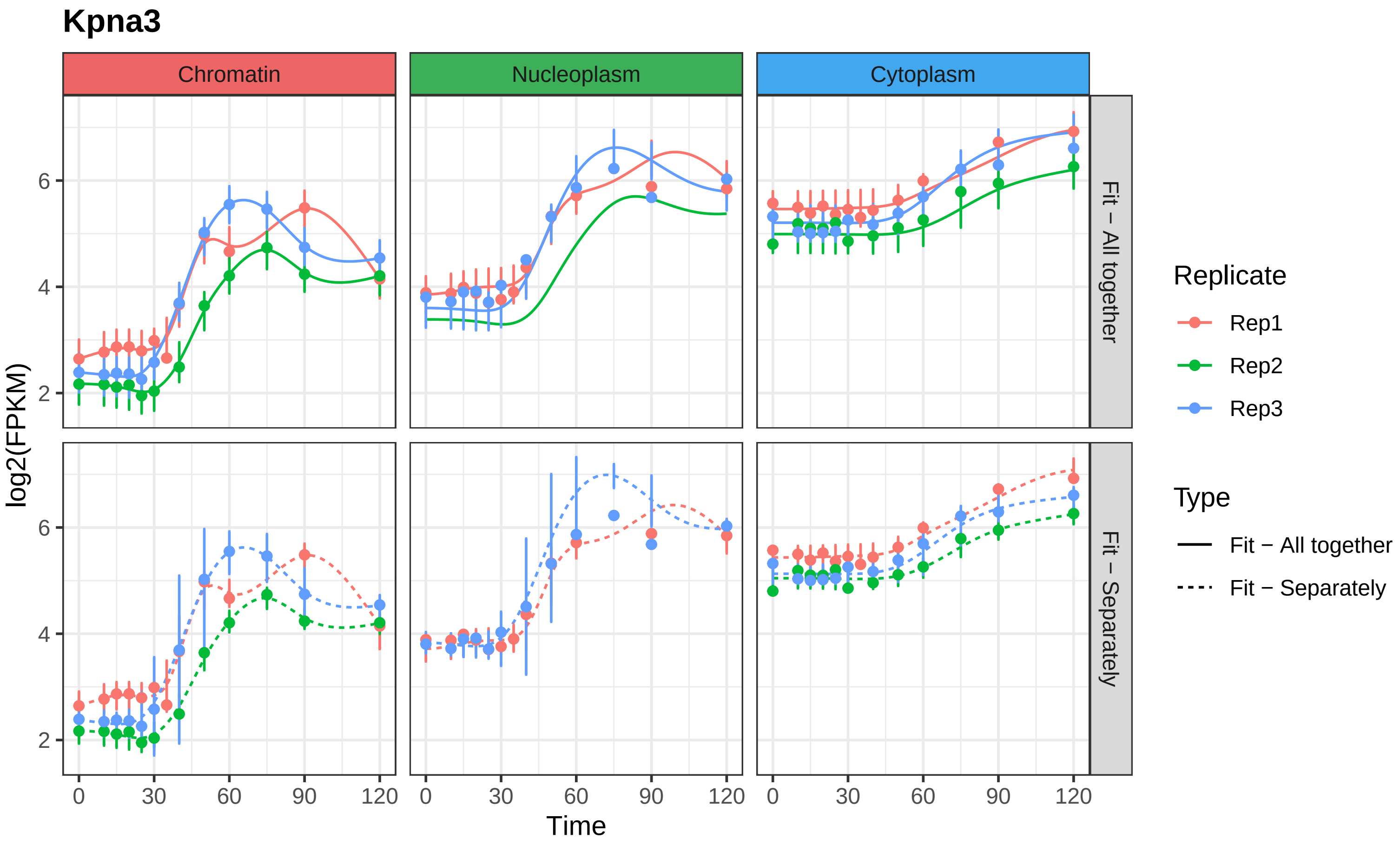

	Together	b1	b2	b3
**−NLL b1 ca**	−6.175	−8.318		
**−NLL b1 np**	−1.235	−2.615		
**−NLL b1 cyto**	−6.695	−9.828		
**−NLL b2 ca**	−8.656		−10.91	
**−NLL b2 np**				
**−NLL b2 cyto**	−4.981		−7.676	
**−NLL b3 ca**	−8.86			−5.6
**−NLL b3 np**	11.59			2.378
**−NLL b3 cyto**	−3.946			−8.037
Total	−28.96	−20.76	−18.58	−11.26
**Total with regul**	−28.41	−20.14	−19.99	−12.08

	Together	b1	b2	b3
spar	3.462e−01	0.33730	0.3904	0.39310
σ_b	1.190e−01	0.07447	0.06319	0.06151
σ_t	4.909e−06	2.53100	0.0003621	5.73700
ca_{0,b_1}				
$\log_{10}(k_1')$	−7.200e−01	−0.69730		−0.36570
$\log_{10}(k_2)$	−1.084e+00	−1.02000	−0.6317 or −2.141	−0.80240
$\log_{10}(k_2')$	−1.606e+00	−1.48800		−1.87800
$\log_{10}(k_{deg})$	−2.090e+00	−2.00500	−2.141 or −0.6317	−2.26800
$\log_{10}(k_1'k_2')$	−2.326e+00	−2.18500	−1.91	−2.24400
$\log_{10}(k_1'/k_2)$	3.645e−01	0.32260		0.43680
transport = $\log_{10}(k_1'k_2'/k_2)$	−1.242e+00	−1.16500	−1.279 or 0.2308	−1.44100

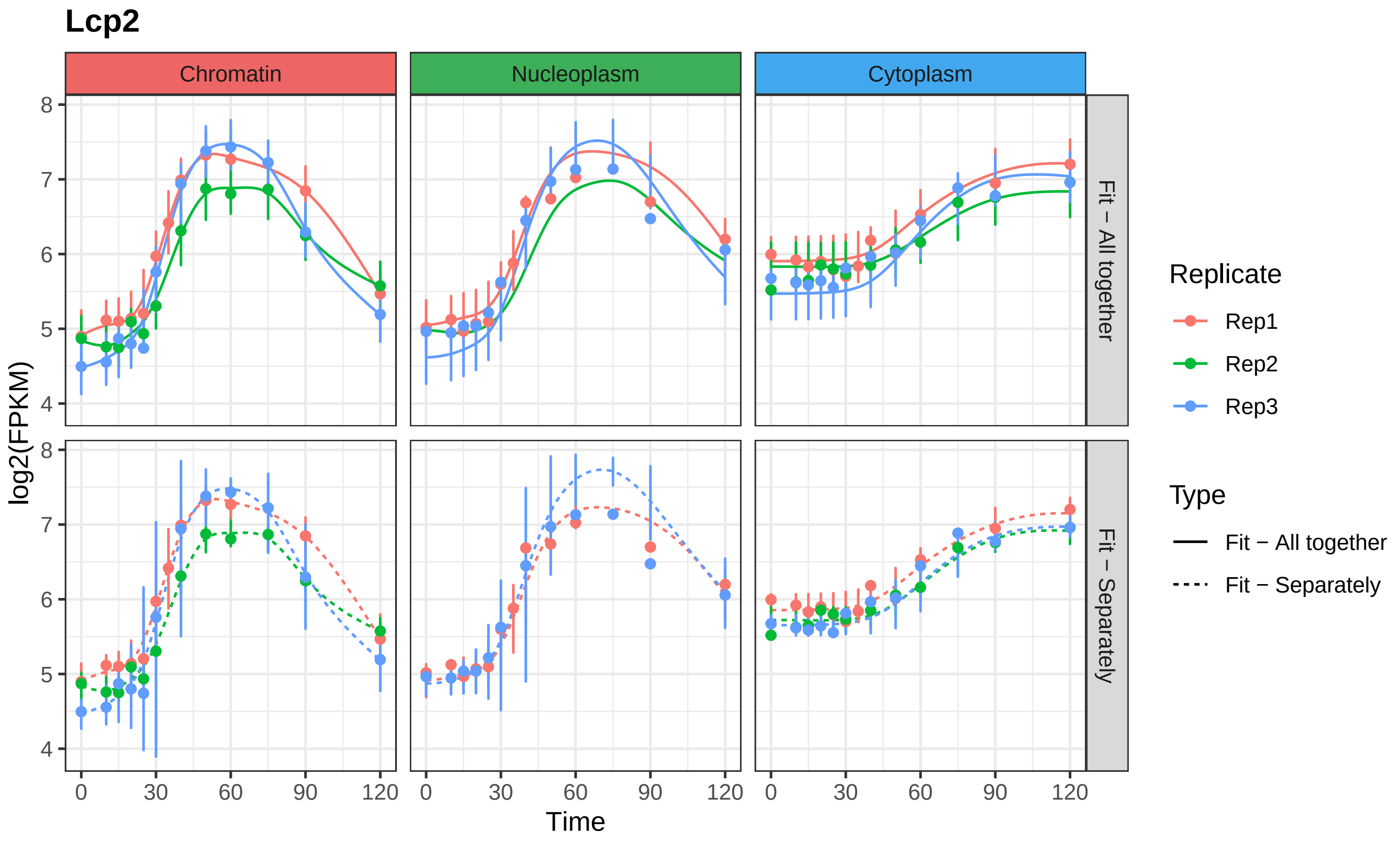

	Together	b1	b2	b3
**−NLL b1 ca**	−8.487	−10.05		
**−NLL b1 np**	0.4058	−1.598		
**−NLL b1 cyto**	−6.59	−9.124		
**−NLL b2 ca**	−8.457		−11.21	
**−NLL b2 np**				
**−NLL b2 cyto**	−6.125		−10.12	
**−NLL b3 ca**	−6.651			−4.284
**−NLL b3 np**	9.536			2.676
**−NLL b3 cyto**	−3.381			−9.798
Total	−29.75	−20.77	−21.33	−11.41
**Total with regul**	−29.8	−21.85	−22.01	−12.01

	Together	b1	b2	b3
spar	0.36120	0.384500	0.3646	0.38410
σ_b	0.11560	0.073540	0.05887	0.04569
σ_t	1.15800	2.370000	1.046e−05	6.08500
ca_{0,b_1}				
$\log_{10}(k_1')$	−0.92500	−1.014000		−0.95510
$\log_{10}(k_2)$	−0.96720	−1.015000	−1.127 or −1.894	−1.07700
$\log_{10}(k_2')$	−1.82100	−1.804000		−2.01700
$\log_{10}(k_{deg})$	−2.07700	−2.087000	−1.894 or −1.127	−2.25200
$\log_{10}(k_1' k_2')$	−2.74600	−2.818000	−2.755	−2.97200
$\log_{10}(k_1'/k_2)$	0.04219	0.001443		0.12180
$\text{transport} = \log_{10}(k_1' k_2'/k_2)$	−1.77900	−1.803000	−1.628 or −0.8609	−1.89500

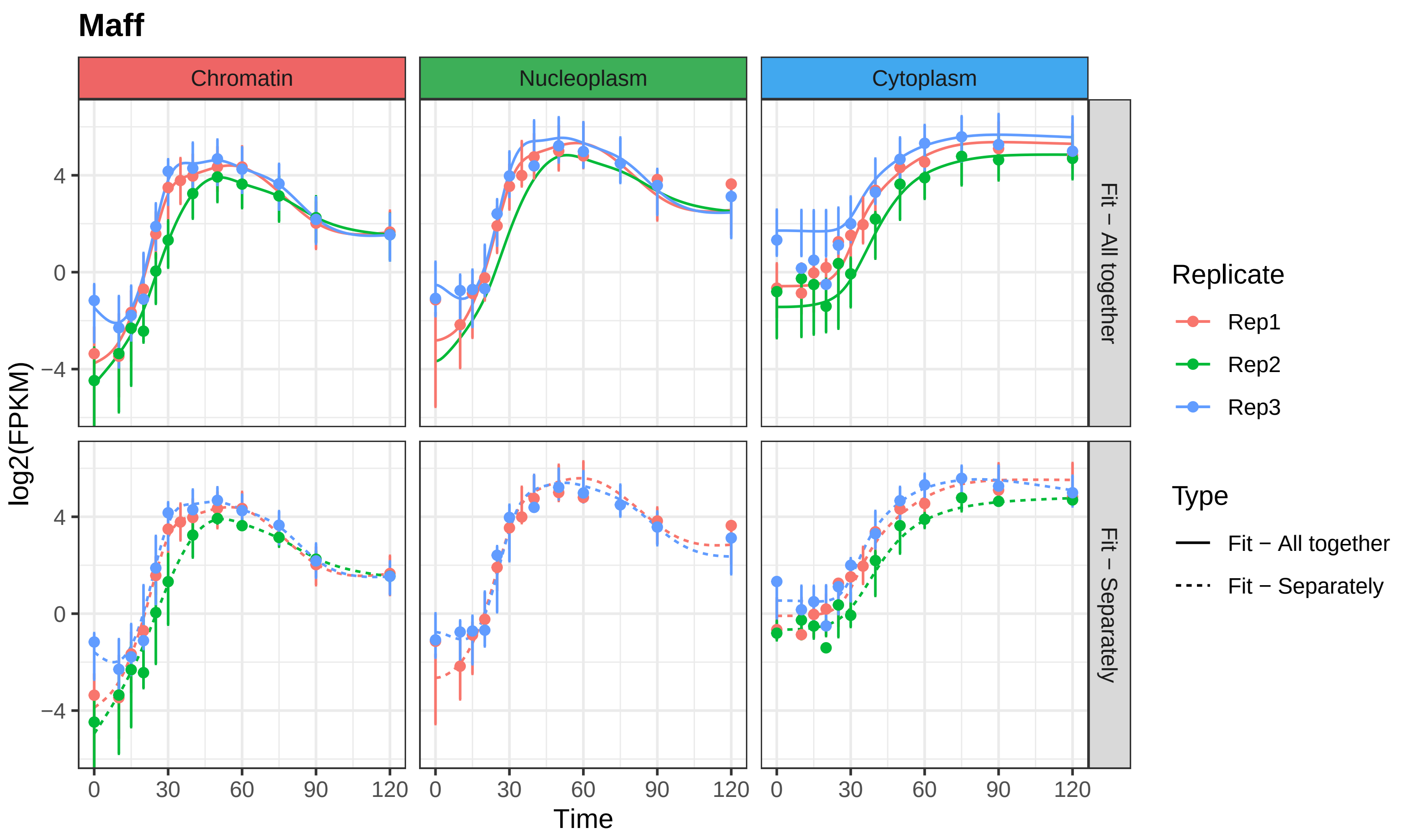

	Together	b1	b2	b3
**−NLL b1 ca**	4.914	3.537		
**−NLL b1 np**	13.2	12.09		
**−NLL b1 cyto**	8.901	9.675		
**−NLL b2 ca**	5.316		1.77	
**−NLL b2 np**				
**−NLL b2 cyto**	12.01		5.425	
**−NLL b3 ca**	5.395			4.769
**−NLL b3 np**	8.987			8.632
**−NLL b3 cyto**	19.95			7.07
Total	78.67	25.31	7.195	20.47
**Total with regul**	85.6	29.25	5.8	23.54

	Together	b1	b2	b3
spar	3.022e−01	3.265e−01	0.3885	0.3275
σ_b	3.122e−01	2.537e−01	0.0196	0.2165
σ_t	5.946e−05	4.319e−05	2.714	1.3850
ca_{0,b_1}				
$\log_{10}(k_1')$	−9.446e−02	−2.827e−01		−0.4460
$\log_{10}(k_2)$	−3.780e−01	−6.535e−01	4.609 or −2.661	−0.6942
$\log_{10}(k_2')$	−1.529e+00	−1.601e+00		−1.3920
$\log_{10}(k_{deg})$	−2.204e+00	−2.370e+00	−2.661 or 4.609	−1.7850
$\log_{10}(k_1'k_2')$	−1.624e+00	−1.883e+00	3.241	−1.8380
$\log_{10}(k_1'/k_2)$	2.836e−01	3.708e−01		0.2482
$transport = \log_{10}(k_1'k_2'/k_2)$	−1.246e+00	−1.230e+00	−1.368 or 5.902	−1.1440

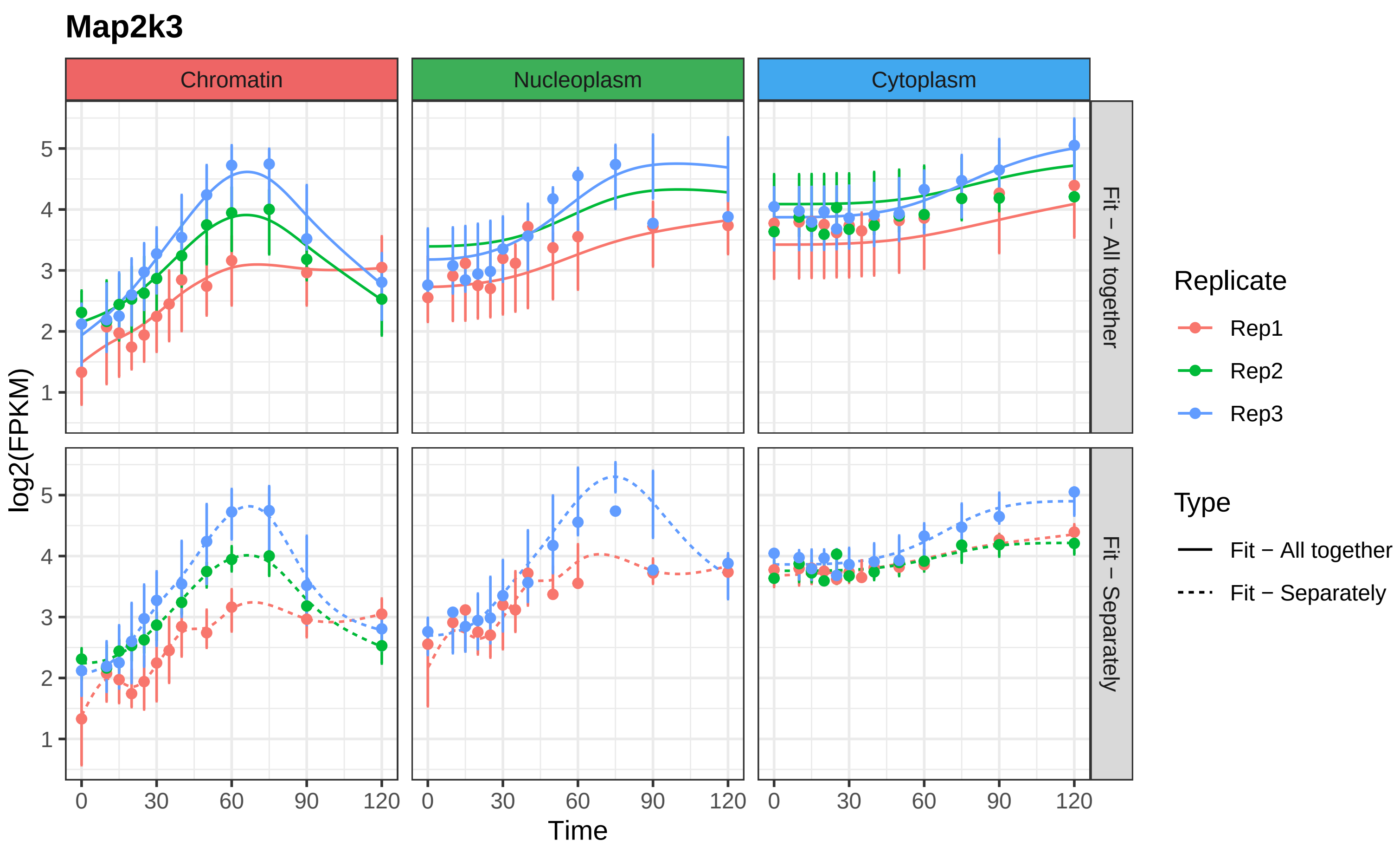

	Together	b1	b2	b3
**−NLL b1 ca**	−1.88	−7.802		
**−NLL b1 np**	2.901	0.8428		
**−NLL b1 cyto**	3.365	−13.49		
**−NLL b2 ca**	−3.452		−10.37	
**−NLL b2 np**				
**−NLL b2 cyto**	6.498		−7.71	
**−NLL b3 ca**	−2.042			−3.757
**−NLL b3 np**	10.59			5.652
**−NLL b3 cyto**	−4.052			−7.005
Total	11.93	−20.45	−18.08	−5.111
**Total with regul**	12.35	−18.86	−20.14	−6.093

	Together	b1	b2	b3
spar	5.270e−01	0.3172	0.4261	0.40030
σ_b	1.759e−01	0.0406	0.05284	0.07698
σ_t	5.117e−06	3.8770	0.0005585	5.22400
ca_{0,b_1}				
$\log_{10}(k_1')$	−1.613e+00	1.0530		−0.82230
$\log_{10}(k_2)$	−1.987e+00	0.8158	−0.6899 or −2.436	−1.01400
$\log_{10}(k_2')$	−1.589e+00	−1.9850		−1.93400
$\log_{10}(k_{deg})$	−1.798e+00	−2.4410	−2.436 or −0.6899	−2.28500
$\log_{10}(k_1'k_2')$	−3.202e+00	−0.9313	−2.668	−2.75600
$\log_{10}(k_1'/k_2)$	3.741e−01	0.2375		0.19200
$\text{transport} = \log_{10}(k_1'k_2'/k_2)$	−1.215e+00	−1.7470	−1.978 or −0.2327	−1.74200

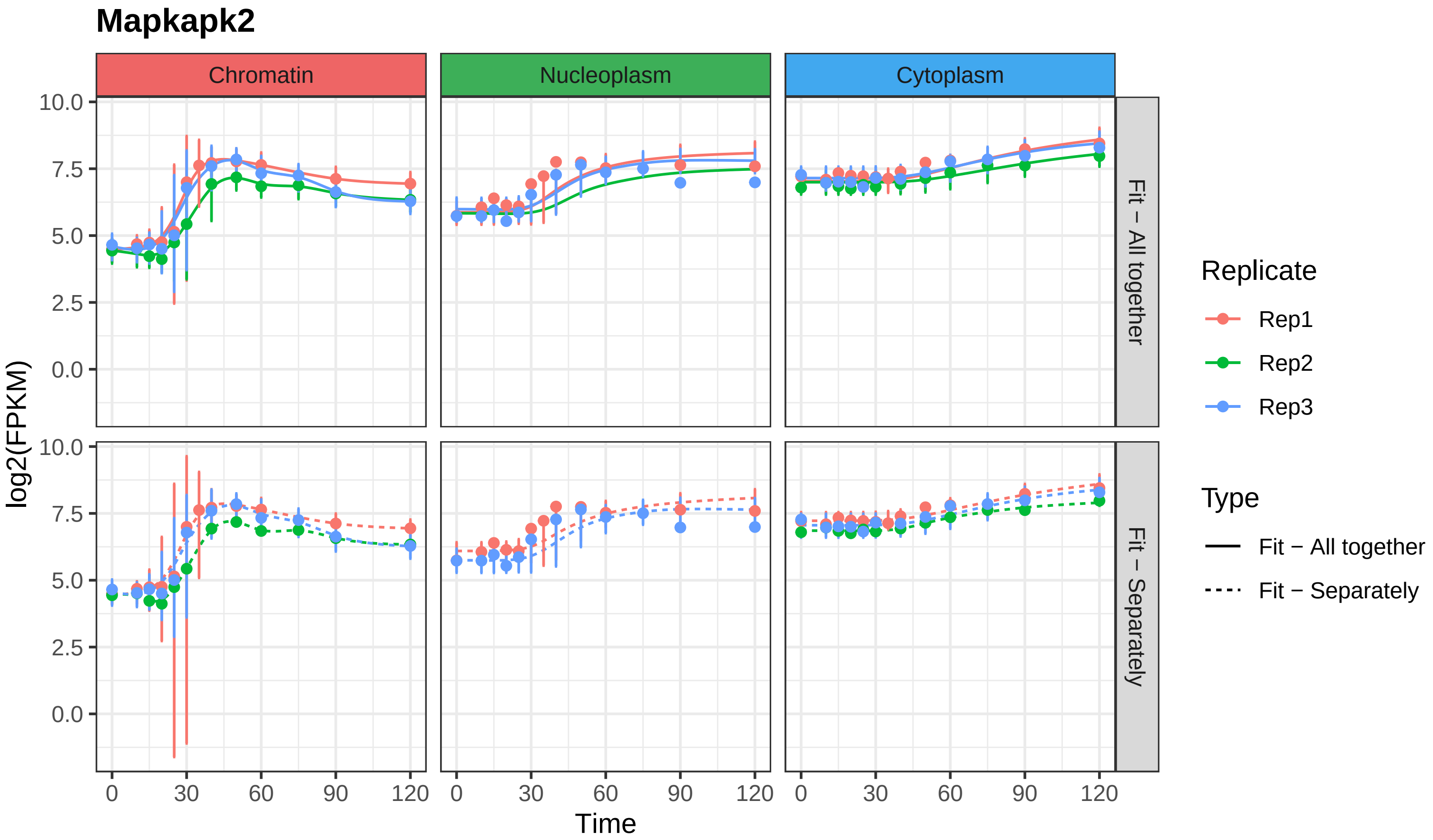

	Together	b1	b2	b3
**−NLL b1 ca**	−0.1136	1.206		
**−NLL b1 np**	13.79	10.56		
**−NLL b1 cyto**	−1.537	−6.766		
**−NLL b2 ca**	−1.788		−15.96	
**−NLL b2 np**				
**−NLL b2 cyto**	−4.232		−14.41	
**−NLL b3 ca**	−0.3524			0.5792
**−NLL b3 np**	13.69			10.55
**−NLL b3 cyto**	−3.445			−3.691
Total	16.01	5	−30.37	7.437
**Total with regul**	17.09	5.576	−26.6	7.841

	Together	b1	b2	b3
spar	0.3591	0.3788	0.2765	0.3835
σ_b	0.1549	0.1156	0.03751	0.1551
σ_t	5.2010	7.7780	0.0001857	5.6300
ca_{0,b_1}				
$\log_{10}(k_1')$	−1.5940	−1.6800		−1.6200
$\log_{10}(k_2)$	−2.0130	−2.1730	−0.67 or −2.41	−1.9780
$\log_{10}(k_2')$	−1.5340	−1.5050		−1.5280
$\log_{10}(k_{deg})$	−1.8840	−1.8450	−2.41 or −0.67	−1.9220
$\log_{10}(k_1'k_2')$	−3.1280	−3.1850	−2.359	−3.1480
$\log_{10}(k_1'/k_2)$	0.4188	0.4932		0.3573
transport $= \log_{10}(k_1'k_2'/k_2)$	−1.1150	−1.0120	−1.689 or 0.05094	−1.1700

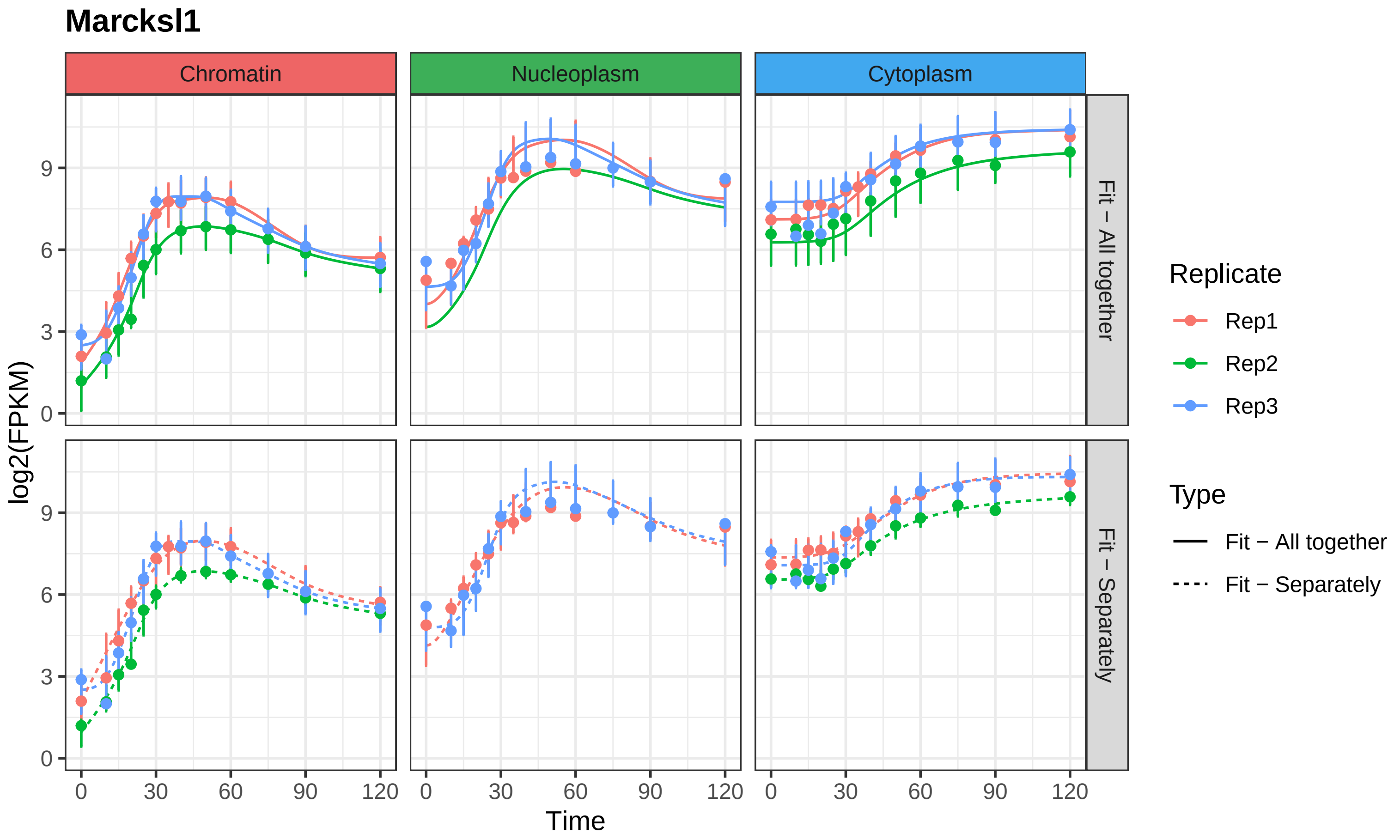

	Together	b1	b2	b3
**−NLL b1 ca**	0.9725	3.48		
**−NLL b1 np**	15.76	11.92		
**−NLL b1 cyto**	2.668	1.167		
**−NLL b2 ca**	1.572		−4.896	
**−NLL b2 np**				
**−NLL b2 cyto**	3.749		−4.035	
**−NLL b3 ca**	3.762			3.521
**−NLL b3 np**	12.26			11.27
**−NLL b3 cyto**	12.67			5.538
Total	53.42	16.57	−8.931	20.33
**Total with regul**	57.31	18.57	−9.242	24.34

	Together	b1	b2	b3
spar	0.3369000	0.5386	0.3603	3.325e−01
σ_b	0.2704000	0.2346	0.08599	2.672e−01
σ_t	0.0002399	0.5336	1.233	7.704e−05
ca_{0,b_1}				
$\log_{10}(k_1')$	−0.0857000	−0.1936		−2.347e−01
$\log_{10}(k_2)$	−0.7337000	−0.8060	3.994 or −2.683	−9.240e−01
$\log_{10}(k_2')$	−1.6080000	−1.5790		−1.590e+00
$\log_{10}(k_{deg})$	−2.5430000	−2.5510	−2.683 or 3.994	−2.276e+00
$\log_{10}(k_1'k_2')$	−1.6940000	−1.7730	2.994	−1.824e+00
$\log_{10}(k_1'/k_2)$	0.6480000	0.6124		6.894e−01
transport = $\log_{10}(k_1'k_2'/k_2)$	−0.9605000	−0.9669	−0.9997 or 5.677	−9.002e−01

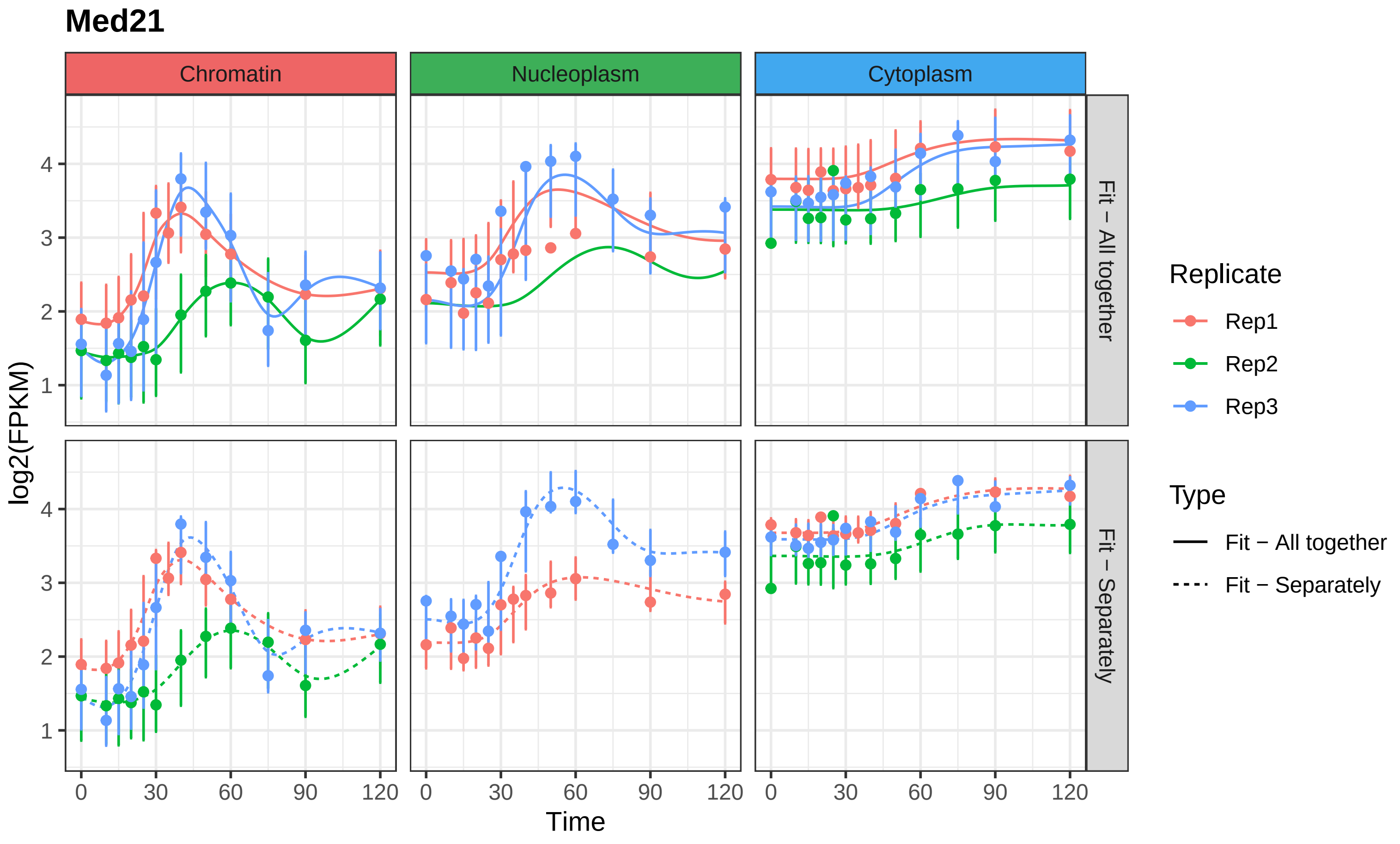

	Together	b1	b2	b3
**−NLL b1 ca**	−2.103	−5.03		
**−NLL b1 np**	13.49	−5.375		
**−NLL b1 cyto**	−4.214	−10.34		
**−NLL b2 ca**	−3.656		−3.109	
**−NLL b2 np**				
**−NLL b2 cyto**	−0.8605		0.3779	
**−NLL b3 ca**	−0.9577			−2.484
**−NLL b3 np**	11.24			−2.173
**−NLL b3 cyto**	−3.255			−8.082
Total	9.69	−20.75	−2.731	−12.74
**Total with regul**	10.43	−22.27	−4.38	−14.2

	Together	b1	b2	b3
spar	0.3548	0.39530	0.4404	0.39760
σ_b	0.1389	0.02812	0.1141	0.05182
σ_t	3.7140	2.86200	4.667e−07	2.90900
ca_{0,b_1}				
$\log_{10}(k_1')$	−1.0460	−1.47600		−0.85410
$\log_{10}(k_2)$	−1.2440	−1.58000	−1.037 or −1.777	−1.17100
$\log_{10}(k_2')$	−1.5490	−1.26700		−1.86000
$\log_{10}(k_{deg})$	−1.9300	−1.71400	−1.777 or −1.037	−2.18700
$\log_{10}(k_1'k_2')$	−2.5940	−2.74300	−2.233	−2.71400
$\log_{10}(k_1'/k_2)$	0.1980	0.10380		0.31680
$\text{transport} = \log_{10}(k_1'k_2'/k_2)$	−1.3510	−1.16300	−1.196 or −0.4558	−1.54300

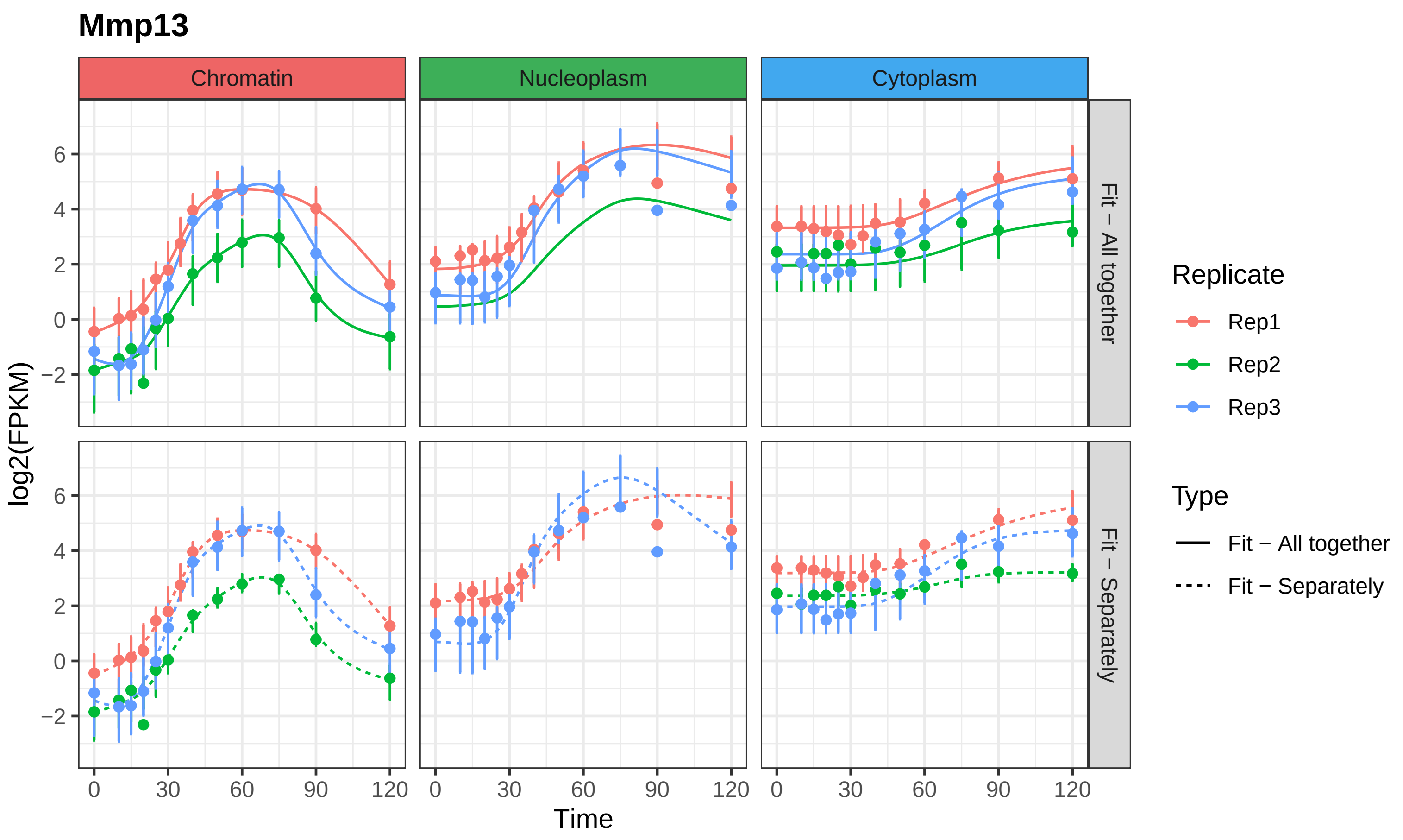

	Together	b1	b2	b3
**−NLL b1 ca**	2.151	−0.181		
**−NLL b1 np**	10.48	11.66		
**−NLL b1 cyto**	3.462	1.548		
**−NLL b2 ca**	5.064		2.279	
**−NLL b2 np**				
**−NLL b2 cyto**	7.27		−2.889	
**−NLL b3 ca**	2.909			3.54
**−NLL b3 np**	19.87			16.38
**−NLL b3 cyto**	9.75			5.287
Total	60.96	13.02	−0.6099	25.21
**Total with regul**	64.01	13.52	−1.745	28.53

	Together	b1	b2	b3
spar	0.3672000	4.004e−01	0.3897	3.677e−01
σ_b	0.2830000	2.154e−01	0.09559	2.927e−01
σ_t	0.0000482	7.297e−05	5.017e−05	1.207e−05
ca_{0,b_1}				
$\log_{10}(k_1')$	−0.9439000	−1.205e+00		−5.798e−01
$\log_{10}(k_2)$	−1.6390000	−2.015e+00	−0.5459 or −3.126	−1.219e+00
$\log_{10}(k_2')$	−2.0790000	−1.876e+00		−2.265e+00
$\log_{10}(k_{deg})$	−2.5280000	−2.183e+00	−3.126 or −0.5459	−2.650e+00
$\log_{10}(k_1'k_2')$	−3.0230000	−3.081e+00	−2.398	−2.844e+00
$\log_{10}(k_1'/k_2)$	0.6953000	8.105e−01		6.396e−01
transport $= \log_{10}(k_1'k_2'/k_2)$	−1.3840000	−1.066e+00	−1.852 or 0.7282	−1.625e+00

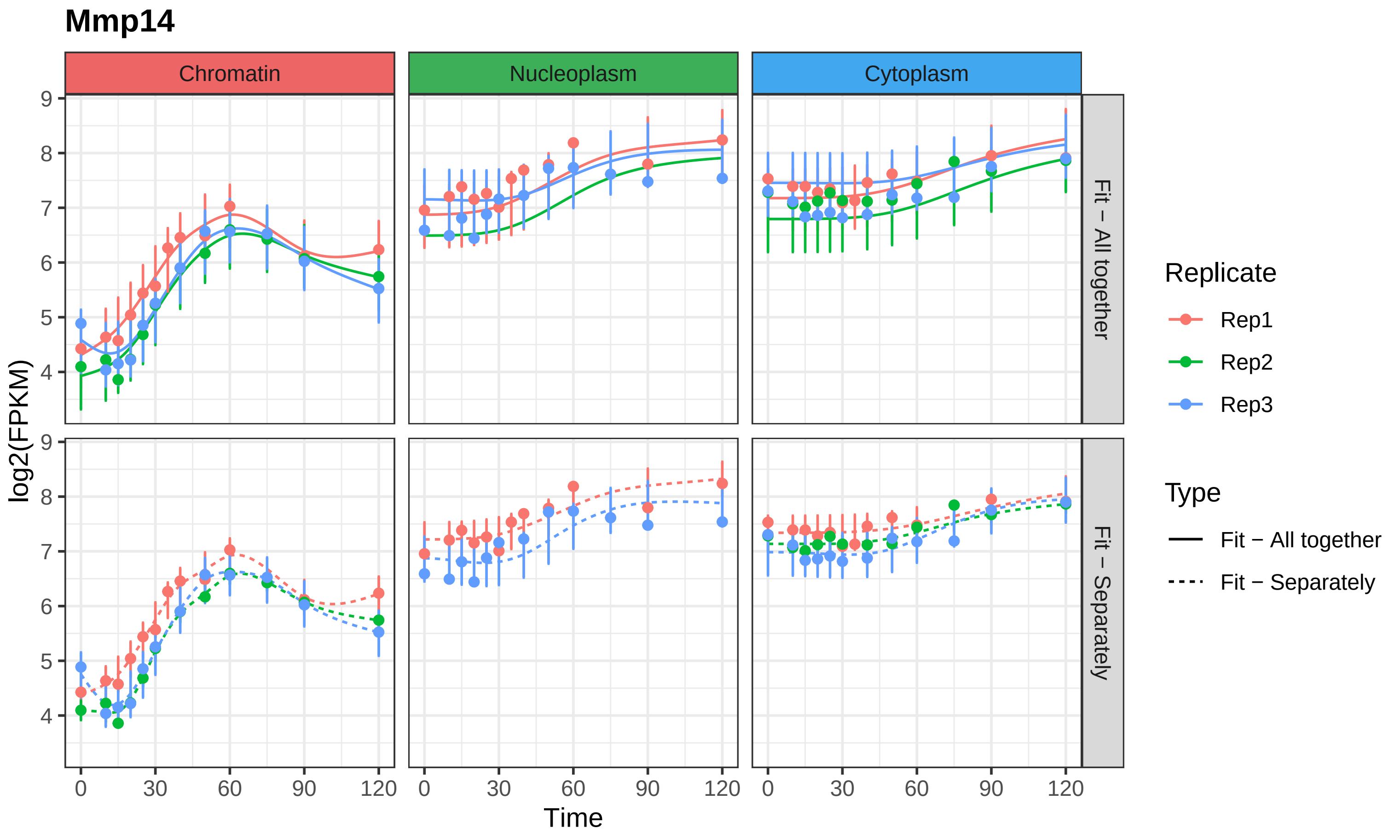

	Together	b1	b2	b3
**−NLL b1 ca**	−2.338	−7.847		
**−NLL b1 np**	3.965	0.3951		
**−NLL b1 cyto**	−1.024	−6.033		
**−NLL b2 ca**	−2.015		−11.29	
**−NLL b2 np**				
**−NLL b2 cyto**	4.069		−11.15	
**−NLL b3 ca**	−1.448			−5.638
**−NLL b3 np**	8.306			6.445
**−NLL b3 cyto**	10.91			−4.628
Total	20.43	−13.48	−22.44	−3.822
**Total with regul**	20.24	−14.9	−22.01	−4.537

	Together	b1	b2	b3
spar	4.782e−01	4.132e−01	0.3361	3.959e−01
σ_b	1.988e−01	1.120e−01	0.05596	1.426e−01
σ_t	7.726e−06	6.815e−06	1.804e−05	1.374e−06
ca_{0,b_1}				
$\log_{10}(k_1')$	−1.394e+00	−1.456e+00		−1.280e+00
$\log_{10}(k_2)$	−2.166e+00	−2.311e+00	−1.013 or −2.548	−1.918e+00
$\log_{10}(k_2')$	−1.514e+00	−1.756e+00		−1.301e+00
$\log_{10}(k_{deg})$	−1.605e+00	−1.793e+00	−2.548 or −1.013	−1.335e+00
$\log_{10}(k_1'k_2')$	−2.908e+00	−3.212e+00	−2.647	−2.582e+00
$\log_{10}(k_1'/k_2)$	7.722e−01	8.552e−01		6.374e−01
transport = $\log_{10}(k_1'k_2'/k_2)$	−7.417e−01	−9.008e−01	−1.634 or −0.09822	−6.639e−01

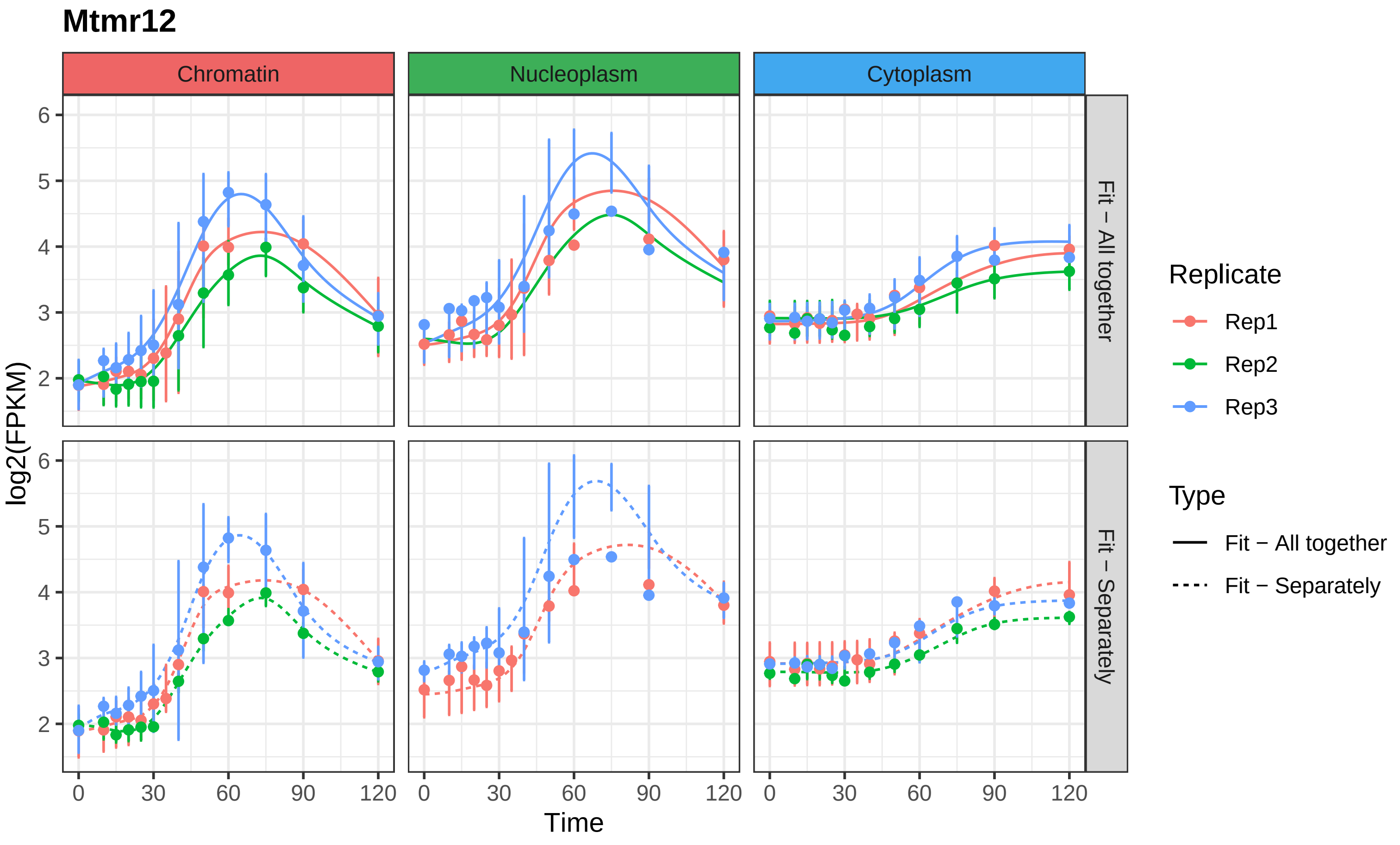

	Together	b1	b2	b3
**−NLL b1 ca**	−4.615	−7.897		
**−NLL b1 np**	5.942	2.968		
**−NLL b1 cyto**	−6.547	−8.384		
**−NLL b2 ca**	−6.529		−13.51	
**−NLL b2 np**				
**−NLL b2 cyto**	−6.825		−13.72	
**−NLL b3 ca**	−3.883			−5.133
**−NLL b3 np**	13.64			1.898
**−NLL b3 cyto**	−7.922			−12.9
Total	−16.74	−13.31	−27.23	−16.14
**Total with regul**	−17.95	−14.82	−28.91	−16.77

	Together	b1	b2	b3
spar	0.45220	0.4146000	0.3934	3.870e−01
σ_b	0.08812	0.1059000	3.212e−05	2.488e−06
σ_t	5.99300	0.0003044	0.0006319	6.679e+00
ca_{0,b_1}				
$\log_{10}(k_1')$	−0.15520	−0.6913000		−3.902e−01
$\log_{10}(k_2)$	−0.34480	−0.8617000	−0.202 or −2.102	−6.526e−01
$\log_{10}(k_2')$	−2.14100	−1.9640000		−2.378e+00
$\log_{10}(k_{deg})$	−2.23600	−2.1040000	−2.102 or −0.202	−2.411e+00
$\log_{10}(k_1'k_2')$	−2.29600	−2.6550000	−2.063	−2.768e+00
$\log_{10}(k_1'/k_2)$	0.18960	0.1704000		2.624e−01
transport $= \log_{10}(k_1'k_2'/k_2)$	−1.95200	−1.7930000	−1.861 or 0.0389	−2.116e+00

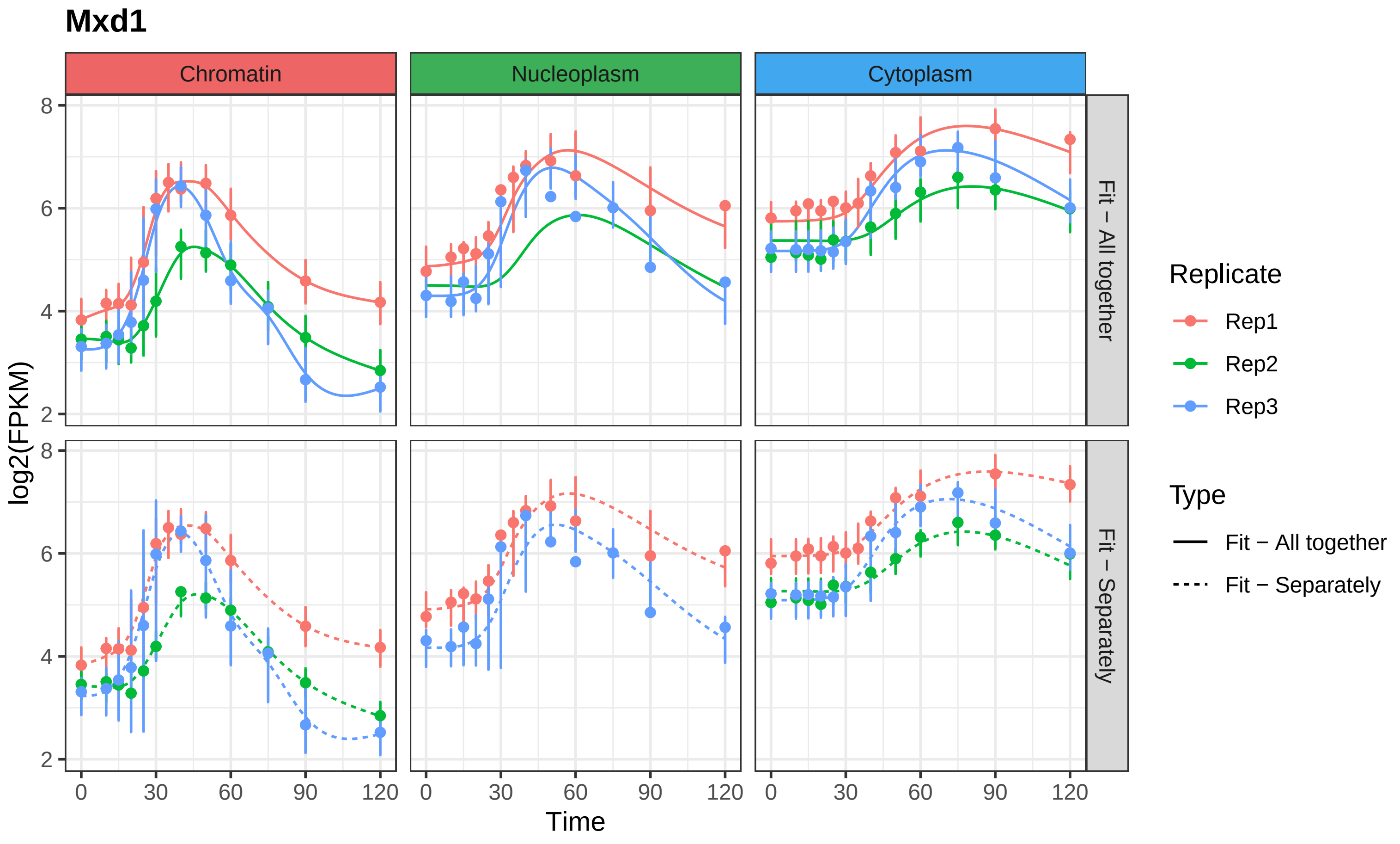

	Together	b1	b2	b3
**−NLL b1 ca**	−4.197	−4.424		
**−NLL b1 np**	5.069	6.375		
**−NLL b1 cyto**	−2.332	−7.029		
**−NLL b2 ca**	−5.945		−8.186	
**−NLL b2 np**				
**−NLL b2 cyto**	−2.587		−4.605	
**−NLL b3 ca**	−2.867			0.2664
**−NLL b3 np**	12.78			9.179
**−NLL b3 cyto**	−4.244			−4.152
Total	−4.319	−5.078	−12.79	5.294
**Total with regul**	−3.32	−5.319	−14.16	5.444

	Together	b1	b2	b3
spar	0.3431	0.3709	0.3969	0.3688
σ_b	0.1344	0.1160	0.08757	0.1195
σ_t	2.5400	2.7730	0.01474	4.6720
ca_{0,b_1}				
$\log_{10}(k_1')$	−0.9944	−0.9937		−1.1040
$\log_{10}(k_2)$	−1.3060	−1.3230	−1.276 or −1.311	−1.3860
$\log_{10}(k_2')$	−1.1680	−1.3370		−1.0930
$\log_{10}(k_{deg})$	−1.4300	−1.6480	−1.311 or −1.276	−1.3710
$\log_{10}(k_1'k_2')$	−2.1620	−2.3300	−2.037	−2.1970
$\log_{10}(k_1'/k_2)$	0.3119	0.3290		0.2824
transport $= \log_{10}(k_1'k_2'/k_2)$	−0.8560	−1.0080	−0.7615 or −0.7263	−0.8103

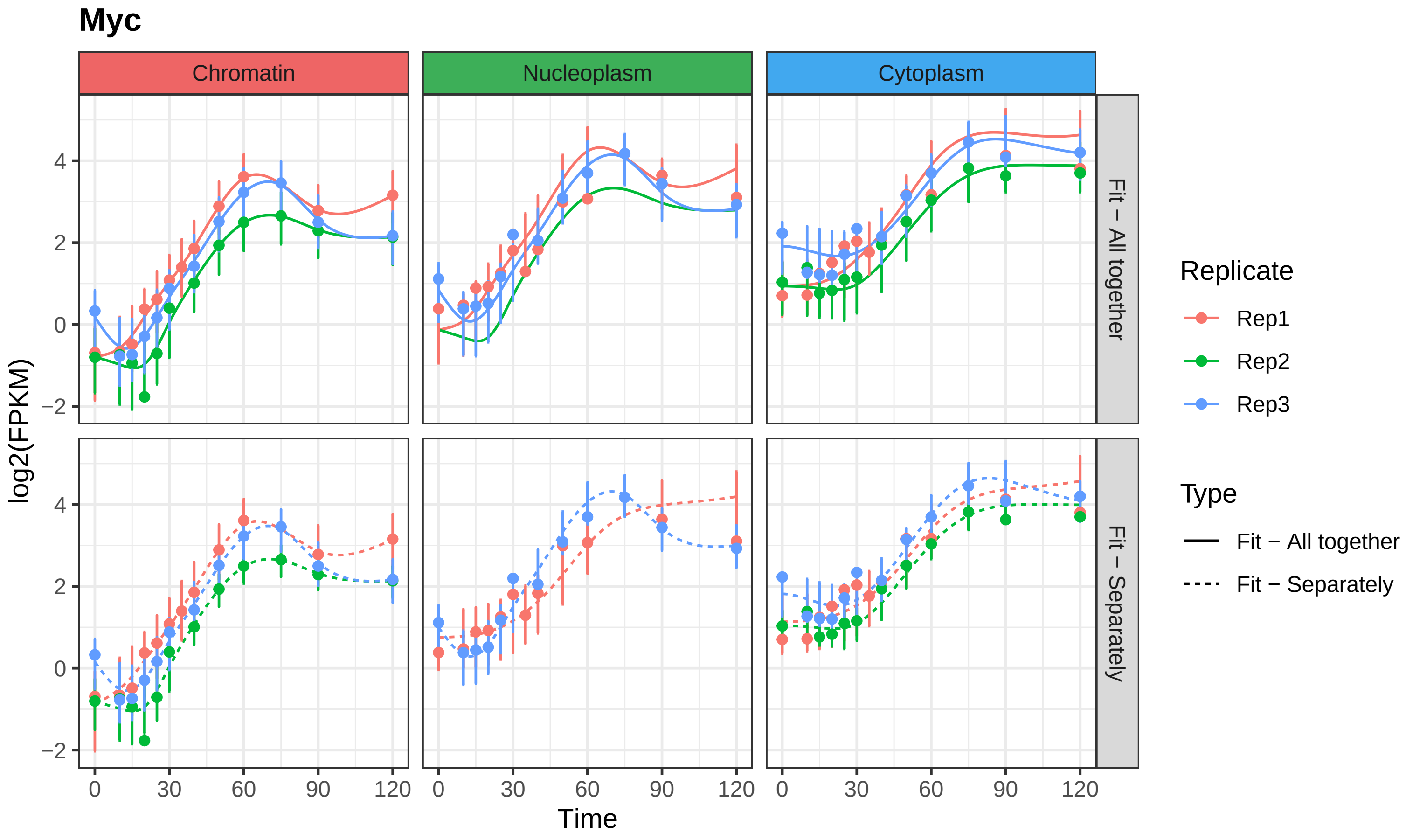

	Together	b1	b2	b3
**−NLL b1 ca**	−0.6852	0.06027		
**−NLL b1 np**	14.83	9.033		
**−NLL b1 cyto**	8.951	6.365		
**−NLL b2 ca**	1.436		−0.2218	
**−NLL b2 np**				
**−NLL b2 cyto**	0.8081		0.223	
**−NLL b3 ca**	−0.7622			−1.388
**−NLL b3 np**	3.517			2.292
**−NLL b3 cyto**	4.798			5.428
Total	32.89	15.46	0.001219	6.331
**Total with regul**	34	15.59	−0.7861	6.252

	Together	b1	b2	b3
spar	0.3711	0.4471000	0.3844	0.3867
σ_b	0.2086	0.2212000	0.1157	0.1694
σ_t	0.5963	0.0001563	0.00978	0.4213
ca_{0,b_1}				
$\log_{10}(k_1')$	5.3090	−1.3670000		4.2210
$\log_{10}(k_2)$	5.1110	−1.8590000	5.56 or −1.519	3.9680
$\log_{10}(k_2')$	−1.1860	5.0790000		−1.1140
$\log_{10}(k_{deg})$	−1.5070	4.9650000	−1.519 or 5.56	−1.3610
$\log_{10}(k_1'k_2')$	4.1240	3.7120000	4.597	3.1070
$\log_{10}(k_1'/k_2)$	0.1987	0.4922000		0.2531
$\text{transport} = \log_{10}(k_1'k_2'/k_2)$	−0.9869	5.5710000	−0.9635 or 6.116	−0.8612

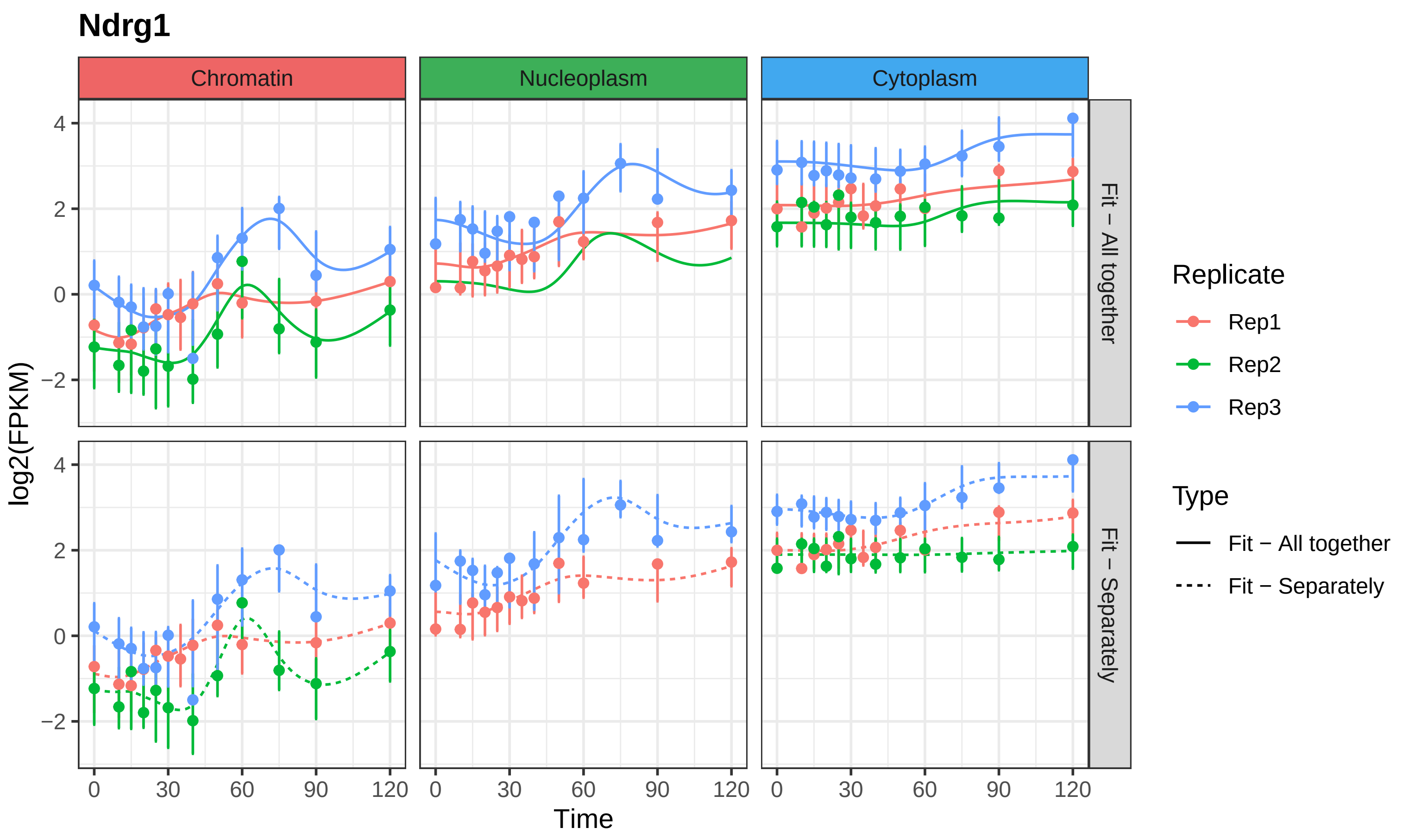

	Together	b1	b2	b3
**−NLL b1 ca**	0.8778	0.06695		
**−NLL b1 np**	1.877	0.5444		
**−NLL b1 cyto**	1.145	0.7332		
**−NLL b2 ca**	5.573		3.717	
**−NLL b2 np**				
**−NLL b2 cyto**	3.64		−0.5966	
**−NLL b3 ca**	4.988			5.715
**−NLL b3 np**	8.22			6.513
**−NLL b3 cyto**	−0.6558			−3.737
Total	25.66	1.345	3.121	8.491
**Total with regul**	25	0.1853	2.261	8.506

	Together	b1	b2	b3
spar	0.4426	0.4894000	0.3906	0.5070
σ_b	0.1706	0.1303000	0.1226	0.1159
σ_t	3.0860	0.0001847	0.1731	7.1170
ca_{0,b_1}				
$\log_{10}(k_1')$	−0.6477	−0.3465000		7.2280
$\log_{10}(k_2)$	−1.1160	−0.7820000	−2.179 or −2.215	6.7300
$\log_{10}(k_2')$	−1.3170	−1.1200000		−1.3600
$\log_{10}(k_{deg})$	−1.7280	−1.5530000	−2.215 or −2.179	−1.7190
$\log_{10}(k_1'k_2')$	−1.9640	−1.4660000	−3.442	5.8680
$\log_{10}(k_1'/k_2)$	0.4680	0.4356000		0.4982
transport = $\log_{10}(k_1'k_2'/k_2)$	−0.8486	−0.6843000	−1.263 or −1.227	−0.8618

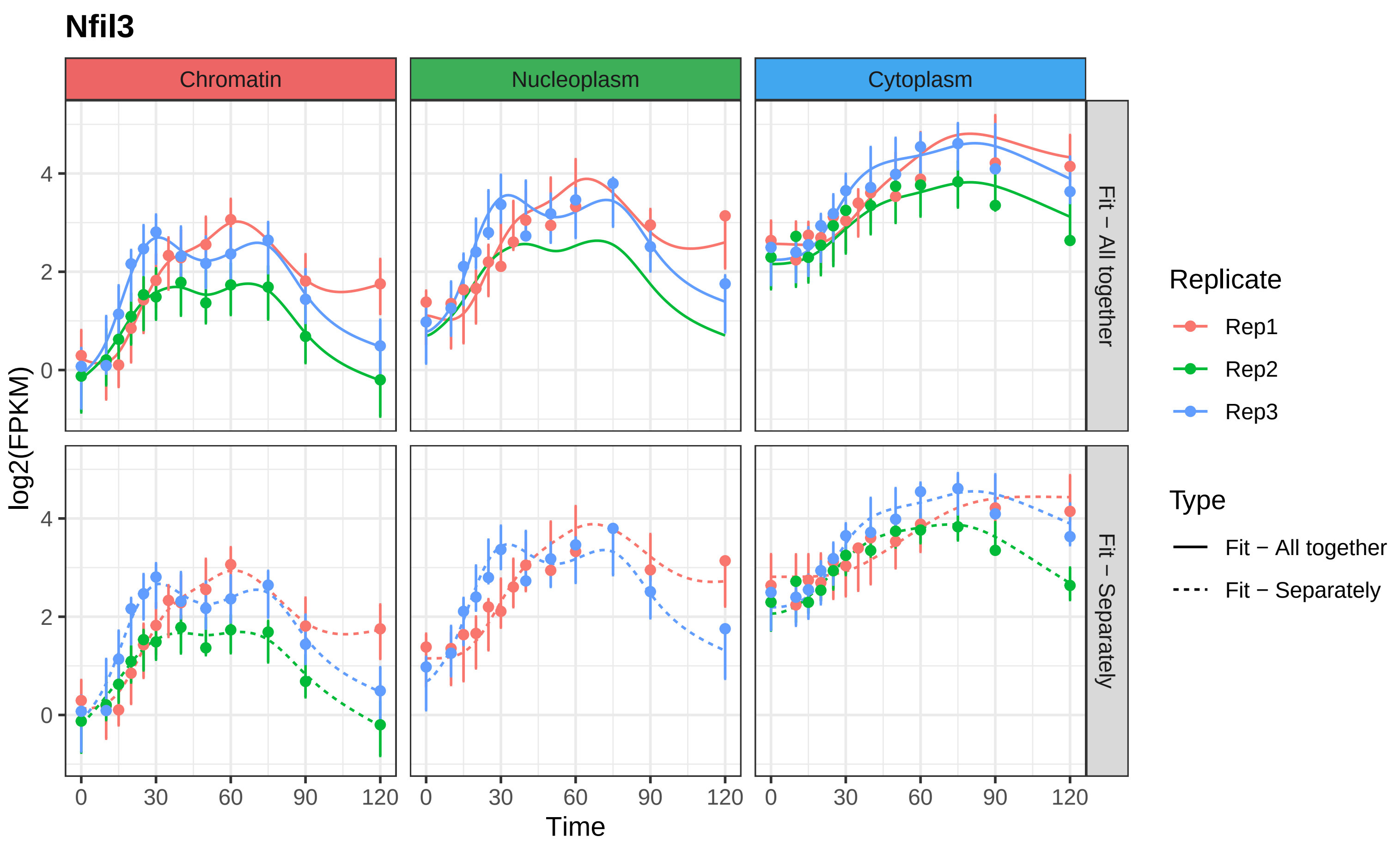

	Together	b1	b2	b3
**−NLL b1 ca**	−3.316	−2.643		
**−NLL b1 np**	7.098	3.466		
**−NLL b1 cyto**	3.488	1.915		
**−NLL b2 ca**	−3.476		−3.607	
**−NLL b2 np**				
**−NLL b2 cyto**	2.206		−1.329	
**−NLL b3 ca**	−2.412			−1.543
**−NLL b3 np**	2.579			2.346
**−NLL b3 cyto**	−0.2889			−0.2255
Total	5.878	2.737	−4.936	0.5775
**Total with regul**	6.58	1.793	−6.655	0.1529

	Together	b1	b2	b3
spar	3.577e−01	0.4268000	0.4324	0.385100
σ_b	1.622e−01	0.1592000	0.1037	0.145000
σ_t	1.654e−05	0.0001403	0.0007129	0.000233
ca_{0,b_1}				
$\log_{10}(k_1')$	2.172e−03	−0.5475000		0.170300
$\log_{10}(k_2)$	−2.627e−01	−0.8549000	5.008 or −1.218	−0.073780
$\log_{10}(k_2')$	−1.057e+00	−1.4760000		−1.081000
$\log_{10}(k_{deg})$	−1.497e+00	−1.9760000	−1.218 or 5.008	−1.535000
$\log_{10}(k_1' k_2')$	−1.055e+00	−2.0240000	4.472	−0.910700
$\log_{10}(k_1'/k_2)$	2.649e−01	0.3074000		0.244100
$\text{transport} = \log_{10}(k_1' k_2'/k_2)$	−7.926e−01	−1.1690000	−0.536 or 5.69	−0.836900

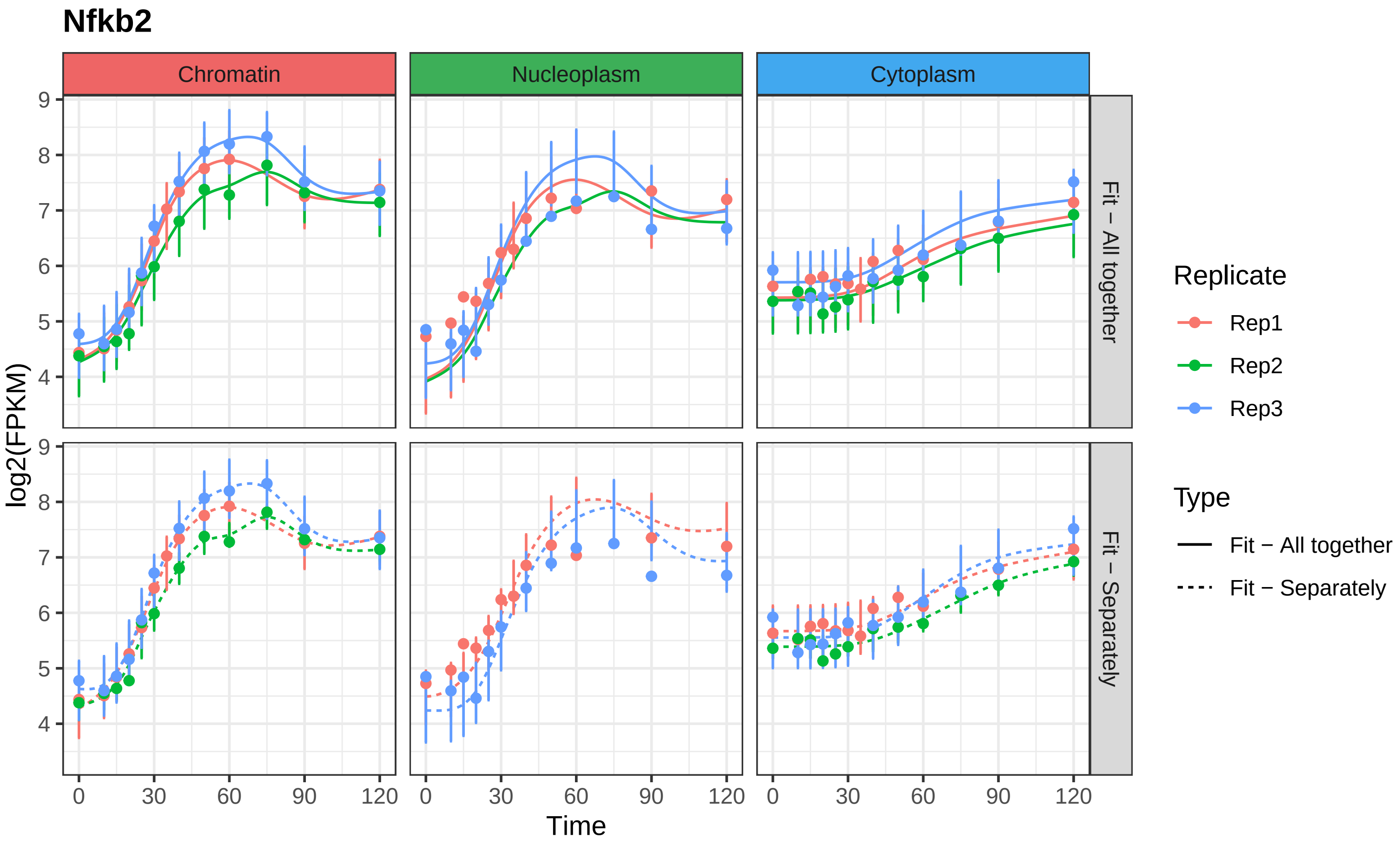

	Together	b1	b2	b3
**−NLL b1 ca**	−3.261	−5.171		
**−NLL b1 np**	13.61	10.79		
**−NLL b1 cyto**	−0.3092	−3.947		
**−NLL b2 ca**	−2.177		−7.929	
**−NLL b2 np**				
**−NLL b2 cyto**	−2.577		−6.665	
**−NLL b3 ca**	−2.566			−3.404
**−NLL b3 np**	16.36			10.15
**−NLL b3 cyto**	1.794			−1.42
Total	20.87	1.669	−14.59	5.327
**Total with regul**	20.81	0.7548	−15.28	5.399

	Together	b1	b2	b3
spar	0.4089	0.4342000	0.368	3.890e−01
σ_b	0.1955	0.1646000	0.07048	1.814e−01
σ_t	0.8244	0.0002087	1.455	6.458e−06
ca_{0,b_1}				
$\log_{10}(k_1')$	4.3810	−0.8433000		−9.575e−01
$\log_{10}(k_2)$	4.4870	−0.9094000	−1.012 or −2.431	−8.406e−01
$\log_{10}(k_2')$	−2.1230	−2.2180000		−2.030e+00
$\log_{10}(k_{deg})$	−2.5650	−2.5730000	−2.431 or −1.012	−2.426e+00
$\log_{10}(k_1'k_2')$	2.2580	−3.0620000	−3.121	−2.988e+00
$\log_{10}(k_1'/k_2)$	−0.1057	0.0660700		−1.169e−01
$\text{transport} = \log_{10}(k_1'k_2'/k_2)$	−2.2290	−2.1520000	−2.108 or −0.6892	−2.147e+00

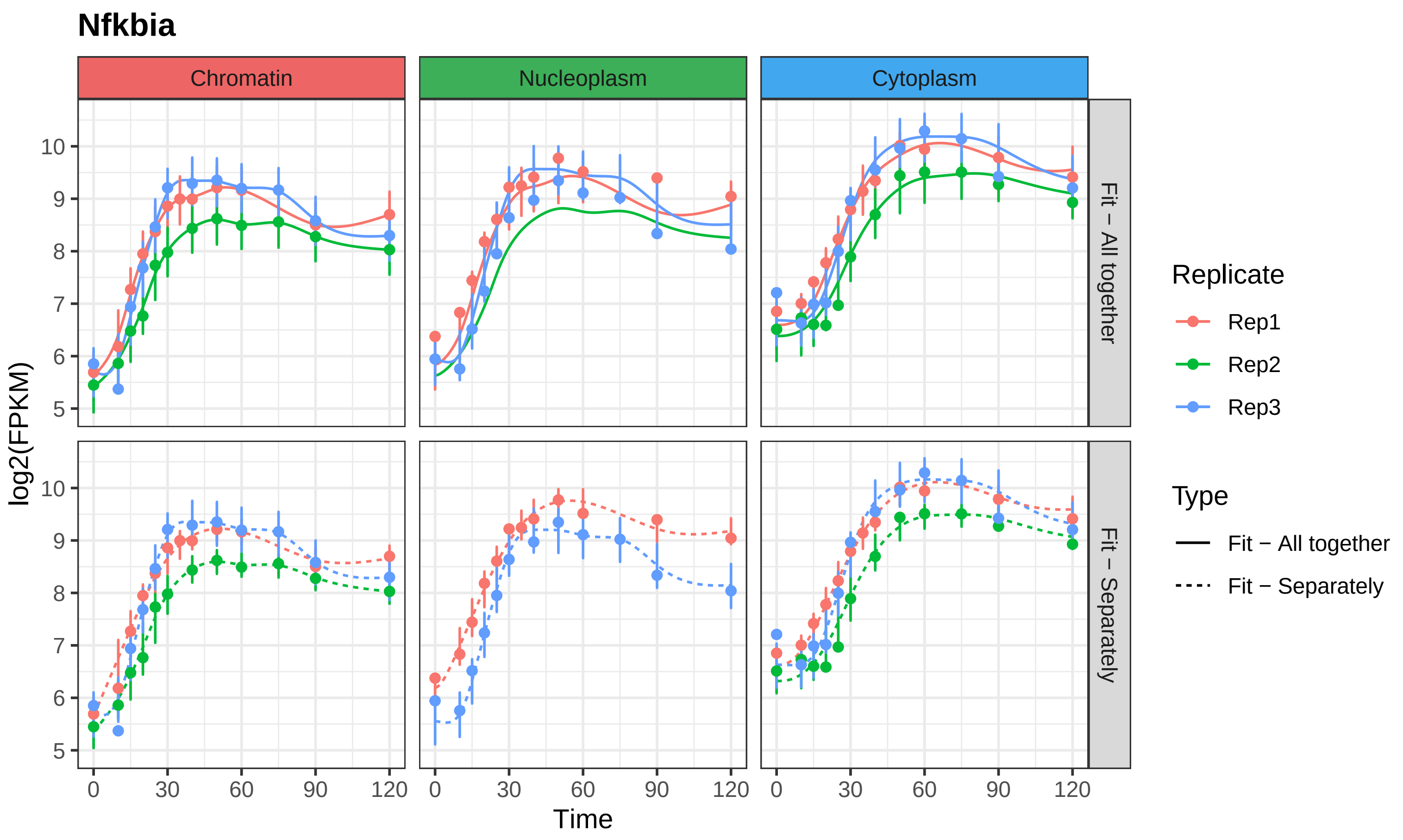

	Together	b1	b2	b3
**−NLL b1 ca**	−5.664	−5.06		
**−NLL b1 np**	6.534	−5.604		
**−NLL b1 cyto**	−2.933	−7.603		
**−NLL b2 ca**	−5.709		−8.893	
**−NLL b2 np**				
**−NLL b2 cyto**	−1.43		0.01782	
**−NLL b3 ca**	−3.015			−2.518
**−NLL b3 np**	10.45			−3.003
**−NLL b3 cyto**	1.411			2.428
Total	−0.3563	−18.27	−8.875	−3.093
**Total with regul**	3.944	−18.93	−9.012	0.3988

	Together	b1	b2	b3
spar	0.28630	0.52960	0.3551	2.958e−01
σ_b	0.15810	0.08536	0.07826	1.452e−01
σ_t	0.69260	1.13500	1.938	7.358e−05
ca_{0,b_1}				
$\log_{10}(k_1')$	−0.22850	−0.34550		−3.458e−01
$\log_{10}(k_2)$	−0.29500	−0.50920	−0.7836 or −0.8013	−3.022e−01
$\log_{10}(k_2')$	−0.87530	−0.85930		−7.383e−01
$\log_{10}(k_{deg})$	−1.10200	−0.98990	−0.8013 or −0.7836	−1.063e+00
$\log_{10}(k_1'k_2')$	−1.10400	−1.20500	−1.297	−1.084e+00
$\log_{10}(k_1'/k_2)$	0.06654	0.16370		−4.361e−02
transport $= \log_{10}(k_1'k_2'/k_2)$	−0.80870	−0.69560	−0.5134 or −0.4957	−7.820e−01

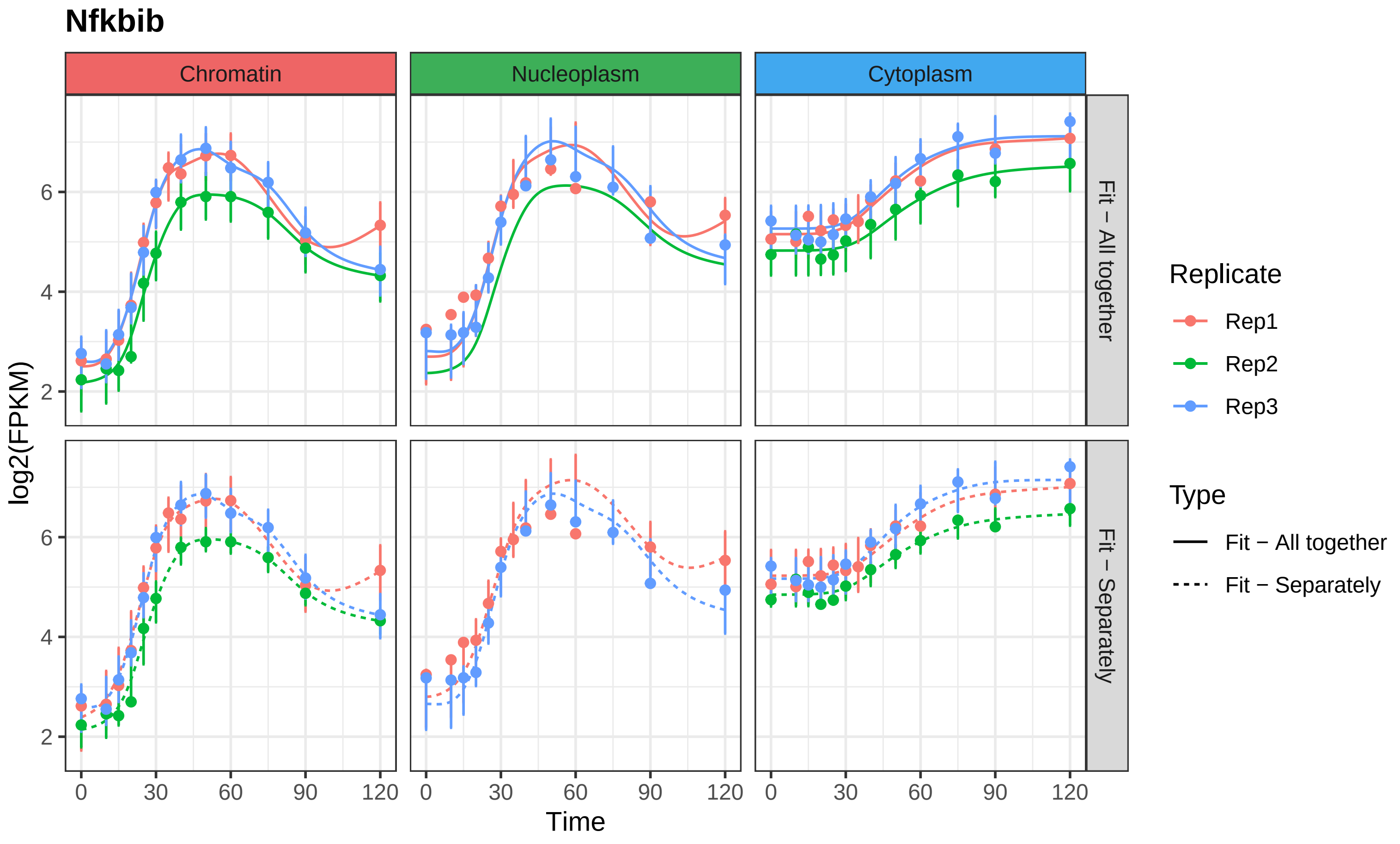

	Together	b1	b2	b3
**−NLL b1 ca**	−4.618	−2.423		
**−NLL b1 np**	18.29	11.97		
**−NLL b1 cyto**	−3.056	−2.596		
**−NLL b2 ca**	−3.543		−6.089	
**−NLL b2 np**				
**−NLL b2 cyto**	−3.704		−5.407	
**−NLL b3 ca**	−4.372			−4.366
**−NLL b3 np**	7.652			6.409
**−NLL b3 cyto**	−2.218			−2.901
Total	4.428	6.949	−11.5	−0.858
**Total with regul**	6.082	7.092	−11.78	0.2355

	Together	b1	b2	b3
spar	3.354e−01	3.890e−01	0.3573	0.3425000
σ_b	1.641e−01	1.854e−01	0.07644	0.1483000
σ_t	1.341e−05	7.506e−07	1.175	0.0004994
ca_{0,b_1}				
$\log_{10}(k_1')$	−4.897e−01	−5.942e−01		−0.5650000
$\log_{10}(k_2)$	−5.482e−01	−7.183e−01	4.785 or −2.505	−0.5814000
$\log_{10}(k_2')$	−1.710e+00	−1.837e+00		−1.6330000
$\log_{10}(k_{deg})$	−2.449e+00	−2.567e+00	−2.505 or 4.785	−2.3890000
$\log_{10}(k_1'k_2')$	−2.200e+00	−2.431e+00	3.095	−2.1980000
$\log_{10}(k_1'/k_2)$	5.843e−02	1.241e−01		0.0164100
$transport = \log_{10}(k_1'k_2'/k_2)$	−1.652e+00	−1.713e+00	−1.69 or 5.6	−1.6170000

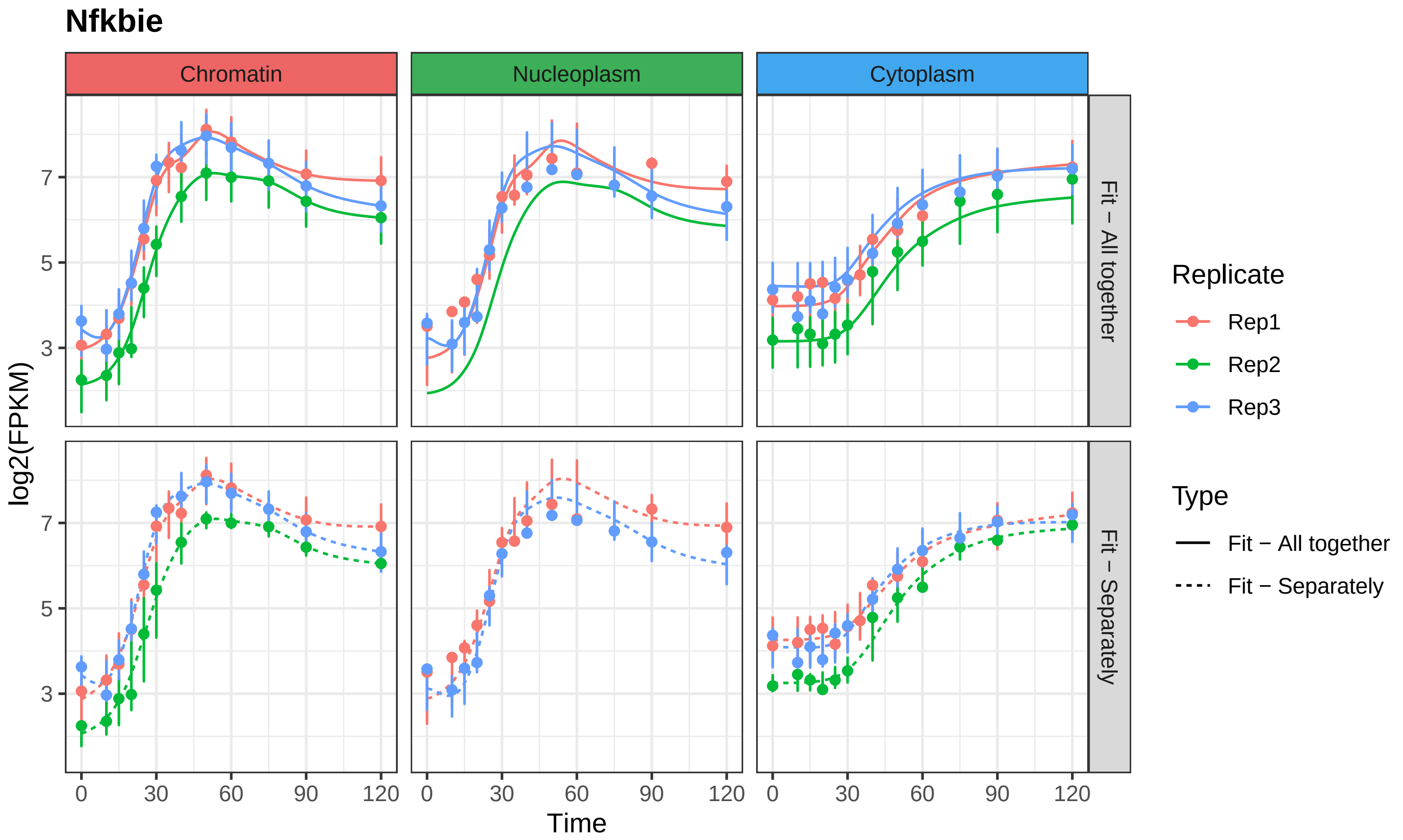

	Together	b1	b2	b3
**−NLL b1 ca**	−2.714	−2.203		
**−NLL b1 np**	12.41	10.73		
**−NLL b1 cyto**	1.444	−1.792		
**−NLL b2 ca**	−2.394		−6.112	
**−NLL b2 np**				
**−NLL b2 cyto**	2.074		−3.003	
**−NLL b3 ca**	−1.845			−3.249
**−NLL b3 np**	6.566			4.997
**−NLL b3 cyto**	5.282			−2.245
Total	20.82	6.73	−9.115	−0.4967
**Total with regul**	23.6	7.418	−9.592	1.627

	Together	b1	b2	b3
spar	3.246e−01	0.3707000	0.3613	0.322300
σ_b	1.977e−01	0.1870000	0.0541	0.153800
σ_t	4.733e−05	0.0004791	2.463	0.001882
ca_{0,b_1}				
$\log_{10}(k_1')$	−1.534e−01	−0.2681000		−0.513900
$\log_{10}(k_2)$	−9.352e−02	−0.2752000	−0.5826 or −2.097	−0.418200
$\log_{10}(k_2')$	−1.821e+00	−1.9920000		−1.798000
$\log_{10}(k_{deg})$	−2.186e+00	−2.4040000	−2.097 or −0.5826	−2.091000
$\log_{10}(k_1'k_2')$	−1.974e+00	−2.2600000	−2.322	−2.312000
$\log_{10}(k_1'/k_2)$	−5.991e−02	0.0070420		−0.095730
$\text{transport} = \log_{10}(k_1'k_2'/k_2)$	−1.881e+00	−1.9850000	−1.739 or −0.225	−1.894000

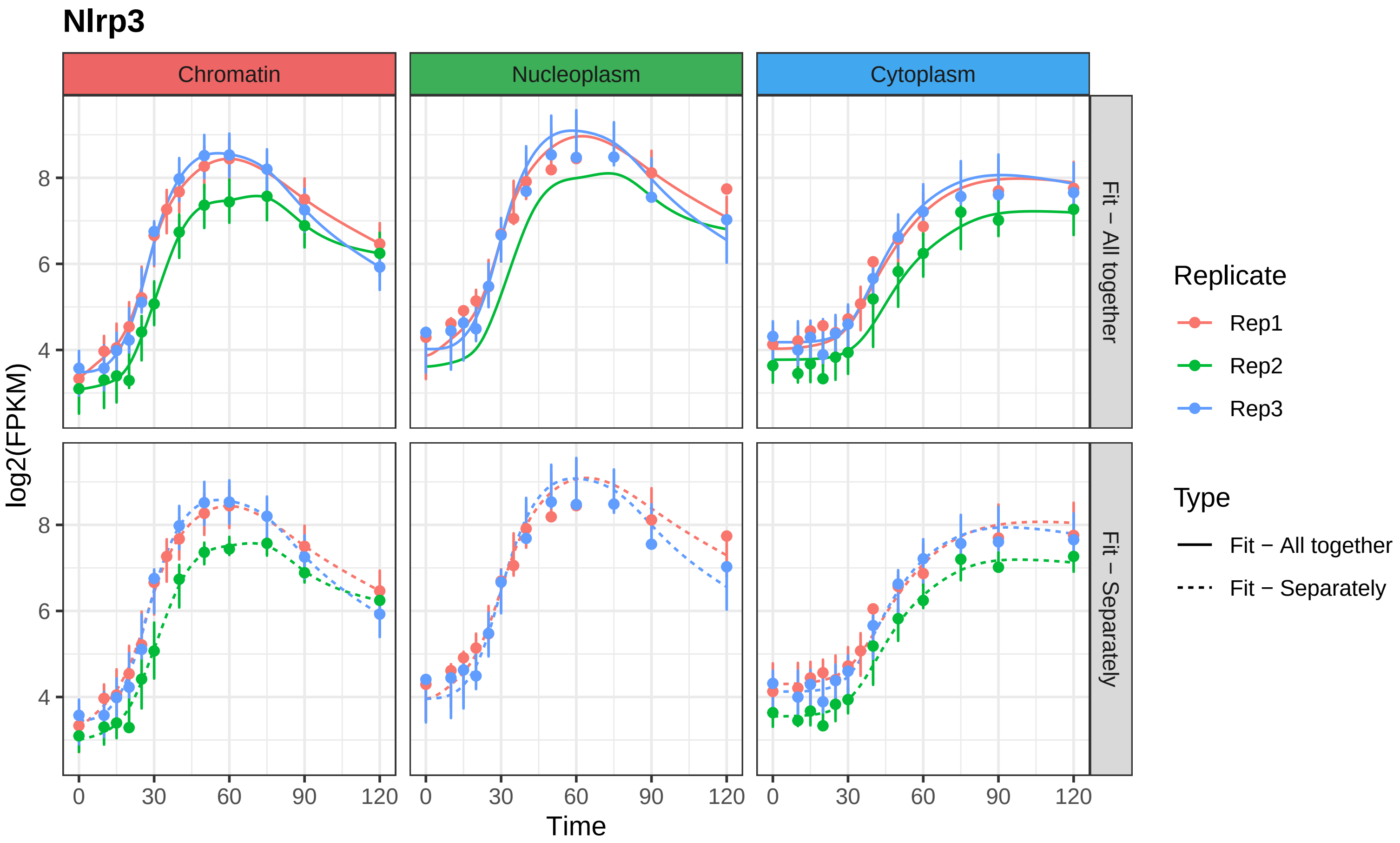

	Together	b1	b2	b3
**−NLL b1 ca**	−4.281	−3.775		
**−NLL b1 np**	7.777	5.942		
**−NLL b1 cyto**	1.181	0.5018		
**−NLL b2 ca**	−4.019		−6.271	
**−NLL b2 np**				
**−NLL b2 cyto**	2.013		−3.197	
**−NLL b3 ca**	−3.572			−2.549
**−NLL b3 np**	8.693			8.013
**−NLL b3 cyto**	−0.7454			−1.911
Total	7.046	2.67	−9.468	3.553
**Total with regul**	9.491	2.936	−10.41	4.911

	Together	b1	b2	b3
spar	0.3221000	3.742e−01	0.3772	0.3457000
σ_b	0.1728000	1.694e−01	0.0704	0.1732000
σ_t	0.0003357	8.462e−05	1.896	0.0002565
ca_{0,b_1}				
$\log_{10}(k_1')$	−0.2369000	−4.329e−01		−0.3309000
$\log_{10}(k_2)$	−0.3979000	−6.381e−01	5.34 or −1.79	−0.4859000
$\log_{10}(k_2')$	−1.8150000	−1.883e+00		−1.8660000
$\log_{10}(k_{deg})$	−1.8630000	−1.985e+00	−1.79 or 5.34	−1.9160000
$\log_{10}(k_1'k_2')$	−2.0520000	−2.316e+00	3.712	−2.1960000
$\log_{10}(k_1'/k_2)$	0.1610000	2.052e−01		0.1550000
$\text{transport} = \log_{10}(k_1'k_2'/k_2)$	−1.6540000	−1.678e+00	−1.627 or 5.503	−1.7110000

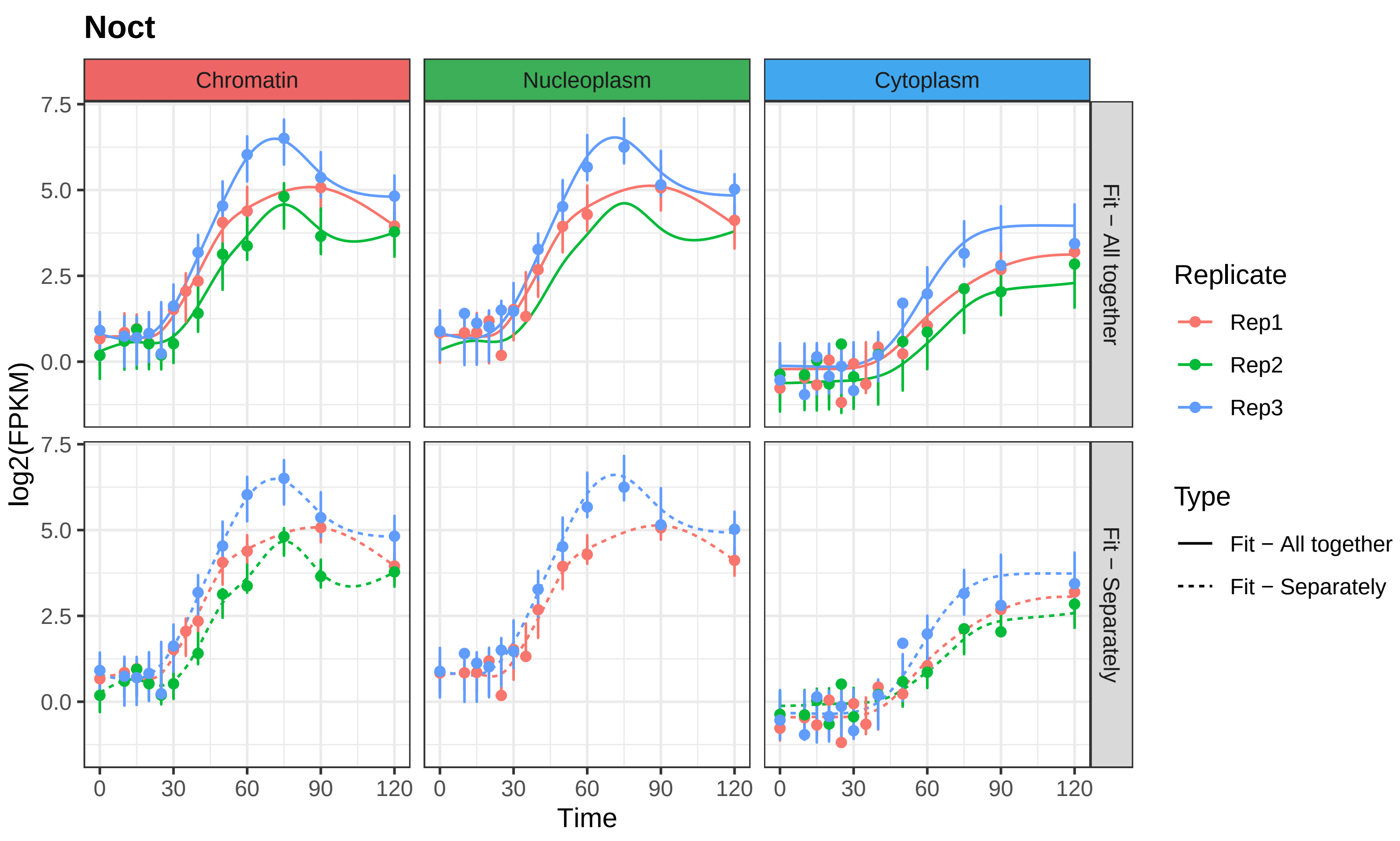

	Together	b1	b2	b3
**−NLL b1 ca**	1.152	−1.855		
**−NLL b1 np**	3.108	1.465		
**−NLL b1 cyto**	7.13	6.195		
**−NLL b2 ca**	1.299		−2.363	
**−NLL b2 np**				
**−NLL b2 cyto**	11.08		4.573	
**−NLL b3 ca**	0.9902			1.254
**−NLL b3 np**	3.535			3.17
**−NLL b3 cyto**	13.27			10.62
Total	41.56	5.805	2.21	15.05
**Total with regul**	41.98	5.472	2.38	15.35

	Together	b1	b2	b3
spar	0.4162000	0.37840	0.3614	0.420300
σ_b	0.2240000	0.13690	0.1355	0.221000
σ_t	0.0004374	1.06100	0.00113	0.004733
ca_{0,b_1}				
$\log_{10}(k_1')$	6.1860000	−0.22530		3.985000
$\log_{10}(k_2)$	6.1750000	−0.24430	9.317 or −2.027	3.949000
$\log_{10}(k_2')$	−2.1900000	−2.16200		−2.291000
$\log_{10}(k_{deg})$	−1.9010000	−1.79300	−2.027 or 9.317	−1.917000
$\log_{10}(k_1'k_2')$	3.9960000	−2.38800	7.175	1.694000
$\log_{10}(k_1'/k_2)$	0.0112800	0.01906		0.036070
transport $= \log_{10}(k_1'k_2'/k_2)$	−2.1790000	−2.14300	−2.142 or 9.203	−2.255000

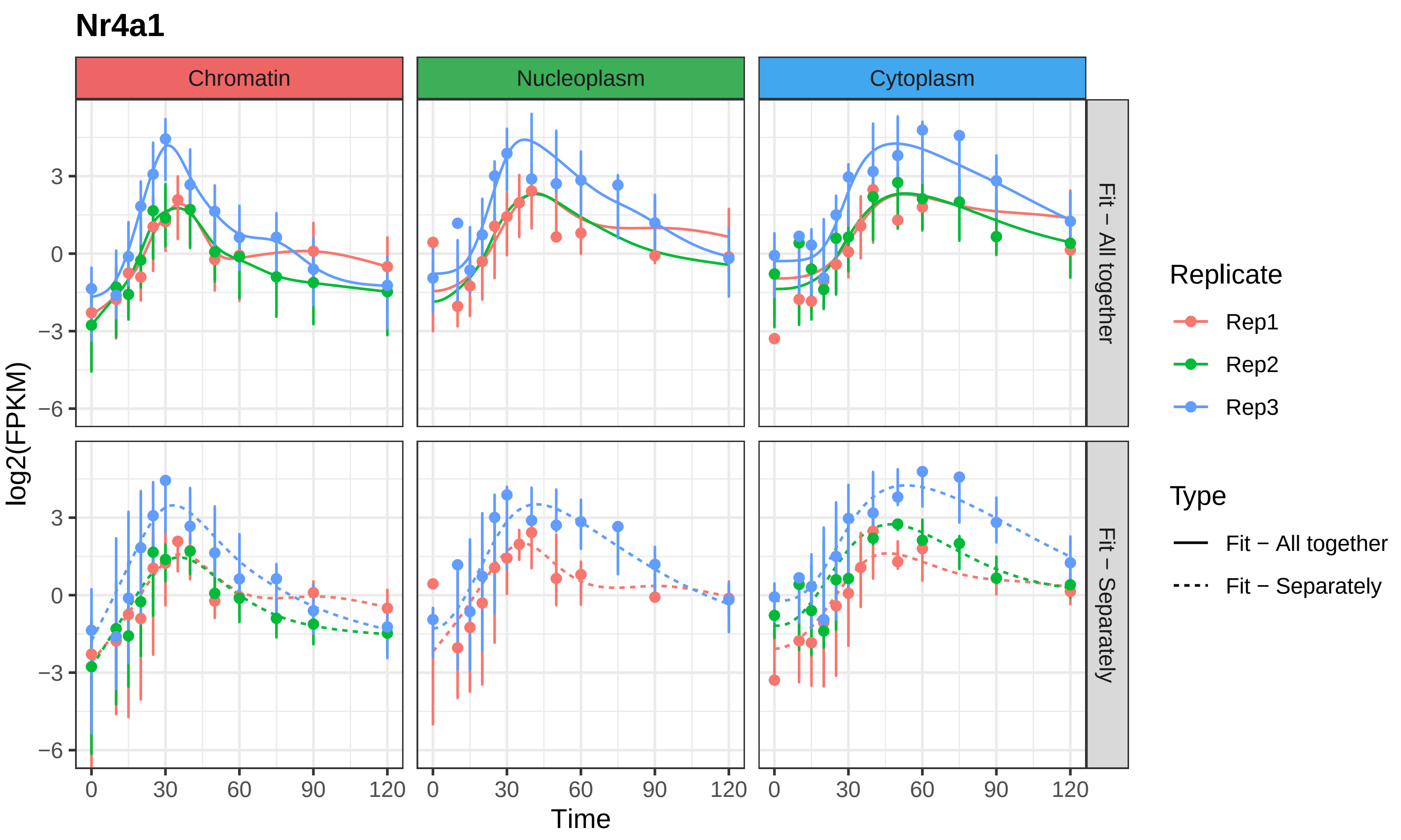

	Together	b1	b2	b3
**−NLL b1 ca**	6.852	9.79		
**−NLL b1 np**	18.45	15.26		
**−NLL b1 cyto**	18.54	13.22		
**−NLL b2 ca**	6.993		7.79	
**−NLL b2 np**				
**−NLL b2 cyto**	12.91		9.302	
**−NLL b3 ca**	6.484			14.06
**−NLL b3 np**	16.41			11.85
**−NLL b3 cyto**	12.64			10.16
Total	99.28	38.26	17.09	36.07
**Total with regul**	107.7	38.32	15.86	37.54

	Together	b1	b2	b3
spar	0.3198000	0.4856	0.4924	0.5071
σ_b	0.3843000	0.1506	1.873e−06	0.2290
σ_t	0.0001375	7.0440	6.012	5.0870
ca_{0,b_1}				
$\log_{10}(k_1')$	−0.7479000	6.3810		−0.9458
$\log_{10}(k_2)$	−1.0200000	6.2590	−0.4897 or −1.045	−1.0920
$\log_{10}(k_2')$	−1.0530000	−1.1410		−0.8045
$\log_{10}(k_{deg})$	−1.2010000	−1.1760	−1.045 or −0.4897	−1.1260
$\log_{10}(k_1'k_2')$	−1.8010000	5.2400	−1.049	−1.7500
$\log_{10}(k_1'/k_2)$	0.2719000	0.1223		0.1466
$\text{transport} = \log_{10}(k_1'k_2'/k_2)$	−0.7811000	−1.0190	−0.5595 or −0.004394	−0.6579

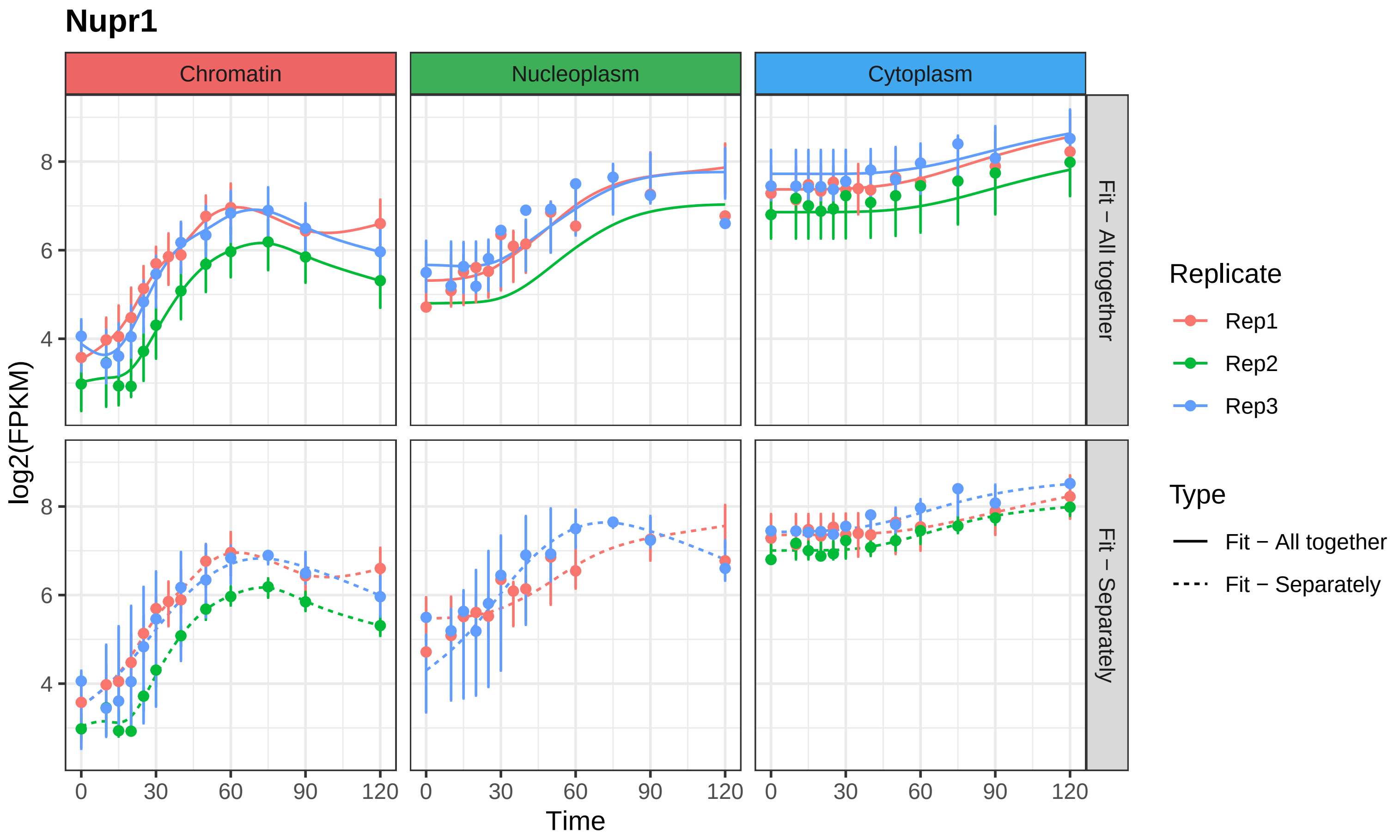

	Together	b1	b2	b3
**−NLL b1 ca**	−2.907	−3.994		
**−NLL b1 np**	10.66	11.54		
**−NLL b1 cyto**	−1.928	−4.576		
**−NLL b2 ca**	−1.648		−6.489	
**−NLL b2 np**				
**−NLL b2 cyto**	1.029		−8.151	
**−NLL b3 ca**	−2.613			4.054
**−NLL b3 np**	16.31			7.848
**−NLL b3 cyto**	0.7576			−12.47
Total	19.67	2.969	−14.64	−0.5641
**Total with regul**	20.03	2.175	−14.76	1.94

	Together	b1	b2	b3
spar	3.878e−01	4.284e−01	0.3509	5.710e−01
σ_b	1.955e−01	1.692e−01	0.06777	4.736e−05
σ_t	2.801e−06	7.189e−07	0.0002859	9.735e+00
ca_{0,b_1}				
$\log_{10}(k_1')$	−1.378e+00	−1.613e+00		4.636e+00
$\log_{10}(k_2)$	−1.915e+00	−2.210e+00	4.803 or −2.659	4.391e+00
$\log_{10}(k_2')$	−1.625e+00	−1.652e+00		−1.774e+00
$\log_{10}(k_{deg})$	−2.245e+00	−2.219e+00	−2.659 or 4.803	−2.715e+00
$\log_{10}(k_1'k_2')$	−3.003e+00	−3.266e+00	3.339	2.862e+00
$\log_{10}(k_1'/k_2)$	5.363e−01	5.961e−01		2.445e−01
$transport = \log_{10}(k_1'k_2'/k_2)$	−1.089e+00	−1.056e+00	−1.464 or 5.997	−1.529e+00

Odc1

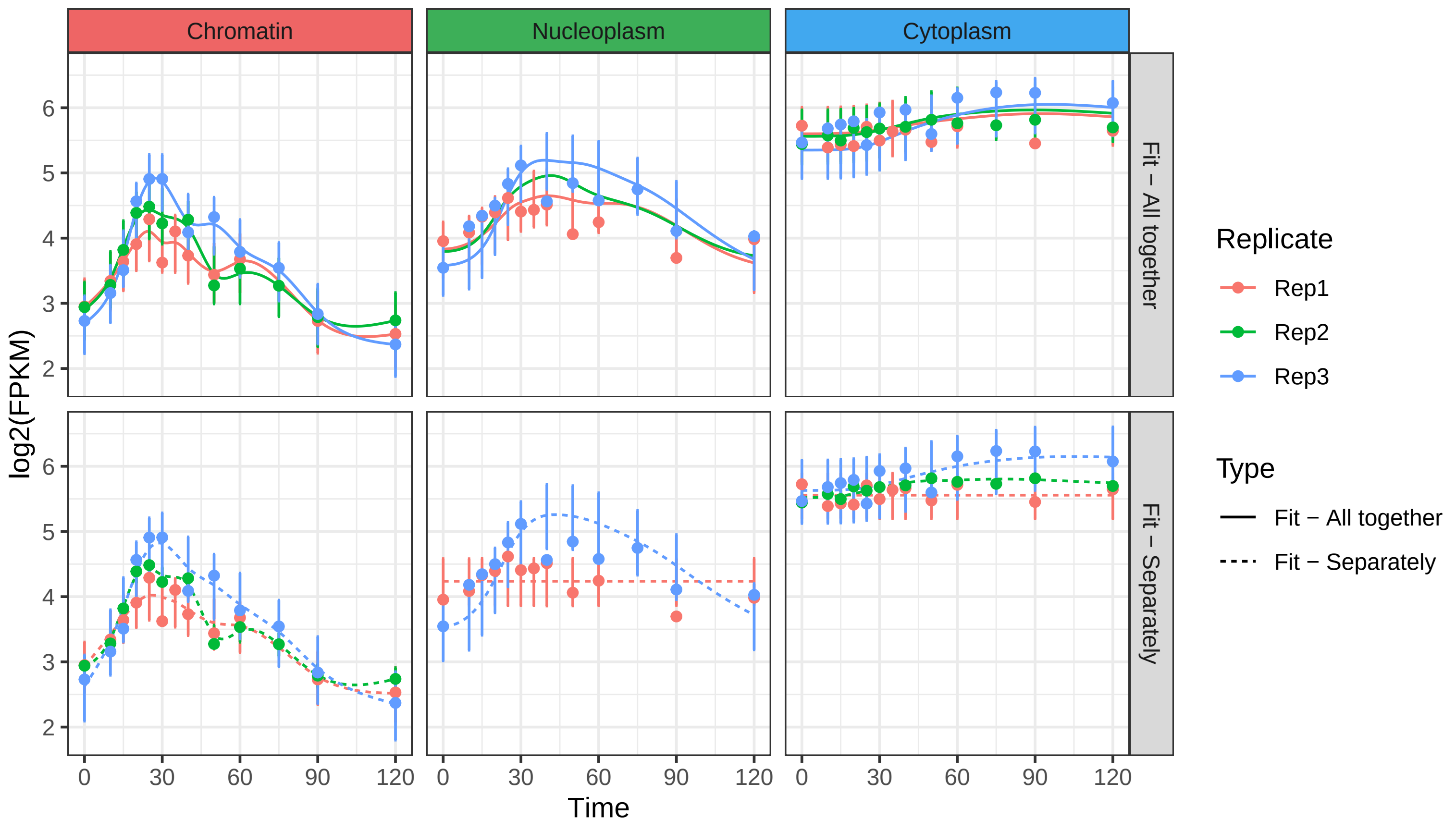

	Together	b1	b2	b3
**−NLL b1 ca**	−4.963	−4.539		
**−NLL b1 np**	2.802	2.395		
**−NLL b1 cyto**	−1.351	−6.728		
**−NLL b2 ca**	−6.036		−12.64	
**−NLL b2 np**				
**−NLL b2 cyto**	−5.393		−17.25	
**−NLL b3 ca**	−5.833			−1.912
**−NLL b3 np**	9.379			7.41
**−NLL b3 cyto**	2.804			−2.659
Total	−8.591	−8.871	−29.9	2.838
**Total with regul**	−4.951	−10.22	−25.39	2.626

	Together	b1	b2	b3
spar	0.2936000	4.157e−01	0.2657	3.914e−01
σ_b	0.1461000	1.210e−01	0.02676	1.685e−01
σ_t	0.0006545	1.122e−05	0.001376	4.375e−05
ca_{0,b_1}				
$\log_{10}(k_1')$	−1.0200000	−1.653e+01		−1.003e+00
$\log_{10}(k_2)$	−1.2880000	−1.693e+01	4.756 or −2.396	−1.280e+00
$\log_{10}(k_2')$	−1.6140000	−3.619e+01		−1.778e+00
$\log_{10}(k_{deg})$	−2.1460000	−3.659e+01	−2.396 or 4.756	−2.406e+00
$\log_{10}(k_1' k_2')$	−2.6340000	−5.273e+01	3.145	−2.781e+00
$\log_{10}(k_1'/k_2)$	0.2679000	3.922e−01		2.769e−01
$\text{transport} = \log_{10}(k_1' k_2'/k_2)$	−1.3460000	−3.580e+01	−1.612 or 5.541	−1.502e+00

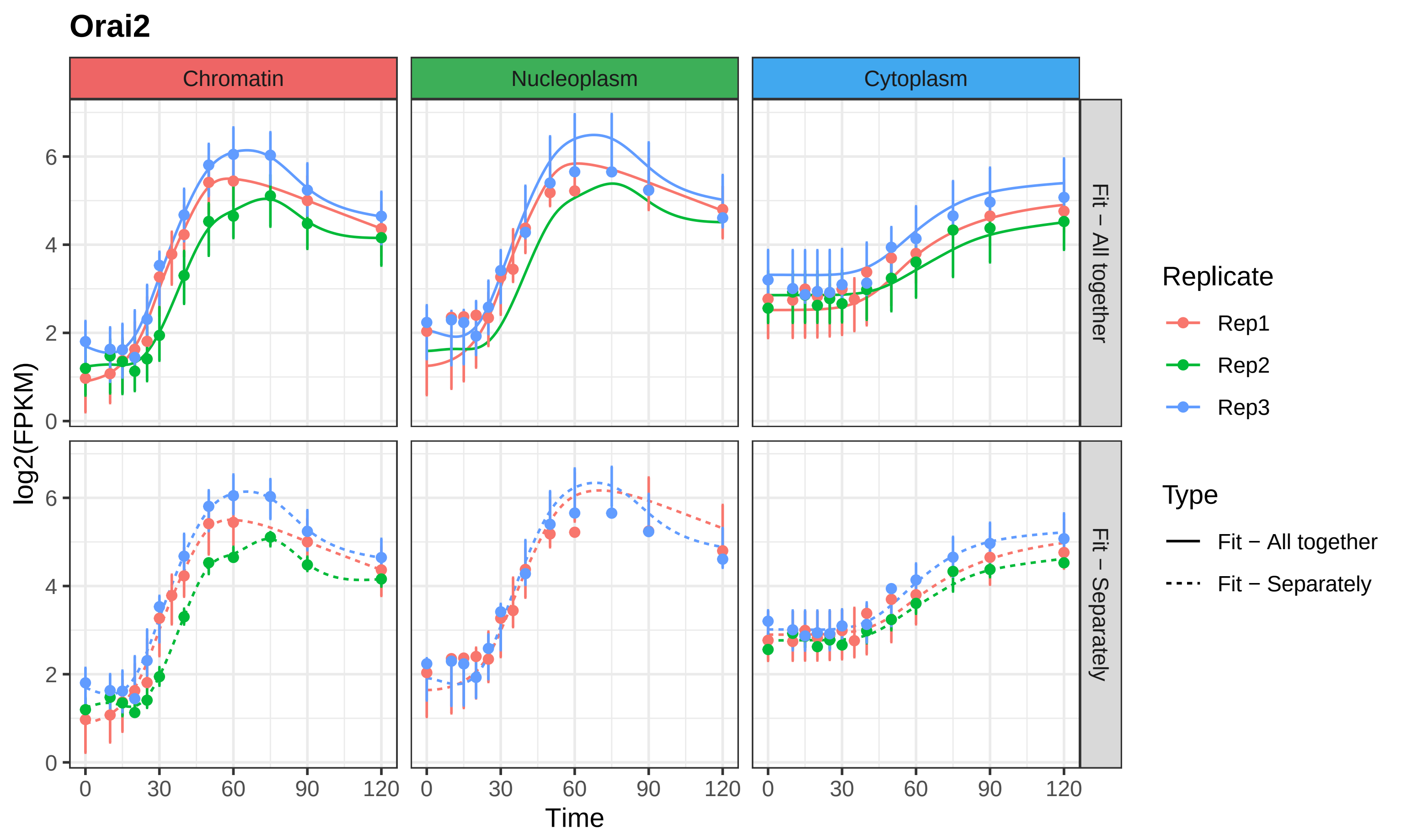

	Together	b1	b2	b3
**−NLL b1 ca**	−1.522	−1.92		
**−NLL b1 np**	15.1	11.28		
**−NLL b1 cyto**	3.214	−1.398		
**−NLL b2 ca**	−2.271		−11.68	
**−NLL b2 np**				
**−NLL b2 cyto**	−0.7909		−8.274	
**−NLL b3 ca**	−1.258			−2.506
**−NLL b3 np**	9.274			7.303
**−NLL b3 cyto**	2.951			−3.674
Total	24.7	7.962	−19.96	1.123
**Total with regul**	25.42	8.094	−19.03	0.9907

	Together	b1	b2	b3
spar	3.805e−01	3.945e−01	0.3253	0.38040
σ_b	2.037e−01	1.915e−01	0.05168	0.15510
σ_t	2.157e−07	2.892e−05	0.001077	0.83850
ca_{0,b_1}				
$\log_{10}(k_1')$	−2.680e−01	−6.832e−01		−0.41650
$\log_{10}(k_2)$	−3.752e−01	−9.130e−01	6.524 or −2.365	−0.48050
$\log_{10}(k_2')$	−2.082e+00	−2.207e+00		−2.07200
$\log_{10}(k_{deg})$	−2.463e+00	−2.585e+00	−2.365 or 6.524	−2.40500
$\log_{10}(k_1'k_2')$	−2.350e+00	−2.890e+00	4.619	−2.48800
$\log_{10}(k_1'/k_2)$	1.072e−01	2.298e−01		0.06395
transport = $\log_{10}(k_1'k_2'/k_2)$	−1.975e+00	−1.977e+00	−1.905 or 6.984	−2.00800

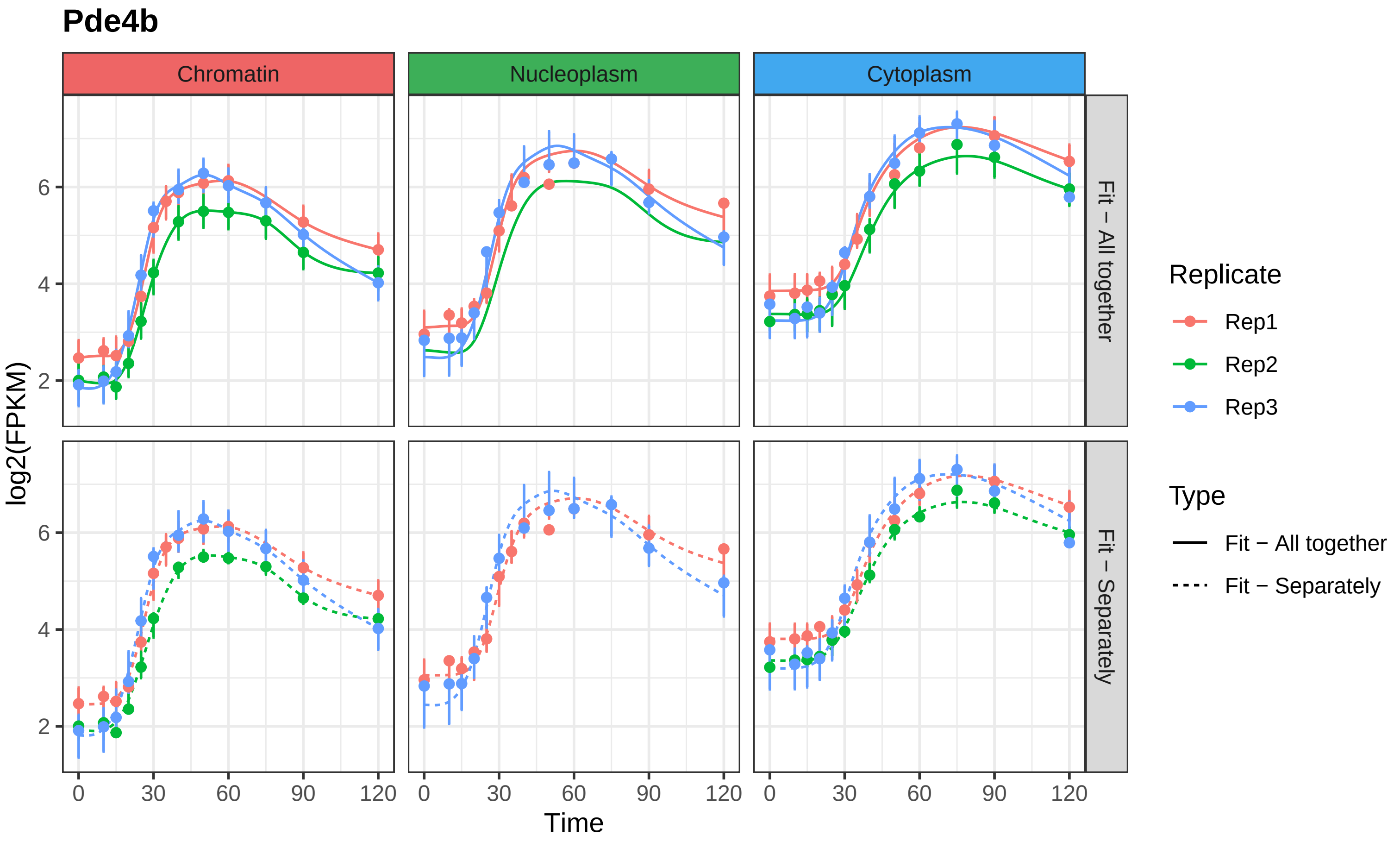

	Together	b1	b2	b3
**−NLL b1 ca**	−8.466	−8.034		
**−NLL b1 np**	2.611	1.921		
**−NLL b1 cyto**	−5.239	−7.117		
**−NLL b2 ca**	−8.727		−10.59	
**−NLL b2 np**				
**−NLL b2 cyto**	−6.473		−11.98	
**−NLL b3 ca**	−8.296			−5.004
**−NLL b3 np**	5.449			2.503
**−NLL b3 cyto**	−0.3723			−0.5916
Total	−29.51	−13.23	−22.57	−3.092
**Total with regul**	−25.49	−11.68	−22.66	−0.7912

	Together	b1	b2	b3
spar	0.2820	0.3220	0.3463	0.3159000
σ_b	0.1176	0.1086	0.03708	0.1429000
σ_t	0.2184	0.3243	0.6892	0.0001223
ca_{0,b_1}				
$\log_{10}(k_1')$	−0.3486	−0.4908		−0.1225000
$\log_{10}(k_2)$	−0.5370	−0.6711	0.1516 or −1.355	−0.3108000
$\log_{10}(k_2')$	−1.0680	−1.0930		−1.1100000
$\log_{10}(k_{deg})$	−1.2950	−1.3180	−1.355 or 0.1516	−1.3370000
$\log_{10}(k_1'k_2')$	−1.4170	−1.5830	−0.7756	−1.2320000
$\log_{10}(k_1'/k_2)$	0.1884	0.1803		0.1883000
transport = $\log_{10}(k_1'k_2'/k_2)$	−0.8798	−0.9123	−0.9272 or 0.5796	−0.9214000

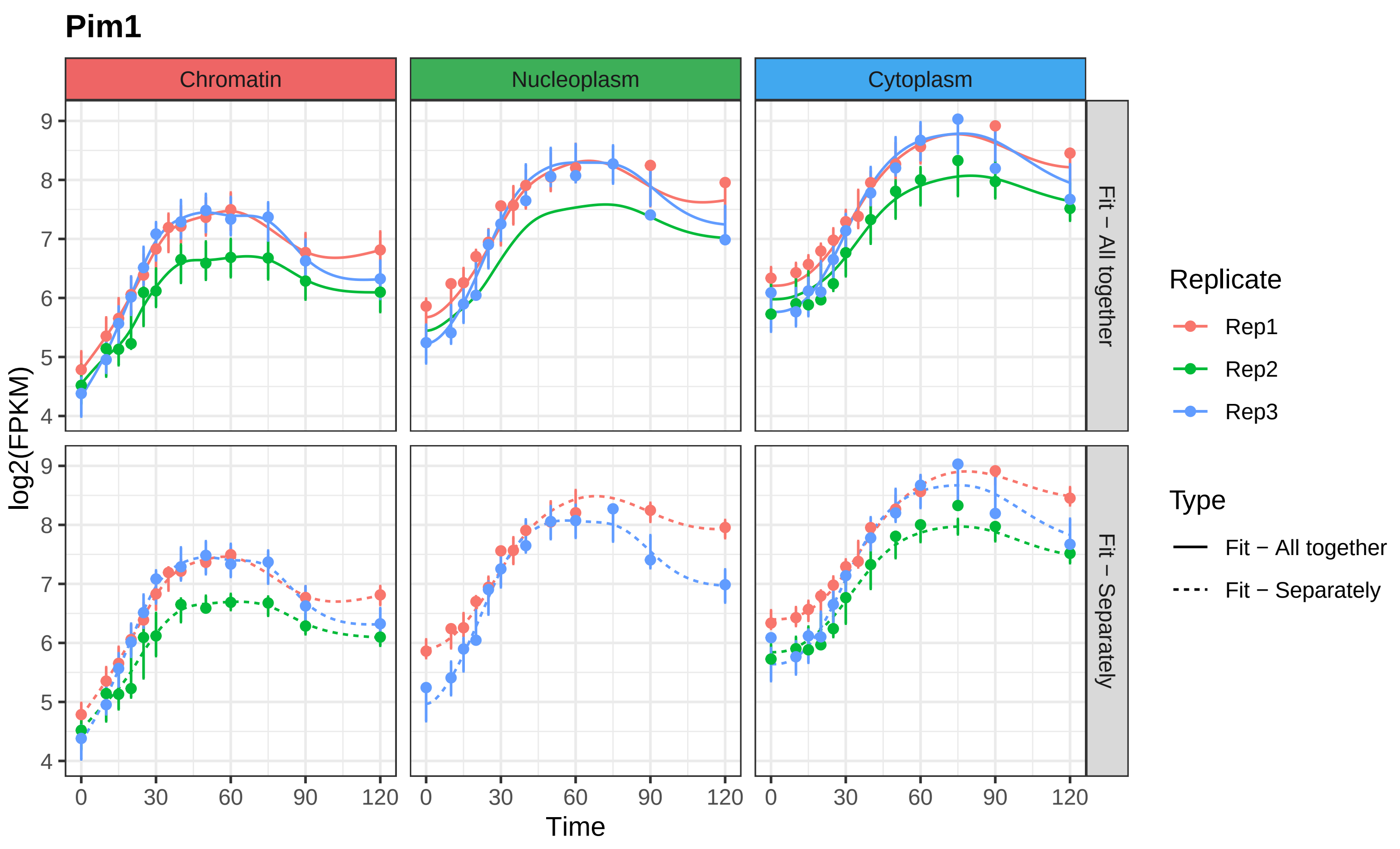

	Together	b1	b2	b3
**−NLL b1 ca**	−10.3	−15.06		
**−NLL b1 np**	−0.6245	−4.695		
**−NLL b1 cyto**	−5.082	−13.34		
**−NLL b2 ca**	−7.8		−9.367	
**−NLL b2 np**				
**−NLL b2 cyto**	−3.021		−6.237	
**−NLL b3 ca**	−9.695			−9.9
**−NLL b3 np**	0.256			−6.253
**−NLL b3 cyto**	0.0002904			−0.618
Total	−36.27	−33.09	−15.6	−16.77
**Total with regul**	−35.98	−34.89	−17.3	−17.33

	Together	b1	b2	b3
spar	3.507e−01	0.40840	0.4098	3.699e−01
σ_b	1.129e−01	0.05346	0.04564	9.766e−02
σ_t	2.167e−05	1.32600	3.143	1.506e−05
ca_{0,b_1}				
$\log_{10}(k_1')$	−6.248e−01	−0.78160		−5.110e−01
$\log_{10}(k_2)$	−8.956e−01	−1.12600	−0.852 or −0.8872	−7.060e−01
$\log_{10}(k_2')$	−8.722e−01	−0.88650		−8.988e−01
$\log_{10}(k_{deg})$	−1.032e+00	−1.03500	−0.8872 or −0.852	−1.101e+00
$\log_{10}(k_1'k_2')$	−1.497e+00	−1.66800	−1.344	−1.410e+00
$\log_{10}(k_1'/k_2)$	2.708e−01	0.34470		1.950e−01
$\text{transport} = \log_{10}(k_1'k_2'/k_2)$	−6.014e−01	−0.54180	−0.4922 or −0.4571	−7.038e−01

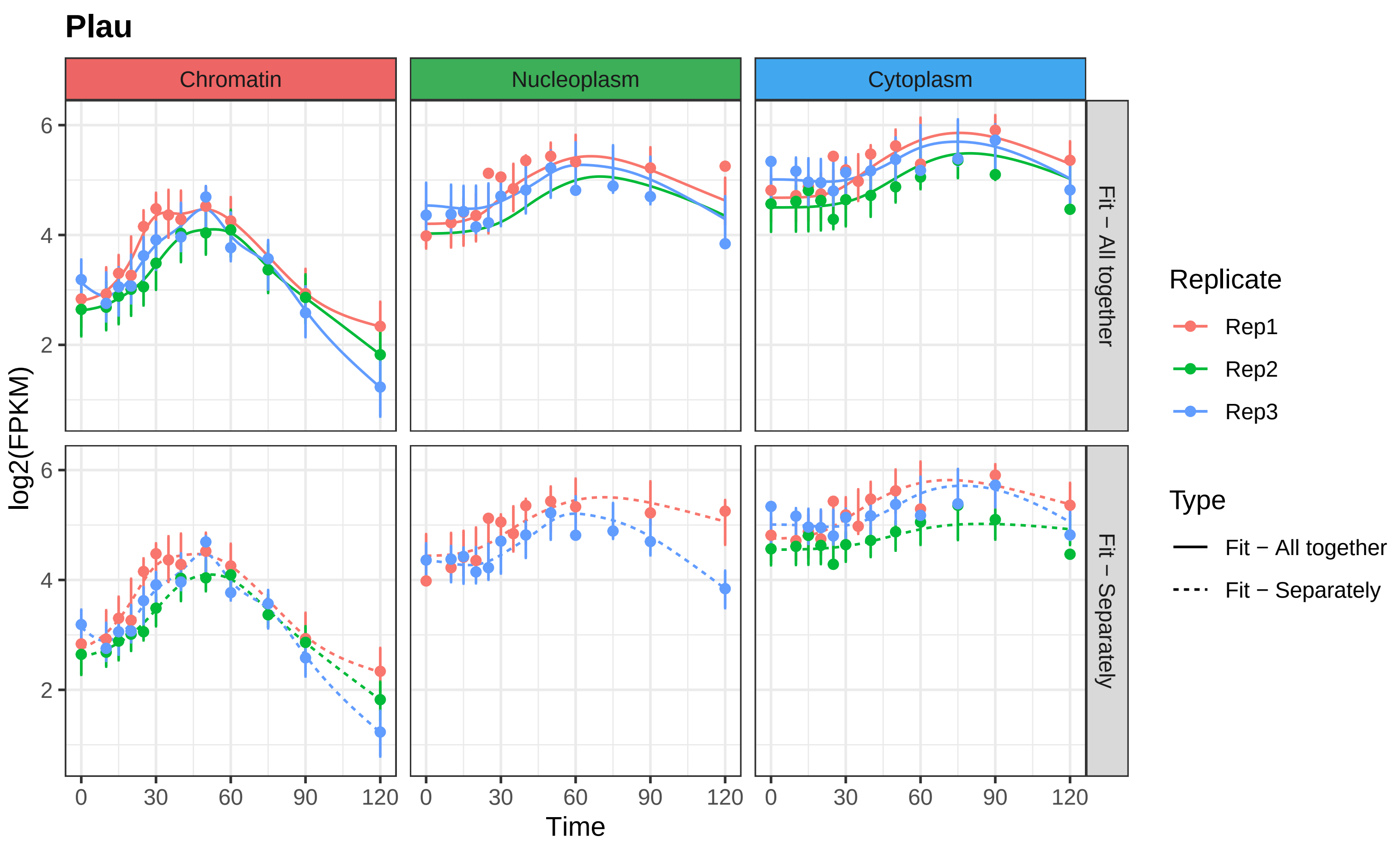

	Together	b1	b2	b3
**−NLL b1 ca**	−5.356	−4.598		
**−NLL b1 np**	5.759	1.282		
**−NLL b1 cyto**	1.867	−0.9723		
**−NLL b2 ca**	−6.493		−8.747	
**−NLL b2 np**				
**−NLL b2 cyto**	0.05518		−2.078	
**−NLL b3 ca**	−4.651			−5.438
**−NLL b3 np**	2.034			−4.021
**−NLL b3 cyto**	−1.941			−2.277
Total	−8.726	−4.289	−10.82	−11.74
**Total with regul**	−7.87	−5.545	−12.53	−11.14

	Together	b1	b2	b3
spar	3.476e−01	0.4303	0.4209	3.425e−01
σ_b	1.468e−01	0.1396	0.0943	1.094e−01
σ_t	6.449e−05	0.7153	0.0007371	5.841e−06
ca_{0,b_1}				
$\log_{10}(k_1')$	−1.145e+00	−1.2730		−1.057e+00
$\log_{10}(k_2)$	−1.567e+00	−1.7850	4.142 or −2.228	−1.422e+00
$\log_{10}(k_2')$	−8.542e−01	6.4900		−1.045e+00
$\log_{10}(k_{deg})$	−9.972e−01	6.3960	−2.228 or 4.142	−1.243e+00
$\log_{10}(k_1'k_2')$	−1.999e+00	5.2170	2.503	−2.102e+00
$\log_{10}(k_1'/k_2)$	4.219e−01	0.5115		3.649e−01
$\text{transport} = \log_{10}(k_1'k_2'/k_2)$	−4.324e−01	7.0010	−1.64 or 4.73	−6.798e−01

Plaur

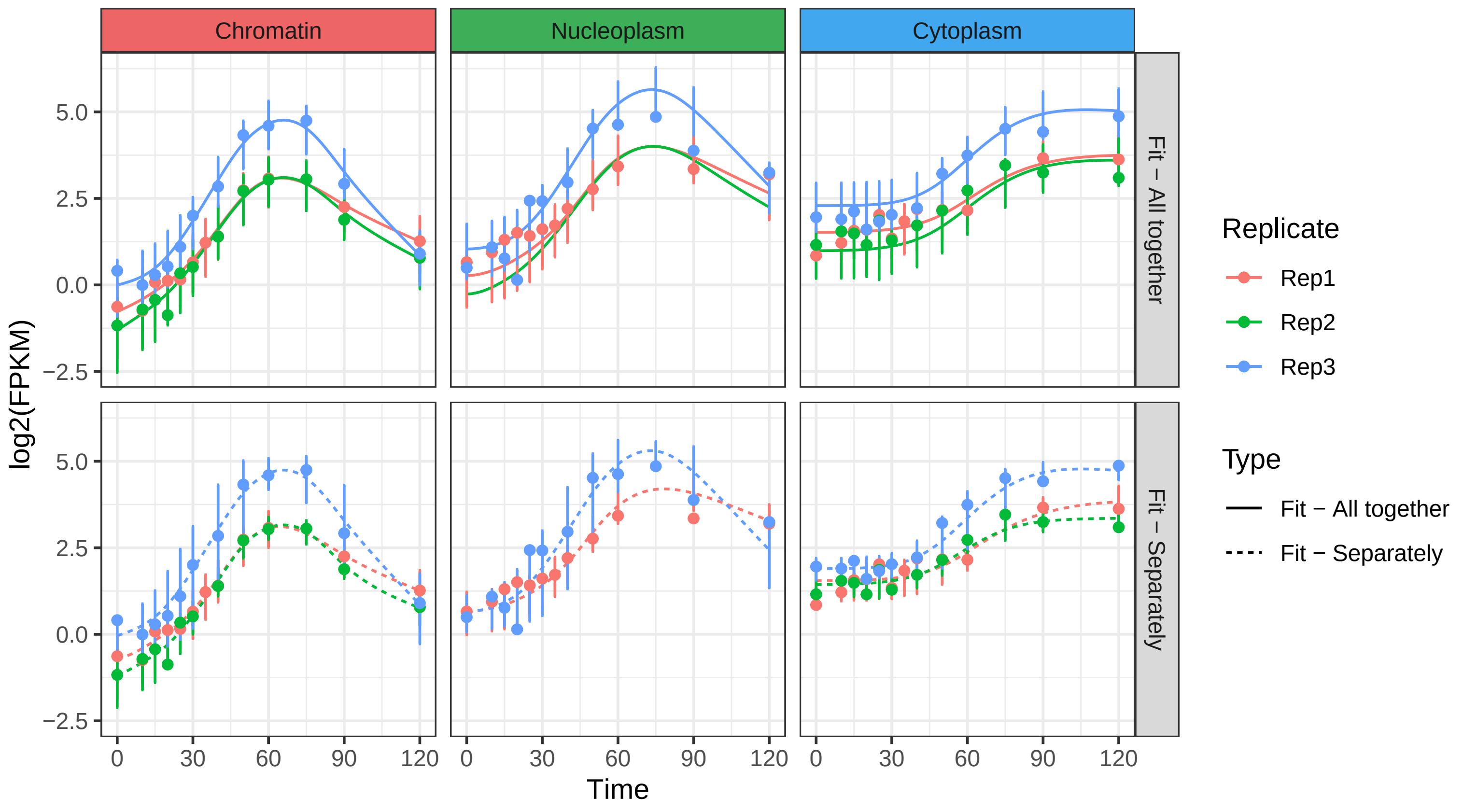

	Together	b1	b2	b3
**−NLL b1 ca**	1.744	−0.7289		
**−NLL b1 np**	7.019	3.898		
**−NLL b1 cyto**	3.256	3.482		
**−NLL b2 ca**	2.058		−0.9892	
**−NLL b2 np**				
**−NLL b2 cyto**	7.488		−0.946	
**−NLL b3 ca**	1.971			4.492
**−NLL b3 np**	16.64			10.64
**−NLL b3 cyto**	5.487			−1.58
Total	45.67	6.652	−1.935	13.55
**Total with regul**	46.25	5.644	−3.699	12.55

	Together	b1	b2	b3
spar	4.795e−01	4.392e−01	0.4228	0.48920
σ_b	2.333e−01	1.605e−01	0.09015	0.08517
σ_t	2.432e−05	1.117e−06	0.002226	5.67500
ca_{0,b_1}				
$\log_{10}(k_1')$	−6.559e−01	−8.119e−01		−0.72160
$\log_{10}(k_2)$	−9.697e−01	−1.236e+00	7.041 or −2.447	−0.93250
$\log_{10}(k_2')$	−1.760e+00	−1.794e+00		−1.73000
$\log_{10}(k_{deg})$	−2.136e+00	−2.058e+00	−2.447 or 7.041	−2.10200
$\log_{10}(k_1'k_2')$	−2.415e+00	−2.606e+00	5.39	−2.45200
$\log_{10}(k_1'/k_2)$	3.138e−01	4.241e−01		0.21100
transport $= \log_{10}(k_1'k_2'/k_2)$	−1.446e+00	−1.370e+00	−1.65 or 7.837	−1.51900

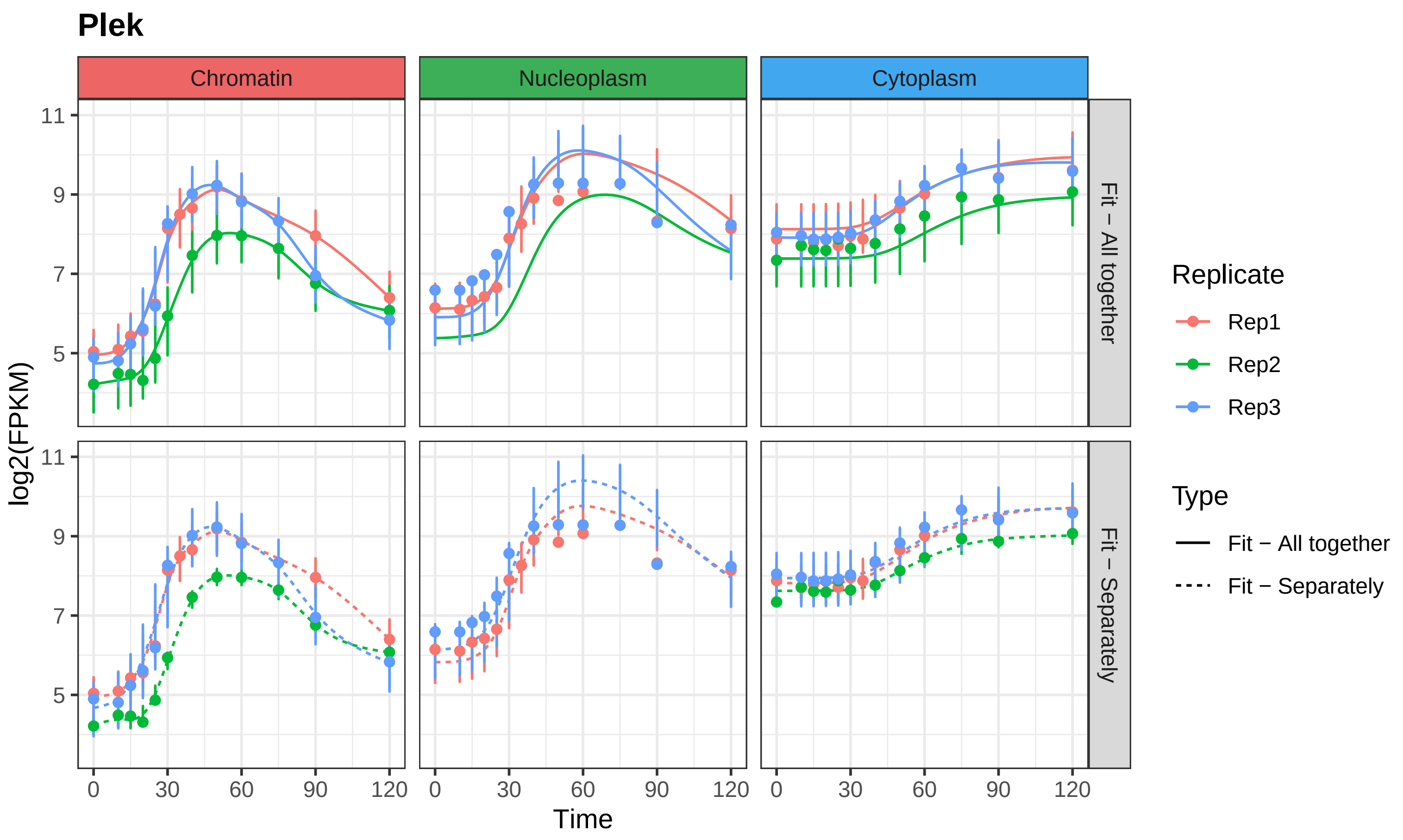

	Together	b1	b2	b3
**−NLL b1 ca**	0.3999	−2.134		
**−NLL b1 np**	11.7	11.41		
**−NLL b1 cyto**	3.099	−4.069		
**−NLL b2 ca**	−0.5815		−10.66	
**−NLL b2 np**				
**−NLL b2 cyto**	2.664		−7.542	
**−NLL b3 ca**	0.7424			1.631
**−NLL b3 np**	22.05			15.85
**−NLL b3 cyto**	−0.8878			−0.7667
Total	39.19	5.205	−18.2	16.71
**Total with regul**	41.14	6.169	−15.96	17.93

	Together	b1	b2	b3
spar	0.3595	0.3561	0.3031	0.3863
σ_b	0.2273	0.1725	0.0703	0.2300
σ_t	1.5760	1.2370	0.0004793	1.9500
ca_{0,b_1}				
$\log_{10}(k_1')$	−0.7408	−0.7767		−0.6694
$\log_{10}(k_2)$	−1.0910	−1.0360	−0.1909 or −2.539	−1.1130
$\log_{10}(k_2')$	−1.8440	−1.8080		−2.0030
$\log_{10}(k_{deg})$	−2.4480	−2.4070	−2.539 or −0.1909	−2.5430
$\log_{10}(k_1'k_2')$	−2.5850	−2.5840	−1.712	−2.6720
$\log_{10}(k_1'/k_2)$	0.3504	0.2594		0.4435
transport $= \log_{10}(k_1'k_2'/k_2)$	−1.4940	−1.5480	−1.521 or 0.8277	−1.5590

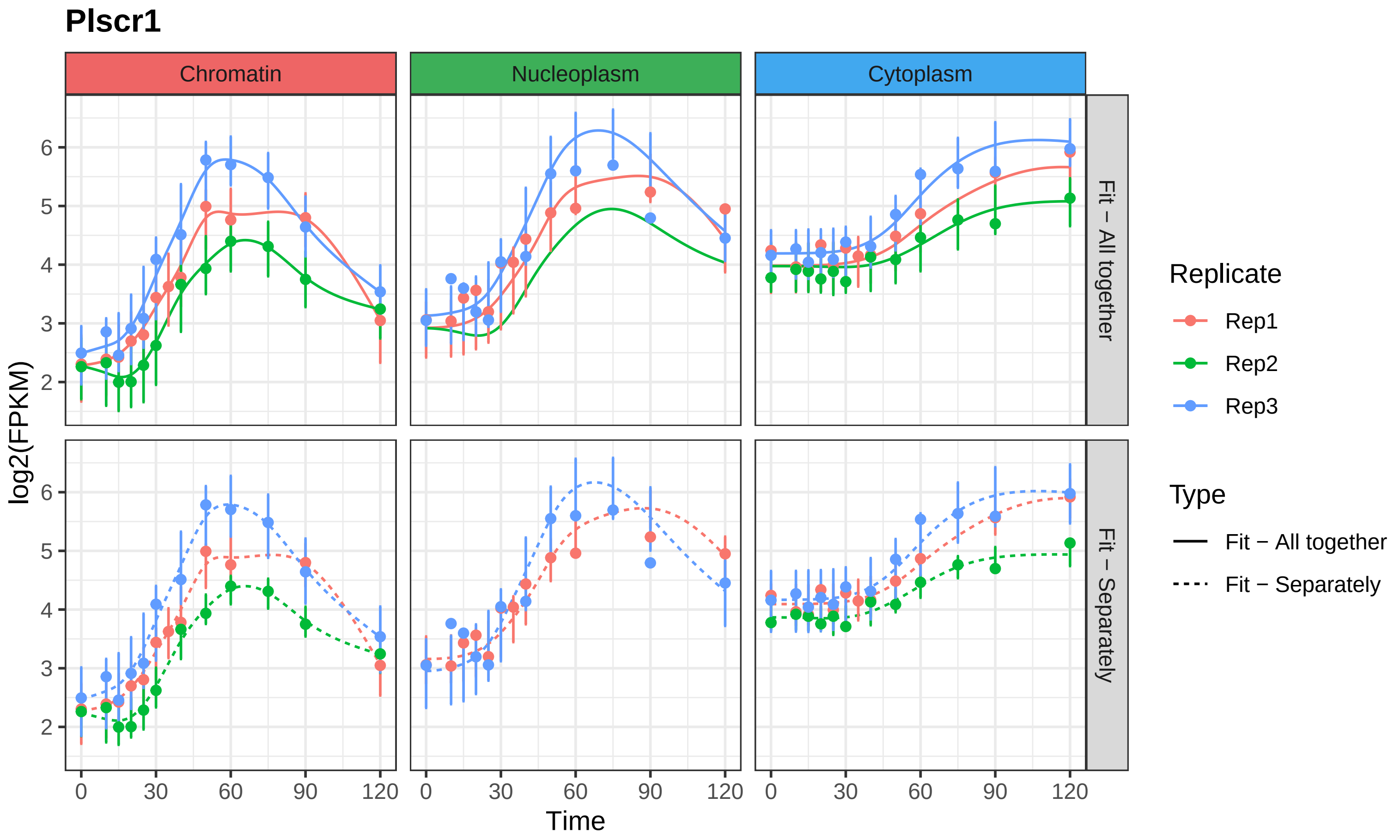

	Together	b1	b2	b3
**−NLL b1 ca**	−3.109	−5.044		
**−NLL b1 np**	5.54	3.839		
**−NLL b1 cyto**	−3.29	−7.023		
**−NLL b2 ca**	−4.268		−6.141	
**−NLL b2 np**				
**−NLL b2 cyto**	−4.345		−7.324	
**−NLL b3 ca**	−2.495			−0.9764
**−NLL b3 np**	15.91			9.886
**−NLL b3 cyto**	−2.624			−1.781
Total	1.323	−8.228	−13.47	7.129
**Total with regul**	1.047	−9.488	−15.52	6.838

	Together	b1	b2	b3
spar	0.3802	0.4020000	0.4242	0.4011
σ_b	0.1362	0.1094000	0.05037	0.1730
σ_t	2.6550	0.0001047	0.0005824	1.6100
ca_{0,b_1}				
$\log_{10}(k_1')$	−0.7975	−0.9146000		−0.7483
$\log_{10}(k_2)$	−0.9906	−1.1810000	3.745 or −2.051	−0.8927
$\log_{10}(k_2')$	−1.6720	−1.6080000		−1.6890
$\log_{10}(k_{deg})$	−1.9900	−1.8890000	−2.051 or 3.745	−2.0540
$\log_{10}(k_1'k_2')$	−2.4690	−2.5220000	2.182	−2.4370
$\log_{10}(k_1'/k_2)$	0.1931	0.2664000		0.1444
$transport = \log_{10}(k_1'k_2'/k_2)$	−1.4780	−1.3420000	−1.563 or 4.233	−1.5440

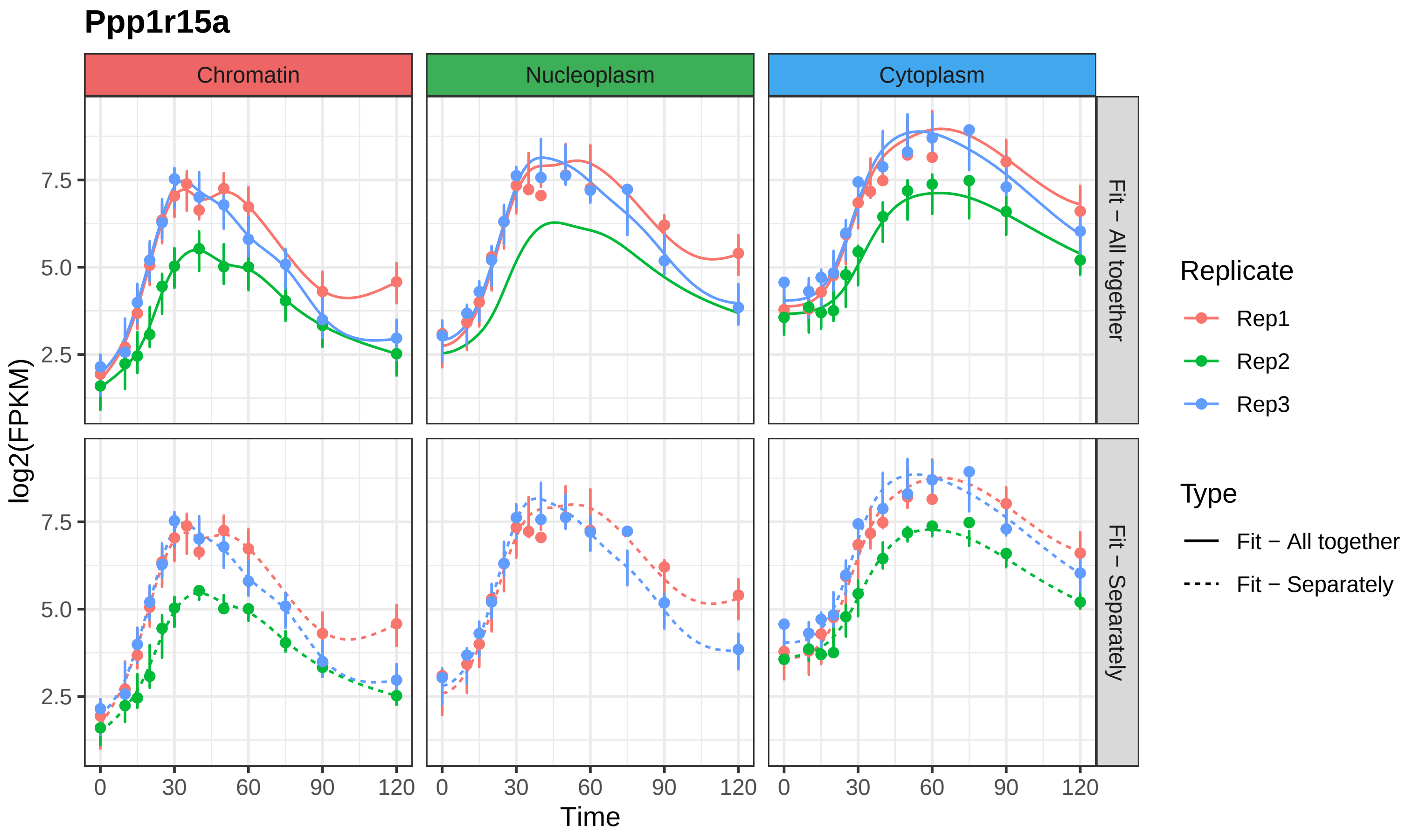

	Together	b1	b2	b3
**−NLL b1 ca**	−2.486	−1.259		
**−NLL b1 np**	7.513	7.318		
**−NLL b1 cyto**	5.722	2.544		
**−NLL b2 ca**	−2.964		−5.888	
**−NLL b2 np**				
**−NLL b2 cyto**	0.7227		−4.282	
**−NLL b3 ca**	−1.801			−2.283
**−NLL b3 np**	4.322			−0.5887
**−NLL b3 cyto**	7.056			5.502
Total	18.09	8.602	−10.17	2.63
**Total with regul**	21.41	10.14	−10.82	5.404

	Together	b1	b2	b3
spar	3.129e−01	0.352100	0.3659	0.3147000
σ_b	1.938e−01	0.196700	0.0622	0.1692000
σ_t	2.354e−05	0.000395	1.738	0.0001467
ca_{0,b_1}				
$\log_{10}(k_1')$	−5.729e−01	−0.563100		−0.4097000
$\log_{10}(k_2)$	−8.679e−01	−0.838200	−0.9237 or −0.9632	−0.6729000
$\log_{10}(k_2')$	−8.112e−01	−0.878700		−0.8575000
$\log_{10}(k_{deg})$	−1.151e+00	−1.181000	−0.9632 or −0.9237	−1.2280000
$\log_{10}(k_1' k_2')$	−1.384e+00	−1.442000	−1.237	−1.2670000
$\log_{10}(k_1'/k_2)$	2.950e−01	0.275100		0.2632000
transport = $\log_{10}(k_1' k_2'/k_2)$	−5.161e−01	−0.603600	−0.313 or −0.2735	−0.5944000

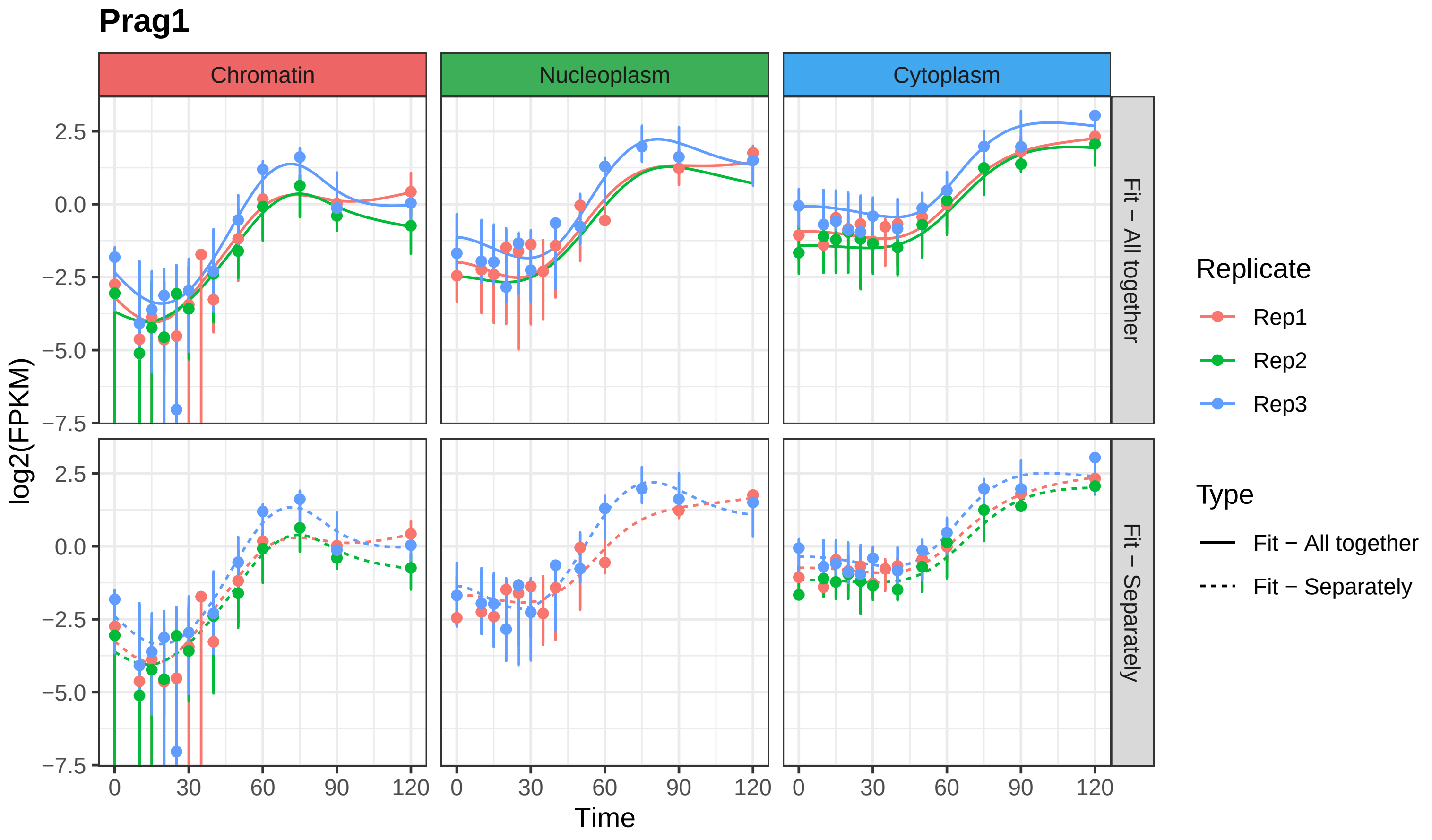

	Together	b1	b2	b3
**−NLL b1 ca**	10.98	10.43		
**−NLL b1 np**	11.69	8.425		
**−NLL b1 cyto**	2.939	−0.8403		
**−NLL b2 ca**	8.087		8.708	
**−NLL b2 np**				
**−NLL b2 cyto**	2.773		0.3312	
**−NLL b3 ca**	11.99			12.79
**−NLL b3 np**	9.512			7.651
**−NLL b3 cyto**	8.484			4.789
Total	66.46	18.02	9.039	25.23
**Total with regul**	66.38	16.73	7.242	25.45

	Together	b1	b2	b3
spar	0.5043000	5.172e−01	0.4888	0.5148000
σ_b	0.1818000	3.988e−07	1.51e−06	0.1844000
σ_t	0.0005547	2.553e+00	3.322	0.0001538
ca_{0,b_1}				
$\log_{10}(k_1')$	−0.7808000	−1.026e+00		−0.6159000
$\log_{10}(k_2)$	−1.1440000	−1.508e+00	−1.278 or −1.504	−0.9284000
$\log_{10}(k_2')$	−1.0840000	−9.363e−01		−1.2140000
$\log_{10}(k_{deg})$	−1.4040000	−1.212e+00	−1.504 or −1.278	−1.5160000
$\log_{10}(k_1' k_2')$	−1.8650000	−1.962e+00	−2.037	−1.8290000
$\log_{10}(k_1'/k_2)$	0.3636000	4.824e−01		0.3125000
transport = $\log_{10}(k_1' k_2'/k_2)$	−0.7203000	−4.539e−01	−0.7594 or −0.5336	−0.9010000

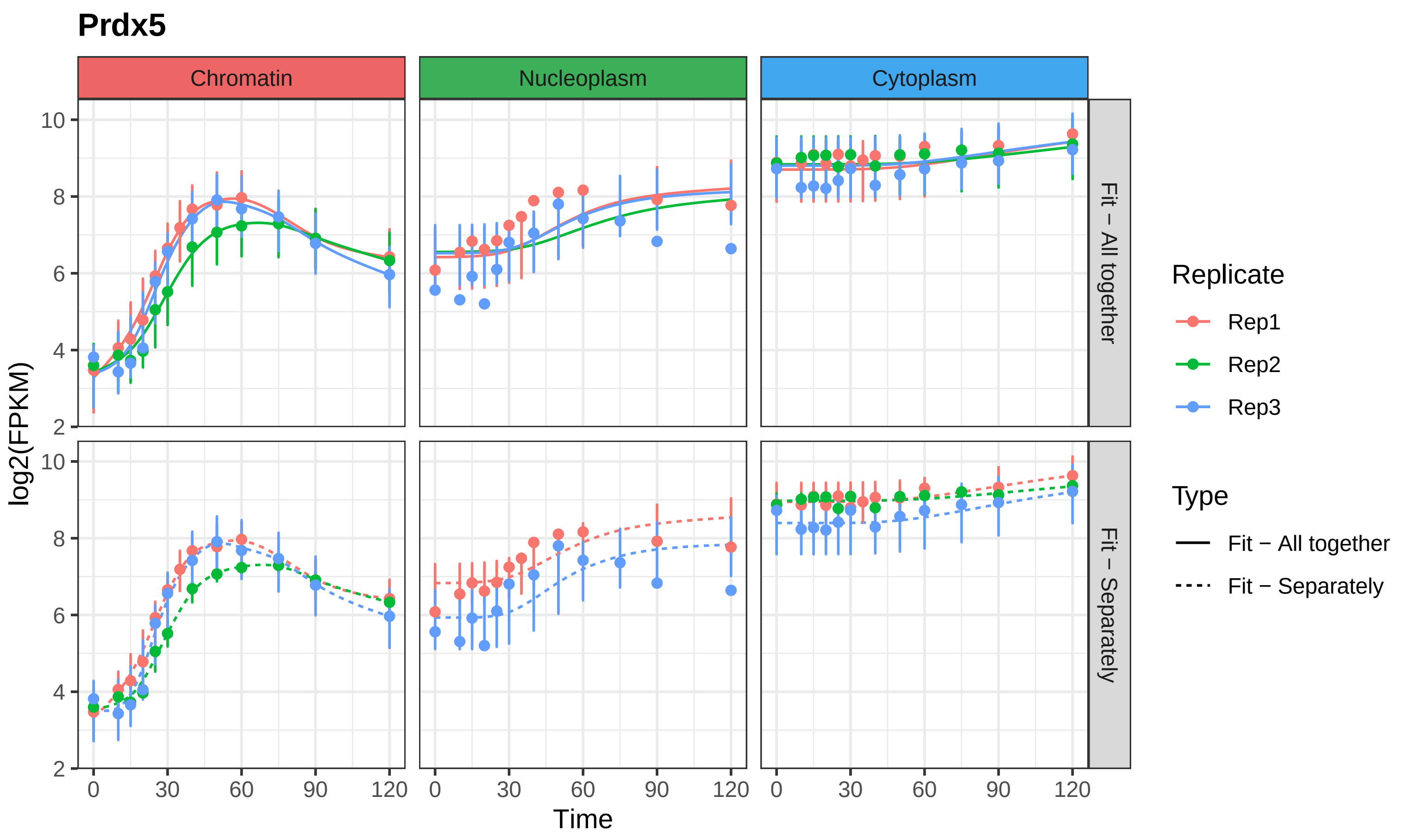

	Together	b1	b2	b3
**−NLL b1 ca**	0.8248	−3.456		
**−NLL b1 np**	13.02	12.28		
**−NLL b1 cyto**	2.131	−3.994		
**−NLL b2 ca**	1.1		−8.02	
**−NLL b2 np**				
**−NLL b2 cyto**	0.604		−8.386	
**−NLL b3 ca**	3.557			1.748
**−NLL b3 np**	24.36			16.55
**−NLL b3 cyto**	5.844			0.7138
Total	51.44	4.831	−16.41	19.02
**Total with regul**	52.7	4.459	−17.16	21.08

	Together	b1	b2	b3
spar	4.326e−01	4.086e−01	0.3687	3.784e−01
σ_b	2.652e−01	1.791e−01	0.06599	2.590e−01
σ_t	2.741e−05	1.864e−06	1.007	7.159e−06
ca_{0,b_1}				
$\log_{10}(k_1')$	−1.793e+00	−1.702e+00		−1.807e+00
$\log_{10}(k_2)$	−2.738e+00	−2.755e+00	−1.887 or −3.08	−2.521e+00
$\log_{10}(k_2')$	−1.558e+00	−1.606e+00		−1.505e+00
$\log_{10}(k_{deg})$	−2.245e+00	−2.243e+00	−3.08 or −1.887	−2.247e+00
$\log_{10}(k_1'k_2')$	−3.351e+00	−3.308e+00	−3.331	−3.312e+00
$\log_{10}(k_1'/k_2)$	9.451e−01	1.053e+00		7.144e−01
$\text{transport} = \log_{10}(k_1'k_2'/k_2)$	−6.132e−01	−5.526e−01	−1.444 or −0.2506	−7.911e−01

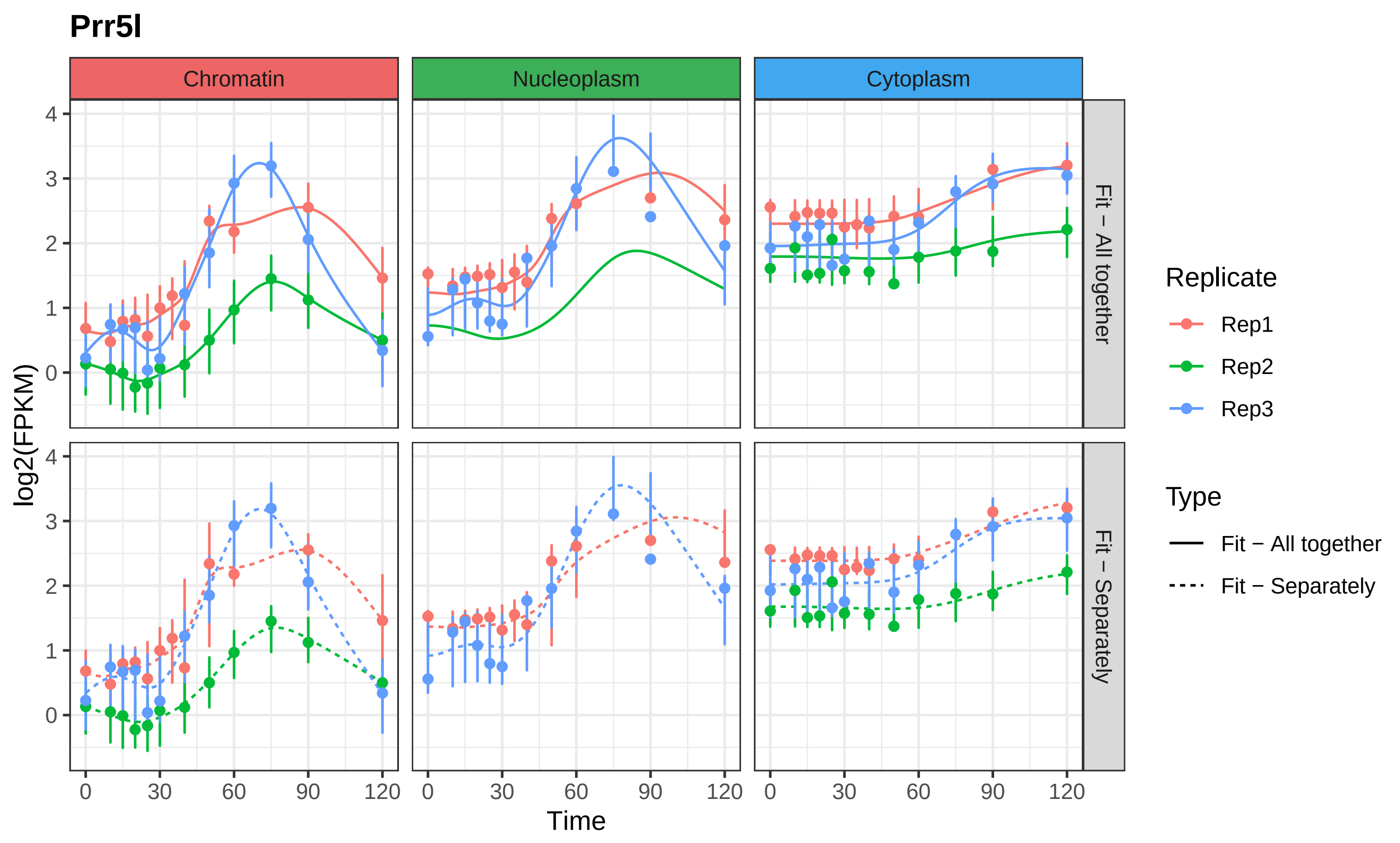

	Together	b1	b2	b3
**−NLL b1 ca**	−3.252	−4.327		
**−NLL b1 np**	−1.626	−1.442		
**−NLL b1 cyto**	−5.88	−9.017		
**−NLL b2 ca**	−6.085		−5.824	
**−NLL b2 np**				
**−NLL b2 cyto**	−1.352		−2.086	
**−NLL b3 ca**	−3.767			−1.703
**−NLL b3 np**	11.1			6.817
**−NLL b3 cyto**	−1.06			−1.262
Total	−11.93	−14.78	−7.91	3.851
**Total with regul**	−12.04	−14.57	−9.785	3.146

	Together	b1	b2	b3
spar	0.3685	0.36580	0.4433	0.4135000
σ_b	0.1238	0.05936	0.09325	0.1641000
σ_t	2.2930	6.84400	0.002471	0.0001543
ca_{0,b_1}				
$\log_{10}(k_1')$	−0.8082	−1.16600		−0.8454000
$\log_{10}(k_2)$	−0.9850	−1.38200	−1.636 or −1.68	−1.0190000
$\log_{10}(k_2')$	−1.8190	−1.72300		−1.9080000
$\log_{10}(k_{deg})$	−2.1400	−2.03000	−1.68 or −1.636	−2.2390000
$\log_{10}(k_1'k_2')$	−2.6280	−2.89000	−2.851	−2.7530000
$\log_{10}(k_1'/k_2)$	0.1768	0.21610		0.1739000
$\text{transport} = \log_{10}(k_1'k_2'/k_2)$	−1.6430	−1.50700	−1.215 or −1.171	−1.7340000

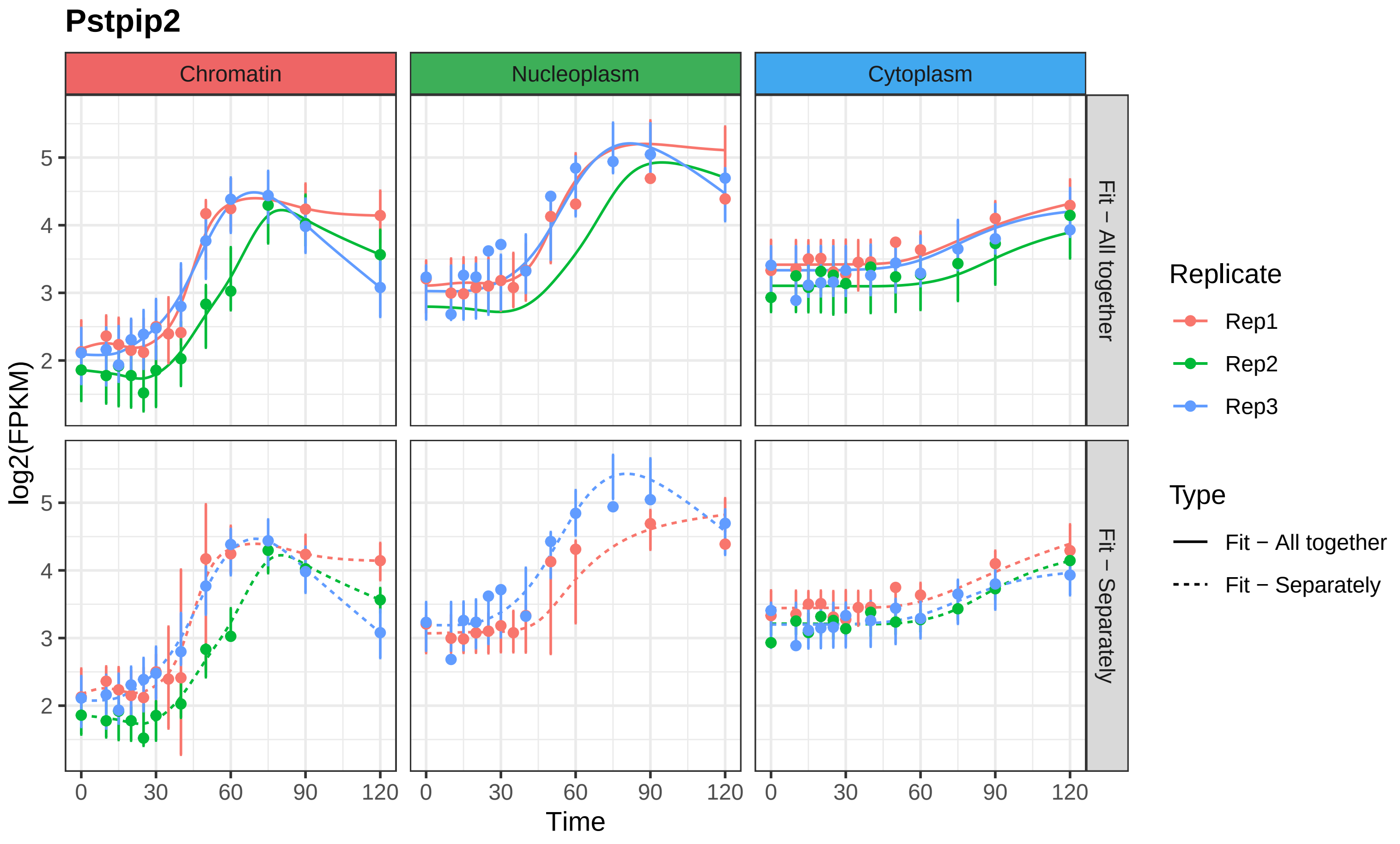

	Together	b1	b2	b3
**−NLL b1 ca**	−4.363	−5.092		
**−NLL b1 np**	4.885	0.316		
**−NLL b1 cyto**	−6.574	−7.598		
**−NLL b2 ca**	−5.504		−7.497	
**−NLL b2 np**				
**−NLL b2 cyto**	−3.997		−8.599	
**−NLL b3 ca**	−6.402			−6.267
**−NLL b3 np**	6.723			2.824
**−NLL b3 cyto**	−2.22			−5.808
Total	−17.45	−12.37	−16.1	−9.251
**Total with regul**	−18.61	−13.1	−17.99	−10.94

	Together	b1	b2	b3
spar	0.4089	0.40450	0.4085	4.350e−01
σ_b	0.1224	0.07863	0.03819	1.079e−01
σ_t	1.9390	6.57300	0.3336	2.663e−06
ca_{0,b_1}				
$\log_{10}(k_1')$	−0.8732	−1.46600		−7.274e−01
$\log_{10}(k_2)$	−1.1550	−1.73600	−1.069 or −2.205	−1.062e+00
$\log_{10}(k_2')$	−2.1990	−1.85000		−2.394e+00
$\log_{10}(k_{deg})$	−2.2920	−1.96300	−2.205 or −1.069	−2.397e+00
$\log_{10}(k_1' k_2')$	−3.0720	−3.31600	−2.867	−3.122e+00
$\log_{10}(k_1'/k_2)$	0.2815	0.27030		3.350e−01
$\text{transport} = \log_{10}(k_1' k_2'/k_2)$	−1.9170	−1.58000	−1.798 or −0.6615	−2.059e+00

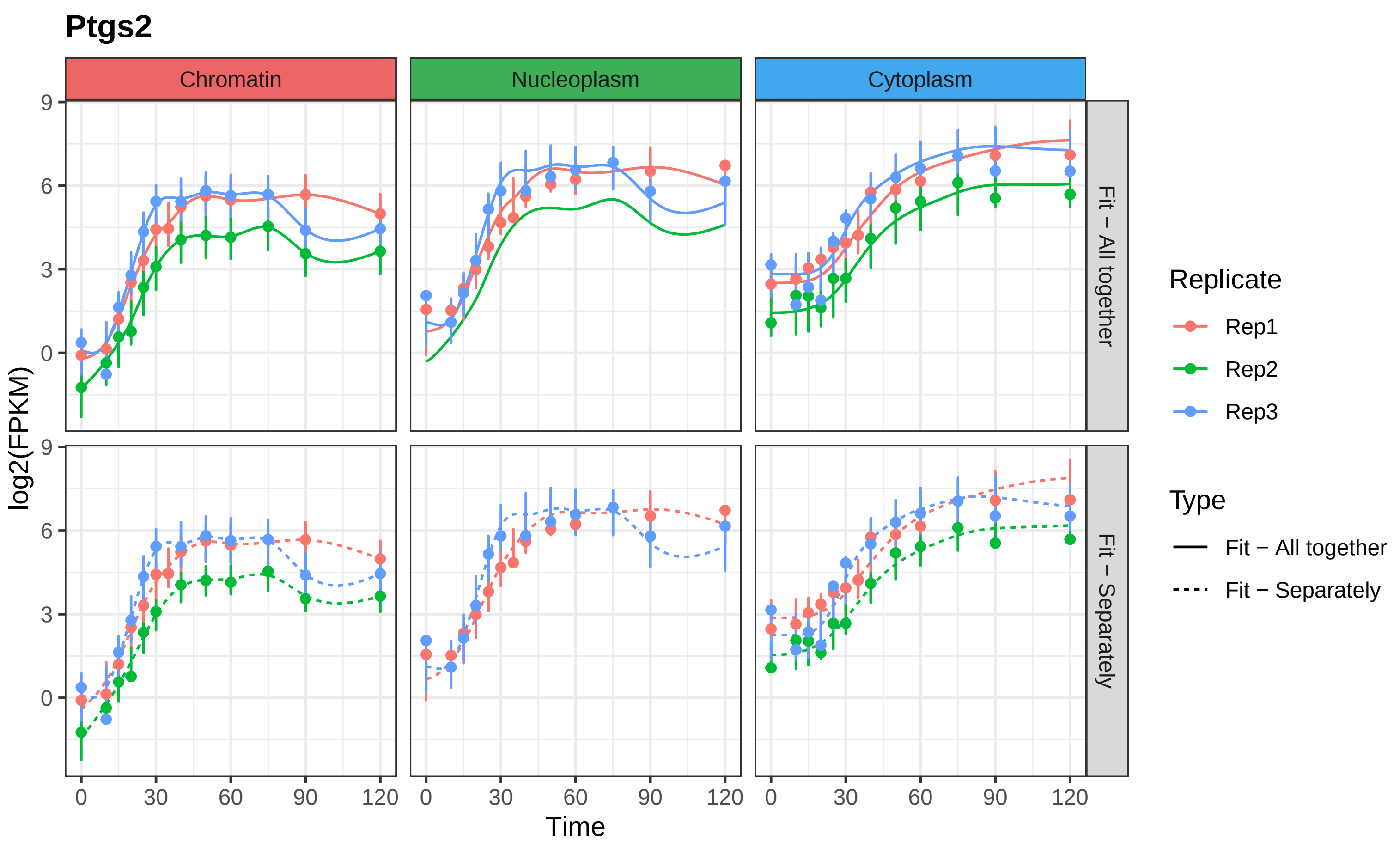

	Together	b1	b2	b3
**−NLL b1 ca**	0.3122	0.508		
**−NLL b1 np**	9.017	7.333		
**−NLL b1 cyto**	6.924	8.146		
**−NLL b2 ca**	0.4793		−1.282	
**−NLL b2 np**				
**−NLL b2 cyto**	5.441		5.779	
**−NLL b3 ca**	3.312			3.804
**−NLL b3 np**	8.593			8.362
**−NLL b3 cyto**	14.53			9.792
Total	48.61	15.99	4.498	21.96
**Total with regul**	55.66	17.52	4.649	29.18

	Together	b1	b2	b3
spar	0.2757000	0.3742000	0.3845	2.800e−01
σ_b	0.2565000	0.2324000	0.1796	2.743e−01
σ_t	0.0001849	0.0001747	0.000134	2.017e−05
ca_{0,b_1}				
$\log_{10}(k_1')$	0.1921000	−0.2796000		2.352e−01
$\log_{10}(k_2)$	−0.1067000	−0.6116000	7.949 or −1.991	−7.480e−02
$\log_{10}(k_2')$	−1.3870000	−1.3960000		−1.372e+00
$\log_{10}(k_{deg})$	−1.9100000	−2.0520000	−1.991 or 7.949	−1.714e+00
$\log_{10}(k_1' k_2')$	−1.1950000	−1.6760000	6.852	−1.137e+00
$\log_{10}(k_1'/k_2)$	0.2988000	0.3320000		3.100e−01
$\text{transport} = \log_{10}(k_1' k_2'/k_2)$	−1.0890000	−1.0640000	−1.097 or 8.843	−1.062e+00

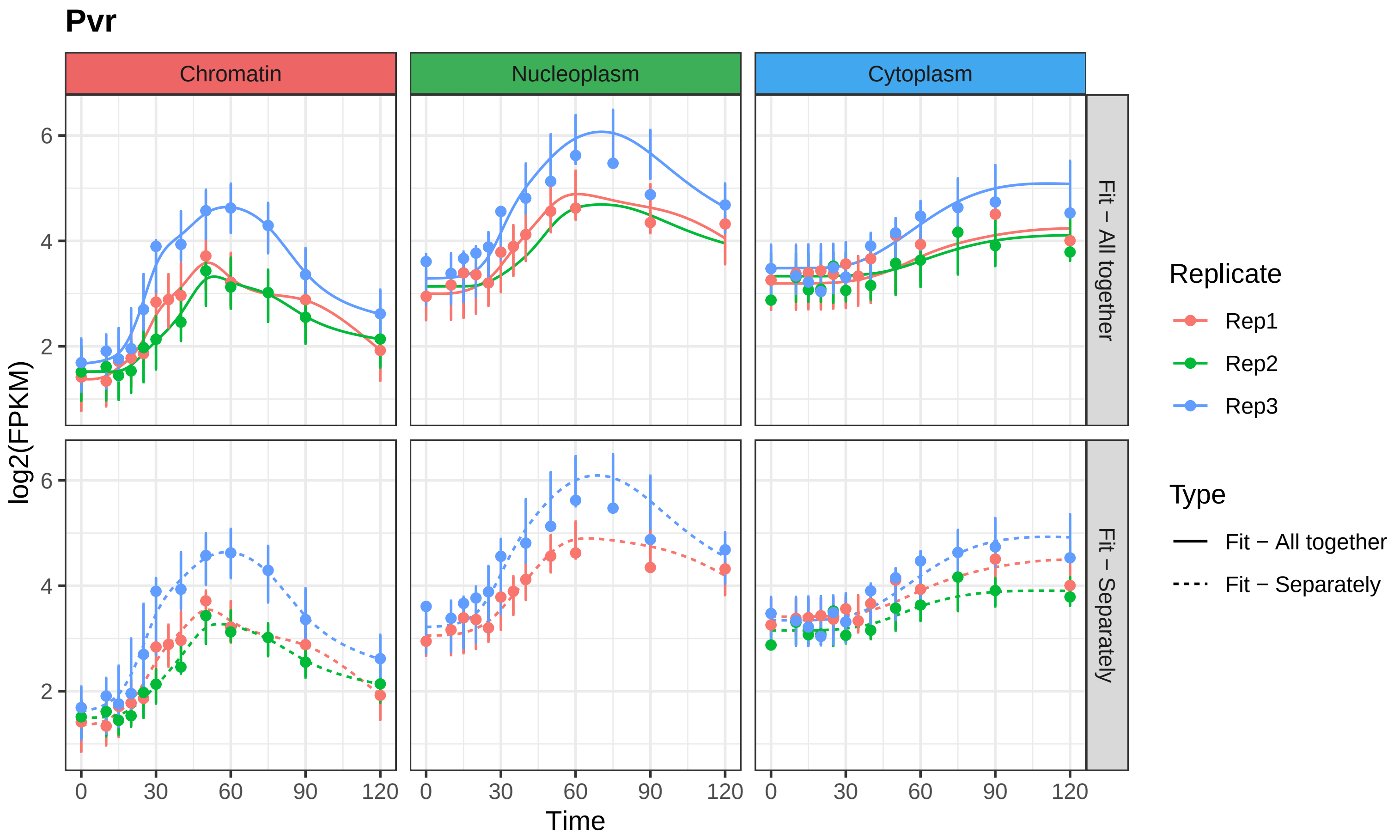

	Together	b1	b2	b3
**−NLL b1 ca**	−3.447	−4.54		
**−NLL b1 np**	−1.745	−2.682		
**−NLL b1 cyto**	3.162	−1.565		
**−NLL b2 ca**	−4.593		−5.932	
**−NLL b2 np**				
**−NLL b2 cyto**	0.3154		−3.758	
**−NLL b3 ca**	−3.143			−1.191
**−NLL b3 np**	8.881			6.223
**−NLL b3 cyto**	1.005			−0.6033
Total	0.4343	−8.786	−9.69	4.429
**Total with regul**	1.49	−9.615	−11.27	4.12

	Together	b1	b2	b3
spar	0.3471	3.871e−01	0.4083	0.3941
σ_b	0.1580	1.182e−01	0.0881	0.1572
σ_t	0.6640	9.391e−05	0.000878	2.7350
ca_{0,b_1}				
$\log_{10}(k_1')$	−0.6676	−7.317e−01		−0.5939
$\log_{10}(k_2)$	−1.1550	−1.242e+00	5.48 or −2.115	−1.0770
$\log_{10}(k_2')$	−1.9910	−1.913e+00		−2.0650
$\log_{10}(k_{deg})$	−2.0500	−2.019e+00	−2.115 or 5.48	−2.1030
$\log_{10}(k_1'k_2')$	−2.6590	−2.644e+00	3.864	−2.6590
$\log_{10}(k_1'/k_2)$	0.4872	5.103e−01		0.4834
$\text{transport} = \log_{10}(k_1'k_2'/k_2)$	−1.5040	−1.402e+00	−1.616 or 5.979	−1.5820

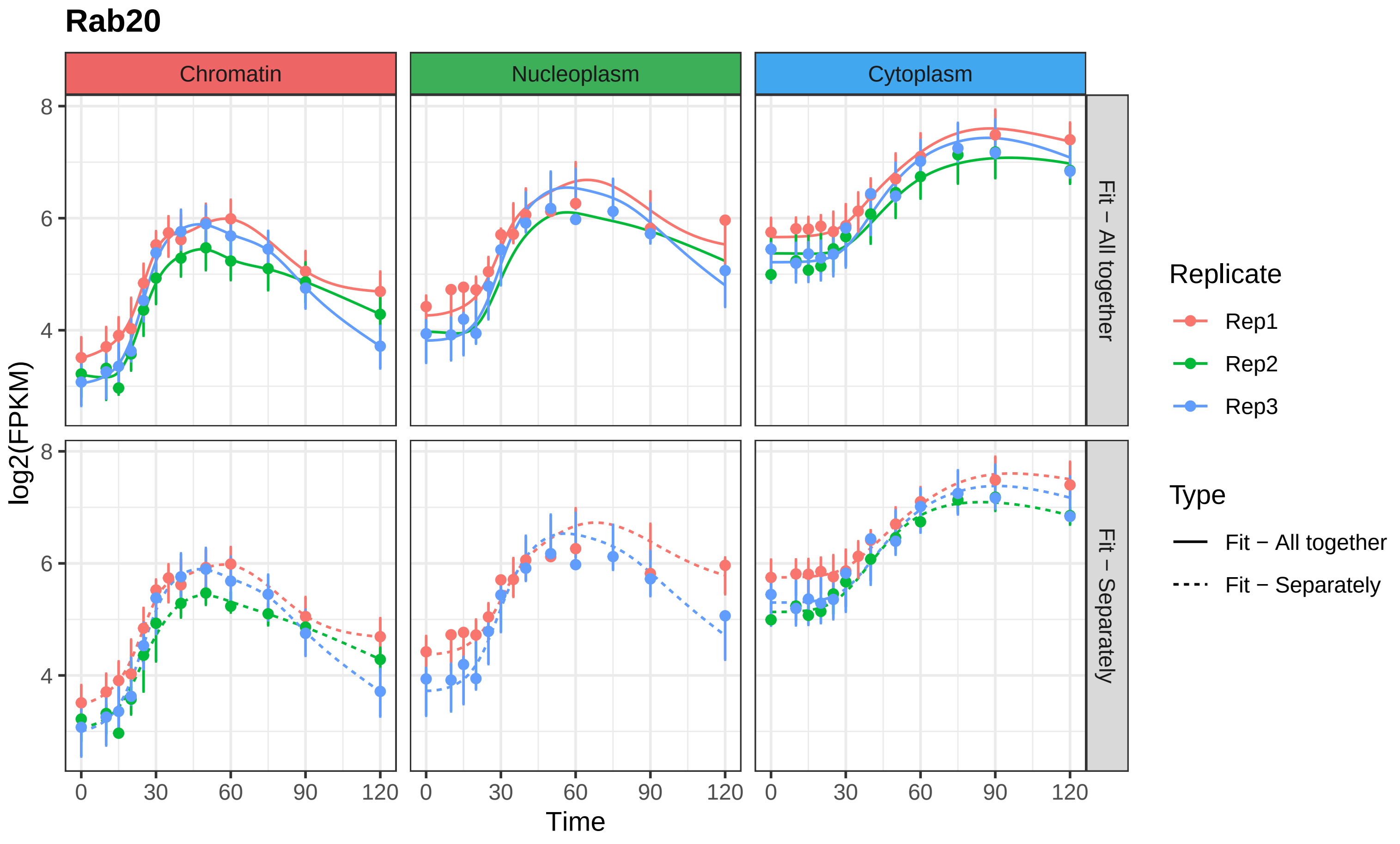

	Together	b1	b2	b3
**−NLL b1 ca**	−8.102	−7.802		
**−NLL b1 np**	5.899	5.299		
**−NLL b1 cyto**	−8.152	−9.393		
**−NLL b2 ca**	−7.369		−5.699	
**−NLL b2 np**				
**−NLL b2 cyto**	−3.272		−8.889	
**−NLL b3 ca**	−7.959			−5.1
**−NLL b3 np**	3.099			2.31
**−NLL b3 cyto**	−0.913			−1.524
Total	−26.77	−11.9	−14.59	−4.314
**Total with regul**	−24.92	−12.28	−16.55	−4.462

	Together	b1	b2	b3
spar	0.318900	0.3694	0.4266	3.717e−01
σ_b	0.120900	0.1115	0.04944	1.373e−01
σ_t	0.001866	0.7453	2.538	2.161e−05
ca_{0,b_1}				
$\log_{10}(k_1')$	−0.717600	−0.8978		−6.650e−01
$\log_{10}(k_2)$	−0.948200	−1.1700	−0.6995 or −1.467	−8.829e−01
$\log_{10}(k_2')$	−1.166000	−1.2270		−1.268e+00
$\log_{10}(k_{deg})$	−1.587000	−1.6410	−1.467 or −0.6995	−1.742e+00
$\log_{10}(k_1' k_2')$	−1.883000	−2.1250	−1.544	−1.933e+00
$\log_{10}(k_1'/k_2)$	0.230500	0.2720		2.179e−01
$\text{transport} = \log_{10}(k_1' k_2'/k_2)$	−0.935300	−0.9553	−0.8449 or −0.07758	−1.050e+00

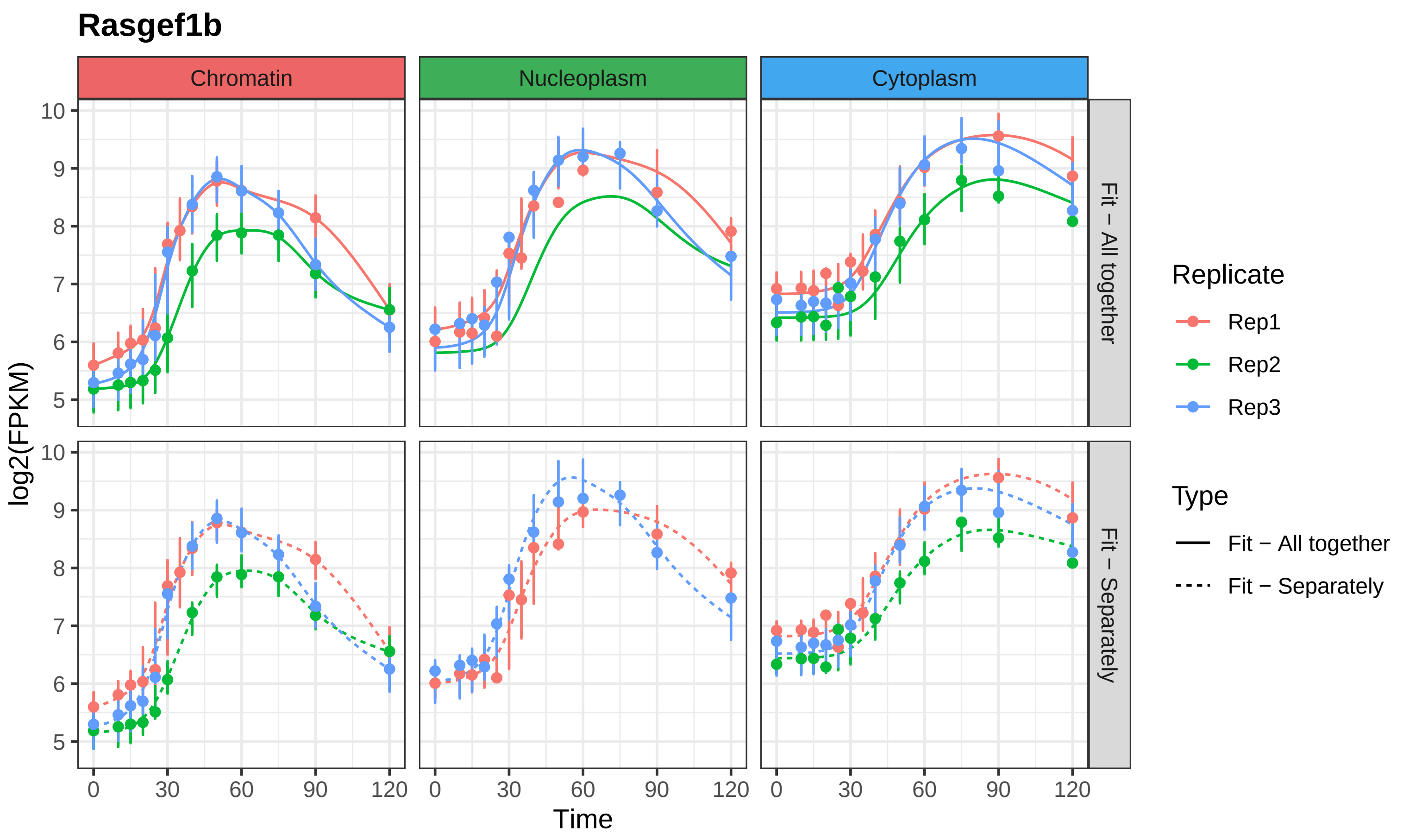

	Together	b1	b2	b3
**−NLL b1 ca**	−5.216	−6.512		
**−NLL b1 np**	7.54	−1.524		
**−NLL b1 cyto**	−2.47	−0.794		
**−NLL b2 ca**	−7.125		−9.858	
**−NLL b2 np**				
**−NLL b2 cyto**	0.1104		−1.784	
**−NLL b3 ca**	−5.217			−4.892
**−NLL b3 np**	4.338			0.05204
**−NLL b3 cyto**	−0.2611			−1.401
Total	−8.301	−8.829	−11.64	−6.241
**Total with regul**	−7.025	−9.296	−12.8	−5.938

	Together	b1	b2	b3
spar	0.3353	0.36970	0.3903	0.3552
σ_b	0.1327	0.09041	0.09507	0.1269
σ_t	2.0080	2.77600	2.036e−06	0.9774
ca_{0,b_1}				
$\log_{10}(k_1')$	−0.6978	−0.92620		−0.3270
$\log_{10}(k_2)$	−0.8872	−1.05600	5.232 or −1.591	−0.5633
$\log_{10}(k_2')$	−1.1970	−1.03600		−1.4030
$\log_{10}(k_{deg})$	−1.3790	−1.28000	−1.591 or 5.232	−1.5460
$\log_{10}(k_1' k_2')$	−1.8940	−1.96300	4.026	−1.7300
$\log_{10}(k_1'/k_2)$	0.1894	0.13010		0.2363
$\text{transport} = \log_{10}(k_1' k_2'/k_2)$	−1.0070	−0.90620	−1.206 or 5.617	−1.1670

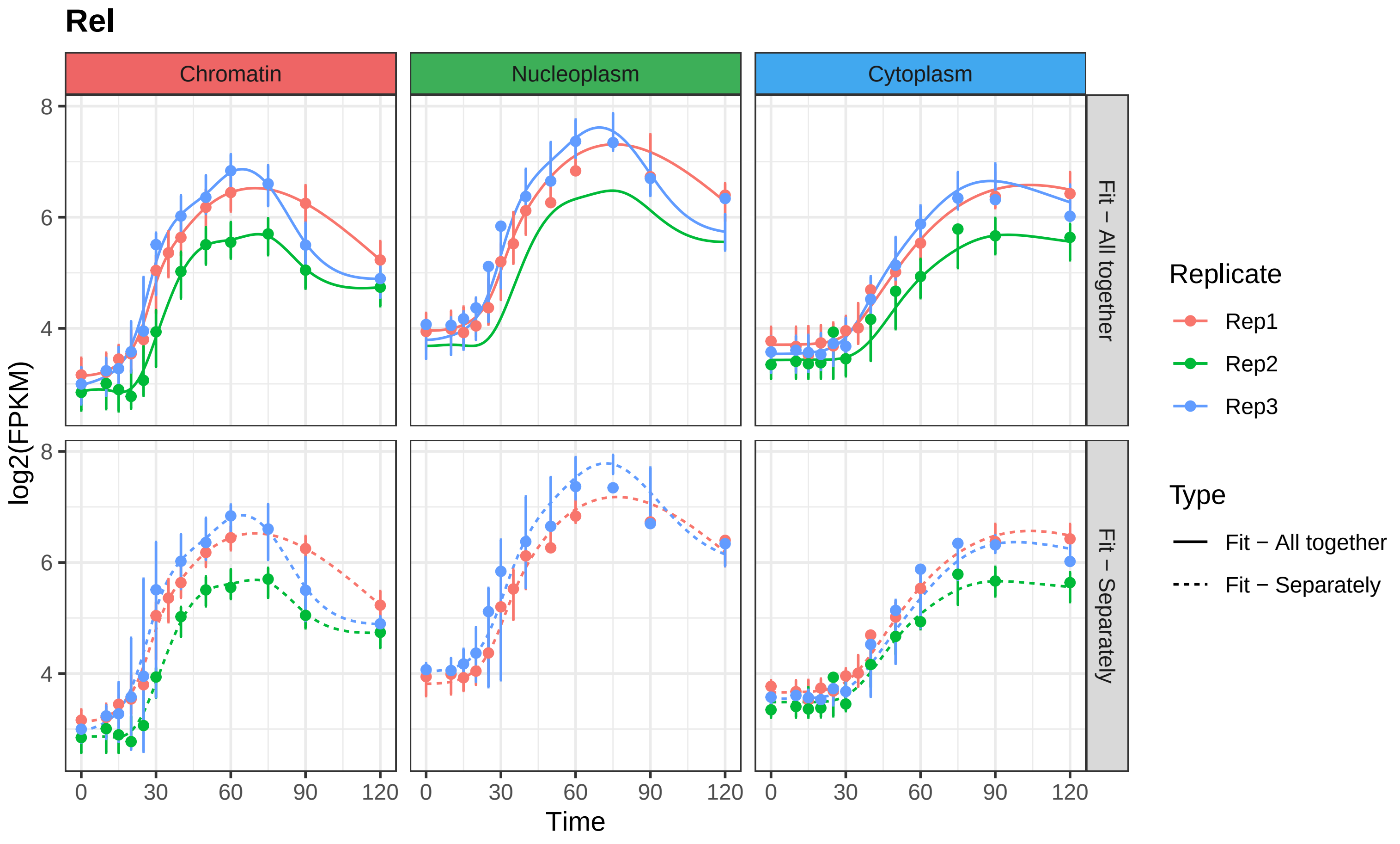

	Together	b1	b2	b3
**−NLL b1 ca**	−7.524	−9.813		
**−NLL b1 np**	0.3218	−1.061		
**−NLL b1 cyto**	−6.971	−8.727		
**−NLL b2 ca**	−8.117		−8.137	
**−NLL b2 np**				
**−NLL b2 cyto**	0.4405		−3.509	
**−NLL b3 ca**	−6.475			−4.603
**−NLL b3 np**	8.193			−0.1773
**−NLL b3 cyto**	−5.371			−4.514
Total	−25.5	−19.6	−11.65	−9.294
**Total with regul**	−24.18	−20.02	−12.04	−9.172

	Together	b1	b2	b3
spar	0.3287	0.36180	0.3636	0.35240
σ_b	0.1137	0.07337	0.09293	0.04847
σ_t	1.5260	1.88000	0.004419	4.56800
ca_{0,b_1}				
$\log_{10}(k_1')$	−0.5139	−0.61570		−0.68770
$\log_{10}(k_2)$	−0.7592	−0.82300	4.155 or −1.682	−1.01400
$\log_{10}(k_2')$	−1.6800	−1.63400		−1.92100
$\log_{10}(k_{deg})$	−1.6040	−1.58700	−1.682 or 4.155	−1.77200
$\log_{10}(k_1' k_2')$	−2.1940	−2.25000	2.662	−2.60800
$\log_{10}(k_1'/k_2)$	0.2452	0.20730		0.32580
$\text{transport} = \log_{10}(k_1' k_2'/k_2)$	−1.4350	−1.42700	−1.493 or 4.344	−1.59500

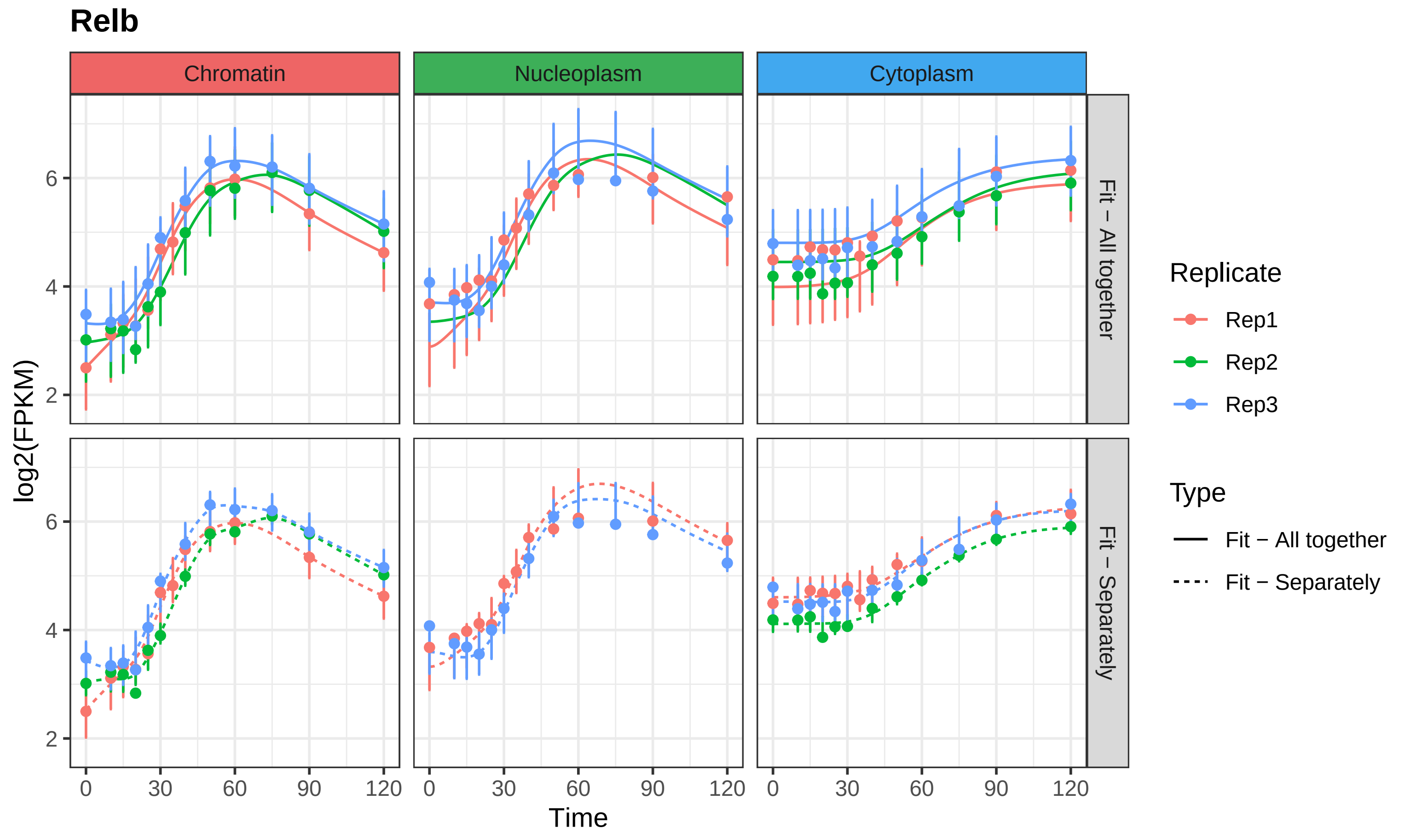

	Together	b1	b2	b3
**−NLL b1 ca**	−0.6545	−5.002		
**−NLL b1 np**	7.346	4.829		
**−NLL b1 cyto**	12.52	−6.992		
**−NLL b2 ca**	−0.9547		−9.382	
**−NLL b2 np**				
**−NLL b2 cyto**	3.171		−10.39	
**−NLL b3 ca**	−0.7094			−5.949
**−NLL b3 np**	7.812			2.446
**−NLL b3 cyto**	3.646			−5.968
Total	32.18	−7.165	−19.77	−9.471
**Total with regul**	32.27	−8.209	−20.2	−9.666

	Together	b1	b2	b3
spar	0.437500	0.3990	0.3535	3.634e−01
σ_b	0.217900	0.1219	0.02659	1.115e−01
σ_t	0.000839	0.7241	0.0001029	5.637e−05
ca_{0,b_1}				
$\log_{10}(k_1')$	−0.409000	−0.6748		−7.444e−01
$\log_{10}(k_2)$	−0.524200	−0.9141	−0.01929 or −2.131	−7.925e−01
$\log_{10}(k_2')$	−1.915000	−1.9820		−1.820e+00
$\log_{10}(k_{deg})$	−2.247000	−2.3670	−2.131 or −0.01929	−2.099e+00
$\log_{10}(k_1'k_2')$	−2.324000	−2.6570	−1.826	−2.564e+00
$\log_{10}(k_1'/k_2)$	0.115200	0.2393		4.808e−02
transport = $\log_{10}(k_1'k_2'/k_2)$	−1.800000	−1.7420	−1.807 or 0.305	−1.771e+00

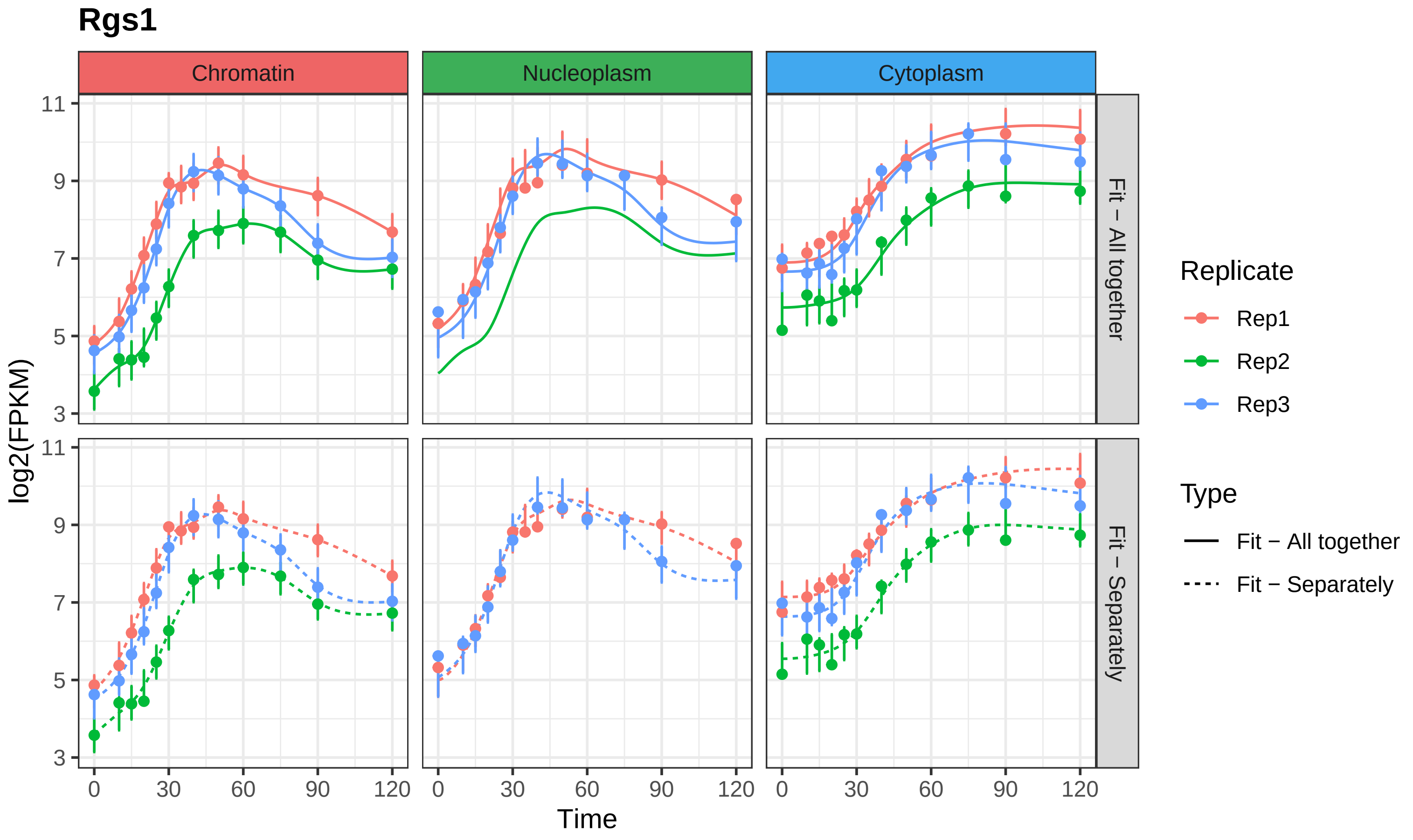

	Together	b1	b2	b3
**−NLL b1 ca**	−5.075	−5.618		
**−NLL b1 np**	8.065	2.632		
**−NLL b1 cyto**	−0.8967	−1.789		
**−NLL b2 ca**	−4.834		−4.08	
**−NLL b2 np**				
**−NLL b2 cyto**	2.377		2.541	
**−NLL b3 ca**	−5.435			−4.832
**−NLL b3 np**	4.958			2.118
**−NLL b3 cyto**	3.018			3.254
Total	2.177	−4.775	−1.539	0.5394
**Total with regul**	5.782	−4.219	−1.454	2.05

	Together	b1	b2	b3
spar	0.299200	0.3529000	0.3678	0.337400
σ_b	0.165900	0.1407000	0.145	0.160800
σ_t	0.000558	0.0004251	0.0006745	0.001449
ca_{0,b_1}				
$\log_{10}(k_1')$	0.539000	−0.2232000		4.281000
$\log_{10}(k_2)$	0.414800	−0.3079000	4.772 or −1.742	4.112000
$\log_{10}(k_2')$	−1.358000	−1.4020000		−1.389000
$\log_{10}(k_{deg})$	−1.869000	−2.0470000	−1.742 or 4.772	−1.863000
$\log_{10}(k_1'k_2')$	−0.818900	−1.6250000	3.616	2.892000
$\log_{10}(k_1'/k_2)$	0.124200	0.0846800		0.168900
$\text{transport} = \log_{10}(k_1'k_2'/k_2)$	−1.234000	−1.3170000	−1.155 or 5.358	−1.220000

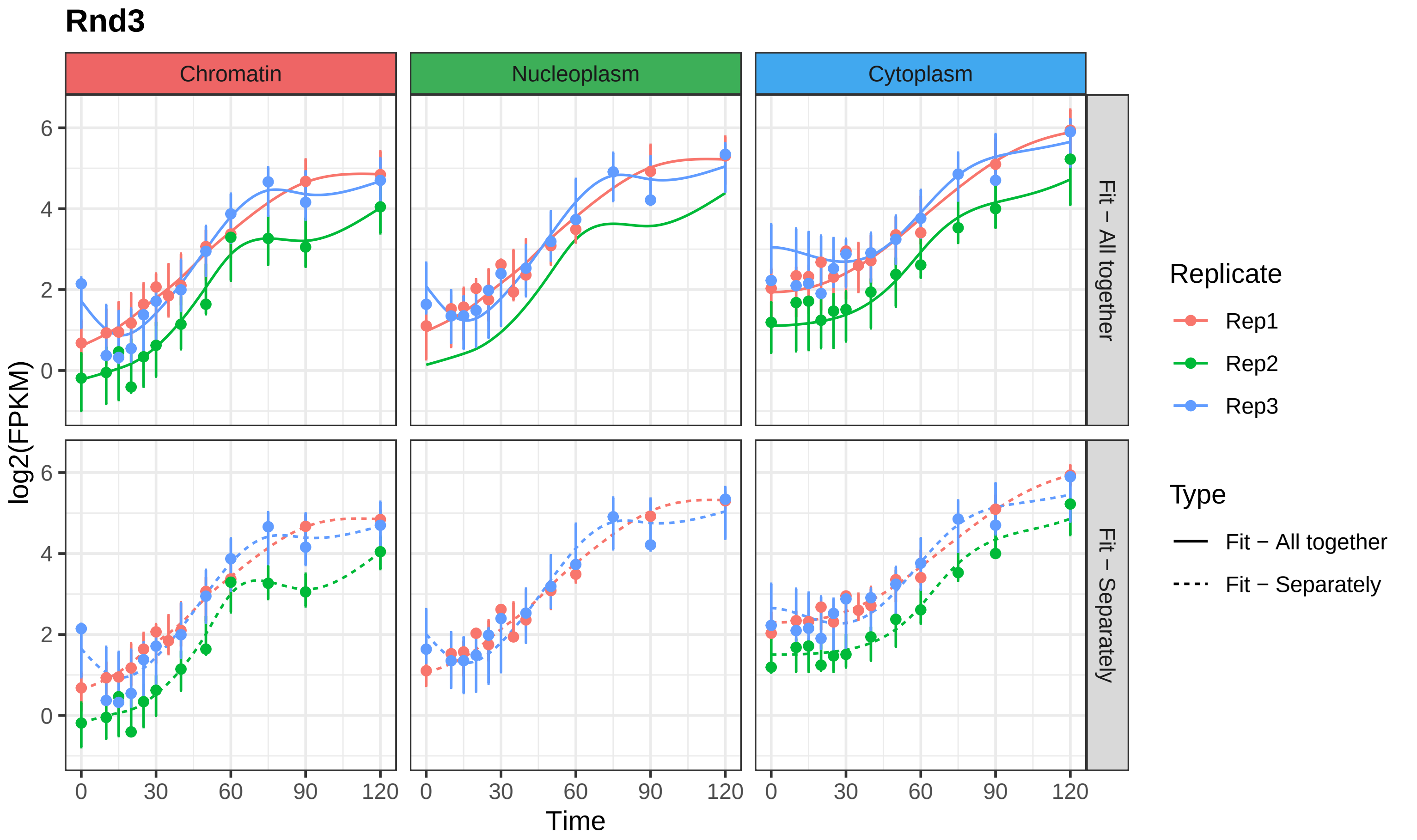

	Together	b1	b2	b3
**−NLL b1 ca**	−1.39	−5.275		
**−NLL b1 np**	1.772	0.3037		
**−NLL b1 cyto**	1.745	−1.965		
**−NLL b2 ca**	1.814		0.1045	
**−NLL b2 np**				
**−NLL b2 cyto**	3.113		0.1877	
**−NLL b3 ca**	3.367			4.307
**−NLL b3 np**	4.516			4.037
**−NLL b3 cyto**	12.18			5.39
Total	27.12	−6.936	0.2922	13.73
**Total with regul**	27.13	−8.688	−1.021	14.43

	Together	b1	b2	b3
spar	0.4865000	0.45260	0.4267	0.507300
σ_b	0.2035000	0.07421	0.1325	0.217400
σ_t	0.0000822	3.80800	0.9798	0.004902
ca_{0,b_1}				
$\log_{10}(k_1')$	5.0530000	−0.31440		6.014000
$\log_{10}(k_2)$	4.9440000	−0.45390	−1.228 or −1.272	5.905000
$\log_{10}(k_2')$	−1.1610000	−1.27700		−1.101000
$\log_{10}(k_{deg})$	−1.4520000	−1.64000	−1.272 or −1.228	−1.295000
$\log_{10}(k_1' k_2')$	3.8920000	−1.59100	−1.995	4.913000
$\log_{10}(k_1'/k_2)$	0.1099000	0.13950		0.109000
$\text{transport} = \log_{10}(k_1' k_2'/k_2)$	−1.0520000	−1.13700	−0.7671 or −0.7225	−0.991700

Rnf149

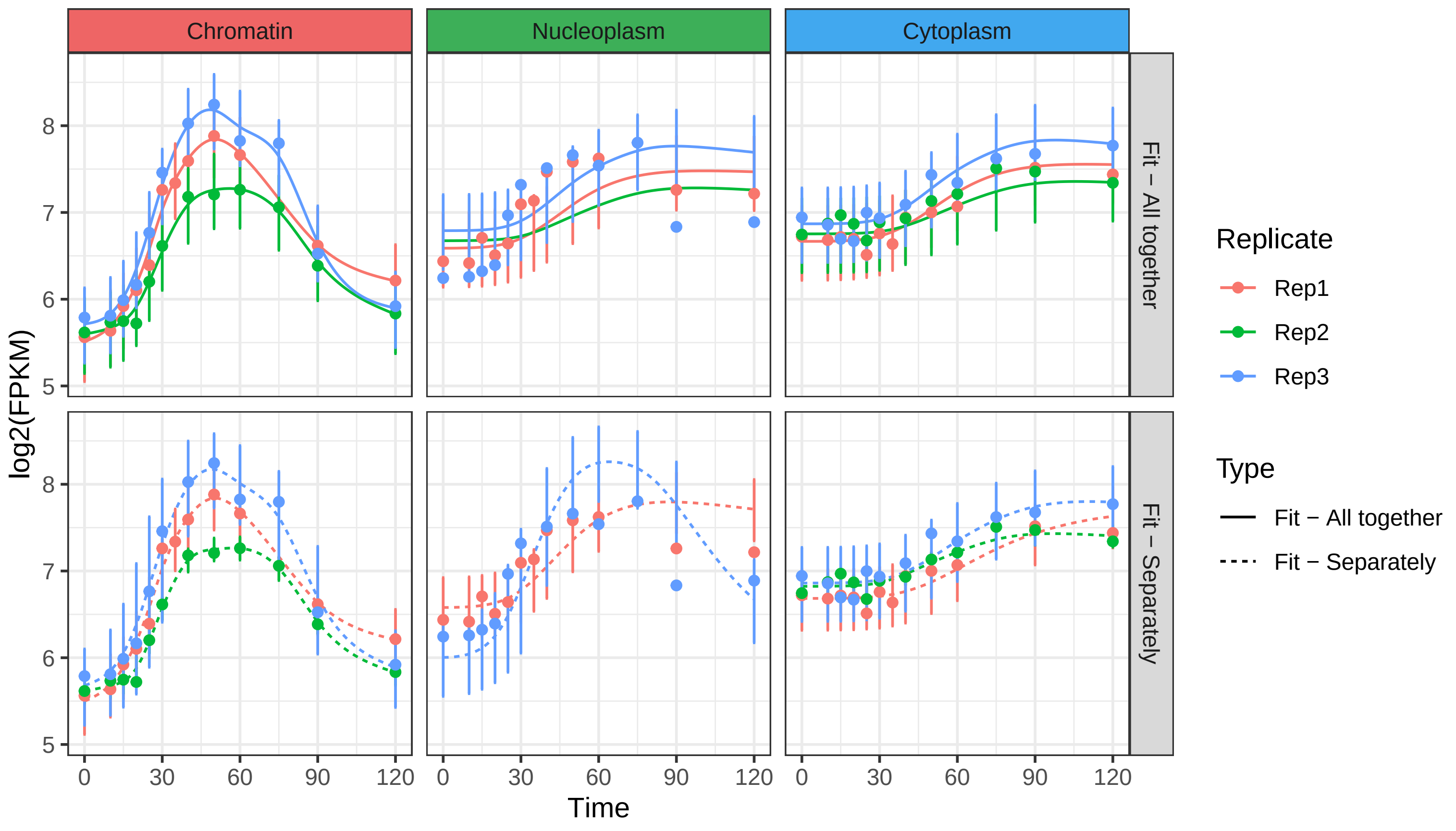

	Together	b1	b2	b3
**−NLL b1 ca**	−5.991	−7.643		
**−NLL b1 np**	5.805	5.346		
**−NLL b1 cyto**	−5.924	−7.411		
**−NLL b2 ca**	−6.495		−15.13	
**−NLL b2 np**				
**−NLL b2 cyto**	−5.032		−12.24	
**−NLL b3 ca**	−5.699			−2.461
**−NLL b3 np**	20.82			10.45
**−NLL b3 cyto**	−5.332			−4.702
Total	−7.851	−9.708	−27.38	3.289
**Total with regul**	−8.252	−10.84	−27.47	2.615

	Together	b1	b2	b3
spar	3.867e−01	4.036e−01	0.3467	0.41470
σ_b	1.494e−01	1.228e−01	0.04256	0.14840
σ_t	3.074e−06	3.115e−05	0.0001411	3.83800
ca_{0,b_1}				
$\log_{10}(k_1')$	−1.926e+00	−1.672e+00		−1.07400
$\log_{10}(k_2)$	−2.250e+00	−1.998e+00	−0.01445 or −2.241	−1.17200
$\log_{10}(k_2')$	−8.022e−01	−1.706e+00		−2.00700
$\log_{10}(k_{deg})$	−8.259e−01	−1.737e+00	−2.241 or −0.01445	−2.26500
$\log_{10}(k_1'k_2')$	−2.728e+00	−3.378e+00	−1.891	−3.08100
$\log_{10}(k_1'/k_2)$	3.241e−01	3.256e−01		0.09784
$transport = \log_{10}(k_1'k_2'/k_2)$	−4.780e−01	−1.380e+00	−1.877 or 0.3494	−1.90900

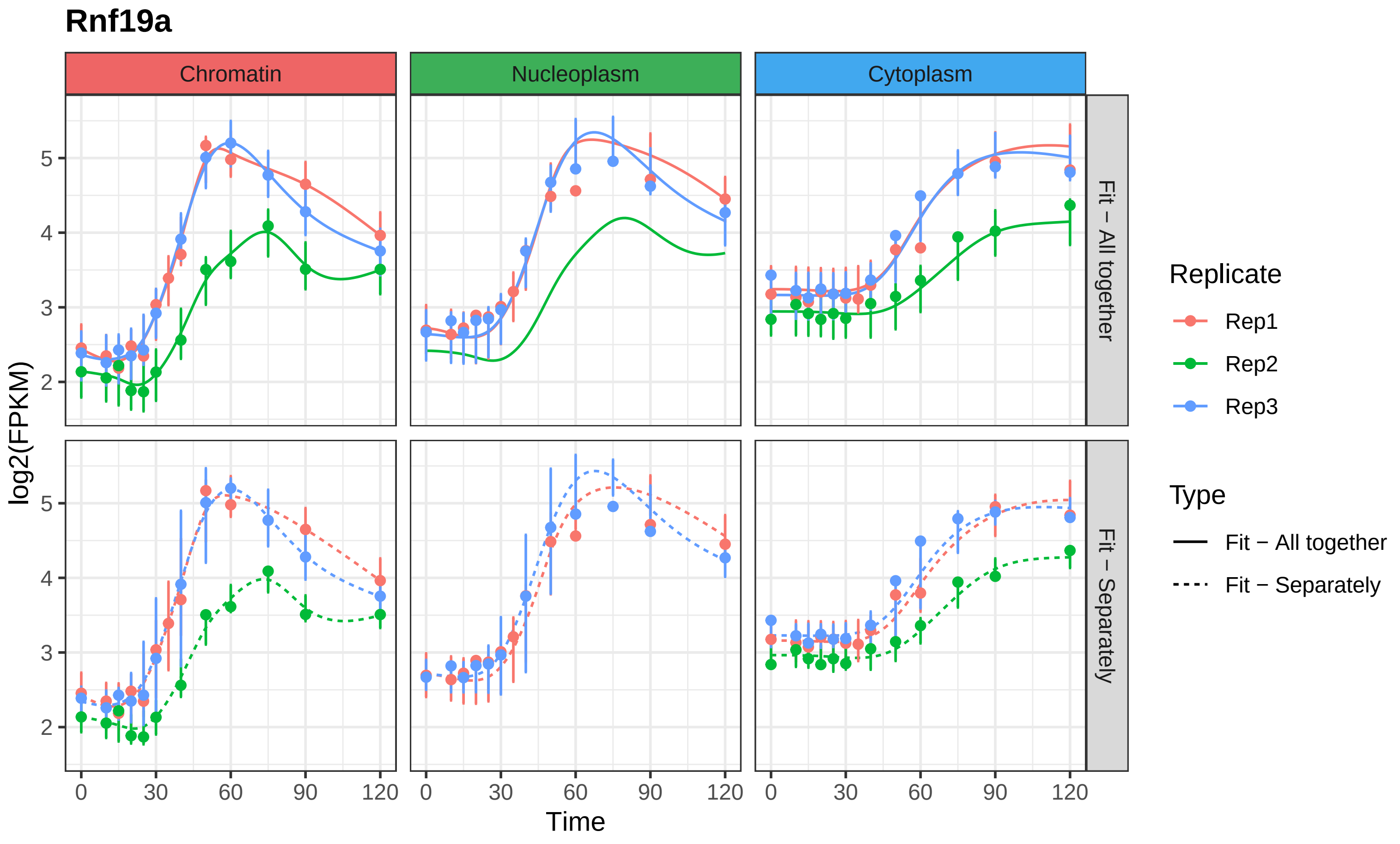

	Together	b1	b2	b3
**−NLL b1 ca**	−7.462	−6.14		
**−NLL b1 np**	2.893	1.458		
**−NLL b1 cyto**	−3.537	−8.233		
**−NLL b2 ca**	−8.542		−8.685	
**−NLL b2 np**				
**−NLL b2 cyto**	−7.263		−11.04	
**−NLL b3 ca**	−9.844			−7.561
**−NLL b3 np**	−2.398			−3.141
**−NLL b3 cyto**	−4.101			−6.251
Total	−40.25	−12.92	−19.73	−16.95
**Total with regul**	−40.85	−14.45	−21.64	−18.4

	Together	b1	b2	b3
spar	0.3729000	0.41500	0.412	0.40870
σ_b	0.1030000	0.08948	0.04191	0.04371
σ_t	0.0006633	2.60200	1.242	4.66500
ca_{0,b_1}				
$\log_{10}(k_1')$	−0.8245000	−1.01700		−0.77790
$\log_{10}(k_2)$	−0.9076000	−1.10400	−1.079 or −1.566	−0.88800
$\log_{10}(k_2')$	−1.5560000	−1.63000		−1.71800
$\log_{10}(k_{deg})$	−1.7140000	−1.76700	−1.566 or −1.079	−1.87500
$\log_{10}(k_1'k_2')$	−2.3800000	−2.64700	−2.397	−2.49600
$\log_{10}(k_1'/k_2)$	0.0830900	0.08657		0.11010
transport $= \log_{10}(k_1'k_2'/k_2)$	−1.4730000	−1.54400	−1.318 or −0.8307	−1.60800

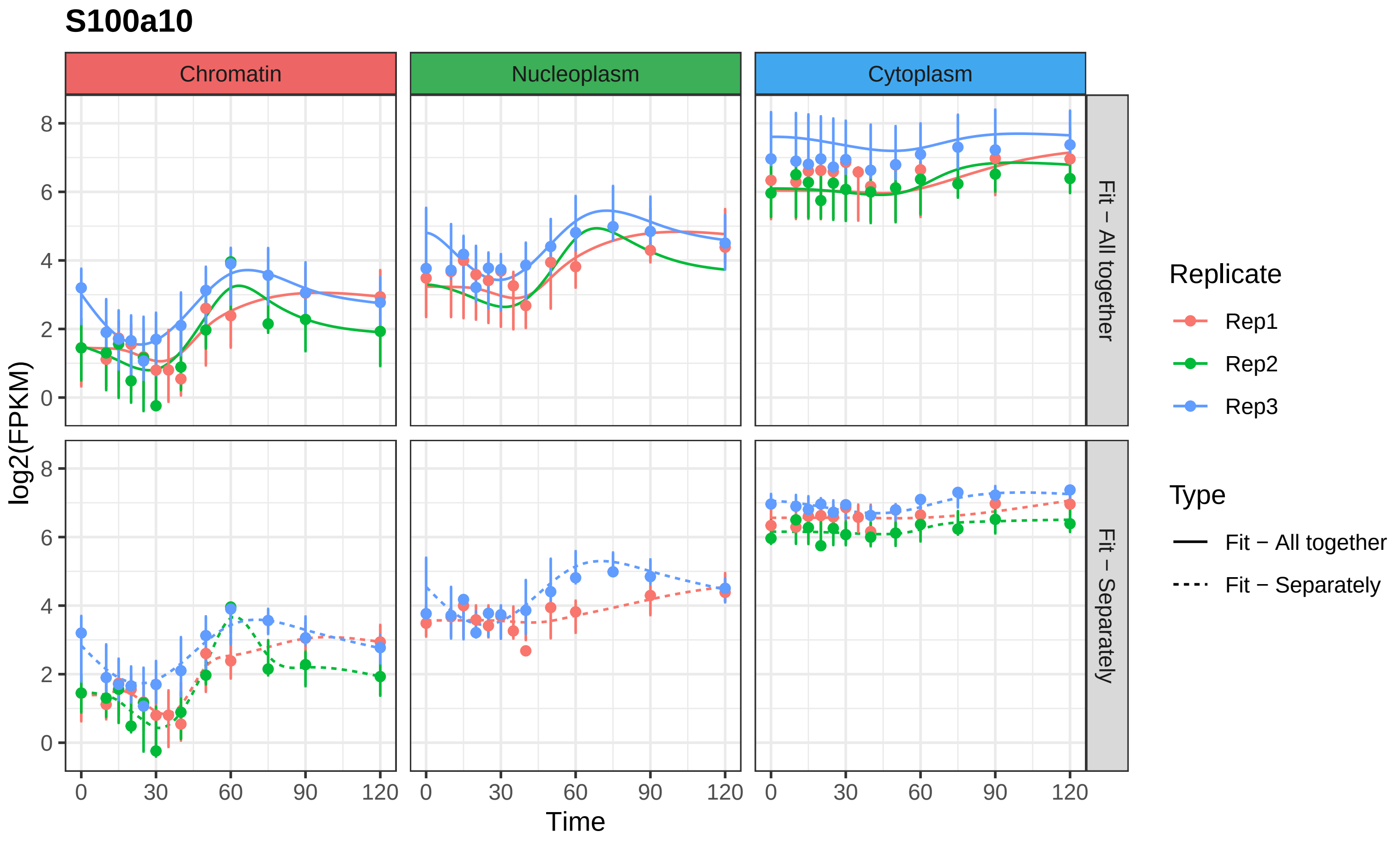

	Together	b1	b2	b3
**−NLL b1 ca**	4.963	0.5656		
**−NLL b1 np**	7.796	3.23		
**−NLL b1 cyto**	10.44	−2.117		
**−NLL b2 ca**	8.924		3.057	
**−NLL b2 np**				
**−NLL b2 cyto**	2.745		−2.727	
**−NLL b3 ca**	2.173			3.725
**−NLL b3 np**	7.109			4.123
**−NLL b3 cyto**	10.89			−8.153
Total	55.04	1.679	0.3306	−0.3048
**Total with regul**	56.36	1.041	0.04259	0.01024

	Together	b1	b2	b3
spar	0.4812000	0.3890000	0.3679	0.54250
σ_b	0.2624000	0.1369000	0.1169	0.07069
σ_t	0.0001007	0.0001311	0.003939	5.79400
ca_{0,b_1}				
$\log_{10}(k_1')$	−0.1953000	−1.3140000		6.70400
$\log_{10}(k_2)$	−0.7333000	−1.9650000	4.676 or −2.367	6.18900
$\log_{10}(k_2')$	−0.9362000	−0.8260000		−0.99240
$\log_{10}(k_{deg})$	−1.7800000	−1.7270000	−2.367 or 4.676	−1.74600
$\log_{10}(k_1'k_2')$	−1.1320000	−2.1400000	3.717	5.71100
$\log_{10}(k_1'/k_2)$	0.5380000	0.6511000		0.51500
transport $= \log_{10}(k_1'k_2'/k_2)$	−0.3982000	−0.1748000	−0.9592 or 6.084	−0.47740

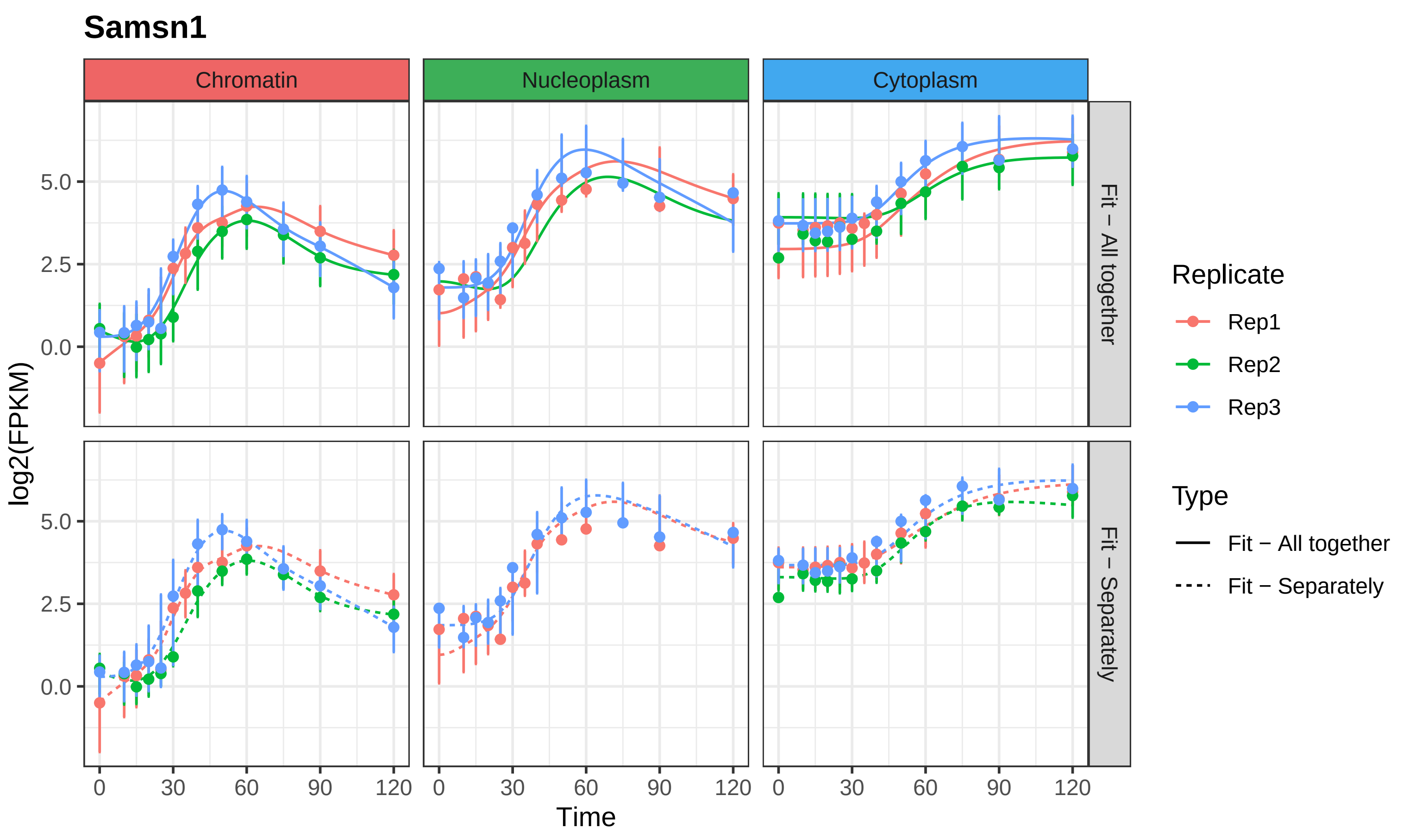

	Together	b1	b2	b3
**−NLL b1 ca**	3.272	1.712		
**−NLL b1 np**	11.85	15.02		
**−NLL b1 cyto**	10.36	−1.119		
**−NLL b2 ca**	1.749		−1.154	
**−NLL b2 np**				
**−NLL b2 cyto**	10.65		0.5741	
**−NLL b3 ca**	3.582			2.334
**−NLL b3 np**	10.3			8.658
**−NLL b3 cyto**	2.358			1.779
Total	54.13	15.62	−0.58	12.77
**Total with regul**	55.81	16.49	−2.026	12.69

	Together	b1	b2	b3
spar	0.397100	0.3796	0.4272	0.4022
σ_b	0.262000	0.2091	0.124	0.1751
σ_t	0.000473	0.7657	0.0002938	3.4750
ca_{0,b_1}				
$\log_{10}(k_1')$	−0.533100	−0.4414		−0.7811
$\log_{10}(k_2)$	−0.982100	−0.8685	−0.4866 or −1.804	−1.2520
$\log_{10}(k_2')$	−1.470000	−1.6070		−1.4920
$\log_{10}(k_{deg})$	−2.054000	−2.4060	−1.804 or −0.4866	−2.0400
$\log_{10}(k_1' k_2')$	−2.003000	−2.0490	−1.423	−2.2730
$\log_{10}(k_1'/k_2)$	0.449100	0.4271		0.4704
$\text{transport} = \log_{10}(k_1' k_2'/k_2)$	−1.021000	−1.1800	−0.9359 or 0.3811	−1.0210

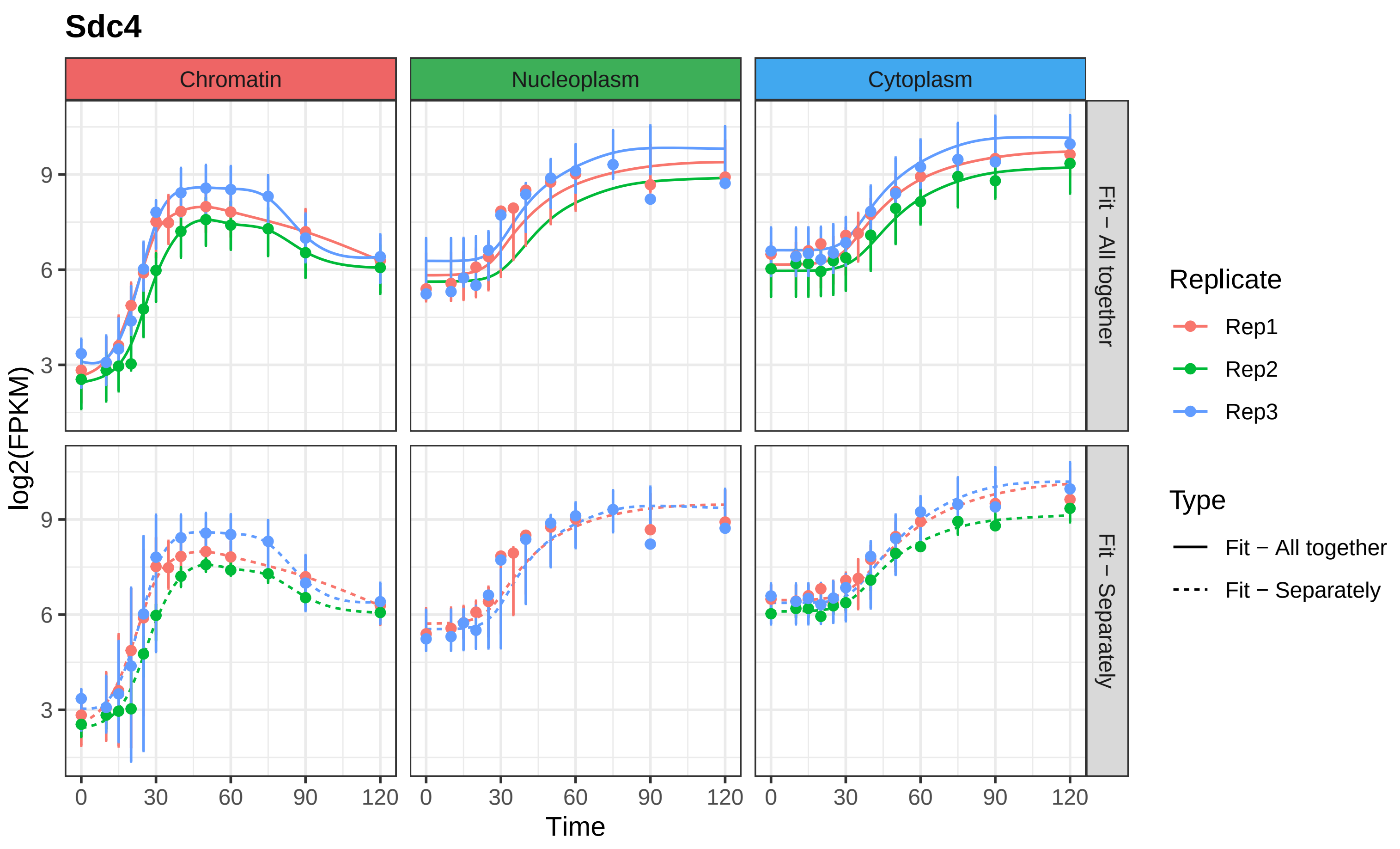

	Together	b1	b2	b3
**−NLL b1 ca**	0.7131	2.649		
**−NLL b1 np**	16.14	11.52		
**−NLL b1 cyto**	2.431	1.35		
**−NLL b2 ca**	1.112		−5.682	
**−NLL b2 np**				
**−NLL b2 cyto**	0.9742		−6.096	
**−NLL b3 ca**	1.253			4.509
**−NLL b3 np**	22.45			14.69
**−NLL b3 cyto**	4.127			2.029
Total	49.2	15.52	−11.78	21.23
**Total with regul**	53.07	16.61	−11.54	23.71

	Together	b1	b2	b3
spar	3.319e−01	0.36150	0.344	0.3490
σ_b	2.607e−01	0.17300	0.07413	0.2207
σ_t	8.044e−06	4.19100	1.35	4.0300
ca_{0,b_1}				
$\log_{10}(k_1')$	−1.281e+00	−1.24200		−1.3710
$\log_{10}(k_2)$	−2.237e+00	−2.19100	0.5718 or −2.359	−2.1260
$\log_{10}(k_2')$	−5.563e−01	−1.00900		−0.9460
$\log_{10}(k_{deg})$	−6.580e−01	−1.23100	−2.359 or 0.5718	−1.1950
$\log_{10}(k_1'k_2')$	−1.838e+00	−2.25000	−0.6784	−2.3170
$\log_{10}(k_1'/k_2)$	9.557e−01	0.94910		0.7545
$transport = \log_{10}(k_1'k_2'/k_2)$	3.995e−01	−0.05973	−1.25 or 1.68	−0.1915

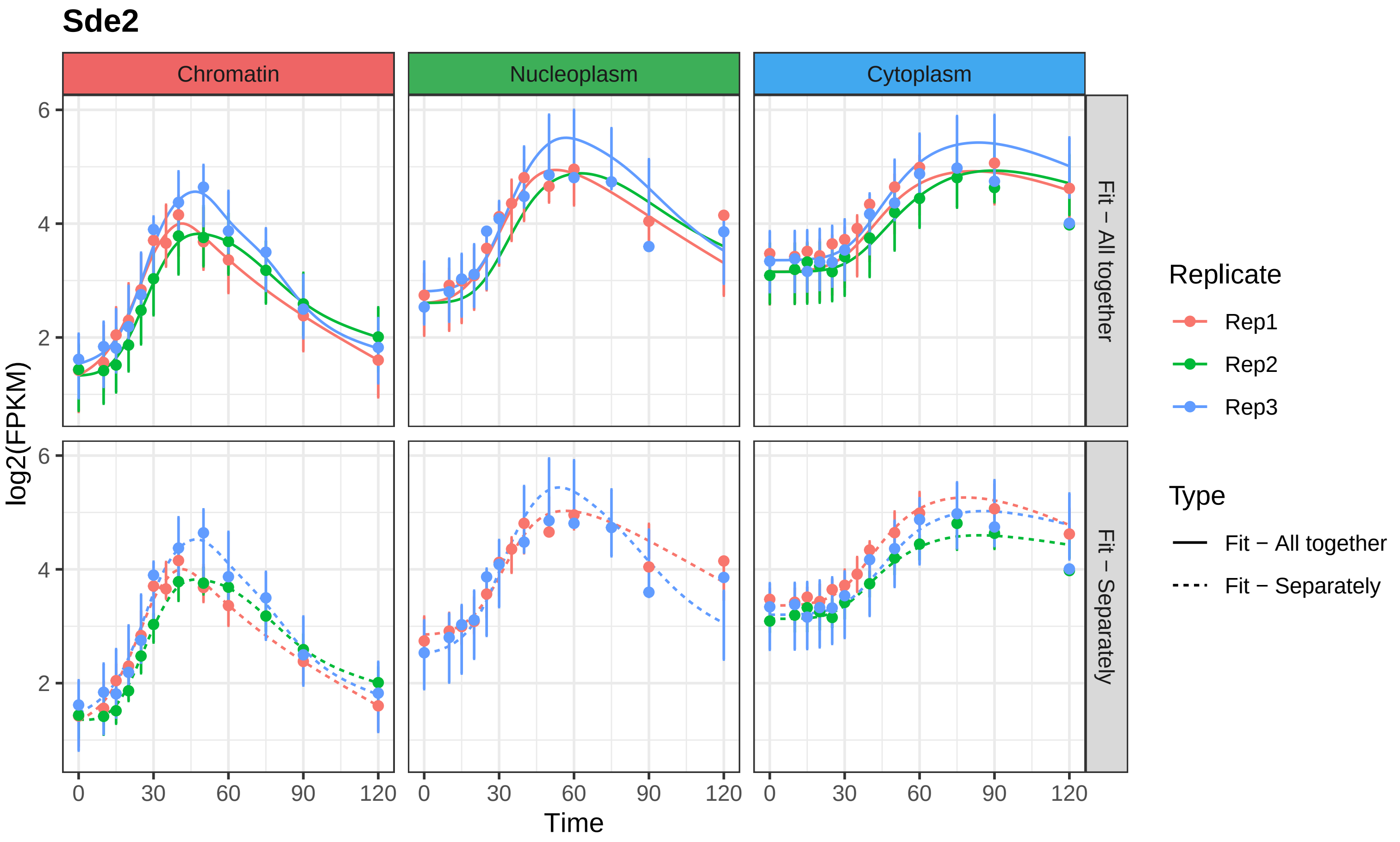

	Together	b1	b2	b3
**−NLL b1 ca**	−2.829	−6.538		
**−NLL b1 np**	2.902	1.172		
**−NLL b1 cyto**	1.527	−8.767		
**−NLL b2 ca**	−3.763		−9.621	
**−NLL b2 np**				
**−NLL b2 cyto**	−0.1341		−4.401	
**−NLL b3 ca**	−2.36			−0.6083
**−NLL b3 np**	11.41			8.26
**−NLL b3 cyto**	6.774			1.538
Total	13.53	−14.13	−14.02	9.189
**Total with regul**	13.56	−15.24	−14.72	8.969

	Together	b1	b2	b3
spar	0.3918000	3.918e−01	0.3683	0.4274000
σ_b	0.1830000	1.032e−01	0.0758	0.1998000
σ_t	0.0001842	7.447e−07	0.0002216	0.0008221
ca_{0,b_1}				
$\log_{10}(k_1')$	−0.7693000	−8.688e−01		−0.5922000
$\log_{10}(k_2)$	−1.1540000	−1.327e+00	9.242 or −1.889	−0.9123000
$\log_{10}(k_2')$	−1.4310000	−1.197e+00		−1.6560000
$\log_{10}(k_{deg})$	−1.5960000	−1.351e+00	−1.889 or 9.242	−1.8560000
$\log_{10}(k_1'k_2')$	−2.2010000	−2.066e+00	7.886	−2.2480000
$\log_{10}(k_1'/k_2)$	0.3843000	4.582e−01		0.3200000
$transport = \log_{10}(k_1'k_2'/k_2)$	−1.0470000	−7.392e−01	−1.356 or 9.775	−1.3360000

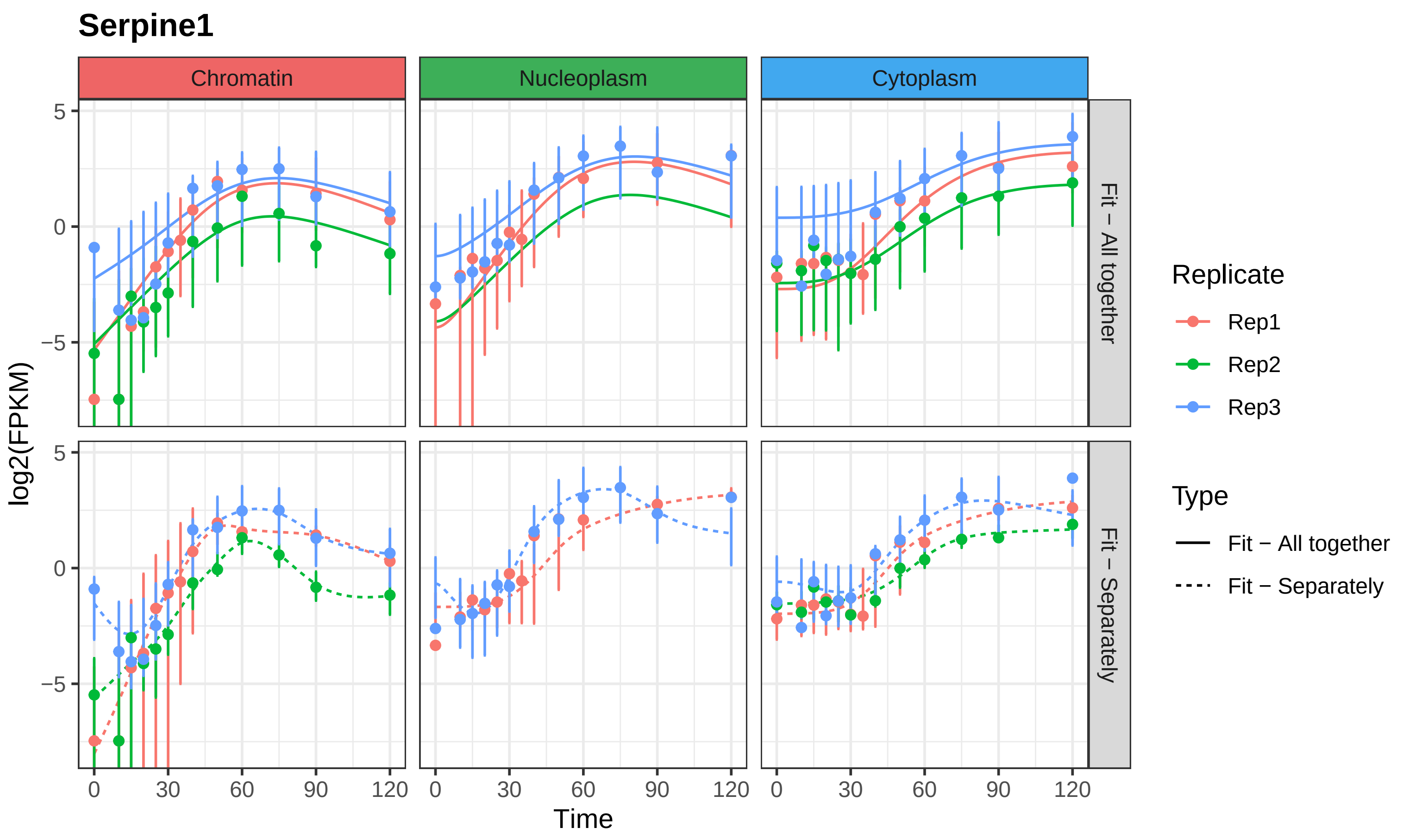

	Together	b1	b2	b3
**−NLL b1 ca**	17.81	12.88		
**−NLL b1 np**	14.03	14.63		
**−NLL b1 cyto**	13.97	7.281		
**−NLL b2 ca**	17.78		10.47	
**−NLL b2 np**				
**−NLL b2 cyto**	11.92		4.666	
**−NLL b3 ca**	24			10.63
**−NLL b3 np**	16.65			14.93
**−NLL b3 cyto**	26.17			16.68
Total	142.3	34.79	15.13	42.24
**Total with regul**	161.9	33.77	13.64	47.37

	Together	b1	b2	b3
spar	0.6768	0.41690	0.4183	0.445600
σ_b	0.4768	0.08335	0.1114	0.385700
σ_t	3.5330	6.05800	0.007489	0.001112
ca_{0,b_1}				
$\log_{10}(k_1')$	−0.5113	−1.39300		−0.039050
$\log_{10}(k_2)$	−0.8034	−3.30300	5.028 or −2.66	−0.298000
$\log_{10}(k_2')$	−1.5120	5.05000		−1.419000
$\log_{10}(k_{deg})$	−2.0110	5.13900	−2.66 or 5.028	−1.442000
$\log_{10}(k_1'k_2')$	−2.0230	3.65700	3.592	−1.458000
$\log_{10}(k_1'/k_2)$	0.2922	1.91000		0.259000
$transport = \log_{10}(k_1'k_2'/k_2)$	−1.2190	6.96000	−1.436 or 6.252	−1.160000

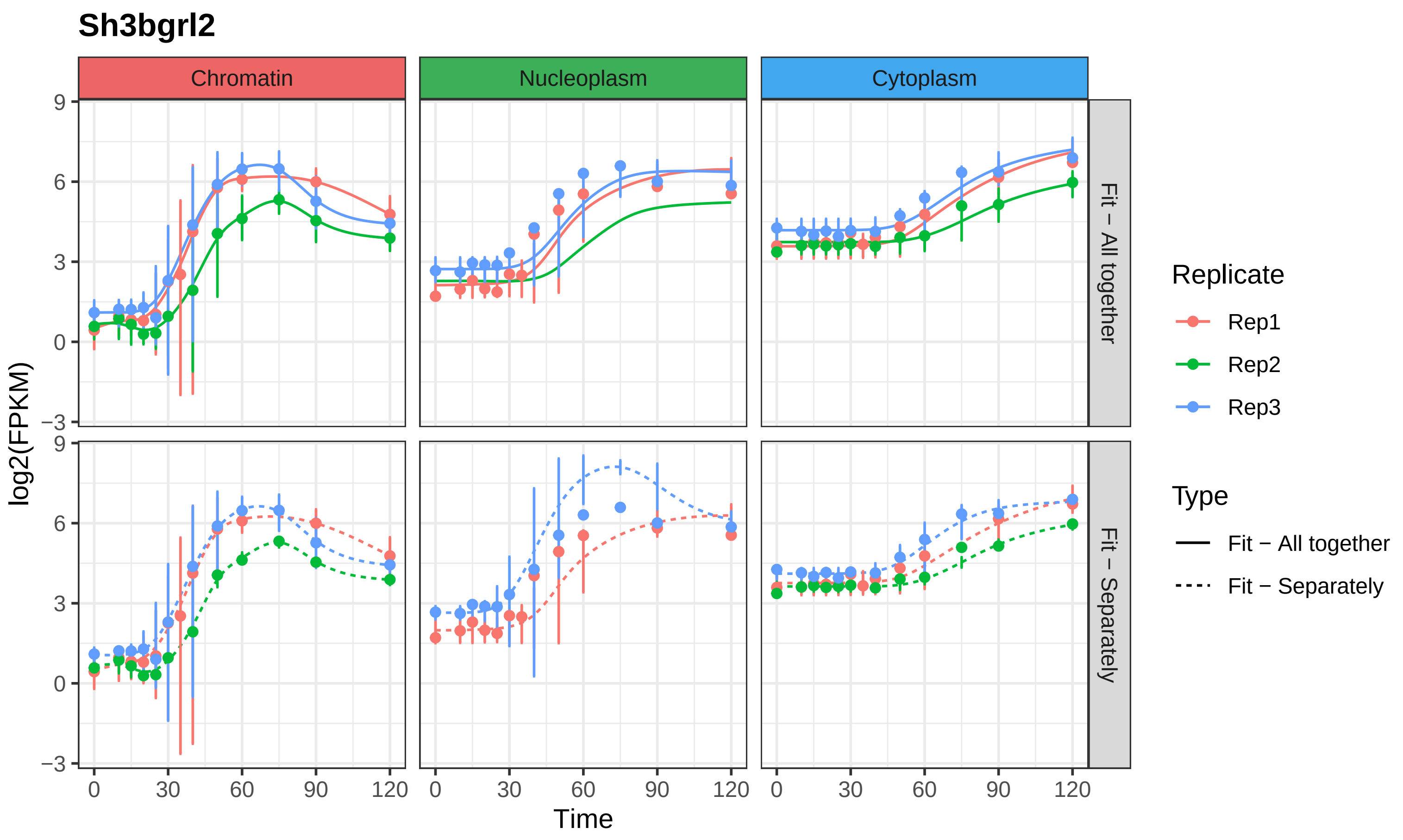

	Together	b1	b2	b3
**−NLL b1 ca**	3.686	4.644		
**−NLL b1 np**	13.92	12.24		
**−NLL b1 cyto**	0.3753	−2.236		
**−NLL b2 ca**	0.5951		−6.824	
**−NLL b2 np**				
**−NLL b2 cyto**	−1.928		−8.671	
**−NLL b3 ca**	2.107			1.28
**−NLL b3 np**	12.04			11.92
**−NLL b3 cyto**	−0.8737			−6.766
Total	29.93	14.65	−15.5	6.432
**Total with regul**	31.52	14.93	−15.19	6.121

	Together	b1	b2	b3
spar	0.3475	0.3902	0.3397	0.38090
σ_b	0.1517	0.1490	0.05969	0.07095
σ_t	5.7470	6.2150	0.7208	6.33300
ca_{0,b_1}				
$\log_{10}(k_1')$	−1.5690	−1.6320		−0.35210
$\log_{10}(k_2)$	−2.0590	−2.0840	−1.6 or −2.118	−0.82940
$\log_{10}(k_2')$	−1.3270	−1.3920		−2.05400
$\log_{10}(k_{deg})$	−1.7660	−1.9250	−2.118 or −1.6	−2.49400
$\log_{10}(k_1'k_2')$	−2.8960	−3.0240	−2.821	−2.40600
$\log_{10}(k_1'/k_2)$	0.4902	0.4520		0.47720
$\text{transport} = \log_{10}(k_1'k_2'/k_2)$	−0.8372	−0.9401	−1.221 or −0.7029	−1.57700

Sh3d21

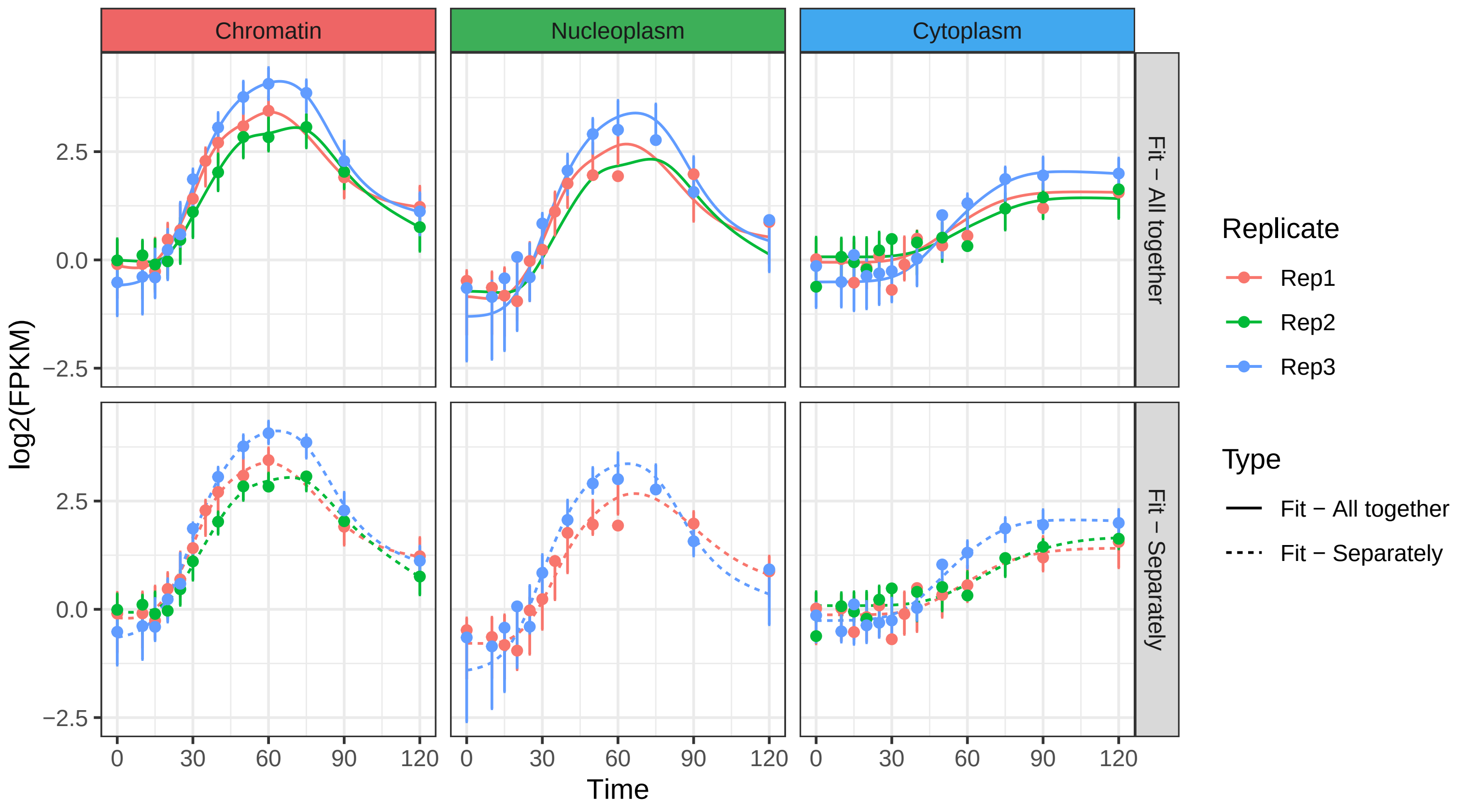

	Together	b1	b2	b3
**−NLL b1 ca**	−4.335	−4.565		
**−NLL b1 np**	8.373	5.042		
**−NLL b1 cyto**	3.294	0.2164		
**−NLL b2 ca**	−5.451		−5.324	
**−NLL b2 np**				
**−NLL b2 cyto**	1.975		0.7936	
**−NLL b3 ca**	−4.932			−4.374
**−NLL b3 np**	8.259			8.035
**−NLL b3 cyto**	0.4331			−2.497
Total	7.617	0.6933	−4.53	1.165
**Total with regul**	8.257	−0.6757	−6.458	0.04746

	Together	b1	b2	b3
spar	3.437e−01	4.029e−01	0.4058	3.823e−01
σ_b	1.183e−01	1.006e−01	1.885e−07	7.540e−02
σ_t	9.951e−05	8.666e−07	2.054e−06	5.673e−05
ca_{0,b_1}				
$\log_{10}(k_1')$	−6.698e−01	−1.117e+00		9.268e−02
$\log_{10}(k_2)$	−4.540e−01	−9.396e−01	−1.295 or −2.109	3.204e−01
$\log_{10}(k_2')$	−2.016e+00	−2.132e+00		−2.034e+00
$\log_{10}(k_{deg})$	−2.256e+00	−2.330e+00	−2.109 or −1.295	−2.378e+00
$\log_{10}(k_1'k_2')$	−2.686e+00	−3.249e+00	−3.361	−1.941e+00
$\log_{10}(k_1'/k_2)$	−2.158e−01	−1.771e−01		−2.277e−01
$\text{transport} = \log_{10}(k_1'k_2'/k_2)$	−2.232e+00	−2.309e+00	−2.066 or −1.252	−2.262e+00

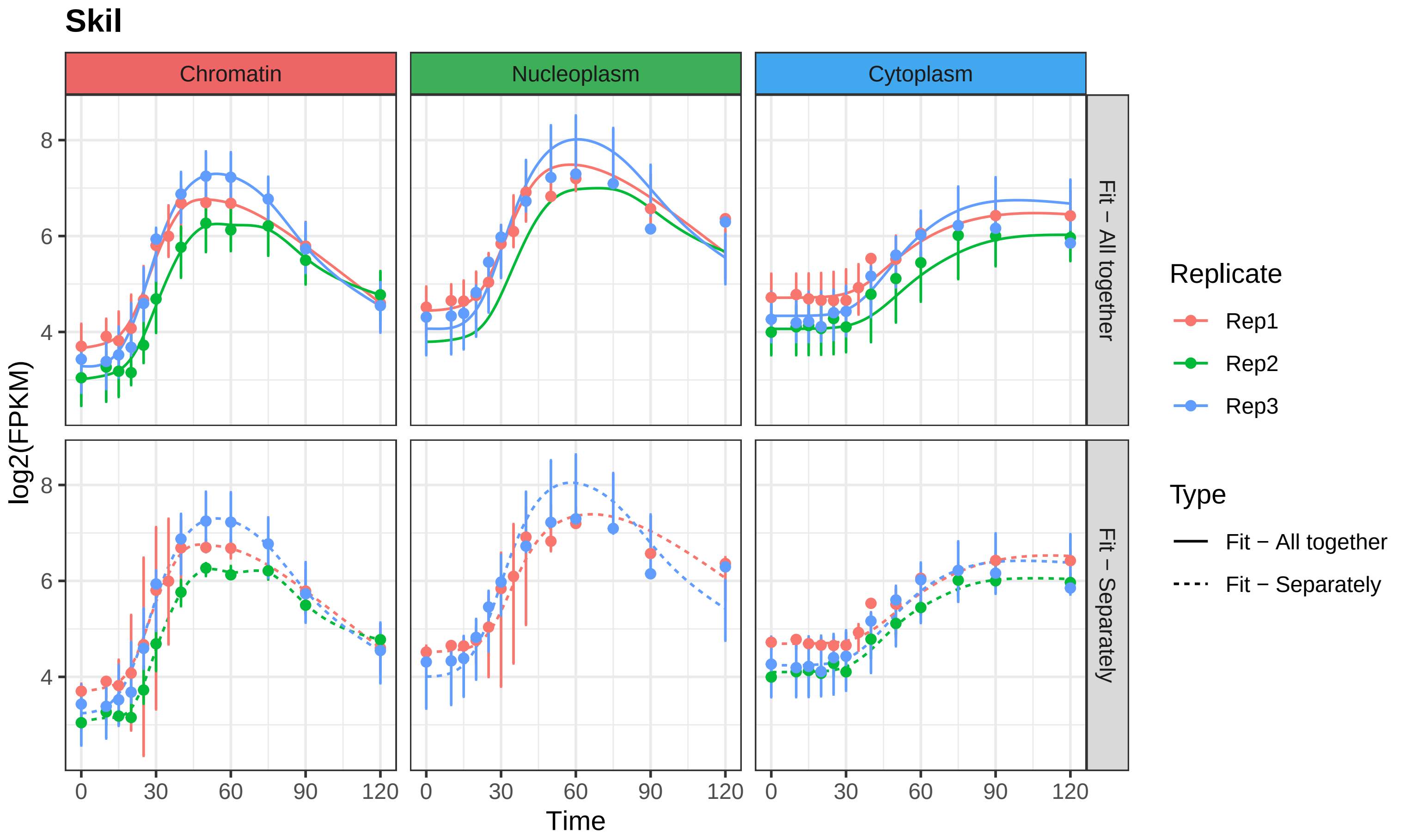

	Together	b1	b2	b3
**−NLL b1 ca**	−3.269	−3.523		
**−NLL b1 np**	3.38	0.9962		
**−NLL b1 cyto**	−2.858	−7.699		
**−NLL b2 ca**	−3.353		−11.65	
**−NLL b2 np**				
**−NLL b2 cyto**	−0.8735		−10.48	
**−NLL b3 ca**	−2.507			−0.3153
**−NLL b3 np**	17.16			11.12
**−NLL b3 cyto**	3.891			0.8897
Total	11.57	−10.23	−22.13	11.69
**Total with regul**	11.8	−9.831	−20.75	12.18

	Together	b1	b2	b3
spar	0.3827	0.35210	0.3164	0.4023000
σ_b	0.1809	0.04067	0.04371	0.2168000
σ_t	0.5405	5.96500	1.409	0.0005246
ca_{0,b_1}				
$\log_{10}(k_1')$	−0.5464	−0.89270		−0.2974000
$\log_{10}(k_2)$	−0.7814	−1.14200	4.432 or −2.052	−0.5282000
$\log_{10}(k_2')$	−1.9910	−1.93700		−2.1420000
$\log_{10}(k_{deg})$	−2.0710	−1.98800	−2.052 or 4.432	−2.2120000
$\log_{10}(k_1'k_2')$	−2.5370	−2.83000	2.693	−2.4390000
$\log_{10}(k_1'/k_2)$	0.2350	0.24970		0.2308000
transport $= \log_{10}(k_1'k_2'/k_2)$	−1.7560	−1.68700	−1.739 or 4.745	−1.9110000

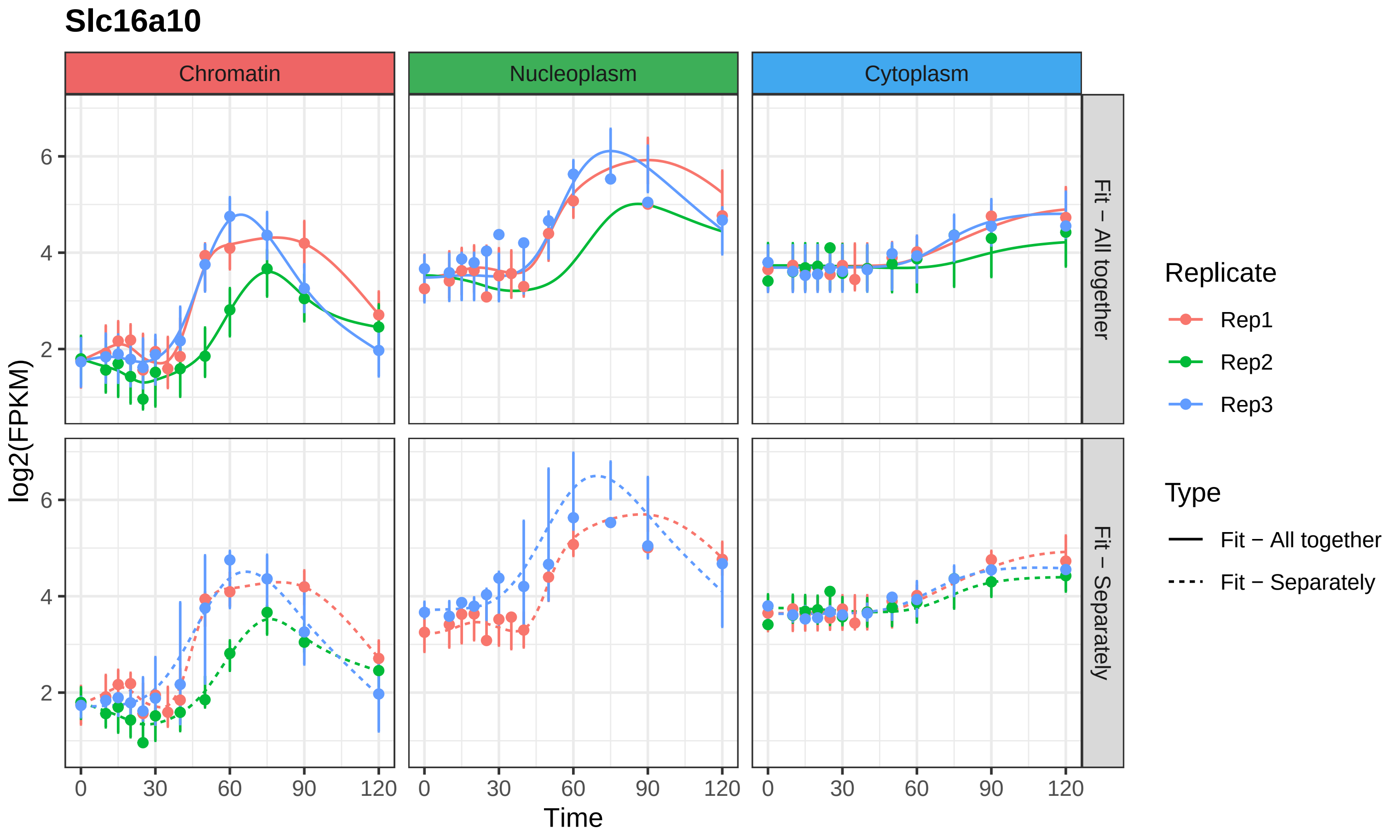

	Together	b1	b2	b3
**−NLL b1 ca**	−2.67	−4.826		
**−NLL b1 np**	6.878	2.422		
**−NLL b1 cyto**	−3.789	−6.658		
**−NLL b2 ca**	−3.9		−5.268	
**−NLL b2 np**				
**−NLL b2 cyto**	0.8013		−3.235	
**−NLL b3 ca**	−4.731			−0.9073
**−NLL b3 np**	15.38			2.726
**−NLL b3 cyto**	−3.953			−10.71
Total	4.014	−9.061	−8.503	−8.886
**Total with regul**	5.078	−8.005	−10.1	−9.719

	Together	b1	b2	b3
spar	3.501e−01	0.3350000	0.4162	0.49690
σ_b	1.664e−01	0.1207000	0.0988	0.04958
σ_t	6.236e−06	0.0004525	0.0004386	6.79200
ca_{0,b_1}				
$\log_{10}(k_1')$	−6.509e−01	−0.5552000		0.15490
$\log_{10}(k_2)$	−1.173e+00	−0.9953000	4.345 or −2.051	−0.45000
$\log_{10}(k_2')$	−2.114e+00	−2.0210000		−2.35000
$\log_{10}(k_{deg})$	−2.176e+00	−2.1500000	−2.051 or 4.345	−2.32500
$\log_{10}(k_1'k_2')$	−2.764e+00	−2.5760000	2.884	−2.19500
$\log_{10}(k_1'/k_2)$	5.222e−01	0.4401000		0.60490
transport $= \log_{10}(k_1'k_2'/k_2)$	−1.591e+00	−1.5810000	−1.462 or 4.935	−1.74500

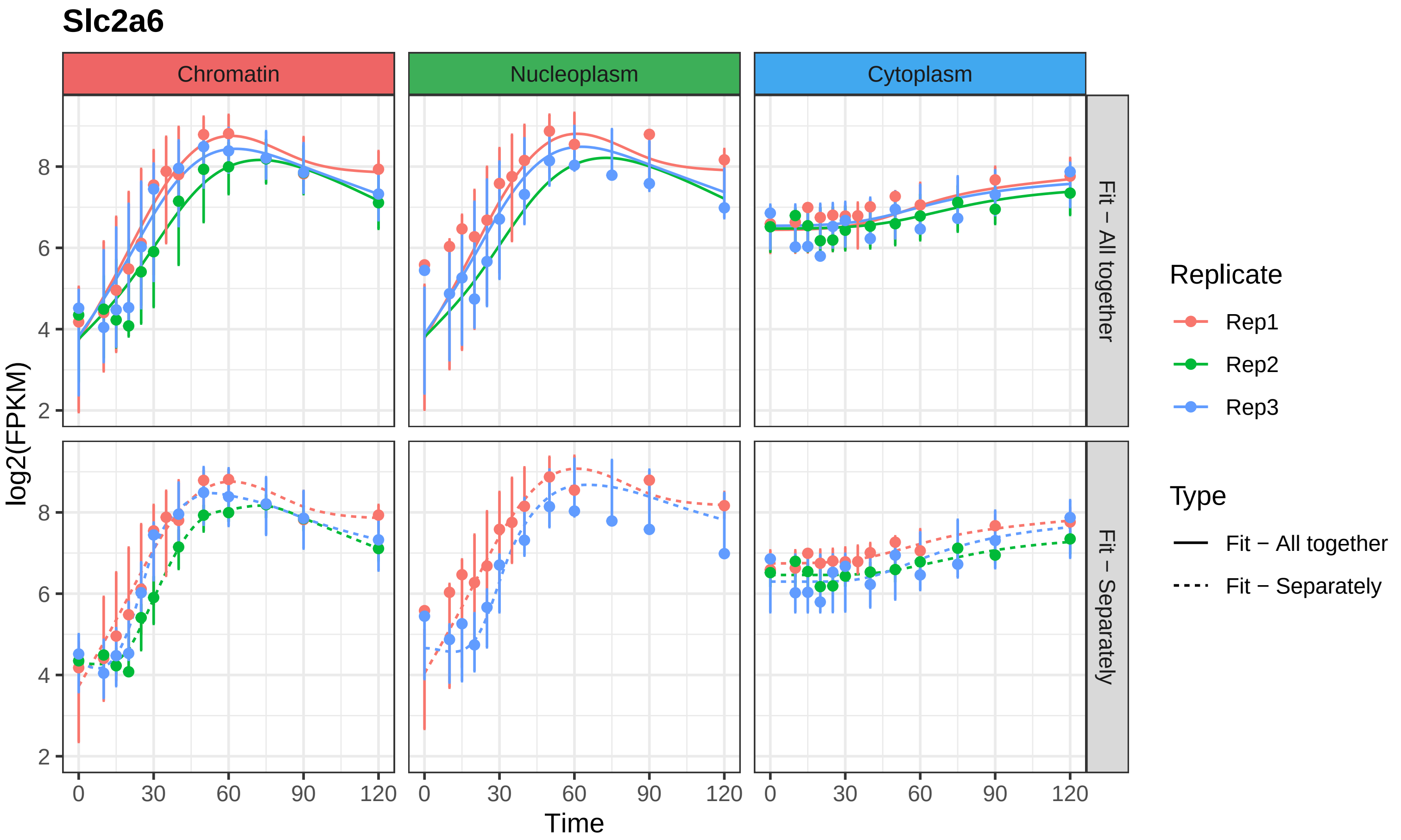

	Together	b1	b2	b3
**−NLL b1 ca**	5.935	3.888		
**−NLL b1 np**	12.49	12.32		
**−NLL b1 cyto**	1.612	−6.874		
**−NLL b2 ca**	4.863		−3.552	
**−NLL b2 np**				
**−NLL b2 cyto**	−1.703		−3.851	
**−NLL b3 ca**	6.531			1.077
**−NLL b3 np**	13.71			11.44
**−NLL b3 cyto**	8.664			3.707
Total	52.1	9.329	−7.403	16.23
**Total with regul**	55.35	11.17	−7.899	18.7

	Together	b1	b2	b3
spar	0.57430	0.57180	0.371	3.540e−01
σ_b	0.18800	0.11450	0.1005	2.428e−01
σ_t	5.97900	5.18100	1.87	3.295e−05
ca_{0,b_1}				
$\log_{10}(k_1')$	6.57200	6.66200		−8.742e−01
$\log_{10}(k_2)$	6.55600	6.56600	−0.6755 or −3.035	−9.717e−01
$\log_{10}(k_2')$	−2.36800	−2.46600		−2.297e+00
$\log_{10}(k_{deg})$	−3.17100	−3.28000	−3.035 or −0.6755	−2.790e+00
$\log_{10}(k_1' k_2')$	4.20400	4.19600	−3.06	−3.172e+00
$\log_{10}(k_1'/k_2)$	0.01532	0.09598		9.747e−02
transport $= \log_{10}(k_1' k_2'/k_2)$	−2.35200	−2.37000	−2.385 or −0.02567	−2.200e+00

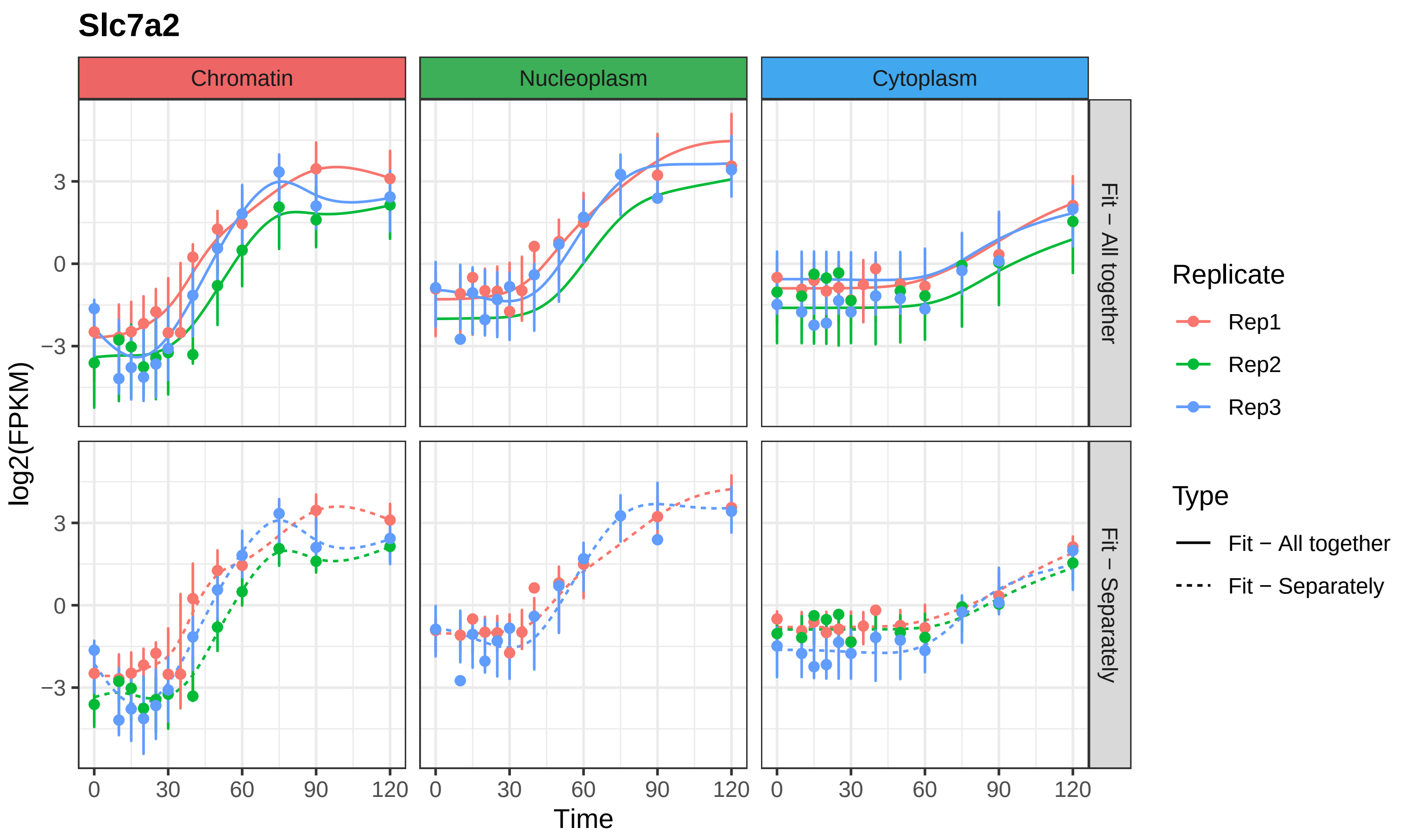

	Together	b1	b2	b3
**−NLL b1 ca**	9.347	4.604		
**−NLL b1 np**	10.01	10.66		
**−NLL b1 cyto**	6.194	2.336		
**−NLL b2 ca**	7.867		2.662	
**−NLL b2 np**				
**−NLL b2 cyto**	14.9		5.135	
**−NLL b3 ca**	9.261			5.749
**−NLL b3 np**	12.71			15.48
**−NLL b3 cyto**	22.41			5.917
Total	92.7	17.6	7.797	27.15
**Total with regul**	97.29	17.62	6.981	28.8

	Together	b1	b2	b3
spar	4.950e−01	0.4093	0.4131	0.4556000
σ_b	3.607e−01	0.1778	0.1568	0.2814000
σ_t	2.721e−05	4.6430	0.0426	0.0001809
ca_{0,b_1}				
$\log_{10}(k_1')$	−1.066e+00	−1.2760		−0.9786000
$\log_{10}(k_2)$	−1.487e+00	−1.7360	−2.007 or −2.053	−1.3660000
$\log_{10}(k_2')$	−2.251e+00	−2.2250		−2.2930000
$\log_{10}(k_{deg})$	−2.371e+00	−2.2950	−2.053 or −2.007	−2.0590000
$\log_{10}(k_1' k_2')$	−3.317e+00	−3.5010	−3.317	−3.2710000
$\log_{10}(k_1'/k_2)$	4.209e−01	0.4600		0.3871000
transport $= \log_{10}(k_1' k_2'/k_2)$	−1.830e+00	−1.7650	−1.31 or −1.264	−1.9050000

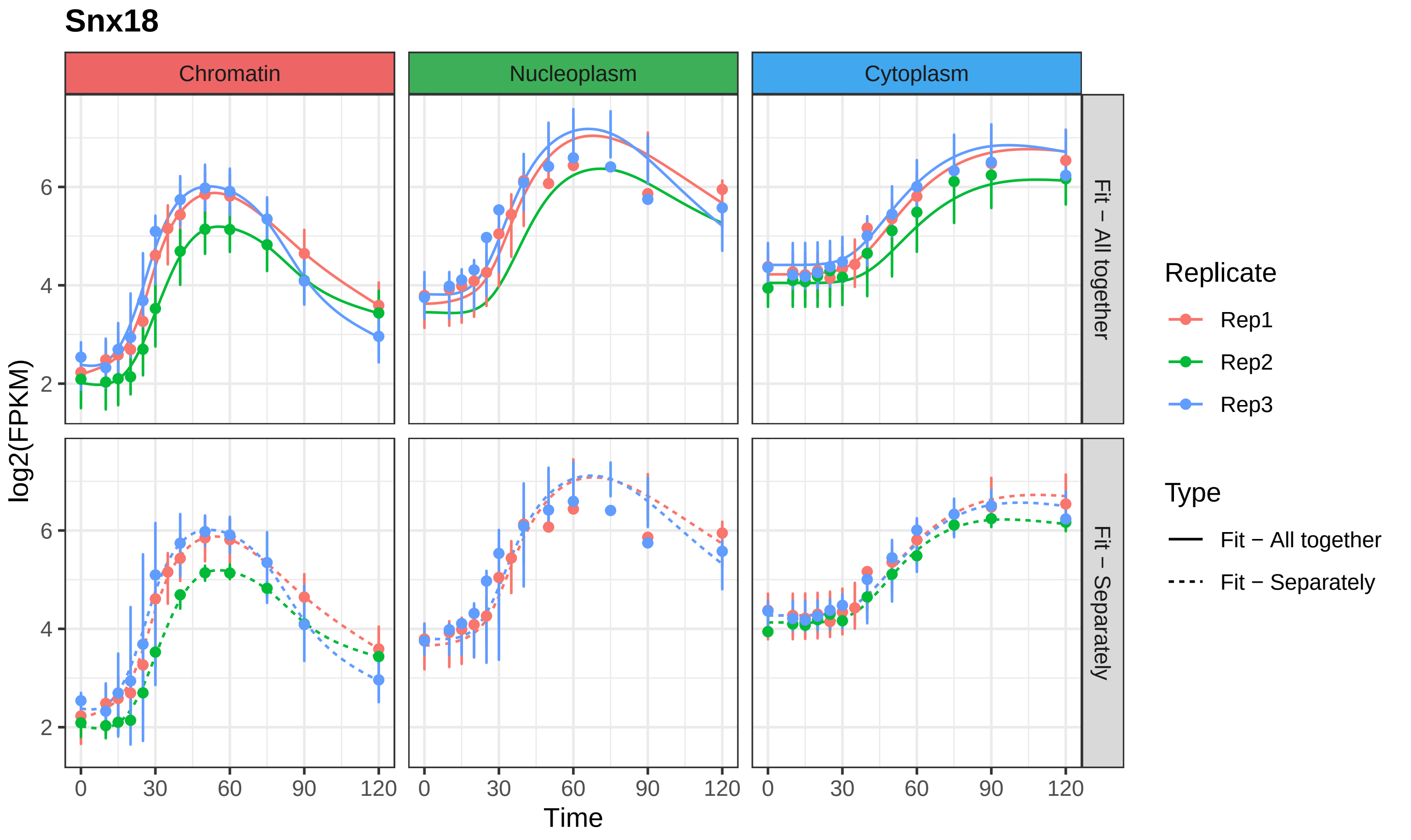

	Together	b1	b2	b3
**−NLL b1 ca**	−3.431	−3.885		
**−NLL b1 np**	7.825	8.207		
**−NLL b1 cyto**	−2.923	−3.083		
**−NLL b2 ca**	−4.492		−11.62	
**−NLL b2 np**				
**−NLL b2 cyto**	−0.9508		−10.51	
**−NLL b3 ca**	−2.95			−0.7805
**−NLL b3 np**	13.54			8.801
**−NLL b3 cyto**	−0.7308			−4.999
Total	5.886	1.24	−22.13	3.022
**Total with regul**	5.963	1.044	−23.22	2.719

	Together	b1	b2	b3
spar	0.3773	0.3850	0.3755	0.3795
σ_b	0.1600	0.1585	0.04762	0.1036
σ_t	1.7140	1.0340	0.5536	4.9170
ca_{0,b_1}				
$\log_{10}(k_1')$	−0.7680	−0.7632		−0.8288
$\log_{10}(k_2)$	−1.1990	−1.2090	−0.6473 or −1.866	−1.2550
$\log_{10}(k_2')$	−1.6360	−1.6970		−1.7470
$\log_{10}(k_{deg})$	−1.8160	−1.8800	−1.866 or −0.6473	−1.8910
$\log_{10}(k_1'k_2')$	−2.4040	−2.4610	−1.878	−2.5760
$\log_{10}(k_1'/k_2)$	0.4315	0.4456		0.4263
$\text{transport} = \log_{10}(k_1'k_2'/k_2)$	−1.2040	−1.2520	−1.231 or −0.01226	−1.3210

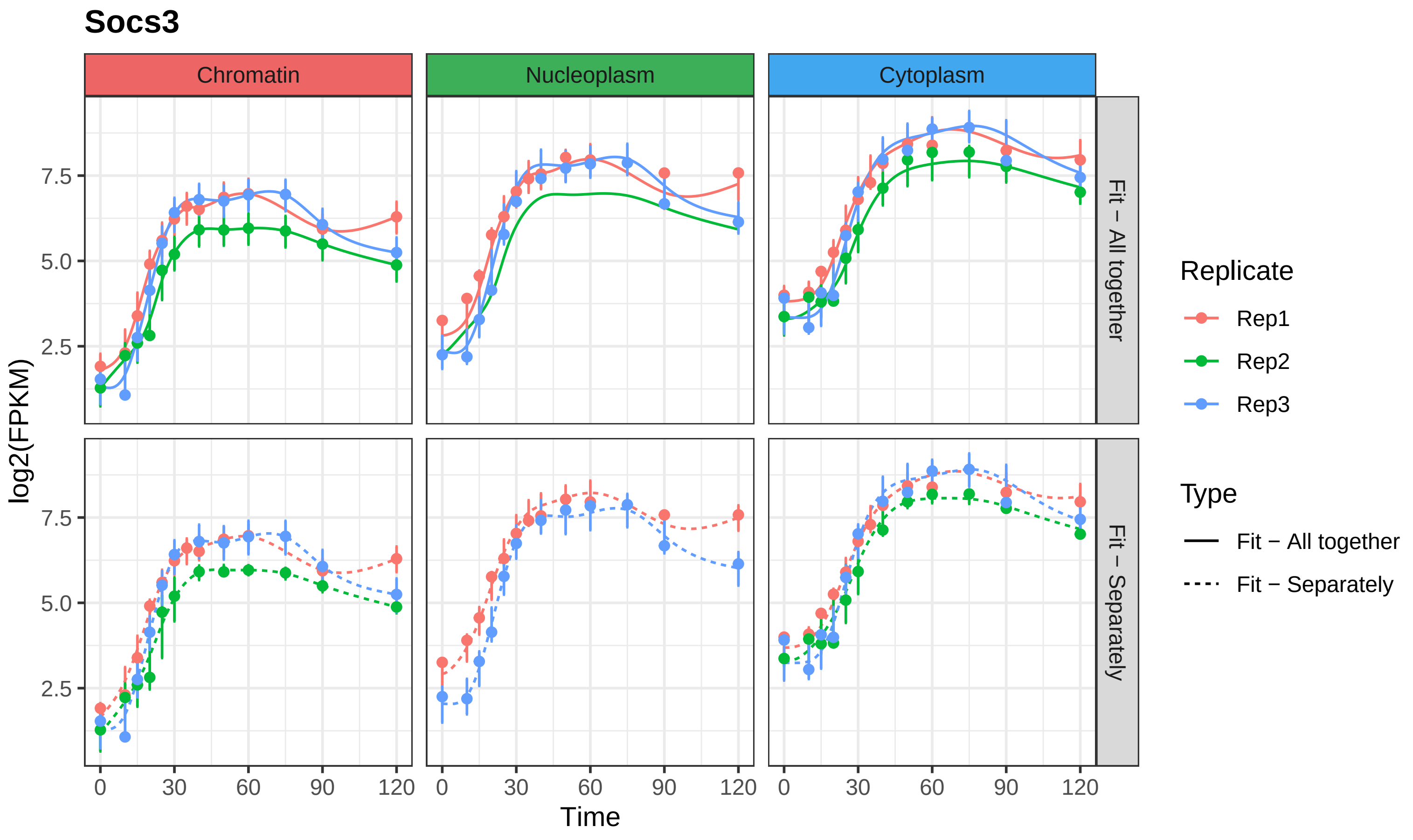

	Together	b1	b2	b3
**−NLL b1 ca**	−4.938	−4.321		
**−NLL b1 np**	5.244	−0.9935		
**−NLL b1 cyto**	−0.3952	−1.147		
**−NLL b2 ca**	−3.816		−6.431	
**−NLL b2 np**				
**−NLL b2 cyto**	−0.4652		−0.3653	
**−NLL b3 ca**	−2.76			−1.897
**−NLL b3 np**	3.674			−2.398
**−NLL b3 cyto**	7.85			7.59
Total	4.394	−6.461	−6.797	3.295
**Total with regul**	10.22	−6.08	−7.299	8.213

	Together	b1	b2	b3
spar	0.26470	0.35360	0.3613	0.2798000
σ_b	0.15900	0.12950	0.0486	0.1697000
σ_t	0.72350	0.36470	2.483	0.0002305
ca_{0,b_1}				
$\log_{10}(k_1')$	0.06609	−0.03749		−0.0999700
$\log_{10}(k_2)$	−0.23930	−0.42220	−0.6058 or −0.6432	−0.3241000
$\log_{10}(k_2')$	−0.73600	−0.83760		−0.5660000
$\log_{10}(k_{deg})$	−1.03700	−1.06800	−0.6432 or −0.6058	−0.9271000
$\log_{10}(k_1'k_2')$	−0.66990	−0.87510	−0.6122	−0.6660000
$\log_{10}(k_1'/k_2)$	0.30540	0.38470		0.2241000
$\text{transport} = \log_{10}(k_1'k_2'/k_2)$	−0.43060	−0.45290	−0.006469 or 0.03098	−0.3419000

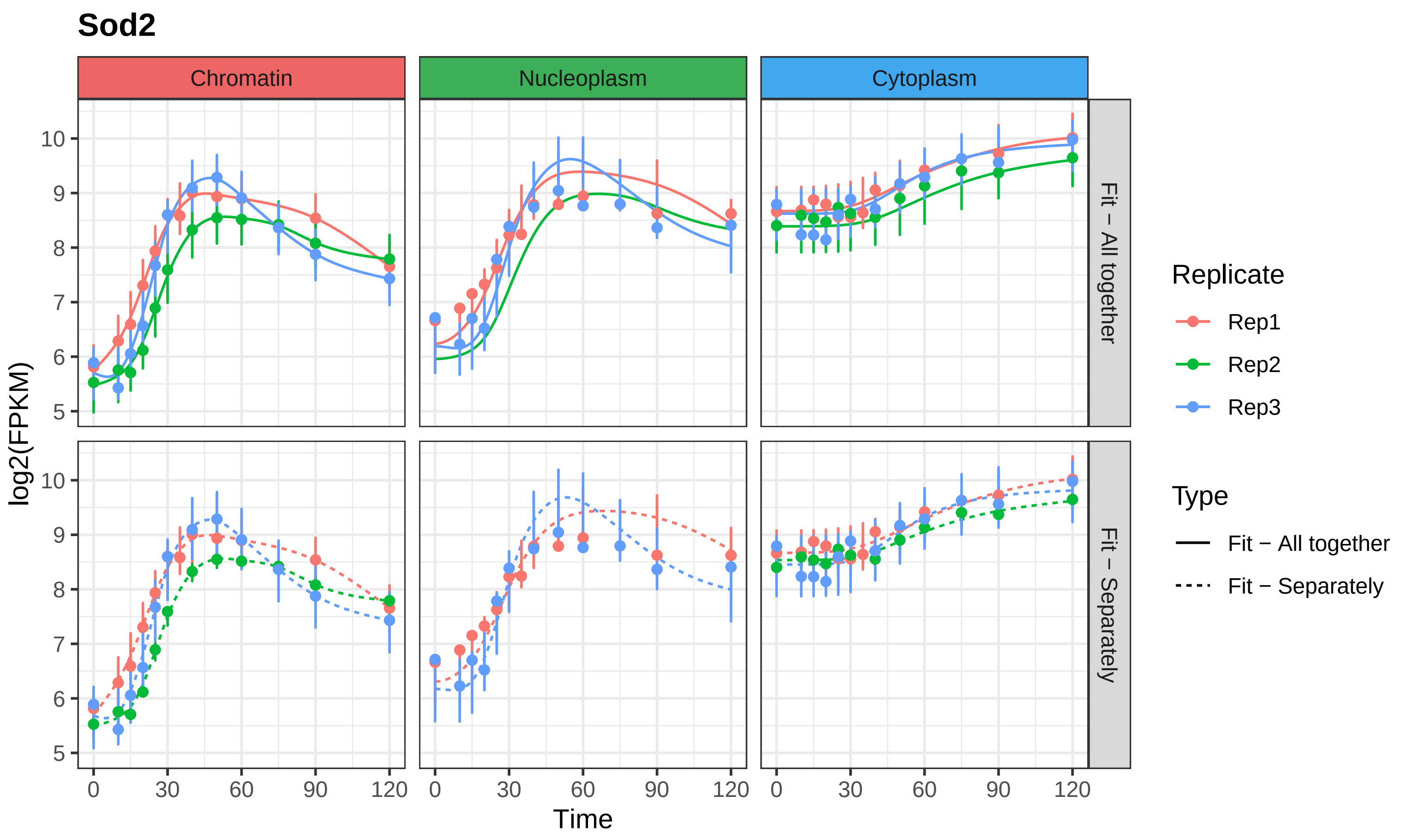

	Together	b1	b2	b3
**−NLL b1 ca**	−5.51	−5.999		
**−NLL b1 np**	8.341	8.579		
**−NLL b1 cyto**	−4.749	−5.402		
**−NLL b2 ca**	−5.534		−13.16	
**−NLL b2 np**				
**−NLL b2 cyto**	−3.641		−10.38	
**−NLL b3 ca**	−4.297			−2.103
**−NLL b3 np**	14.5			9.635
**−NLL b3 cyto**	−0.0996			−0.5474
Total	−0.9916	−2.822	−23.54	6.984
**Total with regul**	−0.3217	−3.614	−23.1	7.767

	Together	b1	b2	b3
spar	0.3574	4.032e−01	0.3357	0.3702000
σ_b	0.1593	1.476e−01	0.05273	0.1913000
σ_t	0.4827	5.840e−06	0.003797	0.0001089
ca_{0,b_1}				
$\log_{10}(k_1')$	−0.7811	−9.756e−01		−0.6448000
$\log_{10}(k_2)$	−0.9271	−1.157e+00	−0.5137 or −2.613	−0.7934000
$\log_{10}(k_2')$	−1.7890	−1.801e+00		−1.7920000
$\log_{10}(k_{deg})$	−2.5210	−2.512e+00	−2.613 or −0.5137	−2.4790000
$\log_{10}(k_1'k_2')$	−2.5700	−2.777e+00	−2.211	−2.4370000
$\log_{10}(k_1'/k_2)$	0.1460	1.816e−01		0.1487000
$\text{transport} = \log_{10}(k_1'k_2'/k_2)$	−1.6430	−1.620e+00	−1.697 or 0.4019	−1.6430000

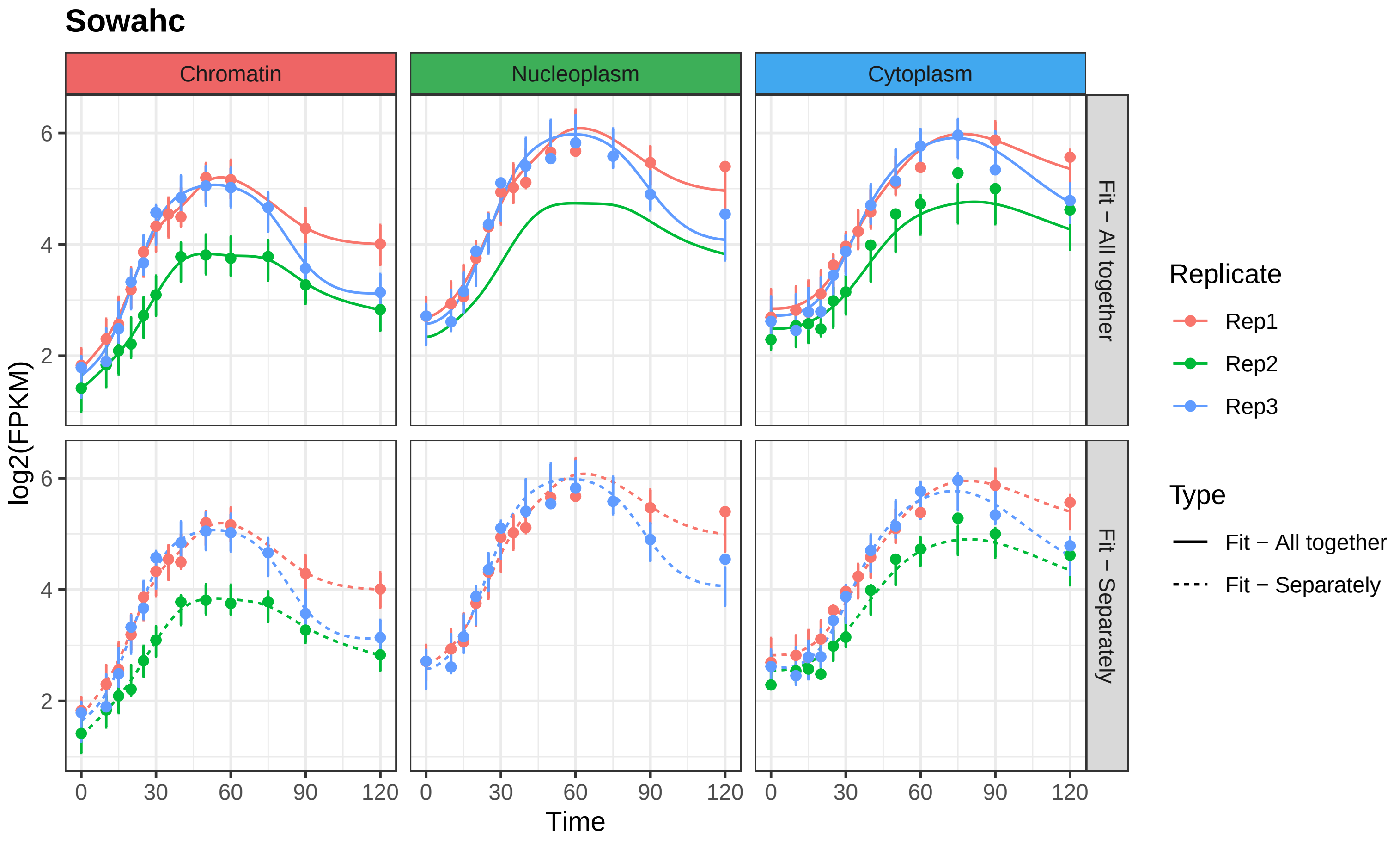

	Together	b1	b2	b3
**−NLL b1 ca**	−8.045	−8.628		
**−NLL b1 np**	−0.4759	0.2091		
**−NLL b1 cyto**	−4.914	−6.413		
**−NLL b2 ca**	−8.755		−9.64	
**−NLL b2 np**				
**−NLL b2 cyto**	3.803		−2.036	
**−NLL b3 ca**	−6.489			−5.962
**−NLL b3 np**	1.138			1.481
**−NLL b3 cyto**	−3.762			−5.721
Total	−27.5	−14.83	−11.68	−10.2
**Total with regul**	−27.93	−16.09	−13.49	−10.63

	Together	b1	b2	b3
spar	0.3743000	0.4004000	0.4276	3.723e−01
σ_b	0.1221000	0.1065000	0.09026	1.167e−01
σ_t	0.0001114	0.0001584	0.001948	6.831e−05
ca_{0,b_1}				
$\log_{10}(k_1')$	−0.4764000	−0.5616000		−3.393e−01
$\log_{10}(k_2)$	−0.7587000	−0.8498000	−0.9915 or −1.034	−6.209e−01
$\log_{10}(k_2')$	−1.2010000	−1.2330000		−1.271e+00
$\log_{10}(k_{deg})$	−1.2440000	−1.2710000	−1.034 or −0.9915	−1.275e+00
$\log_{10}(k_1'k_2')$	−1.6770000	−1.7950000	−1.671	−1.610e+00
$\log_{10}(k_1'/k_2)$	0.2823000	0.2882000		2.816e−01
transport = $\log_{10}(k_1'k_2'/k_2)$	−0.9185000	−0.9451000	−0.6796 or −0.6373	−9.890e−01

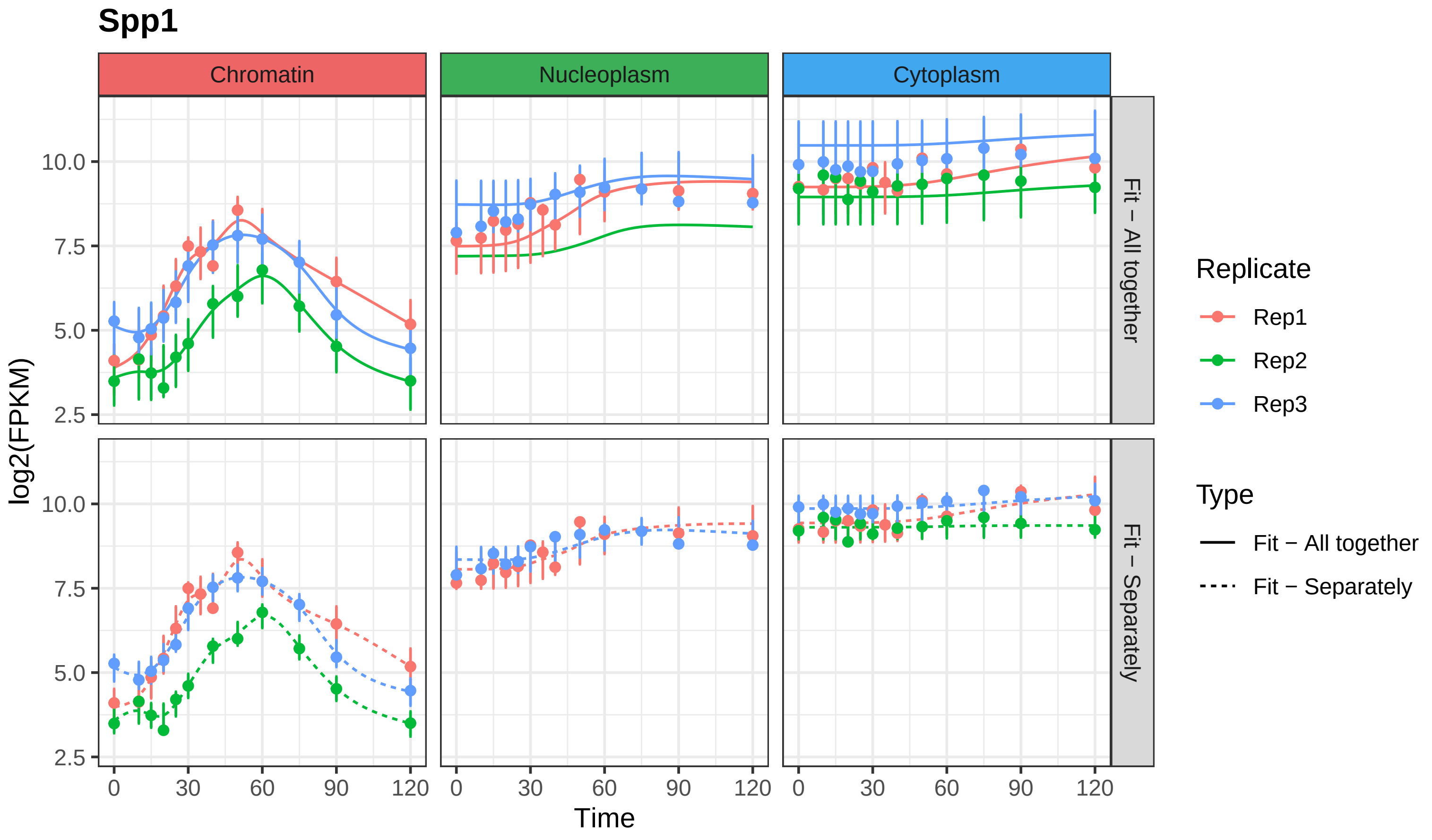

	Together	b1	b2	b3
**−NLL b1 ca**	2.678	−0.8661		
**−NLL b1 np**	10.52	5.404		
**−NLL b1 cyto**	4.325	2.529		
**−NLL b2 ca**	1.565		−3.575	
**−NLL b2 np**				
**−NLL b2 cyto**	5.443		−2.165	
**−NLL b3 ca**	0.3411			−5.411
**−NLL b3 np**	9.246			5.617
**−NLL b3 cyto**	14.01			−5.825
Total	48.13	7.067	−5.74	−5.619
**Total with regul**	49.9	9.009	−4.433	−5.886

	Together	b1	b2	b3
spar	3.888e−01	0.3393000	0.3295	3.747e−01
σ_b	2.583e−01	0.1902000	0.12	1.355e−01
σ_t	1.221e−06	0.0001979	4.411e−07	7.762e−05
ca_{0,b_1}				
$\log_{10}(k_1')$	−1.295e+00	−1.4060000		−1.384e+00
$\log_{10}(k_2)$	−2.379e+00	−2.6350000	4.332 or −3.849	−2.348e+00
$\log_{10}(k_2')$	−1.717e+00	−1.5340000		−1.739e+00
$\log_{10}(k_{deg})$	−2.245e+00	−1.9480000	−3.849 or 4.332	−2.194e+00
$\log_{10}(k_1'k_2')$	−3.012e+00	−2.9400000	2.206	−3.123e+00
$\log_{10}(k_1'/k_2)$	1.084e+00	1.2290000		9.643e−01
$transport = \log_{10}(k_1'k_2'/k_2)$	−6.327e−01	−0.3050000	−2.127 or 6.055	−7.747e−01

Spred1

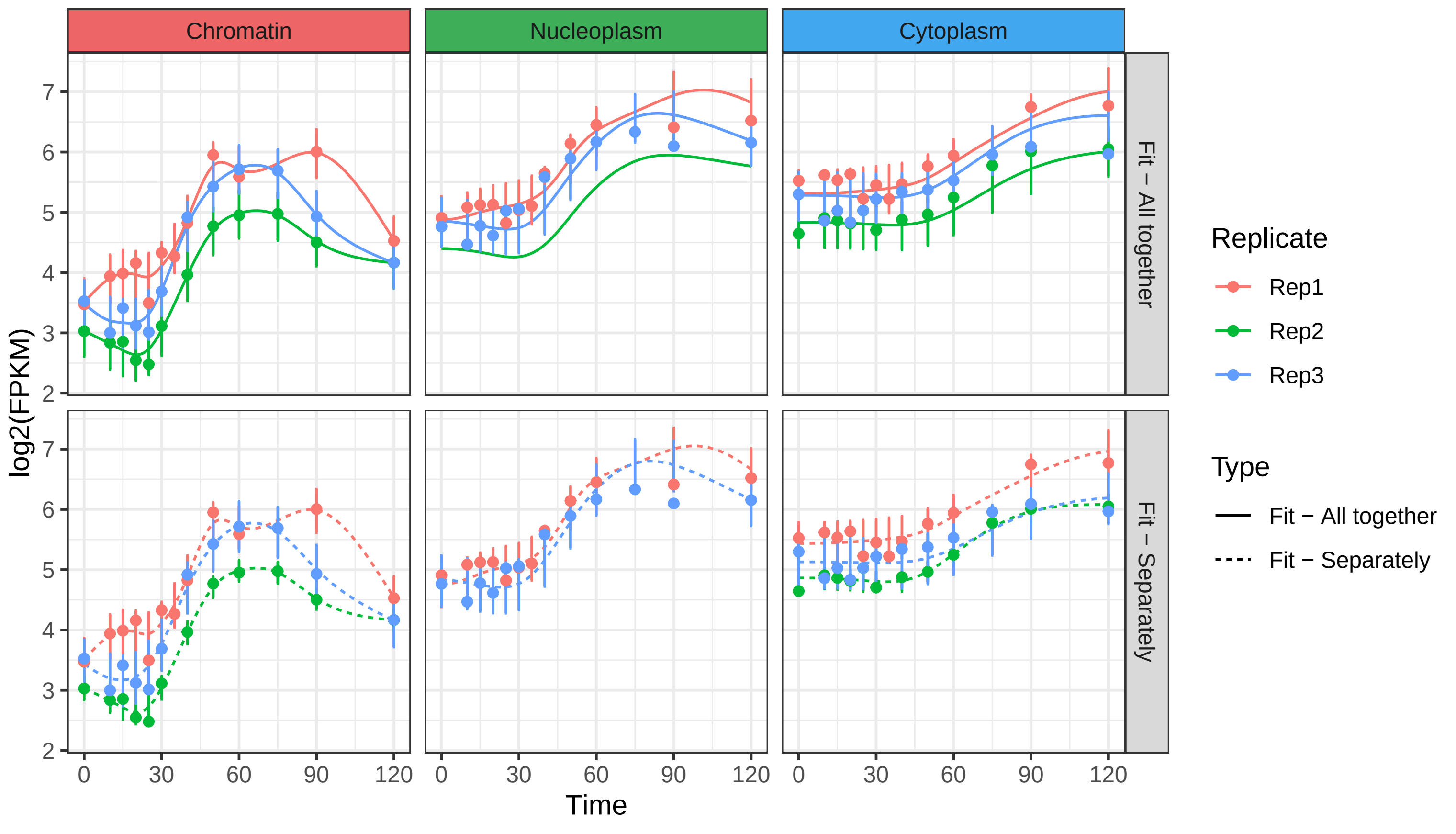

	Together	b1	b2	b3
**−NLL b1 ca**	−3.956	−4.232		
**−NLL b1 np**	−0.441	−0.1802		
**−NLL b1 cyto**	−2.587	−4.698		
**−NLL b2 ca**	−6.714		−10.86	
**−NLL b2 np**				
**−NLL b2 cyto**	−3.57		−9.604	
**−NLL b3 ca**	−5.409			−3.273
**−NLL b3 np**	4.173			2.977
**−NLL b3 cyto**	3.502			−2.65
Total	−15	−9.11	−20.46	−2.945
**Total with regul**	−14.72	−9.124	−20.75	−4.07

	Together	b1	b2	b3
spar	3.599e−01	3.628e−01	0.3538	0.42410
σ_b	1.396e−01	1.253e−01	0.05996	0.14550
σ_t	9.759e−06	2.257e−05	2.219e−07	0.01985
ca_{0,b_1}				
$\log_{10}(k_1')$	−1.100e+00	−9.367e−01		−0.95230
$\log_{10}(k_2)$	−1.512e+00	−1.311e+00	−0.9404 or −1.82	−1.37200
$\log_{10}(k_2')$	−1.564e+00	−1.726e+00		−1.94700
$\log_{10}(k_{deg})$	−1.694e+00	−1.932e+00	−1.82 or −0.9404	−2.03700
$\log_{10}(k_1'k_2')$	−2.664e+00	−2.663e+00	−2.209	−2.89900
$\log_{10}(k_1'/k_2)$	4.115e−01	3.746e−01		0.41970
$\text{transport} = \log_{10}(k_1'k_2'/k_2)$	−1.152e+00	−1.352e+00	−1.269 or −0.3894	−1.52700

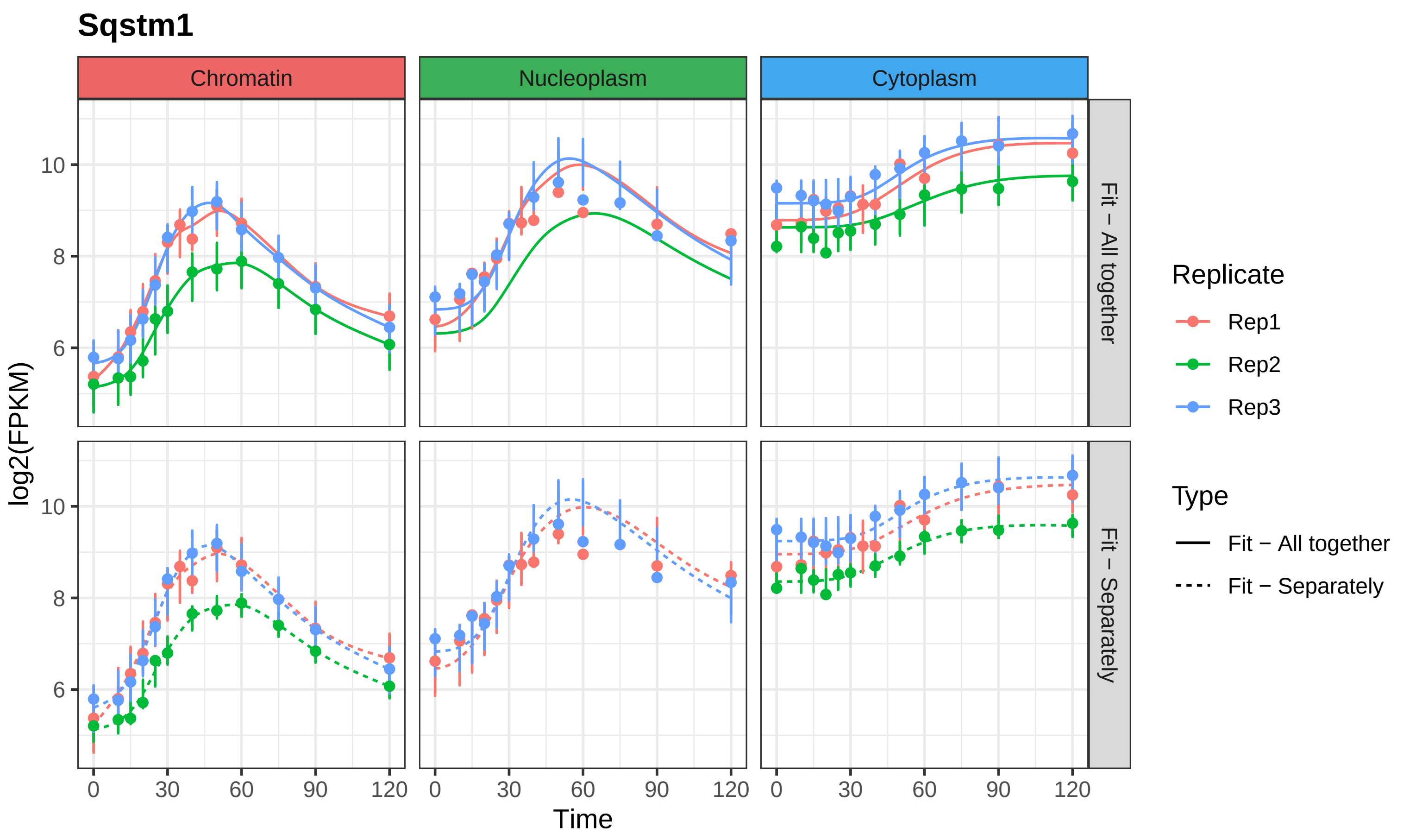

	Together	b1	b2	b3
**−NLL b1 ca**	−3.685	−2.168		
**−NLL b1 np**	13.28	10.65		
**−NLL b1 cyto**	0.2946	−0.2835		
**−NLL b2 ca**	−3.992		−8.337	
**−NLL b2 np**				
**−NLL b2 cyto**	0.02729		−5.053	
**−NLL b3 ca**	−4.06			−3.227
**−NLL b3 np**	10.15			9.422
**−NLL b3 cyto**	−2.665			−2.78
Total	9.353	8.2	−13.39	3.414
**Total with regul**	9.966	7.935	−14.37	3.121

	Together	b1	b2	b3
spar	3.682e−01	4.237e−01	0.3805	3.998e−01
σ_b	1.785e−01	1.957e−01	0.08361	1.745e−01
σ_t	3.443e−05	3.423e−07	1.056	1.527e−07
ca_{0,b_1}				
$\log_{10}(k_1')$	−6.357e−01	−7.303e−01		−6.456e−01
$\log_{10}(k_2)$	−9.892e−01	−1.101e+00	1.085 or −2.245	−1.014e+00
$\log_{10}(k_2')$	−1.609e+00	−1.685e+00		−1.627e+00
$\log_{10}(k_{deg})$	−2.307e+00	−2.436e+00	−2.245 or 1.085	−2.353e+00
$\log_{10}(k_1' k_2')$	−2.245e+00	−2.415e+00	−0.1865	−2.272e+00
$\log_{10}(k_1'/k_2)$	3.535e−01	3.711e−01		3.683e−01
$\text{transport} = \log_{10}(k_1' k_2'/k_2)$	−1.256e+00	−1.313e+00	−1.272 or 2.059	−1.259e+00

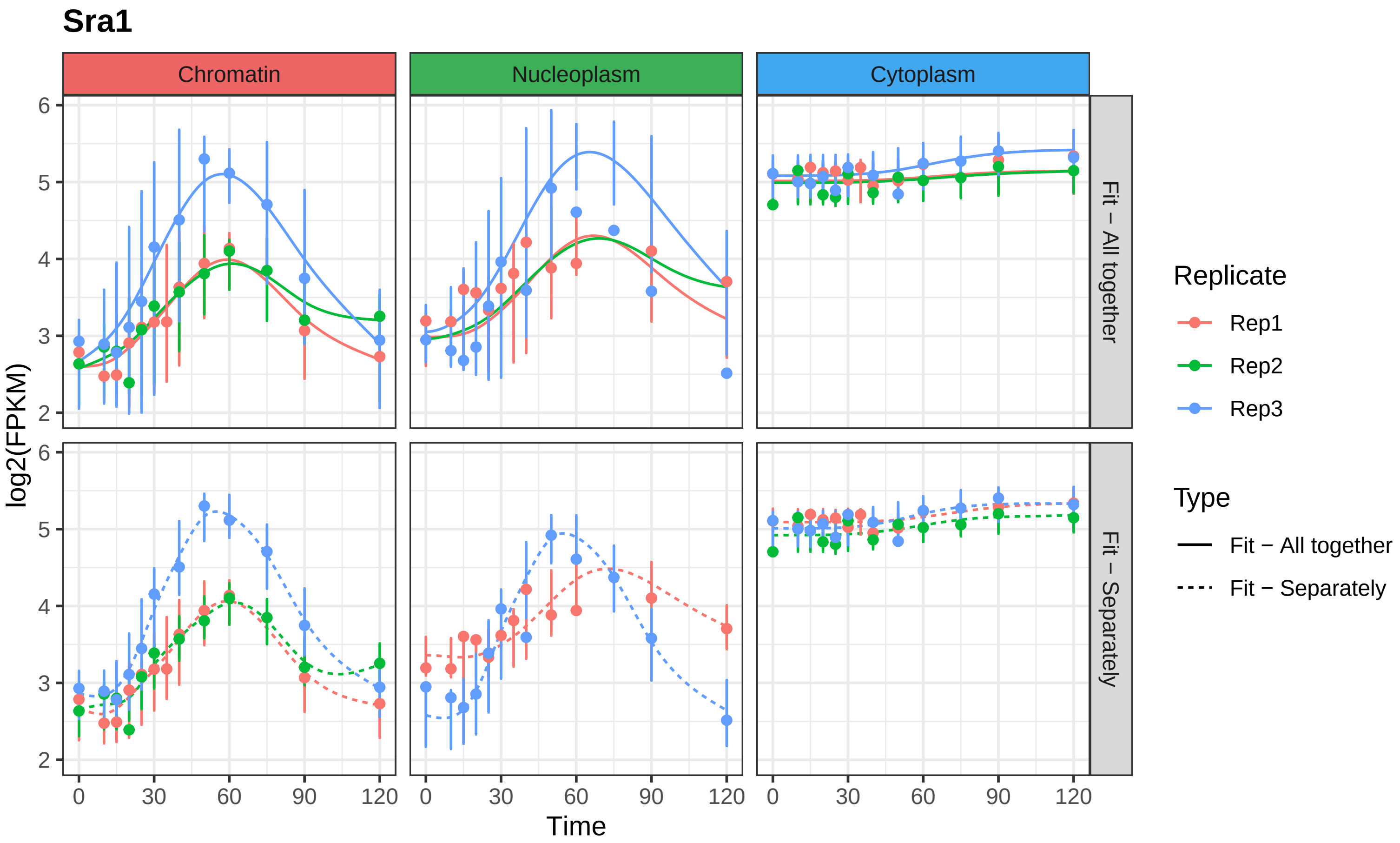

	Together	b1	b2	b3
**−NLL b1 ca**	−1.31	−5.887		
**−NLL b1 np**	7.791	1.493		
**−NLL b1 cyto**	−7.8	−13.61		
**−NLL b2 ca**	−2.31		−5.553	
**−NLL b2 np**				
**−NLL b2 cyto**	−7.186		−7.2	
**−NLL b3 ca**	2.611			−5.394
**−NLL b3 np**	20.51			3.403
**−NLL b3 cyto**	−8.101			−8.262
Total	4.202	−18.01	−12.75	−10.25
**Total with regul**	5.299	−19.64	−14.48	−11.55

	Together	b1	b2	b3
spar	0.51990	0.45280	0.4066	0.39680
σ_b	0.08969	0.04632	0.06607	0.07331
σ_t	10.09000	5.36700	0.002154	3.08200
ca_{0,b_1}				
$\log_{10}(k_1')$	−0.82940	−1.11500		3.87100
$\log_{10}(k_2)$	−0.94560	−1.32200	0.5088 or −2.57	3.95600
$\log_{10}(k_2')$	−2.28800	−1.96500		−2.09700
$\log_{10}(k_{deg})$	−2.89900	−2.48600	−2.57 or 0.5088	−2.82900
$\log_{10}(k_1' k_2')$	−3.11700	−3.08000	−1.379	1.77400
$\log_{10}(k_1'/k_2)$	0.11620	0.20690		−0.08536
transport $= \log_{10}(k_1' k_2'/k_2)$	−2.17200	−1.75800	−1.888 or 1.191	−2.18200

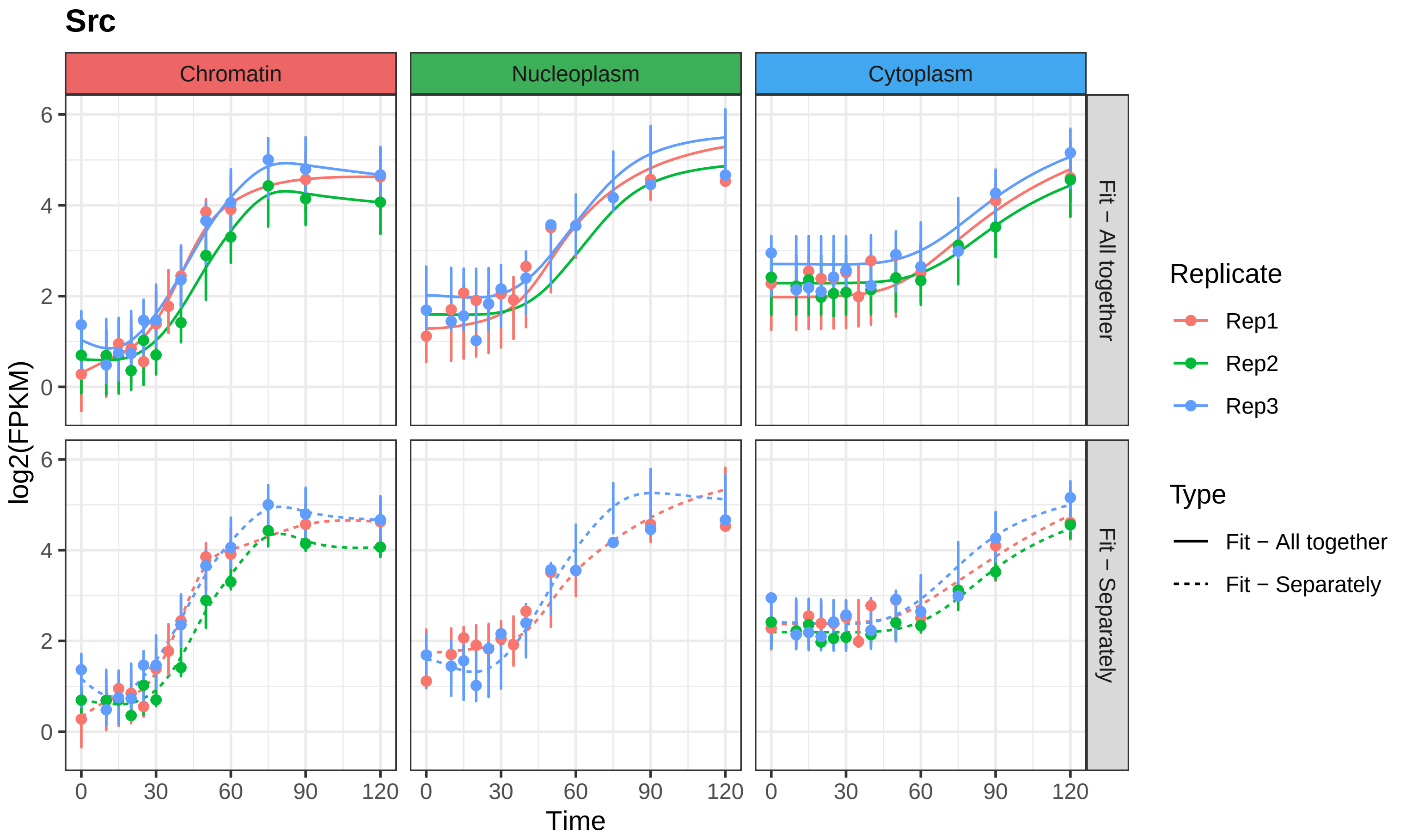

	Together	b1	b2	b3
**−NLL b1 ca**	0.5813	−2.665		
**−NLL b1 np**	9.927	7.608		
**−NLL b1 cyto**	6.753	0.02123		
**−NLL b2 ca**	0.3998		−4.408	
**−NLL b2 np**				
**−NLL b2 cyto**	−0.5225		−6.399	
**−NLL b3 ca**	0.4244			−1.314
**−NLL b3 np**	11.85			7.757
**−NLL b3 cyto**	6.066			1.257
Total	35.48	4.964	−10.81	7.7
**Total with regul**	35.87	4.671	−12.58	7.278

	Together	b1	b2	b3
spar	0.48270	3.991e−01	0.4127	0.4263000
σ_b	0.22360	1.736e−01	0.06665	0.1889000
σ_t	0.00007	2.779e−05	1.445	0.0003162
ca_{0,b_1}				
$\log_{10}(k_1')$	−1.29600	−1.417e+00		−0.8547000
$\log_{10}(k_2)$	−1.59300	−1.845e+00	−1.675 or −1.712	−0.9809000
$\log_{10}(k_2')$	−1.68700	−1.680e+00		−1.7340000
$\log_{10}(k_{deg})$	−1.89500	−1.869e+00	−1.712 or −1.675	−1.9820000
$\log_{10}(k_1'k_2')$	−2.98300	−3.097e+00	−2.933	−2.5890000
$\log_{10}(k_1'/k_2)$	0.29610	4.274e−01		0.1262000
$\text{transport} = \log_{10}(k_1'k_2'/k_2)$	−1.39100	−1.253e+00	−1.258 or −1.221	−1.6080000

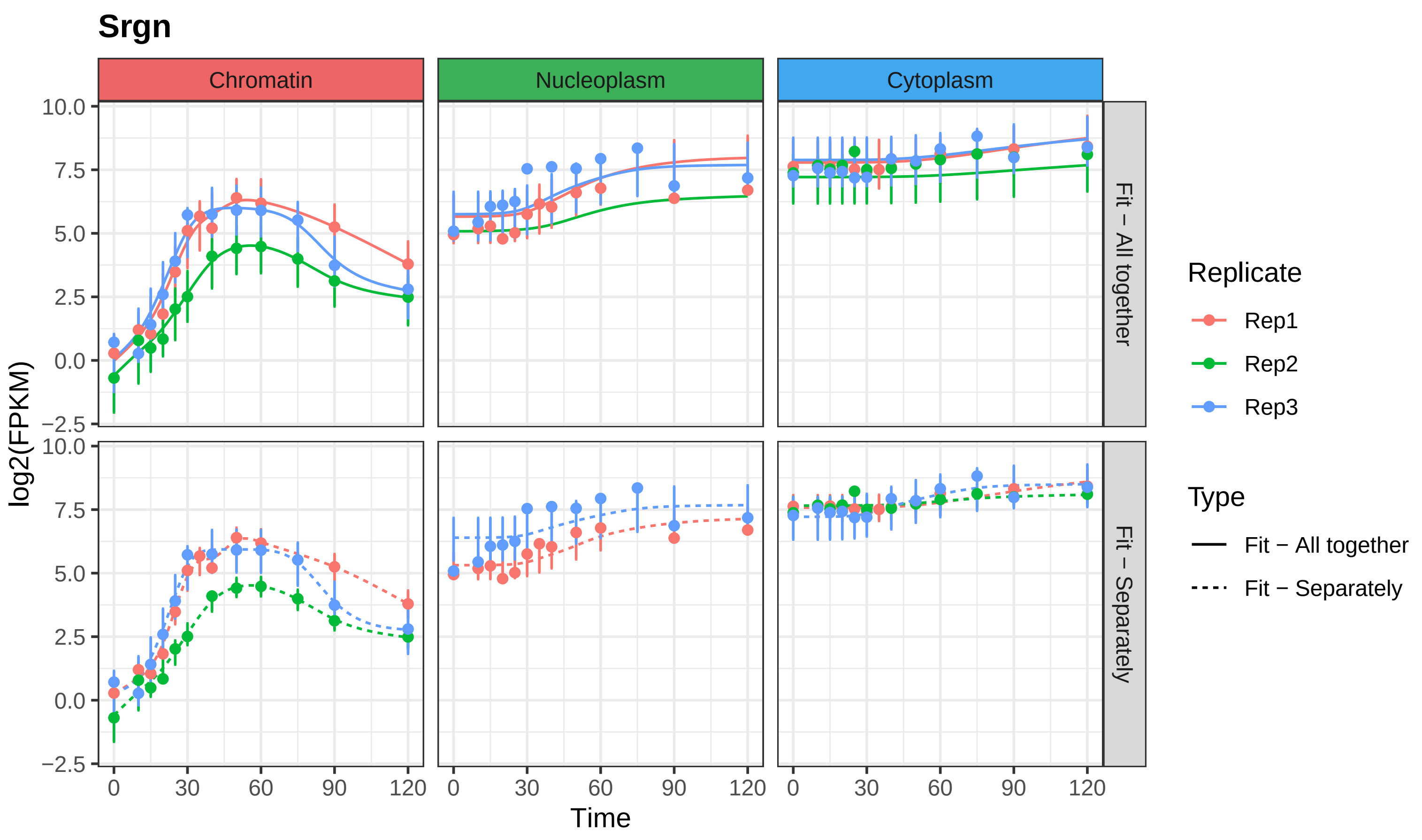

	Together	b1	b2	b3
**−NLL b1 ca**	5.763	−0.1606		
**−NLL b1 np**	14.69	9.417		
**−NLL b1 cyto**	4.261	−3.407		
**−NLL b2 ca**	4.066		−1.267	
**−NLL b2 np**				
**−NLL b2 cyto**	8.867		−1.021	
**−NLL b3 ca**	6.757			3.678
**−NLL b3 np**	20.02			17.34
**−NLL b3 cyto**	8.509			2.149
Total	72.93	5.849	−2.288	23.17
**Total with regul**	76.02	8.191	−3.464	26.56

	Together	b1	b2	b3
spar	4.104e−01	0.3261000	0.4057	3.576e−01
σ_b	3.192e−01	0.1784000	0.1224	2.830e−01
σ_t	5.153e−08	0.0001361	6.723e−05	8.801e−05
ca_{0,b_1}				
$\log_{10}(k_1')$	−1.316e+00	−1.6100000		−1.427e+00
$\log_{10}(k_2)$	−3.025e+00	−3.1340000	−0.7736 or −3.695	−3.265e+00
$\log_{10}(k_2')$	−1.660e+00	−1.3230000		2.877e+00
$\log_{10}(k_{deg})$	−2.302e+00	−2.0040000	−3.695 or −0.7736	2.630e+00
$\log_{10}(k_1' k_2')$	−2.976e+00	−2.9330000	−1.987	1.450e+00
$\log_{10}(k_1'/k_2)$	1.708e+00	1.5250000		1.838e+00
$\text{transport} = \log_{10}(k_1' k_2'/k_2)$	4.831e−02	0.2014000	−1.213 or 1.708	4.715e+00

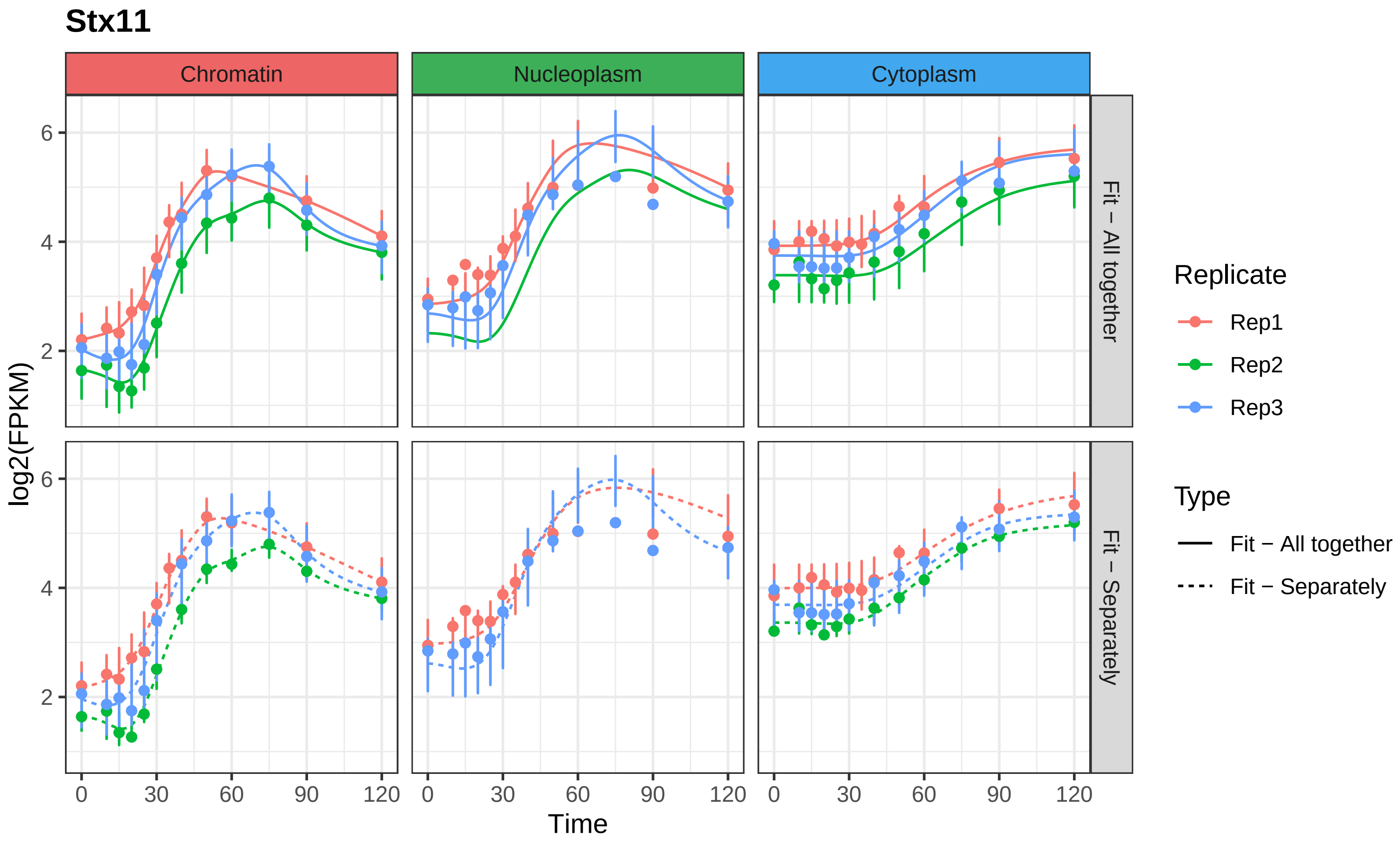

	Together	b1	b2	b3
**−NLL b1 ca**	−4.846	−4.922		
**−NLL b1 np**	8.822	8.698		
**−NLL b1 cyto**	−4.49	−4.792		
**−NLL b2 ca**	−4.501		−8.861	
**−NLL b2 np**				
**−NLL b2 cyto**	−2.941		−7.327	
**−NLL b3 ca**	−3.586			−1.783
**−NLL b3 np**	13.8			8.731
**−NLL b3 cyto**	−1.665			−2.916
Total	0.5937	−1.017	−16.19	4.033
**Total with regul**	1.377	−1.747	−16.42	3.518

	Together	b1	b2	b3
spar	0.354500	4.035e−01	0.352	0.4022
σ_b	0.159800	1.521e−01	0.05828	0.1567
σ_t	0.001295	2.828e−06	0.0001007	2.4400
ca_{0,b_1}				
$\log_{10}(k_1')$	−0.774000	−9.810e−01		−0.5710
$\log_{10}(k_2)$	−0.973200	−1.223e+00	0.2615 or −2.114	−0.7664
$\log_{10}(k_2')$	−1.803000	−1.849e+00		−1.9530
$\log_{10}(k_{deg})$	−2.124000	−2.156e+00	−2.114 or 0.2615	−2.2770
$\log_{10}(k_1' k_2')$	−2.577000	−2.830e+00	−1.34	−2.5240
$\log_{10}(k_1'/k_2)$	0.199200	2.420e−01		0.1954
transport $= \log_{10}(k_1' k_2'/k_2)$	−1.604000	−1.607e+00	−1.602 or 0.7733	−1.7570

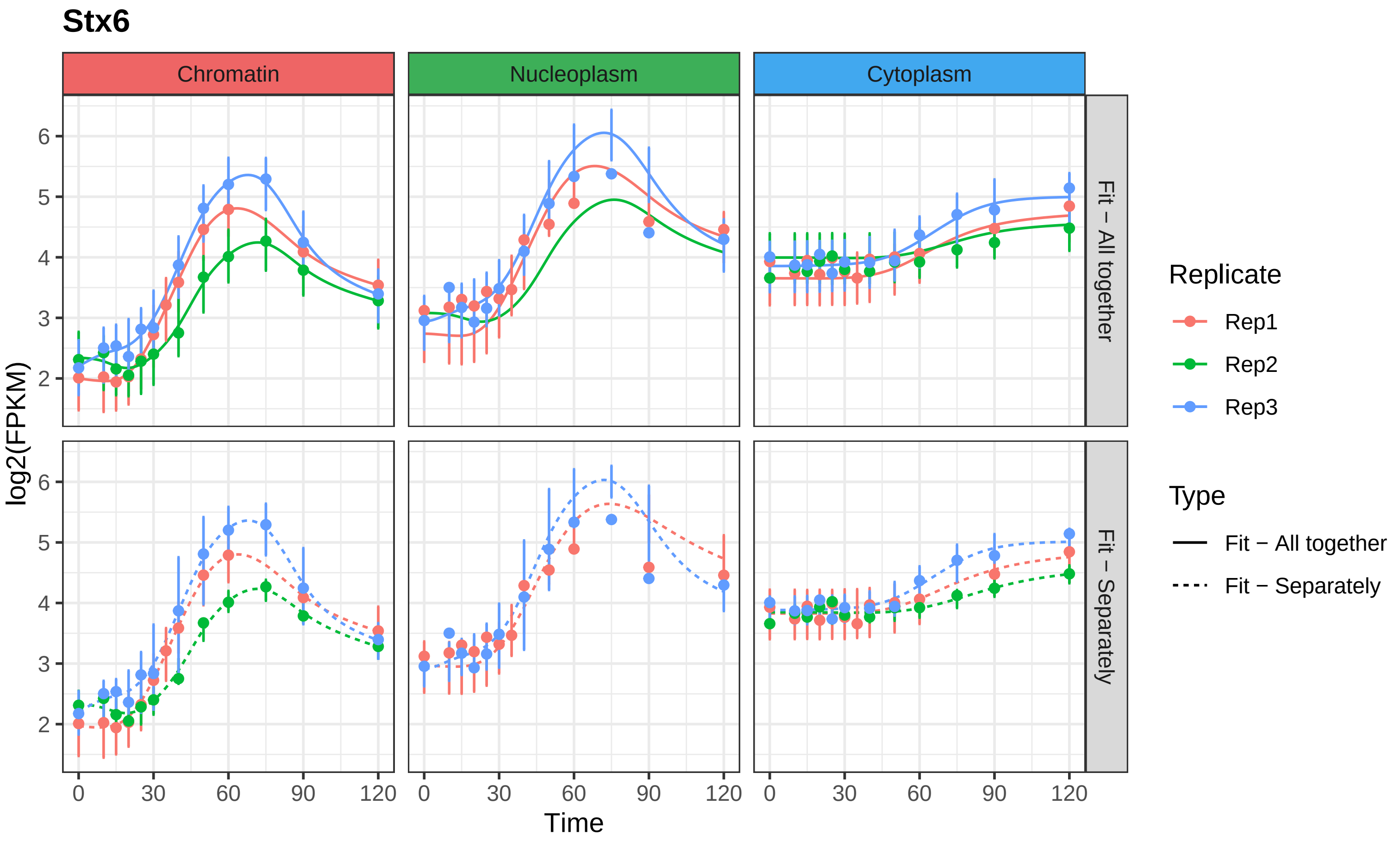

	Together	b1	b2	b3
**−NLL b1 ca**	−5.859	−6.637		
**−NLL b1 np**	11.94	8.468		
**−NLL b1 cyto**	−3.06	−6.343		
**−NLL b2 ca**	−6.078		−10.46	
**−NLL b2 np**				
**−NLL b2 cyto**	−3.01		−10.53	
**−NLL b3 ca**	−5.667			−4.553
**−NLL b3 np**	11.07			5.865
**−NLL b3 cyto**	−6.19			−8.586
Total	−6.852	−4.512	−20.99	−7.274
**Total with regul**	−7.151	−5.795	−22.86	−7.748

	Together	b1	b2	b3
spar	0.3802	0.4276000	0.4071	0.37500
σ_b	0.1420	0.1367000	0.04209	0.07449
σ_t	1.3660	0.0001963	2.387e−05	4.70400
ca_{0,b_1}				
$\log_{10}(k_1')$	−0.4553	−0.7466000		−0.45910
$\log_{10}(k_2)$	−0.6777	−1.0450000	−1.283 or −2.233	−0.67340
$\log_{10}(k_2')$	−2.1230	−2.2070000		−2.11400
$\log_{10}(k_{deg})$	−2.3980	−2.4680000	−2.233 or −1.283	−2.40600
$\log_{10}(k_1' k_2')$	−2.5780	−2.9540000	−3.063	−2.57400
$\log_{10}(k_1'/k_2)$	0.2224	0.2985000		0.21430
$\text{transport} = \log_{10}(k_1' k_2'/k_2)$	−1.9010	−1.9090000	−1.78 or −0.8292	−1.90000

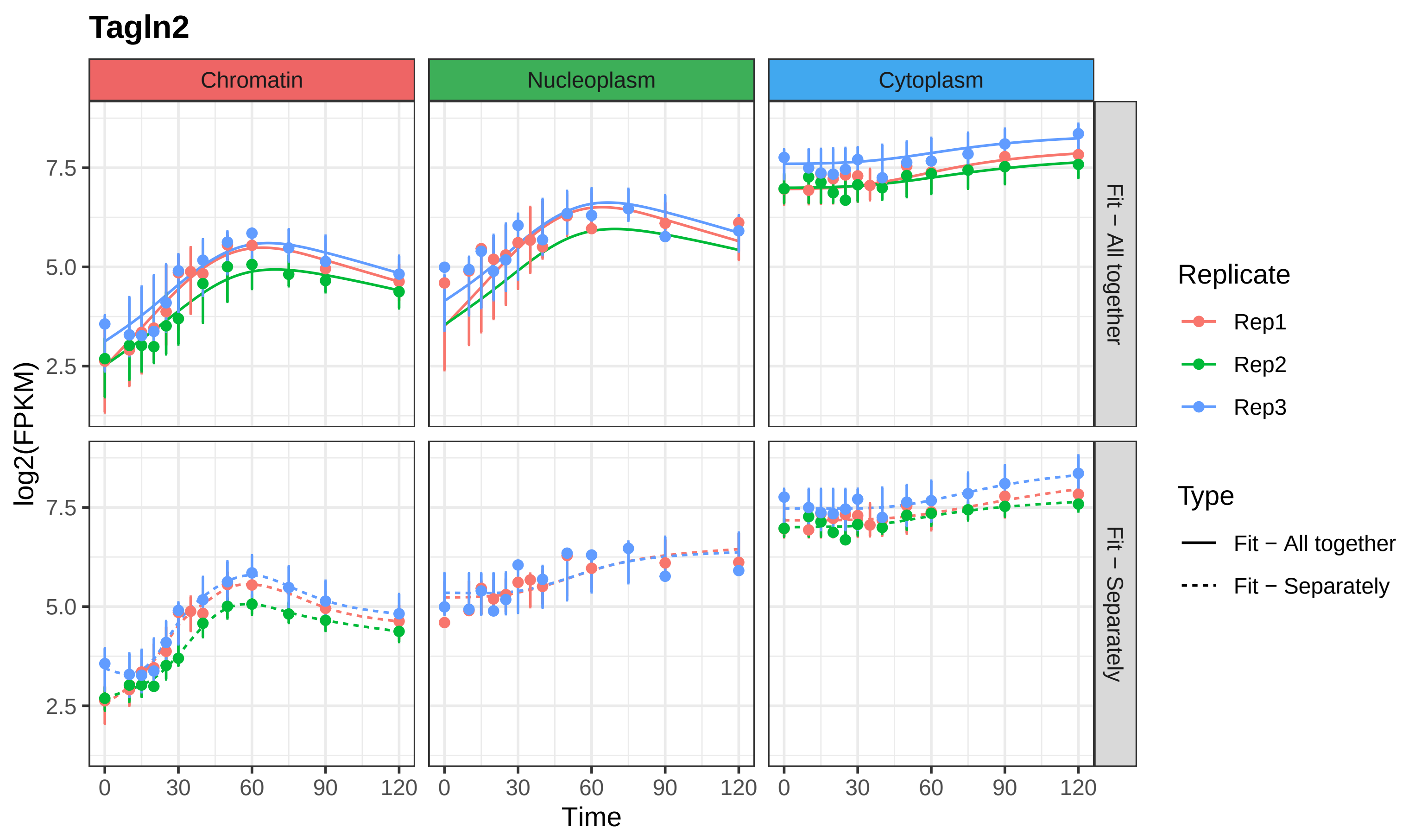

	Together	b1	b2	b3
**−NLL b1 ca**	1.382	−4.198		
**−NLL b1 np**	11.26	5.471		
**−NLL b1 cyto**	−3.087	−4.465		
**−NLL b2 ca**	−0.5974		−9.486	
**−NLL b2 np**				
**−NLL b2 cyto**	−4.991		−4.523	
**−NLL b3 ca**	2.56			−2.63
**−NLL b3 np**	7.912			10.35
**−NLL b3 cyto**	−1.737			−2.886
Total	12.7	−3.192	−14.01	4.83
**Total with regul**	17.65	−4.265	−15.75	4.508

	Together	b1	b2	b3
spar	0.6154	4.182e−01	0.4153	4.062e−01
σ_b	0.1310	1.445e−01	0.08249	1.797e−01
σ_t	6.8640	1.918e−06	0.0002355	6.304e−06
ca_{0,b_1}				
$\log_{10}(k_1')$	10.3200	−1.678e+00		−1.692e+00
$\log_{10}(k_2)$	10.0200	−2.485e+00	5.214 or −2.663	−2.265e+00
$\log_{10}(k_2')$	−1.6980	−1.214e+00		−8.690e−01
$\log_{10}(k_{deg})$	−2.7390	−1.799e+00	−2.663 or 5.214	−1.508e+00
$\log_{10}(k_1'k_2')$	8.6240	−2.892e+00	3.85	−2.561e+00
$\log_{10}(k_1'/k_2)$	0.3073	8.065e−01		5.732e−01
transport $= \log_{10}(k_1'k_2'/k_2)$	−1.3910	−4.073e−01	−1.364 or 6.513	−2.958e−01

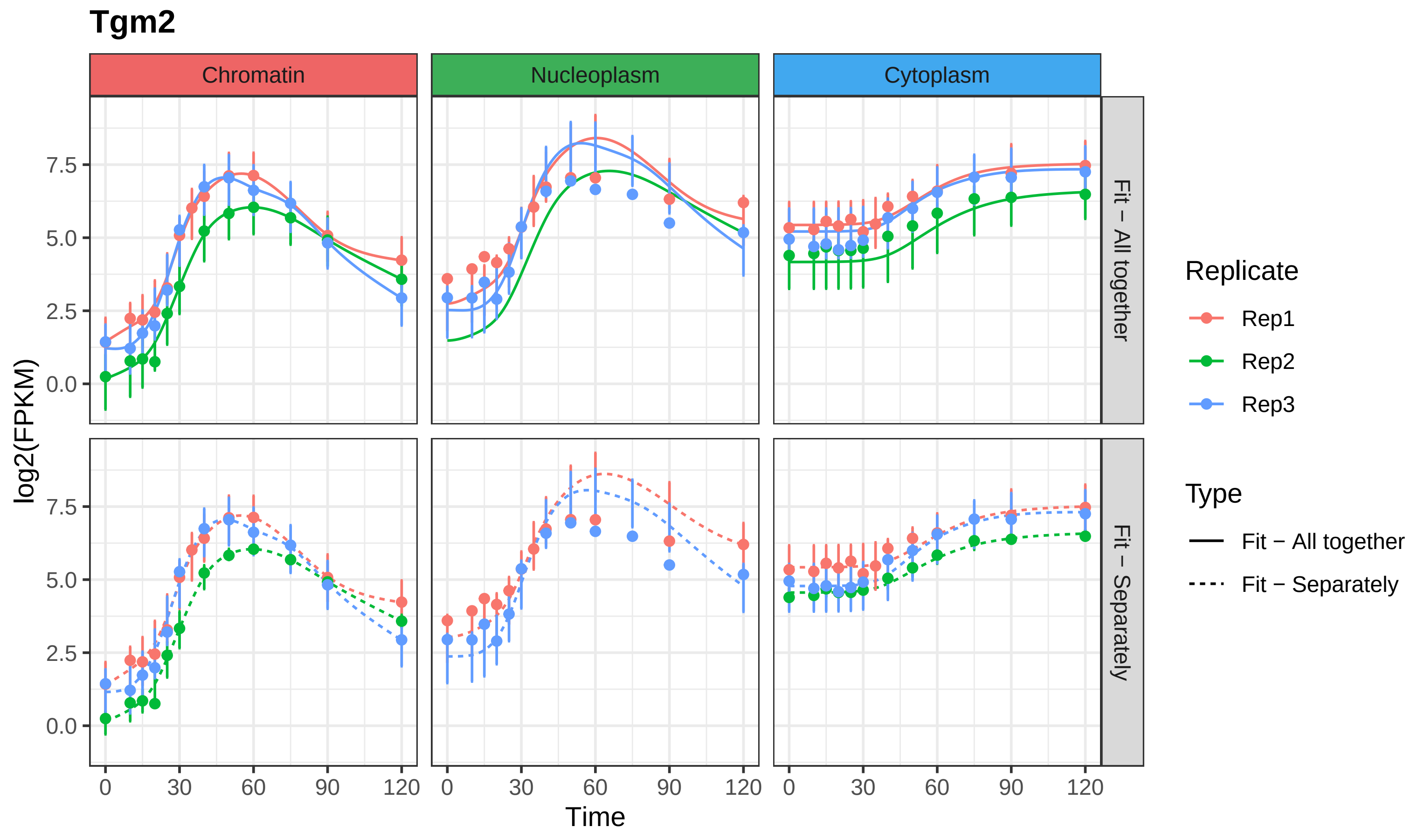

	Together	b1	b2	b3
**−NLL b1 ca**	2.123	2.046		
**−NLL b1 np**	19.62	19.08		
**−NLL b1 cyto**	2.089	1.778		
**−NLL b2 ca**	2.397		−4.816	
**−NLL b2 np**				
**−NLL b2 cyto**	5.167		−9.459	
**−NLL b3 ca**	2.634			2.705
**−NLL b3 np**	21.21			17.6
**−NLL b3 cyto**	5.175			1.488
Total	60.41	22.91	−14.28	21.79
**Total with regul**	64.35	25.42	−14.69	24.74

	Together	b1	b2	b3
spar	3.469e−01	3.767e−01	0.3571	3.646e−01
σ_b	2.874e−01	2.742e−01	0.05106	2.760e−01
σ_t	2.119e−05	7.338e−05	1.442	1.859e−05
ca_{0,b_1}				
$\log_{10}(k_1')$	−3.843e−01	−5.306e−01		−5.580e−01
$\log_{10}(k_2)$	−7.774e−01	−1.024e+00	4.376 or −2.973	−9.238e−01
$\log_{10}(k_2')$	−1.995e+00	−2.099e+00		−1.932e+00
$\log_{10}(k_{deg})$	−2.804e+00	−2.818e+00	−2.973 or 4.376	−2.657e+00
$\log_{10}(k_1' k_2')$	−2.379e+00	−2.629e+00	2.729	−2.490e+00
$\log_{10}(k_1'/k_2)$	3.931e−01	4.938e−01		3.658e−01
$\text{transport} = \log_{10}(k_1' k_2'/k_2)$	−1.602e+00	−1.605e+00	−1.647 or 5.702	−1.566e+00

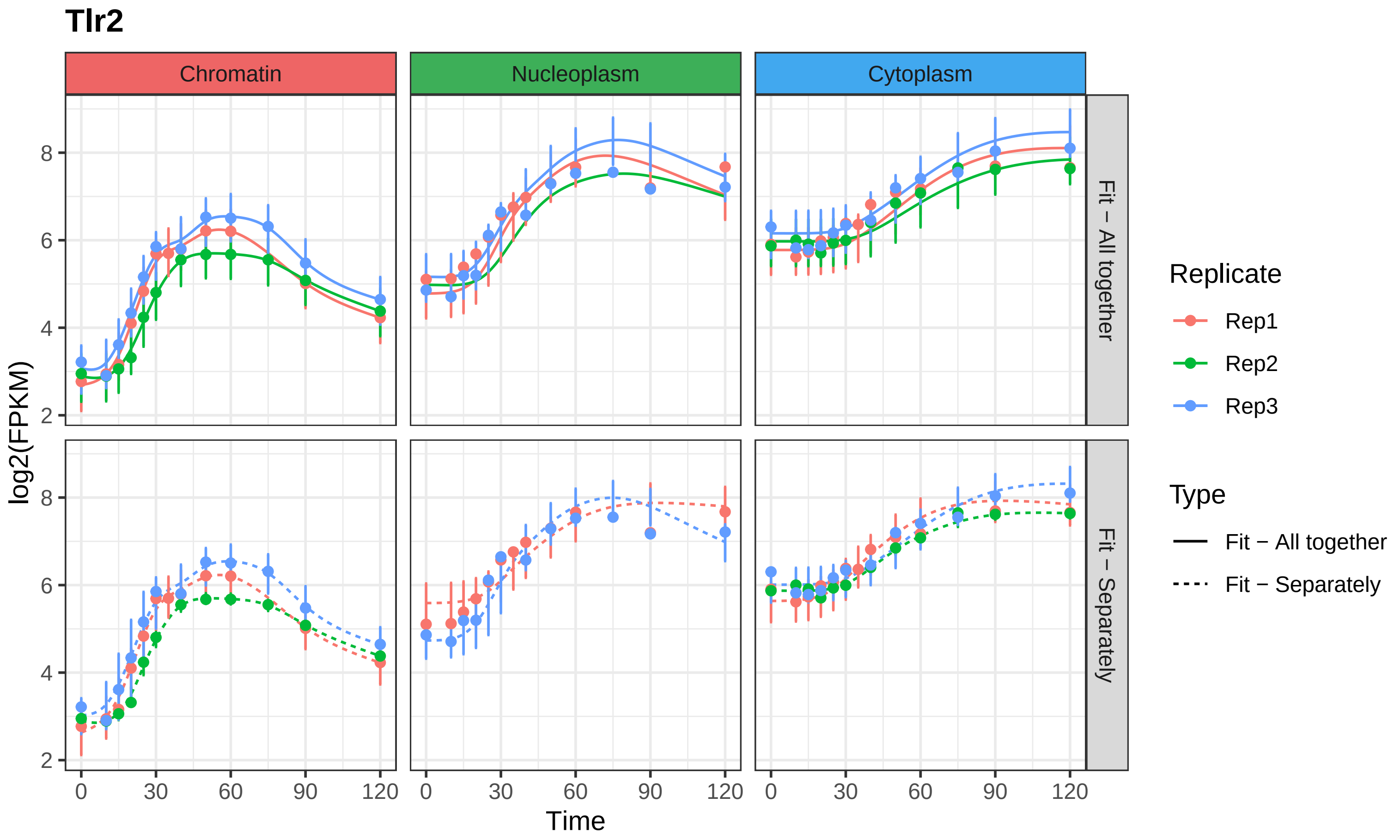

	Together	b1	b2	b3
**−NLL b1 ca**	−3.67	−4.505		
**−NLL b1 np**	9.53	7.474		
**−NLL b1 cyto**	3.159	−2.41		
**−NLL b2 ca**	−4.008		−14.75	
**−NLL b2 np**				
**−NLL b2 cyto**	−1.474		−13.32	
**−NLL b3 ca**	−3.084			−1.753
**−NLL b3 np**	12.81			4.723
**−NLL b3 cyto**	0.8487			−2.183
Total	14.11	0.5589	−28.06	0.7864
**Total with regul**	16.17	0.6166	−27.29	0.9499

	Together	b1	b2	b3
spar	3.353e−01	0.3767000	0.3274	0.3667
σ_b	1.868e−01	0.1613000	0.03856	0.1379
σ_t	3.564e−05	0.0002501	0.526	2.6730
ca_{0,b_1}				
$\log_{10}(k_1')$	−7.640e−01	−1.1260000		−0.7823
$\log_{10}(k_2)$	−1.393e+00	−2.0160000	−0.2885 or −2.008	−1.2980
$\log_{10}(k_2')$	−1.574e+00	8.0940000		−1.5540
$\log_{10}(k_{deg})$	−1.873e+00	8.0800000	−2.008 or −0.2885	−1.9380
$\log_{10}(k_1'k_2')$	−2.338e+00	6.9690000	−1.401	−2.3360
$\log_{10}(k_1'/k_2)$	6.295e−01	0.8909000		0.5159
$\text{transport} = \log_{10}(k_1'k_2'/k_2)$	−9.442e−01	8.9850000	−1.112 or 0.6072	−1.0380

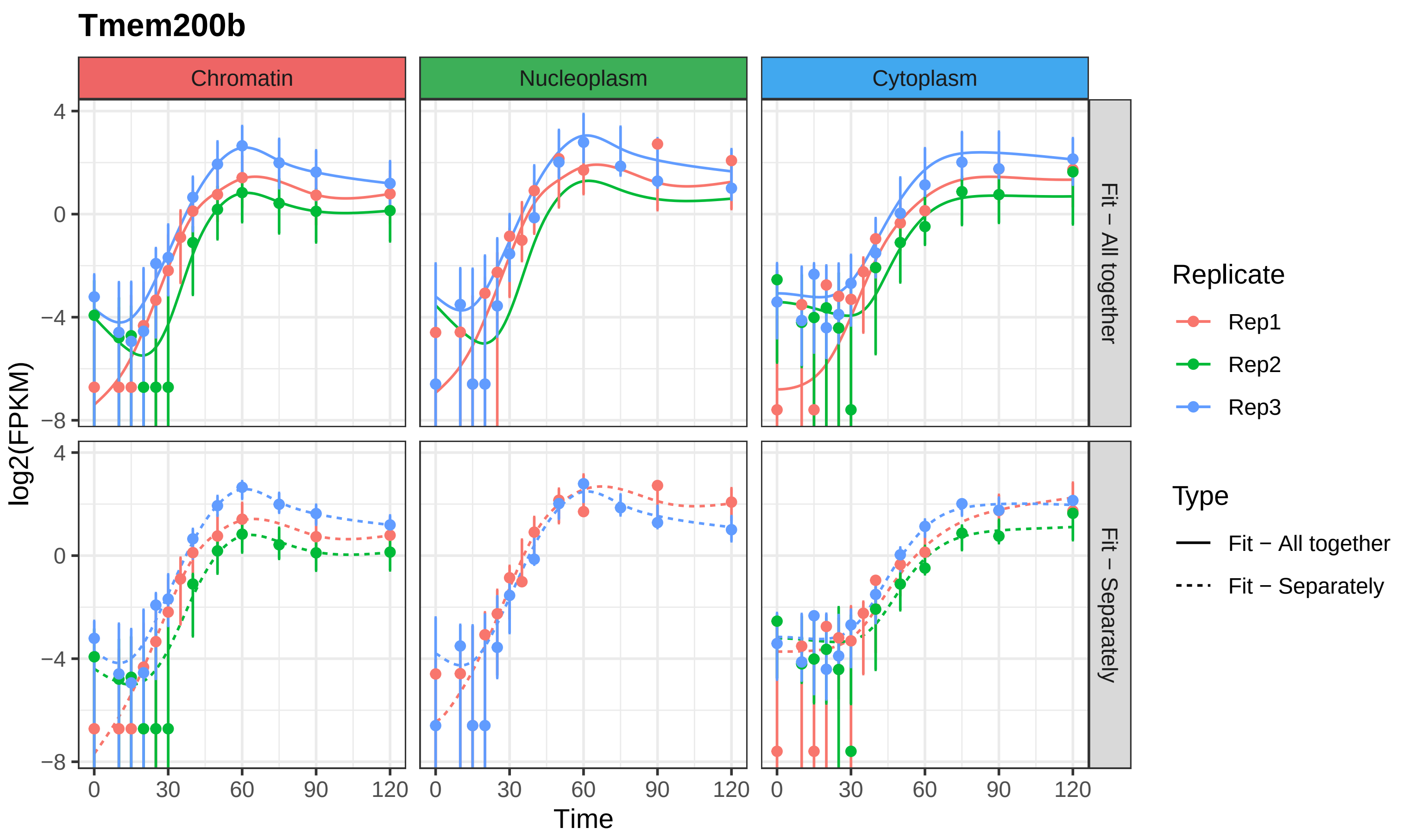

	Together	b1	b2	b3
−NLL b1 ca	11.37	9.962		
−NLL b1 np	20.45	13.36		
−NLL b1 cyto	22.3	17.92		
−NLL b2 ca	12.9		13.61	
−NLL b2 np				
−NLL b2 cyto	15.6		15.14	
−NLL b3 ca	8.16			4.415
−NLL b3 np	24.58			15.61
−NLL b3 cyto	12.1			6.206
Total	127.5	41.24	28.76	26.23
Total with regul	130.4	40.92	27.15	24.79

	Together	b1	b2	b3
spar	0.3851	0.44070	0.4572	3.935e−01
σ_b	0.2988	0.19880	0.1189	6.926e−02
σ_t	0.1142	0.48390	0.0005001	2.171e−05
ca_{0,b_1}				
$\log_{10}(k_1')$	1.2380	0.01305		5.574e−01
$\log_{10}(k_2)$	1.0970	−0.37020	3.214 or −1.696	5.844e−01
$\log_{10}(k_2')$	−1.4320	−1.86900		−1.506e+00
$\log_{10}(k_{deg})$	−1.4710	−2.67900	−1.696 or 3.214	−1.700e+00
$\log_{10}(k_1'k_2')$	−0.1945	−1.85600	1.876	−9.487e−01
$\log_{10}(k_1'/k_2)$	0.1407	0.38330		−2.703e−02
$\text{transport} = \log_{10}(k_1'k_2'/k_2)$	−1.2920	−1.48600	−1.338 or 3.572	−1.533e+00

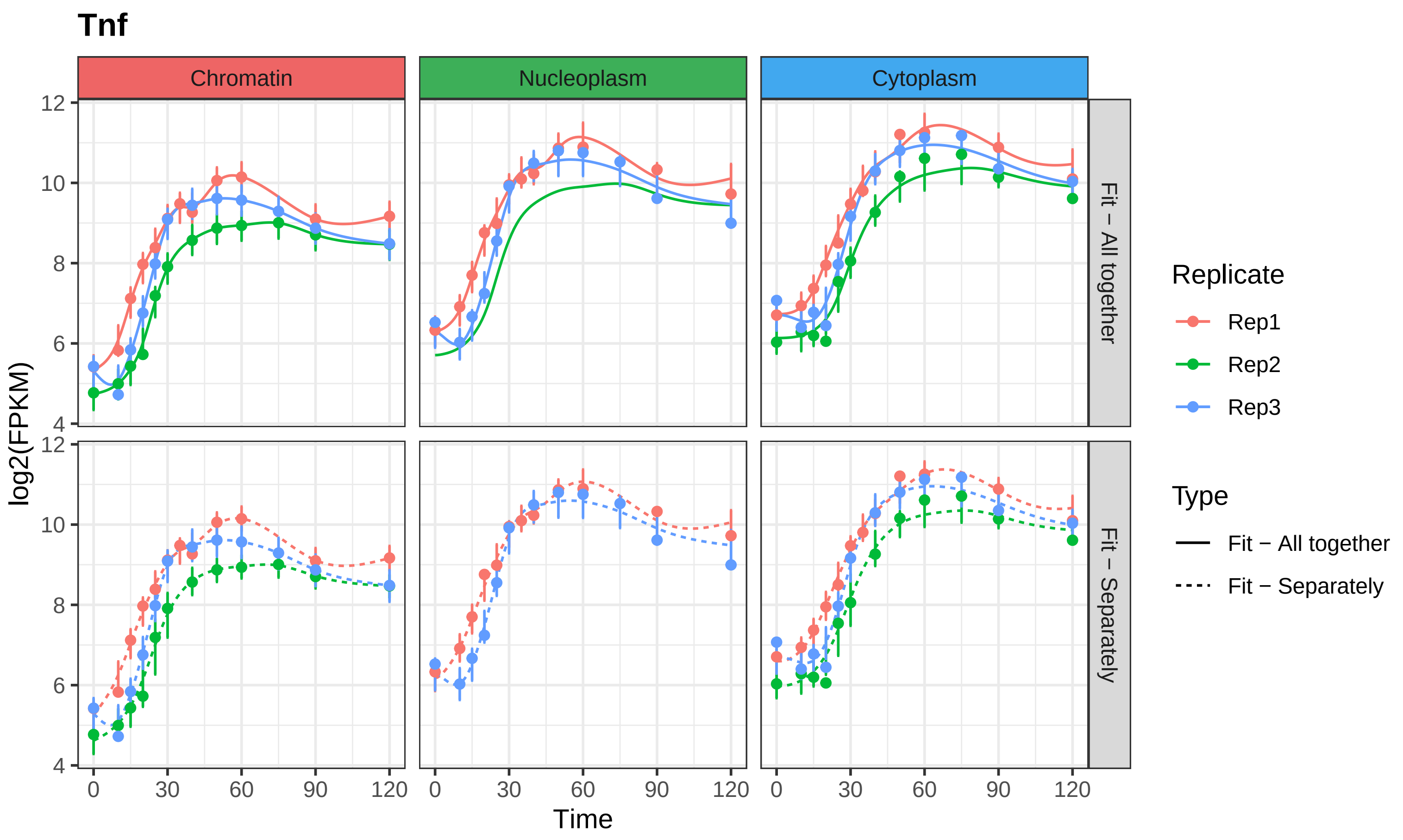

	Together	b1	b2	b3
−NLL b1 ca	−7.186	−5.33		
−NLL b1 np	−3.253	−3.369		
−NLL b1 cyto	−2.354	−4.402		
−NLL b2 ca	−7.37		−5.837	
−NLL b2 np				
−NLL b2 cyto	3.921		2.203	
−NLL b3 ca	−6.753			−5.276
−NLL b3 np	−0.1903			−0.3616
−NLL b3 cyto	1.151			0.475
Total	−22.03	−13.1	−3.634	−5.162
Total with regul	−16.44	−12.63	−3.549	−0.4713

	Together	b1	b2	b3
spar	0.2621000	0.34530	0.3548	2.758e−01
σ_b	0.1305000	0.10860	0.1026	1.371e−01
σ_t	0.0002459	0.53710	1.818	2.134e−05
ca_{0,b_1}				
$\log_{10}(k_1')$	0.0013610	−0.05389		5.828e−02
$\log_{10}(k_2)$	−0.2909000	−0.33210	−0.5478 or −0.5989	−2.390e−01
$\log_{10}(k_2')$	−0.7878000	−0.81060		−7.886e−01
$\log_{10}(k_{deg})$	−0.9161000	−0.93660	−0.5989 or −0.5478	−9.134e−01
$\log_{10}(k_1'k_2')$	−0.7864000	−0.86450	−0.737	−7.303e−01
$\log_{10}(k_1'/k_2)$	0.2923000	0.27820		2.973e−01
transport $= \log_{10}(k_1'k_2'/k_2)$	−0.4955000	−0.53240	−0.1892 or −0.1382	−4.913e−01

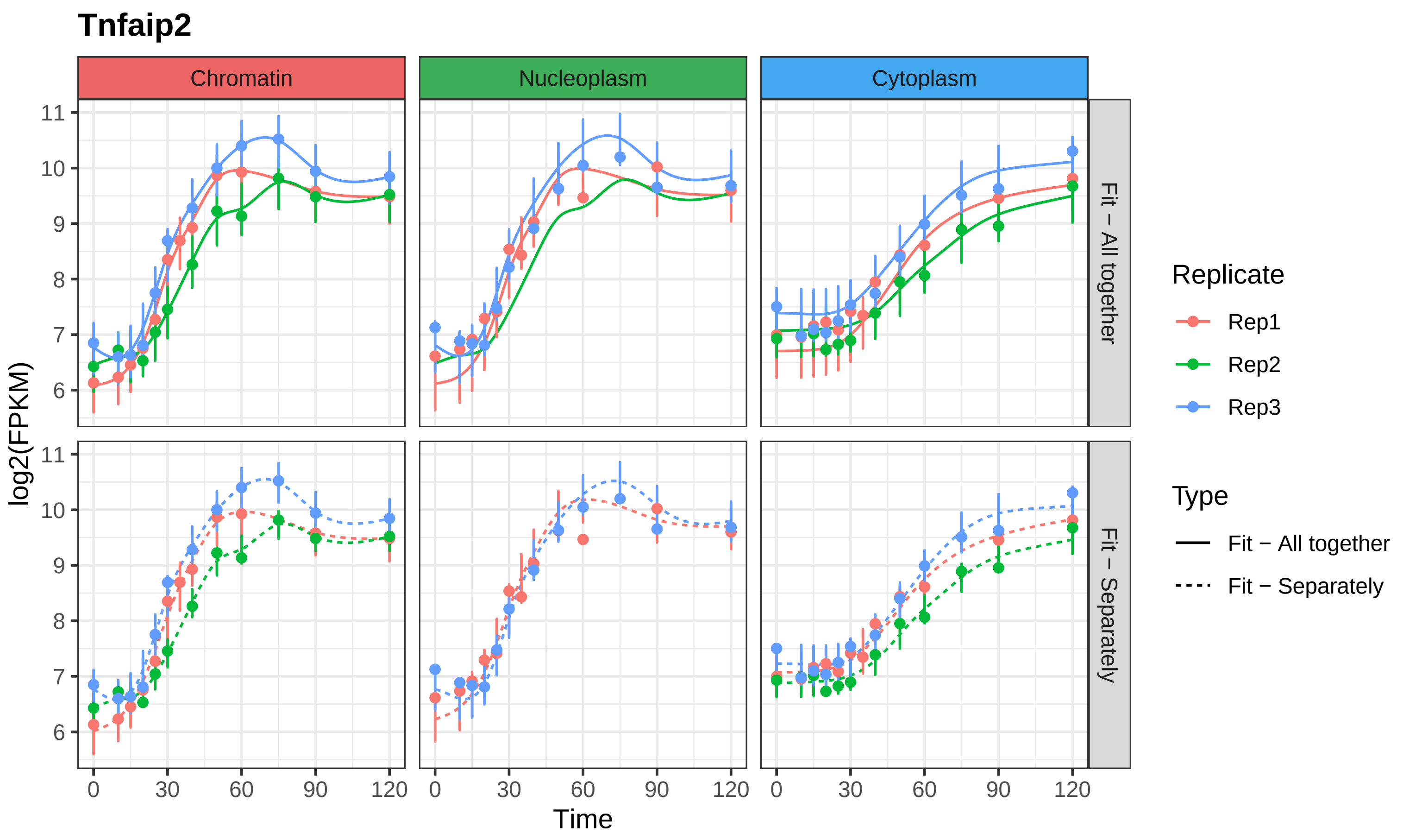

	Together	b1	b2	b3
**−NLL b1 ca**	−5.371	−5.753		
**−NLL b1 np**	8.081	6.848		
**−NLL b1 cyto**	3.473	−5.612		
**−NLL b2 ca**	−5.403		−8.852	
**−NLL b2 np**				
**−NLL b2 cyto**	−1.796		−4.916	
**−NLL b3 ca**	−4.826			−6.19
**−NLL b3 np**	4.619			0.5172
**−NLL b3 cyto**	−0.3777			−3.021
**Total**	−1.6	−4.516	−13.77	−8.694
**Total with regul**	−0.6049	−5.547	−14.27	−8.085

	Together	b1	b2	b3
spar	3.493e−01	0.40940	0.3653	3.462e−01
σ_b	1.603e−01	0.13720	0.08715	1.244e−01
σ_t	2.717e−05	0.91200	0.00136	5.644e−08
ca_{0,b_1}				
$\log_{10}(k_1')$	5.305e−01	0.17780		−5.173e−01
$\log_{10}(k_2)$	5.202e−01	0.11150	4.574 or −1.824	−5.132e−01
$\log_{10}(k_2')$	−1.742e+00	−1.84100		−1.687e+00
$\log_{10}(k_{deg})$	−1.919e+00	−2.09300	−1.824 or 4.574	−1.829e+00
$\log_{10}(k_1'k_2')$	−1.212e+00	−1.66300	2.88	−2.204e+00
$\log_{10}(k_1'/k_2)$	1.025e−02	0.06636		−4.036e−03
$transport = \log_{10}(k_1'k_2'/k_2)$	−1.732e+00	−1.77400	−1.694 or 4.704	−1.691e+00

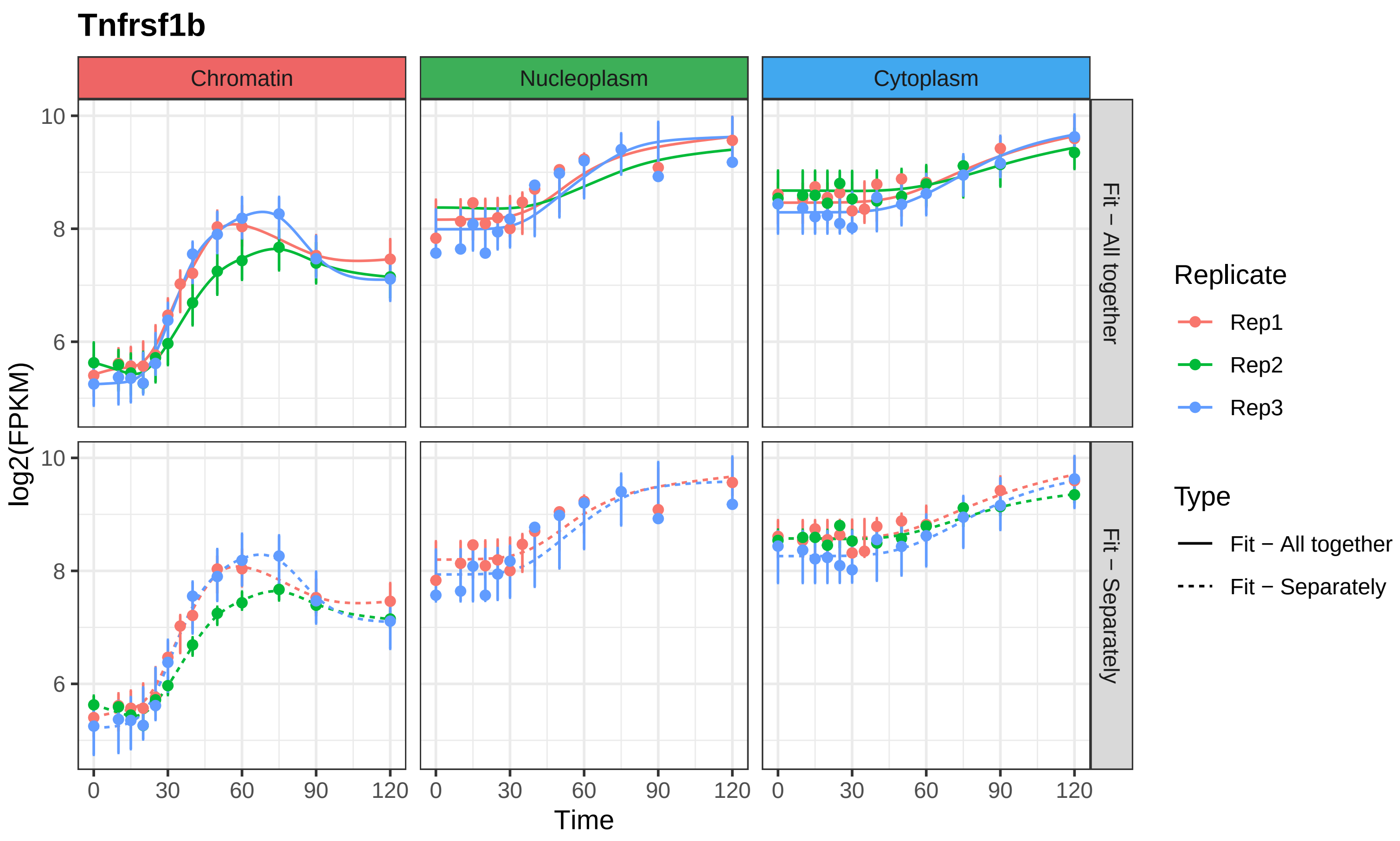

	Together	b1	b2	b3
**−NLL b1 ca**	−7.997	−8.375		
**−NLL b1 np**	1.384	2.273		
**−NLL b1 cyto**	−3.874	−5.964		
**−NLL b2 ca**	−8.324		−13.1	
**−NLL b2 np**				
**−NLL b2 cyto**	−5.954		−10.62	
**−NLL b3 ca**	−7.605			−4.35
**−NLL b3 np**	13.73			8.512
**−NLL b3 cyto**	−5.902			−4.025
Total	−24.54	−12.07	−23.72	0.1369
**Total with regul**	−24.58	−13.17	−24.68	−0.3918

	Together	b1	b2	b3
spar	3.645e−01	3.983e−01	0.3722	3.992e−01
σ_b	1.275e−01	1.166e−01	0.0551	1.596e−01
σ_t	7.178e−05	1.165e−05	6.156e−05	1.001e−06
ca_{0,b_1}				
$\log_{10}(k_1')$	−1.314e+00	−1.305e+00		−1.331e+00
$\log_{10}(k_2)$	−2.140e+00	−2.145e+00	−0.9667 or −2.302	−2.148e+00
$\log_{10}(k_2')$	−1.463e+00	−1.502e+00		−1.515e+00
$\log_{10}(k_{deg})$	−1.553e+00	−1.614e+00	−2.302 or −0.9667	−1.613e+00
$\log_{10}(k_1'k_2')$	−2.777e+00	−2.807e+00	−2.383	−2.846e+00
$\log_{10}(k_1'/k_2)$	8.257e−01	8.395e−01		8.171e−01
transport = $\log_{10}(k_1'k_2'/k_2)$	−6.369e−01	−6.621e−01	−1.416 or −0.08038	−6.980e−01

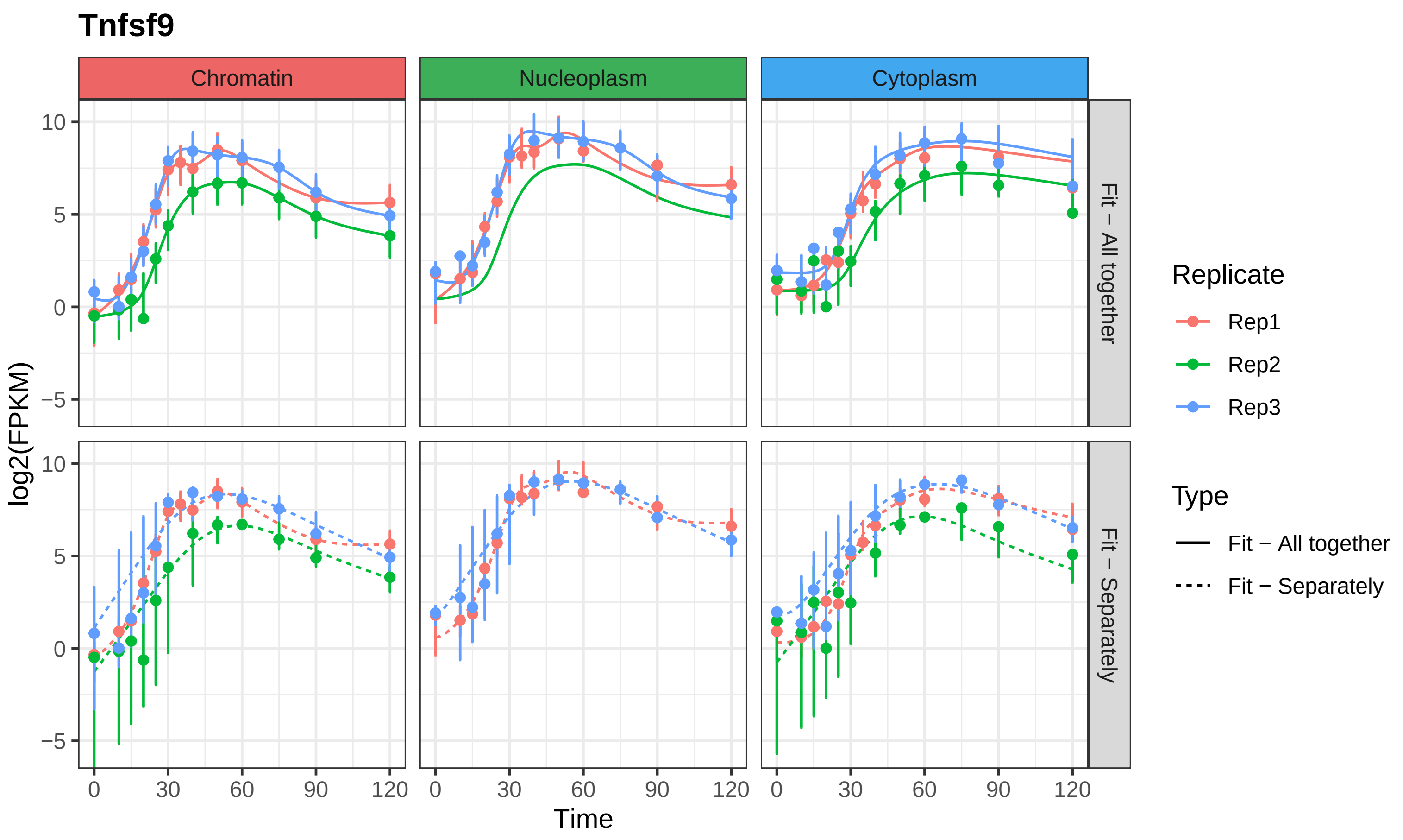

	Together	b1	b2	b3
**−NLL b1 ca**	4.278	1.597		
**−NLL b1 np**	10.87	12.27		
**−NLL b1 cyto**	10.67	8.881		
**−NLL b2 ca**	6.813		10.94	
**−NLL b2 np**				
**−NLL b2 cyto**	22.2		17.06	
**−NLL b3 ca**	4.828			12.48
**−NLL b3 np**	8.291			8.401
**−NLL b3 cyto**	16.31			8.783
Total	84.25	22.75	27.99	29.67
**Total with regul**	94.57	29.17	29.48	31.56

	Together	b1	b2	b3
spar	2.682e−01	0.2878000	0.5697	5.838e−01
σ_b	3.472e−01	0.2637000	0.0007633	9.288e−07
σ_t	1.932e−05	0.0008781	6.856	5.621e+00
ca_{0,b_1}				
$\log_{10}(k_1')$	2.278e−01	−0.1538000		−1.706e−01
$\log_{10}(k_2)$	−6.281e−02	−0.5070000	3.943 or 3.277	−3.838e−01
$\log_{10}(k_2')$	−1.479e+00	−1.4190000		−9.881e−01
$\log_{10}(k_{deg})$	−1.609e+00	−1.3330000	3.277 or 3.943	−9.879e−01
$\log_{10}(k_1'k_2')$	−1.251e+00	−1.5720000	7.371	−1.159e+00
$\log_{10}(k_1'/k_2)$	2.906e−01	0.3532000		2.132e−01
$transport = \log_{10}(k_1'k_2'/k_2)$	−1.189e+00	−1.0650000	3.428 or 4.094	−7.749e−01

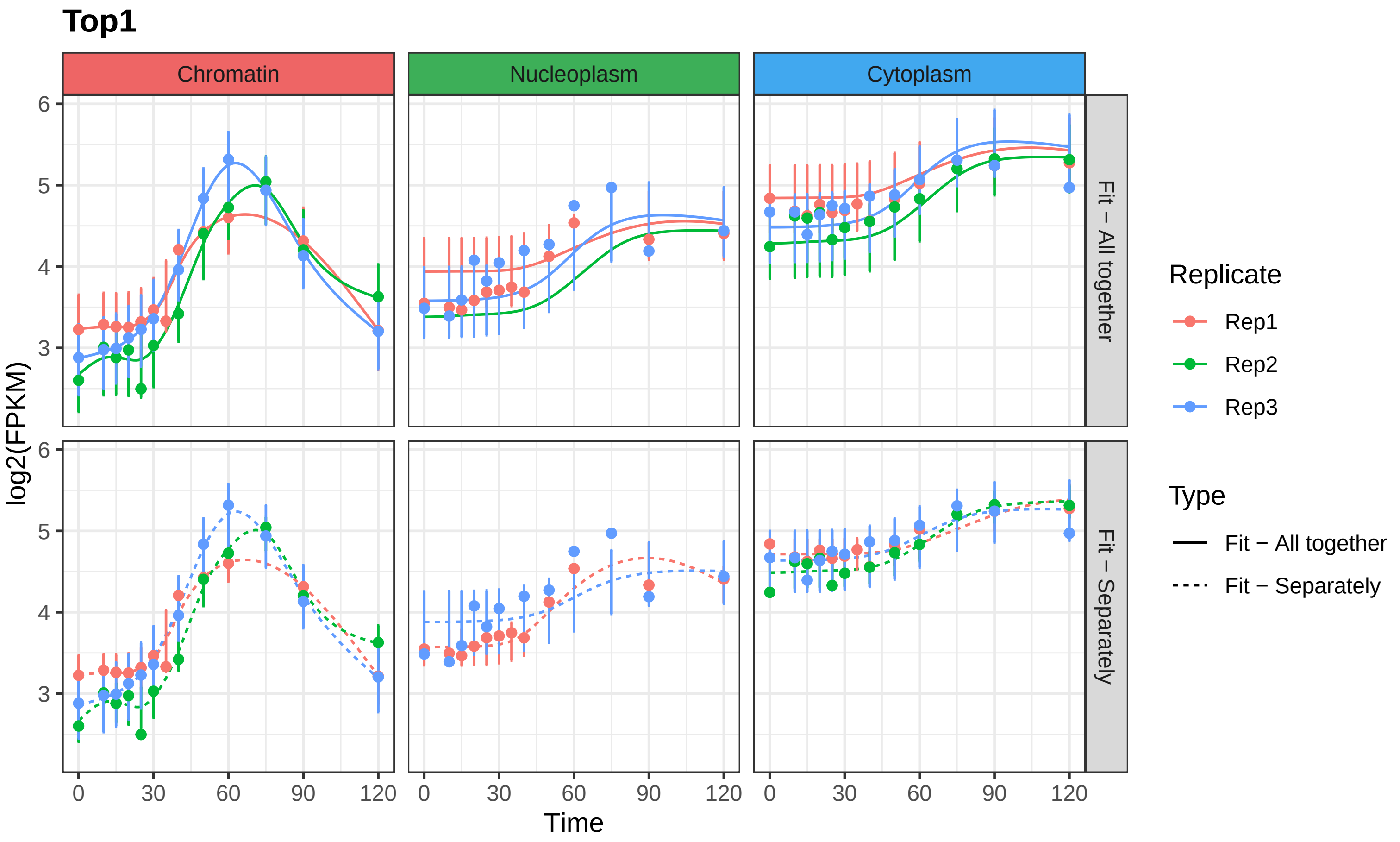

	Together	b1	b2	b3
**−NLL b1 ca**	−5.402	−10.33		
**−NLL b1 np**	4.614	−5.446		
**−NLL b1 cyto**	−3.102	−11.17		
**−NLL b2 ca**	−5.072		−7.537	
**−NLL b2 np**				
**−NLL b2 cyto**	−2.916		−8.088	
**−NLL b3 ca**	−6.975			−6.796
**−NLL b3 np**	10.47			6.193
**−NLL b3 cyto**	−1.138			−5.26
Total	−9.523	−26.94	−15.62	−5.863
**Total with regul**	−10.21	−28.52	−16.58	−7.252

	Together	b1	b2	b3
spar	0.394800	0.40510	0.3744	4.284e−01
σ_b	0.143000	0.06221	0.06628	1.294e−01
σ_t	0.003092	2.62600	0.001713	9.325e−09
ca_{0,b_1}				
$\log_{10}(k_1')$	−1.870000	−1.41300		−2.145e+00
$\log_{10}(k_2)$	−2.083000	−1.51500	4.128 or −2.25	−2.451e+00
$\log_{10}(k_2')$	6.076000	−1.52000		4.089e+00
$\log_{10}(k_{deg})$	5.804000	−1.86500	−2.25 or 4.128	3.861e+00
$\log_{10}(k_1' k_2')$	4.206000	−2.93400	2.427	1.944e+00
$\log_{10}(k_1'/k_2)$	0.212800	0.10180		3.064e−01
transport $= \log_{10}(k_1' k_2'/k_2)$	6.289000	−1.41900	−1.702 or 4.677	4.395e+00

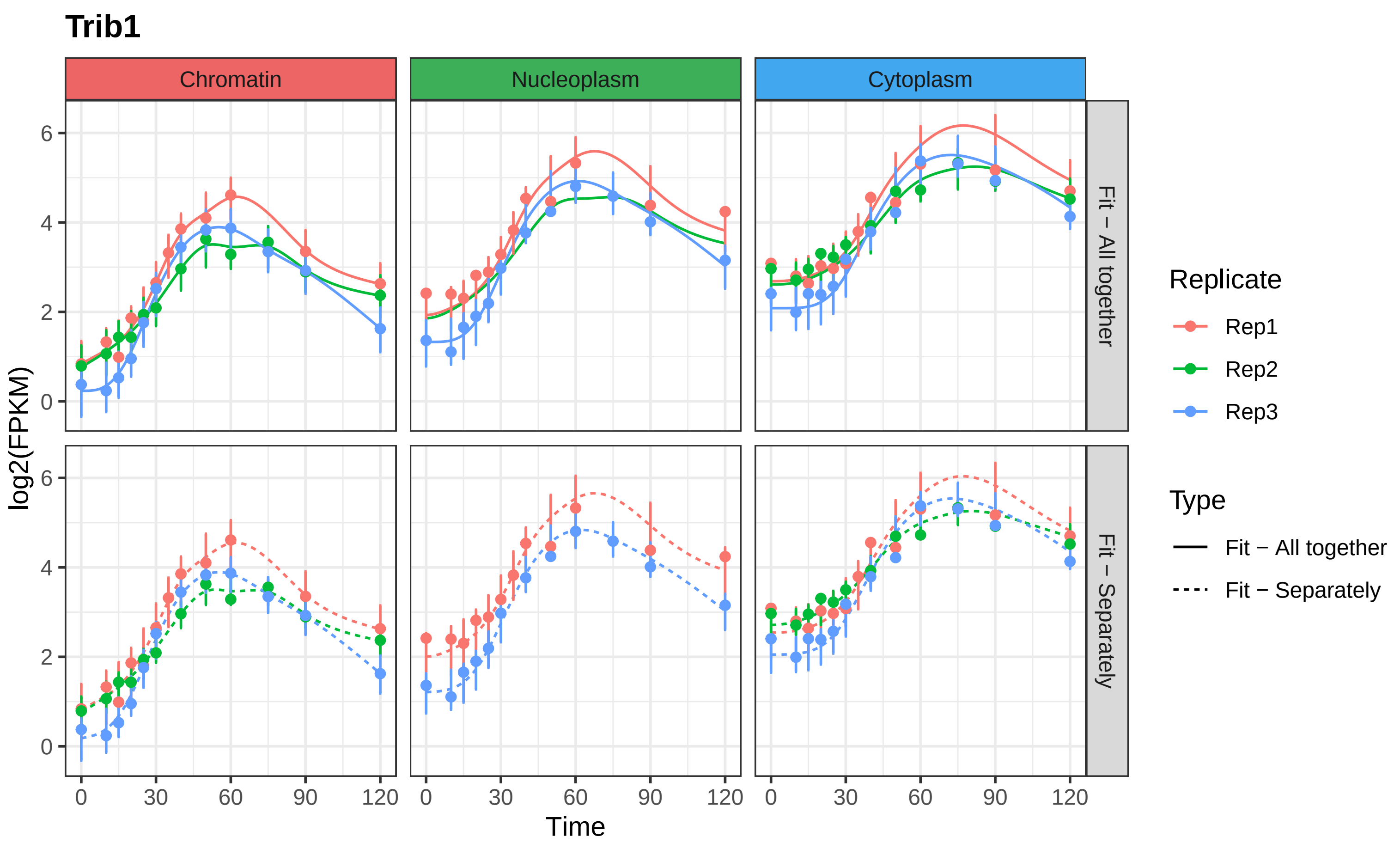

	Together	b1	b2	b3
**−NLL b1 ca**	−3.324	−1.991		
**−NLL b1 np**	4.6	3.068		
**−NLL b1 cyto**	8.443	5.378		
**−NLL b2 ca**	−4.906		−6.936	
**−NLL b2 np**				
**−NLL b2 cyto**	−0.221		−2.682	
**−NLL b3 ca**	−4.946			−5.13
**−NLL b3 np**	−1.721			−3.249
**−NLL b3 cyto**	1.406			3.101
Total	−0.6695	6.454	−9.618	−5.277
**Total with regul**	−0.611	6.286	−10.81	−6.352

	Together	b1	b2	b3
spar	0.3747000	0.4022000	0.3924	4.047e−01
σ_b	0.1577000	0.1840000	0.09623	1.292e−01
σ_t	0.0007702	0.0001978	0.0003171	6.426e−06
ca_{0,b_1}				
$\log_{10}(k_1')$	−0.4497000	−0.4607000		−5.486e−01
$\log_{10}(k_2)$	−0.7782000	−0.8180000	4.426 or −1.31	−8.593e−01
$\log_{10}(k_2')$	−0.8514000	−0.8855000		−7.768e−01
$\log_{10}(k_{deg})$	−1.0790000	−1.0470000	−1.31 or 4.426	−1.029e+00
$\log_{10}(k_1' k_2')$	−1.3010000	−1.3460000	3.704	−1.325e+00
$\log_{10}(k_1'/k_2)$	0.3286000	0.3573000		3.107e−01
$\text{transport} = \log_{10}(k_1' k_2'/k_2)$	−0.5228000	−0.5282000	−0.7224 or 5.014	−4.661e−01

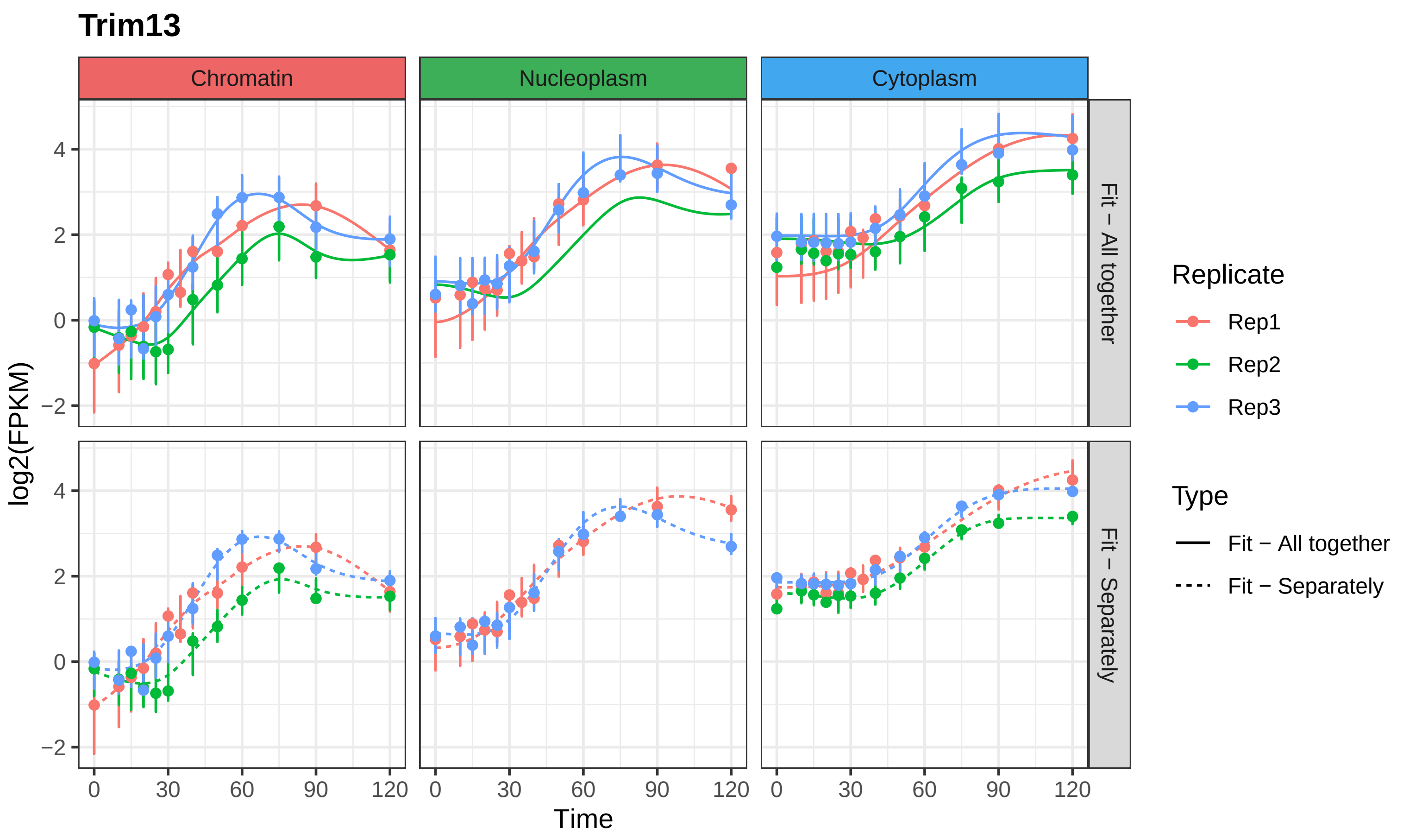

	Together	b1	b2	b3
**−NLL b1 ca**	0.2793	−1.603		
**−NLL b1 np**	4.745	−0.8934		
**−NLL b1 cyto**	12.93	−4.459		
**−NLL b2 ca**	−0.8593		−1.82	
**−NLL b2 np**				
**−NLL b2 cyto**	3.093		−9.007	
**−NLL b3 ca**	−0.004383			−0.9338
**−NLL b3 np**	1.797			−3.741
**−NLL b3 cyto**	0.03541			−11.87
Total	22.01	−6.955	−10.83	−16.54
**Total with regul**	21.39	−8.86	−12.89	−18.78

	Together	b1	b2	b3
spar	0.4241000	0.43740	0.4861	4.618e−01
σ_b	0.1769000	0.07878	3.029e−05	5.591e−07
σ_t	0.0000173	1.90100	0.007787	1.547e+00
ca_{0,b_1}				
$\log_{10}(k_1')$	−0.6962000	−0.88110		−6.827e−01
$\log_{10}(k_2)$	−0.9996000	−1.29800	7.724 or −1.451	−9.261e−01
$\log_{10}(k_2')$	−1.1950000	−1.43600		−1.383e+00
$\log_{10}(k_{deg})$	−1.5180000	−1.86100	−1.451 or 7.724	−1.745e+00
$\log_{10}(k_1'k_2')$	−1.8920000	−2.31700	6.826	−2.066e+00
$\log_{10}(k_1'/k_2)$	0.3034000	0.41660		2.434e−01
$transport = \log_{10}(k_1'k_2'/k_2)$	−0.8919000	−1.01900	−0.8977 or 8.277	−1.140e+00

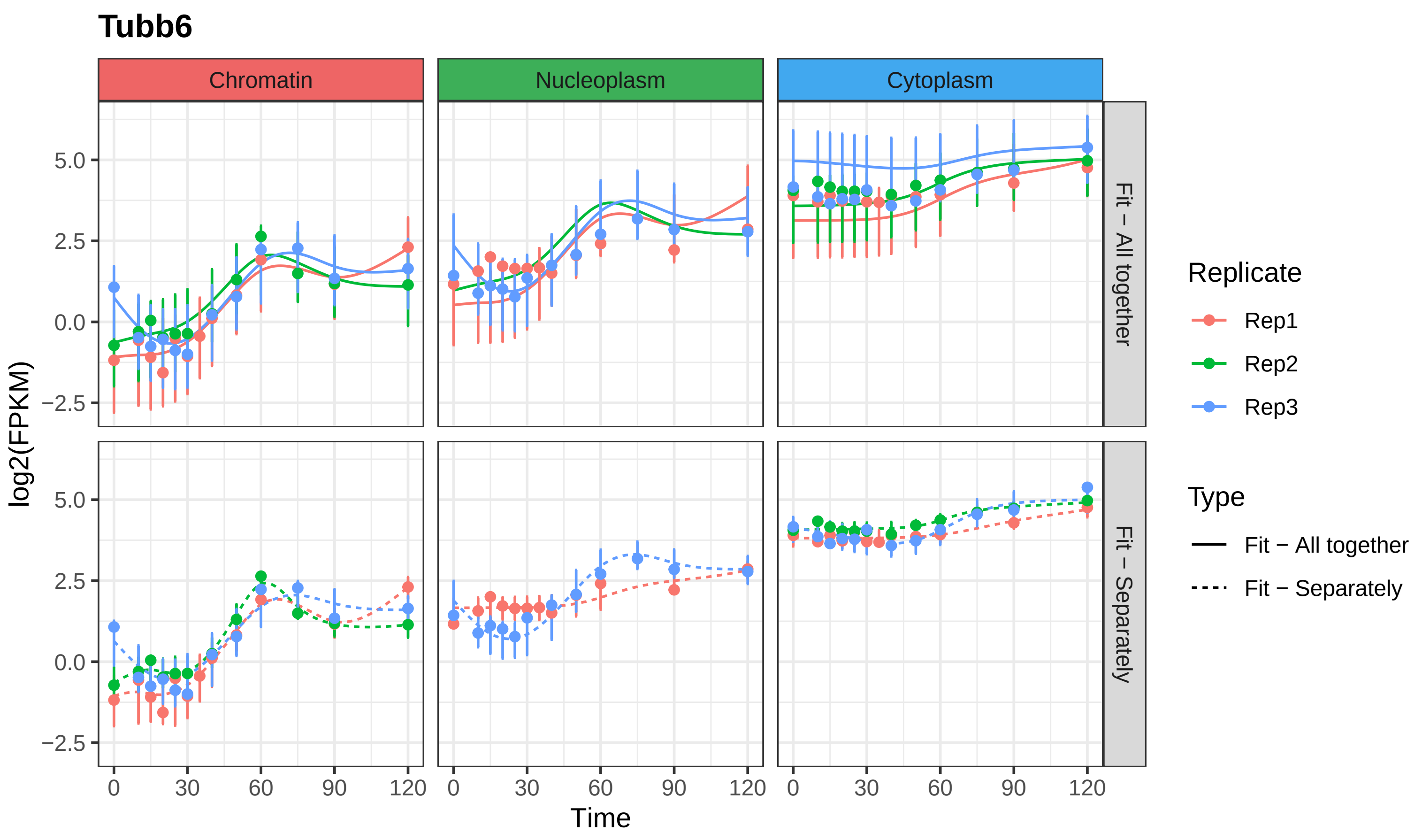

	Together	b1	b2	b3
**−NLL b1 ca**	6.569	0.2017		
**−NLL b1 np**	18.84	2.597		
**−NLL b1 cyto**	9.734	−10.62		
**−NLL b2 ca**	6.17		−2.186	
**−NLL b2 np**				
**−NLL b2 cyto**	5.774		−8.89	
**−NLL b3 ca**	6.013			5.073
**−NLL b3 np**	9.125			1.644
**−NLL b3 cyto**	20.78			−1.631
Total	83	−7.825	−11.08	5.085
**Total with regul**	87.24	−9.756	−12.24	5.379

	Together	b1	b2	b3
spar	0.508900	4.270e−01	0.3805	0.5399
σ_b	0.342400	7.502e−02	0.06151	0.1299
σ_t	0.001273	1.442e−07	4.62e−06	2.7520
ca_{0,b_1}				
$\log_{10}(k_1')$	5.225000	−1.640e+00		5.0400
$\log_{10}(k_2)$	4.741000	−2.459e+00	−0.2769 or −2.504	4.6620
$\log_{10}(k_2')$	−1.278000	−7.491e−01		−0.9677
$\log_{10}(k_{deg})$	−2.063000	−1.397e+00	−2.504 or −0.2769	−1.6320
$\log_{10}(k_1'k_2')$	3.948000	−2.389e+00	−1.359	4.0720
$\log_{10}(k_1'/k_2)$	0.484300	8.189e−01		0.3773
transport $= \log_{10}(k_1'k_2'/k_2)$	−0.793400	6.985e−02	−1.082 or 1.145	−0.5903

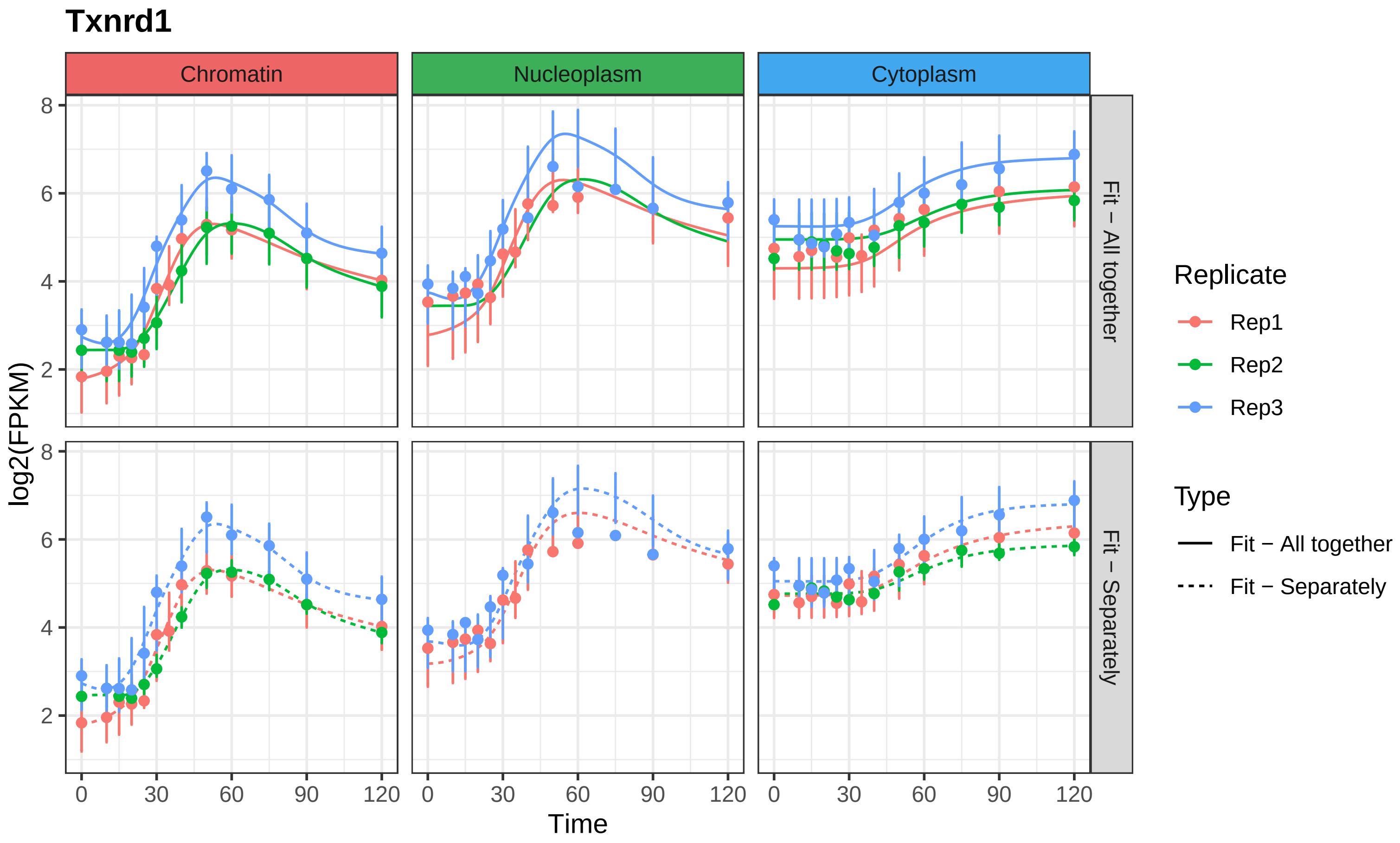

	Together	b1	b2	b3
**−NLL b1 ca**	0.3375	−1.516		
**−NLL b1 np**	10.13	9.099		
**−NLL b1 cyto**	6.137	−2.916		
**−NLL b2 ca**	−1.683		−10.5	
**−NLL b2 np**				
**−NLL b2 cyto**	1.019		−5.902	
**−NLL b3 ca**	0.6262			0.268
**−NLL b3 np**	14.11			10.67
**−NLL b3 cyto**	2.218			−1.198
Total	32.89	4.668	−16.4	9.736
**Total with regul**	33.5	4.197	−17.3	9.711

	Together	b1	b2	b3
spar	0.4015	0.4045	0.3736	0.4053
σ_b	0.2216	0.1661	0.07061	0.1895
σ_t	0.3264	1.6430	0.0003333	2.2260
ca_{0,b_1}				
$\log_{10}(k_1')$	0.1952	−0.4152		−0.6028
$\log_{10}(k_2)$	−0.1067	−0.8360	6.362 or −2.387	−0.8884
$\log_{10}(k_2')$	−1.9160	−1.9310		−1.8390
$\log_{10}(k_{deg})$	−2.3700	−2.3960	−2.387 or 6.362	−2.2510
$\log_{10}(k_1'k_2')$	−1.7210	−2.3470	4.67	−2.4410
$\log_{10}(k_1'/k_2)$	0.3019	0.4208		0.2856
$\text{transport} = \log_{10}(k_1'k_2'/k_2)$	−1.6140	−1.5110	−1.692 or 7.058	−1.5530

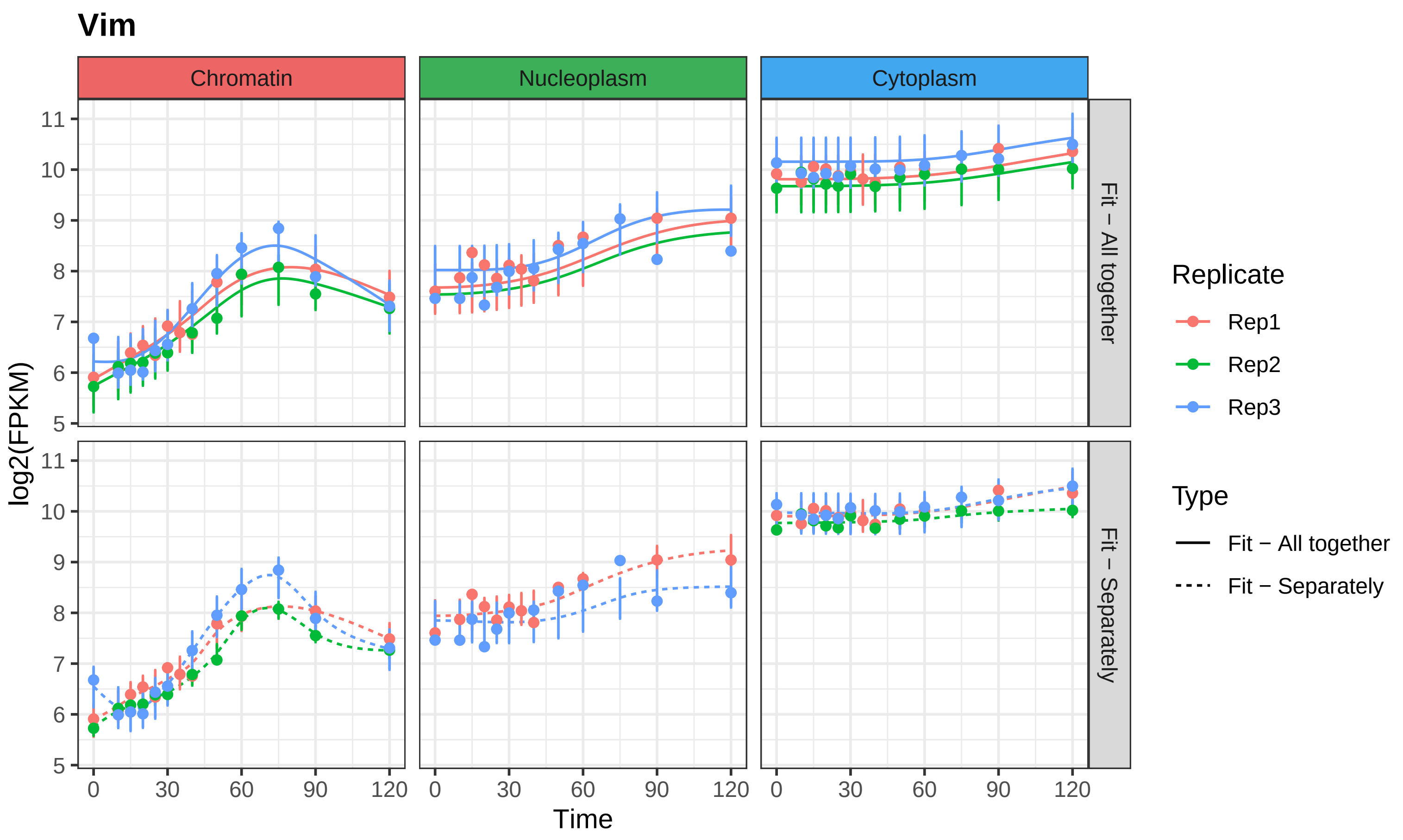

	Together	b1	b2	b3
**−NLL b1 ca**	−2.828	−6.79		
**−NLL b1 np**	4.869	0.07364		
**−NLL b1 cyto**	−3.386	−7.715		
**−NLL b2 ca**	−3.097		−13.91	
**−NLL b2 np**				
**−NLL b2 cyto**	−3.817		−9.491	
**−NLL b3 ca**	0.9618			−6.23
**−NLL b3 np**	14.19			7.801
**−NLL b3 cyto**	−1.785			−6.554
Total	5.109	−14.43	−23.4	−4.984
**Total with regul**	7.181	−15.84	−24.54	−6.26

	Together	b1	b2	b3
spar	5.710e−01	0.4105	0.3787	4.285e−01
σ_b	1.710e−01	0.1085	0.05548	1.378e−01
σ_t	8.584e−06	0.7053	0.518	2.627e−06
ca_{0,b_1}				
$\log_{10}(k_1')$	−1.546e+00	−1.4900		−1.935e+00
$\log_{10}(k_2)$	−2.089e+00	−2.1070	5.267 or −3.001	−2.326e+00
$\log_{10}(k_2')$	−1.490e+00	−1.4430		−9.609e−01
$\log_{10}(k_{deg})$	−2.132e+00	−2.0340	−3.001 or 5.267	−1.600e+00
$\log_{10}(k_1' k_2')$	−3.036e+00	−2.9330	3.475	−2.895e+00
$\log_{10}(k_1'/k_2)$	5.428e−01	0.6167		3.910e−01
transport $= \log_{10}(k_1' k_2'/k_2)$	−9.474e−01	−0.8264	−1.792 or 6.476	−5.699e−01

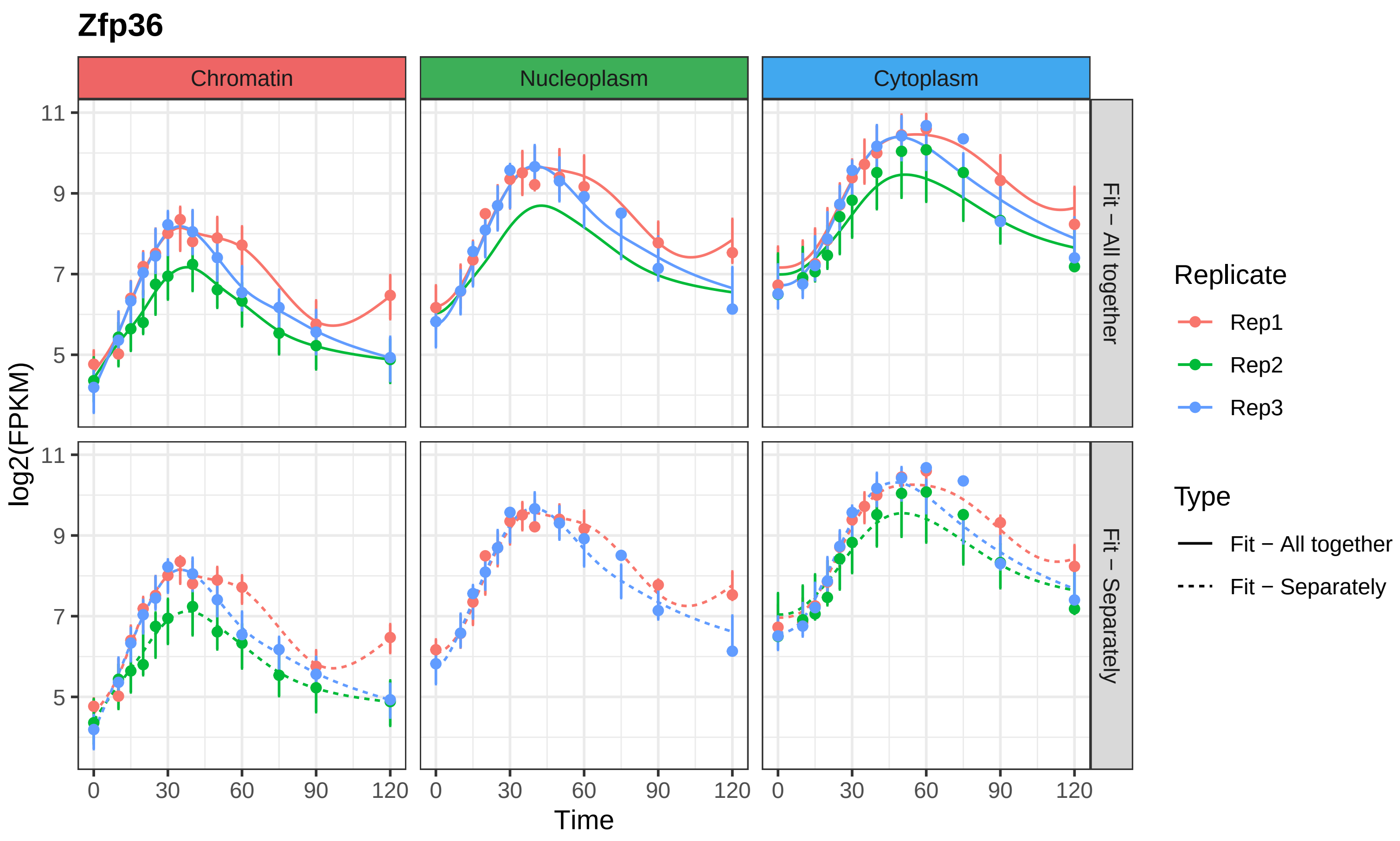

	Together	b1	b2	b3
**−NLL b1 ca**	−1.471	−4.019		
**−NLL b1 np**	0.0768	−2.228		
**−NLL b1 cyto**	1.334	−1.648		
**−NLL b2 ca**	−3.265		−2.416	
**−NLL b2 np**				
**−NLL b2 cyto**	11.39		7.352	
**−NLL b3 ca**	−3.543			−5.239
**−NLL b3 np**	1.651			−1.666
**−NLL b3 cyto**	8.546			3.371
Total	14.72	−7.895	4.937	−3.534
**Total with regul**	15.45	−7.777	4.704	−4.292

	Together	b1	b2	b3
spar	0.370100	0.3574	0.4163	3.987e−01
σ_b	0.188200	0.1180	0.1928	1.439e−01
σ_t	0.001593	1.0690	0.1085	4.717e−05
ca_{0,b_1}				
$\log_{10}(k_1')$	−0.262200	−0.1928		−1.757e−01
$\log_{10}(k_2)$	−0.749200	−0.6436	−0.8061 or −0.8424	−6.585e−01
$\log_{10}(k_2')$	−0.722300	−0.7470		−6.960e−01
$\log_{10}(k_{deg})$	−1.012000	−1.0090	−0.8424 or −0.8061	−9.503e−01
$\log_{10}(k_1' k_2')$	−0.984600	−0.9399	−0.8577	−8.716e−01
$\log_{10}(k_1'/k_2)$	0.487000	0.4508		4.828e−01
$\text{transport} = \log_{10}(k_1' k_2'/k_2)$	−0.235300	−0.2963	−0.05162 or −0.01525	−2.131e−01

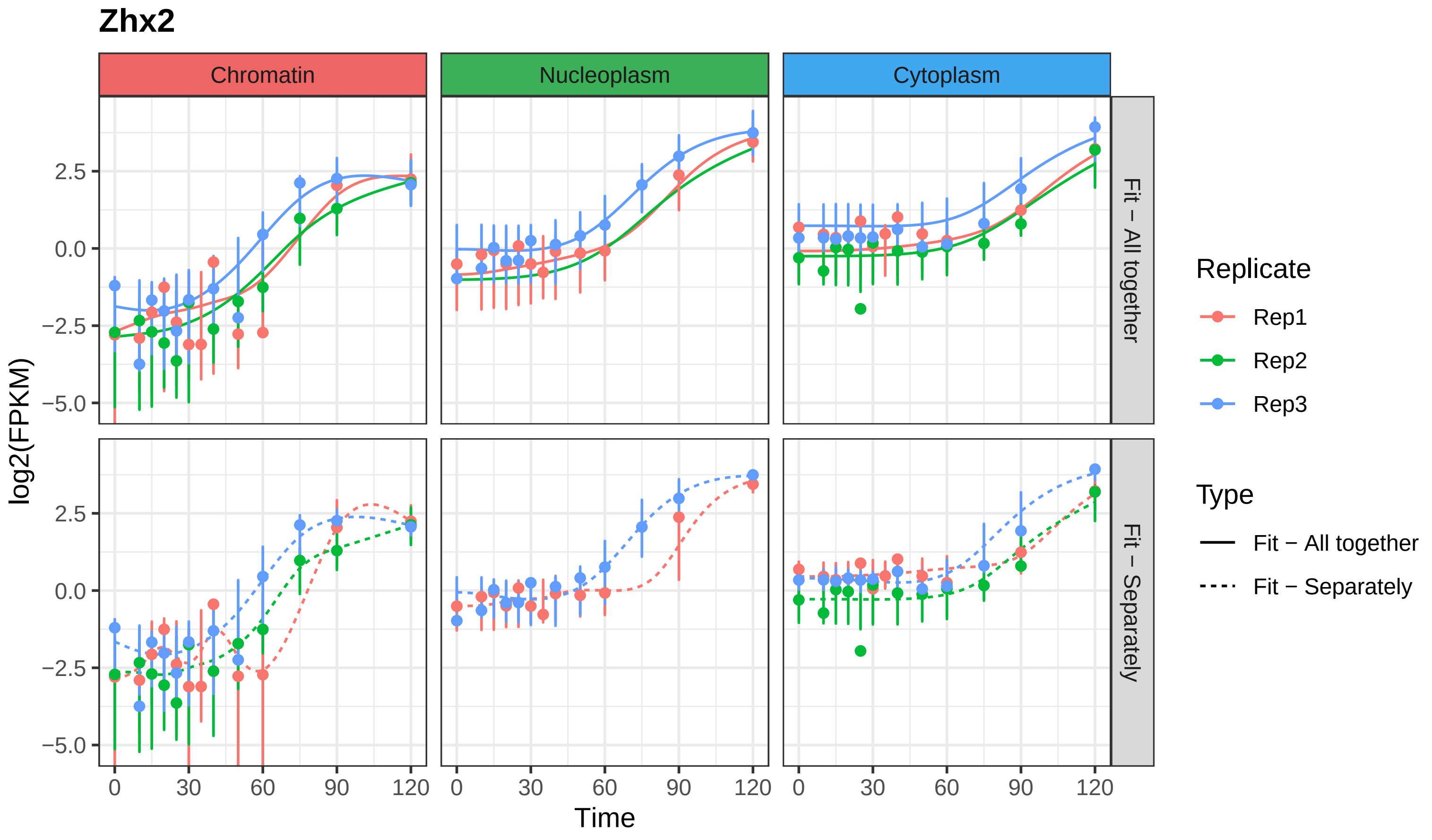

	Together	b1	b2	b3
−NLL b1 ca	16.67	10.6		
−NLL b1 np	5.236	3.125		
−NLL b1 cyto	10.65	2.227		
−NLL b2 ca	8.529		7.828	
−NLL b2 np				
−NLL b2 cyto	7.844		9.84	
−NLL b3 ca	12.2			8.532
−NLL b3 np	4.95			5.585
−NLL b3 cyto	9.071			−0.6848
Total	75.15	15.95	17.67	13.43
Total with regul	79.85	15.55	17.13	13.29

	Together	b1	b2	b3
spar	5.952e−01	0.3723	0.4512	5.428e−01
σ_b	2.365e−01	0.0956	0.1888	4.834e−06
σ_t	1.767e−05	3.5300	0.0279	4.772e+00
ca_{0,b_1}				
$\log_{10}(k_1')$	−9.592e−01	−1.1830		−8.742e−01
$\log_{10}(k_2)$	−1.516e+00	−1.9020	−1.48 or −1.512	−1.355e+00
$\log_{10}(k_2')$	−1.438e+00	−1.3610		−1.209e+00
$\log_{10}(k_{deg})$	−1.666e+00	−1.6480	−1.512 or −1.48	−1.346e+00
$\log_{10}(k_1'k_2')$	−2.397e+00	−2.5430	−2.283	−2.083e+00
$\log_{10}(k_1'/k_2)$	5.568e−01	0.7193		4.811e−01
$\text{transport} = \log_{10}(k_1'k_2'/k_2)$	−8.808e−01	−0.6414	−0.8027 or −0.7711	−7.278e−01

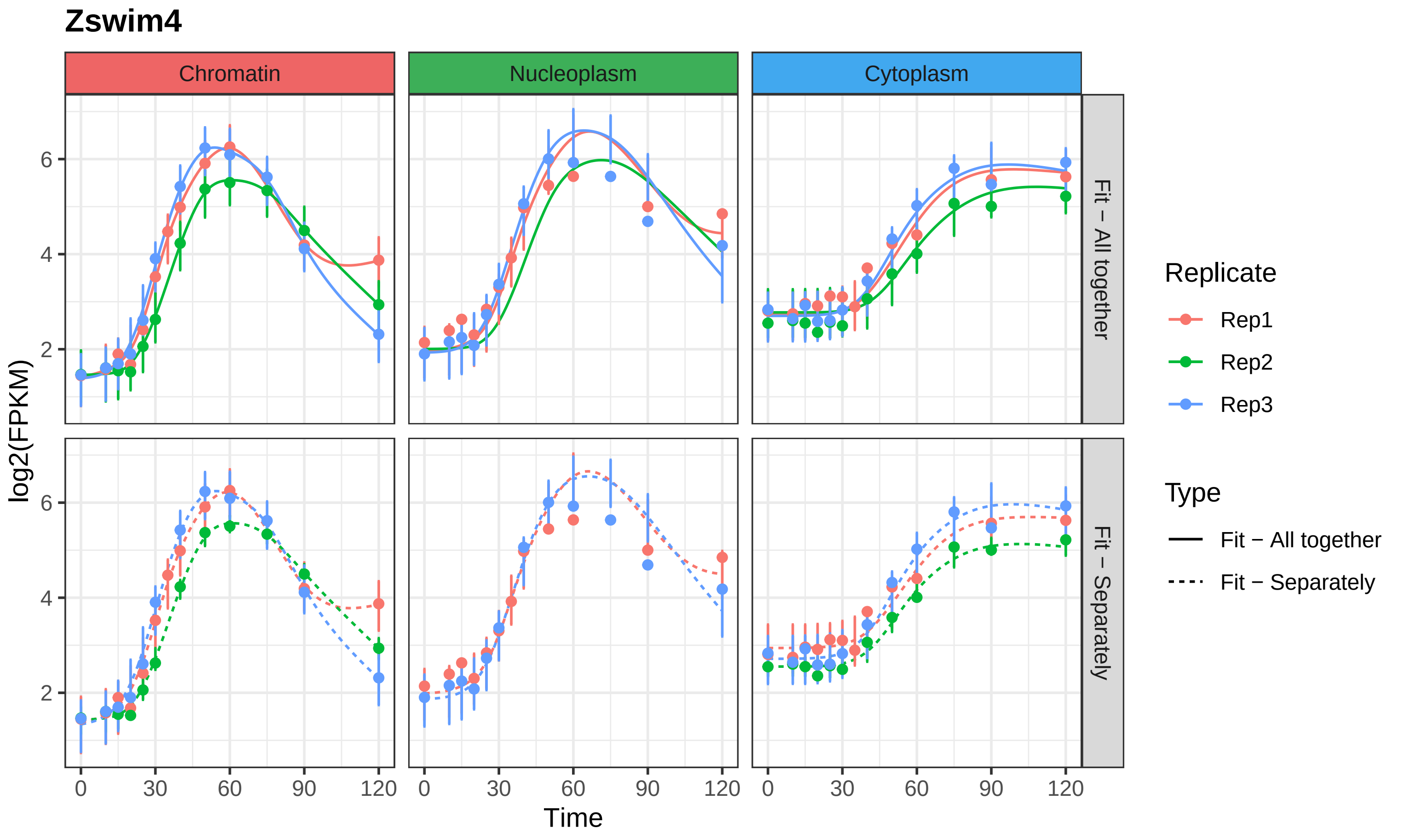

	Together	b1	b2	b3
**−NLL b1 ca**	−3.483	−2.902		
**−NLL b1 np**	9.1	8.673		
**−NLL b1 cyto**	0.923	−1.451		
**−NLL b2 ca**	−4.45		−9.908	
**−NLL b2 np**				
**−NLL b2 cyto**	−0.1645		−7.101	
**−NLL b3 ca**	−3.751			−2.719
**−NLL b3 np**	12.26			7.666
**−NLL b3 cyto**	−2.054			−1.6
Total	8.385	4.32	−17.01	3.347
**Total with regul**	9.368	4.13	−18.14	3.391

	Together	b1	b2	b3
spar	3.550e−01	3.943e−01	0.3787	3.826e−01
σ_b	1.729e−01	1.736e−01	0.05854	1.705e−01
σ_t	9.877e−05	8.184e−08	7.267e−05	4.194e−05
ca_{0,b_1}				
$\log_{10}(k_1')$	−8.061e−01	−7.543e−01		−8.791e−01
$\log_{10}(k_2)$	−9.705e−01	−9.403e−01	−0.4119 or −2.041	−1.041e+00
$\log_{10}(k_2')$	−1.752e+00	−1.859e+00		−1.712e+00
$\log_{10}(k_{deg})$	−1.984e+00	−2.145e+00	−2.041 or −0.4119	−1.965e+00
$\log_{10}(k_1'k_2')$	−2.558e+00	−2.614e+00	−2.118	−2.591e+00
$\log_{10}(k_1'/k_2)$	1.645e−01	1.860e−01		1.619e−01
transport = $\log_{10}(k_1'k_2'/k_2)$	−1.588e+00	−1.673e+00	−1.706 or −0.07639	−1.550e+00
