# Supplementary material for: Kinetics of mRNA nuclear export regulate innate immune response gene expression
Source: Nat Commun. 2022 Nov 23;13:7197. doi: 10.1038/s41467-022-34635-5 (PMC9691726; doi:10.1038/s41467-022-34635-5)
Supplement: Supplementary file 7 — Source Data [file 41467_2022_34635_MOESM7_ESM.zip › Source Data File 5 Profile_dMod_merged_lpa_with_optim_points_v2.pdf]

Abtb2

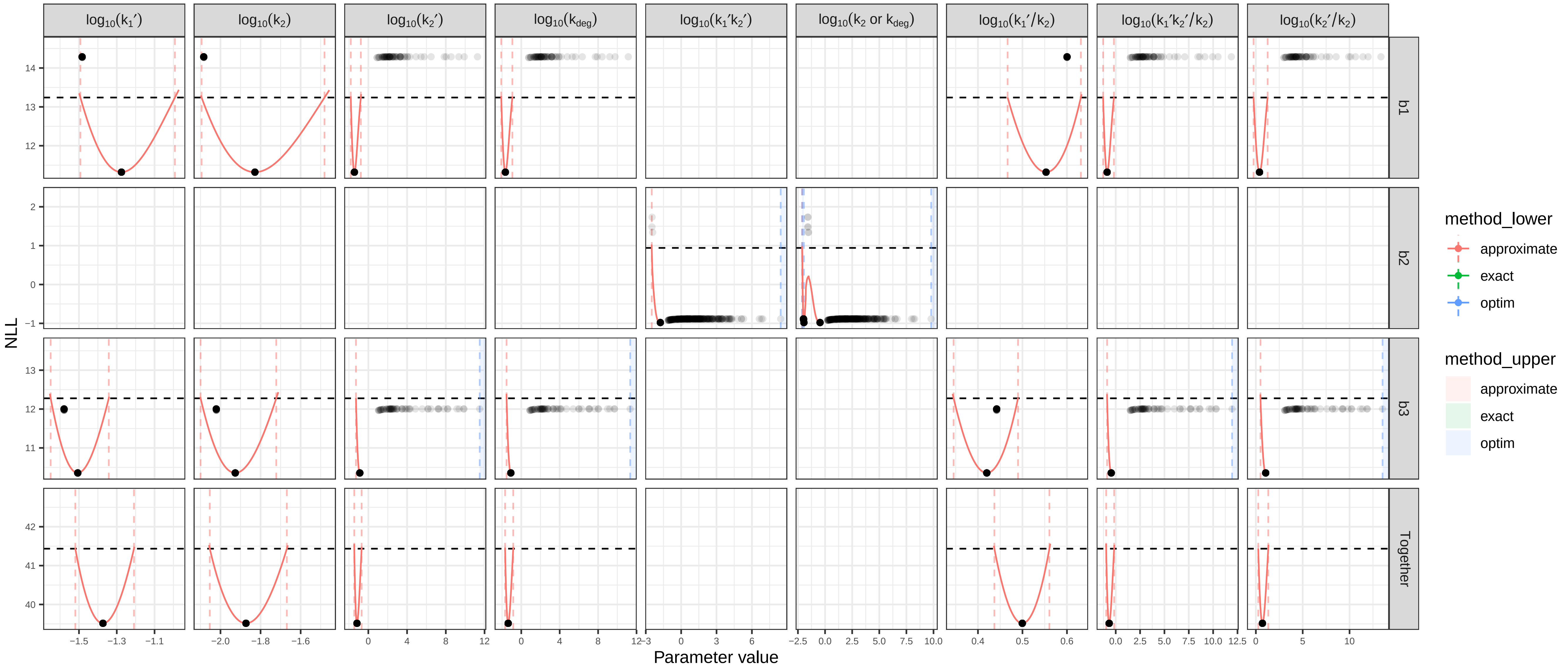

Replicate	Par	Best value	CI95 LB	CI95 UB	Method LB	Method UB
Together	$\log_{10}(k_1')$	−1.373	−1.519	−1.209	approximate	approximate
Together	$\log_{10}(k_2)$	−1.873	−2.053	−1.669	approximate	approximate
Together	$\log_{10}(k_2')$	−1.172	−1.452	−0.6896	approximate	approximate
Together	$\log_{10}(k_{deg})$	−1.362	−1.683	−0.8363	approximate	approximate
Together	$\log_{10}(k_1'/k_2)$	0.4997	0.4369	0.5605	approximate	approximate
Together	$\log_{10}(k_1'k_2'/k_2)$	−0.6721	−0.9805	−0.1627	approximate	approximate
Together	$\log_{10}(k_2'/k_2)$	0.701	0.2535	1.323	approximate	approximate
b1	$\log_{10}(k_1')$	−1.275	−1.493	−0.9923	approximate	approximate
b1	$\log_{10}(k_2)$	−1.828	−2.094	−1.481	approximate	approximate
b1	$\log_{10}(k_2')$	−1.437	−1.811	−0.7725	approximate	approximate
b1	$\log_{10}(k_{deg})$	−1.656	−2.083	−0.9265	approximate	approximate
b1	$\log_{10}(k_1'/k_2)$	0.553	0.4666	0.6312	approximate	approximate
b1	$\log_{10}(k_1'k_2'/k_2)$	−0.8839	−1.294	−0.1838	approximate	approximate
b1	$\log_{10}(k_2'/k_2)$	0.3914	−0.245	1.258	approximate	approximate
b2	$\log_{10}(k_1'k_2')$	−1.762	−2.493	> 8.438	approximate	optim
b2	$\log_{10}(k_2 \text{ or } k_{deg})$	−0.4614	−2.11	> 9.782	approximate	optim
b2	$\log_{10}(k_2 \text{ or } k_{deg})$	−1.949	−2.11	−1.949	approximate	optim
b3	$\log_{10}(k_1')$	−1.507	−1.65	−1.342	approximate	approximate
b3	$\log_{10}(k_2)$	−1.926	−2.099	−1.722	approximate	approximate
b3	$\log_{10}(k_2')$	−0.8789	−1.276	> 11.5	approximate	optim
b3	$\log_{10}(k_{deg})$	−1.083	−1.527	> 11.34	approximate	optim
b3	$\log_{10}(k_1'/k_2)$	0.4198	0.3453	0.49	approximate	approximate
b3	$\log_{10}(k_1'k_2'/k_2)$	−0.4591	−0.8855	> 11.94	approximate	optim
b3	$\log_{10}(k_2'/k_2)$	1.047	0.4911	> 13.52	approximate	optim

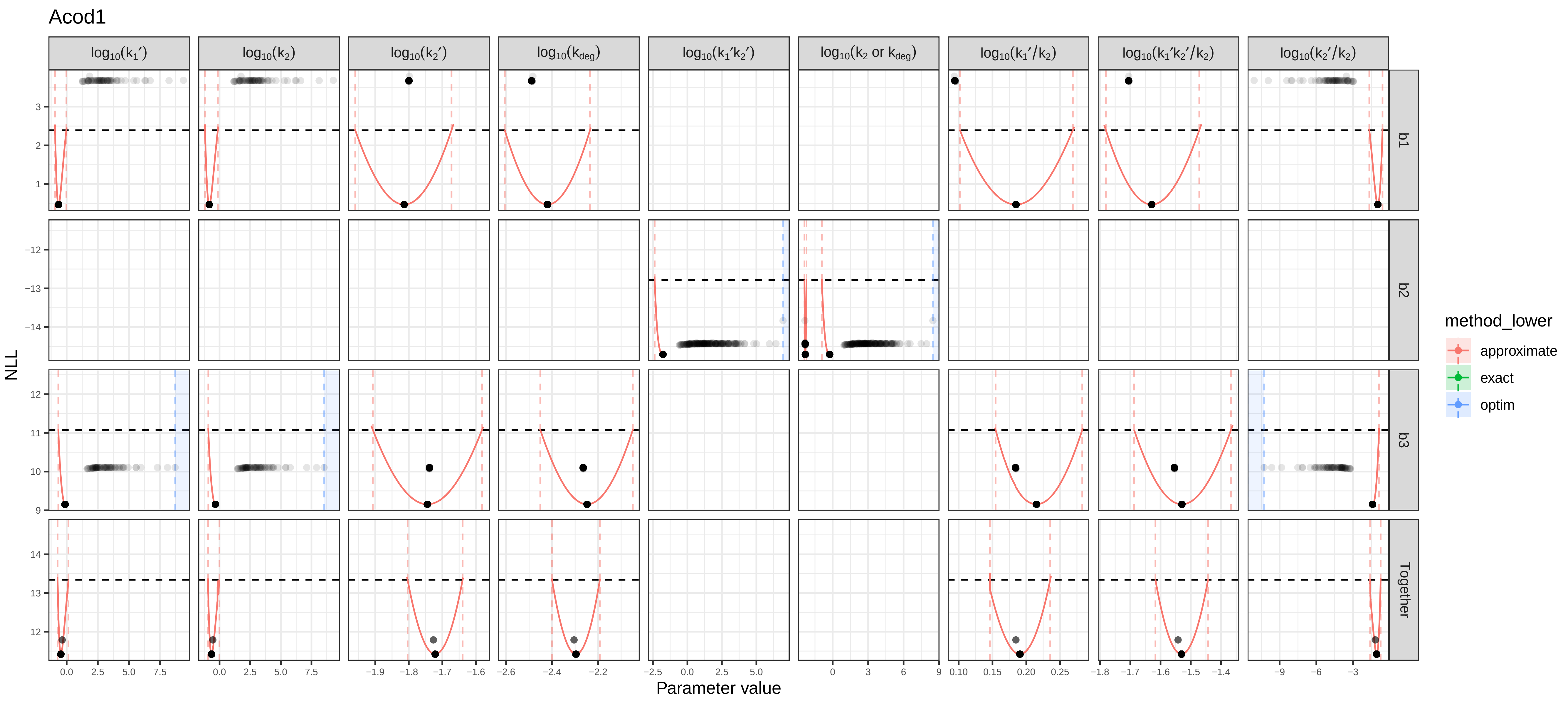

Replicate	Par	Best value	CI95 LB	CI95 UB	Method LB	Method UB
Together	$\log_{10}(k_1')$	−0.4627	−0.7281	0.1449	approximate	approximate
Together	$\log_{10}(k_2)$	−0.6533	−0.9449	−0.00847	approximate	approximate
Together	$\log_{10}(k_2')$	−1.721	−1.804	−1.639	approximate	approximate
Together	$\log_{10}(k_{deg})$	−2.296	−2.4	−2.192	approximate	approximate
Together	$\log_{10}(k_1'/k_2)$	0.1906	0.1463	0.2355	approximate	approximate
Together	$\log_{10}(k_1'k_2'/k_2)$	−1.531	−1.617	−1.443	approximate	approximate
Together	$\log_{10}(k_2'/k_2)$	−1.068	−1.604	−0.7442	approximate	approximate
b1	$\log_{10}(k_1')$	−0.6541	−0.9283	−0.02345	approximate	approximate
b1	$\log_{10}(k_2)$	−0.8388	−1.183	−0.1381	approximate	approximate
b1	$\log_{10}(k_2')$	−1.814	−1.96	−1.673	approximate	approximate
b1	$\log_{10}(k_{deg})$	−2.42	−2.606	−2.235	approximate	approximate
b1	$\log_{10}(k_1'/k_2)$	0.1847	0.1019	0.2689	approximate	approximate
b1	$\log_{10}(k_1'k_2'/k_2)$	−1.629	−1.781	−1.472	approximate	approximate
b1	$\log_{10}(k_2'/k_2)$	−0.9751	−1.671	−0.5913	approximate	approximate
b2	$\log_{10}(k_1'k_2')$	−1.773	−2.368	> 6.932	approximate	optim
b2	$\log_{10}(k_2 \text{ or } k_{deg})$	−0.2582	−0.9227	> 8.483	approximate	optim
b2	$\log_{10}(k_2 \text{ or } k_{deg})$	−2.315	−2.392	−2.22	approximate	approximate
b3	$\log_{10}(k_1')$	−0.1249	−0.6707	> 8.714	approximate	optim
b3	$\log_{10}(k_2)$	−0.3399	−0.917	> 8.53	approximate	optim
b3	$\log_{10}(k_2')$	−1.745	−1.907	−1.582	approximate	approximate
b3	$\log_{10}(k_{deg})$	−2.248	−2.451	−2.049	approximate	approximate
b3	$\log_{10}(k_1'/k_2)$	0.215	0.1546	0.2828	approximate	approximate
b3	$\log_{10}(k_1'k_2'/k_2)$	−1.53	−1.687	−1.367	approximate	approximate
b3	$\log_{10}(k_2'/k_2)$	−1.405	< −10.27	−0.8769	optim	approximate

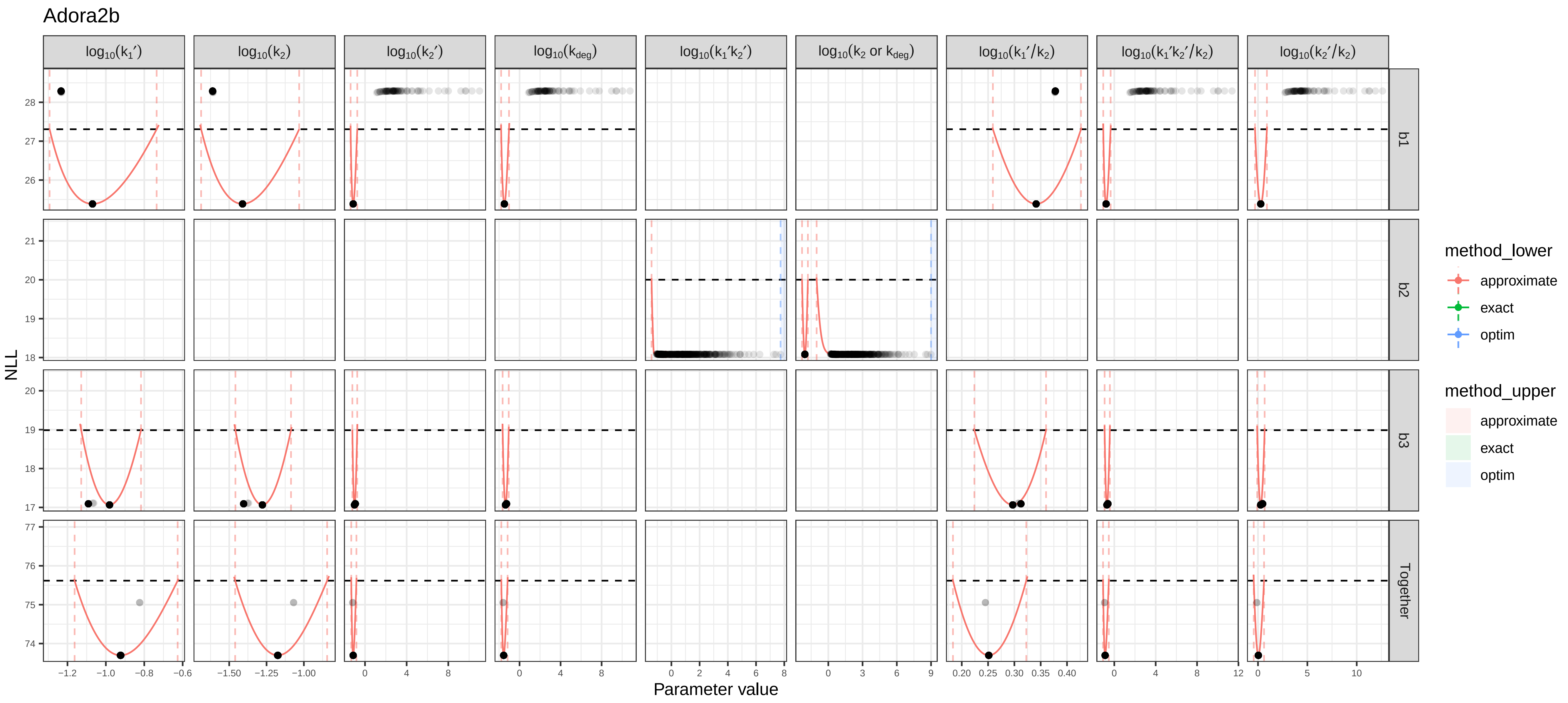

Replicate	Par	Best value	CI95 LB	CI95 UB	Method LB	Method UB
Together	$\log_{10}(k_1')$	−0.9231	−1.163	−0.6261	approximate	approximate
Together	$\log_{10}(k_2)$	−1.174	−1.46	−0.8441	approximate	approximate
Together	$\log_{10}(k_2')$	−1.137	−1.321	−0.8237	approximate	approximate
Together	$\log_{10}(k_{deg})$	−1.545	−1.776	−1.163	approximate	approximate
Together	$\log_{10}(k_1'/k_2)$	0.2513	0.1833	0.3226	approximate	approximate
Together	$\log_{10}(k_1'k_2'/k_2)$	−0.8861	−1.088	−0.5303	approximate	approximate
Together	$\log_{10}(k_2'/k_2)$	0.037	−0.4314	0.6139	approximate	approximate
b1	$\log_{10}(k_1')$	−1.07	−1.293	−0.7354	approximate	approximate
b1	$\log_{10}(k_2)$	−1.411	−1.688	−1.031	approximate	approximate
b1	$\log_{10}(k_2')$	−1.133	−1.385	−0.7426	approximate	approximate
b1	$\log_{10}(k_{deg})$	−1.479	−1.795	−1.024	approximate	approximate
b1	$\log_{10}(k_1'/k_2)$	0.3413	0.2592	0.4266	approximate	approximate
b1	$\log_{10}(k_1'k_2'/k_2)$	−0.792	−1.067	−0.354	approximate	approximate
b1	$\log_{10}(k_2'/k_2)$	0.2776	−0.3009	0.9036	approximate	approximate
b2	$\log_{10}(k_1'k_2')$	−0.4292	−1.406	> 7.737	approximate	optim
b2	$\log_{10}(k_2 \text{ or } k_{deg})$	0.8312	−1.024	> 9	approximate	optim
b2	$\log_{10}(k_2 \text{ or } k_{deg})$	−2.057	−2.302	−1.79	approximate	approximate
b3	$\log_{10}(k_1')$	−0.9809	−1.128	−0.8169	approximate	approximate
b3	$\log_{10}(k_2)$	−1.278	−1.458	−1.086	approximate	approximate
b3	$\log_{10}(k_2')$	−1.008	−1.214	−0.7472	approximate	approximate
b3	$\log_{10}(k_{deg})$	−1.366	−1.649	−1.054	approximate	approximate
b3	$\log_{10}(k_1'/k_2)$	0.2969	0.224	0.3602	approximate	approximate
b3	$\log_{10}(k_1'k_2'/k_2)$	−0.7115	−0.936	−0.4279	approximate	approximate
b3	$\log_{10}(k_2'/k_2)$	0.2694	−0.07917	0.6724	approximate	approximate

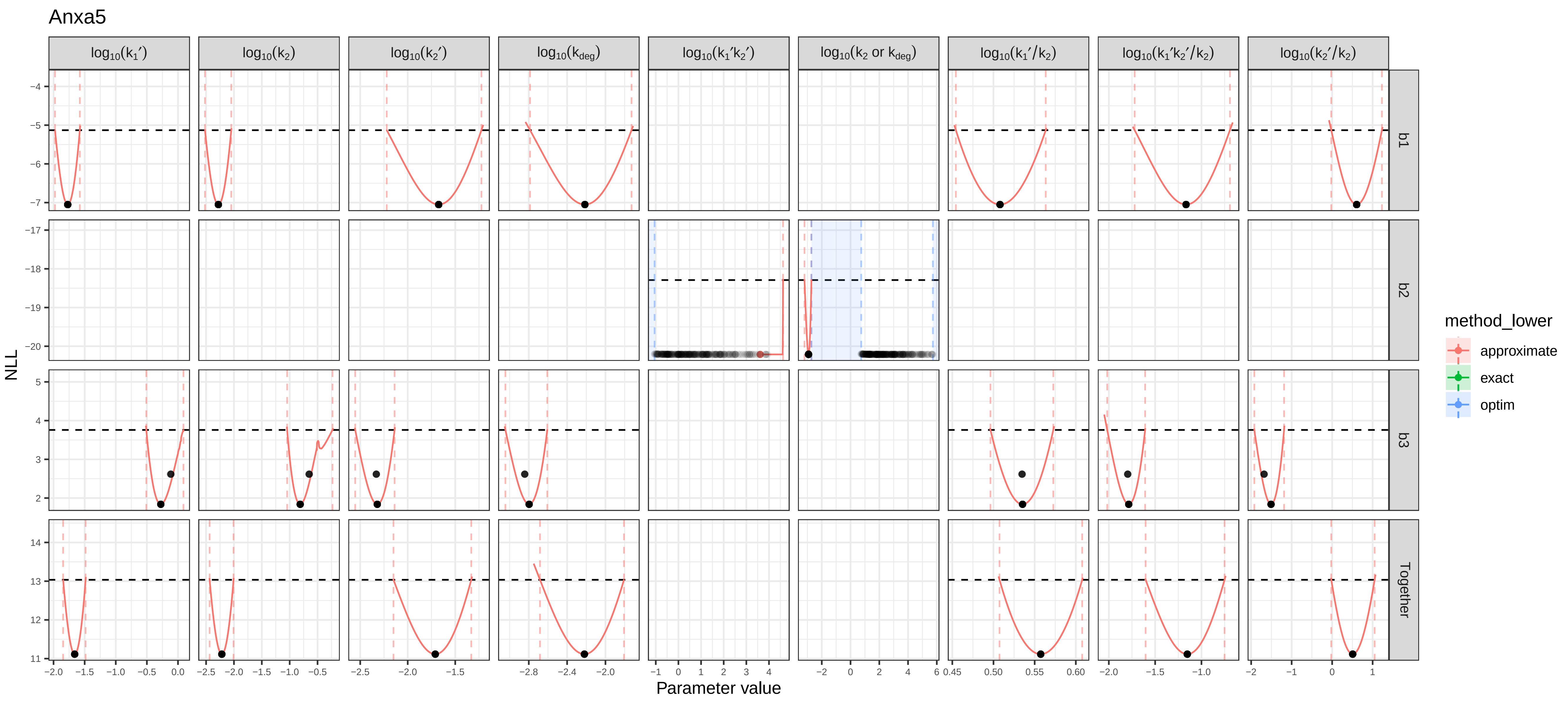

Replicate	Par	Best value	CI95 LB	CI95 UB	Method LB	Method UB
Together	$\log_{10}(k_1')$	−1.66	−1.846	−1.485	approximate	approximate
Together	$\log_{10}(k_2)$	−2.218	−2.437	−2.007	approximate	approximate
Together	$\log_{10}(k_2')$	−1.71	−2.15	−1.331	approximate	approximate
Together	$\log_{10}(k_{deg})$	−2.22	−2.683	−1.806	approximate	approximate
Together	$\log_{10}(k_1'/k_2)$	0.5574	0.5073	0.6077	approximate	approximate
Together	$\log_{10}(k_1'k_2'/k_2)$	−1.153	−1.603	−0.7508	approximate	approximate
Together	$\log_{10}(k_2'/k_2)$	0.5077	−0.02213	1.05	approximate	approximate
b1	$\log_{10}(k_1')$	−1.772	−1.977	−1.578	approximate	approximate
b1	$\log_{10}(k_2)$	−2.28	−2.52	−2.048	approximate	approximate
b1	$\log_{10}(k_2')$	−1.674	−2.221	−1.224	approximate	approximate
b1	$\log_{10}(k_{deg})$	−2.215	−2.787	−1.728	approximate	approximate
b1	$\log_{10}(k_1'/k_2)$	0.5079	0.4543	0.5634	approximate	approximate
b1	$\log_{10}(k_1'k_2'/k_2)$	−1.166	−1.721	−0.6927	approximate	approximate
b1	$\log_{10}(k_2'/k_2)$	0.6054	−0.02221	1.229	approximate	approximate
b2	$\log_{10}(k_1'k_2')$	3.613	< −1.054	4.62	optim	approximate
b2	$\log_{10}(k_2 \text{ or } k_{deg})$	5.408	0.7408	> 5.73	optim	optim
b2	$\log_{10}(k_2 \text{ or } k_{deg})$	−2.922	−3.204	−2.724	approximate	approximate
b3	$\log_{10}(k_1')$	−0.276	−0.5097	0.08779	approximate	approximate
b3	$\log_{10}(k_2)$	−0.8113	−1.046	−0.2319	approximate	approximate
b3	$\log_{10}(k_2')$	−2.32	−2.553	−2.138	approximate	approximate
b3	$\log_{10}(k_{deg})$	−2.796	−3.044	−2.607	approximate	approximate
b3	$\log_{10}(k_1'/k_2)$	0.5353	0.4963	0.5726	approximate	approximate
b3	$\log_{10}(k_1'k_2'/k_2)$	−1.785	−2.018	−1.608	approximate	approximate
b3	$\log_{10}(k_2'/k_2)$	−1.509	−1.921	−1.188	approximate	approximate

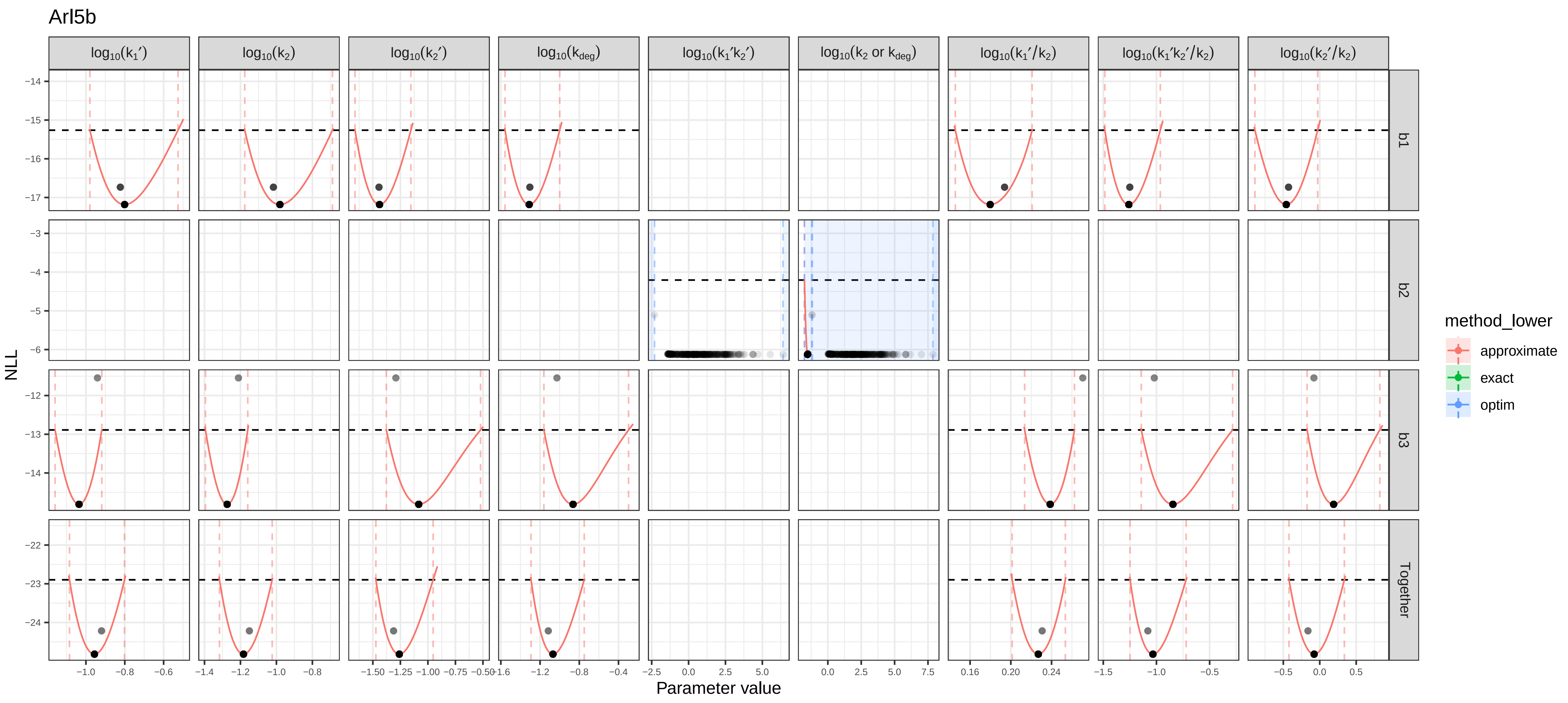

Replicate	Par	Best value	CI95 LB	CI95 UB	Method LB	Method UB
Together	$\log_{10}(k_1')$	−0.9561	−1.085	−0.8013	approximate	approximate
Together	$\log_{10}(k_2)$	−1.183	−1.317	−1.023	approximate	approximate
Together	$\log_{10}(k_2')$	−1.26	−1.473	−0.9524	approximate	approximate
Together	$\log_{10}(k_{deg})$	−1.068	−1.292	−0.7496	approximate	approximate
Together	$\log_{10}(k_1'/k_2)$	0.2269	0.2013	0.2535	approximate	approximate
Together	$\log_{10}(k_1'k_2'/k_2)$	−1.033	−1.248	−0.7208	approximate	approximate
Together	$\log_{10}(k_2'/k_2)$	−0.07701	−0.4217	0.3376	approximate	approximate
b1	$\log_{10}(k_1')$	−0.8007	−0.9796	−0.5258	approximate	approximate
b1	$\log_{10}(k_2)$	−0.9805	−1.176	−0.6884	approximate	approximate
b1	$\log_{10}(k_2')$	−1.439	−1.662	−1.155	approximate	approximate
b1	$\log_{10}(k_{deg})$	−1.311	−1.557	−0.9997	approximate	approximate
b1	$\log_{10}(k_1'/k_2)$	0.1797	0.1455	0.2209	approximate	approximate
b1	$\log_{10}(k_1'k_2'/k_2)$	−1.26	−1.485	−0.9637	approximate	approximate
b1	$\log_{10}(k_2'/k_2)$	−0.459	−0.8893	−0.02795	approximate	approximate
b2	$\log_{10}(k_1'k_2')$	2.569	< -2.306	> 6.402	optim	optim
b2	$\log_{10}(k_2 \text{ or } k_{deg})$	4.036	−1.156	> 7.869	optim	optim
b2	$\log_{10}(k_2 \text{ or } k_{deg})$	−1.518	−1.751	> -1.213	approximate	optim
b3	$\log_{10}(k_1')$	−1.035	−1.159	−0.9183	approximate	approximate
b3	$\log_{10}(k_2)$	−1.274	−1.395	−1.159	approximate	approximate
b3	$\log_{10}(k_2')$	−1.083	−1.378	−0.5218	approximate	approximate
b3	$\log_{10}(k_{deg})$	−0.8634	−1.161	−0.2973	approximate	approximate
b3	$\log_{10}(k_1'/k_2)$	0.2386	0.2136	0.2625	approximate	approximate
b3	$\log_{10}(k_1'k_2'/k_2)$	−0.8448	−1.143	−0.2835	approximate	approximate
b3	$\log_{10}(k_2'/k_2)$	0.1906	−0.1734	0.824	approximate	approximate

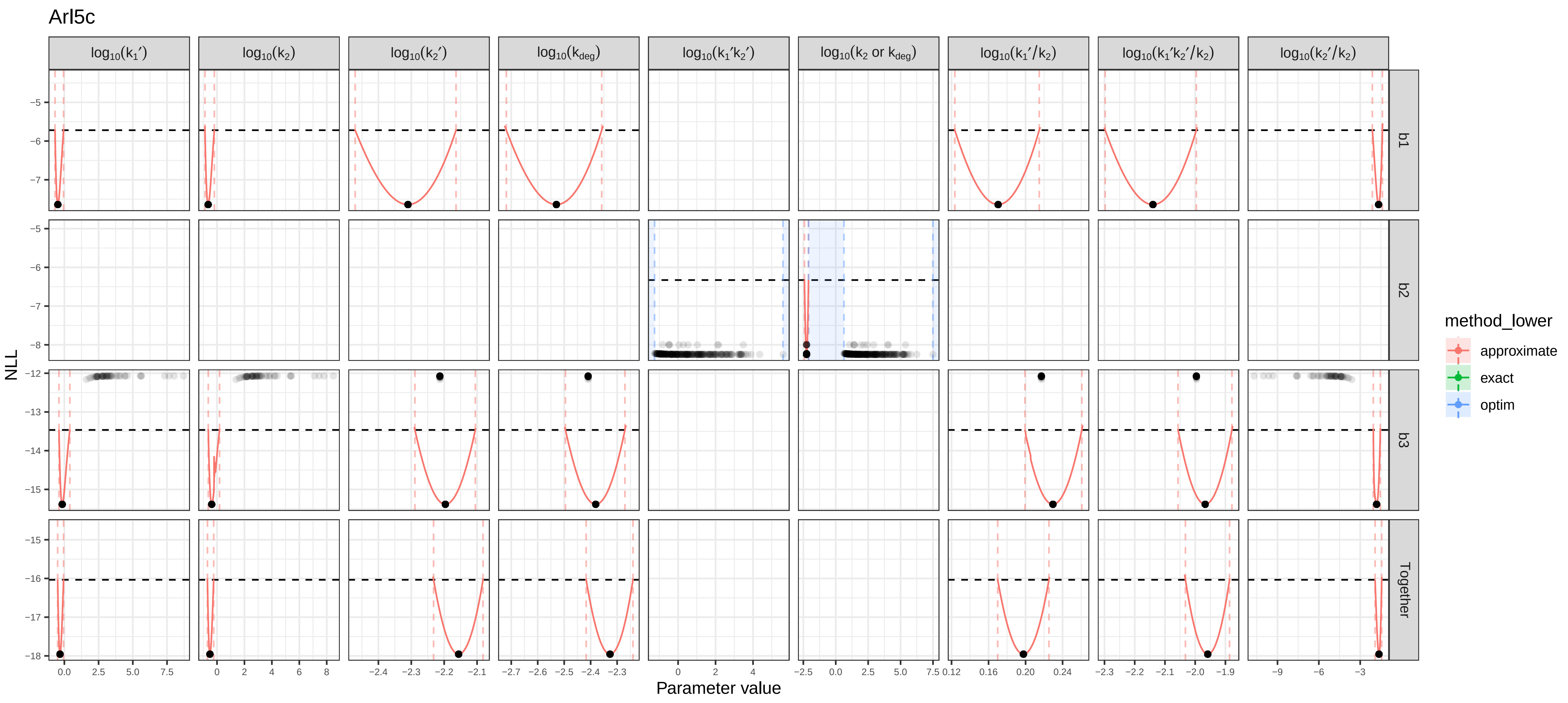

Replicate	Par	Best value	CI95 LB	CI95 UB	Method LB	Method UB
Together	$\log_{10}(k_1')$	−0.3173	−0.4883	−0.05766	approximate	approximate
Together	$\log_{10}(k_2)$	−0.5149	−0.6967	−0.2411	approximate	approximate
Together	$\log_{10}(k_2')$	−2.156	−2.232	−2.082	approximate	approximate
Together	$\log_{10}(k_{deg})$	−2.327	−2.417	−2.24	approximate	approximate
Together	$\log_{10}(k_1'/k_2)$	0.1977	0.1699	0.2253	approximate	approximate
Together	$\log_{10}(k_1'k_2'/k_2)$	−1.958	−2.032	−1.886	approximate	approximate
Together	$\log_{10}(k_2'/k_2)$	−1.641	−1.922	−1.441	approximate	approximate
b1	$\log_{10}(k_1')$	−0.4747	−0.6814	−0.05599	approximate	approximate
b1	$\log_{10}(k_2)$	−0.6451	−0.8768	−0.1938	approximate	approximate
b1	$\log_{10}(k_2')$	−2.31	−2.471	−2.164	approximate	approximate
b1	$\log_{10}(k_{deg})$	−2.529	−2.719	−2.358	approximate	approximate
b1	$\log_{10}(k_1'/k_2)$	0.1704	0.1235	0.2148	approximate	approximate
b1	$\log_{10}(k_1'k_2'/k_2)$	−2.14	−2.298	−1.996	approximate	approximate
b1	$\log_{10}(k_2'/k_2)$	−1.665	−2.118	−1.39	approximate	approximate
b2	$\log_{10}(k_1'k_2')$	2.035	< -1.257	> 5.602	optim	optim
b2	$\log_{10}(k_2 \text{ or } k_{deg})$	3.925	0.6316	> 7.492	optim	optim
b2	$\log_{10}(k_2 \text{ or } k_{deg})$	−2.25	−2.414	−2.101	approximate	approximate
b3	$\log_{10}(k_1')$	−0.1548	−0.3934	0.4029	approximate	approximate
b3	$\log_{10}(k_2)$	−0.3844	−0.6355	0.1865	approximate	approximate
b3	$\log_{10}(k_2')$	−2.196	−2.289	−2.105	approximate	approximate
b3	$\log_{10}(k_{deg})$	−2.381	−2.495	−2.27	approximate	approximate
b3	$\log_{10}(k_1'/k_2)$	0.2296	0.1993	0.2608	approximate	approximate
b3	$\log_{10}(k_1'k_2'/k_2)$	−1.967	−2.057	−1.878	approximate	approximate
b3	$\log_{10}(k_2'/k_2)$	−1.812	−2.051	−1.54	approximate	approximate

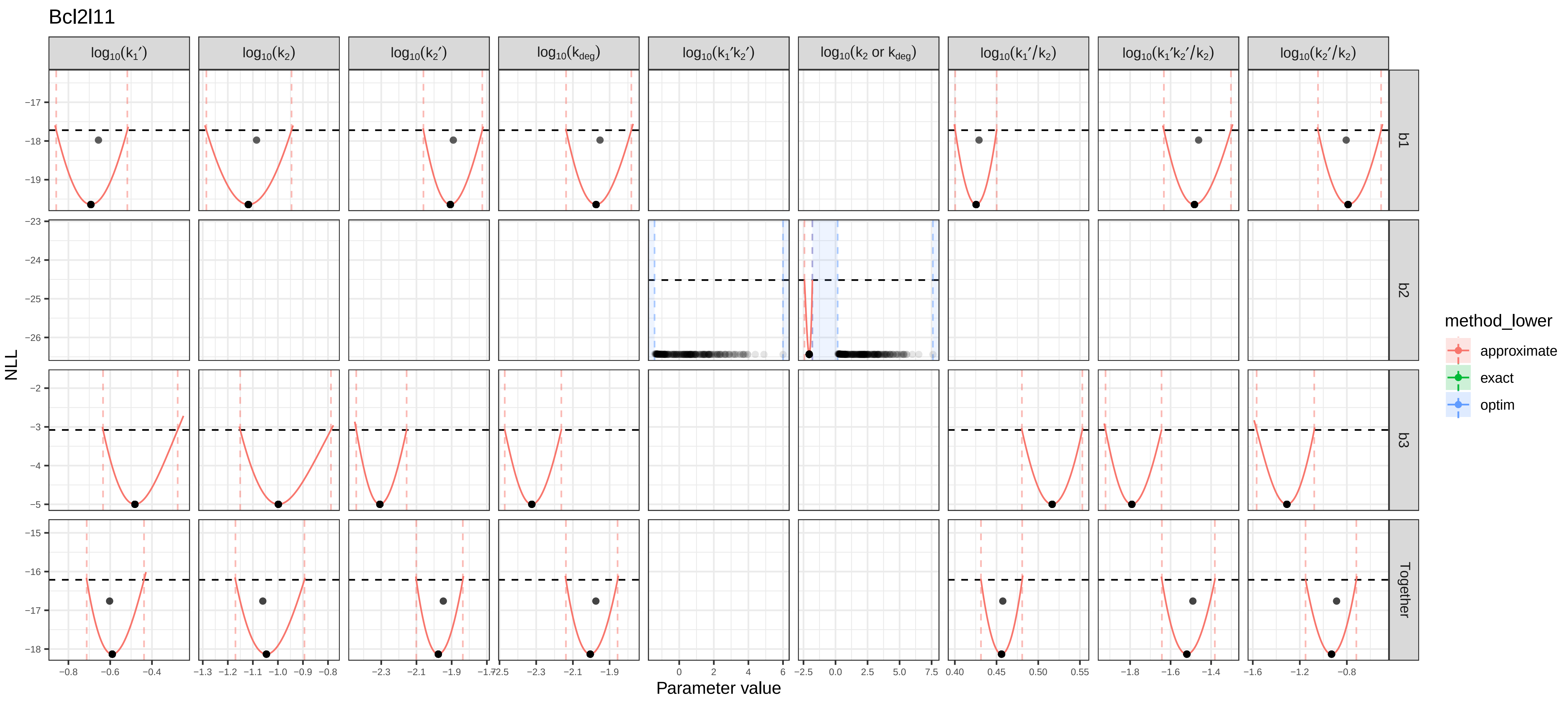

Replicate	Par	Best value	CI95 LB	CI95 UB	Method LB	Method UB
Together	$\log_{10}(k_1')$	−0.5899	−0.7125	−0.4379	approximate	approximate
Together	$\log_{10}(k_2)$	−1.046	−1.17	−0.8939	approximate	approximate
Together	$\log_{10}(k_2')$	−1.976	−2.101	−1.837	approximate	approximate
Together	$\log_{10}(k_{deg})$	−2.005	−2.138	−1.856	approximate	approximate
Together	$\log_{10}(k_1'/k_2)$	0.456	0.4313	0.4808	approximate	approximate
Together	$\log_{10}(k_1'k_2'/k_2)$	−1.52	−1.643	−1.381	approximate	approximate
Together	$\log_{10}(k_2'/k_2)$	−0.9299	−1.151	−0.7193	approximate	approximate
b1	$\log_{10}(k_1')$	−0.6923	−0.8585	−0.5179	approximate	approximate
b1	$\log_{10}(k_2)$	−1.118	−1.286	−0.9455	approximate	approximate
b1	$\log_{10}(k_2')$	−1.908	−2.06	−1.726	approximate	approximate
b1	$\log_{10}(k_{deg})$	−1.974	−2.137	−1.782	approximate	approximate
b1	$\log_{10}(k_1'/k_2)$	0.4255	0.4004	0.4502	approximate	approximate
b1	$\log_{10}(k_1'k_2'/k_2)$	−1.482	−1.633	−1.302	approximate	approximate
b1	$\log_{10}(k_2'/k_2)$	−0.7897	−1.045	−0.5088	approximate	approximate
b2	$\log_{10}(k_1'k_2')$	1.498	< −1.426	> 6.001	optim	optim
b2	$\log_{10}(k_2 \text{ or } k_{deg})$	3.098	0.1691	> 7.601	optim	optim
b2	$\log_{10}(k_2 \text{ or } k_{deg})$	−2.056	−2.425	−1.807	approximate	approximate
b3	$\log_{10}(k_1')$	−0.4815	−0.6343	−0.2767	approximate	approximate
b3	$\log_{10}(k_2)$	−0.9982	−1.151	−0.7882	approximate	approximate
b3	$\log_{10}(k_2')$	−2.308	−2.442	−2.156	approximate	approximate
b3	$\log_{10}(k_{deg})$	−2.324	−2.471	−2.163	approximate	approximate
b3	$\log_{10}(k_1'/k_2)$	0.5167	0.4804	0.5529	approximate	approximate
b3	$\log_{10}(k_1'k_2'/k_2)$	−1.791	−1.921	−1.645	approximate	approximate
b3	$\log_{10}(k_2'/k_2)$	−1.31	−1.567	−1.077	approximate	approximate

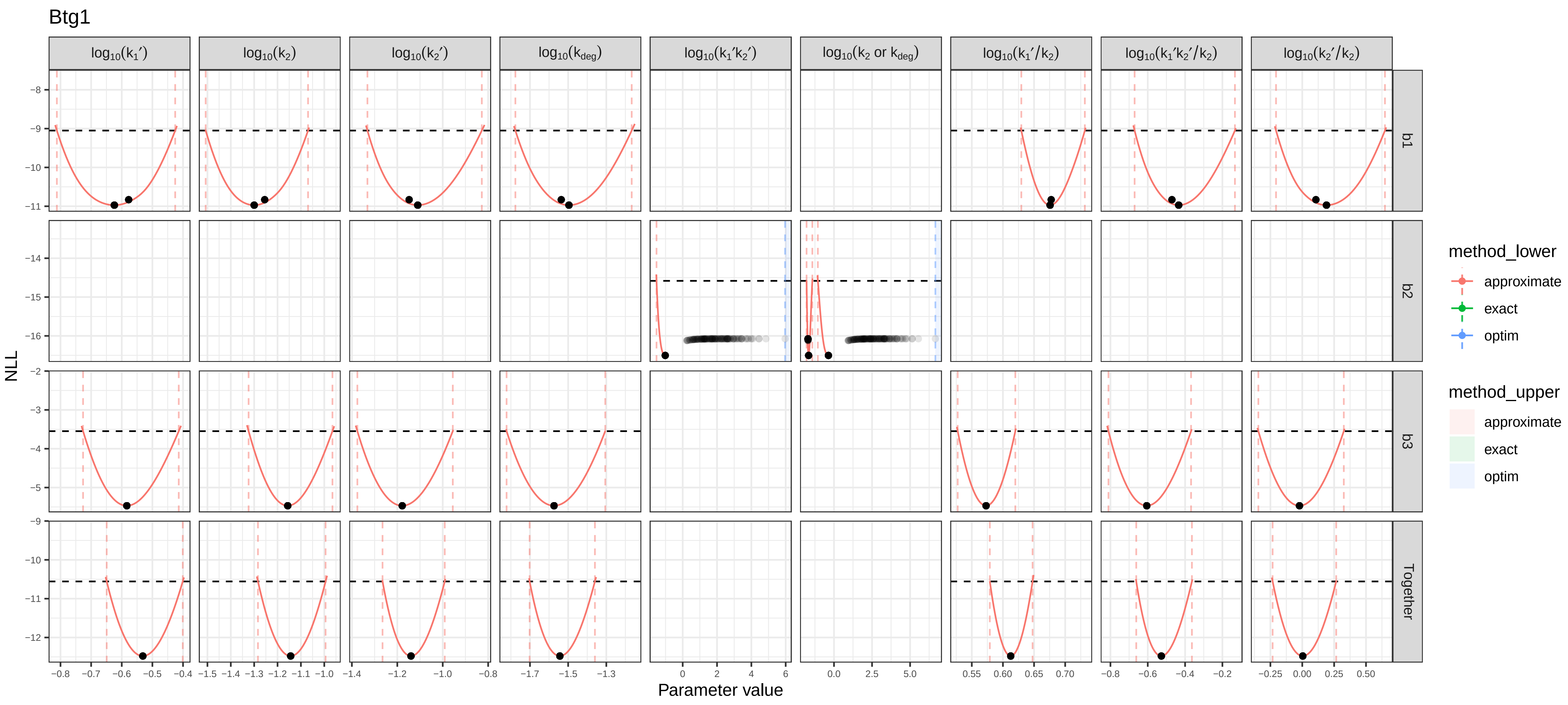

Replicate	Par	Best value	CI95 LB	CI95 UB	Method LB	Method UB
Together	$\log_{10}(k_1')$	−0.5313	−0.6491	−0.4009	approximate	approximate
Together	$\log_{10}(k_2)$	−1.144	−1.284	−0.9944	approximate	approximate
Together	$\log_{10}(k_2')$	−1.14	−1.265	−0.9911	approximate	approximate
Together	$\log_{10}(k_{deg})$	−1.543	−1.702	−1.36	approximate	approximate
Together	$\log_{10}(k_1'/k_2)$	0.6125	0.579	0.6476	approximate	approximate
Together	$\log_{10}(k_1'k_2'/k_2)$	−0.5271	−0.6617	−0.3633	approximate	approximate
Together	$\log_{10}(k_2'/k_2)$	0.004155	−0.2325	0.2662	approximate	approximate
b1	$\log_{10}(k_1')$	−0.6238	−0.8119	−0.4256	approximate	approximate
b1	$\log_{10}(k_2)$	−1.3	−1.508	−1.069	approximate	approximate
b1	$\log_{10}(k_2')$	−1.11	−1.332	−0.8279	approximate	approximate
b1	$\log_{10}(k_{deg})$	−1.496	−1.776	−1.167	approximate	approximate
b1	$\log_{10}(k_1'/k_2)$	0.6757	0.6295	0.7316	approximate	approximate
b1	$\log_{10}(k_1'k_2'/k_2)$	−0.4346	−0.6705	−0.1329	approximate	approximate
b1	$\log_{10}(k_2'/k_2)$	0.1892	−0.2058	0.6486	approximate	approximate
b2	$\log_{10}(k_1'k_2')$	−1.018	−1.525	> 5.965	approximate	optim
b2	$\log_{10}(k_2 \text{ or } k_{deg})$	−0.3672	−1.06	> 6.66	approximate	optim
b2	$\log_{10}(k_2 \text{ or } k_{deg})$	−1.667	−1.802	−1.422	approximate	approximate
b3	$\log_{10}(k_1')$	−0.5835	−0.7263	−0.4139	approximate	approximate
b3	$\log_{10}(k_2)$	−1.156	−1.324	−0.965	approximate	approximate
b3	$\log_{10}(k_2')$	−1.178	−1.376	−0.9557	approximate	approximate
b3	$\log_{10}(k_{deg})$	−1.574	−1.823	−1.306	approximate	approximate
b3	$\log_{10}(k_1'/k_2)$	0.5729	0.5273	0.6199	approximate	approximate
b3	$\log_{10}(k_1'k_2'/k_2)$	−0.6054	−0.8106	−0.3679	approximate	approximate
b3	$\log_{10}(k_2'/k_2)$	−0.02186	−0.3445	0.3259	approximate	approximate

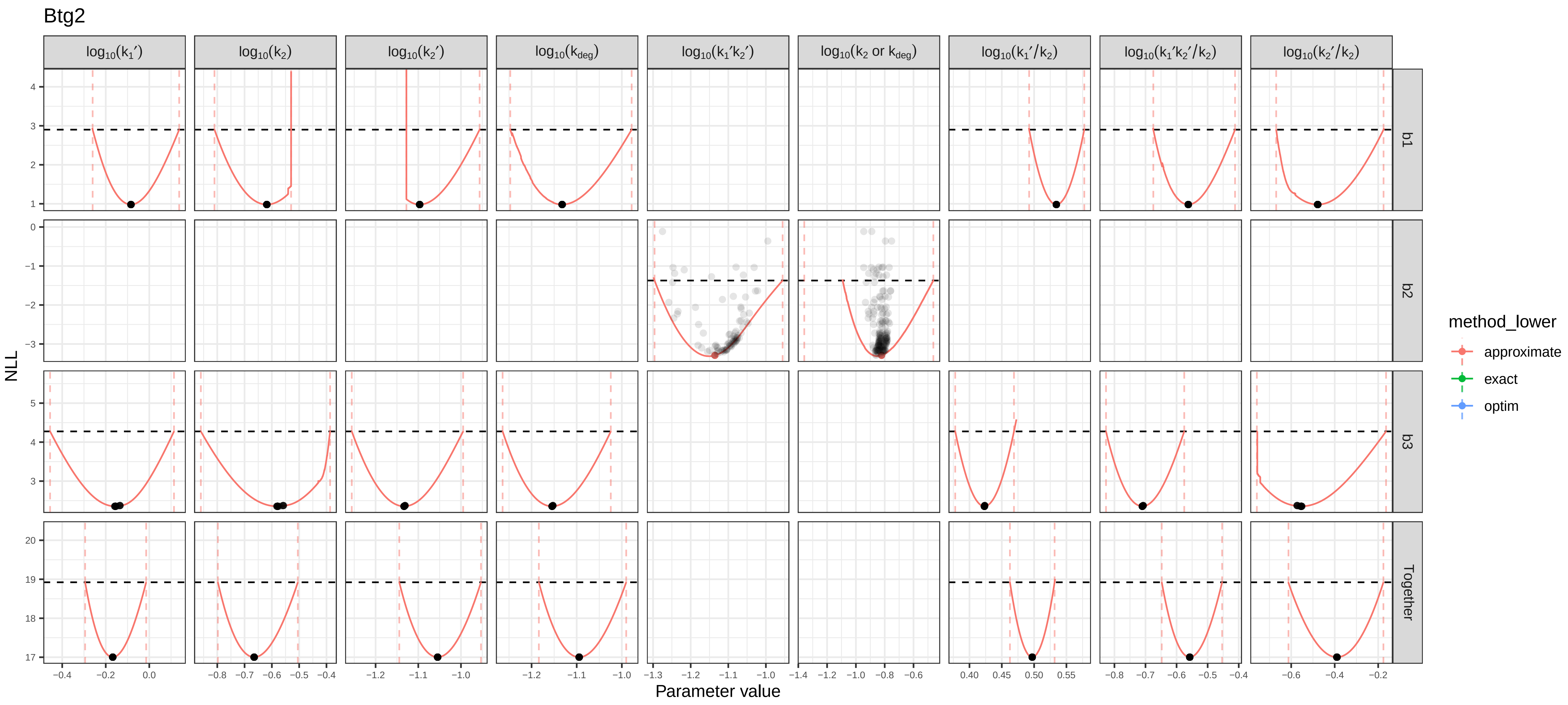

Replicate	Par	Best value	CI95 LB	CI95 UB	Method LB	Method UB
Together	$\log_{10}(k_1')$	−0.1675	−0.2948	−0.01434	approximate	approximate
Together	$\log_{10}(k_2)$	−0.6646	−0.7978	−0.5049	approximate	approximate
Together	$\log_{10}(k_2')$	−1.055	−1.145	−0.9531	approximate	approximate
Together	$\log_{10}(k_{deg})$	−1.095	−1.184	−0.9903	approximate	approximate
Together	$\log_{10}(k_1'/k_2)$	0.4971	0.4626	0.5317	approximate	approximate
Together	$\log_{10}(k_1'k_2'/k_2)$	−0.5578	−0.6477	−0.4534	approximate	approximate
Together	$\log_{10}(k_2'/k_2)$	−0.3902	−0.6126	−0.1759	approximate	approximate
b1	$\log_{10}(k_1')$	−0.08408	−0.2602	0.1373	approximate	approximate
b1	$\log_{10}(k_2)$	−0.6185	−0.8103	−0.5296	approximate	approximate
b1	$\log_{10}(k_2')$	−1.097	−1.128	−0.9563	approximate	approximate
b1	$\log_{10}(k_{deg})$	−1.132	−1.247	−0.9782	approximate	approximate
b1	$\log_{10}(k_1'/k_2)$	0.5344	0.4923	0.5776	approximate	approximate
b1	$\log_{10}(k_1'k_2'/k_2)$	−0.5624	−0.6748	−0.4119	approximate	approximate
b1	$\log_{10}(k_2'/k_2)$	−0.4783	−0.6691	−0.1753	approximate	approximate
b2	$\log_{10}(k_1'k_2')$	−1.136	−1.296	−0.9555	approximate	approximate
b2	$\log_{10}(k_2 \text{ or } k_{deg})$	−0.8218	−1.359	−0.4624	approximate	approximate
b2	$\log_{10}(k_2 \text{ or } k_{deg})$	−0.8581	−1.359	−0.4624	approximate	approximate
b3	$\log_{10}(k_1')$	−0.1561	−0.4558	0.1135	approximate	approximate
b3	$\log_{10}(k_2)$	−0.5792	−0.8602	−0.3867	approximate	approximate
b3	$\log_{10}(k_2')$	−1.133	−1.256	−0.9949	approximate	approximate
b3	$\log_{10}(k_{deg})$	−1.155	−1.264	−1.024	approximate	approximate
b3	$\log_{10}(k_1'/k_2)$	0.4231	0.3779	0.4688	approximate	approximate
b3	$\log_{10}(k_1'k_2'/k_2)$	−0.7102	−0.8271	−0.5753	approximate	approximate
b3	$\log_{10}(k_2'/k_2)$	−0.5541	−0.7579	−0.1638	approximate	approximate

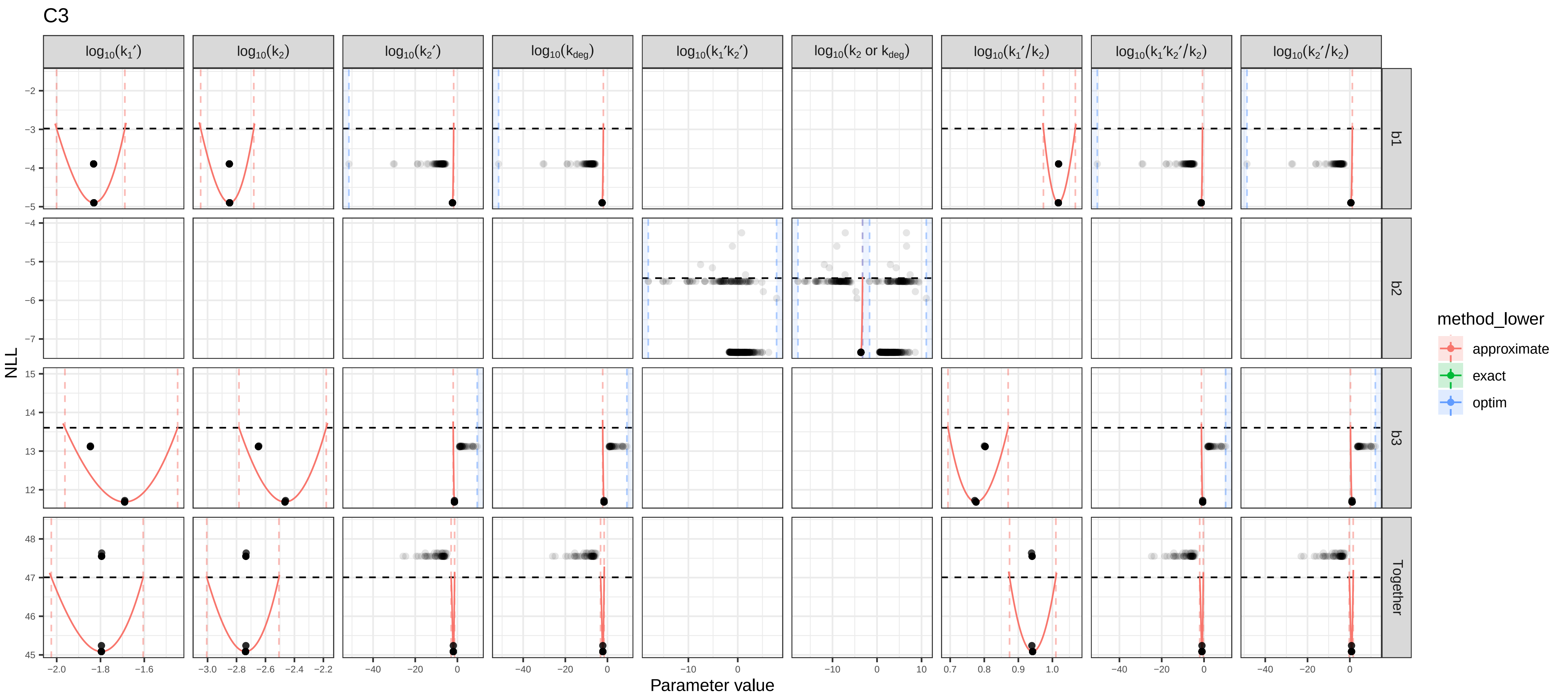

Replicate	Par	Best value	CI95 LB	CI95 UB	Method LB	Method UB
Together	$\log_{10}(k_1')$	−1.796	−2.025	−1.605	approximate	approximate
Together	$\log_{10}(k_2)$	−2.738	−3.006	−2.507	approximate	approximate
Together	$\log_{10}(k_2')$	−1.927	−2.981	−1.323	approximate	approximate
Together	$\log_{10}(k_{deg})$	−2.193	−3.27	−1.548	approximate	approximate
Together	$\log_{10}(k_1'/k_2)$	0.942	0.8742	1.01	approximate	approximate
Together	$\log_{10}(k_1'k_2'/k_2)$	−0.9847	−2.042	−0.3551	approximate	approximate
Together	$\log_{10}(k_2'/k_2)$	0.8112	−0.2681	1.604	approximate	approximate
b1	$\log_{10}(k_1')$	−1.83	−2.001	−1.689	approximate	approximate
b1	$\log_{10}(k_2)$	−2.848	−3.048	−2.681	approximate	approximate
b1	$\log_{10}(k_2')$	−2.333	< −51.46	−1.766	optim	approximate
b1	$\log_{10}(k_{deg})$	−2.526	< −51.67	−1.93	optim	approximate
b1	$\log_{10}(k_1'/k_2)$	1.018	0.9736	1.067	approximate	approximate
b1	$\log_{10}(k_1'k_2'/k_2)$	−1.315	< −50.45	−0.7337	optim	approximate
b1	$\log_{10}(k_2'/k_2)$	0.515	< −48.61	1.194	optim	approximate
b2	$\log_{10}(k_1'k_2')$	1.532	< −18.12	> 7.866	optim	optim
b2	$\log_{10}(k_2 \text{ or } k_{deg})$	3.84	−1.679	> 11.06	optim	optim
b2	$\log_{10}(k_2 \text{ or } k_{deg})$	−3.617	< −17.76	−3.254	optim	approximate
b3	$\log_{10}(k_1')$	−1.69	−1.962	−1.448	approximate	approximate
b3	$\log_{10}(k_2)$	−2.465	−2.783	−2.18	approximate	approximate
b3	$\log_{10}(k_2')$	−1.407	−1.984	> 9.415	approximate	optim
b3	$\log_{10}(k_{deg})$	−1.626	−2.246	> 9.248	approximate	optim
b3	$\log_{10}(k_1'/k_2)$	0.7757	0.6935	0.8701	approximate	approximate
b3	$\log_{10}(k_1'k_2'/k_2)$	−0.6312	−1.227	> 10.22	approximate	optim
b3	$\log_{10}(k_2'/k_2)$	1.058	0.313	> 12.06	approximate	optim

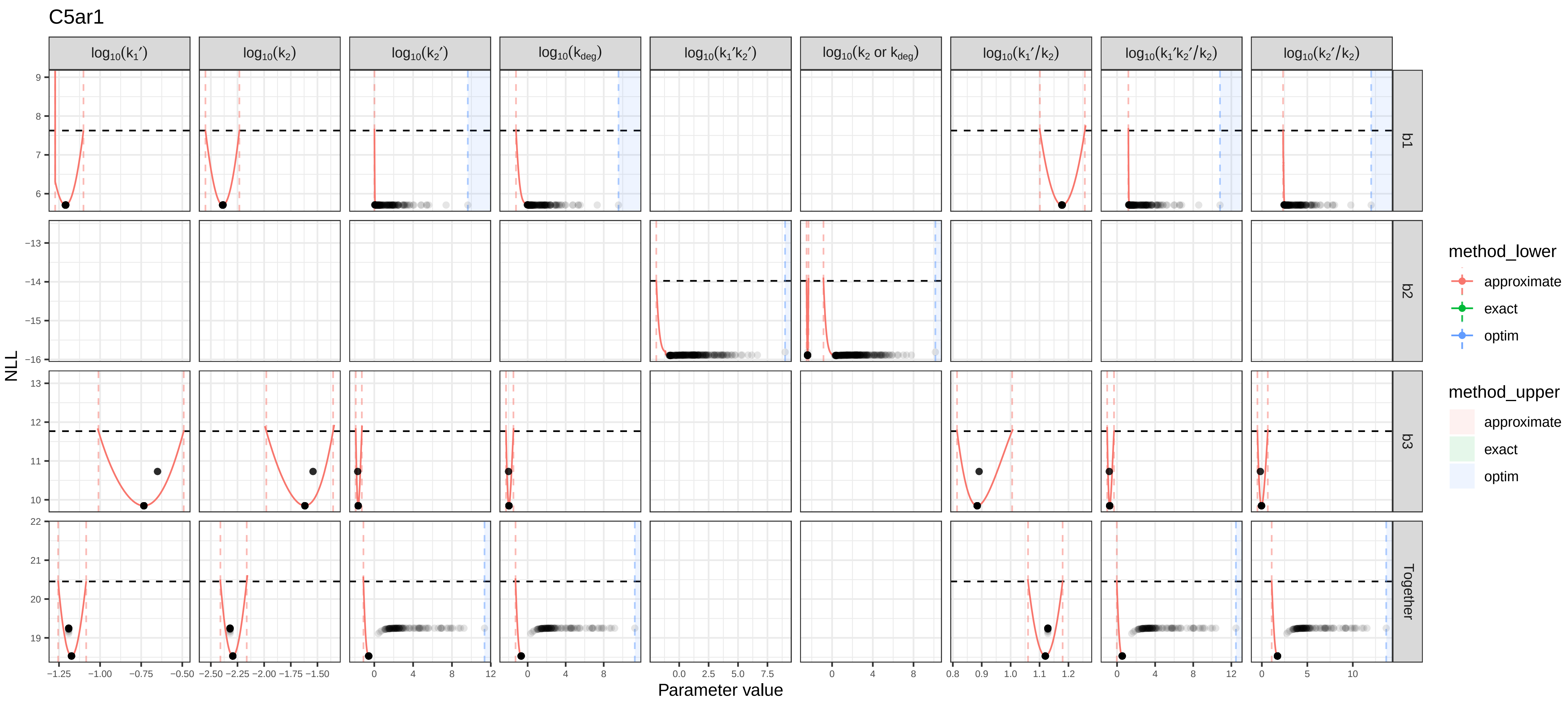

Replicate	Par	Best value	CI95 LB	CI95 UB	Method LB	Method UB
Together	$\log_{10}(k_1')$	−1.174	−1.255	−1.085	approximate	approximate
Together	$\log_{10}(k_2)$	−2.294	−2.41	−2.163	approximate	approximate
Together	$\log_{10}(k_2')$	−0.5757	−1.117	> 11.35	approximate	optim
Together	$\log_{10}(k_{deg})$	−0.6825	−1.275	> 11.27	approximate	optim
Together	$\log_{10}(k_1'/k_2)$	1.12	1.06	1.18	approximate	approximate
Together	$\log_{10}(k_1'k_2'/k_2)$	0.5444	−0.02656	> 12.48	approximate	optim
Together	$\log_{10}(k_2'/k_2)$	1.718	1.082	> 13.67	approximate	optim
b1	$\log_{10}(k_1')$	−1.211	−1.273	−1.101	approximate	approximate
b1	$\log_{10}(k_2)$	−2.389	−2.551	−2.232	approximate	approximate
b1	$\log_{10}(k_2')$	0.5705	0.0177	> 9.631	approximate	optim
b1	$\log_{10}(k_{deg})$	0.4915	−1.226	> 9.555	approximate	optim
b1	$\log_{10}(k_1'/k_2)$	1.178	1.101	1.257	approximate	approximate
b1	$\log_{10}(k_1'k_2'/k_2)$	1.748	1.187	> 10.81	approximate	optim
b1	$\log_{10}(k_2'/k_2)$	2.959	2.335	> 12.02	approximate	optim
b2	$\log_{10}(k_1'k_2')$	−0.7446	−1.945	> 8.998	approximate	optim
b2	$\log_{10}(k_2 \text{ or } k_{deg})$	0.4006	−0.8456	> 10.14	approximate	optim
b2	$\log_{10}(k_2 \text{ or } k_{deg})$	−2.41	−2.509	−2.317	approximate	approximate
b3	$\log_{10}(k_1')$	−0.7335	−1.01	−0.4911	approximate	approximate
b3	$\log_{10}(k_2)$	−1.618	−1.98	−1.353	approximate	approximate
b3	$\log_{10}(k_2')$	−1.656	−1.907	−1.282	approximate	approximate
b3	$\log_{10}(k_{deg})$	−1.969	−2.277	−1.497	approximate	approximate
b3	$\log_{10}(k_1'/k_2)$	0.8844	0.8146	1.005	approximate	approximate
b3	$\log_{10}(k_1'k_2'/k_2)$	−0.7715	−1.034	−0.319	approximate	approximate
b3	$\log_{10}(k_2'/k_2)$	−0.03802	−0.4843	0.6545	approximate	approximate

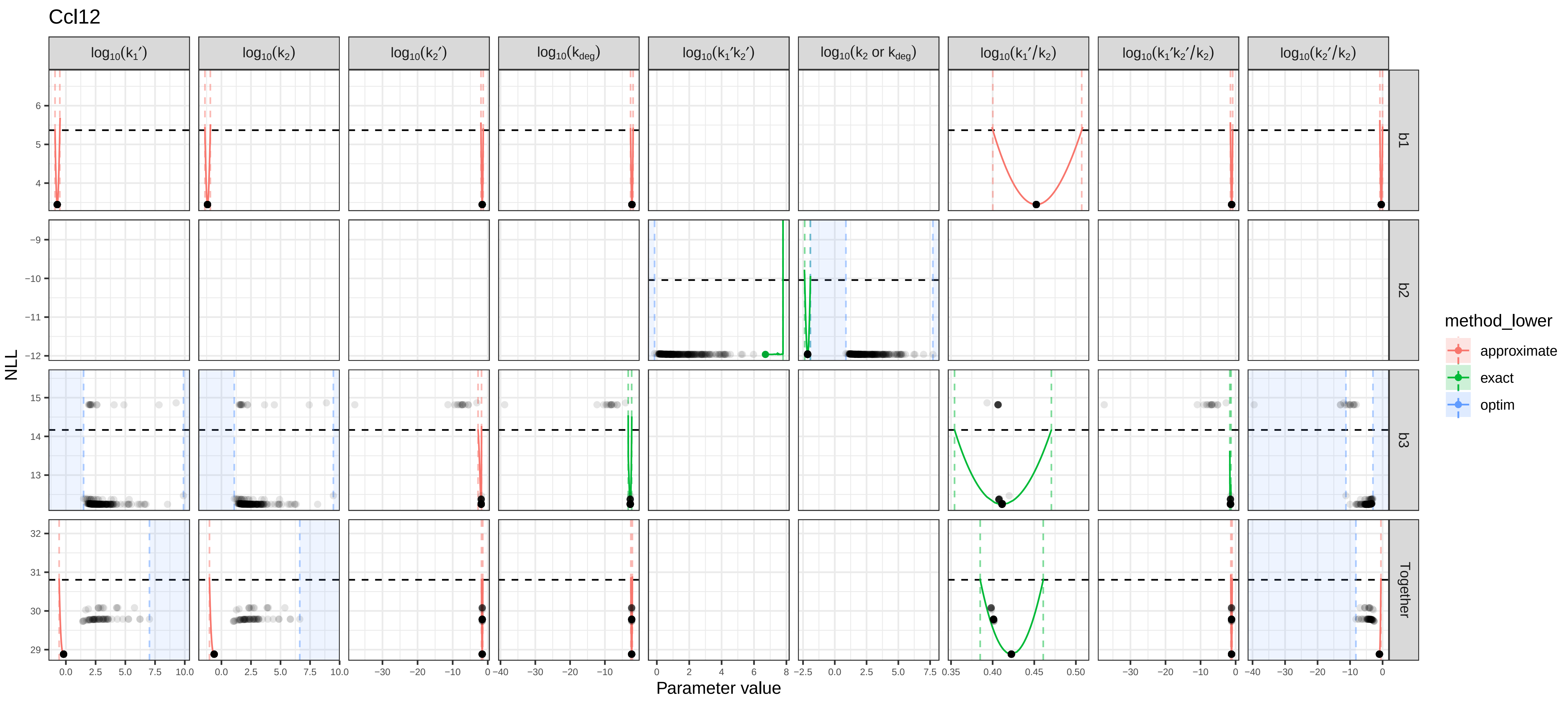

Replicate	Par	Best value	CI95 LB	CI95 UB	Method LB	Method UB
Together	$\log_{10}(k_1')$	−0.1912	−0.5669	> 7.03	approximate	optim
Together	$\log_{10}(k_2)$	−0.6136	−1.013	> 6.629	approximate	optim
Together	$\log_{10}(k_2')$	−1.593	−1.799	−1.415	approximate	approximate
Together	$\log_{10}(k_{deg})$	−2.309	−2.543	−2.1	approximate	approximate
Together	$\log_{10}(k_1'/k_2)$	0.4225	0.385	0.4608	exact	exact
Together	$\log_{10}(k_1'k_2'/k_2)$	−1.17	−1.372	−0.9923	approximate	approximate
Together	$\log_{10}(k_2'/k_2)$	−0.9792	< −8.206	−0.5186	optim	approximate
b1	$\log_{10}(k_1')$	−0.7259	−0.9132	−0.5094	approximate	approximate
b1	$\log_{10}(k_2)$	−1.178	−1.395	−0.9392	approximate	approximate
b1	$\log_{10}(k_2')$	−1.587	−1.935	−1.297	approximate	approximate
b1	$\log_{10}(k_{deg})$	−2.229	−2.623	−1.89	approximate	approximate
b1	$\log_{10}(k_1'/k_2)$	0.4524	0.4002	0.507	approximate	approximate
b1	$\log_{10}(k_1'k_2'/k_2)$	−1.134	−1.482	−0.8411	approximate	approximate
b1	$\log_{10}(k_2'/k_2)$	−0.4085	−0.8198	−0.01795	approximate	approximate
b2	$\log_{10}(k_1'k_2')$	6.704	< −0.1405	7.791	optim	exact
b2	$\log_{10}(k_2 \text{ or } k_{deg})$	7.72	0.8762	> 7.72	optim	optim
b2	$\log_{10}(k_2 \text{ or } k_{deg})$	−2.137	−2.368	−1.921	exact	exact
b3	$\log_{10}(k_1')$	8.552	< 1.492	> 9.89	optim	optim
b3	$\log_{10}(k_2)$	8.141	< 1.084	> 9.47	optim	optim
b3	$\log_{10}(k_2')$	−1.879	−2.77	−1.782	approximate	approximate
b3	$\log_{10}(k_{deg})$	−2.711	−3.294	−2.302	exact	exact
b3	$\log_{10}(k_1'/k_2)$	0.4113	0.3542	0.4706	exact	exact
b3	$\log_{10}(k_1'k_2'/k_2)$	−1.467	−1.657	−1.309	exact	exact
b3	$\log_{10}(k_2'/k_2)$	−10.02	< −11.28	> −2.964	optim	optim

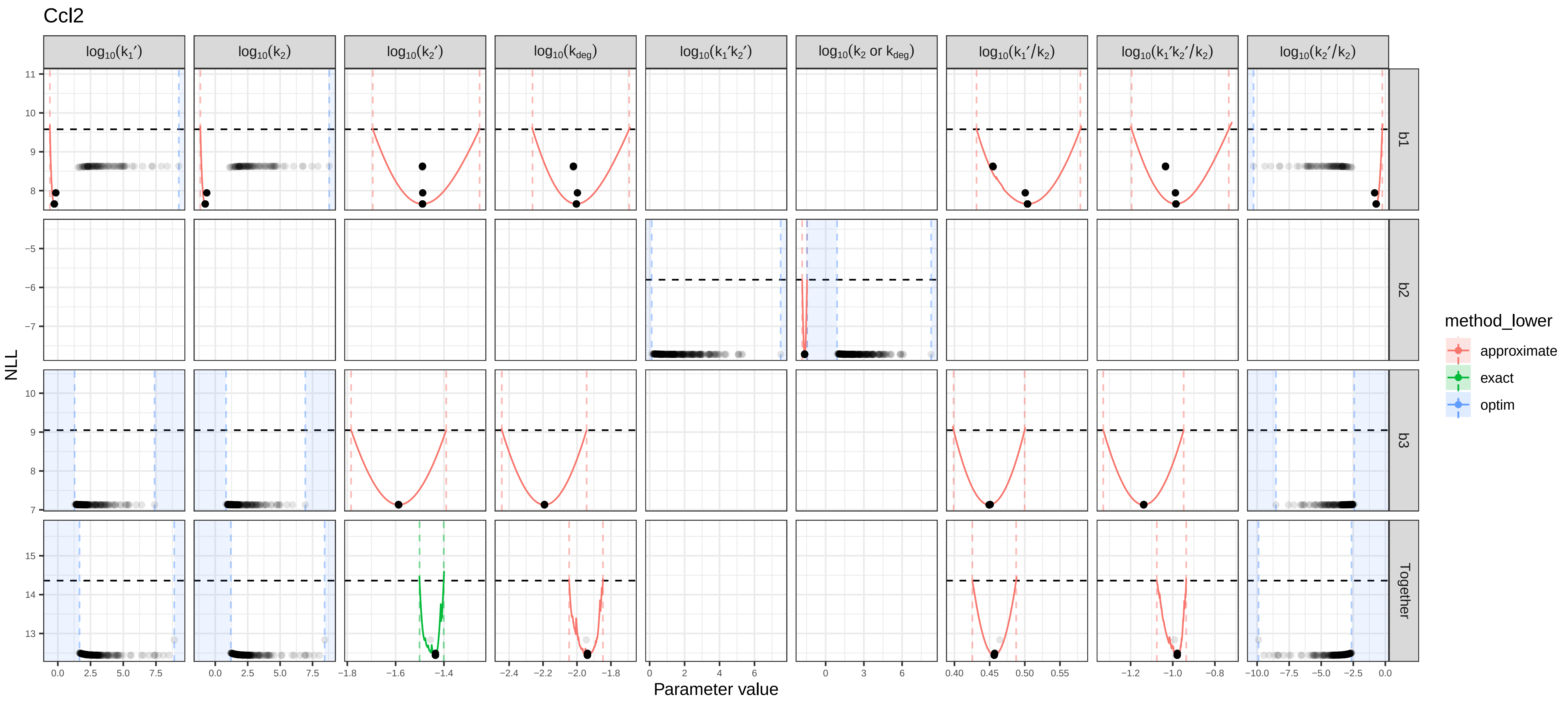

Replicate	Par	Best value	CI95 LB	CI95 UB	Method LB	Method UB
Together	$\log_{10}(k_1')$	8.489	< 1.659	> 8.895	optim	optim
Together	$\log_{10}(k_2)$	8.032	< 1.202	> 8.431	optim	optim
Together	$\log_{10}(k_2')$	−1.435	−1.501	−1.401	exact	exact
Together	$\log_{10}(k_{deg})$	−1.938	−2.046	−1.847	approximate	approximate
Together	$\log_{10}(k_1'/k_2)$	0.4565	0.4255	0.4877	approximate	approximate
Together	$\log_{10}(k_1'k_2'/k_2)$	−0.9788	−1.076	−0.9361	approximate	approximate
Together	$\log_{10}(k_2'/k_2)$	−9.468	< −9.886	> −2.637	optim	optim
b1	$\log_{10}(k_1')$	−0.2697	−0.6045	> 9.243	approximate	optim
b1	$\log_{10}(k_2)$	−0.7736	−1.147	> 8.788	approximate	optim
b1	$\log_{10}(k_2')$	−1.489	−1.695	−1.252	approximate	approximate
b1	$\log_{10}(k_{deg})$	−2.004	−2.262	−1.692	approximate	approximate
b1	$\log_{10}(k_1'/k_2)$	0.5039	0.4314	0.5789	approximate	approximate
b1	$\log_{10}(k_1'k_2'/k_2)$	−0.9848	−1.195	−0.7341	approximate	approximate
b1	$\log_{10}(k_2'/k_2)$	−0.7151	< −10.28	−0.2351	optim	approximate
b2	$\log_{10}(k_1'k_2')$	4.059	< 0.118	> 7.498	optim	optim
b2	$\log_{10}(k_2 \text{ or } k_{deg})$	4.841	0.8974	> 8.279	optim	optim
b2	$\log_{10}(k_2 \text{ or } k_{deg})$	−1.65	−1.844	−1.458	approximate	approximate
b3	$\log_{10}(k_1')$	4.808	< 1.284	> 7.391	optim	optim
b3	$\log_{10}(k_2)$	4.359	< 0.8326	> 6.942	optim	optim
b3	$\log_{10}(k_2')$	−1.588	−1.784	−1.391	approximate	approximate
b3	$\log_{10}(k_{deg})$	−2.192	−2.443	−1.943	approximate	approximate
b3	$\log_{10}(k_1'/k_2)$	0.4493	0.3988	0.4998	approximate	approximate
b3	$\log_{10}(k_1'k_2'/k_2)$	−1.138	−1.33	−0.948	approximate	approximate
b3	$\log_{10}(k_2'/k_2)$	−5.946	< −8.53	> −2.421	optim	optim

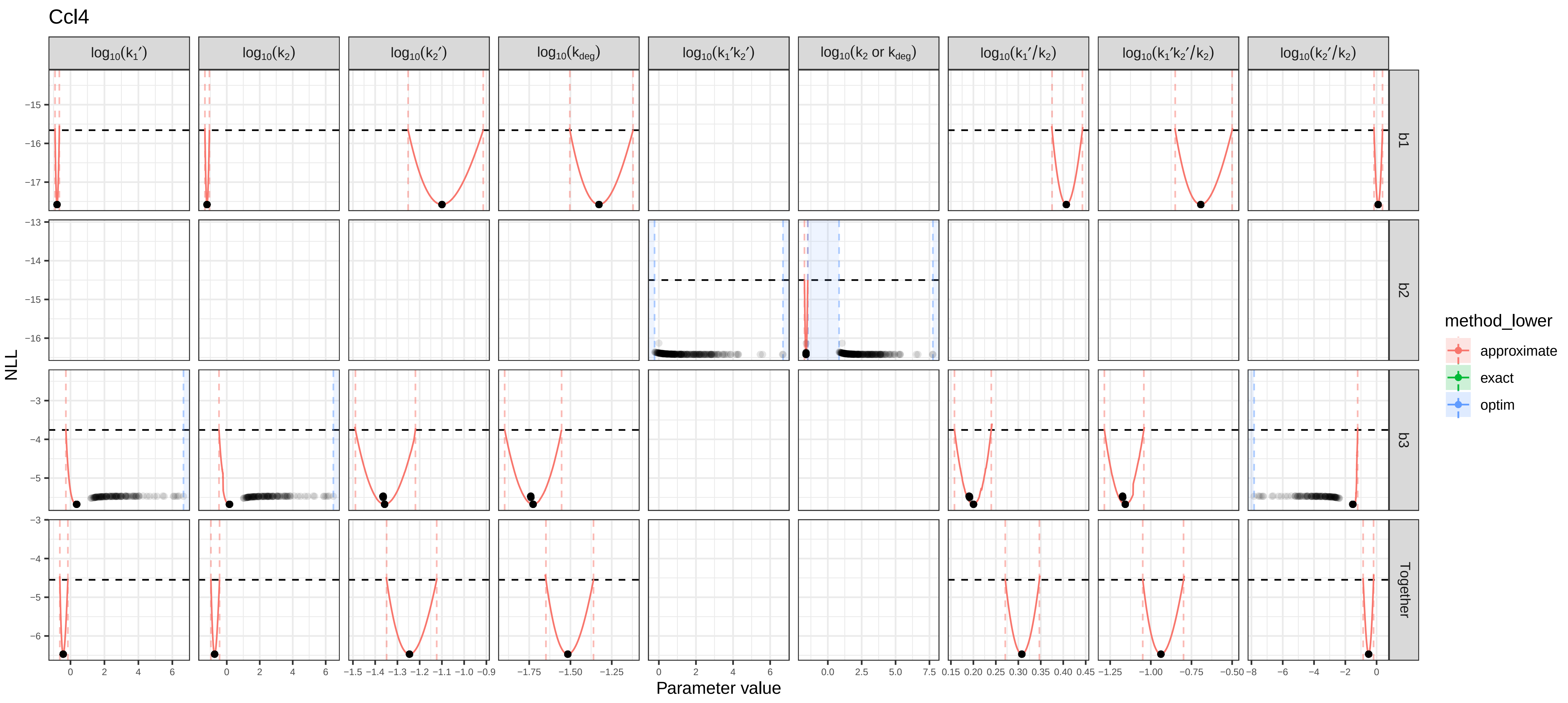

Replicate	Par	Best value	CI95 LB	CI95 UB	Method LB	Method UB
Together	$\log_{10}(k_1')$	−0.4299	−0.6277	−0.1534	approximate	approximate
Together	$\log_{10}(k_2)$	−0.7378	−0.9624	−0.4377	approximate	approximate
Together	$\log_{10}(k_2')$	−1.246	−1.349	−1.123	approximate	approximate
Together	$\log_{10}(k_{deg})$	−1.516	−1.649	−1.36	approximate	approximate
Together	$\log_{10}(k_1'/k_2)$	0.3079	0.271	0.3469	approximate	approximate
Together	$\log_{10}(k_1'k_2'/k_2)$	−0.9379	−1.05	−0.7988	approximate	approximate
Together	$\log_{10}(k_2'/k_2)$	−0.508	−0.8605	−0.1939	approximate	approximate
b1	$\log_{10}(k_1')$	−0.7929	−0.9109	−0.6569	approximate	approximate
b1	$\log_{10}(k_2)$	−1.2	−1.326	−1.053	approximate	approximate
b1	$\log_{10}(k_2')$	−1.1	−1.252	−0.9142	approximate	approximate
b1	$\log_{10}(k_{deg})$	−1.327	−1.504	−1.121	approximate	approximate
b1	$\log_{10}(k_1'/k_2)$	0.4068	0.3751	0.4427	approximate	approximate
b1	$\log_{10}(k_1'k_2'/k_2)$	−0.6928	−0.85	−0.5	approximate	approximate
b1	$\log_{10}(k_2'/k_2)$	0.1001	−0.163	0.3785	approximate	approximate
b2	$\log_{10}(k_1'k_2')$	3.182	< −0.2315	> 6.693	optim	optim
b2	$\log_{10}(k_2 \text{ or } k_{deg})$	4.245	0.8291	> 7.756	optim	optim
b2	$\log_{10}(k_2 \text{ or } k_{deg})$	−1.607	−1.725	−1.481	approximate	approximate
b3	$\log_{10}(k_1')$	0.3622	−0.2715	> 6.656	approximate	optim
b3	$\log_{10}(k_2)$	0.162	−0.4719	> 6.465	approximate	optim
b3	$\log_{10}(k_2')$	−1.357	−1.489	−1.219	approximate	approximate
b3	$\log_{10}(k_{deg})$	−1.726	−1.898	−1.554	approximate	approximate
b3	$\log_{10}(k_1'/k_2)$	0.2003	0.1579	0.2398	approximate	approximate
b3	$\log_{10}(k_1'k_2'/k_2)$	−1.157	−1.286	−1.043	approximate	approximate
b3	$\log_{10}(k_2'/k_2)$	−1.519	< −7.83	−1.211	optim	approximate

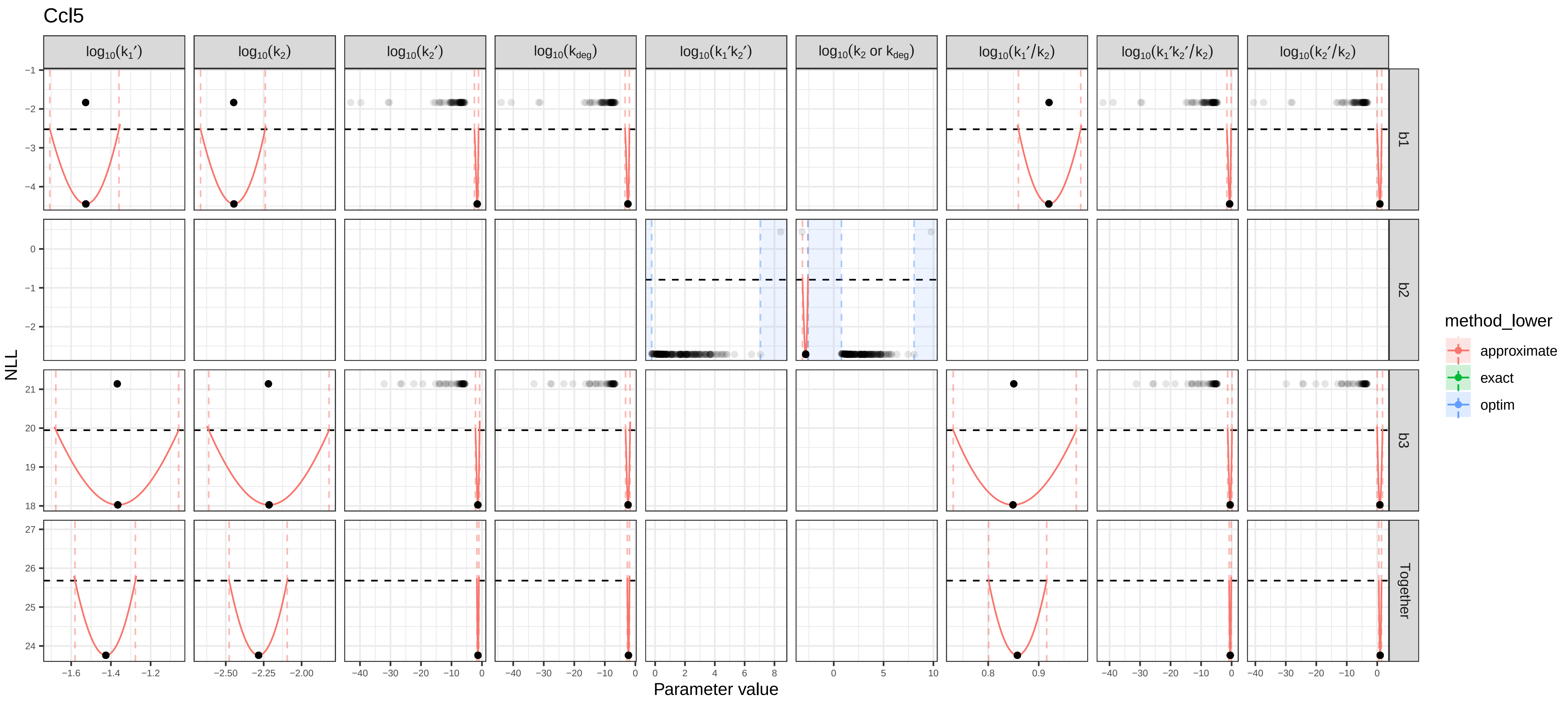

Replicate	Par	Best value	CI95 LB	CI95 UB	Method LB	Method UB
Together	$\log_{10}(k_1')$	−1.426	−1.581	−1.277	approximate	approximate
Together	$\log_{10}(k_2)$	−2.283	−2.478	−2.095	approximate	approximate
Together	$\log_{10}(k_2')$	−1.311	−1.659	−1	approximate	approximate
Together	$\log_{10}(k_{deg})$	−2.269	−2.644	−1.921	approximate	approximate
Together	$\log_{10}(k_1'/k_2)$	0.8578	0.8008	0.9158	approximate	approximate
Together	$\log_{10}(k_1'k_2'/k_2)$	−0.4533	−0.8155	−0.1165	approximate	approximate
Together	$\log_{10}(k_2'/k_2)$	0.9723	0.5299	1.424	approximate	approximate
b1	$\log_{10}(k_1')$	−1.526	−1.707	−1.359	approximate	approximate
b1	$\log_{10}(k_2)$	−2.446	−2.666	−2.24	approximate	approximate
b1	$\log_{10}(k_2')$	−1.563	−2.484	−1.116	approximate	approximate
b1	$\log_{10}(k_{deg})$	−2.435	−3.377	−1.954	approximate	approximate
b1	$\log_{10}(k_1'/k_2)$	0.9199	0.8598	0.9831	approximate	approximate
b1	$\log_{10}(k_1'k_2'/k_2)$	−0.6433	−1.566	−0.1743	approximate	approximate
b1	$\log_{10}(k_2'/k_2)$	0.8829	−0.05747	1.471	approximate	approximate
b2	$\log_{10}(k_1'k_2')$	3.9	< −0.2327	> 7.059	optim	optim
b2	$\log_{10}(k_2 \text{ or } k_{deg})$	4.909	0.7761	> 8.069	optim	optim
b2	$\log_{10}(k_2 \text{ or } k_{deg})$	−2.815	−3.134	−2.585	approximate	approximate
b3	$\log_{10}(k_1')$	−1.365	−1.677	−1.059	approximate	approximate
b3	$\log_{10}(k_2)$	−2.214	−2.612	−1.819	approximate	approximate
b3	$\log_{10}(k_2')$	−1.343	−2.129	−0.7732	approximate	approximate
b3	$\log_{10}(k_{deg})$	−2.383	−3.216	−1.737	approximate	approximate
b3	$\log_{10}(k_1'/k_2)$	0.849	0.7307	0.9744	approximate	approximate
b3	$\log_{10}(k_1'k_2'/k_2)$	−0.4941	−1.294	0.1368	approximate	approximate
b3	$\log_{10}(k_2'/k_2)$	0.8714	−0.02381	1.746	approximate	approximate

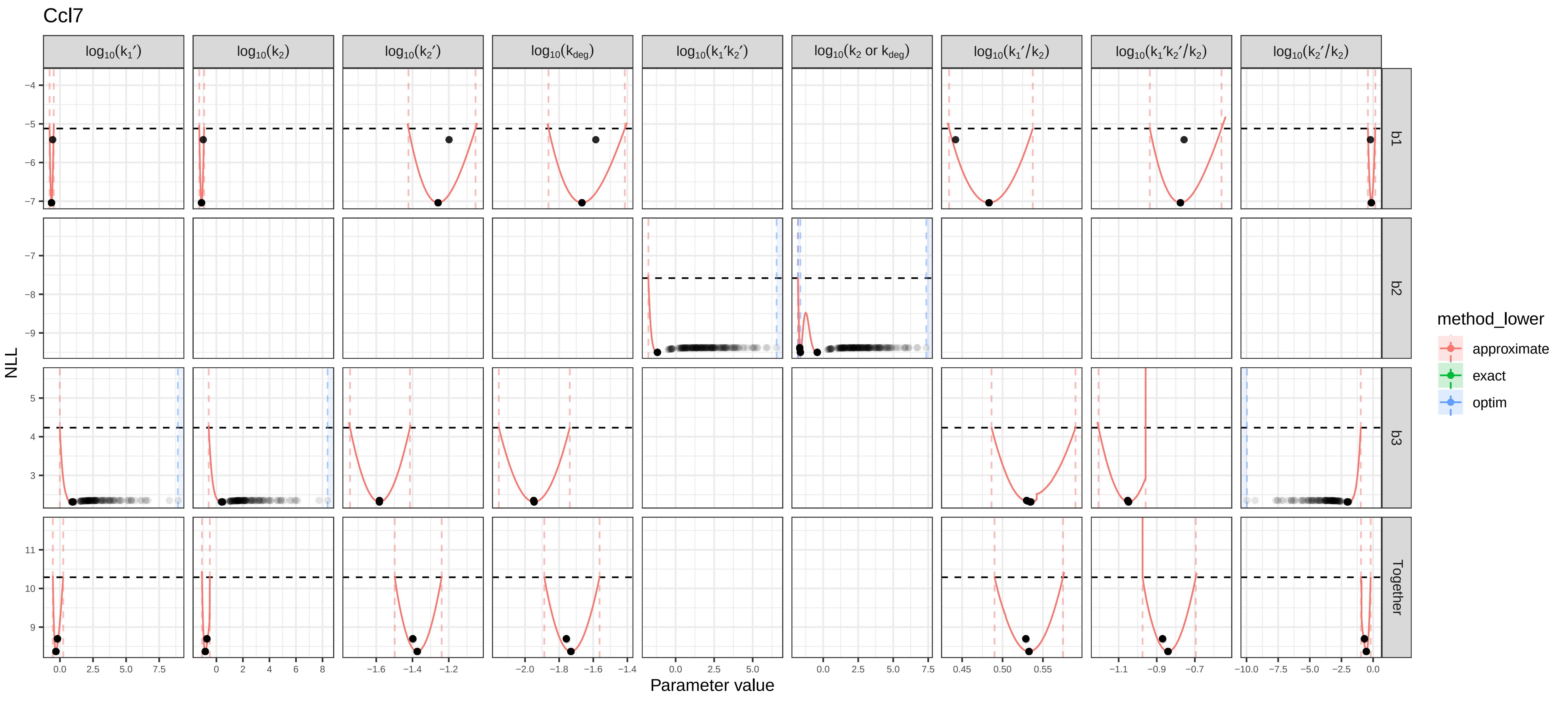

Replicate	Par	Best value	CI95 LB	CI95 UB	Method LB	Method UB
Together	$\log_{10}(k_1')$	−0.3049	−0.5344	0.2478	approximate	approximate
Together	$\log_{10}(k_2)$	−0.8377	−1.088	−0.4938	approximate	approximate
Together	$\log_{10}(k_2')$	−1.374	−1.498	−1.239	approximate	approximate
Together	$\log_{10}(k_{deg})$	−1.731	−1.887	−1.563	approximate	approximate
Together	$\log_{10}(k_1'/k_2)$	0.5328	0.4901	0.5748	approximate	approximate
Together	$\log_{10}(k_1'k_2'/k_2)$	−0.8408	−0.9745	−0.6948	approximate	approximate
Together	$\log_{10}(k_2'/k_2)$	−0.5359	−0.9563	−0.1917	approximate	approximate
b1	$\log_{10}(k_1')$	−0.6366	−0.7815	−0.4679	approximate	approximate
b1	$\log_{10}(k_2)$	−1.12	−1.29	−0.9246	approximate	approximate
b1	$\log_{10}(k_2')$	−1.259	−1.422	−1.052	approximate	approximate
b1	$\log_{10}(k_{deg})$	−1.666	−1.862	−1.415	approximate	approximate
b1	$\log_{10}(k_1'/k_2)$	0.4833	0.4338	0.5375	approximate	approximate
b1	$\log_{10}(k_1'k_2'/k_2)$	−0.7758	−0.9363	−0.5602	approximate	approximate
b1	$\log_{10}(k_2'/k_2)$	−0.1391	−0.4112	0.1741	approximate	approximate
b2	$\log_{10}(k_1'k_2')$	−1.184	−1.77	> 6.552	approximate	optim
b2	$\log_{10}(k_2 \text{ or } k_{deg})$	−0.4243	−1.803	> 7.363	approximate	optim
b2	$\log_{10}(k_2 \text{ or } k_{deg})$	−1.629	−1.803	−1.627	approximate	optim
b3	$\log_{10}(k_1')$	0.968	−0.008422	> 8.909	approximate	optim
b3	$\log_{10}(k_2)$	0.4334	−0.5783	> 8.379	approximate	optim
b3	$\log_{10}(k_2')$	−1.583	−1.745	−1.414	approximate	approximate
b3	$\log_{10}(k_{deg})$	−1.947	−2.154	−1.738	approximate	approximate
b3	$\log_{10}(k_1'/k_2)$	0.5346	0.4864	0.5903	approximate	approximate
b3	$\log_{10}(k_1'k_2'/k_2)$	−1.048	−1.206	−0.9584	approximate	approximate
b3	$\log_{10}(k_2'/k_2)$	−2.016	< −9.962	−0.9727	optim	approximate

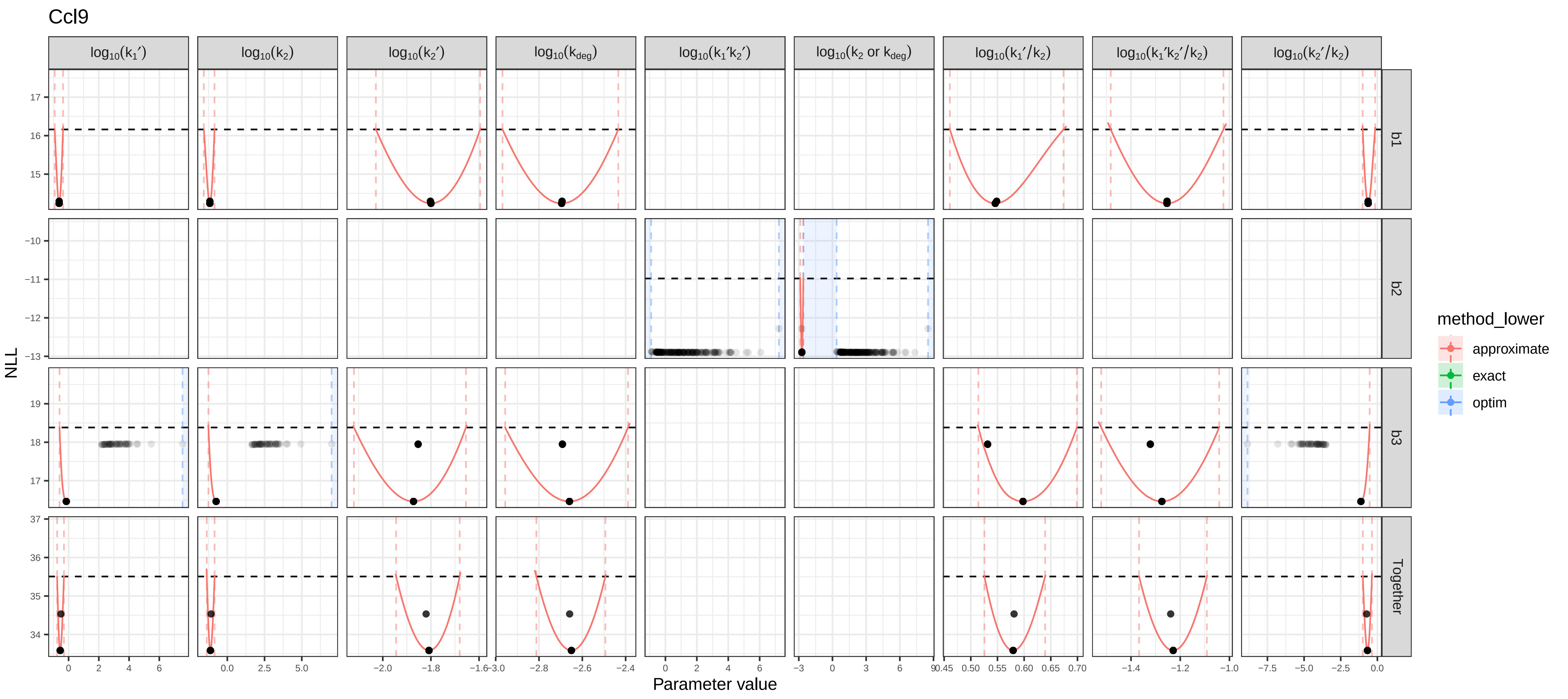

Replicate	Par	Best value	CI95 LB	CI95 UB	Method LB	Method UB
Together	$\log_{10}(k_1')$	−0.5535	−0.7576	−0.307	approximate	approximate
Together	$\log_{10}(k_2)$	−1.133	−1.377	−0.8529	approximate	approximate
Together	$\log_{10}(k_2')$	−1.808	−1.945	−1.68	approximate	approximate
Together	$\log_{10}(k_{deg})$	−2.65	−2.812	−2.494	approximate	approximate
Together	$\log_{10}(k_1'/k_2)$	0.5794	0.5255	0.6394	approximate	approximate
Together	$\log_{10}(k_1'k_2'/k_2)$	−1.229	−1.367	−1.092	approximate	approximate
Together	$\log_{10}(k_2'/k_2)$	−0.6751	−0.9939	−0.3715	approximate	approximate
b1	$\log_{10}(k_1')$	−0.6268	−0.916	−0.3662	approximate	approximate
b1	$\log_{10}(k_2)$	−1.173	−1.576	−0.8577	approximate	approximate
b1	$\log_{10}(k_2')$	−1.8	−2.029	−1.595	approximate	approximate
b1	$\log_{10}(k_{deg})$	−2.695	−2.969	−2.434	approximate	approximate
b1	$\log_{10}(k_1'/k_2)$	0.5459	0.4608	0.6739	approximate	approximate
b1	$\log_{10}(k_1'k_2'/k_2)$	−1.254	−1.482	−1.024	approximate	approximate
b1	$\log_{10}(k_2'/k_2)$	−0.6271	−1.002	−0.1513	approximate	approximate
b2	$\log_{10}(k_1'k_2')$	2.578	< −0.9091	> 7.227	optim	optim
b2	$\log_{10}(k_2 \text{ or } k_{deg})$	3.861	0.3715	> 8.513	optim	optim
b2	$\log_{10}(k_2 \text{ or } k_{deg})$	−2.724	−2.869	−2.595	approximate	approximate
b3	$\log_{10}(k_1')$	−0.1523	−0.5994	> 7.534	approximate	optim
b3	$\log_{10}(k_2)$	−0.7502	−1.269	> 7.002	approximate	optim
b3	$\log_{10}(k_2')$	−1.872	−2.12	−1.655	approximate	approximate
b3	$\log_{10}(k_{deg})$	−2.659	−2.956	−2.389	approximate	approximate
b3	$\log_{10}(k_1'/k_2)$	0.5978	0.5142	0.699	approximate	approximate
b3	$\log_{10}(k_1'k_2'/k_2)$	−1.274	−1.521	−1.042	approximate	approximate
b3	$\log_{10}(k_2'/k_2)$	−1.122	< −8.855	−0.5248	optim	approximate

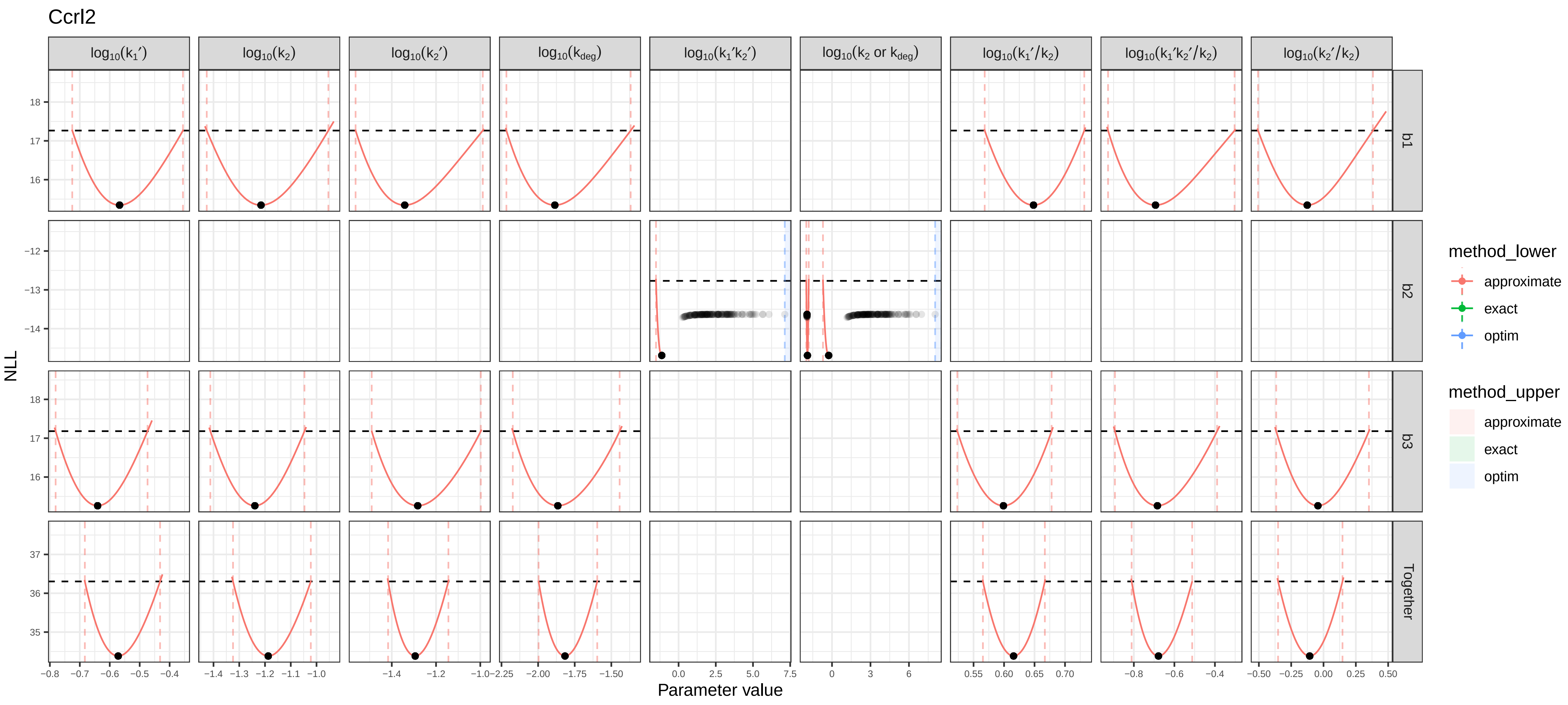

Replicate	Par	Best value	CI95 LB	CI95 UB	Method LB	Method UB
Together	$\log_{10}(k_1')$	−0.5717	−0.6832	−0.4319	approximate	approximate
Together	$\log_{10}(k_2)$	−1.187	−1.324	−1.022	approximate	approximate
Together	$\log_{10}(k_2')$	−1.294	−1.417	−1.144	approximate	approximate
Together	$\log_{10}(k_{deg})$	−1.817	−1.997	−1.596	approximate	approximate
Together	$\log_{10}(k_1'/k_2)$	0.6157	0.5654	0.6669	approximate	approximate
Together	$\log_{10}(k_1'k_2'/k_2)$	−0.6788	−0.811	−0.5121	approximate	approximate
Together	$\log_{10}(k_2'/k_2)$	−0.1071	−0.3515	0.1469	approximate	approximate
b1	$\log_{10}(k_1')$	−0.5672	−0.7252	−0.3555	approximate	approximate
b1	$\log_{10}(k_2)$	−1.215	−1.426	−0.9539	approximate	approximate
b1	$\log_{10}(k_2')$	−1.342	−1.564	−0.9887	approximate	approximate
b1	$\log_{10}(k_{deg})$	−1.886	−2.217	−1.368	approximate	approximate
b1	$\log_{10}(k_1'/k_2)$	0.6483	0.5683	0.7316	approximate	approximate
b1	$\log_{10}(k_1'k_2'/k_2)$	−0.6932	−0.9272	−0.3031	approximate	approximate
b1	$\log_{10}(k_2'/k_2)$	−0.1261	−0.5064	0.3826	approximate	approximate
b2	$\log_{10}(k_1'k_2')$	−1.129	−1.518	> 7.137	approximate	optim
b2	$\log_{10}(k_2 \text{ or } k_{deg})$	−0.2606	−0.7044	> 8.04	approximate	optim
b2	$\log_{10}(k_2 \text{ or } k_{deg})$	−1.916	−2.006	−1.815	approximate	approximate
b3	$\log_{10}(k_1')$	−0.6404	−0.7808	−0.4737	approximate	approximate
b3	$\log_{10}(k_2)$	−1.239	−1.412	−1.047	approximate	approximate
b3	$\log_{10}(k_2')$	−1.283	−1.491	−0.9976	approximate	approximate
b3	$\log_{10}(k_{deg})$	−1.865	−2.173	−1.443	approximate	approximate
b3	$\log_{10}(k_1'/k_2)$	0.599	0.5234	0.678	approximate	approximate
b3	$\log_{10}(k_1'k_2'/k_2)$	−0.6837	−0.8933	−0.3892	approximate	approximate
b3	$\log_{10}(k_2'/k_2)$	−0.04331	−0.366	0.3513	approximate	approximate

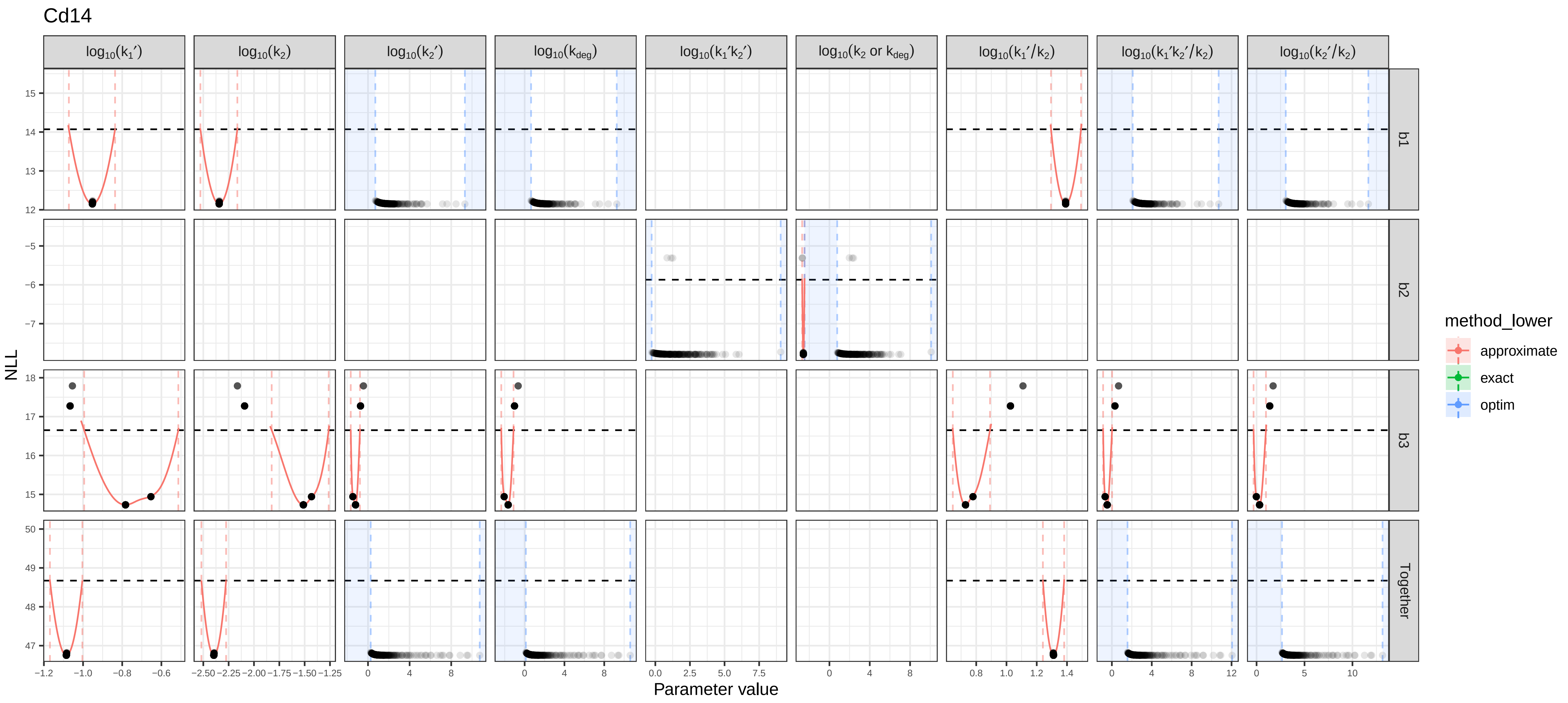

Replicate	Par	**Best value**	**CI95 LB**	**CI95 UB**	**Method LB**	**Method UB**
Together	$log_{10}(k_1')$	−1.085	−1.169	−1.004	approximate	approximate
Together	$log_{10}(k_2)$	−2.396	−2.519	−2.275	approximate	approximate
Together	$log_{10}(k_2')$	6.069	< 0.2611	> 10.74	optim	optim
Together	$log_{10}(k_{deg})$	5.938	< 0.1244	> 10.61	optim	optim
Together	$log_{10}(k_1'/k_2)$	1.311	1.241	1.381	approximate	approximate
Together	$log_{10}(k_1'k_2'/k_2)$	7.38	< 1.57	> 12.05	optim	optim
Together	$log_{10}(k_2'/k_2)$	8.465	< 2.654	> 13.14	optim	optim
b1	$log_{10}(k_1')$	−0.9524	−1.072	−0.8365	approximate	approximate
b1	$log_{10}(k_2)$	−2.344	−2.529	−2.164	approximate	approximate
b1	$log_{10}(k_2')$	4.31	< 0.7042	> 9.315	optim	optim
b1	$log_{10}(k_{deg})$	4.254	< 0.6459	> 9.259	optim	optim
b1	$log_{10}(k_1'/k_2)$	1.391	1.294	1.493	approximate	approximate
b1	$log_{10}(k_1'k_2'/k_2)$	5.701	< 2.094	> 10.71	optim	optim
b1	$log_{10}(k_2'/k_2)$	6.654	< 3.045	> 11.66	optim	optim
b2	$log_{10}(k_1'k_2')$	3.735	< −0.2638	> 9.056	optim	optim
b2	$log_{10}(k_2 \text{ or } k_{deg})$	4.77	0.7702	> 10.09	optim	optim
b2	$log_{10}(k_2 \text{ or } k_{deg})$	−2.589	−2.707	−2.47	approximate	approximate
b3	$log_{10}(k_1')$	−0.7832	−0.9943	−0.5135	approximate	approximate
b3	$log_{10}(k_2)$	−1.512	−1.825	−1.263	approximate	approximate
b3	$log_{10}(k_2')$	−1.198	−1.659	−0.773	approximate	approximate
b3	$log_{10}(k_{deg})$	−1.656	−2.351	−1.116	approximate	approximate
b3	$log_{10}(k_1'/k_2)$	0.7293	0.6453	0.8913	approximate	approximate
b3	$log_{10}(k_1'k_2'/k_2)$	−0.4691	−0.878	0.01979	approximate	approximate
b3	$log_{10}(k_2'/k_2)$	0.3141	−0.3116	0.9794	approximate	approximate

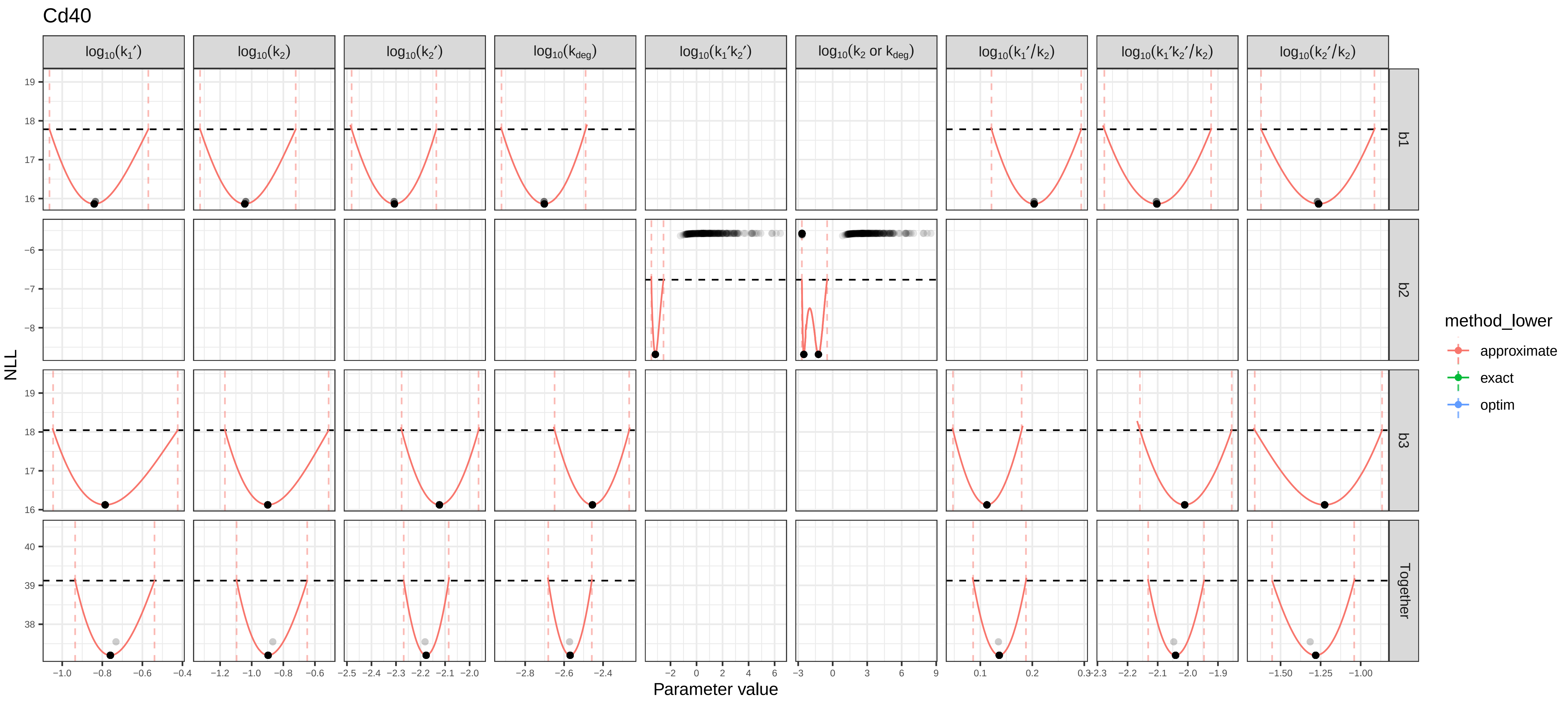

Replicate	Par	Best value	CI95 LB	CI95 UB	Method LB	Method UB
Together	$\log_{10}(k_1')$	−0.7597	−0.9355	−0.5396	approximate	approximate
Together	$\log_{10}(k_2)$	−0.896	−1.096	−0.6488	approximate	approximate
Together	$\log_{10}(k_2')$	−2.177	−2.269	−2.085	approximate	approximate
Together	$\log_{10}(k_{deg})$	−2.569	−2.681	−2.458	approximate	approximate
Together	$\log_{10}(k_1'/k_2)$	0.1364	0.08621	0.1878	approximate	approximate
Together	$\log_{10}(k_1'k_2'/k_2)$	−2.041	−2.132	−1.946	approximate	approximate
Together	$\log_{10}(k_2'/k_2)$	−1.281	−1.553	−1.04	approximate	approximate
b1	$\log_{10}(k_1')$	−0.8403	−1.064	−0.5704	approximate	approximate
b1	$\log_{10}(k_2)$	−1.044	−1.327	−0.7215	approximate	approximate
b1	$\log_{10}(k_2')$	−2.306	−2.481	−2.136	approximate	approximate
b1	$\log_{10}(k_{deg})$	−2.702	−2.921	−2.49	approximate	approximate
b1	$\log_{10}(k_1'/k_2)$	0.2037	0.1215	0.2941	approximate	approximate
b1	$\log_{10}(k_1'k_2'/k_2)$	−2.103	−2.277	−1.923	approximate	approximate
b1	$\log_{10}(k_2'/k_2)$	−1.262	−1.622	−0.9141	approximate	approximate
b2	$\log_{10}(k_1'k_2')$	−3.162	−3.469	−2.537	approximate	approximate
b2	$\log_{10}(k_2 \text{ or } k_{deg})$	−1.238	−2.681	−0.4821	approximate	approximate
b2	$\log_{10}(k_2 \text{ or } k_{deg})$	−2.508	−2.681	−0.4821	approximate	approximate
b3	$\log_{10}(k_1')$	−0.7858	−1.045	−0.4236	approximate	approximate
b3	$\log_{10}(k_2)$	−0.8985	−1.169	−0.5133	approximate	approximate
b3	$\log_{10}(k_2')$	−2.123	−2.277	−1.964	approximate	approximate
b3	$\log_{10}(k_{deg})$	−2.455	−2.649	−2.266	approximate	approximate
b3	$\log_{10}(k_1'/k_2)$	0.1127	0.04743	0.1795	approximate	approximate
b3	$\log_{10}(k_1'k_2'/k_2)$	−2.01	−2.159	−1.854	approximate	approximate
b3	$\log_{10}(k_2'/k_2)$	−1.225	−1.661	−0.8664	approximate	approximate

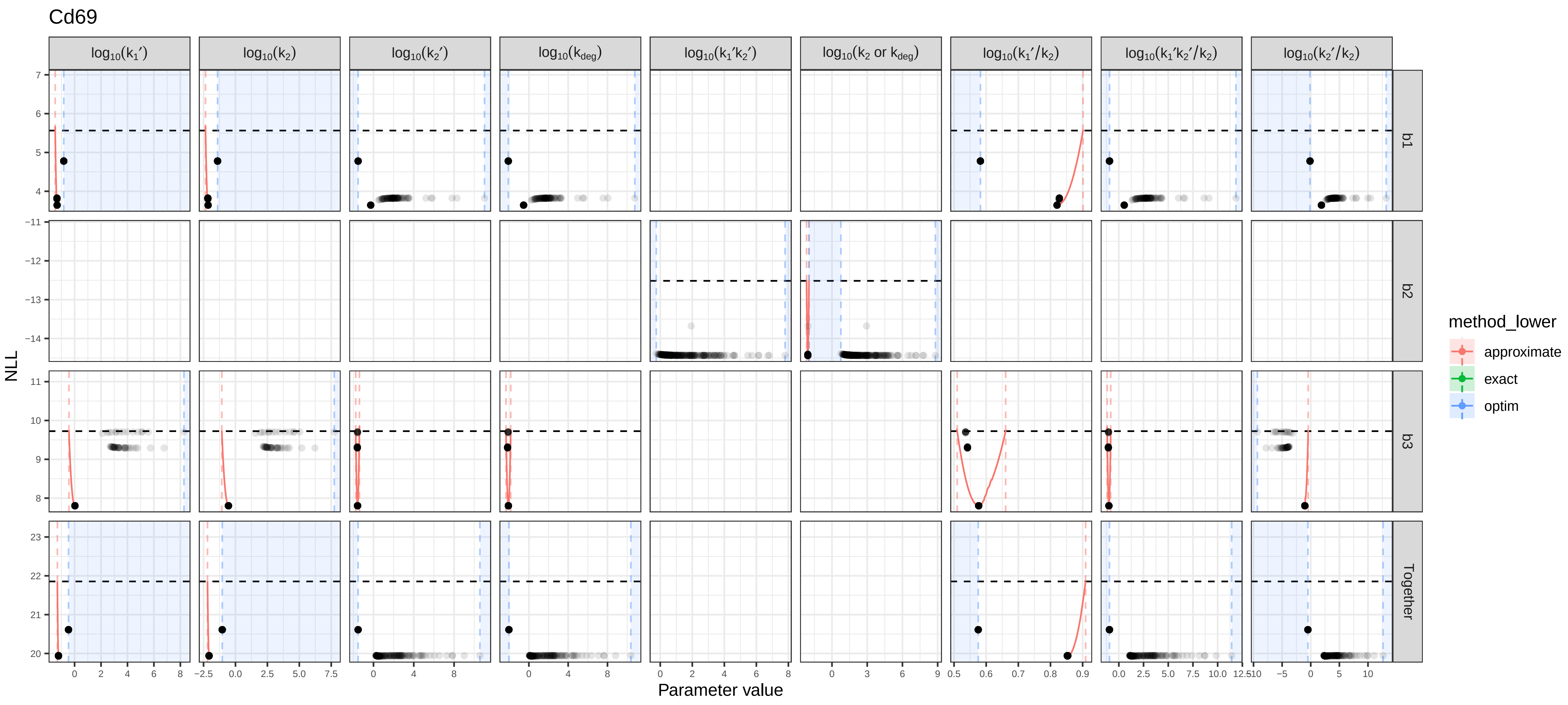

Replicate	Par	Best value	CI95 LB	CI95 UB	Method LB	Method UB
Together	$\log_{10}(k_1')$	−1.216	−1.303	> −0.4586	approximate	optim
Together	$\log_{10}(k_2)$	−2.069	−2.19	> −1.034	approximate	optim
Together	$\log_{10}(k_2')$	0.619	< −1.527	> 10.56	optim	optim
Together	$\log_{10}(k_{deg})$	0.4336	< −2.034	> 10.38	optim	optim
Together	$\log_{10}(k_1'/k_2)$	0.8532	< 0.5752	0.9093	optim	approximate
Together	$\log_{10}(k_1'k_2'/k_2)$	1.472	< −0.9521	> 11.42	optim	optim
Together	$\log_{10}(k_2'/k_2)$	2.688	< −0.4935	> 12.64	optim	optim
b1	$\log_{10}(k_1')$	−1.321	−1.466	> −0.8191	approximate	optim
b1	$\log_{10}(k_2)$	−2.142	−2.345	> −1.401	approximate	optim
b1	$\log_{10}(k_2')$	−0.2781	< −1.527	> 11.02	optim	optim
b1	$\log_{10}(k_{deg})$	−0.5393	< −2.095	> 10.78	optim	optim
b1	$\log_{10}(k_1'/k_2)$	0.8204	< 0.5821	0.9013	optim	approximate
b1	$\log_{10}(k_1'k_2'/k_2)$	0.5422	< −0.9449	> 11.85	optim	optim
b1	$\log_{10}(k_2'/k_2)$	1.863	< −0.1258	> 13.18	optim	optim
b2	$\log_{10}(k_1'k_2')$	3.101	< −0.2486	> 7.794	optim	optim
b2	$\log_{10}(k_2 \text{ or } k_{deg})$	4.111	0.7602	> 8.804	optim	optim
b2	$\log_{10}(k_2 \text{ or } k_{deg})$	−2.059	−2.165	−1.959	approximate	approximate
b3	$\log_{10}(k_1')$	0.02681	−0.4263	> 8.272	approximate	optim
b3	$\log_{10}(k_2)$	−0.5499	−1.069	> 7.736	approximate	optim
b3	$\log_{10}(k_2')$	−1.582	−1.763	−1.397	approximate	approximate
b3	$\log_{10}(k_{deg})$	−2.087	−2.327	−1.846	approximate	approximate
b3	$\log_{10}(k_1'/k_2)$	0.5767	0.5098	0.6606	approximate	approximate
b3	$\log_{10}(k_1'k_2'/k_2)$	−1.005	−1.18	−0.8246	approximate	approximate
b3	$\log_{10}(k_2'/k_2)$	−1.032	< −9.318	−0.4475	optim	approximate

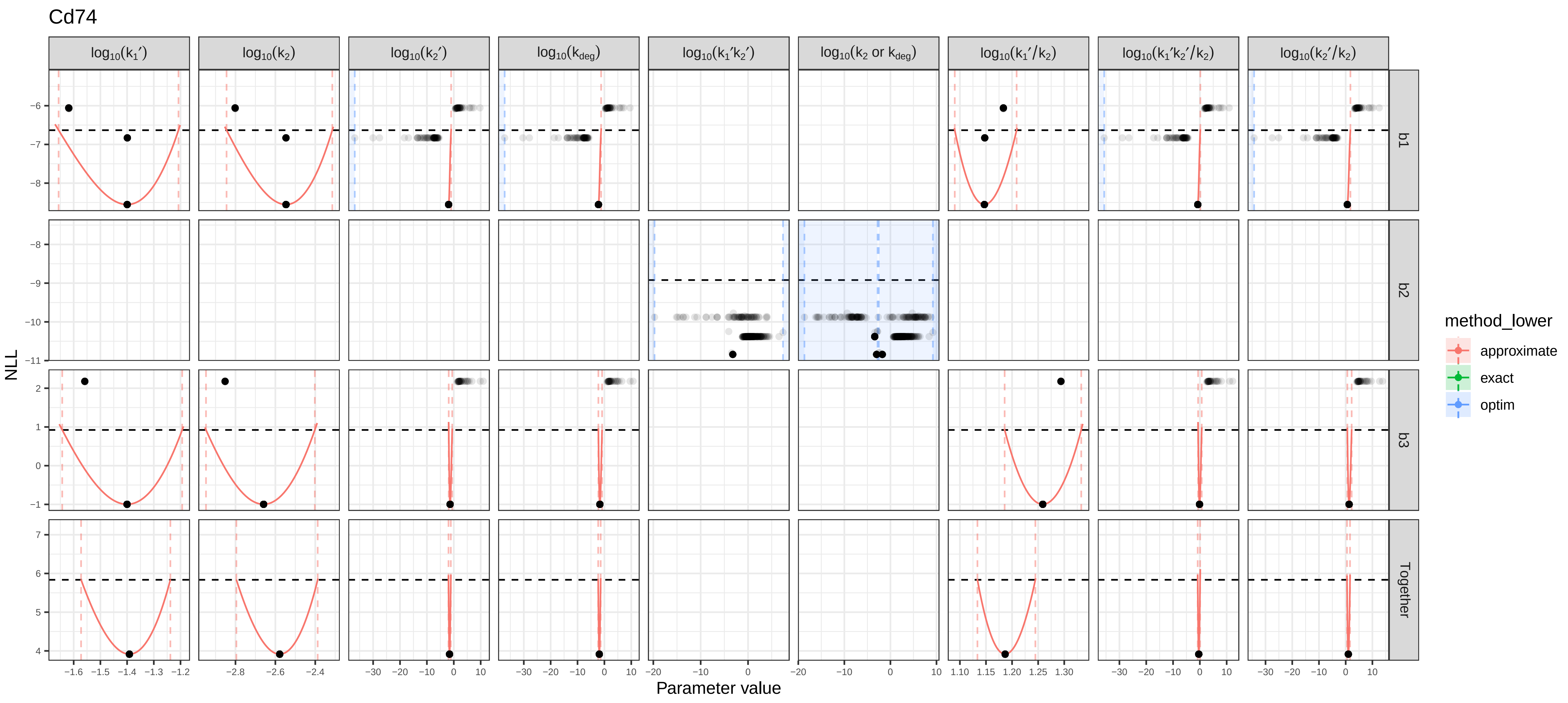

Replicate	Par	Best value	CI95 LB	CI95 UB	Method LB	Method UB
Together	$\log_{10}(k_1')$	−1.391	−1.572	−1.238	approximate	approximate
Together	$\log_{10}(k_2)$	−2.578	−2.795	−2.388	approximate	approximate
Together	$\log_{10}(k_2')$	−1.586	−1.969	−1.108	approximate	approximate
Together	$\log_{10}(k_{deg})$	−1.896	−2.309	−1.381	approximate	approximate
Together	$\log_{10}(k_1'/k_2)$	1.187	1.134	1.245	approximate	approximate
Together	$\log_{10}(k_1'k_2'/k_2)$	−0.3993	−0.7985	0.1055	approximate	approximate
Together	$\log_{10}(k_2'/k_2)$	0.9921	0.5004	1.629	approximate	approximate
b1	$\log_{10}(k_1')$	−1.4	−1.656	−1.208	approximate	approximate
b1	$\log_{10}(k_2)$	−2.547	−2.845	−2.314	approximate	approximate
b1	$\log_{10}(k_2')$	−1.929	< −36.82	−0.9905	optim	approximate
b1	$\log_{10}(k_{deg})$	−2.209	< −37.12	−1.217	optim	approximate
b1	$\log_{10}(k_1'/k_2)$	1.147	1.09	1.209	approximate	approximate
b1	$\log_{10}(k_1'k_2'/k_2)$	−0.7823	< −35.67	0.1874	optim	approximate
b1	$\log_{10}(k_2'/k_2)$	0.6173	< −34.27	1.771	optim	approximate
b2	$\log_{10}(k_1'k_2')$	−3.219	< −19.73	> 7.384	optim	optim
b2	$\log_{10}(k_2 \text{ or } k_{deg})$	−1.758	< −2.793	> 9.224	optim	optim
b2	$\log_{10}(k_2 \text{ or } k_{deg})$	−3	< −18.66	> −2.519	optim	optim
b3	$\log_{10}(k_1')$	−1.4	−1.643	−1.194	approximate	approximate
b3	$\log_{10}(k_2)$	−2.659	−2.948	−2.402	approximate	approximate
b3	$\log_{10}(k_2')$	−1.371	−1.887	−0.5881	approximate	approximate
b3	$\log_{10}(k_{deg})$	−1.681	−2.241	−0.8571	approximate	approximate
b3	$\log_{10}(k_1'/k_2)$	1.259	1.186	1.333	approximate	approximate
b3	$\log_{10}(k_1'k_2'/k_2)$	−0.1124	−0.6484	0.6982	approximate	approximate
b3	$\log_{10}(k_2'/k_2)$	1.288	0.6234	2.235	approximate	approximate

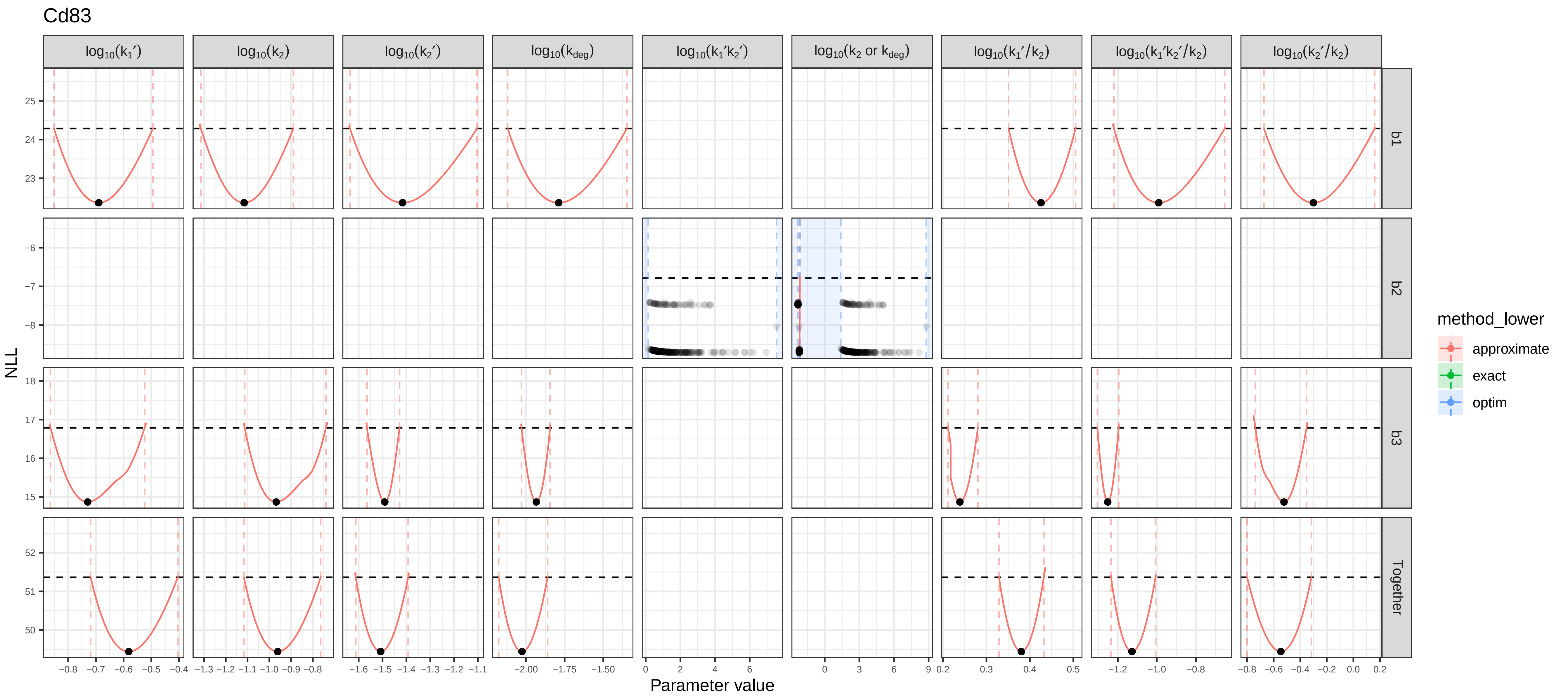

Replicate	Par	Best value	CI95 LB	CI95 UB	Method LB	Method UB
Together	$\log_{10}(k_1')$	−0.5813	−0.7189	−0.4042	approximate	approximate
Together	$\log_{10}(k_2)$	−0.9615	−1.117	−0.764	approximate	approximate
Together	$\log_{10}(k_2')$	−1.508	−1.612	−1.393	approximate	approximate
Together	$\log_{10}(k_{deg})$	−2.027	−2.178	−1.861	approximate	approximate
Together	$\log_{10}(k_1'/k_2)$	0.3802	0.3289	0.4321	approximate	approximate
Together	$\log_{10}(k_1'k_2'/k_2)$	−1.127	−1.235	−1.007	approximate	approximate
Together	$\log_{10}(k_2'/k_2)$	−0.5461	−0.8001	−0.3157	approximate	approximate
b1	$\log_{10}(k_1')$	−0.6899	−0.8511	−0.4939	approximate	approximate
b1	$\log_{10}(k_2)$	−1.115	−1.315	−0.8891	approximate	approximate
b1	$\log_{10}(k_2')$	−1.416	−1.637	−1.104	approximate	approximate
b1	$\log_{10}(k_{deg})$	−1.789	−2.12	−1.346	approximate	approximate
b1	$\log_{10}(k_1'/k_2)$	0.4256	0.3508	0.5052	approximate	approximate
b1	$\log_{10}(k_1'k_2'/k_2)$	−0.9904	−1.22	−0.652	approximate	approximate
b1	$\log_{10}(k_2'/k_2)$	−0.3005	−0.674	0.1605	approximate	approximate
b2	$\log_{10}(k_1'k_2')$	5.197	< 0.149	> 7.551	optim	optim
b2	$\log_{10}(k_2 \text{ or } k_{deg})$	6.432	1.384	> 8.798	optim	optim
b2	$\log_{10}(k_2 \text{ or } k_{deg})$	−2.198	< −2.318	−2.157	optim	approximate
b3	$\log_{10}(k_1')$	−0.7293	−0.8639	−0.5239	approximate	approximate
b3	$\log_{10}(k_2)$	−0.9683	−1.113	−0.7397	approximate	approximate
b3	$\log_{10}(k_2')$	−1.491	−1.565	−1.429	approximate	approximate
b3	$\log_{10}(k_{deg})$	−1.935	−2.03	−1.844	approximate	approximate
b3	$\log_{10}(k_1'/k_2)$	0.239	0.2111	0.2803	approximate	approximate
b3	$\log_{10}(k_1'k_2'/k_2)$	−1.252	−1.305	−1.195	approximate	approximate
b3	$\log_{10}(k_2'/k_2)$	−0.5223	−0.7381	−0.3526	approximate	approximate

Cdc42ep2

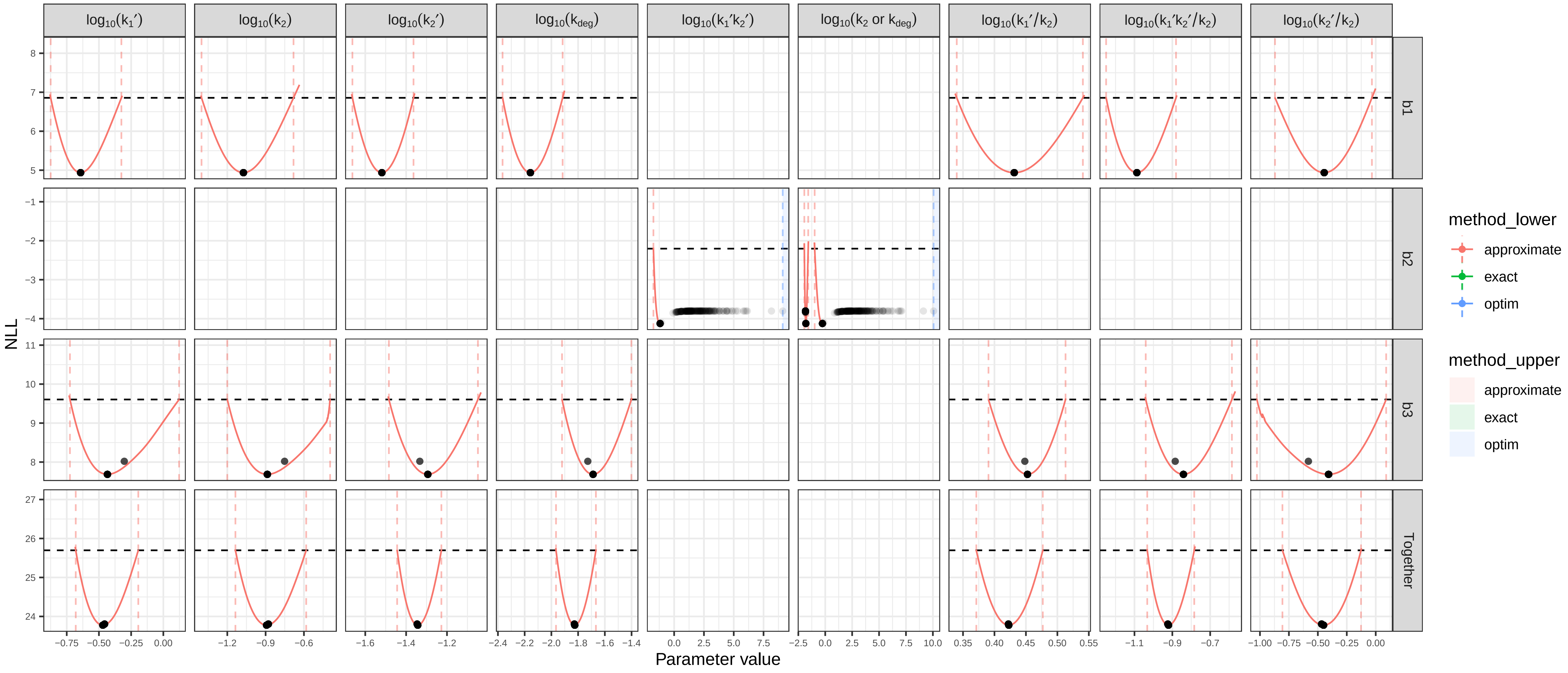

Replicate	Par	Best value	CI95 LB	CI95 UB	Method LB	Method UB
Together	$\log_{10}(k_1')$	−0.4693	−0.68	−0.1944	approximate	approximate
Together	$\log_{10}(k_2)$	−0.892	−1.136	−0.5823	approximate	approximate
Together	$\log_{10}(k_2')$	−1.343	−1.444	−1.227	approximate	approximate
Together	$\log_{10}(k_{deg})$	−1.825	−1.965	−1.667	approximate	approximate
Together	$\log_{10}(k_1'/k_2)$	0.4227	0.3711	0.4769	approximate	approximate
Together	$\log_{10}(k_1'k_2'/k_2)$	−0.9204	−1.033	−0.7842	approximate	approximate
Together	$\log_{10}(k_2'/k_2)$	−0.4511	−0.8059	−0.127	approximate	approximate
b1	$\log_{10}(k_1')$	−0.6421	−0.8748	−0.3257	approximate	approximate
b1	$\log_{10}(k_2)$	−1.074	−1.402	−0.6815	approximate	approximate
b1	$\log_{10}(k_2')$	−1.519	−1.663	−1.364	approximate	approximate
b1	$\log_{10}(k_{deg})$	−2.157	−2.365	−1.916	approximate	approximate
b1	$\log_{10}(k_1'/k_2)$	0.4314	0.3401	0.5406	approximate	approximate
b1	$\log_{10}(k_1'k_2'/k_2)$	−1.087	−1.25	−0.8805	approximate	approximate
b1	$\log_{10}(k_2'/k_2)$	−0.4452	−0.87	−0.0328	approximate	approximate
b2	$\log_{10}(k_1'k_2')$	−1.176	−1.738	> 9.11	approximate	optim
b2	$\log_{10}(k_2 \text{ or } k_{deg})$	−0.2605	−0.9831	> 10.07	approximate	optim
b2	$\log_{10}(k_2 \text{ or } k_{deg})$	−1.8	−1.944	−1.582	approximate	approximate
b3	$\log_{10}(k_1')$	−0.4342	−0.725	0.1225	approximate	approximate
b3	$\log_{10}(k_2)$	−0.8867	−1.2	−0.3949	approximate	approximate
b3	$\log_{10}(k_2')$	−1.294	−1.484	−1.049	approximate	approximate
b3	$\log_{10}(k_{deg})$	−1.688	−1.92	−1.401	approximate	approximate
b3	$\log_{10}(k_1'/k_2)$	0.4525	0.3905	0.513	approximate	approximate
b3	$\log_{10}(k_1'k_2'/k_2)$	−0.8412	−1.041	−0.5852	approximate	approximate
b3	$\log_{10}(k_2'/k_2)$	−0.407	−1.026	0.09027	approximate	approximate

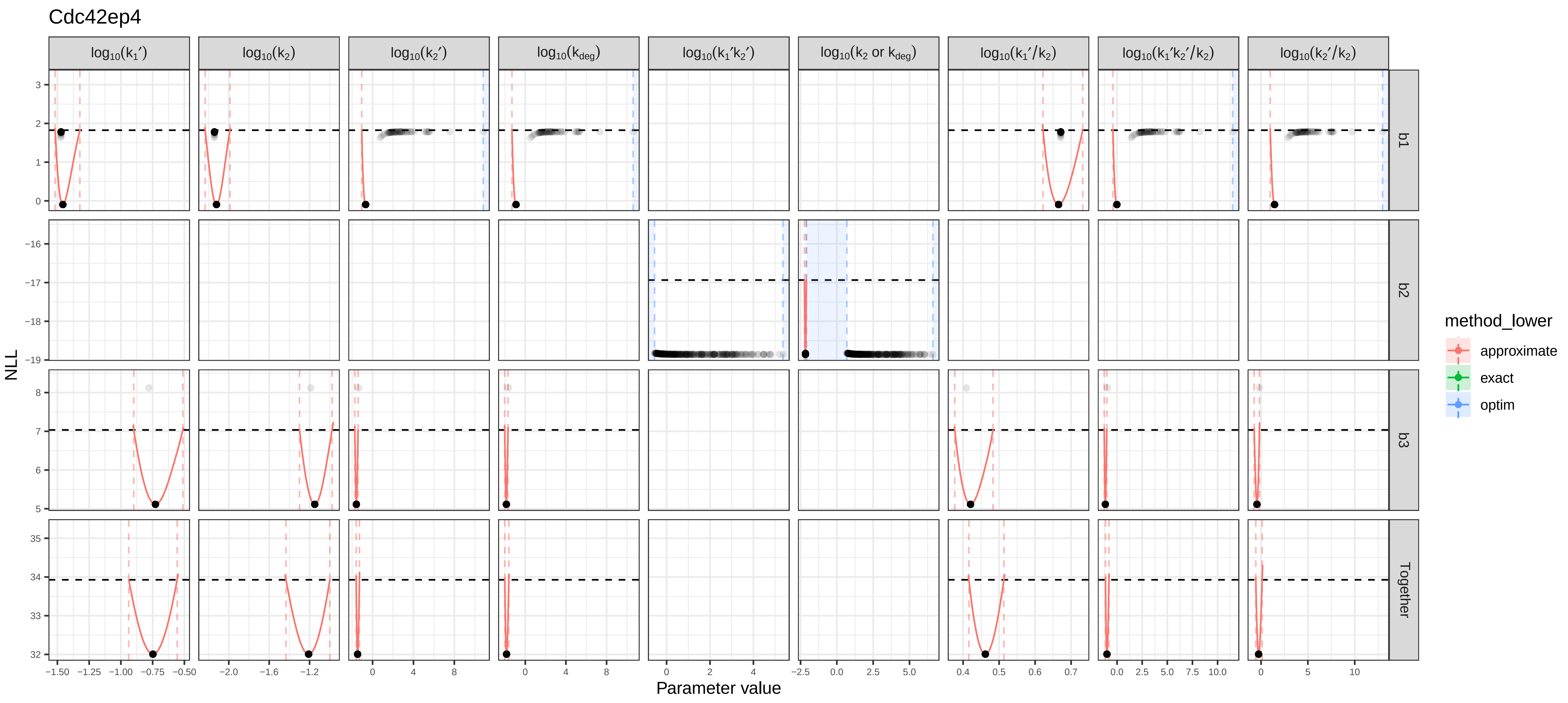

Replicate	Par	**Best value**	**CI95 LB**	**CI95 UB**	**Method LB**	**Method UB**
Together	$\log_{10}(k_1')$	−0.7474	−0.9382	−0.5568	approximate	approximate
Together	$\log_{10}(k_2)$	−1.209	−1.434	−0.9993	approximate	approximate
Together	$\log_{10}(k_2')$	−1.465	−1.604	−1.286	approximate	approximate
Together	$\log_{10}(k_{deg})$	−1.84	−2.013	−1.617	approximate	approximate
Together	$\log_{10}(k_1'/k_2)$	0.4618	0.4161	0.5133	approximate	approximate
Together	$\log_{10}(k_1'k_2'/k_2)$	−1.003	−1.15	−0.8002	approximate	approximate
Together	$\log_{10}(k_2'/k_2)$	−0.2555	−0.5592	0.1192	approximate	approximate
b1	$\log_{10}(k_1')$	−1.457	−1.518	−1.323	approximate	approximate
b1	$\log_{10}(k_2)$	−2.122	−2.234	−1.988	approximate	approximate
b1	$\log_{10}(k_2')$	−0.6809	−1.064	> 10.83	approximate	optim
b1	$\log_{10}(k_{deg})$	−0.9104	−1.309	> 10.61	approximate	optim
b1	$\log_{10}(k_1'/k_2)$	0.6652	0.622	0.7323	approximate	approximate
b1	$\log_{10}(k_1'k_2'/k_2)$	−0.01567	−0.4082	> 11.5	approximate	optim
b1	$\log_{10}(k_2'/k_2)$	1.441	0.9809	> 12.97	approximate	optim
b2	$\log_{10}(k_1'k_2')$	3.126	< −0.5543	> 5.365	optim	optim
b2	$\log_{10}(k_2 \text{ or } k_{deg})$	4.373	0.6897	> 6.611	optim	optim
b2	$\log_{10}(k_2 \text{ or } k_{deg})$	−2.162	−2.23	−2.099	approximate	approximate
b3	$\log_{10}(k_1')$	−0.7279	−0.8984	−0.5121	approximate	approximate
b3	$\log_{10}(k_2)$	−1.149	−1.3	−0.9767	approximate	approximate
b3	$\log_{10}(k_2')$	−1.584	−1.736	−1.413	approximate	approximate
b3	$\log_{10}(k_{deg})$	−1.86	−2.021	−1.697	approximate	approximate
b3	$\log_{10}(k_1'/k_2)$	0.4207	0.3768	0.483	approximate	approximate
b3	$\log_{10}(k_1'k_2'/k_2)$	−1.164	−1.273	−1.01	approximate	approximate
b3	$\log_{10}(k_2'/k_2)$	−0.4357	−0.7337	−0.1687	approximate	approximate

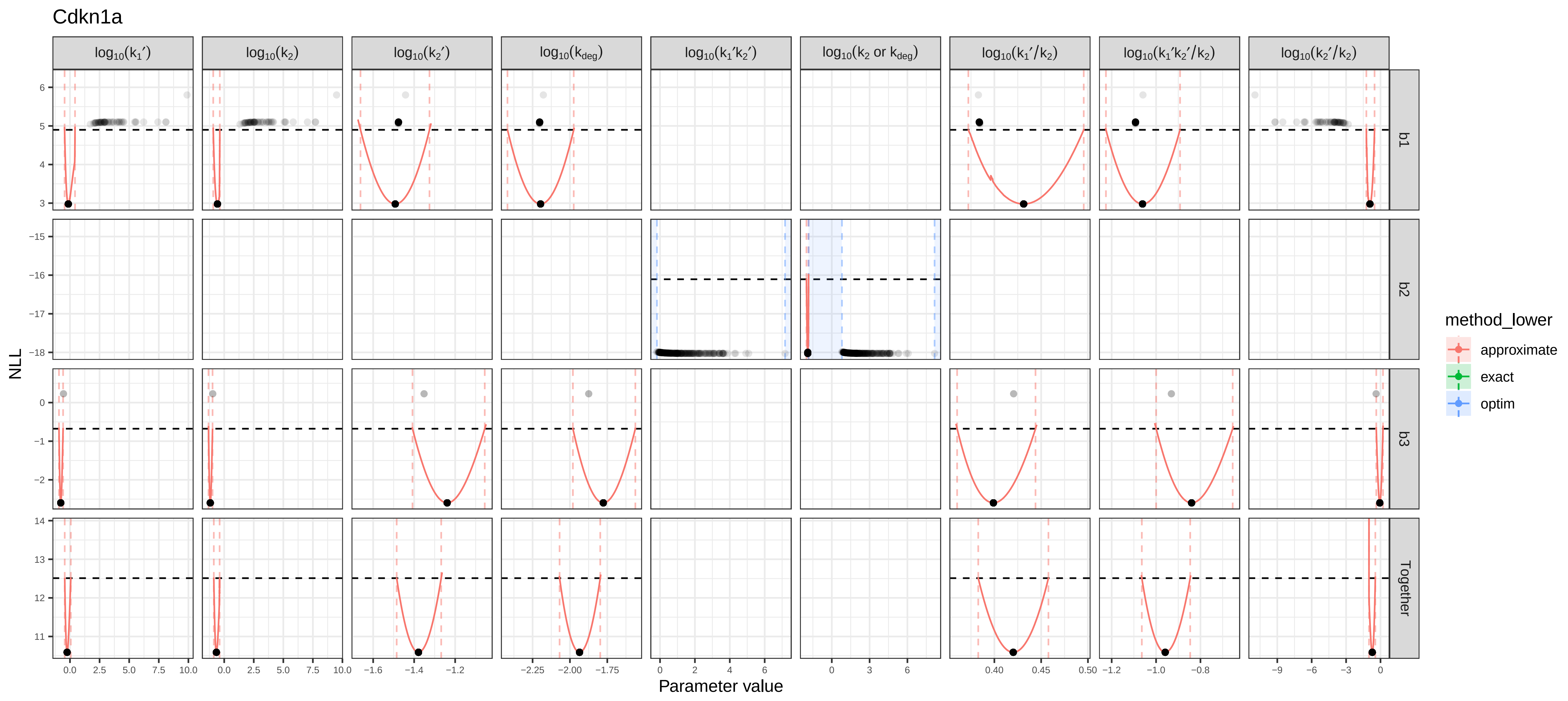

Replicate	Par	Best value	CI95 LB	CI95 UB	Method LB	Method UB
Together	$\log_{10}(k_1')$	−0.2408	−0.4424	0.07168	approximate	approximate
Together	$\log_{10}(k_2)$	−0.661	−0.8803	−0.3879	approximate	approximate
Together	$\log_{10}(k_2')$	−1.379	−1.485	−1.268	approximate	approximate
Together	$\log_{10}(k_{deg})$	−1.936	−2.069	−1.797	approximate	approximate
Together	$\log_{10}(k_1'/k_2)$	0.4202	0.3828	0.4578	approximate	approximate
Together	$\log_{10}(k_1'k_2'/k_2)$	−0.9588	−1.064	−0.8462	approximate	approximate
Together	$\log_{10}(k_2'/k_2)$	−0.718	−1.003	−0.4515	approximate	approximate
b1	$\log_{10}(k_1')$	−0.1394	−0.4539	0.4266	approximate	approximate
b1	$\log_{10}(k_2)$	−0.5707	−0.9287	−0.3712	approximate	approximate
b1	$\log_{10}(k_2')$	−1.492	−1.662	−1.325	approximate	approximate
b1	$\log_{10}(k_{deg})$	−2.197	−2.418	−1.975	approximate	approximate
b1	$\log_{10}(k_1'/k_2)$	0.4313	0.3722	0.4955	approximate	approximate
b1	$\log_{10}(k_1'k_2'/k_2)$	−1.061	−1.226	−0.8919	approximate	approximate
b1	$\log_{10}(k_2'/k_2)$	−0.9216	−1.234	−0.5193	approximate	approximate
b2	$\log_{10}(k_1'k_2')$	3.872	< −0.1769	> 7.169	optim	optim
b2	$\log_{10}(k_2 \text{ or } k_{deg})$	4.858	0.8075	> 8.155	optim	optim
b2	$\log_{10}(k_2 \text{ or } k_{deg})$	−1.906	−2.011	−1.842	approximate	approximate
b3	$\log_{10}(k_1')$	−0.7782	−0.928	−0.5795	approximate	approximate
b3	$\log_{10}(k_2)$	−1.177	−1.329	−0.9857	approximate	approximate
b3	$\log_{10}(k_2')$	−1.239	−1.408	−1.056	approximate	approximate
b3	$\log_{10}(k_{deg})$	−1.777	−1.98	−1.562	approximate	approximate
b3	$\log_{10}(k_1'/k_2)$	0.399	0.3602	0.444	approximate	approximate
b3	$\log_{10}(k_1'k_2'/k_2)$	−0.8399	−0.9996	−0.6547	approximate	approximate
b3	$\log_{10}(k_2'/k_2)$	−0.06164	−0.369	0.2164	approximate	approximate

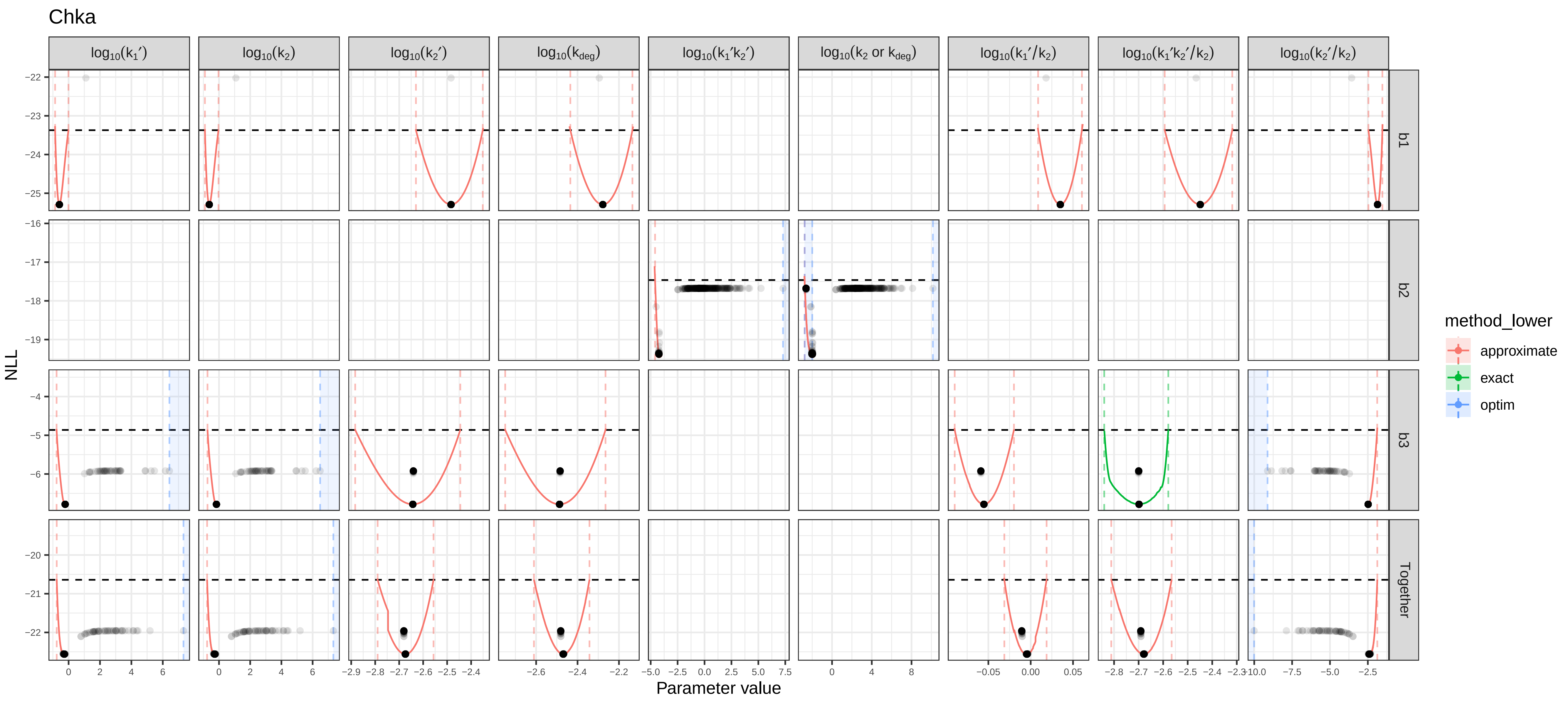

Replicate	Par	Best value	CI95 LB	CI95 UB	Method LB	Method UB
Together	$\log_{10}(k_1')$	−0.278	−0.7561	> 7.317	approximate	optim
Together	$\log_{10}(k_2)$	−0.2735	−0.7615	> 7.328	approximate	optim
Together	$\log_{10}(k_2')$	−2.674	−2.79	−2.557	approximate	approximate
Together	$\log_{10}(k_{deg})$	−2.469	−2.611	−2.342	approximate	approximate
Together	$\log_{10}(k_1'/k_2)$	−0.004506	−0.03115	0.01878	approximate	approximate
Together	$\log_{10}(k_1'k_2'/k_2)$	−2.679	−2.811	−2.565	approximate	approximate
Together	$\log_{10}(k_2'/k_2)$	−2.401	< −10.01	−1.875	optim	approximate
b1	$\log_{10}(k_1')$	−0.5881	−0.8589	−0.00693	approximate	approximate
b1	$\log_{10}(k_2)$	−0.6231	−0.9011	−0.03038	approximate	approximate
b1	$\log_{10}(k_2')$	−2.484	−2.63	−2.351	approximate	approximate
b1	$\log_{10}(k_{deg})$	−2.278	−2.435	−2.135	approximate	approximate
b1	$\log_{10}(k_1'/k_2)$	0.03497	0.008782	0.06049	approximate	approximate
b1	$\log_{10}(k_1'k_2'/k_2)$	−2.449	−2.594	−2.318	approximate	approximate
b1	$\log_{10}(k_2'/k_2)$	−1.861	−2.462	−1.54	approximate	approximate
b2	$\log_{10}(k_1'k_2')$	−4.231	−4.594	> 7.291	approximate	optim
b2	$\log_{10}(k_2 \text{ or } k_{deg})$	−1.989	−2.76	> 10.15	approximate	optim
b2	$\log_{10}(k_2 \text{ or } k_{deg})$	−2	−2.76	−1.991	approximate	optim
b3	$\log_{10}(k_1')$	−0.2196	−0.765	> 6.421	approximate	optim
b3	$\log_{10}(k_2)$	−0.1643	−0.7366	> 6.48	approximate	optim
b3	$\log_{10}(k_2')$	−2.643	−2.883	−2.445	approximate	approximate
b3	$\log_{10}(k_{deg})$	−2.487	−2.749	−2.265	approximate	approximate
b3	$\log_{10}(k_1'/k_2)$	−0.05525	−0.08974	−0.01979	approximate	approximate
b3	$\log_{10}(k_1'k_2'/k_2)$	−2.698	−2.84	−2.579	exact	exact
b3	$\log_{10}(k_2'/k_2)$	−2.479	< −9.121	−1.883	optim	approximate

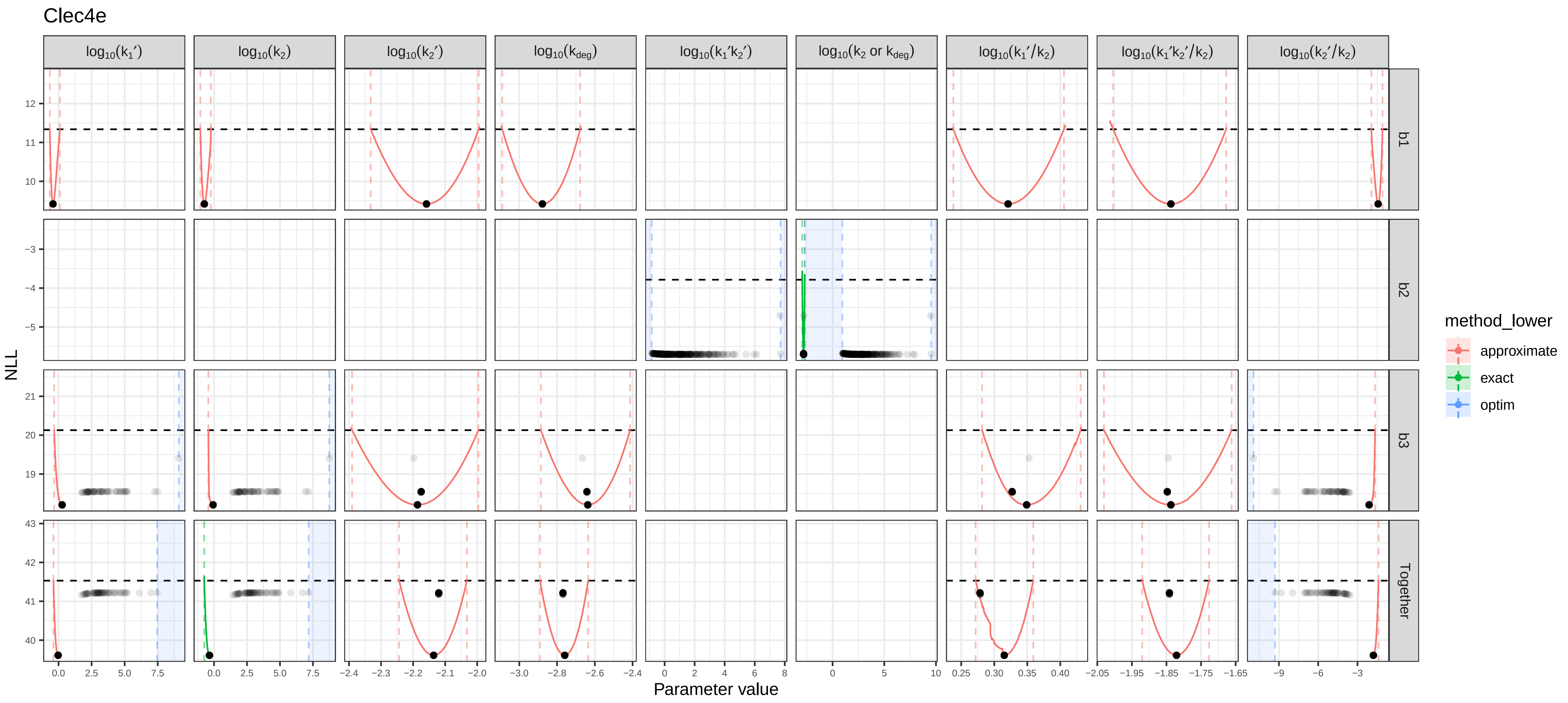

Replicate	Par	Best value	CI95 LB	CI95 UB	Method LB	Method UB
Together	$\log_{10}(k_1')$	−0.03172	−0.383	> 7.458	approximate	optim
Together	$\log_{10}(k_2)$	−0.3469	−0.7457	> 7.179	exact	optim
Together	$\log_{10}(k_2')$	−2.136	−2.244	−2.032	approximate	approximate
Together	$\log_{10}(k_{deg})$	−2.759	−2.89	−2.635	approximate	approximate
Together	$\log_{10}(k_1'/k_2)$	0.3152	0.2718	0.3591	approximate	approximate
Together	$\log_{10}(k_1'k_2'/k_2)$	−1.821	−1.92	−1.727	approximate	approximate
Together	$\log_{10}(k_2'/k_2)$	−1.789	< −9.299	−1.392	optim	approximate
b1	$\log_{10}(k_1')$	−0.4145	−0.6583	0.1067	approximate	approximate
b1	$\log_{10}(k_2)$	−0.7354	−1.041	−0.2408	approximate	approximate
b1	$\log_{10}(k_2')$	−2.158	−2.332	−1.995	approximate	approximate
b1	$\log_{10}(k_{deg})$	−2.877	−3.091	−2.678	approximate	approximate
b1	$\log_{10}(k_1'/k_2)$	0.3209	0.2381	0.4055	approximate	approximate
b1	$\log_{10}(k_1'k_2'/k_2)$	−1.837	−2.004	−1.678	approximate	approximate
b1	$\log_{10}(k_2'/k_2)$	−1.423	−1.94	−1.09	approximate	approximate
b2	$\log_{10}(k_1'k_2')$	2.827	< −0.8623	> 7.728	optim	optim
b2	$\log_{10}(k_2 \text{ or } k_{deg})$	4.631	0.941	> 9.532	optim	optim
b2	$\log_{10}(k_2 \text{ or } k_{deg})$	−2.806	−2.935	−2.69	exact	exact
b3	$\log_{10}(k_1')$	0.2756	−0.332	> 9.091	approximate	optim
b3	$\log_{10}(k_2)$	−0.07345	−0.4322	> 8.738	approximate	optim
b3	$\log_{10}(k_2')$	−2.186	−2.39	−1.996	approximate	approximate
b3	$\log_{10}(k_{deg})$	−2.637	−2.886	−2.413	approximate	approximate
b3	$\log_{10}(k_1'/k_2)$	0.3491	0.2815	0.4307	approximate	approximate
b3	$\log_{10}(k_1'k_2'/k_2)$	−1.837	−2.031	−1.662	approximate	approximate
b3	$\log_{10}(k_2'/k_2)$	−2.113	< −10.94	−1.654	optim	approximate

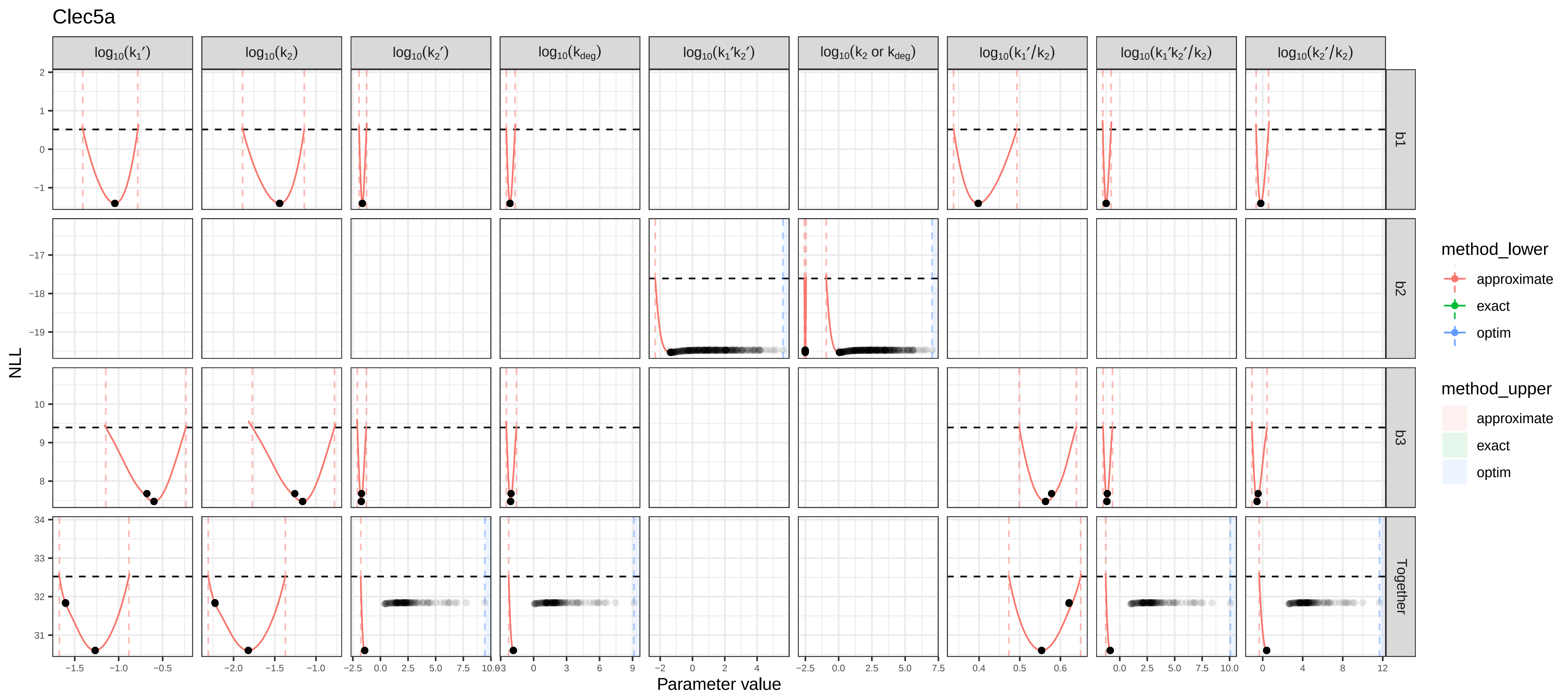

Replicate	Par	Best value	CI95 LB	CI95 UB	Method LB	Method UB
Together	$\log_{10}(k_1')$	−1.267	−1.675	−0.8832	approximate	approximate
Together	$\log_{10}(k_2)$	−1.821	−2.307	−1.372	approximate	approximate
Together	$\log_{10}(k_2')$	−1.425	−1.784	> 9.445	approximate	optim
Together	$\log_{10}(k_{deg})$	−1.847	−2.269	> 9.125	approximate	optim
Together	$\log_{10}(k_1'/k_2)$	0.5538	0.4733	0.6496	approximate	approximate
Together	$\log_{10}(k_1'k_2'/k_2)$	−0.8715	−1.278	> 10.07	approximate	optim
Together	$\log_{10}(k_2'/k_2)$	0.3959	−0.3454	> 11.67	approximate	optim
b1	$\log_{10}(k_1')$	−1.043	−1.408	−0.7826	approximate	approximate
b1	$\log_{10}(k_2)$	−1.441	−1.89	−1.142	approximate	approximate
b1	$\log_{10}(k_2')$	−1.638	−1.932	−1.258	approximate	approximate
b1	$\log_{10}(k_{deg})$	−2.151	−2.484	−1.689	approximate	approximate
b1	$\log_{10}(k_1'/k_2)$	0.3979	0.3374	0.493	approximate	approximate
b1	$\log_{10}(k_1'k_2'/k_2)$	−1.24	−1.546	−0.7911	approximate	approximate
b1	$\log_{10}(k_2'/k_2)$	−0.197	−0.6596	0.5803	approximate	approximate
b2	$\log_{10}(k_1'k_2')$	−1.349	−2.304	> 5.604	approximate	optim
b2	$\log_{10}(k_2 \text{ or } k_{deg})$	0.07125	−0.9305	> 7.029	approximate	optim
b2	$\log_{10}(k_2 \text{ or } k_{deg})$	−2.512	−2.578	−2.444	approximate	approximate
b3	$\log_{10}(k_1')$	−0.5987	−1.146	−0.2352	approximate	approximate
b3	$\log_{10}(k_2)$	−1.162	−1.771	−0.7751	approximate	approximate
b3	$\log_{10}(k_2')$	−1.733	−2.092	−1.274	approximate	approximate
b3	$\log_{10}(k_{deg})$	−2.096	−2.482	−1.559	approximate	approximate
b3	$\log_{10}(k_1'/k_2)$	0.5633	0.4987	0.6392	approximate	approximate
b3	$\log_{10}(k_1'k_2'/k_2)$	−1.17	−1.52	−0.6621	approximate	approximate
b3	$\log_{10}(k_2'/k_2)$	−0.5714	−1.081	0.442	approximate	approximate

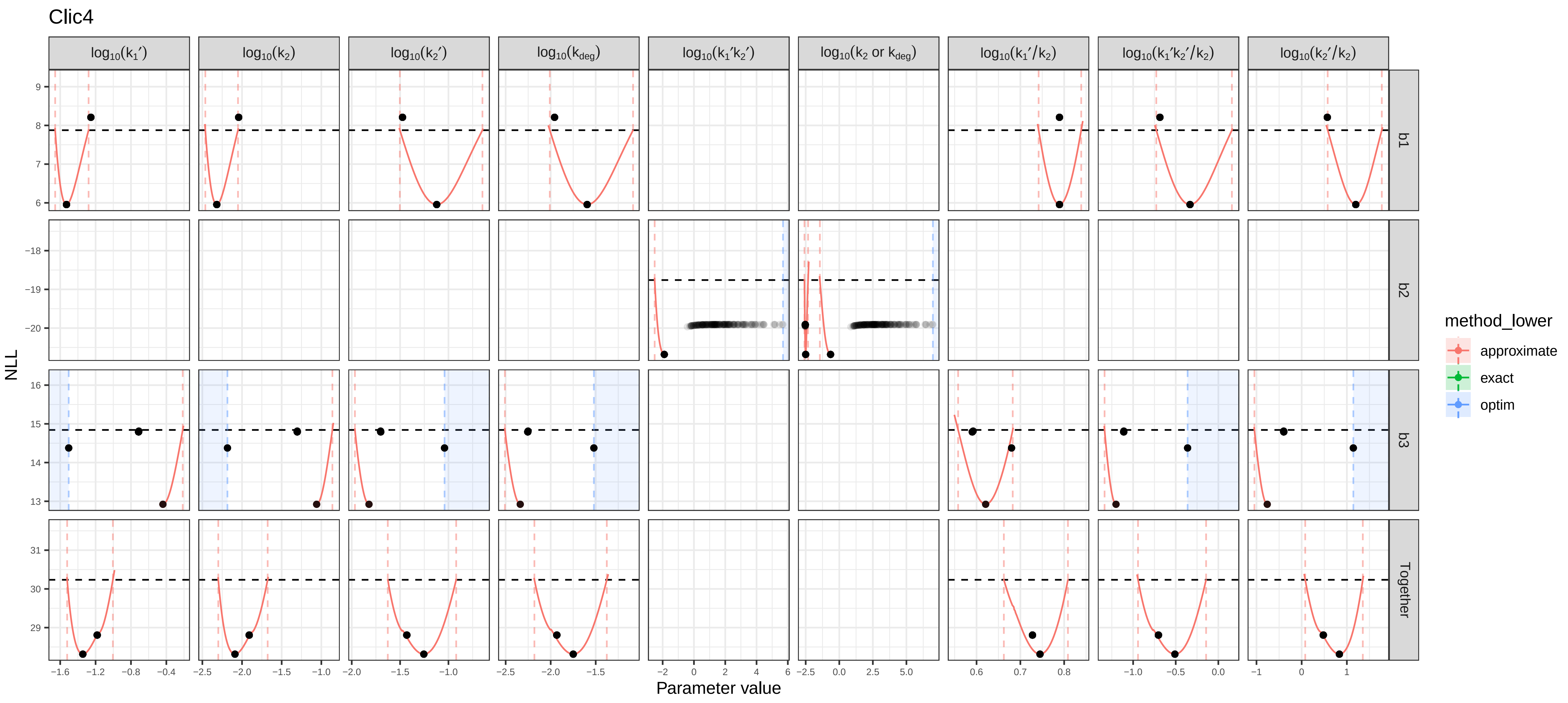

Replicate	Par	Best value	CI95 LB	CI95 UB	Method LB	Method UB
Together	$\log_{10}(k_1')$	−1.344	−1.521	−1.004	approximate	approximate
Together	$\log_{10}(k_2)$	−2.089	−2.299	−1.677	approximate	approximate
Together	$\log_{10}(k_2')$	−1.255	−1.627	−0.92	approximate	approximate
Together	$\log_{10}(k_{deg})$	−1.749	−2.181	−1.377	approximate	approximate
Together	$\log_{10}(k_1'/k_2)$	0.7448	0.6623	0.8086	approximate	approximate
Together	$\log_{10}(k_1'k_2'/k_2)$	−0.5097	−0.9426	−0.1435	approximate	approximate
Together	$\log_{10}(k_2'/k_2)$	0.8345	0.07829	1.353	approximate	approximate
b1	$\log_{10}(k_1')$	−1.529	−1.657	−1.279	approximate	approximate
b1	$\log_{10}(k_2)$	−2.318	−2.463	−2.051	approximate	approximate
b1	$\log_{10}(k_2')$	−1.121	−1.502	−0.6484	approximate	approximate
b1	$\log_{10}(k_{deg})$	−1.595	−2.01	−1.085	approximate	approximate
b1	$\log_{10}(k_1'/k_2)$	0.7893	0.7417	0.839	approximate	approximate
b1	$\log_{10}(k_1'k_2'/k_2)$	−0.332	−0.728	0.1592	approximate	approximate
b1	$\log_{10}(k_2'/k_2)$	1.197	0.5756	1.773	approximate	approximate
b2	$\log_{10}(k_1'k_2')$	−1.895	−2.51	> 5.692	approximate	optim
b2	$\log_{10}(k_2 \text{ or } k_{deg})$	−0.6487	−1.444	> 6.973	approximate	optim
b2	$\log_{10}(k_2 \text{ or } k_{deg})$	−2.504	−2.593	−2.322	approximate	approximate
b3	$\log_{10}(k_1')$	−0.4388	< −1.504	−0.2146	optim	approximate
b3	$\log_{10}(k_2)$	−1.06	< −2.184	−0.8606	optim	approximate
b3	$\log_{10}(k_2')$	−1.822	−1.967	> −1.04	approximate	optim
b3	$\log_{10}(k_{deg})$	−2.338	−2.508	> −1.52	approximate	optim
b3	$\log_{10}(k_1'/k_2)$	0.6208	0.5579	0.6826	approximate	approximate
b3	$\log_{10}(k_1'k_2'/k_2)$	−1.201	−1.335	> −0.3604	approximate	optim
b3	$\log_{10}(k_2'/k_2)$	−0.7622	−1.046	> 1.143	approximate	optim

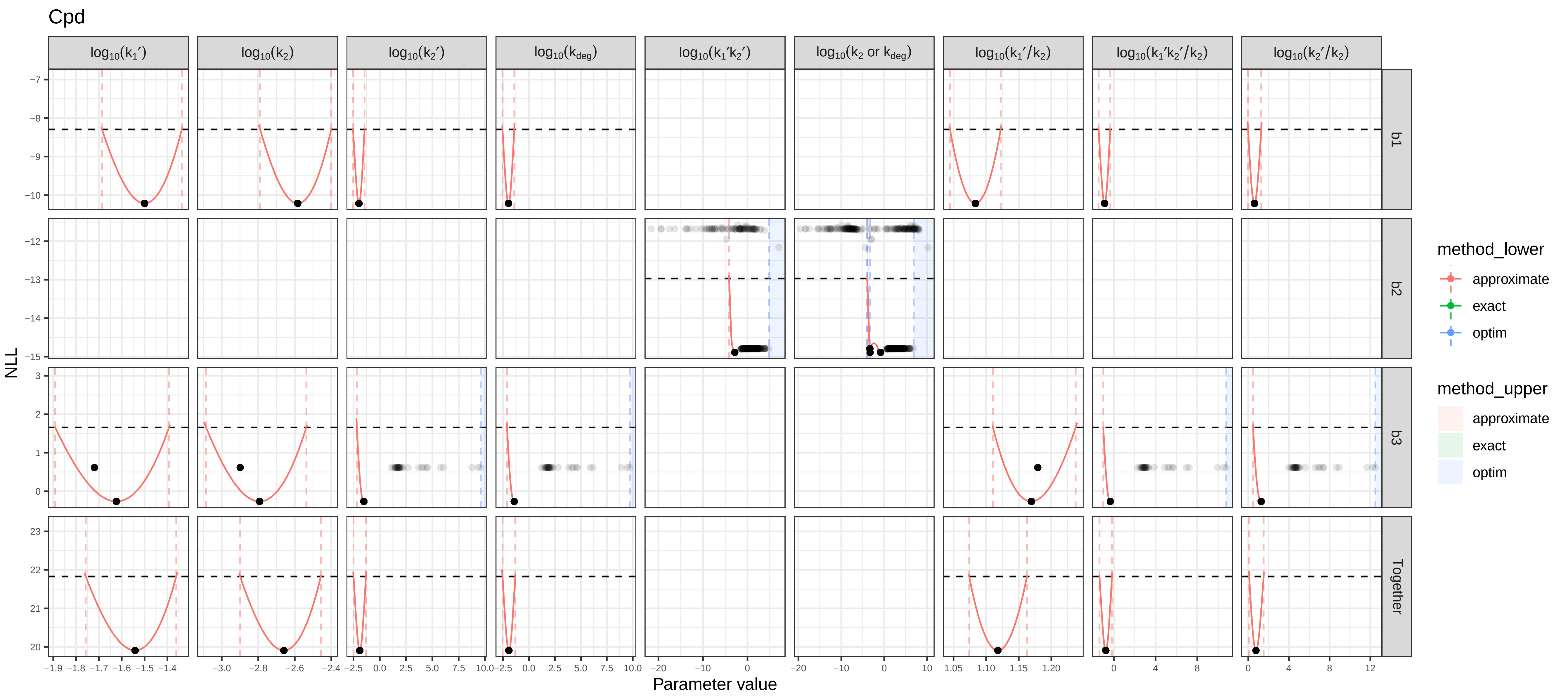

Replicate	Par	Best value	CI95 LB	CI95 UB	Method LB	Method UB
Together	$\log_{10}(k_1')$	−1.541	−1.758	−1.361	approximate	approximate
Together	$\log_{10}(k_2)$	−2.66	−2.899	−2.457	approximate	approximate
Together	$\log_{10}(k_2')$	−1.901	−2.498	−1.316	approximate	approximate
Together	$\log_{10}(k_{deg})$	−1.93	−2.546	−1.316	approximate	approximate
Together	$\log_{10}(k_1'/k_2)$	1.118	1.074	1.163	approximate	approximate
Together	$\log_{10}(k_1'k_2'/k_2)$	−0.7827	−1.385	−0.1806	approximate	approximate
Together	$\log_{10}(k_2'/k_2)$	0.7588	0.09176	1.517	approximate	approximate
b1	$\log_{10}(k_1')$	−1.5	−1.687	−1.337	approximate	approximate
b1	$\log_{10}(k_2)$	−2.584	−2.79	−2.4	approximate	approximate
b1	$\log_{10}(k_2')$	−1.978	−2.544	−1.447	approximate	approximate
b1	$\log_{10}(k_{deg})$	−1.968	−2.553	−1.412	approximate	approximate
b1	$\log_{10}(k_1'/k_2)$	1.084	1.045	1.122	approximate	approximate
b1	$\log_{10}(k_1'k_2'/k_2)$	−0.8945	−1.466	−0.351	approximate	approximate
b1	$\log_{10}(k_2'/k_2)$	0.6055	−0.02108	1.276	approximate	approximate
b2	$\log_{10}(k_1'k_2')$	−2.862	−4.139	> 4.807	approximate	optim
b2	$\log_{10}(k_2 \text{ or } k_{deg})$	−0.8569	−3.975	> 6.877	approximate	optim
b2	$\log_{10}(k_2 \text{ or } k_{deg})$	−3.289	−3.975	−3.287	approximate	optim
b3	$\log_{10}(k_1')$	−1.623	−1.891	−1.393	approximate	approximate
b3	$\log_{10}(k_2)$	−2.793	−3.085	−2.537	approximate	approximate
b3	$\log_{10}(k_2')$	−1.517	−2.18	> 9.597	approximate	optim
b3	$\log_{10}(k_{deg})$	−1.417	−2.109	> 9.718	approximate	optim
b3	$\log_{10}(k_1'/k_2)$	1.169	1.11	1.237	approximate	approximate
b3	$\log_{10}(k_1'k_2'/k_2)$	−0.3472	−1.021	> 10.78	approximate	optim
b3	$\log_{10}(k_2'/k_2)$	1.276	0.4728	> 12.5	approximate	optim

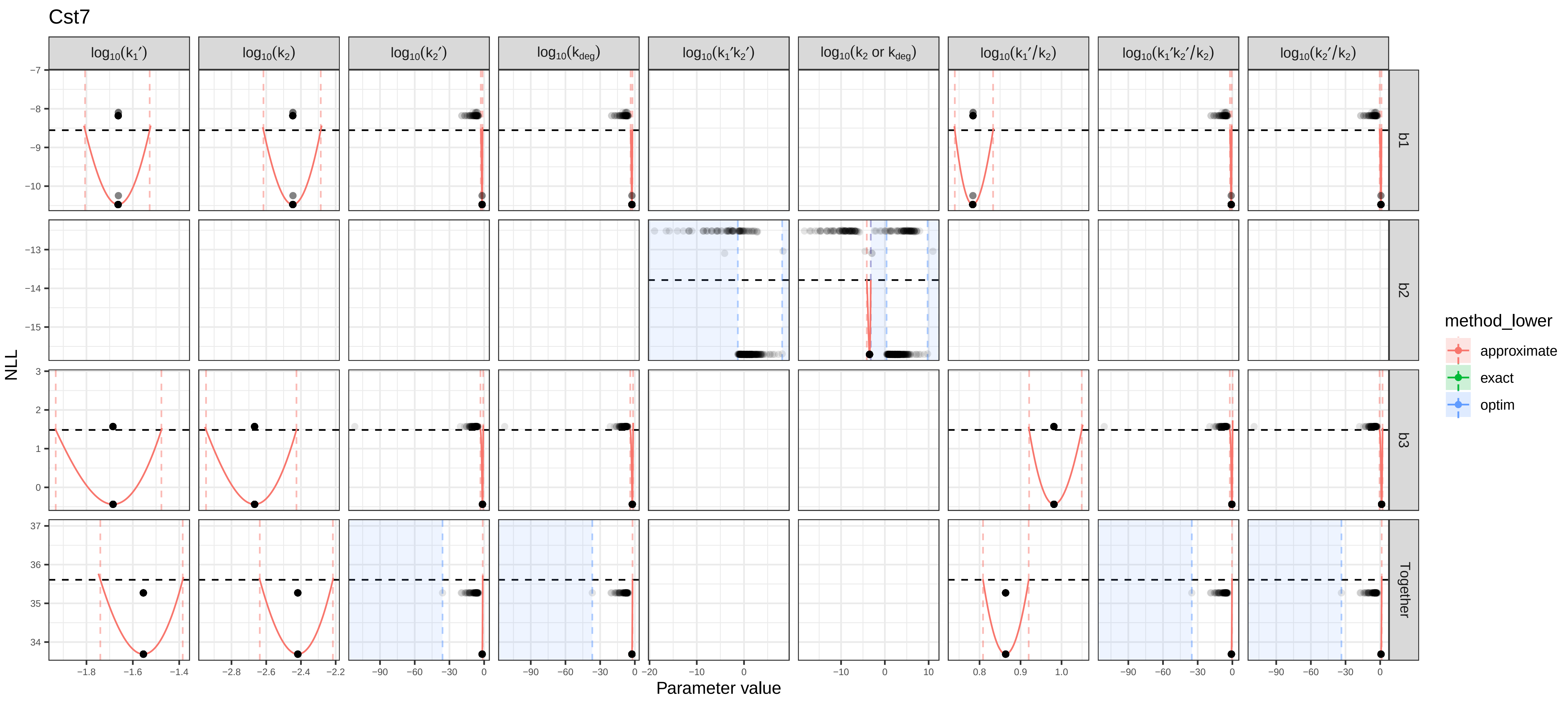

Replicate	Par	Best value	CI95 LB	CI95 UB	Method LB	Method UB
Together	$\log_{10}(k_1')$	−1.554	−1.74	−1.385	approximate	approximate
Together	$\log_{10}(k_2)$	−2.418	−2.637	−2.216	approximate	approximate
Together	$\log_{10}(k_2')$	−1.695	< −35.95	−1.205	optim	approximate
Together	$\log_{10}(k_{deg})$	−2.515	< −36.78	−1.995	optim	approximate
Together	$\log_{10}(k_1'/k_2)$	0.8637	0.8084	0.9199	approximate	approximate
Together	$\log_{10}(k_1'k_2'/k_2)$	−0.8312	< −35.08	−0.3238	optim	approximate
Together	$\log_{10}(k_2'/k_2)$	0.7232	< −33.53	1.348	optim	approximate
b1	$\log_{10}(k_1')$	−1.664	−1.806	−1.527	approximate	approximate
b1	$\log_{10}(k_2)$	−2.447	−2.616	−2.286	approximate	approximate
b1	$\log_{10}(k_2')$	−1.77	−2.883	−1.36	approximate	approximate
b1	$\log_{10}(k_{deg})$	−2.52	−3.646	−2.087	approximate	approximate
b1	$\log_{10}(k_1'/k_2)$	0.7833	0.7396	0.833	approximate	approximate
b1	$\log_{10}(k_1'k_2'/k_2)$	−0.987	−2.1	−0.5649	approximate	approximate
b1	$\log_{10}(k_2'/k_2)$	0.6766	−0.4436	1.181	approximate	approximate
b2	$\log_{10}(k_1'k_2')$	1.638	< −1.308	> 8.077	optim	optim
b2	$\log_{10}(k_2 \text{ or } k_{deg})$	3.348	0.4003	> 9.76	optim	optim
b2	$\log_{10}(k_2 \text{ or } k_{deg})$	−3.495	−4.124	−3.215	approximate	approximate
b3	$\log_{10}(k_1')$	−1.685	−1.933	−1.477	approximate	approximate
b3	$\log_{10}(k_2)$	−2.667	−2.947	−2.426	approximate	approximate
b3	$\log_{10}(k_2')$	−1.418	−3.182	−0.7872	approximate	approximate
b3	$\log_{10}(k_{deg})$	−2.169	−3.953	−1.503	approximate	approximate
b3	$\log_{10}(k_1'/k_2)$	0.9815	0.921	1.049	approximate	approximate
b3	$\log_{10}(k_1'k_2'/k_2)$	−0.4367	−2.201	0.2151	approximate	approximate
b3	$\log_{10}(k_2'/k_2)$	1.249	−0.5148	2.07	approximate	approximate

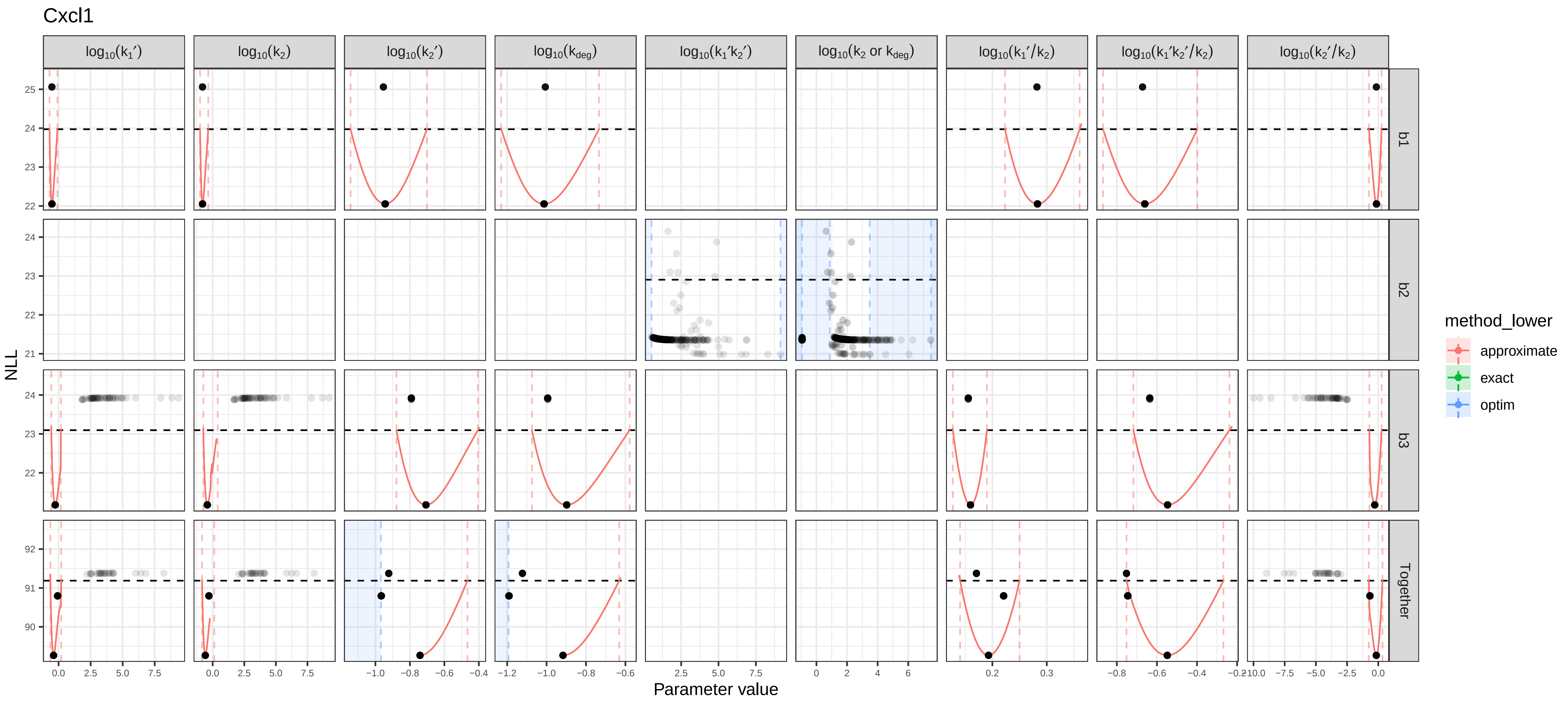

Replicate	Par	Best value	CI95 LB	CI95 UB	Method LB	Method UB
Together	$\log_{10}(k_1')$	−0.3901	−0.6418	0.2014	approximate	approximate
Together	$\log_{10}(k_2)$	−0.5831	−0.8414	0.1281	approximate	approximate
Together	$\log_{10}(k_2')$	−0.7412	< −0.9676	−0.4661	optim	approximate
Together	$\log_{10}(k_{deg})$	−0.9167	< −1.193	−0.6316	optim	approximate
Together	$\log_{10}(k_1'/k_2)$	0.193	0.1407	0.25	approximate	approximate
Together	$\log_{10}(k_1'k_2'/k_2)$	−0.5482	−0.7519	−0.2683	approximate	approximate
Together	$\log_{10}(k_2'/k_2)$	−0.1581	−0.7574	0.3388	approximate	approximate
b1	$\log_{10}(k_1')$	−0.5192	−0.7048	−0.09196	approximate	approximate
b1	$\log_{10}(k_2)$	−0.8022	−0.9989	−0.3516	approximate	approximate
b1	$\log_{10}(k_2')$	−0.9431	−1.144	−0.7011	approximate	approximate
b1	$\log_{10}(k_{deg})$	−1.013	−1.231	−0.7341	approximate	approximate
b1	$\log_{10}(k_1'/k_2)$	0.2829	0.2233	0.3604	approximate	approximate
b1	$\log_{10}(k_1'k_2'/k_2)$	−0.6602	−0.8687	−0.3984	approximate	approximate
b1	$\log_{10}(k_2'/k_2)$	−0.1409	−0.7468	0.2613	approximate	approximate
b2	$\log_{10}(k_1'k_2')$	8.302	< 0.5183	> 9.152	optim	optim
b2	$\log_{10}(k_2 \text{ or } k_{deg})$	4.522	< 0.873	> 7.49	optim	optim
b2	$\log_{10}(k_2 \text{ or } k_{deg})$	3.486	< −0.9404	> 3.486	optim	optim
b3	$\log_{10}(k_1')$	−0.2636	−0.5682	0.1811	approximate	approximate
b3	$\log_{10}(k_2)$	−0.4234	−0.7389	0.3984	approximate	approximate
b3	$\log_{10}(k_2')$	−0.7068	−0.8783	−0.4057	approximate	approximate
b3	$\log_{10}(k_{deg})$	−0.8979	−1.074	−0.5786	approximate	approximate
b3	$\log_{10}(k_1'/k_2)$	0.1598	0.1274	0.1901	approximate	approximate
b3	$\log_{10}(k_1'k_2'/k_2)$	−0.547	−0.7174	−0.2383	approximate	approximate
b3	$\log_{10}(k_2'/k_2)$	−0.2834	−0.7113	0.2697	approximate	approximate

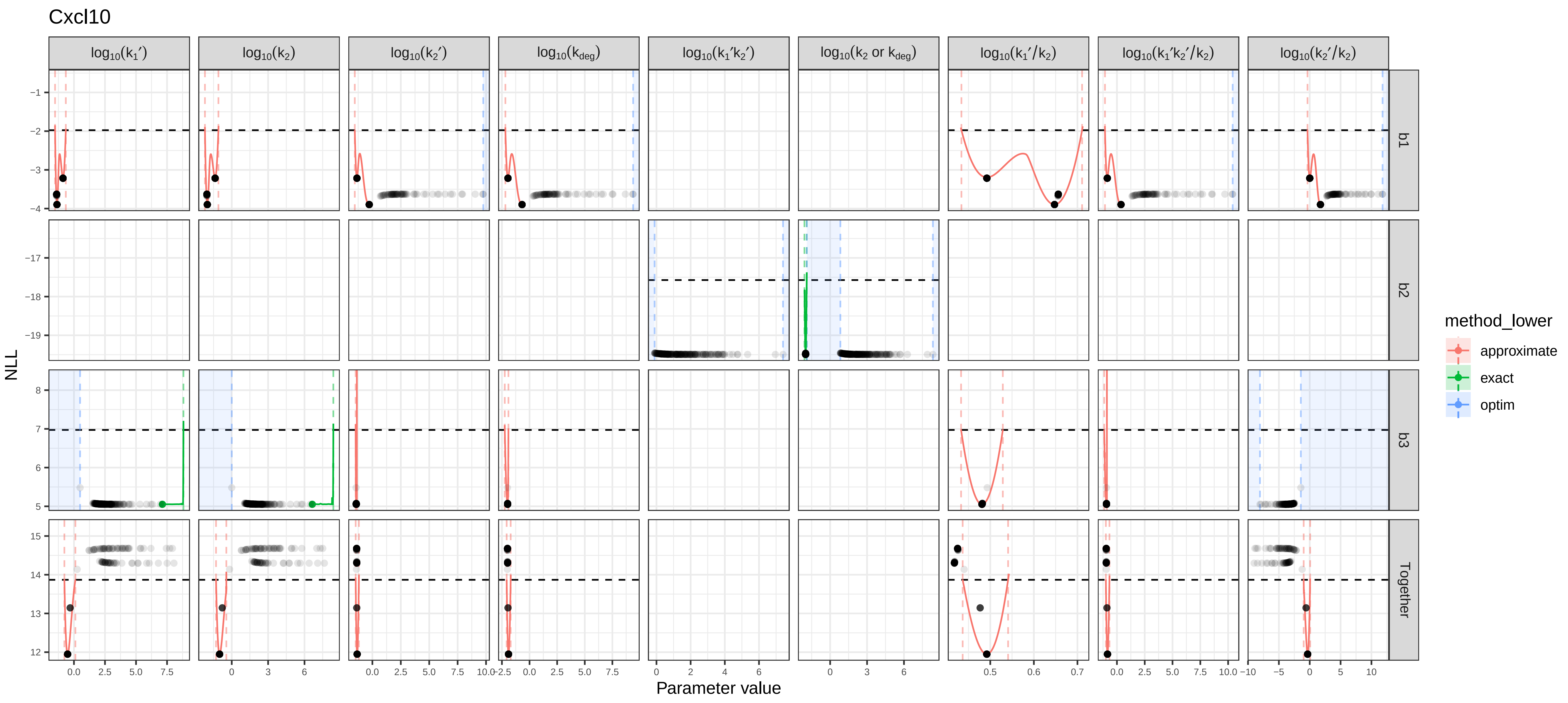

Replicate	Par	Best value	CI95 LB	CI95 UB	Method LB	Method UB
Together	$\log_{10}(k_1')$	−0.5107	−0.7707	0.1164	approximate	approximate
Together	$\log_{10}(k_2)$	−1.003	−1.296	−0.4475	approximate	approximate
Together	$\log_{10}(k_2')$	−1.34	−1.471	−1.186	approximate	approximate
Together	$\log_{10}(k_{deg})$	−1.902	−2.07	−1.707	approximate	approximate
Together	$\log_{10}(k_1'/k_2)$	0.4919	0.4366	0.5409	approximate	approximate
Together	$\log_{10}(k_1'k_2'/k_2)$	−0.848	−0.9949	−0.6724	approximate	approximate
Together	$\log_{10}(k_2'/k_2)$	−0.3373	−0.9789	0.06936	approximate	approximate
b1	$\log_{10}(k_1')$	−1.368	−1.521	−0.6548	approximate	approximate
b1	$\log_{10}(k_2)$	−2.015	−2.211	−1.099	approximate	approximate
b1	$\log_{10}(k_2')$	−0.2795	−1.549	> 9.755	approximate	optim
b1	$\log_{10}(k_{deg})$	−0.6773	−2.186	> 9.379	approximate	optim
b1	$\log_{10}(k_1'/k_2)$	0.6474	0.4338	0.7107	approximate	approximate
b1	$\log_{10}(k_1'k_2'/k_2)$	0.3679	−1.075	> 10.41	approximate	optim
b1	$\log_{10}(k_2'/k_2)$	1.736	−0.3598	> 11.8	approximate	optim
b2	$\log_{10}(k_1'k_2')$	7.407	< −0.1119	> 7.407	optim	optim
b2	$\log_{10}(k_2 \text{ or } k_{deg})$	8.351	0.8313	> 8.351	optim	optim
b2	$\log_{10}(k_2 \text{ or } k_{deg})$	−1.994	−2.089	−1.905	exact	exact
b3	$\log_{10}(k_1')$	7.124	< 0.4938	8.819	optim	exact
b3	$\log_{10}(k_2)$	6.643	< 0.0007875	8.379	optim	exact
b3	$\log_{10}(k_2')$	−1.421	−1.477	−1.367	approximate	approximate
b3	$\log_{10}(k_{deg})$	−1.985	−2.242	−1.91	approximate	approximate
b3	$\log_{10}(k_1'/k_2)$	0.4809	0.433	0.5288	approximate	approximate
b3	$\log_{10}(k_1'k_2'/k_2)$	−0.9401	−1.142	−0.9022	approximate	approximate
b3	$\log_{10}(k_2'/k_2)$	−8.064	< −8.064	> −1.439	optim	optim

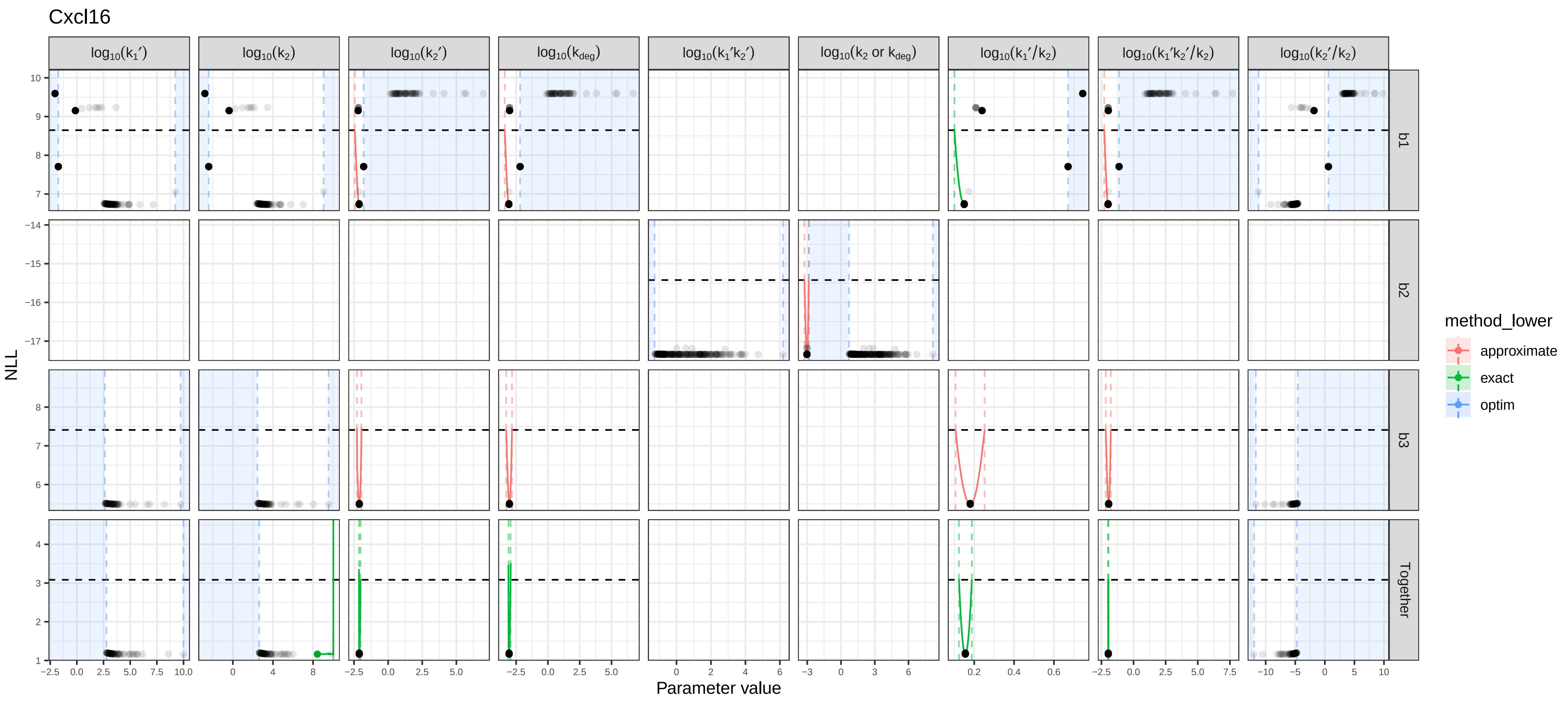

Replicate	Par	Best value	CI95 LB	CI95 UB	Method LB	Method UB
Together	$\log_{10}(k_1')$	8.587	< 2.774	> 9.994	optim	optim
Together	$\log_{10}(k_2)$	8.432	< 2.619	10.03	optim	exact
Together	$\log_{10}(k_2')$	−2.119	−2.137	−2.026	exact	exact
Together	$\log_{10}(k_{deg})$	−3.052	−3.101	−2.936	exact	exact
Together	$\log_{10}(k_1'/k_2)$	0.1554	0.1236	0.1881	exact	exact
Together	$\log_{10}(k_1'k_2'/k_2)$	−1.963	−1.982	−1.947	exact	exact
Together	$\log_{10}(k_2'/k_2)$	−10.55	< −11.96	> −4.737	optim	optim
b1	$\log_{10}(k_1')$	7.171	< −1.738	> 9.23	optim	optim
b1	$\log_{10}(k_2)$	7.021	< −2.41	> 9.056	optim	optim
b1	$\log_{10}(k_2')$	−2.13	−2.444	> −1.795	approximate	optim
b1	$\log_{10}(k_{deg})$	−3.073	−3.396	> −2.187	approximate	optim
b1	$\log_{10}(k_1'/k_2)$	0.1498	0.1007	> 0.6712	exact	optim
b1	$\log_{10}(k_1'k_2'/k_2)$	−1.98	−2.28	> −1.124	approximate	optim
b1	$\log_{10}(k_2'/k_2)$	−9.15	< −11.2	> 0.6144	optim	optim
b2	$\log_{10}(k_1'k_2')$	3.323	< −1.274	> 6.183	optim	optim
b2	$\log_{10}(k_2 \text{ or } k_{deg})$	5.306	0.7088	> 8.166	optim	optim
b2	$\log_{10}(k_2 \text{ or } k_{deg})$	−3.025	−3.246	−2.856	approximate	approximate
b3	$\log_{10}(k_1')$	6.6	< 2.634	> 9.73	optim	optim
b3	$\log_{10}(k_2)$	6.421	< 2.454	> 9.55	optim	optim
b3	$\log_{10}(k_2')$	−2.115	−2.285	−1.959	approximate	approximate
b3	$\log_{10}(k_{deg})$	−3.028	−3.281	−2.823	approximate	approximate
b3	$\log_{10}(k_1'/k_2)$	0.1798	0.1066	0.2522	approximate	approximate
b3	$\log_{10}(k_1'k_2'/k_2)$	−1.936	−2.161	−1.768	approximate	approximate
b3	$\log_{10}(k_2'/k_2)$	−8.536	< −11.67	> −4.569	optim	optim

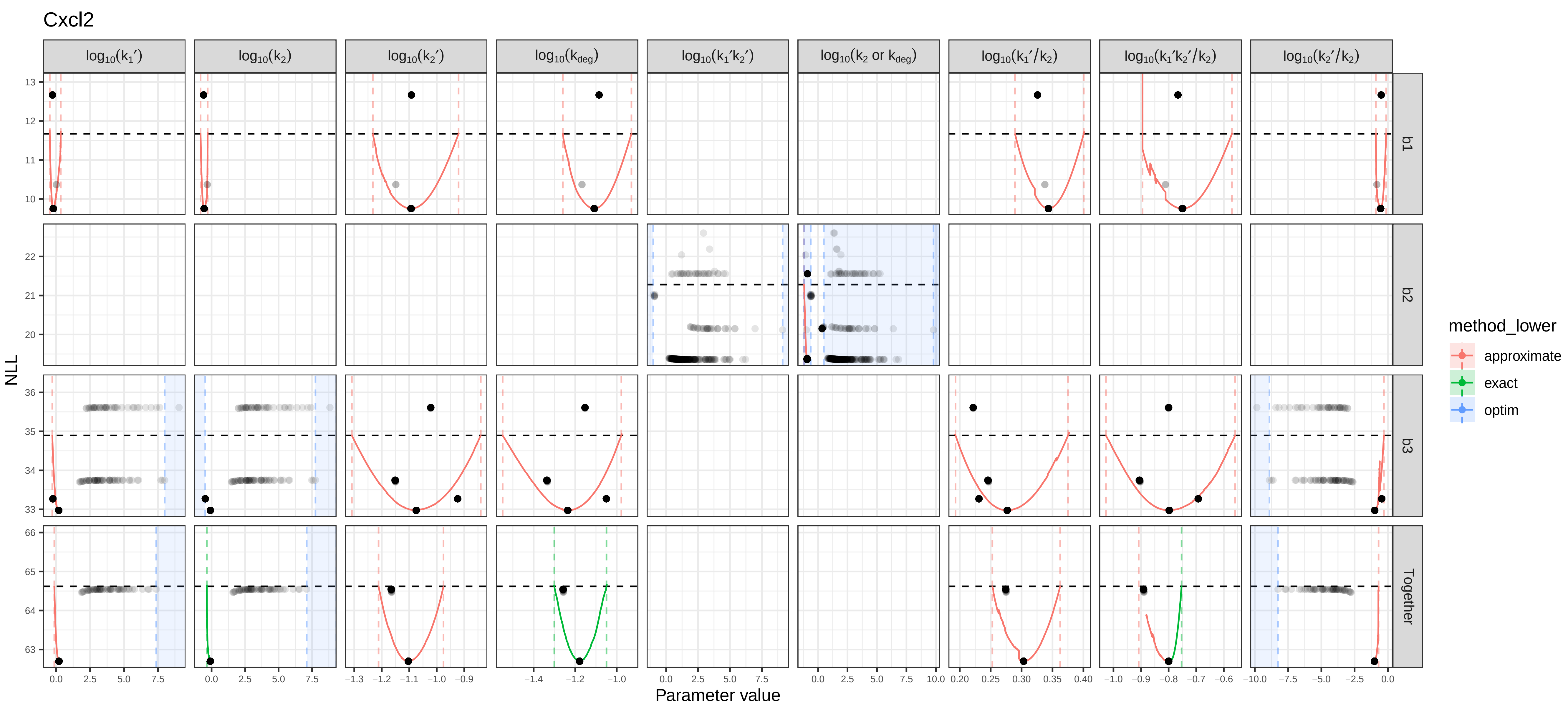

Replicate	Par	Best value	CI95 LB	CI95 UB	Method LB	Method UB
Together	$\log_{10}(k_1')$	0.2092	−0.1353	> 7.371	approximate	optim
Together	$\log_{10}(k_2)$	−0.09413	−0.3543	> 7.097	exact	optim
Together	$\log_{10}(k_2')$	−1.103	−1.212	−0.9754	approximate	approximate
Together	$\log_{10}(k_{deg})$	−1.179	−1.301	−1.049	exact	exact
Together	$\log_{10}(k_1'/k_2)$	0.3033	0.2528	0.3624	approximate	approximate
Together	$\log_{10}(k_1'k_2'/k_2)$	−0.8001	−0.9085	−0.7531	approximate	exact
Together	$\log_{10}(k_2'/k_2)$	−1.009	< −8.261	−0.6981	optim	approximate
b1	$\log_{10}(k_1')$	−0.2113	−0.4708	0.338	approximate	approximate
b1	$\log_{10}(k_2)$	−0.5547	−0.8201	−0.2907	approximate	approximate
b1	$\log_{10}(k_2')$	−1.093	−1.233	−0.9206	approximate	approximate
b1	$\log_{10}(k_{deg})$	−1.108	−1.26	−0.9293	approximate	approximate
b1	$\log_{10}(k_1'/k_2)$	0.3435	0.2893	0.4006	approximate	approximate
b1	$\log_{10}(k_1'k_2'/k_2)$	−0.7498	−0.8945	−0.5703	approximate	approximate
b1	$\log_{10}(k_2'/k_2)$	−0.5386	−0.9031	−0.1278	approximate	approximate
b2	$\log_{10}(k_1'k_2')$	3.482	< −1.005	> 9.122	optim	optim
b2	$\log_{10}(k_2 \text{ or } k_{deg})$	4.094	−0.6196	> 9.797	optim	optim
b2	$\log_{10}(k_2 \text{ or } k_{deg})$	−0.9283	−1.178	> 0.4921	approximate	optim
b3	$\log_{10}(k_1')$	0.1888	−0.284	> 7.997	approximate	optim
b3	$\log_{10}(k_2)$	−0.08802	< −0.4707	> 7.751	optim	optim
b3	$\log_{10}(k_2')$	−1.075	−1.309	−0.8399	approximate	approximate
b3	$\log_{10}(k_{deg})$	−1.236	−1.549	−0.9779	approximate	approximate
b3	$\log_{10}(k_1'/k_2)$	0.2768	0.1931	0.3751	approximate	approximate
b3	$\log_{10}(k_1'k_2'/k_2)$	−0.7978	−1.027	−0.5592	approximate	approximate
b3	$\log_{10}(k_2'/k_2)$	−0.9866	< −8.903	−0.2978	optim	approximate

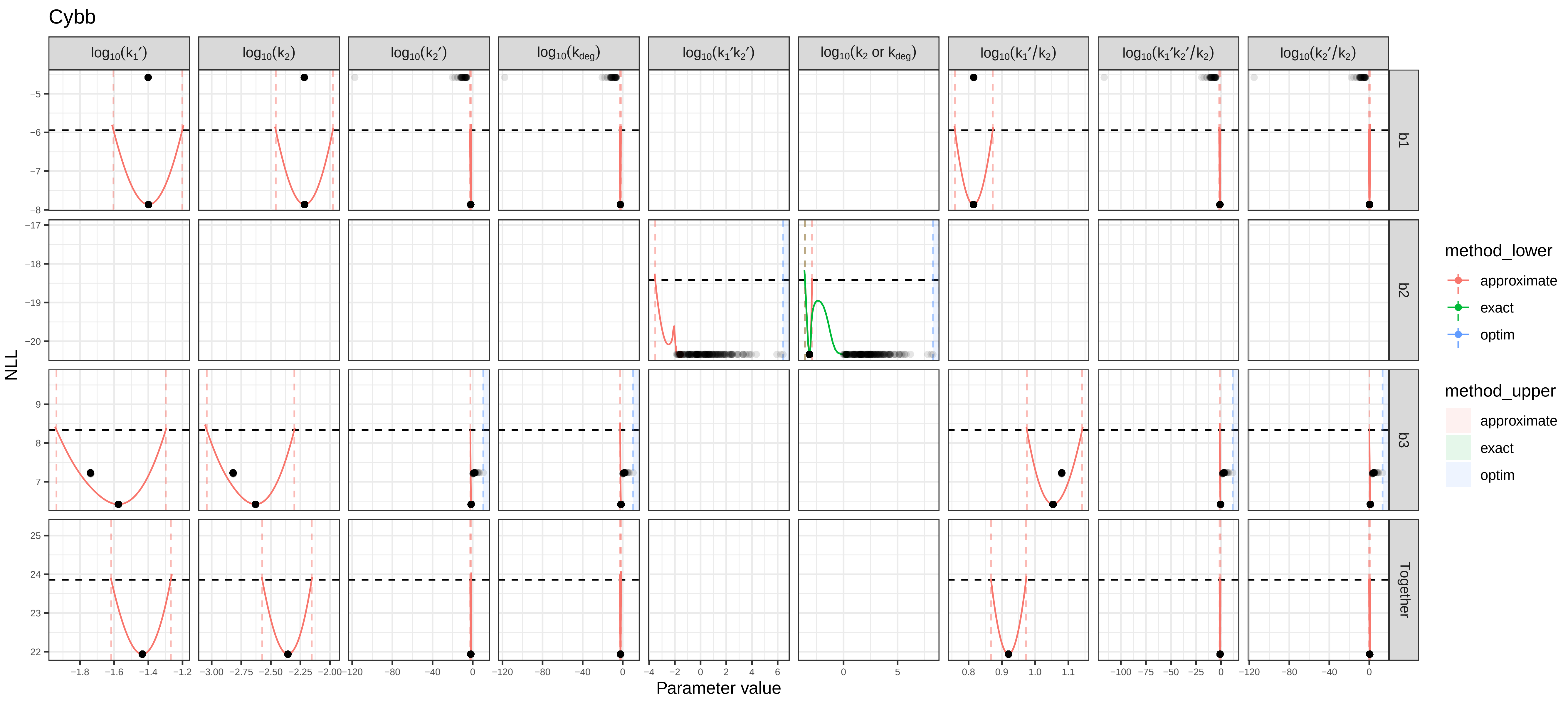

Replicate	Par	Best value	CI95 LB	CI95 UB	Method LB	Method UB
Together	$\log_{10}(k_1')$	−1.435	−1.618	−1.268	approximate	approximate
Together	$\log_{10}(k_2)$	−2.355	−2.572	−2.154	approximate	approximate
Together	$\log_{10}(k_2')$	−1.928	−2.476	−1.504	approximate	approximate
Together	$\log_{10}(k_{deg})$	−2.227	−2.798	−1.766	approximate	approximate
Together	$\log_{10}(k_1'/k_2)$	0.9201	0.8675	0.973	approximate	approximate
Together	$\log_{10}(k_1'k_2'/k_2)$	−1.008	−1.563	−0.5608	approximate	approximate
Together	$\log_{10}(k_2'/k_2)$	0.4267	−0.1834	1.006	approximate	approximate
b1	$\log_{10}(k_1')$	−1.399	−1.604	−1.201	approximate	approximate
b1	$\log_{10}(k_2)$	−2.214	−2.458	−1.975	approximate	approximate
b1	$\log_{10}(k_2')$	−2.01	−2.719	−1.553	approximate	approximate
b1	$\log_{10}(k_{deg})$	−2.339	−3.072	−1.846	approximate	approximate
b1	$\log_{10}(k_1'/k_2)$	0.8149	0.759	0.8728	approximate	approximate
b1	$\log_{10}(k_1'k_2'/k_2)$	−1.195	−1.909	−0.7148	approximate	approximate
b1	$\log_{10}(k_2'/k_2)$	0.2043	−0.5522	0.8301	approximate	approximate
b2	$\log_{10}(k_1'k_2')$	−1.574	−3.529	> 6.42	approximate	optim
b2	$\log_{10}(k_2 \text{ or } k_{deg})$	0.2723	−3.571	> 8.269	exact	optim
b2	$\log_{10}(k_2 \text{ or } k_{deg})$	−3.154	−3.571	−2.918	approximate	approximate
b3	$\log_{10}(k_1')$	−1.575	−1.938	−1.298	approximate	approximate
b3	$\log_{10}(k_2)$	−2.629	−3.042	−2.301	approximate	approximate
b3	$\log_{10}(k_2')$	−1.591	−2.345	> 10.47	approximate	optim
b3	$\log_{10}(k_{deg})$	−1.739	−2.538	> 10.37	approximate	optim
b3	$\log_{10}(k_1'/k_2)$	1.054	0.9753	1.142	approximate	approximate
b3	$\log_{10}(k_1'k_2'/k_2)$	−0.5371	−1.307	> 11.55	approximate	optim
b3	$\log_{10}(k_2'/k_2)$	1.038	0.1263	> 13.28	approximate	optim

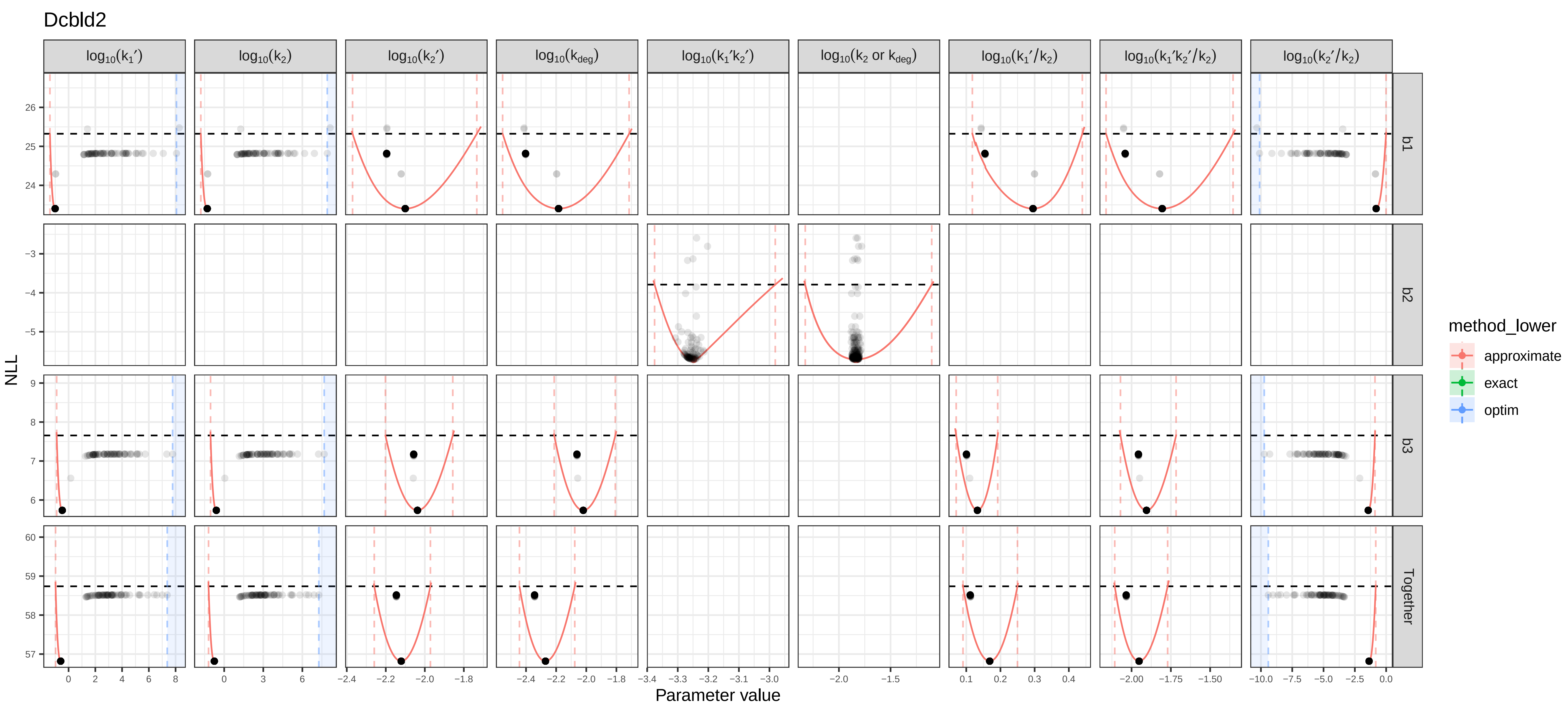

Replicate	Par	Best value	CI95 LB	CI95 UB	Method LB	Method UB
Together	$\log_{10}(k_1')$	−0.5893	−0.9713	> 7.376	approximate	optim
Together	$\log_{10}(k_2)$	−0.7579	−1.201	> 7.264	approximate	optim
Together	$\log_{10}(k_2')$	−2.121	−2.259	−1.971	approximate	approximate
Together	$\log_{10}(k_{deg})$	−2.271	−2.444	−2.076	approximate	approximate
Together	$\log_{10}(k_1'/k_2)$	0.1686	0.09045	0.2498	approximate	approximate
Together	$\log_{10}(k_1'k_2'/k_2)$	−1.952	−2.107	−1.77	approximate	approximate
Together	$\log_{10}(k_2'/k_2)$	−1.363	< −9.41	−0.8306	optim	approximate
b1	$\log_{10}(k_1')$	−1.005	−1.392	> 8.065	approximate	optim
b1	$\log_{10}(k_2)$	−1.3	−1.794	> 7.911	approximate	optim
b1	$\log_{10}(k_2')$	−2.1	−2.37	−1.733	approximate	approximate
b1	$\log_{10}(k_{deg})$	−2.184	−2.555	−1.715	approximate	approximate
b1	$\log_{10}(k_1'/k_2)$	0.2954	0.1179	0.4395	approximate	approximate
b1	$\log_{10}(k_1'k_2'/k_2)$	−1.805	−2.162	−1.354	approximate	approximate
b1	$\log_{10}(k_2'/k_2)$	−0.8001	< −10.11	−0.007112	optim	approximate
b2	$\log_{10}(k_1'k_2')$	−3.248	−3.376	−2.981	approximate	approximate
b2	$\log_{10}(k_2 \text{ or } k_{deg})$	−1.826	−2.326	−1.102	approximate	approximate
b2	$\log_{10}(k_2 \text{ or } k_{deg})$	−1.84	−2.326	−1.102	approximate	approximate
b3	$\log_{10}(k_1')$	−0.4731	−0.8889	> 7.778	approximate	optim
b3	$\log_{10}(k_2)$	−0.6057	−1.047	> 7.677	approximate	optim
b3	$\log_{10}(k_2')$	−2.038	−2.202	−1.857	approximate	approximate
b3	$\log_{10}(k_{deg})$	−2.02	−2.213	−1.807	approximate	approximate
b3	$\log_{10}(k_1'/k_2)$	0.1326	0.07057	0.192	approximate	approximate
b3	$\log_{10}(k_1'k_2'/k_2)$	−1.905	−2.071	−1.717	approximate	approximate
b3	$\log_{10}(k_2'/k_2)$	−1.432	< −9.734	−0.8925	optim	approximate

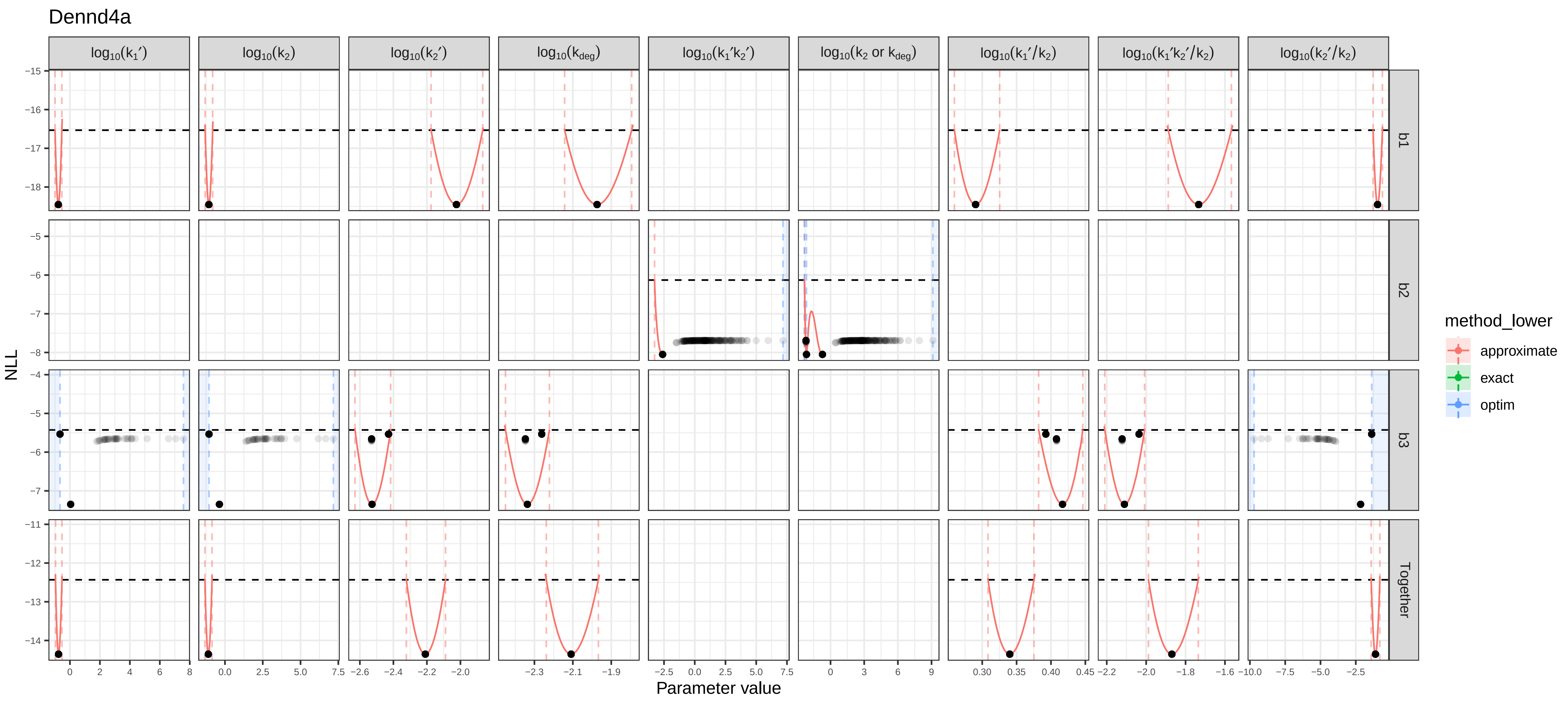

Replicate	Par	Best value	CI95 LB	CI95 UB	Method LB	Method UB
Together	$\log_{10}(k_1')$	−0.7604	−0.9626	−0.5324	approximate	approximate
Together	$\log_{10}(k_2)$	−1.1	−1.327	−0.8549	approximate	approximate
Together	$\log_{10}(k_2')$	−2.209	−2.323	−2.089	approximate	approximate
Together	$\log_{10}(k_{deg})$	−2.109	−2.238	−1.967	approximate	approximate
Together	$\log_{10}(k_1'/k_2)$	0.3401	0.3084	0.3753	approximate	approximate
Together	$\log_{10}(k_1'k_2'/k_2)$	−1.869	−1.988	−1.735	approximate	approximate
Together	$\log_{10}(k_2'/k_2)$	−1.109	−1.411	−0.7974	approximate	approximate
b1	$\log_{10}(k_1')$	−0.7752	−0.9962	−0.5401	approximate	approximate
b1	$\log_{10}(k_2)$	−1.066	−1.311	−0.8154	approximate	approximate
b1	$\log_{10}(k_2')$	−2.024	−2.175	−1.867	approximate	approximate
b1	$\log_{10}(k_{deg})$	−1.974	−2.143	−1.794	approximate	approximate
b1	$\log_{10}(k_1'/k_2)$	0.2904	0.2595	0.3254	approximate	approximate
b1	$\log_{10}(k_1'k_2'/k_2)$	−1.733	−1.888	−1.567	approximate	approximate
b1	$\log_{10}(k_2'/k_2)$	−0.9582	−1.277	−0.6139	approximate	approximate
b2	$\log_{10}(k_1'k_2')$	−2.613	−3.266	> 7.18	approximate	optim
b2	$\log_{10}(k_2 \text{ or } k_{deg})$	−0.7199	−2.325	> 9.122	approximate	optim
b2	$\log_{10}(k_2 \text{ or } k_{deg})$	−2.145	−2.325	−2.144	approximate	optim
b3	$\log_{10}(k_1')$	0.04797	< −0.6622	> 7.579	optim	optim
b3	$\log_{10}(k_2)$	−0.3689	< −1.055	> 7.171	optim	optim
b3	$\log_{10}(k_2')$	−2.527	−2.629	−2.416	approximate	approximate
b3	$\log_{10}(k_{deg})$	−2.337	−2.451	−2.222	approximate	approximate
b3	$\log_{10}(k_1'/k_2)$	0.4168	0.3821	0.4463	approximate	approximate
b3	$\log_{10}(k_1'k_2'/k_2)$	−2.11	−2.21	−2.008	approximate	approximate
b3	$\log_{10}(k_2'/k_2)$	−2.158	< −9.701	> −1.374	optim	optim

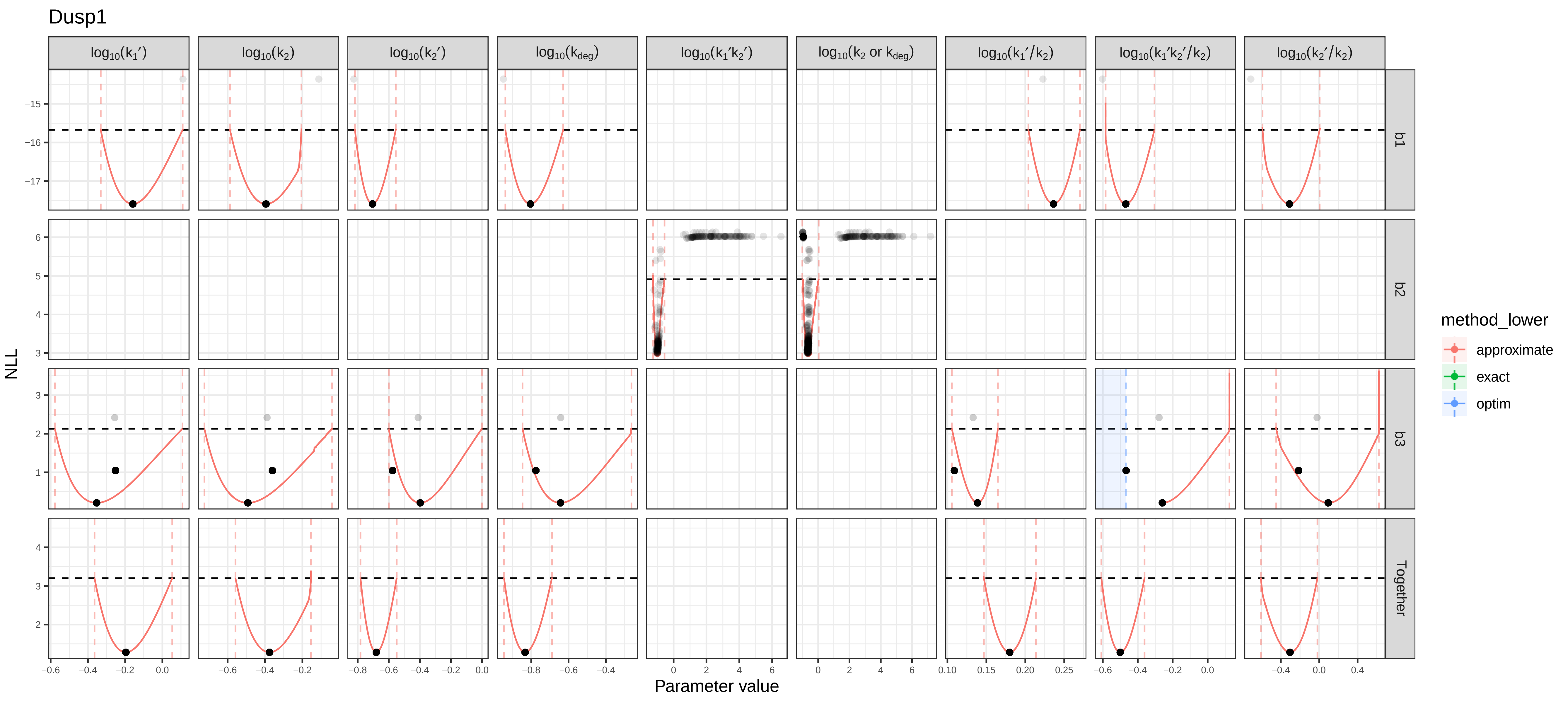

Replicate	Par	Best value	CI95 LB	CI95 UB	Method LB	Method UB
Together	$\log_{10}(k_1')$	−0.1962	−0.3643	0.05311	approximate	approximate
Together	$\log_{10}(k_2)$	−0.3762	−0.5583	−0.1538	approximate	approximate
Together	$\log_{10}(k_2')$	−0.6801	−0.782	−0.5492	approximate	approximate
Together	$\log_{10}(k_{deg})$	−0.8323	−0.9451	−0.6893	approximate	approximate
Together	$\log_{10}(k_1'/k_2)$	0.1799	0.1468	0.2137	approximate	approximate
Together	$\log_{10}(k_1'k_2'/k_2)$	−0.5001	−0.6082	−0.3612	approximate	approximate
Together	$\log_{10}(k_2'/k_2)$	−0.3039	−0.6077	−0.01843	approximate	approximate
b1	$\log_{10}(k_1')$	−0.1584	−0.3309	0.1091	approximate	approximate
b1	$\log_{10}(k_2)$	−0.3947	−0.5876	−0.2053	approximate	approximate
b1	$\log_{10}(k_2')$	−0.7047	−0.8174	−0.5552	approximate	approximate
b1	$\log_{10}(k_{deg})$	−0.8042	−0.938	−0.6288	approximate	approximate
b1	$\log_{10}(k_1'/k_2)$	0.2363	0.204	0.2702	approximate	approximate
b1	$\log_{10}(k_1'k_2'/k_2)$	−0.4684	−0.5838	−0.3043	approximate	approximate
b1	$\log_{10}(k_2'/k_2)$	−0.31	−0.5905	0.004731	approximate	approximate
b2	$\log_{10}(k_1'k_2')$	−0.9853	−1.258	−0.5536	approximate	approximate
b2	$\log_{10}(k_2 \text{ or } k_{deg})$	−0.6316	−1.012	0.03113	approximate	approximate
b2	$\log_{10}(k_2 \text{ or } k_{deg})$	−0.6809	−1.012	0.03113	approximate	approximate
b3	$\log_{10}(k_1')$	−0.3531	−0.5773	0.1078	approximate	approximate
b3	$\log_{10}(k_2)$	−0.4918	−0.7244	−0.04071	approximate	approximate
b3	$\log_{10}(k_2')$	−0.3977	−0.6002	−0.0009712	approximate	approximate
b3	$\log_{10}(k_{deg})$	−0.643	−0.8462	−0.2639	approximate	approximate
b3	$\log_{10}(k_1'/k_2)$	0.1387	0.1057	0.1649	approximate	approximate
b3	$\log_{10}(k_1'k_2'/k_2)$	−0.259	< −0.4673	0.1246	optim	approximate
b3	$\log_{10}(k_2'/k_2)$	0.09414	−0.448	0.6226	approximate	approximate

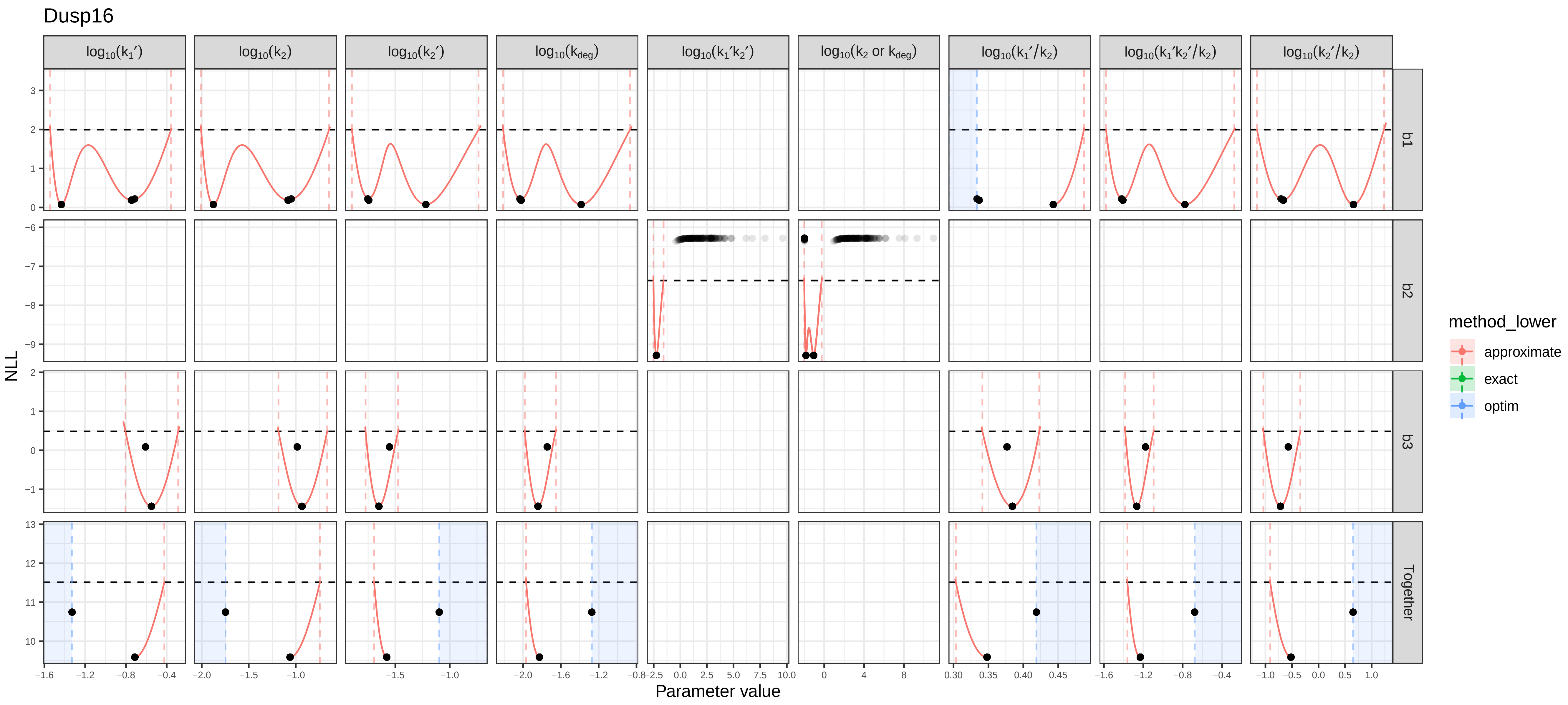

Replicate	Par	Best value	CI95 LB	CI95 UB	Method LB	Method UB
Together	$\log_{10}(k_1')$	−0.7122	< −1.328	−0.4232	optim	approximate
Together	$\log_{10}(k_2)$	−1.06	< −1.747	−0.7421	optim	approximate
Together	$\log_{10}(k_2')$	−1.579	−1.694	> −1.097	approximate	optim
Together	$\log_{10}(k_{deg})$	−1.826	−1.968	> −1.273	approximate	optim
Together	$\log_{10}(k_1'/k_2)$	0.3481	0.3033	> 0.4187	approximate	optim
Together	$\log_{10}(k_1'k_2'/k_2)$	−1.231	−1.361	> −0.6778	approximate	optim
Together	$\log_{10}(k_2'/k_2)$	−0.5186	−0.9107	> 0.6505	approximate	optim
b1	$\log_{10}(k_1')$	−1.434	−1.543	−0.3568	approximate	approximate
b1	$\log_{10}(k_2)$	−1.877	−2.007	−0.6453	approximate	approximate
b1	$\log_{10}(k_2')$	−1.22	−1.899	−0.7353	approximate	approximate
b1	$\log_{10}(k_{deg})$	−1.385	−2.212	−0.8683	approximate	approximate
b1	$\log_{10}(k_1'/k_2)$	0.4429	< 0.3335	0.4869	optim	approximate
b1	$\log_{10}(k_1'k_2'/k_2)$	−0.7769	−1.578	−0.2755	approximate	approximate
b1	$\log_{10}(k_2'/k_2)$	0.6568	−1.16	1.232	approximate	approximate
b2	$\log_{10}(k_1'k_2')$	−2.247	−2.523	−1.571	approximate	approximate
b2	$\log_{10}(k_2 \text{ or } k_{deg})$	−1.057	−1.999	−0.251	approximate	approximate
b2	$\log_{10}(k_2 \text{ or } k_{deg})$	−1.829	−1.999	−0.251	approximate	approximate
b3	$\log_{10}(k_1')$	−0.5499	−0.8047	−0.287	approximate	approximate
b3	$\log_{10}(k_2)$	−0.9342	−1.184	−0.6653	approximate	approximate
b3	$\log_{10}(k_2')$	−1.651	−1.773	−1.474	approximate	approximate
b3	$\log_{10}(k_{deg})$	−1.842	−1.982	−1.653	approximate	approximate
b3	$\log_{10}(k_1'/k_2)$	0.3843	0.3414	0.4229	approximate	approximate
b3	$\log_{10}(k_1'k_2'/k_2)$	−1.266	−1.384	−1.095	approximate	approximate
b3	$\log_{10}(k_2'/k_2)$	−0.7163	−1.039	−0.3429	approximate	approximate

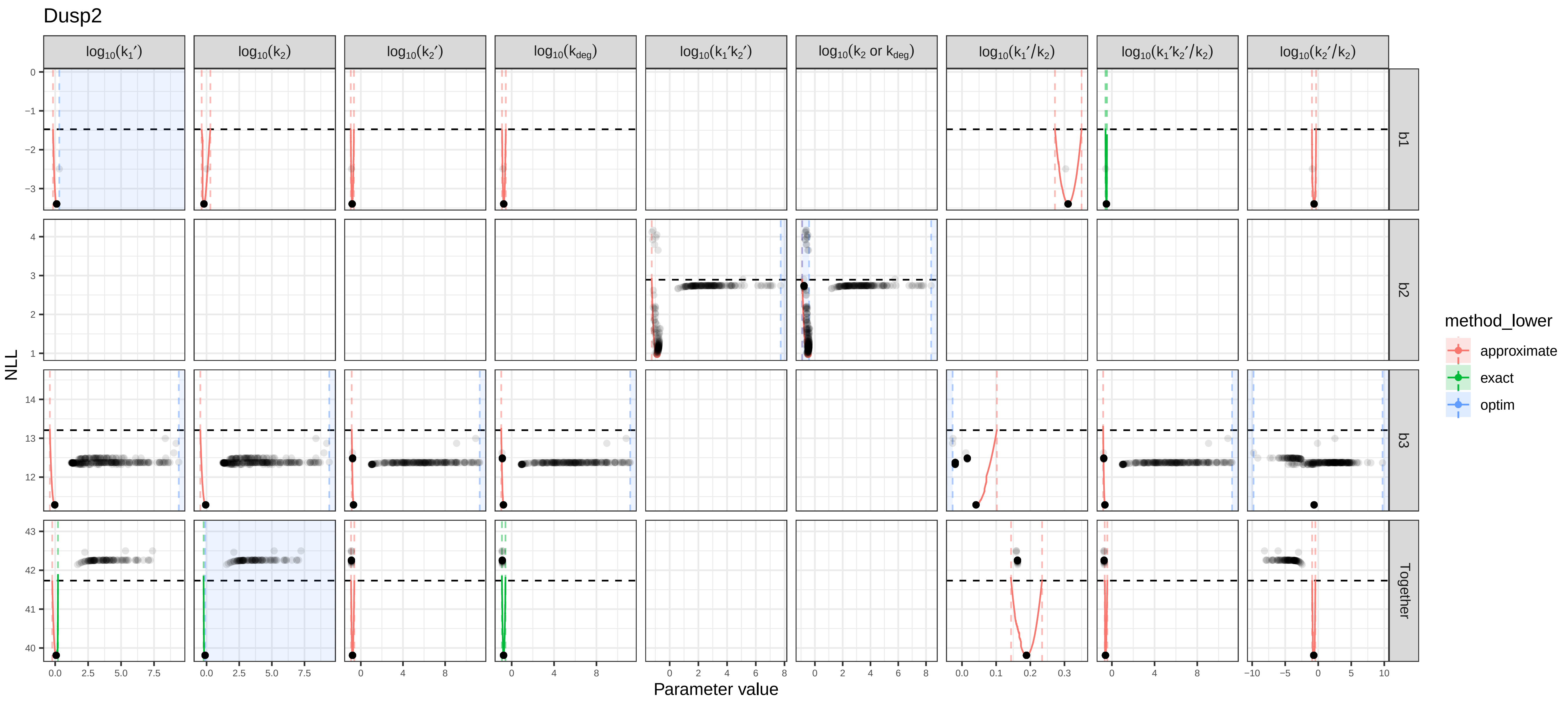

Replicate	Par	**Best value**	**CI95 LB**	**CI95 UB**	**Method LB**	**Method UB**
Together	$\log_{10}(k_1')$	0.07577	−0.2241	0.2119	approximate	exact
Together	$\log_{10}(k_2)$	−0.113	−0.2261	> −0.1099	exact	optim
Together	$\log_{10}(k_2')$	−0.7905	−0.9285	−0.6195	approximate	approximate
Together	$\log_{10}(k_{deg})$	−0.7637	−0.9189	−0.5843	exact	exact
Together	$\log_{10}(k_1'/k_2)$	0.1888	0.1437	0.2344	approximate	approximate
Together	$\log_{10}(k_1'k_2'/k_2)$	−0.6018	−0.6729	−0.4286	approximate	approximate
Together	$\log_{10}(k_2'/k_2)$	−0.6775	−0.9245	−0.4318	approximate	approximate
b1	$\log_{10}(k_1')$	0.1092	−0.1664	> 0.3108	approximate	optim
b1	$\log_{10}(k_2)$	−0.2015	−0.3774	0.2885	approximate	approximate
b1	$\log_{10}(k_2')$	−0.8234	−0.9566	−0.6522	approximate	approximate
b1	$\log_{10}(k_{deg})$	−0.7483	−0.9129	−0.5559	approximate	approximate
b1	$\log_{10}(k_1'/k_2)$	0.3106	0.272	0.3501	approximate	approximate
b1	$\log_{10}(k_1'k_2'/k_2)$	−0.5128	−0.6014	−0.4615	exact	exact
b1	$\log_{10}(k_2'/k_2)$	−0.622	−0.9494	−0.32	approximate	approximate
b2	$\log_{10}(k_1'k_2')$	−0.8806	−1.25	> 7.733	approximate	optim
b2	$\log_{10}(k_2 \text{ or } k_{deg})$	−0.4909	−0.9165	> 8.367	approximate	optim
b2	$\log_{10}(k_2 \text{ or } k_{deg})$	−0.5393	−0.9165	−0.4181	approximate	optim
b3	$\log_{10}(k_1')$	−0.02841	−0.4047	> 9.378	approximate	optim
b3	$\log_{10}(k_2)$	−0.06948	−0.4815	> 9.397	approximate	optim
b3	$\log_{10}(k_2')$	−0.6929	−0.8659	> 11.28	approximate	optim
b3	$\log_{10}(k_{deg})$	−0.7775	−0.984	> 11.19	approximate	optim
b3	$\log_{10}(k_1'/k_2)$	0.04108	< −0.02745	0.102	optim	approximate
b3	$\log_{10}(k_1'k_2'/k_2)$	−0.6518	−0.8199	> 11.26	approximate	optim
b3	$\log_{10}(k_2'/k_2)$	−0.6234	< −9.79	> 9.738	optim	optim

Dusp4

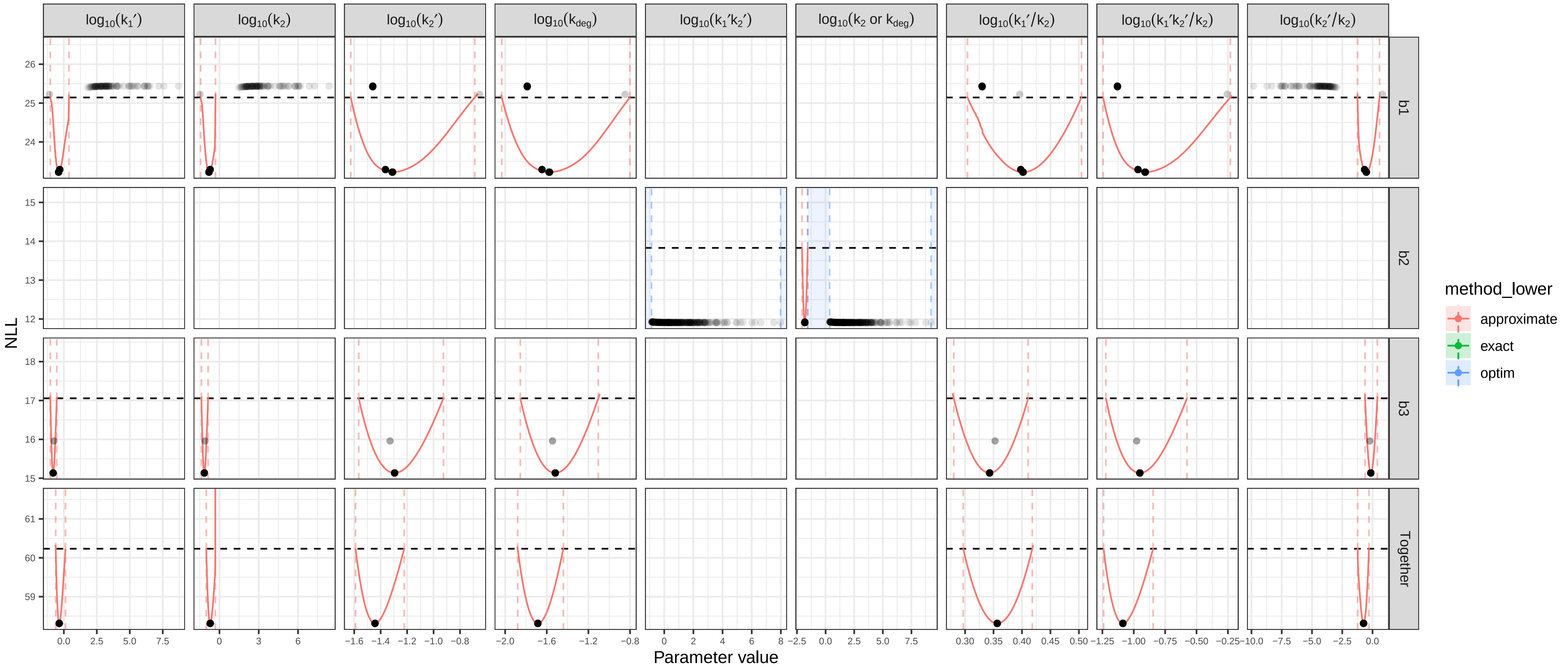

Replicate	Par	Best value	CI95 LB	CI95 UB	Method LB	Method UB
Together	$\log_{10}(k_1')$	−0.3473	−0.6182	0.1252	approximate	approximate
Together	$\log_{10}(k_2)$	−0.7038	−1.007	−0.313	approximate	approximate
Together	$\log_{10}(k_2')$	−1.443	−1.59	−1.222	approximate	approximate
Together	$\log_{10}(k_{deg})$	−1.686	−1.88	−1.441	approximate	approximate
Together	$\log_{10}(k_1'/k_2)$	0.3565	0.2967	0.4181	approximate	approximate
Together	$\log_{10}(k_1'k_2'/k_2)$	−1.087	−1.241	−0.8459	approximate	approximate
Together	$\log_{10}(k_2'/k_2)$	−0.7394	−1.227	−0.2924	approximate	approximate
b1	$\log_{10}(k_1')$	−0.3972	−1.027	0.3837	approximate	approximate
b1	$\log_{10}(k_2)$	−0.799	−1.441	−0.3038	approximate	approximate
b1	$\log_{10}(k_2')$	−1.311	−1.627	−0.6889	approximate	approximate
b1	$\log_{10}(k_{deg})$	−1.576	−2.033	−0.8016	approximate	approximate
b1	$\log_{10}(k_1'/k_2)$	0.4018	0.3041	0.5046	approximate	approximate
b1	$\log_{10}(k_1'k_2'/k_2)$	−0.9095	−1.244	−0.2307	approximate	approximate
b1	$\log_{10}(k_2'/k_2)$	−0.5123	−1.24	0.5769	approximate	approximate
b2	$\log_{10}(k_1'k_2')$	3.58	< −0.8711	> 7.978	optim	optim
b2	$\log_{10}(k_2 \text{ or } k_{deg})$	4.815	0.3534	> 9.213	optim	optim
b2	$\log_{10}(k_2 \text{ or } k_{deg})$	−1.821	−2.059	−1.565	approximate	approximate
b3	$\log_{10}(k_1')$	−0.8117	−1.028	−0.5358	approximate	approximate
b3	$\log_{10}(k_2)$	−1.155	−1.377	−0.8671	approximate	approximate
b3	$\log_{10}(k_2')$	−1.295	−1.566	−0.9255	approximate	approximate
b3	$\log_{10}(k_{deg})$	−1.518	−1.855	−1.105	approximate	approximate
b3	$\log_{10}(k_1'/k_2)$	0.3431	0.28	0.4108	approximate	approximate
b3	$\log_{10}(k_1'k_2'/k_2)$	−0.9517	−1.222	−0.5759	approximate	approximate
b3	$\log_{10}(k_2'/k_2)$	−0.14	−0.6196	0.3984	approximate	approximate

Dusp5

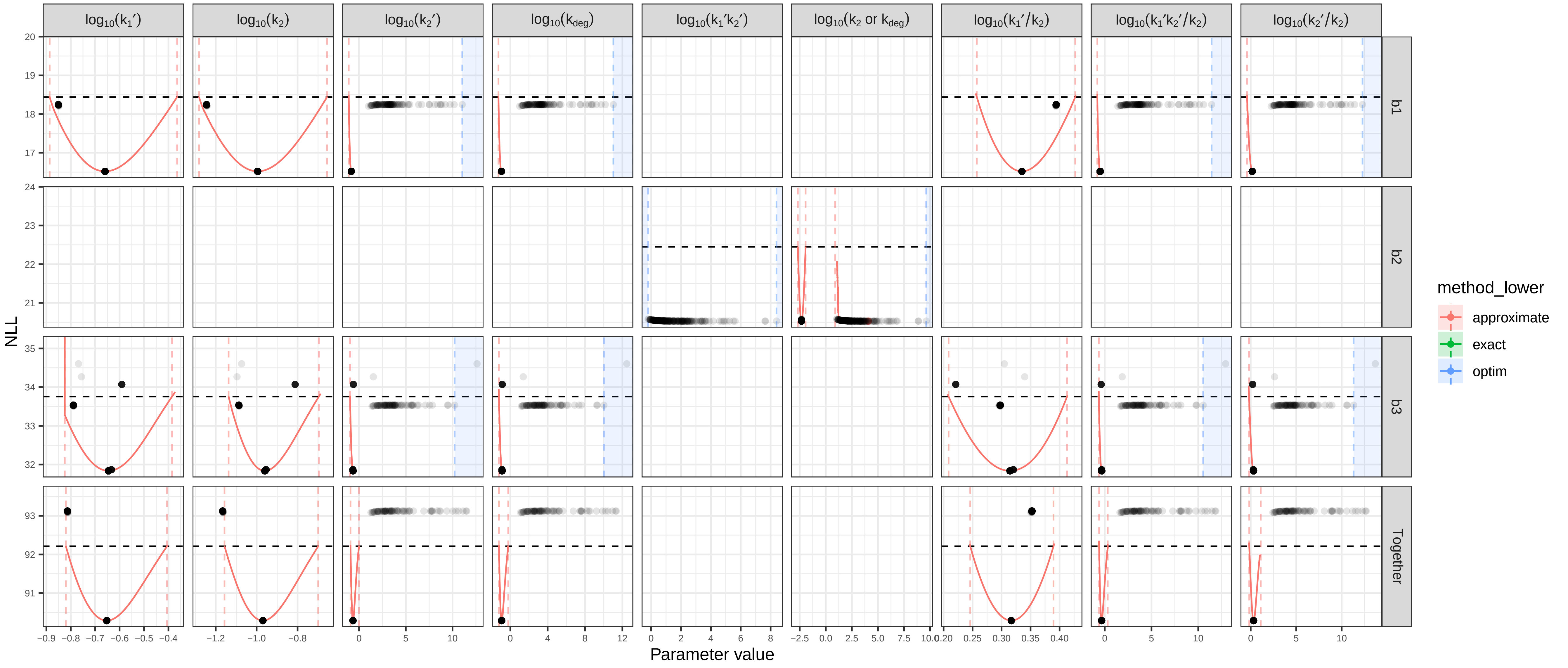

Replicate	Par	Best value	CI95 LB	CI95 UB	Method LB	Method UB
Together	$\log_{10}(k_1')$	−0.6528	−0.82	−0.4058	approximate	approximate
Together	$\log_{10}(k_2)$	−0.9696	−1.157	−0.6991	approximate	approximate
Together	$\log_{10}(k_2')$	−0.6494	−0.9115	−0.002857	approximate	approximate
Together	$\log_{10}(k_{deg})$	−0.9254	−1.217	−0.2238	approximate	approximate
Together	$\log_{10}(k_1'/k_2)$	0.3168	0.246	0.3896	approximate	approximate
Together	$\log_{10}(k_1'k_2'/k_2)$	−0.3326	−0.604	0.321	approximate	approximate
Together	$\log_{10}(k_2'/k_2)$	0.3202	−0.1583	1.104	approximate	approximate
b1	$\log_{10}(k_1')$	−0.6599	−0.8864	−0.3644	approximate	approximate
b1	$\log_{10}(k_2)$	−0.995	−1.282	−0.6556	approximate	approximate
b1	$\log_{10}(k_2')$	−0.8256	−1.101	> 11.04	approximate	optim
b1	$\log_{10}(k_{deg})$	−0.9482	−1.271	> 11.03	approximate	optim
b1	$\log_{10}(k_1'/k_2)$	0.3352	0.2573	0.427	approximate	approximate
b1	$\log_{10}(k_1'k_2'/k_2)$	−0.4904	−0.7947	> 11.43	approximate	optim
b1	$\log_{10}(k_2'/k_2)$	0.1694	−0.3996	> 12.28	approximate	optim
b2	$\log_{10}(k_1'k_2')$	2.838	< −0.1996	> 8.393	optim	optim
b2	$\log_{10}(k_2 \text{ or } k_{deg})$	4.075	0.9019	> 9.629	approximate	optim
b2	$\log_{10}(k_2 \text{ or } k_{deg})$	−2.329	−2.68	−1.936	approximate	approximate
b3	$\log_{10}(k_1')$	−0.6455	−0.8245	−0.3851	approximate	approximate
b3	$\log_{10}(k_2)$	−0.9601	−1.137	−0.6957	approximate	approximate
b3	$\log_{10}(k_2')$	−0.6429	−0.9609	> 10.23	approximate	optim
b3	$\log_{10}(k_{deg})$	−0.8833	−1.222	> 10.02	approximate	optim
b3	$\log_{10}(k_1'/k_2)$	0.3146	0.2086	0.413	approximate	approximate
b3	$\log_{10}(k_1'k_2'/k_2)$	−0.3283	−0.6359	> 10.53	approximate	optim
b3	$\log_{10}(k_2'/k_2)$	0.3172	−0.1809	> 11.32	approximate	optim

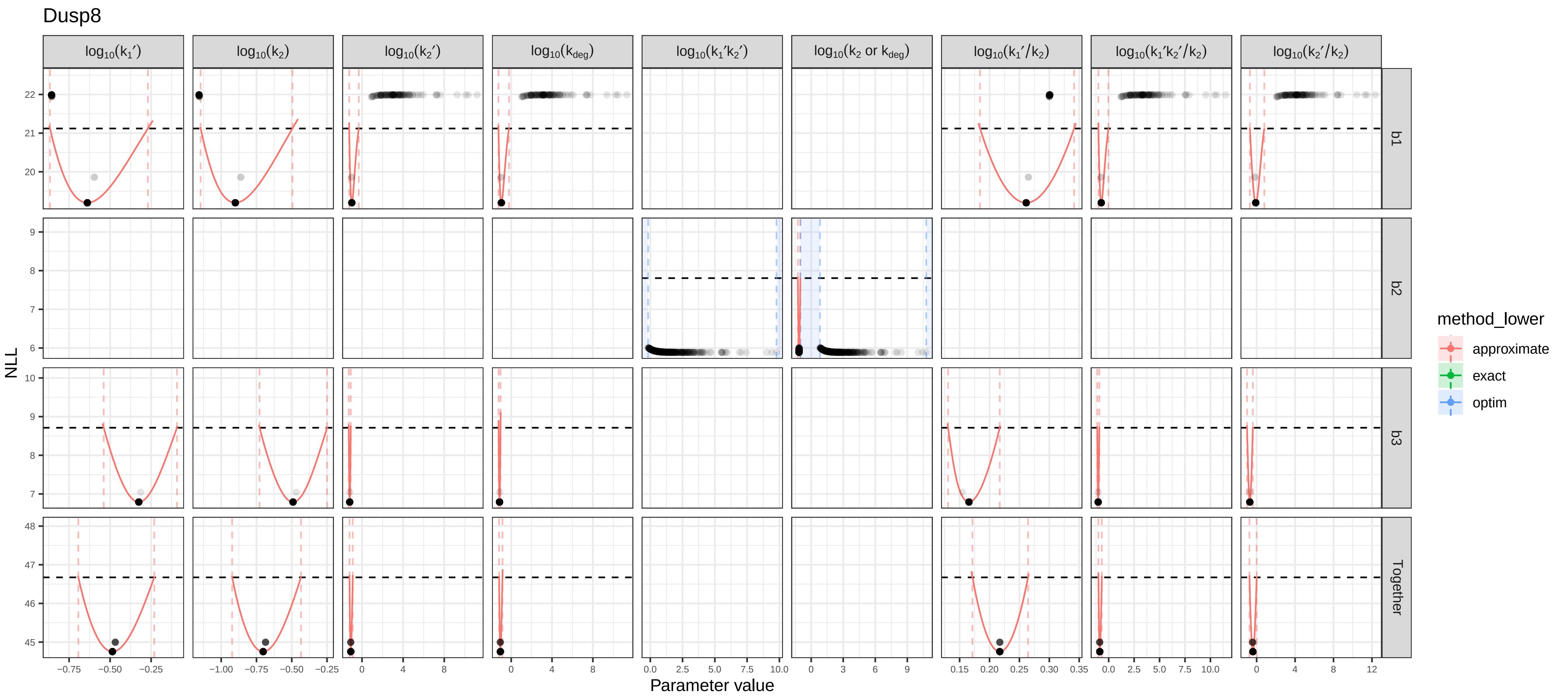

Replicate	Par	Best value	CI95 LB	CI95 UB	Method LB	Method UB
Together	$\log_{10}(k_1')$	−0.4861	−0.6935	−0.2311	approximate	approximate
Together	$\log_{10}(k_2)$	−0.7032	−0.9225	−0.4345	approximate	approximate
Together	$\log_{10}(k_2')$	−1.091	−1.217	−0.9002	approximate	approximate
Together	$\log_{10}(k_{deg})$	−1.057	−1.183	−0.8543	approximate	approximate
Together	$\log_{10}(k_1'/k_2)$	0.2172	0.1715	0.2645	approximate	approximate
Together	$\log_{10}(k_1'k_2'/k_2)$	−0.874	−1.003	−0.6709	approximate	approximate
Together	$\log_{10}(k_2'/k_2)$	−0.3879	−0.7488	0.007627	approximate	approximate
b1	$\log_{10}(k_1')$	−0.6388	−0.8661	−0.2697	approximate	approximate
b1	$\log_{10}(k_2)$	−0.9002	−1.146	−0.4952	approximate	approximate
b1	$\log_{10}(k_2')$	−0.9932	−1.25	−0.3157	approximate	approximate
b1	$\log_{10}(k_{deg})$	−0.9635	−1.245	−0.2143	approximate	approximate
b1	$\log_{10}(k_1'/k_2)$	0.2614	0.1841	0.3415	approximate	approximate
b1	$\log_{10}(k_1'k_2'/k_2)$	−0.7318	−1.008	−0.0248	approximate	approximate
b1	$\log_{10}(k_2'/k_2)$	−0.09299	−0.7018	0.7997	approximate	approximate
b2	$\log_{10}(k_1'k_2')$	3.806	< −0.1666	> 9.775	optim	optim
b2	$\log_{10}(k_2 \text{ or } k_{deg})$	4.802	0.8215	> 10.77	optim	optim
b2	$\log_{10}(k_2 \text{ or } k_{deg})$	−1.138	−1.247	−1.014	approximate	approximate
b3	$\log_{10}(k_1')$	−0.3254	−0.5389	−0.09237	approximate	approximate
b3	$\log_{10}(k_2)$	−0.4911	−0.7284	−0.2496	approximate	approximate
b3	$\log_{10}(k_2')$	−1.199	−1.293	−1.094	approximate	approximate
b3	$\log_{10}(k_{deg})$	−1.144	−1.233	−1.043	approximate	approximate
b3	$\log_{10}(k_1'/k_2)$	0.1657	0.1308	0.2171	approximate	approximate
b3	$\log_{10}(k_1'k_2'/k_2)$	−1.033	−1.119	−0.9135	approximate	approximate
b3	$\log_{10}(k_2'/k_2)$	−0.708	−0.9976	−0.3951	approximate	approximate

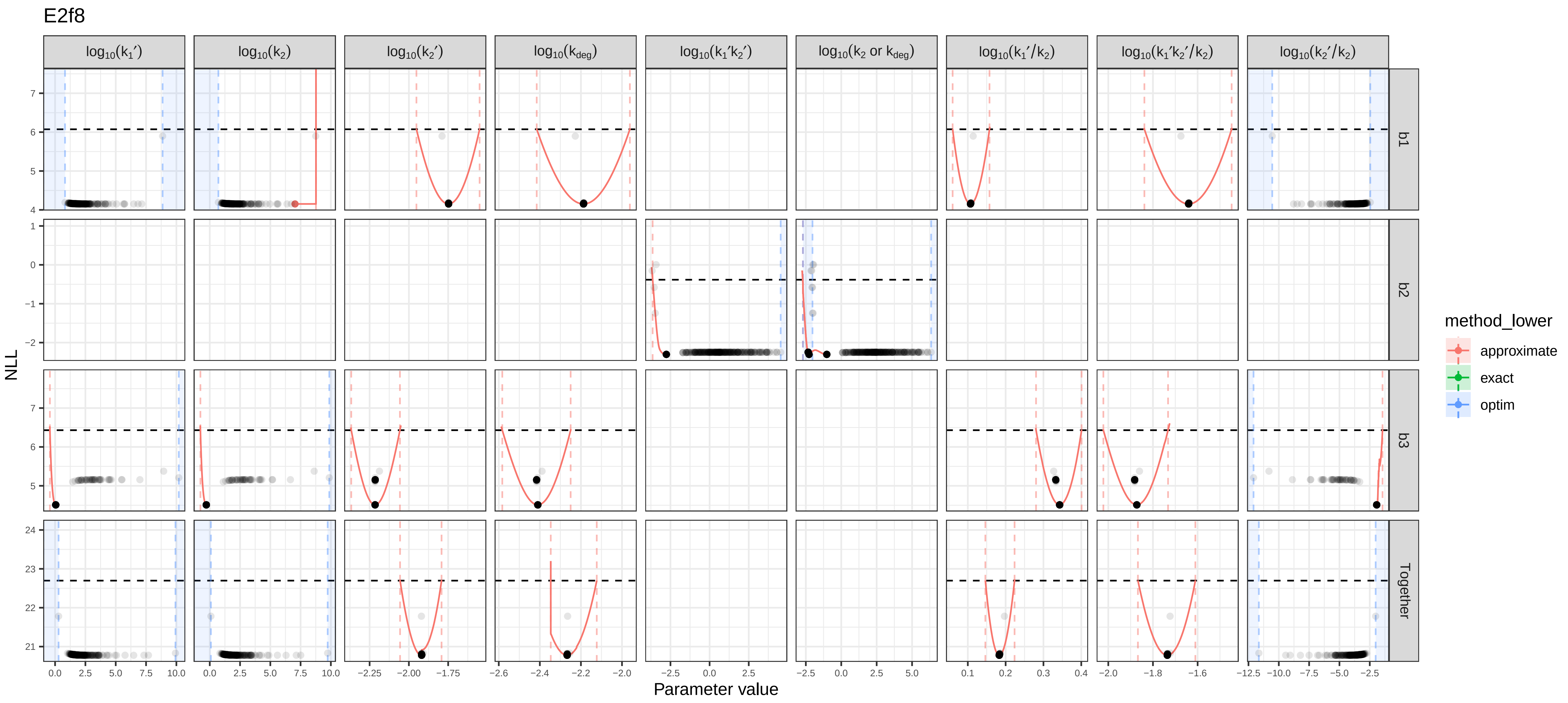

Replicate	Par	Best value	CI95 LB	CI95 UB	Method LB	Method UB
Together	$\log_{10}(k_1')$	4.446	< 0.2898	> 9.923	optim	optim
Together	$\log_{10}(k_2)$	4.263	< 0.09301	> 9.737	optim	optim
Together	$\log_{10}(k_2')$	−1.919	−2.056	−1.792	approximate	approximate
Together	$\log_{10}(k_{deg})$	−2.267	−2.347	−2.123	approximate	approximate
Together	$\log_{10}(k_1'/k_2)$	0.1832	0.1464	0.2236	approximate	approximate
Together	$\log_{10}(k_1'k_2'/k_2)$	−1.736	−1.867	−1.61	approximate	approximate
Together	$\log_{10}(k_2'/k_2)$	−6.182	< −11.64	> −2.013	optim	optim
b1	$\log_{10}(k_1')$	7.147	< 0.8201	> 8.865	optim	optim
b1	$\log_{10}(k_2)$	7.04	< 0.7113	8.771	optim	approximate
b1	$\log_{10}(k_2')$	−1.748	−1.952	−1.55	approximate	approximate
b1	$\log_{10}(k_{deg})$	−2.188	−2.415	−1.962	approximate	approximate
b1	$\log_{10}(k_1'/k_2)$	0.107	0.05984	0.1574	approximate	approximate
b1	$\log_{10}(k_1'k_2'/k_2)$	−1.641	−1.838	−1.448	approximate	approximate
b1	$\log_{10}(k_2'/k_2)$	−8.788	< −10.54	> −2.459	optim	optim
b2	$\log_{10}(k_1'k_2')$	−2.762	−3.638	> 4.539	approximate	optim
b2	$\log_{10}(k_2 \text{ or } k_{deg})$	−1.021	−2.707	> 6.346	approximate	optim
b2	$\log_{10}(k_2 \text{ or } k_{deg})$	−2.273	−2.707	−2.028	approximate	optim
b3	$\log_{10}(k_1')$	0.05751	−0.4255	> 10.2	approximate	optim
b3	$\log_{10}(k_2)$	−0.2852	−0.7739	> 9.868	approximate	optim
b3	$\log_{10}(k_2')$	−2.215	−2.367	−2.057	approximate	approximate
b3	$\log_{10}(k_{deg})$	−2.41	−2.583	−2.25	approximate	approximate
b3	$\log_{10}(k_1'/k_2)$	0.3427	0.2804	0.4009	approximate	approximate
b3	$\log_{10}(k_1'k_2'/k_2)$	−1.872	−2.022	−1.732	approximate	approximate
b3	$\log_{10}(k_2'/k_2)$	−1.93	< −12.08	−1.447	optim	approximate

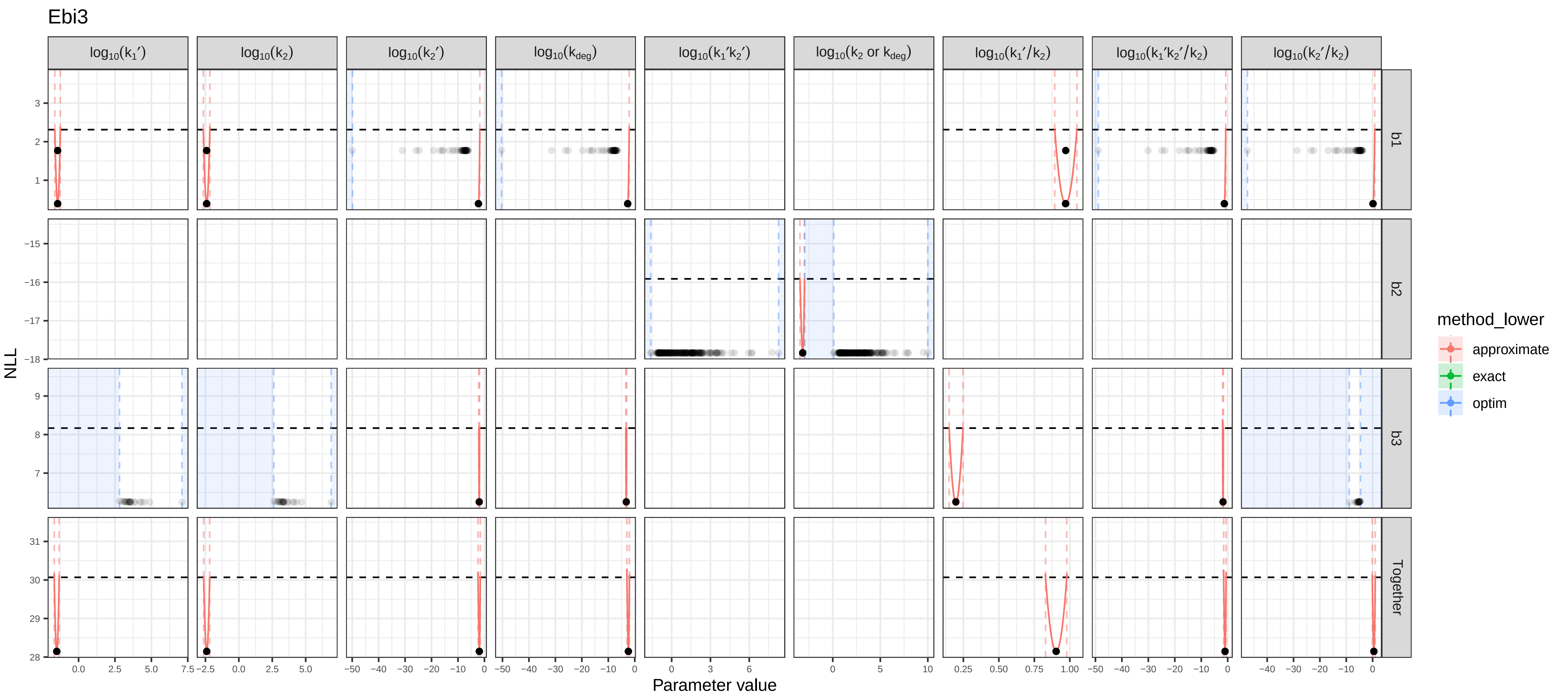

Replicate	Par	Best value	CI95 LB	CI95 UB	Method LB	Method UB
Together	$\log_{10}(k_1')$	−1.499	−1.673	−1.335	approximate	approximate
Together	$\log_{10}(k_2)$	−2.402	−2.63	−2.182	approximate	approximate
Together	$\log_{10}(k_2')$	−1.922	−2.422	−1.554	approximate	approximate
Together	$\log_{10}(k_{deg})$	−2.414	−2.942	−2.001	approximate	approximate
Together	$\log_{10}(k_1'/k_2)$	0.9038	0.8289	0.9786	approximate	approximate
Together	$\log_{10}(k_1'k_2'/k_2)$	−1.019	−1.531	−0.616	approximate	approximate
Together	$\log_{10}(k_2'/k_2)$	0.48	−0.09507	1.01	approximate	approximate
b1	$\log_{10}(k_1')$	−1.437	−1.632	−1.255	approximate	approximate
b1	$\log_{10}(k_2)$	−2.407	−2.655	−2.169	approximate	approximate
b1	$\log_{10}(k_2')$	−2.25	< −49.89	−1.738	optim	approximate
b1	$\log_{10}(k_{deg})$	−2.66	< −50.32	−2.107	optim	approximate
b1	$\log_{10}(k_1'/k_2)$	0.9704	0.8937	1.05	approximate	approximate
b1	$\log_{10}(k_1'k_2'/k_2)$	−1.28	< −48.92	−0.7401	optim	approximate
b1	$\log_{10}(k_2'/k_2)$	0.1568	< −47.48	0.8213	optim	approximate
b2	$\log_{10}(k_1'k_2')$	2.226	< −1.614	> 8.273	optim	optim
b2	$\log_{10}(k_2 \text{ or } k_{deg})$	3.95	0.1058	> 9.998	optim	optim
b2	$\log_{10}(k_2 \text{ or } k_{deg})$	−3.165	−3.451	−2.972	approximate	approximate
b3	$\log_{10}(k_1')$	7.102	< 2.809	> 7.102	optim	optim
b3	$\log_{10}(k_2)$	6.905	< 2.612	> 6.905	optim	optim
b3	$\log_{10}(k_2')$	−1.961	−2.126	−1.831	approximate	approximate
b3	$\log_{10}(k_{deg})$	−3.197	−3.364	−3.064	approximate	approximate
b3	$\log_{10}(k_1'/k_2)$	0.197	0.1497	0.2478	approximate	approximate
b3	$\log_{10}(k_1'k_2'/k_2)$	−1.764	−1.917	−1.643	approximate	approximate
b3	$\log_{10}(k_2'/k_2)$	−8.866	< −8.866	> −4.573	optim	optim

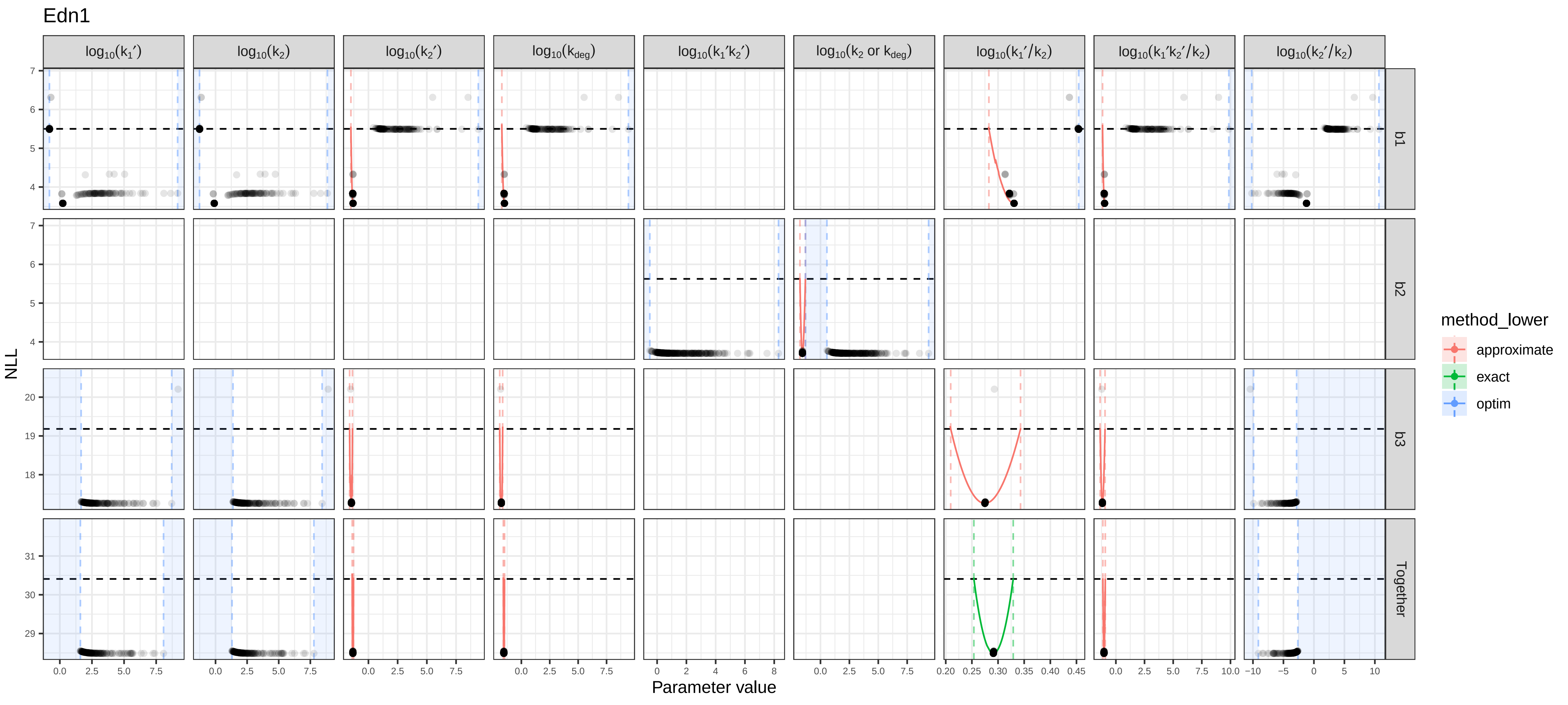

Replicate	Par	Best value	CI95 LB	CI95 UB	Method LB	Method UB
Together	$\log_{10}(k_1')$	5.578	< 1.598	> 8.075	optim	optim
Together	$\log_{10}(k_2)$	5.287	< 1.307	> 7.784	optim	optim
Together	$\log_{10}(k_2')$	−1.329	−1.389	−1.273	approximate	approximate
Together	$\log_{10}(k_{deg})$	−1.543	−1.588	−1.481	approximate	approximate
Together	$\log_{10}(k_1'/k_2)$	0.2911	0.2538	0.3291	exact	exact
Together	$\log_{10}(k_1'k_2'/k_2)$	−1.038	−1.138	−0.9242	approximate	approximate
Together	$\log_{10}(k_2'/k_2)$	−6.615	< −9.112	> −2.634	optim	optim
b1	$\log_{10}(k_1')$	0.2289	< −0.8119	> 9.171	optim	optim
b1	$\log_{10}(k_2)$	−0.1017	< −1.266	> 8.85	optim	optim
b1	$\log_{10}(k_2')$	−1.317	−1.5	> 9.402	approximate	optim
b1	$\log_{10}(k_{deg})$	−1.489	−1.715	> 9.423	approximate	optim
b1	$\log_{10}(k_1'/k_2)$	0.3306	0.2825	> 0.4543	approximate	optim
b1	$\log_{10}(k_1'k_2'/k_2)$	−0.9861	−1.162	> 9.856	approximate	optim
b1	$\log_{10}(k_2'/k_2)$	−1.215	< −10.19	> 10.67	optim	optim
b2	$\log_{10}(k_1'k_2')$	4.629	< −0.4977	> 8.293	optim	optim
b2	$\log_{10}(k_2 \text{ or } k_{deg})$	5.691	0.558	> 9.355	optim	optim
b2	$\log_{10}(k_2 \text{ or } k_{deg})$	−1.563	−1.776	−1.303	approximate	approximate
b3	$\log_{10}(k_1')$	6.242	< 1.656	> 8.708	optim	optim
b3	$\log_{10}(k_2)$	5.967	< 1.381	> 8.433	optim	optim
b3	$\log_{10}(k_2')$	−1.453	−1.609	−1.365	approximate	approximate
b3	$\log_{10}(k_{deg})$	−1.761	−1.903	−1.646	approximate	approximate
b3	$\log_{10}(k_1'/k_2)$	0.2748	0.2095	0.343	approximate	approximate
b3	$\log_{10}(k_1'k_2'/k_2)$	−1.179	−1.373	−0.9461	approximate	approximate
b3	$\log_{10}(k_2'/k_2)$	−7.42	< −9.887	> −2.834	optim	optim

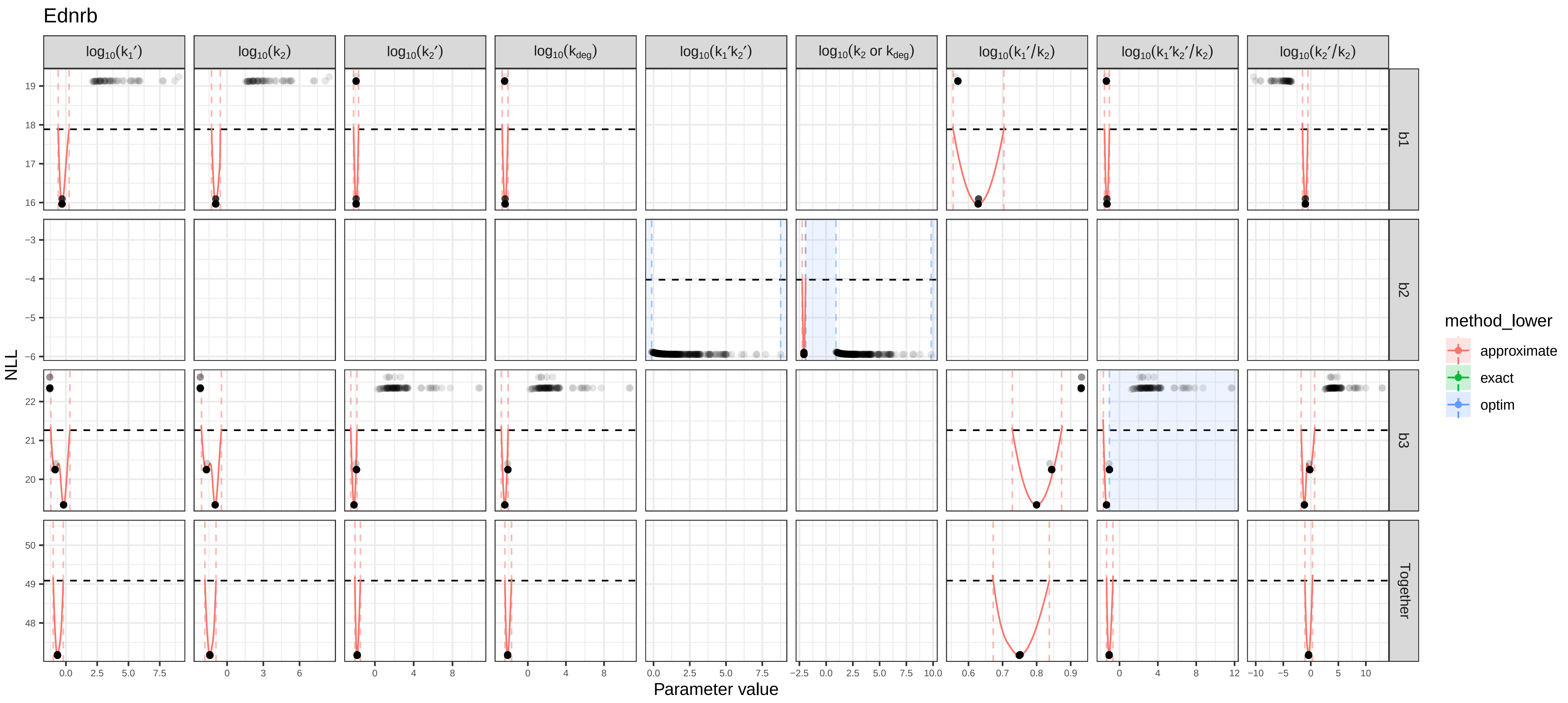

Replicate	Par	Best value	CI95 LB	CI95 UB	Method LB	Method UB
Together	$\log_{10}(k_1')$	−0.6729	−1.015	−0.2108	approximate	approximate
Together	$\log_{10}(k_2)$	−1.422	−1.838	−0.9163	approximate	approximate
Together	$\log_{10}(k_2')$	−1.83	−2.066	−1.494	approximate	approximate
Together	$\log_{10}(k_{deg})$	−2.118	−2.403	−1.708	approximate	approximate
Together	$\log_{10}(k_1'/k_2)$	0.7492	0.6726	0.8373	approximate	approximate
Together	$\log_{10}(k_1'k_2'/k_2)$	−1.081	−1.347	−0.6813	approximate	approximate
Together	$\log_{10}(k_2'/k_2)$	−0.4082	−1.064	0.3108	approximate	approximate
b1	$\log_{10}(k_1')$	−0.3154	−0.6187	0.2541	approximate	approximate
b1	$\log_{10}(k_2)$	−0.9437	−1.287	−0.5583	approximate	approximate
b1	$\log_{10}(k_2')$	−1.934	−2.198	−1.686	approximate	approximate
b1	$\log_{10}(k_{deg})$	−2.384	−2.69	−2.092	approximate	approximate
b1	$\log_{10}(k_1'/k_2)$	0.6283	0.5548	0.7039	approximate	approximate
b1	$\log_{10}(k_1'k_2'/k_2)$	−1.305	−1.567	−1.055	approximate	approximate
b1	$\log_{10}(k_2'/k_2)$	−0.99	−1.503	−0.5361	approximate	approximate
b2	$\log_{10}(k_1'k_2')$	4.492	< −0.1272	> 8.768	optim	optim
b2	$\log_{10}(k_2 \text{ or } k_{deg})$	5.542	0.9232	> 9.819	optim	optim
b2	$\log_{10}(k_2 \text{ or } k_{deg})$	−2.079	−2.244	−1.919	approximate	approximate
b3	$\log_{10}(k_1')$	−0.1878	−1.206	0.3185	approximate	approximate
b3	$\log_{10}(k_2)$	−0.9876	−2.117	−0.4705	approximate	approximate
b3	$\log_{10}(k_2')$	−2.155	−2.483	−1.85	approximate	approximate
b3	$\log_{10}(k_{deg})$	−2.413	−2.78	−2.073	approximate	approximate
b3	$\log_{10}(k_1'/k_2)$	0.7998	0.7288	0.8733	approximate	approximate
b3	$\log_{10}(k_1'k_2'/k_2)$	−1.355	−1.68	> −1.054	approximate	optim
b3	$\log_{10}(k_2'/k_2)$	−1.167	−1.756	0.6917	approximate	approximate

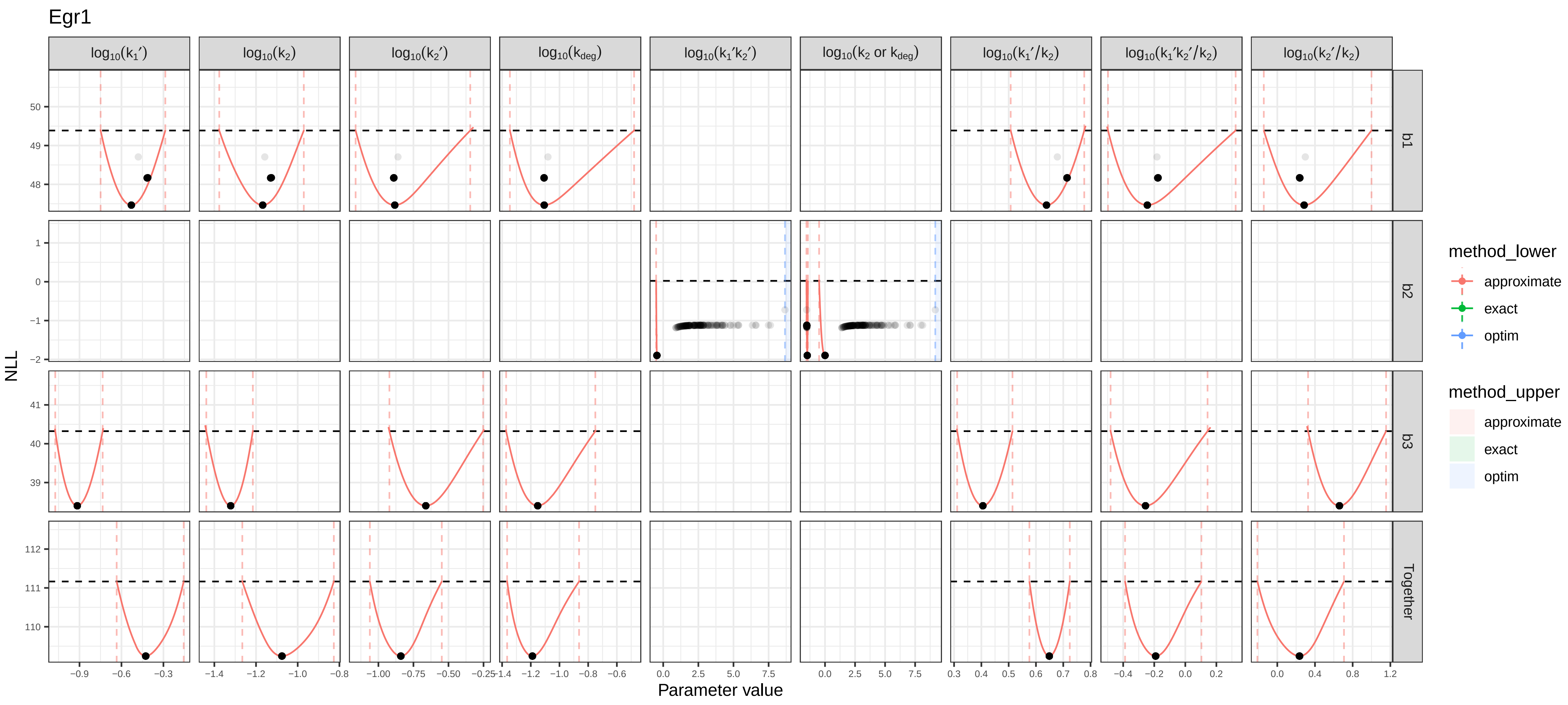

Replicate	Par	Best value	CI95 LB	CI95 UB	Method LB	Method UB
Together	$\log_{10}(k_1')$	−0.4266	−0.6343	−0.155	approximate	approximate
Together	$\log_{10}(k_2)$	−1.075	−1.266	−0.8267	approximate	approximate
Together	$\log_{10}(k_2')$	−0.8399	−1.061	−0.5477	approximate	approximate
Together	$\log_{10}(k_{deg})$	−1.189	−1.365	−0.8635	approximate	approximate
Together	$\log_{10}(k_1'/k_2)$	0.6488	0.576	0.7239	approximate	approximate
Together	$\log_{10}(k_1'k_2'/k_2)$	−0.191	−0.3878	0.1039	approximate	approximate
Together	$\log_{10}(k_2'/k_2)$	0.2356	−0.2111	0.708	approximate	approximate
b1	$\log_{10}(k_1')$	−0.5293	−0.7492	−0.2864	approximate	approximate
b1	$\log_{10}(k_2)$	−1.168	−1.376	−0.9707	approximate	approximate
b1	$\log_{10}(k_2')$	−0.8832	−1.163	−0.3448	approximate	approximate
b1	$\log_{10}(k_{deg})$	−1.107	−1.346	−0.4799	approximate	approximate
b1	$\log_{10}(k_1'/k_2)$	0.6386	0.5074	0.7769	approximate	approximate
b1	$\log_{10}(k_1'k_2'/k_2)$	−0.2447	−0.4976	0.3242	approximate	approximate
b1	$\log_{10}(k_2'/k_2)$	0.2847	−0.1433	1	approximate	approximate
b2	$\log_{10}(k_1'k_2')$	−0.4482	−0.5031	> 8.663	approximate	optim
b2	$\log_{10}(k_2 \text{ or } k_{deg})$	0.002619	−0.4913	> 9.177	approximate	optim
b2	$\log_{10}(k_2 \text{ or } k_{deg})$	−1.479	−1.544	−1.422	approximate	approximate
b3	$\log_{10}(k_1')$	−0.9161	−1.074	−0.7343	approximate	approximate
b3	$\log_{10}(k_2)$	−1.322	−1.439	−1.215	approximate	approximate
b3	$\log_{10}(k_2')$	−0.6619	−0.9214	−0.2529	approximate	approximate
b3	$\log_{10}(k_{deg})$	−1.152	−1.372	−0.7502	approximate	approximate
b3	$\log_{10}(k_1'/k_2)$	0.4055	0.3113	0.514	approximate	approximate
b3	$\log_{10}(k_1'k_2'/k_2)$	−0.2564	−0.481	0.1433	approximate	approximate
b3	$\log_{10}(k_2'/k_2)$	0.6597	0.3267	1.155	approximate	approximate

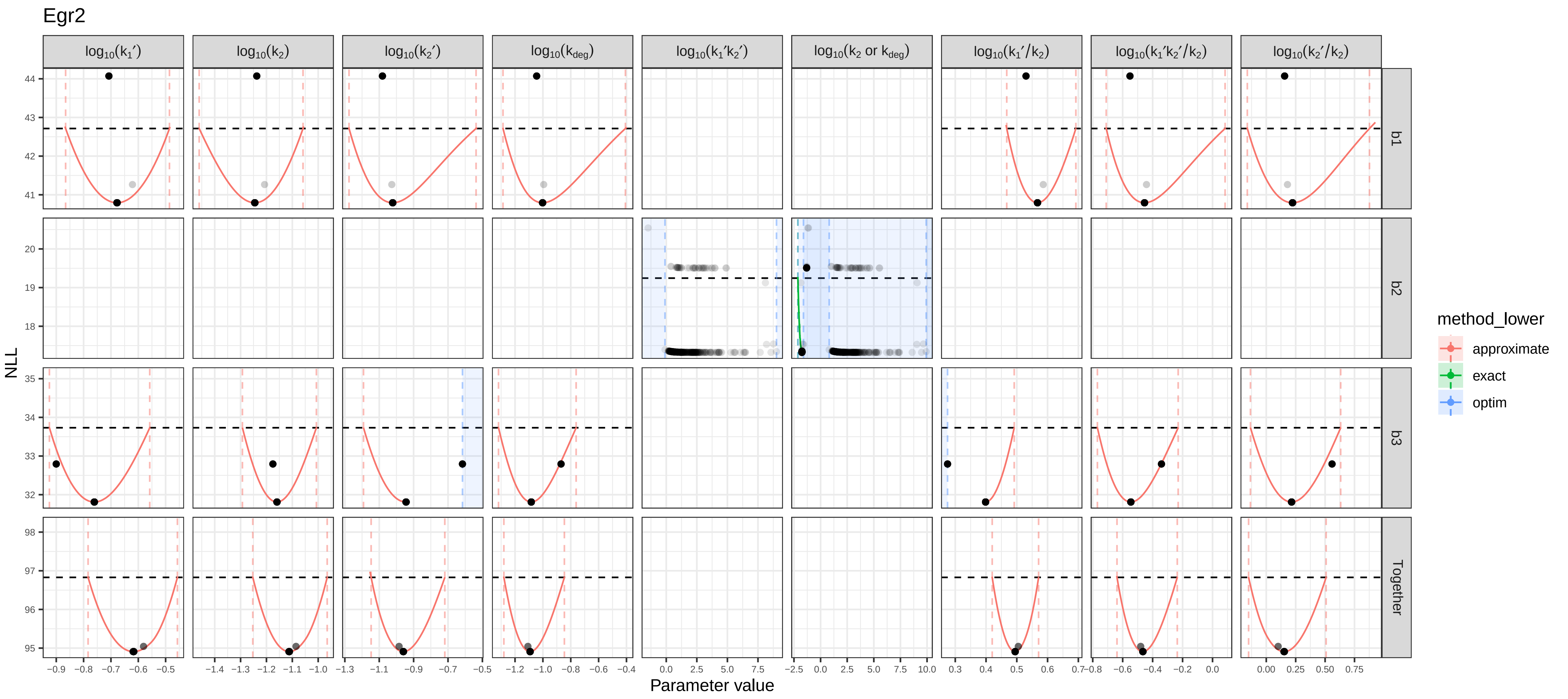

Replicate	Par	Best value	CI95 LB	CI95 UB	Method LB	Method UB
Together	$\log_{10}(k_1')$	−0.6176	−0.7836	−0.4569	approximate	approximate
Together	$\log_{10}(k_2)$	−1.112	−1.253	−0.9651	approximate	approximate
Together	$\log_{10}(k_2')$	−0.9598	−1.147	−0.7186	approximate	approximate
Together	$\log_{10}(k_{deg})$	−1.092	−1.28	−0.8458	approximate	approximate
Together	$\log_{10}(k_1'/k_2)$	0.4946	0.4201	0.5709	approximate	approximate
Together	$\log_{10}(k_1'k_2'/k_2)$	−0.4653	−0.638	−0.2355	approximate	approximate
Together	$\log_{10}(k_2'/k_2)$	0.1523	−0.1501	0.5087	approximate	approximate
b1	$\log_{10}(k_1')$	−0.6777	−0.8658	−0.486	approximate	approximate
b1	$\log_{10}(k_2)$	−1.245	−1.46	−1.059	approximate	approximate
b1	$\log_{10}(k_2')$	−1.022	−1.276	−0.5369	approximate	approximate
b1	$\log_{10}(k_{deg})$	−1.002	−1.286	−0.4077	approximate	approximate
b1	$\log_{10}(k_1'/k_2)$	0.5673	0.4673	0.692	approximate	approximate
b1	$\log_{10}(k_1'k_2'/k_2)$	−0.4544	−0.7099	0.08374	approximate	approximate
b1	$\log_{10}(k_2'/k_2)$	0.2233	−0.1609	0.8772	approximate	approximate
b2	$\log_{10}(k_1'k_2')$	7.683	< −0.1032	> 9.012	optim	optim
b2	$\log_{10}(k_2 \text{ or } k_{deg})$	8.603	0.8084	> 9.919	optim	optim
b2	$\log_{10}(k_2 \text{ or } k_{deg})$	−1.753	−2.132	> −1.607	exact	optim
b3	$\log_{10}(k_1')$	−0.7609	−0.9251	−0.5581	approximate	approximate
b3	$\log_{10}(k_2)$	−1.159	−1.293	−1.007	approximate	approximate
b3	$\log_{10}(k_2')$	−0.9438	−1.191	> −0.6159	approximate	optim
b3	$\log_{10}(k_{deg})$	−1.083	−1.319	−0.7614	approximate	approximate
b3	$\log_{10}(k_1'/k_2)$	0.3985	< 0.275	0.4916	optim	approximate
b3	$\log_{10}(k_1'k_2'/k_2)$	−0.5453	−0.7688	−0.2295	approximate	approximate
b3	$\log_{10}(k_2'/k_2)$	0.2156	−0.1344	0.6325	approximate	approximate

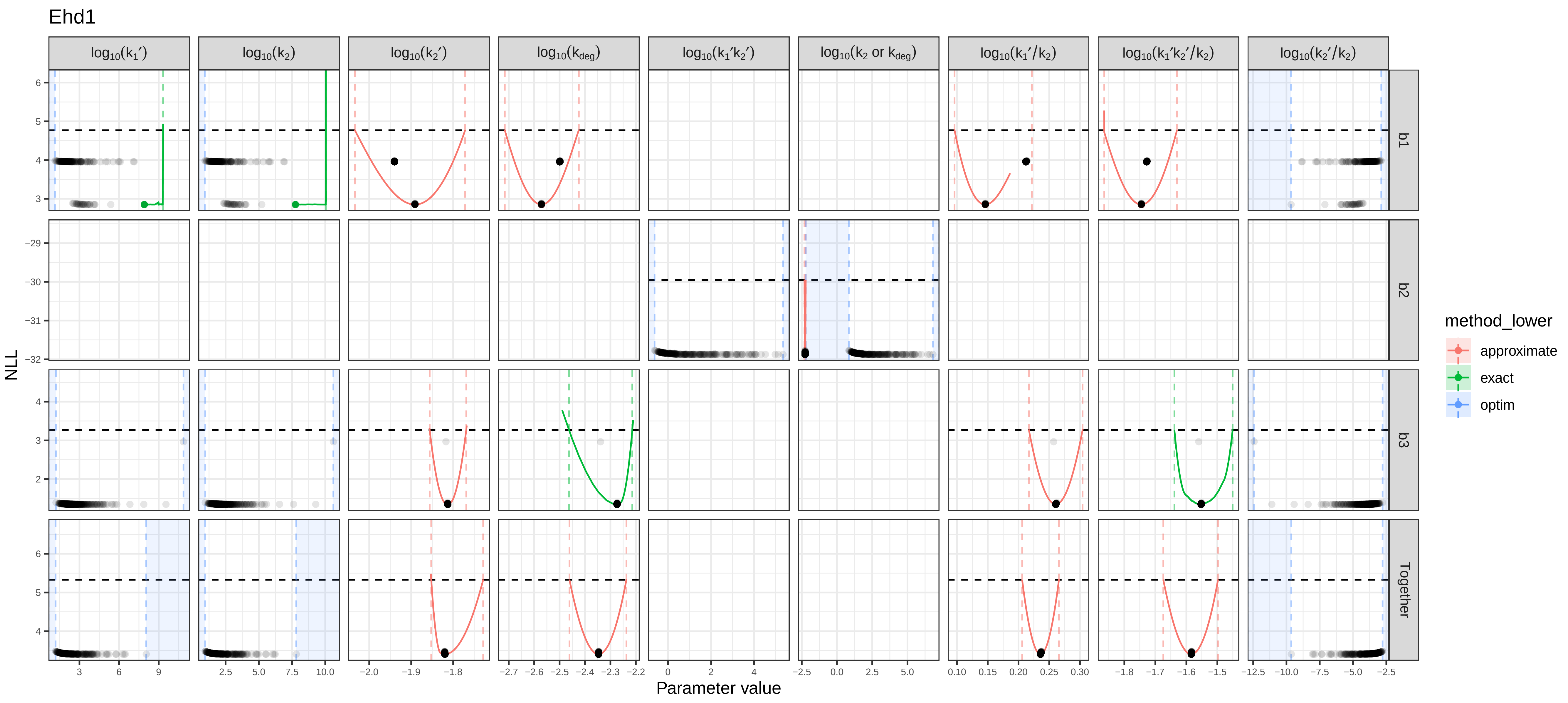

Replicate	Par	Best value	CI95 LB	CI95 UB	Method LB	Method UB
Together	$\log_{10}(k_1')$	5.008	< 1.198	> 8.053	optim	optim
Together	$\log_{10}(k_2)$	4.772	< 0.9609	> 7.817	optim	optim
Together	$\log_{10}(k_2')$	−1.82	−1.852	−1.729	approximate	approximate
Together	$\log_{10}(k_{deg})$	−2.347	−2.461	−2.237	approximate	approximate
Together	$\log_{10}(k_1'/k_2)$	0.2358	0.206	0.2658	approximate	approximate
Together	$\log_{10}(k_1'k_2'/k_2)$	−1.584	−1.674	−1.498	approximate	approximate
Together	$\log_{10}(k_2'/k_2)$	−6.592	< −9.637	> −2.781	optim	optim
b1	$\log_{10}(k_1')$	7.907	< 1.153	9.327	optim	exact
b1	$\log_{10}(k_2)$	7.761	< 0.9389	10.05	optim	exact
b1	$\log_{10}(k_2')$	−1.891	−2.034	−1.771	approximate	approximate
b1	$\log_{10}(k_{deg})$	−2.571	−2.716	−2.424	approximate	approximate
b1	$\log_{10}(k_1'/k_2)$	0.146	0.09591	0.2219	approximate	approximate
b1	$\log_{10}(k_1'k_2'/k_2)$	−1.745	−1.865	−1.63	approximate	approximate
b1	$\log_{10}(k_2'/k_2)$	−9.652	< −9.652	> −2.879	optim	optim
b2	$\log_{10}(k_1'k_2')$	3.202	< −0.6232	> 5.341	optim	optim
b2	$\log_{10}(k_2 \text{ or } k_{deg})$	4.667	0.841	> 6.806	optim	optim
b2	$\log_{10}(k_2 \text{ or } k_{deg})$	−2.262	−2.31	−2.22	approximate	approximate
b3	$\log_{10}(k_1')$	5.511	< 1.229	> 10.87	optim	optim
b3	$\log_{10}(k_2)$	5.25	< 0.9673	> 10.61	optim	optim
b3	$\log_{10}(k_2')$	−1.813	−1.856	−1.769	approximate	approximate
b3	$\log_{10}(k_{deg})$	−2.274	−2.463	−2.214	exact	exact
b3	$\log_{10}(k_1'/k_2)$	0.2607	0.217	0.3043	approximate	approximate
b3	$\log_{10}(k_1'k_2'/k_2)$	−1.552	−1.638	−1.451	exact	exact
b3	$\log_{10}(k_2'/k_2)$	−7.063	< −12.43	> −2.781	optim	optim

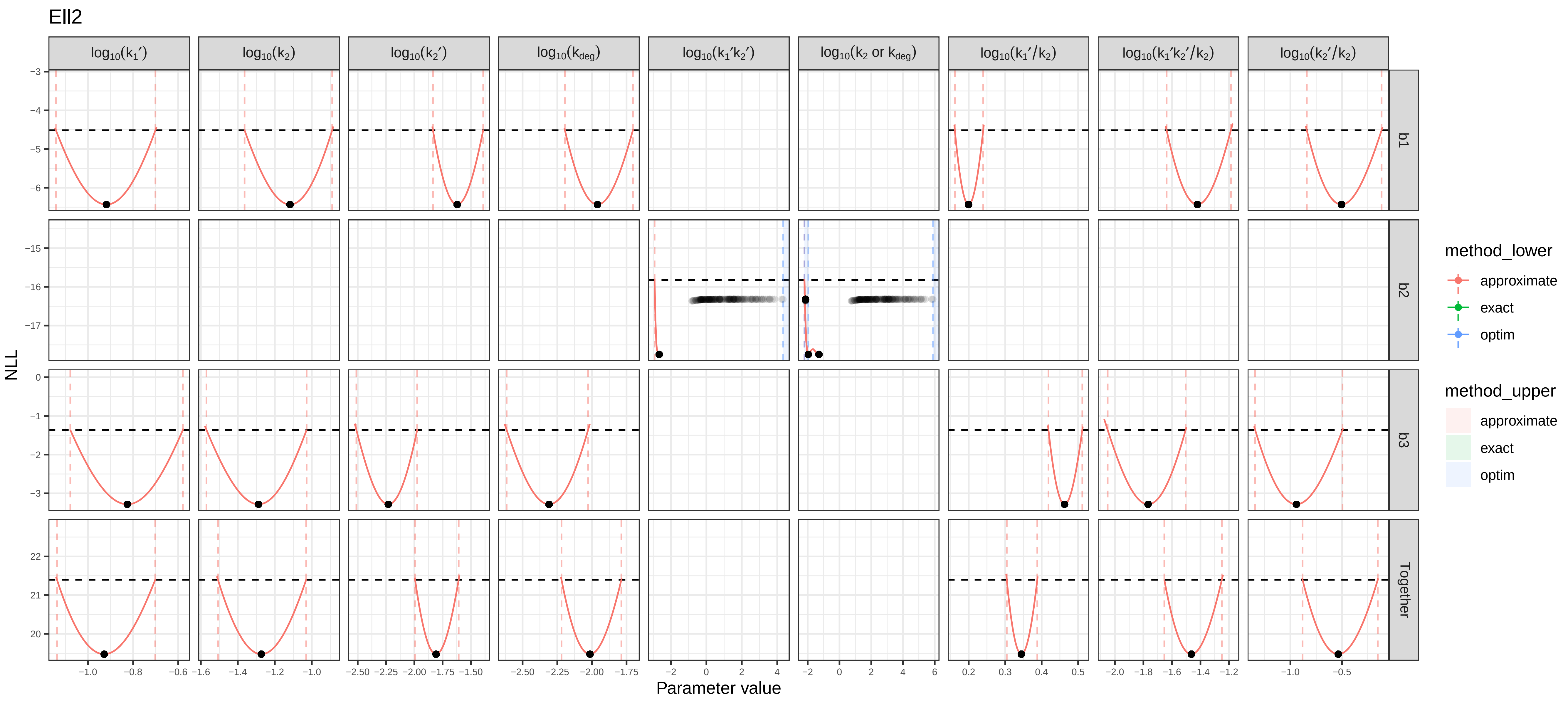

Replicate	Par	Best value	CI95 LB	CI95 UB	Method LB	Method UB
Together	$\log_{10}(k_1')$	−0.9283	−1.137	−0.7017	approximate	approximate
Together	$\log_{10}(k_2)$	−1.273	−1.507	−1.03	approximate	approximate
Together	$\log_{10}(k_2')$	−1.808	−1.993	−1.608	approximate	approximate
Together	$\log_{10}(k_{deg})$	−2.014	−2.219	−1.787	approximate	approximate
Together	$\log_{10}(k_1'/k_2)$	0.3444	0.3043	0.388	approximate	approximate
Together	$\log_{10}(k_1'k_2'/k_2)$	−1.464	−1.653	−1.25	approximate	approximate
Together	$\log_{10}(k_2'/k_2)$	−0.5355	−0.8816	−0.1523	approximate	approximate
b1	$\log_{10}(k_1')$	−0.9182	−1.142	−0.7009	approximate	approximate
b1	$\log_{10}(k_2)$	−1.118	−1.364	−0.8897	approximate	approximate
b1	$\log_{10}(k_2')$	−1.621	−1.836	−1.391	approximate	approximate
b1	$\log_{10}(k_{deg})$	−1.96	−2.195	−1.704	approximate	approximate
b1	$\log_{10}(k_1'/k_2)$	0.1996	0.1619	0.2398	approximate	approximate
b1	$\log_{10}(k_1'k_2'/k_2)$	−1.422	−1.637	−1.187	approximate	approximate
b1	$\log_{10}(k_2'/k_2)$	−0.5035	−0.8406	−0.1152	approximate	approximate
b2	$\log_{10}(k_1'k_2')$	−2.663	−2.924	> 4.328	approximate	optim
b2	$\log_{10}(k_2 \text{ or } k_{deg})$	−1.291	−2.204	> 5.885	approximate	optim
b2	$\log_{10}(k_2 \text{ or } k_{deg})$	−1.96	−2.204	−1.96	approximate	optim
b3	$\log_{10}(k_1')$	−0.8254	−1.078	−0.5792	approximate	approximate
b3	$\log_{10}(k_2)$	−1.288	−1.569	−1.028	approximate	approximate
b3	$\log_{10}(k_2')$	−2.23	−2.512	−1.975	approximate	approximate
b3	$\log_{10}(k_{deg})$	−2.31	−2.615	−2.027	approximate	approximate
b3	$\log_{10}(k_1'/k_2)$	0.4628	0.4186	0.5115	approximate	approximate
b3	$\log_{10}(k_1'k_2'/k_2)$	−1.767	−2.051	−1.504	approximate	approximate
b3	$\log_{10}(k_2'/k_2)$	−0.9419	−1.341	−0.4943	approximate	approximate

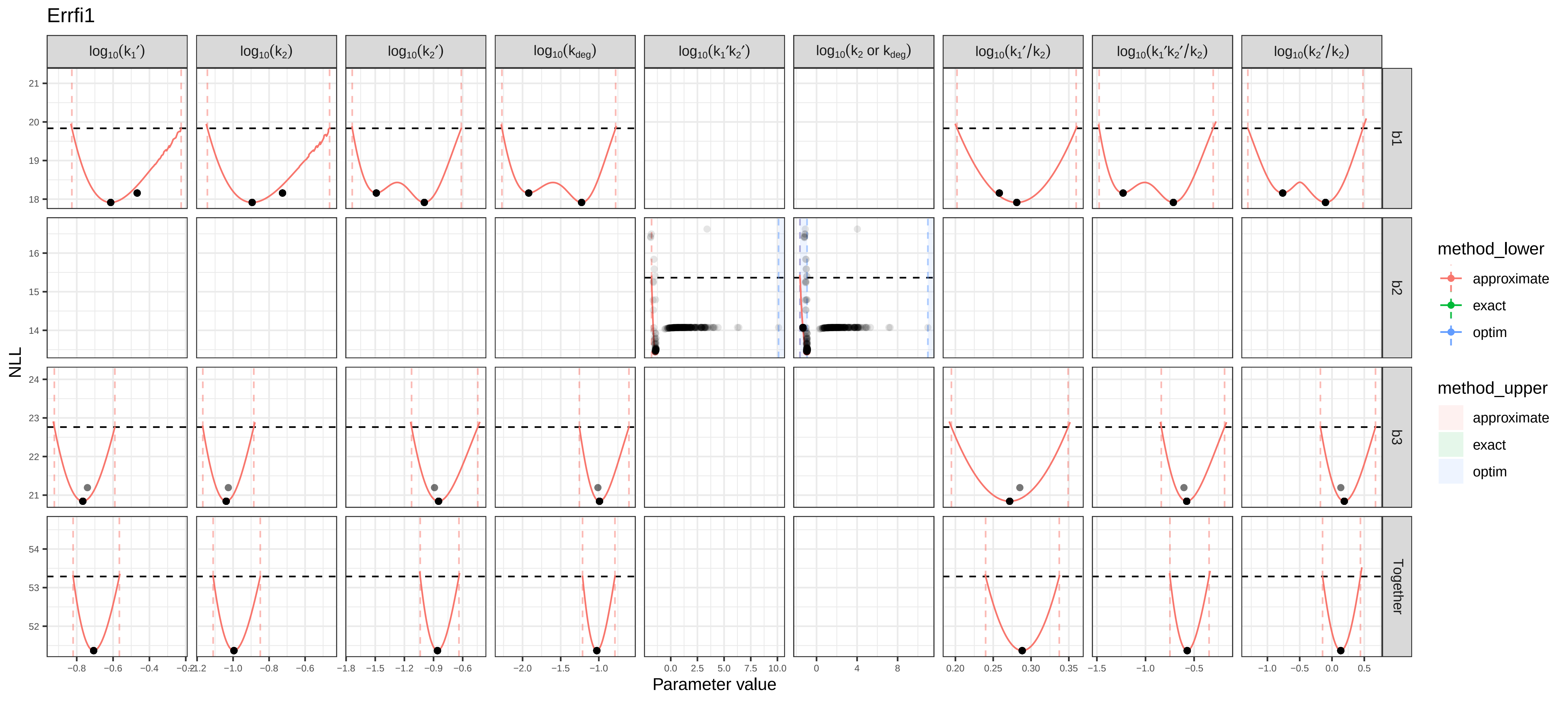

Replicate	Par	Best value	CI95 LB	CI95 UB	Method LB	Method UB
Together	$\log_{10}(k_1')$	−0.7068	−0.8199	−0.5654	approximate	approximate
Together	$\log_{10}(k_2)$	−0.9952	−1.111	−0.849	approximate	approximate
Together	$\log_{10}(k_2')$	−0.8596	−1.037	−0.6383	approximate	approximate
Together	$\log_{10}(k_{deg})$	−1.026	−1.213	−0.7883	approximate	approximate
Together	$\log_{10}(k_1'/k_2)$	0.2883	0.24	0.3373	approximate	approximate
Together	$\log_{10}(k_1'k_2'/k_2)$	−0.5713	−0.7486	−0.3462	approximate	approximate
Together	$\log_{10}(k_2'/k_2)$	0.1356	−0.1462	0.4404	approximate	approximate
b1	$\log_{10}(k_1')$	−0.613	−0.8268	−0.2253	approximate	approximate
b1	$\log_{10}(k_2)$	−0.8941	−1.143	−0.4641	approximate	approximate
b1	$\log_{10}(k_2')$	−0.9946	−1.737	−0.6142	approximate	approximate
b1	$\log_{10}(k_{deg})$	−1.226	−2.271	−0.7792	approximate	approximate
b1	$\log_{10}(k_1'/k_2)$	0.2811	0.2022	0.3597	approximate	approximate
b1	$\log_{10}(k_1'k_2'/k_2)$	−0.7135	−1.477	−0.3036	approximate	approximate
b1	$\log_{10}(k_2'/k_2)$	−0.1005	−1.303	0.4797	approximate	approximate
b2	$\log_{10}(k_1'k_2')$	−1.452	−1.797	> 10.1	approximate	optim
b2	$\log_{10}(k_2 \text{ or } k_{deg})$	−0.9479	−1.637	> 10.98	approximate	optim
b2	$\log_{10}(k_2 \text{ or } k_{deg})$	−0.9792	−1.637	−0.9355	approximate	optim
b3	$\log_{10}(k_1')$	−0.7666	−0.9233	−0.59	approximate	approximate
b3	$\log_{10}(k_2)$	−1.038	−1.168	−0.8849	approximate	approximate
b3	$\log_{10}(k_2')$	−0.8481	−1.126	−0.4446	approximate	approximate
b3	$\log_{10}(k_{deg})$	−0.9911	−1.255	−0.6046	approximate	approximate
b3	$\log_{10}(k_1'/k_2)$	0.2718	0.1947	0.3491	approximate	approximate
b3	$\log_{10}(k_1'k_2'/k_2)$	−0.5763	−0.8379	−0.1868	approximate	approximate
b3	$\log_{10}(k_2'/k_2)$	0.1903	−0.1795	0.6738	approximate	approximate

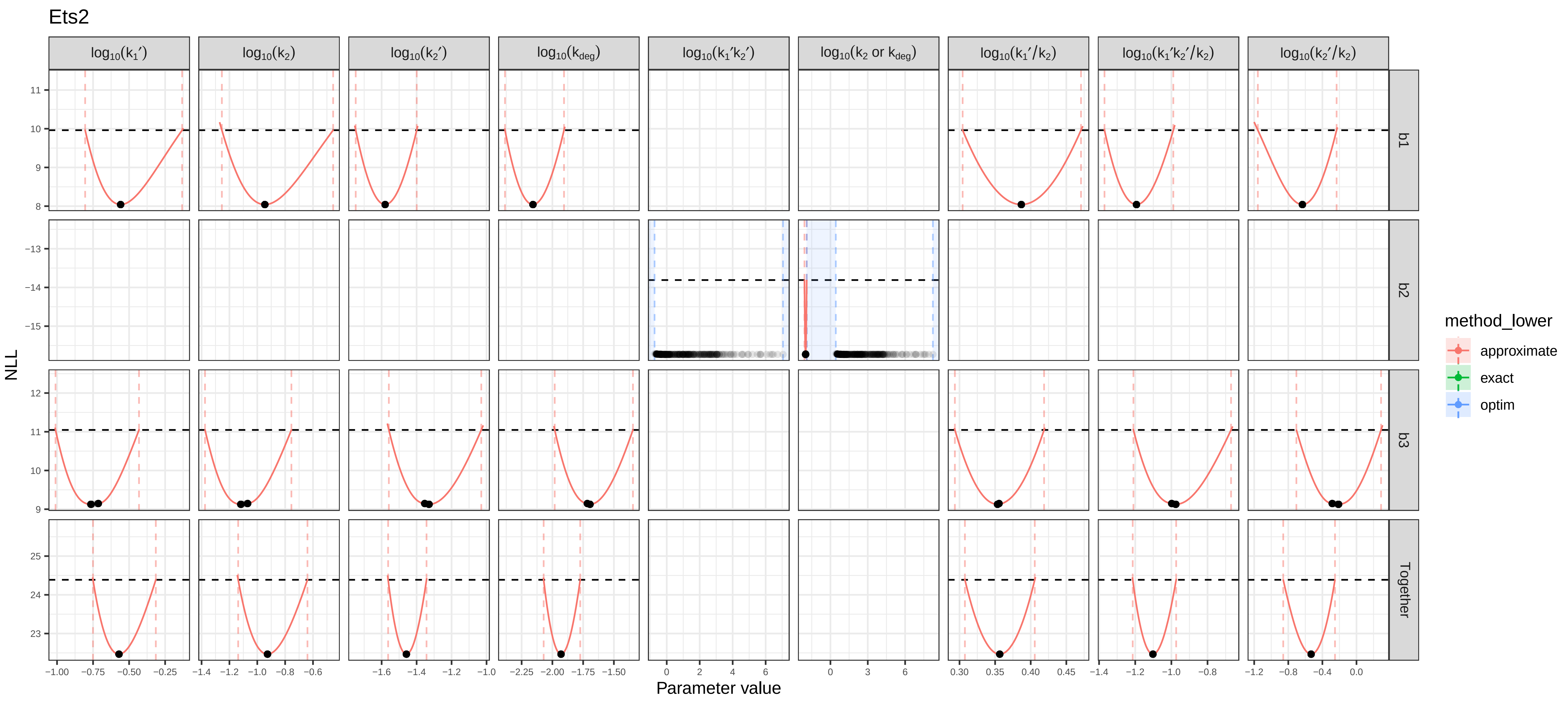

Replicate	Par	**Best value**	**CI95 LB**	**CI95 UB**	**Method LB**	**Method UB**
Together	$\log_{10}(k_1')$	−0.5705	−0.7506	−0.3146	approximate	approximate
Together	$\log_{10}(k_2)$	−0.927	−1.137	−0.6397	approximate	approximate
Together	$\log_{10}(k_2')$	−1.458	−1.563	−1.343	approximate	approximate
Together	$\log_{10}(k_{deg})$	−1.93	−2.071	−1.774	approximate	approximate
Together	$\log_{10}(k_1'/k_2)$	0.3565	0.3076	0.4057	approximate	approximate
Together	$\log_{10}(k_1'k_2'/k_2)$	−1.102	−1.214	−0.9735	approximate	approximate
Together	$\log_{10}(k_2'/k_2)$	−0.5314	−0.8592	−0.2521	approximate	approximate
b1	$\log_{10}(k_1')$	−0.5589	−0.805	−0.1321	approximate	approximate
b1	$\log_{10}(k_2)$	−0.9457	−1.254	−0.4553	approximate	approximate
b1	$\log_{10}(k_2')$	−1.58	−1.748	−1.399	approximate	approximate
b1	$\log_{10}(k_{deg})$	−2.158	−2.385	−1.905	approximate	approximate
b1	$\log_{10}(k_1'/k_2)$	0.3868	0.3044	0.4706	approximate	approximate
b1	$\log_{10}(k_1'k_2'/k_2)$	−1.193	−1.37	−0.9891	approximate	approximate
b1	$\log_{10}(k_2'/k_2)$	−0.6343	−1.157	−0.2332	approximate	approximate
b2	$\log_{10}(k_1'k_2')$	0.07252	< −0.7381	> 7.054	optim	optim
b2	$\log_{10}(k_2 \text{ or } k_{deg})$	1.248	0.433	> 8.23	optim	optim
b2	$\log_{10}(k_2 \text{ or } k_{deg})$	−1.994	−2.086	−1.904	approximate	approximate
b3	$\log_{10}(k_1')$	−0.7642	−1.011	−0.4313	approximate	approximate
b3	$\log_{10}(k_2)$	−1.118	−1.375	−0.7549	approximate	approximate
b3	$\log_{10}(k_2')$	−1.329	−1.559	−1.03	approximate	approximate
b3	$\log_{10}(k_{deg})$	−1.694	−1.981	−1.345	approximate	approximate
b3	$\log_{10}(k_1'/k_2)$	0.3534	0.2935	0.4188	approximate	approximate
b3	$\log_{10}(k_1'k_2'/k_2)$	−0.9757	−1.21	−0.6698	approximate	approximate
b3	$\log_{10}(k_2'/k_2)$	−0.2116	−0.705	0.2883	approximate	approximate

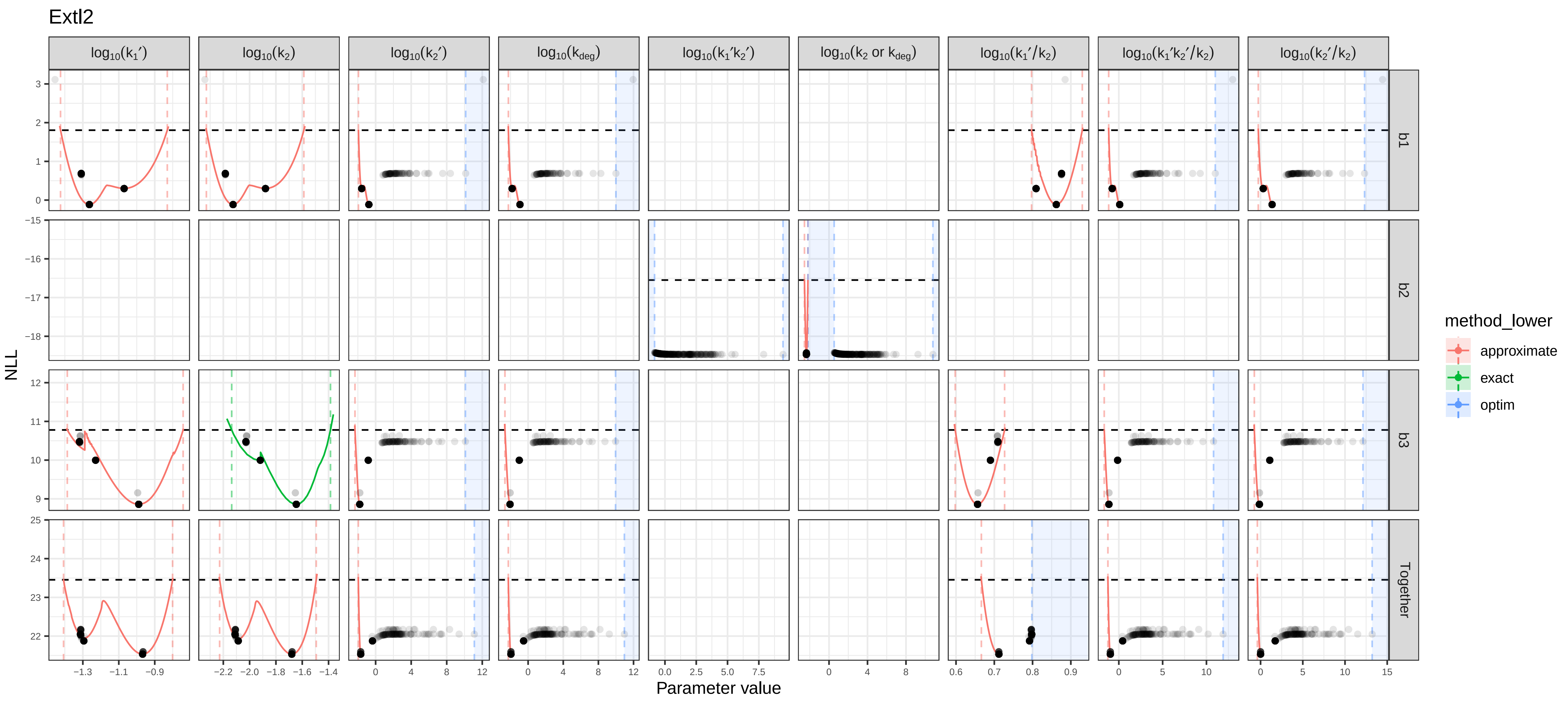

Replicate	Par	Best value	CI95 LB	CI95 UB	Method LB	Method UB
Together	$\log_{10}(k_1')$	−0.9674	−1.407	−0.8008	approximate	approximate
Together	$\log_{10}(k_2)$	−1.679	−2.228	−1.494	approximate	approximate
Together	$\log_{10}(k_2')$	−1.678	−1.949	> 11.11	approximate	optim
Together	$\log_{10}(k_{deg})$	−1.952	−2.253	> 10.96	approximate	optim
Together	$\log_{10}(k_1'/k_2)$	0.712	0.6662	> 0.7982	approximate	optim
Together	$\log_{10}(k_1'k_2'/k_2)$	−0.9662	−1.246	> 11.9	approximate	optim
Together	$\log_{10}(k_2'/k_2)$	0.001173	−0.3803	> 13.22	approximate	optim
b1	$\log_{10}(k_1')$	−1.264	−1.424	−0.8302	approximate	approximate
b1	$\log_{10}(k_2)$	−2.126	−2.329	−1.588	approximate	approximate
b1	$\log_{10}(k_2')$	−0.7617	−1.935	> 10.13	approximate	optim
b1	$\log_{10}(k_{deg})$	−0.9262	−2.246	> 9.995	approximate	optim
b1	$\log_{10}(k_1'/k_2)$	0.862	0.7974	0.93	approximate	approximate
b1	$\log_{10}(k_1'k_2'/k_2)$	0.1003	−1.158	> 11.01	approximate	optim
b1	$\log_{10}(k_2'/k_2)$	1.364	−0.27	> 12.32	approximate	optim
b2	$\log_{10}(k_1'k_2')$	3.491	< −0.8294	> 9.431	optim	optim
b2	$\log_{10}(k_2 \text{ or } k_{deg})$	4.857	0.5358	> 10.8	optim	optim
b2	$\log_{10}(k_2 \text{ or } k_{deg})$	−2.352	−2.554	−2.193	approximate	approximate
b3	$\log_{10}(k_1')$	−0.9891	−1.386	−0.7422	approximate	approximate
b3	$\log_{10}(k_2)$	−1.645	−2.136	−1.385	exact	exact
b3	$\log_{10}(k_2')$	−1.788	−2.323	> 10.1	approximate	optim
b3	$\log_{10}(k_{deg})$	−2.056	−2.636	> 9.953	approximate	optim
b3	$\log_{10}(k_1'/k_2)$	0.6563	0.5978	0.727	approximate	approximate
b3	$\log_{10}(k_1'k_2'/k_2)$	−1.132	−1.666	> 10.81	approximate	optim
b3	$\log_{10}(k_2'/k_2)$	−0.1428	−0.7408	> 12.13	approximate	optim

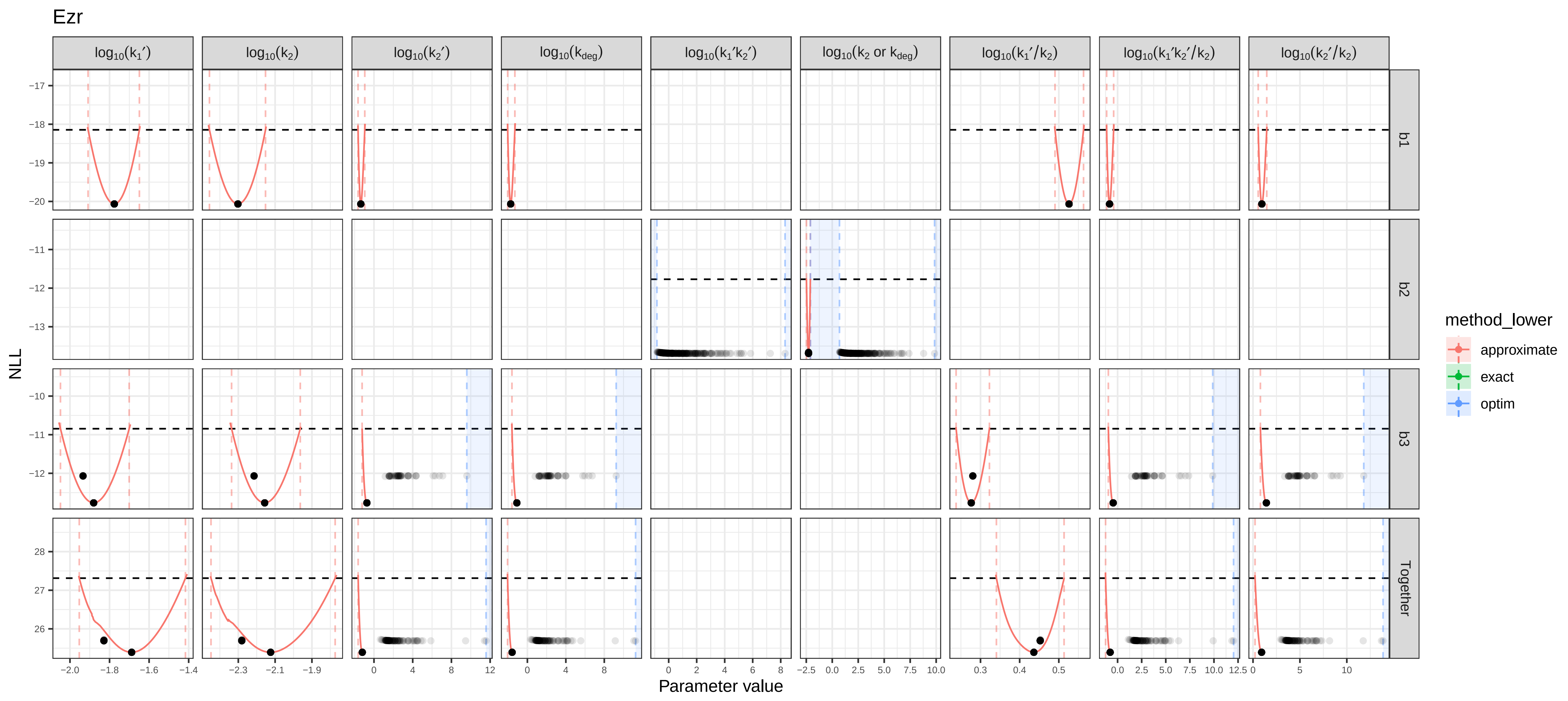

Replicate	Par	Best value	CI95 LB	CI95 UB	Method LB	Method UB
Together	$\log_{10}(k_1')$	−1.688	−1.953	−1.416	approximate	approximate
Together	$\log_{10}(k_2)$	−2.124	−2.448	−1.774	approximate	approximate
Together	$\log_{10}(k_2')$	−1.207	−1.637	> 11.58	approximate	optim
Together	$\log_{10}(k_{deg})$	−1.588	−2.062	> 11.26	approximate	optim
Together	$\log_{10}(k_1'/k_2)$	0.4362	0.3406	0.5134	approximate	approximate
Together	$\log_{10}(k_1'k_2'/k_2)$	−0.7709	−1.246	> 12.03	approximate	optim
Together	$\log_{10}(k_2'/k_2)$	0.9172	0.2187	> 13.86	approximate	optim
b1	$\log_{10}(k_1')$	−1.776	−1.908	−1.649	approximate	approximate
b1	$\log_{10}(k_2)$	−2.302	−2.457	−2.152	approximate	approximate
b1	$\log_{10}(k_2')$	−1.36	−1.647	−0.9516	approximate	approximate
b1	$\log_{10}(k_{deg})$	−1.737	−2.048	−1.3	approximate	approximate
b1	$\log_{10}(k_1'/k_2)$	0.5262	0.4903	0.5632	approximate	approximate
b1	$\log_{10}(k_1'k_2'/k_2)$	−0.8335	−1.134	−0.4084	approximate	approximate
b1	$\log_{10}(k_2'/k_2)$	0.9424	0.5544	1.467	approximate	approximate
b2	$\log_{10}(k_1'k_2')$	3.904	< −0.8379	> 8.311	optim	optim
b2	$\log_{10}(k_2 \text{ or } k_{deg})$	5.439	0.6962	> 9.846	optim	optim
b2	$\log_{10}(k_2 \text{ or } k_{deg})$	−2.284	−2.483	−2.107	approximate	approximate
b3	$\log_{10}(k_1')$	−1.88	−2.048	−1.701	approximate	approximate
b3	$\log_{10}(k_2)$	−2.157	−2.336	−1.963	approximate	approximate
b3	$\log_{10}(k_2')$	−0.7338	−1.227	> 9.59	approximate	optim
b3	$\log_{10}(k_{deg})$	−1.096	−1.605	> 9.238	approximate	optim
b3	$\log_{10}(k_1'/k_2)$	0.2764	0.2377	0.3228	approximate	approximate
b3	$\log_{10}(k_1'k_2'/k_2)$	−0.4574	−0.9592	> 9.87	approximate	optim
b3	$\log_{10}(k_2'/k_2)$	1.423	0.7882	> 11.8	approximate	optim

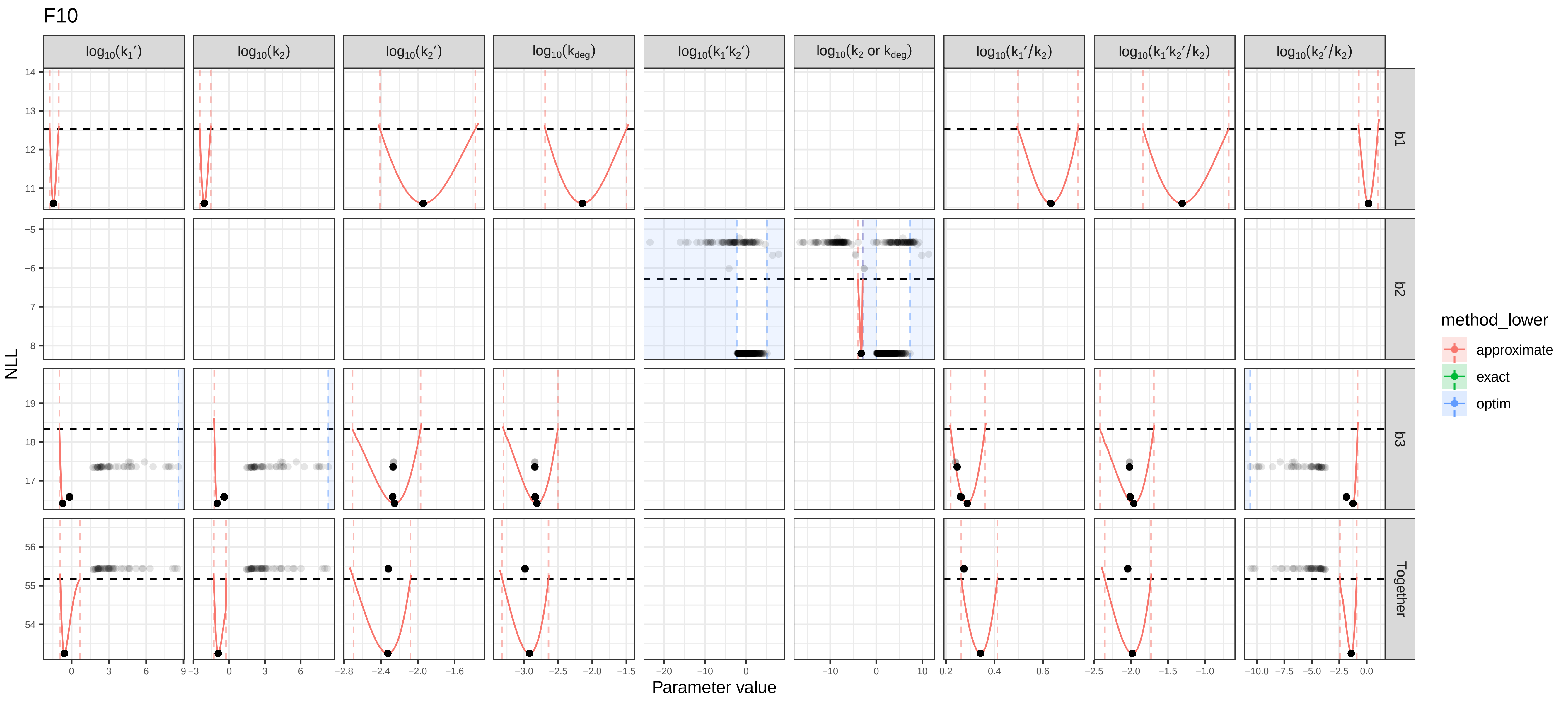

Replicate	Par	Best value	CI95 LB	CI95 UB	Method LB	Method UB
Together	$\log_{10}(k_1')$	−0.5779	−0.9083	0.6568	approximate	approximate
Together	$\log_{10}(k_2)$	−0.9205	−1.298	−0.2631	approximate	approximate
Together	$\log_{10}(k_2')$	−2.325	−2.696	−2.08	approximate	approximate
Together	$\log_{10}(k_{deg})$	−2.92	−3.319	−2.641	approximate	approximate
Together	$\log_{10}(k_1'/k_2)$	0.3426	0.2635	0.412	approximate	approximate
Together	$\log_{10}(k_1'k_2'/k_2)$	−1.983	−2.355	−1.731	approximate	approximate
Together	$\log_{10}(k_2'/k_2)$	−1.405	−2.447	−0.9164	approximate	approximate
b1	$\log_{10}(k_1')$	−1.466	−1.762	−1.038	approximate	approximate
b1	$\log_{10}(k_2)$	−2.099	−2.475	−1.544	approximate	approximate
b1	$\log_{10}(k_2')$	−1.941	−2.411	−1.375	approximate	approximate
b1	$\log_{10}(k_{deg})$	−2.145	−2.69	−1.498	approximate	approximate
b1	$\log_{10}(k_1'/k_2)$	0.6329	0.4968	0.7448	approximate	approximate
b1	$\log_{10}(k_1'k_2'/k_2)$	−1.309	−1.838	−0.6796	approximate	approximate
b1	$\log_{10}(k_2'/k_2)$	0.1576	−0.7243	1.034	approximate	approximate
b2	$\log_{10}(k_1'k_2')$	0.5648	< -2.176	> 5.127	optim	optim
b2	$\log_{10}(k_2 \text{ or } k_{deg})$	2.766	0.02274	> 7.328	optim	optim
b2	$\log_{10}(k_2 \text{ or } k_{deg})$	−3.275	−4.006	−2.975	approximate	approximate
b3	$\log_{10}(k_1')$	−0.7146	−0.9778	> 8.582	approximate	optim
b3	$\log_{10}(k_2)$	−1.003	−1.256	> 8.336	approximate	optim
b3	$\log_{10}(k_2')$	−2.252	−2.708	−1.97	approximate	approximate
b3	$\log_{10}(k_{deg})$	−2.811	−3.3	−2.505	approximate	approximate
b3	$\log_{10}(k_1'/k_2)$	0.288	0.2193	0.3613	approximate	approximate
b3	$\log_{10}(k_1'k_2'/k_2)$	−1.964	−2.417	−1.692	approximate	approximate
b3	$\log_{10}(k_2'/k_2)$	−1.249	< -10.6	−0.8343	optim	approximate

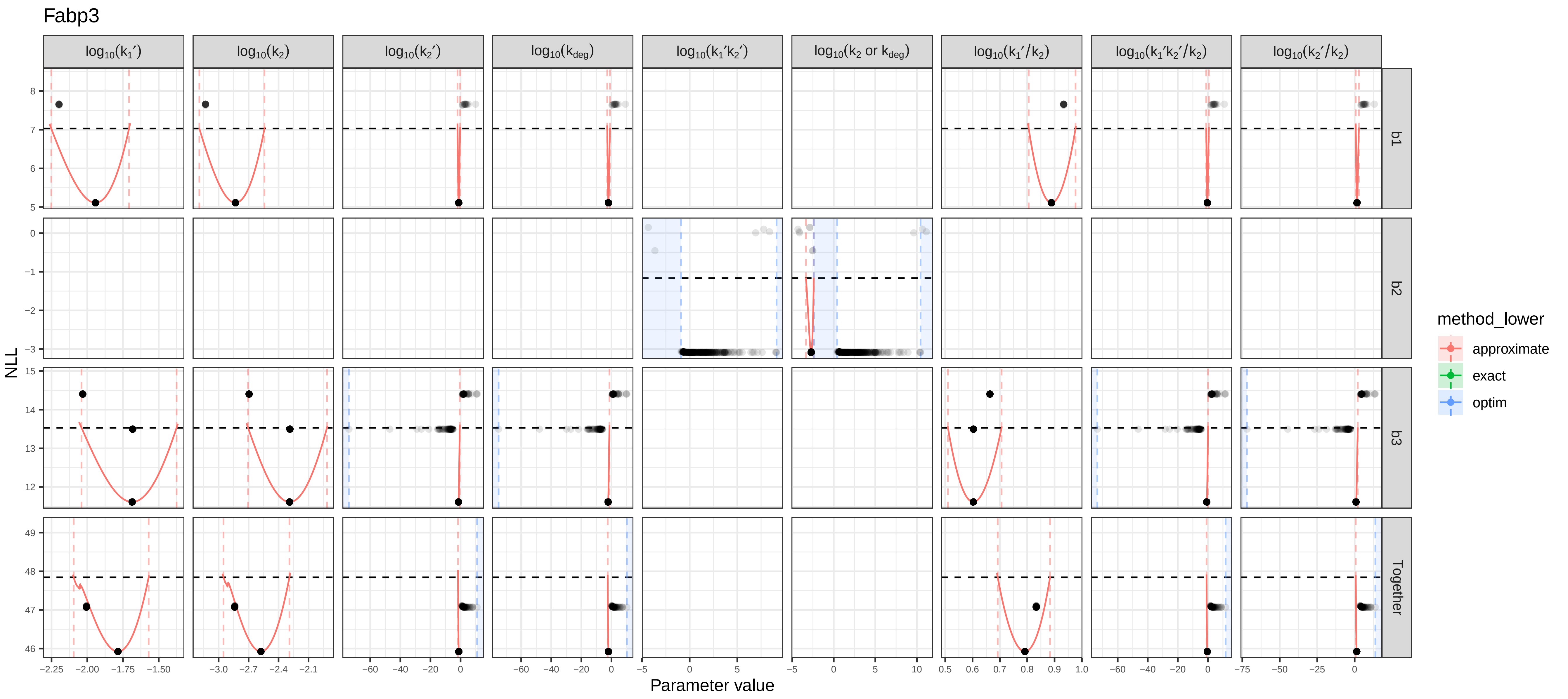

Replicate	Par	Best value	CI95 LB	CI95 UB	Method LB	Method UB
Together	$\log_{10}(k_1')$	−1.785	−2.095	−1.57	approximate	approximate
Together	$\log_{10}(k_2)$	−2.577	−2.951	−2.291	approximate	approximate
Together	$\log_{10}(k_2')$	−1.186	−1.68	> 10.96	approximate	optim
Together	$\log_{10}(k_{deg})$	−1.944	−2.477	> 10.28	approximate	optim
Together	$\log_{10}(k_1'/k_2)$	0.7915	0.692	0.8835	approximate	approximate
Together	$\log_{10}(k_1'k_2'/k_2)$	−0.3942	−0.9142	> 11.79	approximate	optim
Together	$\log_{10}(k_2'/k_2)$	1.391	0.7341	> 13.8	approximate	optim
b1	$\log_{10}(k_1')$	−1.943	−2.252	−1.708	approximate	approximate
b1	$\log_{10}(k_2)$	−2.832	−3.193	−2.541	approximate	approximate
b1	$\log_{10}(k_2')$	−1.255	−2.012	−0.2784	approximate	approximate
b1	$\log_{10}(k_{deg})$	−2.009	−2.799	−0.9754	approximate	approximate
b1	$\log_{10}(k_1'/k_2)$	0.8889	0.8054	0.9768	approximate	approximate
b1	$\log_{10}(k_1'k_2'/k_2)$	−0.3663	−1.134	0.6491	approximate	approximate
b1	$\log_{10}(k_2'/k_2)$	1.576	0.7259	2.816	approximate	approximate
b2	$\log_{10}(k_1'k_2')$	6.608	< −0.9058	> 9.131	optim	optim
b2	$\log_{10}(k_2 \text{ or } k_{deg})$	7.927	0.4137	> 10.45	optim	optim
b2	$\log_{10}(k_2 \text{ or } k_{deg})$	−2.718	−3.345	−2.401	approximate	approximate
b3	$\log_{10}(k_1')$	−1.686	−2.04	−1.375	approximate	approximate
b3	$\log_{10}(k_2)$	−2.289	−2.705	−1.915	approximate	approximate
b3	$\log_{10}(k_2')$	−1.378	< −74.15	−0.5381	optim	approximate
b3	$\log_{10}(k_{deg})$	−2.226	< −75.02	−1.323	optim	approximate
b3	$\log_{10}(k_1'/k_2)$	0.6028	0.5099	0.7063	approximate	approximate
b3	$\log_{10}(k_1'k_2'/k_2)$	−0.7751	< −73.54	0.1119	optim	approximate
b3	$\log_{10}(k_2'/k_2)$	0.9109	< −71.86	2.071	optim	approximate

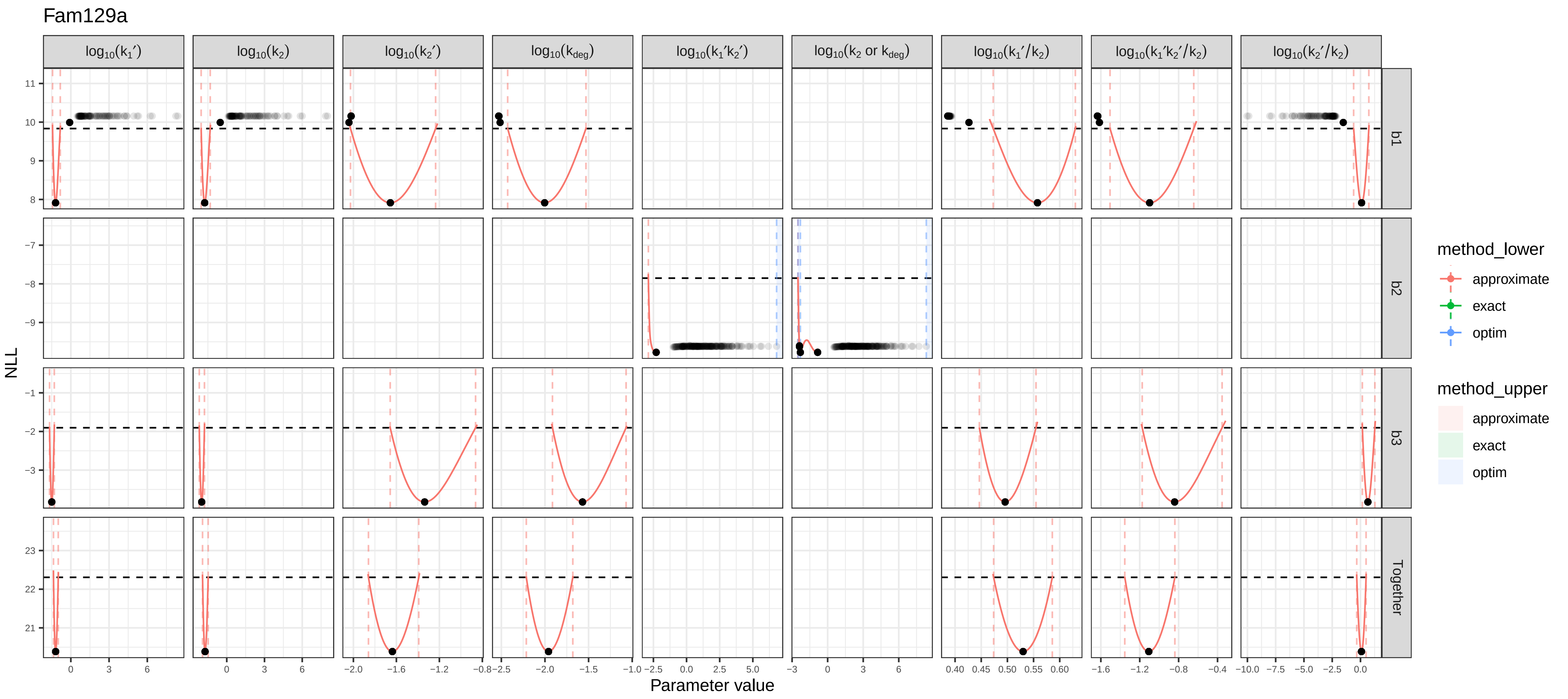

Replicate	Par	Best value	CI95 LB	CI95 UB	Method LB	Method UB
Together	$\log_{10}(k_1')$	−1.194	−1.361	−0.9878	approximate	approximate
Together	$\log_{10}(k_2)$	−1.724	−1.925	−1.478	approximate	approximate
Together	$\log_{10}(k_2')$	−1.637	−1.86	−1.392	approximate	approximate
Together	$\log_{10}(k_{deg})$	−1.959	−2.214	−1.681	approximate	approximate
Together	$\log_{10}(k_1'/k_2)$	0.53	0.4737	0.5858	approximate	approximate
Together	$\log_{10}(k_1'k_2'/k_2)$	−1.107	−1.354	−0.8386	approximate	approximate
Together	$\log_{10}(k_2'/k_2)$	0.08686	−0.3309	0.4942	approximate	approximate
b1	$\log_{10}(k_1')$	−1.197	−1.434	−0.8295	approximate	approximate
b1	$\log_{10}(k_2)$	−1.755	−2.037	−1.316	approximate	approximate
b1	$\log_{10}(k_2')$	−1.656	−2.027	−1.235	approximate	approximate
b1	$\log_{10}(k_{deg})$	−2.004	−2.427	−1.53	approximate	approximate
b1	$\log_{10}(k_1'/k_2)$	0.5575	0.4729	0.6298	approximate	approximate
b1	$\log_{10}(k_1'k_2'/k_2)$	−1.098	−1.505	−0.6445	approximate	approximate
b1	$\log_{10}(k_2'/k_2)$	0.09887	−0.6058	0.7386	approximate	approximate
b2	$\log_{10}(k_1'k_2')$	−2.29	−2.881	> 6.793	approximate	optim
b2	$\log_{10}(k_2 \text{ or } k_{deg})$	−0.8427	−2.503	> 8.322	approximate	optim
b2	$\log_{10}(k_2 \text{ or } k_{deg})$	−2.302	−2.503	−2.3	approximate	optim
b3	$\log_{10}(k_1')$	−1.497	−1.67	−1.299	approximate	approximate
b3	$\log_{10}(k_2)$	−1.992	−2.186	−1.774	approximate	approximate
b3	$\log_{10}(k_2')$	−1.337	−1.658	−0.863	approximate	approximate
b3	$\log_{10}(k_{deg})$	−1.569	−1.914	−1.07	approximate	approximate
b3	$\log_{10}(k_1'/k_2)$	0.4957	0.4464	0.5546	approximate	approximate
b3	$\log_{10}(k_1'k_2'/k_2)$	−0.8411	−1.175	−0.353	approximate	approximate
b3	$\log_{10}(k_2'/k_2)$	0.6554	0.1649	1.272	approximate	approximate

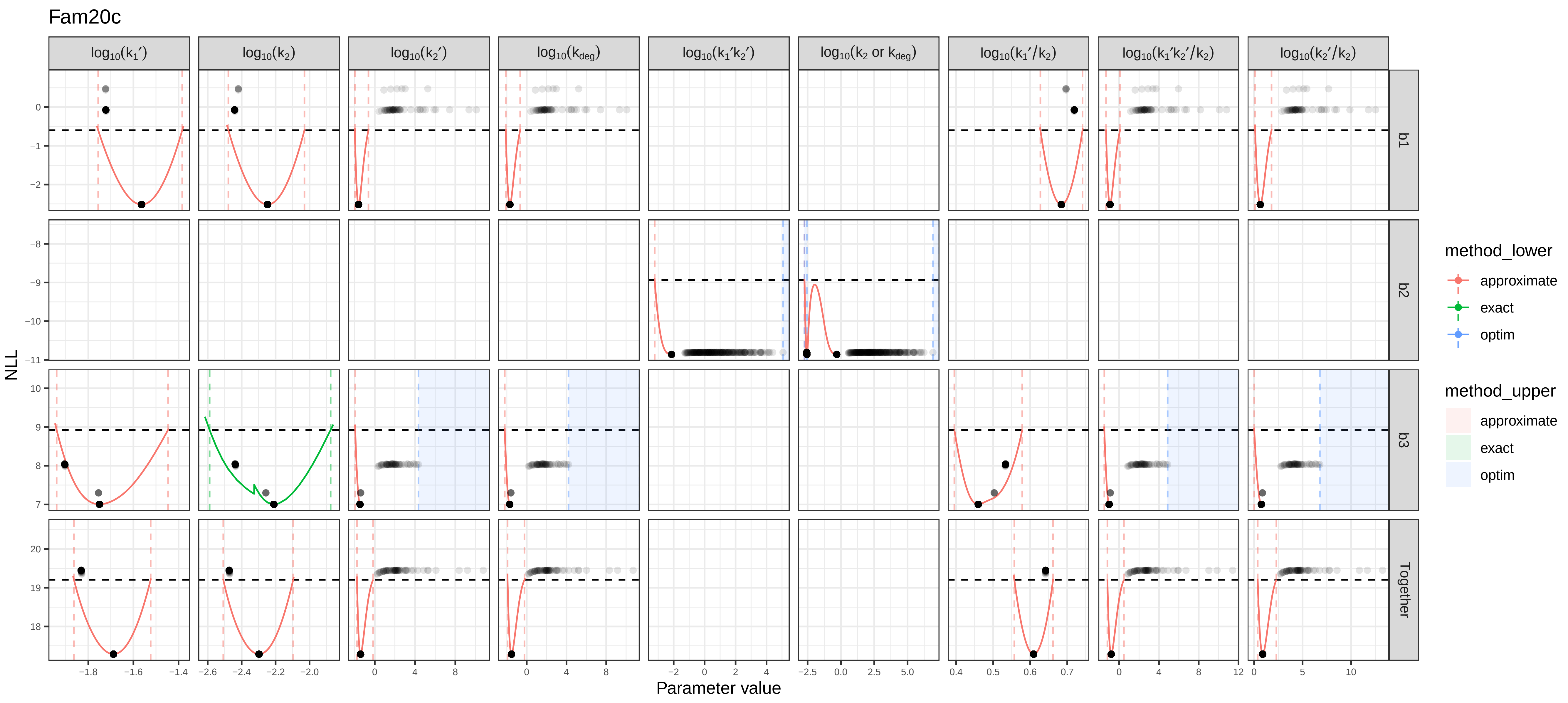

Replicate	Par	Best value	CI95 LB	CI95 UB	Method LB	Method UB
Together	$\log_{10}(k_1')$	−1.688	−1.864	−1.524	approximate	approximate
Together	$\log_{10}(k_2)$	−2.298	−2.508	−2.097	approximate	approximate
Together	$\log_{10}(k_2')$	−1.406	−1.762	−0.165	approximate	approximate
Together	$\log_{10}(k_{deg})$	−1.532	−1.93	−0.2294	approximate	approximate
Together	$\log_{10}(k_1'/k_2)$	0.6093	0.5571	0.6617	approximate	approximate
Together	$\log_{10}(k_1'k_2'/k_2)$	−0.7969	−1.18	0.4786	approximate	approximate
Together	$\log_{10}(k_2'/k_2)$	0.8915	0.3735	2.298	approximate	approximate
b1	$\log_{10}(k_1')$	−1.564	−1.756	−1.384	approximate	approximate
b1	$\log_{10}(k_2)$	−2.248	−2.478	−2.03	approximate	approximate
b1	$\log_{10}(k_2')$	−1.605	−1.978	−0.6246	approximate	approximate
b1	$\log_{10}(k_{deg})$	−1.697	−2.112	−0.6567	approximate	approximate
b1	$\log_{10}(k_1'/k_2)$	0.684	0.6275	0.7413	approximate	approximate
b1	$\log_{10}(k_1'k_2'/k_2)$	−0.9211	−1.317	0.09023	approximate	approximate
b1	$\log_{10}(k_2'/k_2)$	0.6425	0.1127	1.798	approximate	approximate
b2	$\log_{10}(k_1'k_2')$	−2.138	−3.225	> 5.065	approximate	optim
b2	$\log_{10}(k_2 \text{ or } k_{deg})$	−0.3153	−2.733	> 6.902	approximate	optim
b2	$\log_{10}(k_2 \text{ or } k_{deg})$	−2.552	−2.733	−2.552	approximate	optim
b3	$\log_{10}(k_1')$	−1.75	−1.94	−1.446	approximate	approximate
b3	$\log_{10}(k_2)$	−2.21	−2.589	−1.876	exact	exact
b3	$\log_{10}(k_2')$	−1.463	−1.938	> 4.343	approximate	optim
b3	$\log_{10}(k_{deg})$	−1.71	−2.214	> 4.204	approximate	optim
b3	$\log_{10}(k_1'/k_2)$	0.4596	0.3951	0.5785	approximate	approximate
b3	$\log_{10}(k_1'k_2'/k_2)$	−1.004	−1.488	> 4.876	approximate	optim
b3	$\log_{10}(k_2'/k_2)$	0.7466	0.01604	> 6.78	approximate	optim

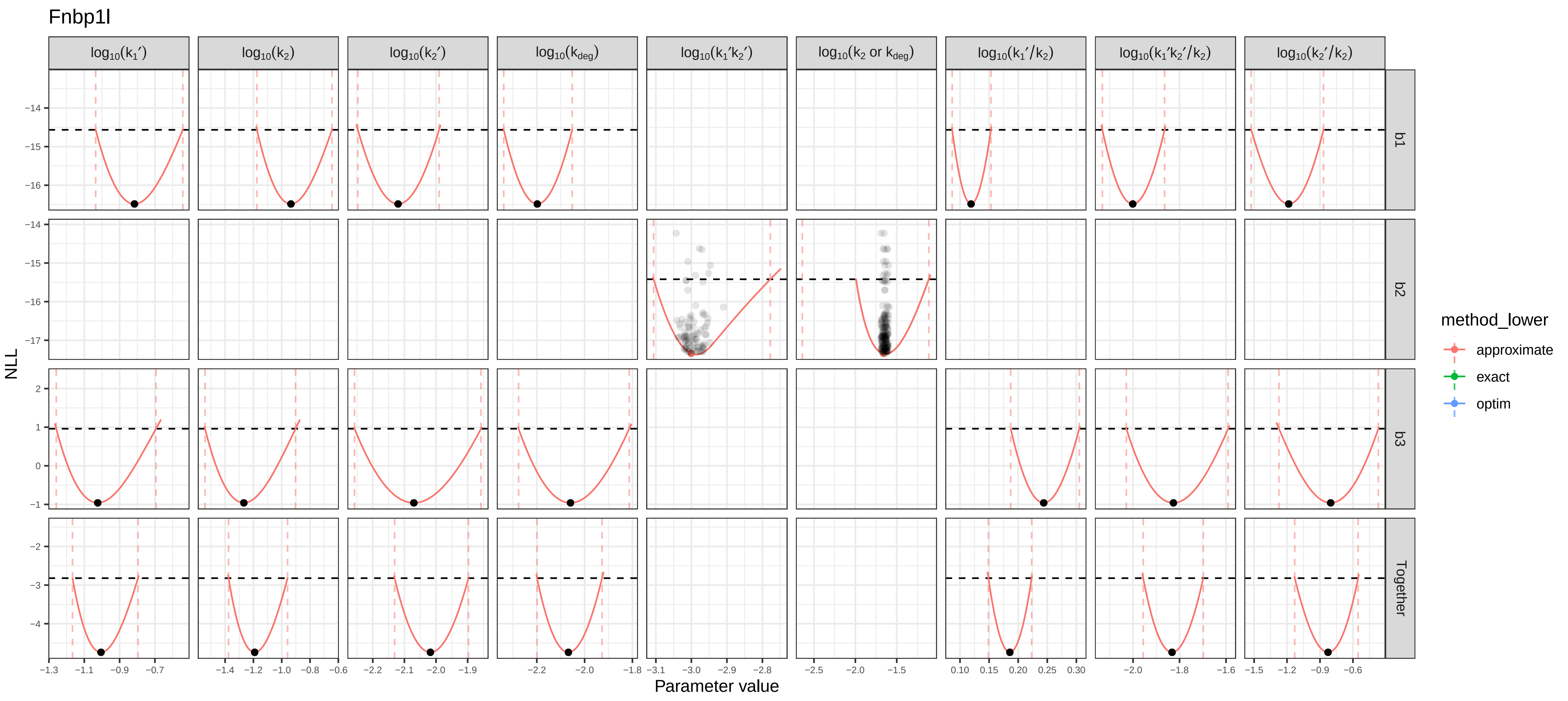

Replicate	Par	Best value	CI95 LB	CI95 UB	Method LB	Method UB
Together	$\log_{10}(k_1')$	−1.005	−1.167	−0.7959	approximate	approximate
Together	$\log_{10}(k_2)$	−1.191	−1.375	−0.9587	approximate	approximate
Together	$\log_{10}(k_2')$	−2.018	−2.131	−1.897	approximate	approximate
Together	$\log_{10}(k_{deg})$	−2.068	−2.199	−1.927	approximate	approximate
Together	$\log_{10}(k_1'/k_2)$	0.1856	0.1488	0.2232	approximate	approximate
Together	$\log_{10}(k_1'k_2'/k_2)$	−1.832	−1.956	−1.698	approximate	approximate
Together	$\log_{10}(k_2'/k_2)$	−0.827	−1.13	−0.5528	approximate	approximate
b1	$\log_{10}(k_1')$	−0.8157	−1.036	−0.5423	approximate	approximate
b1	$\log_{10}(k_2)$	−0.9346	−1.175	−0.645	approximate	approximate
b1	$\log_{10}(k_2')$	−2.12	−2.247	−1.99	approximate	approximate
b1	$\log_{10}(k_{deg})$	−2.198	−2.338	−2.052	approximate	approximate
b1	$\log_{10}(k_1'/k_2)$	0.1189	0.08654	0.1532	approximate	approximate
b1	$\log_{10}(k_1'k_2'/k_2)$	−2.001	−2.132	−1.864	approximate	approximate
b1	$\log_{10}(k_2'/k_2)$	−1.185	−1.526	−0.8682	approximate	approximate
b2	$\log_{10}(k_1'k_2')$	−3.001	−3.106	−2.778	approximate	approximate
b2	$\log_{10}(k_2 \text{ or } k_{deg})$	−1.633	−2.64	−1.113	approximate	approximate
b2	$\log_{10}(k_2 \text{ or } k_{deg})$	−1.661	−2.64	−1.113	approximate	approximate
b3	$\log_{10}(k_1')$	−1.024	−1.259	−0.6941	approximate	approximate
b3	$\log_{10}(k_2)$	−1.268	−1.542	−0.9022	approximate	approximate
b3	$\log_{10}(k_2')$	−2.07	−2.258	−1.858	approximate	approximate
b3	$\log_{10}(k_{deg})$	−2.059	−2.276	−1.813	approximate	approximate
b3	$\log_{10}(k_1'/k_2)$	0.2439	0.1871	0.305	approximate	approximate
b3	$\log_{10}(k_1'k_2'/k_2)$	−1.826	−2.029	−1.591	approximate	approximate
b3	$\log_{10}(k_2'/k_2)$	−0.8025	−1.274	−0.3688	approximate	approximate

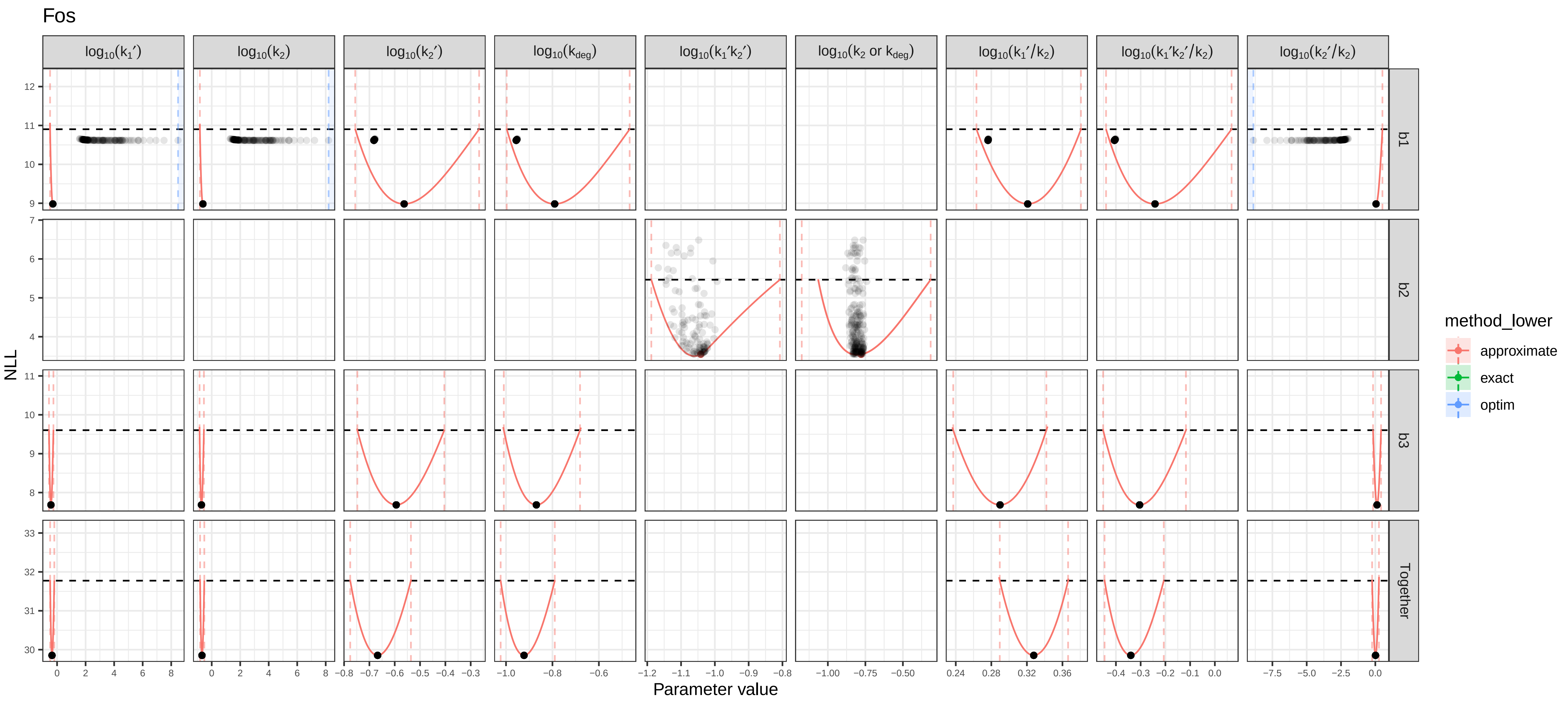

Replicate	Par	Best value	CI95 LB	CI95 UB	Method LB	Method UB
Together	$\log_{10}(k_1')$	−0.3555	−0.4857	−0.1946	approximate	approximate
Together	$\log_{10}(k_2)$	−0.6832	−0.8158	−0.5197	approximate	approximate
Together	$\log_{10}(k_2')$	−0.6674	−0.7757	−0.5361	approximate	approximate
Together	$\log_{10}(k_{deg})$	−0.9224	−1.023	−0.7899	approximate	approximate
Together	$\log_{10}(k_1'/k_2)$	0.3277	0.2895	0.3663	approximate	approximate
Together	$\log_{10}(k_1'k_2'/k_2)$	−0.3397	−0.446	−0.2065	approximate	approximate
Together	$\log_{10}(k_2'/k_2)$	0.01573	−0.2343	0.2635	approximate	approximate
b1	$\log_{10}(k_1')$	−0.2963	−0.49	> 8.478	approximate	optim
b1	$\log_{10}(k_2)$	−0.6172	−0.8292	> 8.202	approximate	optim
b1	$\log_{10}(k_2')$	−0.5627	−0.7559	−0.2667	approximate	approximate
b1	$\log_{10}(k_{deg})$	−0.7902	−0.9968	−0.4675	approximate	approximate
b1	$\log_{10}(k_1'/k_2)$	0.3209	0.2633	0.3808	approximate	approximate
b1	$\log_{10}(k_1'k_2'/k_2)$	−0.2418	−0.4399	0.06768	approximate	approximate
b1	$\log_{10}(k_2'/k_2)$	0.05456	< −8.884	0.5191	optim	approximate
b2	$\log_{10}(k_1'k_2')$	−1.042	−1.188	−0.8076	approximate	approximate
b2	$\log_{10}(k_2 \text{ or } k_{deg})$	−0.7782	−1.174	−0.315	approximate	approximate
b2	$\log_{10}(k_2 \text{ or } k_{deg})$	−0.8168	−1.174	−0.315	approximate	approximate
b3	$\log_{10}(k_1')$	−0.4291	−0.5667	−0.2551	approximate	approximate
b3	$\log_{10}(k_2)$	−0.7189	−0.852	−0.5475	approximate	approximate
b3	$\log_{10}(k_2')$	−0.594	−0.7476	−0.4043	approximate	approximate
b3	$\log_{10}(k_{deg})$	−0.8693	−1.01	−0.6812	approximate	approximate
b3	$\log_{10}(k_1'/k_2)$	0.2898	0.2372	0.3419	approximate	approximate
b3	$\log_{10}(k_1'k_2'/k_2)$	−0.3042	−0.4517	−0.1169	approximate	approximate
b3	$\log_{10}(k_2'/k_2)$	0.1249	−0.1648	0.4192	approximate	approximate

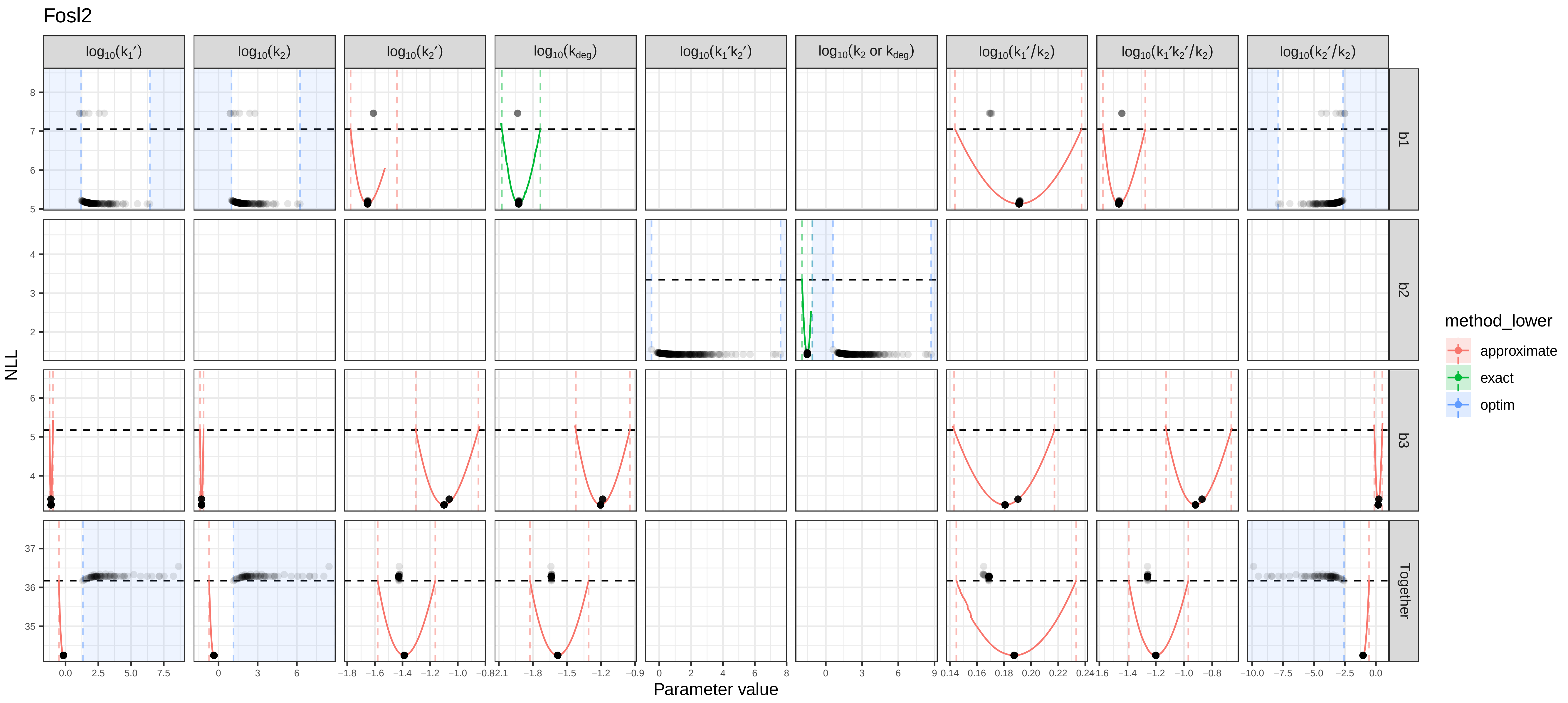

Replicate	Par	Best value	CI95 LB	CI95 UB	Method LB	Method UB
Together	$\log_{10}(k_1')$	−0.1622	−0.5097	> 1.32	approximate	optim
Together	$\log_{10}(k_2)$	−0.3497	−0.716	> 1.151	approximate	optim
Together	$\log_{10}(k_2')$	−1.387	−1.58	−1.162	approximate	approximate
Together	$\log_{10}(k_{deg})$	−1.581	−1.825	−1.309	approximate	approximate
Together	$\log_{10}(k_1'/k_2)$	0.1875	0.1448	0.2333	approximate	approximate
Together	$\log_{10}(k_1'k_2'/k_2)$	−1.2	−1.392	−0.9689	approximate	approximate
Together	$\log_{10}(k_2'/k_2)$	−1.038	< −2.578	−0.5433	optim	approximate
b1	$\log_{10}(k_1')$	6.231	< 1.19	> 6.434	optim	optim
b1	$\log_{10}(k_2)$	6.04	< 0.9983	> 6.243	optim	optim
b1	$\log_{10}(k_2')$	−1.653	−1.776	−1.441	approximate	approximate
b1	$\log_{10}(k_{deg})$	−1.925	−2.074	−1.733	exact	exact
b1	$\log_{10}(k_1'/k_2)$	0.1911	0.1439	0.2372	approximate	approximate
b1	$\log_{10}(k_1'k_2'/k_2)$	−1.462	−1.574	−1.274	approximate	approximate
b1	$\log_{10}(k_2'/k_2)$	−7.692	< −7.896	> −2.649	optim	optim
b2	$\log_{10}(k_1'k_2')$	7.316	< −0.4832	> 7.619	optim	optim
b2	$\log_{10}(k_2 \text{ or } k_{deg})$	8.413	0.6064	> 8.715	optim	optim
b2	$\log_{10}(k_2 \text{ or } k_{deg})$	−1.533	−1.962	−1.106	exact	exact
b3	$\log_{10}(k_1')$	−1.107	−1.221	−0.9672	approximate	approximate
b3	$\log_{10}(k_2)$	−1.287	−1.41	−1.142	approximate	approximate
b3	$\log_{10}(k_2')$	−1.099	−1.304	−0.8509	approximate	approximate
b3	$\log_{10}(k_{deg})$	−1.204	−1.422	−0.946	approximate	approximate
b3	$\log_{10}(k_1'/k_2)$	0.1808	0.1432	0.2172	approximate	approximate
b3	$\log_{10}(k_1'k_2'/k_2)$	−0.9187	−1.126	−0.6642	approximate	approximate
b3	$\log_{10}(k_2'/k_2)$	0.1879	−0.1224	0.5209	approximate	approximate

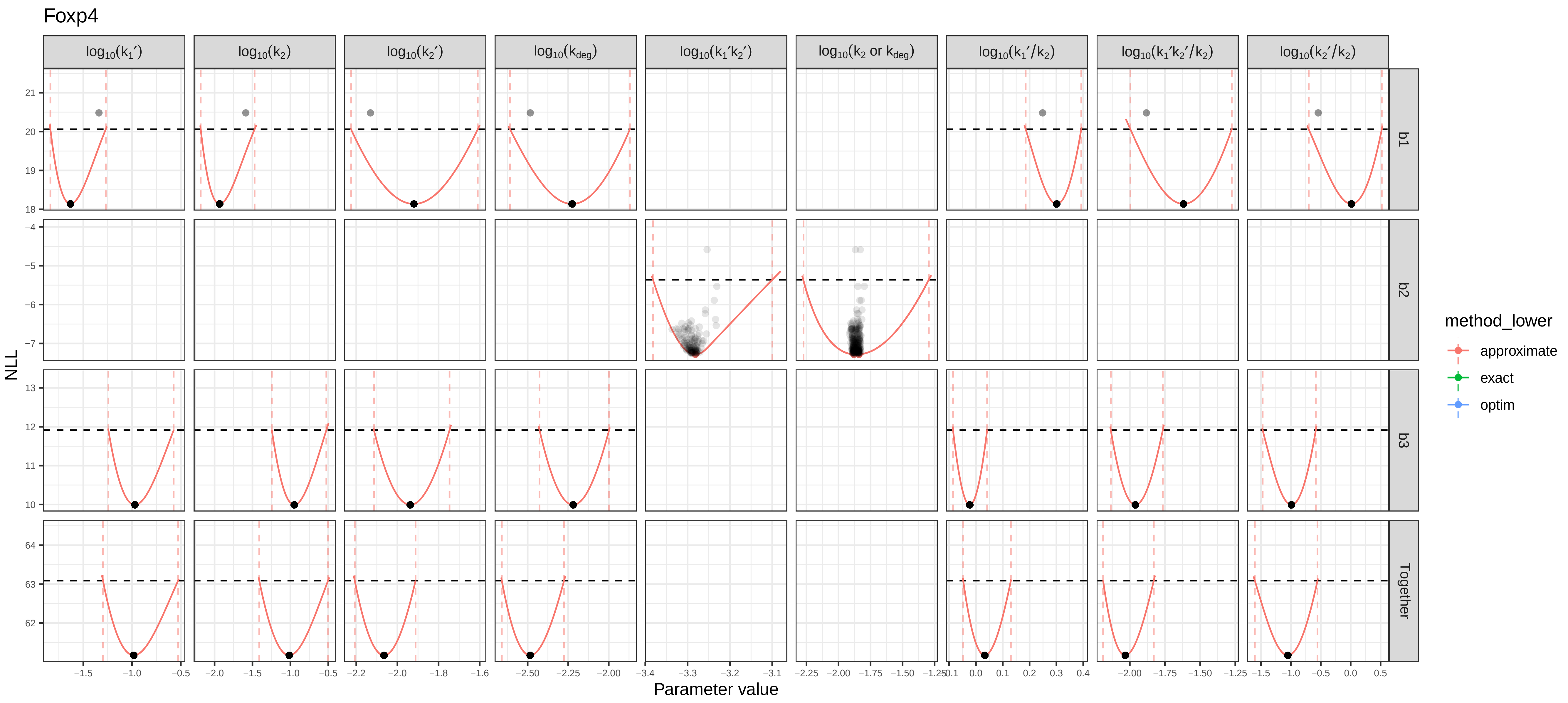

Replicate	Par	Best value	CI95 LB	CI95 UB	Method LB	Method UB
Together	$\log_{10}(k_1')$	−0.9821	−1.298	−0.5277	approximate	approximate
Together	$\log_{10}(k_2)$	−1.015	−1.41	−0.503	approximate	approximate
Together	$\log_{10}(k_2')$	−2.065	−2.207	−1.912	approximate	approximate
Together	$\log_{10}(k_{deg})$	−2.484	−2.659	−2.275	approximate	approximate
Together	$\log_{10}(k_1'/k_2)$	0.03309	−0.04739	0.1303	approximate	approximate
Together	$\log_{10}(k_1'k_2'/k_2)$	−2.032	−2.19	−1.829	approximate	approximate
Together	$\log_{10}(k_2'/k_2)$	−1.05	−1.602	−0.5543	approximate	approximate
b1	$\log_{10}(k_1')$	−1.631	−1.837	−1.269	approximate	approximate
b1	$\log_{10}(k_2)$	−1.932	−2.183	−1.472	approximate	approximate
b1	$\log_{10}(k_2')$	−1.92	−2.226	−1.61	approximate	approximate
b1	$\log_{10}(k_{deg})$	−2.226	−2.609	−1.87	approximate	approximate
b1	$\log_{10}(k_1'/k_2)$	0.3012	0.1854	0.3927	approximate	approximate
b1	$\log_{10}(k_1'k_2'/k_2)$	−1.618	−1.996	−1.275	approximate	approximate
b1	$\log_{10}(k_2'/k_2)$	0.01261	−0.701	0.5205	approximate	approximate
b2	$\log_{10}(k_1'k_2')$	−3.281	−3.382	−3.1	approximate	approximate
b2	$\log_{10}(k_2 \text{ or } k_{deg})$	−1.84	−2.273	−1.295	approximate	approximate
b2	$\log_{10}(k_2 \text{ or } k_{deg})$	−1.88	−2.273	−1.295	approximate	approximate
b3	$\log_{10}(k_1')$	−0.9709	−1.243	−0.5732	approximate	approximate
b3	$\log_{10}(k_2)$	−0.948	−1.244	−0.5255	approximate	approximate
b3	$\log_{10}(k_2')$	−1.937	−2.114	−1.747	approximate	approximate
b3	$\log_{10}(k_{deg})$	−2.219	−2.427	−1.998	approximate	approximate
b3	$\log_{10}(k_1'/k_2)$	−0.02284	−0.08528	0.04181	approximate	approximate
b3	$\log_{10}(k_1'k_2'/k_2)$	−1.96	−2.135	−1.766	approximate	approximate
b3	$\log_{10}(k_2'/k_2)$	−0.9893	−1.471	−0.5825	approximate	approximate

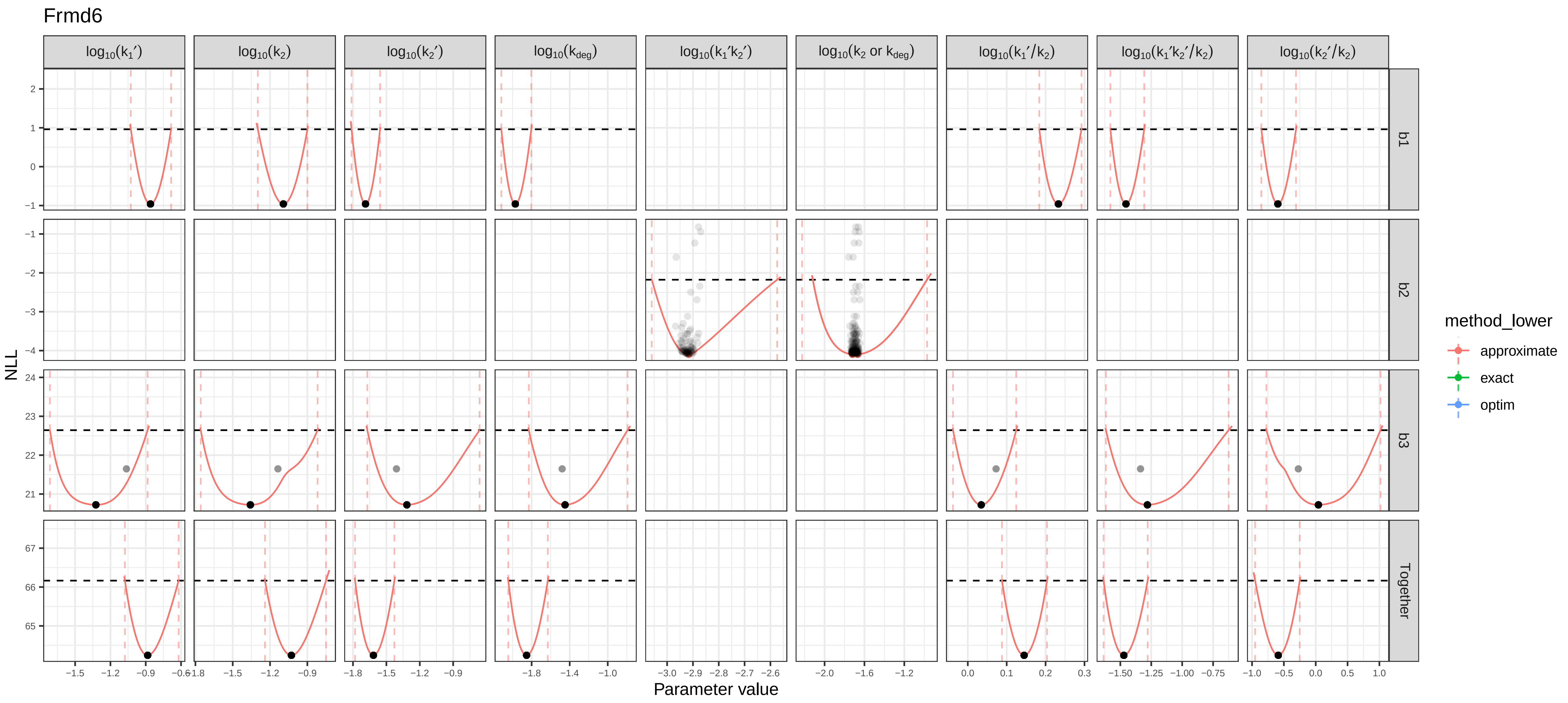

Replicate	Par	Best value	CI95 LB	CI95 UB	Method LB	Method UB
Together	$\log_{10}(k_1')$	−0.8835	−1.077	−0.6203	approximate	approximate
Together	$\log_{10}(k_2)$	−1.028	−1.241	−0.7501	approximate	approximate
Together	$\log_{10}(k_2')$	−1.616	−1.781	−1.428	approximate	approximate
Together	$\log_{10}(k_{deg})$	−1.853	−2.046	−1.63	approximate	approximate
Together	$\log_{10}(k_1'/k_2)$	0.1449	0.08779	0.2038	approximate	approximate
Together	$\log_{10}(k_1'k_2'/k_2)$	−1.472	−1.635	−1.279	approximate	approximate
Together	$\log_{10}(k_2'/k_2)$	−0.5881	−0.9521	−0.2513	approximate	approximate
b1	$\log_{10}(k_1')$	−0.8599	−1.027	−0.6843	approximate	approximate
b1	$\log_{10}(k_2)$	−1.093	−1.298	−0.8994	approximate	approximate
b1	$\log_{10}(k_2')$	−1.687	−1.813	−1.557	approximate	approximate
b1	$\log_{10}(k_{deg})$	−1.971	−2.118	−1.803	approximate	approximate
b1	$\log_{10}(k_1'/k_2)$	0.233	0.1837	0.2923	approximate	approximate
b1	$\log_{10}(k_1'k_2'/k_2)$	−1.454	−1.581	−1.307	approximate	approximate
b1	$\log_{10}(k_2'/k_2)$	−0.5944	−0.8541	−0.3093	approximate	approximate
b2	$\log_{10}(k_1'k_2')$	−2.916	−3.06	−2.574	approximate	approximate
b2	$\log_{10}(k_2 \text{ or } k_{deg})$	−1.667	−2.225	−0.9718	approximate	approximate
b2	$\log_{10}(k_2 \text{ or } k_{deg})$	−1.721	−2.225	−0.9718	approximate	approximate
b3	$\log_{10}(k_1')$	−1.324	−1.714	−0.8838	approximate	approximate
b3	$\log_{10}(k_2)$	−1.358	−1.757	−0.8179	approximate	approximate
b3	$\log_{10}(k_2')$	−1.316	−1.671	−0.6627	approximate	approximate
b3	$\log_{10}(k_{deg})$	−1.448	−1.829	−0.7918	approximate	approximate
b3	$\log_{10}(k_1'/k_2)$	0.0346	−0.03793	0.1244	approximate	approximate
b3	$\log_{10}(k_1'k_2'/k_2)$	−1.281	−1.617	−0.6245	approximate	approximate
b3	$\log_{10}(k_2'/k_2)$	0.04274	−0.7753	1.018	approximate	approximate

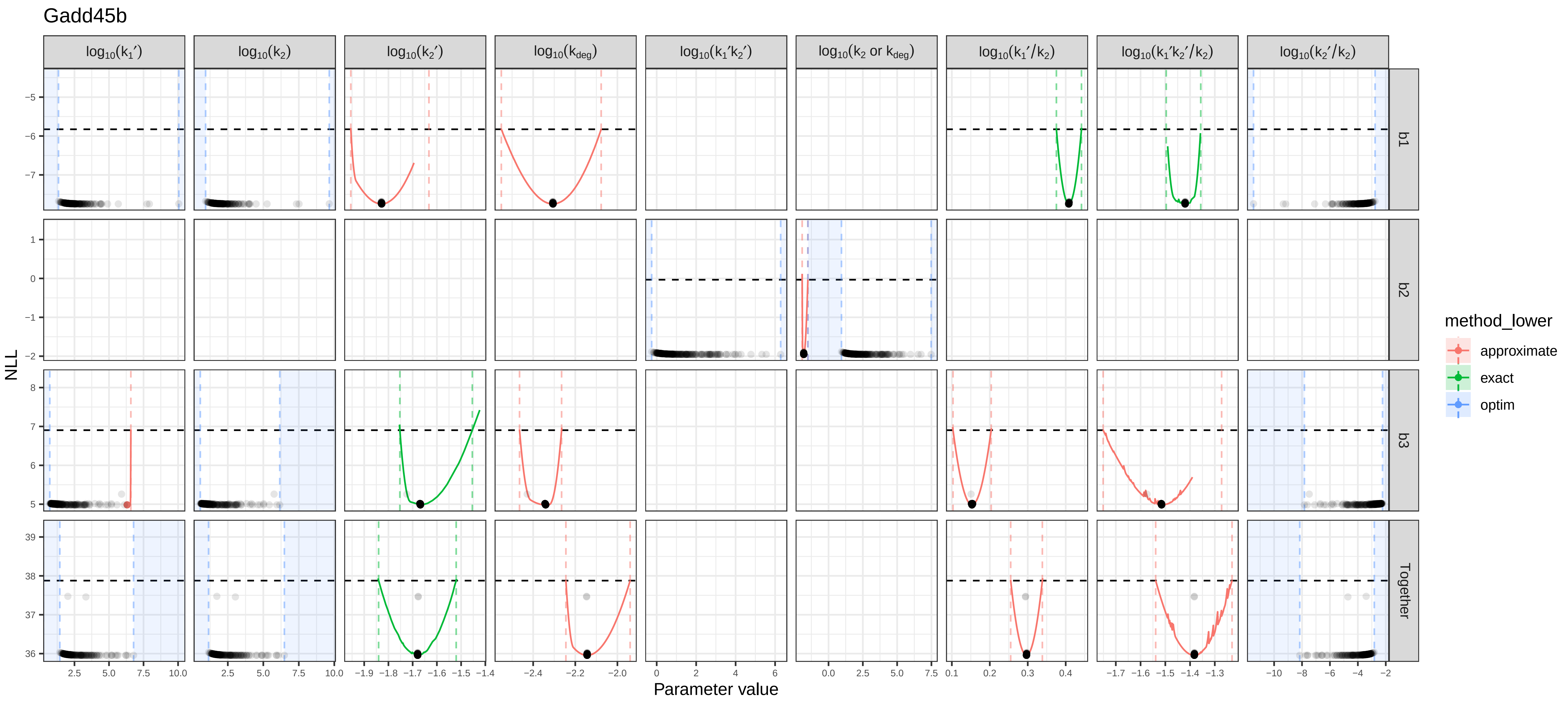

Replicate	Par	Best value	CI95 LB	CI95 UB	Method LB	Method UB
Together	$\log_{10}(k_1')$	5.654	< 1.44	> 6.78	optim	optim
Together	$\log_{10}(k_2)$	5.358	< 1.143	> 6.483	optim	optim
Together	$\log_{10}(k_2')$	−1.679	−1.841	−1.52	exact	exact
Together	$\log_{10}(k_{deg})$	−2.144	−2.245	−1.94	approximate	approximate
Together	$\log_{10}(k_1'/k_2)$	0.2966	0.255	0.3385	approximate	approximate
Together	$\log_{10}(k_1'k_2'/k_2)$	−1.383	−1.539	−1.23	approximate	approximate
Together	$\log_{10}(k_2'/k_2)$	−7.037	< −8.163	> −2.823	optim	optim
b1	$\log_{10}(k_1')$	7.926	< 1.344	> 10.05	optim	optim
b1	$\log_{10}(k_2)$	7.518	< 0.9351	> 9.646	optim	optim
b1	$\log_{10}(k_2')$	−1.828	−1.955	−1.633	approximate	approximate
b1	$\log_{10}(k_{deg})$	−2.306	−2.551	−2.077	approximate	approximate
b1	$\log_{10}(k_1'/k_2)$	0.4081	0.3754	0.4416	exact	exact
b1	$\log_{10}(k_1'k_2'/k_2)$	−1.42	−1.496	−1.357	exact	exact
b1	$\log_{10}(k_2'/k_2)$	−9.346	< −11.47	> −2.764	optim	optim
b2	$\log_{10}(k_1'k_2')$	3.521	< −0.254	> 6.283	optim	optim
b2	$\log_{10}(k_2 \text{ or } k_{deg})$	4.716	0.9432	> 7.478	optim	optim
b2	$\log_{10}(k_2 \text{ or } k_{deg})$	−1.805	−1.922	−1.506	approximate	approximate
b3	$\log_{10}(k_1')$	6.313	< 0.715	6.582	optim	approximate
b3	$\log_{10}(k_2)$	6.16	< 0.56	> 6.16	optim	optim
b3	$\log_{10}(k_2')$	−1.668	−1.753	−1.454	exact	exact
b3	$\log_{10}(k_{deg})$	−2.341	−2.465	−2.265	approximate	approximate
b3	$\log_{10}(k_1'/k_2)$	0.1529	0.1031	0.2037	approximate	approximate
b3	$\log_{10}(k_1'k_2'/k_2)$	−1.515	−1.751	−1.272	approximate	approximate
b3	$\log_{10}(k_2'/k_2)$	−7.828	< −7.828	> −2.231	optim	optim

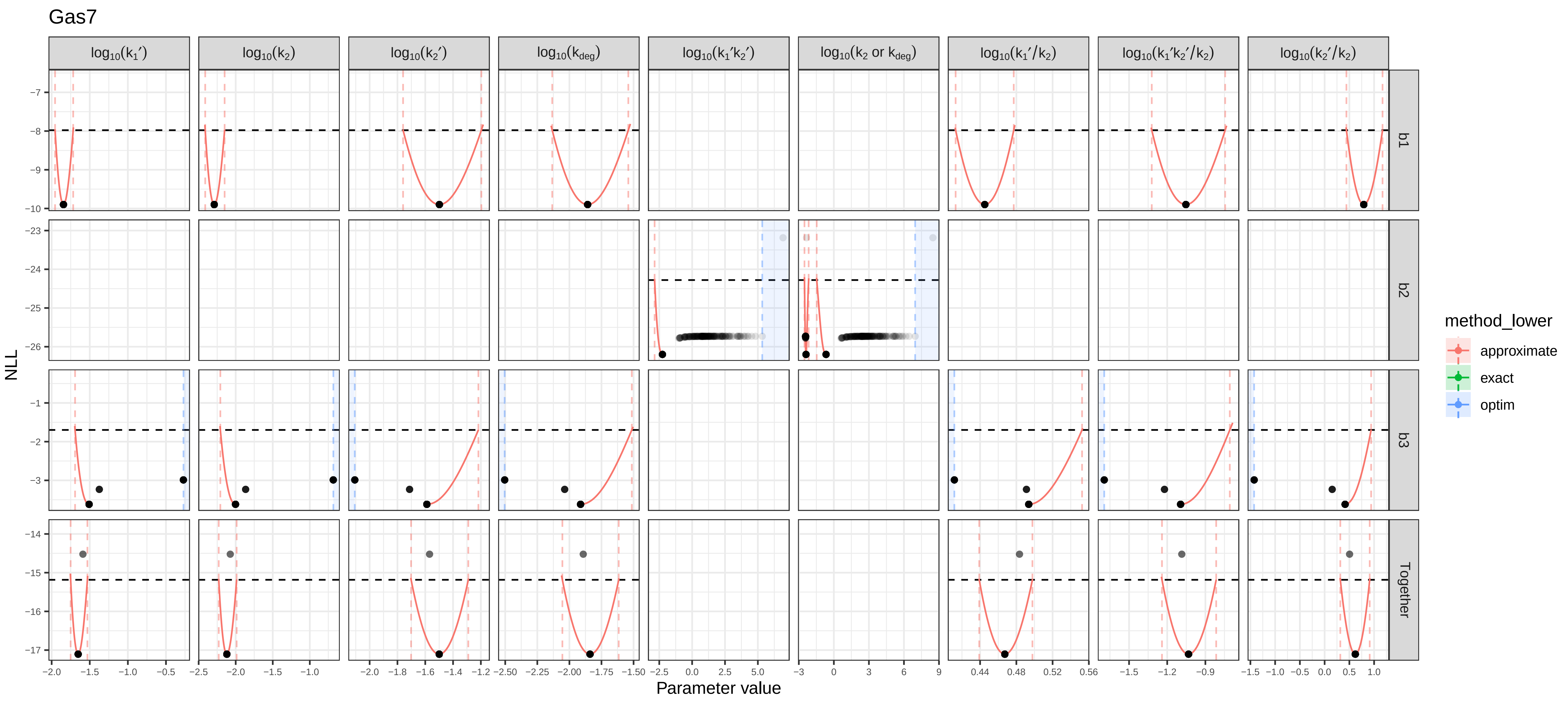

Replicate	Par	Best value	CI95 LB	CI95 UB	Method LB	Method UB
Together	$\log_{10}(k_1')$	−1.652	−1.751	−1.531	approximate	approximate
Together	$\log_{10}(k_2)$	−2.12	−2.229	−1.987	approximate	approximate
Together	$\log_{10}(k_2')$	−1.5	−1.702	−1.29	approximate	approximate
Together	$\log_{10}(k_{deg})$	−1.839	−2.055	−1.616	approximate	approximate
Together	$\log_{10}(k_1'/k_2)$	0.4673	0.439	0.4977	approximate	approximate
Together	$\log_{10}(k_1'k_2'/k_2)$	−1.032	−1.241	−0.8149	approximate	approximate
Together	$\log_{10}(k_2'/k_2)$	0.6201	0.317	0.9111	approximate	approximate
b1	$\log_{10}(k_1')$	−1.845	−1.955	−1.718	approximate	approximate
b1	$\log_{10}(k_2)$	−2.29	−2.412	−2.15	approximate	approximate
b1	$\log_{10}(k_2')$	−1.498	−1.76	−1.196	approximate	approximate
b1	$\log_{10}(k_{deg})$	−1.858	−2.134	−1.541	approximate	approximate
b1	$\log_{10}(k_1'/k_2)$	0.4451	0.413	0.4771	approximate	approximate
b1	$\log_{10}(k_1'k_2'/k_2)$	−1.053	−1.322	−0.7445	approximate	approximate
b1	$\log_{10}(k_2'/k_2)$	0.7915	0.4403	1.171	approximate	approximate
b2	$\log_{10}(k_1'k_2')$	−2.258	−2.854	> 5.333	approximate	optim
b2	$\log_{10}(k_2 \text{ or } k_{deg})$	−0.6685	−1.467	> 6.959	approximate	optim
b2	$\log_{10}(k_2 \text{ or } k_{deg})$	−2.385	−2.516	−2.158	approximate	approximate
b3	$\log_{10}(k_1')$	−1.509	−1.693	> −0.2704	approximate	optim
b3	$\log_{10}(k_2)$	−2.003	−2.207	> −0.682	approximate	optim
b3	$\log_{10}(k_2')$	−1.588	< −2.107	−1.218	optim	approximate
b3	$\log_{10}(k_{deg})$	−1.913	< −2.504	−1.514	optim	approximate
b3	$\log_{10}(k_1'/k_2)$	0.4937	< 0.4116	0.5525	optim	approximate
b3	$\log_{10}(k_1'k_2'/k_2)$	−1.095	< −1.695	−0.7079	optim	approximate
b3	$\log_{10}(k_2'/k_2)$	0.4141	< −1.425	0.94	optim	approximate

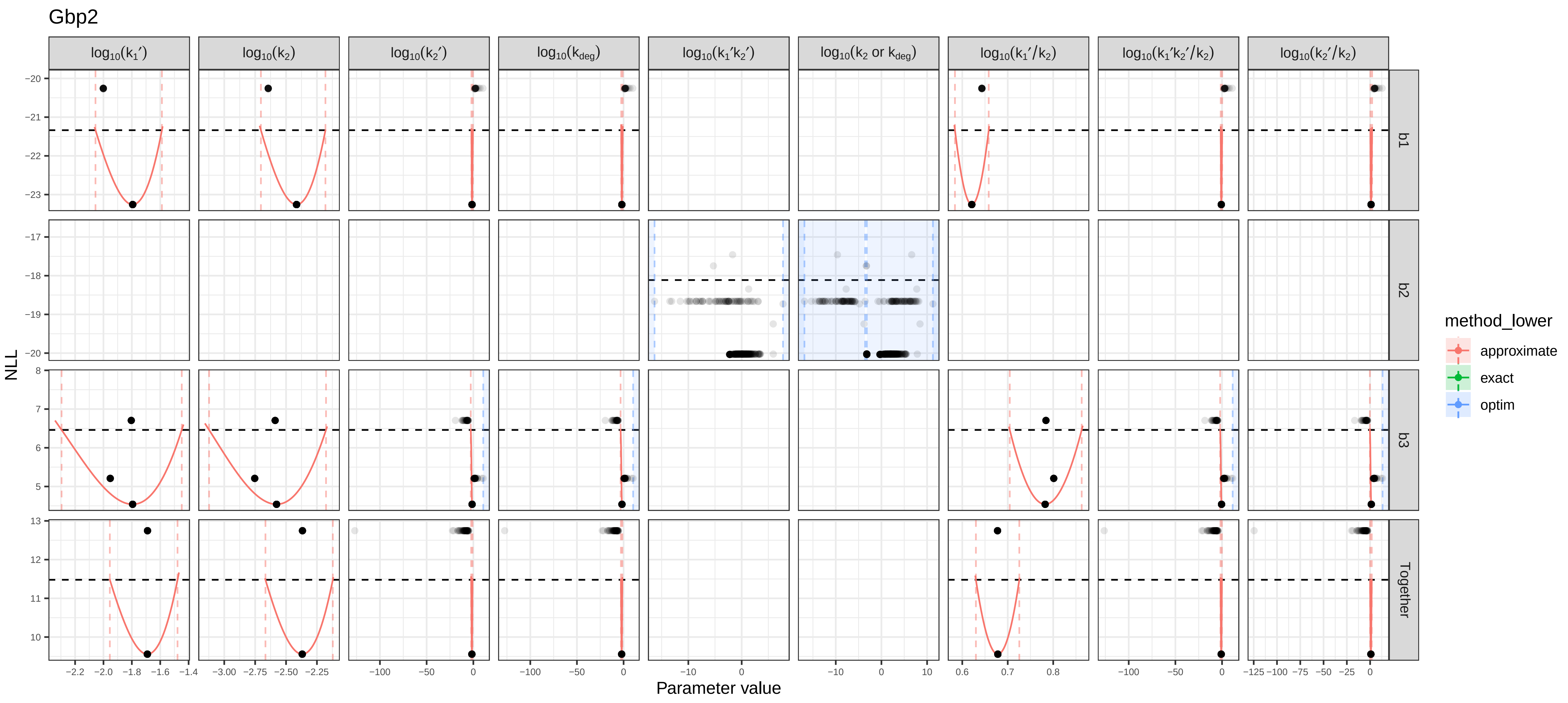

Replicate	Par	Best value	CI95 LB	CI95 UB	Method LB	Method UB
Together	$\log_{10}(k_1')$	−1.691	−1.955	−1.478	approximate	approximate
Together	$\log_{10}(k_2)$	−2.369	−2.667	−2.122	approximate	approximate
Together	$\log_{10}(k_2')$	−1.56	−2.339	−0.8573	approximate	approximate
Together	$\log_{10}(k_{deg})$	−2.064	−2.864	−1.321	approximate	approximate
Together	$\log_{10}(k_1'/k_2)$	0.6782	0.63	0.7254	approximate	approximate
Together	$\log_{10}(k_1'k_2'/k_2)$	−0.8818	−1.666	−0.1535	approximate	approximate
Together	$\log_{10}(k_2'/k_2)$	0.8088	−0.02646	1.737	approximate	approximate
b1	$\log_{10}(k_1')$	−1.794	−2.056	−1.588	approximate	approximate
b1	$\log_{10}(k_2)$	−2.415	−2.703	−2.181	approximate	approximate
b1	$\log_{10}(k_2')$	−1.406	−2.035	−0.473	approximate	approximate
b1	$\log_{10}(k_{deg})$	−1.877	−2.527	−0.9122	approximate	approximate
b1	$\log_{10}(k_1'/k_2)$	0.621	0.5839	0.6582	approximate	approximate
b1	$\log_{10}(k_1'k_2'/k_2)$	−0.7852	−1.421	0.1662	approximate	approximate
b1	$\log_{10}(k_2'/k_2)$	1.009	0.2916	2.139	approximate	approximate
b2	$\log_{10}(k_1'k_2')$	−2.247	< −16.33	> 7.745	optim	optim
b2	$\log_{10}(k_2 \text{ or } k_{deg})$	−0.3233	< −3.572	> 11.27	optim	optim
b2	$\log_{10}(k_2 \text{ or } k_{deg})$	−3.205	< −16.86	> −3.203	optim	optim
b3	$\log_{10}(k_1')$	−1.795	−2.295	−1.448	approximate	approximate
b3	$\log_{10}(k_2)$	−2.577	−3.123	−2.177	approximate	approximate
b3	$\log_{10}(k_2')$	−1.315	−2.79	> 10.6	approximate	optim
b3	$\log_{10}(k_{deg})$	−1.724	−3.232	> 10.23	approximate	optim
b3	$\log_{10}(k_1'/k_2)$	0.7823	0.7045	0.8626	approximate	approximate
b3	$\log_{10}(k_1'k_2'/k_2)$	−0.5322	−2.009	> 11.4	approximate	optim
b3	$\log_{10}(k_2'/k_2)$	1.262	−0.2423	> 13.36	approximate	optim

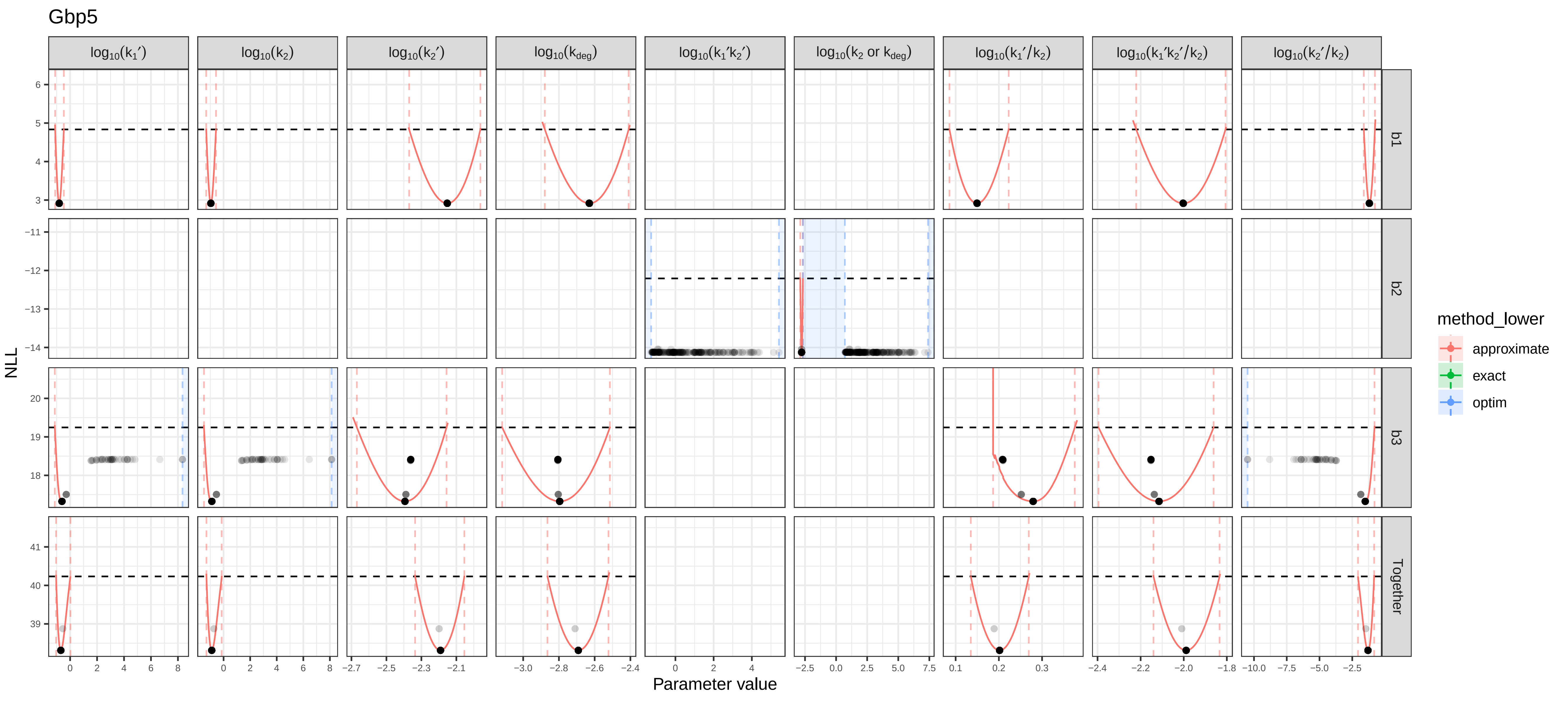

Replicate	Par	Best value	CI95 LB	CI95 UB	Method LB	Method UB
Together	$\log_{10}(k_1')$	−0.6891	−1.041	0.02282	approximate	approximate
Together	$\log_{10}(k_2)$	−0.8908	−1.291	−0.1449	approximate	approximate
Together	$\log_{10}(k_2')$	−2.19	−2.336	−2.054	approximate	approximate
Together	$\log_{10}(k_{deg})$	−2.691	−2.864	−2.523	approximate	approximate
Together	$\log_{10}(k_1'/k_2)$	0.2017	0.1351	0.2693	approximate	approximate
Together	$\log_{10}(k_1'k_2'/k_2)$	−1.989	−2.14	−1.834	approximate	approximate
Together	$\log_{10}(k_2'/k_2)$	−1.3	−2.061	−0.8305	approximate	approximate
b1	$\log_{10}(k_1')$	−0.81	−1.109	−0.4651	approximate	approximate
b1	$\log_{10}(k_2)$	−0.9596	−1.309	−0.5707	approximate	approximate
b1	$\log_{10}(k_2')$	−2.152	−2.37	−1.962	approximate	approximate
b1	$\log_{10}(k_{deg})$	−2.63	−2.879	−2.41	approximate	approximate
b1	$\log_{10}(k_1'/k_2)$	0.1496	0.08563	0.2227	approximate	approximate
b1	$\log_{10}(k_1'k_2'/k_2)$	−2.002	−2.221	−1.806	approximate	approximate
b1	$\log_{10}(k_2'/k_2)$	−1.192	−1.619	−0.7709	approximate	approximate
b2	$\log_{10}(k_1'k_2')$	1.858	< -1.27	> 5.417	optim	optim
b2	$\log_{10}(k_2 \text{ or } k_{deg})$	3.836	0.7063	> 7.395	optim	optim
b2	$\log_{10}(k_2 \text{ or } k_{deg})$	−2.773	−2.883	−2.671	approximate	approximate
b3	$\log_{10}(k_1')$	−0.6048	−1.135	> 8.339	approximate	optim
b3	$\log_{10}(k_2)$	−0.884	−1.477	> 8.13	approximate	optim
b3	$\log_{10}(k_2')$	−2.394	−2.669	−2.156	approximate	approximate
b3	$\log_{10}(k_{deg})$	−2.796	−3.118	−2.515	approximate	approximate
b3	$\log_{10}(k_1'/k_2)$	0.2793	0.1867	0.3757	approximate	approximate
b3	$\log_{10}(k_1'k_2'/k_2)$	−2.115	−2.396	−1.861	approximate	approximate
b3	$\log_{10}(k_2'/k_2)$	−1.51	< -10.49	−0.8022	optim	approximate

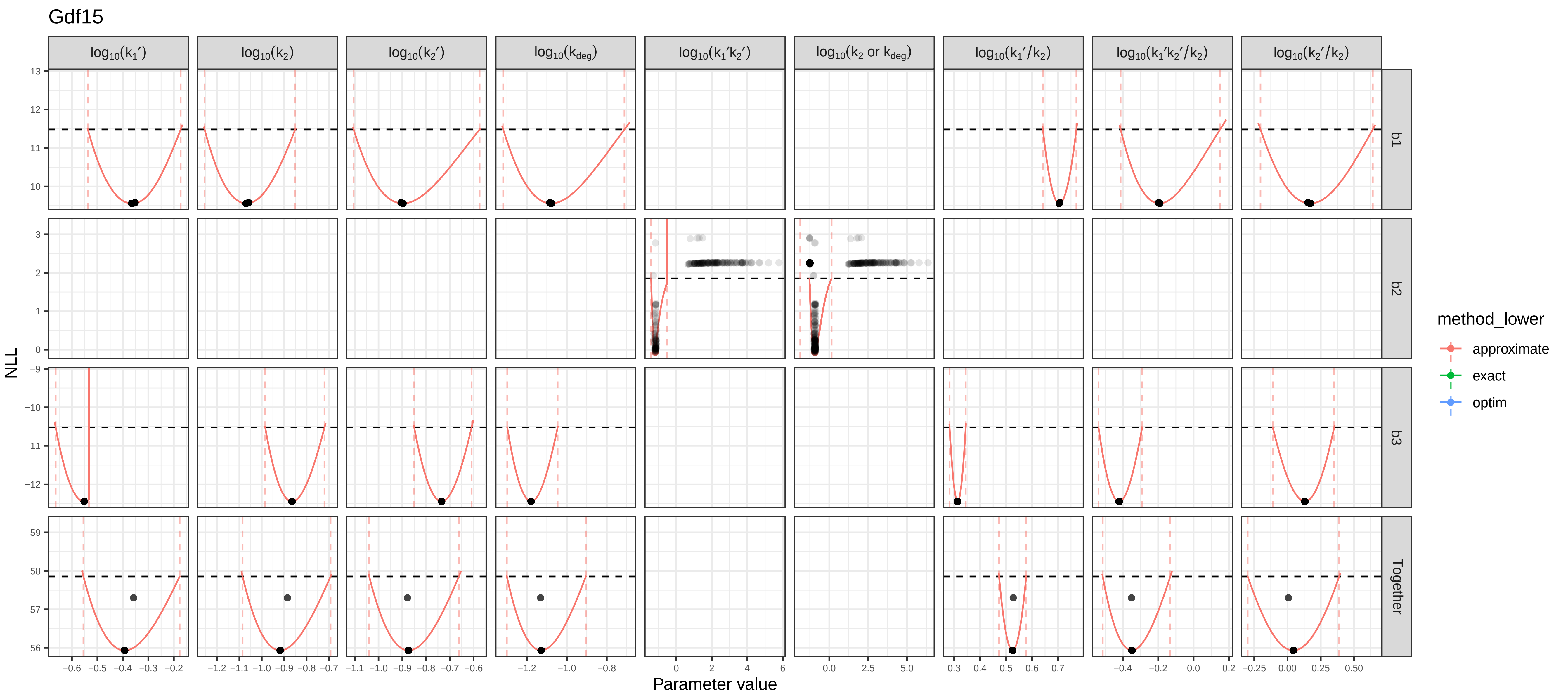

Replicate	Par	Best value	CI95 LB	CI95 UB	Method LB	Method UB
Together	$\log_{10}(k_1')$	−0.393	−0.5548	−0.1771	approximate	approximate
Together	$\log_{10}(k_2)$	−0.9177	−1.086	−0.6929	approximate	approximate
Together	$\log_{10}(k_2')$	−0.8736	−1.039	−0.6622	approximate	approximate
Together	$\log_{10}(k_{deg})$	−1.129	−1.301	−0.9049	approximate	approximate
Together	$\log_{10}(k_1'/k_2)$	0.5248	0.4729	0.5775	approximate	approximate
Together	$\log_{10}(k_1'k_2'/k_2)$	−0.3488	−0.5139	−0.1315	approximate	approximate
Together	$\log_{10}(k_2'/k_2)$	0.04415	−0.2993	0.3888	approximate	approximate
b1	$\log_{10}(k_1')$	−0.365	−0.5375	−0.1731	approximate	approximate
b1	$\log_{10}(k_2)$	−1.07	−1.255	−0.8505	approximate	approximate
b1	$\log_{10}(k_2')$	−0.8976	−1.105	−0.5745	approximate	approximate
b1	$\log_{10}(k_{deg})$	−1.078	−1.319	−0.7116	approximate	approximate
b1	$\log_{10}(k_1'/k_2)$	0.7046	0.6417	0.7707	approximate	approximate
b1	$\log_{10}(k_1'k_2'/k_2)$	−0.193	−0.4125	0.1496	approximate	approximate
b1	$\log_{10}(k_2'/k_2)$	0.172	−0.2028	0.6412	approximate	approximate
b2	$\log_{10}(k_1'k_2')$	−1.177	−1.411	−0.5167	approximate	approximate
b2	$\log_{10}(k_2 \text{ or } k_{deg})$	−0.9275	−1.868	0.1476	approximate	approximate
b2	$\log_{10}(k_2 \text{ or } k_{deg})$	−0.9345	−1.868	0.1476	approximate	approximate
b3	$\log_{10}(k_1')$	−0.5515	−0.6637	−0.5333	approximate	approximate
b3	$\log_{10}(k_2)$	−0.865	−0.9846	−0.7199	approximate	approximate
b3	$\log_{10}(k_2')$	−0.7344	−0.8498	−0.6082	approximate	approximate
b3	$\log_{10}(k_{deg})$	−1.179	−1.298	−1.046	approximate	approximate
b3	$\log_{10}(k_1'/k_2)$	0.3134	0.2823	0.3444	approximate	approximate
b3	$\log_{10}(k_1'k_2'/k_2)$	−0.421	−0.5376	−0.2905	approximate	approximate
b3	$\log_{10}(k_2'/k_2)$	0.1305	−0.1104	0.3512	approximate	approximate

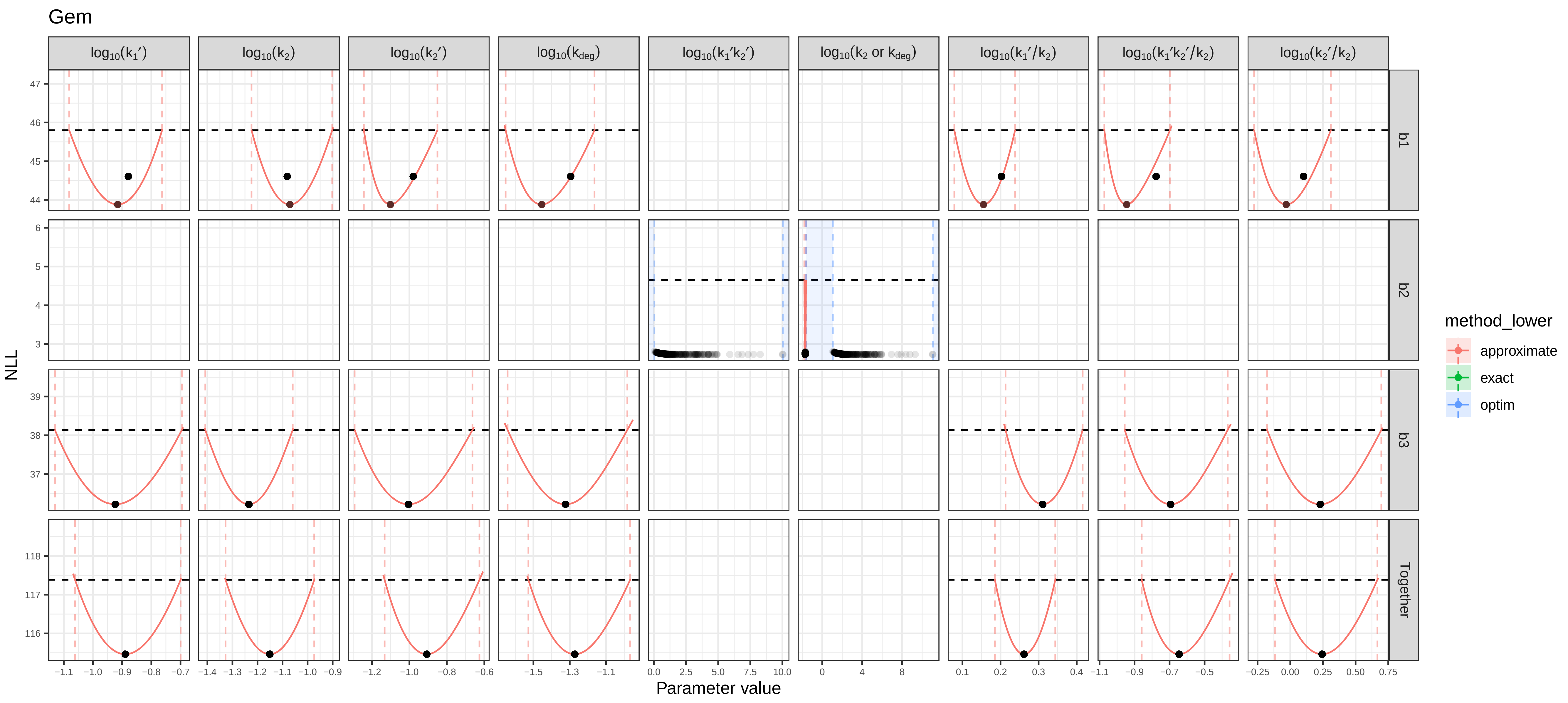

Replicate	Par	Best value	CI95 LB	CI95 UB	Method LB	Method UB
Together	$\log_{10}(k_1')$	−0.8892	−1.061	−0.6996	approximate	approximate
Together	$\log_{10}(k_2)$	−1.151	−1.327	−0.9734	approximate	approximate
Together	$\log_{10}(k_2')$	−0.9068	−1.132	−0.6262	approximate	approximate
Together	$\log_{10}(k_{deg})$	−1.272	−1.528	−0.9673	approximate	approximate
Together	$\log_{10}(k_1'/k_2)$	0.2616	0.1853	0.3437	approximate	approximate
Together	$\log_{10}(k_1'k_2'/k_2)$	−0.6452	−0.8592	−0.3573	approximate	approximate
Together	$\log_{10}(k_2'/k_2)$	0.244	−0.1174	0.6669	approximate	approximate
b1	$\log_{10}(k_1')$	−0.9153	−1.082	−0.763	approximate	approximate
b1	$\log_{10}(k_2)$	−1.071	−1.224	−0.9016	approximate	approximate
b1	$\log_{10}(k_2')$	−1.101	−1.243	−0.8502	approximate	approximate
b1	$\log_{10}(k_{deg})$	−1.454	−1.653	−1.164	approximate	approximate
b1	$\log_{10}(k_1'/k_2)$	0.1553	0.07876	0.2383	approximate	approximate
b1	$\log_{10}(k_1'k_2'/k_2)$	−0.9456	−1.073	−0.6979	approximate	approximate
b1	$\log_{10}(k_2'/k_2)$	−0.0303	−0.2765	0.3109	approximate	approximate
b2	$\log_{10}(k_1'k_2')$	6.558	< 0.03823	> 10.04	optim	optim
b2	$\log_{10}(k_2 \text{ or } k_{deg})$	7.586	1.065	> 11.07	optim	optim
b2	$\log_{10}(k_2 \text{ or } k_{deg})$	−1.695	−1.788	−1.629	approximate	approximate
b3	$\log_{10}(k_1')$	−0.9237	−1.13	−0.6954	approximate	approximate
b3	$\log_{10}(k_2)$	−1.234	−1.408	−1.059	approximate	approximate
b3	$\log_{10}(k_2')$	−1.005	−1.292	−0.6634	approximate	approximate
b3	$\log_{10}(k_{deg})$	−1.323	−1.642	−0.9829	approximate	approximate
b3	$\log_{10}(k_1'/k_2)$	0.3107	0.2132	0.4156	approximate	approximate
b3	$\log_{10}(k_1'k_2'/k_2)$	−0.694	−0.9563	−0.3677	approximate	approximate
b3	$\log_{10}(k_2'/k_2)$	0.2297	−0.1771	0.6971	approximate	approximate

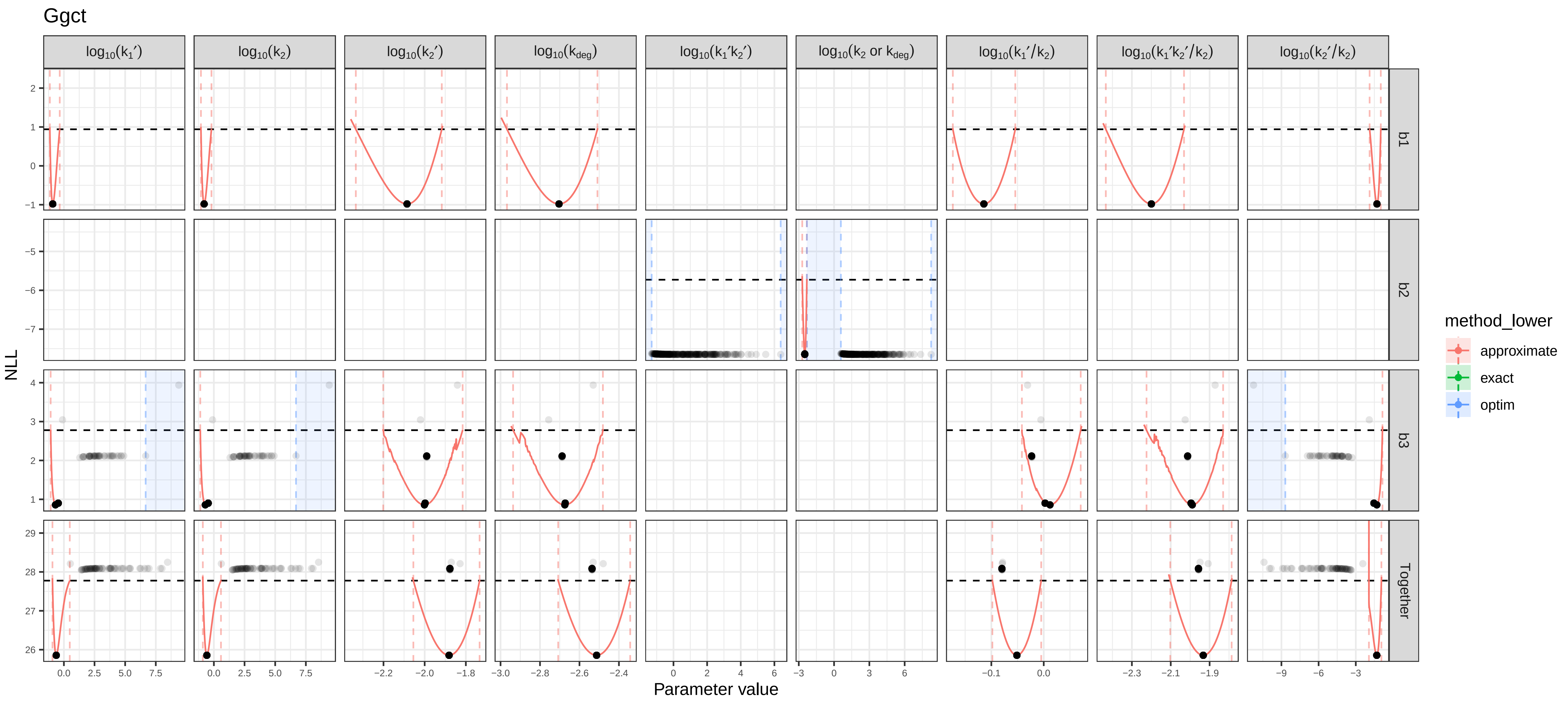

Replicate	Par	Best value	CI95 LB	CI95 UB	Method LB	Method UB
Together	$\log_{10}(k_1')$	−0.6253	−0.9332	0.4802	approximate	approximate
Together	$\log_{10}(k_2)$	−0.5741	−0.9005	0.5783	approximate	approximate
Together	$\log_{10}(k_2')$	−1.881	−2.055	−1.733	approximate	approximate
Together	$\log_{10}(k_{deg})$	−2.513	−2.708	−2.343	approximate	approximate
Together	$\log_{10}(k_1'/k_2)$	−0.05125	−0.098	−0.004806	approximate	approximate
Together	$\log_{10}(k_1'k_2'/k_2)$	−1.933	−2.102	−1.787	approximate	approximate
Together	$\log_{10}(k_2'/k_2)$	−1.307	−1.94	−0.9327	approximate	approximate
b1	$\log_{10}(k_1')$	−0.9092	−1.147	−0.3385	approximate	approximate
b1	$\log_{10}(k_2)$	−0.7949	−1.055	−0.197	approximate	approximate
b1	$\log_{10}(k_2')$	−2.086	−2.334	−1.917	approximate	approximate
b1	$\log_{10}(k_{deg})$	−2.704	−2.967	−2.509	approximate	approximate
b1	$\log_{10}(k_1'/k_2)$	−0.1143	−0.1735	−0.05432	approximate	approximate
b1	$\log_{10}(k_1'k_2'/k_2)$	−2.2	−2.434	−2.032	approximate	approximate
b1	$\log_{10}(k_2'/k_2)$	−1.291	−1.885	−0.982	approximate	approximate
b2	$\log_{10}(k_1'k_2')$	2.329	< −1.297	> 6.374	optim	optim
b2	$\log_{10}(k_2 \text{ or } k_{deg})$	4.207	0.5806	> 8.252	optim	optim
b2	$\log_{10}(k_2 \text{ or } k_{deg})$	−2.491	−2.706	−2.314	approximate	approximate
b3	$\log_{10}(k_1')$	−0.6885	−1.079	> 6.674	approximate	optim
b3	$\log_{10}(k_2)$	−0.7005	−1.109	> 6.697	approximate	optim
b3	$\log_{10}(k_2')$	−2.001	−2.201	−1.815	approximate	approximate
b3	$\log_{10}(k_{deg})$	−2.674	−2.936	−2.481	approximate	approximate
b3	$\log_{10}(k_1'/k_2)$	0.01195	−0.04164	0.07057	approximate	approximate
b3	$\log_{10}(k_1'k_2'/k_2)$	−1.989	−2.225	−1.83	approximate	approximate
b3	$\log_{10}(k_2'/k_2)$	−1.301	< −8.687	−0.8479	optim	approximate

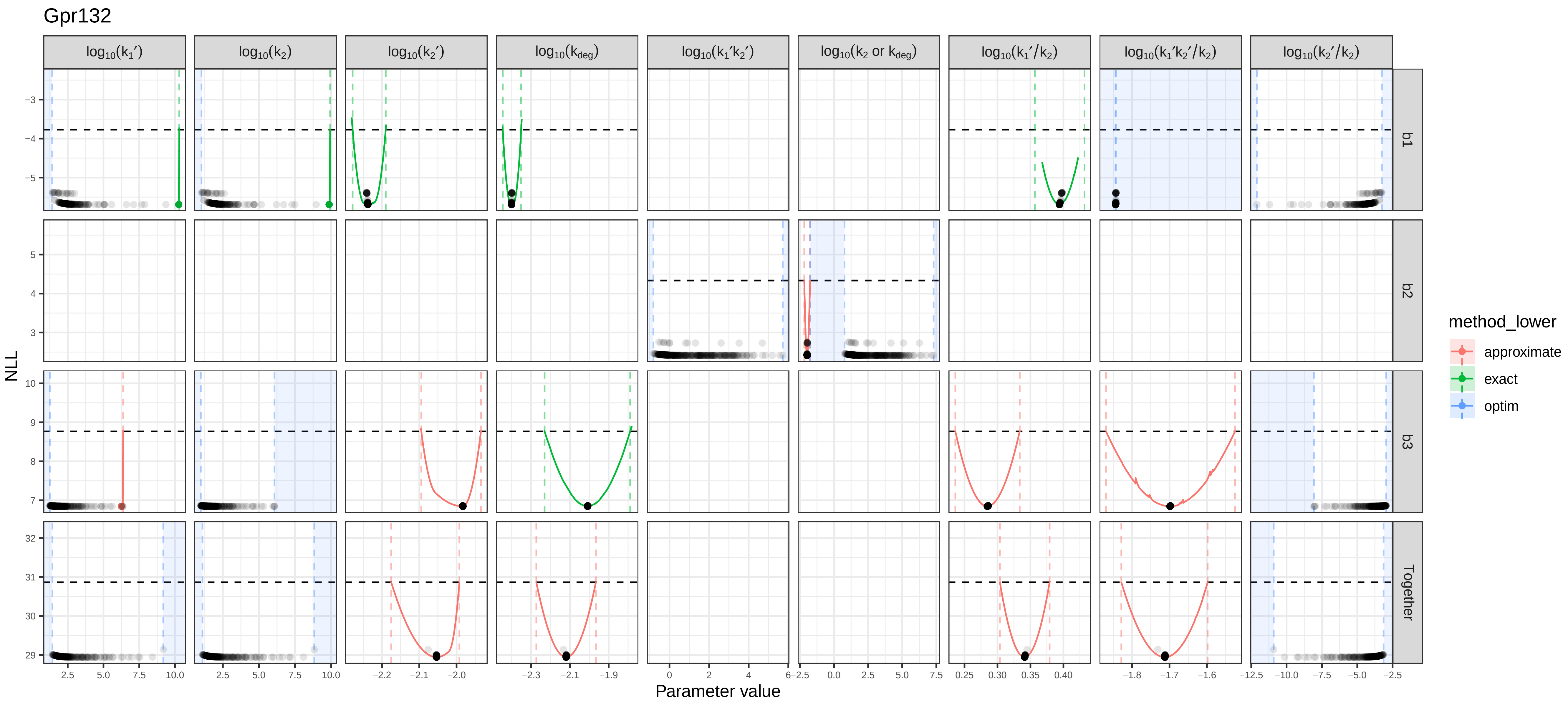

Replicate	Par	**Best value**	**CI95 LB**	**CI95 UB**	**Method LB**	**Method UB**
Together	$\log_{10}(k_1')$	5.067	< 1.429	> 9.179	optim	optim
Together	$\log_{10}(k_2)$	4.726	< 1.086	> 8.834	optim	optim
Together	$\log_{10}(k_2')$	−2.053	−2.175	−1.992	approximate	approximate
Together	$\log_{10}(k_{deg})$	−2.119	−2.273	−1.966	approximate	approximate
Together	$\log_{10}(k_1'/k_2)$	0.3411	0.3036	0.379	approximate	approximate
Together	$\log_{10}(k_1'k_2'/k_2)$	−1.712	−1.829	−1.598	approximate	approximate
Together	$\log_{10}(k_2'/k_2)$	−6.779	< −10.91	> −3.141	optim	optim
b1	$\log_{10}(k_1')$	10.26	< 1.416	10.3	optim	exact
b1	$\log_{10}(k_2)$	9.865	< 1.018	9.933	optim	exact
b1	$\log_{10}(k_2')$	−2.238	−2.279	−2.19	exact	exact
b1	$\log_{10}(k_{deg})$	−2.401	−2.446	−2.351	exact	exact
b1	$\log_{10}(k_1'/k_2)$	0.3939	0.3566	0.4319	exact	exact
b1	$\log_{10}(k_1'k_2'/k_2)$	−1.844	< −1.844	> −1.842	optim	optim
b1	$\log_{10}(k_2'/k_2)$	−12.1	< −12.1	> −3.258	optim	optim
b2	$\log_{10}(k_1'k_2')$	3.339	< −0.814	> 5.718	optim	optim
b2	$\log_{10}(k_2 \text{ or } k_{deg})$	4.923	0.7678	> 7.301	optim	optim
b2	$\log_{10}(k_2 \text{ or } k_{deg})$	−1.974	−2.181	−1.754	approximate	approximate
b3	$\log_{10}(k_1')$	6.25	< 1.257	6.375	optim	approximate
b3	$\log_{10}(k_2)$	5.965	< 0.9712	> 6.075	optim	optim
b3	$\log_{10}(k_2')$	−1.983	−2.095	−1.935	approximate	approximate
b3	$\log_{10}(k_{deg})$	−2.009	−2.231	−1.789	exact	exact
b3	$\log_{10}(k_1'/k_2)$	0.2845	0.2361	0.3337	approximate	approximate
b3	$\log_{10}(k_1'k_2'/k_2)$	−1.699	−1.869	−1.525	approximate	approximate
b3	$\log_{10}(k_2'/k_2)$	−7.948	< −8.059	> −2.955	optim	optim

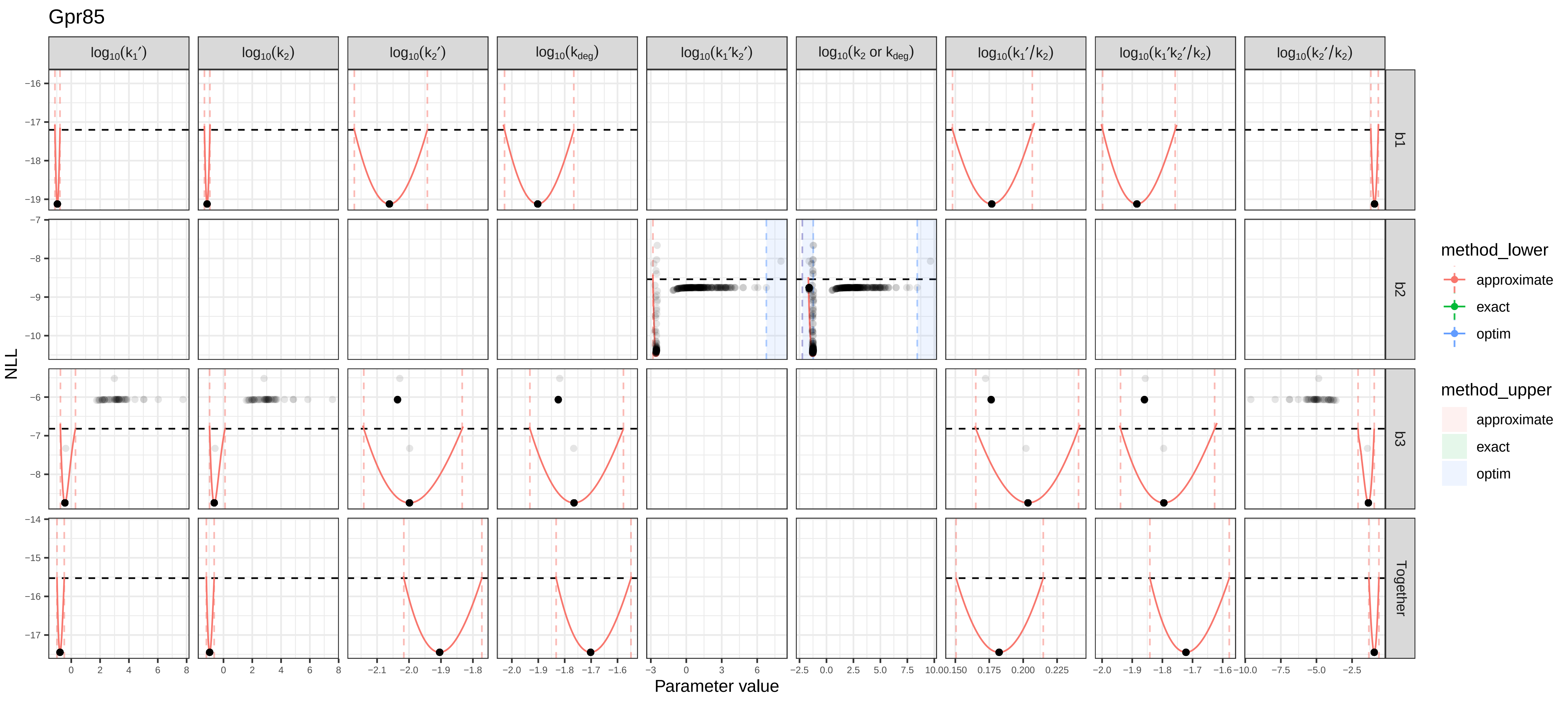

Replicate	Par	Best value	CI95 LB	CI95 UB	Method LB	Method UB
Together	$\log_{10}(k_1')$	−0.7788	−0.9878	−0.4844	approximate	approximate
Together	$\log_{10}(k_2)$	−0.9611	−1.188	−0.6472	approximate	approximate
Together	$\log_{10}(k_2')$	−1.904	−2.016	−1.772	approximate	approximate
Together	$\log_{10}(k_{deg})$	−1.702	−1.832	−1.549	approximate	approximate
Together	$\log_{10}(k_1'/k_2)$	0.1823	0.1505	0.2148	approximate	approximate
Together	$\log_{10}(k_1'k_2'/k_2)$	−1.722	−1.841	−1.578	approximate	approximate
Together	$\log_{10}(k_2'/k_2)$	−0.9431	−1.321	−0.6148	approximate	approximate
b1	$\log_{10}(k_1')$	−0.9619	−1.13	−0.7787	approximate	approximate
b1	$\log_{10}(k_2)$	−1.139	−1.323	−0.9419	approximate	approximate
b1	$\log_{10}(k_2')$	−2.062	−2.171	−1.943	approximate	approximate
b1	$\log_{10}(k_{deg})$	−1.902	−2.028	−1.766	approximate	approximate
b1	$\log_{10}(k_1'/k_2)$	0.1768	0.1479	0.2067	approximate	approximate
b1	$\log_{10}(k_1'k_2'/k_2)$	−1.885	−1.998	−1.758	approximate	approximate
b1	$\log_{10}(k_2'/k_2)$	−0.9228	−1.181	−0.6583	approximate	approximate
b2	$\log_{10}(k_1'k_2')$	−2.574	−2.82	> 6.775	approximate	optim
b2	$\log_{10}(k_2 \text{ or } k_{deg})$	−1.272	−2.238	> 8.419	approximate	optim
b2	$\log_{10}(k_2 \text{ or } k_{deg})$	−1.277	−2.238	−1.226	approximate	optim
b3	$\log_{10}(k_1')$	−0.4396	−0.7496	0.2937	approximate	approximate
b3	$\log_{10}(k_2)$	−0.6431	−0.9652	0.1104	approximate	approximate
b3	$\log_{10}(k_2')$	−1.999	−2.142	−1.833	approximate	approximate
b3	$\log_{10}(k_{deg})$	−1.765	−1.932	−1.578	approximate	approximate
b3	$\log_{10}(k_1'/k_2)$	0.2036	0.1651	0.2408	approximate	approximate
b3	$\log_{10}(k_1'k_2'/k_2)$	−1.795	−1.939	−1.627	approximate	approximate
b3	$\log_{10}(k_2'/k_2)$	−1.356	−2.079	−0.9431	approximate	approximate

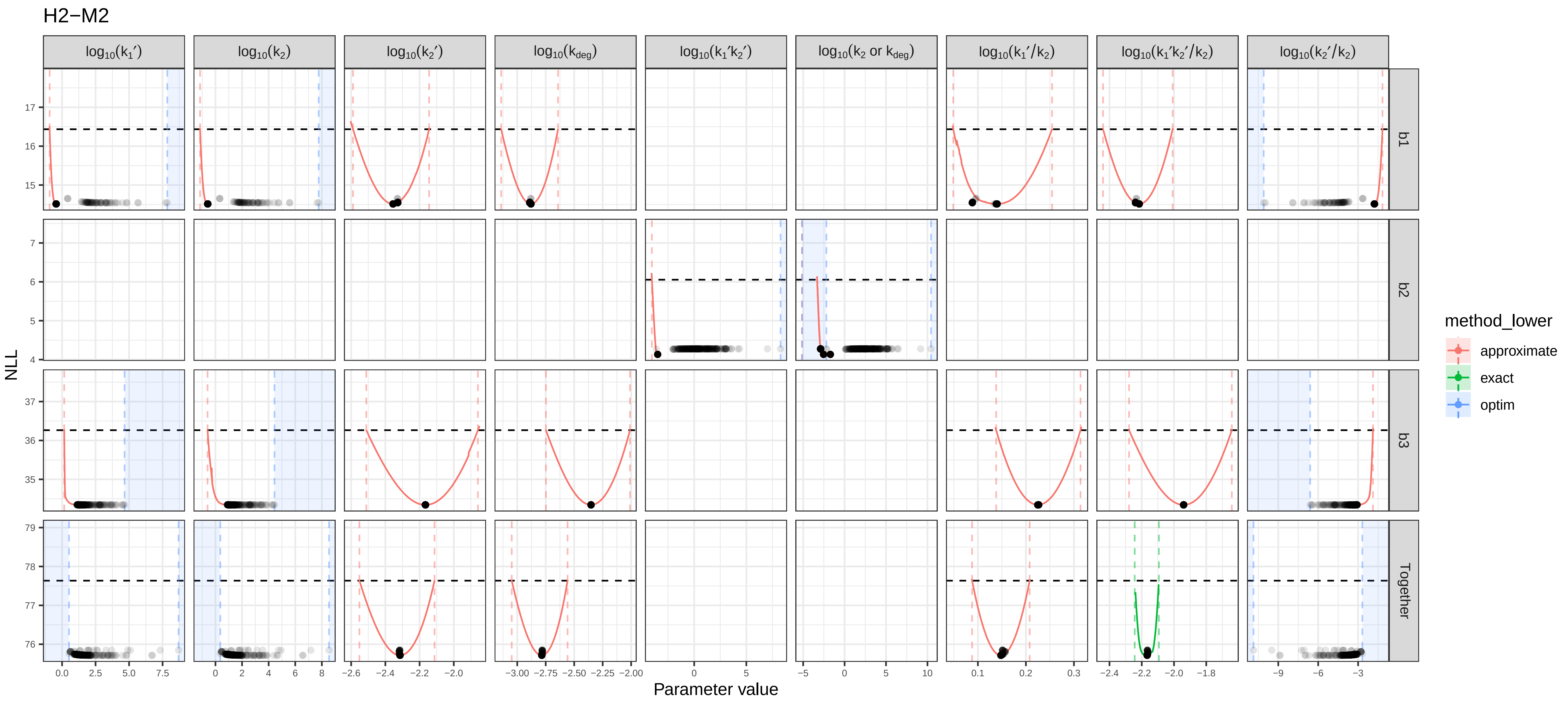

Replicate	Par	Best value	CI95 LB	CI95 UB	Method LB	Method UB
Together	$\log_{10}(k_1')$	4.771	< 0.5189	> 8.693	optim	optim
Together	$\log_{10}(k_2)$	4.623	< 0.3627	> 8.542	optim	optim
Together	$\log_{10}(k_2')$	−2.314	−2.552	−2.112	approximate	approximate
Together	$\log_{10}(k_{deg})$	−2.785	−3.048	−2.558	approximate	approximate
Together	$\log_{10}(k_1'/k_2)$	0.1479	0.08795	0.2078	approximate	approximate
Together	$\log_{10}(k_1'k_2'/k_2)$	−2.166	−2.244	−2.094	exact	exact
Together	$\log_{10}(k_2'/k_2)$	−6.937	< −10.86	> −2.682	optim	optim
b1	$\log_{10}(k_1')$	−0.4429	−0.9283	> 7.853	approximate	optim
b1	$\log_{10}(k_2)$	−0.5822	−1.156	> 7.764	approximate	optim
b1	$\log_{10}(k_2')$	−2.355	−2.589	−2.144	approximate	approximate
b1	$\log_{10}(k_{deg})$	−2.88	−3.141	−2.642	approximate	approximate
b1	$\log_{10}(k_1'/k_2)$	0.1393	0.0487	0.2545	approximate	approximate
b1	$\log_{10}(k_1'k_2'/k_2)$	−2.216	−2.44	−2.008	approximate	approximate
b1	$\log_{10}(k_2'/k_2)$	−1.773	< −10.09	−1.167	optim	approximate
b2	$\log_{10}(k_1'k_2')$	−3.504	−4.058	> 8.282	approximate	optim
b2	$\log_{10}(k_2 \text{ or } k_{deg})$	−1.717	−5.135	> 10.43	approximate	optim
b2	$\log_{10}(k_2 \text{ or } k_{deg})$	−2.541	−5.135	−2.197	approximate	optim
b3	$\log_{10}(k_1')$	1.738	0.1589	> 4.663	approximate	optim
b3	$\log_{10}(k_2)$	1.513	−0.5929	> 4.438	approximate	optim
b3	$\log_{10}(k_2')$	−2.166	−2.511	−1.859	approximate	approximate
b3	$\log_{10}(k_{deg})$	−2.352	−2.748	−2.01	approximate	approximate
b3	$\log_{10}(k_1'/k_2)$	0.2251	0.1381	0.3137	approximate	approximate
b3	$\log_{10}(k_1'k_2'/k_2)$	−1.941	−2.279	−1.641	approximate	approximate
b3	$\log_{10}(k_2'/k_2)$	−3.679	< −6.603	−1.883	optim	approximate

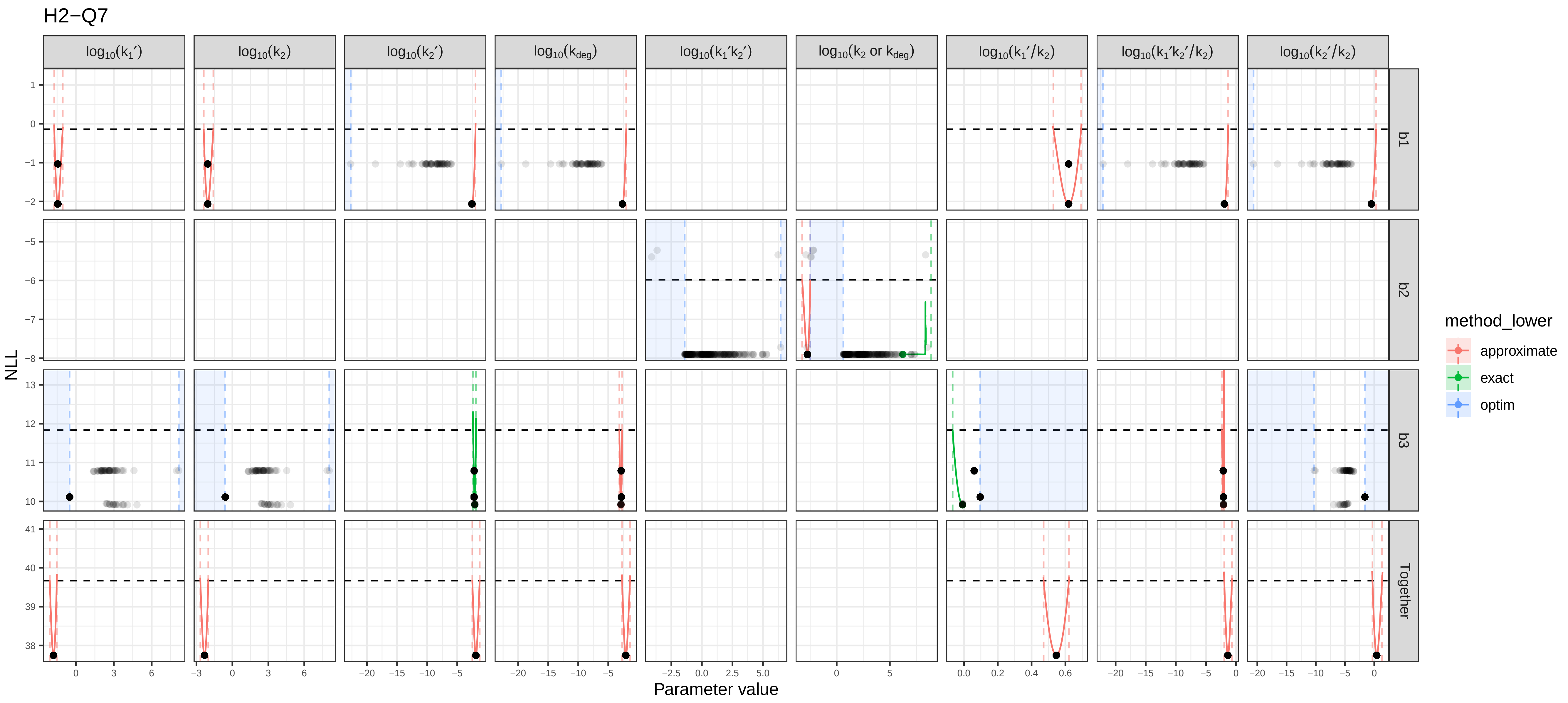

Replicate	Par	Best value	CI95 LB	CI95 UB	Method LB	Method UB
Together	$\log_{10}(k_1')$	−1.779	−2.073	−1.523	approximate	approximate
Together	$\log_{10}(k_2)$	−2.326	−2.672	−2.011	approximate	approximate
Together	$\log_{10}(k_2')$	−1.904	−2.488	−1.265	approximate	approximate
Together	$\log_{10}(k_{deg})$	−2.081	−2.698	−1.388	approximate	approximate
Together	$\log_{10}(k_1'/k_2)$	0.5464	0.4713	0.621	approximate	approximate
Together	$\log_{10}(k_1'k_2'/k_2)$	−1.358	−1.959	−0.6773	approximate	approximate
Together	$\log_{10}(k_2'/k_2)$	0.4219	−0.3074	1.338	approximate	approximate
b1	$\log_{10}(k_1')$	−1.437	−1.721	−1.051	approximate	approximate
b1	$\log_{10}(k_2)$	−2.056	−2.392	−1.587	approximate	approximate
b1	$\log_{10}(k_2')$	−2.551	< −22.7	−1.961	optim	approximate
b1	$\log_{10}(k_{deg})$	−2.646	< −22.81	−2.009	optim	approximate
b1	$\log_{10}(k_1'/k_2)$	0.6192	0.5289	0.6932	approximate	approximate
b1	$\log_{10}(k_1'k_2'/k_2)$	−1.932	< −22.08	−1.309	optim	approximate
b1	$\log_{10}(k_2'/k_2)$	−0.4949	< −20.64	0.3303	optim	approximate
b2	$\log_{10}(k_1'k_2')$	4.179	< −1.406	> 6.448	optim	optim
b2	$\log_{10}(k_2 \text{ or } k_{deg})$	6.211	0.6254	8.886	optim	exact
b2	$\log_{10}(k_2 \text{ or } k_{deg})$	−2.752	−3.246	−2.477	approximate	approximate
b3	$\log_{10}(k_1')$	4.832	< −0.5055	> 8.147	optim	optim
b3	$\log_{10}(k_2)$	4.841	< −0.6016	> 8.086	optim	optim
b3	$\log_{10}(k_2')$	−2.074	−2.357	−1.891	exact	exact
b3	$\log_{10}(k_{deg})$	−2.884	−3.161	−2.693	approximate	approximate
b3	$\log_{10}(k_1'/k_2)$	−0.008352	−0.06696	> 0.09606	exact	optim
b3	$\log_{10}(k_1'k_2'/k_2)$	−2.083	−2.341	−2.005	approximate	approximate
b3	$\log_{10}(k_2'/k_2)$	−6.915	< −10.27	> −1.595	optim	optim

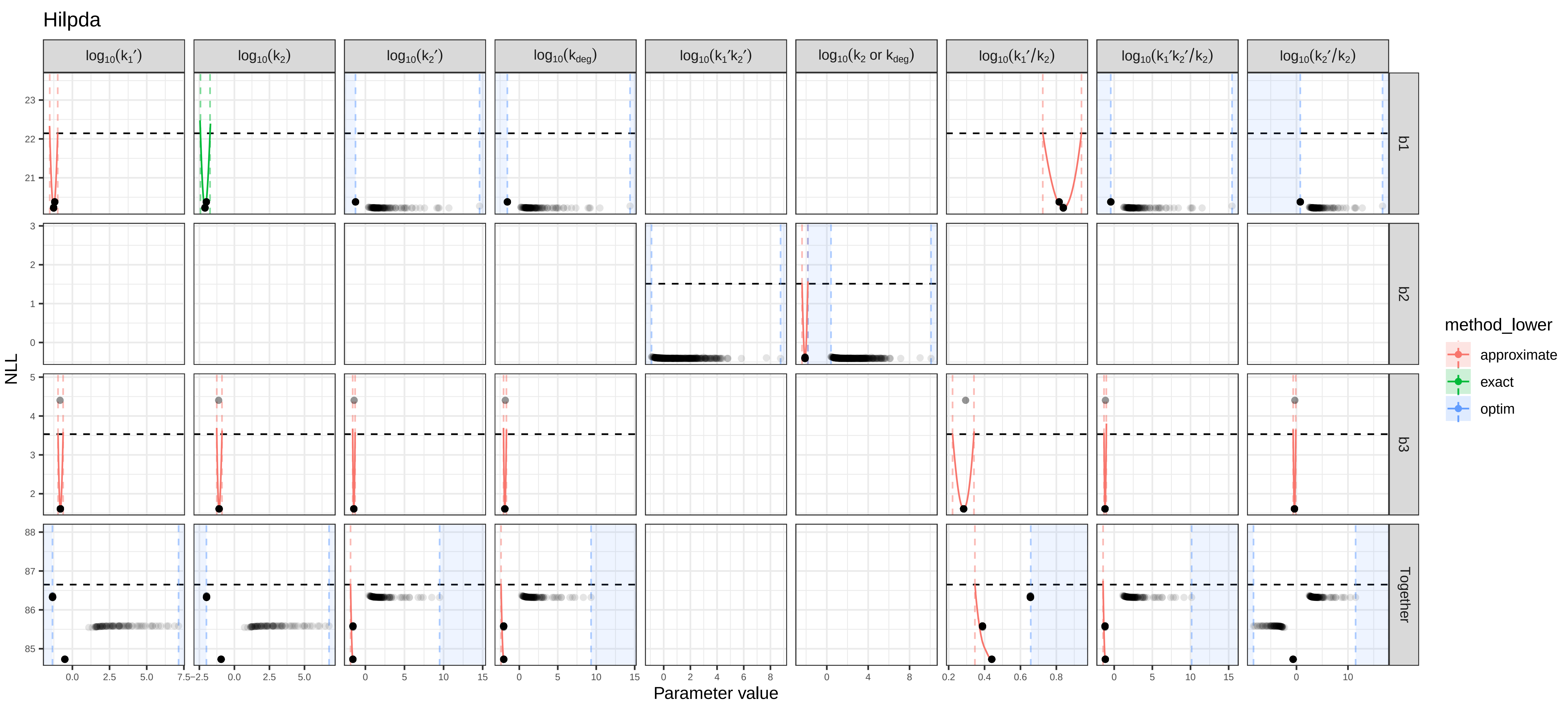

Replicate	Par	Best value	CI95 LB	CI95 UB	Method LB	Method UB
Together	$\log_{10}(k_1')$	−0.5068	< −1.339	> 7.141	optim	optim
Together	$\log_{10}(k_2)$	−0.947	< −1.996	> 6.753	optim	optim
Together	$\log_{10}(k_2')$	−1.602	−1.897	> 9.494	approximate	optim
Together	$\log_{10}(k_{deg})$	−2.02	−2.37	> 9.331	approximate	optim
Together	$\log_{10}(k_1'/k_2)$	0.4402	0.3463	> 0.6574	approximate	optim
Together	$\log_{10}(k_1'k_2'/k_2)$	−1.162	−1.454	> 10.15	approximate	optim
Together	$\log_{10}(k_2'/k_2)$	−0.6552	< −8.341	> 11.48	optim	optim
b1	$\log_{10}(k_1')$	−1.26	−1.522	−0.9856	approximate	approximate
b1	$\log_{10}(k_2)$	−2.099	−2.422	−1.738	exact	exact
b1	$\log_{10}(k_2')$	4.56	< −1.263	> 14.6	optim	optim
b1	$\log_{10}(k_{deg})$	4.356	< −1.551	> 14.4	optim	optim
b1	$\log_{10}(k_1'/k_2)$	0.8386	0.7244	0.9399	approximate	approximate
b1	$\log_{10}(k_1'k_2'/k_2)$	5.398	< −0.4476	> 15.44	optim	optim
b1	$\log_{10}(k_2'/k_2)$	6.659	< 0.7384	> 16.71	optim	optim
b2	$\log_{10}(k_1'k_2')$	3.134	< −0.9109	> 8.758	optim	optim
b2	$\log_{10}(k_2 \text{ or } k_{deg})$	4.454	0.408	> 10.08	optim	optim
b2	$\log_{10}(k_2 \text{ or } k_{deg})$	−2.107	−2.394	−1.831	approximate	approximate
b3	$\log_{10}(k_1')$	−0.8076	−0.9663	−0.6232	approximate	approximate
b3	$\log_{10}(k_2)$	−1.091	−1.259	−0.8864	approximate	approximate
b3	$\log_{10}(k_2')$	−1.481	−1.623	−1.313	approximate	approximate
b3	$\log_{10}(k_{deg})$	−1.877	−2.044	−1.674	approximate	approximate
b3	$\log_{10}(k_1'/k_2)$	0.2835	0.2217	0.341	approximate	approximate
b3	$\log_{10}(k_1'k_2'/k_2)$	−1.198	−1.333	−1.031	approximate	approximate
b3	$\log_{10}(k_2'/k_2)$	−0.3899	−0.6424	−0.133	approximate	approximate

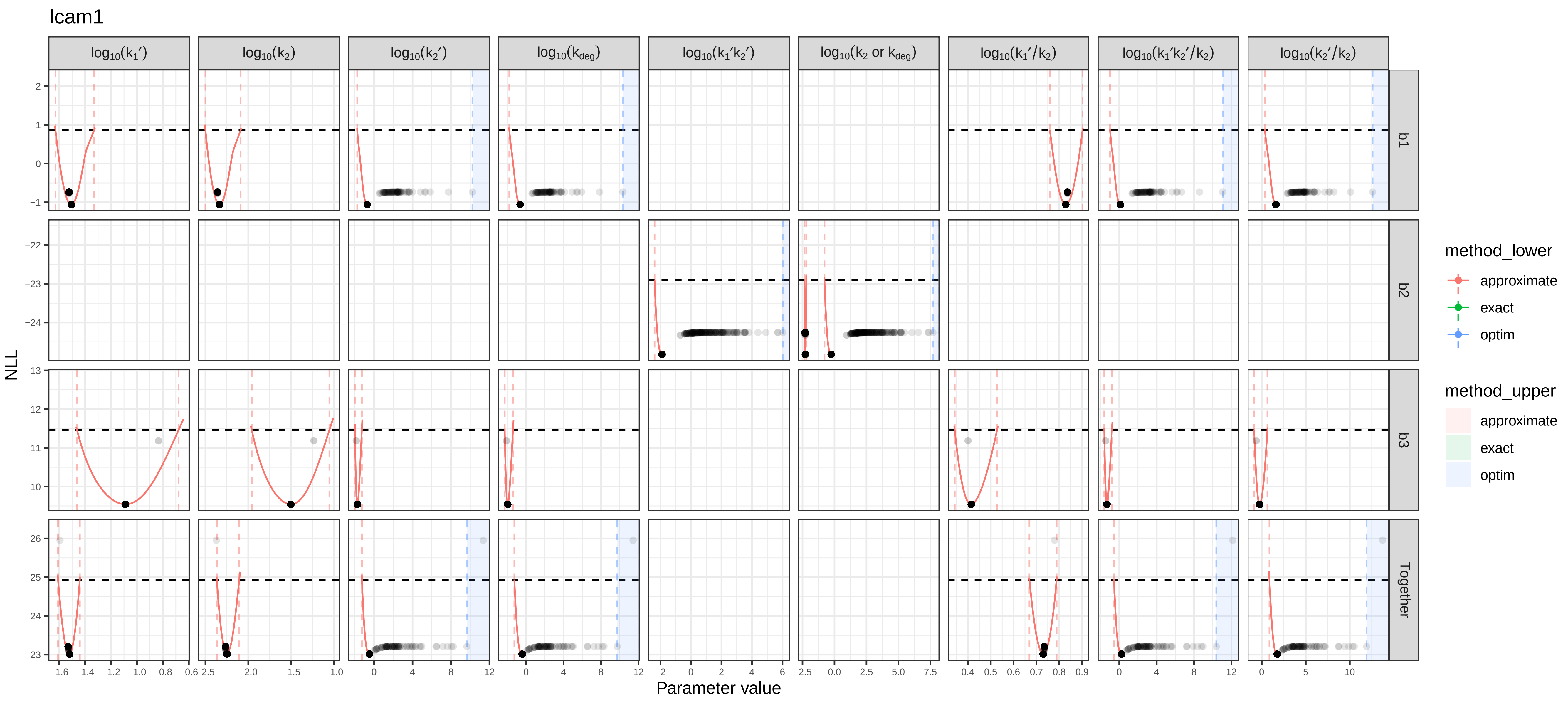

Replicate	Par	Best value	CI95 LB	CI95 UB	Method LB	Method UB
Together	$\log_{10}(k_1')$	−1.519	−1.608	−1.44	approximate	approximate
Together	$\log_{10}(k_2)$	−2.249	−2.367	−2.105	approximate	approximate
Together	$\log_{10}(k_2')$	−0.4824	−1.264	> 9.646	approximate	optim
Together	$\log_{10}(k_{deg})$	−0.4148	−1.25	> 9.725	approximate	optim
Together	$\log_{10}(k_1'/k_2)$	0.7294	0.6695	0.7886	approximate	approximate
Together	$\log_{10}(k_1'k_2'/k_2)$	0.247	−0.566	> 10.38	approximate	optim
Together	$\log_{10}(k_2'/k_2)$	1.766	0.8758	> 11.91	approximate	optim
b1	$\log_{10}(k_1')$	−1.506	−1.628	−1.33	approximate	approximate
b1	$\log_{10}(k_2)$	−2.335	−2.5	−2.09	approximate	approximate
b1	$\log_{10}(k_2')$	−0.7161	−1.751	> 10.23	approximate	optim
b1	$\log_{10}(k_{deg})$	−0.6325	−1.788	> 10.34	approximate	optim
b1	$\log_{10}(k_1'/k_2)$	0.8285	0.759	0.9019	approximate	approximate
b1	$\log_{10}(k_1'k_2'/k_2)$	0.1124	−0.9838	> 11.07	approximate	optim
b1	$\log_{10}(k_2'/k_2)$	1.619	0.3601	> 12.59	approximate	optim
b2	$\log_{10}(k_1'k_2')$	−1.898	−2.387	> 6.035	approximate	optim
b2	$\log_{10}(k_2 \text{ or } k_{deg})$	−0.2569	−0.7807	> 7.694	approximate	optim
b2	$\log_{10}(k_2 \text{ or } k_{deg})$	−2.277	−2.348	−2.206	approximate	approximate
b3	$\log_{10}(k_1')$	−1.089	−1.462	−0.6787	approximate	approximate
b3	$\log_{10}(k_2)$	−1.504	−1.96	−1.054	approximate	approximate
b3	$\log_{10}(k_2')$	−1.735	−1.998	−1.273	approximate	approximate
b3	$\log_{10}(k_{deg})$	−1.958	−2.28	−1.397	approximate	approximate
b3	$\log_{10}(k_1'/k_2)$	0.4147	0.3423	0.5278	approximate	approximate
b3	$\log_{10}(k_1'k_2'/k_2)$	−1.32	−1.604	−0.7804	approximate	approximate
b3	$\log_{10}(k_2'/k_2)$	−0.2313	−0.8621	0.6464	approximate	approximate

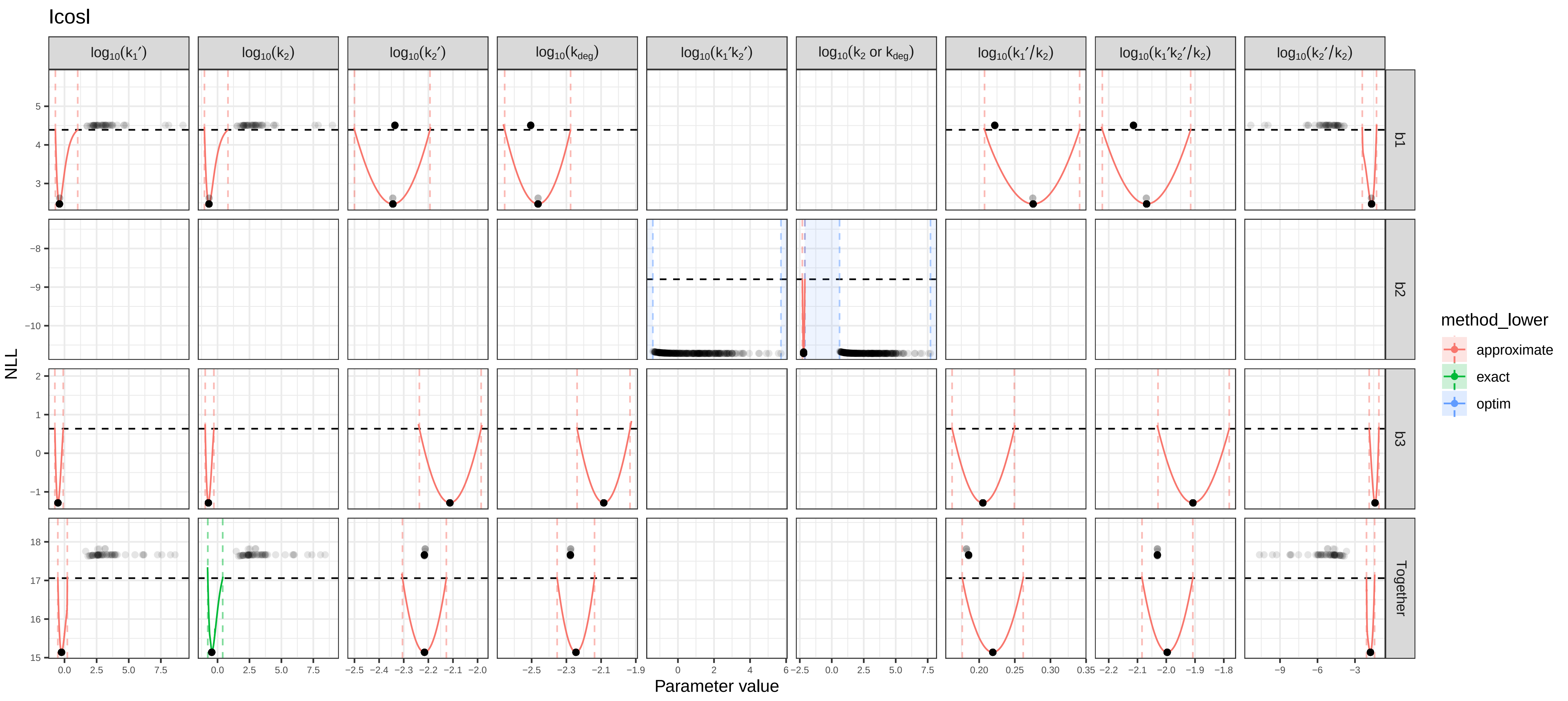

Replicate	Par	Best value	CI95 LB	CI95 UB	Method LB	Method UB
Together	$\log_{10}(k_1')$	−0.2326	−0.5206	0.218	approximate	approximate
Together	$\log_{10}(k_2)$	−0.4518	−0.7618	0.4053	exact	exact
Together	$\log_{10}(k_2')$	−2.216	−2.305	−2.127	approximate	approximate
Together	$\log_{10}(k_{deg})$	−2.245	−2.353	−2.138	approximate	approximate
Together	$\log_{10}(k_1'/k_2)$	0.2191	0.1763	0.2618	approximate	approximate
Together	$\log_{10}(k_1'k_2'/k_2)$	−1.997	−2.084	−1.908	approximate	approximate
Together	$\log_{10}(k_2'/k_2)$	−1.764	−2.079	−1.43	approximate	approximate
b1	$\log_{10}(k_1')$	−0.3959	−0.7107	1.029	approximate	approximate
b1	$\log_{10}(k_2)$	−0.6717	−1.025	0.8176	approximate	approximate
b1	$\log_{10}(k_2')$	−2.344	−2.5	−2.193	approximate	approximate
b1	$\log_{10}(k_{deg})$	−2.463	−2.655	−2.276	approximate	approximate
b1	$\log_{10}(k_1'/k_2)$	0.2757	0.2075	0.3409	approximate	approximate
b1	$\log_{10}(k_1'k_2'/k_2)$	−2.068	−2.222	−1.915	approximate	approximate
b1	$\log_{10}(k_2'/k_2)$	−1.672	−2.416	−1.276	approximate	approximate
b2	$\log_{10}(k_1'k_2')$	2.234	< -1.392	> 5.713	optim	optim
b2	$\log_{10}(k_2 \text{ or } k_{deg})$	4.237	0.6092	> 7.717	optim	optim
b2	$\log_{10}(k_2 \text{ or } k_{deg})$	−2.203	−2.302	−2.104	approximate	approximate
b3	$\log_{10}(k_1')$	−0.5116	−0.7502	−0.1001	approximate	approximate
b3	$\log_{10}(k_2)$	−0.7168	−0.9641	−0.2861	approximate	approximate
b3	$\log_{10}(k_2')$	−2.113	−2.236	−1.985	approximate	approximate
b3	$\log_{10}(k_{deg})$	−2.085	−2.238	−1.933	approximate	approximate
b3	$\log_{10}(k_1'/k_2)$	0.2052	0.162	0.2493	approximate	approximate
b3	$\log_{10}(k_1'k_2'/k_2)$	−1.907	−2.029	−1.781	approximate	approximate
b3	$\log_{10}(k_2'/k_2)$	−1.396	−1.863	−1.086	approximate	approximate

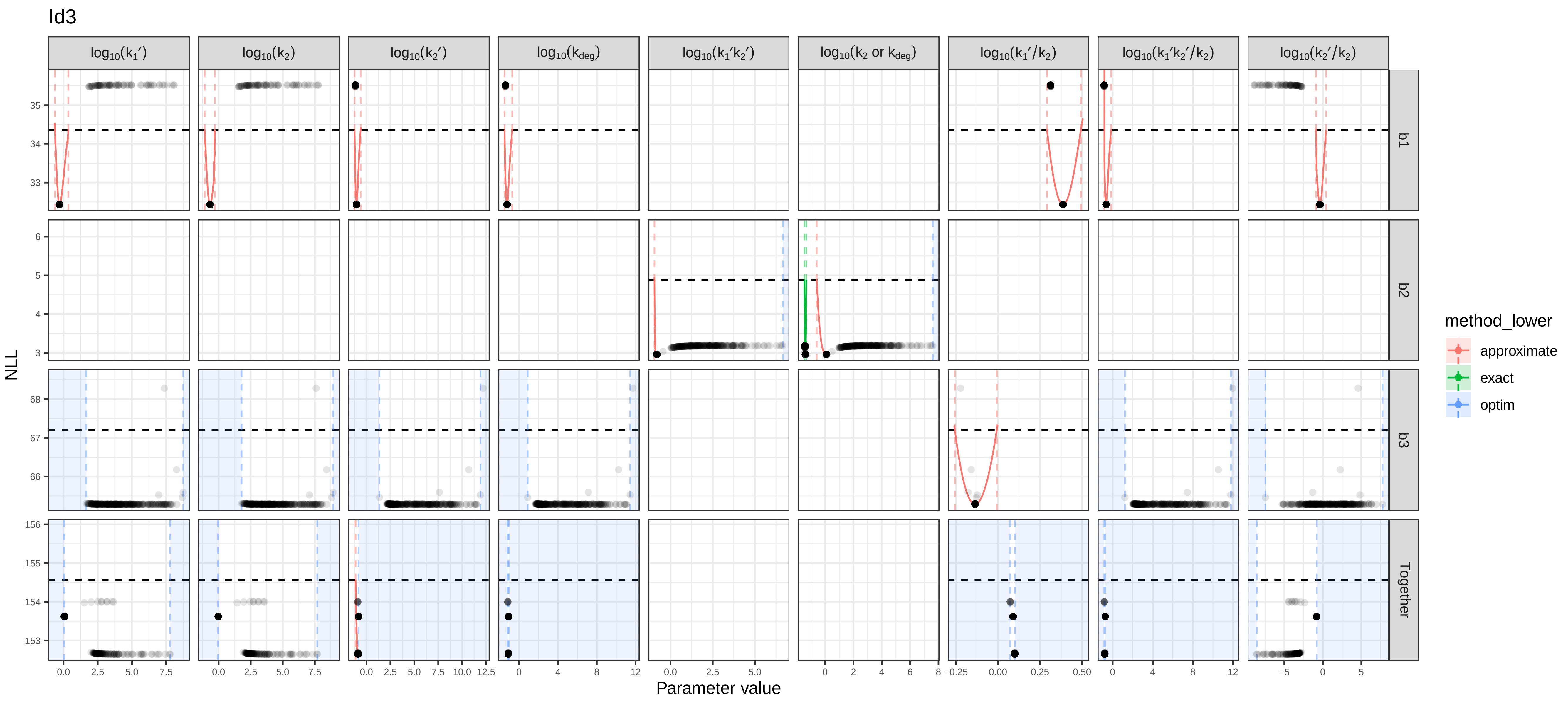

Replicate	Par	Best value	CI95 LB	CI95 UB	Method LB	Method UB
Together	$\log_{10}(k_1')$	4.344	< 0.04919	> 7.794	optim	optim
Together	$\log_{10}(k_2)$	4.244	< −0.04048	> 7.694	optim	optim
Together	$\log_{10}(k_2')$	−0.8859	−1.138	> −0.8248	approximate	optim
Together	$\log_{10}(k_{deg})$	−1.111	< −1.164	> −1.065	optim	optim
Together	$\log_{10}(k_1'/k_2)$	0.1001	< 0.07243	> 0.1006	optim	optim
Together	$\log_{10}(k_1'k_2'/k_2)$	−0.7858	< −0.8357	> −0.7351	optim	optim
Together	$\log_{10}(k_2'/k_2)$	−5.13	< −8.582	> −0.7843	optim	optim
b1	$\log_{10}(k_1')$	−0.2824	−0.622	0.3507	approximate	approximate
b1	$\log_{10}(k_2)$	−0.6691	−1.087	−0.297	approximate	approximate
b1	$\log_{10}(k_2')$	−1.039	−1.244	−0.6102	approximate	approximate
b1	$\log_{10}(k_{deg})$	−1.25	−1.504	−0.7052	approximate	approximate
b1	$\log_{10}(k_1'/k_2)$	0.3867	0.2917	0.4929	approximate	approximate
b1	$\log_{10}(k_1'k_2'/k_2)$	−0.6527	−0.8252	−0.1525	approximate	approximate
b1	$\log_{10}(k_2'/k_2)$	−0.3703	−0.8696	0.4418	approximate	approximate
b2	$\log_{10}(k_1'k_2')$	−0.8159	−0.9544	> 6.66	approximate	optim
b2	$\log_{10}(k_2 \text{ or } k_{deg})$	0.09554	−0.5924	> 7.602	approximate	optim
b2	$\log_{10}(k_2 \text{ or } k_{deg})$	−1.393	−1.465	−1.325	exact	exact
b3	$\log_{10}(k_1')$	7.895	< 1.658	> 8.751	optim	optim
b3	$\log_{10}(k_2)$	8.031	< 1.794	> 8.928	optim	optim
b3	$\log_{10}(k_2')$	11.39	< 1.349	> 11.91	optim	optim
b3	$\log_{10}(k_{deg})$	10.93	< 0.8827	> 11.45	optim	optim
b3	$\log_{10}(k_1'/k_2)$	−0.1366	−0.2568	−0.007045	approximate	approximate
b3	$\log_{10}(k_1'k_2'/k_2)$	11.25	< 1.219	> 11.78	optim	optim
b3	$\log_{10}(k_2'/k_2)$	3.358	< −7.459	> 7.765	optim	optim

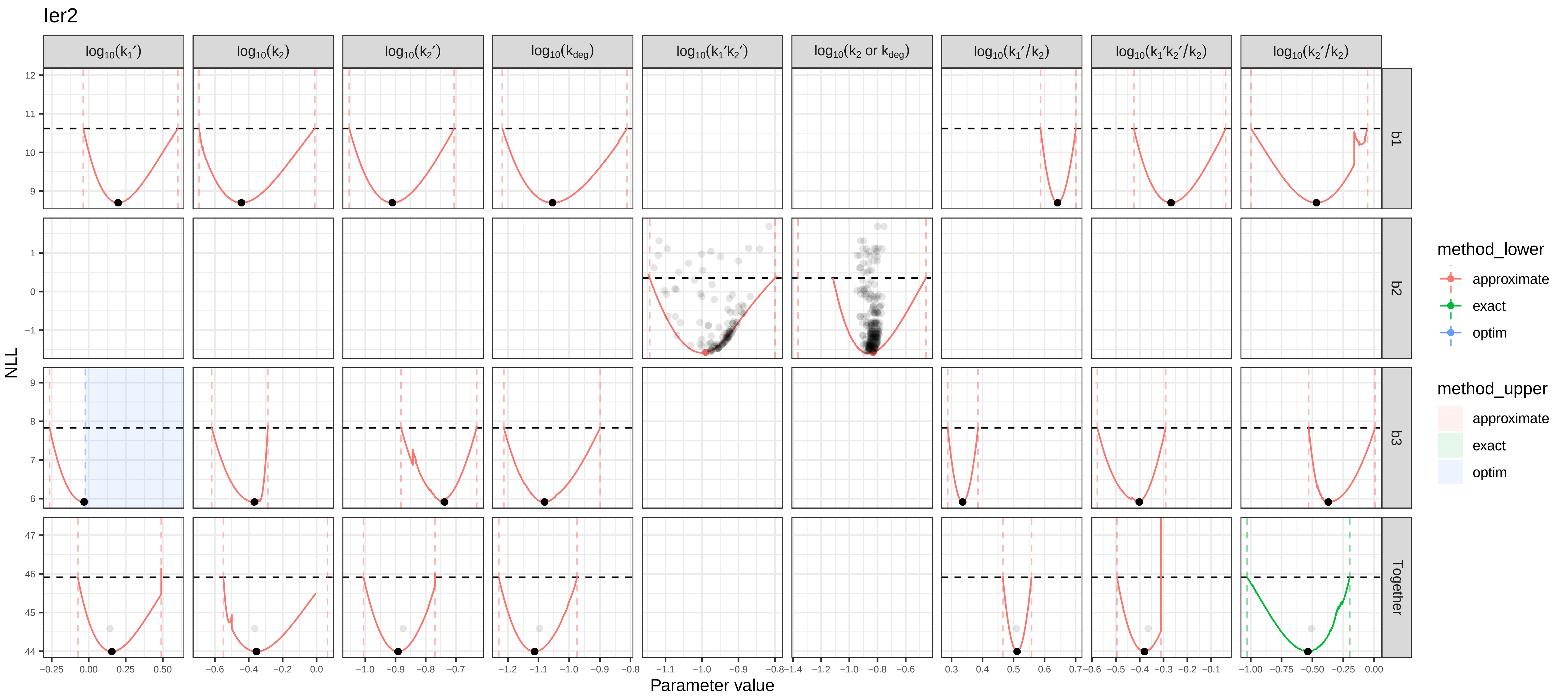

Replicate	Par	Best value	CI95 LB	CI95 UB	Method LB	Method UB
Together	$\log_{10}(k_1')$	0.1564	−0.07382	0.4902	approximate	approximate
Together	$\log_{10}(k_2)$	−0.3548	−0.5494	0.06466	approximate	approximate
Together	$\log_{10}(k_2')$	−0.8912	−1.006	−0.7689	approximate	approximate
Together	$\log_{10}(k_{deg})$	−1.113	−1.23	−0.974	approximate	approximate
Together	$\log_{10}(k_1'/k_2)$	0.5112	0.4653	0.5579	approximate	approximate
Together	$\log_{10}(k_1'k_2'/k_2)$	−0.38	−0.4949	−0.3112	approximate	approximate
Together	$\log_{10}(k_2'/k_2)$	−0.5364	−1.028	−0.1977	exact	exact
b1	$\log_{10}(k_1')$	0.1989	−0.03643	0.6019	approximate	approximate
b1	$\log_{10}(k_2)$	−0.4431	−0.6921	−0.01082	approximate	approximate
b1	$\log_{10}(k_2')$	−0.9102	−1.053	−0.706	approximate	approximate
b1	$\log_{10}(k_{deg})$	−1.054	−1.218	−0.8118	approximate	approximate
b1	$\log_{10}(k_1'/k_2)$	0.642	0.587	0.7012	approximate	approximate
b1	$\log_{10}(k_1'k_2'/k_2)$	−0.2682	−0.4247	−0.03952	approximate	approximate
b1	$\log_{10}(k_2'/k_2)$	−0.4671	−0.9967	−0.05234	approximate	approximate
b2	$\log_{10}(k_1'k_2')$	−0.9899	−1.143	−0.8001	approximate	approximate
b2	$\log_{10}(k_2 \text{ or } k_{deg})$	−0.8308	−1.364	−0.4561	approximate	approximate
b2	$\log_{10}(k_2 \text{ or } k_{deg})$	−0.8868	−1.364	−0.4561	approximate	approximate
b3	$\log_{10}(k_1')$	−0.03166	−0.2643	> −0.02231	approximate	optim
b3	$\log_{10}(k_2)$	−0.3675	−0.6188	−0.2875	approximate	approximate
b3	$\log_{10}(k_2')$	−0.7373	−0.8816	−0.631	approximate	approximate
b3	$\log_{10}(k_{deg})$	−1.08	−1.213	−0.8986	approximate	approximate
b3	$\log_{10}(k_1'/k_2)$	0.3358	0.2876	0.3856	approximate	approximate
b3	$\log_{10}(k_1'k_2'/k_2)$	−0.4014	−0.5775	−0.2911	approximate	approximate
b3	$\log_{10}(k_2'/k_2)$	−0.3698	−0.5306	0.00884	approximate	approximate

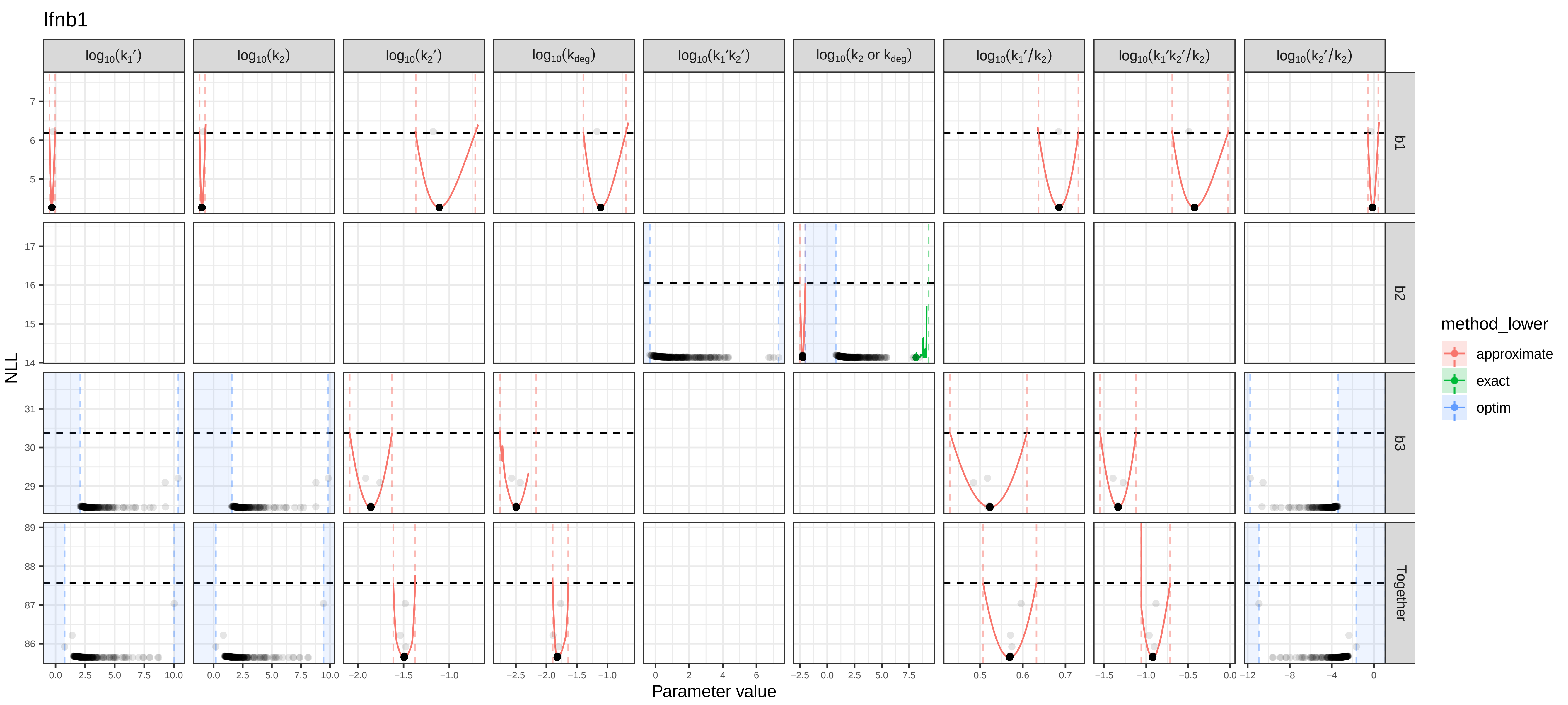

Replicate	Par	**Best value**	**CI95 LB**	**CI95 UB**	**Method LB**	**Method UB**
Together	$\log_{10}(k_1')$	5.84	< 0.765	> 10.04	optim	optim
Together	$\log_{10}(k_2)$	5.271	< 0.1905	> 9.441	optim	optim
Together	$\log_{10}(k_2')$	−1.493	−1.612	−1.374	approximate	approximate
Together	$\log_{10}(k_{deg})$	−1.822	−1.897	−1.642	approximate	approximate
Together	$\log_{10}(k_1'/k_2)$	0.5691	0.5069	0.6321	approximate	approximate
Together	$\log_{10}(k_1'k_2'/k_2)$	−0.9236	−1.058	−0.7145	approximate	approximate
Together	$\log_{10}(k_2'/k_2)$	−6.763	< −10.92	> −1.668	optim	optim
b1	$\log_{10}(k_1')$	−0.3117	−0.5126	−0.04522	approximate	approximate
b1	$\log_{10}(k_2)$	−0.9965	−1.207	−0.7134	approximate	approximate
b1	$\log_{10}(k_2')$	−1.111	−1.368	−0.7168	approximate	approximate
b1	$\log_{10}(k_{deg})$	−1.113	−1.394	−0.7007	approximate	approximate
b1	$\log_{10}(k_1'/k_2)$	0.6848	0.6367	0.7303	approximate	approximate
b1	$\log_{10}(k_1'k_2'/k_2)$	−0.4257	−0.6895	−0.02612	approximate	approximate
b1	$\log_{10}(k_2'/k_2)$	−0.114	−0.5796	0.4234	approximate	approximate
b2	$\log_{10}(k_1'k_2')$	7.023	< −0.3367	> 7.309	optim	optim
b2	$\log_{10}(k_2 \text{ or } k_{deg})$	8.139	0.7797	9.285	optim	exact
b2	$\log_{10}(k_2 \text{ or } k_{deg})$	−2.259	−2.503	−2.004	approximate	approximate
b3	$\log_{10}(k_1')$	7.494	< 2.092	> 10.36	optim	optim
b3	$\log_{10}(k_2)$	6.971	< 1.569	> 9.842	optim	optim
b3	$\log_{10}(k_2')$	−1.857	−2.089	−1.626	approximate	approximate
b3	$\log_{10}(k_{deg})$	−2.493	−2.761	−2.165	approximate	approximate
b3	$\log_{10}(k_1'/k_2)$	0.5227	0.4296	0.6095	approximate	approximate
b3	$\log_{10}(k_1'k_2'/k_2)$	−1.334	−1.549	−1.119	approximate	approximate
b3	$\log_{10}(k_2'/k_2)$	−8.828	< −11.75	> −3.426	optim	optim

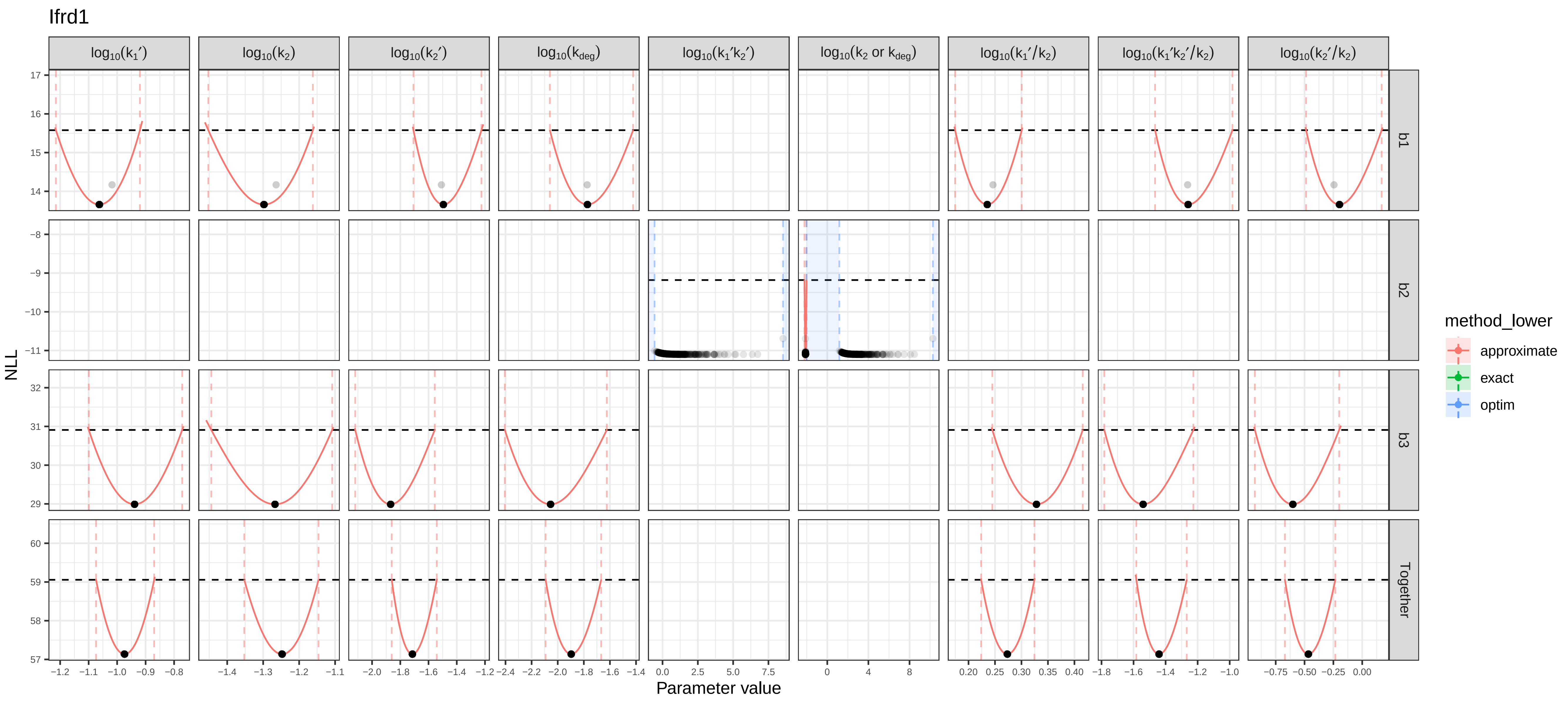

Replicate	Par	**Best value**	**CI95 LB**	**CI95 UB**	**Method LB**	**Method UB**
Together	$\log_{10}(k_1')$	−0.9739	−1.074	−0.87	approximate	approximate
Together	$\log_{10}(k_2)$	−1.247	−1.353	−1.146	approximate	approximate
Together	$\log_{10}(k_2')$	−1.714	−1.861	−1.541	approximate	approximate
Together	$\log_{10}(k_{deg})$	−1.897	−2.093	−1.667	approximate	approximate
Together	$\log_{10}(k_1'/k_2)$	0.2734	0.2238	0.3244	approximate	approximate
Together	$\log_{10}(k_1'k_2'/k_2)$	−1.441	−1.583	−1.268	approximate	approximate
Together	$\log_{10}(k_2'/k_2)$	−0.4669	−0.6701	−0.2333	approximate	approximate
b1	$\log_{10}(k_1')$	−1.063	−1.215	−0.9201	approximate	approximate
b1	$\log_{10}(k_2)$	−1.298	−1.452	−1.162	approximate	approximate
b1	$\log_{10}(k_2')$	−1.494	−1.707	−1.225	approximate	approximate
b1	$\log_{10}(k_{deg})$	−1.772	−2.06	−1.423	approximate	approximate
b1	$\log_{10}(k_1'/k_2)$	0.2354	0.1747	0.3005	approximate	approximate
b1	$\log_{10}(k_1'k_2'/k_2)$	−1.259	−1.466	−0.9829	approximate	approximate
b1	$\log_{10}(k_2'/k_2)$	−0.1965	−0.4866	0.1684	approximate	approximate
b2	$\log_{10}(k_1'k_2')$	3.239	< -0.5649	> 8.531	optim	optim
b2	$\log_{10}(k_2 \text{ or } k_{deg})$	4.977	1.172	> 10.27	optim	optim
b2	$\log_{10}(k_2 \text{ or } k_{deg})$	−2.117	−2.221	−2.017	approximate	approximate
b3	$\log_{10}(k_1')$	−0.9389	−1.1	−0.7717	approximate	approximate
b3	$\log_{10}(k_2)$	−1.267	−1.444	−1.108	approximate	approximate
b3	$\log_{10}(k_2')$	−1.868	−2.119	−1.556	approximate	approximate
b3	$\log_{10}(k_{deg})$	−2.055	−2.405	−1.624	approximate	approximate
b3	$\log_{10}(k_1'/k_2)$	0.3282	0.2449	0.4157	approximate	approximate
b3	$\log_{10}(k_1'k_2'/k_2)$	−1.54	−1.783	−1.227	approximate	approximate
b3	$\log_{10}(k_2'/k_2)$	−0.6013	−0.9305	−0.1997	approximate	approximate

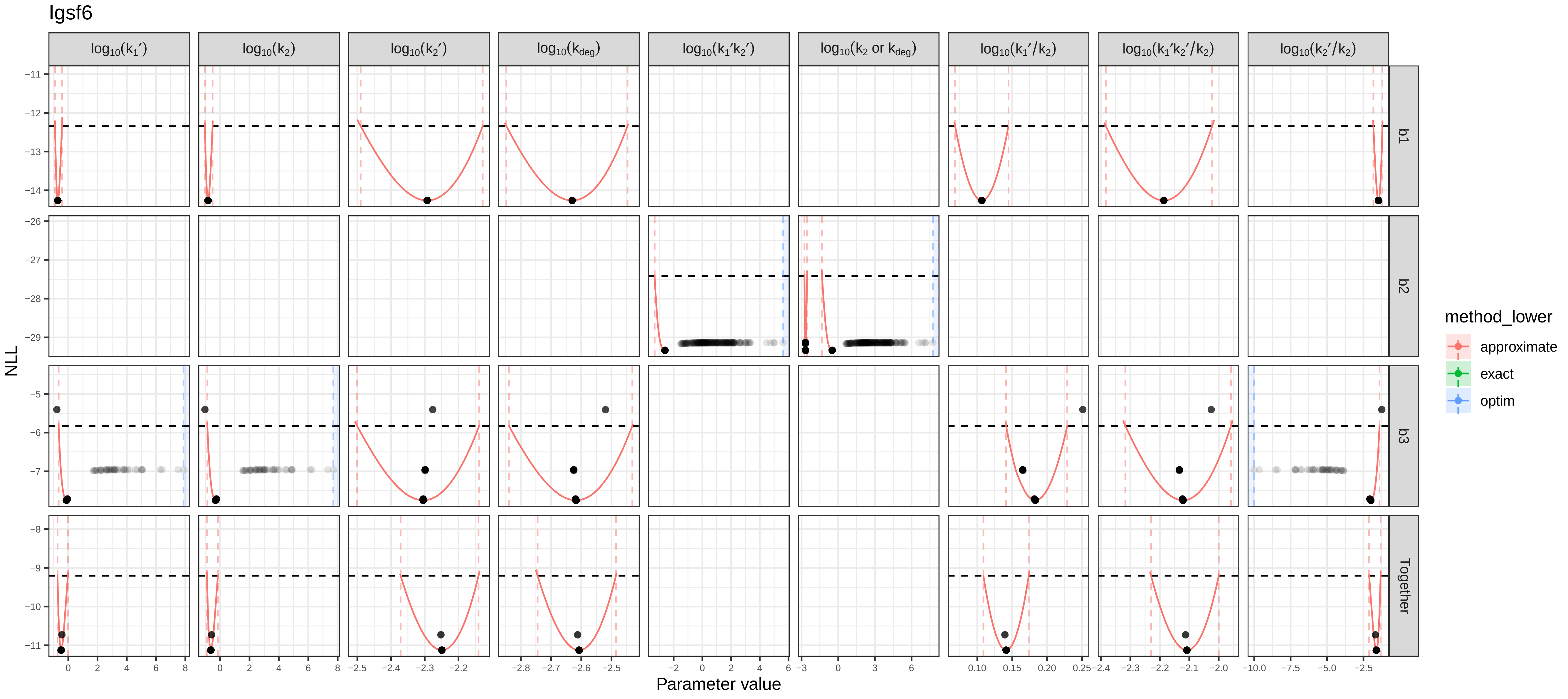

Replicate	Par	Best value	CI95 LB	CI95 UB	Method LB	Method UB
Together	$\log_{10}(k_1')$	−0.4889	−0.7338	−0.02605	approximate	approximate
Together	$\log_{10}(k_2)$	−0.6303	−0.8906	−0.148	approximate	approximate
Together	$\log_{10}(k_2')$	−2.249	−2.371	−2.14	approximate	approximate
Together	$\log_{10}(k_{deg})$	−2.607	−2.744	−2.485	approximate	approximate
Together	$\log_{10}(k_1'/k_2)$	0.1414	0.1088	0.1737	approximate	approximate
Together	$\log_{10}(k_1'k_2'/k_2)$	−2.108	−2.23	−2	approximate	approximate
Together	$\log_{10}(k_2'/k_2)$	−1.619	−2.111	−1.324	approximate	approximate
b1	$\log_{10}(k_1')$	−0.7146	−0.9006	−0.4298	approximate	approximate
b1	$\log_{10}(k_2)$	−0.8211	−1.029	−0.5122	approximate	approximate
b1	$\log_{10}(k_2')$	−2.293	−2.49	−2.128	approximate	approximate
b1	$\log_{10}(k_{deg})$	−2.63	−2.848	−2.447	approximate	approximate
b1	$\log_{10}(k_1'/k_2)$	0.1065	0.06823	0.1447	approximate	approximate
b1	$\log_{10}(k_1'k_2'/k_2)$	−2.186	−2.383	−2.022	approximate	approximate
b1	$\log_{10}(k_2'/k_2)$	−1.472	−1.819	−1.197	approximate	approximate
b2	$\log_{10}(k_1'k_2')$	−2.605	−3.324	> 5.621	approximate	optim
b2	$\log_{10}(k_2 \text{ or } k_{deg})$	−0.494	−1.328	> 7.748	approximate	optim
b2	$\log_{10}(k_2 \text{ or } k_{deg})$	−2.673	−2.764	−2.545	approximate	approximate
b3	$\log_{10}(k_1')$	−0.1247	−0.6585	> 7.872	approximate	optim
b3	$\log_{10}(k_2)$	−0.308	−0.8668	> 7.707	approximate	optim
b3	$\log_{10}(k_2')$	−2.304	−2.501	−2.139	approximate	approximate
b3	$\log_{10}(k_{deg})$	−2.617	−2.839	−2.431	approximate	approximate
b3	$\log_{10}(k_1'/k_2)$	0.1833	0.1413	0.2289	approximate	approximate
b3	$\log_{10}(k_1'k_2'/k_2)$	−2.121	−2.317	−1.959	approximate	approximate
b3	$\log_{10}(k_2'/k_2)$	−1.996	< −10.01	−1.398	optim	approximate

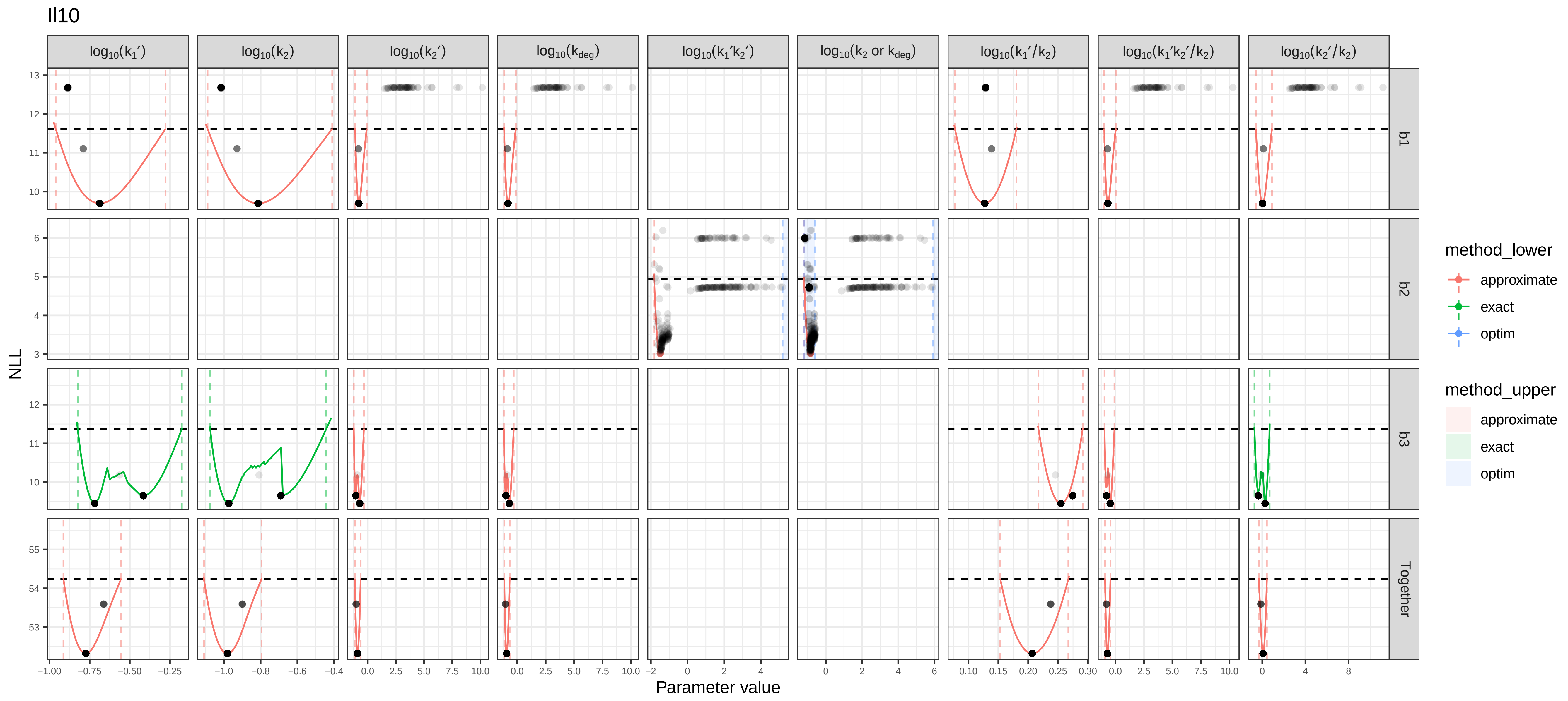

Replicate	Par	Best value	CI95 LB	CI95 UB	Method LB	Method UB
Together	$\log_{10}(k_1')$	−0.7739	−0.9131	−0.5547	approximate	approximate
Together	$\log_{10}(k_2)$	−0.9808	−1.109	−0.7946	approximate	approximate
Together	$\log_{10}(k_2')$	−0.9069	−1.141	−0.6292	approximate	approximate
Together	$\log_{10}(k_{deg})$	−0.9331	−1.138	−0.6658	approximate	approximate
Together	$\log_{10}(k_1'/k_2)$	0.2068	0.1533	0.2672	approximate	approximate
Together	$\log_{10}(k_1'k_2'/k_2)$	−0.7	−0.906	−0.4363	approximate	approximate
Together	$\log_{10}(k_2'/k_2)$	0.07388	−0.3162	0.4227	approximate	approximate
b1	$\log_{10}(k_1')$	−0.6865	−0.9614	−0.2772	approximate	approximate
b1	$\log_{10}(k_2)$	−0.8138	−1.089	−0.4102	approximate	approximate
b1	$\log_{10}(k_2')$	−0.7925	−1.117	−0.08793	approximate	approximate
b1	$\log_{10}(k_{deg})$	−0.8183	−1.15	−0.1245	approximate	approximate
b1	$\log_{10}(k_1'/k_2)$	0.1272	0.07738	0.1803	approximate	approximate
b1	$\log_{10}(k_1'k_2'/k_2)$	−0.6653	−0.98	0.0394	approximate	approximate
b1	$\log_{10}(k_2'/k_2)$	0.02124	−0.5968	0.8998	approximate	approximate
b2	$\log_{10}(k_1'k_2')$	−1.487	−1.819	> 5.185	approximate	optim
b2	$\log_{10}(k_2 \text{ or } k_{deg})$	−0.8451	−1.203	> 5.903	approximate	optim
b2	$\log_{10}(k_2 \text{ or } k_{deg})$	−0.8908	−1.203	−0.5992	approximate	optim
b3	$\log_{10}(k_1')$	−0.7184	−0.8244	−0.1758	exact	exact
b3	$\log_{10}(k_2)$	−0.9732	−1.075	−0.4428	exact	exact
b3	$\log_{10}(k_2')$	−0.7113	−1.241	−0.3429	approximate	approximate
b3	$\log_{10}(k_{deg})$	−0.6927	−1.181	−0.3104	approximate	approximate
b3	$\log_{10}(k_1'/k_2)$	0.2548	0.2172	0.2912	approximate	approximate
b3	$\log_{10}(k_1'k_2'/k_2)$	−0.4566	−0.9572	−0.08469	approximate	approximate
b3	$\log_{10}(k_2'/k_2)$	0.2619	−0.73	0.6786	exact	exact

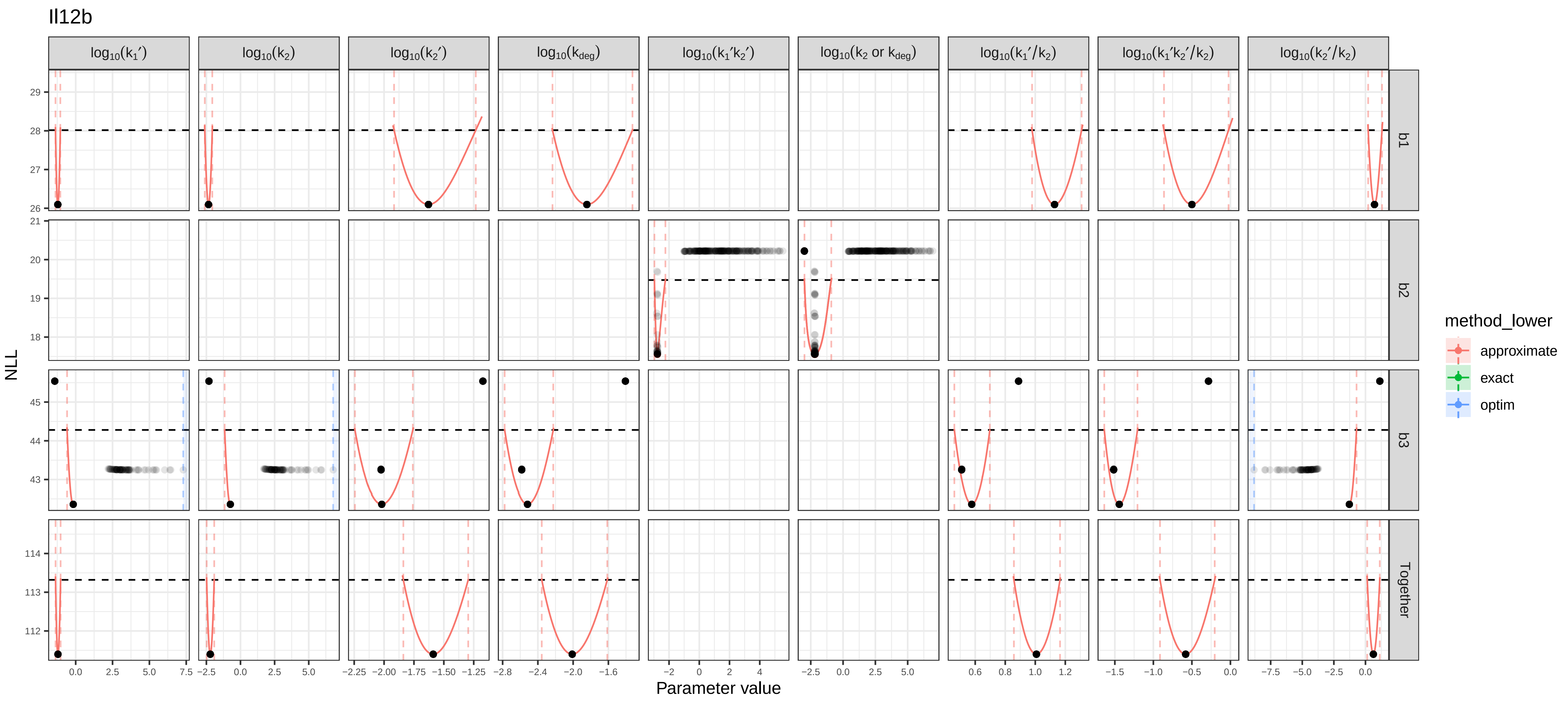

Replicate	Par	Best value	CI95 LB	CI95 UB	Method LB	Method UB
Together	$\log_{10}(k_1')$	−1.206	−1.367	−1.024	approximate	approximate
Together	$\log_{10}(k_2)$	−2.213	−2.478	−1.925	approximate	approximate
Together	$\log_{10}(k_2')$	−1.589	−1.839	−1.296	approximate	approximate
Together	$\log_{10}(k_{deg})$	−2.01	−2.356	−1.612	approximate	approximate
Together	$\log_{10}(k_1'/k_2)$	1.008	0.8578	1.165	approximate	approximate
Together	$\log_{10}(k_1'k_2'/k_2)$	−0.581	−0.9139	−0.2039	approximate	approximate
Together	$\log_{10}(k_2'/k_2)$	0.6246	0.1385	1.13	approximate	approximate
b1	$\log_{10}(k_1')$	−1.213	−1.371	−1.043	approximate	approximate
b1	$\log_{10}(k_2)$	−2.341	−2.615	−2.067	approximate	approximate
b1	$\log_{10}(k_2')$	−1.629	−1.916	−1.233	approximate	approximate
b1	$\log_{10}(k_{deg})$	−1.844	−2.234	−1.327	approximate	approximate
b1	$\log_{10}(k_1'/k_2)$	1.129	0.9787	1.309	approximate	approximate
b1	$\log_{10}(k_1'k_2'/k_2)$	−0.5001	−0.8621	−0.02416	approximate	approximate
b1	$\log_{10}(k_2'/k_2)$	0.7128	0.2126	1.31	approximate	approximate
b2	$\log_{10}(k_1'k_2')$	−2.769	−2.966	−2.24	approximate	approximate
b2	$\log_{10}(k_2 \text{ or } k_{deg})$	−2.167	−2.986	−0.9108	approximate	approximate
b2	$\log_{10}(k_2 \text{ or } k_{deg})$	−2.206	−2.986	−0.9108	approximate	approximate
b3	$\log_{10}(k_1')$	−0.1659	−0.5851	> 7.297	approximate	optim
b3	$\log_{10}(k_2)$	−0.7429	−1.161	> 6.787	approximate	optim
b3	$\log_{10}(k_2')$	−2.019	−2.244	−1.759	approximate	approximate
b3	$\log_{10}(k_{deg})$	−2.518	−2.777	−2.226	approximate	approximate
b3	$\log_{10}(k_1'/k_2)$	0.5769	0.461	0.6973	approximate	approximate
b3	$\log_{10}(k_1'k_2'/k_2)$	−1.443	−1.637	−1.206	approximate	approximate
b3	$\log_{10}(k_2'/k_2)$	−1.277	< −8.812	−0.7056	optim	approximate

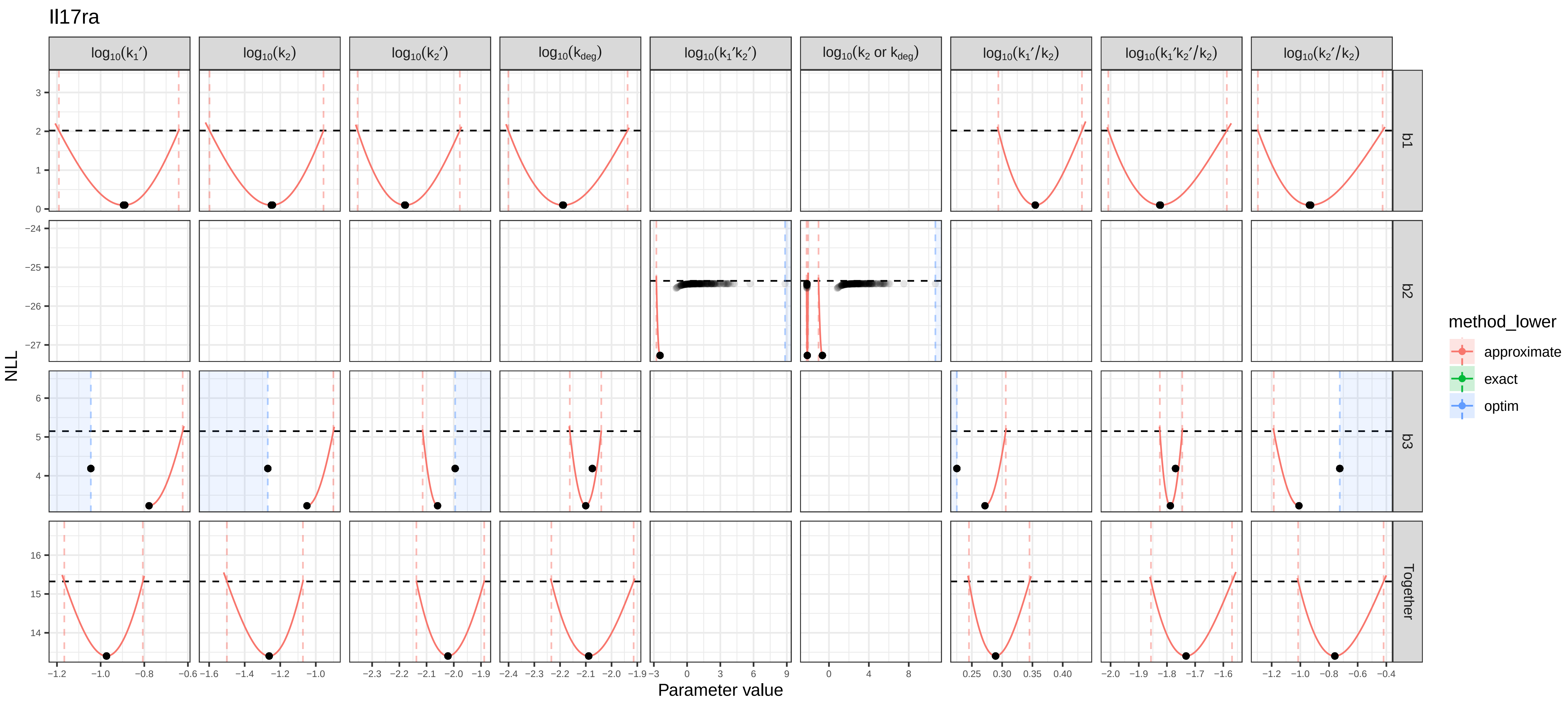

Replicate	Par	Best value	CI95 LB	CI95 UB	Method LB	Method UB
Together	$\log_{10}(k_1')$	−0.9731	−1.166	−0.8067	approximate	approximate
Together	$\log_{10}(k_2)$	−1.262	−1.5	−1.072	approximate	approximate
Together	$\log_{10}(k_2')$	−2.021	−2.137	−1.888	approximate	approximate
Together	$\log_{10}(k_{deg})$	−2.089	−2.234	−1.914	approximate	approximate
Together	$\log_{10}(k_1'/k_2)$	0.2891	0.2452	0.3452	approximate	approximate
Together	$\log_{10}(k_1'k_2'/k_2)$	−1.732	−1.857	−1.569	approximate	approximate
Together	$\log_{10}(k_2'/k_2)$	−0.7592	−1.015	−0.419	approximate	approximate
b1	$\log_{10}(k_1')$	−0.8948	−1.191	−0.6422	approximate	approximate
b1	$\log_{10}(k_2)$	−1.25	−1.598	−0.9565	approximate	approximate
b1	$\log_{10}(k_2')$	−2.178	−2.353	−1.978	approximate	approximate
b1	$\log_{10}(k_{deg})$	−2.188	−2.401	−1.938	approximate	approximate
b1	$\log_{10}(k_1'/k_2)$	0.3547	0.2937	0.4317	approximate	approximate
b1	$\log_{10}(k_1'k_2'/k_2)$	−1.823	−2.008	−1.588	approximate	approximate
b1	$\log_{10}(k_2'/k_2)$	−0.9287	−1.296	−0.4259	approximate	approximate
b2	$\log_{10}(k_1'k_2')$	−2.441	−2.772	> 8.845	approximate	optim
b2	$\log_{10}(k_2 \text{ or } k_{deg})$	−0.6584	−1.048	> 10.67	approximate	optim
b2	$\log_{10}(k_2 \text{ or } k_{deg})$	−2.176	−2.246	−2.098	approximate	approximate
b3	$\log_{10}(k_1')$	−0.7781	< −1.045	−0.624	optim	approximate
b3	$\log_{10}(k_2)$	−1.05	< −1.27	−0.9011	optim	approximate
b3	$\log_{10}(k_2')$	−2.06	−2.114	> −1.995	approximate	optim
b3	$\log_{10}(k_{deg})$	−2.1	−2.162	−2.04	approximate	approximate
b3	$\log_{10}(k_1'/k_2)$	0.2716	< 0.2252	0.306	optim	approximate
b3	$\log_{10}(k_1'k_2'/k_2)$	−1.788	−1.825	−1.746	approximate	approximate
b3	$\log_{10}(k_2'/k_2)$	−1.01	−1.185	> −0.7247	approximate	optim

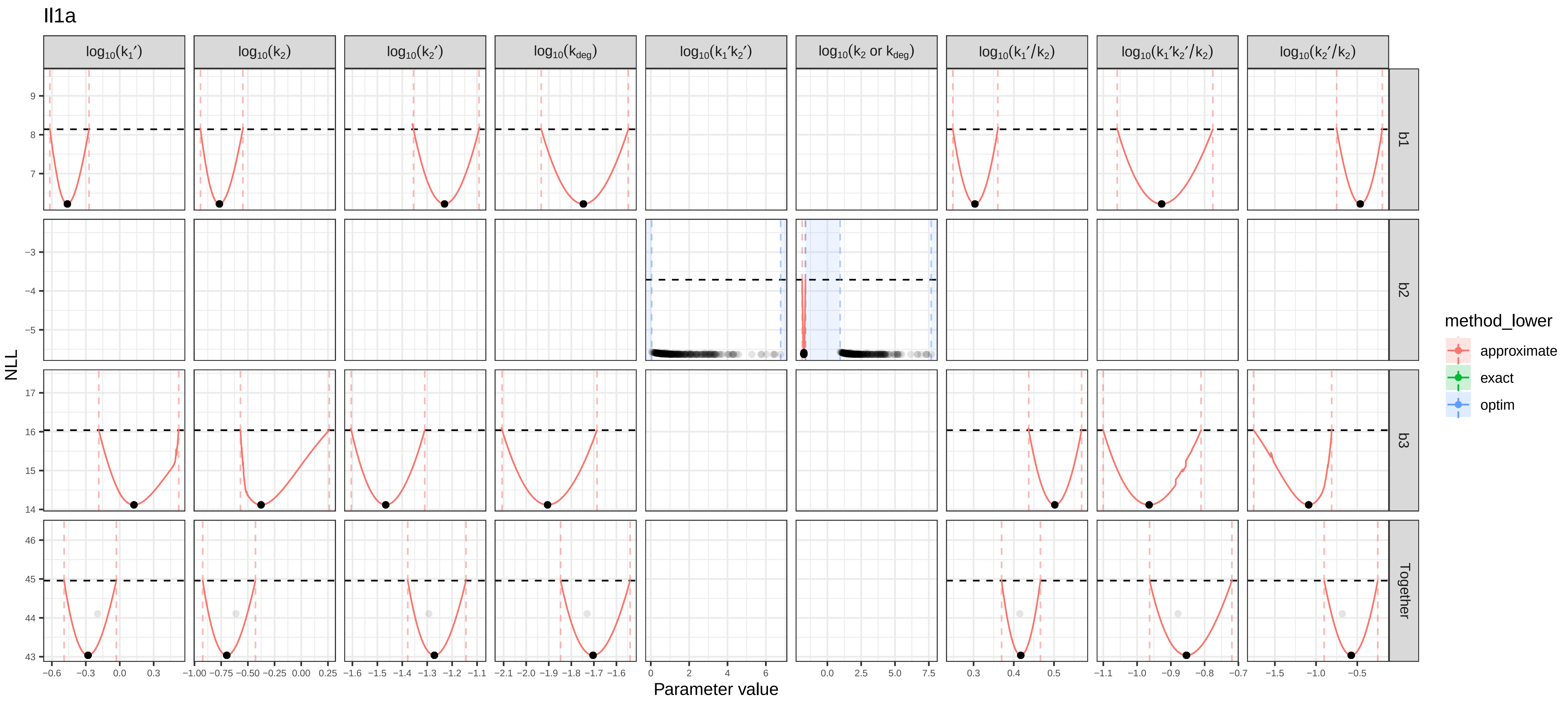

Replicate	Par	Best value	CI95 LB	CI95 UB	Method LB	Method UB
Together	$\log_{10}(k_1')$	−0.2802	−0.4919	−0.03007	approximate	approximate
Together	$\log_{10}(k_2)$	−0.6976	−0.9227	−0.4287	approximate	approximate
Together	$\log_{10}(k_2')$	−1.271	−1.378	−1.144	approximate	approximate
Together	$\log_{10}(k_{deg})$	−1.703	−1.847	−1.539	approximate	approximate
Together	$\log_{10}(k_1'/k_2)$	0.4174	0.3696	0.4662	approximate	approximate
Together	$\log_{10}(k_1'k_2'/k_2)$	−0.8538	−0.963	−0.7192	approximate	approximate
Together	$\log_{10}(k_2'/k_2)$	−0.5736	−0.9022	−0.2518	approximate	approximate
b1	$\log_{10}(k_1')$	−0.4631	−0.618	−0.2716	approximate	approximate
b1	$\log_{10}(k_2)$	−0.7665	−0.9438	−0.5469	approximate	approximate
b1	$\log_{10}(k_2')$	−1.231	−1.355	−1.092	approximate	approximate
b1	$\log_{10}(k_{deg})$	−1.746	−1.933	−1.547	approximate	approximate
b1	$\log_{10}(k_1'/k_2)$	0.3034	0.2483	0.3602	approximate	approximate
b1	$\log_{10}(k_1'k_2'/k_2)$	−0.9271	−1.059	−0.7757	approximate	approximate
b1	$\log_{10}(k_2'/k_2)$	−0.464	−0.7505	−0.1963	approximate	approximate
b2	$\log_{10}(k_1'k_2')$	4.224	< 0.047	> 6.77	optim	optim
b2	$\log_{10}(k_2 \text{ or } k_{deg})$	5.125	0.9448	> 7.671	optim	optim
b2	$\log_{10}(k_2 \text{ or } k_{deg})$	−1.739	−1.864	−1.619	approximate	approximate
b3	$\log_{10}(k_1')$	0.1254	−0.1855	0.5213	approximate	approximate
b3	$\log_{10}(k_2)$	−0.3767	−0.5694	0.2623	approximate	approximate
b3	$\log_{10}(k_2')$	−1.466	−1.605	−1.31	approximate	approximate
b3	$\log_{10}(k_{deg})$	−1.905	−2.107	−1.686	approximate	approximate
b3	$\log_{10}(k_1'/k_2)$	0.502	0.4372	0.5686	approximate	approximate
b3	$\log_{10}(k_1'k_2'/k_2)$	−0.9642	−1.1	−0.8107	approximate	approximate
b3	$\log_{10}(k_2'/k_2)$	−1.09	−1.758	−0.8108	approximate	approximate

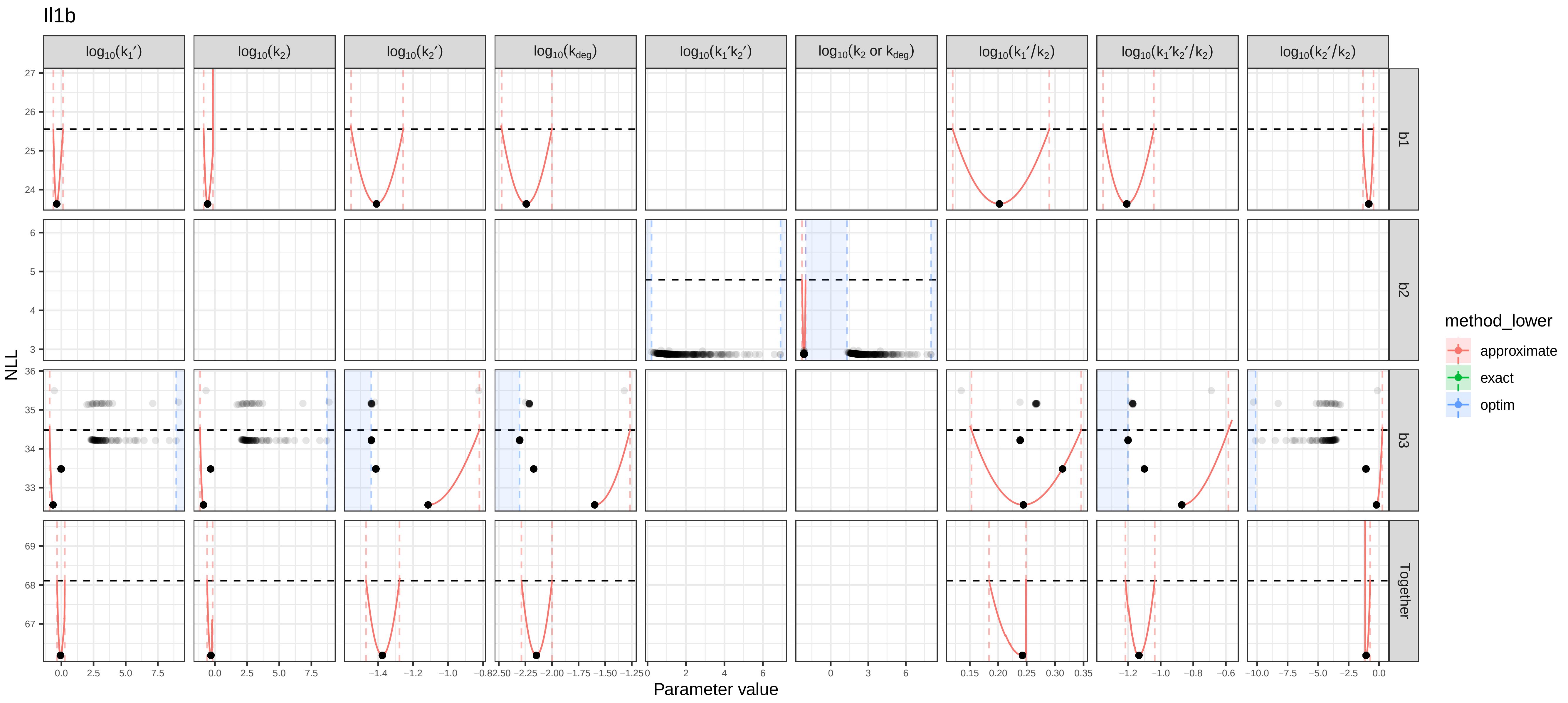

Replicate	Par	Best value	CI95 LB	CI95 UB	Method LB	Method UB
Together	$\log_{10}(k_1')$	−0.06685	−0.3375	0.2607	approximate	approximate
Together	$\log_{10}(k_2)$	−0.3094	−0.6066	−0.1765	approximate	approximate
Together	$\log_{10}(k_2')$	−1.376	−1.469	−1.278	approximate	approximate
Together	$\log_{10}(k_{deg})$	−2.147	−2.287	−1.999	approximate	approximate
Together	$\log_{10}(k_1'/k_2)$	0.2426	0.1839	0.2488	approximate	approximate
Together	$\log_{10}(k_1'k_2'/k_2)$	−1.133	−1.216	−1.037	approximate	approximate
Together	$\log_{10}(k_2'/k_2)$	−1.066	−1.145	−0.7348	approximate	approximate
b1	$\log_{10}(k_1')$	−0.3646	−0.6261	0.1347	approximate	approximate
b1	$\log_{10}(k_2)$	−0.5667	−0.8765	−0.157	approximate	approximate
b1	$\log_{10}(k_2')$	−1.41	−1.554	−1.257	approximate	approximate
b1	$\log_{10}(k_{deg})$	−2.241	−2.473	−2.001	approximate	approximate
b1	$\log_{10}(k_1'/k_2)$	0.2021	0.1199	0.2898	approximate	approximate
b1	$\log_{10}(k_1'k_2'/k_2)$	−1.208	−1.353	−1.042	approximate	approximate
b1	$\log_{10}(k_2'/k_2)$	−0.8432	−1.335	−0.4602	approximate	approximate
b2	$\log_{10}(k_1'k_2')$	5.32	< 0.2084	> 6.919	optim	optim
b2	$\log_{10}(k_2 \text{ or } k_{deg})$	6.42	1.307	> 8.019	optim	optim
b2	$\log_{10}(k_2 \text{ or } k_{deg})$	−2.135	−2.292	−2.012	approximate	approximate
b3	$\log_{10}(k_1')$	−0.6478	−0.9151	> 8.931	approximate	optim
b3	$\log_{10}(k_2)$	−0.892	−1.151	> 8.693	approximate	optim
b3	$\log_{10}(k_2')$	−1.115	< −1.439	−0.8212	optim	approximate
b3	$\log_{10}(k_{deg})$	−1.598	< −2.306	−1.266	optim	approximate
b3	$\log_{10}(k_1'/k_2)$	0.2442	0.153	0.3458	approximate	approximate
b3	$\log_{10}(k_1'k_2'/k_2)$	−0.8704	< −1.201	−0.5845	optim	approximate
b3	$\log_{10}(k_2'/k_2)$	−0.2226	< −10.13	0.2694	optim	approximate

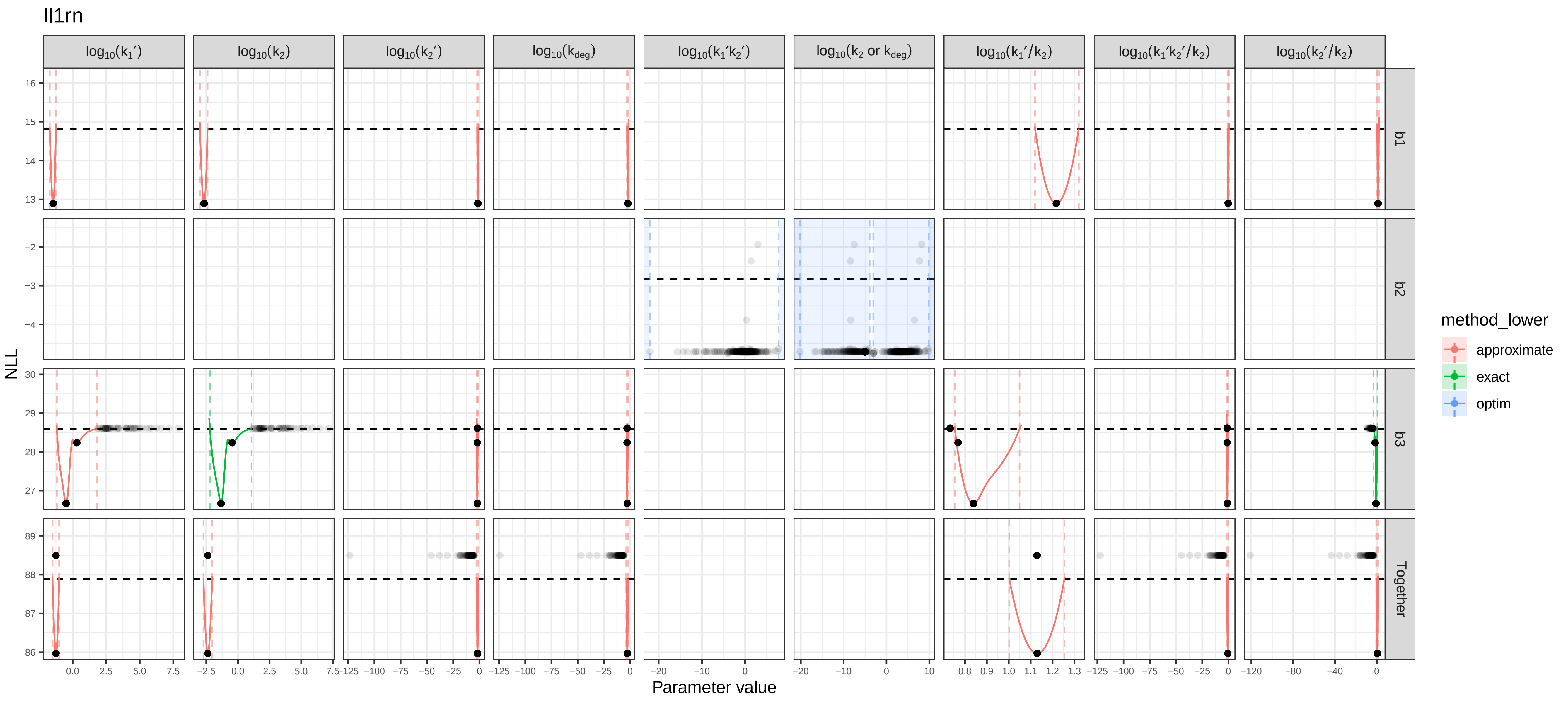

Replicate	Par	Best value	CI95 LB	CI95 UB	Method LB	Method UB
Together	$\log_{10}(k_1')$	−1.245	−1.499	−1.006	approximate	approximate
Together	$\log_{10}(k_2)$	−2.375	−2.72	−2.037	approximate	approximate
Together	$\log_{10}(k_2')$	−1.807	−2.803	−1.3	approximate	approximate
Together	$\log_{10}(k_{deg})$	−2.56	−3.594	−1.972	approximate	approximate
Together	$\log_{10}(k_1'/k_2)$	1.13	1.002	1.254	approximate	approximate
Together	$\log_{10}(k_1'k_2'/k_2)$	−0.6769	−1.681	−0.1088	approximate	approximate
Together	$\log_{10}(k_2'/k_2)$	0.5685	−0.4677	1.328	approximate	approximate
b1	$\log_{10}(k_1')$	−1.459	−1.711	−1.248	approximate	approximate
b1	$\log_{10}(k_2)$	−2.676	−2.997	−2.397	approximate	approximate
b1	$\log_{10}(k_2')$	−1.526	−2.107	−0.9367	approximate	approximate
b1	$\log_{10}(k_{deg})$	−2.151	−2.779	−1.492	approximate	approximate
b1	$\log_{10}(k_1'/k_2)$	1.217	1.12	1.32	approximate	approximate
b1	$\log_{10}(k_1'k_2'/k_2)$	−0.3091	−0.9091	0.3298	approximate	approximate
b1	$\log_{10}(k_2'/k_2)$	1.15	0.4507	1.979	approximate	approximate
b2	$\log_{10}(k_1'k_2')$	−3.974	< −22.01	> 7.749	optim	optim
b2	$\log_{10}(k_2 \text{ or } k_{deg})$	−3.006	< −3.933	> 9.853	optim	optim
b2	$\log_{10}(k_2 \text{ or } k_{deg})$	−3.062	< −20.17	> −3.062	optim	optim
b3	$\log_{10}(k_1')$	−0.4885	−1.179	1.812	approximate	approximate
b3	$\log_{10}(k_2)$	−1.327	−2.207	1.081	exact	exact
b3	$\log_{10}(k_2')$	−2.011	−2.46	−1.481	approximate	approximate
b3	$\log_{10}(k_{deg})$	−2.663	−3.17	−1.951	approximate	approximate
b3	$\log_{10}(k_1'/k_2)$	0.8388	0.7538	1.049	approximate	approximate
b3	$\log_{10}(k_1'k_2'/k_2)$	−1.172	−1.624	−0.4764	approximate	approximate
b3	$\log_{10}(k_2'/k_2)$	−0.6836	−3.129	0.6653	exact	exact

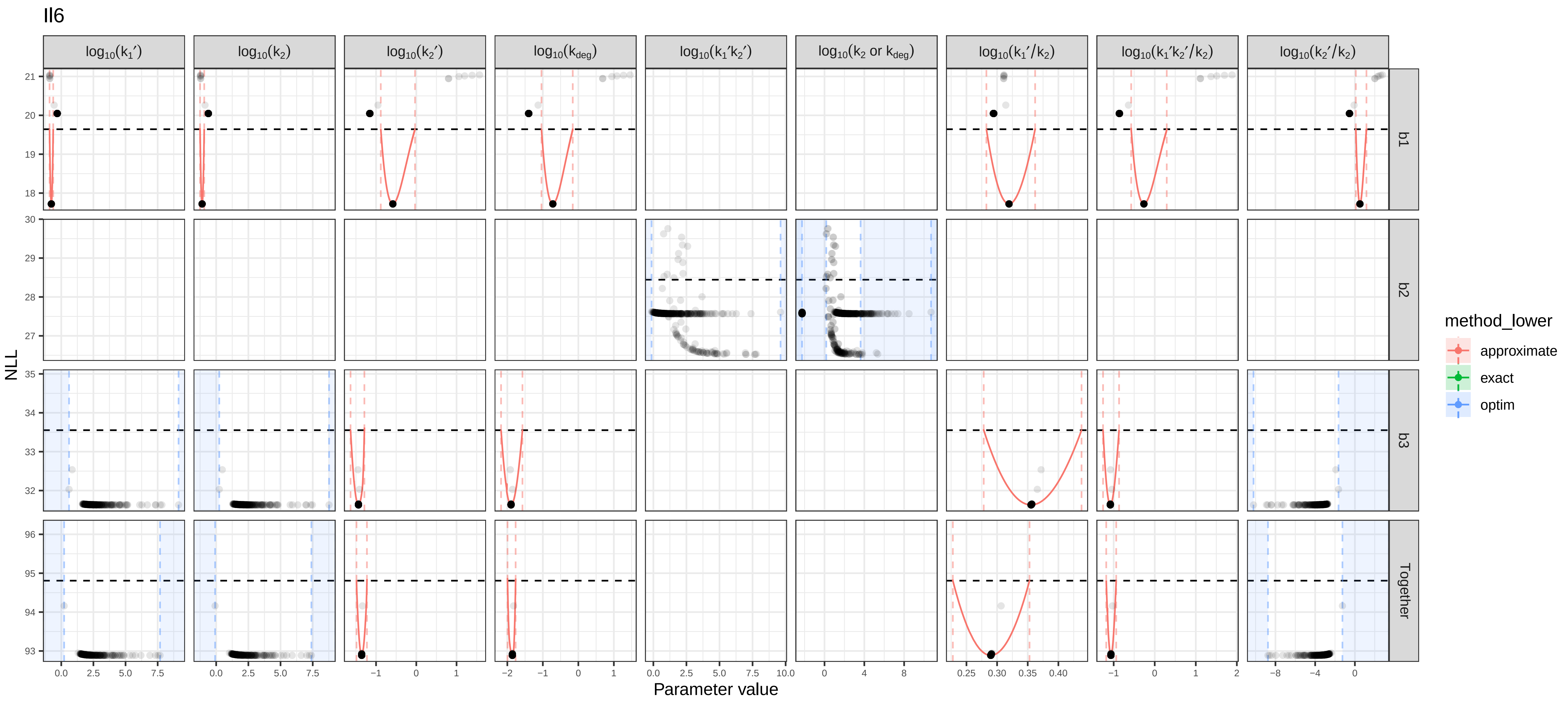

Replicate	Par	Best value	CI95 LB	CI95 UB	Method LB	Method UB
Together	$\log_{10}(k_1')$	5.551	< 0.2231	> 7.685	optim	optim
Together	$\log_{10}(k_2)$	5.261	< −0.0835	> 7.395	optim	optim
Together	$\log_{10}(k_2')$	−1.357	−1.485	−1.226	approximate	approximate
Together	$\log_{10}(k_{deg})$	−1.858	−1.995	−1.766	approximate	approximate
Together	$\log_{10}(k_1'/k_2)$	0.2897	0.2278	0.3529	approximate	approximate
Together	$\log_{10}(k_1'k_2'/k_2)$	−1.067	−1.181	−0.9382	approximate	approximate
Together	$\log_{10}(k_2'/k_2)$	−6.618	< −8.752	> −1.255	optim	optim
b1	$\log_{10}(k_1')$	−0.7729	−0.9121	−0.6269	approximate	approximate
b1	$\log_{10}(k_2)$	−1.092	−1.252	−0.9285	approximate	approximate
b1	$\log_{10}(k_2')$	−0.5832	−0.8823	−0.03156	approximate	approximate
b1	$\log_{10}(k_{deg})$	−0.719	−1.038	−0.1575	approximate	approximate
b1	$\log_{10}(k_1'/k_2)$	0.3195	0.2825	0.3621	approximate	approximate
b1	$\log_{10}(k_1'k_2'/k_2)$	−0.2637	−0.5733	0.2929	approximate	approximate
b1	$\log_{10}(k_2'/k_2)$	0.5092	0.09519	1.164	approximate	approximate
b2	$\log_{10}(k_1'k_2')$	7.7	< −0.1369	> 9.616	optim	optim
b2	$\log_{10}(k_2 \text{ or } k_{deg})$	3.703	< 0.1762	> 10.71	optim	optim
b2	$\log_{10}(k_2 \text{ or } k_{deg})$	3.627	< −2.258	> 3.627	optim	optim
b3	$\log_{10}(k_1')$	4.547	< 0.6046	> 9.125	optim	optim
b3	$\log_{10}(k_2)$	4.192	< 0.239	> 8.769	optim	optim
b3	$\log_{10}(k_2')$	−1.436	−1.634	−1.29	approximate	approximate
b3	$\log_{10}(k_{deg})$	−1.895	−2.177	−1.574	approximate	approximate
b3	$\log_{10}(k_1'/k_2)$	0.3554	0.2782	0.4378	approximate	approximate
b3	$\log_{10}(k_1'k_2'/k_2)$	−1.081	−1.259	−0.8684	approximate	approximate
b3	$\log_{10}(k_2'/k_2)$	−5.628	< −10.21	> −1.649	optim	optim

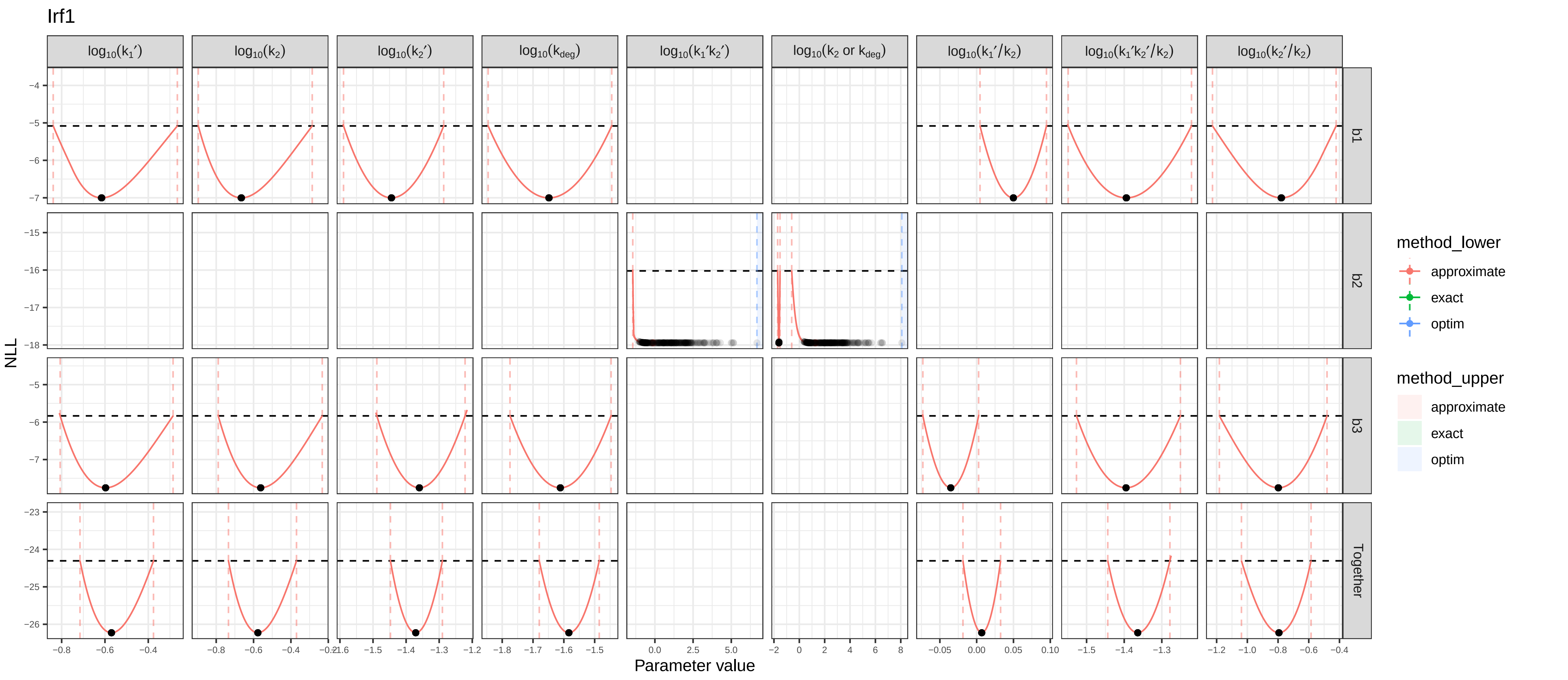

Replicate	Par	Best value	CI95 LB	CI95 UB	Method LB	Method UB
Together	$\log_{10}(k_1')$	−0.5699	−0.7152	−0.3759	approximate	approximate
Together	$\log_{10}(k_2)$	−0.5767	−0.7343	−0.3702	approximate	approximate
Together	$\log_{10}(k_2')$	−1.371	−1.447	−1.29	approximate	approximate
Together	$\log_{10}(k_{deg})$	−1.584	−1.68	−1.485	approximate	approximate
Together	$\log_{10}(k_1'/k_2)$	0.006884	−0.01861	0.03251	approximate	approximate
Together	$\log_{10}(k_1'k_2'/k_2)$	−1.364	−1.444	−1.278	approximate	approximate
Together	$\log_{10}(k_2'/k_2)$	−0.7942	−1.038	−0.5851	approximate	approximate
b1	$\log_{10}(k_1')$	−0.6155	−0.839	−0.2658	approximate	approximate
b1	$\log_{10}(k_2)$	−0.6653	−0.8958	−0.2859	approximate	approximate
b1	$\log_{10}(k_2')$	−1.444	−1.589	−1.286	approximate	approximate
b1	$\log_{10}(k_{deg})$	−1.648	−1.846	−1.445	approximate	approximate
b1	$\log_{10}(k_1'/k_2)$	0.04988	0.004551	0.09484	approximate	approximate
b1	$\log_{10}(k_1'k_2'/k_2)$	−1.394	−1.549	−1.221	approximate	approximate
b1	$\log_{10}(k_2'/k_2)$	−0.7789	−1.227	−0.4219	approximate	approximate
b2	$\log_{10}(k_1'k_2')$	−0.1178	−1.448	> 6.679	approximate	optim
b2	$\log_{10}(k_2 \text{ or } k_{deg})$	1.286	−0.6	> 8.084	approximate	optim
b2	$\log_{10}(k_2 \text{ or } k_{deg})$	−1.611	−1.708	−1.52	approximate	approximate
b3	$\log_{10}(k_1')$	−0.597	−0.807	−0.2854	approximate	approximate
b3	$\log_{10}(k_2)$	−0.5618	−0.789	−0.2324	approximate	approximate
b3	$\log_{10}(k_2')$	−1.36	−1.488	−1.222	approximate	approximate
b3	$\log_{10}(k_{deg})$	−1.611	−1.775	−1.447	approximate	approximate
b3	$\log_{10}(k_1'/k_2)$	−0.03522	−0.07311	0.002642	approximate	approximate
b3	$\log_{10}(k_1'k_2'/k_2)$	−1.395	−1.527	−1.251	approximate	approximate
b3	$\log_{10}(k_2'/k_2)$	−0.7981	−1.182	−0.4811	approximate	approximate

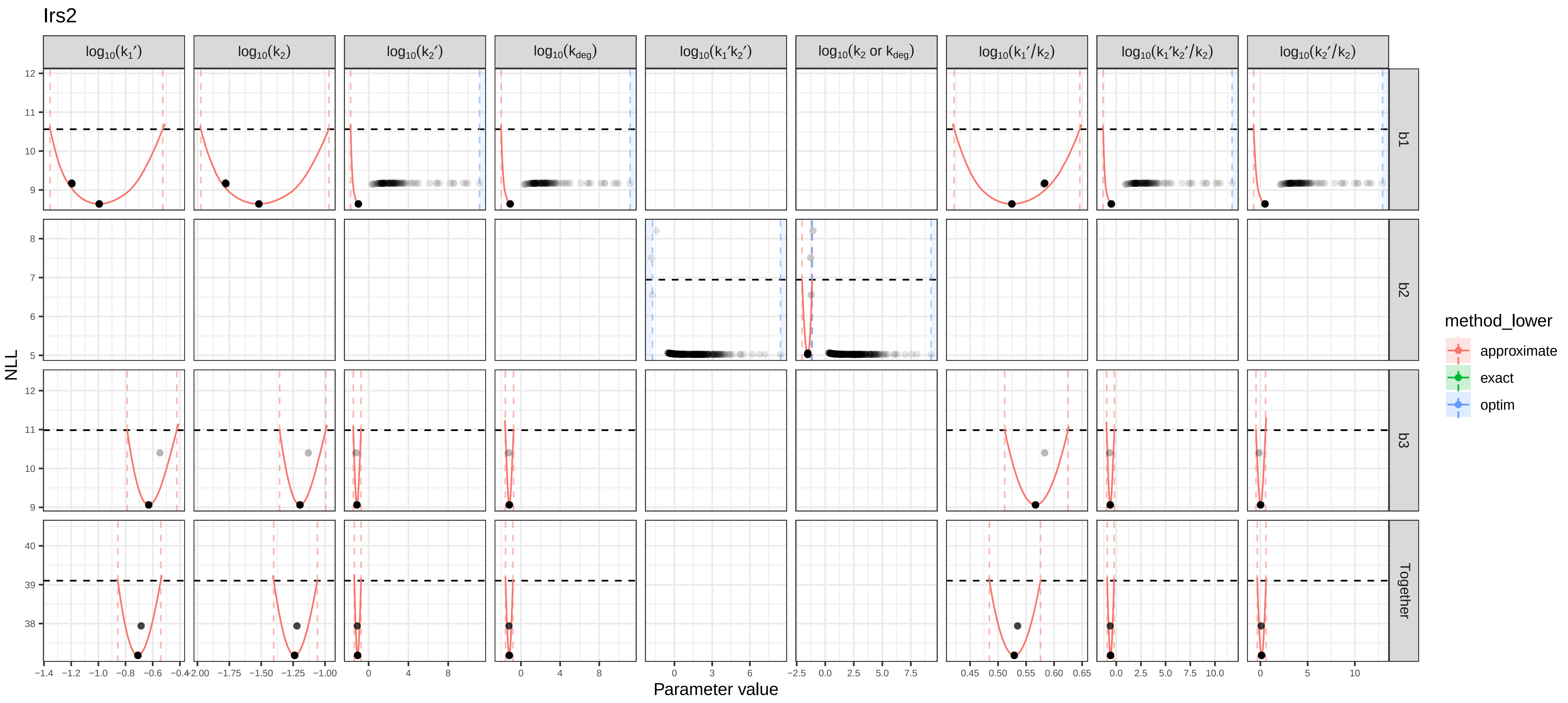

Replicate	Par	Best value	CI95 LB	CI95 UB	Method LB	Method UB
Together	$\log_{10}(k_1')$	−0.7091	−0.8569	−0.5405	approximate	approximate
Together	$\log_{10}(k_2)$	−1.238	−1.402	−1.06	approximate	approximate
Together	$\log_{10}(k_2')$	−1.108	−1.441	−0.7578	approximate	approximate
Together	$\log_{10}(k_{deg})$	−1.198	−1.577	−0.8223	approximate	approximate
Together	$\log_{10}(k_1'/k_2)$	0.5289	0.4844	0.5758	approximate	approximate
Together	$\log_{10}(k_1'k_2'/k_2)$	−0.5793	−0.9177	−0.218	approximate	approximate
Together	$\log_{10}(k_2'/k_2)$	0.1298	−0.3248	0.598	approximate	approximate
b1	$\log_{10}(k_1')$	−0.9935	−1.355	−0.5261	approximate	approximate
b1	$\log_{10}(k_2)$	−1.518	−1.973	−0.9695	approximate	approximate
b1	$\log_{10}(k_2')$	−1.031	−1.805	> 11.15	approximate	optim
b1	$\log_{10}(k_{deg})$	−1.104	−2.023	> 11.14	approximate	optim
b1	$\log_{10}(k_1'/k_2)$	0.5246	0.4216	0.646	approximate	approximate
b1	$\log_{10}(k_1'k_2'/k_2)$	−0.5066	−1.332	> 11.73	approximate	optim
b1	$\log_{10}(k_2'/k_2)$	0.4869	−0.7073	> 12.93	approximate	optim
b2	$\log_{10}(k_1'k_2')$	3.479	< −1.743	> 8.436	optim	optim
b2	$\log_{10}(k_2 \text{ or } k_{deg})$	4.315	−1.207	> 9.271	optim	optim
b2	$\log_{10}(k_2 \text{ or } k_{deg})$	−1.538	−2.034	−1.147	approximate	approximate
b3	$\log_{10}(k_1')$	−0.6295	−0.7881	−0.4227	approximate	approximate
b3	$\log_{10}(k_2)$	−1.196	−1.356	−0.9939	approximate	approximate
b3	$\log_{10}(k_2')$	−1.177	−1.541	−0.7615	approximate	approximate
b3	$\log_{10}(k_{deg})$	−1.191	−1.599	−0.7557	approximate	approximate
b3	$\log_{10}(k_1'/k_2)$	0.5668	0.5118	0.6249	approximate	approximate
b3	$\log_{10}(k_1'k_2'/k_2)$	−0.6103	−0.9723	−0.1906	approximate	approximate
b3	$\log_{10}(k_2'/k_2)$	0.01916	−0.4646	0.5461	approximate	approximate

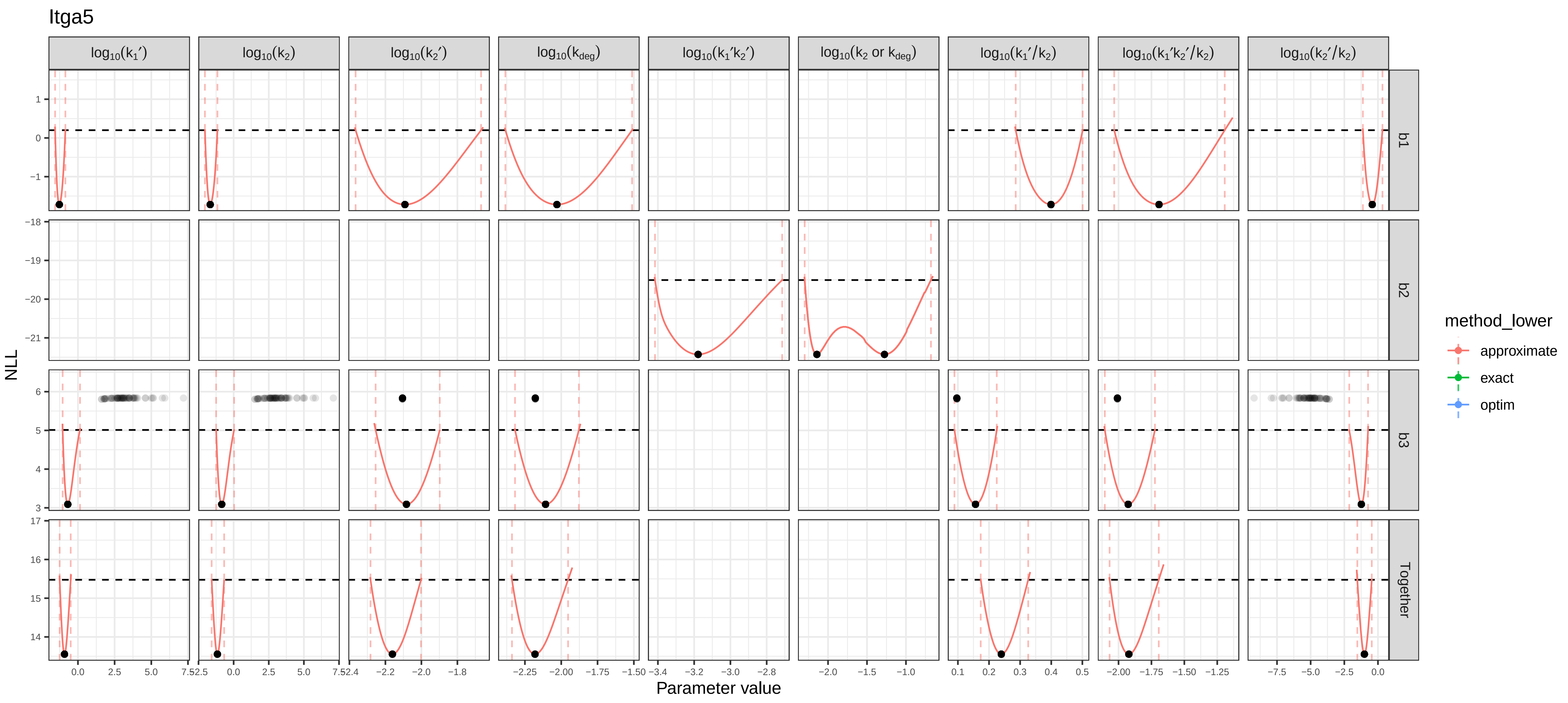

Replicate	Par	Best value	CI95 LB	CI95 UB	Method LB	Method UB
Together	$\log_{10}(k_1')$	−0.9162	−1.246	−0.493	approximate	approximate
Together	$\log_{10}(k_2)$	−1.156	−1.565	−0.6762	approximate	approximate
Together	$\log_{10}(k_2')$	−2.161	−2.283	−2.002	approximate	approximate
Together	$\log_{10}(k_{deg})$	−2.18	−2.339	−1.953	approximate	approximate
Together	$\log_{10}(k_1'/k_2)$	0.2395	0.1733	0.3259	approximate	approximate
Together	$\log_{10}(k_1'k_2'/k_2)$	−1.922	−2.068	−1.694	approximate	approximate
Together	$\log_{10}(k_2'/k_2)$	−1.005	−1.534	−0.4594	approximate	approximate
b1	$\log_{10}(k_1')$	−1.268	−1.557	−0.8564	approximate	approximate
b1	$\log_{10}(k_2)$	−1.667	−2.044	−1.154	approximate	approximate
b1	$\log_{10}(k_2')$	−2.091	−2.366	−1.668	approximate	approximate
b1	$\log_{10}(k_{deg})$	−2.03	−2.384	−1.512	approximate	approximate
b1	$\log_{10}(k_1'/k_2)$	0.3991	0.2858	0.5009	approximate	approximate
b1	$\log_{10}(k_1'k_2'/k_2)$	−1.692	−2.033	−1.193	approximate	approximate
b1	$\log_{10}(k_2'/k_2)$	−0.4246	−1.119	0.3358	approximate	approximate
b2	$\log_{10}(k_1'k_2')$	−3.178	−3.416	−2.715	approximate	approximate
b2	$\log_{10}(k_2 \text{ or } k_{deg})$	−1.276	−2.3	−0.6791	approximate	approximate
b2	$\log_{10}(k_2 \text{ or } k_{deg})$	−2.143	−2.3	−0.6791	approximate	approximate
b3	$\log_{10}(k_1')$	−0.6917	−1.043	0.1418	approximate	approximate
b3	$\log_{10}(k_2)$	−0.8482	−1.246	0.03388	approximate	approximate
b3	$\log_{10}(k_2')$	−2.083	−2.254	−1.898	approximate	approximate
b3	$\log_{10}(k_{deg})$	−2.108	−2.318	−1.878	approximate	approximate
b3	$\log_{10}(k_1'/k_2)$	0.1566	0.08883	0.2249	approximate	approximate
b3	$\log_{10}(k_1'k_2'/k_2)$	−1.927	−2.104	−1.723	approximate	approximate
b3	$\log_{10}(k_2'/k_2)$	−1.235	−2.133	−0.7363	approximate	approximate

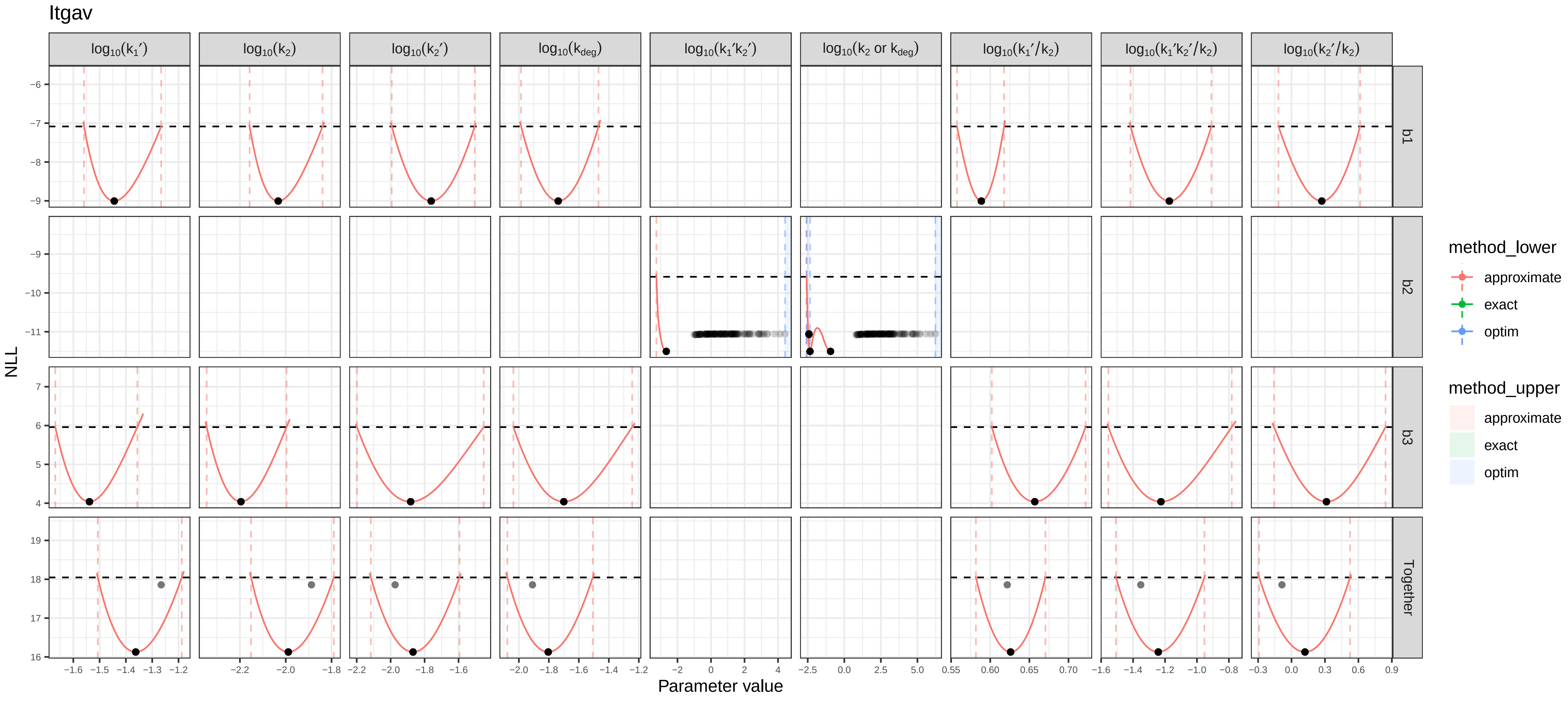

Replicate	Par	Best value	CI95 LB	CI95 UB	Method LB	Method UB
Together	$\log_{10}(k_1')$	−1.363	−1.507	−1.188	approximate	approximate
Together	$\log_{10}(k_2)$	−1.989	−2.152	−1.79	approximate	approximate
Together	$\log_{10}(k_2')$	−1.868	−2.117	−1.595	approximate	approximate
Together	$\log_{10}(k_{deg})$	−1.804	−2.077	−1.507	approximate	approximate
Together	$\log_{10}(k_1'/k_2)$	0.6261	0.5817	0.6705	approximate	approximate
Together	$\log_{10}(k_1'k_2'/k_2)$	−1.242	−1.507	−0.9532	approximate	approximate
Together	$\log_{10}(k_2'/k_2)$	0.1207	−0.2911	0.5253	approximate	approximate
b1	$\log_{10}(k_1')$	−1.444	−1.559	−1.266	approximate	approximate
b1	$\log_{10}(k_2)$	−2.033	−2.158	−1.839	approximate	approximate
b1	$\log_{10}(k_2')$	−1.761	−1.994	−1.505	approximate	approximate
b1	$\log_{10}(k_{deg})$	−1.738	−1.985	−1.469	approximate	approximate
b1	$\log_{10}(k_1'/k_2)$	0.5887	0.5576	0.6177	approximate	approximate
b1	$\log_{10}(k_1'k_2'/k_2)$	−1.173	−1.416	−0.9091	approximate	approximate
b1	$\log_{10}(k_2'/k_2)$	0.2716	−0.1173	0.6157	approximate	approximate
b2	$\log_{10}(k_1'k_2')$	−2.667	−3.258	> 4.42	approximate	optim
b2	$\log_{10}(k_2 \text{ or } k_{deg})$	−0.9409	−2.58	> 6.227	approximate	optim
b2	$\log_{10}(k_2 \text{ or } k_{deg})$	−2.342	−2.58	−2.341	approximate	optim
b3	$\log_{10}(k_1')$	−1.539	−1.668	−1.357	approximate	approximate
b3	$\log_{10}(k_2)$	−2.196	−2.345	−1.997	approximate	approximate
b3	$\log_{10}(k_2')$	−1.882	−2.199	−1.452	approximate	approximate
b3	$\log_{10}(k_{deg})$	−1.7	−2.037	−1.244	approximate	approximate
b3	$\log_{10}(k_1'/k_2)$	0.6569	0.6021	0.7219	approximate	approximate
b3	$\log_{10}(k_1'k_2'/k_2)$	−1.225	−1.555	−0.7821	approximate	approximate
b3	$\log_{10}(k_2'/k_2)$	0.3133	−0.1569	0.8436	approximate	approximate

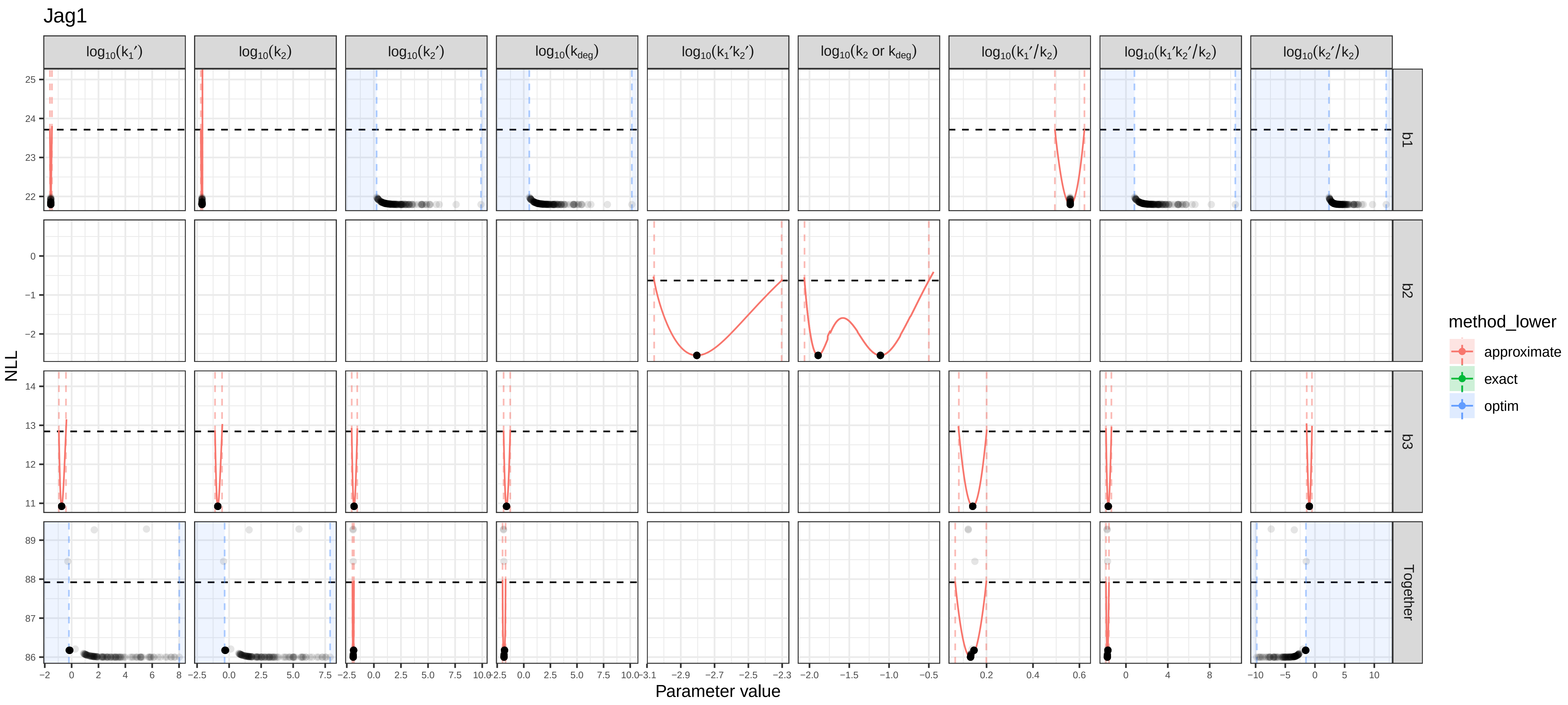

Replicate	Par	**Best value**	**CI95 LB**	**CI95 UB**	**Method LB**	**Method UB**
Together	$\log_{10}(k_1')$	6.142	< −0.2015	> 8.017	optim	optim
Together	$\log_{10}(k_2)$	6.011	< −0.3485	> 7.886	optim	optim
Together	$\log_{10}(k_2')$	−1.905	−1.98	−1.84	approximate	approximate
Together	$\log_{10}(k_{deg})$	−1.855	−1.995	−1.71	approximate	approximate
Together	$\log_{10}(k_1'/k_2)$	0.1311	0.06485	0.1988	approximate	approximate
Together	$\log_{10}(k_1'k_2'/k_2)$	−1.774	−1.906	−1.621	approximate	approximate
Together	$\log_{10}(k_2'/k_2)$	−7.917	< −9.792	> −1.516	optim	optim
b1	$\log_{10}(k_1')$	−1.554	−1.615	−1.466	approximate	approximate
b1	$\log_{10}(k_2)$	−2.114	−2.21	−2.082	approximate	approximate
b1	$\log_{10}(k_2')$	5.172	< 0.2521	> 9.888	optim	optim
b1	$\log_{10}(k_{deg})$	5.44	< 0.5193	> 10.16	optim	optim
b1	$\log_{10}(k_1'/k_2)$	0.5605	0.4944	0.6217	approximate	approximate
b1	$\log_{10}(k_1'k_2'/k_2)$	5.732	< 0.8119	> 10.45	optim	optim
b1	$\log_{10}(k_2'/k_2)$	7.286	< 2.365	> 12	optim	optim
b2	$\log_{10}(k_1'k_2')$	−2.805	−3.058	−2.303	approximate	approximate
b2	$\log_{10}(k_2 \text{ or } k_{deg})$	−1.109	−2.063	−0.4999	approximate	approximate
b2	$\log_{10}(k_2 \text{ or } k_{deg})$	−1.892	−2.063	−0.4999	approximate	approximate
b3	$\log_{10}(k_1')$	−0.745	−0.9495	−0.4219	approximate	approximate
b3	$\log_{10}(k_2)$	−0.8853	−1.1	−0.5515	approximate	approximate
b3	$\log_{10}(k_2')$	−1.828	−2.045	−1.534	approximate	approximate
b3	$\log_{10}(k_{deg})$	−1.622	−1.894	−1.273	approximate	approximate
b3	$\log_{10}(k_1'/k_2)$	0.1403	0.08057	0.2003	approximate	approximate
b3	$\log_{10}(k_1'k_2'/k_2)$	−1.687	−1.902	−1.391	approximate	approximate
b3	$\log_{10}(k_2'/k_2)$	−0.9422	−1.387	−0.5095	approximate	approximate

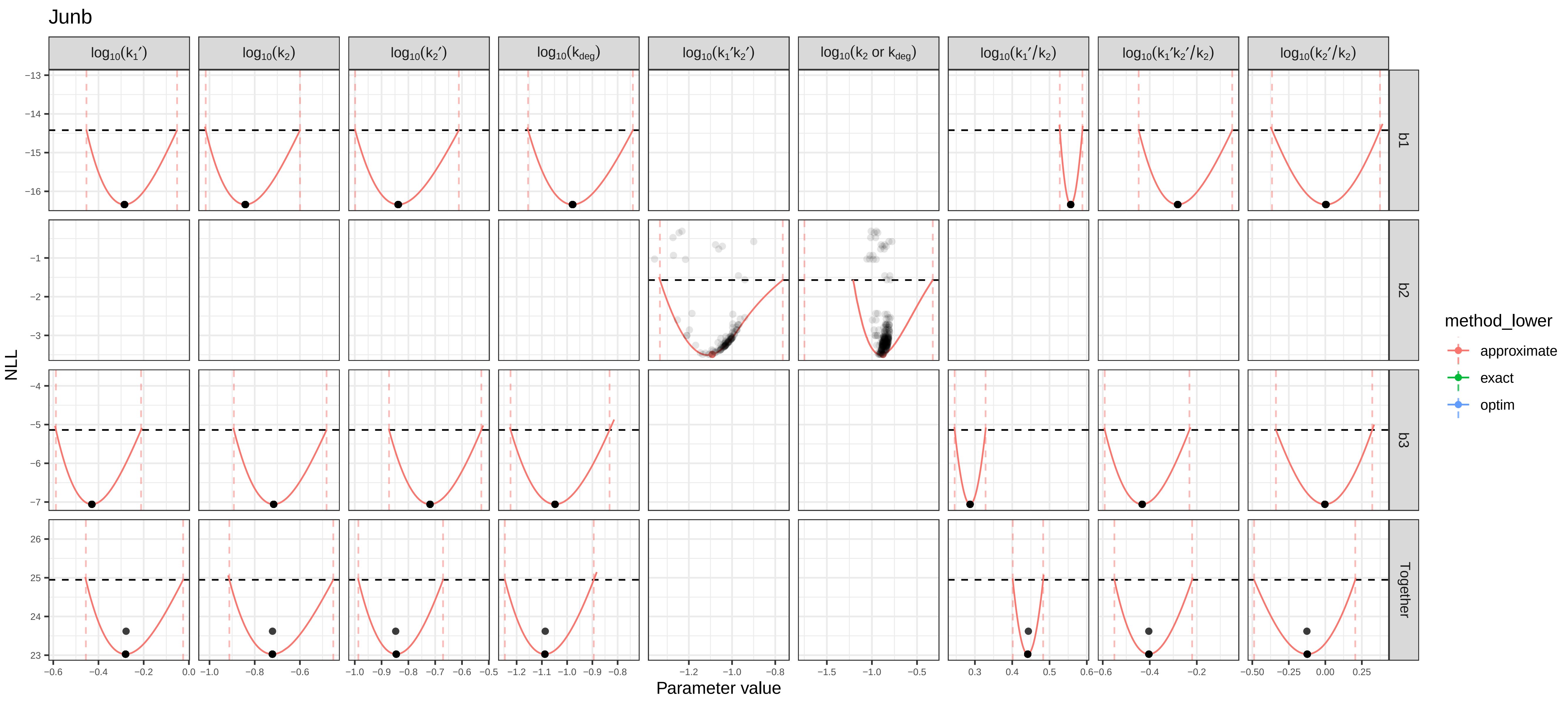

Replicate	Par	Best value	CI95 LB	CI95 UB	Method LB	Method UB
Together	$\log_{10}(k_1')$	−0.2804	−0.4558	−0.02552	approximate	approximate
Together	$\log_{10}(k_2)$	−0.7221	−0.9123	−0.4524	approximate	approximate
Together	$\log_{10}(k_2')$	−0.8452	−0.9867	−0.6706	approximate	approximate
Together	$\log_{10}(k_{deg})$	−1.088	−1.246	−0.8945	approximate	approximate
Together	$\log_{10}(k_1'/k_2)$	0.4416	0.4015	0.483	approximate	approximate
Together	$\log_{10}(k_1'k_2'/k_2)$	−0.4036	−0.5505	−0.2198	approximate	approximate
Together	$\log_{10}(k_2'/k_2)$	−0.1232	−0.4875	0.205	approximate	approximate
b1	$\log_{10}(k_1')$	−0.2849	−0.4532	−0.05222	approximate	approximate
b1	$\log_{10}(k_2)$	−0.8417	−1.017	−0.5995	approximate	approximate
b1	$\log_{10}(k_2')$	−0.838	−0.9986	−0.6117	approximate	approximate
b1	$\log_{10}(k_{deg})$	−0.9784	−1.155	−0.7396	approximate	approximate
b1	$\log_{10}(k_1'/k_2)$	0.5568	0.5275	0.5882	approximate	approximate
b1	$\log_{10}(k_1'k_2'/k_2)$	−0.2812	−0.4471	−0.04927	approximate	approximate
b1	$\log_{10}(k_2'/k_2)$	0.003697	−0.3649	0.3742	approximate	approximate
b2	$\log_{10}(k_1'k_2')$	−1.092	−1.334	−0.765	approximate	approximate
b2	$\log_{10}(k_2 \text{ or } k_{deg})$	−0.8771	−1.748	−0.325	approximate	approximate
b2	$\log_{10}(k_2 \text{ or } k_{deg})$	−0.9006	−1.748	−0.325	approximate	approximate
b3	$\log_{10}(k_1')$	−0.429	−0.5882	−0.2116	approximate	approximate
b3	$\log_{10}(k_2)$	−0.716	−0.8923	−0.4821	approximate	approximate
b3	$\log_{10}(k_2')$	−0.7193	−0.8722	−0.5278	approximate	approximate
b3	$\log_{10}(k_{deg})$	−1.048	−1.224	−0.8319	approximate	approximate
b3	$\log_{10}(k_1'/k_2)$	0.287	0.2457	0.3286	approximate	approximate
b3	$\log_{10}(k_1'k_2'/k_2)$	−0.4323	−0.5914	−0.2313	approximate	approximate
b3	$\log_{10}(k_2'/k_2)$	−0.003237	−0.3381	0.3213	approximate	approximate

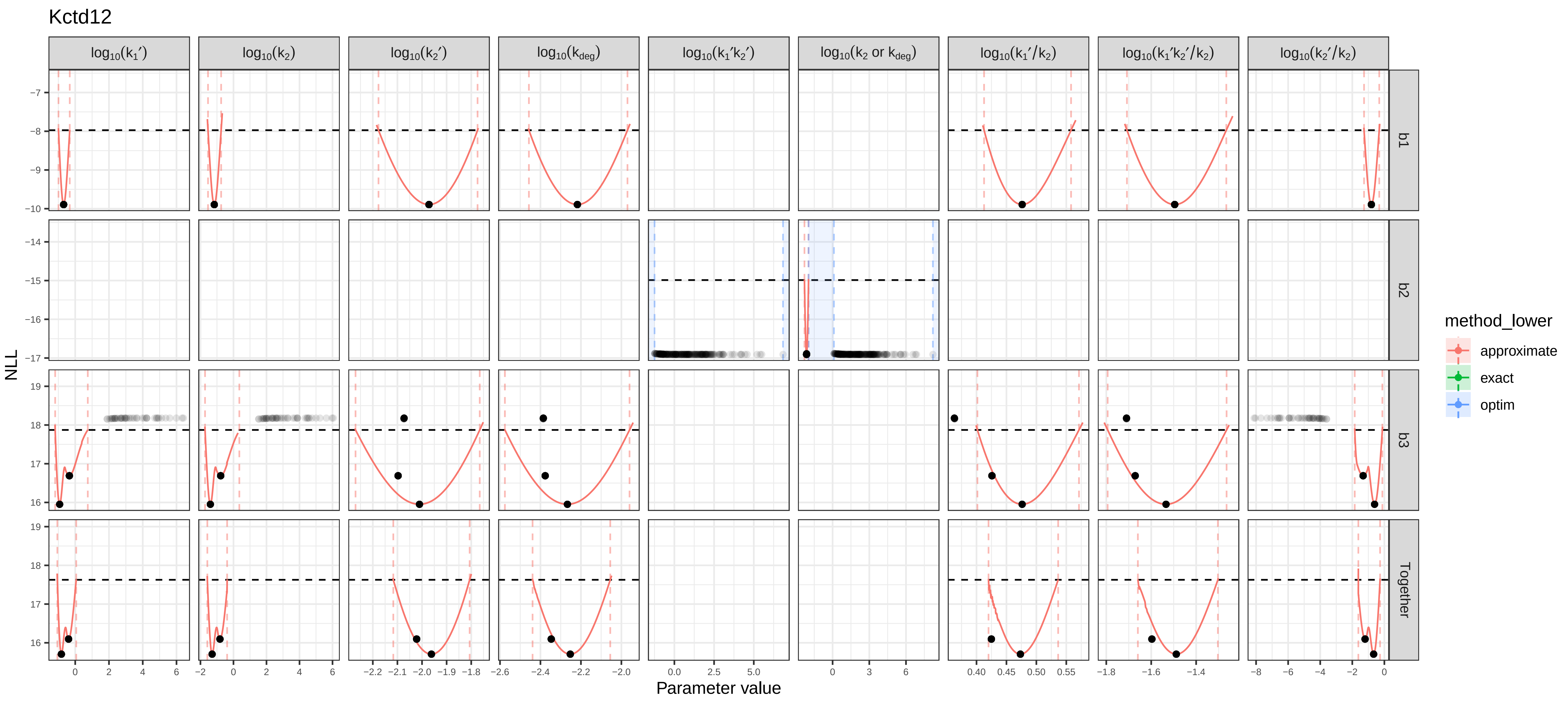

Replicate	Par	Best value	CI95 LB	CI95 UB	Method LB	Method UB
Together	$\log_{10}(k_1')$	−0.8198	−1.059	0.05956	approximate	approximate
Together	$\log_{10}(k_2)$	−1.293	−1.583	−0.3848	approximate	approximate
Together	$\log_{10}(k_2')$	−1.962	−2.117	−1.806	approximate	approximate
Together	$\log_{10}(k_{deg})$	−2.252	−2.439	−2.055	approximate	approximate
Together	$\log_{10}(k_1'/k_2)$	0.4734	0.4201	0.5364	approximate	approximate
Together	$\log_{10}(k_1'k_2'/k_2)$	−1.488	−1.659	−1.303	approximate	approximate
Together	$\log_{10}(k_2'/k_2)$	−0.6685	−1.623	−0.2674	approximate	approximate
b1	$\log_{10}(k_1')$	−0.6856	−0.9907	−0.3239	approximate	approximate
b1	$\log_{10}(k_2)$	−1.162	−1.542	−0.7468	approximate	approximate
b1	$\log_{10}(k_2')$	−1.972	−2.177	−1.774	approximate	approximate
b1	$\log_{10}(k_{deg})$	−2.218	−2.457	−1.97	approximate	approximate
b1	$\log_{10}(k_1'/k_2)$	0.4763	0.4126	0.558	approximate	approximate
b1	$\log_{10}(k_1'k_2'/k_2)$	−1.495	−1.708	−1.266	approximate	approximate
b1	$\log_{10}(k_2'/k_2)$	−0.8097	−1.267	−0.3196	approximate	approximate
b2	$\log_{10}(k_1'k_2')$	2.318	< -1.247	> 6.84	optim	optim
b2	$\log_{10}(k_2 \text{ or } k_{deg})$	3.679	0.1104	> 8.202	optim	optim
b2	$\log_{10}(k_2 \text{ or } k_{deg})$	−2.132	−2.303	−1.97	approximate	approximate
b3	$\log_{10}(k_1')$	−0.9232	−1.188	0.7445	approximate	approximate
b3	$\log_{10}(k_2)$	−1.4	−1.722	0.3548	approximate	approximate
b3	$\log_{10}(k_2')$	−2.01	−2.27	−1.765	approximate	approximate
b3	$\log_{10}(k_{deg})$	−2.267	−2.576	−1.96	approximate	approximate
b3	$\log_{10}(k_1'/k_2)$	0.4764	0.4015	0.5712	approximate	approximate
b3	$\log_{10}(k_1'k_2'/k_2)$	−1.534	−1.794	−1.264	approximate	approximate
b3	$\log_{10}(k_2'/k_2)$	−0.6108	−1.847	−0.1251	approximate	approximate

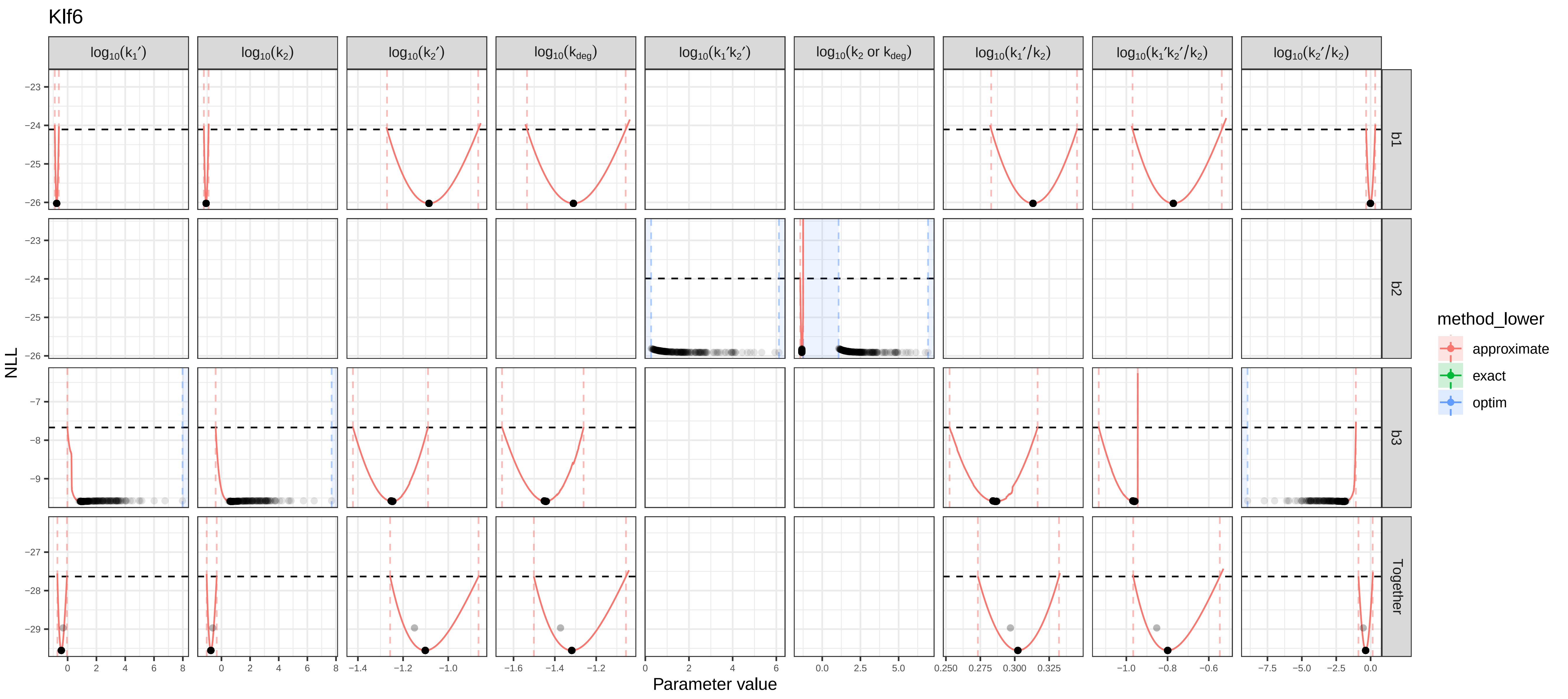

Replicate	Par	Best value	CI95 LB	CI95 UB	Method LB	Method UB
Together	$\log_{10}(k_1')$	−0.4367	−0.7109	−0.04728	approximate	approximate
Together	$\log_{10}(k_2)$	−0.7389	−1.032	−0.3307	approximate	approximate
Together	$\log_{10}(k_2')$	−1.102	−1.257	−0.8659	approximate	approximate
Together	$\log_{10}(k_{deg})$	−1.318	−1.502	−1.056	approximate	approximate
Together	$\log_{10}(k_1'/k_2)$	0.3022	0.2732	0.3321	approximate	approximate
Together	$\log_{10}(k_1'k_2'/k_2)$	−0.7996	−0.9666	−0.5466	approximate	approximate
Together	$\log_{10}(k_2'/k_2)$	−0.3629	−0.8844	0.1506	approximate	approximate
b1	$\log_{10}(k_1')$	−0.7587	−0.8912	−0.6075	approximate	approximate
b1	$\log_{10}(k_2)$	−1.072	−1.228	−0.9002	approximate	approximate
b1	$\log_{10}(k_2')$	−1.085	−1.271	−0.8673	approximate	approximate
b1	$\log_{10}(k_{deg})$	−1.31	−1.535	−1.057	approximate	approximate
b1	$\log_{10}(k_1'/k_2)$	0.3133	0.2829	0.3452	approximate	approximate
b1	$\log_{10}(k_1'k_2'/k_2)$	−0.772	−0.9697	−0.537	approximate	approximate
b1	$\log_{10}(k_2'/k_2)$	−0.01323	−0.3279	0.3319	approximate	approximate
b2	$\log_{10}(k_1'k_2')$	3.973	< 0.2674	> 6.12	optim	optim
b2	$\log_{10}(k_2 \text{ or } k_{deg})$	4.78	1.072	> 6.927	optim	optim
b2	$\log_{10}(k_2 \text{ or } k_{deg})$	−1.332	−1.439	−1.251	approximate	approximate
b3	$\log_{10}(k_1')$	1.044	−0.01669	> 7.978	approximate	optim
b3	$\log_{10}(k_2)$	0.7577	−0.4072	> 7.693	approximate	optim
b3	$\log_{10}(k_2')$	−1.248	−1.422	−1.09	approximate	approximate
b3	$\log_{10}(k_{deg})$	−1.445	−1.656	−1.261	approximate	approximate
b3	$\log_{10}(k_1'/k_2)$	0.286	0.2526	0.3166	approximate	approximate
b3	$\log_{10}(k_1'k_2'/k_2)$	−0.9616	−1.134	−0.9446	approximate	approximate
b3	$\log_{10}(k_2'/k_2)$	−2.005	< −8.946	−1.072	optim	approximate

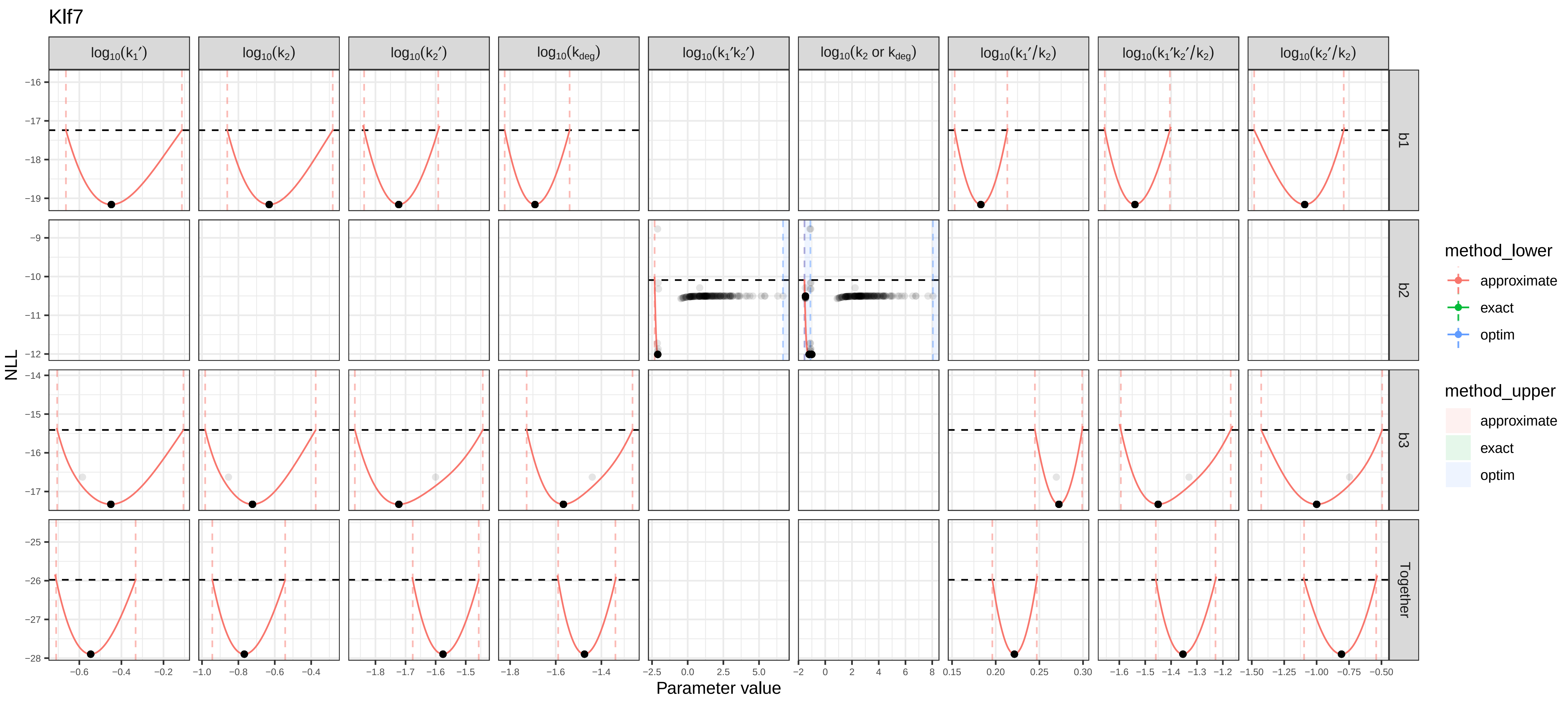

Replicate	Par	Best value	CI95 LB	CI95 UB	Method LB	Method UB
Together	$\log_{10}(k_1')$	−0.546	−0.7102	−0.3325	approximate	approximate
Together	$\log_{10}(k_2)$	−0.7674	−0.9433	−0.5428	approximate	approximate
Together	$\log_{10}(k_2')$	−1.576	−1.676	−1.457	approximate	approximate
Together	$\log_{10}(k_{deg})$	−1.474	−1.589	−1.338	approximate	approximate
Together	$\log_{10}(k_1'/k_2)$	0.2214	0.1961	0.2471	approximate	approximate
Together	$\log_{10}(k_1'k_2'/k_2)$	−1.354	−1.459	−1.228	approximate	approximate
Together	$\log_{10}(k_2'/k_2)$	−0.8081	−1.097	−0.5406	approximate	approximate
b1	$\log_{10}(k_1')$	−0.4473	−0.6637	−0.1125	approximate	approximate
b1	$\log_{10}(k_2)$	−0.6303	−0.8611	−0.2815	approximate	approximate
b1	$\log_{10}(k_2')$	−1.723	−1.838	−1.591	approximate	approximate
b1	$\log_{10}(k_{deg})$	−1.691	−1.824	−1.539	approximate	approximate
b1	$\log_{10}(k_1'/k_2)$	0.183	0.153	0.2133	approximate	approximate
b1	$\log_{10}(k_1'k_2'/k_2)$	−1.54	−1.656	−1.405	approximate	approximate
b1	$\log_{10}(k_2'/k_2)$	−1.092	−1.482	−0.7909	approximate	approximate
b2	$\log_{10}(k_1'k_2')$	−2.096	−2.318	> 6.682	approximate	optim
b2	$\log_{10}(k_2 \text{ or } k_{deg})$	−1.003	−1.551	> 8.052	approximate	optim
b2	$\log_{10}(k_2 \text{ or } k_{deg})$	−1.204	−1.551	−1.109	approximate	optim
b3	$\log_{10}(k_1')$	−0.4502	−0.7052	−0.1052	approximate	approximate
b3	$\log_{10}(k_2)$	−0.7226	−0.9825	−0.375	approximate	approximate
b3	$\log_{10}(k_2')$	−1.722	−1.868	−1.443	approximate	approximate
b3	$\log_{10}(k_{deg})$	−1.566	−1.727	−1.264	approximate	approximate
b3	$\log_{10}(k_1'/k_2)$	0.2724	0.2449	0.299	approximate	approximate
b3	$\log_{10}(k_1'k_2'/k_2)$	−1.45	−1.594	−1.17	approximate	approximate
b3	$\log_{10}(k_2'/k_2)$	−0.9997	−1.427	−0.4945	approximate	approximate

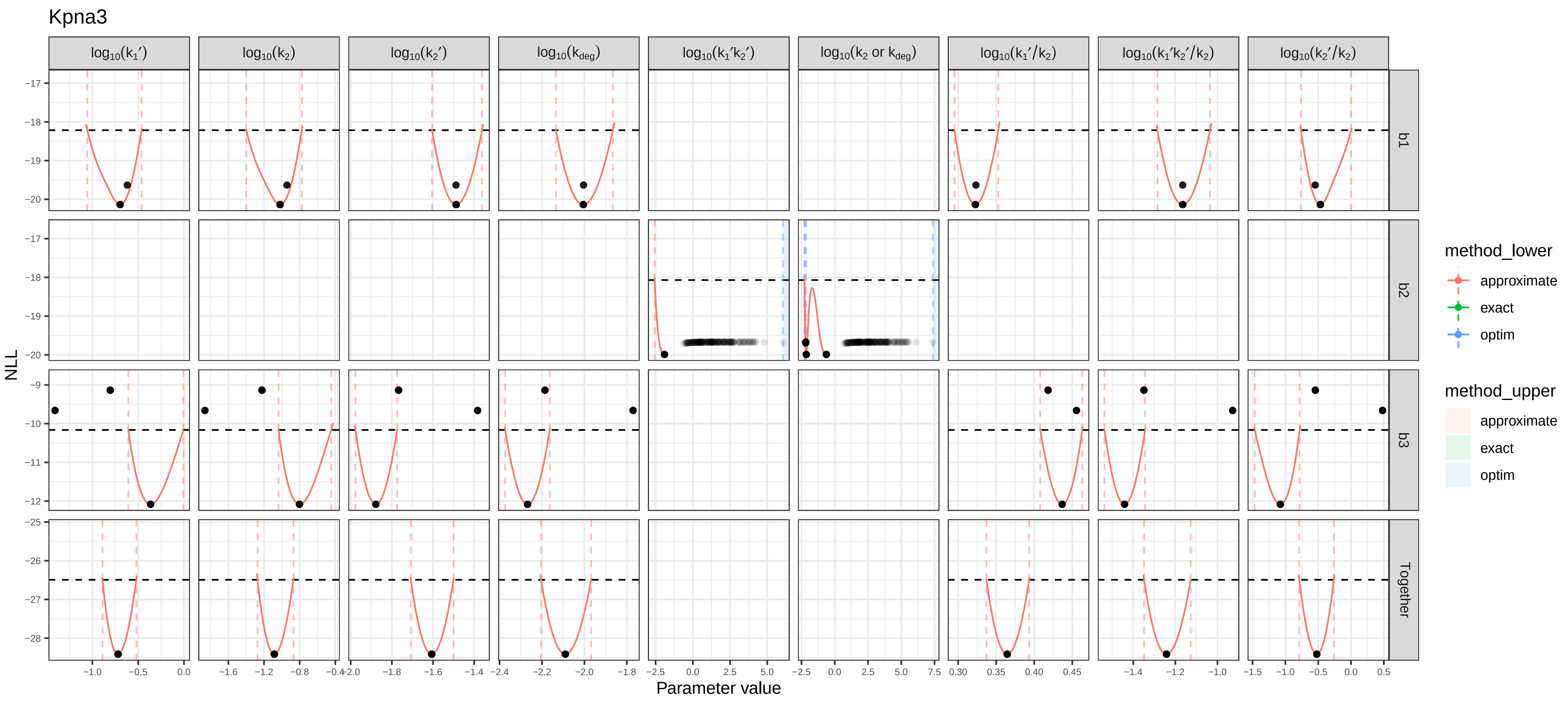

Replicate	Par	Best value	CI95 LB	CI95 UB	Method LB	Method UB
Together	$\log_{10}(k_1')$	−0.72	−0.8898	−0.5199	approximate	approximate
Together	$\log_{10}(k_2)$	−1.084	−1.273	−0.8697	approximate	approximate
Together	$\log_{10}(k_2')$	−1.606	−1.708	−1.5	approximate	approximate
Together	$\log_{10}(k_{deg})$	−2.09	−2.204	−1.968	approximate	approximate
Together	$\log_{10}(k_1'/k_2)$	0.3645	0.3372	0.3934	approximate	approximate
Together	$\log_{10}(k_1'k_2'/k_2)$	−1.242	−1.348	−1.127	approximate	approximate
Together	$\log_{10}(k_2'/k_2)$	−0.5219	−0.7891	−0.2624	approximate	approximate
b1	$\log_{10}(k_1')$	−0.6973	−1.056	−0.4642	approximate	approximate
b1	$\log_{10}(k_2)$	−1.02	−1.399	−0.7753	approximate	approximate
b1	$\log_{10}(k_2')$	−1.488	−1.605	−1.362	approximate	approximate
b1	$\log_{10}(k_{deg})$	−2.005	−2.135	−1.866	approximate	approximate
b1	$\log_{10}(k_1'/k_2)$	0.3226	0.2952	0.3528	approximate	approximate
b1	$\log_{10}(k_1'k_2'/k_2)$	−1.165	−1.285	−1.035	approximate	approximate
b1	$\log_{10}(k_2'/k_2)$	−0.4676	−0.7627	0.002517	approximate	approximate
b2	$\log_{10}(k_1'k_2')$	−1.91	−2.564	> 6.057	approximate	optim
b2	$\log_{10}(k_2 \text{ or } k_{deg})$	−0.6317	−2.274	> 7.375	approximate	optim
b2	$\log_{10}(k_2 \text{ or } k_{deg})$	−2.141	−2.274	−2.141	approximate	optim
b3	$\log_{10}(k_1')$	−0.3657	−0.6077	−0.005775	approximate	approximate
b3	$\log_{10}(k_2)$	−0.8024	−1.037	−0.4467	approximate	approximate
b3	$\log_{10}(k_2')$	−1.878	−1.977	−1.774	approximate	approximate
b3	$\log_{10}(k_{deg})$	−2.268	−2.374	−2.163	approximate	approximate
b3	$\log_{10}(k_1'/k_2)$	0.4368	0.4079	0.4632	approximate	approximate
b3	$\log_{10}(k_1'k_2'/k_2)$	−1.441	−1.537	−1.344	approximate	approximate
b3	$\log_{10}(k_2'/k_2)$	−1.075	−1.467	−0.7819	approximate	approximate

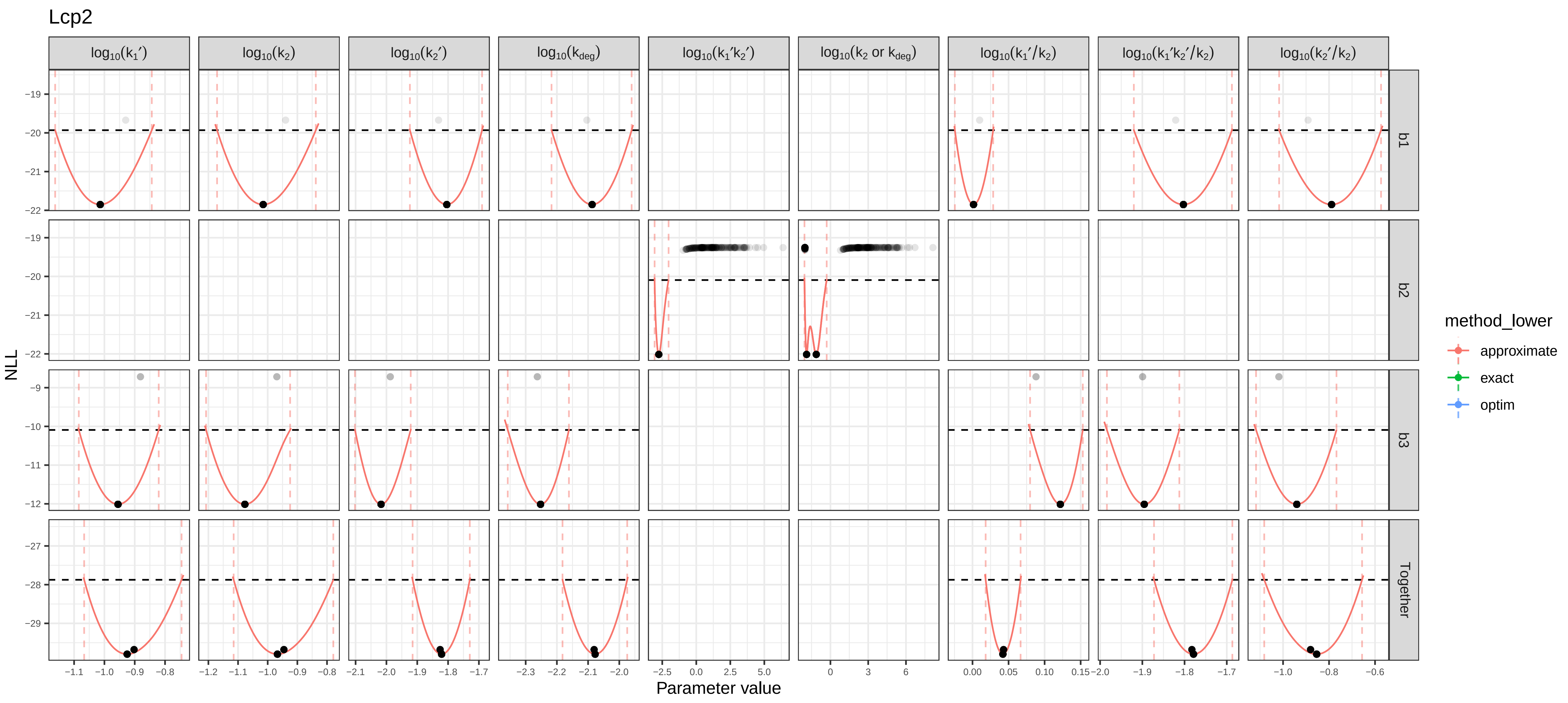

Replicate	Par	**Best value**	**CI95 LB**	**CI95 UB**	**Method LB**	**Method UB**
Together	$\log_{10}(k_1')$	−0.925	−1.067	−0.7454	approximate	approximate
Together	$\log_{10}(k_2)$	−0.9672	−1.115	−0.7788	approximate	approximate
Together	$\log_{10}(k_2')$	−1.821	−1.915	−1.73	approximate	approximate
Together	$\log_{10}(k_{deg})$	−2.077	−2.182	−1.975	approximate	approximate
Together	$\log_{10}(k_1'/k_2)$	0.04219	0.01822	0.0667	approximate	approximate
Together	$\log_{10}(k_1'k_2'/k_2)$	−1.779	−1.872	−1.687	approximate	approximate
Together	$\log_{10}(k_2'/k_2)$	−0.8537	−1.082	−0.6563	approximate	approximate
b1	$\log_{10}(k_1')$	−1.014	−1.162	−0.8437	approximate	approximate
b1	$\log_{10}(k_2)$	−1.015	−1.171	−0.8378	approximate	approximate
b1	$\log_{10}(k_2')$	−1.804	−1.924	−1.69	approximate	approximate
b1	$\log_{10}(k_{deg})$	−2.087	−2.217	−1.96	approximate	approximate
b1	$\log_{10}(k_1'/k_2)$	0.001443	−0.02449	0.02865	approximate	approximate
b1	$\log_{10}(k_1'k_2'/k_2)$	−1.803	−1.92	−1.688	approximate	approximate
b1	$\log_{10}(k_2'/k_2)$	−0.7889	−1.017	−0.5738	approximate	approximate
b2	$\log_{10}(k_1'k_2')$	−2.755	−3.062	−2.034	approximate	approximate
b2	$\log_{10}(k_2 \text{ or } k_{deg})$	−1.127	−2.061	−0.2958	approximate	approximate
b2	$\log_{10}(k_2 \text{ or } k_{deg})$	−1.894	−2.061	−0.2958	approximate	approximate
b3	$\log_{10}(k_1')$	−0.9551	−1.084	−0.8209	approximate	approximate
b3	$\log_{10}(k_2)$	−1.077	−1.208	−0.9244	approximate	approximate
b3	$\log_{10}(k_2')$	−2.017	−2.102	−1.921	approximate	approximate
b3	$\log_{10}(k_{deg})$	−2.252	−2.357	−2.161	approximate	approximate
b3	$\log_{10}(k_1'/k_2)$	0.1218	0.07986	0.153	approximate	approximate
b3	$\log_{10}(k_1'k_2'/k_2)$	−1.895	−1.984	−1.812	approximate	approximate
b3	$\log_{10}(k_2'/k_2)$	−0.9403	−1.117	−0.7676	approximate	approximate

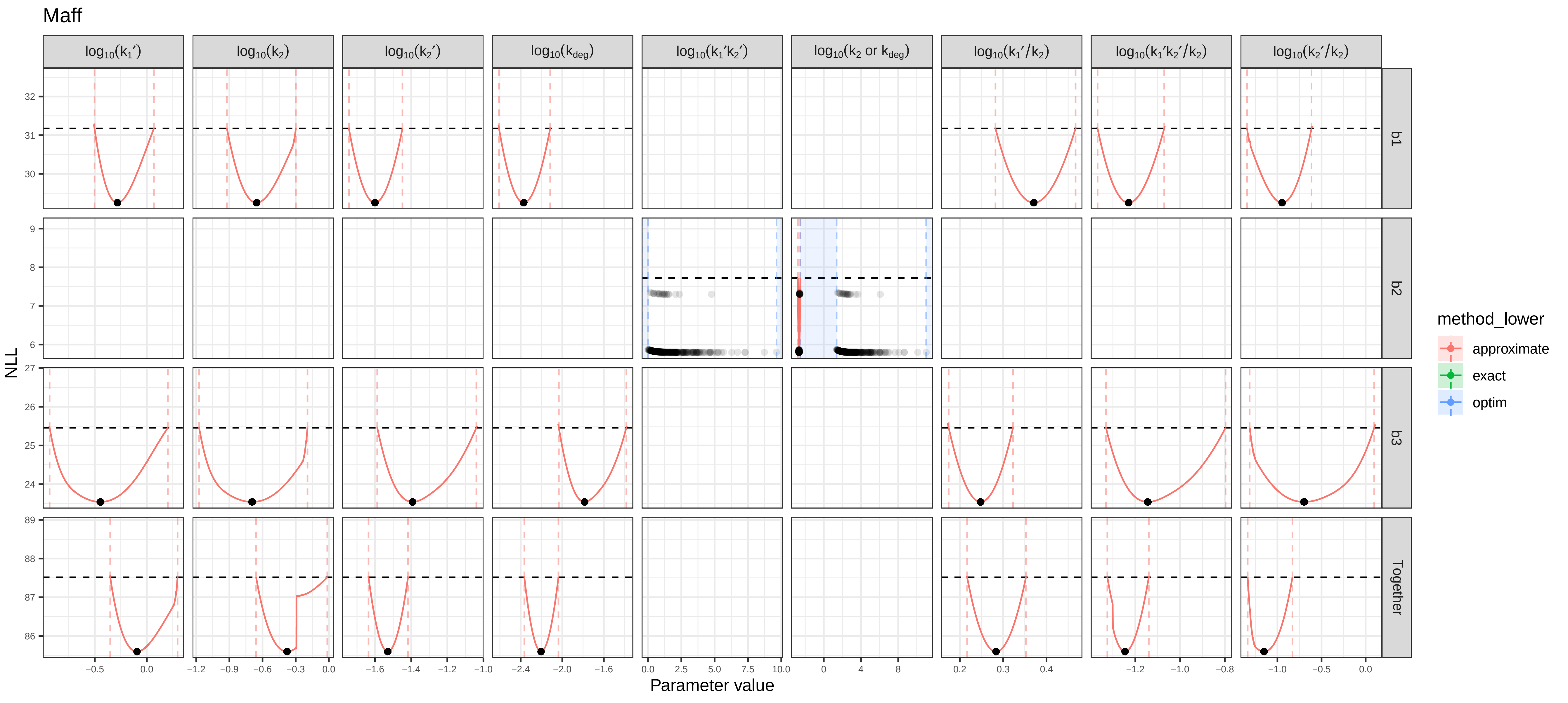

Replicate	Par	Best value	CI95 LB	CI95 UB	Method LB	Method UB
Together	$\log_{10}(k_1')$	−0.09446	−0.3519	0.2952	approximate	approximate
Together	$\log_{10}(k_2)$	−0.378	−0.6577	−0.01395	approximate	approximate
Together	$\log_{10}(k_2')$	−1.529	−1.636	−1.418	approximate	approximate
Together	$\log_{10}(k_{deg})$	−2.204	−2.365	−2.037	approximate	approximate
Together	$\log_{10}(k_1'/k_2)$	0.2836	0.2169	0.3526	approximate	approximate
Together	$\log_{10}(k_1'k_2'/k_2)$	−1.246	−1.325	−1.14	approximate	approximate
Together	$\log_{10}(k_2'/k_2)$	−1.151	−1.337	−0.8297	approximate	approximate
b1	$\log_{10}(k_1')$	−0.2827	−0.5029	0.06774	approximate	approximate
b1	$\log_{10}(k_2)$	−0.6535	−0.9221	−0.2988	approximate	approximate
b1	$\log_{10}(k_2')$	−1.601	−1.745	−1.448	approximate	approximate
b1	$\log_{10}(k_{deg})$	−2.37	−2.609	−2.116	approximate	approximate
b1	$\log_{10}(k_1'/k_2)$	0.3708	0.2823	0.4671	approximate	approximate
b1	$\log_{10}(k_1'k_2'/k_2)$	−1.23	−1.369	−1.071	approximate	approximate
b1	$\log_{10}(k_2'/k_2)$	−0.9471	−1.345	−0.6136	approximate	approximate
b2	$\log_{10}(k_1'k_2')$	3.241	< 0.01039	> 9.641	optim	optim
b2	$\log_{10}(k_2 \text{ or } k_{deg})$	4.609	1.378	> 11.01	optim	optim
b2	$\log_{10}(k_2 \text{ or } k_{deg})$	−2.661	−2.788	−2.521	approximate	approximate
b3	$\log_{10}(k_1')$	−0.446	−0.9346	0.2018	approximate	approximate
b3	$\log_{10}(k_2)$	−0.6942	−1.174	−0.193	approximate	approximate
b3	$\log_{10}(k_2')$	−1.392	−1.588	−1.039	approximate	approximate
b3	$\log_{10}(k_{deg})$	−1.785	−2.033	−1.383	approximate	approximate
b3	$\log_{10}(k_1'/k_2)$	0.2482	0.1743	0.3229	approximate	approximate
b3	$\log_{10}(k_1'k_2'/k_2)$	−1.144	−1.331	−0.7968	approximate	approximate
b3	$\log_{10}(k_2'/k_2)$	−0.6981	−1.315	0.09699	approximate	approximate

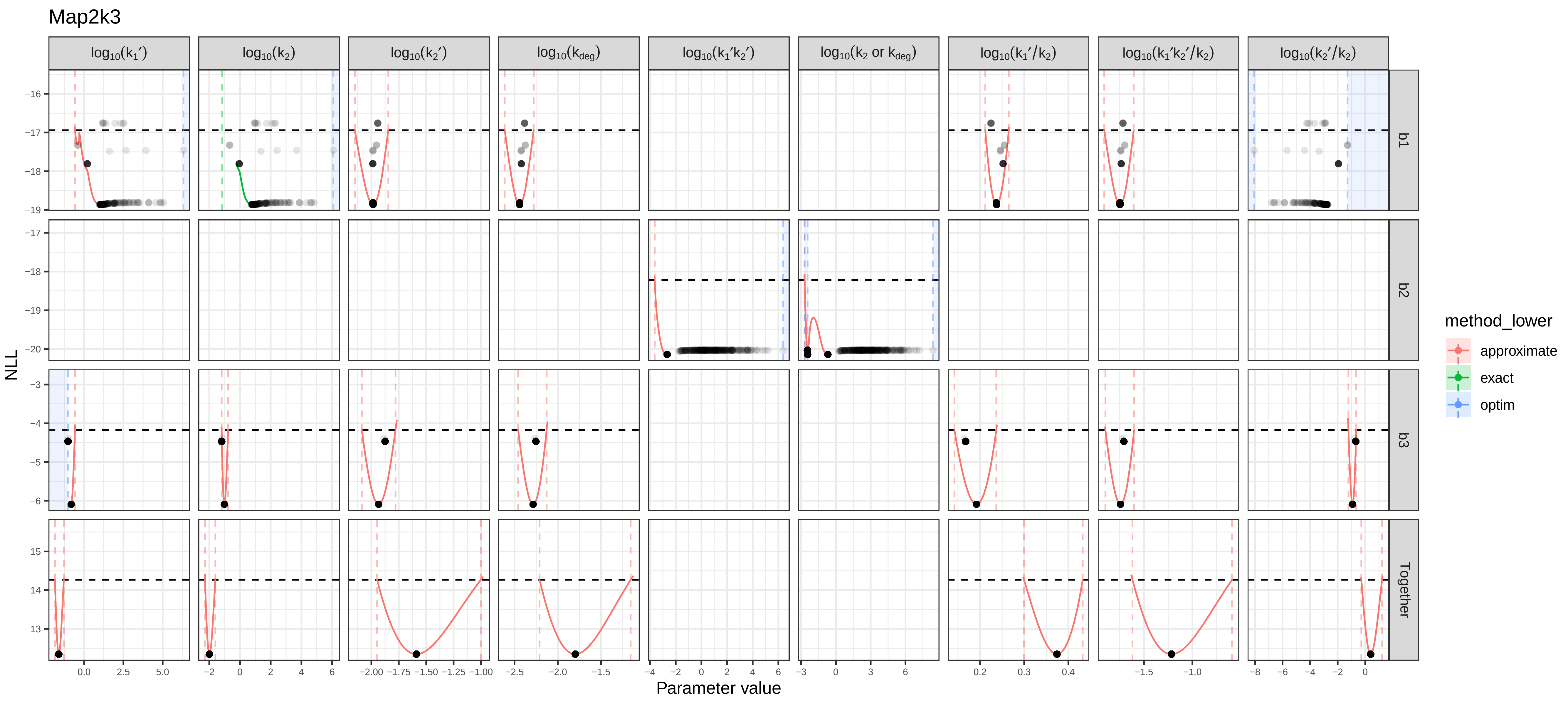

Replicate	Par	Best value	CI95 LB	CI95 UB	Method LB	Method UB
Together	$\log_{10}(k_1')$	−1.613	−1.854	−1.296	approximate	approximate
Together	$\log_{10}(k_2)$	−1.987	−2.273	−1.602	approximate	approximate
Together	$\log_{10}(k_2')$	−1.589	−1.947	−1.002	approximate	approximate
Together	$\log_{10}(k_{deg})$	−1.798	−2.211	−1.159	approximate	approximate
Together	$\log_{10}(k_1'/k_2)$	0.3741	0.2994	0.4325	approximate	approximate
Together	$\log_{10}(k_1'k_2'/k_2)$	−1.215	−1.618	−0.5909	approximate	approximate
Together	$\log_{10}(k_2'/k_2)$	0.3973	−0.2834	1.224	approximate	approximate
b1	$\log_{10}(k_1')$	1.053	−0.5797	> 6.326	approximate	optim
b1	$\log_{10}(k_2)$	0.8158	−1.152	> 6.08	exact	optim
b1	$\log_{10}(k_2')$	−1.985	−2.151	−1.846	approximate	approximate
b1	$\log_{10}(k_{deg})$	−2.441	−2.613	−2.279	approximate	approximate
b1	$\log_{10}(k_1'/k_2)$	0.2375	0.2119	0.2651	approximate	approximate
b1	$\log_{10}(k_1'k_2'/k_2)$	−1.747	−1.91	−1.606	approximate	approximate
b1	$\log_{10}(k_2'/k_2)$	−2.8	< −8.065	> −1.284	optim	optim
b2	$\log_{10}(k_1'k_2')$	−2.668	−3.637	> 6.364	approximate	optim
b2	$\log_{10}(k_2 \text{ or } k_{deg})$	−0.6899	−2.696	> 8.364	approximate	optim
b2	$\log_{10}(k_2 \text{ or } k_{deg})$	−2.436	−2.696	−2.435	approximate	optim
b3	$\log_{10}(k_1')$	−0.8223	< −1.024	−0.5859	optim	approximate
b3	$\log_{10}(k_2)$	−1.014	−1.192	−0.7752	approximate	approximate
b3	$\log_{10}(k_2')$	−1.934	−2.087	−1.78	approximate	approximate
b3	$\log_{10}(k_{deg})$	−2.285	−2.458	−2.129	approximate	approximate
b3	$\log_{10}(k_1'/k_2)$	0.192	0.142	0.2368	approximate	approximate
b3	$\log_{10}(k_1'k_2'/k_2)$	−1.742	−1.898	−1.601	approximate	approximate
b3	$\log_{10}(k_2'/k_2)$	−0.9196	−1.22	−0.6531	approximate	approximate

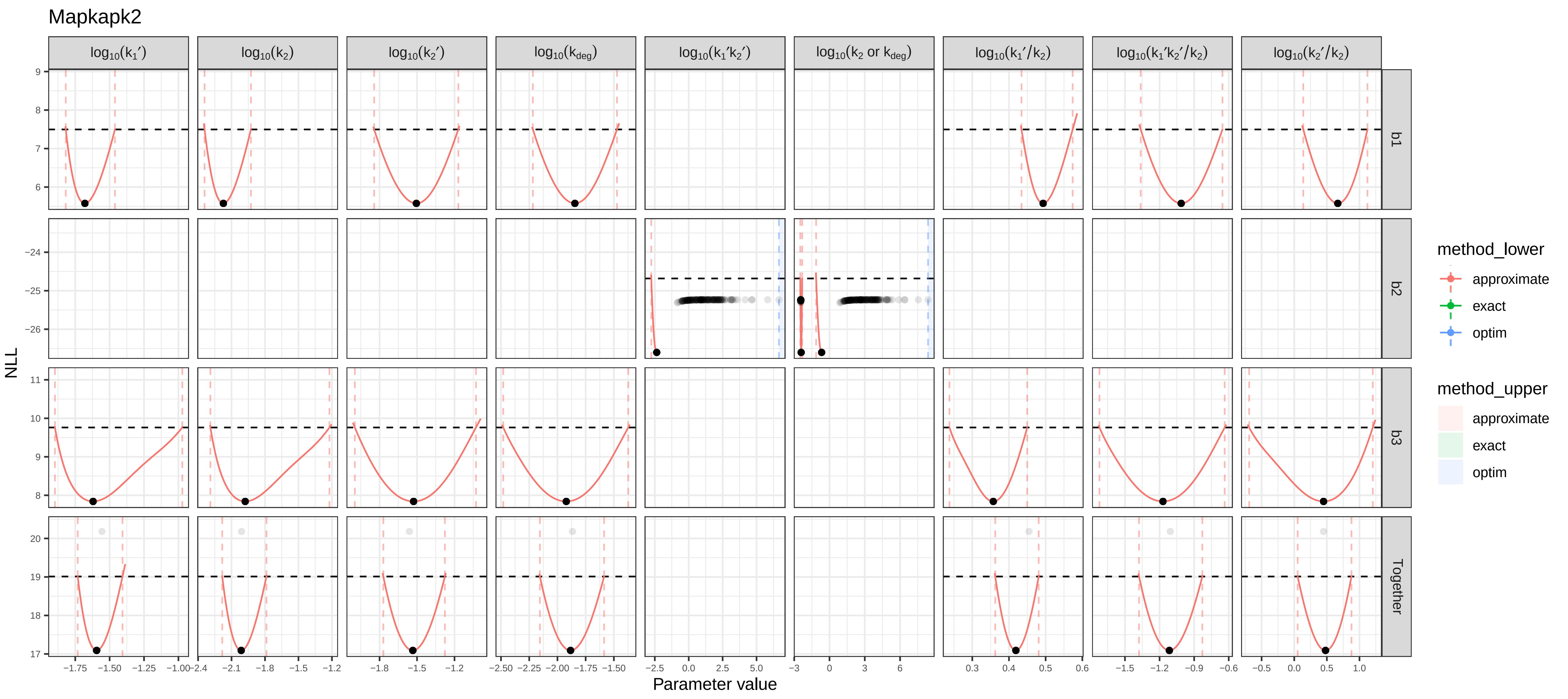

Replicate	Par	Best value	CI95 LB	CI95 UB	Method LB	Method UB
Together	$\log_{10}(k_1')$	−1.594	−1.732	−1.406	approximate	approximate
Together	$\log_{10}(k_2)$	−2.013	−2.183	−1.787	approximate	approximate
Together	$\log_{10}(k_2')$	−1.534	−1.77	−1.277	approximate	approximate
Together	$\log_{10}(k_{deg})$	−1.884	−2.155	−1.587	approximate	approximate
Together	$\log_{10}(k_1'/k_2)$	0.4188	0.3626	0.4811	approximate	approximate
Together	$\log_{10}(k_1'k_2'/k_2)$	−1.115	−1.378	−0.829	approximate	approximate
Together	$\log_{10}(k_2'/k_2)$	0.4795	0.05229	0.8772	approximate	approximate
b1	$\log_{10}(k_1')$	−1.68	−1.819	−1.461	approximate	approximate
b1	$\log_{10}(k_2)$	−2.173	−2.342	−1.926	approximate	approximate
b1	$\log_{10}(k_2')$	−1.505	−1.843	−1.17	approximate	approximate
b1	$\log_{10}(k_{deg})$	−1.845	−2.218	−1.472	approximate	approximate
b1	$\log_{10}(k_1'/k_2)$	0.4932	0.4342	0.5737	approximate	approximate
b1	$\log_{10}(k_1'k_2'/k_2)$	−1.012	−1.363	−0.6543	approximate	approximate
b1	$\log_{10}(k_2'/k_2)$	0.6677	0.1386	1.122	approximate	approximate
b2	$\log_{10}(k_1'k_2')$	−2.359	−2.761	> 6.65	approximate	optim
b2	$\log_{10}(k_2 \text{ or } k_{deg})$	−0.67	−1.137	> 8.377	approximate	optim
b2	$\log_{10}(k_2 \text{ or } k_{deg})$	−2.41	−2.487	−2.322	approximate	approximate
b3	$\log_{10}(k_1')$	−1.62	−1.898	−0.9717	approximate	approximate
b3	$\log_{10}(k_2)$	−1.978	−2.29	−1.222	approximate	approximate
b3	$\log_{10}(k_2')$	−1.528	−1.998	−1.028	approximate	approximate
b3	$\log_{10}(k_{deg})$	−1.922	−2.48	−1.373	approximate	approximate
b3	$\log_{10}(k_1'/k_2)$	0.3573	0.2377	0.4498	approximate	approximate
b3	$\log_{10}(k_1'k_2'/k_2)$	−1.17	−1.721	−0.6349	approximate	approximate
b3	$\log_{10}(k_2'/k_2)$	0.4499	−0.6915	1.204	approximate	approximate

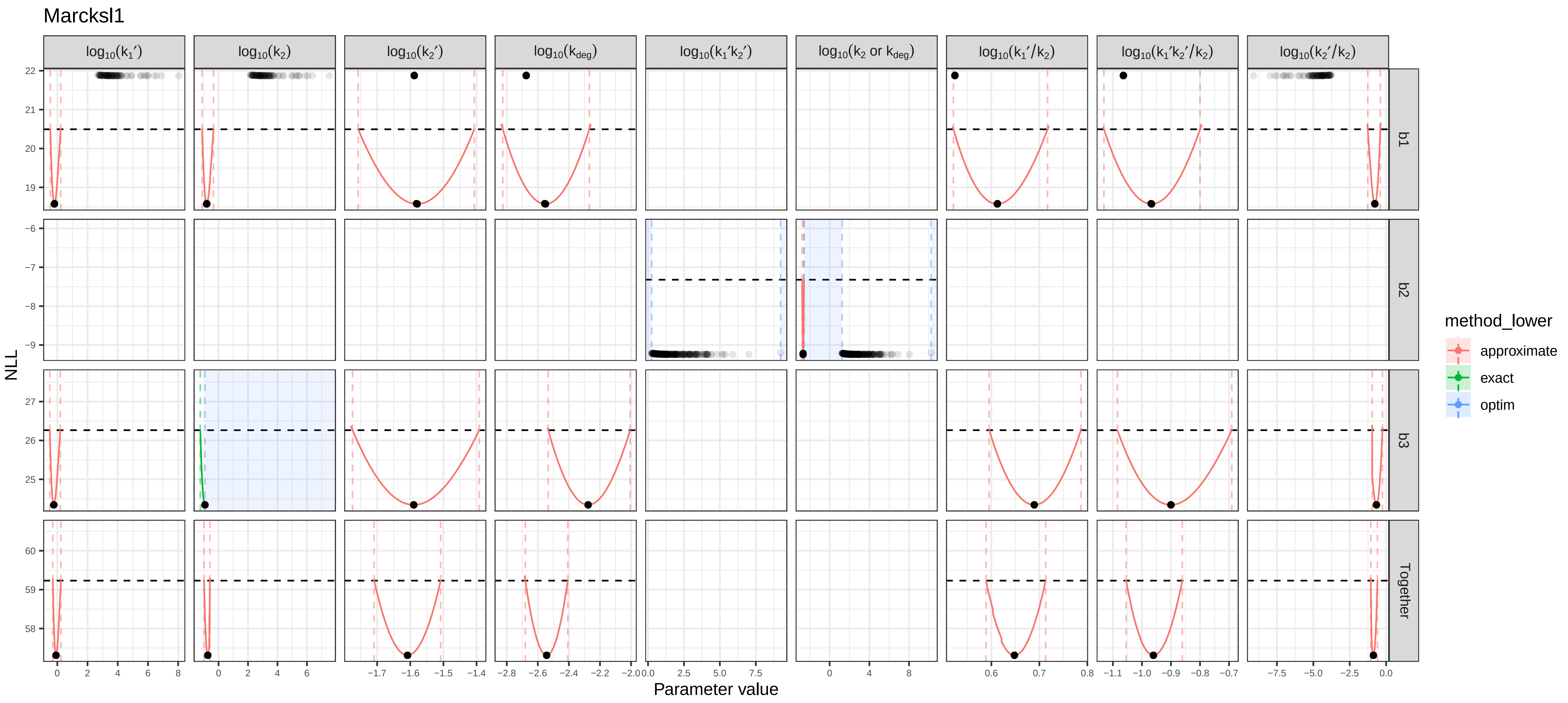

Replicate	Par	Best value	CI95 LB	CI95 UB	Method LB	Method UB
Together	$\log_{10}(k_1')$	−0.0857	−0.301	0.2372	approximate	approximate
Together	$\log_{10}(k_2)$	−0.7337	−0.9868	−0.5879	approximate	approximate
Together	$\log_{10}(k_2')$	−1.608	−1.709	−1.508	approximate	approximate
Together	$\log_{10}(k_{deg})$	−2.543	−2.681	−2.407	approximate	approximate
Together	$\log_{10}(k_1'/k_2)$	0.648	0.589	0.7132	approximate	approximate
Together	$\log_{10}(k_1'k_2'/k_2)$	−0.9605	−1.055	−0.861	approximate	approximate
Together	$\log_{10}(k_2'/k_2)$	−0.8748	−1.055	−0.5968	approximate	approximate
b1	$\log_{10}(k_1')$	−0.1936	−0.4643	0.2244	approximate	approximate
b1	$\log_{10}(k_2)$	−0.806	−1.116	−0.3409	approximate	approximate
b1	$\log_{10}(k_2')$	−1.579	−1.757	−1.407	approximate	approximate
b1	$\log_{10}(k_{deg})$	−2.551	−2.826	−2.268	approximate	approximate
b1	$\log_{10}(k_1'/k_2)$	0.6124	0.521	0.7171	approximate	approximate
b1	$\log_{10}(k_1'k_2'/k_2)$	−0.9669	−1.131	−0.7998	approximate	approximate
b1	$\log_{10}(k_2'/k_2)$	−0.7733	−1.262	−0.3994	approximate	approximate
b2	$\log_{10}(k_1'k_2')$	2.994	< 0.2531	> 9.232	optim	optim
b2	$\log_{10}(k_2 \text{ or } k_{deg})$	3.994	1.252	> 10.23	optim	optim
b2	$\log_{10}(k_2 \text{ or } k_{deg})$	−2.683	−2.777	−2.593	approximate	approximate
b3	$\log_{10}(k_1')$	−0.2347	−0.4971	0.1974	approximate	approximate
b3	$\log_{10}(k_2)$	−0.924	−1.241	> −0.9217	exact	optim
b3	$\log_{10}(k_2')$	−1.59	−1.774	−1.392	approximate	approximate
b3	$\log_{10}(k_{deg})$	−2.276	−2.533	−2.006	approximate	approximate
b3	$\log_{10}(k_1'/k_2)$	0.6894	0.5955	0.7867	approximate	approximate
b3	$\log_{10}(k_1'k_2'/k_2)$	−0.9002	−1.084	−0.6907	approximate	approximate
b3	$\log_{10}(k_2'/k_2)$	−0.6655	−0.958	−0.2544	approximate	approximate

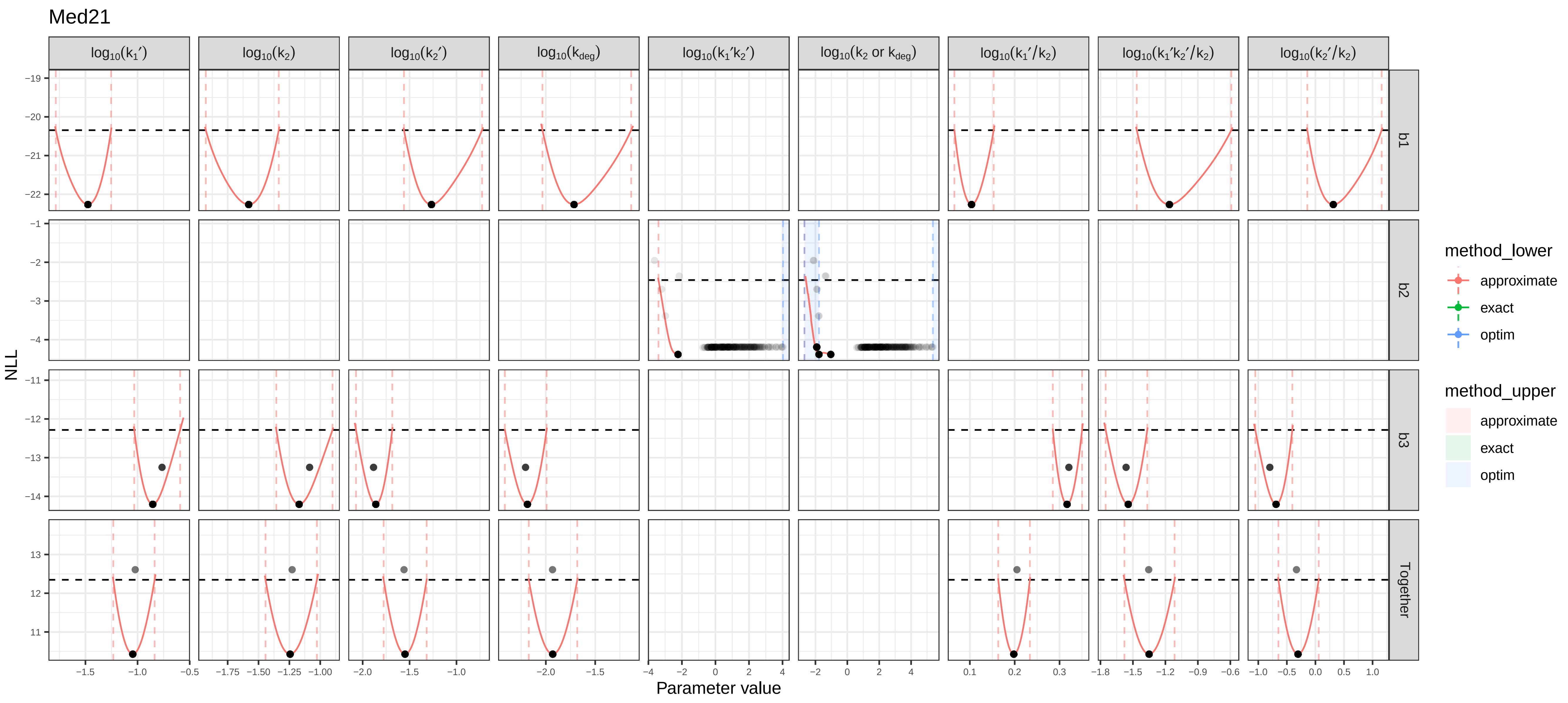

Replicate	Par	Best value	CI95 LB	CI95 UB	Method LB	Method UB
Together	$\log_{10}(k_1')$	−1.046	−1.233	−0.8365	approximate	approximate
Together	$\log_{10}(k_2)$	−1.244	−1.443	−1.027	approximate	approximate
Together	$\log_{10}(k_2')$	−1.549	−1.776	−1.318	approximate	approximate
Together	$\log_{10}(k_{deg})$	−1.93	−2.173	−1.681	approximate	approximate
Together	$\log_{10}(k_1'/k_2)$	0.198	0.1633	0.2339	approximate	approximate
Together	$\log_{10}(k_1'k_2'/k_2)$	−1.351	−1.578	−1.115	approximate	approximate
Together	$\log_{10}(k_2'/k_2)$	−0.3048	−0.6479	0.0576	approximate	approximate
b1	$\log_{10}(k_1')$	−1.476	−1.784	−1.254	approximate	approximate
b1	$\log_{10}(k_2)$	−1.58	−1.927	−1.336	approximate	approximate
b1	$\log_{10}(k_2')$	−1.267	−1.56	−0.7273	approximate	approximate
b1	$\log_{10}(k_{deg})$	−1.714	−2.034	−1.136	approximate	approximate
b1	$\log_{10}(k_1'/k_2)$	0.1038	0.06549	0.1532	approximate	approximate
b1	$\log_{10}(k_1'k_2'/k_2)$	−1.163	−1.464	−0.5909	approximate	approximate
b1	$\log_{10}(k_2'/k_2)$	0.3132	−0.1423	1.159	approximate	approximate
b2	$\log_{10}(k_1'k_2')$	−2.233	−3.399	> 4.029	approximate	optim
b2	$\log_{10}(k_2 \text{ or } k_{deg})$	−1.037	−2.689	> 5.368	approximate	optim
b2	$\log_{10}(k_2 \text{ or } k_{deg})$	−1.777	−2.689	−1.775	approximate	optim
b3	$\log_{10}(k_1')$	−0.8541	−1.032	−0.5919	approximate	approximate
b3	$\log_{10}(k_2)$	−1.171	−1.356	−0.9003	approximate	approximate
b3	$\log_{10}(k_2')$	−1.86	−2.071	−1.684	approximate	approximate
b3	$\log_{10}(k_{deg})$	−2.187	−2.415	−1.992	approximate	approximate
b3	$\log_{10}(k_1'/k_2)$	0.3168	0.285	0.3503	approximate	approximate
b3	$\log_{10}(k_1'k_2'/k_2)$	−1.543	−1.752	−1.367	approximate	approximate
b3	$\log_{10}(k_2'/k_2)$	−0.6891	−1.053	−0.4041	approximate	approximate

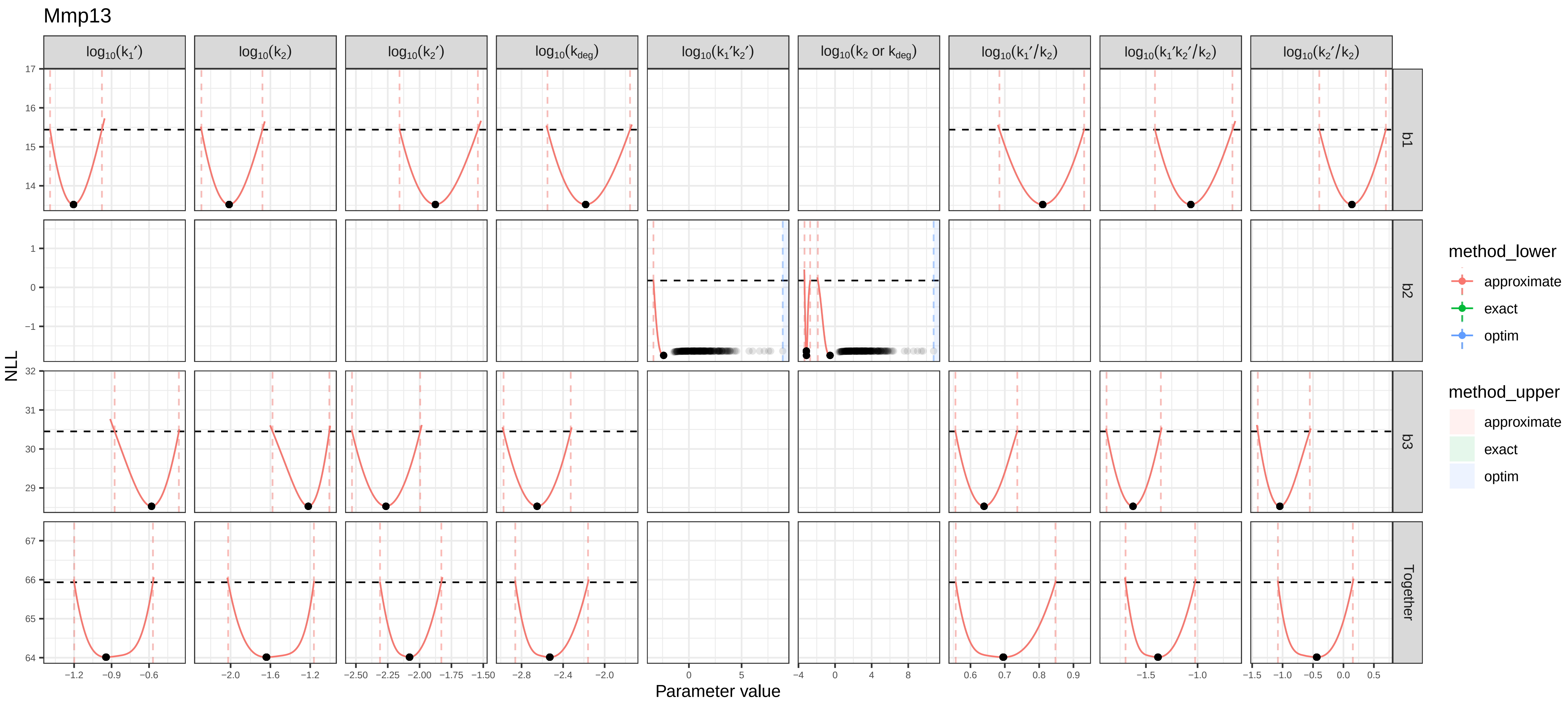

Replicate	Par	Best value	CI95 LB	CI95 UB	Method LB	Method UB
Together	$\log_{10}(k_1')$	−0.9439	−1.2	−0.5688	approximate	approximate
Together	$\log_{10}(k_2)$	−1.639	−2.025	−1.163	approximate	approximate
Together	$\log_{10}(k_2')$	−2.079	−2.311	−1.829	approximate	approximate
Together	$\log_{10}(k_{deg})$	−2.528	−2.861	−2.16	approximate	approximate
Together	$\log_{10}(k_1'/k_2)$	0.6953	0.557	0.8474	approximate	approximate
Together	$\log_{10}(k_1'k_2'/k_2)$	−1.384	−1.698	−1.024	approximate	approximate
Together	$\log_{10}(k_2'/k_2)$	−0.4398	−1.077	0.1538	approximate	approximate
b1	$\log_{10}(k_1')$	−1.205	−1.393	−0.9775	approximate	approximate
b1	$\log_{10}(k_2)$	−2.015	−2.294	−1.68	approximate	approximate
b1	$\log_{10}(k_2')$	−1.876	−2.158	−1.542	approximate	approximate
b1	$\log_{10}(k_{deg})$	−2.183	−2.55	−1.756	approximate	approximate
b1	$\log_{10}(k_1'/k_2)$	0.8105	0.6842	0.931	approximate	approximate
b1	$\log_{10}(k_1'k_2'/k_2)$	−1.066	−1.413	−0.6628	approximate	approximate
b1	$\log_{10}(k_2'/k_2)$	0.139	−0.3979	0.6964	approximate	approximate
b2	$\log_{10}(k_1'k_2')$	−2.398	−3.367	> 8.901	approximate	optim
b2	$\log_{10}(k_2 \text{ or } k_{deg})$	−0.5459	−1.884	> 10.77	approximate	optim
b2	$\log_{10}(k_2 \text{ or } k_{deg})$	−3.126	−3.346	−2.725	approximate	approximate
b3	$\log_{10}(k_1')$	−0.5798	−0.8755	−0.3612	approximate	approximate
b3	$\log_{10}(k_2)$	−1.219	−1.579	−1.008	approximate	approximate
b3	$\log_{10}(k_2')$	−2.265	−2.531	−1.995	approximate	approximate
b3	$\log_{10}(k_{deg})$	−2.65	−2.974	−2.327	approximate	approximate
b3	$\log_{10}(k_1'/k_2)$	0.6396	0.556	0.7363	approximate	approximate
b3	$\log_{10}(k_1'k_2'/k_2)$	−1.625	−1.882	−1.356	approximate	approximate
b3	$\log_{10}(k_2'/k_2)$	−1.045	−1.407	−0.5533	approximate	approximate

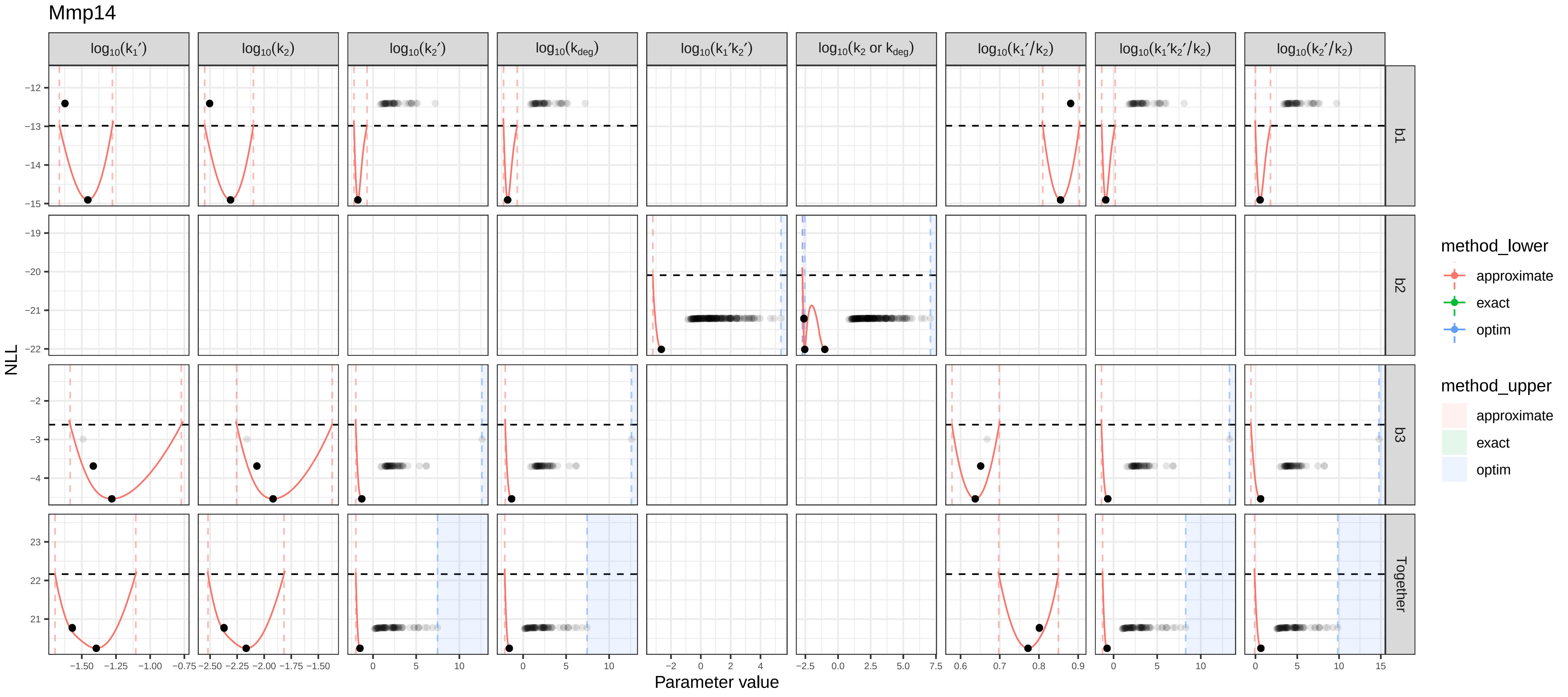

Replicate	Par	Best value	CI95 LB	CI95 UB	Method LB	Method UB
Together	$\log_{10}(k_1')$	−1.394	−1.696	−1.105	approximate	approximate
Together	$\log_{10}(k_2)$	−2.166	−2.519	−1.816	approximate	approximate
Together	$\log_{10}(k_2')$	−1.514	−1.99	> 7.469	approximate	optim
Together	$\log_{10}(k_{deg})$	−1.605	−2.132	> 7.432	approximate	optim
Together	$\log_{10}(k_1'/k_2)$	0.7722	0.6973	0.8497	approximate	approximate
Together	$\log_{10}(k_1'k_2'/k_2)$	−0.7417	−1.257	> 8.271	approximate	optim
Together	$\log_{10}(k_2'/k_2)$	0.6526	−0.1062	> 9.841	approximate	optim
b1	$\log_{10}(k_1')$	−1.456	−1.664	−1.276	approximate	approximate
b1	$\log_{10}(k_2)$	−2.311	−2.55	−2.1	approximate	approximate
b1	$\log_{10}(k_2')$	−1.756	−2.203	−0.687	approximate	approximate
b1	$\log_{10}(k_{deg})$	−1.793	−2.269	−0.6769	approximate	approximate
b1	$\log_{10}(k_1'/k_2)$	0.8552	0.8096	0.9032	approximate	approximate
b1	$\log_{10}(k_1'k_2'/k_2)$	−0.9008	−1.361	0.1916	approximate	approximate
b1	$\log_{10}(k_2'/k_2)$	0.5549	−0.01263	1.799	approximate	approximate
b2	$\log_{10}(k_1'k_2')$	−2.647	−3.215	> 5.373	approximate	optim
b2	$\log_{10}(k_2 \text{ or } k_{deg})$	−1.013	−2.724	> 7.078	approximate	optim
b2	$\log_{10}(k_2 \text{ or } k_{deg})$	−2.548	−2.724	−2.548	approximate	optim
b3	$\log_{10}(k_1')$	−1.28	−1.585	−0.7723	approximate	approximate
b3	$\log_{10}(k_2)$	−1.918	−2.255	−1.371	approximate	approximate
b3	$\log_{10}(k_2')$	−1.301	−1.994	> 12.62	approximate	optim
b3	$\log_{10}(k_{deg})$	−1.335	−2.074	> 12.6	approximate	optim
b3	$\log_{10}(k_1'/k_2)$	0.6374	0.5781	0.6986	approximate	approximate
b3	$\log_{10}(k_1'k_2'/k_2)$	−0.6639	−1.385	> 13.29	approximate	optim
b3	$\log_{10}(k_2'/k_2)$	0.6166	−0.5382	> 14.78	approximate	optim

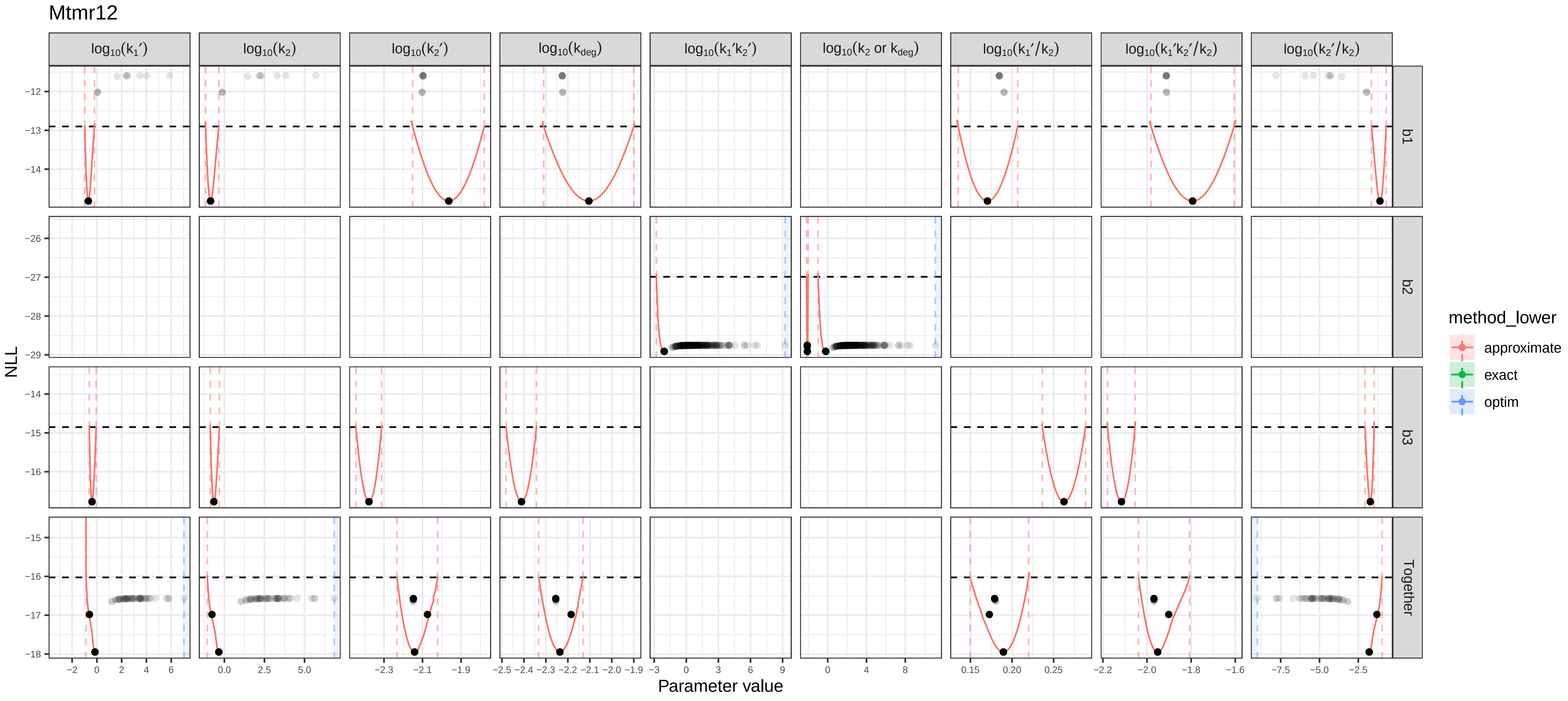

Replicate	Par	Best value	CI95 LB	CI95 UB	Method LB	Method UB
Together	$\log_{10}(k_1')$	−0.1552	−0.8717	> 7.031	approximate	optim
Together	$\log_{10}(k_2)$	−0.3448	−1.063	> 6.852	approximate	optim
Together	$\log_{10}(k_2')$	−2.141	−2.233	−2.021	approximate	approximate
Together	$\log_{10}(k_{deg})$	−2.236	−2.333	−2.131	approximate	approximate
Together	$\log_{10}(k_1'/k_2)$	0.1896	0.1497	0.2199	approximate	approximate
Together	$\log_{10}(k_1'k_2'/k_2)$	−1.952	−2.039	−1.807	approximate	approximate
Together	$\log_{10}(k_2'/k_2)$	−1.796	< −8.999	−0.9676	optim	approximate
b1	$\log_{10}(k_1')$	−0.6913	−0.9791	−0.1965	approximate	approximate
b1	$\log_{10}(k_2)$	−0.8617	−1.171	−0.3471	approximate	approximate
b1	$\log_{10}(k_2')$	−1.964	−2.152	−1.779	approximate	approximate
b1	$\log_{10}(k_{deg})$	−2.104	−2.31	−1.9	approximate	approximate
b1	$\log_{10}(k_1'/k_2)$	0.1704	0.1353	0.2068	approximate	approximate
b1	$\log_{10}(k_1'k_2'/k_2)$	−1.793	−1.982	−1.606	approximate	approximate
b1	$\log_{10}(k_2'/k_2)$	−1.102	−1.652	−0.7049	approximate	approximate
b2	$\log_{10}(k_1'k_2')$	−2.063	−2.797	> 9.231	approximate	optim
b2	$\log_{10}(k_2 \text{ or } k_{deg})$	−0.202	−0.9937	> 11.1	approximate	optim
b2	$\log_{10}(k_2 \text{ or } k_{deg})$	−2.102	−2.182	−2.026	approximate	approximate
b3	$\log_{10}(k_1')$	−0.3902	−0.6145	−0.05742	approximate	approximate
b3	$\log_{10}(k_2)$	−0.6526	−0.8806	−0.3145	approximate	approximate
b3	$\log_{10}(k_2')$	−2.378	−2.446	−2.313	approximate	approximate
b3	$\log_{10}(k_{deg})$	−2.411	−2.481	−2.343	approximate	approximate
b3	$\log_{10}(k_1'/k_2)$	0.2624	0.2364	0.2883	approximate	approximate
b3	$\log_{10}(k_1'k_2'/k_2)$	−2.116	−2.179	−2.054	approximate	approximate
b3	$\log_{10}(k_2'/k_2)$	−1.725	−2.071	−1.481	approximate	approximate

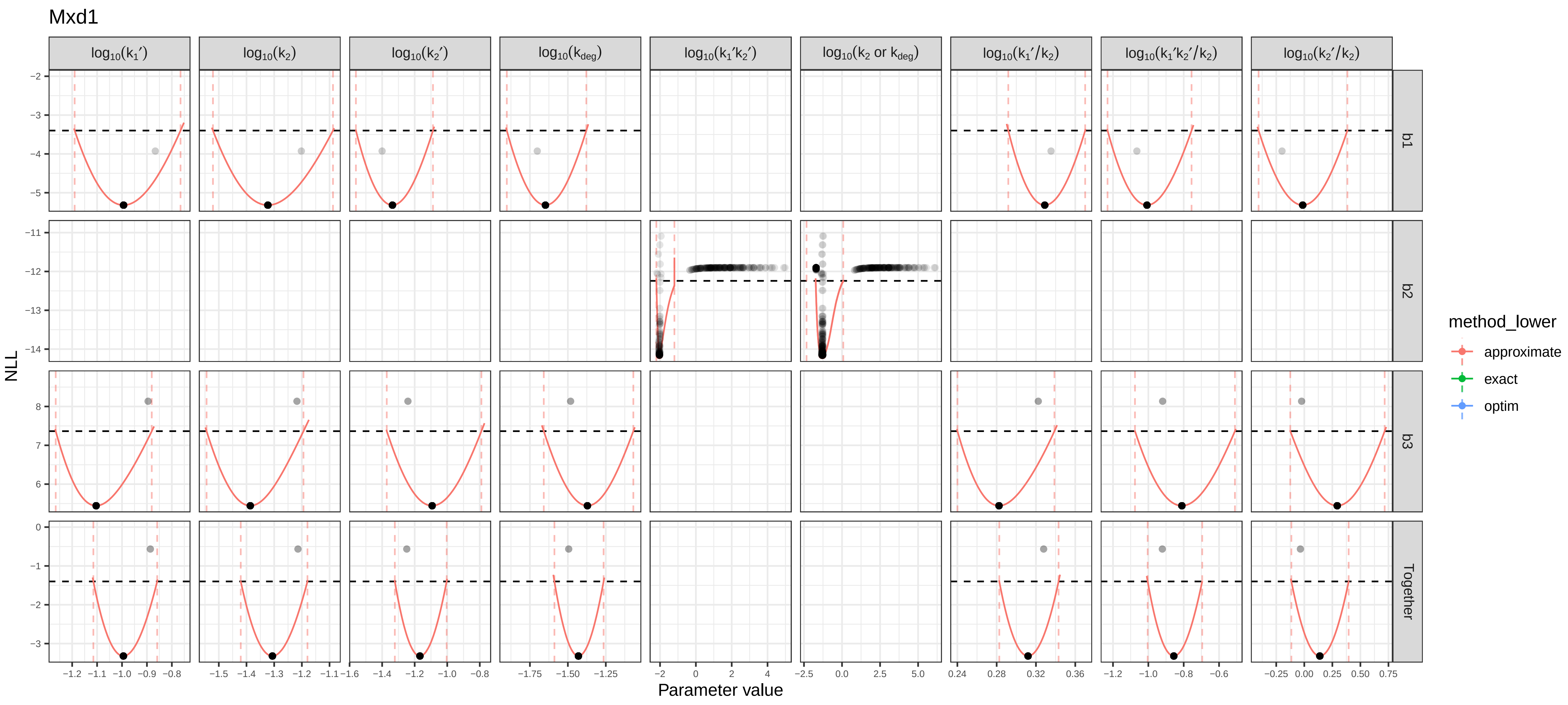

Replicate	Par	Best value	CI95 LB	CI95 UB	Method LB	Method UB
Together	$\log_{10}(k_1')$	−0.9944	−1.115	−0.8589	approximate	approximate
Together	$\log_{10}(k_2)$	−1.306	−1.421	−1.18	approximate	approximate
Together	$\log_{10}(k_2')$	−1.168	−1.322	−1.003	approximate	approximate
Together	$\log_{10}(k_{deg})$	−1.43	−1.589	−1.265	approximate	approximate
Together	$\log_{10}(k_1'/k_2)$	0.3119	0.2827	0.343	approximate	approximate
Together	$\log_{10}(k_1'k_2'/k_2)$	−0.856	−1.004	−0.6953	approximate	approximate
Together	$\log_{10}(k_2'/k_2)$	0.1384	−0.1151	0.3954	approximate	approximate
b1	$\log_{10}(k_1')$	−0.9937	−1.19	−0.7653	approximate	approximate
b1	$\log_{10}(k_2)$	−1.323	−1.521	−1.088	approximate	approximate
b1	$\log_{10}(k_2')$	−1.337	−1.561	−1.088	approximate	approximate
b1	$\log_{10}(k_{deg})$	−1.648	−1.902	−1.378	approximate	approximate
b1	$\log_{10}(k_1'/k_2)$	0.329	0.2918	0.3701	approximate	approximate
b1	$\log_{10}(k_1'k_2'/k_2)$	−1.008	−1.231	−0.7555	approximate	approximate
b1	$\log_{10}(k_2'/k_2)$	−0.01402	−0.4072	0.384	approximate	approximate
b2	$\log_{10}(k_1'k_2')$	−2.037	−2.206	−1.198	approximate	approximate
b2	$\log_{10}(k_2 \text{ or } k_{deg})$	−1.276	−2.33	0.08279	approximate	approximate
b2	$\log_{10}(k_2 \text{ or } k_{deg})$	−1.311	−2.33	0.08279	approximate	approximate
b3	$\log_{10}(k_1')$	−1.104	−1.266	−0.8806	approximate	approximate
b3	$\log_{10}(k_2)$	−1.386	−1.544	−1.194	approximate	approximate
b3	$\log_{10}(k_2')$	−1.093	−1.372	−0.7901	approximate	approximate
b3	$\log_{10}(k_{deg})$	−1.371	−1.659	−1.069	approximate	approximate
b3	$\log_{10}(k_1'/k_2)$	0.2824	0.2401	0.3388	approximate	approximate
b3	$\log_{10}(k_1'k_2'/k_2)$	−0.8103	−1.076	−0.51	approximate	approximate
b3	$\log_{10}(k_2'/k_2)$	0.2936	−0.1246	0.7163	approximate	approximate

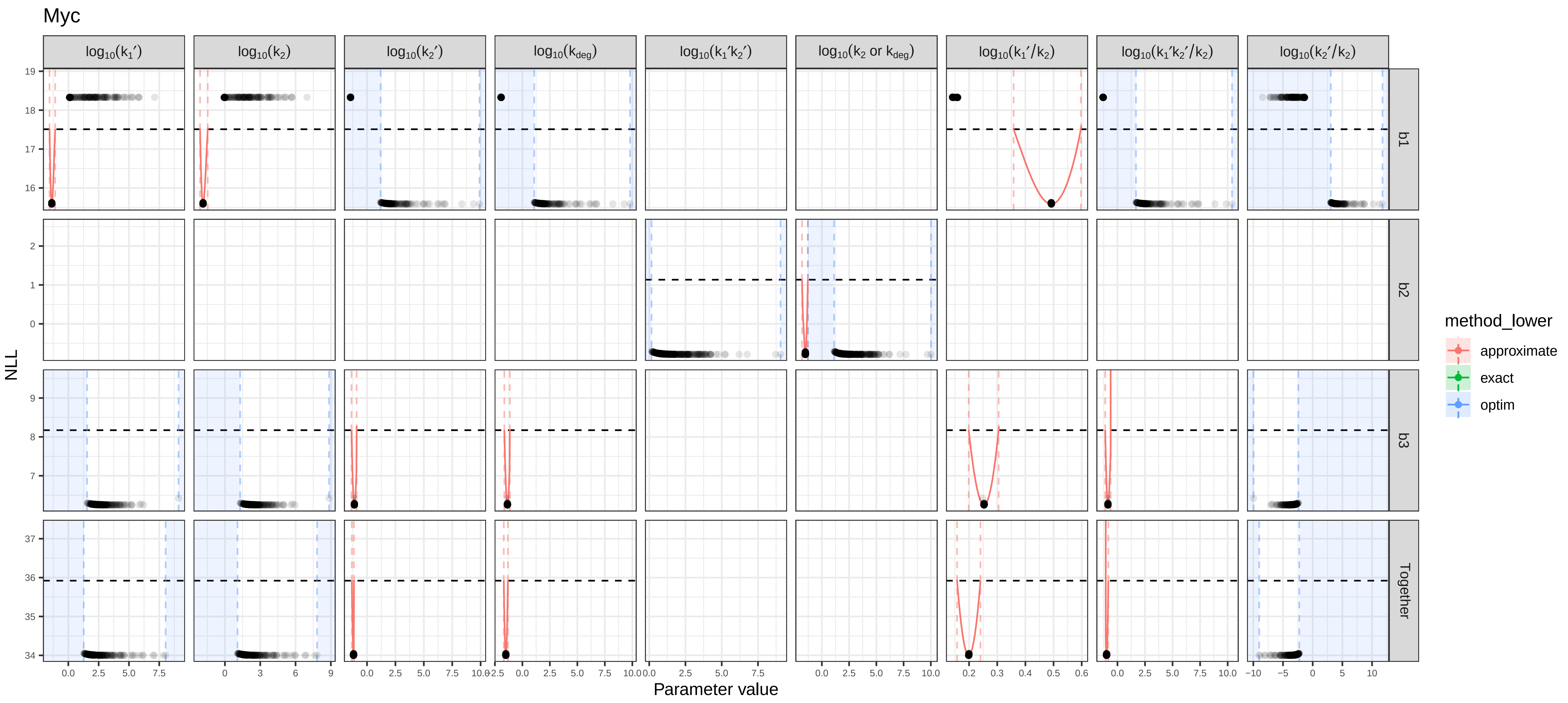

Replicate	Par	Best value	CI95 LB	CI95 UB	Method LB	Method UB
Together	$\log_{10}(k_1')$	5.309	< 1.268	> 8.028	optim	optim
Together	$\log_{10}(k_2)$	5.111	< 1.069	> 7.829	optim	optim
Together	$\log_{10}(k_2')$	−1.186	−1.335	−1.148	approximate	approximate
Together	$\log_{10}(k_{deg})$	−1.507	−1.691	−1.317	approximate	approximate
Together	$\log_{10}(k_1'/k_2)$	0.1987	0.1574	0.2405	approximate	approximate
Together	$\log_{10}(k_1'k_2'/k_2)$	−0.9869	−1.061	−0.8287	approximate	approximate
Together	$\log_{10}(k_2'/k_2)$	−6.296	< −9.014	> −2.252	optim	optim
b1	$\log_{10}(k_1')$	−1.367	−1.551	−1.085	approximate	approximate
b1	$\log_{10}(k_2)$	−1.859	−2.112	−1.457	approximate	approximate
b1	$\log_{10}(k_2')$	5.079	< 1.191	> 9.906	optim	optim
b1	$\log_{10}(k_{deg})$	4.965	< 1.076	> 9.792	optim	optim
b1	$\log_{10}(k_1'/k_2)$	0.4922	0.3581	0.597	approximate	approximate
b1	$\log_{10}(k_1'k_2'/k_2)$	5.571	< 1.682	> 10.4	optim	optim
b1	$\log_{10}(k_2'/k_2)$	6.938	< 3.048	> 11.77	optim	optim
b2	$\log_{10}(k_1'k_2')$	4.597	< 0.1603	> 9.056	optim	optim
b2	$\log_{10}(k_2 \text{ or } k_{deg})$	5.56	1.122	> 10.02	optim	optim
b2	$\log_{10}(k_2 \text{ or } k_{deg})$	−1.519	−1.819	−1.285	approximate	approximate
b3	$\log_{10}(k_1')$	4.221	< 1.548	> 9.094	optim	optim
b3	$\log_{10}(k_2)$	3.968	< 1.294	> 8.846	optim	optim
b3	$\log_{10}(k_2')$	−1.114	−1.366	−0.9139	approximate	approximate
b3	$\log_{10}(k_{deg})$	−1.361	−1.643	−1.141	approximate	approximate
b3	$\log_{10}(k_1'/k_2)$	0.2531	0.1987	0.305	approximate	approximate
b3	$\log_{10}(k_1'k_2'/k_2)$	−0.8612	−1.112	−0.619	approximate	approximate
b3	$\log_{10}(k_2'/k_2)$	−5.082	< −9.963	> −2.406	optim	optim

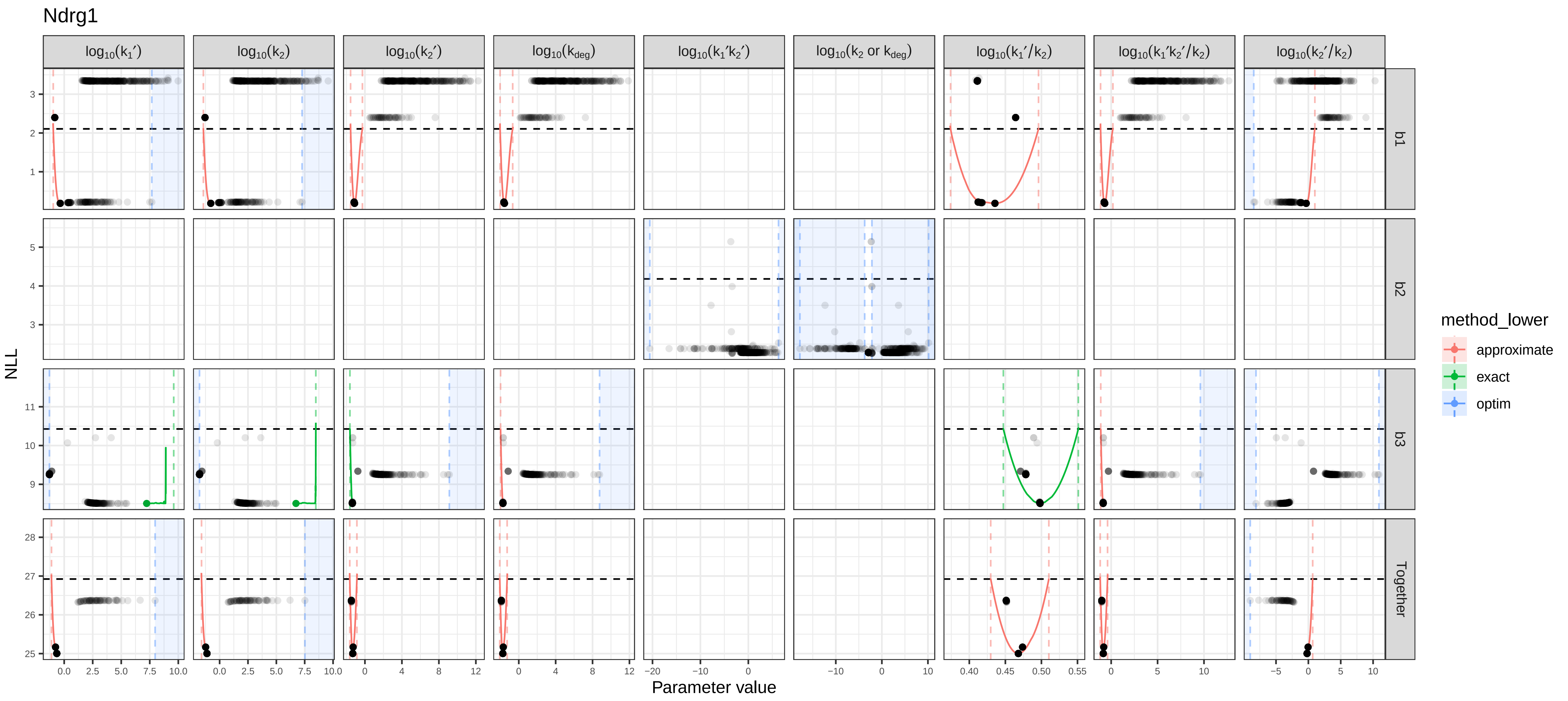

Replicate	Par	Best value	CI95 LB	CI95 UB	Method LB	Method UB
Together	$\log_{10}(k_1')$	−0.6477	−1.112	> 7.969	approximate	optim
Together	$\log_{10}(k_2)$	−1.116	−1.595	> 7.517	approximate	optim
Together	$\log_{10}(k_2')$	−1.317	−1.641	−0.8696	approximate	approximate
Together	$\log_{10}(k_{deg})$	−1.728	−2.064	−1.262	approximate	approximate
Together	$\log_{10}(k_1'/k_2)$	0.468	0.4297	0.5103	approximate	approximate
Together	$\log_{10}(k_1'k_2'/k_2)$	−0.8486	−1.18	−0.3881	approximate	approximate
Together	$\log_{10}(k_2'/k_2)$	−0.201	< −8.98	0.6672	optim	approximate
b1	$\log_{10}(k_1')$	−0.3465	−0.96	> 7.681	approximate	optim
b1	$\log_{10}(k_2)$	−0.782	−1.433	> 7.269	approximate	optim
b1	$\log_{10}(k_2')$	−1.12	−1.554	−0.2728	approximate	approximate
b1	$\log_{10}(k_{deg})$	−1.553	−2.027	−0.66	approximate	approximate
b1	$\log_{10}(k_1'/k_2)$	0.4356	0.3741	0.496	approximate	approximate
b1	$\log_{10}(k_1'k_2'/k_2)$	−0.6843	−1.139	0.1894	approximate	approximate
b1	$\log_{10}(k_2'/k_2)$	−0.3378	< −8.437	1.002	optim	approximate
b2	$\log_{10}(k_1'k_2')$	−3.442	< −20.56	> 6.299	optim	optim
b2	$\log_{10}(k_2 \text{ or } k_{deg})$	−2.179	< −3.728	> 10.15	optim	optim
b2	$\log_{10}(k_2 \text{ or } k_{deg})$	−2.215	< −17.79	> −2.161	optim	optim
b3	$\log_{10}(k_1')$	7.228	< −1.294	9.6	optim	exact
b3	$\log_{10}(k_2)$	6.73	< −1.773	8.481	optim	exact
b3	$\log_{10}(k_2')$	−1.36	−1.623	> 9.13	exact	optim
b3	$\log_{10}(k_{deg})$	−1.719	−1.975	> 8.763	approximate	optim
b3	$\log_{10}(k_1'/k_2)$	0.4982	0.4472	0.5511	exact	exact
b3	$\log_{10}(k_1'k_2'/k_2)$	−0.8618	−1.121	> 9.609	approximate	optim
b3	$\log_{10}(k_2'/k_2)$	−8.09	< −8.09	> 10.9	optim	optim

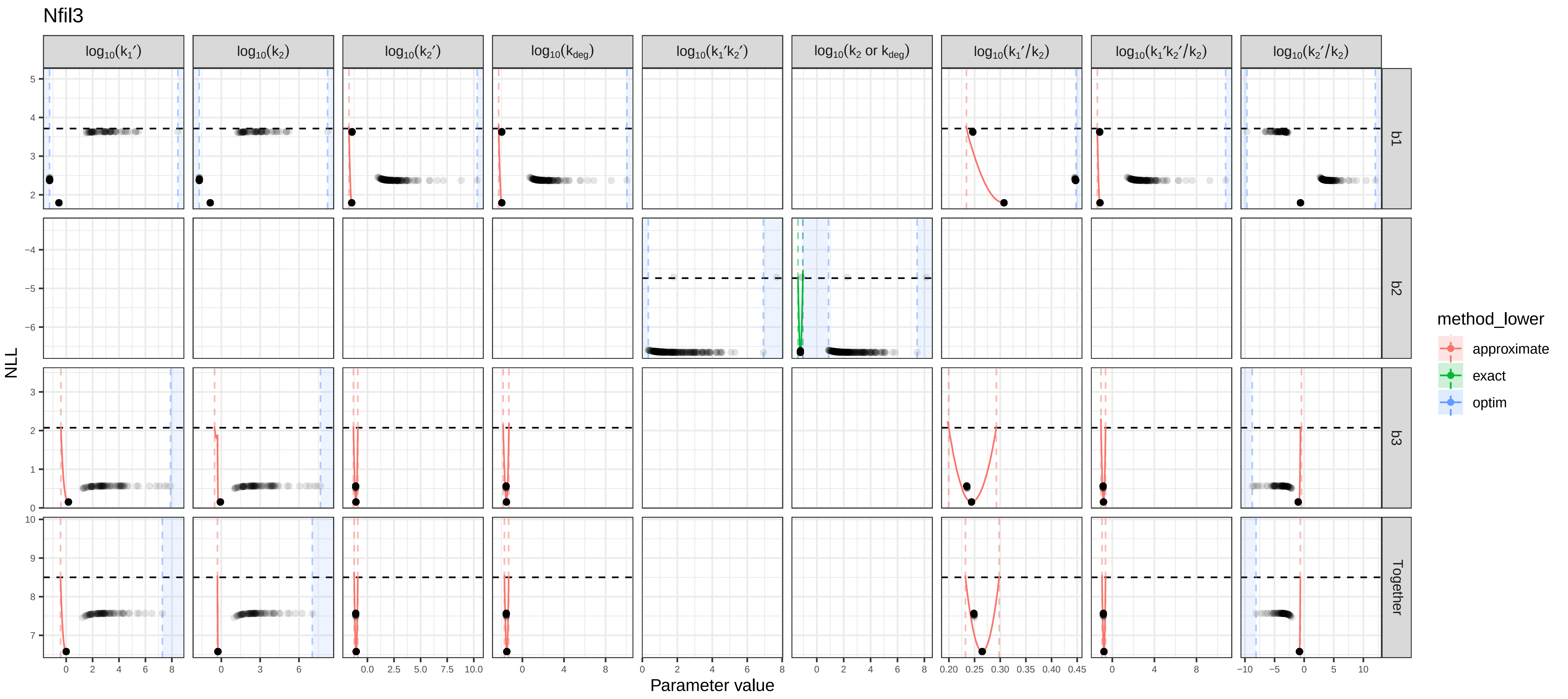

Replicate	Par	Best value	CI95 LB	CI95 UB	Method LB	Method UB
Together	$\log_{10}(k_1')$	0.002172	−0.4281	> 7.28	approximate	optim
Together	$\log_{10}(k_2)$	−0.2627	−0.2982	> 7.031	approximate	optim
Together	$\log_{10}(k_2')$	−1.057	−1.244	−0.9037	approximate	approximate
Together	$\log_{10}(k_{deg})$	−1.497	−1.729	−1.318	approximate	approximate
Together	$\log_{10}(k_1'/k_2)$	0.2649	0.2322	0.298	approximate	approximate
Together	$\log_{10}(k_1'k_2'/k_2)$	−0.7926	−0.9773	−0.6418	approximate	approximate
Together	$\log_{10}(k_2'/k_2)$	−0.7947	< −8.128	−0.6436	optim	approximate
b1	$\log_{10}(k_1')$	−0.5475	< −1.258	> 8.454	optim	optim
b1	$\log_{10}(k_2)$	−0.8549	< −1.705	> 8.208	optim	optim
b1	$\log_{10}(k_2')$	−1.476	−1.735	> 10.32	approximate	optim
b1	$\log_{10}(k_{deg})$	−1.976	−2.278	> 10.05	approximate	optim
b1	$\log_{10}(k_1'/k_2)$	0.3074	0.2342	> 0.4476	approximate	optim
b1	$\log_{10}(k_1'k_2'/k_2)$	−1.169	−1.422	> 10.77	approximate	optim
b1	$\log_{10}(k_2'/k_2)$	−0.6215	< −9.653	> 12.02	optim	optim
b2	$\log_{10}(k_1'k_2')$	4.472	< 0.3403	> 6.923	optim	optim
b2	$\log_{10}(k_2 \text{ or } k_{deg})$	5.008	0.872	> 7.459	optim	optim
b2	$\log_{10}(k_2 \text{ or } k_{deg})$	−1.218	−1.397	−1.038	exact	exact
b3	$\log_{10}(k_1')$	0.1703	−0.3985	> 7.897	approximate	optim
b3	$\log_{10}(k_2)$	−0.07378	−0.5202	> 7.662	approximate	optim
b3	$\log_{10}(k_2')$	−1.081	−1.316	−0.8983	approximate	approximate
b3	$\log_{10}(k_{deg})$	−1.535	−1.85	−1.296	approximate	approximate
b3	$\log_{10}(k_1'/k_2)$	0.2441	0.1996	0.2925	approximate	approximate
b3	$\log_{10}(k_1'k_2'/k_2)$	−0.8369	−1.07	−0.6412	approximate	approximate
b3	$\log_{10}(k_2'/k_2)$	−1.007	< −8.769	−0.4616	optim	approximate

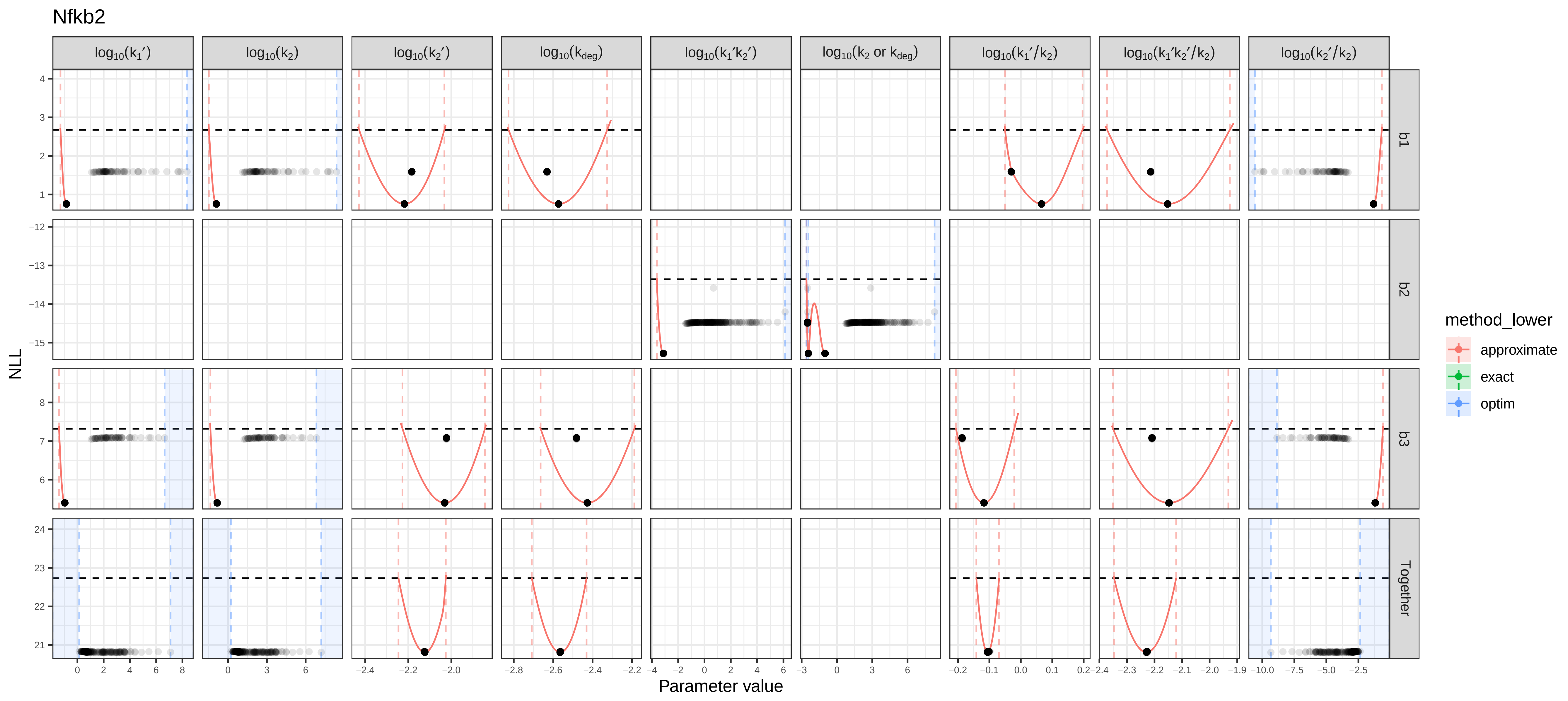

Replicate	Par	Best value	CI95 LB	CI95 UB	Method LB	Method UB
Together	$\log_{10}(k_1')$	4.381	< 0.137	> 7.099	optim	optim
Together	$\log_{10}(k_2)$	4.487	< 0.235	> 7.204	optim	optim
Together	$\log_{10}(k_2')$	−2.123	−2.246	−2.026	approximate	approximate
Together	$\log_{10}(k_{deg})$	−2.565	−2.709	−2.431	approximate	approximate
Together	$\log_{10}(k_1'/k_2)$	−0.1057	−0.1413	−0.06902	approximate	approximate
Together	$\log_{10}(k_1'k_2'/k_2)$	−2.229	−2.347	−2.121	approximate	approximate
Together	$\log_{10}(k_2'/k_2)$	−6.61	< −9.328	> −2.362	optim	optim
b1	$\log_{10}(k_1')$	−0.8433	−1.292	> 8.358	approximate	optim
b1	$\log_{10}(k_2)$	−0.9094	−1.482	> 8.388	approximate	optim
b1	$\log_{10}(k_2')$	−2.218	−2.429	−2.032	approximate	approximate
b1	$\log_{10}(k_{deg})$	−2.573	−2.826	−2.326	approximate	approximate
b1	$\log_{10}(k_1'/k_2)$	0.06607	−0.05014	0.1963	approximate	approximate
b1	$\log_{10}(k_1'k_2'/k_2)$	−2.152	−2.372	−1.927	approximate	approximate
b1	$\log_{10}(k_2'/k_2)$	−1.309	< −10.57	−0.6742	optim	approximate
b2	$\log_{10}(k_1'k_2')$	−3.121	−3.609	> 6.124	approximate	optim
b2	$\log_{10}(k_2 \text{ or } k_{deg})$	−1.012	−2.591	> 8.296	approximate	optim
b2	$\log_{10}(k_2 \text{ or } k_{deg})$	−2.431	−2.591	−2.431	approximate	optim
b3	$\log_{10}(k_1')$	−0.9575	−1.397	> 6.645	approximate	optim
b3	$\log_{10}(k_2)$	−0.8406	−1.361	> 6.832	approximate	optim
b3	$\log_{10}(k_2')$	−2.03	−2.227	−1.844	approximate	approximate
b3	$\log_{10}(k_{deg})$	−2.426	−2.664	−2.188	approximate	approximate
b3	$\log_{10}(k_1'/k_2)$	−0.1169	−0.2059	−0.02072	approximate	approximate
b3	$\log_{10}(k_1'k_2'/k_2)$	−2.147	−2.351	−1.932	approximate	approximate
b3	$\log_{10}(k_2'/k_2)$	−1.19	< −8.854	−0.5872	optim	approximate

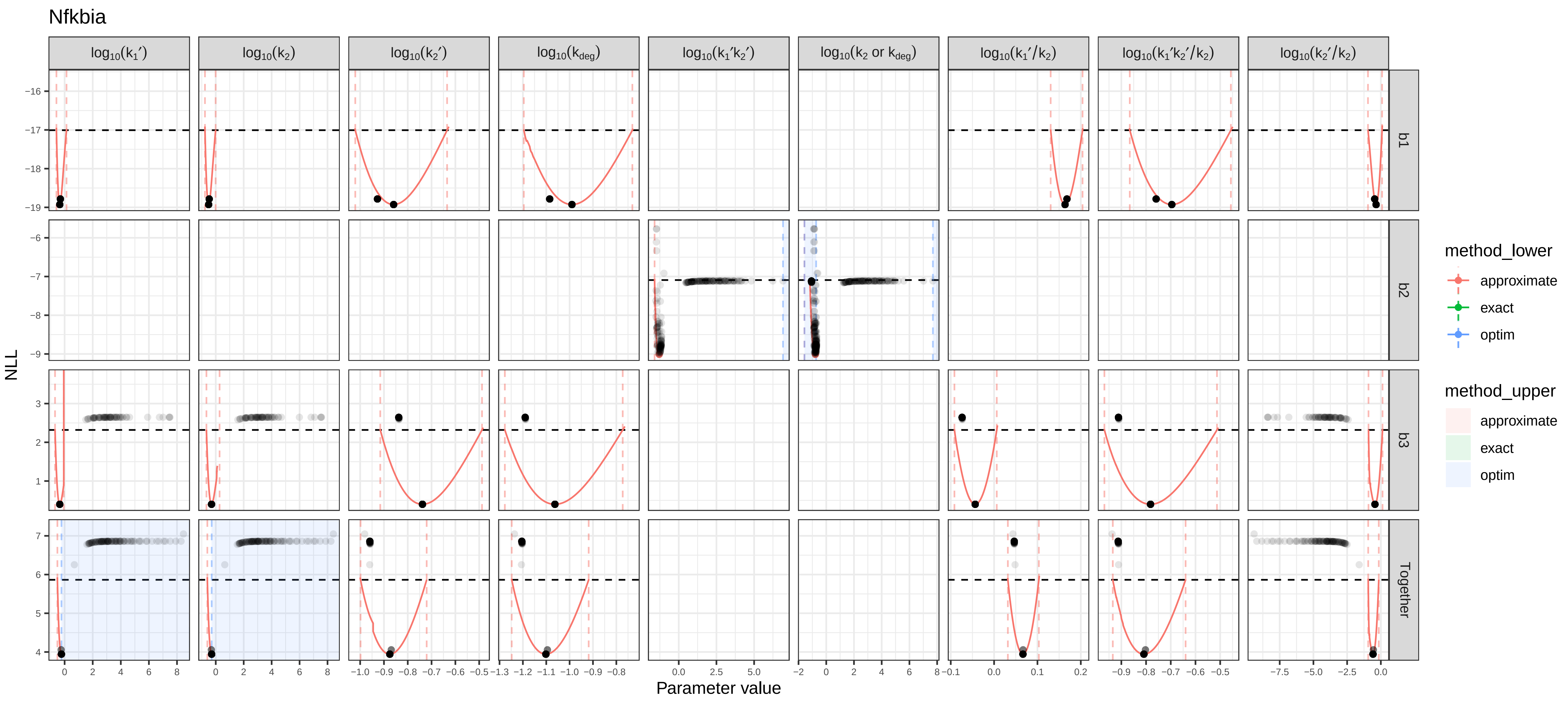

Replicate	Par	Best value	CI95 LB	CI95 UB	Method LB	Method UB
Together	$\log_{10}(k_1')$	−0.2285	−0.5138	> −0.2246	approximate	optim
Together	$\log_{10}(k_2)$	−0.295	−0.6021	> −0.291	approximate	optim
Together	$\log_{10}(k_2')$	−0.8753	−0.9983	−0.7205	approximate	approximate
Together	$\log_{10}(k_{deg})$	−1.102	−1.248	−0.9183	approximate	approximate
Together	$\log_{10}(k_1'/k_2)$	0.06654	0.03175	0.1033	approximate	approximate
Together	$\log_{10}(k_1'k_2'/k_2)$	−0.8087	−0.9346	−0.64	approximate	approximate
Together	$\log_{10}(k_2'/k_2)$	−0.5802	−0.9376	−0.1538	approximate	approximate
b1	$\log_{10}(k_1')$	−0.3455	−0.5824	0.1349	approximate	approximate
b1	$\log_{10}(k_2)$	−0.5092	−0.7712	−0.007658	approximate	approximate
b1	$\log_{10}(k_2')$	−0.8593	−1.02	−0.6347	approximate	approximate
b1	$\log_{10}(k_{deg})$	−0.9899	−1.196	−0.7313	approximate	approximate
b1	$\log_{10}(k_1'/k_2)$	0.1637	0.1305	0.2041	approximate	approximate
b1	$\log_{10}(k_1'k_2'/k_2)$	−0.6956	−0.8659	−0.4567	approximate	approximate
b1	$\log_{10}(k_2'/k_2)$	−0.3501	−0.9545	0.09181	approximate	approximate
b2	$\log_{10}(k_1'k_2')$	−1.297	−1.603	> 6.918	approximate	optim
b2	$\log_{10}(k_2 \text{ or } k_{deg})$	−0.7836	−1.573	> 7.695	approximate	optim
b2	$\log_{10}(k_2 \text{ or } k_{deg})$	−0.8013	−1.573	−0.7287	approximate	optim
b3	$\log_{10}(k_1')$	−0.3458	−0.6824	−0.05962	approximate	approximate
b3	$\log_{10}(k_2)$	−0.3022	−0.6675	0.2733	approximate	approximate
b3	$\log_{10}(k_2')$	−0.7383	−0.9155	−0.4881	approximate	approximate
b3	$\log_{10}(k_{deg})$	−1.063	−1.277	−0.7727	approximate	approximate
b3	$\log_{10}(k_1'/k_2)$	−0.04361	−0.09153	0.006162	approximate	approximate
b3	$\log_{10}(k_1'k_2'/k_2)$	−0.782	−0.9686	−0.513	approximate	approximate
b3	$\log_{10}(k_2'/k_2)$	−0.4361	−0.9162	0.1257	approximate	approximate

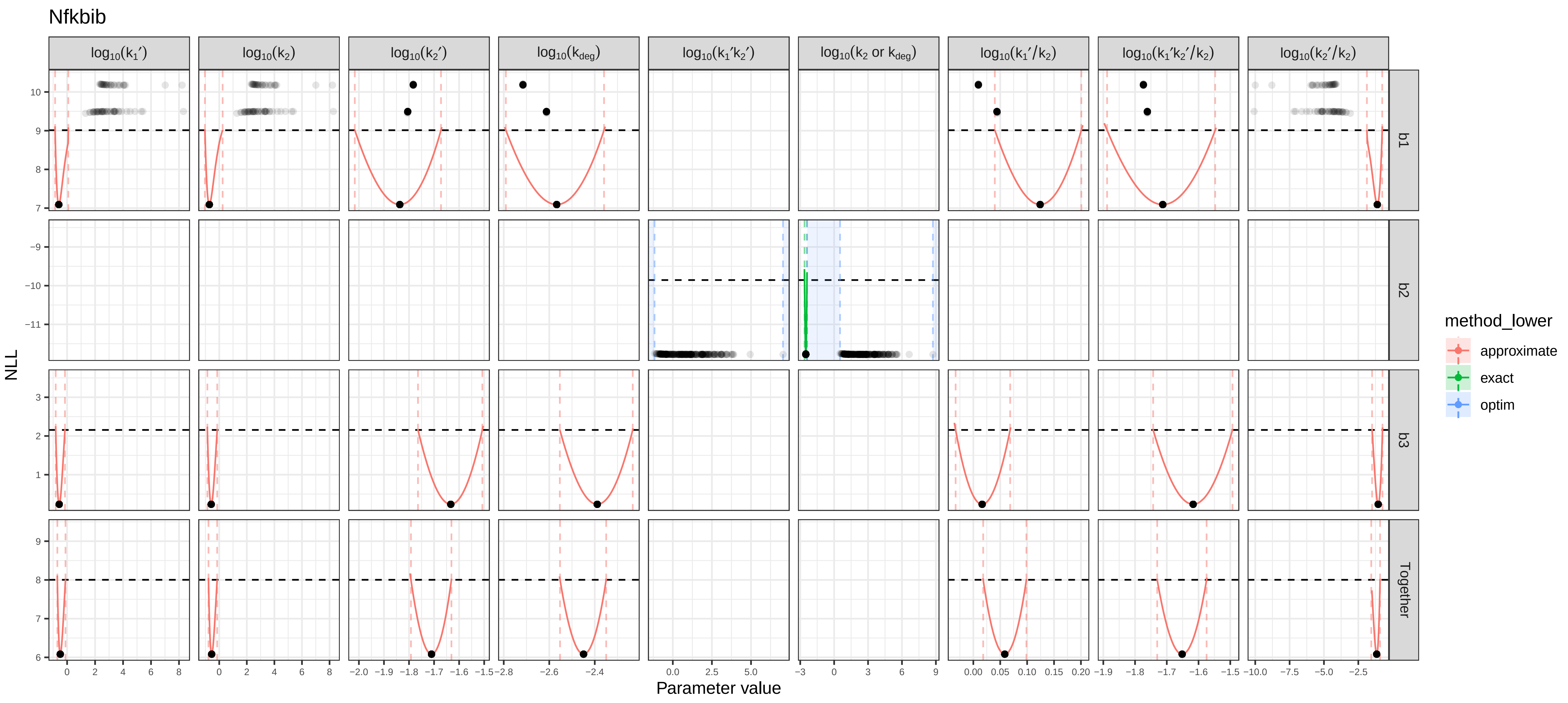

Replicate	Par	Best value	CI95 LB	CI95 UB	Method LB	Method UB
Together	$\log_{10}(k_1')$	−0.4897	−0.6978	−0.1174	approximate	approximate
Together	$\log_{10}(k_2)$	−0.5482	−0.7797	−0.1502	approximate	approximate
Together	$\log_{10}(k_2')$	−1.71	−1.792	−1.631	approximate	approximate
Together	$\log_{10}(k_{deg})$	−2.449	−2.553	−2.35	approximate	approximate
Together	$\log_{10}(k_1'/k_2)$	0.05843	0.01831	0.09865	approximate	approximate
Together	$\log_{10}(k_1'k_2'/k_2)$	−1.652	−1.73	−1.575	approximate	approximate
Together	$\log_{10}(k_2'/k_2)$	−1.162	−1.558	−0.9174	approximate	approximate
b1	$\log_{10}(k_1')$	−0.5942	−0.8598	0.08496	approximate	approximate
b1	$\log_{10}(k_2)$	−0.7183	−1.038	0.2475	approximate	approximate
b1	$\log_{10}(k_2')$	−1.837	−2.016	−1.672	approximate	approximate
b1	$\log_{10}(k_{deg})$	−2.567	−2.791	−2.359	approximate	approximate
b1	$\log_{10}(k_1'/k_2)$	0.1241	0.03995	0.2008	approximate	approximate
b1	$\log_{10}(k_1'k_2'/k_2)$	−1.713	−1.888	−1.548	approximate	approximate
b1	$\log_{10}(k_2'/k_2)$	−1.118	−1.876	−0.7608	approximate	approximate
b2	$\log_{10}(k_1'k_2')$	3.095	< −1.167	> 7.046	optim	optim
b2	$\log_{10}(k_2 \text{ or } k_{deg})$	4.785	0.5215	> 8.737	optim	optim
b2	$\log_{10}(k_2 \text{ or } k_{deg})$	−2.505	−2.617	−2.405	exact	exact
b3	$\log_{10}(k_1')$	−0.565	−0.8154	−0.1542	approximate	approximate
b3	$\log_{10}(k_2)$	−0.5814	−0.859	−0.1456	approximate	approximate
b3	$\log_{10}(k_2')$	−1.633	−1.764	−1.507	approximate	approximate
b3	$\log_{10}(k_{deg})$	−2.389	−2.553	−2.233	approximate	approximate
b3	$\log_{10}(k_1'/k_2)$	0.01641	−0.03289	0.06847	approximate	approximate
b3	$\log_{10}(k_1'k_2'/k_2)$	−1.617	−1.743	−1.493	approximate	approximate
b3	$\log_{10}(k_2'/k_2)$	−1.052	−1.498	−0.7403	approximate	approximate

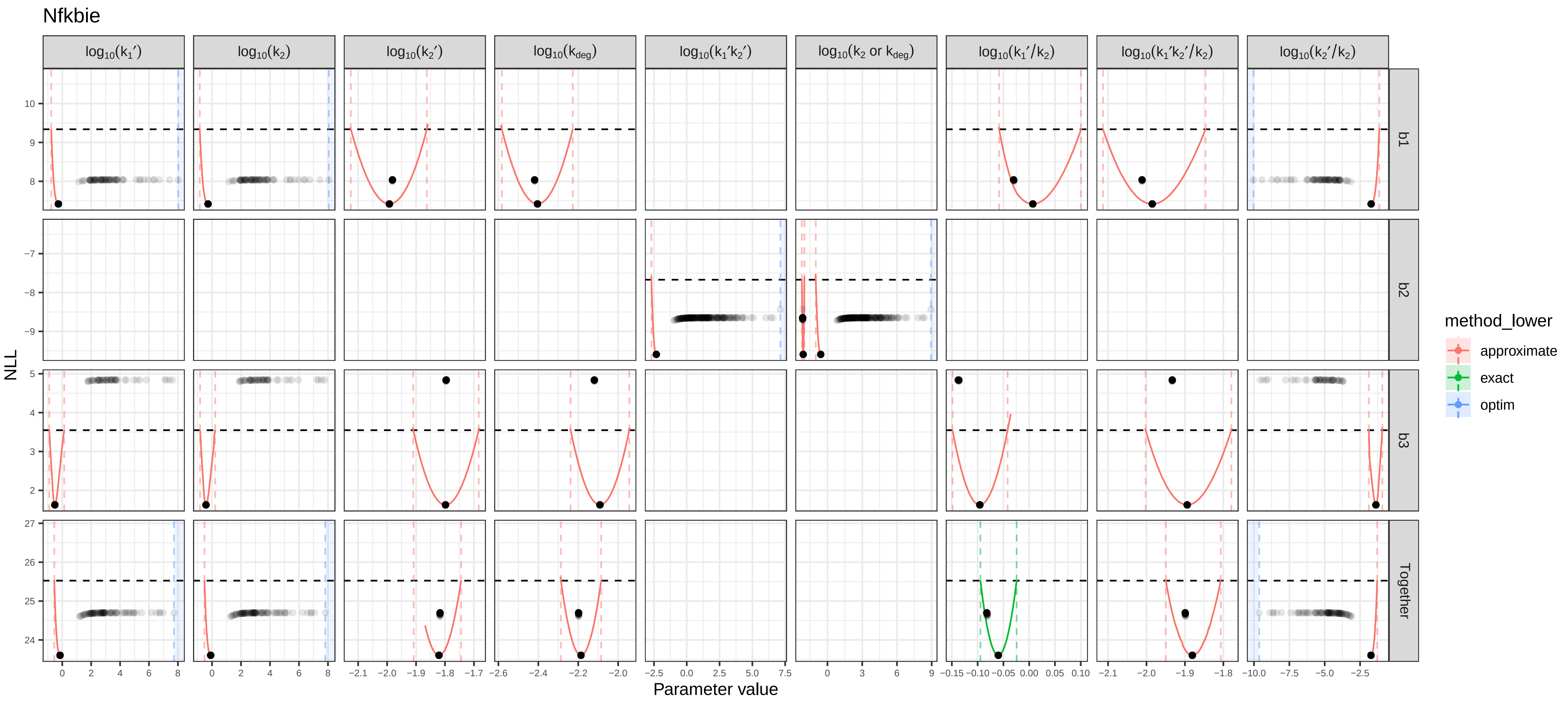

Replicate	Par	Best value	CI95 LB	CI95 UB	Method LB	Method UB
Together	$\log_{10}(k_1')$	−0.1534	−0.5522	> 7.729	approximate	optim
Together	$\log_{10}(k_2)$	−0.09352	−0.5224	> 7.811	approximate	optim
Together	$\log_{10}(k_2')$	−1.821	−1.908	−1.744	approximate	approximate
Together	$\log_{10}(k_{deg})$	−2.186	−2.287	−2.085	approximate	approximate
Together	$\log_{10}(k_1'/k_2)$	−0.05991	−0.09462	−0.02428	exact	exact
Together	$\log_{10}(k_1'k_2'/k_2)$	−1.881	−1.95	−1.807	approximate	approximate
Together	$\log_{10}(k_2'/k_2)$	−1.727	< −9.628	−1.291	optim	approximate
b1	$\log_{10}(k_1')$	−0.2681	−0.7646	> 8.027	approximate	optim
b1	$\log_{10}(k_2)$	−0.2752	−0.8452	> 8.057	approximate	optim
b1	$\log_{10}(k_2')$	−1.992	−2.125	−1.863	approximate	approximate
b1	$\log_{10}(k_{deg})$	−2.404	−2.583	−2.227	approximate	approximate
b1	$\log_{10}(k_1'/k_2)$	0.007042	−0.0584	0.1007	approximate	approximate
b1	$\log_{10}(k_1'k_2'/k_2)$	−1.985	−2.113	−1.846	approximate	approximate
b1	$\log_{10}(k_2'/k_2)$	−1.717	< −10.04	−1.136	optim	approximate
b2	$\log_{10}(k_1'k_2')$	−2.322	−2.694	> 7.136	approximate	optim
b2	$\log_{10}(k_2 \text{ or } k_{deg})$	−0.5826	−1.012	> 8.93	approximate	optim
b2	$\log_{10}(k_2 \text{ or } k_{deg})$	−2.097	−2.212	−1.996	approximate	approximate
b3	$\log_{10}(k_1')$	−0.5139	−0.8991	0.1368	approximate	approximate
b3	$\log_{10}(k_2)$	−0.4182	−0.8246	0.2196	approximate	approximate
b3	$\log_{10}(k_2')$	−1.798	−1.91	−1.683	approximate	approximate
b3	$\log_{10}(k_{deg})$	−2.091	−2.238	−1.944	approximate	approximate
b3	$\log_{10}(k_1'/k_2)$	−0.09573	−0.1488	−0.04181	approximate	approximate
b3	$\log_{10}(k_1'k_2'/k_2)$	−1.894	−2.002	−1.779	approximate	approximate
b3	$\log_{10}(k_2'/k_2)$	−1.38	−1.874	−0.9276	approximate	approximate

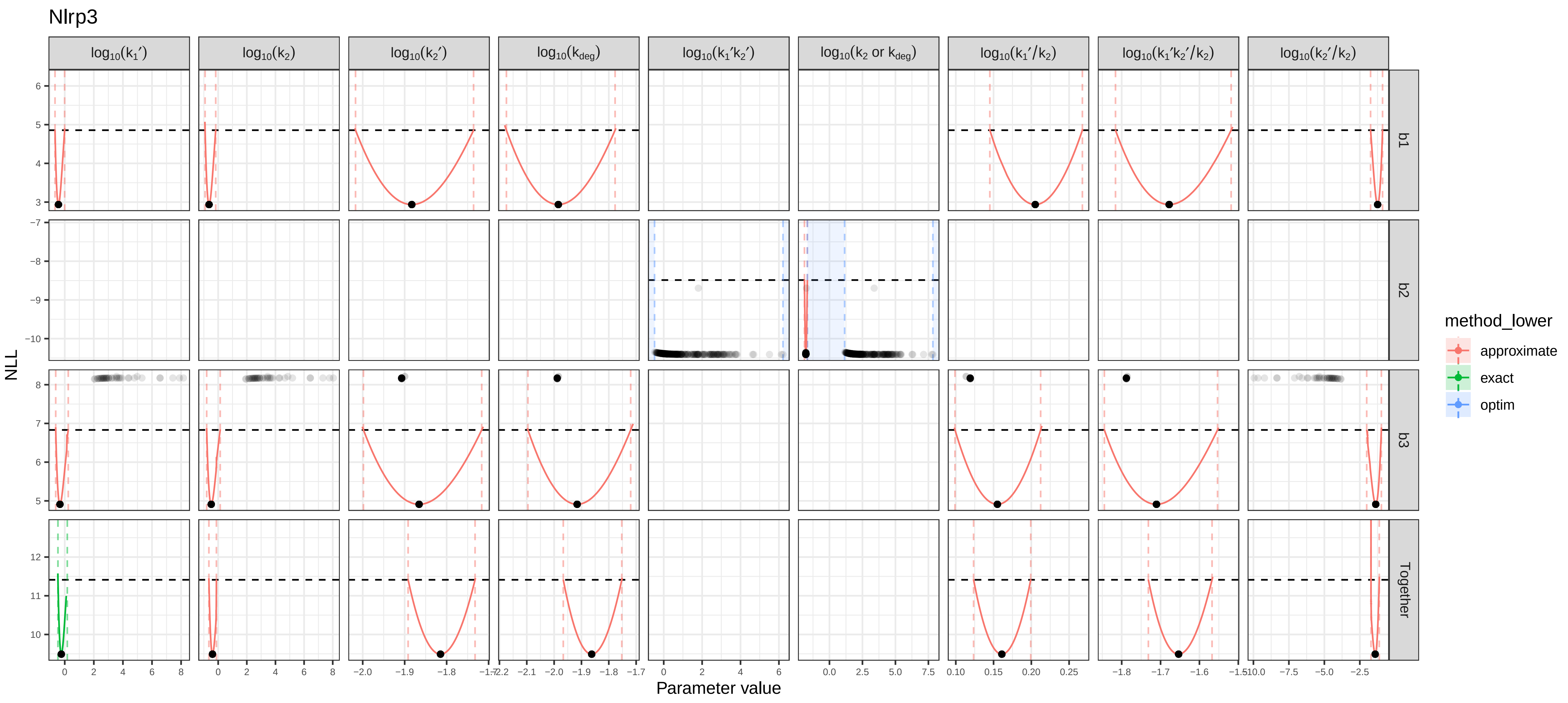

Replicate	Par	**Best value**	**CI95 LB**	**CI95 UB**	**Method LB**	**Method UB**
Together	$\log_{10}(k_1')$	−0.2369	−0.4674	0.1756	exact	exact
Together	$\log_{10}(k_2)$	−0.3979	−0.6486	−0.1242	approximate	approximate
Together	$\log_{10}(k_2')$	−1.815	−1.892	−1.732	approximate	approximate
Together	$\log_{10}(k_{deg})$	−1.863	−1.966	−1.752	approximate	approximate
Together	$\log_{10}(k_1'/k_2)$	0.161	0.1236	0.1992	approximate	approximate
Together	$\log_{10}(k_1'k_2'/k_2)$	−1.654	−1.731	−1.568	approximate	approximate
Together	$\log_{10}(k_2'/k_2)$	−1.417	−1.712	−1.13	approximate	approximate
b1	$\log_{10}(k_1')$	−0.4329	−0.6693	−0.007017	approximate	approximate
b1	$\log_{10}(k_2)$	−0.6381	−0.9144	−0.1727	approximate	approximate
b1	$\log_{10}(k_2')$	−1.883	−2.017	−1.736	approximate	approximate
b1	$\log_{10}(k_{deg})$	−1.985	−2.174	−1.777	approximate	approximate
b1	$\log_{10}(k_1'/k_2)$	0.2052	0.1451	0.2676	approximate	approximate
b1	$\log_{10}(k_1'k_2'/k_2)$	−1.678	−1.816	−1.518	approximate	approximate
b1	$\log_{10}(k_2'/k_2)$	−1.245	−1.742	−0.8975	approximate	approximate
b2	$\log_{10}(k_1'k_2')$	3.712	< −0.4775	> 6.214	optim	optim
b2	$\log_{10}(k_2 \text{ or } k_{deg})$	5.34	1.149	> 7.841	optim	optim
b2	$\log_{10}(k_2 \text{ or } k_{deg})$	−1.79	−1.896	−1.678	approximate	approximate
b3	$\log_{10}(k_1')$	−0.3309	−0.6204	0.2411	approximate	approximate
b3	$\log_{10}(k_2)$	−0.4859	−0.8038	0.1412	approximate	approximate
b3	$\log_{10}(k_2')$	−1.866	−1.998	−1.716	approximate	approximate
b3	$\log_{10}(k_{deg})$	−1.916	−2.096	−1.72	approximate	approximate
b3	$\log_{10}(k_1'/k_2)$	0.155	0.09894	0.2123	approximate	approximate
b3	$\log_{10}(k_1'k_2'/k_2)$	−1.711	−1.845	−1.553	approximate	approximate
b3	$\log_{10}(k_2'/k_2)$	−1.38	−2.014	−0.9818	approximate	approximate

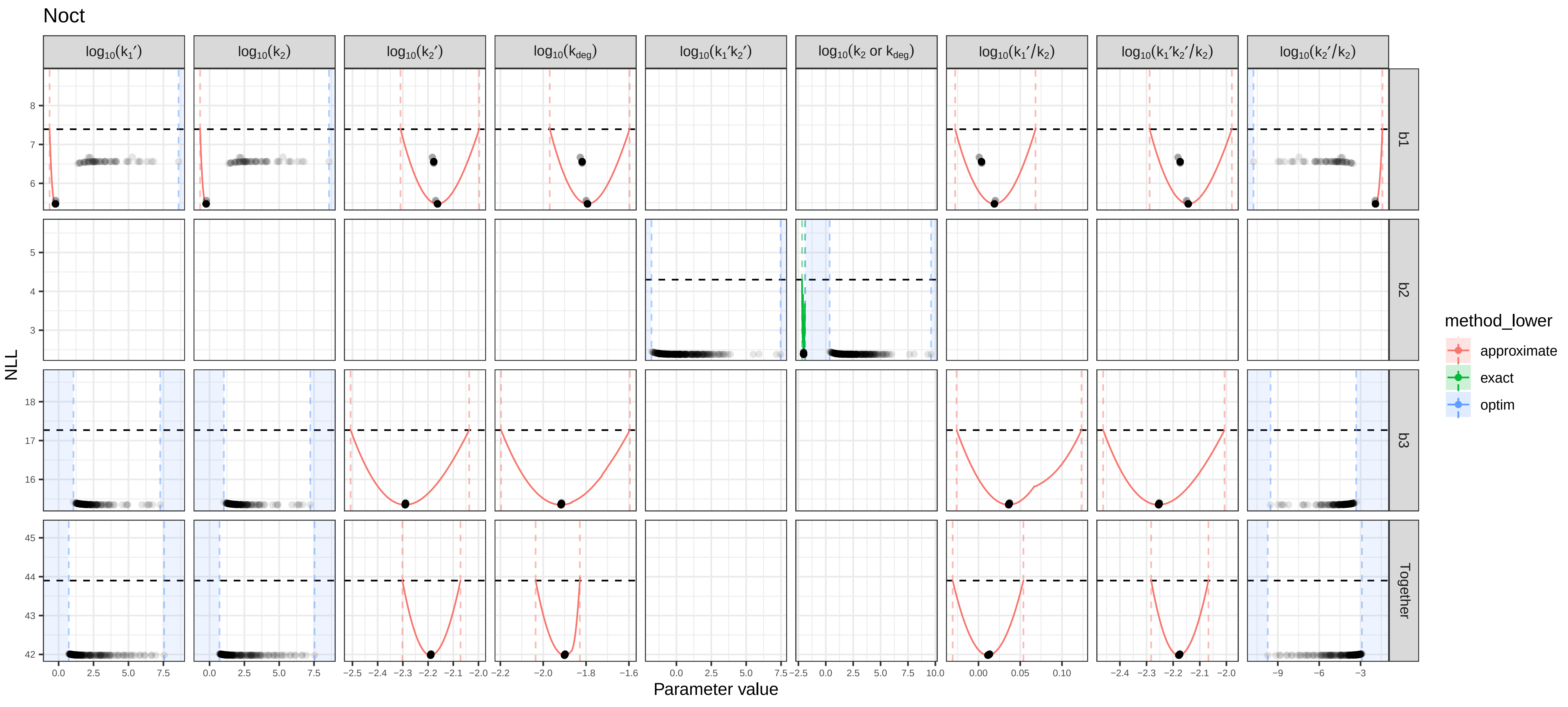

Replicate	Par	Best value	CI95 LB	CI95 UB	Method LB	Method UB
Together	$\log_{10}(k_1')$	6.186	< 0.7285	> 7.552	optim	optim
Together	$\log_{10}(k_2)$	6.175	< 0.7149	> 7.541	optim	optim
Together	$\log_{10}(k_2')$	−2.19	−2.302	−2.071	approximate	approximate
Together	$\log_{10}(k_{deg})$	−1.901	−2.035	−1.829	approximate	approximate
Together	$\log_{10}(k_1'/k_2)$	0.01128	−0.03095	0.05381	approximate	approximate
Together	$\log_{10}(k_1'k_2'/k_2)$	−2.179	−2.283	−2.068	approximate	approximate
Together	$\log_{10}(k_2'/k_2)$	−8.365	< −9.731	> −2.903	optim	optim
b1	$\log_{10}(k_1')$	−0.2253	−0.6455	> 8.586	approximate	optim
b1	$\log_{10}(k_2)$	−0.2443	−0.6789	> 8.582	approximate	optim
b1	$\log_{10}(k_2')$	−2.162	−2.309	−1.997	approximate	approximate
b1	$\log_{10}(k_{deg})$	−1.793	−1.969	−1.597	approximate	approximate
b1	$\log_{10}(k_1'/k_2)$	0.01906	−0.02794	0.0683	approximate	approximate
b1	$\log_{10}(k_1'k_2'/k_2)$	−2.143	−2.288	−1.979	approximate	approximate
b1	$\log_{10}(k_2'/k_2)$	−1.918	< −10.76	−1.41	optim	approximate
b2	$\log_{10}(k_1'k_2')$	7.175	< −1.784	> 7.464	optim	optim
b2	$\log_{10}(k_2 \text{ or } k_{deg})$	9.317	0.3539	> 9.606	optim	optim
b2	$\log_{10}(k_2 \text{ or } k_{deg})$	−2.027	−2.168	−1.887	exact	exact
b3	$\log_{10}(k_1')$	3.985	< 1.065	> 7.271	optim	optim
b3	$\log_{10}(k_2)$	3.949	< 1.027	> 7.235	optim	optim
b3	$\log_{10}(k_2')$	−2.291	−2.507	−2.037	approximate	approximate
b3	$\log_{10}(k_{deg})$	−1.917	−2.197	−1.595	approximate	approximate
b3	$\log_{10}(k_1'/k_2)$	0.03607	−0.02626	0.1233	approximate	approximate
b3	$\log_{10}(k_1'k_2'/k_2)$	−2.255	−2.463	−2.007	approximate	approximate
b3	$\log_{10}(k_2'/k_2)$	−6.24	< −9.526	> −3.316	optim	optim

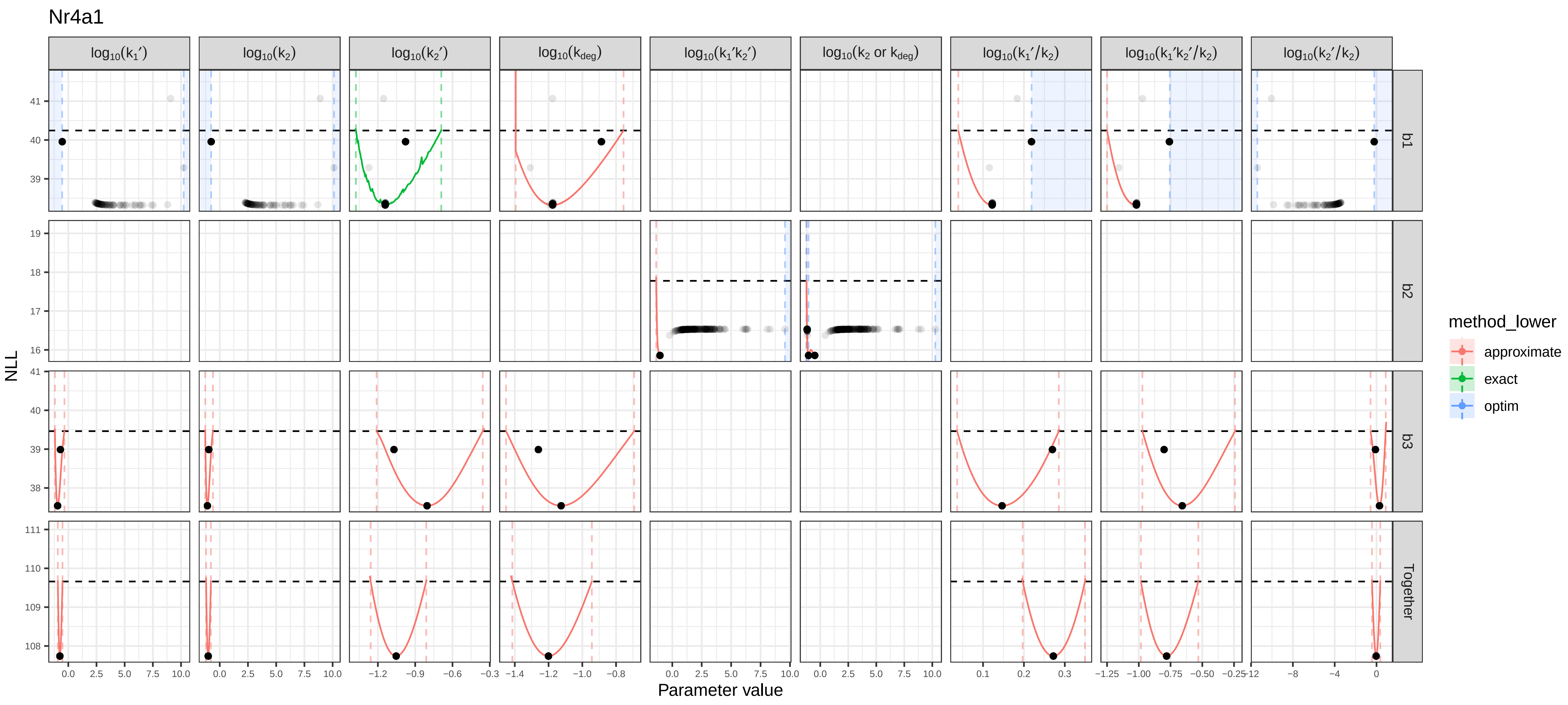

Replicate	Par	Best value	CI95 LB	CI95 UB	Method LB	Method UB
Together	$\log_{10}(k_1')$	−0.7479	−0.9275	−0.5137	approximate	approximate
Together	$\log_{10}(k_2)$	−1.02	−1.208	−0.7802	approximate	approximate
Together	$\log_{10}(k_2')$	−1.053	−1.258	−0.8111	approximate	approximate
Together	$\log_{10}(k_{deg})$	−1.201	−1.415	−0.9432	approximate	approximate
Together	$\log_{10}(k_1'/k_2)$	0.2719	0.197	0.3493	approximate	approximate
Together	$\log_{10}(k_1'k_2'/k_2)$	−0.7811	−0.9834	−0.5311	approximate	approximate
Together	$\log_{10}(k_2'/k_2)$	−0.03316	−0.4303	0.3613	approximate	approximate
b1	$\log_{10}(k_1')$	6.381	< −0.5413	> 10.24	optim	optim
b1	$\log_{10}(k_2)$	6.259	< −0.7601	> 10.12	optim	optim
b1	$\log_{10}(k_2')$	−1.141	−1.377	−0.6902	exact	exact
b1	$\log_{10}(k_{deg})$	−1.176	−1.395	−0.7555	approximate	approximate
b1	$\log_{10}(k_1'/k_2)$	0.1223	0.03945	> 0.2188	approximate	optim
b1	$\log_{10}(k_1'k_2'/k_2)$	−1.019	−1.25	> −0.7559	approximate	optim
b1	$\log_{10}(k_2'/k_2)$	−7.4	< −11.4	> −0.2146	optim	optim
b2	$\log_{10}(k_1'k_2')$	−1.049	−1.37	> 9.574	approximate	optim
b2	$\log_{10}(k_2 \text{ or } k_{deg})$	−0.4897	−1.233	> 10.27	approximate	optim
b2	$\log_{10}(k_2 \text{ or } k_{deg})$	−1.045	−1.233	−1.044	approximate	optim
b3	$\log_{10}(k_1')$	−0.9458	−1.187	−0.3425	approximate	approximate
b3	$\log_{10}(k_2)$	−1.092	−1.285	−0.6009	approximate	approximate
b3	$\log_{10}(k_2')$	−0.8045	−1.21	−0.3567	approximate	approximate
b3	$\log_{10}(k_{deg})$	−1.126	−1.452	−0.6927	approximate	approximate
b3	$\log_{10}(k_1'/k_2)$	0.1466	0.03668	0.2854	approximate	approximate
b3	$\log_{10}(k_1'k_2'/k_2)$	−0.6579	−0.9717	−0.242	approximate	approximate
b3	$\log_{10}(k_2'/k_2)$	0.2879	−0.5694	0.8814	approximate	approximate

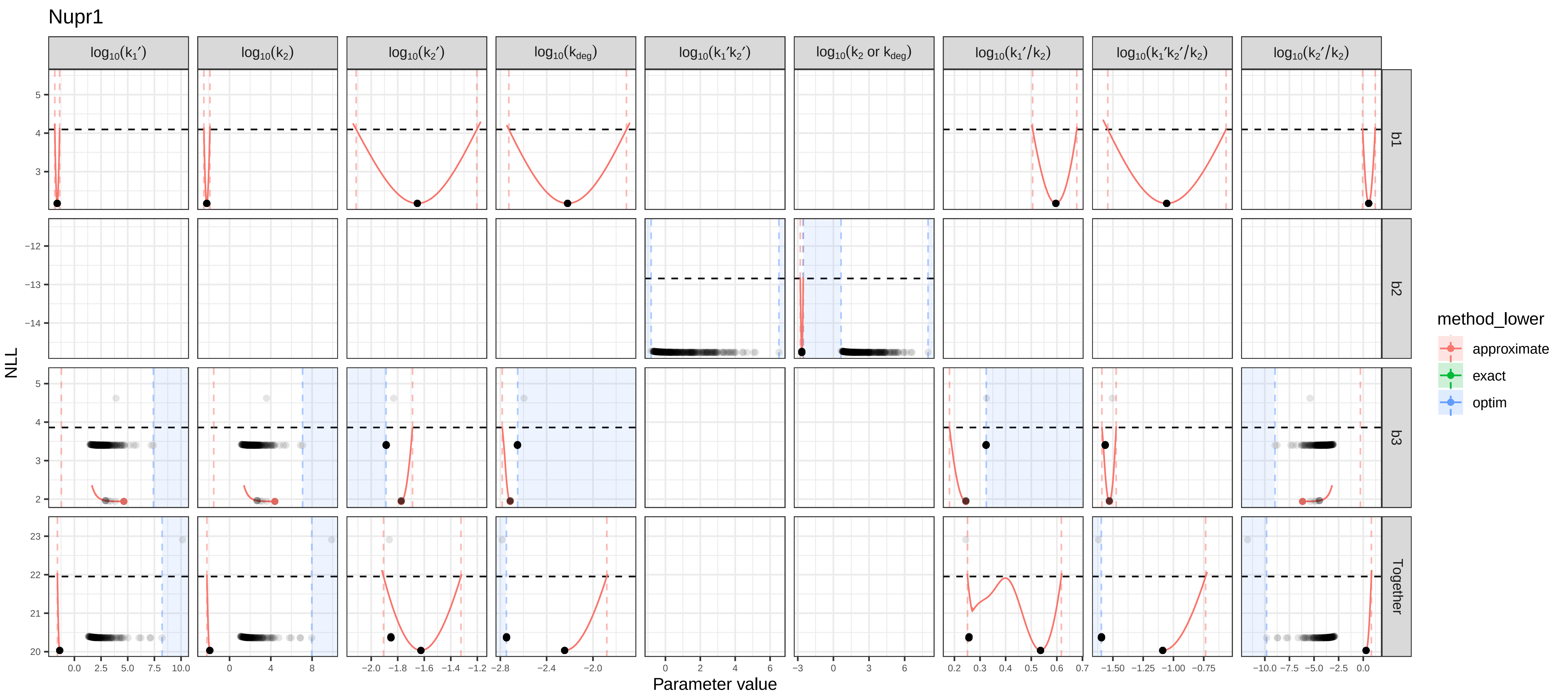

Replicate	Par	Best value	CI95 LB	CI95 UB	Method LB	Method UB
Together	$\log_{10}(k_1')$	−1.378	−1.59	> 8.226	approximate	optim
Together	$\log_{10}(k_2)$	−1.915	−2.192	> 7.969	approximate	optim
Together	$\log_{10}(k_2')$	−1.625	−1.907	−1.323	approximate	approximate
Together	$\log_{10}(k_{deg})$	−2.245	< −2.748	−1.88	optim	approximate
Together	$\log_{10}(k_1'/k_2)$	0.5363	0.2512	0.6176	approximate	approximate
Together	$\log_{10}(k_1'k_2'/k_2)$	−1.089	< −1.595	−0.7342	optim	approximate
Together	$\log_{10}(k_2'/k_2)$	0.2896	< −9.82	0.8275	optim	approximate
b1	$\log_{10}(k_1')$	−1.613	−1.834	−1.382	approximate	approximate
b1	$\log_{10}(k_2)$	−2.21	−2.49	−1.902	approximate	approximate
b1	$\log_{10}(k_2')$	−1.652	−2.113	−1.204	approximate	approximate
b1	$\log_{10}(k_{deg})$	−2.219	−2.726	−1.711	approximate	approximate
b1	$\log_{10}(k_1'/k_2)$	0.5961	0.5054	0.6778	approximate	approximate
b1	$\log_{10}(k_1'k_2'/k_2)$	−1.056	−1.542	−0.5653	approximate	approximate
b1	$\log_{10}(k_2'/k_2)$	0.5574	−0.06293	1.218	approximate	approximate
b2	$\log_{10}(k_1'k_2')$	3.339	< −0.818	> 6.507	optim	optim
b2	$\log_{10}(k_2 \text{ or } k_{deg})$	4.803	0.6452	> 7.971	optim	optim
b2	$\log_{10}(k_2 \text{ or } k_{deg})$	−2.659	−2.792	−2.542	approximate	approximate
b3	$\log_{10}(k_1')$	4.636	−1.226	> 7.403	approximate	optim
b3	$\log_{10}(k_2)$	4.391	−1.535	> 7.08	approximate	optim
b3	$\log_{10}(k_2')$	−1.774	< −1.888	−1.688	optim	approximate
b3	$\log_{10}(k_{deg})$	−2.715	−2.784	> −2.65	approximate	optim
b3	$\log_{10}(k_1'/k_2)$	0.2445	0.1806	> 0.3242	approximate	optim
b3	$\log_{10}(k_1'k_2'/k_2)$	−1.529	−1.591	−1.473	approximate	approximate
b3	$\log_{10}(k_2'/k_2)$	−6.165	< −8.967	−0.2904	optim	approximate

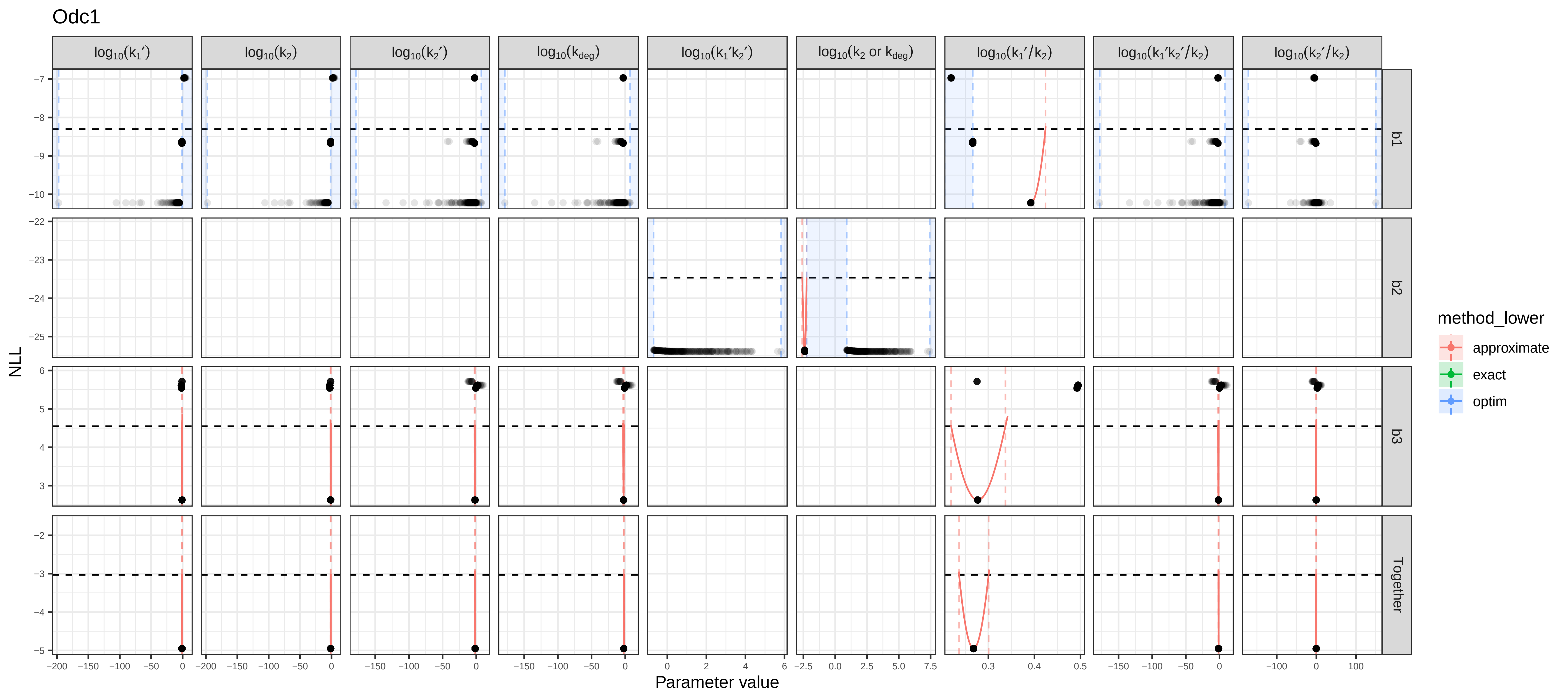

Replicate	Par	Best value	CI95 LB	CI95 UB	Method LB	Method UB
Together	$\log_{10}(k_1')$	−1.02	−1.209	−0.7787	approximate	approximate
Together	$\log_{10}(k_2)$	−1.288	−1.496	−1.031	approximate	approximate
Together	$\log_{10}(k_2')$	−1.614	−1.856	−1.394	approximate	approximate
Together	$\log_{10}(k_{deg})$	−2.146	−2.41	−1.902	approximate	approximate
Together	$\log_{10}(k_1'/k_2)$	0.2679	0.2364	0.3006	approximate	approximate
Together	$\log_{10}(k_1'k_2'/k_2)$	−1.346	−1.59	−1.122	approximate	approximate
Together	$\log_{10}(k_2'/k_2)$	−0.3253	−0.679	0.01838	approximate	approximate
b1	$\log_{10}(k_1')$	−16.53	< −197.3	> −1.112	optim	optim
b1	$\log_{10}(k_2)$	−16.93	< −197.6	> −1.378	optim	optim
b1	$\log_{10}(k_2')$	−36.19	< −178.4	> 7.573	optim	optim
b1	$\log_{10}(k_{deg})$	−36.59	< −178.8	> 7.176	optim	optim
b1	$\log_{10}(k_1'/k_2)$	0.3922	< 0.266	0.4241	optim	approximate
b1	$\log_{10}(k_1'k_2'/k_2)$	−35.8	< −178	> 7.965	optim	optim
b1	$\log_{10}(k_2'/k_2)$	−19.27	< −171.6	> 150.3	optim	optim
b2	$\log_{10}(k_1'k_2')$	3.145	< −0.7074	> 5.82	optim	optim
b2	$\log_{10}(k_2 \text{ or } k_{deg})$	4.756	0.9058	> 7.431	optim	optim
b2	$\log_{10}(k_2 \text{ or } k_{deg})$	−2.396	−2.59	−2.246	approximate	approximate
b3	$\log_{10}(k_1')$	−1.003	−1.262	−0.6617	approximate	approximate
b3	$\log_{10}(k_2)$	−1.28	−1.581	−0.9059	approximate	approximate
b3	$\log_{10}(k_2')$	−1.778	−2.51	−1.399	approximate	approximate
b3	$\log_{10}(k_{deg})$	−2.406	−3.185	−1.976	approximate	approximate
b3	$\log_{10}(k_1'/k_2)$	0.2769	0.2191	0.3371	approximate	approximate
b3	$\log_{10}(k_1'k_2'/k_2)$	−1.502	−2.235	−1.116	approximate	approximate
b3	$\log_{10}(k_2'/k_2)$	−0.4989	−1.263	0.02597	approximate	approximate

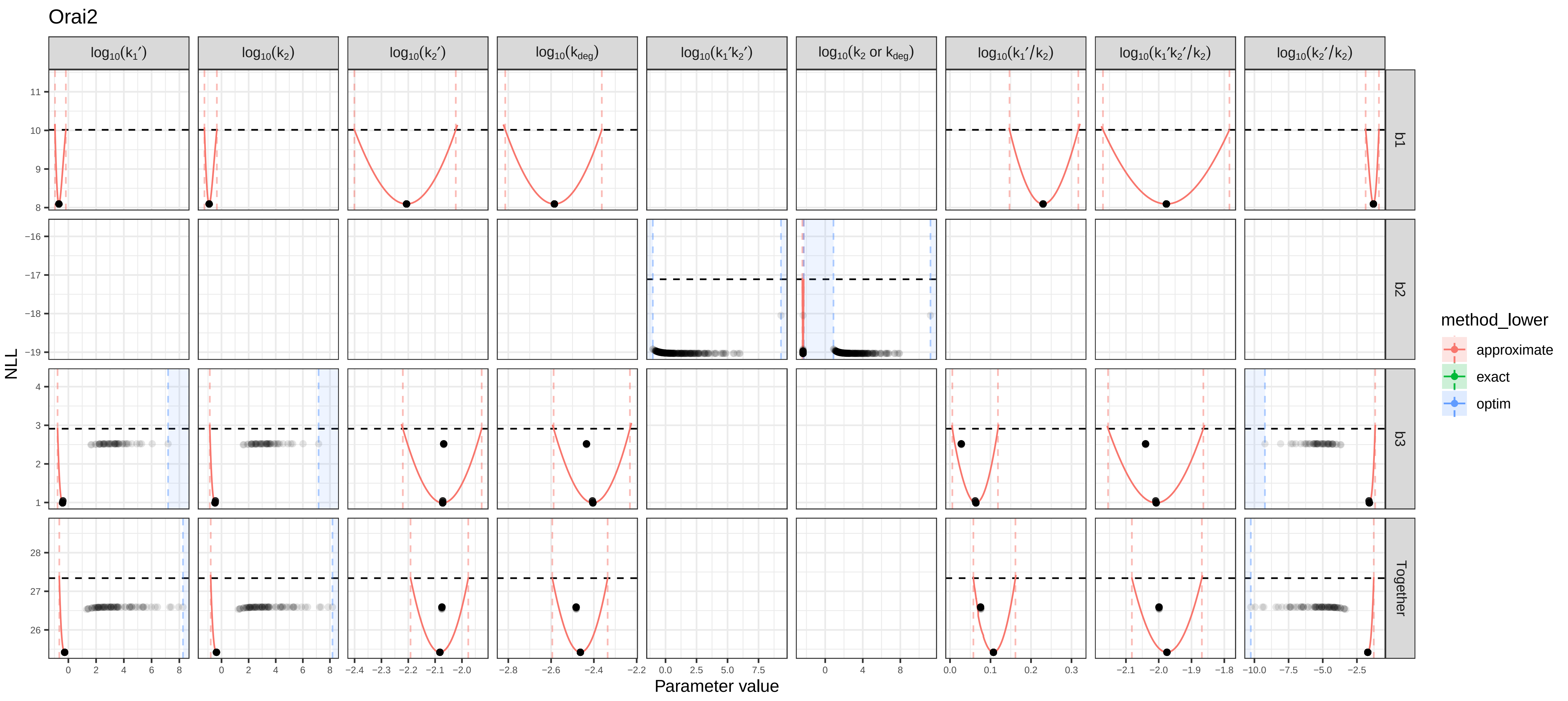

Replicate	Par	Best value	CI95 LB	CI95 UB	Method LB	Method UB
Together	$\log_{10}(k_1')$	−0.268	−0.6523	> 8.263	approximate	optim
Together	$\log_{10}(k_2)$	−0.3752	−0.7965	> 8.188	approximate	optim
Together	$\log_{10}(k_2')$	−2.082	−2.192	−1.977	approximate	approximate
Together	$\log_{10}(k_{deg})$	−2.463	−2.594	−2.336	approximate	approximate
Together	$\log_{10}(k_1'/k_2)$	0.1072	0.05768	0.1615	approximate	approximate
Together	$\log_{10}(k_1'k_2'/k_2)$	−1.975	−2.082	−1.868	approximate	approximate
Together	$\log_{10}(k_2'/k_2)$	−1.707	< −10.26	−1.268	optim	approximate
b1	$\log_{10}(k_1')$	−0.6832	−0.9623	−0.1811	approximate	approximate
b1	$\log_{10}(k_2)$	−0.913	−1.26	−0.3449	approximate	approximate
b1	$\log_{10}(k_2')$	−2.207	−2.401	−2.023	approximate	approximate
b1	$\log_{10}(k_{deg})$	−2.585	−2.814	−2.363	approximate	approximate
b1	$\log_{10}(k_1'/k_2)$	0.2298	0.147	0.3168	approximate	approximate
b1	$\log_{10}(k_1'k_2'/k_2)$	−1.977	−2.17	−1.784	approximate	approximate
b1	$\log_{10}(k_2'/k_2)$	−1.294	−1.863	−0.8895	approximate	approximate
b2	$\log_{10}(k_1'k_2')$	4.619	< −1.023	> 9.309	optim	optim
b2	$\log_{10}(k_2 \text{ or } k_{deg})$	6.524	0.8815	> 11.21	optim	optim
b2	$\log_{10}(k_2 \text{ or } k_{deg})$	−2.365	−2.435	−2.293	approximate	approximate
b3	$\log_{10}(k_1')$	−0.4165	−0.7779	> 7.19	approximate	optim
b3	$\log_{10}(k_2)$	−0.4805	−0.8641	> 7.162	approximate	optim
b3	$\log_{10}(k_2')$	−2.072	−2.22	−1.926	approximate	approximate
b3	$\log_{10}(k_{deg})$	−2.405	−2.588	−2.231	approximate	approximate
b3	$\log_{10}(k_1'/k_2)$	0.06395	0.005784	0.1184	approximate	approximate
b3	$\log_{10}(k_1'k_2'/k_2)$	−2.008	−2.154	−1.864	approximate	approximate
b3	$\log_{10}(k_2'/k_2)$	−1.591	< −9.23	−1.165	optim	approximate

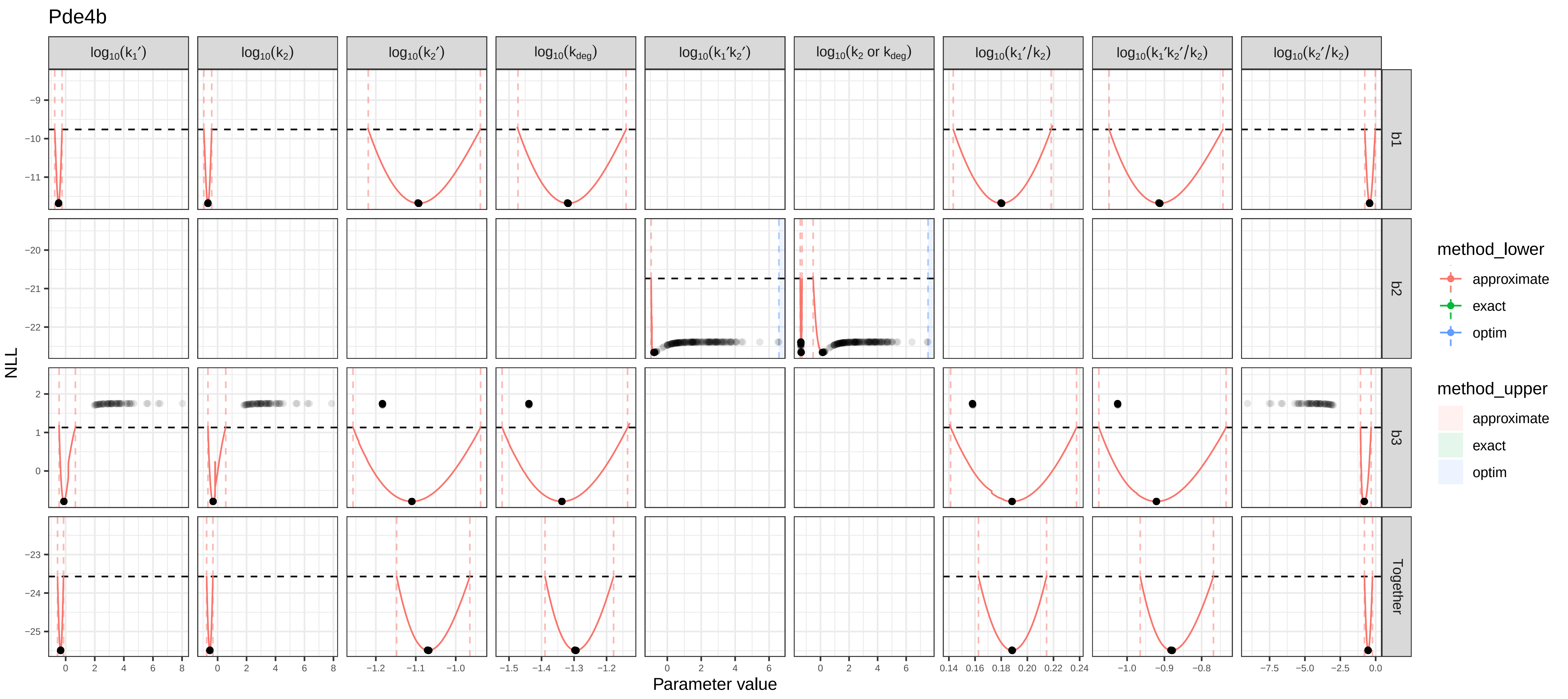

Replicate	Par	Best value	CI95 LB	CI95 UB	Method LB	Method UB
Together	$\log_{10}(k_1')$	−0.3486	−0.5589	−0.1487	approximate	approximate
Together	$\log_{10}(k_2)$	−0.537	−0.7573	−0.3242	approximate	approximate
Together	$\log_{10}(k_2')$	−1.068	−1.148	−0.9646	approximate	approximate
Together	$\log_{10}(k_{deg})$	−1.295	−1.389	−1.179	approximate	approximate
Together	$\log_{10}(k_1'/k_2)$	0.1884	0.1626	0.2147	approximate	approximate
Together	$\log_{10}(k_1'k_2'/k_2)$	−0.8798	−0.9648	−0.7684	approximate	approximate
Together	$\log_{10}(k_2'/k_2)$	−0.5313	−0.7944	−0.2229	approximate	approximate
b1	$\log_{10}(k_1')$	−0.4908	−0.7525	−0.2479	approximate	approximate
b1	$\log_{10}(k_2)$	−0.6711	−0.9491	−0.4069	approximate	approximate
b1	$\log_{10}(k_2')$	−1.093	−1.219	−0.9384	approximate	approximate
b1	$\log_{10}(k_{deg})$	−1.318	−1.473	−1.14	approximate	approximate
b1	$\log_{10}(k_1'/k_2)$	0.1803	0.1433	0.2183	approximate	approximate
b1	$\log_{10}(k_1'k_2'/k_2)$	−0.9123	−1.048	−0.7429	approximate	approximate
b1	$\log_{10}(k_2'/k_2)$	−0.4215	−0.7667	−0.02036	approximate	approximate
b2	$\log_{10}(k_1'k_2')$	−0.7756	−0.9527	> 6.582	approximate	optim
b2	$\log_{10}(k_2 \text{ or } k_{deg})$	0.1516	−0.5159	> 7.531	approximate	optim
b2	$\log_{10}(k_2 \text{ or } k_{deg})$	−1.355	−1.419	−1.292	approximate	approximate
b3	$\log_{10}(k_1')$	−0.1225	−0.4514	0.668	approximate	approximate
b3	$\log_{10}(k_2)$	−0.3108	−0.6673	0.5639	approximate	approximate
b3	$\log_{10}(k_2')$	−1.11	−1.257	−0.9377	approximate	approximate
b3	$\log_{10}(k_{deg})$	−1.337	−1.521	−1.136	approximate	approximate
b3	$\log_{10}(k_1'/k_2)$	0.1883	0.1413	0.2376	approximate	approximate
b3	$\log_{10}(k_1'k_2'/k_2)$	−0.9214	−1.076	−0.7343	approximate	approximate
b3	$\log_{10}(k_2'/k_2)$	−0.799	−1.072	−0.3167	approximate	approximate

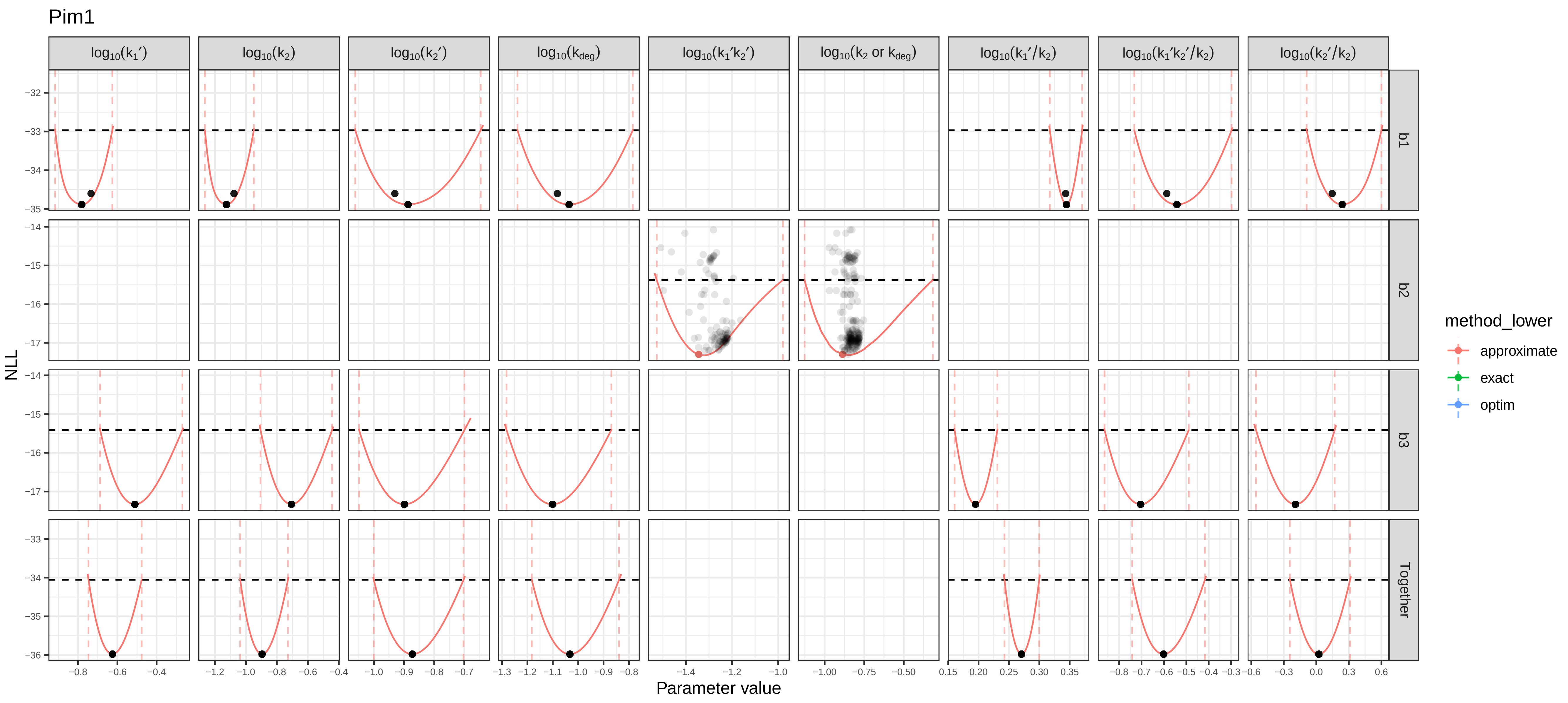

Replicate	Par	Best value	CI95 LB	CI95 UB	Method LB	Method UB
Together	$\log_{10}(k_1')$	−0.6248	−0.7469	−0.4763	approximate	approximate
Together	$\log_{10}(k_2)$	−0.8956	−1.037	−0.7288	approximate	approximate
Together	$\log_{10}(k_2')$	−0.8722	−1	−0.7024	approximate	approximate
Together	$\log_{10}(k_{deg})$	−1.032	−1.182	−0.8392	approximate	approximate
Together	$\log_{10}(k_1'/k_2)$	0.2708	0.2427	0.2998	approximate	approximate
Together	$\log_{10}(k_1'k_2'/k_2)$	−0.6014	−0.7415	−0.417	approximate	approximate
Together	$\log_{10}(k_2'/k_2)$	0.02347	−0.2441	0.3112	approximate	approximate
b1	$\log_{10}(k_1')$	−0.7816	−0.9165	−0.6257	approximate	approximate
b1	$\log_{10}(k_2)$	−1.126	−1.265	−0.9492	approximate	approximate
b1	$\log_{10}(k_2')$	−0.8865	−1.062	−0.6455	approximate	approximate
b1	$\log_{10}(k_{deg})$	−1.035	−1.239	−0.785	approximate	approximate
b1	$\log_{10}(k_1'/k_2)$	0.3447	0.3172	0.3705	approximate	approximate
b1	$\log_{10}(k_1'k_2'/k_2)$	−0.5418	−0.7321	−0.2978	approximate	approximate
b1	$\log_{10}(k_2'/k_2)$	0.2398	−0.08911	0.6002	approximate	approximate
b2	$\log_{10}(k_1'k_2')$	−1.344	−1.526	−0.9795	approximate	approximate
b2	$\log_{10}(k_2 \text{ or } k_{deg})$	−0.852	−1.127	−0.3162	approximate	approximate
b2	$\log_{10}(k_2 \text{ or } k_{deg})$	−0.8872	−1.127	−0.3162	approximate	approximate
b3	$\log_{10}(k_1')$	−0.511	−0.6879	−0.2688	approximate	approximate
b3	$\log_{10}(k_2)$	−0.706	−0.9059	−0.4434	approximate	approximate
b3	$\log_{10}(k_2')$	−0.8988	−1.049	−0.6992	approximate	approximate
b3	$\log_{10}(k_{deg})$	−1.101	−1.282	−0.8695	approximate	approximate
b3	$\log_{10}(k_1'/k_2)$	0.195	0.1606	0.231	approximate	approximate
b3	$\log_{10}(k_1'k_2'/k_2)$	−0.7038	−0.8649	−0.4888	approximate	approximate
b3	$\log_{10}(k_2'/k_2)$	−0.1928	−0.5567	0.1696	approximate	approximate

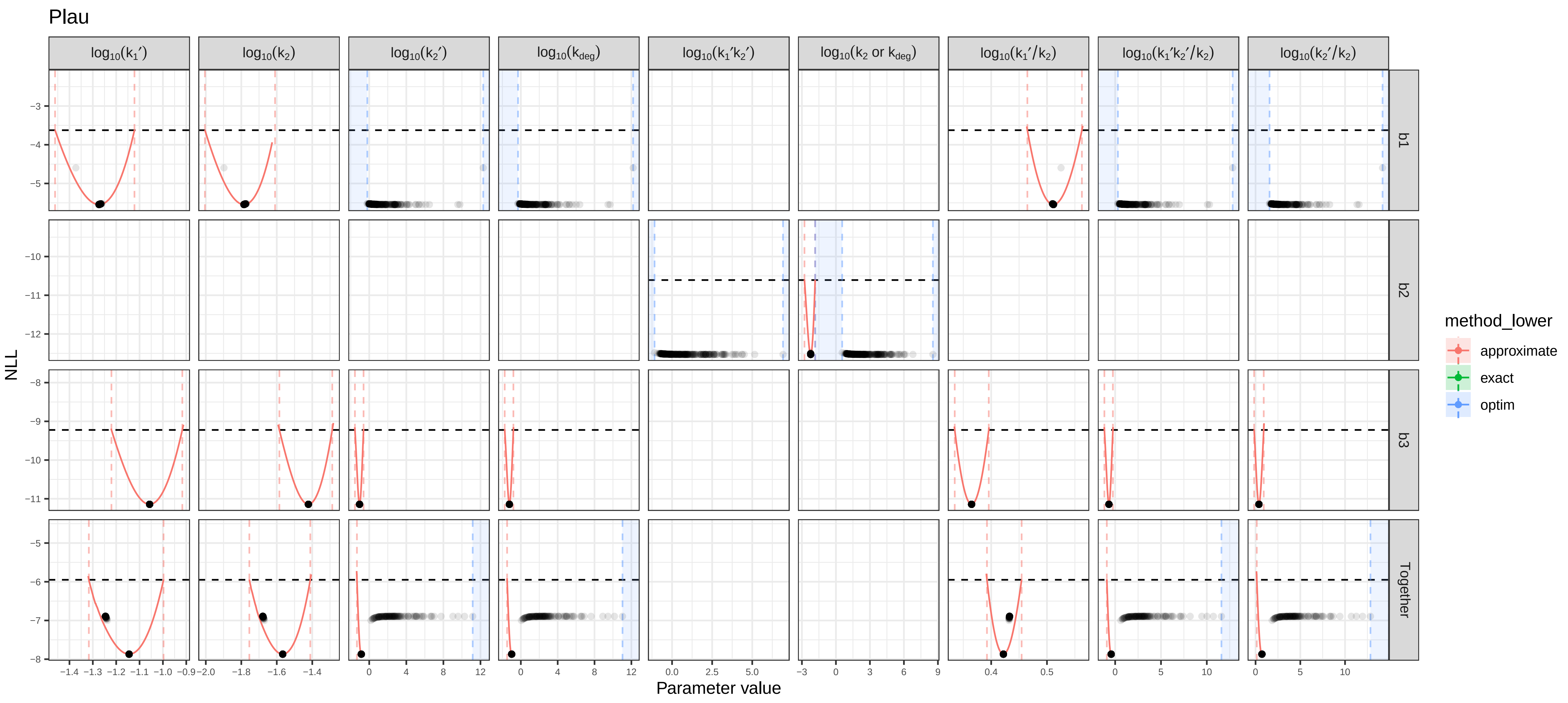

Replicate	Par	Best value	CI95 LB	CI95 UB	Method LB	Method UB
Together	$\log_{10}(k_1')$	−1.145	−1.317	−0.9971	approximate	approximate
Together	$\log_{10}(k_2)$	−1.567	−1.755	−1.411	approximate	approximate
Together	$\log_{10}(k_2')$	−0.8542	−1.325	> 11.16	approximate	optim
Together	$\log_{10}(k_{deg})$	−0.9972	−1.492	> 11.04	approximate	optim
Together	$\log_{10}(k_1'/k_2)$	0.4219	0.3924	0.4546	approximate	approximate
Together	$\log_{10}(k_1'k_2'/k_2)$	−0.4324	−0.9097	> 11.6	approximate	optim
Together	$\log_{10}(k_2'/k_2)$	0.7123	0.1378	> 12.84	approximate	optim
b1	$\log_{10}(k_1')$	−1.273	−1.462	−1.122	approximate	approximate
b1	$\log_{10}(k_2)$	−1.785	−2.005	−1.61	approximate	approximate
b1	$\log_{10}(k_2')$	6.49	< −0.2069	> 12.3	optim	optim
b1	$\log_{10}(k_{deg})$	6.396	< −0.3083	> 12.19	optim	optim
b1	$\log_{10}(k_1'/k_2)$	0.5115	0.4648	0.5626	approximate	approximate
b1	$\log_{10}(k_1'k_2'/k_2)$	7.001	< 0.3024	> 12.82	optim	optim
b1	$\log_{10}(k_2'/k_2)$	8.274	< 1.564	> 14.2	optim	optim
b2	$\log_{10}(k_1'k_2')$	2.503	< −1.091	> 6.919	optim	optim
b2	$\log_{10}(k_2 \text{ or } k_{deg})$	4.142	0.5526	> 8.558	optim	optim
b2	$\log_{10}(k_2 \text{ or } k_{deg})$	−2.228	−2.775	−1.833	approximate	approximate
b3	$\log_{10}(k_1')$	−1.057	−1.22	−0.9169	approximate	approximate
b3	$\log_{10}(k_2)$	−1.422	−1.585	−1.287	approximate	approximate
b3	$\log_{10}(k_2')$	−1.045	−1.541	−0.6106	approximate	approximate
b3	$\log_{10}(k_{deg})$	−1.243	−1.752	−0.8082	approximate	approximate
b3	$\log_{10}(k_1'/k_2)$	0.3649	0.3347	0.3957	approximate	approximate
b3	$\log_{10}(k_1'k_2'/k_2)$	−0.6798	−1.175	−0.2454	approximate	approximate
b3	$\log_{10}(k_2'/k_2)$	0.3771	−0.1605	0.9141	approximate	approximate

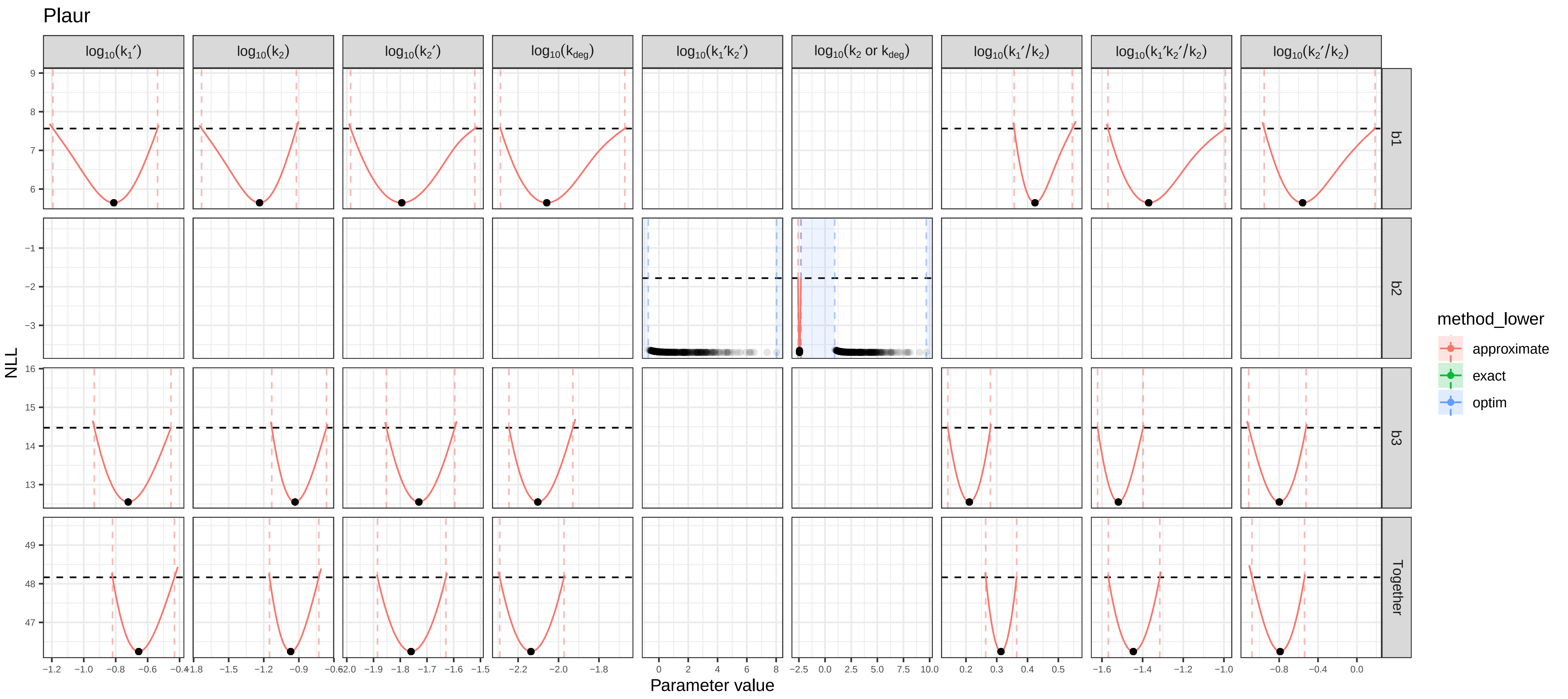

Replicate	Par	Best value	CI95 LB	CI95 UB	Method LB	Method UB
Together	$\log_{10}(k_1')$	−0.6559	−0.82	−0.4321	approximate	approximate
Together	$\log_{10}(k_2)$	−0.9697	−1.151	−0.729	approximate	approximate
Together	$\log_{10}(k_2')$	−1.76	−1.885	−1.629	approximate	approximate
Together	$\log_{10}(k_{deg})$	−2.136	−2.291	−1.973	approximate	approximate
Together	$\log_{10}(k_1'/k_2)$	0.3138	0.2642	0.3646	approximate	approximate
Together	$\log_{10}(k_1'k_2'/k_2)$	−1.446	−1.569	−1.315	approximate	approximate
Together	$\log_{10}(k_2'/k_2)$	−0.7898	−1.08	−0.5389	approximate	approximate
b1	$\log_{10}(k_1')$	−0.8119	−1.193	−0.5381	approximate	approximate
b1	$\log_{10}(k_2)$	−1.236	−1.732	−0.9212	approximate	approximate
b1	$\log_{10}(k_2')$	−1.794	−1.986	−1.521	approximate	approximate
b1	$\log_{10}(k_{deg})$	−2.058	−2.288	−1.672	approximate	approximate
b1	$\log_{10}(k_1'/k_2)$	0.4241	0.3567	0.5453	approximate	approximate
b1	$\log_{10}(k_1'k_2'/k_2)$	−1.37	−1.571	−0.993	approximate	approximate
b1	$\log_{10}(k_2'/k_2)$	−0.5584	−0.954	0.1872	approximate	approximate
b2	$\log_{10}(k_1'k_2')$	5.39	< -0.7266	> 8.03	optim	optim
b2	$\log_{10}(k_2 \text{ or } k_{deg})$	7.041	0.9231	> 9.68	optim	optim
b2	$\log_{10}(k_2 \text{ or } k_{deg})$	−2.447	−2.589	−2.306	approximate	approximate
b3	$\log_{10}(k_1')$	−0.7216	−0.9341	−0.454	approximate	approximate
b3	$\log_{10}(k_2)$	−0.9325	−1.132	−0.6637	approximate	approximate
b3	$\log_{10}(k_2')$	−1.73	−1.852	−1.596	approximate	approximate
b3	$\log_{10}(k_{deg})$	−2.102	−2.245	−1.929	approximate	approximate
b3	$\log_{10}(k_1'/k_2)$	0.211	0.1412	0.2795	approximate	approximate
b3	$\log_{10}(k_1'k_2'/k_2)$	−1.519	−1.622	−1.397	approximate	approximate
b3	$\log_{10}(k_2'/k_2)$	−0.7979	−1.113	−0.5229	approximate	approximate

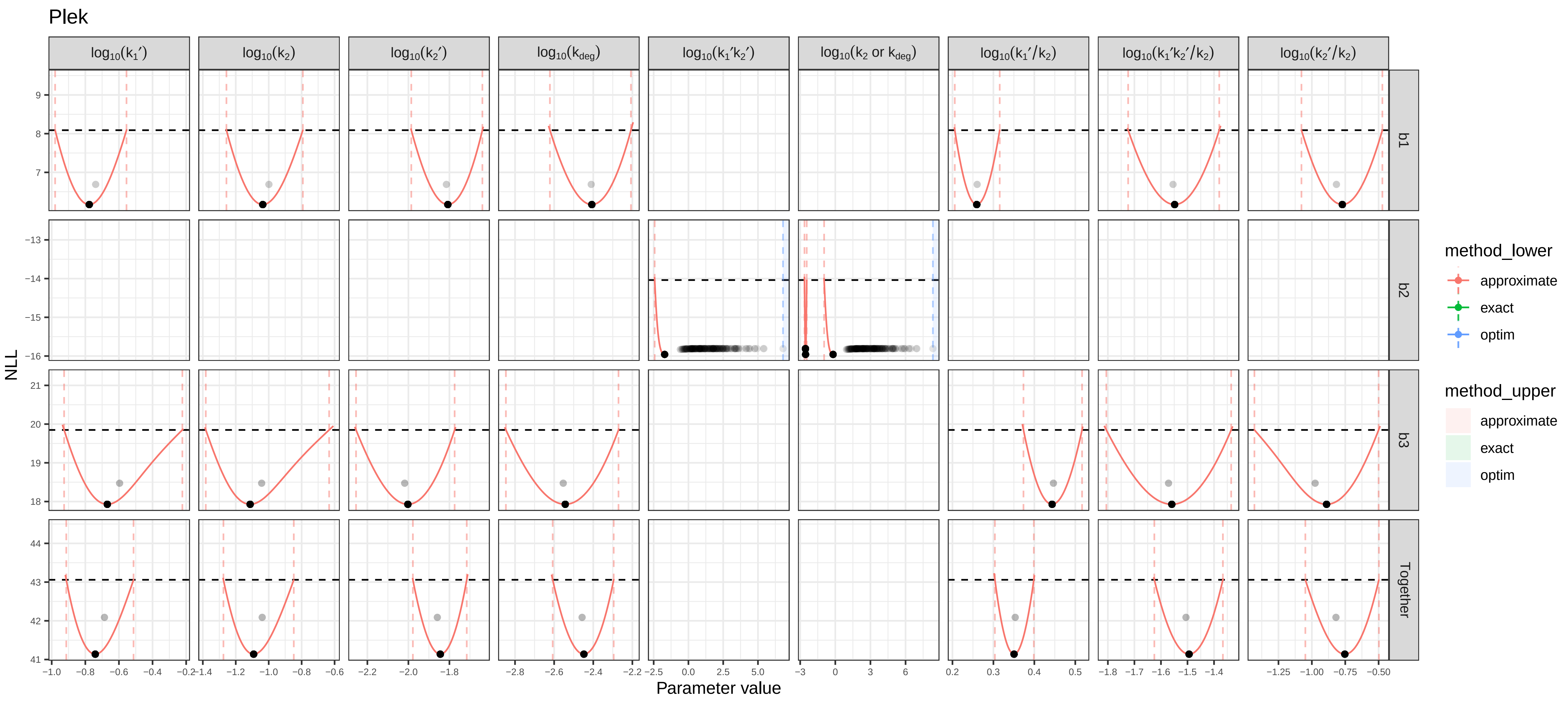

Replicate	Par	Best value	CI95 LB	CI95 UB	Method LB	Method UB
Together	$\log_{10}(k_1')$	−0.7408	−0.9135	−0.5132	approximate	approximate
Together	$\log_{10}(k_2)$	−1.091	−1.275	−0.8477	approximate	approximate
Together	$\log_{10}(k_2')$	−1.844	−1.978	−1.716	approximate	approximate
Together	$\log_{10}(k_{deg})$	−2.448	−2.607	−2.295	approximate	approximate
Together	$\log_{10}(k_1'/k_2)$	0.3504	0.3037	0.3988	approximate	approximate
Together	$\log_{10}(k_1'k_2'/k_2)$	−1.494	−1.625	−1.367	approximate	approximate
Together	$\log_{10}(k_2'/k_2)$	−0.7531	−1.049	−0.4979	approximate	approximate
b1	$\log_{10}(k_1')$	−0.7767	−0.9797	−0.555	approximate	approximate
b1	$\log_{10}(k_2)$	−1.036	−1.257	−0.7926	approximate	approximate
b1	$\log_{10}(k_2')$	−1.808	−1.986	−1.639	approximate	approximate
b1	$\log_{10}(k_{deg})$	−2.407	−2.622	−2.207	approximate	approximate
b1	$\log_{10}(k_1'/k_2)$	0.2594	0.2057	0.3154	approximate	approximate
b1	$\log_{10}(k_1'k_2'/k_2)$	−1.548	−1.724	−1.38	approximate	approximate
b1	$\log_{10}(k_2'/k_2)$	−0.7717	−1.078	−0.4718	approximate	approximate
b2	$\log_{10}(k_1'k_2')$	−1.712	−2.426	> 6.802	approximate	optim
b2	$\log_{10}(k_2 \text{ or } k_{deg})$	−0.1909	−0.955	> 8.336	approximate	optim
b2	$\log_{10}(k_2 \text{ or } k_{deg})$	−2.539	−2.633	−2.44	approximate	approximate
b3	$\log_{10}(k_1')$	−0.6694	−0.9263	−0.2226	approximate	approximate
b3	$\log_{10}(k_2)$	−1.113	−1.382	−0.6331	approximate	approximate
b3	$\log_{10}(k_2')$	−2.003	−2.255	−1.775	approximate	approximate
b3	$\log_{10}(k_{deg})$	−2.543	−2.847	−2.271	approximate	approximate
b3	$\log_{10}(k_1'/k_2)$	0.4435	0.3735	0.5166	approximate	approximate
b3	$\log_{10}(k_1'k_2'/k_2)$	−1.559	−1.806	−1.335	approximate	approximate
b3	$\log_{10}(k_2'/k_2)$	−0.8897	−1.43	−0.4999	approximate	approximate

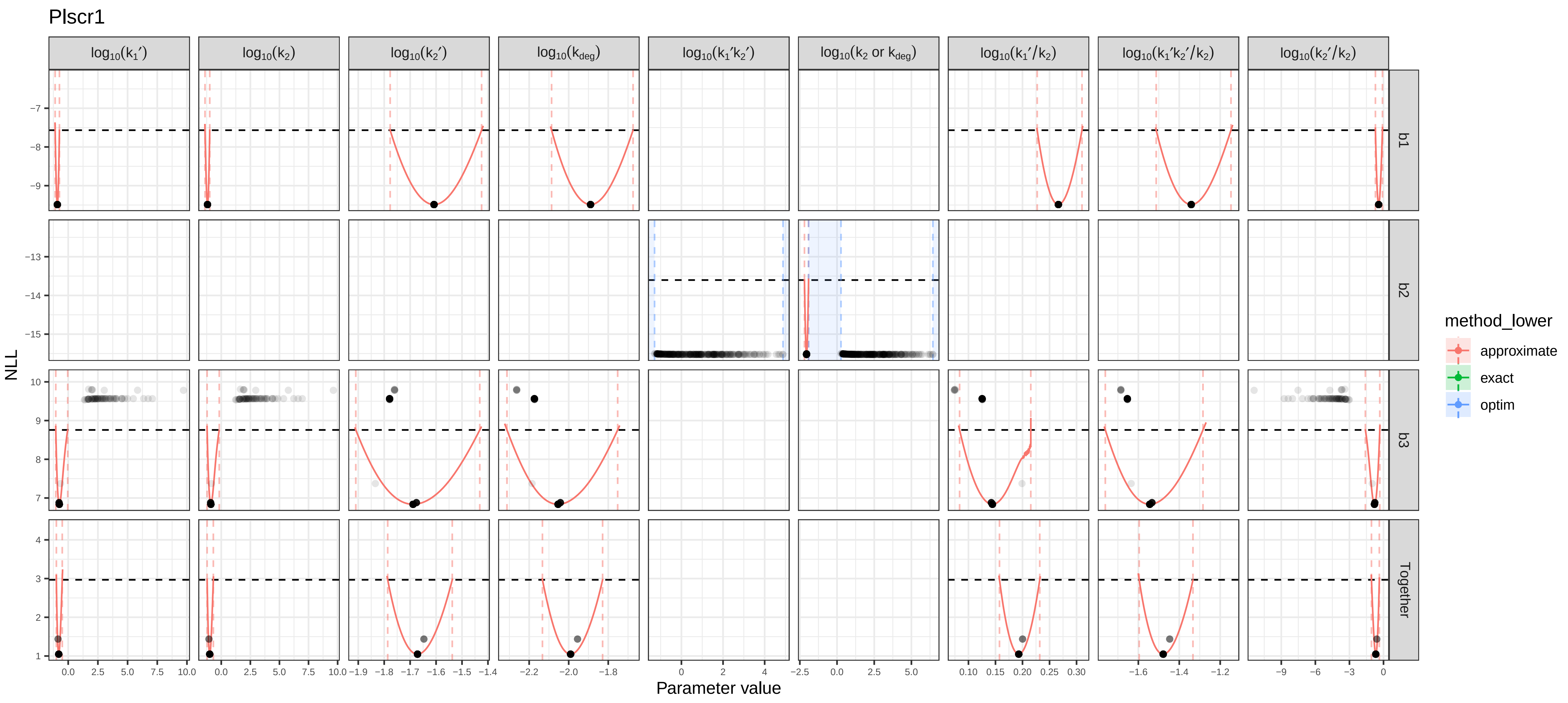

Replicate	Par	Best value	CI95 LB	CI95 UB	Method LB	Method UB
Together	$\log_{10}(k_1')$	−0.7975	−0.9983	−0.5019	approximate	approximate
Together	$\log_{10}(k_2)$	−0.9906	−1.214	−0.6804	approximate	approximate
Together	$\log_{10}(k_2')$	−1.672	−1.786	−1.538	approximate	approximate
Together	$\log_{10}(k_{deg})$	−1.99	−2.133	−1.829	approximate	approximate
Together	$\log_{10}(k_1'/k_2)$	0.1931	0.1574	0.2319	approximate	approximate
Together	$\log_{10}(k_1'k_2'/k_2)$	−1.478	−1.596	−1.333	approximate	approximate
Together	$\log_{10}(k_2'/k_2)$	−0.681	−1.06	−0.3683	approximate	approximate
b1	$\log_{10}(k_1')$	−0.9146	−1.1	−0.7393	approximate	approximate
b1	$\log_{10}(k_2)$	−1.181	−1.395	−0.9902	approximate	approximate
b1	$\log_{10}(k_2')$	−1.608	−1.777	−1.424	approximate	approximate
b1	$\log_{10}(k_{deg})$	−1.889	−2.087	−1.674	approximate	approximate
b1	$\log_{10}(k_1'/k_2)$	0.2664	0.2272	0.3101	approximate	approximate
b1	$\log_{10}(k_1'k_2'/k_2)$	−1.342	−1.513	−1.147	approximate	approximate
b1	$\log_{10}(k_2'/k_2)$	−0.4269	−0.7113	−0.09155	approximate	approximate
b2	$\log_{10}(k_1'k_2')$	2.182	< −1.291	> 4.882	optim	optim
b2	$\log_{10}(k_2 \text{ or } k_{deg})$	3.745	0.2677	> 6.445	optim	optim
b2	$\log_{10}(k_2 \text{ or } k_{deg})$	−2.051	−2.189	−1.913	approximate	approximate
b3	$\log_{10}(k_1')$	−0.7483	−1.046	−0.04044	approximate	approximate
b3	$\log_{10}(k_2)$	−0.8927	−1.215	−0.1717	approximate	approximate
b3	$\log_{10}(k_2')$	−1.689	−1.909	−1.431	approximate	approximate
b3	$\log_{10}(k_{deg})$	−2.054	−2.312	−1.752	approximate	approximate
b3	$\log_{10}(k_1'/k_2)$	0.1444	0.08365	0.2154	approximate	approximate
b3	$\log_{10}(k_1'k_2'/k_2)$	−1.544	−1.761	−1.284	approximate	approximate
b3	$\log_{10}(k_2'/k_2)$	−0.796	−1.597	−0.3263	approximate	approximate

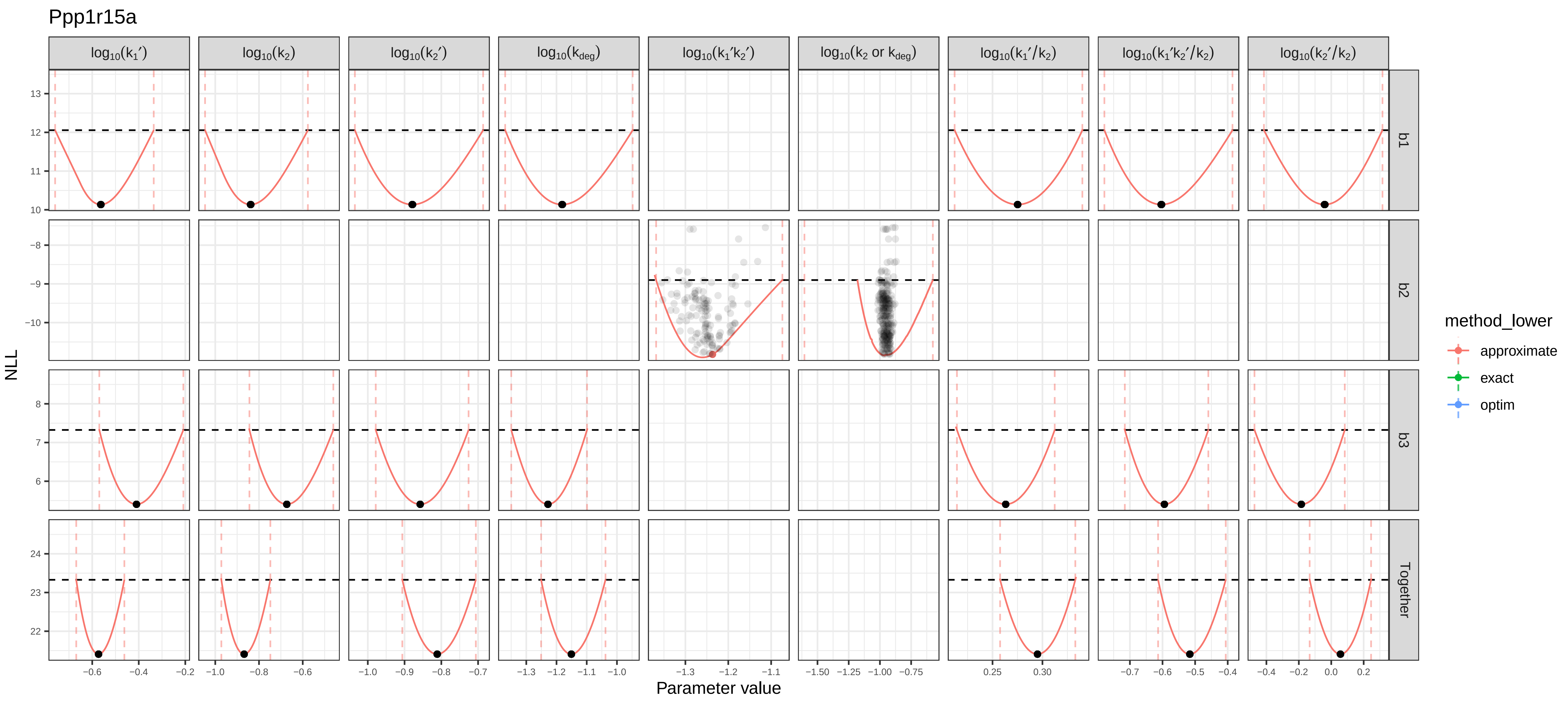

Replicate	Par	**Best value**	**CI95 LB**	**CI95 UB**	**Method LB**	**Method UB**
Together	$\log_{10}(k_1')$	−0.5729	−0.6688	−0.4619	approximate	approximate
Together	$\log_{10}(k_2)$	−0.8679	−0.9722	−0.7481	approximate	approximate
Together	$\log_{10}(k_2')$	−0.8112	−0.9061	−0.7061	approximate	approximate
Together	$\log_{10}(k_{deg})$	−1.151	−1.251	−1.038	approximate	approximate
Together	$\log_{10}(k_1'/k_2)$	0.295	0.2576	0.3329	approximate	approximate
Together	$\log_{10}(k_1'k_2'/k_2)$	−0.5161	−0.6136	−0.406	approximate	approximate
Together	$\log_{10}(k_2'/k_2)$	0.05675	−0.1332	0.2457	approximate	approximate
b1	$\log_{10}(k_1')$	−0.5631	−0.7595	−0.3356	approximate	approximate
b1	$\log_{10}(k_2)$	−0.8382	−1.047	−0.5764	approximate	approximate
b1	$\log_{10}(k_2')$	−0.8787	−1.035	−0.6862	approximate	approximate
b1	$\log_{10}(k_{deg})$	−1.181	−1.37	−0.9479	approximate	approximate
b1	$\log_{10}(k_1'/k_2)$	0.2751	0.212	0.34	approximate	approximate
b1	$\log_{10}(k_1'k_2'/k_2)$	−0.6036	−0.7784	−0.3853	approximate	approximate
b1	$\log_{10}(k_2'/k_2)$	−0.04054	−0.4152	0.3161	approximate	approximate
b2	$\log_{10}(k_1'k_2')$	−1.237	−1.368	−1.074	approximate	approximate
b2	$\log_{10}(k_2 \text{ or } k_{deg})$	−0.9237	−1.604	−0.5762	approximate	approximate
b2	$\log_{10}(k_2 \text{ or } k_{deg})$	−0.9632	−1.604	−0.5762	approximate	approximate
b3	$\log_{10}(k_1')$	−0.4097	−0.5696	−0.2083	approximate	approximate
b3	$\log_{10}(k_2)$	−0.6729	−0.8438	−0.4601	approximate	approximate
b3	$\log_{10}(k_2')$	−0.8575	−0.9783	−0.7258	approximate	approximate
b3	$\log_{10}(k_{deg})$	−1.228	−1.35	−1.099	approximate	approximate
b3	$\log_{10}(k_1'/k_2)$	0.2632	0.2143	0.3123	approximate	approximate
b3	$\log_{10}(k_1'k_2'/k_2)$	−0.5944	−0.7156	−0.4595	approximate	approximate
b3	$\log_{10}(k_2'/k_2)$	−0.1847	−0.4736	0.08338	approximate	approximate

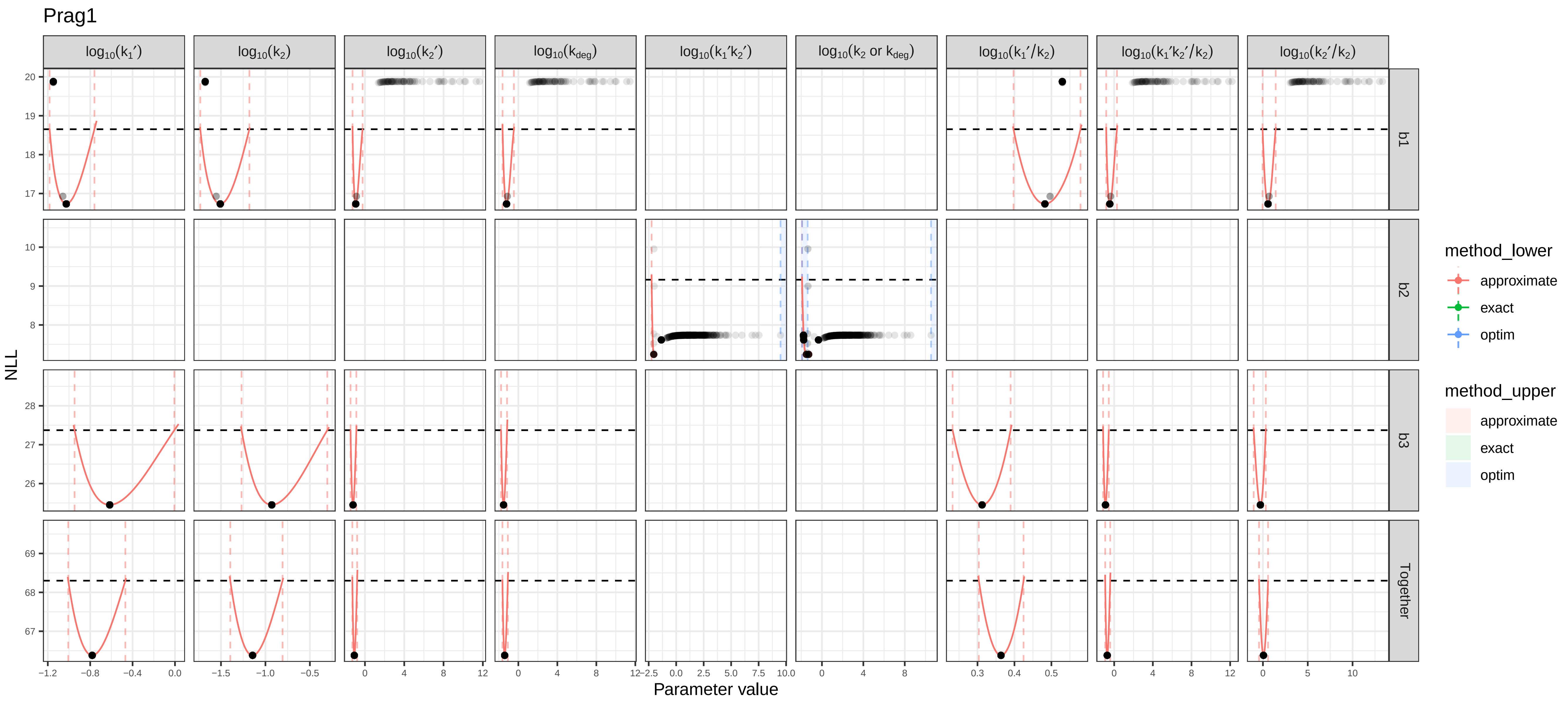

Replicate	Par	Best value	CI95 LB	CI95 UB	Method LB	Method UB
Together	$\log_{10}(k_1')$	−0.7808	−1.007	−0.4682	approximate	approximate
Together	$\log_{10}(k_2)$	−1.144	−1.395	−0.8073	approximate	approximate
Together	$\log_{10}(k_2')$	−1.084	−1.28	−0.7955	approximate	approximate
Together	$\log_{10}(k_{deg})$	−1.404	−1.632	−1.079	approximate	approximate
Together	$\log_{10}(k_1'/k_2)$	0.3636	0.3037	0.4247	approximate	approximate
Together	$\log_{10}(k_1'k_2'/k_2)$	−0.7203	−0.9292	−0.4098	approximate	approximate
Together	$\log_{10}(k_2'/k_2)$	0.06056	−0.4341	0.5751	approximate	approximate
b1	$\log_{10}(k_1')$	−1.026	−1.183	−0.7597	approximate	approximate
b1	$\log_{10}(k_2)$	−1.508	−1.733	−1.181	approximate	approximate
b1	$\log_{10}(k_2')$	−0.9363	−1.261	−0.2387	approximate	approximate
b1	$\log_{10}(k_{deg})$	−1.212	−1.62	−0.4559	approximate	approximate
b1	$\log_{10}(k_1'/k_2)$	0.4824	0.3975	0.5787	approximate	approximate
b1	$\log_{10}(k_1'k_2'/k_2)$	−0.4539	−0.8251	0.2882	approximate	approximate
b1	$\log_{10}(k_2'/k_2)$	0.5717	−0.04177	1.427	approximate	approximate
b2	$\log_{10}(k_1'k_2')$	−2.037	−2.235	> 9.494	approximate	optim
b2	$\log_{10}(k_2 \text{ or } k_{deg})$	−1.278	−1.918	> 10.54	approximate	optim
b2	$\log_{10}(k_2 \text{ or } k_{deg})$	−1.504	−1.918	−1.379	approximate	optim
b3	$\log_{10}(k_1')$	−0.6159	−0.9473	−0.006028	approximate	approximate
b3	$\log_{10}(k_2)$	−0.9284	−1.269	−0.3041	approximate	approximate
b3	$\log_{10}(k_2')$	−1.214	−1.466	−0.8734	approximate	approximate
b3	$\log_{10}(k_{deg})$	−1.516	−1.776	−1.161	approximate	approximate
b3	$\log_{10}(k_1'/k_2)$	0.3125	0.2327	0.3896	approximate	approximate
b3	$\log_{10}(k_1'k_2'/k_2)$	−0.901	−1.148	−0.558	approximate	approximate
b3	$\log_{10}(k_2'/k_2)$	−0.2851	−1.03	0.3209	approximate	approximate

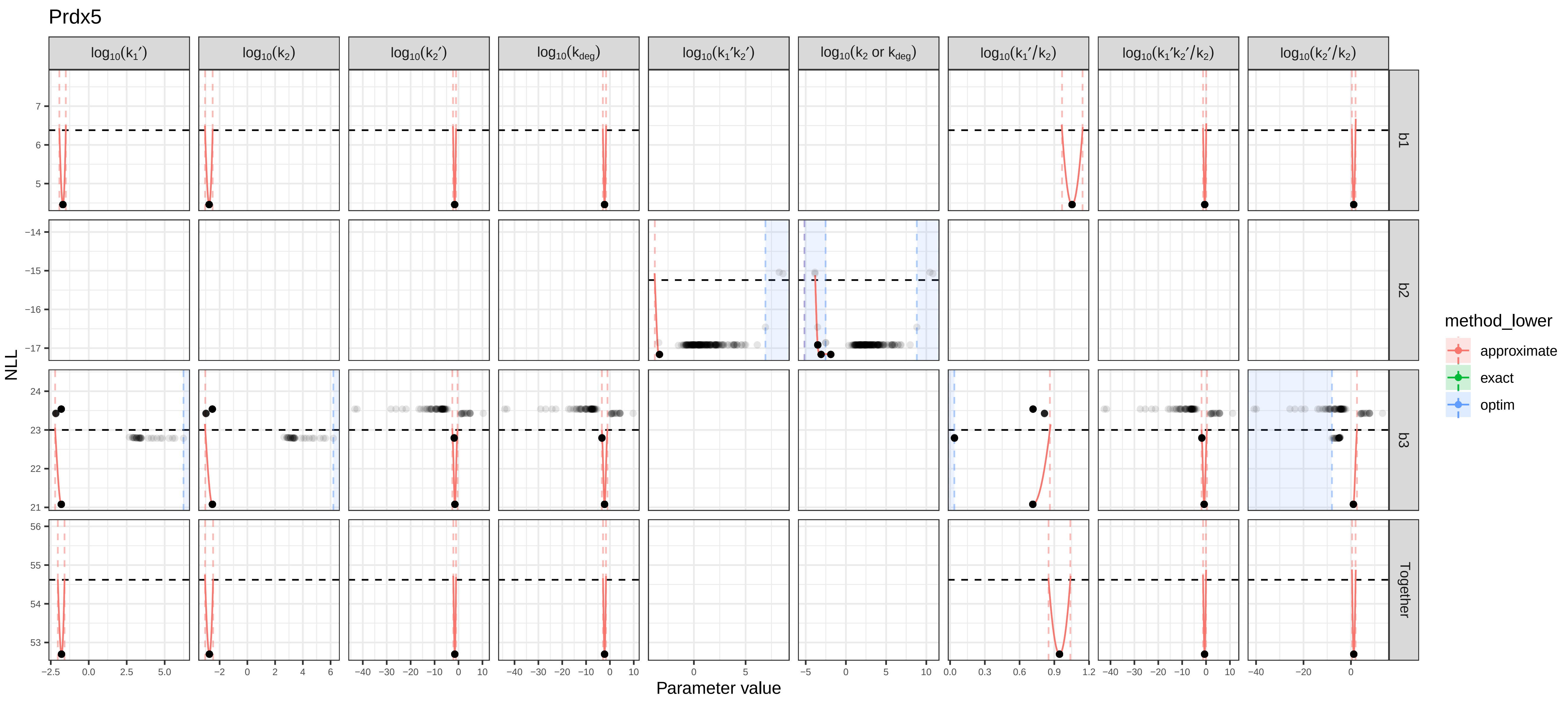

Replicate	Par	Best value	CI95 LB	CI95 UB	Method LB	Method UB
Together	$\log_{10}(k_1')$	−1.793	−2.034	−1.595	approximate	approximate
Together	$\log_{10}(k_2)$	−2.738	−3.046	−2.47	approximate	approximate
Together	$\log_{10}(k_2')$	−1.558	−2.172	−1.028	approximate	approximate
Together	$\log_{10}(k_{deg})$	−2.245	−2.896	−1.65	approximate	approximate
Together	$\log_{10}(k_1'/k_2)$	0.9451	0.8504	1.039	approximate	approximate
Together	$\log_{10}(k_1'k_2'/k_2)$	−0.6132	−1.244	−0.03325	approximate	approximate
Together	$\log_{10}(k_2'/k_2)$	1.179	0.4712	1.948	approximate	approximate
b1	$\log_{10}(k_1')$	−1.702	−1.935	−1.51	approximate	approximate
b1	$\log_{10}(k_2)$	−2.755	−3.049	−2.502	approximate	approximate
b1	$\log_{10}(k_2')$	−1.606	−2.292	−1.037	approximate	approximate
b1	$\log_{10}(k_{deg})$	−2.243	−2.965	−1.613	approximate	approximate
b1	$\log_{10}(k_1'/k_2)$	1.053	0.9669	1.144	approximate	approximate
b1	$\log_{10}(k_1'k_2'/k_2)$	−0.5526	−1.25	0.05857	approximate	approximate
b1	$\log_{10}(k_2'/k_2)$	1.15	0.3927	1.933	approximate	approximate
b2	$\log_{10}(k_1'k_2')$	−3.331	−3.777	> 6.918	approximate	optim
b2	$\log_{10}(k_2 \text{ or } k_{deg})$	−1.887	−5.158	> 8.822	approximate	optim
b2	$\log_{10}(k_2 \text{ or } k_{deg})$	−3.08	−5.158	−2.53	approximate	optim
b3	$\log_{10}(k_1')$	−1.807	−2.212	> 6.237	approximate	optim
b3	$\log_{10}(k_2)$	−2.521	−3.035	> 6.2	approximate	optim
b3	$\log_{10}(k_2')$	−1.505	−2.583	−0.4004	approximate	approximate
b3	$\log_{10}(k_{deg})$	−2.247	−3.376	−1.024	approximate	approximate
b3	$\log_{10}(k_1'/k_2)$	0.7144	< 0.03656	0.8621	optim	approximate
b3	$\log_{10}(k_1'k_2'/k_2)$	−0.7911	−1.878	0.4035	approximate	approximate
b3	$\log_{10}(k_2'/k_2)$	1.016	< −8.007	2.532	optim	approximate

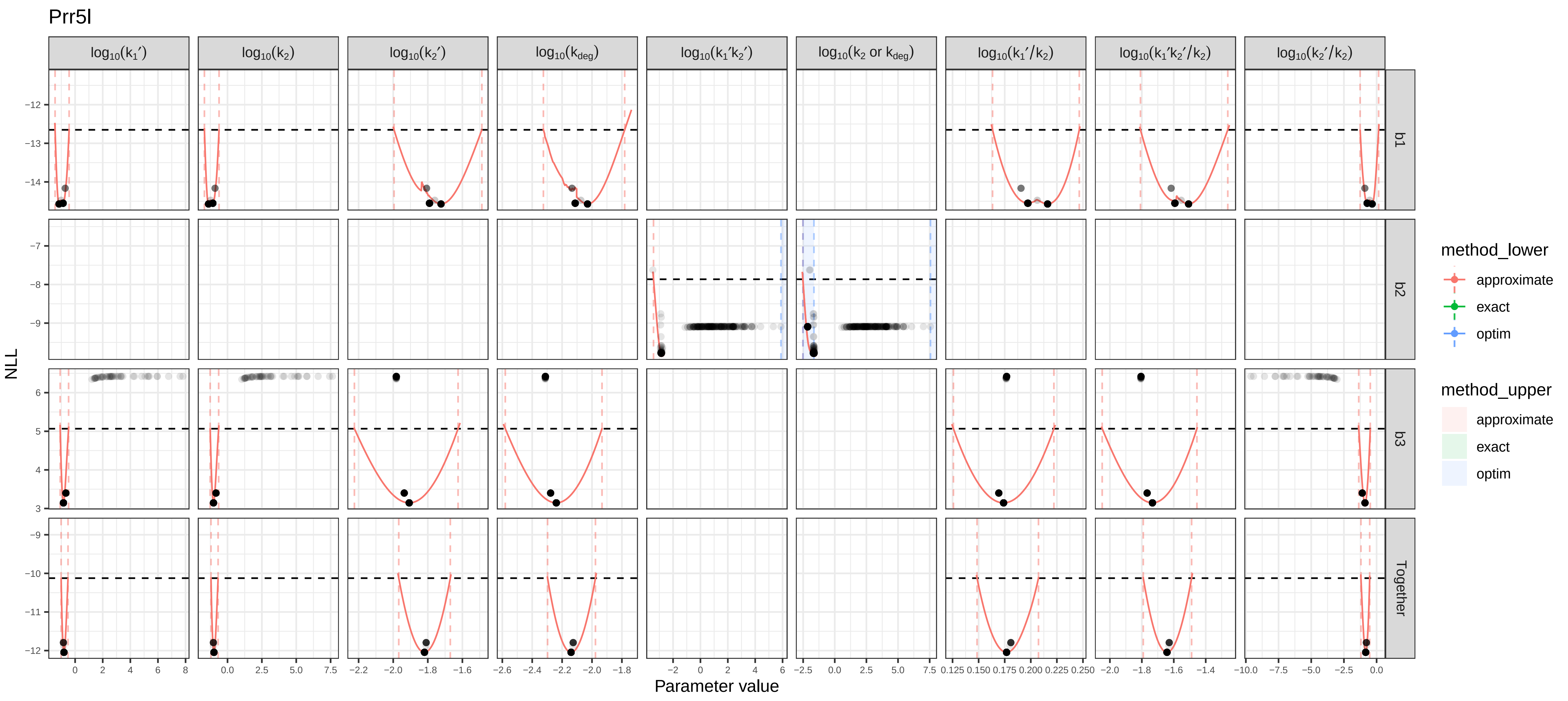

Replicate	Par	Best value	CI95 LB	CI95 UB	Method LB	Method UB
Together	$\log_{10}(k_1')$	−0.8082	−1.013	−0.5137	approximate	approximate
Together	$\log_{10}(k_2)$	−0.985	−1.201	−0.6833	approximate	approximate
Together	$\log_{10}(k_2')$	−1.819	−1.968	−1.67	approximate	approximate
Together	$\log_{10}(k_{deg})$	−2.14	−2.297	−1.977	approximate	approximate
Together	$\log_{10}(k_1'/k_2)$	0.1768	0.1485	0.2074	approximate	approximate
Together	$\log_{10}(k_1'k_2'/k_2)$	−1.643	−1.791	−1.488	approximate	approximate
Together	$\log_{10}(k_2'/k_2)$	−0.8344	−1.197	−0.5275	approximate	approximate
b1	$\log_{10}(k_1')$	−1.166	−1.451	−0.434	approximate	approximate
b1	$\log_{10}(k_2)$	−1.382	−1.68	−0.615	approximate	approximate
b1	$\log_{10}(k_2')$	−1.723	−1.995	−1.487	approximate	approximate
b1	$\log_{10}(k_{deg})$	−2.03	−2.325	−1.781	approximate	approximate
b1	$\log_{10}(k_1'/k_2)$	0.2161	0.1634	0.2465	approximate	approximate
b1	$\log_{10}(k_1'k_2'/k_2)$	−1.507	−1.809	−1.262	approximate	approximate
b1	$\log_{10}(k_2'/k_2)$	−0.3412	−1.26	0.1553	approximate	approximate
b2	$\log_{10}(k_1'k_2')$	−2.851	−3.428	> 5.883	approximate	optim
b2	$\log_{10}(k_2 \text{ or } k_{deg})$	−1.636	−2.513	> 7.564	approximate	optim
b2	$\log_{10}(k_2 \text{ or } k_{deg})$	−1.68	−2.513	−1.645	approximate	optim
b3	$\log_{10}(k_1')$	−0.8454	−1.081	−0.4718	approximate	approximate
b3	$\log_{10}(k_2)$	−1.019	−1.261	−0.643	approximate	approximate
b3	$\log_{10}(k_2')$	−1.908	−2.224	−1.625	approximate	approximate
b3	$\log_{10}(k_{deg})$	−2.239	−2.58	−1.933	approximate	approximate
b3	$\log_{10}(k_1'/k_2)$	0.1739	0.1258	0.2222	approximate	approximate
b3	$\log_{10}(k_1'k_2'/k_2)$	−1.734	−2.048	−1.455	approximate	approximate
b3	$\log_{10}(k_2'/k_2)$	−0.8883	−1.366	−0.4831	approximate	approximate

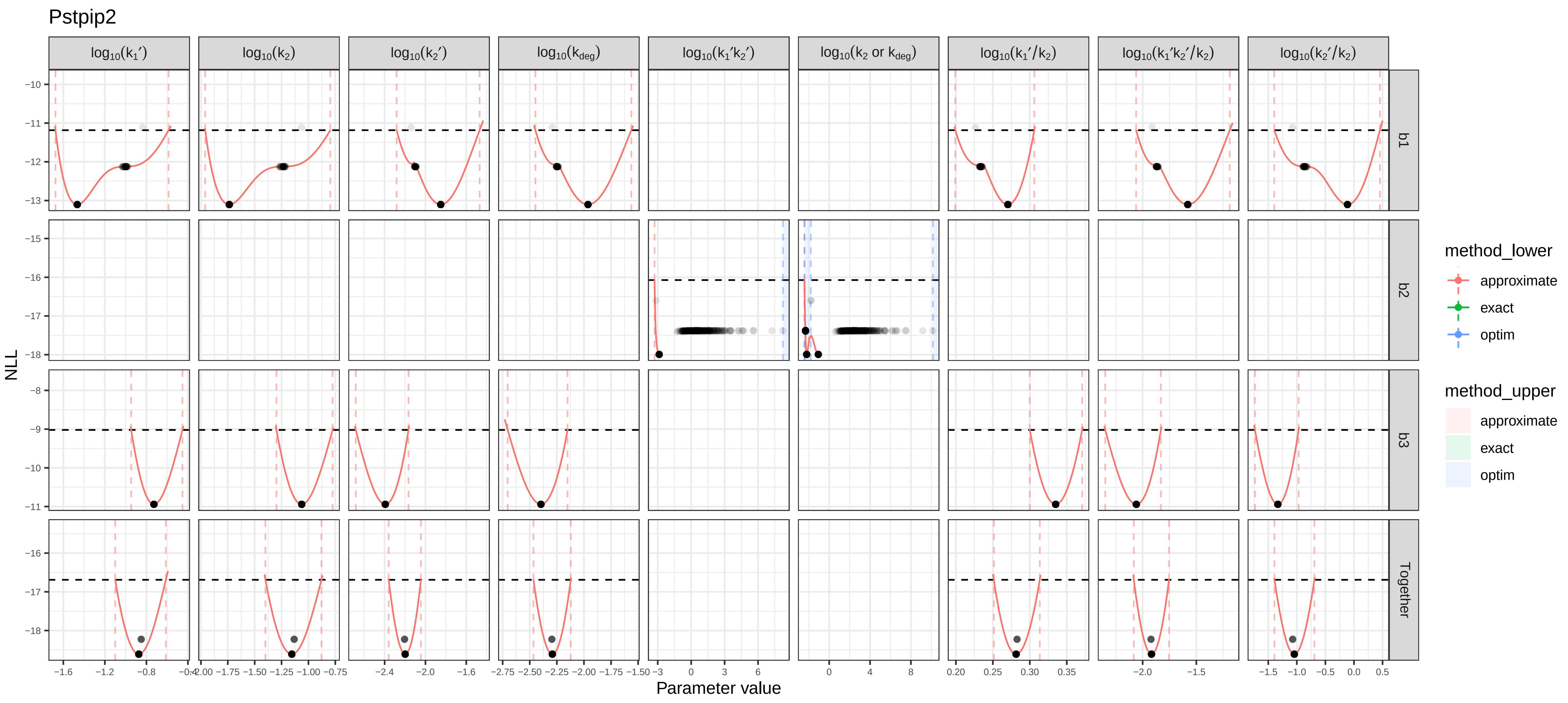

Replicate	Par	**Best value**	**CI95 LB**	**CI95 UB**	**Method LB**	**Method UB**
Together	$\log_{10}(k_1')$	−0.8732	−1.1	−0.6126	approximate	approximate
Together	$\log_{10}(k_2)$	−1.155	−1.4	−0.8785	approximate	approximate
Together	$\log_{10}(k_2')$	−2.199	−2.361	−2.045	approximate	approximate
Together	$\log_{10}(k_{deg})$	−2.292	−2.466	−2.122	approximate	approximate
Together	$\log_{10}(k_1'/k_2)$	0.2815	0.2513	0.3135	approximate	approximate
Together	$\log_{10}(k_1'k_2'/k_2)$	−1.917	−2.082	−1.754	approximate	approximate
Together	$\log_{10}(k_2'/k_2)$	−1.044	−1.392	−0.6927	approximate	approximate
b1	$\log_{10}(k_1')$	−1.466	−1.676	−0.5862	approximate	approximate
b1	$\log_{10}(k_2)$	−1.736	−1.96	−0.7967	approximate	approximate
b1	$\log_{10}(k_2')$	−1.85	−2.283	−1.469	approximate	approximate
b1	$\log_{10}(k_{deg})$	−1.963	−2.448	−1.563	approximate	approximate
b1	$\log_{10}(k_1'/k_2)$	0.2703	0.1992	0.306	approximate	approximate
b1	$\log_{10}(k_1'k_2'/k_2)$	−1.58	−2.061	−1.188	approximate	approximate
b1	$\log_{10}(k_2'/k_2)$	−0.1139	−1.395	0.455	approximate	approximate
b2	$\log_{10}(k_1'k_2')$	−2.867	−3.283	> 8.239	approximate	optim
b2	$\log_{10}(k_2 \text{ or } k_{deg})$	−1.069	−2.424	> 10.16	approximate	optim
b2	$\log_{10}(k_2 \text{ or } k_{deg})$	−2.205	−2.424	−1.804	approximate	optim
b3	$\log_{10}(k_1')$	−0.7274	−0.9468	−0.4527	approximate	approximate
b3	$\log_{10}(k_2)$	−1.062	−1.298	−0.775	approximate	approximate
b3	$\log_{10}(k_2')$	−2.394	−2.683	−2.166	approximate	approximate
b3	$\log_{10}(k_{deg})$	−2.397	−2.704	−2.151	approximate	approximate
b3	$\log_{10}(k_1'/k_2)$	0.335	0.3	0.3708	approximate	approximate
b3	$\log_{10}(k_1'k_2'/k_2)$	−2.059	−2.348	−1.83	approximate	approximate
b3	$\log_{10}(k_2'/k_2)$	−1.332	−1.732	−0.9682	approximate	approximate

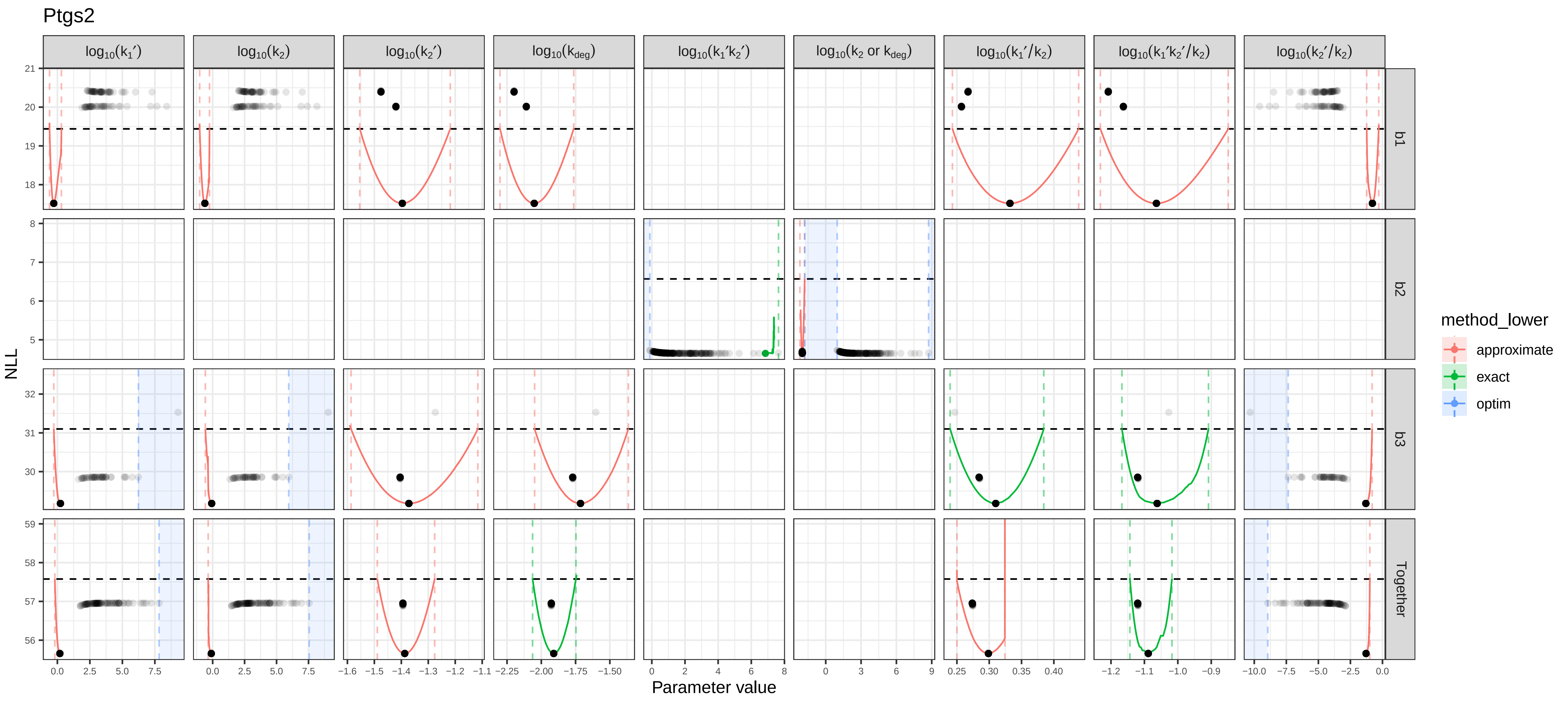

Replicate	Par	Best value	CI95 LB	CI95 UB	Method LB	Method UB
Together	$\log_{10}(k_1')$	0.1921	−0.1955	> 7.827	approximate	optim
Together	$\log_{10}(k_2)$	−0.1067	−0.3494	> 7.553	approximate	optim
Together	$\log_{10}(k_2')$	−1.387	−1.489	−1.276	approximate	approximate
Together	$\log_{10}(k_{deg})$	−1.91	−2.064	−1.747	exact	exact
Together	$\log_{10}(k_1'/k_2)$	0.2988	0.25	0.3242	approximate	approximate
Together	$\log_{10}(k_1'k_2'/k_2)$	−1.089	−1.143	−1.018	exact	exact
Together	$\log_{10}(k_2'/k_2)$	−1.281	< −8.947	−0.9838	optim	approximate
b1	$\log_{10}(k_1')$	−0.2796	−0.6009	0.3096	approximate	approximate
b1	$\log_{10}(k_2)$	−0.6116	−1.015	−0.2462	approximate	approximate
b1	$\log_{10}(k_2')$	−1.396	−1.554	−1.218	approximate	approximate
b1	$\log_{10}(k_{deg})$	−2.052	−2.301	−1.764	approximate	approximate
b1	$\log_{10}(k_1'/k_2)$	0.332	0.243	0.4384	approximate	approximate
b1	$\log_{10}(k_1'k_2'/k_2)$	−1.064	−1.232	−0.849	approximate	approximate
b1	$\log_{10}(k_2'/k_2)$	−0.7846	−1.231	−0.2906	approximate	approximate
b2	$\log_{10}(k_1'k_2')$	6.852	< −0.1199	7.642	optim	exact
b2	$\log_{10}(k_2 \text{ or } k_{deg})$	7.949	0.975	> 8.734	optim	optim
b2	$\log_{10}(k_2 \text{ or } k_{deg})$	−1.991	−2.189	−1.789	approximate	approximate
b3	$\log_{10}(k_1')$	0.2352	−0.2752	> 6.236	approximate	optim
b3	$\log_{10}(k_2)$	−0.0748	−0.572	> 5.951	approximate	optim
b3	$\log_{10}(k_2')$	−1.372	−1.586	−1.116	approximate	approximate
b3	$\log_{10}(k_{deg})$	−1.714	−2.049	−1.365	approximate	approximate
b3	$\log_{10}(k_1'/k_2)$	0.31	0.2395	0.3844	exact	exact
b3	$\log_{10}(k_1'k_2'/k_2)$	−1.062	−1.167	−0.9082	exact	exact
b3	$\log_{10}(k_2'/k_2)$	−1.297	< −7.355	−0.8067	optim	approximate

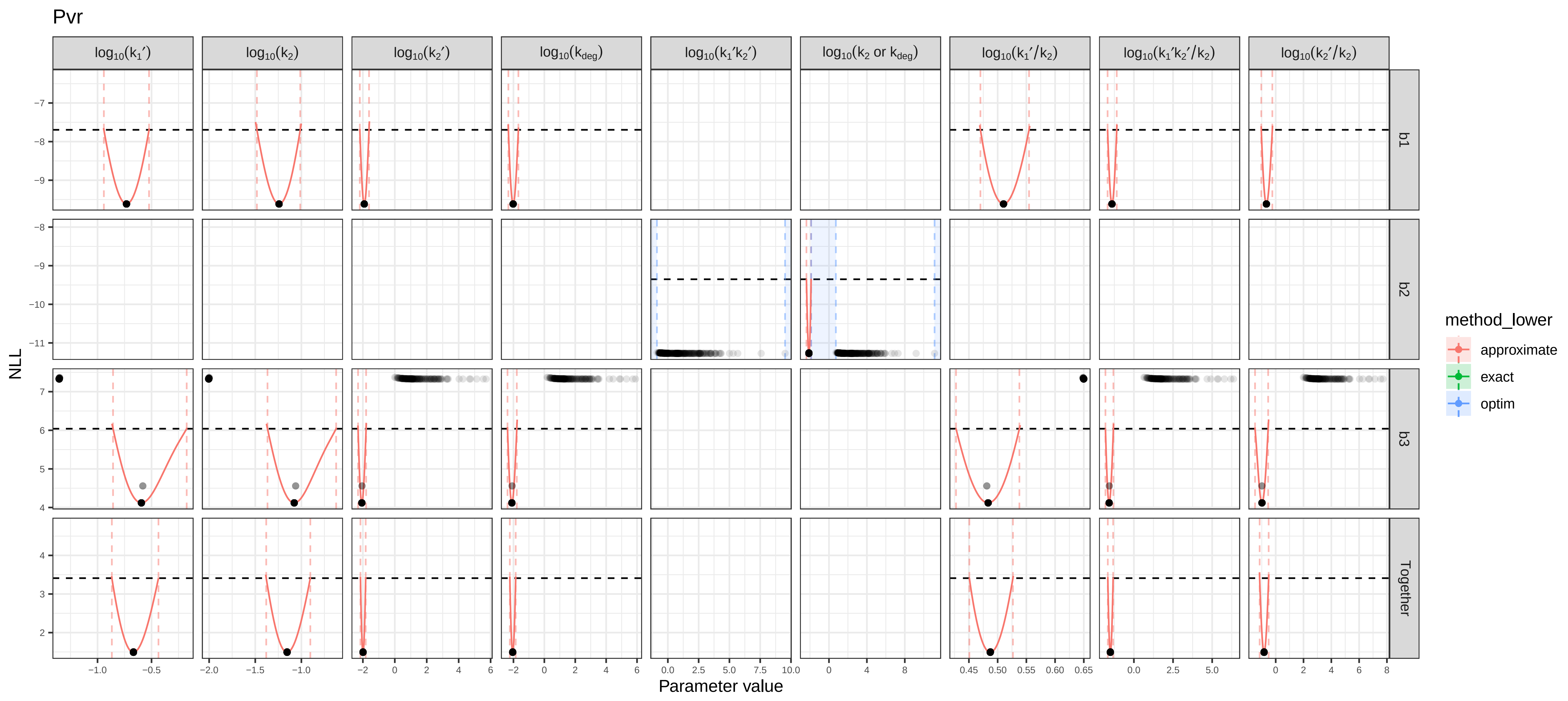

Replicate	Par	Best value	CI95 LB	CI95 UB	Method LB	Method UB
Together	$\log_{10}(k_1')$	−0.6676	−0.8673	−0.4358	approximate	approximate
Together	$\log_{10}(k_2)$	−1.155	−1.381	−0.9029	approximate	approximate
Together	$\log_{10}(k_2')$	−1.991	−2.152	−1.823	approximate	approximate
Together	$\log_{10}(k_{deg})$	−2.05	−2.234	−1.854	approximate	approximate
Together	$\log_{10}(k_1'/k_2)$	0.4872	0.4507	0.5266	approximate	approximate
Together	$\log_{10}(k_1'k_2'/k_2)$	−1.504	−1.668	−1.326	approximate	approximate
Together	$\log_{10}(k_2'/k_2)$	−0.8364	−1.155	−0.5064	approximate	approximate
b1	$\log_{10}(k_1')$	−0.7317	−0.9412	−0.523	approximate	approximate
b1	$\log_{10}(k_2)$	−1.242	−1.482	−1.012	approximate	approximate
b1	$\log_{10}(k_2')$	−1.913	−2.191	−1.614	approximate	approximate
b1	$\log_{10}(k_{deg})$	−2.019	−2.329	−1.681	approximate	approximate
b1	$\log_{10}(k_1'/k_2)$	0.5103	0.47	0.5547	approximate	approximate
b1	$\log_{10}(k_1'k_2'/k_2)$	−1.402	−1.681	−1.096	approximate	approximate
b1	$\log_{10}(k_2'/k_2)$	−0.6706	−1.037	−0.2426	approximate	approximate
b2	$\log_{10}(k_1'k_2')$	3.864	< −0.8861	> 9.52	optim	optim
b2	$\log_{10}(k_2 \text{ or } k_{deg})$	5.48	0.7291	> 11.14	optim	optim
b2	$\log_{10}(k_2 \text{ or } k_{deg})$	−2.115	−2.375	−1.881	approximate	approximate
b3	$\log_{10}(k_1')$	−0.5939	−0.8564	−0.1745	approximate	approximate
b3	$\log_{10}(k_2)$	−1.077	−1.367	−0.6228	approximate	approximate
b3	$\log_{10}(k_2')$	−2.065	−2.302	−1.798	approximate	approximate
b3	$\log_{10}(k_{deg})$	−2.103	−2.385	−1.783	approximate	approximate
b3	$\log_{10}(k_1'/k_2)$	0.4834	0.4275	0.5379	approximate	approximate
b3	$\log_{10}(k_1'k_2'/k_2)$	−1.582	−1.818	−1.31	approximate	approximate
b3	$\log_{10}(k_2'/k_2)$	−0.9879	−1.487	−0.5429	approximate	approximate

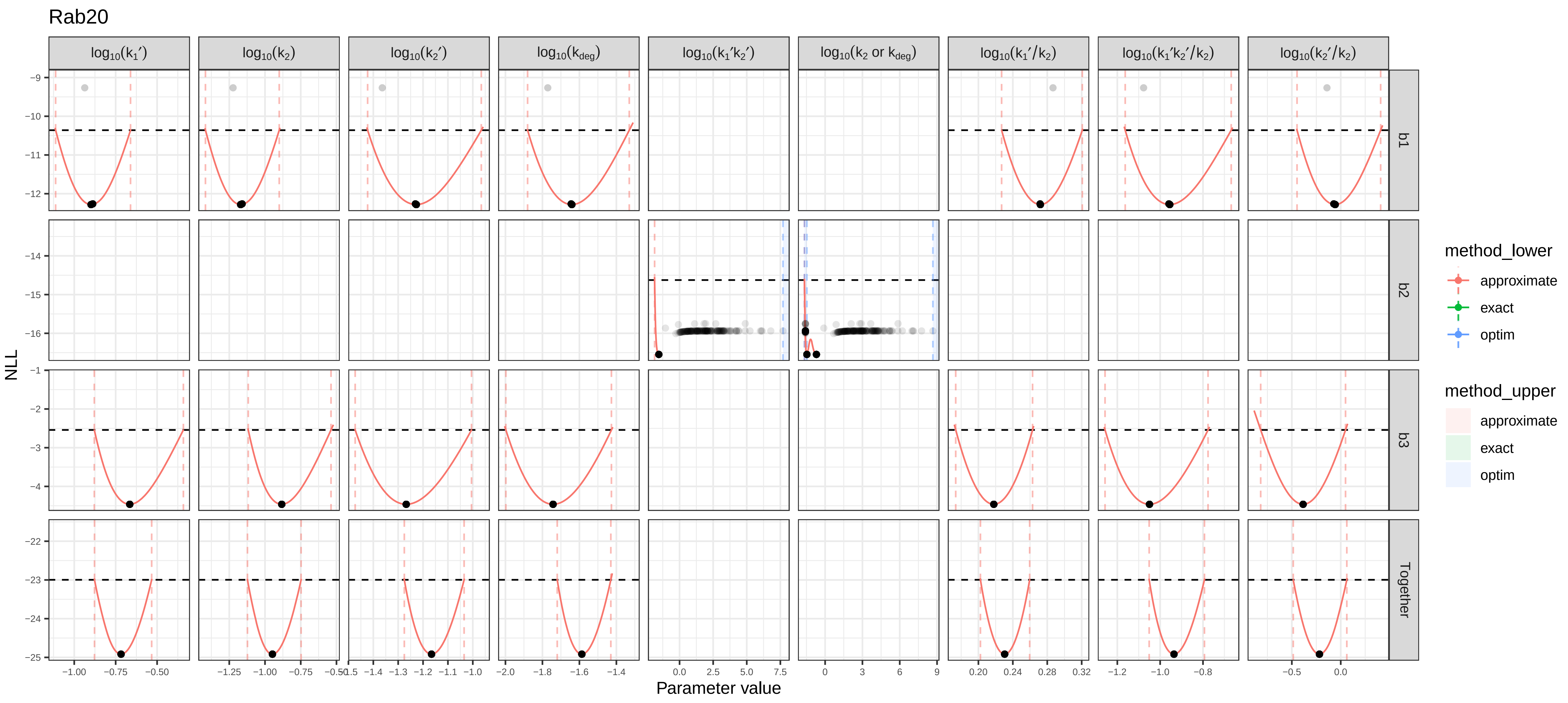

Replicate	Par	Best value	CI95 LB	CI95 UB	Method LB	Method UB
Together	$\log_{10}(k_1')$	−0.7176	−0.8777	−0.5327	approximate	approximate
Together	$\log_{10}(k_2)$	−0.9482	−1.123	−0.7474	approximate	approximate
Together	$\log_{10}(k_2')$	−1.166	−1.275	−1.035	approximate	approximate
Together	$\log_{10}(k_{deg})$	−1.587	−1.72	−1.43	approximate	approximate
Together	$\log_{10}(k_1'/k_2)$	0.2305	0.2023	0.2596	approximate	approximate
Together	$\log_{10}(k_1'k_2'/k_2)$	−0.9353	−1.051	−0.7932	approximate	approximate
Together	$\log_{10}(k_2'/k_2)$	−0.2176	−0.4858	0.06223	approximate	approximate
b1	$\log_{10}(k_1')$	−0.8978	−1.112	−0.6604	approximate	approximate
b1	$\log_{10}(k_2)$	−1.17	−1.417	−0.9004	approximate	approximate
b1	$\log_{10}(k_2')$	−1.227	−1.423	−0.9658	approximate	approximate
b1	$\log_{10}(k_{deg})$	−1.641	−1.88	−1.33	approximate	approximate
b1	$\log_{10}(k_1'/k_2)$	0.272	0.227	0.3207	approximate	approximate
b1	$\log_{10}(k_1'k_2'/k_2)$	−0.9553	−1.163	−0.6682	approximate	approximate
b1	$\log_{10}(k_2'/k_2)$	−0.0575	−0.4469	0.4082	approximate	approximate
b2	$\log_{10}(k_1'k_2')$	−1.544	−1.857	> 7.699	approximate	optim
b2	$\log_{10}(k_2 \text{ or } k_{deg})$	−0.6995	−1.661	> 8.667	approximate	optim
b2	$\log_{10}(k_2 \text{ or } k_{deg})$	−1.467	−1.661	−1.464	approximate	optim
b3	$\log_{10}(k_1')$	−0.665	−0.8789	−0.3415	approximate	approximate
b3	$\log_{10}(k_2)$	−0.8829	−1.117	−0.5382	approximate	approximate
b3	$\log_{10}(k_2')$	−1.268	−1.473	−1.005	approximate	approximate
b3	$\log_{10}(k_{deg})$	−1.742	−1.998	−1.427	approximate	approximate
b3	$\log_{10}(k_1'/k_2)$	0.2179	0.1737	0.2629	approximate	approximate
b3	$\log_{10}(k_1'k_2'/k_2)$	−1.05	−1.257	−0.7764	approximate	approximate
b3	$\log_{10}(k_2'/k_2)$	−0.3848	−0.8187	0.04903	approximate	approximate

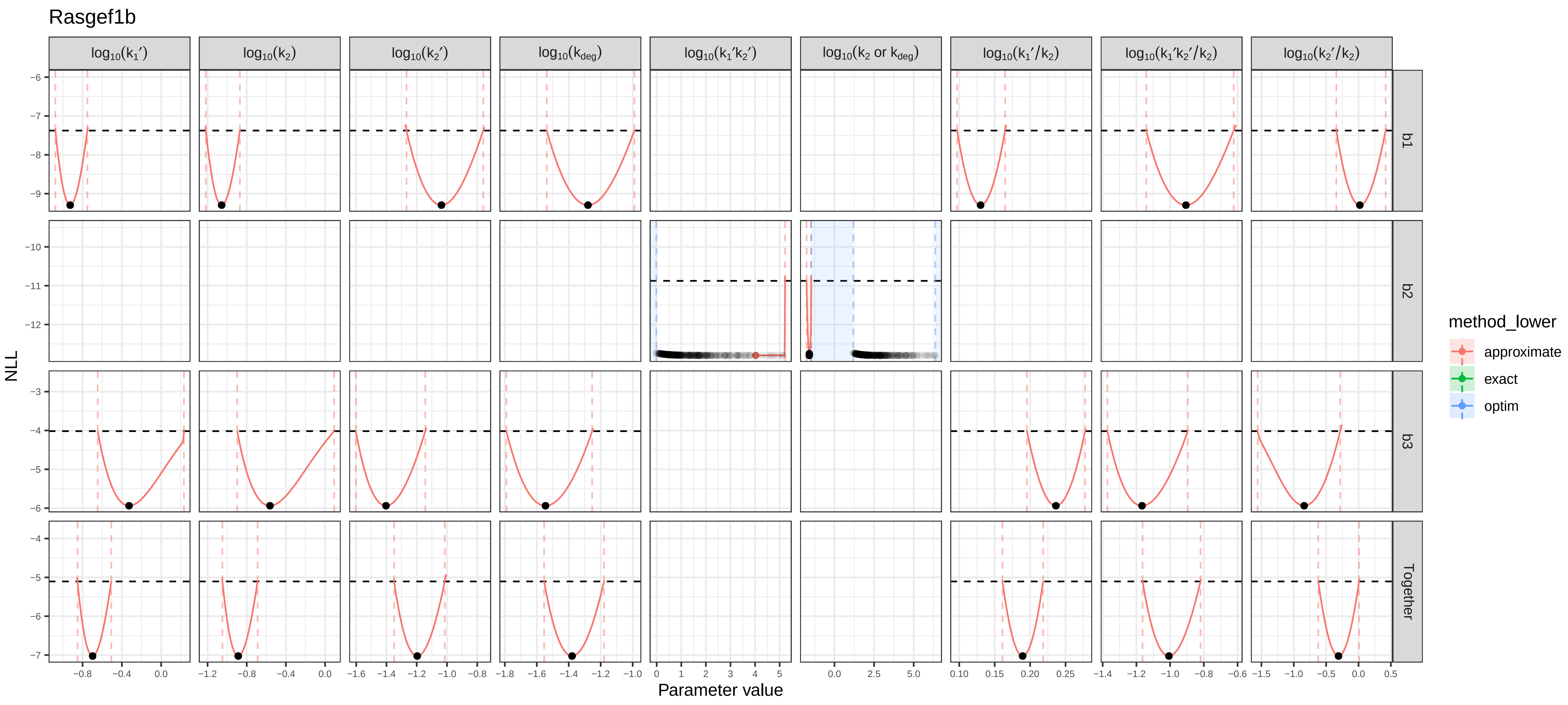

Replicate	Par	Best value	CI95 LB	CI95 UB	Method LB	Method UB
Together	$\log_{10}(k_1')$	−0.6978	−0.8514	−0.5073	approximate	approximate
Together	$\log_{10}(k_2)$	−0.8872	−1.048	−0.6891	approximate	approximate
Together	$\log_{10}(k_2')$	−1.197	−1.349	−1.014	approximate	approximate
Together	$\log_{10}(k_{deg})$	−1.379	−1.554	−1.179	approximate	approximate
Together	$\log_{10}(k_1'/k_2)$	0.1894	0.161	0.2183	approximate	approximate
Together	$\log_{10}(k_1'k_2'/k_2)$	−1.007	−1.163	−0.8197	approximate	approximate
Together	$\log_{10}(k_2'/k_2)$	−0.3093	−0.6223	0.008216	approximate	approximate
b1	$\log_{10}(k_1')$	−0.9262	−1.077	−0.7508	approximate	approximate
b1	$\log_{10}(k_2)$	−1.056	−1.218	−0.87	approximate	approximate
b1	$\log_{10}(k_2')$	−1.036	−1.268	−0.76	approximate	approximate
b1	$\log_{10}(k_{deg})$	−1.28	−1.538	−0.9889	approximate	approximate
b1	$\log_{10}(k_1'/k_2)$	0.1301	0.09695	0.1648	approximate	approximate
b1	$\log_{10}(k_1'k_2'/k_2)$	−0.9062	−1.142	−0.6231	approximate	approximate
b1	$\log_{10}(k_2'/k_2)$	0.02005	−0.3438	0.4194	approximate	approximate
b2	$\log_{10}(k_1'k_2')$	4.026	< −0.01639	5.219	optim	approximate
b2	$\log_{10}(k_2 \text{ or } k_{deg})$	5.232	1.189	> 6.368	optim	optim
b2	$\log_{10}(k_2 \text{ or } k_{deg})$	−1.591	−1.761	−1.473	approximate	approximate
b3	$\log_{10}(k_1')$	−0.327	−0.6459	0.2318	approximate	approximate
b3	$\log_{10}(k_2)$	−0.5633	−0.8977	0.09266	approximate	approximate
b3	$\log_{10}(k_2')$	−1.403	−1.603	−1.143	approximate	approximate
b3	$\log_{10}(k_{deg})$	−1.546	−1.79	−1.254	approximate	approximate
b3	$\log_{10}(k_1'/k_2)$	0.2363	0.1955	0.2776	approximate	approximate
b3	$\log_{10}(k_1'k_2'/k_2)$	−1.167	−1.372	−0.8954	approximate	approximate
b3	$\log_{10}(k_2'/k_2)$	−0.8398	−1.558	−0.2829	approximate	approximate

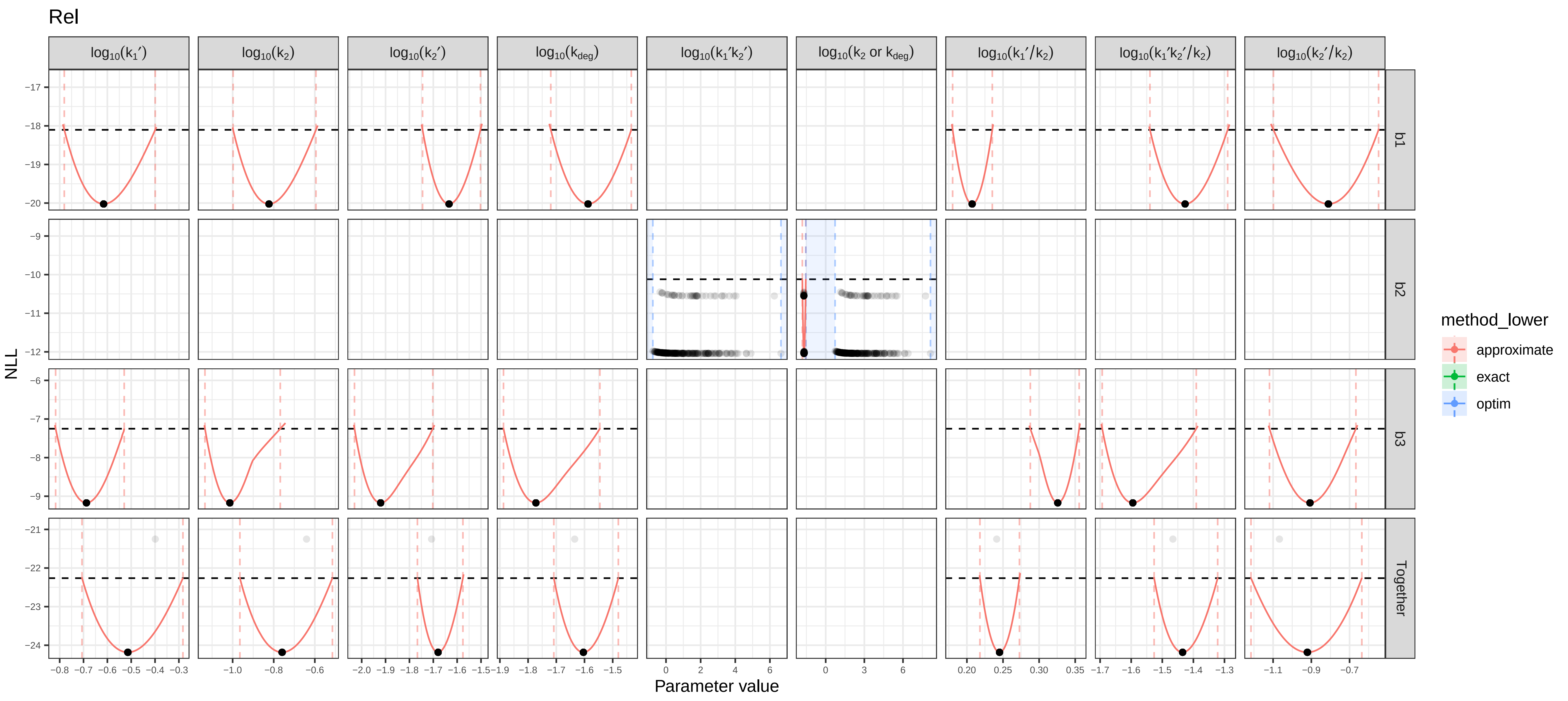

Replicate	Par	Best value	CI95 LB	CI95 UB	Method LB	Method UB
Together	$\log_{10}(k_1')$	−0.5139	−0.7063	−0.283	approximate	approximate
Together	$\log_{10}(k_2)$	−0.7592	−0.9647	−0.5144	approximate	approximate
Together	$\log_{10}(k_2')$	−1.68	−1.766	−1.576	approximate	approximate
Together	$\log_{10}(k_{deg})$	−1.604	−1.709	−1.48	approximate	approximate
Together	$\log_{10}(k_1'/k_2)$	0.2452	0.2181	0.2728	approximate	approximate
Together	$\log_{10}(k_1'k_2'/k_2)$	−1.435	−1.526	−1.322	approximate	approximate
Together	$\log_{10}(k_2'/k_2)$	−0.9208	−1.216	−0.6359	approximate	approximate
b1	$\log_{10}(k_1')$	−0.6157	−0.7807	−0.3993	approximate	approximate
b1	$\log_{10}(k_2)$	−0.823	−0.9977	−0.5944	approximate	approximate
b1	$\log_{10}(k_2')$	−1.634	−1.745	−1.502	approximate	approximate
b1	$\log_{10}(k_{deg})$	−1.587	−1.72	−1.434	approximate	approximate
b1	$\log_{10}(k_1'/k_2)$	0.2073	0.1802	0.2352	approximate	approximate
b1	$\log_{10}(k_1'k_2'/k_2)$	−1.427	−1.54	−1.29	approximate	approximate
b1	$\log_{10}(k_2'/k_2)$	−0.8109	−1.098	−0.5483	approximate	approximate
b2	$\log_{10}(k_1'k_2')$	2.662	< −0.76	> 6.639	optim	optim
b2	$\log_{10}(k_2 \text{ or } k_{deg})$	4.155	0.7299	> 8.132	optim	optim
b2	$\log_{10}(k_2 \text{ or } k_{deg})$	−1.682	−1.813	−1.546	approximate	approximate
b3	$\log_{10}(k_1')$	−0.6877	−0.8171	−0.5294	approximate	approximate
b3	$\log_{10}(k_2)$	−1.014	−1.135	−0.7681	approximate	approximate
b3	$\log_{10}(k_2')$	−1.921	−2.03	−1.702	approximate	approximate
b3	$\log_{10}(k_{deg})$	−1.772	−1.887	−1.546	approximate	approximate
b3	$\log_{10}(k_1'/k_2)$	0.3258	0.2878	0.3555	approximate	approximate
b3	$\log_{10}(k_1'k_2'/k_2)$	−1.595	−1.693	−1.391	approximate	approximate
b3	$\log_{10}(k_2'/k_2)$	−0.907	−1.119	−0.6669	approximate	approximate

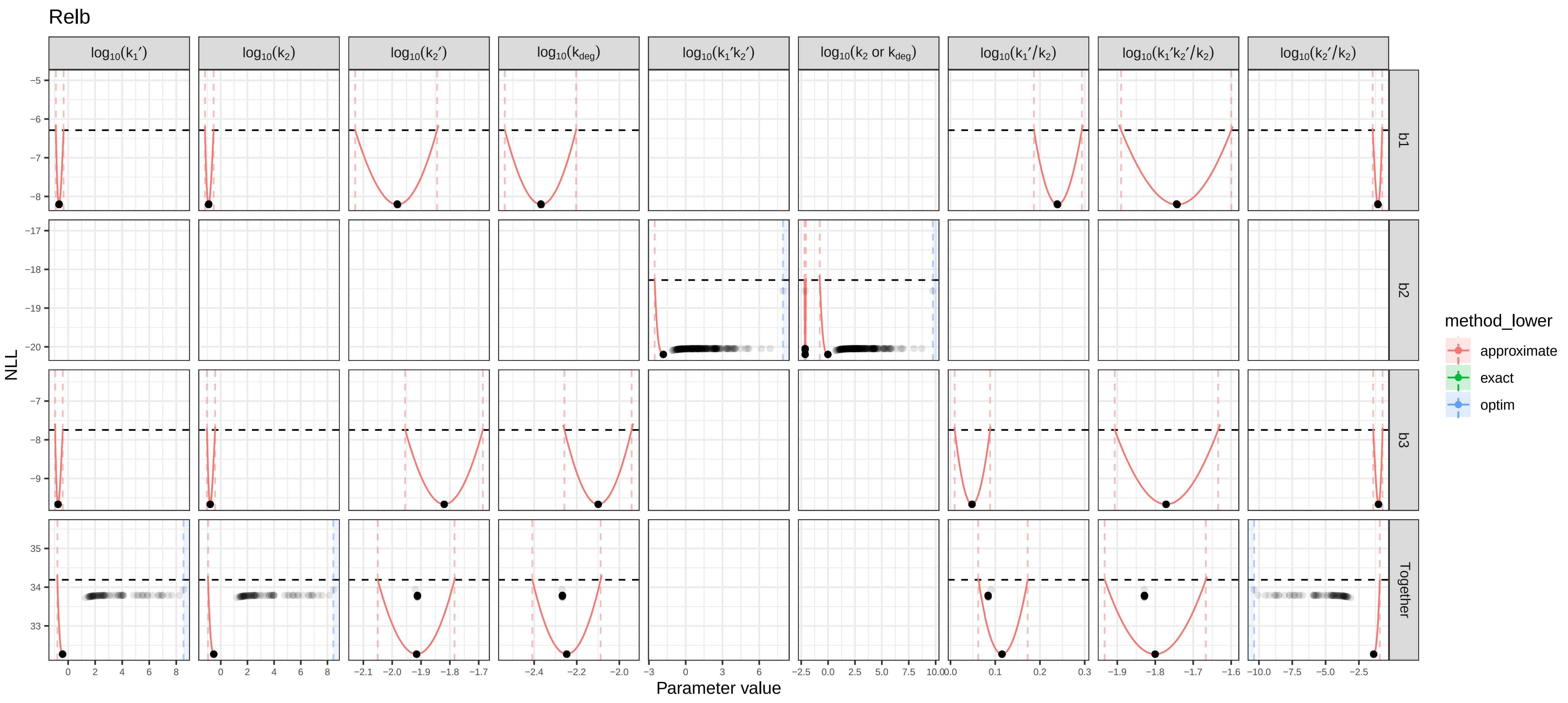

Replicate	Par	**Best value**	**CI95 LB**	**CI95 UB**	**Method LB**	**Method UB**
Together	$\log_{10}(k_1')$	−0.409	−0.7974	> 8.534	approximate	optim
Together	$\log_{10}(k_2)$	−0.5242	−0.9519	> 8.443	approximate	optim
Together	$\log_{10}(k_2')$	−1.915	−2.05	−1.784	approximate	approximate
Together	$\log_{10}(k_{deg})$	−2.247	−2.409	−2.088	approximate	approximate
Together	$\log_{10}(k_1'/k_2)$	0.1152	0.06189	0.1725	approximate	approximate
Together	$\log_{10}(k_1'k_2'/k_2)$	−1.8	−1.933	−1.667	approximate	approximate
Together	$\log_{10}(k_2'/k_2)$	−1.391	< −10.36	−0.9308	optim	approximate
b1	$\log_{10}(k_1')$	−0.6748	−0.9084	−0.3354	approximate	approximate
b1	$\log_{10}(k_2)$	−0.9141	−1.183	−0.5353	approximate	approximate
b1	$\log_{10}(k_2')$	−1.982	−2.129	−1.844	approximate	approximate
b1	$\log_{10}(k_{deg})$	−2.367	−2.538	−2.203	approximate	approximate
b1	$\log_{10}(k_1'/k_2)$	0.2393	0.1864	0.2938	approximate	approximate
b1	$\log_{10}(k_1'k_2'/k_2)$	−1.742	−1.89	−1.6	approximate	approximate
b1	$\log_{10}(k_2'/k_2)$	−1.068	−1.464	−0.7498	approximate	approximate
b2	$\log_{10}(k_1'k_2')$	−1.826	−2.536	> 7.943	approximate	optim
b2	$\log_{10}(k_2 \text{ or } k_{deg})$	−0.01929	−0.7726	> 9.747	approximate	optim
b2	$\log_{10}(k_2 \text{ or } k_{deg})$	−2.131	−2.198	−2.066	approximate	approximate
b3	$\log_{10}(k_1')$	−0.7444	−0.963	−0.3977	approximate	approximate
b3	$\log_{10}(k_2)$	−0.7925	−1.031	−0.4253	approximate	approximate
b3	$\log_{10}(k_2')$	−1.82	−1.955	−1.686	approximate	approximate
b3	$\log_{10}(k_{deg})$	−2.099	−2.258	−1.942	approximate	approximate
b3	$\log_{10}(k_1'/k_2)$	0.04808	0.009326	0.08846	approximate	approximate
b3	$\log_{10}(k_1'k_2'/k_2)$	−1.771	−1.907	−1.634	approximate	approximate
b3	$\log_{10}(k_2'/k_2)$	−1.027	−1.429	−0.7268	approximate	approximate

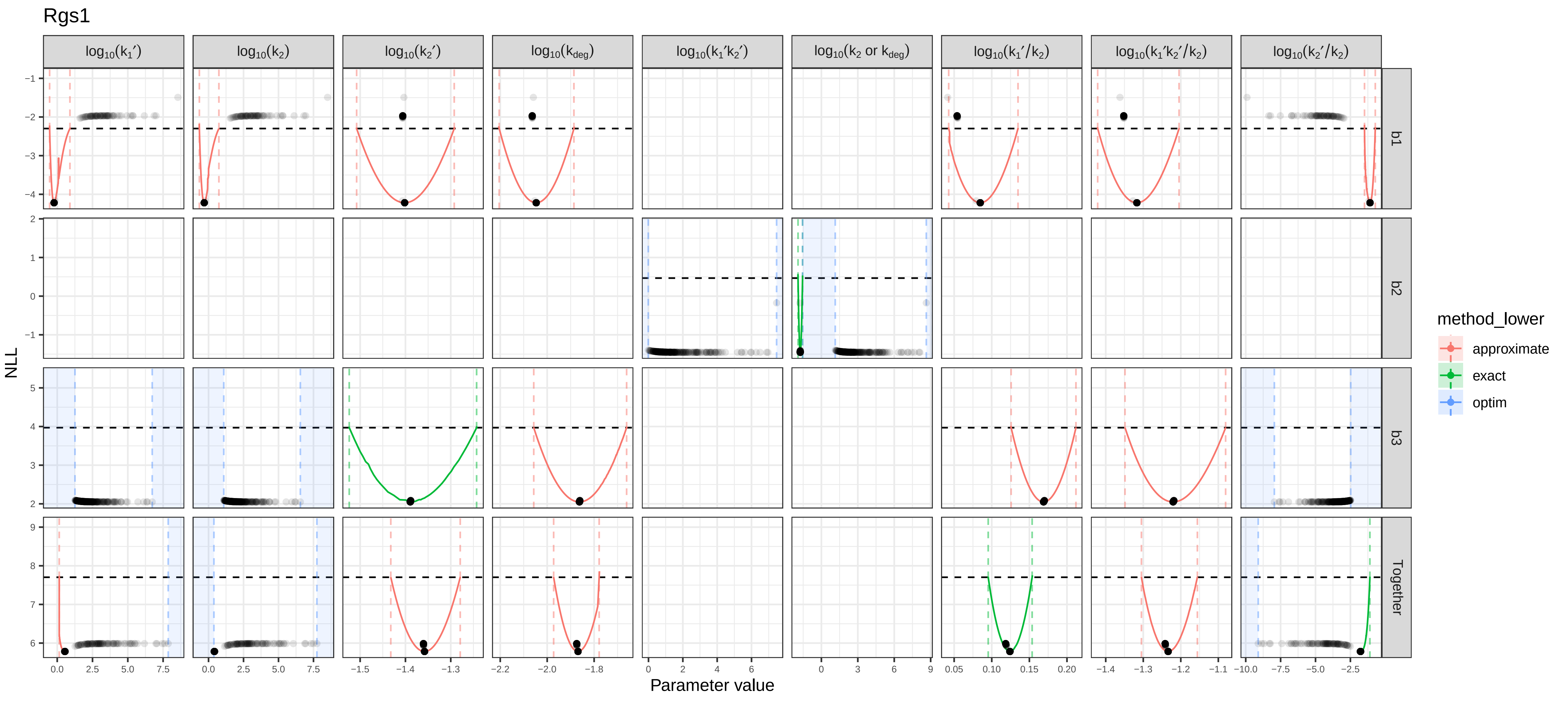

Replicate	Par	Best value	CI95 LB	CI95 UB	Method LB	Method UB
Together	$\log_{10}(k_1')$	0.539	0.1428	> 7.862	approximate	optim
Together	$\log_{10}(k_2)$	0.4148	< 0.3838	> 7.744	optim	optim
Together	$\log_{10}(k_2')$	−1.358	−1.432	−1.279	approximate	approximate
Together	$\log_{10}(k_{deg})$	−1.869	−1.972	−1.778	approximate	approximate
Together	$\log_{10}(k_1'/k_2)$	0.1242	0.09528	0.1537	exact	exact
Together	$\log_{10}(k_1'k_2'/k_2)$	−1.234	−1.305	−1.156	approximate	approximate
Together	$\log_{10}(k_2'/k_2)$	−1.773	< −9.104	−1.103	optim	exact
b1	$\log_{10}(k_1')$	−0.2232	−0.5384	0.9006	approximate	approximate
b1	$\log_{10}(k_2)$	−0.3079	−0.6561	0.7404	approximate	approximate
b1	$\log_{10}(k_2')$	−1.402	−1.508	−1.292	approximate	approximate
b1	$\log_{10}(k_{deg})$	−2.047	−2.204	−1.886	approximate	approximate
b1	$\log_{10}(k_1'/k_2)$	0.08468	0.04275	0.1349	approximate	approximate
b1	$\log_{10}(k_1'k_2'/k_2)$	−1.317	−1.421	−1.205	approximate	approximate
b1	$\log_{10}(k_2'/k_2)$	−1.094	−1.49	−0.717	approximate	approximate
b2	$\log_{10}(k_1'k_2')$	3.616	< −0.01238	> 7.46	optim	optim
b2	$\log_{10}(k_2 \text{ or } k_{deg})$	4.772	1.142	> 8.639	optim	optim
b2	$\log_{10}(k_2 \text{ or } k_{deg})$	−1.742	−1.923	−1.55	exact	exact
b3	$\log_{10}(k_1')$	4.281	< 1.258	> 6.729	optim	optim
b3	$\log_{10}(k_2)$	4.112	< 1.088	> 6.56	optim	optim
b3	$\log_{10}(k_2')$	−1.389	−1.524	−1.243	exact	exact
b3	$\log_{10}(k_{deg})$	−1.863	−2.057	−1.662	approximate	approximate
b3	$\log_{10}(k_1'/k_2)$	0.1689	0.1257	0.2119	approximate	approximate
b3	$\log_{10}(k_1'k_2'/k_2)$	−1.22	−1.349	−1.081	approximate	approximate
b3	$\log_{10}(k_2'/k_2)$	−5.502	< −7.95	> −2.477	optim	optim

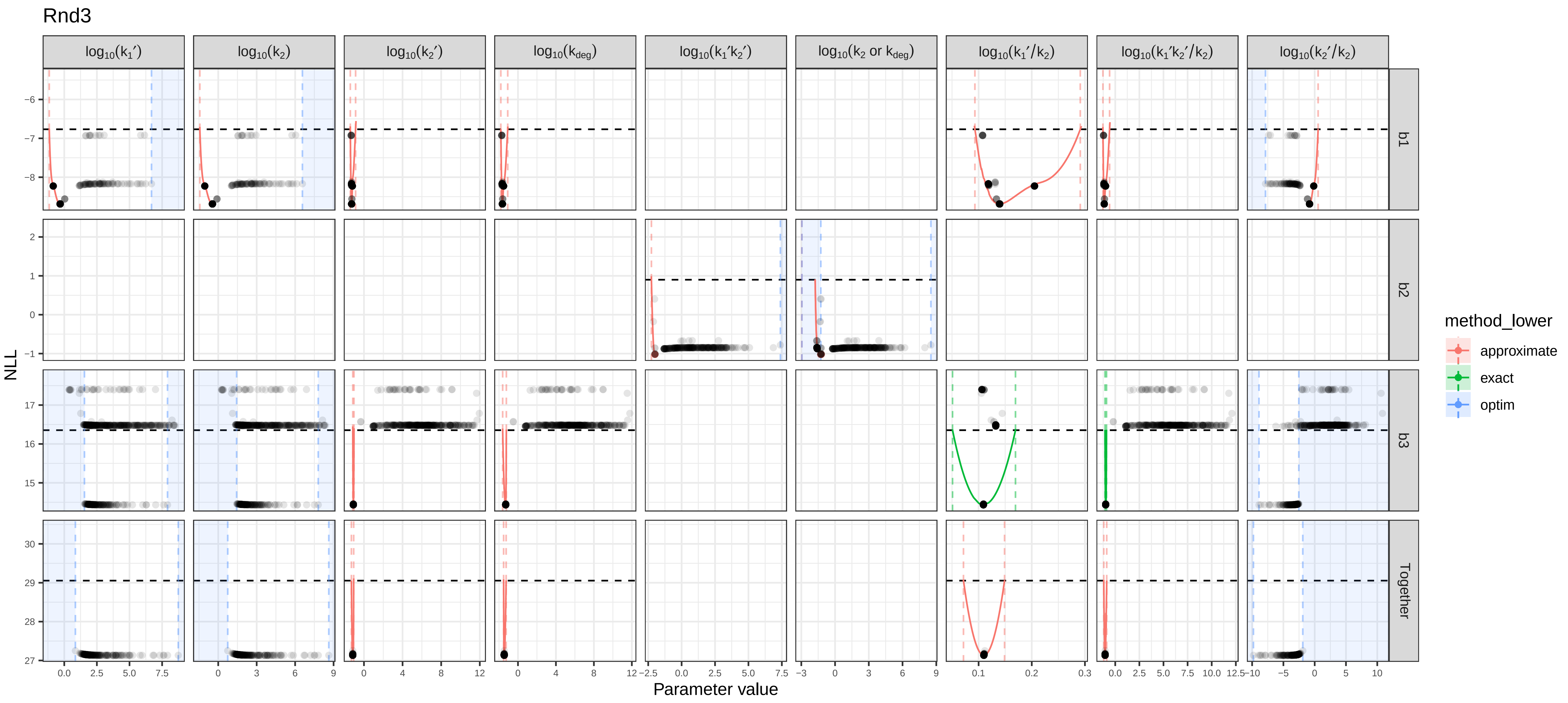

Replicate	Par	Best value	CI95 LB	CI95 UB	Method LB	Method UB
Together	$\log_{10}(k_1')$	5.053	< 0.8532	> 8.737	optim	optim
Together	$\log_{10}(k_2)$	4.944	< 0.7415	> 8.627	optim	optim
Together	$\log_{10}(k_2')$	−1.161	−1.302	−1.066	approximate	approximate
Together	$\log_{10}(k_{deg})$	−1.452	−1.512	−1.247	approximate	approximate
Together	$\log_{10}(k_1'/k_2)$	0.1099	0.07155	0.1488	approximate	approximate
Together	$\log_{10}(k_1'k_2'/k_2)$	−1.052	−1.188	−0.8817	approximate	approximate
Together	$\log_{10}(k_2'/k_2)$	−6.105	< −9.789	> −1.9	optim	optim
b1	$\log_{10}(k_1')$	−0.3144	−1.149	> 6.686	approximate	optim
b1	$\log_{10}(k_2)$	−0.4539	−1.435	> 6.568	approximate	optim
b1	$\log_{10}(k_2')$	−1.277	−1.415	−0.8661	approximate	approximate
b1	$\log_{10}(k_{deg})$	−1.64	−1.808	−1.08	approximate	approximate
b1	$\log_{10}(k_1'/k_2)$	0.1395	0.09305	0.2912	approximate	approximate
b1	$\log_{10}(k_1'k_2'/k_2)$	−1.137	−1.274	−0.5845	approximate	approximate
b1	$\log_{10}(k_2'/k_2)$	−0.8229	< −7.862	0.5503	optim	approximate
b2	$\log_{10}(k_1'k_2')$	−1.995	−2.261	> 7.39	approximate	optim
b2	$\log_{10}(k_2 \text{ or } k_{deg})$	−1.228	−2.951	> 8.522	approximate	optim
b2	$\log_{10}(k_2 \text{ or } k_{deg})$	−1.272	−2.951	−1.265	approximate	optim
b3	$\log_{10}(k_1')$	6.014	< 1.547	> 7.914	optim	optim
b3	$\log_{10}(k_2)$	5.905	< 1.438	> 7.805	optim	optim
b3	$\log_{10}(k_2')$	−1.101	−1.158	−1.035	approximate	approximate
b3	$\log_{10}(k_{deg})$	−1.295	−1.613	−1.226	approximate	approximate
b3	$\log_{10}(k_1'/k_2)$	0.109	0.05098	0.1694	exact	exact
b3	$\log_{10}(k_1'k_2'/k_2)$	−0.9917	−1.058	−0.9036	exact	exact
b3	$\log_{10}(k_2'/k_2)$	−7.006	< −8.905	> −2.537	optim	optim

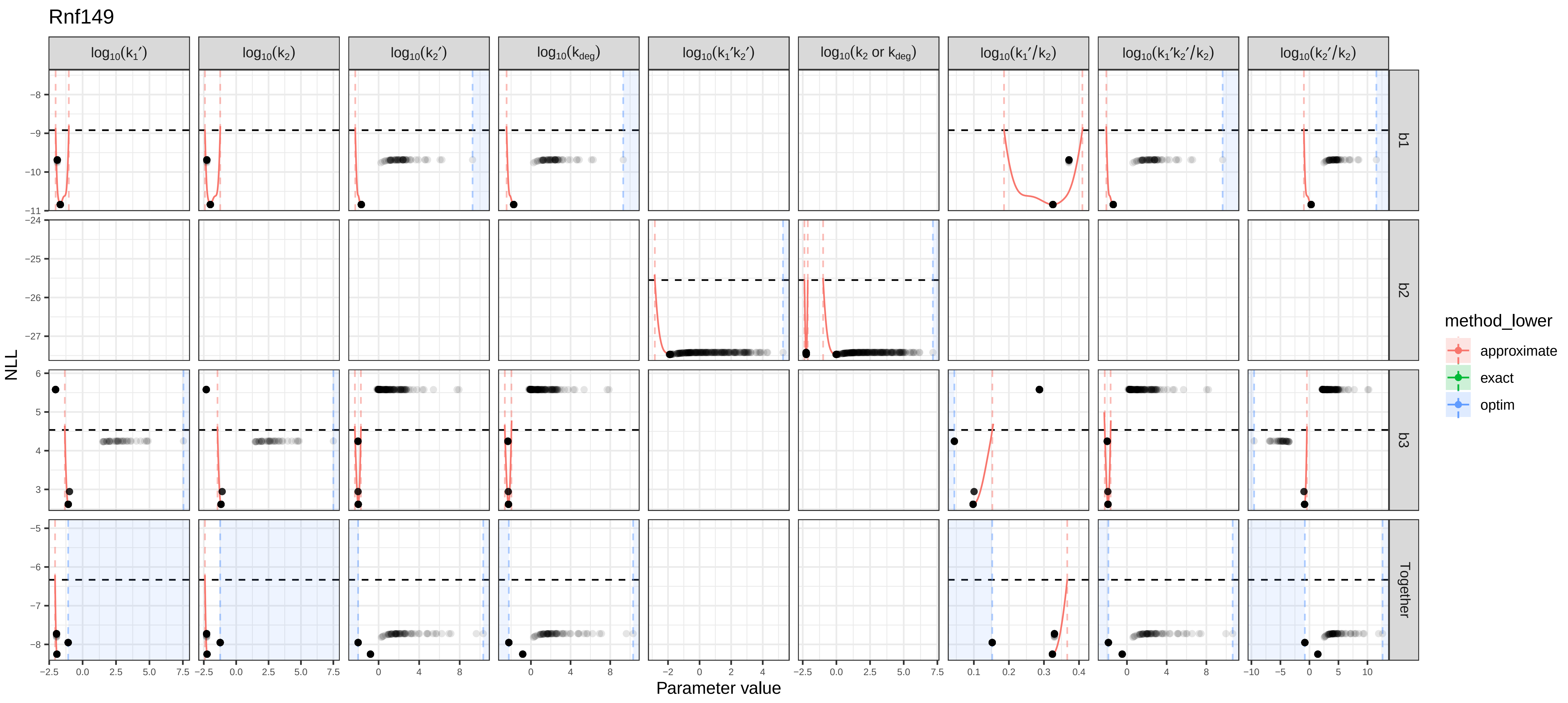

Replicate	Par	Best value	CI95 LB	CI95 UB	Method LB	Method UB
Together	$\log_{10}(k_1')$	−1.926	−2.061	> −1.081	approximate	optim
Together	$\log_{10}(k_2)$	−2.25	−2.411	> −1.233	approximate	optim
Together	$\log_{10}(k_2')$	−0.8022	< −2.022	> 10.32	optim	optim
Together	$\log_{10}(k_{deg})$	−0.8259	< −2.228	> 10.31	optim	optim
Together	$\log_{10}(k_1'/k_2)$	0.3241	< 0.1524	0.3664	optim	approximate
Together	$\log_{10}(k_1'k_2'/k_2)$	−0.478	< −1.869	> 10.65	optim	optim
Together	$\log_{10}(k_2'/k_2)$	1.447	< −0.7883	> 12.6	optim	optim
b1	$\log_{10}(k_1')$	−1.672	−2.019	−1.033	approximate	approximate
b1	$\log_{10}(k_2)$	−1.998	−2.411	−1.232	approximate	approximate
b1	$\log_{10}(k_2')$	−1.706	−2.303	> 9.267	approximate	optim
b1	$\log_{10}(k_{deg})$	−1.737	−2.453	> 9.311	approximate	optim
b1	$\log_{10}(k_1'/k_2)$	0.3256	0.1861	0.4096	approximate	approximate
b1	$\log_{10}(k_1'k_2'/k_2)$	−1.38	−2.078	> 9.639	approximate	optim
b1	$\log_{10}(k_2'/k_2)$	0.2918	−0.9546	> 11.53	approximate	optim
b2	$\log_{10}(k_1'k_2')$	−1.891	−2.832	> 5.285	approximate	optim
b2	$\log_{10}(k_2 \text{ or } k_{deg})$	−0.01445	−0.9861	> 7.168	approximate	optim
b2	$\log_{10}(k_2 \text{ or } k_{deg})$	−2.241	−2.372	−2.124	approximate	approximate
b3	$\log_{10}(k_1')$	−1.074	−1.33	> 7.546	approximate	optim
b3	$\log_{10}(k_2)$	−1.172	−1.442	> 7.502	approximate	optim
b3	$\log_{10}(k_2')$	−2.007	−2.337	−1.745	approximate	approximate
b3	$\log_{10}(k_{deg})$	−2.265	−2.632	−1.967	approximate	approximate
b3	$\log_{10}(k_1'/k_2)$	0.09784	< 0.04431	0.1527	optim	approximate
b3	$\log_{10}(k_1'k_2'/k_2)$	−1.909	−2.237	−1.645	approximate	approximate
b3	$\log_{10}(k_2'/k_2)$	−0.8352	< −9.534	−0.435	optim	approximate

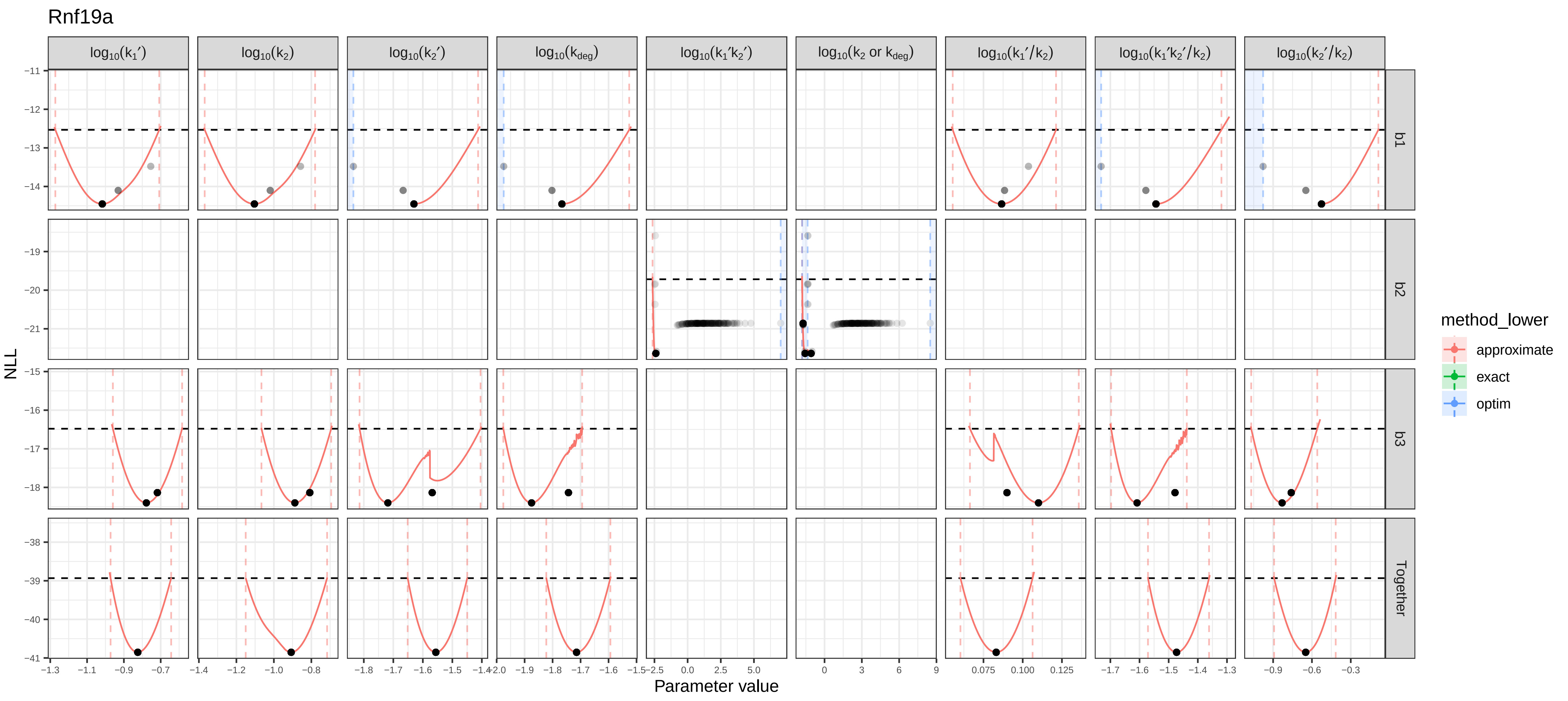

Replicate	Par	Best value	CI95 LB	CI95 UB	Method LB	Method UB
Together	$\log_{10}(k_1')$	−0.8245	−0.9722	−0.6432	approximate	approximate
Together	$\log_{10}(k_2)$	−0.9076	−1.15	−0.7159	approximate	approximate
Together	$\log_{10}(k_2')$	−1.556	−1.651	−1.45	approximate	approximate
Together	$\log_{10}(k_{deg})$	−1.714	−1.823	−1.593	approximate	approximate
Together	$\log_{10}(k_1'/k_2)$	0.08309	0.06032	0.1063	approximate	approximate
Together	$\log_{10}(k_1'k_2'/k_2)$	−1.473	−1.571	−1.361	approximate	approximate
Together	$\log_{10}(k_2'/k_2)$	−0.6483	−0.8925	−0.4163	approximate	approximate
b1	$\log_{10}(k_1')$	−1.017	−1.272	−0.708	approximate	approximate
b1	$\log_{10}(k_2)$	−1.104	−1.369	−0.78	approximate	approximate
b1	$\log_{10}(k_2')$	−1.63	< −1.835	−1.413	optim	approximate
b1	$\log_{10}(k_{deg})$	−1.767	< −1.975	−1.526	optim	approximate
b1	$\log_{10}(k_1'/k_2)$	0.08657	0.05541	0.1212	approximate	approximate
b1	$\log_{10}(k_1'k_2'/k_2)$	−1.544	< −1.732	−1.319	optim	approximate
b1	$\log_{10}(k_2'/k_2)$	−0.5262	< −0.9775	−0.08652	optim	approximate
b2	$\log_{10}(k_1'k_2')$	−2.397	−2.645	> 7.015	approximate	optim
b2	$\log_{10}(k_2 \text{ or } k_{deg})$	−1.079	−1.805	> 8.5	approximate	optim
b2	$\log_{10}(k_2 \text{ or } k_{deg})$	−1.566	−1.805	−1.358	approximate	optim
b3	$\log_{10}(k_1')$	−0.7779	−0.9598	−0.5837	approximate	approximate
b3	$\log_{10}(k_2)$	−0.888	−1.066	−0.6941	approximate	approximate
b3	$\log_{10}(k_2')$	−1.718	−1.814	−1.404	approximate	approximate
b3	$\log_{10}(k_{deg})$	−1.875	−1.976	−1.694	approximate	approximate
b3	$\log_{10}(k_1'/k_2)$	0.1101	0.06638	0.1357	approximate	approximate
b3	$\log_{10}(k_1'k_2'/k_2)$	−1.608	−1.698	−1.438	approximate	approximate
b3	$\log_{10}(k_2'/k_2)$	−0.8305	−1.069	−0.5588	approximate	approximate

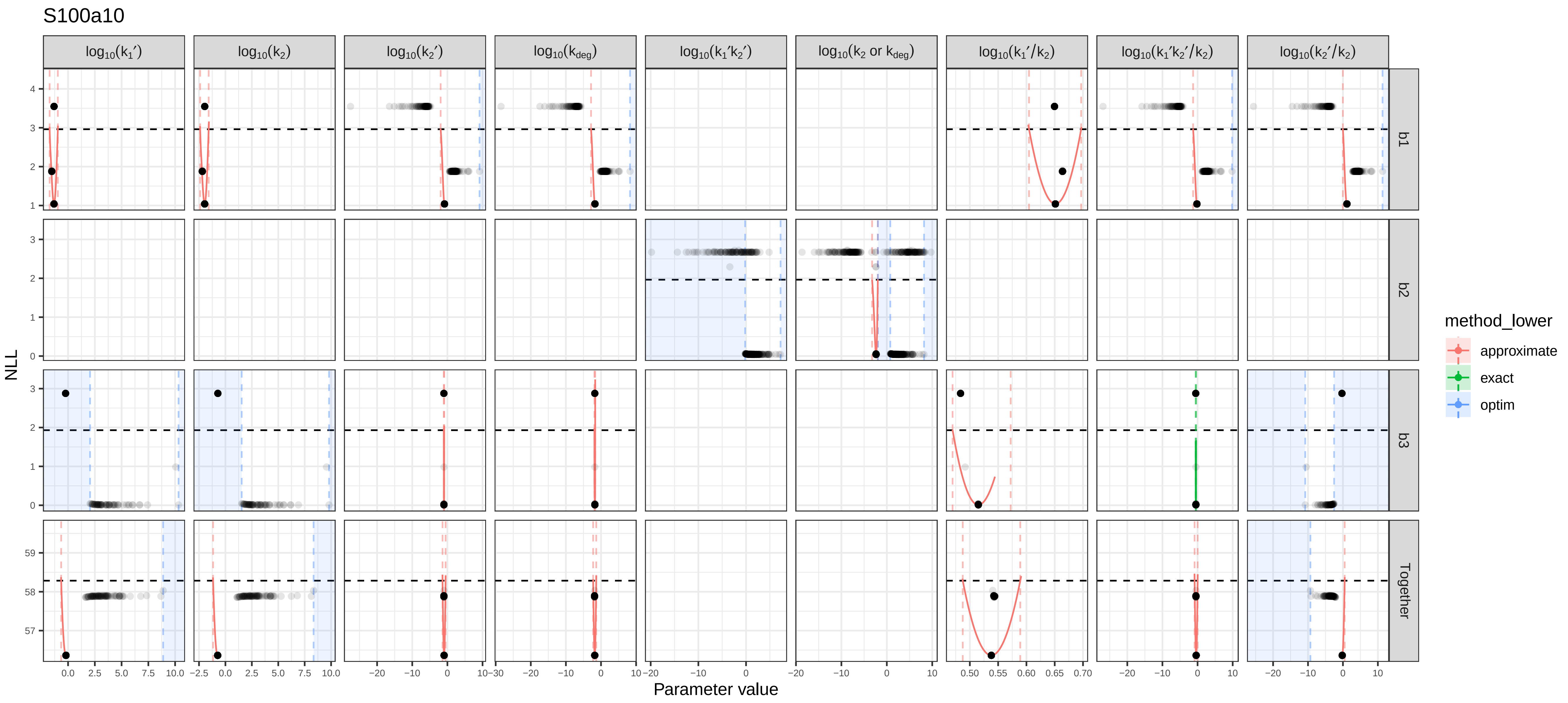

Replicate	Par	Best value	CI95 LB	CI95 UB	Method LB	Method UB
Together	$\log_{10}(k_1')$	−0.1953	−0.6451	> 8.883	approximate	optim
Together	$\log_{10}(k_2)$	−0.7333	−1.168	> 8.343	approximate	optim
Together	$\log_{10}(k_2')$	−0.9362	−1.386	−0.5081	approximate	approximate
Together	$\log_{10}(k_{deg})$	−1.78	−2.24	−1.359	approximate	approximate
Together	$\log_{10}(k_1'/k_2)$	0.538	0.4875	0.5891	approximate	approximate
Together	$\log_{10}(k_1'k_2'/k_2)$	−0.3982	−0.8462	0.0235	approximate	approximate
Together	$\log_{10}(k_2'/k_2)$	−0.2028	< −9.315	0.4955	optim	approximate
b1	$\log_{10}(k_1')$	−1.314	−1.723	−0.9537	approximate	approximate
b1	$\log_{10}(k_2)$	−1.965	−2.393	−1.584	approximate	approximate
b1	$\log_{10}(k_2')$	−0.826	−1.921	> 9.151	approximate	optim
b1	$\log_{10}(k_{deg})$	−1.727	−2.84	> 8.275	approximate	optim
b1	$\log_{10}(k_1'/k_2)$	0.6511	0.6046	0.6967	approximate	approximate
b1	$\log_{10}(k_1'k_2'/k_2)$	−0.1748	−1.273	> 9.815	approximate	optim
b1	$\log_{10}(k_2'/k_2)$	1.139	−0.0191	> 11.34	approximate	optim
b2	$\log_{10}(k_1'k_2')$	3.717	< −0.194	> 7.235	optim	optim
b2	$\log_{10}(k_2 \text{ or } k_{deg})$	4.676	0.7676	> 8.194	optim	optim
b2	$\log_{10}(k_2 \text{ or } k_{deg})$	−2.367	−3.244	−1.98	approximate	approximate
b3	$\log_{10}(k_1')$	6.704	< 2.054	> 10.32	optim	optim
b3	$\log_{10}(k_2)$	6.189	< 1.539	> 9.807	optim	optim
b3	$\log_{10}(k_2')$	−0.9924	−1.02	−0.9081	approximate	approximate
b3	$\log_{10}(k_{deg})$	−1.746	−1.871	−1.661	approximate	approximate
b3	$\log_{10}(k_1'/k_2)$	0.515	0.4695	0.5722	approximate	approximate
b3	$\log_{10}(k_1'k_2'/k_2)$	−0.4774	−0.5358	−0.4242	exact	exact
b3	$\log_{10}(k_2'/k_2)$	−7.181	< −10.8	> −2.531	optim	optim

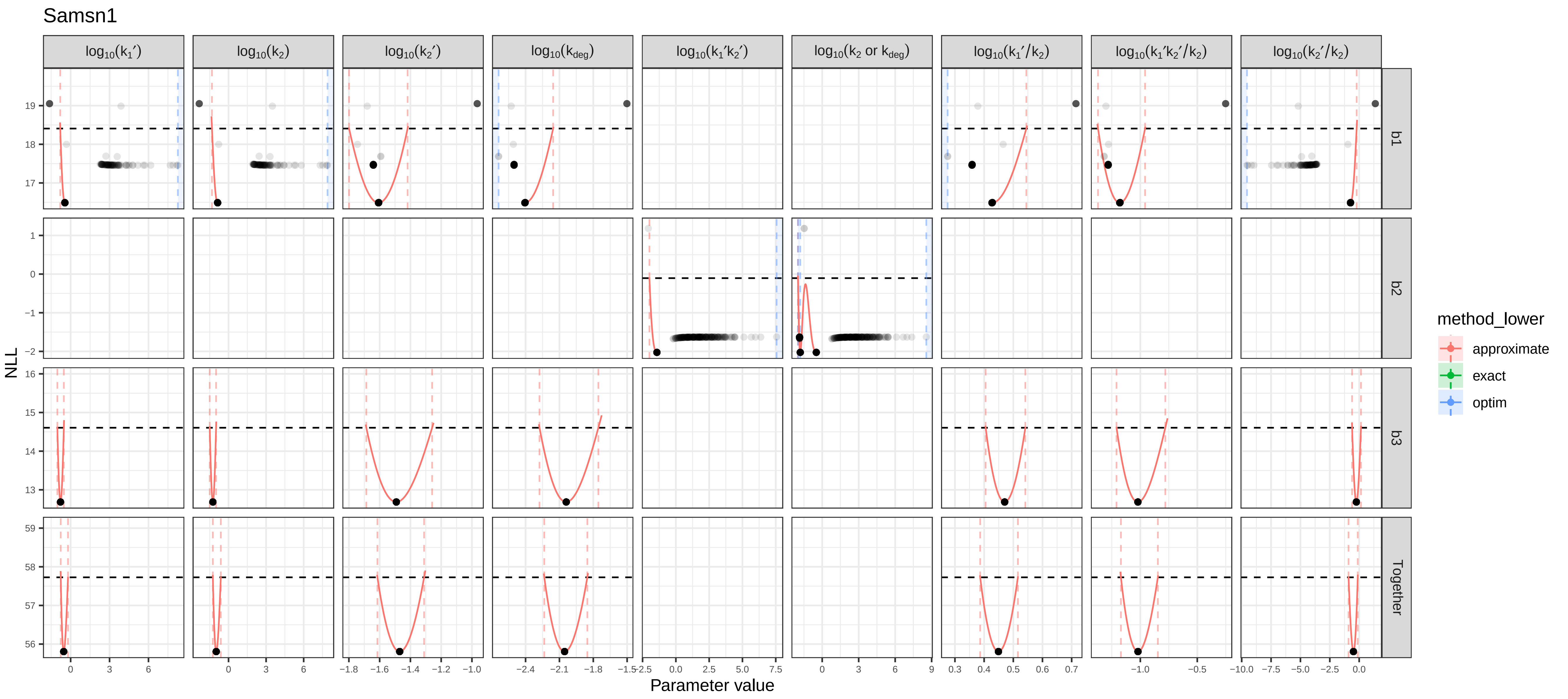

Replicate	Par	Best value	CI95 LB	CI95 UB	Method LB	Method UB
Together	$\log_{10}(k_1')$	−0.5331	−0.7634	−0.2022	approximate	approximate
Together	$\log_{10}(k_2)$	−0.9821	−1.253	−0.6167	approximate	approximate
Together	$\log_{10}(k_2')$	−1.47	−1.614	−1.311	approximate	approximate
Together	$\log_{10}(k_{deg})$	−2.054	−2.234	−1.853	approximate	approximate
Together	$\log_{10}(k_1'/k_2)$	0.4491	0.3865	0.5157	approximate	approximate
Together	$\log_{10}(k_1'k_2'/k_2)$	−1.021	−1.17	−0.846	approximate	approximate
Together	$\log_{10}(k_2'/k_2)$	−0.4876	−0.906	−0.1219	approximate	approximate
b1	$\log_{10}(k_1')$	−0.4414	−0.8018	> 8.264	approximate	optim
b1	$\log_{10}(k_2)$	−0.8685	−1.323	> 7.906	approximate	optim
b1	$\log_{10}(k_2')$	−1.607	−1.799	−1.418	approximate	approximate
b1	$\log_{10}(k_{deg})$	−2.406	< −2.639	−2.156	optim	approximate
b1	$\log_{10}(k_1'/k_2)$	0.4271	< 0.275	0.5447	optim	approximate
b1	$\log_{10}(k_1'k_2'/k_2)$	−1.18	−1.371	−0.9583	approximate	approximate
b1	$\log_{10}(k_2'/k_2)$	−0.7388	< −9.547	−0.2145	optim	approximate
b2	$\log_{10}(k_1'k_2')$	−1.423	−1.984	> 7.563	approximate	optim
b2	$\log_{10}(k_2 \text{ or } k_{deg})$	−0.4866	−1.989	> 8.558	approximate	optim
b2	$\log_{10}(k_2 \text{ or } k_{deg})$	−1.804	−1.989	−1.803	approximate	optim
b3	$\log_{10}(k_1')$	−0.7811	−1.023	−0.5236	approximate	approximate
b3	$\log_{10}(k_2)$	−1.252	−1.503	−0.994	approximate	approximate
b3	$\log_{10}(k_2')$	−1.492	−1.687	−1.259	approximate	approximate
b3	$\log_{10}(k_{deg})$	−2.04	−2.277	−1.755	approximate	approximate
b3	$\log_{10}(k_1'/k_2)$	0.4704	0.4052	0.5413	approximate	approximate
b3	$\log_{10}(k_1'k_2'/k_2)$	−1.021	−1.209	−0.7805	approximate	approximate
b3	$\log_{10}(k_2'/k_2)$	−0.2402	−0.6037	0.1529	approximate	approximate

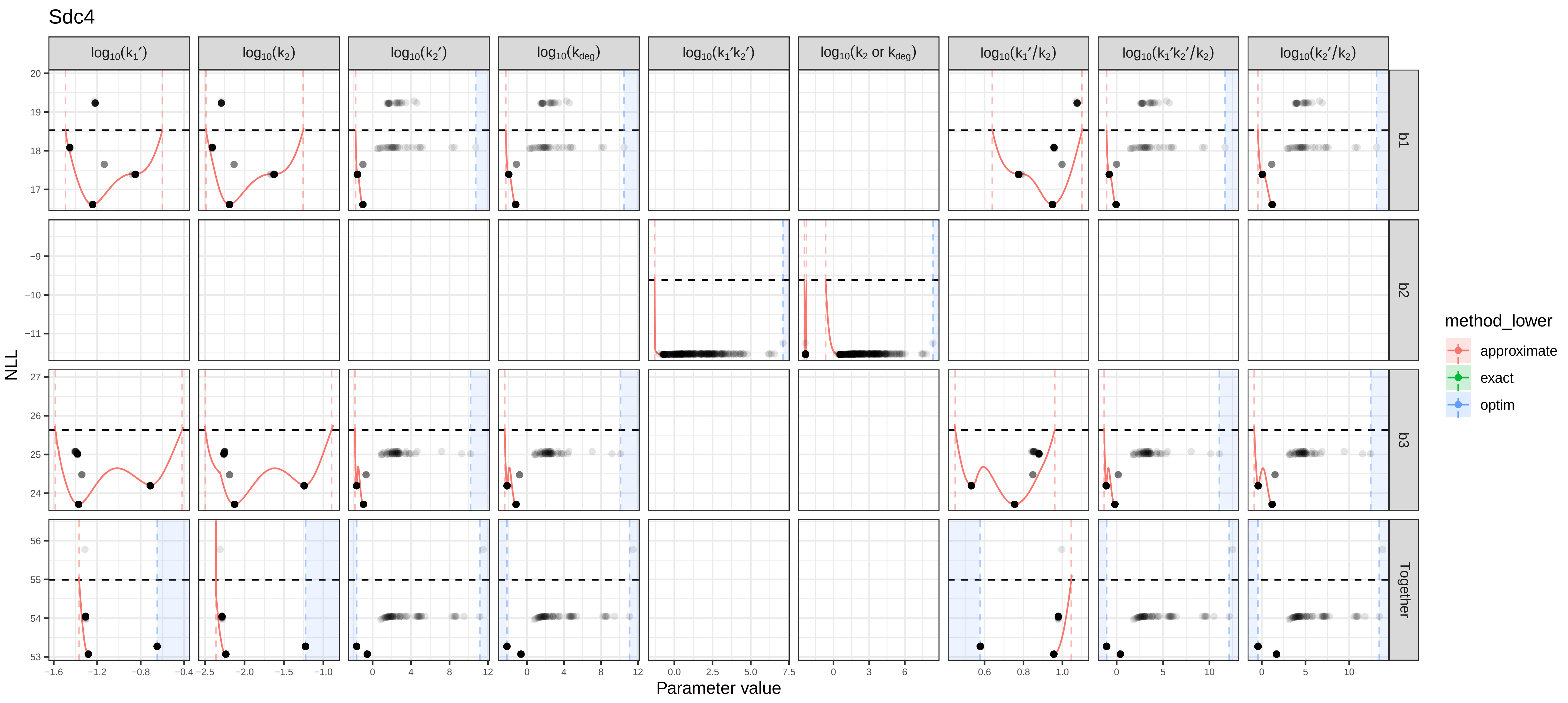

Replicate	Par	Best value	CI95 LB	CI95 UB	Method LB	Method UB
Together	$\log_{10}(k_1')$	−1.281	−1.366	> −0.6475	approximate	optim
Together	$\log_{10}(k_2)$	−2.237	−2.362	> −1.225	approximate	optim
Together	$\log_{10}(k_2')$	−0.5563	< −1.659	> 11.15	optim	optim
Together	$\log_{10}(k_{deg})$	−0.658	< −2.182	> 11.09	optim	optim
Together	$\log_{10}(k_1'/k_2)$	0.9557	< 0.5778	1.046	optim	approximate
Together	$\log_{10}(k_1'k_2'/k_2)$	0.3995	< −1.081	> 12.13	optim	optim
Together	$\log_{10}(k_2'/k_2)$	1.681	< −0.4334	> 13.43	optim	optim
b1	$\log_{10}(k_1')$	−1.242	−1.492	−0.6003	approximate	approximate
b1	$\log_{10}(k_2)$	−2.191	−2.488	−1.256	approximate	approximate
b1	$\log_{10}(k_2')$	−1.009	−1.772	> 10.72	approximate	optim
b1	$\log_{10}(k_{deg})$	−1.231	−2.313	> 10.49	approximate	optim
b1	$\log_{10}(k_1'/k_2)$	0.9491	0.6399	1.102	approximate	approximate
b1	$\log_{10}(k_1'k_2'/k_2)$	−0.05973	−1.086	> 11.68	approximate	optim
b1	$\log_{10}(k_2'/k_2)$	1.182	−0.4605	> 13.13	approximate	optim
b2	$\log_{10}(k_1'k_2')$	−0.6784	−1.293	> 7.108	approximate	optim
b2	$\log_{10}(k_2 \text{ or } k_{deg})$	0.5718	−0.6657	> 8.366	approximate	optim
b2	$\log_{10}(k_2 \text{ or } k_{deg})$	−2.359	−2.447	−2.278	approximate	approximate
b3	$\log_{10}(k_1')$	−1.371	−1.585	−0.4184	approximate	approximate
b3	$\log_{10}(k_2)$	−2.126	−2.496	−0.8964	approximate	approximate
b3	$\log_{10}(k_2')$	−0.946	−1.849	> 10.19	approximate	optim
b3	$\log_{10}(k_{deg})$	−1.195	−2.42	> 10.11	approximate	optim
b3	$\log_{10}(k_1'/k_2)$	0.7545	0.4493	0.9605	approximate	approximate
b3	$\log_{10}(k_1'k_2'/k_2)$	−0.1915	−1.335	> 11.07	approximate	optim
b3	$\log_{10}(k_2'/k_2)$	1.18	−0.8656	> 12.45	approximate	optim

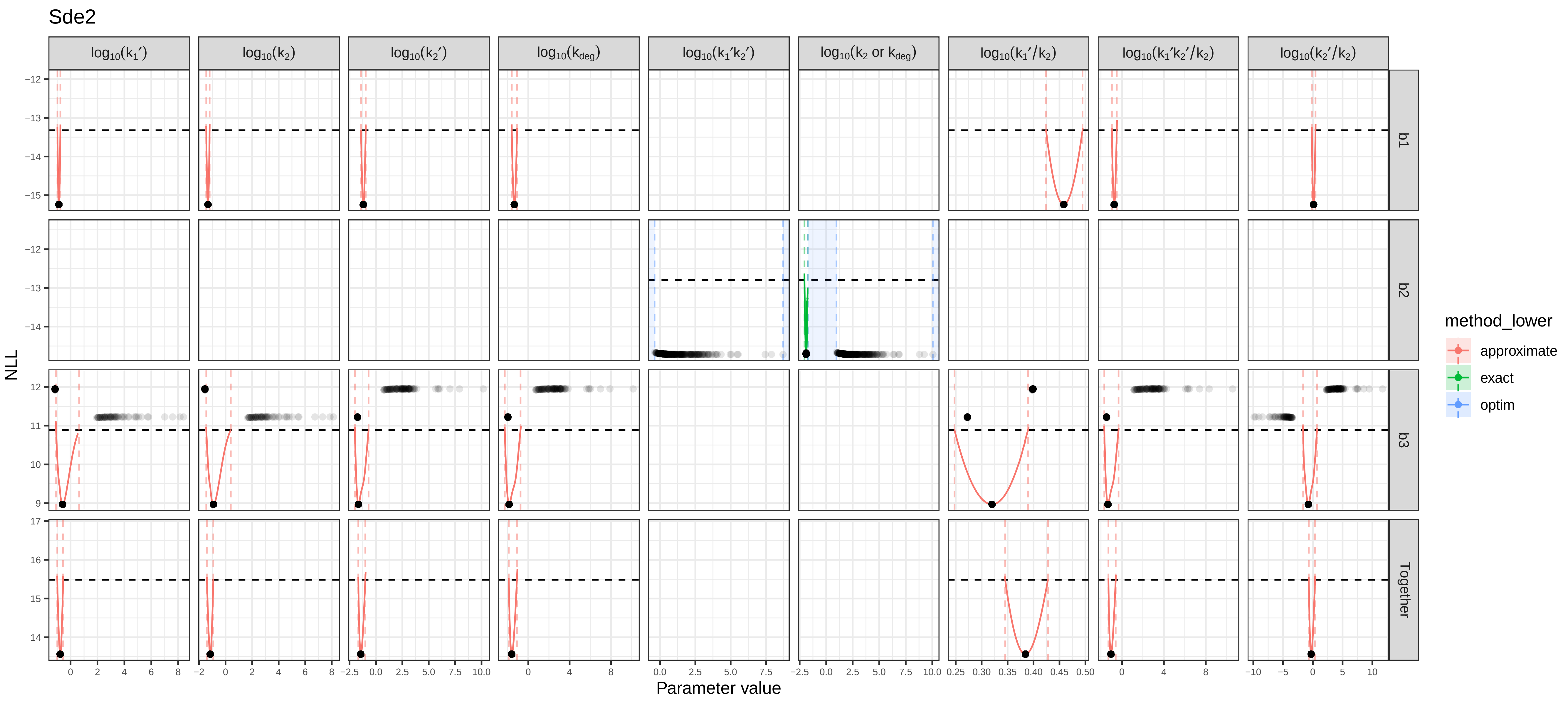

Replicate	Par	Best value	CI95 LB	CI95 UB	Method LB	Method UB
Together	$\log_{10}(k_1')$	−0.7693	−0.9878	−0.557	approximate	approximate
Together	$\log_{10}(k_2)$	−1.154	−1.403	−0.924	approximate	approximate
Together	$\log_{10}(k_2')$	−1.431	−1.668	−1.006	approximate	approximate
Together	$\log_{10}(k_{deg})$	−1.596	−1.887	−1.103	approximate	approximate
Together	$\log_{10}(k_1'/k_2)$	0.3843	0.3454	0.4278	approximate	approximate
Together	$\log_{10}(k_1'k_2'/k_2)$	−1.047	−1.29	−0.5949	approximate	approximate
Together	$\log_{10}(k_2'/k_2)$	−0.2777	−0.6748	0.3751	approximate	approximate
b1	$\log_{10}(k_1')$	−0.8688	−0.9774	−0.7587	approximate	approximate
b1	$\log_{10}(k_2)$	−1.327	−1.455	−1.202	approximate	approximate
b1	$\log_{10}(k_2')$	−1.197	−1.409	−0.966	approximate	approximate
b1	$\log_{10}(k_{deg})$	−1.351	−1.6	−1.089	approximate	approximate
b1	$\log_{10}(k_1'/k_2)$	0.4582	0.4241	0.4945	approximate	approximate
b1	$\log_{10}(k_1'k_2'/k_2)$	−0.7392	−0.9589	−0.4945	approximate	approximate
b1	$\log_{10}(k_2'/k_2)$	0.1296	−0.1625	0.4556	approximate	approximate
b2	$\log_{10}(k_1'k_2')$	7.886	< −0.382	> 8.704	optim	optim
b2	$\log_{10}(k_2 \text{ or } k_{deg})$	9.242	0.9722	> 10.06	optim	optim
b2	$\log_{10}(k_2 \text{ or } k_{deg})$	−1.889	−2.045	−1.741	exact	exact
b3	$\log_{10}(k_1')$	−0.5922	−1.076	0.635	approximate	approximate
b3	$\log_{10}(k_2)$	−0.9123	−1.453	0.3927	approximate	approximate
b3	$\log_{10}(k_2')$	−1.656	−1.997	−0.6932	approximate	approximate
b3	$\log_{10}(k_{deg})$	−1.856	−2.277	−0.7511	approximate	approximate
b3	$\log_{10}(k_1'/k_2)$	0.32	0.2476	0.3895	approximate	approximate
b3	$\log_{10}(k_1'k_2'/k_2)$	−1.336	−1.681	−0.3237	approximate	approximate
b3	$\log_{10}(k_2'/k_2)$	−0.7433	−1.626	0.709	approximate	approximate

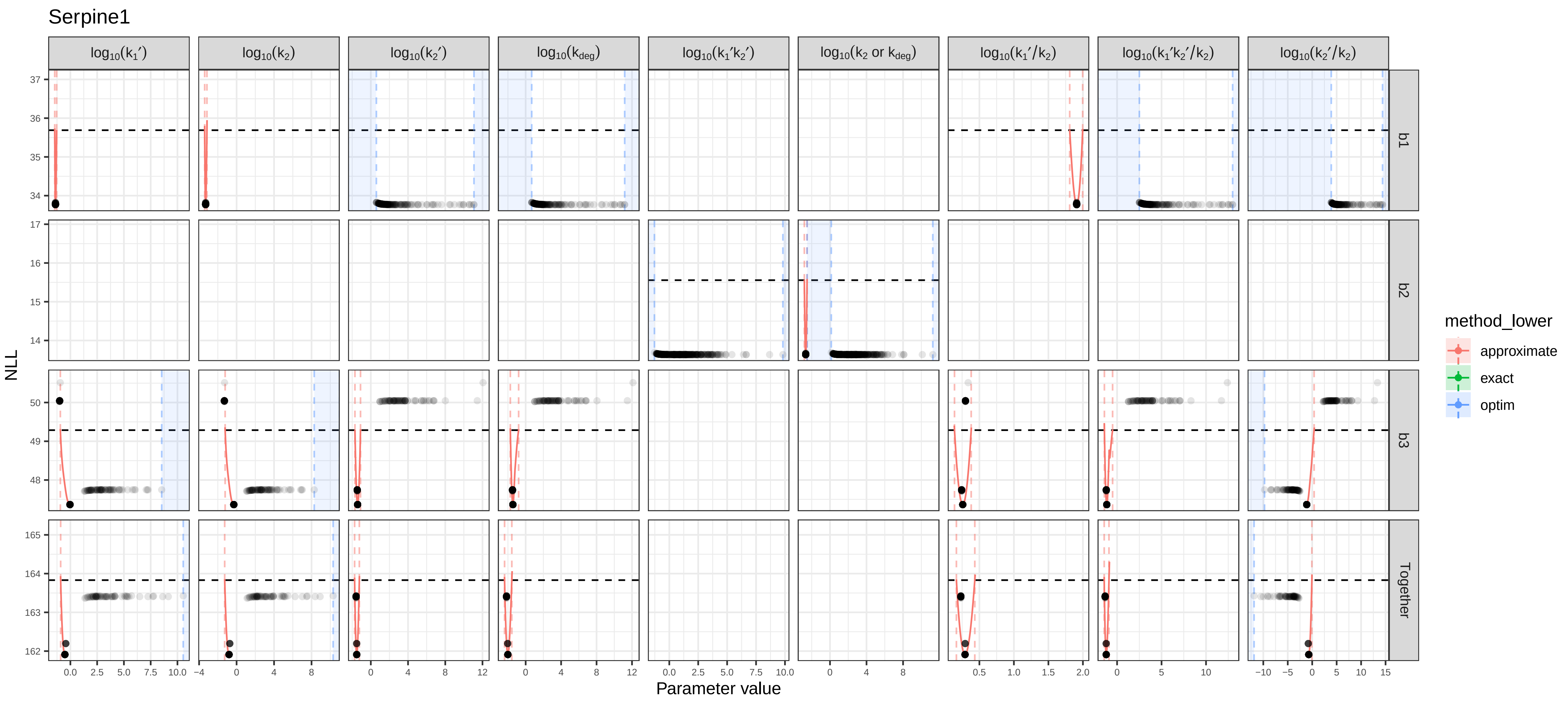

Replicate	Par	Best value	CI95 LB	CI95 UB	Method LB	Method UB
Together	$\log_{10}(k_1')$	−0.5113	−0.9094	> 10.54	approximate	optim
Together	$\log_{10}(k_2)$	−0.8034	−1.27	> 10.31	approximate	optim
Together	$\log_{10}(k_2')$	−1.512	−1.74	−1.233	approximate	approximate
Together	$\log_{10}(k_{deg})$	−2.011	−2.381	−1.563	approximate	approximate
Together	$\log_{10}(k_1'/k_2)$	0.2922	0.1669	0.4336	approximate	approximate
Together	$\log_{10}(k_1'k_2'/k_2)$	−1.219	−1.448	−0.912	approximate	approximate
Together	$\log_{10}(k_2'/k_2)$	−0.7081	< −11.88	−0.06692	optim	approximate
b1	$\log_{10}(k_1')$	−1.393	−1.46	−1.279	approximate	approximate
b1	$\log_{10}(k_2)$	−3.303	−3.404	−3.165	approximate	approximate
b1	$\log_{10}(k_2')$	5.05	< 0.5958	> 11.09	optim	optim
b1	$\log_{10}(k_{deg})$	5.139	< 0.6822	> 11.17	optim	optim
b1	$\log_{10}(k_1'/k_2)$	1.91	1.809	1.997	approximate	approximate
b1	$\log_{10}(k_1'k_2'/k_2)$	6.96	< 2.505	> 13	optim	optim
b1	$\log_{10}(k_2'/k_2)$	8.353	< 3.897	> 14.39	optim	optim
b2	$\log_{10}(k_1'k_2')$	3.592	< −1.285	> 9.821	optim	optim
b2	$\log_{10}(k_2 \text{ or } k_{deg})$	5.028	0.1416	> 11.26	optim	optim
b2	$\log_{10}(k_2 \text{ or } k_{deg})$	−2.66	−2.808	−2.507	approximate	approximate
b3	$\log_{10}(k_1')$	−0.03905	−0.9386	> 8.53	approximate	optim
b3	$\log_{10}(k_2)$	−0.298	−1.221	> 8.287	approximate	optim
b3	$\log_{10}(k_2')$	−1.419	−1.705	−1.107	approximate	approximate
b3	$\log_{10}(k_{deg})$	−1.442	−1.732	−0.7939	approximate	approximate
b3	$\log_{10}(k_1'/k_2)$	0.259	0.1395	0.3813	approximate	approximate
b3	$\log_{10}(k_1'k_2'/k_2)$	−1.16	−1.427	−0.5043	approximate	approximate
b3	$\log_{10}(k_2'/k_2)$	−1.121	< −9.746	0.392	optim	approximate

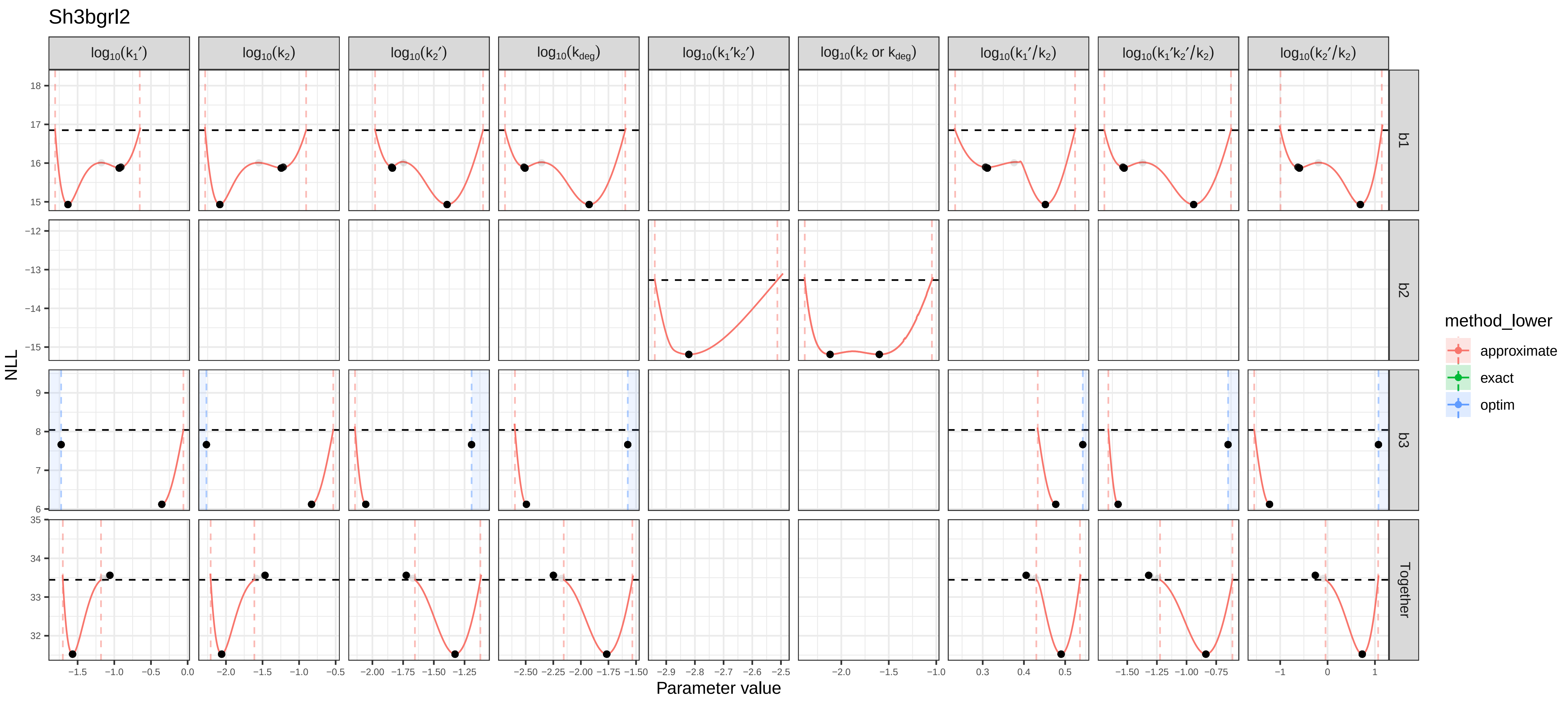

Replicate	Par	Best value	CI95 LB	CI95 UB	Method LB	Method UB
Together	$\log_{10}(k_1')$	−1.569	−1.701	−1.181	approximate	approximate
Together	$\log_{10}(k_2)$	−2.059	−2.209	−1.612	approximate	approximate
Together	$\log_{10}(k_2')$	−1.327	−1.654	−1.121	approximate	approximate
Together	$\log_{10}(k_{deg})$	−1.766	−2.155	−1.533	approximate	approximate
Together	$\log_{10}(k_1'/k_2)$	0.4902	0.4301	0.5359	approximate	approximate
Together	$\log_{10}(k_1'k_2'/k_2)$	−0.8372	−1.223	−0.6132	approximate	approximate
Together	$\log_{10}(k_2'/k_2)$	0.7316	−0.04293	1.068	approximate	approximate
b1	$\log_{10}(k_1')$	−1.632	−1.806	−0.6529	approximate	approximate
b1	$\log_{10}(k_2)$	−2.084	−2.284	−0.9037	approximate	approximate
b1	$\log_{10}(k_2')$	−1.392	−1.978	−1.099	approximate	approximate
b1	$\log_{10}(k_{deg})$	−1.925	−2.687	−1.598	approximate	approximate
b1	$\log_{10}(k_1'/k_2)$	0.452	0.2332	0.524	approximate	approximate
b1	$\log_{10}(k_1'k_2'/k_2)$	−0.9401	−1.693	−0.6255	approximate	approximate
b1	$\log_{10}(k_2'/k_2)$	0.6919	−0.9925	1.145	approximate	approximate
b2	$\log_{10}(k_1'k_2')$	−2.821	−2.94	−2.512	approximate	approximate
b2	$\log_{10}(k_2 \text{ or } k_{deg})$	−1.6	−2.385	−1.046	approximate	approximate
b2	$\log_{10}(k_2 \text{ or } k_{deg})$	−2.118	−2.385	−1.046	approximate	approximate
b3	$\log_{10}(k_1')$	−0.3521	< −1.724	−0.05778	optim	approximate
b3	$\log_{10}(k_2)$	−0.8294	< −2.267	−0.5327	optim	approximate
b3	$\log_{10}(k_2')$	−2.054	−2.141	> −1.193	approximate	optim
b3	$\log_{10}(k_{deg})$	−2.494	−2.597	> −1.575	approximate	optim
b3	$\log_{10}(k_1'/k_2)$	0.4772	0.4335	> 0.5428	approximate	optim
b3	$\log_{10}(k_1'k_2'/k_2)$	−1.577	−1.659	> −0.6499	approximate	optim
b3	$\log_{10}(k_2'/k_2)$	−1.225	−1.548	> 1.075	approximate	optim

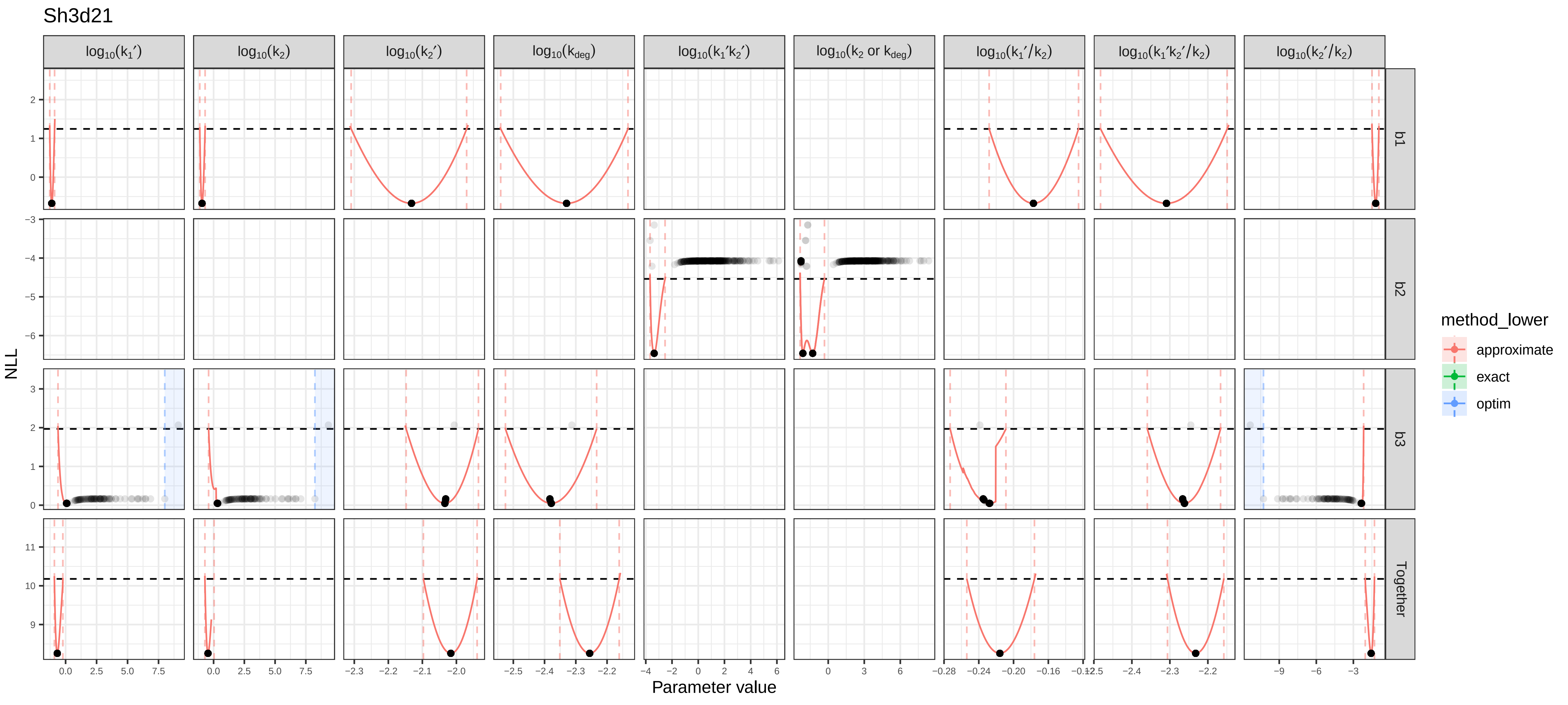

Replicate	Par	Best value	CI95 LB	CI95 UB	Method LB	Method UB
Together	$\log_{10}(k_1')$	−0.6698	−0.9052	−0.2212	approximate	approximate
Together	$\log_{10}(k_2)$	−0.454	−0.7065	0.04004	approximate	approximate
Together	$\log_{10}(k_2')$	−2.016	−2.097	−1.939	approximate	approximate
Together	$\log_{10}(k_{deg})$	−2.256	−2.351	−2.161	approximate	approximate
Together	$\log_{10}(k_1'/k_2)$	−0.2158	−0.2538	−0.176	approximate	approximate
Together	$\log_{10}(k_1'k_2'/k_2)$	−2.232	−2.306	−2.158	approximate	approximate
Together	$\log_{10}(k_2'/k_2)$	−1.562	−2.039	−1.293	approximate	approximate
b1	$\log_{10}(k_1')$	−1.117	−1.282	−0.8886	approximate	approximate
b1	$\log_{10}(k_2)$	−0.9396	−1.124	−0.69	approximate	approximate
b1	$\log_{10}(k_2')$	−2.132	−2.309	−1.97	approximate	approximate
b1	$\log_{10}(k_{deg})$	−2.33	−2.54	−2.133	approximate	approximate
b1	$\log_{10}(k_1'/k_2)$	−0.1771	−0.2281	−0.1252	approximate	approximate
b1	$\log_{10}(k_1'k_2'/k_2)$	−2.309	−2.482	−2.149	approximate	approximate
b1	$\log_{10}(k_2'/k_2)$	−1.192	−1.491	−0.937	approximate	approximate
b2	$\log_{10}(k_1'k_2')$	−3.361	−3.675	−2.53	approximate	approximate
b2	$\log_{10}(k_2 \text{ or } k_{deg})$	−1.295	−2.338	−0.3139	approximate	approximate
b2	$\log_{10}(k_2 \text{ or } k_{deg})$	−2.109	−2.338	−0.3139	approximate	approximate
b3	$\log_{10}(k_1')$	0.09268	−0.6138	> 8.005	approximate	optim
b3	$\log_{10}(k_2)$	0.3204	−0.4054	> 8.24	approximate	optim
b3	$\log_{10}(k_2')$	−2.034	−2.148	−1.935	approximate	approximate
b3	$\log_{10}(k_{deg})$	−2.378	−2.525	−2.233	approximate	approximate
b3	$\log_{10}(k_1'/k_2)$	−0.2277	−0.2729	−0.2088	approximate	approximate
b3	$\log_{10}(k_1'k_2'/k_2)$	−2.262	−2.359	−2.167	approximate	approximate
b3	$\log_{10}(k_2'/k_2)$	−2.354	< −10.27	−2.168	optim	approximate

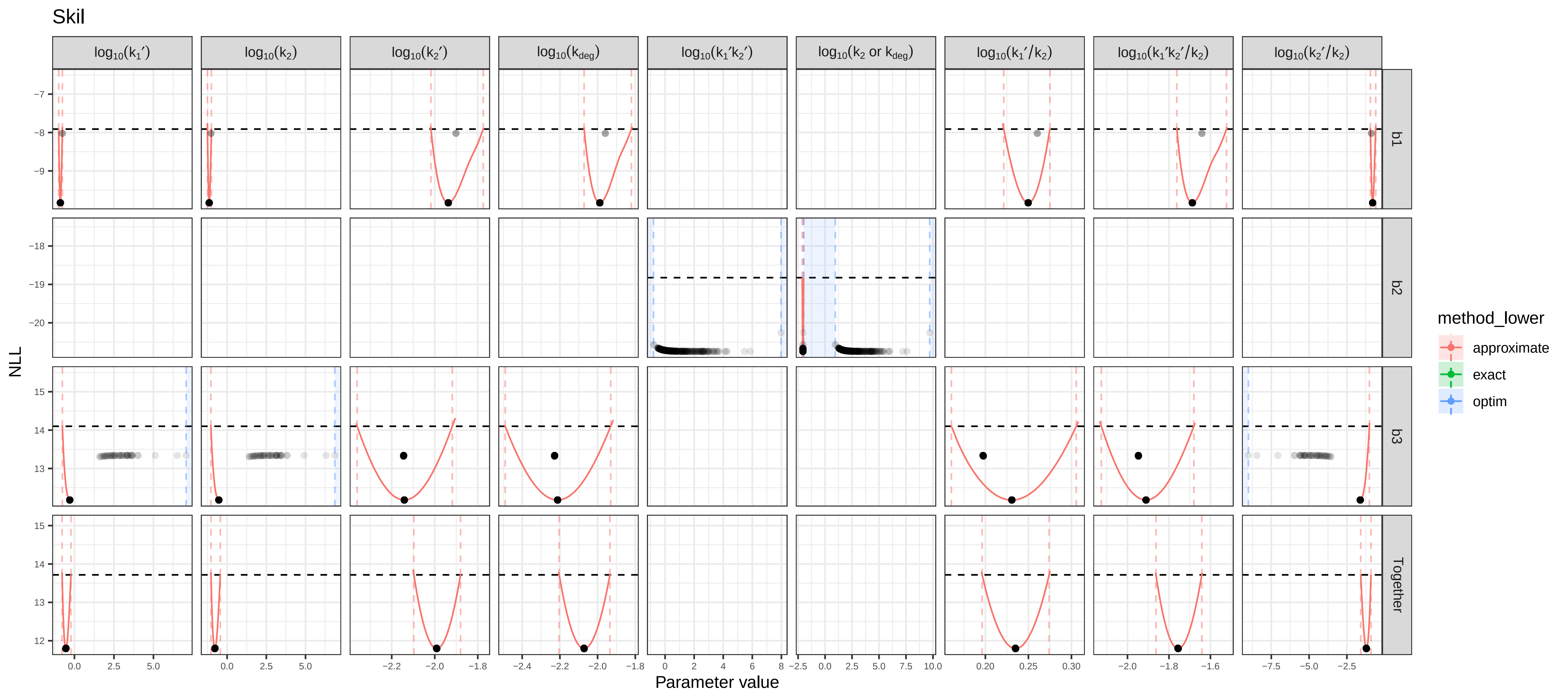

Replicate	Par	Best value	CI95 LB	CI95 UB	Method LB	Method UB
Together	$\log_{10}(k_1')$	−0.5464	−0.7803	−0.218	approximate	approximate
Together	$\log_{10}(k_2)$	−0.7814	−1.028	−0.4333	approximate	approximate
Together	$\log_{10}(k_2')$	−1.991	−2.097	−1.88	approximate	approximate
Together	$\log_{10}(k_{deg})$	−2.071	−2.204	−1.935	approximate	approximate
Together	$\log_{10}(k_1'/k_2)$	0.235	0.1963	0.274	approximate	approximate
Together	$\log_{10}(k_1'k_2'/k_2)$	−1.756	−1.863	−1.642	approximate	approximate
Together	$\log_{10}(k_2'/k_2)$	−1.209	−1.586	−0.9041	approximate	approximate
b1	$\log_{10}(k_1')$	−0.8927	−0.9995	−0.7618	approximate	approximate
b1	$\log_{10}(k_2)$	−1.142	−1.254	−1.002	approximate	approximate
b1	$\log_{10}(k_2')$	−1.937	−2.018	−1.775	approximate	approximate
b1	$\log_{10}(k_{deg})$	−1.988	−2.072	−1.821	approximate	approximate
b1	$\log_{10}(k_1'/k_2)$	0.2497	0.2213	0.2749	approximate	approximate
b1	$\log_{10}(k_1'k_2'/k_2)$	−1.687	−1.762	−1.523	approximate	approximate
b1	$\log_{10}(k_2'/k_2)$	−0.7946	−0.9462	−0.5907	approximate	approximate
b2	$\log_{10}(k_1'k_2')$	2.693	< −0.7931	> 7.973	optim	optim
b2	$\log_{10}(k_2 \text{ or } k_{deg})$	4.432	0.9452	> 9.697	optim	optim
b2	$\log_{10}(k_2 \text{ or } k_{deg})$	−2.052	−2.114	−1.983	approximate	approximate
b3	$\log_{10}(k_1')$	−0.2974	−0.7632	> 7.067	approximate	optim
b3	$\log_{10}(k_2)$	−0.5282	−1.035	> 6.87	approximate	optim
b3	$\log_{10}(k_2')$	−2.142	−2.361	−1.918	approximate	approximate
b3	$\log_{10}(k_{deg})$	−2.212	−2.49	−1.93	approximate	approximate
b3	$\log_{10}(k_1'/k_2)$	0.2308	0.1605	0.3055	approximate	approximate
b3	$\log_{10}(k_1'k_2'/k_2)$	−1.911	−2.126	−1.68	approximate	approximate
b3	$\log_{10}(k_2'/k_2)$	−1.613	< −9.015	−1.01	optim	approximate

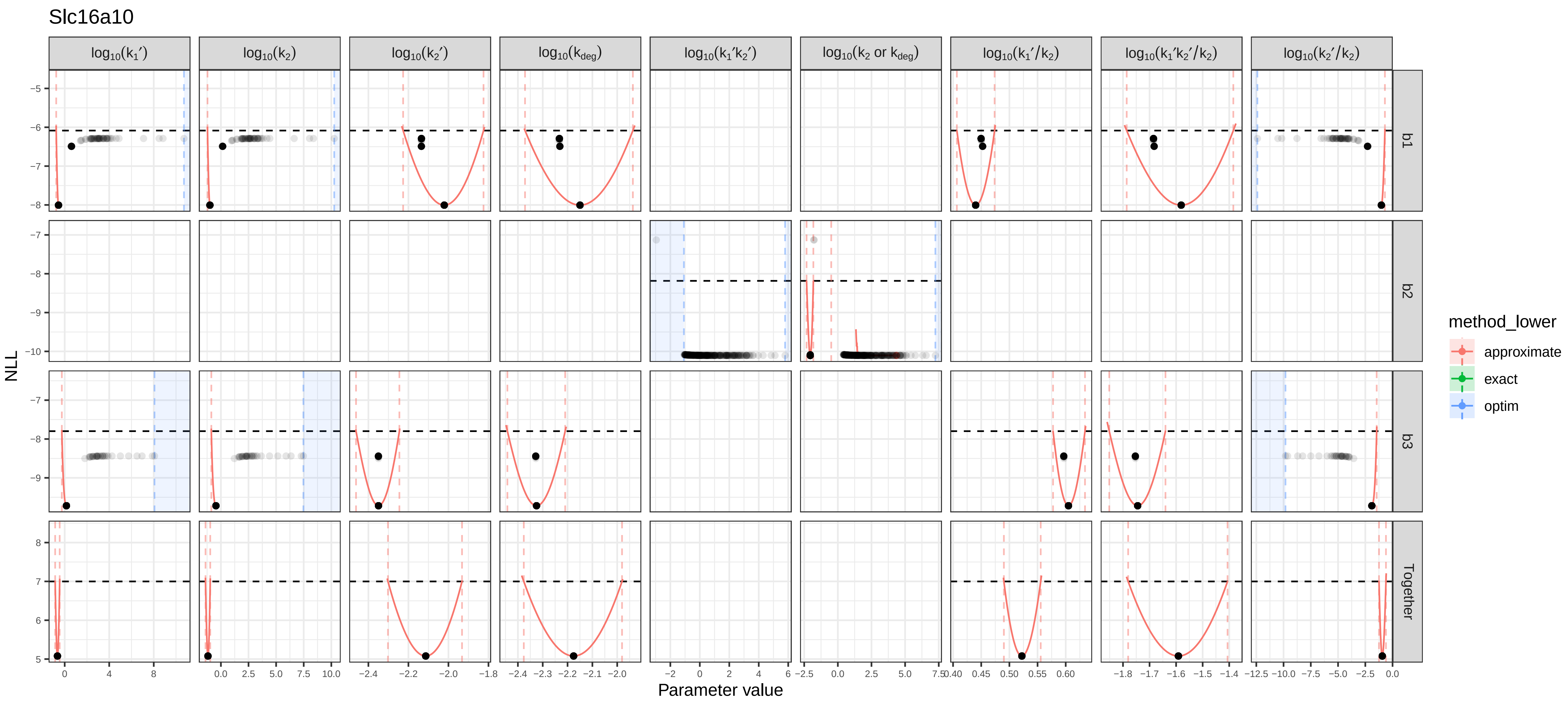

Replicate	Par	Best value	CI95 LB	CI95 UB	Method LB	Method UB
Together	$\log_{10}(k_1')$	−0.6509	−0.8558	−0.4505	approximate	approximate
Together	$\log_{10}(k_2)$	−1.173	−1.395	−0.9655	approximate	approximate
Together	$\log_{10}(k_2')$	−2.114	−2.302	−1.932	approximate	approximate
Together	$\log_{10}(k_{deg})$	−2.176	−2.376	−1.981	approximate	approximate
Together	$\log_{10}(k_1'/k_2)$	0.5222	0.4902	0.5556	approximate	approximate
Together	$\log_{10}(k_1'k_2'/k_2)$	−1.591	−1.78	−1.406	approximate	approximate
Together	$\log_{10}(k_2'/k_2)$	−0.9404	−1.242	−0.6044	approximate	approximate
b1	$\log_{10}(k_1')$	−0.5552	−0.762	> 10.71	approximate	optim
b1	$\log_{10}(k_2)$	−0.9953	−1.214	> 10.26	approximate	optim
b1	$\log_{10}(k_2')$	−2.021	−2.226	−1.825	approximate	approximate
b1	$\log_{10}(k_{deg})$	−2.15	−2.371	−1.937	approximate	approximate
b1	$\log_{10}(k_1'/k_2)$	0.4401	0.4068	0.4738	approximate	approximate
b1	$\log_{10}(k_1'k_2'/k_2)$	−1.581	−1.786	−1.385	approximate	approximate
b1	$\log_{10}(k_2'/k_2)$	−1.026	< −12.4	−0.7015	optim	approximate
b2	$\log_{10}(k_1'k_2')$	2.884	< −1.079	> 5.787	optim	optim
b2	$\log_{10}(k_2 \text{ or } k_{deg})$	4.345	−0.4912	> 7.249	approximate	optim
b2	$\log_{10}(k_2 \text{ or } k_{deg})$	−2.051	−2.325	−1.82	approximate	approximate
b3	$\log_{10}(k_1')$	0.1549	−0.2567	> 8.067	approximate	optim
b3	$\log_{10}(k_2)$	−0.45	−0.8683	> 7.47	approximate	optim
b3	$\log_{10}(k_2')$	−2.35	−2.462	−2.245	approximate	approximate
b3	$\log_{10}(k_{deg})$	−2.325	−2.442	−2.21	approximate	approximate
b3	$\log_{10}(k_1'/k_2)$	0.6049	0.5775	0.6342	approximate	approximate
b3	$\log_{10}(k_1'k_2'/k_2)$	−1.745	−1.852	−1.64	approximate	approximate
b3	$\log_{10}(k_2'/k_2)$	−1.9	< −9.82	−1.454	optim	approximate

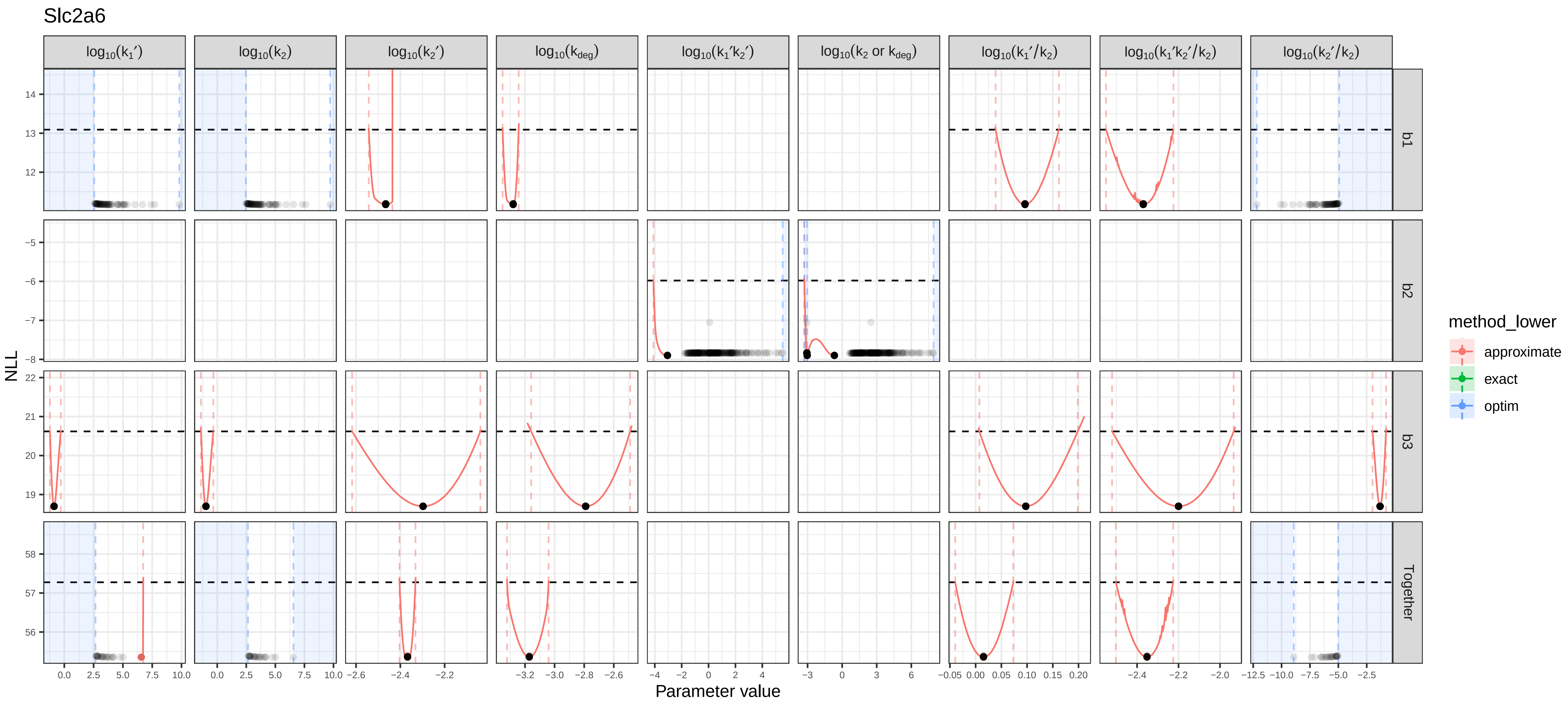

Replicate	Par	Best value	CI95 LB	CI95 UB	Method LB	Method UB
Together	$\log_{10}(k_1')$	6.572	< 2.663	6.731	optim	approximate
Together	$\log_{10}(k_2)$	6.556	< 2.648	> 6.556	optim	optim
Together	$\log_{10}(k_2')$	−2.368	−2.404	−2.332	approximate	approximate
Together	$\log_{10}(k_{deg})$	−3.171	−3.321	−3.041	approximate	approximate
Together	$\log_{10}(k_1'/k_2)$	0.01532	−0.03973	0.07317	approximate	approximate
Together	$\log_{10}(k_1'k_2'/k_2)$	−2.352	−2.503	−2.226	approximate	approximate
Together	$\log_{10}(k_2'/k_2)$	−8.924	< −8.924	> −5.015	optim	optim
b1	$\log_{10}(k_1')$	6.662	< 2.558	> 9.807	optim	optim
b1	$\log_{10}(k_2)$	6.566	< 2.462	> 9.712	optim	optim
b1	$\log_{10}(k_2')$	−2.466	−2.543	−2.436	approximate	approximate
b1	$\log_{10}(k_{deg})$	−3.28	−3.351	−3.243	approximate	approximate
b1	$\log_{10}(k_1'/k_2)$	0.09598	0.03886	0.162	approximate	approximate
b1	$\log_{10}(k_1'k_2'/k_2)$	−2.37	−2.55	−2.225	approximate	approximate
b1	$\log_{10}(k_2'/k_2)$	−9.032	< −12.18	> −4.928	optim	optim
b2	$\log_{10}(k_1'k_2')$	−3.06	−4.102	> 5.519	approximate	optim
b2	$\log_{10}(k_2 \text{ or } k_{deg})$	−0.6755	−3.282	> 7.932	approximate	optim
b2	$\log_{10}(k_2 \text{ or } k_{deg})$	−3.035	−3.282	−3.034	approximate	optim
b3	$\log_{10}(k_1')$	−0.8742	−1.223	−0.2974	approximate	approximate
b3	$\log_{10}(k_2)$	−0.9717	−1.397	−0.3325	approximate	approximate
b3	$\log_{10}(k_2')$	−2.297	−2.618	−2.039	approximate	approximate
b3	$\log_{10}(k_{deg})$	−2.79	−3.159	−2.489	approximate	approximate
b3	$\log_{10}(k_1'/k_2)$	0.09747	0.007255	0.1986	approximate	approximate
b3	$\log_{10}(k_1'k_2'/k_2)$	−2.2	−2.521	−1.934	approximate	approximate
b3	$\log_{10}(k_2'/k_2)$	−1.326	−1.992	−0.8011	approximate	approximate

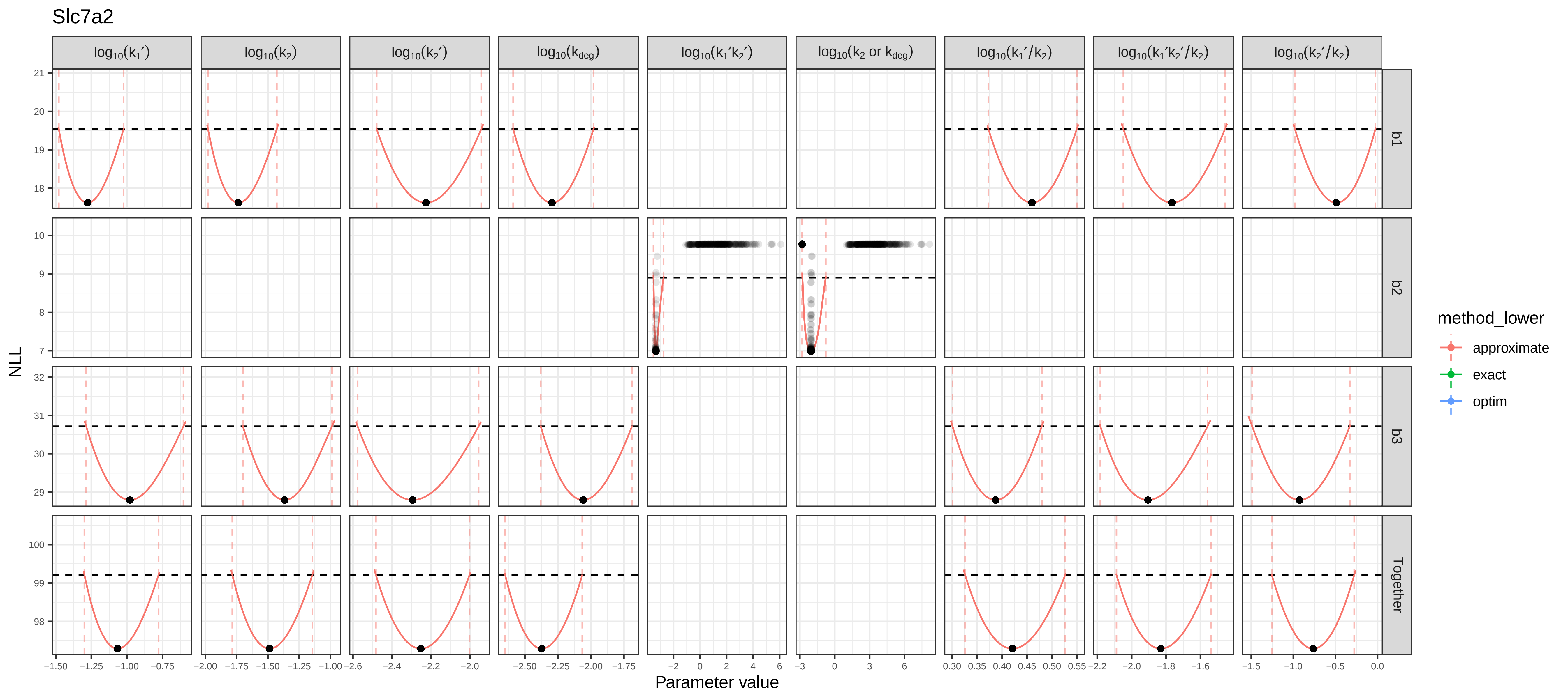

Replicate	Par	Best value	CI95 LB	CI95 UB	Method LB	Method UB
Together	$\log_{10}(k_1')$	−1.066	−1.299	−0.7779	approximate	approximate
Together	$\log_{10}(k_2)$	−1.487	−1.785	−1.144	approximate	approximate
Together	$\log_{10}(k_2')$	−2.251	−2.482	−2.001	approximate	approximate
Together	$\log_{10}(k_{deg})$	−2.371	−2.649	−2.065	approximate	approximate
Together	$\log_{10}(k_1'/k_2)$	0.4209	0.326	0.5263	approximate	approximate
Together	$\log_{10}(k_1'k_2'/k_2)$	−1.83	−2.088	−1.539	approximate	approximate
Together	$\log_{10}(k_2'/k_2)$	−0.7646	−1.257	−0.2763	approximate	approximate
b1	$\log_{10}(k_1')$	−1.276	−1.479	−1.024	approximate	approximate
b1	$\log_{10}(k_2)$	−1.736	−1.98	−1.429	approximate	approximate
b1	$\log_{10}(k_2')$	−2.225	−2.478	−1.941	approximate	approximate
b1	$\log_{10}(k_{deg})$	−2.295	−2.588	−1.98	approximate	approximate
b1	$\log_{10}(k_1'/k_2)$	0.46	0.3725	0.5496	approximate	approximate
b1	$\log_{10}(k_1'k_2'/k_2)$	−1.765	−2.048	−1.457	approximate	approximate
b1	$\log_{10}(k_2'/k_2)$	−0.4889	−0.9818	−0.02515	approximate	approximate
b2	$\log_{10}(k_1'k_2')$	−3.317	−3.511	−2.749	approximate	approximate
b2	$\log_{10}(k_2 \text{ or } k_{deg})$	−2.007	−2.796	−0.767	approximate	approximate
b2	$\log_{10}(k_2 \text{ or } k_{deg})$	−2.053	−2.796	−0.767	approximate	approximate
b3	$\log_{10}(k_1')$	−0.9786	−1.286	−0.6031	approximate	approximate
b3	$\log_{10}(k_2)$	−1.366	−1.7	−0.9871	approximate	approximate
b3	$\log_{10}(k_2')$	−2.293	−2.576	−1.955	approximate	approximate
b3	$\log_{10}(k_{deg})$	−2.059	−2.38	−1.688	approximate	approximate
b3	$\log_{10}(k_1'/k_2)$	0.3871	0.3008	0.4795	approximate	approximate
b3	$\log_{10}(k_1'k_2'/k_2)$	−1.905	−2.182	−1.559	approximate	approximate
b3	$\log_{10}(k_2'/k_2)$	−0.9268	−1.489	−0.3296	approximate	approximate

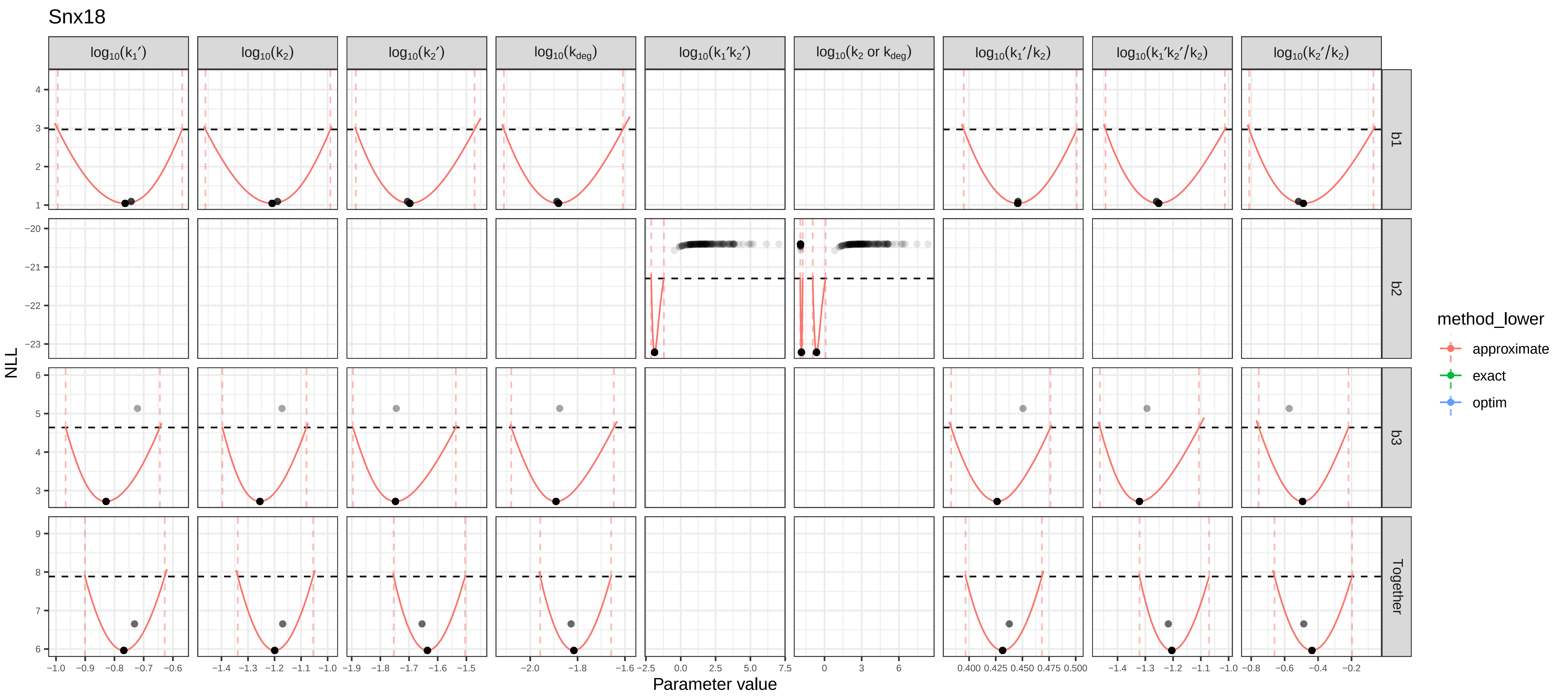

Replicate	Par	Best value	CI95 LB	CI95 UB	Method LB	Method UB
Together	$\log_{10}(k_1')$	−0.768	−0.9011	−0.628	approximate	approximate
Together	$\log_{10}(k_2)$	−1.199	−1.339	−1.054	approximate	approximate
Together	$\log_{10}(k_2')$	−1.636	−1.754	−1.505	approximate	approximate
Together	$\log_{10}(k_{deg})$	−1.816	−1.958	−1.658	approximate	approximate
Together	$\log_{10}(k_1'/k_2)$	0.4315	0.3967	0.4683	approximate	approximate
Together	$\log_{10}(k_1'k_2'/k_2)$	−1.204	−1.321	−1.07	approximate	approximate
Together	$\log_{10}(k_2'/k_2)$	−0.4364	−0.6604	−0.1968	approximate	approximate
b1	$\log_{10}(k_1')$	−0.7632	−0.9938	−0.5682	approximate	approximate
b1	$\log_{10}(k_2)$	−1.209	−1.46	−0.9898	approximate	approximate
b1	$\log_{10}(k_2')$	−1.697	−1.886	−1.471	approximate	approximate
b1	$\log_{10}(k_{deg})$	−1.88	−2.111	−1.609	approximate	approximate
b1	$\log_{10}(k_1'/k_2)$	0.4456	0.395	0.5009	approximate	approximate
b1	$\log_{10}(k_1'k_2'/k_2)$	−1.252	−1.443	−1.013	approximate	approximate
b1	$\log_{10}(k_2'/k_2)$	−0.4885	−0.8115	−0.06931	approximate	approximate
b2	$\log_{10}(k_1'k_2')$	−1.878	−2.12	−1.205	approximate	approximate
b2	$\log_{10}(k_2 \text{ or } k_{deg})$	−0.6473	−0.9517	0.08904	approximate	approximate
b2	$\log_{10}(k_2 \text{ or } k_{deg})$	−1.866	−1.958	−1.761	approximate	approximate
b3	$\log_{10}(k_1')$	−0.8288	−0.967	−0.6445	approximate	approximate
b3	$\log_{10}(k_2)$	−1.255	−1.397	−1.08	approximate	approximate
b3	$\log_{10}(k_2')$	−1.747	−1.896	−1.537	approximate	approximate
b3	$\log_{10}(k_{deg})$	−1.891	−2.08	−1.647	approximate	approximate
b3	$\log_{10}(k_1'/k_2)$	0.4263	0.3832	0.4763	approximate	approximate
b3	$\log_{10}(k_1'k_2'/k_2)$	−1.321	−1.463	−1.107	approximate	approximate
b3	$\log_{10}(k_2'/k_2)$	−0.4923	−0.7547	−0.2183	approximate	approximate

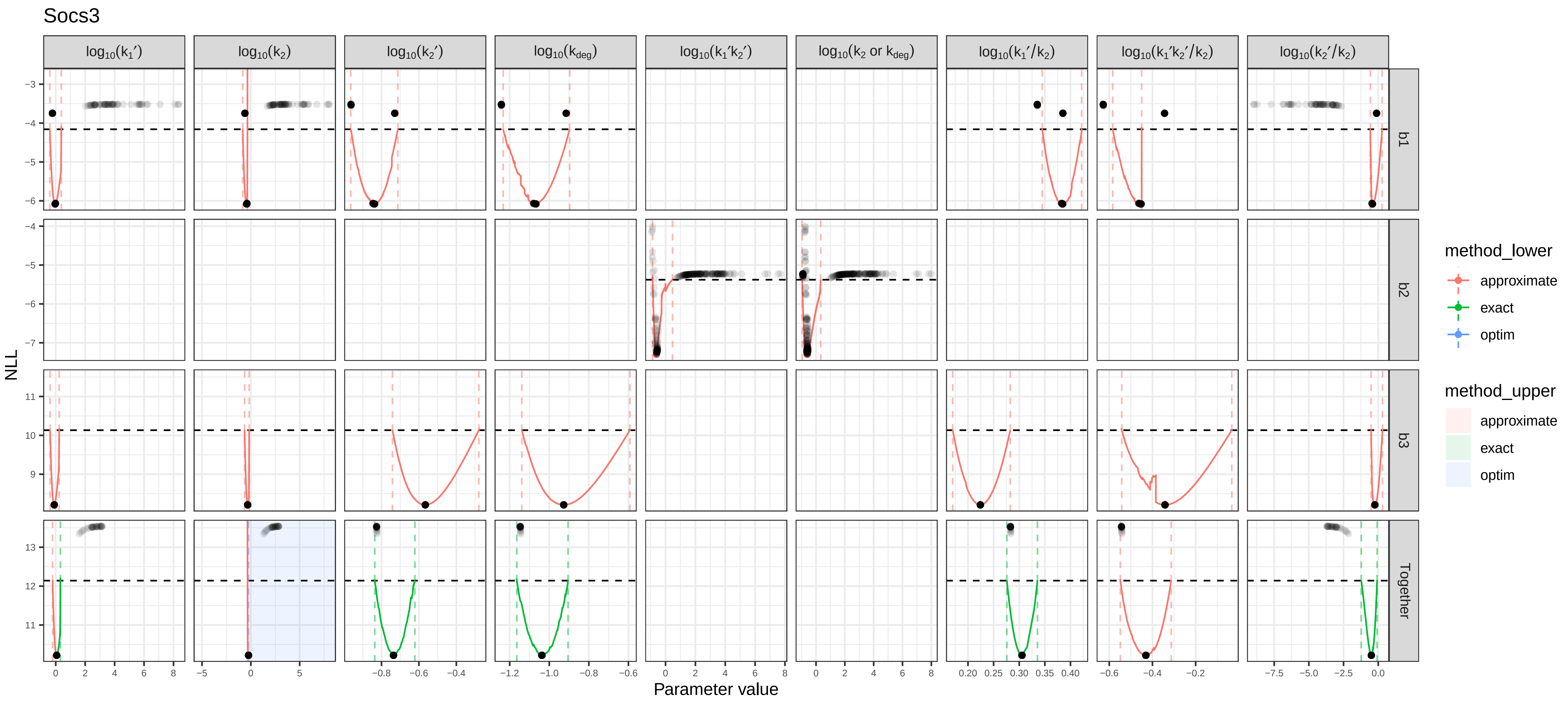

Replicate	Par	Best value	CI95 LB	CI95 UB	Method LB	Method UB
Together	$\log_{10}(k_1')$	0.06609	−0.2111	0.3185	approximate	exact
Together	$\log_{10}(k_2)$	−0.2393	−0.3406	> −0.2343	approximate	optim
Together	$\log_{10}(k_2')$	−0.736	−0.8361	−0.6215	exact	exact
Together	$\log_{10}(k_{deg})$	−1.037	−1.164	−0.9054	exact	exact
Together	$\log_{10}(k_1'/k_2)$	0.3054	0.2759	0.3356	exact	exact
Together	$\log_{10}(k_1'k_2'/k_2)$	−0.4306	−0.5487	−0.313	approximate	approximate
Together	$\log_{10}(k_2'/k_2)$	−0.4967	−1.223	−0.08099	exact	exact
b1	$\log_{10}(k_1')$	−0.03749	−0.4005	0.3802	approximate	approximate
b1	$\log_{10}(k_2)$	−0.4222	−0.8314	−0.3473	approximate	approximate
b1	$\log_{10}(k_2')$	−0.8376	−0.9644	−0.7125	approximate	approximate
b1	$\log_{10}(k_{deg})$	−1.068	−1.233	−0.8971	approximate	approximate
b1	$\log_{10}(k_1'/k_2)$	0.3847	0.3448	0.4217	approximate	approximate
b1	$\log_{10}(k_1'k_2'/k_2)$	−0.4529	−0.584	−0.4498	approximate	approximate
b1	$\log_{10}(k_2'/k_2)$	−0.4154	−0.5667	0.2823	approximate	approximate
b2	$\log_{10}(k_1'k_2')$	−0.6122	−0.8702	0.4672	approximate	approximate
b2	$\log_{10}(k_2 \text{ or } k_{deg})$	−0.6058	−0.9643	0.3225	approximate	approximate
b2	$\log_{10}(k_2 \text{ or } k_{deg})$	−0.6432	−0.9643	0.3225	approximate	approximate
b3	$\log_{10}(k_1')$	−0.09997	−0.3832	0.2373	approximate	approximate
b3	$\log_{10}(k_2)$	−0.3241	−0.6394	−0.1697	approximate	approximate
b3	$\log_{10}(k_2')$	−0.566	−0.7414	−0.2797	approximate	approximate
b3	$\log_{10}(k_{deg})$	−0.9271	−1.139	−0.5926	approximate	approximate
b3	$\log_{10}(k_1'/k_2)$	0.2241	0.1702	0.2825	approximate	approximate
b3	$\log_{10}(k_1'k_2'/k_2)$	−0.3419	−0.5421	−0.03276	approximate	approximate
b3	$\log_{10}(k_2'/k_2)$	−0.2419	−0.5152	0.3086	approximate	approximate

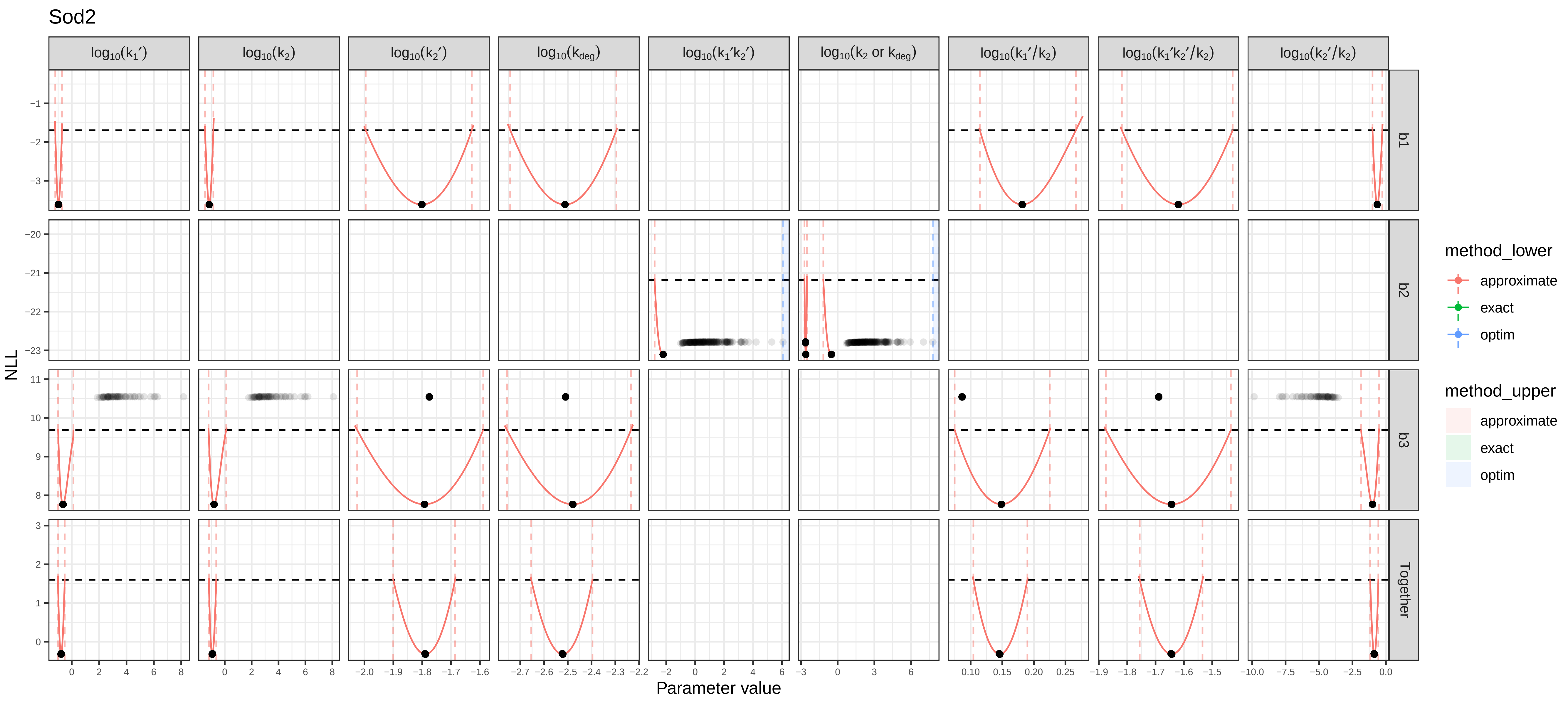

Replicate	Par	Best value	CI95 LB	CI95 UB	Method LB	Method UB
Together	$\log_{10}(k_1')$	−0.7811	−1.01	−0.5164	approximate	approximate
Together	$\log_{10}(k_2)$	−0.9271	−1.183	−0.6339	approximate	approximate
Together	$\log_{10}(k_2')$	−1.789	−1.9	−1.686	approximate	approximate
Together	$\log_{10}(k_{deg})$	−2.521	−2.654	−2.396	approximate	approximate
Together	$\log_{10}(k_1'/k_2)$	0.146	0.1044	0.1898	approximate	approximate
Together	$\log_{10}(k_1'k_2'/k_2)$	−1.643	−1.756	−1.534	approximate	approximate
Together	$\log_{10}(k_2'/k_2)$	−0.8618	−1.174	−0.5634	approximate	approximate
b1	$\log_{10}(k_1')$	−0.9756	−1.21	−0.7178	approximate	approximate
b1	$\log_{10}(k_2)$	−1.157	−1.467	−0.847	approximate	approximate
b1	$\log_{10}(k_2')$	−1.801	−1.996	−1.628	approximate	approximate
b1	$\log_{10}(k_{deg})$	−2.512	−2.742	−2.296	approximate	approximate
b1	$\log_{10}(k_1'/k_2)$	0.1816	0.1145	0.2665	approximate	approximate
b1	$\log_{10}(k_1'k_2'/k_2)$	−1.62	−1.819	−1.428	approximate	approximate
b1	$\log_{10}(k_2'/k_2)$	−0.6439	−0.9996	−0.2668	approximate	approximate
b2	$\log_{10}(k_1'k_2')$	−2.211	−2.799	> 6.069	approximate	optim
b2	$\log_{10}(k_2 \text{ or } k_{deg})$	−0.5137	−1.176	> 7.789	approximate	optim
b2	$\log_{10}(k_2 \text{ or } k_{deg})$	−2.613	−2.718	−2.509	approximate	approximate
b3	$\log_{10}(k_1')$	−0.6448	−0.9996	0.1223	approximate	approximate
b3	$\log_{10}(k_2)$	−0.7934	−1.201	0.1114	approximate	approximate
b3	$\log_{10}(k_2')$	−1.792	−2.025	−1.588	approximate	approximate
b3	$\log_{10}(k_{deg})$	−2.479	−2.756	−2.234	approximate	approximate
b3	$\log_{10}(k_1'/k_2)$	0.1487	0.07468	0.2252	approximate	approximate
b3	$\log_{10}(k_1'k_2'/k_2)$	−1.643	−1.875	−1.434	approximate	approximate
b3	$\log_{10}(k_2'/k_2)$	−0.9987	−1.848	−0.5197	approximate	approximate

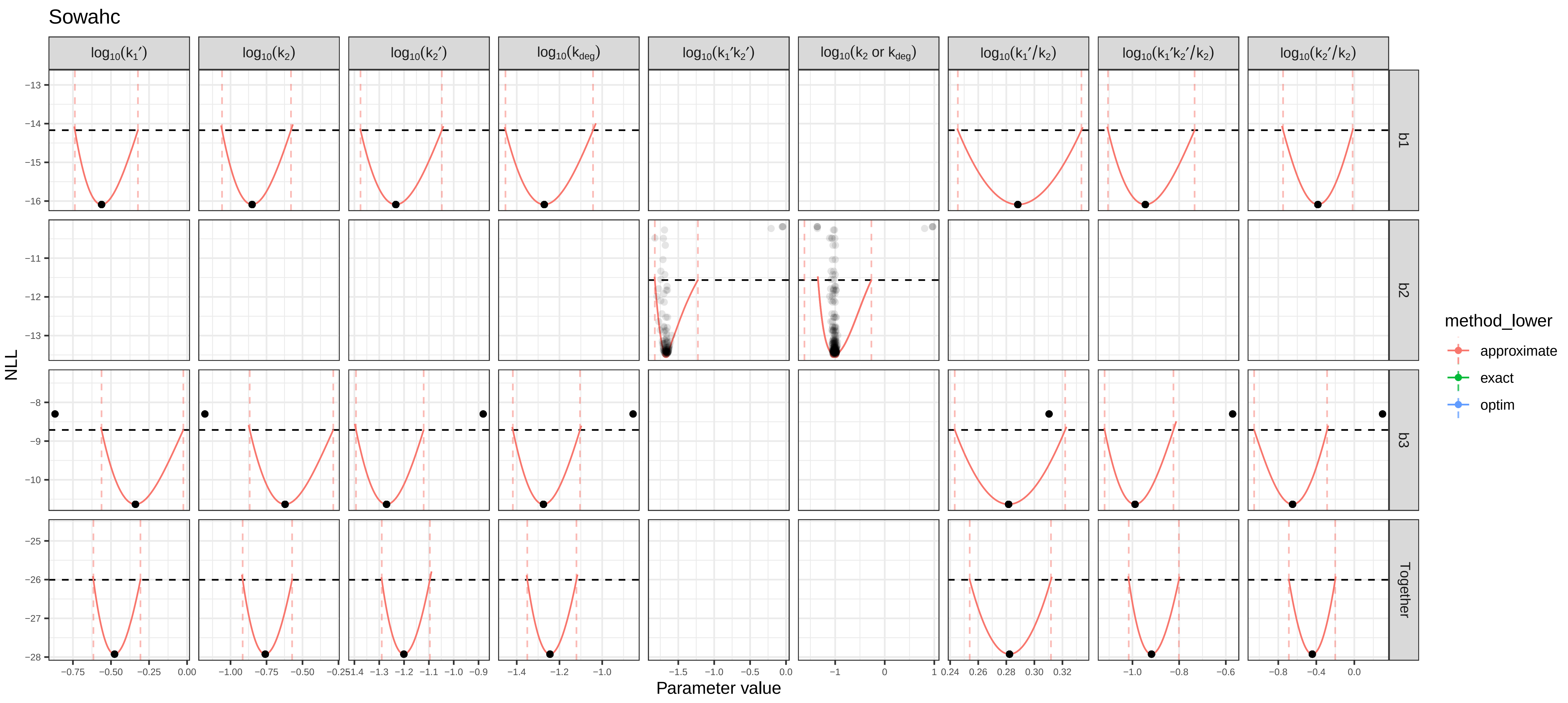

Replicate	Par	Best value	CI95 LB	CI95 UB	Method LB	Method UB
Together	$\log_{10}(k_1')$	−0.4764	−0.6154	−0.3064	approximate	approximate
Together	$\log_{10}(k_2)$	−0.7587	−0.9158	−0.5723	approximate	approximate
Together	$\log_{10}(k_2')$	−1.201	−1.29	−1.096	approximate	approximate
Together	$\log_{10}(k_{deg})$	−1.244	−1.351	−1.121	approximate	approximate
Together	$\log_{10}(k_1'/k_2)$	0.2823	0.2539	0.3118	approximate	approximate
Together	$\log_{10}(k_1'k_2'/k_2)$	−0.9185	−1.016	−0.8011	approximate	approximate
Together	$\log_{10}(k_2'/k_2)$	−0.4421	−0.6885	−0.2011	approximate	approximate
b1	$\log_{10}(k_1')$	−0.5616	−0.7371	−0.3229	approximate	approximate
b1	$\log_{10}(k_2)$	−0.8498	−1.059	−0.58	approximate	approximate
b1	$\log_{10}(k_2')$	−1.233	−1.375	−1.048	approximate	approximate
b1	$\log_{10}(k_{deg})$	−1.271	−1.453	−1.043	approximate	approximate
b1	$\log_{10}(k_1'/k_2)$	0.2882	0.2455	0.3335	approximate	approximate
b1	$\log_{10}(k_1'k_2'/k_2)$	−0.9451	−1.105	−0.7337	approximate	approximate
b1	$\log_{10}(k_2'/k_2)$	−0.3835	−0.7494	−0.01776	approximate	approximate
b2	$\log_{10}(k_1'k_2')$	−1.671	−1.826	−1.227	approximate	approximate
b2	$\log_{10}(k_2 \text{ or } k_{deg})$	−0.9915	−1.62	−0.2692	approximate	approximate
b2	$\log_{10}(k_2 \text{ or } k_{deg})$	−1.034	−1.62	−0.2692	approximate	approximate
b3	$\log_{10}(k_1')$	−0.3393	−0.562	−0.02464	approximate	approximate
b3	$\log_{10}(k_2)$	−0.6209	−0.8668	−0.2862	approximate	approximate
b3	$\log_{10}(k_2')$	−1.271	−1.393	−1.121	approximate	approximate
b3	$\log_{10}(k_{deg})$	−1.275	−1.418	−1.104	approximate	approximate
b3	$\log_{10}(k_1'/k_2)$	0.2816	0.2433	0.322	approximate	approximate
b3	$\log_{10}(k_1'k_2'/k_2)$	−0.989	−1.119	−0.8243	approximate	approximate
b3	$\log_{10}(k_2'/k_2)$	−0.6497	−1.054	−0.2864	approximate	approximate

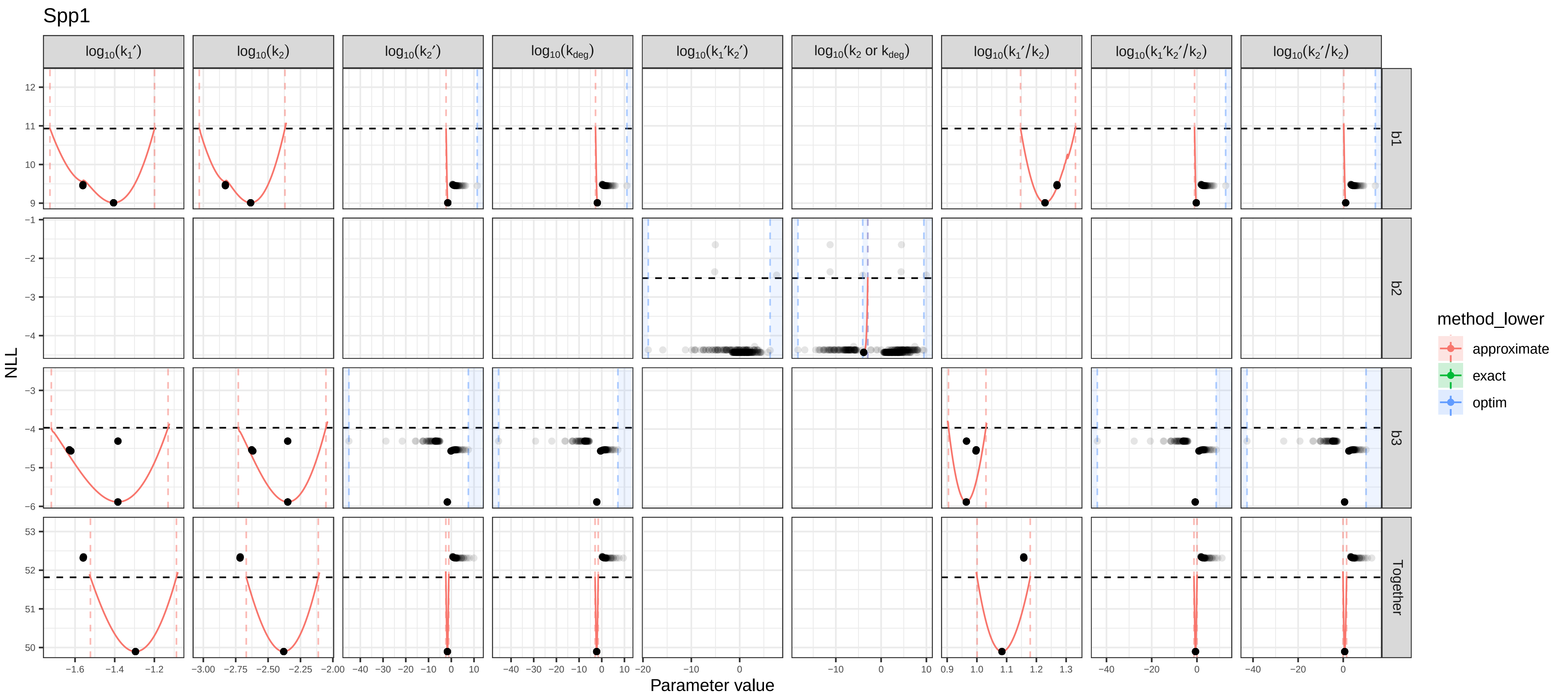

Replicate	Par	Best value	CI95 LB	CI95 UB	Method LB	Method UB
Together	$\log_{10}(k_1')$	−1.295	−1.522	−1.089	approximate	approximate
Together	$\log_{10}(k_2)$	−2.379	−2.669	−2.113	approximate	approximate
Together	$\log_{10}(k_2')$	−1.717	−2.386	−1.09	approximate	approximate
Together	$\log_{10}(k_{deg})$	−2.245	−2.947	−1.553	approximate	approximate
Together	$\log_{10}(k_1'/k_2)$	1.084	1.001	1.179	approximate	approximate
Together	$\log_{10}(k_1'k_2'/k_2)$	−0.6327	−1.317	0.0397	approximate	approximate
Together	$\log_{10}(k_2'/k_2)$	0.6624	−0.08903	1.509	approximate	approximate
b1	$\log_{10}(k_1')$	−1.406	−1.727	−1.199	approximate	approximate
b1	$\log_{10}(k_2)$	−2.635	−3.031	−2.37	approximate	approximate
b1	$\log_{10}(k_2')$	−1.534	−2.29	> 11.41	approximate	optim
b1	$\log_{10}(k_{deg})$	−1.948	−2.753	> 11.09	approximate	optim
b1	$\log_{10}(k_1'/k_2)$	1.229	1.147	1.331	approximate	approximate
b1	$\log_{10}(k_1'k_2'/k_2)$	−0.305	−1.074	> 12.68	approximate	optim
b1	$\log_{10}(k_2'/k_2)$	1.101	0.256	> 14.24	approximate	optim
b2	$\log_{10}(k_1'k_2')$	2.206	< −18.9	> 6.292	optim	optim
b2	$\log_{10}(k_2 \text{ or } k_{deg})$	4.332	−4.063	> 9.448	optim	optim
b2	$\log_{10}(k_2 \text{ or } k_{deg})$	−3.849	< −18.36	−2.942	optim	approximate
b3	$\log_{10}(k_1')$	−1.384	−1.72	−1.131	approximate	approximate
b3	$\log_{10}(k_2)$	−2.348	−2.73	−2.054	approximate	approximate
b3	$\log_{10}(k_2')$	−1.739	< −45.01	> 7.509	optim	optim
b3	$\log_{10}(k_{deg})$	−2.194	< −45.49	> 7.119	optim	optim
b3	$\log_{10}(k_1'/k_2)$	0.9643	0.9045	1.03	approximate	approximate
b3	$\log_{10}(k_1'k_2'/k_2)$	−0.7747	< −44.05	> 8.507	optim	optim
b3	$\log_{10}(k_2'/k_2)$	0.6092	< −42.66	> 10.14	optim	optim

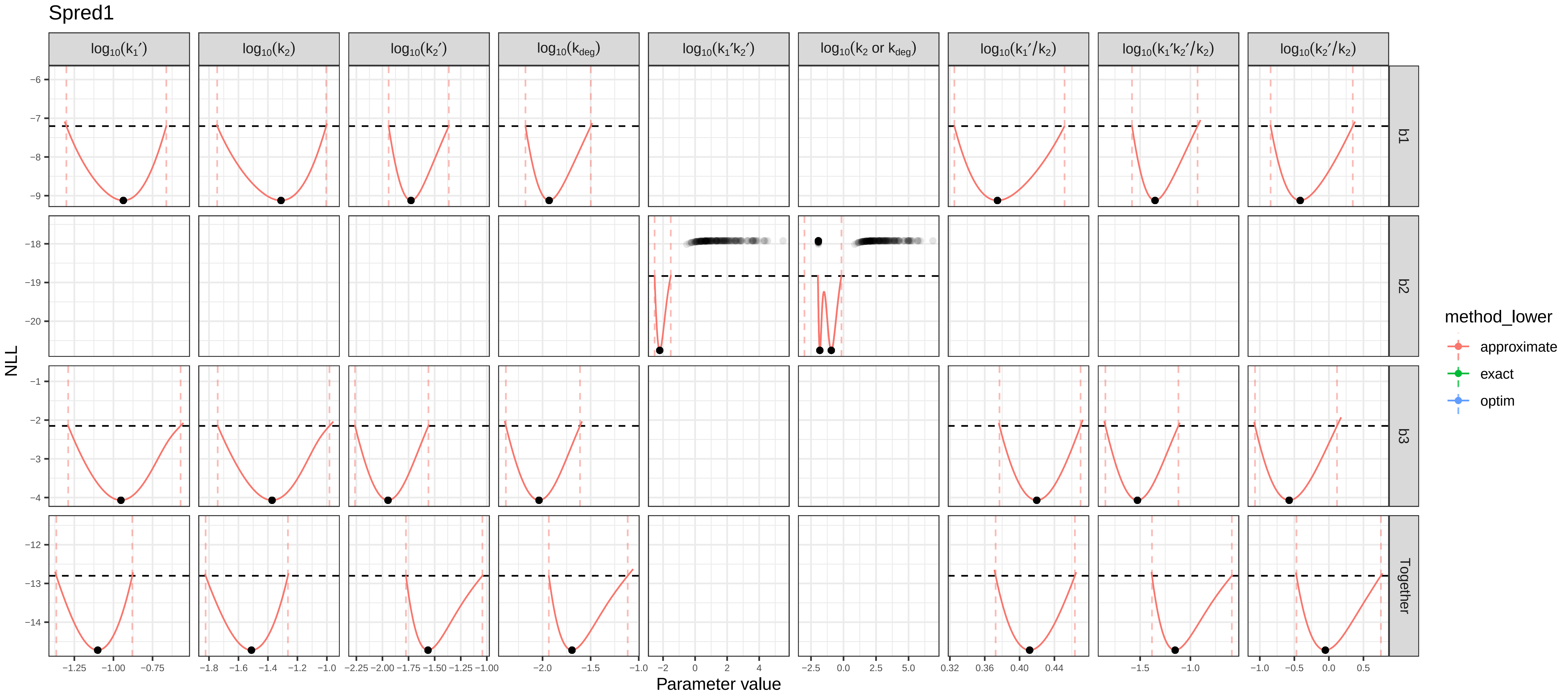

Replicate	Par	Best value	CI95 LB	CI95 UB	Method LB	Method UB
Together	$\log_{10}(k_1')$	−1.1	−1.365	−0.8795	approximate	approximate
Together	$\log_{10}(k_2)$	−1.512	−1.823	−1.264	approximate	approximate
Together	$\log_{10}(k_2')$	−1.564	−1.775	−1.043	approximate	approximate
Together	$\log_{10}(k_{deg})$	−1.694	−1.935	−1.115	approximate	approximate
Together	$\log_{10}(k_1'/k_2)$	0.4115	0.3725	0.4635	approximate	approximate
Together	$\log_{10}(k_1'k_2'/k_2)$	−1.152	−1.382	−0.5881	approximate	approximate
Together	$\log_{10}(k_2'/k_2)$	−0.0515	−0.4692	0.7534	approximate	approximate
b1	$\log_{10}(k_1')$	−0.9367	−1.301	−0.6621	approximate	approximate
b1	$\log_{10}(k_2)$	−1.311	−1.744	−1.005	approximate	approximate
b1	$\log_{10}(k_2')$	−1.726	−1.941	−1.364	approximate	approximate
b1	$\log_{10}(k_{deg})$	−1.932	−2.178	−1.498	approximate	approximate
b1	$\log_{10}(k_1'/k_2)$	0.3746	0.3251	0.4516	approximate	approximate
b1	$\log_{10}(k_1'k_2'/k_2)$	−1.352	−1.581	−0.9288	approximate	approximate
b1	$\log_{10}(k_2'/k_2)$	−0.4151	−0.8435	0.346	approximate	approximate
b2	$\log_{10}(k_1'k_2')$	−2.209	−2.53	−1.52	approximate	approximate
b2	$\log_{10}(k_2 \text{ or } k_{deg})$	−0.9404	−3.005	−0.1599	approximate	approximate
b2	$\log_{10}(k_2 \text{ or } k_{deg})$	−1.82	−3.005	−0.1599	approximate	approximate
b3	$\log_{10}(k_1')$	−0.9523	−1.289	−0.5707	approximate	approximate
b3	$\log_{10}(k_2)$	−1.372	−1.741	−0.9825	approximate	approximate
b3	$\log_{10}(k_2')$	−1.947	−2.263	−1.56	approximate	approximate
b3	$\log_{10}(k_{deg})$	−2.037	−2.381	−1.61	approximate	approximate
b3	$\log_{10}(k_1'/k_2)$	0.4197	0.3769	0.47	approximate	approximate
b3	$\log_{10}(k_1'k_2'/k_2)$	−1.527	−1.846	−1.119	approximate	approximate
b3	$\log_{10}(k_2'/k_2)$	−0.5745	−1.069	0.1175	approximate	approximate

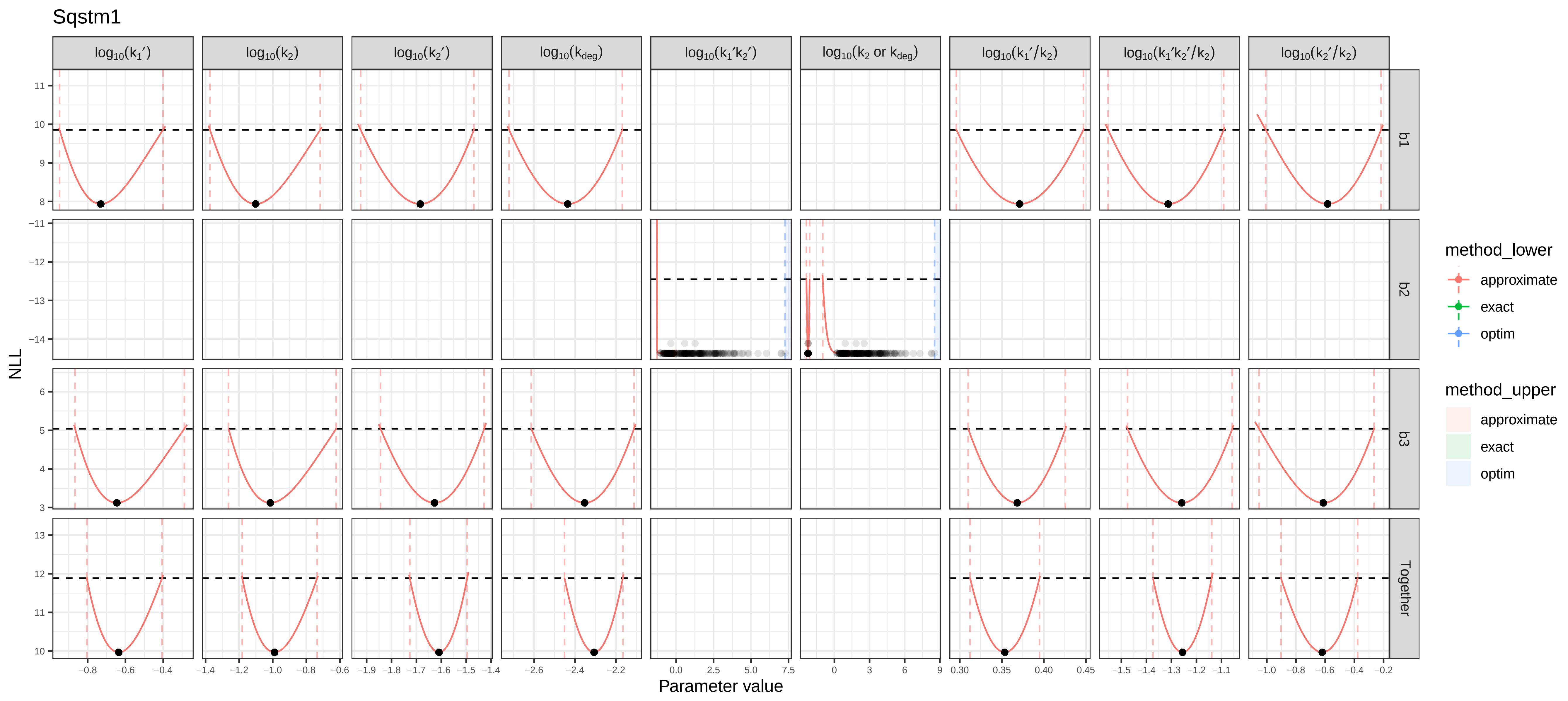

Replicate	Par	Best value	CI95 LB	CI95 UB	Method LB	Method UB
Together	$\log_{10}(k_1')$	−0.6357	−0.8047	−0.4063	approximate	approximate
Together	$\log_{10}(k_2)$	−0.9892	−1.182	−0.7351	approximate	approximate
Together	$\log_{10}(k_2')$	−1.609	−1.727	−1.496	approximate	approximate
Together	$\log_{10}(k_{deg})$	−2.307	−2.451	−2.167	approximate	approximate
Together	$\log_{10}(k_1'/k_2)$	0.3535	0.3123	0.3948	approximate	approximate
Together	$\log_{10}(k_1'k_2'/k_2)$	−1.256	−1.374	−1.139	approximate	approximate
Together	$\log_{10}(k_2'/k_2)$	−0.6199	−0.9033	−0.3777	approximate	approximate
b1	$\log_{10}(k_1')$	−0.7303	−0.9488	−0.4011	approximate	approximate
b1	$\log_{10}(k_2)$	−1.101	−1.374	−0.7173	approximate	approximate
b1	$\log_{10}(k_2')$	−1.685	−1.924	−1.469	approximate	approximate
b1	$\log_{10}(k_{deg})$	−2.436	−2.722	−2.169	approximate	approximate
b1	$\log_{10}(k_1'/k_2)$	0.3711	0.296	0.4471	approximate	approximate
b1	$\log_{10}(k_1'k_2'/k_2)$	−1.313	−1.553	−1.091	approximate	approximate
b1	$\log_{10}(k_2'/k_2)$	−0.5831	−1.008	−0.2172	approximate	approximate
b2	$\log_{10}(k_1'k_2')$	−0.1865	−1.278	> 7.272	approximate	optim
b2	$\log_{10}(k_2 \text{ or } k_{deg})$	1.085	−0.9974	> 8.547	approximate	optim
b2	$\log_{10}(k_2 \text{ or } k_{deg})$	−2.245	−2.384	−2.116	approximate	approximate
b3	$\log_{10}(k_1')$	−0.6456	−0.8667	−0.288	approximate	approximate
b3	$\log_{10}(k_2)$	−1.014	−1.263	−0.6216	approximate	approximate
b3	$\log_{10}(k_2')$	−1.627	−1.844	−1.428	approximate	approximate
b3	$\log_{10}(k_{deg})$	−2.353	−2.614	−2.112	approximate	approximate
b3	$\log_{10}(k_1'/k_2)$	0.3683	0.31	0.4256	approximate	approximate
b3	$\log_{10}(k_1'k_2'/k_2)$	−1.259	−1.475	−1.057	approximate	approximate
b3	$\log_{10}(k_2'/k_2)$	−0.613	−1.053	−0.265	approximate	approximate

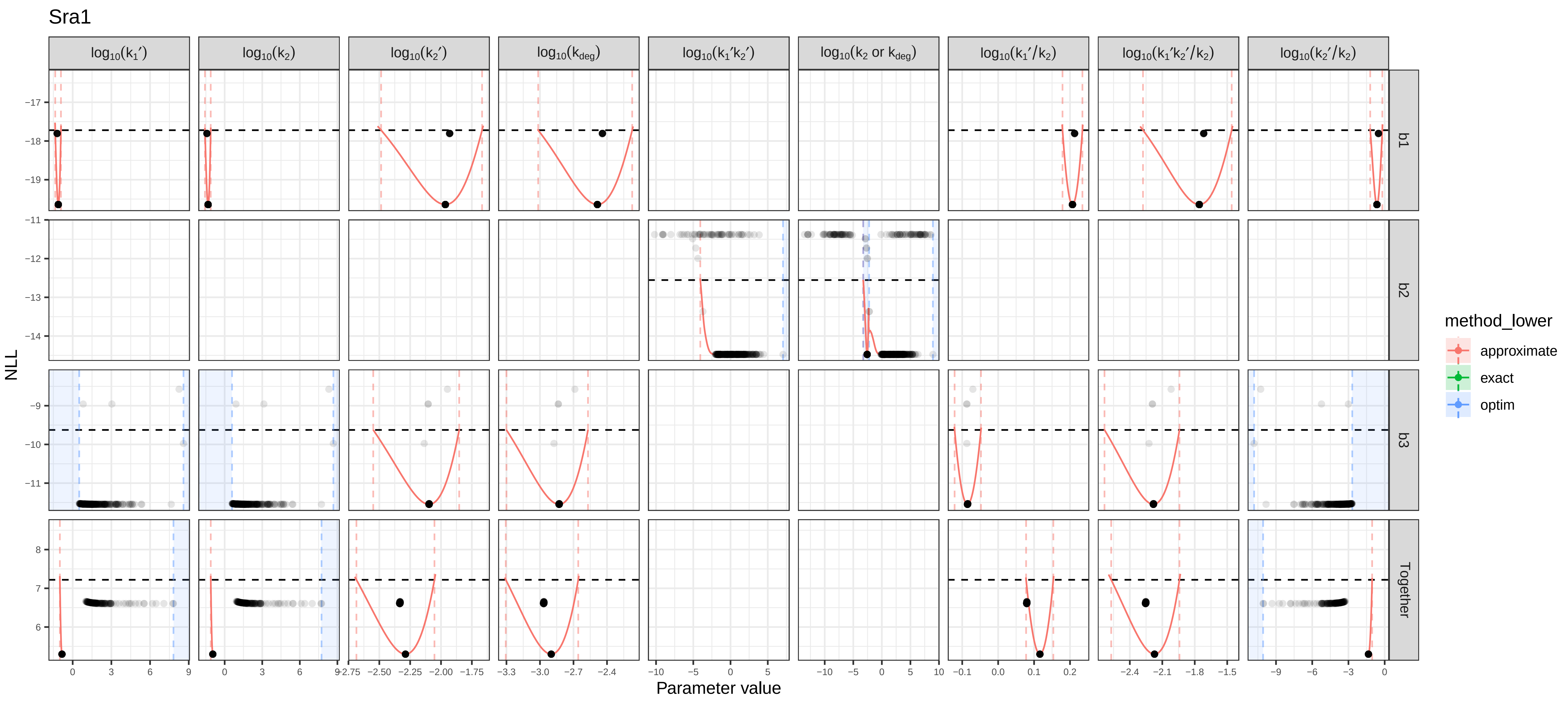

Replicate	Par	Best value	CI95 LB	CI95 UB	Method LB	Method UB
Together	$\log_{10}(k_1')$	−0.8294	−0.9939	> 7.814	approximate	optim
Together	$\log_{10}(k_2)$	−0.9456	−1.11	> 7.734	approximate	optim
Together	$\log_{10}(k_2')$	−2.288	−2.685	−2.053	approximate	approximate
Together	$\log_{10}(k_{deg})$	−2.899	−3.306	−2.658	approximate	approximate
Together	$\log_{10}(k_1'/k_2)$	0.1162	0.07776	0.1536	approximate	approximate
Together	$\log_{10}(k_1'k_2'/k_2)$	−2.172	−2.572	−1.941	approximate	approximate
Together	$\log_{10}(k_2'/k_2)$	−1.342	< −10.07	−1.039	optim	approximate
b1	$\log_{10}(k_1')$	−1.115	−1.355	−0.9102	approximate	approximate
b1	$\log_{10}(k_2)$	−1.322	−1.573	−1.112	approximate	approximate
b1	$\log_{10}(k_2')$	−1.965	−2.485	−1.667	approximate	approximate
b1	$\log_{10}(k_{deg})$	−2.486	−3.016	−2.175	approximate	approximate
b1	$\log_{10}(k_1'/k_2)$	0.2069	0.1791	0.2345	approximate	approximate
b1	$\log_{10}(k_1'k_2'/k_2)$	−1.758	−2.278	−1.459	approximate	approximate
b1	$\log_{10}(k_2'/k_2)$	−0.6433	−1.198	−0.1953	approximate	approximate
b2	$\log_{10}(k_1'k_2')$	−1.379	−4.062	> 7.046	approximate	optim
b2	$\log_{10}(k_2 \text{ or } k_{deg})$	0.5088	−3.258	> 8.935	approximate	optim
b2	$\log_{10}(k_2 \text{ or } k_{deg})$	−2.57	−3.258	−2.227	approximate	optim
b3	$\log_{10}(k_1')$	3.871	< 0.5053	> 8.59	optim	optim
b3	$\log_{10}(k_2)$	3.956	< 0.5898	> 8.677	optim	optim
b3	$\log_{10}(k_2')$	−2.097	−2.549	−1.853	approximate	approximate
b3	$\log_{10}(k_{deg})$	−2.829	−3.3	−2.57	approximate	approximate
b3	$\log_{10}(k_1'/k_2)$	−0.08536	−0.1212	−0.04778	approximate	approximate
b3	$\log_{10}(k_1'k_2'/k_2)$	−2.182	−2.634	−1.941	approximate	approximate
b3	$\log_{10}(k_2'/k_2)$	−6.053	< −10.81	> −2.685	optim	optim

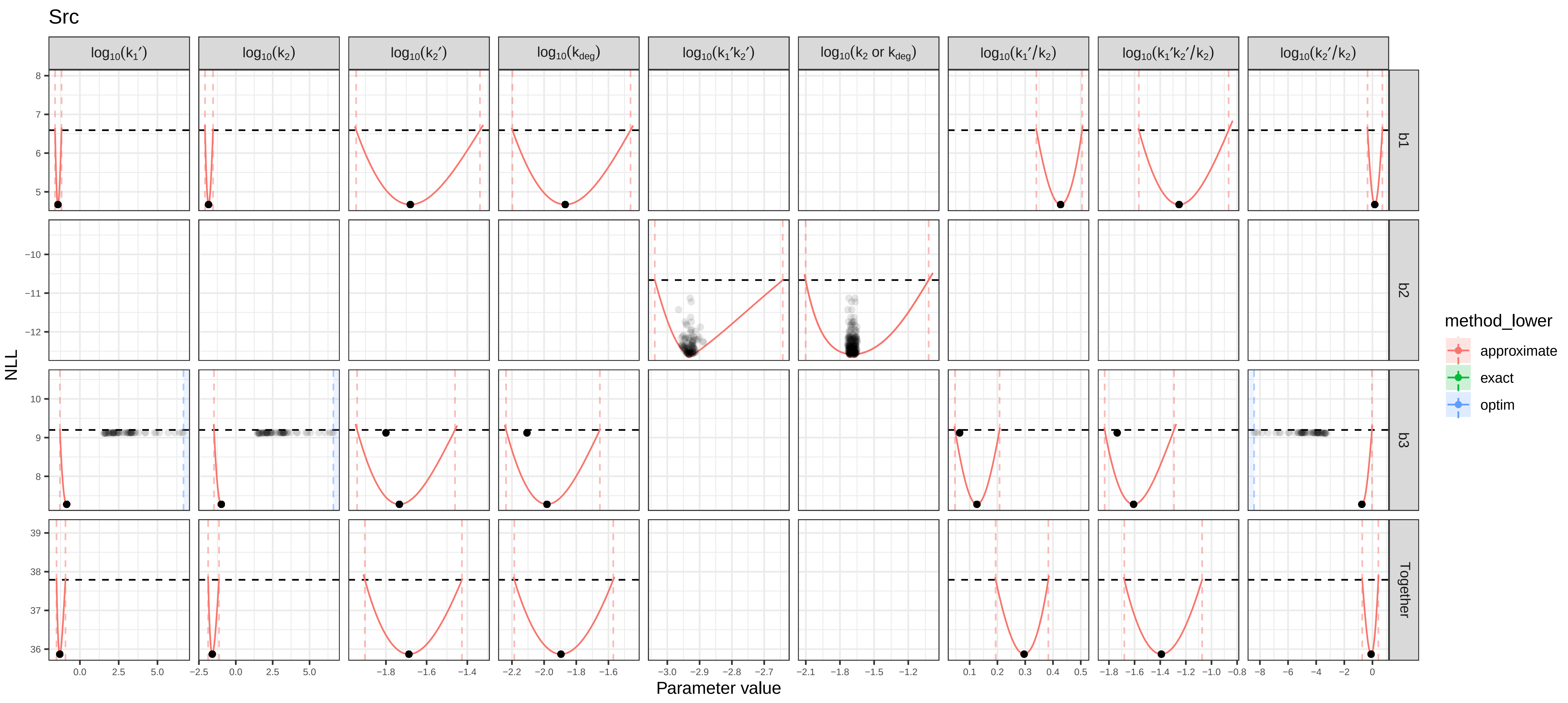

Replicate	Par	**Best value**	**CI95 LB**	**CI95 UB**	**Method LB**	**Method UB**
Together	$\log_{10}(k_1')$	−1.296	−1.51	−0.9328	approximate	approximate
Together	$\log_{10}(k_2)$	−1.593	−1.875	−1.136	approximate	approximate
Together	$\log_{10}(k_2')$	−1.687	−1.904	−1.426	approximate	approximate
Together	$\log_{10}(k_{deg})$	−1.895	−2.185	−1.57	approximate	approximate
Together	$\log_{10}(k_1'/k_2)$	0.2961	0.1937	0.3832	approximate	approximate
Together	$\log_{10}(k_1'k_2'/k_2)$	−1.391	−1.68	−1.073	approximate	approximate
Together	$\log_{10}(k_2'/k_2)$	−0.09442	−0.7279	0.4181	approximate	approximate
b1	$\log_{10}(k_1')$	−1.417	−1.604	−1.186	approximate	approximate
b1	$\log_{10}(k_2)$	−1.845	−2.087	−1.542	approximate	approximate
b1	$\log_{10}(k_2')$	−1.68	−1.946	−1.338	approximate	approximate
b1	$\log_{10}(k_{deg})$	−1.869	−2.197	−1.463	approximate	approximate
b1	$\log_{10}(k_1'/k_2)$	0.4274	0.3402	0.505	approximate	approximate
b1	$\log_{10}(k_1'k_2'/k_2)$	−1.253	−1.567	−0.8662	approximate	approximate
b1	$\log_{10}(k_2'/k_2)$	0.1646	−0.3484	0.7047	approximate	approximate
b2	$\log_{10}(k_1'k_2')$	−2.933	−3.039	−2.643	approximate	approximate
b2	$\log_{10}(k_2 \text{ or } k_{deg})$	−1.675	−2.102	−1.021	approximate	approximate
b2	$\log_{10}(k_2 \text{ or } k_{deg})$	−1.712	−2.102	−1.021	approximate	approximate
b3	$\log_{10}(k_1')$	−0.8547	−1.292	> 6.677	approximate	optim
b3	$\log_{10}(k_2)$	−0.9809	−1.478	> 6.613	approximate	optim
b3	$\log_{10}(k_2')$	−1.734	−1.942	−1.461	approximate	approximate
b3	$\log_{10}(k_{deg})$	−1.982	−2.237	−1.653	approximate	approximate
b3	$\log_{10}(k_1'/k_2)$	0.1262	0.0471	0.2078	approximate	approximate
b3	$\log_{10}(k_1'k_2'/k_2)$	−1.608	−1.834	−1.292	approximate	approximate
b3	$\log_{10}(k_2'/k_2)$	−0.753	< −8.413	−0.03618	optim	approximate

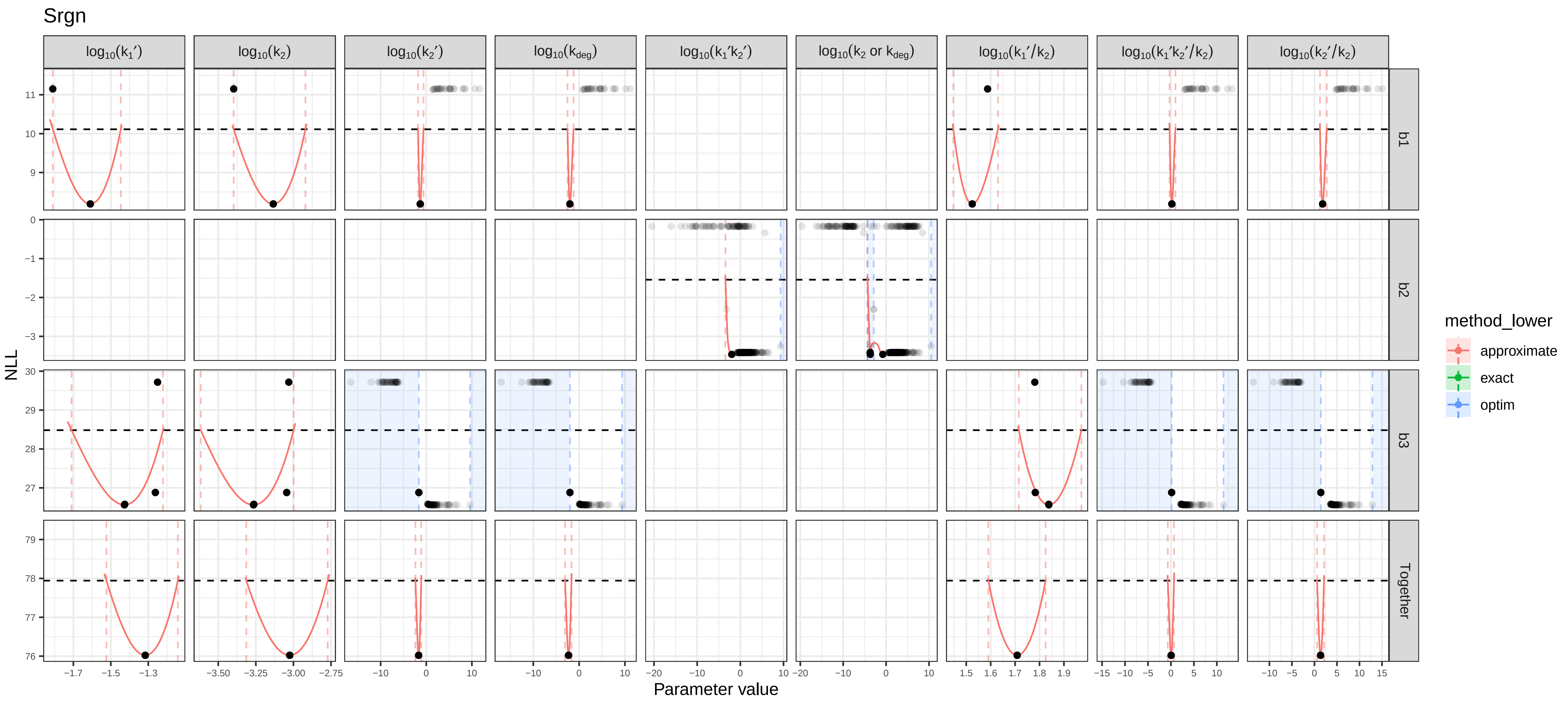

Replicate	Par	Best value	CI95 LB	CI95 UB	Method LB	Method UB
Together	$\log_{10}(k_1')$	−1.316	−1.523	−1.142	approximate	approximate
Together	$\log_{10}(k_2)$	−3.025	−3.313	−2.772	approximate	approximate
Together	$\log_{10}(k_2')$	−1.66	−2.377	−1.104	approximate	approximate
Together	$\log_{10}(k_{deg})$	−2.302	−3.065	−1.663	approximate	approximate
Together	$\log_{10}(k_1'/k_2)$	1.708	1.591	1.825	approximate	approximate
Together	$\log_{10}(k_1'k_2'/k_2)$	0.04831	−0.6861	0.6631	approximate	approximate
Together	$\log_{10}(k_2'/k_2)$	1.365	0.5813	2.132	approximate	approximate
b1	$\log_{10}(k_1')$	−1.61	−1.81	−1.447	approximate	approximate
b1	$\log_{10}(k_2)$	−3.134	−3.398	−2.92	approximate	approximate
b1	$\log_{10}(k_2')$	−1.323	−1.802	−0.5905	approximate	approximate
b1	$\log_{10}(k_{deg})$	−2.004	−2.527	−1.198	approximate	approximate
b1	$\log_{10}(k_1'/k_2)$	1.525	1.447	1.63	approximate	approximate
b1	$\log_{10}(k_1'k_2'/k_2)$	0.2014	−0.2949	0.9809	approximate	approximate
b1	$\log_{10}(k_2'/k_2)$	1.811	1.226	2.744	approximate	approximate
b2	$\log_{10}(k_1'k_2')$	−1.987	−3.455	> 9.34	approximate	optim
b2	$\log_{10}(k_2 \text{ or } k_{deg})$	−0.7736	−4.346	> 10.49	approximate	optim
b2	$\log_{10}(k_2 \text{ or } k_{deg})$	−3.695	−4.346	−2.88	approximate	optim
b3	$\log_{10}(k_1')$	−1.427	−1.71	−1.221	approximate	approximate
b3	$\log_{10}(k_2)$	−3.265	−3.616	−2.999	approximate	approximate
b3	$\log_{10}(k_2')$	2.877	< −1.628	> 9.61	optim	optim
b3	$\log_{10}(k_{deg})$	2.63	< −1.999	> 9.362	optim	optim
b3	$\log_{10}(k_1'/k_2)$	1.838	1.715	1.971	approximate	approximate
b3	$\log_{10}(k_1'k_2'/k_2)$	4.715	< 0.1541	> 11.45	optim	optim
b3	$\log_{10}(k_2'/k_2)$	6.143	< 1.416	> 12.88	optim	optim

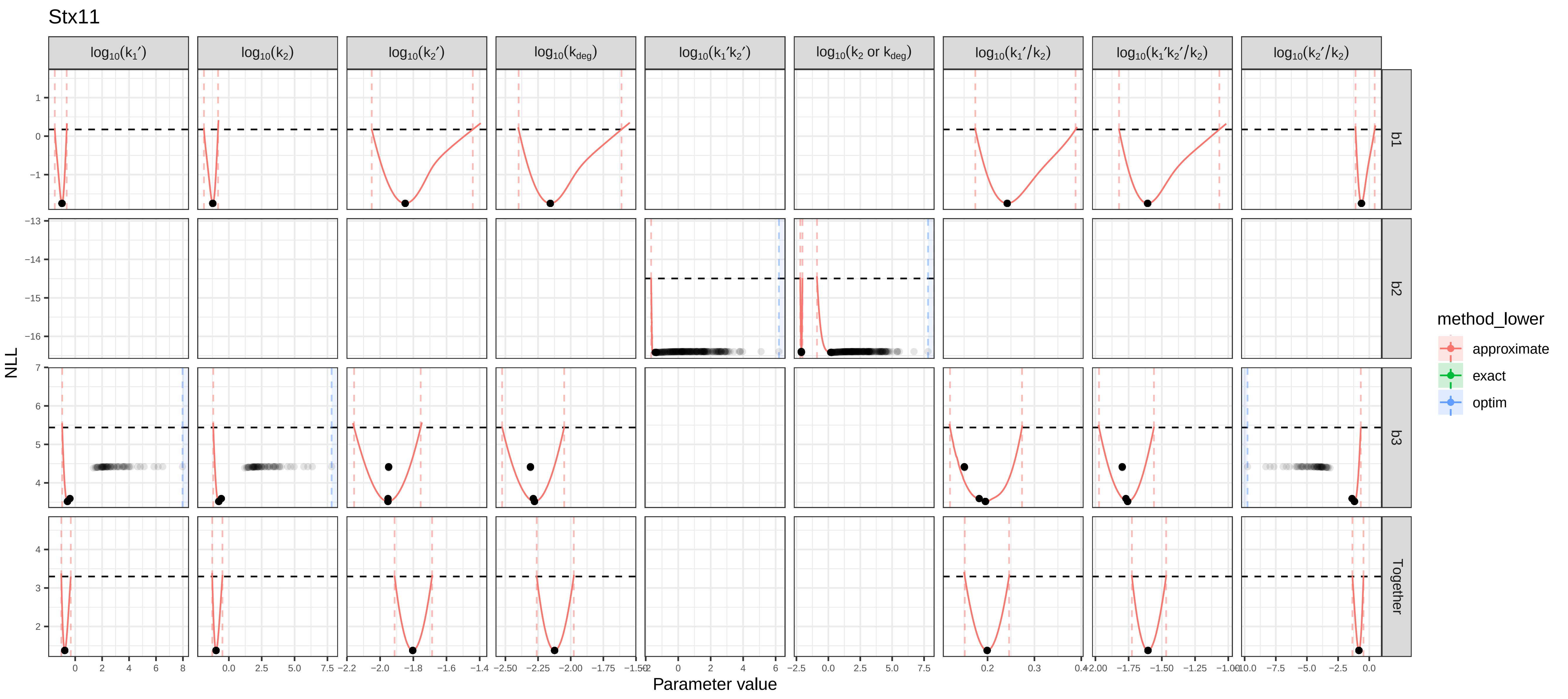

Replicate	Par	Best value	CI95 LB	CI95 UB	Method LB	Method UB
Together	$\log_{10}(k_1')$	−0.774	−1.03	−0.3263	approximate	approximate
Together	$\log_{10}(k_2)$	−0.9732	−1.265	−0.4877	approximate	approximate
Together	$\log_{10}(k_2')$	−1.803	−1.913	−1.687	approximate	approximate
Together	$\log_{10}(k_{deg})$	−2.124	−2.26	−1.977	approximate	approximate
Together	$\log_{10}(k_1'/k_2)$	0.1992	0.1514	0.246	approximate	approximate
Together	$\log_{10}(k_1'k_2'/k_2)$	−1.604	−1.725	−1.468	approximate	approximate
Together	$\log_{10}(k_2'/k_2)$	−0.8303	−1.354	−0.4623	approximate	approximate
b1	$\log_{10}(k_1')$	−0.981	−1.513	−0.6306	approximate	approximate
b1	$\log_{10}(k_2)$	−1.223	−1.894	−0.8199	approximate	approximate
b1	$\log_{10}(k_2')$	−1.849	−2.05	−1.442	approximate	approximate
b1	$\log_{10}(k_{deg})$	−2.156	−2.399	−1.611	approximate	approximate
b1	$\log_{10}(k_1'/k_2)$	0.242	0.1738	0.3878	approximate	approximate
b1	$\log_{10}(k_1'k_2'/k_2)$	−1.607	−1.822	−1.067	approximate	approximate
b1	$\log_{10}(k_2'/k_2)$	−0.6258	−1.101	0.4366	approximate	approximate
b2	$\log_{10}(k_1'k_2')$	−1.34	−1.666	> 6.203	approximate	optim
b2	$\log_{10}(k_2 \text{ or } k_{deg})$	0.2615	−0.8898	> 7.811	approximate	optim
b2	$\log_{10}(k_2 \text{ or } k_{deg})$	−2.114	−2.202	−2.03	approximate	approximate
b3	$\log_{10}(k_1')$	−0.571	−0.9641	> 7.968	approximate	optim
b3	$\log_{10}(k_2)$	−0.7664	−1.184	> 7.818	approximate	optim
b3	$\log_{10}(k_2')$	−1.953	−2.157	−1.755	approximate	approximate
b3	$\log_{10}(k_{deg})$	−2.277	−2.525	−2.05	approximate	approximate
b3	$\log_{10}(k_1'/k_2)$	0.1954	0.1199	0.2735	approximate	approximate
b3	$\log_{10}(k_1'k_2'/k_2)$	−1.757	−1.973	−1.559	approximate	approximate
b3	$\log_{10}(k_2'/k_2)$	−1.186	< −9.766	−0.6784	optim	approximate

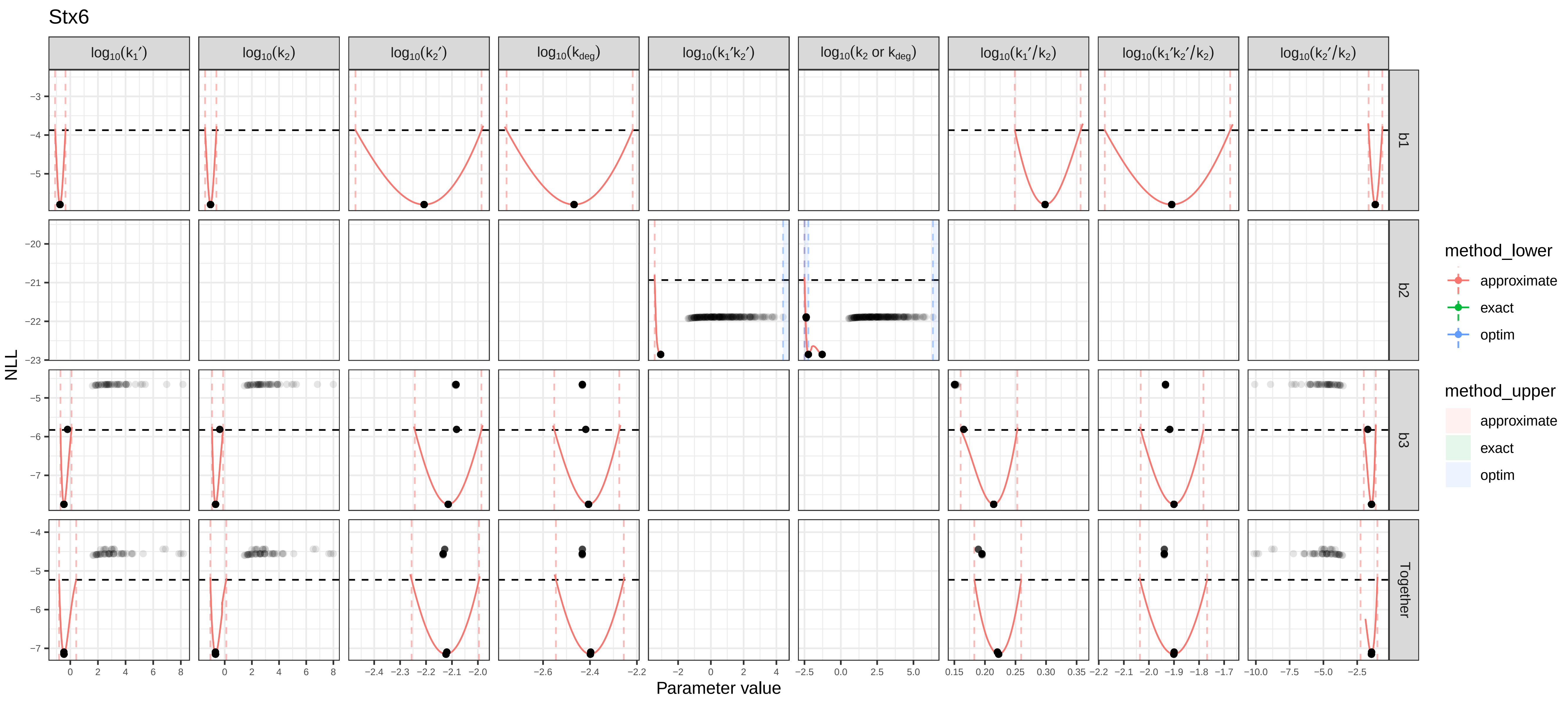

Replicate	Par	Best value	CI95 LB	CI95 UB	Method LB	Method UB
Together	$\log_{10}(k_1')$	−0.4553	−0.8115	0.4278	approximate	approximate
Together	$\log_{10}(k_2)$	−0.6777	−1.06	0.1162	approximate	approximate
Together	$\log_{10}(k_2')$	−2.123	−2.256	−1.996	approximate	approximate
Together	$\log_{10}(k_{deg})$	−2.398	−2.545	−2.256	approximate	approximate
Together	$\log_{10}(k_1'/k_2)$	0.2224	0.1826	0.2593	approximate	approximate
Together	$\log_{10}(k_1'k_2'/k_2)$	−1.901	−2.036	−1.768	approximate	approximate
Together	$\log_{10}(k_2'/k_2)$	−1.445	−2.256	−1.009	approximate	approximate
b1	$\log_{10}(k_1')$	−0.7466	−1.102	−0.3473	approximate	approximate
b1	$\log_{10}(k_2)$	−1.045	−1.448	−0.613	approximate	approximate
b1	$\log_{10}(k_2')$	−2.207	−2.472	−1.986	approximate	approximate
b1	$\log_{10}(k_{deg})$	−2.468	−2.755	−2.218	approximate	approximate
b1	$\log_{10}(k_1'/k_2)$	0.2985	0.2488	0.3565	approximate	approximate
b1	$\log_{10}(k_1'k_2'/k_2)$	−1.909	−2.176	−1.676	approximate	approximate
b1	$\log_{10}(k_2'/k_2)$	−1.162	−1.66	−0.6418	approximate	approximate
b2	$\log_{10}(k_1'k_2')$	−3.063	−3.429	> 4.404	approximate	optim
b2	$\log_{10}(k_2 \text{ or } k_{deg})$	−1.283	−2.498	> 6.339	approximate	optim
b2	$\log_{10}(k_2 \text{ or } k_{deg})$	−2.233	−2.498	−2.233	approximate	optim
b3	$\log_{10}(k_1')$	−0.4591	−0.7149	0.08864	approximate	approximate
b3	$\log_{10}(k_2)$	−0.6734	−0.935	−0.1229	approximate	approximate
b3	$\log_{10}(k_2')$	−2.114	−2.244	−1.986	approximate	approximate
b3	$\log_{10}(k_{deg})$	−2.406	−2.552	−2.275	approximate	approximate
b3	$\log_{10}(k_1'/k_2)$	0.2143	0.1603	0.2529	approximate	approximate
b3	$\log_{10}(k_1'k_2'/k_2)$	−1.9	−2.033	−1.783	approximate	approximate
b3	$\log_{10}(k_2'/k_2)$	−1.441	−2.014	−1.134	approximate	approximate

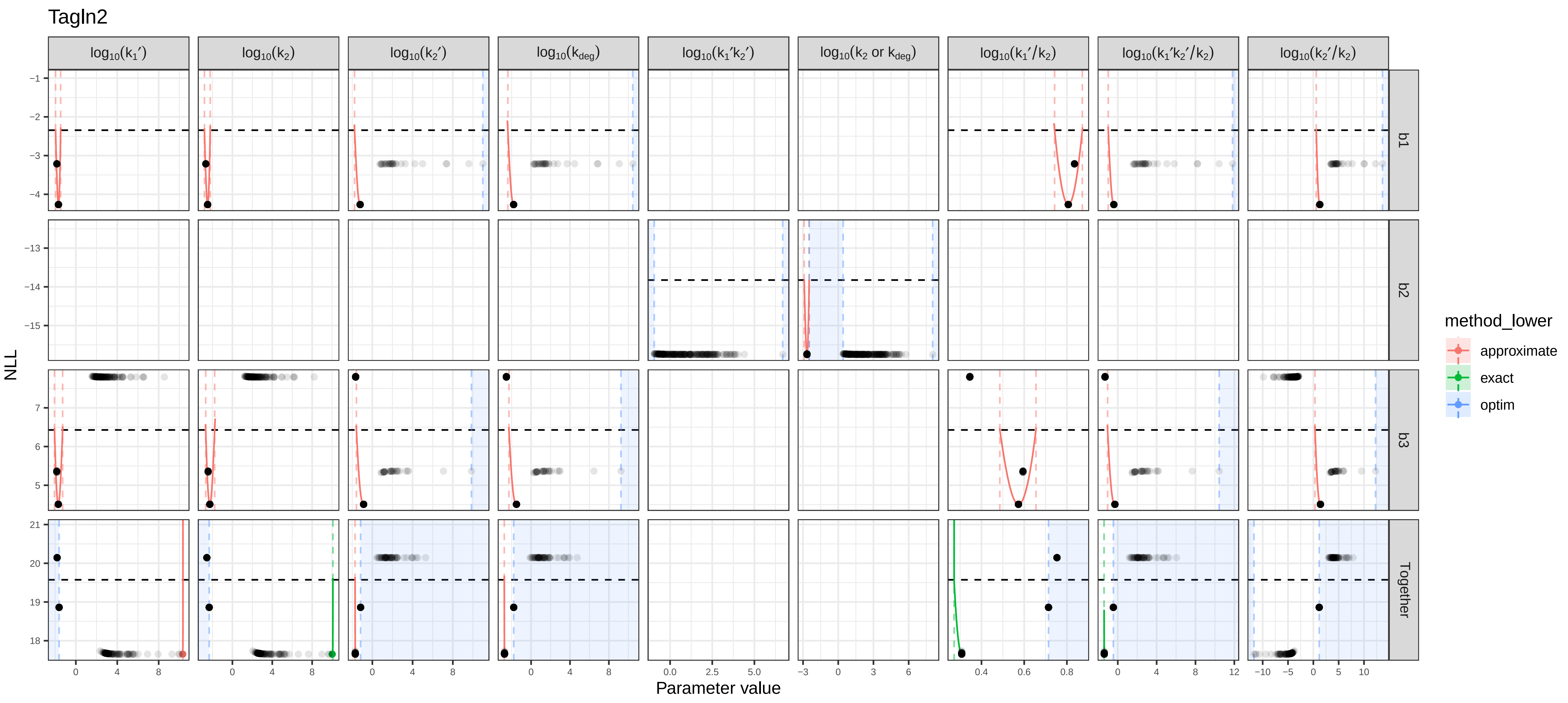

Replicate	Par	Best value	CI95 LB	CI95 UB	Method LB	Method UB
Together	$\log_{10}(k_1')$	10.32	< −1.615	10.34	optim	approximate
Together	$\log_{10}(k_2)$	10.02	< −2.329	10.08	optim	exact
Together	$\log_{10}(k_2')$	−1.698	−1.717	> −1.168	approximate	optim
Together	$\log_{10}(k_{deg})$	−2.739	−2.76	> −1.788	approximate	optim
Together	$\log_{10}(k_1'/k_2)$	0.3073	0.2718	> 0.7141	exact	optim
Together	$\log_{10}(k_1'k_2'/k_2)$	−1.391	−1.412	> −0.4537	exact	optim
Together	$\log_{10}(k_2'/k_2)$	−11.71	< −11.71	> 1.161	optim	optim
b1	$\log_{10}(k_1')$	−1.678	−1.954	−1.465	approximate	approximate
b1	$\log_{10}(k_2)$	−2.485	−2.806	−2.224	approximate	approximate
b1	$\log_{10}(k_2')$	−1.214	−1.768	> 10.98	approximate	optim
b1	$\log_{10}(k_{deg})$	−1.799	−2.393	> 10.46	approximate	optim
b1	$\log_{10}(k_1'/k_2)$	0.8065	0.7424	0.8723	approximate	approximate
b1	$\log_{10}(k_1'k_2'/k_2)$	−0.4073	−0.9797	> 11.82	approximate	optim
b1	$\log_{10}(k_2'/k_2)$	1.271	0.5744	> 13.65	approximate	optim
b2	$\log_{10}(k_1'k_2')$	3.85	< −0.9431	> 6.681	optim	optim
b2	$\log_{10}(k_2 \text{ or } k_{deg})$	5.214	0.4195	> 8.045	optim	optim
b2	$\log_{10}(k_2 \text{ or } k_{deg})$	−2.663	−2.903	−2.477	approximate	approximate
b3	$\log_{10}(k_1')$	−1.692	−2.06	−1.275	approximate	approximate
b3	$\log_{10}(k_2)$	−2.265	−2.679	−1.777	approximate	approximate
b3	$\log_{10}(k_2')$	−0.869	−1.583	> 9.849	approximate	optim
b3	$\log_{10}(k_{deg})$	−1.508	−2.279	> 9.245	approximate	optim
b3	$\log_{10}(k_1'/k_2)$	0.5732	0.4858	0.655	approximate	approximate
b3	$\log_{10}(k_1'k_2'/k_2)$	−0.2958	−1.044	> 10.44	approximate	optim
b3	$\log_{10}(k_2'/k_2)$	1.396	0.3292	> 12.28	approximate	optim

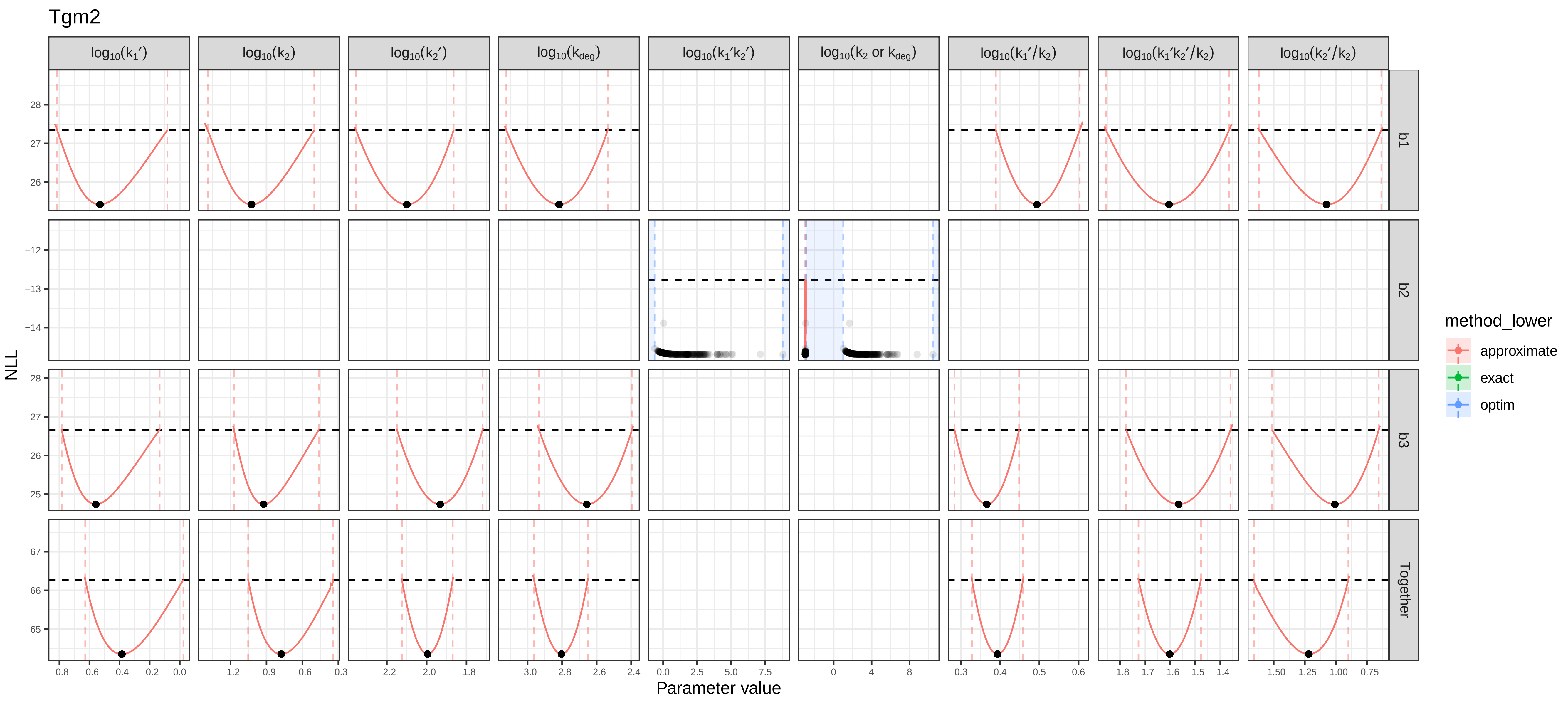

Replicate	Par	Best value	CI95 LB	CI95 UB	Method LB	Method UB
Together	$\log_{10}(k_1')$	−0.3843	−0.6281	0.0247	approximate	approximate
Together	$\log_{10}(k_2)$	−0.7774	−1.053	−0.3425	approximate	approximate
Together	$\log_{10}(k_2')$	−1.995	−2.124	−1.869	approximate	approximate
Together	$\log_{10}(k_{deg})$	−2.804	−2.963	−2.651	approximate	approximate
Together	$\log_{10}(k_1'/k_2)$	0.3931	0.3278	0.4584	approximate	approximate
Together	$\log_{10}(k_1'k_2'/k_2)$	−1.602	−1.726	−1.477	approximate	approximate
Together	$\log_{10}(k_2'/k_2)$	−1.217	−1.657	−0.8999	approximate	approximate
b1	$\log_{10}(k_1')$	−0.5306	−0.815	−0.08193	approximate	approximate
b1	$\log_{10}(k_2)$	−1.024	−1.391	−0.5009	approximate	approximate
b1	$\log_{10}(k_2')$	−2.099	−2.354	−1.865	approximate	approximate
b1	$\log_{10}(k_{deg})$	−2.818	−3.123	−2.536	approximate	approximate
b1	$\log_{10}(k_1'/k_2)$	0.4938	0.3888	0.6029	approximate	approximate
b1	$\log_{10}(k_1'k_2'/k_2)$	−1.605	−1.856	−1.365	approximate	approximate
b1	$\log_{10}(k_2'/k_2)$	−1.075	−1.615	−0.6346	approximate	approximate
b2	$\log_{10}(k_1'k_2')$	2.729	< −0.6306	> 8.807	optim	optim
b2	$\log_{10}(k_2 \text{ or } k_{deg})$	4.376	1.016	> 10.45	optim	optim
b2	$\log_{10}(k_2 \text{ or } k_{deg})$	−2.973	−3.07	−2.898	approximate	approximate
b3	$\log_{10}(k_1')$	−0.558	−0.7849	−0.1337	approximate	approximate
b3	$\log_{10}(k_2)$	−0.9238	−1.171	−0.4633	approximate	approximate
b3	$\log_{10}(k_2')$	−1.932	−2.149	−1.719	approximate	approximate
b3	$\log_{10}(k_{deg})$	−2.657	−2.934	−2.395	approximate	approximate
b3	$\log_{10}(k_1'/k_2)$	0.3658	0.2834	0.4484	approximate	approximate
b3	$\log_{10}(k_1'k_2'/k_2)$	−1.566	−1.776	−1.359	approximate	approximate
b3	$\log_{10}(k_2'/k_2)$	−1.008	−1.514	−0.6561	approximate	approximate

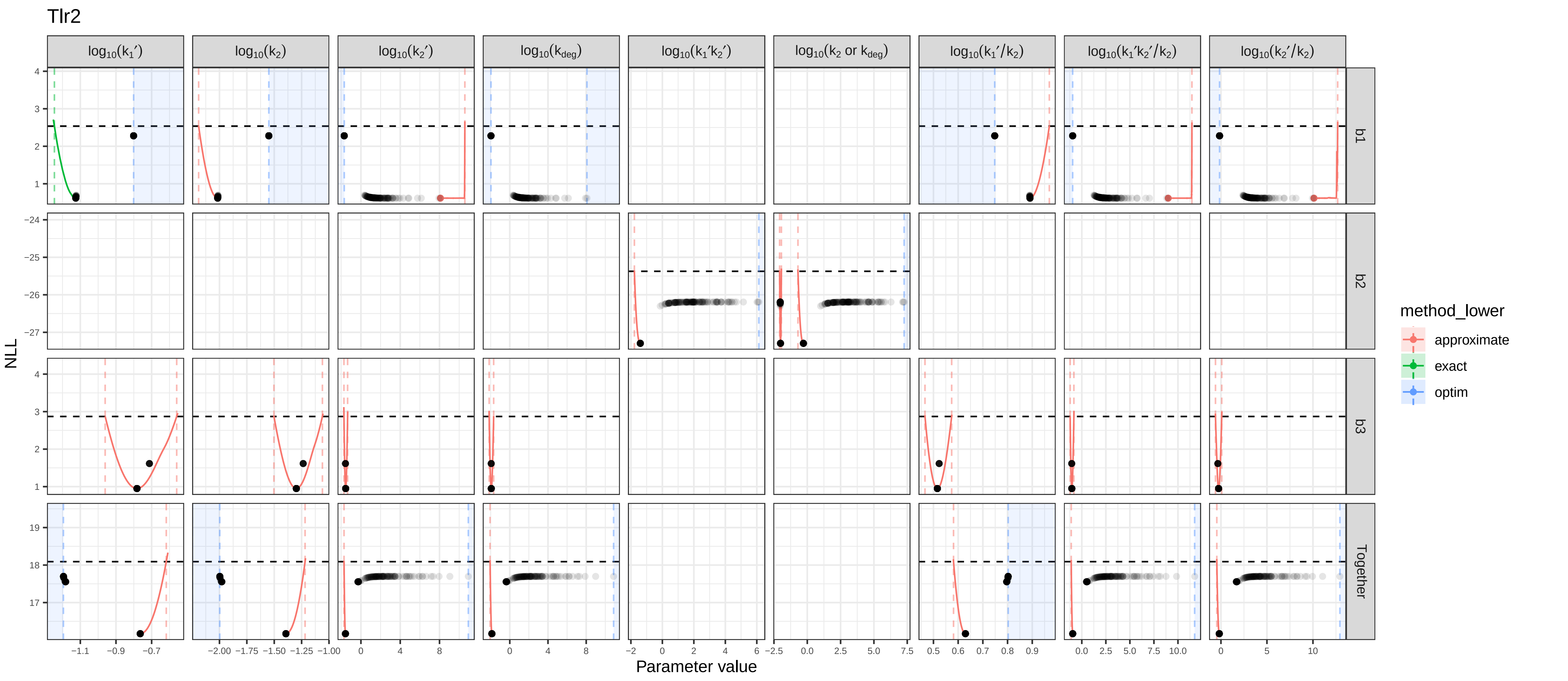

Replicate	Par	Best value	CI95 LB	CI95 UB	Method LB	Method UB
Together	$\log_{10}(k_1')$	−0.764	< −1.195	−0.618	optim	approximate
Together	$\log_{10}(k_2)$	−1.393	< −1.997	−1.218	optim	approximate
Together	$\log_{10}(k_2')$	−1.574	−1.72	> 10.93	approximate	optim
Together	$\log_{10}(k_{deg})$	−1.873	−2.06	> 10.87	approximate	optim
Together	$\log_{10}(k_1'/k_2)$	0.6295	0.5813	> 0.8026	approximate	optim
Together	$\log_{10}(k_1'k_2'/k_2)$	−0.9442	−1.103	> 11.74	approximate	optim
Together	$\log_{10}(k_2'/k_2)$	−0.1802	−0.4495	> 12.93	approximate	optim
b1	$\log_{10}(k_1')$	−1.126	−1.245	> −0.8011	exact	optim
b1	$\log_{10}(k_2)$	−2.016	−2.19	> −1.549	approximate	optim
b1	$\log_{10}(k_2')$	8.094	< −1.699	10.58	optim	approximate
b1	$\log_{10}(k_{deg})$	8.08	< −1.969	> 8.08	optim	optim
b1	$\log_{10}(k_1'/k_2)$	0.8909	< 0.7481	0.9692	optim	approximate
b1	$\log_{10}(k_1'k_2'/k_2)$	8.985	< −0.9505	11.45	optim	approximate
b1	$\log_{10}(k_2'/k_2)$	10.11	< −0.1494	12.69	optim	approximate
b2	$\log_{10}(k_1'k_2')$	−1.401	−1.779	> 6.13	approximate	optim
b2	$\log_{10}(k_2 \text{ or } k_{deg})$	−0.2885	−0.6991	> 7.266	approximate	optim
b2	$\log_{10}(k_2 \text{ or } k_{deg})$	−2.008	−2.075	−1.943	approximate	approximate
b3	$\log_{10}(k_1')$	−0.7823	−0.9607	−0.5595	approximate	approximate
b3	$\log_{10}(k_2)$	−1.298	−1.503	−1.06	approximate	approximate
b3	$\log_{10}(k_2')$	−1.554	−1.728	−1.355	approximate	approximate
b3	$\log_{10}(k_{deg})$	−1.938	−2.156	−1.687	approximate	approximate
b3	$\log_{10}(k_1'/k_2)$	0.5159	0.4657	0.5738	approximate	approximate
b3	$\log_{10}(k_1'k_2'/k_2)$	−1.038	−1.213	−0.8247	approximate	approximate
b3	$\log_{10}(k_2'/k_2)$	−0.2557	−0.5937	0.08567	approximate	approximate

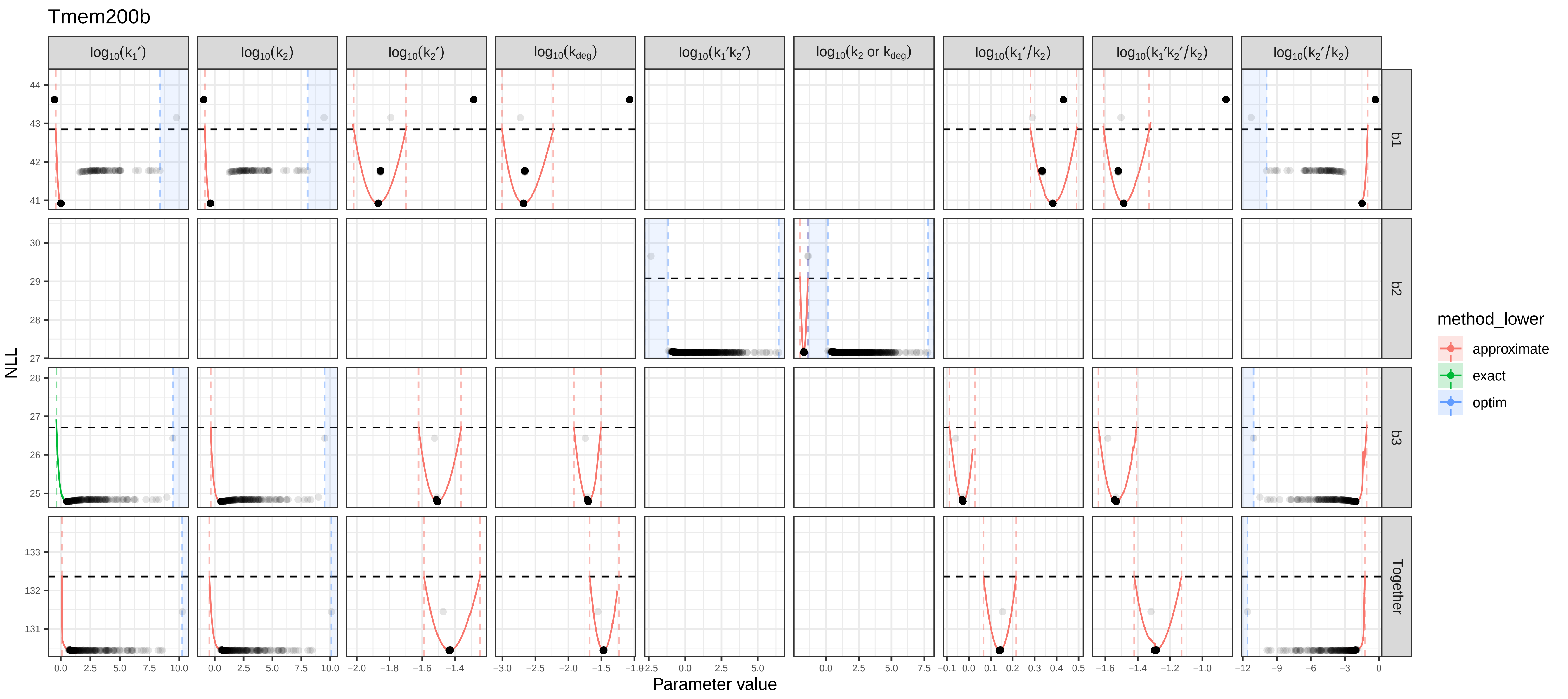

Replicate	Par	Best value	CI95 LB	CI95 UB	Method LB	Method UB
Together	$\log_{10}(k_1')$	1.238	0.08458	> 10.26	approximate	optim
Together	$\log_{10}(k_2)$	1.097	−0.4636	> 10.11	approximate	optim
Together	$\log_{10}(k_2')$	−1.432	−1.59	−1.247	approximate	approximate
Together	$\log_{10}(k_{deg})$	−1.471	−1.679	−1.233	approximate	approximate
Together	$\log_{10}(k_1'/k_2)$	0.1407	0.06669	0.2157	approximate	approximate
Together	$\log_{10}(k_1'k_2'/k_2)$	−1.292	−1.421	−1.129	approximate	approximate
Together	$\log_{10}(k_2'/k_2)$	−2.529	< −11.58	−1.249	optim	approximate
b1	$\log_{10}(k_1')$	0.01305	−0.4226	> 8.38	approximate	optim
b1	$\log_{10}(k_2)$	−0.3702	−0.8559	> 8.045	approximate	optim
b1	$\log_{10}(k_2')$	−1.869	−2.018	−1.699	approximate	approximate
b1	$\log_{10}(k_{deg})$	−2.679	−3.006	−2.228	approximate	approximate
b1	$\log_{10}(k_1'/k_2)$	0.3833	0.2806	0.4912	approximate	approximate
b1	$\log_{10}(k_1'k_2'/k_2)$	−1.486	−1.61	−1.328	approximate	approximate
b1	$\log_{10}(k_2'/k_2)$	−1.499	< −9.9	−0.9973	optim	approximate
b2	$\log_{10}(k_1'k_2')$	1.876	< −1.168	> 6.448	optim	optim
b2	$\log_{10}(k_2 \text{ or } k_{deg})$	3.214	0.155	> 7.787	optim	optim
b2	$\log_{10}(k_2 \text{ or } k_{deg})$	−1.696	−1.97	−1.386	approximate	approximate
b3	$\log_{10}(k_1')$	0.5574	−0.363	> 9.468	exact	optim
b3	$\log_{10}(k_2)$	0.5844	−0.3537	> 9.528	approximate	optim
b3	$\log_{10}(k_2')$	−1.506	−1.622	−1.361	approximate	approximate
b3	$\log_{10}(k_{deg})$	−1.7	−1.919	−1.51	approximate	approximate
b3	$\log_{10}(k_1'/k_2)$	−0.02703	−0.08841	0.02832	approximate	approximate
b3	$\log_{10}(k_1'k_2'/k_2)$	−1.533	−1.643	−1.407	approximate	approximate
b3	$\log_{10}(k_2'/k_2)$	−2.09	< −11.05	−1.097	optim	approximate

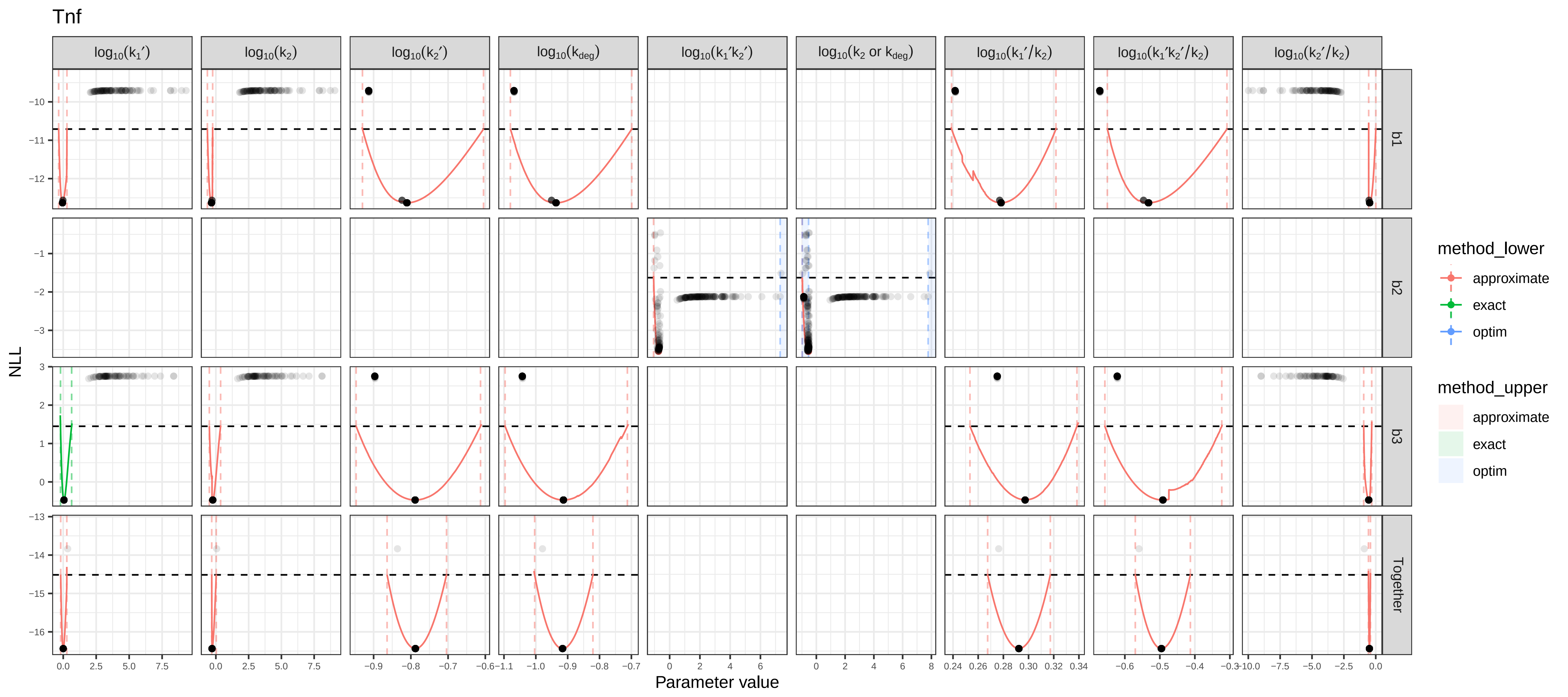

Replicate	Par	Best value	CI95 LB	CI95 UB	Method LB	Method UB
Together	$\log_{10}(k_1')$	0.001361	−0.1912	0.2721	approximate	approximate
Together	$\log_{10}(k_2)$	−0.2909	−0.3157	0.02962	approximate	approximate
Together	$\log_{10}(k_2')$	−0.7878	−0.8638	−0.7041	approximate	approximate
Together	$\log_{10}(k_{deg})$	−0.9161	−1.004	−0.8207	approximate	approximate
Together	$\log_{10}(k_1'/k_2)$	0.2923	0.2675	0.3173	approximate	approximate
Together	$\log_{10}(k_1'k_2'/k_2)$	−0.4955	−0.5702	−0.4128	approximate	approximate
Together	$\log_{10}(k_2'/k_2)$	−0.4969	−0.5713	−0.414	approximate	approximate
b1	$\log_{10}(k_1')$	−0.05389	−0.3444	0.283	approximate	approximate
b1	$\log_{10}(k_2)$	−0.3321	−0.648	−0.2453	approximate	approximate
b1	$\log_{10}(k_2')$	−0.8106	−0.93	−0.6048	approximate	approximate
b1	$\log_{10}(k_{deg})$	−0.9366	−1.08	−0.6988	approximate	approximate
b1	$\log_{10}(k_1'/k_2)$	0.2782	0.2389	0.3218	approximate	approximate
b1	$\log_{10}(k_1'k_2'/k_2)$	−0.5324	−0.6499	−0.3084	approximate	approximate
b1	$\log_{10}(k_2'/k_2)$	−0.4785	−0.5535	0.007299	approximate	approximate
b2	$\log_{10}(k_1'k_2')$	−0.737	−1.062	> 7.305	approximate	optim
b2	$\log_{10}(k_2 \text{ or } k_{deg})$	−0.5478	−0.9802	> 7.772	approximate	optim
b2	$\log_{10}(k_2 \text{ or } k_{deg})$	−0.5989	−0.9802	−0.5389	approximate	optim
b3	$\log_{10}(k_1')$	0.05828	−0.2062	0.6396	exact	exact
b3	$\log_{10}(k_2)$	−0.239	−0.4998	0.362	approximate	approximate
b3	$\log_{10}(k_2')$	−0.7886	−0.947	−0.6129	approximate	approximate
b3	$\log_{10}(k_{deg})$	−0.9134	−1.096	−0.7126	approximate	approximate
b3	$\log_{10}(k_1'/k_2)$	0.2973	0.2535	0.3384	approximate	approximate
b3	$\log_{10}(k_1'k_2'/k_2)$	−0.4913	−0.657	−0.3232	approximate	approximate
b3	$\log_{10}(k_2'/k_2)$	−0.5496	−0.9436	−0.3152	approximate	approximate

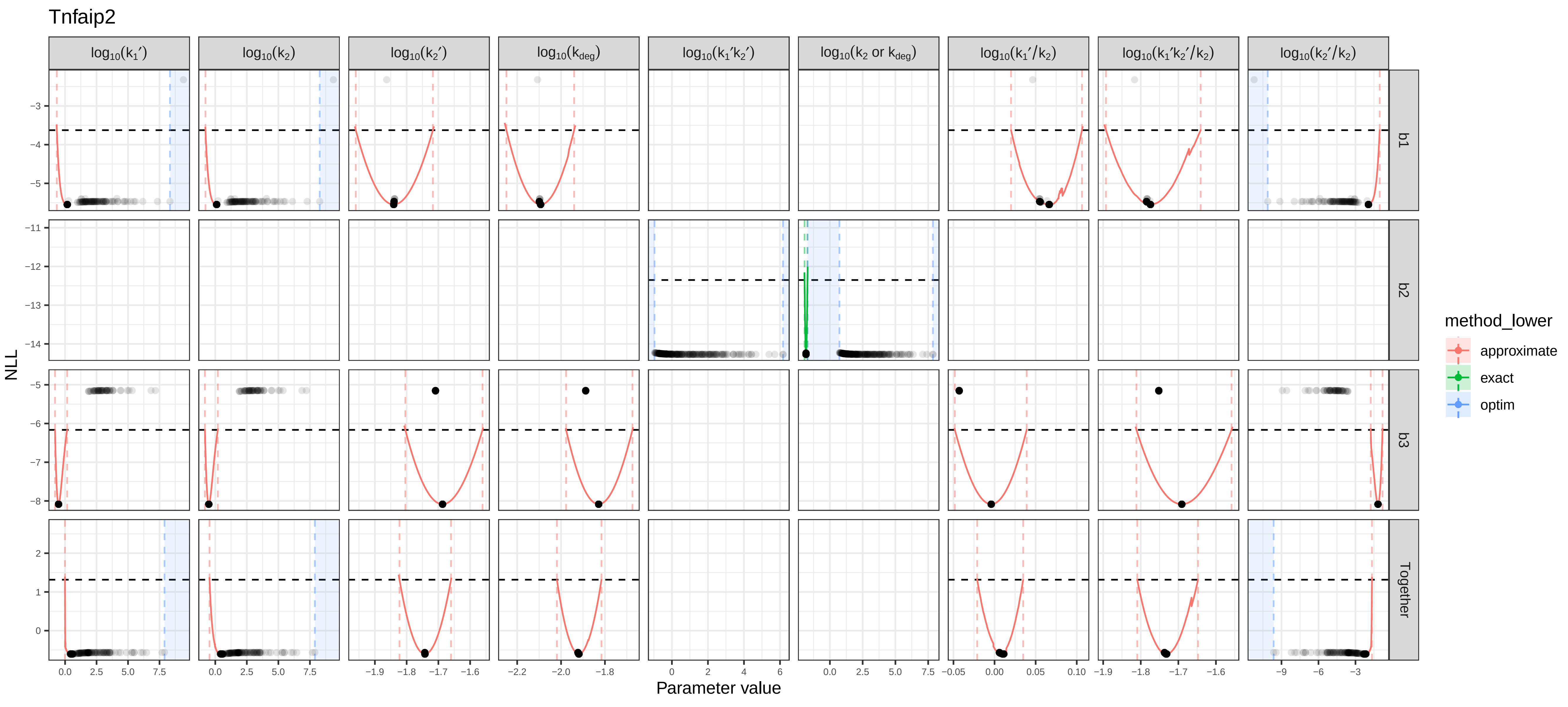

Replicate	Par	Best value	CI95 LB	CI95 UB	Method LB	Method UB
Together	$\log_{10}(k_1')$	0.5305	−0.007085	> 7.9	approximate	optim
Together	$\log_{10}(k_2)$	0.5202	−0.4561	> 7.894	approximate	optim
Together	$\log_{10}(k_2')$	−1.742	−1.822	−1.66	approximate	approximate
Together	$\log_{10}(k_{deg})$	−1.919	−2.019	−1.816	approximate	approximate
Together	$\log_{10}(k_1'/k_2)$	0.01025	−0.02114	0.03481	approximate	approximate
Together	$\log_{10}(k_1'k_2'/k_2)$	−1.732	−1.809	−1.648	approximate	approximate
Together	$\log_{10}(k_2'/k_2)$	−2.263	< −9.637	−1.663	optim	approximate
b1	$\log_{10}(k_1')$	0.1778	−0.6562	> 8.332	approximate	optim
b1	$\log_{10}(k_2)$	0.1115	−0.779	> 8.277	approximate	optim
b1	$\log_{10}(k_2')$	−1.841	−1.96	−1.717	approximate	approximate
b1	$\log_{10}(k_{deg})$	−2.093	−2.249	−1.94	approximate	approximate
b1	$\log_{10}(k_1'/k_2)$	0.06636	0.02004	0.1063	approximate	approximate
b1	$\log_{10}(k_1'k_2'/k_2)$	−1.774	−1.893	−1.641	approximate	approximate
b1	$\log_{10}(k_2'/k_2)$	−1.952	< −10.12	−1.037	optim	approximate
b2	$\log_{10}(k_1'k_2')$	2.88	< −0.9637	> 6.167	optim	optim
b2	$\log_{10}(k_2 \text{ or } k_{deg})$	4.574	0.7271	> 7.861	optim	optim
b2	$\log_{10}(k_2 \text{ or } k_{deg})$	−1.824	−1.936	−1.71	exact	exact
b3	$\log_{10}(k_1')$	−0.5173	−0.7999	0.1645	approximate	approximate
b3	$\log_{10}(k_2)$	−0.5132	−0.8214	0.2029	approximate	approximate
b3	$\log_{10}(k_2')$	−1.687	−1.804	−1.561	approximate	approximate
b3	$\log_{10}(k_{deg})$	−1.829	−1.977	−1.674	approximate	approximate
b3	$\log_{10}(k_1'/k_2)$	−0.004036	−0.04833	0.0391	approximate	approximate
b3	$\log_{10}(k_1'k_2'/k_2)$	−1.691	−1.811	−1.559	approximate	approximate
b3	$\log_{10}(k_2'/k_2)$	−1.174	−1.763	−0.8076	approximate	approximate

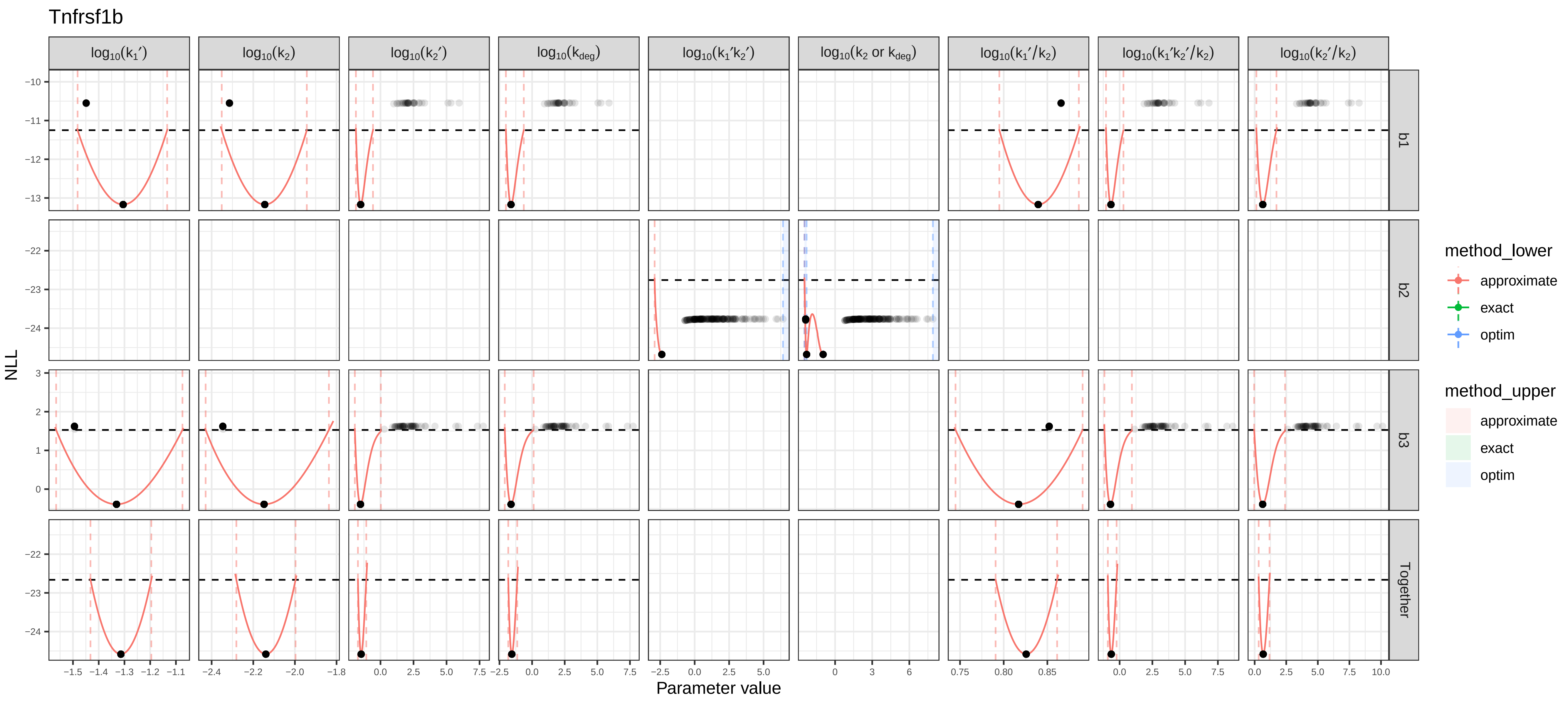

Replicate	Par	Best value	CI95 LB	CI95 UB	Method LB	Method UB
Together	$\log_{10}(k_1')$	−1.314	−1.432	−1.196	approximate	approximate
Together	$\log_{10}(k_2)$	−2.14	−2.281	−1.997	approximate	approximate
Together	$\log_{10}(k_2')$	−1.463	−1.708	−1.076	approximate	approximate
Together	$\log_{10}(k_{deg})$	−1.553	−1.825	−1.136	approximate	approximate
Together	$\log_{10}(k_1'/k_2)$	0.8257	0.7907	0.8612	approximate	approximate
Together	$\log_{10}(k_1'k_2'/k_2)$	−0.6369	−0.8996	−0.2309	approximate	approximate
Together	$\log_{10}(k_2'/k_2)$	0.677	0.3187	1.177	approximate	approximate
b1	$\log_{10}(k_1')$	−1.305	−1.482	−1.134	approximate	approximate
b1	$\log_{10}(k_2)$	−2.145	−2.351	−1.943	approximate	approximate
b1	$\log_{10}(k_2')$	−1.502	−1.861	−0.5683	approximate	approximate
b1	$\log_{10}(k_{deg})$	−1.614	−2.006	−0.6355	approximate	approximate
b1	$\log_{10}(k_1'/k_2)$	0.8395	0.795	0.886	approximate	approximate
b1	$\log_{10}(k_1'k_2'/k_2)$	−0.6621	−1.039	0.2933	approximate	approximate
b1	$\log_{10}(k_2'/k_2)$	0.643	0.1389	1.727	approximate	approximate
b2	$\log_{10}(k_1'k_2')$	−2.383	−2.905	> 6.412	approximate	optim
b2	$\log_{10}(k_2 \text{ or } k_{deg})$	−0.9667	−2.475	> 7.906	approximate	optim
b2	$\log_{10}(k_2 \text{ or } k_{deg})$	−2.302	−2.475	−2.302	approximate	optim
b3	$\log_{10}(k_1')$	−1.331	−1.565	−1.075	approximate	approximate
b3	$\log_{10}(k_2)$	−2.148	−2.429	−1.837	approximate	approximate
b3	$\log_{10}(k_2')$	−1.515	−1.941	0.02944	approximate	approximate
b3	$\log_{10}(k_{deg})$	−1.613	−2.093	0.1221	approximate	approximate
b3	$\log_{10}(k_1'/k_2)$	0.8171	0.7448	0.8904	approximate	approximate
b3	$\log_{10}(k_1'k_2'/k_2)$	−0.698	−1.16	0.9361	approximate	approximate
b3	$\log_{10}(k_2'/k_2)$	0.6329	−0.03891	2.406	approximate	approximate

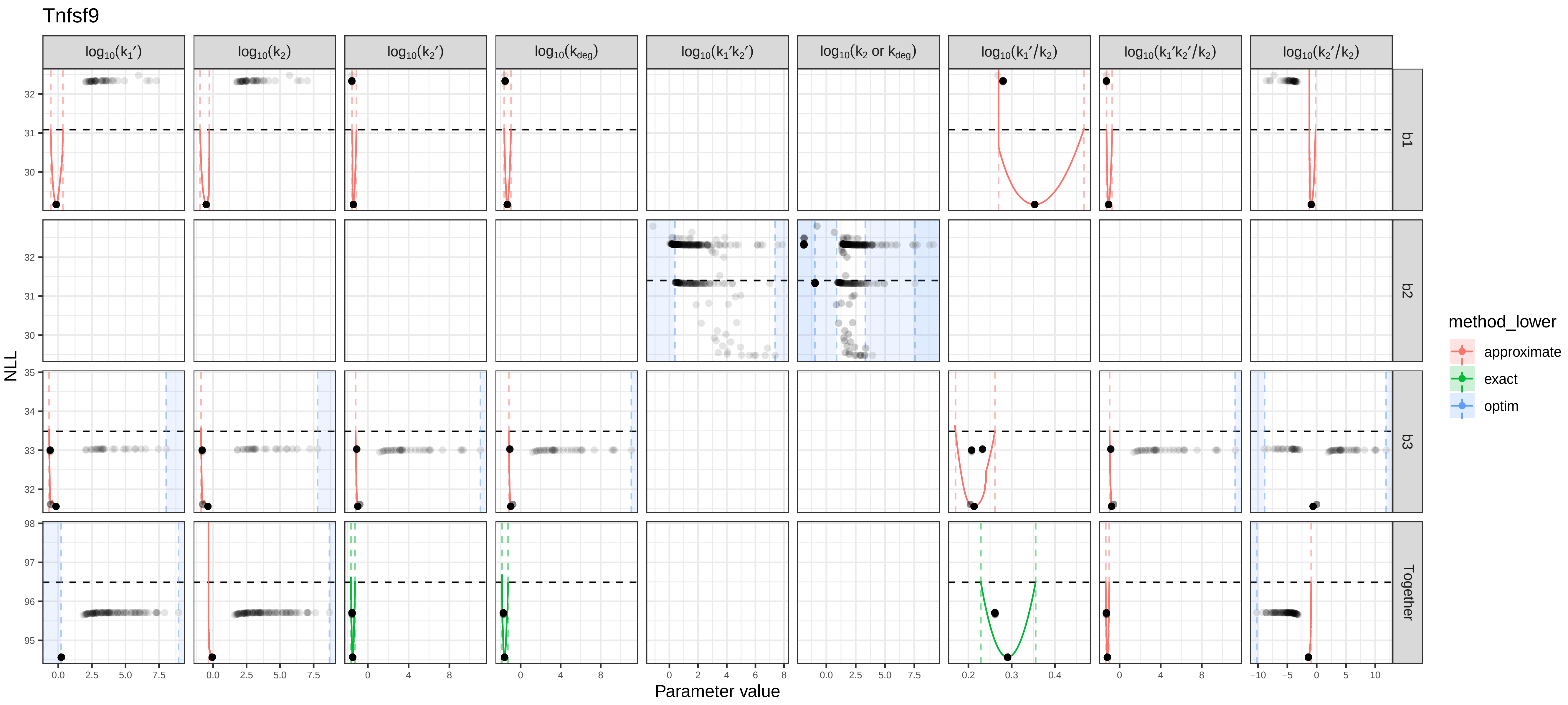

Replicate	Par	Best value	CI95 LB	CI95 UB	Method LB	Method UB
Together	$\log_{10}(k_1')$	0.2278	< 0.221	> 8.945	optim	optim
Together	$\log_{10}(k_2)$	−0.06281	−0.3389	> 8.682	approximate	optim
Together	$\log_{10}(k_2')$	−1.479	−1.645	−1.258	exact	exact
Together	$\log_{10}(k_{deg})$	−1.609	−1.86	−1.267	exact	exact
Together	$\log_{10}(k_1'/k_2)$	0.2906	0.2287	0.3554	exact	exact
Together	$\log_{10}(k_1'k_2'/k_2)$	−1.189	−1.345	−1.007	approximate	approximate
Together	$\log_{10}(k_2'/k_2)$	−1.416	< −10.23	−0.9291	optim	approximate
b1	$\log_{10}(k_1')$	−0.1538	−0.5638	0.3361	approximate	approximate
b1	$\log_{10}(k_2)$	−0.507	−0.9674	−0.2765	approximate	approximate
b1	$\log_{10}(k_2')$	−1.419	−1.544	−1.118	approximate	approximate
b1	$\log_{10}(k_{deg})$	−1.333	−1.644	−0.9662	approximate	approximate
b1	$\log_{10}(k_1'/k_2)$	0.3532	0.2696	0.4666	approximate	approximate
b1	$\log_{10}(k_1'k_2'/k_2)$	−1.065	−1.273	−0.7186	approximate	approximate
b1	$\log_{10}(k_2'/k_2)$	−0.9117	−1.24	−0.1879	approximate	approximate
b2	$\log_{10}(k_1'k_2')$	7.371	< 0.3971	> 7.371	optim	optim
b2	$\log_{10}(k_2 \text{ or } k_{deg})$	3.943	< 0.8684	> 7.565	optim	optim
b2	$\log_{10}(k_2 \text{ or } k_{deg})$	3.277	< −0.9674	> 3.325	optim	optim
b3	$\log_{10}(k_1')$	−0.1706	−0.6802	> 8.029	approximate	optim
b3	$\log_{10}(k_2)$	−0.3838	−0.8944	> 7.797	approximate	optim
b3	$\log_{10}(k_2')$	−0.9881	−1.195	> 11.04	approximate	optim
b3	$\log_{10}(k_{deg})$	−0.9879	−1.185	> 11.06	approximate	optim
b3	$\log_{10}(k_1'/k_2)$	0.2132	0.1701	0.2617	approximate	approximate
b3	$\log_{10}(k_1'k_2'/k_2)$	−0.7749	−0.9724	> 11.25	approximate	optim
b3	$\log_{10}(k_2'/k_2)$	−0.6043	< −8.884	> 11.85	optim	optim

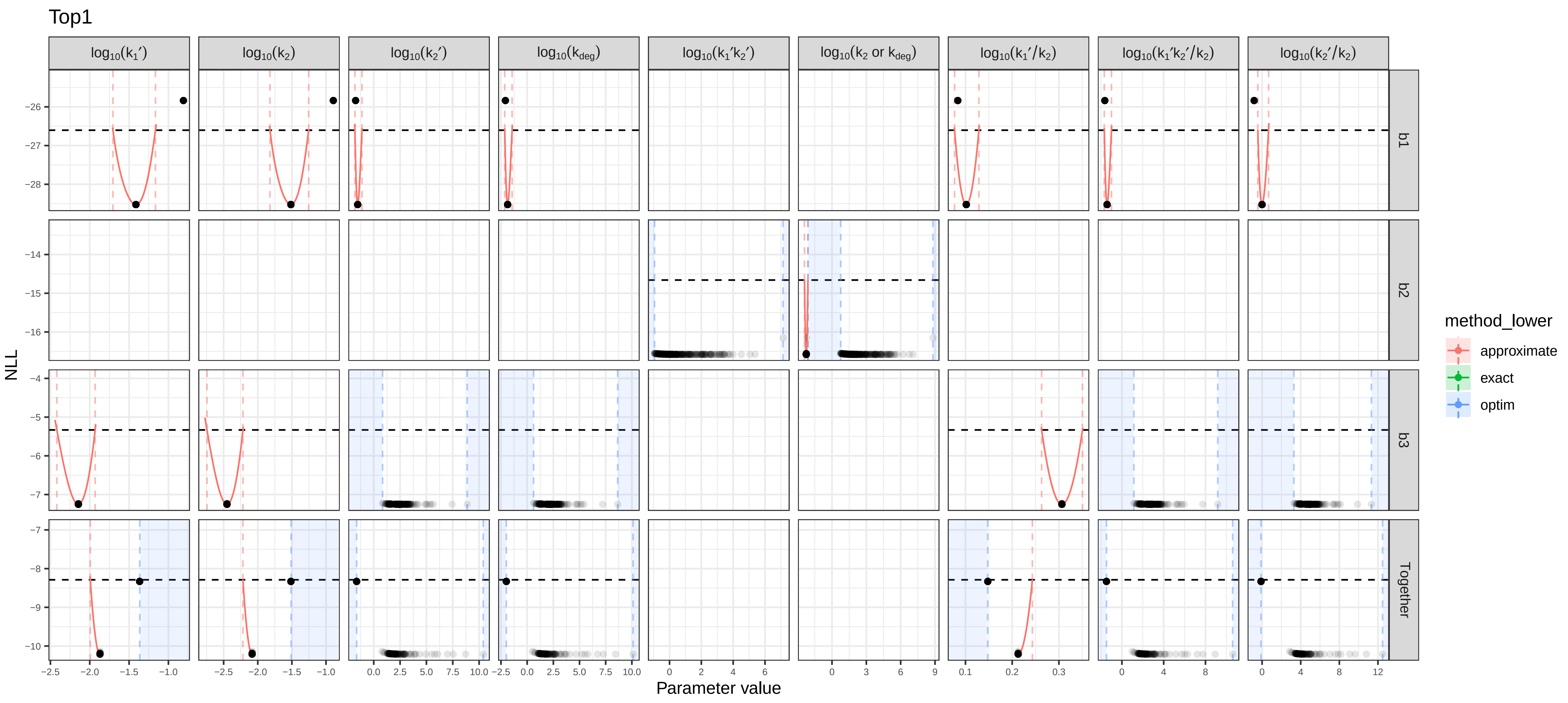

Replicate	Par	**Best value**	**CI95 LB**	**CI95 UB**	**Method LB**	**Method UB**
Together	$\log_{10}(k_1')$	−1.87	−1.993	> −1.364	approximate	optim
Together	$\log_{10}(k_2)$	−2.083	−2.218	> −1.512	approximate	optim
Together	$\log_{10}(k_2')$	6.076	< −1.625	> 10.39	optim	optim
Together	$\log_{10}(k_{deg})$	5.804	< −1.991	> 10.12	optim	optim
Together	$\log_{10}(k_1'/k_2)$	0.2128	< 0.1475	0.2432	optim	approximate
Together	$\log_{10}(k_1'k_2'/k_2)$	6.289	< −1.478	> 10.61	optim	optim
Together	$\log_{10}(k_2'/k_2)$	8.159	< −0.1135	> 12.48	optim	optim
b1	$\log_{10}(k_1')$	−1.413	−1.705	−1.165	approximate	approximate
b1	$\log_{10}(k_2)$	−1.515	−1.821	−1.254	approximate	approximate
b1	$\log_{10}(k_2')$	−1.52	−1.786	−1.113	approximate	approximate
b1	$\log_{10}(k_{deg})$	−1.865	−2.144	−1.436	approximate	approximate
b1	$\log_{10}(k_1'/k_2)$	0.1018	0.07663	0.1287	approximate	approximate
b1	$\log_{10}(k_1'k_2'/k_2)$	−1.419	−1.689	−0.9985	approximate	approximate
b1	$\log_{10}(k_2'/k_2)$	−0.005415	−0.4427	0.6698	approximate	approximate
b2	$\log_{10}(k_1'k_2')$	2.427	< −0.9409	> 7.133	optim	optim
b2	$\log_{10}(k_2 \text{ or } k_{deg})$	4.128	0.7601	> 8.818	optim	optim
b2	$\log_{10}(k_2 \text{ or } k_{deg})$	−2.25	−2.406	−2.101	approximate	approximate
b3	$\log_{10}(k_1')$	−2.145	−2.418	−1.931	approximate	approximate
b3	$\log_{10}(k_2)$	−2.451	−2.744	−2.217	approximate	approximate
b3	$\log_{10}(k_2')$	4.089	< 0.8402	> 8.871	optim	optim
b3	$\log_{10}(k_{deg})$	3.861	< 0.612	> 8.643	optim	optim
b3	$\log_{10}(k_1'/k_2)$	0.3064	0.263	0.3505	approximate	approximate
b3	$\log_{10}(k_1'k_2'/k_2)$	4.395	< 1.147	> 9.177	optim	optim
b3	$\log_{10}(k_2'/k_2)$	6.54	< 3.292	> 11.32	optim	optim

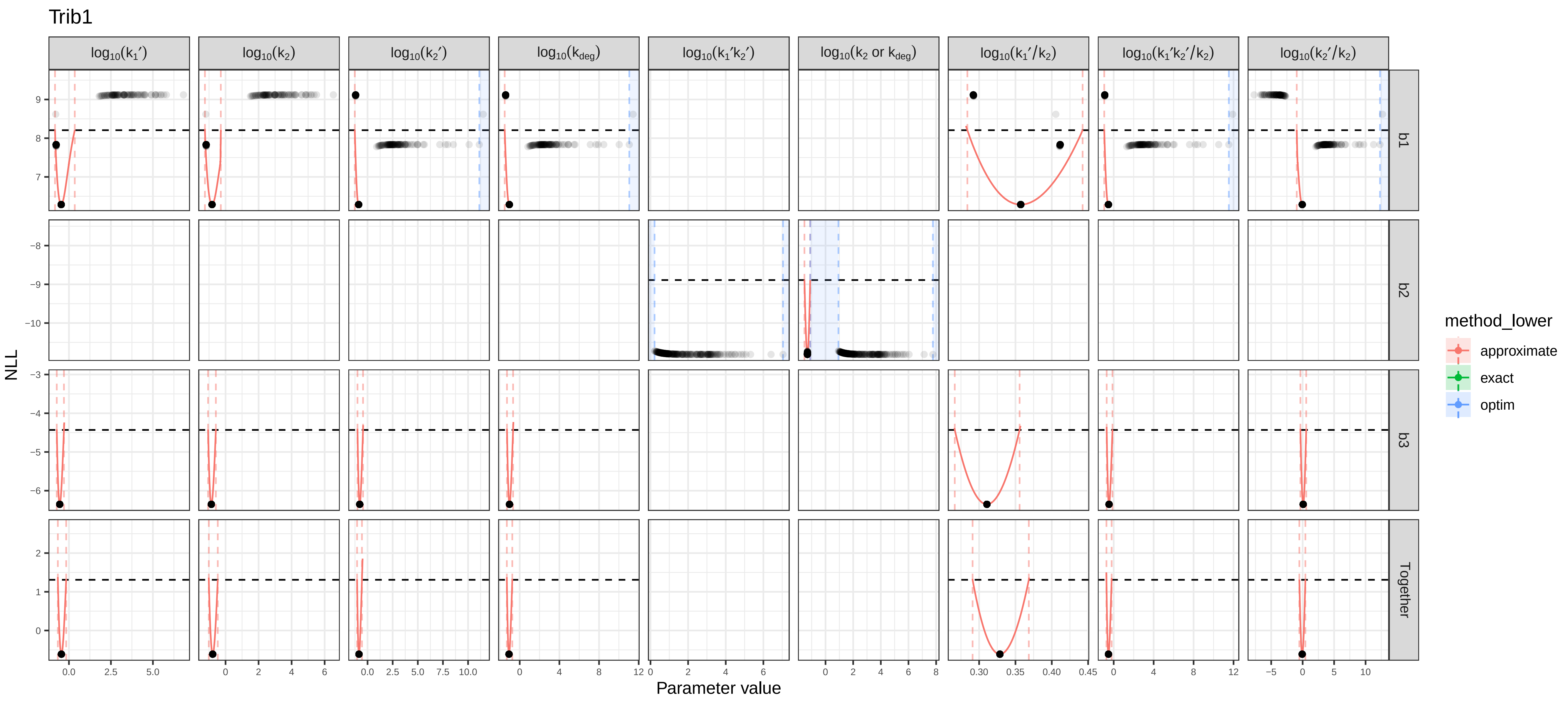

Replicate	Par	Best value	CI95 LB	CI95 UB	Method LB	Method UB
Together	$\log_{10}(k_1')$	−0.4497	−0.6602	−0.1642	approximate	approximate
Together	$\log_{10}(k_2)$	−0.7782	−1.014	−0.4709	approximate	approximate
Together	$\log_{10}(k_2')$	−0.8514	−1.04	−0.5562	approximate	approximate
Together	$\log_{10}(k_{deg})$	−1.079	−1.297	−0.748	approximate	approximate
Together	$\log_{10}(k_1'/k_2)$	0.3286	0.291	0.3684	approximate	approximate
Together	$\log_{10}(k_1'k_2'/k_2)$	−0.5228	−0.7252	−0.2055	approximate	approximate
Together	$\log_{10}(k_2'/k_2)$	−0.07315	−0.5263	0.4334	approximate	approximate
b1	$\log_{10}(k_1')$	−0.4607	−0.8287	0.3472	approximate	approximate
b1	$\log_{10}(k_2)$	−0.818	−1.248	−0.2863	approximate	approximate
b1	$\log_{10}(k_2')$	−0.8855	−1.269	> 11.12	approximate	optim
b1	$\log_{10}(k_{deg})$	−1.047	−1.514	> 11.05	approximate	optim
b1	$\log_{10}(k_1'/k_2)$	0.3573	0.2838	0.4424	approximate	approximate
b1	$\log_{10}(k_1'k_2'/k_2)$	−0.5282	−0.9461	> 11.53	approximate	optim
b1	$\log_{10}(k_2'/k_2)$	−0.06749	−0.9391	> 12.29	approximate	optim
b2	$\log_{10}(k_1'k_2')$	3.704	< 0.2165	> 7.044	optim	optim
b2	$\log_{10}(k_2 \text{ or } k_{deg})$	4.426	0.9347	> 7.766	optim	optim
b2	$\log_{10}(k_2 \text{ or } k_{deg})$	−1.31	−1.523	−1.107	approximate	approximate
b3	$\log_{10}(k_1')$	−0.5486	−0.7247	−0.2972	approximate	approximate
b3	$\log_{10}(k_2)$	−0.8593	−1.057	−0.5857	approximate	approximate
b3	$\log_{10}(k_2')$	−0.7768	−0.9998	−0.4484	approximate	approximate
b3	$\log_{10}(k_{deg})$	−1.029	−1.283	−0.6668	approximate	approximate
b3	$\log_{10}(k_1'/k_2)$	0.3107	0.2665	0.3557	approximate	approximate
b3	$\log_{10}(k_1'k_2'/k_2)$	−0.4661	−0.7011	−0.1213	approximate	approximate
b3	$\log_{10}(k_2'/k_2)$	0.08255	−0.3586	0.567	approximate	approximate

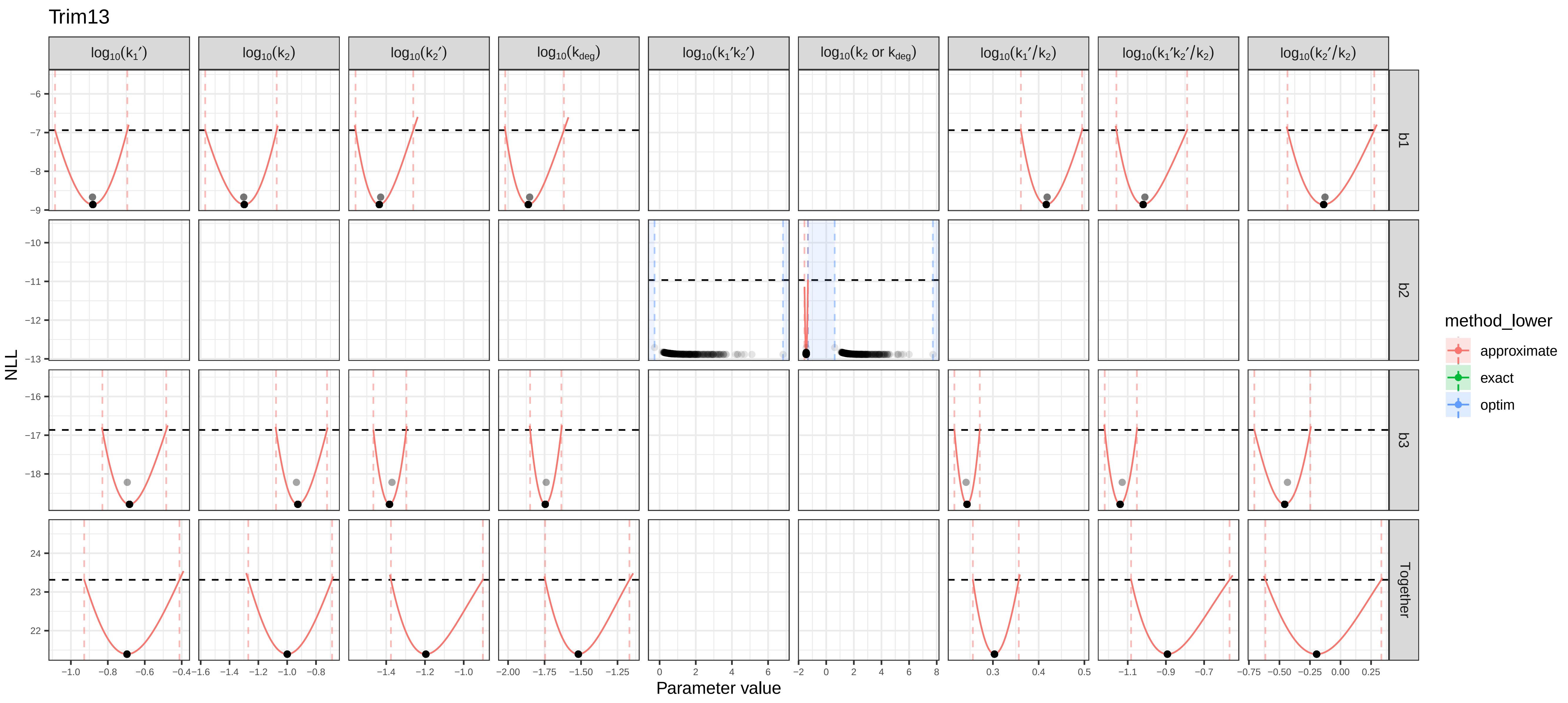

Replicate	Par	Best value	CI95 LB	CI95 UB	Method LB	Method UB
Together	$log_{10}(k_1')$	−0.6962	−0.9279	−0.4122	approximate	approximate
Together	$log_{10}(k_2)$	−0.9996	−1.27	−0.6886	approximate	approximate
Together	$log_{10}(k_2')$	−1.195	−1.376	−0.9011	approximate	approximate
Together	$log_{10}(k_{deg})$	−1.518	−1.746	−1.168	approximate	approximate
Together	$log_{10}(k_1'/k_2)$	0.3034	0.2562	0.3566	approximate	approximate
Together	$log_{10}(k_1'k_2'/k_2)$	−0.8919	−1.082	−0.5669	approximate	approximate
Together	$log_{10}(k_2'/k_2)$	−0.1957	−0.6153	0.3342	approximate	approximate
b1	$log_{10}(k_1')$	−0.8811	−1.086	−0.6946	approximate	approximate
b1	$log_{10}(k_2)$	−1.298	−1.569	−1.073	approximate	approximate
b1	$log_{10}(k_2')$	−1.436	−1.558	−1.26	approximate	approximate
b1	$log_{10}(k_{deg})$	−1.861	−2.019	−1.617	approximate	approximate
b1	$log_{10}(k_1'/k_2)$	0.4166	0.3612	0.4949	approximate	approximate
b1	$log_{10}(k_1'k_2'/k_2)$	−1.019	−1.159	−0.789	approximate	approximate
b1	$log_{10}(k_2'/k_2)$	−0.1381	−0.4342	0.2762	approximate	approximate
b2	$log_{10}(k_1'k_2')$	6.826	< −0.2791	> 6.826	optim	optim
b2	$log_{10}(k_2$ or $k_{deg})$	7.724	0.6144	> 7.724	optim	optim
b2	$log_{10}(k_2$ or $k_{deg})$	−1.451	−1.577	−1.324	approximate	approximate
b3	$log_{10}(k_1')$	−0.6827	−0.8293	−0.4838	approximate	approximate
b3	$log_{10}(k_2)$	−0.9261	−1.077	−0.7236	approximate	approximate
b3	$log_{10}(k_2')$	−1.383	−1.466	−1.296	approximate	approximate
b3	$log_{10}(k_{deg})$	−1.745	−1.849	−1.635	approximate	approximate
b3	$log_{10}(k_1'/k_2)$	0.2434	0.2157	0.2713	approximate	approximate
b3	$log_{10}(k_1'k_2'/k_2)$	−1.14	−1.22	−1.052	approximate	approximate
b3	$log_{10}(k_2'/k_2)$	−0.4571	−0.7056	−0.247	approximate	approximate

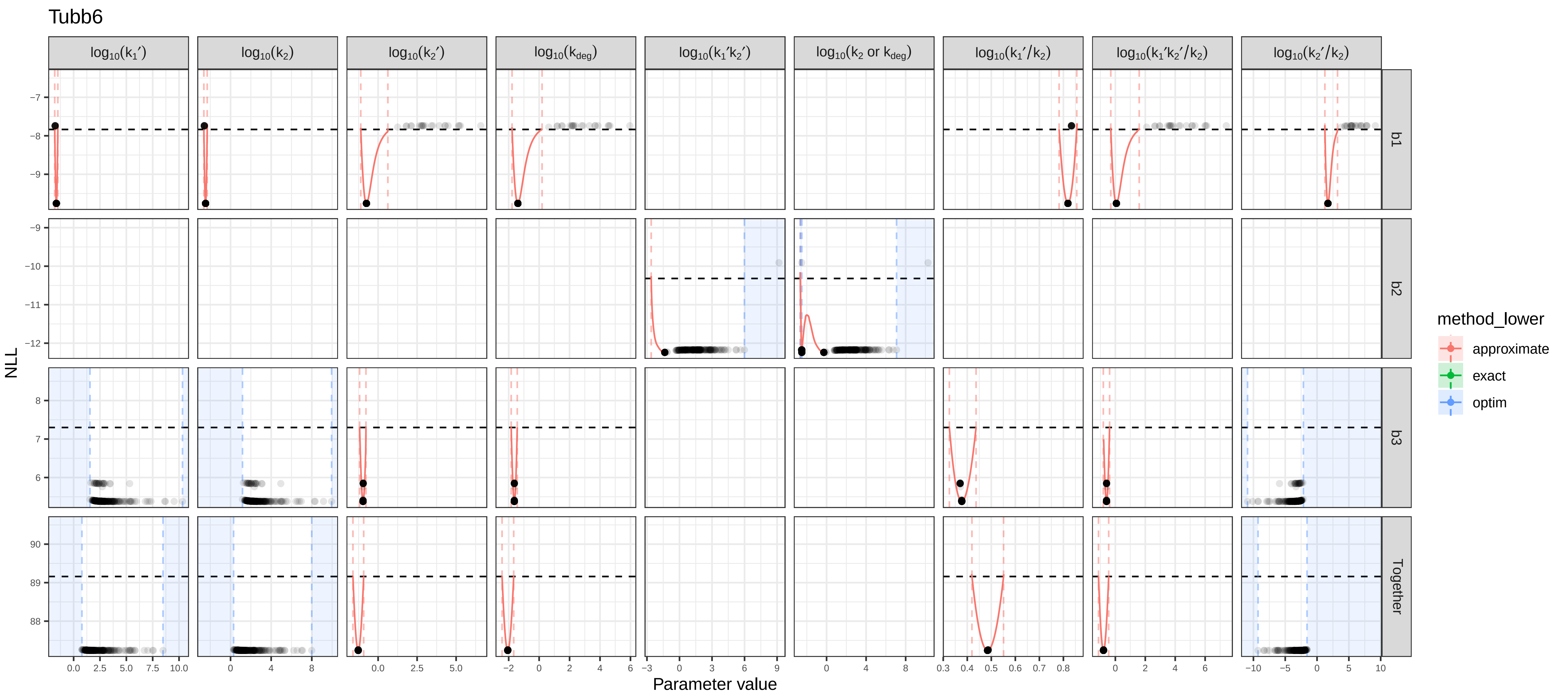

Replicate	Par	Best value	CI95 LB	CI95 UB	Method LB	Method UB
Together	$\log_{10}(k_1')$	5.225	< 0.7877	> 8.479	optim	optim
Together	$\log_{10}(k_2)$	4.741	< 0.3	> 7.995	optim	optim
Together	$\log_{10}(k_2')$	−1.278	−1.614	−0.9244	approximate	approximate
Together	$\log_{10}(k_{deg})$	−2.063	−2.435	−1.67	approximate	approximate
Together	$\log_{10}(k_1'/k_2)$	0.4843	0.4195	0.5512	approximate	approximate
Together	$\log_{10}(k_1'k_2'/k_2)$	−0.7934	−1.127	−0.4432	approximate	approximate
Together	$\log_{10}(k_2'/k_2)$	−6.019	< −9.273	> −1.573	optim	optim
b1	$\log_{10}(k_1')$	−1.64	−1.792	−1.498	approximate	approximate
b1	$\log_{10}(k_2)$	−2.459	−2.628	−2.298	approximate	approximate
b1	$\log_{10}(k_2')$	−0.7491	−1.112	0.625	approximate	approximate
b1	$\log_{10}(k_{deg})$	−1.397	−1.779	0.1931	approximate	approximate
b1	$\log_{10}(k_1'/k_2)$	0.8189	0.7822	0.8557	approximate	approximate
b1	$\log_{10}(k_1'k_2'/k_2)$	0.06985	−0.3041	1.595	approximate	approximate
b1	$\log_{10}(k_2'/k_2)$	1.71	1.232	3.218	approximate	approximate
b2	$\log_{10}(k_1'k_2')$	−1.359	−2.61	> 5.987	approximate	optim
b2	$\log_{10}(k_2 \text{ or } k_{deg})$	−0.2769	−2.655	> 7.081	approximate	optim
b2	$\log_{10}(k_2 \text{ or } k_{deg})$	−2.504	−2.655	−2.503	approximate	optim
b3	$\log_{10}(k_1')$	5.04	< 1.553	> 10.33	optim	optim
b3	$\log_{10}(k_2)$	4.662	< 1.176	> 9.951	optim	optim
b3	$\log_{10}(k_2')$	−0.9677	−1.186	−0.7794	approximate	approximate
b3	$\log_{10}(k_{deg})$	−1.632	−1.837	−1.437	approximate	approximate
b3	$\log_{10}(k_1'/k_2)$	0.3773	0.3255	0.4367	approximate	approximate
b3	$\log_{10}(k_1'k_2'/k_2)$	−0.5903	−0.8034	−0.3837	approximate	approximate
b3	$\log_{10}(k_2'/k_2)$	−5.63	< −10.92	> −2.142	optim	optim

Txnrd1

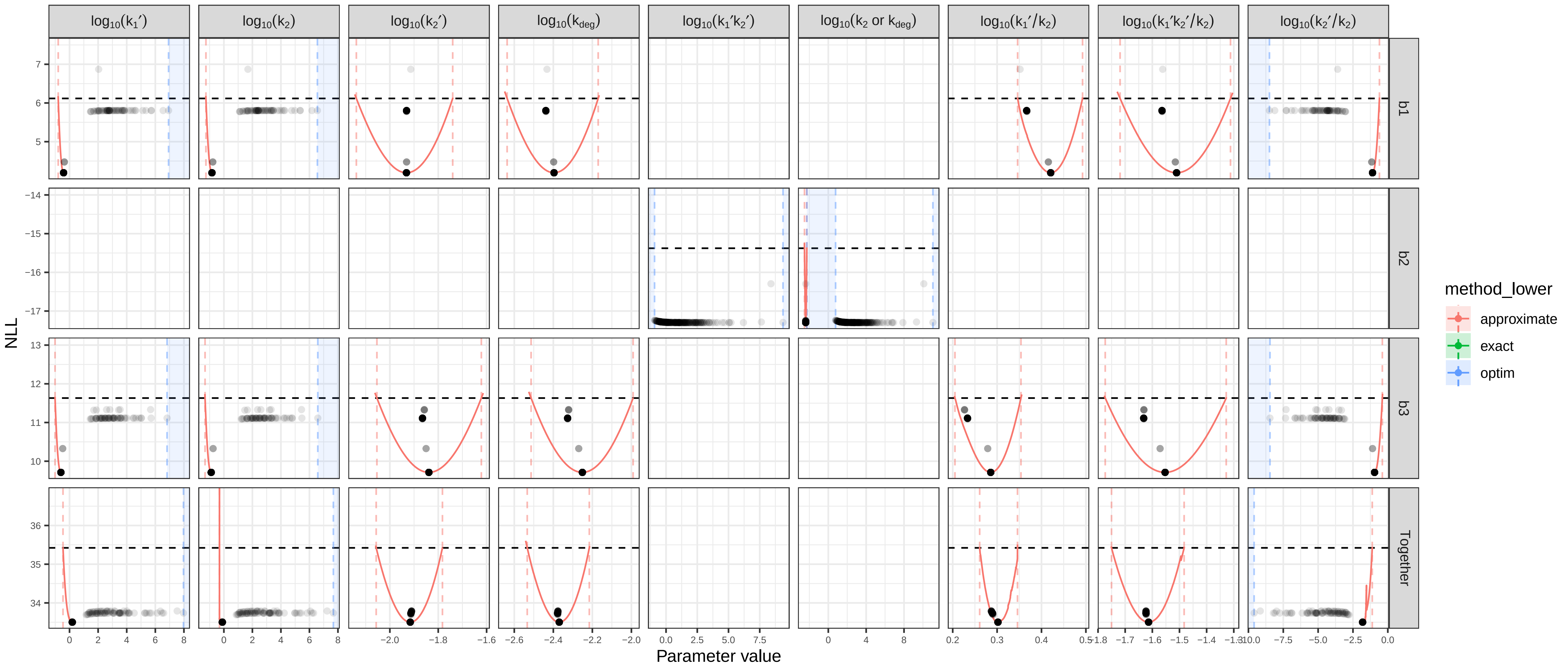

Replicate	Par	Best value	CI95 LB	CI95 UB	Method LB	Method UB
Together	$\log_{10}(k_1')$	0.1952	−0.4556	> 7.968	approximate	optim
Together	$\log_{10}(k_2)$	−0.1067	−0.308	> 7.679	approximate	optim
Together	$\log_{10}(k_2')$	−1.916	−2.057	−1.783	approximate	approximate
Together	$\log_{10}(k_{deg})$	−2.37	−2.534	−2.216	approximate	approximate
Together	$\log_{10}(k_1'/k_2)$	0.3019	0.2607	0.3459	approximate	approximate
Together	$\log_{10}(k_1'k_2'/k_2)$	−1.614	−1.75	−1.483	approximate	approximate
Together	$\log_{10}(k_2'/k_2)$	−1.809	< −9.592	−1.117	optim	approximate
b1	$\log_{10}(k_1')$	−0.4152	−0.7901	> 6.926	approximate	optim
b1	$\log_{10}(k_2)$	−0.836	−1.259	> 6.559	approximate	optim
b1	$\log_{10}(k_2')$	−1.931	−2.139	−1.74	approximate	approximate
b1	$\log_{10}(k_{deg})$	−2.396	−2.637	−2.17	approximate	approximate
b1	$\log_{10}(k_1'/k_2)$	0.4208	0.3462	0.4925	approximate	approximate
b1	$\log_{10}(k_1'k_2'/k_2)$	−1.511	−1.719	−1.312	approximate	approximate
b1	$\log_{10}(k_2'/k_2)$	−1.095	< −8.49	−0.6013	optim	approximate
b2	$\log_{10}(k_1'k_2')$	4.67	< −0.9187	> 9.363	optim	optim
b2	$\log_{10}(k_2 \text{ or } k_{deg})$	6.362	0.7724	> 11.05	optim	optim
b2	$\log_{10}(k_2 \text{ or } k_{deg})$	−2.387	−2.515	−2.27	approximate	approximate
b3	$\log_{10}(k_1')$	−0.6028	−1.012	> 6.821	approximate	optim
b3	$\log_{10}(k_2)$	−0.8884	−1.321	> 6.587	approximate	optim
b3	$\log_{10}(k_2')$	−1.839	−2.054	−1.622	approximate	approximate
b3	$\log_{10}(k_{deg})$	−2.251	−2.514	−1.991	approximate	approximate
b3	$\log_{10}(k_1'/k_2)$	0.2856	0.205	0.3537	approximate	approximate
b3	$\log_{10}(k_1'k_2'/k_2)$	−1.553	−1.773	−1.328	approximate	approximate
b3	$\log_{10}(k_2'/k_2)$	−0.9502	< −8.453	−0.3923	optim	approximate

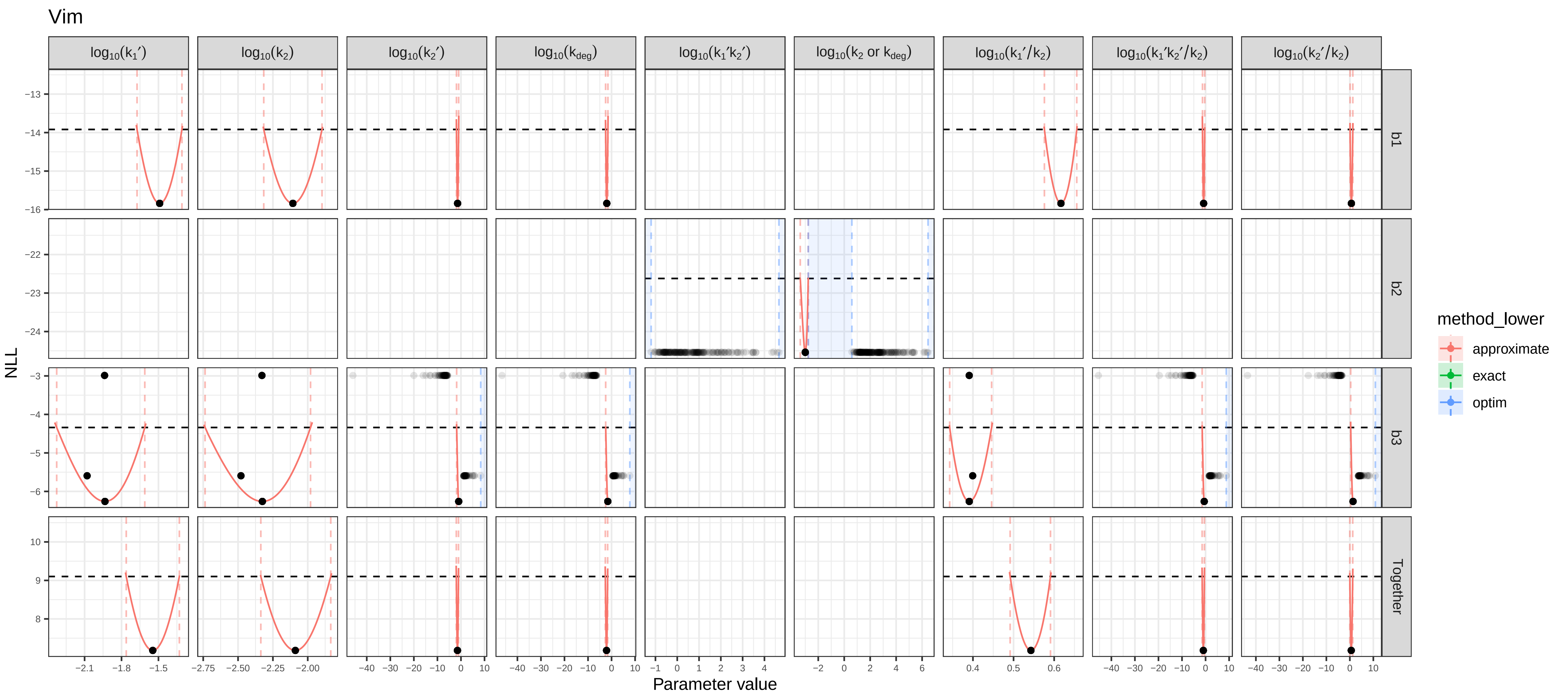

Replicate	Par	Best value	CI95 LB	CI95 UB	Method LB	Method UB
Together	$\log_{10}(k_1')$	−1.546	−1.762	−1.33	approximate	approximate
Together	$\log_{10}(k_2)$	−2.089	−2.337	−1.835	approximate	approximate
Together	$\log_{10}(k_2')$	−1.49	−1.985	−1.051	approximate	approximate
Together	$\log_{10}(k_{deg})$	−2.132	−2.65	−1.66	approximate	approximate
Together	$\log_{10}(k_1'/k_2)$	0.5428	0.4916	0.591	approximate	approximate
Together	$\log_{10}(k_1'k_2'/k_2)$	−0.9474	−1.452	−0.4866	approximate	approximate
Together	$\log_{10}(k_2'/k_2)$	0.5987	0.001126	1.224	approximate	approximate
b1	$\log_{10}(k_1')$	−1.49	−1.674	−1.309	approximate	approximate
b1	$\log_{10}(k_2)$	−2.107	−2.316	−1.898	approximate	approximate
b1	$\log_{10}(k_2')$	−1.443	−1.917	−0.9843	approximate	approximate
b1	$\log_{10}(k_{deg})$	−2.034	−2.53	−1.544	approximate	approximate
b1	$\log_{10}(k_1'/k_2)$	0.6167	0.5759	0.6557	approximate	approximate
b1	$\log_{10}(k_1'k_2'/k_2)$	−0.8264	−1.308	−0.3509	approximate	approximate
b1	$\log_{10}(k_2'/k_2)$	0.6637	0.1062	1.269	approximate	approximate
b2	$\log_{10}(k_1'k_2')$	3.475	< −1.198	> 4.665	optim	optim
b2	$\log_{10}(k_2 \text{ or } k_{deg})$	5.267	0.5908	> 6.457	optim	optim
b2	$\log_{10}(k_2 \text{ or } k_{deg})$	−3.001	−3.386	−2.77	approximate	approximate
b3	$\log_{10}(k_1')$	−1.935	−2.327	−1.611	approximate	approximate
b3	$\log_{10}(k_2)$	−2.326	−2.737	−1.98	approximate	approximate
b3	$\log_{10}(k_2')$	−0.9609	−1.836	> 8.384	approximate	optim
b3	$\log_{10}(k_{deg})$	−1.6	−2.496	> 7.762	approximate	optim
b3	$\log_{10}(k_1'/k_2)$	0.391	0.3427	0.446	approximate	approximate
b3	$\log_{10}(k_1'k_2'/k_2)$	−0.5699	−1.45	> 8.783	approximate	optim
b3	$\log_{10}(k_2'/k_2)$	1.365	0.3648	> 10.86	approximate	optim

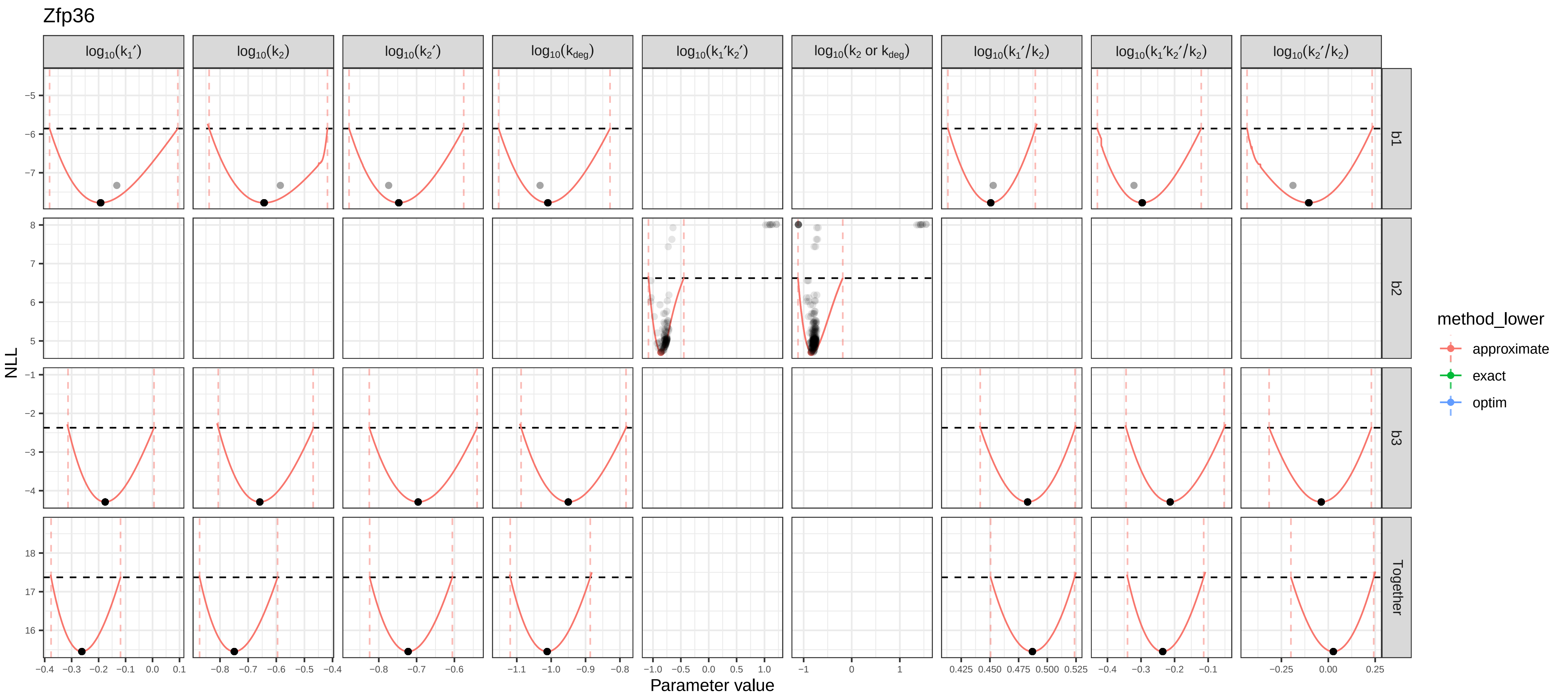

Replicate	Par	Best value	CI95 LB	CI95 UB	Method LB	Method UB
Together	$\log_{10}(k_1')$	−0.2622	−0.3763	−0.1188	approximate	approximate
Together	$\log_{10}(k_2)$	−0.7492	−0.8725	−0.5953	approximate	approximate
Together	$\log_{10}(k_2')$	−0.7223	−0.8242	−0.6054	approximate	approximate
Together	$\log_{10}(k_{deg})$	−1.012	−1.119	−0.8863	approximate	approximate
Together	$\log_{10}(k_1'/k_2)$	0.487	0.4506	0.5237	approximate	approximate
Together	$\log_{10}(k_1'k_2'/k_2)$	−0.2353	−0.3402	−0.1133	approximate	approximate
Together	$\log_{10}(k_2'/k_2)$	0.02688	−0.1992	0.2423	approximate	approximate
b1	$\log_{10}(k_1')$	−0.1928	−0.3818	0.09369	approximate	approximate
b1	$\log_{10}(k_2)$	−0.6436	−0.8387	−0.4181	approximate	approximate
b1	$\log_{10}(k_2')$	−0.747	−0.8787	−0.5753	approximate	approximate
b1	$\log_{10}(k_{deg})$	−1.009	−1.153	−0.8288	approximate	approximate
b1	$\log_{10}(k_1'/k_2)$	0.4508	0.4134	0.4895	approximate	approximate
b1	$\log_{10}(k_1'k_2'/k_2)$	−0.2963	−0.4299	−0.1206	approximate	approximate
b1	$\log_{10}(k_2'/k_2)$	−0.1034	−0.433	0.2338	approximate	approximate
b2	$\log_{10}(k_1'k_2')$	−0.8577	−1.081	−0.4462	approximate	approximate
b2	$\log_{10}(k_2 \text{ or } k_{deg})$	−0.8061	−1.117	−0.1864	approximate	approximate
b2	$\log_{10}(k_2 \text{ or } k_{deg})$	−0.8424	−1.117	−0.1864	approximate	approximate
b3	$\log_{10}(k_1')$	−0.1757	−0.3135	0.005446	approximate	approximate
b3	$\log_{10}(k_2)$	−0.6585	−0.8062	−0.4689	approximate	approximate
b3	$\log_{10}(k_2')$	−0.696	−0.8252	−0.5399	approximate	approximate
b3	$\log_{10}(k_{deg})$	−0.9503	−1.087	−0.7823	approximate	approximate
b3	$\log_{10}(k_1'/k_2)$	0.4828	0.4416	0.5242	approximate	approximate
b3	$\log_{10}(k_1'k_2'/k_2)$	−0.2131	−0.3445	−0.05244	approximate	approximate
b3	$\log_{10}(k_2'/k_2)$	−0.03745	−0.3155	0.2298	approximate	approximate

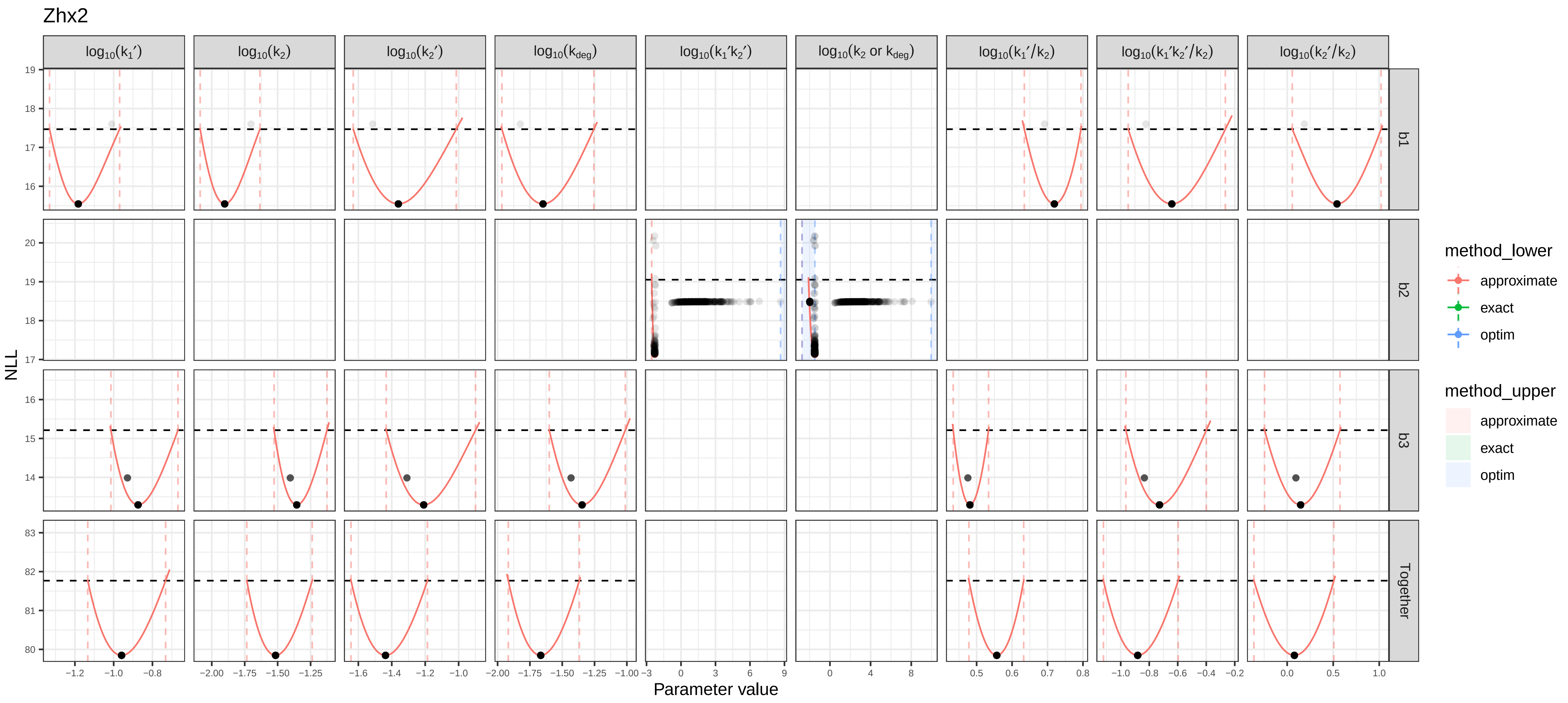

Replicate	Par	Best value	CI95 LB	CI95 UB	Method LB	Method UB
Together	$\log_{10}(k_1')$	−0.9592	−1.134	−0.732	approximate	approximate
Together	$\log_{10}(k_2)$	−1.516	−1.734	−1.238	approximate	approximate
Together	$\log_{10}(k_2')$	−1.438	−1.644	−1.187	approximate	approximate
Together	$\log_{10}(k_{deg})$	−1.666	−1.917	−1.368	approximate	approximate
Together	$\log_{10}(k_1'/k_2)$	0.5568	0.4783	0.6328	approximate	approximate
Together	$\log_{10}(k_1'k_2'/k_2)$	−0.8808	−1.121	−0.5972	approximate	approximate
Together	$\log_{10}(k_2'/k_2)$	0.07841	−0.3611	0.5073	approximate	approximate
b1	$\log_{10}(k_1')$	−1.183	−1.331	−0.9692	approximate	approximate
b1	$\log_{10}(k_2)$	−1.902	−2.088	−1.635	approximate	approximate
b1	$\log_{10}(k_2')$	−1.361	−1.63	−1.014	approximate	approximate
b1	$\log_{10}(k_{deg})$	−1.648	−1.967	−1.255	approximate	approximate
b1	$\log_{10}(k_1'/k_2)$	0.7193	0.6345	0.7944	approximate	approximate
b1	$\log_{10}(k_1'k_2'/k_2)$	−0.6414	−0.9476	−0.266	approximate	approximate
b1	$\log_{10}(k_2'/k_2)$	0.5411	0.05574	1.02	approximate	approximate
b2	$\log_{10}(k_1'k_2')$	−2.283	−2.553	> 8.659	approximate	optim
b2	$\log_{10}(k_2 \text{ or } k_{deg})$	−1.48	−2.746	> 9.946	approximate	optim
b2	$\log_{10}(k_2 \text{ or } k_{deg})$	−1.512	−2.746	−1.476	approximate	optim
b3	$\log_{10}(k_1')$	−0.8742	−1.014	−0.6682	approximate	approximate
b3	$\log_{10}(k_2)$	−1.355	−1.527	−1.126	approximate	approximate
b3	$\log_{10}(k_2')$	−1.209	−1.433	−0.8987	approximate	approximate
b3	$\log_{10}(k_{deg})$	−1.346	−1.6	−1.012	approximate	approximate
b3	$\log_{10}(k_1'/k_2)$	0.4811	0.4339	0.5336	approximate	approximate
b3	$\log_{10}(k_1'k_2'/k_2)$	−0.7278	−0.9637	−0.3994	approximate	approximate
b3	$\log_{10}(k_2'/k_2)$	0.1463	−0.2454	0.574	approximate	approximate

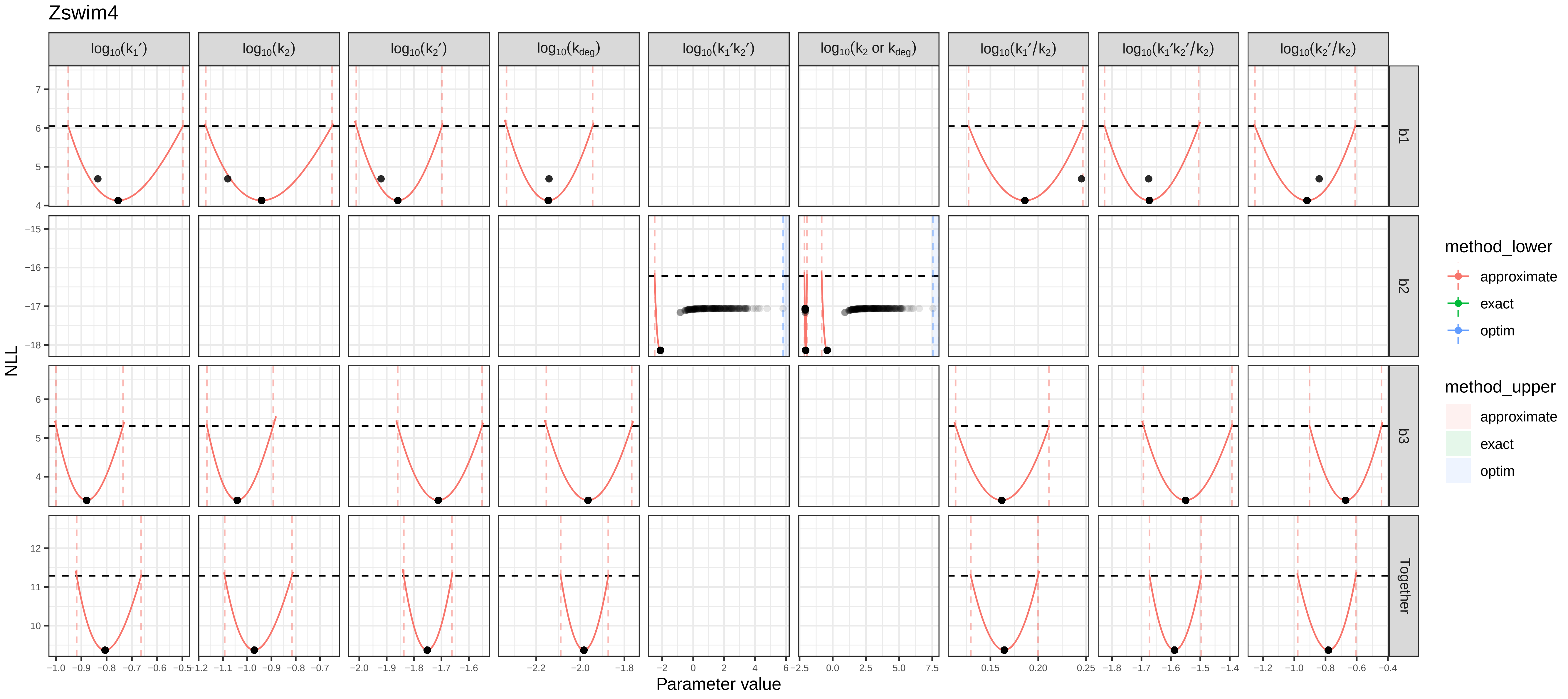

Replicate	Par	**Best value**	**CI95 LB**	**CI95 UB**	**Method LB**	**Method UB**
Together	$\log_{10}(k_1')$	−0.8061	−0.9186	−0.6632	approximate	approximate
Together	$\log_{10}(k_2)$	−0.9705	−1.093	−0.8157	approximate	approximate
Together	$\log_{10}(k_2')$	−1.752	−1.838	−1.662	approximate	approximate
Together	$\log_{10}(k_{deg})$	−1.984	−2.089	−1.873	approximate	approximate
Together	$\log_{10}(k_1'/k_2)$	0.1645	0.1294	0.1999	approximate	approximate
Together	$\log_{10}(k_1'k_2'/k_2)$	−1.588	−1.673	−1.497	approximate	approximate
Together	$\log_{10}(k_2'/k_2)$	−0.7815	−0.9784	−0.6057	approximate	approximate
b1	$\log_{10}(k_1')$	−0.7543	−0.9519	−0.498	approximate	approximate
b1	$\log_{10}(k_2)$	−0.9403	−1.171	−0.6509	approximate	approximate
b1	$\log_{10}(k_2')$	−1.859	−2.011	−1.698	approximate	approximate
b1	$\log_{10}(k_{deg})$	−2.145	−2.335	−1.944	approximate	approximate
b1	$\log_{10}(k_1'/k_2)$	0.186	0.1272	0.2466	approximate	approximate
b1	$\log_{10}(k_1'k_2'/k_2)$	−1.673	−1.825	−1.506	approximate	approximate
b1	$\log_{10}(k_2'/k_2)$	−0.9189	−1.253	−0.6093	approximate	approximate
b2	$\log_{10}(k_1'k_2')$	−2.118	−2.49	> 5.807	approximate	optim
b2	$\log_{10}(k_2 \text{ or } k_{deg})$	−0.4119	−0.8362	> 7.55	approximate	optim
b2	$\log_{10}(k_2 \text{ or } k_{deg})$	−2.041	−2.13	−1.947	approximate	approximate
b3	$\log_{10}(k_1')$	−0.8791	−1	−0.7346	approximate	approximate
b3	$\log_{10}(k_2)$	−1.041	−1.166	−0.8925	approximate	approximate
b3	$\log_{10}(k_2')$	−1.712	−1.86	−1.552	approximate	approximate
b3	$\log_{10}(k_{deg})$	−1.965	−2.154	−1.767	approximate	approximate
b3	$\log_{10}(k_1'/k_2)$	0.1619	0.1135	0.2113	approximate	approximate
b3	$\log_{10}(k_1'k_2'/k_2)$	−1.55	−1.693	−1.393	approximate	approximate
b3	$\log_{10}(k_2'/k_2)$	−0.6708	−0.9025	−0.4408	approximate	approximate
