# Supplementary figures and images for: Kinetics of mRNA nuclear export regulate innate immune response gene expression
Source: Nat Commun. 2022 Nov 23;13:7197. doi: 10.1038/s41467-022-34635-5 (PMC9691726; doi:10.1038/s41467-022-34635-5)

Source Data File 1: uncropped Western blot images for Fig. S1A

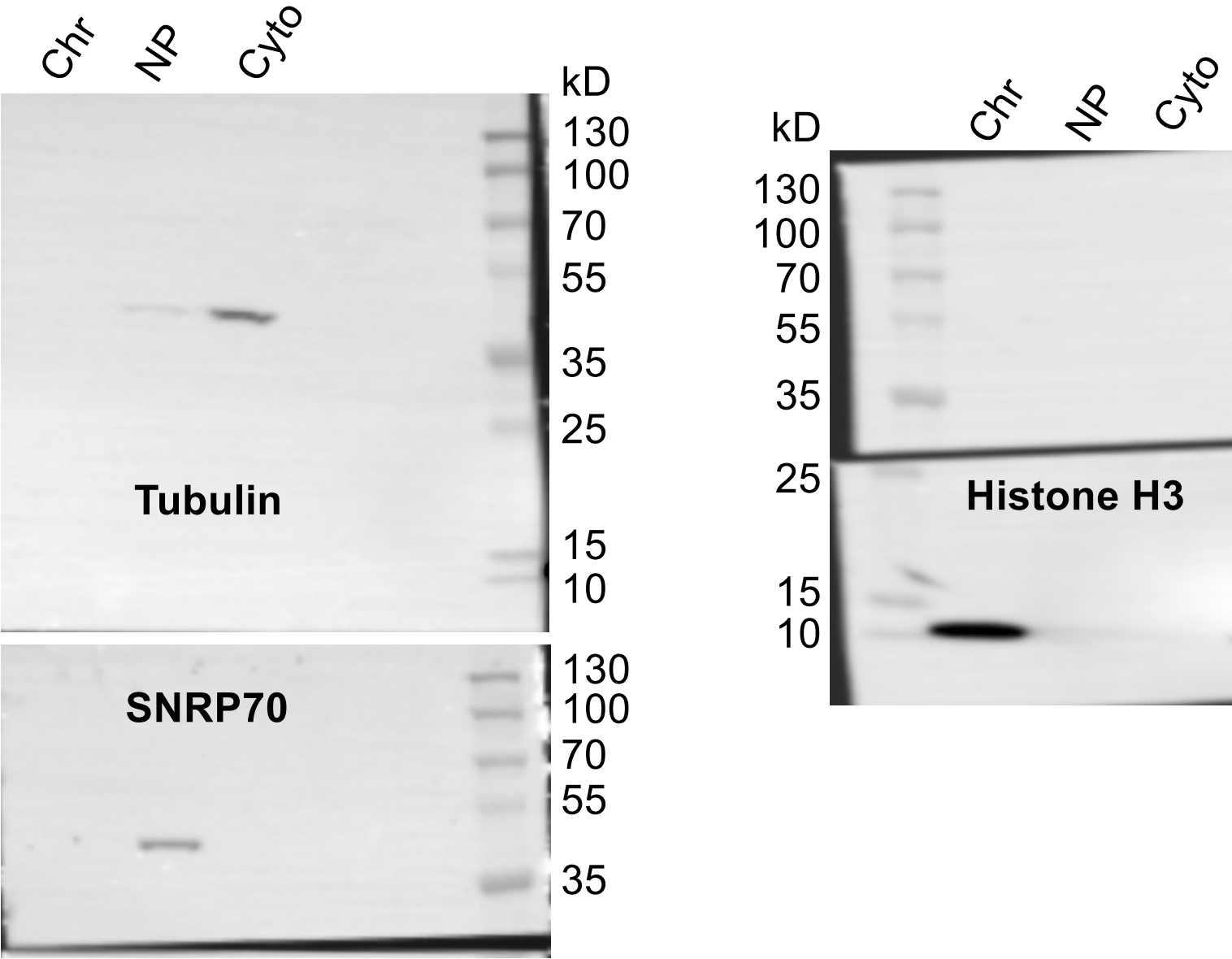

Supplement: Supplementary file 7 — Source Data [file 41467_2022_34635_MOESM7_ESM.zip › Source Data File 1 uncropped immunoblots.pdf]
