# Supplementary material for: Kinetics of mRNA nuclear export regulate innate immune response gene expression
Source: Nat Commun. 2022 Nov 23;13:7197. doi: 10.1038/s41467-022-34635-5 (PMC9691726; doi:10.1038/s41467-022-34635-5)
Supplement: Supplementary file 7 — Source Data [file Source Data File 4 Profile_dMod_merged_naive_with_optim_points_v2.pdf › Source Data File 4 Profile_dMod_merged_naive_with_optim_points_v2.pdf]

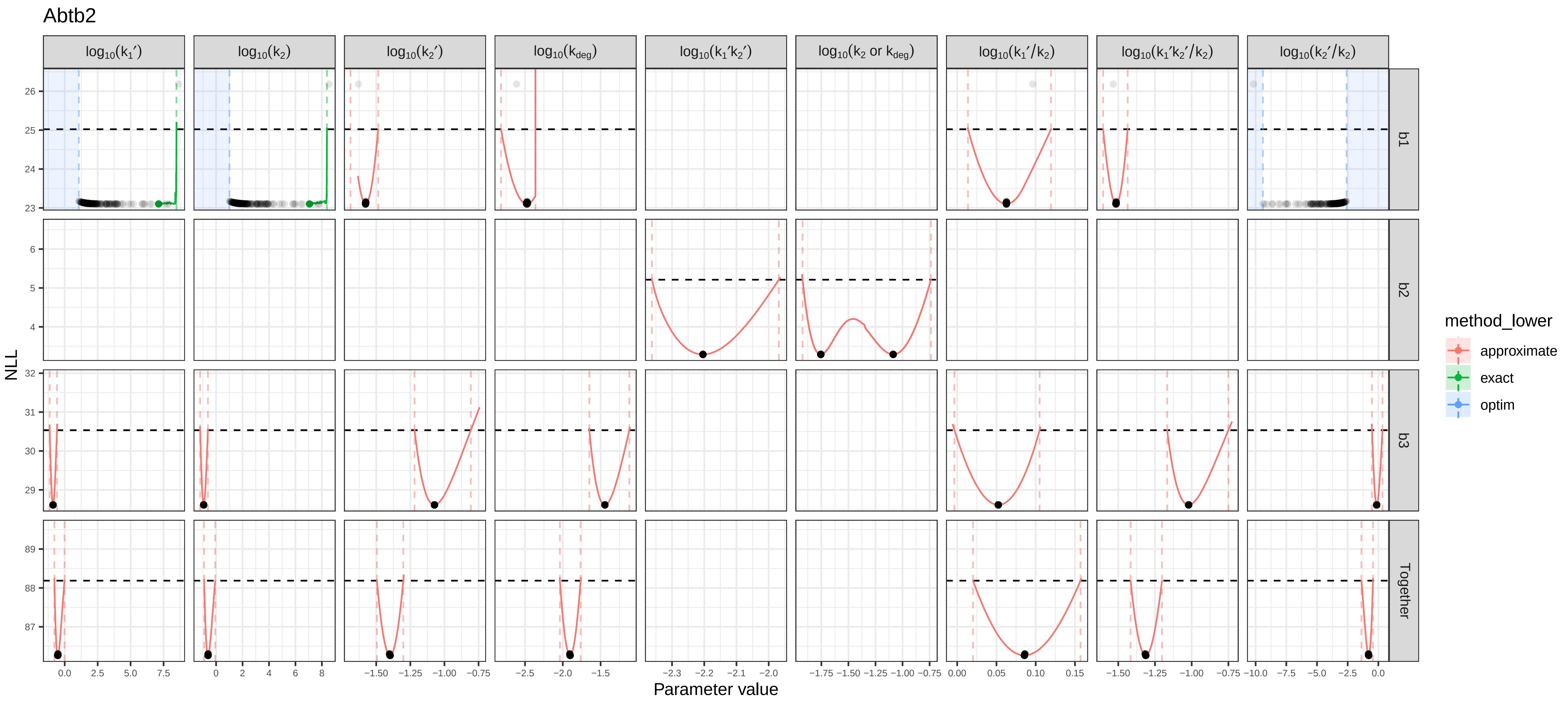

Replicate	Par	Best value	CI95 LB	CI95 UB	Method LB	Method UB
Together	$\log_{10}(k_1')$	−0.5242	−0.7825	−0.01361	approximate	approximate
Together	$\log_{10}(k_2)$	−0.6098	−0.9051	−0.06322	approximate	approximate
Together	$\log_{10}(k_2')$	−1.398	−1.495	−1.301	approximate	approximate
Together	$\log_{10}(k_{deg})$	−1.903	−2.038	−1.764	approximate	approximate
Together	$\log_{10}(k_1'/k_2)$	0.08559	0.02014	0.1571	approximate	approximate
Together	$\log_{10}(k_1'k_2'/k_2)$	−1.313	−1.416	−1.202	approximate	approximate
Together	$\log_{10}(k_2'/k_2)$	−0.7887	−1.38	−0.4358	approximate	approximate
b1	$\log_{10}(k_1')$	7.123	< 1.072	8.48	optim	exact
b1	$\log_{10}(k_2)$	7.06	< 1.01	8.384	optim	exact
b1	$\log_{10}(k_2')$	−1.578	−1.688	−1.485	approximate	approximate
b1	$\log_{10}(k_{deg})$	−2.474	−2.816	−2.361	approximate	approximate
b1	$\log_{10}(k_1'/k_2)$	0.06264	0.01374	0.1195	approximate	approximate
b1	$\log_{10}(k_1'k_2'/k_2)$	−1.515	−1.603	−1.436	approximate	approximate
b1	$\log_{10}(k_2'/k_2)$	−8.638	< −9.406	> −2.586	optim	optim
b2	$\log_{10}(k_1'k_2')$	−2.204	−2.362	−1.968	approximate	approximate
b2	$\log_{10}(k_2 \text{ or } k_{deg})$	−1.086	−1.923	−0.7375	approximate	approximate
b2	$\log_{10}(k_2 \text{ or } k_{deg})$	−1.755	−1.923	−0.7375	approximate	approximate
b3	$\log_{10}(k_1')$	−0.8858	−1.14	−0.5844	approximate	approximate
b3	$\log_{10}(k_2)$	−0.9381	−1.206	−0.6168	approximate	approximate
b3	$\log_{10}(k_2')$	−1.073	−1.219	−0.8065	approximate	approximate
b3	$\log_{10}(k_{deg})$	−1.443	−1.649	−1.12	approximate	approximate
b3	$\log_{10}(k_1'/k_2)$	0.05237	−0.003488	0.105	approximate	approximate
b3	$\log_{10}(k_1'k_2'/k_2)$	−1.021	−1.167	−0.7499	approximate	approximate
b3	$\log_{10}(k_2'/k_2)$	−0.1351	−0.5195	0.3404	approximate	approximate

Acod1

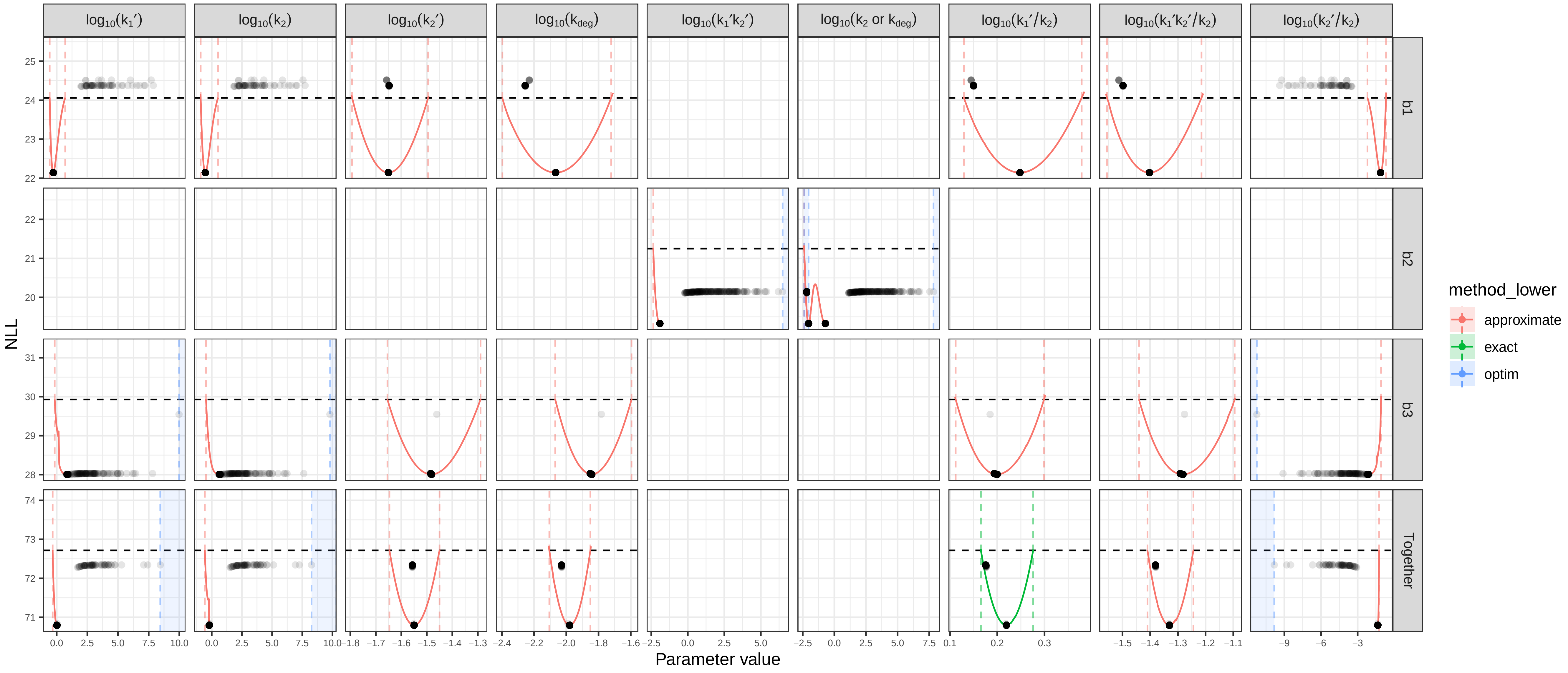

Replicate	Par	Best value	CI95 LB	CI95 UB	Method LB	Method UB
Together	$\log_{10}(k_1')$	0.01929	−0.3296	> 8.445	approximate	optim
Together	$\log_{10}(k_2)$	−0.2003	−0.5679	> 8.269	approximate	optim
Together	$\log_{10}(k_2')$	−1.55	−1.647	−1.45	approximate	approximate
Together	$\log_{10}(k_{deg})$	−1.979	−2.105	−1.851	approximate	approximate
Together	$\log_{10}(k_1'/k_2)$	0.2196	0.1654	0.276	exact	exact
Together	$\log_{10}(k_1'k_2'/k_2)$	−1.33	−1.41	−1.244	approximate	approximate
Together	$\log_{10}(k_2'/k_2)$	−1.35	< −9.826	−1.245	optim	approximate
b1	$\log_{10}(k_1')$	−0.2802	−0.5715	0.6945	approximate	approximate
b1	$\log_{10}(k_2)$	−0.5282	−0.9136	0.5275	approximate	approximate
b1	$\log_{10}(k_2')$	−1.651	−1.793	−1.495	approximate	approximate
b1	$\log_{10}(k_{deg})$	−2.066	−2.396	−1.721	approximate	approximate
b1	$\log_{10}(k_1'/k_2)$	0.248	0.1294	0.3786	approximate	approximate
b1	$\log_{10}(k_1'k_2'/k_2)$	−1.403	−1.556	−1.215	approximate	approximate
b1	$\log_{10}(k_2'/k_2)$	−1.122	−2.2	−0.6784	approximate	approximate
b2	$\log_{10}(k_1'k_2')$	−1.91	−2.363	> 6.492	approximate	optim
b2	$\log_{10}(k_2 \text{ or } k_{deg})$	−0.7083	−2.38	> 7.838	approximate	optim
b2	$\log_{10}(k_2 \text{ or } k_{deg})$	−2.035	−2.38	−2.034	approximate	optim
b3	$\log_{10}(k_1')$	0.8692	−0.1628	> 9.975	approximate	optim
b3	$\log_{10}(k_2)$	0.6697	−0.4701	> 9.79	approximate	optim
b3	$\log_{10}(k_2')$	−1.482	−1.654	−1.289	approximate	approximate
b3	$\log_{10}(k_{deg})$	−1.842	−2.069	−1.595	approximate	approximate
b3	$\log_{10}(k_1'/k_2)$	0.1995	0.1121	0.299	approximate	approximate
b3	$\log_{10}(k_1'k_2'/k_2)$	−1.282	−1.44	−1.095	approximate	approximate
b3	$\log_{10}(k_2'/k_2)$	−2.151	< −11.25	−1.085	optim	approximate

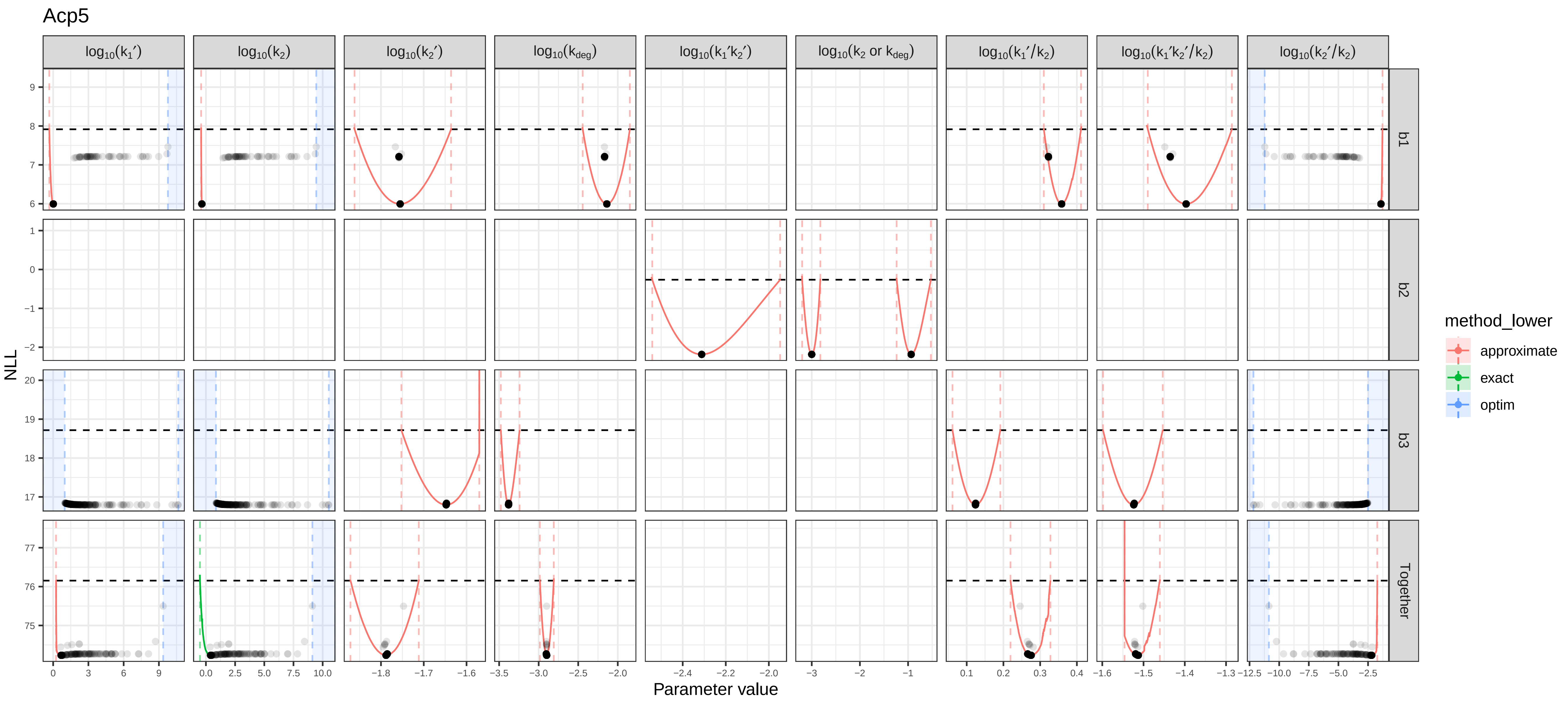

Replicate	Par	Best value	CI95 LB	CI95 UB	Method LB	Method UB
Together	$\log_{10}(k_1')$	0.7184	0.2441	> 9.364	approximate	optim
Together	$\log_{10}(k_2)$	0.4436	−0.5092	> 9.118	exact	optim
Together	$\log_{10}(k_2')$	−1.788	−1.871	−1.712	approximate	approximate
Together	$\log_{10}(k_{deg})$	−2.899	−2.985	−2.809	approximate	approximate
Together	$\log_{10}(k_1'/k_2)$	0.2748	0.2192	0.328	approximate	approximate
Together	$\log_{10}(k_1'k_2'/k_2)$	−1.513	−1.546	−1.46	approximate	approximate
Together	$\log_{10}(k_2'/k_2)$	−2.232	< −10.87	−1.721	optim	approximate
b1	$\log_{10}(k_1')$	0.01403	−0.3314	> 9.767	approximate	optim
b1	$\log_{10}(k_2)$	−0.3444	−0.4112	> 9.449	approximate	optim
b1	$\log_{10}(k_2')$	−1.755	−1.862	−1.636	approximate	approximate
b1	$\log_{10}(k_{deg})$	−2.138	−2.443	−1.847	approximate	approximate
b1	$\log_{10}(k_1'/k_2)$	0.3584	0.31	0.4112	approximate	approximate
b1	$\log_{10}(k_1'k_2'/k_2)$	−1.397	−1.49	−1.286	approximate	approximate
b1	$\log_{10}(k_2'/k_2)$	−1.411	< −11.22	−1.282	optim	approximate
b2	$\log_{10}(k_1'k_2')$	−2.312	−2.541	−1.948	approximate	approximate
b2	$\log_{10}(k_2 \text{ or } k_{deg})$	−0.9358	−1.238	−0.5263	approximate	approximate
b2	$\log_{10}(k_2 \text{ or } k_{deg})$	−3	−3.2	−2.822	approximate	approximate
b3	$\log_{10}(k_1')$	5.728	< 0.9847	> 10.65	optim	optim
b3	$\log_{10}(k_2)$	5.605	< 0.8601	> 10.52	optim	optim
b3	$\log_{10}(k_2')$	−1.648	−1.752	−1.57	approximate	approximate
b3	$\log_{10}(k_{deg})$	−3.38	−3.479	−3.241	approximate	approximate
b3	$\log_{10}(k_1'/k_2)$	0.1239	0.06128	0.1915	approximate	approximate
b3	$\log_{10}(k_1'k_2'/k_2)$	−1.524	−1.598	−1.453	approximate	approximate
b3	$\log_{10}(k_2'/k_2)$	−7.252	< −12.17	> −2.507	optim	optim

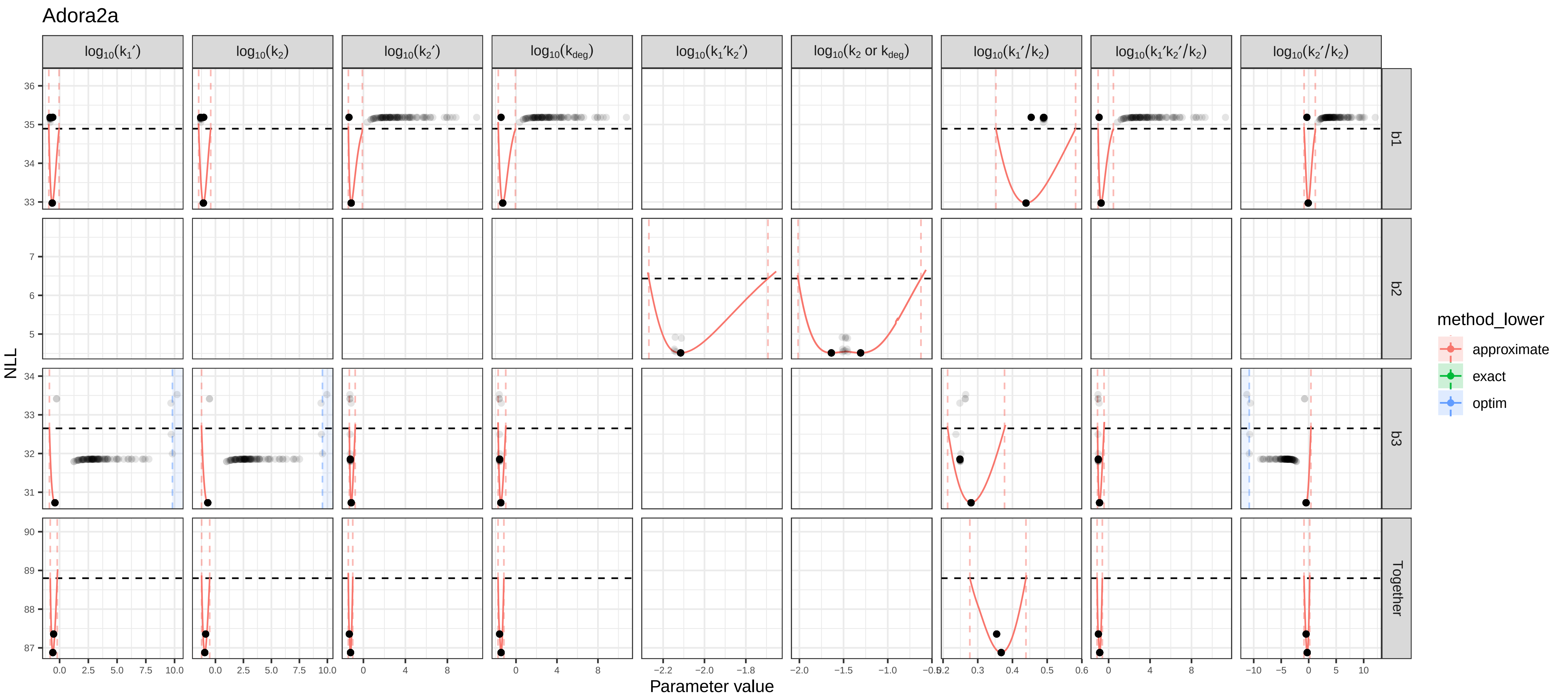

Replicate	Par	Best value	CI95 LB	CI95 UB	Method LB	Method UB
Together	$\log_{10}(k_1')$	−0.5873	−0.8137	−0.2039	approximate	approximate
Together	$\log_{10}(k_2)$	−0.9549	−1.235	−0.5124	approximate	approximate
Together	$\log_{10}(k_2')$	−1.225	−1.438	−1.014	approximate	approximate
Together	$\log_{10}(k_{deg})$	−1.456	−1.753	−1.184	approximate	approximate
Together	$\log_{10}(k_1'/k_2)$	0.3676	0.2772	0.4389	approximate	approximate
Together	$\log_{10}(k_1'k_2'/k_2)$	−0.8569	−1.111	−0.6057	approximate	approximate
Together	$\log_{10}(k_2'/k_2)$	−0.2696	−0.8518	0.1889	approximate	approximate
b1	$\log_{10}(k_1')$	−0.6386	−0.9384	−0.04398	approximate	approximate
b1	$\log_{10}(k_2)$	−1.078	−1.5	−0.4225	approximate	approximate
b1	$\log_{10}(k_2')$	−1.162	−1.431	−0.09831	approximate	approximate
b1	$\log_{10}(k_{deg})$	−1.294	−1.73	−0.05845	approximate	approximate
b1	$\log_{10}(k_1'/k_2)$	0.4391	0.3523	0.5819	approximate	approximate
b1	$\log_{10}(k_1'k_2'/k_2)$	−0.7228	−1.015	0.4621	approximate	approximate
b1	$\log_{10}(k_2'/k_2)$	−0.08423	−0.8376	1.206	approximate	approximate
b2	$\log_{10}(k_1'k_2')$	−2.115	−2.269	−1.693	approximate	approximate
b2	$\log_{10}(k_2 \text{ or } k_{deg})$	−1.307	−2.015	−0.6243	approximate	approximate
b2	$\log_{10}(k_2 \text{ or } k_{deg})$	−1.638	−2.015	−0.6243	approximate	approximate
b3	$\log_{10}(k_1')$	−0.4046	−0.8861	> 9.812	approximate	optim
b3	$\log_{10}(k_2)$	−0.6854	−1.242	> 9.56	approximate	optim
b3	$\log_{10}(k_2')$	−1.158	−1.34	−0.7818	approximate	approximate
b3	$\log_{10}(k_{deg})$	−1.48	−1.744	−1.001	approximate	approximate
b3	$\log_{10}(k_1'/k_2)$	0.2808	0.2132	0.3771	approximate	approximate
b3	$\log_{10}(k_1'k_2'/k_2)$	−0.8769	−1.069	−0.4416	approximate	approximate
b3	$\log_{10}(k_2'/k_2)$	−0.4723	< −10.81	0.4071	optim	approximate

Adora2b

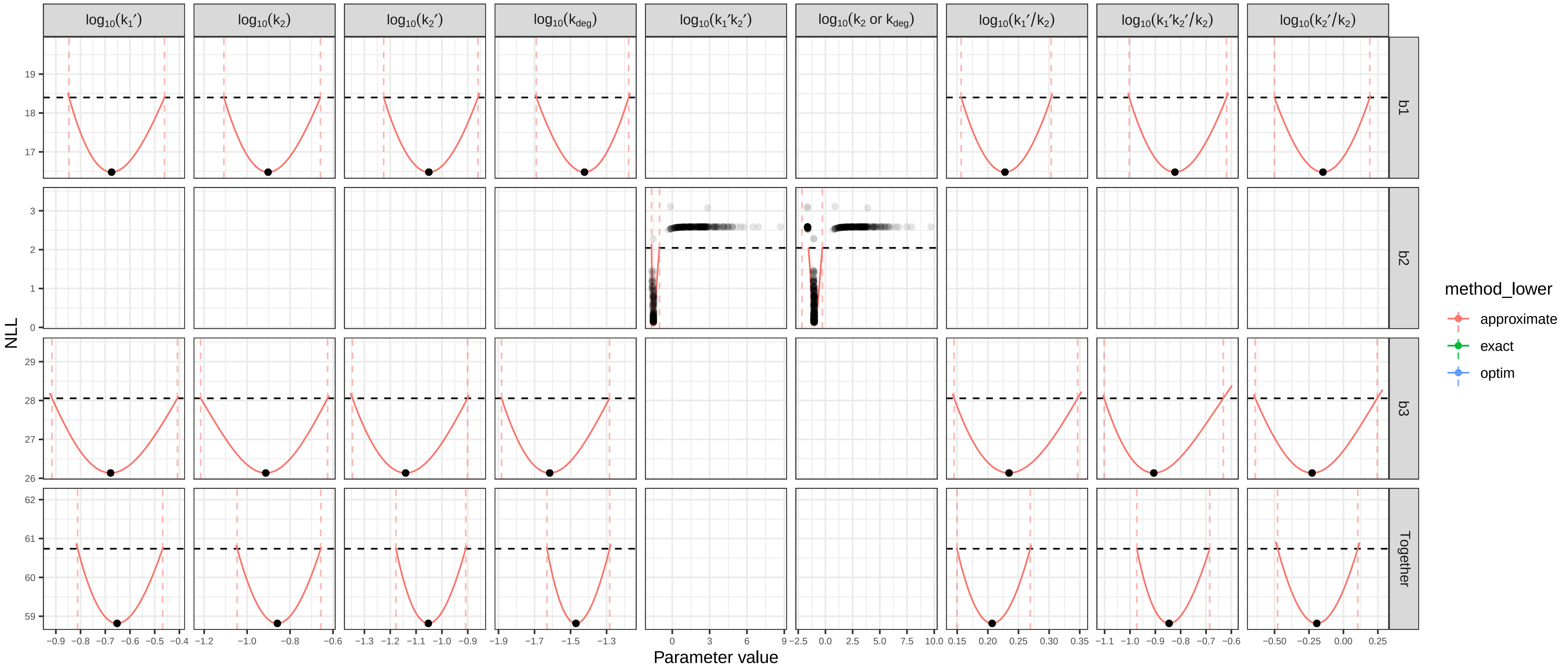

Replicate	Par	Best value	CI95 LB	CI95 UB	Method LB	Method UB
Together	$\log_{10}(k_1')$	−0.652	−0.8119	−0.4678	approximate	approximate
Together	$\log_{10}(k_2)$	−0.859	−1.046	−0.6569	approximate	approximate
Together	$\log_{10}(k_2')$	−1.053	−1.178	−0.9078	approximate	approximate
Together	$\log_{10}(k_{deg})$	−1.47	−1.631	−1.283	approximate	approximate
Together	$\log_{10}(k_1'/k_2)$	0.207	0.1496	0.2693	approximate	approximate
Together	$\log_{10}(k_1'k_2'/k_2)$	−0.8456	−0.9731	−0.6852	approximate	approximate
Together	$\log_{10}(k_2'/k_2)$	−0.1936	−0.478	0.1044	approximate	approximate
b1	$\log_{10}(k_1')$	−0.6742	−0.8465	−0.4603	approximate	approximate
b1	$\log_{10}(k_2)$	−0.902	−1.108	−0.6584	approximate	approximate
b1	$\log_{10}(k_2')$	−1.051	−1.225	−0.8604	approximate	approximate
b1	$\log_{10}(k_{deg})$	−1.423	−1.69	−1.178	approximate	approximate
b1	$\log_{10}(k_1'/k_2)$	0.2279	0.1564	0.3035	approximate	approximate
b1	$\log_{10}(k_1'k_2'/k_2)$	−0.8226	−1.003	−0.6187	approximate	approximate
b1	$\log_{10}(k_2'/k_2)$	−0.1485	−0.5007	0.1918	approximate	approximate
b2	$\log_{10}(k_1'k_2')$	−1.514	−1.663	−1.026	approximate	approximate
b2	$\log_{10}(k_2 \text{ or } k_{deg})$	−1.03	−2.149	−0.2777	approximate	approximate
b2	$\log_{10}(k_2 \text{ or } k_{deg})$	−1.045	−2.149	−0.2777	approximate	approximate
b3	$\log_{10}(k_1')$	−0.6785	−0.9157	−0.4079	approximate	approximate
b3	$\log_{10}(k_2)$	−0.9131	−1.216	−0.6252	approximate	approximate
b3	$\log_{10}(k_2')$	−1.141	−1.346	−0.9006	approximate	approximate
b3	$\log_{10}(k_{deg})$	−1.616	−1.883	−1.284	approximate	approximate
b3	$\log_{10}(k_1'/k_2)$	0.2346	0.145	0.3465	approximate	approximate
b3	$\log_{10}(k_1'k_2'/k_2)$	−0.9059	−1.102	−0.6315	approximate	approximate
b3	$\log_{10}(k_2'/k_2)$	−0.2274	−0.6404	0.2454	approximate	approximate

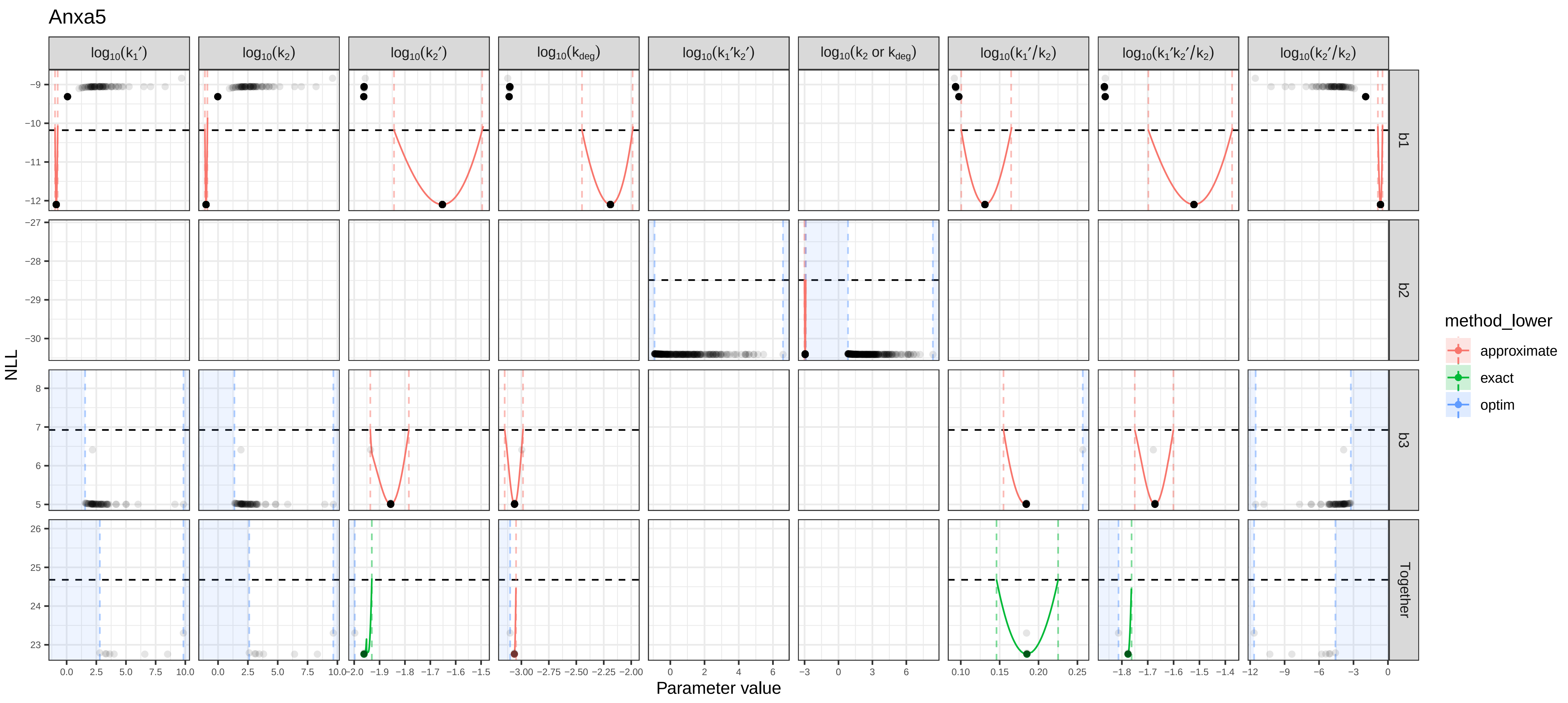

Replicate	Par	Best value	CI95 LB	CI95 UB	Method LB	Method UB
Together	$\log_{10}(k_1')$	8.508	< 2.798	> 9.84	optim	optim
Together	$\log_{10}(k_2)$	8.323	< 2.613	> 9.655	optim	optim
Together	$\log_{10}(k_2')$	−1.961	< −1.998	−1.93	optim	exact
Together	$\log_{10}(k_{deg})$	−3.064	< −3.102	−3.048	optim	approximate
Together	$\log_{10}(k_1'/k_2)$	0.185	0.146	0.2252	exact	exact
Together	$\log_{10}(k_1'k_2'/k_2)$	−1.776	< −1.813	−1.762	optim	exact
Together	$\log_{10}(k_2'/k_2)$	−10.28	< −11.65	> −4.574	optim	optim
b1	$\log_{10}(k_1')$	−0.8736	−0.9776	−0.7526	approximate	approximate
b1	$\log_{10}(k_2)$	−1.005	−1.101	−0.8909	approximate	approximate
b1	$\log_{10}(k_2')$	−1.652	−1.843	−1.496	approximate	approximate
b1	$\log_{10}(k_{deg})$	−2.189	−2.448	−1.987	approximate	approximate
b1	$\log_{10}(k_1'/k_2)$	0.1311	0.1006	0.1648	approximate	approximate
b1	$\log_{10}(k_1'k_2'/k_2)$	−1.521	−1.698	−1.374	approximate	approximate
b1	$\log_{10}(k_2'/k_2)$	−0.6477	−0.8758	−0.4734	approximate	approximate
b2	$\log_{10}(k_1'k_2')$	4.881	< −0.9217	> 6.589	optim	optim
b2	$\log_{10}(k_2 \text{ or } k_{deg})$	6.644	0.8402	> 8.352	optim	optim
b2	$\log_{10}(k_2 \text{ or } k_{deg})$	−2.95	−3.012	−2.897	approximate	approximate
b3	$\log_{10}(k_1')$	5.019	< 1.558	> 9.845	optim	optim
b3	$\log_{10}(k_2)$	4.835	< 1.374	> 9.661	optim	optim
b3	$\log_{10}(k_2')$	−1.856	−1.936	−1.785	approximate	approximate
b3	$\log_{10}(k_{deg})$	−3.062	−3.152	−2.985	approximate	approximate
b3	$\log_{10}(k_1'/k_2)$	0.1842	0.1549	> 0.2569	approximate	optim
b3	$\log_{10}(k_1'k_2'/k_2)$	−1.672	−1.75	−1.601	approximate	approximate
b3	$\log_{10}(k_2'/k_2)$	−6.691	< −11.52	> −3.23	optim	optim

Arl5b

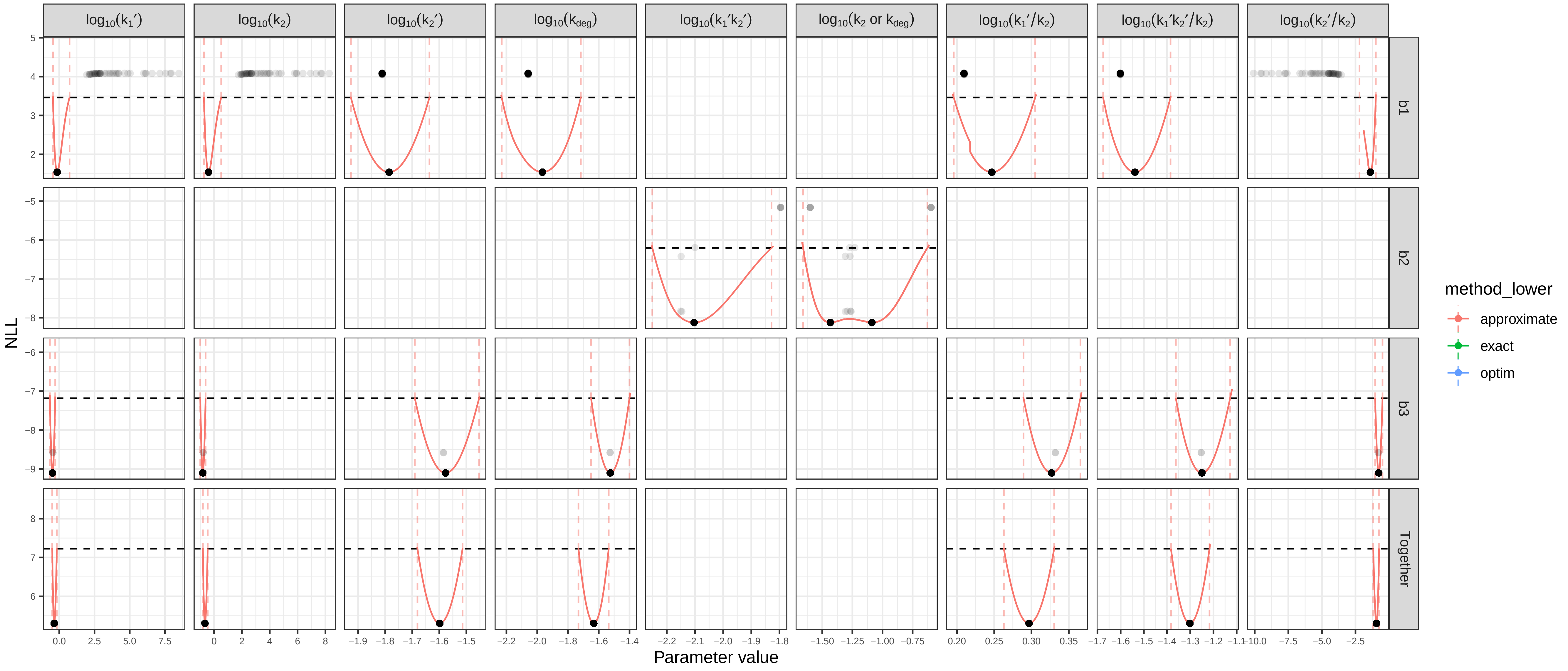

Replicate	Par	Best value	CI95 LB	CI95 UB	Method LB	Method UB
Together	$\log_{10}(k_1')$	−0.3591	−0.4996	−0.174	approximate	approximate
Together	$\log_{10}(k_2)$	−0.6558	−0.8057	−0.4597	approximate	approximate
Together	$\log_{10}(k_2')$	−1.598	−1.681	−1.514	approximate	approximate
Together	$\log_{10}(k_{deg})$	−1.632	−1.732	−1.535	approximate	approximate
Together	$\log_{10}(k_1'/k_2)$	0.2967	0.2629	0.3304	approximate	approximate
Together	$\log_{10}(k_1'k_2'/k_2)$	−1.302	−1.383	−1.217	approximate	approximate
Together	$\log_{10}(k_2'/k_2)$	−0.9424	−1.177	−0.7418	approximate	approximate
b1	$\log_{10}(k_1')$	−0.1493	−0.448	0.7269	approximate	approximate
b1	$\log_{10}(k_2)$	−0.3962	−0.7271	0.512	approximate	approximate
b1	$\log_{10}(k_2')$	−1.785	−1.926	−1.636	approximate	approximate
b1	$\log_{10}(k_{deg})$	−1.966	−2.231	−1.717	approximate	approximate
b1	$\log_{10}(k_1'/k_2)$	0.2469	0.1958	0.305	approximate	approximate
b1	$\log_{10}(k_1'k_2'/k_2)$	−1.538	−1.675	−1.385	approximate	approximate
b1	$\log_{10}(k_2'/k_2)$	−1.389	−2.207	−0.9885	approximate	approximate
b2	$\log_{10}(k_1'k_2')$	−2.103	−2.251	−1.828	approximate	approximate
b2	$\log_{10}(k_2 \text{ or } k_{deg})$	−1.088	−1.658	−0.6275	approximate	approximate
b2	$\log_{10}(k_2 \text{ or } k_{deg})$	−1.433	−1.658	−0.6275	approximate	approximate
b3	$\log_{10}(k_1')$	−0.4827	−0.6689	−0.2887	approximate	approximate
b3	$\log_{10}(k_2)$	−0.8096	−0.9925	−0.6091	approximate	approximate
b3	$\log_{10}(k_2')$	−1.577	−1.69	−1.453	approximate	approximate
b3	$\log_{10}(k_{deg})$	−1.525	−1.65	−1.4	approximate	approximate
b3	$\log_{10}(k_1'/k_2)$	0.327	0.2894	0.3656	approximate	approximate
b3	$\log_{10}(k_1'k_2'/k_2)$	−1.25	−1.362	−1.128	approximate	approximate
b3	$\log_{10}(k_2'/k_2)$	−0.7669	−1.038	−0.4886	approximate	approximate

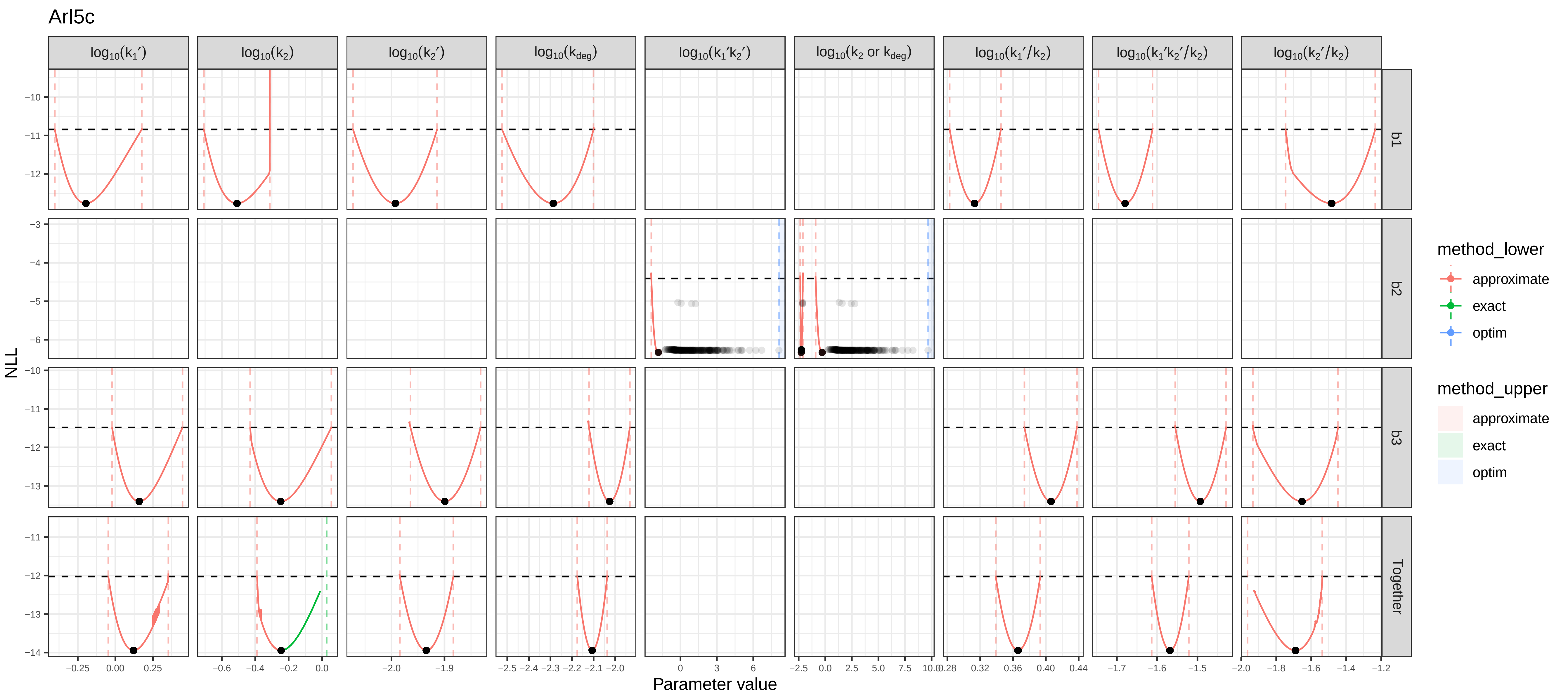

Replicate	Par	Best value	CI95 LB	CI95 UB	Method LB	Method UB
Together	$\log_{10}(k_1')$	0.1212	−0.04699	0.3528	approximate	approximate
Together	$\log_{10}(k_2)$	−0.2449	−0.3889	0.02731	approximate	exact
Together	$\log_{10}(k_2')$	−1.934	−1.984	−1.883	approximate	approximate
Together	$\log_{10}(k_{deg})$	−2.107	−2.176	−2.037	approximate	approximate
Together	$\log_{10}(k_1'/k_2)$	0.3661	0.3389	0.3932	approximate	approximate
Together	$\log_{10}(k_1'k_2'/k_2)$	−1.568	−1.614	−1.522	approximate	approximate
Together	$\log_{10}(k_2'/k_2)$	−1.689	−1.964	−1.537	approximate	approximate
b1	$\log_{10}(k_1')$	−0.1962	−0.4031	0.1756	approximate	approximate
b1	$\log_{10}(k_2)$	−0.5093	−0.7074	−0.3128	approximate	approximate
b1	$\log_{10}(k_2')$	−1.993	−2.073	−1.914	approximate	approximate
b1	$\log_{10}(k_{deg})$	−2.287	−2.524	−2.1	approximate	approximate
b1	$\log_{10}(k_1'/k_2)$	0.3131	0.2828	0.3452	approximate	approximate
b1	$\log_{10}(k_1'k_2'/k_2)$	−1.68	−1.745	−1.612	approximate	approximate
b1	$\log_{10}(k_2'/k_2)$	−1.483	−1.746	−1.234	approximate	approximate
b2	$\log_{10}(k_1'k_2')$	−1.816	−2.4	> 8.117	approximate	optim
b2	$\log_{10}(k_2 \text{ or } k_{deg})$	−0.2839	−0.9057	> 9.667	approximate	optim
b2	$\log_{10}(k_2 \text{ or } k_{deg})$	−2.25	−2.348	−2.106	approximate	approximate
b3	$\log_{10}(k_1')$	0.159	−0.02201	0.4469	approximate	approximate
b3	$\log_{10}(k_2)$	−0.2474	−0.4301	0.05576	approximate	approximate
b3	$\log_{10}(k_2')$	−1.899	−1.964	−1.832	approximate	approximate
b3	$\log_{10}(k_{deg})$	−2.025	−2.121	−1.932	approximate	approximate
b3	$\log_{10}(k_1'/k_2)$	0.4063	0.3739	0.4379	approximate	approximate
b3	$\log_{10}(k_1'k_2'/k_2)$	−1.493	−1.555	−1.429	approximate	approximate
b3	$\log_{10}(k_2'/k_2)$	−1.652	−1.933	−1.447	approximate	approximate

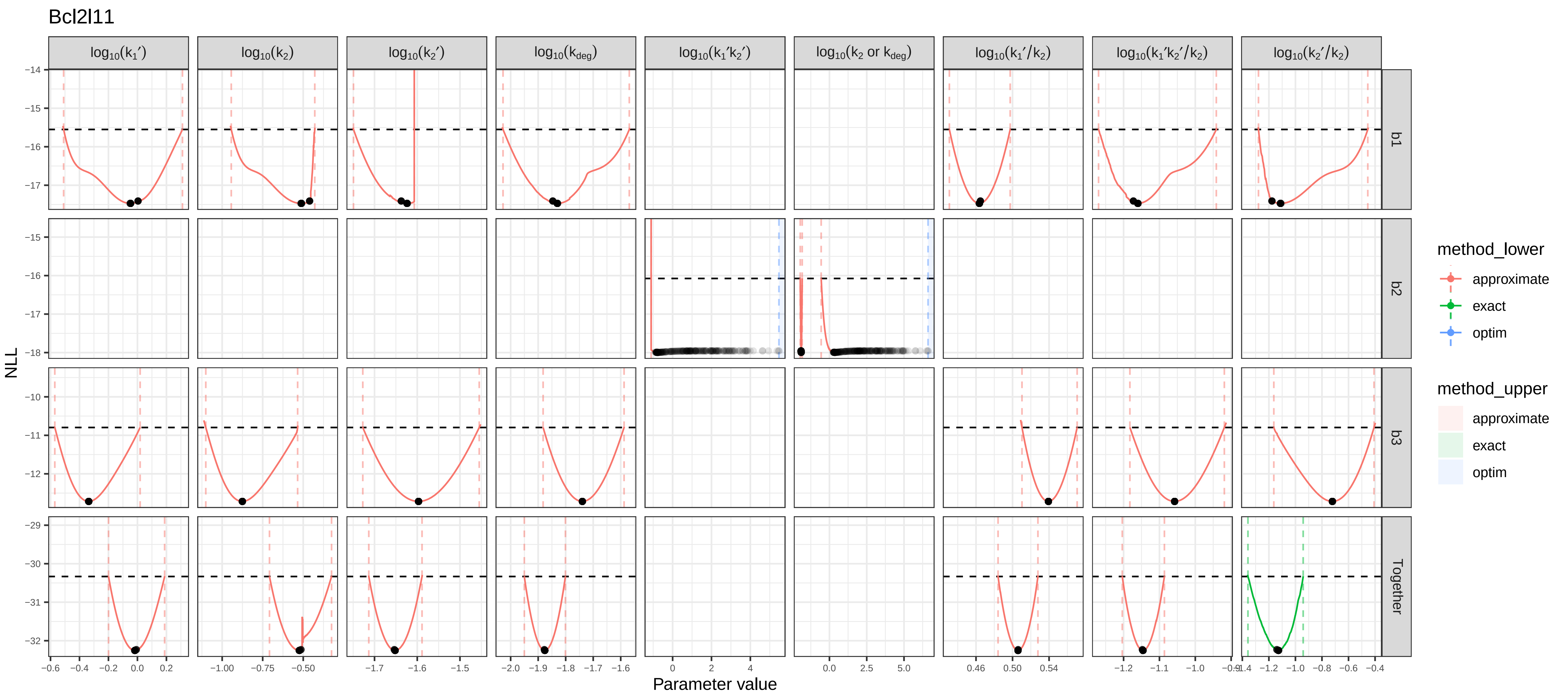

Replicate	Par	Best value	CI95 LB	CI95 UB	Method LB	Method UB
Together	$\log_{10}(k_1')$	−0.01751	−0.1995	0.1877	approximate	approximate
Together	$\log_{10}(k_2)$	−0.5235	−0.7082	−0.3252	approximate	approximate
Together	$\log_{10}(k_2')$	−1.652	−1.713	−1.589	approximate	approximate
Together	$\log_{10}(k_{deg})$	−1.876	−1.95	−1.801	approximate	approximate
Together	$\log_{10}(k_1'/k_2)$	0.506	0.4841	0.5278	approximate	approximate
Together	$\log_{10}(k_1'k_2'/k_2)$	−1.146	−1.204	−1.086	approximate	approximate
Together	$\log_{10}(k_2'/k_2)$	−1.128	−1.358	−0.9416	exact	exact
b1	$\log_{10}(k_1')$	−0.04808	−0.5095	0.3108	approximate	approximate
b1	$\log_{10}(k_2)$	−0.5117	−0.944	−0.4287	approximate	approximate
b1	$\log_{10}(k_2')$	−1.624	−1.749	−1.607	approximate	approximate
b1	$\log_{10}(k_{deg})$	−1.83	−2.028	−1.569	approximate	approximate
b1	$\log_{10}(k_1'/k_2)$	0.4637	0.4308	0.4974	approximate	approximate
b1	$\log_{10}(k_1'k_2'/k_2)$	−1.16	−1.27	−0.9414	approximate	approximate
b1	$\log_{10}(k_2'/k_2)$	−1.112	−1.278	−0.4548	approximate	approximate
b2	$\log_{10}(k_1'k_2')$	−0.7473	−1.106	> 5.471	approximate	optim
b2	$\log_{10}(k_2 \text{ or } k_{deg})$	0.3856	−0.555	> 6.612	approximate	optim
b2	$\log_{10}(k_2 \text{ or } k_{deg})$	−1.897	−1.958	−1.828	approximate	approximate
b3	$\log_{10}(k_1')$	−0.3358	−0.5703	0.01863	approximate	approximate
b3	$\log_{10}(k_2)$	−0.8751	−1.101	−0.5345	approximate	approximate
b3	$\log_{10}(k_2')$	−1.597	−1.727	−1.455	approximate	approximate
b3	$\log_{10}(k_{deg})$	−1.739	−1.882	−1.588	approximate	approximate
b3	$\log_{10}(k_1'/k_2)$	0.5393	0.5103	0.5707	approximate	approximate
b3	$\log_{10}(k_1'k_2'/k_2)$	−1.058	−1.182	−0.919	approximate	approximate
b3	$\log_{10}(k_2'/k_2)$	−0.722	−1.163	−0.4079	approximate	approximate

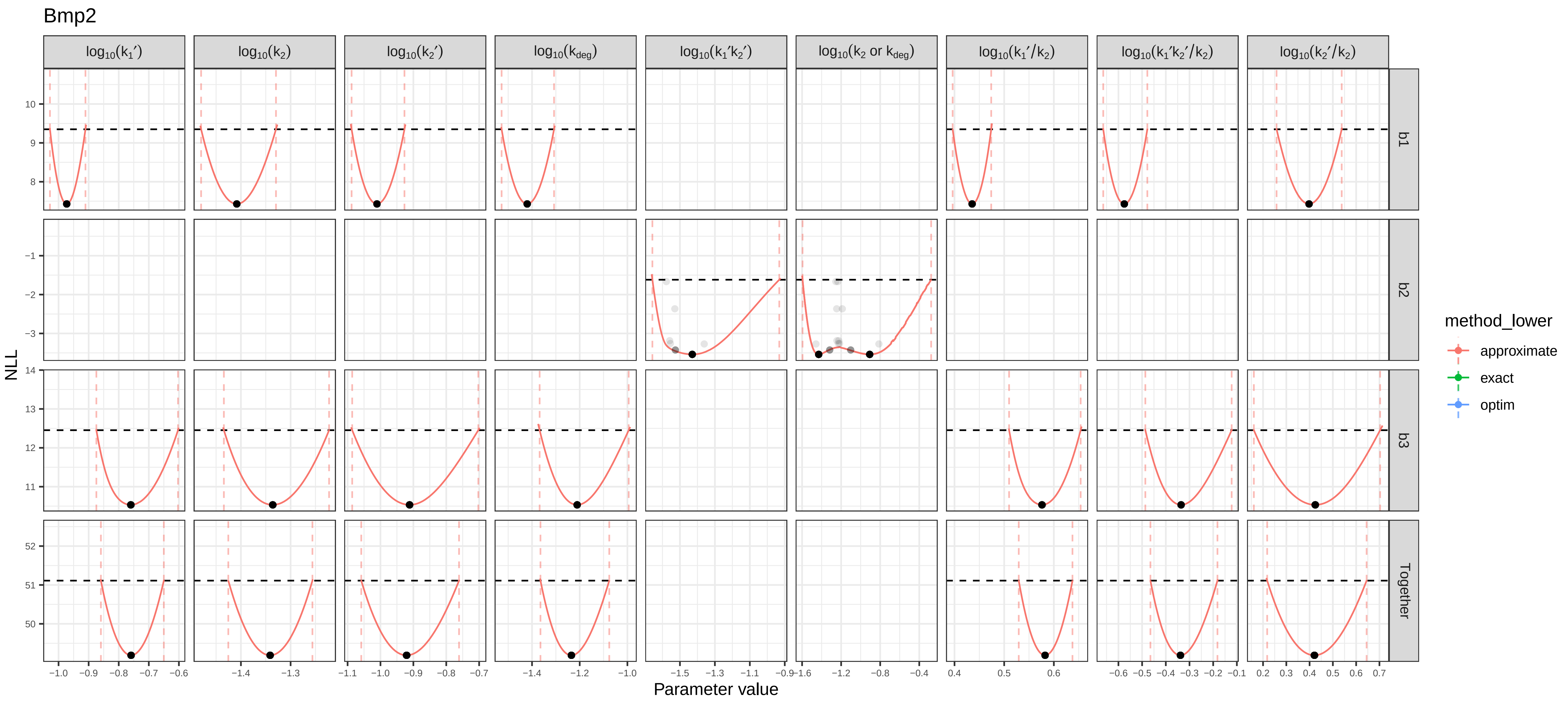

Replicate	Par	Best value	CI95 LB	CI95 UB	Method LB	Method UB
Together	$\log_{10}(k_1')$	−0.7591	−0.8593	−0.6501	approximate	approximate
Together	$\log_{10}(k_2)$	−1.341	−1.425	−1.256	approximate	approximate
Together	$\log_{10}(k_2')$	−0.9202	−1.058	−0.7611	approximate	approximate
Together	$\log_{10}(k_{deg})$	−1.235	−1.365	−1.076	approximate	approximate
Together	$\log_{10}(k_1'/k_2)$	0.5823	0.5293	0.6375	approximate	approximate
Together	$\log_{10}(k_1'k_2'/k_2)$	−0.338	−0.4649	−0.1819	approximate	approximate
Together	$\log_{10}(k_2'/k_2)$	0.4212	0.2179	0.6449	approximate	approximate
b1	$\log_{10}(k_1')$	−0.9728	−1.029	−0.9106	approximate	approximate
b1	$\log_{10}(k_2)$	−1.408	−1.48	−1.329	approximate	approximate
b1	$\log_{10}(k_2')$	−1.011	−1.088	−0.9271	approximate	approximate
b1	$\log_{10}(k_{deg})$	−1.42	−1.528	−1.308	approximate	approximate
b1	$\log_{10}(k_1'/k_2)$	0.4355	0.3962	0.4739	approximate	approximate
b1	$\log_{10}(k_1'k_2'/k_2)$	−0.5752	−0.6641	−0.478	approximate	approximate
b1	$\log_{10}(k_2'/k_2)$	0.3977	0.2582	0.538	approximate	approximate
b2	$\log_{10}(k_1'k_2')$	−1.429	−1.658	−0.9314	approximate	approximate
b2	$\log_{10}(k_2 \text{ or } k_{deg})$	−0.9079	−1.595	−0.2782	approximate	approximate
b2	$\log_{10}(k_2 \text{ or } k_{deg})$	−1.43	−1.595	−0.2782	approximate	approximate
b3	$\log_{10}(k_1')$	−0.7598	−0.8743	−0.6029	approximate	approximate
b3	$\log_{10}(k_2)$	−1.336	−1.434	−1.223	approximate	approximate
b3	$\log_{10}(k_2')$	−0.9115	−1.086	−0.7025	approximate	approximate
b3	$\log_{10}(k_{deg})$	−1.211	−1.368	−0.9944	approximate	approximate
b3	$\log_{10}(k_1'/k_2)$	0.5761	0.5099	0.654	approximate	approximate
b3	$\log_{10}(k_1'k_2'/k_2)$	−0.3354	−0.4864	−0.122	approximate	approximate
b3	$\log_{10}(k_2'/k_2)$	0.4244	0.1606	0.7029	approximate	approximate

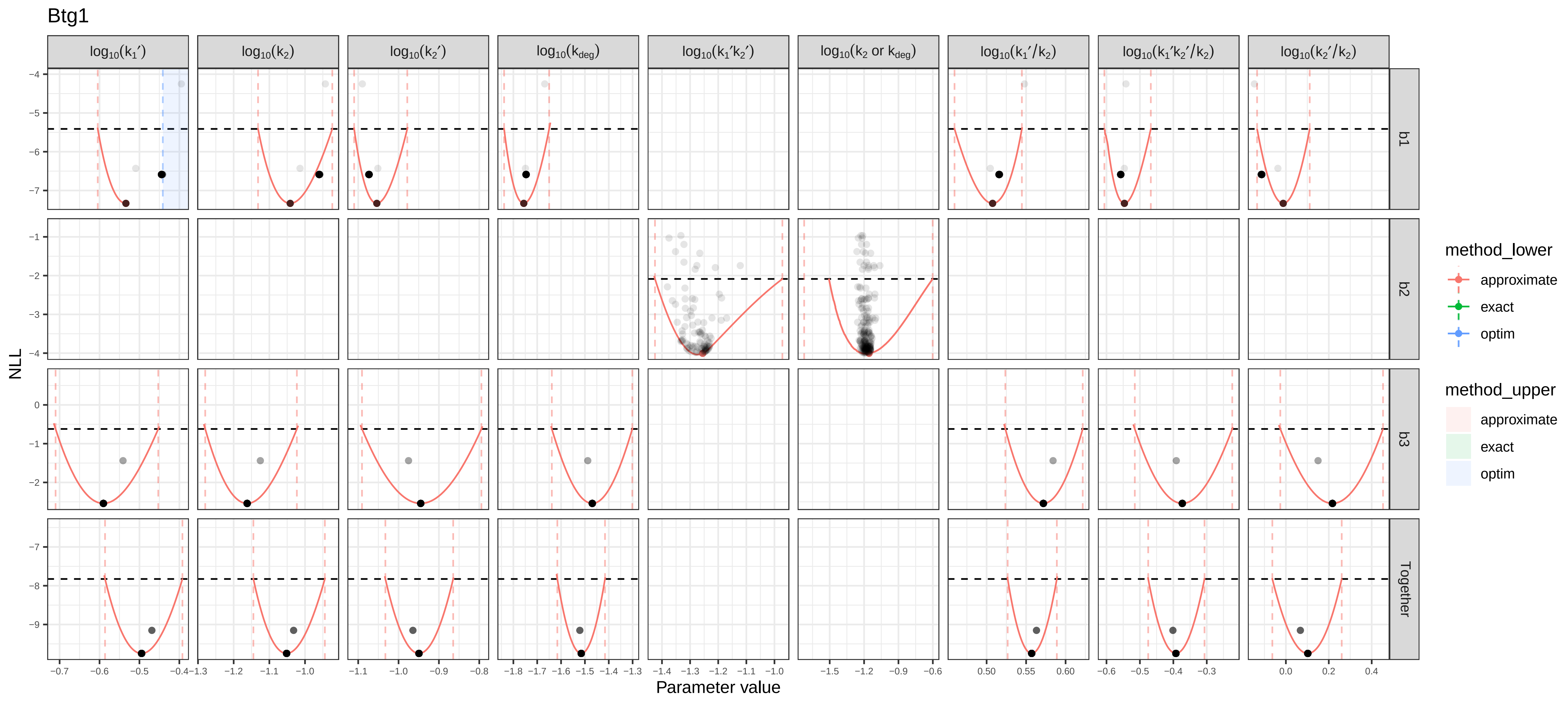

Replicate	Par	**Best value**	**CI95 LB**	**CI95 UB**	**Method LB**	**Method UB**
Together	$\log_{10}(k_1')$	−0.4946	−0.5862	−0.3923	approximate	approximate
Together	$\log_{10}(k_2)$	−1.052	−1.145	−0.9444	approximate	approximate
Together	$\log_{10}(k_2')$	−0.9494	−1.033	−0.8646	approximate	approximate
Together	$\log_{10}(k_{deg})$	−1.515	−1.616	−1.416	approximate	approximate
Together	$\log_{10}(k_1'/k_2)$	0.5571	0.5267	0.5889	approximate	approximate
Together	$\log_{10}(k_1'k_2'/k_2)$	−0.3924	−0.4752	−0.3068	approximate	approximate
Together	$\log_{10}(k_2'/k_2)$	0.1022	−0.06313	0.2603	approximate	approximate
b1	$\log_{10}(k_1')$	−0.5338	−0.6044	> −0.4414	approximate	optim
b1	$\log_{10}(k_2)$	−1.041	−1.132	−0.9235	approximate	approximate
b1	$\log_{10}(k_2')$	−1.054	−1.11	−0.9784	approximate	approximate
b1	$\log_{10}(k_{deg})$	−1.756	−1.839	−1.65	approximate	approximate
b1	$\log_{10}(k_1'/k_2)$	0.5075	0.4593	0.5447	approximate	approximate
b1	$\log_{10}(k_1'k_2'/k_2)$	−0.5464	−0.6063	−0.4673	approximate	approximate
b1	$\log_{10}(k_2'/k_2)$	−0.01256	−0.1342	0.1112	approximate	approximate
b2	$\log_{10}(k_1'k_2')$	−1.255	−1.425	−0.9718	approximate	approximate
b2	$\log_{10}(k_2 \text{ or } k_{deg})$	−1.156	−1.723	−0.6012	approximate	approximate
b2	$\log_{10}(k_2 \text{ or } k_{deg})$	−1.194	−1.723	−0.6012	approximate	approximate
b3	$\log_{10}(k_1')$	−0.5901	−0.7101	−0.4528	approximate	approximate
b3	$\log_{10}(k_2)$	−1.162	−1.28	−1.023	approximate	approximate
b3	$\log_{10}(k_2')$	−0.9453	−1.091	−0.7947	approximate	approximate
b3	$\log_{10}(k_{deg})$	−1.469	−1.639	−1.301	approximate	approximate
b3	$\log_{10}(k_1'/k_2)$	0.5719	0.5237	0.6217	approximate	approximate
b3	$\log_{10}(k_1'k_2'/k_2)$	−0.3734	−0.5153	−0.2237	approximate	approximate
b3	$\log_{10}(k_2'/k_2)$	0.2167	−0.02598	0.4528	approximate	approximate

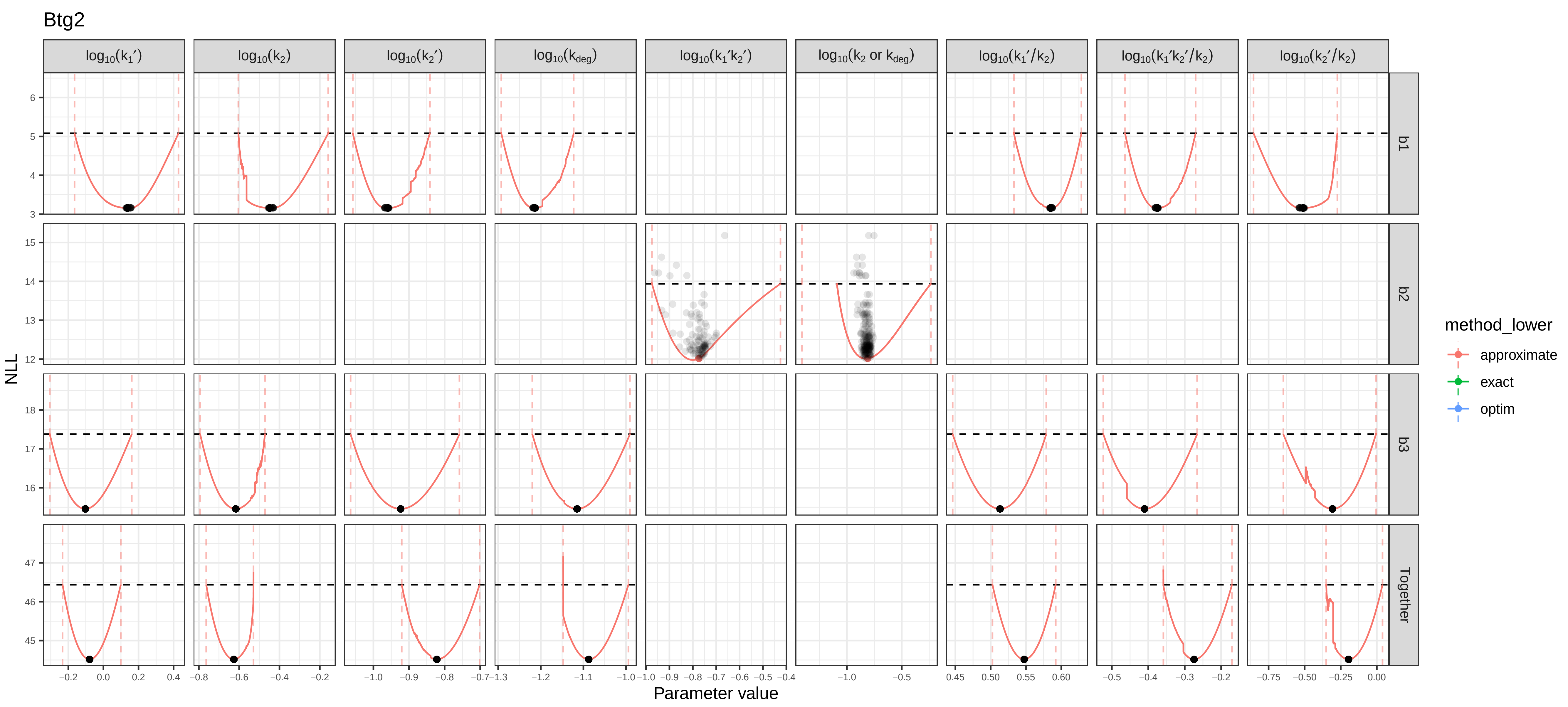

Replicate	Par	Best value	CI95 LB	CI95 UB	Method LB	Method UB
Together	$\log_{10}(k_1')$	−0.07858	−0.2329	0.09896	approximate	approximate
Together	$\log_{10}(k_2)$	−0.6261	−0.7633	−0.5288	approximate	approximate
Together	$\log_{10}(k_2')$	−0.8219	−0.9205	−0.7018	approximate	approximate
Together	$\log_{10}(k_{deg})$	−1.088	−1.147	−0.9944	approximate	approximate
Together	$\log_{10}(k_1'/k_2)$	0.5475	0.5025	0.5921	approximate	approximate
Together	$\log_{10}(k_1'k_2'/k_2)$	−0.2744	−0.3585	−0.1702	approximate	approximate
Together	$\log_{10}(k_2'/k_2)$	−0.1958	−0.3509	0.03968	approximate	approximate
b1	$\log_{10}(k_1')$	0.1365	−0.1646	0.4278	approximate	approximate
b1	$\log_{10}(k_2)$	−0.4482	−0.6039	−0.1582	approximate	approximate
b1	$\log_{10}(k_2')$	−0.9594	−1.058	−0.8412	approximate	approximate
b1	$\log_{10}(k_{deg})$	−1.214	−1.293	−1.123	approximate	approximate
b1	$\log_{10}(k_1'/k_2)$	0.5847	0.5329	0.6285	approximate	approximate
b1	$\log_{10}(k_1'k_2'/k_2)$	−0.3747	−0.4637	−0.2701	approximate	approximate
b1	$\log_{10}(k_2'/k_2)$	−0.5112	−0.8543	−0.2733	approximate	approximate
b2	$\log_{10}(k_1'k_2')$	−0.7742	−0.9747	−0.4251	approximate	approximate
b2	$\log_{10}(k_2 \text{ or } k_{deg})$	−0.8106	−1.409	−0.2341	approximate	approximate
b2	$\log_{10}(k_2 \text{ or } k_{deg})$	−0.8257	−1.409	−0.2341	approximate	approximate
b3	$\log_{10}(k_1')$	−0.1033	−0.3056	0.1615	approximate	approximate
b3	$\log_{10}(k_2)$	−0.6166	−0.7932	−0.4718	approximate	approximate
b3	$\log_{10}(k_2')$	−0.9233	−1.064	−0.7584	approximate	approximate
b3	$\log_{10}(k_{deg})$	−1.115	−1.22	−0.9909	approximate	approximate
b3	$\log_{10}(k_1'/k_2)$	0.5133	0.4462	0.5787	approximate	approximate
b3	$\log_{10}(k_1'k_2'/k_2)$	−0.41	−0.5234	−0.2659	approximate	approximate
b3	$\log_{10}(k_2'/k_2)$	−0.3067	−0.6477	−0.005848	approximate	approximate

C3

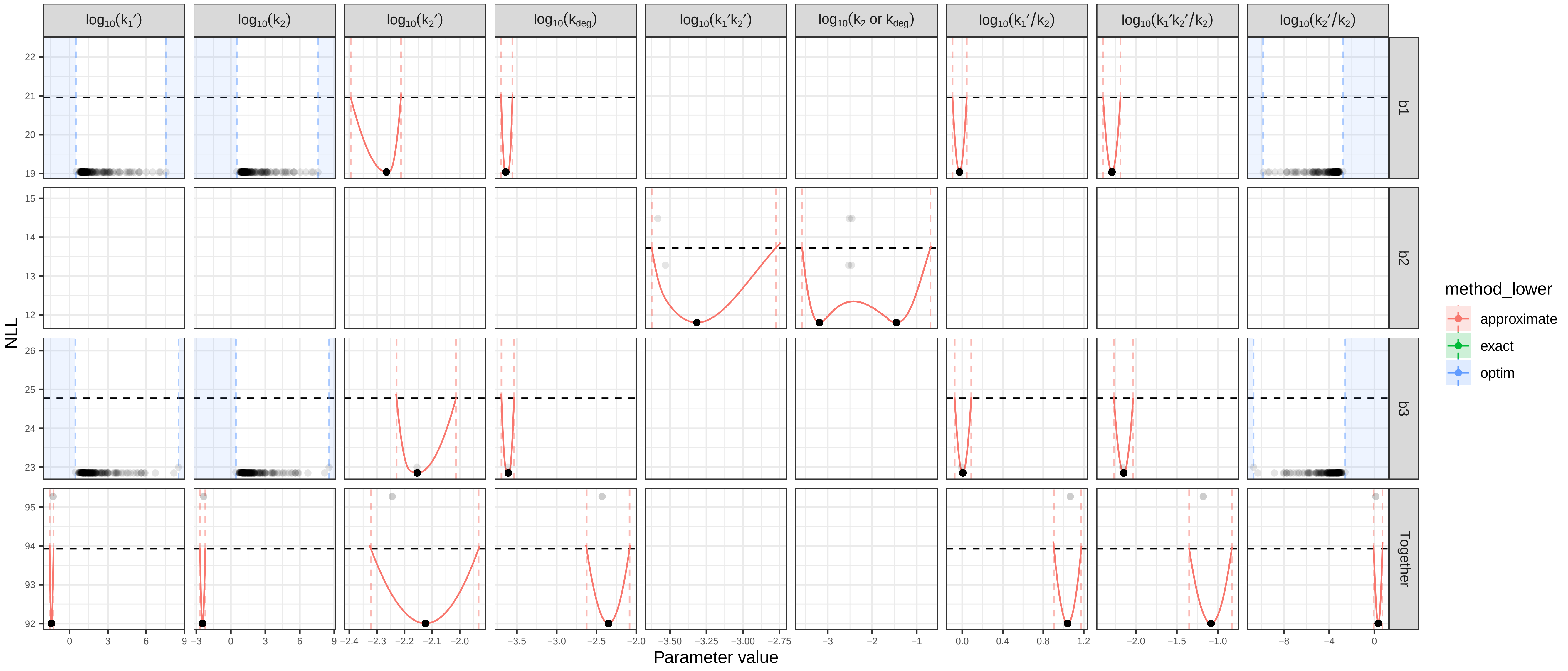

Replicate	Par	Best value	CI95 LB	CI95 UB	Method LB	Method UB
Together	$\log_{10}(k_1')$	−1.425	−1.57	−1.264	approximate	approximate
Together	$\log_{10}(k_2)$	−2.467	−2.677	−2.221	approximate	approximate
Together	$\log_{10}(k_2')$	−2.124	−2.322	−1.931	approximate	approximate
Together	$\log_{10}(k_{deg})$	−2.35	−2.624	−2.085	approximate	approximate
Together	$\log_{10}(k_1'/k_2)$	1.041	0.906	1.176	approximate	approximate
Together	$\log_{10}(k_1'k_2'/k_2)$	−1.082	−1.349	−0.8288	approximate	approximate
Together	$\log_{10}(k_2'/k_2)$	0.3428	−0.05966	0.7067	approximate	approximate
b1	$\log_{10}(k_1')$	5.433	< 0.507	> 7.555	optim	optim
b1	$\log_{10}(k_2)$	5.46	< 0.5311	> 7.583	optim	optim
b1	$\log_{10}(k_2')$	−2.265	−2.395	−2.213	approximate	approximate
b1	$\log_{10}(k_{deg})$	−3.643	−3.698	−3.556	approximate	approximate
b1	$\log_{10}(k_1'/k_2)$	−0.02777	−0.09605	0.04426	approximate	approximate
b1	$\log_{10}(k_1'k_2'/k_2)$	−2.293	−2.401	−2.189	approximate	approximate
b1	$\log_{10}(k_2'/k_2)$	−7.725	< −9.848	> −2.796	optim	optim
b2	$\log_{10}(k_1'k_2')$	−3.316	−3.625	−2.775	approximate	approximate
b2	$\log_{10}(k_2 \text{ or } k_{deg})$	−1.457	−3.575	−0.6912	approximate	approximate
b2	$\log_{10}(k_2 \text{ or } k_{deg})$	−3.187	−3.575	−0.6912	approximate	approximate
b3	$\log_{10}(k_1')$	5.6	< 0.4508	> 8.538	optim	optim
b3	$\log_{10}(k_2)$	5.596	< 0.442	> 8.547	optim	optim
b3	$\log_{10}(k_2')$	−2.154	−2.229	−2.013	approximate	approximate
b3	$\log_{10}(k_{deg})$	−3.609	−3.694	−3.536	approximate	approximate
b3	$\log_{10}(k_1'/k_2)$	0.004409	−0.07497	0.08906	approximate	approximate
b3	$\log_{10}(k_1'k_2'/k_2)$	−2.15	−2.268	−2.033	approximate	approximate
b3	$\log_{10}(k_2'/k_2)$	−7.75	< −10.7	> −2.596	optim	optim

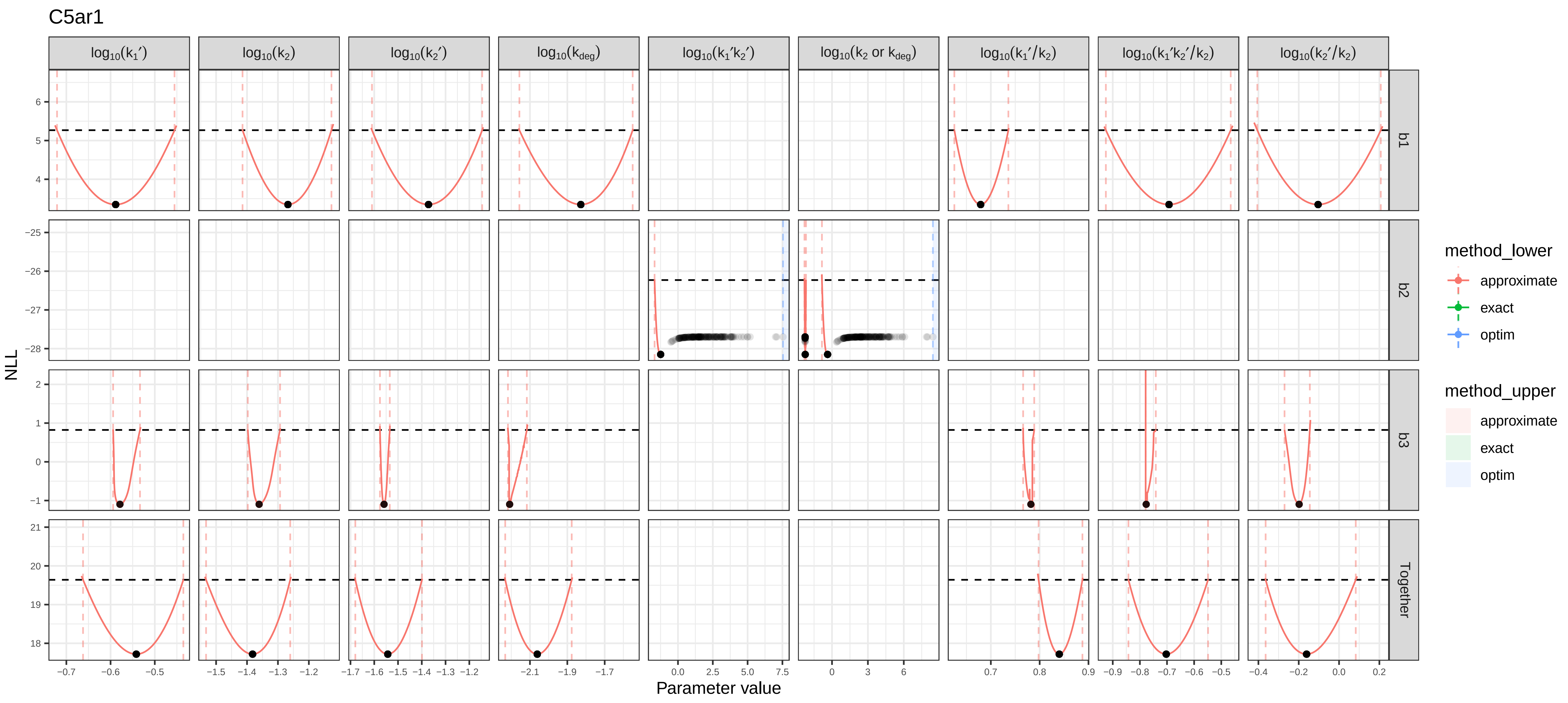

Replicate	Par	Best value	CI95 LB	CI95 UB	Method LB	Method UB
Together	$\log_{10}(k_1')$	−0.5419	−0.6623	−0.4354	approximate	approximate
Together	$\log_{10}(k_2)$	−1.382	−1.532	−1.26	approximate	approximate
Together	$\log_{10}(k_2')$	−1.543	−1.68	−1.399	approximate	approximate
Together	$\log_{10}(k_{deg})$	−2.061	−2.233	−1.876	approximate	approximate
Together	$\log_{10}(k_1'/k_2)$	0.8402	0.7978	0.8876	approximate	approximate
Together	$\log_{10}(k_1'k_2'/k_2)$	−0.7029	−0.8422	−0.5487	approximate	approximate
Together	$\log_{10}(k_2'/k_2)$	−0.161	−0.3642	0.08291	approximate	approximate
b1	$\log_{10}(k_1')$	−0.5885	−0.7211	−0.4554	approximate	approximate
b1	$\log_{10}(k_2)$	−1.268	−1.414	−1.127	approximate	approximate
b1	$\log_{10}(k_2')$	−1.372	−1.61	−1.146	approximate	approximate
b1	$\log_{10}(k_{deg})$	−1.828	−2.157	−1.55	approximate	approximate
b1	$\log_{10}(k_1'/k_2)$	0.6792	0.6252	0.7359	approximate	approximate
b1	$\log_{10}(k_1'k_2'/k_2)$	−0.6925	−0.9253	−0.4651	approximate	approximate
b1	$\log_{10}(k_2'/k_2)$	−0.104	−0.4055	0.2071	approximate	approximate
b2	$\log_{10}(k_1'k_2')$	−1.242	−1.68	> 7.543	approximate	optim
b2	$\log_{10}(k_2 \text{ or } k_{deg})$	−0.3672	−0.8418	> 8.435	approximate	optim
b2	$\log_{10}(k_2 \text{ or } k_{deg})$	−2.239	−2.303	−2.174	approximate	approximate
b3	$\log_{10}(k_1')$	−0.5789	−0.5943	−0.5334	approximate	approximate
b3	$\log_{10}(k_2)$	−1.361	−1.397	−1.293	approximate	approximate
b3	$\log_{10}(k_2')$	−1.559	−1.576	−1.535	approximate	approximate
b3	$\log_{10}(k_{deg})$	−2.209	−2.218	−2.117	approximate	approximate
b3	$\log_{10}(k_1'/k_2)$	0.782	0.7662	0.7888	approximate	approximate
b3	$\log_{10}(k_1'k_2'/k_2)$	−0.7767	−0.7788	−0.7413	approximate	approximate
b3	$\log_{10}(k_2'/k_2)$	−0.1979	−0.2705	−0.1449	approximate	approximate

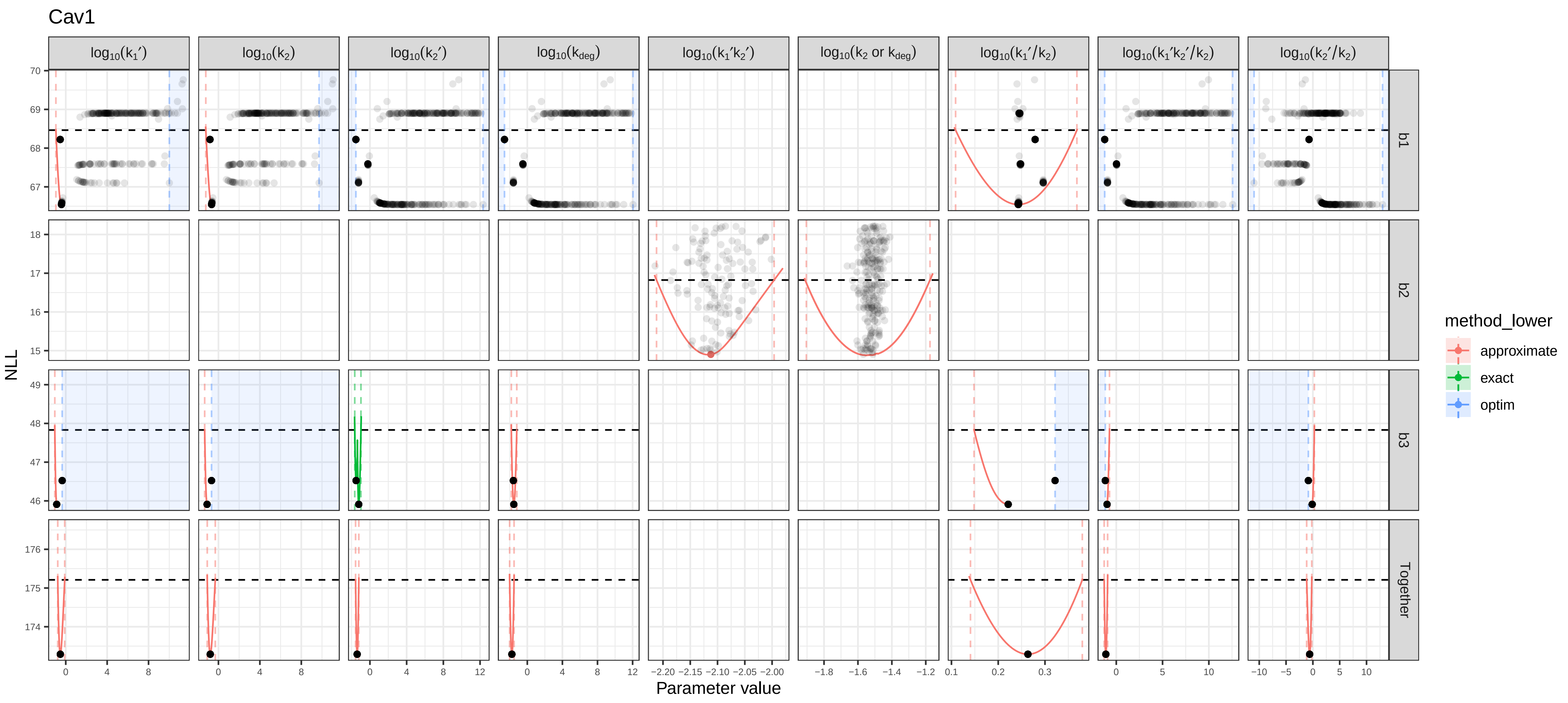

Replicate	Par	Best value	CI95 LB	CI95 UB	Method LB	Method UB
Together	$\log_{10}(k_1')$	−0.5253	−0.774	−0.09947	approximate	approximate
Together	$\log_{10}(k_2)$	−0.7889	−1.08	−0.3051	approximate	approximate
Together	$\log_{10}(k_2')$	−1.392	−1.563	−1.211	approximate	approximate
Together	$\log_{10}(k_{deg})$	−1.758	−2.011	−1.509	approximate	approximate
Together	$\log_{10}(k_1'/k_2)$	0.2635	0.1407	0.3799	approximate	approximate
Together	$\log_{10}(k_1'k_2'/k_2)$	−1.128	−1.306	−0.9332	approximate	approximate
Together	$\log_{10}(k_2'/k_2)$	−0.6028	−1.156	−0.2026	approximate	approximate
b1	$\log_{10}(k_1')$	−0.4169	−0.9531	> 10.01	approximate	optim
b1	$\log_{10}(k_2)$	−0.6598	−1.219	> 9.714	approximate	optim
b1	$\log_{10}(k_2')$	5.988	< −1.529	> 12.33	optim	optim
b1	$\log_{10}(k_{deg})$	5.702	< −2.598	> 12.04	optim	optim
b1	$\log_{10}(k_1'/k_2)$	0.2429	0.1088	0.3683	approximate	approximate
b1	$\log_{10}(k_1'k_2'/k_2)$	6.23	< −1.25	> 12.57	optim	optim
b1	$\log_{10}(k_2'/k_2)$	6.647	< −10.96	> 12.99	optim	optim
b2	$\log_{10}(k_1'k_2')$	−2.112	−2.212	−1.996	approximate	approximate
b2	$\log_{10}(k_2 \text{ or } k_{deg})$	−1.512	−1.904	−1.175	approximate	approximate
b2	$\log_{10}(k_2 \text{ or } k_{deg})$	−1.568	−1.904	−1.175	approximate	approximate
b3	$\log_{10}(k_1')$	−0.8756	−1.059	> −0.3444	approximate	optim
b3	$\log_{10}(k_2)$	−1.097	−1.328	> −0.6661	approximate	optim
b3	$\log_{10}(k_2')$	−1.219	−1.653	−0.9773	exact	exact
b3	$\log_{10}(k_{deg})$	−1.534	−1.82	−1.198	approximate	approximate
b3	$\log_{10}(k_1'/k_2)$	0.2214	0.1483	> 0.3217	approximate	optim
b3	$\log_{10}(k_1'k_2'/k_2)$	−0.9974	< −1.186	−0.7324	optim	approximate
b3	$\log_{10}(k_2'/k_2)$	−0.1219	< −0.842	0.2736	optim	approximate

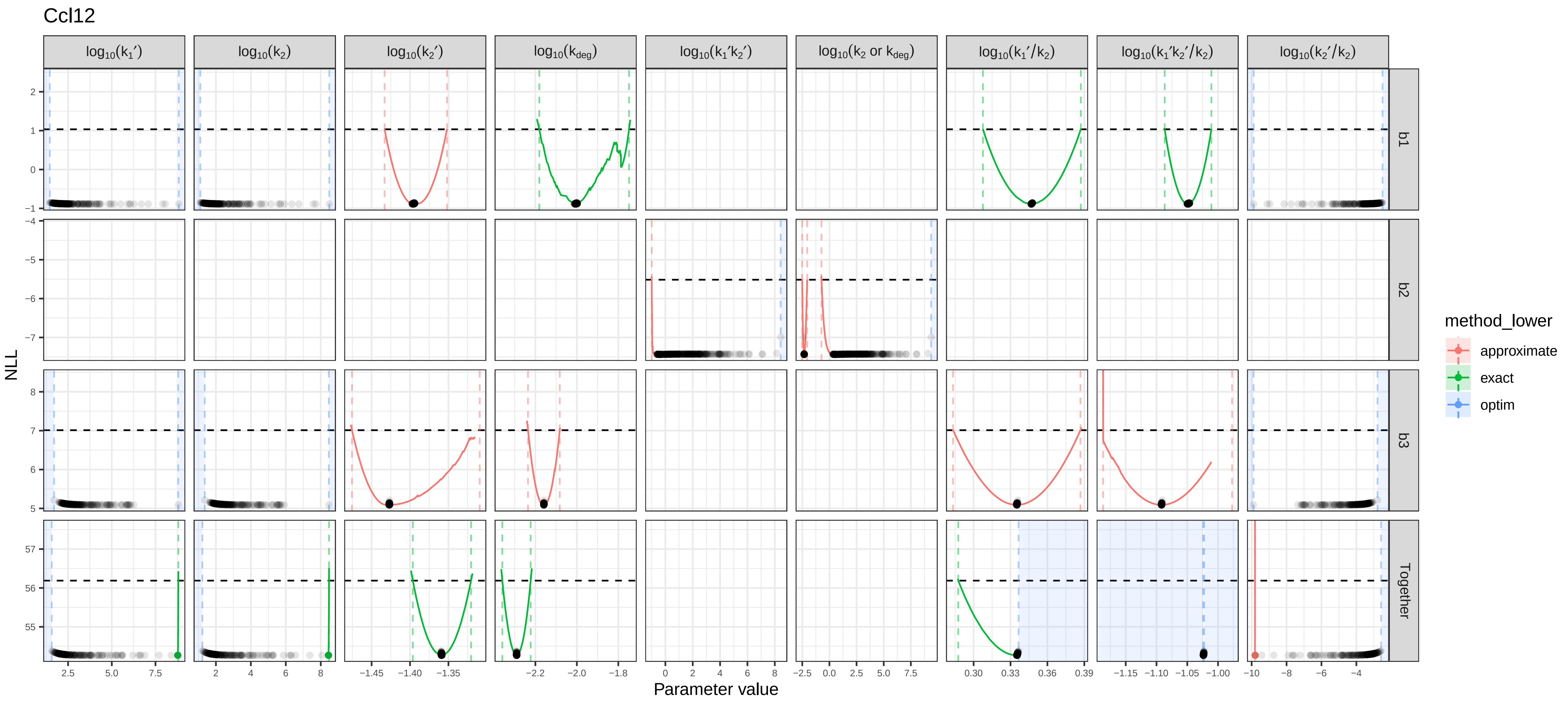

Replicate	Par	Best value	CI95 LB	CI95 UB	Method LB	Method UB
Together	$\log_{10}(k_1')$	8.777	< 1.561	8.807	optim	exact
Together	$\log_{10}(k_2)$	8.442	< 1.225	8.471	optim	exact
Together	$\log_{10}(k_2')$	−1.359	−1.396	−1.321	exact	exact
Together	$\log_{10}(k_{deg})$	−2.29	−2.36	−2.223	exact	exact
Together	$\log_{10}(k_1'/k_2)$	0.3354	0.2875	> 0.3366	exact	optim
Together	$\log_{10}(k_1'k_2'/k_2)$	−1.024	< −1.024	> −1.023	optim	optim
Together	$\log_{10}(k_2'/k_2)$	−9.8	−9.809	> −2.584	approximate	optim
b1	$\log_{10}(k_1')$	6.692	< 1.453	> 8.834	optim	optim
b1	$\log_{10}(k_2)$	6.345	< 1.105	> 8.487	optim	optim
b1	$\log_{10}(k_2')$	−1.396	−1.433	−1.352	approximate	approximate
b1	$\log_{10}(k_{deg})$	−2.006	−2.181	−1.748	exact	exact
b1	$\log_{10}(k_1'/k_2)$	0.3469	0.3076	0.3871	exact	exact
b1	$\log_{10}(k_1'k_2'/k_2)$	−1.049	−1.087	−1.011	exact	exact
b1	$\log_{10}(k_2'/k_2)$	−7.741	< −9.883	> −2.499	optim	optim
b2	$\log_{10}(k_1'k_2')$	−0.3862	−0.9995	> 8.43	approximate	optim
b2	$\log_{10}(k_2 \text{ or } k_{deg})$	0.5576	−0.7348	> 9.368	approximate	optim
b2	$\log_{10}(k_2 \text{ or } k_{deg})$	−2.324	−2.51	−2.049	approximate	approximate
b3	$\log_{10}(k_1')$	5.621	< 1.697	> 8.8	optim	optim
b3	$\log_{10}(k_2)$	5.286	< 1.361	> 8.465	optim	optim
b3	$\log_{10}(k_2')$	−1.427	−1.476	−1.309	approximate	approximate
b3	$\log_{10}(k_{deg})$	−2.159	−2.236	−2.083	approximate	approximate
b3	$\log_{10}(k_1'/k_2)$	0.3352	0.2833	0.3868	approximate	approximate
b3	$\log_{10}(k_1'k_2'/k_2)$	−1.092	−1.187	−0.9772	approximate	approximate
b3	$\log_{10}(k_2'/k_2)$	−6.713	< −9.892	> −2.788	optim	optim

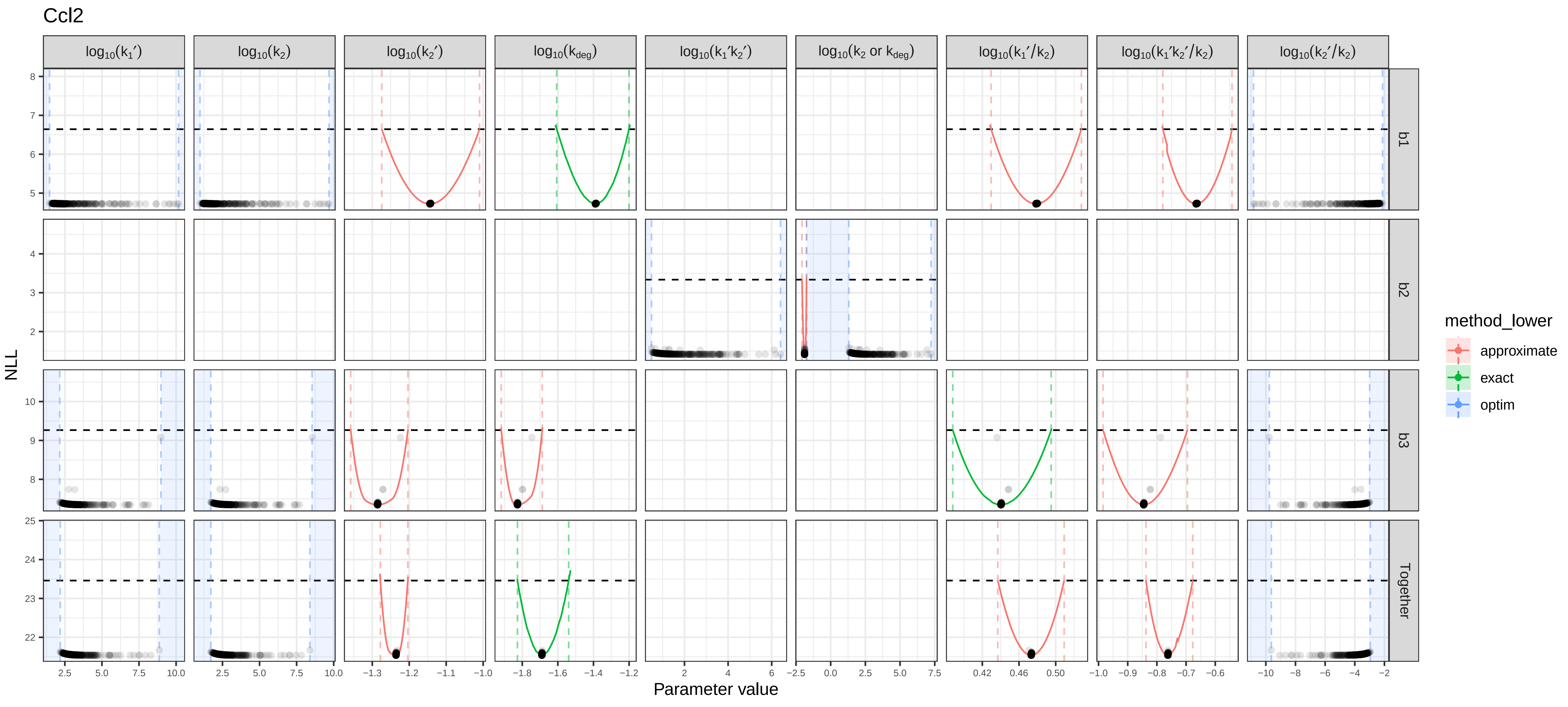

Replicate	Par	Best value	CI95 LB	CI95 UB	Method LB	Method UB
Together	$\log_{10}(k_1')$	5.512	< 2.19	> 8.867	optim	optim
Together	$\log_{10}(k_2)$	5.038	< 1.716	> 8.397	optim	optim
Together	$\log_{10}(k_2')$	−1.236	−1.278	−1.204	approximate	approximate
Together	$\log_{10}(k_{deg})$	−1.69	−1.827	−1.539	exact	exact
Together	$\log_{10}(k_1'/k_2)$	0.4734	0.4369	0.5092	approximate	approximate
Together	$\log_{10}(k_1'k_2'/k_2)$	−0.7625	−0.8374	−0.6771	approximate	approximate
Together	$\log_{10}(k_2'/k_2)$	−6.274	< −9.63	> −2.951	optim	optim
b1	$\log_{10}(k_1')$	4.943	< 1.468	> 10.17	optim	optim
b1	$\log_{10}(k_2)$	4.464	< 0.987	> 9.687	optim	optim
b1	$\log_{10}(k_2')$	−1.144	−1.274	−1.01	approximate	approximate
b1	$\log_{10}(k_{deg})$	−1.389	−1.607	−1.2	exact	exact
b1	$\log_{10}(k_1'/k_2)$	0.4785	0.4296	0.528	approximate	approximate
b1	$\log_{10}(k_1'k_2'/k_2)$	−0.6656	−0.7797	−0.5426	approximate	approximate
b1	$\log_{10}(k_2'/k_2)$	−5.608	< −10.83	> −2.128	optim	optim
b2	$\log_{10}(k_1'k_2')$	3.878	< 0.4895	> 6.406	optim	optim
b2	$\log_{10}(k_2 \text{ or } k_{deg})$	4.706	1.311	> 7.233	optim	optim
b2	$\log_{10}(k_2 \text{ or } k_{deg})$	−1.883	−2.065	−1.742	approximate	approximate
b3	$\log_{10}(k_1')$	5.682	< 2.152	> 8.977	optim	optim
b3	$\log_{10}(k_2)$	5.242	< 1.712	> 8.541	optim	optim
b3	$\log_{10}(k_2')$	−1.286	−1.358	−1.204	approximate	approximate
b3	$\log_{10}(k_{deg})$	−1.827	−1.918	−1.688	approximate	approximate
b3	$\log_{10}(k_1'/k_2)$	0.4403	0.3878	0.4951	exact	exact
b3	$\log_{10}(k_1'k_2'/k_2)$	−0.8453	−0.9845	−0.6952	approximate	approximate
b3	$\log_{10}(k_2'/k_2)$	−6.527	< −9.765	> −2.997	optim	optim

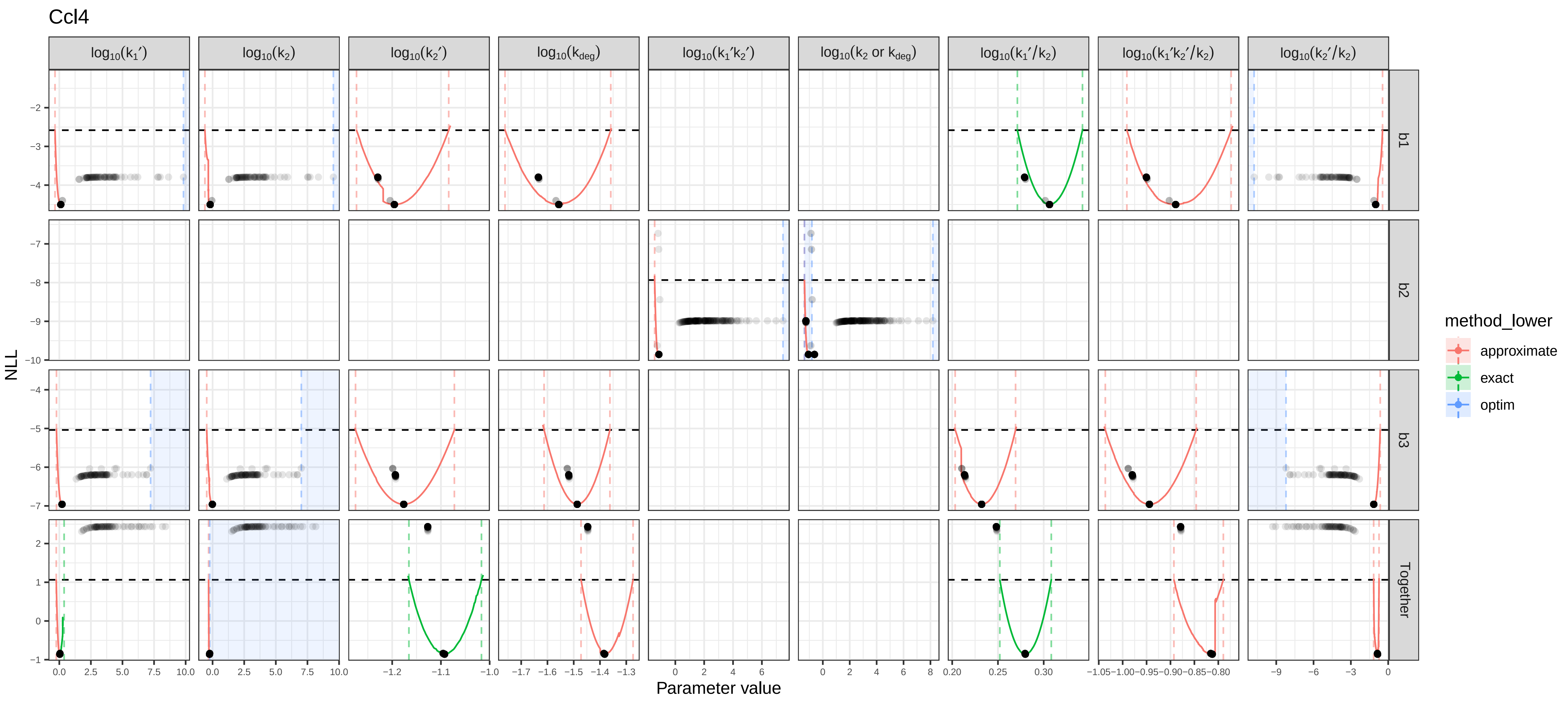

Replicate	Par	**Best value**	**CI95 LB**	**CI95 UB**	**Method LB**	**Method UB**
Together	$\log_{10}(k_1')$	0.03964	−0.2412	0.3805	approximate	exact
Together	$\log_{10}(k_2)$	−0.2402	−0.313	> −0.2245	approximate	optim
Together	$\log_{10}(k_2')$	−1.093	−1.166	−1.017	exact	exact
Together	$\log_{10}(k_{deg})$	−1.382	−1.472	−1.274	approximate	approximate
Together	$\log_{10}(k_1'/k_2)$	0.2799	0.2521	0.3083	exact	exact
Together	$\log_{10}(k_1'k_2'/k_2)$	−0.8127	−0.8928	−0.7894	approximate	approximate
Together	$\log_{10}(k_2'/k_2)$	−0.8524	−1.168	−0.7364	approximate	approximate
b1	$\log_{10}(k_1')$	0.1172	−0.3398	> 9.828	approximate	optim
b1	$\log_{10}(k_2)$	−0.1891	−0.605	> 9.549	approximate	optim
b1	$\log_{10}(k_2')$	−1.196	−1.273	−1.084	approximate	approximate
b1	$\log_{10}(k_{deg})$	−1.557	−1.761	−1.359	approximate	approximate
b1	$\log_{10}(k_1'/k_2)$	0.3063	0.2712	0.3424	exact	exact
b1	$\log_{10}(k_1'k_2'/k_2)$	−0.8892	−0.9912	−0.7731	approximate	approximate
b1	$\log_{10}(k_2'/k_2)$	−1.006	< −10.78	−0.4523	optim	approximate
b2	$\log_{10}(k_1'k_2')$	−1.142	−1.432	> 7.465	approximate	optim
b2	$\log_{10}(k_2 \text{ or } k_{deg})$	−0.6207	−1.366	> 8.182	approximate	optim
b2	$\log_{10}(k_2 \text{ or } k_{deg})$	−1.069	−1.366	−0.81	approximate	optim
b3	$\log_{10}(k_1')$	0.2147	−0.2225	> 7.225	approximate	optim
b3	$\log_{10}(k_2)$	−0.0175	−0.462	> 7.015	approximate	optim
b3	$\log_{10}(k_2')$	−1.176	−1.275	−1.072	approximate	approximate
b3	$\log_{10}(k_{deg})$	−1.487	−1.613	−1.362	approximate	approximate
b3	$\log_{10}(k_1'/k_2)$	0.2322	0.2032	0.2693	approximate	approximate
b3	$\log_{10}(k_1'k_2'/k_2)$	−0.9441	−1.036	−0.8463	approximate	approximate
b3	$\log_{10}(k_2'/k_2)$	−1.159	< −8.214	−0.6406	optim	approximate

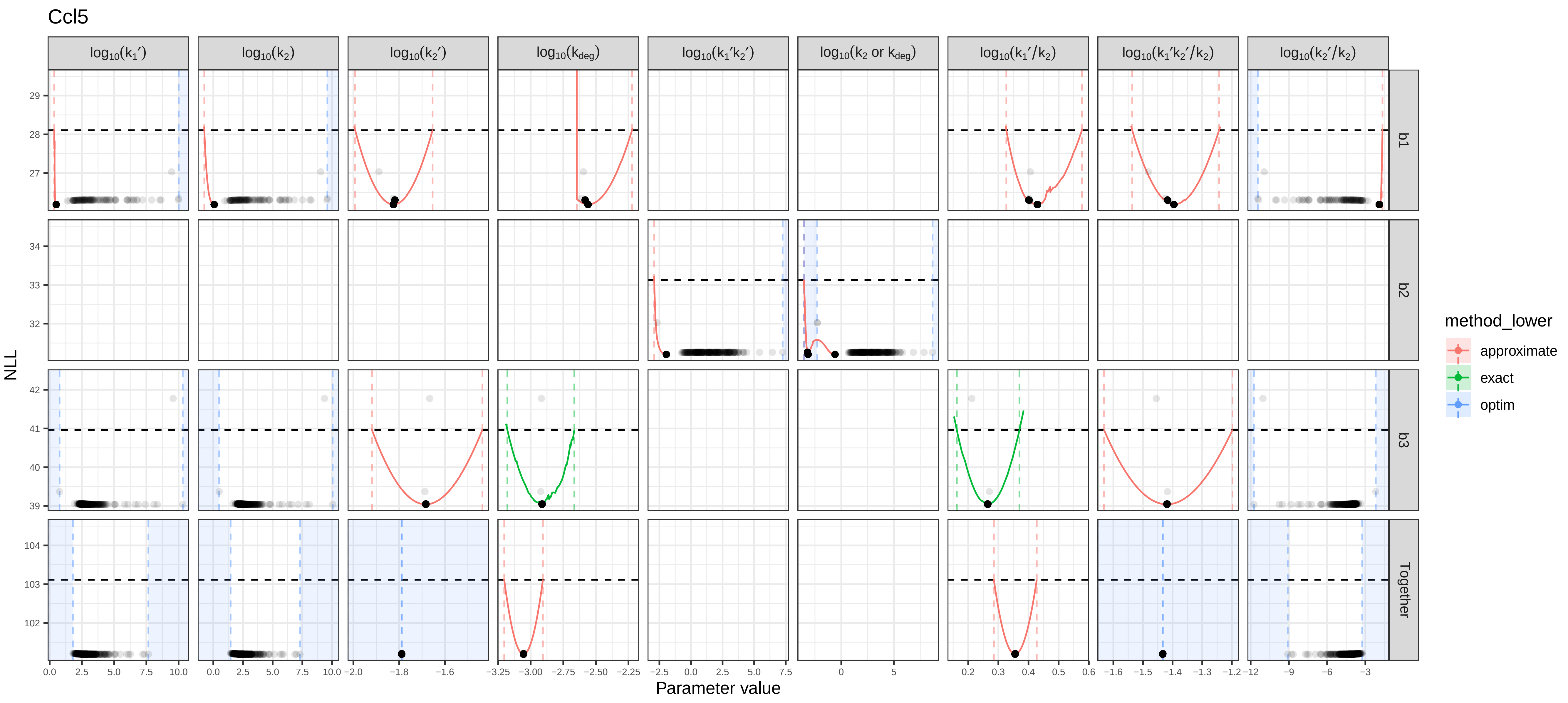

Replicate	Par	Best value	CI95 LB	CI95 UB	Method LB	Method UB
Together	$\log_{10}(k_1')$	4.723	< 1.817	> 7.656	optim	optim
Together	$\log_{10}(k_2)$	4.367	< 1.46	> 7.301	optim	optim
Together	$\log_{10}(k_2')$	−1.789	< −1.79	> −1.788	optim	optim
Together	$\log_{10}(k_{deg})$	−3.055	−3.203	−2.906	approximate	approximate
Together	$\log_{10}(k_1'/k_2)$	0.3553	0.2851	0.4273	approximate	approximate
Together	$\log_{10}(k_1'k_2'/k_2)$	−1.433	< −1.434	> −1.433	optim	optim
Together	$\log_{10}(k_2'/k_2)$	−6.156	< −9.09	> −3.249	optim	optim
b1	$\log_{10}(k_1')$	0.5088	0.3489	> 10.01	approximate	optim
b1	$\log_{10}(k_2)$	0.07942	−0.7571	> 9.608	approximate	optim
b1	$\log_{10}(k_2')$	−1.825	−1.992	−1.654	approximate	approximate
b1	$\log_{10}(k_{deg})$	−2.56	−2.646	−2.222	approximate	approximate
b1	$\log_{10}(k_1'/k_2)$	0.4293	0.3265	0.5775	approximate	approximate
b1	$\log_{10}(k_1'k_2'/k_2)$	−1.395	−1.536	−1.243	approximate	approximate
b1	$\log_{10}(k_2'/k_2)$	−1.904	< −11.43	−1.656	optim	approximate
b2	$\log_{10}(k_1'k_2')$	−1.951	−2.913	> 7.251	approximate	optim
b2	$\log_{10}(k_2 \text{ or } k_{deg})$	−0.593	−3.535	> 8.675	approximate	optim
b2	$\log_{10}(k_2 \text{ or } k_{deg})$	−3.151	−3.535	−2.285	approximate	optim
b3	$\log_{10}(k_1')$	4.497	< 0.7667	> 10.32	optim	optim
b3	$\log_{10}(k_2)$	4.232	< 0.4964	> 10.06	optim	optim
b3	$\log_{10}(k_2')$	−1.684	−1.919	−1.437	approximate	approximate
b3	$\log_{10}(k_{deg})$	−2.913	−3.179	−2.665	exact	exact
b3	$\log_{10}(k_1'/k_2)$	0.265	0.1623	0.3699	exact	exact
b3	$\log_{10}(k_1'k_2'/k_2)$	−1.419	−1.631	−1.198	approximate	approximate
b3	$\log_{10}(k_2'/k_2)$	−5.916	< −11.74	> −2.184	optim	optim

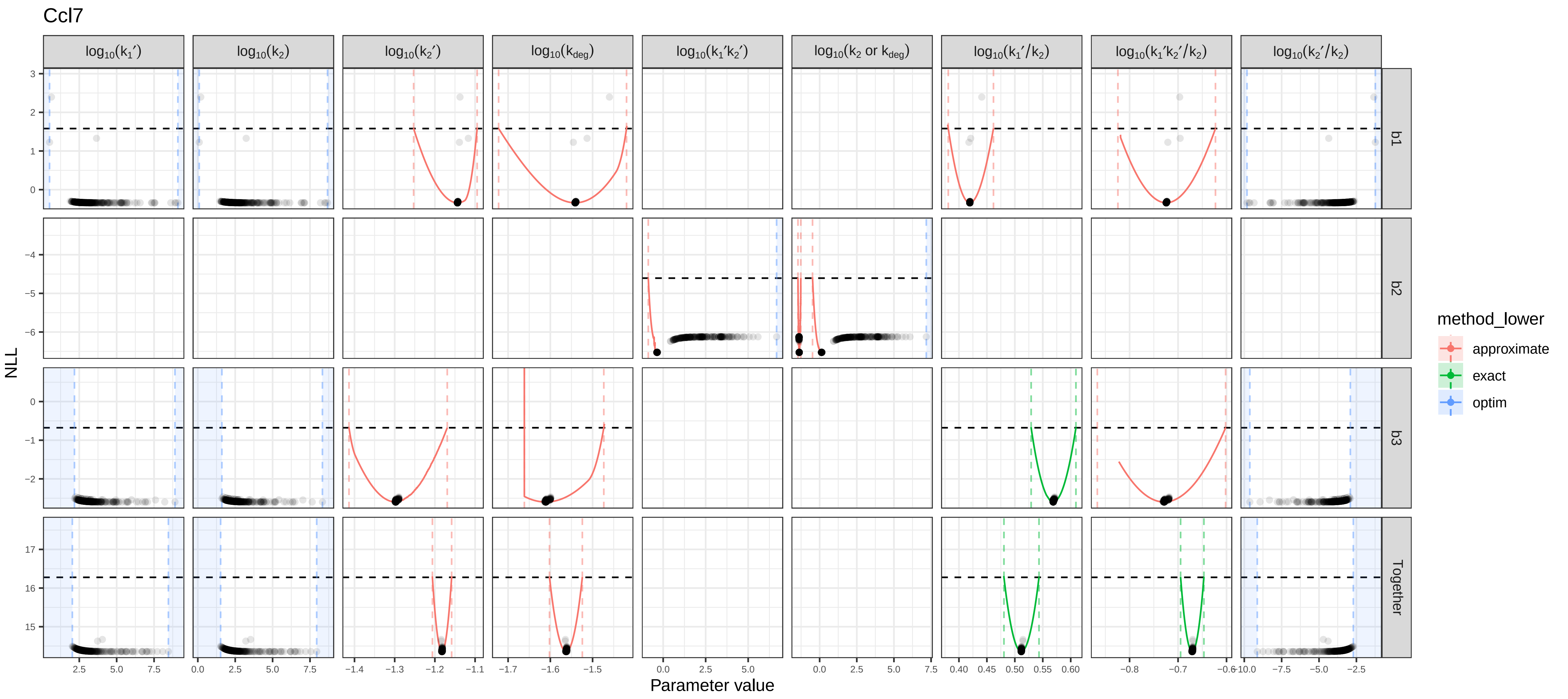

Replicate	Par	Best value	CI95 LB	CI95 UB	Method LB	Method UB
Together	$\log_{10}(k_1')$	6.286	< 2.036	> 8.459	optim	optim
Together	$\log_{10}(k_2)$	5.775	< 1.524	> 7.948	optim	optim
Together	$\log_{10}(k_2')$	−1.182	−1.206	−1.158	approximate	approximate
Together	$\log_{10}(k_{deg})$	−1.563	−1.602	−1.524	approximate	approximate
Together	$\log_{10}(k_1'/k_2)$	0.5115	0.4805	0.5432	exact	exact
Together	$\log_{10}(k_1'k_2'/k_2)$	−0.6709	−0.6948	−0.6467	exact	exact
Together	$\log_{10}(k_2'/k_2)$	−6.957	< −9.13	> −2.706	optim	optim
b1	$\log_{10}(k_1')$	5.324	< 0.5125	> 9.092	optim	optim
b1	$\log_{10}(k_2)$	4.904	< 0.09464	> 8.672	optim	optim
b1	$\log_{10}(k_2')$	−1.144	−1.253	−1.095	approximate	approximate
b1	$\log_{10}(k_{deg})$	−1.541	−1.722	−1.42	approximate	approximate
b1	$\log_{10}(k_1'/k_2)$	0.4194	0.3808	0.4618	approximate	approximate
b1	$\log_{10}(k_1'k_2'/k_2)$	−0.7244	−0.8241	−0.6228	approximate	approximate
b1	$\log_{10}(k_2'/k_2)$	−6.048	< −9.816	> −1.234	optim	optim
b2	$\log_{10}(k_1'k_2')$	−0.368	−0.8762	> 6.672	approximate	optim
b2	$\log_{10}(k_2 \text{ or } k_{deg})$	0.1241	−0.4779	> 7.176	approximate	optim
b2	$\log_{10}(k_2 \text{ or } k_{deg})$	−1.375	−1.459	−1.267	approximate	approximate
b3	$\log_{10}(k_1')$	5.742	< 2.178	> 8.898	optim	optim
b3	$\log_{10}(k_2)$	5.173	< 1.609	> 8.33	optim	optim
b3	$\log_{10}(k_2')$	−1.297	−1.414	−1.169	approximate	approximate
b3	$\log_{10}(k_{deg})$	−1.611	−1.662	−1.473	approximate	approximate
b3	$\log_{10}(k_1'/k_2)$	0.5687	0.5292	0.6093	exact	exact
b3	$\log_{10}(k_1'k_2'/k_2)$	−0.7288	−0.8665	−0.6019	approximate	approximate
b3	$\log_{10}(k_2'/k_2)$	−6.471	< −9.628	> −2.905	optim	optim

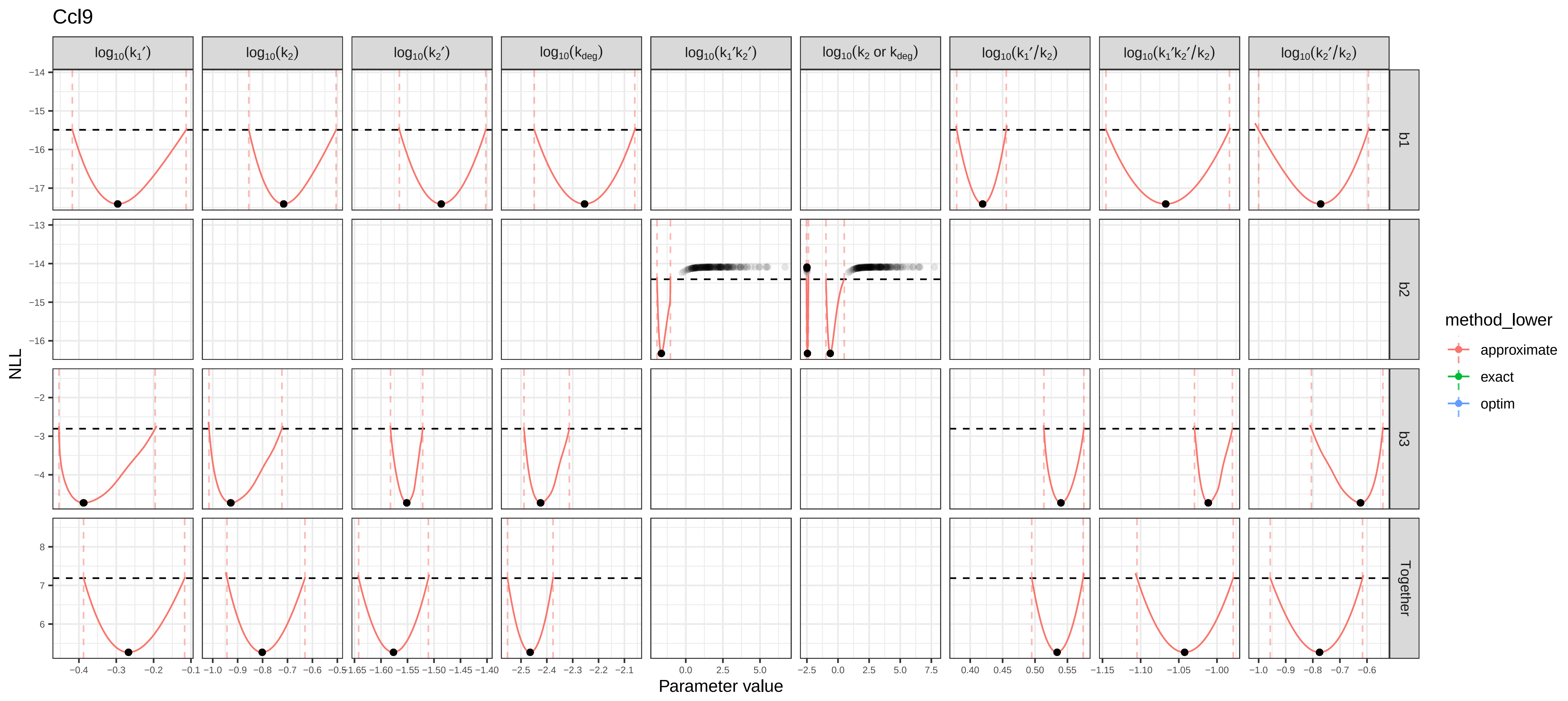

Replicate	Par	Best value	CI95 LB	CI95 UB	Method LB	Method UB
Together	$\log_{10}(k_1')$	−0.2672	−0.3876	−0.1168	approximate	approximate
Together	$\log_{10}(k_2)$	−0.8013	−0.9429	−0.63	approximate	approximate
Together	$\log_{10}(k_2')$	−1.576	−1.642	−1.511	approximate	approximate
Together	$\log_{10}(k_{deg})$	−2.465	−2.552	−2.376	approximate	approximate
Together	$\log_{10}(k_1'/k_2)$	0.5341	0.4949	0.5742	approximate	approximate
Together	$\log_{10}(k_1'k_2'/k_2)$	−1.042	−1.105	−0.9787	approximate	approximate
Together	$\log_{10}(k_2'/k_2)$	−0.7752	−0.9571	−0.6164	approximate	approximate
b1	$\log_{10}(k_1')$	−0.2962	−0.4177	−0.1128	approximate	approximate
b1	$\log_{10}(k_2)$	−0.7154	−0.8553	−0.5048	approximate	approximate
b1	$\log_{10}(k_2')$	−1.486	−1.565	−1.402	approximate	approximate
b1	$\log_{10}(k_{deg})$	−2.254	−2.449	−2.06	approximate	approximate
b1	$\log_{10}(k_1'/k_2)$	0.4192	0.3791	0.4556	approximate	approximate
b1	$\log_{10}(k_1'k_2'/k_2)$	−1.067	−1.145	−0.9837	approximate	approximate
b1	$\log_{10}(k_2'/k_2)$	−0.771	−1	−0.5948	approximate	approximate
b2	$\log_{10}(k_1'k_2')$	−1.644	−1.938	−1.035	approximate	approximate
b2	$\log_{10}(k_2 \text{ or } k_{deg})$	−0.6235	−0.9703	0.5009	approximate	approximate
b2	$\log_{10}(k_2 \text{ or } k_{deg})$	−2.458	−2.537	−2.38	approximate	approximate
b3	$\log_{10}(k_1')$	−0.3863	−0.4534	−0.1957	approximate	approximate
b3	$\log_{10}(k_2)$	−0.9264	−1.015	−0.7222	approximate	approximate
b3	$\log_{10}(k_2')$	−1.551	−1.582	−1.521	approximate	approximate
b3	$\log_{10}(k_{deg})$	−2.423	−2.488	−2.313	approximate	approximate
b3	$\log_{10}(k_1'/k_2)$	0.54	0.5137	0.5754	approximate	approximate
b3	$\log_{10}(k_1'k_2'/k_2)$	−1.011	−1.029	−0.9798	approximate	approximate
b3	$\log_{10}(k_2'/k_2)$	−0.6248	−0.8052	−0.5414	approximate	approximate

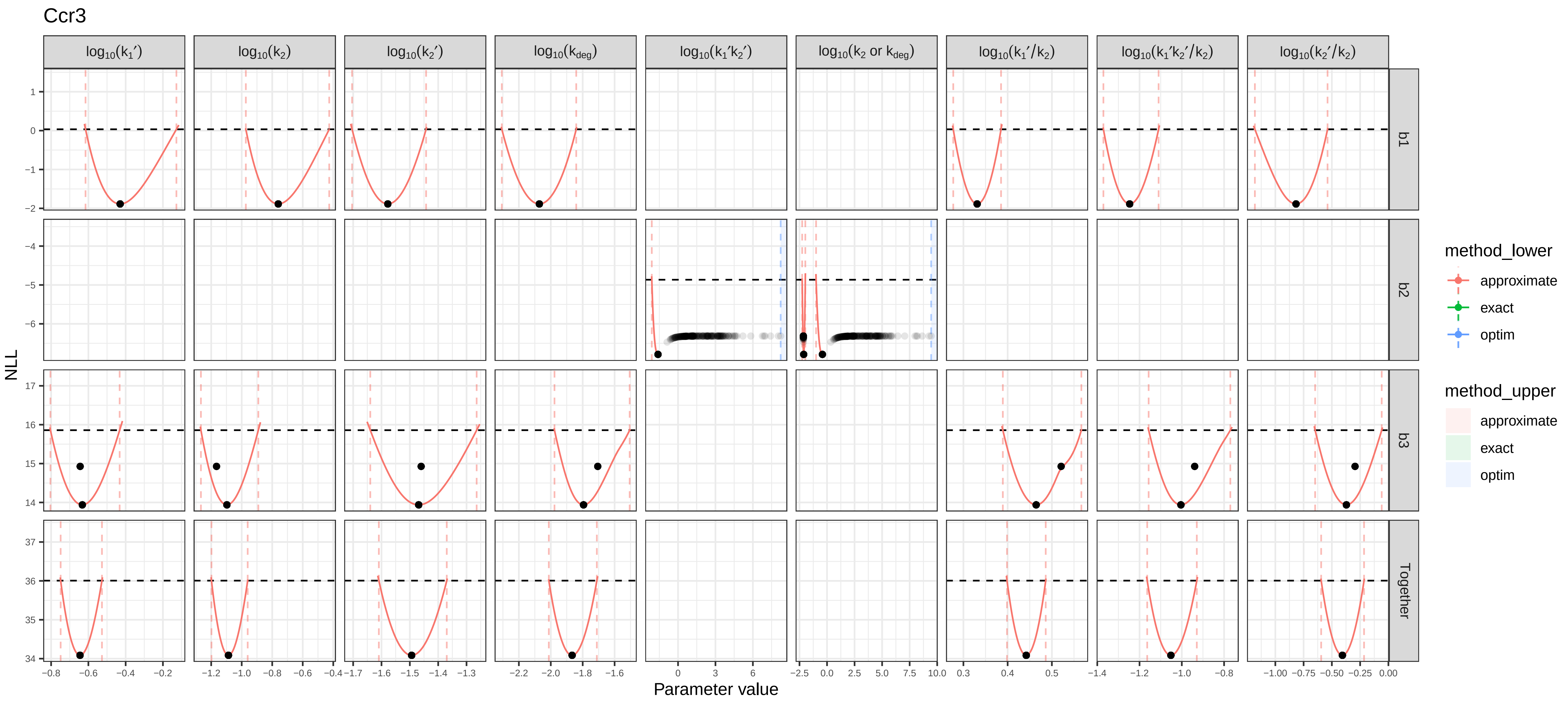

Replicate	Par	Best value	CI95 LB	CI95 UB	Method LB	Method UB
Together	$\log_{10}(k_1')$	−0.6448	−0.7488	−0.5272	approximate	approximate
Together	$\log_{10}(k_2)$	−1.087	−1.198	−0.9607	approximate	approximate
Together	$\log_{10}(k_2')$	−1.494	−1.609	−1.37	approximate	approximate
Together	$\log_{10}(k_{deg})$	−1.866	−2.012	−1.711	approximate	approximate
Together	$\log_{10}(k_1'/k_2)$	0.442	0.3986	0.4861	approximate	approximate
Together	$\log_{10}(k_1'k_2'/k_2)$	−1.052	−1.164	−0.929	approximate	approximate
Together	$\log_{10}(k_2'/k_2)$	−0.4067	−0.5947	−0.2154	approximate	approximate
b1	$\log_{10}(k_1')$	−0.4295	−0.6154	−0.1276	approximate	approximate
b1	$\log_{10}(k_2)$	−0.7606	−0.9729	−0.4264	approximate	approximate
b1	$\log_{10}(k_2')$	−1.578	−1.704	−1.443	approximate	approximate
b1	$\log_{10}(k_{deg})$	−2.072	−2.307	−1.841	approximate	approximate
b1	$\log_{10}(k_1'/k_2)$	0.3311	0.277	0.3851	approximate	approximate
b1	$\log_{10}(k_1'k_2'/k_2)$	−1.247	−1.371	−1.11	approximate	approximate
b1	$\log_{10}(k_2'/k_2)$	−0.8171	−1.181	−0.5381	approximate	approximate
b2	$\log_{10}(k_1'k_2')$	−1.606	−2.105	> 8.228	approximate	optim
b2	$\log_{10}(k_2 \text{ or } k_{deg})$	−0.4198	−0.9941	> 9.451	approximate	optim
b2	$\log_{10}(k_2 \text{ or } k_{deg})$	−2.117	−2.257	−1.971	approximate	approximate
b3	$\log_{10}(k_1')$	−0.6326	−0.8037	−0.4322	approximate	approximate
b3	$\log_{10}(k_2)$	−1.097	−1.267	−0.891	approximate	approximate
b3	$\log_{10}(k_2')$	−1.469	−1.64	−1.264	approximate	approximate
b3	$\log_{10}(k_{deg})$	−1.795	−1.977	−1.506	approximate	approximate
b3	$\log_{10}(k_1'/k_2)$	0.4645	0.3891	0.5667	approximate	approximate
b3	$\log_{10}(k_1'k_2'/k_2)$	−1.004	−1.157	−0.7704	approximate	approximate
b3	$\log_{10}(k_2'/k_2)$	−0.3717	−0.648	−0.05911	approximate	approximate

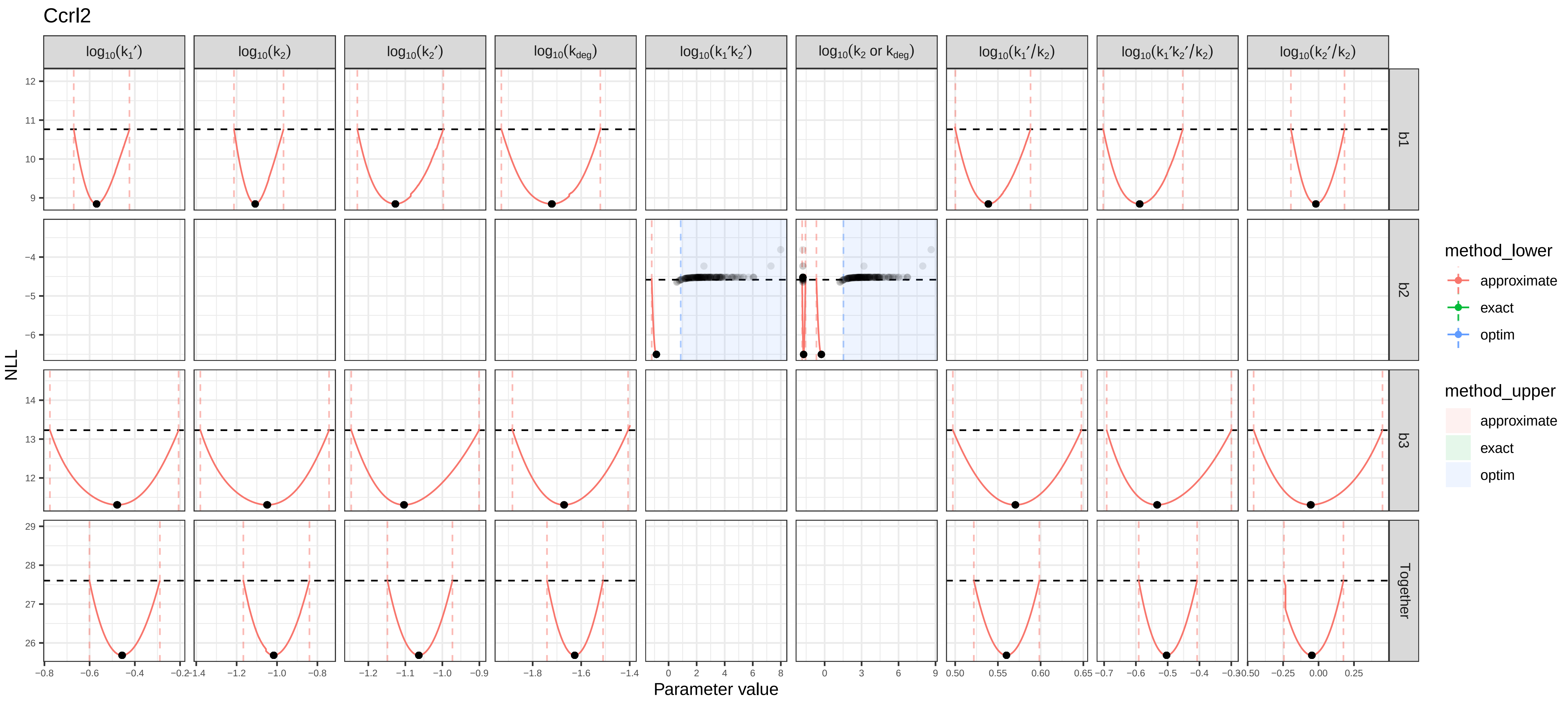

Replicate	Par	Best value	CI95 LB	CI95 UB	Method LB	Method UB
Together	$\log_{10}(k_1')$	−0.4564	−0.6008	−0.2892	approximate	approximate
Together	$\log_{10}(k_2)$	−1.016	−1.167	−0.8393	approximate	approximate
Together	$\log_{10}(k_2')$	−1.064	−1.148	−0.9727	approximate	approximate
Together	$\log_{10}(k_{deg})$	−1.627	−1.741	−1.51	approximate	approximate
Together	$\log_{10}(k_1'/k_2)$	0.56	0.5218	0.5985	approximate	approximate
Together	$\log_{10}(k_1'k_2'/k_2)$	−0.5035	−0.5911	−0.4074	approximate	approximate
Together	$\log_{10}(k_2'/k_2)$	−0.04714	−0.2441	0.1757	approximate	approximate
b1	$\log_{10}(k_1')$	−0.5693	−0.6702	−0.4239	approximate	approximate
b1	$\log_{10}(k_2)$	−1.108	−1.213	−0.9677	approximate	approximate
b1	$\log_{10}(k_2')$	−1.127	−1.23	−0.9973	approximate	approximate
b1	$\log_{10}(k_{deg})$	−1.721	−1.929	−1.521	approximate	approximate
b1	$\log_{10}(k_1'/k_2)$	0.5388	0.5002	0.5882	approximate	approximate
b1	$\log_{10}(k_1'k_2'/k_2)$	−0.5883	−0.7029	−0.4527	approximate	approximate
b1	$\log_{10}(k_2'/k_2)$	−0.01898	−0.195	0.1833	approximate	approximate
b2	$\log_{10}(k_1'k_2')$	−0.8692	−1.201	> 0.8633	approximate	optim
b2	$\log_{10}(k_2 \text{ or } k_{deg})$	−0.2649	−0.6684	> 1.527	approximate	optim
b2	$\log_{10}(k_2 \text{ or } k_{deg})$	−1.708	−1.826	−1.556	approximate	approximate
b3	$\log_{10}(k_1')$	−0.4781	−0.7757	−0.2064	approximate	approximate
b3	$\log_{10}(k_2)$	−1.049	−1.38	−0.7422	approximate	approximate
b3	$\log_{10}(k_2')$	−1.104	−1.246	−0.9008	approximate	approximate
b3	$\log_{10}(k_{deg})$	−1.671	−1.883	−1.407	approximate	approximate
b3	$\log_{10}(k_1'/k_2)$	0.5704	0.4973	0.6477	approximate	approximate
b3	$\log_{10}(k_1'k_2'/k_2)$	−0.5332	−0.6919	−0.2996	approximate	approximate
b3	$\log_{10}(k_2'/k_2)$	−0.05513	−0.4578	0.4513	approximate	approximate

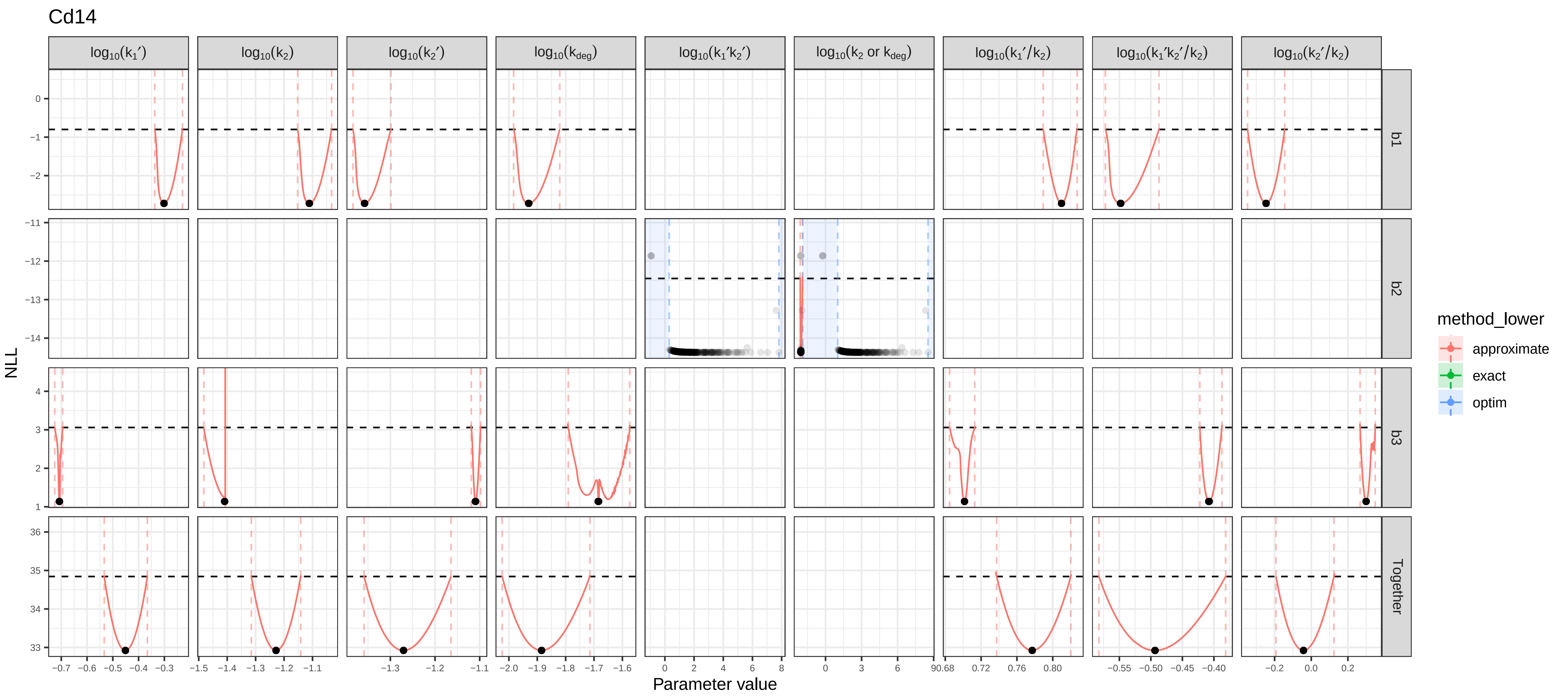

Replicate	Par	Best value	CI95 LB	CI95 UB	Method LB	Method UB
Together	$\log_{10}(k_1')$	−0.4511	−0.5333	−0.3659	approximate	approximate
Together	$\log_{10}(k_2)$	−1.228	−1.315	−1.142	approximate	approximate
Together	$\log_{10}(k_2')$	−1.271	−1.359	−1.164	approximate	approximate
Together	$\log_{10}(k_{deg})$	−1.885	−2.024	−1.715	approximate	approximate
Together	$\log_{10}(k_1'/k_2)$	0.7771	0.7374	0.8202	approximate	approximate
Together	$\log_{10}(k_1'k_2'/k_2)$	−0.4935	−0.5825	−0.3814	approximate	approximate
Together	$\log_{10}(k_2'/k_2)$	−0.04233	−0.1925	0.1239	approximate	approximate
b1	$\log_{10}(k_1')$	−0.3013	−0.3373	−0.2296	approximate	approximate
b1	$\log_{10}(k_2)$	−1.111	−1.152	−1.033	approximate	approximate
b1	$\log_{10}(k_2')$	−1.358	−1.384	−1.299	approximate	approximate
b1	$\log_{10}(k_{deg})$	−1.93	−1.983	−1.821	approximate	approximate
b1	$\log_{10}(k_1'/k_2)$	0.8098	0.7893	0.8272	approximate	approximate
b1	$\log_{10}(k_1'k_2'/k_2)$	−0.548	−0.5721	−0.4872	approximate	approximate
b1	$\log_{10}(k_2'/k_2)$	−0.2467	−0.3474	−0.1449	approximate	approximate
b2	$\log_{10}(k_1'k_2')$	4.325	< 0.2964	> 7.815	optim	optim
b2	$\log_{10}(k_2 \text{ or } k_{deg})$	5.043	1.014	> 8.533	optim	optim
b2	$\log_{10}(k_2 \text{ or } k_{deg})$	−2.055	−2.104	−1.908	approximate	approximate
b3	$\log_{10}(k_1')$	−0.7073	−0.7253	−0.6946	approximate	approximate
b3	$\log_{10}(k_2)$	−1.409	−1.482	−1.407	approximate	approximate
b3	$\log_{10}(k_2')$	−1.109	−1.119	−1.098	approximate	approximate
b3	$\log_{10}(k_{deg})$	−1.684	−1.791	−1.574	approximate	approximate
b3	$\log_{10}(k_1'/k_2)$	0.7014	0.6848	0.7129	approximate	approximate
b3	$\log_{10}(k_1'k_2'/k_2)$	−0.4076	−0.4224	−0.3874	approximate	approximate
b3	$\log_{10}(k_2'/k_2)$	0.2997	0.2667	0.3493	approximate	approximate

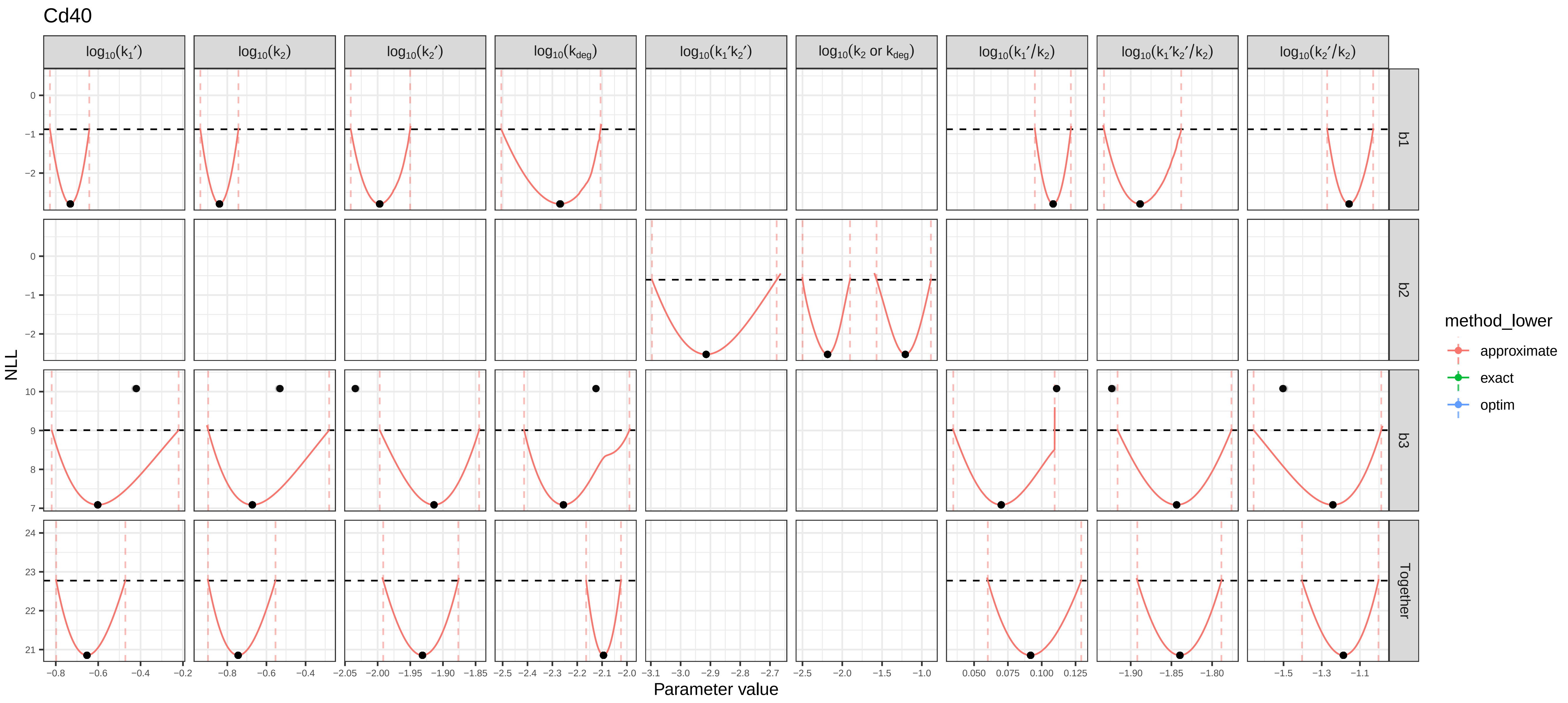

Replicate	Par	Best value	CI95 LB	CI95 UB	Method LB	Method UB
Together	$\log_{10}(k_1')$	−0.6528	−0.7981	−0.4719	approximate	approximate
Together	$\log_{10}(k_2)$	−0.7445	−0.8985	−0.5538	approximate	approximate
Together	$\log_{10}(k_2')$	−1.931	−1.991	−1.876	approximate	approximate
Together	$\log_{10}(k_{deg})$	−2.094	−2.164	−2.024	approximate	approximate
Together	$\log_{10}(k_1'/k_2)$	0.09178	0.06016	0.1292	approximate	approximate
Together	$\log_{10}(k_1'k_2'/k_2)$	−1.84	−1.892	−1.789	approximate	approximate
Together	$\log_{10}(k_2'/k_2)$	−1.187	−1.404	−1.003	approximate	approximate
b1	$\log_{10}(k_1')$	−0.7316	−0.8275	−0.6419	approximate	approximate
b1	$\log_{10}(k_2)$	−0.84	−0.9376	−0.7432	approximate	approximate
b1	$\log_{10}(k_2')$	−1.997	−2.041	−1.95	approximate	approximate
b1	$\log_{10}(k_{deg})$	−2.269	−2.506	−2.106	approximate	approximate
b1	$\log_{10}(k_1'/k_2)$	0.1084	0.09493	0.1215	approximate	approximate
b1	$\log_{10}(k_1'k_2'/k_2)$	−1.889	−1.933	−1.838	approximate	approximate
b1	$\log_{10}(k_2'/k_2)$	−1.157	−1.272	−1.031	approximate	approximate
b2	$\log_{10}(k_1'k_2')$	−2.914	−3.096	−2.678	approximate	approximate
b2	$\log_{10}(k_2 \text{ or } k_{deg})$	−1.208	−1.569	−0.886	approximate	approximate
b2	$\log_{10}(k_2 \text{ or } k_{deg})$	−2.185	−2.499	−1.903	approximate	approximate
b3	$\log_{10}(k_1')$	−0.602	−0.8193	−0.2204	approximate	approximate
b3	$\log_{10}(k_2)$	−0.672	−0.8975	−0.2796	approximate	approximate
b3	$\log_{10}(k_2')$	−1.914	−1.997	−1.845	approximate	approximate
b3	$\log_{10}(k_{deg})$	−2.255	−2.414	−1.99	approximate	approximate
b3	$\log_{10}(k_1'/k_2)$	0.07006	0.03464	0.1096	approximate	approximate
b3	$\log_{10}(k_1'k_2'/k_2)$	−1.844	−1.916	−1.776	approximate	approximate
b3	$\log_{10}(k_2'/k_2)$	−1.242	−1.657	−0.9884	approximate	approximate

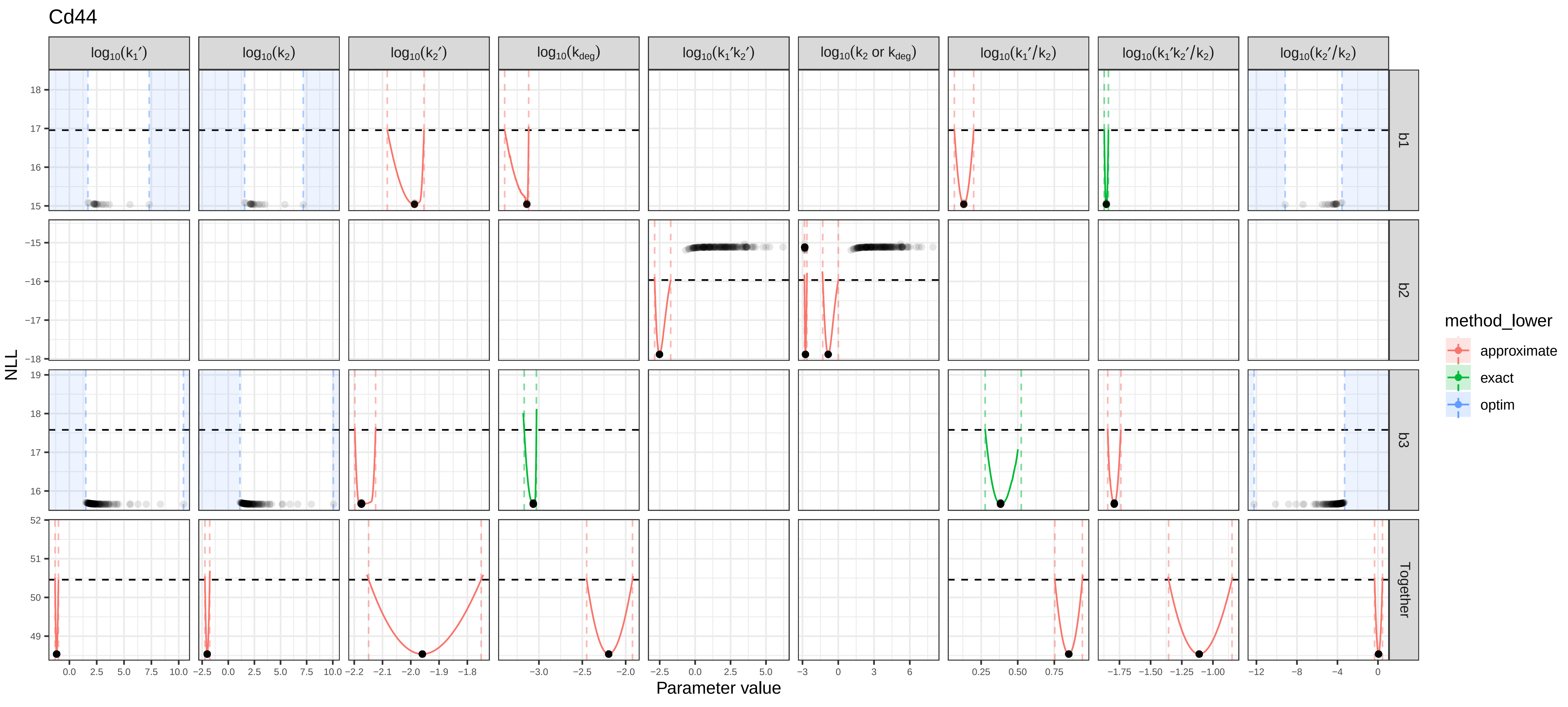

Replicate	Par	Best value	CI95 LB	CI95 UB	Method LB	Method UB
Together	$\log_{10}(k_1')$	−1.168	−1.314	−1.002	approximate	approximate
Together	$\log_{10}(k_2)$	−2.017	−2.232	−1.775	approximate	approximate
Together	$\log_{10}(k_2')$	−1.959	−2.149	−1.751	approximate	approximate
Together	$\log_{10}(k_{deg})$	−2.196	−2.45	−1.921	approximate	approximate
Together	$\log_{10}(k_1'/k_2)$	0.8489	0.7536	0.9419	approximate	approximate
Together	$\log_{10}(k_1'k_2'/k_2)$	−1.11	−1.356	−0.8467	approximate	approximate
Together	$\log_{10}(k_2'/k_2)$	0.05795	−0.3345	0.4481	approximate	approximate
b1	$\log_{10}(k_1')$	5.547	< 1.695	> 7.304	optim	optim
b1	$\log_{10}(k_2)$	5.415	< 1.564	> 7.173	optim	optim
b1	$\log_{10}(k_2')$	−1.987	−2.083	−1.953	approximate	approximate
b1	$\log_{10}(k_{deg})$	−3.14	−3.395	−3.118	approximate	approximate
b1	$\log_{10}(k_1'/k_2)$	0.1313	0.06678	0.199	approximate	approximate
b1	$\log_{10}(k_1'k_2'/k_2)$	−1.856	−1.872	−1.839	exact	exact
b1	$\log_{10}(k_2'/k_2)$	−7.402	< −9.16	> −3.551	optim	optim
b2	$\log_{10}(k_1'k_2')$	−2.513	−2.856	−1.723	approximate	approximate
b2	$\log_{10}(k_2 \text{ or } k_{deg})$	−0.845	−1.302	0.006962	approximate	approximate
b2	$\log_{10}(k_2 \text{ or } k_{deg})$	−2.75	−2.833	−2.627	approximate	approximate
b3	$\log_{10}(k_1')$	6.285	< 1.499	> 10.44	optim	optim
b3	$\log_{10}(k_2)$	5.902	< 1.116	> 10.05	optim	optim
b3	$\log_{10}(k_2')$	−2.175	−2.198	−2.124	approximate	approximate
b3	$\log_{10}(k_{deg})$	−3.065	−3.17	−3.029	exact	exact
b3	$\log_{10}(k_1'/k_2)$	0.3827	0.2775	0.5239	exact	exact
b3	$\log_{10}(k_1'k_2'/k_2)$	−1.792	−1.844	−1.739	approximate	approximate
b3	$\log_{10}(k_2'/k_2)$	−8.077	< −12.23	> −3.29	optim	optim

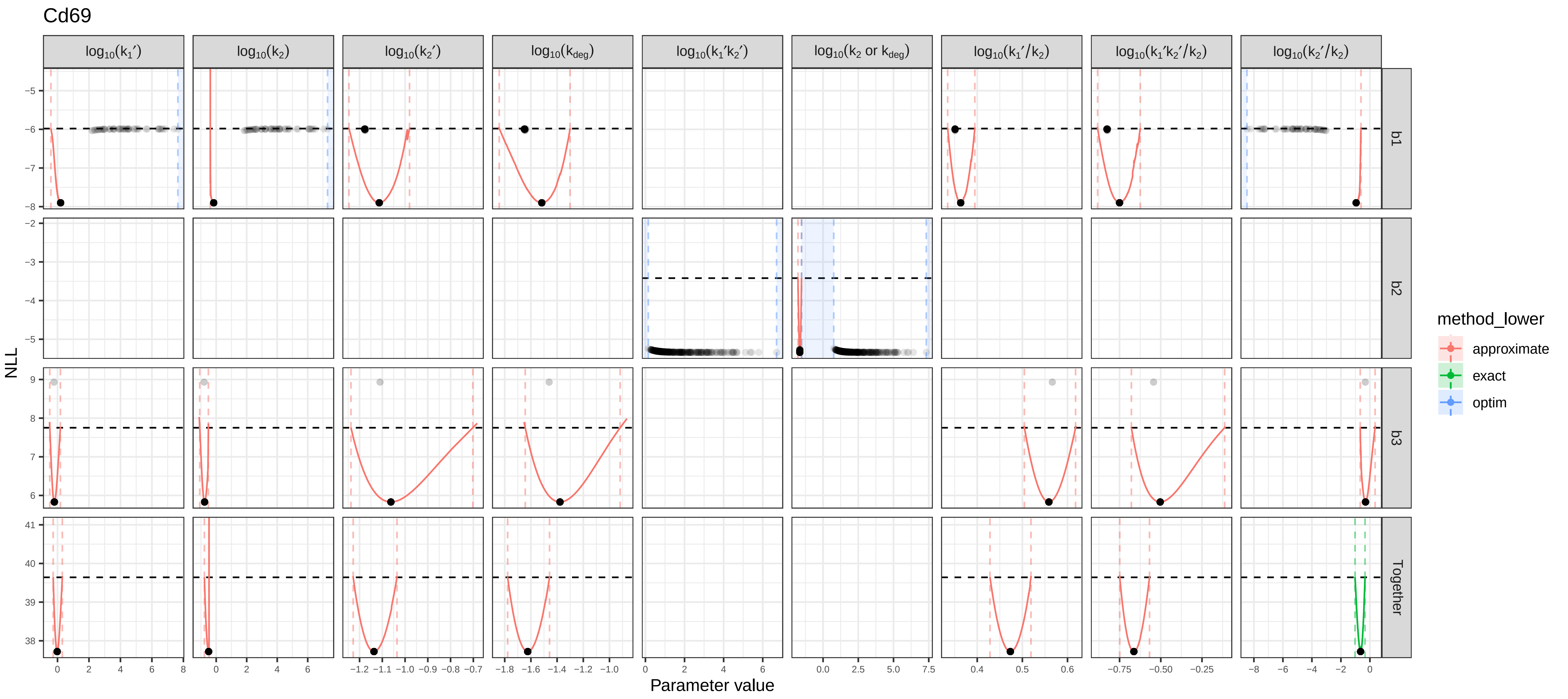

Replicate	Par	Best value	CI95 LB	CI95 UB	Method LB	Method UB
Together	$\log_{10}(k_1')$	−0.02066	−0.2744	0.306	approximate	approximate
Together	$\log_{10}(k_2)$	−0.4941	−0.7728	−0.4694	approximate	approximate
Together	$\log_{10}(k_2')$	−1.137	−1.228	−1.035	approximate	approximate
Together	$\log_{10}(k_{deg})$	−1.624	−1.778	−1.458	approximate	approximate
Together	$\log_{10}(k_1'/k_2)$	0.4734	0.4283	0.5191	approximate	approximate
Together	$\log_{10}(k_1'k_2'/k_2)$	−0.6631	−0.749	−0.5684	approximate	approximate
Together	$\log_{10}(k_2'/k_2)$	−0.6424	−1.028	−0.3378	exact	exact
b1	$\log_{10}(k_1')$	0.1994	−0.419	> 7.649	approximate	optim
b1	$\log_{10}(k_2)$	−0.164	−0.3877	> 7.299	approximate	optim
b1	$\log_{10}(k_2')$	−1.113	−1.246	−0.9803	approximate	approximate
b1	$\log_{10}(k_{deg})$	−1.517	−1.842	−1.301	approximate	approximate
b1	$\log_{10}(k_1'/k_2)$	0.3634	0.3345	0.3947	approximate	approximate
b1	$\log_{10}(k_1'k_2'/k_2)$	−0.7501	−0.883	−0.6243	approximate	approximate
b1	$\log_{10}(k_2'/k_2)$	−0.9495	< −8.476	−0.6157	optim	approximate
b2	$\log_{10}(k_1'k_2')$	4.082	< 0.1458	> 6.709	optim	optim
b2	$\log_{10}(k_2 \text{ or } k_{deg})$	4.698	0.7577	> 7.325	optim	optim
b2	$\log_{10}(k_2 \text{ or } k_{deg})$	−1.649	−1.78	−1.526	approximate	approximate
b3	$\log_{10}(k_1')$	−0.2012	−0.4908	0.182	approximate	approximate
b3	$\log_{10}(k_2)$	−0.76	−1.079	−0.5116	approximate	approximate
b3	$\log_{10}(k_2')$	−1.062	−1.238	−0.7022	approximate	approximate
b3	$\log_{10}(k_{deg})$	−1.377	−1.643	−0.9186	approximate	approximate
b3	$\log_{10}(k_1'/k_2)$	0.5587	0.5044	0.6179	approximate	approximate
b3	$\log_{10}(k_1'k_2'/k_2)$	−0.5033	−0.6783	−0.1128	approximate	approximate
b3	$\log_{10}(k_2'/k_2)$	−0.3021	−0.6688	0.3575	approximate	approximate

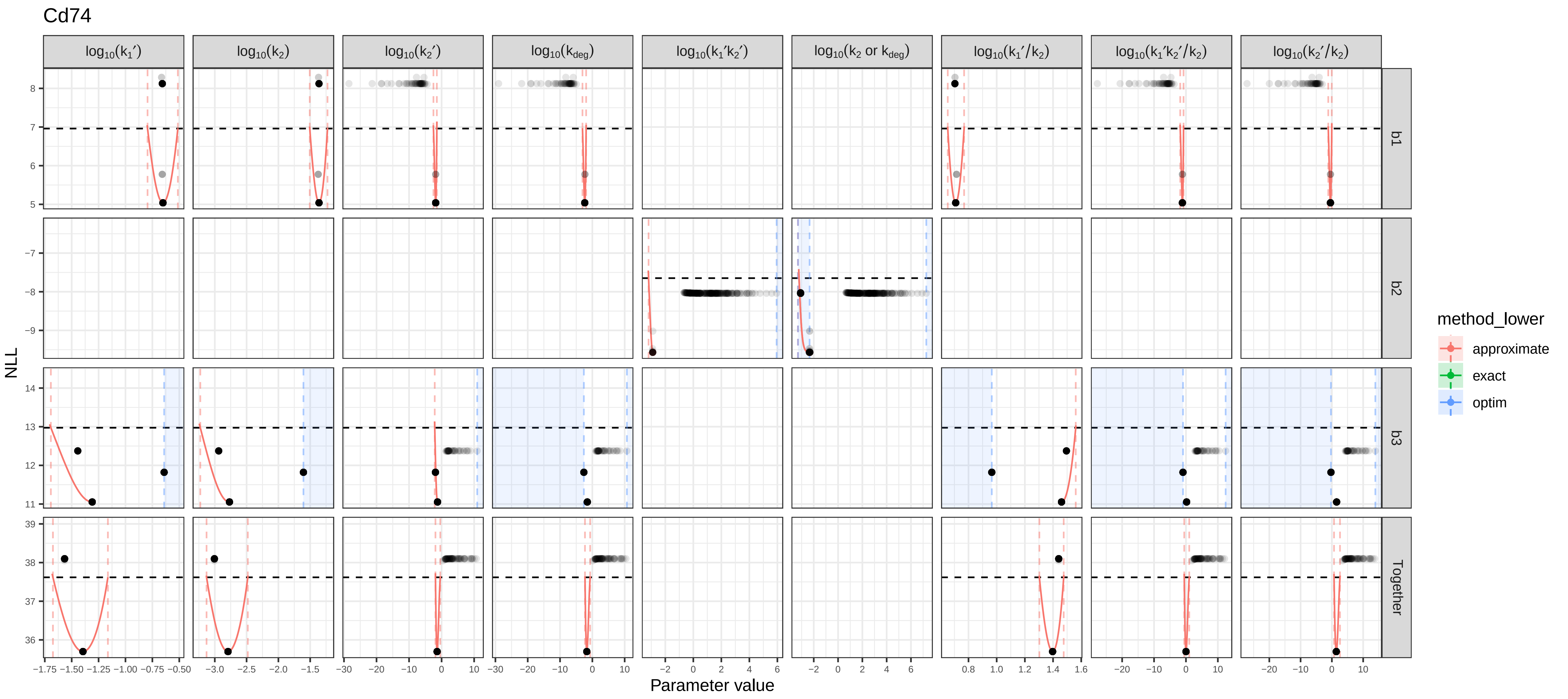

Replicate	Par	Best value	CI95 LB	CI95 UB	Method LB	Method UB
Together	$\log_{10}(k_1')$	−1.396	−1.674	−1.163	approximate	approximate
Together	$\log_{10}(k_2)$	−2.793	−3.13	−2.481	approximate	approximate
Together	$\log_{10}(k_2')$	−1.377	−1.889	−0.4028	approximate	approximate
Together	$\log_{10}(k_{deg})$	−1.713	−2.286	−0.6835	approximate	approximate
Together	$\log_{10}(k_1'/k_2)$	1.397	1.303	1.476	approximate	approximate
Together	$\log_{10}(k_1'k_2'/k_2)$	0.01956	−0.5338	1.034	approximate	approximate
Together	$\log_{10}(k_2'/k_2)$	1.415	0.6896	2.595	approximate	approximate
b1	$\log_{10}(k_1')$	−0.6515	−0.7948	−0.513	approximate	approximate
b1	$\log_{10}(k_2)$	−1.361	−1.507	−1.227	approximate	approximate
b1	$\log_{10}(k_2')$	−1.816	−2.505	−1.462	approximate	approximate
b1	$\log_{10}(k_{deg})$	−2.35	−3.059	−1.925	approximate	approximate
b1	$\log_{10}(k_1'/k_2)$	0.7091	0.6527	0.7688	approximate	approximate
b1	$\log_{10}(k_1'k_2'/k_2)$	−1.107	−1.797	−0.756	approximate	approximate
b1	$\log_{10}(k_2'/k_2)$	−0.4558	−1.157	−0.0396	approximate	approximate
b2	$\log_{10}(k_1'k_2')$	−2.898	−3.192	> 5.944	approximate	optim
b2	$\log_{10}(k_2 \text{ or } k_{deg})$	−2.334	−3.3	> 7.261	approximate	optim
b2	$\log_{10}(k_2 \text{ or } k_{deg})$	−2.351	−3.3	−2.342	approximate	optim
b3	$\log_{10}(k_1')$	−1.309	−1.694	> −0.6405	approximate	optim
b3	$\log_{10}(k_2)$	−2.769	−3.228	> −1.606	approximate	optim
b3	$\log_{10}(k_2')$	−1.251	−2.109	> 10.95	approximate	optim
b3	$\log_{10}(k_{deg})$	−1.565	< −2.632	> 10.69	optim	optim
b3	$\log_{10}(k_1'/k_2)$	1.46	< 0.9652	1.561	optim	approximate
b3	$\log_{10}(k_1'k_2'/k_2)$	0.2097	< −0.9278	> 12.45	optim	optim
b3	$\log_{10}(k_2'/k_2)$	1.519	< −0.2873	> 13.89	optim	optim

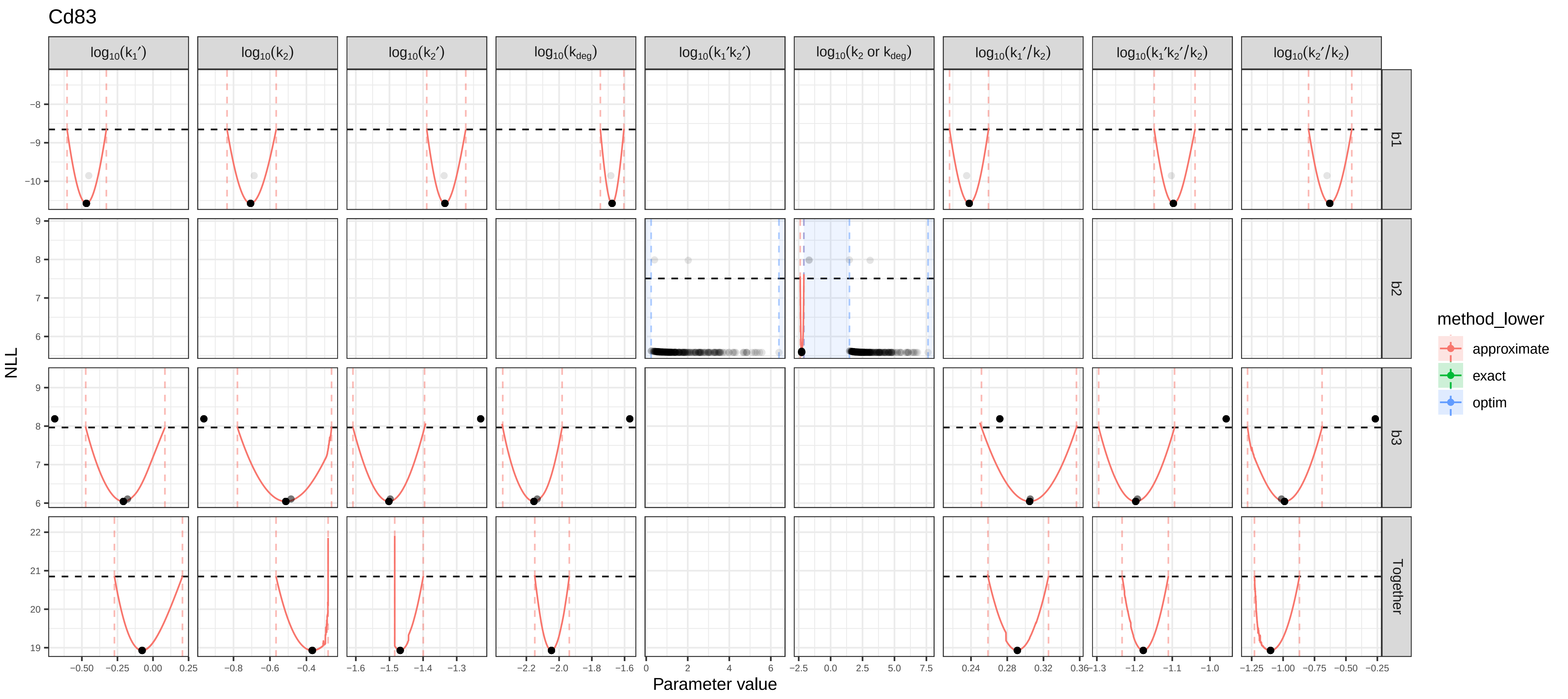

Replicate	Par	Best value	CI95 LB	CI95 UB	Method LB	Method UB
Together	$\log_{10}(k_1')$	−0.07643	−0.2714	0.2069	approximate	approximate
Together	$\log_{10}(k_2)$	−0.3676	−0.5667	−0.2812	approximate	approximate
Together	$\log_{10}(k_2')$	−1.468	−1.484	−1.4	approximate	approximate
Together	$\log_{10}(k_{deg})$	−2.047	−2.148	−1.938	approximate	approximate
Together	$\log_{10}(k_1'/k_2)$	0.2912	0.2587	0.3256	approximate	approximate
Together	$\log_{10}(k_1'k_2'/k_2)$	−1.177	−1.233	−1.11	approximate	approximate
Together	$\log_{10}(k_2'/k_2)$	−1.1	−1.228	−0.8699	approximate	approximate
b1	$\log_{10}(k_1')$	−0.4683	−0.6041	−0.3281	approximate	approximate
b1	$\log_{10}(k_2)$	−0.7064	−0.8355	−0.5658	approximate	approximate
b1	$\log_{10}(k_2')$	−1.335	−1.389	−1.273	approximate	approximate
b1	$\log_{10}(k_{deg})$	−1.676	−1.748	−1.604	approximate	approximate
b1	$\log_{10}(k_1'/k_2)$	0.2381	0.2163	0.2593	approximate	approximate
b1	$\log_{10}(k_1'k_2'/k_2)$	−1.097	−1.148	−1.04	approximate	approximate
b1	$\log_{10}(k_2'/k_2)$	−0.6288	−0.7975	−0.4535	approximate	approximate
b2	$\log_{10}(k_1'k_2')$	4.172	< 0.2375	> 6.391	optim	optim
b2	$\log_{10}(k_2 \text{ or } k_{deg})$	5.411	1.476	> 7.629	optim	optim
b2	$\log_{10}(k_2 \text{ or } k_{deg})$	−2.268	−2.378	−2.097	approximate	approximate
b3	$\log_{10}(k_1')$	−0.2085	−0.473	0.08372	approximate	approximate
b3	$\log_{10}(k_2)$	−0.5132	−0.7786	−0.2622	approximate	approximate
b3	$\log_{10}(k_2')$	−1.502	−1.609	−1.396	approximate	approximate
b3	$\log_{10}(k_{deg})$	−2.154	−2.344	−1.981	approximate	approximate
b3	$\log_{10}(k_1'/k_2)$	0.3047	0.2516	0.3564	approximate	approximate
b3	$\log_{10}(k_1'k_2'/k_2)$	−1.197	−1.295	−1.094	approximate	approximate
b3	$\log_{10}(k_2'/k_2)$	−0.9885	−1.283	−0.6899	approximate	approximate

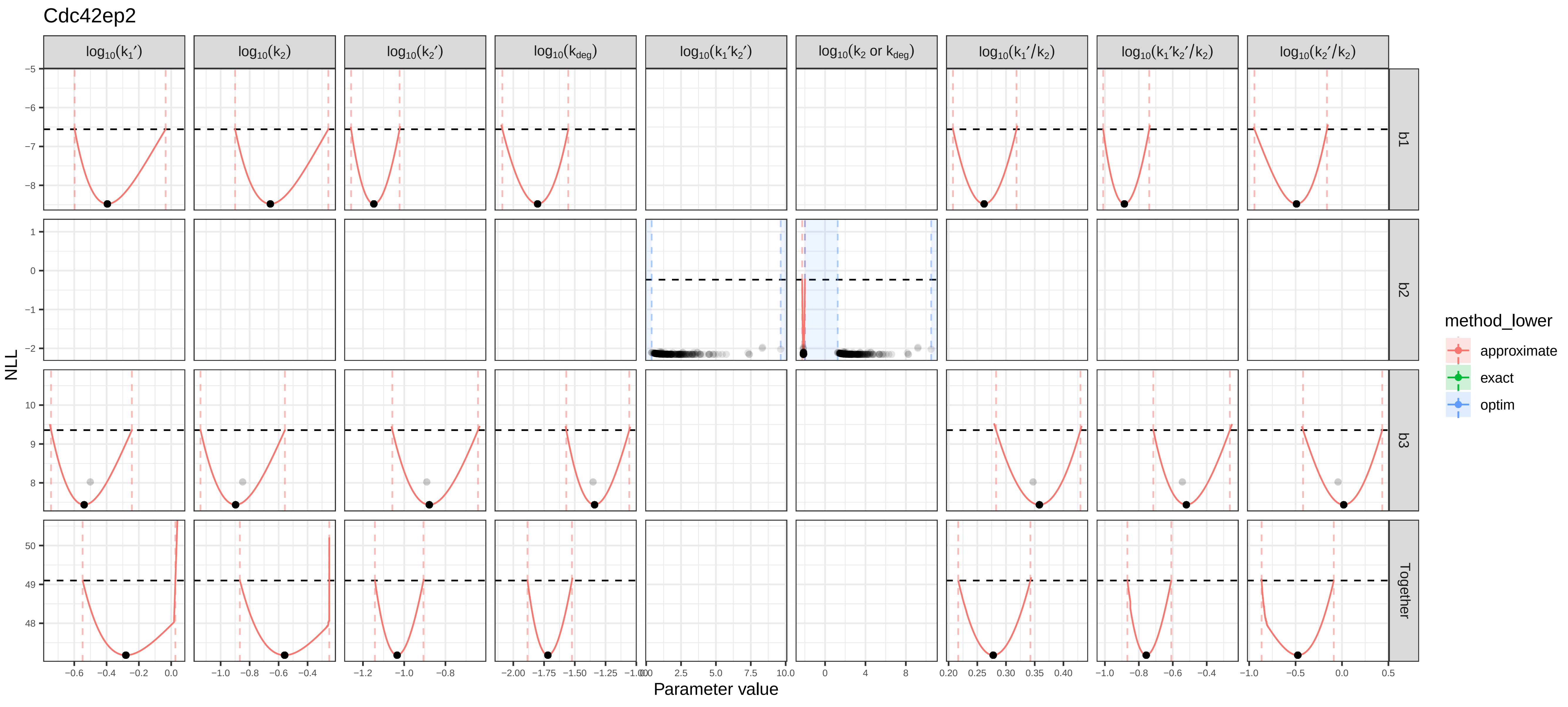

Replicate	Par	Best value	CI95 LB	CI95 UB	Method LB	Method UB
Together	$\log_{10}(k_1')$	−0.2804	−0.5483	0.02589	approximate	approximate
Together	$\log_{10}(k_2)$	−0.5581	−0.8673	−0.2505	approximate	approximate
Together	$\log_{10}(k_2')$	−1.034	−1.142	−0.9066	approximate	approximate
Together	$\log_{10}(k_{deg})$	−1.719	−1.885	−1.523	approximate	approximate
Together	$\log_{10}(k_1'/k_2)$	0.2777	0.2168	0.3424	approximate	approximate
Together	$\log_{10}(k_1'k_2'/k_2)$	−0.7565	−0.8676	−0.609	approximate	approximate
Together	$\log_{10}(k_2'/k_2)$	−0.4761	−0.8661	−0.08842	approximate	approximate
b1	$\log_{10}(k_1')$	−0.395	−0.5979	−0.03457	approximate	approximate
b1	$\log_{10}(k_2)$	−0.6569	−0.9003	−0.257	approximate	approximate
b1	$\log_{10}(k_2')$	−1.147	−1.258	−1.022	approximate	approximate
b1	$\log_{10}(k_{deg})$	−1.803	−2.089	−1.553	approximate	approximate
b1	$\log_{10}(k_1'/k_2)$	0.2619	0.2076	0.3183	approximate	approximate
b1	$\log_{10}(k_1'k_2'/k_2)$	−0.8852	−1.01	−0.7388	approximate	approximate
b1	$\log_{10}(k_2'/k_2)$	−0.4902	−0.9434	−0.1621	approximate	approximate
b2	$\log_{10}(k_1'k_2')$	5.739	< 0.3945	> 9.642	optim	optim
b2	$\log_{10}(k_2 \text{ or } k_{deg})$	6.596	1.252	> 10.51	optim	optim
b2	$\log_{10}(k_2 \text{ or } k_{deg})$	−2.141	−2.27	−1.997	approximate	approximate
b3	$\log_{10}(k_1')$	−0.5388	−0.7437	−0.2434	approximate	approximate
b3	$\log_{10}(k_2)$	−0.897	−1.139	−0.5566	approximate	approximate
b3	$\log_{10}(k_2')$	−0.8782	−1.058	−0.6421	approximate	approximate
b3	$\log_{10}(k_{deg})$	−1.339	−1.569	−1.057	approximate	approximate
b3	$\log_{10}(k_1'/k_2)$	0.3581	0.2827	0.4296	approximate	approximate
b3	$\log_{10}(k_1'k_2'/k_2)$	−0.5201	−0.7147	−0.2638	approximate	approximate
b3	$\log_{10}(k_2'/k_2)$	0.01878	−0.4197	0.4332	approximate	approximate

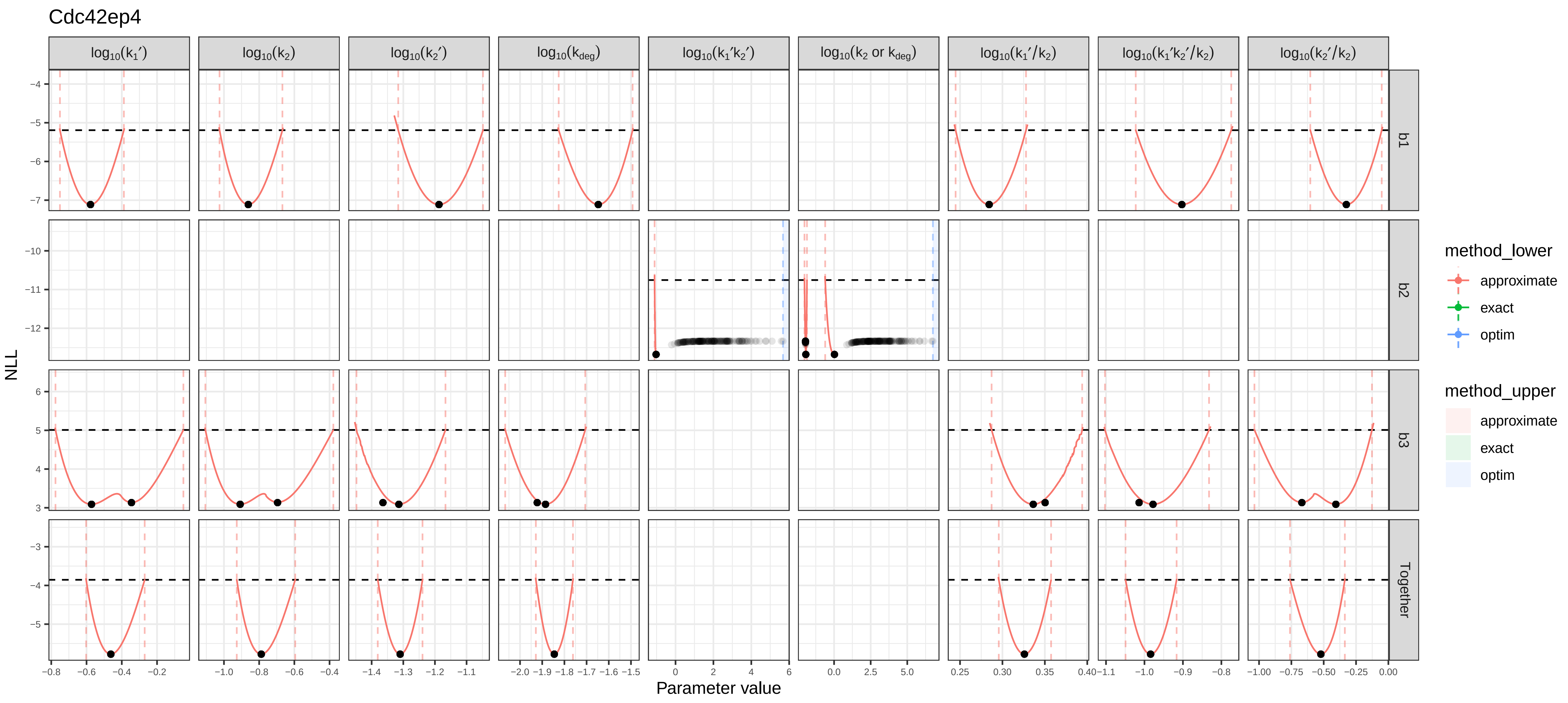

Replicate	Par	Best value	CI95 LB	CI95 UB	Method LB	Method UB
Together	$\log_{10}(k_1')$	−0.462	−0.6021	−0.2701	approximate	approximate
Together	$\log_{10}(k_2)$	−0.7879	−0.9272	−0.5952	approximate	approximate
Together	$\log_{10}(k_2')$	−1.31	−1.381	−1.239	approximate	approximate
Together	$\log_{10}(k_{deg})$	−1.846	−1.929	−1.761	approximate	approximate
Together	$\log_{10}(k_1'/k_2)$	0.3259	0.2957	0.3574	approximate	approximate
Together	$\log_{10}(k_1'k_2'/k_2)$	−0.984	−1.049	−0.9164	approximate	approximate
Together	$\log_{10}(k_2'/k_2)$	−0.522	−0.7607	−0.3365	approximate	approximate
b1	$\log_{10}(k_1')$	−0.5775	−0.7508	−0.3881	approximate	approximate
b1	$\log_{10}(k_2)$	−0.8618	−1.025	−0.6678	approximate	approximate
b1	$\log_{10}(k_2')$	−1.187	−1.316	−1.048	approximate	approximate
b1	$\log_{10}(k_{deg})$	−1.647	−1.826	−1.493	approximate	approximate
b1	$\log_{10}(k_1'/k_2)$	0.2843	0.2448	0.3278	approximate	approximate
b1	$\log_{10}(k_1'k_2'/k_2)$	−0.9027	−1.023	−0.7744	approximate	approximate
b1	$\log_{10}(k_2'/k_2)$	−0.3253	−0.6038	−0.05126	approximate	approximate
b2	$\log_{10}(k_1'k_2')$	−1.028	−1.106	> 5.675	approximate	optim
b2	$\log_{10}(k_2 \text{ or } k_{deg})$	0.02481	−0.6078	> 6.751	approximate	optim
b2	$\log_{10}(k_2 \text{ or } k_{deg})$	−1.929	−2.022	−1.852	approximate	approximate
b3	$\log_{10}(k_1')$	−0.5717	−0.7761	−0.05056	approximate	approximate
b3	$\log_{10}(k_2)$	−0.908	−1.106	−0.3784	approximate	approximate
b3	$\log_{10}(k_2')$	−1.314	−1.448	−1.167	approximate	approximate
b3	$\log_{10}(k_{deg})$	−1.885	−2.067	−1.706	approximate	approximate
b3	$\log_{10}(k_1'/k_2)$	0.3363	0.2872	0.394	approximate	approximate
b3	$\log_{10}(k_1'k_2'/k_2)$	−0.9777	−1.103	−0.832	approximate	approximate
b3	$\log_{10}(k_2'/k_2)$	−0.406	−1.036	−0.1267	approximate	approximate

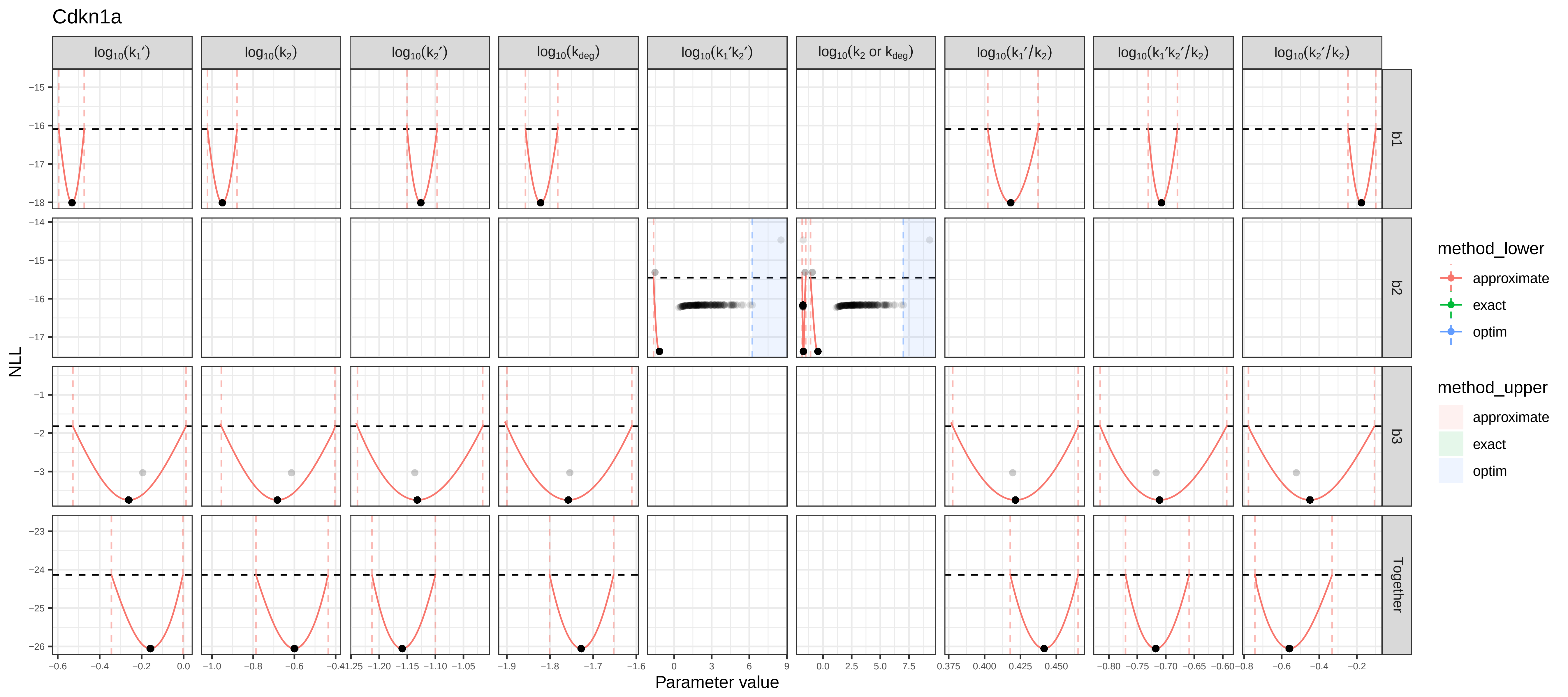

Replicate	Par	Best value	CI95 LB	CI95 UB	Method LB	Method UB
Together	$\log_{10}(k_1')$	−0.1586	−0.3441	−0.003625	approximate	approximate
Together	$\log_{10}(k_2)$	−0.6001	−0.787	−0.4356	approximate	approximate
Together	$\log_{10}(k_2')$	−1.159	−1.213	−1.1	approximate	approximate
Together	$\log_{10}(k_{deg})$	−1.728	−1.801	−1.653	approximate	approximate
Together	$\log_{10}(k_1'/k_2)$	0.4415	0.4179	0.4652	approximate	approximate
Together	$\log_{10}(k_1'k_2'/k_2)$	−0.7175	−0.7707	−0.6589	approximate	approximate
Together	$\log_{10}(k_2'/k_2)$	−0.5589	−0.7426	−0.3312	approximate	approximate
b1	$\log_{10}(k_1')$	−0.5318	−0.5952	−0.4735	approximate	approximate
b1	$\log_{10}(k_2)$	−0.9501	−1.022	−0.8784	approximate	approximate
b1	$\log_{10}(k_2')$	−1.126	−1.15	−1.097	approximate	approximate
b1	$\log_{10}(k_{deg})$	−1.821	−1.857	−1.782	approximate	approximate
b1	$\log_{10}(k_1'/k_2)$	0.4183	0.4022	0.4373	approximate	approximate
b1	$\log_{10}(k_1'k_2'/k_2)$	−0.7075	−0.7308	−0.6795	approximate	approximate
b1	$\log_{10}(k_2'/k_2)$	−0.1757	−0.2472	−0.09912	approximate	approximate
b2	$\log_{10}(k_1'k_2')$	−1.169	−1.641	> 6.239	approximate	optim
b2	$\log_{10}(k_2 \text{ or } k_{deg})$	−0.4454	−1.09	> 7.013	approximate	optim
b2	$\log_{10}(k_2 \text{ or } k_{deg})$	−1.71	−1.813	−1.513	approximate	approximate
b3	$\log_{10}(k_1')$	−0.2613	−0.5279	0.01165	approximate	approximate
b3	$\log_{10}(k_2)$	−0.6827	−0.9547	−0.4038	approximate	approximate
b3	$\log_{10}(k_2')$	−1.132	−1.239	−1.016	approximate	approximate
b3	$\log_{10}(k_{deg})$	−1.758	−1.9	−1.611	approximate	approximate
b3	$\log_{10}(k_1'/k_2)$	0.4214	0.3778	0.4652	approximate	approximate
b3	$\log_{10}(k_1'k_2'/k_2)$	−0.7108	−0.8151	−0.5932	approximate	approximate
b3	$\log_{10}(k_2'/k_2)$	−0.4495	−0.7767	−0.1063	approximate	approximate

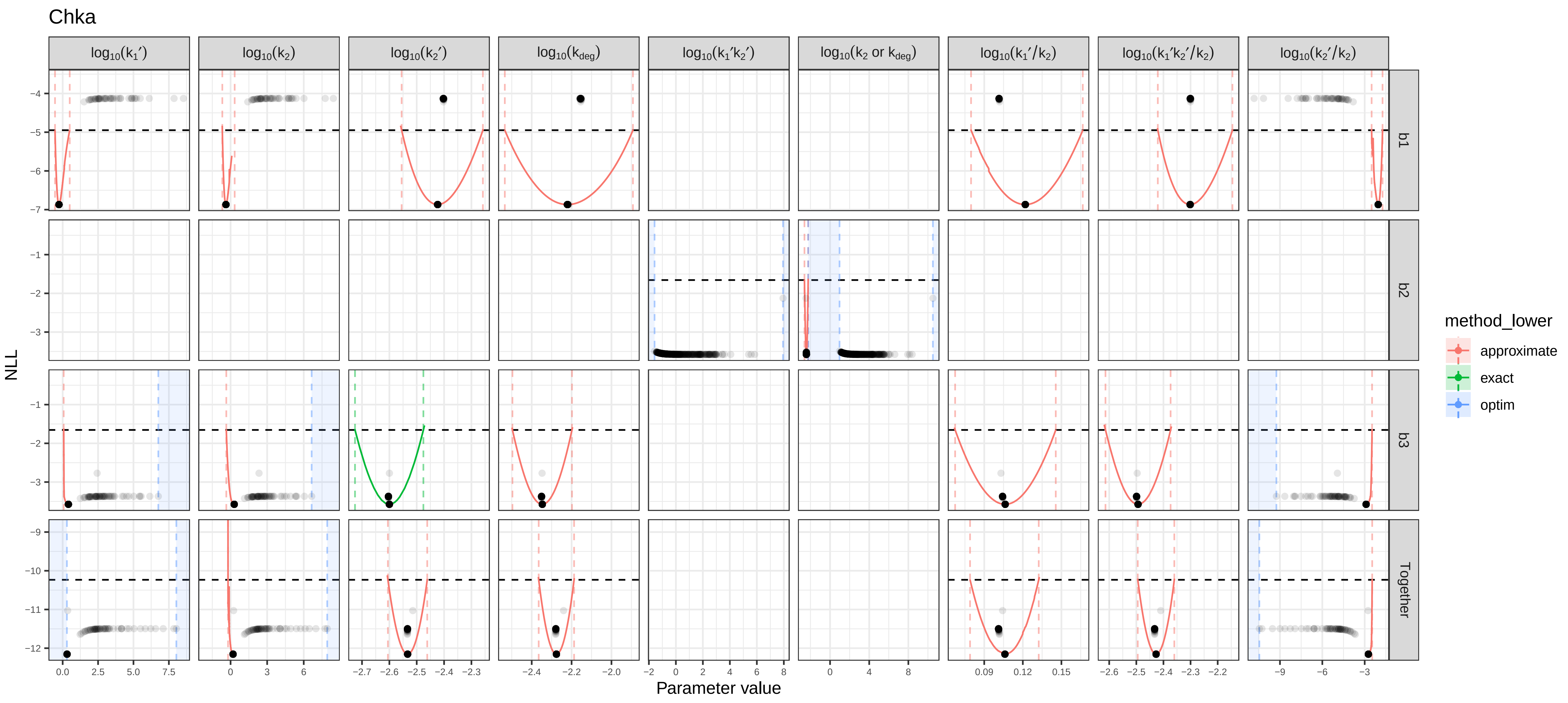

Replicate	Par	Best value	CI95 LB	CI95 UB	Method LB	Method UB
Together	$\log_{10}(k_1')$	0.31	< 0.3001	> 8.028	optim	optim
Together	$\log_{10}(k_2)$	0.2039	−0.2087	> 7.927	approximate	optim
Together	$\log_{10}(k_2')$	−2.533	−2.605	−2.462	approximate	approximate
Together	$\log_{10}(k_{deg})$	−2.276	−2.365	−2.188	approximate	approximate
Together	$\log_{10}(k_1'/k_2)$	0.1061	0.07897	0.1324	approximate	approximate
Together	$\log_{10}(k_1'k_2'/k_2)$	−2.427	−2.495	−2.36	approximate	approximate
Together	$\log_{10}(k_2'/k_2)$	−2.737	< −10.46	−2.468	optim	approximate
b1	$\log_{10}(k_1')$	−0.2607	−0.541	0.4986	approximate	approximate
b1	$\log_{10}(k_2)$	−0.3827	−0.6786	0.3344	approximate	approximate
b1	$\log_{10}(k_2')$	−2.423	−2.555	−2.258	approximate	approximate
b1	$\log_{10}(k_{deg})$	−2.22	−2.535	−1.892	approximate	approximate
b1	$\log_{10}(k_1'/k_2)$	0.1219	0.07975	0.1666	approximate	approximate
b1	$\log_{10}(k_1'k_2'/k_2)$	−2.302	−2.421	−2.146	approximate	approximate
b1	$\log_{10}(k_2'/k_2)$	−2.041	−2.508	−1.735	approximate	approximate
b2	$\log_{10}(k_1'k_2')$	2.891	< −1.58	> 7.94	optim	optim
b2	$\log_{10}(k_2 \text{ or } k_{deg})$	5.439	0.9685	> 10.51	optim	optim
b2	$\log_{10}(k_2 \text{ or } k_{deg})$	−2.418	−2.615	−2.235	approximate	approximate
b3	$\log_{10}(k_1')$	0.4049	0.07393	> 6.754	approximate	optim
b3	$\log_{10}(k_2)$	0.2986	−0.3523	> 6.65	approximate	optim
b3	$\log_{10}(k_2')$	−2.6	−2.726	−2.476	exact	exact
b3	$\log_{10}(k_{deg})$	−2.347	−2.497	−2.198	approximate	approximate
b3	$\log_{10}(k_1'/k_2)$	0.1063	0.06737	0.1456	approximate	approximate
b3	$\log_{10}(k_1'k_2'/k_2)$	−2.494	−2.614	−2.374	approximate	approximate
b3	$\log_{10}(k_2'/k_2)$	−2.899	< −9.254	−2.477	optim	approximate

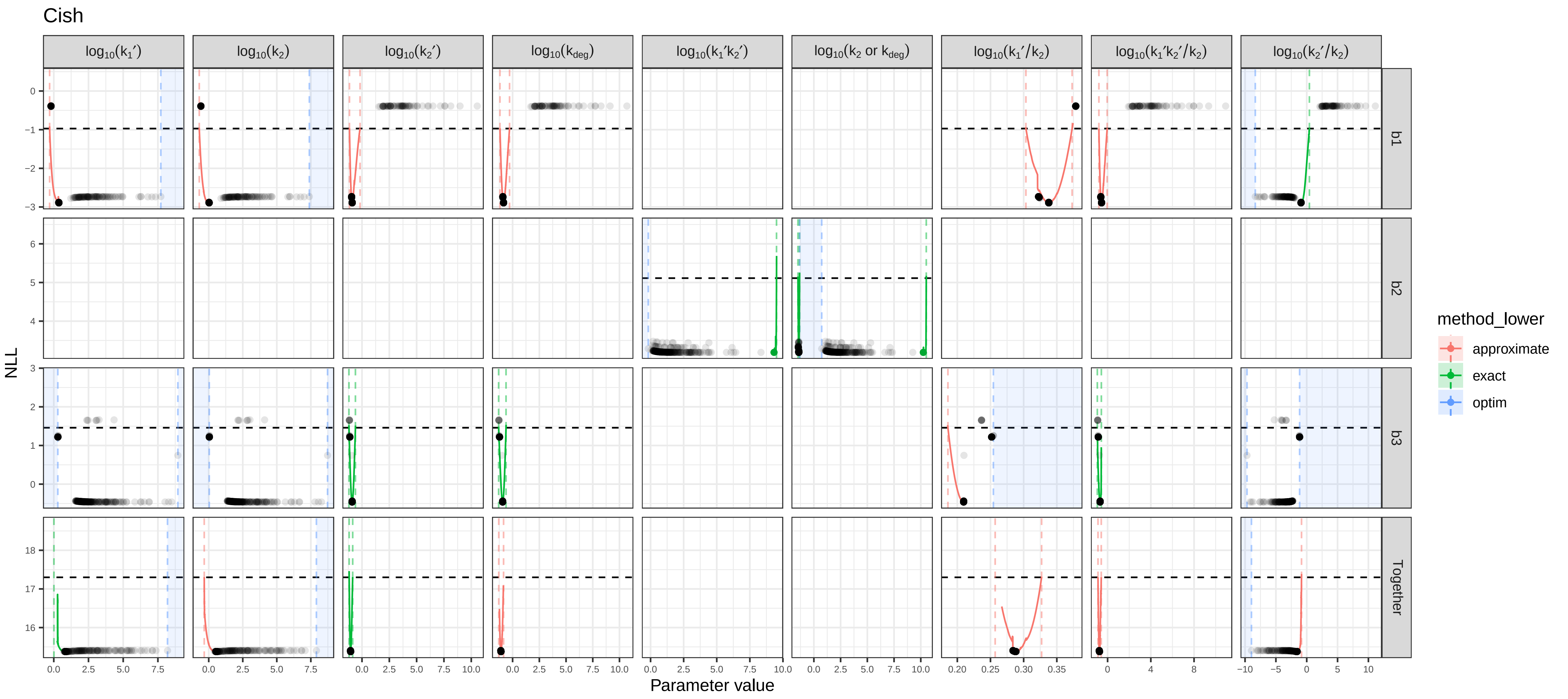

Replicate	Par	Best value	CI95 LB	CI95 UB	Method LB	Method UB
Together	$\log_{10}(k_1')$	0.8672	0.01096	> 8.193	exact	optim
Together	$\log_{10}(k_2)$	0.5795	−0.3309	> 7.909	approximate	optim
Together	$\log_{10}(k_2')$	−1.041	−1.173	−0.8521	exact	exact
Together	$\log_{10}(k_{deg})$	−1.089	−1.296	−0.8464	approximate	approximate
Together	$\log_{10}(k_1'/k_2)$	0.2877	0.2571	0.327	approximate	approximate
Together	$\log_{10}(k_1'k_2'/k_2)$	−0.7529	−0.8879	−0.5857	approximate	approximate
Together	$\log_{10}(k_2'/k_2)$	−1.62	< −8.961	−0.8428	optim	approximate
b1	$\log_{10}(k_1')$	0.3664	−0.2996	> 7.712	approximate	optim
b1	$\log_{10}(k_2)$	0.02856	−0.697	> 7.39	approximate	optim
b1	$\log_{10}(k_2')$	−0.8965	−1.147	−0.1763	approximate	approximate
b1	$\log_{10}(k_{deg})$	−0.847	−1.182	−0.2864	approximate	approximate
b1	$\log_{10}(k_1'/k_2)$	0.3378	0.3034	0.3731	approximate	approximate
b1	$\log_{10}(k_1'k_2'/k_2)$	−0.5587	−0.8058	−0.03624	approximate	approximate
b1	$\log_{10}(k_2'/k_2)$	−0.9251	< −8.339	0.4315	optim	exact
b2	$\log_{10}(k_1'k_2')$	9.326	< −0.1727	9.514	optim	exact
b2	$\log_{10}(k_2 \text{ or } k_{deg})$	10.24	0.7338	10.51	optim	exact
b2	$\log_{10}(k_2 \text{ or } k_{deg})$	−1.413	−1.488	−1.341	exact	exact
b3	$\log_{10}(k_1')$	8.011	< 0.291	> 8.939	optim	optim
b3	$\log_{10}(k_2)$	7.802	< 0.03881	> 8.729	optim	optim
b3	$\log_{10}(k_2')$	−0.9029	−1.192	−0.6021	exact	exact
b3	$\log_{10}(k_{deg})$	−0.9348	−1.307	−0.6147	exact	exact
b3	$\log_{10}(k_1'/k_2)$	0.2095	0.1858	> 0.2544	approximate	optim
b3	$\log_{10}(k_1'k_2'/k_2)$	−0.6934	−0.9488	−0.5775	exact	exact
b3	$\log_{10}(k_2'/k_2)$	−8.705	< −9.68	> −1.152	optim	optim

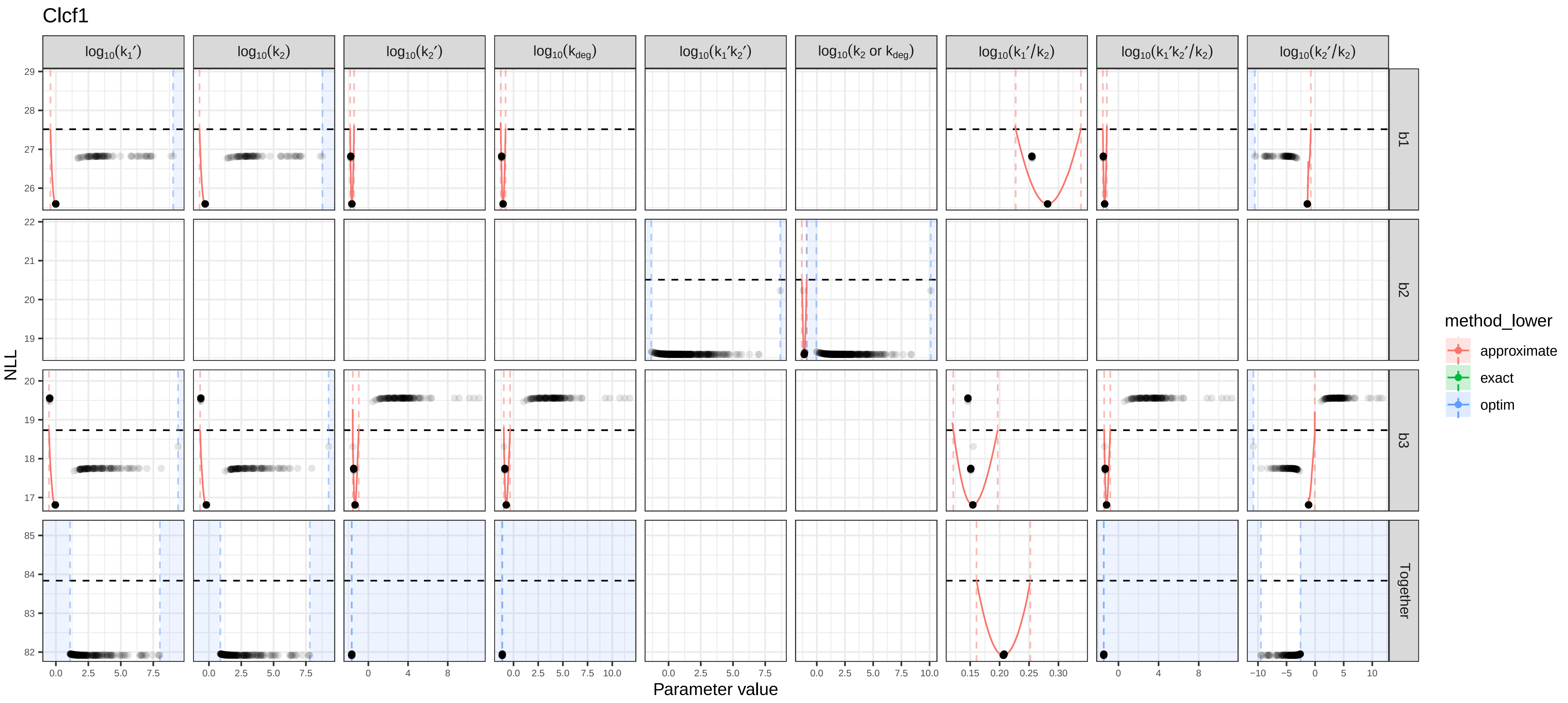

Replicate	Par	Best value	CI95 LB	CI95 UB	Method LB	Method UB
Together	$\log_{10}(k_1')$	6.662	< 1.092	> 8.011	optim	optim
Together	$\log_{10}(k_2)$	6.456	< 0.8835	> 7.805	optim	optim
Together	$\log_{10}(k_2')$	−1.677	< −1.679	> −1.668	optim	optim
Together	$\log_{10}(k_{deg})$	−1.141	< −1.144	> −1.13	optim	optim
Together	$\log_{10}(k_1'/k_2)$	0.2064	0.1608	0.2523	approximate	approximate
Together	$\log_{10}(k_1'k_2'/k_2)$	−1.47	< −1.473	> −1.46	optim	optim
Together	$\log_{10}(k_2'/k_2)$	−8.133	< −9.481	> −2.552	optim	optim
b1	$\log_{10}(k_1')$	−0.009547	−0.4182	> 9.033	approximate	optim
b1	$\log_{10}(k_2)$	−0.2912	−0.7238	> 8.778	approximate	optim
b1	$\log_{10}(k_2')$	−1.657	−1.839	−1.438	approximate	approximate
b1	$\log_{10}(k_{deg})$	−1.057	−1.292	−0.8031	approximate	approximate
b1	$\log_{10}(k_1'/k_2)$	0.2816	0.2274	0.3383	approximate	approximate
b1	$\log_{10}(k_1'k_2'/k_2)$	−1.375	−1.554	−1.154	approximate	approximate
b1	$\log_{10}(k_2'/k_2)$	−1.366	< −10.55	−0.7435	optim	approximate
b2	$\log_{10}(k_1'k_2')$	2.468	< −1.347	> 8.668	optim	optim
b2	$\log_{10}(k_2 \text{ or } k_{deg})$	3.836	−0.03832	> 10.11	optim	optim
b2	$\log_{10}(k_2 \text{ or } k_{deg})$	−1.117	−1.333	−0.8944	approximate	approximate
b3	$\log_{10}(k_1')$	−0.04437	−0.5313	> 9.408	approximate	optim
b3	$\log_{10}(k_2)$	−0.1989	−0.6798	> 9.253	approximate	optim
b3	$\log_{10}(k_2')$	−1.342	−1.57	−0.968	approximate	approximate
b3	$\log_{10}(k_{deg})$	−0.7364	−0.9822	−0.3462	approximate	approximate
b3	$\log_{10}(k_1'/k_2)$	0.1546	0.1213	0.1968	approximate	approximate
b3	$\log_{10}(k_1'k_2'/k_2)$	−1.187	−1.423	−0.8117	approximate	approximate
b3	$\log_{10}(k_2'/k_2)$	−1.143	< −10.8	−0.06316	optim	approximate

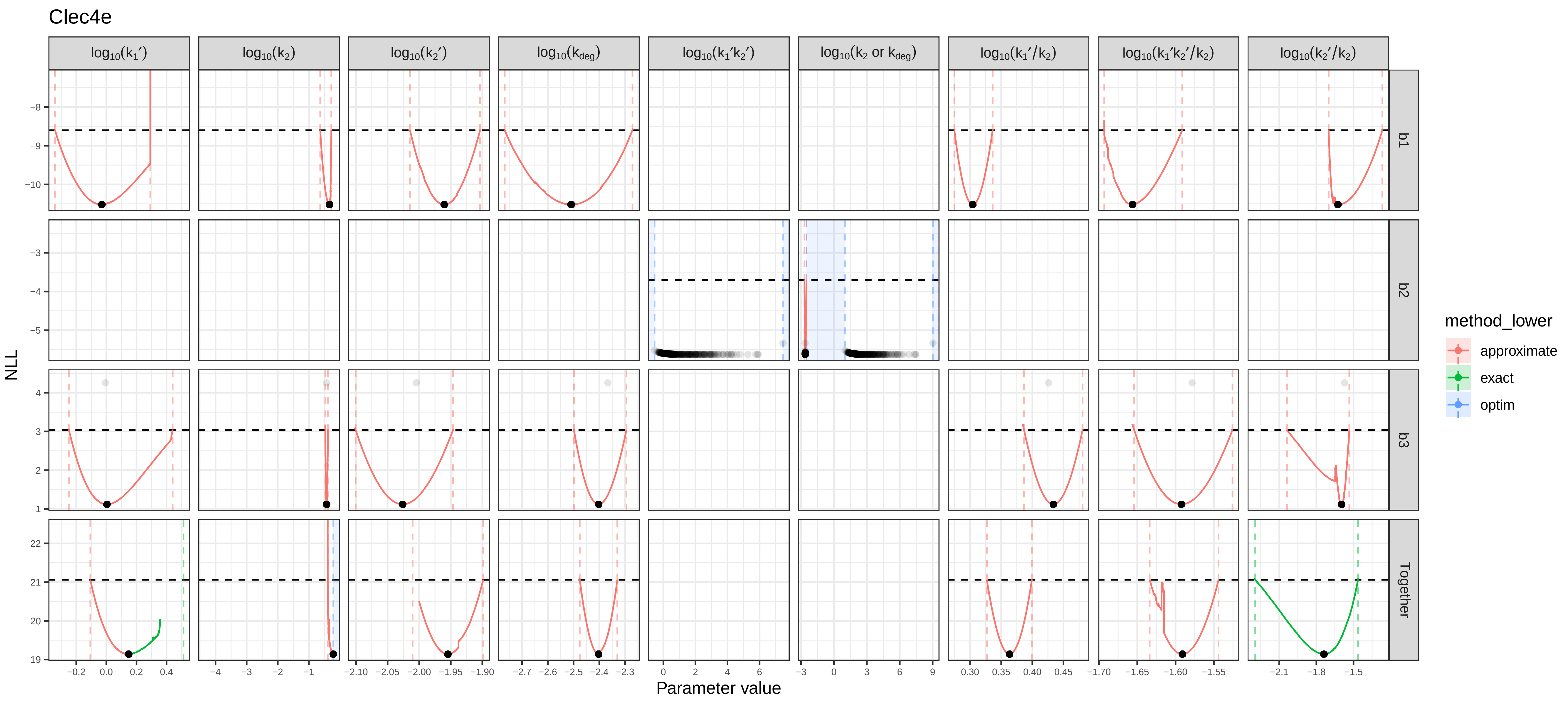

Replicate	Par	Best value	CI95 LB	CI95 UB	Method LB	Method UB
Together	$\log_{10}(k_1')$	0.1482	−0.1067	0.5122	approximate	exact
Together	$\log_{10}(k_2)$	−0.2152	−0.3923	> −0.2124	approximate	optim
Together	$\log_{10}(k_2')$	−1.954	−2.01	−1.899	approximate	approximate
Together	$\log_{10}(k_{deg})$	−2.403	−2.476	−2.33	approximate	approximate
Together	$\log_{10}(k_1'/k_2)$	0.3635	0.3269	0.3992	approximate	approximate
Together	$\log_{10}(k_1'k_2'/k_2)$	−1.591	−1.634	−1.544	approximate	approximate
Together	$\log_{10}(k_2'/k_2)$	−1.739	−2.294	−1.464	exact	exact
b1	$\log_{10}(k_1')$	−0.03026	−0.341	0.2921	approximate	approximate
b1	$\log_{10}(k_2)$	−0.3345	−0.6345	−0.278	approximate	approximate
b1	$\log_{10}(k_2')$	−1.96	−2.015	−1.903	approximate	approximate
b1	$\log_{10}(k_{deg})$	−2.509	−2.767	−2.271	approximate	approximate
b1	$\log_{10}(k_1'/k_2)$	0.3043	0.2747	0.3362	approximate	approximate
b1	$\log_{10}(k_1'k_2'/k_2)$	−1.656	−1.693	−1.591	approximate	approximate
b1	$\log_{10}(k_2'/k_2)$	−1.626	−1.701	−1.267	approximate	approximate
b2	$\log_{10}(k_1'k_2')$	3.455	< −0.5507	> 7.464	optim	optim
b2	$\log_{10}(k_2 \text{ or } k_{deg})$	5.014	1.007	> 9.024	optim	optim
b2	$\log_{10}(k_2 \text{ or } k_{deg})$	−2.616	−2.693	−2.512	approximate	approximate
b3	$\log_{10}(k_1')$	0.004082	−0.249	0.4402	approximate	approximate
b3	$\log_{10}(k_2)$	−0.4297	−0.4706	−0.3848	approximate	approximate
b3	$\log_{10}(k_2')$	−2.026	−2.101	−1.946	approximate	approximate
b3	$\log_{10}(k_{deg})$	−2.403	−2.499	−2.295	approximate	approximate
b3	$\log_{10}(k_1'/k_2)$	0.4338	0.3866	0.4807	approximate	approximate
b3	$\log_{10}(k_1'k_2'/k_2)$	−1.592	−1.654	−1.526	approximate	approximate
b3	$\log_{10}(k_2'/k_2)$	−1.596	−2.038	−1.534	approximate	approximate

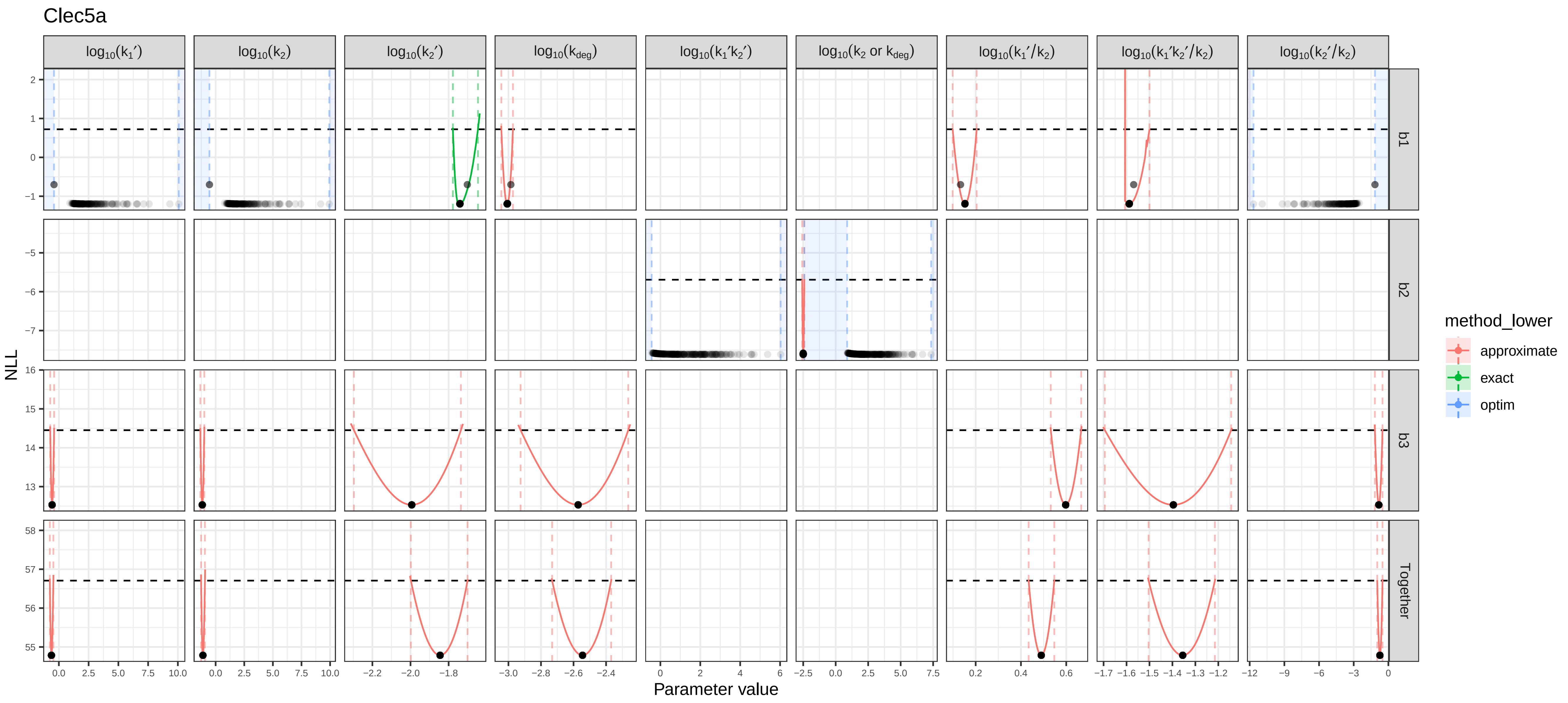

Replicate	Par	**Best value**	**CI95 LB**	**CI95 UB**	**Method LB**	**Method UB**
Together	$\log_{10}(k_1')$	−0.6231	−0.7596	−0.4641	approximate	approximate
Together	$\log_{10}(k_2)$	−1.113	−1.271	−0.9358	approximate	approximate
Together	$\log_{10}(k_2')$	−1.845	−1.998	−1.701	approximate	approximate
Together	$\log_{10}(k_{deg})$	−2.545	−2.732	−2.369	approximate	approximate
Together	$\log_{10}(k_1'/k_2)$	0.4897	0.4338	0.5472	approximate	approximate
Together	$\log_{10}(k_1'k_2'/k_2)$	−1.355	−1.504	−1.215	approximate	approximate
Together	$\log_{10}(k_2'/k_2)$	−0.732	−0.9659	−0.507	approximate	approximate
b1	$\log_{10}(k_1')$	5.836	< −0.4086	> 10.08	optim	optim
b1	$\log_{10}(k_2)$	5.683	< −0.5417	> 9.926	optim	optim
b1	$\log_{10}(k_2')$	−1.741	−1.778	−1.646	exact	exact
b1	$\log_{10}(k_{deg})$	−3.008	−3.044	−2.973	approximate	approximate
b1	$\log_{10}(k_1'/k_2)$	0.1525	0.09821	0.2046	approximate	approximate
b1	$\log_{10}(k_1'k_2'/k_2)$	−1.588	−1.606	−1.5	approximate	approximate
b1	$\log_{10}(k_2'/k_2)$	−7.424	< −11.67	> −1.16	optim	optim
b2	$\log_{10}(k_1'k_2')$	2.799	< −0.4353	> 6.03	optim	optim
b2	$\log_{10}(k_2 \text{ or } k_{deg})$	4.115	0.88	> 7.346	optim	optim
b2	$\log_{10}(k_2 \text{ or } k_{deg})$	−2.499	−2.581	−2.417	approximate	approximate
b3	$\log_{10}(k_1')$	−0.5688	−0.7258	−0.4012	approximate	approximate
b3	$\log_{10}(k_2)$	−1.166	−1.34	−0.992	approximate	approximate
b3	$\log_{10}(k_2')$	−1.993	−2.297	−1.736	approximate	approximate
b3	$\log_{10}(k_{deg})$	−2.572	−2.925	−2.264	approximate	approximate
b3	$\log_{10}(k_1'/k_2)$	0.5976	0.5317	0.6659	approximate	approximate
b3	$\log_{10}(k_1'k_2'/k_2)$	−1.396	−1.695	−1.144	approximate	approximate
b3	$\log_{10}(k_2'/k_2)$	−0.8271	−1.167	−0.5091	approximate	approximate

Clic4

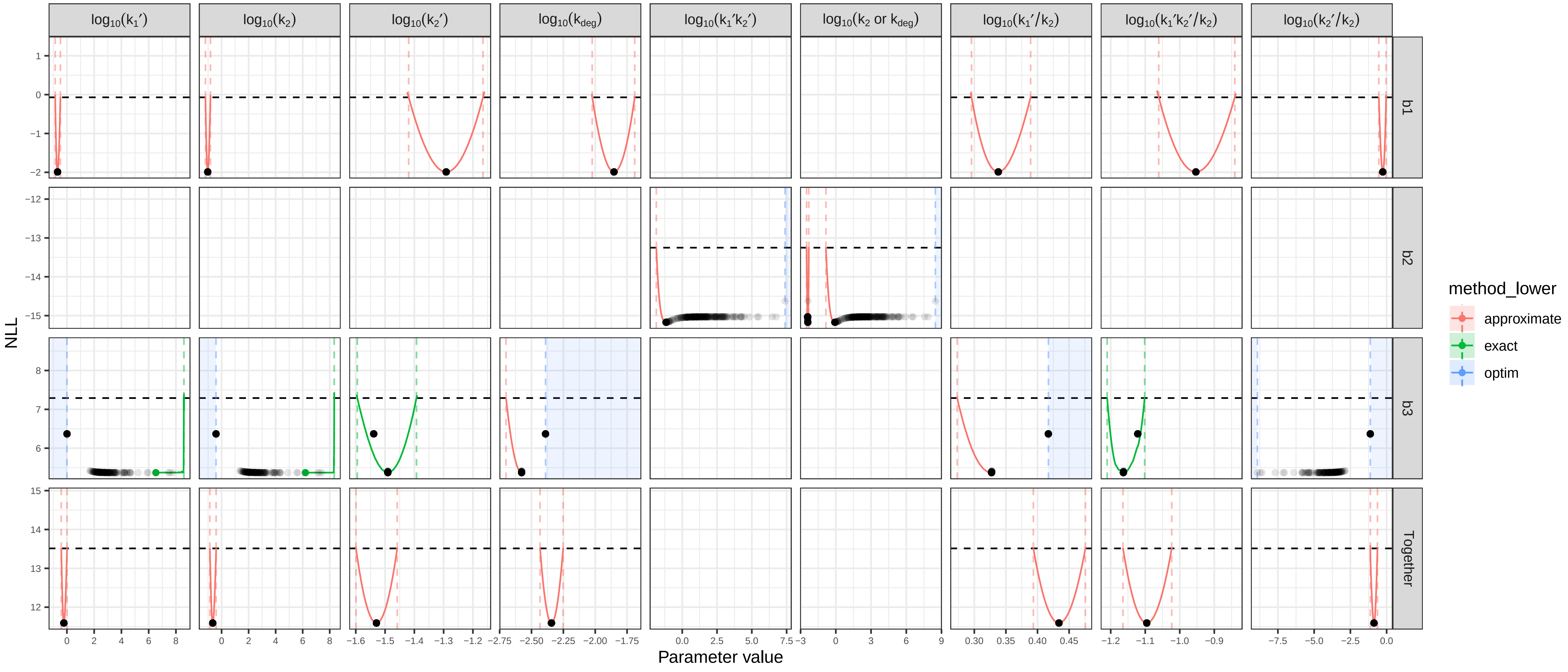

Replicate	Par	Best value	CI95 LB	CI95 UB	Method LB	Method UB
Together	$\log_{10}(k_1')$	−0.2241	−0.4148	0.01147	approximate	approximate
Together	$\log_{10}(k_2)$	−0.658	−0.8695	−0.4054	approximate	approximate
Together	$\log_{10}(k_2')$	−1.529	−1.599	−1.458	approximate	approximate
Together	$\log_{10}(k_{deg})$	−2.343	−2.433	−2.252	approximate	approximate
Together	$\log_{10}(k_1'/k_2)$	0.4339	0.3934	0.4757	approximate	approximate
Together	$\log_{10}(k_1'k_2'/k_2)$	−1.096	−1.164	−1.023	approximate	approximate
Together	$\log_{10}(k_2'/k_2)$	−0.8715	−1.124	−0.634	approximate	approximate
b1	$\log_{10}(k_1')$	−0.6871	−0.8601	−0.4763	approximate	approximate
b1	$\log_{10}(k_2)$	−1.025	−1.195	−0.8257	approximate	approximate
b1	$\log_{10}(k_2')$	−1.291	−1.419	−1.165	approximate	approximate
b1	$\log_{10}(k_{deg})$	−1.853	−2.024	−1.69	approximate	approximate
b1	$\log_{10}(k_1'/k_2)$	0.3379	0.2956	0.3891	approximate	approximate
b1	$\log_{10}(k_1'k_2'/k_2)$	−0.9532	−1.061	−0.8405	approximate	approximate
b1	$\log_{10}(k_2'/k_2)$	−0.2662	−0.5404	−0.0338	approximate	approximate
b2	$\log_{10}(k_1'k_2')$	−1.153	−1.865	> 7.39	approximate	optim
b2	$\log_{10}(k_2 \text{ or } k_{deg})$	−0.07079	−0.8434	> 8.477	approximate	optim
b2	$\log_{10}(k_2 \text{ or } k_{deg})$	−2.384	−2.49	−2.297	approximate	approximate
b3	$\log_{10}(k_1')$	6.526	< 0.004809	8.587	optim	exact
b3	$\log_{10}(k_2)$	6.199	< −0.4125	8.333	optim	exact
b3	$\log_{10}(k_2')$	−1.491	−1.595	−1.393	exact	exact
b3	$\log_{10}(k_{deg})$	−2.578	−2.7	> −2.39	approximate	optim
b3	$\log_{10}(k_1'/k_2)$	0.3274	0.2729	> 0.4173	approximate	optim
b3	$\log_{10}(k_1'k_2'/k_2)$	−1.163	−1.21	−1.102	exact	exact
b3	$\log_{10}(k_2'/k_2)$	−7.69	< −8.914	> −1.126	optim	optim

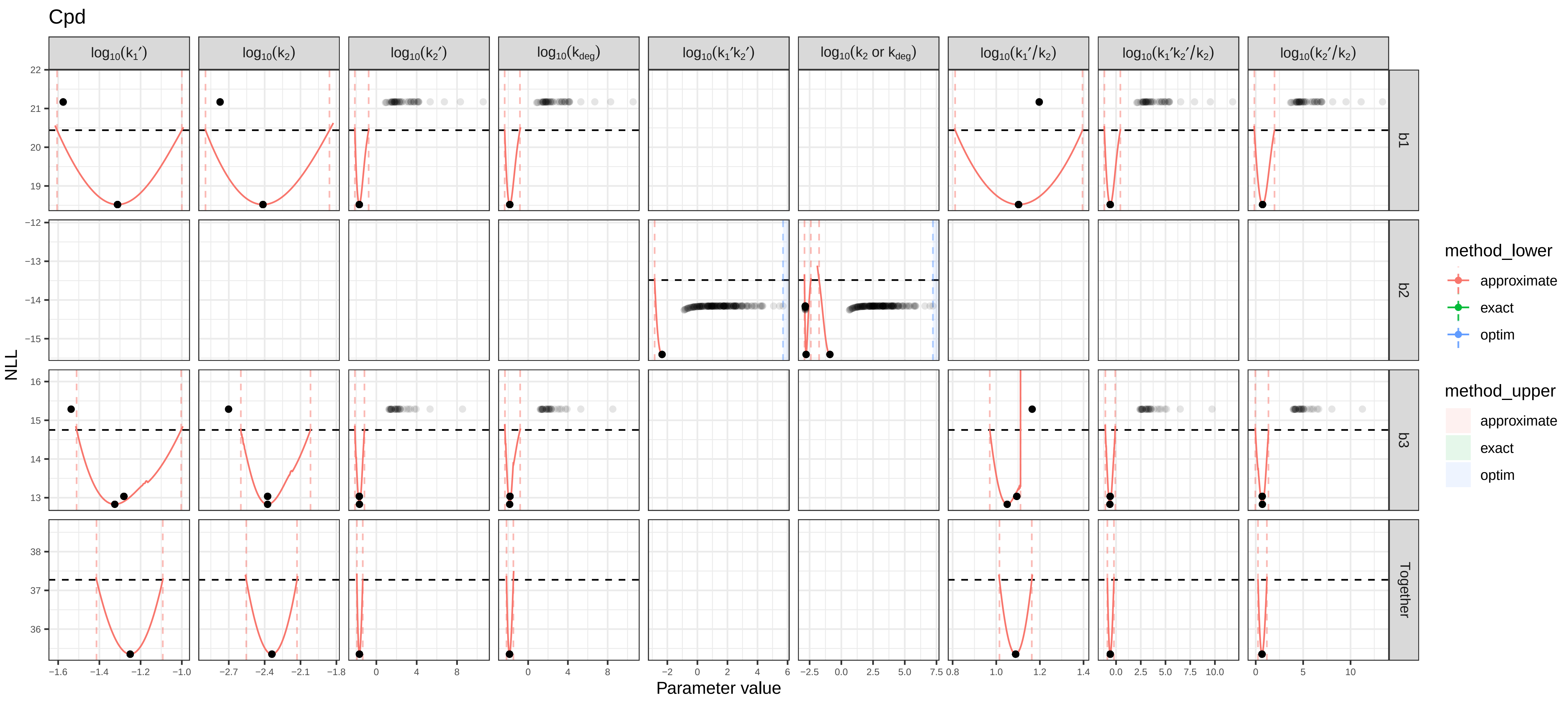

Replicate	Par	Best value	CI95 LB	CI95 UB	Method LB	Method UB
Together	$\log_{10}(k_1')$	−1.251	−1.414	−1.092	approximate	approximate
Together	$\log_{10}(k_2)$	−2.339	−2.554	−2.129	approximate	approximate
Together	$\log_{10}(k_2')$	−1.679	−1.932	−1.347	approximate	approximate
Together	$\log_{10}(k_{deg})$	−1.876	−2.177	−1.488	approximate	approximate
Together	$\log_{10}(k_1'/k_2)$	1.089	1.015	1.163	approximate	approximate
Together	$\log_{10}(k_1'k_2'/k_2)$	−0.5903	−0.8802	−0.2155	approximate	approximate
Together	$\log_{10}(k_2'/k_2)$	0.6602	0.2372	1.176	approximate	approximate
b1	$\log_{10}(k_1')$	−1.312	−1.605	−0.9998	approximate	approximate
b1	$\log_{10}(k_2)$	−2.414	−2.895	−1.858	approximate	approximate
b1	$\log_{10}(k_2')$	−1.694	−2.132	−0.7637	approximate	approximate
b1	$\log_{10}(k_{deg})$	−1.85	−2.361	−0.824	approximate	approximate
b1	$\log_{10}(k_1'/k_2)$	1.102	0.8115	1.395	approximate	approximate
b1	$\log_{10}(k_1'k_2'/k_2)$	−0.5922	−1.183	0.4328	approximate	approximate
b1	$\log_{10}(k_2'/k_2)$	0.7197	−0.1433	1.98	approximate	approximate
b2	$\log_{10}(k_1'k_2')$	−2.352	−2.845	> 5.696	approximate	optim
b2	$\log_{10}(k_2 \text{ or } k_{deg})$	−0.901	−1.753	> 7.217	approximate	optim
b2	$\log_{10}(k_2 \text{ or } k_{deg})$	−2.778	−2.903	−2.403	approximate	approximate
b3	$\log_{10}(k_1')$	−1.325	−1.51	−1.003	approximate	approximate
b3	$\log_{10}(k_2)$	−2.375	−2.599	−2.017	approximate	approximate
b3	$\log_{10}(k_2')$	−1.677	−2.124	−1.181	approximate	approximate
b3	$\log_{10}(k_{deg})$	−1.859	−2.334	−0.8031	approximate	approximate
b3	$\log_{10}(k_1'/k_2)$	1.05	0.9703	1.111	approximate	approximate
b3	$\log_{10}(k_1'k_2'/k_2)$	−0.6274	−1.085	−0.08193	approximate	approximate
b3	$\log_{10}(k_2'/k_2)$	0.6979	−0.04138	1.343	approximate	approximate

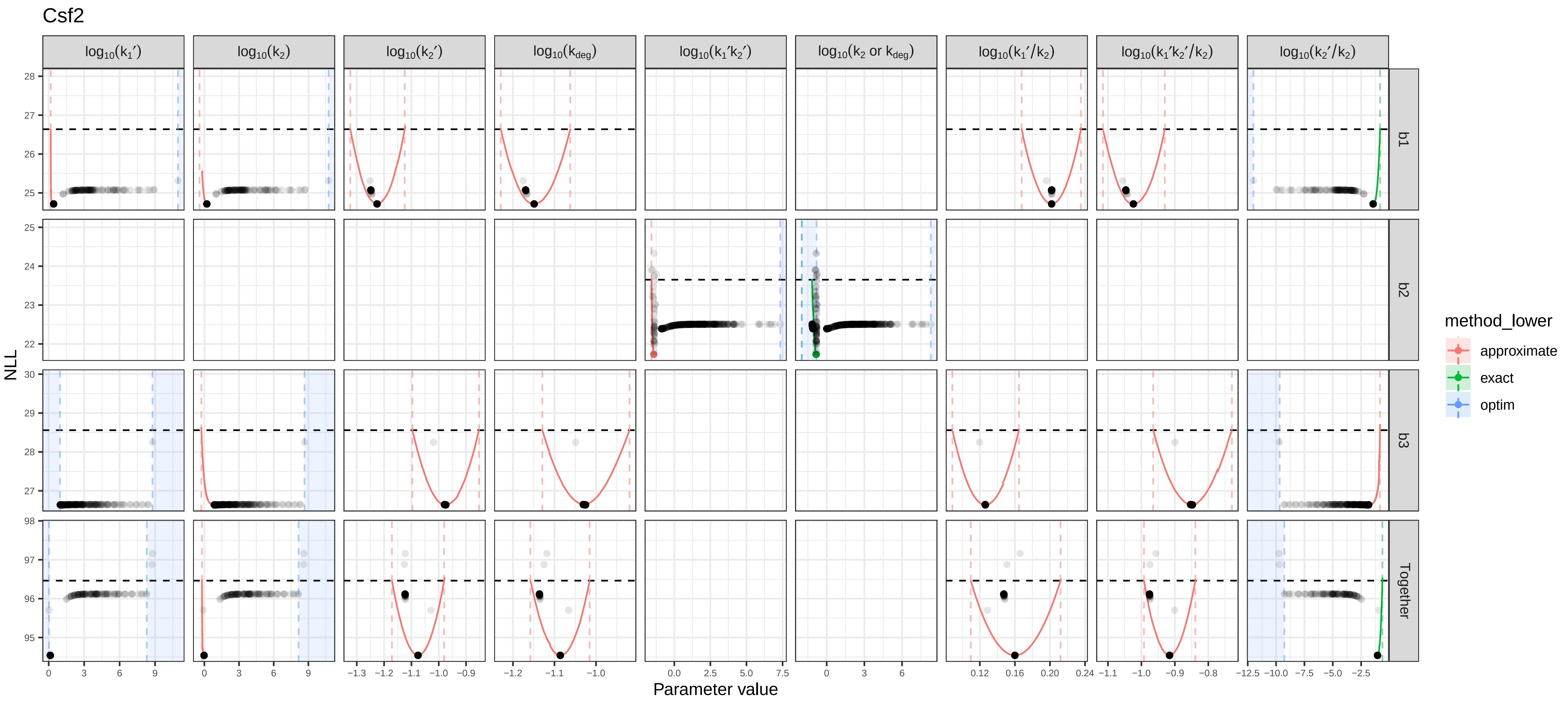

Replicate	Par	Best value	CI95 LB	CI95 UB	Method LB	Method UB
Together	$\log_{10}(k_1')$	0.1366	< 0.02589	> 8.298	optim	optim
Together	$\log_{10}(k_2)$	−0.02332	−0.2076	> 8.15	approximate	optim
Together	$\log_{10}(k_2')$	−1.076	−1.171	−0.9804	approximate	approximate
Together	$\log_{10}(k_{deg})$	−1.086	−1.158	−1.015	approximate	approximate
Together	$\log_{10}(k_1'/k_2)$	0.16	0.1096	0.2121	approximate	approximate
Together	$\log_{10}(k_1'k_2'/k_2)$	−0.9159	−0.9924	−0.8389	approximate	approximate
Together	$\log_{10}(k_2'/k_2)$	−1.053	< −9.274	−0.6236	optim	exact
b1	$\log_{10}(k_1')$	0.4172	0.1703	> 10.94	approximate	optim
b1	$\log_{10}(k_2)$	0.2153	−0.4169	> 10.75	approximate	optim
b1	$\log_{10}(k_2')$	−1.226	−1.324	−1.125	approximate	approximate
b1	$\log_{10}(k_{deg})$	−1.149	−1.229	−1.062	approximate	approximate
b1	$\log_{10}(k_1'/k_2)$	0.2019	0.1677	0.2354	approximate	approximate
b1	$\log_{10}(k_1'k_2'/k_2)$	−1.024	−1.115	−0.9301	approximate	approximate
b1	$\log_{10}(k_2'/k_2)$	−1.441	< −12	−0.8282	optim	exact
b2	$\log_{10}(k_1'k_2')$	−1.419	−1.58	> 7.339	approximate	optim
b2	$\log_{10}(k_2 \text{ or } k_{deg})$	−0.8259	−1.987	> 8.29	exact	optim
b2	$\log_{10}(k_2 \text{ or } k_{deg})$	−0.8565	−1.987	−0.8067	exact	optim
b3	$\log_{10}(k_1')$	1.128	< 0.9586	> 8.78	optim	optim
b3	$\log_{10}(k_2)$	1.002	−0.2574	> 8.661	approximate	optim
b3	$\log_{10}(k_2')$	−0.9754	−1.096	−0.8525	approximate	approximate
b3	$\log_{10}(k_{deg})$	−1.027	−1.129	−0.9194	approximate	approximate
b3	$\log_{10}(k_1'/k_2)$	0.1263	0.08863	0.1646	approximate	approximate
b3	$\log_{10}(k_1'k_2'/k_2)$	−0.8491	−0.9648	−0.7295	approximate	approximate
b3	$\log_{10}(k_2'/k_2)$	−1.977	< −9.68	−0.8373	optim	approximate

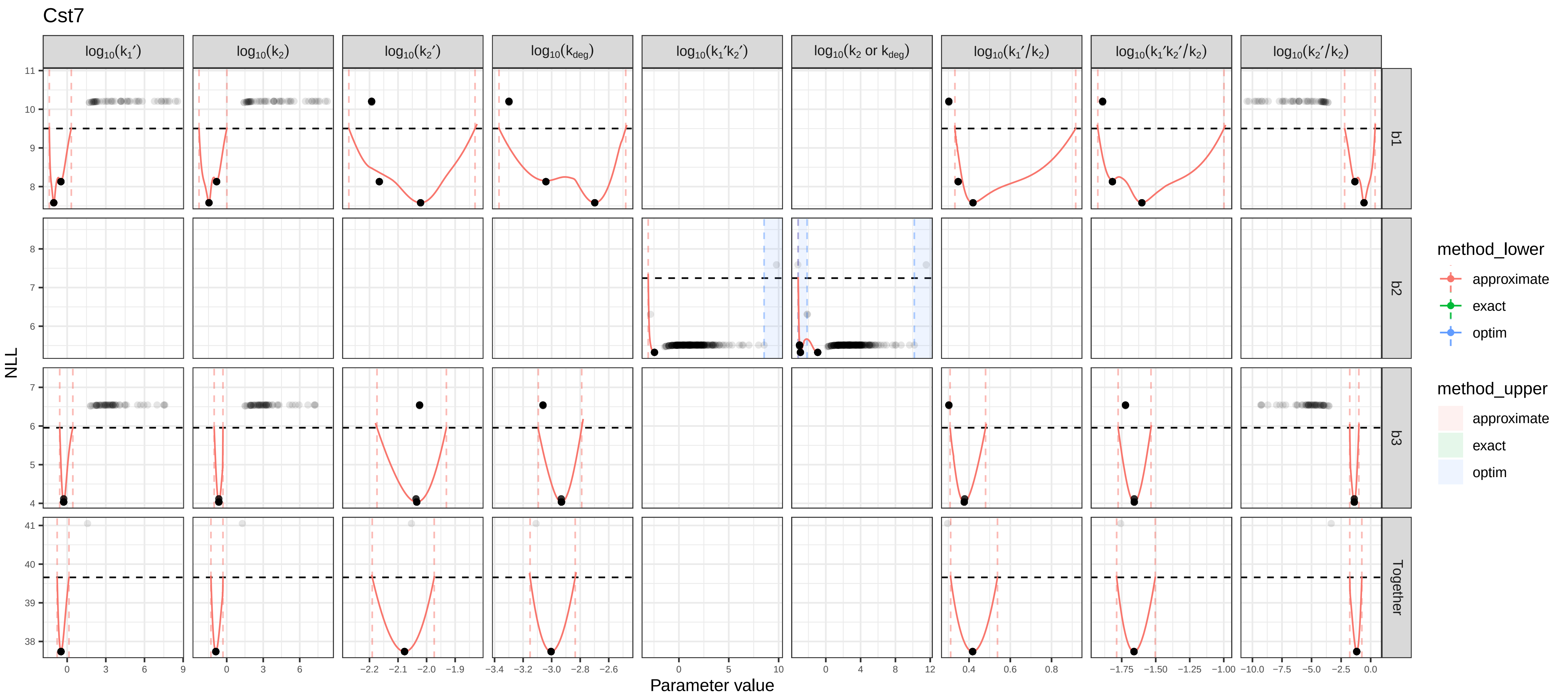

Replicate	Par	Best value	CI95 LB	CI95 UB	Method LB	Method UB
Together	$\log_{10}(k_1')$	−0.4786	−0.7766	0.1409	approximate	approximate
Together	$\log_{10}(k_2)$	−0.8962	−1.297	−0.3081	approximate	approximate
Together	$\log_{10}(k_2')$	−2.077	−2.19	−1.973	approximate	approximate
Together	$\log_{10}(k_{deg})$	−3.004	−3.151	−2.835	approximate	approximate
Together	$\log_{10}(k_1'/k_2)$	0.4176	0.311	0.538	approximate	approximate
Together	$\log_{10}(k_1'k_2'/k_2)$	−1.659	−1.787	−1.503	approximate	approximate
Together	$\log_{10}(k_2'/k_2)$	−1.181	−1.769	−0.7472	approximate	approximate
b1	$\log_{10}(k_1')$	−1.039	−1.385	0.321	approximate	approximate
b1	$\log_{10}(k_2)$	−1.459	−2.276	0.01253	approximate	approximate
b1	$\log_{10}(k_2')$	−2.021	−2.272	−1.83	approximate	approximate
b1	$\log_{10}(k_{deg})$	−2.698	−3.369	−2.481	approximate	approximate
b1	$\log_{10}(k_1'/k_2)$	0.4193	0.3317	0.917	approximate	approximate
b1	$\log_{10}(k_1'k_2'/k_2)$	−1.602	−1.926	−0.9984	approximate	approximate
b1	$\log_{10}(k_2'/k_2)$	−0.5625	−2.205	0.374	approximate	approximate
b2	$\log_{10}(k_1'k_2')$	−2.453	−3.082	> 8.541	approximate	optim
b2	$\log_{10}(k_2 \text{ or } k_{deg})$	−0.9299	−3.182	> 10.17	approximate	optim
b2	$\log_{10}(k_2 \text{ or } k_{deg})$	−2.918	−3.182	−2.153	approximate	optim
b3	$\log_{10}(k_1')$	−0.2712	−0.5766	0.4401	approximate	approximate
b3	$\log_{10}(k_2)$	−0.6479	−1.03	−0.3083	approximate	approximate
b3	$\log_{10}(k_2')$	−2.035	−2.174	−1.931	approximate	approximate
b3	$\log_{10}(k_{deg})$	−2.931	−3.094	−2.789	approximate	approximate
b3	$\log_{10}(k_1'/k_2)$	0.3767	0.3082	0.4802	approximate	approximate
b3	$\log_{10}(k_1'k_2'/k_2)$	−1.658	−1.776	−1.535	approximate	approximate
b3	$\log_{10}(k_2'/k_2)$	−1.387	−1.771	−1.001	approximate	approximate

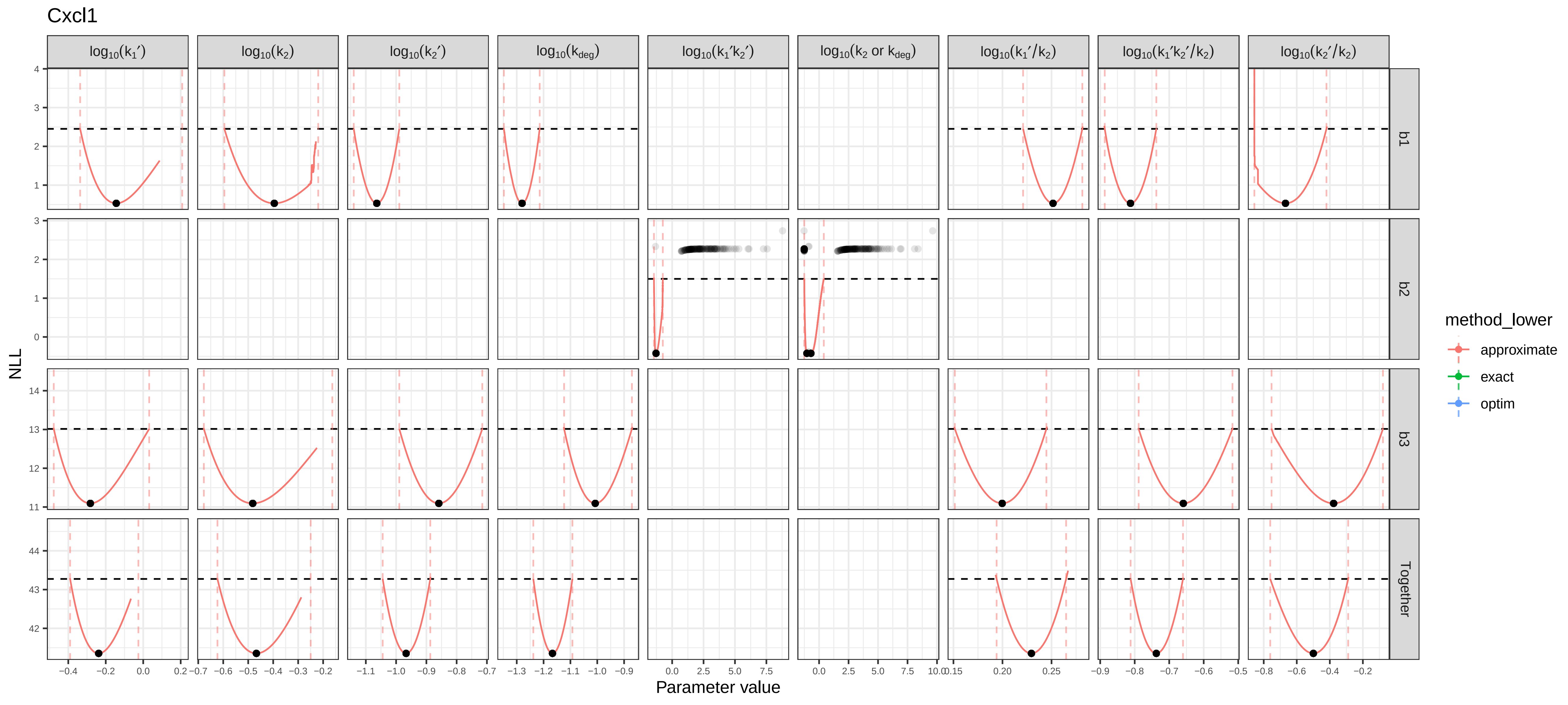

Replicate	Par	**Best value**	**CI95 LB**	**CI95 UB**	**Method LB**	**Method UB**
Together	$\log_{10}(k_1')$	−0.238	−0.3902	−0.02582	approximate	approximate
Together	$\log_{10}(k_2)$	−0.4675	−0.6231	−0.2499	approximate	approximate
Together	$\log_{10}(k_2')$	−0.9668	−1.044	−0.887	approximate	approximate
Together	$\log_{10}(k_{deg})$	−1.167	−1.238	−1.093	approximate	approximate
Together	$\log_{10}(k_1'/k_2)$	0.2295	0.194	0.2649	approximate	approximate
Together	$\log_{10}(k_1'k_2'/k_2)$	−0.7373	−0.8119	−0.66	approximate	approximate
Together	$\log_{10}(k_2'/k_2)$	−0.4994	−0.7607	−0.2889	approximate	approximate
b1	$\log_{10}(k_1')$	−0.144	−0.3366	0.2081	approximate	approximate
b1	$\log_{10}(k_2)$	−0.3956	−0.5954	−0.2198	approximate	approximate
b1	$\log_{10}(k_2')$	−1.064	−1.14	−0.9894	approximate	approximate
b1	$\log_{10}(k_{deg})$	−1.28	−1.348	−1.215	approximate	approximate
b1	$\log_{10}(k_1'/k_2)$	0.2516	0.221	0.2814	approximate	approximate
b1	$\log_{10}(k_1'k_2'/k_2)$	−0.8122	−0.8868	−0.7373	approximate	approximate
b1	$\log_{10}(k_2'/k_2)$	−0.6682	−0.8566	−0.4203	approximate	approximate
b2	$\log_{10}(k_1'k_2')$	−1.283	−1.448	−0.7418	approximate	approximate
b2	$\log_{10}(k_2 \text{ or } k_{deg})$	−0.691	−1.257	0.4003	approximate	approximate
b2	$\log_{10}(k_2 \text{ or } k_{deg})$	−1.033	−1.257	0.4003	approximate	approximate
b3	$\log_{10}(k_1')$	−0.2822	−0.4774	0.0319	approximate	approximate
b3	$\log_{10}(k_2)$	−0.4819	−0.6776	−0.1633	approximate	approximate
b3	$\log_{10}(k_2')$	−0.8588	−0.9894	−0.7153	approximate	approximate
b3	$\log_{10}(k_{deg})$	−1.008	−1.124	−0.8709	approximate	approximate
b3	$\log_{10}(k_1'/k_2)$	0.1997	0.1514	0.2448	approximate	approximate
b3	$\log_{10}(k_1'k_2'/k_2)$	−0.6591	−0.7885	−0.5166	approximate	approximate
b3	$\log_{10}(k_2'/k_2)$	−0.3769	−0.7526	−0.07847	approximate	approximate

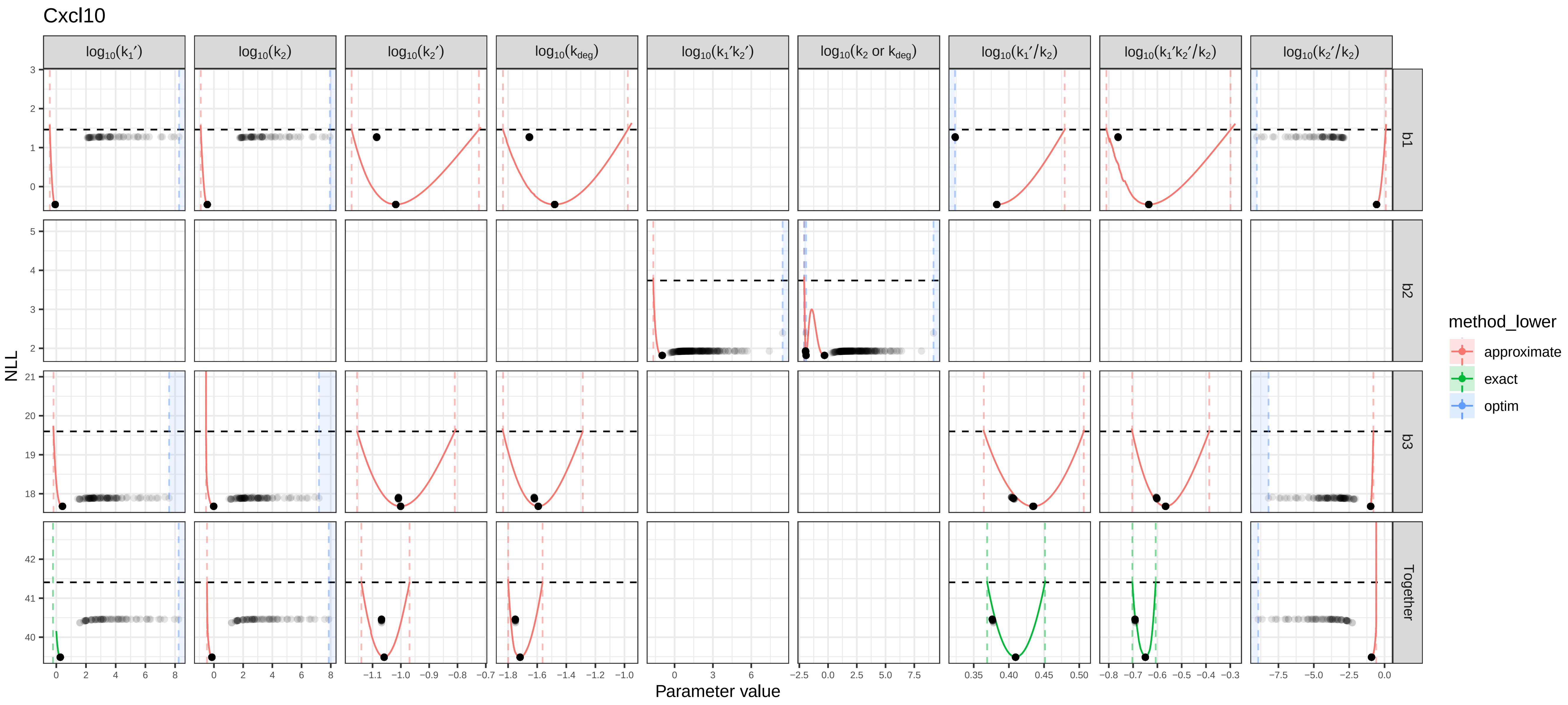

Replicate	Par	Best value	CI95 LB	CI95 UB	Method LB	Method UB
Together	$\log_{10}(k_1')$	0.2698	−0.2235	> 8.237	exact	optim
Together	$\log_{10}(k_2)$	−0.1398	−0.4761	> 7.86	approximate	optim
Together	$\log_{10}(k_2')$	−1.059	−1.14	−0.9693	approximate	approximate
Together	$\log_{10}(k_{deg})$	−1.718	−1.8	−1.563	approximate	approximate
Together	$\log_{10}(k_1'/k_2)$	0.4096	0.369	0.4513	exact	exact
Together	$\log_{10}(k_1'k_2'/k_2)$	−0.6495	−0.7032	−0.607	exact	exact
Together	$\log_{10}(k_2'/k_2)$	−0.9193	< −8.928	−0.5921	optim	approximate
b1	$\log_{10}(k_1')$	−0.07198	−0.4349	> 8.265	approximate	optim
b1	$\log_{10}(k_2)$	−0.4547	−0.9027	> 7.942	approximate	optim
b1	$\log_{10}(k_2')$	−1.018	−1.174	−0.7243	approximate	approximate
b1	$\log_{10}(k_{deg})$	−1.48	−1.836	−0.9776	approximate	approximate
b1	$\log_{10}(k_1'/k_2)$	0.3827	< 0.3234	0.4793	optim	approximate
b1	$\log_{10}(k_1'k_2'/k_2)$	−0.6354	−0.8098	−0.2989	approximate	approximate
b1	$\log_{10}(k_2'/k_2)$	−0.5635	< −9.027	0.08697	optim	approximate
b2	$\log_{10}(k_1'k_2')$	−0.9728	−1.663	> 8.452	approximate	optim
b2	$\log_{10}(k_2 \text{ or } k_{deg})$	−0.3163	−2.076	> 9.17	approximate	optim
b2	$\log_{10}(k_2 \text{ or } k_{deg})$	−1.91	−2.076	−1.908	approximate	optim
b3	$\log_{10}(k_1')$	0.4175	−0.1884	> 7.598	approximate	optim
b3	$\log_{10}(k_2)$	−0.01693	−0.5431	> 7.192	approximate	optim
b3	$\log_{10}(k_2')$	−1.001	−1.155	−0.8096	approximate	approximate
b3	$\log_{10}(k_{deg})$	−1.593	−1.835	−1.287	approximate	approximate
b3	$\log_{10}(k_1'/k_2)$	0.4345	0.3643	0.5065	approximate	approximate
b3	$\log_{10}(k_1'k_2'/k_2)$	−0.5665	−0.7036	−0.3865	approximate	approximate
b3	$\log_{10}(k_2'/k_2)$	−0.984	< −8.201	−0.7916	optim	approximate

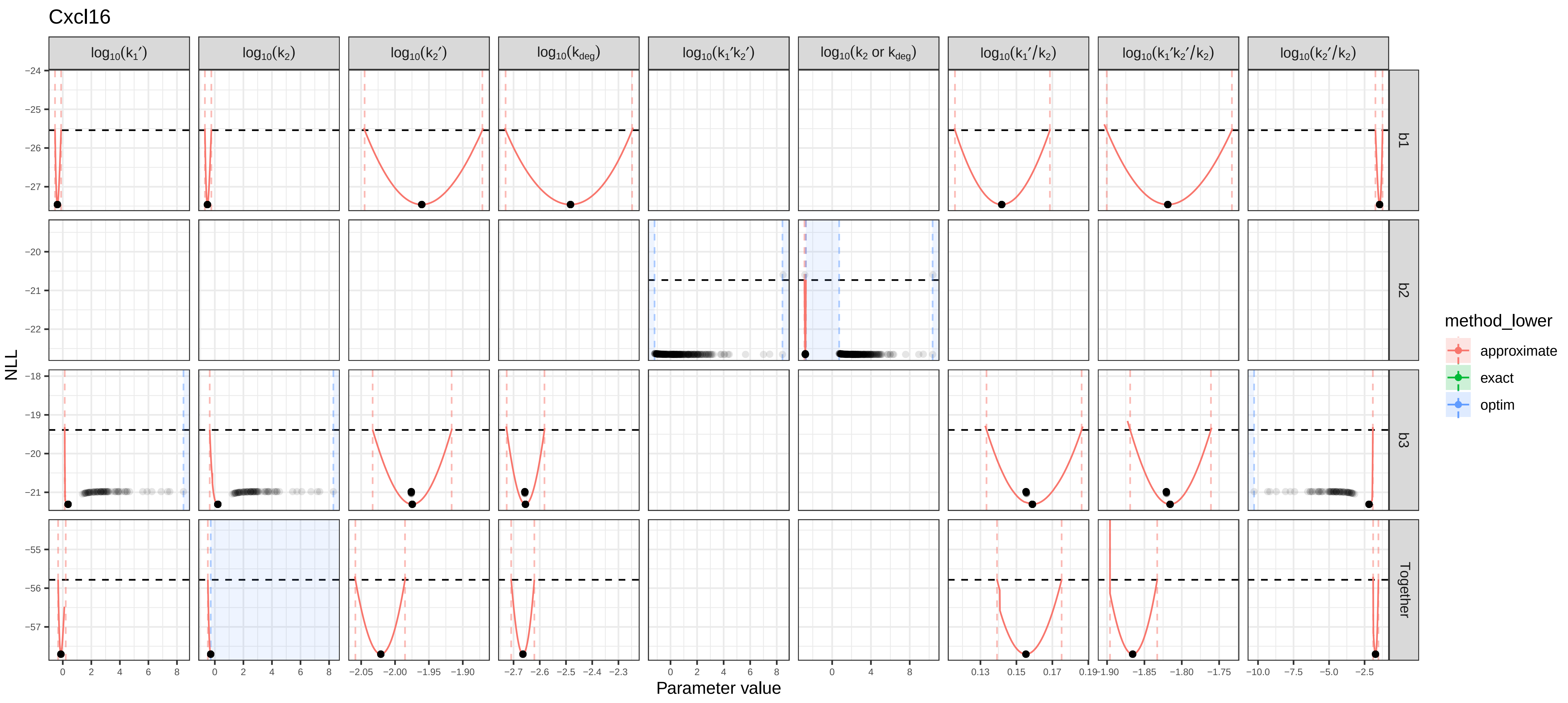

Replicate	Par	Best value	CI95 LB	CI95 UB	Method LB	Method UB
Together	$\log_{10}(k_1')$	−0.1341	−0.3272	0.2076	approximate	approximate
Together	$\log_{10}(k_2)$	−0.2894	−0.4893	> −0.2866	approximate	optim
Together	$\log_{10}(k_2')$	−2.021	−2.059	−1.985	approximate	approximate
Together	$\log_{10}(k_{deg})$	−2.664	−2.709	−2.621	approximate	approximate
Together	$\log_{10}(k_1'/k_2)$	0.1553	0.1392	0.1752	approximate	approximate
Together	$\log_{10}(k_1'k_2'/k_2)$	−1.866	−1.896	−1.833	approximate	approximate
Together	$\log_{10}(k_2'/k_2)$	−1.732	−1.898	−1.527	approximate	approximate
b1	$\log_{10}(k_1')$	−0.3765	−0.5439	−0.1167	approximate	approximate
b1	$\log_{10}(k_2)$	−0.5183	−0.6945	−0.2448	approximate	approximate
b1	$\log_{10}(k_2')$	−1.961	−2.045	−1.871	approximate	approximate
b1	$\log_{10}(k_{deg})$	−2.483	−2.73	−2.248	approximate	approximate
b1	$\log_{10}(k_1'/k_2)$	0.1418	0.1158	0.1686	approximate	approximate
b1	$\log_{10}(k_1'k_2'/k_2)$	−1.819	−1.9	−1.733	approximate	approximate
b1	$\log_{10}(k_2'/k_2)$	−1.442	−1.737	−1.235	approximate	approximate
b2	$\log_{10}(k_1'k_2')$	2.13	< −1.203	> 8.431	optim	optim
b2	$\log_{10}(k_2 \text{ or } k_{deg})$	4.059	0.7244	> 10.36	optim	optim
b2	$\log_{10}(k_2 \text{ or } k_{deg})$	−2.768	−2.856	−2.707	approximate	approximate
b3	$\log_{10}(k_1')$	0.3663	0.137	> 8.455	approximate	optim
b3	$\log_{10}(k_2)$	0.2074	−0.3579	> 8.3	approximate	optim
b3	$\log_{10}(k_2')$	−1.975	−2.033	−1.916	approximate	approximate
b3	$\log_{10}(k_{deg})$	−2.655	−2.726	−2.582	approximate	approximate
b3	$\log_{10}(k_1'/k_2)$	0.1589	0.1334	0.1863	approximate	approximate
b3	$\log_{10}(k_1'k_2'/k_2)$	−1.816	−1.869	−1.761	approximate	approximate
b3	$\log_{10}(k_2'/k_2)$	−2.182	< −10.28	−1.918	optim	approximate

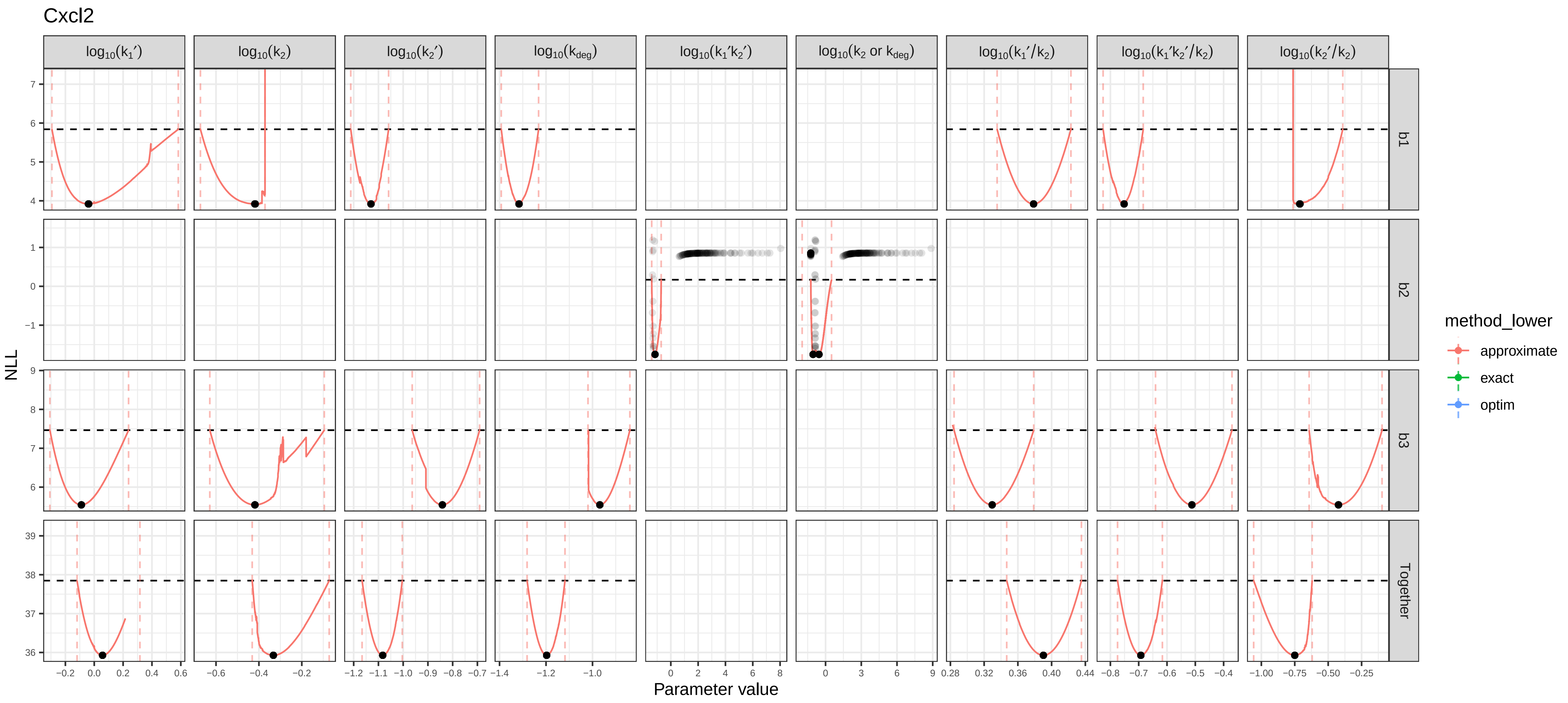

Replicate	Par	Best value	CI95 LB	CI95 UB	Method LB	Method UB
Together	$\log_{10}(k_1')$	0.05754	−0.119	0.3166	approximate	approximate
Together	$\log_{10}(k_2)$	−0.3327	−0.4313	−0.07231	approximate	approximate
Together	$\log_{10}(k_2')$	−1.083	−1.166	−1.004	approximate	approximate
Together	$\log_{10}(k_{deg})$	−1.197	−1.281	−1.118	approximate	approximate
Together	$\log_{10}(k_1'/k_2)$	0.3903	0.3468	0.4354	approximate	approximate
Together	$\log_{10}(k_1'k_2'/k_2)$	−0.6923	−0.7747	−0.6163	approximate	approximate
Together	$\log_{10}(k_2'/k_2)$	−0.7498	−1.057	−0.6198	approximate	approximate
b1	$\log_{10}(k_1')$	−0.0394	−0.2932	0.5823	approximate	approximate
b1	$\log_{10}(k_2)$	−0.418	−0.6727	−0.3715	approximate	approximate
b1	$\log_{10}(k_2')$	−1.13	−1.212	−1.059	approximate	approximate
b1	$\log_{10}(k_{deg})$	−1.316	−1.393	−1.232	approximate	approximate
b1	$\log_{10}(k_1'/k_2)$	0.3786	0.3354	0.4229	approximate	approximate
b1	$\log_{10}(k_1'k_2'/k_2)$	−0.7514	−0.8257	−0.684	approximate	approximate
b1	$\log_{10}(k_2'/k_2)$	−0.712	−0.7624	−0.3907	approximate	approximate
b2	$\log_{10}(k_1'k_2')$	−1.158	−1.4	−0.7054	approximate	approximate
b2	$\log_{10}(k_2 \text{ or } k_{deg})$	−0.5495	−1.966	0.5023	approximate	approximate
b2	$\log_{10}(k_2 \text{ or } k_{deg})$	−1.048	−1.966	0.5023	approximate	approximate
b3	$\log_{10}(k_1')$	−0.08906	−0.3072	0.2379	approximate	approximate
b3	$\log_{10}(k_2)$	−0.4186	−0.6289	−0.09522	approximate	approximate
b3	$\log_{10}(k_2')$	−0.8415	−0.9635	−0.691	approximate	approximate
b3	$\log_{10}(k_{deg})$	−0.9685	−1.019	−0.8393	approximate	approximate
b3	$\log_{10}(k_1'/k_2)$	0.3295	0.2843	0.3788	approximate	approximate
b3	$\log_{10}(k_1'k_2'/k_2)$	−0.512	−0.6405	−0.3704	approximate	approximate
b3	$\log_{10}(k_2'/k_2)$	−0.423	−0.6425	−0.09702	approximate	approximate

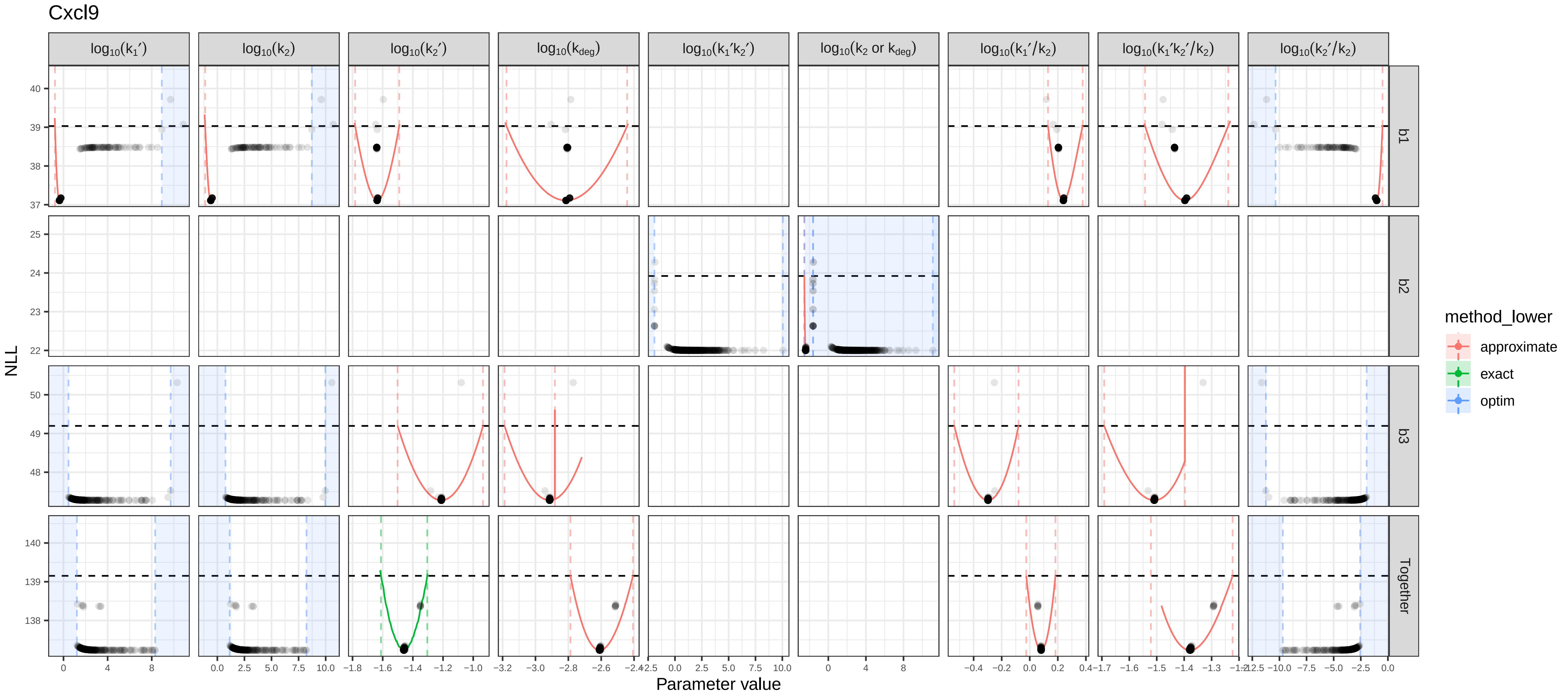

Replicate	Par	Best value	CI95 LB	CI95 UB	Method LB	Method UB
Together	$\log_{10}(k_1')$	7.318	< 1.216	> 8.312	optim	optim
Together	$\log_{10}(k_2)$	7.237	< 1.137	> 8.23	optim	optim
Together	$\log_{10}(k_2')$	−1.459	−1.611	−1.305	exact	exact
Together	$\log_{10}(k_{deg})$	−2.609	−2.787	−2.407	approximate	approximate
Together	$\log_{10}(k_1'/k_2)$	0.08091	−0.02501	0.1829	approximate	approximate
Together	$\log_{10}(k_1'k_2'/k_2)$	−1.378	−1.521	−1.223	approximate	approximate
Together	$\log_{10}(k_2'/k_2)$	−8.696	< −9.69	> −2.557	optim	optim
b1	$\log_{10}(k_1')$	−0.366	−0.7742	> 8.913	approximate	optim
b1	$\log_{10}(k_2)$	−0.6056	−1.124	> 8.72	approximate	optim
b1	$\log_{10}(k_2')$	−1.637	−1.783	−1.49	approximate	approximate
b1	$\log_{10}(k_{deg})$	−2.814	−3.176	−2.442	approximate	approximate
b1	$\log_{10}(k_1'/k_2)$	0.2396	0.13	0.3772	approximate	approximate
b1	$\log_{10}(k_1'k_2'/k_2)$	−1.397	−1.543	−1.239	approximate	approximate
b1	$\log_{10}(k_2'/k_2)$	−1.031	< −10.36	−0.4957	optim	approximate
b2	$\log_{10}(k_1'k_2')$	4.14	< −1.915	> 10.05	optim	optim
b2	$\log_{10}(k_2 \text{ or } k_{deg})$	5.203	−1.615	> 11.12	optim	optim
b2	$\log_{10}(k_2 \text{ or } k_{deg})$	−2.413	−2.55	> −1.628	approximate	optim
b3	$\log_{10}(k_1')$	4.515	< 0.4526	> 9.715	optim	optim
b3	$\log_{10}(k_2)$	4.812	< 0.7502	> 9.965	optim	optim
b3	$\log_{10}(k_2')$	−1.212	−1.501	−0.9347	approximate	approximate
b3	$\log_{10}(k_{deg})$	−2.914	−3.188	−2.882	approximate	approximate
b3	$\log_{10}(k_1'/k_2)$	−0.297	−0.538	−0.08014	approximate	approximate
b3	$\log_{10}(k_1'k_2'/k_2)$	−1.509	−1.692	−1.397	approximate	approximate
b3	$\log_{10}(k_2'/k_2)$	−6.025	< −11.25	> −1.96	optim	optim

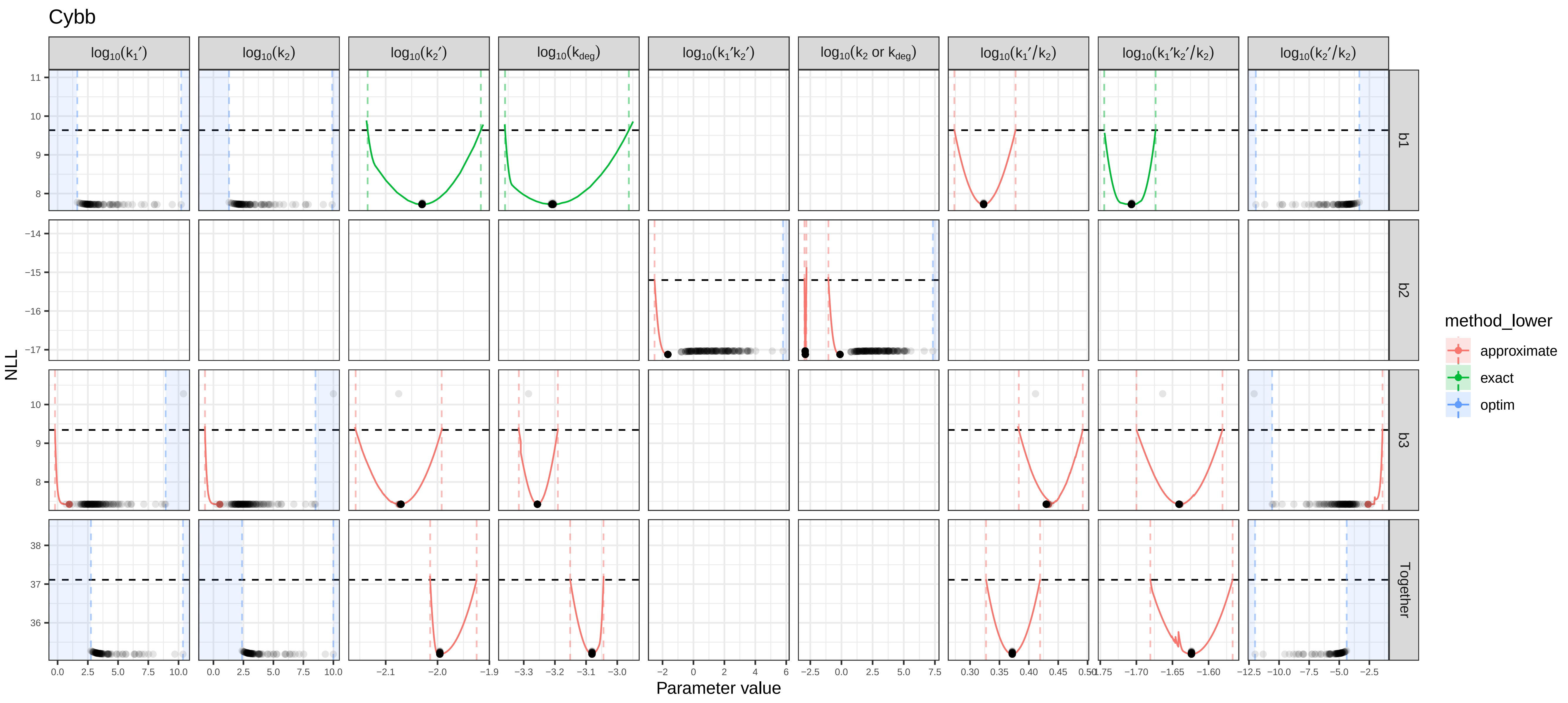

Replicate	Par	Best value	CI95 LB	CI95 UB	Method LB	Method UB
Together	$\log_{10}(k_1')$	7.523	< 2.759	> 10.37	optim	optim
Together	$\log_{10}(k_2)$	7.151	< 2.387	> 9.997	optim	optim
Together	$\log_{10}(k_2')$	−1.996	−2.014	−1.925	approximate	approximate
Together	$\log_{10}(k_{deg})$	−3.081	−3.151	−3.044	approximate	approximate
Together	$\log_{10}(k_1'/k_2)$	0.3718	0.3272	0.4192	approximate	approximate
Together	$\log_{10}(k_1'k_2'/k_2)$	−1.624	−1.681	−1.567	approximate	approximate
Together	$\log_{10}(k_2'/k_2)$	−9.147	< −11.99	> −4.383	optim	optim
b1	$\log_{10}(k_1')$	7.183	< 1.627	> 10.22	optim	optim
b1	$\log_{10}(k_2)$	6.86	< 1.304	> 9.898	optim	optim
b1	$\log_{10}(k_2')$	−2.03	−2.135	−1.916	exact	exact
b1	$\log_{10}(k_{deg})$	−3.207	−3.36	−2.963	exact	exact
b1	$\log_{10}(k_1'/k_2)$	0.323	0.2733	0.3775	approximate	approximate
b1	$\log_{10}(k_1'k_2'/k_2)$	−1.707	−1.745	−1.673	exact	exact
b1	$\log_{10}(k_2'/k_2)$	−8.89	< −11.93	> −3.333	optim	optim
b2	$\log_{10}(k_1'k_2')$	−1.657	−2.515	> 5.792	approximate	optim
b2	$\log_{10}(k_2 \text{ or } k_{deg})$	−0.119	−1.047	> 7.34	approximate	optim
b2	$\log_{10}(k_2 \text{ or } k_{deg})$	−2.903	−2.971	−2.815	approximate	approximate
b3	$\log_{10}(k_1')$	0.9592	−0.2135	> 8.935	approximate	optim
b3	$\log_{10}(k_2)$	0.5255	−0.7029	> 8.505	approximate	optim
b3	$\log_{10}(k_2')$	−2.073	−2.158	−1.992	approximate	approximate
b3	$\log_{10}(k_{deg})$	−3.256	−3.316	−3.19	approximate	approximate
b3	$\log_{10}(k_1'/k_2)$	0.4337	0.3829	0.4918	approximate	approximate
b3	$\log_{10}(k_1'k_2'/k_2)$	−1.64	−1.7	−1.581	approximate	approximate
b3	$\log_{10}(k_2'/k_2)$	−2.599	< −10.58	−1.404	optim	approximate

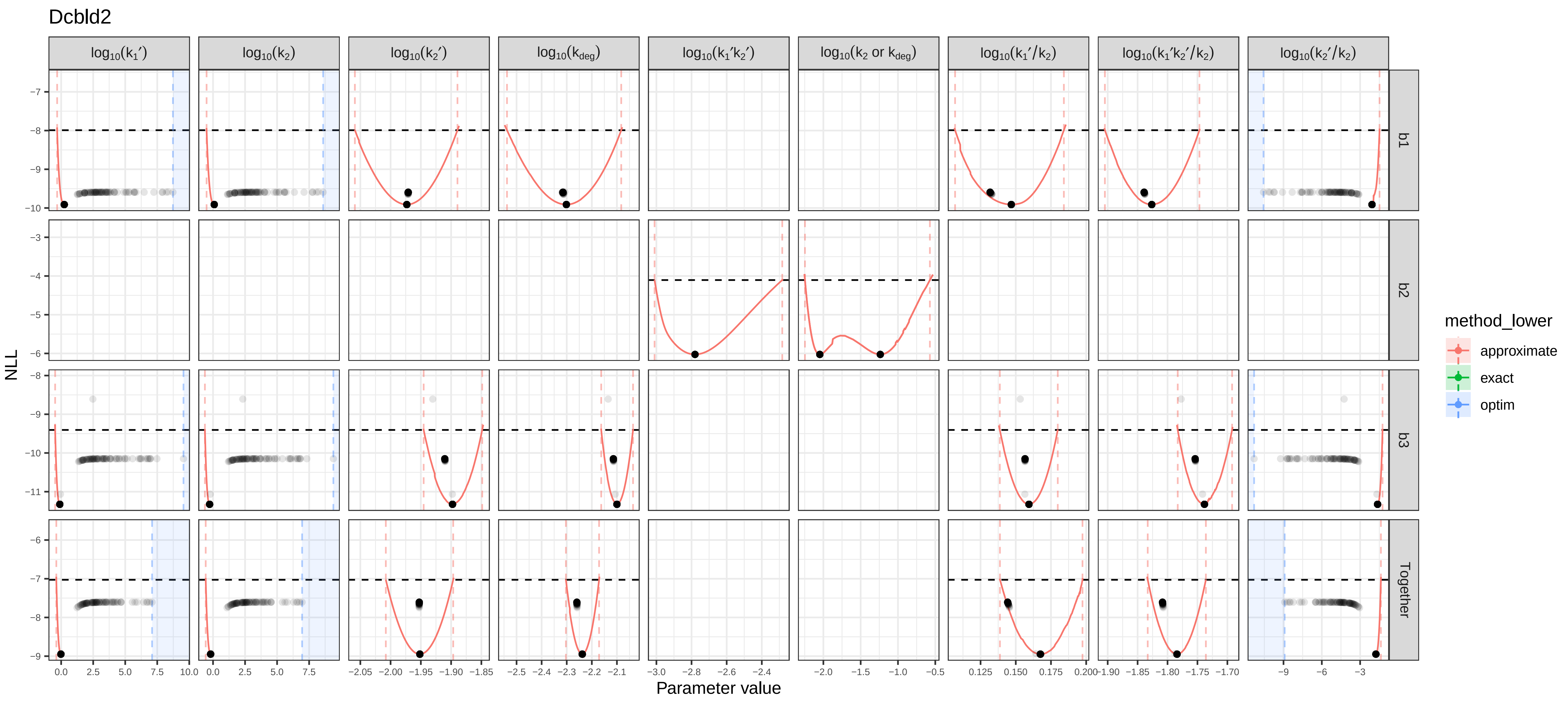

Replicate	Par	Best value	CI95 LB	CI95 UB	Method LB	Method UB
Together	$\log_{10}(k_1')$	−0.02212	−0.3782	> 7.098	approximate	optim
Together	$\log_{10}(k_2)$	−0.1897	−0.5702	> 6.953	approximate	optim
Together	$\log_{10}(k_2')$	−1.952	−2.008	−1.897	approximate	approximate
Together	$\log_{10}(k_{deg})$	−2.238	−2.303	−2.171	approximate	approximate
Together	$\log_{10}(k_1'/k_2)$	0.1676	0.1388	0.1974	approximate	approximate
Together	$\log_{10}(k_1'k_2'/k_2)$	−1.784	−1.833	−1.736	approximate	approximate
Together	$\log_{10}(k_2'/k_2)$	−1.762	< −8.906	−1.373	optim	approximate
b1	$\log_{10}(k_1')$	0.2428	−0.3128	> 8.726	approximate	optim
b1	$\log_{10}(k_2)$	0.09598	−0.5028	> 8.594	approximate	optim
b1	$\log_{10}(k_2')$	−1.973	−2.059	−1.889	approximate	approximate
b1	$\log_{10}(k_{deg})$	−2.301	−2.538	−2.083	approximate	approximate
b1	$\log_{10}(k_1'/k_2)$	0.1468	0.1069	0.1842	approximate	approximate
b1	$\log_{10}(k_1'k_2'/k_2)$	−1.826	−1.905	−1.747	approximate	approximate
b1	$\log_{10}(k_2'/k_2)$	−2.069	< −10.56	−1.48	optim	approximate
b2	$\log_{10}(k_1'k_2')$	−2.78	−3.011	−2.284	approximate	approximate
b2	$\log_{10}(k_2 \text{ or } k_{deg})$	−1.237	−2.247	−0.5722	approximate	approximate
b2	$\log_{10}(k_2 \text{ or } k_{deg})$	−2.047	−2.247	−0.5722	approximate	approximate
b3	$\log_{10}(k_1')$	−0.1071	−0.4699	> 9.548	approximate	optim
b3	$\log_{10}(k_2)$	−0.2665	−0.633	> 9.391	approximate	optim
b3	$\log_{10}(k_2')$	−1.898	−1.945	−1.849	approximate	approximate
b3	$\log_{10}(k_{deg})$	−2.1	−2.163	−2.036	approximate	approximate
b3	$\log_{10}(k_1'/k_2)$	0.1594	0.1387	0.1798	approximate	approximate
b3	$\log_{10}(k_1'k_2'/k_2)$	−1.738	−1.783	−1.692	approximate	approximate
b3	$\log_{10}(k_2'/k_2)$	−1.631	< −11.3	−1.245	optim	approximate

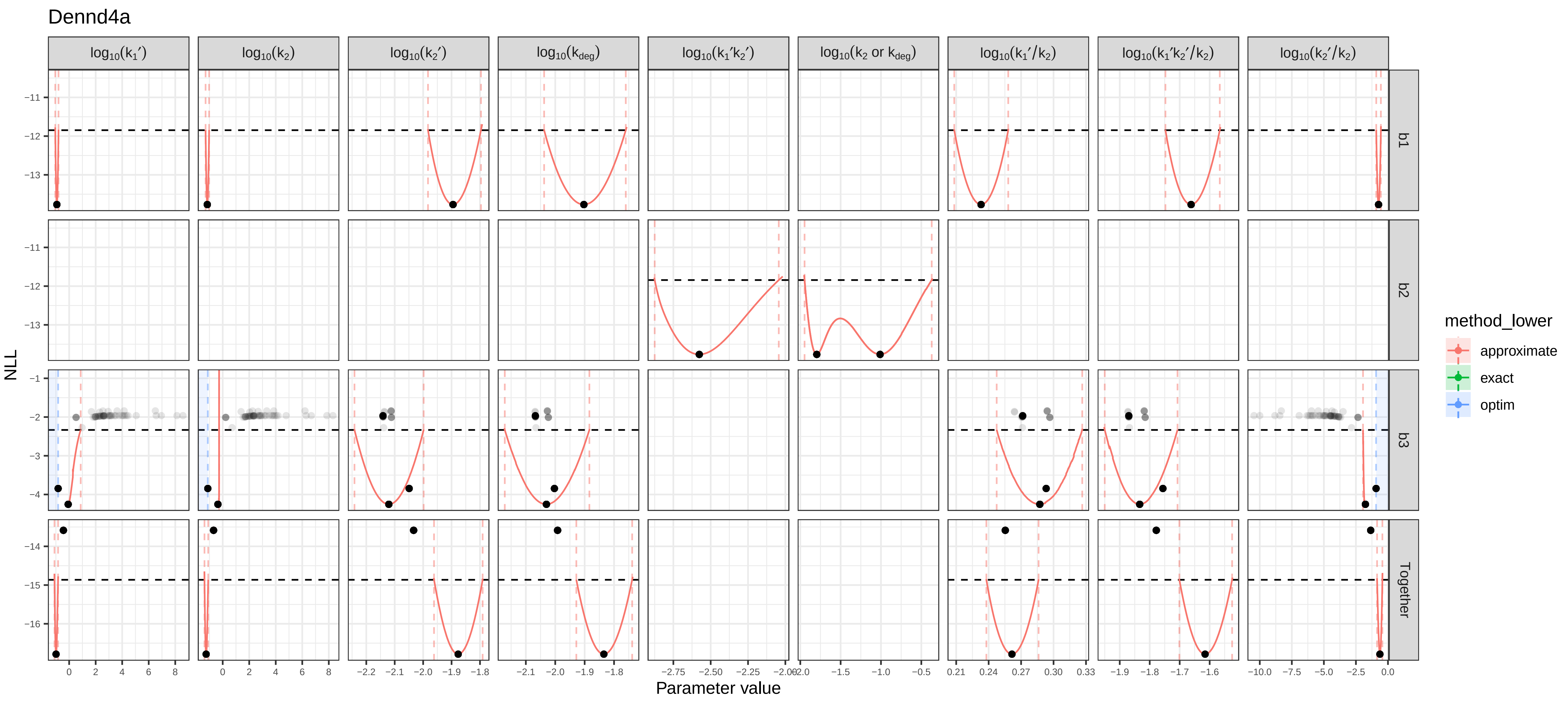

Replicate	Par	Best value	CI95 LB	CI95 UB	Method LB	Method UB
Together	$\log_{10}(k_1')$	−0.9868	−1.104	−0.8351	approximate	approximate
Together	$\log_{10}(k_2)$	−1.248	−1.374	−1.092	approximate	approximate
Together	$\log_{10}(k_2')$	−1.877	−1.962	−1.791	approximate	approximate
Together	$\log_{10}(k_{deg})$	−1.835	−1.928	−1.738	approximate	approximate
Together	$\log_{10}(k_1'/k_2)$	0.2615	0.2379	0.2862	approximate	approximate
Together	$\log_{10}(k_1'k_2'/k_2)$	−1.615	−1.701	−1.525	approximate	approximate
Together	$\log_{10}(k_2'/k_2)$	−0.6285	−0.8498	−0.4355	approximate	approximate
b1	$\log_{10}(k_1')$	−0.9283	−1.047	−0.8009	approximate	approximate
b1	$\log_{10}(k_2)$	−1.161	−1.291	−1.024	approximate	approximate
b1	$\log_{10}(k_2')$	−1.895	−1.983	−1.797	approximate	approximate
b1	$\log_{10}(k_{deg})$	−1.903	−2.038	−1.76	approximate	approximate
b1	$\log_{10}(k_1'/k_2)$	0.233	0.2082	0.2581	approximate	approximate
b1	$\log_{10}(k_1'k_2'/k_2)$	−1.662	−1.748	−1.566	approximate	approximate
b1	$\log_{10}(k_2'/k_2)$	−0.7336	−0.915	−0.5524	approximate	approximate
b2	$\log_{10}(k_1'k_2')$	−2.576	−2.875	−2.044	approximate	approximate
b2	$\log_{10}(k_2 \text{ or } k_{deg})$	−1.011	−1.947	−0.3711	approximate	approximate
b2	$\log_{10}(k_2 \text{ or } k_{deg})$	−1.796	−1.947	−0.3711	approximate	approximate
b3	$\log_{10}(k_1')$	−0.07308	< −0.8294	0.8703	optim	approximate
b3	$\log_{10}(k_2)$	−0.3603	< −1.123	−0.2725	optim	approximate
b3	$\log_{10}(k_2')$	−2.121	−2.242	−1.998	approximate	approximate
b3	$\log_{10}(k_{deg})$	−2.03	−2.172	−1.884	approximate	approximate
b3	$\log_{10}(k_1'/k_2)$	0.2873	0.2474	0.3265	approximate	approximate
b3	$\log_{10}(k_1'k_2'/k_2)$	−1.833	−1.95	−1.707	approximate	approximate
b3	$\log_{10}(k_2'/k_2)$	−1.76	−1.946	> −0.9264	approximate	optim

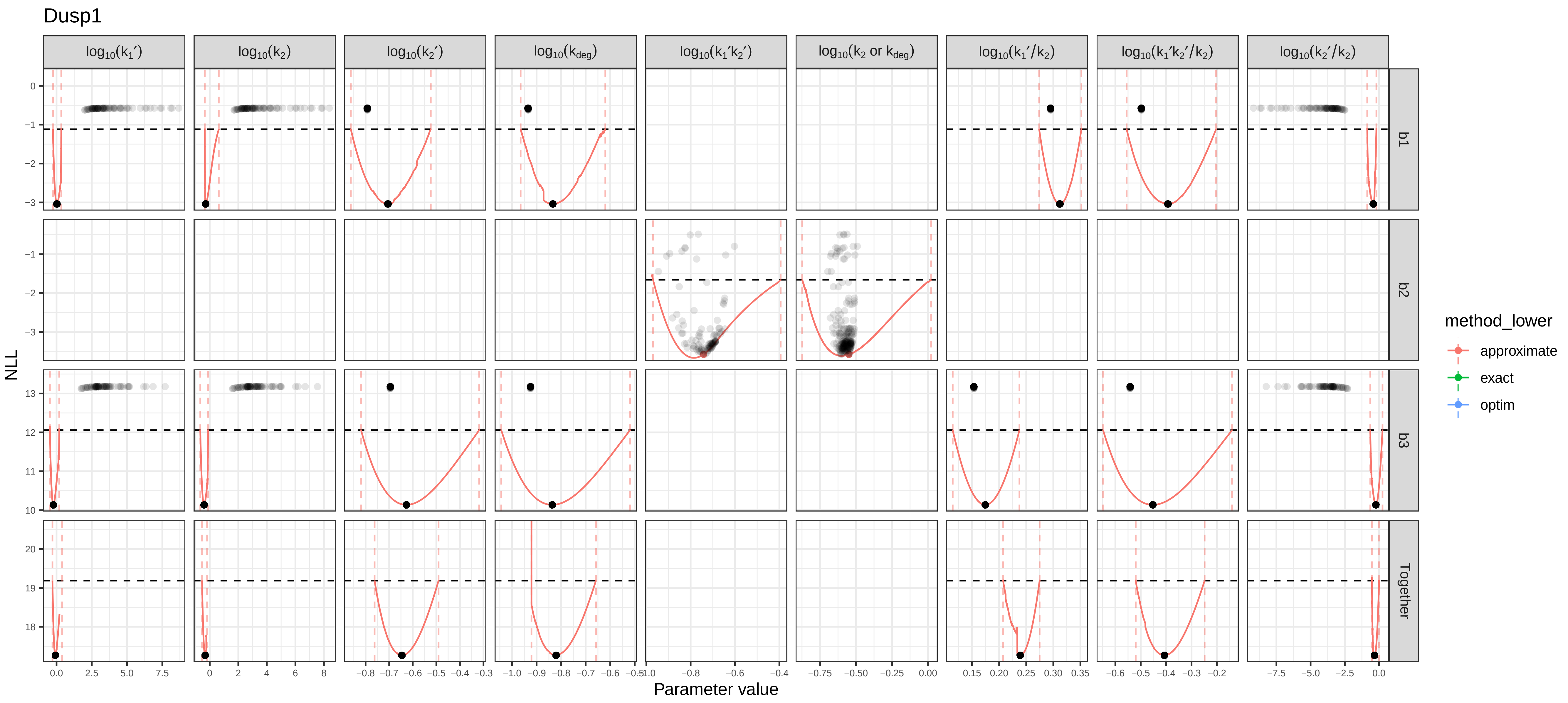

Replicate	Par	Best value	CI95 LB	CI95 UB	Method LB	Method UB
Together	$\log_{10}(k_1')$	−0.08093	−0.2891	0.3982	approximate	approximate
Together	$\log_{10}(k_2)$	−0.3199	−0.5386	−0.1872	approximate	approximate
Together	$\log_{10}(k_2')$	−0.646	−0.7618	−0.4905	approximate	approximate
Together	$\log_{10}(k_{deg})$	−0.8207	−0.921	−0.6581	approximate	approximate
Together	$\log_{10}(k_1'/k_2)$	0.239	0.2074	0.2748	approximate	approximate
Together	$\log_{10}(k_1'k_2'/k_2)$	−0.407	−0.5198	−0.2485	approximate	approximate
Together	$\log_{10}(k_2'/k_2)$	−0.3261	−0.5077	0.005042	approximate	approximate
b1	$\log_{10}(k_1')$	0.02784	−0.2571	0.3366	approximate	approximate
b1	$\log_{10}(k_2)$	−0.2844	−0.3481	0.633	approximate	approximate
b1	$\log_{10}(k_2')$	−0.7051	−0.8626	−0.5235	approximate	approximate
b1	$\log_{10}(k_{deg})$	−0.8338	−0.9653	−0.6199	approximate	approximate
b1	$\log_{10}(k_1'/k_2)$	0.3122	0.2739	0.3519	approximate	approximate
b1	$\log_{10}(k_1'k_2'/k_2)$	−0.3929	−0.5554	−0.2034	approximate	approximate
b1	$\log_{10}(k_2'/k_2)$	−0.4207	−0.8615	−0.1922	approximate	approximate
b2	$\log_{10}(k_1'k_2')$	−0.7418	−0.9697	−0.394	approximate	approximate
b2	$\log_{10}(k_2 \text{ or } k_{deg})$	−0.5502	−0.8737	0.02112	approximate	approximate
b2	$\log_{10}(k_2 \text{ or } k_{deg})$	−0.6017	−0.8737	0.02112	approximate	approximate
b3	$\log_{10}(k_1')$	−0.2232	−0.4708	0.1929	approximate	approximate
b3	$\log_{10}(k_2)$	−0.3977	−0.6636	−0.1189	approximate	approximate
b3	$\log_{10}(k_2')$	−0.6268	−0.8192	−0.3183	approximate	approximate
b3	$\log_{10}(k_{deg})$	−0.8363	−1.044	−0.5193	approximate	approximate
b3	$\log_{10}(k_1'/k_2)$	0.1745	0.1143	0.2374	approximate	approximate
b3	$\log_{10}(k_1'k_2'/k_2)$	−0.4523	−0.6482	−0.1407	approximate	approximate
b3	$\log_{10}(k_2'/k_2)$	−0.2291	−0.6391	0.2557	approximate	approximate

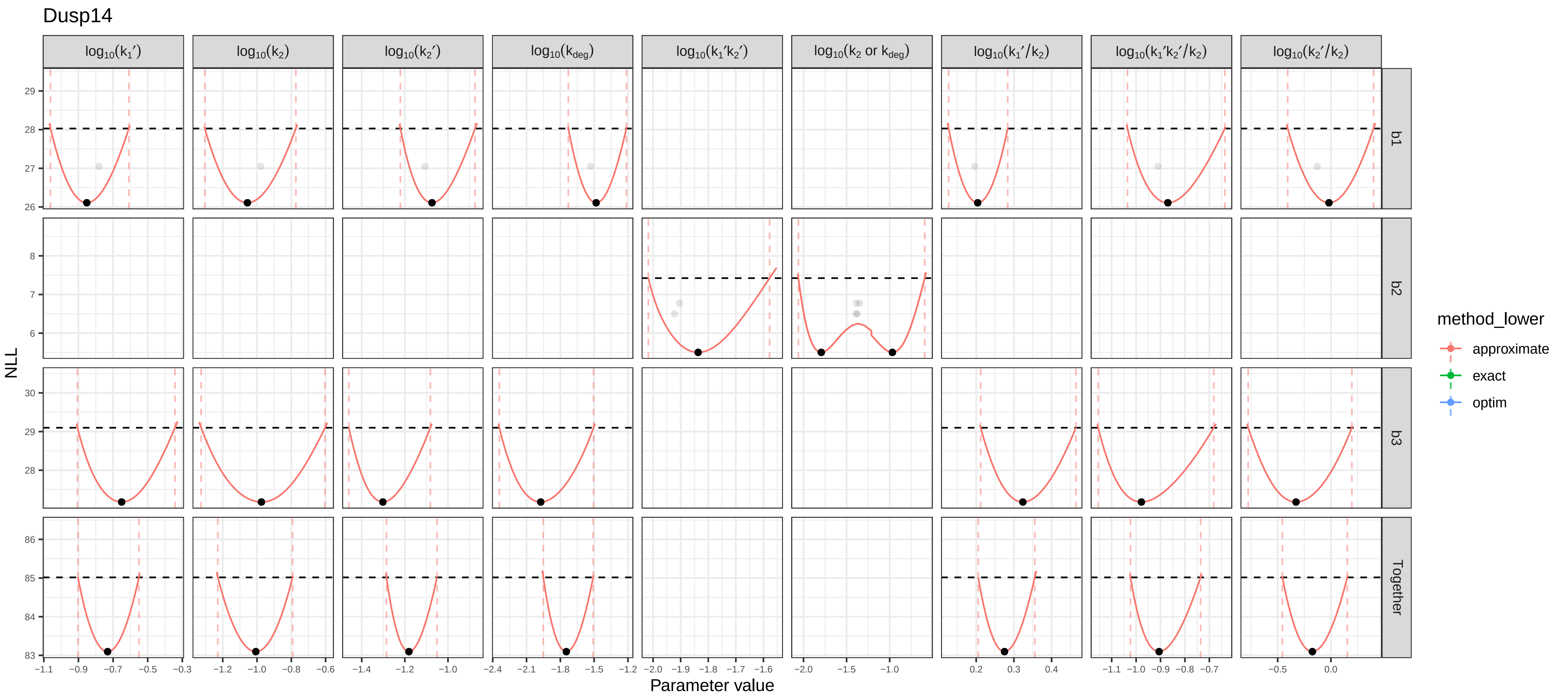

Replicate	Par	Best value	CI95 LB	CI95 UB	Method LB	Method UB
Together	$\log_{10}(k_1')$	−0.7317	−0.9028	−0.5505	approximate	approximate
Together	$\log_{10}(k_2)$	−1.007	−1.229	−0.7923	approximate	approximate
Together	$\log_{10}(k_2')$	−1.181	−1.285	−1.051	approximate	approximate
Together	$\log_{10}(k_{deg})$	−1.747	−1.951	−1.508	approximate	approximate
Together	$\log_{10}(k_1'/k_2)$	0.2752	0.2054	0.3555	approximate	approximate
Together	$\log_{10}(k_1'k_2'/k_2)$	−0.9058	−1.023	−0.7357	approximate	approximate
Together	$\log_{10}(k_2'/k_2)$	−0.174	−0.4557	0.1537	approximate	approximate
b1	$\log_{10}(k_1')$	−0.8518	−1.062	−0.6087	approximate	approximate
b1	$\log_{10}(k_2)$	−1.056	−1.304	−0.7734	approximate	approximate
b1	$\log_{10}(k_2')$	−1.074	−1.221	−0.8743	approximate	approximate
b1	$\log_{10}(k_{deg})$	−1.482	−1.729	−1.213	approximate	approximate
b1	$\log_{10}(k_1'/k_2)$	0.2041	0.127	0.2837	approximate	approximate
b1	$\log_{10}(k_1'k_2'/k_2)$	−0.8699	−1.036	−0.6363	approximate	approximate
b1	$\log_{10}(k_2'/k_2)$	−0.01808	−0.406	0.4002	approximate	approximate
b2	$\log_{10}(k_1'k_2')$	−1.837	−2.017	−1.578	approximate	approximate
b2	$\log_{10}(k_2 \text{ or } k_{deg})$	−0.9685	−2.06	−0.5933	approximate	approximate
b2	$\log_{10}(k_2 \text{ or } k_{deg})$	−1.794	−2.06	−0.5933	approximate	approximate
b3	$\log_{10}(k_1')$	−0.6505	−0.9056	−0.3425	approximate	approximate
b3	$\log_{10}(k_2)$	−0.9743	−1.326	−0.6036	approximate	approximate
b3	$\log_{10}(k_2')$	−1.302	−1.46	−1.082	approximate	approximate
b3	$\log_{10}(k_{deg})$	−1.974	−2.341	−1.505	approximate	approximate
b3	$\log_{10}(k_1'/k_2)$	0.3238	0.2119	0.4645	approximate	approximate
b3	$\log_{10}(k_1'k_2'/k_2)$	−0.9782	−1.156	−0.6819	approximate	approximate
b3	$\log_{10}(k_2'/k_2)$	−0.3277	−0.7767	0.1967	approximate	approximate

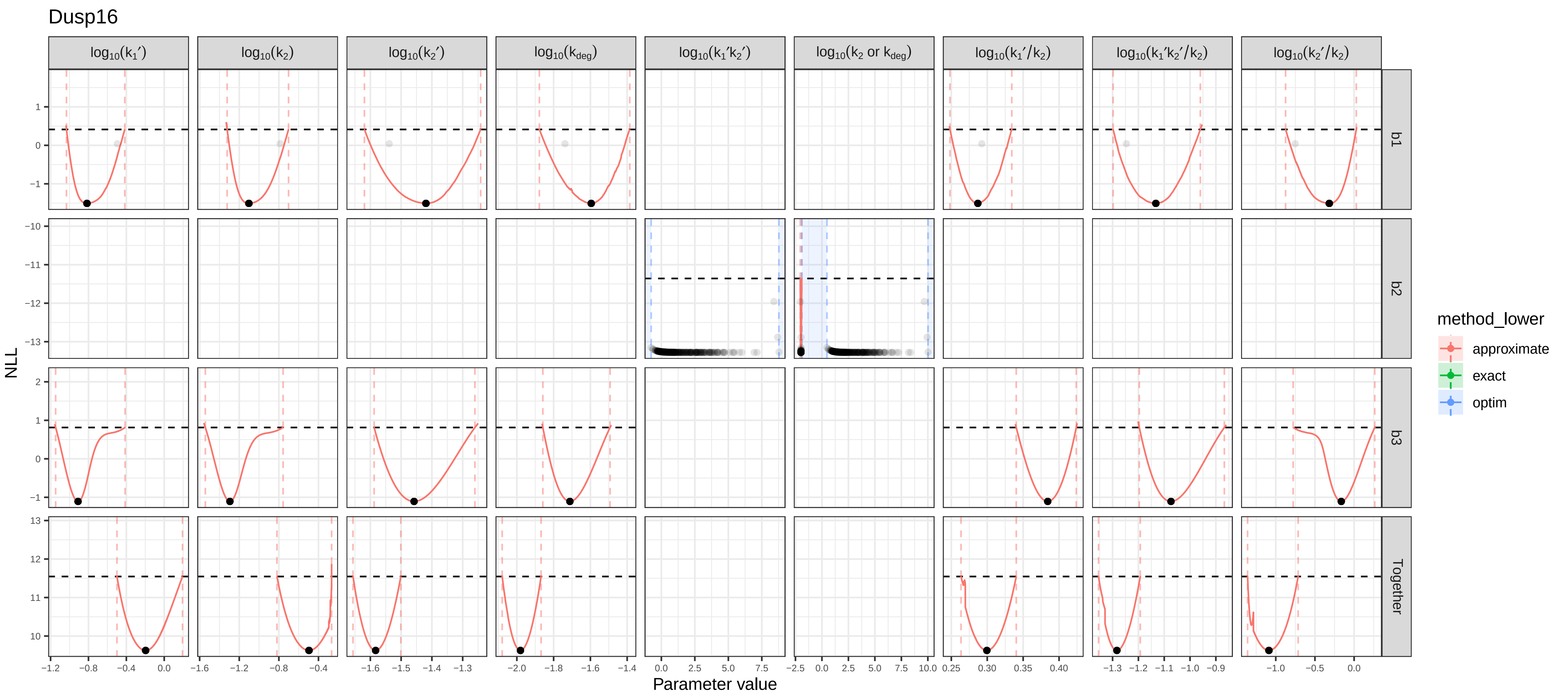

Replicate	Par	Best value	CI95 LB	CI95 UB	Method LB	Method UB
Together	$\log_{10}(k_1')$	−0.1963	−0.4991	0.1933	approximate	approximate
Together	$\log_{10}(k_2)$	−0.4958	−0.8194	−0.2674	approximate	approximate
Together	$\log_{10}(k_2')$	−1.583	−1.656	−1.501	approximate	approximate
Together	$\log_{10}(k_{deg})$	−1.98	−2.08	−1.868	approximate	approximate
Together	$\log_{10}(k_1'/k_2)$	0.2995	0.2635	0.3404	approximate	approximate
Together	$\log_{10}(k_1'k_2'/k_2)$	−1.283	−1.354	−1.193	approximate	approximate
Together	$\log_{10}(k_2'/k_2)$	−1.087	−1.36	−0.7143	approximate	approximate
b1	$\log_{10}(k_1')$	−0.8155	−1.032	−0.4165	approximate	approximate
b1	$\log_{10}(k_2)$	−1.102	−1.322	−0.7022	approximate	approximate
b1	$\log_{10}(k_2')$	−1.42	−1.619	−1.242	approximate	approximate
b1	$\log_{10}(k_{deg})$	−1.596	−1.878	−1.386	approximate	approximate
b1	$\log_{10}(k_1'/k_2)$	0.287	0.2482	0.3342	approximate	approximate
b1	$\log_{10}(k_1'k_2'/k_2)$	−1.133	−1.298	−0.9602	approximate	approximate
b1	$\log_{10}(k_2'/k_2)$	−0.3171	−0.8746	0.02797	approximate	approximate
b2	$\log_{10}(k_1'k_2')$	3.436	< −0.762	> 8.777	optim	optim
b2	$\log_{10}(k_2 \text{ or } k_{deg})$	4.678	0.4746	> 10.02	optim	optim
b2	$\log_{10}(k_2 \text{ or } k_{deg})$	−1.979	−2.048	−1.901	approximate	approximate
b3	$\log_{10}(k_1')$	−0.9099	−1.147	−0.4133	approximate	approximate
b3	$\log_{10}(k_2)$	−1.294	−1.543	−0.7574	approximate	approximate
b3	$\log_{10}(k_2')$	−1.458	−1.588	−1.26	approximate	approximate
b3	$\log_{10}(k_{deg})$	−1.711	−1.859	−1.493	approximate	approximate
b3	$\log_{10}(k_1'/k_2)$	0.3842	0.3404	0.424	approximate	approximate
b3	$\log_{10}(k_1'k_2'/k_2)$	−1.074	−1.197	−0.8671	approximate	approximate
b3	$\log_{10}(k_2'/k_2)$	−0.1638	−0.778	0.2634	approximate	approximate

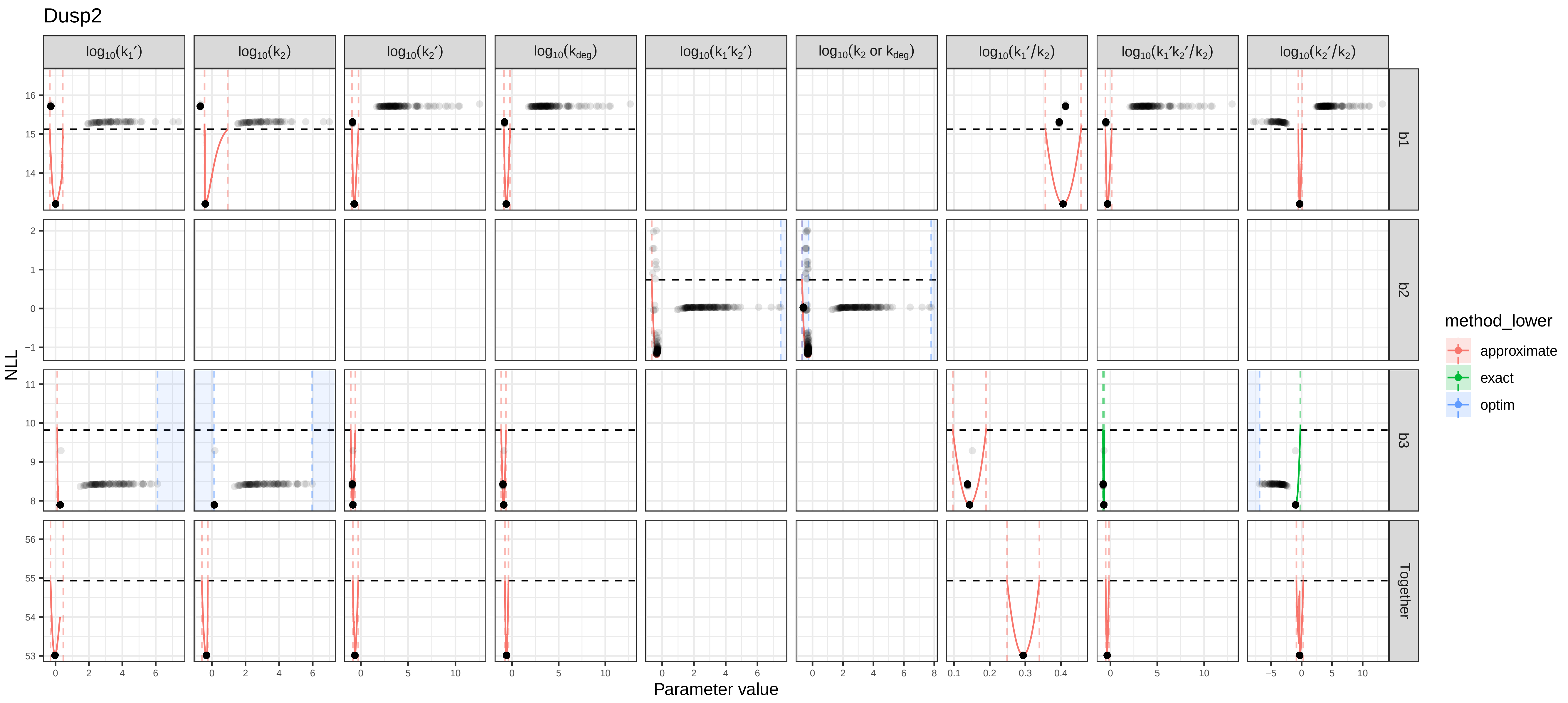

Replicate	Par	Best value	CI95 LB	CI95 UB	Method LB	Method UB
Together	$\log_{10}(k_1')$	−0.03566	−0.3004	0.4654	approximate	approximate
Together	$\log_{10}(k_2)$	−0.3295	−0.6026	−0.2495	approximate	approximate
Together	$\log_{10}(k_2')$	−0.6368	−0.8497	−0.2731	approximate	approximate
Together	$\log_{10}(k_{deg})$	−0.591	−0.768	−0.3778	approximate	approximate
Together	$\log_{10}(k_1'/k_2)$	0.2939	0.2487	0.3395	approximate	approximate
Together	$\log_{10}(k_1'k_2'/k_2)$	−0.3429	−0.5108	−0.1663	approximate	approximate
Together	$\log_{10}(k_2'/k_2)$	−0.3072	−0.8417	0.282	approximate	approximate
b1	$\log_{10}(k_1')$	0.005215	−0.3449	0.4292	approximate	approximate
b1	$\log_{10}(k_2)$	−0.4007	−0.4443	0.9374	approximate	approximate
b1	$\log_{10}(k_2')$	−0.7058	−0.9387	−0.2544	approximate	approximate
b1	$\log_{10}(k_{deg})$	−0.6187	−0.8664	−0.2117	approximate	approximate
b1	$\log_{10}(k_1'/k_2)$	0.406	0.3562	0.4567	approximate	approximate
b1	$\log_{10}(k_1'k_2'/k_2)$	−0.2999	−0.5329	0.1373	approximate	approximate
b1	$\log_{10}(k_2'/k_2)$	−0.3051	−0.5339	0.1098	approximate	approximate
b2	$\log_{10}(k_1'k_2')$	−0.3454	−0.6594	> 7.461	approximate	optim
b2	$\log_{10}(k_2 \text{ or } k_{deg})$	−0.3039	−0.6805	> 7.793	approximate	optim
b2	$\log_{10}(k_2 \text{ or } k_{deg})$	−0.3178	−0.6805	−0.2581	approximate	optim
b3	$\log_{10}(k_1')$	0.2773	0.1036	> 6.109	approximate	optim
b3	$\log_{10}(k_2)$	0.1337	< 0.1297	> 5.971	optim	optim
b3	$\log_{10}(k_2')$	−0.8462	−1.072	−0.5835	approximate	approximate
b3	$\log_{10}(k_{deg})$	−0.8907	−1.157	−0.6562	approximate	approximate
b3	$\log_{10}(k_1'/k_2)$	0.1436	0.09611	0.1899	approximate	approximate
b3	$\log_{10}(k_1'k_2'/k_2)$	−0.7026	−0.7834	−0.6458	exact	exact
b3	$\log_{10}(k_2'/k_2)$	−0.9799	< −6.888	−0.208	optim	exact

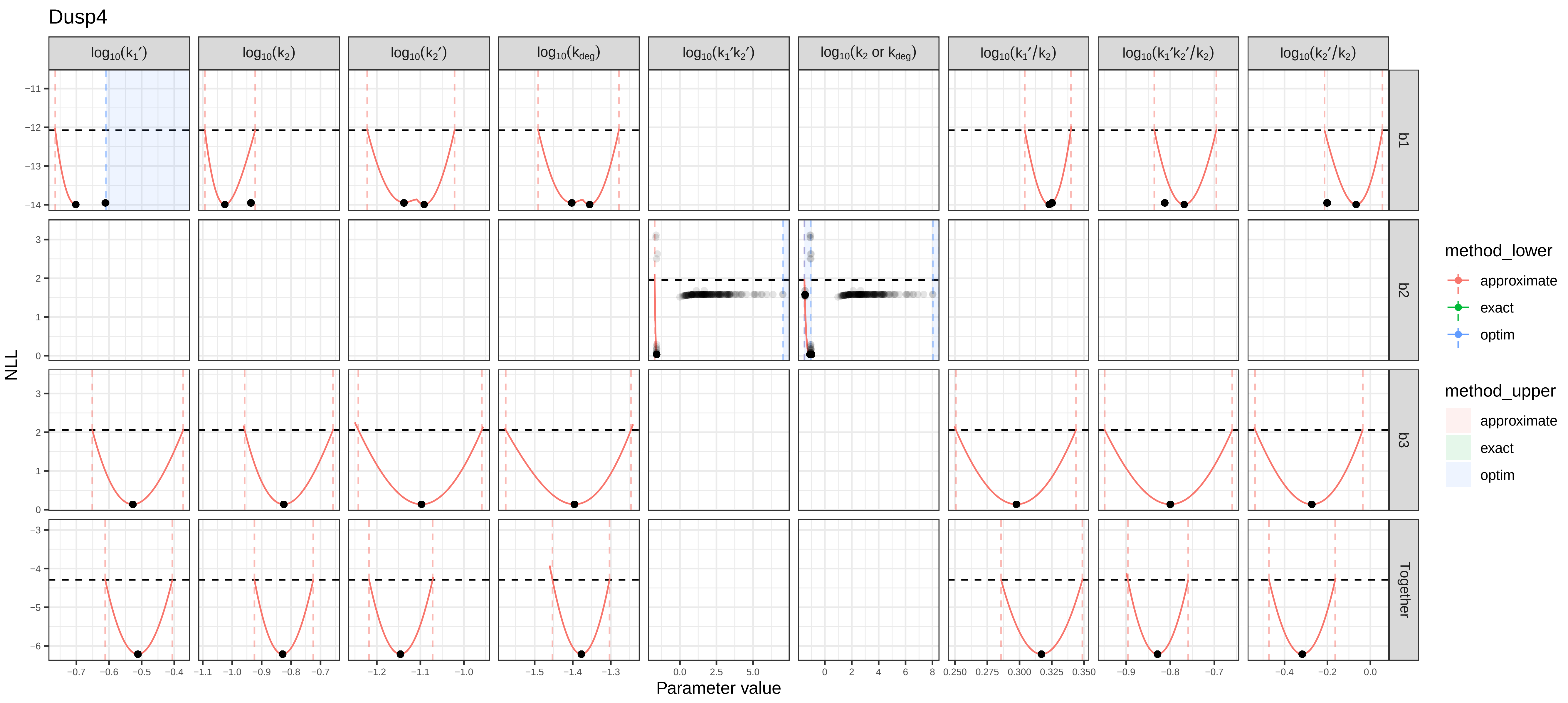

Replicate	Par	Best value	CI95 LB	CI95 UB	Method LB	Method UB
Together	$\log_{10}(k_1')$	−0.5114	−0.6117	−0.4059	approximate	approximate
Together	$\log_{10}(k_2)$	−0.8284	−0.9244	−0.7246	approximate	approximate
Together	$\log_{10}(k_2')$	−1.146	−1.218	−1.072	approximate	approximate
Together	$\log_{10}(k_{deg})$	−1.377	−1.453	−1.303	approximate	approximate
Together	$\log_{10}(k_1'/k_2)$	0.317	0.2857	0.3487	approximate	approximate
Together	$\log_{10}(k_1'k_2'/k_2)$	−0.8288	−0.8962	−0.7592	approximate	approximate
Together	$\log_{10}(k_2'/k_2)$	−0.3174	−0.4723	−0.1639	approximate	approximate
b1	$\log_{10}(k_1')$	−0.702	−0.7648	> −0.6096	approximate	optim
b1	$\log_{10}(k_2)$	−1.025	−1.093	−0.9217	approximate	approximate
b1	$\log_{10}(k_2')$	−1.091	−1.222	−1.022	approximate	approximate
b1	$\log_{10}(k_{deg})$	−1.355	−1.491	−1.279	approximate	approximate
b1	$\log_{10}(k_1'/k_2)$	0.323	0.304	0.3398	approximate	approximate
b1	$\log_{10}(k_1'k_2'/k_2)$	−0.7683	−0.8361	−0.6955	approximate	approximate
b1	$\log_{10}(k_2'/k_2)$	−0.06631	−0.2138	0.05623	approximate	approximate
b2	$\log_{10}(k_1'k_2')$	−1.589	−1.726	> 7.051	approximate	optim
b2	$\log_{10}(k_2 \text{ or } k_{deg})$	−0.9828	−1.512	> 8.032	approximate	optim
b2	$\log_{10}(k_2 \text{ or } k_{deg})$	−1.109	−1.512	−1.049	approximate	optim
b3	$\log_{10}(k_1')$	−0.527	−0.6514	−0.3725	approximate	approximate
b3	$\log_{10}(k_2)$	−0.8246	−0.9578	−0.6574	approximate	approximate
b3	$\log_{10}(k_2')$	−1.097	−1.242	−0.9591	approximate	approximate
b3	$\log_{10}(k_{deg})$	−1.395	−1.576	−1.247	approximate	approximate
b3	$\log_{10}(k_1'/k_2)$	0.2976	0.2506	0.3438	approximate	approximate
b3	$\log_{10}(k_1'k_2'/k_2)$	−0.7998	−0.9486	−0.6595	approximate	approximate
b3	$\log_{10}(k_2'/k_2)$	−0.2728	−0.5368	−0.03556	approximate	approximate

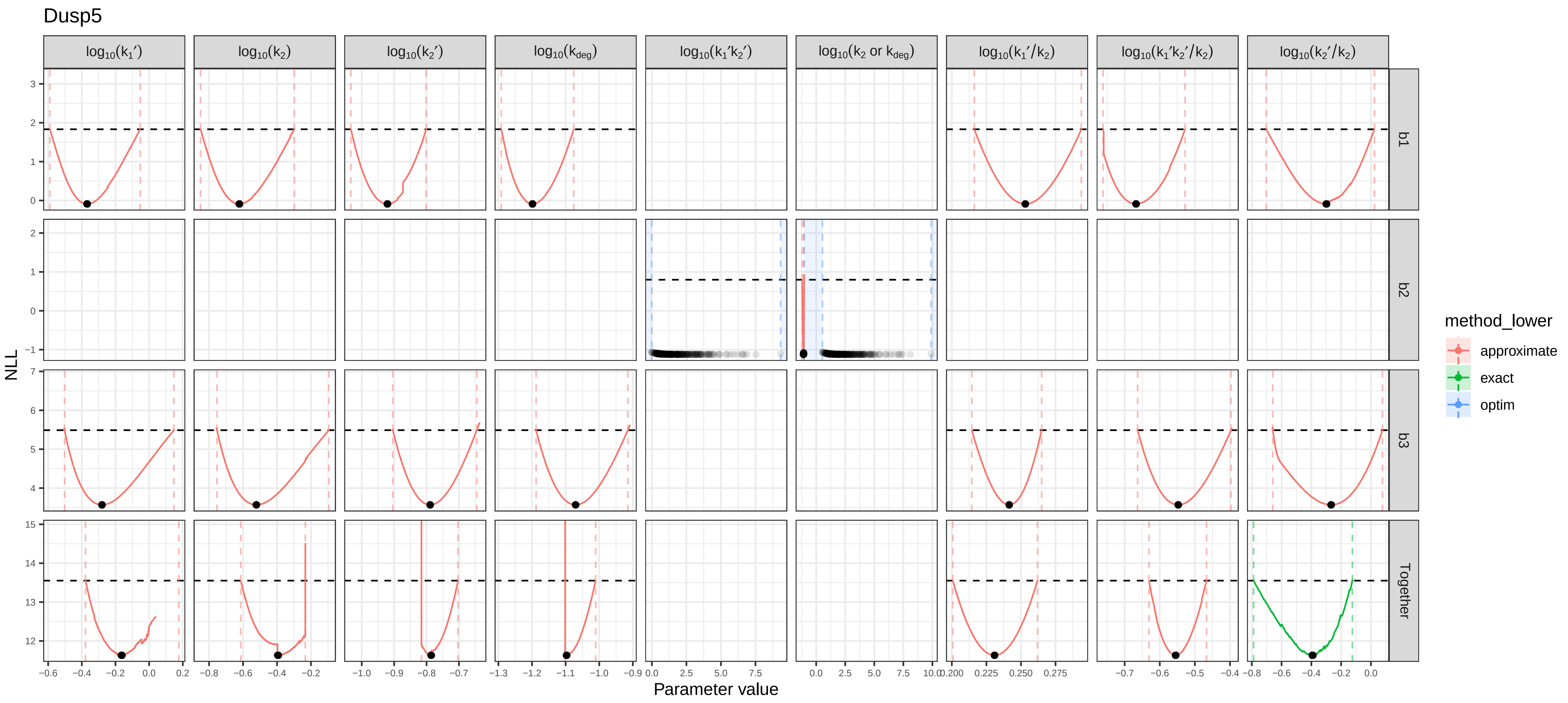

Replicate	Par	Best value	CI95 LB	CI95 UB	Method LB	Method UB
Together	$\log_{10}(k_1')$	−0.1625	−0.378	0.1763	approximate	approximate
Together	$\log_{10}(k_2)$	−0.3934	−0.6136	−0.2331	approximate	approximate
Together	$\log_{10}(k_2')$	−0.7858	−0.8157	−0.7028	approximate	approximate
Together	$\log_{10}(k_{deg})$	−1.097	−1.101	−1.011	approximate	approximate
Together	$\log_{10}(k_1'/k_2)$	0.2309	0.2007	0.262	approximate	approximate
Together	$\log_{10}(k_1'k_2'/k_2)$	−0.5549	−0.6307	−0.4668	approximate	approximate
Together	$\log_{10}(k_2'/k_2)$	−0.3924	−0.788	−0.1246	exact	exact
b1	$\log_{10}(k_1')$	−0.3682	−0.5898	−0.05267	approximate	approximate
b1	$\log_{10}(k_2)$	−0.6213	−0.8504	−0.2979	approximate	approximate
b1	$\log_{10}(k_2')$	−0.9208	−1.034	−0.8013	approximate	approximate
b1	$\log_{10}(k_{deg})$	−1.199	−1.292	−1.076	approximate	approximate
b1	$\log_{10}(k_1'/k_2)$	0.2531	0.2163	0.2936	approximate	approximate
b1	$\log_{10}(k_1'k_2'/k_2)$	−0.6677	−0.7615	−0.528	approximate	approximate
b1	$\log_{10}(k_2'/k_2)$	−0.2995	−0.7031	0.02368	approximate	approximate
b2	$\log_{10}(k_1'k_2')$	6.217	< −0.01434	> 9.324	optim	optim
b2	$\log_{10}(k_2 \text{ or } k_{deg})$	6.783	0.5395	> 9.893	optim	optim
b2	$\log_{10}(k_2 \text{ or } k_{deg})$	−1.09	−1.208	−1.044	approximate	approximate
b3	$\log_{10}(k_1')$	−0.2797	−0.5025	0.1471	approximate	approximate
b3	$\log_{10}(k_2)$	−0.5212	−0.7532	−0.09492	approximate	approximate
b3	$\log_{10}(k_2')$	−0.789	−0.9042	−0.6452	approximate	approximate
b3	$\log_{10}(k_{deg})$	−1.07	−1.188	−0.9148	approximate	approximate
b3	$\log_{10}(k_1'/k_2)$	0.2415	0.2144	0.265	approximate	approximate
b3	$\log_{10}(k_1'k_2'/k_2)$	−0.5475	−0.6631	−0.397	approximate	approximate
b3	$\log_{10}(k_2'/k_2)$	−0.2678	−0.6591	0.07736	approximate	approximate

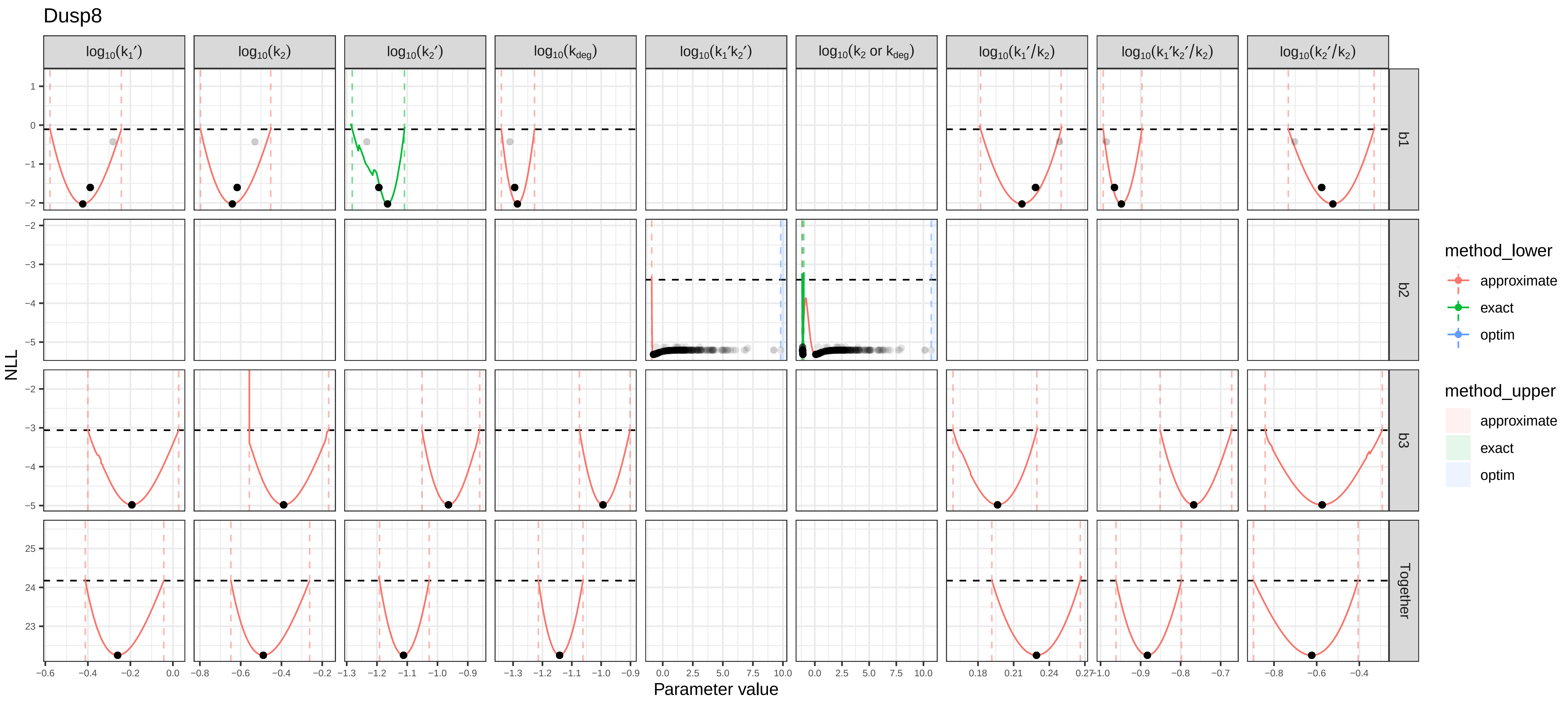

Replicate	Par	Best value	CI95 LB	CI95 UB	Method LB	Method UB
Together	$\log_{10}(k_1')$	−0.2599	−0.4121	−0.04291	approximate	approximate
Together	$\log_{10}(k_2)$	−0.4891	−0.6482	−0.2616	approximate	approximate
Together	$\log_{10}(k_2')$	−1.112	−1.192	−1.027	approximate	approximate
Together	$\log_{10}(k_{deg})$	−1.141	−1.214	−1.062	approximate	approximate
Together	$\log_{10}(k_1'/k_2)$	0.2293	0.1916	0.2661	approximate	approximate
Together	$\log_{10}(k_1'k_2'/k_2)$	−0.8831	−0.9616	−0.7979	approximate	approximate
Together	$\log_{10}(k_2'/k_2)$	−0.6232	−0.8958	−0.4058	approximate	approximate
b1	$\log_{10}(k_1')$	−0.424	−0.5778	−0.2424	approximate	approximate
b1	$\log_{10}(k_2)$	−0.641	−0.7972	−0.4525	approximate	approximate
b1	$\log_{10}(k_2')$	−1.165	−1.282	−1.109	exact	exact
b1	$\log_{10}(k_{deg})$	−1.285	−1.34	−1.227	approximate	approximate
b1	$\log_{10}(k_1'/k_2)$	0.217	0.1821	0.25	approximate	approximate
b1	$\log_{10}(k_1'k_2'/k_2)$	−0.948	−0.9934	−0.8965	approximate	approximate
b1	$\log_{10}(k_2'/k_2)$	−0.524	−0.7331	−0.3307	approximate	approximate
b2	$\log_{10}(k_1'k_2')$	−0.758	−0.9184	> 9.811	approximate	optim
b2	$\log_{10}(k_2 \text{ or } k_{deg})$	0.1059	−1.162	> 10.71	approximate	optim
b2	$\log_{10}(k_2 \text{ or } k_{deg})$	−1.099	−1.165	−1.015	exact	exact
b3	$\log_{10}(k_1')$	−0.1929	−0.4	0.02723	approximate	approximate
b3	$\log_{10}(k_2)$	−0.3893	−0.5576	−0.1684	approximate	approximate
b3	$\log_{10}(k_2')$	−0.9638	−1.051	−0.8605	approximate	approximate
b3	$\log_{10}(k_{deg})$	−0.9937	−1.074	−0.9015	approximate	approximate
b3	$\log_{10}(k_1'/k_2)$	0.1964	0.159	0.2296	approximate	approximate
b3	$\log_{10}(k_1'k_2'/k_2)$	−0.7673	−0.8514	−0.6724	approximate	approximate
b3	$\log_{10}(k_2'/k_2)$	−0.5745	−0.8416	−0.2925	approximate	approximate

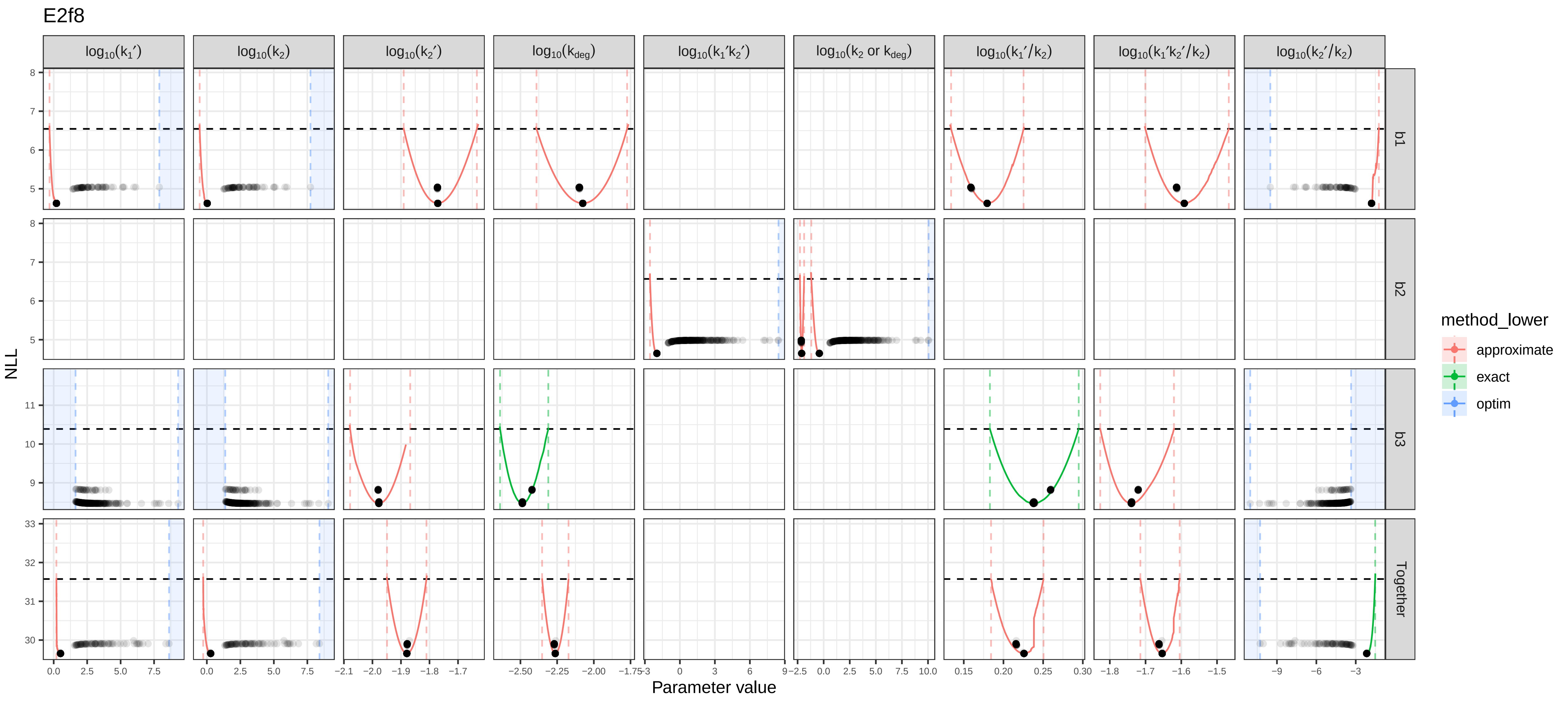

Replicate	Par	Best value	CI95 LB	CI95 UB	Method LB	Method UB
Together	$\log_{10}(k_1')$	0.51	0.2009	> 8.622	approximate	optim
Together	$\log_{10}(k_2)$	0.2841	−0.273	> 8.406	approximate	optim
Together	$\log_{10}(k_2')$	−1.879	−1.949	−1.811	approximate	approximate
Together	$\log_{10}(k_{deg})$	−2.263	−2.352	−2.172	approximate	approximate
Together	$\log_{10}(k_1'/k_2)$	0.2259	0.1844	0.2505	approximate	approximate
Together	$\log_{10}(k_1'k_2'/k_2)$	−1.653	−1.714	−1.604	approximate	approximate
Together	$\log_{10}(k_2'/k_2)$	−2.163	< −10.28	−1.52	optim	exact
b1	$\log_{10}(k_1')$	0.2054	−0.3129	> 7.893	approximate	optim
b1	$\log_{10}(k_2)$	0.02597	−0.5361	> 7.734	approximate	optim
b1	$\log_{10}(k_2')$	−1.771	−1.89	−1.634	approximate	approximate
b1	$\log_{10}(k_{deg})$	−2.075	−2.391	−1.773	approximate	approximate
b1	$\log_{10}(k_1'/k_2)$	0.1795	0.134	0.2254	approximate	approximate
b1	$\log_{10}(k_1'k_2'/k_2)$	−1.592	−1.701	−1.467	approximate	approximate
b1	$\log_{10}(k_2'/k_2)$	−1.797	< −9.506	−1.239	optim	approximate
b2	$\log_{10}(k_1'k_2')$	−1.972	−2.561	> 8.455	approximate	optim
b2	$\log_{10}(k_2 \text{ or } k_{deg})$	−0.4168	−1.185	> 10.06	approximate	optim
b2	$\log_{10}(k_2 \text{ or } k_{deg})$	−2.107	−2.27	−1.873	approximate	approximate
b3	$\log_{10}(k_1')$	7.966	< 1.633	> 9.294	optim	optim
b3	$\log_{10}(k_2)$	7.728	< 1.373	> 9.056	optim	optim
b3	$\log_{10}(k_2')$	−1.978	−2.078	−1.868	approximate	approximate
b3	$\log_{10}(k_{deg})$	−2.487	−2.639	−2.31	exact	exact
b3	$\log_{10}(k_1'/k_2)$	0.2379	0.183	0.2948	exact	exact
b3	$\log_{10}(k_1'k_2'/k_2)$	−1.74	−1.827	−1.621	approximate	approximate
b3	$\log_{10}(k_2'/k_2)$	−9.706	< −11.03	> −3.354	optim	optim

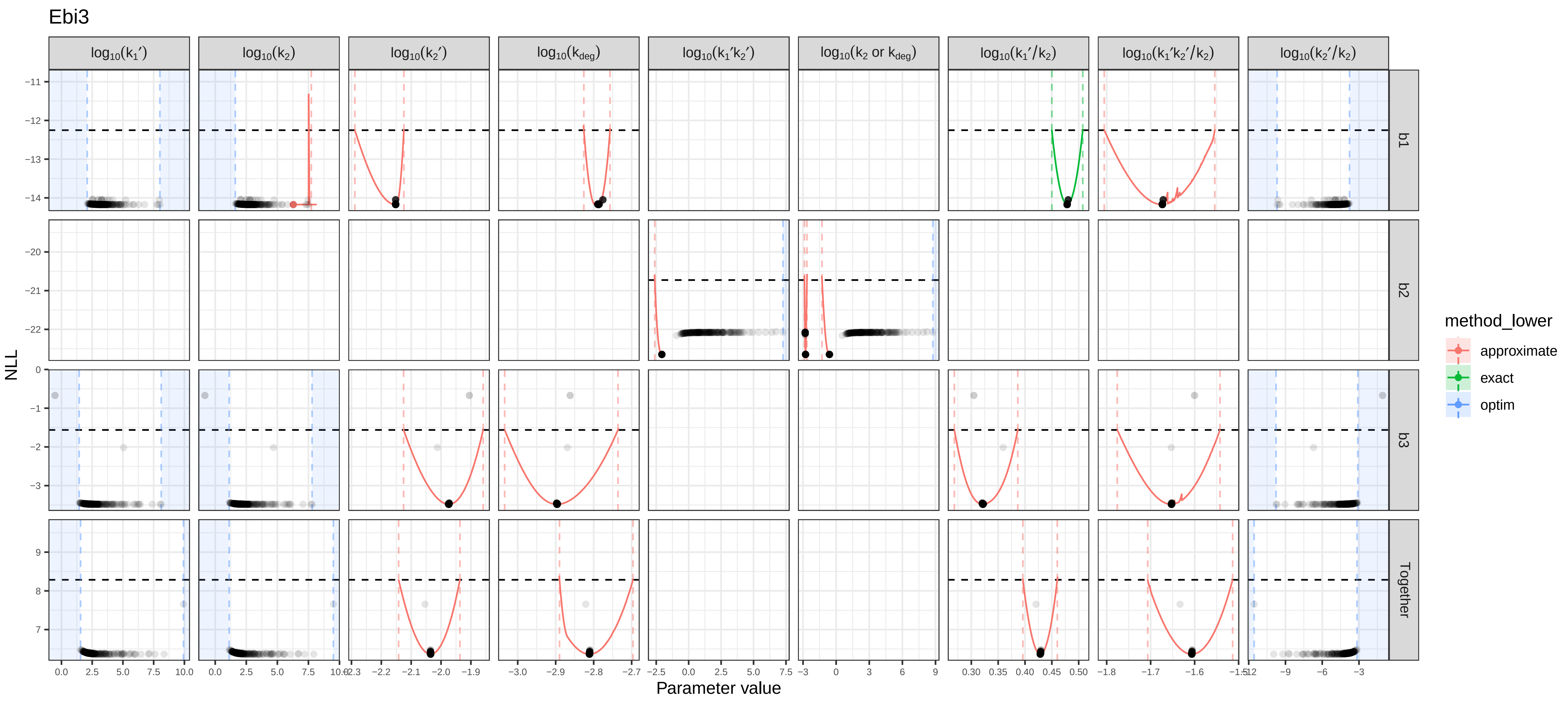

Replicate	Par	Best value	CI95 LB	CI95 UB	Method LB	Method UB
Together	$\log_{10}(k_1')$	5.466	< 1.555	> 9.925	optim	optim
Together	$\log_{10}(k_2)$	5.038	< 1.125	> 9.505	optim	optim
Together	$\log_{10}(k_2')$	−2.035	−2.142	−1.937	approximate	approximate
Together	$\log_{10}(k_{deg})$	−2.811	−2.89	−2.696	approximate	approximate
Together	$\log_{10}(k_1'/k_2)$	0.4284	0.396	0.4602	approximate	approximate
Together	$\log_{10}(k_1'k_2'/k_2)$	−1.607	−1.707	−1.514	approximate	approximate
Together	$\log_{10}(k_2'/k_2)$	−7.073	< −11.56	> −3.16	optim	optim
b1	$\log_{10}(k_1')$	6.762	< 2.095	> 8.009	optim	optim
b1	$\log_{10}(k_2)$	6.283	< 1.616	7.731	optim	approximate
b1	$\log_{10}(k_2')$	−2.152	−2.289	−2.124	approximate	approximate
b1	$\log_{10}(k_{deg})$	−2.788	−2.826	−2.757	approximate	approximate
b1	$\log_{10}(k_1'/k_2)$	0.4785	0.45	0.5076	exact	exact
b1	$\log_{10}(k_1'k_2'/k_2)$	−1.673	−1.806	−1.554	approximate	approximate
b1	$\log_{10}(k_2'/k_2)$	−8.435	< −9.683	> −3.768	optim	optim
b2	$\log_{10}(k_1'k_2')$	−2.066	−2.613	> 7.271	approximate	optim
b2	$\log_{10}(k_2 \text{ or } k_{deg})$	−0.6099	−1.282	> 8.764	approximate	optim
b2	$\log_{10}(k_2 \text{ or } k_{deg})$	−2.771	−2.869	−2.643	approximate	approximate
b3	$\log_{10}(k_1')$	5.124	< 1.443	> 8.115	optim	optim
b3	$\log_{10}(k_2)$	4.802	< 1.122	> 7.793	optim	optim
b3	$\log_{10}(k_2')$	−1.974	−2.125	−1.859	approximate	approximate
b3	$\log_{10}(k_{deg})$	−2.896	−3.034	−2.736	approximate	approximate
b3	$\log_{10}(k_1'/k_2)$	0.3216	0.2684	0.3866	approximate	approximate
b3	$\log_{10}(k_1'k_2'/k_2)$	−1.653	−1.776	−1.542	approximate	approximate
b3	$\log_{10}(k_2'/k_2)$	−6.776	< −9.767	> −3.095	optim	optim

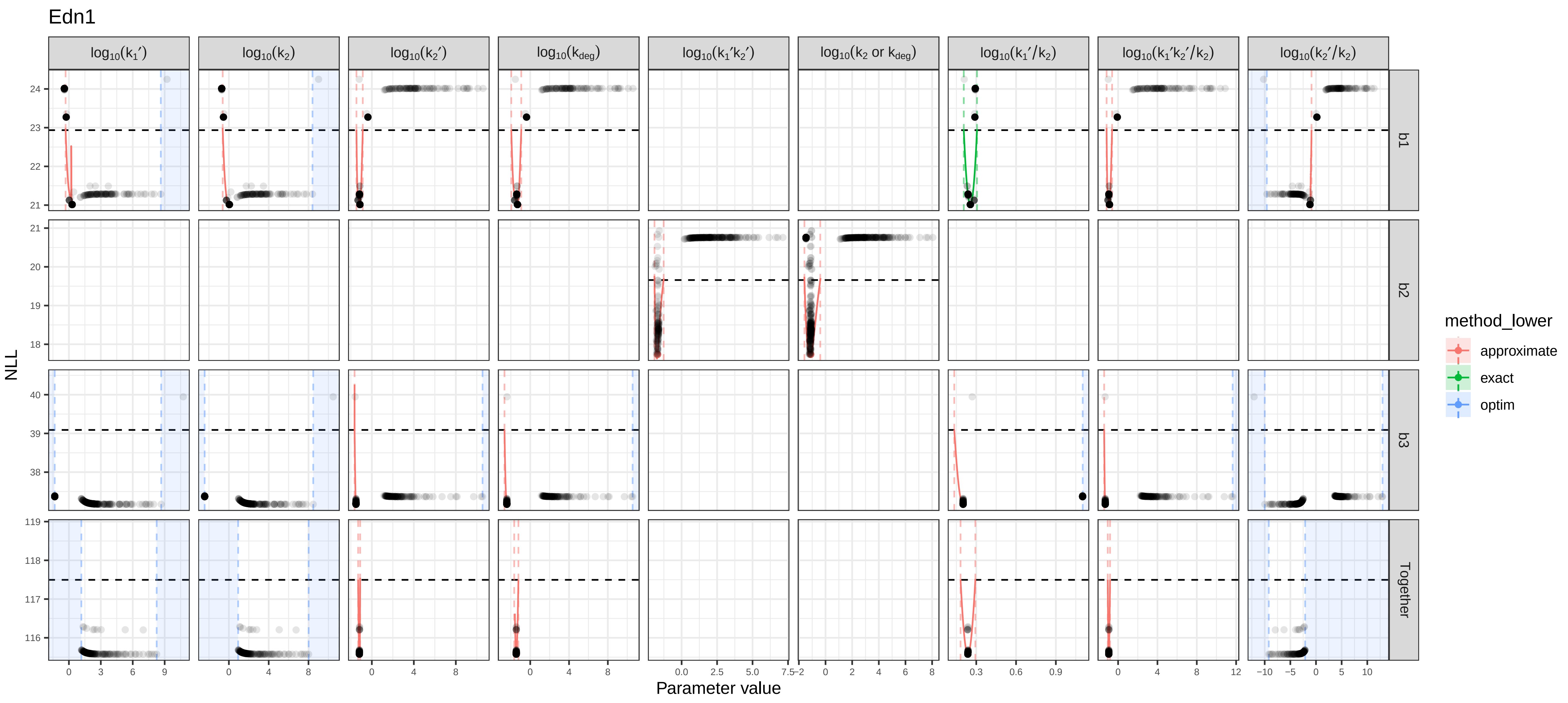

Replicate	Par	Best value	CI95 LB	CI95 UB	Method LB	Method UB
Together	$\log_{10}(k_1')$	6.976	< 1.17	> 8.252	optim	optim
Together	$\log_{10}(k_2)$	6.739	< 0.931	> 8.015	optim	optim
Together	$\log_{10}(k_2')$	−1.186	−1.306	−1.113	approximate	approximate
Together	$\log_{10}(k_{deg})$	−1.443	−1.653	−1.22	approximate	approximate
Together	$\log_{10}(k_1'/k_2)$	0.2363	0.1816	0.2931	approximate	approximate
Together	$\log_{10}(k_1'k_2'/k_2)$	−0.9496	−1.053	−0.8186	approximate	approximate
Together	$\log_{10}(k_2'/k_2)$	−7.925	< −9.202	> −2.114	optim	optim
b1	$\log_{10}(k_1')$	0.3036	−0.3097	> 8.64	approximate	optim
b1	$\log_{10}(k_2)$	0.04824	−0.6197	> 8.402	approximate	optim
b1	$\log_{10}(k_2')$	−1.133	−1.465	−0.8639	approximate	approximate
b1	$\log_{10}(k_{deg})$	−1.317	−1.954	−0.9278	approximate	approximate
b1	$\log_{10}(k_1'/k_2)$	0.2554	0.206	0.3056	exact	exact
b1	$\log_{10}(k_1'k_2'/k_2)$	−0.8775	−1.17	−0.6108	approximate	approximate
b1	$\log_{10}(k_2'/k_2)$	−1.181	< −9.578	−0.8701	optim	approximate
b2	$\log_{10}(k_1'k_2')$	−1.688	−1.913	−1.265	approximate	approximate
b2	$\log_{10}(k_2 \text{ or } k_{deg})$	−1.109	−1.584	−0.3953	approximate	approximate
b2	$\log_{10}(k_2 \text{ or } k_{deg})$	−1.166	−1.584	−0.3953	approximate	approximate
b3	$\log_{10}(k_1')$	4.05	< −1.331	> 8.675	optim	optim
b3	$\log_{10}(k_2)$	3.849	< −2.433	> 8.474	optim	optim
b3	$\log_{10}(k_2')$	−1.51	−1.644	> 10.55	approximate	optim
b3	$\log_{10}(k_{deg})$	−2.413	−2.655	> 10.55	approximate	optim
b3	$\log_{10}(k_1'/k_2)$	0.2007	0.1342	> 1.103	approximate	optim
b3	$\log_{10}(k_1'k_2'/k_2)$	−1.309	−1.417	> 11.65	approximate	optim
b3	$\log_{10}(k_2'/k_2)$	−5.359	< −9.985	> 12.98	optim	optim

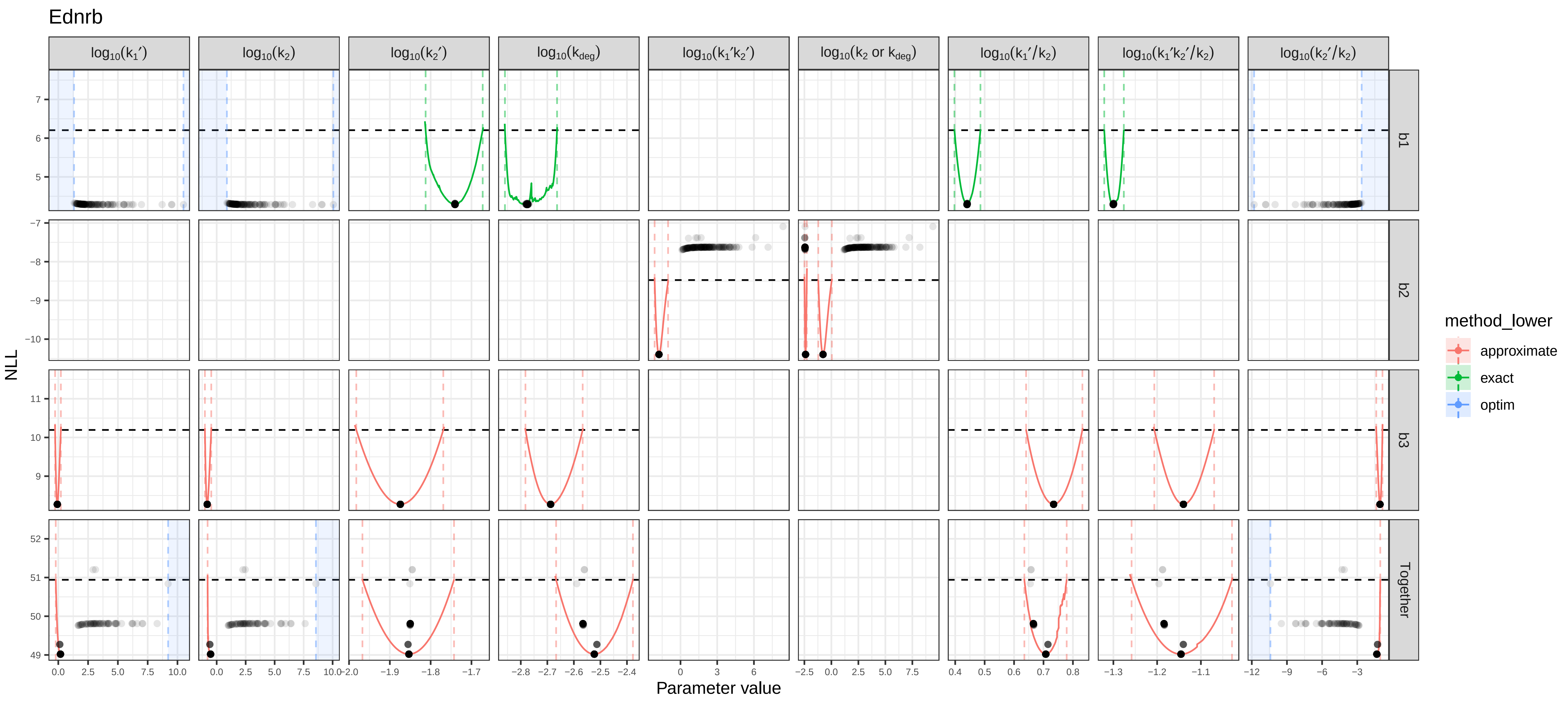

Replicate	Par	Best value	CI95 LB	CI95 UB	Method LB	Method UB
Together	$\log_{10}(k_1')$	0.1835	−0.2126	> 9.216	approximate	optim
Together	$\log_{10}(k_2)$	−0.5244	−0.7834	> 8.561	approximate	optim
Together	$\log_{10}(k_2')$	−1.853	−1.967	−1.743	approximate	approximate
Together	$\log_{10}(k_{deg})$	−2.523	−2.667	−2.377	approximate	approximate
Together	$\log_{10}(k_1'/k_2)$	0.7079	0.635	0.7789	approximate	approximate
Together	$\log_{10}(k_1'k_2'/k_2)$	−1.145	−1.258	−1.029	approximate	approximate
Together	$\log_{10}(k_2'/k_2)$	−1.329	< −10.41	−1.036	optim	approximate
b1	$\log_{10}(k_1')$	9.53	< 1.324	> 10.5	optim	optim
b1	$\log_{10}(k_2)$	9.09	< 0.8824	> 10.06	optim	optim
b1	$\log_{10}(k_2')$	−1.74	−1.812	−1.672	exact	exact
b1	$\log_{10}(k_{deg})$	−2.776	−2.86	−2.663	exact	exact
b1	$\log_{10}(k_1'/k_2)$	0.4401	0.3973	0.4858	exact	exact
b1	$\log_{10}(k_1'k_2'/k_2)$	−1.3	−1.321	−1.276	exact	exact
b1	$\log_{10}(k_2'/k_2)$	−10.83	< −11.8	> −2.623	optim	optim
b2	$\log_{10}(k_1'k_2')$	−1.759	−2.115	−1.015	approximate	approximate
b2	$\log_{10}(k_2 \text{ or } k_{deg})$	−0.7731	−1.214	0.04444	approximate	approximate
b2	$\log_{10}(k_2 \text{ or } k_{deg})$	−2.391	−2.491	−2.277	approximate	approximate
b3	$\log_{10}(k_1')$	−0.08318	−0.2687	0.2163	approximate	approximate
b3	$\log_{10}(k_2)$	−0.8176	−1.014	−0.4672	approximate	approximate
b3	$\log_{10}(k_2')$	−1.874	−1.982	−1.769	approximate	approximate
b3	$\log_{10}(k_{deg})$	−2.688	−2.782	−2.567	approximate	approximate
b3	$\log_{10}(k_1'/k_2)$	0.7345	0.6409	0.8322	approximate	approximate
b3	$\log_{10}(k_1'k_2'/k_2)$	−1.14	−1.206	−1.07	approximate	approximate
b3	$\log_{10}(k_2'/k_2)$	−1.057	−1.377	−0.847	approximate	approximate

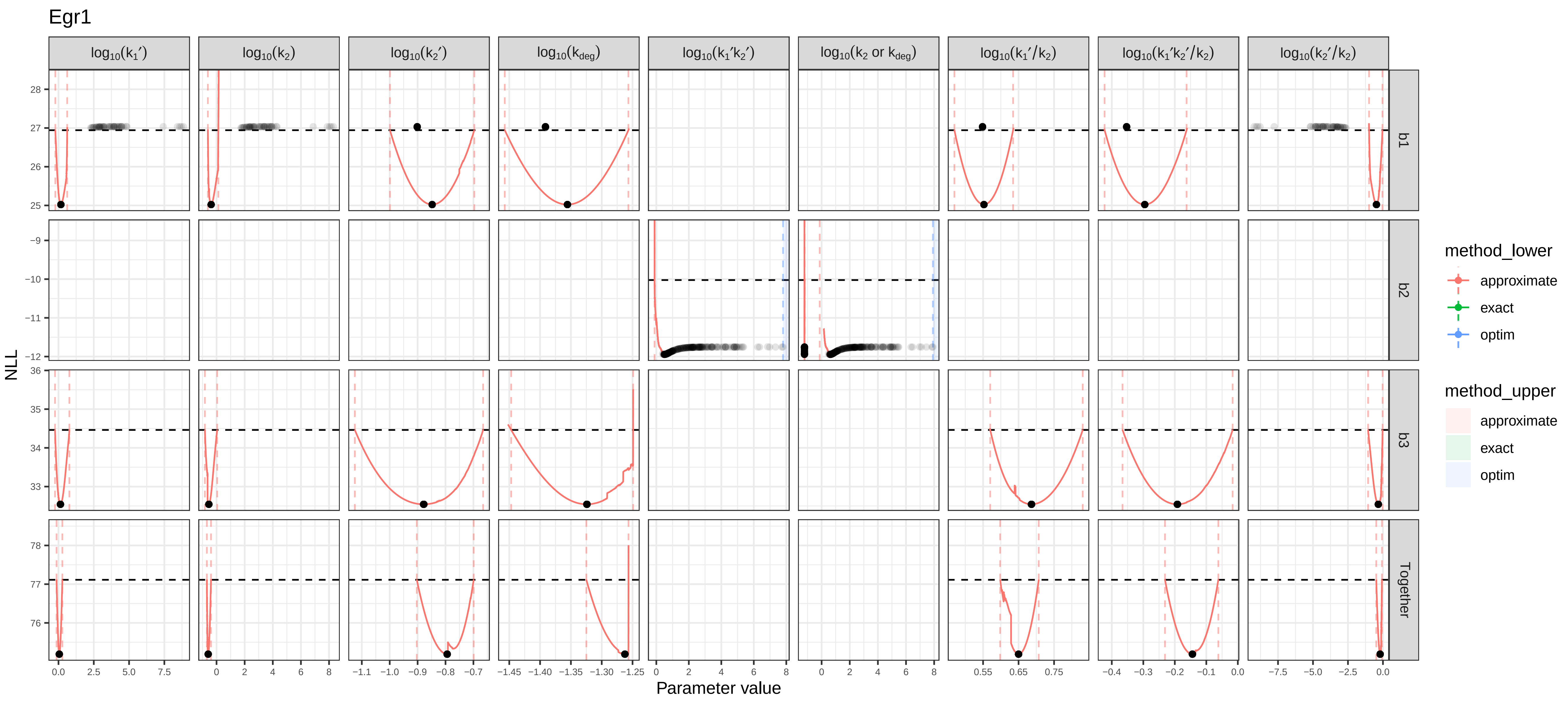

Replicate	Par	Best value	CI95 LB	CI95 UB	Method LB	Method UB
Together	$\log_{10}(k_1')$	0.05704	−0.1353	0.2728	approximate	approximate
Together	$\log_{10}(k_2)$	−0.593	−0.6869	−0.3939	approximate	approximate
Together	$\log_{10}(k_2')$	−0.794	−0.9029	−0.699	approximate	approximate
Together	$\log_{10}(k_{deg})$	−1.262	−1.325	−1.257	approximate	approximate
Together	$\log_{10}(k_1'/k_2)$	0.65	0.5982	0.7069	approximate	approximate
Together	$\log_{10}(k_1'k_2'/k_2)$	−0.144	−0.2311	−0.06186	approximate	approximate
Together	$\log_{10}(k_2'/k_2)$	−0.2011	−0.4784	−0.07065	approximate	approximate
b1	$\log_{10}(k_1')$	0.1701	−0.2246	0.6168	approximate	approximate
b1	$\log_{10}(k_2)$	−0.3824	−0.6171	0.1285	approximate	approximate
b1	$\log_{10}(k_2')$	−0.8478	−0.9992	−0.6969	approximate	approximate
b1	$\log_{10}(k_{deg})$	−1.356	−1.457	−1.257	approximate	approximate
b1	$\log_{10}(k_1'/k_2)$	0.5525	0.4693	0.6346	approximate	approximate
b1	$\log_{10}(k_1'k_2'/k_2)$	−0.2954	−0.4228	−0.1627	approximate	approximate
b1	$\log_{10}(k_2'/k_2)$	−0.4654	−0.9927	−0.03459	approximate	approximate
b2	$\log_{10}(k_1'k_2')$	0.4965	−0.1084	> 7.799	approximate	optim
b2	$\log_{10}(k_2 \text{ or } k_{deg})$	0.6092	−0.1364	> 7.911	approximate	optim
b2	$\log_{10}(k_2 \text{ or } k_{deg})$	−1.22	−1.222	−1.208	approximate	approximate
b3	$\log_{10}(k_1')$	0.139	−0.2465	0.7698	approximate	approximate
b3	$\log_{10}(k_2)$	−0.5475	−0.8274	0.04628	approximate	approximate
b3	$\log_{10}(k_2')$	−0.8784	−1.125	−0.6652	approximate	approximate
b3	$\log_{10}(k_{deg})$	−1.324	−1.447	−1.249	approximate	approximate
b3	$\log_{10}(k_1'/k_2)$	0.6866	0.5701	0.8308	approximate	approximate
b3	$\log_{10}(k_1'k_2'/k_2)$	−0.1919	−0.3659	−0.01642	approximate	approximate
b3	$\log_{10}(k_2'/k_2)$	−0.3309	−1.062	−0.02705	approximate	approximate

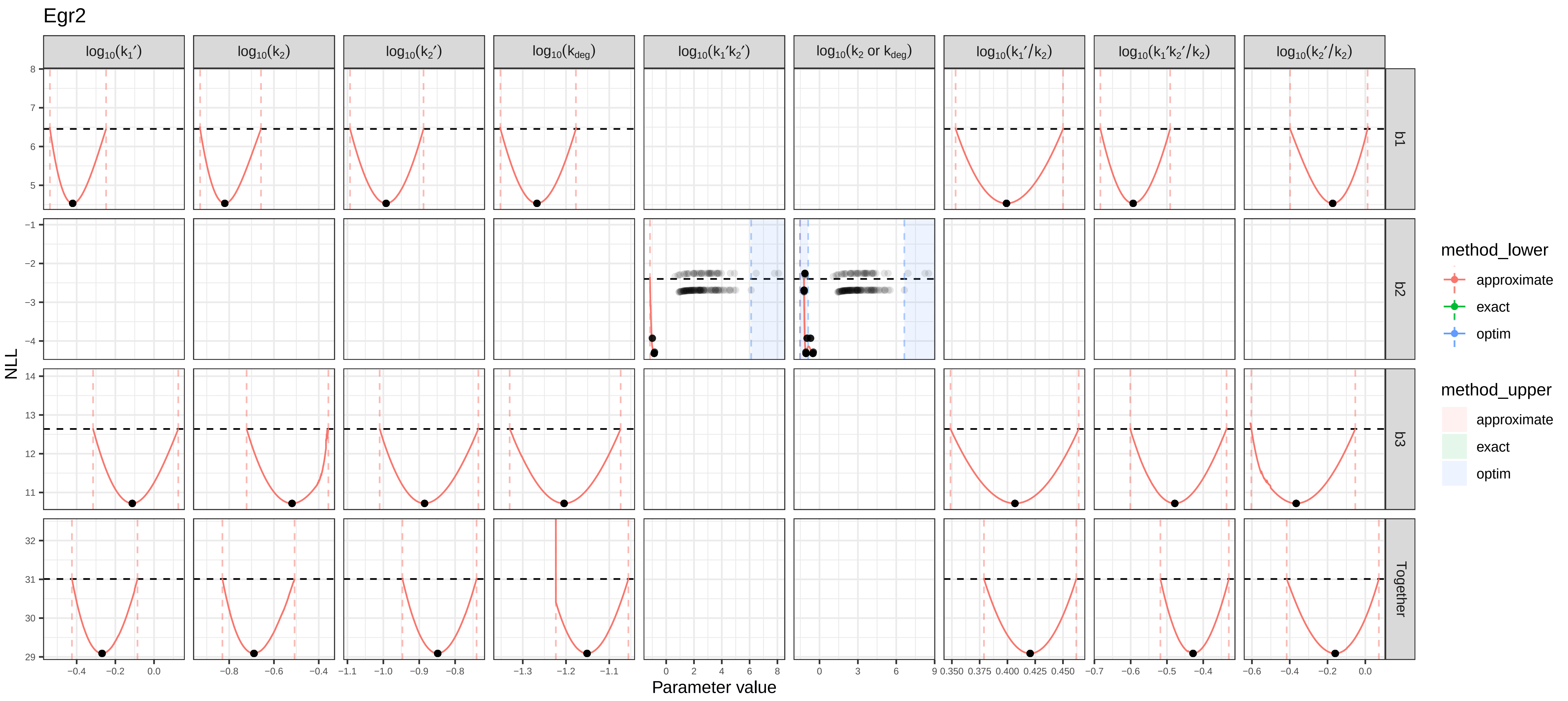

Replicate	Par	**Best value**	**CI95 LB**	**CI95 UB**	**Method LB**	**Method UB**
Together	$\log_{10}(k_1')$	−0.2684	−0.4245	−0.08553	approximate	approximate
Together	$\log_{10}(k_2)$	−0.6889	−0.8302	−0.5078	approximate	approximate
Together	$\log_{10}(k_2')$	−0.8485	−0.9468	−0.7402	approximate	approximate
Together	$\log_{10}(k_{deg})$	−1.151	−1.223	−1.056	approximate	approximate
Together	$\log_{10}(k_1'/k_2)$	0.4205	0.3789	0.462	approximate	approximate
Together	$\log_{10}(k_1'k_2'/k_2)$	−0.428	−0.5181	−0.3294	approximate	approximate
Together	$\log_{10}(k_2'/k_2)$	−0.1596	−0.4161	0.07169	approximate	approximate
b1	$\log_{10}(k_1')$	−0.4206	−0.5382	−0.2479	approximate	approximate
b1	$\log_{10}(k_2)$	−0.8197	−0.9297	−0.6578	approximate	approximate
b1	$\log_{10}(k_2')$	−0.9922	−1.093	−0.888	approximate	approximate
b1	$\log_{10}(k_{deg})$	−1.267	−1.352	−1.177	approximate	approximate
b1	$\log_{10}(k_1'/k_2)$	0.3991	0.3534	0.4501	approximate	approximate
b1	$\log_{10}(k_1'k_2'/k_2)$	−0.5931	−0.6836	−0.4911	approximate	approximate
b1	$\log_{10}(k_2'/k_2)$	−0.1725	−0.398	0.01208	approximate	approximate
b2	$\log_{10}(k_1'k_2')$	−0.8631	−1.163	> 6.113	approximate	optim
b2	$\log_{10}(k_2 \text{ or } k_{deg})$	−0.5155	−1.521	> 6.629	approximate	optim
b2	$\log_{10}(k_2 \text{ or } k_{deg})$	−1.055	−1.521	−0.8852	approximate	optim
b3	$\log_{10}(k_1')$	−0.1123	−0.3152	0.1252	approximate	approximate
b3	$\log_{10}(k_2)$	−0.5191	−0.7222	−0.357	approximate	approximate
b3	$\log_{10}(k_2')$	−0.8851	−1.01	−0.7353	approximate	approximate
b3	$\log_{10}(k_{deg})$	−1.204	−1.33	−1.074	approximate	approximate
b3	$\log_{10}(k_1'/k_2)$	0.4068	0.3488	0.4642	approximate	approximate
b3	$\log_{10}(k_1'k_2'/k_2)$	−0.4784	−0.6012	−0.3357	approximate	approximate
b3	$\log_{10}(k_2'/k_2)$	−0.366	−0.6033	−0.05312	approximate	approximate

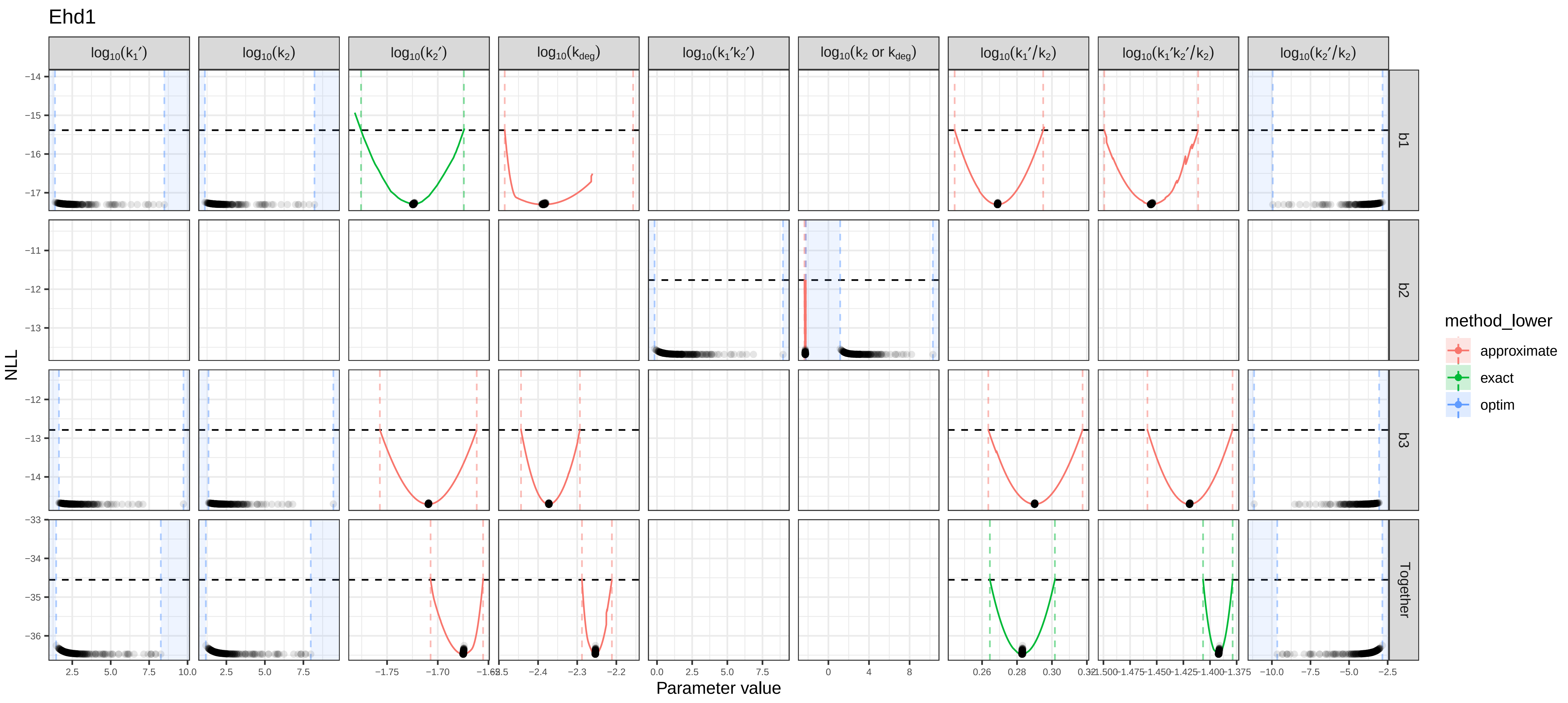

Replicate	Par	**Best value**	**CI95 LB**	**CI95 UB**	**Method LB**	**Method UB**
Together	$log_{10}(k_1')$	7.628	< 1.452	> 8.26	optim	optim
Together	$log_{10}(k_2)$	7.345	< 1.169	> 7.977	optim	optim
Together	$log_{10}(k_2')$	−1.675	−1.707	−1.655	approximate	approximate
Together	$log_{10}(k_{deg})$	−2.254	−2.288	−2.211	approximate	approximate
Together	$log_{10}(k_1'/k_2)$	0.2829	0.2645	0.3017	exact	exact
Together	$log_{10}(k_1'k_2'/k_2)$	−1.392	−1.407	−1.379	exact	exact
Together	$log_{10}(k_2'/k_2)$	−9.02	< −9.652	> −2.843	optim	optim
b1	$log_{10}(k_1')$	5.276	< 1.376	> 8.489	optim	optim
b1	$log_{10}(k_2)$	5.007	< 1.107	> 8.22	optim	optim
b1	$log_{10}(k_2')$	−1.725	−1.776	−1.674	exact	exact
b1	$log_{10}(k_{deg})$	−2.387	−2.485	−2.157	approximate	approximate
b1	$log_{10}(k_1'/k_2)$	0.2689	0.2443	0.295	approximate	approximate
b1	$log_{10}(k_1'k_2'/k_2)$	−1.456	−1.499	−1.411	approximate	approximate
b1	$log_{10}(k_2'/k_2)$	−6.732	< −9.944	> −2.83	optim	optim
b2	$log_{10}(k_1'k_2')$	3.569	< −0.1744	> 8.96	optim	optim
b2	$log_{10}(k_2 \text{ or } k_{deg})$	4.905	1.161	> 10.3	optim	optim
b2	$log_{10}(k_2 \text{ or } k_{deg})$	−2.279	−2.359	−2.227	approximate	approximate
b3	$log_{10}(k_1')$	4.817	< 1.633	> 9.734	optim	optim
b3	$log_{10}(k_2)$	4.527	< 1.343	> 9.445	optim	optim
b3	$log_{10}(k_2')$	−1.709	−1.757	−1.662	approximate	approximate
b3	$log_{10}(k_{deg})$	−2.372	−2.444	−2.293	approximate	approximate
b3	$log_{10}(k_1'/k_2)$	0.2901	0.2636	0.3176	approximate	approximate
b3	$log_{10}(k_1'k_2'/k_2)$	−1.419	−1.459	−1.379	approximate	approximate
b3	$log_{10}(k_2'/k_2)$	−6.236	< −11.15	> −3.052	optim	optim

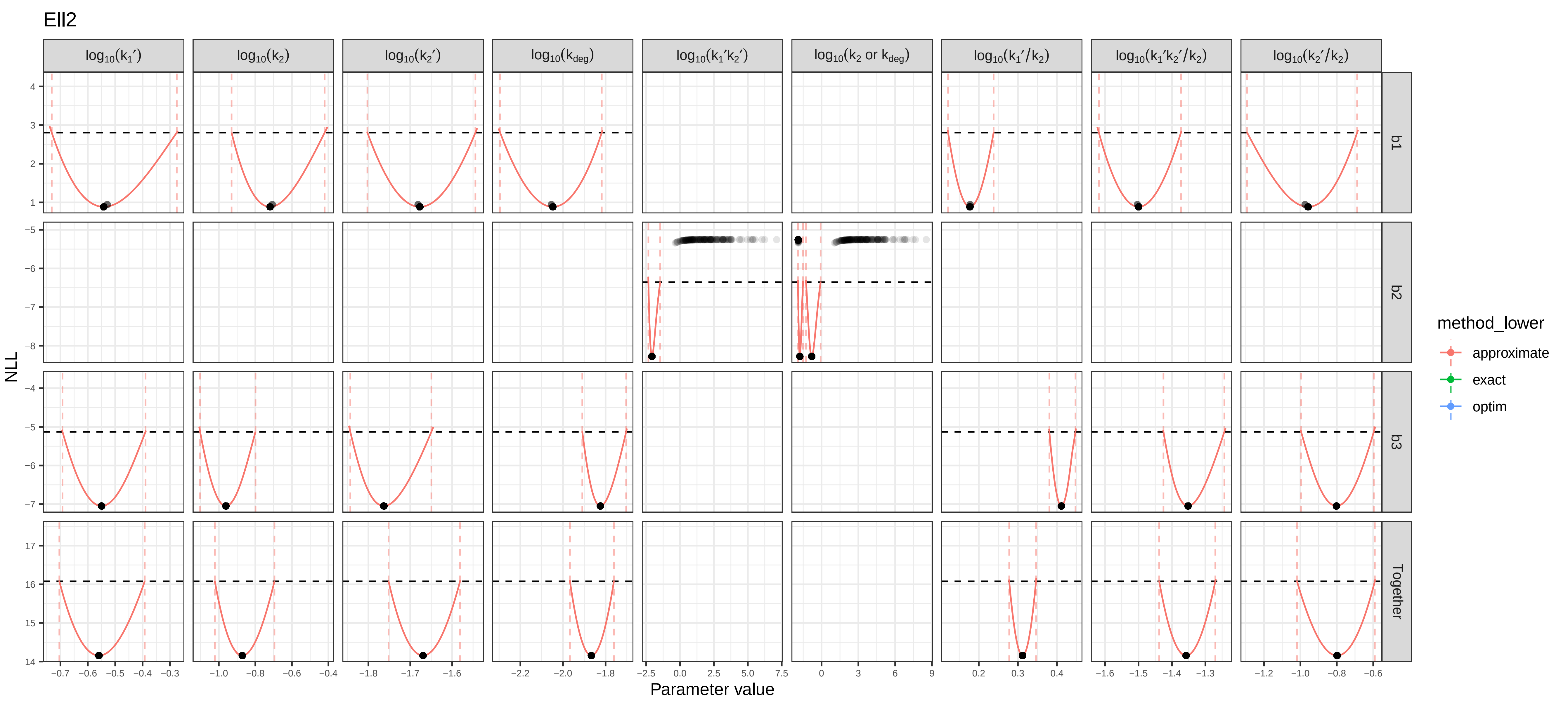

Replicate	Par	Best value	CI95 LB	CI95 UB	Method LB	Method UB
Together	$\log_{10}(k_1')$	−0.5592	−0.7038	−0.3922	approximate	approximate
Together	$\log_{10}(k_2)$	−0.8711	−1.022	−0.6953	approximate	approximate
Together	$\log_{10}(k_2')$	−1.669	−1.752	−1.581	approximate	approximate
Together	$\log_{10}(k_{deg})$	−1.867	−1.967	−1.761	approximate	approximate
Together	$\log_{10}(k_1'/k_2)$	0.3119	0.2778	0.3464	approximate	approximate
Together	$\log_{10}(k_1'k_2'/k_2)$	−1.358	−1.438	−1.27	approximate	approximate
Together	$\log_{10}(k_2'/k_2)$	−0.7984	−1.019	−0.5912	approximate	approximate
b1	$\log_{10}(k_1')$	−0.5418	−0.7317	−0.2754	approximate	approximate
b1	$\log_{10}(k_2)$	−0.7193	−0.9301	−0.4206	approximate	approximate
b1	$\log_{10}(k_2')$	−1.677	−1.802	−1.544	approximate	approximate
b1	$\log_{10}(k_{deg})$	−2.047	−2.295	−1.818	approximate	approximate
b1	$\log_{10}(k_1'/k_2)$	0.1775	0.1216	0.238	approximate	approximate
b1	$\log_{10}(k_1'k_2'/k_2)$	−1.5	−1.619	−1.373	approximate	approximate
b1	$\log_{10}(k_2'/k_2)$	−0.9577	−1.293	−0.6883	approximate	approximate
b2	$\log_{10}(k_1'k_2')$	−2.076	−2.337	−1.465	approximate	approximate
b2	$\log_{10}(k_2 \text{ or } k_{deg})$	−0.8002	−1.267	−0.07993	approximate	approximate
b2	$\log_{10}(k_2 \text{ or } k_{deg})$	−1.771	−1.923	−1.509	approximate	approximate
b3	$\log_{10}(k_1')$	−0.5497	−0.6923	−0.3893	approximate	approximate
b3	$\log_{10}(k_2)$	−0.9609	−1.102	−0.799	approximate	approximate
b3	$\log_{10}(k_2')$	−1.763	−1.843	−1.649	approximate	approximate
b3	$\log_{10}(k_{deg})$	−1.824	−1.909	−1.703	approximate	approximate
b3	$\log_{10}(k_1'/k_2)$	0.4113	0.3805	0.4473	approximate	approximate
b3	$\log_{10}(k_1'k_2'/k_2)$	−1.352	−1.425	−1.243	approximate	approximate
b3	$\log_{10}(k_2'/k_2)$	−0.802	−0.9961	−0.5967	approximate	approximate

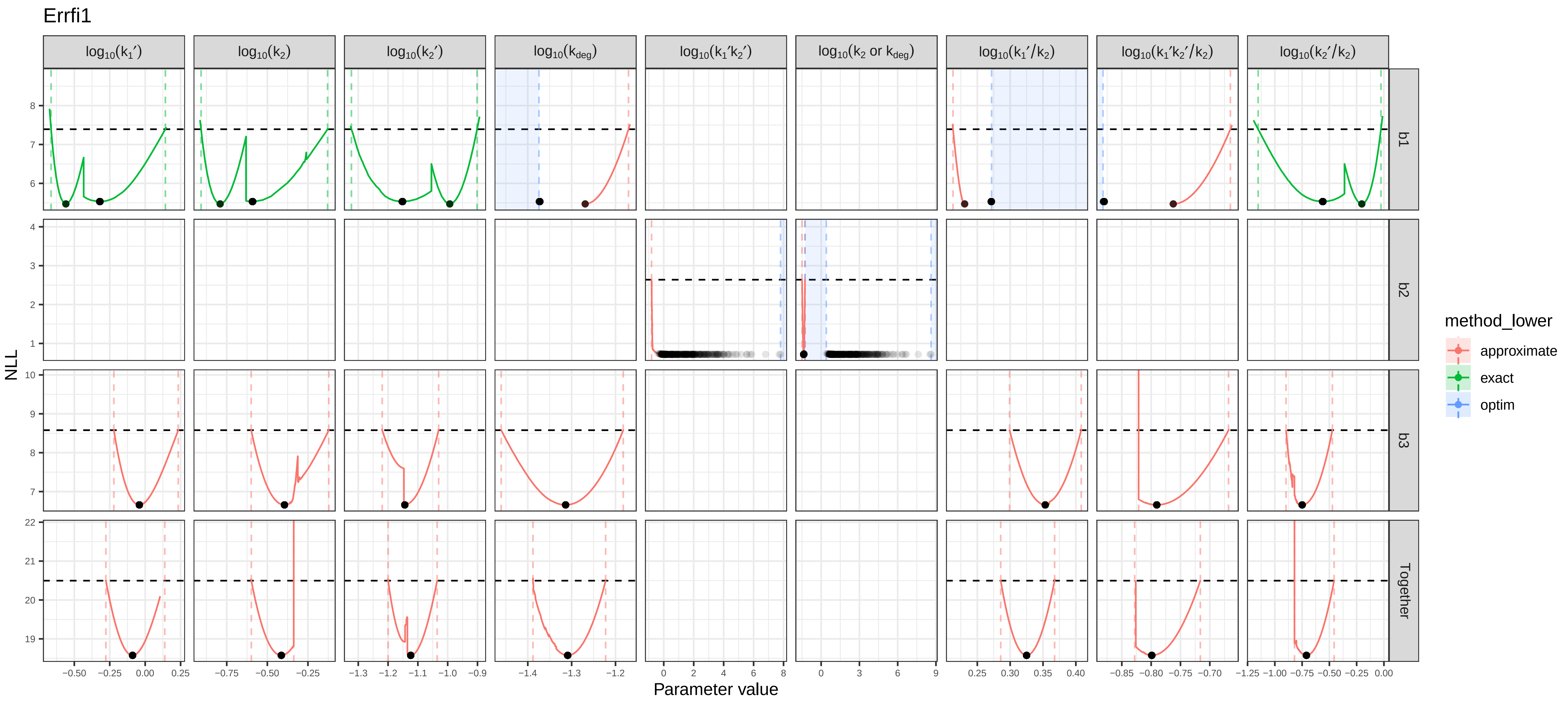

Replicate	Par	Best value	CI95 LB	CI95 UB	Method LB	Method UB
Together	$\log_{10}(k_1')$	−0.08861	−0.2773	0.1384	approximate	approximate
Together	$\log_{10}(k_2)$	−0.4136	−0.5987	−0.3374	approximate	approximate
Together	$\log_{10}(k_2')$	−1.124	−1.2	−1.035	approximate	approximate
Together	$\log_{10}(k_{deg})$	−1.309	−1.388	−1.222	approximate	approximate
Together	$\log_{10}(k_1'/k_2)$	0.325	0.2857	0.3676	approximate	approximate
Together	$\log_{10}(k_1'k_2'/k_2)$	−0.799	−0.828	−0.7161	approximate	approximate
Together	$\log_{10}(k_2'/k_2)$	−0.7104	−0.8198	−0.4565	approximate	approximate
b1	$\log_{10}(k_1')$	−0.5597	−0.6639	0.1426	exact	exact
b1	$\log_{10}(k_2)$	−0.7905	−0.908	−0.129	exact	exact
b1	$\log_{10}(k_2')$	−0.9931	−1.323	−0.9014	exact	exact
b1	$\log_{10}(k_{deg})$	−1.269	< −1.375	−1.17	optim	approximate
b1	$\log_{10}(k_1'/k_2)$	0.2308	0.213	> 0.2717	approximate	optim
b1	$\log_{10}(k_1'k_2'/k_2)$	−0.7623	< −0.882	−0.665	optim	approximate
b1	$\log_{10}(k_2'/k_2)$	−0.2026	−1.153	−0.02709	exact	exact
b2	$\log_{10}(k_1'k_2')$	0.5707	−0.8146	> 7.799	approximate	optim
b2	$\log_{10}(k_2 \text{ or } k_{deg})$	1.384	0.402	> 8.616	optim	optim
b2	$\log_{10}(k_2 \text{ or } k_{deg})$	−1.368	−1.498	−1.265	approximate	approximate
b3	$\log_{10}(k_1')$	−0.04115	−0.2209	0.2334	approximate	approximate
b3	$\log_{10}(k_2)$	−0.3946	−0.5995	−0.1216	approximate	approximate
b3	$\log_{10}(k_2')$	−1.144	−1.22	−1.03	approximate	approximate
b3	$\log_{10}(k_{deg})$	−1.314	−1.461	−1.182	approximate	approximate
b3	$\log_{10}(k_1'/k_2)$	0.3534	0.2991	0.4082	approximate	approximate
b3	$\log_{10}(k_1'k_2'/k_2)$	−0.7904	−0.8213	−0.6681	approximate	approximate
b3	$\log_{10}(k_2'/k_2)$	−0.7492	−0.8972	−0.4723	approximate	approximate

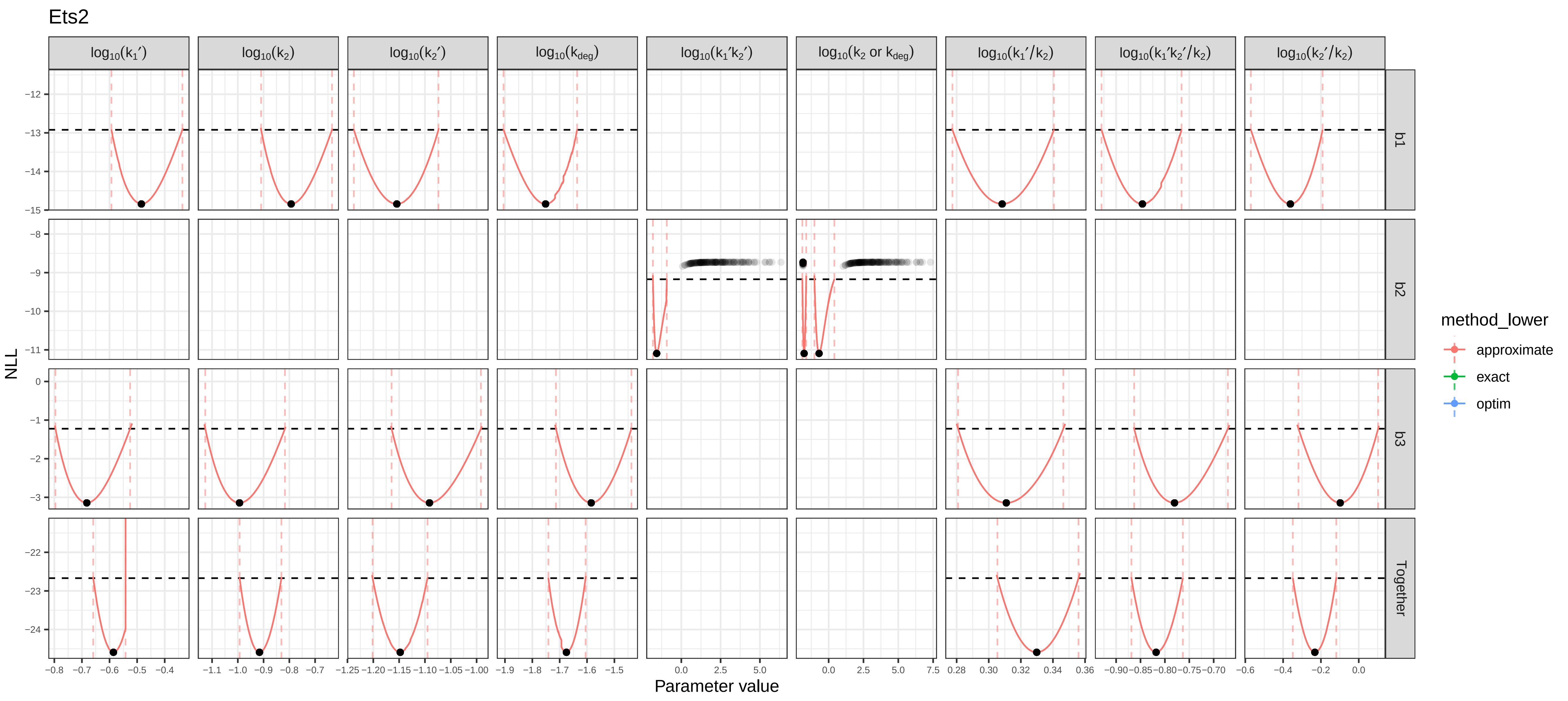

Replicate	Par	**Best value**	**CI95 LB**	**CI95 UB**	**Method LB**	**Method UB**
Together	$\log_{10}(k_1')$	−0.586	−0.6591	−0.5419	approximate	approximate
Together	$\log_{10}(k_2)$	−0.9159	−0.993	−0.8307	approximate	approximate
Together	$\log_{10}(k_2')$	−1.148	−1.201	−1.095	approximate	approximate
Together	$\log_{10}(k_{deg})$	−1.675	−1.741	−1.605	approximate	approximate
Together	$\log_{10}(k_1'/k_2)$	0.3299	0.3054	0.3559	approximate	approximate
Together	$\log_{10}(k_1'k_2'/k_2)$	−0.818	−0.8686	−0.7631	approximate	approximate
Together	$\log_{10}(k_2'/k_2)$	−0.232	−0.3486	−0.1188	approximate	approximate
b1	$\log_{10}(k_1')$	−0.4845	−0.5931	−0.3359	approximate	approximate
b1	$\log_{10}(k_2)$	−0.7929	−0.9095	−0.6342	approximate	approximate
b1	$\log_{10}(k_2')$	−1.154	−1.238	−1.074	approximate	approximate
b1	$\log_{10}(k_{deg})$	−1.752	−1.905	−1.636	approximate	approximate
b1	$\log_{10}(k_1'/k_2)$	0.3084	0.2774	0.3406	approximate	approximate
b1	$\log_{10}(k_1'k_2'/k_2)$	−0.8461	−0.9299	−0.7658	approximate	approximate
b1	$\log_{10}(k_2'/k_2)$	−0.3616	−0.5705	−0.191	approximate	approximate
b2	$\log_{10}(k_1'k_2')$	−1.566	−1.805	−0.9237	approximate	approximate
b2	$\log_{10}(k_2 \text{ or } k_{deg})$	−0.6869	−1.02	0.411	approximate	approximate
b2	$\log_{10}(k_2 \text{ or } k_{deg})$	−1.759	−1.888	−1.61	approximate	approximate
b3	$\log_{10}(k_1')$	−0.6824	−0.796	−0.5253	approximate	approximate
b3	$\log_{10}(k_2)$	−0.9934	−1.126	−0.817	approximate	approximate
b3	$\log_{10}(k_2')$	−1.091	−1.165	−0.9916	approximate	approximate
b3	$\log_{10}(k_{deg})$	−1.585	−1.714	−1.438	approximate	approximate
b3	$\log_{10}(k_1'/k_2)$	0.3109	0.2809	0.3465	approximate	approximate
b3	$\log_{10}(k_1'k_2'/k_2)$	−0.7803	−0.863	−0.6709	approximate	approximate
b3	$\log_{10}(k_2'/k_2)$	−0.09783	−0.3186	0.1027	approximate	approximate

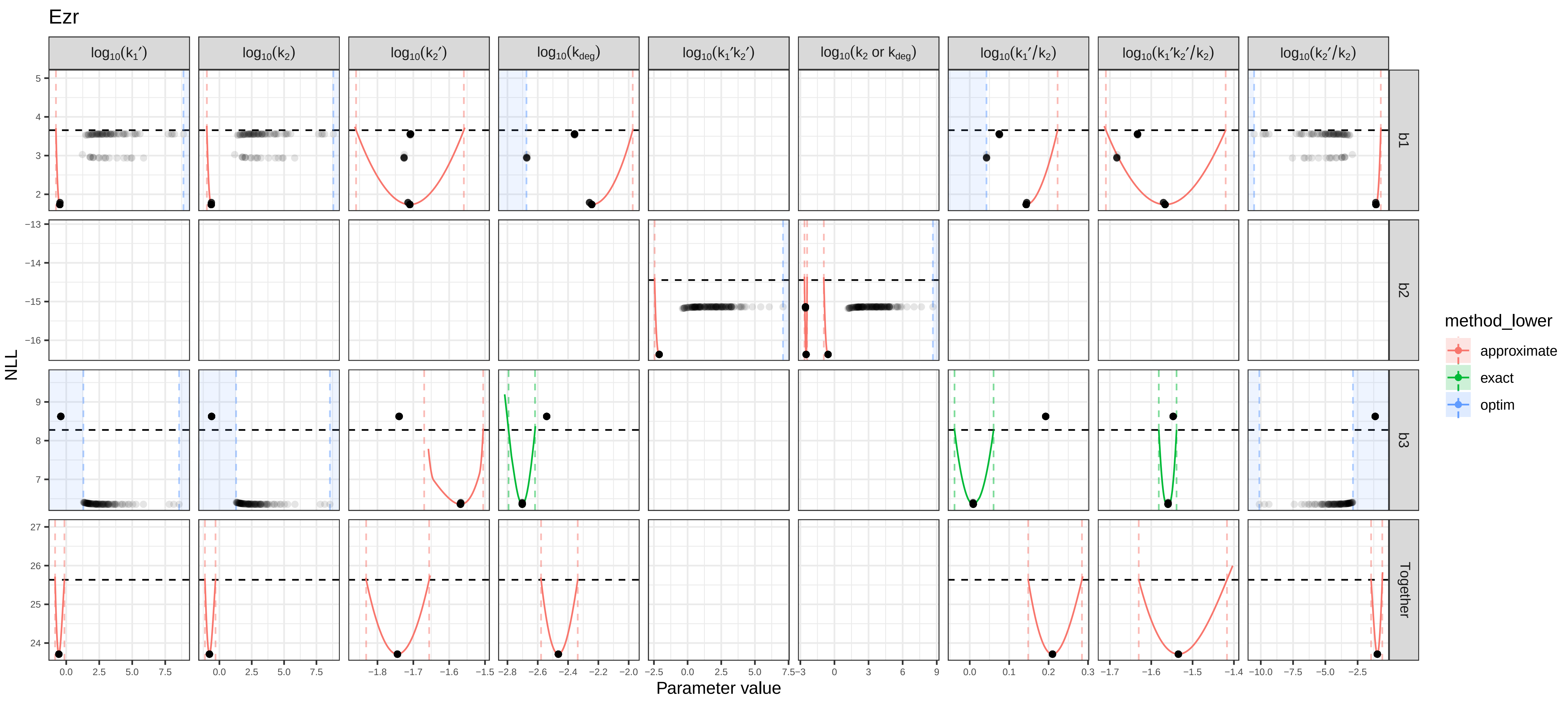

Replicate	Par	Best value	CI95 LB	CI95 UB	Method LB	Method UB
Together	$\log_{10}(k_1')$	−0.5612	−0.8439	−0.1375	approximate	approximate
Together	$\log_{10}(k_2)$	−0.7711	−1.117	−0.3	approximate	approximate
Together	$\log_{10}(k_2')$	−1.744	−1.832	−1.656	approximate	approximate
Together	$\log_{10}(k_{deg})$	−2.464	−2.578	−2.336	approximate	approximate
Together	$\log_{10}(k_1'/k_2)$	0.2099	0.1482	0.2846	approximate	approximate
Together	$\log_{10}(k_1'k_2'/k_2)$	−1.534	−1.63	−1.417	approximate	approximate
Together	$\log_{10}(k_2'/k_2)$	−0.9732	−1.456	−0.5898	approximate	approximate
b1	$\log_{10}(k_1')$	−0.487	−0.7784	> 8.882	approximate	optim
b1	$\log_{10}(k_2)$	−0.6302	−0.9686	> 8.807	approximate	optim
b1	$\log_{10}(k_2')$	−1.71	−1.86	−1.559	approximate	approximate
b1	$\log_{10}(k_{deg})$	−2.243	< −2.674	−1.973	optim	approximate
b1	$\log_{10}(k_1'/k_2)$	0.1432	< 0.04246	0.2229	optim	approximate
b1	$\log_{10}(k_1'k_2'/k_2)$	−1.567	−1.71	−1.42	approximate	approximate
b1	$\log_{10}(k_2'/k_2)$	−1.08	< −10.52	−0.7046	optim	approximate
b2	$\log_{10}(k_1'k_2')$	−2.111	−2.449	> 7.089	approximate	optim
b2	$\log_{10}(k_2 \text{ or } k_{deg})$	−0.5629	−0.9353	> 8.661	approximate	optim
b2	$\log_{10}(k_2 \text{ or } k_{deg})$	−2.493	−2.638	−2.407	approximate	approximate
b3	$\log_{10}(k_1')$	7.808	< 1.301	> 8.552	optim	optim
b3	$\log_{10}(k_2)$	7.8	< 1.292	> 8.543	optim	optim
b3	$\log_{10}(k_2')$	−1.568	−1.67	−1.505	approximate	approximate
b3	$\log_{10}(k_{deg})$	−2.702	−2.793	−2.618	exact	exact
b3	$\log_{10}(k_1'/k_2)$	0.008843	−0.0386	0.06045	exact	exact
b3	$\log_{10}(k_1'k_2'/k_2)$	−1.559	−1.582	−1.539	exact	exact
b3	$\log_{10}(k_2'/k_2)$	−9.368	< −10.11	> −2.86	optim	optim

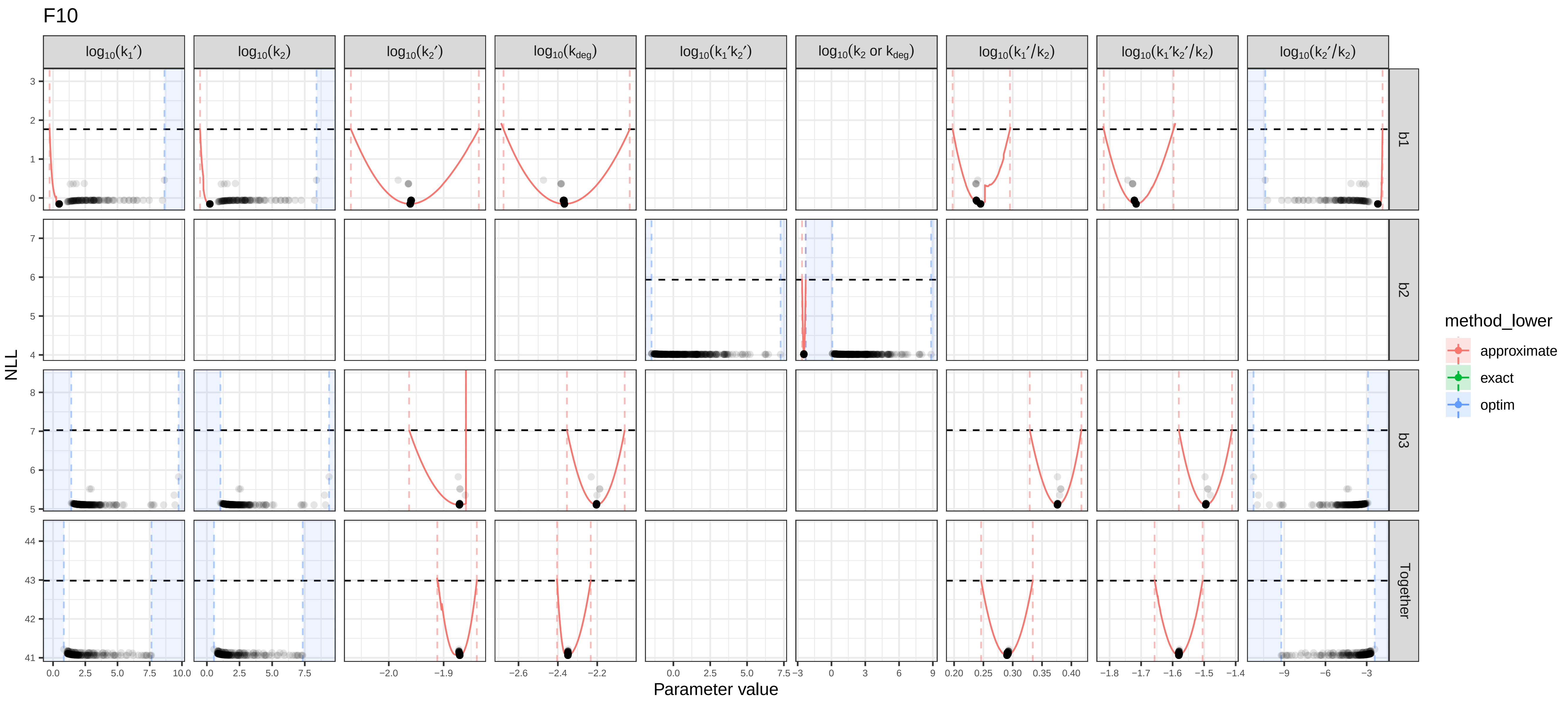

Replicate	Par	Best value	CI95 LB	CI95 UB	Method LB	Method UB
Together	$\log_{10}(k_1')$	5.747	< 0.8361	> 7.636	optim	optim
Together	$\log_{10}(k_2)$	5.456	< 0.5426	> 7.346	optim	optim
Together	$\log_{10}(k_2')$	−1.87	−1.912	−1.839	approximate	approximate
Together	$\log_{10}(k_{deg})$	−2.349	−2.404	−2.233	approximate	approximate
Together	$\log_{10}(k_1'/k_2)$	0.2901	0.2461	0.3341	approximate	approximate
Together	$\log_{10}(k_1'k_2'/k_2)$	−1.58	−1.657	−1.505	approximate	approximate
Together	$\log_{10}(k_2'/k_2)$	−7.327	< −9.216	> −2.415	optim	optim
b1	$\log_{10}(k_1')$	0.4725	−0.2687	> 8.64	approximate	optim
b1	$\log_{10}(k_2)$	0.2274	−0.5234	> 8.399	approximate	optim
b1	$\log_{10}(k_2')$	−1.961	−2.069	−1.836	approximate	approximate
b1	$\log_{10}(k_{deg})$	−2.367	−2.676	−2.035	approximate	approximate
b1	$\log_{10}(k_1'/k_2)$	0.2451	0.1974	0.2953	approximate	approximate
b1	$\log_{10}(k_1'k_2'/k_2)$	−1.716	−1.819	−1.597	approximate	approximate
b1	$\log_{10}(k_2'/k_2)$	−2.188	< −10.38	−1.846	optim	approximate
b2	$\log_{10}(k_1'k_2')$	2.218	< −1.482	> 7.273	optim	optim
b2	$\log_{10}(k_2 \text{ or } k_{deg})$	3.784	0.07552	> 8.84	optim	optim
b2	$\log_{10}(k_2 \text{ or } k_{deg})$	−2.452	−2.613	−2.286	approximate	approximate
b3	$\log_{10}(k_1')$	5.384	< 1.412	> 9.737	optim	optim
b3	$\log_{10}(k_2)$	5.008	< 1.035	> 9.36	optim	optim
b3	$\log_{10}(k_2')$	−1.871	−1.963	−1.859	approximate	approximate
b3	$\log_{10}(k_{deg})$	−2.204	−2.354	−2.06	approximate	approximate
b3	$\log_{10}(k_1'/k_2)$	0.3762	0.3289	0.4171	approximate	approximate
b3	$\log_{10}(k_1'k_2'/k_2)$	−1.495	−1.58	−1.412	approximate	approximate
b3	$\log_{10}(k_2'/k_2)$	−6.879	< −11.23	> −2.906	optim	optim

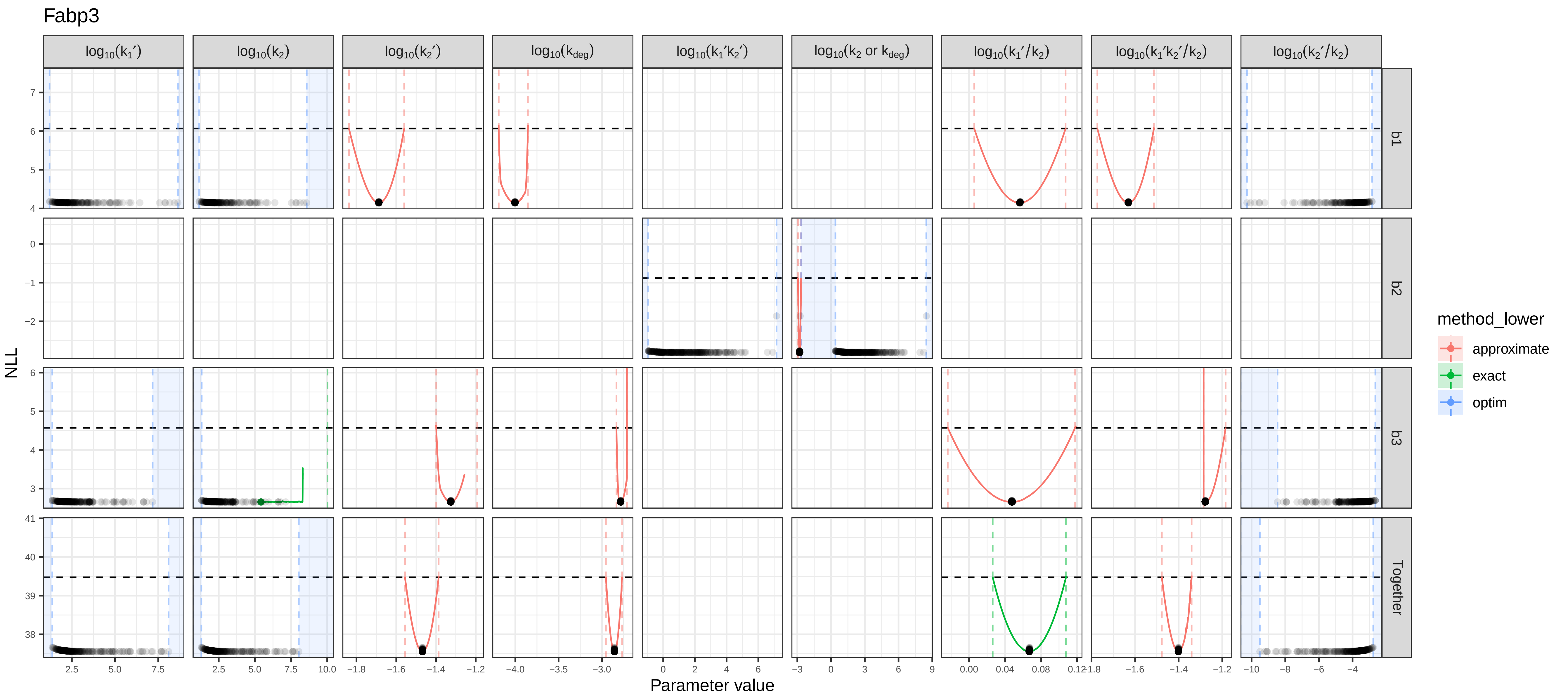

Replicate	Par	Best value	CI95 LB	CI95 UB	Method LB	Method UB
Together	$\log_{10}(k_1')$	7.106	< 1.369	> 8.102	optim	optim
Together	$\log_{10}(k_2)$	7.039	< 1.302	> 8.035	optim	optim
Together	$\log_{10}(k_2')$	−1.468	−1.556	−1.387	approximate	approximate
Together	$\log_{10}(k_{deg})$	−2.855	−2.953	−2.766	approximate	approximate
Together	$\log_{10}(k_1'/k_2)$	0.06692	0.02625	0.1078	exact	exact
Together	$\log_{10}(k_1'k_2'/k_2)$	−1.401	−1.477	−1.341	approximate	approximate
Together	$\log_{10}(k_2'/k_2)$	−8.507	< −9.504	> −2.77	optim	optim
b1	$\log_{10}(k_1')$	4.82	< 1.208	> 8.639	optim	optim
b1	$\log_{10}(k_2)$	4.764	< 1.151	> 8.583	optim	optim
b1	$\log_{10}(k_2')$	−1.688	−1.838	−1.56	approximate	approximate
b1	$\log_{10}(k_{deg})$	−4.004	−4.192	−3.855	approximate	approximate
b1	$\log_{10}(k_1'/k_2)$	0.05642	0.005746	0.1073	approximate	approximate
b1	$\log_{10}(k_1'k_2'/k_2)$	−1.631	−1.773	−1.514	approximate	approximate
b1	$\log_{10}(k_2'/k_2)$	−6.452	< −10.27	> −2.839	optim	optim
b2	$\log_{10}(k_1'k_2')$	3.544	< −0.9411	> 7.156	optim	optim
b2	$\log_{10}(k_2 \text{ or } k_{deg})$	4.876	0.3877	> 8.466	optim	optim
b2	$\log_{10}(k_2 \text{ or } k_{deg})$	−2.799	−2.942	−2.662	approximate	approximate
b3	$\log_{10}(k_1')$	5.466	< 1.369	> 7.179	optim	optim
b3	$\log_{10}(k_2)$	5.419	< 1.322	10.02	optim	exact
b3	$\log_{10}(k_2')$	−1.326	−1.399	−1.193	approximate	approximate
b3	$\log_{10}(k_{deg})$	−2.782	−2.832	−2.711	approximate	approximate
b3	$\log_{10}(k_1'/k_2)$	0.04761	−0.02373	0.1179	approximate	approximate
b3	$\log_{10}(k_1'k_2'/k_2)$	−1.278	−1.285	−1.184	approximate	approximate
b3	$\log_{10}(k_2'/k_2)$	−6.744	< −8.457	> −2.647	optim	optim

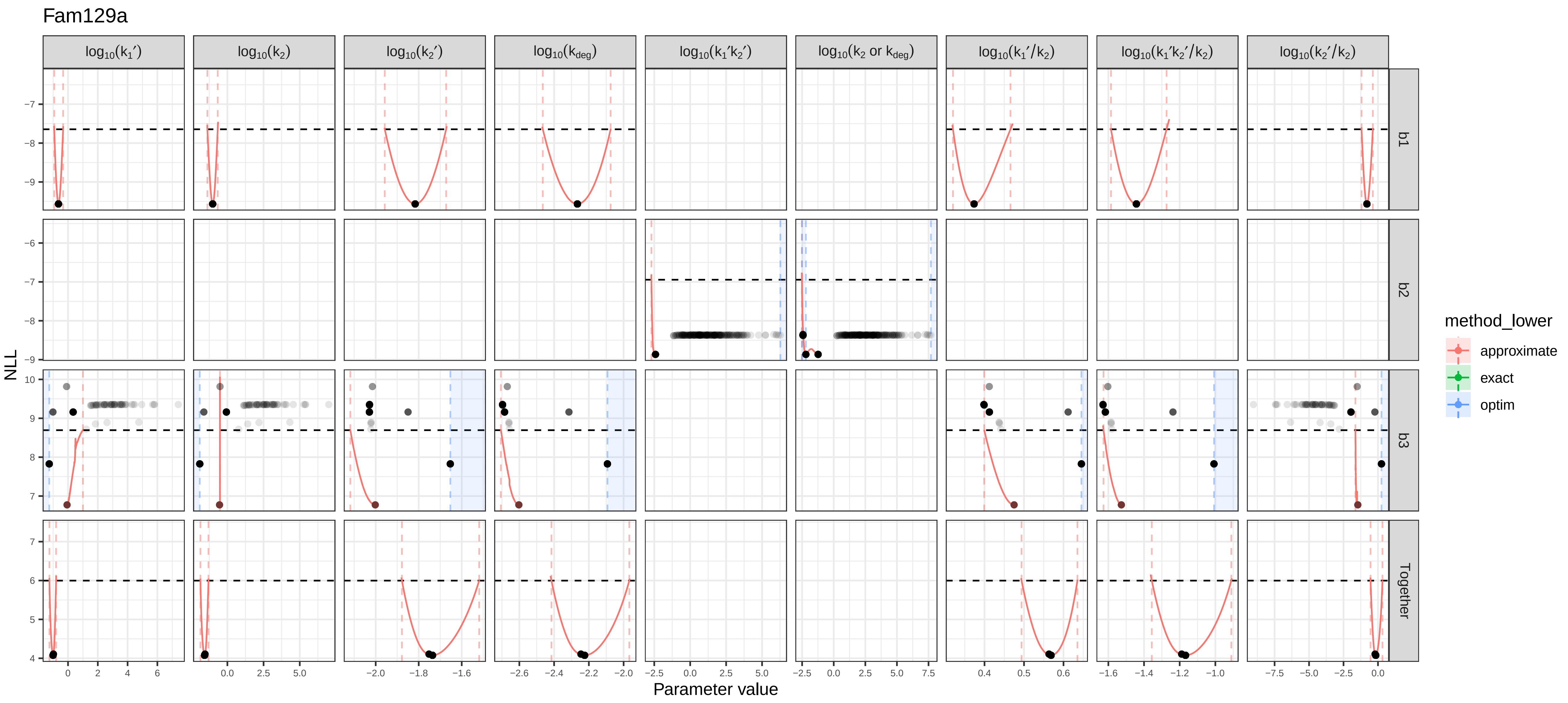

Replicate	Par	Best value	CI95 LB	CI95 UB	Method LB	Method UB
Together	$\log_{10}(k_1')$	−1.006	−1.249	−0.8005	approximate	approximate
Together	$\log_{10}(k_2)$	−1.575	−1.861	−1.304	approximate	approximate
Together	$\log_{10}(k_2')$	−1.736	−1.878	−1.519	approximate	approximate
Together	$\log_{10}(k_{deg})$	−2.224	−2.415	−1.967	approximate	approximate
Together	$\log_{10}(k_1'/k_2)$	0.5686	0.4938	0.6353	approximate	approximate
Together	$\log_{10}(k_1'k_2'/k_2)$	−1.167	−1.356	−0.9104	approximate	approximate
Together	$\log_{10}(k_2'/k_2)$	−0.1607	−0.5397	0.3256	approximate	approximate
b1	$\log_{10}(k_1')$	−0.6405	−0.9264	−0.3289	approximate	approximate
b1	$\log_{10}(k_2)$	−1.014	−1.38	−0.6656	approximate	approximate
b1	$\log_{10}(k_2')$	−1.816	−1.958	−1.672	approximate	approximate
b1	$\log_{10}(k_{deg})$	−2.266	−2.465	−2.075	approximate	approximate
b1	$\log_{10}(k_1'/k_2)$	0.3736	0.3201	0.4658	approximate	approximate
b1	$\log_{10}(k_1'k_2'/k_2)$	−1.443	−1.585	−1.274	approximate	approximate
b1	$\log_{10}(k_2'/k_2)$	−0.8023	−1.191	−0.3723	approximate	approximate
b2	$\log_{10}(k_1'k_2')$	−2.413	−2.697	> 6.273	approximate	optim
b2	$\log_{10}(k_2 \text{ or } k_{deg})$	−1.234	−2.514	> 7.688	approximate	optim
b2	$\log_{10}(k_2 \text{ or } k_{deg})$	−2.203	−2.514	−2.201	approximate	optim
b3	$\log_{10}(k_1')$	−0.06529	< −1.261	1.001	optim	approximate
b3	$\log_{10}(k_2)$	−0.5402	< −1.907	−0.5116	optim	approximate
b3	$\log_{10}(k_2')$	−2.002	−2.118	> −1.653	approximate	optim
b3	$\log_{10}(k_{deg})$	−2.603	−2.706	> −2.093	approximate	optim
b3	$\log_{10}(k_1'/k_2)$	0.4749	0.3992	> 0.6454	approximate	optim
b3	$\log_{10}(k_1'k_2'/k_2)$	−1.527	−1.627	> −1.007	approximate	optim
b3	$\log_{10}(k_2'/k_2)$	−1.462	−1.638	> 0.2539	approximate	optim

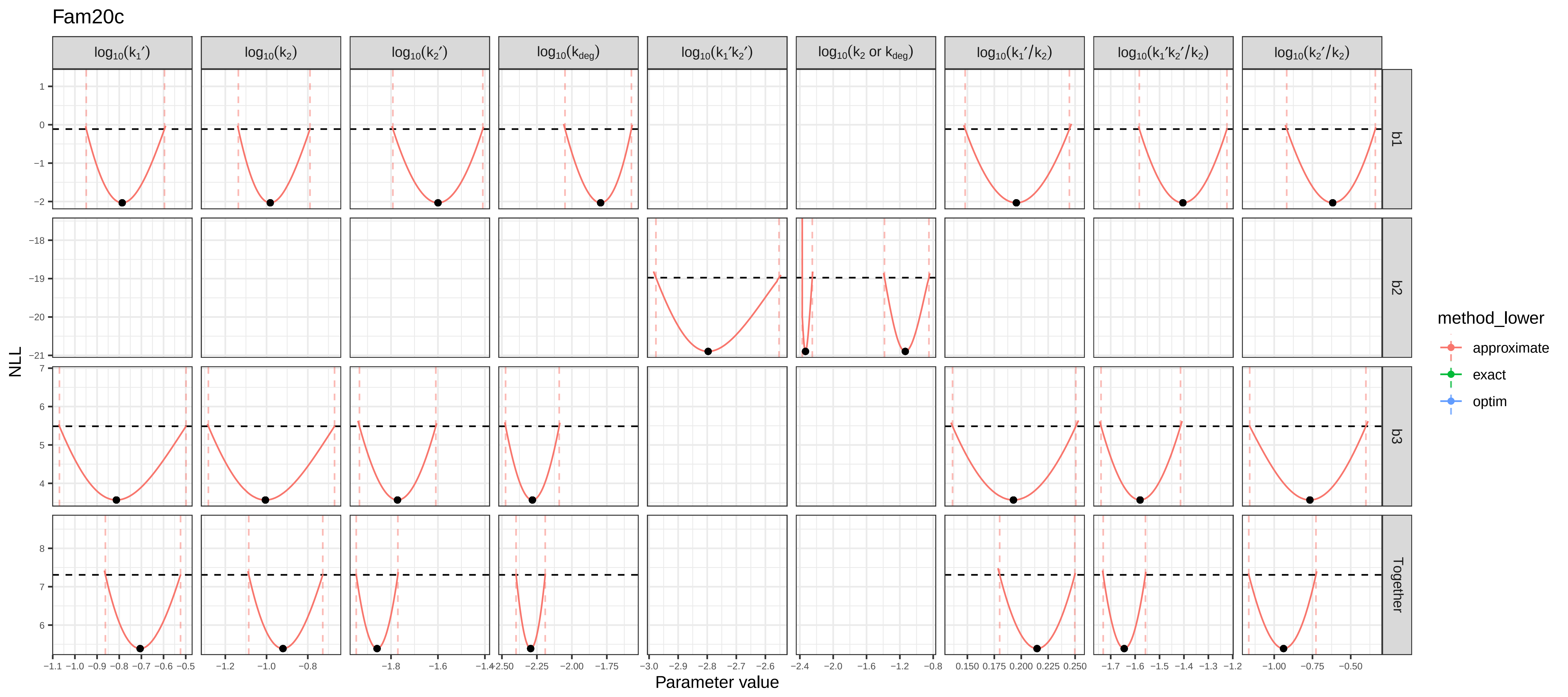

Replicate	Par	Best value	CI95 LB	CI95 UB	Method LB	Method UB
Together	$\log_{10}(k_1')$	−0.7056	−0.8625	−0.5233	approximate	approximate
Together	$\log_{10}(k_2)$	−0.9203	−1.086	−0.7274	approximate	approximate
Together	$\log_{10}(k_2')$	−1.859	−1.948	−1.771	approximate	approximate
Together	$\log_{10}(k_{deg})$	−2.295	−2.399	−2.19	approximate	approximate
Together	$\log_{10}(k_1'/k_2)$	0.2147	0.18	0.2497	approximate	approximate
Together	$\log_{10}(k_1'k_2'/k_2)$	−1.645	−1.731	−1.558	approximate	approximate
Together	$\log_{10}(k_2'/k_2)$	−0.9391	−1.166	−0.7272	approximate	approximate
b1	$\log_{10}(k_1')$	−0.7866	−0.9488	−0.5958	approximate	approximate
b1	$\log_{10}(k_2)$	−0.9821	−1.137	−0.7894	approximate	approximate
b1	$\log_{10}(k_2')$	−1.6	−1.792	−1.409	approximate	approximate
b1	$\log_{10}(k_{deg})$	−1.794	−2.049	−1.574	approximate	approximate
b1	$\log_{10}(k_1'/k_2)$	0.1955	0.148	0.2447	approximate	approximate
b1	$\log_{10}(k_1'k_2'/k_2)$	−1.404	−1.583	−1.224	approximate	approximate
b1	$\log_{10}(k_2'/k_2)$	−0.6177	−0.9184	−0.3393	approximate	approximate
b2	$\log_{10}(k_1'k_2')$	−2.797	−2.976	−2.553	approximate	approximate
b2	$\log_{10}(k_2 \text{ or } k_{deg})$	−1.135	−1.384	−0.8511	approximate	approximate
b2	$\log_{10}(k_2 \text{ or } k_{deg})$	−2.333	−2.371	−2.251	approximate	approximate
b3	$\log_{10}(k_1')$	−0.8129	−1.07	−0.4987	approximate	approximate
b3	$\log_{10}(k_2)$	−1.006	−1.282	−0.6704	approximate	approximate
b3	$\log_{10}(k_2')$	−1.772	−1.934	−1.609	approximate	approximate
b3	$\log_{10}(k_{deg})$	−2.282	−2.474	−2.09	approximate	approximate
b3	$\log_{10}(k_1'/k_2)$	0.1928	0.1362	0.2507	approximate	approximate
b3	$\log_{10}(k_1'k_2'/k_2)$	−1.58	−1.74	−1.414	approximate	approximate
b3	$\log_{10}(k_2'/k_2)$	−0.7667	−1.16	−0.4008	approximate	approximate

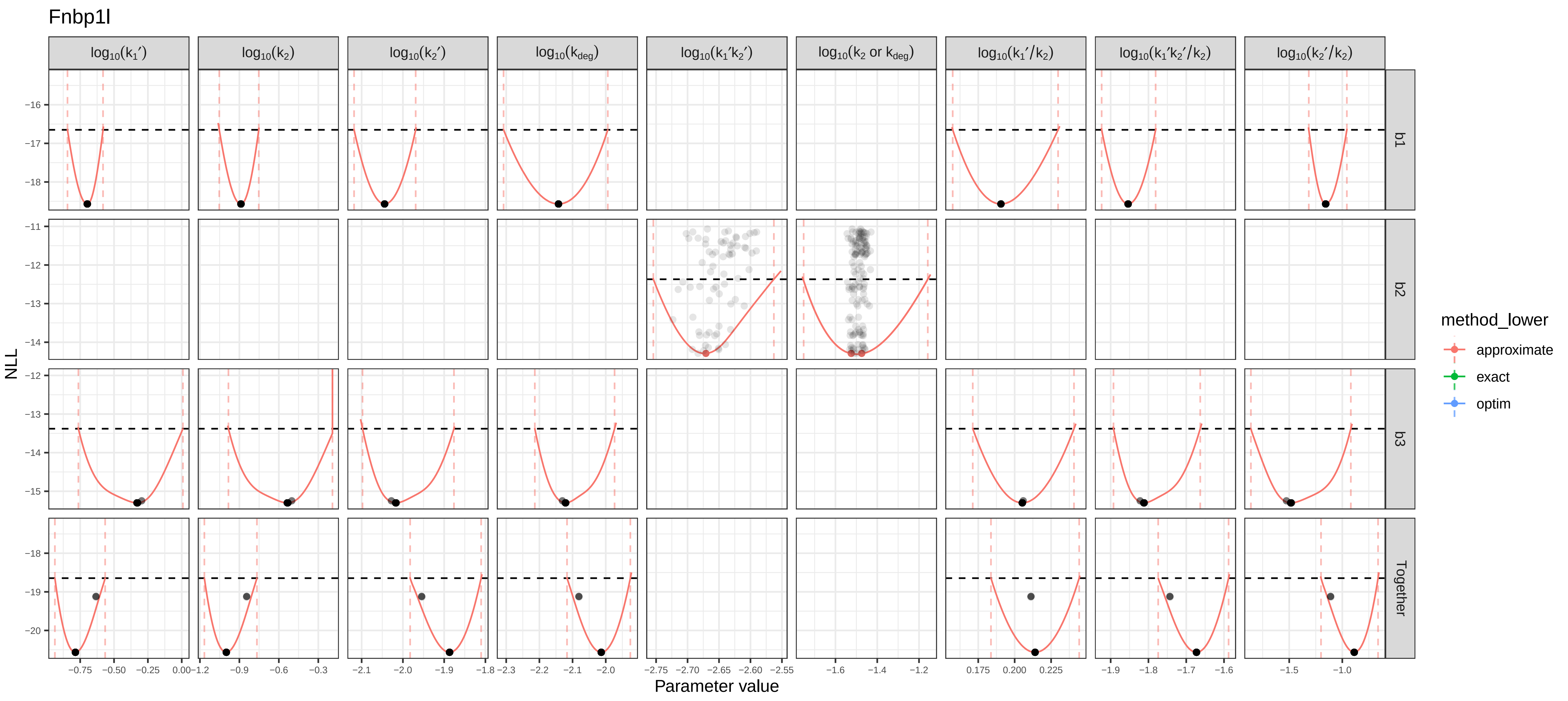

Replicate	Par	Best value	CI95 LB	CI95 UB	Method LB	Method UB
Together	$\log_{10}(k_1')$	−0.7853	−0.9369	−0.5656	approximate	approximate
Together	$\log_{10}(k_2)$	−0.9993	−1.166	−0.7665	approximate	approximate
Together	$\log_{10}(k_2')$	−1.887	−1.982	−1.81	approximate	approximate
Together	$\log_{10}(k_{deg})$	−2.014	−2.117	−1.926	approximate	approximate
Together	$\log_{10}(k_1'/k_2)$	0.214	0.1837	0.2443	approximate	approximate
Together	$\log_{10}(k_1'k_2'/k_2)$	−1.673	−1.774	−1.588	approximate	approximate
Together	$\log_{10}(k_2'/k_2)$	−0.8877	−1.2	−0.6631	approximate	approximate
b1	$\log_{10}(k_1')$	−0.6975	−0.8438	−0.5812	approximate	approximate
b1	$\log_{10}(k_2)$	−0.8881	−1.053	−0.7525	approximate	approximate
b1	$\log_{10}(k_2')$	−2.044	−2.118	−1.969	approximate	approximate
b1	$\log_{10}(k_{deg})$	−2.142	−2.308	−1.994	approximate	approximate
b1	$\log_{10}(k_1'/k_2)$	0.1906	0.1575	0.2298	approximate	approximate
b1	$\log_{10}(k_1'k_2'/k_2)$	−1.854	−1.924	−1.781	approximate	approximate
b1	$\log_{10}(k_2'/k_2)$	−1.156	−1.316	−0.957	approximate	approximate
b2	$\log_{10}(k_1'k_2')$	−2.671	−2.754	−2.563	approximate	approximate
b2	$\log_{10}(k_2 \text{ or } k_{deg})$	−1.474	−1.752	−1.158	approximate	approximate
b2	$\log_{10}(k_2 \text{ or } k_{deg})$	−1.525	−1.752	−1.158	approximate	approximate
b3	$\log_{10}(k_1')$	−0.3288	−0.7634	0.008876	approximate	approximate
b3	$\log_{10}(k_2)$	−0.534	−0.9832	−0.1923	approximate	approximate
b3	$\log_{10}(k_2')$	−2.017	−2.098	−1.876	approximate	approximate
b3	$\log_{10}(k_{deg})$	−2.121	−2.214	−1.973	approximate	approximate
b3	$\log_{10}(k_1'/k_2)$	0.2053	0.1713	0.2407	approximate	approximate
b3	$\log_{10}(k_1'k_2'/k_2)$	−1.812	−1.892	−1.663	approximate	approximate
b3	$\log_{10}(k_2'/k_2)$	−1.483	−1.861	−0.9194	approximate	approximate

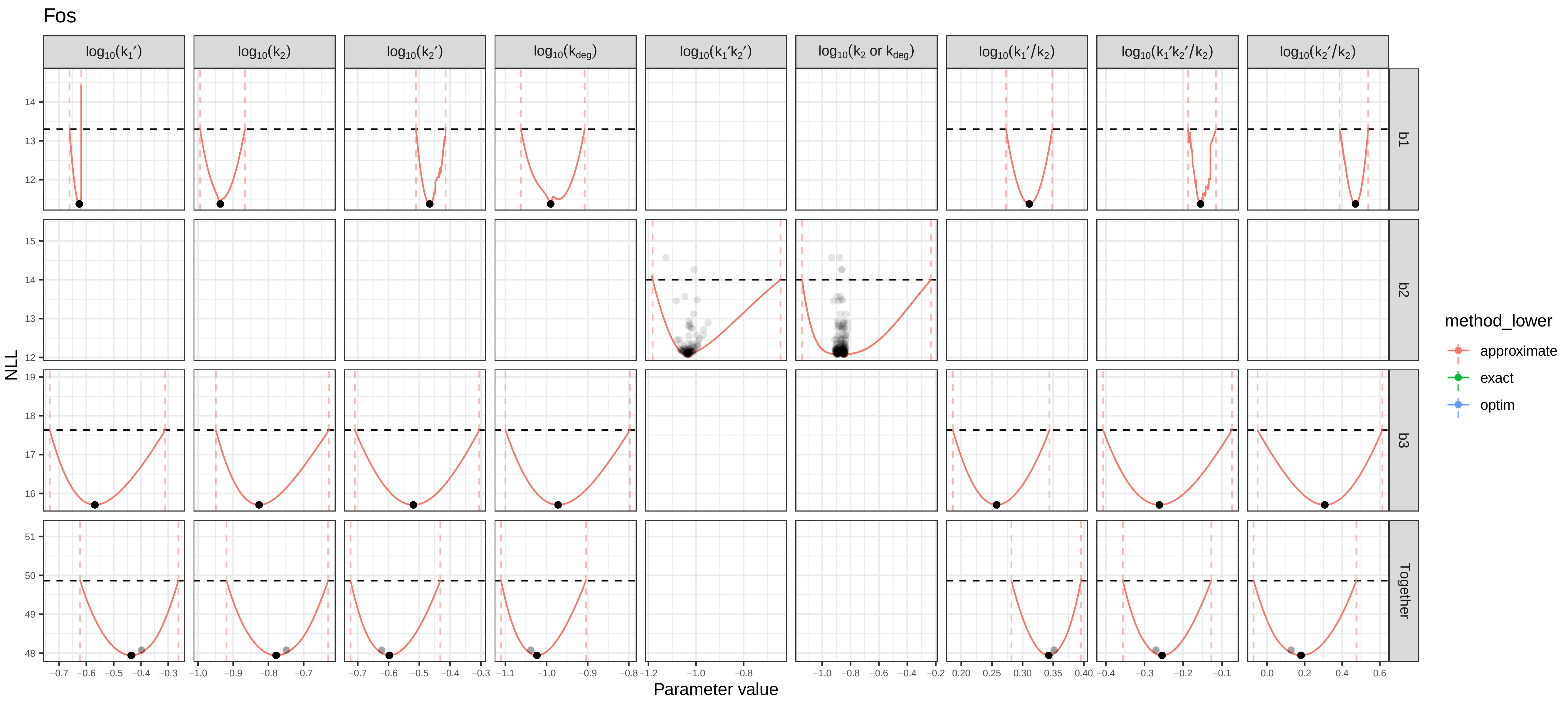

Replicate	Par	**Best value**	**CI95 LB**	**CI95 UB**	**Method LB**	**Method UB**
Together	$\log_{10}(k_1')$	−0.4355	−0.6228	−0.263	approximate	approximate
Together	$\log_{10}(k_2)$	−0.7781	−0.9191	−0.6304	approximate	approximate
Together	$\log_{10}(k_2')$	−0.5972	−0.7231	−0.4318	approximate	approximate
Together	$\log_{10}(k_{deg})$	−1.023	−1.11	−0.9039	approximate	approximate
Together	$\log_{10}(k_1'/k_2)$	0.3426	0.2818	0.3953	approximate	approximate
Together	$\log_{10}(k_1'k_2'/k_2)$	−0.2545	−0.3559	−0.1274	approximate	approximate
Together	$\log_{10}(k_2'/k_2)$	0.1809	−0.0721	0.4755	approximate	approximate
b1	$\log_{10}(k_1')$	−0.6258	−0.6619	−0.619	approximate	approximate
b1	$\log_{10}(k_2)$	−0.9367	−0.994	−0.8666	approximate	approximate
b1	$\log_{10}(k_2')$	−0.4661	−0.5108	−0.4146	approximate	approximate
b1	$\log_{10}(k_{deg})$	−0.9898	−1.062	−0.9072	approximate	approximate
b1	$\log_{10}(k_1'/k_2)$	0.3109	0.2728	0.3484	approximate	approximate
b1	$\log_{10}(k_1'k_2'/k_2)$	−0.1552	−0.1872	−0.1153	approximate	approximate
b1	$\log_{10}(k_2'/k_2)$	0.4706	0.386	0.5385	approximate	approximate
b2	$\log_{10}(k_1'k_2')$	−1.031	−1.182	−0.6444	approximate	approximate
b2	$\log_{10}(k_2 \text{ or } k_{deg})$	−0.8429	−1.142	−0.2337	approximate	approximate
b2	$\log_{10}(k_2 \text{ or } k_{deg})$	−0.8974	−1.142	−0.2337	approximate	approximate
b3	$\log_{10}(k_1')$	−0.5685	−0.7342	−0.3114	approximate	approximate
b3	$\log_{10}(k_2)$	−0.8262	−0.9494	−0.6281	approximate	approximate
b3	$\log_{10}(k_2')$	−0.5196	−0.7089	−0.3054	approximate	approximate
b3	$\log_{10}(k_{deg})$	−0.9719	−1.1	−0.7972	approximate	approximate
b3	$\log_{10}(k_1'/k_2)$	0.2577	0.1862	0.3438	approximate	approximate
b3	$\log_{10}(k_1'k_2'/k_2)$	−0.2619	−0.4069	−0.07389	approximate	approximate
b3	$\log_{10}(k_2'/k_2)$	0.3066	−0.05099	0.614	approximate	approximate

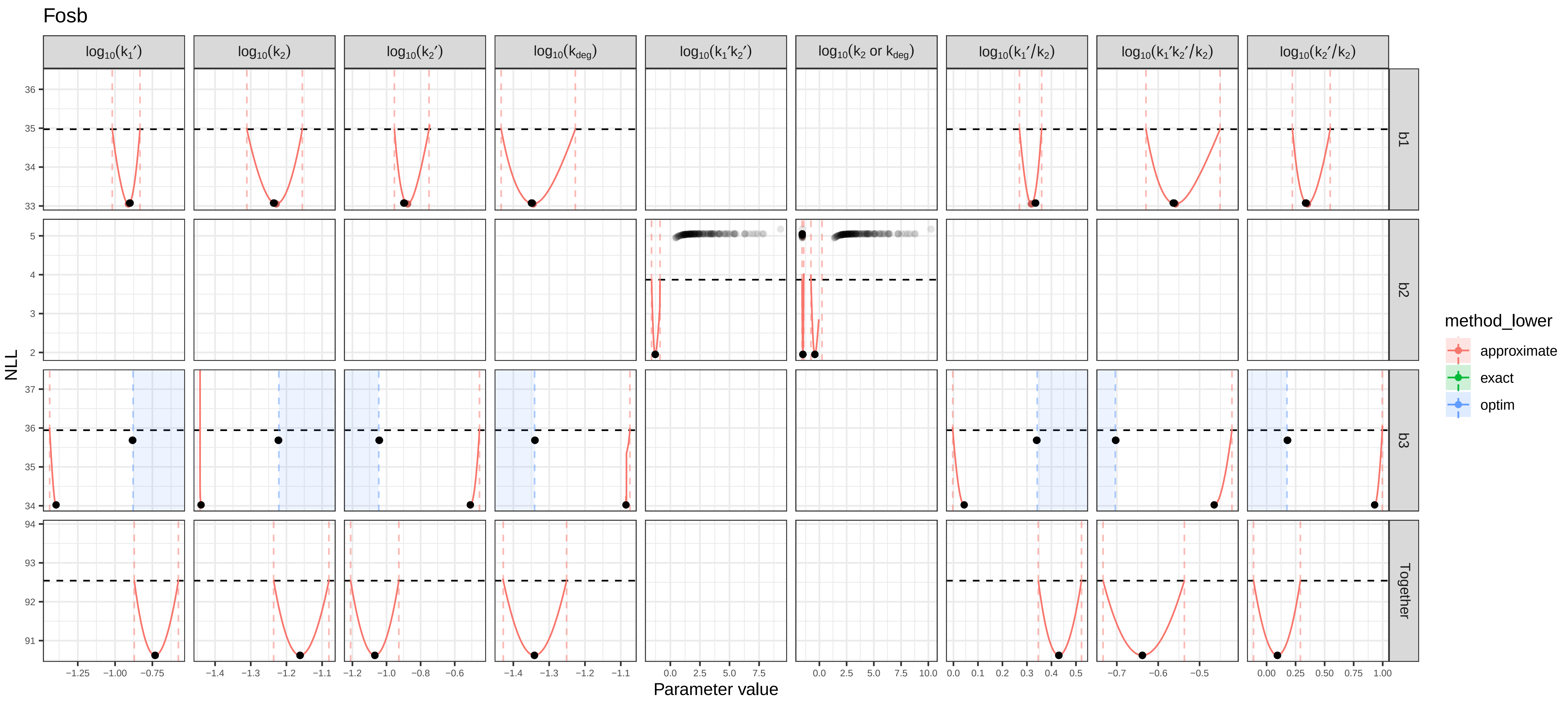

Replicate	Par	Best value	CI95 LB	CI95 UB	Method LB	Method UB
Together	$\log_{10}(k_1')$	−0.7318	−0.8714	−0.5756	approximate	approximate
Together	$\log_{10}(k_2)$	−1.162	−1.236	−1.081	approximate	approximate
Together	$\log_{10}(k_2')$	−1.068	−1.211	−0.9278	approximate	approximate
Together	$\log_{10}(k_{deg})$	−1.341	−1.428	−1.251	approximate	approximate
Together	$\log_{10}(k_1'/k_2)$	0.4301	0.3462	0.5227	approximate	approximate
Together	$\log_{10}(k_1'k_2'/k_2)$	−0.6382	−0.7332	−0.5368	approximate	approximate
Together	$\log_{10}(k_2'/k_2)$	0.09365	−0.1121	0.2911	approximate	approximate
b1	$\log_{10}(k_1')$	−0.9109	−1.019	−0.8327	approximate	approximate
b1	$\log_{10}(k_2)$	−1.229	−1.311	−1.155	approximate	approximate
b1	$\log_{10}(k_2')$	−0.8762	−0.9538	−0.7509	approximate	approximate
b1	$\log_{10}(k_{deg})$	−1.345	−1.434	−1.227	approximate	approximate
b1	$\log_{10}(k_1'/k_2)$	0.3178	0.2695	0.36	approximate	approximate
b1	$\log_{10}(k_1'k_2'/k_2)$	−0.5584	−0.6295	−0.4506	approximate	approximate
b1	$\log_{10}(k_2'/k_2)$	0.3525	0.2222	0.5478	approximate	approximate
b2	$\log_{10}(k_1'k_2')$	−1.276	−1.587	−0.8672	approximate	approximate
b2	$\log_{10}(k_2 \text{ or } k_{deg})$	−0.4113	−0.7699	0.2443	approximate	approximate
b2	$\log_{10}(k_2 \text{ or } k_{deg})$	−1.511	−1.59	−1.434	approximate	approximate
b3	$\log_{10}(k_1')$	−1.395	−1.438	> −0.8792	approximate	optim
b3	$\log_{10}(k_2)$	−1.439	−1.442	> −1.221	approximate	optim
b3	$\log_{10}(k_2')$	−0.5089	< −1.046	−0.455	optim	approximate
b3	$\log_{10}(k_{deg})$	−1.086	< −1.341	−1.075	optim	approximate
b3	$\log_{10}(k_1'/k_2)$	0.04388	−0.002687	> 0.342	approximate	optim
b3	$\log_{10}(k_1'k_2'/k_2)$	−0.465	< −0.7036	−0.4218	optim	approximate
b3	$\log_{10}(k_2'/k_2)$	0.9302	< 0.1757	0.9965	optim	approximate

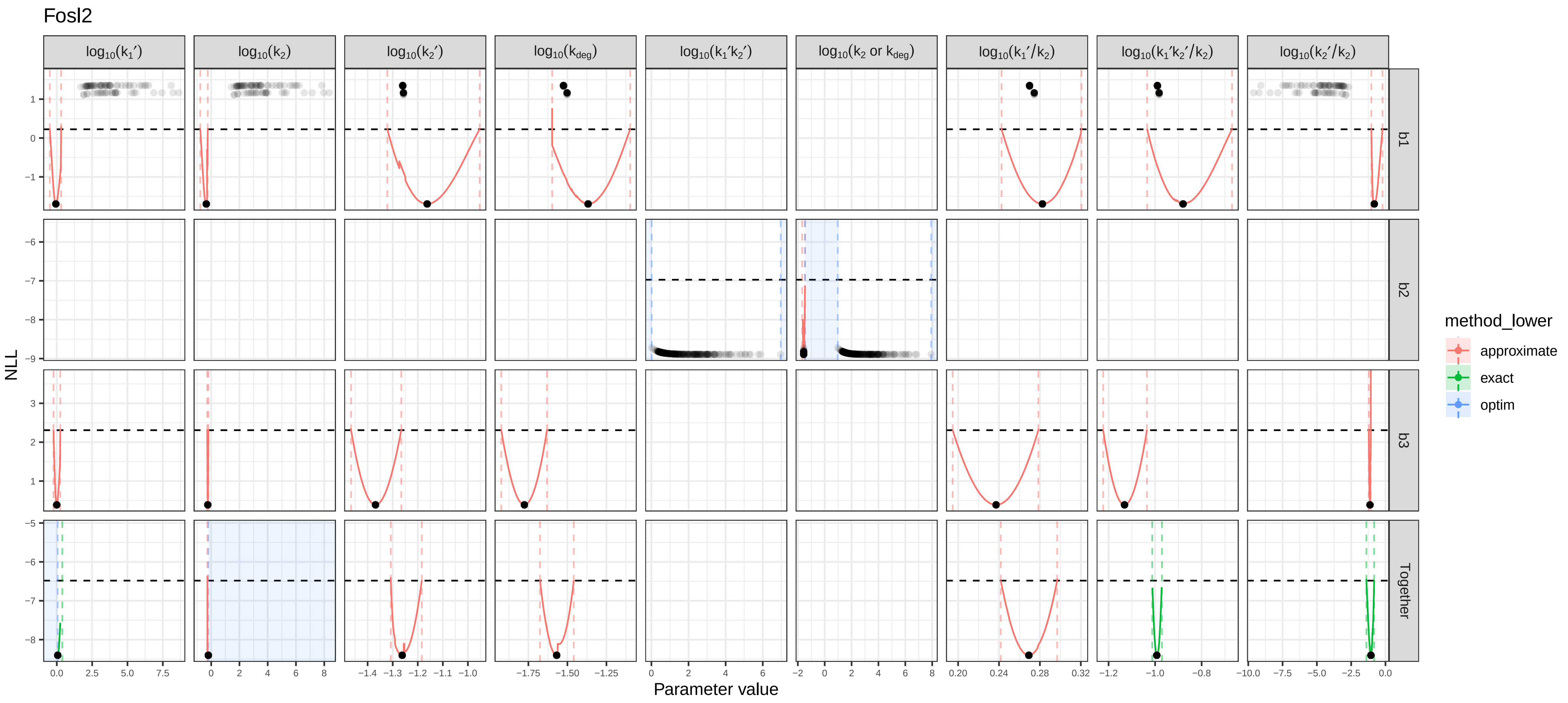

Replicate	Par	Best value	CI95 LB	CI95 UB	Method LB	Method UB
Together	$\log_{10}(k_1')$	0.06601	< 0.06269	0.3877	optim	exact
Together	$\log_{10}(k_2)$	−0.2032	−0.2702	> −0.1975	approximate	optim
Together	$\log_{10}(k_2')$	−1.262	−1.308	−1.184	approximate	approximate
Together	$\log_{10}(k_{deg})$	−1.569	−1.676	−1.459	approximate	approximate
Together	$\log_{10}(k_1'/k_2)$	0.2692	0.2417	0.2968	approximate	approximate
Together	$\log_{10}(k_1'k_2'/k_2)$	−0.9928	−1.012	−0.9708	exact	exact
Together	$\log_{10}(k_2'/k_2)$	−1.059	−1.39	−0.8253	exact	exact
b1	$\log_{10}(k_1')$	−0.06723	−0.4936	0.3043	approximate	approximate
b1	$\log_{10}(k_2)$	−0.3497	−0.7721	−0.2454	approximate	approximate
b1	$\log_{10}(k_2')$	−1.162	−1.321	−0.9529	approximate	approximate
b1	$\log_{10}(k_{deg})$	−1.367	−1.598	−1.098	approximate	approximate
b1	$\log_{10}(k_1'/k_2)$	0.2825	0.2425	0.3207	approximate	approximate
b1	$\log_{10}(k_1'k_2'/k_2)$	−0.8798	−1.034	−0.6694	approximate	approximate
b1	$\log_{10}(k_2'/k_2)$	−0.8125	−1.029	−0.2147	approximate	approximate
b2	$\log_{10}(k_1'k_2')$	5.764	< 0.01479	> 6.964	optim	optim
b2	$\log_{10}(k_2 \text{ or } k_{deg})$	6.722	0.9691	> 7.923	optim	optim
b2	$\log_{10}(k_2 \text{ or } k_{deg})$	−1.564	−1.678	−1.464	approximate	approximate
b3	$\log_{10}(k_1')$	−0.004252	−0.2345	0.2498	approximate	approximate
b3	$\log_{10}(k_2)$	−0.2414	−0.2799	−0.1956	approximate	approximate
b3	$\log_{10}(k_2')$	−1.369	−1.466	−1.266	approximate	approximate
b3	$\log_{10}(k_{deg})$	−1.776	−1.924	−1.631	approximate	approximate
b3	$\log_{10}(k_1'/k_2)$	0.2371	0.1947	0.2785	approximate	approximate
b3	$\log_{10}(k_1'k_2'/k_2)$	−1.132	−1.223	−1.035	approximate	approximate
b3	$\log_{10}(k_2'/k_2)$	−1.127	−1.22	−1.072	approximate	approximate

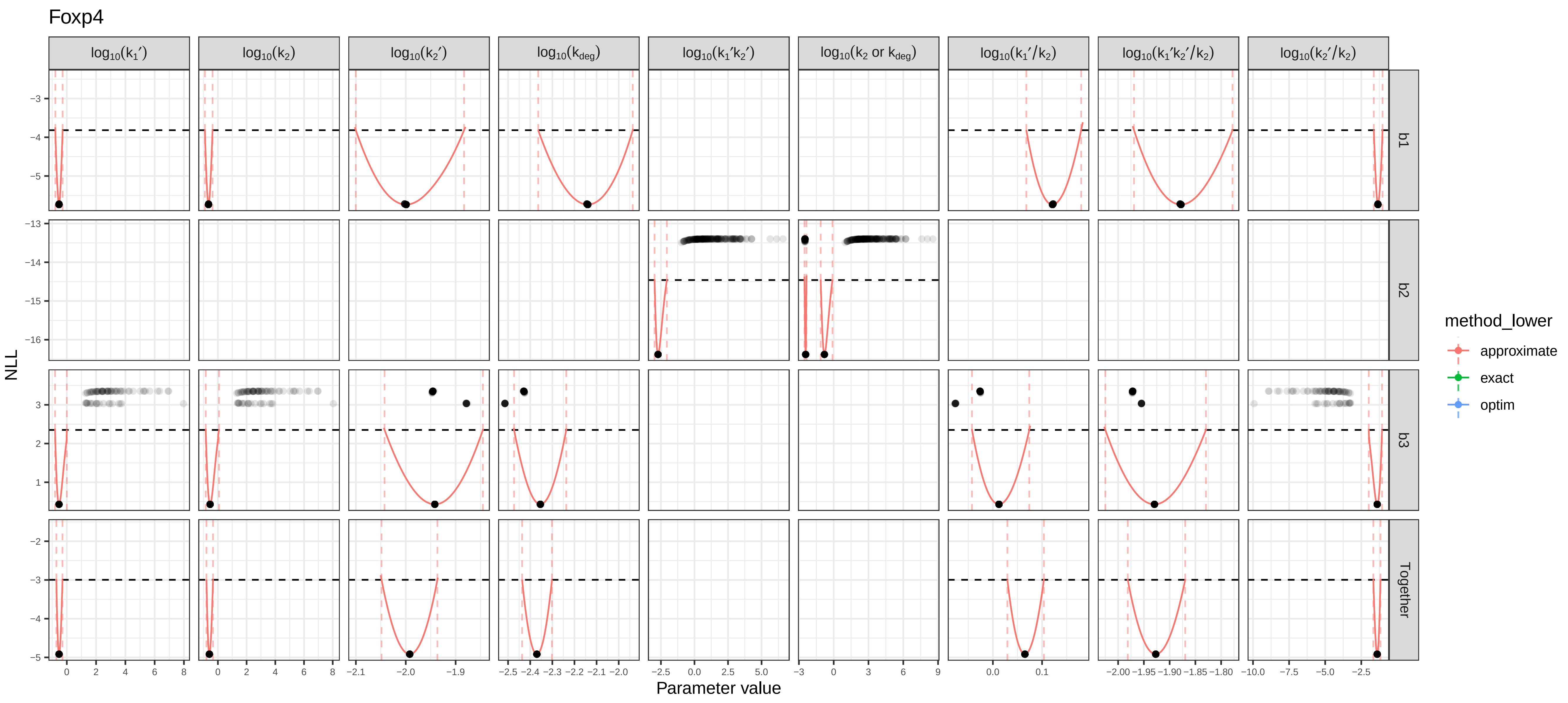

Replicate	Par	Best value	CI95 LB	CI95 UB	Method LB	Method UB
Together	$\log_{10}(k_1')$	−0.5281	−0.713	−0.2947	approximate	approximate
Together	$\log_{10}(k_2)$	−0.5933	−0.7952	−0.3398	approximate	approximate
Together	$\log_{10}(k_2')$	−1.992	−2.049	−1.937	approximate	approximate
Together	$\log_{10}(k_{deg})$	−2.37	−2.437	−2.302	approximate	approximate
Together	$\log_{10}(k_1'/k_2)$	0.06511	0.02945	0.1037	approximate	approximate
Together	$\log_{10}(k_1'k_2'/k_2)$	−1.927	−1.981	−1.87	approximate	approximate
Together	$\log_{10}(k_2'/k_2)$	−1.399	−1.666	−1.172	approximate	approximate
b1	$\log_{10}(k_1')$	−0.5333	−0.7831	−0.2841	approximate	approximate
b1	$\log_{10}(k_2)$	−0.6545	−0.9049	−0.3715	approximate	approximate
b1	$\log_{10}(k_2')$	−1.999	−2.1	−1.883	approximate	approximate
b1	$\log_{10}(k_{deg})$	−2.14	−2.364	−1.937	approximate	approximate
b1	$\log_{10}(k_1'/k_2)$	0.1213	0.068	0.1795	approximate	approximate
b1	$\log_{10}(k_1'k_2'/k_2)$	−1.878	−1.969	−1.778	approximate	approximate
b1	$\log_{10}(k_2'/k_2)$	−1.345	−1.634	−1.028	approximate	approximate
b2	$\log_{10}(k_1'k_2')$	−2.712	−2.972	−2.054	approximate	approximate
b2	$\log_{10}(k_2 \text{ or } k_{deg})$	−0.8009	−1.123	−0.1049	approximate	approximate
b2	$\log_{10}(k_2 \text{ or } k_{deg})$	−2.42	−2.52	−2.351	approximate	approximate
b3	$\log_{10}(k_1')$	−0.532	−0.8036	0.001082	approximate	approximate
b3	$\log_{10}(k_2)$	−0.5443	−0.8481	0.07971	approximate	approximate
b3	$\log_{10}(k_2')$	−1.942	−2.042	−1.845	approximate	approximate
b3	$\log_{10}(k_{deg})$	−2.354	−2.473	−2.237	approximate	approximate
b3	$\log_{10}(k_1'/k_2)$	0.01235	−0.04251	0.07399	approximate	approximate
b3	$\log_{10}(k_1'k_2'/k_2)$	−1.929	−2.025	−1.83	approximate	approximate
b3	$\log_{10}(k_2'/k_2)$	−1.397	−1.983	−1.063	approximate	approximate

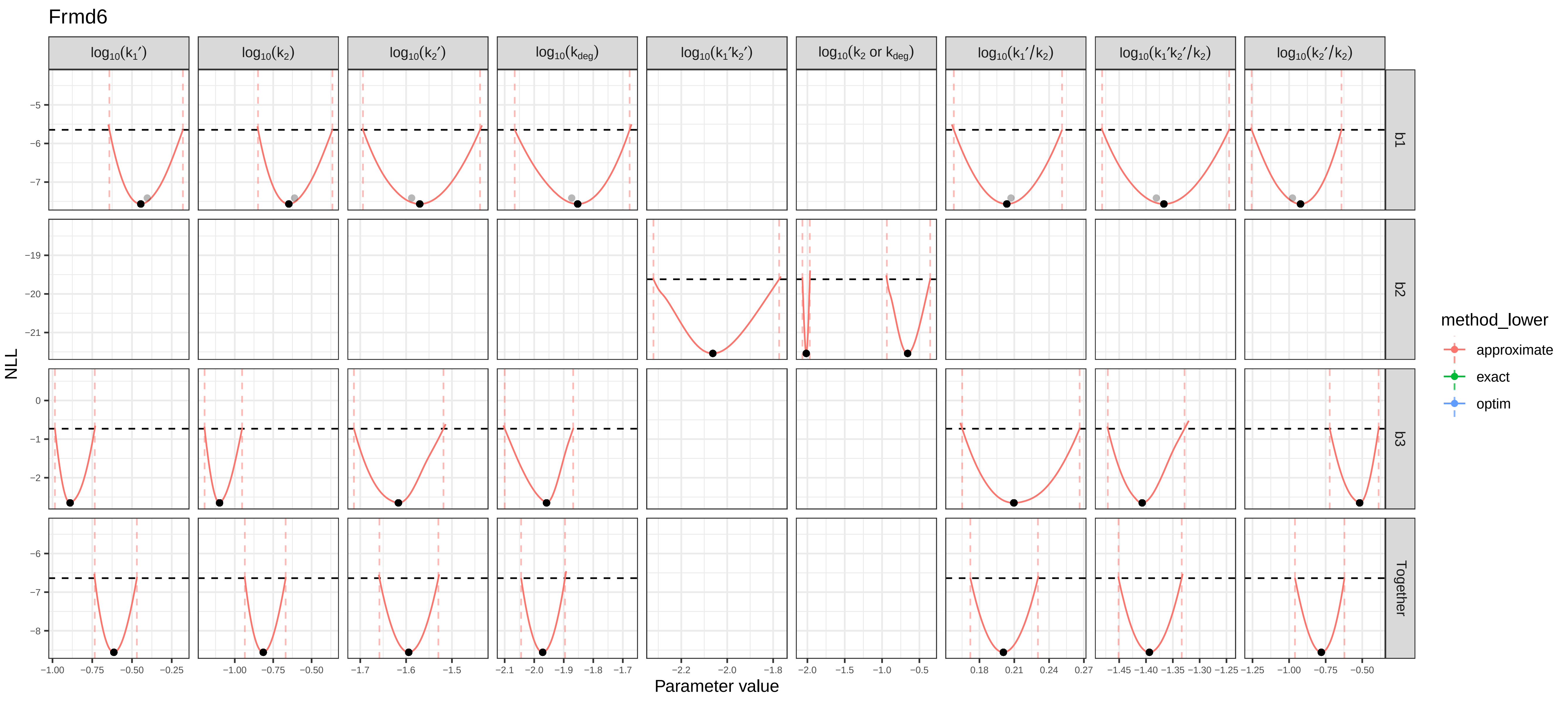

Replicate	Par	Best value	CI95 LB	CI95 UB	Method LB	Method UB
Together	$\log_{10}(k_1')$	−0.6141	−0.7342	−0.4694	approximate	approximate
Together	$\log_{10}(k_2)$	−0.8148	−0.9346	−0.669	approximate	approximate
Together	$\log_{10}(k_2')$	−1.594	−1.658	−1.53	approximate	approximate
Together	$\log_{10}(k_{deg})$	−1.971	−2.044	−1.896	approximate	approximate
Together	$\log_{10}(k_1'/k_2)$	0.2006	0.1721	0.2304	approximate	approximate
Together	$\log_{10}(k_1'k_2'/k_2)$	−1.394	−1.451	−1.333	approximate	approximate
Together	$\log_{10}(k_2'/k_2)$	−0.7795	−0.9598	−0.6219	approximate	approximate
b1	$\log_{10}(k_1')$	−0.4448	−0.6423	−0.1798	approximate	approximate
b1	$\log_{10}(k_2)$	−0.6483	−0.8486	−0.3647	approximate	approximate
b1	$\log_{10}(k_2')$	−1.57	−1.694	−1.439	approximate	approximate
b1	$\log_{10}(k_{deg})$	−1.853	−2.066	−1.677	approximate	approximate
b1	$\log_{10}(k_1'/k_2)$	0.2036	0.1579	0.2512	approximate	approximate
b1	$\log_{10}(k_1'k_2'/k_2)$	−1.367	−1.482	−1.245	approximate	approximate
b1	$\log_{10}(k_2'/k_2)$	−0.9219	−1.256	−0.642	approximate	approximate
b2	$\log_{10}(k_1'k_2')$	−2.063	−2.321	−1.774	approximate	approximate
b2	$\log_{10}(k_2 \text{ or } k_{deg})$	−0.6575	−0.9359	−0.3554	approximate	approximate
b2	$\log_{10}(k_2 \text{ or } k_{deg})$	−2.015	−2.067	−1.968	approximate	approximate
b3	$\log_{10}(k_1')$	−0.8893	−0.9848	−0.7342	approximate	approximate
b3	$\log_{10}(k_2)$	−1.099	−1.196	−0.9525	approximate	approximate
b3	$\log_{10}(k_2')$	−1.617	−1.714	−1.519	approximate	approximate
b3	$\log_{10}(k_{deg})$	−1.958	−2.1	−1.868	approximate	approximate
b3	$\log_{10}(k_1'/k_2)$	0.2097	0.1651	0.2663	approximate	approximate
b3	$\log_{10}(k_1'k_2'/k_2)$	−1.407	−1.471	−1.328	approximate	approximate
b3	$\log_{10}(k_2'/k_2)$	−0.5178	−0.7225	−0.3882	approximate	approximate

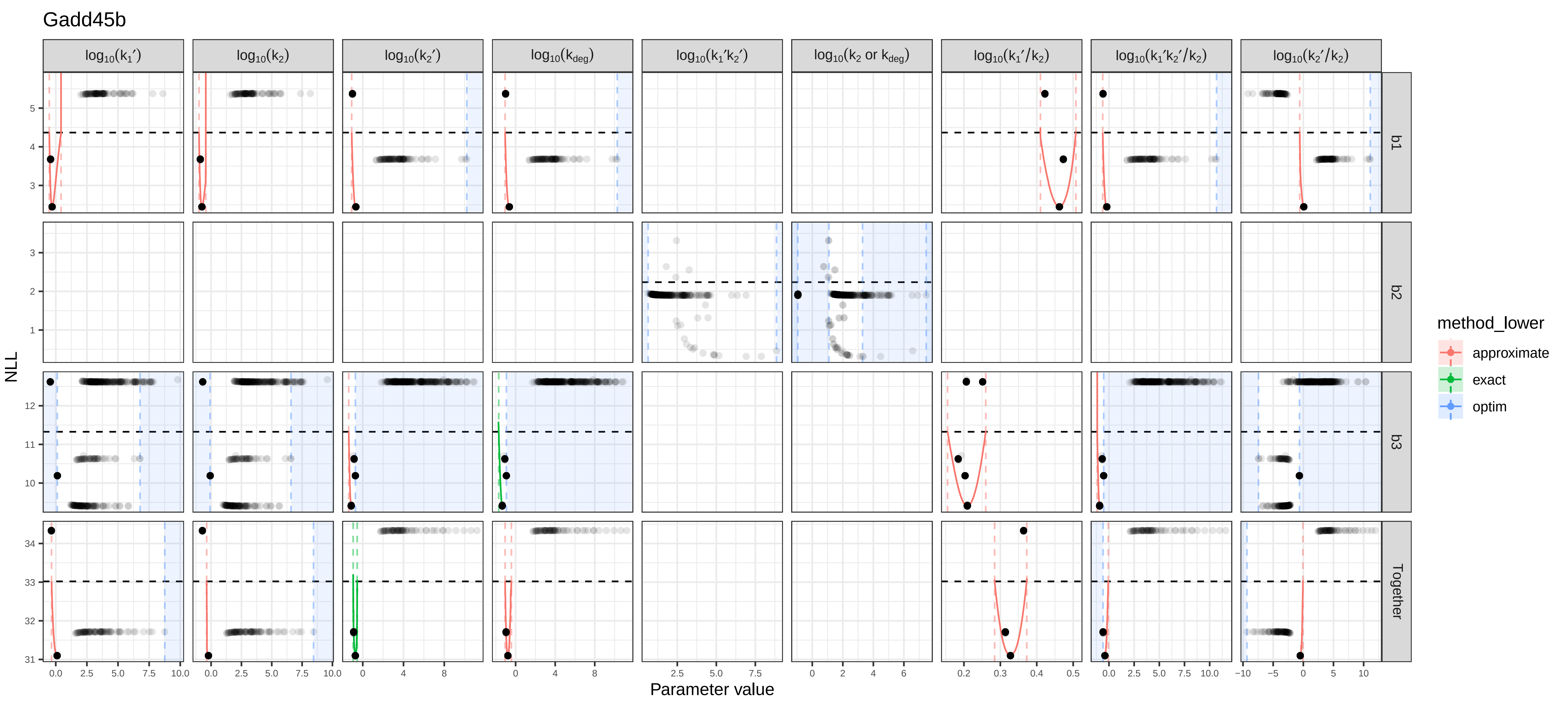

Replicate	Par	Best value	CI95 LB	CI95 UB	Method LB	Method UB
Together	$\log_{10}(k_1')$	0.1083	−0.3393	> 8.755	approximate	optim
Together	$\log_{10}(k_2)$	−0.2195	−0.3672	> 8.442	approximate	optim
Together	$\log_{10}(k_2')$	−0.7311	−0.9413	−0.5456	exact	exact
Together	$\log_{10}(k_{deg})$	−0.7809	−1.063	−0.4236	approximate	approximate
Together	$\log_{10}(k_1'/k_2)$	0.3277	0.2843	0.3724	approximate	approximate
Together	$\log_{10}(k_1'k_2'/k_2)$	−0.4034	< −0.5855	−0.06195	optim	approximate
Together	$\log_{10}(k_2'/k_2)$	−0.5116	< −9.341	−0.06184	optim	approximate
b1	$\log_{10}(k_1')$	−0.2944	−0.5207	0.4231	approximate	approximate
b1	$\log_{10}(k_2)$	−0.7566	−0.9982	−0.4386	approximate	approximate
b1	$\log_{10}(k_2')$	−0.6787	−1.085	> 10.21	approximate	optim
b1	$\log_{10}(k_{deg})$	−0.638	−1.064	> 10.27	approximate	optim
b1	$\log_{10}(k_1'/k_2)$	0.4622	0.4101	0.5075	approximate	approximate
b1	$\log_{10}(k_1'k_2'/k_2)$	−0.2165	−0.6207	> 10.68	approximate	optim
b1	$\log_{10}(k_2'/k_2)$	0.0779	−0.602	> 11.1	approximate	optim
b2	$\log_{10}(k_1'k_2')$	7.881	< 0.6318	> 8.855	optim	optim
b2	$\log_{10}(k_2 \text{ or } k_{deg})$	4.476	< 1.094	> 7.46	optim	optim
b2	$\log_{10}(k_2 \text{ or } k_{deg})$	3.118	< −0.944	> 3.292	optim	optim
b3	$\log_{10}(k_1')$	5.149	< 0.12	> 6.772	optim	optim
b3	$\log_{10}(k_2)$	4.94	< −0.08349	> 6.587	optim	optim
b3	$\log_{10}(k_2')$	−1.135	−1.379	> −0.7239	approximate	optim
b3	$\log_{10}(k_{deg})$	−1.353	−1.71	> −0.9327	exact	optim
b3	$\log_{10}(k_1'/k_2)$	0.2093	0.1551	0.26	approximate	approximate
b3	$\log_{10}(k_1'k_2'/k_2)$	−0.926	−1.16	> −0.5204	approximate	optim
b3	$\log_{10}(k_2'/k_2)$	−6.075	< −7.44	> −0.6404	optim	optim

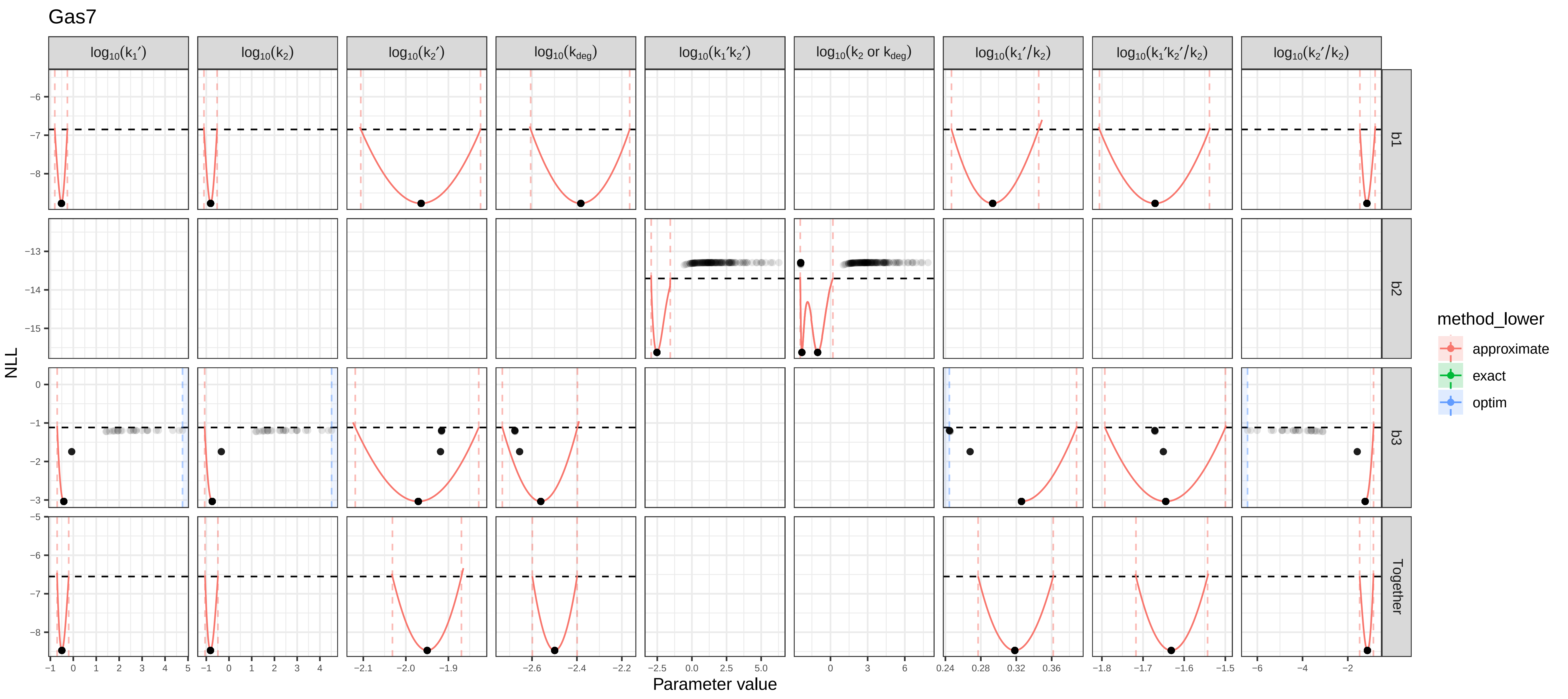

Replicate	Par	Best value	CI95 LB	CI95 UB	Method LB	Method UB
Together	$\log_{10}(k_1')$	−0.4982	−0.7041	−0.1998	approximate	approximate
Together	$\log_{10}(k_2)$	−0.8167	−1.055	−0.4877	approximate	approximate
Together	$\log_{10}(k_2')$	−1.95	−2.031	−1.869	approximate	approximate
Together	$\log_{10}(k_{deg})$	−2.499	−2.598	−2.399	approximate	approximate
Together	$\log_{10}(k_1'/k_2)$	0.3184	0.2769	0.3618	approximate	approximate
Together	$\log_{10}(k_1'k_2'/k_2)$	−1.631	−1.717	−1.543	approximate	approximate
Together	$\log_{10}(k_2'/k_2)$	−1.133	−1.475	−0.8665	approximate	approximate
b1	$\log_{10}(k_1')$	−0.5167	−0.8057	−0.2575	approximate	approximate
b1	$\log_{10}(k_2)$	−0.8101	−1.103	−0.5241	approximate	approximate
b1	$\log_{10}(k_2')$	−1.964	−2.106	−1.824	approximate	approximate
b1	$\log_{10}(k_{deg})$	−2.383	−2.606	−2.165	approximate	approximate
b1	$\log_{10}(k_1'/k_2)$	0.2934	0.2469	0.3454	approximate	approximate
b1	$\log_{10}(k_1'k_2'/k_2)$	−1.671	−1.806	−1.539	approximate	approximate
b1	$\log_{10}(k_2'/k_2)$	−1.154	−1.464	−0.7909	approximate	approximate
b2	$\log_{10}(k_1'k_2')$	−2.523	−2.938	−1.56	approximate	approximate
b2	$\log_{10}(k_2 \text{ or } k_{deg})$	−1.034	−2.44	0.1957	approximate	approximate
b2	$\log_{10}(k_2 \text{ or } k_{deg})$	−2.308	−2.44	0.1957	approximate	approximate
b3	$\log_{10}(k_1')$	−0.413	−0.7008	> 4.76	approximate	optim
b3	$\log_{10}(k_2)$	−0.739	−1.071	> 4.516	approximate	optim
b3	$\log_{10}(k_2')$	−1.971	−2.119	−1.829	approximate	approximate
b3	$\log_{10}(k_{deg})$	−2.561	−2.732	−2.398	approximate	approximate
b3	$\log_{10}(k_1'/k_2)$	0.3259	< 0.2445	0.3881	optim	approximate
b3	$\log_{10}(k_1'k_2'/k_2)$	−1.645	−1.793	−1.5	approximate	approximate
b3	$\log_{10}(k_2'/k_2)$	−1.232	< −6.432	−0.86	optim	approximate

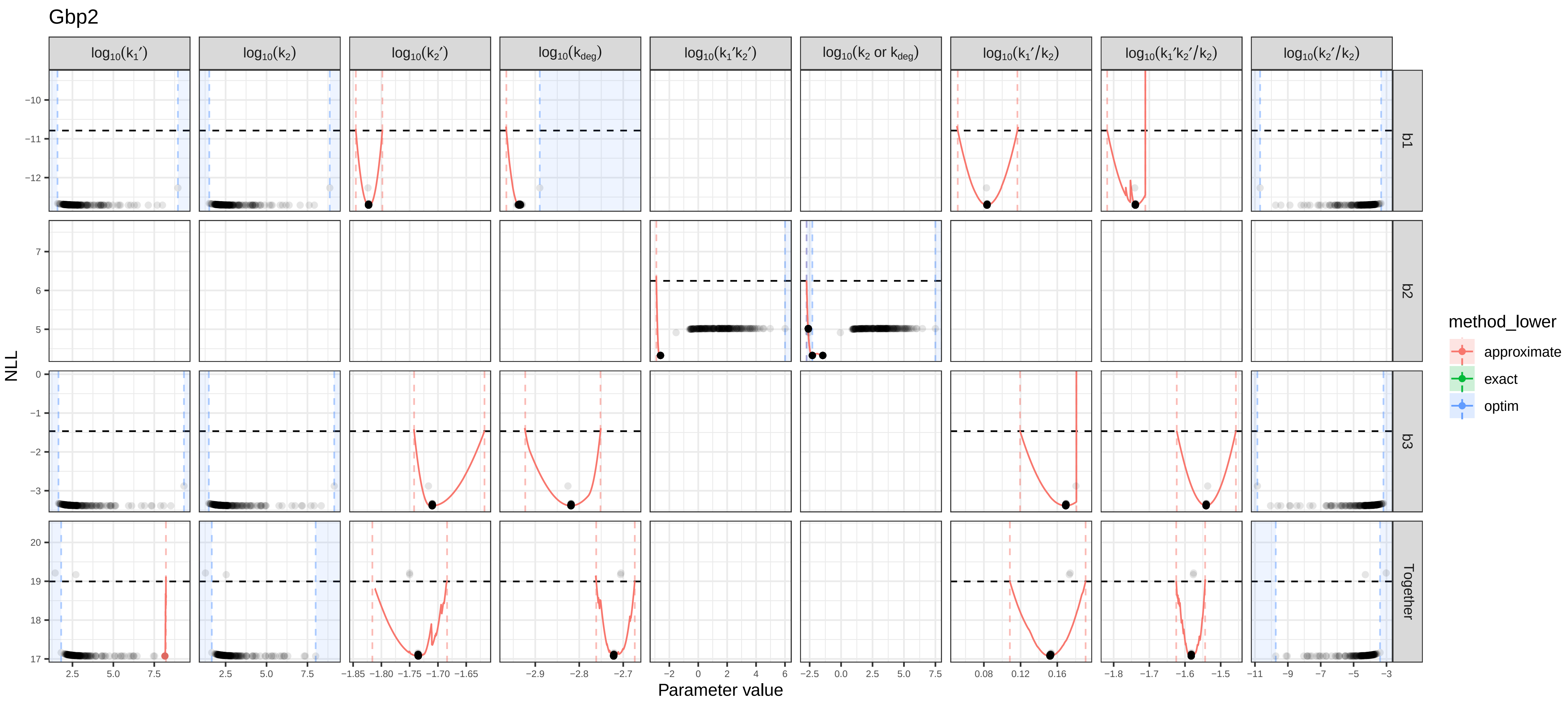

Replicate	Par	Best value	CI95 LB	CI95 UB	Method LB	Method UB
Together	$\log_{10}(k_1')$	8.153	< 1.809	8.214	optim	approximate
Together	$\log_{10}(k_2)$	8.001	< 1.656	> 8.001	optim	optim
Together	$\log_{10}(k_2')$	−1.735	−1.816	−1.684	approximate	approximate
Together	$\log_{10}(k_{deg})$	−2.722	−2.761	−2.674	approximate	approximate
Together	$\log_{10}(k_1'/k_2)$	0.1521	0.1083	0.1909	approximate	approximate
Together	$\log_{10}(k_1'k_2'/k_2)$	−1.583	−1.625	−1.544	approximate	approximate
Together	$\log_{10}(k_2'/k_2)$	−9.735	< −9.735	> −3.391	optim	optim
b1	$\log_{10}(k_1')$	6.461	< 1.586	> 8.946	optim	optim
b1	$\log_{10}(k_2)$	6.378	< 1.502	> 8.864	optim	optim
b1	$\log_{10}(k_2')$	−1.823	−1.845	−1.798	approximate	approximate
b1	$\log_{10}(k_{deg})$	−2.936	−2.966	> −2.89	approximate	optim
b1	$\log_{10}(k_1'/k_2)$	0.08339	0.05155	0.1165	approximate	approximate
b1	$\log_{10}(k_1'k_2'/k_2)$	−1.739	−1.818	−1.711	approximate	approximate
b1	$\log_{10}(k_2'/k_2)$	−8.201	< −10.69	> −3.324	optim	optim
b2	$\log_{10}(k_1'k_2')$	−2.611	−2.896	> 6.007	approximate	optim
b2	$\log_{10}(k_2 \text{ or } k_{deg})$	−1.456	−2.746	> 7.497	approximate	optim
b2	$\log_{10}(k_2 \text{ or } k_{deg})$	−2.285	−2.746	−2.284	approximate	optim
b3	$\log_{10}(k_1')$	6.122	< 1.644	> 9.315	optim	optim
b3	$\log_{10}(k_2)$	5.952	< 1.475	> 9.135	optim	optim
b3	$\log_{10}(k_2')$	−1.71	−1.742	−1.618	approximate	approximate
b3	$\log_{10}(k_{deg})$	−2.819	−2.923	−2.752	approximate	approximate
b3	$\log_{10}(k_1'/k_2)$	0.1692	0.1195	0.1808	approximate	approximate
b3	$\log_{10}(k_1'k_2'/k_2)$	−1.541	−1.623	−1.457	approximate	approximate
b3	$\log_{10}(k_2'/k_2)$	−7.663	< −10.85	> −3.185	optim	optim

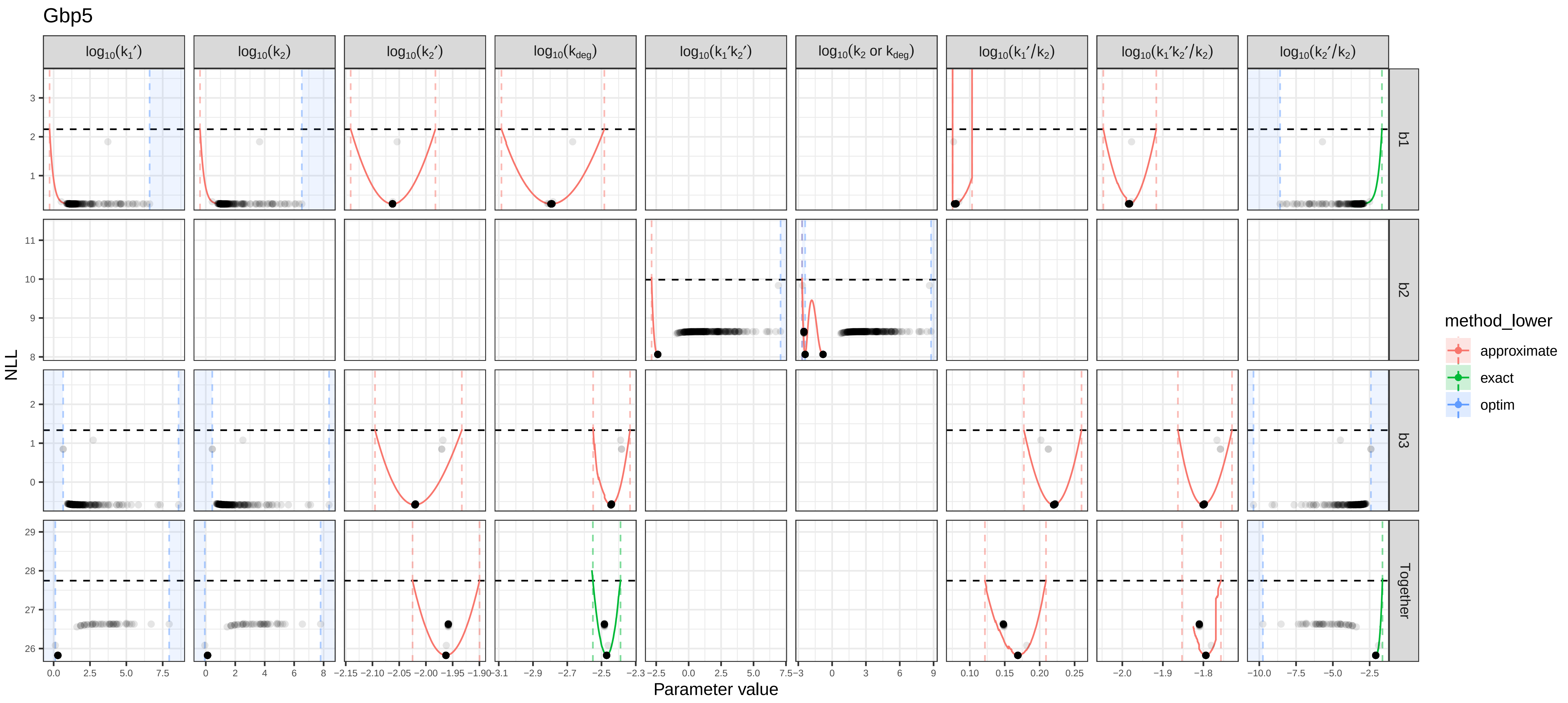

Replicate	Par	**Best value**	**CI95 LB**	**CI95 UB**	**Method LB**	**Method UB**
Together	$\log_{10}(k_1')$	0.2911	< 0.1172	> 7.944	optim	optim
Together	$\log_{10}(k_2)$	0.1223	< −0.06421	> 7.796	optim	optim
Together	$\log_{10}(k_2')$	−1.963	−2.025	−1.9	approximate	approximate
Together	$\log_{10}(k_{deg})$	−2.47	−2.55	−2.389	exact	exact
Together	$\log_{10}(k_1'/k_2)$	0.1688	0.1216	0.2091	approximate	approximate
Together	$\log_{10}(k_1'k_2'/k_2)$	−1.794	−1.853	−1.757	approximate	approximate
Together	$\log_{10}(k_2'/k_2)$	−2.085	< −9.754	−1.629	optim	exact
b1	$\log_{10}(k_1')$	1.512	−0.2786	> 6.6	approximate	optim
b1	$\log_{10}(k_2)$	1.433	−0.3894	> 6.522	approximate	optim
b1	$\log_{10}(k_2')$	−2.063	−2.141	−1.982	approximate	approximate
b1	$\log_{10}(k_{deg})$	−2.793	−3.084	−2.483	approximate	approximate
b1	$\log_{10}(k_1'/k_2)$	0.07882	0.07535	0.1032	approximate	approximate
b1	$\log_{10}(k_1'k_2'/k_2)$	−1.984	−2.047	−1.916	approximate	approximate
b1	$\log_{10}(k_2'/k_2)$	−3.496	< −8.585	−1.664	optim	exact
b2	$\log_{10}(k_1'k_2')$	−2.377	−2.853	> 7.081	approximate	optim
b2	$\log_{10}(k_2 \text{ or } k_{deg})$	−0.8048	−2.666	> 8.776	approximate	optim
b2	$\log_{10}(k_2 \text{ or } k_{deg})$	−2.395	−2.666	−2.394	approximate	optim
b3	$\log_{10}(k_1')$	3.398	< 0.6548	> 8.59	optim	optim
b3	$\log_{10}(k_2)$	3.178	< 0.4423	> 8.37	optim	optim
b3	$\log_{10}(k_2')$	−2.021	−2.095	−1.933	approximate	approximate
b3	$\log_{10}(k_{deg})$	−2.444	−2.549	−2.333	approximate	approximate
b3	$\log_{10}(k_1'/k_2)$	0.2201	0.1774	0.2599	approximate	approximate
b3	$\log_{10}(k_1'k_2'/k_2)$	−1.801	−1.863	−1.729	approximate	approximate
b3	$\log_{10}(k_2'/k_2)$	−5.199	< −10.39	> −2.413	optim	optim

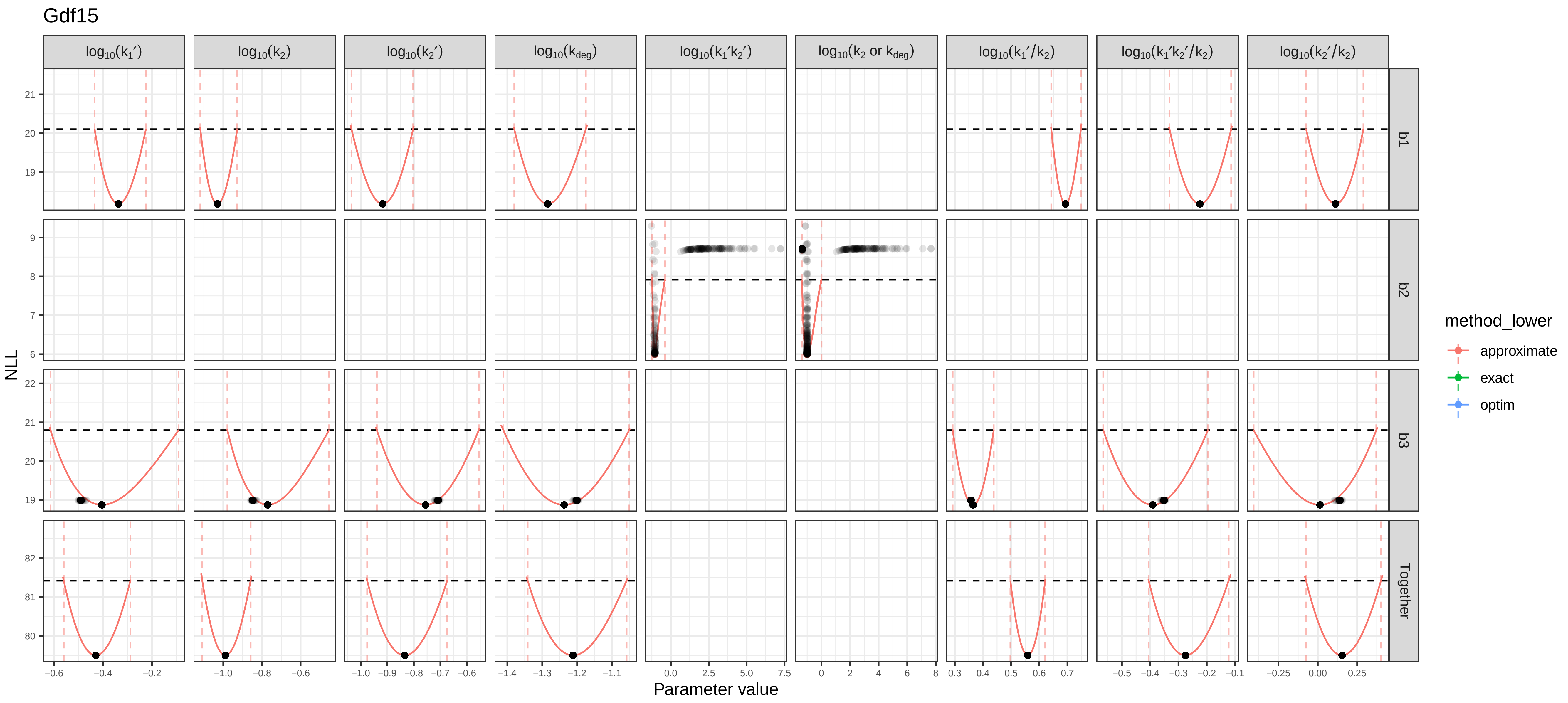

Replicate	Par	Best value	CI95 LB	CI95 UB	Method LB	Method UB
Together	$\log_{10}(k_1')$	−0.4298	−0.5606	−0.2886	approximate	approximate
Together	$\log_{10}(k_2)$	−0.989	−1.109	−0.8586	approximate	approximate
Together	$\log_{10}(k_2')$	−0.8345	−0.9757	−0.6742	approximate	approximate
Together	$\log_{10}(k_{deg})$	−1.212	−1.342	−1.058	approximate	approximate
Together	$\log_{10}(k_1'/k_2)$	0.5591	0.4974	0.6211	approximate	approximate
Together	$\log_{10}(k_1'k_2'/k_2)$	−0.2754	−0.4054	−0.1226	approximate	approximate
Together	$\log_{10}(k_2'/k_2)$	0.1544	−0.07462	0.4014	approximate	approximate
b1	$\log_{10}(k_1')$	−0.3372	−0.4346	−0.2251	approximate	approximate
b1	$\log_{10}(k_2)$	−1.03	−1.119	−0.9277	approximate	approximate
b1	$\log_{10}(k_2')$	−0.9172	−1.035	−0.8028	approximate	approximate
b1	$\log_{10}(k_{deg})$	−1.284	−1.38	−1.175	approximate	approximate
b1	$\log_{10}(k_1'/k_2)$	0.6928	0.6428	0.7477	approximate	approximate
b1	$\log_{10}(k_1'k_2'/k_2)$	−0.2243	−0.3318	−0.1136	approximate	approximate
b1	$\log_{10}(k_2'/k_2)$	0.1129	−0.07399	0.2898	approximate	approximate
b2	$\log_{10}(k_1'k_2')$	−1.059	−1.232	−0.3864	approximate	approximate
b2	$\log_{10}(k_2 \text{ or } k_{deg})$	−0.9791	−1.354	0.007829	approximate	approximate
b2	$\log_{10}(k_2 \text{ or } k_{deg})$	−1.017	−1.354	0.007829	approximate	approximate
b3	$\log_{10}(k_1')$	−0.4049	−0.6145	−0.09198	approximate	approximate
b3	$\log_{10}(k_2)$	−0.7698	−0.9787	−0.4532	approximate	approximate
b3	$\log_{10}(k_2')$	−0.7556	−0.9393	−0.5554	approximate	approximate
b3	$\log_{10}(k_{deg})$	−1.237	−1.411	−1.051	approximate	approximate
b3	$\log_{10}(k_1'/k_2)$	0.3649	0.2924	0.4382	approximate	approximate
b3	$\log_{10}(k_1'k_2'/k_2)$	−0.3907	−0.5659	−0.1957	approximate	approximate
b3	$\log_{10}(k_2'/k_2)$	0.01418	−0.4075	0.3715	approximate	approximate

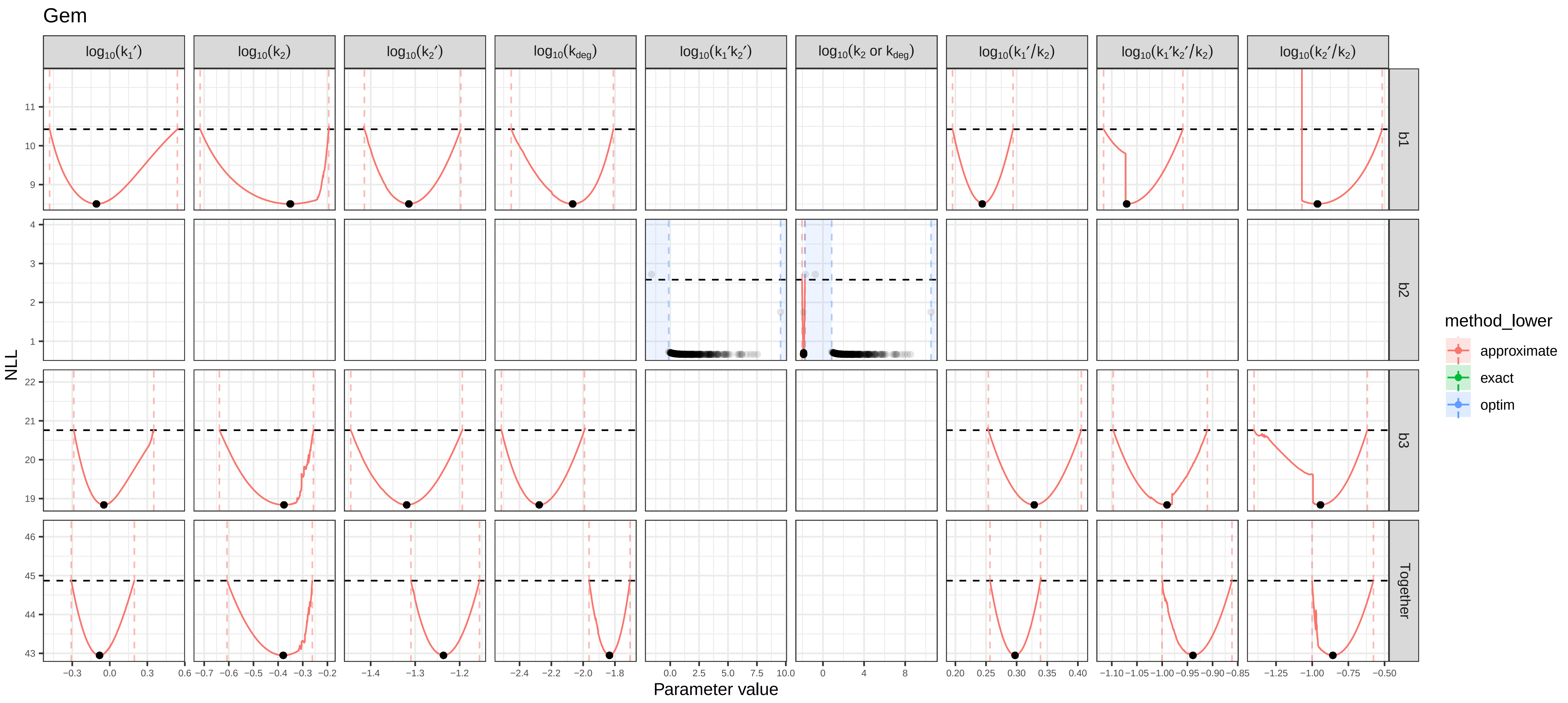

Replicate	Par	Best value	CI95 LB	CI95 UB	Method LB	Method UB
Together	$\log_{10}(k_1')$	−0.08125	−0.308	0.1965	approximate	approximate
Together	$\log_{10}(k_2)$	−0.3786	−0.6074	−0.2611	approximate	approximate
Together	$\log_{10}(k_2')$	−1.236	−1.31	−1.155	approximate	approximate
Together	$\log_{10}(k_{deg})$	−1.835	−1.963	−1.705	approximate	approximate
Together	$\log_{10}(k_1'/k_2)$	0.2974	0.2565	0.339	approximate	approximate
Together	$\log_{10}(k_1'k_2'/k_2)$	−0.939	−1	−0.8615	approximate	approximate
Together	$\log_{10}(k_2'/k_2)$	−0.8578	−1.001	−0.577	approximate	approximate
b1	$\log_{10}(k_1')$	−0.107	−0.4809	0.5404	approximate	approximate
b1	$\log_{10}(k_2)$	−0.3508	−0.7165	−0.1943	approximate	approximate
b1	$\log_{10}(k_2')$	−1.314	−1.414	−1.198	approximate	approximate
b1	$\log_{10}(k_{deg})$	−2.066	−2.453	−1.81	approximate	approximate
b1	$\log_{10}(k_1'/k_2)$	0.2438	0.1953	0.2942	approximate	approximate
b1	$\log_{10}(k_1'k_2'/k_2)$	−1.07	−1.116	−0.9589	approximate	approximate
b1	$\log_{10}(k_2'/k_2)$	−0.9634	−1.071	−0.5161	approximate	approximate
b2	$\log_{10}(k_1'k_2')$	3.36	< −0.1261	> 9.543	optim	optim
b2	$\log_{10}(k_2 \text{ or } k_{deg})$	4.341	0.8516	> 10.52	optim	optim
b2	$\log_{10}(k_2 \text{ or } k_{deg})$	−1.883	−2.035	−1.752	approximate	approximate
b3	$\log_{10}(k_1')$	−0.04732	−0.2878	0.3518	approximate	approximate
b3	$\log_{10}(k_2)$	−0.376	−0.6385	−0.2567	approximate	approximate
b3	$\log_{10}(k_2')$	−1.319	−1.445	−1.194	approximate	approximate
b3	$\log_{10}(k_{deg})$	−2.276	−2.514	−1.992	approximate	approximate
b3	$\log_{10}(k_1'/k_2)$	0.3287	0.2538	0.4056	approximate	approximate
b3	$\log_{10}(k_1'k_2'/k_2)$	−0.9904	−1.097	−0.9103	approximate	approximate
b3	$\log_{10}(k_2'/k_2)$	−0.9431	−1.402	−0.6193	approximate	approximate

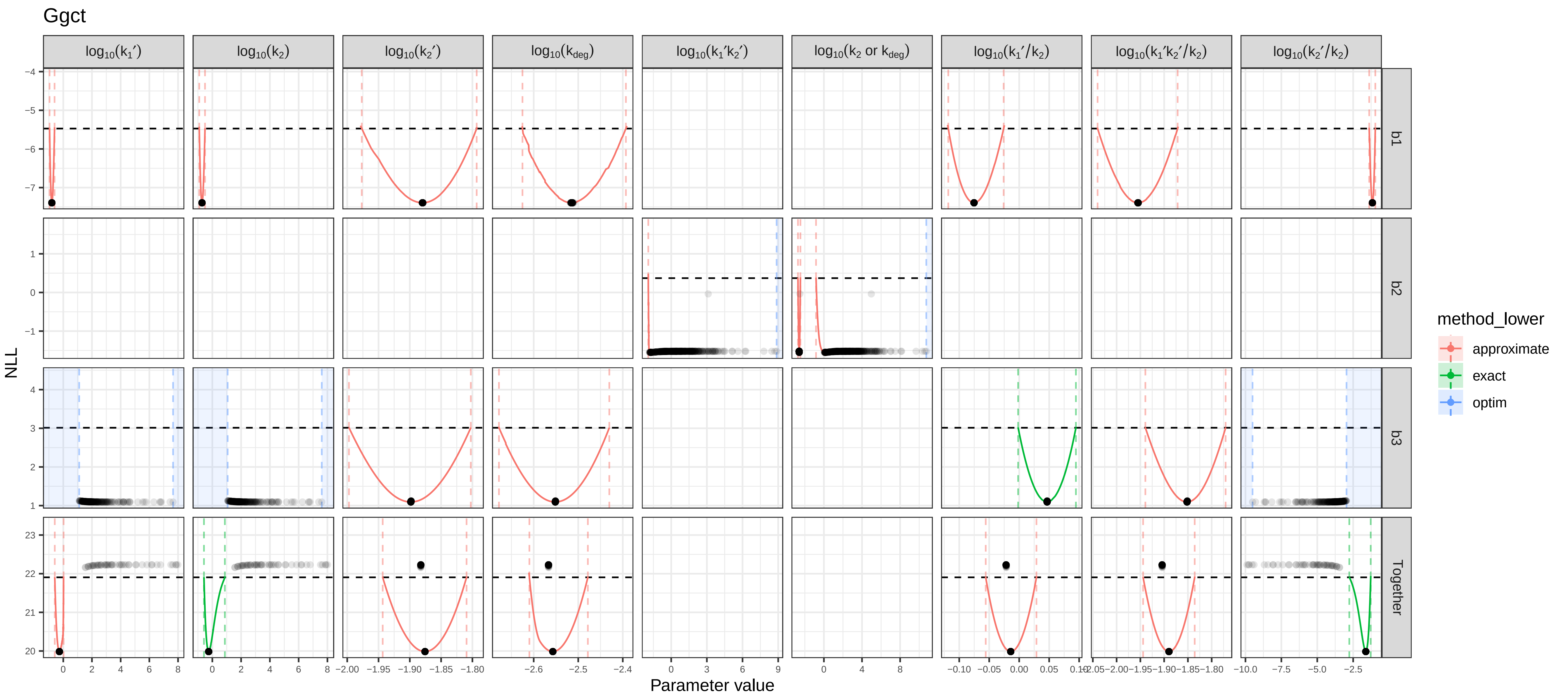

Replicate	Par	Best value	CI95 LB	CI95 UB	Method LB	Method UB
Together	$\log_{10}(k_1')$	−0.2689	−0.5909	0.02311	approximate	approximate
Together	$\log_{10}(k_2)$	−0.2547	−0.5835	0.8722	exact	exact
Together	$\log_{10}(k_2')$	−1.876	−1.943	−1.809	approximate	approximate
Together	$\log_{10}(k_{deg})$	−2.557	−2.61	−2.478	approximate	approximate
Together	$\log_{10}(k_1'/k_2)$	−0.01411	−0.05582	0.02878	approximate	approximate
Together	$\log_{10}(k_1'k_2'/k_2)$	−1.89	−1.944	−1.836	approximate	approximate
Together	$\log_{10}(k_2'/k_2)$	−1.621	−2.769	−1.278	exact	exact
b1	$\log_{10}(k_1')$	−0.795	−0.9549	−0.6067	approximate	approximate
b1	$\log_{10}(k_2)$	−0.7197	−0.9091	−0.5097	approximate	approximate
b1	$\log_{10}(k_2')$	−1.88	−1.977	−1.793	approximate	approximate
b1	$\log_{10}(k_{deg})$	−2.514	−2.625	−2.393	approximate	approximate
b1	$\log_{10}(k_1'/k_2)$	−0.07532	−0.1183	−0.02589	approximate	approximate
b1	$\log_{10}(k_1'k_2'/k_2)$	−1.955	−2.04	−1.872	approximate	approximate
b1	$\log_{10}(k_2'/k_2)$	−1.16	−1.383	−0.9568	approximate	approximate
b2	$\log_{10}(k_1'k_2')$	−1.705	−1.927	> 8.866	approximate	optim
b2	$\log_{10}(k_2 \text{ or } k_{deg})$	0.1675	−0.8254	> 10.74	approximate	optim
b2	$\log_{10}(k_2 \text{ or } k_{deg})$	−2.594	−2.721	−2.464	approximate	approximate
b3	$\log_{10}(k_1')$	4.665	< 1.112	> 7.654	optim	optim
b3	$\log_{10}(k_2)$	4.619	< 1.065	> 7.607	optim	optim
b3	$\log_{10}(k_2')$	−1.898	−1.997	−1.803	approximate	approximate
b3	$\log_{10}(k_{deg})$	−2.551	−2.678	−2.43	approximate	approximate
b3	$\log_{10}(k_1'/k_2)$	0.04654	−0.001738	0.09463	exact	exact
b3	$\log_{10}(k_1'k_2'/k_2)$	−1.852	−1.939	−1.771	approximate	approximate
b3	$\log_{10}(k_2'/k_2)$	−6.517	< −9.506	> −2.963	optim	optim

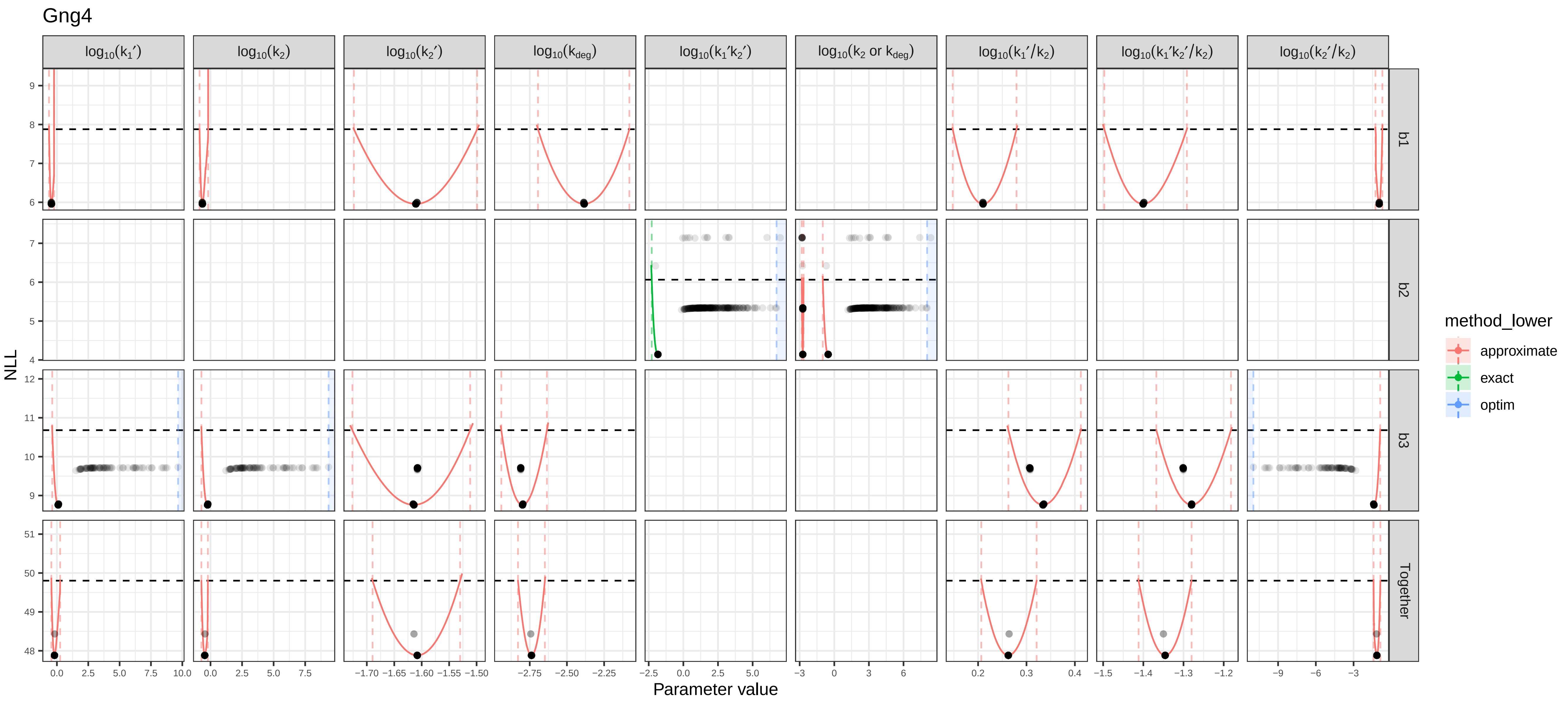

Replicate	Par	Best value	CI95 LB	CI95 UB	Method LB	Method UB
Together	$\log_{10}(k_1')$	−0.1951	−0.442	0.2601	approximate	approximate
Together	$\log_{10}(k_2)$	−0.4576	−0.7231	−0.2073	approximate	approximate
Together	$\log_{10}(k_2')$	−1.608	−1.69	−1.53	approximate	approximate
Together	$\log_{10}(k_{deg})$	−2.738	−2.829	−2.648	approximate	approximate
Together	$\log_{10}(k_1'/k_2)$	0.2624	0.2066	0.321	approximate	approximate
Together	$\log_{10}(k_1'k_2'/k_2)$	−1.346	−1.412	−1.28	approximate	approximate
Together	$\log_{10}(k_2'/k_2)$	−1.151	−1.41	−0.8692	approximate	approximate
b1	$\log_{10}(k_1')$	−0.4352	−0.6282	−0.2278	approximate	approximate
b1	$\log_{10}(k_2)$	−0.6454	−0.8692	−0.1875	approximate	approximate
b1	$\log_{10}(k_2')$	−1.611	−1.723	−1.499	approximate	approximate
b1	$\log_{10}(k_{deg})$	−2.384	−2.695	−2.08	approximate	approximate
b1	$\log_{10}(k_1'/k_2)$	0.2102	0.1476	0.2794	approximate	approximate
b1	$\log_{10}(k_1'k_2'/k_2)$	−1.4	−1.497	−1.292	approximate	approximate
b1	$\log_{10}(k_2'/k_2)$	−0.9653	−1.261	−0.7112	approximate	approximate
b2	$\log_{10}(k_1'k_2')$	−1.837	−2.282	> 6.726	exact	optim
b2	$\log_{10}(k_2 \text{ or } k_{deg})$	−0.5303	−0.9961	> 8.042	approximate	optim
b2	$\log_{10}(k_2 \text{ or } k_{deg})$	−2.732	−2.819	−2.661	approximate	approximate
b3	$\log_{10}(k_1')$	0.1014	−0.3731	> 9.659	approximate	optim
b3	$\log_{10}(k_2)$	−0.2329	−0.7175	> 9.356	approximate	optim
b3	$\log_{10}(k_2')$	−1.614	−1.726	−1.512	approximate	approximate
b3	$\log_{10}(k_{deg})$	−2.798	−2.941	−2.635	approximate	approximate
b3	$\log_{10}(k_1'/k_2)$	0.3343	0.2627	0.4124	approximate	approximate
b3	$\log_{10}(k_1'k_2'/k_2)$	−1.28	−1.368	−1.182	approximate	approximate
b3	$\log_{10}(k_2'/k_2)$	−1.381	< −10.96	−0.8858	optim	approximate

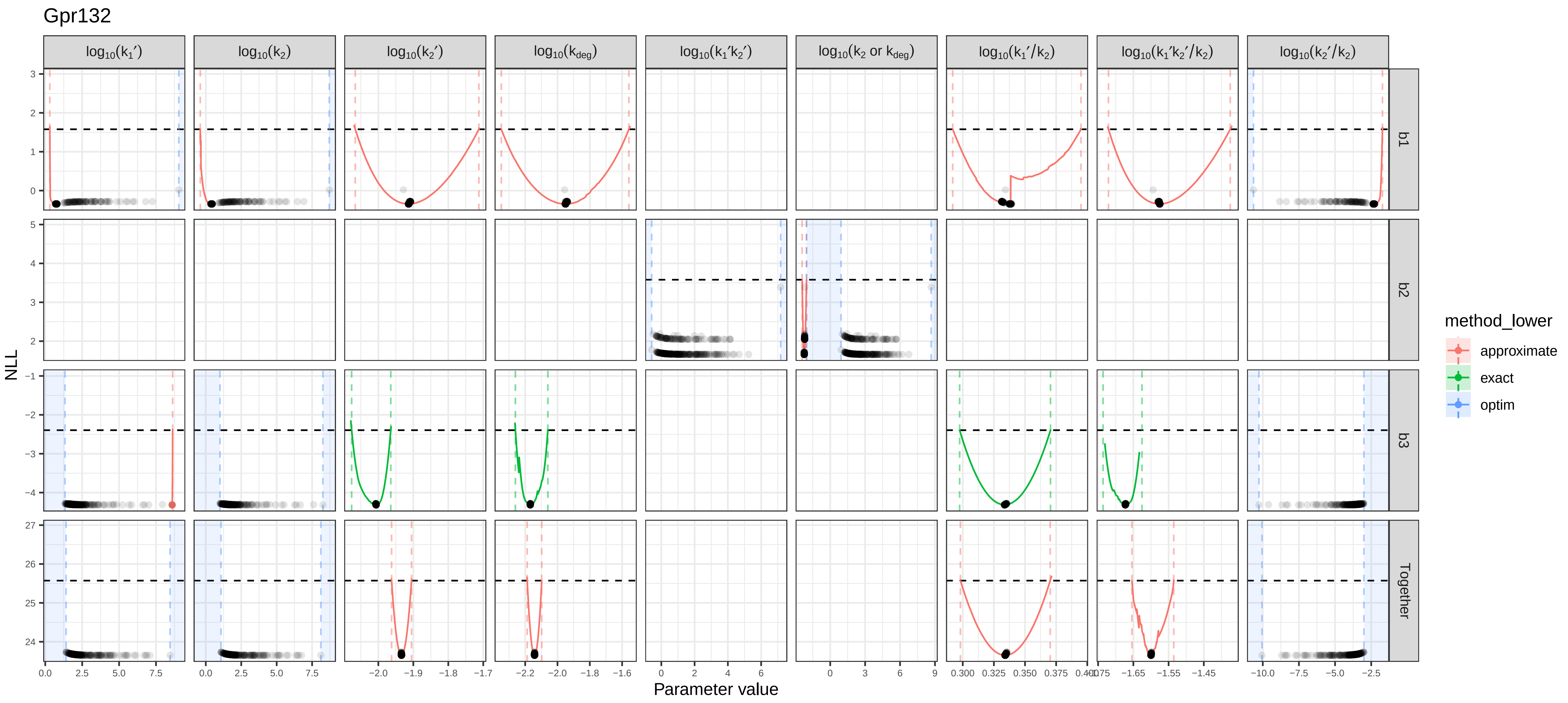

Replicate	Par	Best value	CI95 LB	CI95 UB	Method LB	Method UB
Together	$\log_{10}(k_1')$	6.689	< 1.408	> 8.452	optim	optim
Together	$\log_{10}(k_2)$	6.355	< 1.073	> 8.118	optim	optim
Together	$\log_{10}(k_2')$	−1.934	−1.962	−1.905	approximate	approximate
Together	$\log_{10}(k_{deg})$	−2.142	−2.188	−2.098	approximate	approximate
Together	$\log_{10}(k_1'/k_2)$	0.3339	0.2981	0.3702	approximate	approximate
Together	$\log_{10}(k_1'k_2'/k_2)$	−1.6	−1.654	−1.535	approximate	approximate
Together	$\log_{10}(k_2'/k_2)$	−8.289	< −10.05	> −3.007	optim	optim
b1	$\log_{10}(k_1')$	0.7402	0.308	> 9.044	approximate	optim
b1	$\log_{10}(k_2)$	0.4021	−0.3888	> 8.709	approximate	optim
b1	$\log_{10}(k_2')$	−1.913	−2.066	−1.713	approximate	approximate
b1	$\log_{10}(k_{deg})$	−1.95	−2.348	−1.558	approximate	approximate
b1	$\log_{10}(k_1'/k_2)$	0.3381	0.292	0.395	approximate	approximate
b1	$\log_{10}(k_1'k_2'/k_2)$	−1.575	−1.721	−1.375	approximate	approximate
b1	$\log_{10}(k_2'/k_2)$	−2.315	< −10.64	−1.721	optim	approximate
b2	$\log_{10}(k_1'k_2')$	3.471	< −0.5792	> 7.174	optim	optim
b2	$\log_{10}(k_2 \text{ or } k_{deg})$	4.981	0.9306	> 8.671	optim	optim
b2	$\log_{10}(k_2 \text{ or } k_{deg})$	−2.232	−2.411	−2.035	approximate	approximate
b3	$\log_{10}(k_1')$	8.594	< 1.337	8.628	optim	approximate
b3	$\log_{10}(k_2)$	8.261	< 1.002	> 8.261	optim	optim
b3	$\log_{10}(k_2')$	−2.006	−2.076	−1.964	exact	exact
b3	$\log_{10}(k_{deg})$	−2.169	−2.261	−2.06	exact	exact
b3	$\log_{10}(k_1'/k_2)$	0.3336	0.2975	0.3705	exact	exact
b3	$\log_{10}(k_1'k_2'/k_2)$	−1.673	−1.736	−1.625	exact	exact
b3	$\log_{10}(k_2'/k_2)$	−10.27	< −10.27	> −3.009	optim	optim

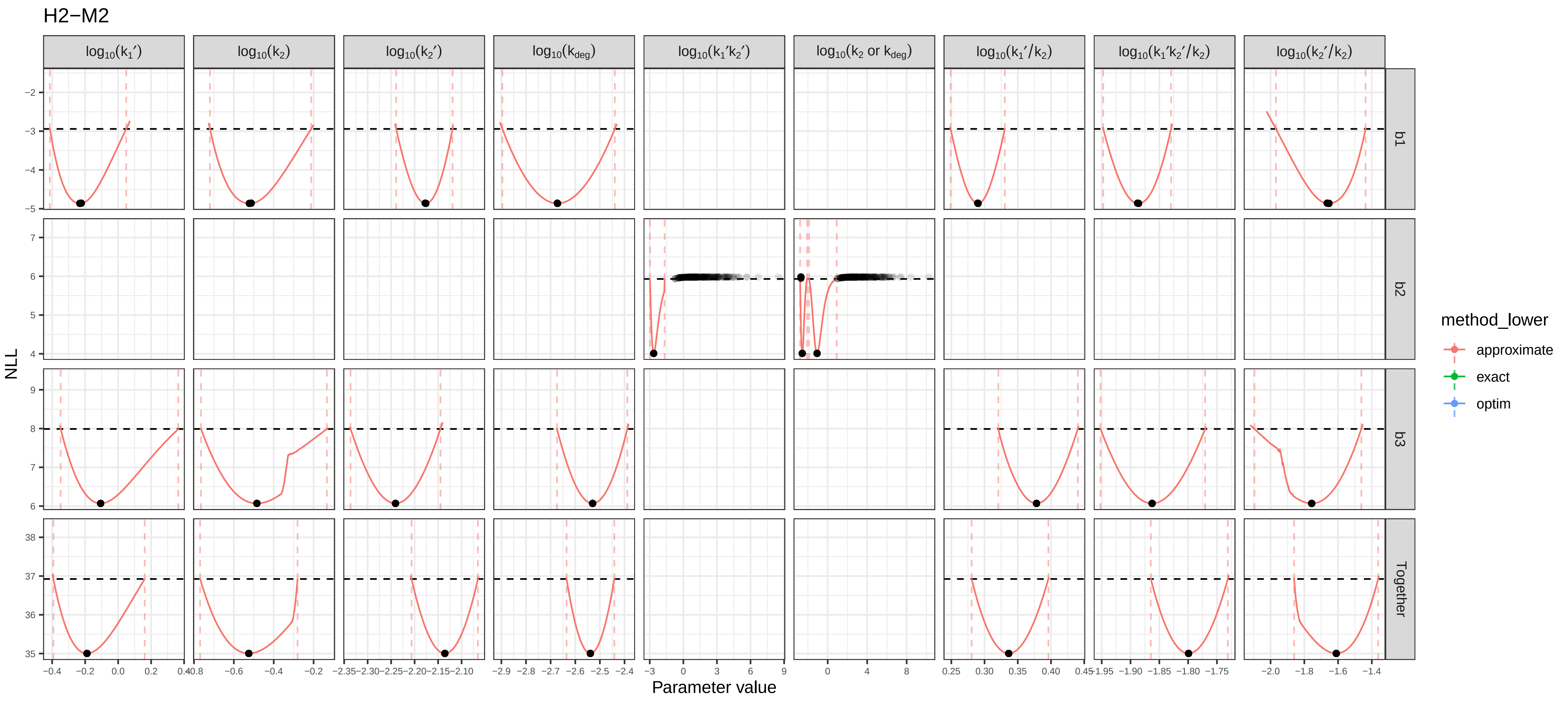

Replicate	Par	Best value	CI95 LB	CI95 UB	Method LB	Method UB
Together	$\log_{10}(k_1')$	−0.1888	−0.3924	0.161	approximate	approximate
Together	$\log_{10}(k_2)$	−0.5252	−0.7692	−0.2805	approximate	approximate
Together	$\log_{10}(k_2')$	−2.136	−2.207	−2.065	approximate	approximate
Together	$\log_{10}(k_{deg})$	−2.539	−2.635	−2.441	approximate	approximate
Together	$\log_{10}(k_1'/k_2)$	0.3364	0.2806	0.396	approximate	approximate
Together	$\log_{10}(k_1'k_2'/k_2)$	−1.799	−1.865	−1.731	approximate	approximate
Together	$\log_{10}(k_2'/k_2)$	−1.61	−1.86	−1.362	approximate	approximate
b1	$\log_{10}(k_1')$	−0.2321	−0.4138	0.04918	approximate	approximate
b1	$\log_{10}(k_2)$	−0.5223	−0.72	−0.2122	approximate	approximate
b1	$\log_{10}(k_2')$	−2.176	−2.239	−2.119	approximate	approximate
b1	$\log_{10}(k_{deg})$	−2.672	−2.896	−2.439	approximate	approximate
b1	$\log_{10}(k_1'/k_2)$	0.2902	0.2489	0.3304	approximate	approximate
b1	$\log_{10}(k_1'k_2'/k_2)$	−1.886	−1.947	−1.83	approximate	approximate
b1	$\log_{10}(k_2'/k_2)$	−1.654	−1.968	−1.437	approximate	approximate
b2	$\log_{10}(k_1'k_2')$	−2.647	−2.979	−1.673	approximate	approximate
b2	$\log_{10}(k_2 \text{ or } k_{deg})$	−1.078	−1.884	0.9158	approximate	approximate
b2	$\log_{10}(k_2 \text{ or } k_{deg})$	−2.576	−2.793	−2.089	approximate	approximate
b3	$\log_{10}(k_1')$	−0.1061	−0.3484	0.3646	approximate	approximate
b3	$\log_{10}(k_2)$	−0.4843	−0.7649	−0.1334	approximate	approximate
b3	$\log_{10}(k_2')$	−2.241	−2.336	−2.145	approximate	approximate
b3	$\log_{10}(k_{deg})$	−2.53	−2.674	−2.389	approximate	approximate
b3	$\log_{10}(k_1'/k_2)$	0.3782	0.3207	0.4404	approximate	approximate
b3	$\log_{10}(k_1'k_2'/k_2)$	−1.863	−1.952	−1.77	approximate	approximate
b3	$\log_{10}(k_2'/k_2)$	−1.756	−2.096	−1.462	approximate	approximate

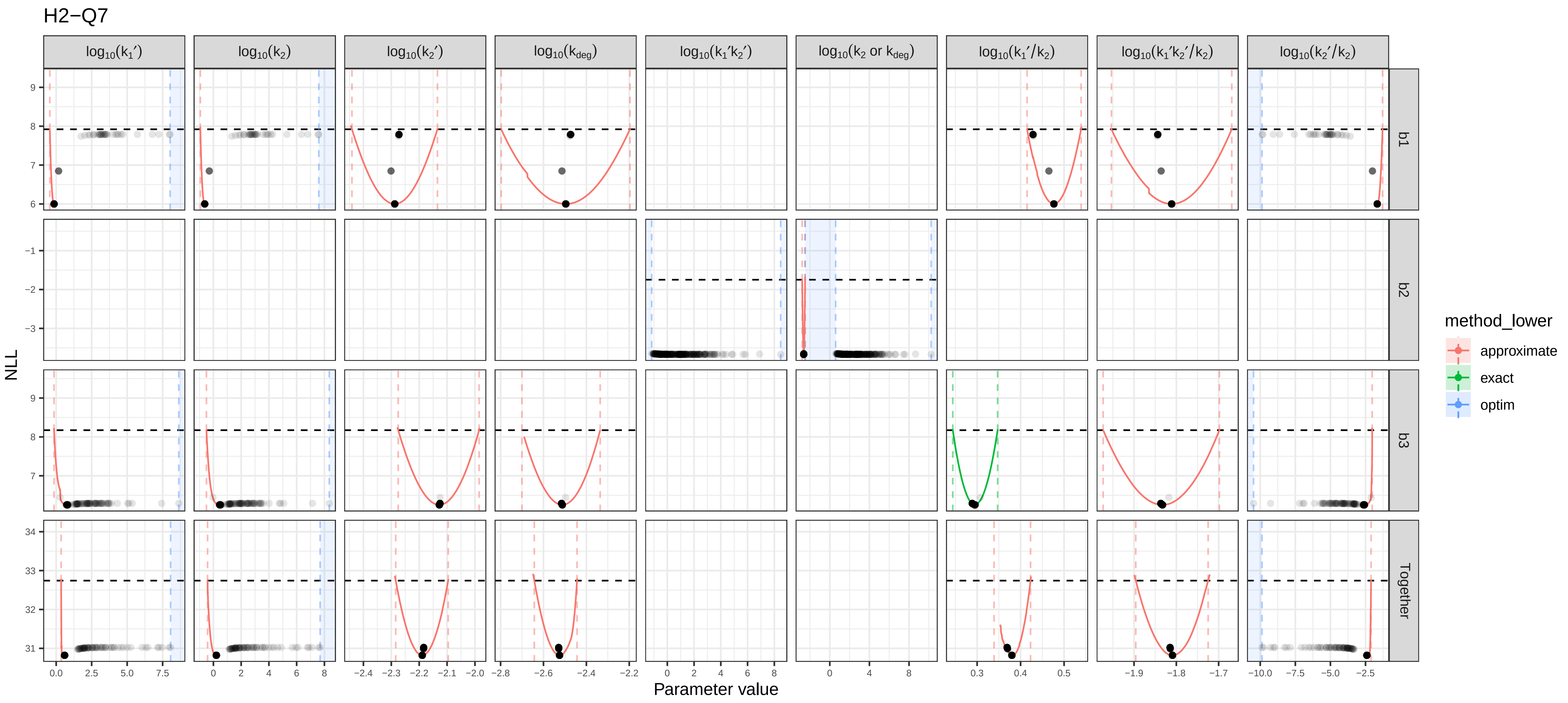

Replicate	Par	Best value	CI95 LB	CI95 UB	Method LB	Method UB
Together	$\log_{10}(k_1')$	0.5926	0.3482	> 8.061	approximate	optim
Together	$\log_{10}(k_2)$	0.2127	−0.4204	> 7.692	approximate	optim
Together	$\log_{10}(k_2')$	−2.189	−2.284	−2.096	approximate	approximate
Together	$\log_{10}(k_{deg})$	−2.525	−2.643	−2.443	approximate	approximate
Together	$\log_{10}(k_1'/k_2)$	0.38	0.3389	0.4229	approximate	approximate
Together	$\log_{10}(k_1'k_2'/k_2)$	−1.809	−1.896	−1.725	approximate	approximate
Together	$\log_{10}(k_2'/k_2)$	−2.402	< −9.876	−2.105	optim	approximate
b1	$\log_{10}(k_1')$	−0.1491	−0.4479	> 8.027	approximate	optim
b1	$\log_{10}(k_2)$	−0.6256	−0.942	> 7.598	approximate	optim
b1	$\log_{10}(k_2')$	−2.288	−2.441	−2.135	approximate	approximate
b1	$\log_{10}(k_{deg})$	−2.496	−2.797	−2.197	approximate	approximate
b1	$\log_{10}(k_1'/k_2)$	0.4765	0.4149	0.5392	approximate	approximate
b1	$\log_{10}(k_1'k_2'/k_2)$	−1.811	−1.953	−1.669	approximate	approximate
b1	$\log_{10}(k_2'/k_2)$	−1.662	< −9.871	−1.288	optim	approximate
b2	$\log_{10}(k_1'k_2')$	2.228	< −1.168	> 8.475	optim	optim
b2	$\log_{10}(k_2 \text{ or } k_{deg})$	3.988	0.5895	> 10.23	optim	optim
b2	$\log_{10}(k_2 \text{ or } k_{deg})$	−2.633	−2.792	−2.497	approximate	approximate
b3	$\log_{10}(k_1')$	0.7793	−0.1506	> 8.638	approximate	optim
b3	$\log_{10}(k_2)$	0.4845	−0.502	> 8.35	approximate	optim
b3	$\log_{10}(k_2')$	−2.128	−2.275	−1.985	approximate	approximate
b3	$\log_{10}(k_{deg})$	−2.512	−2.701	−2.336	approximate	approximate
b3	$\log_{10}(k_1'/k_2)$	0.2948	0.2441	0.3472	exact	exact
b3	$\log_{10}(k_1'k_2'/k_2)$	−1.833	−1.973	−1.698	approximate	approximate
b3	$\log_{10}(k_2'/k_2)$	−2.612	< −10.48	−2.025	optim	approximate

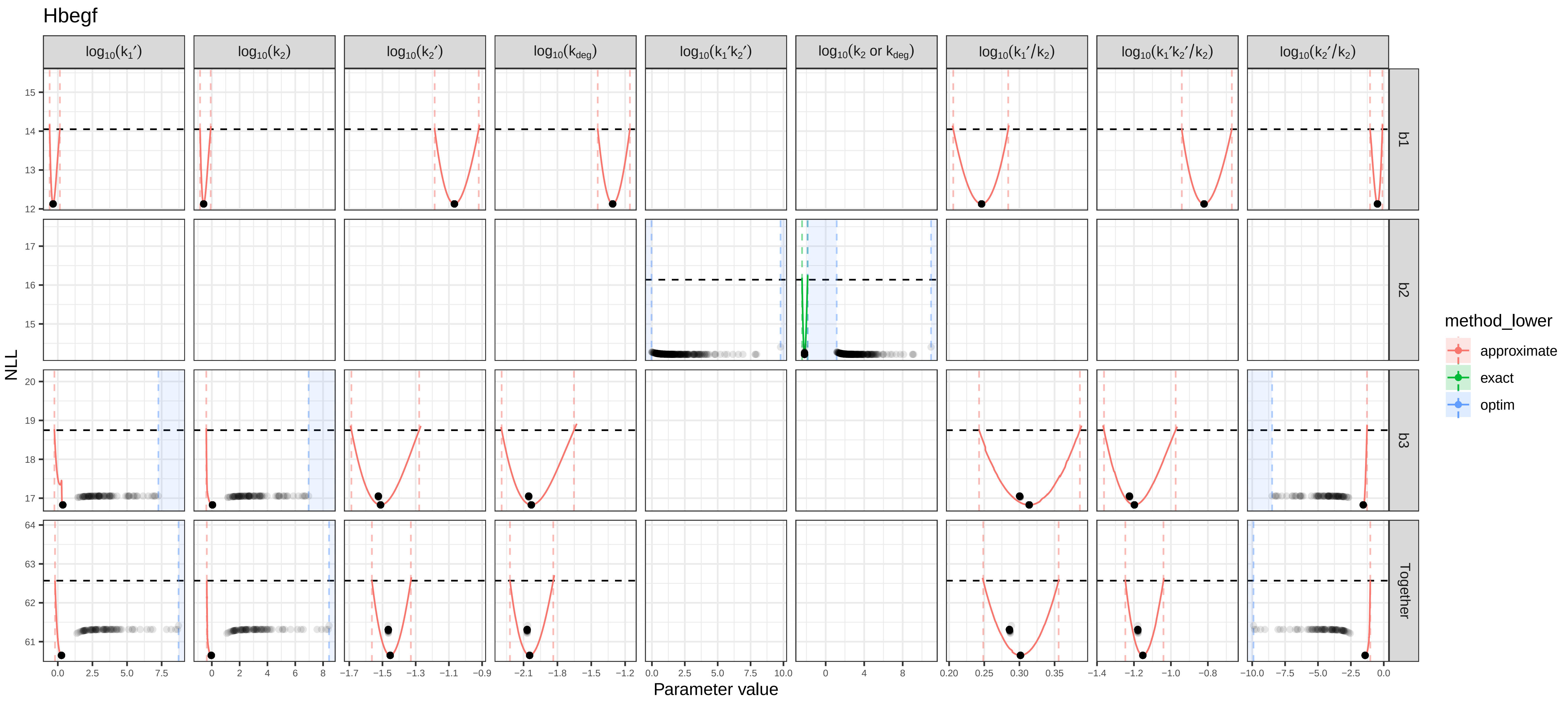

Replicate	Par	**Best value**	**CI95 LB**	**CI95 UB**	**Method LB**	**Method UB**
Together	$\log_{10}(k_1')$	0.2689	−0.2016	> 8.72	approximate	optim
Together	$\log_{10}(k_2)$	−0.03247	−0.3618	> 8.431	approximate	optim
Together	$\log_{10}(k_2')$	−1.454	−1.564	−1.329	approximate	approximate
Together	$\log_{10}(k_{deg})$	−2.047	−2.221	−1.837	approximate	approximate
Together	$\log_{10}(k_1'/k_2)$	0.3014	0.2486	0.3558	approximate	approximate
Together	$\log_{10}(k_1'k_2'/k_2)$	−1.152	−1.247	−1.04	approximate	approximate
Together	$\log_{10}(k_2'/k_2)$	−1.421	< −9.899	−1.024	optim	approximate
b1	$\log_{10}(k_1')$	−0.3414	−0.5897	0.1522	approximate	approximate
b1	$\log_{10}(k_2)$	−0.5876	−0.8453	−0.08101	approximate	approximate
b1	$\log_{10}(k_2')$	−1.067	−1.186	−0.9204	approximate	approximate
b1	$\log_{10}(k_{deg})$	−1.31	−1.443	−1.157	approximate	approximate
b1	$\log_{10}(k_1'/k_2)$	0.2462	0.2059	0.2841	approximate	approximate
b1	$\log_{10}(k_1'k_2'/k_2)$	−0.8206	−0.9411	−0.6703	approximate	approximate
b1	$\log_{10}(k_2'/k_2)$	−0.4792	−1.044	−0.1051	approximate	approximate
b2	$\log_{10}(k_1'k_2')$	6.919	< −0.02999	> 9.779	optim	optim
b2	$\log_{10}(k_2 \text{ or } k_{deg})$	8.094	1.144	> 10.95	optim	optim
b2	$\log_{10}(k_2 \text{ or } k_{deg})$	−2.199	−2.454	−1.88	exact	exact
b3	$\log_{10}(k_1')$	0.3605	−0.2462	> 7.261	approximate	optim
b3	$\log_{10}(k_2)$	0.04649	−0.3979	> 6.961	approximate	optim
b3	$\log_{10}(k_2')$	−1.511	−1.687	−1.279	approximate	approximate
b3	$\log_{10}(k_{deg})$	−2.031	−2.294	−1.653	approximate	approximate
b3	$\log_{10}(k_1'/k_2)$	0.314	0.2427	0.386	approximate	approximate
b3	$\log_{10}(k_1'k_2'/k_2)$	−1.197	−1.362	−0.9743	approximate	approximate
b3	$\log_{10}(k_2'/k_2)$	−1.558	< −8.485	−1.276	optim	approximate

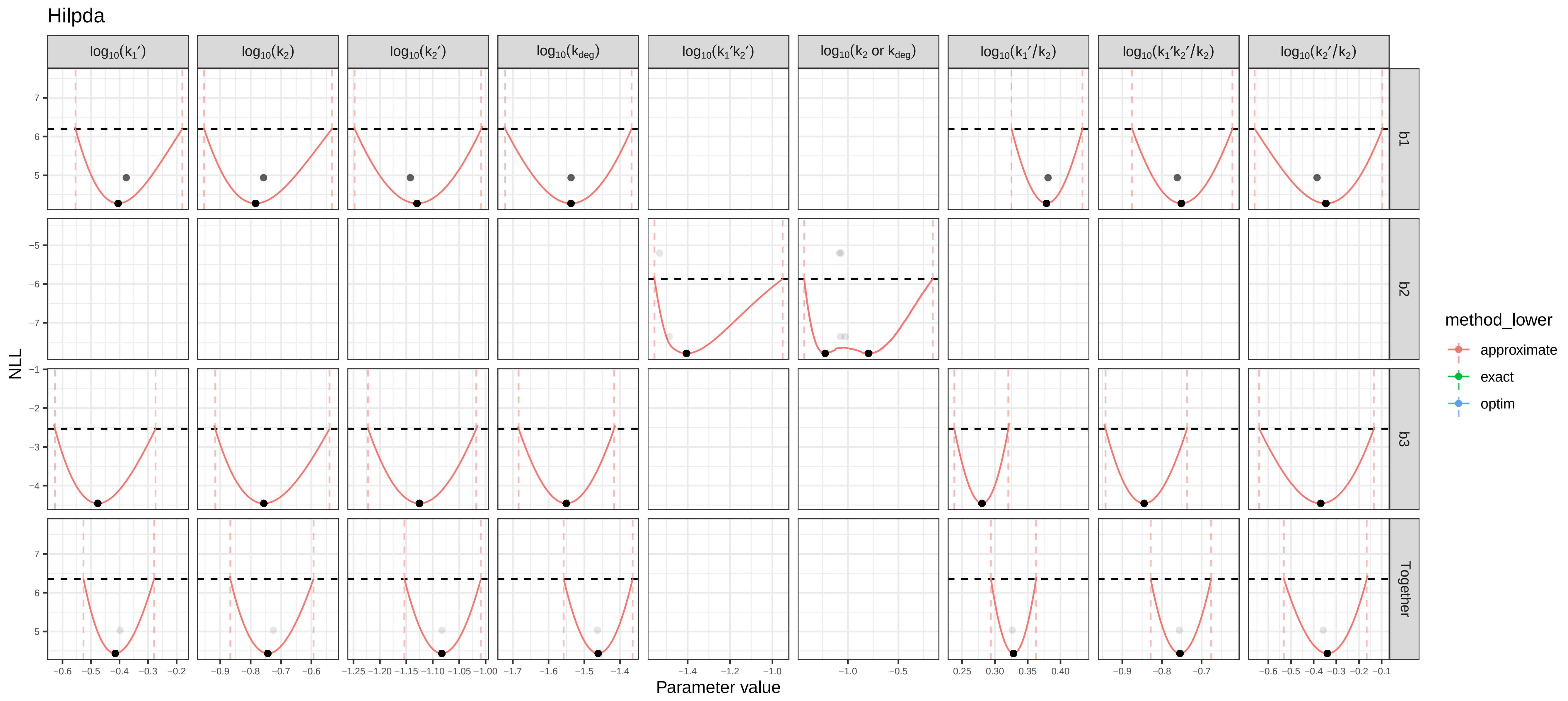

Replicate	Par	Best value	CI95 LB	CI95 UB	Method LB	Method UB
Together	$\log_{10}(k_1')$	−0.415	−0.5265	−0.2789	approximate	approximate
Together	$\log_{10}(k_2)$	−0.7432	−0.867	−0.5931	approximate	approximate
Together	$\log_{10}(k_2')$	−1.083	−1.154	−1.009	approximate	approximate
Together	$\log_{10}(k_{deg})$	−1.461	−1.558	−1.365	approximate	approximate
Together	$\log_{10}(k_1'/k_2)$	0.3282	0.2938	0.3627	approximate	approximate
Together	$\log_{10}(k_1'k_2'/k_2)$	−0.7545	−0.8286	−0.6758	approximate	approximate
Together	$\log_{10}(k_2'/k_2)$	−0.3395	−0.5317	−0.1658	approximate	approximate
b1	$\log_{10}(k_1')$	−0.4052	−0.5546	−0.1801	approximate	approximate
b1	$\log_{10}(k_2)$	−0.7838	−0.9532	−0.5325	approximate	approximate
b1	$\log_{10}(k_2')$	−1.13	−1.248	−1.008	approximate	approximate
b1	$\log_{10}(k_{deg})$	−1.537	−1.721	−1.368	approximate	approximate
b1	$\log_{10}(k_1'/k_2)$	0.3786	0.3252	0.4334	approximate	approximate
b1	$\log_{10}(k_1'k_2'/k_2)$	−0.7511	−0.8755	−0.6223	approximate	approximate
b1	$\log_{10}(k_2'/k_2)$	−0.3458	−0.6604	−0.09665	approximate	approximate
b2	$\log_{10}(k_1'k_2')$	−1.405	−1.557	−0.9518	approximate	approximate
b2	$\log_{10}(k_2 \text{ or } k_{deg})$	−0.7963	−1.433	−0.1596	approximate	approximate
b2	$\log_{10}(k_2 \text{ or } k_{deg})$	−1.224	−1.433	−0.1596	approximate	approximate
b3	$\log_{10}(k_1')$	−0.4763	−0.6265	−0.2741	approximate	approximate
b3	$\log_{10}(k_2)$	−0.7565	−0.9167	−0.5407	approximate	approximate
b3	$\log_{10}(k_2')$	−1.125	−1.222	−1.018	approximate	approximate
b3	$\log_{10}(k_{deg})$	−1.551	−1.684	−1.417	approximate	approximate
b3	$\log_{10}(k_1'/k_2)$	0.2803	0.2379	0.3203	approximate	approximate
b3	$\log_{10}(k_1'k_2'/k_2)$	−0.845	−0.9424	−0.7369	approximate	approximate
b3	$\log_{10}(k_2'/k_2)$	−0.3687	−0.6403	−0.1346	approximate	approximate

Hivep1

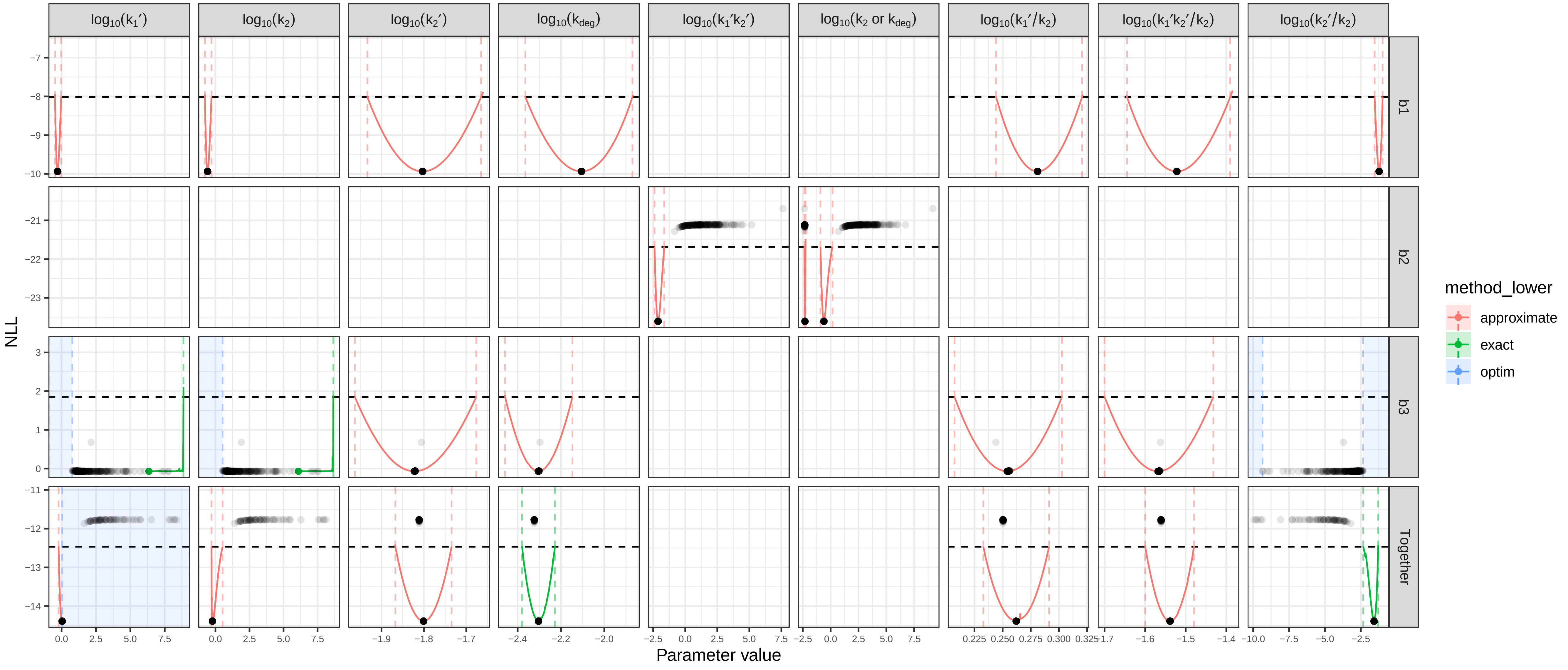

Replicate	Par	Best value	CI95 LB	CI95 UB	Method LB	Method UB
Together	$\log_{10}(k_1')$	0.04018	−0.2172	> 0.04274	approximate	optim
Together	$\log_{10}(k_2)$	−0.2219	−0.2897	0.5231	approximate	approximate
Together	$\log_{10}(k_2')$	−1.801	−1.867	−1.735	approximate	approximate
Together	$\log_{10}(k_{deg})$	−2.304	−2.38	−2.228	exact	exact
Together	$\log_{10}(k_1'/k_2)$	0.2621	0.2329	0.2913	approximate	approximate
Together	$\log_{10}(k_1'k_2'/k_2)$	−1.539	−1.6	−1.48	approximate	approximate
Together	$\log_{10}(k_2'/k_2)$	−1.579	−2.332	−1.294	exact	exact
b1	$\log_{10}(k_1')$	−0.2968	−0.4745	−0.02991	approximate	approximate
b1	$\log_{10}(k_2)$	−0.5779	−0.7709	−0.2898	approximate	approximate
b1	$\log_{10}(k_2')$	−1.803	−1.933	−1.665	approximate	approximate
b1	$\log_{10}(k_{deg})$	−2.105	−2.364	−1.87	approximate	approximate
b1	$\log_{10}(k_1'/k_2)$	0.2811	0.2442	0.3205	approximate	approximate
b1	$\log_{10}(k_1'k_2'/k_2)$	−1.522	−1.645	−1.39	approximate	approximate
b1	$\log_{10}(k_2'/k_2)$	−1.225	−1.54	−0.9857	approximate	approximate
b2	$\log_{10}(k_1'k_2')$	−2.118	−2.392	−1.64	approximate	approximate
b2	$\log_{10}(k_2 \text{ or } k_{deg})$	−0.6076	−0.9205	0.1644	approximate	approximate
b2	$\log_{10}(k_2 \text{ or } k_{deg})$	−2.308	−2.354	−2.254	approximate	approximate
b3	$\log_{10}(k_1')$	6.349	< 0.7801	8.863	optim	exact
b3	$\log_{10}(k_2)$	6.095	< 0.5235	8.656	optim	exact
b3	$\log_{10}(k_2')$	−1.822	−1.963	−1.676	approximate	approximate
b3	$\log_{10}(k_{deg})$	−2.305	−2.458	−2.147	approximate	approximate
b3	$\log_{10}(k_1'/k_2)$	0.2542	0.2072	0.3027	approximate	approximate
b3	$\log_{10}(k_1'k_2'/k_2)$	−1.568	−1.7	−1.432	approximate	approximate
b3	$\log_{10}(k_2'/k_2)$	−7.918	< −9.351	> −2.343	optim	optim

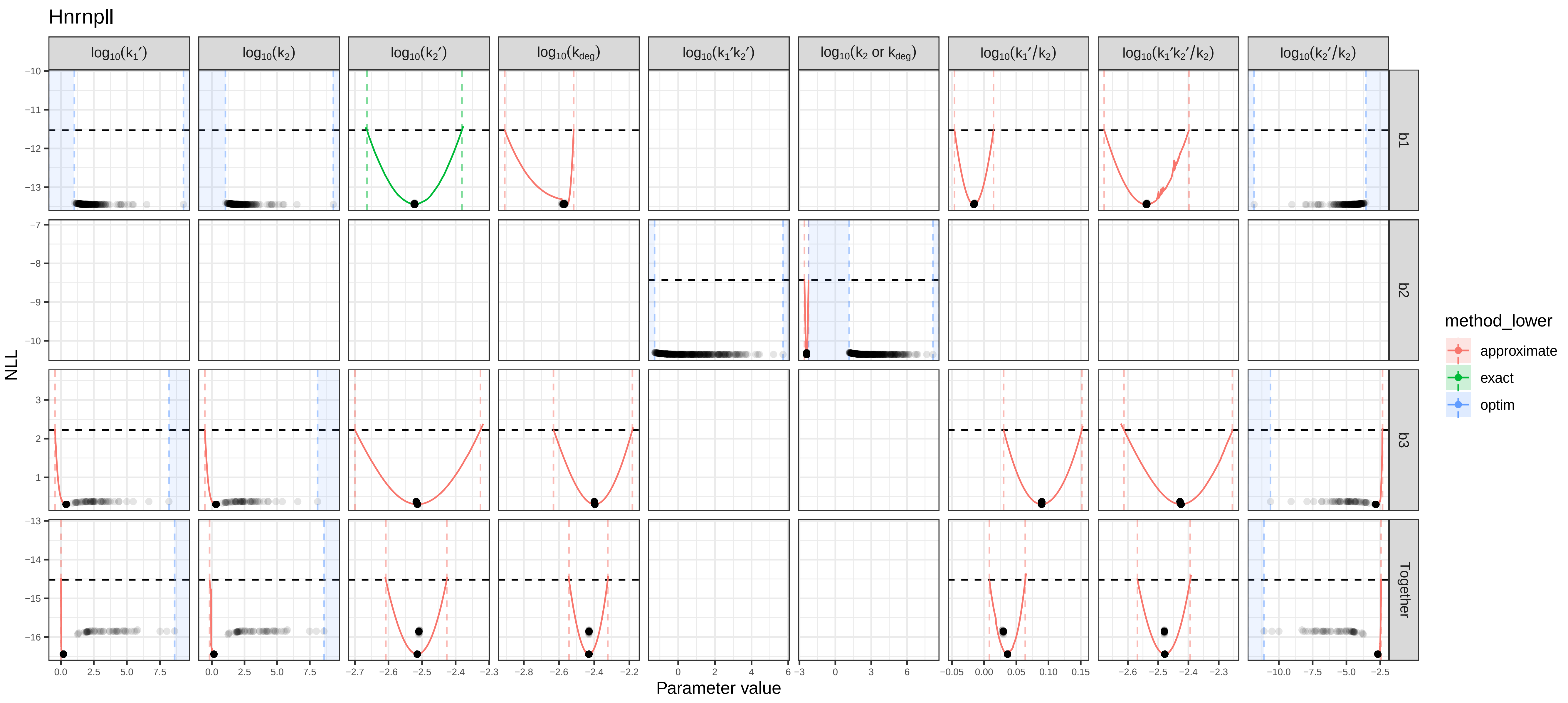

Replicate	Par	Best value	CI95 LB	CI95 UB	Method LB	Method UB
Together	$\log_{10}(k_1')$	0.1973	0.01687	> 8.617	approximate	optim
Together	$\log_{10}(k_2)$	0.161	−0.1772	> 8.587	approximate	optim
Together	$\log_{10}(k_2')$	−2.514	−2.608	−2.426	approximate	approximate
Together	$\log_{10}(k_{deg})$	−2.43	−2.543	−2.323	approximate	approximate
Together	$\log_{10}(k_1'/k_2)$	0.03636	0.008201	0.06395	approximate	approximate
Together	$\log_{10}(k_1'k_2'/k_2)$	−2.478	−2.568	−2.394	approximate	approximate
Together	$\log_{10}(k_2'/k_2)$	−2.675	< −11.1	−2.437	optim	approximate
b1	$\log_{10}(k_1')$	4.806	< 1.025	> 9.289	optim	optim
b1	$\log_{10}(k_2)$	4.822	< 1.039	> 9.304	optim	optim
b1	$\log_{10}(k_2')$	−2.522	−2.664	−2.381	exact	exact
b1	$\log_{10}(k_{deg})$	−2.572	−2.909	−2.517	approximate	approximate
b1	$\log_{10}(k_1'/k_2)$	−0.01595	−0.04596	0.01458	approximate	approximate
b1	$\log_{10}(k_1'k_2'/k_2)$	−2.538	−2.678	−2.398	approximate	approximate
b1	$\log_{10}(k_2'/k_2)$	−7.344	< −11.83	> −3.561	optim	optim
b2	$\log_{10}(k_1'k_2')$	3.194	< −1.288	> 5.715	optim	optim
b2	$\log_{10}(k_2 \text{ or } k_{deg})$	5.64	1.158	> 8.16	optim	optim
b2	$\log_{10}(k_2 \text{ or } k_{deg})$	−2.4	−2.584	−2.242	approximate	approximate
b3	$\log_{10}(k_1')$	0.4035	−0.439	> 8.188	approximate	optim
b3	$\log_{10}(k_2)$	0.3142	−0.5334	> 8.098	approximate	optim
b3	$\log_{10}(k_2')$	−2.514	−2.7	−2.326	approximate	approximate
b3	$\log_{10}(k_{deg})$	−2.397	−2.631	−2.183	approximate	approximate
b3	$\log_{10}(k_1'/k_2)$	0.08935	0.03028	0.1517	approximate	approximate
b3	$\log_{10}(k_1'k_2'/k_2)$	−2.424	−2.613	−2.255	approximate	approximate
b3	$\log_{10}(k_2'/k_2)$	−2.828	< −10.62	−2.33	optim	approximate

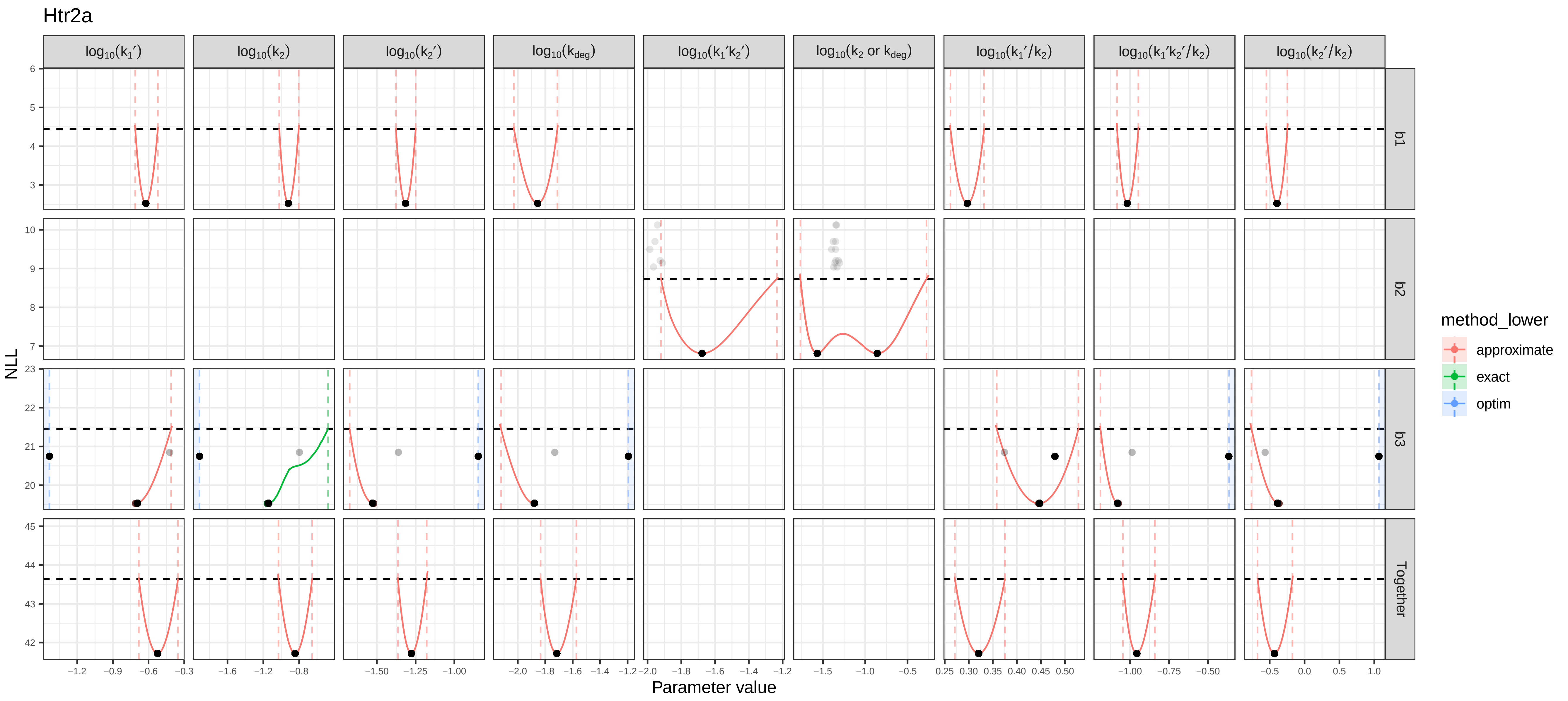

Replicate	Par	**Best value**	**CI95 LB**	**CI95 UB**	**Method LB**	**Method UB**
Together	$\log_{10}(k_1')$	−0.5244	−0.6821	−0.3523	approximate	approximate
Together	$\log_{10}(k_2)$	−0.845	−1.031	−0.6543	approximate	approximate
Together	$\log_{10}(k_2')$	−1.277	−1.365	−1.178	approximate	approximate
Together	$\log_{10}(k_{deg})$	−1.716	−1.834	−1.573	approximate	approximate
Together	$\log_{10}(k_1'/k_2)$	0.3205	0.271	0.3752	approximate	approximate
Together	$\log_{10}(k_1'k_2'/k_2)$	−0.9567	−1.047	−0.8409	approximate	approximate
Together	$\log_{10}(k_2'/k_2)$	−0.4323	−0.6736	−0.1739	approximate	approximate
b1	$\log_{10}(k_1')$	−0.623	−0.7123	−0.522	approximate	approximate
b1	$\log_{10}(k_2)$	−0.9201	−1.023	−0.8051	approximate	approximate
b1	$\log_{10}(k_2')$	−1.315	−1.377	−1.25	approximate	approximate
b1	$\log_{10}(k_{deg})$	−1.855	−2.028	−1.711	approximate	approximate
b1	$\log_{10}(k_1'/k_2)$	0.2971	0.2619	0.3319	approximate	approximate
b1	$\log_{10}(k_1'k_2'/k_2)$	−1.018	−1.084	−0.9467	approximate	approximate
b1	$\log_{10}(k_2'/k_2)$	−0.3947	−0.5471	−0.2465	approximate	approximate
b2	$\log_{10}(k_1'k_2')$	−1.677	−1.92	−1.234	approximate	approximate
b2	$\log_{10}(k_2 \text{ or } k_{deg})$	−0.8578	−1.766	−0.2767	approximate	approximate
b2	$\log_{10}(k_2 \text{ or } k_{deg})$	−1.567	−1.766	−0.2767	approximate	approximate
b3	$\log_{10}(k_1')$	−0.7097	< −1.433	−0.4103	optim	approximate
b3	$\log_{10}(k_2)$	−1.155	< −1.913	−0.4767	optim	exact
b3	$\log_{10}(k_2')$	−1.521	−1.676	> −0.8455	approximate	optim
b3	$\log_{10}(k_{deg})$	−1.881	−2.122	> −1.194	approximate	optim
b3	$\log_{10}(k_1'/k_2)$	0.4454	0.3581	0.5279	approximate	approximate
b3	$\log_{10}(k_1'k_2'/k_2)$	−1.075	−1.19	> −0.3664	approximate	optim
b3	$\log_{10}(k_2'/k_2)$	−0.3654	−0.7614	> 1.067	approximate	optim

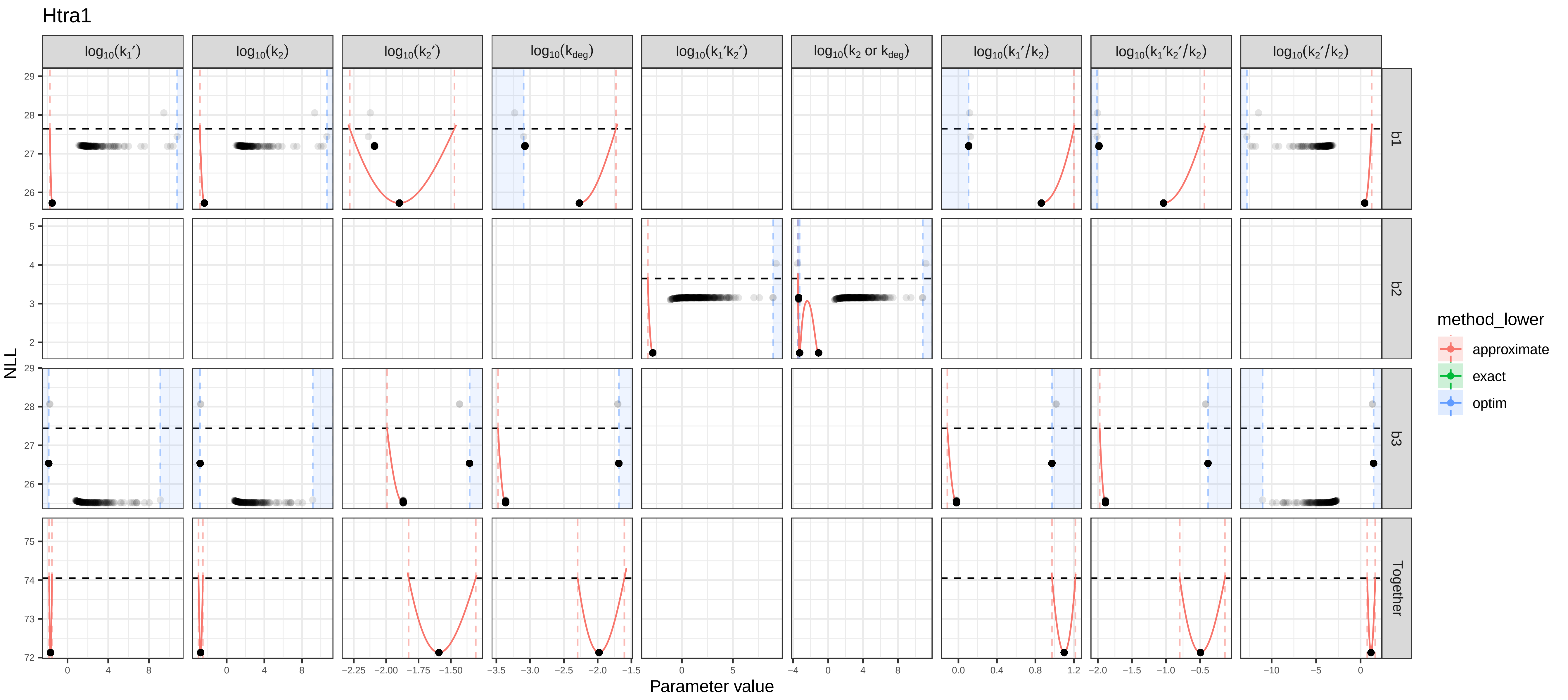

Replicate	Par	Best value	CI95 LB	CI95 UB	Method LB	Method UB
Together	$\log_{10}(k_1')$	−1.667	−1.807	−1.527	approximate	approximate
Together	$\log_{10}(k_2)$	−2.766	−2.989	−2.535	approximate	approximate
Together	$\log_{10}(k_2')$	−1.592	−1.826	−1.308	approximate	approximate
Together	$\log_{10}(k_{deg})$	−1.979	−2.296	−1.604	approximate	approximate
Together	$\log_{10}(k_1'/k_2)$	1.099	0.9726	1.216	approximate	approximate
Together	$\log_{10}(k_1'k_2'/k_2)$	−0.4933	−0.7984	−0.1354	approximate	approximate
Together	$\log_{10}(k_2'/k_2)$	1.173	0.7522	1.65	approximate	approximate
b1	$\log_{10}(k_1')$	−1.509	−1.733	> 10.77	approximate	optim
b1	$\log_{10}(k_2)$	−2.371	−2.845	> 10.65	approximate	optim
b1	$\log_{10}(k_2')$	−1.898	−2.281	−1.472	approximate	approximate
b1	$\log_{10}(k_{deg})$	−2.271	< −3.096	−1.728	optim	approximate
b1	$\log_{10}(k_1'/k_2)$	0.8615	< 0.105	1.199	optim	approximate
b1	$\log_{10}(k_1'k_2'/k_2)$	−1.037	< −2.012	−0.4363	optim	approximate
b1	$\log_{10}(k_2'/k_2)$	0.4723	< −12.78	1.248	optim	approximate
b2	$\log_{10}(k_1'k_2')$	−2.847	−3.336	> 8.947	approximate	optim
b2	$\log_{10}(k_2 \text{ or } k_{deg})$	−1.087	−3.462	> 10.83	approximate	optim
b2	$\log_{10}(k_2 \text{ or } k_{deg})$	−3.259	−3.462	−3.259	approximate	optim
b3	$\log_{10}(k_1')$	5.29	< −1.846	> 9.118	optim	optim
b3	$\log_{10}(k_2)$	5.31	< −2.818	> 9.144	optim	optim
b3	$\log_{10}(k_2')$	−1.868	−1.993	> −1.355	approximate	optim
b3	$\log_{10}(k_{deg})$	−3.363	−3.475	> −1.686	approximate	optim
b3	$\log_{10}(k_1'/k_2)$	−0.02052	−0.1151	> 0.9718	approximate	optim
b3	$\log_{10}(k_1'k_2'/k_2)$	−1.889	−1.973	> −0.3832	approximate	optim
b3	$\log_{10}(k_2'/k_2)$	−7.179	< −11.01	> 1.463	optim	optim

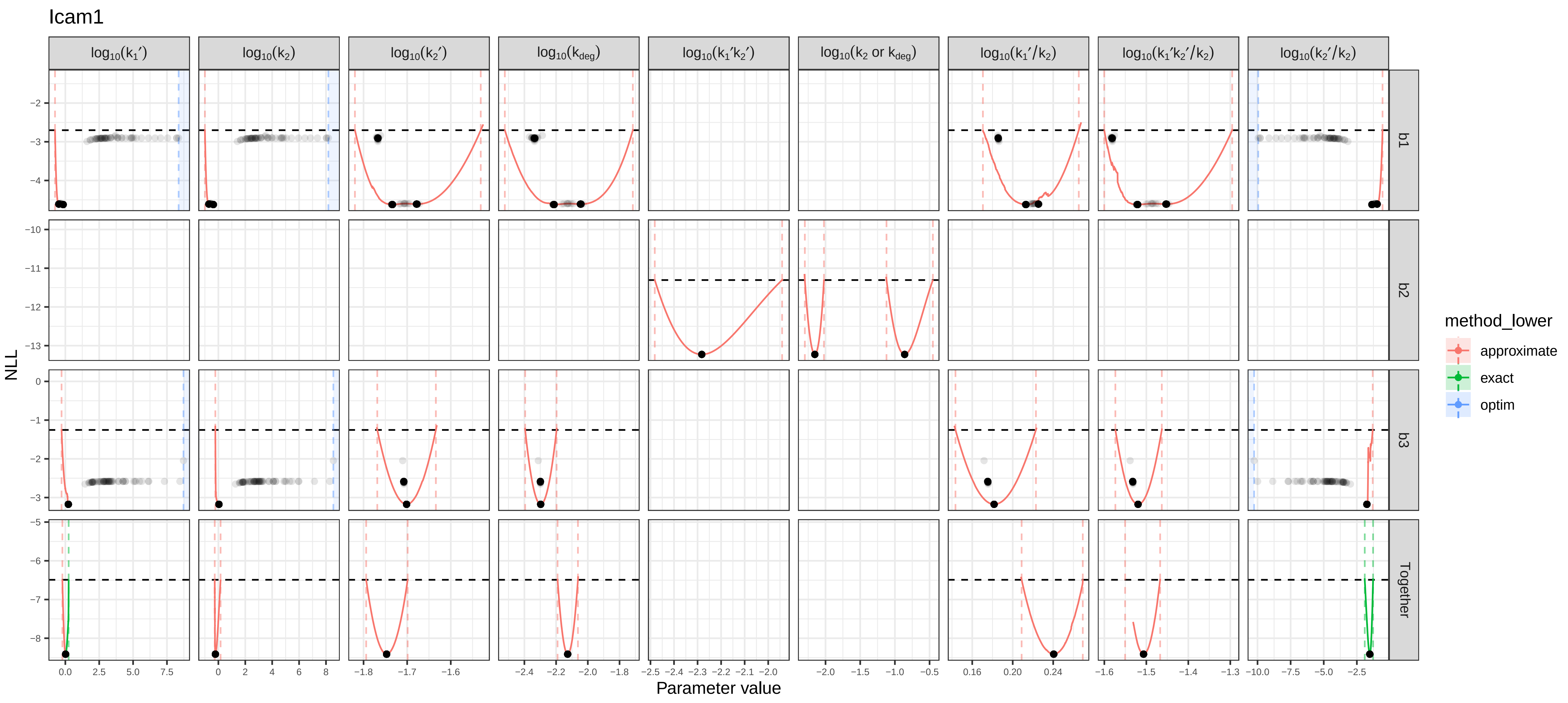

Replicate	Par	Best value	CI95 LB	CI95 UB	Method LB	Method UB
Together	$\log_{10}(k_1')$	0.01784	−0.2182	0.2454	approximate	exact
Together	$\log_{10}(k_2)$	−0.2227	−0.2694	0.1662	approximate	approximate
Together	$\log_{10}(k_2')$	−1.747	−1.794	−1.699	approximate	approximate
Together	$\log_{10}(k_{deg})$	−2.127	−2.191	−2.062	approximate	approximate
Together	$\log_{10}(k_1'/k_2)$	0.2406	0.2088	0.2692	approximate	approximate
Together	$\log_{10}(k_1'k_2'/k_2)$	−1.507	−1.551	−1.467	approximate	approximate
Together	$\log_{10}(k_2'/k_2)$	−1.524	−1.905	−1.277	exact	exact
b1	$\log_{10}(k_1')$	−0.162	−0.7611	> 8.363	approximate	optim
b1	$\log_{10}(k_2)$	−0.3751	−1	> 8.178	approximate	optim
b1	$\log_{10}(k_2')$	−1.734	−1.82	−1.531	approximate	approximate
b1	$\log_{10}(k_{deg})$	−2.213	−2.523	−1.716	approximate	approximate
b1	$\log_{10}(k_1'/k_2)$	0.213	0.1706	0.2653	approximate	approximate
b1	$\log_{10}(k_1'k_2'/k_2)$	−1.521	−1.6	−1.295	approximate	approximate
b1	$\log_{10}(k_2'/k_2)$	−1.359	< −9.945	−0.5605	optim	approximate
b2	$\log_{10}(k_1'k_2')$	−2.282	−2.482	−1.941	approximate	approximate
b2	$\log_{10}(k_2 \text{ or } k_{deg})$	−0.8594	−1.121	−0.4521	approximate	approximate
b2	$\log_{10}(k_2 \text{ or } k_{deg})$	−2.154	−2.298	−2.023	approximate	approximate
b3	$\log_{10}(k_1')$	0.223	−0.2777	> 8.719	approximate	optim
b3	$\log_{10}(k_2)$	0.04136	−0.2257	> 8.547	approximate	optim
b3	$\log_{10}(k_2')$	−1.701	−1.769	−1.634	approximate	approximate
b3	$\log_{10}(k_{deg})$	−2.297	−2.395	−2.197	approximate	approximate
b3	$\log_{10}(k_1'/k_2)$	0.1816	0.1434	0.2229	approximate	approximate
b3	$\log_{10}(k_1'k_2'/k_2)$	−1.52	−1.574	−1.463	approximate	approximate
b3	$\log_{10}(k_2'/k_2)$	−1.743	< −10.26	−1.304	optim	approximate

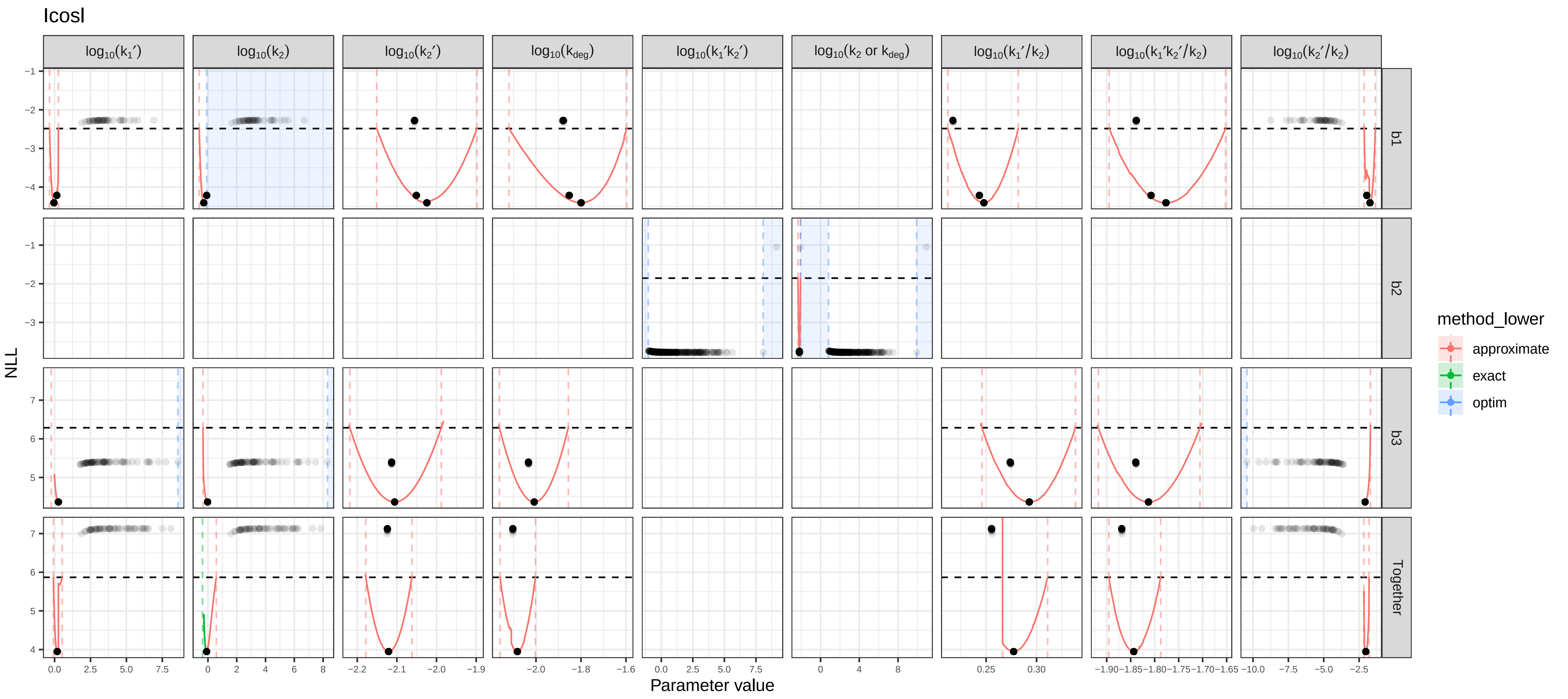

Replicate	Par	Best value	CI95 LB	CI95 UB	Method LB	Method UB
Together	$\log_{10}(k_1')$	0.1829	−0.08533	0.5297	approximate	approximate
Together	$\log_{10}(k_2)$	−0.09446	−0.3782	0.5814	exact	approximate
Together	$\log_{10}(k_2')$	−2.121	−2.178	−2.062	approximate	approximate
Together	$\log_{10}(k_{deg})$	−2.083	−2.159	−2.002	approximate	approximate
Together	$\log_{10}(k_1'/k_2)$	0.2773	0.2663	0.311	approximate	approximate
Together	$\log_{10}(k_1'k_2'/k_2)$	−1.844	−1.896	−1.787	approximate	approximate
Together	$\log_{10}(k_2'/k_2)$	−2.026	−2.172	−1.797	approximate	approximate
b1	$\log_{10}(k_1')$	−0.04396	−0.3509	0.267	approximate	approximate
b1	$\log_{10}(k_2)$	−0.2918	−0.6056	> −0.08729	approximate	optim
b1	$\log_{10}(k_2')$	−2.024	−2.151	−1.898	approximate	approximate
b1	$\log_{10}(k_{deg})$	−1.8	−2.12	−1.597	approximate	approximate
b1	$\log_{10}(k_1'/k_2)$	0.2478	0.212	0.2818	approximate	approximate
b1	$\log_{10}(k_1'k_2'/k_2)$	−1.777	−1.895	−1.652	approximate	approximate
b1	$\log_{10}(k_2'/k_2)$	−1.733	−2.141	−1.355	approximate	approximate
b2	$\log_{10}(k_1'k_2')$	4.23	< −1.029	> 8.069	optim	optim
b2	$\log_{10}(k_2 \text{ or } k_{deg})$	6.082	0.8218	> 9.922	optim	optim
b2	$\log_{10}(k_2 \text{ or } k_{deg})$	−2.192	−2.333	−2.077	approximate	approximate
b3	$\log_{10}(k_1')$	0.2663	−0.2323	> 8.583	approximate	optim
b3	$\log_{10}(k_2)$	−0.0265	−0.3451	> 8.309	approximate	optim
b3	$\log_{10}(k_2')$	−2.106	−2.219	−1.988	approximate	approximate
b3	$\log_{10}(k_{deg})$	−2.008	−2.163	−1.856	approximate	approximate
b3	$\log_{10}(k_1'/k_2)$	0.2928	0.2459	0.3385	approximate	approximate
b3	$\log_{10}(k_1'k_2'/k_2)$	−1.813	−1.917	−1.706	approximate	approximate
b3	$\log_{10}(k_2'/k_2)$	−2.079	< −10.42	−1.699	optim	approximate

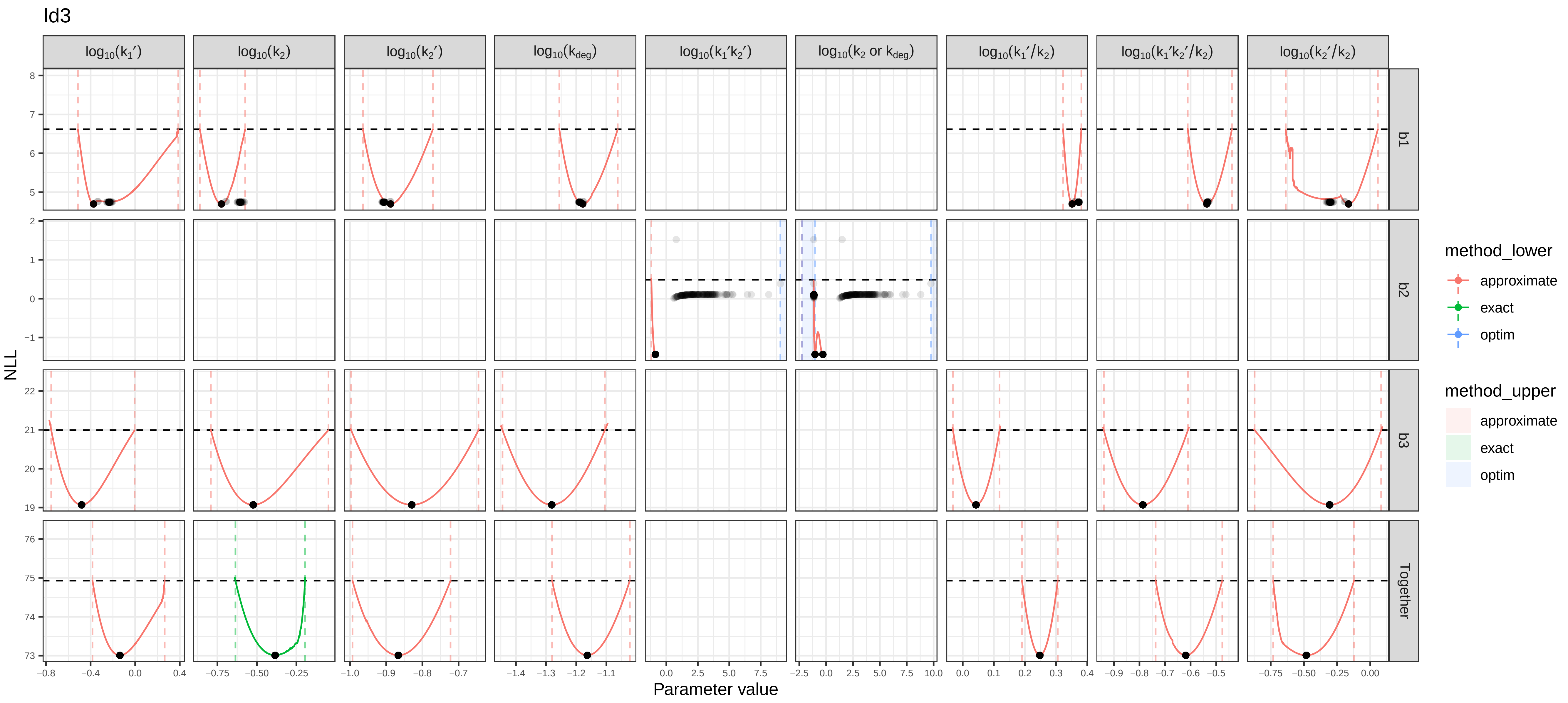

Replicate	Par	Best value	CI95 LB	CI95 UB	Method LB	Method UB
Together	$\log_{10}(k_1')$	−0.1367	−0.383	0.2647	approximate	approximate
Together	$\log_{10}(k_2)$	−0.3843	−0.6358	−0.1954	exact	exact
Together	$\log_{10}(k_2')$	−0.8665	−0.993	−0.7222	approximate	approximate
Together	$\log_{10}(k_{deg})$	−1.163	−1.28	−1.022	approximate	approximate
Together	$\log_{10}(k_1'/k_2)$	0.2476	0.1903	0.3053	approximate	approximate
Together	$\log_{10}(k_1'k_2'/k_2)$	−0.6189	−0.7367	−0.4759	approximate	approximate
Together	$\log_{10}(k_2'/k_2)$	−0.4822	−0.7314	−0.1221	approximate	approximate
b1	$\log_{10}(k_1')$	−0.3733	−0.5141	0.3866	approximate	approximate
b1	$\log_{10}(k_2)$	−0.7247	−0.8615	−0.5743	approximate	approximate
b1	$\log_{10}(k_2')$	−0.8878	−0.9642	−0.7709	approximate	approximate
b1	$\log_{10}(k_{deg})$	−1.178	−1.256	−1.062	approximate	approximate
b1	$\log_{10}(k_1'/k_2)$	0.3514	0.3224	0.3811	approximate	approximate
b1	$\log_{10}(k_1'k_2'/k_2)$	−0.5364	−0.6112	−0.4383	approximate	approximate
b1	$\log_{10}(k_2'/k_2)$	−0.1631	−0.6368	0.05728	approximate	approximate
b2	$\log_{10}(k_1'k_2')$	−0.8687	−1.188	> 9.063	approximate	optim
b2	$\log_{10}(k_2 \text{ or } k_{deg})$	−0.3077	−2.259	> 9.748	approximate	optim
b2	$\log_{10}(k_2 \text{ or } k_{deg})$	−1.031	−2.259	−1.03	approximate	optim
b3	$\log_{10}(k_1')$	−0.4803	−0.7533	−0.004468	approximate	approximate
b3	$\log_{10}(k_2)$	−0.523	−0.7915	−0.04678	approximate	approximate
b3	$\log_{10}(k_2')$	−0.8293	−0.9969	−0.645	approximate	approximate
b3	$\log_{10}(k_{deg})$	−1.281	−1.444	−1.105	approximate	approximate
b3	$\log_{10}(k_1'/k_2)$	0.04266	−0.03144	0.1182	approximate	approximate
b3	$\log_{10}(k_1'k_2'/k_2)$	−0.7867	−0.9395	−0.6103	approximate	approximate
b3	$\log_{10}(k_2'/k_2)$	−0.3063	−0.8712	0.08215	approximate	approximate

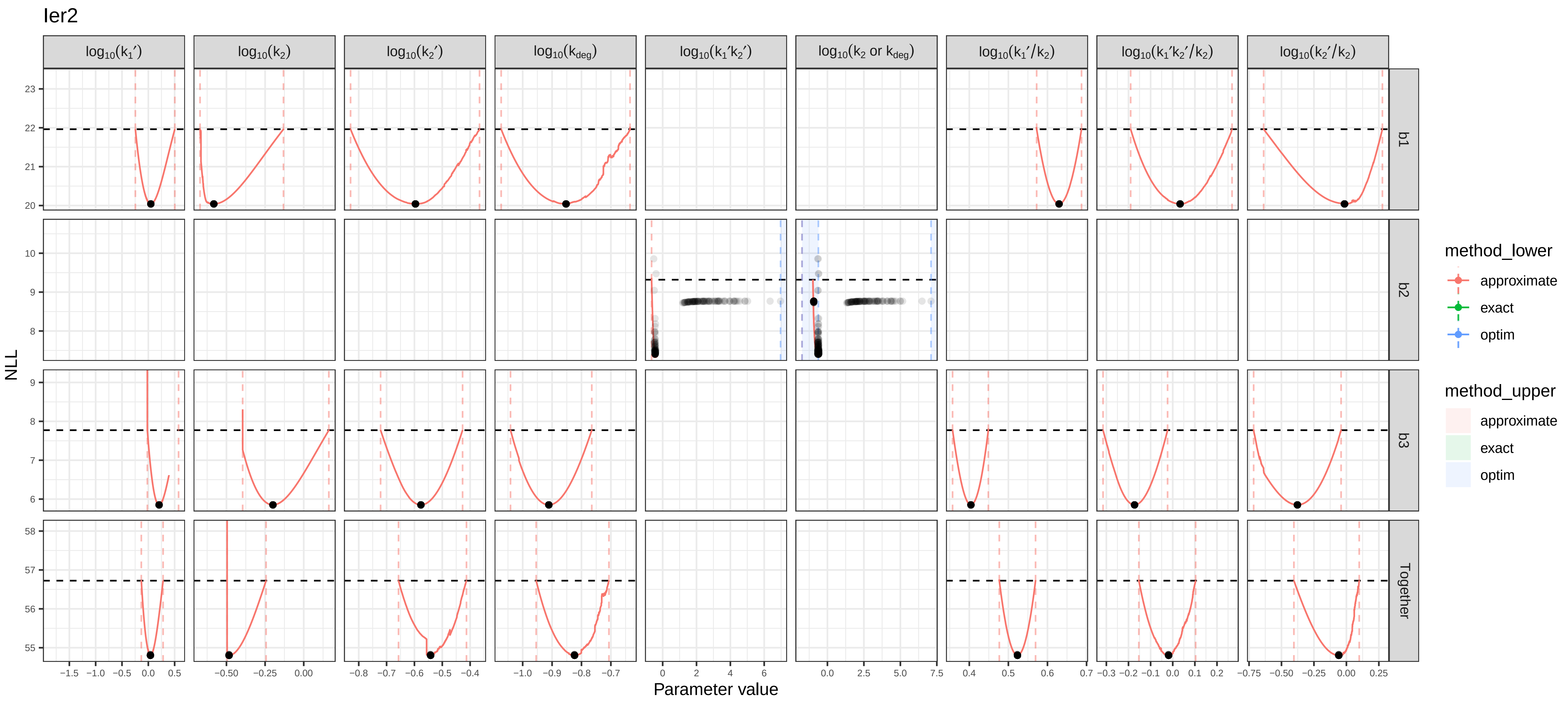

Replicate	Par	Best value	CI95 LB	CI95 UB	Method LB	Method UB
Together	$\log_{10}(k_1')$	0.03984	−0.1331	0.2807	approximate	approximate
Together	$\log_{10}(k_2)$	−0.4835	−0.4957	−0.2436	approximate	approximate
Together	$\log_{10}(k_2')$	−0.5417	−0.6571	−0.4131	approximate	approximate
Together	$\log_{10}(k_{deg})$	−0.8234	−0.9541	−0.7066	approximate	approximate
Together	$\log_{10}(k_1'/k_2)$	0.5233	0.4768	0.5695	approximate	approximate
Together	$\log_{10}(k_1'k_2'/k_2)$	−0.01839	−0.1532	0.1035	approximate	approximate
Together	$\log_{10}(k_2'/k_2)$	−0.05823	−0.4044	0.09881	approximate	approximate
b1	$\log_{10}(k_1')$	0.0476	−0.2459	0.5031	approximate	approximate
b1	$\log_{10}(k_2)$	−0.5821	−0.6708	−0.1311	approximate	approximate
b1	$\log_{10}(k_2')$	−0.5961	−0.8294	−0.3662	approximate	approximate
b1	$\log_{10}(k_{deg})$	−0.8522	−1.073	−0.6349	approximate	approximate
b1	$\log_{10}(k_1'/k_2)$	0.6297	0.5723	0.6871	approximate	approximate
b1	$\log_{10}(k_1'k_2'/k_2)$	0.03361	−0.1902	0.268	approximate	approximate
b1	$\log_{10}(k_2'/k_2)$	−0.01399	−0.6375	0.2774	approximate	approximate
b2	$\log_{10}(k_1'k_2')$	−0.4514	−0.6556	> 6.967	approximate	optim
b2	$\log_{10}(k_2 \text{ or } k_{deg})$	−0.6084	−1.741	> 7.104	approximate	optim
b2	$\log_{10}(k_2 \text{ or } k_{deg})$	−0.6488	−1.741	−0.6214	approximate	optim
b3	$\log_{10}(k_1')$	0.2048	−0.01855	0.5744	approximate	approximate
b3	$\log_{10}(k_2)$	−0.1994	−0.3951	0.1624	approximate	approximate
b3	$\log_{10}(k_2')$	−0.5767	−0.7215	−0.4271	approximate	approximate
b3	$\log_{10}(k_{deg})$	−0.9111	−1.041	−0.7649	approximate	approximate
b3	$\log_{10}(k_1'/k_2)$	0.4042	0.3573	0.4487	approximate	approximate
b3	$\log_{10}(k_1'k_2'/k_2)$	−0.1725	−0.315	−0.02328	approximate	approximate
b3	$\log_{10}(k_2'/k_2)$	−0.3773	−0.7145	−0.0408	approximate	approximate

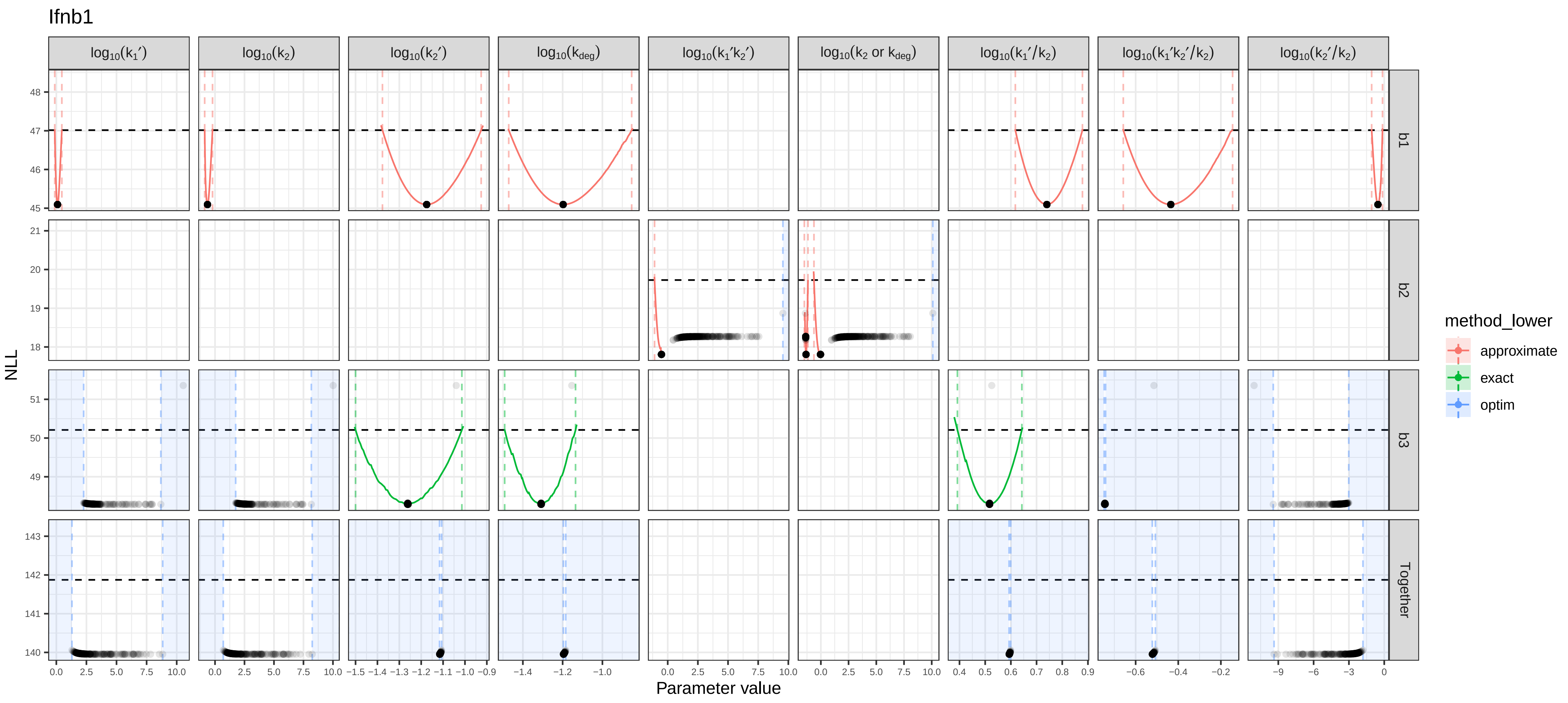

Replicate	Par	Best value	CI95 LB	CI95 UB	Method LB	Method UB
Together	$\log_{10}(k_1')$	3.903	< 1.299	> 8.838	optim	optim
Together	$\log_{10}(k_2)$	3.309	< 0.7002	> 8.244	optim	optim
Together	$\log_{10}(k_2')$	−1.115	< −1.116	> −1.107	optim	optim
Together	$\log_{10}(k_{deg})$	−1.195	< −1.196	> −1.184	optim	optim
Together	$\log_{10}(k_1'/k_2)$	0.5938	< 0.5931	> 0.5992	optim	optim
Together	$\log_{10}(k_1'k_2'/k_2)$	−0.5213	< −0.5218	> −0.5074	optim	optim
Together	$\log_{10}(k_2'/k_2)$	−4.424	< −9.359	> −1.807	optim	optim
b1	$\log_{10}(k_1')$	0.09924	−0.1307	0.4573	approximate	approximate
b1	$\log_{10}(k_2)$	−0.6408	−0.8767	−0.2105	approximate	approximate
b1	$\log_{10}(k_2')$	−1.175	−1.377	−0.9259	approximate	approximate
b1	$\log_{10}(k_{deg})$	−1.197	−1.471	−0.8529	approximate	approximate
b1	$\log_{10}(k_1'/k_2)$	0.74	0.6174	0.8787	approximate	approximate
b1	$\log_{10}(k_1'k_2'/k_2)$	−0.435	−0.658	−0.145	approximate	approximate
b1	$\log_{10}(k_2'/k_2)$	−0.5343	−1.068	−0.1453	approximate	approximate
b2	$\log_{10}(k_1'k_2')$	−0.522	−1.091	> 9.55	approximate	optim
b2	$\log_{10}(k_2 \text{ or } k_{deg})$	−0.03362	−0.6216	> 10.1	approximate	optim
b2	$\log_{10}(k_2 \text{ or } k_{deg})$	−1.338	−1.49	−1.16	approximate	approximate
b3	$\log_{10}(k_1')$	7.437	< 2.27	> 8.684	optim	optim
b3	$\log_{10}(k_2)$	6.92	< 1.753	> 8.167	optim	optim
b3	$\log_{10}(k_2')$	−1.261	−1.5	−1.014	exact	exact
b3	$\log_{10}(k_{deg})$	−1.308	−1.492	−1.135	exact	exact
b3	$\log_{10}(k_1'/k_2)$	0.5169	0.3919	0.6428	exact	exact
b3	$\log_{10}(k_1'k_2'/k_2)$	−0.7443	< −0.7477	> −0.741	optim	optim
b3	$\log_{10}(k_2'/k_2)$	−8.181	< −9.429	> −3.014	optim	optim

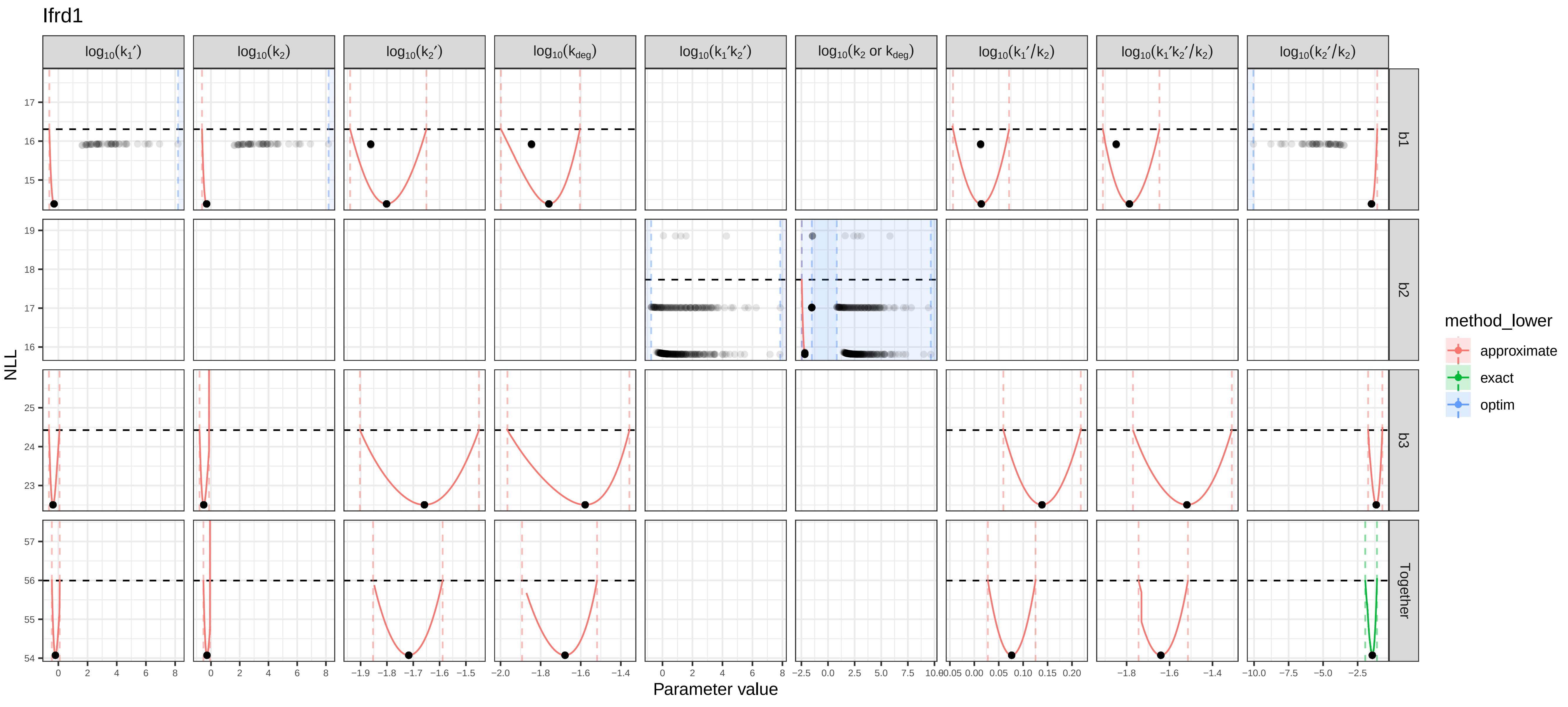

Replicate	Par	Best value	CI95 LB	CI95 UB	Method LB	Method UB
Together	$\log_{10}(k_1')$	−0.2031	−0.4445	0.09789	approximate	approximate
Together	$\log_{10}(k_2)$	−0.2796	−0.5278	−0.06921	approximate	approximate
Together	$\log_{10}(k_2')$	−1.717	−1.853	−1.588	approximate	approximate
Together	$\log_{10}(k_{deg})$	−1.678	−1.893	−1.519	approximate	approximate
Together	$\log_{10}(k_1'/k_2)$	0.0765	0.02771	0.1253	approximate	approximate
Together	$\log_{10}(k_1'k_2'/k_2)$	−1.641	−1.744	−1.514	approximate	approximate
Together	$\log_{10}(k_2'/k_2)$	−1.437	−1.946	−1.1	exact	exact
b1	$\log_{10}(k_1')$	−0.2902	−0.6175	> 8.198	approximate	optim
b1	$\log_{10}(k_2)$	−0.3045	−0.6247	> 8.185	approximate	optim
b1	$\log_{10}(k_2')$	−1.801	−1.94	−1.65	approximate	approximate
b1	$\log_{10}(k_{deg})$	−1.759	−1.998	−1.604	approximate	approximate
b1	$\log_{10}(k_1'/k_2)$	0.01432	−0.04359	0.0713	approximate	approximate
b1	$\log_{10}(k_1'k_2'/k_2)$	−1.787	−1.91	−1.647	approximate	approximate
b1	$\log_{10}(k_2'/k_2)$	−1.497	< −10.05	−1.078	optim	approximate
b2	$\log_{10}(k_1'k_2')$	3.208	< −0.7534	> 7.854	optim	optim
b2	$\log_{10}(k_2 \text{ or } k_{deg})$	5.035	0.8233	> 9.657	optim	optim
b2	$\log_{10}(k_2 \text{ or } k_{deg})$	−2.175	−2.466	> −1.519	approximate	optim
b3	$\log_{10}(k_1')$	−0.3669	−0.6408	0.0826	approximate	approximate
b3	$\log_{10}(k_2)$	−0.5055	−0.7975	−0.1343	approximate	approximate
b3	$\log_{10}(k_2')$	−1.658	−1.902	−1.45	approximate	approximate
b3	$\log_{10}(k_{deg})$	−1.578	−1.966	−1.357	approximate	approximate
b3	$\log_{10}(k_1'/k_2)$	0.1386	0.0595	0.2181	approximate	approximate
b3	$\log_{10}(k_1'k_2'/k_2)$	−1.519	−1.77	−1.31	approximate	approximate
b3	$\log_{10}(k_2'/k_2)$	−1.152	−1.737	−0.7086	approximate	approximate

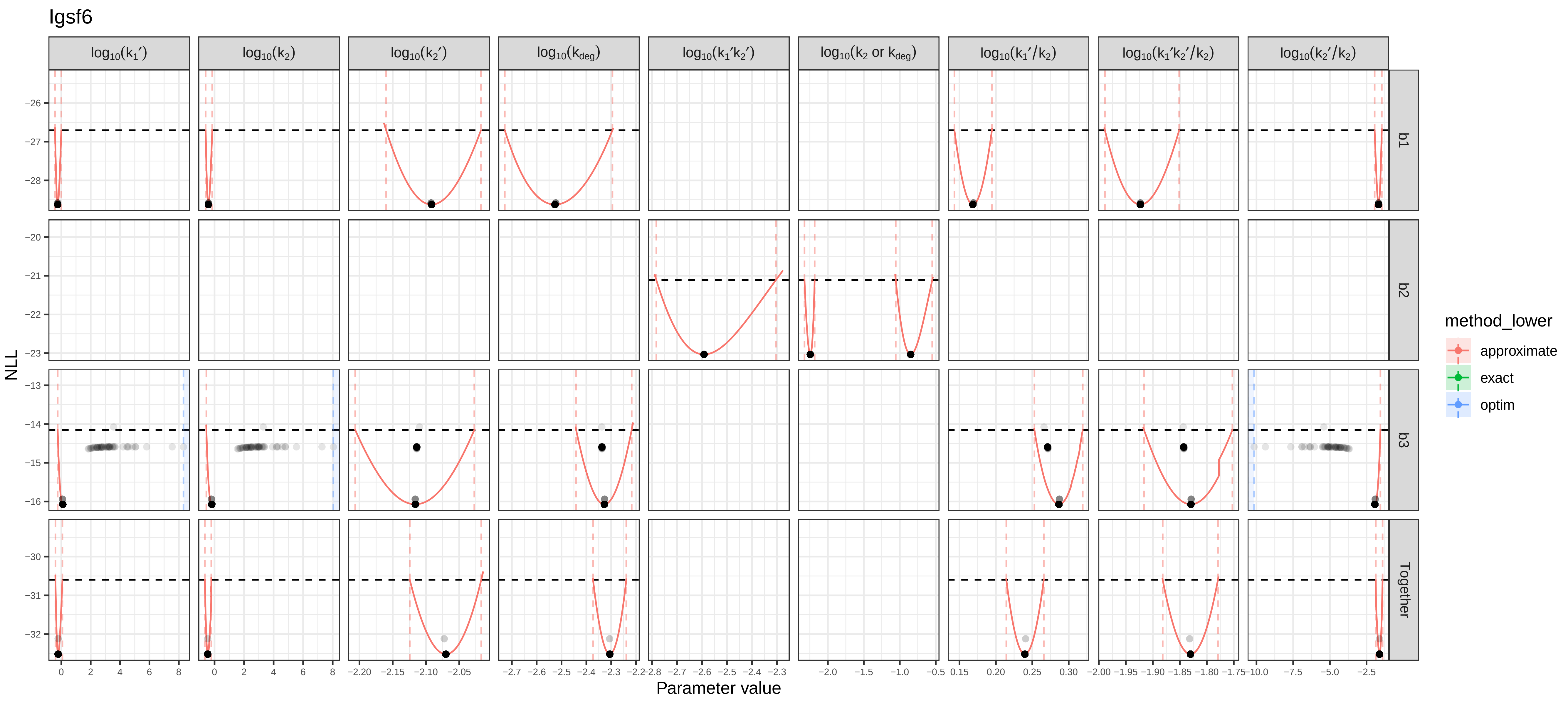

Replicate	Par	Best value	CI95 LB	CI95 UB	Method LB	Method UB
Together	$\log_{10}(k_1')$	−0.2161	−0.4003	0.07524	approximate	approximate
Together	$\log_{10}(k_2)$	−0.4559	−0.6511	−0.2182	approximate	approximate
Together	$\log_{10}(k_2')$	−2.07	−2.124	−2.017	approximate	approximate
Together	$\log_{10}(k_{deg})$	−2.305	−2.373	−2.239	approximate	approximate
Together	$\log_{10}(k_1'/k_2)$	0.2398	0.2143	0.2658	approximate	approximate
Together	$\log_{10}(k_1'k_2'/k_2)$	−1.83	−1.882	−1.779	approximate	approximate
Together	$\log_{10}(k_2'/k_2)$	−1.614	−1.869	−1.406	approximate	approximate
b1	$\log_{10}(k_1')$	−0.2477	−0.427	−0.0007911	approximate	approximate
b1	$\log_{10}(k_2)$	−0.4161	−0.6011	−0.1542	approximate	approximate
b1	$\log_{10}(k_2')$	−2.092	−2.16	−2.017	approximate	approximate
b1	$\log_{10}(k_{deg})$	−2.526	−2.728	−2.295	approximate	approximate
b1	$\log_{10}(k_1'/k_2)$	0.1684	0.143	0.1946	approximate	approximate
b1	$\log_{10}(k_1'k_2'/k_2)$	−1.923	−1.989	−1.851	approximate	approximate
b1	$\log_{10}(k_2'/k_2)$	−1.675	−1.947	−1.46	approximate	approximate
b2	$\log_{10}(k_1'k_2')$	−2.592	−2.783	−2.304	approximate	approximate
b2	$\log_{10}(k_2 \text{ or } k_{deg})$	−0.8487	−1.057	−0.5488	approximate	approximate
b2	$\log_{10}(k_2 \text{ or } k_{deg})$	−2.244	−2.325	−2.184	approximate	approximate
b3	$\log_{10}(k_1')$	0.1004	−0.2488	> 8.318	approximate	optim
b3	$\log_{10}(k_2)$	−0.1861	−0.5518	> 8.047	approximate	optim
b3	$\log_{10}(k_2')$	−2.116	−2.206	−2.027	approximate	approximate
b3	$\log_{10}(k_{deg})$	−2.327	−2.441	−2.217	approximate	approximate
b3	$\log_{10}(k_1'/k_2)$	0.2866	0.2529	0.3192	approximate	approximate
b3	$\log_{10}(k_1'k_2'/k_2)$	−1.829	−1.916	−1.753	approximate	approximate
b3	$\log_{10}(k_2'/k_2)$	−1.93	< −10.16	−1.553	optim	approximate

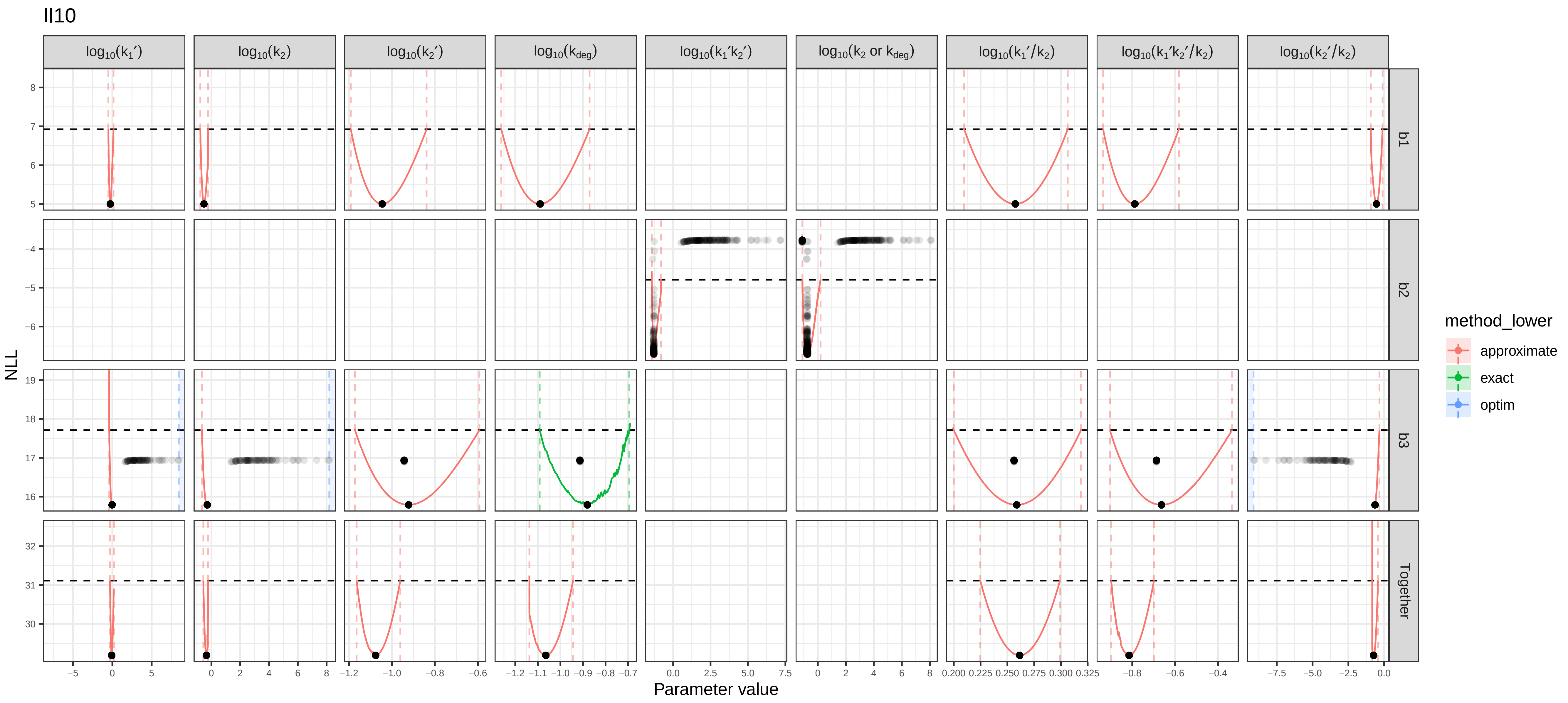

Replicate	Par	**Best value**	**CI95 LB**	**CI95 UB**	**Method LB**	**Method UB**
Together	$\log_{10}(k_1')$	−0.08029	−0.2902	0.2065	approximate	approximate
Together	$\log_{10}(k_2)$	−0.3418	−0.5583	−0.2264	approximate	approximate
Together	$\log_{10}(k_2')$	−1.076	−1.164	−0.961	approximate	approximate
Together	$\log_{10}(k_{deg})$	−1.065	−1.137	−0.9436	approximate	approximate
Together	$\log_{10}(k_1'/k_2)$	0.2615	0.2247	0.2991	approximate	approximate
Together	$\log_{10}(k_1'k_2'/k_2)$	−0.8143	−0.8982	−0.6984	approximate	approximate
Together	$\log_{10}(k_2'/k_2)$	−0.734	−0.8308	−0.4269	approximate	approximate
b1	$\log_{10}(k_1')$	−0.2558	−0.507	0.1539	approximate	approximate
b1	$\log_{10}(k_2)$	−0.5131	−0.7696	−0.2204	approximate	approximate
b1	$\log_{10}(k_2')$	−1.045	−1.192	−0.8388	approximate	approximate
b1	$\log_{10}(k_{deg})$	−1.09	−1.262	−0.8707	approximate	approximate
b1	$\log_{10}(k_1'/k_2)$	0.2574	0.2097	0.3064	approximate	approximate
b1	$\log_{10}(k_1'k_2'/k_2)$	−0.7879	−0.9358	−0.582	approximate	approximate
b1	$\log_{10}(k_2'/k_2)$	−0.5322	−0.9313	−0.1099	approximate	approximate
b2	$\log_{10}(k_1'k_2')$	−1.29	−1.424	−0.8057	approximate	approximate
b2	$\log_{10}(k_2 \text{ or } k_{deg})$	−0.7264	−1.106	0.1965	approximate	approximate
b2	$\log_{10}(k_2 \text{ or } k_{deg})$	−0.7826	−1.106	0.1965	approximate	approximate
b3	$\log_{10}(k_1')$	−0.03058	−0.393	> 8.439	approximate	optim
b3	$\log_{10}(k_2)$	−0.2893	−0.6558	> 8.183	approximate	optim
b3	$\log_{10}(k_2')$	−0.9223	−1.172	−0.5928	approximate	approximate
b3	$\log_{10}(k_{deg})$	−0.8807	−1.092	−0.6949	exact	exact
b3	$\log_{10}(k_1'/k_2)$	0.2587	0.2	0.3188	approximate	approximate
b3	$\log_{10}(k_1'k_2'/k_2)$	−0.6635	−0.9043	−0.3343	approximate	approximate
b3	$\log_{10}(k_2'/k_2)$	−0.6329	< −9.126	−0.3296	optim	approximate

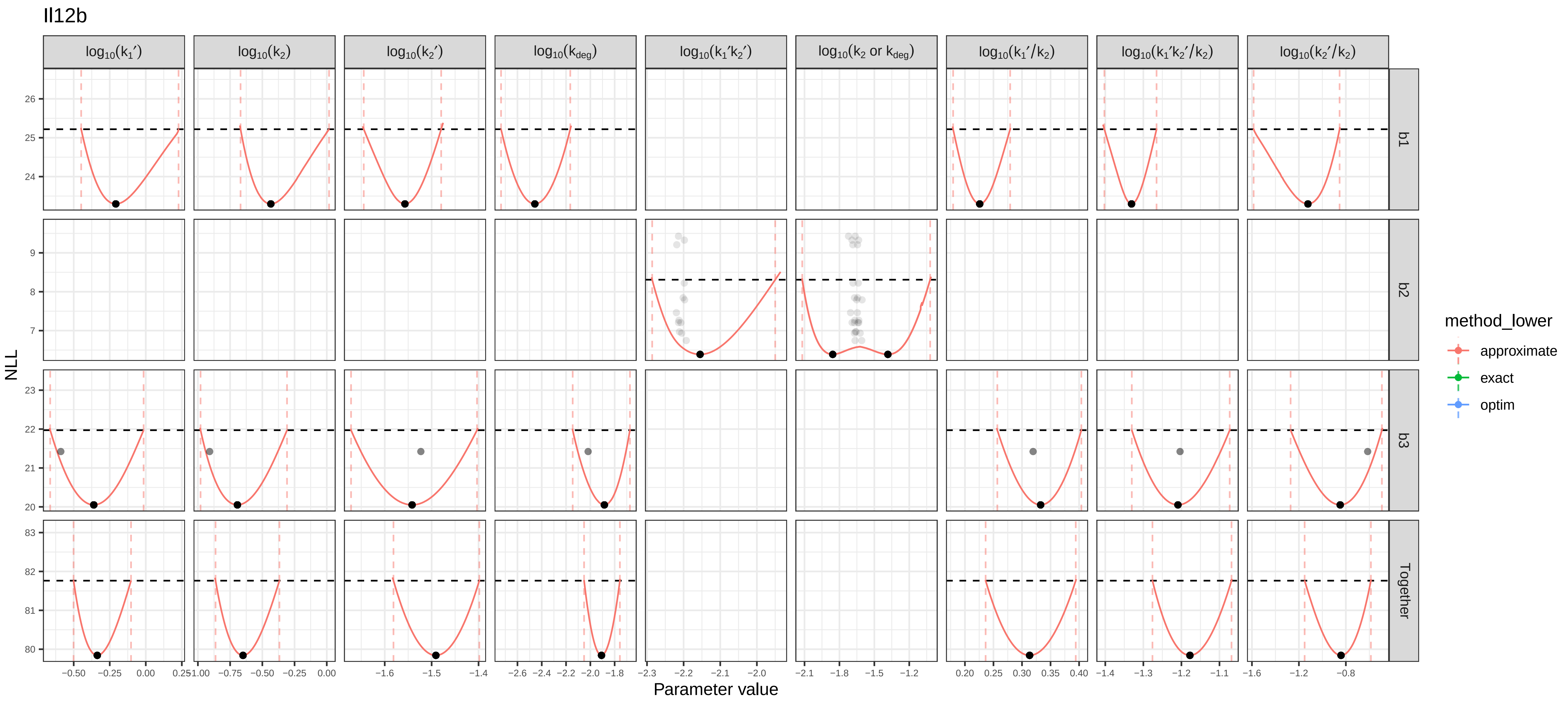

Replicate	Par	Best value	CI95 LB	CI95 UB	Method LB	Method UB
Together	$\log_{10}(k_1')$	−0.3364	−0.5009	−0.1023	approximate	approximate
Together	$\log_{10}(k_2)$	−0.6497	−0.8631	−0.3674	approximate	approximate
Together	$\log_{10}(k_2')$	−1.491	−1.581	−1.398	approximate	approximate
Together	$\log_{10}(k_{deg})$	−1.908	−2.052	−1.757	approximate	approximate
Together	$\log_{10}(k_1'/k_2)$	0.3133	0.2363	0.3942	approximate	approximate
Together	$\log_{10}(k_1'k_2'/k_2)$	−1.178	−1.276	−1.068	approximate	approximate
Together	$\log_{10}(k_2'/k_2)$	−0.8413	−1.151	−0.5868	approximate	approximate
b1	$\log_{10}(k_1')$	−0.2084	−0.4482	0.2273	approximate	approximate
b1	$\log_{10}(k_2)$	−0.4343	−0.669	0.01778	approximate	approximate
b1	$\log_{10}(k_2')$	−1.557	−1.645	−1.48	approximate	approximate
b1	$\log_{10}(k_{deg})$	−2.457	−2.735	−2.165	approximate	approximate
b1	$\log_{10}(k_1'/k_2)$	0.2259	0.1793	0.2793	approximate	approximate
b1	$\log_{10}(k_1'k_2'/k_2)$	−1.331	−1.402	−1.265	approximate	approximate
b1	$\log_{10}(k_2'/k_2)$	−1.123	−1.586	−0.8534	approximate	approximate
b2	$\log_{10}(k_1'k_2')$	−2.155	−2.286	−1.95	approximate	approximate
b2	$\log_{10}(k_2 \text{ or } k_{deg})$	−1.384	−2.12	−1.02	approximate	approximate
b2	$\log_{10}(k_2 \text{ or } k_{deg})$	−1.858	−2.12	−1.02	approximate	approximate
b3	$\log_{10}(k_1')$	−0.3607	−0.6631	−0.01513	approximate	approximate
b3	$\log_{10}(k_2)$	−0.6933	−0.9794	−0.3085	approximate	approximate
b3	$\log_{10}(k_2')$	−1.542	−1.672	−1.403	approximate	approximate
b3	$\log_{10}(k_{deg})$	−1.884	−2.145	−1.674	approximate	approximate
b3	$\log_{10}(k_1'/k_2)$	0.3326	0.2568	0.404	approximate	approximate
b3	$\log_{10}(k_1'k_2'/k_2)$	−1.209	−1.33	−1.073	approximate	approximate
b3	$\log_{10}(k_2'/k_2)$	−0.8484	−1.271	−0.493	approximate	approximate

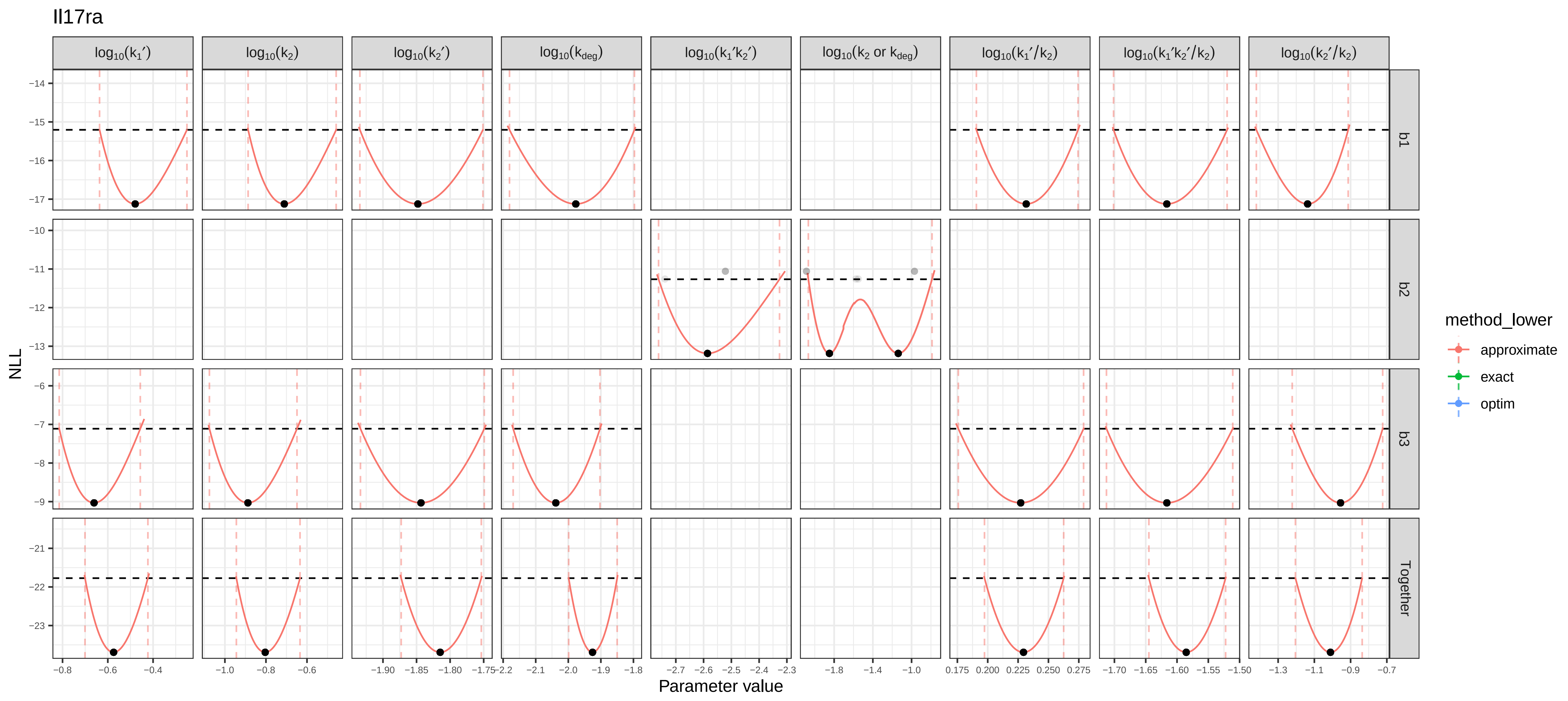

Replicate	Par	Best value	CI95 LB	CI95 UB	Method LB	Method UB
Together	$\log_{10}(k_1')$	−0.5743	−0.7007	−0.4229	approximate	approximate
Together	$\log_{10}(k_2)$	−0.8038	−0.9441	−0.634	approximate	approximate
Together	$\log_{10}(k_2')$	−1.815	−1.873	−1.754	approximate	approximate
Together	$\log_{10}(k_{deg})$	−1.926	−1.999	−1.85	approximate	approximate
Together	$\log_{10}(k_1'/k_2)$	0.2295	0.1973	0.2625	approximate	approximate
Together	$\log_{10}(k_1'k_2'/k_2)$	−1.585	−1.645	−1.522	approximate	approximate
Together	$\log_{10}(k_2'/k_2)$	−1.011	−1.205	−0.8361	approximate	approximate
b1	$\log_{10}(k_1')$	−0.4788	−0.6364	−0.2503	approximate	approximate
b1	$\log_{10}(k_2)$	−0.7104	−0.8869	−0.4579	approximate	approximate
b1	$\log_{10}(k_2')$	−1.848	−1.934	−1.751	approximate	approximate
b1	$\log_{10}(k_{deg})$	−1.977	−2.18	−1.797	approximate	approximate
b1	$\log_{10}(k_1'/k_2)$	0.2316	0.1907	0.2744	approximate	approximate
b1	$\log_{10}(k_1'k_2'/k_2)$	−1.616	−1.701	−1.52	approximate	approximate
b1	$\log_{10}(k_2'/k_2)$	−1.138	−1.42	−0.9133	approximate	approximate
b2	$\log_{10}(k_1'k_2')$	−2.586	−2.762	−2.327	approximate	approximate
b2	$\log_{10}(k_2 \text{ or } k_{deg})$	−1.138	−2.067	−0.7887	approximate	approximate
b2	$\log_{10}(k_2 \text{ or } k_{deg})$	−1.849	−2.067	−0.7887	approximate	approximate
b3	$\log_{10}(k_1')$	−0.6607	−0.8147	−0.4564	approximate	approximate
b3	$\log_{10}(k_2)$	−0.8879	−1.075	−0.6488	approximate	approximate
b3	$\log_{10}(k_2')$	−1.843	−1.934	−1.749	approximate	approximate
b3	$\log_{10}(k_{deg})$	−2.038	−2.169	−1.903	approximate	approximate
b3	$\log_{10}(k_1'/k_2)$	0.2272	0.1758	0.2789	approximate	approximate
b3	$\log_{10}(k_1'k_2'/k_2)$	−1.616	−1.713	−1.511	approximate	approximate
b3	$\log_{10}(k_2'/k_2)$	−0.9555	−1.222	−0.7229	approximate	approximate

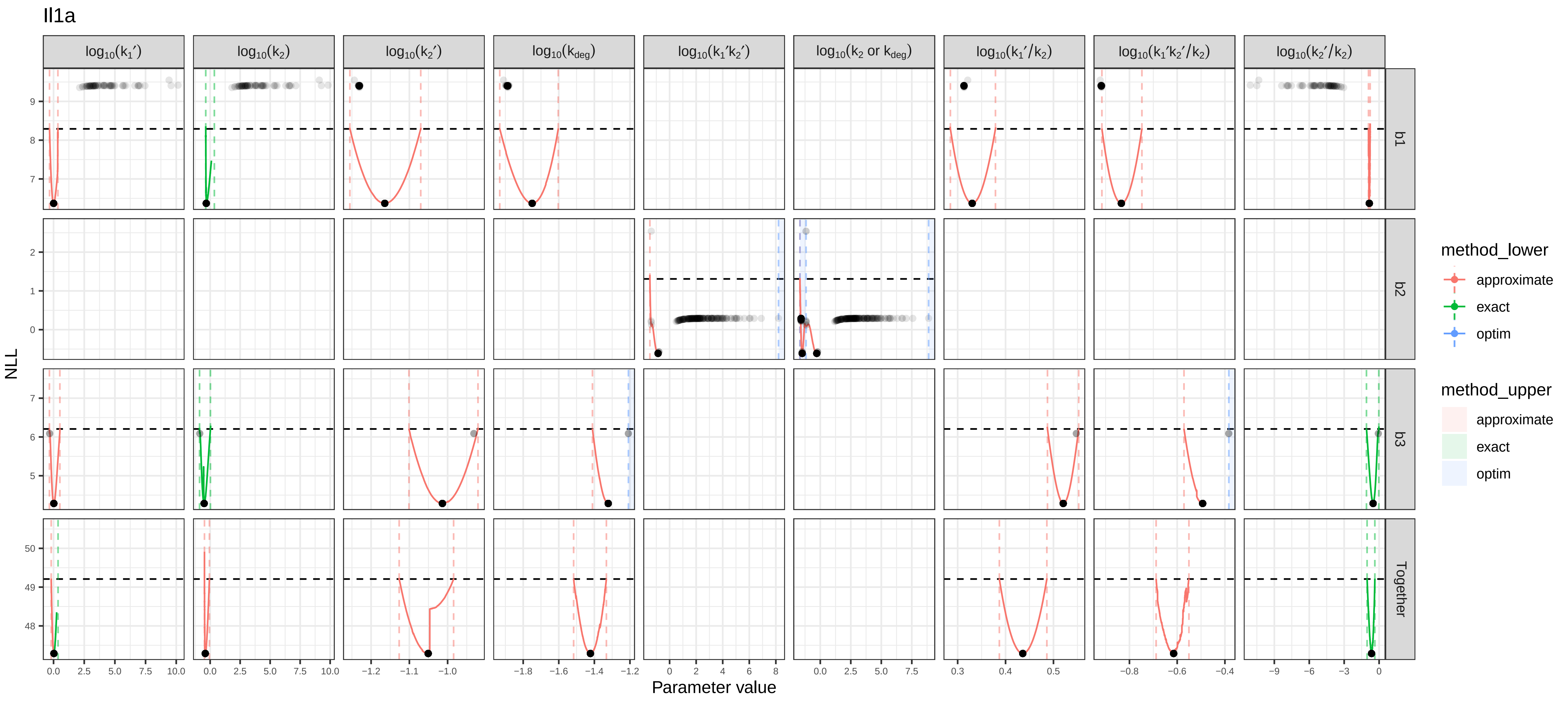

Replicate	Par	Best value	CI95 LB	CI95 UB	Method LB	Method UB
Together	$\log_{10}(k_1')$	0.0313	−0.1917	0.3671	approximate	exact
Together	$\log_{10}(k_2)$	−0.4046	−0.4725	−0.05959	approximate	approximate
Together	$\log_{10}(k_2')$	−1.051	−1.127	−0.9843	approximate	approximate
Together	$\log_{10}(k_{deg})$	−1.422	−1.517	−1.332	approximate	approximate
Together	$\log_{10}(k_1'/k_2)$	0.4359	0.387	0.4862	approximate	approximate
Together	$\log_{10}(k_1'k_2'/k_2)$	−0.6149	−0.6882	−0.5508	approximate	approximate
Together	$\log_{10}(k_2'/k_2)$	−0.6462	−1.032	−0.3647	exact	exact
b1	$\log_{10}(k_1')$	0.007322	−0.3277	0.355	approximate	approximate
b1	$\log_{10}(k_2)$	−0.323	−0.365	0.351	exact	exact
b1	$\log_{10}(k_2')$	−1.164	−1.255	−1.07	approximate	approximate
b1	$\log_{10}(k_{deg})$	−1.75	−1.932	−1.603	approximate	approximate
b1	$\log_{10}(k_1'/k_2)$	0.3304	0.2848	0.3789	approximate	approximate
b1	$\log_{10}(k_1'k_2'/k_2)$	−0.8339	−0.9154	−0.7478	approximate	approximate
b1	$\log_{10}(k_2'/k_2)$	−0.8413	−0.913	−0.7683	approximate	approximate
b2	$\log_{10}(k_1'k_2')$	−0.8659	−1.483	> 8.201	approximate	optim
b2	$\log_{10}(k_2 \text{ or } k_{deg})$	−0.2904	−1.666	> 8.878	approximate	optim
b2	$\log_{10}(k_2 \text{ or } k_{deg})$	−1.481	−1.666	−1.163	approximate	optim
b3	$\log_{10}(k_1')$	0.0227	−0.3364	0.5165	approximate	approximate
b3	$\log_{10}(k_2)$	−0.4977	−0.882	0.0242	exact	exact
b3	$\log_{10}(k_2')$	−1.014	−1.101	−0.9206	approximate	approximate
b3	$\log_{10}(k_{deg})$	−1.322	−1.411	> −1.209	approximate	optim
b3	$\log_{10}(k_1'/k_2)$	0.5204	0.4875	0.5527	approximate	approximate
b3	$\log_{10}(k_1'k_2'/k_2)$	−0.4932	−0.5714	> −0.3836	approximate	optim
b3	$\log_{10}(k_2'/k_2)$	−0.5159	−1.085	−0.02589	exact	exact

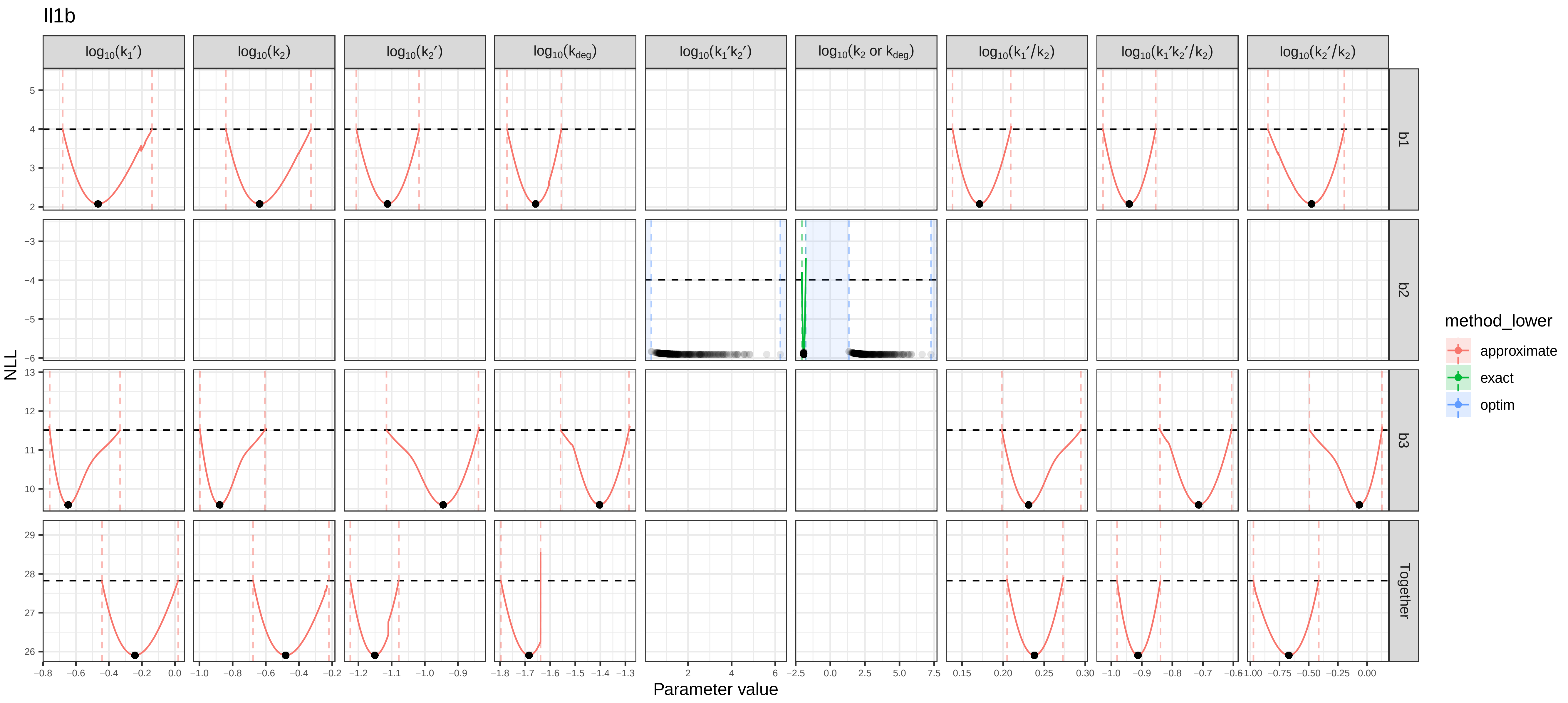

Replicate	Par	Best value	CI95 LB	CI95 UB	Method LB	Method UB
Together	$\log_{10}(k_1')$	−0.2425	−0.4421	0.02036	approximate	approximate
Together	$\log_{10}(k_2)$	−0.4806	−0.6769	−0.2205	approximate	approximate
Together	$\log_{10}(k_2')$	−1.15	−1.224	−1.078	approximate	approximate
Together	$\log_{10}(k_{deg})$	−1.684	−1.797	−1.639	approximate	approximate
Together	$\log_{10}(k_1'/k_2)$	0.238	0.2049	0.2727	approximate	approximate
Together	$\log_{10}(k_1'k_2'/k_2)$	−0.9125	−0.9809	−0.8389	approximate	approximate
Together	$\log_{10}(k_2'/k_2)$	−0.6699	−0.9731	−0.4142	approximate	approximate
b1	$\log_{10}(k_1')$	−0.4662	−0.6806	−0.1382	approximate	approximate
b1	$\log_{10}(k_2)$	−0.6375	−0.8413	−0.3284	approximate	approximate
b1	$\log_{10}(k_2')$	−1.112	−1.206	−1.017	approximate	approximate
b1	$\log_{10}(k_{deg})$	−1.658	−1.772	−1.556	approximate	approximate
b1	$\log_{10}(k_1'/k_2)$	0.1713	0.1384	0.2091	approximate	approximate
b1	$\log_{10}(k_1'k_2'/k_2)$	−0.9411	−1.027	−0.8546	approximate	approximate
b1	$\log_{10}(k_2'/k_2)$	−0.4749	−0.8505	−0.195	approximate	approximate
b2	$\log_{10}(k_1'k_2')$	6.236	< 0.314	> 6.236	optim	optim
b2	$\log_{10}(k_2 \text{ or } k_{deg})$	7.267	1.344	> 7.267	optim	optim
b2	$\log_{10}(k_2 \text{ or } k_{deg})$	−1.919	−2.046	−1.784	exact	exact
b3	$\log_{10}(k_1')$	−0.647	−0.7593	−0.332	approximate	approximate
b3	$\log_{10}(k_2)$	−0.878	−0.9967	−0.6063	approximate	approximate
b3	$\log_{10}(k_2')$	−0.9448	−1.115	−0.8384	approximate	approximate
b3	$\log_{10}(k_{deg})$	−1.403	−1.559	−1.286	approximate	approximate
b3	$\log_{10}(k_1'/k_2)$	0.2309	0.1984	0.2945	approximate	approximate
b3	$\log_{10}(k_1'k_2'/k_2)$	−0.7138	−0.8403	−0.6059	approximate	approximate
b3	$\log_{10}(k_2'/k_2)$	−0.06683	−0.493	0.1273	approximate	approximate

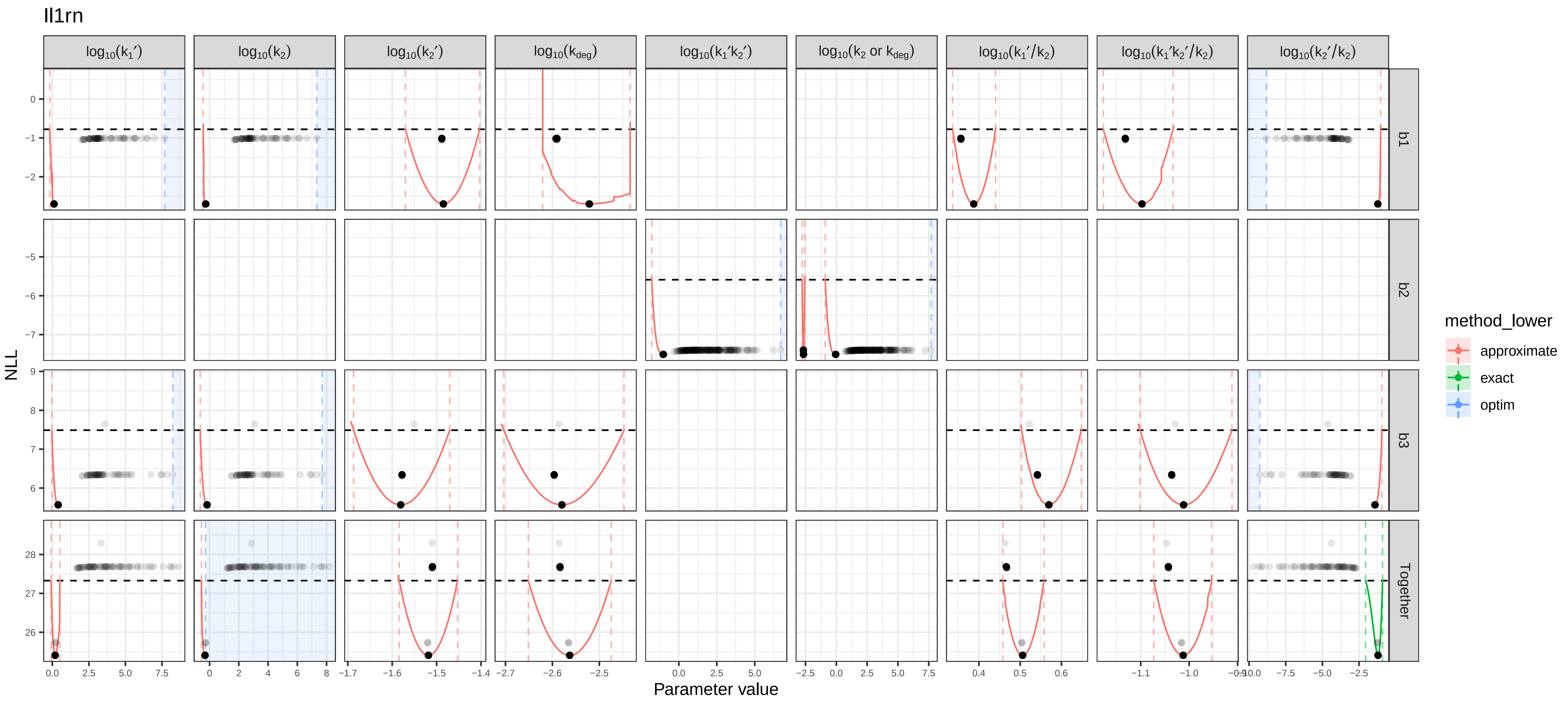

Replicate	Par	Best value	CI95 LB	CI95 UB	Method LB	Method UB
Together	$\log_{10}(k_1')$	0.192	−0.08469	0.5132	approximate	approximate
Together	$\log_{10}(k_2)$	−0.314	−0.5547	> −0.2768	approximate	optim
Together	$\log_{10}(k_2')$	−1.518	−1.584	−1.453	approximate	approximate
Together	$\log_{10}(k_{deg})$	−2.563	−2.651	−2.475	approximate	approximate
Together	$\log_{10}(k_1'/k_2)$	0.506	0.4578	0.5581	approximate	approximate
Together	$\log_{10}(k_1'k_2'/k_2)$	−1.012	−1.073	−0.9536	approximate	approximate
Together	$\log_{10}(k_2'/k_2)$	−1.204	−2.051	−0.8963	exact	exact
b1	$\log_{10}(k_1')$	0.1185	−0.1728	> 7.688	approximate	optim
b1	$\log_{10}(k_2)$	−0.269	−0.4396	> 7.332	approximate	optim
b1	$\log_{10}(k_2')$	−1.485	−1.57	−1.403	approximate	approximate
b1	$\log_{10}(k_{deg})$	−2.521	−2.621	−2.434	approximate	approximate
b1	$\log_{10}(k_1'/k_2)$	0.3874	0.3363	0.4404	approximate	approximate
b1	$\log_{10}(k_1'k_2'/k_2)$	−1.097	−1.177	−1.033	approximate	approximate
b1	$\log_{10}(k_2'/k_2)$	−1.216	< −8.82	−1.027	optim	approximate
b2	$\log_{10}(k_1'k_2')$	−1.03	−1.783	> 6.706	approximate	optim
b2	$\log_{10}(k_2 \text{ or } k_{deg})$	−0.05935	−0.9005	> 7.695	approximate	optim
b2	$\log_{10}(k_2 \text{ or } k_{deg})$	−2.661	−2.773	−2.562	approximate	approximate
b3	$\log_{10}(k_1')$	0.3962	−0.03575	> 8.241	approximate	optim
b3	$\log_{10}(k_2)$	−0.1732	−0.6371	> 7.699	approximate	optim
b3	$\log_{10}(k_2')$	−1.581	−1.687	−1.47	approximate	approximate
b3	$\log_{10}(k_{deg})$	−2.58	−2.704	−2.447	approximate	approximate
b3	$\log_{10}(k_1'/k_2)$	0.5694	0.5035	0.6482	approximate	approximate
b3	$\log_{10}(k_1'k_2'/k_2)$	−1.011	−1.101	−0.9117	approximate	approximate
b3	$\log_{10}(k_2'/k_2)$	−1.408	< −9.277	−0.9392	optim	approximate

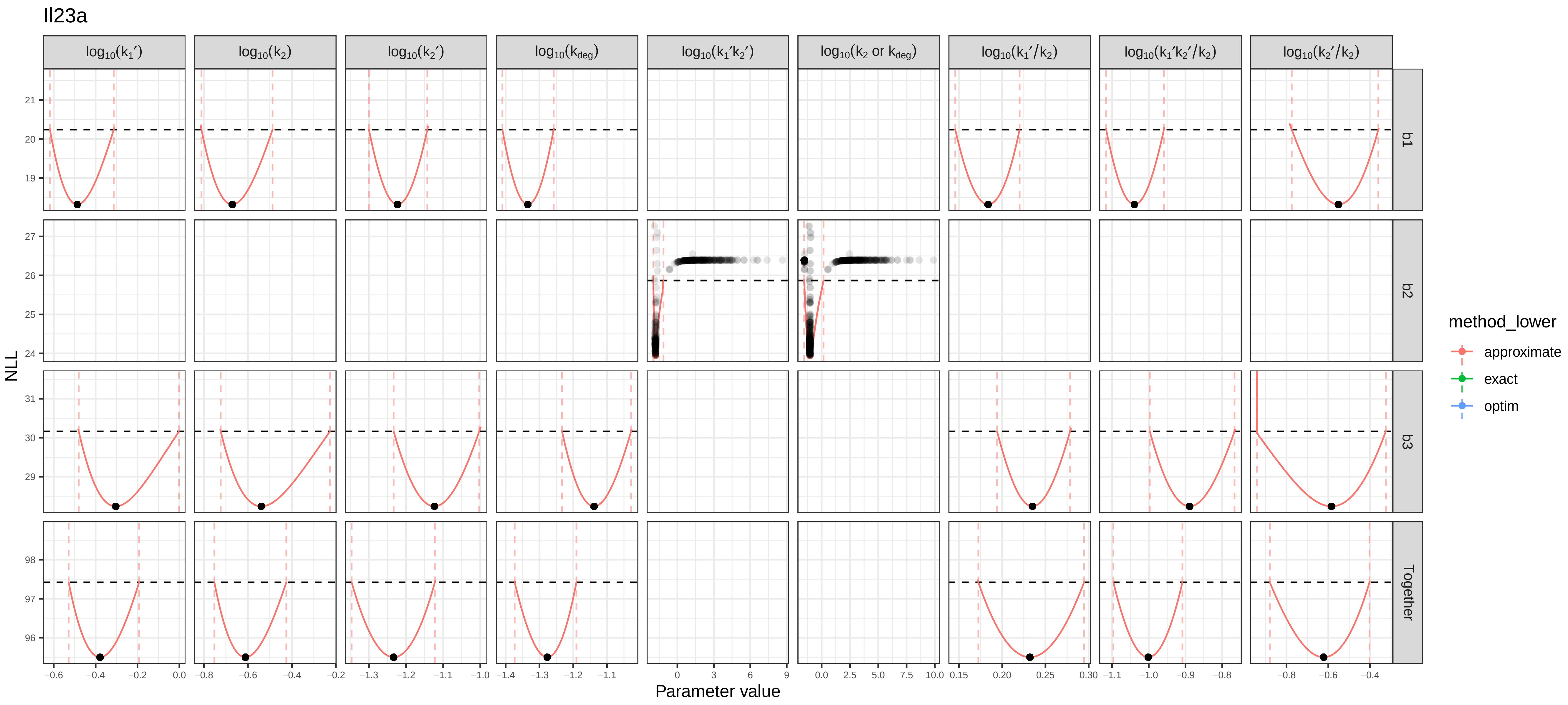

Replicate	Par	Best value	CI95 LB	CI95 UB	Method LB	Method UB
Together	$\log_{10}(k_1')$	−0.3792	−0.5289	−0.1924	approximate	approximate
Together	$\log_{10}(k_2)$	−0.6113	−0.7526	−0.4254	approximate	approximate
Together	$\log_{10}(k_2')$	−1.234	−1.347	−1.122	approximate	approximate
Together	$\log_{10}(k_{deg})$	−1.277	−1.373	−1.191	approximate	approximate
Together	$\log_{10}(k_1'/k_2)$	0.232	0.1724	0.2946	approximate	approximate
Together	$\log_{10}(k_1'k_2'/k_2)$	−1.001	−1.096	−0.9083	approximate	approximate
Together	$\log_{10}(k_2'/k_2)$	−0.6222	−0.8799	−0.4031	approximate	approximate
b1	$\log_{10}(k_1')$	−0.4876	−0.6185	−0.3133	approximate	approximate
b1	$\log_{10}(k_2)$	−0.6714	−0.8114	−0.488	approximate	approximate
b1	$\log_{10}(k_2')$	−1.223	−1.3	−1.143	approximate	approximate
b1	$\log_{10}(k_{deg})$	−1.334	−1.41	−1.258	approximate	approximate
b1	$\log_{10}(k_1'/k_2)$	0.1838	0.1458	0.2201	approximate	approximate
b1	$\log_{10}(k_1'k_2'/k_2)$	−1.039	−1.116	−0.9588	approximate	approximate
b1	$\log_{10}(k_2'/k_2)$	−0.5516	−0.7748	−0.3606	approximate	approximate
b2	$\log_{10}(k_1'k_2')$	−1.791	−1.98	−1.143	approximate	approximate
b2	$\log_{10}(k_2 \text{ or } k_{deg})$	−1.014	−1.542	0.167	approximate	approximate
b2	$\log_{10}(k_2 \text{ or } k_{deg})$	−1.063	−1.542	0.167	approximate	approximate
b3	$\log_{10}(k_1')$	−0.304	−0.4806	−0.001832	approximate	approximate
b3	$\log_{10}(k_2)$	−0.539	−0.7237	−0.2271	approximate	approximate
b3	$\log_{10}(k_2')$	−1.124	−1.233	−1.003	approximate	approximate
b3	$\log_{10}(k_{deg})$	−1.138	−1.233	−1.029	approximate	approximate
b3	$\log_{10}(k_1'/k_2)$	0.235	0.1941	0.2785	approximate	approximate
b3	$\log_{10}(k_1'k_2'/k_2)$	−0.8886	−0.9973	−0.7663	approximate	approximate
b3	$\log_{10}(k_2'/k_2)$	−0.5846	−0.9418	−0.325	approximate	approximate

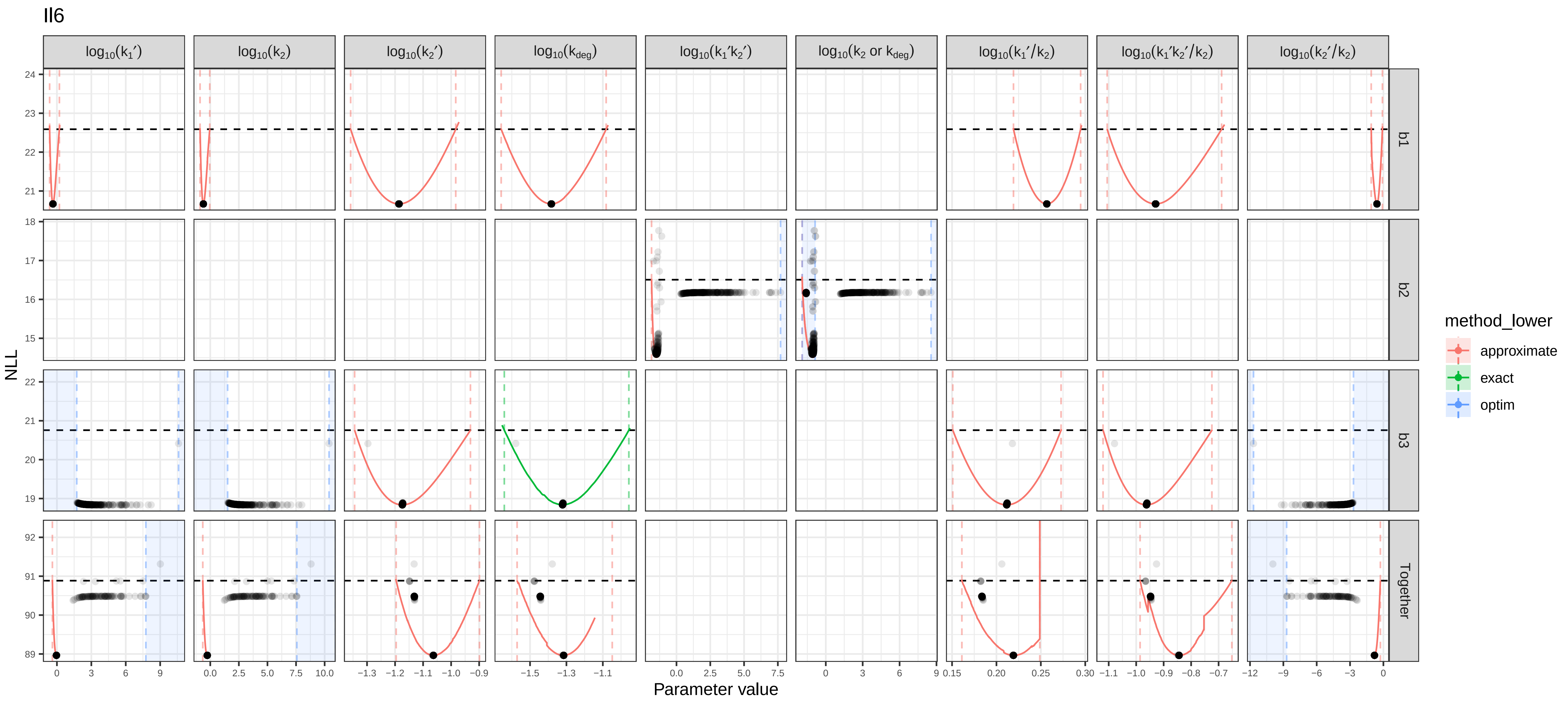

Replicate	Par	Best value	CI95 LB	CI95 UB	Method LB	Method UB
Together	$\log_{10}(k_1')$	−0.04275	−0.3962	> 7.755	approximate	optim
Together	$\log_{10}(k_2)$	−0.2618	−0.6543	> 7.571	approximate	optim
Together	$\log_{10}(k_2')$	−1.063	−1.196	−0.8989	approximate	approximate
Together	$\log_{10}(k_{deg})$	−1.316	−1.571	−1.048	approximate	approximate
Together	$\log_{10}(k_1'/k_2)$	0.219	0.1611	0.2488	approximate	approximate
Together	$\log_{10}(k_1'k_2'/k_2)$	−0.8444	−0.9857	−0.6517	approximate	approximate
Together	$\log_{10}(k_2'/k_2)$	−0.8016	< −8.703	−0.2722	optim	approximate
b1	$\log_{10}(k_1')$	−0.3471	−0.6366	0.2151	approximate	approximate
b1	$\log_{10}(k_2)$	−0.6038	−0.892	−0.04443	approximate	approximate
b1	$\log_{10}(k_2')$	−1.186	−1.359	−0.9835	approximate	approximate
b1	$\log_{10}(k_{deg})$	−1.383	−1.659	−1.082	approximate	approximate
b1	$\log_{10}(k_1'/k_2)$	0.2567	0.2192	0.2948	approximate	approximate
b1	$\log_{10}(k_1'k_2'/k_2)$	−0.9293	−1.106	−0.689	approximate	approximate
b1	$\log_{10}(k_2'/k_2)$	−0.5822	−1.094	−0.08662	approximate	approximate
b2	$\log_{10}(k_1'k_2')$	−1.486	−1.845	> 7.7	approximate	optim
b2	$\log_{10}(k_2 \text{ or } k_{deg})$	−1.053	−1.921	> 8.559	approximate	optim
b2	$\log_{10}(k_2 \text{ or } k_{deg})$	−1.091	−1.921	−0.8682	approximate	optim
b3	$\log_{10}(k_1')$	5.655	< 1.731	> 10.6	optim	optim
b3	$\log_{10}(k_2)$	5.443	< 1.518	> 10.39	optim	optim
b3	$\log_{10}(k_2')$	−1.174	−1.344	−0.9311	approximate	approximate
b3	$\log_{10}(k_{deg})$	−1.322	−1.641	−0.9569	exact	exact
b3	$\log_{10}(k_1'/k_2)$	0.2114	0.1508	0.2728	approximate	approximate
b3	$\log_{10}(k_1'k_2'/k_2)$	−0.9627	−1.121	−0.7244	approximate	approximate
b3	$\log_{10}(k_2'/k_2)$	−6.617	< −11.68	> −2.691	optim	optim

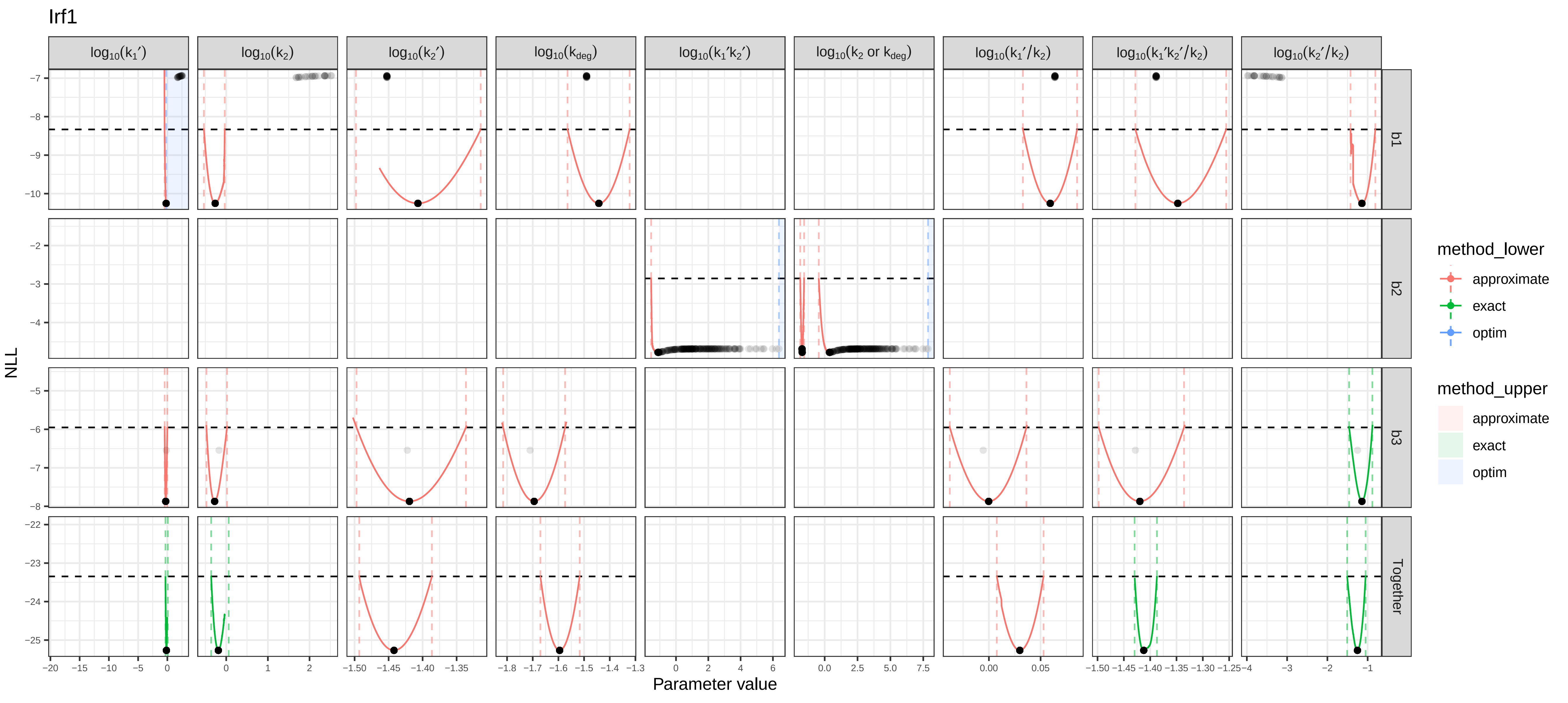

Replicate	Par	Best value	CI95 LB	CI95 UB	Method LB	Method UB
Together	$\log_{10}(k_1')$	−0.159	−0.325	0.08548	exact	exact
Together	$\log_{10}(k_2)$	−0.1888	−0.3612	0.05955	exact	exact
Together	$\log_{10}(k_2')$	−1.442	−1.493	−1.386	approximate	approximate
Together	$\log_{10}(k_{deg})$	−1.595	−1.67	−1.517	approximate	approximate
Together	$\log_{10}(k_1'/k_2)$	0.02989	0.007635	0.05287	approximate	approximate
Together	$\log_{10}(k_1'k_2'/k_2)$	−1.412	−1.43	−1.387	exact	exact
Together	$\log_{10}(k_2'/k_2)$	−1.253	−1.509	−1.051	exact	exact
b1	$\log_{10}(k_1')$	−0.2057	−0.4724	> −0.2003	approximate	optim
b1	$\log_{10}(k_2)$	−0.265	−0.5355	−0.03579	approximate	approximate
b1	$\log_{10}(k_2')$	−1.407	−1.498	−1.315	approximate	approximate
b1	$\log_{10}(k_{deg})$	−1.442	−1.564	−1.323	approximate	approximate
b1	$\log_{10}(k_1'/k_2)$	0.05931	0.03289	0.08533	approximate	approximate
b1	$\log_{10}(k_1'k_2'/k_2)$	−1.348	−1.428	−1.256	approximate	approximate
b1	$\log_{10}(k_2'/k_2)$	−1.142	−1.425	−0.809	approximate	approximate
b2	$\log_{10}(k_1'k_2')$	−1.101	−1.538	> 6.373	approximate	optim
b2	$\log_{10}(k_2 \text{ or } k_{deg})$	0.3775	−0.4496	> 7.863	approximate	optim
b2	$\log_{10}(k_2 \text{ or } k_{deg})$	−1.709	−1.857	−1.565	approximate	approximate
b3	$\log_{10}(k_1')$	−0.2778	−0.4591	−0.006856	approximate	approximate
b3	$\log_{10}(k_2)$	−0.2776	−0.4746	0.02001	approximate	approximate
b3	$\log_{10}(k_2')$	−1.419	−1.497	−1.336	approximate	approximate
b3	$\log_{10}(k_{deg})$	−1.694	−1.815	−1.574	approximate	approximate
b3	$\log_{10}(k_1'/k_2)$	−0.0002268	−0.03782	0.03628	approximate	approximate
b3	$\log_{10}(k_1'k_2'/k_2)$	−1.42	−1.498	−1.336	approximate	approximate
b3	$\log_{10}(k_2'/k_2)$	−1.142	−1.461	−0.8832	exact	exact

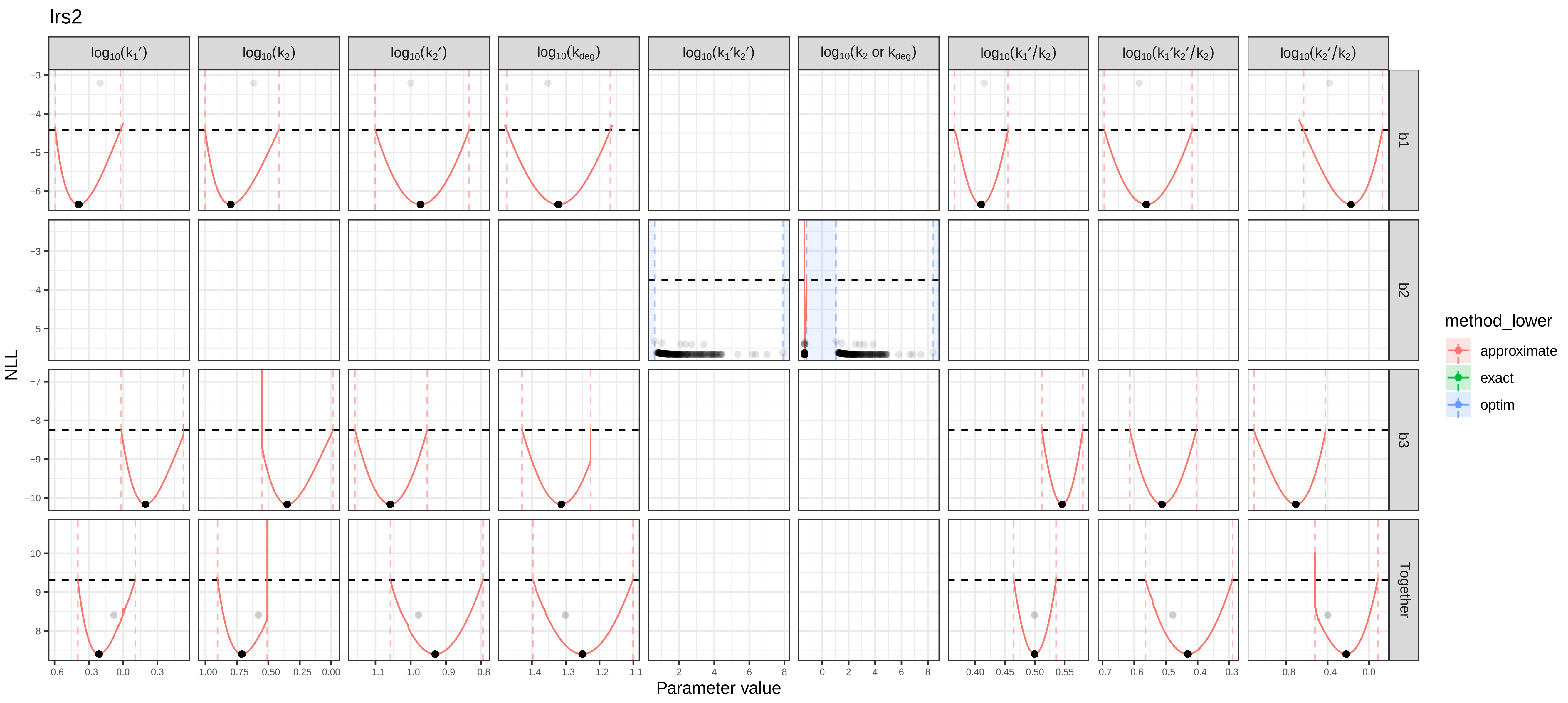

Replicate	Par	Best value	CI95 LB	CI95 UB	Method LB	Method UB
Together	$\log_{10}(k_1')$	−0.2104	−0.3968	0.1075	approximate	approximate
Together	$\log_{10}(k_2)$	−0.7102	−0.9035	−0.5082	approximate	approximate
Together	$\log_{10}(k_2')$	−0.9305	−1.057	−0.7945	approximate	approximate
Together	$\log_{10}(k_{deg})$	−1.251	−1.397	−1.101	approximate	approximate
Together	$\log_{10}(k_1'/k_2)$	0.4997	0.4643	0.5356	approximate	approximate
Together	$\log_{10}(k_1'k_2'/k_2)$	−0.4307	−0.5645	−0.2894	approximate	approximate
Together	$\log_{10}(k_2'/k_2)$	−0.2203	−0.522	0.08477	approximate	approximate
b1	$\log_{10}(k_1')$	−0.388	−0.592	−0.02289	approximate	approximate
b1	$\log_{10}(k_2)$	−0.7978	−1.002	−0.4165	approximate	approximate
b1	$\log_{10}(k_2')$	−0.972	−1.1	−0.8343	approximate	approximate
b1	$\log_{10}(k_{deg})$	−1.322	−1.474	−1.168	approximate	approximate
b1	$\log_{10}(k_1'/k_2)$	0.4098	0.3653	0.455	approximate	approximate
b1	$\log_{10}(k_1'k_2'/k_2)$	−0.5622	−0.6945	−0.4164	approximate	approximate
b1	$\log_{10}(k_2'/k_2)$	−0.1742	−0.6327	0.1297	approximate	approximate
b2	$\log_{10}(k_1'k_2')$	4.084	< 0.5924	> 7.919	optim	optim
b2	$\log_{10}(k_2 \text{ or } k_{deg})$	4.555	1.044	> 8.382	optim	optim
b2	$\log_{10}(k_2 \text{ or } k_{deg})$	−1.339	−1.347	−1.188	approximate	approximate
b3	$\log_{10}(k_1')$	0.1957	−0.01711	0.5276	approximate	approximate
b3	$\log_{10}(k_2)$	−0.3501	−0.5495	0.01692	approximate	approximate
b3	$\log_{10}(k_2')$	−1.058	−1.158	−0.953	approximate	approximate
b3	$\log_{10}(k_{deg})$	−1.313	−1.429	−1.226	approximate	approximate
b3	$\log_{10}(k_1'/k_2)$	0.5458	0.5116	0.5802	approximate	approximate
b3	$\log_{10}(k_1'k_2'/k_2)$	−0.512	−0.6142	−0.4036	approximate	approximate
b3	$\log_{10}(k_2'/k_2)$	−0.7078	−1.111	−0.4206	approximate	approximate

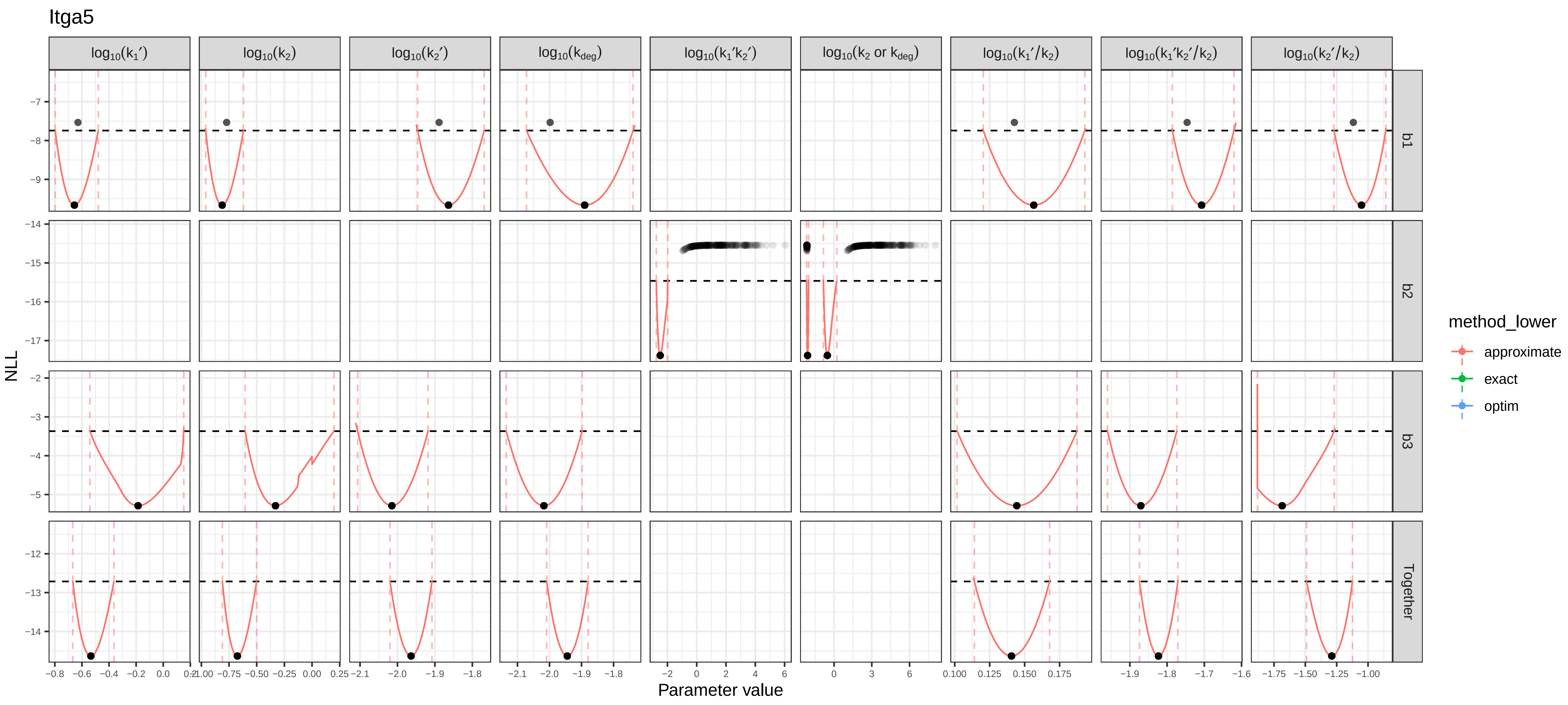

Replicate	Par	Best value	CI95 LB	CI95 UB	Method LB	Method UB
Together	$\log_{10}(k_1')$	−0.534	−0.6674	−0.3638	approximate	approximate
Together	$\log_{10}(k_2)$	−0.6746	−0.8105	−0.5007	approximate	approximate
Together	$\log_{10}(k_2')$	−1.964	−2.02	−1.908	approximate	approximate
Together	$\log_{10}(k_{deg})$	−1.945	−2.009	−1.879	approximate	approximate
Together	$\log_{10}(k_1'/k_2)$	0.1406	0.114	0.1679	approximate	approximate
Together	$\log_{10}(k_1'k_2'/k_2)$	−1.823	−1.874	−1.771	approximate	approximate
Together	$\log_{10}(k_2'/k_2)$	−1.289	−1.49	−1.124	approximate	approximate
b1	$\log_{10}(k_1')$	−0.6554	−0.7975	−0.4792	approximate	approximate
b1	$\log_{10}(k_2)$	−0.8119	−0.9611	−0.6197	approximate	approximate
b1	$\log_{10}(k_2')$	−1.864	−1.946	−1.768	approximate	approximate
b1	$\log_{10}(k_{deg})$	−1.89	−2.072	−1.739	approximate	approximate
b1	$\log_{10}(k_1'/k_2)$	0.1565	0.1204	0.193	approximate	approximate
b1	$\log_{10}(k_1'k_2'/k_2)$	−1.707	−1.786	−1.62	approximate	approximate
b1	$\log_{10}(k_2'/k_2)$	−1.052	−1.273	−0.8583	approximate	approximate
b2	$\log_{10}(k_1'k_2')$	−2.492	−2.76	−1.981	approximate	approximate
b2	$\log_{10}(k_2 \text{ or } k_{deg})$	−0.5296	−0.8441	0.2259	approximate	approximate
b2	$\log_{10}(k_2 \text{ or } k_{deg})$	−2.1	−2.183	−2.022	approximate	approximate
b3	$\log_{10}(k_1')$	−0.1859	−0.5415	0.1516	approximate	approximate
b3	$\log_{10}(k_2)$	−0.3302	−0.6044	0.1979	approximate	approximate
b3	$\log_{10}(k_2')$	−2.015	−2.106	−1.918	approximate	approximate
b3	$\log_{10}(k_{deg})$	−2.018	−2.135	−1.898	approximate	approximate
b3	$\log_{10}(k_1'/k_2)$	0.1444	0.1017	0.1874	approximate	approximate
b3	$\log_{10}(k_1'k_2'/k_2)$	−1.871	−1.96	−1.774	approximate	approximate
b3	$\log_{10}(k_2'/k_2)$	−1.685	−1.882	−1.27	approximate	approximate

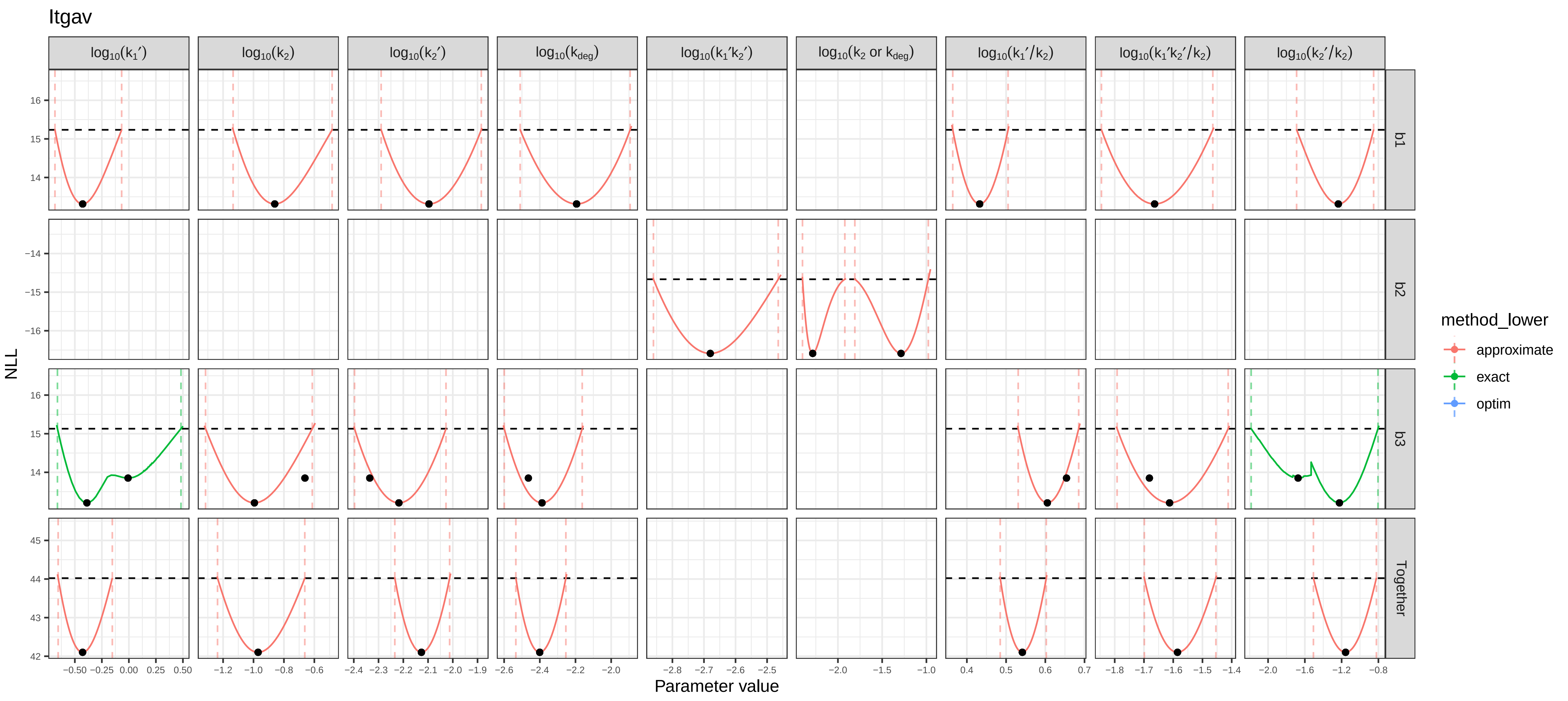

Replicate	Par	Best value	CI95 LB	CI95 UB	Method LB	Method UB
Together	$\log_{10}(k_1')$	−0.4283	−0.6546	−0.1533	approximate	approximate
Together	$\log_{10}(k_2)$	−0.9698	−1.236	−0.6628	approximate	approximate
Together	$\log_{10}(k_2')$	−2.127	−2.234	−2.013	approximate	approximate
Together	$\log_{10}(k_{deg})$	−2.4	−2.534	−2.254	approximate	approximate
Together	$\log_{10}(k_1'/k_2)$	0.5414	0.485	0.6025	approximate	approximate
Together	$\log_{10}(k_1'k_2'/k_2)$	−1.585	−1.699	−1.454	approximate	approximate
Together	$\log_{10}(k_2'/k_2)$	−1.157	−1.507	−0.8217	approximate	approximate
b1	$\log_{10}(k_1')$	−0.4276	−0.6841	−0.06691	approximate	approximate
b1	$\log_{10}(k_2)$	−0.8602	−1.134	−0.4832	approximate	approximate
b1	$\log_{10}(k_2')$	−2.096	−2.289	−1.885	approximate	approximate
b1	$\log_{10}(k_{deg})$	−2.194	−2.51	−1.894	approximate	approximate
b1	$\log_{10}(k_1'/k_2)$	0.4326	0.364	0.5051	approximate	approximate
b1	$\log_{10}(k_1'k_2'/k_2)$	−1.664	−1.845	−1.465	approximate	approximate
b1	$\log_{10}(k_2'/k_2)$	−1.236	−1.689	−0.8517	approximate	approximate
b2	$\log_{10}(k_1'k_2')$	−2.68	−2.86	−2.465	approximate	approximate
b2	$\log_{10}(k_2 \text{ or } k_{deg})$	−1.287	−1.809	−0.9781	approximate	approximate
b2	$\log_{10}(k_2 \text{ or } k_{deg})$	−2.285	−2.4	−1.922	approximate	approximate
b3	$\log_{10}(k_1')$	−0.3885	−0.6611	0.4822	exact	exact
b3	$\log_{10}(k_2)$	−0.9938	−1.315	−0.6136	approximate	approximate
b3	$\log_{10}(k_2')$	−2.218	−2.397	−2.027	approximate	approximate
b3	$\log_{10}(k_{deg})$	−2.387	−2.599	−2.162	approximate	approximate
b3	$\log_{10}(k_1'/k_2)$	0.6053	0.5308	0.6851	approximate	approximate
b3	$\log_{10}(k_1'k_2'/k_2)$	−1.612	−1.792	−1.412	approximate	approximate
b3	$\log_{10}(k_2'/k_2)$	−1.224	−2.184	−0.8045	exact	exact

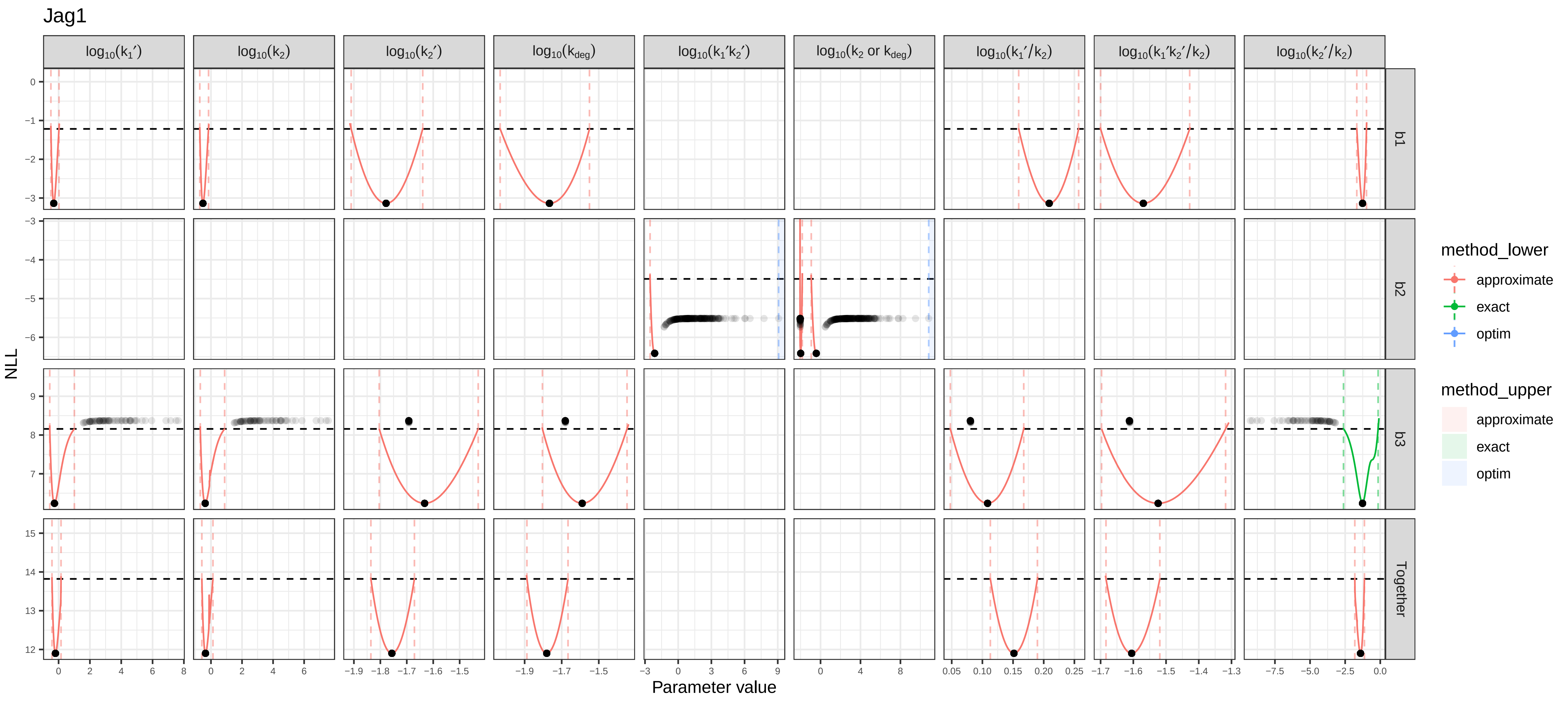

Replicate	Par	Best value	CI95 LB	CI95 UB	Method LB	Method UB
Together	$\log_{10}(k_1')$	−0.2003	−0.4216	0.1562	approximate	approximate
Together	$\log_{10}(k_2)$	−0.3518	−0.5874	0.1291	approximate	approximate
Together	$\log_{10}(k_2')$	−1.756	−1.836	−1.671	approximate	approximate
Together	$\log_{10}(k_{deg})$	−1.781	−1.888	−1.666	approximate	approximate
Together	$\log_{10}(k_1'/k_2)$	0.1515	0.113	0.1897	approximate	approximate
Together	$\log_{10}(k_1'k_2'/k_2)$	−1.605	−1.684	−1.519	approximate	approximate
Together	$\log_{10}(k_2'/k_2)$	−1.405	−1.812	−1.129	approximate	approximate
b1	$\log_{10}(k_1')$	−0.309	−0.4904	0.02741	approximate	approximate
b1	$\log_{10}(k_2)$	−0.5179	−0.7206	−0.1558	approximate	approximate
b1	$\log_{10}(k_2')$	−1.778	−1.91	−1.64	approximate	approximate
b1	$\log_{10}(k_{deg})$	−1.766	−2.032	−1.55	approximate	approximate
b1	$\log_{10}(k_1'/k_2)$	0.2089	0.1592	0.2569	approximate	approximate
b1	$\log_{10}(k_1'k_2'/k_2)$	−1.569	−1.7	−1.428	approximate	approximate
b1	$\log_{10}(k_2'/k_2)$	−1.26	−1.671	−0.9792	approximate	approximate
b2	$\log_{10}(k_1'k_2')$	−2.126	−2.54	> 9.079	approximate	optim
b2	$\log_{10}(k_2 \text{ or } k_{deg})$	−0.4265	−0.9292	> 10.83	approximate	optim
b2	$\log_{10}(k_2 \text{ or } k_{deg})$	−1.988	−2.052	−1.829	approximate	approximate
b3	$\log_{10}(k_1')$	−0.2638	−0.5627	1.008	approximate	approximate
b3	$\log_{10}(k_2)$	−0.3722	−0.6882	0.8807	approximate	approximate
b3	$\log_{10}(k_2')$	−1.633	−1.804	−1.43	approximate	approximate
b3	$\log_{10}(k_{deg})$	−1.589	−1.804	−1.347	approximate	approximate
b3	$\log_{10}(k_1'/k_2)$	0.1085	0.04772	0.1674	approximate	approximate
b3	$\log_{10}(k_1'k_2'/k_2)$	−1.524	−1.697	−1.318	approximate	approximate
b3	$\log_{10}(k_2'/k_2)$	−1.26	−2.618	−0.153	exact	exact

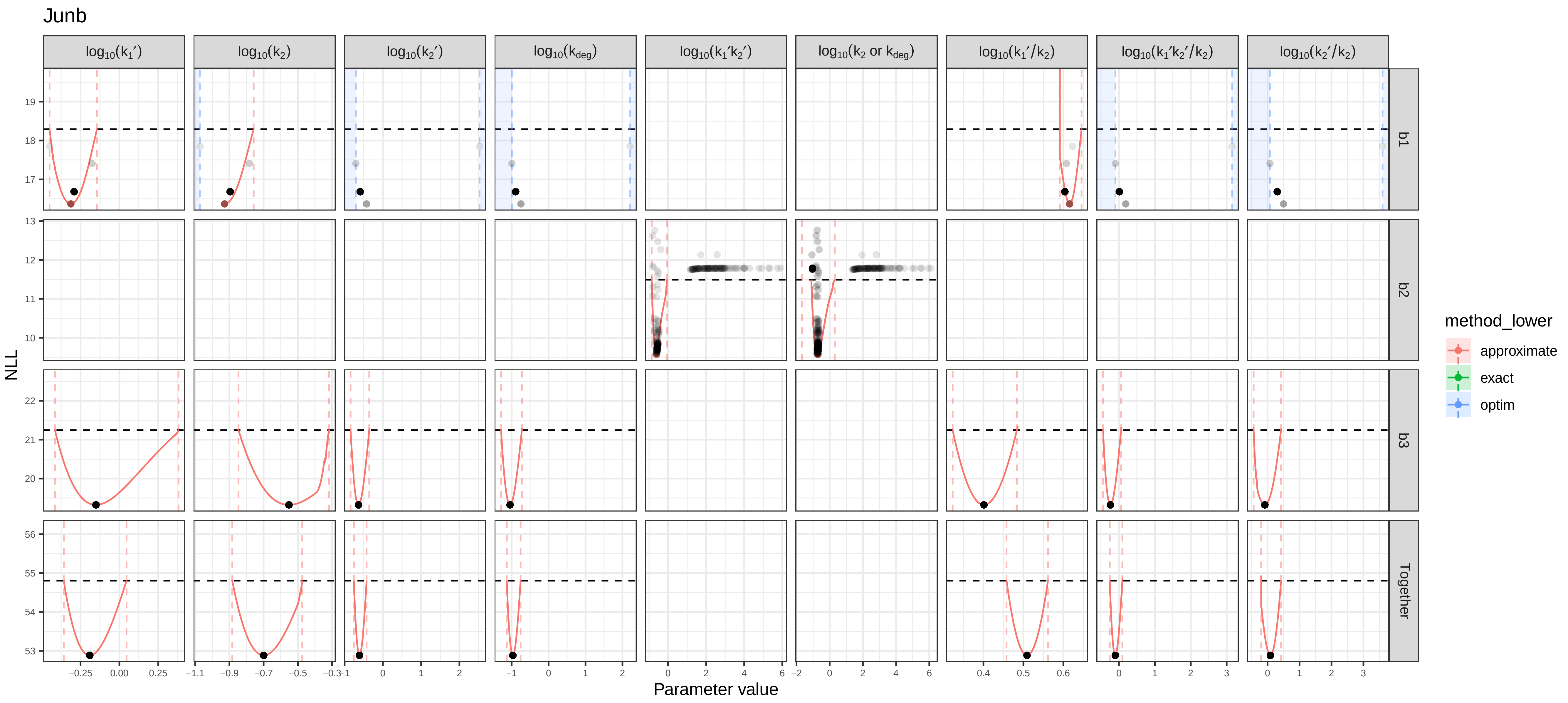

Replicate	Par	Best value	CI95 LB	CI95 UB	Method LB	Method UB
Together	$\log_{10}(k_1')$	−0.1905	−0.358	0.04605	approximate	approximate
Together	$\log_{10}(k_2)$	−0.6995	−0.8833	−0.4744	approximate	approximate
Together	$\log_{10}(k_2')$	−0.612	−0.7589	−0.4262	approximate	approximate
Together	$\log_{10}(k_{deg})$	−0.9673	−1.131	−0.7579	approximate	approximate
Together	$\log_{10}(k_1'/k_2)$	0.509	0.4578	0.5615	approximate	approximate
Together	$\log_{10}(k_1'k_2'/k_2)$	−0.1029	−0.2552	0.09416	approximate	approximate
Together	$\log_{10}(k_2'/k_2)$	0.08756	−0.2002	0.4228	approximate	approximate
b1	$\log_{10}(k_1')$	−0.3123	−0.4494	−0.1444	approximate	approximate
b1	$\log_{10}(k_2)$	−0.928	< −1.072	−0.7582	optim	approximate
b1	$\log_{10}(k_2')$	−0.4265	< −0.7069	> 2.527	optim	optim
b1	$\log_{10}(k_{deg})$	−0.7491	< −0.9927	> 2.206	optim	optim
b1	$\log_{10}(k_1'/k_2)$	0.6158	0.5912	0.6455	approximate	approximate
b1	$\log_{10}(k_1'k_2'/k_2)$	0.1892	< −0.09892	> 3.15	optim	optim
b1	$\log_{10}(k_2'/k_2)$	0.5015	< 0.07515	> 3.598	optim	optim
b2	$\log_{10}(k_1'k_2')$	−0.6008	−0.8637	−0.05898	approximate	approximate
b2	$\log_{10}(k_2 \text{ or } k_{deg})$	−0.7001	−1.665	0.3091	approximate	approximate
b2	$\log_{10}(k_2 \text{ or } k_{deg})$	−0.7429	−1.665	0.3091	approximate	approximate
b3	$\log_{10}(k_1')$	−0.1508	−0.4149	0.3808	approximate	approximate
b3	$\log_{10}(k_2)$	−0.5521	−0.8466	−0.3178	approximate	approximate
b3	$\log_{10}(k_2')$	−0.6368	−0.8444	−0.357	approximate	approximate
b3	$\log_{10}(k_{deg})$	−1.044	−1.286	−0.7229	approximate	approximate
b3	$\log_{10}(k_1'/k_2)$	0.4013	0.3229	0.4836	approximate	approximate
b3	$\log_{10}(k_1'k_2'/k_2)$	−0.2356	−0.4416	0.06118	approximate	approximate
b3	$\log_{10}(k_2'/k_2)$	−0.08477	−0.4363	0.4222	approximate	approximate

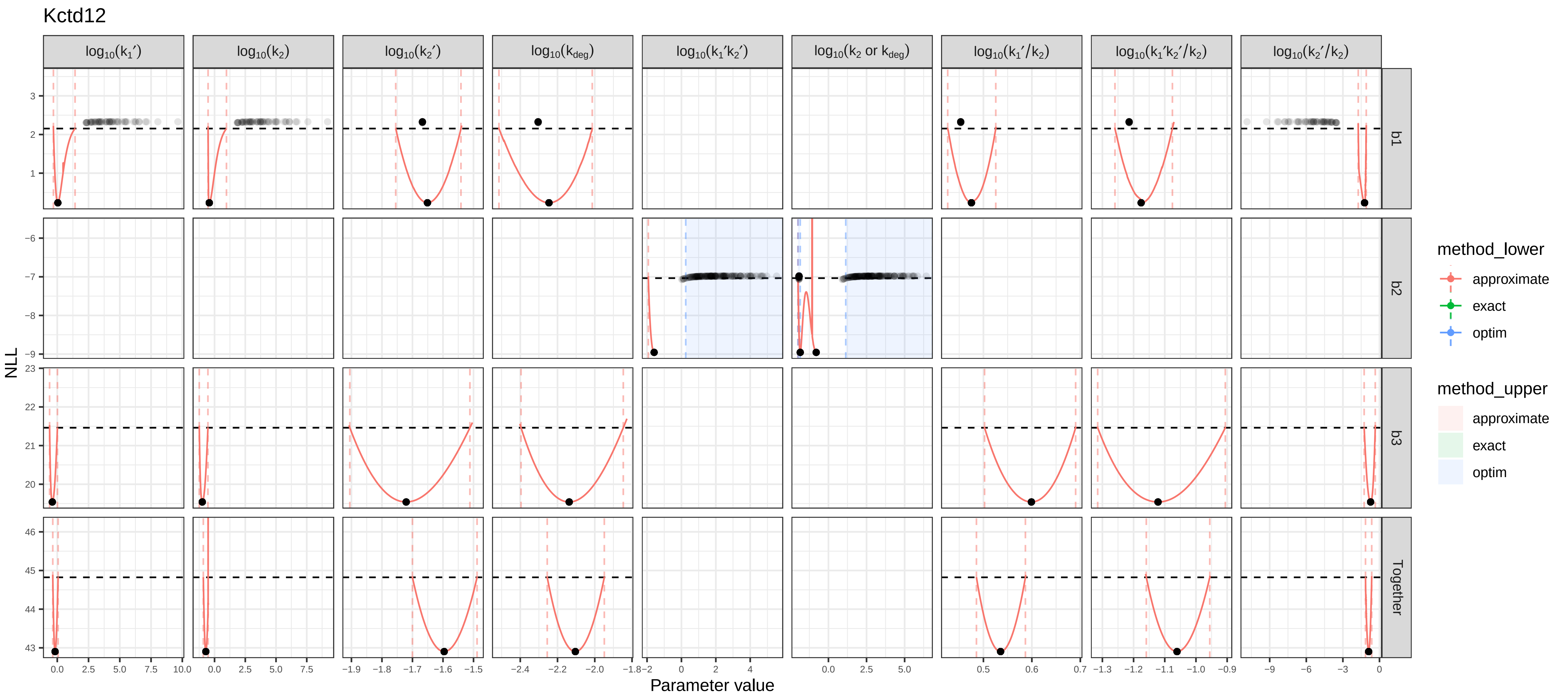

Replicate	Par	Best value	CI95 LB	CI95 UB	Method LB	Method UB
Together	$\log_{10}(k_1')$	−0.1739	−0.3592	0.06726	approximate	approximate
Together	$\log_{10}(k_2)$	−0.7093	−0.9131	−0.5216	approximate	approximate
Together	$\log_{10}(k_2')$	−1.596	−1.7	−1.488	approximate	approximate
Together	$\log_{10}(k_{deg})$	−2.104	−2.255	−1.949	approximate	approximate
Together	$\log_{10}(k_1'/k_2)$	0.5354	0.4856	0.5867	approximate	approximate
Together	$\log_{10}(k_1'k_2'/k_2)$	−1.061	−1.159	−0.9557	approximate	approximate
Together	$\log_{10}(k_2'/k_2)$	−0.8868	−1.145	−0.6359	approximate	approximate
b1	$\log_{10}(k_1')$	0.04641	−0.3086	1.423	approximate	approximate
b1	$\log_{10}(k_2)$	−0.4289	−0.5249	0.9702	approximate	approximate
b1	$\log_{10}(k_2')$	−1.651	−1.755	−1.541	approximate	approximate
b1	$\log_{10}(k_{deg})$	−2.246	−2.514	−2.014	approximate	approximate
b1	$\log_{10}(k_1'/k_2)$	0.4753	0.4263	0.5255	approximate	approximate
b1	$\log_{10}(k_1'k_2'/k_2)$	−1.176	−1.26	−1.075	approximate	approximate
b1	$\log_{10}(k_2'/k_2)$	−1.222	−1.741	−1.085	approximate	approximate
b2	$\log_{10}(k_1'k_2')$	−1.588	−1.925	> 0.2507	approximate	optim
b2	$\log_{10}(k_2 \text{ or } k_{deg})$	−0.8027	−1.997	> 1.136	approximate	optim
b2	$\log_{10}(k_2 \text{ or } k_{deg})$	−1.851	−1.997	−1.85	approximate	optim
b3	$\log_{10}(k_1')$	−0.3965	−0.615	0.01309	approximate	approximate
b3	$\log_{10}(k_2)$	−0.9955	−1.242	−0.5378	approximate	approximate
b3	$\log_{10}(k_2')$	−1.721	−1.905	−1.512	approximate	approximate
b3	$\log_{10}(k_{deg})$	−2.137	−2.396	−1.847	approximate	approximate
b3	$\log_{10}(k_1'/k_2)$	0.599	0.5023	0.6903	approximate	approximate
b3	$\log_{10}(k_1'k_2'/k_2)$	−1.122	−1.315	−0.9059	approximate	approximate
b3	$\log_{10}(k_2'/k_2)$	−0.7251	−1.255	−0.3458	approximate	approximate

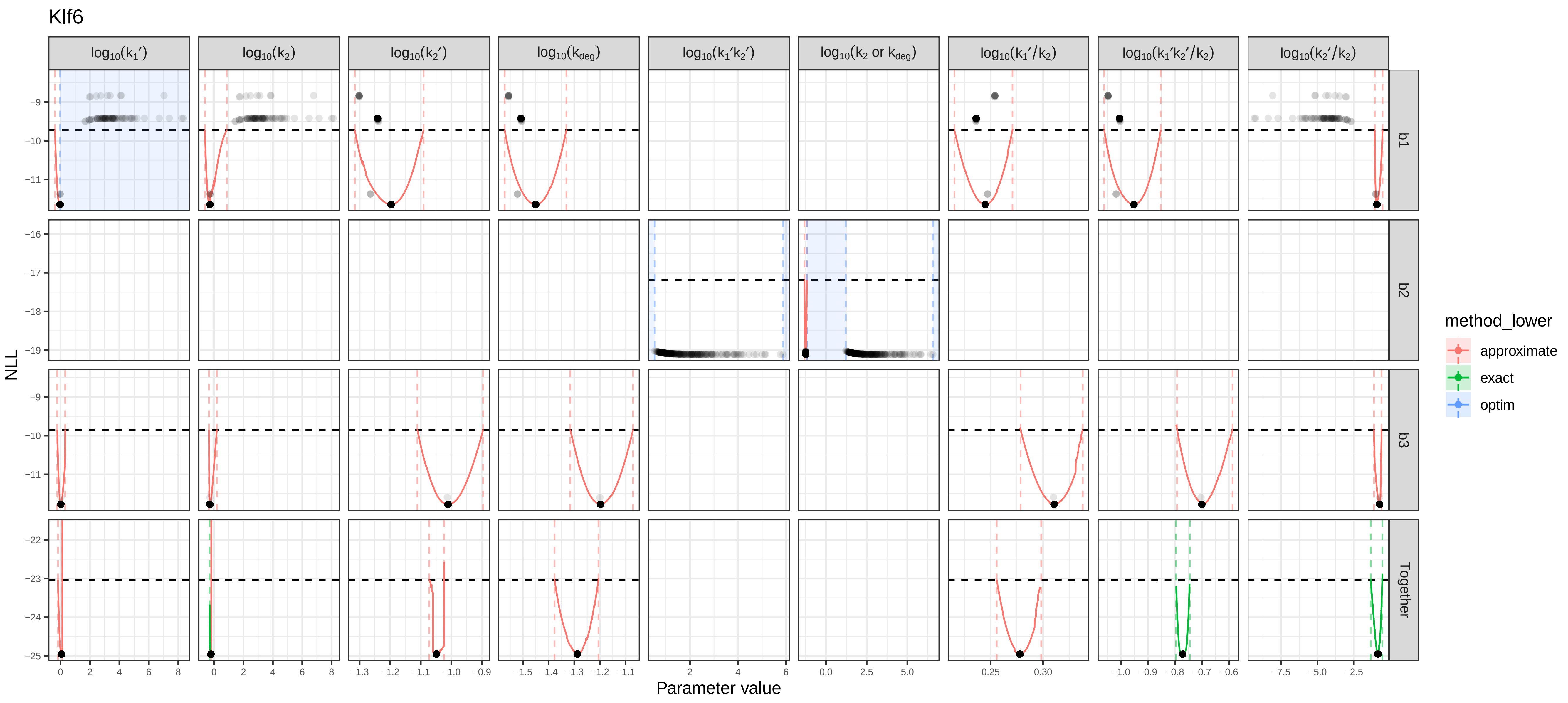

Replicate	Par	Best value	CI95 LB	CI95 UB	Method LB	Method UB
Together	$\log_{10}(k_1')$	0.06897	−0.1708	0.1215	approximate	approximate
Together	$\log_{10}(k_2)$	−0.2089	−0.302	−0.1883	exact	approximate
Together	$\log_{10}(k_2')$	−1.049	−1.072	−1.024	approximate	approximate
Together	$\log_{10}(k_{deg})$	−1.289	−1.377	−1.206	approximate	approximate
Together	$\log_{10}(k_1'/k_2)$	0.2778	0.2558	0.2982	approximate	approximate
Together	$\log_{10}(k_1'k_2'/k_2)$	−0.7709	−0.7959	−0.7449	exact	exact
Together	$\log_{10}(k_2'/k_2)$	−0.8399	−1.339	−0.5394	exact	exact
b1	$\log_{10}(k_1')$	−0.03789	−0.373	> −0.02658	approximate	optim
b1	$\log_{10}(k_2)$	−0.2826	−0.623	0.8631	approximate	approximate
b1	$\log_{10}(k_2')$	−1.197	−1.316	−1.091	approximate	approximate
b1	$\log_{10}(k_{deg})$	−1.451	−1.571	−1.331	approximate	approximate
b1	$\log_{10}(k_1'/k_2)$	0.2448	0.2155	0.2708	approximate	approximate
b1	$\log_{10}(k_1'k_2'/k_2)$	−0.9523	−1.062	−0.8521	approximate	approximate
b1	$\log_{10}(k_2'/k_2)$	−0.9144	−1.059	−0.5207	approximate	approximate
b2	$\log_{10}(k_1'k_2')$	4.027	< 0.5255	> 5.874	optim	optim
b2	$\log_{10}(k_2 \text{ or } k_{deg})$	4.721	1.217	> 6.568	optim	optim
b2	$\log_{10}(k_2 \text{ or } k_{deg})$	−1.246	−1.322	−1.171	approximate	approximate
b3	$\log_{10}(k_1')$	0.02365	−0.2212	0.32	approximate	approximate
b3	$\log_{10}(k_2)$	−0.2868	−0.3411	0.1945	approximate	approximate
b3	$\log_{10}(k_2')$	−1.011	−1.111	−0.8959	approximate	approximate
b3	$\log_{10}(k_{deg})$	−1.198	−1.316	−1.071	approximate	approximate
b3	$\log_{10}(k_1'/k_2)$	0.3105	0.2786	0.3378	approximate	approximate
b3	$\log_{10}(k_1'k_2'/k_2)$	−0.7002	−0.7918	−0.5867	approximate	approximate
b3	$\log_{10}(k_2'/k_2)$	−0.7239	−1.108	−0.5883	approximate	approximate

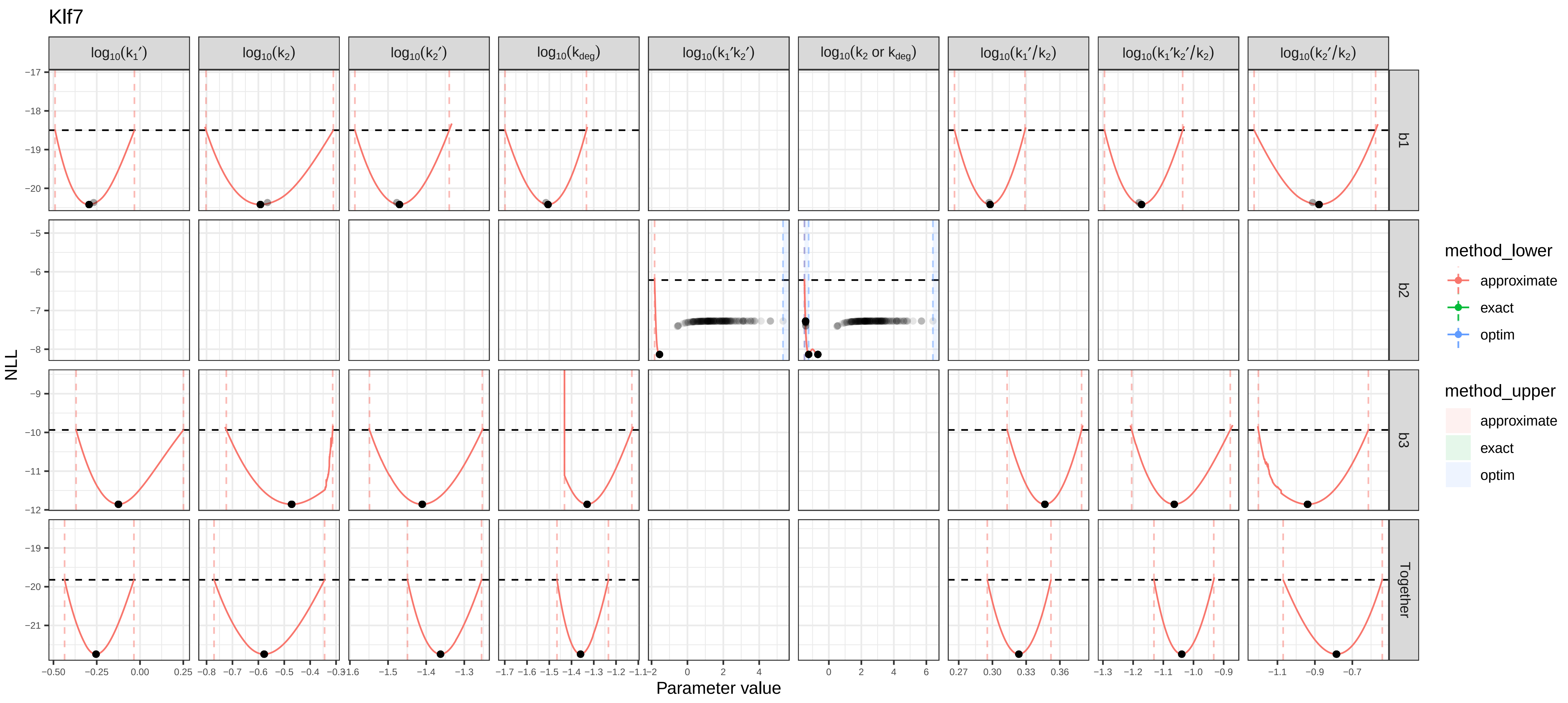

Replicate	Par	Best value	CI95 LB	CI95 UB	Method LB	Method UB
Together	$\log_{10}(k_1')$	−0.2541	−0.4358	−0.03521	approximate	approximate
Together	$\log_{10}(k_2)$	−0.5776	−0.7711	−0.3442	approximate	approximate
Together	$\log_{10}(k_2')$	−1.362	−1.449	−1.255	approximate	approximate
Together	$\log_{10}(k_{deg})$	−1.359	−1.465	−1.234	approximate	approximate
Together	$\log_{10}(k_1'/k_2)$	0.3235	0.2954	0.3521	approximate	approximate
Together	$\log_{10}(k_1'k_2'/k_2)$	−1.039	−1.131	−0.9328	approximate	approximate
Together	$\log_{10}(k_2'/k_2)$	−0.7847	−1.069	−0.54	approximate	approximate
b1	$\log_{10}(k_1')$	−0.2942	−0.4906	−0.03233	approximate	approximate
b1	$\log_{10}(k_2)$	−0.5921	−0.8021	−0.3105	approximate	approximate
b1	$\log_{10}(k_2')$	−1.47	−1.587	−1.339	approximate	approximate
b1	$\log_{10}(k_{deg})$	−1.506	−1.699	−1.333	approximate	approximate
b1	$\log_{10}(k_1'/k_2)$	0.2979	0.2663	0.3289	approximate	approximate
b1	$\log_{10}(k_1'k_2'/k_2)$	−1.172	−1.295	−1.036	approximate	approximate
b1	$\log_{10}(k_2'/k_2)$	−0.8778	−1.224	−0.5757	approximate	approximate
b2	$\log_{10}(k_1'k_2')$	−1.555	−1.83	> 5.329	approximate	optim
b2	$\log_{10}(k_2 \text{ or } k_{deg})$	−0.6745	−1.5	> 6.403	approximate	optim
b2	$\log_{10}(k_2 \text{ or } k_{deg})$	−1.243	−1.5	−1.242	approximate	optim
b3	$\log_{10}(k_1')$	−0.1243	−0.3693	0.251	approximate	approximate
b3	$\log_{10}(k_2)$	−0.471	−0.7237	−0.3131	approximate	approximate
b3	$\log_{10}(k_2')$	−1.41	−1.549	−1.253	approximate	approximate
b3	$\log_{10}(k_{deg})$	−1.33	−1.432	−1.129	approximate	approximate
b3	$\log_{10}(k_1'/k_2)$	0.3467	0.3131	0.3793	approximate	approximate
b3	$\log_{10}(k_1'k_2'/k_2)$	−1.064	−1.205	−0.8781	approximate	approximate
b3	$\log_{10}(k_2'/k_2)$	−0.9394	−1.202	−0.6143	approximate	approximate

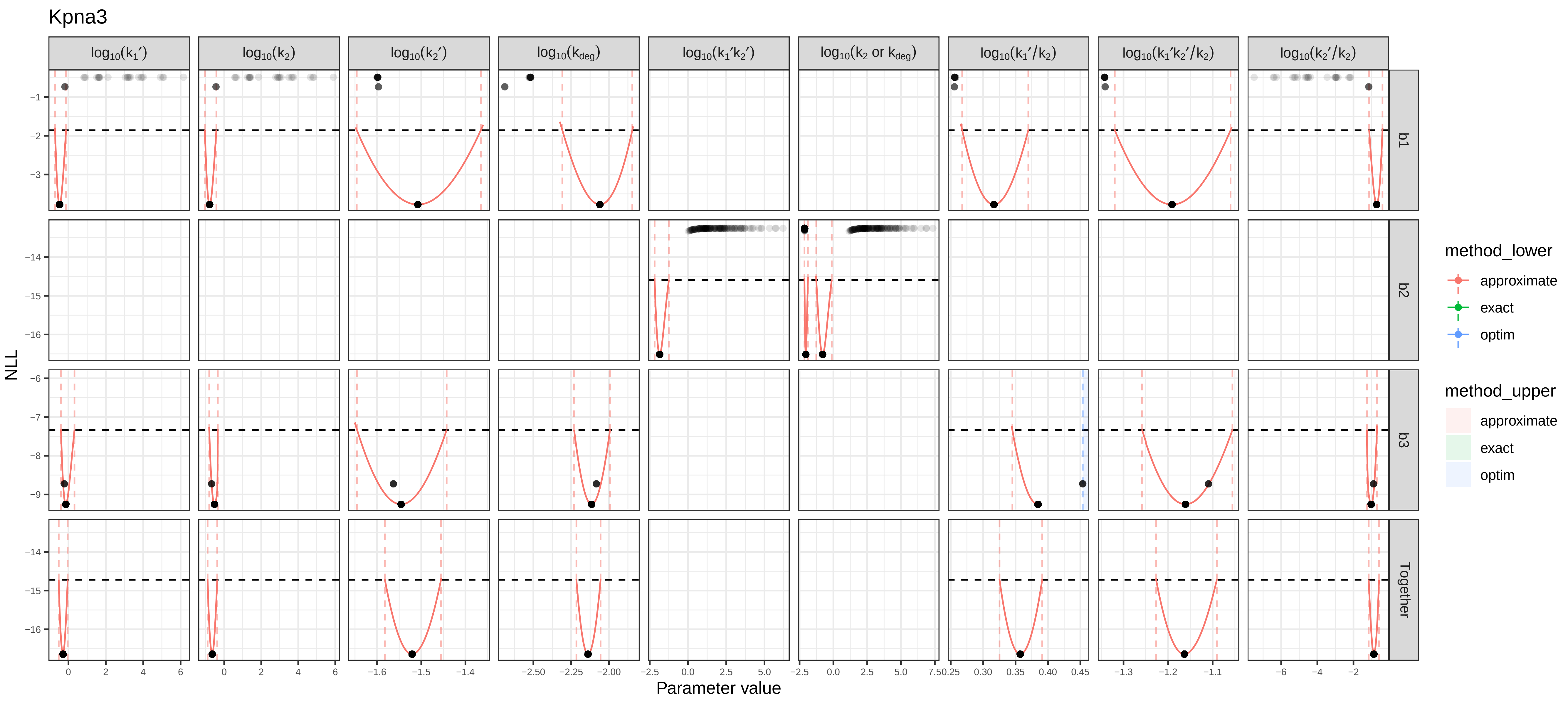

Replicate	Par	Best value	CI95 LB	CI95 UB	Method LB	Method UB
Together	$\log_{10}(k_1')$	−0.2911	−0.5203	−0.03949	approximate	approximate
Together	$\log_{10}(k_2)$	−0.6484	−0.8975	−0.377	approximate	approximate
Together	$\log_{10}(k_2')$	−1.521	−1.582	−1.455	approximate	approximate
Together	$\log_{10}(k_{deg})$	−2.139	−2.215	−2.056	approximate	approximate
Together	$\log_{10}(k_1'/k_2)$	0.3573	0.3253	0.3911	approximate	approximate
Together	$\log_{10}(k_1'k_2'/k_2)$	−1.163	−1.227	−1.091	approximate	approximate
Together	$\log_{10}(k_2'/k_2)$	−0.8722	−1.159	−0.589	approximate	approximate
b1	$\log_{10}(k_1')$	−0.472	−0.7149	−0.1278	approximate	approximate
b1	$\log_{10}(k_2)$	−0.7887	−1.044	−0.4242	approximate	approximate
b1	$\log_{10}(k_2')$	−1.508	−1.646	−1.365	approximate	approximate
b1	$\log_{10}(k_{deg})$	−2.06	−2.309	−1.846	approximate	approximate
b1	$\log_{10}(k_1'/k_2)$	0.3167	0.2677	0.3697	approximate	approximate
b1	$\log_{10}(k_1'k_2'/k_2)$	−1.191	−1.32	−1.06	approximate	approximate
b1	$\log_{10}(k_2'/k_2)$	−0.719	−1.134	−0.3969	approximate	approximate
b2	$\log_{10}(k_1'k_2')$	−1.851	−2.181	−1.247	approximate	approximate
b2	$\log_{10}(k_2 \text{ or } k_{deg})$	−0.7951	−1.265	−0.1214	approximate	approximate
b2	$\log_{10}(k_2 \text{ or } k_{deg})$	−2.04	−2.138	−1.884	approximate	approximate
b3	$\log_{10}(k_1')$	−0.1403	−0.3997	0.3231	approximate	approximate
b3	$\log_{10}(k_2)$	−0.525	−0.8091	−0.3471	approximate	approximate
b3	$\log_{10}(k_2')$	−1.546	−1.645	−1.443	approximate	approximate
b3	$\log_{10}(k_{deg})$	−2.115	−2.231	−1.993	approximate	approximate
b3	$\log_{10}(k_1'/k_2)$	0.3847	0.3451	> 0.4538	approximate	optim
b3	$\log_{10}(k_1'k_2'/k_2)$	−1.161	−1.258	−1.056	approximate	approximate
b3	$\log_{10}(k_2'/k_2)$	−1.021	−1.259	−0.7028	approximate	approximate

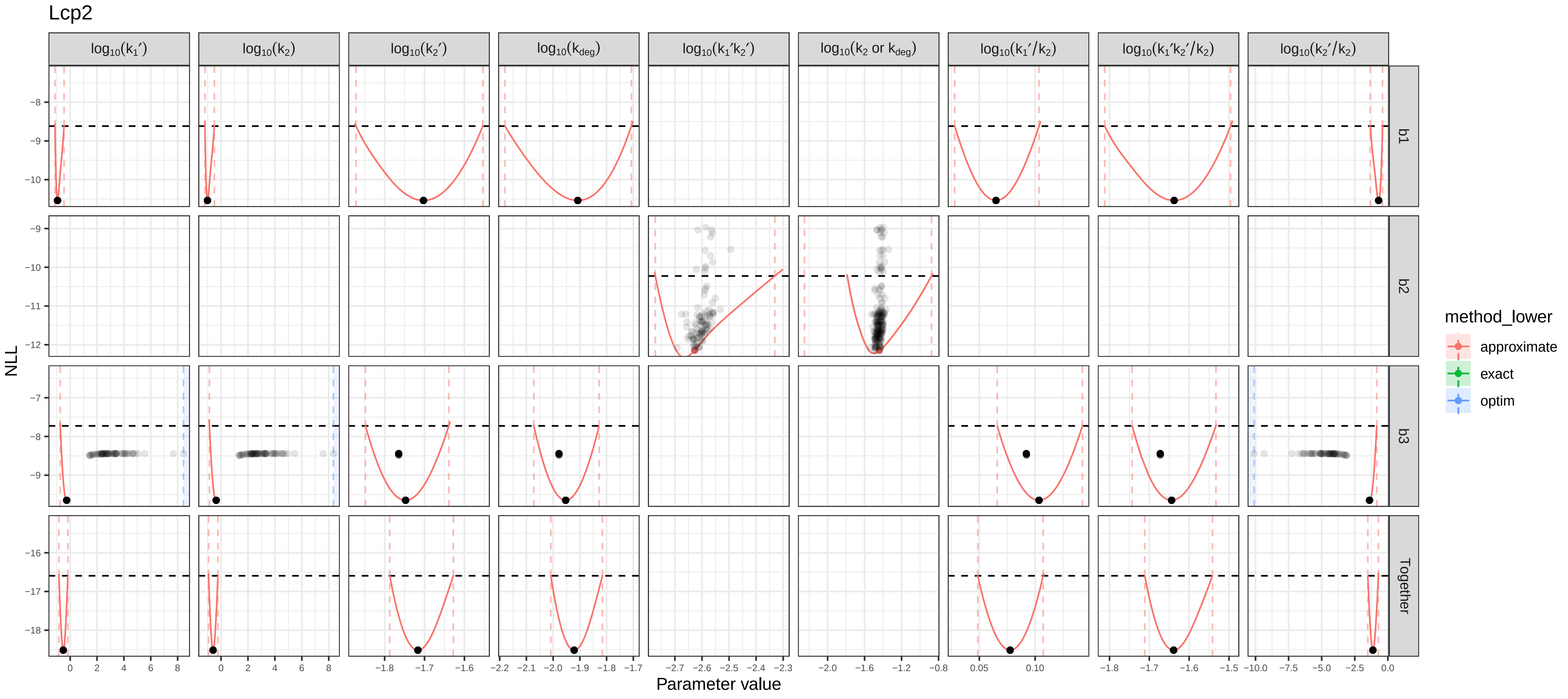

Replicate	Par	Best value	CI95 LB	CI95 UB	Method LB	Method UB
Together	$\log_{10}(k_1')$	−0.5112	−0.8379	−0.1701	approximate	approximate
Together	$\log_{10}(k_2)$	−0.5888	−0.9288	−0.2321	approximate	approximate
Together	$\log_{10}(k_2')$	−1.717	−1.787	−1.628	approximate	approximate
Together	$\log_{10}(k_{deg})$	−1.921	−2.009	−1.816	approximate	approximate
Together	$\log_{10}(k_1'/k_2)$	0.07763	0.04865	0.1072	approximate	approximate
Together	$\log_{10}(k_1'k_2'/k_2)$	−1.639	−1.711	−1.541	approximate	approximate
Together	$\log_{10}(k_2'/k_2)$	−1.128	−1.513	−0.7144	approximate	approximate
b1	$\log_{10}(k_1')$	−0.9416	−1.125	−0.4537	approximate	approximate
b1	$\log_{10}(k_2)$	−1.007	−1.194	−0.4957	approximate	approximate
b1	$\log_{10}(k_2')$	−1.703	−1.872	−1.554	approximate	approximate
b1	$\log_{10}(k_{deg})$	−1.908	−2.18	−1.707	approximate	approximate
b1	$\log_{10}(k_1'/k_2)$	0.06499	0.02794	0.1035	approximate	approximate
b1	$\log_{10}(k_1'k_2'/k_2)$	−1.638	−1.812	−1.496	approximate	approximate
b1	$\log_{10}(k_2'/k_2)$	−0.6962	−1.331	−0.4003	approximate	approximate
b2	$\log_{10}(k_1'k_2')$	−2.627	−2.773	−2.331	approximate	approximate
b2	$\log_{10}(k_2 \text{ or } k_{deg})$	−1.441	−2.25	−0.8762	approximate	approximate
b2	$\log_{10}(k_2 \text{ or } k_{deg})$	−1.488	−2.25	−0.8762	approximate	approximate
b3	$\log_{10}(k_1')$	−0.2601	−0.7402	> 8.435	approximate	optim
b3	$\log_{10}(k_2)$	−0.3636	−0.8644	> 8.342	approximate	optim
b3	$\log_{10}(k_2')$	−1.747	−1.848	−1.639	approximate	approximate
b3	$\log_{10}(k_{deg})$	−1.953	−2.072	−1.828	approximate	approximate
b3	$\log_{10}(k_1'/k_2)$	0.1035	0.06605	0.1424	approximate	approximate
b3	$\log_{10}(k_1'k_2'/k_2)$	−1.644	−1.743	−1.533	approximate	approximate
b3	$\log_{10}(k_2'/k_2)$	−1.384	< −10.11	−0.8358	optim	approximate

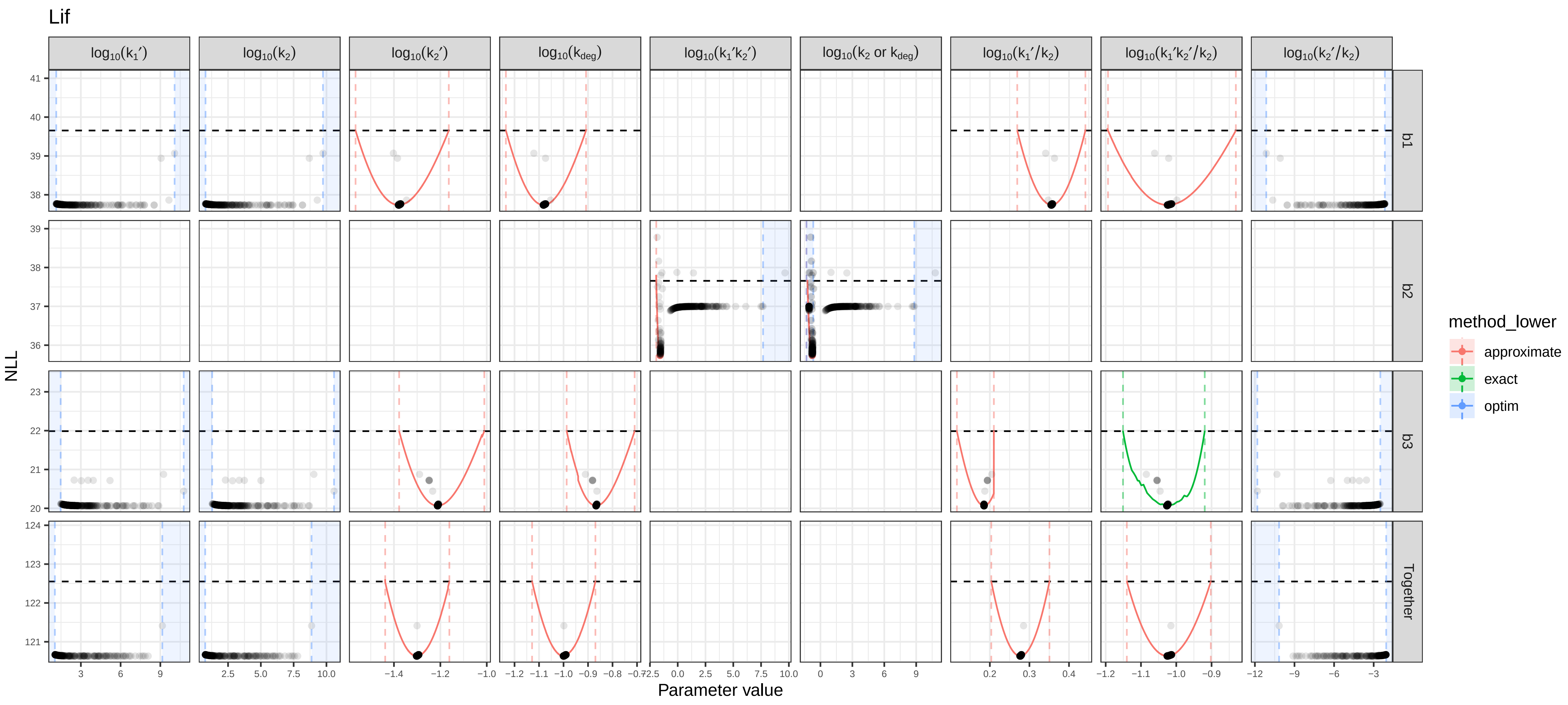

Replicate	Par	Best value	CI95 LB	CI95 UB	Method LB	Method UB
Together	$\log_{10}(k_1')$	5.887	< 1.038	> 9.151	optim	optim
Together	$\log_{10}(k_2)$	5.61	< 0.7583	> 8.865	optim	optim
Together	$\log_{10}(k_2')$	−1.302	−1.438	−1.161	approximate	approximate
Together	$\log_{10}(k_{deg})$	−0.9998	−1.128	−0.8698	approximate	approximate
Together	$\log_{10}(k_1'/k_2)$	0.2768	0.2036	0.3506	approximate	approximate
Together	$\log_{10}(k_1'k_2'/k_2)$	−1.025	−1.14	−0.9021	approximate	approximate
Together	$\log_{10}(k_2'/k_2)$	−6.912	< −10.17	> −2.051	optim	optim
b1	$\log_{10}(k_1')$	4.121	< 1.141	> 10.08	optim	optim
b1	$\log_{10}(k_2)$	3.766	< 0.7831	> 9.736	optim	optim
b1	$\log_{10}(k_2')$	−1.379	−1.565	−1.164	approximate	approximate
b1	$\log_{10}(k_{deg})$	−1.081	−1.235	−0.9081	approximate	approximate
b1	$\log_{10}(k_1'/k_2)$	0.3556	0.2692	0.4414	approximate	approximate
b1	$\log_{10}(k_1'k_2'/k_2)$	−1.023	−1.193	−0.8311	approximate	approximate
b1	$\log_{10}(k_2'/k_2)$	−5.145	< −11.14	> −2.154	optim	optim
b2	$\log_{10}(k_1'k_2')$	−1.588	−1.93	> 7.682	approximate	optim
b2	$\log_{10}(k_2 \text{ or } k_{deg})$	−0.7711	−1.315	> 8.819	approximate	optim
b2	$\log_{10}(k_2 \text{ or } k_{deg})$	−0.8039	−1.315	−0.6733	approximate	optim
b3	$\log_{10}(k_1')$	5.926	< 1.474	> 10.77	optim	optim
b3	$\log_{10}(k_2)$	5.741	< 1.289	> 10.58	optim	optim
b3	$\log_{10}(k_2')$	−1.212	−1.378	−1.011	approximate	approximate
b3	$\log_{10}(k_{deg})$	−0.8673	−0.9871	−0.7098	approximate	approximate
b3	$\log_{10}(k_1'/k_2)$	0.1853	0.1167	0.2101	approximate	approximate
b3	$\log_{10}(k_1'k_2'/k_2)$	−1.027	−1.151	−0.9193	exact	exact
b3	$\log_{10}(k_2'/k_2)$	−6.953	< −11.81	> −2.497	optim	optim

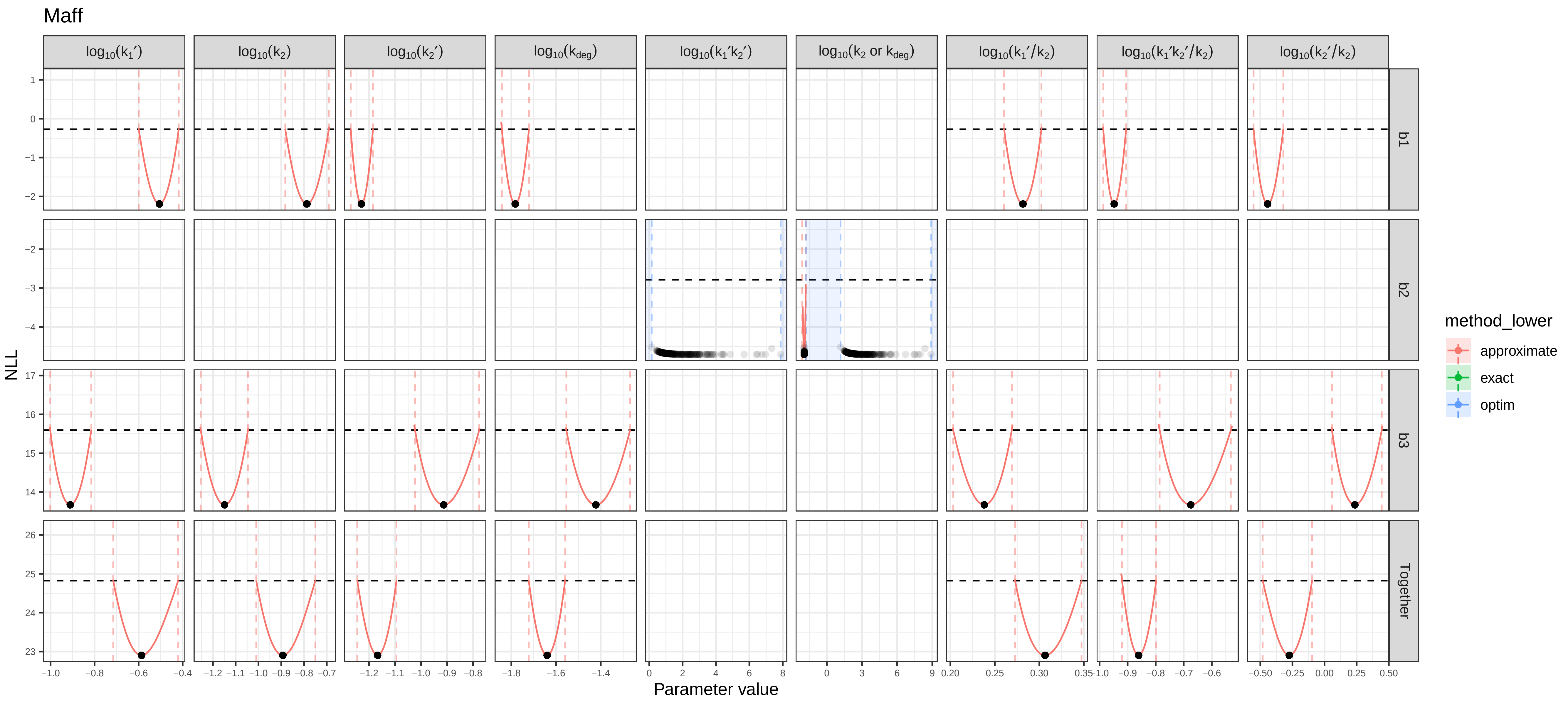

Replicate	Par	Best value	CI95 LB	CI95 UB	Method LB	Method UB
Together	$\log_{10}(k_1')$	−0.5865	−0.7154	−0.4202	approximate	approximate
Together	$\log_{10}(k_2)$	−0.8929	−1.01	−0.7503	approximate	approximate
Together	$\log_{10}(k_2')$	−1.167	−1.245	−1.094	approximate	approximate
Together	$\log_{10}(k_{deg})$	−1.64	−1.722	−1.559	approximate	approximate
Together	$\log_{10}(k_1'/k_2)$	0.3064	0.2727	0.3474	approximate	approximate
Together	$\log_{10}(k_1'k_2'/k_2)$	−0.8607	−0.9199	−0.7981	approximate	approximate
Together	$\log_{10}(k_2'/k_2)$	−0.2742	−0.4815	−0.0968	approximate	approximate
b1	$\log_{10}(k_1')$	−0.5058	−0.5995	−0.4177	approximate	approximate
b1	$\log_{10}(k_2)$	−0.7874	−0.8823	−0.6909	approximate	approximate
b1	$\log_{10}(k_2')$	−1.23	−1.27	−1.185	approximate	approximate
b1	$\log_{10}(k_{deg})$	−1.783	−1.842	−1.721	approximate	approximate
b1	$\log_{10}(k_1'/k_2)$	0.2816	0.2604	0.3023	approximate	approximate
b1	$\log_{10}(k_1'k_2'/k_2)$	−0.948	−0.9869	−0.9054	approximate	approximate
b1	$\log_{10}(k_2'/k_2)$	−0.4423	−0.5524	−0.321	approximate	approximate
b2	$\log_{10}(k_1'k_2')$	6.756	< 0.1476	> 7.876	optim	optim
b2	$\log_{10}(k_2 \text{ or } k_{deg})$	7.781	1.171	> 8.901	optim	optim
b2	$\log_{10}(k_2 \text{ or } k_{deg})$	−1.925	−2.106	−1.793	approximate	approximate
b3	$\log_{10}(k_1')$	−0.9107	−1.002	−0.815	approximate	approximate
b3	$\log_{10}(k_2)$	−1.149	−1.253	−1.046	approximate	approximate
b3	$\log_{10}(k_2')$	−0.9128	−1.023	−0.7763	approximate	approximate
b3	$\log_{10}(k_{deg})$	−1.422	−1.554	−1.269	approximate	approximate
b3	$\log_{10}(k_1'/k_2)$	0.2381	0.2032	0.269	approximate	approximate
b3	$\log_{10}(k_1'k_2'/k_2)$	−0.6747	−0.7858	−0.5322	approximate	approximate
b3	$\log_{10}(k_2'/k_2)$	0.236	0.05757	0.4451	approximate	approximate

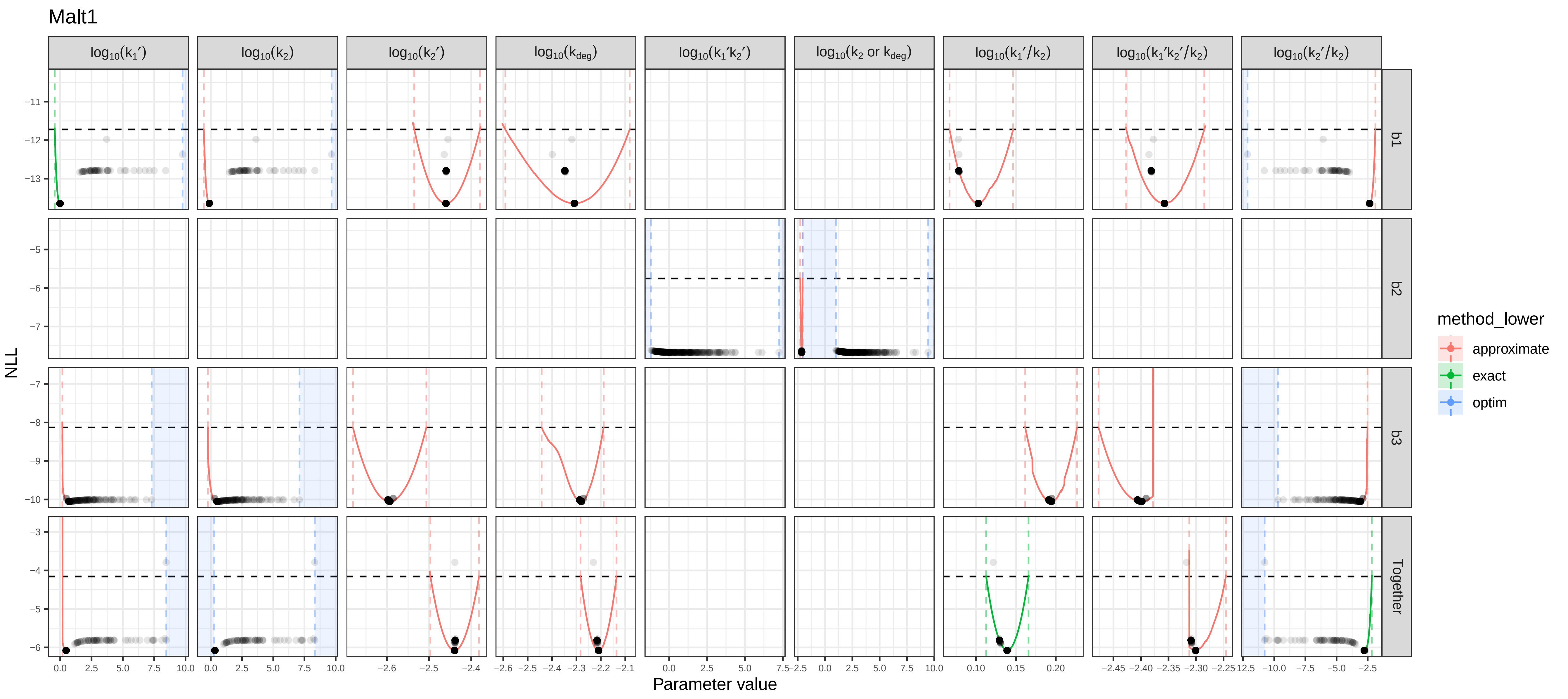

Replicate	Par	Best value	CI95 LB	CI95 UB	Method LB	Method UB
Together	$\log_{10}(k_1')$	0.47	0.1831	> 8.459	approximate	optim
Together	$\log_{10}(k_2)$	0.331	< 0.2654	> 8.33	optim	optim
Together	$\log_{10}(k_2')$	−2.44	−2.497	−2.381	approximate	approximate
Together	$\log_{10}(k_{deg})$	−2.209	−2.283	−2.136	approximate	approximate
Together	$\log_{10}(k_1'/k_2)$	0.139	0.1127	0.1658	exact	exact
Together	$\log_{10}(k_1'k_2'/k_2)$	−2.301	−2.312	−2.246	approximate	approximate
Together	$\log_{10}(k_2'/k_2)$	−2.771	< −10.77	−2.169	optim	exact
b1	$\log_{10}(k_1')$	−0.01754	−0.4317	> 9.756	exact	optim
b1	$\log_{10}(k_2)$	−0.1203	−0.5593	> 9.677	approximate	optim
b1	$\log_{10}(k_2')$	−2.46	−2.535	−2.379	approximate	approximate
b1	$\log_{10}(k_{deg})$	−2.308	−2.592	−2.082	approximate	approximate
b1	$\log_{10}(k_1'/k_2)$	0.1028	0.06672	0.1466	approximate	approximate
b1	$\log_{10}(k_1'k_2'/k_2)$	−2.357	−2.427	−2.285	approximate	approximate
b1	$\log_{10}(k_2'/k_2)$	−2.34	< −12.14	−1.887	optim	approximate
b2	$\log_{10}(k_1'k_2')$	2.653	< −1.185	> 7.238	optim	optim
b2	$\log_{10}(k_2 \text{ or } k_{deg})$	4.844	1.006	> 9.43	optim	optim
b2	$\log_{10}(k_2 \text{ or } k_{deg})$	−2.142	−2.269	−2.046	approximate	approximate
b3	$\log_{10}(k_1')$	0.7428	0.1728	> 7.298	approximate	optim
b3	$\log_{10}(k_2)$	0.5485	−0.2341	> 7.107	approximate	optim
b3	$\log_{10}(k_2')$	−2.594	−2.681	−2.507	approximate	approximate
b3	$\log_{10}(k_{deg})$	−2.28	−2.443	−2.188	approximate	approximate
b3	$\log_{10}(k_1'/k_2)$	0.1943	0.1616	0.2268	approximate	approximate
b3	$\log_{10}(k_1'k_2'/k_2)$	−2.4	−2.477	−2.378	approximate	approximate
b3	$\log_{10}(k_2'/k_2)$	−3.142	< −9.704	−2.519	optim	approximate

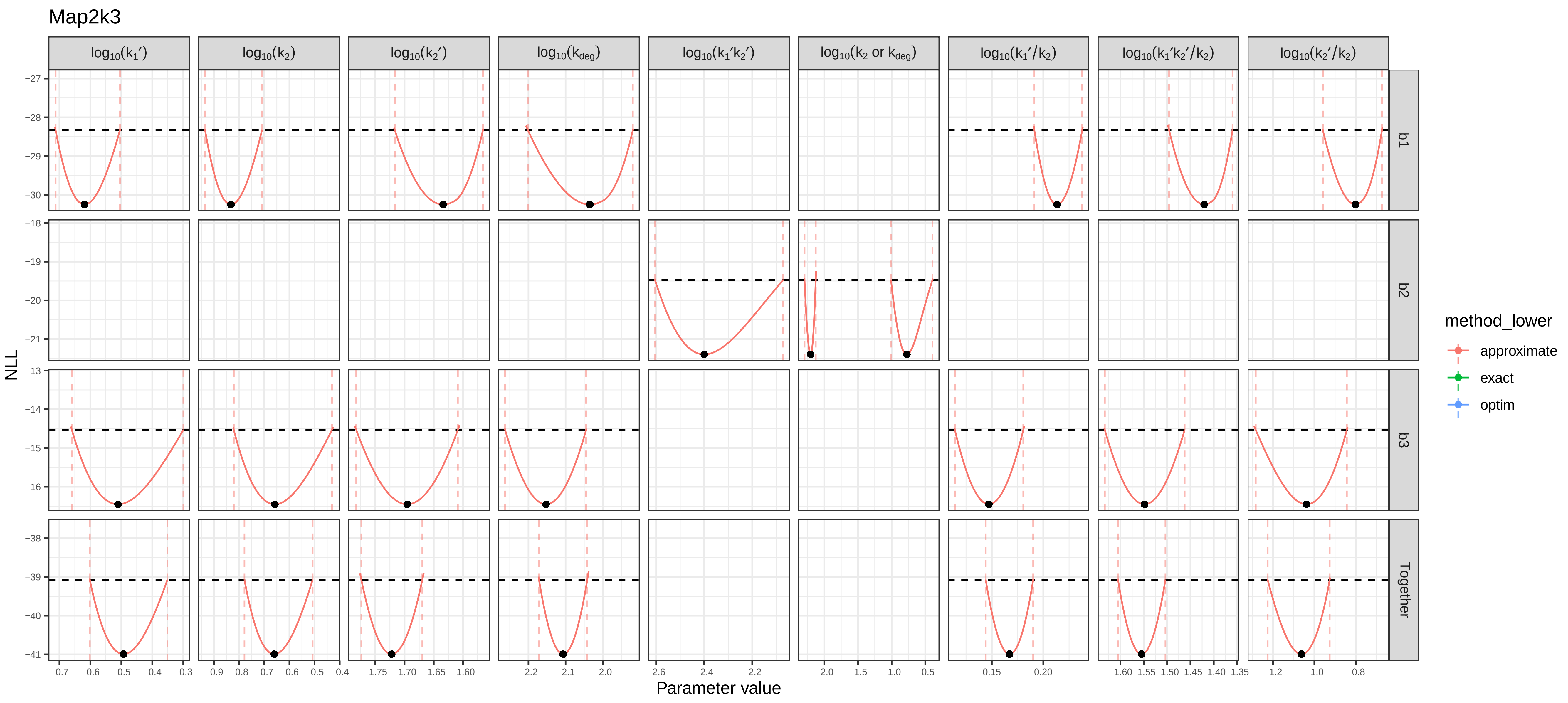

Replicate	Par	Best value	CI95 LB	CI95 UB	Method LB	Method UB
Together	$\log_{10}(k_1')$	−0.4927	−0.602	−0.3513	approximate	approximate
Together	$\log_{10}(k_2)$	−0.66	−0.7788	−0.5078	approximate	approximate
Together	$\log_{10}(k_2')$	−1.722	−1.774	−1.67	approximate	approximate
Together	$\log_{10}(k_{deg})$	−2.107	−2.172	−2.042	approximate	approximate
Together	$\log_{10}(k_1'/k_2)$	0.1673	0.1441	0.1902	approximate	approximate
Together	$\log_{10}(k_1'k_2'/k_2)$	−1.555	−1.605	−1.504	approximate	approximate
Together	$\log_{10}(k_2'/k_2)$	−1.062	−1.226	−0.926	approximate	approximate
b1	$\log_{10}(k_1')$	−0.6188	−0.7124	−0.5046	approximate	approximate
b1	$\log_{10}(k_2)$	−0.8321	−0.9354	−0.7091	approximate	approximate
b1	$\log_{10}(k_2')$	−1.634	−1.717	−1.566	approximate	approximate
b1	$\log_{10}(k_{deg})$	−2.035	−2.201	−1.919	approximate	approximate
b1	$\log_{10}(k_1'/k_2)$	0.2134	0.1913	0.2378	approximate	approximate
b1	$\log_{10}(k_1'k_2'/k_2)$	−1.42	−1.496	−1.36	approximate	approximate
b1	$\log_{10}(k_2'/k_2)$	−0.8016	−0.9591	−0.6732	approximate	approximate
b2	$\log_{10}(k_1'k_2')$	−2.4	−2.605	−2.072	approximate	approximate
b2	$\log_{10}(k_2 \text{ or } k_{deg})$	−0.7741	−1.01	−0.3945	approximate	approximate
b2	$\log_{10}(k_2 \text{ or } k_{deg})$	−2.204	−2.293	−2.127	approximate	approximate
b3	$\log_{10}(k_1')$	−0.5106	−0.6598	−0.2997	approximate	approximate
b3	$\log_{10}(k_2)$	−0.6578	−0.8212	−0.4309	approximate	approximate
b3	$\log_{10}(k_2')$	−1.696	−1.783	−1.609	approximate	approximate
b3	$\log_{10}(k_{deg})$	−2.153	−2.263	−2.045	approximate	approximate
b3	$\log_{10}(k_1'/k_2)$	0.1471	0.1142	0.1806	approximate	approximate
b3	$\log_{10}(k_1'k_2'/k_2)$	−1.548	−1.633	−1.462	approximate	approximate
b3	$\log_{10}(k_2'/k_2)$	−1.038	−1.283	−0.8431	approximate	approximate

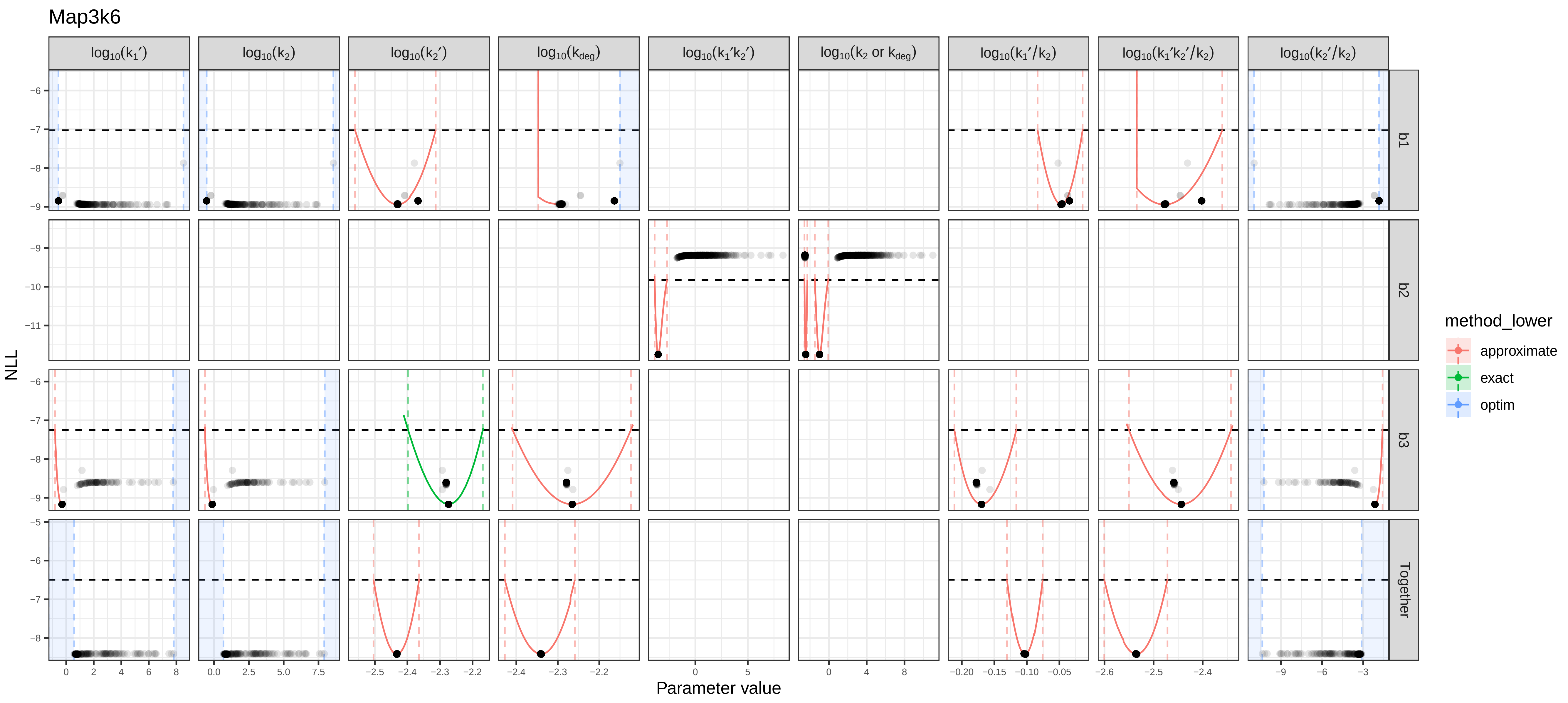

Replicate	Par	Best value	CI95 LB	CI95 UB	Method LB	Method UB
Together	$\log_{10}(k_1')$	0.7809	< 0.5788	> 7.809	optim	optim
Together	$\log_{10}(k_2)$	0.8834	< 0.6802	> 7.914	optim	optim
Together	$\log_{10}(k_2')$	−2.432	−2.504	−2.364	approximate	approximate
Together	$\log_{10}(k_{deg})$	−2.34	−2.428	−2.259	approximate	approximate
Together	$\log_{10}(k_1'/k_2)$	−0.1025	−0.1305	−0.07566	approximate	approximate
Together	$\log_{10}(k_1'k_2'/k_2)$	−2.535	−2.6	−2.472	approximate	approximate
Together	$\log_{10}(k_2'/k_2)$	−3.316	< −10.35	> −3.113	optim	optim
b1	$\log_{10}(k_1')$	4.27	< −0.5637	> 8.515	optim	optim
b1	$\log_{10}(k_2)$	4.317	< −0.5292	> 8.567	optim	optim
b1	$\log_{10}(k_2')$	−2.43	−2.561	−2.313	approximate	approximate
b1	$\log_{10}(k_{deg})$	−2.294	−2.347	> −2.15	approximate	optim
b1	$\log_{10}(k_1'/k_2)$	−0.04743	−0.08351	−0.01432	approximate	approximate
b1	$\log_{10}(k_1'k_2'/k_2)$	−2.478	−2.534	−2.36	approximate	approximate
b1	$\log_{10}(k_2'/k_2)$	−6.748	< −10.95	> −1.838	optim	optim
b2	$\log_{10}(k_1'k_2')$	−3.544	−3.883	−2.703	approximate	approximate
b2	$\log_{10}(k_2 \text{ or } k_{deg})$	−0.9895	−1.486	−0.0673	approximate	approximate
b2	$\log_{10}(k_2 \text{ or } k_{deg})$	−2.457	−2.586	−2.28	approximate	approximate
b3	$\log_{10}(k_1')$	−0.3032	−0.8101	> 7.773	approximate	optim
b3	$\log_{10}(k_2)$	−0.1336	−0.6472	> 7.95	approximate	optim
b3	$\log_{10}(k_2')$	−2.274	−2.398	−2.169	exact	exact
b3	$\log_{10}(k_{deg})$	−2.265	−2.409	−2.124	approximate	approximate
b3	$\log_{10}(k_1'/k_2)$	−0.1696	−0.2114	−0.1162	approximate	approximate
b3	$\log_{10}(k_1'k_2'/k_2)$	−2.444	−2.55	−2.342	approximate	approximate
b3	$\log_{10}(k_2'/k_2)$	−2.14	< −10.23	−1.587	optim	approximate

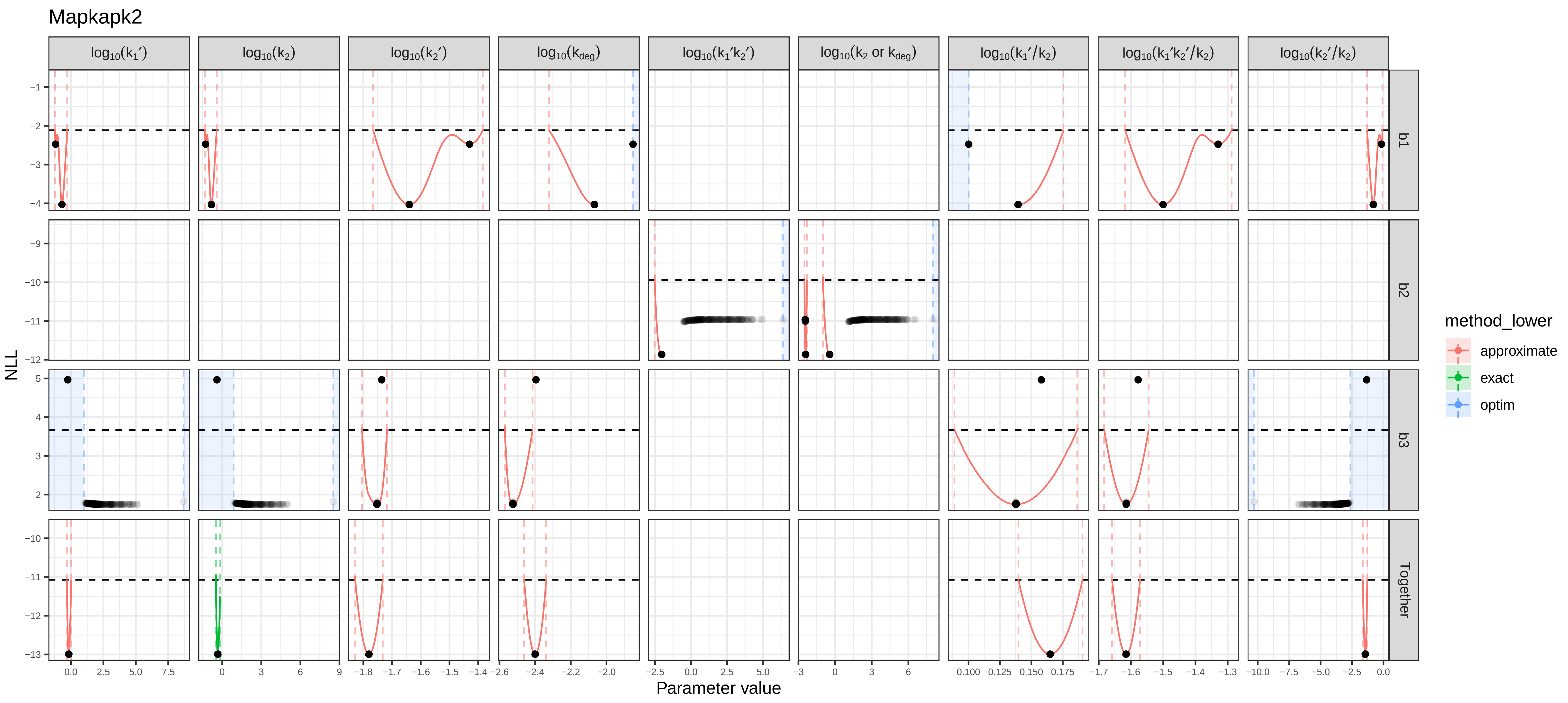

Replicate	Par	Best value	CI95 LB	CI95 UB	Method LB	Method UB
Together	$\log_{10}(k_1')$	−0.1688	−0.3103	0.01722	approximate	approximate
Together	$\log_{10}(k_2)$	−0.3338	−0.4845	−0.1481	exact	exact
Together	$\log_{10}(k_2')$	−1.78	−1.828	−1.732	approximate	approximate
Together	$\log_{10}(k_{deg})$	−2.4	−2.462	−2.338	approximate	approximate
Together	$\log_{10}(k_1'/k_2)$	0.165	0.1397	0.1907	approximate	approximate
Together	$\log_{10}(k_1'k_2'/k_2)$	−1.615	−1.659	−1.572	approximate	approximate
Together	$\log_{10}(k_2'/k_2)$	−1.447	−1.637	−1.288	approximate	approximate
b1	$\log_{10}(k_1')$	−0.6915	−1.226	−0.2836	approximate	approximate
b1	$\log_{10}(k_2)$	−0.831	−1.326	−0.4212	approximate	approximate
b1	$\log_{10}(k_2')$	−1.64	−1.766	−1.384	approximate	approximate
b1	$\log_{10}(k_{deg})$	−2.068	−2.323	> −1.851	approximate	optim
b1	$\log_{10}(k_1'/k_2)$	0.1395	< 0.1001	0.1756	optim	approximate
b1	$\log_{10}(k_1'k_2'/k_2)$	−1.5	−1.618	−1.288	approximate	approximate
b1	$\log_{10}(k_2'/k_2)$	−0.8089	−1.311	−0.07434	approximate	approximate
b2	$\log_{10}(k_1'k_2')$	−2.044	−2.524	> 6.385	approximate	optim
b2	$\log_{10}(k_2 \text{ or } k_{deg})$	−0.4469	−0.992	> 8.007	approximate	optim
b2	$\log_{10}(k_2 \text{ or } k_{deg})$	−2.41	−2.506	−2.303	approximate	approximate
b3	$\log_{10}(k_1')$	5.148	< 1.015	> 8.671	optim	optim
b3	$\log_{10}(k_2)$	5.01	< 0.8776	> 8.534	optim	optim
b3	$\log_{10}(k_2')$	−1.753	−1.805	−1.717	approximate	approximate
b3	$\log_{10}(k_{deg})$	−2.524	−2.57	−2.414	approximate	approximate
b3	$\log_{10}(k_1'/k_2)$	0.1378	0.08882	0.1865	approximate	approximate
b3	$\log_{10}(k_1'k_2'/k_2)$	−1.615	−1.683	−1.545	approximate	approximate
b3	$\log_{10}(k_2'/k_2)$	−6.763	< −10.29	> −2.629	optim	optim

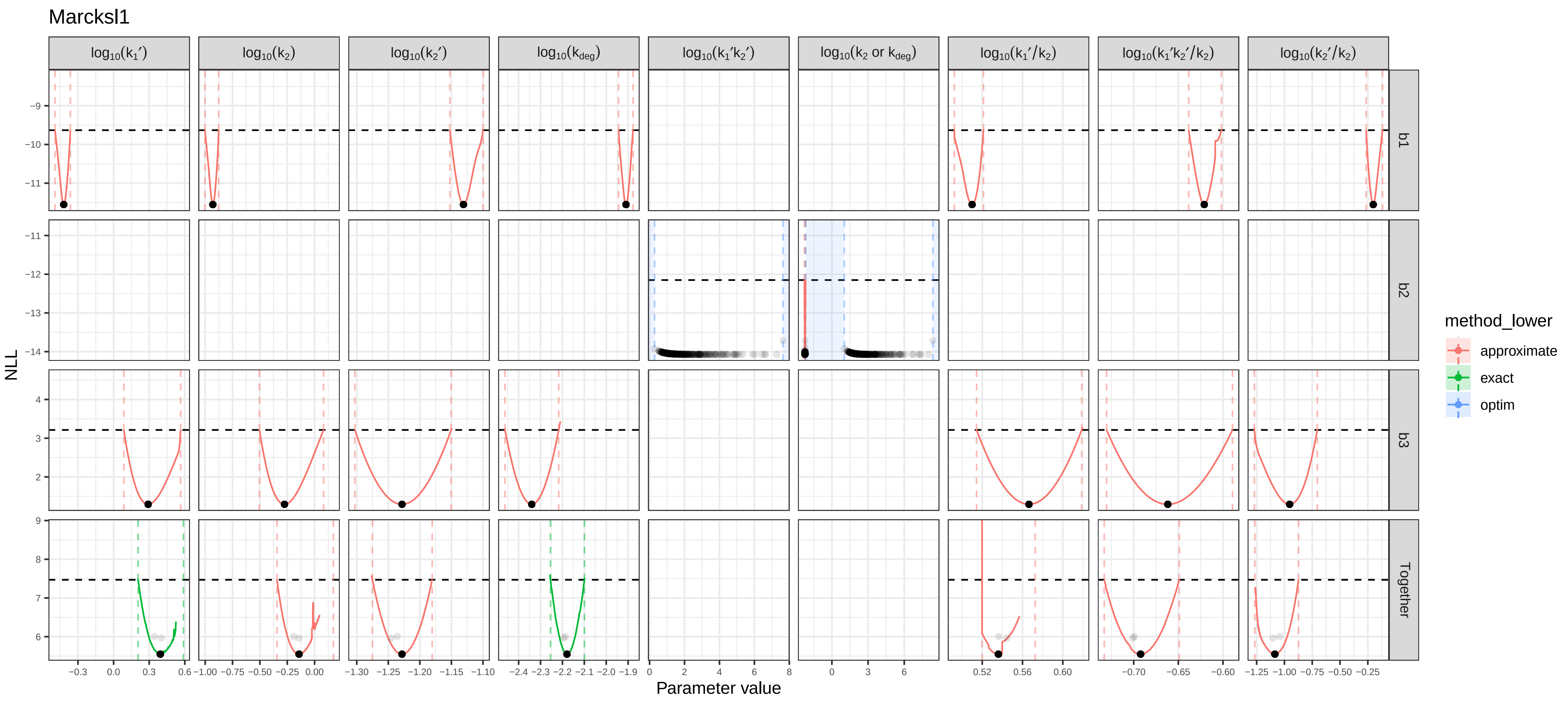

Replicate	Par	Best value	CI95 LB	CI95 UB	Method LB	Method UB
Together	$\log_{10}(k_1')$	0.3939	0.2066	0.5888	exact	exact
Together	$\log_{10}(k_2)$	−0.1422	−0.3438	0.1713	approximate	approximate
Together	$\log_{10}(k_2')$	−1.228	−1.275	−1.18	approximate	approximate
Together	$\log_{10}(k_{deg})$	−2.179	−2.255	−2.1	exact	exact
Together	$\log_{10}(k_1'/k_2)$	0.5361	0.5198	0.5726	approximate	approximate
Together	$\log_{10}(k_1'k_2'/k_2)$	−0.6922	−0.733	−0.649	approximate	approximate
Together	$\log_{10}(k_2'/k_2)$	−1.086	−1.267	−0.873	approximate	approximate
b1	$\log_{10}(k_1')$	−0.4199	−0.4933	−0.3642	approximate	approximate
b1	$\log_{10}(k_2)$	−0.9298	−1.002	−0.8769	approximate	approximate
b1	$\log_{10}(k_2')$	−1.131	−1.152	−1.1	approximate	approximate
b1	$\log_{10}(k_{deg})$	−1.909	−1.944	−1.877	approximate	approximate
b1	$\log_{10}(k_1'/k_2)$	0.5099	0.4923	0.5212	approximate	approximate
b1	$\log_{10}(k_1'k_2'/k_2)$	−0.621	−0.6384	−0.6018	approximate	approximate
b1	$\log_{10}(k_2'/k_2)$	−0.2011	−0.2649	−0.1178	approximate	approximate
b2	$\log_{10}(k_1'k_2')$	4.228	< 0.285	> 7.638	optim	optim
b2	$\log_{10}(k_2 \text{ or } k_{deg})$	4.968	1.025	> 8.376	optim	optim
b2	$\log_{10}(k_2 \text{ or } k_{deg})$	−2.233	−2.276	−2.182	approximate	approximate
b3	$\log_{10}(k_1')$	0.2914	0.08611	0.5651	approximate	approximate
b3	$\log_{10}(k_2)$	−0.2749	−0.5041	0.08196	approximate	approximate
b3	$\log_{10}(k_2')$	−1.228	−1.303	−1.15	approximate	approximate
b3	$\log_{10}(k_{deg})$	−2.34	−2.462	−2.217	approximate	approximate
b3	$\log_{10}(k_1'/k_2)$	0.5664	0.5144	0.6188	approximate	approximate
b3	$\log_{10}(k_1'k_2'/k_2)$	−0.6618	−0.7304	−0.5893	approximate	approximate
b3	$\log_{10}(k_2'/k_2)$	−0.9533	−1.271	−0.7045	approximate	approximate

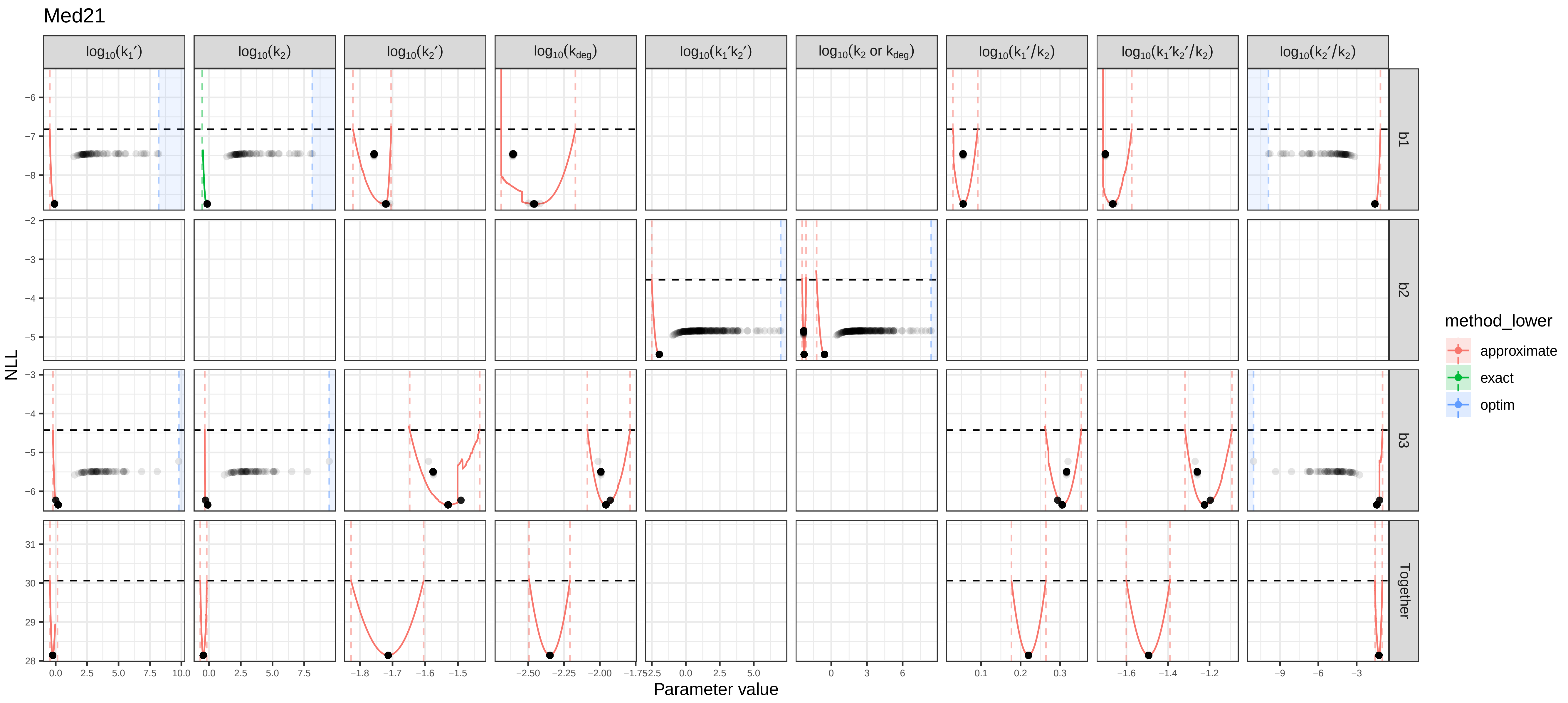

Replicate	Par	Best value	CI95 LB	CI95 UB	Method LB	Method UB
Together	$\log_{10}(k_1')$	−0.2348	−0.4568	0.1444	approximate	approximate
Together	$\log_{10}(k_2)$	−0.4547	−0.6916	−0.1862	approximate	approximate
Together	$\log_{10}(k_2')$	−1.713	−1.827	−1.605	approximate	approximate
Together	$\log_{10}(k_{deg})$	−2.348	−2.493	−2.208	approximate	approximate
Together	$\log_{10}(k_1'/k_2)$	0.2199	0.1769	0.2642	approximate	approximate
Together	$\log_{10}(k_1'k_2'/k_2)$	−1.493	−1.601	−1.39	approximate	approximate
Together	$\log_{10}(k_2'/k_2)$	−1.258	−1.558	−0.9922	approximate	approximate
b1	$\log_{10}(k_1')$	−0.0951	−0.468	> 8.192	approximate	optim
b1	$\log_{10}(k_2)$	−0.1494	−0.5389	> 8.138	exact	optim
b1	$\log_{10}(k_2')$	−1.72	−1.821	−1.704	approximate	approximate
b1	$\log_{10}(k_{deg})$	−2.459	−2.688	−2.17	approximate	approximate
b1	$\log_{10}(k_1'/k_2)$	0.05425	0.02791	0.0912	approximate	approximate
b1	$\log_{10}(k_1'k_2'/k_2)$	−1.666	−1.713	−1.575	approximate	approximate
b1	$\log_{10}(k_2'/k_2)$	−1.571	< −9.895	−1.139	optim	approximate
b2	$\log_{10}(k_1'k_2')$	−1.947	−2.508	> 6.98	approximate	optim
b2	$\log_{10}(k_2 \text{ or } k_{deg})$	−0.5679	−1.229	> 8.395	approximate	optim
b2	$\log_{10}(k_2 \text{ or } k_{deg})$	−2.278	−2.444	−2.11	approximate	approximate
b3	$\log_{10}(k_1')$	0.1956	−0.2247	> 9.795	approximate	optim
b3	$\log_{10}(k_2)$	−0.11	−0.3422	> 9.474	approximate	optim
b3	$\log_{10}(k_2')$	−1.53	−1.648	−1.433	approximate	approximate
b3	$\log_{10}(k_{deg})$	−1.957	−2.087	−1.789	approximate	approximate
b3	$\log_{10}(k_1'/k_2)$	0.3056	0.2631	0.3543	approximate	approximate
b3	$\log_{10}(k_1'k_2'/k_2)$	−1.224	−1.318	−1.092	approximate	approximate
b3	$\log_{10}(k_2'/k_2)$	−1.42	< −11.06	−0.975	optim	approximate

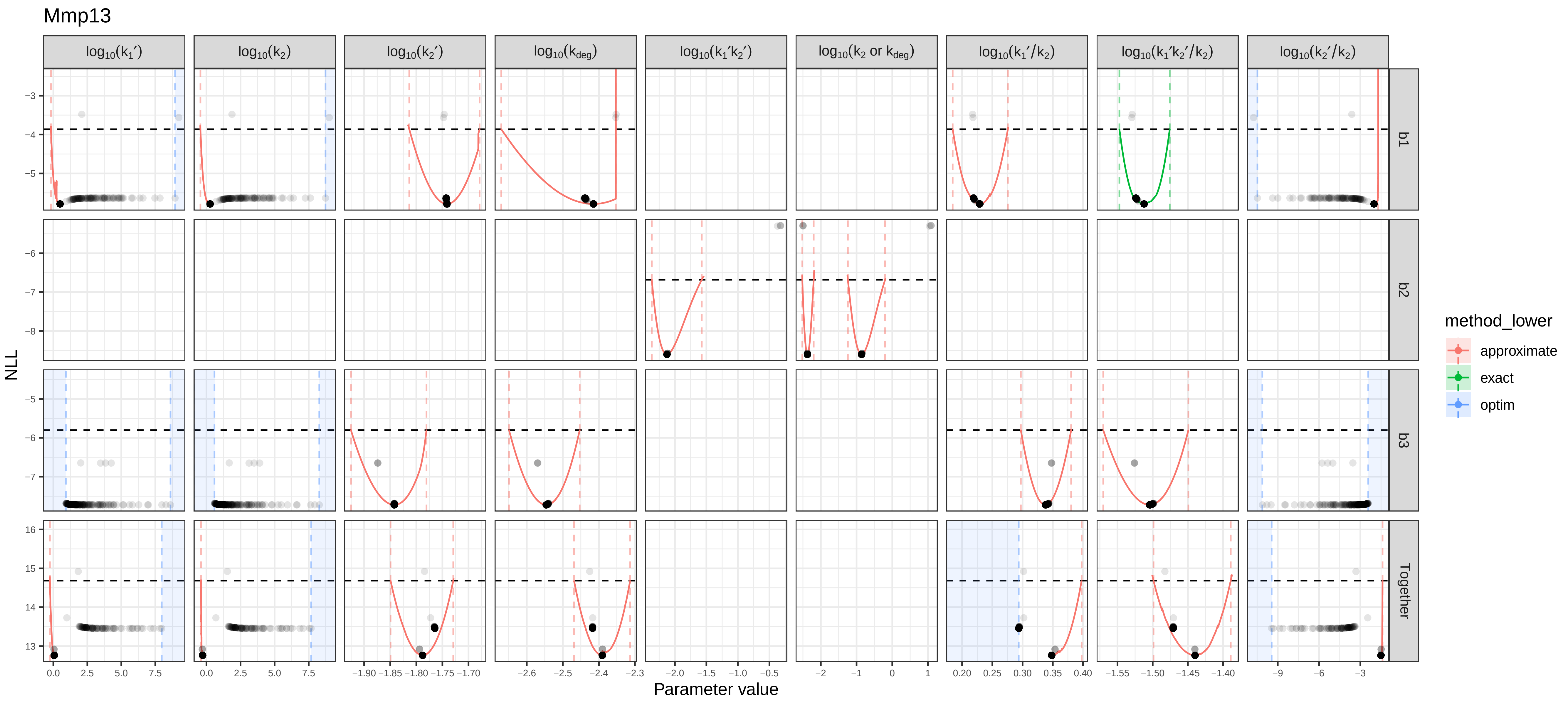

Replicate	Par	Best value	CI95 LB	CI95 UB	Method LB	Method UB
Together	$\log_{10}(k_1')$	0.06556	−0.2524	> 7.972	approximate	optim
Together	$\log_{10}(k_2)$	−0.2826	−0.3967	> 7.678	approximate	optim
Together	$\log_{10}(k_2')$	−1.788	−1.849	−1.729	approximate	approximate
Together	$\log_{10}(k_{deg})$	−2.39	−2.469	−2.313	approximate	approximate
Together	$\log_{10}(k_1'/k_2)$	0.3482	< 0.2936	0.3977	optim	approximate
Together	$\log_{10}(k_1'k_2'/k_2)$	−1.44	−1.499	−1.389	approximate	approximate
Together	$\log_{10}(k_2'/k_2)$	−1.505	< −9.442	−1.389	optim	approximate
b1	$\log_{10}(k_1')$	0.496	−0.1744	> 8.952	approximate	optim
b1	$\log_{10}(k_2)$	0.2669	−0.4427	> 8.733	approximate	optim
b1	$\log_{10}(k_2')$	−1.741	−1.814	−1.679	approximate	approximate
b1	$\log_{10}(k_{deg})$	−2.415	−2.671	−2.353	approximate	approximate
b1	$\log_{10}(k_1'/k_2)$	0.2291	0.1844	0.2757	approximate	approximate
b1	$\log_{10}(k_1'k_2'/k_2)$	−1.512	−1.547	−1.476	exact	exact
b1	$\log_{10}(k_2'/k_2)$	−2.008	< −10.48	−1.702	optim	approximate
b2	$\log_{10}(k_1'k_2')$	−2.126	−2.367	−1.574	approximate	approximate
b2	$\log_{10}(k_2 \text{ or } k_{deg})$	−0.8598	−1.243	−0.2002	approximate	approximate
b2	$\log_{10}(k_2 \text{ or } k_{deg})$	−2.374	−2.518	−2.199	approximate	approximate
b3	$\log_{10}(k_1')$	3.608	< 0.9296	> 8.614	optim	optim
b3	$\log_{10}(k_2)$	3.27	< 0.5859	> 8.276	optim	optim
b3	$\log_{10}(k_2')$	−1.842	−1.925	−1.781	approximate	approximate
b3	$\log_{10}(k_{deg})$	−2.546	−2.649	−2.453	approximate	approximate
b3	$\log_{10}(k_1'/k_2)$	0.3378	0.2971	0.3804	approximate	approximate
b3	$\log_{10}(k_1'k_2'/k_2)$	−1.504	−1.57	−1.449	approximate	approximate
b3	$\log_{10}(k_2'/k_2)$	−5.112	< −10.12	> −2.428	optim	optim

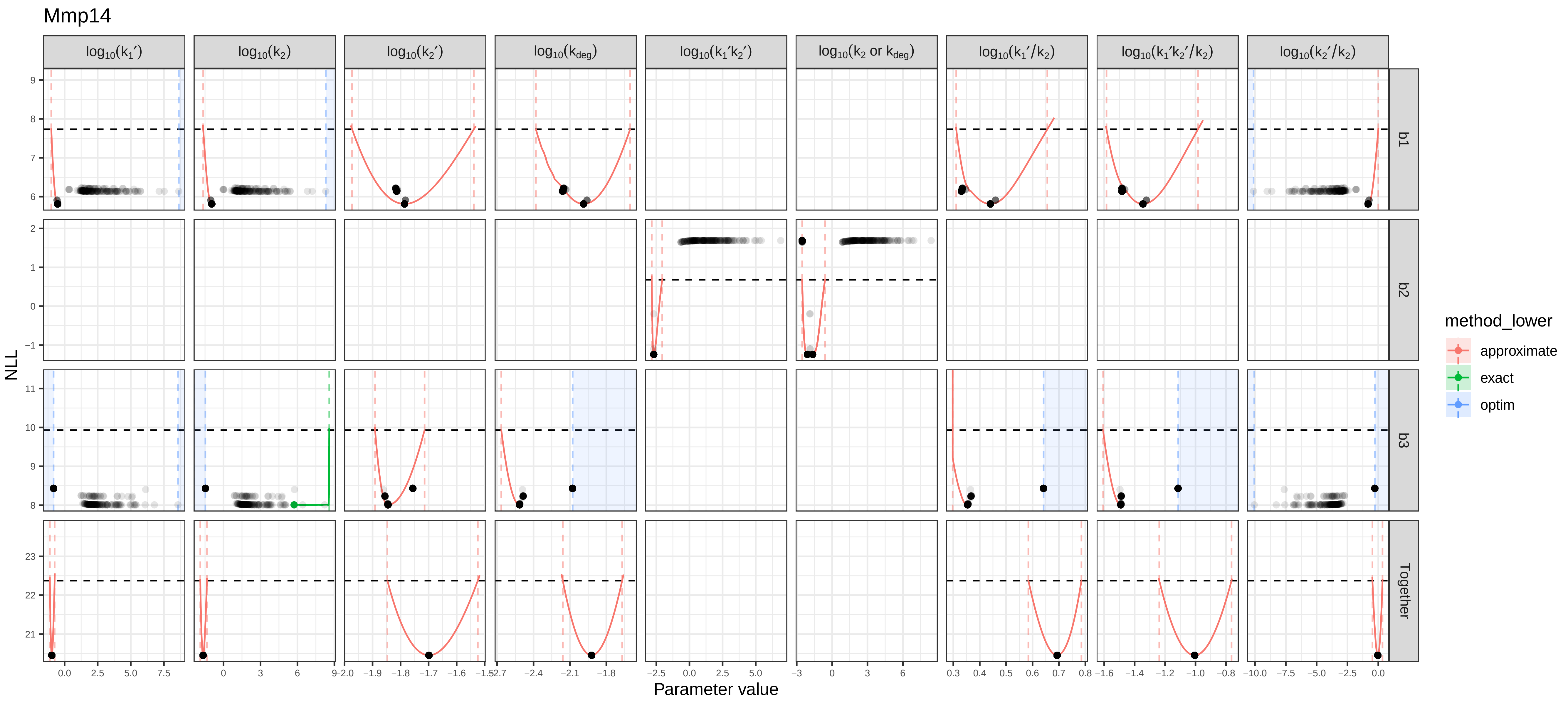

Replicate	Par	Best value	CI95 LB	CI95 UB	Method LB	Method UB
Together	$\log_{10}(k_1')$	−0.9645	−1.113	−0.7515	approximate	approximate
Together	$\log_{10}(k_2)$	−1.658	−1.884	−1.344	approximate	approximate
Together	$\log_{10}(k_2')$	−1.699	−1.847	−1.525	approximate	approximate
Together	$\log_{10}(k_{deg})$	−1.922	−2.159	−1.67	approximate	approximate
Together	$\log_{10}(k_1'/k_2)$	0.6931	0.5842	0.7852	approximate	approximate
Together	$\log_{10}(k_1'k_2'/k_2)$	−1.006	−1.238	−0.7631	approximate	approximate
Together	$\log_{10}(k_2'/k_2)$	−0.04126	−0.4779	0.3391	approximate	approximate
b1	$\log_{10}(k_1')$	−0.5148	−1.005	> 8.628	approximate	optim
b1	$\log_{10}(k_2)$	−0.9551	−1.647	> 8.298	approximate	optim
b1	$\log_{10}(k_2')$	−1.786	−1.973	−1.539	approximate	approximate
b1	$\log_{10}(k_{deg})$	−1.987	−2.381	−1.604	approximate	approximate
b1	$\log_{10}(k_1'/k_2)$	0.4403	0.3111	0.656	approximate	approximate
b1	$\log_{10}(k_1'k_2'/k_2)$	−1.345	−1.585	−0.9836	approximate	approximate
b1	$\log_{10}(k_2'/k_2)$	−0.8304	< −10.11	0.001479	optim	approximate
b2	$\log_{10}(k_1'k_2')$	−2.696	−2.847	−2.053	approximate	approximate
b2	$\log_{10}(k_2 \text{ or } k_{deg})$	−1.652	−2.535	−0.5971	approximate	approximate
b2	$\log_{10}(k_2 \text{ or } k_{deg})$	−2.088	−2.535	−0.5971	approximate	approximate
b3	$\log_{10}(k_1')$	6.083	< −0.8297	> 8.564	optim	optim
b3	$\log_{10}(k_2)$	5.729	< −1.472	8.583	optim	exact
b3	$\log_{10}(k_2')$	−1.845	−1.891	−1.714	approximate	approximate
b3	$\log_{10}(k_{deg})$	−2.515	−2.666	> −2.078	approximate	optim
b3	$\log_{10}(k_1'/k_2)$	0.3541	0.2974	> 0.6419	approximate	optim
b3	$\log_{10}(k_1'k_2'/k_2)$	−1.491	−1.607	> −1.114	approximate	optim
b3	$\log_{10}(k_2'/k_2)$	−7.574	< −10.05	> −0.284	optim	optim

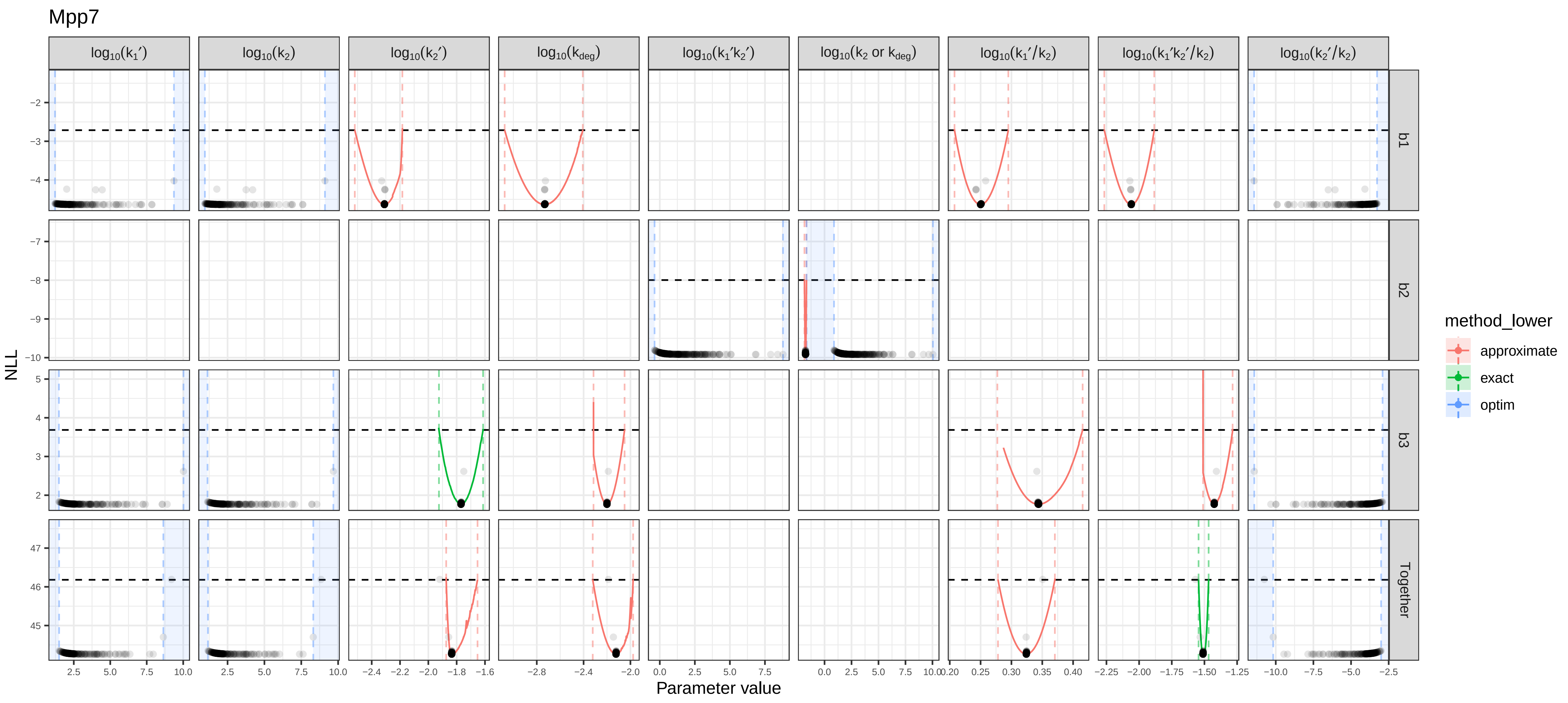

Replicate	Par	Best value	CI95 LB	CI95 UB	Method LB	Method UB
Together	$\log_{10}(k_1')$	7.949	< 1.494	> 8.637	optim	optim
Together	$\log_{10}(k_2)$	7.625	< 1.169	> 8.313	optim	optim
Together	$\log_{10}(k_2')$	−1.835	−1.874	−1.652	approximate	approximate
Together	$\log_{10}(k_{deg})$	−2.123	−2.322	−1.977	approximate	approximate
Together	$\log_{10}(k_1'/k_2)$	0.3241	0.2782	0.3704	approximate	approximate
Together	$\log_{10}(k_1'k_2'/k_2)$	−1.511	−1.545	−1.468	exact	exact
Together	$\log_{10}(k_2'/k_2)$	−9.459	< −10.17	> −3.004	optim	optim
b1	$\log_{10}(k_1')$	4.502	< 1.213	> 9.366	optim	optim
b1	$\log_{10}(k_2)$	4.252	< 0.9618	> 9.108	optim	optim
b1	$\log_{10}(k_2')$	−2.31	−2.519	−2.183	approximate	approximate
b1	$\log_{10}(k_{deg})$	−2.732	−3.074	−2.407	approximate	approximate
b1	$\log_{10}(k_1'/k_2)$	0.25	0.2077	0.295	approximate	approximate
b1	$\log_{10}(k_1'k_2'/k_2)$	−2.06	−2.266	−1.884	approximate	approximate
b1	$\log_{10}(k_2'/k_2)$	−6.562	< −11.44	> −3.271	optim	optim
b2	$\log_{10}(k_1'k_2')$	3.567	< −0.3736	> 8.79	optim	optim
b2	$\log_{10}(k_2 \text{ or } k_{deg})$	4.819	0.8778	> 10.04	optim	optim
b2	$\log_{10}(k_2 \text{ or } k_{deg})$	−1.763	−1.862	−1.67	approximate	approximate
b3	$\log_{10}(k_1')$	6.453	< 1.481	> 10.02	optim	optim
b3	$\log_{10}(k_2)$	6.109	< 1.138	> 9.677	optim	optim
b3	$\log_{10}(k_2')$	−1.769	−1.925	−1.613	exact	exact
b3	$\log_{10}(k_{deg})$	−2.201	−2.315	−2.05	approximate	approximate
b3	$\log_{10}(k_1'/k_2)$	0.3439	0.2772	0.4155	approximate	approximate
b3	$\log_{10}(k_1'k_2'/k_2)$	−1.425	−1.511	−1.284	approximate	approximate
b3	$\log_{10}(k_2'/k_2)$	−7.878	< −11.43	> −2.906	optim	optim

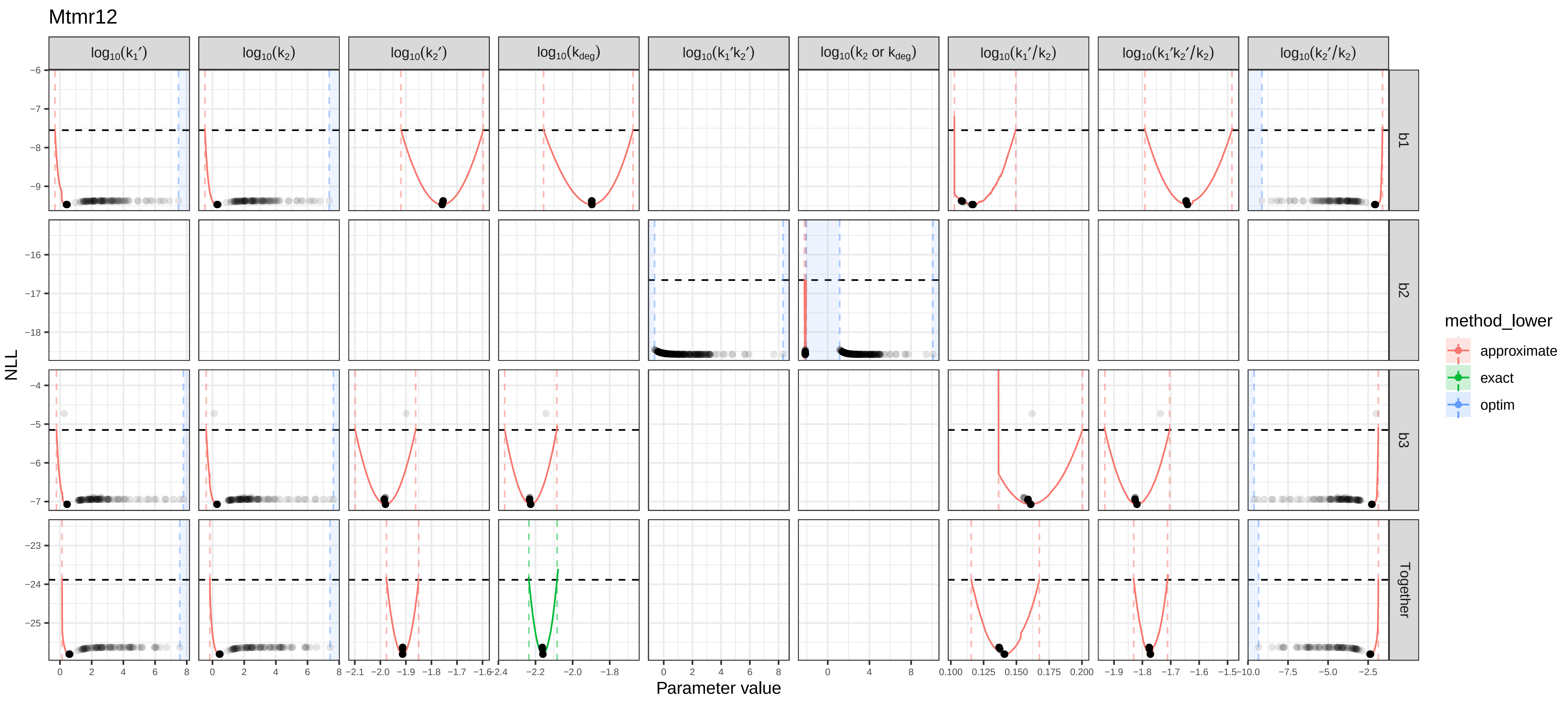

Replicate	Par	Best value	CI95 LB	CI95 UB	Method LB	Method UB
Together	$\log_{10}(k_1')$	0.5936	0.1292	> 7.559	approximate	optim
Together	$\log_{10}(k_2)$	0.4527	−0.1637	> 7.422	approximate	optim
Together	$\log_{10}(k_2')$	−1.913	−1.976	−1.85	approximate	approximate
Together	$\log_{10}(k_{deg})$	−2.16	−2.236	−2.084	exact	exact
Together	$\log_{10}(k_1'/k_2)$	0.1409	0.1155	0.1675	approximate	approximate
Together	$\log_{10}(k_1'k_2'/k_2)$	−1.772	−1.83	−1.711	approximate	approximate
Together	$\log_{10}(k_2'/k_2)$	−2.366	< −9.335	−1.86	optim	approximate
b1	$\log_{10}(k_1')$	0.4139	−0.3146	> 7.48	approximate	optim
b1	$\log_{10}(k_2)$	0.2972	−0.4755	> 7.372	approximate	optim
b1	$\log_{10}(k_2')$	−1.758	−1.919	−1.597	approximate	approximate
b1	$\log_{10}(k_{deg})$	−1.896	−2.156	−1.674	approximate	approximate
b1	$\log_{10}(k_1'/k_2)$	0.1167	0.1027	0.1495	approximate	approximate
b1	$\log_{10}(k_1'k_2'/k_2)$	−1.641	−1.791	−1.485	approximate	approximate
b1	$\log_{10}(k_2'/k_2)$	−2.055	< −9.127	−1.602	optim	approximate
b2	$\log_{10}(k_1'k_2')$	3.559	< −0.6374	> 8.309	optim	optim
b2	$\log_{10}(k_2 \text{ or } k_{deg})$	5.343	1.145	> 10.09	optim	optim
b2	$\log_{10}(k_2 \text{ or } k_{deg})$	−2.161	−2.239	−2.083	approximate	approximate
b3	$\log_{10}(k_1')$	0.4467	−0.2233	> 7.791	approximate	optim
b3	$\log_{10}(k_2)$	0.2857	−0.3972	> 7.632	approximate	optim
b3	$\log_{10}(k_2')$	−1.98	−2.098	−1.862	approximate	approximate
b3	$\log_{10}(k_{deg})$	−2.226	−2.366	−2.085	approximate	approximate
b3	$\log_{10}(k_1'/k_2)$	0.1609	0.1364	0.2005	approximate	approximate
b3	$\log_{10}(k_1'k_2'/k_2)$	−1.819	−1.932	−1.704	approximate	approximate
b3	$\log_{10}(k_2'/k_2)$	−2.266	< −9.617	−1.865	optim	approximate

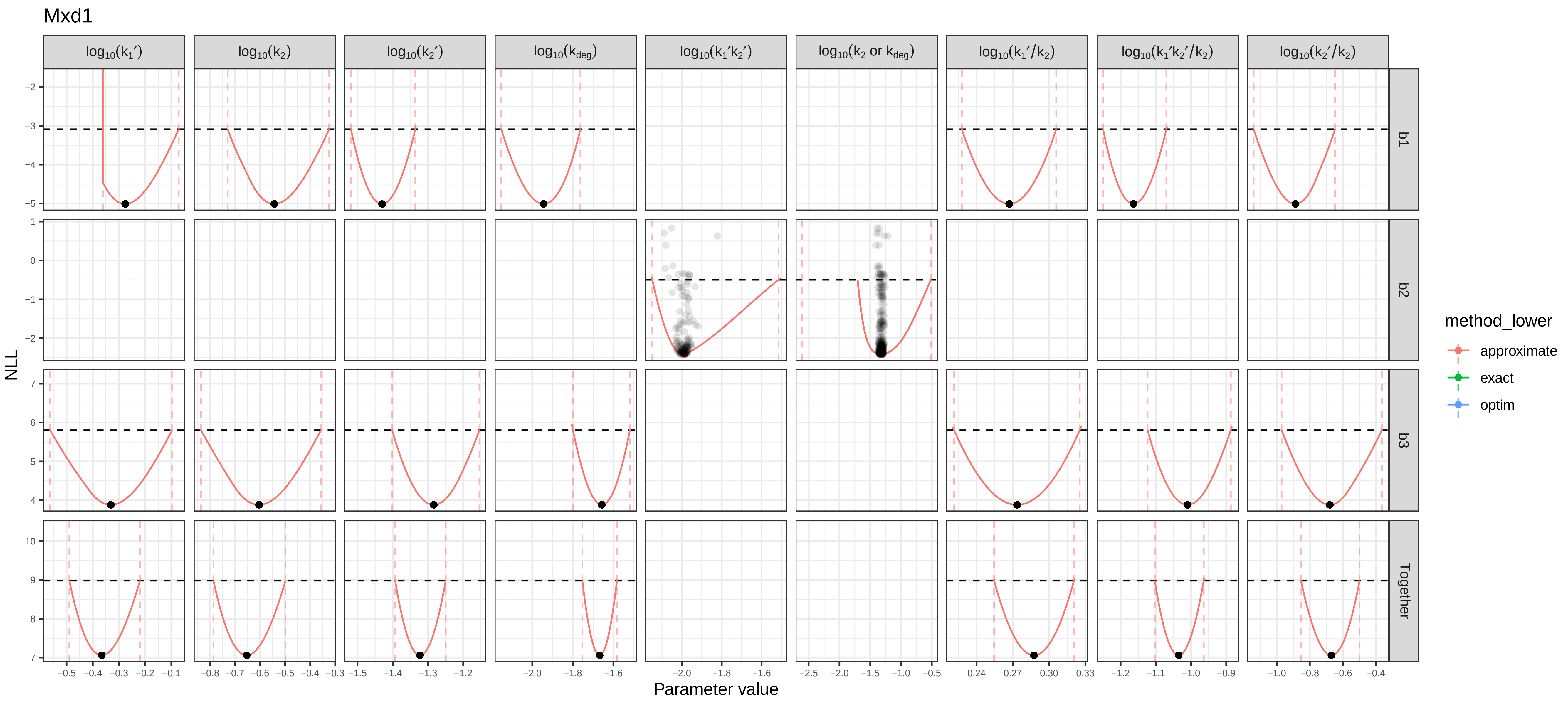

Replicate	Par	Best value	CI95 LB	CI95 UB	Method LB	Method UB
Together	$\log_{10}(k_1')$	−0.3658	−0.4895	−0.2199	approximate	approximate
Together	$\log_{10}(k_2)$	−0.6532	−0.786	−0.4984	approximate	approximate
Together	$\log_{10}(k_2')$	−1.322	−1.393	−1.25	approximate	approximate
Together	$\log_{10}(k_{deg})$	−1.668	−1.753	−1.582	approximate	approximate
Together	$\log_{10}(k_1'/k_2)$	0.2875	0.2545	0.3206	approximate	approximate
Together	$\log_{10}(k_1'k_2'/k_2)$	−1.035	−1.103	−0.9639	approximate	approximate
Together	$\log_{10}(k_2'/k_2)$	−0.6692	−0.854	−0.4988	approximate	approximate
b1	$\log_{10}(k_1')$	−0.2762	−0.3617	−0.07167	approximate	approximate
b1	$\log_{10}(k_2)$	−0.5431	−0.7292	−0.324	approximate	approximate
b1	$\log_{10}(k_2')$	−1.43	−1.519	−1.336	approximate	approximate
b1	$\log_{10}(k_{deg})$	−1.944	−2.153	−1.763	approximate	approximate
b1	$\log_{10}(k_1'/k_2)$	0.267	0.2278	0.3059	approximate	approximate
b1	$\log_{10}(k_1'k_2'/k_2)$	−1.163	−1.25	−1.07	approximate	approximate
b1	$\log_{10}(k_2'/k_2)$	−0.8872	−1.14	−0.6472	approximate	approximate
b2	$\log_{10}(k_1'k_2')$	−1.986	−2.149	−1.515	approximate	approximate
b2	$\log_{10}(k_2 \text{ or } k_{deg})$	−1.309	−2.606	−0.5131	approximate	approximate
b2	$\log_{10}(k_2 \text{ or } k_{deg})$	−1.331	−2.606	−0.5131	approximate	approximate
b3	$\log_{10}(k_1')$	−0.331	−0.5631	−0.09668	approximate	approximate
b3	$\log_{10}(k_2)$	−0.6045	−0.8358	−0.3569	approximate	approximate
b3	$\log_{10}(k_2')$	−1.283	−1.402	−1.154	approximate	approximate
b3	$\log_{10}(k_{deg})$	−1.657	−1.8	−1.518	approximate	approximate
b3	$\log_{10}(k_1'/k_2)$	0.2735	0.2214	0.3253	approximate	approximate
b3	$\log_{10}(k_1'k_2'/k_2)$	−1.01	−1.123	−0.8869	approximate	approximate
b3	$\log_{10}(k_2'/k_2)$	−0.6789	−0.9707	−0.3639	approximate	approximate

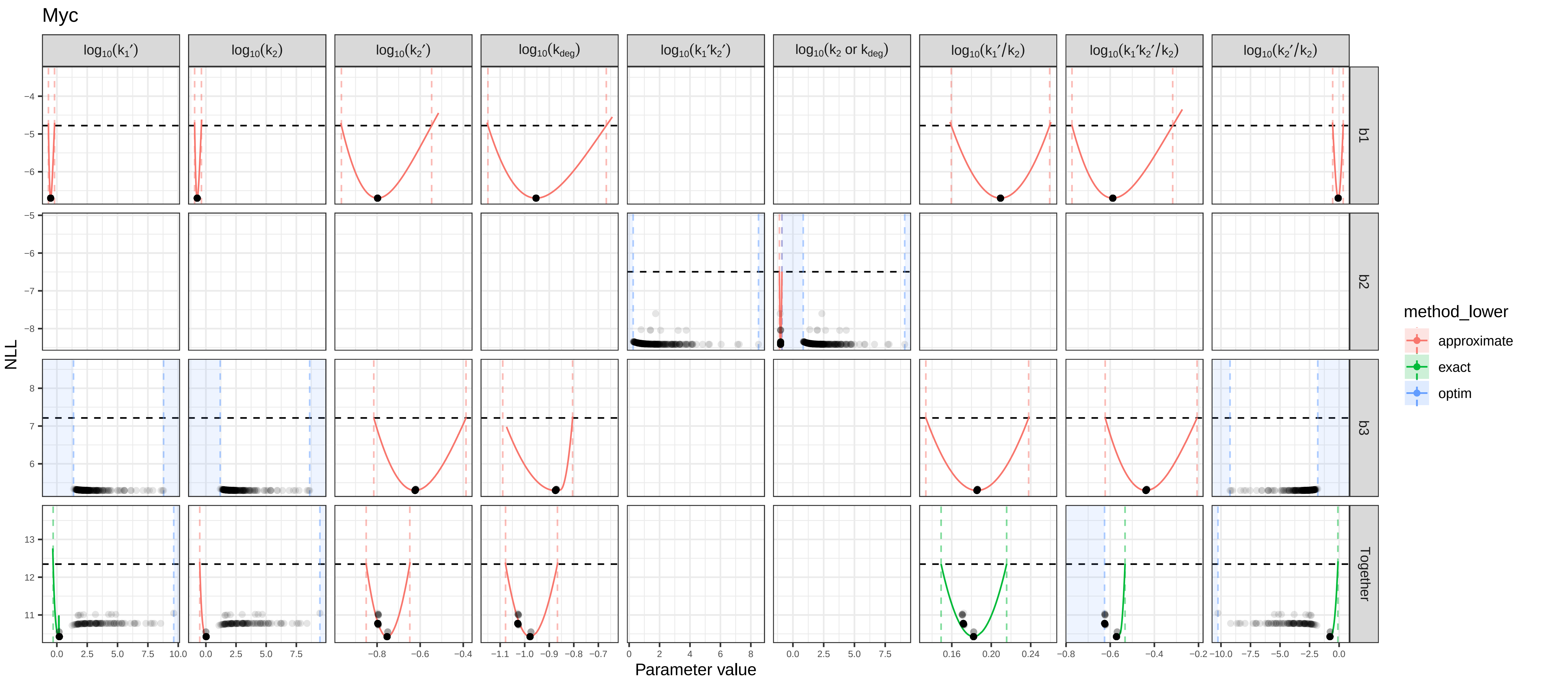

Replicate	Par	**Best value**	**CI95 LB**	**CI95 UB**	**Method LB**	**Method UB**
Together	$\log_{10}(k_1')$	0.2029	−0.3059	> 9.629	exact	optim
Together	$\log_{10}(k_2)$	0.0208	−0.508	> 9.457	approximate	optim
Together	$\log_{10}(k_2')$	−0.7537	−0.8505	−0.648	approximate	approximate
Together	$\log_{10}(k_{deg})$	−0.9782	−1.078	−0.8666	approximate	approximate
Together	$\log_{10}(k_1'/k_2)$	0.1821	0.1492	0.2156	exact	exact
Together	$\log_{10}(k_1'k_2'/k_2)$	−0.5716	< −0.6256	−0.5328	optim	exact
Together	$\log_{10}(k_2'/k_2)$	−0.7745	< −10.25	−0.1032	optim	exact
b1	$\log_{10}(k_1')$	−0.511	−0.6949	−0.1927	approximate	approximate
b1	$\log_{10}(k_2)$	−0.7204	−0.9318	−0.3701	approximate	approximate
b1	$\log_{10}(k_2')$	−0.7976	−0.9655	−0.5466	approximate	approximate
b1	$\log_{10}(k_{deg})$	−0.9538	−1.15	−0.6676	approximate	approximate
b1	$\log_{10}(k_1'/k_2)$	0.2094	0.1595	0.2592	approximate	approximate
b1	$\log_{10}(k_1'k_2'/k_2)$	−0.5882	−0.773	−0.3169	approximate	approximate
b1	$\log_{10}(k_2'/k_2)$	−0.07725	−0.5443	0.3466	approximate	approximate
b2	$\log_{10}(k_1'k_2')$	4.168	< 0.2653	> 8.504	optim	optim
b2	$\log_{10}(k_2 \text{ or } k_{deg})$	4.749	0.8384	> 9.085	optim	optim
b2	$\log_{10}(k_2 \text{ or } k_{deg})$	−1.003	−1.1	−0.8919	approximate	approximate
b3	$\log_{10}(k_1')$	5.575	< 1.375	> 8.793	optim	optim
b3	$\log_{10}(k_2)$	5.389	< 1.189	> 8.608	optim	optim
b3	$\log_{10}(k_2')$	−0.6239	−0.8153	−0.3871	approximate	approximate
b3	$\log_{10}(k_{deg})$	−0.8748	−1.089	−0.8049	approximate	approximate
b3	$\log_{10}(k_1'/k_2)$	0.1855	0.1337	0.2379	approximate	approximate
b3	$\log_{10}(k_1'k_2'/k_2)$	−0.4384	−0.6227	−0.2066	approximate	approximate
b3	$\log_{10}(k_2'/k_2)$	−6.013	< −9.232	> −1.808	optim	optim

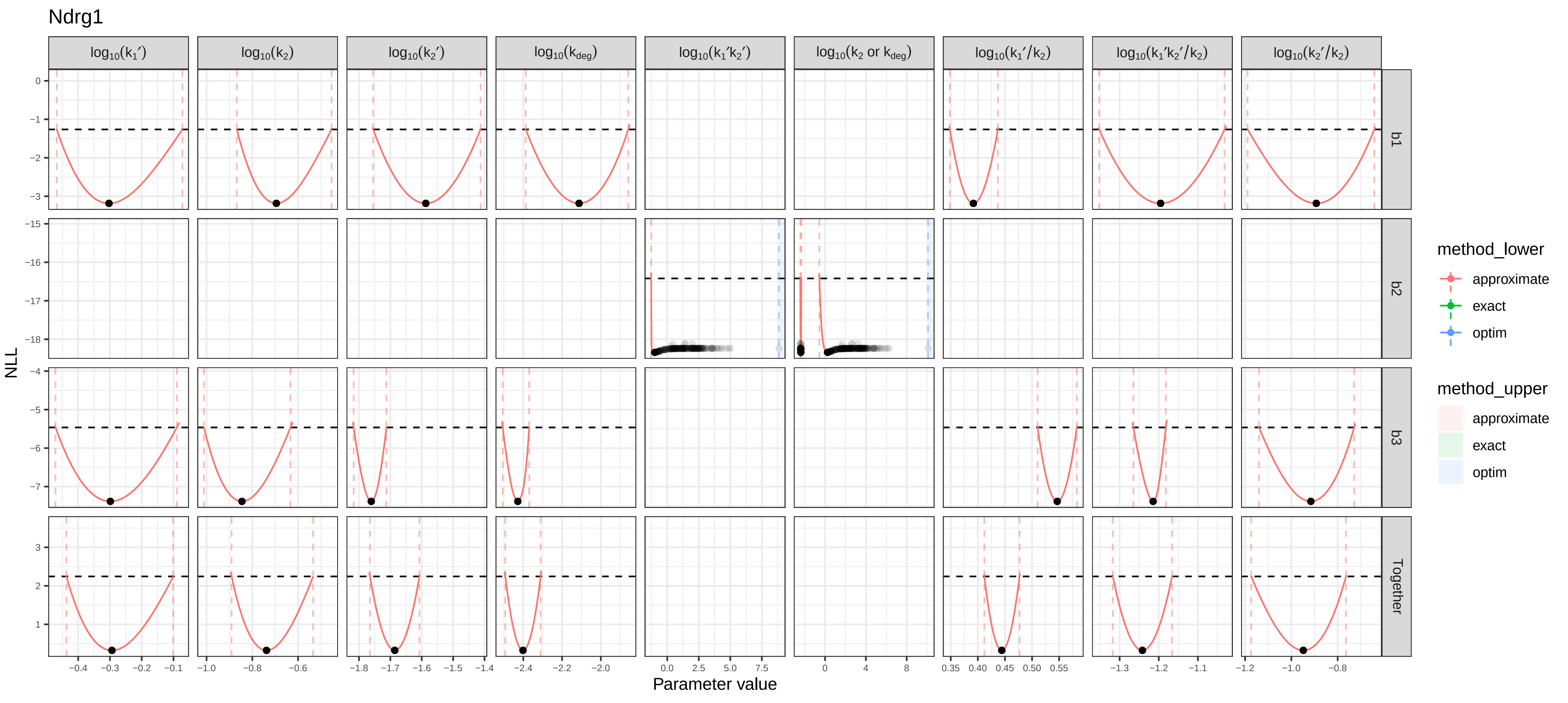

Replicate	Par	Best value	CI95 LB	CI95 UB	Method LB	Method UB
Together	$\log_{10}(k_1')$	−0.2936	−0.437	−0.1008	approximate	approximate
Together	$\log_{10}(k_2)$	−0.7378	−0.8905	−0.534	approximate	approximate
Together	$\log_{10}(k_2')$	−1.686	−1.765	−1.607	approximate	approximate
Together	$\log_{10}(k_{deg})$	−2.402	−2.493	−2.31	approximate	approximate
Together	$\log_{10}(k_1'/k_2)$	0.4442	0.412	0.4769	approximate	approximate
Together	$\log_{10}(k_1'k_2'/k_2)$	−1.242	−1.317	−1.166	approximate	approximate
Together	$\log_{10}(k_2'/k_2)$	−0.9483	−1.174	−0.764	approximate	approximate
b1	$\log_{10}(k_1')$	−0.3031	−0.4674	−0.07179	approximate	approximate
b1	$\log_{10}(k_2)$	−0.6948	−0.8677	−0.4534	approximate	approximate
b1	$\log_{10}(k_2')$	−1.587	−1.755	−1.413	approximate	approximate
b1	$\log_{10}(k_{deg})$	−2.112	−2.387	−1.858	approximate	approximate
b1	$\log_{10}(k_1'/k_2)$	0.3918	0.3484	0.437	approximate	approximate
b1	$\log_{10}(k_1'k_2'/k_2)$	−1.195	−1.352	−1.032	approximate	approximate
b1	$\log_{10}(k_2'/k_2)$	−0.8923	−1.189	−0.6415	approximate	approximate
b2	$\log_{10}(k_1'k_2')$	−0.9942	−1.273	> 8.857	approximate	optim
b2	$\log_{10}(k_2 \text{ or } k_{deg})$	0.2368	−0.5584	> 10.09	approximate	optim
b2	$\log_{10}(k_2 \text{ or } k_{deg})$	−2.38	−2.433	−2.327	approximate	approximate
b3	$\log_{10}(k_1')$	−0.2985	−0.4714	−0.08939	approximate	approximate
b3	$\log_{10}(k_2)$	−0.845	−1.012	−0.6329	approximate	approximate
b3	$\log_{10}(k_2')$	−1.761	−1.817	−1.713	approximate	approximate
b3	$\log_{10}(k_{deg})$	−2.428	−2.506	−2.37	approximate	approximate
b3	$\log_{10}(k_1'/k_2)$	0.5465	0.5103	0.5826	approximate	approximate
b3	$\log_{10}(k_1'k_2'/k_2)$	−1.214	−1.265	−1.182	approximate	approximate
b3	$\log_{10}(k_2'/k_2)$	−0.9159	−1.14	−0.7288	approximate	approximate

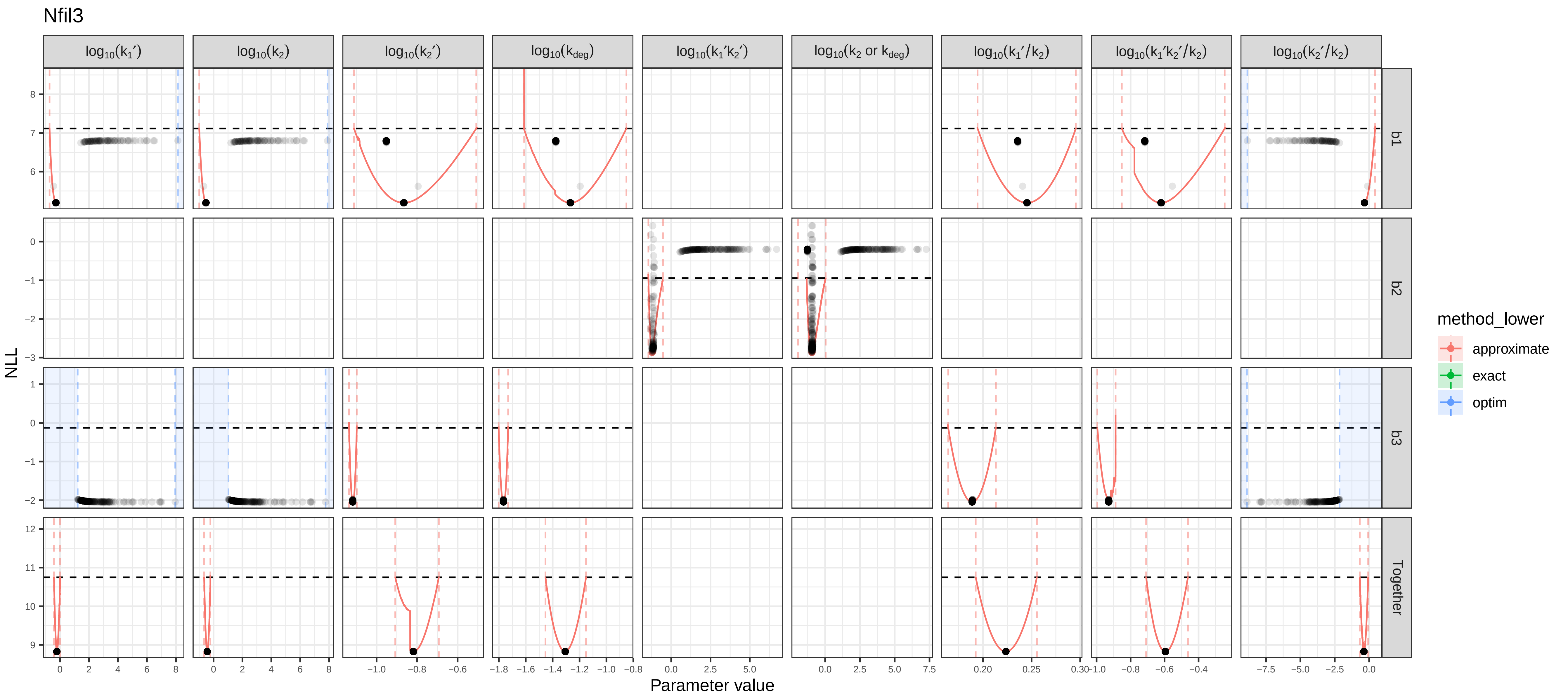

Replicate	Par	Best value	CI95 LB	CI95 UB	Method LB	Method UB
Together	$\log_{10}(k_1')$	−0.2144	−0.4139	0.004732	approximate	approximate
Together	$\log_{10}(k_2)$	−0.4379	−0.6511	−0.2206	approximate	approximate
Together	$\log_{10}(k_2')$	−0.8186	−0.9079	−0.6928	approximate	approximate
Together	$\log_{10}(k_{deg})$	−1.306	−1.453	−1.152	approximate	approximate
Together	$\log_{10}(k_1'/k_2)$	0.2235	0.1926	0.2555	approximate	approximate
Together	$\log_{10}(k_1'k_2'/k_2)$	−0.595	−0.7085	−0.4626	approximate	approximate
Together	$\log_{10}(k_2'/k_2)$	−0.3807	−0.678	−0.07051	approximate	approximate
b1	$\log_{10}(k_1')$	−0.2828	−0.7193	> 8.134	approximate	optim
b1	$\log_{10}(k_2)$	−0.5281	−0.9916	> 7.898	approximate	optim
b1	$\log_{10}(k_2')$	−0.8656	−1.112	−0.5079	approximate	approximate
b1	$\log_{10}(k_{deg})$	−1.268	−1.612	−0.8514	approximate	approximate
b1	$\log_{10}(k_1'/k_2)$	0.2453	0.1945	0.2955	approximate	approximate
b1	$\log_{10}(k_1'k_2'/k_2)$	−0.6203	−0.8516	−0.2463	approximate	approximate
b1	$\log_{10}(k_2'/k_2)$	−0.3375	< −8.851	0.4345	optim	approximate
b2	$\log_{10}(k_1'k_2')$	−1.206	−1.45	−0.5158	approximate	approximate
b2	$\log_{10}(k_2 \text{ or } k_{deg})$	−0.9418	−1.951	0.0416	approximate	approximate
b2	$\log_{10}(k_2 \text{ or } k_{deg})$	−0.977	−1.951	0.0416	approximate	approximate
b3	$\log_{10}(k_1')$	6.901	< 1.221	> 7.95	optim	optim
b3	$\log_{10}(k_2)$	6.712	< 1.031	> 7.761	optim	optim
b3	$\log_{10}(k_2')$	−1.118	−1.136	−1.098	approximate	approximate
b3	$\log_{10}(k_{deg})$	−1.765	−1.802	−1.731	approximate	approximate
b3	$\log_{10}(k_1'/k_2)$	0.1889	0.1643	0.2133	approximate	approximate
b3	$\log_{10}(k_1'k_2'/k_2)$	−0.9288	−0.9956	−0.8882	approximate	approximate
b3	$\log_{10}(k_2'/k_2)$	−7.83	< −8.879	> −2.149	optim	optim

Nfkb2

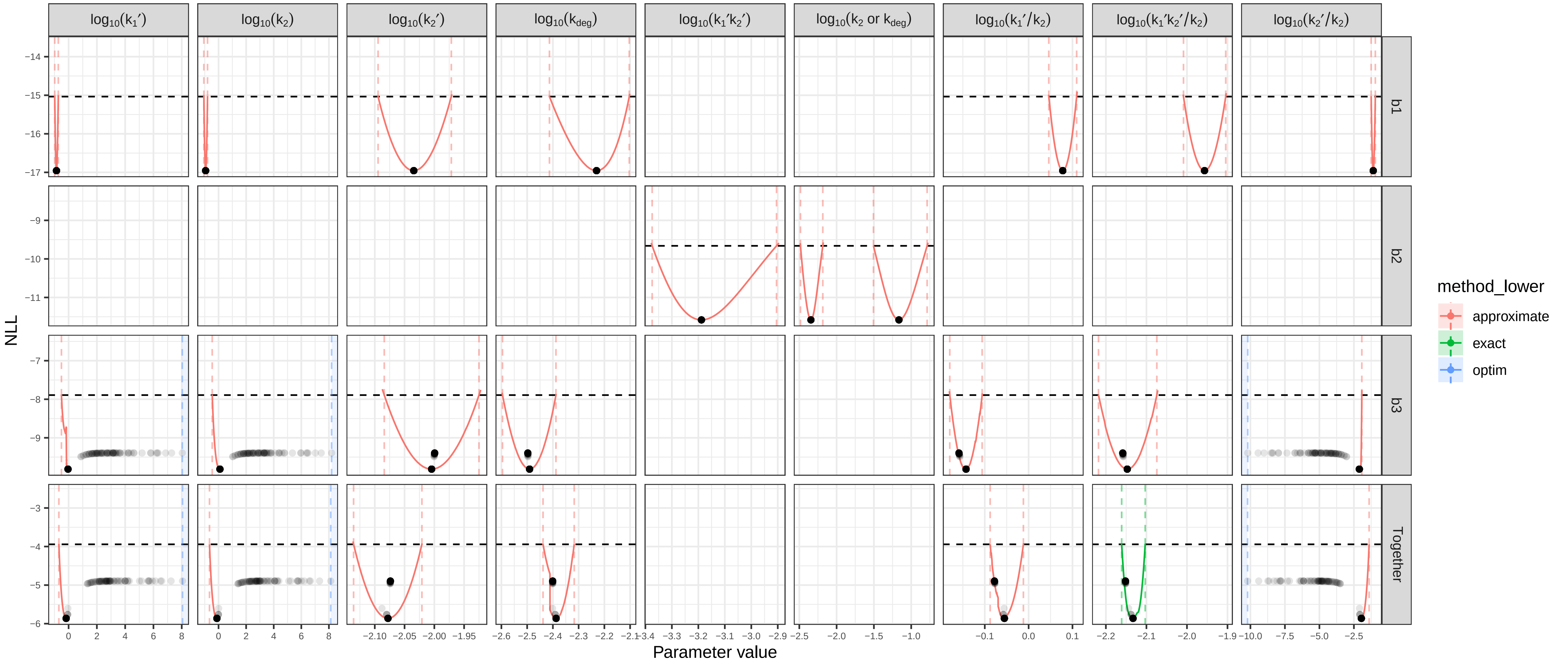

Replicate	Par	Best value	CI95 LB	CI95 UB	Method LB	Method UB
Together	$\log_{10}(k_1')$	−0.1737	−0.6867	> 8.055	approximate	optim
Together	$\log_{10}(k_2)$	−0.1184	−0.6553	> 8.133	approximate	optim
Together	$\log_{10}(k_2')$	−2.078	−2.136	−2.021	approximate	approximate
Together	$\log_{10}(k_{deg})$	−2.387	−2.438	−2.317	approximate	approximate
Together	$\log_{10}(k_1'/k_2)$	−0.05528	−0.08774	−0.01189	approximate	approximate
Together	$\log_{10}(k_1'k_2'/k_2)$	−2.133	−2.161	−2.103	exact	exact
Together	$\log_{10}(k_2'/k_2)$	−1.959	< −10.21	−1.397	optim	approximate
b1	$\log_{10}(k_1')$	−0.8574	−0.9801	−0.7286	approximate	approximate
b1	$\log_{10}(k_2)$	−0.9352	−1.065	−0.7971	approximate	approximate
b1	$\log_{10}(k_2')$	−2.034	−2.094	−1.971	approximate	approximate
b1	$\log_{10}(k_{deg})$	−2.23	−2.413	−2.103	approximate	approximate
b1	$\log_{10}(k_1'/k_2)$	0.07784	0.04605	0.1096	approximate	approximate
b1	$\log_{10}(k_1'k_2'/k_2)$	−1.957	−2.009	−1.904	approximate	approximate
b1	$\log_{10}(k_2'/k_2)$	−1.099	−1.25	−0.9471	approximate	approximate
b2	$\log_{10}(k_1'k_2')$	−3.188	−3.374	−2.905	approximate	approximate
b2	$\log_{10}(k_2 \text{ or } k_{deg})$	−1.165	−1.506	−0.7864	approximate	approximate
b2	$\log_{10}(k_2 \text{ or } k_{deg})$	−2.344	−2.484	−2.185	approximate	approximate
b3	$\log_{10}(k_1')$	−0.04165	−0.506	> 8.037	approximate	optim
b3	$\log_{10}(k_2)$	0.1011	−0.4585	> 8.196	approximate	optim
b3	$\log_{10}(k_2')$	−2.005	−2.084	−1.925	approximate	approximate
b3	$\log_{10}(k_{deg})$	−2.49	−2.596	−2.388	approximate	approximate
b3	$\log_{10}(k_1'/k_2)$	−0.1427	−0.1798	−0.106	approximate	approximate
b3	$\log_{10}(k_1'k_2'/k_2)$	−2.147	−2.218	−2.074	approximate	approximate
b3	$\log_{10}(k_2'/k_2)$	−2.106	< −10.2	−1.927	optim	approximate

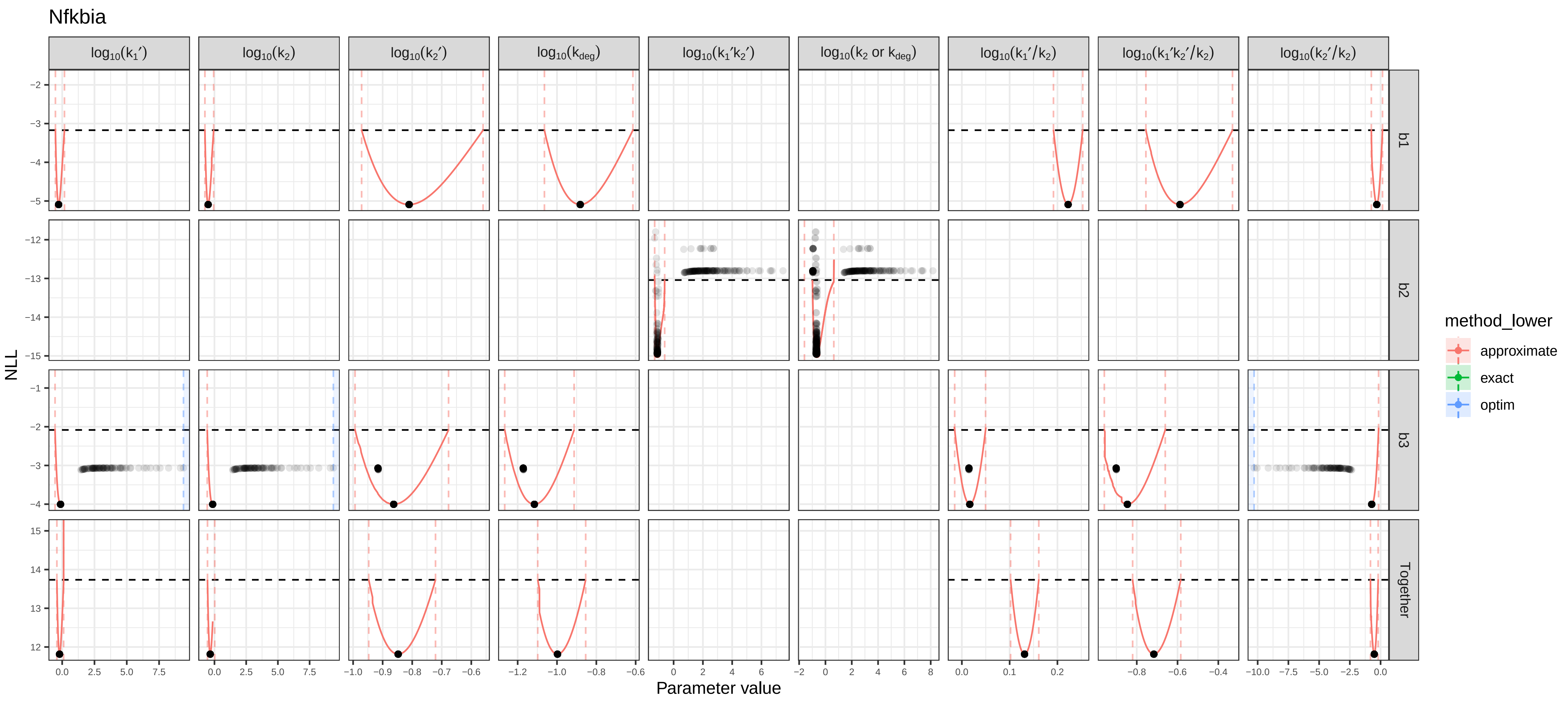

Replicate	Par	Best value	CI95 LB	CI95 UB	Method LB	Method UB
Together	$\log_{10}(k_1')$	−0.2033	−0.4096	0.1128	approximate	approximate
Together	$\log_{10}(k_2)$	−0.3345	−0.5503	0.01894	approximate	approximate
Together	$\log_{10}(k_2')$	−0.8475	−0.9471	−0.7211	approximate	approximate
Together	$\log_{10}(k_{deg})$	−0.9976	−1.098	−0.8541	approximate	approximate
Together	$\log_{10}(k_1'/k_2)$	0.1312	0.102	0.161	approximate	approximate
Together	$\log_{10}(k_1'k_2'/k_2)$	−0.7163	−0.8203	−0.5841	approximate	approximate
Together	$\log_{10}(k_2'/k_2)$	−0.513	−0.8225	−0.1961	approximate	approximate
b1	$\log_{10}(k_1')$	−0.2759	−0.5222	0.1777	approximate	approximate
b1	$\log_{10}(k_2)$	−0.4983	−0.7541	−0.06157	approximate	approximate
b1	$\log_{10}(k_2')$	−0.8104	−0.9712	−0.56	approximate	approximate
b1	$\log_{10}(k_{deg})$	−0.8817	−1.064	−0.6141	approximate	approximate
b1	$\log_{10}(k_1'/k_2)$	0.2224	0.1918	0.253	approximate	approximate
b1	$\log_{10}(k_1'k_2'/k_2)$	−0.588	−0.7552	−0.3304	approximate	approximate
b1	$\log_{10}(k_2'/k_2)$	−0.3121	−0.7524	0.1578	approximate	approximate
b2	$\log_{10}(k_1'k_2')$	−1.111	−1.293	−0.6092	approximate	approximate
b2	$\log_{10}(k_2 \text{ or } k_{deg})$	−0.6553	−1.596	0.6355	approximate	approximate
b2	$\log_{10}(k_2 \text{ or } k_{deg})$	−0.711	−1.596	0.6355	approximate	approximate
b3	$\log_{10}(k_1')$	−0.1266	−0.552	> 9.387	approximate	optim
b3	$\log_{10}(k_2)$	−0.1431	−0.5704	> 9.373	approximate	optim
b3	$\log_{10}(k_2')$	−0.8626	−0.9934	−0.6769	approximate	approximate
b3	$\log_{10}(k_{deg})$	−1.115	−1.266	−0.913	approximate	approximate
b3	$\log_{10}(k_1'/k_2)$	0.01655	−0.01502	0.04961	approximate	approximate
b3	$\log_{10}(k_1'k_2'/k_2)$	−0.8461	−0.9592	−0.6603	approximate	approximate
b3	$\log_{10}(k_2'/k_2)$	−0.7195	< −10.29	−0.1623	optim	approximate

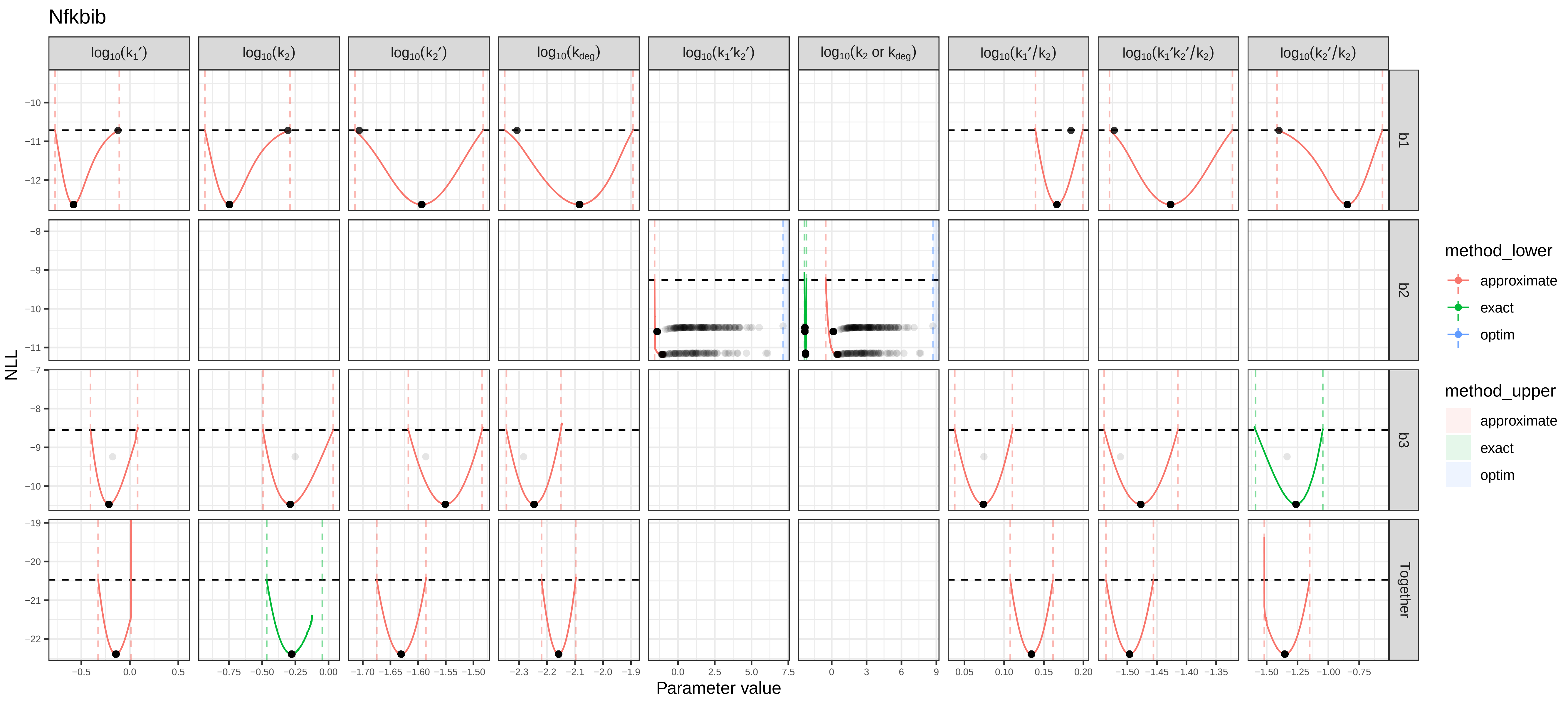

Replicate	Par	Best value	CI95 LB	CI95 UB	Method LB	Method UB
Together	$\log_{10}(k_1')$	−0.1439	−0.3266	0.01208	approximate	approximate
Together	$\log_{10}(k_2)$	−0.2784	−0.466	−0.04646	exact	exact
Together	$\log_{10}(k_2')$	−1.631	−1.675	−1.586	approximate	approximate
Together	$\log_{10}(k_{deg})$	−2.159	−2.22	−2.098	approximate	approximate
Together	$\log_{10}(k_1'/k_2)$	0.1345	0.1077	0.1615	approximate	approximate
Together	$\log_{10}(k_1'k_2'/k_2)$	−1.496	−1.536	−1.456	approximate	approximate
Together	$\log_{10}(k_2'/k_2)$	−1.352	−1.519	−1.151	approximate	approximate
b1	$\log_{10}(k_1')$	−0.5805	−0.7711	−0.1081	approximate	approximate
b1	$\log_{10}(k_2)$	−0.7471	−0.9313	−0.2907	approximate	approximate
b1	$\log_{10}(k_2')$	−1.594	−1.714	−1.482	approximate	approximate
b1	$\log_{10}(k_{deg})$	−2.084	−2.352	−1.893	approximate	approximate
b1	$\log_{10}(k_1'/k_2)$	0.1666	0.1395	0.1991	approximate	approximate
b1	$\log_{10}(k_1'k_2'/k_2)$	−1.427	−1.53	−1.322	approximate	approximate
b1	$\log_{10}(k_2'/k_2)$	−0.8464	−1.416	−0.5615	approximate	approximate
b2	$\log_{10}(k_1'k_2')$	−1.043	−1.587	> 7.138	approximate	optim
b2	$\log_{10}(k_2 \text{ or } k_{deg})$	0.5106	−0.5116	> 8.702	approximate	optim
b2	$\log_{10}(k_2 \text{ or } k_{deg})$	−2.244	−2.334	−2.16	exact	exact
b3	$\log_{10}(k_1')$	−0.2152	−0.4054	0.07988	approximate	approximate
b3	$\log_{10}(k_2)$	−0.2889	−0.4945	0.03566	approximate	approximate
b3	$\log_{10}(k_2')$	−1.551	−1.618	−1.484	approximate	approximate
b3	$\log_{10}(k_{deg})$	−2.246	−2.345	−2.151	approximate	approximate
b3	$\log_{10}(k_1'/k_2)$	0.07372	0.03761	0.1104	approximate	approximate
b3	$\log_{10}(k_1'k_2'/k_2)$	−1.477	−1.539	−1.415	approximate	approximate
b3	$\log_{10}(k_2'/k_2)$	−1.262	−1.59	−1.045	exact	exact

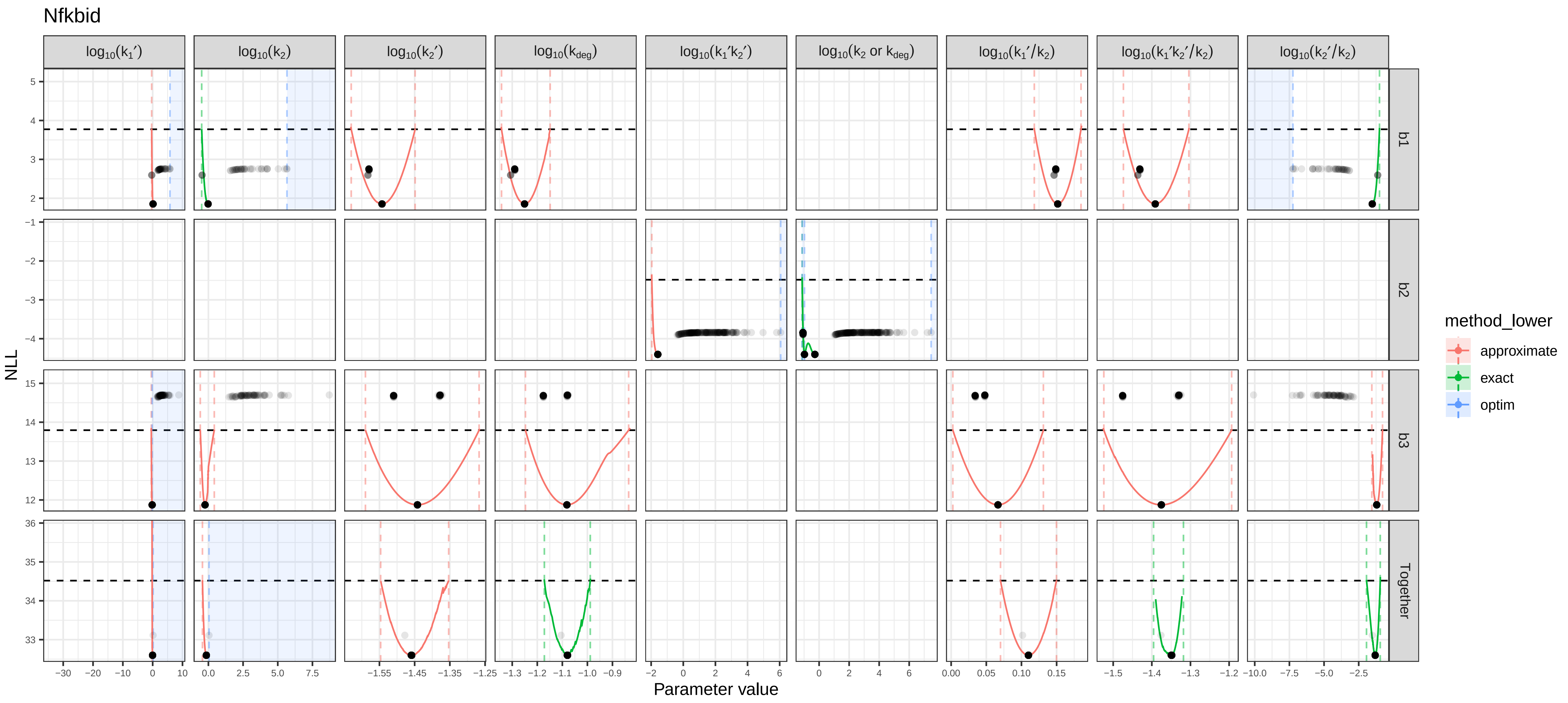

Replicate	Par	Best value	CI95 LB	CI95 UB	Method LB	Method UB
Together	$\log_{10}(k_1')$	−0.03855	−0.175	> 0.1466	approximate	optim
Together	$\log_{10}(k_2)$	−0.1484	−0.428	> 0.04504	approximate	optim
Together	$\log_{10}(k_2')$	−1.459	−1.547	−1.353	approximate	approximate
Together	$\log_{10}(k_{deg})$	−1.08	−1.172	−0.9885	exact	exact
Together	$\log_{10}(k_1'/k_2)$	0.1099	0.07018	0.1498	approximate	approximate
Together	$\log_{10}(k_1'k_2'/k_2)$	−1.349	−1.395	−1.318	exact	exact
Together	$\log_{10}(k_2'/k_2)$	−1.31	−1.937	−0.9468	exact	exact
b1	$\log_{10}(k_1')$	0.1308	−0.3357	> 5.813	approximate	optim
b1	$\log_{10}(k_2)$	−0.02075	−0.4829	> 5.664	exact	optim
b1	$\log_{10}(k_2')$	−1.543	−1.63	−1.449	approximate	approximate
b1	$\log_{10}(k_{deg})$	−1.251	−1.343	−1.149	approximate	approximate
b1	$\log_{10}(k_1'/k_2)$	0.1516	0.1183	0.1847	approximate	approximate
b1	$\log_{10}(k_1'k_2'/k_2)$	−1.391	−1.474	−1.304	approximate	approximate
b1	$\log_{10}(k_2'/k_2)$	−1.522	< −7.244	−1.001	optim	exact
b2	$\log_{10}(k_1'k_2')$	−1.585	−1.96	> 6.063	approximate	optim
b2	$\log_{10}(k_2 \text{ or } k_{deg})$	−0.2813	−1.128	> 7.462	exact	optim
b2	$\log_{10}(k_2 \text{ or } k_{deg})$	−0.9755	−1.128	−0.9724	exact	optim
b3	$\log_{10}(k_1')$	−0.1759	−0.4633	> −0.1742	approximate	optim
b3	$\log_{10}(k_2)$	−0.2425	−0.5842	0.4251	approximate	approximate
b3	$\log_{10}(k_2')$	−1.442	−1.59	−1.267	approximate	approximate
b3	$\log_{10}(k_{deg})$	−1.082	−1.247	−0.8354	approximate	approximate
b3	$\log_{10}(k_1'/k_2)$	0.06659	0.00244	0.1312	approximate	approximate
b3	$\log_{10}(k_1'k_2'/k_2)$	−1.375	−1.524	−1.193	approximate	approximate
b3	$\log_{10}(k_2'/k_2)$	−1.2	−1.554	−0.7816	approximate	approximate

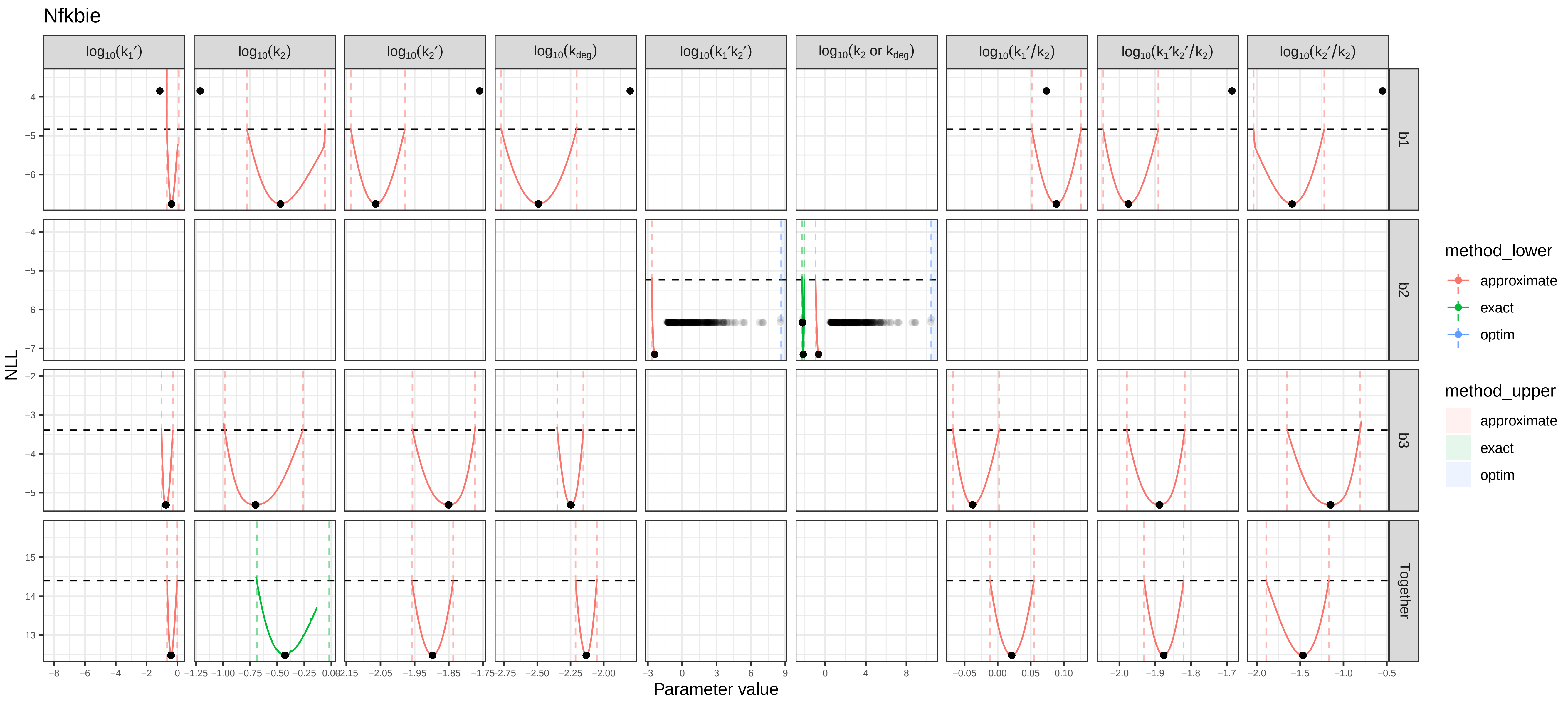

Replicate	Par	Best value	CI95 LB	CI95 UB	Method LB	Method UB
Together	$\log_{10}(k_1')$	−0.4068	−0.6654	−0.01539	approximate	approximate
Together	$\log_{10}(k_2)$	−0.4282	−0.6893	−0.01924	exact	exact
Together	$\log_{10}(k_2')$	−1.898	−1.958	−1.837	approximate	approximate
Together	$\log_{10}(k_{deg})$	−2.131	−2.212	−2.052	approximate	approximate
Together	$\log_{10}(k_1'/k_2)$	0.02132	−0.0114	0.05487	approximate	approximate
Together	$\log_{10}(k_1'k_2'/k_2)$	−1.876	−1.931	−1.821	approximate	approximate
Together	$\log_{10}(k_2'/k_2)$	−1.47	−1.891	−1.169	approximate	approximate
b1	$\log_{10}(k_1')$	−0.382	−0.6871	0.09358	approximate	approximate
b1	$\log_{10}(k_2)$	−0.4706	−0.7801	−0.05818	approximate	approximate
b1	$\log_{10}(k_2')$	−2.064	−2.137	−1.979	approximate	approximate
b1	$\log_{10}(k_{deg})$	−2.492	−2.772	−2.204	approximate	approximate
b1	$\log_{10}(k_1'/k_2)$	0.08862	0.05171	0.1262	approximate	approximate
b1	$\log_{10}(k_1'k_2'/k_2)$	−1.975	−2.046	−1.891	approximate	approximate
b1	$\log_{10}(k_2'/k_2)$	−1.593	−2.038	−1.221	approximate	approximate
b2	$\log_{10}(k_1'k_2')$	−2.404	−2.661	> 8.594	approximate	optim
b2	$\log_{10}(k_2 \text{ or } k_{deg})$	−0.6525	−0.9437	> 10.43	approximate	optim
b2	$\log_{10}(k_2 \text{ or } k_{deg})$	−2.16	−2.266	−2.06	exact	exact
b3	$\log_{10}(k_1')$	−0.7393	−1.026	−0.2996	approximate	approximate
b3	$\log_{10}(k_2)$	−0.7014	−0.985	−0.2617	approximate	approximate
b3	$\log_{10}(k_2')$	−1.85	−1.956	−1.773	approximate	approximate
b3	$\log_{10}(k_{deg})$	−2.247	−2.349	−2.153	approximate	approximate
b3	$\log_{10}(k_1'/k_2)$	−0.03784	−0.0676	0.002376	approximate	approximate
b3	$\log_{10}(k_1'k_2'/k_2)$	−1.888	−1.979	−1.818	approximate	approximate
b3	$\log_{10}(k_2'/k_2)$	−1.149	−1.651	−0.8083	approximate	approximate

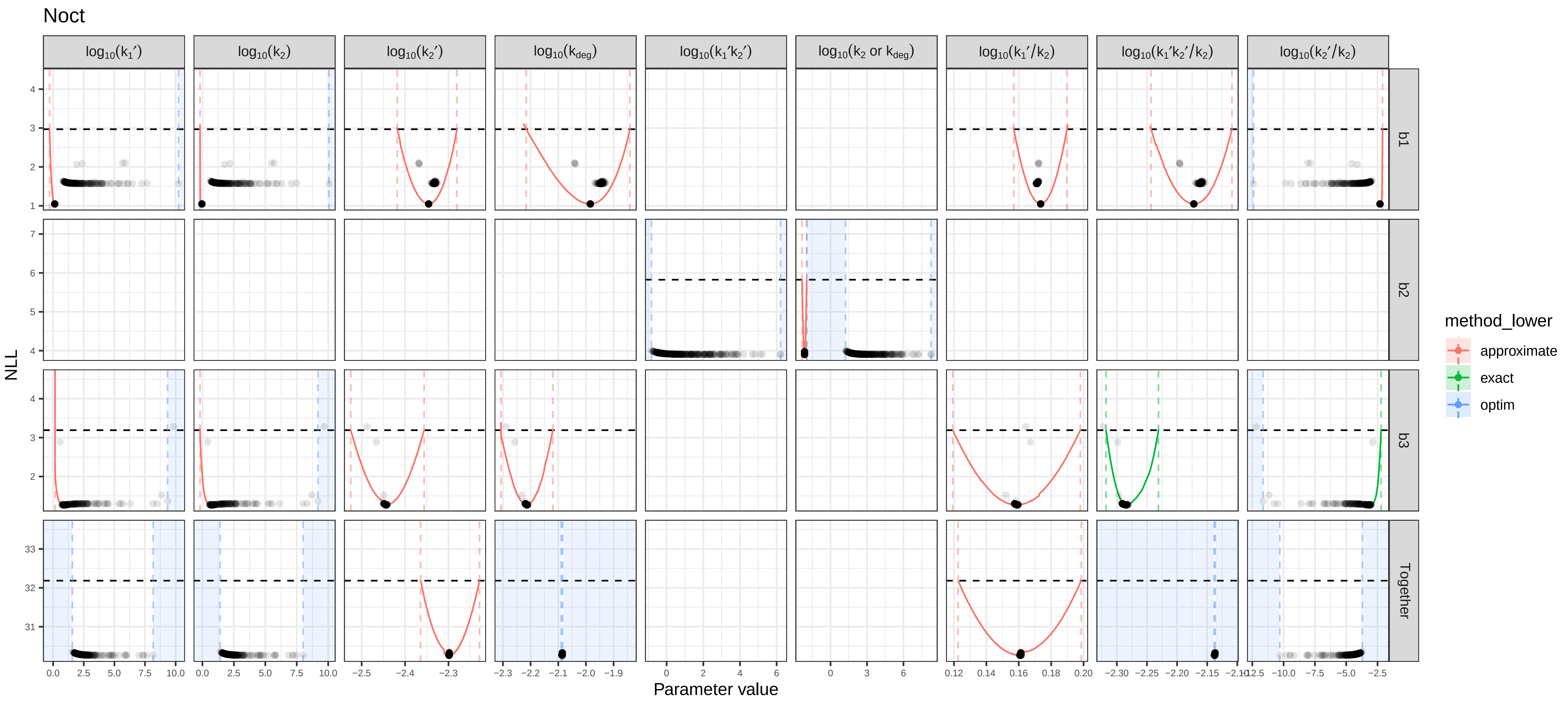

Replicate	Par	**Best value**	**CI95 LB**	**CI95 UB**	**Method LB**	**Method UB**
Together	$\log_{10}(k_1')$	3.443	< 1.571	> 8.165	optim	optim
Together	$\log_{10}(k_2)$	3.282	< 1.41	> 8.004	optim	optim
Together	$\log_{10}(k_2')$	−2.299	−2.364	−2.228	approximate	approximate
Together	$\log_{10}(k_{deg})$	−2.086	< −2.089	> −2.085	optim	optim
Together	$\log_{10}(k_1'/k_2)$	0.1612	0.1226	0.1985	approximate	approximate
Together	$\log_{10}(k_1'k_2'/k_2)$	−2.138	< −2.139	> −2.136	optim	optim
Together	$\log_{10}(k_2'/k_2)$	−5.581	< −10.3	> −3.708	optim	optim
b1	$\log_{10}(k_1')$	0.1304	−0.2923	> 10.24	approximate	optim
b1	$\log_{10}(k_2)$	−0.04307	−0.1904	> 10.07	approximate	optim
b1	$\log_{10}(k_2')$	−2.346	−2.418	−2.28	approximate	approximate
b1	$\log_{10}(k_{deg})$	−1.984	−2.216	−1.841	approximate	approximate
b1	$\log_{10}(k_1'/k_2)$	0.1735	0.1569	0.1897	approximate	approximate
b1	$\log_{10}(k_1'k_2'/k_2)$	−2.172	−2.243	−2.109	approximate	approximate
b1	$\log_{10}(k_2'/k_2)$	−2.302	< −12.4	−2.1	optim	approximate
b2	$\log_{10}(k_1'k_2')$	3.747	< −0.8234	> 6.212	optim	optim
b2	$\log_{10}(k_2 \text{ or } k_{deg})$	5.803	1.233	> 8.269	optim	optim
b2	$\log_{10}(k_2 \text{ or } k_{deg})$	−2.138	−2.344	−1.964	approximate	approximate
b3	$\log_{10}(k_1')$	0.9024	0.1604	> 9.343	approximate	optim
b3	$\log_{10}(k_2)$	0.7435	−0.1883	> 9.185	approximate	optim
b3	$\log_{10}(k_2')$	−2.444	−2.525	−2.356	approximate	approximate
b3	$\log_{10}(k_{deg})$	−2.213	−2.307	−2.119	approximate	approximate
b3	$\log_{10}(k_1'/k_2)$	0.1589	0.1193	0.198	approximate	approximate
b3	$\log_{10}(k_1'k_2'/k_2)$	−2.285	−2.318	−2.231	exact	exact
b3	$\log_{10}(k_2'/k_2)$	−3.187	< −11.64	−2.218	optim	exact

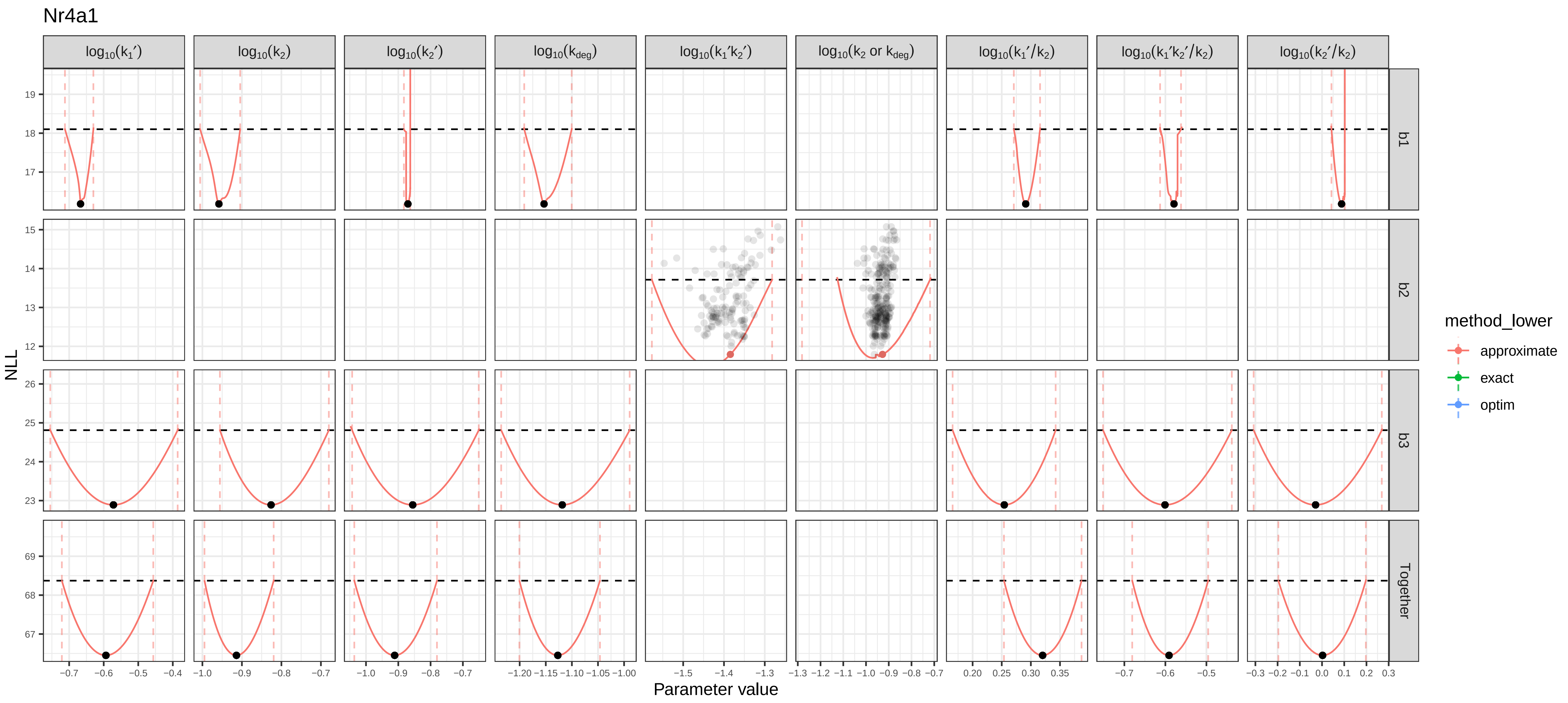

Replicate	Par	**Best value**	**CI95 LB**	**CI95 UB**	**Method LB**	**Method UB**
Together	$\log_{10}(k_1')$	−0.5937	−0.7212	−0.4565	approximate	approximate
Together	$\log_{10}(k_2)$	−0.9139	−0.9947	−0.8196	approximate	approximate
Together	$\log_{10}(k_2')$	−0.9112	−1.036	−0.7802	approximate	approximate
Together	$\log_{10}(k_{deg})$	−1.127	−1.201	−1.046	approximate	approximate
Together	$\log_{10}(k_1'/k_2)$	0.3202	0.2538	0.387	approximate	approximate
Together	$\log_{10}(k_1'k_2'/k_2)$	−0.5911	−0.6808	−0.4957	approximate	approximate
Together	$\log_{10}(k_2'/k_2)$	0.00262	−0.1966	0.1973	approximate	approximate
b1	$\log_{10}(k_1')$	−0.6671	−0.7122	−0.6299	approximate	approximate
b1	$\log_{10}(k_2)$	−0.9583	−1.006	−0.9043	approximate	approximate
b1	$\log_{10}(k_2')$	−0.8702	−0.8826	−0.8629	approximate	approximate
b1	$\log_{10}(k_{deg})$	−1.153	−1.191	−1.1	approximate	approximate
b1	$\log_{10}(k_1'/k_2)$	0.2912	0.2707	0.3158	approximate	approximate
b1	$\log_{10}(k_1'k_2'/k_2)$	−0.579	−0.6129	−0.5619	approximate	approximate
b1	$\log_{10}(k_2'/k_2)$	0.08805	0.04244	0.1025	approximate	approximate
b2	$\log_{10}(k_1'k_2')$	−1.384	−1.577	−1.282	approximate	approximate
b2	$\log_{10}(k_2 \text{ or } k_{deg})$	−0.9275	−1.281	−0.7184	approximate	approximate
b2	$\log_{10}(k_2 \text{ or } k_{deg})$	−0.9635	−1.281	−0.7184	approximate	approximate
b3	$\log_{10}(k_1')$	−0.5719	−0.7547	−0.3859	approximate	approximate
b3	$\log_{10}(k_2)$	−0.8262	−0.9557	−0.6799	approximate	approximate
b3	$\log_{10}(k_2')$	−0.8552	−1.043	−0.6509	approximate	approximate
b3	$\log_{10}(k_{deg})$	−1.118	−1.235	−0.9893	approximate	approximate
b3	$\log_{10}(k_1'/k_2)$	0.2543	0.1657	0.3425	approximate	approximate
b3	$\log_{10}(k_1'k_2'/k_2)$	−0.6009	−0.7519	−0.4381	approximate	approximate
b3	$\log_{10}(k_2'/k_2)$	−0.02899	−0.3079	0.2691	approximate	approximate

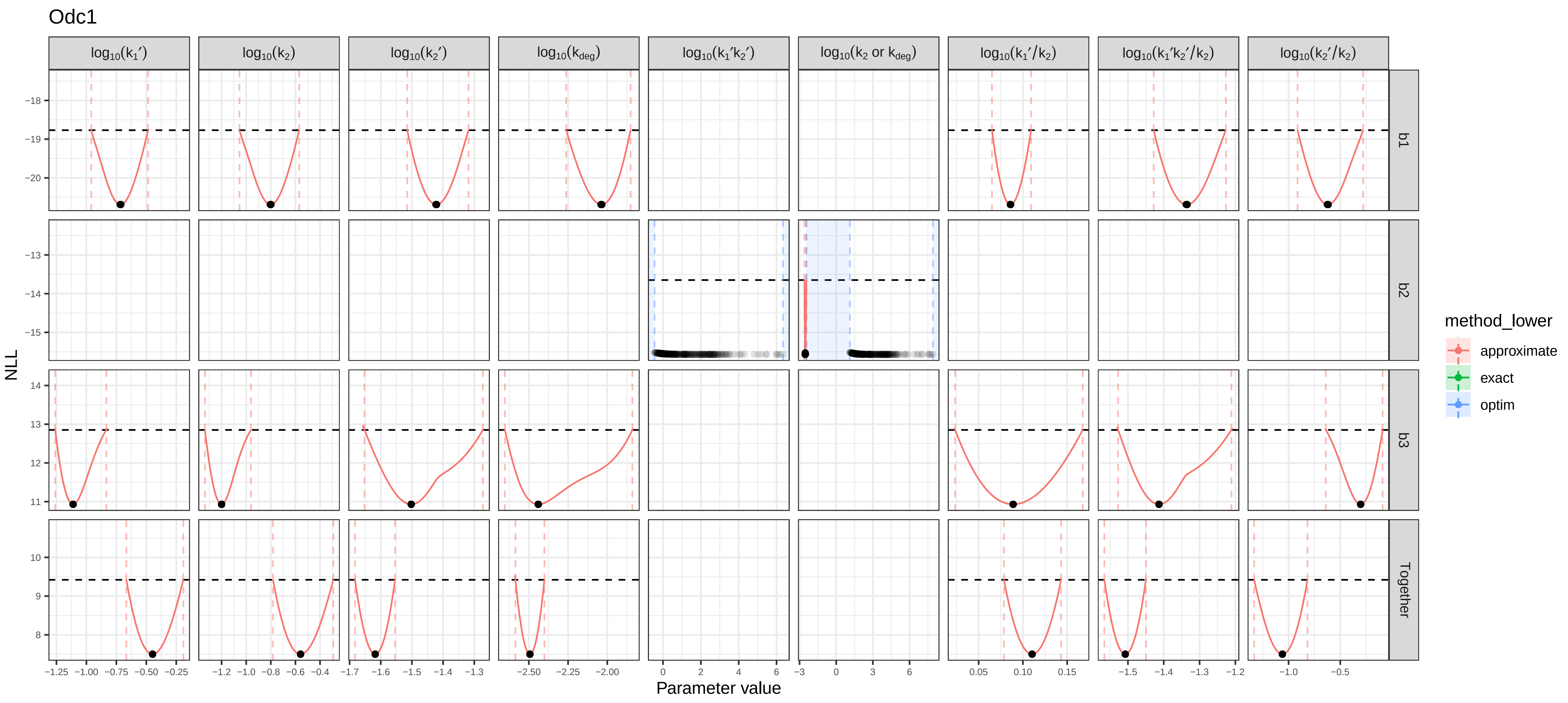

Replicate	Par	Best value	CI95 LB	CI95 UB	Method LB	Method UB
Together	$\log_{10}(k_1')$	−0.4478	−0.6666	−0.1907	approximate	approximate
Together	$\log_{10}(k_2)$	−0.5582	−0.7824	−0.2919	approximate	approximate
Together	$\log_{10}(k_2')$	−1.618	−1.683	−1.554	approximate	approximate
Together	$\log_{10}(k_{deg})$	−2.493	−2.586	−2.401	approximate	approximate
Together	$\log_{10}(k_1'/k_2)$	0.1103	0.07848	0.1431	approximate	approximate
Together	$\log_{10}(k_1'k_2'/k_2)$	−1.508	−1.566	−1.45	approximate	approximate
Together	$\log_{10}(k_2'/k_2)$	−1.06	−1.334	−0.8171	approximate	approximate
b1	$\log_{10}(k_1')$	−0.7158	−0.9598	−0.4851	approximate	approximate
b1	$\log_{10}(k_2)$	−0.8018	−1.055	−0.5692	approximate	approximate
b1	$\log_{10}(k_2')$	−1.422	−1.515	−1.319	approximate	approximate
b1	$\log_{10}(k_{deg})$	−2.037	−2.262	−1.851	approximate	approximate
b1	$\log_{10}(k_1'/k_2)$	0.08595	0.06503	0.1092	approximate	approximate
b1	$\log_{10}(k_1'k_2'/k_2)$	−1.336	−1.428	−1.226	approximate	approximate
b1	$\log_{10}(k_2'/k_2)$	−0.6199	−0.9131	−0.2781	approximate	approximate
b2	$\log_{10}(k_1'k_2')$	3.229	< −0.4481	> 6.343	optim	optim
b2	$\log_{10}(k_2 \text{ or } k_{deg})$	4.797	1.119	> 7.911	optim	optim
b2	$\log_{10}(k_2 \text{ or } k_{deg})$	−2.534	−2.599	−2.44	approximate	approximate
b3	$\log_{10}(k_1')$	−1.111	−1.26	−0.834	approximate	approximate
b3	$\log_{10}(k_2)$	−1.2	−1.336	−0.9617	approximate	approximate
b3	$\log_{10}(k_2')$	−1.502	−1.652	−1.273	approximate	approximate
b3	$\log_{10}(k_{deg})$	−2.44	−2.653	−1.84	approximate	approximate
b3	$\log_{10}(k_1'/k_2)$	0.08894	0.02337	0.1676	approximate	approximate
b3	$\log_{10}(k_1'k_2'/k_2)$	−1.413	−1.528	−1.211	approximate	approximate
b3	$\log_{10}(k_2'/k_2)$	−0.3021	−0.6396	−0.08995	approximate	approximate

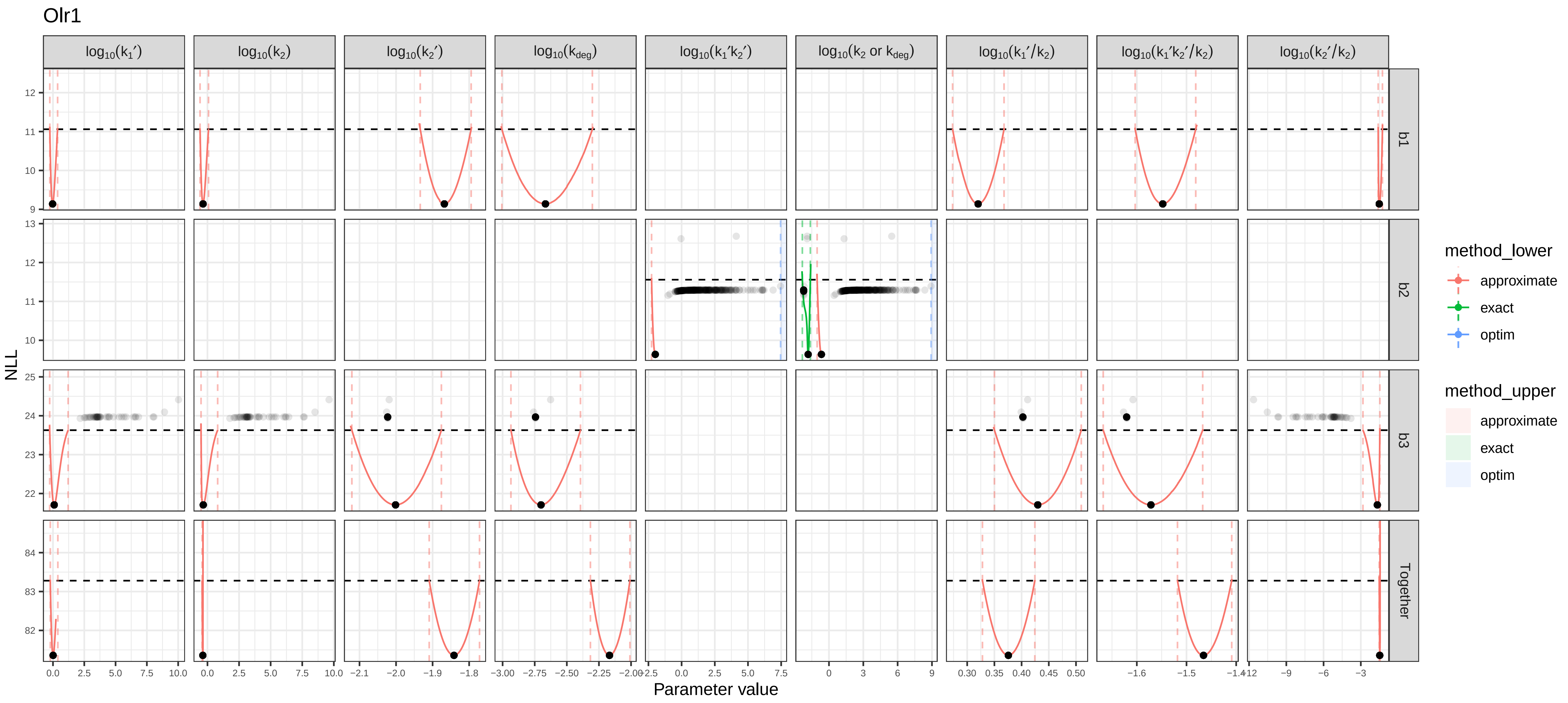

Replicate	Par	Best value	CI95 LB	CI95 UB	Method LB	Method UB
Together	$\log_{10}(k_1')$	0.009809	−0.2151	0.3893	approximate	approximate
Together	$\log_{10}(k_2)$	−0.3659	−0.4232	−0.3558	approximate	approximate
Together	$\log_{10}(k_2')$	−1.841	−1.909	−1.772	approximate	approximate
Together	$\log_{10}(k_{deg})$	−2.168	−2.317	−2.009	approximate	approximate
Together	$\log_{10}(k_1'/k_2)$	0.3757	0.3281	0.4243	approximate	approximate
Together	$\log_{10}(k_1'k_2'/k_2)$	−1.466	−1.518	−1.409	approximate	approximate
Together	$\log_{10}(k_2'/k_2)$	−1.476	−1.518	−1.449	approximate	approximate
b1	$\log_{10}(k_1')$	−0.03122	−0.2529	0.3699	approximate	approximate
b1	$\log_{10}(k_2)$	−0.3513	−0.5859	0.0858	approximate	approximate
b1	$\log_{10}(k_2')$	−1.868	−1.934	−1.794	approximate	approximate
b1	$\log_{10}(k_{deg})$	−2.666	−3.006	−2.301	approximate	approximate
b1	$\log_{10}(k_1'/k_2)$	0.3201	0.2732	0.3677	approximate	approximate
b1	$\log_{10}(k_1'k_2'/k_2)$	−1.548	−1.603	−1.481	approximate	approximate
b1	$\log_{10}(k_2'/k_2)$	−1.517	−1.602	−1.259	approximate	approximate
b2	$\log_{10}(k_1'k_2')$	−1.984	−2.263	> 7.454	approximate	optim
b2	$\log_{10}(k_2 \text{ or } k_{deg})$	−0.6602	−1.035	> 8.92	approximate	optim
b2	$\log_{10}(k_2 \text{ or } k_{deg})$	−1.819	−2.326	−1.627	exact	exact
b3	$\log_{10}(k_1')$	0.09751	−0.2628	1.211	approximate	approximate
b3	$\log_{10}(k_2)$	−0.3323	−0.508	0.8068	approximate	approximate
b3	$\log_{10}(k_2')$	−2.001	−2.121	−1.876	approximate	approximate
b3	$\log_{10}(k_{deg})$	−2.701	−2.935	−2.394	approximate	approximate
b3	$\log_{10}(k_1'/k_2)$	0.4298	0.3501	0.5097	approximate	approximate
b3	$\log_{10}(k_1'k_2'/k_2)$	−1.572	−1.668	−1.467	approximate	approximate
b3	$\log_{10}(k_2'/k_2)$	−1.669	−2.827	−1.468	approximate	approximate

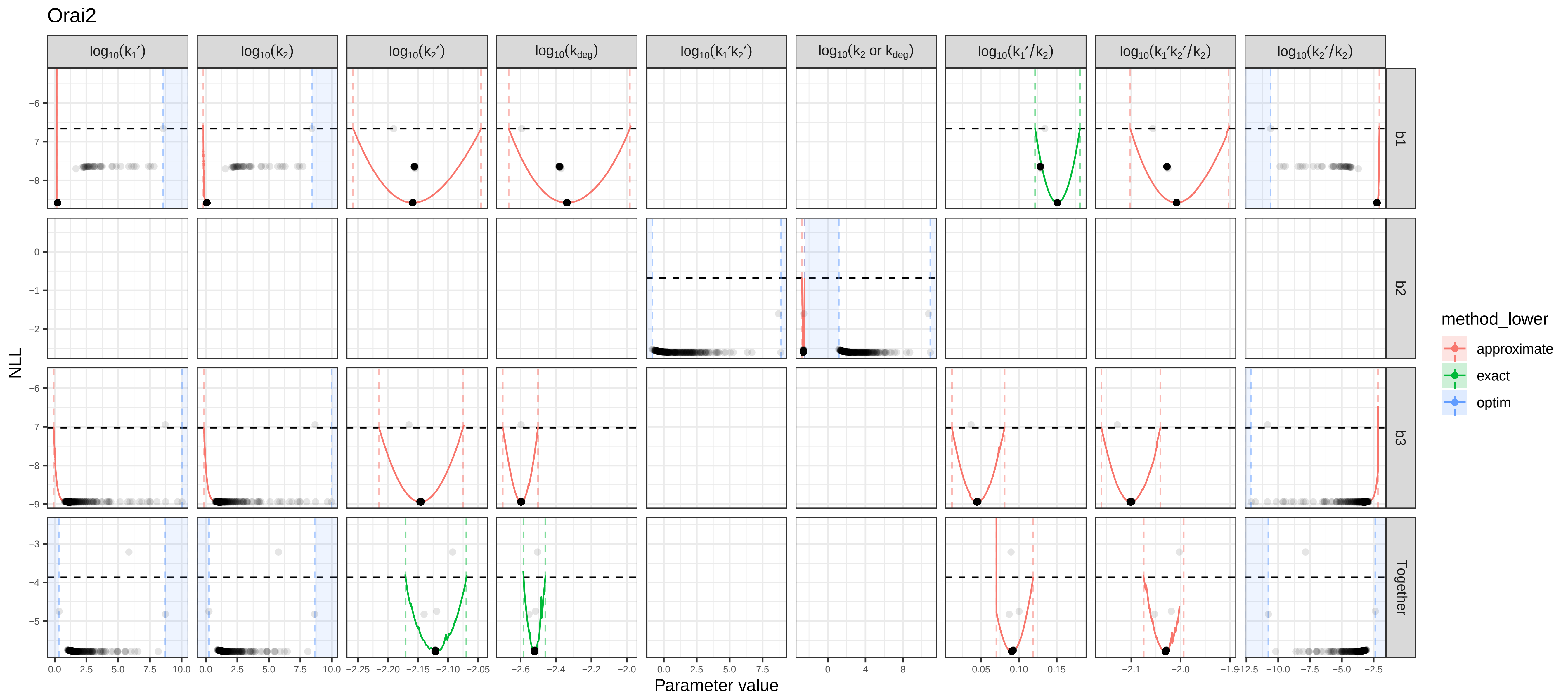

Replicate	Par	Best value	CI95 LB	CI95 UB	Method LB	Method UB
Together	$\log_{10}(k_1')$	8.17	< 0.3482	> 8.72	optim	optim
Together	$\log_{10}(k_2)$	8.079	< 0.248	> 8.633	optim	optim
Together	$\log_{10}(k_2')$	−2.121	−2.171	−2.07	exact	exact
Together	$\log_{10}(k_{deg})$	−2.522	−2.583	−2.46	exact	exact
Together	$\log_{10}(k_1'/k_2)$	0.09059	0.07018	0.1189	approximate	approximate
Together	$\log_{10}(k_1'k_2'/k_2)$	−2.031	−2.075	−1.994	approximate	approximate
Together	$\log_{10}(k_2'/k_2)$	−10.2	< −10.77	> −2.367	optim	optim
b1	$\log_{10}(k_1')$	0.2329	0.1583	> 8.541	approximate	optim
b1	$\log_{10}(k_2)$	0.08209	−0.1957	> 8.407	approximate	optim
b1	$\log_{10}(k_2')$	−2.159	−2.258	−2.045	approximate	approximate
b1	$\log_{10}(k_{deg})$	−2.338	−2.668	−1.983	approximate	approximate
b1	$\log_{10}(k_1'/k_2)$	0.1508	0.1214	0.1808	exact	exact
b1	$\log_{10}(k_1'k_2'/k_2)$	−2.008	−2.102	−1.903	approximate	approximate
b1	$\log_{10}(k_2'/k_2)$	−2.241	< −10.6	−2.046	optim	approximate
b2	$\log_{10}(k_1'k_2')$	3.886	< −0.8649	> 8.87	optim	optim
b2	$\log_{10}(k_2 \text{ or } k_{deg})$	5.914	1.163	> 10.9	optim	optim
b2	$\log_{10}(k_2 \text{ or } k_{deg})$	−2.591	−2.738	−2.466	approximate	approximate
b3	$\log_{10}(k_1')$	1.248	−0.08088	> 10.03	approximate	optim
b3	$\log_{10}(k_2)$	1.203	−0.1454	> 9.983	approximate	optim
b3	$\log_{10}(k_2')$	−2.146	−2.215	−2.075	approximate	approximate
b3	$\log_{10}(k_{deg})$	−2.597	−2.701	−2.502	approximate	approximate
b3	$\log_{10}(k_1'/k_2)$	0.04465	0.01128	0.08096	approximate	approximate
b3	$\log_{10}(k_1'k_2'/k_2)$	−2.101	−2.161	−2.041	approximate	approximate
b3	$\log_{10}(k_2'/k_2)$	−3.349	< −12.13	−2.158	optim	approximate

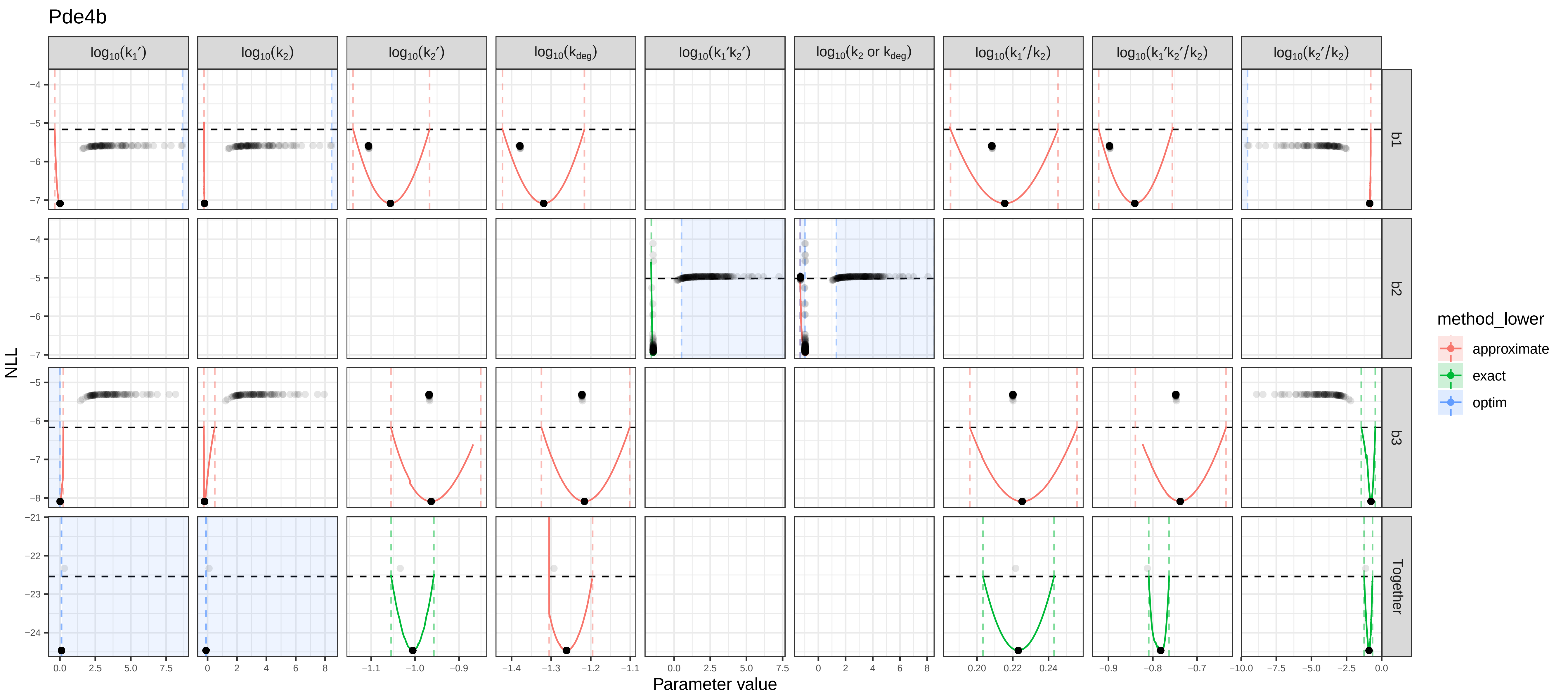

Replicate	Par	Best value	CI95 LB	CI95 UB	Method LB	Method UB
Together	$\log_{10}(k_1')$	0.1165	< 0.1087	> 0.1221	optim	optim
Together	$\log_{10}(k_2)$	−0.1067	< −0.1146	> −0.1011	optim	optim
Together	$\log_{10}(k_2')$	−1.005	−1.054	−0.9574	exact	exact
Together	$\log_{10}(k_{deg})$	−1.261	−1.305	−1.195	approximate	approximate
Together	$\log_{10}(k_1'/k_2)$	0.2231	0.2034	0.2431	exact	exact
Together	$\log_{10}(k_1'k_2'/k_2)$	−0.7822	−0.8092	−0.7631	exact	exact
Together	$\log_{10}(k_2'/k_2)$	−0.8987	−1.25	−0.6484	exact	exact
b1	$\log_{10}(k_1')$	0.009303	−0.3622	> 8.649	approximate	optim
b1	$\log_{10}(k_2)$	−0.2062	−0.2349	> 8.441	approximate	optim
b1	$\log_{10}(k_2')$	−1.056	−1.141	−0.9671	approximate	approximate
b1	$\log_{10}(k_{deg})$	−1.319	−1.423	−1.216	approximate	approximate
b1	$\log_{10}(k_1'/k_2)$	0.2155	0.1852	0.2453	approximate	approximate
b1	$\log_{10}(k_1'k_2'/k_2)$	−0.8407	−0.9222	−0.7558	approximate	approximate
b1	$\log_{10}(k_2'/k_2)$	−0.85	< −9.548	−0.7816	optim	approximate
b2	$\log_{10}(k_1'k_2')$	−1.449	−1.575	> 0.5106	exact	optim
b2	$\log_{10}(k_2 \text{ or } k_{deg})$	−0.961	−1.335	> 1.325	approximate	optim
b2	$\log_{10}(k_2 \text{ or } k_{deg})$	−0.984	−1.335	−0.9747	approximate	optim
b3	$\log_{10}(k_1')$	0.01966	< 0.01818	0.241	optim	approximate
b3	$\log_{10}(k_2)$	−0.2056	−0.2544	0.4887	approximate	approximate
b3	$\log_{10}(k_2')$	−0.9633	−1.055	−0.8506	approximate	approximate
b3	$\log_{10}(k_{deg})$	−1.216	−1.324	−1.102	approximate	approximate
b3	$\log_{10}(k_1'/k_2)$	0.2253	0.196	0.2559	approximate	approximate
b3	$\log_{10}(k_1'k_2'/k_2)$	−0.7381	−0.8392	−0.6346	approximate	approximate
b3	$\log_{10}(k_2'/k_2)$	−0.7577	−1.451	−0.4522	exact	exact

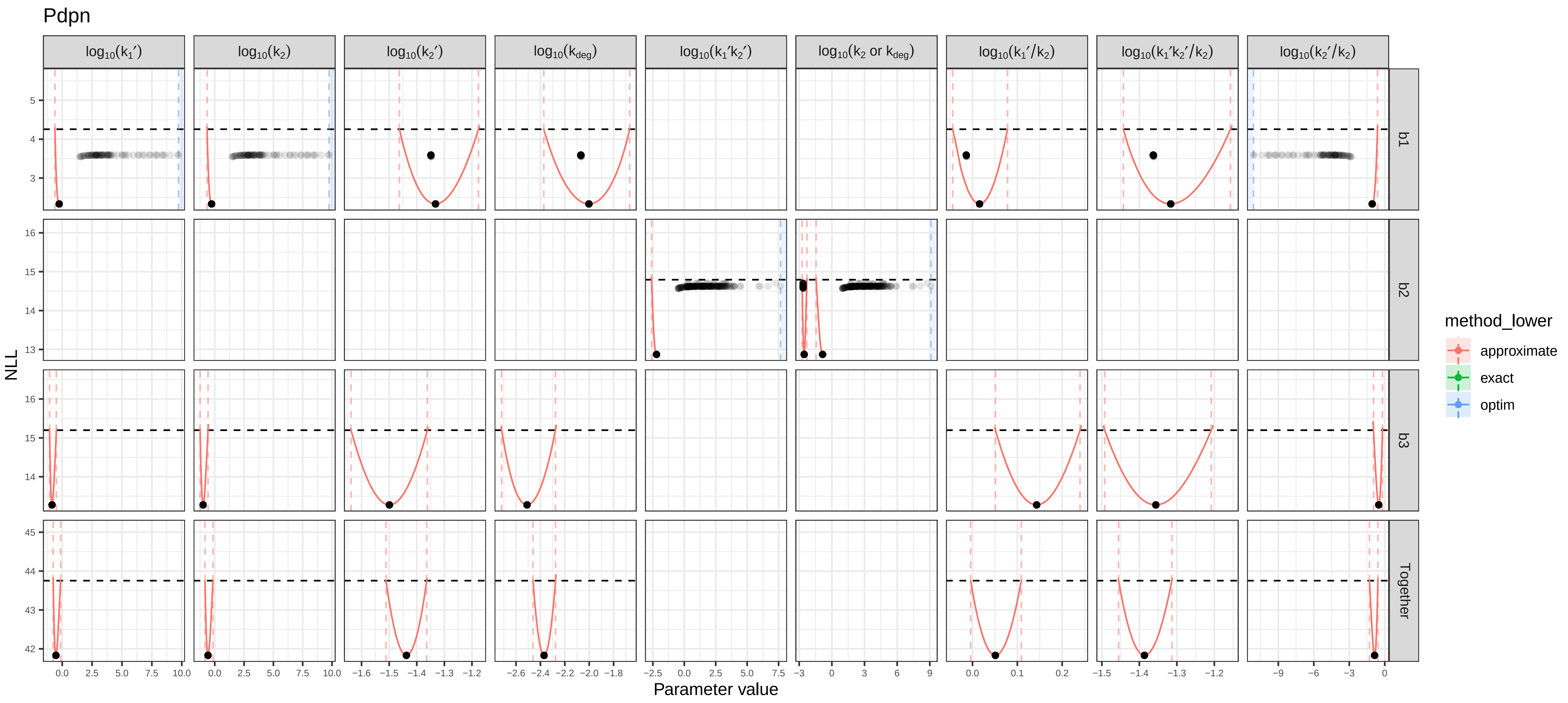

Replicate	Par	Best value	CI95 LB	CI95 UB	Method LB	Method UB
Together	$\log_{10}(k_1')$	−0.5251	−0.753	−0.1299	approximate	approximate
Together	$\log_{10}(k_2)$	−0.5761	−0.8355	−0.1504	approximate	approximate
Together	$\log_{10}(k_2')$	−1.437	−1.512	−1.364	approximate	approximate
Together	$\log_{10}(k_{deg})$	−2.37	−2.461	−2.276	approximate	approximate
Together	$\log_{10}(k_1'/k_2)$	0.05104	−0.004182	0.1089	approximate	approximate
Together	$\log_{10}(k_1'k_2'/k_2)$	−1.386	−1.455	−1.313	approximate	approximate
Together	$\log_{10}(k_2'/k_2)$	−0.8613	−1.295	−0.5822	approximate	approximate
b1	$\log_{10}(k_1')$	−0.2529	−0.6078	> 9.734	approximate	optim
b1	$\log_{10}(k_2)$	−0.2686	−0.655	> 9.748	approximate	optim
b1	$\log_{10}(k_2')$	−1.332	−1.463	−1.176	approximate	approximate
b1	$\log_{10}(k_{deg})$	−2.002	−2.373	−1.668	approximate	approximate
b1	$\log_{10}(k_1'/k_2)$	0.01577	−0.04398	0.07814	approximate	approximate
b1	$\log_{10}(k_1'k_2'/k_2)$	−1.316	−1.443	−1.157	approximate	approximate
b1	$\log_{10}(k_2'/k_2)$	−1.063	< −11.1	−0.6145	optim	approximate
b2	$\log_{10}(k_1'k_2')$	−2.209	−2.597	> 7.656	approximate	optim
b2	$\log_{10}(k_2 \text{ or } k_{deg})$	−0.8498	−1.445	> 9.115	approximate	optim
b2	$\log_{10}(k_2 \text{ or } k_{deg})$	−2.544	−2.738	−2.291	approximate	approximate
b3	$\log_{10}(k_1')$	−0.8488	−1.055	−0.4871	approximate	approximate
b3	$\log_{10}(k_2)$	−0.9919	−1.252	−0.5738	approximate	approximate
b3	$\log_{10}(k_2')$	−1.499	−1.638	−1.362	approximate	approximate
b3	$\log_{10}(k_{deg})$	−2.509	−2.718	−2.277	approximate	approximate
b3	$\log_{10}(k_1'/k_2)$	0.1431	0.05121	0.2399	approximate	approximate
b3	$\log_{10}(k_1'k_2'/k_2)$	−1.356	−1.492	−1.208	approximate	approximate
b3	$\log_{10}(k_2'/k_2)$	−0.5073	−0.9485	−0.1934	approximate	approximate

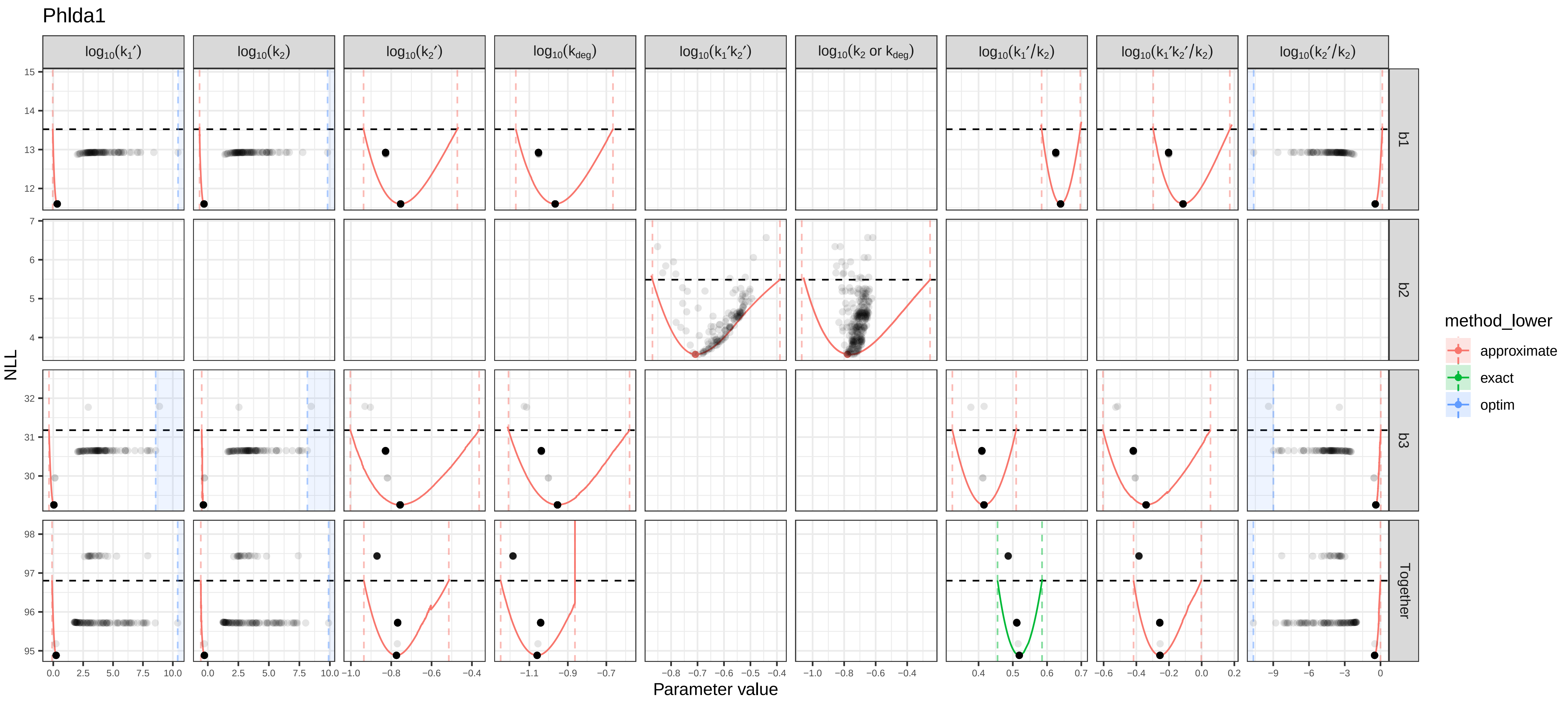

Replicate	Par	Best value	CI95 LB	CI95 UB	Method LB	Method UB
Together	$\log_{10}(k_1')$	0.2371	−0.1023	> 10.41	approximate	optim
Together	$\log_{10}(k_2)$	−0.2818	−0.5744	> 9.894	approximate	optim
Together	$\log_{10}(k_2')$	−0.7741	−0.9356	−0.5145	approximate	approximate
Together	$\log_{10}(k_{deg})$	−1.059	−1.249	−0.8629	approximate	approximate
Together	$\log_{10}(k_1'/k_2)$	0.5189	0.4555	0.5854	exact	exact
Together	$\log_{10}(k_1'k_2'/k_2)$	−0.2552	−0.4169	−0.002451	approximate	approximate
Together	$\log_{10}(k_2'/k_2)$	−0.4923	< −10.66	−0.001539	optim	approximate
b1	$\log_{10}(k_1')$	0.331	−0.04381	> 10.44	approximate	optim
b1	$\log_{10}(k_2)$	−0.3085	−0.6774	> 9.81	approximate	optim
b1	$\log_{10}(k_2')$	−0.7535	−0.9372	−0.4723	approximate	approximate
b1	$\log_{10}(k_{deg})$	−0.9656	−1.17	−0.6656	approximate	approximate
b1	$\log_{10}(k_1'/k_2)$	0.6395	0.5842	0.6971	approximate	approximate
b1	$\log_{10}(k_1'k_2'/k_2)$	−0.114	−0.2965	0.1723	approximate	approximate
b1	$\log_{10}(k_2'/k_2)$	−0.445	< −10.64	0.1552	optim	approximate
b2	$\log_{10}(k_1'k_2')$	−0.7081	−0.8704	−0.3884	approximate	approximate
b2	$\log_{10}(k_2 \text{ or } k_{deg})$	−0.752	−1.069	−0.2549	approximate	approximate
b2	$\log_{10}(k_2 \text{ or } k_{deg})$	−0.7789	−1.069	−0.2549	approximate	approximate
b3	$\log_{10}(k_1')$	0.05184	−0.3572	> 8.566	approximate	optim
b3	$\log_{10}(k_2)$	−0.3639	−0.5001	> 8.156	approximate	optim
b3	$\log_{10}(k_2')$	−0.7561	−1.003	−0.3642	approximate	approximate
b3	$\log_{10}(k_{deg})$	−0.9537	−1.208	−0.5795	approximate	approximate
b3	$\log_{10}(k_1'/k_2)$	0.4158	0.3234	0.5098	approximate	approximate
b3	$\log_{10}(k_1'k_2'/k_2)$	−0.3404	−0.6043	0.05393	approximate	approximate
b3	$\log_{10}(k_2'/k_2)$	−0.3922	< −8.984	0.0264	optim	approximate

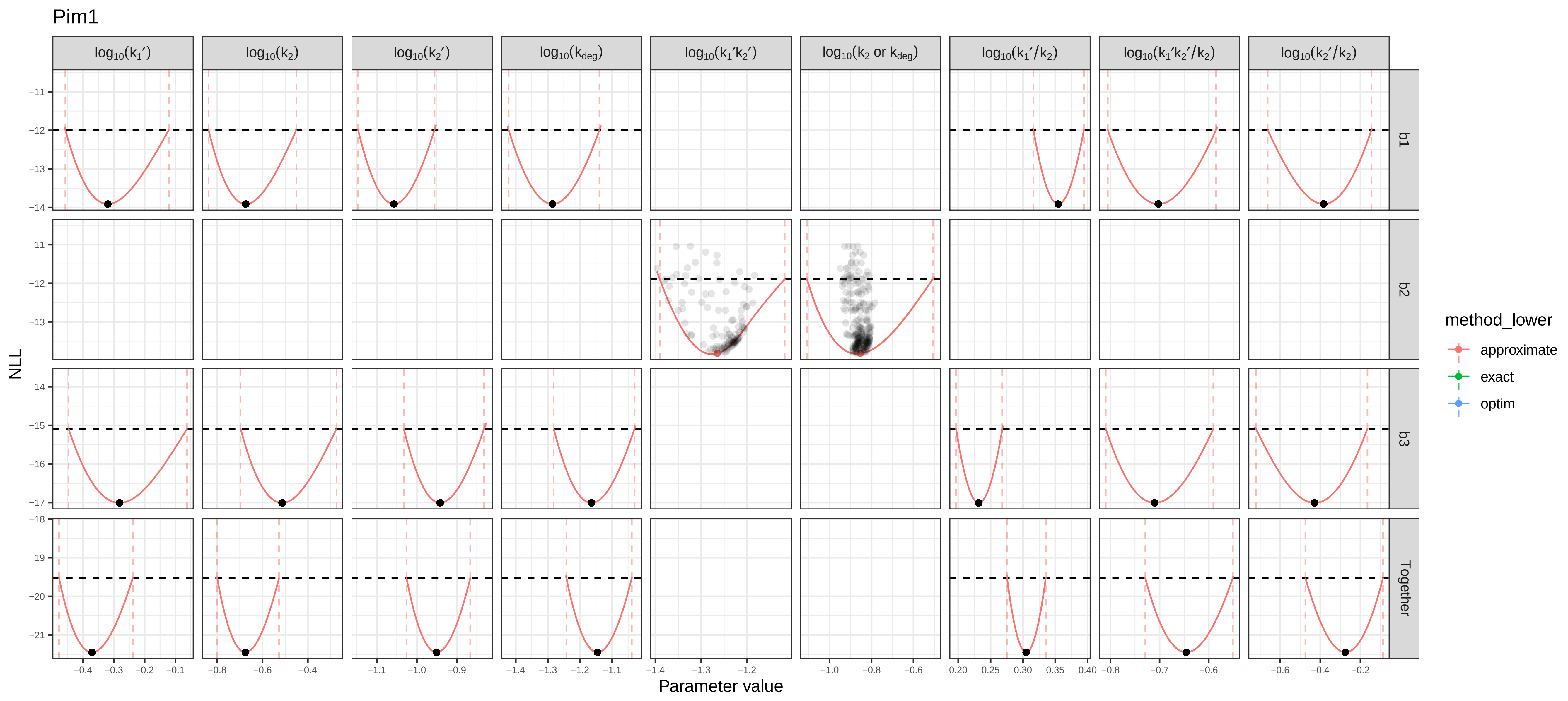

Replicate	Par	Best value	CI95 LB	CI95 UB	Method LB	Method UB
Together	$\log_{10}(k_1')$	−0.3705	−0.4778	−0.2385	approximate	approximate
Together	$\log_{10}(k_2)$	−0.6755	−0.8	−0.527	approximate	approximate
Together	$\log_{10}(k_2')$	−0.9507	−1.026	−0.8665	approximate	approximate
Together	$\log_{10}(k_{deg})$	−1.145	−1.242	−1.039	approximate	approximate
Together	$\log_{10}(k_1'/k_2)$	0.305	0.2755	0.3353	approximate	approximate
Together	$\log_{10}(k_1'k_2'/k_2)$	−0.6457	−0.729	−0.551	approximate	approximate
Together	$\log_{10}(k_2'/k_2)$	−0.2752	−0.4743	−0.08764	approximate	approximate
b1	$\log_{10}(k_1')$	−0.3192	−0.4574	−0.1212	approximate	approximate
b1	$\log_{10}(k_2)$	−0.6738	−0.8384	−0.4505	approximate	approximate
b1	$\log_{10}(k_2')$	−1.057	−1.147	−0.9559	approximate	approximate
b1	$\log_{10}(k_{deg})$	−1.286	−1.422	−1.139	approximate	approximate
b1	$\log_{10}(k_1'/k_2)$	0.3546	0.3162	0.3942	approximate	approximate
b1	$\log_{10}(k_1'k_2'/k_2)$	−0.7027	−0.8055	−0.5851	approximate	approximate
b1	$\log_{10}(k_2'/k_2)$	−0.3835	−0.6628	−0.1448	approximate	approximate
b2	$\log_{10}(k_1'k_2')$	−1.265	−1.391	−1.118	approximate	approximate
b2	$\log_{10}(k_2 \text{ or } k_{deg})$	−0.8533	−1.108	−0.5079	approximate	approximate
b2	$\log_{10}(k_2 \text{ or } k_{deg})$	−0.8714	−1.108	−0.5079	approximate	approximate
b3	$\log_{10}(k_1')$	−0.2813	−0.447	−0.06218	approximate	approximate
b3	$\log_{10}(k_2)$	−0.5131	−0.697	−0.2731	approximate	approximate
b3	$\log_{10}(k_2')$	−0.9419	−1.033	−0.8319	approximate	approximate
b3	$\log_{10}(k_{deg})$	−1.164	−1.282	−1.03	approximate	approximate
b3	$\log_{10}(k_1'/k_2)$	0.2318	0.1966	0.2683	approximate	approximate
b3	$\log_{10}(k_1'k_2'/k_2)$	−0.71	−0.8097	−0.5904	approximate	approximate
b3	$\log_{10}(k_2'/k_2)$	−0.4287	−0.7215	−0.165	approximate	approximate

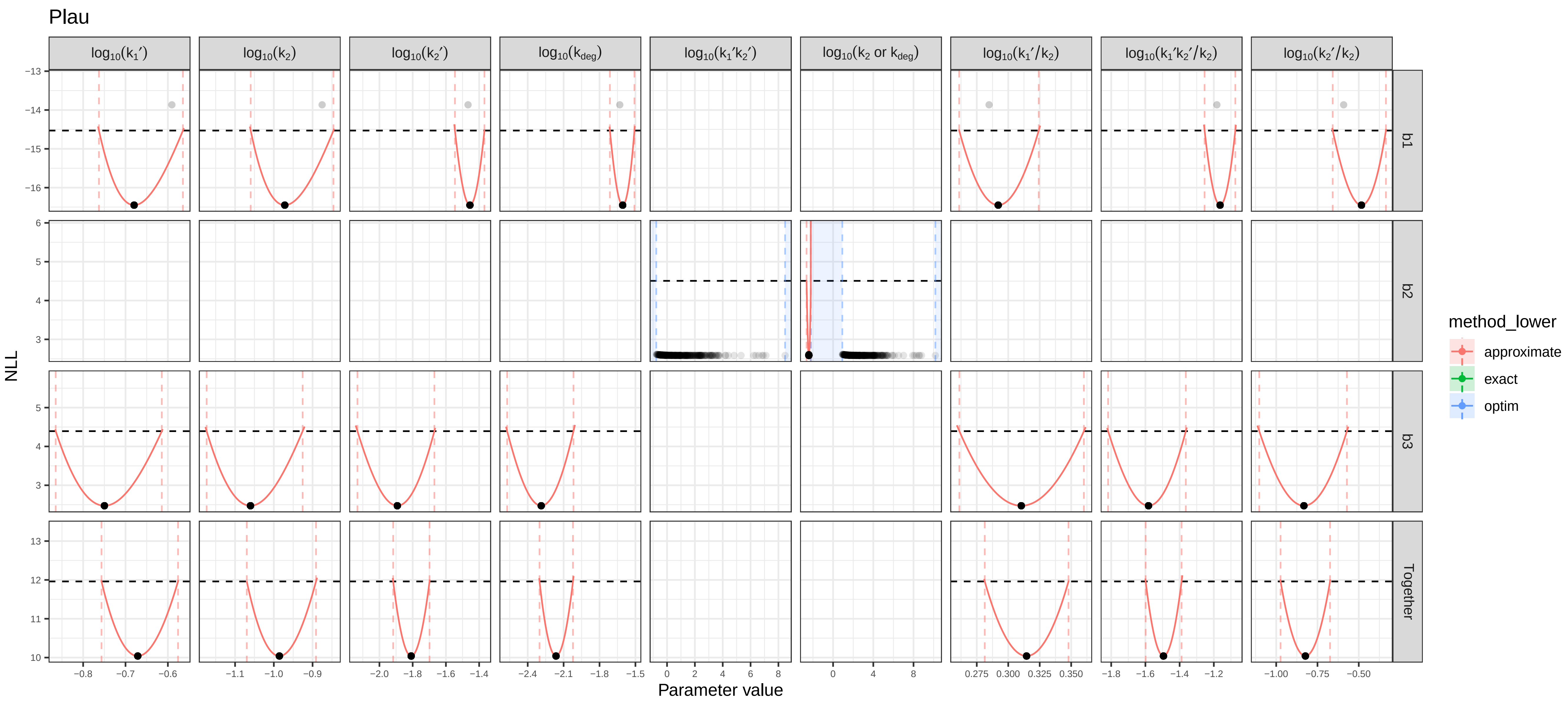

Replicate	Par	Best value	CI95 LB	CI95 UB	Method LB	Method UB
Together	$\log_{10}(k_1')$	−0.6711	−0.7566	−0.576	approximate	approximate
Together	$\log_{10}(k_2)$	−0.9856	−1.07	−0.8911	approximate	approximate
Together	$\log_{10}(k_2')$	−1.809	−1.917	−1.698	approximate	approximate
Together	$\log_{10}(k_{deg})$	−2.164	−2.301	−2.022	approximate	approximate
Together	$\log_{10}(k_1'/k_2)$	0.3146	0.2814	0.3479	approximate	approximate
Together	$\log_{10}(k_1'k_2'/k_2)$	−1.494	−1.598	−1.388	approximate	approximate
Together	$\log_{10}(k_2'/k_2)$	−0.8233	−0.9734	−0.6738	approximate	approximate
b1	$\log_{10}(k_1')$	−0.6797	−0.7627	−0.5645	approximate	approximate
b1	$\log_{10}(k_2)$	−0.9717	−1.06	−0.8461	approximate	approximate
b1	$\log_{10}(k_2')$	−1.455	−1.546	−1.368	approximate	approximate
b1	$\log_{10}(k_{deg})$	−1.607	−1.713	−1.508	approximate	approximate
b1	$\log_{10}(k_1'/k_2)$	0.292	0.261	0.3242	approximate	approximate
b1	$\log_{10}(k_1'k_2'/k_2)$	−1.163	−1.254	−1.074	approximate	approximate
b1	$\log_{10}(k_2'/k_2)$	−0.4838	−0.6578	−0.3354	approximate	approximate
b2	$\log_{10}(k_1'k_2')$	3.68	< −0.7728	> 8.478	optim	optim
b2	$\log_{10}(k_2 \text{ or } k_{deg})$	5.374	0.9199	> 10.17	optim	optim
b2	$\log_{10}(k_2 \text{ or } k_{deg})$	−2.411	−2.637	−2.218	approximate	approximate
b3	$\log_{10}(k_1')$	−0.7498	−0.8647	−0.6143	approximate	approximate
b3	$\log_{10}(k_2)$	−1.06	−1.173	−0.9256	approximate	approximate
b3	$\log_{10}(k_2')$	−1.892	−2.132	−1.669	approximate	approximate
b3	$\log_{10}(k_{deg})$	−2.287	−2.572	−2.018	approximate	approximate
b3	$\log_{10}(k_1'/k_2)$	0.3104	0.2612	0.3601	approximate	approximate
b3	$\log_{10}(k_1'k_2'/k_2)$	−1.582	−1.818	−1.364	approximate	approximate
b3	$\log_{10}(k_2'/k_2)$	−0.8322	−1.103	−0.5719	approximate	approximate

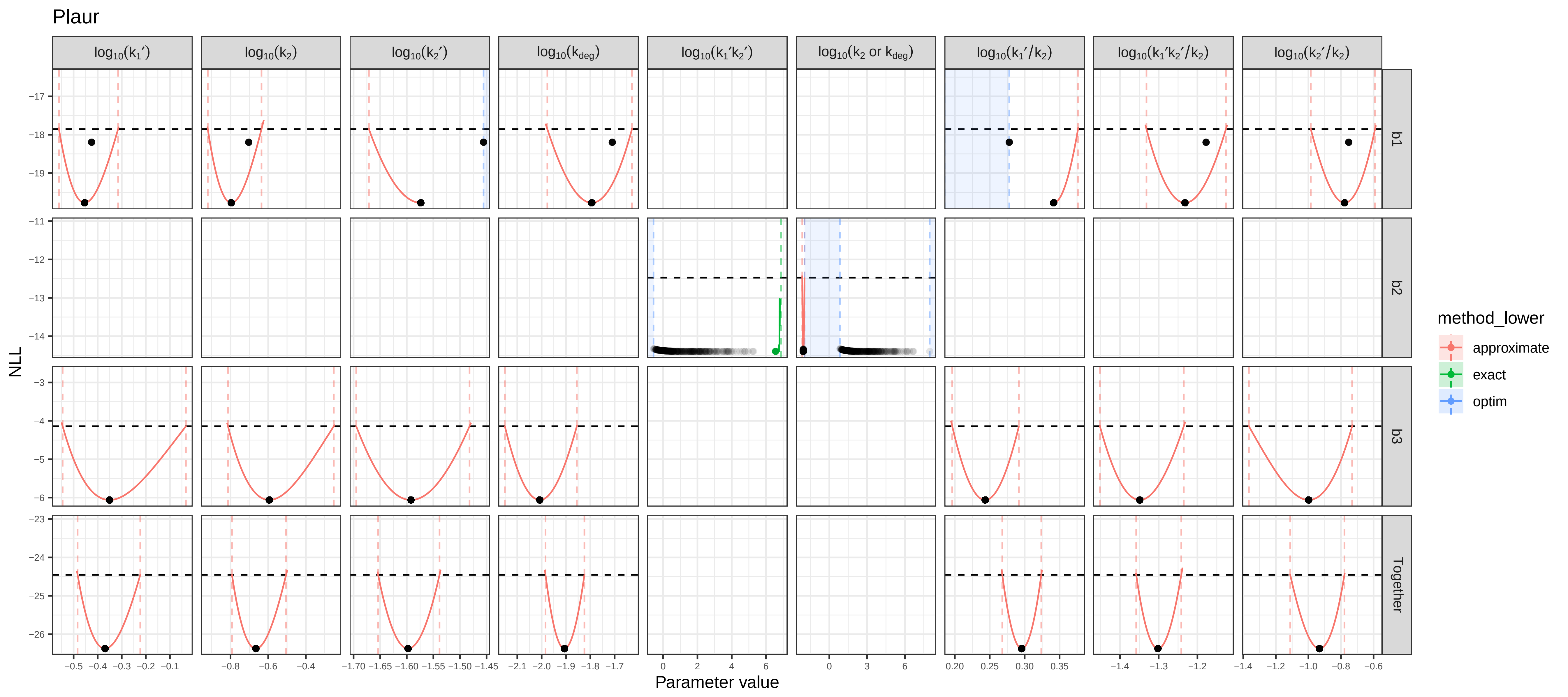

Replicate	Par	Best value	CI95 LB	CI95 UB	Method LB	Method UB
Together	$\log_{10}(k_1')$	−0.3696	−0.4834	−0.2231	approximate	approximate
Together	$\log_{10}(k_2)$	−0.6654	−0.7923	−0.5049	approximate	approximate
Together	$\log_{10}(k_2')$	−1.598	−1.654	−1.538	approximate	approximate
Together	$\log_{10}(k_{deg})$	−1.906	−1.984	−1.824	approximate	approximate
Together	$\log_{10}(k_1'/k_2)$	0.2958	0.2679	0.3238	approximate	approximate
Together	$\log_{10}(k_1'k_2'/k_2)$	−1.302	−1.358	−1.242	approximate	approximate
Together	$\log_{10}(k_2'/k_2)$	−0.9323	−1.112	−0.7788	approximate	approximate
b1	$\log_{10}(k_1')$	−0.4543	−0.561	−0.3153	approximate	approximate
b1	$\log_{10}(k_2)$	−0.7958	−0.9207	−0.6354	approximate	approximate
b1	$\log_{10}(k_2')$	−1.574	−1.671	> −1.456	approximate	optim
b1	$\log_{10}(k_{deg})$	−1.794	−1.977	−1.629	approximate	approximate
b1	$\log_{10}(k_1'/k_2)$	0.3415	< 0.2779	0.3764	optim	approximate
b1	$\log_{10}(k_1'k_2'/k_2)$	−1.232	−1.331	−1.127	approximate	approximate
b1	$\log_{10}(k_2'/k_2)$	−0.7778	−0.9855	−0.5908	approximate	approximate
b2	$\log_{10}(k_1'k_2')$	6.557	< −0.5621	6.873	optim	exact
b2	$\log_{10}(k_2 \text{ or } k_{deg})$	7.967	0.8455	> 7.967	optim	optim
b2	$\log_{10}(k_2 \text{ or } k_{deg})$	−2.063	−2.147	−1.967	approximate	approximate
b3	$\log_{10}(k_1')$	−0.3507	−0.5456	−0.03347	approximate	approximate
b3	$\log_{10}(k_2)$	−0.5941	−0.8135	−0.2518	approximate	approximate
b3	$\log_{10}(k_2')$	−1.592	−1.695	−1.482	approximate	approximate
b3	$\log_{10}(k_{deg})$	−2.008	−2.151	−1.855	approximate	approximate
b3	$\log_{10}(k_1'/k_2)$	0.2434	0.1963	0.2917	approximate	approximate
b3	$\log_{10}(k_1'k_2'/k_2)$	−1.349	−1.452	−1.235	approximate	approximate
b3	$\log_{10}(k_2'/k_2)$	−0.9979	−1.365	−0.7315	approximate	approximate

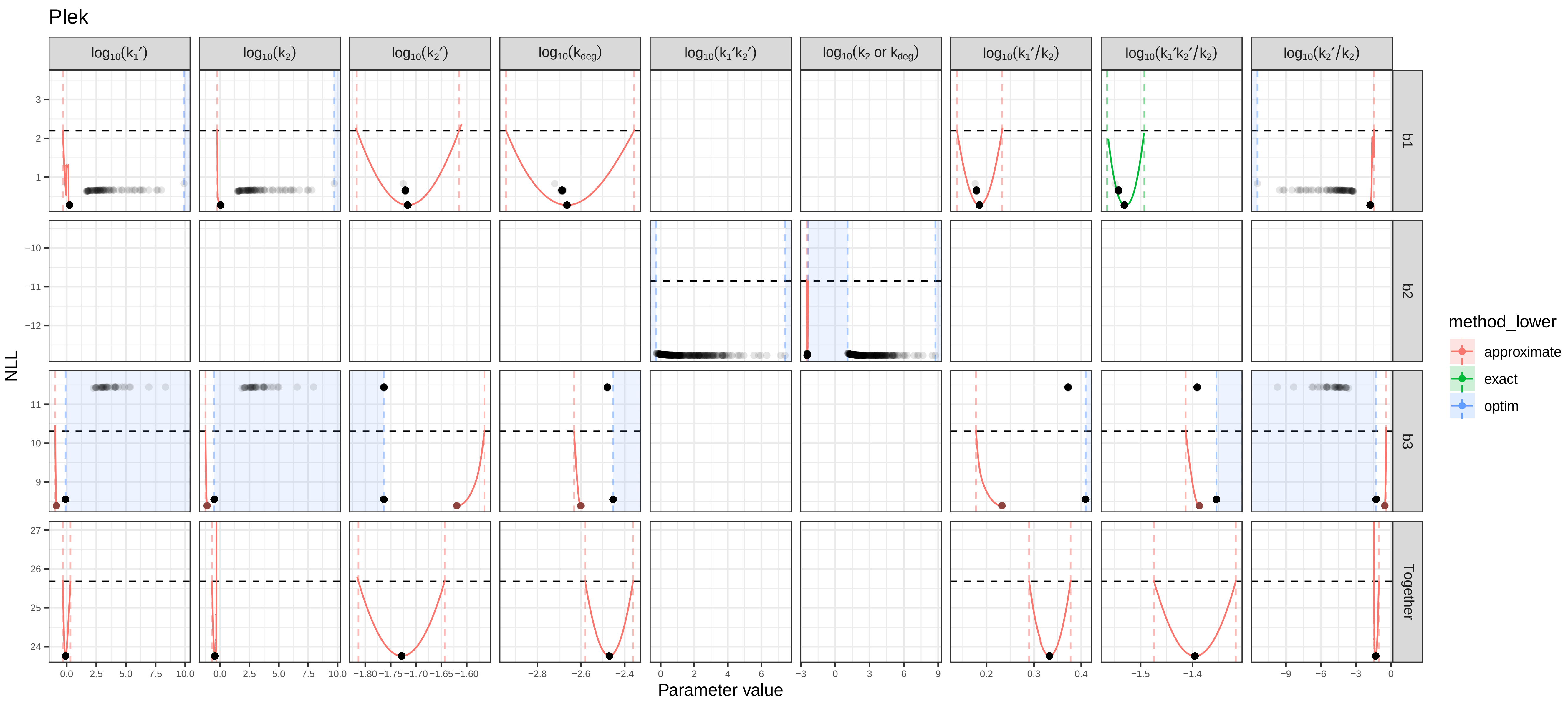

Replicate	Par	Best value	CI95 LB	CI95 UB	Method LB	Method UB
Together	$\log_{10}(k_1')$	−0.09262	−0.3221	0.3224	approximate	approximate
Together	$\log_{10}(k_2)$	−0.4255	−0.6747	−0.3012	approximate	approximate
Together	$\log_{10}(k_2')$	−1.728	−1.814	−1.644	approximate	approximate
Together	$\log_{10}(k_{deg})$	−2.47	−2.581	−2.362	approximate	approximate
Together	$\log_{10}(k_1'/k_2)$	0.3329	0.29	0.3774	approximate	approximate
Together	$\log_{10}(k_1'k_2'/k_2)$	−1.395	−1.474	−1.317	approximate	approximate
Together	$\log_{10}(k_2'/k_2)$	−1.303	−1.456	−1.038	approximate	approximate
b1	$\log_{10}(k_1')$	0.2445	−0.3173	> 9.893	approximate	optim
b1	$\log_{10}(k_2)$	0.05939	−0.2391	> 9.717	approximate	optim
b1	$\log_{10}(k_2')$	−1.716	−1.817	−1.615	approximate	approximate
b1	$\log_{10}(k_{deg})$	−2.664	−2.944	−2.357	approximate	approximate
b1	$\log_{10}(k_1'/k_2)$	0.1851	0.1378	0.233	approximate	approximate
b1	$\log_{10}(k_1'k_2'/k_2)$	−1.531	−1.564	−1.493	exact	exact
b1	$\log_{10}(k_2'/k_2)$	−1.776	< −11.44	−1.446	optim	approximate
b2	$\log_{10}(k_1'k_2')$	3.673	< −0.2624	> 7.409	optim	optim
b2	$\log_{10}(k_2 \text{ or } k_{deg})$	5.017	1.081	> 8.753	optim	optim
b2	$\log_{10}(k_2 \text{ or } k_{deg})$	−2.454	−2.509	−2.366	approximate	approximate
b3	$\log_{10}(k_1')$	−0.8661	−0.9653	> −0.08936	approximate	optim
b3	$\log_{10}(k_2)$	−1.099	−1.24	> −0.4984	approximate	optim
b3	$\log_{10}(k_2')$	−1.619	< −1.763	−1.565	optim	approximate
b3	$\log_{10}(k_{deg})$	−2.601	−2.632	> −2.453	approximate	optim
b3	$\log_{10}(k_1'/k_2)$	0.2326	0.1778	> 0.4092	approximate	optim
b3	$\log_{10}(k_1'k_2'/k_2)$	−1.387	−1.413	> −1.354	approximate	optim
b3	$\log_{10}(k_2'/k_2)$	−0.5206	< −1.265	−0.4067	optim	approximate

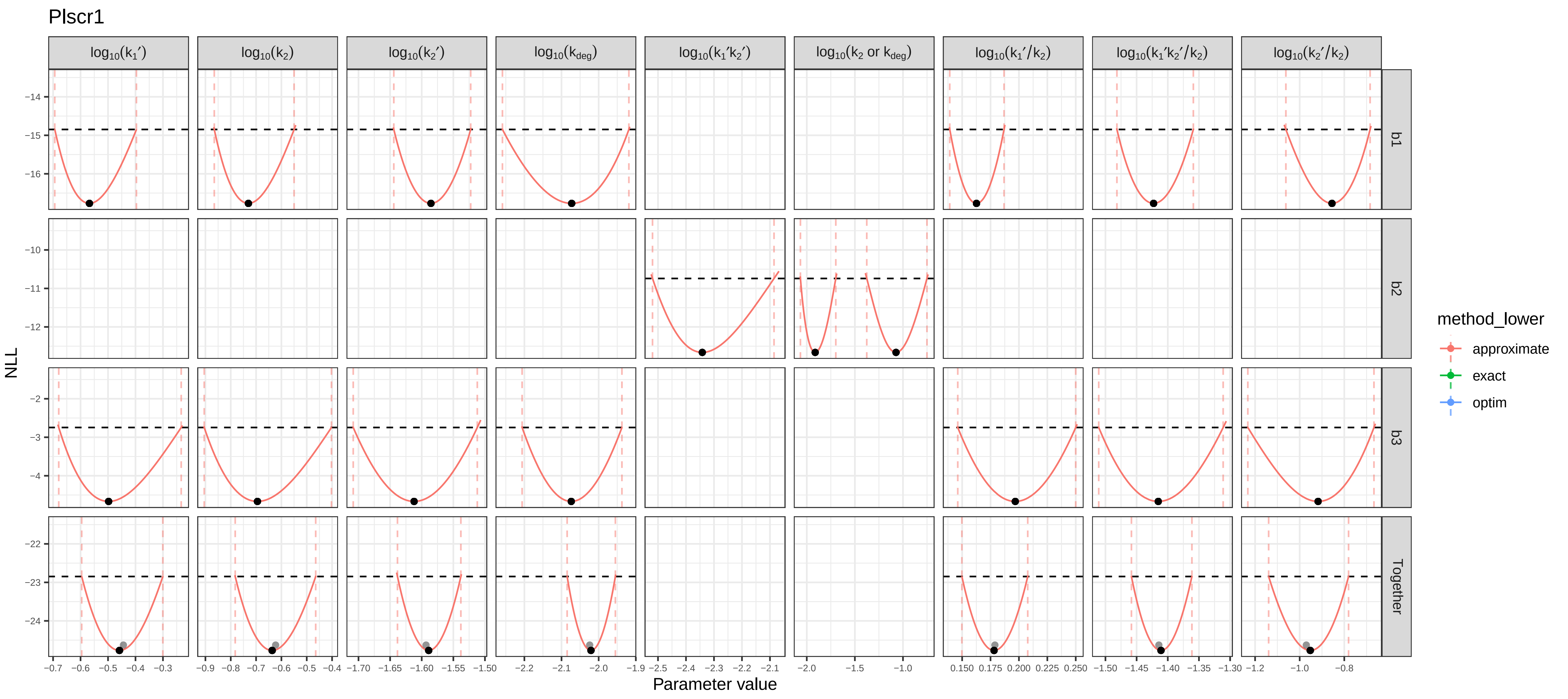

Replicate	Par	Best value	CI95 LB	CI95 UB	Method LB	Method UB
Together	$\log_{10}(k_1')$	−0.4585	−0.5958	−0.3004	approximate	approximate
Together	$\log_{10}(k_2)$	−0.6366	−0.7824	−0.4646	approximate	approximate
Together	$\log_{10}(k_2')$	−1.589	−1.638	−1.538	approximate	approximate
Together	$\log_{10}(k_{deg})$	−2.021	−2.085	−1.955	approximate	approximate
Together	$\log_{10}(k_1'/k_2)$	0.1781	0.1498	0.2077	approximate	approximate
Together	$\log_{10}(k_1'k_2'/k_2)$	−1.411	−1.458	−1.361	approximate	approximate
Together	$\log_{10}(k_2'/k_2)$	−0.9524	−1.139	−0.7808	approximate	approximate
b1	$\log_{10}(k_1')$	−0.5676	−0.6938	−0.3965	approximate	approximate
b1	$\log_{10}(k_2)$	−0.7302	−0.865	−0.55	approximate	approximate
b1	$\log_{10}(k_2')$	−1.585	−1.644	−1.523	approximate	approximate
b1	$\log_{10}(k_{deg})$	−2.073	−2.259	−1.919	approximate	approximate
b1	$\log_{10}(k_1'/k_2)$	0.1626	0.1391	0.1869	approximate	approximate
b1	$\log_{10}(k_1'k_2'/k_2)$	−1.423	−1.482	−1.359	approximate	approximate
b1	$\log_{10}(k_2'/k_2)$	−0.8551	−1.062	−0.6841	approximate	approximate
b2	$\log_{10}(k_1'k_2')$	−2.342	−2.52	−2.086	approximate	approximate
b2	$\log_{10}(k_2 \text{ or } k_{deg})$	−1.072	−1.376	−0.7506	approximate	approximate
b2	$\log_{10}(k_2 \text{ or } k_{deg})$	−1.913	−2.066	−1.698	approximate	approximate
b3	$\log_{10}(k_1')$	−0.4979	−0.6794	−0.2336	approximate	approximate
b3	$\log_{10}(k_2)$	−0.6947	−0.9047	−0.4024	approximate	approximate
b3	$\log_{10}(k_2')$	−1.612	−1.708	−1.512	approximate	approximate
b3	$\log_{10}(k_{deg})$	−2.074	−2.206	−1.937	approximate	approximate
b3	$\log_{10}(k_1'/k_2)$	0.1968	0.1461	0.25	approximate	approximate
b3	$\log_{10}(k_1'k_2'/k_2)$	−1.415	−1.511	−1.311	approximate	approximate
b3	$\log_{10}(k_2'/k_2)$	−0.9173	−1.233	−0.6669	approximate	approximate

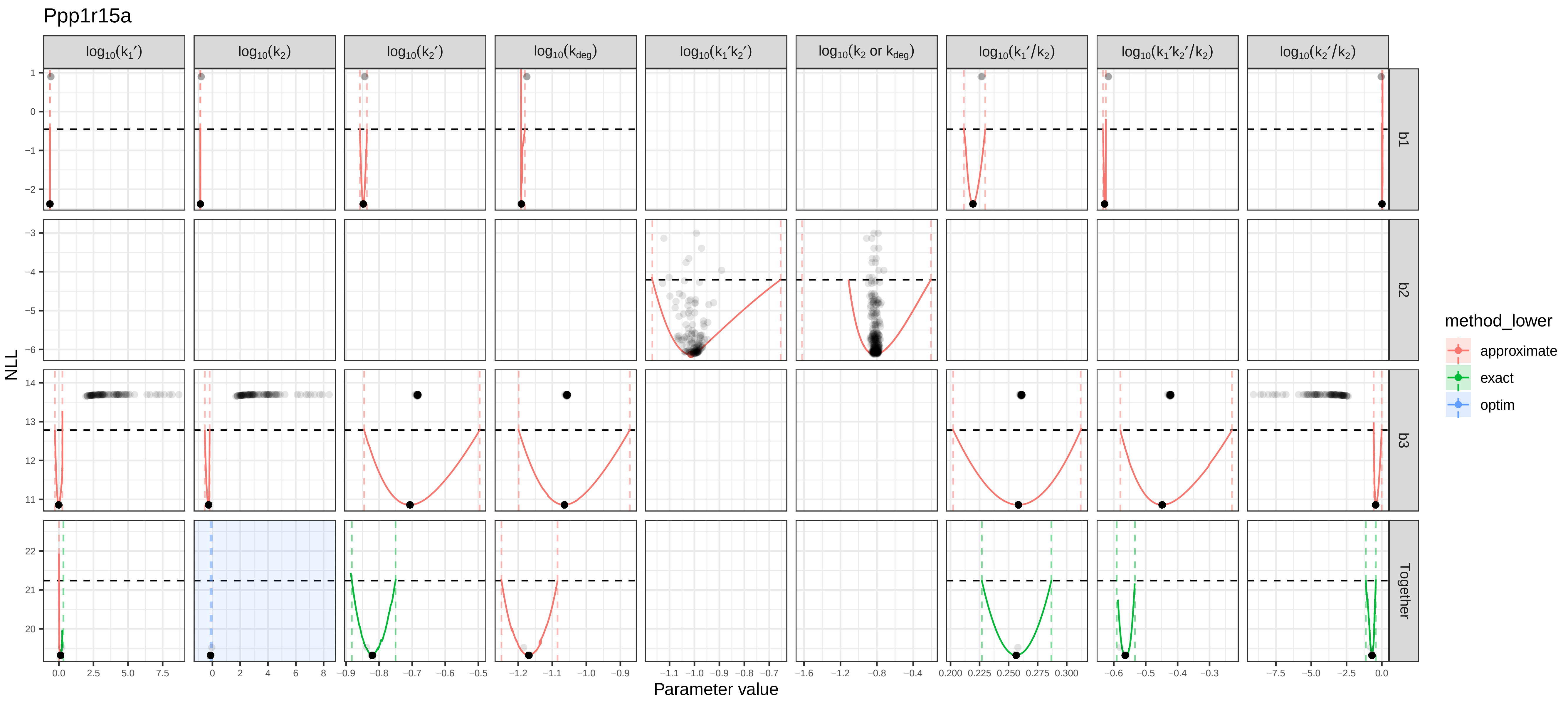

Replicate	Par	Best value	CI95 LB	CI95 UB	Method LB	Method UB
Together	$\log_{10}(k_1')$	0.1267	0.0136	0.3253	approximate	exact
Together	$\log_{10}(k_2)$	−0.1298	< −0.1395	> −0.0449	optim	optim
Together	$\log_{10}(k_2')$	−0.8206	−0.8826	−0.7504	exact	exact
Together	$\log_{10}(k_{deg})$	−1.169	−1.249	−1.085	approximate	approximate
Together	$\log_{10}(k_1'/k_2)$	0.2565	0.2269	0.2868	exact	exact
Together	$\log_{10}(k_1'k_2'/k_2)$	−0.5641	−0.5907	−0.5338	exact	exact
Together	$\log_{10}(k_2'/k_2)$	−0.6908	−1.13	−0.424	exact	exact
b1	$\log_{10}(k_1')$	−0.6473	−0.6563	−0.6408	approximate	approximate
b1	$\log_{10}(k_2)$	−0.8666	−0.8742	−0.8639	approximate	approximate
b1	$\log_{10}(k_2')$	−0.848	−0.8582	−0.8367	approximate	approximate
b1	$\log_{10}(k_{deg})$	−1.191	−1.192	−1.18	approximate	approximate
b1	$\log_{10}(k_1'/k_2)$	0.2193	0.2114	0.2299	approximate	approximate
b1	$\log_{10}(k_1'k_2'/k_2)$	−0.6287	−0.6334	−0.6249	approximate	approximate
b1	$\log_{10}(k_2'/k_2)$	0.01856	0.01623	0.05448	approximate	approximate
b2	$\log_{10}(k_1'k_2')$	−1.014	−1.169	−0.6545	approximate	approximate
b2	$\log_{10}(k_2 \text{ or } k_{deg})$	−0.8152	−1.621	−0.2047	approximate	approximate
b2	$\log_{10}(k_2 \text{ or } k_{deg})$	−0.8284	−1.621	−0.2047	approximate	approximate
b3	$\log_{10}(k_1')$	−0.01289	−0.2896	0.2531	approximate	approximate
b3	$\log_{10}(k_2)$	−0.2713	−0.5533	−0.2002	approximate	approximate
b3	$\log_{10}(k_2')$	−0.7067	−0.8453	−0.4959	approximate	approximate
b3	$\log_{10}(k_{deg})$	−1.064	−1.199	−0.8726	approximate	approximate
b3	$\log_{10}(k_1'/k_2)$	0.2584	0.2023	0.312	approximate	approximate
b3	$\log_{10}(k_1'k_2'/k_2)$	−0.4482	−0.5791	−0.2294	approximate	approximate
b3	$\log_{10}(k_2'/k_2)$	−0.4354	−0.5715	−0.005864	approximate	approximate

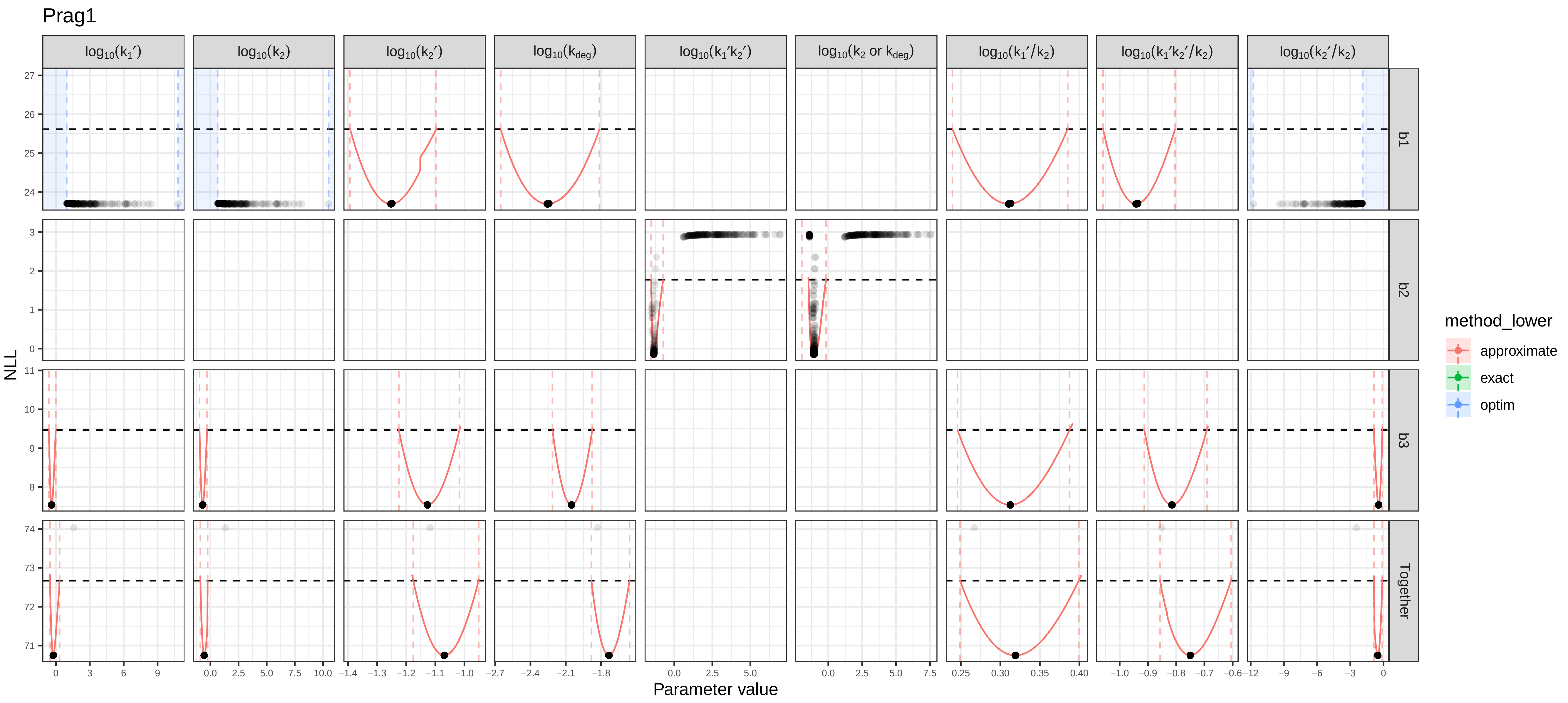

Replicate	Par	Best value	CI95 LB	CI95 UB	Method LB	Method UB
Together	$\log_{10}(k_1')$	−0.2331	−0.5199	0.3237	approximate	approximate
Together	$\log_{10}(k_2)$	−0.5522	−0.8918	−0.2477	approximate	approximate
Together	$\log_{10}(k_2')$	−1.069	−1.176	−0.9511	approximate	approximate
Together	$\log_{10}(k_{deg})$	−1.733	−1.882	−1.558	approximate	approximate
Together	$\log_{10}(k_1'/k_2)$	0.3191	0.249	0.3992	approximate	approximate
Together	$\log_{10}(k_1'k_2'/k_2)$	−0.75	−0.8573	−0.6055	approximate	approximate
Together	$\log_{10}(k_2'/k_2)$	−0.5169	−0.8612	−0.1069	approximate	approximate
b1	$\log_{10}(k_1')$	5.217	< 0.9514	> 10.81	optim	optim
b1	$\log_{10}(k_2)$	4.907	< 0.638	> 10.5	optim	optim
b1	$\log_{10}(k_2')$	−1.252	−1.393	−1.097	approximate	approximate
b1	$\log_{10}(k_{deg})$	−2.256	−2.653	−1.814	approximate	approximate
b1	$\log_{10}(k_1'/k_2)$	0.3104	0.2394	0.3851	approximate	approximate
b1	$\log_{10}(k_1'k_2'/k_2)$	−0.942	−1.059	−0.8039	approximate	approximate
b1	$\log_{10}(k_2'/k_2)$	−6.159	< −11.75	> −1.888	optim	optim
b2	$\log_{10}(k_1'k_2')$	−1.357	−1.518	−0.7263	approximate	approximate
b2	$\log_{10}(k_2 \text{ or } k_{deg})$	−1.047	−1.958	−0.1593	approximate	approximate
b2	$\log_{10}(k_2 \text{ or } k_{deg})$	−1.09	−1.958	−0.1593	approximate	approximate
b3	$\log_{10}(k_1')$	−0.3765	−0.6115	−0.0112	approximate	approximate
b3	$\log_{10}(k_2)$	−0.689	−0.9654	−0.2926	approximate	approximate
b3	$\log_{10}(k_2')$	−1.127	−1.225	−1.017	approximate	approximate
b3	$\log_{10}(k_{deg})$	−2.05	−2.213	−1.875	approximate	approximate
b3	$\log_{10}(k_1'/k_2)$	0.3125	0.2458	0.3875	approximate	approximate
b3	$\log_{10}(k_1'k_2'/k_2)$	−0.8148	−0.9127	−0.6914	approximate	approximate
b3	$\log_{10}(k_2'/k_2)$	−0.4383	−0.8725	−0.1026	approximate	approximate

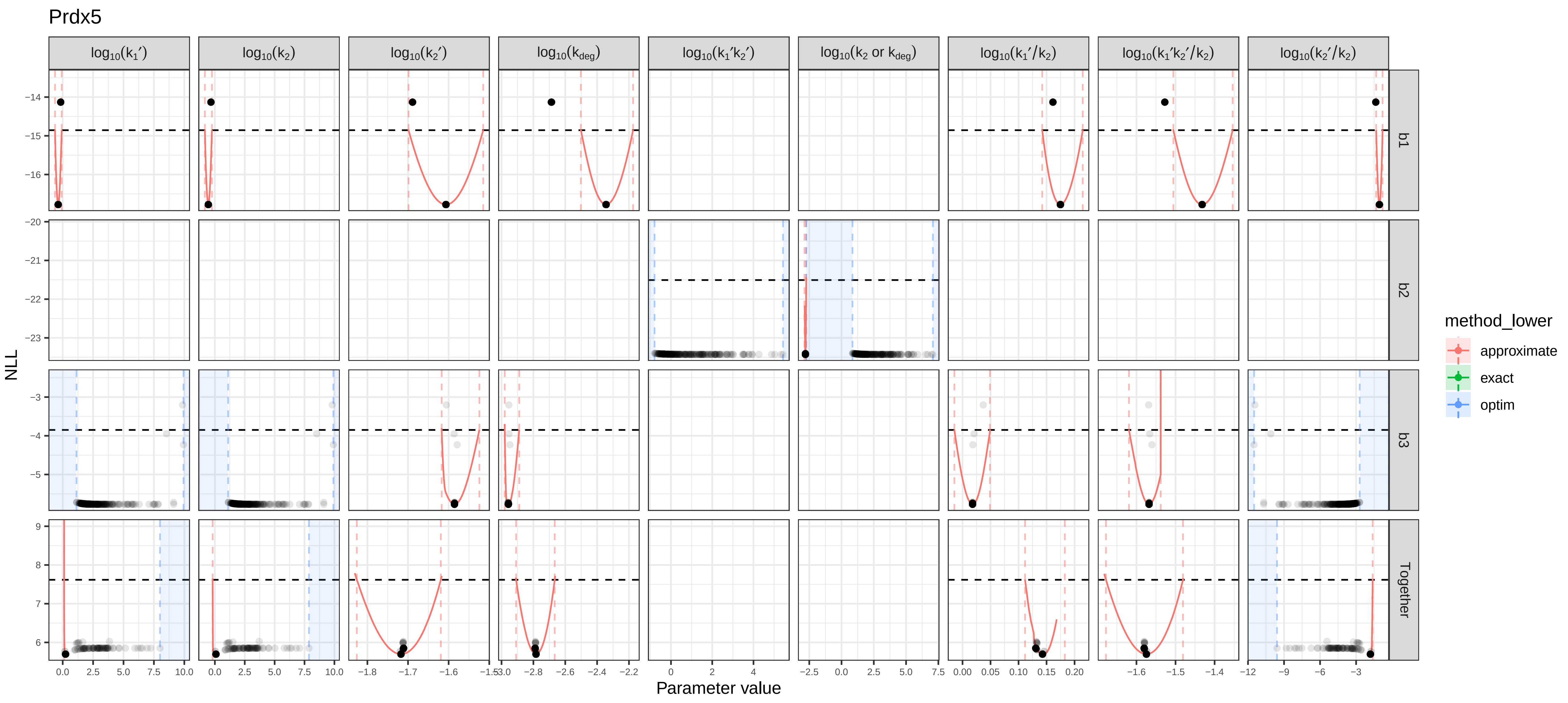

Replicate	Par	Best value	CI95 LB	CI95 UB	Method LB	Method UB
Together	$\log_{10}(k_1')$	0.2339	0.1162	> 8.002	approximate	optim
Together	$\log_{10}(k_2)$	0.09115	−0.1846	> 7.871	approximate	optim
Together	$\log_{10}(k_2')$	−1.717	−1.826	−1.619	approximate	approximate
Together	$\log_{10}(k_{deg})$	−2.783	−2.908	−2.666	approximate	approximate
Together	$\log_{10}(k_1'/k_2)$	0.1428	0.1115	0.1826	approximate	approximate
Together	$\log_{10}(k_1'k_2'/k_2)$	−1.575	−1.678	−1.481	approximate	approximate
Together	$\log_{10}(k_2'/k_2)$	−1.808	< −9.582	−1.621	optim	approximate
b1	$\log_{10}(k_1')$	−0.3717	−0.6331	−0.08152	approximate	approximate
b1	$\log_{10}(k_2)$	−0.5465	−0.8326	−0.2427	approximate	approximate
b1	$\log_{10}(k_2')$	−1.607	−1.699	−1.515	approximate	approximate
b1	$\log_{10}(k_{deg})$	−2.344	−2.502	−2.174	approximate	approximate
b1	$\log_{10}(k_1'/k_2)$	0.1748	0.1423	0.2147	approximate	approximate
b1	$\log_{10}(k_1'k_2'/k_2)$	−1.432	−1.506	−1.354	approximate	approximate
b1	$\log_{10}(k_2'/k_2)$	−1.06	−1.331	−0.797	approximate	approximate
b2	$\log_{10}(k_1'k_2')$	5.432	< −0.7969	> 5.432	optim	optim
b2	$\log_{10}(k_2 \text{ or } k_{deg})$	7.079	0.8488	> 7.079	optim	optim
b2	$\log_{10}(k_2 \text{ or } k_{deg})$	−2.799	−2.873	−2.734	approximate	approximate
b3	$\log_{10}(k_1')$	6.293	< 1.133	> 9.931	optim	optim
b3	$\log_{10}(k_2)$	6.275	< 1.115	> 9.912	optim	optim
b3	$\log_{10}(k_2')$	−1.586	−1.617	−1.524	approximate	approximate
b3	$\log_{10}(k_{deg})$	−2.957	−2.98	−2.889	approximate	approximate
b3	$\log_{10}(k_1'/k_2)$	0.01792	−0.01459	0.04901	approximate	approximate
b3	$\log_{10}(k_1'k_2'/k_2)$	−1.568	−1.619	−1.538	approximate	approximate
b3	$\log_{10}(k_2'/k_2)$	−7.861	< −11.49	> −2.7	optim	optim

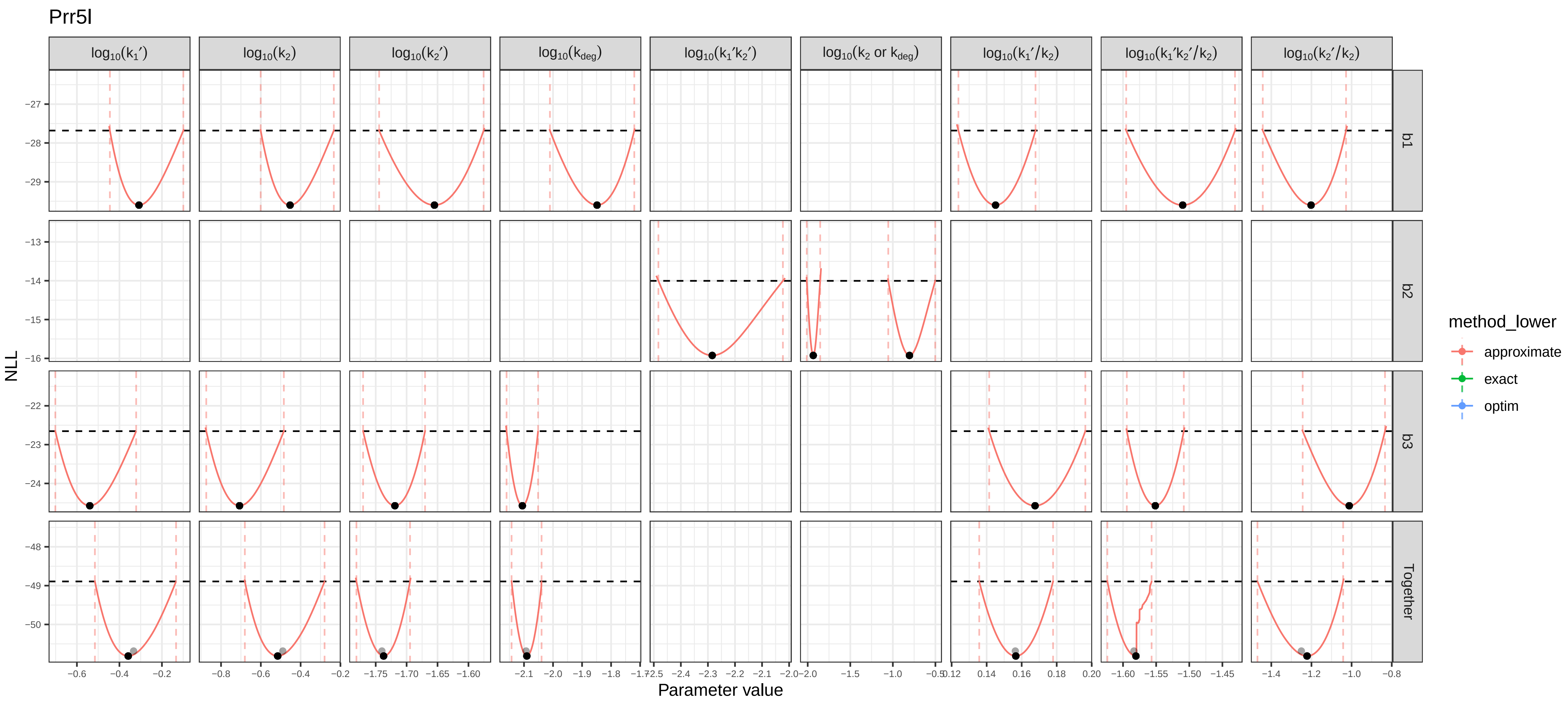

Replicate	Par	Best value	CI95 LB	CI95 UB	Method LB	Method UB
Together	$\log_{10}(k_1')$	−0.3589	−0.5153	−0.1335	approximate	approximate
Together	$\log_{10}(k_2)$	−0.5156	−0.6802	−0.2797	approximate	approximate
Together	$\log_{10}(k_2')$	−1.737	−1.781	−1.694	approximate	approximate
Together	$\log_{10}(k_{deg})$	−2.09	−2.143	−2.039	approximate	approximate
Together	$\log_{10}(k_1'/k_2)$	0.1567	0.1358	0.1779	approximate	approximate
Together	$\log_{10}(k_1'k_2'/k_2)$	−1.581	−1.624	−1.557	approximate	approximate
Together	$\log_{10}(k_2'/k_2)$	−1.222	−1.469	−1.042	approximate	approximate
b1	$\log_{10}(k_1')$	−0.308	−0.4448	−0.09927	approximate	approximate
b1	$\log_{10}(k_2)$	−0.4531	−0.6007	−0.2332	approximate	approximate
b1	$\log_{10}(k_2')$	−1.655	−1.744	−1.575	approximate	approximate
b1	$\log_{10}(k_{deg})$	−1.848	−2.011	−1.72	approximate	approximate
b1	$\log_{10}(k_1'/k_2)$	0.1451	0.1239	0.1679	approximate	approximate
b1	$\log_{10}(k_1'k_2'/k_2)$	−1.51	−1.595	−1.431	approximate	approximate
b1	$\log_{10}(k_2'/k_2)$	−1.202	−1.442	−1.028	approximate	approximate
b2	$\log_{10}(k_1'k_2')$	−2.284	−2.483	−2.023	approximate	approximate
b2	$\log_{10}(k_2 \text{ or } k_{deg})$	−0.8045	−1.054	−0.5021	approximate	approximate
b2	$\log_{10}(k_2 \text{ or } k_{deg})$	−1.935	−2.011	−1.853	approximate	approximate
b3	$\log_{10}(k_1')$	−0.5391	−0.7015	−0.3213	approximate	approximate
b3	$\log_{10}(k_2)$	−0.7067	−0.8742	−0.4843	approximate	approximate
b3	$\log_{10}(k_2')$	−1.719	−1.77	−1.67	approximate	approximate
b3	$\log_{10}(k_{deg})$	−2.106	−2.16	−2.051	approximate	approximate
b3	$\log_{10}(k_1'/k_2)$	0.1676	0.1414	0.1963	approximate	approximate
b3	$\log_{10}(k_1'k_2'/k_2)$	−1.551	−1.594	−1.508	approximate	approximate
b3	$\log_{10}(k_2'/k_2)$	−1.012	−1.244	−0.8337	approximate	approximate

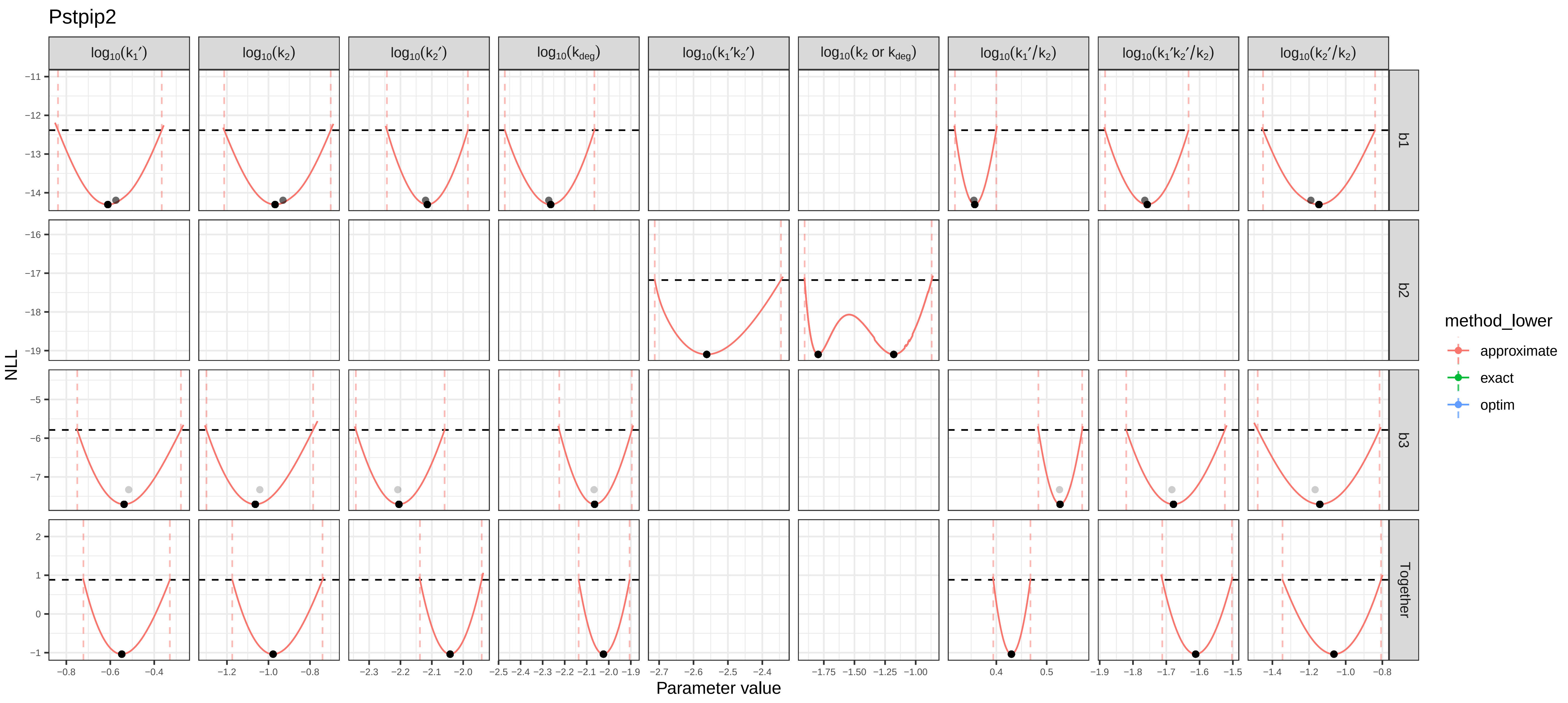

Replicate	Par	Best value	CI95 LB	CI95 UB	Method LB	Method UB
Together	$\log_{10}(k_1')$	−0.5476	−0.7222	−0.3289	approximate	approximate
Together	$\log_{10}(k_2)$	−0.9776	−1.174	−0.7396	approximate	approximate
Together	$\log_{10}(k_2')$	−2.042	−2.138	−1.941	approximate	approximate
Together	$\log_{10}(k_{deg})$	−2.024	−2.137	−1.905	approximate	approximate
Together	$\log_{10}(k_1'/k_2)$	0.43	0.394	0.4677	approximate	approximate
Together	$\log_{10}(k_1'k_2'/k_2)$	−1.612	−1.712	−1.503	approximate	approximate
Together	$\log_{10}(k_2'/k_2)$	−1.064	−1.345	−0.8072	approximate	approximate
b1	$\log_{10}(k_1')$	−0.6111	−0.8379	−0.3658	approximate	approximate
b1	$\log_{10}(k_2)$	−0.9682	−1.213	−0.7002	approximate	approximate
b1	$\log_{10}(k_2')$	−2.114	−2.243	−1.985	approximate	approximate
b1	$\log_{10}(k_{deg})$	−2.263	−2.472	−2.065	approximate	approximate
b1	$\log_{10}(k_1'/k_2)$	0.3572	0.3179	0.4	approximate	approximate
b1	$\log_{10}(k_1'k_2'/k_2)$	−1.757	−1.884	−1.633	approximate	approximate
b1	$\log_{10}(k_2'/k_2)$	−1.146	−1.451	−0.8395	approximate	approximate
b2	$\log_{10}(k_1'k_2')$	−2.562	−2.713	−2.346	approximate	approximate
b2	$\log_{10}(k_2 \text{ or } k_{deg})$	−1.18	−1.907	−0.8693	approximate	approximate
b2	$\log_{10}(k_2 \text{ or } k_{deg})$	−1.796	−1.907	−0.8693	approximate	approximate
b3	$\log_{10}(k_1')$	−0.5371	−0.7502	−0.2786	approximate	approximate
b3	$\log_{10}(k_2)$	−1.064	−1.299	−0.7849	approximate	approximate
b3	$\log_{10}(k_2')$	−2.205	−2.342	−2.059	approximate	approximate
b3	$\log_{10}(k_{deg})$	−2.064	−2.225	−1.895	approximate	approximate
b3	$\log_{10}(k_1'/k_2)$	0.5264	0.4835	0.5704	approximate	approximate
b3	$\log_{10}(k_1'k_2'/k_2)$	−1.678	−1.82	−1.524	approximate	approximate
b3	$\log_{10}(k_2'/k_2)$	−1.141	−1.48	−0.8152	approximate	approximate

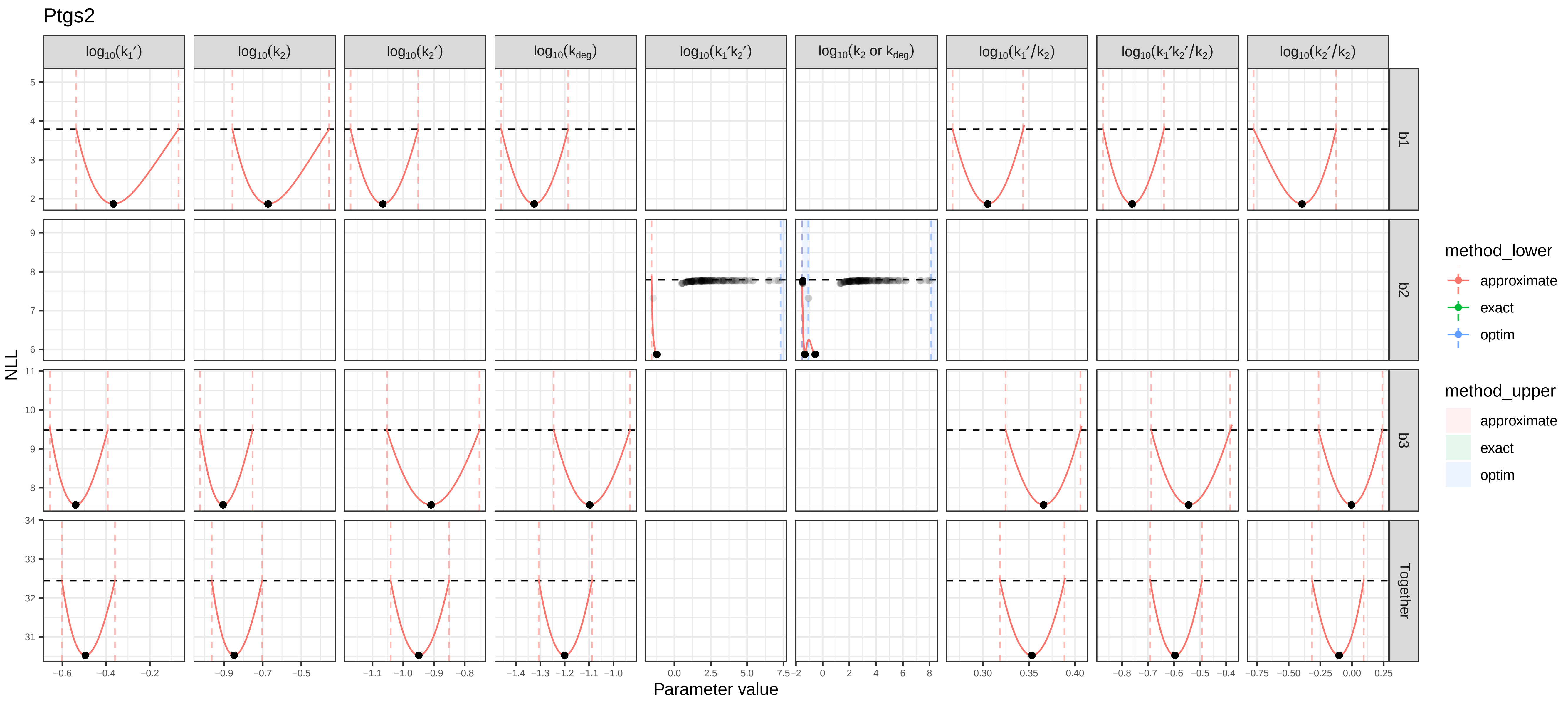

Replicate	Par	Best value	CI95 LB	CI95 UB	Method LB	Method UB
Together	$\log_{10}(k_1')$	−0.4948	−0.6021	−0.3595	approximate	approximate
Together	$\log_{10}(k_2)$	−0.8479	−0.9636	−0.7033	approximate	approximate
Together	$\log_{10}(k_2')$	−0.9493	−1.041	−0.8509	approximate	approximate
Together	$\log_{10}(k_{deg})$	−1.2	−1.306	−1.088	approximate	approximate
Together	$\log_{10}(k_1'/k_2)$	0.3531	0.3185	0.3884	approximate	approximate
Together	$\log_{10}(k_1'k_2'/k_2)$	−0.5963	−0.6911	−0.4927	approximate	approximate
Together	$\log_{10}(k_2'/k_2)$	−0.1015	−0.3157	0.09255	approximate	approximate
b1	$\log_{10}(k_1')$	−0.3668	−0.5368	−0.06847	approximate	approximate
b1	$\log_{10}(k_2)$	−0.6721	−0.8567	−0.3567	approximate	approximate
b1	$\log_{10}(k_2')$	−1.066	−1.171	−0.9514	approximate	approximate
b1	$\log_{10}(k_{deg})$	−1.325	−1.461	−1.186	approximate	approximate
b1	$\log_{10}(k_1'/k_2)$	0.3054	0.2672	0.3437	approximate	approximate
b1	$\log_{10}(k_1'k_2'/k_2)$	−0.761	−0.8725	−0.6386	approximate	approximate
b1	$\log_{10}(k_2'/k_2)$	−0.3943	−0.777	−0.1253	approximate	approximate
b2	$\log_{10}(k_1'k_2')$	−1.214	−1.567	> 7.297	approximate	optim
b2	$\log_{10}(k_2 \text{ or } k_{deg})$	−0.5542	−1.539	> 8.111	approximate	optim
b2	$\log_{10}(k_2 \text{ or } k_{deg})$	−1.325	−1.539	−1.073	approximate	optim
b3	$\log_{10}(k_1')$	−0.5392	−0.6561	−0.3925	approximate	approximate
b3	$\log_{10}(k_2)$	−0.905	−1.024	−0.7526	approximate	approximate
b3	$\log_{10}(k_2')$	−0.9095	−1.053	−0.7521	approximate	approximate
b3	$\log_{10}(k_{deg})$	−1.097	−1.245	−0.9325	approximate	approximate
b3	$\log_{10}(k_1'/k_2)$	0.3659	0.3247	0.4057	approximate	approximate
b3	$\log_{10}(k_1'k_2'/k_2)$	−0.5437	−0.6876	−0.3843	approximate	approximate
b3	$\log_{10}(k_2'/k_2)$	−0.004483	−0.264	0.2393	approximate	approximate

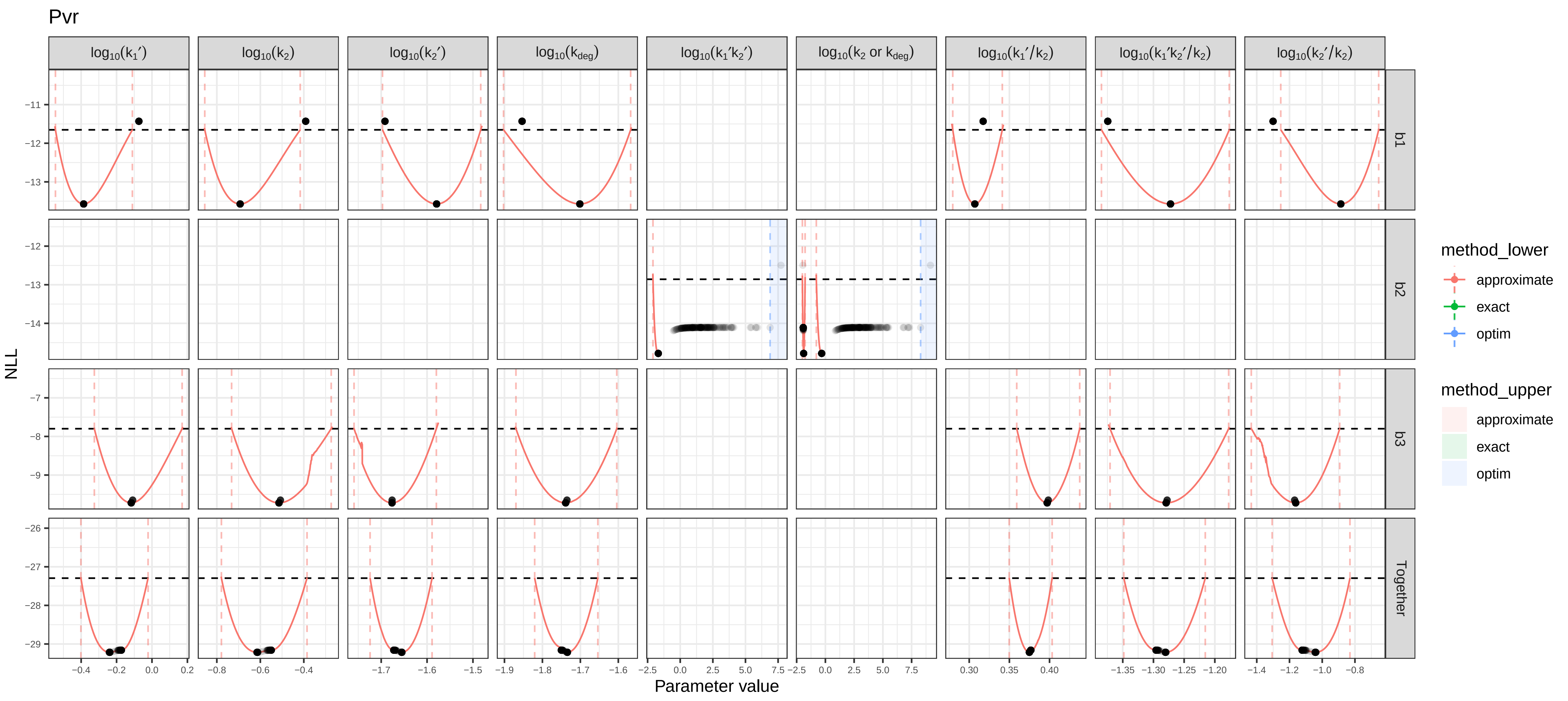

Replicate	Par	Best value	CI95 LB	CI95 UB	Method LB	Method UB
Together	$\log_{10}(k_1')$	−0.2391	−0.4013	−0.02205	approximate	approximate
Together	$\log_{10}(k_2)$	−0.614	−0.779	−0.3852	approximate	approximate
Together	$\log_{10}(k_2')$	−1.655	−1.724	−1.589	approximate	approximate
Together	$\log_{10}(k_{deg})$	−1.734	−1.82	−1.654	approximate	approximate
Together	$\log_{10}(k_1'/k_2)$	0.3749	0.3498	0.4033	approximate	approximate
Together	$\log_{10}(k_1'k_2'/k_2)$	−1.28	−1.348	−1.216	approximate	approximate
Together	$\log_{10}(k_2'/k_2)$	−1.041	−1.306	−0.8305	approximate	approximate
b1	$\log_{10}(k_1')$	−0.3863	−0.5451	−0.1107	approximate	approximate
b1	$\log_{10}(k_2)$	−0.6933	−0.8555	−0.4165	approximate	approximate
b1	$\log_{10}(k_2')$	−1.579	−1.697	−1.483	approximate	approximate
b1	$\log_{10}(k_{deg})$	−1.701	−1.902	−1.567	approximate	approximate
b1	$\log_{10}(k_1'/k_2)$	0.307	0.2795	0.3412	approximate	approximate
b1	$\log_{10}(k_1'k_2'/k_2)$	−1.272	−1.385	−1.176	approximate	approximate
b1	$\log_{10}(k_2'/k_2)$	−0.8858	−1.253	−0.655	approximate	approximate
b2	$\log_{10}(k_1'k_2')$	−1.676	−2.088	> 6.888	approximate	optim
b2	$\log_{10}(k_2 \text{ or } k_{deg})$	−0.3177	−0.7884	> 8.282	approximate	optim
b2	$\log_{10}(k_2 \text{ or } k_{deg})$	−1.889	−2.002	−1.764	approximate	approximate
b3	$\log_{10}(k_1')$	−0.117	−0.3255	0.1702	approximate	approximate
b3	$\log_{10}(k_2)$	−0.5141	−0.732	−0.274	approximate	approximate
b3	$\log_{10}(k_2')$	−1.676	−1.759	−1.58	approximate	approximate
b3	$\log_{10}(k_{deg})$	−1.739	−1.87	−1.604	approximate	approximate
b3	$\log_{10}(k_1'/k_2)$	0.3971	0.3592	0.4376	approximate	approximate
b3	$\log_{10}(k_1'k_2'/k_2)$	−1.279	−1.371	−1.177	approximate	approximate
b3	$\log_{10}(k_2'/k_2)$	−1.162	−1.434	−0.8933	approximate	approximate

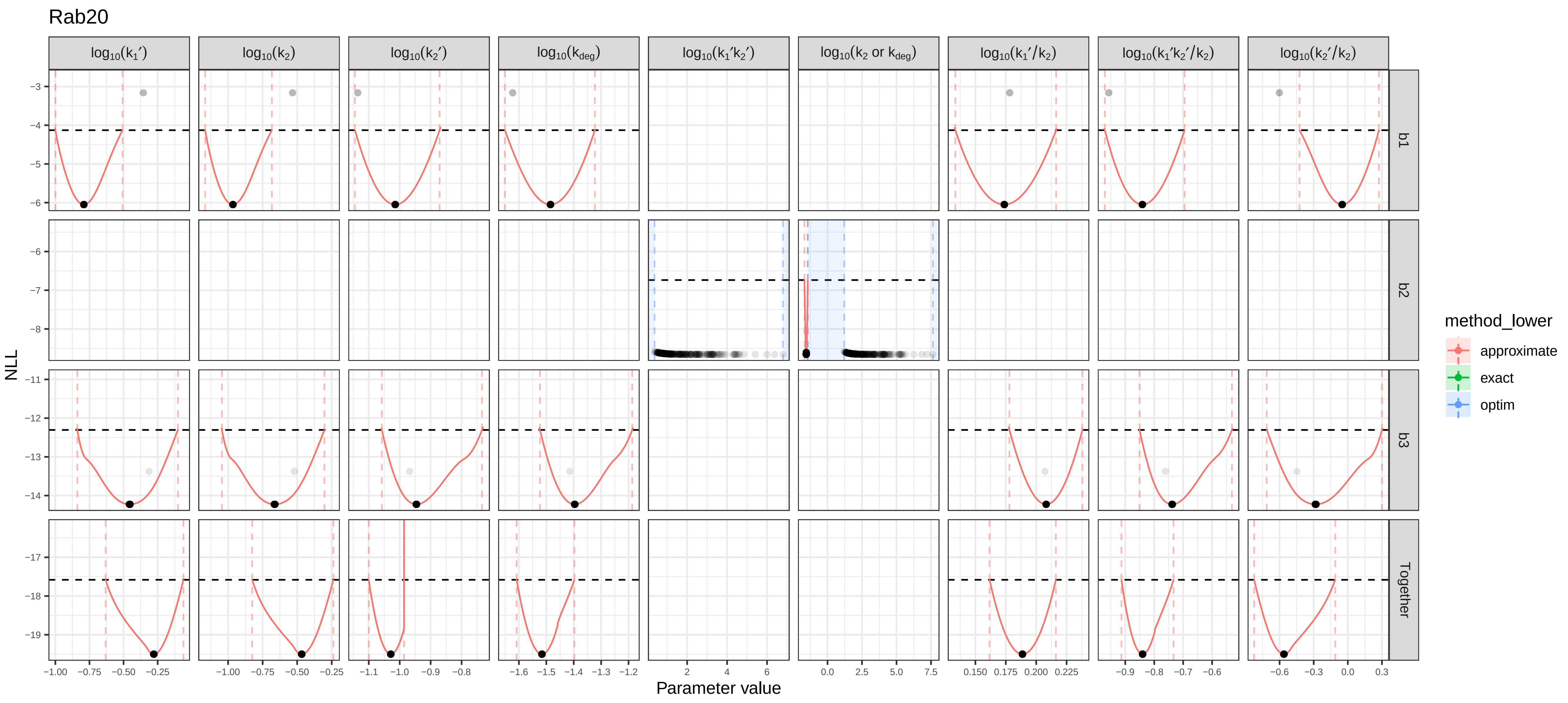

Replicate	Par	**Best value**	**CI95 LB**	**CI95 UB**	**Method LB**	**Method UB**
Together	$\log_{10}(k_1')$	−0.2779	−0.6315	−0.06108	approximate	approximate
Together	$\log_{10}(k_2)$	−0.4666	−0.8258	−0.2381	approximate	approximate
Together	$\log_{10}(k_2')$	−1.029	−1.1	−0.986	approximate	approximate
Together	$\log_{10}(k_{deg})$	−1.516	−1.608	−1.398	approximate	approximate
Together	$\log_{10}(k_1'/k_2)$	0.1887	0.1616	0.2162	approximate	approximate
Together	$\log_{10}(k_1'k_2'/k_2)$	−0.8402	−0.9133	−0.7332	approximate	approximate
Together	$\log_{10}(k_2'/k_2)$	−0.5622	−0.8239	−0.1109	approximate	approximate
b1	$\log_{10}(k_1')$	−0.7912	−0.9996	−0.5071	approximate	approximate
b1	$\log_{10}(k_2)$	−0.965	−1.166	−0.6828	approximate	approximate
b1	$\log_{10}(k_2')$	−1.015	−1.145	−0.8712	approximate	approximate
b1	$\log_{10}(k_{deg})$	−1.485	−1.651	−1.323	approximate	approximate
b1	$\log_{10}(k_1'/k_2)$	0.1738	0.1335	0.2164	approximate	approximate
b1	$\log_{10}(k_1'k_2'/k_2)$	−0.8408	−0.9707	−0.695	approximate	approximate
b1	$\log_{10}(k_2'/k_2)$	−0.04953	−0.4252	0.2734	approximate	approximate
b2	$\log_{10}(k_1'k_2')$	4.36	< 0.3695	> 6.799	optim	optim
b2	$\log_{10}(k_2 \text{ or } k_{deg})$	5.198	1.206	> 7.637	optim	optim
b2	$\log_{10}(k_2 \text{ or } k_{deg})$	−1.541	−1.671	−1.431	approximate	approximate
b3	$\log_{10}(k_1')$	−0.4543	−0.8391	−0.1018	approximate	approximate
b3	$\log_{10}(k_2)$	−0.6625	−1.045	−0.3024	approximate	approximate
b3	$\log_{10}(k_2')$	−0.9462	−1.058	−0.7342	approximate	approximate
b3	$\log_{10}(k_{deg})$	−1.396	−1.523	−1.187	approximate	approximate
b3	$\log_{10}(k_1'/k_2)$	0.2082	0.178	0.238	approximate	approximate
b3	$\log_{10}(k_1'k_2'/k_2)$	−0.738	−0.8507	−0.5315	approximate	approximate
b3	$\log_{10}(k_2'/k_2)$	−0.2837	−0.7133	0.3007	approximate	approximate

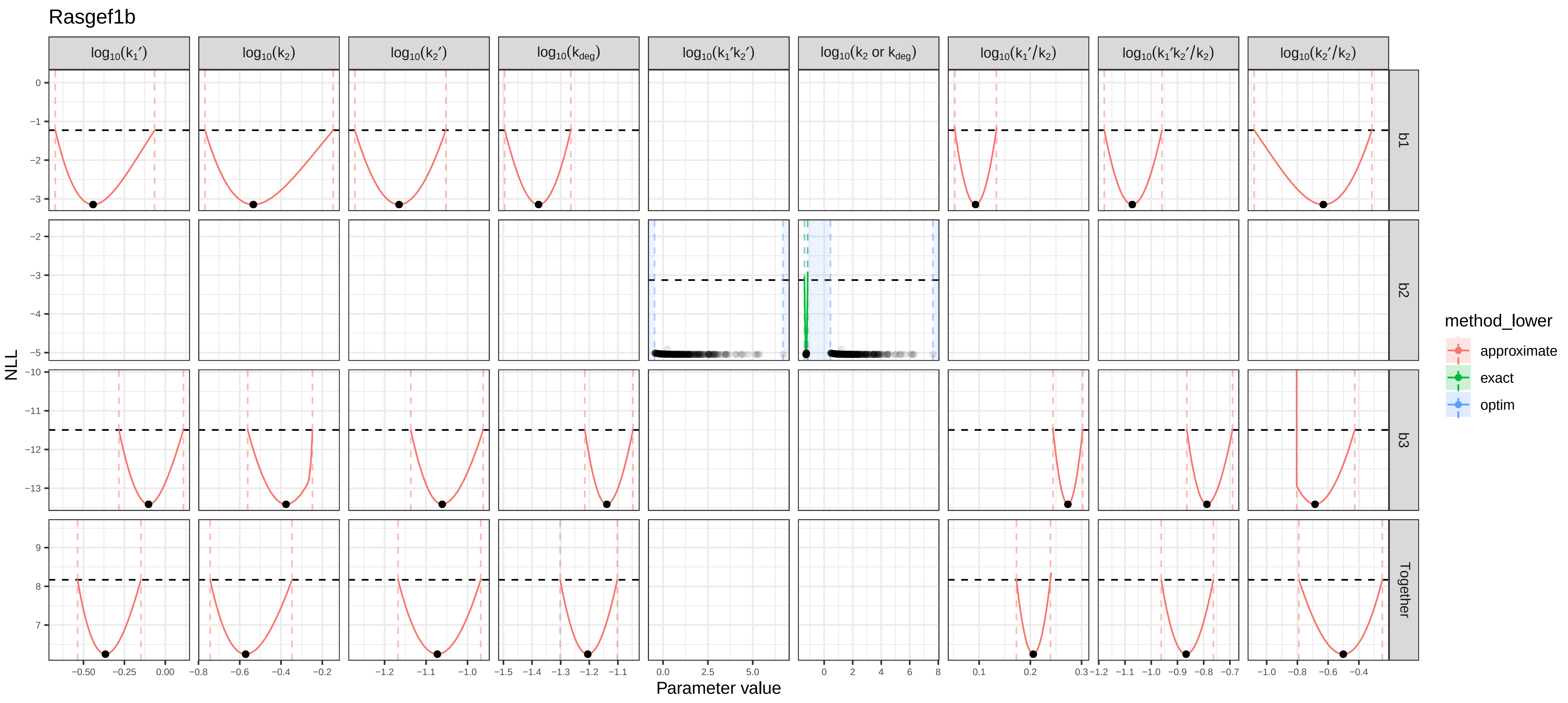

Replicate	Par	Best value	CI95 LB	CI95 UB	Method LB	Method UB
Together	$\log_{10}(k_1')$	−0.3659	−0.536	−0.1489	approximate	approximate
Together	$\log_{10}(k_2)$	−0.5715	−0.7435	−0.3467	approximate	approximate
Together	$\log_{10}(k_2')$	−1.073	−1.168	−0.9682	approximate	approximate
Together	$\log_{10}(k_{deg})$	−1.205	−1.302	−1.102	approximate	approximate
Together	$\log_{10}(k_1'/k_2)$	0.2057	0.1728	0.2392	approximate	approximate
Together	$\log_{10}(k_1'k_2'/k_2)$	−0.867	−0.9619	−0.7631	approximate	approximate
Together	$\log_{10}(k_2'/k_2)$	−0.5011	−0.7902	−0.2473	approximate	approximate
b1	$\log_{10}(k_1')$	−0.4411	−0.6722	−0.06516	approximate	approximate
b1	$\log_{10}(k_2)$	−0.534	−0.7684	−0.1469	approximate	approximate
b1	$\log_{10}(k_2')$	−1.165	−1.272	−1.052	approximate	approximate
b1	$\log_{10}(k_{deg})$	−1.377	−1.495	−1.264	approximate	approximate
b1	$\log_{10}(k_1'/k_2)$	0.09297	0.05252	0.1336	approximate	approximate
b1	$\log_{10}(k_1'k_2'/k_2)$	−1.072	−1.179	−0.959	approximate	approximate
b1	$\log_{10}(k_2'/k_2)$	−0.631	−1.081	−0.3153	approximate	approximate
b2	$\log_{10}(k_1'k_2')$	5.286	< −0.4758	> 6.69	optim	optim
b2	$\log_{10}(k_2 \text{ or } k_{deg})$	6.219	0.4408	> 7.624	optim	optim
b2	$\log_{10}(k_2 \text{ or } k_{deg})$	−1.264	−1.379	−1.156	exact	exact
b3	$\log_{10}(k_1')$	−0.1024	−0.2836	0.1109	approximate	approximate
b3	$\log_{10}(k_2)$	−0.3756	−0.5615	−0.2477	approximate	approximate
b3	$\log_{10}(k_2')$	−1.061	−1.137	−0.9622	approximate	approximate
b3	$\log_{10}(k_{deg})$	−1.139	−1.216	−1.047	approximate	approximate
b3	$\log_{10}(k_1'/k_2)$	0.2732	0.2442	0.3022	approximate	approximate
b3	$\log_{10}(k_1'k_2'/k_2)$	−0.7877	−0.8644	−0.6899	approximate	approximate
b3	$\log_{10}(k_2'/k_2)$	−0.6854	−0.8047	−0.4271	approximate	approximate

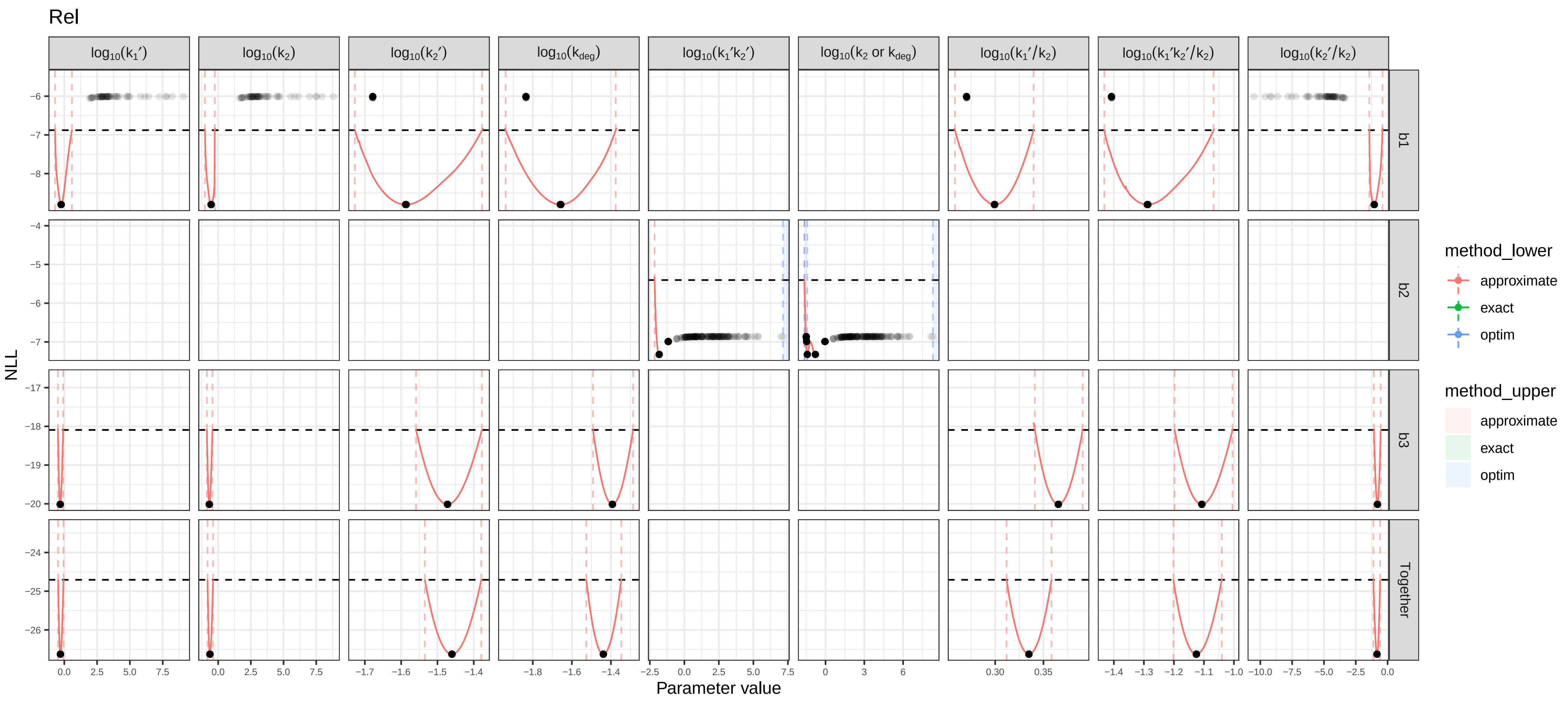

Replicate	Par	Best value	CI95 LB	CI95 UB	Method LB	Method UB
Together	$\log_{10}(k_1')$	−0.2922	−0.4679	−0.06149	approximate	approximate
Together	$\log_{10}(k_2)$	−0.6271	−0.8106	−0.3882	approximate	approximate
Together	$\log_{10}(k_2')$	−1.46	−1.535	−1.379	approximate	approximate
Together	$\log_{10}(k_{deg})$	−1.439	−1.526	−1.347	approximate	approximate
Together	$\log_{10}(k_1'/k_2)$	0.3349	0.3117	0.3584	approximate	approximate
Together	$\log_{10}(k_1'k_2'/k_2)$	−1.125	−1.202	−1.041	approximate	approximate
Together	$\log_{10}(k_2'/k_2)$	−0.833	−1.121	−0.5887	approximate	approximate
b1	$\log_{10}(k_1')$	−0.2374	−0.7027	0.5872	approximate	approximate
b1	$\log_{10}(k_2)$	−0.5367	−1.011	−0.2572	approximate	approximate
b1	$\log_{10}(k_2')$	−1.587	−1.728	−1.377	approximate	approximate
b1	$\log_{10}(k_{deg})$	−1.658	−1.939	−1.376	approximate	approximate
b1	$\log_{10}(k_1'/k_2)$	0.2994	0.2583	0.3398	approximate	approximate
b1	$\log_{10}(k_1'k_2'/k_2)$	−1.288	−1.432	−1.068	approximate	approximate
b1	$\log_{10}(k_2'/k_2)$	−1.05	−1.435	−0.4021	approximate	approximate
b2	$\log_{10}(k_1'k_2')$	−1.821	−2.152	> 7.147	approximate	optim
b2	$\log_{10}(k_2$ or $k_{deg})$	−0.7843	−1.629	> 8.305	approximate	optim
b2	$\log_{10}(k_2$ or $k_{deg})$	−1.408	−1.629	−1.408	approximate	optim
b3	$\log_{10}(k_1')$	−0.308	−0.4834	−0.08414	approximate	approximate
b3	$\log_{10}(k_2)$	−0.6734	−0.8627	−0.4372	approximate	approximate
b3	$\log_{10}(k_2')$	−1.472	−1.559	−1.378	approximate	approximate
b3	$\log_{10}(k_{deg})$	−1.393	−1.492	−1.286	approximate	approximate
b3	$\log_{10}(k_1'/k_2)$	0.3654	0.3411	0.3906	approximate	approximate
b3	$\log_{10}(k_1'k_2'/k_2)$	−1.107	−1.198	−1.005	approximate	approximate
b3	$\log_{10}(k_2'/k_2)$	−0.7989	−1.081	−0.5434	approximate	approximate

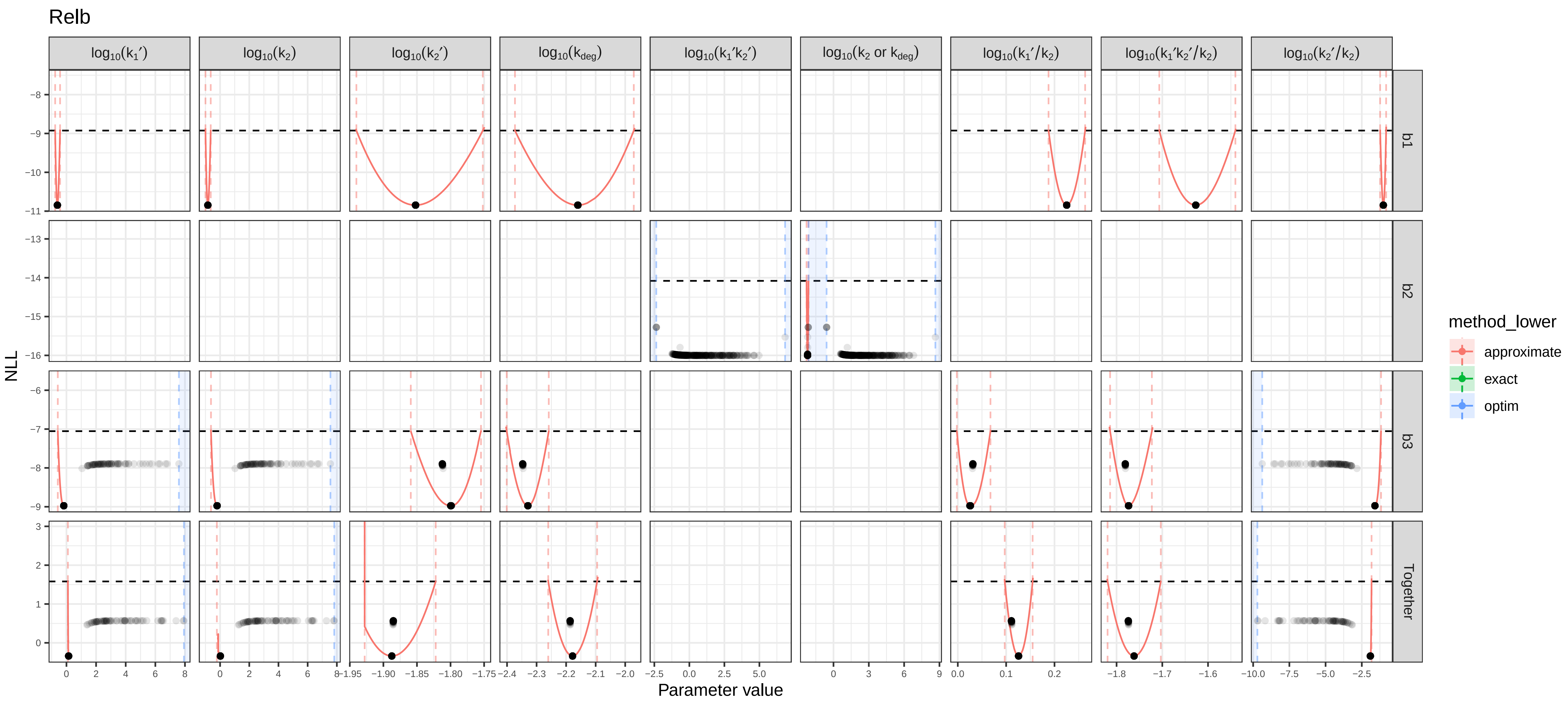

Replicate	Par	Best value	CI95 LB	CI95 UB	Method LB	Method UB
Together	$\log_{10}(k_1')$	0.147	0.09769	> 7.932	approximate	optim
Together	$\log_{10}(k_2)$	0.02132	−0.2199	> 7.821	approximate	optim
Together	$\log_{10}(k_2')$	−1.887	−1.928	−1.822	approximate	approximate
Together	$\log_{10}(k_{deg})$	−2.178	−2.261	−2.095	approximate	approximate
Together	$\log_{10}(k_1'/k_2)$	0.1257	0.09709	0.1548	approximate	approximate
Together	$\log_{10}(k_1'k_2'/k_2)$	−1.762	−1.82	−1.703	approximate	approximate
Together	$\log_{10}(k_2'/k_2)$	−1.909	< −9.706	−1.827	optim	approximate
b1	$\log_{10}(k_1')$	−0.6128	−0.763	−0.4306	approximate	approximate
b1	$\log_{10}(k_2)$	−0.8381	−1.001	−0.6406	approximate	approximate
b1	$\log_{10}(k_2')$	−1.852	−1.94	−1.752	approximate	approximate
b1	$\log_{10}(k_{deg})$	−2.16	−2.373	−1.971	approximate	approximate
b1	$\log_{10}(k_1'/k_2)$	0.2253	0.1877	0.2634	approximate	approximate
b1	$\log_{10}(k_1'k_2'/k_2)$	−1.627	−1.707	−1.54	approximate	approximate
b1	$\log_{10}(k_2'/k_2)$	−1.014	−1.233	−0.8202	approximate	approximate
b2	$\log_{10}(k_1'k_2')$	3.78	< −2.361	> 6.834	optim	optim
b2	$\log_{10}(k_2 \text{ or } k_{deg})$	5.613	−0.5853	> 8.653	optim	optim
b2	$\log_{10}(k_2 \text{ or } k_{deg})$	−2.202	−2.288	−2.123	approximate	approximate
b3	$\log_{10}(k_1')$	−0.1809	−0.5848	> 7.591	approximate	optim
b3	$\log_{10}(k_2)$	−0.2069	−0.6238	> 7.56	approximate	optim
b3	$\log_{10}(k_2')$	−1.8	−1.859	−1.755	approximate	approximate
b3	$\log_{10}(k_{deg})$	−2.329	−2.401	−2.259	approximate	approximate
b3	$\log_{10}(k_1'/k_2)$	0.02601	−0.001946	0.06755	approximate	approximate
b3	$\log_{10}(k_1'k_2'/k_2)$	−1.774	−1.814	−1.723	approximate	approximate
b3	$\log_{10}(k_2'/k_2)$	−1.593	< −9.372	−1.171	optim	approximate

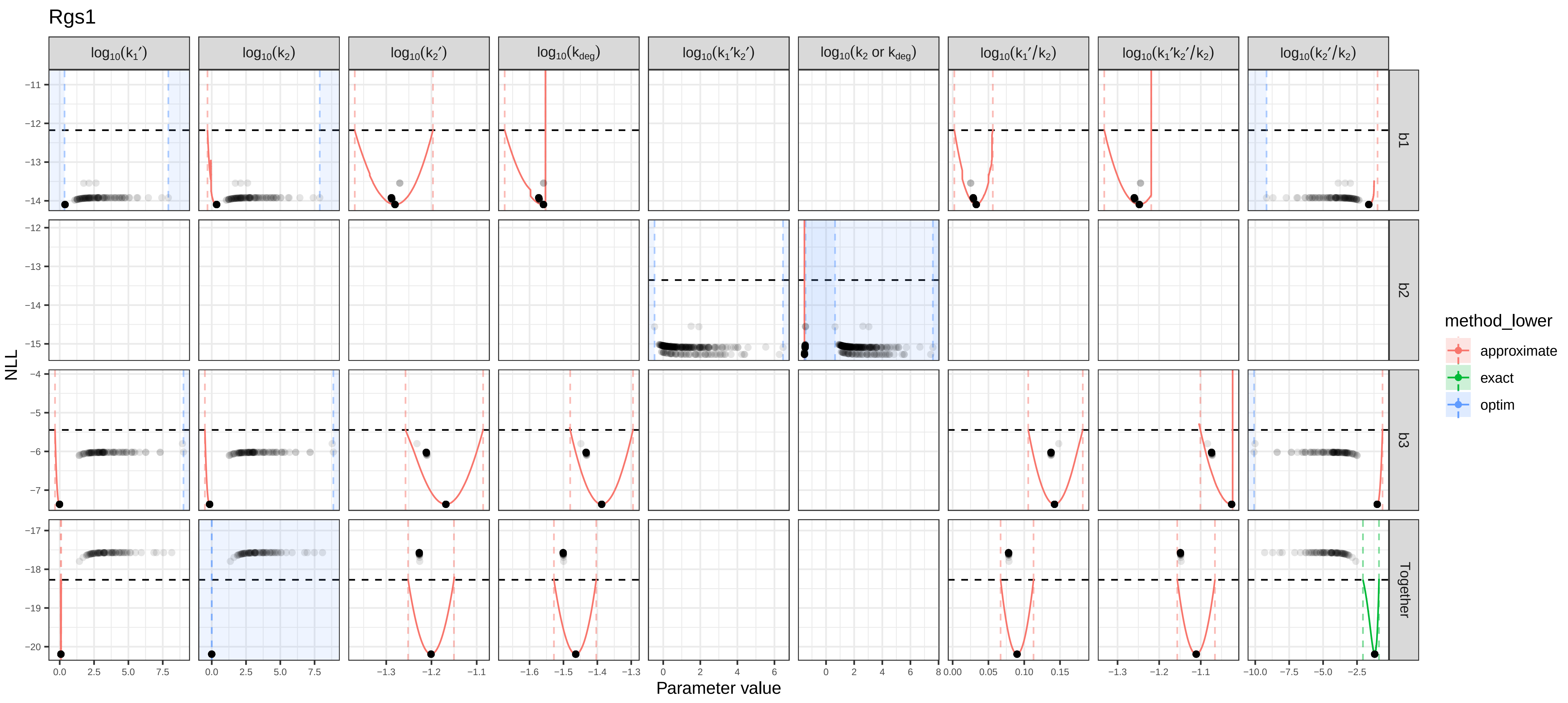

Replicate	Par	Best value	CI95 LB	CI95 UB	Method LB	Method UB
Together	$\log_{10}(k_1')$	0.08641	0.06594	0.1093	approximate	approximate
Together	$\log_{10}(k_2)$	−0.003559	< −0.007388	> 0.003964	optim	optim
Together	$\log_{10}(k_2')$	−1.201	−1.252	−1.15	approximate	approximate
Together	$\log_{10}(k_{deg})$	−1.464	−1.528	−1.403	approximate	approximate
Together	$\log_{10}(k_1'/k_2)$	0.08997	0.06699	0.113	approximate	approximate
Together	$\log_{10}(k_1'k_2'/k_2)$	−1.111	−1.156	−1.066	approximate	approximate
Together	$\log_{10}(k_2'/k_2)$	−1.197	−2.063	−0.8812	exact	exact
b1	$\log_{10}(k_1')$	0.388	< 0.3522	> 7.906	optim	optim
b1	$\log_{10}(k_2)$	0.3551	−0.3108	> 7.878	approximate	optim
b1	$\log_{10}(k_2')$	−1.281	−1.37	−1.197	approximate	approximate
b1	$\log_{10}(k_{deg})$	−1.559	−1.673	−1.553	approximate	approximate
b1	$\log_{10}(k_1'/k_2)$	0.03297	0.002382	0.05614	approximate	approximate
b1	$\log_{10}(k_1'k_2'/k_2)$	−1.248	−1.332	−1.219	approximate	approximate
b1	$\log_{10}(k_2'/k_2)$	−1.636	< −9.166	−0.9857	optim	approximate
b2	$\log_{10}(k_1'k_2')$	6.266	< −0.4676	> 6.462	optim	optim
b2	$\log_{10}(k_2 \text{ or } k_{deg})$	7.441	0.6409	> 7.598	optim	optim
b2	$\log_{10}(k_2 \text{ or } k_{deg})$	−1.534	−1.535	> −1.457	approximate	optim
b3	$\log_{10}(k_1')$	−0.01553	−0.3418	> 9.008	approximate	optim
b3	$\log_{10}(k_2)$	−0.1579	−0.5004	> 8.87	approximate	optim
b3	$\log_{10}(k_2')$	−1.168	−1.257	−1.086	approximate	approximate
b3	$\log_{10}(k_{deg})$	−1.387	−1.48	−1.295	approximate	approximate
b3	$\log_{10}(k_1'/k_2)$	0.1423	0.1056	0.1818	approximate	approximate
b3	$\log_{10}(k_1'k_2'/k_2)$	−1.026	−1.101	−1.023	approximate	approximate
b3	$\log_{10}(k_2'/k_2)$	−1.01	< −10.08	−0.6198	optim	approximate

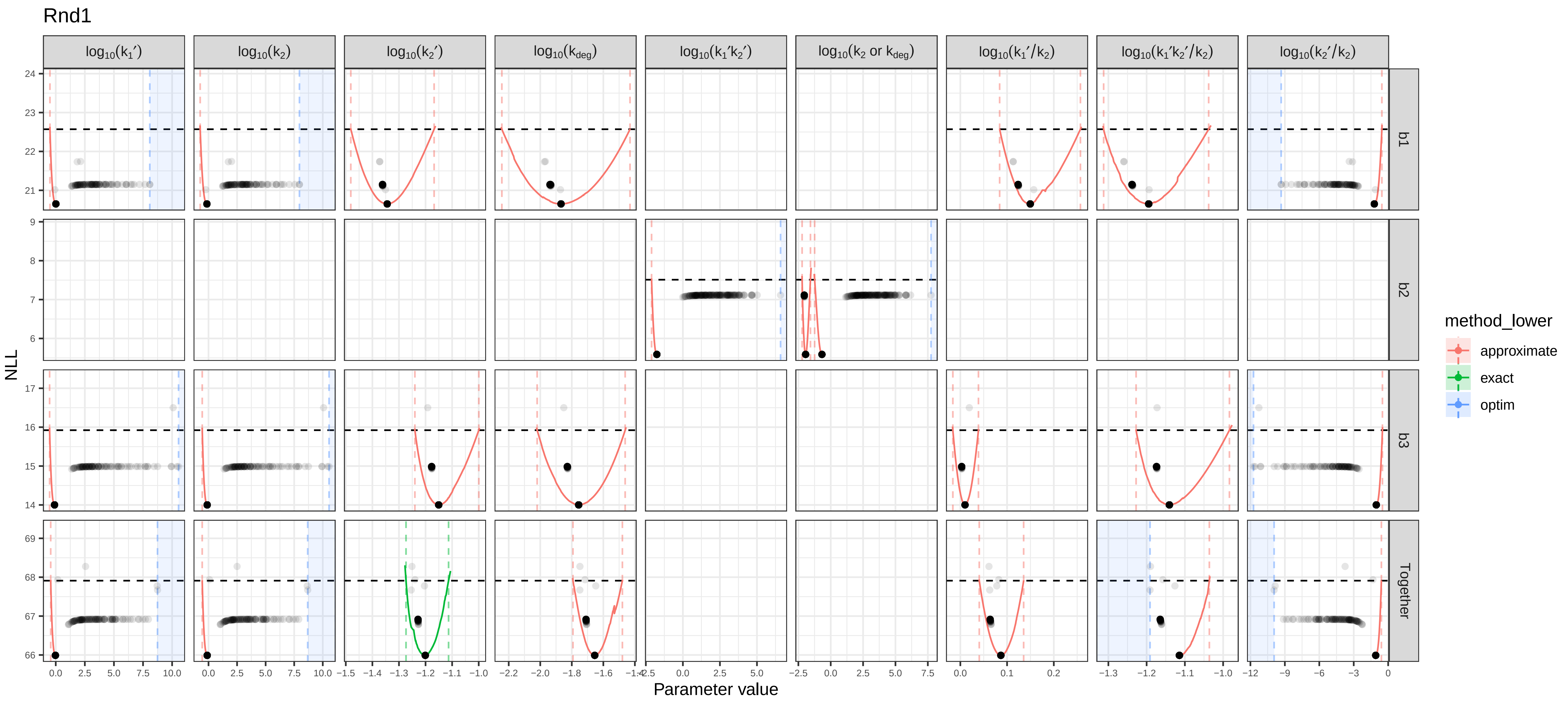

Replicate	Par	Best value	CI95 LB	CI95 UB	Method LB	Method UB
Together	$\log_{10}(k_1')$	−0.02946	−0.4334	> 8.744	approximate	optim
Together	$\log_{10}(k_2)$	−0.1163	−0.5524	> 8.681	approximate	optim
Together	$\log_{10}(k_2')$	−1.201	−1.273	−1.113	exact	exact
Together	$\log_{10}(k_{deg})$	−1.654	−1.792	−1.479	approximate	approximate
Together	$\log_{10}(k_1'/k_2)$	0.08681	0.0405	0.1355	approximate	approximate
Together	$\log_{10}(k_1'k_2'/k_2)$	−1.114	< −1.191	−1.036	optim	approximate
Together	$\log_{10}(k_2'/k_2)$	−1.085	< −9.935	−0.5916	optim	approximate
b1	$\log_{10}(k_1')$	0.01099	−0.4978	> 8.082	approximate	optim
b1	$\log_{10}(k_2)$	−0.1386	−0.7289	> 7.958	approximate	optim
b1	$\log_{10}(k_2')$	−1.344	−1.481	−1.168	approximate	approximate
b1	$\log_{10}(k_{deg})$	−1.868	−2.243	−1.431	approximate	approximate
b1	$\log_{10}(k_1'/k_2)$	0.1496	0.0841	0.257	approximate	approximate
b1	$\log_{10}(k_1'k_2'/k_2)$	−1.195	−1.312	−1.038	approximate	approximate
b1	$\log_{10}(k_2'/k_2)$	−1.206	< −9.321	−0.5488	optim	approximate
b2	$\log_{10}(k_1'k_2')$	−1.756	−2.112	> 6.591	approximate	optim
b2	$\log_{10}(k_2 \text{ or } k_{deg})$	−0.6768	−1.243	> 7.749	approximate	optim
b2	$\log_{10}(k_2 \text{ or } k_{deg})$	−1.94	−2.21	−1.565	approximate	approximate
b3	$\log_{10}(k_1')$	−0.1054	−0.5282	> 10.55	approximate	optim
b3	$\log_{10}(k_2)$	−0.1154	−0.5489	> 10.55	approximate	optim
b3	$\log_{10}(k_2')$	−1.151	−1.24	−0.9996	approximate	approximate
b3	$\log_{10}(k_{deg})$	−1.756	−2.02	−1.462	approximate	approximate
b3	$\log_{10}(k_1'/k_2)$	0.01002	−0.01602	0.03875	approximate	approximate
b3	$\log_{10}(k_1'k_2'/k_2)$	−1.141	−1.228	−0.9835	approximate	approximate
b3	$\log_{10}(k_2'/k_2)$	−1.035	< −11.73	−0.5001	optim	approximate

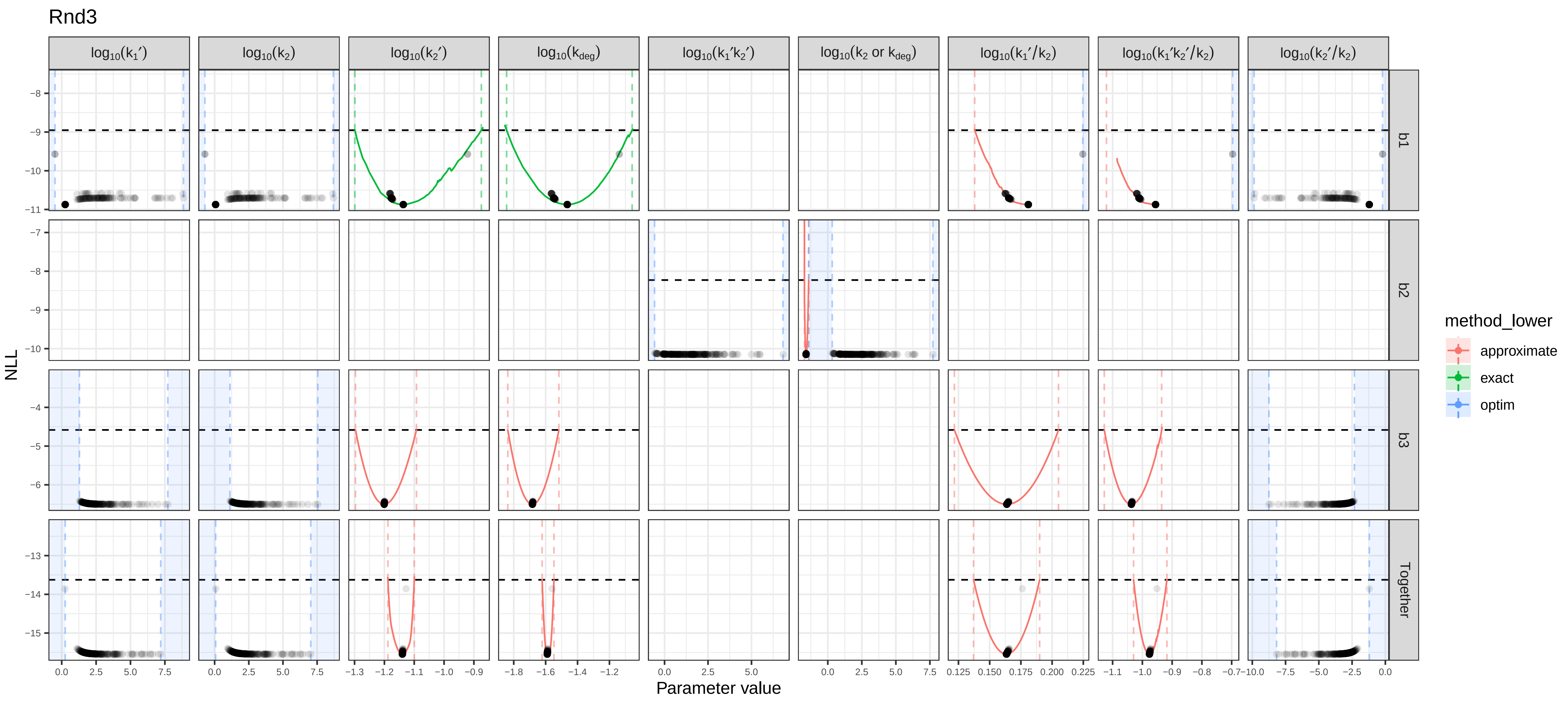

Replicate	Par	Best value	CI95 LB	CI95 UB	Method LB	Method UB
Together	$\log_{10}(k_1')$	5.461	< 0.2533	> 7.198	optim	optim
Together	$\log_{10}(k_2)$	5.298	< 0.07706	> 7.035	optim	optim
Together	$\log_{10}(k_2')$	−1.14	−1.188	−1.1	approximate	approximate
Together	$\log_{10}(k_{deg})$	−1.59	−1.622	−1.548	approximate	approximate
Together	$\log_{10}(k_1'/k_2)$	0.1634	0.1371	0.19	approximate	approximate
Together	$\log_{10}(k_1'k_2'/k_2)$	−0.9761	−1.03	−0.9175	approximate	approximate
Together	$\log_{10}(k_2'/k_2)$	−6.437	< −8.175	> −1.204	optim	optim
b1	$\log_{10}(k_1')$	0.252	< −0.4874	> 8.852	optim	optim
b1	$\log_{10}(k_2)$	0.07104	< −0.712	> 8.687	optim	optim
b1	$\log_{10}(k_2')$	−1.137	−1.299	−0.875	exact	exact
b1	$\log_{10}(k_{deg})$	−1.464	−1.845	−1.058	exact	exact
b1	$\log_{10}(k_1'/k_2)$	0.181	0.138	> 0.2246	approximate	optim
b1	$\log_{10}(k_1'k_2'/k_2)$	−0.9556	−1.121	> −0.6966	approximate	optim
b1	$\log_{10}(k_2'/k_2)$	−1.208	< −9.863	> −0.2092	optim	optim
b2	$\log_{10}(k_1'k_2')$	3.996	< −0.5744	> 6.812	optim	optim
b2	$\log_{10}(k_2 \text{ or } k_{deg})$	4.907	0.3244	> 7.722	optim	optim
b2	$\log_{10}(k_2 \text{ or } k_{deg})$	−1.598	−1.715	−1.403	approximate	approximate
b3	$\log_{10}(k_1')$	5.532	< 1.288	> 7.714	optim	optim
b3	$\log_{10}(k_2)$	5.369	< 1.123	> 7.55	optim	optim
b3	$\log_{10}(k_2')$	−1.201	−1.297	−1.092	approximate	approximate
b3	$\log_{10}(k_{deg})$	−1.683	−1.837	−1.517	approximate	approximate
b3	$\log_{10}(k_1'/k_2)$	0.1637	0.1217	0.2052	approximate	approximate
b3	$\log_{10}(k_1'k_2'/k_2)$	−1.037	−1.128	−0.9352	approximate	approximate
b3	$\log_{10}(k_2'/k_2)$	−6.569	< −8.751	> −2.322	optim	optim

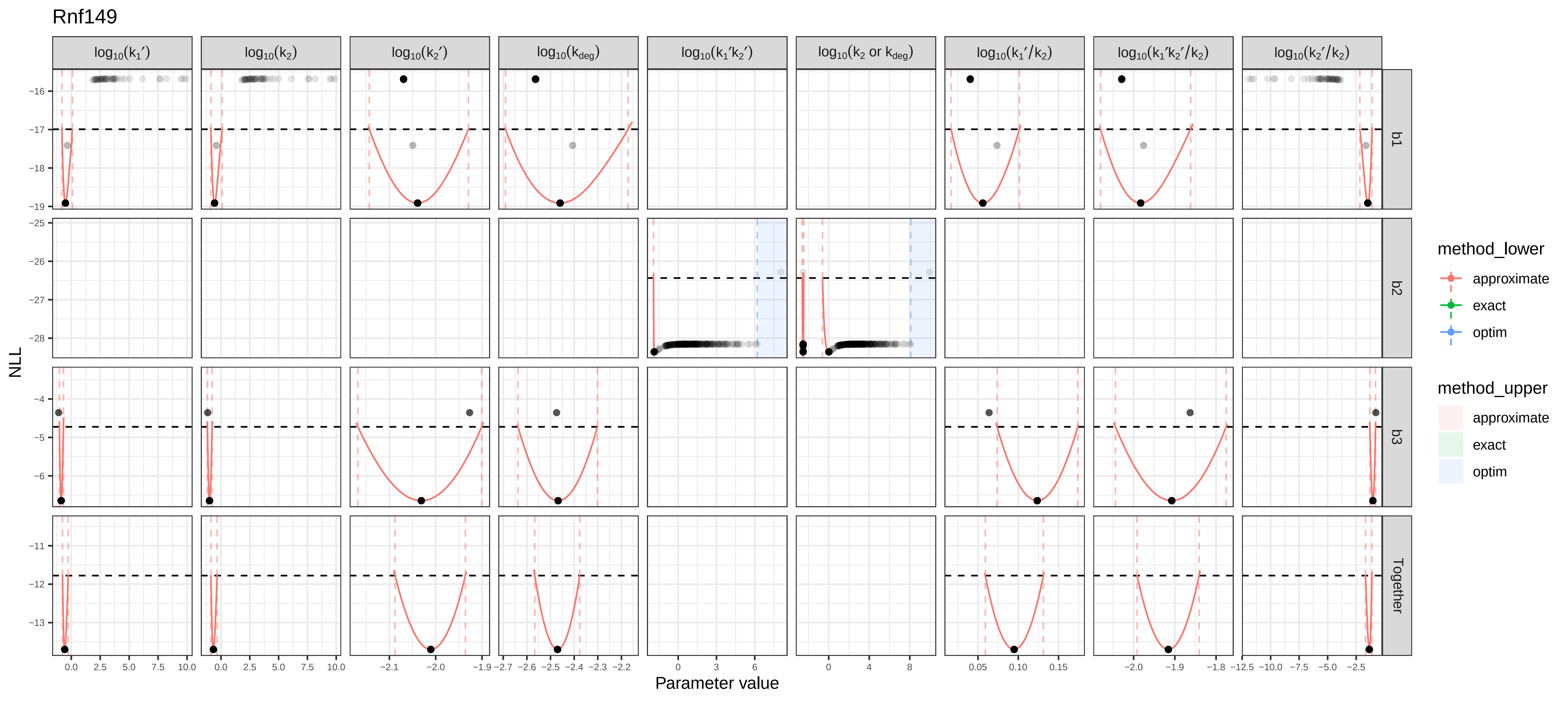

Replicate	Par	Best value	CI95 LB	CI95 UB	Method LB	Method UB
Together	$\log_{10}(k_1')$	−0.5667	−0.7563	−0.2638	approximate	approximate
Together	$\log_{10}(k_2)$	−0.6617	−0.8741	−0.3353	approximate	approximate
Together	$\log_{10}(k_2')$	−2.011	−2.088	−1.936	approximate	approximate
Together	$\log_{10}(k_{deg})$	−2.47	−2.567	−2.376	approximate	approximate
Together	$\log_{10}(k_1'/k_2)$	0.09491	0.05911	0.1312	approximate	approximate
Together	$\log_{10}(k_1'k_2'/k_2)$	−1.916	−1.992	−1.841	approximate	approximate
Together	$\log_{10}(k_2'/k_2)$	−1.349	−1.68	−1.12	approximate	approximate
b1	$\log_{10}(k_1')$	−0.5009	−0.8065	0.1032	approximate	approximate
b1	$\log_{10}(k_2)$	−0.5569	−0.8726	0.1056	approximate	approximate
b1	$\log_{10}(k_2')$	−2.039	−2.143	−1.929	approximate	approximate
b1	$\log_{10}(k_{deg})$	−2.459	−2.691	−2.172	approximate	approximate
b1	$\log_{10}(k_1'/k_2)$	0.05606	0.01661	0.1014	approximate	approximate
b1	$\log_{10}(k_1'k_2'/k_2)$	−1.983	−2.08	−1.862	approximate	approximate
b1	$\log_{10}(k_2'/k_2)$	−1.482	−2.175	−1.103	approximate	approximate
b2	$\log_{10}(k_1'k_2')$	−1.881	−1.928	> 6.194	approximate	optim
b2	$\log_{10}(k_2 \text{ or } k_{deg})$	0.01141	−0.6109	> 8.094	approximate	optim
b2	$\log_{10}(k_2 \text{ or } k_{deg})$	−2.52	−2.607	−2.461	approximate	approximate
b3	$\log_{10}(k_1')$	−0.8696	−1.023	−0.6726	approximate	approximate
b3	$\log_{10}(k_2)$	−0.9932	−1.176	−0.7672	approximate	approximate
b3	$\log_{10}(k_2')$	−2.031	−2.168	−1.901	approximate	approximate
b3	$\log_{10}(k_{deg})$	−2.468	−2.638	−2.302	approximate	approximate
b3	$\log_{10}(k_1'/k_2)$	0.1235	0.0737	0.1738	approximate	approximate
b3	$\log_{10}(k_1'k_2'/k_2)$	−1.907	−2.044	−1.776	approximate	approximate
b3	$\log_{10}(k_2'/k_2)$	−1.038	−1.299	−0.8019	approximate	approximate

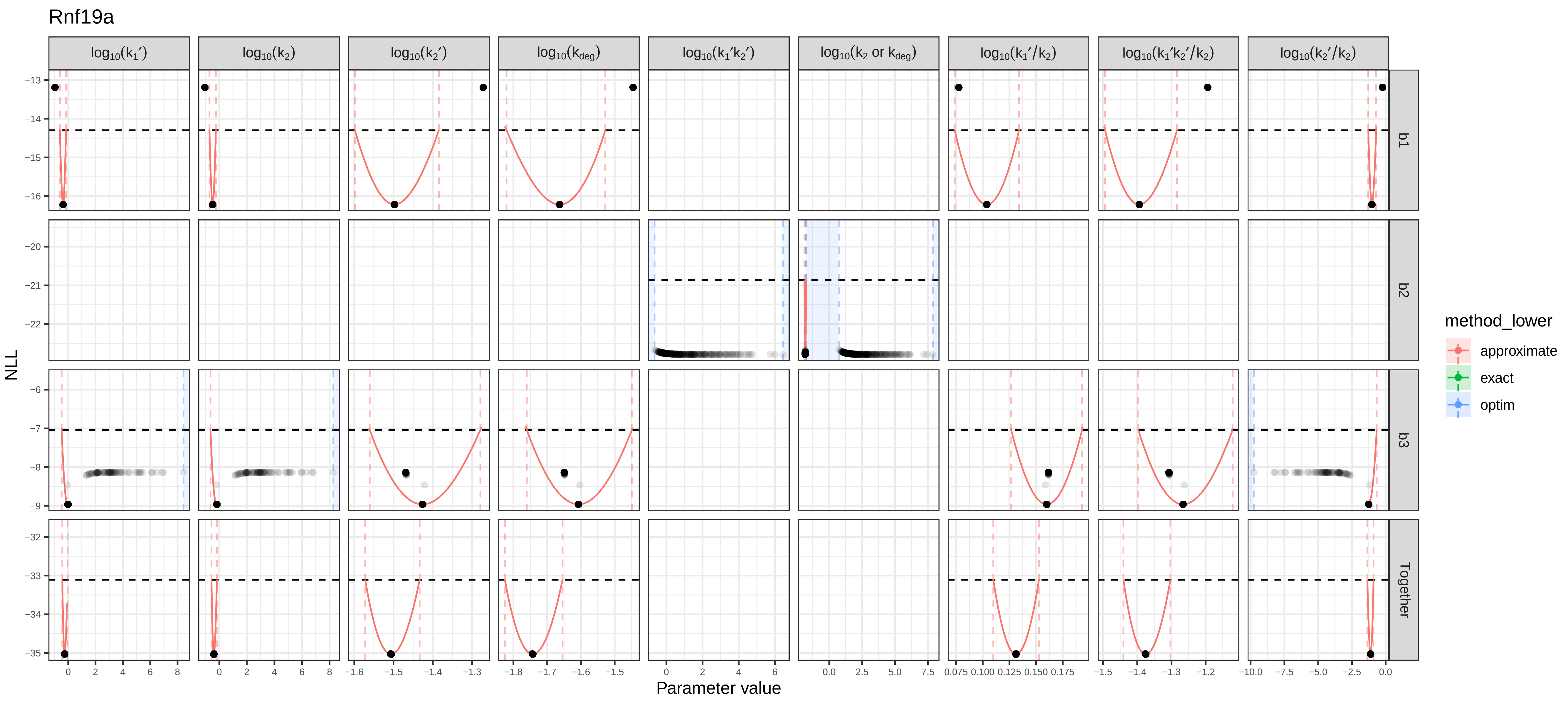

Replicate	Par	Best value	CI95 LB	CI95 UB	Method LB	Method UB
Together	$\log_{10}(k_1')$	−0.2586	−0.4309	−0.0437	approximate	approximate
Together	$\log_{10}(k_2)$	−0.3898	−0.5659	−0.1722	approximate	approximate
Together	$\log_{10}(k_2')$	−1.506	−1.572	−1.434	approximate	approximate
Together	$\log_{10}(k_{deg})$	−1.743	−1.825	−1.654	approximate	approximate
Together	$\log_{10}(k_1'/k_2)$	0.1312	0.1099	0.1527	approximate	approximate
Together	$\log_{10}(k_1'k_2'/k_2)$	−1.375	−1.44	−1.303	approximate	approximate
Together	$\log_{10}(k_2'/k_2)$	−1.116	−1.357	−0.8983	approximate	approximate
b1	$\log_{10}(k_1')$	−0.372	−0.6091	−0.1557	approximate	approximate
b1	$\log_{10}(k_2)$	−0.4757	−0.7116	−0.2465	approximate	approximate
b1	$\log_{10}(k_2')$	−1.498	−1.598	−1.385	approximate	approximate
b1	$\log_{10}(k_{deg})$	−1.663	−1.82	−1.528	approximate	approximate
b1	$\log_{10}(k_1'/k_2)$	0.1037	0.07364	0.134	approximate	approximate
b1	$\log_{10}(k_1'k_2'/k_2)$	−1.394	−1.494	−1.284	approximate	approximate
b1	$\log_{10}(k_2'/k_2)$	−1.022	−1.289	−0.7029	approximate	approximate
b2	$\log_{10}(k_1'k_2')$	3.709	< −0.6559	> 6.446	optim	optim
b2	$\log_{10}(k_2 \text{ or } k_{deg})$	5.135	0.7663	> 7.872	optim	optim
b2	$\log_{10}(k_2 \text{ or } k_{deg})$	−1.814	−1.878	−1.751	approximate	approximate
b3	$\log_{10}(k_1')$	−0.01445	−0.4772	> 8.434	approximate	optim
b3	$\log_{10}(k_2)$	−0.1744	−0.6376	> 8.273	approximate	optim
b3	$\log_{10}(k_2')$	−1.426	−1.561	−1.279	approximate	approximate
b3	$\log_{10}(k_{deg})$	−1.607	−1.76	−1.449	approximate	approximate
b3	$\log_{10}(k_1'/k_2)$	0.16	0.1266	0.1932	approximate	approximate
b3	$\log_{10}(k_1'k_2'/k_2)$	−1.266	−1.397	−1.121	approximate	approximate
b3	$\log_{10}(k_2'/k_2)$	−1.252	< −9.741	−0.6617	optim	approximate

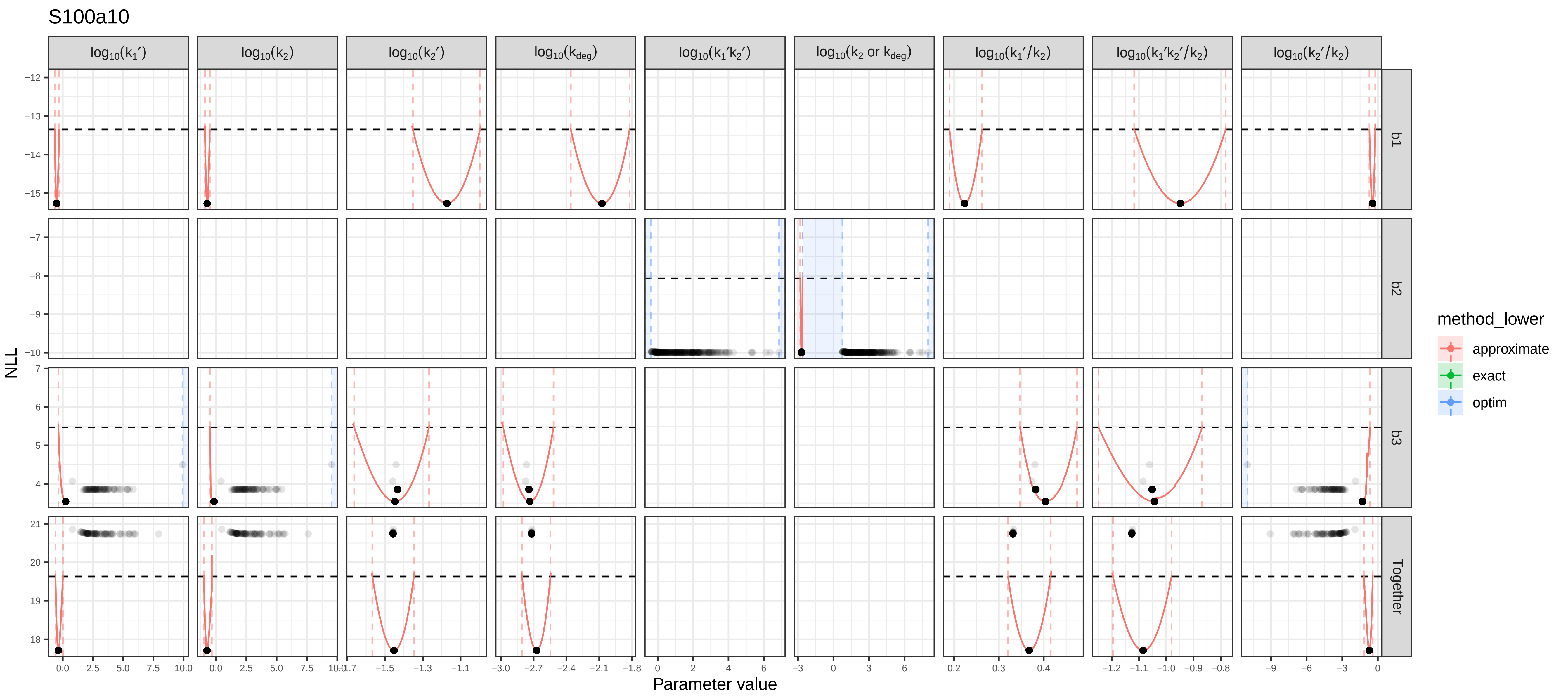

Replicate	Par	Best value	CI95 LB	CI95 UB	Method LB	Method UB
Together	$\log_{10}(k_1')$	−0.3652	−0.6015	0.01589	approximate	approximate
Together	$\log_{10}(k_2)$	−0.733	−0.9973	−0.3431	approximate	approximate
Together	$\log_{10}(k_2')$	−1.452	−1.567	−1.347	approximate	approximate
Together	$\log_{10}(k_{deg})$	−2.672	−2.807	−2.546	approximate	approximate
Together	$\log_{10}(k_1'/k_2)$	0.3678	0.3203	0.4156	approximate	approximate
Together	$\log_{10}(k_1'k_2'/k_2)$	−1.085	−1.195	−0.9804	approximate	approximate
Together	$\log_{10}(k_2'/k_2)$	−0.7194	−1.144	−0.431	approximate	approximate
b1	$\log_{10}(k_1')$	−0.5013	−0.6644	−0.2989	approximate	approximate
b1	$\log_{10}(k_2)$	−0.7252	−0.9072	−0.5082	approximate	approximate
b1	$\log_{10}(k_2')$	−1.172	−1.353	−0.9971	approximate	approximate
b1	$\log_{10}(k_{deg})$	−2.075	−2.36	−1.823	approximate	approximate
b1	$\log_{10}(k_1'/k_2)$	0.224	0.19	0.2628	approximate	approximate
b1	$\log_{10}(k_1'k_2'/k_2)$	−0.9484	−1.117	−0.7818	approximate	approximate
b1	$\log_{10}(k_2'/k_2)$	−0.4472	−0.7098	−0.214	approximate	approximate
b2	$\log_{10}(k_1'k_2')$	2.888	< −0.371	> 6.854	optim	optim
b2	$\log_{10}(k_2 \text{ or } k_{deg})$	4.013	0.7525	> 7.98	optim	optim
b2	$\log_{10}(k_2 \text{ or } k_{deg})$	−2.698	−2.801	−2.61	approximate	approximate
b3	$\log_{10}(k_1')$	0.2406	−0.359	> 9.914	approximate	optim
b3	$\log_{10}(k_2)$	−0.1635	−0.4756	> 9.533	approximate	optim
b3	$\log_{10}(k_2')$	−1.447	−1.663	−1.268	approximate	approximate
b3	$\log_{10}(k_{deg})$	−2.735	−2.979	−2.518	approximate	approximate
b3	$\log_{10}(k_1'/k_2)$	0.404	0.3472	0.4744	approximate	approximate
b3	$\log_{10}(k_1'k_2'/k_2)$	−1.043	−1.248	−0.8683	approximate	approximate
b3	$\log_{10}(k_2'/k_2)$	−1.284	< −10.97	−0.657	optim	approximate

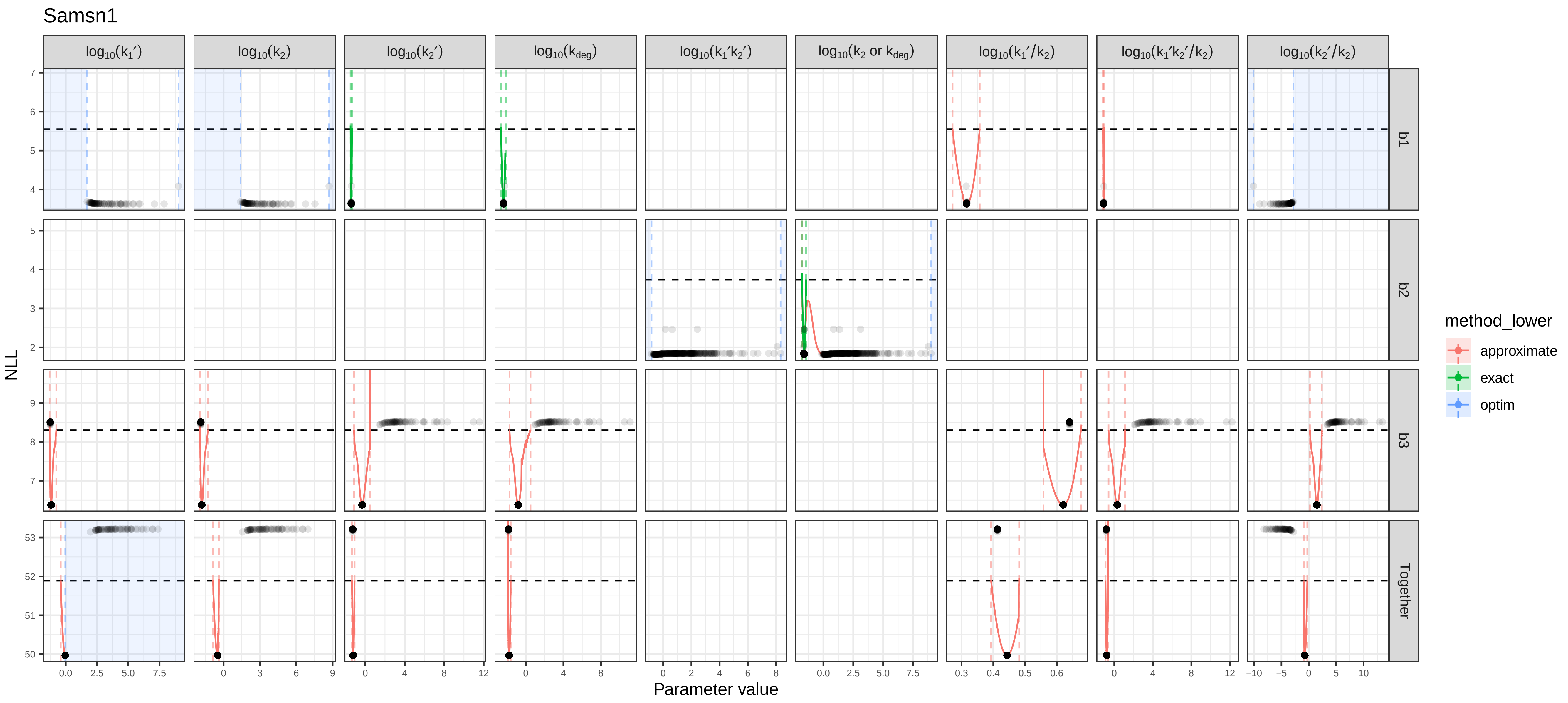

Replicate	Par	Best value	CI95 LB	CI95 UB	Method LB	Method UB
Together	$\log_{10}(k_1')$	−0.03939	−0.4005	> −0.03545	approximate	optim
Together	$\log_{10}(k_2)$	−0.4828	−0.8689	−0.3928	approximate	approximate
Together	$\log_{10}(k_2')$	−1.222	−1.342	−1.08	approximate	approximate
Together	$\log_{10}(k_{deg})$	−1.778	−1.871	−1.611	approximate	approximate
Together	$\log_{10}(k_1'/k_2)$	0.4434	0.393	0.4816	approximate	approximate
Together	$\log_{10}(k_1'k_2'/k_2)$	−0.7783	−0.907	−0.6438	approximate	approximate
Together	$\log_{10}(k_2'/k_2)$	−0.7389	−0.9125	−0.2769	approximate	approximate
b1	$\log_{10}(k_1')$	5.413	< 1.715	> 9.022	optim	optim
b1	$\log_{10}(k_2)$	5.097	< 1.398	> 8.707	optim	optim
b1	$\log_{10}(k_2')$	−1.418	−1.489	−1.348	exact	exact
b1	$\log_{10}(k_{deg})$	−2.371	−2.639	−2.135	exact	exact
b1	$\log_{10}(k_1'/k_2)$	0.3162	0.2718	0.3573	approximate	approximate
b1	$\log_{10}(k_1'k_2'/k_2)$	−1.101	−1.165	−1.057	approximate	approximate
b1	$\log_{10}(k_2'/k_2)$	−6.515	< −10.1	> −2.818	optim	optim
b2	$\log_{10}(k_1'k_2')$	−0.5801	< −0.8201	> 8.314	optim	optim
b2	$\log_{10}(k_2 \text{ or } k_{deg})$	0.1049	−1.786	> 9.014	approximate	optim
b2	$\log_{10}(k_2 \text{ or } k_{deg})$	−1.615	−1.781	−1.453	exact	exact
b3	$\log_{10}(k_1')$	−1.176	−1.29	−0.7533	approximate	approximate
b3	$\log_{10}(k_2)$	−1.795	−1.94	−1.296	approximate	approximate
b3	$\log_{10}(k_2')$	−0.3253	−1.138	0.4592	approximate	approximate
b3	$\log_{10}(k_{deg})$	−0.8213	−1.735	0.5027	approximate	approximate
b3	$\log_{10}(k_1'/k_2)$	0.6196	0.558	0.6759	approximate	approximate
b3	$\log_{10}(k_1'k_2'/k_2)$	0.2943	−0.5907	1.122	approximate	approximate
b3	$\log_{10}(k_2'/k_2)$	1.47	0.1887	2.363	approximate	approximate

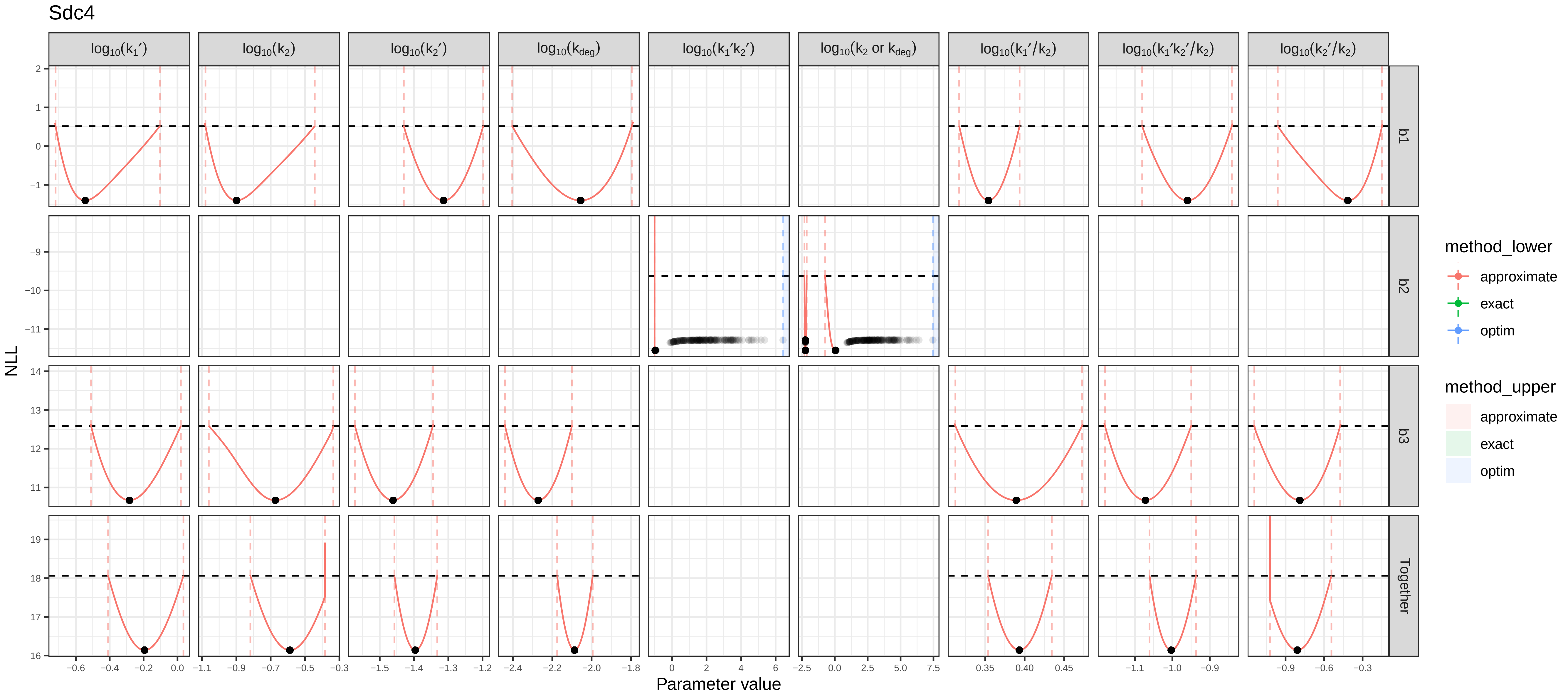

Replicate	Par	Best value	CI95 LB	CI95 UB	Method LB	Method UB
Together	$\log_{10}(k_1')$	−0.1943	−0.4095	0.0351	approximate	approximate
Together	$\log_{10}(k_2)$	−0.5877	−0.8182	−0.3844	approximate	approximate
Together	$\log_{10}(k_2')$	−1.396	−1.457	−1.332	approximate	approximate
Together	$\log_{10}(k_{deg})$	−2.087	−2.175	−1.994	approximate	approximate
Together	$\log_{10}(k_1'/k_2)$	0.3934	0.3537	0.4344	approximate	approximate
Together	$\log_{10}(k_1'k_2'/k_2)$	−1.003	−1.061	−0.9367	approximate	approximate
Together	$\log_{10}(k_2'/k_2)$	−0.8085	−1.021	−0.5427	approximate	approximate
b1	$\log_{10}(k_1')$	−0.5444	−0.7194	−0.1043	approximate	approximate
b1	$\log_{10}(k_2)$	−0.8985	−1.08	−0.4433	approximate	approximate
b1	$\log_{10}(k_2')$	−1.314	−1.43	−1.198	approximate	approximate
b1	$\log_{10}(k_{deg})$	−2.056	−2.404	−1.795	approximate	approximate
b1	$\log_{10}(k_1'/k_2)$	0.3542	0.3171	0.3936	approximate	approximate
b1	$\log_{10}(k_1'k_2'/k_2)$	−0.9595	−1.08	−0.8411	approximate	approximate
b1	$\log_{10}(k_2'/k_2)$	−0.4151	−0.9609	−0.1483	approximate	approximate
b2	$\log_{10}(k_1'k_2')$	−0.9696	−1.007	> 6.428	approximate	optim
b2	$\log_{10}(k_2 \text{ or } k_{deg})$	0.04789	−0.7399	> 7.457	approximate	optim
b2	$\log_{10}(k_2 \text{ or } k_{deg})$	−2.23	−2.306	−2.135	approximate	approximate
b3	$\log_{10}(k_1')$	−0.2831	−0.5101	0.01985	approximate	approximate
b3	$\log_{10}(k_2)$	−0.6726	−1.06	−0.3353	approximate	approximate
b3	$\log_{10}(k_2')$	−1.461	−1.572	−1.345	approximate	approximate
b3	$\log_{10}(k_{deg})$	−2.271	−2.44	−2.1	approximate	approximate
b3	$\log_{10}(k_1'/k_2)$	0.3894	0.3122	0.4727	approximate	approximate
b3	$\log_{10}(k_1'k_2'/k_2)$	−1.072	−1.18	−0.9495	approximate	approximate
b3	$\log_{10}(k_2'/k_2)$	−0.7886	−1.144	−0.4746	approximate	approximate

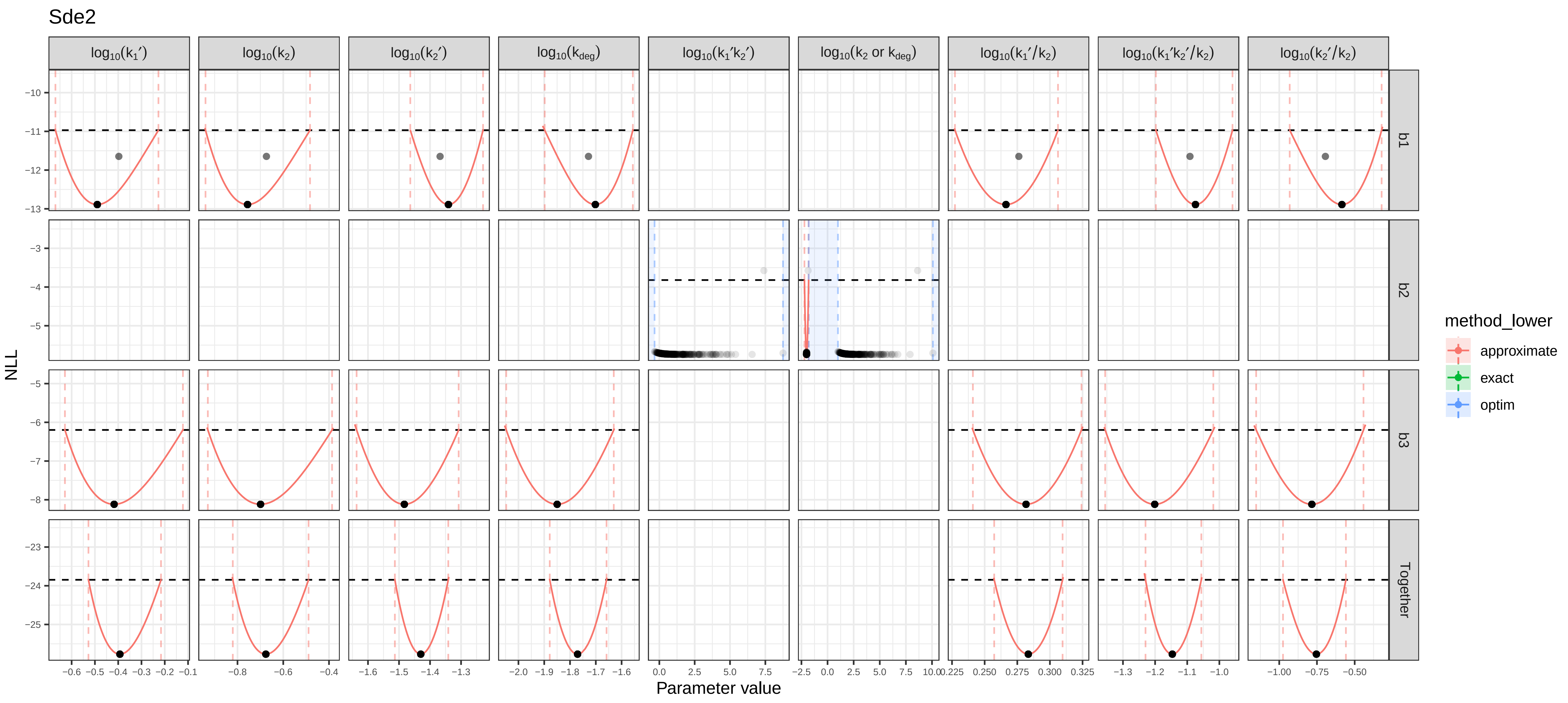

Replicate	Par	Best value	CI95 LB	CI95 UB	Method LB	Method UB
Together	$\log_{10}(k_1')$	−0.3926	−0.5278	−0.2161	approximate	approximate
Together	$\log_{10}(k_2)$	−0.676	−0.8205	−0.4894	approximate	approximate
Together	$\log_{10}(k_2')$	−1.43	−1.513	−1.341	approximate	approximate
Together	$\log_{10}(k_{deg})$	−1.771	−1.879	−1.658	approximate	approximate
Together	$\log_{10}(k_1'/k_2)$	0.2834	0.2573	0.3097	approximate	approximate
Together	$\log_{10}(k_1'k_2'/k_2)$	−1.146	−1.23	−1.056	approximate	approximate
Together	$\log_{10}(k_2'/k_2)$	−0.7538	−0.9749	−0.5581	approximate	approximate
b1	$\log_{10}(k_1')$	−0.4896	−0.6697	−0.2271	approximate	approximate
b1	$\log_{10}(k_2)$	−0.756	−0.9398	−0.4829	approximate	approximate
b1	$\log_{10}(k_2')$	−1.341	−1.464	−1.229	approximate	approximate
b1	$\log_{10}(k_{deg})$	−1.702	−1.898	−1.556	approximate	approximate
b1	$\log_{10}(k_1'/k_2)$	0.2663	0.2273	0.3062	approximate	approximate
b1	$\log_{10}(k_1'k_2'/k_2)$	−1.074	−1.198	−0.959	approximate	approximate
b1	$\log_{10}(k_2'/k_2)$	−0.5848	−0.9306	−0.3214	approximate	approximate
b2	$\log_{10}(k_1'k_2')$	4.777	< −0.3381	> 8.751	optim	optim
b2	$\log_{10}(k_2 \text{ or } k_{deg})$	6.112	0.9962	> 10.09	optim	optim
b2	$\log_{10}(k_2 \text{ or } k_{deg})$	−2.011	−2.212	−1.811	approximate	approximate
b3	$\log_{10}(k_1')$	−0.4174	−0.6288	−0.122	approximate	approximate
b3	$\log_{10}(k_2)$	−0.6991	−0.9296	−0.3865	approximate	approximate
b3	$\log_{10}(k_2')$	−1.483	−1.637	−1.308	approximate	approximate
b3	$\log_{10}(k_{deg})$	−1.85	−2.047	−1.63	approximate	approximate
b3	$\log_{10}(k_1'/k_2)$	0.2817	0.241	0.3242	approximate	approximate
b3	$\log_{10}(k_1'k_2'/k_2)$	−1.201	−1.355	−1.019	approximate	approximate
b3	$\log_{10}(k_2'/k_2)$	−0.7837	−1.153	−0.4415	approximate	approximate

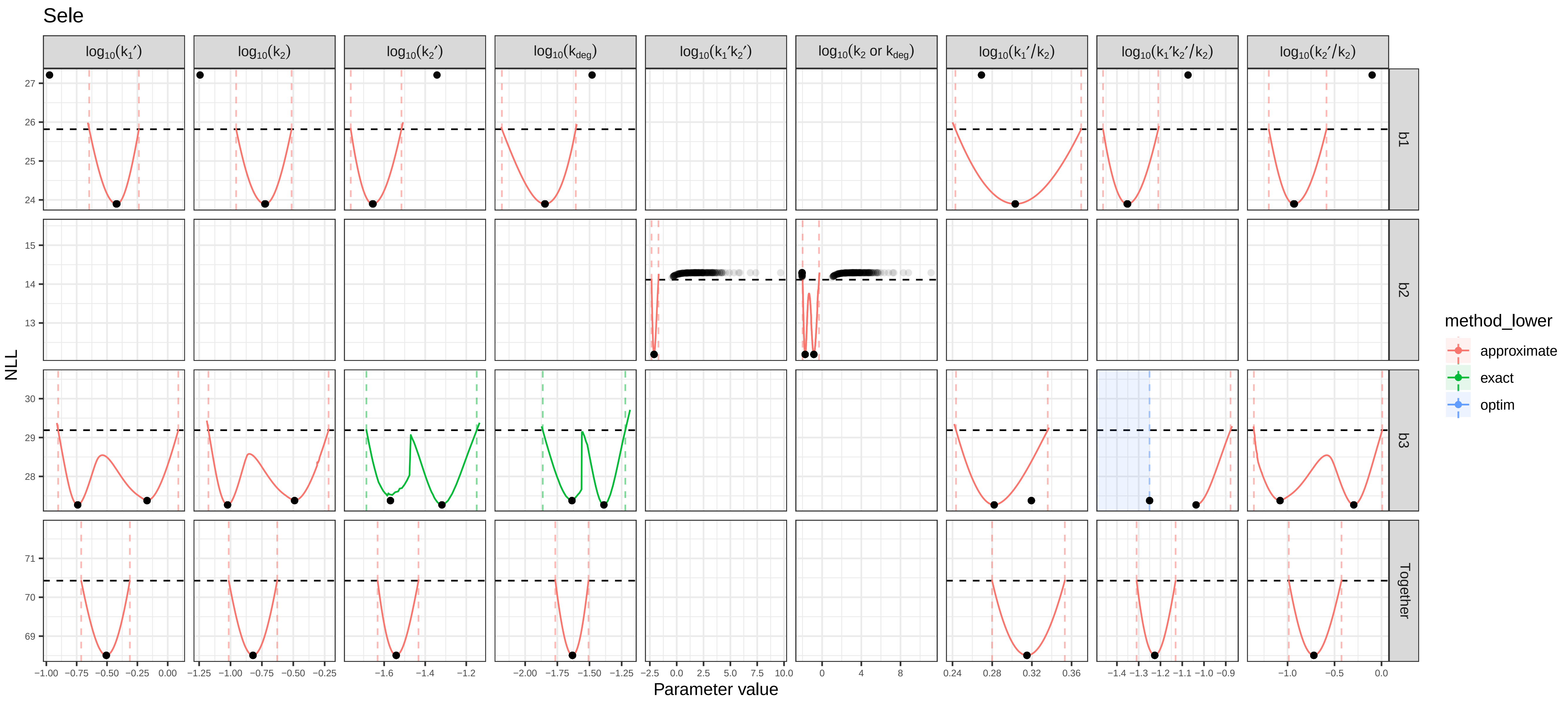

Replicate	Par	Best value	CI95 LB	CI95 UB	Method LB	Method UB
Together	$\log_{10}(k_1')$	−0.5056	−0.713	−0.3119	approximate	approximate
Together	$\log_{10}(k_2)$	−0.8206	−1.014	−0.6282	approximate	approximate
Together	$\log_{10}(k_2')$	−1.541	−1.632	−1.433	approximate	approximate
Together	$\log_{10}(k_{deg})$	−1.633	−1.766	−1.508	approximate	approximate
Together	$\log_{10}(k_1'/k_2)$	0.315	0.2799	0.3532	approximate	approximate
Together	$\log_{10}(k_1'k_2'/k_2)$	−1.226	−1.31	−1.13	approximate	approximate
Together	$\log_{10}(k_2'/k_2)$	−0.7209	−0.9868	−0.4261	approximate	approximate
b1	$\log_{10}(k_1')$	−0.4223	−0.6471	−0.2372	approximate	approximate
b1	$\log_{10}(k_2)$	−0.7255	−0.9554	−0.5133	approximate	approximate
b1	$\log_{10}(k_2')$	−1.655	−1.763	−1.516	approximate	approximate
b1	$\log_{10}(k_{deg})$	−1.845	−2.181	−1.607	approximate	approximate
b1	$\log_{10}(k_1'/k_2)$	0.3032	0.2429	0.3697	approximate	approximate
b1	$\log_{10}(k_1'k_2'/k_2)$	−1.351	−1.464	−1.21	approximate	approximate
b1	$\log_{10}(k_2'/k_2)$	−0.9292	−1.2	−0.5857	approximate	approximate
b2	$\log_{10}(k_1'k_2')$	−2.108	−2.343	−1.703	approximate	approximate
b2	$\log_{10}(k_2 \text{ or } k_{deg})$	−0.8441	−1.989	−0.3178	approximate	approximate
b2	$\log_{10}(k_2 \text{ or } k_{deg})$	−1.73	−1.989	−0.3178	approximate	approximate
b3	$\log_{10}(k_1')$	−0.7412	−0.9029	0.08738	approximate	approximate
b3	$\log_{10}(k_2)$	−1.023	−1.176	−0.2187	approximate	approximate
b3	$\log_{10}(k_2')$	−1.319	−1.687	−1.149	exact	exact
b3	$\log_{10}(k_{deg})$	−1.388	−1.864	−1.222	exact	exact
b3	$\log_{10}(k_1'/k_2)$	0.282	0.2435	0.336	approximate	approximate
b3	$\log_{10}(k_1'k_2'/k_2)$	−1.037	< -1.25	−0.8783	optim	approximate
b3	$\log_{10}(k_2'/k_2)$	−0.2953	−1.358	0.007836	approximate	approximate

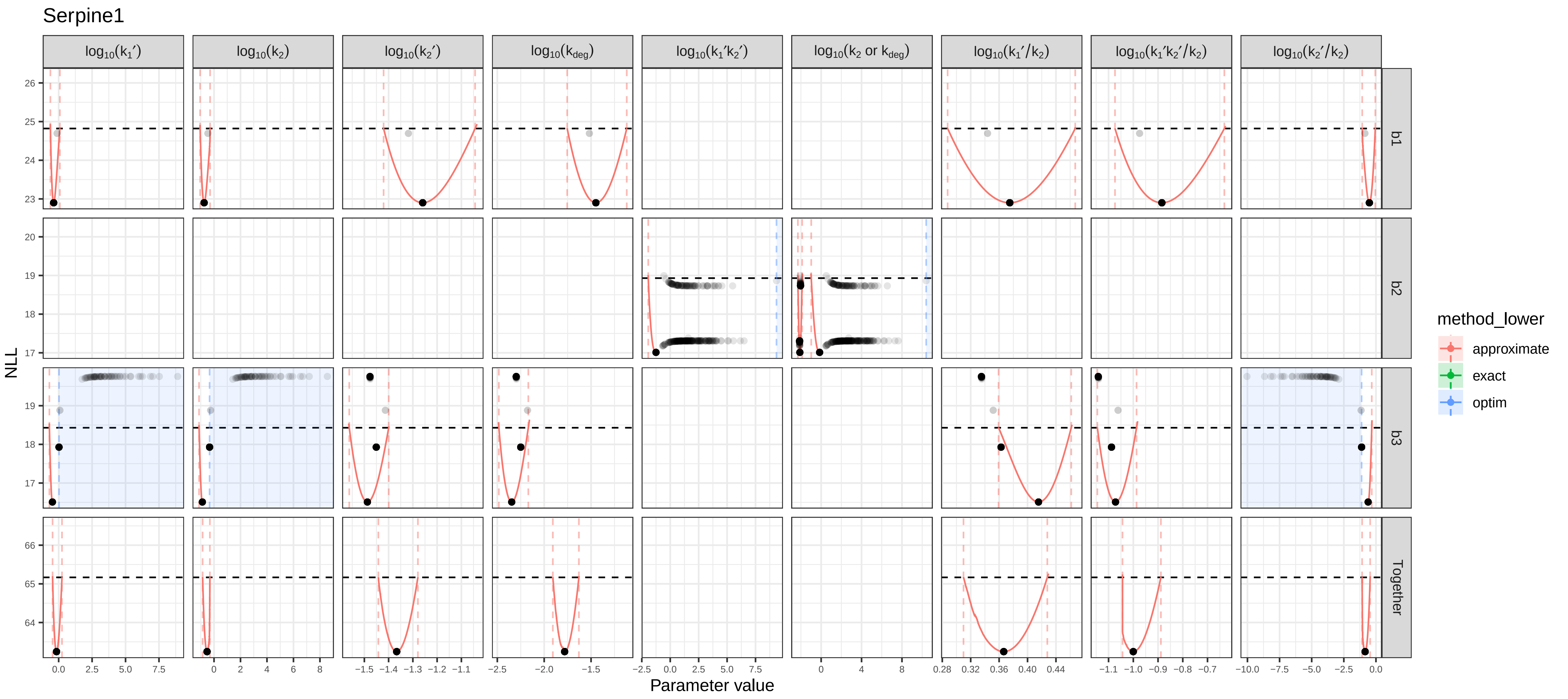

Replicate	Par	Best value	CI95 LB	CI95 UB	Method LB	Method UB
Together	$\log_{10}(k_1')$	−0.1588	−0.4533	0.2491	approximate	approximate
Together	$\log_{10}(k_2)$	−0.5252	−0.8602	−0.3124	approximate	approximate
Together	$\log_{10}(k_2')$	−1.367	−1.442	−1.279	approximate	approximate
Together	$\log_{10}(k_{deg})$	−1.782	−1.908	−1.63	approximate	approximate
Together	$\log_{10}(k_1'/k_2)$	0.3665	0.3099	0.4278	approximate	approximate
Together	$\log_{10}(k_1'k_2'/k_2)$	−1	−1.044	−0.8884	approximate	approximate
Together	$\log_{10}(k_2'/k_2)$	−0.8414	−1.077	−0.4506	approximate	approximate
b1	$\log_{10}(k_1')$	−0.3722	−0.6172	0.09282	approximate	approximate
b1	$\log_{10}(k_2)$	−0.7472	−1.05	−0.2971	approximate	approximate
b1	$\log_{10}(k_2')$	−1.259	−1.421	−1.043	approximate	approximate
b1	$\log_{10}(k_{deg})$	−1.448	−1.755	−1.119	approximate	approximate
b1	$\log_{10}(k_1'/k_2)$	0.375	0.2875	0.4671	approximate	approximate
b1	$\log_{10}(k_1'k_2'/k_2)$	−0.8844	−1.074	−0.6319	approximate	approximate
b1	$\log_{10}(k_2'/k_2)$	−0.5121	−1.069	−0.05057	approximate	approximate
b2	$\log_{10}(k_1'k_2')$	−1.254	−1.933	> 9.342	approximate	optim
b2	$\log_{10}(k_2 \text{ or } k_{deg})$	−0.1519	−0.9815	> 10.4	approximate	optim
b2	$\log_{10}(k_2 \text{ or } k_{deg})$	−2.107	−2.301	−1.878	approximate	approximate
b3	$\log_{10}(k_1')$	−0.4645	−0.6922	> 0.02834	approximate	optim
b3	$\log_{10}(k_2)$	−0.88	−1.13	> −0.3343	approximate	optim
b3	$\log_{10}(k_2')$	−1.487	−1.562	−1.4	approximate	approximate
b3	$\log_{10}(k_{deg})$	−2.346	−2.483	−2.169	approximate	approximate
b3	$\log_{10}(k_1'/k_2)$	0.4155	0.3592	0.4615	approximate	approximate
b3	$\log_{10}(k_1'k_2'/k_2)$	−1.072	−1.145	−0.9868	approximate	approximate
b3	$\log_{10}(k_2'/k_2)$	−0.6074	< −1.117	−0.3258	optim	approximate

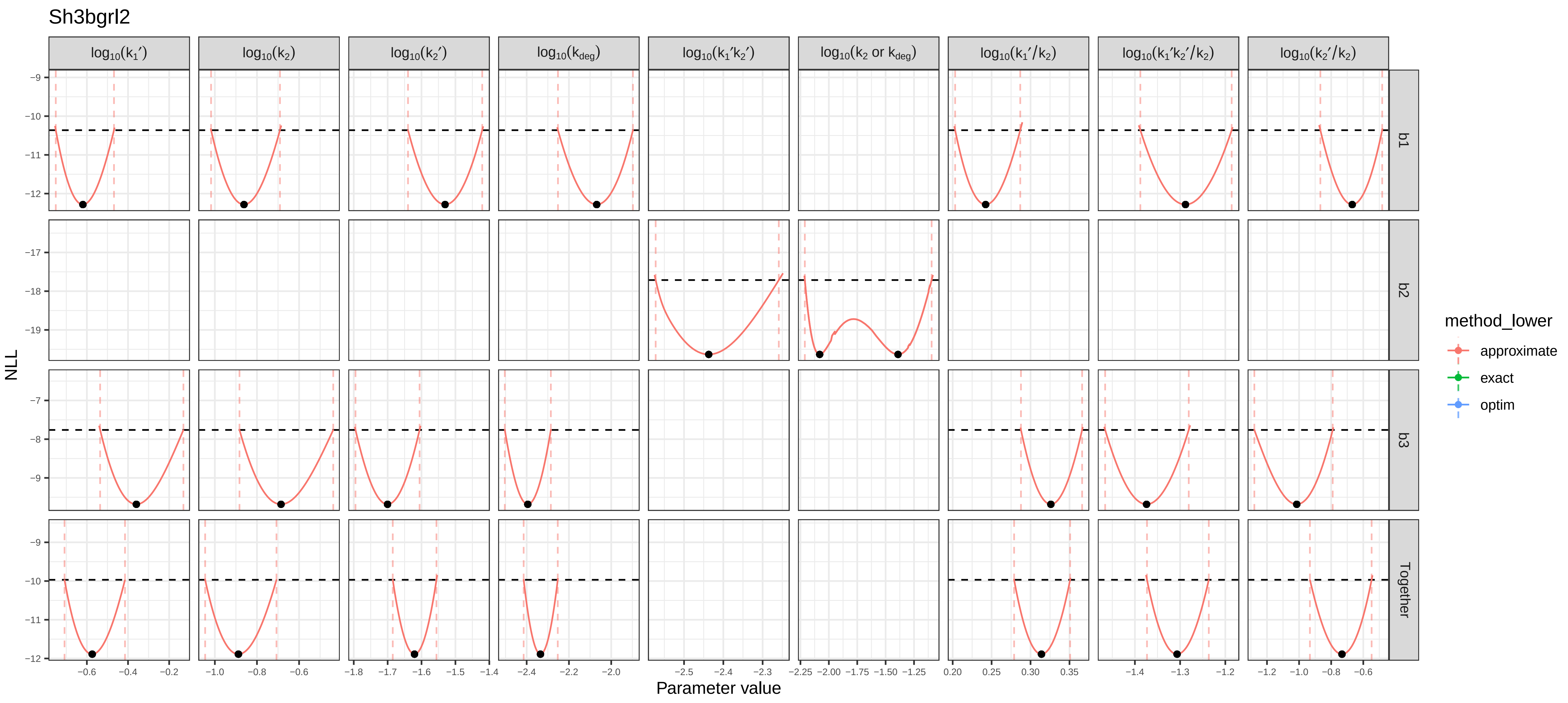

Replicate	Par	Best value	CI95 LB	CI95 UB	Method LB	Method UB
Together	$log_{10}(k_1')$	−0.5739	−0.7087	−0.4147	approximate	approximate
Together	$log_{10}(k_2)$	−0.8879	−1.046	−0.7073	approximate	approximate
Together	$log_{10}(k_2')$	−1.621	−1.685	−1.556	approximate	approximate
Together	$log_{10}(k_{deg})$	−2.335	−2.414	−2.253	approximate	approximate
Together	$log_{10}(k_1'/k_2)$	0.314	0.2789	0.3508	approximate	approximate
Together	$log_{10}(k_1'k_2'/k_2)$	−1.307	−1.373	−1.237	approximate	approximate
Together	$log_{10}(k_2'/k_2)$	−0.7329	−0.9325	−0.548	approximate	approximate
b1	$log_{10}(k_1')$	−0.6193	−0.7511	−0.4682	approximate	approximate
b1	$log_{10}(k_2)$	−0.8616	−1.018	−0.6907	approximate	approximate
b1	$log_{10}(k_2')$	−1.53	−1.64	−1.421	approximate	approximate
b1	$log_{10}(k_{deg})$	−2.069	−2.252	−1.897	approximate	approximate
b1	$log_{10}(k_1'/k_2)$	0.2423	0.2032	0.2868	approximate	approximate
b1	$log_{10}(k_1'k_2'/k_2)$	−1.288	−1.388	−1.186	approximate	approximate
b1	$log_{10}(k_2'/k_2)$	−0.6688	−0.8674	−0.4821	approximate	approximate
b2	$log_{10}(k_1'k_2')$	−2.437	−2.572	−2.259	approximate	approximate
b2	$log_{10}(k_2$ or $k_{deg})$	−1.391	−2.211	−1.095	approximate	approximate
b2	$log_{10}(k_2$ or $k_{deg})$	−2.081	−2.211	−1.095	approximate	approximate
b3	$log_{10}(k_1')$	−0.3596	−0.5356	−0.1307	approximate	approximate
b3	$log_{10}(k_2)$	−0.6857	−0.883	−0.4378	approximate	approximate
b3	$log_{10}(k_2')$	−1.7	−1.795	−1.606	approximate	approximate
b3	$log_{10}(k_{deg})$	−2.395	−2.503	−2.286	approximate	approximate
b3	$log_{10}(k_1'/k_2)$	0.3261	0.2877	0.3662	approximate	approximate
b3	$log_{10}(k_1'k_2'/k_2)$	−1.374	−1.466	−1.281	approximate	approximate
b3	$log_{10}(k_2'/k_2)$	−1.015	−1.279	−0.7904	approximate	approximate

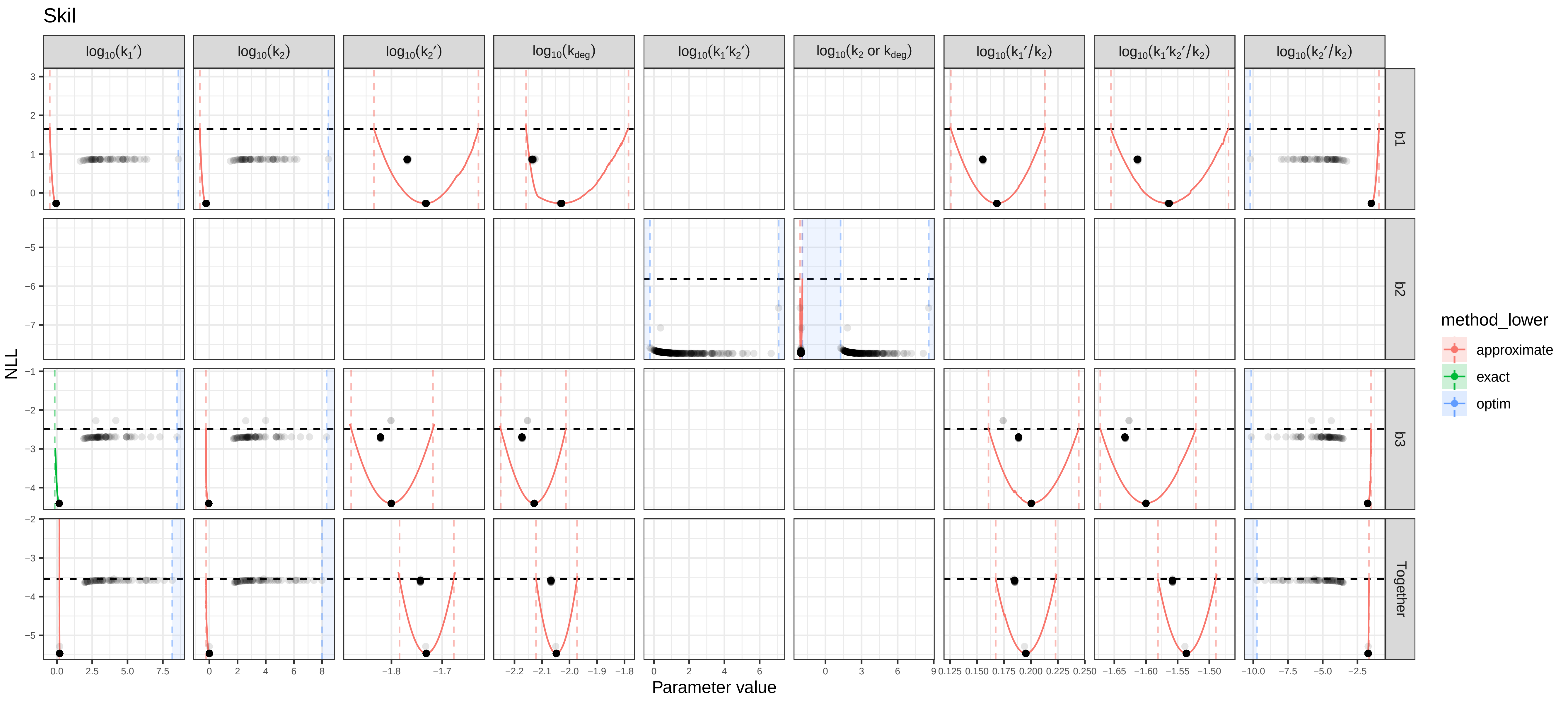

Replicate	Par	Best value	CI95 LB	CI95 UB	Method LB	Method UB
Together	$\log_{10}(k_1')$	0.1964	0.1742	> 8.169	approximate	optim
Together	$\log_{10}(k_2)$	0.001171	−0.2201	> 7.985	approximate	optim
Together	$\log_{10}(k_2')$	−1.731	−1.784	−1.677	approximate	approximate
Together	$\log_{10}(k_{deg})$	−2.048	−2.122	−1.972	approximate	approximate
Together	$\log_{10}(k_1'/k_2)$	0.1953	0.1673	0.2228	approximate	approximate
Together	$\log_{10}(k_1'k_2'/k_2)$	−1.536	−1.581	−1.49	approximate	approximate
Together	$\log_{10}(k_2'/k_2)$	−1.733	< −9.728	−1.677	optim	approximate
b1	$\log_{10}(k_1')$	−0.05808	−0.5084	> 8.599	approximate	optim
b1	$\log_{10}(k_2)$	−0.2265	−0.6758	> 8.444	approximate	optim
b1	$\log_{10}(k_2')$	−1.732	−1.835	−1.628	approximate	approximate
b1	$\log_{10}(k_{deg})$	−2.029	−2.158	−1.785	approximate	approximate
b1	$\log_{10}(k_1'/k_2)$	0.1684	0.1257	0.2131	approximate	approximate
b1	$\log_{10}(k_1'k_2'/k_2)$	−1.564	−1.655	−1.47	approximate	approximate
b1	$\log_{10}(k_2'/k_2)$	−1.505	< −10.21	−0.9566	optim	approximate
b2	$\log_{10}(k_1'k_2')$	3.768	< −0.2273	> 7.078	optim	optim
b2	$\log_{10}(k_2 \text{ or } k_{deg})$	5.249	1.254	> 8.584	optim	optim
b2	$\log_{10}(k_2 \text{ or } k_{deg})$	−2.039	−2.116	−1.921	approximate	approximate
b3	$\log_{10}(k_1')$	0.1609	−0.158	> 8.505	exact	optim
b3	$\log_{10}(k_2)$	−0.0394	−0.243	> 8.316	approximate	optim
b3	$\log_{10}(k_2')$	−1.8	−1.879	−1.718	approximate	approximate
b3	$\log_{10}(k_{deg})$	−2.129	−2.25	−2.013	approximate	approximate
b3	$\log_{10}(k_1'/k_2)$	0.2003	0.1607	0.2443	approximate	approximate
b3	$\log_{10}(k_1'k_2'/k_2)$	−1.6	−1.672	−1.521	approximate	approximate
b3	$\log_{10}(k_2'/k_2)$	−1.761	< −10.14	−1.528	optim	approximate

Slc2a6

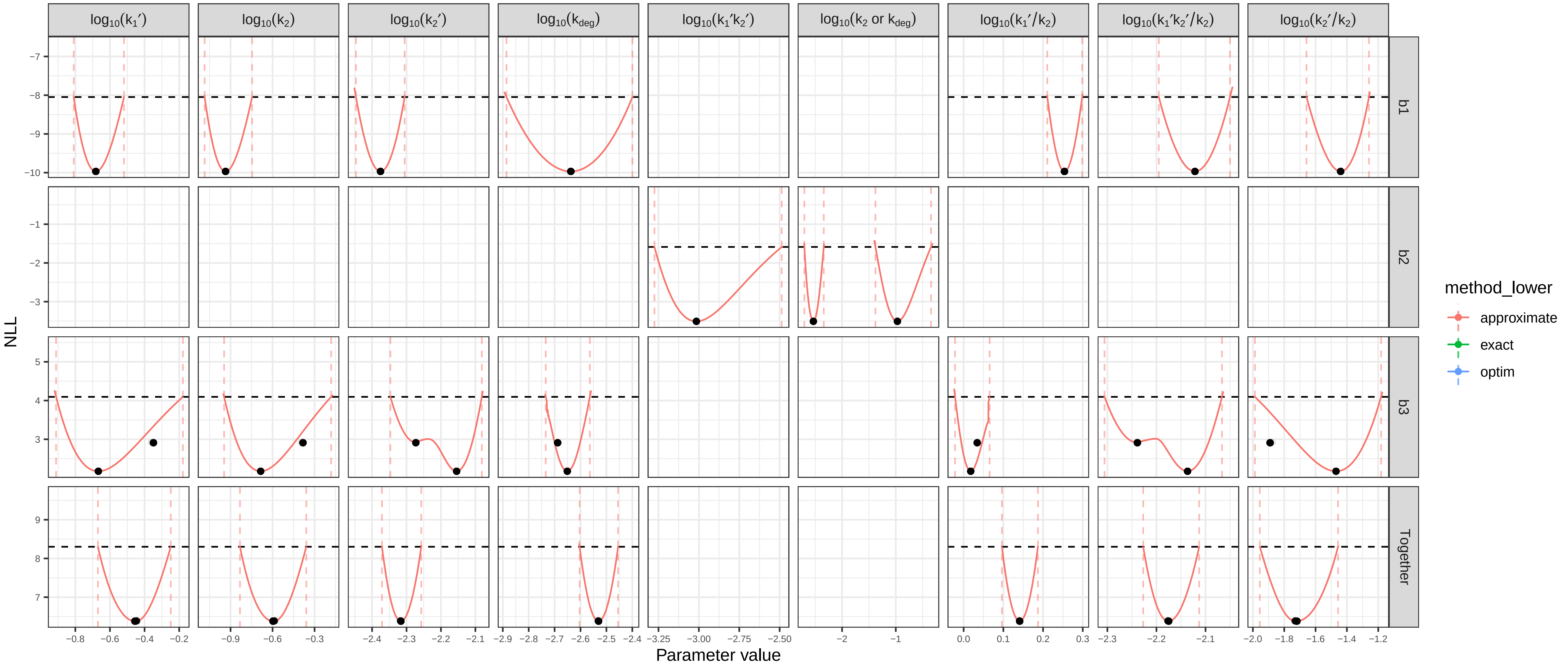

Replicate	Par	Best value	CI95 LB	CI95 UB	Method LB	Method UB
Together	$\log_{10}(k_1')$	−0.4554	−0.6694	−0.2485	approximate	approximate
Together	$\log_{10}(k_2)$	−0.5966	−0.8328	−0.3593	approximate	approximate
Together	$\log_{10}(k_2')$	−2.316	−2.371	−2.257	approximate	approximate
Together	$\log_{10}(k_{deg})$	−2.53	−2.604	−2.455	approximate	approximate
Together	$\log_{10}(k_1'/k_2)$	0.1412	0.09648	0.1871	approximate	approximate
Together	$\log_{10}(k_1'k_2'/k_2)$	−2.175	−2.227	−2.113	approximate	approximate
Together	$\log_{10}(k_2'/k_2)$	−1.72	−1.956	−1.456	approximate	approximate
b1	$\log_{10}(k_1')$	−0.6816	−0.8095	−0.5186	approximate	approximate
b1	$\log_{10}(k_2)$	−0.9356	−1.085	−0.746	approximate	approximate
b1	$\log_{10}(k_2')$	−2.375	−2.447	−2.305	approximate	approximate
b1	$\log_{10}(k_{deg})$	−2.637	−2.886	−2.4	approximate	approximate
b1	$\log_{10}(k_1'/k_2)$	0.254	0.2107	0.2985	approximate	approximate
b1	$\log_{10}(k_1'k_2'/k_2)$	−2.122	−2.195	−2.05	approximate	approximate
b1	$\log_{10}(k_2'/k_2)$	−1.44	−1.658	−1.259	approximate	approximate
b2	$\log_{10}(k_1'k_2')$	−3.016	−3.275	−2.487	approximate	approximate
b2	$\log_{10}(k_2 \text{ or } k_{deg})$	−0.9694	−1.379	−0.3426	approximate	approximate
b2	$\log_{10}(k_2 \text{ or } k_{deg})$	−2.534	−2.702	−2.341	approximate	approximate
b3	$\log_{10}(k_1')$	−0.6672	−0.9112	−0.1785	approximate	approximate
b3	$\log_{10}(k_2)$	−0.685	−0.9461	−0.1816	approximate	approximate
b3	$\log_{10}(k_2')$	−2.155	−2.347	−2.081	approximate	approximate
b3	$\log_{10}(k_{deg})$	−2.651	−2.734	−2.564	approximate	approximate
b3	$\log_{10}(k_1'/k_2)$	0.01786	−0.02161	0.06552	approximate	approximate
b3	$\log_{10}(k_1'k_2'/k_2)$	−2.137	−2.306	−2.066	approximate	approximate
b3	$\log_{10}(k_2'/k_2)$	−1.469	−1.987	−1.182	approximate	approximate

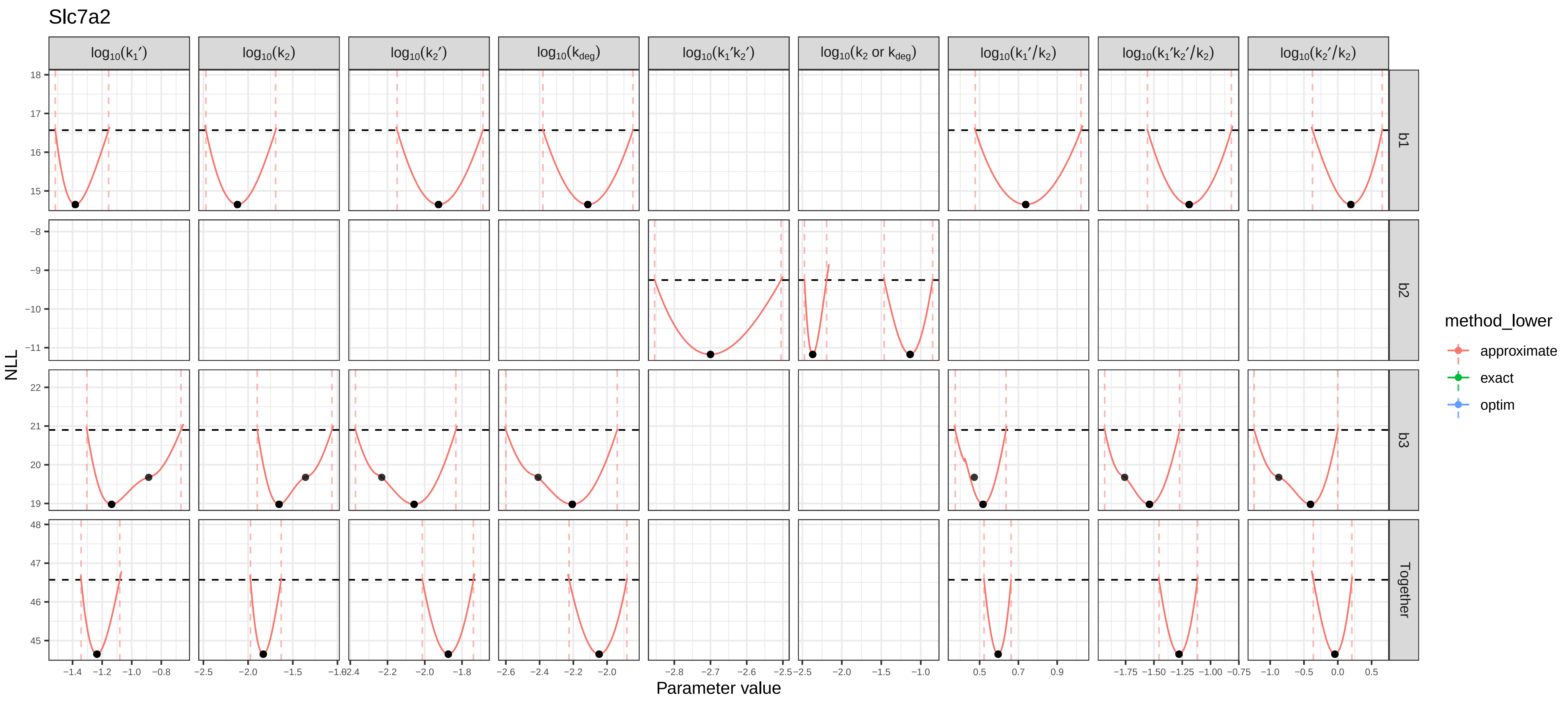

Replicate	Par	Best value	CI95 LB	CI95 UB	Method LB	Method UB
Together	$\log_{10}(k_1')$	−1.235	−1.342	−1.081	approximate	approximate
Together	$\log_{10}(k_2)$	−1.831	−1.973	−1.63	approximate	approximate
Together	$\log_{10}(k_2')$	−1.874	−2.013	−1.739	approximate	approximate
Together	$\log_{10}(k_{deg})$	−2.047	−2.225	−1.883	approximate	approximate
Together	$\log_{10}(k_1'/k_2)$	0.5956	0.5227	0.6624	approximate	approximate
Together	$\log_{10}(k_1'k_2'/k_2)$	−1.278	−1.455	−1.115	approximate	approximate
Together	$\log_{10}(k_2'/k_2)$	−0.0428	−0.3619	0.208	approximate	approximate
b1	$\log_{10}(k_1')$	−1.382	−1.517	−1.156	approximate	approximate
b1	$\log_{10}(k_2)$	−2.119	−2.472	−1.692	approximate	approximate
b1	$\log_{10}(k_2')$	−1.926	−2.148	−1.688	approximate	approximate
b1	$\log_{10}(k_{deg})$	−2.114	−2.38	−1.846	approximate	approximate
b1	$\log_{10}(k_1'/k_2)$	0.7377	0.4774	1.023	approximate	approximate
b1	$\log_{10}(k_1'k_2'/k_2)$	−1.189	−1.557	−0.8158	approximate	approximate
b1	$\log_{10}(k_2'/k_2)$	0.193	−0.3746	0.6573	approximate	approximate
b2	$\log_{10}(k_1'k_2')$	−2.7	−2.854	−2.505	approximate	approximate
b2	$\log_{10}(k_2 \text{ or } k_{deg})$	−1.135	−1.463	−0.8496	approximate	approximate
b2	$\log_{10}(k_2 \text{ or } k_{deg})$	−2.369	−2.473	−2.192	approximate	approximate
b3	$\log_{10}(k_1')$	−1.136	−1.303	−0.6669	approximate	approximate
b3	$\log_{10}(k_2)$	−1.654	−1.898	−1.062	approximate	approximate
b3	$\log_{10}(k_2')$	−2.057	−2.372	−1.833	approximate	approximate
b3	$\log_{10}(k_{deg})$	−2.206	−2.6	−1.94	approximate	approximate
b3	$\log_{10}(k_1'/k_2)$	0.5177	0.3742	0.6361	approximate	approximate
b3	$\log_{10}(k_1'k_2'/k_2)$	−1.54	−1.935	−1.273	approximate	approximate
b3	$\log_{10}(k_2'/k_2)$	−0.4036	−1.237	−0.0008368	approximate	approximate

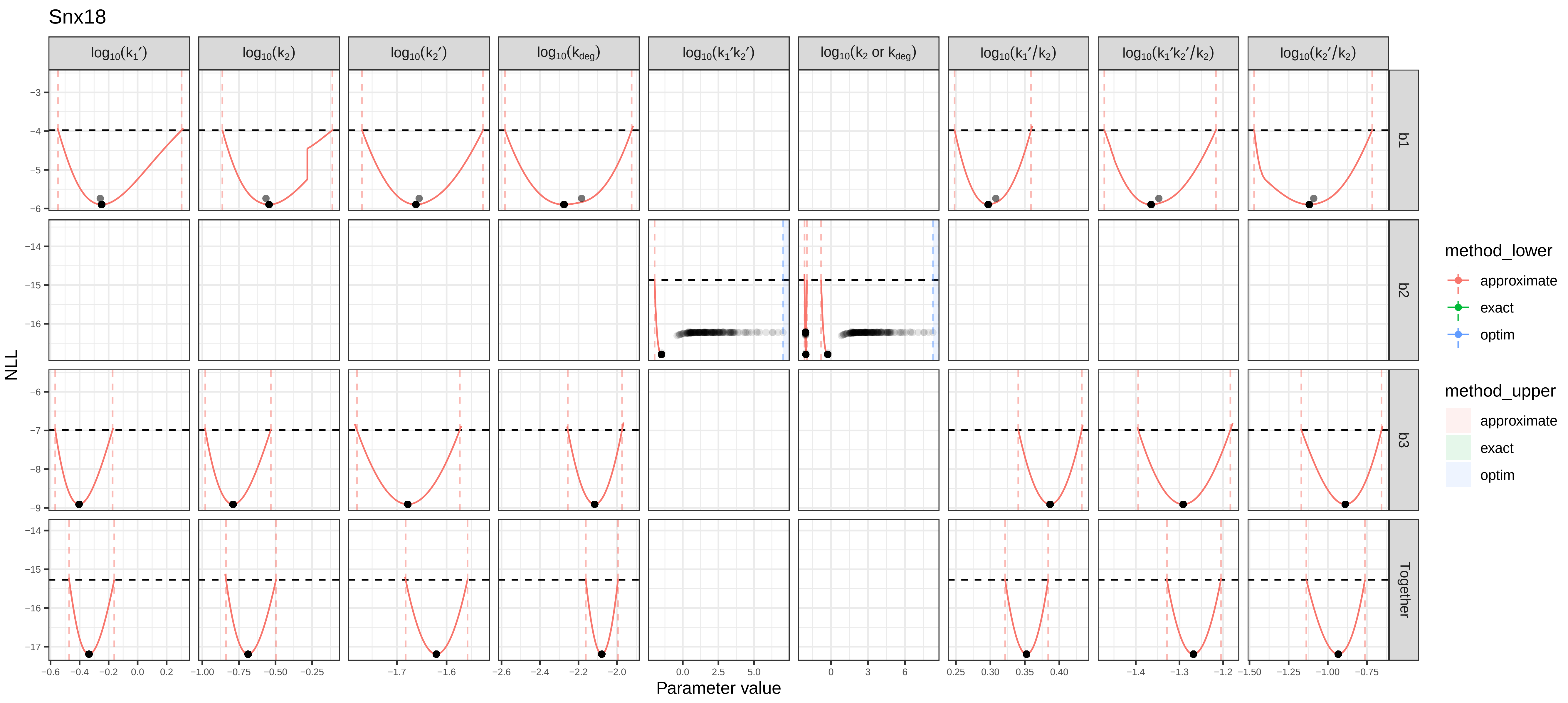

Replicate	Par	Best value	CI95 LB	CI95 UB	Method LB	Method UB
Together	$\log_{10}(k_1')$	−0.3351	−0.4719	−0.1609	approximate	approximate
Together	$\log_{10}(k_2)$	−0.6876	−0.8383	−0.4967	approximate	approximate
Together	$\log_{10}(k_2')$	−1.621	−1.682	−1.558	approximate	approximate
Together	$\log_{10}(k_{deg})$	−2.079	−2.162	−1.995	approximate	approximate
Together	$\log_{10}(k_1'/k_2)$	0.3525	0.3214	0.3839	approximate	approximate
Together	$\log_{10}(k_1'k_2'/k_2)$	−1.268	−1.329	−1.205	approximate	approximate
Together	$\log_{10}(k_2'/k_2)$	−0.9331	−1.137	−0.7623	approximate	approximate
b1	$\log_{10}(k_1')$	−0.2477	−0.5481	0.3035	approximate	approximate
b1	$\log_{10}(k_2)$	−0.5446	−0.863	−0.1122	approximate	approximate
b1	$\log_{10}(k_2')$	−1.662	−1.77	−1.527	approximate	approximate
b1	$\log_{10}(k_{deg})$	−2.275	−2.582	−1.924	approximate	approximate
b1	$\log_{10}(k_1'/k_2)$	0.2969	0.2481	0.3591	approximate	approximate
b1	$\log_{10}(k_1'k_2'/k_2)$	−1.365	−1.472	−1.217	approximate	approximate
b1	$\log_{10}(k_2'/k_2)$	−1.117	−1.47	−0.7159	approximate	approximate
b2	$\log_{10}(k_1'k_2')$	−1.492	−1.976	> 7.031	approximate	optim
b2	$\log_{10}(k_2 \text{ or } k_{deg})$	−0.2733	−0.807	> 8.275	approximate	optim
b2	$\log_{10}(k_2 \text{ or } k_{deg})$	−2.054	−2.157	−1.969	approximate	approximate
b3	$\log_{10}(k_1')$	−0.4036	−0.5678	−0.1724	approximate	approximate
b3	$\log_{10}(k_2)$	−0.7902	−0.9797	−0.5324	approximate	approximate
b3	$\log_{10}(k_2')$	−1.678	−1.781	−1.573	approximate	approximate
b3	$\log_{10}(k_{deg})$	−2.116	−2.256	−1.974	approximate	approximate
b3	$\log_{10}(k_1'/k_2)$	0.3866	0.3405	0.4326	approximate	approximate
b3	$\log_{10}(k_1'k_2'/k_2)$	−1.292	−1.395	−1.184	approximate	approximate
b3	$\log_{10}(k_2'/k_2)$	−0.888	−1.169	−0.6564	approximate	approximate

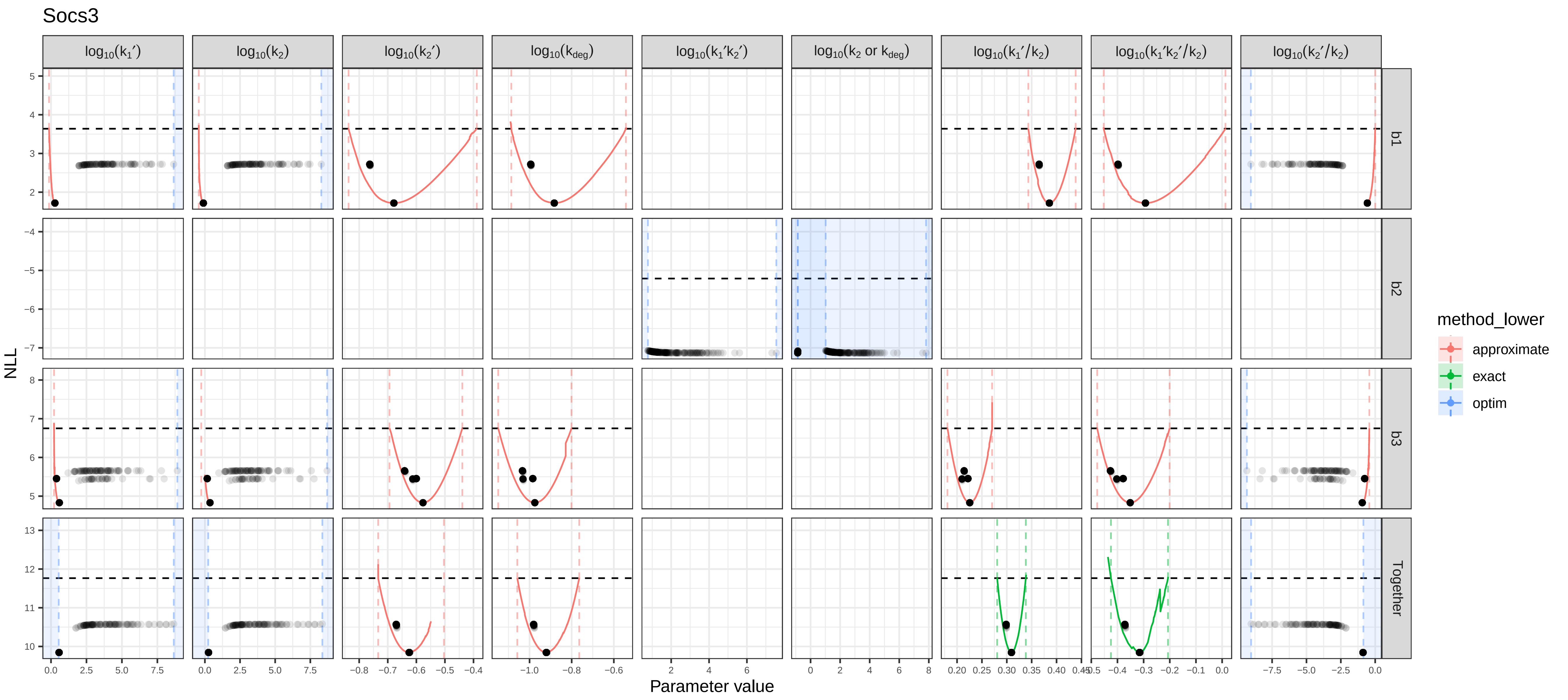

Replicate	Par	Best value	CI95 LB	CI95 UB	Method LB	Method UB
Together	$\log_{10}(k_1')$	0.5714	< 0.5452	> 8.656	optim	optim
Together	$\log_{10}(k_2)$	0.2621	< 0.2352	> 8.356	optim	optim
Together	$\log_{10}(k_2')$	−0.6252	−0.7335	−0.5033	approximate	approximate
Together	$\log_{10}(k_{deg})$	−0.9207	−1.058	−0.7642	approximate	approximate
Together	$\log_{10}(k_1'/k_2)$	0.3093	0.2806	0.3383	exact	exact
Together	$\log_{10}(k_1'k_2'/k_2)$	−0.3159	−0.4249	−0.207	exact	exact
Together	$\log_{10}(k_2'/k_2)$	−0.8873	< −9.028	> −0.8573	optim	optim
b1	$\log_{10}(k_1')$	0.2766	−0.1405	> 8.648	approximate	optim
b1	$\log_{10}(k_2)$	−0.109	−0.438	> 8.283	approximate	optim
b1	$\log_{10}(k_2')$	−0.6788	−0.8371	−0.3884	approximate	approximate
b1	$\log_{10}(k_{deg})$	−0.8828	−1.087	−0.5426	approximate	approximate
b1	$\log_{10}(k_1'/k_2)$	0.3856	0.3432	0.4384	approximate	approximate
b1	$\log_{10}(k_1'k_2'/k_2)$	−0.2932	−0.4525	0.01279	approximate	approximate
b1	$\log_{10}(k_2'/k_2)$	−0.5698	< −9.046	0.004444	optim	approximate
b2	$\log_{10}(k_1'k_2')$	4.45	< 0.7725	> 7.552	optim	optim
b2	$\log_{10}(k_2 \text{ or } k_{deg})$	4.712	< 1.025	> 7.813	optim	optim
b2	$\log_{10}(k_2 \text{ or } k_{deg})$	−0.8709	< −0.8722	> −0.8604	optim	optim
b3	$\log_{10}(k_1')$	0.5891	0.2146	> 8.908	approximate	optim
b3	$\log_{10}(k_2)$	0.3636	−0.2602	> 8.694	approximate	optim
b3	$\log_{10}(k_2')$	−0.5768	−0.6939	−0.4392	approximate	approximate
b3	$\log_{10}(k_{deg})$	−0.975	−1.149	−0.8009	approximate	approximate
b3	$\log_{10}(k_1'/k_2)$	0.2255	0.1806	0.2704	approximate	approximate
b3	$\log_{10}(k_1'k_2'/k_2)$	−0.3513	−0.4775	−0.2009	approximate	approximate
b3	$\log_{10}(k_2'/k_2)$	−0.9404	< −9.334	−0.4255	optim	approximate

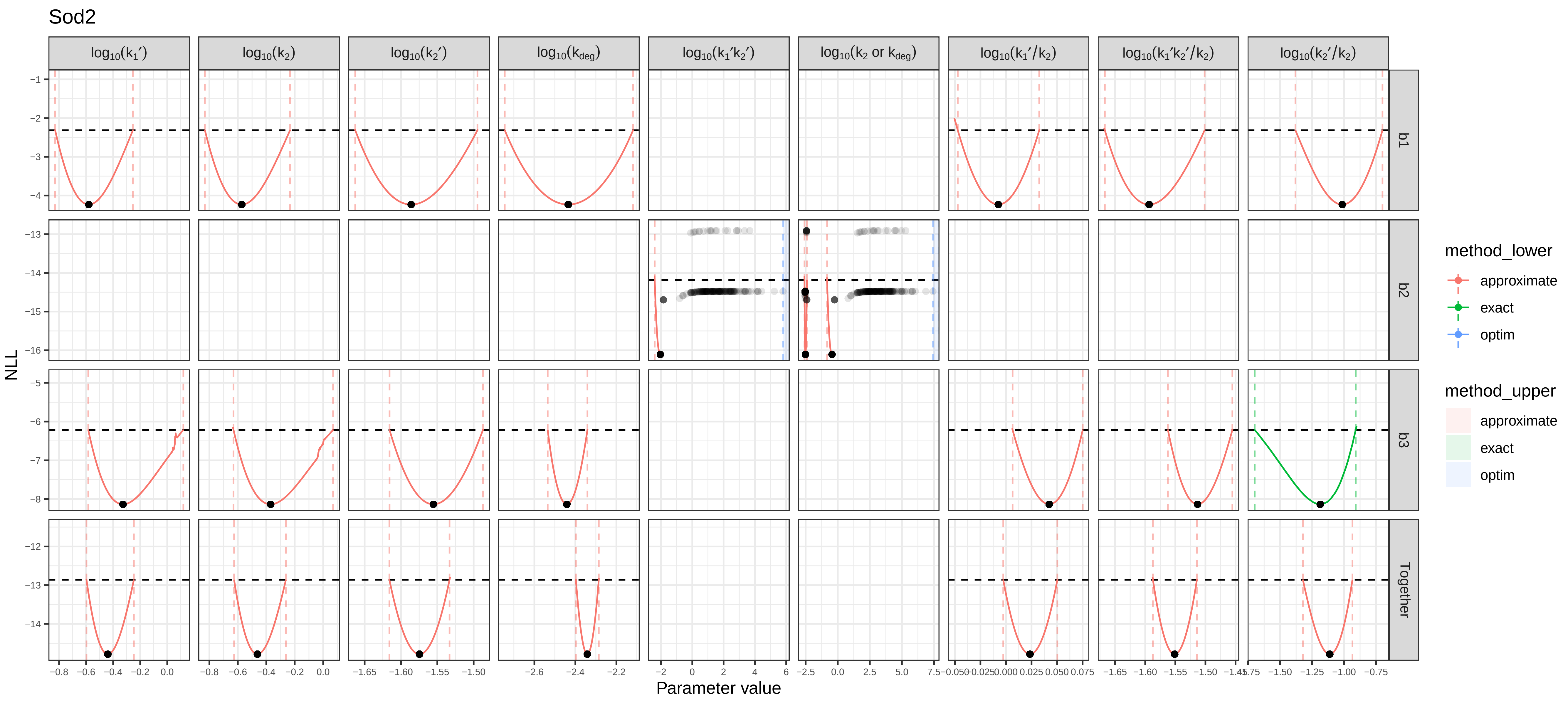

Replicate	Par	Best value	CI95 LB	CI95 UB	Method LB	Method UB
Together	$\log_{10}(k_1')$	−0.4389	−0.597	−0.2467	approximate	approximate
Together	$\log_{10}(k_2)$	−0.4623	−0.6262	−0.2621	approximate	approximate
Together	$\log_{10}(k_2')$	−1.575	−1.616	−1.533	approximate	approximate
Together	$\log_{10}(k_{deg})$	−2.342	−2.398	−2.286	approximate	approximate
Together	$\log_{10}(k_1'/k_2)$	0.02344	−0.002665	0.05029	approximate	approximate
Together	$\log_{10}(k_1'k_2'/k_2)$	−1.551	−1.587	−1.514	approximate	approximate
Together	$\log_{10}(k_2'/k_2)$	−1.112	−1.322	−0.9346	approximate	approximate
b1	$\log_{10}(k_1')$	−0.5796	−0.8288	−0.2539	approximate	approximate
b1	$\log_{10}(k_2)$	−0.572	−0.8314	−0.2328	approximate	approximate
b1	$\log_{10}(k_2')$	−1.586	−1.663	−1.495	approximate	approximate
b1	$\log_{10}(k_{deg})$	−2.434	−2.745	−2.118	approximate	approximate
b1	$\log_{10}(k_1'/k_2)$	−0.007523	−0.0472	0.03255	approximate	approximate
b1	$\log_{10}(k_1'k_2'/k_2)$	−1.593	−1.667	−1.501	approximate	approximate
b1	$\log_{10}(k_2'/k_2)$	−1.014	−1.38	−0.6998	approximate	approximate
b2	$\log_{10}(k_1'k_2')$	−2.04	−2.401	> 5.797	approximate	optim
b2	$\log_{10}(k_2 \text{ or } k_{deg})$	−0.4453	−0.8317	> 7.416	approximate	optim
b2	$\log_{10}(k_2 \text{ or } k_{deg})$	−2.518	−2.595	−2.398	approximate	approximate
b3	$\log_{10}(k_1')$	−0.3268	−0.5839	0.1193	approximate	approximate
b3	$\log_{10}(k_2)$	−0.3691	−0.6302	0.06958	approximate	approximate
b3	$\log_{10}(k_2')$	−1.555	−1.616	−1.487	approximate	approximate
b3	$\log_{10}(k_{deg})$	−2.441	−2.535	−2.341	approximate	approximate
b3	$\log_{10}(k_1'/k_2)$	0.04232	0.00646	0.07509	approximate	approximate
b3	$\log_{10}(k_1'k_2'/k_2)$	−1.513	−1.562	−1.455	approximate	approximate
b3	$\log_{10}(k_2'/k_2)$	−1.186	−1.699	−0.909	exact	exact

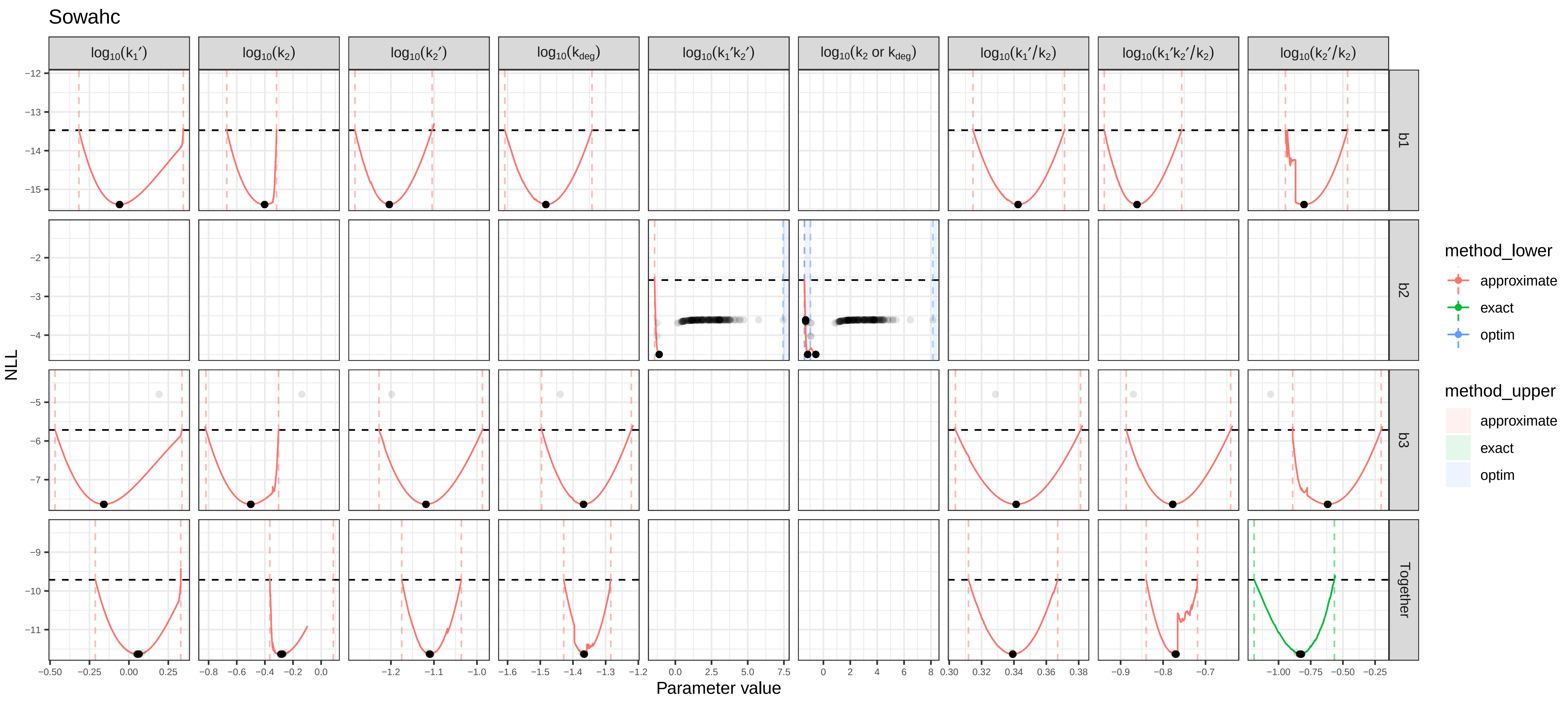

Replicate	Par	Best value	CI95 LB	CI95 UB	Method LB	Method UB
Together	$\log_{10}(k_1')$	0.05658	−0.213	0.3292	approximate	approximate
Together	$\log_{10}(k_2)$	−0.2827	−0.3649	0.08688	approximate	approximate
Together	$\log_{10}(k_2')$	−1.109	−1.175	−1.036	approximate	approximate
Together	$\log_{10}(k_{deg})$	−1.366	−1.428	−1.284	approximate	approximate
Together	$\log_{10}(k_1'/k_2)$	0.3393	0.3119	0.3671	approximate	approximate
Together	$\log_{10}(k_1'k_2'/k_2)$	−0.7699	−0.8398	−0.7188	approximate	approximate
Together	$\log_{10}(k_2'/k_2)$	−0.8265	−1.19	−0.5662	exact	exact
b1	$\log_{10}(k_1')$	−0.05937	−0.317	0.3453	approximate	approximate
b1	$\log_{10}(k_2)$	−0.4019	−0.671	−0.3166	approximate	approximate
b1	$\log_{10}(k_2')$	−1.204	−1.284	−1.104	approximate	approximate
b1	$\log_{10}(k_{deg})$	−1.483	−1.608	−1.342	approximate	approximate
b1	$\log_{10}(k_1'/k_2)$	0.3426	0.3147	0.3712	approximate	approximate
b1	$\log_{10}(k_1'k_2'/k_2)$	−0.8612	−0.9386	−0.756	approximate	approximate
b1	$\log_{10}(k_2'/k_2)$	−0.8018	−0.9467	−0.4635	approximate	approximate
b2	$\log_{10}(k_1'k_2')$	−1.115	−1.43	> 7.438	approximate	optim
b2	$\log_{10}(k_2 \text{ or } k_{deg})$	−0.5633	−1.41	> 8.146	approximate	optim
b2	$\log_{10}(k_2 \text{ or } k_{deg})$	−1.171	−1.41	−0.9627	approximate	optim
b3	$\log_{10}(k_1')$	−0.1588	−0.4684	0.3365	approximate	approximate
b3	$\log_{10}(k_2)$	−0.5001	−0.8191	−0.3028	approximate	approximate
b3	$\log_{10}(k_2')$	−1.119	−1.228	−0.9872	approximate	approximate
b3	$\log_{10}(k_{deg})$	−1.368	−1.496	−1.222	approximate	approximate
b3	$\log_{10}(k_1'/k_2)$	0.3413	0.3038	0.3812	approximate	approximate
b3	$\log_{10}(k_1'k_2'/k_2)$	−0.7773	−0.8868	−0.6408	approximate	approximate
b3	$\log_{10}(k_2'/k_2)$	−0.6185	−0.8903	−0.2026	approximate	approximate

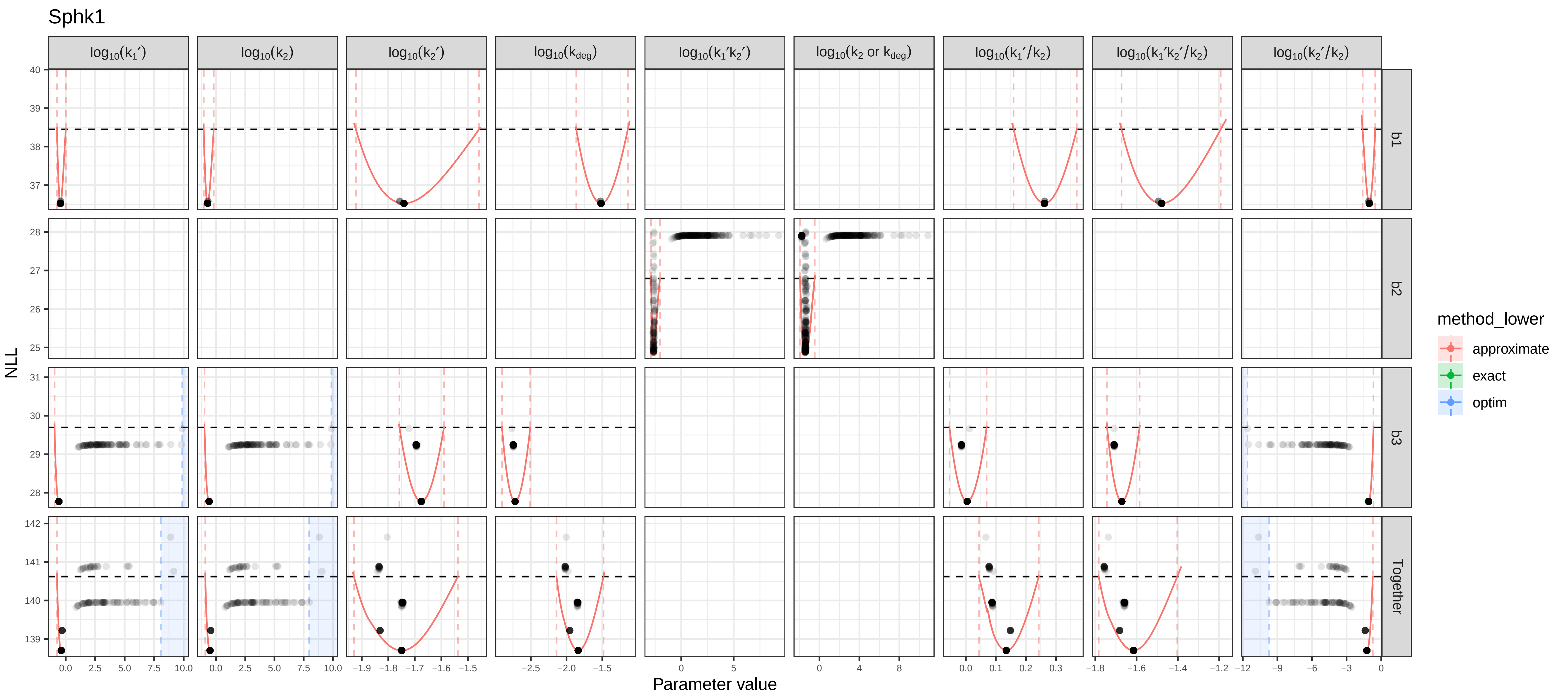

Replicate	Par	Best value	CI95 LB	CI95 UB	Method LB	Method UB
Together	$\log_{10}(k_1')$	−0.3715	−0.7346	> 8.054	approximate	optim
Together	$\log_{10}(k_2)$	−0.5062	−0.9126	> 7.966	approximate	optim
Together	$\log_{10}(k_2')$	−1.749	−1.93	−1.538	approximate	approximate
Together	$\log_{10}(k_{deg})$	−1.837	−2.141	−1.482	approximate	approximate
Together	$\log_{10}(k_1'/k_2)$	0.1347	0.0441	0.243	approximate	approximate
Together	$\log_{10}(k_1'k_2'/k_2)$	−1.615	−1.784	−1.403	approximate	approximate
Together	$\log_{10}(k_2'/k_2)$	−1.243	< −9.712	−0.729	optim	approximate
b1	$\log_{10}(k_1')$	−0.4488	−0.7372	0.006407	approximate	approximate
b1	$\log_{10}(k_2)$	−0.7108	−1.043	−0.1902	approximate	approximate
b1	$\log_{10}(k_2')$	−1.741	−1.922	−1.458	approximate	approximate
b1	$\log_{10}(k_{deg})$	−1.517	−1.864	−1.142	approximate	approximate
b1	$\log_{10}(k_1'/k_2)$	0.262	0.1591	0.3697	approximate	approximate
b1	$\log_{10}(k_1'k_2'/k_2)$	−1.479	−1.673	−1.193	approximate	approximate
b1	$\log_{10}(k_2'/k_2)$	−1.03	−1.617	−0.5163	approximate	approximate
b2	$\log_{10}(k_1'k_2')$	−2.646	−2.881	−2.024	approximate	approximate
b2	$\log_{10}(k_2 \text{ or } k_{deg})$	−1.397	−1.921	−0.4695	approximate	approximate
b2	$\log_{10}(k_2 \text{ or } k_{deg})$	−1.429	−1.921	−0.4695	approximate	approximate
b3	$\log_{10}(k_1')$	−0.5792	−0.944	> 9.879	approximate	optim
b3	$\log_{10}(k_2)$	−0.5835	−0.9763	> 9.867	approximate	optim
b3	$\log_{10}(k_2')$	−1.676	−1.758	−1.59	approximate	approximate
b3	$\log_{10}(k_{deg})$	−2.721	−2.904	−2.508	approximate	approximate
b3	$\log_{10}(k_1'/k_2)$	0.00431	−0.05462	0.06922	approximate	approximate
b3	$\log_{10}(k_1'k_2'/k_2)$	−1.671	−1.743	−1.586	approximate	approximate
b3	$\log_{10}(k_2'/k_2)$	−1.092	< −11.59	−0.6695	optim	approximate

Spp1

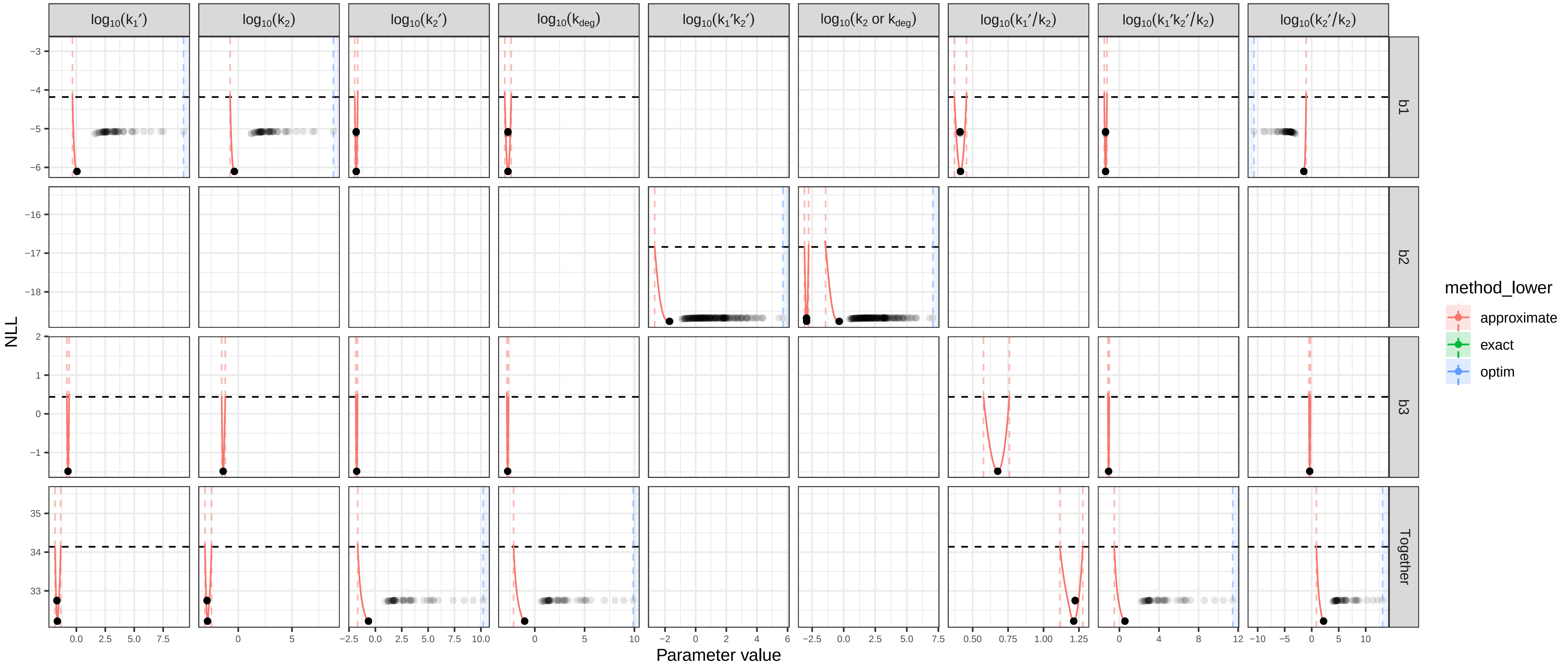

Replicate	Par	Best value	CI95 LB	CI95 UB	Method LB	Method UB
Together	$\log_{10}(k_1')$	−1.64	−1.844	−1.352	approximate	approximate
Together	$\log_{10}(k_2)$	−2.853	−3.093	−2.48	approximate	approximate
Together	$\log_{10}(k_2')$	−0.6545	−1.67	> 10.22	approximate	optim
Together	$\log_{10}(k_{deg})$	−1.022	−2.136	> 9.873	approximate	optim
Together	$\log_{10}(k_1'/k_2)$	1.213	1.115	1.277	approximate	approximate
Together	$\log_{10}(k_1'k_2'/k_2)$	0.5586	−0.5208	> 11.44	approximate	optim
Together	$\log_{10}(k_2'/k_2)$	2.198	0.8704	> 13.12	approximate	optim
b1	$\log_{10}(k_1')$	0.05592	−0.3344	> 9.255	approximate	optim
b1	$\log_{10}(k_2)$	−0.3584	−0.7542	> 8.844	approximate	optim
b1	$\log_{10}(k_2')$	−1.812	−1.949	−1.669	approximate	approximate
b1	$\log_{10}(k_{deg})$	−2.691	−3.015	−2.366	approximate	approximate
b1	$\log_{10}(k_1'/k_2)$	0.4143	0.371	0.4565	approximate	approximate
b1	$\log_{10}(k_1'k_2'/k_2)$	−1.398	−1.531	−1.256	approximate	approximate
b1	$\log_{10}(k_2'/k_2)$	−1.454	< −10.66	−1.027	optim	approximate
b2	$\log_{10}(k_1'k_2')$	−1.713	−2.678	> 5.703	approximate	optim
b2	$\log_{10}(k_2 \text{ or } k_{deg})$	−0.3626	−1.435	> 7.067	approximate	optim
b2	$\log_{10}(k_2 \text{ or } k_{deg})$	−2.939	−3.112	−2.781	approximate	approximate
b3	$\log_{10}(k_1')$	−0.7218	−0.8174	−0.6203	approximate	approximate
b3	$\log_{10}(k_2)$	−1.399	−1.534	−1.205	approximate	approximate
b3	$\log_{10}(k_2')$	−1.769	−1.854	−1.688	approximate	approximate
b3	$\log_{10}(k_{deg})$	−2.727	−2.805	−2.645	approximate	approximate
b3	$\log_{10}(k_1'/k_2)$	0.6768	0.5767	0.7593	approximate	approximate
b3	$\log_{10}(k_1'k_2'/k_2)$	−1.093	−1.161	−1.023	approximate	approximate
b3	$\log_{10}(k_2'/k_2)$	−0.3709	−0.5096	−0.2319	approximate	approximate

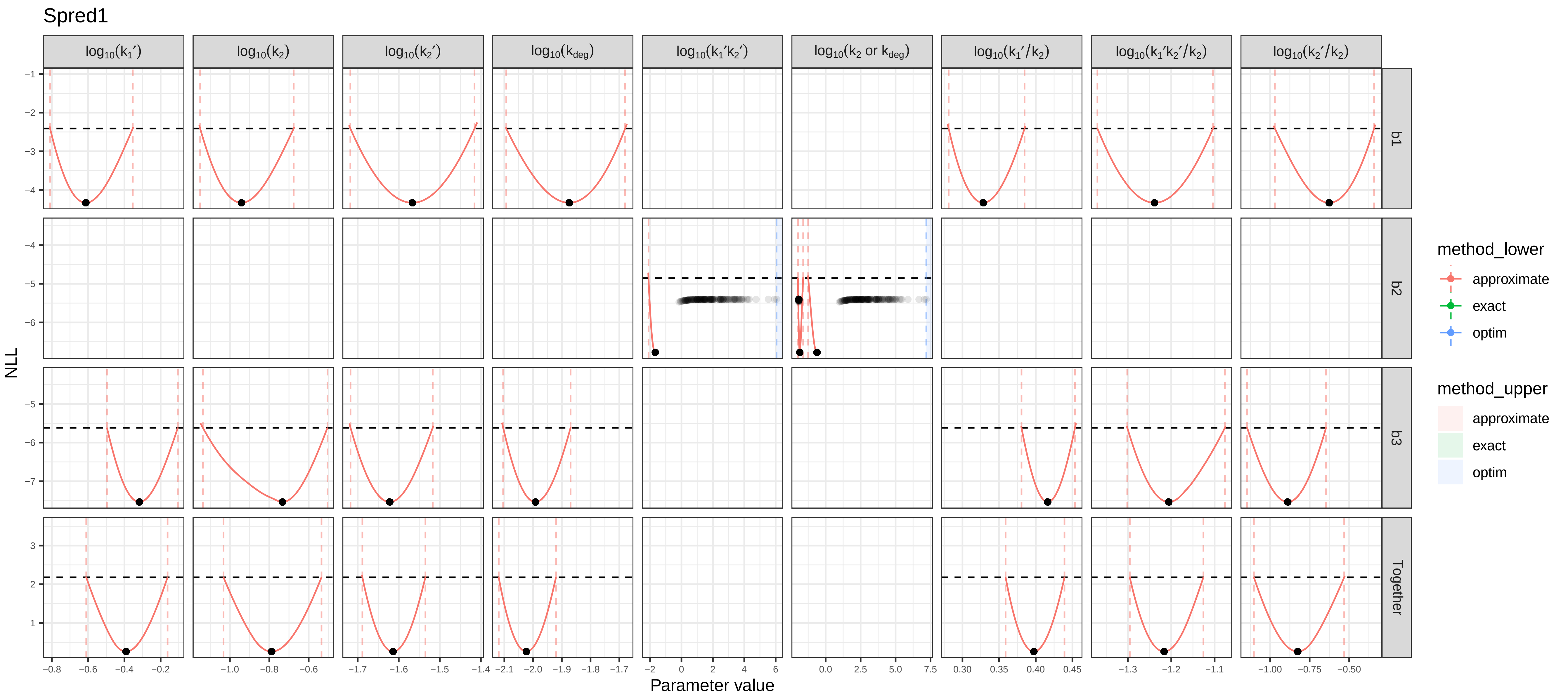

Replicate	Par	Best value	CI95 LB	CI95 UB	Method LB	Method UB
Together	$\log_{10}(k_1')$	−0.3907	−0.6104	−0.1618	approximate	approximate
Together	$\log_{10}(k_2)$	−0.7881	−1.032	−0.535	approximate	approximate
Together	$\log_{10}(k_2')$	−1.614	−1.689	−1.535	approximate	approximate
Together	$\log_{10}(k_{deg})$	−2.023	−2.12	−1.92	approximate	approximate
Together	$\log_{10}(k_1'/k_2)$	0.3974	0.3588	0.4392	approximate	approximate
Together	$\log_{10}(k_1'k_2'/k_2)$	−1.216	−1.296	−1.126	approximate	approximate
Together	$\log_{10}(k_2'/k_2)$	−0.8258	−1.103	−0.5314	approximate	approximate
b1	$\log_{10}(k_1')$	−0.6126	−0.8098	−0.3539	approximate	approximate
b1	$\log_{10}(k_2)$	−0.941	−1.151	−0.6761	approximate	approximate
b1	$\log_{10}(k_2')$	−1.567	−1.718	−1.415	approximate	approximate
b1	$\log_{10}(k_{deg})$	−1.874	−2.093	−1.68	approximate	approximate
b1	$\log_{10}(k_1'/k_2)$	0.3283	0.2812	0.3847	approximate	approximate
b1	$\log_{10}(k_1'k_2'/k_2)$	−1.239	−1.37	−1.104	approximate	approximate
b1	$\log_{10}(k_2'/k_2)$	−0.6259	−0.9698	−0.3424	approximate	approximate
b2	$\log_{10}(k_1'k_2')$	−1.67	−2.1	> 6.061	approximate	optim
b2	$\log_{10}(k_2 \text{ or } k_{deg})$	−0.618	−1.25	> 7.194	approximate	optim
b2	$\log_{10}(k_2 \text{ or } k_{deg})$	−1.846	−1.978	−1.606	approximate	approximate
b3	$\log_{10}(k_1')$	−0.3175	−0.4962	−0.1052	approximate	approximate
b3	$\log_{10}(k_2)$	−0.7338	−1.137	−0.5047	approximate	approximate
b3	$\log_{10}(k_2')$	−1.622	−1.718	−1.517	approximate	approximate
b3	$\log_{10}(k_{deg})$	−1.992	−2.104	−1.869	approximate	approximate
b3	$\log_{10}(k_1'/k_2)$	0.4163	0.3805	0.4536	approximate	approximate
b3	$\log_{10}(k_1'k_2'/k_2)$	−1.206	−1.301	−1.076	approximate	approximate
b3	$\log_{10}(k_2'/k_2)$	−0.8881	−1.145	−0.646	approximate	approximate

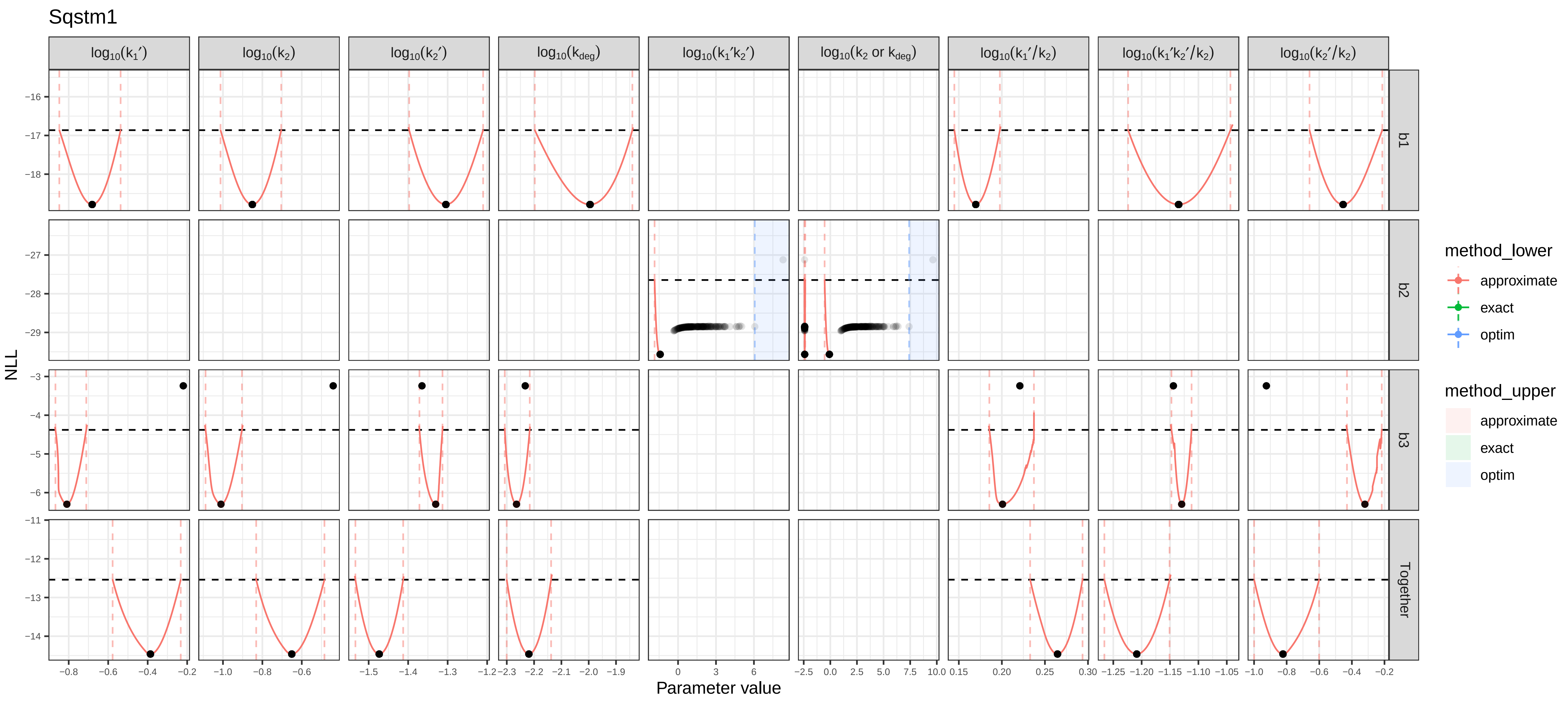

Replicate	Par	Best value	CI95 LB	CI95 UB	Method LB	Method UB
Together	$\log_{10}(k_1')$	−0.3861	−0.5776	−0.2325	approximate	approximate
Together	$\log_{10}(k_2)$	−0.6508	−0.8313	−0.485	approximate	approximate
Together	$\log_{10}(k_2')$	−1.473	−1.534	−1.413	approximate	approximate
Together	$\log_{10}(k_{deg})$	−2.219	−2.3	−2.138	approximate	approximate
Together	$\log_{10}(k_1'/k_2)$	0.2647	0.2328	0.2937	approximate	approximate
Together	$\log_{10}(k_1'k_2'/k_2)$	−1.209	−1.266	−1.151	approximate	approximate
Together	$\log_{10}(k_2'/k_2)$	−0.8225	−0.9996	−0.6011	approximate	approximate
b1	$\log_{10}(k_1')$	−0.6812	−0.8476	−0.5371	approximate	approximate
b1	$\log_{10}(k_2)$	−0.8509	−1.013	−0.7043	approximate	approximate
b1	$\log_{10}(k_2')$	−1.304	−1.397	−1.21	approximate	approximate
b1	$\log_{10}(k_{deg})$	−1.995	−2.197	−1.84	approximate	approximate
b1	$\log_{10}(k_1'/k_2)$	0.1697	0.1448	0.1977	approximate	approximate
b1	$\log_{10}(k_1'k_2'/k_2)$	−1.135	−1.224	−1.044	approximate	approximate
b1	$\log_{10}(k_2'/k_2)$	−0.4536	−0.6598	−0.2133	approximate	approximate
b2	$\log_{10}(k_1'k_2')$	−1.422	−1.856	> 6.068	approximate	optim
b2	$\log_{10}(k_2 \text{ or } k_{deg})$	−0.09567	−0.5368	> 7.398	approximate	optim
b2	$\log_{10}(k_2 \text{ or } k_{deg})$	−2.405	−2.43	−2.356	approximate	approximate
b3	$\log_{10}(k_1')$	−0.8094	−0.8673	−0.7111	approximate	approximate
b3	$\log_{10}(k_2)$	−1.01	−1.088	−0.9035	approximate	approximate
b3	$\log_{10}(k_2')$	−1.33	−1.371	−1.313	approximate	approximate
b3	$\log_{10}(k_{deg})$	−2.264	−2.307	−2.216	approximate	approximate
b3	$\log_{10}(k_1'/k_2)$	0.2008	0.1854	0.2372	approximate	approximate
b3	$\log_{10}(k_1'k_2'/k_2)$	−1.129	−1.147	−1.112	approximate	approximate
b3	$\log_{10}(k_2'/k_2)$	−0.32	−0.4301	−0.2167	approximate	approximate

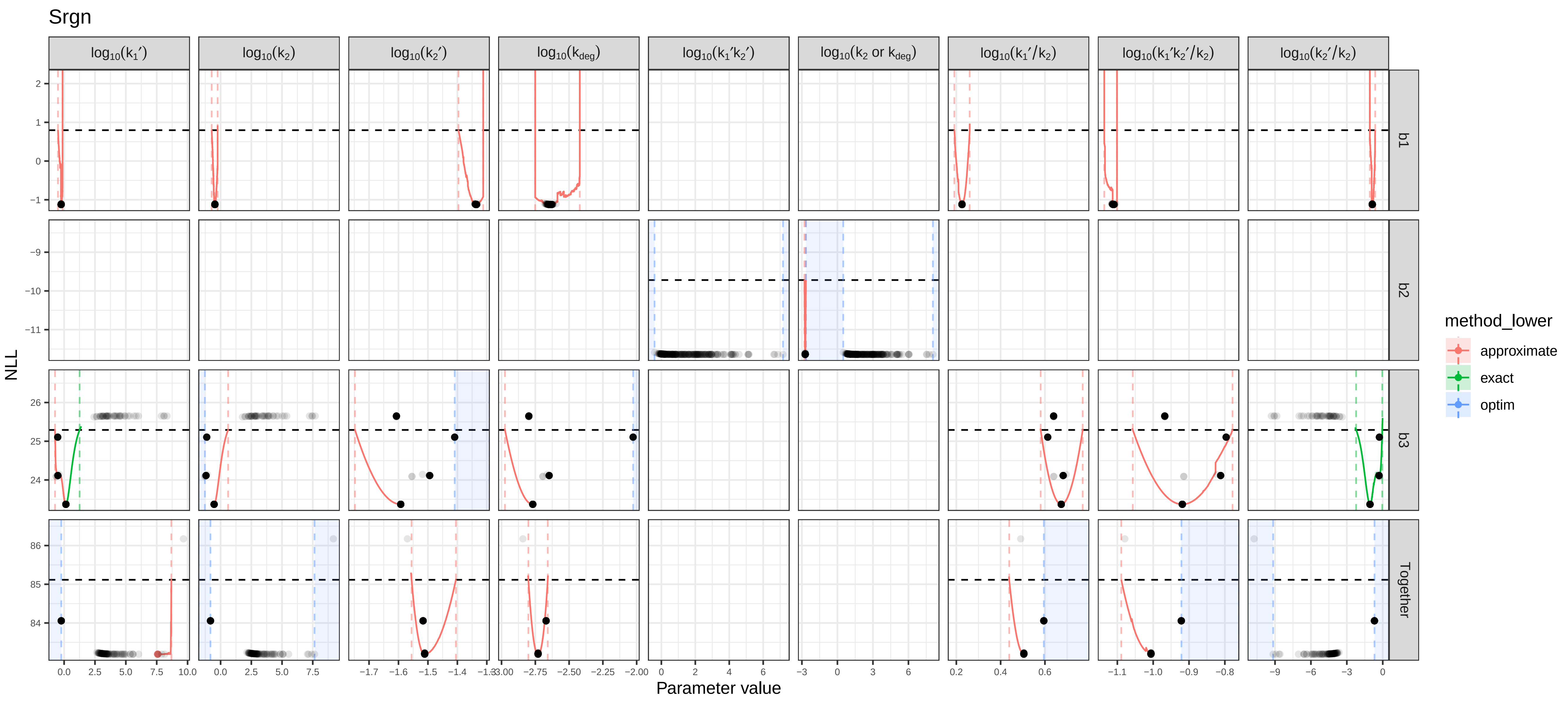

Replicate	Par	**Best value**	**CI95 LB**	**CI95 UB**	**Method LB**	**Method UB**
Together	$\log_{10}(k_1')$	7.575	< −0.23	8.683	optim	approximate
Together	$\log_{10}(k_2)$	7.07	< −0.8251	> 7.645	optim	optim
Together	$\log_{10}(k_2')$	−1.511	−1.556	−1.405	approximate	approximate
Together	$\log_{10}(k_{deg})$	−2.73	−2.801	−2.658	approximate	approximate
Together	$\log_{10}(k_1'/k_2)$	0.5049	0.4387	> 0.5951	approximate	optim
Together	$\log_{10}(k_1'k_2'/k_2)$	−1.006	−1.089	> −0.9211	approximate	optim
Together	$\log_{10}(k_2'/k_2)$	−8.581	< −9.156	> −0.6911	optim	optim
b1	$\log_{10}(k_1')$	−0.2409	−0.4955	−0.1234	approximate	approximate
b1	$\log_{10}(k_2)$	−0.4665	−0.7253	−0.241	approximate	approximate
b1	$\log_{10}(k_2')$	−1.336	−1.396	−1.312	approximate	approximate
b1	$\log_{10}(k_{deg})$	−2.639	−2.751	−2.421	approximate	approximate
b1	$\log_{10}(k_1'/k_2)$	0.2256	0.191	0.2602	approximate	approximate
b1	$\log_{10}(k_1'k_2'/k_2)$	−1.111	−1.136	−1.101	approximate	approximate
b1	$\log_{10}(k_2'/k_2)$	−0.8697	−1.08	−0.6297	approximate	approximate
b2	$\log_{10}(k_1'k_2')$	2.855	< −0.4003	> 7.169	optim	optim
b2	$\log_{10}(k_2 \text{ or } k_{deg})$	3.751	0.4929	> 8.066	optim	optim
b2	$\log_{10}(k_2 \text{ or } k_{deg})$	−2.719	−2.785	−2.663	approximate	approximate
b3	$\log_{10}(k_1')$	0.1452	−0.7366	1.256	approximate	exact
b3	$\log_{10}(k_2)$	−0.5285	< −1.28	0.6125	optim	approximate
b3	$\log_{10}(k_2')$	−1.592	−1.748	> −1.409	approximate	optim
b3	$\log_{10}(k_{deg})$	−2.769	−2.975	> −2.026	approximate	optim
b3	$\log_{10}(k_1'/k_2)$	0.6737	0.5807	0.7706	approximate	approximate
b3	$\log_{10}(k_1'k_2'/k_2)$	−0.9186	−1.057	−0.7785	approximate	approximate
b3	$\log_{10}(k_2'/k_2)$	−1.064	−2.225	−0.0411	exact	exact

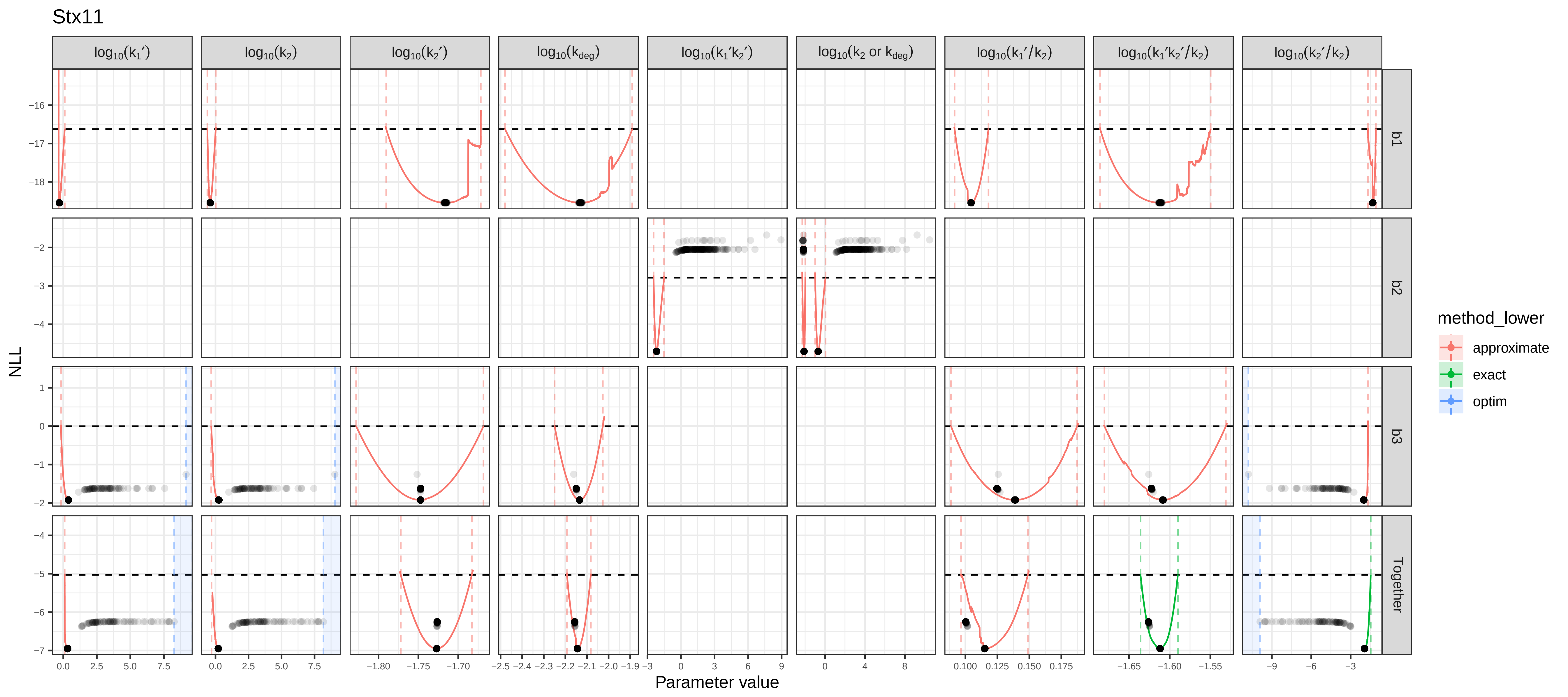

Replicate	Par	Best value	CI95 LB	CI95 UB	Method LB	Method UB
Together	$\log_{10}(k_1')$	0.3265	0.1018	> 8.272	approximate	optim
Together	$\log_{10}(k_2)$	0.2114	−0.3007	> 8.172	approximate	optim
Together	$\log_{10}(k_2')$	−1.727	−1.772	−1.683	approximate	approximate
Together	$\log_{10}(k_{deg})$	−2.144	−2.193	−2.082	approximate	approximate
Together	$\log_{10}(k_1'/k_2)$	0.1151	0.09662	0.1488	approximate	approximate
Together	$\log_{10}(k_1'k_2'/k_2)$	−1.612	−1.636	−1.59	exact	exact
Together	$\log_{10}(k_2'/k_2)$	−1.938	< −9.898	−1.472	optim	exact
b1	$\log_{10}(k_1')$	−0.2939	−0.3441	0.1086	approximate	approximate
b1	$\log_{10}(k_2)$	−0.3984	−0.6138	0.01951	approximate	approximate
b1	$\log_{10}(k_2')$	−1.716	−1.79	−1.672	approximate	approximate
b1	$\log_{10}(k_{deg})$	−2.13	−2.479	−1.891	approximate	approximate
b1	$\log_{10}(k_1'/k_2)$	0.1045	0.09154	0.1181	approximate	approximate
b1	$\log_{10}(k_1'k_2'/k_2)$	−1.611	−1.686	−1.55	approximate	approximate
b1	$\log_{10}(k_2'/k_2)$	−1.317	−1.678	−1.08	approximate	approximate
b2	$\log_{10}(k_1'k_2')$	−2.186	−2.45	−1.532	approximate	approximate
b2	$\log_{10}(k_2 \text{ or } k_{deg})$	−0.6864	−0.9888	0.05308	approximate	approximate
b2	$\log_{10}(k_2 \text{ or } k_{deg})$	−2.108	−2.28	−1.978	approximate	approximate
b3	$\log_{10}(k_1')$	0.3877	−0.1745	> 9.161	approximate	optim
b3	$\log_{10}(k_2)$	0.2489	−0.3199	> 9.036	approximate	optim
b3	$\log_{10}(k_2')$	−1.747	−1.828	−1.668	approximate	approximate
b3	$\log_{10}(k_{deg})$	−2.135	−2.25	−2.027	approximate	approximate
b3	$\log_{10}(k_1'/k_2)$	0.1388	0.08877	0.1873	approximate	approximate
b3	$\log_{10}(k_1'k_2'/k_2)$	−1.608	−1.68	−1.531	approximate	approximate
b3	$\log_{10}(k_2'/k_2)$	−1.996	< −10.79	−1.672	optim	approximate

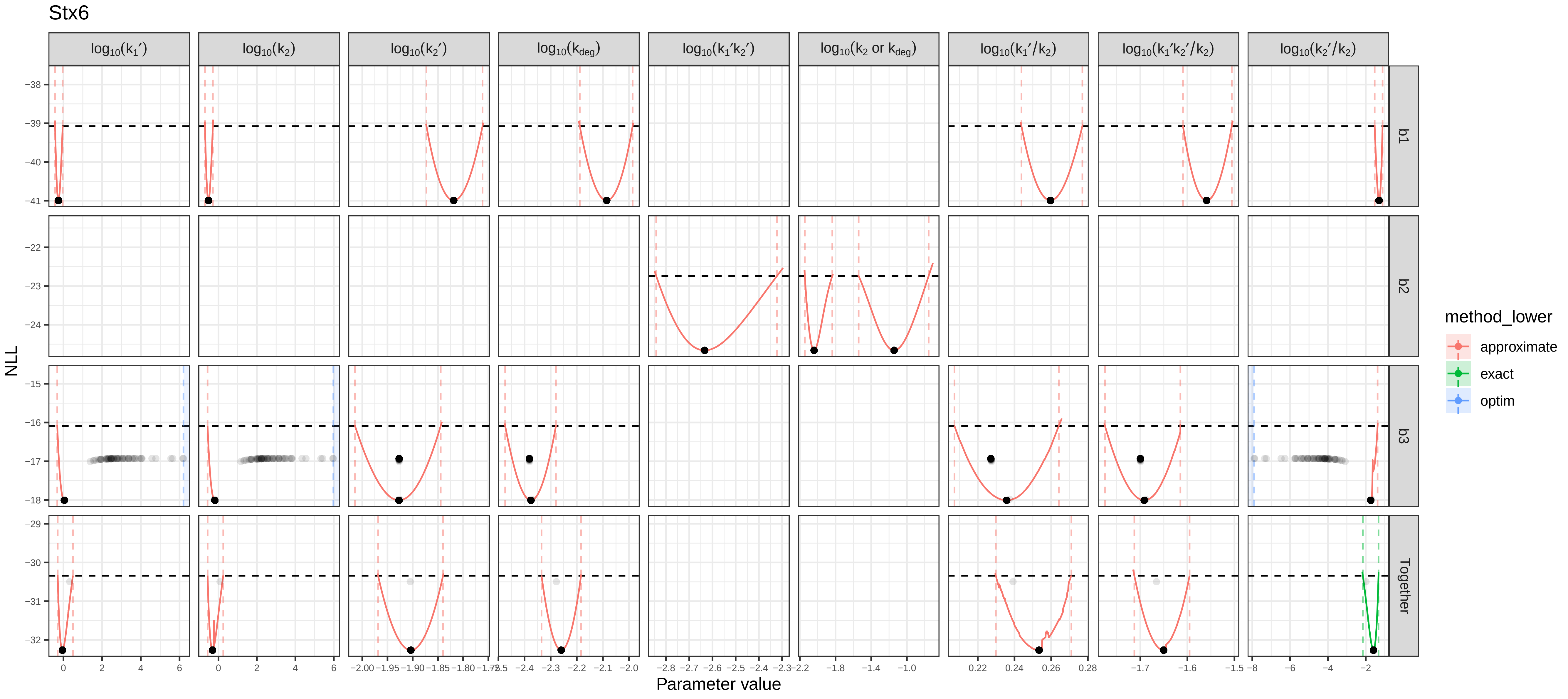

Replicate	Par	**Best value**	**CI95 LB**	**CI95 UB**	**Method LB**	**Method UB**
Together	$\log_{10}(k_1')$	−0.05607	−0.2987	0.4885	approximate	approximate
Together	$\log_{10}(k_2)$	−0.3095	−0.5653	0.2492	approximate	approximate
Together	$\log_{10}(k_2')$	−1.904	−1.969	−1.84	approximate	approximate
Together	$\log_{10}(k_{deg})$	−2.259	−2.335	−2.184	approximate	approximate
Together	$\log_{10}(k_1'/k_2)$	0.2534	0.2298	0.271	approximate	approximate
Together	$\log_{10}(k_1'k_2'/k_2)$	−1.65	−1.713	−1.595	approximate	approximate
Together	$\log_{10}(k_2'/k_2)$	−1.594	−2.155	−1.327	exact	exact
b1	$\log_{10}(k_1')$	−0.2619	−0.4324	−0.04004	approximate	approximate
b1	$\log_{10}(k_2)$	−0.5215	−0.7011	−0.2925	approximate	approximate
b1	$\log_{10}(k_2')$	−1.819	−1.873	−1.761	approximate	approximate
b1	$\log_{10}(k_{deg})$	−2.086	−2.189	−1.986	approximate	approximate
b1	$\log_{10}(k_1'/k_2)$	0.2596	0.2438	0.277	approximate	approximate
b1	$\log_{10}(k_1'k_2'/k_2)$	−1.559	−1.609	−1.506	approximate	approximate
b1	$\log_{10}(k_2'/k_2)$	−1.297	−1.53	−1.113	approximate	approximate
b2	$\log_{10}(k_1'k_2')$	−2.634	−2.842	−2.322	approximate	approximate
b2	$\log_{10}(k_2 \text{ or } k_{deg})$	−1.143	−1.541	−0.7562	approximate	approximate
b2	$\log_{10}(k_2 \text{ or } k_{deg})$	−2.041	−2.144	−1.836	approximate	approximate
b3	$\log_{10}(k_1')$	0.04707	−0.3205	> 6.204	approximate	optim
b3	$\log_{10}(k_2)$	−0.1887	−0.5682	> 5.976	approximate	optim
b3	$\log_{10}(k_2')$	−1.927	−2.014	−1.844	approximate	approximate
b3	$\log_{10}(k_{deg})$	−2.375	−2.474	−2.28	approximate	approximate
b3	$\log_{10}(k_1'/k_2)$	0.2357	0.2073	0.2642	approximate	approximate
b3	$\log_{10}(k_1'k_2'/k_2)$	−1.692	−1.775	−1.615	approximate	approximate
b3	$\log_{10}(k_2'/k_2)$	−1.739	< −7.903	−1.376	optim	approximate

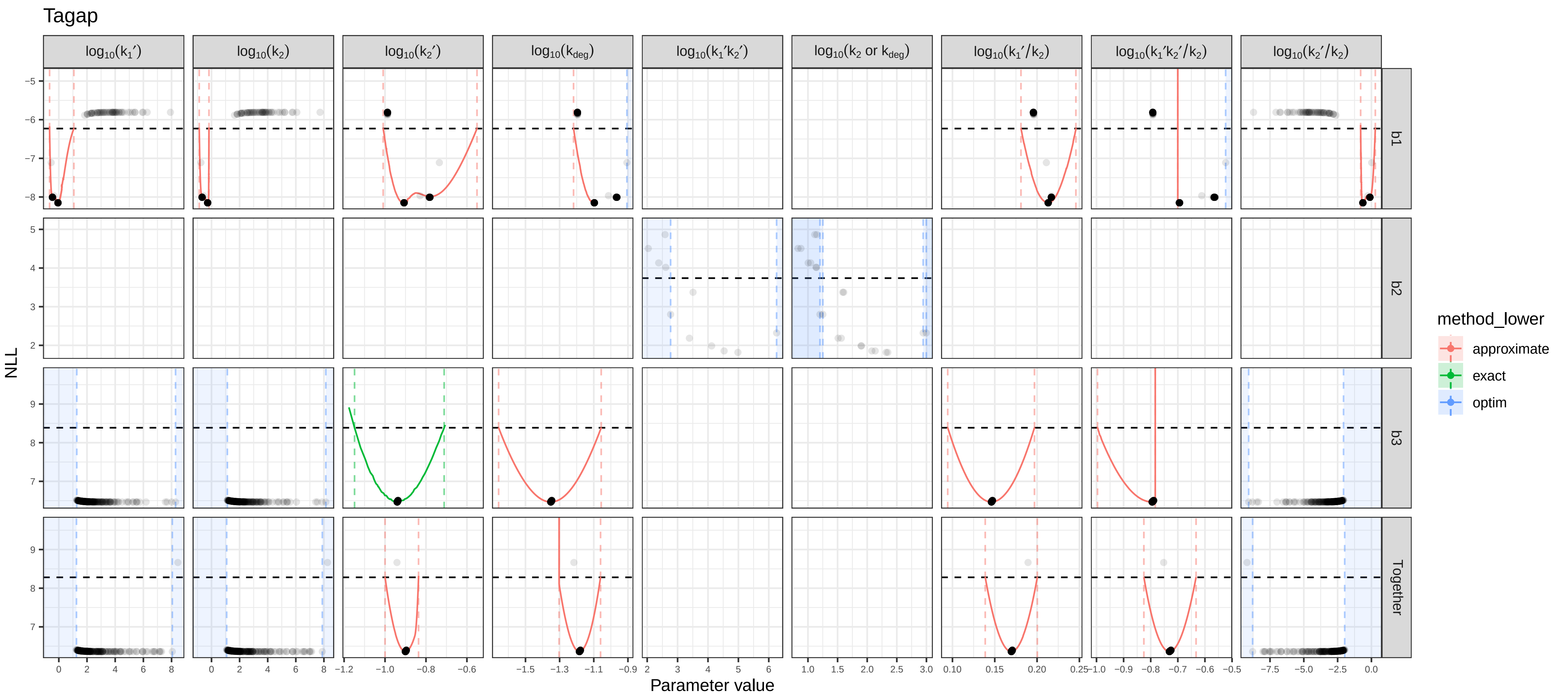

Replicate	Par	Best value	CI95 LB	CI95 UB	Method LB	Method UB
Together	$\log_{10}(k_1')$	5.016	< 1.252	> 8.053	optim	optim
Together	$\log_{10}(k_2)$	4.847	< 1.081	> 7.884	optim	optim
Together	$\log_{10}(k_2')$	−0.9004	−0.9999	−0.8363	approximate	approximate
Together	$\log_{10}(k_{deg})$	−1.184	−1.303	−1.06	approximate	approximate
Together	$\log_{10}(k_1'/k_2)$	0.1696	0.1387	0.2003	approximate	approximate
Together	$\log_{10}(k_1'k_2'/k_2)$	−0.7308	−0.8253	−0.6327	approximate	approximate
Together	$\log_{10}(k_2'/k_2)$	−5.747	< −8.784	> −1.976	optim	optim
b1	$\log_{10}(k_1')$	−0.06186	−0.656	1.069	approximate	approximate
b1	$\log_{10}(k_2)$	−0.275	−0.8733	−0.1802	approximate	approximate
b1	$\log_{10}(k_2')$	−0.907	−1.009	−0.5507	approximate	approximate
b1	$\log_{10}(k_{deg})$	−1.097	−1.218	> −0.9059	approximate	optim
b1	$\log_{10}(k_1'/k_2)$	0.2131	0.181	0.2459	approximate	approximate
b1	$\log_{10}(k_1'k_2'/k_2)$	−0.6939	−0.7002	> −0.5236	approximate	optim
b1	$\log_{10}(k_2'/k_2)$	−0.6321	−0.798	0.2874	approximate	approximate
b2	$\log_{10}(k_1'k_2')$	4.984	< 2.771	> 6.257	optim	optim
b2	$\log_{10}(k_2 \text{ or } k_{deg})$	2.348	< 1.252	> 2.997	optim	optim
b2	$\log_{10}(k_2 \text{ or } k_{deg})$	2.319	< 1.203	> 2.945	optim	optim
b3	$\log_{10}(k_1')$	5.285	< 1.275	> 8.283	optim	optim
b3	$\log_{10}(k_2)$	5.139	< 1.128	> 8.137	optim	optim
b3	$\log_{10}(k_2')$	−0.9402	−1.149	−0.7112	exact	exact
b3	$\log_{10}(k_{deg})$	−1.351	−1.657	−1.056	approximate	approximate
b3	$\log_{10}(k_1'/k_2)$	0.146	0.0944	0.1969	approximate	approximate
b3	$\log_{10}(k_1'k_2'/k_2)$	−0.7942	−0.9962	−0.7833	approximate	approximate
b3	$\log_{10}(k_2'/k_2)$	−6.08	< −9.077	> −2.063	optim	optim

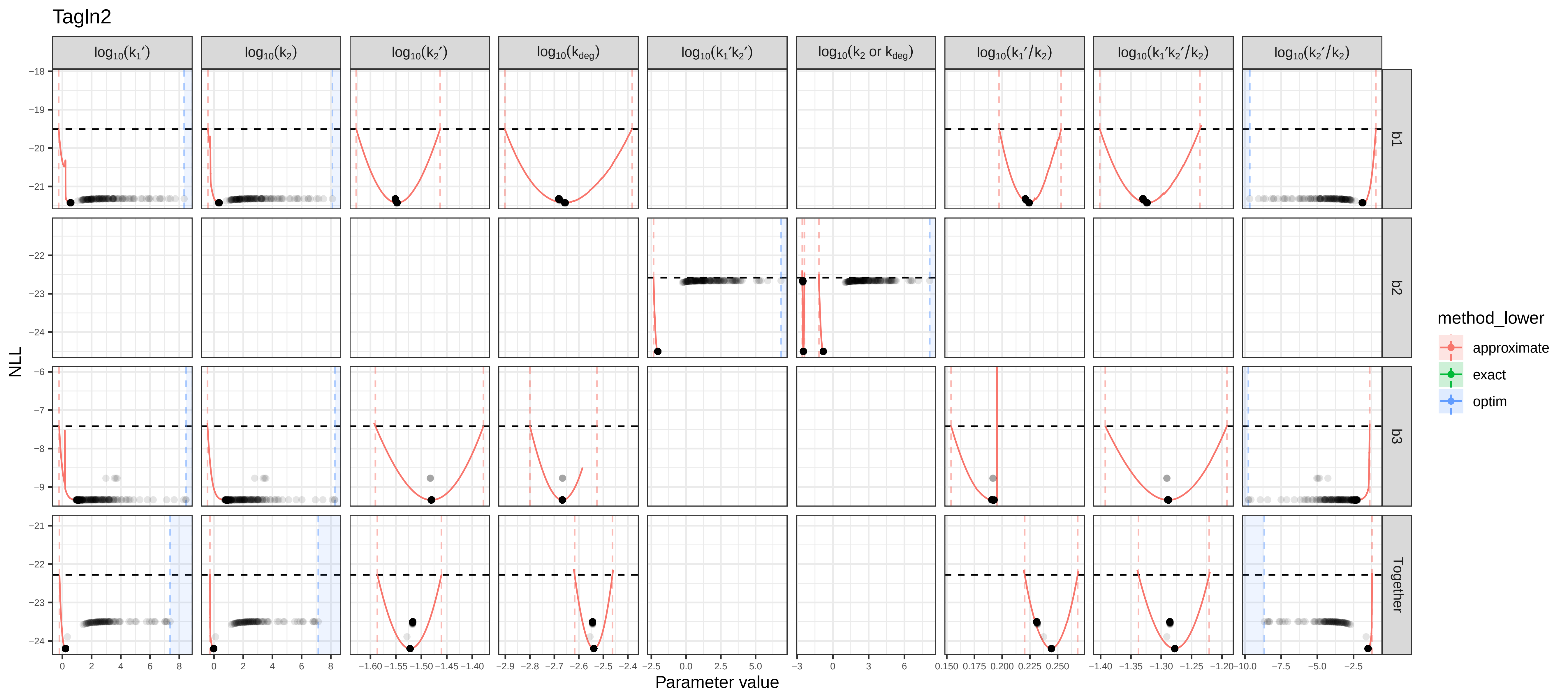

Replicate	Par	Best value	CI95 LB	CI95 UB	Method LB	Method UB
Together	$\log_{10}(k_1')$	0.2212	−0.1999	> 7.375	approximate	optim
Together	$\log_{10}(k_2)$	−0.0232	−0.2726	> 7.143	approximate	optim
Together	$\log_{10}(k_2')$	−1.522	−1.586	−1.461	approximate	approximate
Together	$\log_{10}(k_{deg})$	−2.539	−2.617	−2.463	approximate	approximate
Together	$\log_{10}(k_1'/k_2)$	0.2444	0.2203	0.2681	approximate	approximate
Together	$\log_{10}(k_1'k_2'/k_2)$	−1.278	−1.337	−1.221	approximate	approximate
Together	$\log_{10}(k_2'/k_2)$	−1.499	< −8.66	−1.221	optim	approximate
b1	$\log_{10}(k_1')$	0.566	−0.2558	> 8.333	approximate	optim
b1	$\log_{10}(k_2)$	0.3418	−0.4218	> 8.113	approximate	optim
b1	$\log_{10}(k_2')$	−1.548	−1.628	−1.463	approximate	approximate
b1	$\log_{10}(k_{deg})$	−2.657	−2.902	−2.382	approximate	approximate
b1	$\log_{10}(k_1'/k_2)$	0.2242	0.1972	0.2532	approximate	approximate
b1	$\log_{10}(k_1'k_2'/k_2)$	−1.324	−1.401	−1.236	approximate	approximate
b1	$\log_{10}(k_2'/k_2)$	−1.89	< −9.664	−0.9603	optim	approximate
b2	$\log_{10}(k_1'k_2')$	−2.035	−2.341	> 6.83	approximate	optim
b2	$\log_{10}(k_2 \text{ or } k_{deg})$	−0.7986	−1.169	> 8.119	approximate	optim
b2	$\log_{10}(k_2 \text{ or } k_{deg})$	−2.469	−2.56	−2.382	approximate	approximate
b3	$\log_{10}(k_1')$	1.137	−0.2247	> 8.468	approximate	optim
b3	$\log_{10}(k_2)$	0.9452	−0.4452	> 8.278	approximate	optim
b3	$\log_{10}(k_2')$	−1.48	−1.59	−1.378	approximate	approximate
b3	$\log_{10}(k_{deg})$	−2.667	−2.8	−2.526	approximate	approximate
b3	$\log_{10}(k_1'/k_2)$	0.1921	0.154	0.1954	approximate	approximate
b3	$\log_{10}(k_1'k_2'/k_2)$	−1.288	−1.392	−1.192	approximate	approximate
b3	$\log_{10}(k_2'/k_2)$	−2.426	< −9.757	−1.385	optim	approximate

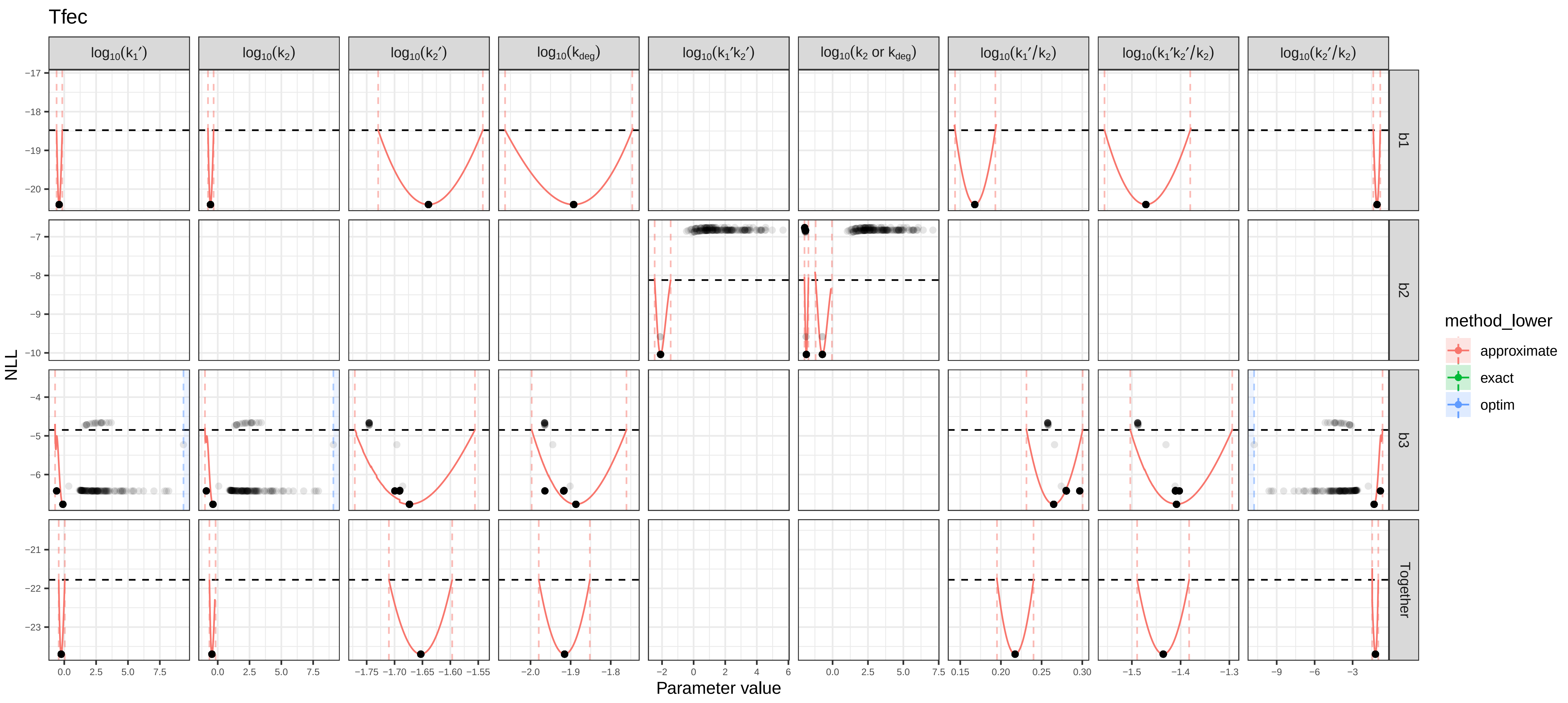

Replicate	Par	Best value	CI95 LB	CI95 UB	Method LB	Method UB
Together	$\log_{10}(k_1')$	−0.2386	−0.4335	0.04337	approximate	approximate
Together	$\log_{10}(k_2)$	−0.456	−0.6515	−0.1677	approximate	approximate
Together	$\log_{10}(k_2')$	−1.653	−1.71	−1.597	approximate	approximate
Together	$\log_{10}(k_{deg})$	−1.915	−1.98	−1.852	approximate	approximate
Together	$\log_{10}(k_1'/k_2)$	0.2174	0.1952	0.2401	approximate	approximate
Together	$\log_{10}(k_1'k_2'/k_2)$	−1.436	−1.489	−1.383	approximate	approximate
Together	$\log_{10}(k_2'/k_2)$	−1.197	−1.45	−0.9707	approximate	approximate
b1	$\log_{10}(k_1')$	−0.4008	−0.6006	−0.1554	approximate	approximate
b1	$\log_{10}(k_2)$	−0.5687	−0.7655	−0.3204	approximate	approximate
b1	$\log_{10}(k_2')$	−1.639	−1.729	−1.542	approximate	approximate
b1	$\log_{10}(k_{deg})$	−1.892	−2.063	−1.746	approximate	approximate
b1	$\log_{10}(k_1'/k_2)$	0.1679	0.1436	0.1932	approximate	approximate
b1	$\log_{10}(k_1'k_2'/k_2)$	−1.471	−1.556	−1.38	approximate	approximate
b1	$\log_{10}(k_2'/k_2)$	−1.07	−1.366	−0.8143	approximate	approximate
b2	$\log_{10}(k_1'k_2')$	−2.095	−2.467	−1.453	approximate	approximate
b2	$\log_{10}(k_2 \text{ or } k_{deg})$	−0.723	−1.197	−0.04472	approximate	approximate
b2	$\log_{10}(k_2 \text{ or } k_{deg})$	−1.855	−1.986	−1.708	approximate	approximate
b3	$\log_{10}(k_1')$	−0.1085	−0.7204	> 9.354	approximate	optim
b3	$\log_{10}(k_2)$	−0.3734	−1.002	> 9.088	approximate	optim
b3	$\log_{10}(k_2')$	−1.673	−1.771	−1.556	approximate	approximate
b3	$\log_{10}(k_{deg})$	−1.887	−1.997	−1.761	approximate	approximate
b3	$\log_{10}(k_1'/k_2)$	0.2648	0.2314	0.3005	approximate	approximate
b3	$\log_{10}(k_1'k_2'/k_2)$	−1.408	−1.503	−1.294	approximate	approximate
b3	$\log_{10}(k_2'/k_2)$	−1.3	< −10.78	−0.633	optim	approximate

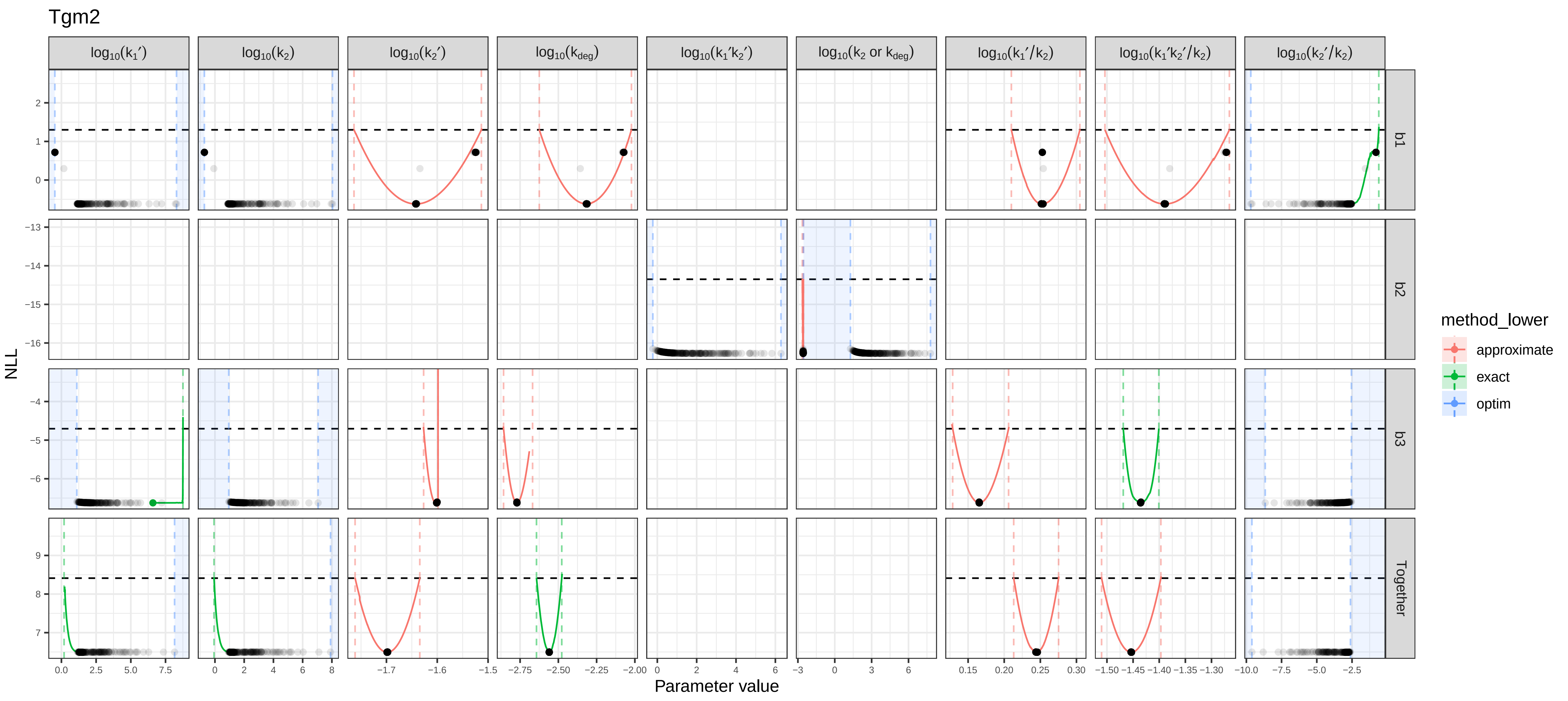

Replicate	Par	Best value	CI95 LB	CI95 UB	Method LB	Method UB
Together	$\log_{10}(k_1')$	1.516	0.191	> 8.151	exact	optim
Together	$\log_{10}(k_2)$	1.271	−0.06267	> 7.907	exact	optim
Together	$\log_{10}(k_2')$	−1.698	−1.762	−1.634	approximate	approximate
Together	$\log_{10}(k_{deg})$	−2.56	−2.643	−2.478	exact	exact
Together	$\log_{10}(k_1'/k_2)$	0.245	0.2131	0.2753	approximate	approximate
Together	$\log_{10}(k_1'k_2'/k_2)$	−1.453	−1.51	−1.397	approximate	approximate
Together	$\log_{10}(k_2'/k_2)$	−2.969	< −9.605	> −2.612	optim	optim
b1	$\log_{10}(k_1')$	1.441	< −0.4722	> 8.292	optim	optim
b1	$\log_{10}(k_2)$	1.188	< −0.7249	> 8.041	optim	optim
b1	$\log_{10}(k_2')$	−1.642	−1.764	−1.513	approximate	approximate
b1	$\log_{10}(k_{deg})$	−2.315	−2.624	−2.022	approximate	approximate
b1	$\log_{10}(k_1'/k_2)$	0.2527	0.2099	0.3047	approximate	approximate
b1	$\log_{10}(k_1'k_2'/k_2)$	−1.389	−1.504	−1.266	approximate	approximate
b1	$\log_{10}(k_2'/k_2)$	−2.83	< −9.682	−0.6011	optim	exact
b2	$\log_{10}(k_1'k_2')$	4.885	< −0.227	> 6.285	optim	optim
b2	$\log_{10}(k_2 \text{ or } k_{deg})$	6.386	1.274	> 7.786	optim	optim
b2	$\log_{10}(k_2 \text{ or } k_{deg})$	−2.571	−2.638	−2.55	approximate	approximate
b3	$\log_{10}(k_1')$	6.591	< 1.111	8.756	optim	exact
b3	$\log_{10}(k_2)$	6.426	< 0.9457	> 7.059	optim	optim
b3	$\log_{10}(k_2')$	−1.601	−1.627	−1.598	approximate	approximate
b3	$\log_{10}(k_{deg})$	−2.771	−2.858	−2.668	approximate	approximate
b3	$\log_{10}(k_1'/k_2)$	0.1655	0.1286	0.206	approximate	approximate
b3	$\log_{10}(k_1'k_2'/k_2)$	−1.436	−1.469	−1.401	exact	exact
b3	$\log_{10}(k_2'/k_2)$	−8.027	< −8.66	> −2.546	optim	optim

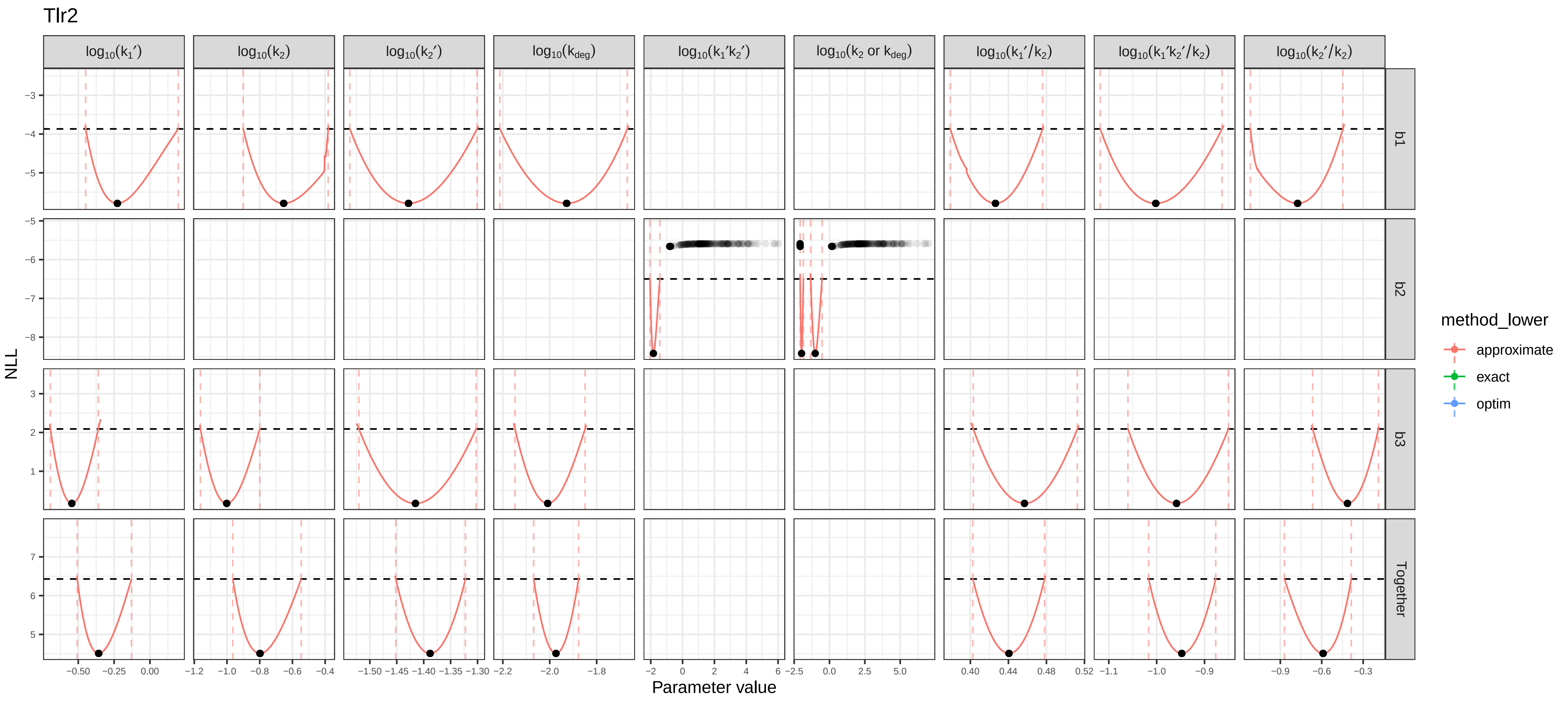

Replicate	Par	Best value	CI95 LB	CI95 UB	Method LB	Method UB
Together	$\log_{10}(k_1')$	−0.3572	−0.5074	−0.1286	approximate	approximate
Together	$\log_{10}(k_2)$	−0.7977	−0.9647	−0.5464	approximate	approximate
Together	$\log_{10}(k_2')$	−1.388	−1.452	−1.322	approximate	approximate
Together	$\log_{10}(k_{deg})$	−1.973	−2.068	−1.877	approximate	approximate
Together	$\log_{10}(k_1'/k_2)$	0.4405	0.4026	0.4781	approximate	approximate
Together	$\log_{10}(k_1'k_2'/k_2)$	−0.9476	−1.017	−0.8767	approximate	approximate
Together	$\log_{10}(k_2'/k_2)$	−0.5904	−0.8703	−0.3858	approximate	approximate
b1	$\log_{10}(k_1')$	−0.227	−0.4482	0.1973	approximate	approximate
b1	$\log_{10}(k_2)$	−0.6534	−0.9011	−0.3814	approximate	approximate
b1	$\log_{10}(k_2')$	−1.428	−1.537	−1.301	approximate	approximate
b1	$\log_{10}(k_{deg})$	−1.928	−2.212	−1.67	approximate	approximate
b1	$\log_{10}(k_1'/k_2)$	0.4264	0.3788	0.476	approximate	approximate
b1	$\log_{10}(k_1'k_2'/k_2)$	−1.002	−1.118	−0.8635	approximate	approximate
b1	$\log_{10}(k_2'/k_2)$	−0.7748	−1.117	−0.4468	approximate	approximate
b2	$\log_{10}(k_1'k_2')$	−1.834	−2.049	−1.429	approximate	approximate
b2	$\log_{10}(k_2 \text{ or } k_{deg})$	−1.008	−1.333	−0.5207	approximate	approximate
b2	$\log_{10}(k_2 \text{ or } k_{deg})$	−1.971	−2.072	−1.843	approximate	approximate
b3	$\log_{10}(k_1')$	−0.5448	−0.6944	−0.36	approximate	approximate
b3	$\log_{10}(k_2)$	−1.002	−1.162	−0.7996	approximate	approximate
b3	$\log_{10}(k_2')$	−1.415	−1.52	−1.303	approximate	approximate
b3	$\log_{10}(k_{deg})$	−2.009	−2.148	−1.849	approximate	approximate
b3	$\log_{10}(k_1'/k_2)$	0.4569	0.4031	0.5125	approximate	approximate
b3	$\log_{10}(k_1'k_2'/k_2)$	−0.9585	−1.06	−0.85	approximate	approximate
b3	$\log_{10}(k_2'/k_2)$	−0.4136	−0.6658	−0.1892	approximate	approximate

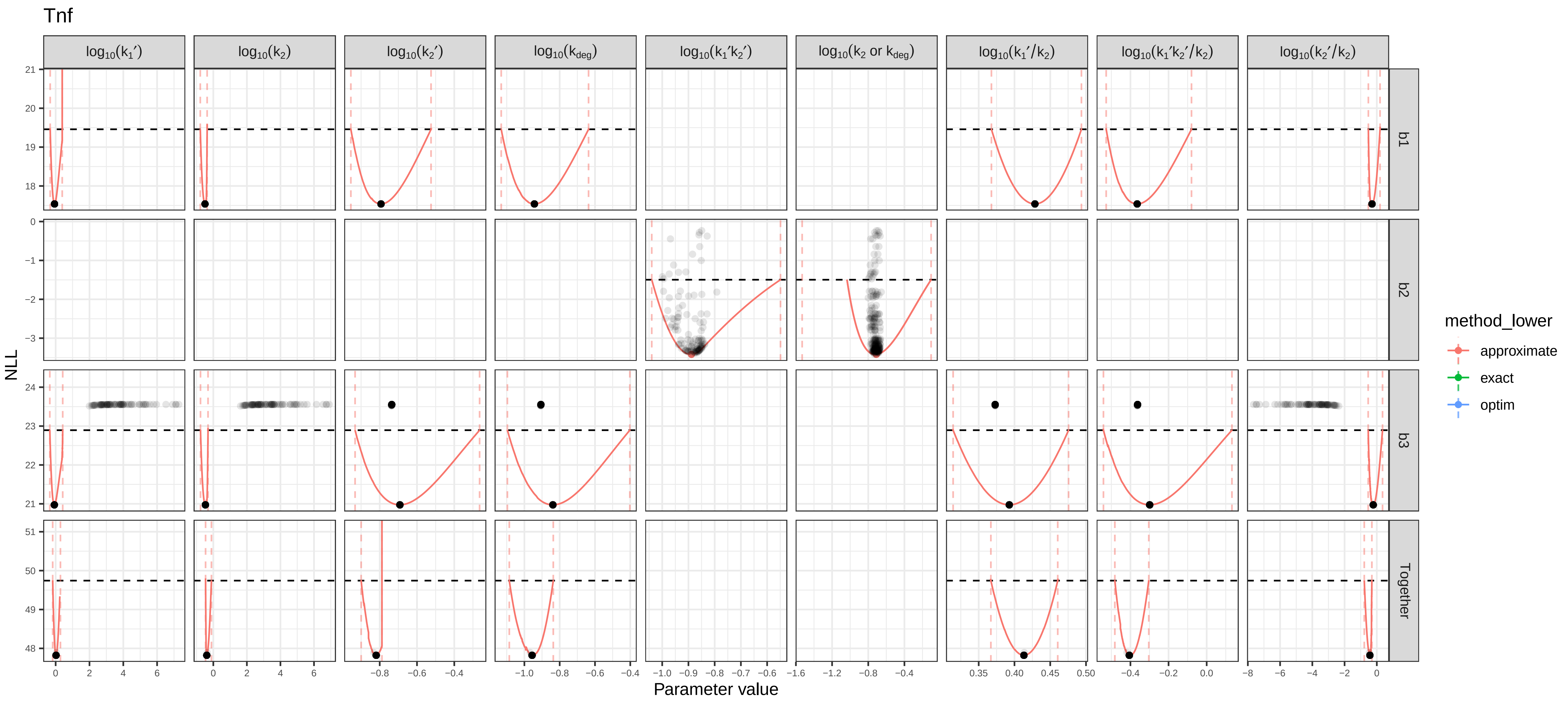

Replicate	Par	Best value	CI95 LB	CI95 UB	Method LB	Method UB
Together	$\log_{10}(k_1')$	0.02254	−0.175	0.2827	approximate	approximate
Together	$\log_{10}(k_2)$	−0.3907	−0.4612	−0.1116	approximate	approximate
Together	$\log_{10}(k_2')$	−0.8189	−0.8995	−0.7881	approximate	approximate
Together	$\log_{10}(k_{deg})$	−0.9562	−1.086	−0.8363	approximate	approximate
Together	$\log_{10}(k_1'/k_2)$	0.4132	0.367	0.4605	approximate	approximate
Together	$\log_{10}(k_1'k_2'/k_2)$	−0.4057	−0.4817	−0.3037	approximate	approximate
Together	$\log_{10}(k_2'/k_2)$	−0.4282	−0.7791	−0.3076	approximate	approximate
b1	$\log_{10}(k_1')$	−0.06675	−0.323	0.3885	approximate	approximate
b1	$\log_{10}(k_2)$	−0.4953	−0.7769	−0.3676	approximate	approximate
b1	$\log_{10}(k_2')$	−0.7931	−0.9548	−0.5246	approximate	approximate
b1	$\log_{10}(k_{deg})$	−0.9433	−1.131	−0.6364	approximate	approximate
b1	$\log_{10}(k_1'/k_2)$	0.4286	0.3678	0.4936	approximate	approximate
b1	$\log_{10}(k_1'k_2'/k_2)$	−0.3645	−0.5272	−0.07961	approximate	approximate
b1	$\log_{10}(k_2'/k_2)$	−0.2977	−0.5254	0.1993	approximate	approximate
b2	$\log_{10}(k_1'k_2')$	−0.8885	−1.04	−0.549	approximate	approximate
b2	$\log_{10}(k_2 \text{ or } k_{deg})$	−0.7108	−1.531	−0.105	approximate	approximate
b2	$\log_{10}(k_2 \text{ or } k_{deg})$	−0.731	−1.531	−0.105	approximate	approximate
b3	$\log_{10}(k_1')$	−0.07739	−0.3457	0.4176	approximate	approximate
b3	$\log_{10}(k_2)$	−0.4701	−0.7636	−0.3149	approximate	approximate
b3	$\log_{10}(k_2')$	−0.6917	−0.9322	−0.2641	approximate	approximate
b3	$\log_{10}(k_{deg})$	−0.8385	−1.096	−0.4025	approximate	approximate
b3	$\log_{10}(k_1'/k_2)$	0.3928	0.3142	0.4756	approximate	approximate
b3	$\log_{10}(k_1'k_2'/k_2)$	−0.2989	−0.5414	0.1325	approximate	approximate
b3	$\log_{10}(k_2'/k_2)$	−0.2215	−0.5353	0.3549	approximate	approximate

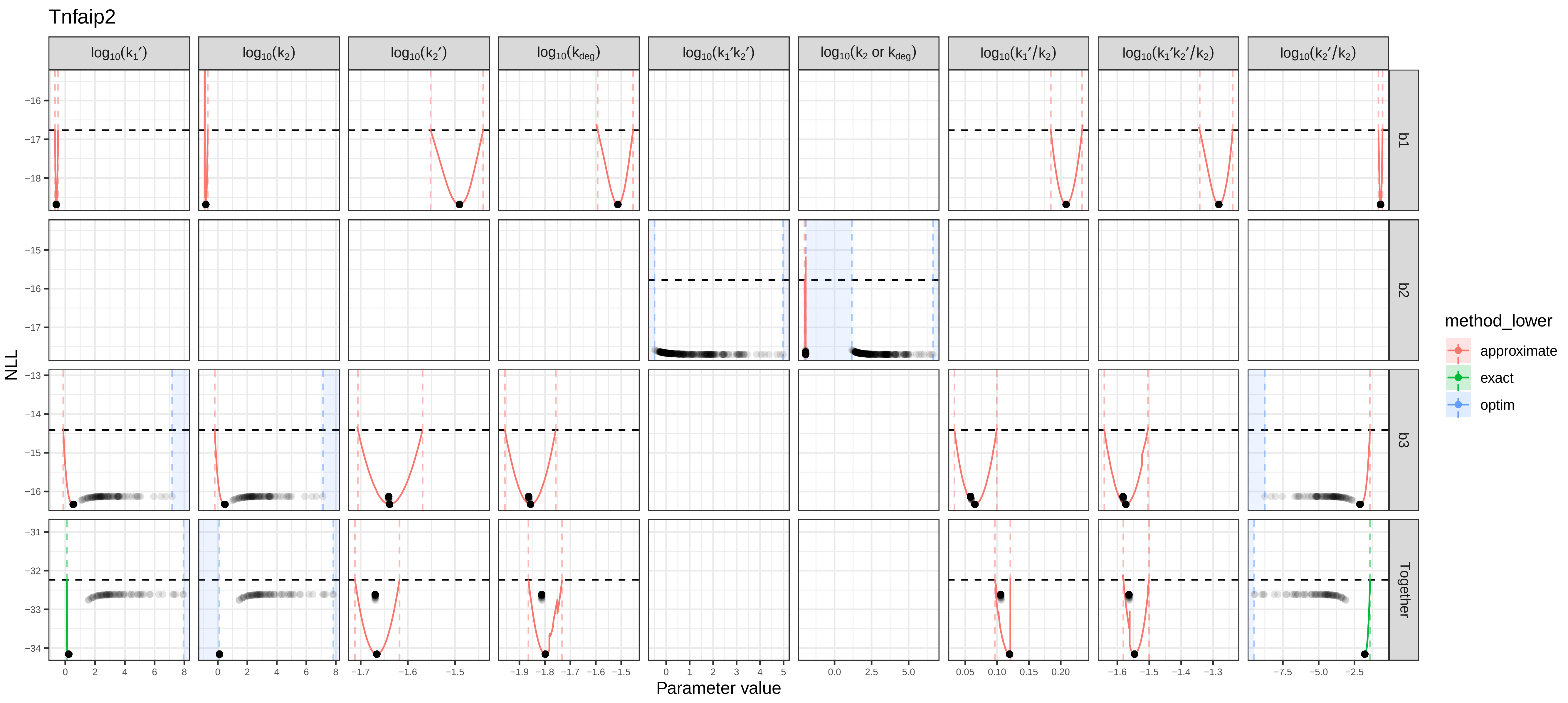

Replicate	Par	Best value	CI95 LB	CI95 UB	Method LB	Method UB
Together	$\log_{10}(k_1')$	0.2344	0.1092	> 7.938	exact	optim
Together	$\log_{10}(k_2)$	0.1147	< 0.1071	> 7.833	optim	optim
Together	$\log_{10}(k_2')$	−1.666	−1.712	−1.618	approximate	approximate
Together	$\log_{10}(k_{deg})$	−1.798	−1.865	−1.732	approximate	approximate
Together	$\log_{10}(k_1'/k_2)$	0.1197	0.09637	0.1209	approximate	approximate
Together	$\log_{10}(k_1'k_2'/k_2)$	−1.546	−1.581	−1.501	approximate	approximate
Together	$\log_{10}(k_2'/k_2)$	−1.78	< −9.502	−1.414	optim	exact
b1	$\log_{10}(k_1')$	−0.6056	−0.6895	−0.4794	approximate	approximate
b1	$\log_{10}(k_2)$	−0.814	−0.8774	−0.6808	approximate	approximate
b1	$\log_{10}(k_2')$	−1.491	−1.552	−1.44	approximate	approximate
b1	$\log_{10}(k_{deg})$	−1.513	−1.593	−1.453	approximate	approximate
b1	$\log_{10}(k_1'/k_2)$	0.2085	0.1844	0.2338	approximate	approximate
b1	$\log_{10}(k_1'k_2'/k_2)$	−1.282	−1.342	−1.239	approximate	approximate
b1	$\log_{10}(k_2'/k_2)$	−0.6766	−0.8289	−0.5435	approximate	approximate
b2	$\log_{10}(k_1'k_2')$	3.125	< −0.4961	> 4.977	optim	optim
b2	$\log_{10}(k_2 \text{ or } k_{deg})$	4.789	1.167	> 6.641	optim	optim
b2	$\log_{10}(k_2 \text{ or } k_{deg})$	−1.953	−2.03	−1.946	approximate	approximate
b3	$\log_{10}(k_1')$	0.5351	−0.1343	> 7.175	approximate	optim
b3	$\log_{10}(k_2)$	0.4699	−0.2108	> 7.117	approximate	optim
b3	$\log_{10}(k_2')$	−1.639	−1.706	−1.569	approximate	approximate
b3	$\log_{10}(k_{deg})$	−1.856	−1.957	−1.757	approximate	approximate
b3	$\log_{10}(k_1'/k_2)$	0.06519	0.03295	0.09944	approximate	approximate
b3	$\log_{10}(k_1'k_2'/k_2)$	−1.573	−1.641	−1.505	approximate	approximate
b3	$\log_{10}(k_2'/k_2)$	−2.109	< −8.757	−1.421	optim	approximate

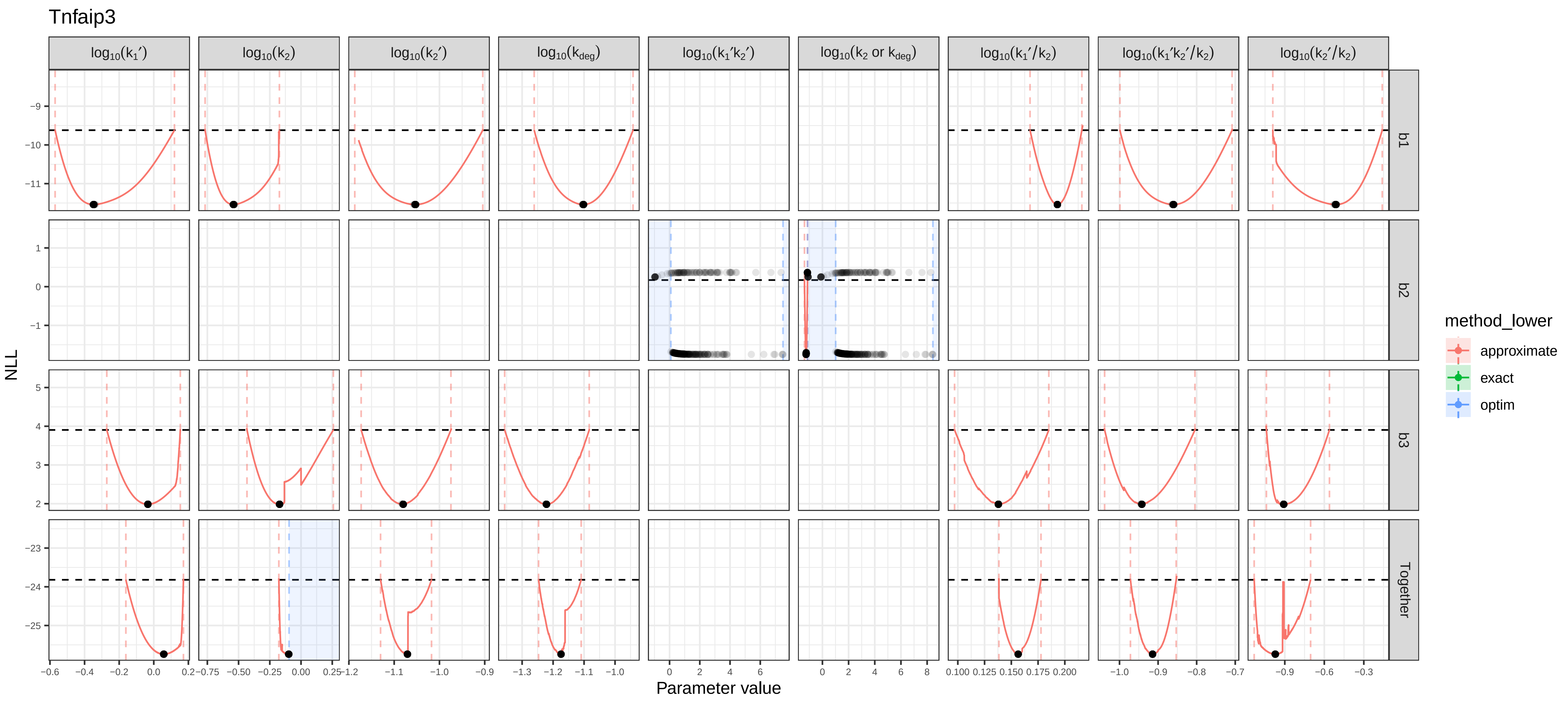

Replicate	Par	Best value	CI95 LB	CI95 UB	Method LB	Method UB
Together	$\log_{10}(k_1')$	0.05811	−0.1612	0.1715	approximate	approximate
Together	$\log_{10}(k_2)$	−0.09831	−0.1775	> −0.09591	approximate	optim
Together	$\log_{10}(k_2')$	−1.071	−1.13	−1.017	approximate	approximate
Together	$\log_{10}(k_{deg})$	−1.174	−1.247	−1.109	approximate	approximate
Together	$\log_{10}(k_1'/k_2)$	0.1564	0.1384	0.1777	approximate	approximate
Together	$\log_{10}(k_1'k_2'/k_2)$	−0.9143	−0.9717	−0.853	approximate	approximate
Together	$\log_{10}(k_2'/k_2)$	−0.9724	−1.132	−0.7039	approximate	approximate
b1	$\log_{10}(k_1')$	−0.3473	−0.5703	0.1195	approximate	approximate
b1	$\log_{10}(k_2)$	−0.5403	−0.7678	−0.1732	approximate	approximate
b1	$\log_{10}(k_2')$	−1.053	−1.187	−0.9043	approximate	approximate
b1	$\log_{10}(k_{deg})$	−1.102	−1.261	−0.9418	approximate	approximate
b1	$\log_{10}(k_1'/k_2)$	0.1931	0.1675	0.2159	approximate	approximate
b1	$\log_{10}(k_1'k_2'/k_2)$	−0.8602	−0.9989	−0.7082	approximate	approximate
b1	$\log_{10}(k_2'/k_2)$	−0.513	−0.9909	−0.1599	approximate	approximate
b2	$\log_{10}(k_1'k_2')$	3.724	< 0.08747	> 7.506	optim	optim
b2	$\log_{10}(k_2 \text{ or } k_{deg})$	4.658	1.014	> 8.442	optim	optim
b2	$\log_{10}(k_2 \text{ or } k_{deg})$	−1.249	−1.373	−1.139	approximate	approximate
b3	$\log_{10}(k_1')$	−0.0342	−0.2715	0.1534	approximate	approximate
b3	$\log_{10}(k_2)$	−0.1721	−0.4326	0.2587	approximate	approximate
b3	$\log_{10}(k_2')$	−1.08	−1.173	−0.9746	approximate	approximate
b3	$\log_{10}(k_{deg})$	−1.221	−1.356	−1.083	approximate	approximate
b3	$\log_{10}(k_1'/k_2)$	0.1379	0.09697	0.1851	approximate	approximate
b3	$\log_{10}(k_1'k_2'/k_2)$	−0.9421	−1.039	−0.8045	approximate	approximate
b3	$\log_{10}(k_2'/k_2)$	−0.9079	−1.04	−0.5609	approximate	approximate

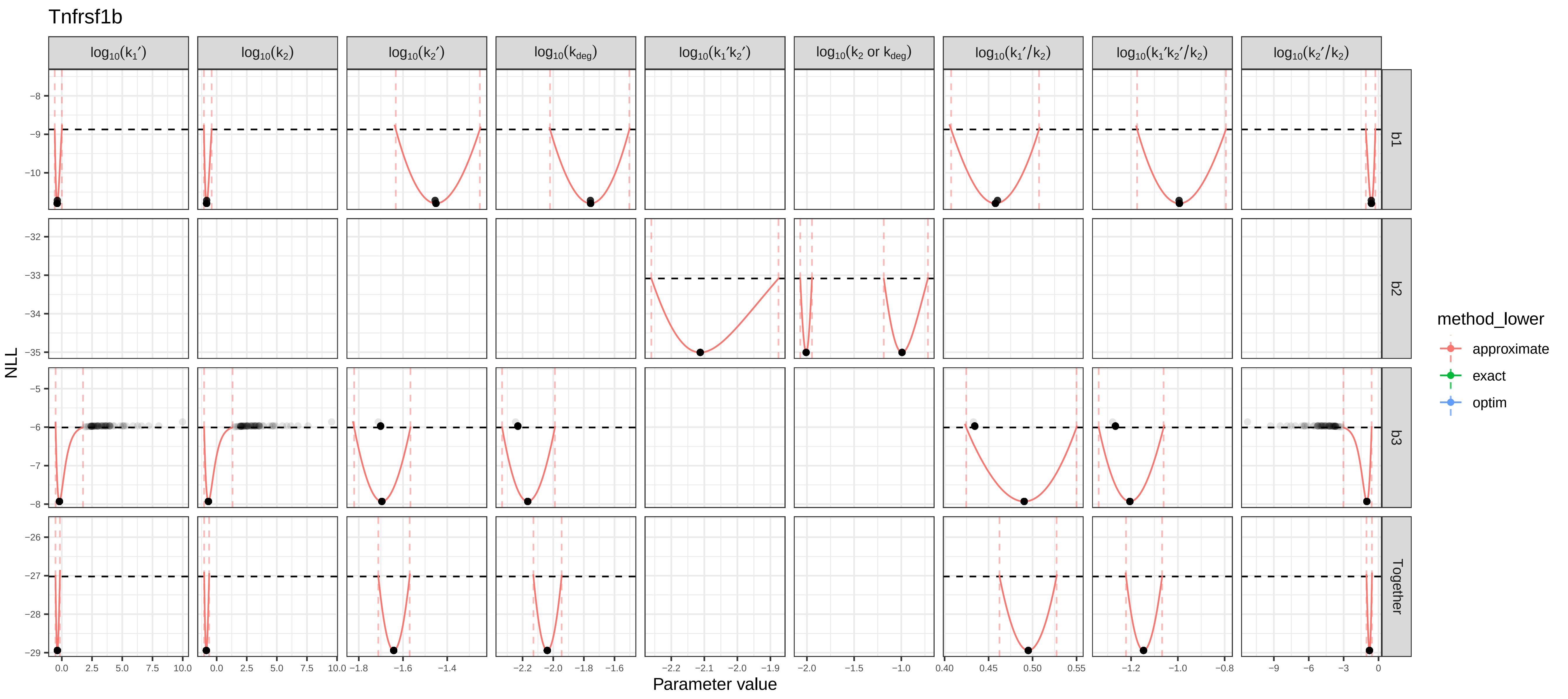

Replicate	Par	Best value	CI95 LB	CI95 UB	Method LB	Method UB
Together	$\log_{10}(k_1')$	−0.3669	−0.5228	−0.1565	approximate	approximate
Together	$\log_{10}(k_2)$	−0.8621	−1.039	−0.6284	approximate	approximate
Together	$\log_{10}(k_2')$	−1.642	−1.712	−1.57	approximate	approximate
Together	$\log_{10}(k_{deg})$	−2.039	−2.129	−1.945	approximate	approximate
Together	$\log_{10}(k_1'/k_2)$	0.4951	0.4624	0.5273	approximate	approximate
Together	$\log_{10}(k_1'k_2'/k_2)$	−1.147	−1.222	−1.067	approximate	approximate
Together	$\log_{10}(k_2'/k_2)$	−0.7799	−1.036	−0.5664	approximate	approximate
b1	$\log_{10}(k_1')$	−0.3799	−0.5849	0.004024	approximate	approximate
b1	$\log_{10}(k_2)$	−0.8377	−1.062	−0.4167	approximate	approximate
b1	$\log_{10}(k_2')$	−1.451	−1.633	−1.253	approximate	approximate
b1	$\log_{10}(k_{deg})$	−1.756	−2.021	−1.503	approximate	approximate
b1	$\log_{10}(k_1'/k_2)$	0.4577	0.4074	0.5074	approximate	approximate
b1	$\log_{10}(k_1'k_2'/k_2)$	−0.9934	−1.174	−0.7945	approximate	approximate
b1	$\log_{10}(k_2'/k_2)$	−0.6134	−1.091	−0.2755	approximate	approximate
b2	$\log_{10}(k_1'k_2')$	−2.112	−2.26	−1.876	approximate	approximate
b2	$\log_{10}(k_2 \text{ or } k_{deg})$	−0.9923	−1.186	−0.7182	approximate	approximate
b2	$\log_{10}(k_2 \text{ or } k_{deg})$	−2.009	−2.071	−1.947	approximate	approximate
b3	$\log_{10}(k_1')$	−0.1992	−0.5117	1.754	approximate	approximate
b3	$\log_{10}(k_2)$	−0.6897	−1.046	1.32	approximate	approximate
b3	$\log_{10}(k_2')$	−1.696	−1.821	−1.566	approximate	approximate
b3	$\log_{10}(k_{deg})$	−2.166	−2.334	−1.989	approximate	approximate
b3	$\log_{10}(k_1'/k_2)$	0.4905	0.4246	0.5499	approximate	approximate
b3	$\log_{10}(k_1'k_2'/k_2)$	−1.205	−1.338	−1.061	approximate	approximate
b3	$\log_{10}(k_2'/k_2)$	−1.006	−3.021	−0.595	approximate	approximate

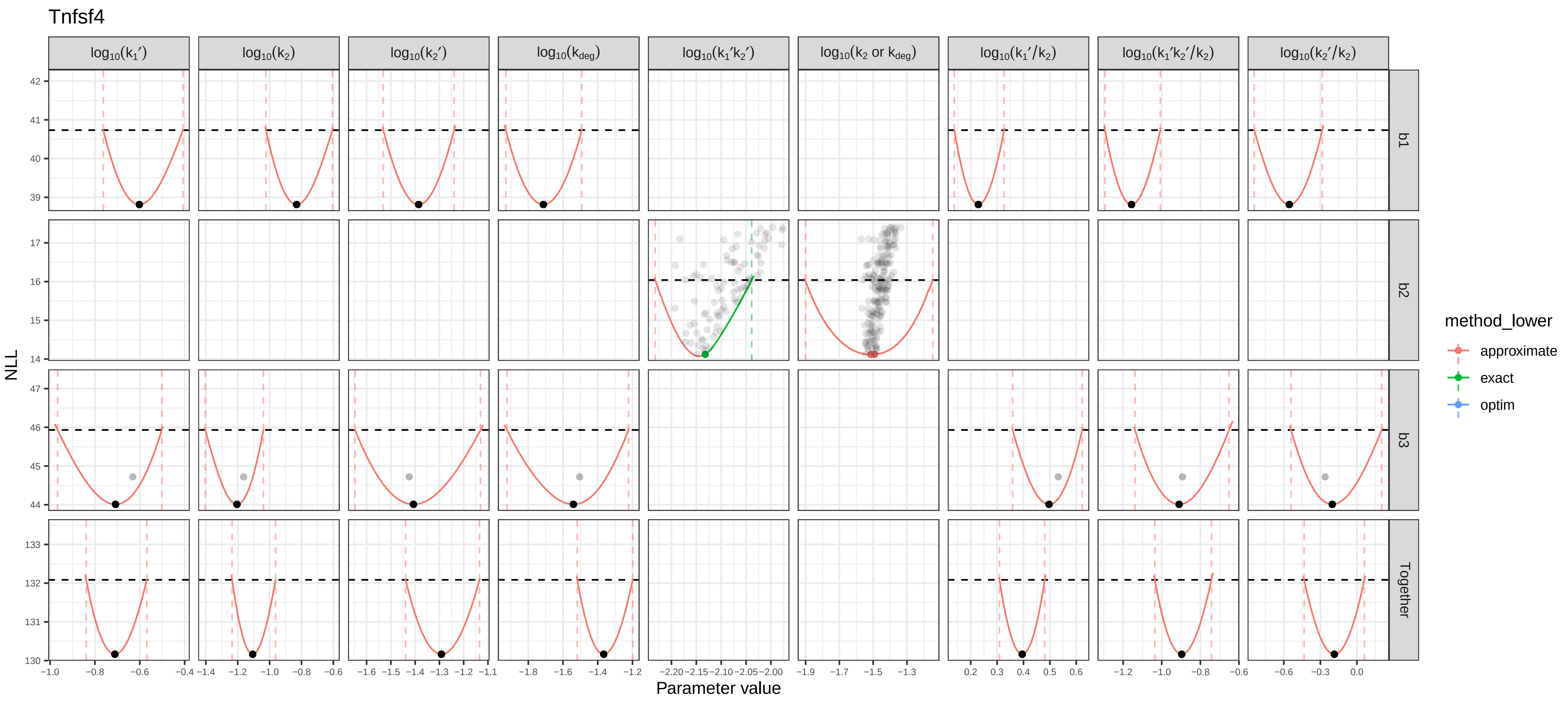

Replicate	Par	Best value	CI95 LB	CI95 UB	Method LB	Method UB
Together	$\log_{10}(k_1')$	−0.7111	−0.8391	−0.5687	approximate	approximate
Together	$\log_{10}(k_2)$	−1.106	−1.235	−0.9623	approximate	approximate
Together	$\log_{10}(k_2')$	−1.292	−1.439	−1.135	approximate	approximate
Together	$\log_{10}(k_{deg})$	−1.366	−1.518	−1.199	approximate	approximate
Together	$\log_{10}(k_1'/k_2)$	0.3951	0.309	0.4806	approximate	approximate
Together	$\log_{10}(k_1'k_2'/k_2)$	−0.8966	−1.036	−0.7426	approximate	approximate
Together	$\log_{10}(k_2'/k_2)$	−0.1854	−0.4334	0.06086	approximate	approximate
b1	$\log_{10}(k_1')$	−0.6019	−0.7627	−0.4076	approximate	approximate
b1	$\log_{10}(k_2)$	−0.8307	−1.023	−0.606	approximate	approximate
b1	$\log_{10}(k_2')$	−1.385	−1.531	−1.239	approximate	approximate
b1	$\log_{10}(k_{deg})$	−1.713	−1.929	−1.492	approximate	approximate
b1	$\log_{10}(k_1'/k_2)$	0.2288	0.1375	0.3266	approximate	approximate
b1	$\log_{10}(k_1'k_2'/k_2)$	−1.157	−1.294	−1.008	approximate	approximate
b1	$\log_{10}(k_2'/k_2)$	−0.5548	−0.8424	−0.2841	approximate	approximate
b2	$\log_{10}(k_1'k_2')$	−2.132	−2.232	−2.039	approximate	exact
b2	$\log_{10}(k_2 \text{ or } k_{deg})$	−1.493	−1.9	−1.147	approximate	approximate
b2	$\log_{10}(k_2 \text{ or } k_{deg})$	−1.513	−1.9	−1.147	approximate	approximate
b3	$\log_{10}(k_1')$	−0.7082	−0.9664	−0.502	approximate	approximate
b3	$\log_{10}(k_2)$	−1.205	−1.404	−1.039	approximate	approximate
b3	$\log_{10}(k_2')$	−1.407	−1.648	−1.131	approximate	approximate
b3	$\log_{10}(k_{deg})$	−1.54	−1.923	−1.223	approximate	approximate
b3	$\log_{10}(k_1'/k_2)$	0.4967	0.359	0.6227	approximate	approximate
b3	$\log_{10}(k_1'k_2'/k_2)$	−0.9101	−1.139	−0.6511	approximate	approximate
b3	$\log_{10}(k_2'/k_2)$	−0.2019	−0.541	0.2031	approximate	approximate

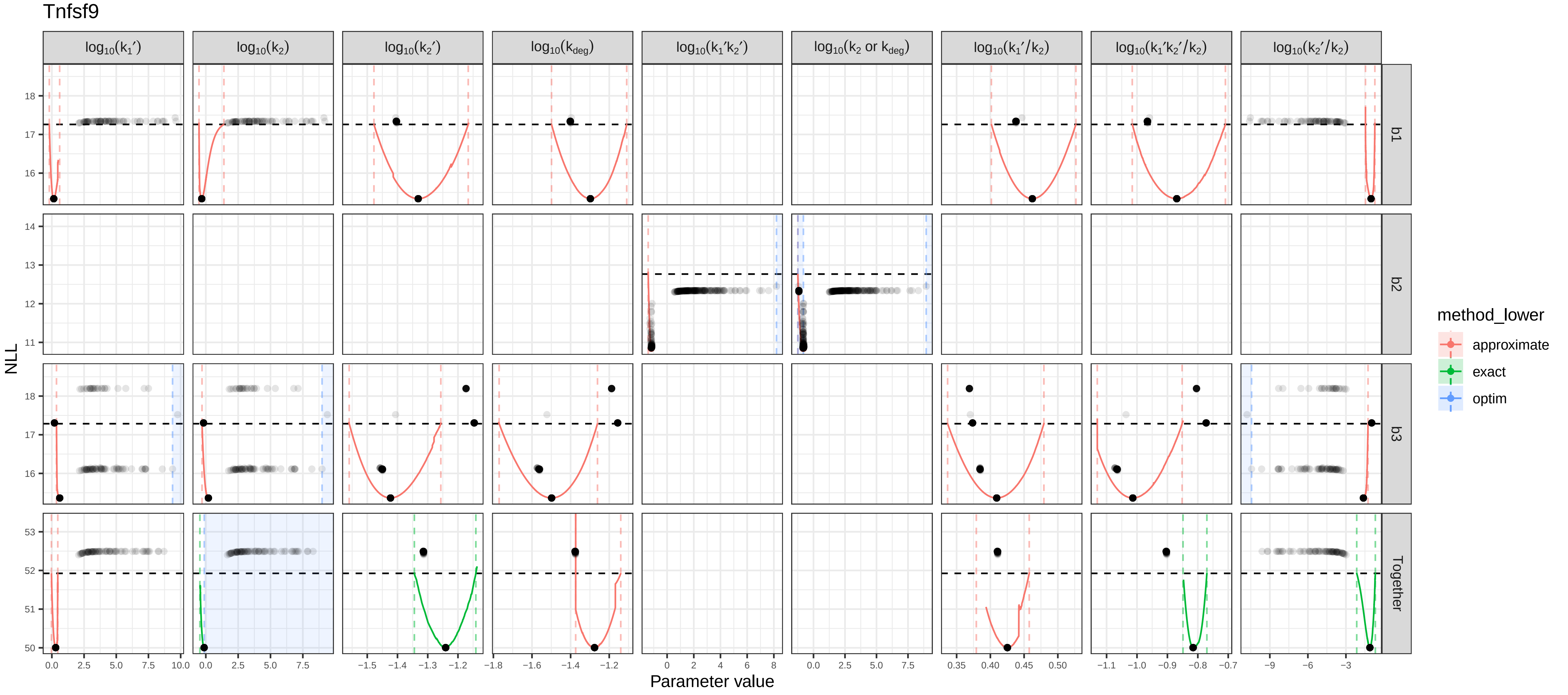

Replicate	Par	Best value	CI95 LB	CI95 UB	Method LB	Method UB
Together	$\log_{10}(k_1')$	0.2953	−0.03405	0.4608	approximate	approximate
Together	$\log_{10}(k_2)$	−0.13	−0.4586	> −0.1217	exact	optim
Together	$\log_{10}(k_2')$	−1.241	−1.344	−1.141	exact	exact
Together	$\log_{10}(k_{deg})$	−1.276	−1.374	−1.14	approximate	approximate
Together	$\log_{10}(k_1'/k_2)$	0.4253	0.3792	0.4576	approximate	approximate
Together	$\log_{10}(k_1'k_2'/k_2)$	−0.8154	−0.8487	−0.7702	exact	exact
Together	$\log_{10}(k_2'/k_2)$	−1.111	−2.149	−0.6822	exact	exact
b1	$\log_{10}(k_1')$	0.1479	−0.1986	0.6045	approximate	approximate
b1	$\log_{10}(k_2)$	−0.3143	−0.5258	1.4	approximate	approximate
b1	$\log_{10}(k_2')$	−1.331	−1.478	−1.166	approximate	approximate
b1	$\log_{10}(k_{deg})$	−1.298	−1.499	−1.109	approximate	approximate
b1	$\log_{10}(k_1'/k_2)$	0.4622	0.4015	0.5268	approximate	approximate
b1	$\log_{10}(k_1'k_2'/k_2)$	−0.8692	−1.016	−0.7093	approximate	approximate
b1	$\log_{10}(k_2'/k_2)$	−1.017	−1.459	−0.7189	approximate	approximate
b2	$\log_{10}(k_1'k_2')$	−1.188	−1.418	> 8.197	approximate	optim
b2	$\log_{10}(k_2 \text{ or } k_{deg})$	−0.7981	−1.241	> 8.944	approximate	optim
b2	$\log_{10}(k_2 \text{ or } k_{deg})$	−0.8321	−1.241	−0.8012	approximate	optim
b3	$\log_{10}(k_1')$	0.6078	0.3593	> 9.372	approximate	optim
b3	$\log_{10}(k_2)$	0.1984	−0.2938	> 8.987	approximate	optim
b3	$\log_{10}(k_2')$	−1.423	−1.56	−1.257	approximate	approximate
b3	$\log_{10}(k_{deg})$	−1.498	−1.77	−1.261	approximate	approximate
b3	$\log_{10}(k_1'/k_2)$	0.4094	0.3367	0.4793	approximate	approximate
b3	$\log_{10}(k_1'k_2'/k_2)$	−1.014	−1.131	−0.8517	approximate	approximate
b3	$\log_{10}(k_2'/k_2)$	−1.621	< −10.44	−1.257	optim	approximate

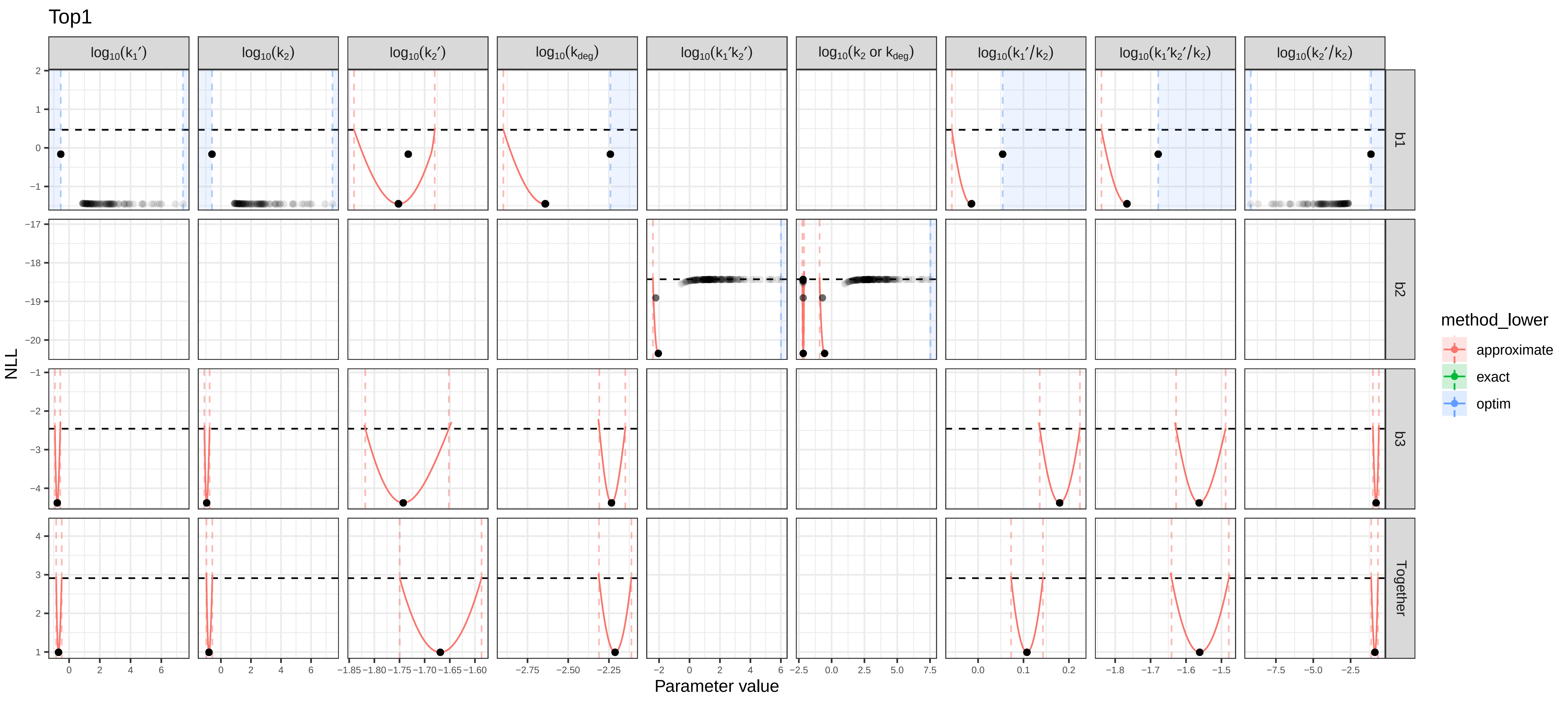

Replicate	Par	Best value	CI95 LB	CI95 UB	Method LB	Method UB
Together	$\log_{10}(k_1')$	−0.6874	−0.8417	−0.48	approximate	approximate
Together	$\log_{10}(k_2)$	−0.795	−0.9641	−0.5714	approximate	approximate
Together	$\log_{10}(k_2')$	−1.669	−1.75	−1.587	approximate	approximate
Together	$\log_{10}(k_{deg})$	−2.212	−2.311	−2.112	approximate	approximate
Together	$\log_{10}(k_1'/k_2)$	0.1076	0.07267	0.1428	approximate	approximate
Together	$\log_{10}(k_1'k_2'/k_2)$	−1.561	−1.641	−1.479	approximate	approximate
Together	$\log_{10}(k_2'/k_2)$	−0.8739	−1.122	−0.6681	approximate	approximate
b1	$\log_{10}(k_1')$	4.858	< −0.5412	> 7.412	optim	optim
b1	$\log_{10}(k_2)$	4.873	< −0.5954	> 7.427	optim	optim
b1	$\log_{10}(k_2')$	−1.752	−1.84	−1.68	approximate	approximate
b1	$\log_{10}(k_{deg})$	−2.641	−2.898	> −2.24	approximate	optim
b1	$\log_{10}(k_1'/k_2)$	−0.01497	−0.0578	> 0.05419	approximate	optim
b1	$\log_{10}(k_1'k_2'/k_2)$	−1.767	−1.839	> −1.678	approximate	optim
b1	$\log_{10}(k_2'/k_2)$	−6.625	< −9.179	> −1.137	optim	optim
b2	$\log_{10}(k_1'k_2')$	−2.049	−2.404	> 6.007	approximate	optim
b2	$\log_{10}(k_2 \text{ or } k_{deg})$	−0.5383	−0.9184	> 7.533	approximate	optim
b2	$\log_{10}(k_2 \text{ or } k_{deg})$	−2.166	−2.234	−2.105	approximate	approximate
b3	$\log_{10}(k_1')$	−0.769	−0.9225	−0.5742	approximate	approximate
b3	$\log_{10}(k_2)$	−0.9486	−1.1	−0.7522	approximate	approximate
b3	$\log_{10}(k_2')$	−1.743	−1.818	−1.652	approximate	approximate
b3	$\log_{10}(k_{deg})$	−2.234	−2.309	−2.15	approximate	approximate
b3	$\log_{10}(k_1'/k_2)$	0.1796	0.1358	0.2242	approximate	approximate
b3	$\log_{10}(k_1'k_2'/k_2)$	−1.563	−1.628	−1.488	approximate	approximate
b3	$\log_{10}(k_2'/k_2)$	−0.794	−1.005	−0.6069	approximate	approximate

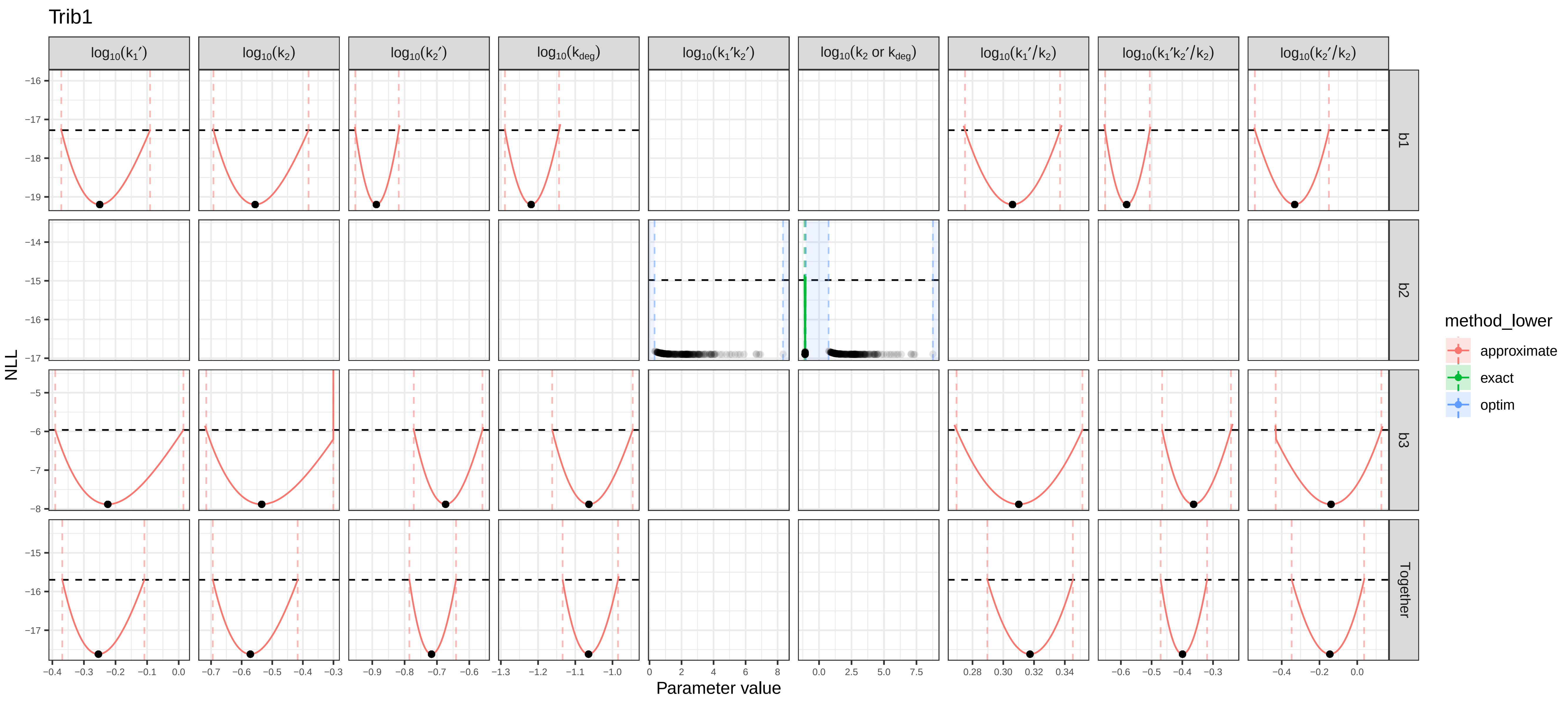

Replicate	Par	Best value	CI95 LB	CI95 UB	Method LB	Method UB
Together	$\log_{10}(k_1')$	−0.2541	−0.3686	−0.1087	approximate	approximate
Together	$\log_{10}(k_2)$	−0.5715	−0.6943	−0.4169	approximate	approximate
Together	$\log_{10}(k_2')$	−0.7167	−0.7853	−0.641	approximate	approximate
Together	$\log_{10}(k_{deg})$	−1.064	−1.134	−0.9851	approximate	approximate
Together	$\log_{10}(k_1'/k_2)$	0.3174	0.2896	0.3452	approximate	approximate
Together	$\log_{10}(k_1'k_2'/k_2)$	−0.3993	−0.4706	−0.3193	approximate	approximate
Together	$\log_{10}(k_2'/k_2)$	−0.1452	−0.3473	0.03635	approximate	approximate
b1	$\log_{10}(k_1')$	−0.2501	−0.3717	−0.09059	approximate	approximate
b1	$\log_{10}(k_2)$	−0.5561	−0.6922	−0.3814	approximate	approximate
b1	$\log_{10}(k_2')$	−0.8873	−0.9526	−0.8181	approximate	approximate
b1	$\log_{10}(k_{deg})$	−1.219	−1.289	−1.144	approximate	approximate
b1	$\log_{10}(k_1'/k_2)$	0.306	0.2751	0.3369	approximate	approximate
b1	$\log_{10}(k_1'k_2'/k_2)$	−0.5813	−0.6514	−0.506	approximate	approximate
b1	$\log_{10}(k_2'/k_2)$	−0.3312	−0.5422	−0.1502	approximate	approximate
b2	$\log_{10}(k_1'k_2')$	6.934	< 0.3146	> 8.338	optim	optim
b2	$\log_{10}(k_2 \text{ or } k_{deg})$	7.362	0.7347	> 8.765	optim	optim
b2	$\log_{10}(k_2 \text{ or } k_{deg})$	−1.078	−1.123	−1.03	exact	exact
b3	$\log_{10}(k_1')$	−0.2243	−0.3909	0.01494	approximate	approximate
b3	$\log_{10}(k_2)$	−0.5344	−0.7156	−0.3004	approximate	approximate
b3	$\log_{10}(k_2')$	−0.6733	−0.7717	−0.5593	approximate	approximate
b3	$\log_{10}(k_{deg})$	−1.063	−1.162	−0.9456	approximate	approximate
b3	$\log_{10}(k_1'/k_2)$	0.3101	0.2696	0.3514	approximate	approximate
b3	$\log_{10}(k_1'k_2'/k_2)$	−0.3632	−0.4657	−0.2416	approximate	approximate
b3	$\log_{10}(k_2'/k_2)$	−0.1389	−0.4321	0.1278	approximate	approximate

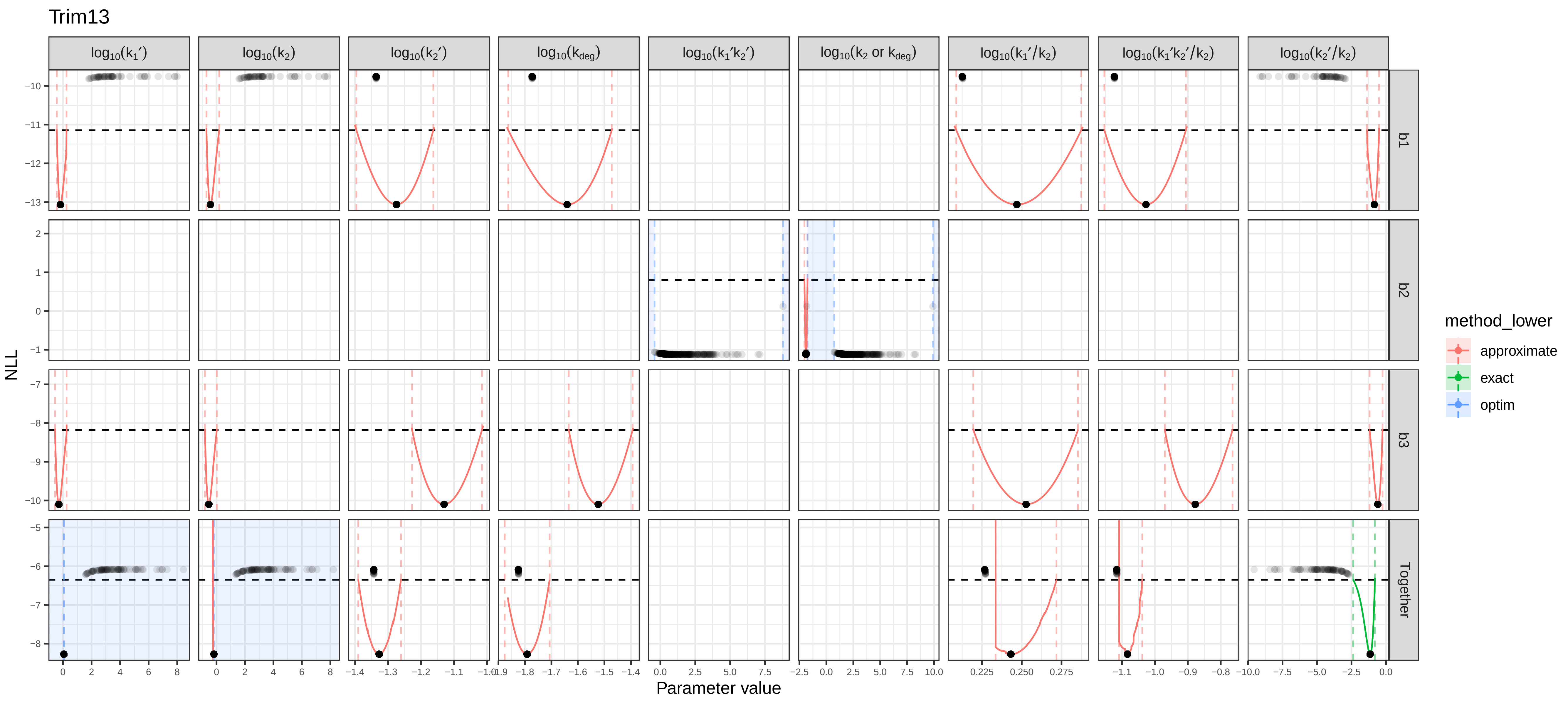

Replicate	Par	Best value	CI95 LB	CI95 UB	Method LB	Method UB
Together	$\log_{10}(k_1')$	0.06259	< 0.05976	> 0.06824	optim	optim
Together	$\log_{10}(k_2)$	−0.1806	−0.2525	> −0.1747	approximate	optim
Together	$\log_{10}(k_2')$	−1.327	−1.39	−1.261	approximate	approximate
Together	$\log_{10}(k_{deg})$	−1.792	−1.876	−1.707	approximate	approximate
Together	$\log_{10}(k_1'/k_2)$	0.2432	0.2335	0.2718	approximate	approximate
Together	$\log_{10}(k_1'k_2'/k_2)$	−1.084	−1.109	−1.039	approximate	approximate
Together	$\log_{10}(k_2'/k_2)$	−1.146	−2.38	−0.8051	exact	exact
b1	$\log_{10}(k_1')$	−0.182	−0.4348	0.2443	approximate	approximate
b1	$\log_{10}(k_2)$	−0.4289	−0.7073	0.1919	approximate	approximate
b1	$\log_{10}(k_2')$	−1.274	−1.396	−1.163	approximate	approximate
b1	$\log_{10}(k_{deg})$	−1.641	−1.863	−1.472	approximate	approximate
b1	$\log_{10}(k_1'/k_2)$	0.2469	0.2088	0.2874	approximate	approximate
b1	$\log_{10}(k_1'k_2'/k_2)$	−1.027	−1.154	−0.9061	approximate	approximate
b1	$\log_{10}(k_2'/k_2)$	−0.8455	−1.373	−0.5024	approximate	approximate
b2	$\log_{10}(k_1'k_2')$	4.725	< −0.413	> 8.789	optim	optim
b2	$\log_{10}(k_2 \text{ or } k_{deg})$	5.87	0.7277	> 9.893	optim	optim
b2	$\log_{10}(k_2 \text{ or } k_{deg})$	−1.89	−2.034	−1.751	approximate	approximate
b3	$\log_{10}(k_1')$	−0.2896	−0.5584	0.2513	approximate	approximate
b3	$\log_{10}(k_2)$	−0.5424	−0.8152	0.0227	approximate	approximate
b3	$\log_{10}(k_2')$	−1.13	−1.227	−1.015	approximate	approximate
b3	$\log_{10}(k_{deg})$	−1.523	−1.635	−1.393	approximate	approximate
b3	$\log_{10}(k_1'/k_2)$	0.2527	0.2195	0.2854	approximate	approximate
b3	$\log_{10}(k_1'k_2'/k_2)$	−0.8774	−0.9702	−0.7641	approximate	approximate
b3	$\log_{10}(k_2'/k_2)$	−0.5878	−1.19	−0.2482	approximate	approximate

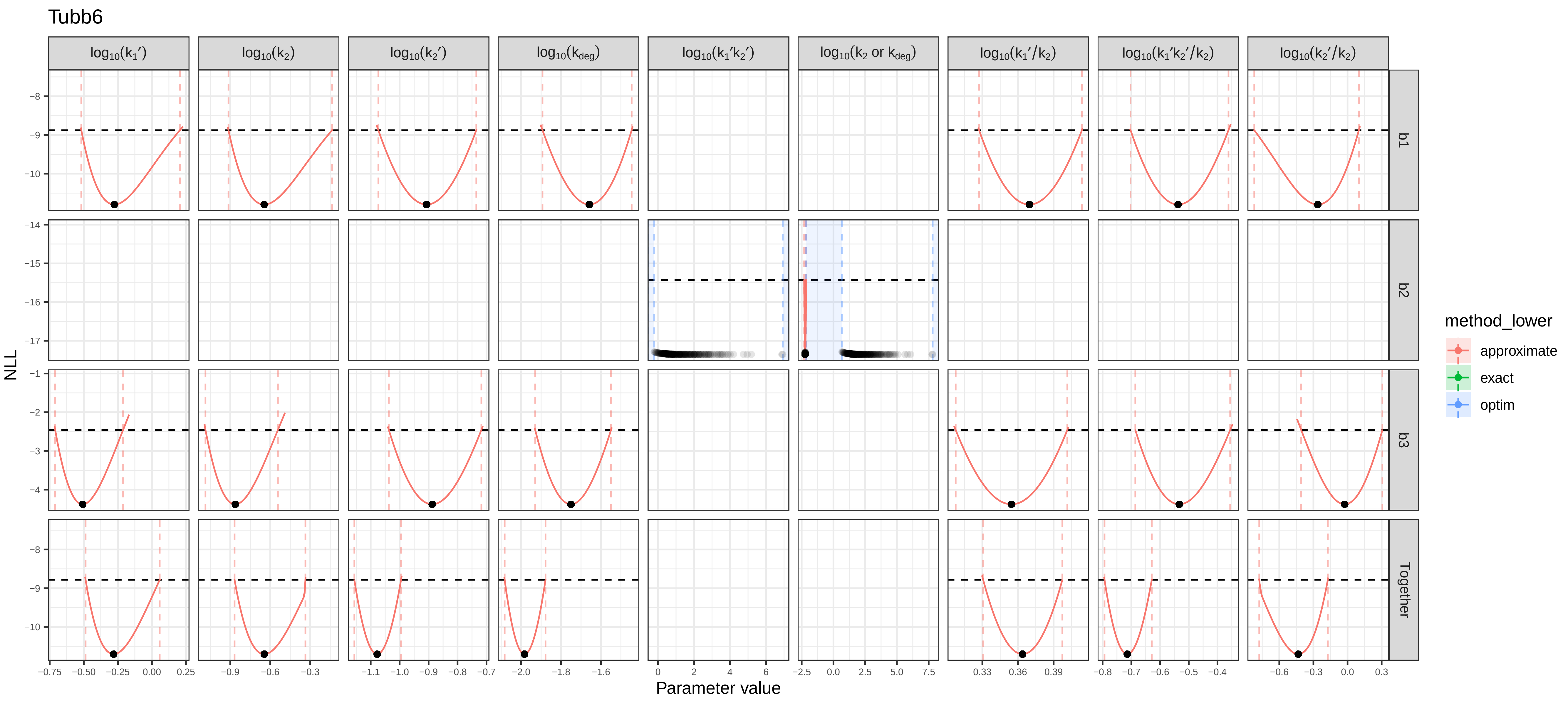

Replicate	Par	Best value	CI95 LB	CI95 UB	Method LB	Method UB
Together	$\log_{10}(k_1')$	−0.2812	−0.4867	0.0573	approximate	approximate
Together	$\log_{10}(k_2)$	−0.6451	−0.8676	−0.3359	approximate	approximate
Together	$\log_{10}(k_2')$	−1.078	−1.157	−0.9952	approximate	approximate
Together	$\log_{10}(k_{deg})$	−1.983	−2.083	−1.878	approximate	approximate
Together	$\log_{10}(k_1'/k_2)$	0.3639	0.3307	0.3973	approximate	approximate
Together	$\log_{10}(k_1'k_2'/k_2)$	−0.7139	−0.7933	−0.6288	approximate	approximate
Together	$\log_{10}(k_2'/k_2)$	−0.4326	−0.7761	−0.1734	approximate	approximate
b1	$\log_{10}(k_1')$	−0.2758	−0.5183	0.2056	approximate	approximate
b1	$\log_{10}(k_2)$	−0.6455	−0.9132	−0.1359	approximate	approximate
b1	$\log_{10}(k_2')$	−0.9067	−1.074	−0.7352	approximate	approximate
b1	$\log_{10}(k_{deg})$	−1.658	−1.893	−1.446	approximate	approximate
b1	$\log_{10}(k_1'/k_2)$	0.3696	0.3273	0.4138	approximate	approximate
b1	$\log_{10}(k_1'k_2'/k_2)$	−0.5371	−0.7026	−0.3615	approximate	approximate
b1	$\log_{10}(k_2'/k_2)$	−0.2612	−0.8199	0.09952	approximate	approximate
b2	$\log_{10}(k_1'k_2')$	3.25	< −0.2114	> 6.927	optim	optim
b2	$\log_{10}(k_2 \text{ or } k_{deg})$	4.138	0.6747	> 7.816	optim	optim
b2	$\log_{10}(k_2 \text{ or } k_{deg})$	−2.228	−2.306	−2.15	approximate	approximate
b3	$\log_{10}(k_1')$	−0.5077	−0.71	−0.2114	approximate	approximate
b3	$\log_{10}(k_2)$	−0.8622	−1.086	−0.5424	approximate	approximate
b3	$\log_{10}(k_2')$	−0.8874	−1.037	−0.7176	approximate	approximate
b3	$\log_{10}(k_{deg})$	−1.75	−1.93	−1.55	approximate	approximate
b3	$\log_{10}(k_1'/k_2)$	0.3545	0.3076	0.4015	approximate	approximate
b3	$\log_{10}(k_1'k_2'/k_2)$	−0.5329	−0.686	−0.3556	approximate	approximate
b3	$\log_{10}(k_2'/k_2)$	−0.02521	−0.4074	0.3065	approximate	approximate

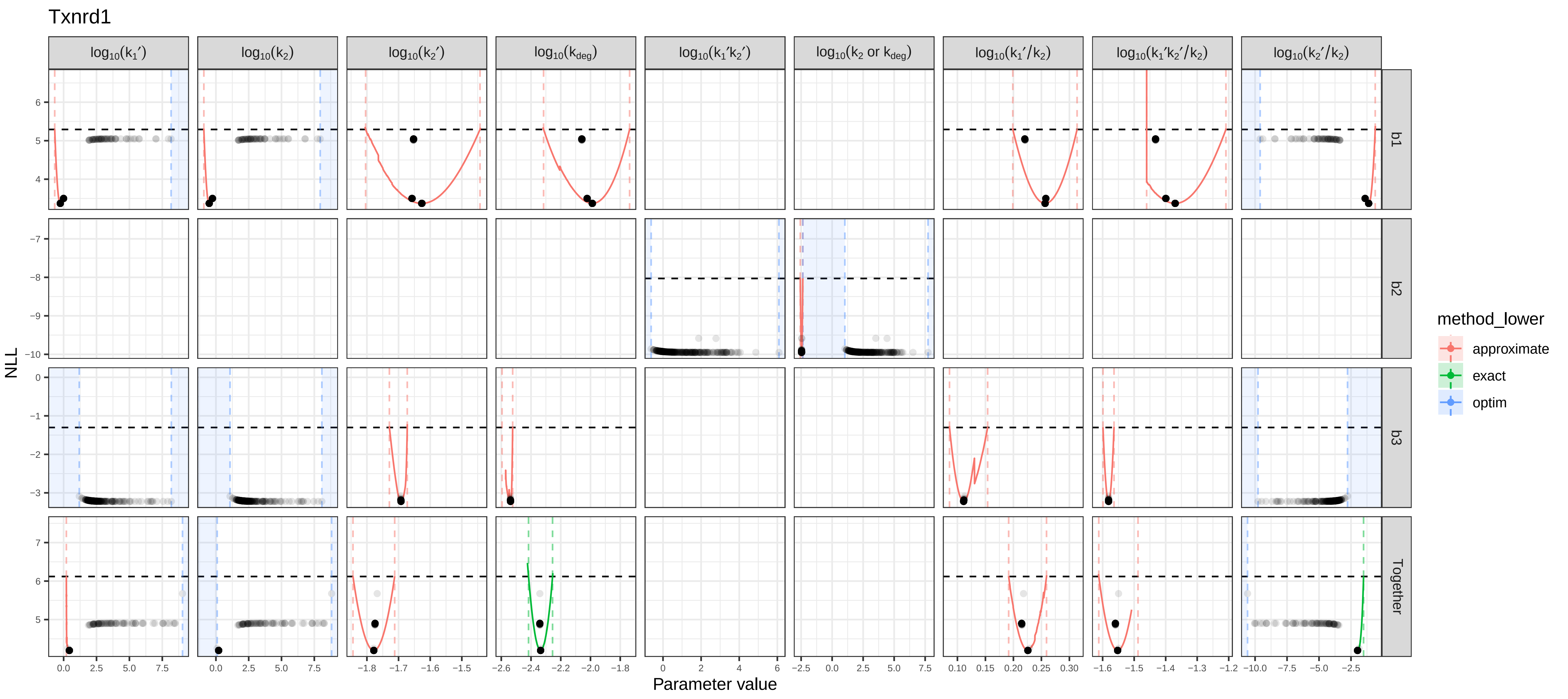

Replicate	Par	**Best value**	**CI95 LB**	**CI95 UB**	**Method LB**	**Method UB**
Together	$\log_{10}(k_1')$	0.4333	0.2063	> 9.038	approximate	optim
Together	$\log_{10}(k_2)$	0.2075	< 0.09613	> 8.82	optim	optim
Together	$\log_{10}(k_2')$	−1.778	−1.844	−1.712	approximate	approximate
Together	$\log_{10}(k_{deg})$	−2.336	−2.417	−2.255	exact	exact
Together	$\log_{10}(k_1'/k_2)$	0.2259	0.1915	0.2592	approximate	approximate
Together	$\log_{10}(k_1'k_2'/k_2)$	−1.552	−1.612	−1.488	approximate	approximate
Together	$\log_{10}(k_2'/k_2)$	−1.986	< −10.59	−1.516	optim	exact
b1	$\log_{10}(k_1')$	−0.2534	−0.677	> 8.17	approximate	optim
b1	$\log_{10}(k_2)$	−0.5101	−0.9193	> 7.95	approximate	optim
b1	$\log_{10}(k_2')$	−1.627	−1.804	−1.443	approximate	approximate
b1	$\log_{10}(k_{deg})$	−1.988	−2.315	−1.737	approximate	approximate
b1	$\log_{10}(k_1'/k_2)$	0.2566	0.1989	0.3138	approximate	approximate
b1	$\log_{10}(k_1'k_2'/k_2)$	−1.37	−1.46	−1.209	approximate	approximate
b1	$\log_{10}(k_2'/k_2)$	−1.117	< −9.603	−0.6055	optim	approximate
b2	$\log_{10}(k_1'k_2')$	3.594	< −0.6222	> 6.083	optim	optim
b2	$\log_{10}(k_2 \text{ or } k_{deg})$	5.244	1.027	> 7.733	optim	optim
b2	$\log_{10}(k_2 \text{ or } k_{deg})$	−2.462	−2.569	−2.364	approximate	approximate
b3	$\log_{10}(k_1')$	7.09	< 1.188	> 8.188	optim	optim
b3	$\log_{10}(k_2)$	6.979	< 1.076	> 8.077	optim	optim
b3	$\log_{10}(k_2')$	−1.692	−1.729	−1.672	approximate	approximate
b3	$\log_{10}(k_{deg})$	−2.538	−2.595	−2.523	approximate	approximate
b3	$\log_{10}(k_1'/k_2)$	0.1111	0.08585	0.154	approximate	approximate
b3	$\log_{10}(k_1'k_2'/k_2)$	−1.581	−1.599	−1.564	approximate	approximate
b3	$\log_{10}(k_2'/k_2)$	−8.671	< −9.769	> −2.77	optim	optim

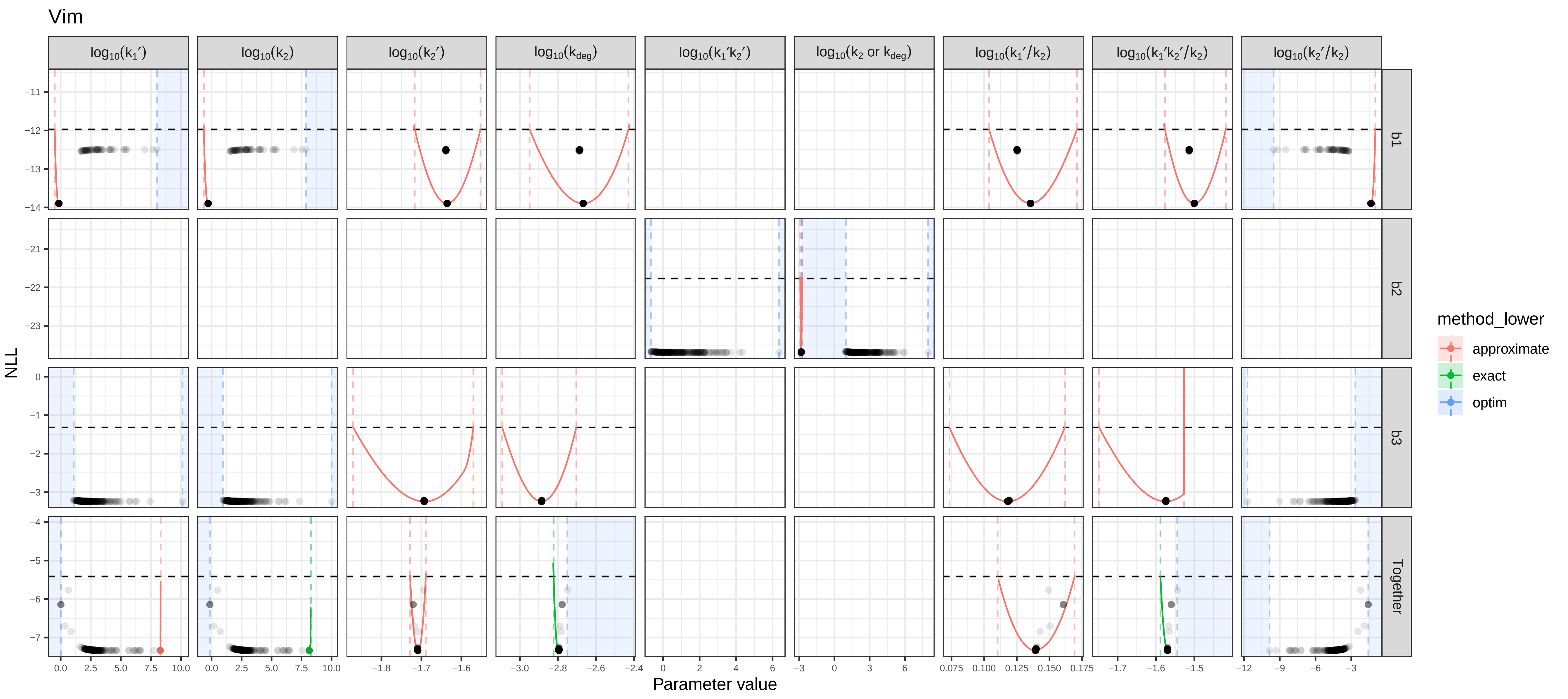

Replicate	Par	**Best value**	**CI95 LB**	**CI95 UB**	**Method LB**	**Method UB**
Together	$\log_{10}(k_1')$	8.287	< 0.01277	8.305	optim	approximate
Together	$\log_{10}(k_2)$	8.148	< −0.1479	8.277	optim	exact
Together	$\log_{10}(k_2')$	−1.709	−1.728	−1.689	approximate	approximate
Together	$\log_{10}(k_{deg})$	−2.795	−2.822	> −2.75	exact	optim
Together	$\log_{10}(k_1'/k_2)$	0.1393	0.1103	0.1692	approximate	approximate
Together	$\log_{10}(k_1'k_2'/k_2)$	−1.57	−1.589	> −1.545	exact	optim
Together	$\log_{10}(k_2'/k_2)$	−9.857	< −9.857	> −1.573	optim	optim
b1	$\log_{10}(k_1')$	−0.1554	−0.4944	> 8.001	approximate	optim
b1	$\log_{10}(k_2)$	−0.2909	−0.6417	> 7.876	approximate	optim
b1	$\log_{10}(k_2')$	−1.636	−1.717	−1.552	approximate	approximate
b1	$\log_{10}(k_{deg})$	−2.667	−2.948	−2.429	approximate	approximate
b1	$\log_{10}(k_1'/k_2)$	0.1355	0.1037	0.1711	approximate	approximate
b1	$\log_{10}(k_1'k_2'/k_2)$	−1.5	−1.577	−1.418	approximate	approximate
b1	$\log_{10}(k_2'/k_2)$	−1.345	< −9.515	−0.9793	optim	approximate
b2	$\log_{10}(k_1'k_2')$	3.262	< −0.6593	> 6.351	optim	optim
b2	$\log_{10}(k_2 \text{ or } k_{deg})$	4.89	0.9678	> 7.978	optim	optim
b2	$\log_{10}(k_2 \text{ or } k_{deg})$	−2.834	−2.912	−2.763	approximate	approximate
b3	$\log_{10}(k_1')$	5.011	< 1.082	> 10.13	optim	optim
b3	$\log_{10}(k_2)$	4.893	< 0.9618	> 10.01	optim	optim
b3	$\log_{10}(k_2')$	−1.693	−1.87	−1.57	approximate	approximate
b3	$\log_{10}(k_{deg})$	−2.886	−3.092	−2.703	approximate	approximate
b3	$\log_{10}(k_1'/k_2)$	0.118	0.07344	0.1618	approximate	approximate
b3	$\log_{10}(k_1'k_2'/k_2)$	−1.575	−1.748	−1.527	approximate	approximate
b3	$\log_{10}(k_2'/k_2)$	−6.586	< −11.7	> −2.655	optim	optim

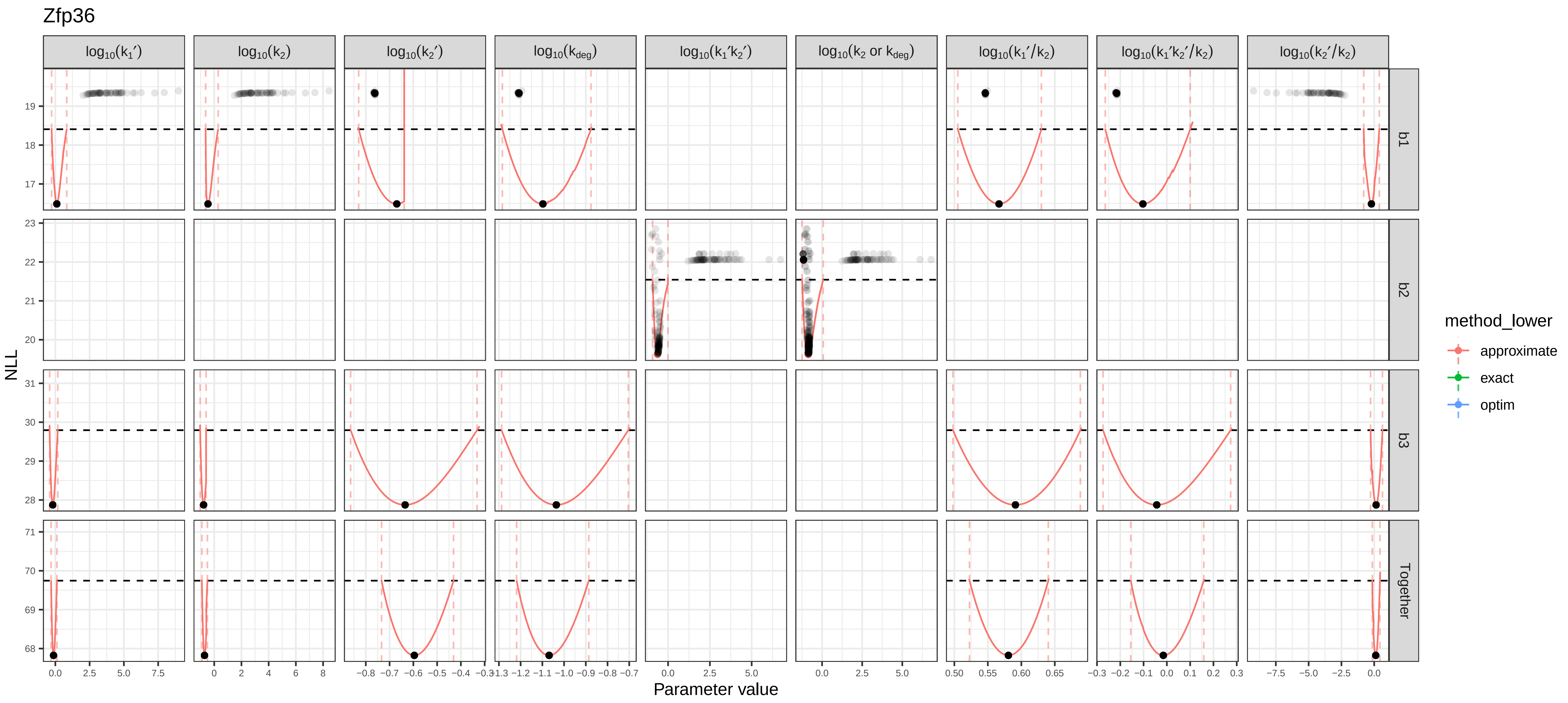

Replicate	Par	Best value	CI95 LB	CI95 UB	Method LB	Method UB
Together	$\log_{10}(k_1')$	−0.1326	−0.3102	0.1178	approximate	approximate
Together	$\log_{10}(k_2)$	−0.7132	−0.9065	−0.5013	approximate	approximate
Together	$\log_{10}(k_2')$	−0.596	−0.7338	−0.4309	approximate	approximate
Together	$\log_{10}(k_{deg})$	−1.069	−1.218	−0.8863	approximate	approximate
Together	$\log_{10}(k_1'/k_2)$	0.5807	0.5226	0.6403	approximate	approximate
Together	$\log_{10}(k_1'k_2'/k_2)$	−0.01535	−0.155	0.1584	approximate	approximate
Together	$\log_{10}(k_2'/k_2)$	0.1172	−0.1457	0.4378	approximate	approximate
b1	$\log_{10}(k_1')$	0.1097	−0.2718	0.8299	approximate	approximate
b1	$\log_{10}(k_2)$	−0.457	−0.6312	0.2895	approximate	approximate
b1	$\log_{10}(k_2')$	−0.6695	−0.8299	−0.6385	approximate	approximate
b1	$\log_{10}(k_{deg})$	−1.097	−1.284	−0.876	approximate	approximate
b1	$\log_{10}(k_1'/k_2)$	0.5667	0.5051	0.63	approximate	approximate
b1	$\log_{10}(k_1'k_2'/k_2)$	−0.1028	−0.2644	0.1002	approximate	approximate
b1	$\log_{10}(k_2'/k_2)$	−0.2125	−0.8013	0.3878	approximate	approximate
b2	$\log_{10}(k_1'k_2')$	−0.6316	−0.9269	−0.01017	approximate	approximate
b2	$\log_{10}(k_2 \text{ or } k_{deg})$	−0.8529	−1.246	0.07083	approximate	approximate
b2	$\log_{10}(k_2 \text{ or } k_{deg})$	−0.889	−1.246	0.07083	approximate	approximate
b3	$\log_{10}(k_1')$	−0.19	−0.4168	0.1784	approximate	approximate
b3	$\log_{10}(k_2)$	−0.7812	−1.033	−0.5965	approximate	approximate
b3	$\log_{10}(k_2')$	−0.6347	−0.8638	−0.3321	approximate	approximate
b3	$\log_{10}(k_{deg})$	−1.036	−1.288	−0.7042	approximate	approximate
b3	$\log_{10}(k_1'/k_2)$	0.5912	0.4979	0.6881	approximate	approximate
b3	$\log_{10}(k_1'k_2'/k_2)$	−0.04347	−0.2739	0.2738	approximate	approximate
b3	$\log_{10}(k_2'/k_2)$	0.1465	−0.2787	0.6323	approximate	approximate

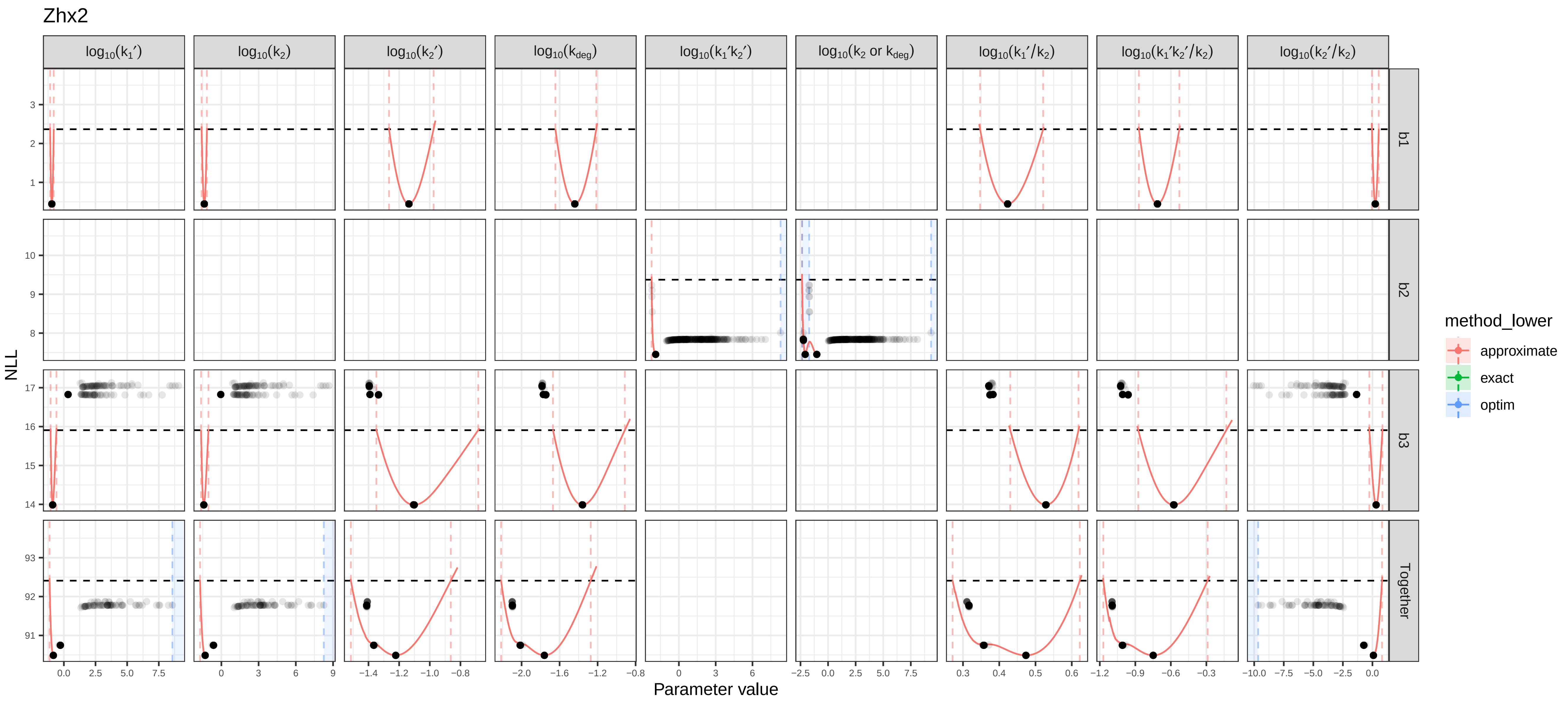

Replicate	Par	Best value	CI95 LB	CI95 UB	Method LB	Method UB
Together	$\log_{10}(k_1')$	−0.8307	−1.128	> 8.571	approximate	optim
Together	$\log_{10}(k_2)$	−1.304	−1.718	> 8.255	approximate	optim
Together	$\log_{10}(k_2')$	−1.222	−1.515	−0.8629	approximate	approximate
Together	$\log_{10}(k_{deg})$	−1.76	−2.216	−1.273	approximate	approximate
Together	$\log_{10}(k_1'/k_2)$	0.4734	0.2713	0.622	approximate	approximate
Together	$\log_{10}(k_1'k_2'/k_2)$	−0.7489	−1.17	−0.2892	approximate	approximate
Together	$\log_{10}(k_2'/k_2)$	0.08177	< −9.667	0.8114	optim	approximate
b1	$\log_{10}(k_1')$	−0.9529	−1.086	−0.7995	approximate	approximate
b1	$\log_{10}(k_2)$	−1.376	−1.593	−1.167	approximate	approximate
b1	$\log_{10}(k_2')$	−1.136	−1.266	−0.9754	approximate	approximate
b1	$\log_{10}(k_{deg})$	−1.44	−1.644	−1.214	approximate	approximate
b1	$\log_{10}(k_1'/k_2)$	0.4231	0.3472	0.5211	approximate	approximate
b1	$\log_{10}(k_1'k_2'/k_2)$	−0.7131	−0.8698	−0.528	approximate	approximate
b1	$\log_{10}(k_2'/k_2)$	0.2398	−0.04197	0.5329	approximate	approximate
b2	$\log_{10}(k_1'k_2')$	−1.891	−2.226	> 8.288	approximate	optim
b2	$\log_{10}(k_2 \text{ or } k_{deg})$	−1.019	−2.354	> 9.334	approximate	optim
b2	$\log_{10}(k_2 \text{ or } k_{deg})$	−2.073	−2.354	−1.712	approximate	optim
b3	$\log_{10}(k_1')$	−0.8867	−1.053	−0.5874	approximate	approximate
b3	$\log_{10}(k_2)$	−1.415	−1.637	−1.041	approximate	approximate
b3	$\log_{10}(k_2')$	−1.102	−1.349	−0.6842	approximate	approximate
b3	$\log_{10}(k_{deg})$	−1.359	−1.67	−0.9137	approximate	approximate
b3	$\log_{10}(k_1'/k_2)$	0.5282	0.4303	0.6185	approximate	approximate
b3	$\log_{10}(k_1'k_2'/k_2)$	−0.5737	−0.8751	−0.1316	approximate	approximate
b3	$\log_{10}(k_2'/k_2)$	0.313	−0.2636	0.8462	approximate	approximate

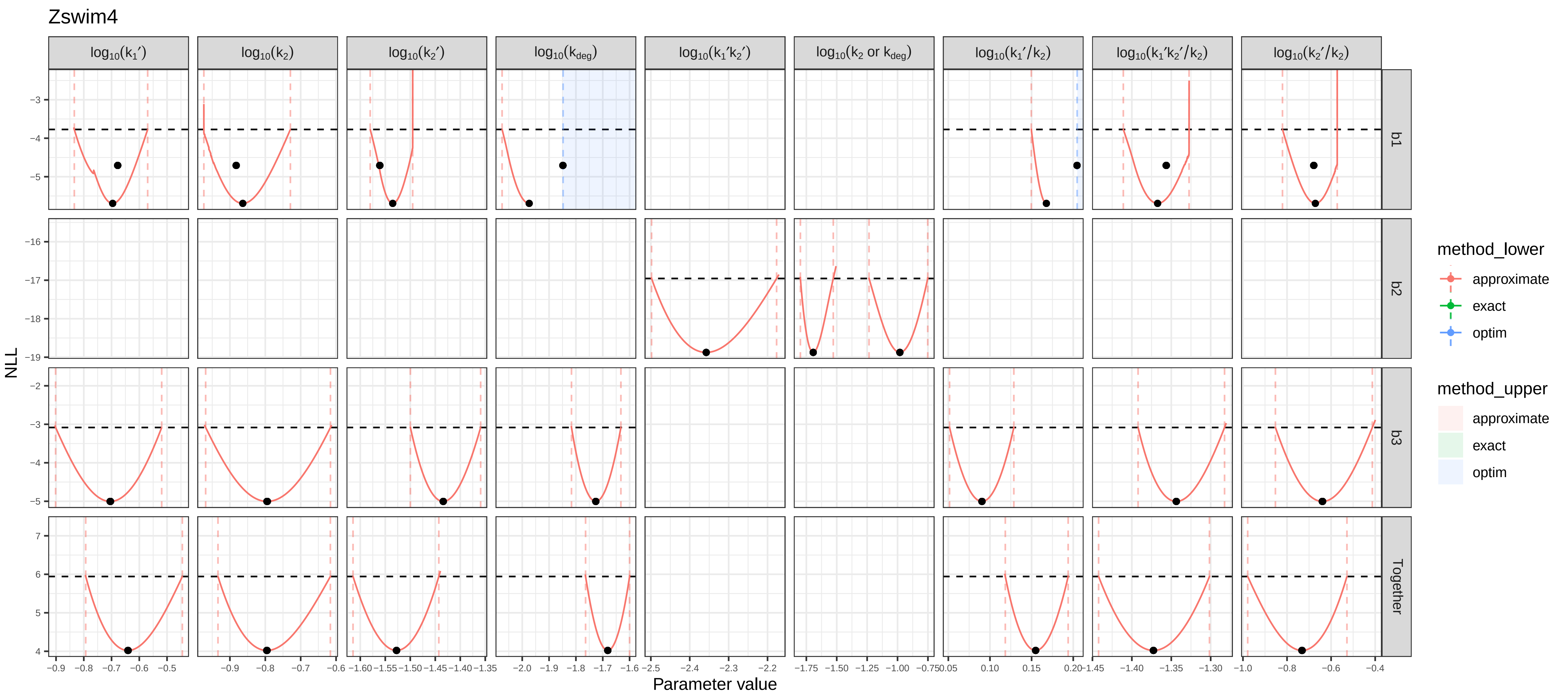

Replicate	Par	Best value	CI95 LB	CI95 UB	Method LB	Method UB
Together	$\log_{10}(k_1')$	−0.6407	−0.793	−0.4449	approximate	approximate
Together	$\log_{10}(k_2)$	−0.7955	−0.9337	−0.6161	approximate	approximate
Together	$\log_{10}(k_2')$	−1.527	−1.614	−1.443	approximate	approximate
Together	$\log_{10}(k_{deg})$	−1.681	−1.764	−1.6	approximate	approximate
Together	$\log_{10}(k_1'/k_2)$	0.1548	0.1184	0.1939	approximate	approximate
Together	$\log_{10}(k_1'k_2'/k_2)$	−1.373	−1.442	−1.302	approximate	approximate
Together	$\log_{10}(k_2'/k_2)$	−0.7319	−0.9785	−0.5281	approximate	approximate
b1	$\log_{10}(k_1')$	−0.6959	−0.8342	−0.5698	approximate	approximate
b1	$\log_{10}(k_2)$	−0.8636	−0.9736	−0.7291	approximate	approximate
b1	$\log_{10}(k_2')$	−1.535	−1.58	−1.495	approximate	approximate
b1	$\log_{10}(k_{deg})$	−1.974	−2.075	> −1.848	approximate	optim
b1	$\log_{10}(k_1'/k_2)$	0.1677	0.1497	> 0.2047	approximate	optim
b1	$\log_{10}(k_1'k_2'/k_2)$	−1.367	−1.411	−1.327	approximate	approximate
b1	$\log_{10}(k_2'/k_2)$	−0.6715	−0.8206	−0.5722	approximate	approximate
b2	$\log_{10}(k_1'k_2')$	−2.358	−2.498	−2.177	approximate	approximate
b2	$\log_{10}(k_2 \text{ or } k_{deg})$	−0.9809	−1.234	−0.7516	approximate	approximate
b2	$\log_{10}(k_2 \text{ or } k_{deg})$	−1.694	−1.799	−1.528	approximate	approximate
b3	$\log_{10}(k_1')$	−0.7042	−0.9015	−0.5193	approximate	approximate
b3	$\log_{10}(k_2)$	−0.7946	−0.9686	−0.6158	approximate	approximate
b3	$\log_{10}(k_2')$	−1.434	−1.5	−1.359	approximate	approximate
b3	$\log_{10}(k_{deg})$	−1.726	−1.816	−1.632	approximate	approximate
b3	$\log_{10}(k_1'/k_2)$	0.09039	0.05149	0.1287	approximate	approximate
b3	$\log_{10}(k_1'k_2'/k_2)$	−1.344	−1.392	−1.282	approximate	approximate
b3	$\log_{10}(k_2'/k_2)$	−0.6394	−0.8528	−0.4129	approximate	approximate
