# Supplementary material for: Kinetics of mRNA nuclear export regulate innate immune response gene expression
Source: Nat Commun. 2022 Nov 23;13:7197. doi: 10.1038/s41467-022-34635-5 (PMC9691726; doi:10.1038/s41467-022-34635-5)
Supplement: Supplementary file 7 — Source Data [file Source Data File 2 all_genes_optim_naive.pdf › Source Data File 2 all_genes_optim_naive.pdf]

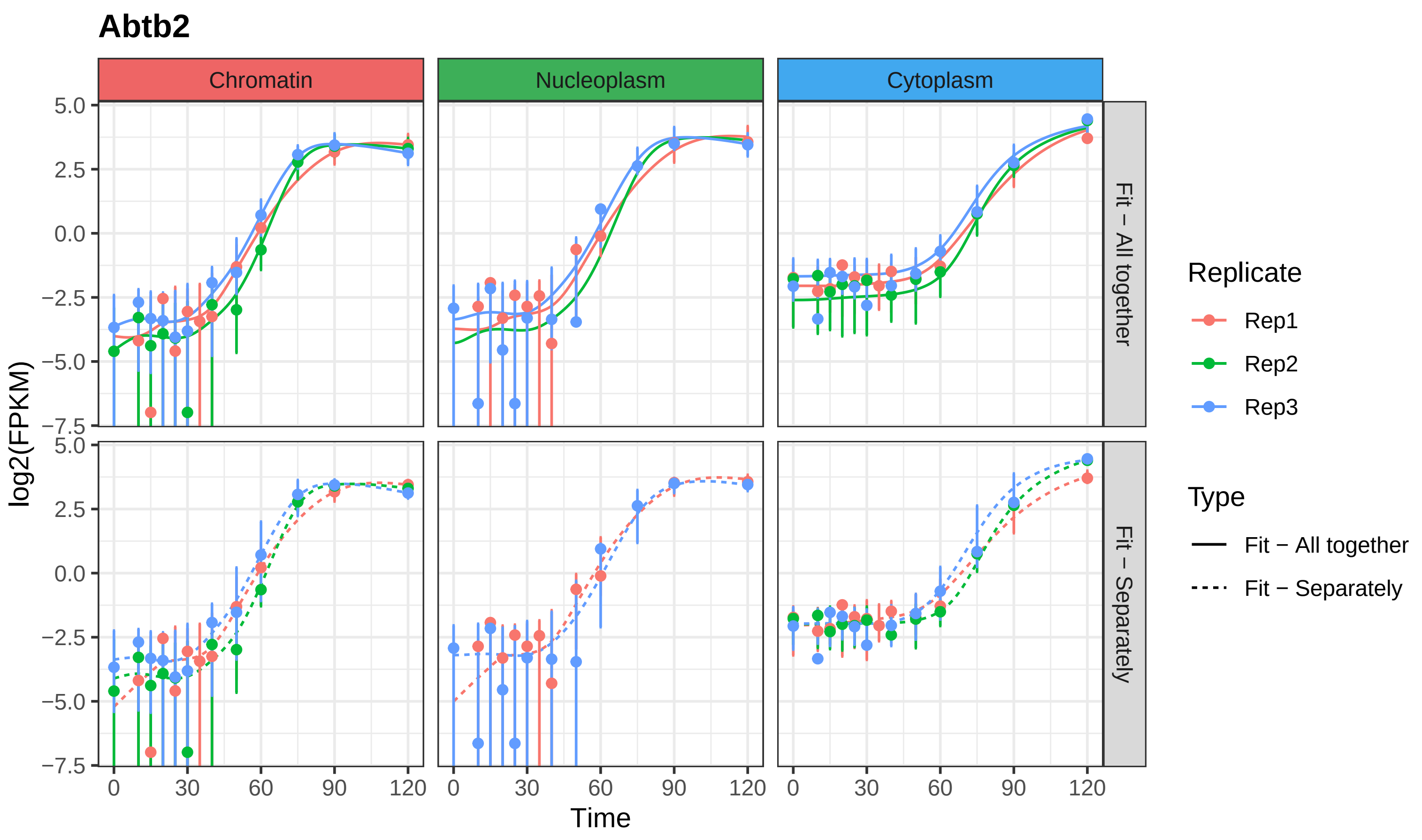

	Together	b1	b2	b3
**−NLL b1 ca**	10.15	9.601		
**−NLL b1 np**	13.06	11.17		
**−NLL b1 cyto**	5.114	3.926		
**−NLL b2 ca**	9.174		7.136	
**−NLL b2 np**				
**−NLL b2 cyto**	6.711		−1.682	
**−NLL b3 ca**	4.707			4.63
**−NLL b3 np**	20			19.01
**−NLL b3 cyto**	14.55			6.936
Total	83.46	24.7	5.454	30.58
**Total with regul**	86.27	23.1	3.291	28.61

	Together	b1	b2	b3
spar	0.41130	4.142e−01	0.4221	4.289e−01
σ_b	0.14040	2.952e−05	1.386e−06	3.840e−07
σ_t	0.72510	3.224e+00	2.86e−05	3.648e+00
ca_{0,b_1}	−3.99600	−5.217e+00		
$\log_{10}(k_1')$	−0.52420	7.123e+00		−8.858e−01
$\log_{10}(k_2)$	−0.60980	7.060e+00	−1.086 or −1.755	−9.381e−01
$\log_{10}(k_2')$	−1.39800	−1.578e+00		−1.073e+00
$\log_{10}(k_{deg})$	−1.90300	−2.474e+00	−1.755 or −1.086	−1.443e+00
$\log_{10}(k_1' k_2')$	−1.92300	5.545e+00	−2.204	−1.959e+00
$\log_{10}(k_1'/k_2)$	0.08559	6.264e−02		5.237e−02
transport $= \log_{10}(k_1' k_2'/k_2)$	−1.31300	−1.515e+00	−1.117 or −0.4492	−1.021e+00

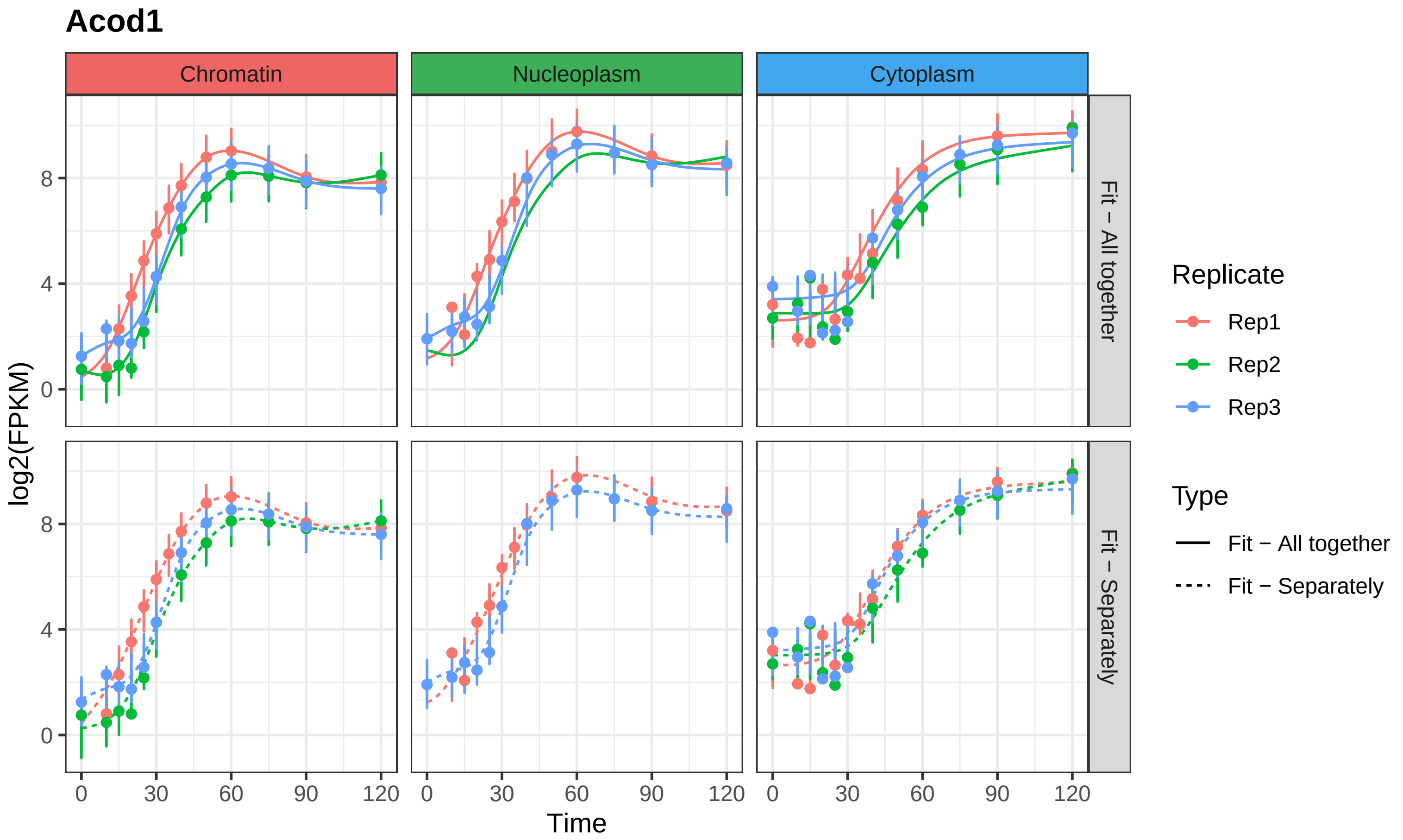

	Together	b1	b2	b3
**−NLL b1 ca**	2.35	2.723		
**−NLL b1 np**	7.895	6.511		
**−NLL b1 cyto**	12.87	11.46		
**−NLL b2 ca**	3.617		3.81	
**−NLL b2 np**				
**−NLL b2 cyto**	12.98		12.62	
**−NLL b3 ca**	3.577			3.014
**−NLL b3 np**	5.058			3.748
**−NLL b3 cyto**	16.88			16.27
Total	65.23	20.69	16.43	23.03
**Total with regul**	70.8	22.14	19.33	28.01

	Together	b1	b2	b3
spar	0.3252000	0.4252000	0.3717	3.264e−01
σ_b	0.3013000	0.2610000	0.2826	2.911e−01
σ_t	0.0001994	0.0001213	0.001746	4.799e−05
ca_{0,b_1}	0.4867000	0.5064000		
$\log_{10}(k_1')$	0.0192900	−0.2802000		8.692e−01
$\log_{10}(k_2)$	−0.2003000	−0.5282000	−0.7083 or −2.035	6.697e−01
$\log_{10}(k_2')$	−1.5500000	−1.6510000		−1.482e+00
$\log_{10}(k_{deg})$	−1.9790000	−2.0660000	−2.035 or −0.7083	−1.842e+00
$\log_{10}(k_1' k_2')$	−1.5310000	−1.9310000	−1.91	−6.125e−01
$\log_{10}(k_1'/k_2)$	0.2196000	0.2480000		1.995e−01
transport = $\log_{10}(k_1' k_2'/k_2)$	−1.3300000	−1.4030000	−1.202 or 0.1248	−1.282e+00

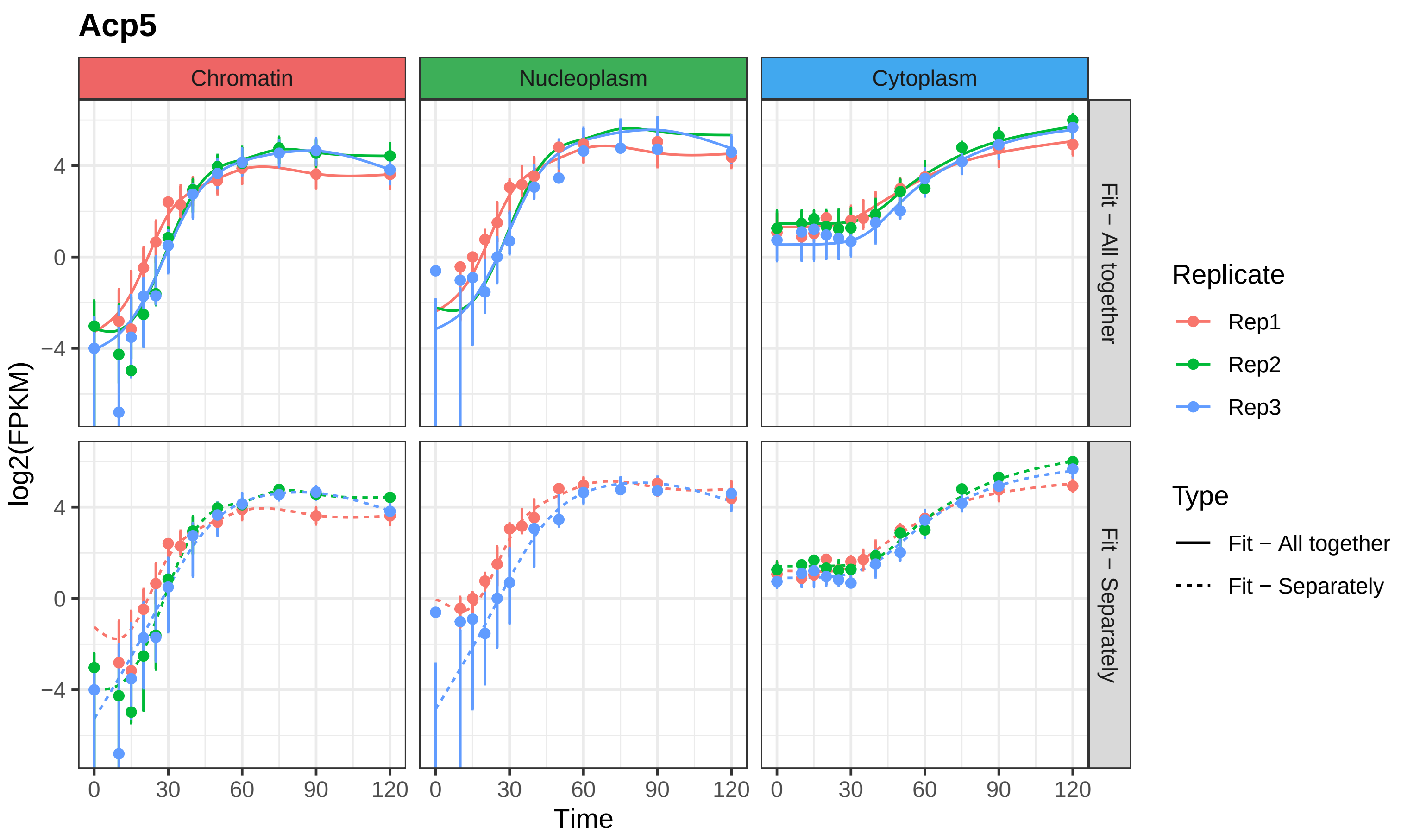

	Together	b1	b2	b3
**−NLL b1 ca**	4.275	5.11		
**−NLL b1 np**	7.334	3.143		
**−NLL b1 cyto**	1.32	−1.395		
**−NLL b2 ca**	8.267		1.114	
**−NLL b2 np**				
**−NLL b2 cyto**	1.5		−2.3	
**−NLL b3 ca**	8.041			7.435
**−NLL b3 np**	28.84			11.74
**−NLL b3 cyto**	2.75			−1.422
**Total**	62.32	6.858	−1.186	17.75
**Total with regul**	74.23	5.995	−2.184	16.8

	Together	b1	b2	b3
spar	0.4045	0.40840	0.3719	0.50890
σ_b	0.1988	0.11500	1.146e−06	0.09454
σ_t	1.0730	1.48000	2.252	3.32300
ca_{0,b_1}	−3.1990	−1.09000		
$\log_{10}(k_1')$	0.7184	0.01403		5.72800
$\log_{10}(k_2)$	0.4436	−0.34440	−0.9358 or −3	5.60500
$\log_{10}(k_2')$	−1.7880	−1.75500		−1.64800
$\log_{10}(k_{deg})$	−2.8990	−2.13800	−3 or −0.9358	−3.38000
$\log_{10}(k_1'k_2')$	−1.0700	−1.74100	−2.312	4.08100
$\log_{10}(k_1'/k_2)$	0.2748	0.35840		0.12390
transport = $\log_{10}(k_1'k_2'/k_2)$	−1.5130	−1.39700	−1.376 or 0.6882	−1.52400

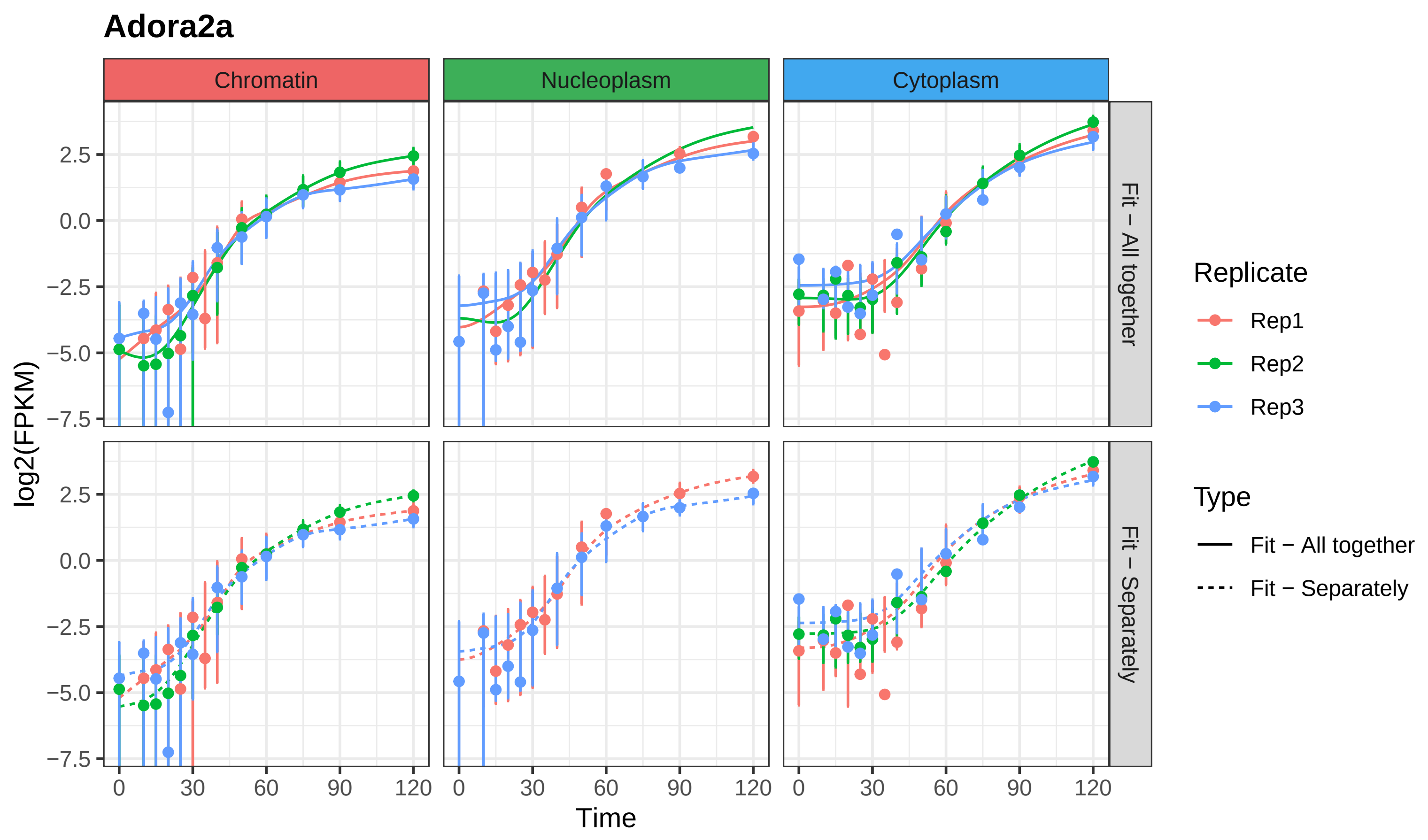

	Together	b1	b2	b3
**−NLL b1 ca**	7.486	7.717		
**−NLL b1 np**	7.709	6.199		
**−NLL b1 cyto**	21.4	19.93		
**−NLL b2 ca**	6.064		3.742	
**−NLL b2 np**				
**−NLL b2 cyto**	3.908		3.066	
**−NLL b3 ca**	7.817			8.512
**−NLL b3 np**	12.86			9.209
**−NLL b3 cyto**	20.04			14.64
Total	87.29	33.85	6.809	32.37
**Total with regul**	86.88	32.97	4.514	30.73

	Together	b1	b2	b3
spar	0.4122	4.466e−01	0.4455	4.150e−01
σ_b	0.0759	2.737e−08	1.029e−05	5.997e−06
σ_t	3.9460	5.204e+00	0.9869	4.677e+00
ca_{0,b_1}	−5.2520	−5.201e+00		
$\log_{10}(k_1')$	−0.5873	−6.386e−01		−4.046e−01
$\log_{10}(k_2)$	−0.9549	−1.078e+00	−1.307 or −1.638	−6.854e−01
$\log_{10}(k_2')$	−1.2250	−1.162e+00		−1.158e+00
$\log_{10}(k_{deg})$	−1.4560	−1.294e+00	−1.638 or −1.307	−1.480e+00
$\log_{10}(k_1' k_2')$	−1.8120	−1.800e+00	−2.115	−1.562e+00
$\log_{10}(k_1'/k_2)$	0.3676	4.391e−01		2.808e−01
$\text{transport} = \log_{10}(k_1' k_2'/k_2)$	−0.8569	−7.228e−01	−0.8082 or −0.477	−8.769e−01

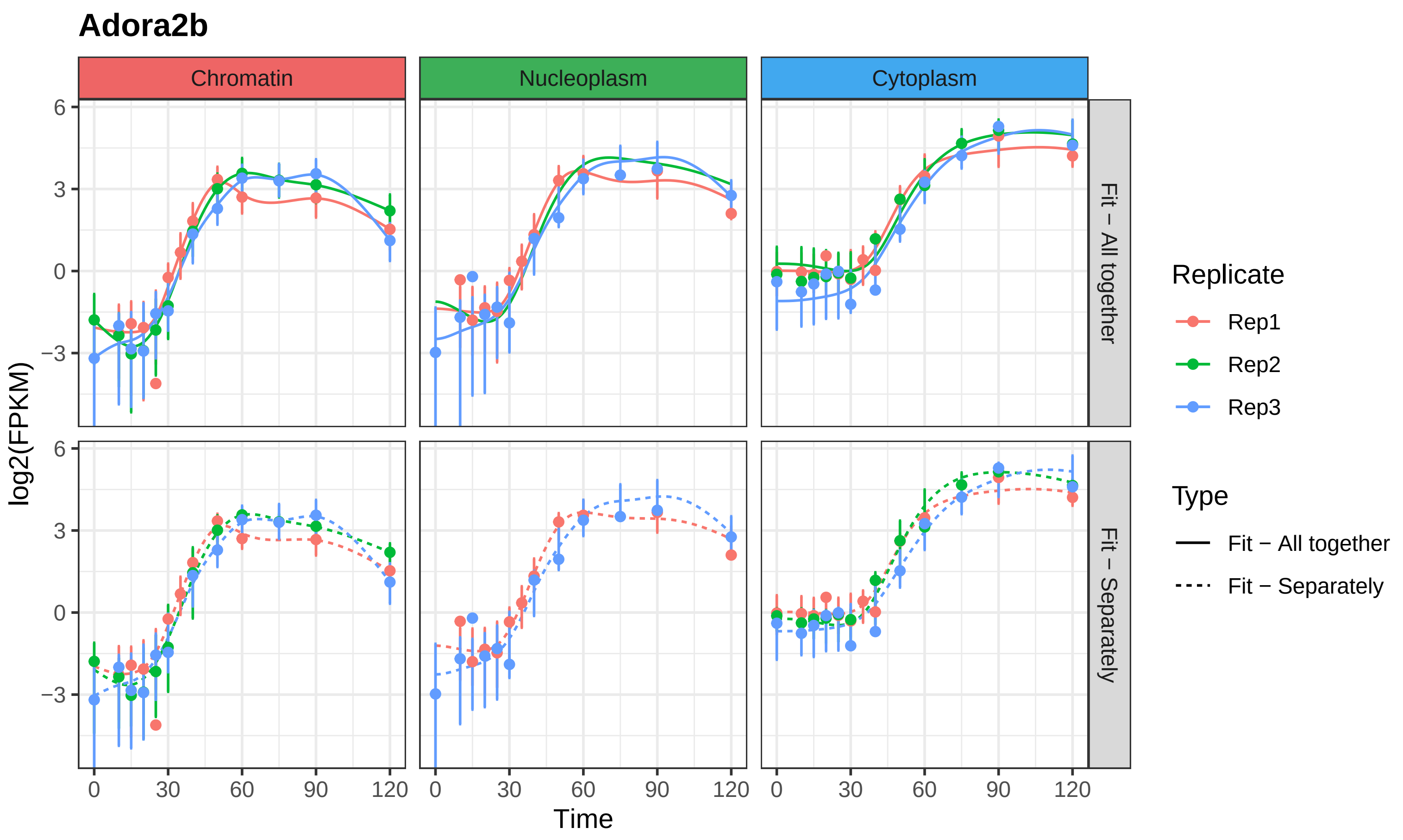

	Together	b1	b2	b3
**−NLL b1 ca**	5.609	5.717		
**−NLL b1 np**	5.978	5.45		
**−NLL b1 cyto**	4.82	4.989		
**−NLL b2 ca**	2.193		1.489	
**−NLL b2 np**				
**−NLL b2 cyto**	6.72		0.3504	
**−NLL b3 ca**	3.937			4.537
**−NLL b3 np**	14.73			12.37
**−NLL b3 cyto**	12.45			7.992
Total	56.43	16.16	1.839	24.9
**Total with regul**	58.82	16.48	0.1244	26.14

	Together	b1	b2	b3
spar	0.3509000	4.035e−01	0.4024	0.3697000
σ_b	0.1981000	1.523e−01	2.945e−05	0.2129000
σ_t	0.0004936	2.067e−08	2.74	0.0001188
ca_{0,b_1}	−2.0570000	−1.941e+00		
$\log_{10}(k_1')$	−0.6520000	−6.742e−01		−0.6785000
$\log_{10}(k_2)$	−0.8590000	−9.020e−01	−1.03 or −1.045	−0.9131000
$\log_{10}(k_2')$	−1.0530000	−1.051e+00		−1.1410000
$\log_{10}(k_{deg})$	−1.4700000	−1.423e+00	−1.045 or −1.03	−1.6160000
$\log_{10}(k_1'k_2')$	−1.7050000	−1.725e+00	−1.514	−1.8190000
$\log_{10}(k_1'/k_2)$	0.2070000	2.279e−01		0.2346000
$\text{transport} = \log_{10}(k_1'k_2'/k_2)$	−0.8456000	−8.226e−01	−0.4834 or −0.4685	−0.9059000

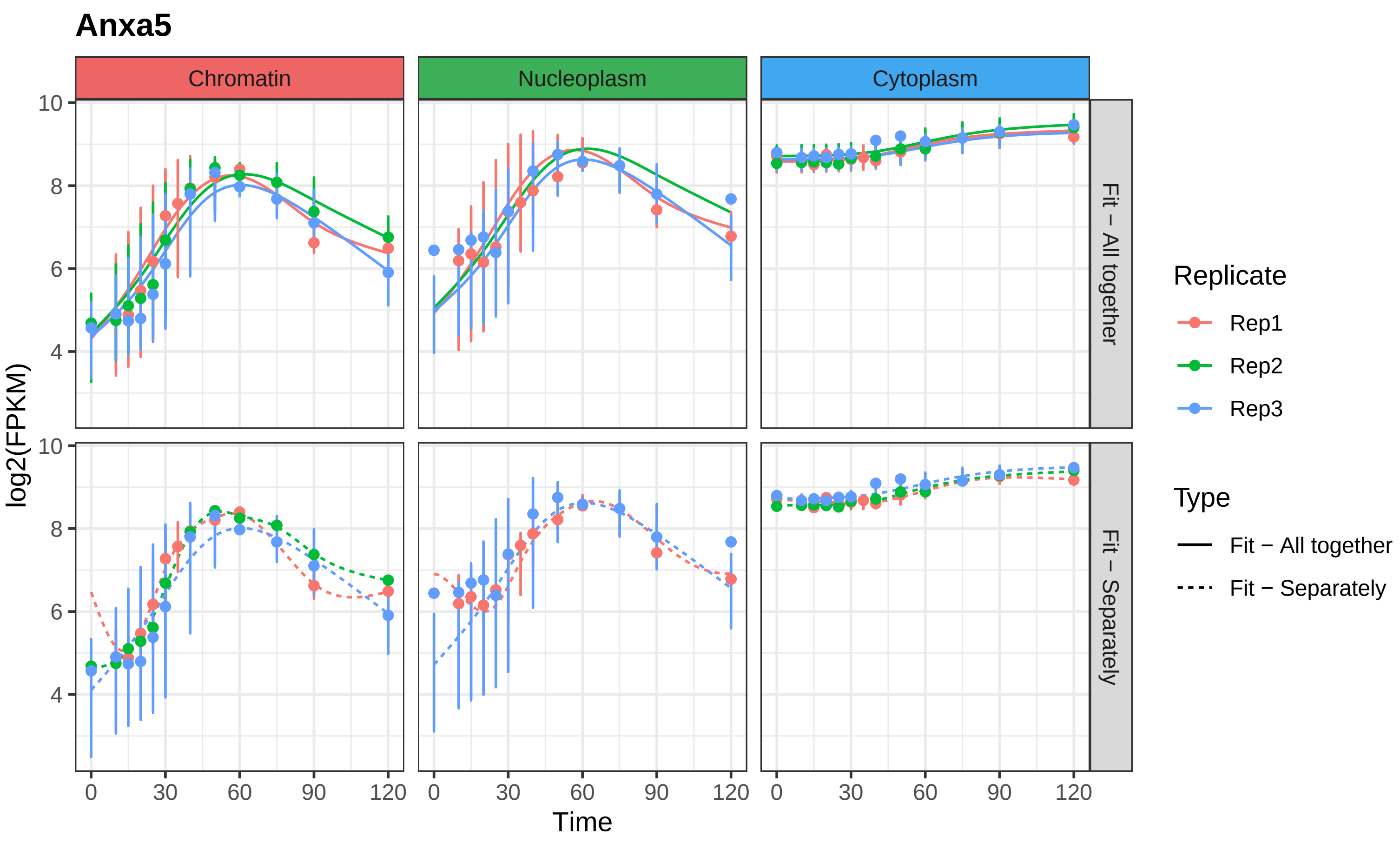

	Together	b1	b2	b3
**−NLL b1 ca**	4.861	−5.051		
**−NLL b1 np**	9.323	1.868		
**−NLL b1 cyto**	−9.819	−15.41		
**−NLL b2 ca**	3.646		−11.77	
**−NLL b2 np**				
**−NLL b2 cyto**	−6.204		−18.44	
**−NLL b3 ca**	4.761			5.193
**−NLL b3 np**	16.86			8.467
**−NLL b3 cyto**	−3.188			−11.42
Total	20.24	−18.6	−30.21	2.242
**Total with regul**	22.76	−12.1	−30.41	5.004

	Together	b1	b2	b3
spar	0.56970	0.37310	0.3486	0.57650
σ_b	0.09012	0.04768	0.02693	0.02949
σ_t	7.93900	3.26300	1.06	9.58800
ca_{0,b_1}	4.49300	6.57800		
$\log_{10}(k_1')$	8.50800	−0.87360		5.01900
$\log_{10}(k_2)$	8.32300	−1.00500	6.644 or −2.95	4.83500
$\log_{10}(k_2')$	−1.96100	−1.65200		−1.85600
$\log_{10}(k_{deg})$	−3.06400	−2.18900	−2.95 or 6.644	−3.06200
$\log_{10}(k_1' k_2')$	6.54700	−2.52600	4.881	3.16300
$\log_{10}(k_1'/k_2)$	0.18500	0.13110		0.18420
transport $= \log_{10}(k_1' k_2'/k_2)$	−1.77600	−1.52100	−1.763 or 7.831	−1.67200

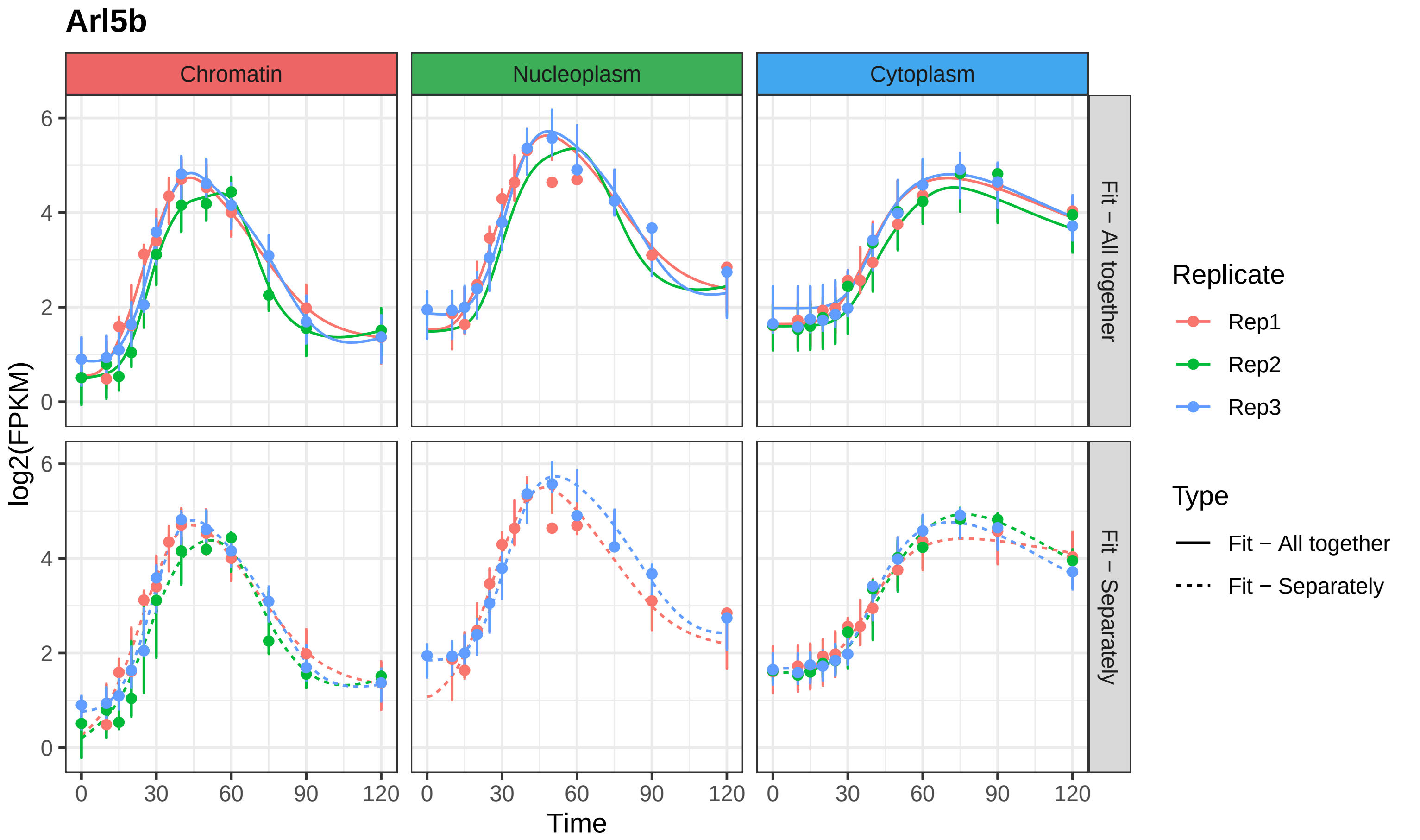

	Together	b1	b2	b3
**−NLL b1 ca**	−1.644	−1.022		
**−NLL b1 np**	8.248	7.125		
**−NLL b1 cyto**	−0.6384	−4.155		
**−NLL b2 ca**	−3.456		0.851	
**−NLL b2 np**				
**−NLL b2 cyto**	3.651		−6.972	
**−NLL b3 ca**	−4.078			−4.259
**−NLL b3 np**	0.858			2.731
**−NLL b3 cyto**	0.3234			−6.927
Total	3.263	1.948	−6.121	−8.454
**Total with regul**	5.306	1.541	−8.125	−9.104

	Together	b1	b2	b3
spar	3.353e−01	4.079e−01	0.4437	0.3769
σ_b	1.653e−01	1.632e−01	0.04611	0.1073
σ_t	1.692e−06	9.517e−05	3.167	1.0740
ca_{0,b_1}	5.861e−01	3.302e−01		
$\log_{10}(k_1')$	−3.591e−01	−1.493e−01		−0.4827
$\log_{10}(k_2)$	−6.558e−01	−3.962e−01	−1.088 or −1.433	−0.8096
$\log_{10}(k_2')$	−1.598e+00	−1.785e+00		−1.5770
$\log_{10}(k_{deg})$	−1.632e+00	−1.966e+00	−1.433 or −1.088	−1.5250
$\log_{10}(k_1'k_2')$	−1.957e+00	−1.935e+00	−2.103	−2.0590
$\log_{10}(k_1'/k_2)$	2.967e−01	2.469e−01		0.3270
transport = $\log_{10}(k_1'k_2'/k_2)$	−1.302e+00	−1.538e+00	−1.015 or −0.6706	−1.2500

Arl5c

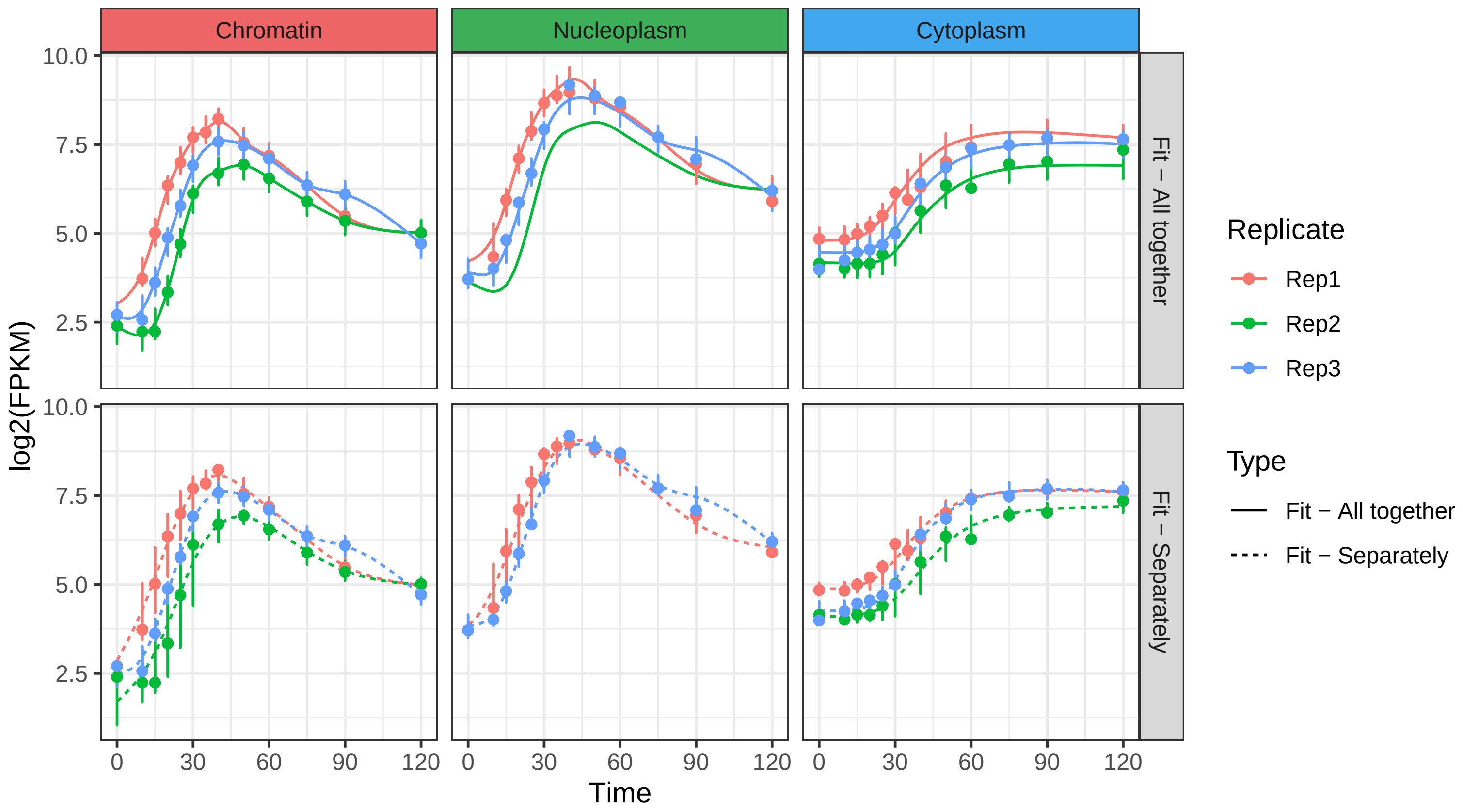

	Together	b1	b2	b3
**−NLL b1 ca**	−6.919	−3.278		
**−NLL b1 np**	0.4239	−0.1925		
**−NLL b1 cyto**	3.054	−9.545		
**−NLL b2 ca**	−7.129		−0.9853	
**−NLL b2 np**				
**−NLL b2 cyto**	1.282		−3.436	
**−NLL b3 ca**	−7.188			−6.353
**−NLL b3 np**	−1.218			−1.955
**−NLL b3 cyto**	−3.122			−7.079
Total	−20.82	−13.02	−4.422	−15.39
**Total with regul**	−13.94	−12.76	−6.329	−13.4

	Together	b1	b2	b3
spar	2.667e−01	0.40910	0.4438	3.111e−01
σ_b	1.291e−01	0.04867	0.05743	9.394e−02
σ_t	3.826e−05	2.27500	3.455	8.423e−06
ca_{0,b_1}	3.038e+00	2.93300		
$\log_{10}(k_1')$	1.212e−01	−0.19620		1.590e−01
$\log_{10}(k_2)$	−2.449e−01	−0.50930	−0.2839 or −2.25	−2.474e−01
$\log_{10}(k_2')$	−1.934e+00	−1.99300		−1.899e+00
$\log_{10}(k_{deg})$	−2.107e+00	−2.28700	−2.25 or −0.2839	−2.025e+00
$\log_{10}(k_1'k_2')$	−1.813e+00	−2.18900	−1.816	−1.740e+00
$\log_{10}(k_1'/k_2)$	3.661e−01	0.31310		4.063e−01
transport $= \log_{10}(k_1'k_2'/k_2)$	−1.568e+00	−1.68000	−1.532 or 0.4347	−1.493e+00

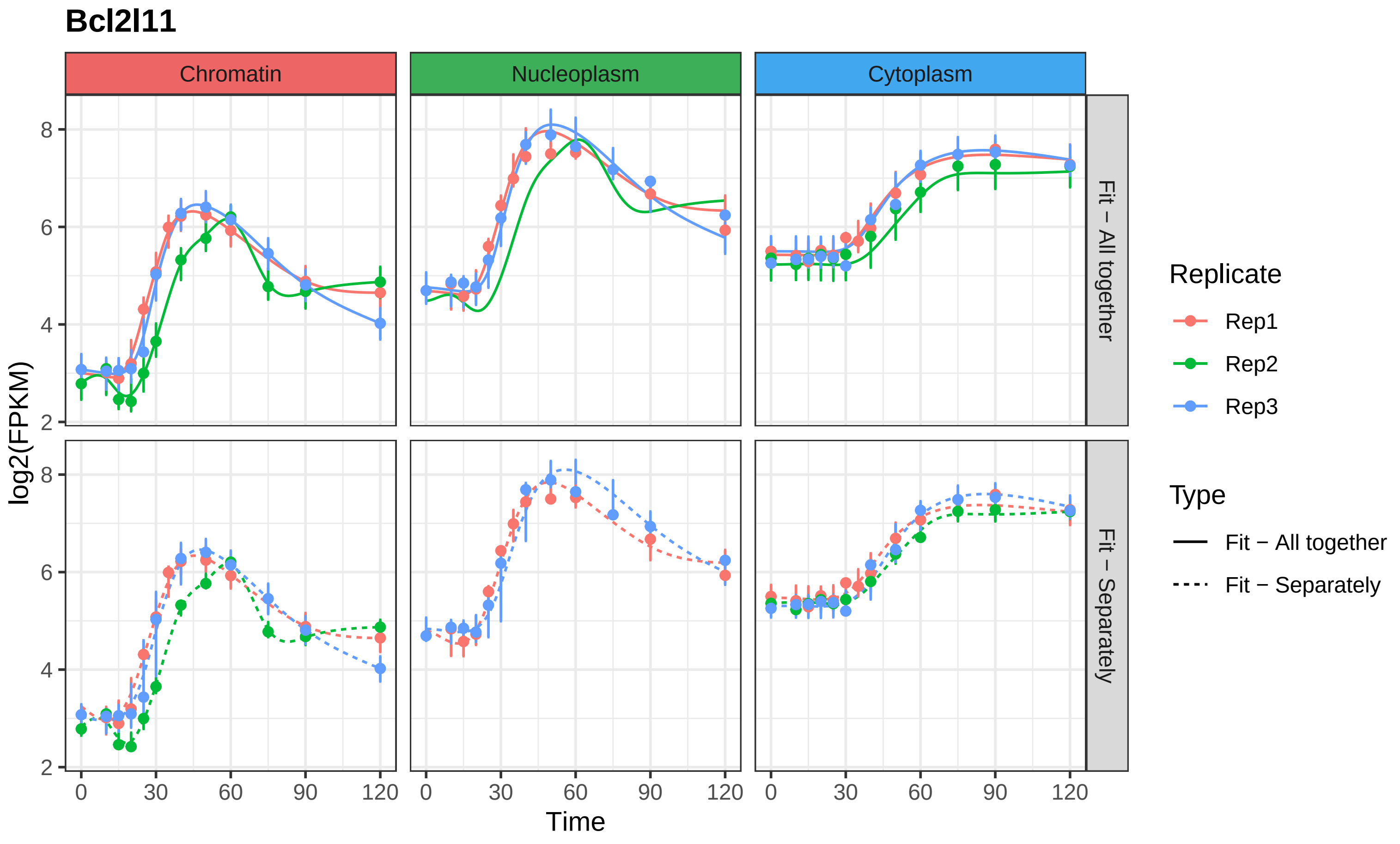

	Together	b1	b2	b3
**−NLL b1 ca**	−8.683	−7.219		
**−NLL b1 np**	1.336	−1.384		
**−NLL b1 cyto**	−7.245	−8.377		
**−NLL b2 ca**	−8.601		−11.45	
**−NLL b2 np**				
**−NLL b2 cyto**	−3.475		−12.12	
**−NLL b3 ca**	−8.073			−5.972
**−NLL b3 np**	1.145			1.413
**−NLL b3 cyto**	−3.113			−8.415
Total	−36.71	−16.98	−23.57	−12.97
**Total with regul**	−32.25	−17.47	−18	−12.72

	Together	b1	b2	b3
spar	0.27810	0.38690	0.2529	0.3462
σ_b	0.11010	0.09335	0.04971	0.0803
σ_t	0.30060	0.65010	0.0004249	2.2020
ca_{0,b_1}	3.00100	3.30200		
$\log_{10}(k_1')$	−0.01751	−0.04808		−0.3358
$\log_{10}(k_2)$	−0.52350	−0.51170	0.3856 or −1.897	−0.8751
$\log_{10}(k_2')$	−1.65200	−1.62400		−1.5970
$\log_{10}(k_{deg})$	−1.87600	−1.83000	−1.897 or 0.3856	−1.7390
$\log_{10}(k_1' k_2')$	−1.66900	−1.67200	−0.7473	−1.9330
$\log_{10}(k_1'/k_2)$	0.50600	0.46370		0.5393
$\text{transport} = \log_{10}(k_1' k_2'/k_2)$	−1.14600	−1.16000	−1.133 or 1.149	−1.0580

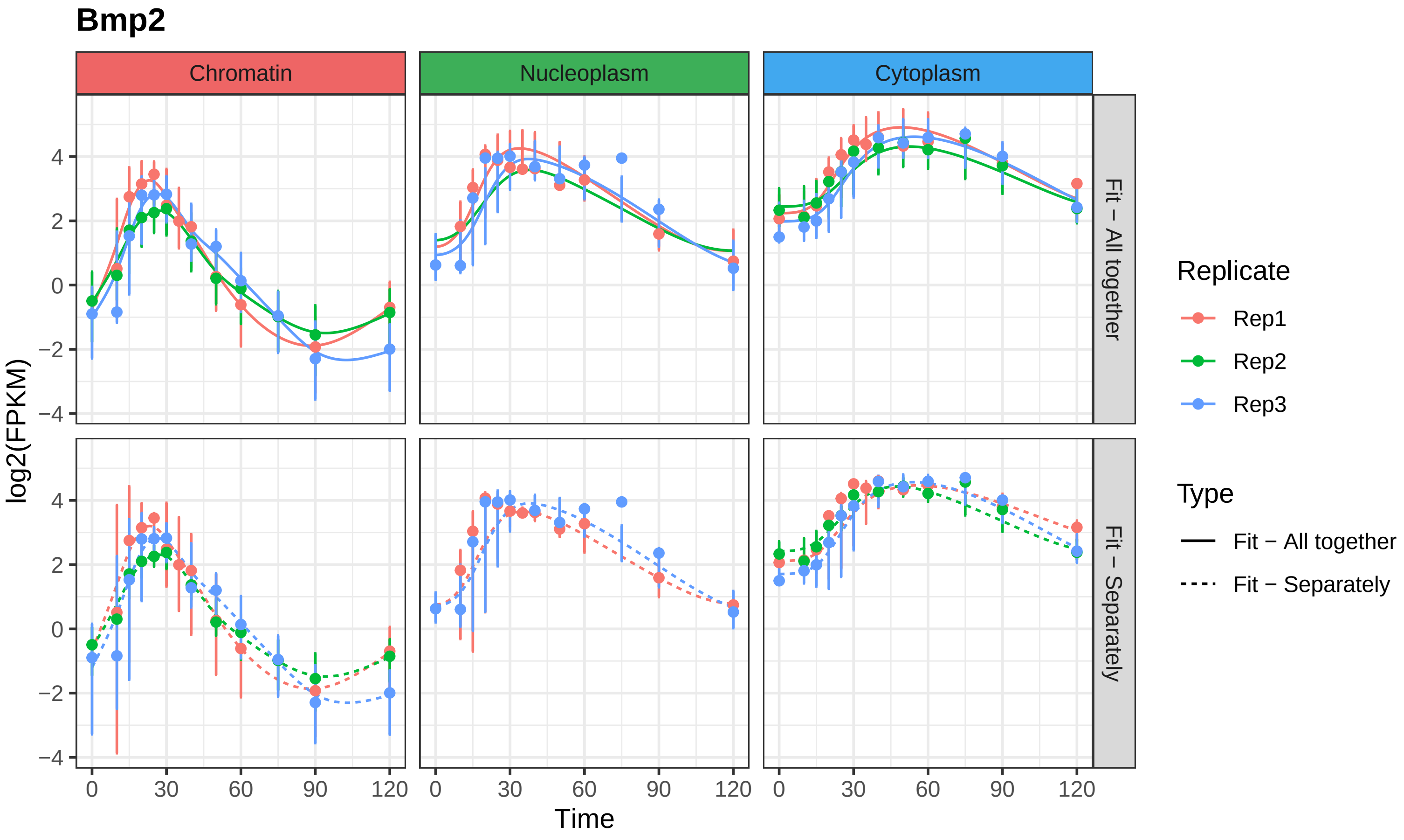

	Together	b1	b2	b3
**−NLL b1 ca**	4.045	6.734		
**−NLL b1 np**	9.039	0.8439		
**−NLL b1 cyto**	3.643	−0.2063		
**−NLL b2 ca**	2.127		−1.357	
**−NLL b2 np**				
**−NLL b2 cyto**	2.977		−1.34	
**−NLL b3 ca**	5.187			4.634
**−NLL b3 np**	17.63			8.14
**−NLL b3 cyto**	1.975			−1.465
Total	46.62	7.371	−2.697	11.31
**Total with regul**	49.19	7.429	−3.539	10.53

	Together	b1	b2	b3
spar	0.3732	4.058e−01	0.3823	0.38770
σ_b	0.2048	1.137e−06	0.1014	0.08307
σ_t	2.8780	5.692e+00	1.519	4.59000
ca_{0,b_1}	−0.6959	−6.614e−01		
$\log_{10}(k_1')$	−0.7591	−9.728e−01		−0.75980
$\log_{10}(k_2)$	−1.3410	−1.408e+00	−0.9079 or −1.43	−1.33600
$\log_{10}(k_2')$	−0.9202	−1.011e+00		−0.91150
$\log_{10}(k_{deg})$	−1.2350	−1.420e+00	−1.43 or −0.9079	−1.21100
$\log_{10}(k_1'k_2')$	−1.6790	−1.984e+00	−1.429	−1.67100
$\log_{10}(k_1'/k_2)$	0.5823	4.355e−01		0.57610
transport $= \log_{10}(k_1'k_2'/k_2)$	−0.3380	−5.752e−01	−0.5216 or 0.0002497	−0.33540

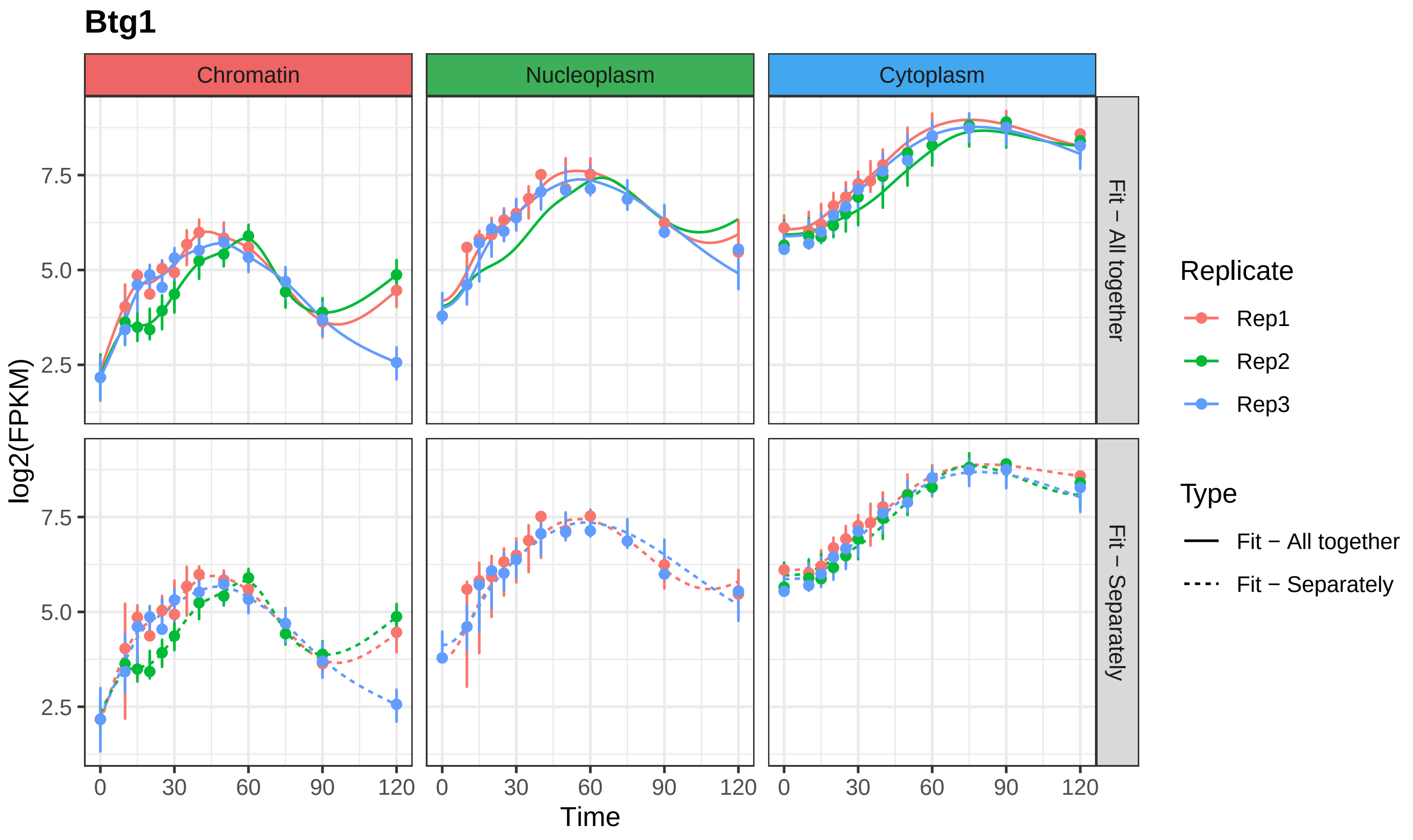

	Together	b1	b2	b3
**−NLL b1 ca**	−4.305	−1.911		
**−NLL b1 np**	3.554	5.937		
**−NLL b1 cyto**	−4.338	−11.22		
**−NLL b2 ca**	−6.366		−6.162	
**−NLL b2 np**				
**−NLL b2 cyto**	1.524		−0.04316	
**−NLL b3 ca**	−4.924			−2.498
**−NLL b3 np**	4.298			2.972
**−NLL b3 cyto**	−3.652			−2.829
Total	−14.21	−7.196	−6.205	−2.355
**Total with regul**	−9.747	−7.332	−4.006	−2.541

	Together	b1	b2	b3
spar	0.2851	4.020e−01	0.3119	0.3697
σ_b	0.1298	1.648e−05	0.1185	0.1208
σ_t	1.3200	4.783e+00	0.008695	2.3660
ca_{0,b_1}	2.3280	1.957e+00		
$\log_{10}(k_1')$	−0.4946	−5.338e−01		−0.5901
$\log_{10}(k_2)$	−1.0520	−1.041e+00	−1.156 or −1.194	−1.1620
$\log_{10}(k_2')$	−0.9494	−1.054e+00		−0.9453
$\log_{10}(k_{deg})$	−1.5150	−1.756e+00	−1.194 or −1.156	−1.4690
$\log_{10}(k_1'k_2')$	−1.4440	−1.588e+00	−1.255	−1.5350
$\log_{10}(k_1'/k_2)$	0.5571	5.075e−01		0.5719
transport $= \log_{10}(k_1'k_2'/k_2)$	−0.3924	−5.464e−01	−0.09896 or −0.061	−0.3734

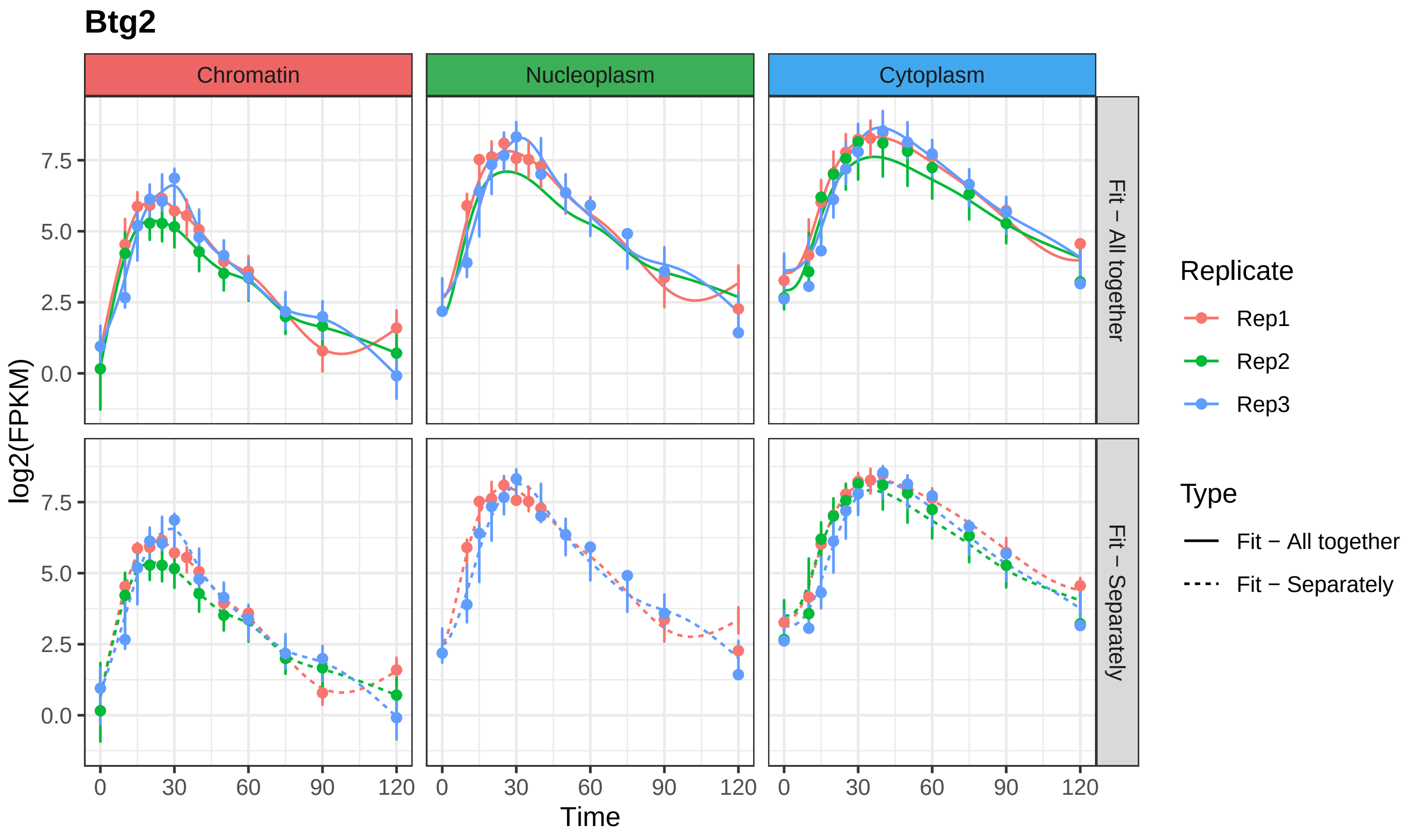

	Together	b1	b2	b3
**−NLL b1 ca**	−1.124	−4.131		
**−NLL b1 np**	5.134	8.456		
**−NLL b1 cyto**	1.631	−4.552		
**−NLL b2 ca**	−0.9109		−1.824	
**−NLL b2 np**				
**−NLL b2 cyto**	11.15		10.08	
**−NLL b3 ca**	1.506			2.456
**−NLL b3 np**	7.396			4.78
**−NLL b3 cyto**	13			6.126
Total	37.78	−0.2272	8.253	13.36
**Total with regul**	44.52	3.159	12.02	15.45

	Together	b1	b2	b3
spar	0.29130	0.3565	0.3064	0.3383
σ_b	0.21510	0.1491	0.1959	0.1938
σ_t	1.09500	0.3807	1.383	1.3930
ca_{0,b_1}	0.79840	0.5064		
$\log_{10}(k_1')$	−0.07858	0.1365		−0.1033
$\log_{10}(k_2)$	−0.62610	−0.4482	−0.8106 or −0.8257	−0.6166
$\log_{10}(k_2')$	−0.82190	−0.9594		−0.9233
$\log_{10}(k_{deg})$	−1.08800	−1.2140	−0.8257 or −0.8106	−1.1150
$\log_{10}(k_1' k_2')$	−0.90050	−0.8229	−0.7742	−1.0270
$\log_{10}(k_1'/k_2)$	0.54750	0.5847		0.5133
transport = $\log_{10}(k_1' k_2'/k_2)$	−0.27440	−0.3747	0.03641 or 0.05152	−0.4100

C3

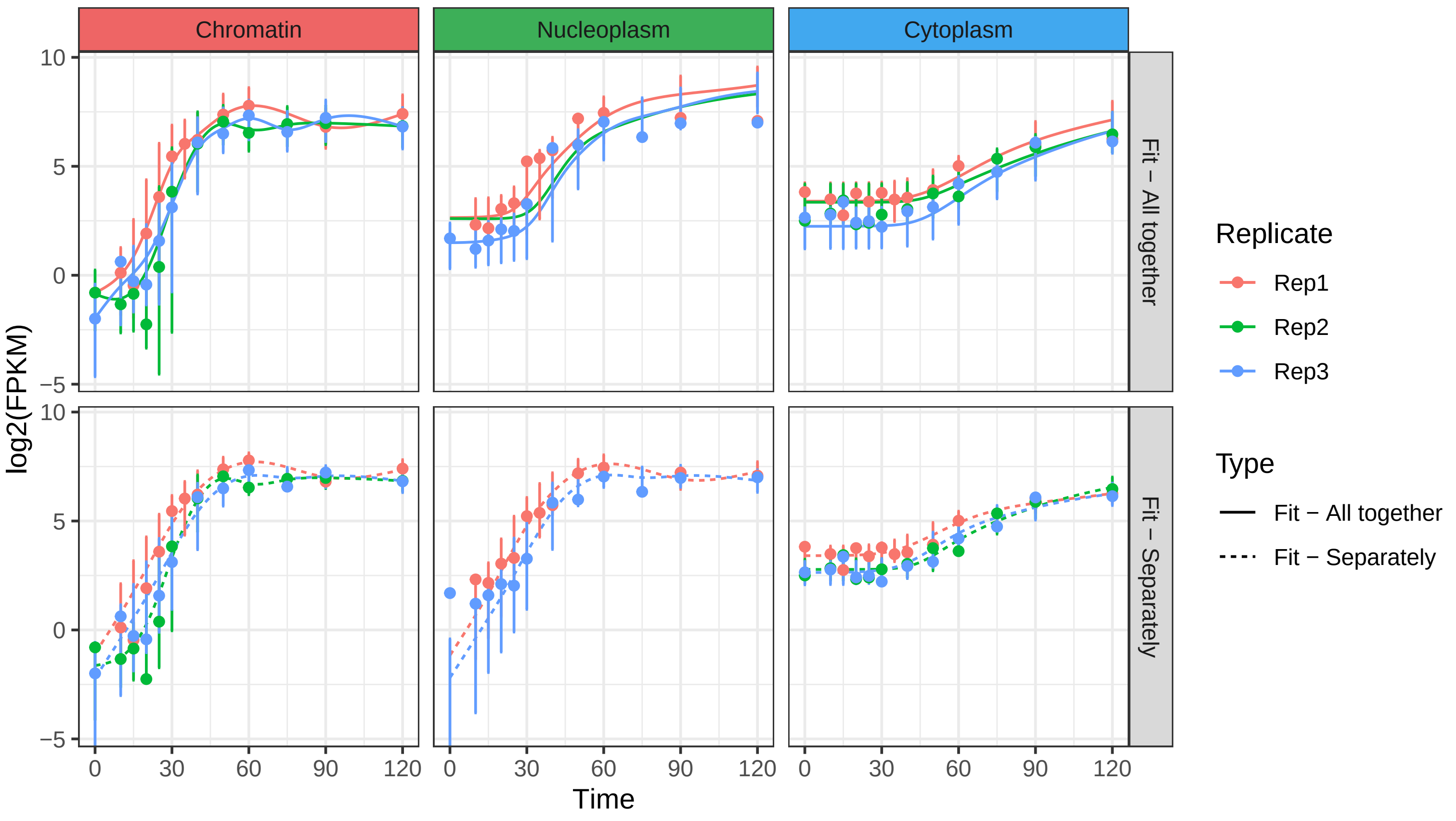

	Together	b1	b2	b3
**−NLL b1 ca**	8.155	7.293		
**−NLL b1 np**	17.11	7.86		
**−NLL b1 cyto**	6.028	3.44		
**−NLL b2 ca**	9.804		6.426	
**−NLL b2 np**				
**−NLL b2 cyto**	11.03		4.943	
**−NLL b3 ca**	9.832			8.37
**−NLL b3 np**	16.32			7.851
**−NLL b3 cyto**	9.19			6.471
Total	87.48	18.59	11.37	22.69
**Total with regul**	92	19.03	11.8	22.85

	Together	b1	b2	b3
spar	0.3592	0.52570	0.371	0.507000
σ_b	0.3058	0.15120	0.1626	0.172900
σ_t	3.5440	3.45900	3.021	4.077000
ca_{0,b_1}	−0.7749	−0.97540		
$\log_{10}(k_1')$	−1.4250	5.43300		5.600000
$\log_{10}(k_2)$	−2.4670	5.46000	−1.457 or −3.187	5.596000
$\log_{10}(k_2')$	−2.1240	−2.26500		−2.154000
$\log_{10}(k_{deg})$	−2.3500	−3.64300	−3.187 or −1.457	−3.609000
$\log_{10}(k_1'k_2')$	−3.5490	3.16700	−3.316	3.446000
$\log_{10}(k_1'/k_2)$	1.0410	−0.02777		0.004409
transport $= \log_{10}(k_1'k_2'/k_2)$	−1.0820	−2.29300	−1.859 or −0.1293	−2.150000

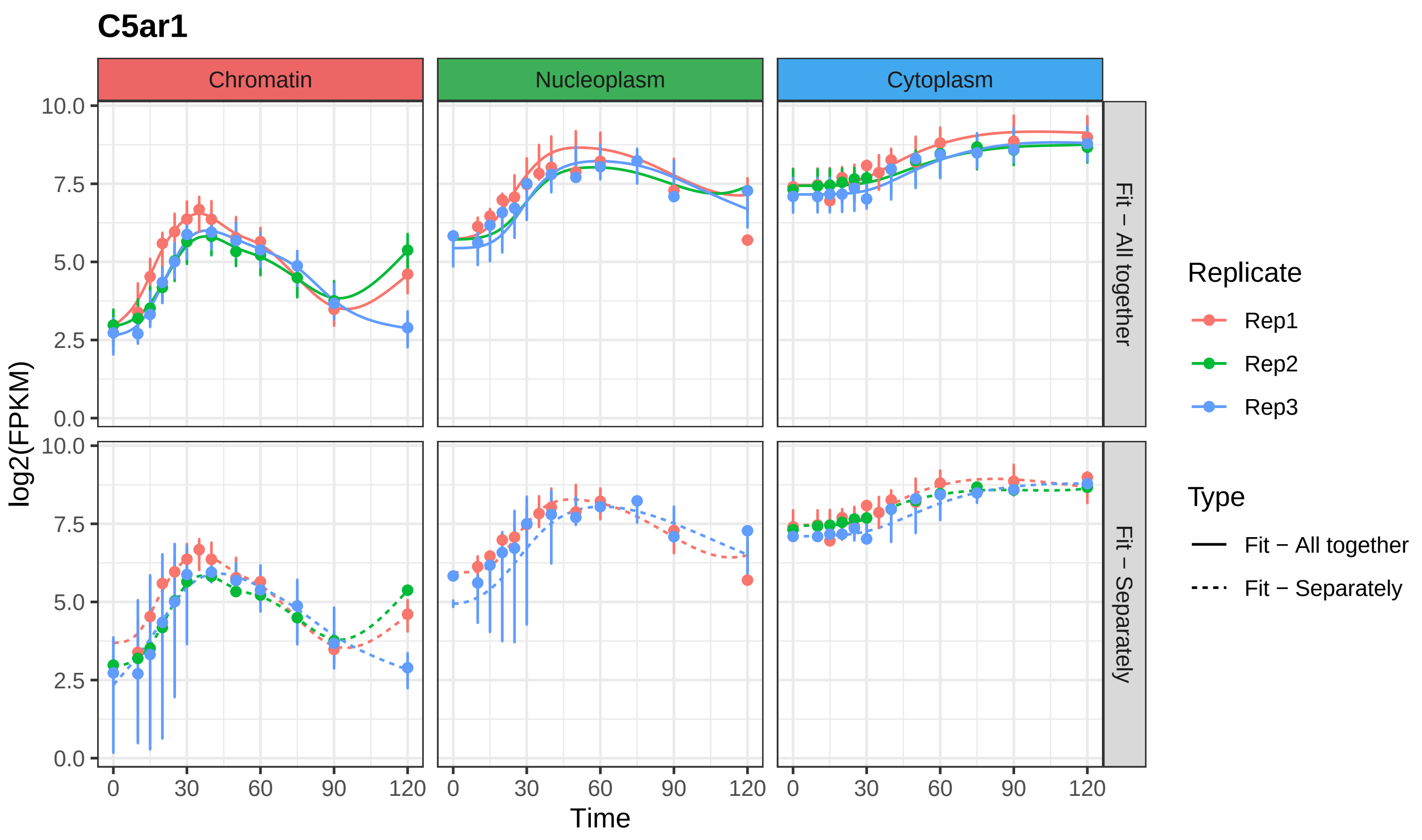

	Together	b1	b2	b3
**−NLL b1 ca**	−2.205	−1.255		
**−NLL b1 np**	17.15	4.043		
**−NLL b1 cyto**	0.2436	−0.1882		
**−NLL b2 ca**	−3.481		−13.97	
**−NLL b2 np**				
**−NLL b2 cyto**	−3.011		−15.51	
**−NLL b3 ca**	−2.814			4.4
**−NLL b3 np**	11.6			6.232
**−NLL b3 cyto**	−1.343			−11.26
Total	16.14	2.599	−29.48	−0.6267
**Total with regul**	17.72	3.347	−28.15	−1.097

	Together	b1	b2	b3
spar	0.3580000	3.836e−01	0.3161	4.829e−01
σ_b	0.1905000	1.692e−01	0.0356	2.132e−06
σ_t	0.0004423	5.435e−05	9.3e−06	9.031e+00
ca_{0,b_1}	3.0090000	3.808e+00		
$\log_{10}(k_1')$	−0.5419000	−5.885e−01		−5.789e−01
$\log_{10}(k_2)$	−1.3820000	−1.268e+00	−0.3672 or −2.239	−1.361e+00
$\log_{10}(k_2')$	−1.5430000	−1.372e+00		−1.559e+00
$\log_{10}(k_{deg})$	−2.0610000	−1.828e+00	−2.239 or −0.3672	−2.209e+00
$\log_{10}(k_1'k_2')$	−2.0850000	−1.960e+00	−1.242	−2.138e+00
$\log_{10}(k_1'/k_2)$	0.8402000	6.792e−01		7.820e−01
$\text{transport} = \log_{10}(k_1'k_2'/k_2)$	−0.7029000	−6.925e−01	−0.8744 or 0.9973	−7.767e−01

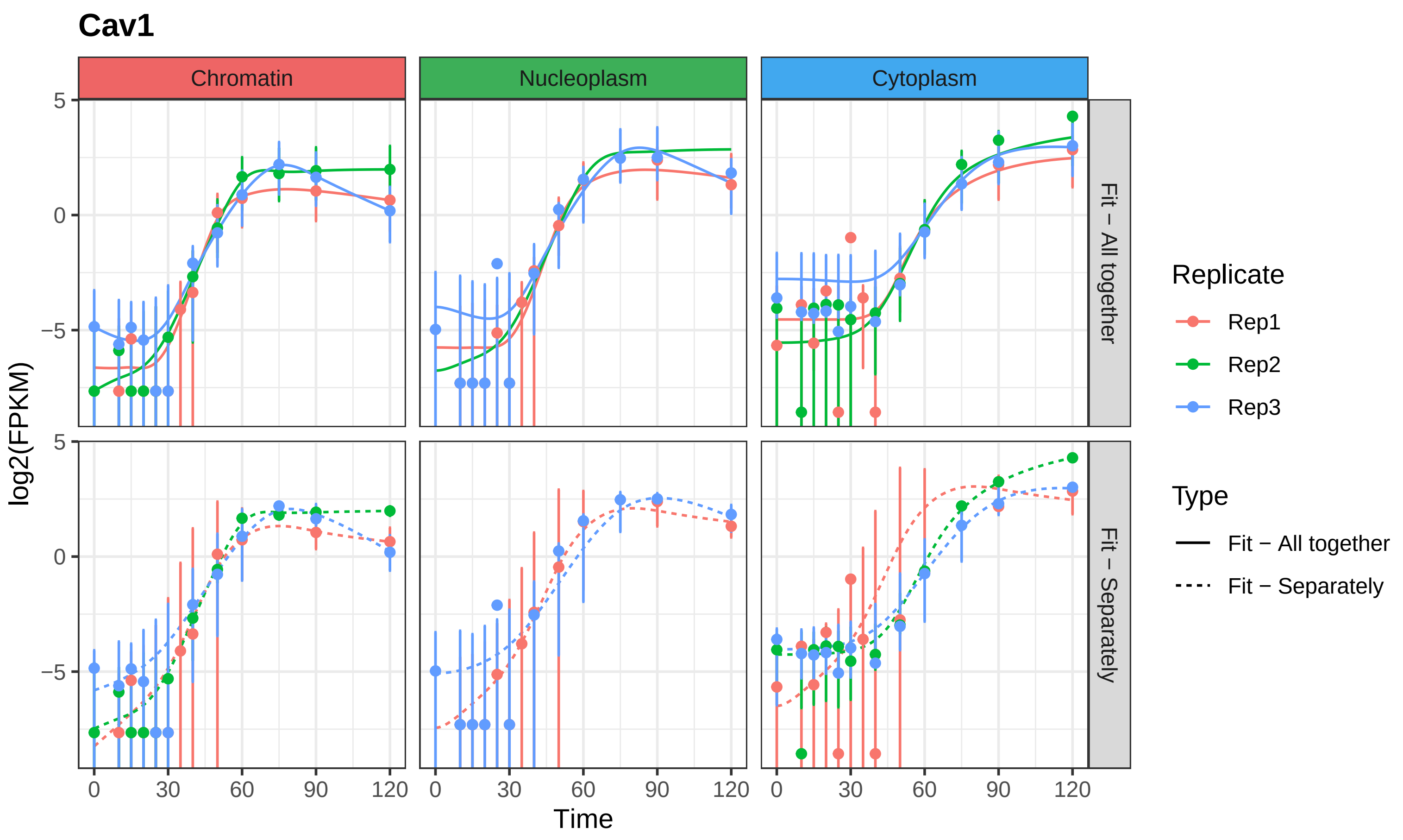

	Together	b1	b2	b3
**−NLL b1 ca**	14.61	17.81		
**−NLL b1 np**	15.28	18.07		
**−NLL b1 cyto**	40.04	29.19		
**−NLL b2 ca**	14		7.99	
**−NLL b2 np**				
**−NLL b2 cyto**	17.46		9.034	
**−NLL b3 ca**	14.17			14.94
**−NLL b3 np**	25.67			22.76
**−NLL b3 cyto**	22.11			8.806
Total	163.3	65.06	17.02	46.51
**Total with regul**	173.3	66.54	14.9	45.91

	Together	b1	b2	b3
spar	3.988e−01	0.5124	0.4198	5.256e−01
σ_b	3.698e−01	0.1803	0.003751	1.255e−07
σ_t	3.457e−06	7.6010	0.8273	5.386e+00
ca_{0,b_1}	−6.622e+00	−8.2350		
$\log_{10}(k_1')$	−5.253e−01	−0.4169		−8.756e−01
$\log_{10}(k_2)$	−7.889e−01	−0.6598	−1.512 or −1.568	−1.097e+00
$\log_{10}(k_2')$	−1.392e+00	5.9880		−1.219e+00
$\log_{10}(k_{deg})$	−1.758e+00	5.7020	−1.568 or −1.512	−1.534e+00
$\log_{10}(k_1'k_2')$	−1.917e+00	5.5710	−2.112	−2.094e+00
$\log_{10}(k_1'/k_2)$	2.635e−01	0.2429		2.214e−01
$transport = \log_{10}(k_1'k_2'/k_2)$	−1.128e+00	6.2300	−0.6001 or −0.5443	−9.974e−01

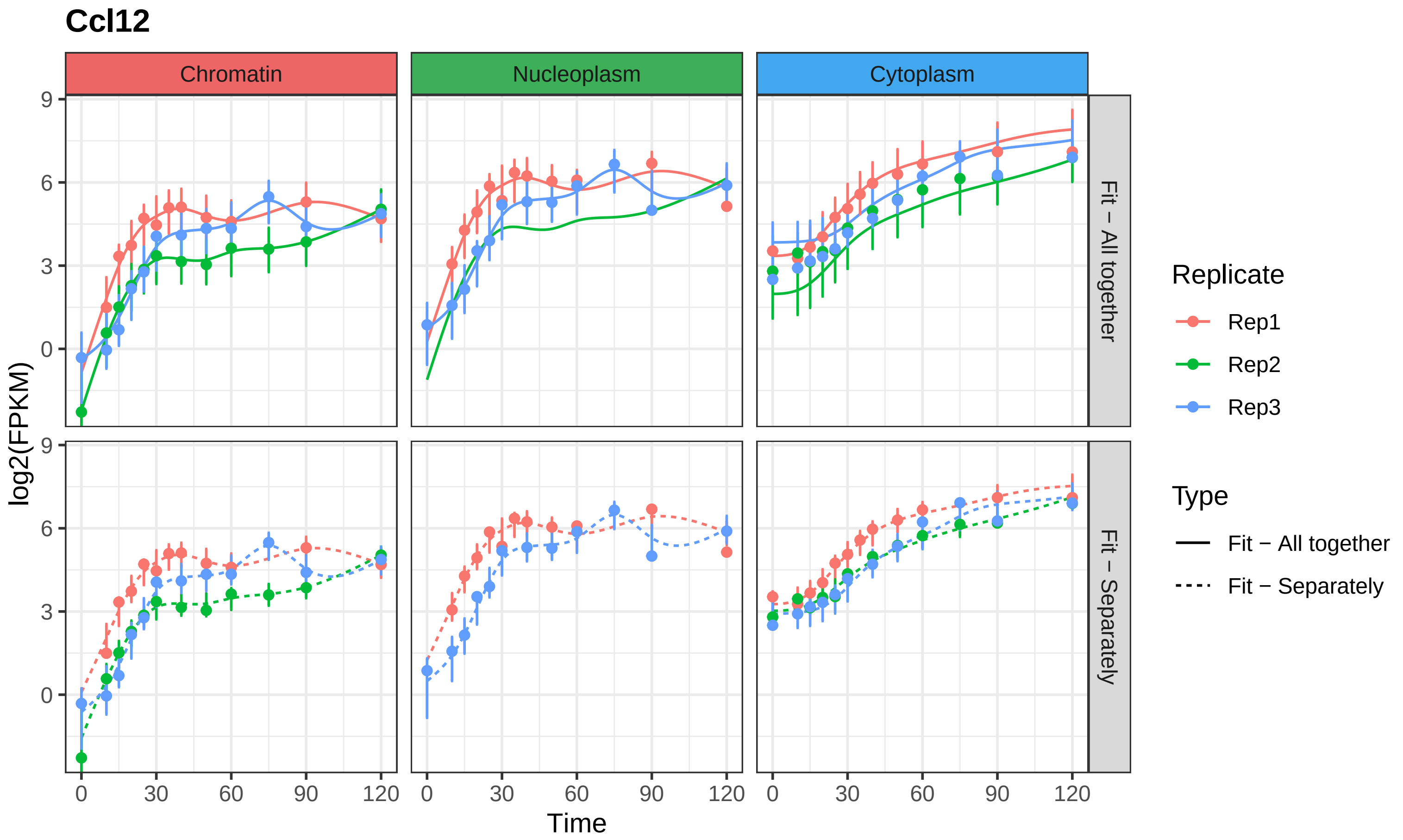

	Together	b1	b2	b3
**−NLL b1 ca**	1.28	−1.097		
**−NLL b1 np**	3.233	4.471		
**−NLL b1 cyto**	3.225	−3.678		
**−NLL b2 ca**	1.618		−4.215	
**−NLL b2 np**				
**−NLL b2 cyto**	17.92		−1.52	
**−NLL b3 ca**	2.826			−0.3521
**−NLL b3 np**	3.188			2.123
**−NLL b3 cyto**	17.38			2.634
Total	50.68	−0.3044	−5.735	4.404
**Total with regul**	54.27	−0.8857	−7.436	5.092

	Together	b1	b2	b3
spar	0.37670	0.4060000	0.4241	0.359100
σ_b	0.25690	0.1461000	0.09839	0.164600
σ_t	0.00492	0.0006156	9.584e−05	0.004122
ca_{0,b_1}	−0.87780	0.0994600		
$\log_{10}(k_1')$	8.77700	6.6920000		5.621000
$\log_{10}(k_2)$	8.44200	6.3450000	0.5576 or −2.324	5.286000
$\log_{10}(k_2')$	−1.35900	−1.3960000		−1.427000
$\log_{10}(k_{deg})$	−2.29000	−2.0060000	−2.324 or 0.5576	−2.159000
$\log_{10}(k_1'k_2')$	7.41800	5.2960000	−0.3862	4.194000
$\log_{10}(k_1'/k_2)$	0.33540	0.3469000		0.335200
transport = $\log_{10}(k_1'k_2'/k_2)$	−1.02400	−1.0490000	−0.9438 or 1.938	−1.092000

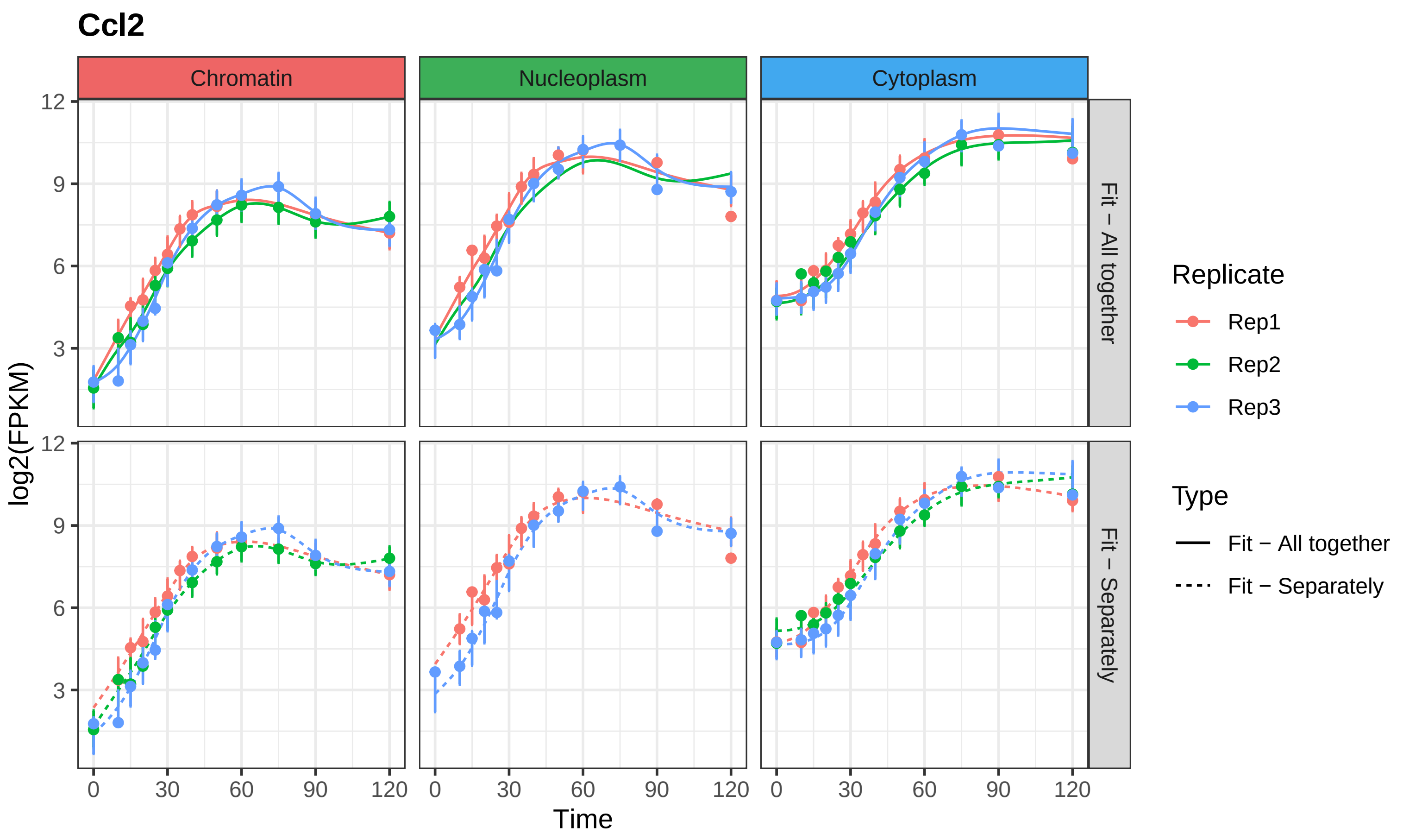

	Together	b1	b2	b3
−NLL b1 ca	−2.627	−2.384		
−NLL b1 np	8.521	8.672		
−NLL b1 cyto	2.139	−1.114		
−NLL b2 ca	−0.9886		−0.4565	
−NLL b2 np				
−NLL b2 cyto	6.018		2.728	
−NLL b3 ca	−0.6002			−0.1976
−NLL b3 np	4.298			3.529
−NLL b3 cyto	1.627			3.479
Total	18.39	5.174	2.272	6.81
Total with regul	21.54	4.723	1.415	7.345

	Together	b1	b2	b3
spar	0.335100	0.4641	0.4199	0.3684
σ_b	0.193300	0.1785	0.1589	0.1731
σ_t	0.006864	0.4630	0.9619	1.1200
ca_{0,b_1}	1.827000	2.3930		
$\log_{10}(k_1')$	5.512000	4.9430		5.6820
$\log_{10}(k_2)$	5.038000	4.4640	4.706 or −1.883	5.2420
$\log_{10}(k_2')$	−1.236000	−1.1440		−1.2860
$\log_{10}(k_{deg})$	−1.690000	−1.3890	−1.883 or 4.706	−1.8270
$\log_{10}(k_1' k_2')$	4.276000	3.7990	3.878	4.3960
$\log_{10}(k_1'/k_2)$	0.473400	0.4785		0.4403
transport = $\log_{10}(k_1' k_2'/k_2)$	−0.762500	−0.6656	−0.8276 or 5.762	−0.8453

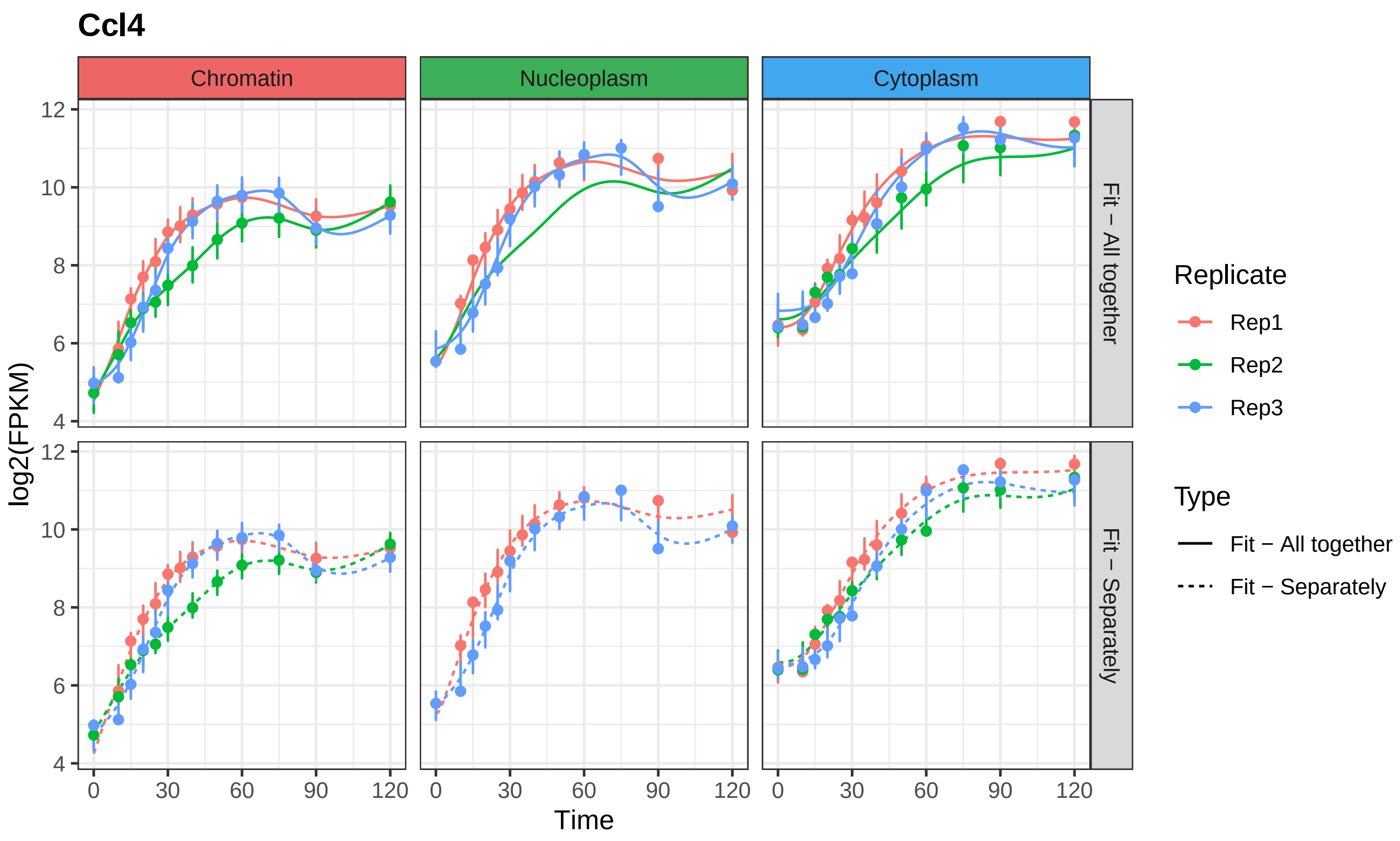

	Together	b1	b2	b3
**−NLL b1 ca**	−4.927	−5.985		
**−NLL b1 np**	2.707	2.792		
**−NLL b1 cyto**	0.595	−2.417		
**−NLL b2 ca**	−6.136		−7.773	
**−NLL b2 np**				
**−NLL b2 cyto**	2.612		−0.7593	
**−NLL b3 ca**	−4.569			−3.928
**−NLL b3 np**	0.7774			−0.3302
**−NLL b3 cyto**	5.164			−2.007
Total	−3.776	−5.61	−8.532	−6.265
**Total with regul**	−0.8573	−4.502	−9.855	−6.958

	Together	b1	b2	b3
spar	0.33490	0.4456	0.4037	0.3820
σ_b	0.15430	0.1291	0.1065	0.1164
σ_t	0.42300	0.7585	0.002715	1.0380
ca_{0,b_1}	4.53100	4.2110		
$\log_{10}(k_1')$	0.03964	0.1172		0.2147
$\log_{10}(k_2)$	−0.24020	−0.1891	−0.6207 or −1.069	−0.0175
$\log_{10}(k_2')$	−1.09300	−1.1960		−1.1760
$\log_{10}(k_{deg})$	−1.38200	−1.5570	−1.069 or −0.6207	−1.4870
$\log_{10}(k_1'k_2')$	−1.05300	−1.0780	−1.142	−0.9616
$\log_{10}(k_1'/k_2)$	0.27990	0.3063		0.2322
$\text{transport} = \log_{10}(k_1'k_2'/k_2)$	−0.81270	−0.8892	−0.5218 or −0.07397	−0.9441

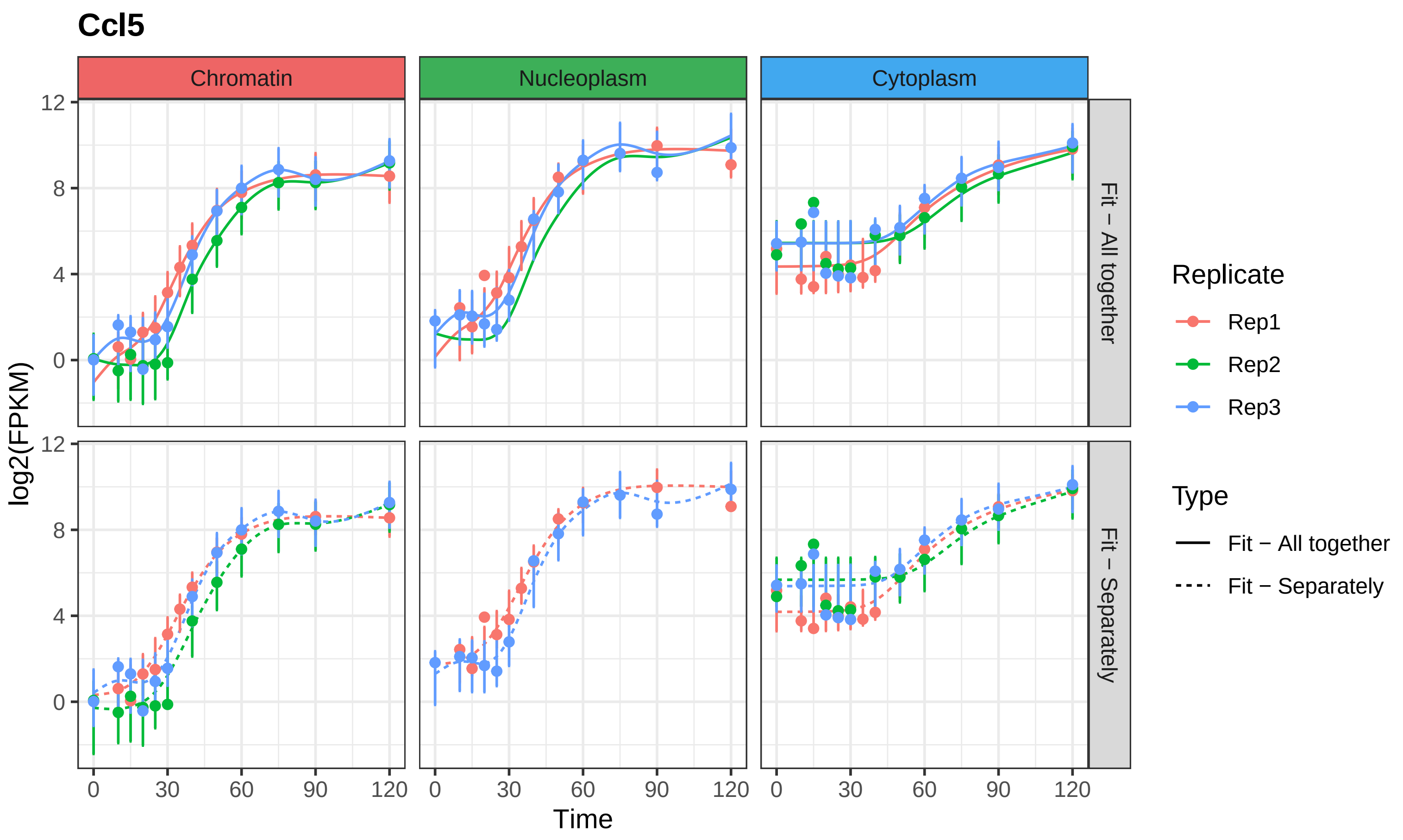

	Together	b1	b2	b3
**−NLL b1 ca**	5.209	3.481		
**−NLL b1 np**	12.55	11.33		
**−NLL b1 cyto**	9.898	9.486		
**−NLL b2 ca**	6.929		7.489	
**−NLL b2 np**				
**−NLL b2 cyto**	19.96		18.63	
**−NLL b3 ca**	7.577			6.816
**−NLL b3 np**	9.723			7.037
**−NLL b3 cyto**	17.92			18.29
Total	89.76	24.3	26.12	32.14
**Total with regul**	101.2	26.19	31.21	39.04

	Together	b1	b2	b3
spar	0.3051000	0.4442000	0.4017	0.32760
σ_b	0.3687000	0.2766000	0.3726	0.35270
σ_t	0.0005144	0.0004695	7.684e−05	0.02984
ca_{0,b_1}	−1.0440000	0.3529000		
$\log_{10}(k_1')$	4.7230000	0.5088000		4.49700
$\log_{10}(k_2)$	4.3670000	0.0794200	−0.593 or −3.151	4.23200
$\log_{10}(k_2')$	−1.7890000	−1.8250000		−1.68400
$\log_{10}(k_{deg})$	−3.0550000	−2.5600000	−3.151 or −0.593	−2.91300
$\log_{10}(k_1'k_2')$	2.9340000	−1.3160000	−1.951	2.81300
$\log_{10}(k_1'/k_2)$	0.3553000	0.4293000		0.26500
$\text{transport} = \log_{10}(k_1'k_2'/k_2)$	−1.4330000	−1.3950000	−1.358 or 1.201	−1.41900

Ccl7

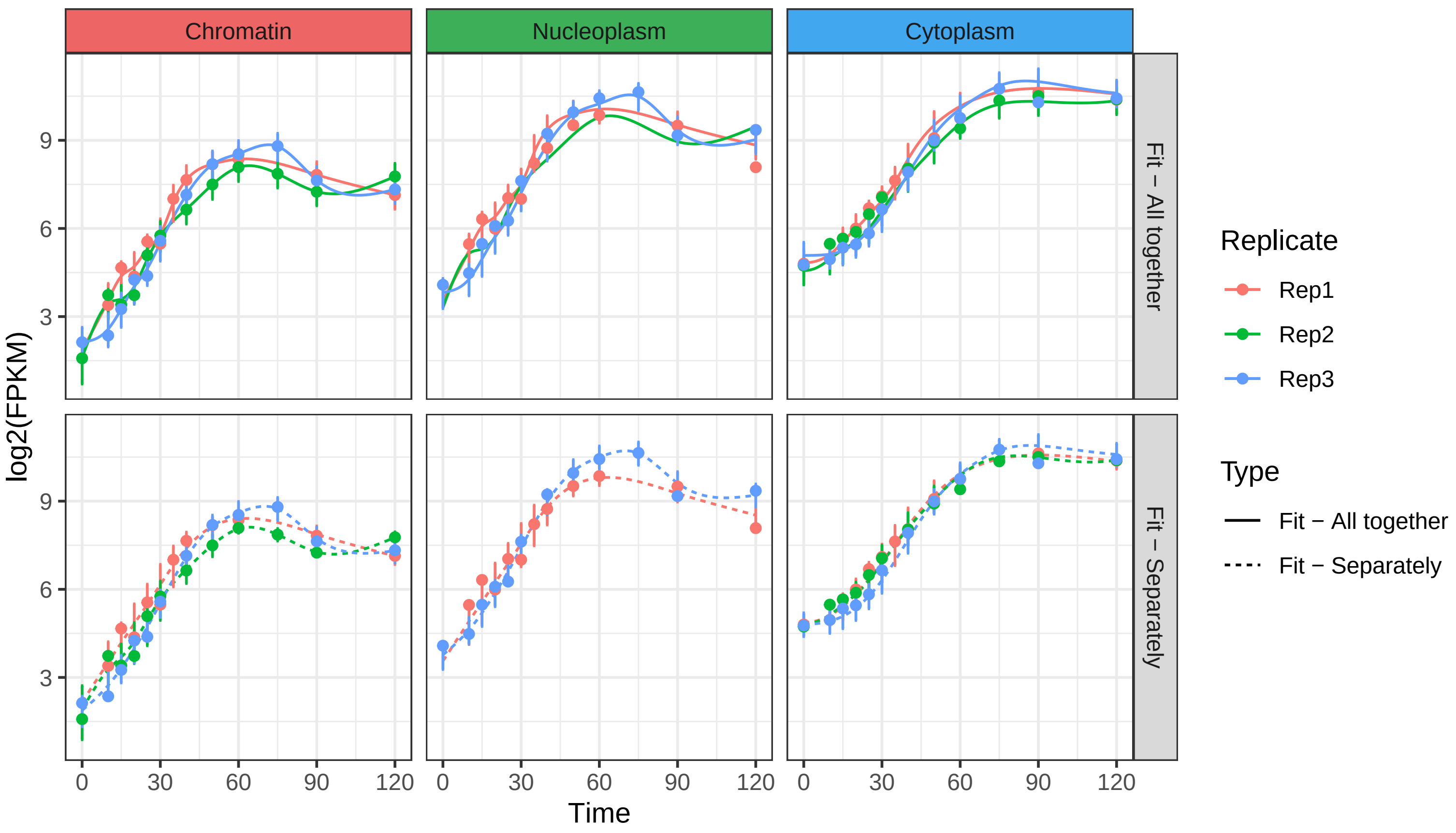

	Together	b1	b2	b3
**−NLL b1 ca**	−1.874	−0.8033		
**−NLL b1 np**	8.593	5.884		
**−NLL b1 cyto**	−0.3853	−4.325		
**−NLL b2 ca**	−3.433		−3.75	
**−NLL b2 np**				
**−NLL b2 cyto**	3.256		−3.377	
**−NLL b3 ca**	−3.803			−2.737
**−NLL b3 np**	1.798			−0.06648
**−NLL b3 cyto**	1.687			0.3246
Total	5.838	0.7557	−7.127	−2.479
**Total with regul**	14.36	−0.3399	−6.527	−2.598

	Together	b1	b2	b3
spar	0.2366	0.49920	0.332	0.3708
σ_b	0.1592	0.08718	4.334e−06	0.1367
σ_t	0.9416	2.51400	2.588	0.6794
ca_{0,b_1}	1.8710	2.14900		
$\log_{10}(k_1')$	6.2860	5.32400		5.7420
$\log_{10}(k_2)$	5.7750	4.90400	0.1241 or −1.375	5.1730
$\log_{10}(k_2')$	−1.1820	−1.14400		−1.2970
$\log_{10}(k_{deg})$	−1.5630	−1.54100	−1.375 or 0.1241	−1.6110
$\log_{10}(k_1'k_2')$	5.1040	4.18000	−0.368	4.4450
$\log_{10}(k_1'/k_2)$	0.5115	0.41940		0.5687
transport $= \log_{10}(k_1'k_2'/k_2)$	−0.6709	−0.72440	−0.4921 or 1.007	−0.7288

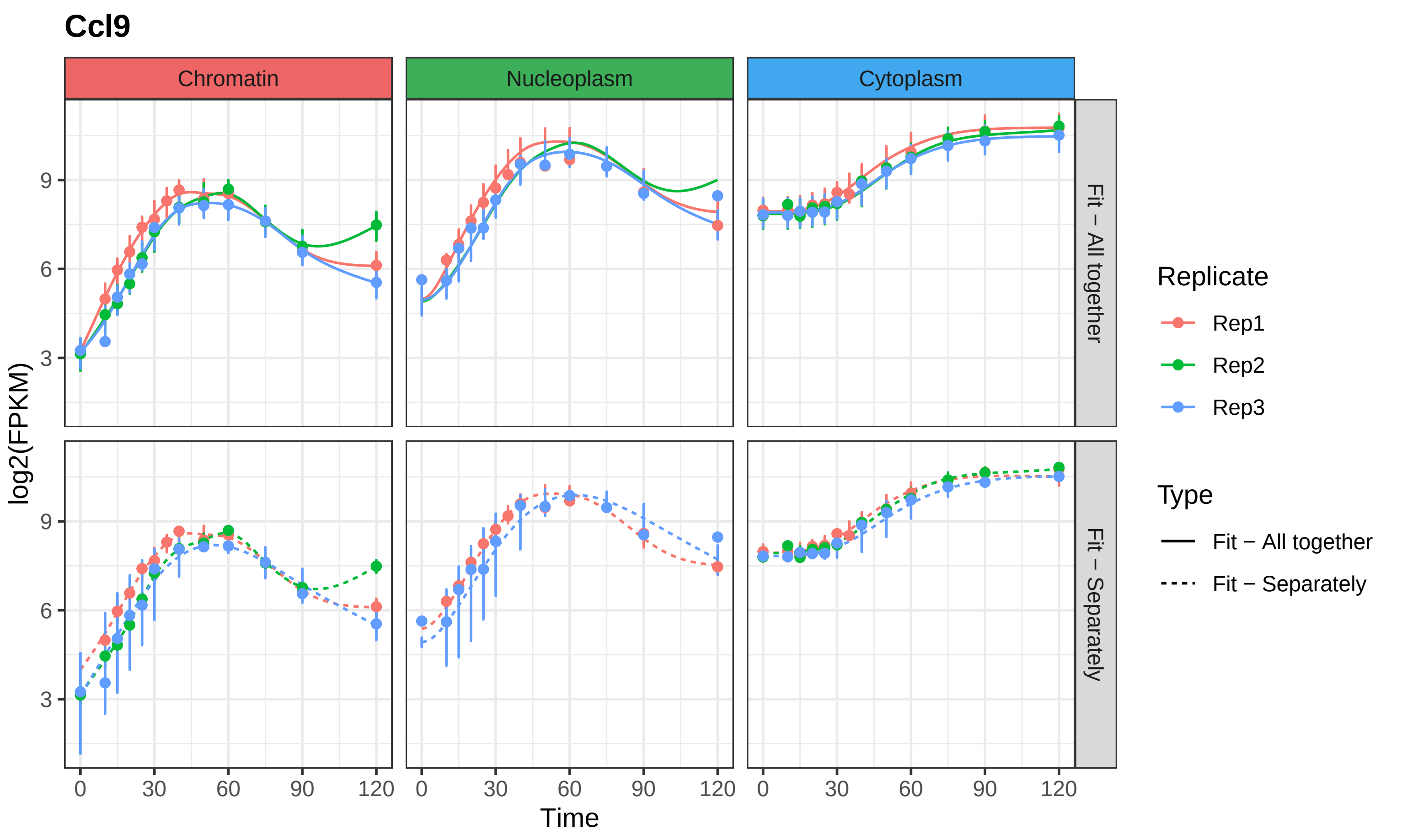

	Together	b1	b2	b3
**−NLL b1 ca**	−4.31	−7.033		
**−NLL b1 np**	8.362	−2.452		
**−NLL b1 cyto**	−3.892	−6.79		
**−NLL b2 ca**	−4.301		−9.989	
**−NLL b2 np**				
**−NLL b2 cyto**	−3.372		−6.982	
**−NLL b3 ca**	−0.6343			1.177
**−NLL b3 np**	16.72			7.894
**−NLL b3 cyto**	−5.033			−13.75
Total	3.539	−16.28	−16.97	−4.684
**Total with regul**	5.268	−17.41	−16.33	−4.727

	Together	b1	b2	b3
spar	4.005e−01	0.4053000	0.3337	5.415e−01
σ_b	1.674e−01	0.1008000	0.07179	2.201e−06
σ_t	1.966e−06	0.0003981	3.169e−06	5.490e+00
ca_{0,b_1}	3.198e+00	4.0210000		
$\log_{10}(k_1')$	−2.672e−01	−0.2962000		−3.863e−01
$\log_{10}(k_2)$	−8.013e−01	−0.7154000	−0.6235 or −2.458	−9.264e−01
$\log_{10}(k_2')$	−1.576e+00	−1.4860000		−1.551e+00
$\log_{10}(k_{deg})$	−2.465e+00	−2.2540000	−2.458 or −0.6235	−2.423e+00
$\log_{10}(k_1' k_2')$	−1.844e+00	−1.7830000	−1.644	−1.937e+00
$\log_{10}(k_1'/k_2)$	5.341e−01	0.4192000		5.400e−01
transport $= \log_{10}(k_1' k_2'/k_2)$	−1.042e+00	−1.0670000	−1.02 or 0.8142	−1.011e+00

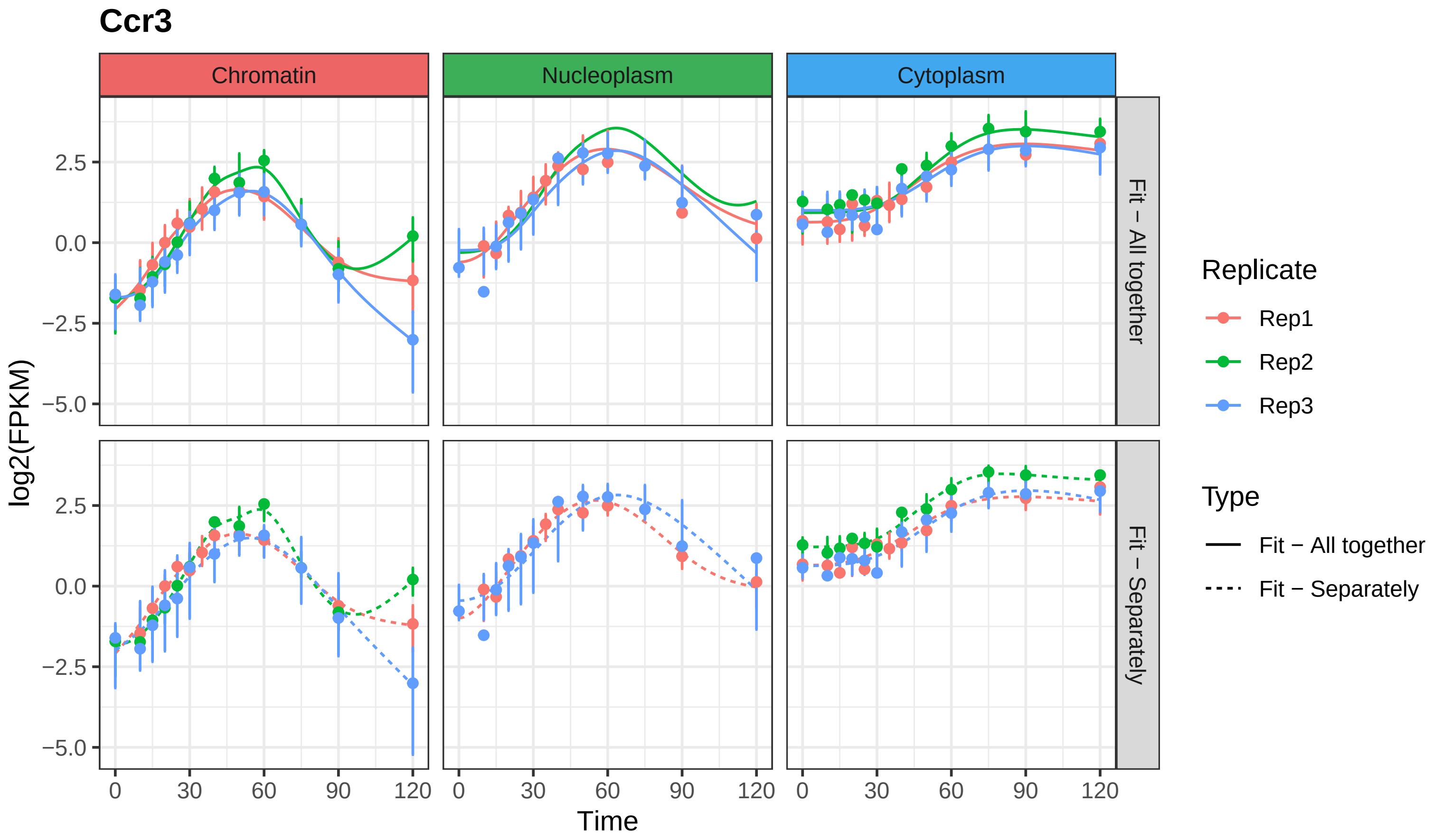

	Together	b1	b2	b3
**−NLL b1 ca**	−0.4552	−2.444		
**−NLL b1 np**	5.646	−0.8213		
**−NLL b1 cyto**	1.462	1.539		
**−NLL b2 ca**	0.2376		−2.652	
**−NLL b2 np**				
**−NLL b2 cyto**	2.842		−3.825	
**−NLL b3 ca**	0.2467			3.633
**−NLL b3 np**	18.14			10.86
**−NLL b3 cyto**	4.331			−0.07914
Total	32.46	−1.726	−6.478	14.41
**Total with regul**	34.09	−1.886	−6.786	13.94

	Together	b1	b2	b3
spar	3.945e−01	4.430e−01	0.361	0.5044
σ_b	2.013e−01	1.276e−01	0.09227	0.1035
σ_t	2.745e−05	2.164e−06	7.454e−06	5.9790
ca_{0,b_1}	−2.025e+00	−2.035e+00		
$\log_{10}(k_1')$	−6.448e−01	−4.295e−01		−0.6326
$\log_{10}(k_2)$	−1.087e+00	−7.606e−01	−0.4198 or −2.117	−1.0970
$\log_{10}(k_2')$	−1.494e+00	−1.578e+00		−1.4690
$\log_{10}(k_{deg})$	−1.866e+00	−2.072e+00	−2.117 or −0.4198	−1.7950
$\log_{10}(k_1' k_2')$	−2.138e+00	−2.007e+00	−1.606	−2.1010
$\log_{10}(k_1'/k_2)$	4.420e−01	3.311e−01		0.4645
transport $= \log_{10}(k_1' k_2'/k_2)$	−1.052e+00	−1.247e+00	−1.187 or 0.5106	−1.0040

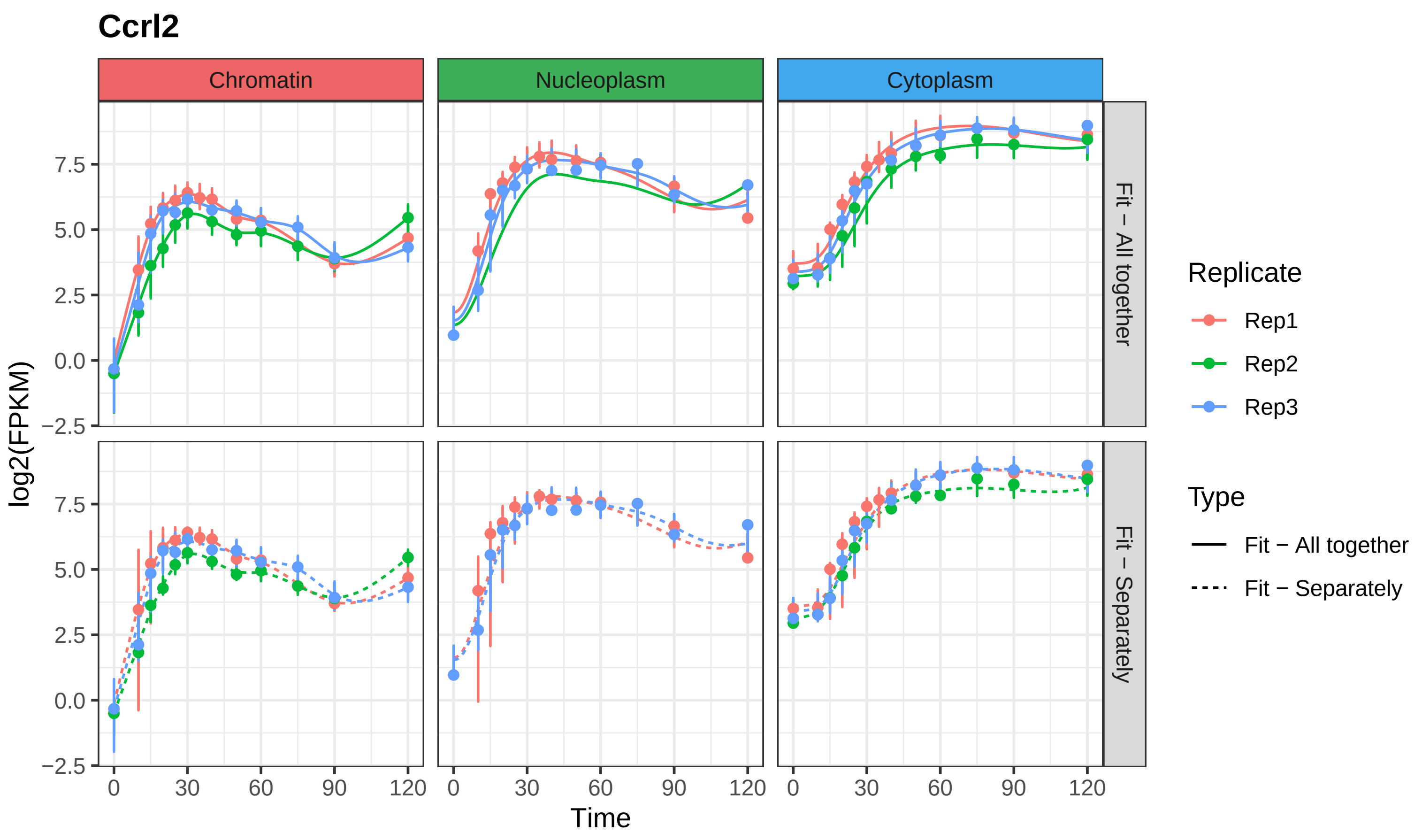

	Together	b1	b2	b3
**−NLL b1 ca**	−2.954	−3.3		
**−NLL b1 np**	6.844	7.195		
**−NLL b1 cyto**	3.406	1.056		
**−NLL b2 ca**	−2.022		−6.016	
**−NLL b2 np**				
**−NLL b2 cyto**	3.527		−1.607	
**−NLL b3 ca**	−0.6594			−0.3695
**−NLL b3 np**	10.37			7.734
**−NLL b3 cyto**	2.038			1.807
**Total**	20.55	4.95	−7.623	9.171
**Total with regul**	25.68	8.844	−6.503	11.31

	Together	b1	b2	b3
spar	0.32160	0.34930	0.3293	0.3286
σ_b	0.16180	0.08762	0.102	0.1706
σ_t	1.67100	3.33900	0.4925	1.6220
ca_{0,b_1}	−0.04996	−0.21340		
$\log_{10}(k_1')$	−0.45640	−0.56930		−0.4781
$\log_{10}(k_2)$	−1.01600	−1.10800	−0.2649 or −1.708	−1.0490
$\log_{10}(k_2')$	−1.06400	−1.12700		−1.1040
$\log_{10}(k_{deg})$	−1.62700	−1.72100	−1.708 or −0.2649	−1.6710
$\log_{10}(k_1'k_2')$	−1.52000	−1.69600	−0.8692	−1.5820
$\log_{10}(k_1'/k_2)$	0.56000	0.53880		0.5704
transport $= \log_{10}(k_1'k_2'/k_2)$	−0.50350	−0.58830	−0.6044 or 0.8387	−0.5332

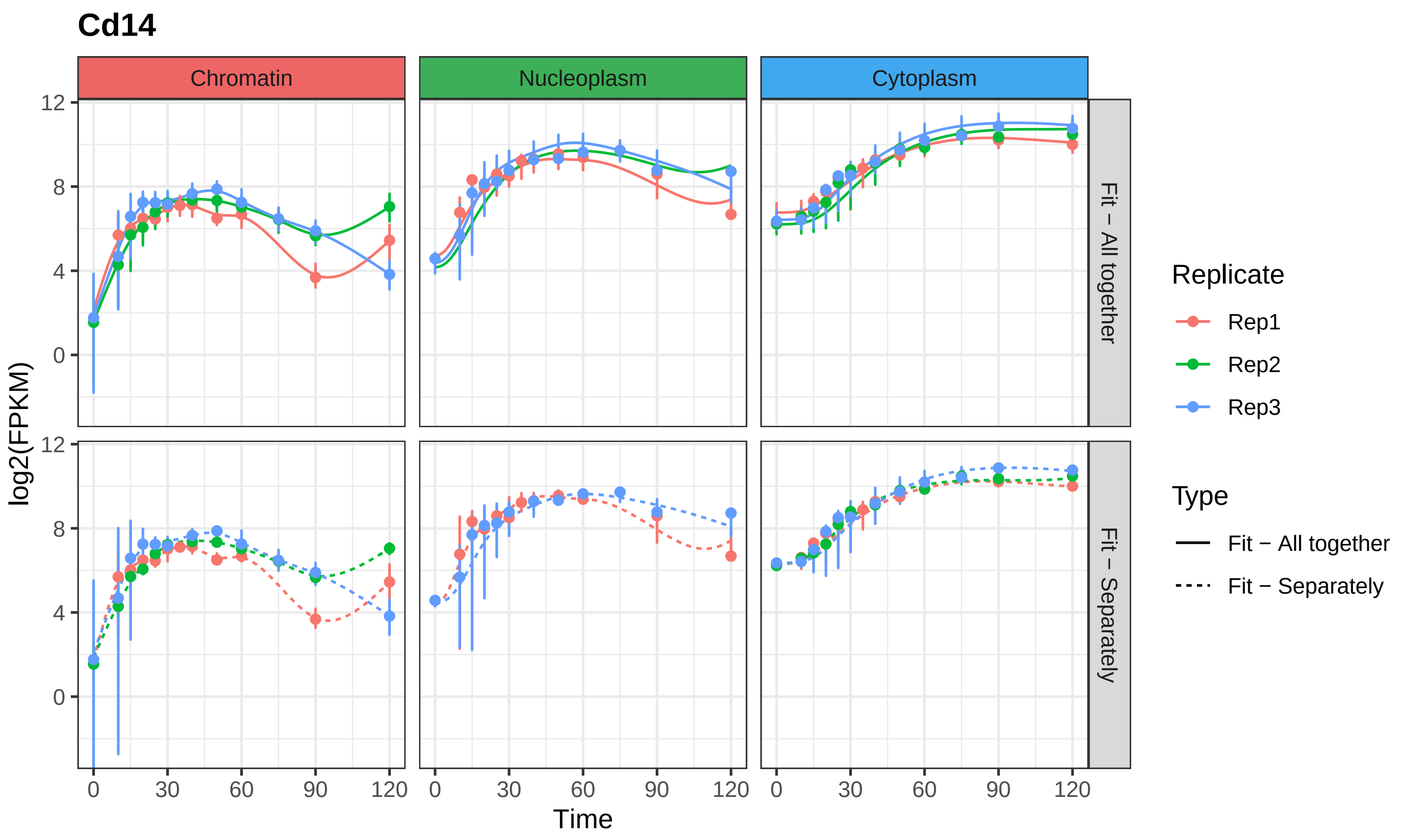

	Together	b1	b2	b3
**−NLL b1 ca**	−1.827	−5.89		
**−NLL b1 np**	6.991	3.673		
**−NLL b1 cyto**	1.331	−7.062		
**−NLL b2 ca**	−0.1266		−9.002	
**−NLL b2 np**				
**−NLL b2 cyto**	6.992		−5.037	
**−NLL b3 ca**	0.2354			1.108
**−NLL b3 np**	13.22			6.043
**−NLL b3 cyto**	2.617			−5.815
Total	29.43	−9.279	−14.04	1.336
**Total with regul**	32.92	−2.72	−14.37	1.139

	Together	b1	b2	b3
spar	0.3326	2.862e−01	0.3553	3.614e−01
σ_b	0.1582	2.683e−06	0.05612	2.718e−07
σ_t	3.0470	4.027e+00	1.317	5.248e+00
ca_{0,b_1}	2.0360	1.652e+00		
$\log_{10}(k_1')$	−0.4511	−3.013e−01		−7.073e−01
$\log_{10}(k_2)$	−1.2280	−1.111e+00	5.043 or −2.055	−1.409e+00
$\log_{10}(k_2')$	−1.2710	−1.358e+00		−1.109e+00
$\log_{10}(k_{deg})$	−1.8850	−1.930e+00	−2.055 or 5.043	−1.684e+00
$\log_{10}(k_1'k_2')$	−1.7220	−1.659e+00	4.325	−1.816e+00
$\log_{10}(k_1'/k_2)$	0.7771	8.098e−01		7.014e−01
$transport = \log_{10}(k_1'k_2'/k_2)$	−0.4935	−5.480e−01	−0.7183 or 6.38	−4.076e−01

Cd40

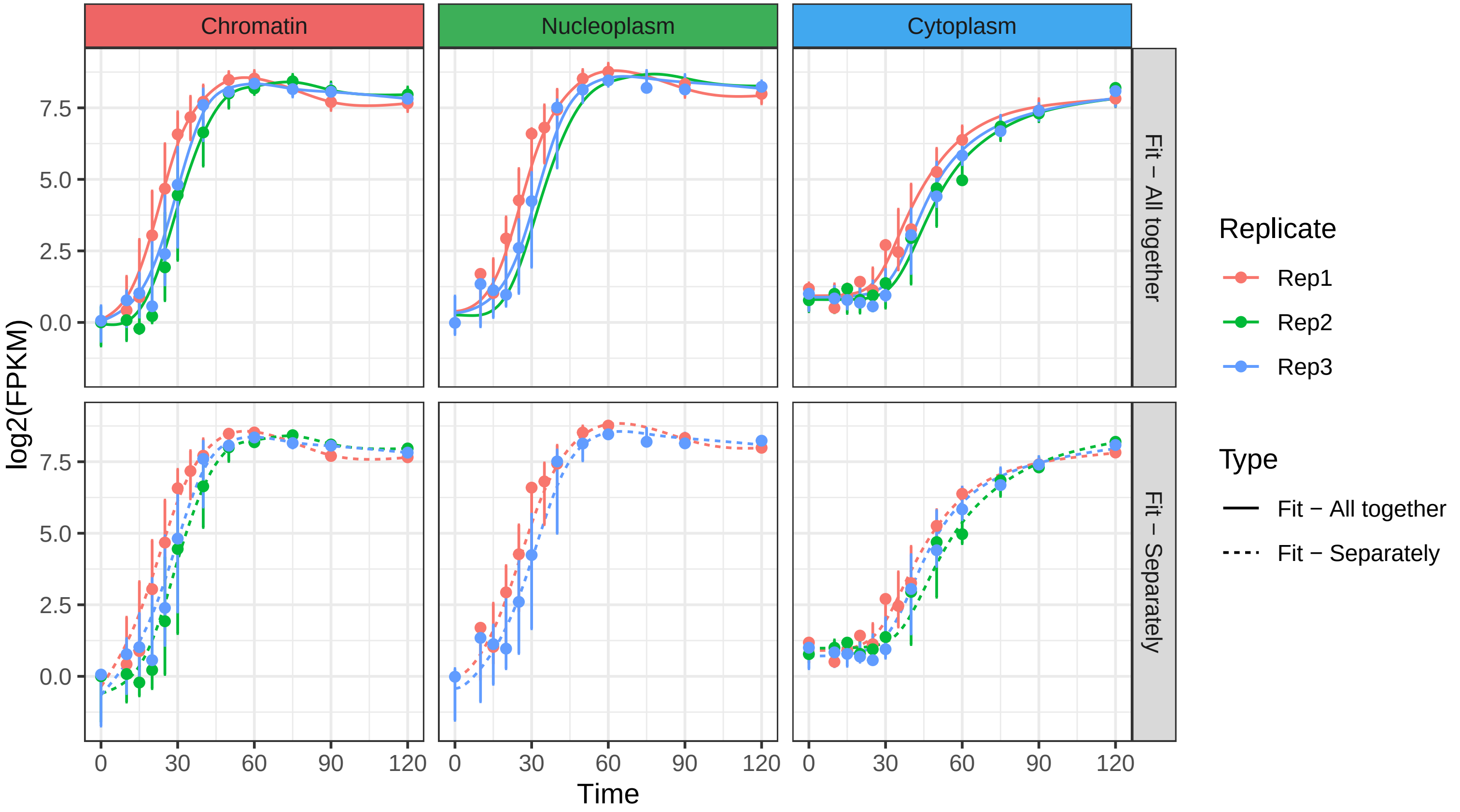

	Together	b1	b2	b3
**−NLL b1 ca**	0.2058	−2.347		
**−NLL b1 np**	5.614	1.052		
**−NLL b1 cyto**	2.674	0.2044		
**−NLL b2 ca**	0.5153		−0.8904	
**−NLL b2 np**				
**−NLL b2 cyto**	3.469		−1.184	
**−NLL b3 ca**	−0.1514			1.495
**−NLL b3 np**	6.42			6.784
**−NLL b3 cyto**	2.044			0.422
Total	20.79	−1.09	−2.074	8.702
**Total with regul**	20.85	−2.794	−2.526	7.088

	Together	b1	b2	b3
spar	0.36150	4.298e−01	0.3579	0.40940
σ_b	0.09496	8.657e−07	7.452e−07	0.06147
σ_t	2.36000	2.525e+00	2.928	3.05200
ca_{0,b_1}	0.14420	−2.717e−01		
$\log_{10}(k_1')$	−0.65280	−7.316e−01		−0.60200
$\log_{10}(k_2)$	−0.74450	−8.400e−01	−1.208 or −2.185	−0.67200
$\log_{10}(k_2')$	−1.93100	−1.997e+00		−1.91400
$\log_{10}(k_{deg})$	−2.09400	−2.269e+00	−2.185 or −1.208	−2.25500
$\log_{10}(k_1'k_2')$	−2.58400	−2.729e+00	−2.914	−2.51600
$\log_{10}(k_1'/k_2)$	0.09178	1.084e−01		0.07006
$\text{transport} = \log_{10}(k_1'k_2'/k_2)$	−1.84000	−1.889e+00	−1.707 or −0.7298	−1.84400

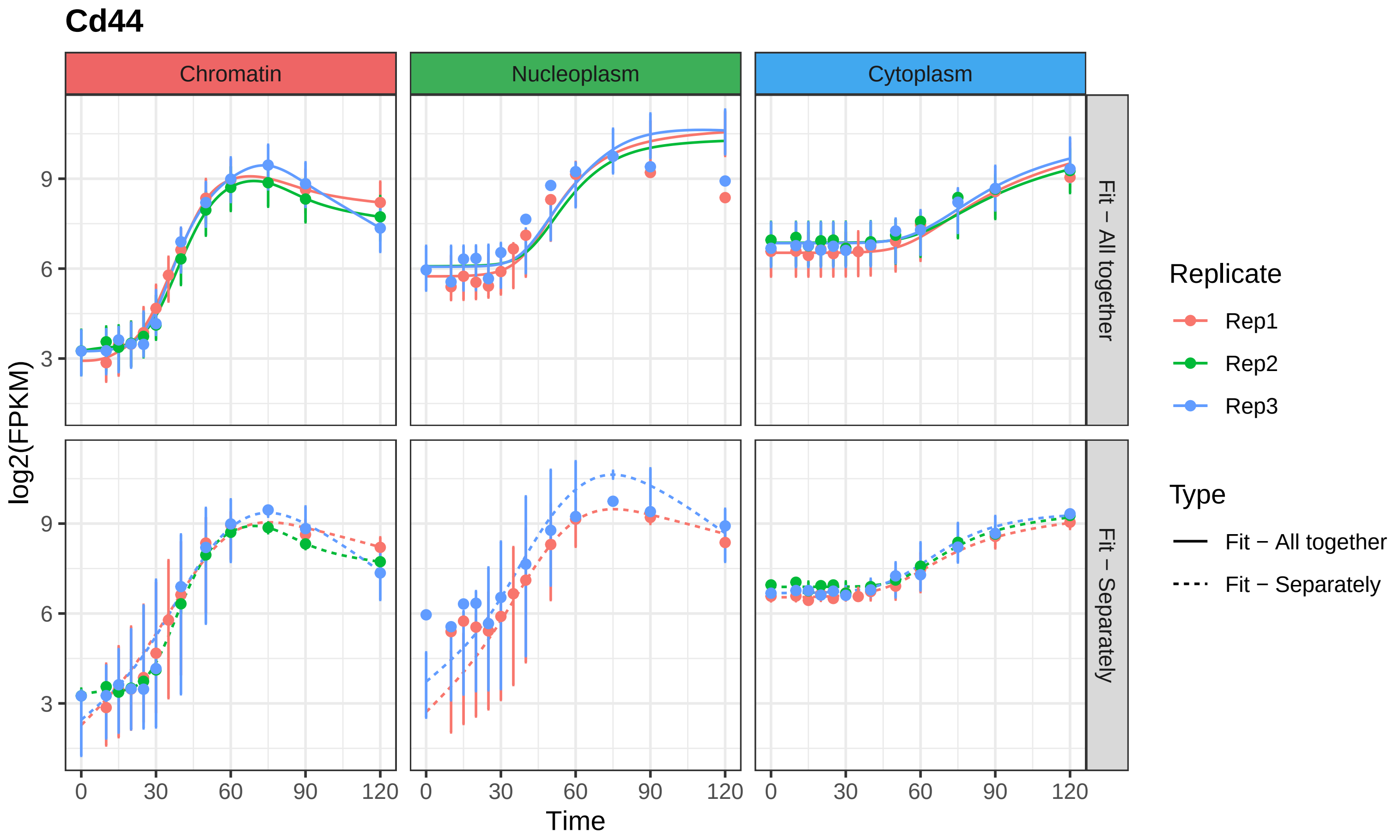

	Together	b1	b2	b3
**−NLL b1 ca**	−0.06924	7.67		
**−NLL b1 np**	18.99	15.12		
**−NLL b1 cyto**	0.919	−11.56		
**−NLL b2 ca**	−0.0466		−10.1	
**−NLL b2 np**				
**−NLL b2 cyto**	1.31		−9.031	
**−NLL b3 ca**	1.027			7.78
**−NLL b3 np**	22.23			16.32
**−NLL b3 cyto**	1.116			−8.846
Total	45.48	11.23	−19.13	15.25
**Total with regul**	48.54	15.04	−17.88	15.66

	Together	b1	b2	b3
spar	3.548e−01	0.60380	0.3201	0.5387
σ_b	2.541e−01	0.04524	0.06126	0.0454
σ_t	5.661e−06	6.90200	0.474	7.2350
ca_{0,b_1}	2.937e+00	2.47100		
$\log_{10}(k_1')$	−1.168e+00	5.54700		6.2850
$\log_{10}(k_2)$	−2.017e+00	5.41500	−0.845 or −2.75	5.9020
$\log_{10}(k_2')$	−1.959e+00	−1.98700		−2.1750
$\log_{10}(k_{deg})$	−2.196e+00	−3.14000	−2.75 or −0.845	−3.0650
$\log_{10}(k_1'k_2')$	−3.127e+00	3.56000	−2.513	4.1100
$\log_{10}(k_1'/k_2)$	8.489e−01	0.13130		0.3827
$transport = \log_{10}(k_1'k_2'/k_2)$	−1.110e+00	−1.85600	−1.668 or 0.2372	−1.7920

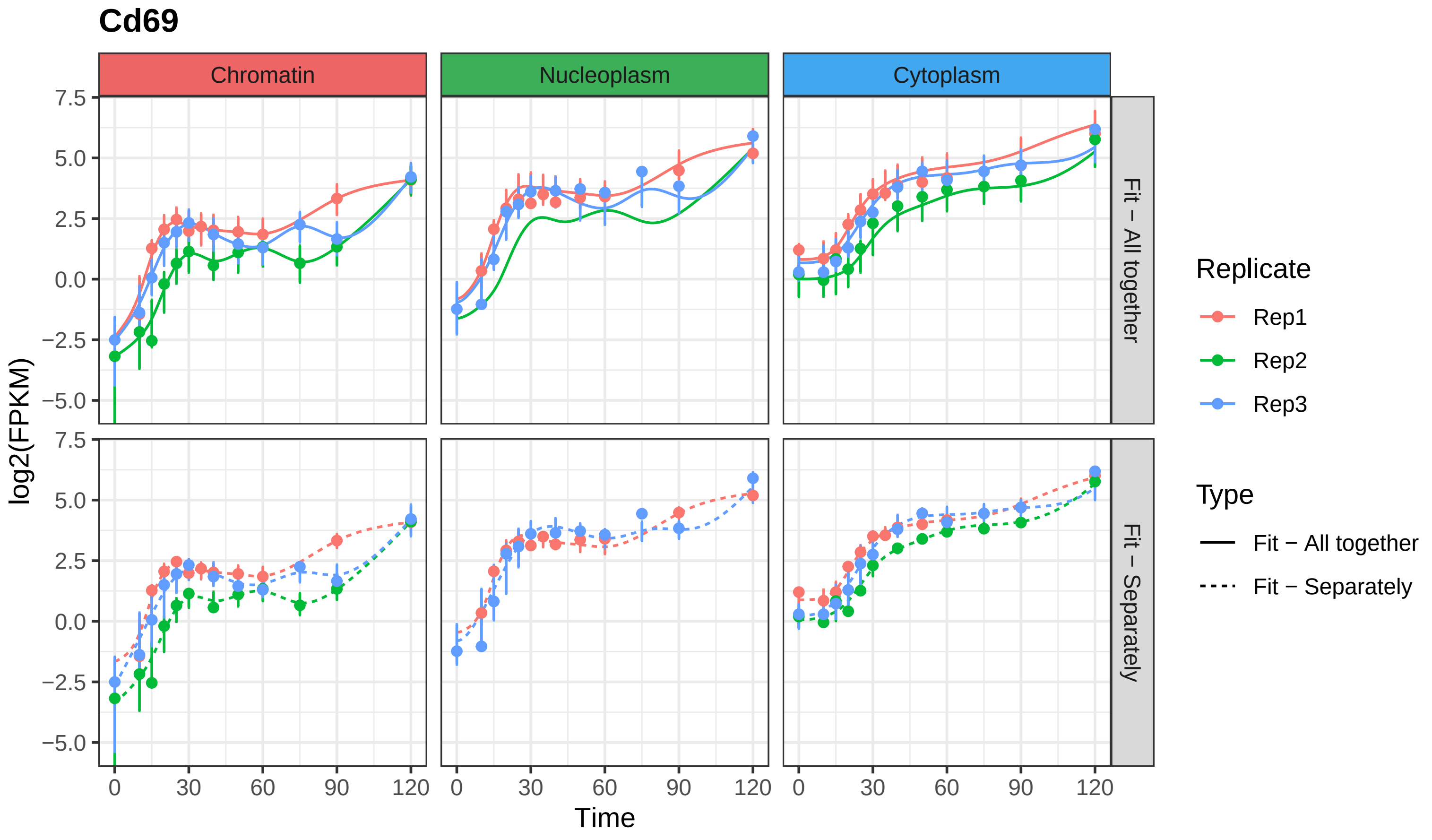

	Together	b1	b2	b3
**−NLL b1 ca**	0.7541	−3.501		
**−NLL b1 np**	4.589	−0.4035		
**−NLL b1 cyto**	4.071	−8.213		
**−NLL b2 ca**	2.301		−1.333	
**−NLL b2 np**				
**−NLL b2 cyto**	4.903		−3.983	
**−NLL b3 ca**	−0.3964			0.8986
**−NLL b3 np**	12.36			2.695
**−NLL b3 cyto**	4.64			3.545
Total	33.22	−12.12	−5.316	7.139
**Total with regul**	37.72	−7.9	−5.34	5.831

	Together	b1	b2	b3
spar	0.3008000	0.2997	0.3433	0.4138
σ_b	0.2045000	0.0682	0.0002342	0.1117
σ_t	0.0004453	0.6862	0.9771	2.4930
ca_{0,b_1}	−2.3450000	−1.6230		
$\log_{10}(k_1')$	−0.0206600	0.1994		−0.2012
$\log_{10}(k_2)$	−0.4941000	−0.1640	4.698 or −1.649	−0.7600
$\log_{10}(k_2')$	−1.1370000	−1.1130		−1.0620
$\log_{10}(k_{deg})$	−1.6240000	−1.5170	−1.649 or 4.698	−1.3770
$\log_{10}(k_1'k_2')$	−1.1570000	−0.9141	4.082	−1.2630
$\log_{10}(k_1'/k_2)$	0.4734000	0.3634		0.5587
transport = $\log_{10}(k_1'k_2'/k_2)$	−0.6631000	−0.7501	−0.6159 or 5.732	−0.5033

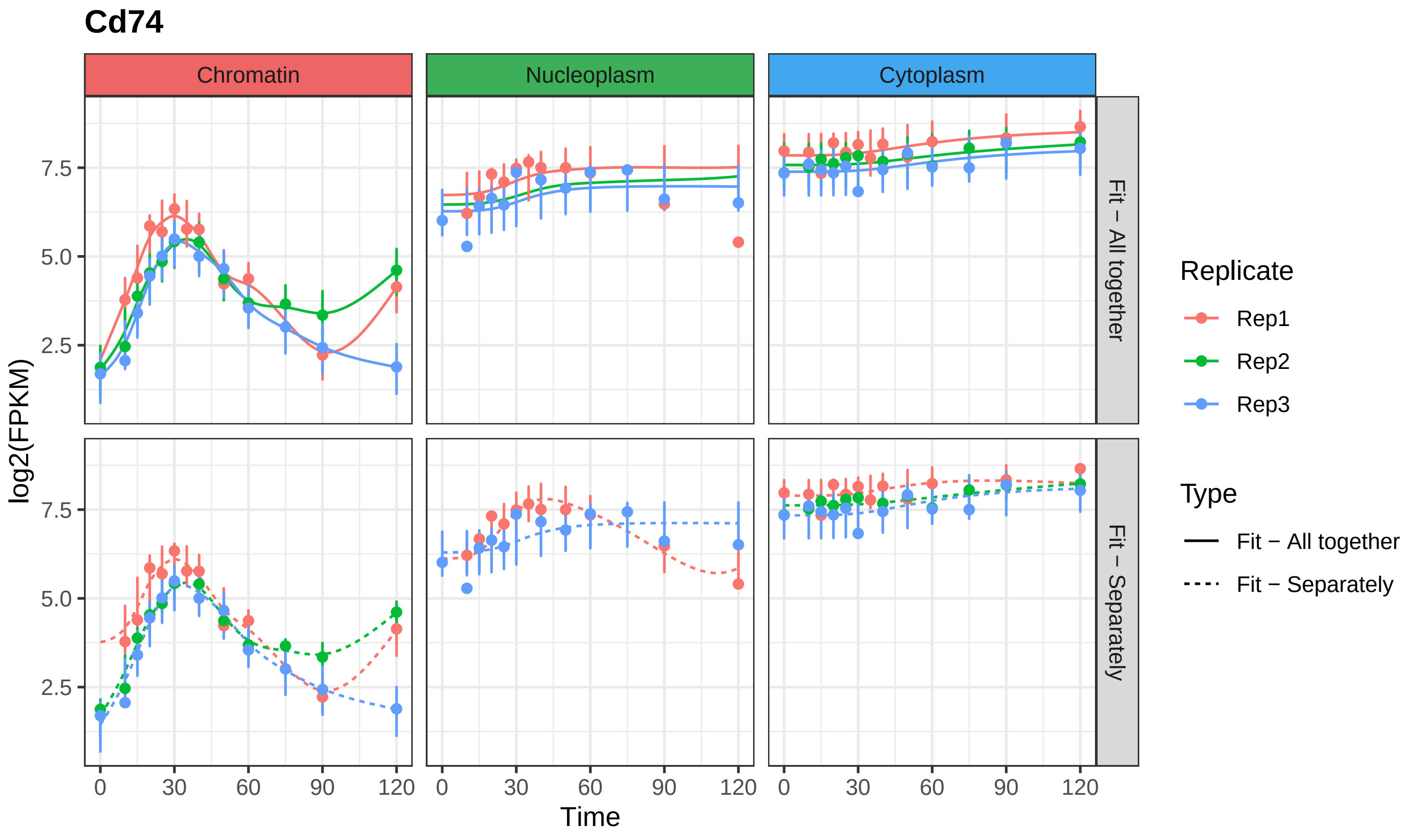

	Together	b1	b2	b3
**−NLL b1 ca**	0.342	1.794		
**−NLL b1 np**	20.62	1.791		
**−NLL b1 cyto**	0.337	0.8704		
**−NLL b2 ca**	−0.965		−4.049	
**−NLL b2 np**				
**−NLL b2 cyto**	−1.29		−4.646	
**−NLL b3 ca**	−0.7102			0.3553
**−NLL b3 np**	10.7			9.912
**−NLL b3 cyto**	0.9188			0.2559
Total	29.95	4.456	−8.695	10.52
**Total with regul**	35.7	5.04	−9.569	11.05

	Together	b1	b2	b3
spar	3.368e−01	0.3915	0.3797	3.942e−01
σ_b	2.174e−01	0.1567	0.09278	2.110e−01
σ_t	1.837e−05	2.5900	1.153	1.782e−05
ca_{0,b_1}	2.097e+00	3.8650		
$\log_{10}(k_1')$	−1.396e+00	−0.6515		−1.309e+00
$\log_{10}(k_2)$	−2.793e+00	−1.3610	−2.334 or −2.351	−2.769e+00
$\log_{10}(k_2')$	−1.377e+00	−1.8160		−1.251e+00
$\log_{10}(k_{deg})$	−1.713e+00	−2.3500	−2.351 or −2.334	−1.565e+00
$\log_{10}(k_1'k_2')$	−2.773e+00	−2.4680	−2.898	−2.560e+00
$\log_{10}(k_1'/k_2)$	1.397e+00	0.7091		1.460e+00
$\text{transport} = \log_{10}(k_1'k_2'/k_2)$	1.956e−02	−1.1070	−0.5642 or −0.5473	2.097e−01

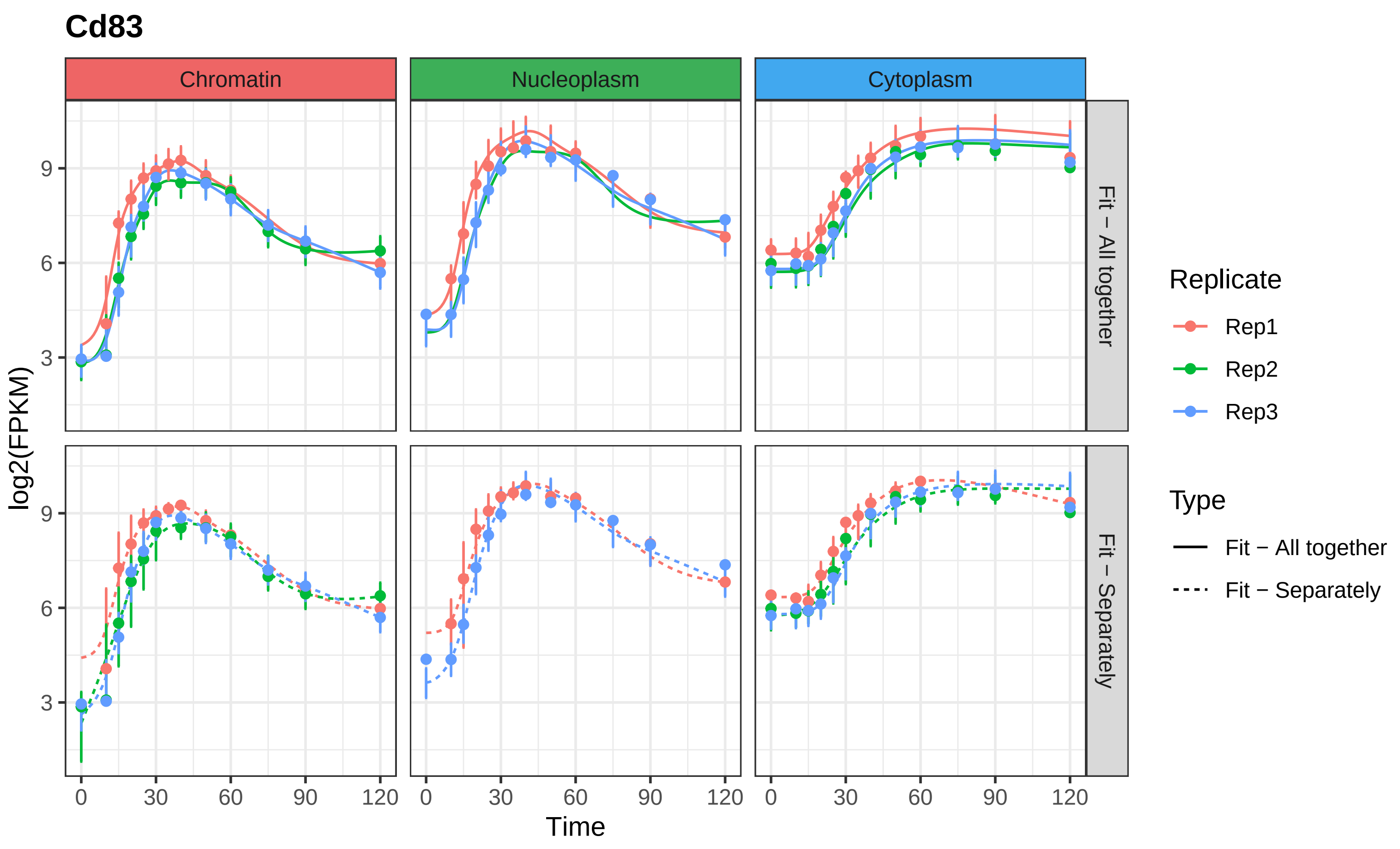

	Together	b1	b2	b3
**−NLL b1 ca**	−1.905	−6.981		
**−NLL b1 np**	2.4	0.007676		
**−NLL b1 cyto**	1.659	−7.352		
**−NLL b2 ca**	−2.448		1.25	
**−NLL b2 np**				
**−NLL b2 cyto**	8.499		5.039	
**−NLL b3 ca**	−2.661			−0.2391
**−NLL b3 np**	5.86			1.641
**−NLL b3 cyto**	−1.809			0.2056
Total	9.596	−14.32	6.29	1.608
**Total with regul**	18.93	−10.58	5.586	6.044

	Together	b1	b2	b3
spar	0.23160	0.29140	0.4136	0.2833
σ_b	0.16670	0.02324	0.1575	0.1539
σ_t	0.77830	2.68000	2.339	0.6826
ca_{0,b_1}	3.42900	4.48800		
$\log_{10}(k_1')$	−0.07643	−0.46830		−0.2085
$\log_{10}(k_2)$	−0.36760	−0.70640	5.411 or −2.268	−0.5132
$\log_{10}(k_2')$	−1.46800	−1.33500		−1.5020
$\log_{10}(k_{deg})$	−2.04700	−1.67600	−2.268 or 5.411	−2.1540
$\log_{10}(k_1' k_2')$	−1.54400	−1.80300	4.172	−1.7100
$\log_{10}(k_1'/k_2)$	0.29120	0.23810		0.3047
transport $= \log_{10}(k_1' k_2'/k_2)$	−1.17700	−1.09700	−1.239 or 6.441	−1.1970

Cdc42ep2

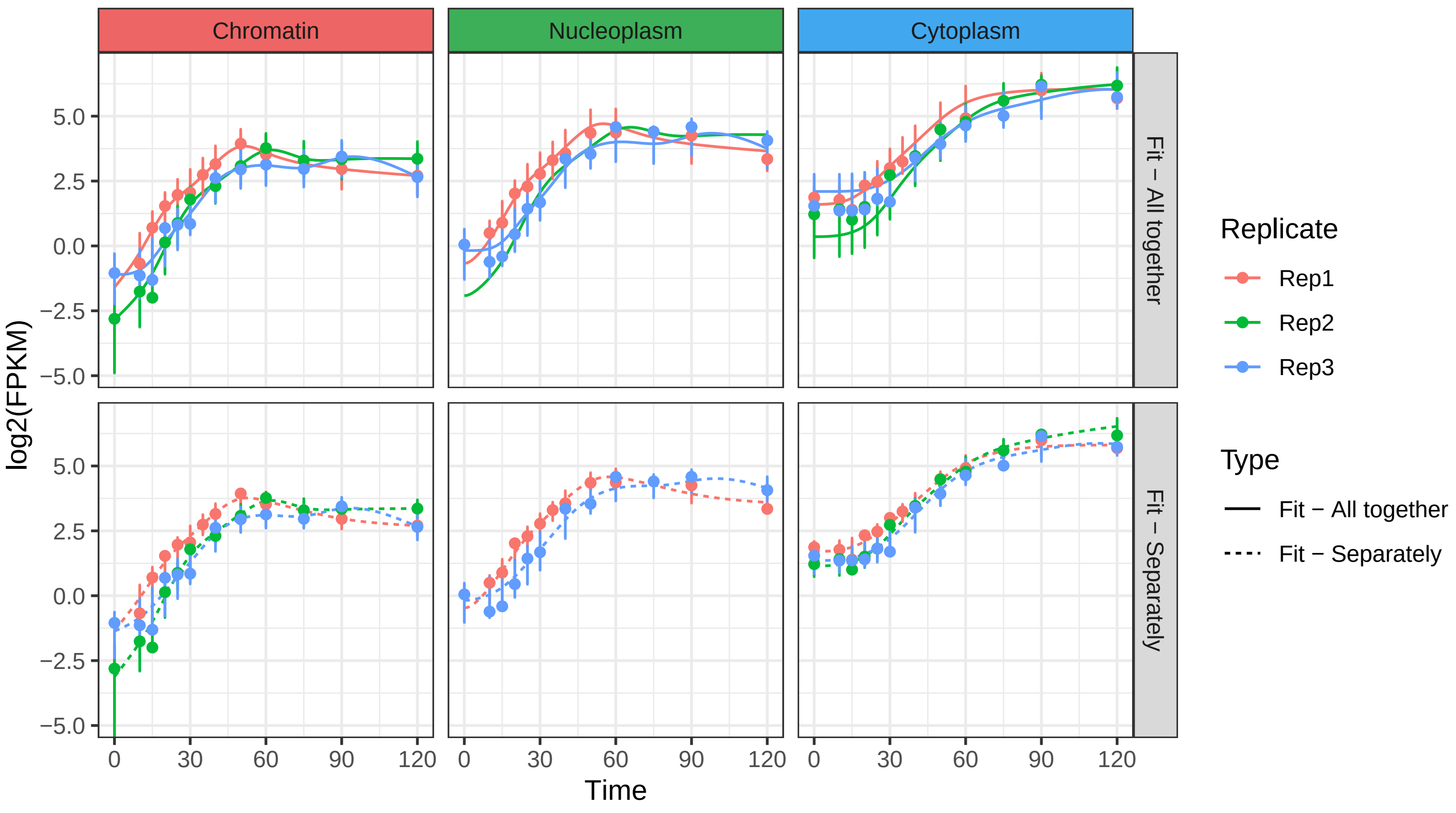

	Together	b1	b2	b3
**−NLL b1 ca**	0.2425	−2.747		
**−NLL b1 np**	1.389	−2.221		
**−NLL b1 cyto**	4.127	−2.132		
**−NLL b2 ca**	2.663		−0.9207	
**−NLL b2 np**				
**−NLL b2 cyto**	15.42		−0.3409	
**−NLL b3 ca**	3.186			2.135
**−NLL b3 np**	5.261			4.914
**−NLL b3 cyto**	12.17			1.364
Total	44.46	−7.1	−1.262	8.412
**Total with regul**	47.18	−8.478	−2.154	7.436

	Together	b1	b2	b3
spar	3.573e−01	0.4284000	0.3862	0.4187
σ_b	2.347e−01	0.1080000	0.1089	0.1447
σ_t	2.075e−05	0.0002363	0.9355	2.2130
ca_{0,b_1}	−1.564e+00	−1.2930000		
$\log_{10}(k_1')$	−2.804e−01	−0.3950000		−0.5388
$\log_{10}(k_2)$	−5.581e−01	−0.6569000	6.596 or −2.141	−0.8970
$\log_{10}(k_2')$	−1.034e+00	−1.1470000		−0.8782
$\log_{10}(k_{deg})$	−1.719e+00	−1.8030000	−2.141 or 6.596	−1.3390
$\log_{10}(k_1'k_2')$	−1.315e+00	−1.5420000	5.739	−1.4170
$\log_{10}(k_1'/k_2)$	2.777e−01	0.2619000		0.3581
$\text{transport} = \log_{10}(k_1'k_2'/k_2)$	−7.565e−01	−0.8852000	−0.8573 or 7.88	−0.5201

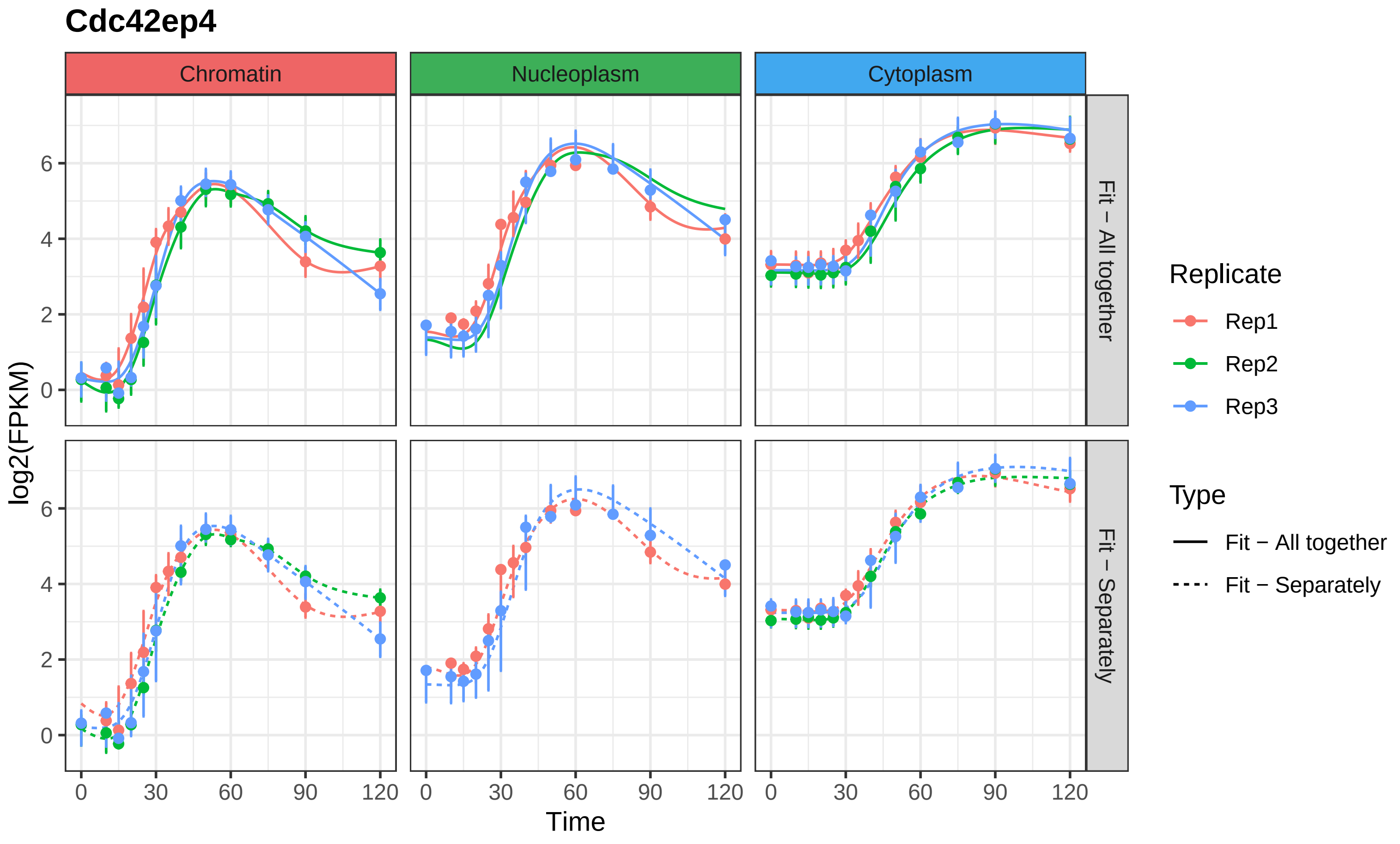

	Together	b1	b2	b3
**−NLL b1 ca**	−3.054	−1.778		
**−NLL b1 np**	6.882	2.821		
**−NLL b1 cyto**	−5.75	−7.672		
**−NLL b2 ca**	−4.28		−6.311	
**−NLL b2 np**				
**−NLL b2 cyto**	−4.697		−7.289	
**−NLL b3 ca**	−2.01			0.8994
**−NLL b3 np**	8.077			4.408
**−NLL b3 cyto**	−2.208			−2.428
Total	−7.04	−6.629	−13.6	2.879
**Total with regul**	−5.775	−7.113	−12.68	3.09

	Together	b1	b2	b3
spar	0.3374	0.39920	0.3277	0.3590
σ_b	0.1220	0.08281	0.07024	0.1234
σ_t	1.4580	1.90900	0.4776	2.3620
ca_{0,b_1}	0.4859	0.93680		
$\log_{10}(k_1')$	−0.4620	−0.57750		−0.5717
$\log_{10}(k_2)$	−0.7879	−0.86180	0.02481 or −1.929	−0.9080
$\log_{10}(k_2')$	−1.3100	−1.18700		−1.3140
$\log_{10}(k_{deg})$	−1.8460	−1.64700	−1.929 or 0.02481	−1.8850
$\log_{10}(k_1'k_2')$	−1.7720	−1.76500	−1.028	−1.8860
$\log_{10}(k_1'/k_2)$	0.3259	0.28430		0.3363
transport = $\log_{10}(k_1'k_2'/k_2)$	−0.9840	−0.90270	−1.053 or 0.9007	−0.9777

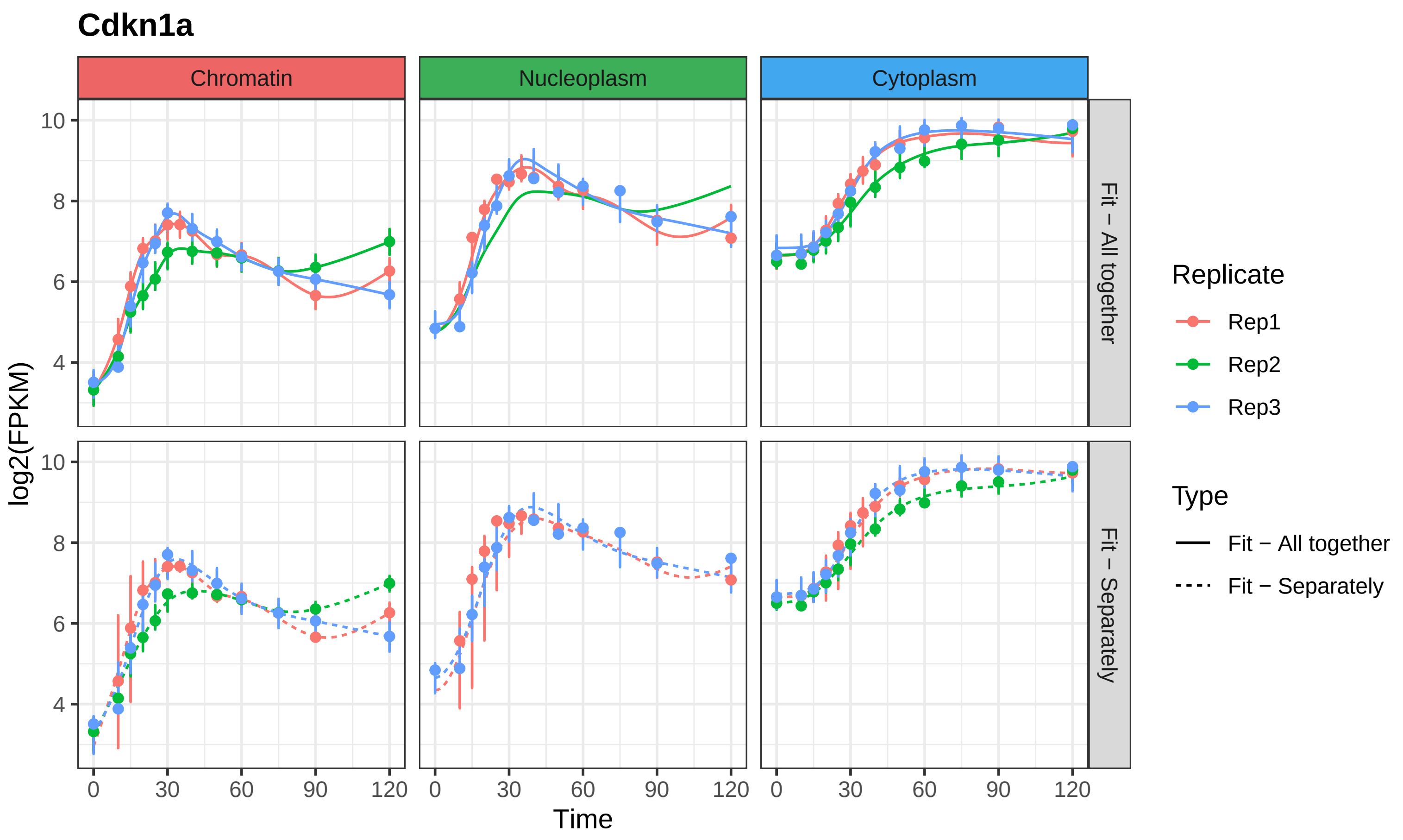

	Together	b1	b2	b3
**−NLL b1 ca**	−8.789	−7.977		
**−NLL b1 np**	3.219	3.152		
**−NLL b1 cyto**	−6.958	−15.88		
**−NLL b2 ca**	−9.315		−8.356	
**−NLL b2 np**				
**−NLL b2 cyto**	−6.358		−7.362	
**−NLL b3 ca**	−8.062			−2.524
**−NLL b3 np**	7.632			4.529
**−NLL b3 cyto**	−5.204			−5.596
Total	−33.84	−20.71	−15.72	−3.591
**Total with regul**	−26.06	−18.01	−17.37	−3.741

	Together	b1	b2	b3
spar	0.2455	3.373e−01	0.4028	0.3672
σ_b	0.1109	2.538e−08	0.06019	0.1238
σ_t	0.5050	3.568e+00	1.373	1.2670
ca_{0,b_1}	3.3050	2.972e+00		
$\log_{10}(k_1')$	−0.1586	−5.318e−01		−0.2613
$\log_{10}(k_2)$	−0.6001	−9.501e−01	−0.4454 or −1.71	−0.6827
$\log_{10}(k_2')$	−1.1590	−1.126e+00		−1.1320
$\log_{10}(k_{deg})$	−1.7280	−1.821e+00	−1.71 or −0.4454	−1.7580
$\log_{10}(k_1'k_2')$	−1.3180	−1.658e+00	−1.169	−1.3940
$\log_{10}(k_1'/k_2)$	0.4415	4.183e−01		0.4214
$transport = \log_{10}(k_1'k_2'/k_2)$	−0.7175	−7.075e−01	−0.7232 or 0.5419	−0.7108

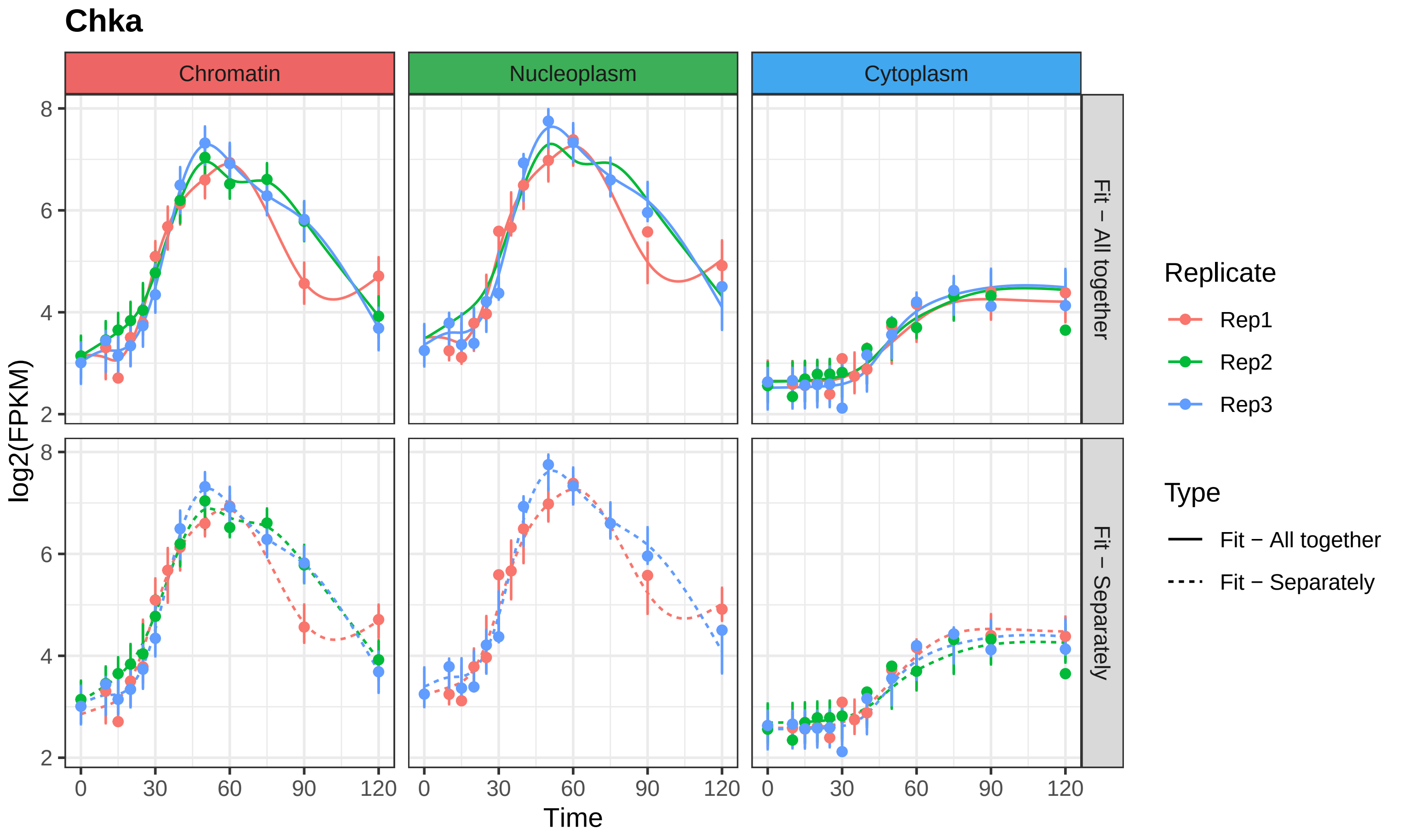

	Together	b1	b2	b3
**−NLL b1 ca**	−4.305	−1.811		
**−NLL b1 np**	3.146	0.1421		
**−NLL b1 cyto**	−1.735	−4.534		
**−NLL b2 ca**	−7.618		−5.523	
**−NLL b2 np**				
**−NLL b2 cyto**	3.418		2.301	
**−NLL b3 ca**	−6.967			−5.424
**−NLL b3 np**	−0.49			1.099
**−NLL b3 cyto**	−0.4579			−0.6195
Total	−15.01	−6.203	−3.222	−4.944
**Total with regul**	−12.15	−6.869	−3.577	−3.575

	Together	b1	b2	b3
spar	0.3083	0.38430	0.3743	0.3282
σ_b	0.1286	0.09839	0.1279	0.1184
σ_t	0.7834	1.83000	0.0008135	1.0230
ca_{0,b_1}	3.1470	2.85500		
$\log_{10}(k_1')$	0.3100	−0.26070		0.4049
$\log_{10}(k_2)$	0.2039	−0.38270	5.439 or −2.418	0.2986
$\log_{10}(k_2')$	−2.5330	−2.42300		−2.6000
$\log_{10}(k_{deg})$	−2.2760	−2.22000	−2.418 or 5.439	−2.3470
$\log_{10}(k_1' k_2')$	−2.2230	−2.68400	2.891	−2.1950
$\log_{10}(k_1'/k_2)$	0.1061	0.12190		0.1063
transport $= \log_{10}(k_1' k_2'/k_2)$	−2.4270	−2.30200	−2.548 or 5.31	−2.4940

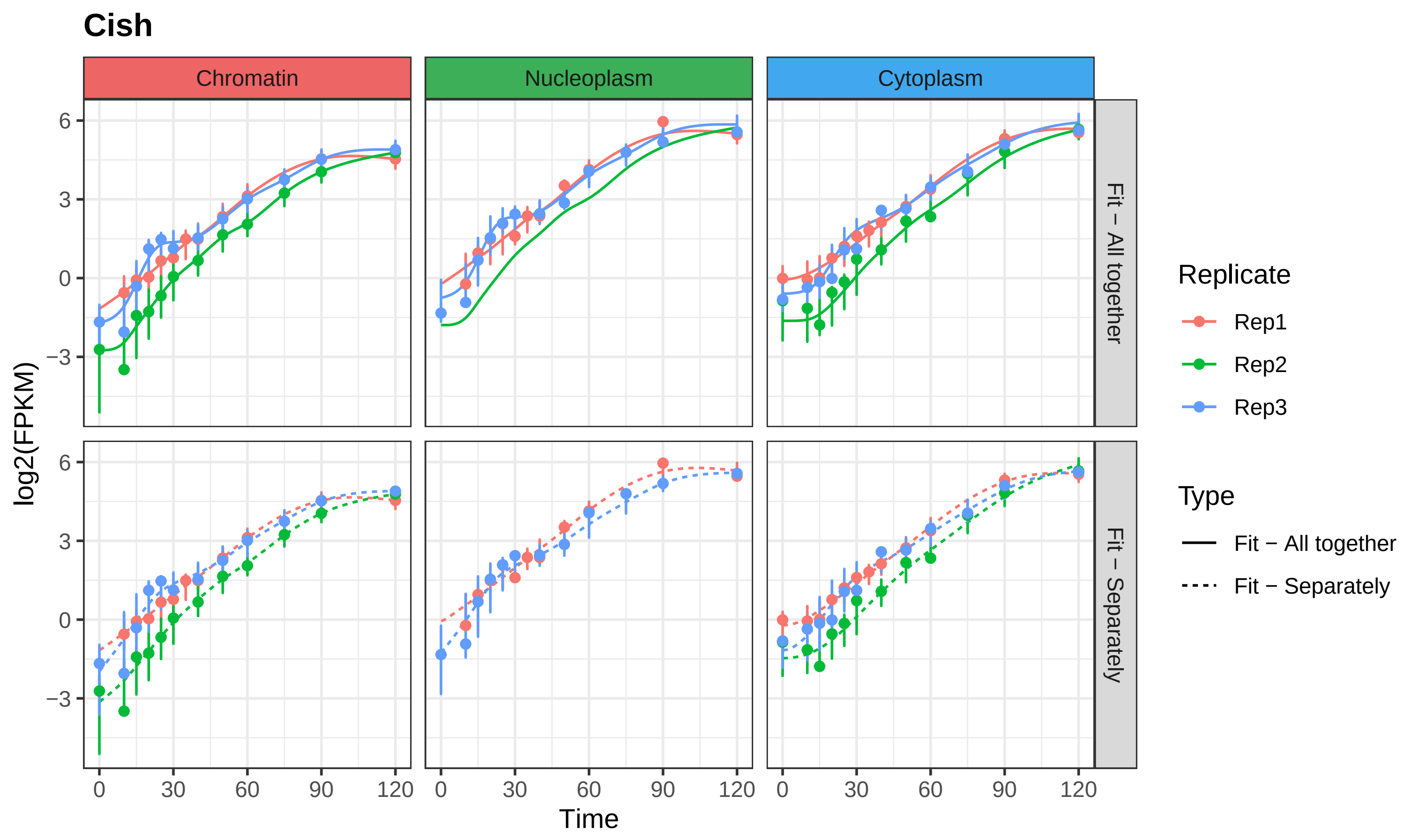

	Together	b1	b2	b3
**−NLL b1 ca**	−3.961	−5.155		
**−NLL b1 np**	5.833	6.05		
**−NLL b1 cyto**	−3.309	−5.076		
**−NLL b2 ca**	−0.01292		0.08366	
**−NLL b2 np**				
**−NLL b2 cyto**	6.693		4.794	
**−NLL b3 ca**	−0.9854			0.508
**−NLL b3 np**	1.879			0.3611
**−NLL b3 cyto**	4.476			0.6829
Total	10.61	−4.182	4.878	1.552
**Total with regul**	15.38	−2.891	3.192	−0.4614

	Together	b1	b2	b3
spar	0.3216	0.43660	0.4157	4.361e−01
σ_b	0.1168	0.10190	0.07794	2.477e−06
σ_t	1.7760	0.76390	2.155	3.650e+00
ca_{0,b_1}	−1.1680	−1.16800		
$\log_{10}(k_1')$	0.8672	0.36640		8.011e+00
$\log_{10}(k_2)$	0.5795	0.02856	10.24 or −1.413	7.802e+00
$\log_{10}(k_2')$	−1.0410	−0.89650		−9.029e−01
$\log_{10}(k_{deg})$	−1.0890	−0.84700	−1.413 or 10.24	−9.348e−01
$\log_{10}(k_1'k_2')$	−0.1735	−0.53010	9.326	7.109e+00
$\log_{10}(k_1'/k_2)$	0.2877	0.33780		2.095e−01
transport $= \log_{10}(k_1'k_2'/k_2)$	−0.7529	−0.55870	−0.9114 or 10.74	−6.934e−01

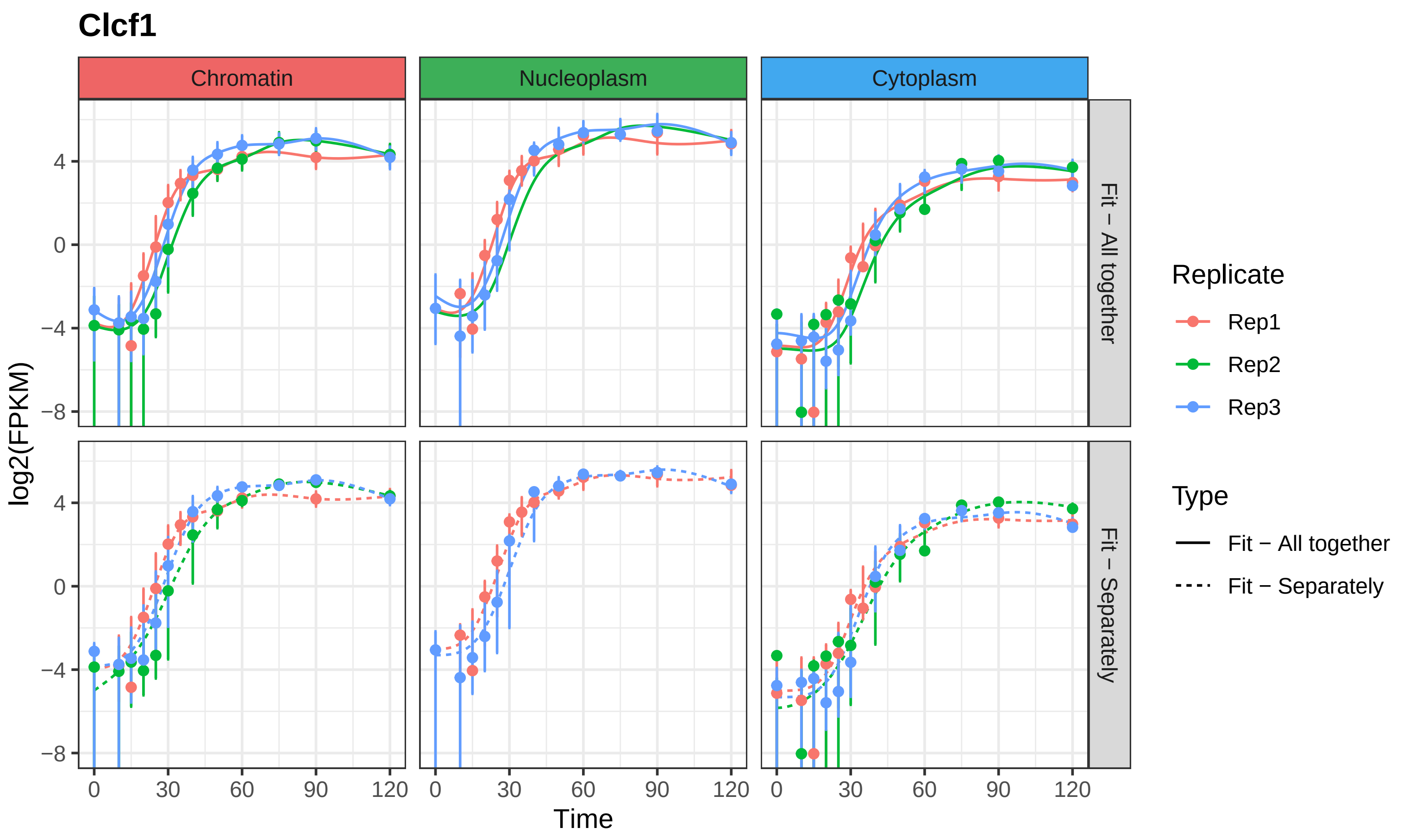

	Together	b1	b2	b3
**−NLL b1 ca**	3.127	3.03		
**−NLL b1 np**	6.858	7.18		
**−NLL b1 cyto**	16.59	14.7		
**−NLL b2 ca**	4.249		4.817	
**−NLL b2 np**				
**−NLL b2 cyto**	22.49		15.72	
**−NLL b3 ca**	3.207			2.003
**−NLL b3 np**	7.677			6.164
**−NLL b3 cyto**	13.76			9.239
Total	77.95	24.91	20.54	17.41
**Total with regul**	81.92	25.6	18.59	16.81

	Together	b1	b2	b3
spar	0.3042	0.364000	0.4275	0.36400
σ_b	0.1752	0.116700	7.449e−05	0.04454
σ_t	1.5880	2.102000	3.637	2.70900
ca_{0,b_1}	−3.7250	−3.903000		
$\log_{10}(k_1')$	6.6620	−0.009547		−0.04437
$\log_{10}(k_2)$	6.4560	−0.291200	3.836 or −1.117	−0.19890
$\log_{10}(k_2')$	−1.6770	−1.657000		−1.34200
$\log_{10}(k_{deg})$	−1.1410	−1.057000	−1.117 or 3.836	−0.73640
$\log_{10}(k_1'k_2')$	4.9860	−1.667000	2.468	−1.38600
$\log_{10}(k_1'/k_2)$	0.2064	0.281600		0.15460
transport $= \log_{10}(k_1'k_2'/k_2)$	−1.4700	−1.375000	−1.368 or 3.585	−1.18700

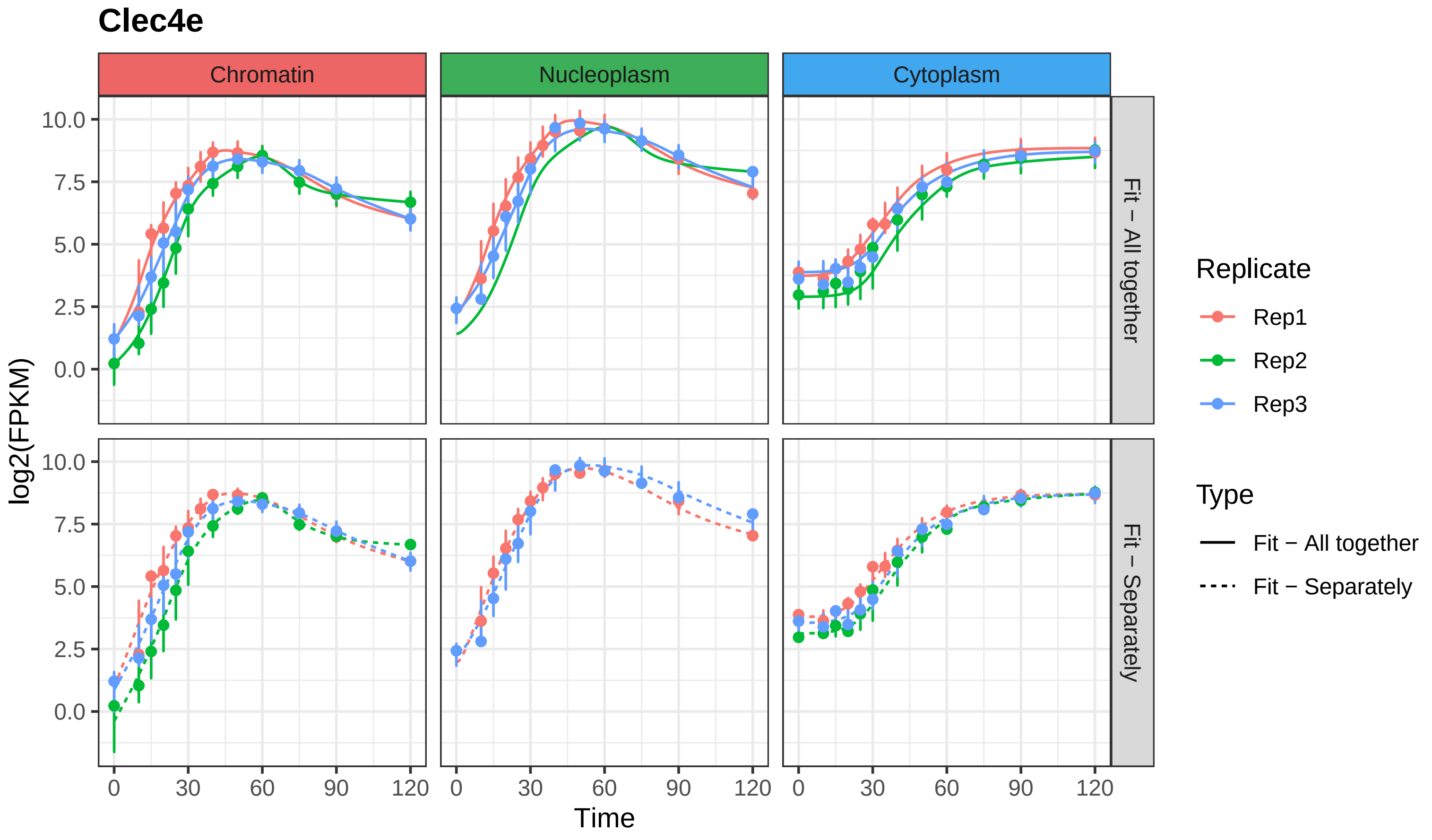

	Together	b1	b2	b3
**−NLL b1 ca**	0.048	−1.659		
**−NLL b1 np**	1.485	−1.284		
**−NLL b1 cyto**	−0.9716	−6.186		
**−NLL b2 ca**	−1.936		−2.598	
**−NLL b2 np**				
**−NLL b2 cyto**	8.812		−1.763	
**−NLL b3 ca**	−1.859			−1.826
**−NLL b3 np**	5.308			3.837
**−NLL b3 cyto**	4.688			−0.2717
Total	15.57	−9.13	−4.361	1.739
**Total with regul**	19.14	−10.52	−5.625	1.119

	Together	b1	b2	b3
spar	0.3036	0.43940	0.3853	0.379600
σ_b	0.1480	0.06123	0.04831	0.113600
σ_t	1.6160	1.77500	2.249	1.744000
ca_{0,b_1}	1.0730	0.94210		
$\log_{10}(k_1')$	0.1482	−0.03026		0.004082
$\log_{10}(k_2)$	−0.2152	−0.33450	5.014 or −2.616	−0.429700
$\log_{10}(k_2')$	−1.9540	−1.96000		−2.026000
$\log_{10}(k_{deg})$	−2.4030	−2.50900	−2.616 or 5.014	−2.403000
$\log_{10}(k_1'k_2')$	−1.8060	−1.99100	3.455	−2.022000
$\log_{10}(k_1'/k_2)$	0.3635	0.30430		0.433800
transport = $\log_{10}(k_1'k_2'/k_2)$	−1.5910	−1.65600	−1.559 or 6.07	−1.592000

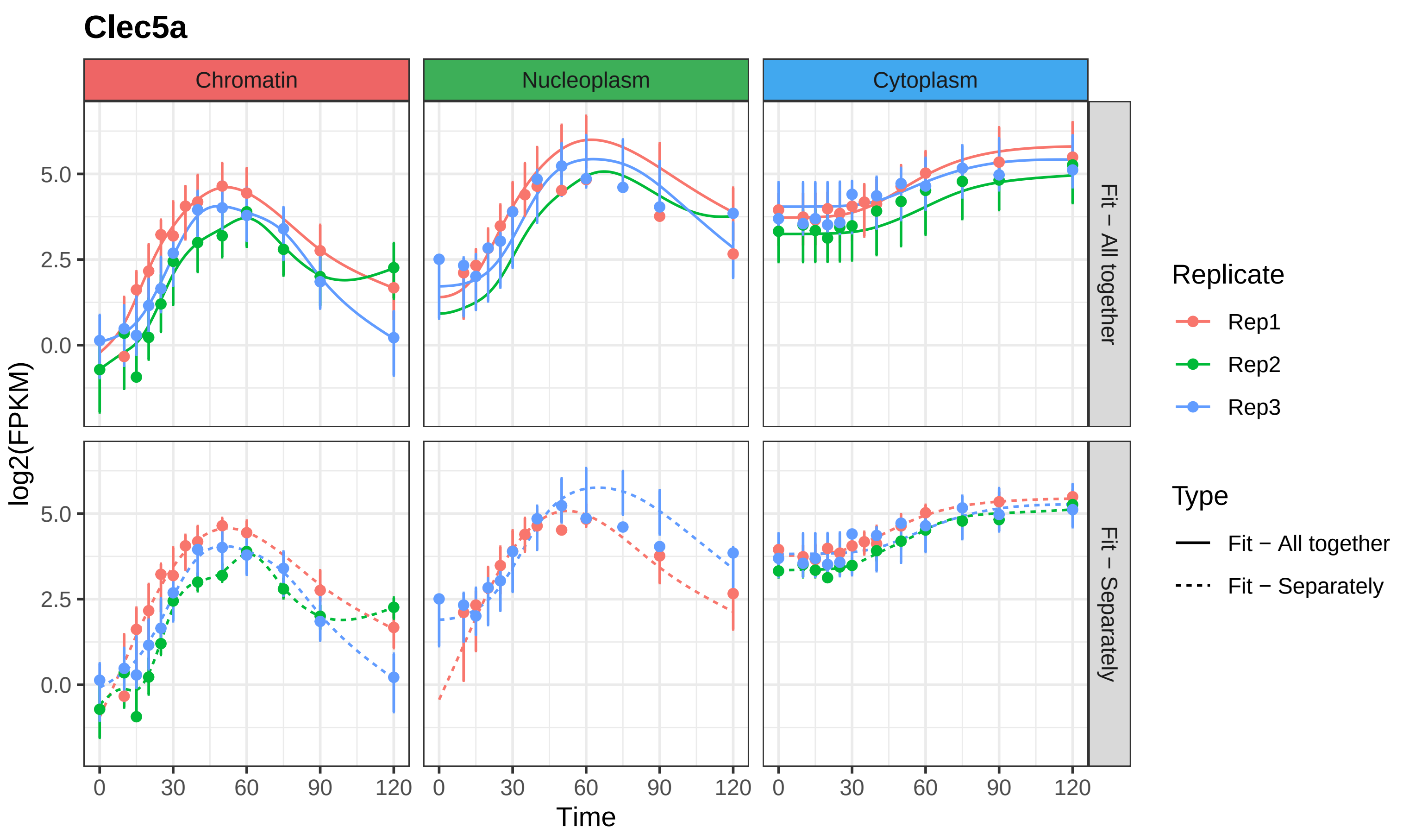

	Together	b1	b2	b3
**−NLL b1 ca**	3.083	−0.3371		
**−NLL b1 np**	18.87	8.385		
**−NLL b1 cyto**	0.7979	−8.505		
**−NLL b2 ca**	4.855		−2.035	
**−NLL b2 np**				
**−NLL b2 cyto**	2.478		−8.083	
**−NLL b3 ca**	1.334			0.6271
**−NLL b3 np**	14.91			9.113
**−NLL b3 cyto**	4.799			2.665
Total	51.13	−0.457	−10.12	12.4
**Total with regul**	54.79	−1.198	−7.613	12.53

	Together	b1	b2	b3
spar	3.872e−01	0.50960	0.2975	0.427300
σ_b	2.575e−01	0.09969	0.05861	0.216400
σ_t	3.701e−05	2.39100	0.004145	0.000247
ca_{0,b_1}	−1.317e−01	−0.91580		
$\log_{10}(k_1')$	−6.231e−01	5.83600		−0.568800
$\log_{10}(k_2)$	−1.113e+00	5.68300	4.115 or −2.499	−1.166000
$\log_{10}(k_2')$	−1.845e+00	−1.74100		−1.993000
$\log_{10}(k_{deg})$	−2.545e+00	−3.00800	−2.499 or 4.115	−2.572000
$\log_{10}(k_1'k_2')$	−2.468e+00	4.09500	2.799	−2.562000
$\log_{10}(k_1'/k_2)$	4.897e−01	0.15250		0.597600
transport $= \log_{10}(k_1'k_2'/k_2)$	−1.355e+00	−1.58800	−1.316 or 5.298	−1.396000

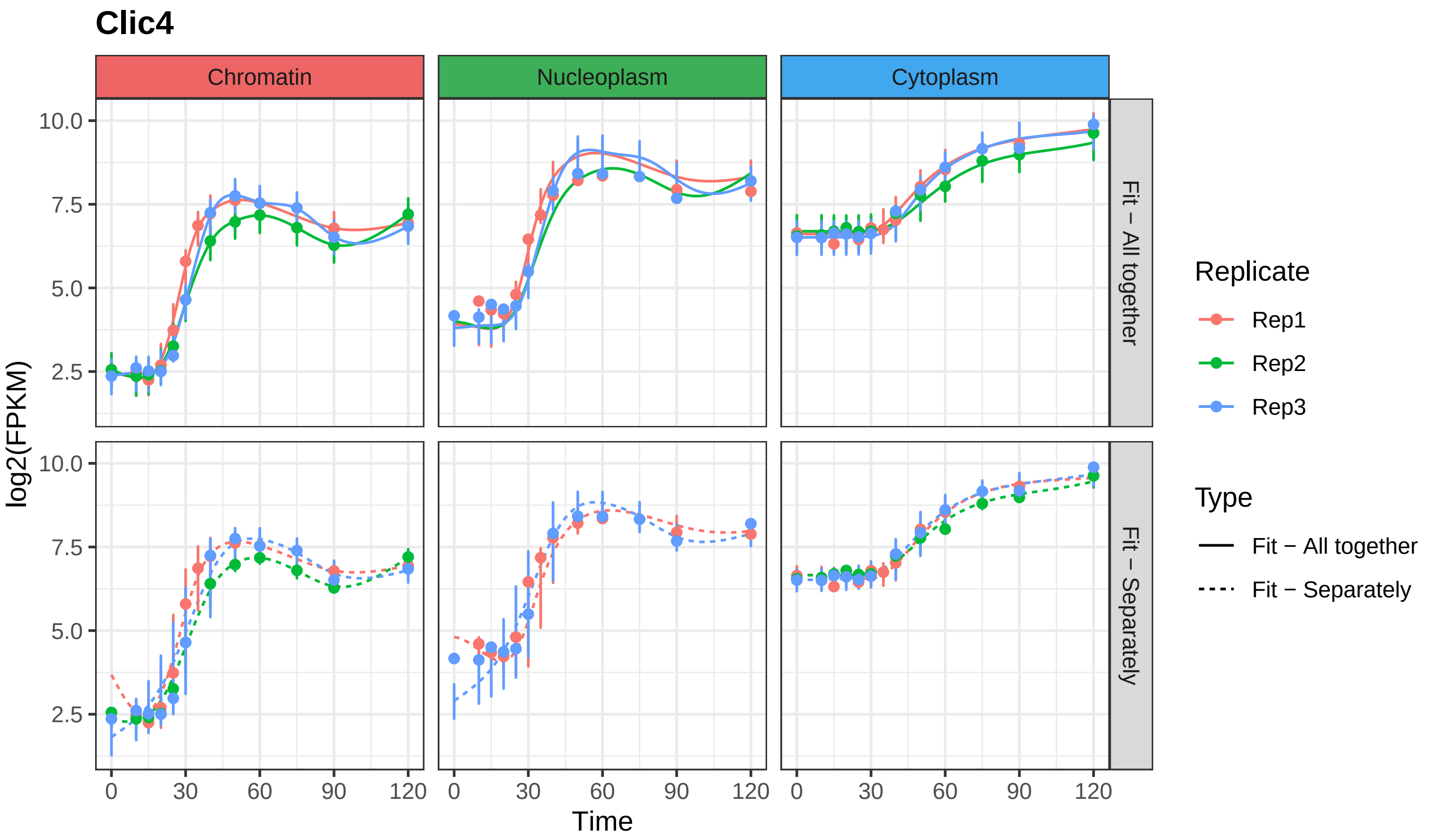

	Together	b1	b2	b3
**−NLL b1 ca**	−3.551	−2.188		
**−NLL b1 np**	16.72	2.251		
**−NLL b1 cyto**	−3.166	−4.942		
**−NLL b2 ca**	−4.685		−6.21	
**−NLL b2 np**				
**−NLL b2 cyto**	−3.424		−7.642	
**−NLL b3 ca**	−3.884			3.601
**−NLL b3 np**	11.98			8.725
**−NLL b3 cyto**	−3.225			−5.618
Total	6.757	−4.878	−13.85	6.708
**Total with regul**	11.59	−1.99	−15.17	5.37

	Together	b1	b2	b3
spar	0.2853000	0.37940	0.3881	0.4677
σ_b	0.1735000	0.09063	0.05697	0.1138
σ_t	0.0005938	2.54500	1.871	3.6740
ca_{0,b_1}	2.4680000	3.76700		
$\log_{10}(k_1')$	−0.2241000	−0.68710		6.5260
$\log_{10}(k_2)$	−0.6580000	−1.02500	−0.07079 or −2.384	6.1990
$\log_{10}(k_2')$	−1.5290000	−1.29100		−1.4910
$\log_{10}(k_{deg})$	−2.3430000	−1.85300	−2.384 or −0.07079	−2.5780
$\log_{10}(k_1'k_2')$	−1.7540000	−1.97800	−1.153	5.0360
$\log_{10}(k_1'/k_2)$	0.4339000	0.33790		0.3274
transport $= \log_{10}(k_1'k_2'/k_2)$	−1.0960000	−0.95320	−1.082 or 1.231	−1.1630

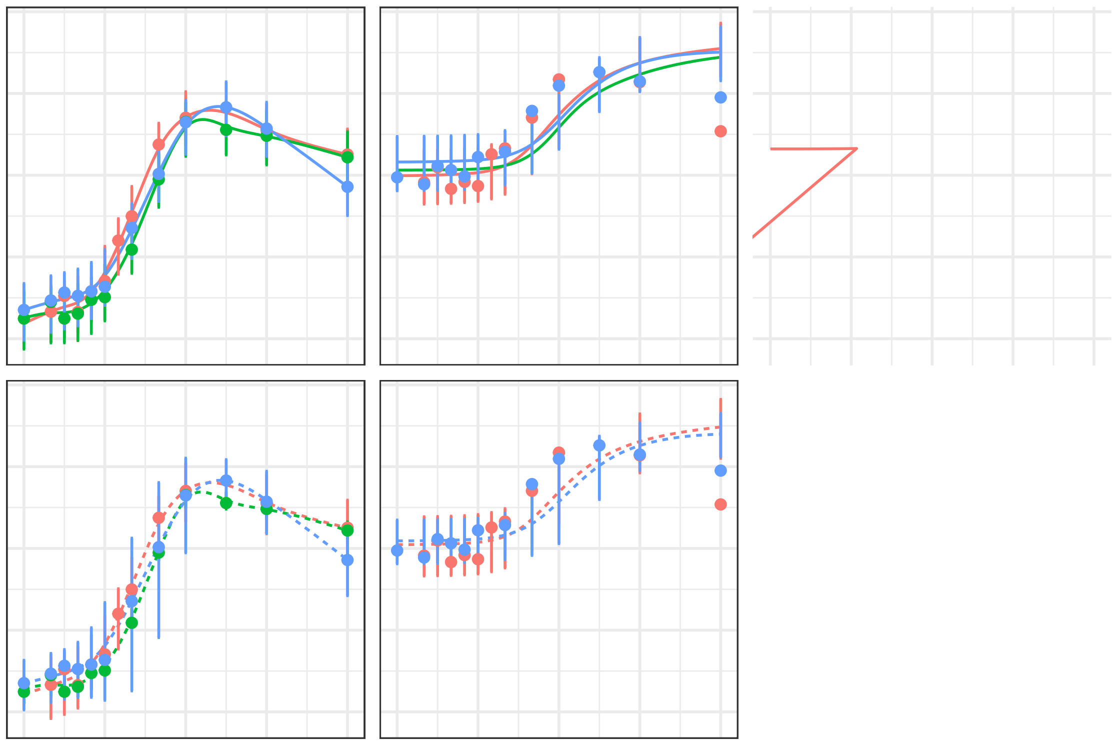

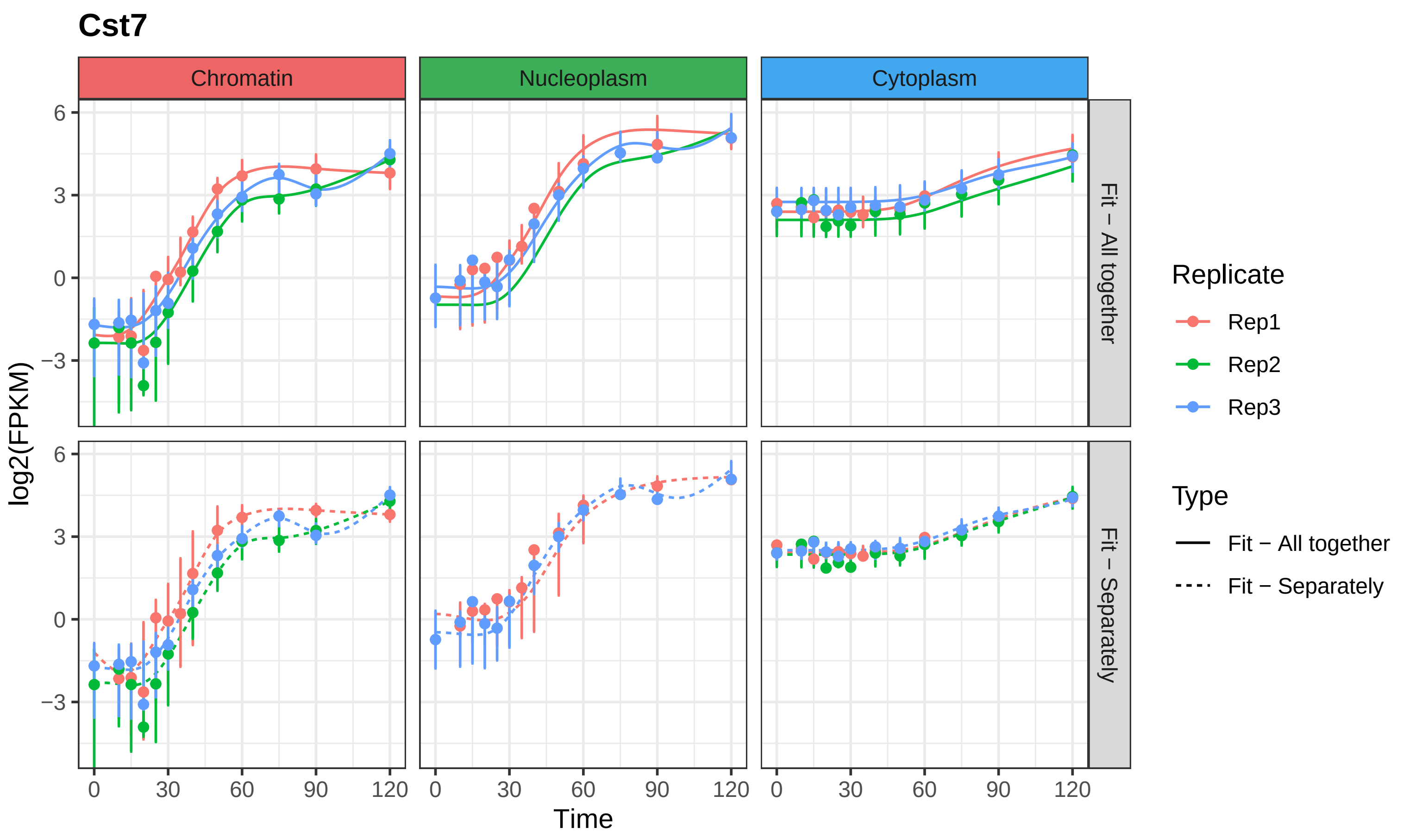

	Together	b1	b2	b3
**−NLL b1 ca**	4.912	4.393		
**−NLL b1 np**	10.89	6.498		
**−NLL b1 cyto**	−0.8867	−5.659		
**−NLL b2 ca**	4.59		4.092	
**−NLL b2 np**				
**−NLL b2 cyto**	6.847		2.46	
**−NLL b3 ca**	3.593			1.915
**−NLL b3 np**	7.425			9.651
**−NLL b3 cyto**	0.4519			−6.411
Total	37.83	5.232	6.552	5.155
**Total with regul**	37.74	7.582	5.323	4.035

	Together	b1	b2	b3
spar	0.4308	0.40370	0.4158	0.38460
σ_b	0.1747	0.04011	0.1308	0.08046
σ_t	0.4474	4.66600	7.563e−06	0.98600
ca_{0,b_1}	−2.0150	−1.14000		
$\log_{10}(k_1')$	−0.4786	−1.03900		−0.27120
$\log_{10}(k_2)$	−0.8962	−1.45900	−0.9299 or −2.918	−0.64790
$\log_{10}(k_2')$	−2.0770	−2.02100		−2.03500
$\log_{10}(k_{deg})$	−3.0040	−2.69800	−2.918 or −0.9299	−2.93100
$\log_{10}(k_1'k_2')$	−2.5560	−3.06000	−2.453	−2.30600
$\log_{10}(k_1'/k_2)$	0.4176	0.41930		0.37670
transport $= \log_{10}(k_1'k_2'/k_2)$	−1.6590	−1.60200	−1.523 or 0.4652	−1.65800

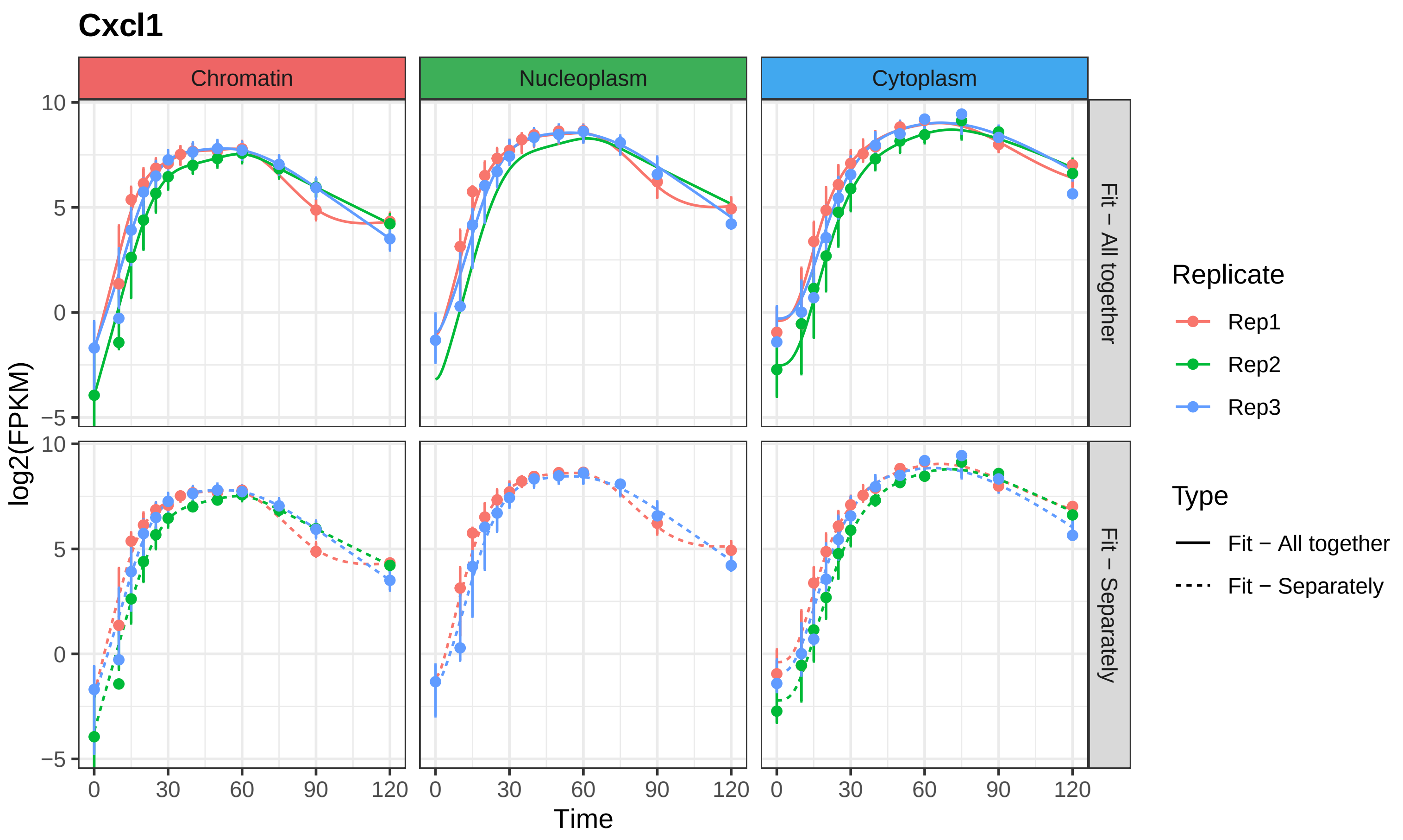

	Together	b1	b2	b3
**−NLL b1 ca**	−1.498	−4.321		
**−NLL b1 np**	−0.594	−3.762		
**−NLL b1 cyto**	7.577	5.4		
**−NLL b2 ca**	1.294		−1.376	
**−NLL b2 np**				
**−NLL b2 cyto**	4.422		1.523	
**−NLL b3 ca**	1.711			−0.138
**−NLL b3 np**	3.67			1.934
**−NLL b3 cyto**	19.19			7.932
Total	35.77	−2.683	0.1473	9.727
**Total with regul**	41.35	0.5309	−0.4229	11.09

	Together	b1	b2	b3
spar	0.3157	0.37510	0.3658	0.3264
σ_b	0.1445	0.06878	0.07832	0.1067
σ_t	1.5510	1.58200	1.112	1.7260
ca_{0,b_1}	−1.8220	−1.99900		
$\log_{10}(k_1')$	−0.2380	−0.14400		−0.2822
$\log_{10}(k_2)$	−0.4675	−0.39560	−0.691 or −1.033	−0.4819
$\log_{10}(k_2')$	−0.9668	−1.06400		−0.8588
$\log_{10}(k_{deg})$	−1.1670	−1.28000	−1.033 or −0.691	−1.0080
$\log_{10}(k_1'k_2')$	−1.2050	−1.20800	−1.283	−1.1410
$\log_{10}(k_1'/k_2)$	0.2295	0.25160		0.1997
$\text{transport} = \log_{10}(k_1'k_2'/k_2)$	−0.7373	−0.81220	−0.5915 or −0.2493	−0.6591

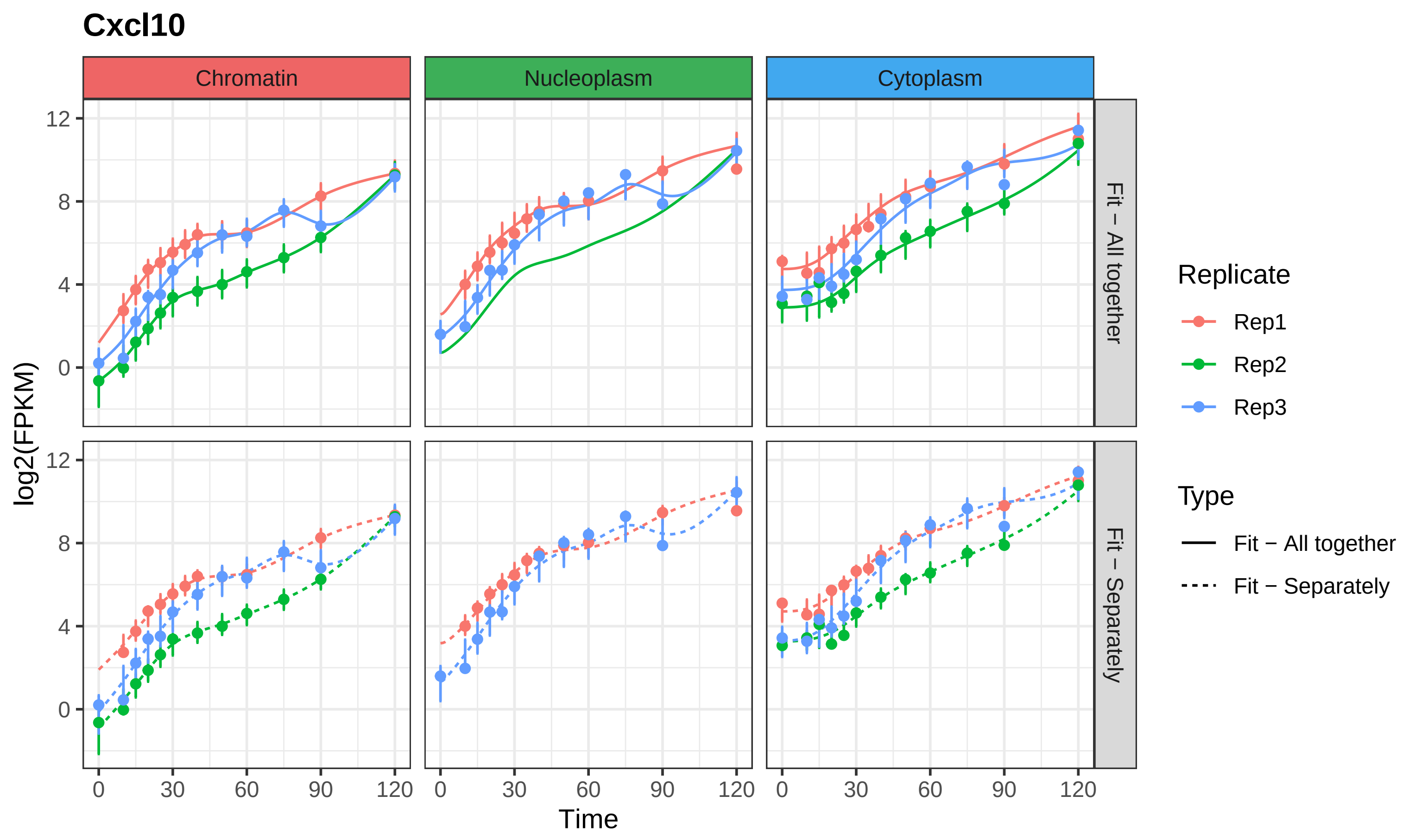

	Together	b1	b2	b3
**−NLL b1 ca**	−1.498	−3.774		
**−NLL b1 np**	4.565	3.648		
**−NLL b1 cyto**	5.049	0.09141		
**−NLL b2 ca**	−0.3553		−2.336	
**−NLL b2 np**				
**−NLL b2 cyto**	5.77		4.86	
**−NLL b3 ca**	2.422			3.126
**−NLL b3 np**	6.749			4.988
**−NLL b3 cyto**	12.06			8.307
Total	34.76	−0.03507	2.524	16.42
**Total with regul**	39.49	−0.459	1.818	17.68

	Together	b1	b2	b3
spar	3.545e−01	4.239e−01	0.4127	0.39530
σ_b	2.253e−01	1.573e−01	0.1633	0.24400
σ_t	7.211e−06	1.071e−05	0.0002614	0.00410
ca_{0,b_1}	1.200e+00	1.923e+00		
$\log_{10}(k_1')$	2.698e−01	−7.198e−02		0.41750
$\log_{10}(k_2)$	−1.398e−01	−4.547e−01	−0.3163 or −1.91	−0.01693
$\log_{10}(k_2')$	−1.059e+00	−1.018e+00		−1.00100
$\log_{10}(k_{deg})$	−1.718e+00	−1.480e+00	−1.91 or −0.3163	−1.59300
$\log_{10}(k_1'k_2')$	−7.893e−01	−1.090e+00	−0.9728	−0.58340
$\log_{10}(k_1'/k_2)$	4.096e−01	3.827e−01		0.43450
$\text{transport} = \log_{10}(k_1'k_2'/k_2)$	−6.495e−01	−6.354e−01	−0.6566 or 0.9372	−0.56650

Cxcl16

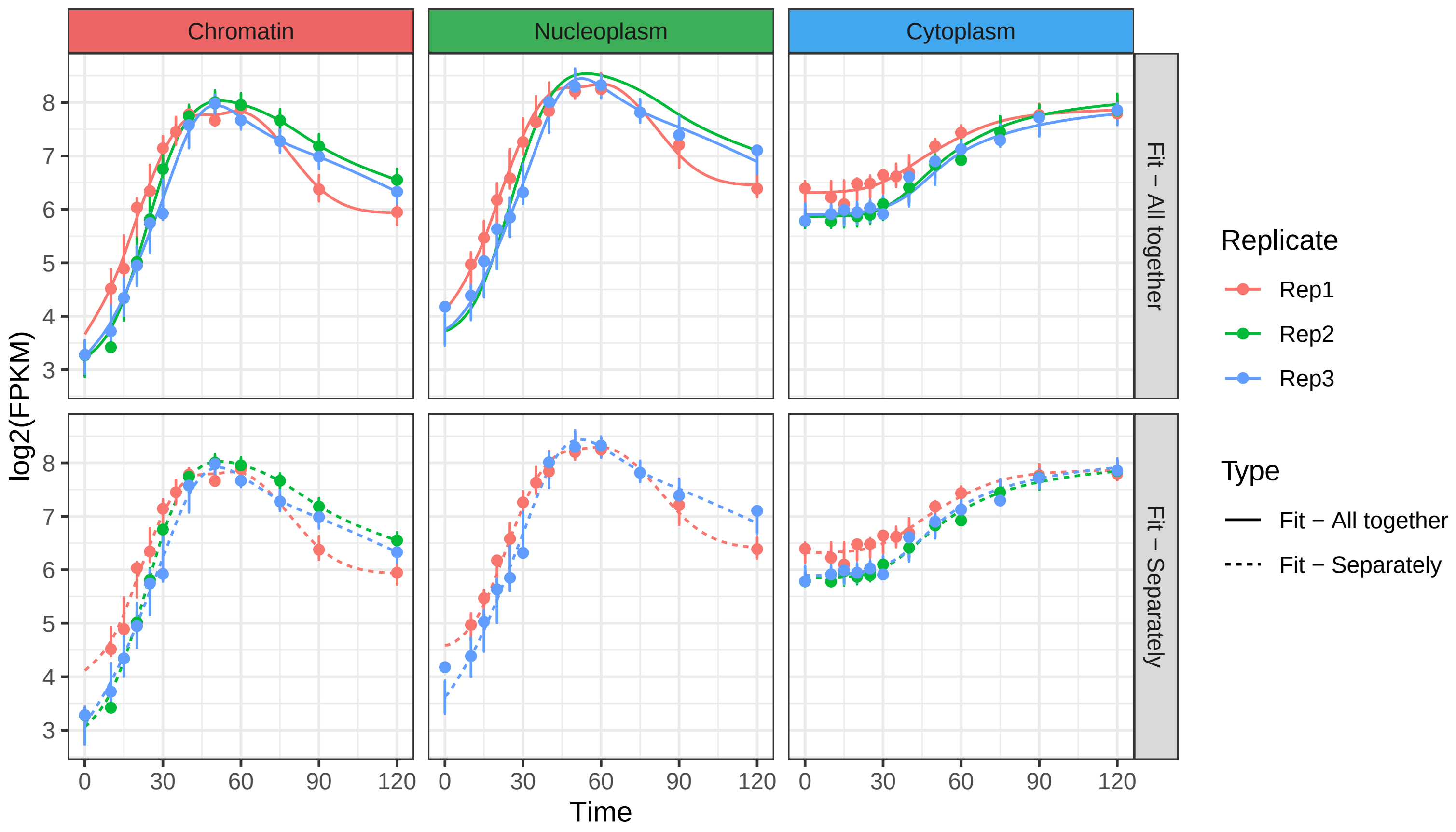

	Together	b1	b2	b3
**−NLL b1 ca**	−8.818	−7.873		
**−NLL b1 np**	−3.557	−8.824		
**−NLL b1 cyto**	−10.03	−9.675		
**−NLL b2 ca**	−9.532		−11.26	
**−NLL b2 np**				
**−NLL b2 cyto**	−10.56		−11.46	
**−NLL b3 ca**	−9.864			−6.779
**−NLL b3 np**	0.9726			−2.504
**−NLL b3 cyto**	−6.785			−10.29
Total	−58.16	−26.37	−22.72	−19.58
**Total with regul**	−57.7	−27.46	−22.65	−21.31

	Together	b1	b2	b3
spar	0.35760	0.38690	0.3433	0.40720
σ_b	0.06942	0.06063	0.04665	0.05911
σ_t	1.18800	1.01200	0.004021	1.50100
ca_{0,b_1}	3.68700	4.17700		
$\log_{10}(k_1')$	−0.13410	−0.37650		0.36630
$\log_{10}(k_2)$	−0.28940	−0.51830	4.059 or −2.768	0.20740
$\log_{10}(k_2')$	−2.02100	−1.96100		−1.97500
$\log_{10}(k_{deg})$	−2.66400	−2.48300	−2.768 or 4.059	−2.65500
$\log_{10}(k_1' k_2')$	−2.15500	−2.33700	2.13	−1.60800
$\log_{10}(k_1'/k_2)$	0.15530	0.14180		0.15890
transport $= \log_{10}(k_1' k_2'/k_2)$	−1.86600	−1.81900	−1.928 or 4.898	−1.81600

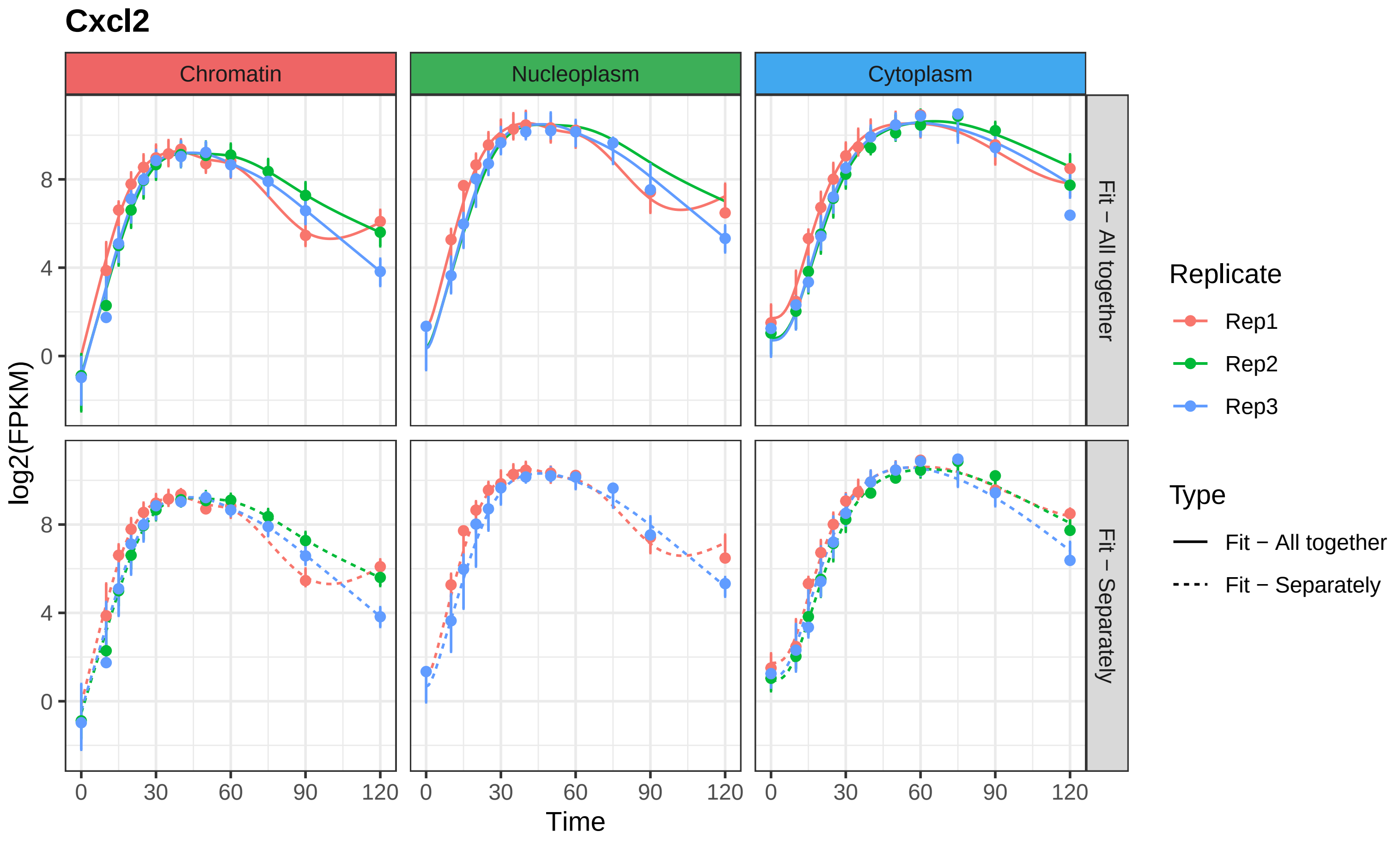

	Together	b1	b2	b3
**−NLL b1 ca**	−0.6095	−3.336		
**−NLL b1 np**	3.936	5.272		
**−NLL b1 cyto**	4.486	−0.632		
**−NLL b2 ca**	−0.1109		−2.364	
**−NLL b2 np**				
**−NLL b2 cyto**	3.359		1.314	
**−NLL b3 ca**	3.166			0.4837
**−NLL b3 np**	6.192			1.57
**−NLL b3 cyto**	12.39			4.657
Total	32.81	1.304	−1.051	6.71
**Total with regul**	35.93	3.919	−1.752	5.544

	Together	b1	b2	b3
spar	0.364800	0.3647	0.3838	0.39930
σ_b	0.202500	0.1231	0.1209	0.10870
σ_t	0.643500	1.1340	0.7149	1.53700
ca_{0,b_1}	0.001226	−0.1871		
$\log_{10}(k_1')$	0.057540	−0.0394		−0.08906
$\log_{10}(k_2)$	−0.332700	−0.4180	−0.5495 or −1.048	−0.41860
$\log_{10}(k_2')$	−1.083000	−1.1300		−0.84150
$\log_{10}(k_{deg})$	−1.197000	−1.3160	−1.048 or −0.5495	−0.96850
$\log_{10}(k_1'k_2')$	−1.025000	−1.1690	−1.158	−0.93060
$\log_{10}(k_1'/k_2)$	0.390300	0.3786		0.32950
transport $= \log_{10}(k_1'k_2'/k_2)$	−0.692300	−0.7514	−0.6089 or −0.1106	−0.51200

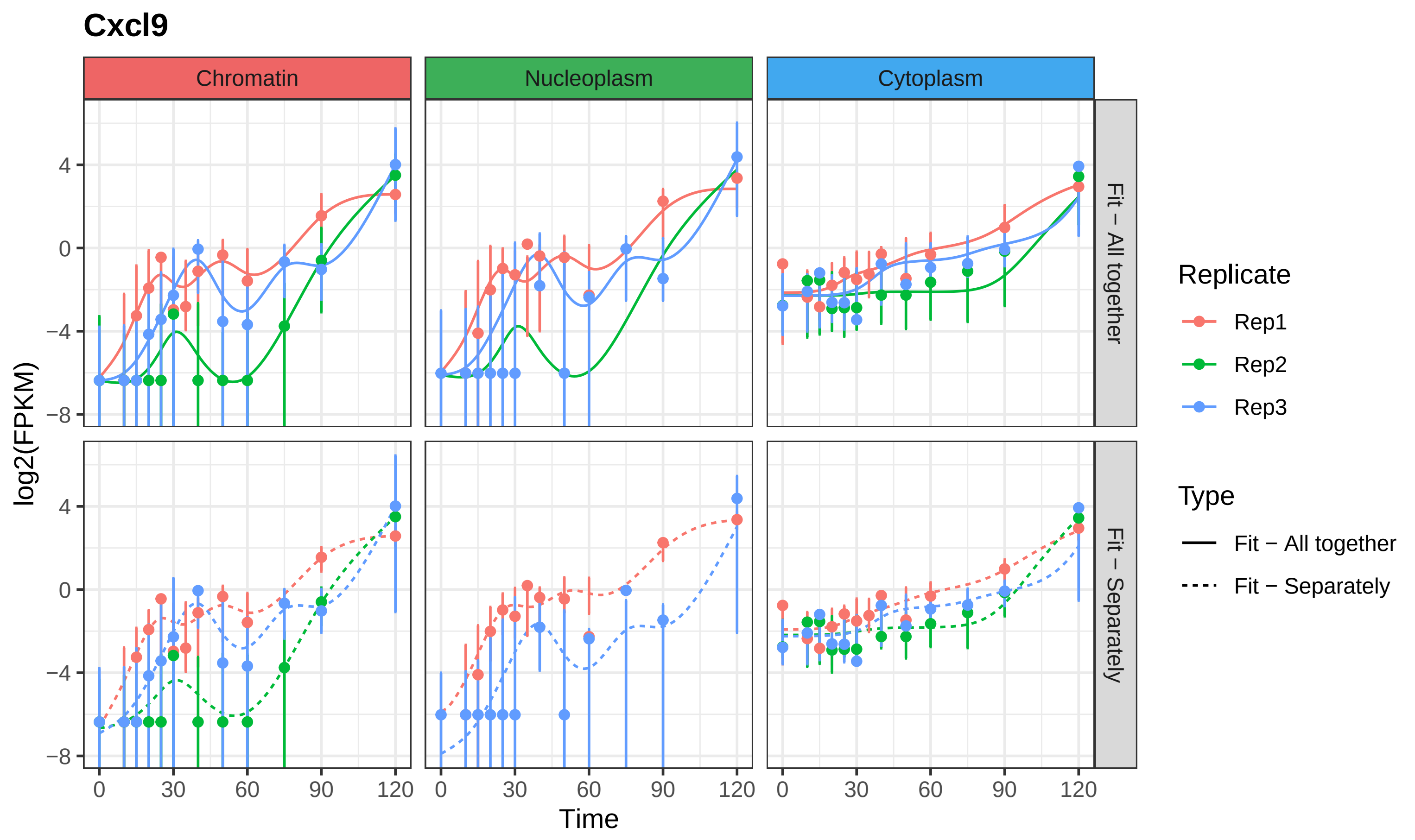

	Together	b1	b2	b3
**−NLL b1 ca**	12.57	11.6		
**−NLL b1 np**	17.57	15.92		
**−NLL b1 cyto**	11.09	10.26		
**−NLL b2 ca**	20.46		16.53	
**−NLL b2 np**				
**−NLL b2 cyto**	13.6		7.726	
**−NLL b3 ca**	15.88			15.85
**−NLL b3 np**	28.02			18.5
**−NLL b3 cyto**	14.99			13.86
Total	134.2	37.77	24.26	48.21
**Total with regul**	137.2	37.11	22	47.28

	Together	b1	b2	b3
spar	0.36720	0.4007000	0.4323	3.866e−01
σ_b	0.26340	0.1290000	4.264e−06	1.638e−05
σ_t	3.51700	0.0003343	0.0002201	5.404e+00
ca_{0,b_1}	−6.20200	−6.6050000		
$\log_{10}(k_1')$	7.31800	−0.3660000		4.515e+00
$\log_{10}(k_2)$	7.23700	−0.6056000	5.203 or −2.413	4.812e+00
$\log_{10}(k_2')$	−1.45900	−1.6370000		−1.212e+00
$\log_{10}(k_{deg})$	−2.60900	−2.8140000	−2.413 or 5.203	−2.914e+00
$\log_{10}(k_1'k_2')$	5.85900	−2.0030000	4.14	3.303e+00
$\log_{10}(k_1'/k_2)$	0.08091	0.2396000		−2.970e−01
$\text{transport} = \log_{10}(k_1'k_2'/k_2)$	−1.37800	−1.3970000	−1.063 or 6.553	−1.509e+00

Cybb

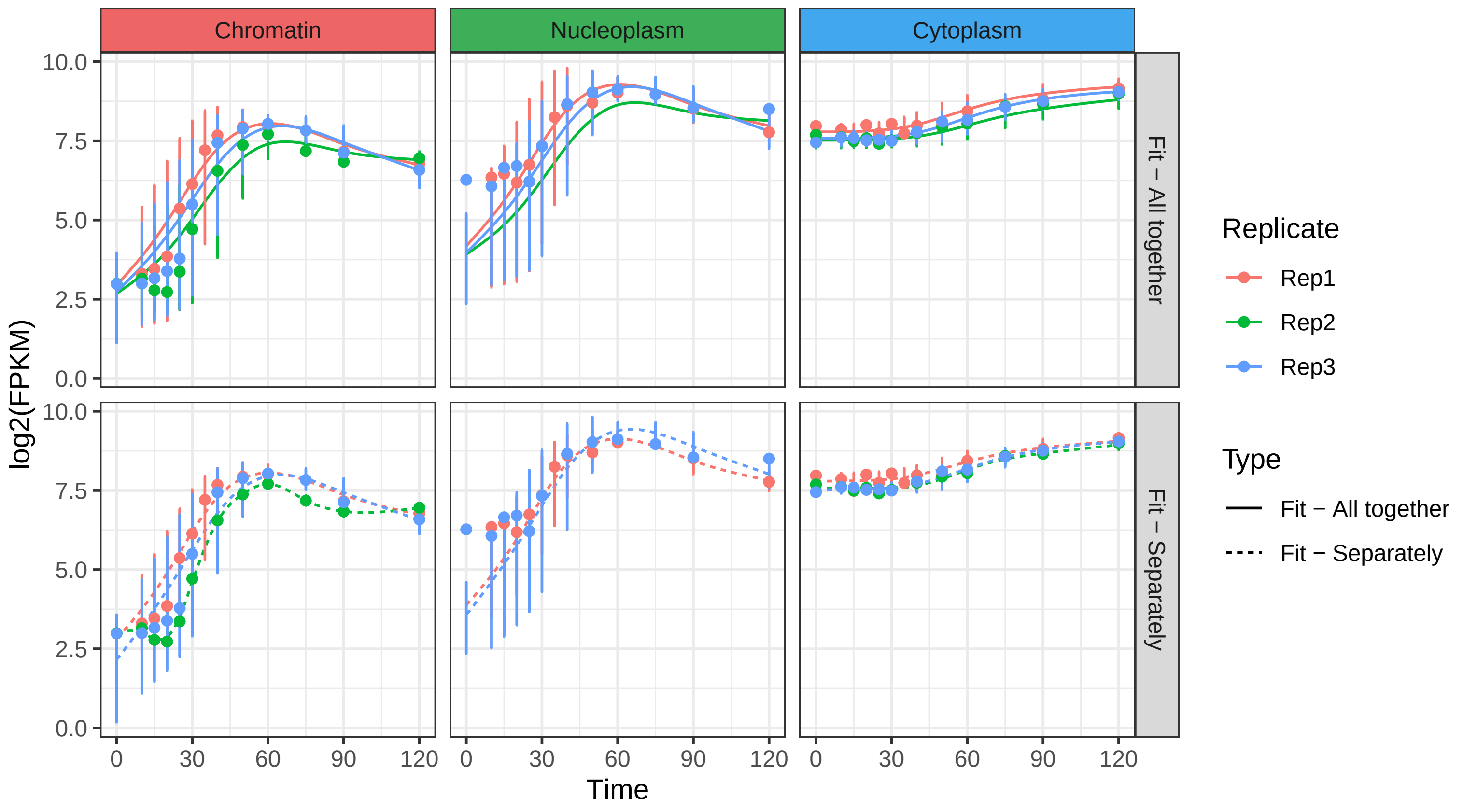

	Together	b1	b2	b3
**−NLL b1 ca**	6.462	3.806		
**−NLL b1 np**	8.944	7.077		
**−NLL b1 cyto**	−3.057	−4.728		
**−NLL b2 ca**	8.457		−11.28	
**−NLL b2 np**				
**−NLL b2 cyto**	−6.955		−11.41	
**−NLL b3 ca**	7.736			6.336
**−NLL b3 np**	20.24			12.2
**−NLL b3 cyto**	−9.424			−12.86
Total	32.41	6.155	−22.69	5.679
**Total with regul**	35.19	7.716	−17.12	7.423

	Together	b1	b2	b3
spar	0.57650	0.56430	0.253	0.57170
σ_b	0.08544	0.09003	0.05081	0.03859
σ_t	8.10400	5.40600	0.2635	7.15800
ca_{0,b_1}	3.22500	3.05700		
$\log_{10}(k_1')$	7.52300	7.18300		0.95920
$\log_{10}(k_2)$	7.15100	6.86000	−0.119 or −2.903	0.52550
$\log_{10}(k_2')$	−1.99600	−2.03000		−2.07300
$\log_{10}(k_{deg})$	−3.08100	−3.20700	−2.903 or −0.119	−3.25600
$\log_{10}(k_1'k_2')$	5.52700	5.15400	−1.657	−1.11400
$\log_{10}(k_1'/k_2)$	0.37180	0.32300		0.43370
transport = $\log_{10}(k_1'k_2'/k_2)$	−1.62400	−1.70700	−1.538 or 1.246	−1.64000

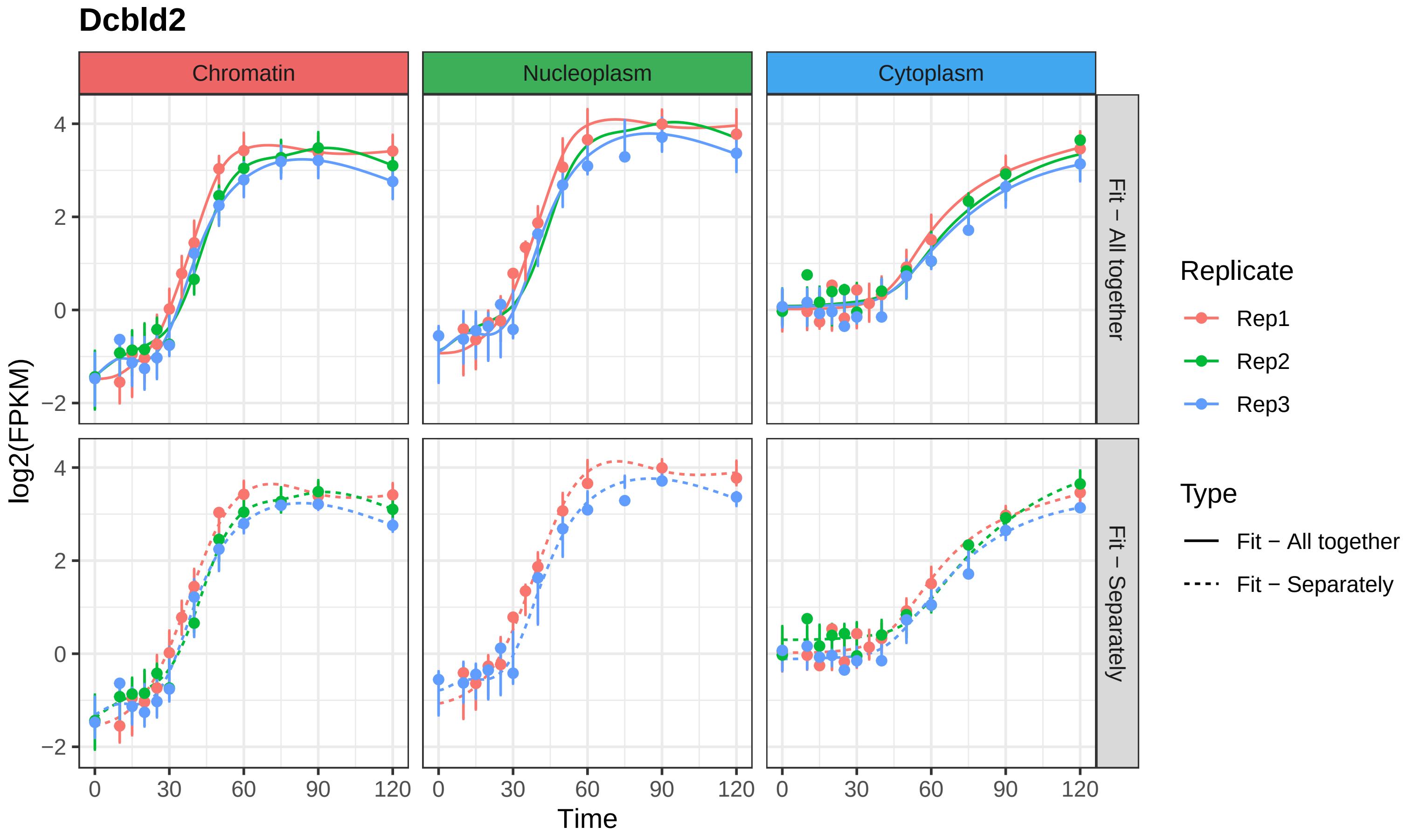

	Together	b1	b2	b3
**−NLL b1 ca**	−5.192	−4.858		
**−NLL b1 np**	0.4252	−2.023		
**−NLL b1 cyto**	−2.022	−2.5		
**−NLL b2 ca**	−4.994		−4.465	
**−NLL b2 np**				
**−NLL b2 cyto**	4.071		−0.5568	
**−NLL b3 ca**	−4.047			−3.765
**−NLL b3 np**	1.443			−1.887
**−NLL b3 cyto**	−0.03495			−4.211
Total	−10.35	−9.38	−5.022	−9.863
**Total with regul**	−8.948	−9.911	−6.026	−11.33

	Together	b1	b2	b3
spar	0.3598000	0.4761000	0.3814	3.877e−01
σ_b	0.1208000	0.0844900	0.08627	1.461e−06
σ_t	0.0009135	0.0001125	3.973e−05	1.999e+00
ca_{0,b_1}	−1.4720000	−1.4960000		
$\log_{10}(k_1')$	−0.0221200	0.2428000		−1.071e−01
$\log_{10}(k_2)$	−0.1897000	0.0959800	−1.237 or −2.047	−2.665e−01
$\log_{10}(k_2')$	−1.9520000	−1.9730000		−1.898e+00
$\log_{10}(k_{deg})$	−2.2380000	−2.3010000	−2.047 or −1.237	−2.100e+00
$\log_{10}(k_1'k_2')$	−1.9740000	−1.7300000	−2.78	−2.005e+00
$\log_{10}(k_1'/k_2)$	0.1676000	0.1468000		1.594e−01
$\text{transport} = \log_{10}(k_1'k_2'/k_2)$	−1.7840000	−1.8260000	−1.543 or −0.7329	−1.738e+00

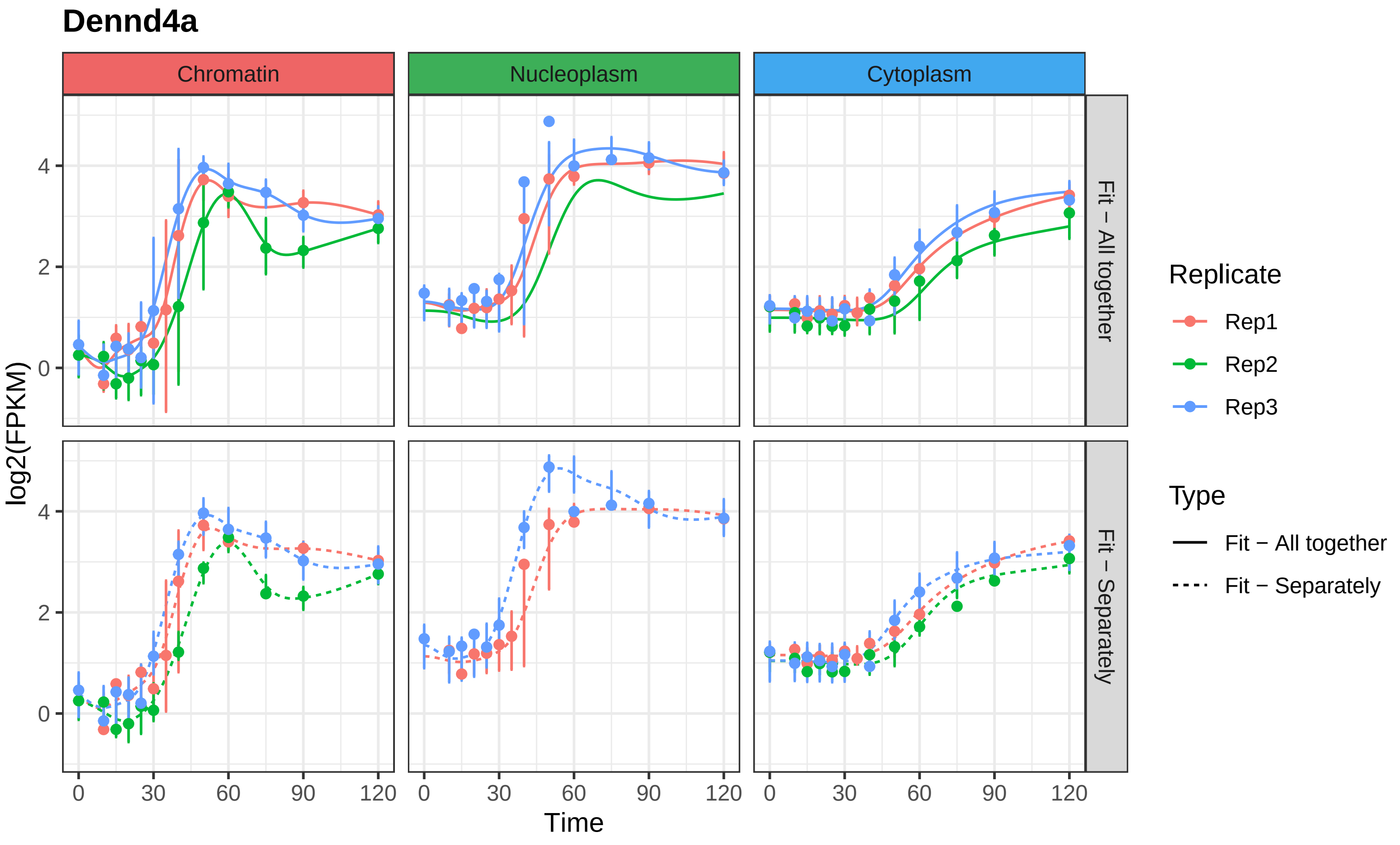

	Together	b1	b2	b3
**−NLL b1 ca**	−1.312	−0.7298		
**−NLL b1 np**	−1.46	−2.388		
**−NLL b1 cyto**	−8.401	−10.41		
**−NLL b2 ca**	−4.466		−6.44	
**−NLL b2 np**				
**−NLL b2 cyto**	−2.849		−6.394	
**−NLL b3 ca**	−3.763			−3.708
**−NLL b3 np**	6.992			3.476
**−NLL b3 cyto**	−4.367			−5.481
Total	−19.63	−13.53	−12.83	−5.713
**Total with regul**	−16.78	−13.77	−13.76	−4.254

	Together	b1	b2	b3
spar	0.30990	3.703e−01	0.3698	0.3261000
σ_b	0.07102	1.311e−06	0.04516	0.1190000
σ_t	4.17200	3.572e+00	9.7e−07	0.0002004
ca_{0,b_1}	0.45080	4.044e−01		
$\log_{10}(k_1')$	−0.98680	−9.283e−01		−0.0730800
$\log_{10}(k_2)$	−1.24800	−1.161e+00	−1.011 or −1.796	−0.3603000
$\log_{10}(k_2')$	−1.87700	−1.895e+00		−2.1210000
$\log_{10}(k_{deg})$	−1.83500	−1.903e+00	−1.796 or −1.011	−2.0300000
$\log_{10}(k_1'k_2')$	−2.86400	−2.823e+00	−2.576	−2.1940000
$\log_{10}(k_1'/k_2)$	0.26150	2.330e−01		0.2873000
$\text{transport} = \log_{10}(k_1'k_2'/k_2)$	−1.61500	−1.662e+00	−1.566 or −0.7808	−1.8330000

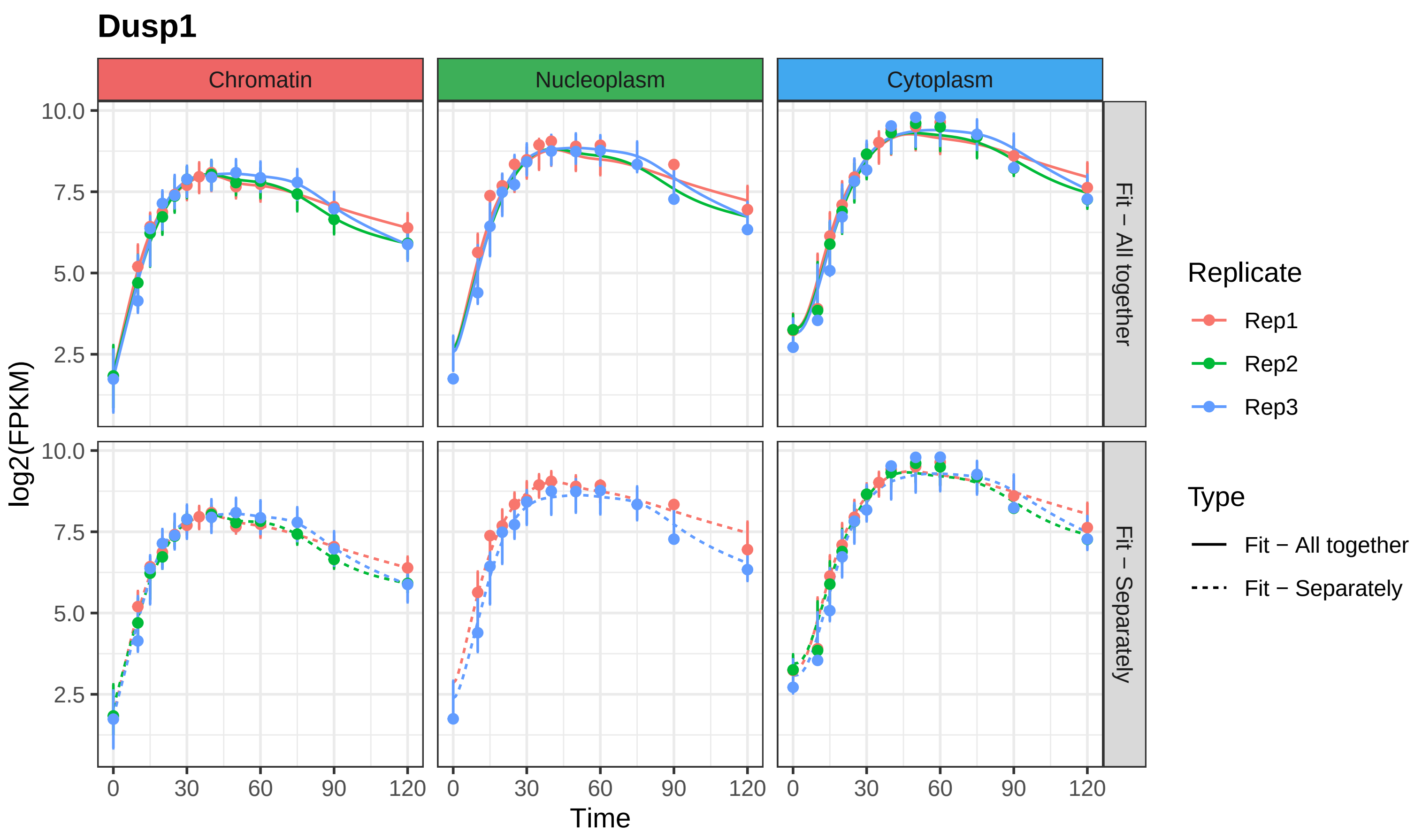

	Together	b1	b2	b3
**−NLL b1 ca**	−4.007	−6.082		
**−NLL b1 np**	4.834	0.2633		
**−NLL b1 cyto**	2.424	2.053		
**−NLL b2 ca**	−3.504		−7.017	
**−NLL b2 np**				
**−NLL b2 cyto**	0.2783		2.565	
**−NLL b3 ca**	−2.277			−1.522
**−NLL b3 np**	7.4			0.5788
**−NLL b3 cyto**	10.56			9.354
Total	15.71	−3.766	−4.451	8.411
**Total with regul**	17.27	−3.039	−3.579	10.14

	Together	b1	b2	b3
spar	0.37140	0.40380	0.334	0.3398
σ_b	0.16120	0.12150	0.09724	0.1781
σ_t	1.28600	1.14100	1.192	1.1340
ca_{0,b_1}	1.82700	1.66000		
$\log_{10}(k_1')$	−0.08093	0.02784		−0.2232
$\log_{10}(k_2)$	−0.31990	−0.28440	−0.5502 or −0.6017	−0.3977
$\log_{10}(k_2')$	−0.64600	−0.70510		−0.6268
$\log_{10}(k_{deg})$	−0.82070	−0.83380	−0.6017 or −0.5502	−0.8363
$\log_{10}(k_1'k_2')$	−0.72690	−0.67720	−0.7418	−0.8501
$\log_{10}(k_1'/k_2)$	0.23900	0.31220		0.1745
$\text{transport} = \log_{10}(k_1'k_2'/k_2)$	−0.40700	−0.39290	−0.1916 or −0.1402	−0.4523

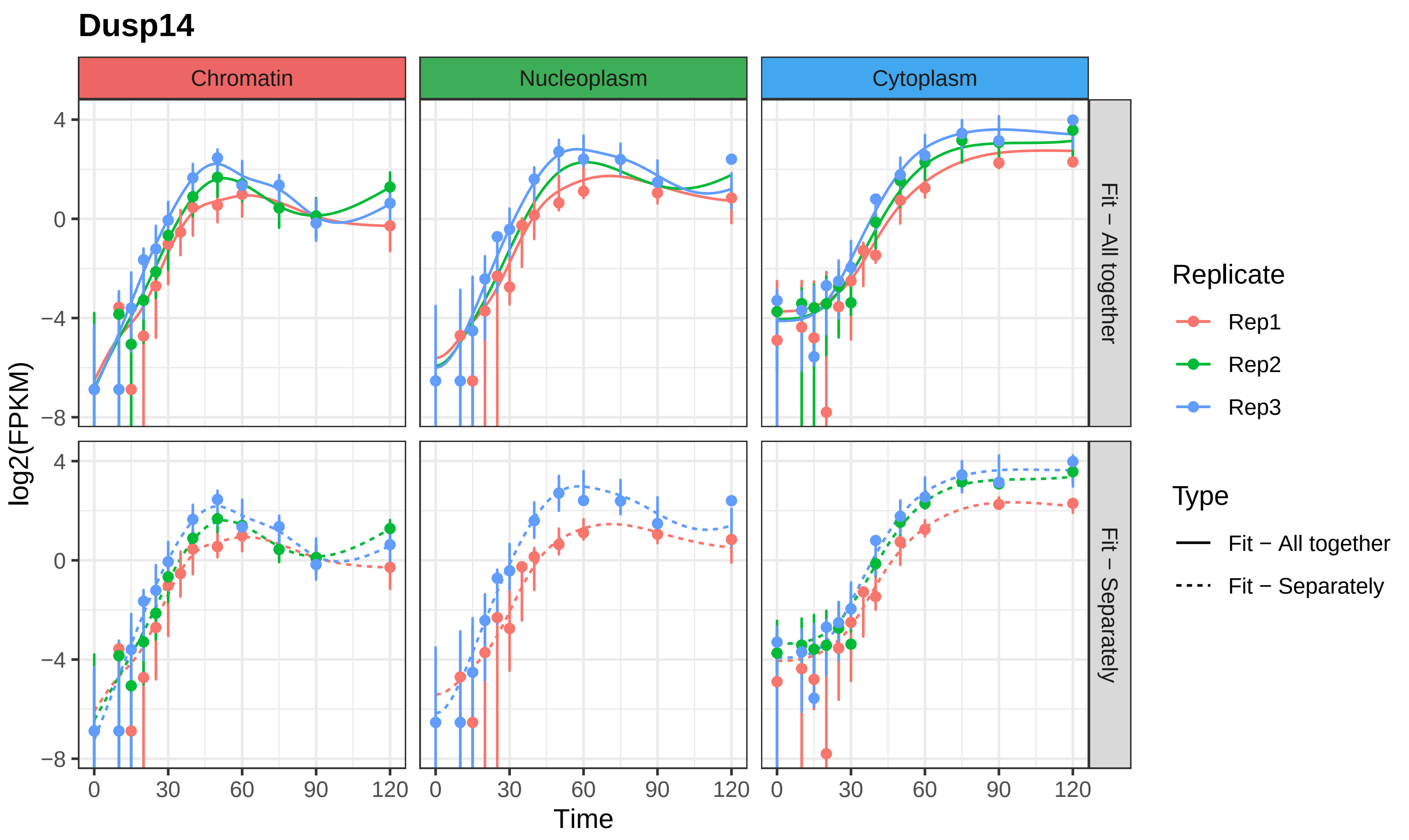

	Together	b1	b2	b3
**−NLL b1 ca**	8.169	7.147		
**−NLL b1 np**	10.17	8.327		
**−NLL b1 cyto**	15.76	10.15		
**−NLL b2 ca**	5.743		2.628	
**−NLL b2 np**				
**−NLL b2 cyto**	6.101		4.946	
**−NLL b3 ca**	6.831			6.364
**−NLL b3 np**	16.07			11.93
**−NLL b3 cyto**	9.371			8.859
Total	78.22	25.62	7.574	27.15
**Total with regul**	83.1	26.11	5.503	27.18

	Together	b1	b2	b3
spar	3.978e−01	4.143e−01	0.4345	4.288e−01
σ_b	1.886e−01	4.238e−08	0.06321	2.122e−01
σ_t	2.060e−06	1.685e+00	7.096e−06	5.007e−05
ca_{0,b_1}	−6.612e+00	−6.153e+00		
$\log_{10}(k_1')$	−7.317e−01	−8.518e−01		−6.505e−01
$\log_{10}(k_2)$	−1.007e+00	−1.056e+00	−0.9685 or −1.794	−9.743e−01
$\log_{10}(k_2')$	−1.181e+00	−1.074e+00		−1.302e+00
$\log_{10}(k_{deg})$	−1.747e+00	−1.482e+00	−1.794 or −0.9685	−1.974e+00
$\log_{10}(k_1'k_2')$	−1.913e+00	−1.926e+00	−1.837	−1.952e+00
$\log_{10}(k_1'/k_2)$	2.752e−01	2.041e−01		3.238e−01
transport $= \log_{10}(k_1'k_2'/k_2)$	−9.058e−01	−8.699e−01	−0.8682 or −0.04252	−9.782e−01

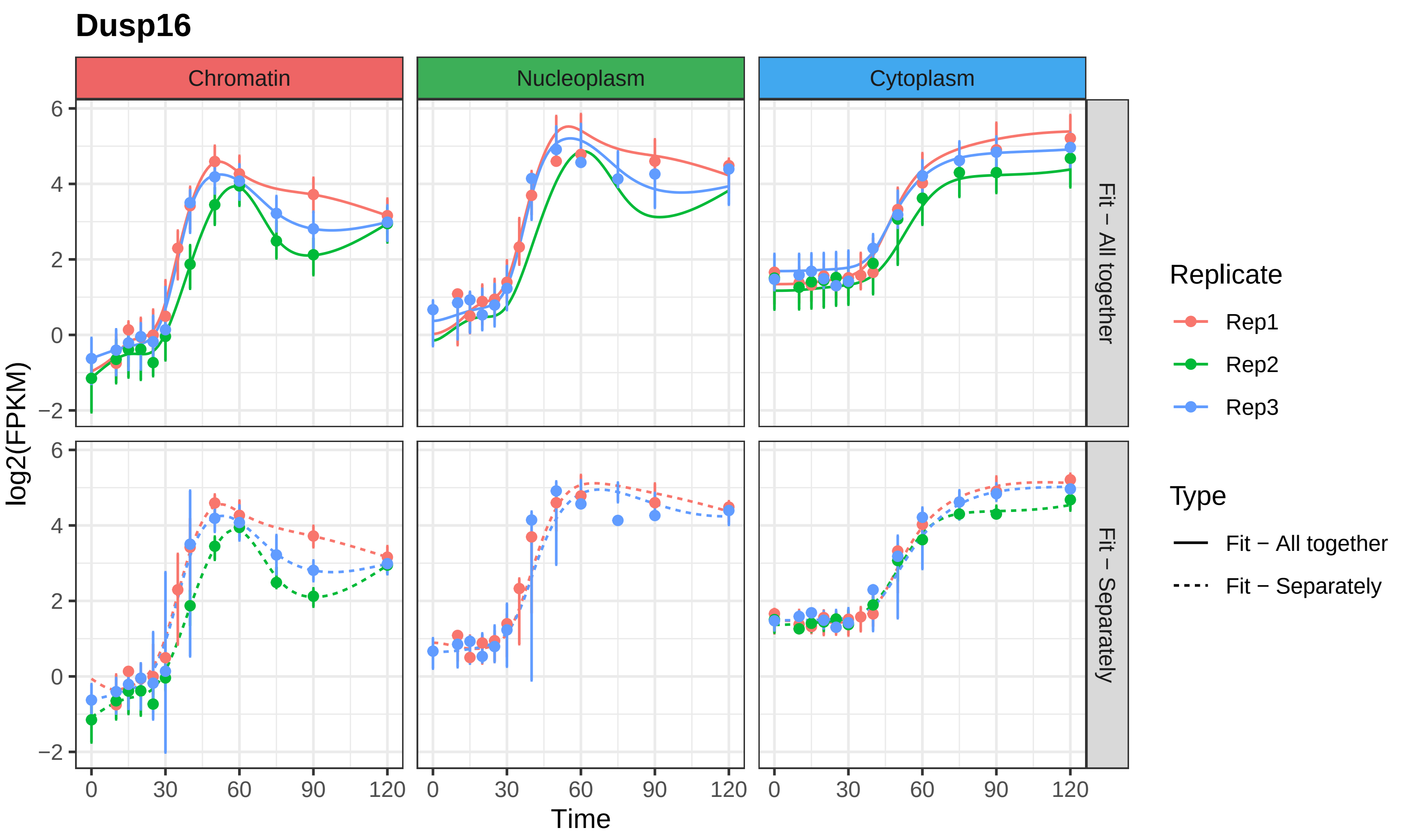

	Together	b1	b2	b3
**−NLL b1 ca**	−2.147	−0.4049		
**−NLL b1 np**	8.971	3.094		
**−NLL b1 cyto**	−0.4737	−6.175		
**−NLL b2 ca**	−3.385		−5.244	
**−NLL b2 np**				
**−NLL b2 cyto**	2.193		−7.38	
**−NLL b3 ca**	−2.519			−1.269
**−NLL b3 np**	5.282			3.65
**−NLL b3 cyto**	−1.753			−3.371
Total	6.167	−3.486	−12.62	−0.9905
**Total with regul**	9.626	−1.509	−13.28	−1.107

	Together	b1	b2	b3
spar	0.3040	0.37140	0.3625	0.3626
σ_b	0.1568	0.08634	0.04642	0.0673
σ_t	0.7390	2.25400	1.109	4.7320
ca_{0,b_1}	−0.9627	−0.02892		
$\log_{10}(k_1')$	−0.1963	−0.81550		−0.9099
$\log_{10}(k_2)$	−0.4958	−1.10200	4.678 or −1.979	−1.2940
$\log_{10}(k_2')$	−1.5830	−1.42000		−1.4580
$\log_{10}(k_{deg})$	−1.9800	−1.59600	−1.979 or 4.678	−1.7110
$\log_{10}(k_1'k_2')$	−1.7790	−2.23500	3.436	−2.3680
$\log_{10}(k_1'/k_2)$	0.2995	0.28700		0.3842
$transport = \log_{10}(k_1'k_2'/k_2)$	−1.2830	−1.13300	−1.242 or 5.415	−1.0740

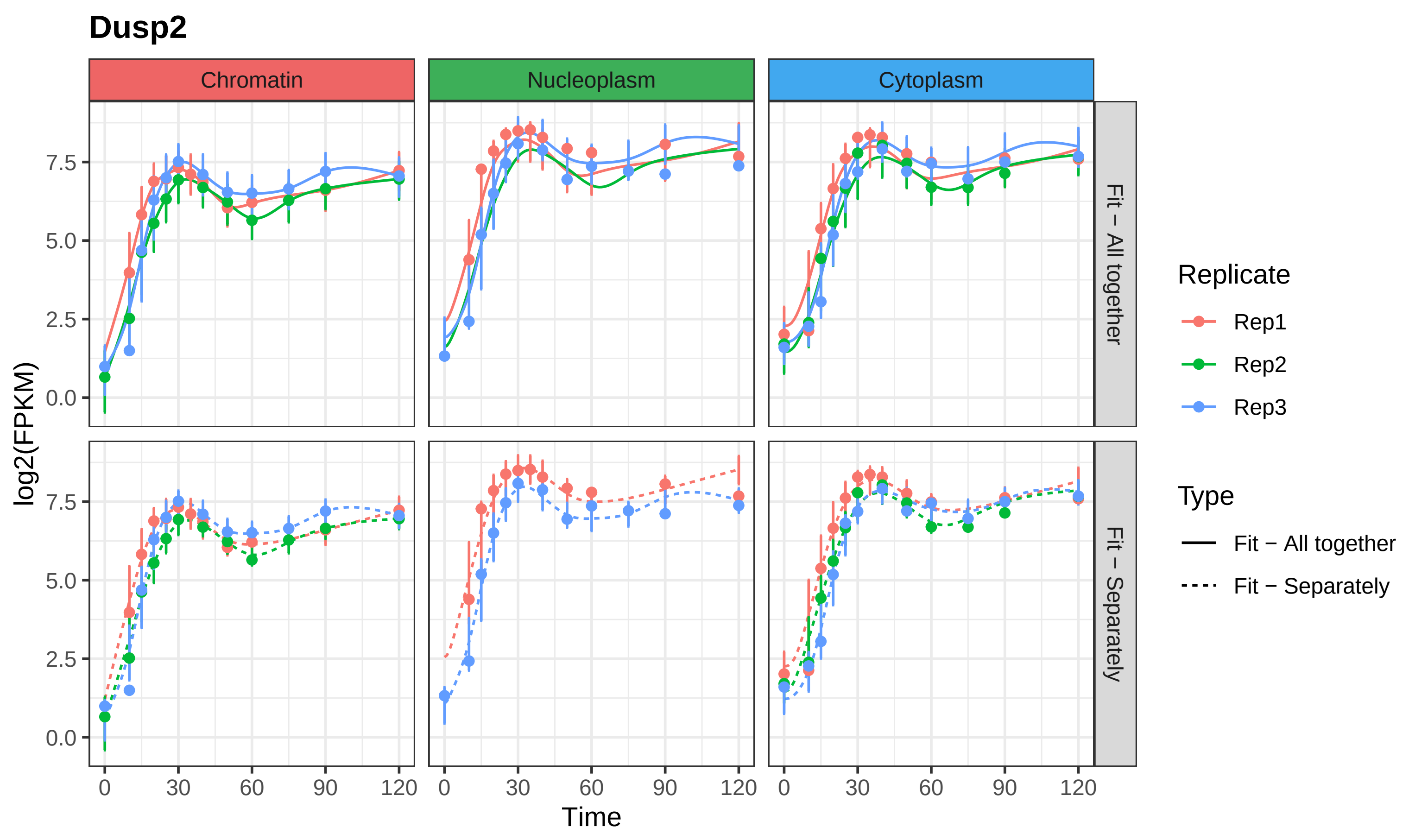

	Together	b1	b2	b3
**−NLL b1 ca**	−0.8653	−2.349		
**−NLL b1 np**	11.38	5.895		
**−NLL b1 cyto**	8.338	4.66		
**−NLL b2 ca**	−0.204		−3.793	
**−NLL b2 np**				
**−NLL b2 cyto**	3.358		2.979	
**−NLL b3 ca**	1.782			−0.8028
**−NLL b3 np**	13.3			4.065
**−NLL b3 cyto**	6.106			0.151
Total	43.19	8.206	−0.814	3.413
**Total with regul**	53.01	13.2	−1.181	7.895

	Together	b1	b2	b3
spar	0.27480	0.399500	0.3696	0.2783
σ_b	0.21230	0.152400	0.1122	0.1325
σ_t	1.45300	1.876000	1.243	1.1680
ca_{0,b_1}	1.47100	1.154000		
$\log_{10}(k_1')$	−0.03566	0.005215		0.2773
$\log_{10}(k_2)$	−0.32950	−0.400700	−0.3039 or −0.3178	0.1337
$\log_{10}(k_2')$	−0.63680	−0.705800		−0.8462
$\log_{10}(k_{deg})$	−0.59100	−0.618700	−0.3178 or −0.3039	−0.8907
$\log_{10}(k_1'k_2')$	−0.67240	−0.700600	−0.3454	−0.5689
$\log_{10}(k_1'/k_2)$	0.29390	0.406000		0.1436
$\text{transport} = \log_{10}(k_1'k_2'/k_2)$	−0.34290	−0.299900	−0.04151 or −0.02758	−0.7026

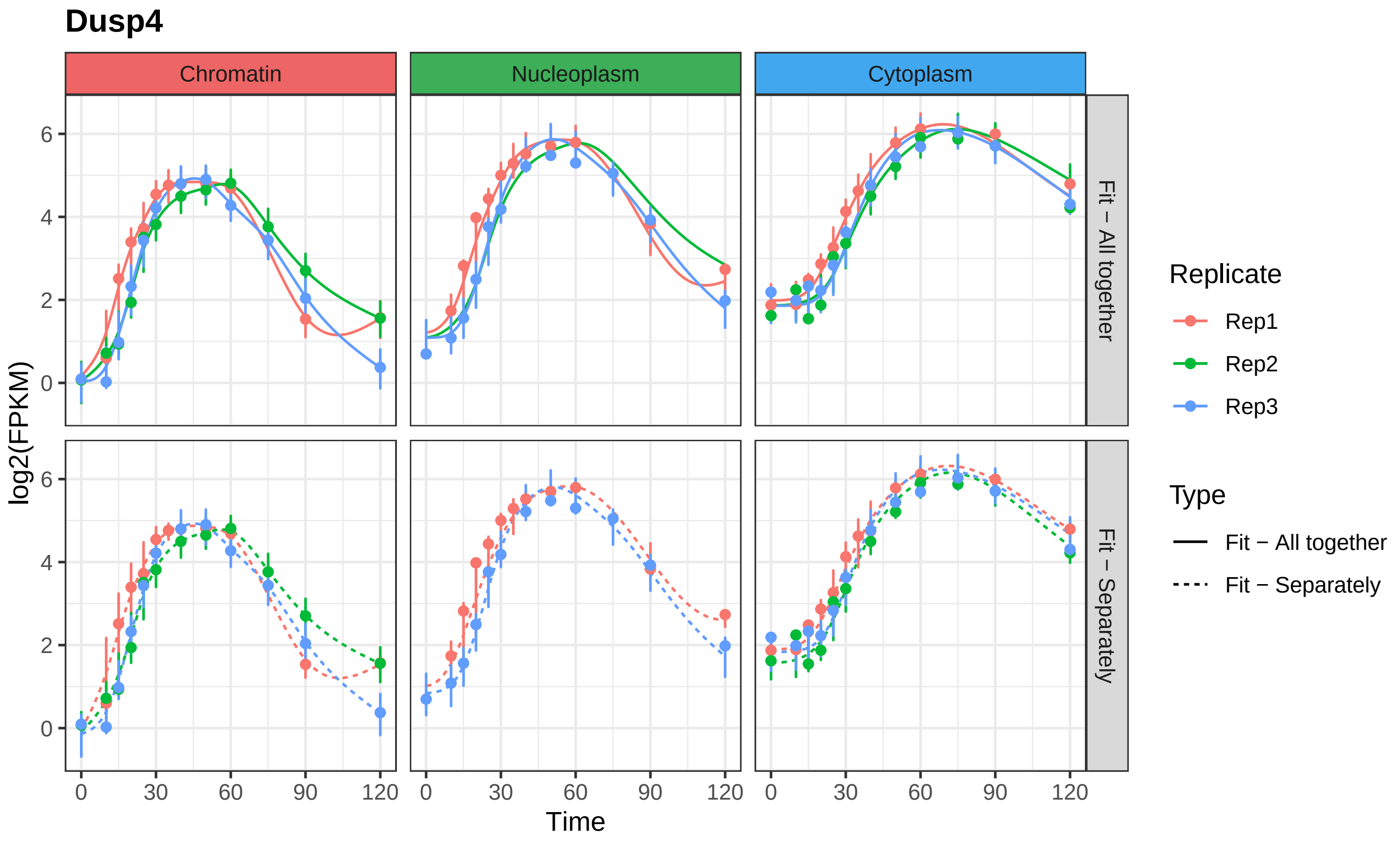

	Together	b1	b2	b3
**−NLL b1 ca**	−3.043	−5.925		
**−NLL b1 np**	−0.2388	−0.6638		
**−NLL b1 cyto**	−2.792	−7.186		
**−NLL b2 ca**	−4.911		−3.362	
**−NLL b2 np**				
**−NLL b2 cyto**	6.476		2.419	
**−NLL b3 ca**	−4.799			−3.882
**−NLL b3 np**	1.204			−0.1196
**−NLL b3 cyto**	−0.6367			2.067
Total	−8.74	−13.77	−0.9432	−1.934
**Total with regul**	−6.211	−14	0.03283	0.1397

	Together	b1	b2	b3
spar	0.3137	3.615e−01	0.3395	0.3207000
σ_b	0.1320	2.727e−05	0.1286	0.1449000
σ_t	0.9290	2.438e+00	1.095	0.0000657
ca_{0,b_1}	0.2166	8.087e−03		
$\log_{10}(k_1')$	−0.5114	−7.020e−01		−0.5270000
$\log_{10}(k_2)$	−0.8284	−1.025e+00	−0.9828 or −1.109	−0.8246000
$\log_{10}(k_2')$	−1.1460	−1.091e+00		−1.0970000
$\log_{10}(k_{deg})$	−1.3770	−1.355e+00	−1.109 or −0.9828	−1.3950000
$\log_{10}(k_1' k_2')$	−1.6570	−1.793e+00	−1.589	−1.6240000
$\log_{10}(k_1'/k_2)$	0.3170	3.230e−01		0.2976000
$transport = \log_{10}(k_1' k_2'/k_2)$	−0.8288	−7.683e−01	−0.606 or −0.4793	−0.7998000

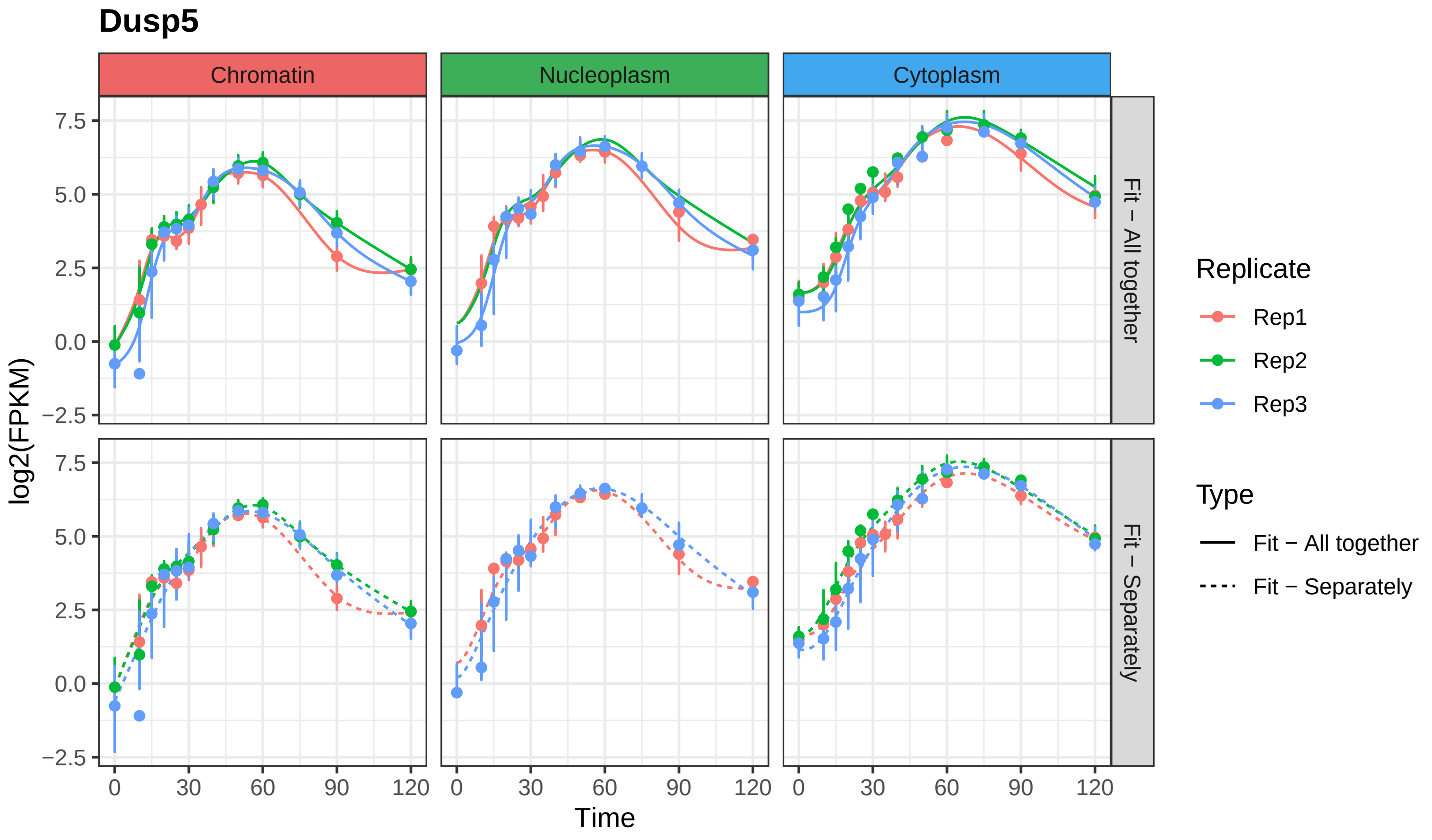

	Together	b1	b2	b3
**−NLL b1 ca**	−4.134	−2.269		
**−NLL b1 np**	−0.3742	−0.9396		
**−NLL b1 cyto**	4.595	1.097		
**−NLL b2 ca**	−4.275		−1.392	
**−NLL b2 np**				
**−NLL b2 cyto**	5.303		1.436	
**−NLL b3 ca**	−0.4626			3.578
**−NLL b3 np**	−0.275			0.916
**−NLL b3 cyto**	2.503			0.9027
Total	2.881	−2.111	0.04452	5.396
**Total with regul**	11.63	−0.08895	−1.122	3.568

	Together	b1	b2	b3
spar	0.2574	0.37620	0.3916	4.882e−01
σ_b	0.1189	0.05066	0.08531	3.006e−06
σ_t	1.6130	2.55600	2.294	3.150e+00
ca_{0,b_1}	−0.1241	−0.14640		
$\log_{10}(k_1')$	−0.1625	−0.36820		−2.797e−01
$\log_{10}(k_2)$	−0.3934	−0.62130	6.783 or −1.09	−5.212e−01
$\log_{10}(k_2')$	−0.7858	−0.92080		−7.890e−01
$\log_{10}(k_{deg})$	−1.0970	−1.19900	−1.09 or 6.783	−1.070e+00
$\log_{10}(k_1'k_2')$	−0.9483	−1.28900	6.217	−1.069e+00
$\log_{10}(k_1'/k_2)$	0.2309	0.25310		2.415e−01
transport $= \log_{10}(k_1'k_2'/k_2)$	−0.5549	−0.66770	−0.5656 or 7.307	−5.475e−01

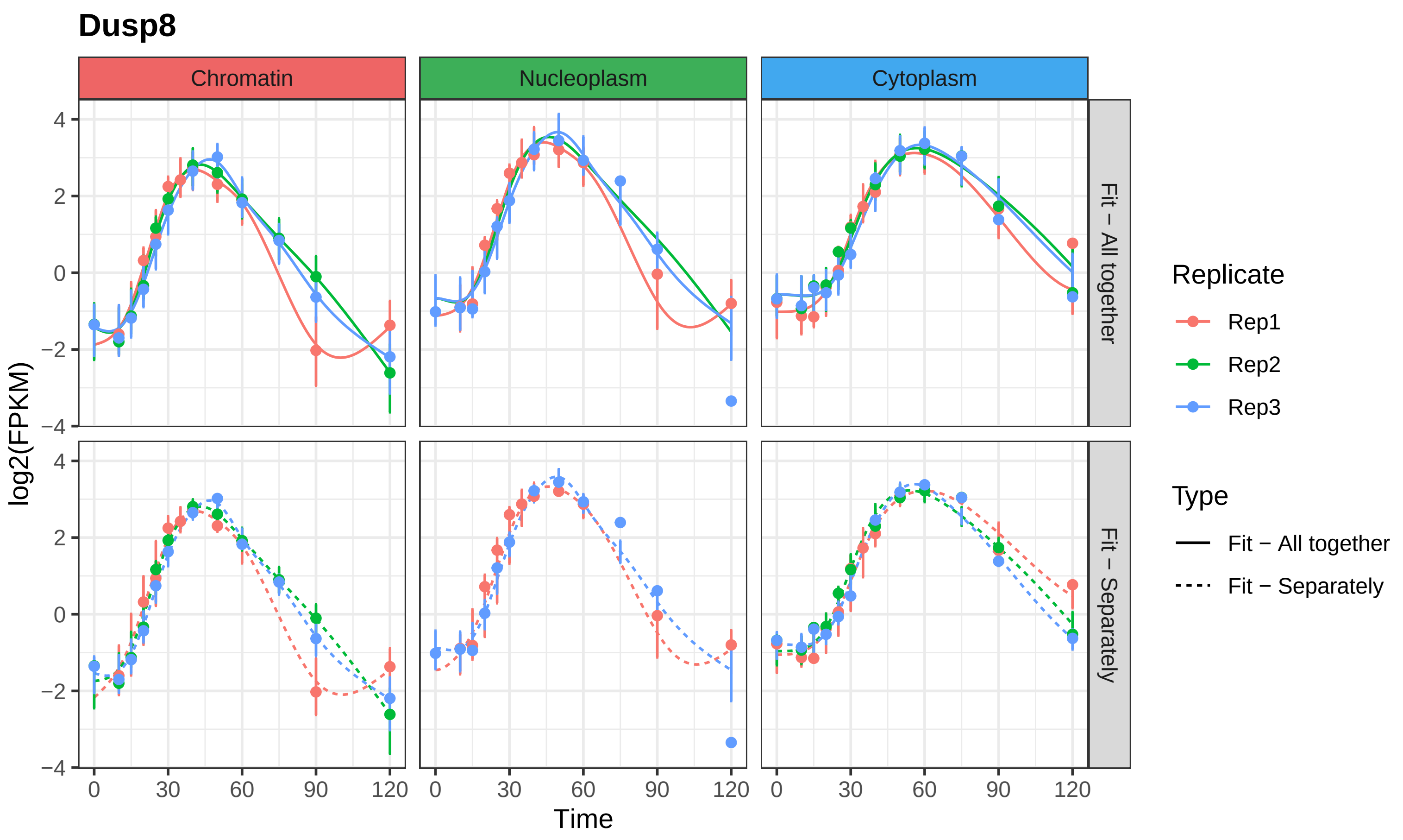

	Together	b1	b2	b3
**−NLL b1 ca**	−1.368	−2.108		
**−NLL b1 np**	2.146	−1.106		
**−NLL b1 cyto**	9.846	0.9142		
**−NLL b2 ca**	−2.163		−3.163	
**−NLL b2 np**				
**−NLL b2 cyto**	2.518		−1.195	
**−NLL b3 ca**	−2.537			−5.136
**−NLL b3 np**	8.996			4.087
**−NLL b3 cyto**	2.748			−4.238
Total	20.19	−2.3	−4.358	−5.286
**Total with regul**	22.25	−2.027	−5.316	−4.981

	Together	b1	b2	b3
spar	3.499e−01	3.928e−01	0.3762	0.33880
σ_b	1.660e−01	2.202e−06	0.07154	0.05381
σ_t	8.942e−05	2.140e+00	0.8666	0.52530
ca_{0,b_1}	−1.826e+00	−2.103e+00		
$\log_{10}(k_1')$	−2.599e−01	−4.240e−01		−0.19290
$\log_{10}(k_2)$	−4.891e−01	−6.410e−01	0.1059 or −1.099	−0.38930
$\log_{10}(k_2')$	−1.112e+00	−1.165e+00		−0.96380
$\log_{10}(k_{deg})$	−1.141e+00	−1.285e+00	−1.099 or 0.1059	−0.99370
$\log_{10}(k_1'k_2')$	−1.372e+00	−1.589e+00	−0.758	−1.15700
$\log_{10}(k_1'/k_2)$	2.293e−01	2.170e−01		0.19640
$\text{transport} = \log_{10}(k_1'k_2'/k_2)$	−8.831e−01	−9.480e−01	−0.8639 or 0.3405	−0.76730

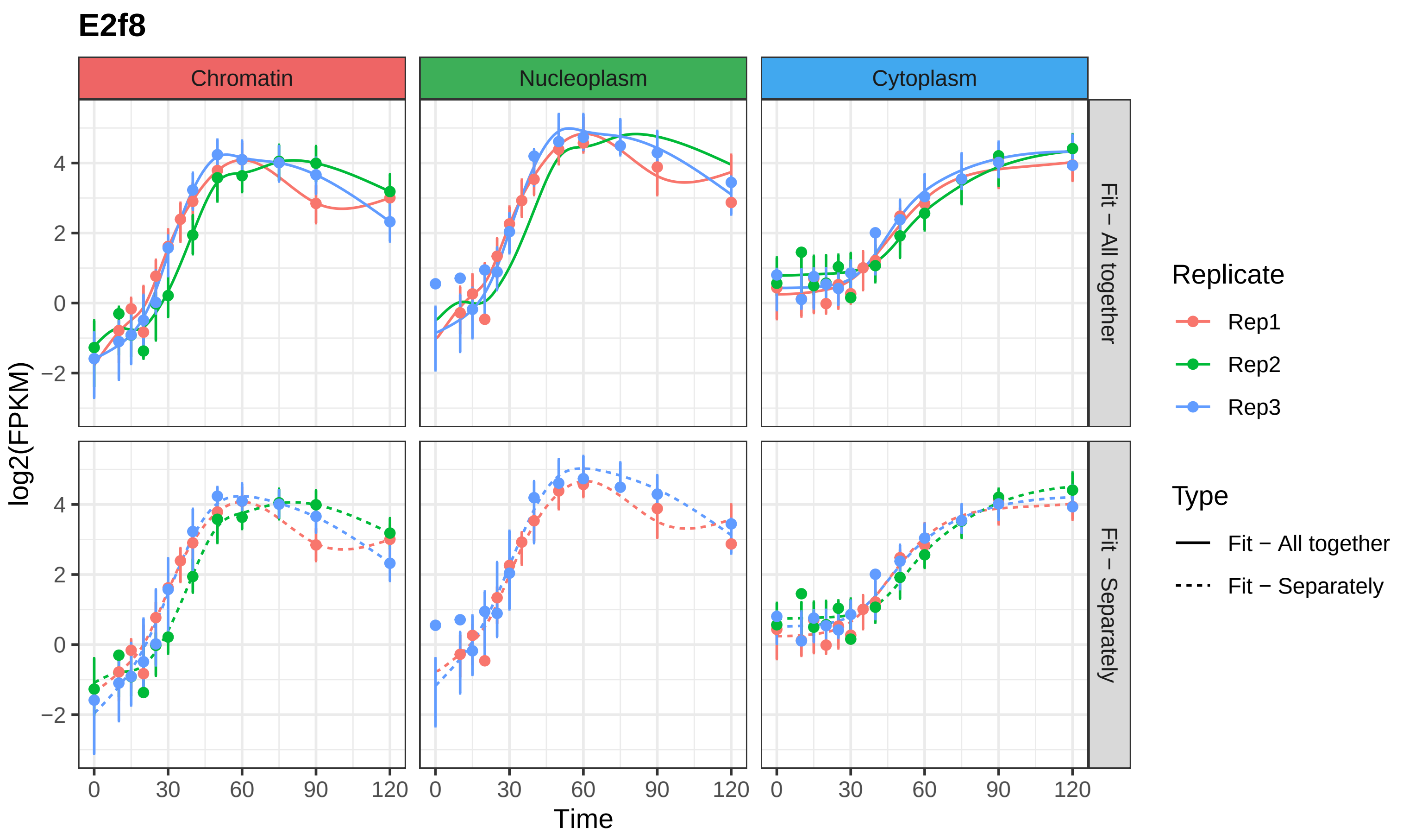

	Together	b1	b2	b3
**−NLL b1 ca**	−0.8283	−0.9787		
**−NLL b1 np**	6.809	6.177		
**−NLL b1 cyto**	0.3957	−0.2362		
**−NLL b2 ca**	−0.1471		0.8889	
**−NLL b2 np**				
**−NLL b2 cyto**	4.103		3.995	
**−NLL b3 ca**	−2.231			0.8224
**−NLL b3 np**	16.43			7.847
**−NLL b3 cyto**	2.376			1.177
Total	26.9	4.962	4.884	9.846
**Total with regul**	29.65	4.624	4.647	8.468

	Together	b1	b2	b3
spar	0.3236000	0.3937000	0.378	0.4462
σ_b	0.1730000	0.1463000	0.1445	0.1246
σ_t	0.0002631	0.0001398	6.409e−05	2.8290
ca_{0,b_1}	−1.7820000	−1.3530000		
$\log_{10}(k_1')$	0.5100000	0.2054000		7.9660
$\log_{10}(k_2)$	0.2841000	0.0259700	−0.4168 or −2.107	7.7280
$\log_{10}(k_2')$	−1.8790000	−1.7710000		−1.9780
$\log_{10}(k_{deg})$	−2.2630000	−2.0750000	−2.107 or −0.4168	−2.4870
$\log_{10}(k_1'k_2')$	−1.3690000	−1.5660000	−1.972	5.9890
$\log_{10}(k_1'/k_2)$	0.2259000	0.1795000		0.2379
$transport = \log_{10}(k_1'k_2'/k_2)$	−1.6530000	−1.5920000	−1.556 or 0.1348	−1.7400

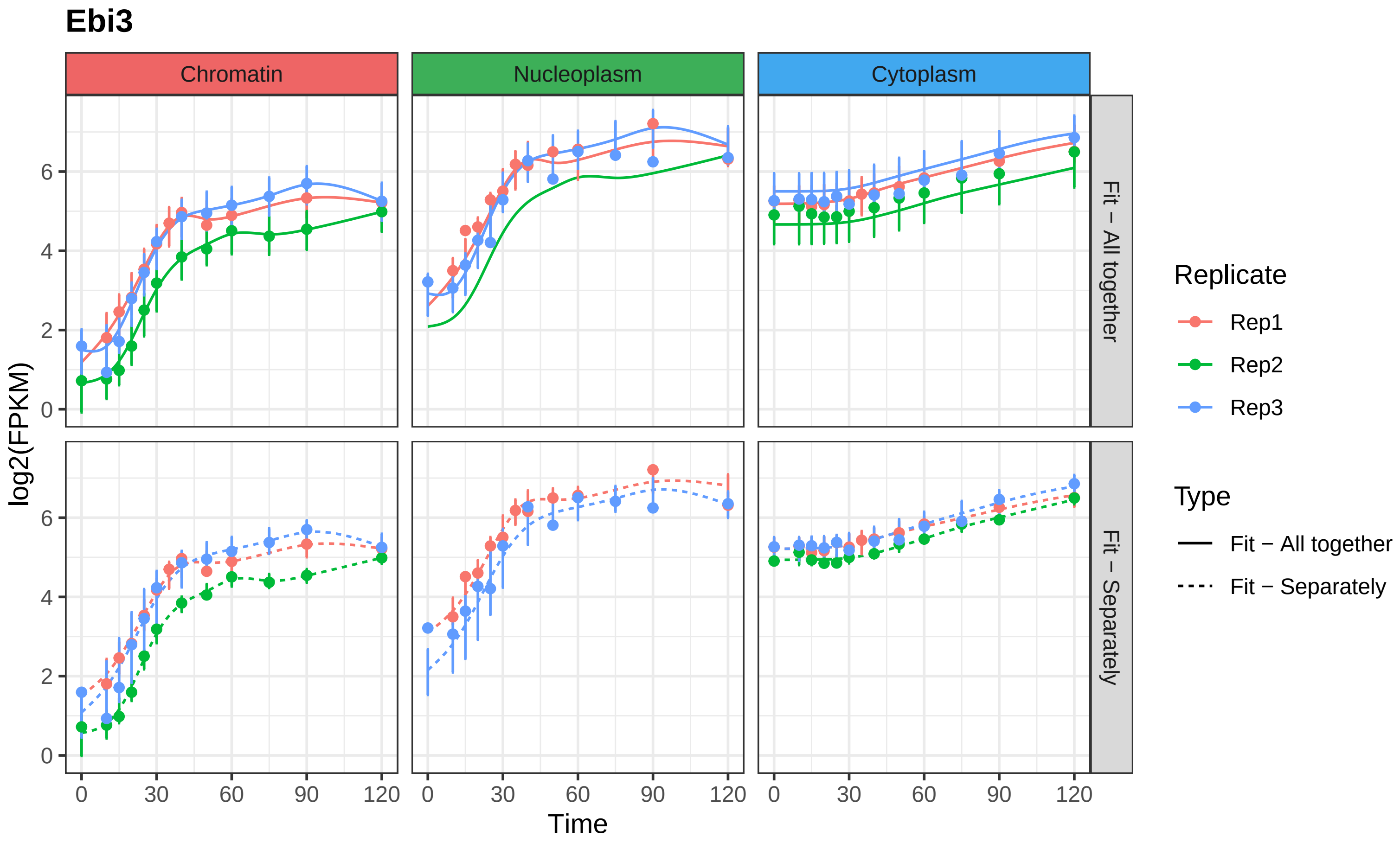

	Together	b1	b2	b3
**−NLL b1 ca**	−4.258	−5.96		
**−NLL b1 np**	4.484	3.573		
**−NLL b1 cyto**	−5.058	−10.76		
**−NLL b2 ca**	−3.622		−9.646	
**−NLL b2 np**				
**−NLL b2 cyto**	2.643		−12.52	
**−NLL b3 ca**	−1.722			0.04913
**−NLL b3 np**	9.574			6.244
**−NLL b3 cyto**	2.56			−8.221
Total	4.601	−13.15	−22.16	−1.928
**Total with regul**	6.365	−14.17	−22.65	−3.482

	Together	b1	b2	b3
spar	0.3842	0.4439	0.3561	0.4614
σ_b	0.1641	0.0990	0.03783	0.1027
σ_t	0.5216	0.9265	0.0006415	3.2480
ca_{0,b_1}	1.2100	1.5890		
$\log_{10}(k_1')$	5.4660	6.7620		5.1240
$\log_{10}(k_2)$	5.0380	6.2830	−0.6099 or −2.771	4.8020
$\log_{10}(k_2')$	−2.0350	−2.1520		−1.9740
$\log_{10}(k_{deg})$	−2.8110	−2.7880	−2.771 or −0.6099	−2.8960
$\log_{10}(k_1'k_2')$	3.4310	4.6100	−2.066	3.1500
$\log_{10}(k_1'/k_2)$	0.4284	0.4785		0.3216
$\text{transport} = \log_{10}(k_1'k_2'/k_2)$	−1.6070	−1.6730	−1.456 or 0.7057	−1.6530

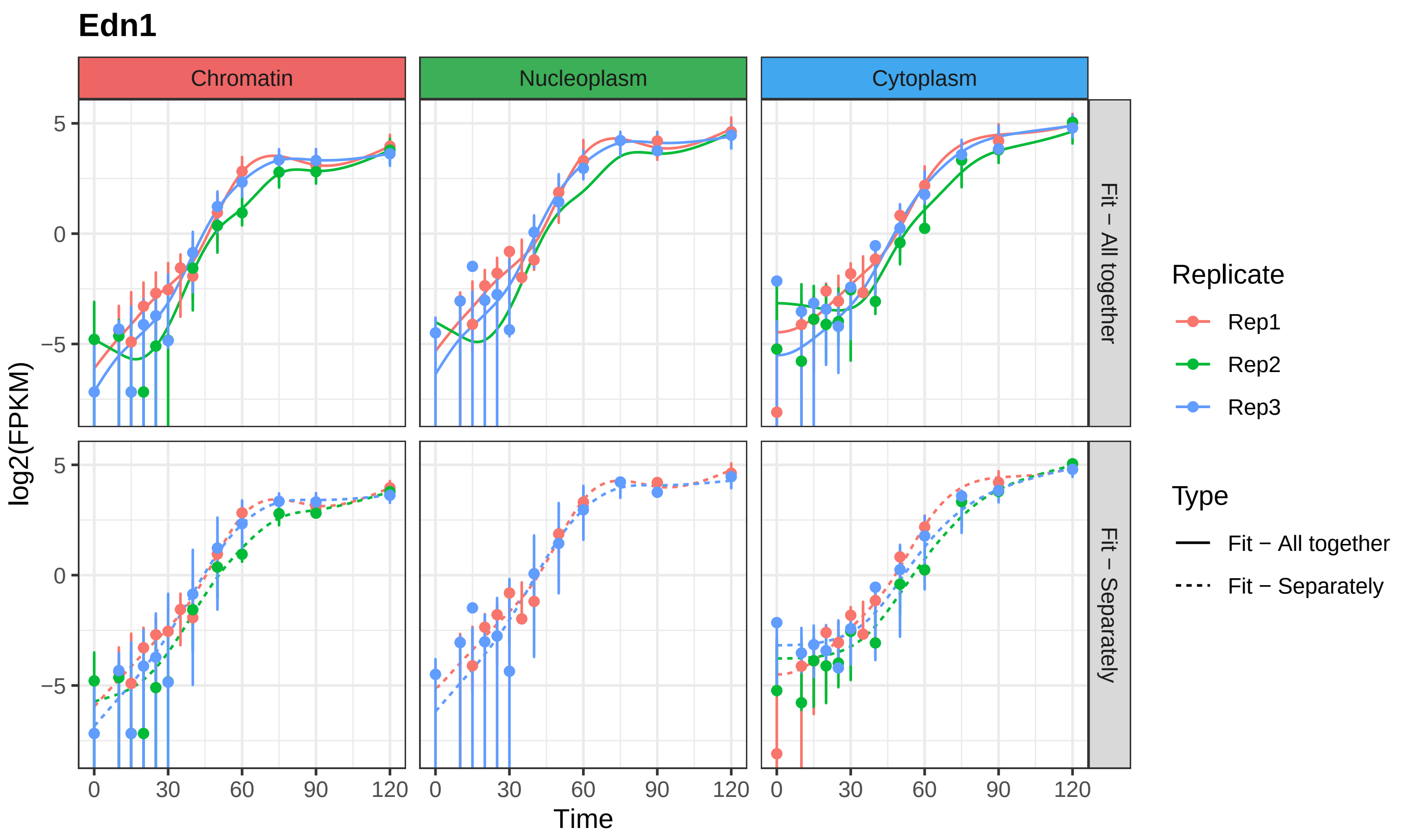

	Together	b1	b2	b3
**−NLL b1 ca**	5.372	3.944		
**−NLL b1 np**	8.308	8.657		
**−NLL b1 cyto**	10.35	10.03		
**−NLL b2 ca**	9.333		10.27	
**−NLL b2 np**				
**−NLL b2 cyto**	20.19		9.234	
**−NLL b3 ca**	9.468			10.06
**−NLL b3 np**	19.93			18.26
**−NLL b3 cyto**	31.67			10
Total	114.6	22.63	19.51	38.33
**Total with regul**	115.6	21.02	17.74	37.17

	Together	b1	b2	b3
spar	0.3663	0.45340	0.4987	0.47890
σ_b	0.1715	0.09328	9.425e−06	0.09631
σ_t	1.7870	1.14200	1.929	5.14100
ca_{0,b_1}	−6.1130	−5.96400		
$\log_{10}(k_1')$	6.9760	0.30360		4.05000
$\log_{10}(k_2)$	6.7390	0.04824	−1.109 or −1.166	3.84900
$\log_{10}(k_2')$	−1.1860	−1.13300		−1.51000
$\log_{10}(k_{deg})$	−1.4430	−1.31700	−1.166 or −1.109	−2.41300
$\log_{10}(k_1'k_2')$	5.7900	−0.82920	−1.688	2.53900
$\log_{10}(k_1'/k_2)$	0.2363	0.25540		0.20070
$\text{transport} = \log_{10}(k_1'k_2'/k_2)$	−0.9496	−0.87750	−0.5798 or −0.5228	−1.30900

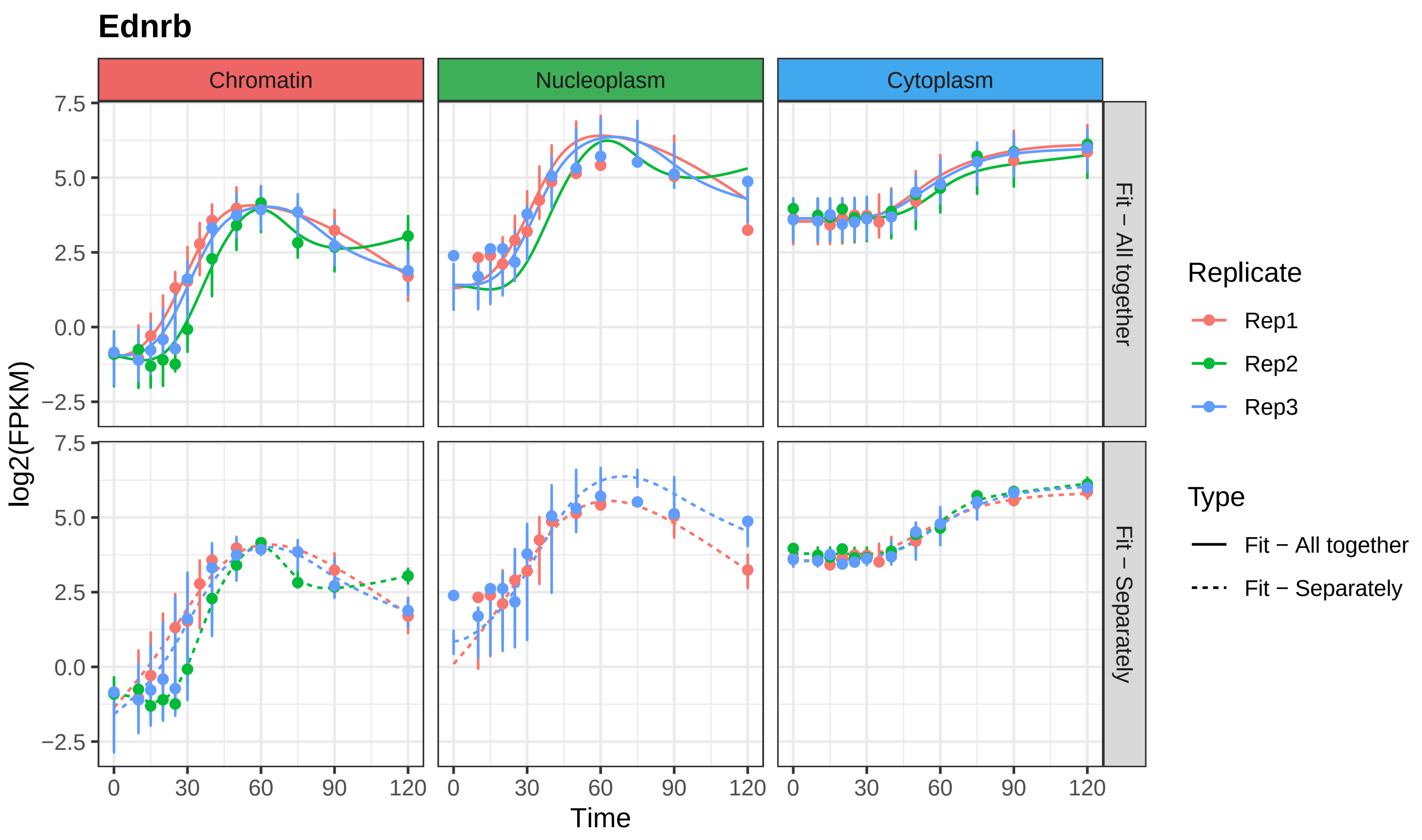

	Together	b1	b2	b3
**−NLL b1 ca**	2.459	4.247		
**−NLL b1 np**	17.75	6.073		
**−NLL b1 cyto**	1.092	−7.951		
**−NLL b2 ca**	3.502		−3.045	
**−NLL b2 np**				
**−NLL b2 cyto**	1.937		−6.81	
**−NLL b3 ca**	4.392			4.771
**−NLL b3 np**	17.28			11.66
**−NLL b3 cyto**	−0.3157			−7.413
Total	48.1	2.369	−9.855	9.02
**Total with regul**	49.02	4.285	−10.4	8.274

	Together	b1	b2	b3
spar	0.4339	0.58070	0.363	0.50800
σ_b	0.2416	0.05131	0.06864	0.06034
σ_t	1.4520	4.59800	1.211	5.98500
ca_{0,b_1}	−0.9502	−1.12700		
$\log_{10}(k_1')$	0.1835	9.53000		−0.08318
$\log_{10}(k_2)$	−0.5244	9.09000	−0.7731 or −2.391	−0.81760
$\log_{10}(k_2')$	−1.8530	−1.74000		−1.87400
$\log_{10}(k_{deg})$	−2.5230	−2.77600	−2.391 or −0.7731	−2.68800
$\log_{10}(k_1' k_2')$	−1.6700	7.79000	−1.759	−1.95800
$\log_{10}(k_1'/k_2)$	0.7079	0.44010		0.73450
$\text{transport} = \log_{10}(k_1' k_2'/k_2)$	−1.1450	−1.30000	−0.9859 or 0.6315	−1.14000

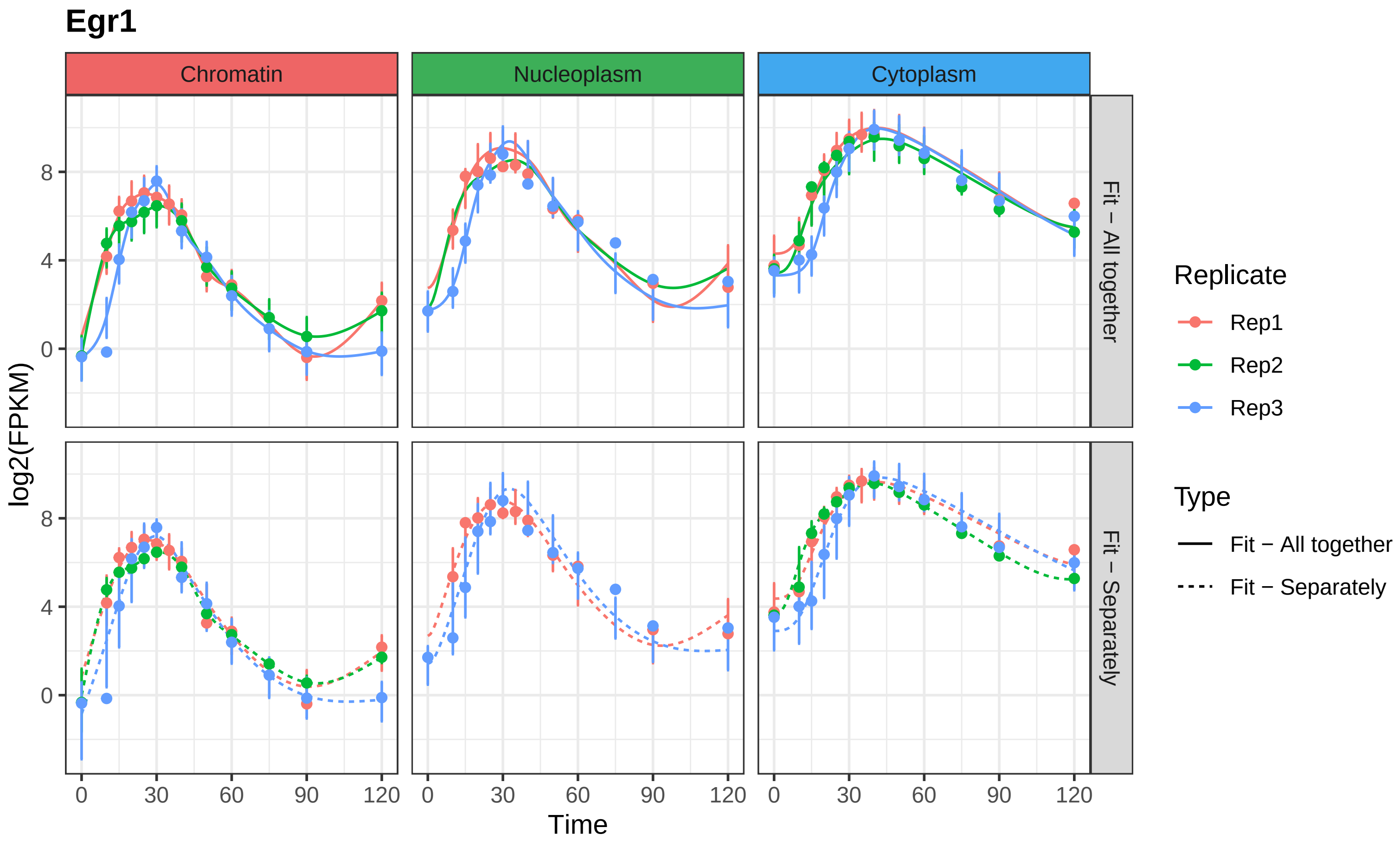

	Together	b1	b2	b3
**−NLL b1 ca**	1.888	5.335		
**−NLL b1 np**	12.13	9.733		
**−NLL b1 cyto**	9.273	7.236		
**−NLL b2 ca**	1.65		−11.1	
**−NLL b2 np**				
**−NLL b2 cyto**	7.458		−9.763	
**−NLL b3 ca**	6.922			8.162
**−NLL b3 np**	17.4			15.3
**−NLL b3 cyto**	5.943			6.98
Total	62.66	22.3	−20.86	30.44
**Total with regul**	75.19	25.02	−11.94	32.54

	Together	b1	b2	b3
spar	0.2434000	0.4503	0.2133	0.3960
σ_b	0.2953000	0.2520	3.26e−06	0.2742
σ_t	0.0003952	1.2600	1.114	1.9230
ca_{0,b_1}	0.5888000	0.6234		
$\log_{10}(k_1')$	0.0570400	0.1701		0.1390
$\log_{10}(k_2)$	−0.5930000	−0.3824	0.6092 or −1.22	−0.5475
$\log_{10}(k_2')$	−0.7940000	−0.8478		−0.8784
$\log_{10}(k_{deg})$	−1.2620000	−1.3560	−1.22 or 0.6092	−1.3240
$\log_{10}(k_1'k_2')$	−0.7370000	−0.6777	0.4965	−0.7394
$\log_{10}(k_1'/k_2)$	0.6500000	0.5525		0.6866
transport = $\log_{10}(k_1'k_2'/k_2)$	−0.1440000	−0.2954	−0.1127 or 1.717	−0.1919

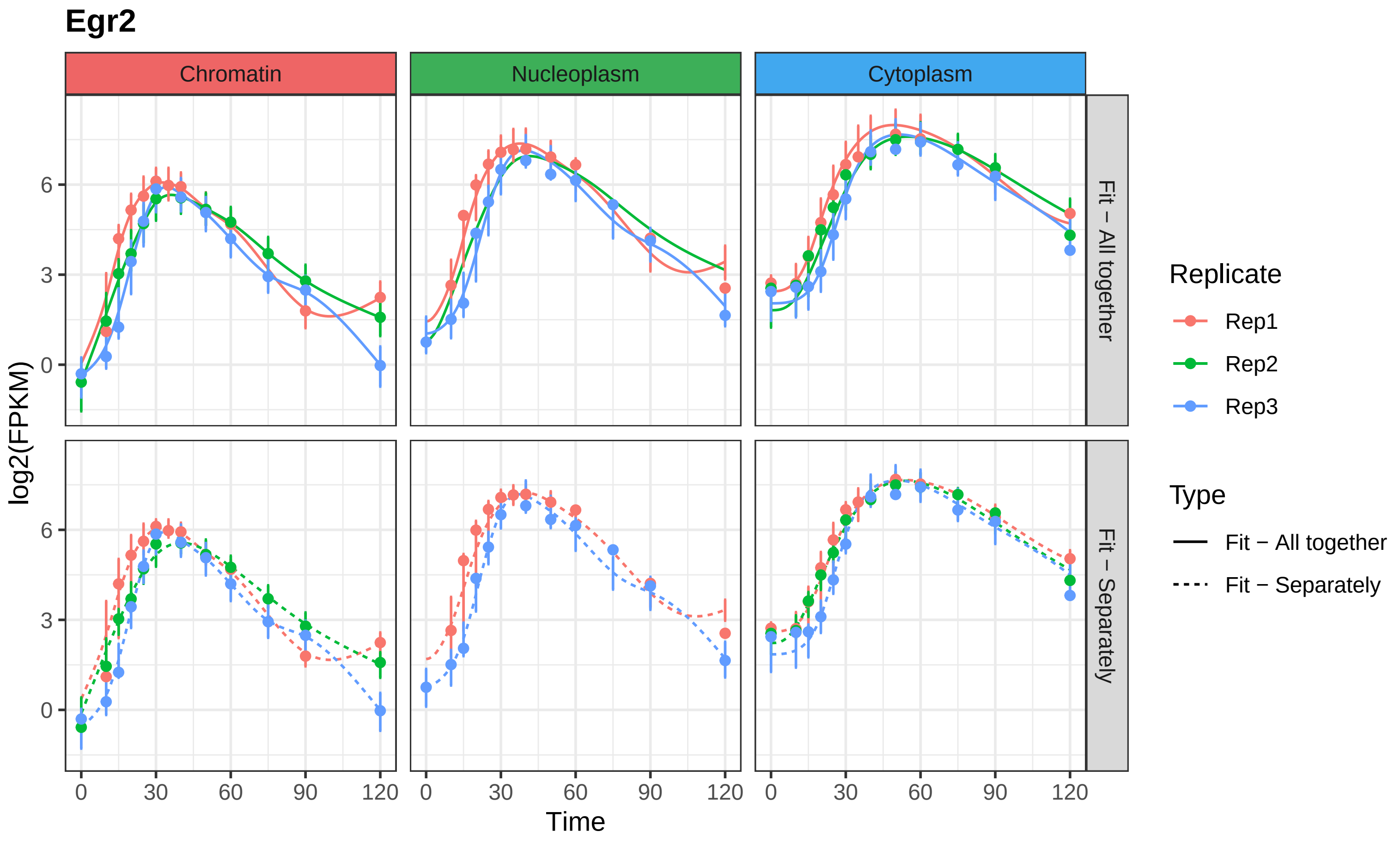

	Together	b1	b2	b3
**−NLL b1 ca**	0.8441	−1.588		
**−NLL b1 np**	6.123	8.536		
**−NLL b1 cyto**	4.562	−3.372		
**−NLL b2 ca**	−2.132		−1.699	
**−NLL b2 np**				
**−NLL b2 cyto**	9.335		−1.287	
**−NLL b3 ca**	−0.5293			−1.705
**−NLL b3 np**	3.986			1.281
**−NLL b3 cyto**	3.636			6.663
Total	25.82	3.576	−2.986	6.239
**Total with regul**	29.09	4.533	−4.32	10.72

	Together	b1	b2	b3
spar	0.3189	0.3589	0.479	0.2906000
σ_b	0.1860	0.1002	0.1273	0.1851000
σ_t	1.0610	2.2130	0.5124	0.0005507
ca_{0,b_1}	0.0949	0.4333		
$\log_{10}(k_1')$	−0.2684	−0.4206		−0.1123000
$\log_{10}(k_2)$	−0.6889	−0.8197	−0.5155 or −1.055	−0.5191000
$\log_{10}(k_2')$	−0.8485	−0.9922		−0.8851000
$\log_{10}(k_{deg})$	−1.1510	−1.2670	−1.055 or −0.5155	−1.2040000
$\log_{10}(k_1'k_2')$	−1.1170	−1.4130	−0.8631	−0.9975000
$\log_{10}(k_1'/k_2)$	0.4205	0.3991		0.4068000
transport $= \log_{10}(k_1'k_2'/k_2)$	−0.4280	−0.5931	−0.3476 or 0.1915	−0.4784000

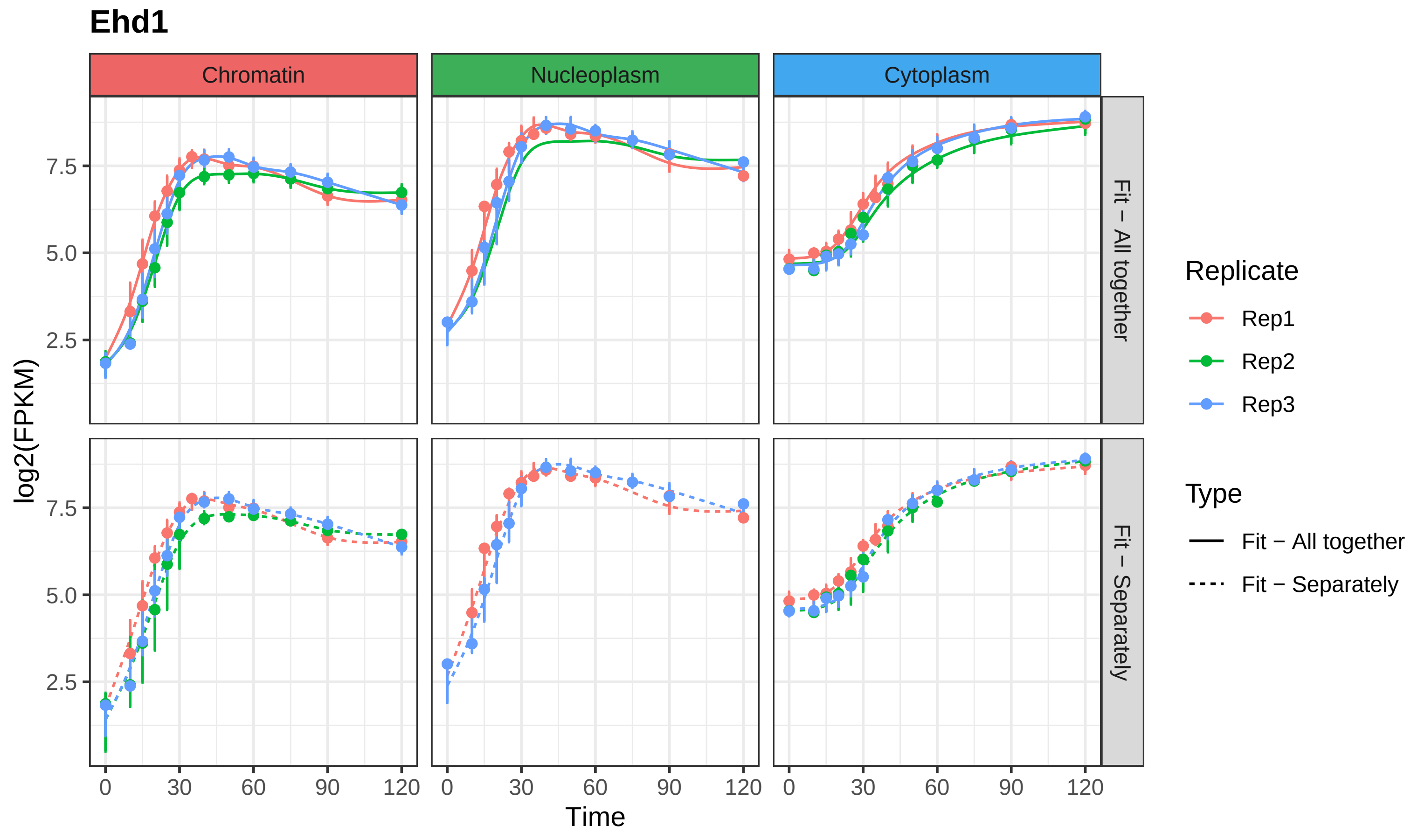

	Together	b1	b2	b3
**−NLL b1 ca**	−8.508	−8.127		
**−NLL b1 np**	0.001211	0.2793		
**−NLL b1 cyto**	−5.545	−10.03		
**−NLL b2 ca**	−8.688		−6.46	
**−NLL b2 np**				
**−NLL b2 cyto**	−0.1395		−5.676	
**−NLL b3 ca**	−7.442			−5.26
**−NLL b3 np**	−1.975			−0.758
**−NLL b3 cyto**	−7.214			−7.998
Total	−39.51	−17.88	−12.14	−14.02
**Total with regul**	−36.47	−17.31	−13.68	−14.71

	Together	b1	b2	b3
spar	0.31560	0.39660	0.396	0.3675
σ_b	0.08166	0.07148	0.02175	0.0678
σ_t	1.25700	1.21900	2.858	1.3380
ca_{0,b_1}	2.00900	1.81800		
$\log_{10}(k_1')$	7.62800	5.27600		4.8170
$\log_{10}(k_2)$	7.34500	5.00700	4.905 or −2.279	4.5270
$\log_{10}(k_2')$	−1.67500	−1.72500		−1.7090
$\log_{10}(k_{deg})$	−2.25400	−2.38700	−2.279 or 4.905	−2.3720
$\log_{10}(k_1' k_2')$	5.95300	3.55100	3.569	3.1080
$\log_{10}(k_1'/k_2)$	0.28290	0.26890		0.2901
transport $= \log_{10}(k_1' k_2'/k_2)$	−1.39200	−1.45600	−1.336 or 5.848	−1.4190

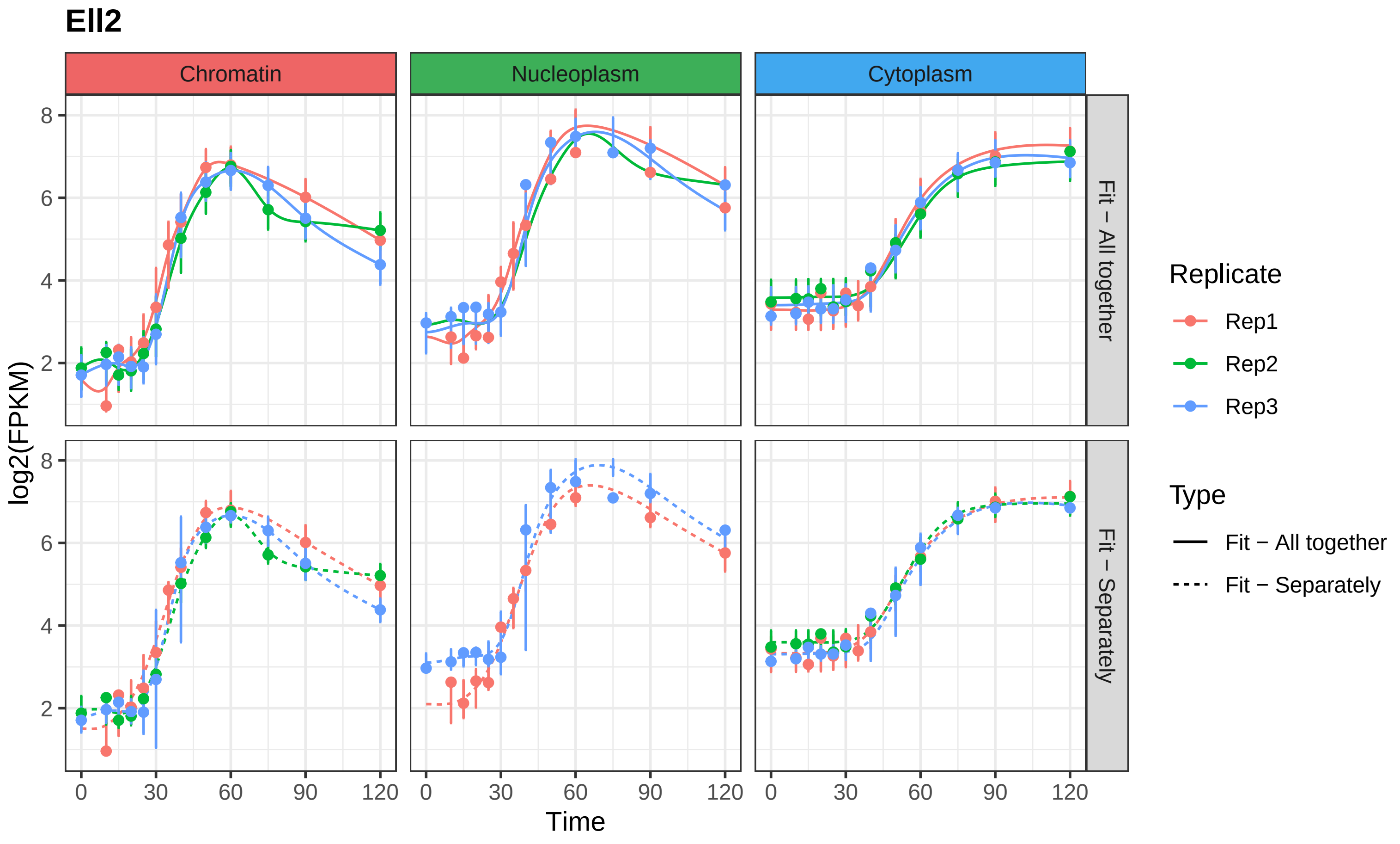

	Together	b1	b2	b3
**−NLL b1 ca**	−0.6077	2.221		
**−NLL b1 np**	12.69	1.334		
**−NLL b1 cyto**	−1.405	−3.51		
**−NLL b2 ca**	−4.163		−5.131	
**−NLL b2 np**				
**−NLL b2 cyto**	−2.515		−3.402	
**−NLL b3 ca**	−4.168			−4.043
**−NLL b3 np**	8.923			−1.829
**−NLL b3 cyto**	−1.882			−2.995
Total	6.869	0.04549	−8.533	−8.867
**Total with regul**	14.16	0.8845	−8.277	−7.048

	Together	b1	b2	b3
spar	0.2732	0.4220	0.3476	0.31310
σ_b	0.1537	0.1436	0.09744	0.05329
σ_t	1.5500	0.6543	0.37	3.52000
ca_{0,b_1}	1.6180	1.5750		
$\log_{10}(k_1')$	−0.5592	−0.5418		−0.54970
$\log_{10}(k_2)$	−0.8711	−0.7193	−0.8002 or −1.771	−0.96090
$\log_{10}(k_2')$	−1.6690	−1.6770		−1.76300
$\log_{10}(k_{deg})$	−1.8670	−2.0470	−1.771 or −0.8002	−1.82400
$\log_{10}(k_1'k_2')$	−2.2290	−2.2190	−2.076	−2.31300
$\log_{10}(k_1'/k_2)$	0.3119	0.1775		0.41130
transport $= \log_{10}(k_1'k_2'/k_2)$	−1.3580	−1.5000	−1.275 or −0.3046	−1.35200

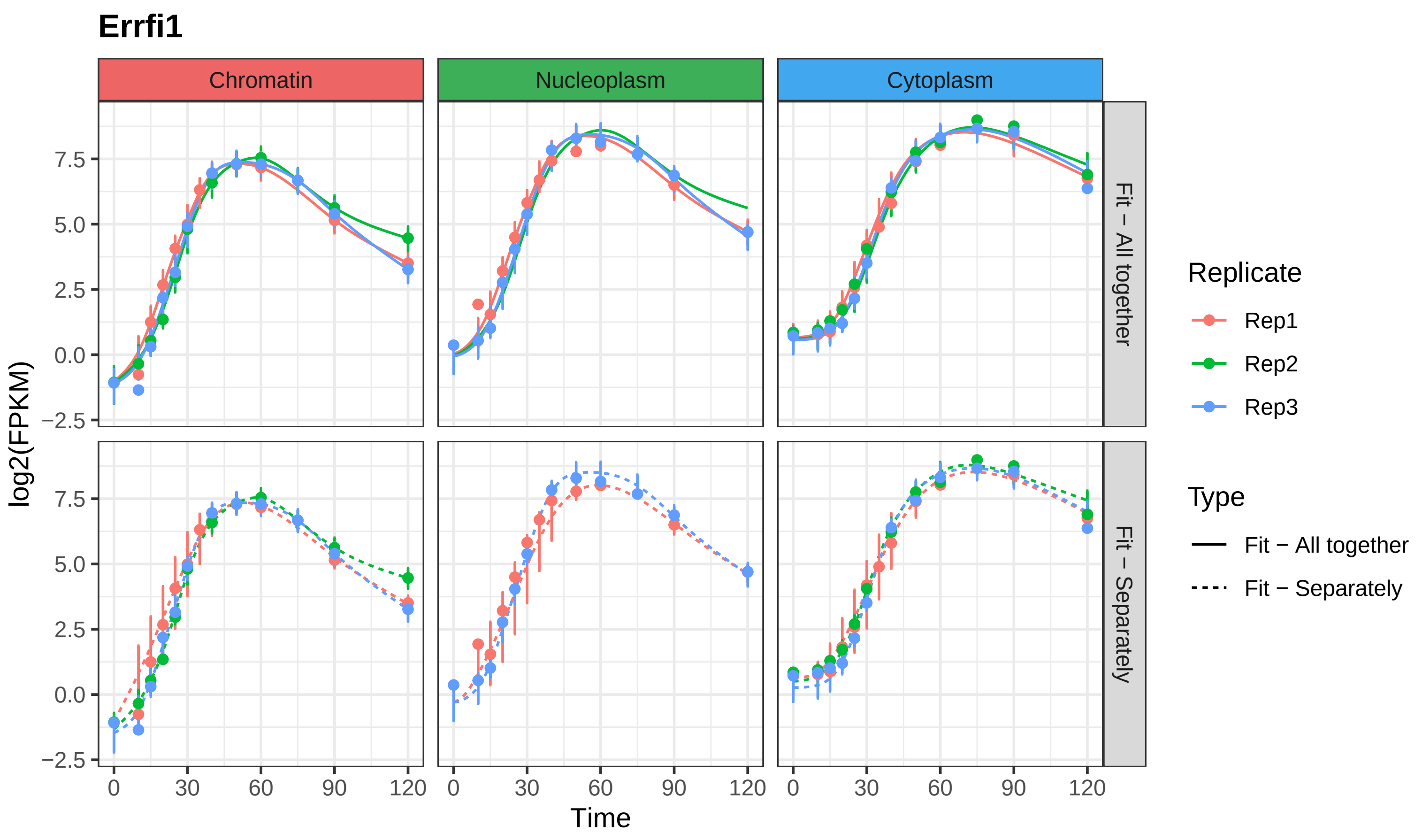

	Together	b1	b2	b3
**−NLL b1 ca**	−0.2804	1.892		
**−NLL b1 np**	9.483	3.217		
**−NLL b1 cyto**	3.751	1.98		
**−NLL b2 ca**	−2.774		−4.577	
**−NLL b2 np**				
**−NLL b2 cyto**	4.206		2.05	
**−NLL b3 ca**	1.688			−1.076
**−NLL b3 np**	−0.5259			−2.408
**−NLL b3 cyto**	0.6441			5.013
Total	16.19	7.089	−2.527	1.529
**Total with regul**	18.58	5.472	0.7185	6.658

	Together	b1	b2	b3
spar	0.32410	4.892e−01	0.2972	2.725e−01
σ_b	0.16070	2.560e−06	0.1338	1.513e−01
σ_t	0.82420	2.827e+00	0.0001909	3.707e−05
ca_{0,b_1}	−0.98380	−9.172e−01		
$\log_{10}(k_1')$	−0.08861	−5.597e−01		−4.115e−02
$\log_{10}(k_2)$	−0.41360	−7.905e−01	1.384 or −1.368	−3.946e−01
$\log_{10}(k_2')$	−1.12400	−9.931e−01		−1.144e+00
$\log_{10}(k_{deg})$	−1.30900	−1.269e+00	−1.368 or 1.384	−1.314e+00
$\log_{10}(k_1'k_2')$	−1.21300	−1.553e+00	0.5707	−1.185e+00
$\log_{10}(k_1'/k_2)$	0.32500	2.308e−01		3.534e−01
transport $= \log_{10}(k_1'k_2'/k_2)$	−0.79900	−7.623e−01	−0.8136 or 1.939	−7.904e−01

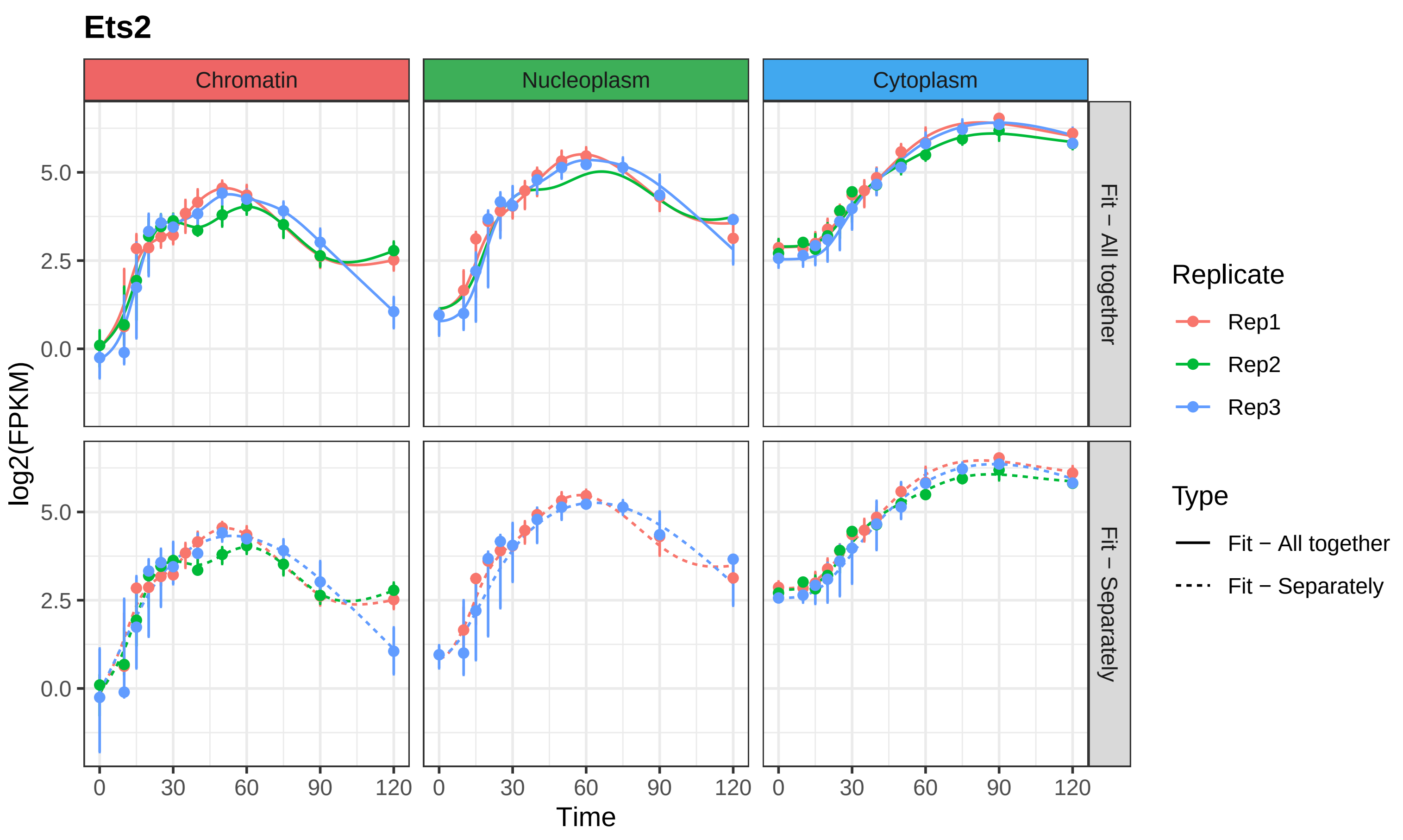

	Together	b1	b2	b3
**−NLL b1 ca**	−5.981	−5.633		
**−NLL b1 np**	0.4047	−0.3862		
**−NLL b1 cyto**	−7.262	−9.199		
**−NLL b2 ca**	−7.555		−6.472	
**−NLL b2 np**				
**−NLL b2 cyto**	−6.144		−5.202	
**−NLL b3 ca**	−5.092			2.167
**−NLL b3 np**	8.536			3.857
**−NLL b3 cyto**	−4.708			−8.235
Total	−27.8	−15.22	−11.67	−2.212
**Total with regul**	−24.59	−14.84	−11.09	−3.144

	Together	b1	b2	b3
spar	0.29350	0.35160	0.3343	5.206e−01
σ_b	0.06771	0.05324	0.05528	2.978e−06
σ_t	2.31000	1.92700	1.85	4.409e+00
ca_{0,b_1}	0.07624	−0.13390		
$\log_{10}(k_1')$	−0.58600	−0.48450		−6.824e−01
$\log_{10}(k_2)$	−0.91590	−0.79290	−0.6869 or −1.759	−9.934e−01
$\log_{10}(k_2')$	−1.14800	−1.15400		−1.091e+00
$\log_{10}(k_{deg})$	−1.67500	−1.75200	−1.759 or −0.6869	−1.585e+00
$\log_{10}(k_1'k_2')$	−1.73400	−1.63900	−1.566	−1.774e+00
$\log_{10}(k_1'/k_2)$	0.32990	0.30840		3.109e−01
$\text{transport} = \log_{10}(k_1'k_2'/k_2)$	−0.81800	−0.84610	−0.8791 or 0.1932	−7.803e−01

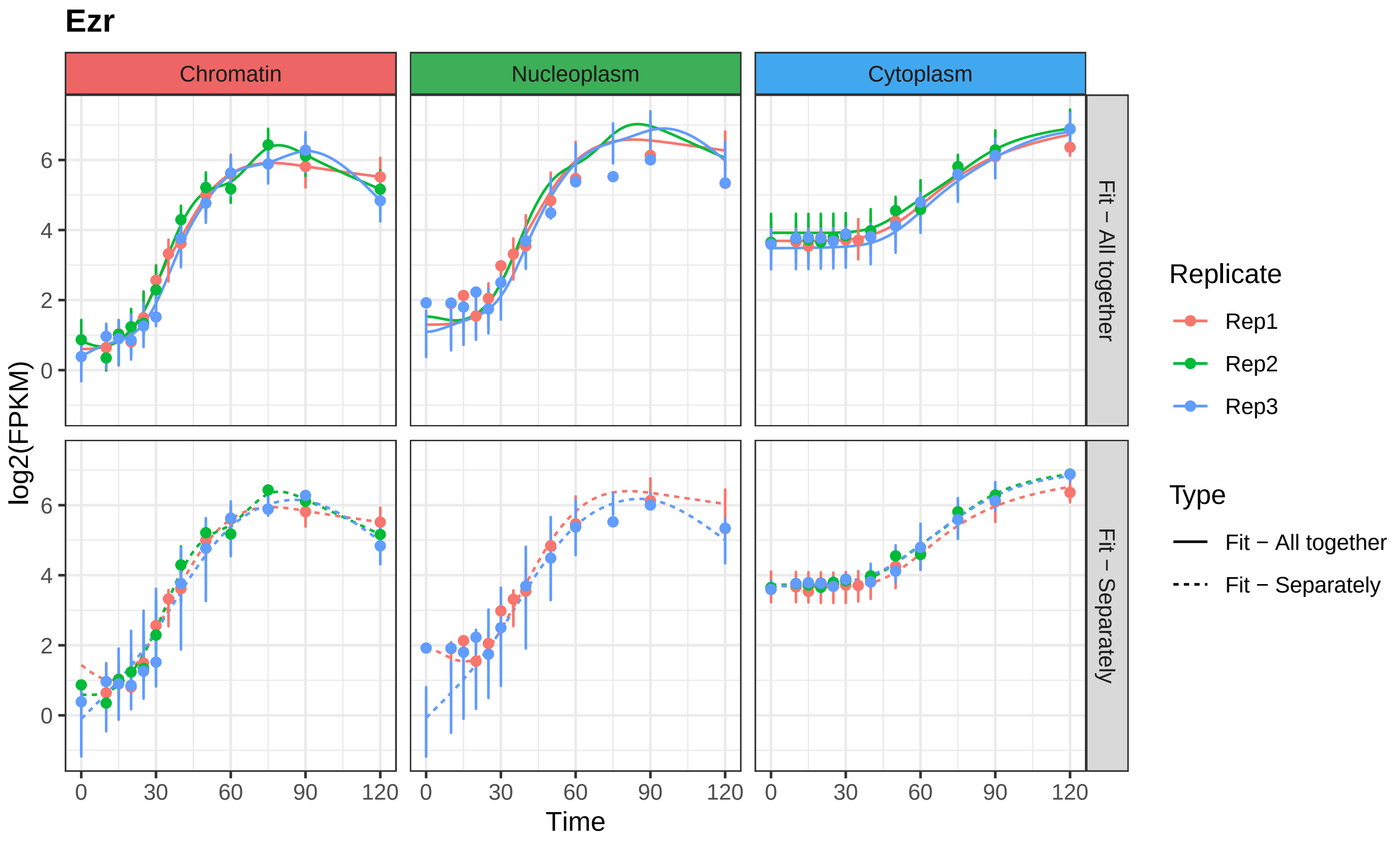

	Together	b1	b2	b3
**−NLL b1 ca**	−1.759	−1.133		
**−NLL b1 np**	11.41	8.114		
**−NLL b1 cyto**	−2.532	−6.055		
**−NLL b2 ca**	−1.362		−1.426	
**−NLL b2 np**				
**−NLL b2 cyto**	−1.237		−13.34	
**−NLL b3 ca**	−1.74			4.978
**−NLL b3 np**	20.57			9.602
**−NLL b3 cyto**	−0.9689			−9.043
Total	22.39	0.9258	−14.77	5.537
**Total with regul**	23.71	1.735	−16.37	6.356

	Together	b1	b2	b3
spar	0.3633000	0.4663	0.3965	0.558800
σ_b	0.1956000	0.1464	2.007e−05	0.043370
σ_t	0.0008315	0.8726	2.735	5.810000
ca_{0,b_1}	0.6107000	1.5530		
$\log_{10}(k_1')$	−0.5612000	−0.4870		7.808000
$\log_{10}(k_2)$	−0.7711000	−0.6302	−0.5629 or −2.493	7.800000
$\log_{10}(k_2')$	−1.7440000	−1.7100		−1.568000
$\log_{10}(k_{deg})$	−2.4640000	−2.2430	−2.493 or −0.5629	−2.702000
$\log_{10}(k_1'k_2')$	−2.3050000	−2.1970	−2.111	6.240000
$\log_{10}(k_1'/k_2)$	0.2099000	0.1432		0.008843
$\text{transport} = \log_{10}(k_1'k_2'/k_2)$	−1.5340000	−1.5670	−1.548 or 0.3817	−1.559000

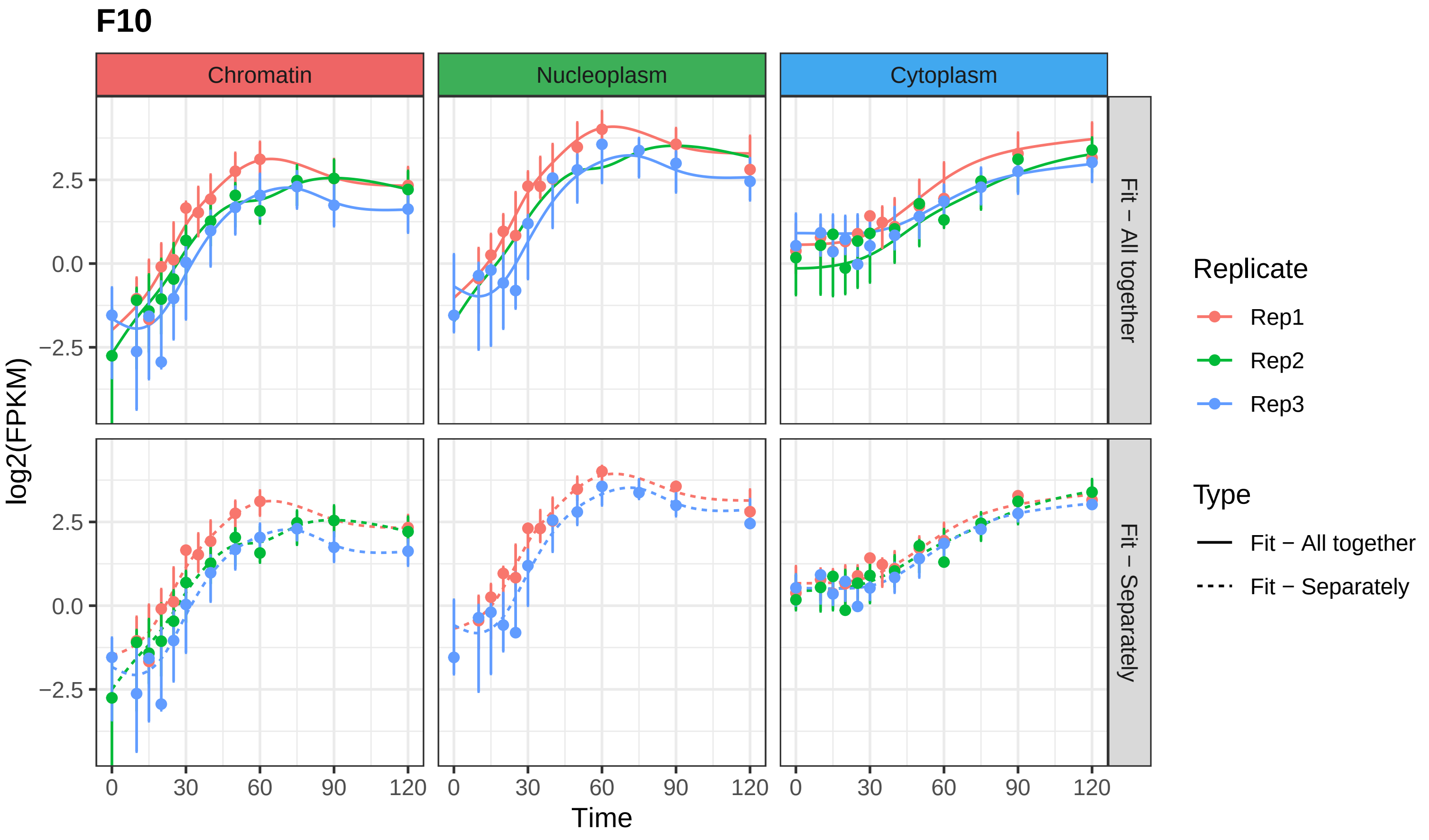

	Together	b1	b2	b3
−NLL b1 ca	2.269	0.443		
−NLL b1 np	3.255	0.7132		
−NLL b1 cyto	4.088	0.3733		
−NLL b2 ca	2.78		1.491	
−NLL b2 np				
−NLL b2 cyto	11.17		3.908	
−NLL b3 ca	4.298			3.407
−NLL b3 np	8.665			5.869
−NLL b3 cyto	4.262			−2.337
Total	40.79	1.53	5.399	6.939
Total with regul	41.06	−0.1535	4.013	5.106

	Together	b1	b2	b3
spar	0.4261	0.42610	0.4193	4.039e−01
σ_b	0.1702	0.06754	0.122	2.329e−05
σ_t	1.1570	1.56900	0.009424	1.735e+00
ca_{0,b_1}	−1.9600	−1.43200		
$\log_{10}(k_1')$	5.7470	0.47250		5.384e+00
$\log_{10}(k_2)$	5.4560	0.22740	3.784 or −2.452	5.008e+00
$\log_{10}(k_2')$	−1.8700	−1.96100		−1.871e+00
$\log_{10}(k_{deg})$	−2.3490	−2.36700	−2.452 or 3.784	−2.204e+00
$\log_{10}(k_1'k_2')$	3.8760	−1.48800	2.218	3.513e+00
$\log_{10}(k_1'/k_2)$	0.2901	0.24510		3.762e−01
$\text{transport} = \log_{10}(k_1'k_2'/k_2)$	−1.5800	−1.71600	−1.566 or 4.671	−1.495e+00

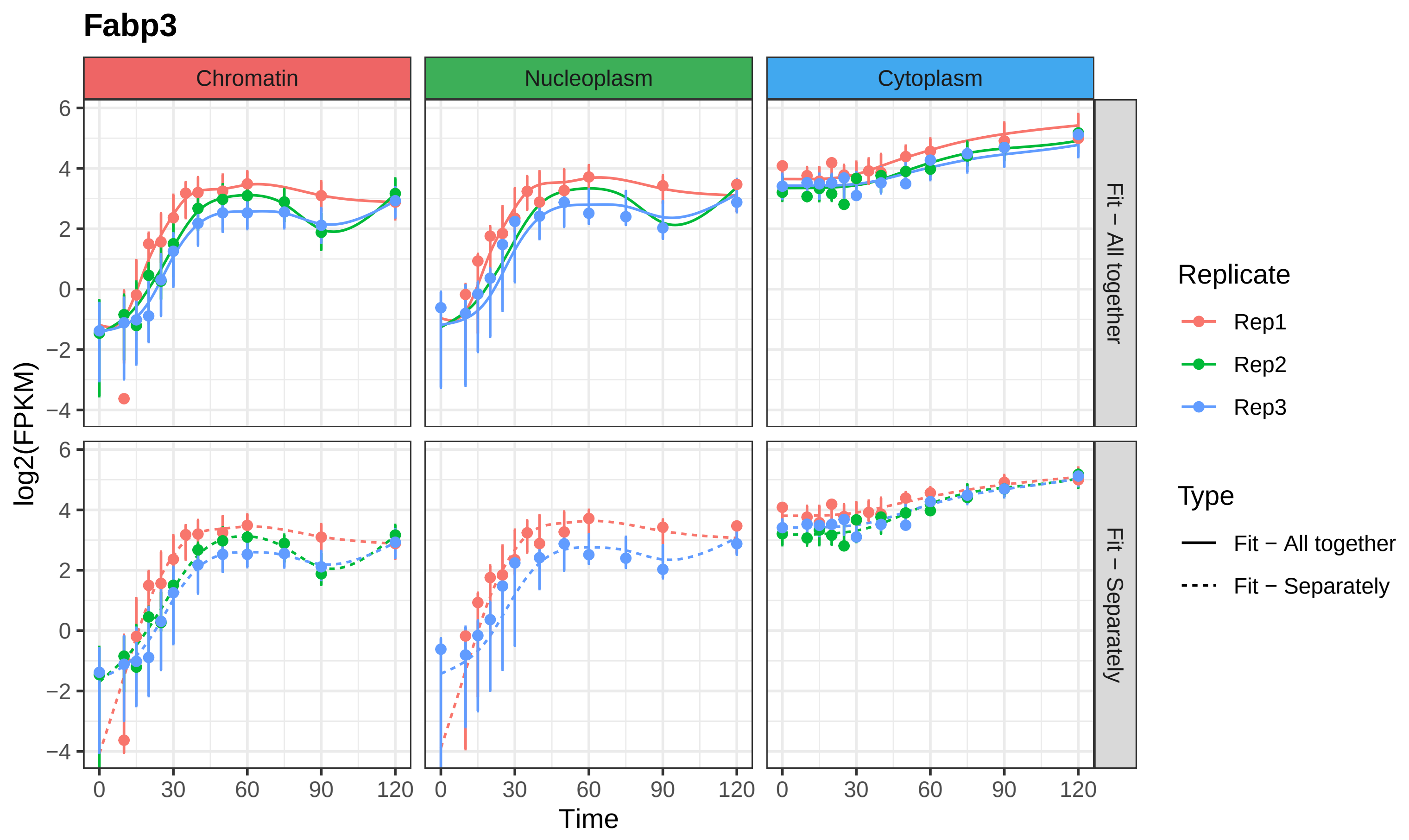

	Together	b1	b2	b3
**−NLL b1 ca**	4.709	2.178		
**−NLL b1 np**	4.686	6.354		
**−NLL b1 cyto**	1.416	−3.025		
**−NLL b2 ca**	1.091		0.4637	
**−NLL b2 np**				
**−NLL b2 cyto**	−0.2787		−1.536	
**−NLL b3 ca**	0.07687			1.446
**−NLL b3 np**	8.125			5.286
**−NLL b3 cyto**	−0.8494			−2.593
Total	18.98	5.506	−1.072	4.139
**Total with regul**	37.55	4.146	−2.804	2.654

	Together	b1	b2	b3
spar	0.36240	0.42820	0.4282	0.41420
σ_b	0.13030	0.10260	0.09906	0.07256
σ_t	1.93300	2.33000	0.0002901	3.95800
ca_{0,b_1}	−1.05300	−4.04700		
$\log_{10}(k_1')$	7.10600	4.82000		5.46600
$\log_{10}(k_2)$	7.03900	4.76400	4.876 or −2.799	5.41900
$\log_{10}(k_2')$	−1.46800	−1.68800		−1.32600
$\log_{10}(k_{deg})$	−2.85500	−4.00400	−2.799 or 4.876	−2.78200
$\log_{10}(k_1'k_2')$	5.63800	3.13300	3.544	4.14000
$\log_{10}(k_1'/k_2)$	0.06692	0.05642		0.04761
$\text{transport} = \log_{10}(k_1'k_2'/k_2)$	−1.40100	−1.63100	−1.332 or 6.343	−1.27800

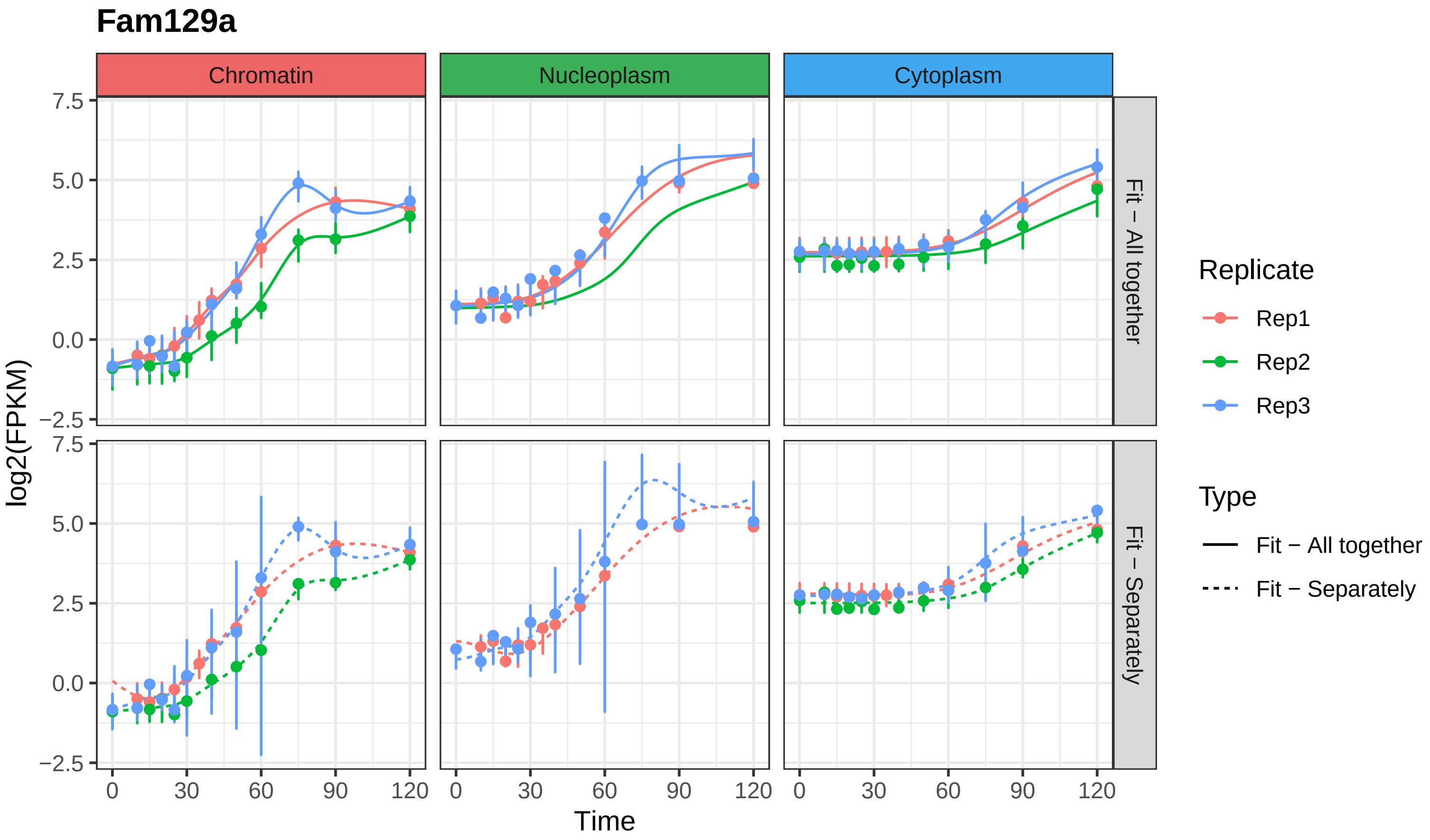

	Together	b1	b2	b3
**−NLL b1 ca**	−3.885	−6.209		
**−NLL b1 np**	4.666	4.23		
**−NLL b1 cyto**	−3.91	−7.103		
**−NLL b2 ca**	−3.022		−4.001	
**−NLL b2 np**				
**−NLL b2 cyto**	−0.6915		−3.477	
**−NLL b3 ca**	0.188			5.927
**−NLL b3 np**	14.88			10.67
**−NLL b3 cyto**	−4.304			−10.4
Total	3.923	−9.082	−7.479	6.19
**Total with regul**	4.076	−9.565	−8.865	6.773

	Together	b1	b2	b3
spar	0.3837	4.433e−01	0.4018	0.36430
σ_b	0.1589	1.154e−01	0.09661	0.02162
σ_t	1.0180	5.992e−05	0.0002801	8.39200
ca_{0,b_1}	−0.7814	1.059e−01		
$\log_{10}(k_1')$	−1.0060	−6.405e−01		−0.06529
$\log_{10}(k_2)$	−1.5750	−1.014e+00	−1.234 or −2.203	−0.54020
$\log_{10}(k_2')$	−1.7360	−1.816e+00		−2.00200
$\log_{10}(k_{deg})$	−2.2240	−2.266e+00	−2.203 or −1.234	−2.60300
$\log_{10}(k_1'k_2')$	−2.7420	−2.457e+00	−2.413	−2.06700
$\log_{10}(k_1'/k_2)$	0.5686	3.736e−01		0.47490
transport = $\log_{10}(k_1'k_2'/k_2)$	−1.1670	−1.443e+00	−1.18 or −0.2103	−1.52700

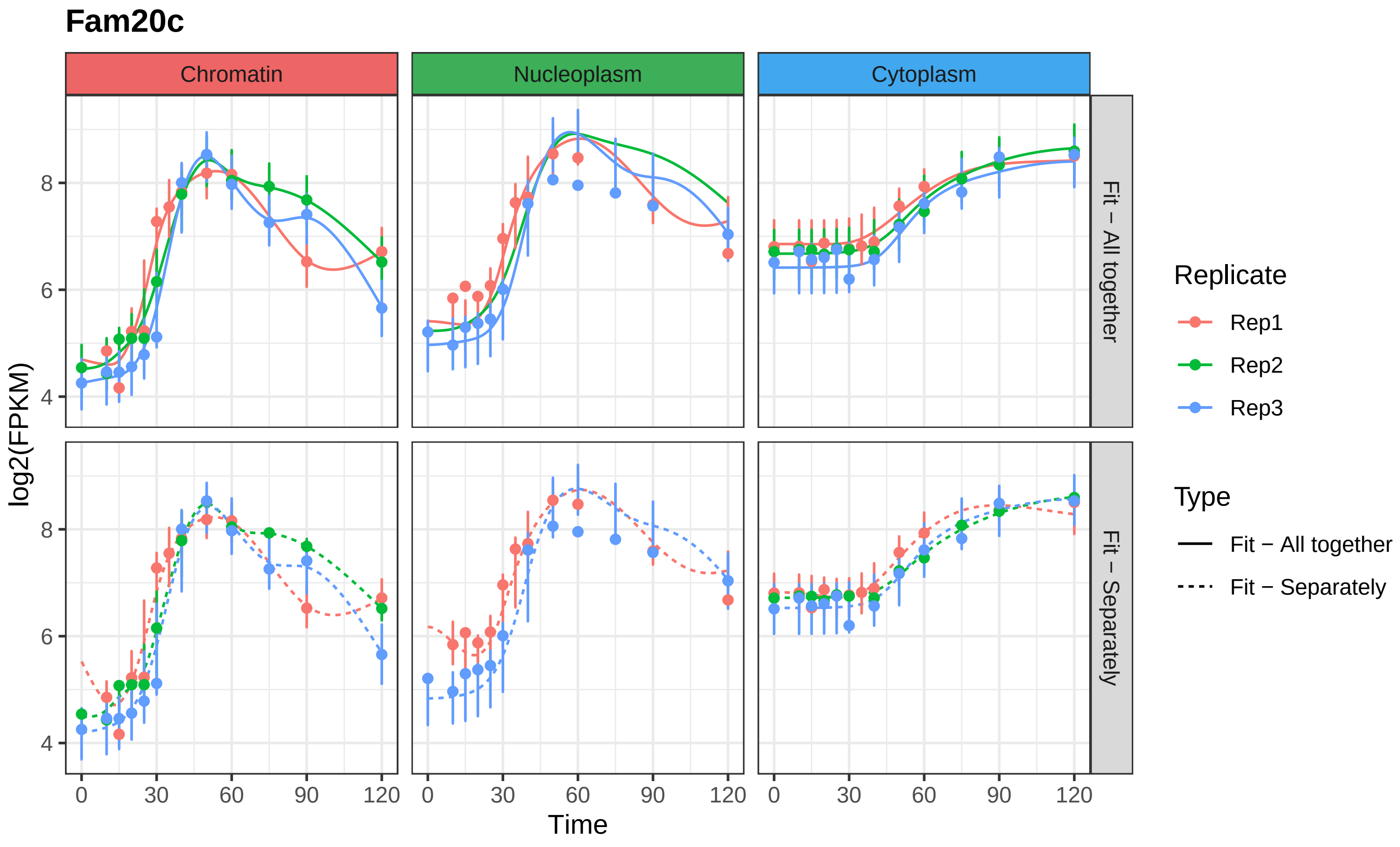

	Together	b1	b2	b3
**−NLL b1 ca**	0.4136	0.3729		
**−NLL b1 np**	9.885	3.641		
**−NLL b1 cyto**	−4.15	−6.844		
**−NLL b2 ca**	−3.338		−6.632	
**−NLL b2 np**				
**−NLL b2 cyto**	−5.391		−17.31	
**−NLL b3 ca**	−3.328			−0.5083
**−NLL b3 np**	12.55			8.054
**−NLL b3 cyto**	−2.303			−3.427
Total	4.342	−2.83	−23.94	4.118
**Total with regul**	5.388	−2.034	−20.9	3.566

	Together	b1	b2	b3
spar	0.3551	0.3878	0.2882	0.4060
σ_b	0.1601	0.1244	0.02109	0.1614
σ_t	1.3420	1.9830	2.275	1.9600
ca_{0,b_1}	4.7040	5.6000		
$\log_{10}(k_1')$	−0.7056	−0.7866		−0.8129
$\log_{10}(k_2)$	−0.9203	−0.9821	−1.135 or −2.333	−1.0060
$\log_{10}(k_2')$	−1.8590	−1.6000		−1.7720
$\log_{10}(k_{deg})$	−2.2950	−1.7940	−2.333 or −1.135	−2.2820
$\log_{10}(k_1' k_2')$	−2.5650	−2.3860	−2.797	−2.5850
$\log_{10}(k_1'/k_2)$	0.2147	0.1955		0.1928
$transport = \log_{10}(k_1' k_2'/k_2)$	−1.6450	−1.4040	−1.662 or −0.4641	−1.5800

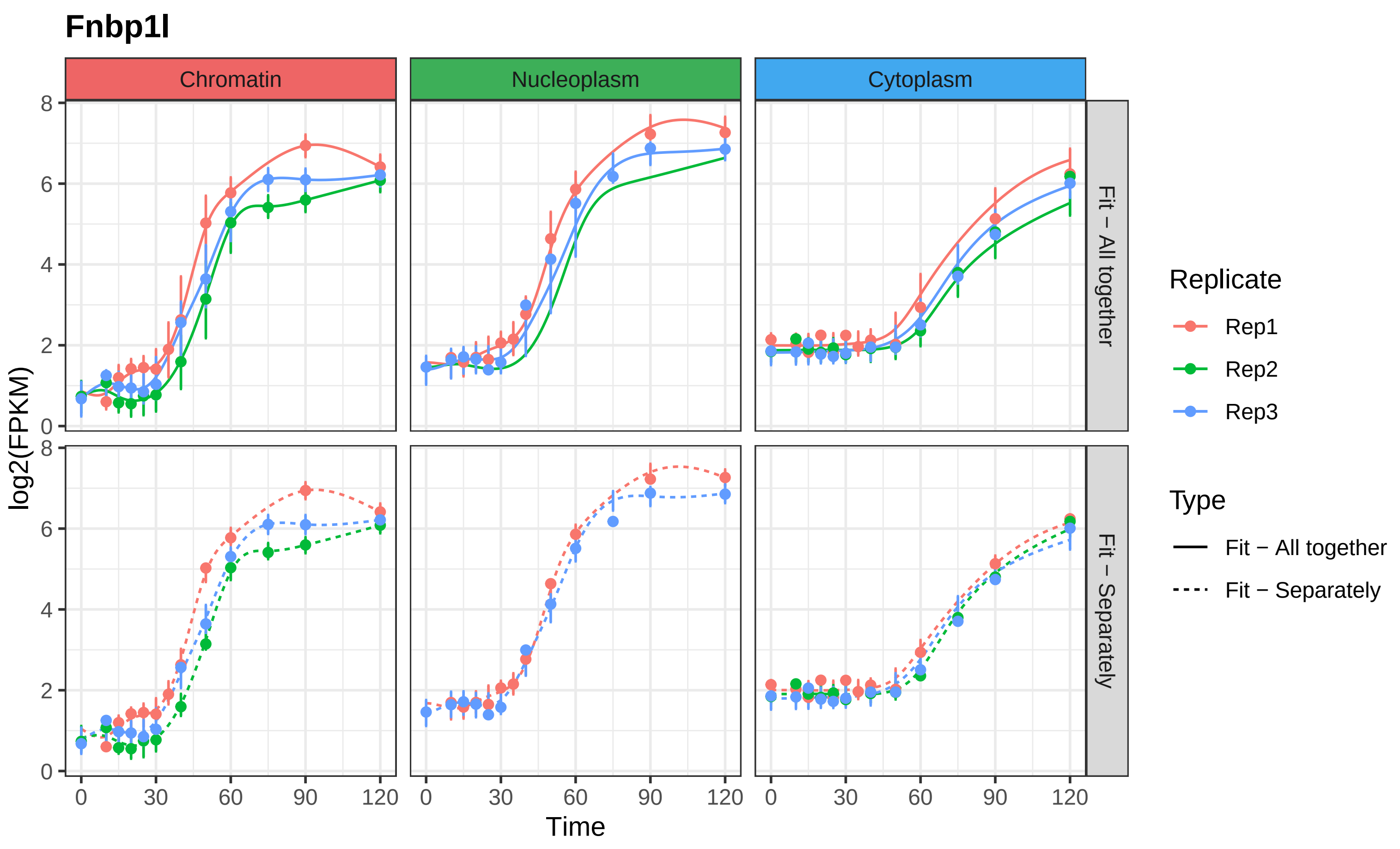

	Together	b1	b2	b3
**−NLL b1 ca**	−4.943	−8.654		
**−NLL b1 np**	−4.556	−8.415		
**−NLL b1 cyto**	1.123	−4.396		
**−NLL b2 ca**	−6.322		−8.105	
**−NLL b2 np**				
**−NLL b2 cyto**	3.779		−6.806	
**−NLL b3 ca**	−6.494			−6.711
**−NLL b3 np**	−0.6734			−6.399
**−NLL b3 cyto**	−5.611			−2.874
Total	−23.7	−21.47	−14.91	−15.98
**Total with regul**	−20.57	−18.57	−14.29	−15.3

	Together	b1	b2	b3
spar	0.31590	0.32640	0.3335	0.3347
σ_b	0.09532	0.07251	0.06713	0.0813
σ_t	2.34000	0.11730	0.0277	0.7807
ca_{0,b_1}	0.87310	1.05700		
$\log_{10}(k_1')$	−0.78530	−0.69750		−0.3288
$\log_{10}(k_2)$	−0.99930	−0.88810	−1.474 or −1.525	−0.5340
$\log_{10}(k_2')$	−1.88700	−2.04400		−2.0170
$\log_{10}(k_{deg})$	−2.01400	−2.14200	−1.525 or −1.474	−2.1210
$\log_{10}(k_1'k_2')$	−2.67200	−2.74200	−2.671	−2.3460
$\log_{10}(k_1'/k_2)$	0.21400	0.19060		0.2053
$\text{transport} = \log_{10}(k_1'k_2'/k_2)$	−1.67300	−1.85400	−1.197 or −1.146	−1.8120

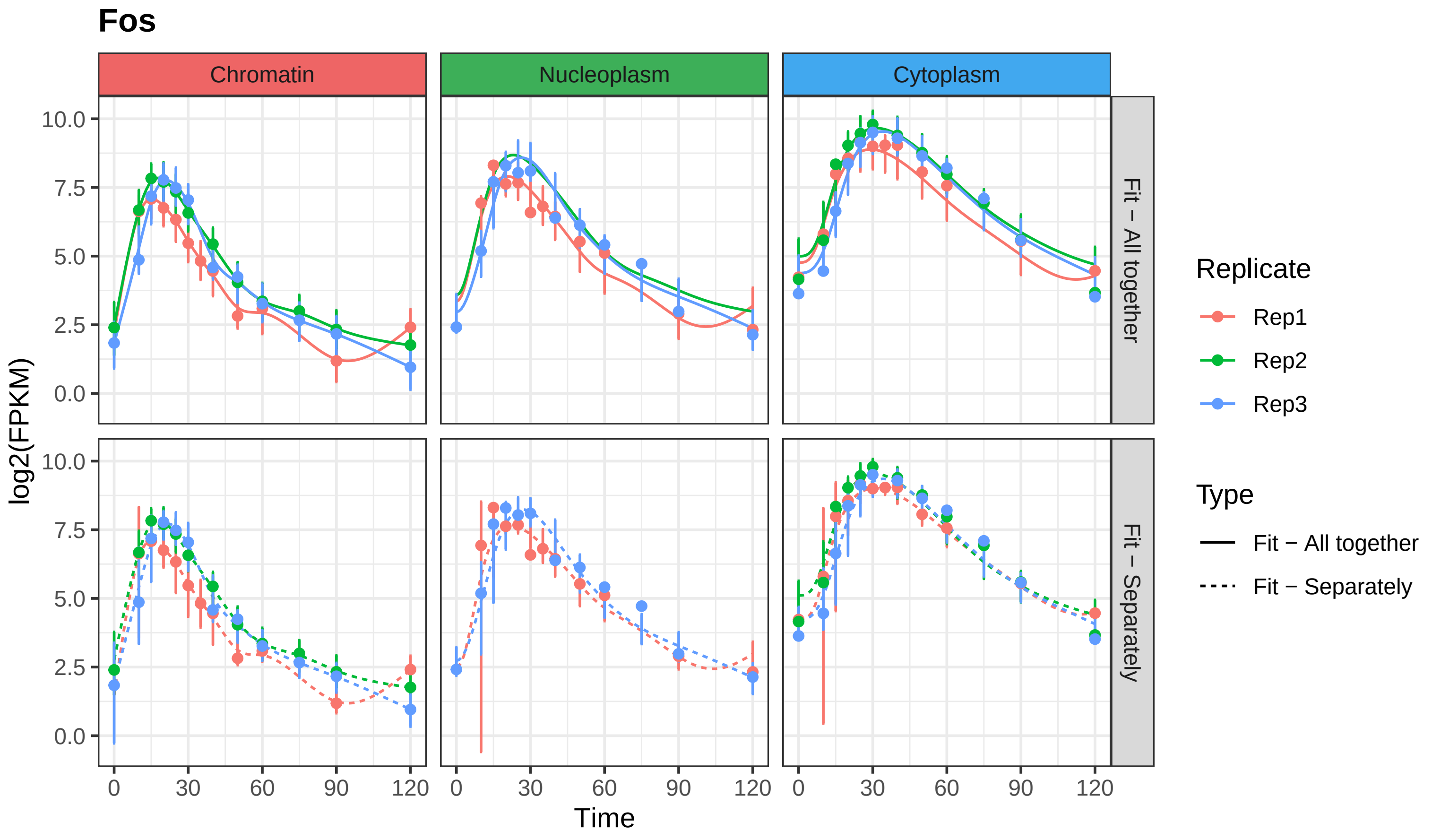

	Together	b1	b2	b3
**−NLL b1 ca**	−0.414	−1.754		
**−NLL b1 np**	10.33	9.011		
**−NLL b1 cyto**	5.157	−4.498		
**−NLL b2 ca**	−0.7205		−1.984	
**−NLL b2 np**				
**−NLL b2 cyto**	8.202		9.42	
**−NLL b3 ca**	0.5079			0.8416
**−NLL b3 np**	10.71			5.806
**−NLL b3 cyto**	6.739			7.053
Total	40.51	2.759	7.436	13.7
**Total with regul**	47.94	11.38	12.08	15.71

	Together	b1	b2	b3
spar	0.2850	2.839e−01	0.2907	0.3296
σ_b	0.2328	2.168e−05	0.1932	0.1633
σ_t	0.6638	3.551e+00	1.038	2.1140
ca_{0,b_1}	2.1530	1.407e+00		
$\log_{10}(k_1')$	−0.4355	−6.258e−01		−0.5685
$\log_{10}(k_2)$	−0.7781	−9.367e−01	−0.8429 or −0.8974	−0.8262
$\log_{10}(k_2')$	−0.5972	−4.661e−01		−0.5196
$\log_{10}(k_{deg})$	−1.0230	−9.898e−01	−0.8974 or −0.8429	−0.9719
$\log_{10}(k_1'k_2')$	−1.0330	−1.092e+00	−1.031	−1.0880
$\log_{10}(k_1'/k_2)$	0.3426	3.109e−01		0.2577
$\text{transport} = \log_{10}(k_1'k_2'/k_2)$	−0.2545	−1.552e−01	−0.1886 or −0.1341	−0.2619

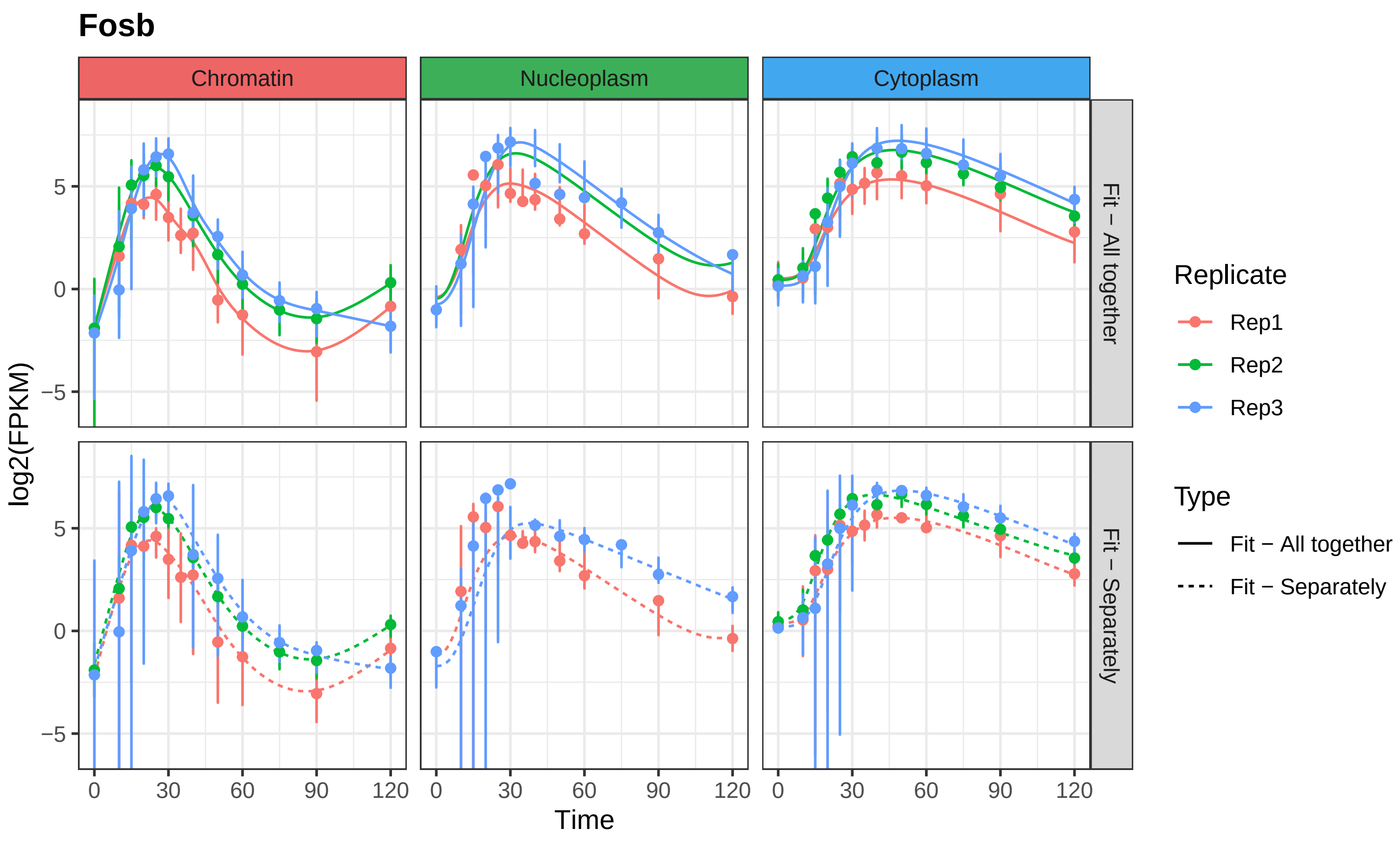

	Together	b1	b2	b3
**−NLL b1 ca**	6.85	11.2		
**−NLL b1 np**	18.78	16.02		
**−NLL b1 cyto**	9.384	5.195		
**−NLL b2 ca**	6.727		−0.9536	
**−NLL b2 np**				
**−NLL b2 cyto**	7.636		1.902	
**−NLL b3 ca**	8.079			12.04
**−NLL b3 np**	21.64			21.08
**−NLL b3 cyto**	6.25			2.266
Total	85.34	32.41	0.9481	35.38
**Total with regul**	90.62	33.05	1.951	34.02

	Together	b1	b2	b3
spar	0.3255	0.4057000	0.3365	4.126e−01
σ_b	0.2779	0.0002168	0.1201	6.075e−06
σ_t	2.3760	5.7060000	1.038	5.820e+00
ca_{0,b_1}	−1.8220	−2.3540000		
$\log_{10}(k_1')$	−0.7318	−0.9109000		−1.395e+00
$\log_{10}(k_2)$	−1.1620	−1.2290000	−0.4113 or −1.511	−1.439e+00
$\log_{10}(k_2')$	−1.0680	−0.8762000		−5.089e−01
$\log_{10}(k_{deg})$	−1.3410	−1.3450000	−1.511 or −0.4113	−1.086e+00
$\log_{10}(k_1'k_2')$	−1.8000	−1.7870000	−1.276	−1.904e+00
$\log_{10}(k_1'/k_2)$	0.4301	0.3178000		4.388e−02
transport $= \log_{10}(k_1'k_2'/k_2)$	−0.6382	−0.5584000	−0.8643 or 0.2358	−4.650e−01

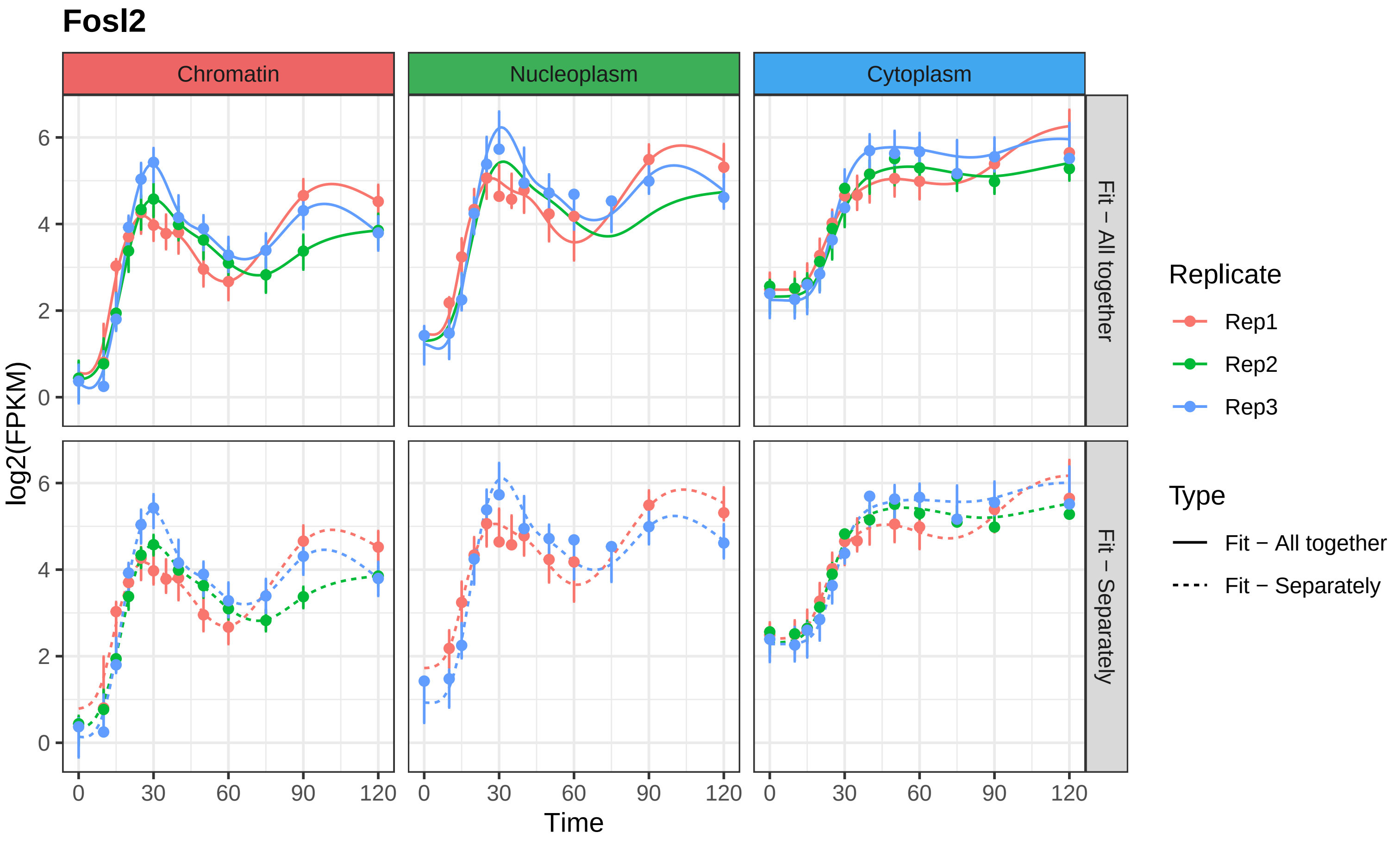

	Together	b1	b2	b3
**−NLL b1 ca**	−4.104	−2.367		
**−NLL b1 np**	1.425	1.065		
**−NLL b1 cyto**	−2.469	−3.409		
**−NLL b2 ca**	−7.3		−10.11	
**−NLL b2 np**				
**−NLL b2 cyto**	−1.001		−3.862	
**−NLL b3 ca**	−5.419			−3.341
**−NLL b3 np**	2.616			1.068
**−NLL b3 cyto**	1.281			−2.285
Total	−14.97	−4.711	−13.97	−4.558
**Total with regul**	−8.403	−1.696	−8.892	0.3882

	Together	b1	b2	b3
spar	0.25360	0.30500	0.2619	0.2718000
σ_b	0.13610	0.13000	0.07975	0.1351000
σ_t	0.21500	0.73080	0.001652	0.0006738
ca_{0,b_1}	0.60910	0.85210		
$\log_{10}(k_1')$	0.06601	−0.06723		−0.0042520
$\log_{10}(k_2)$	−0.20320	−0.34970	6.722 or −1.564	−0.2414000
$\log_{10}(k_2')$	−1.26200	−1.16200		−1.3690000
$\log_{10}(k_{deg})$	−1.56900	−1.36700	−1.564 or 6.722	−1.7760000
$\log_{10}(k_1'k_2')$	−1.19600	−1.22900	5.764	−1.3730000
$\log_{10}(k_1'/k_2)$	0.26920	0.28250		0.2371000
transport $= \log_{10}(k_1'k_2'/k_2)$	−0.99280	−0.87980	−0.9583 or 7.328	−1.1320000

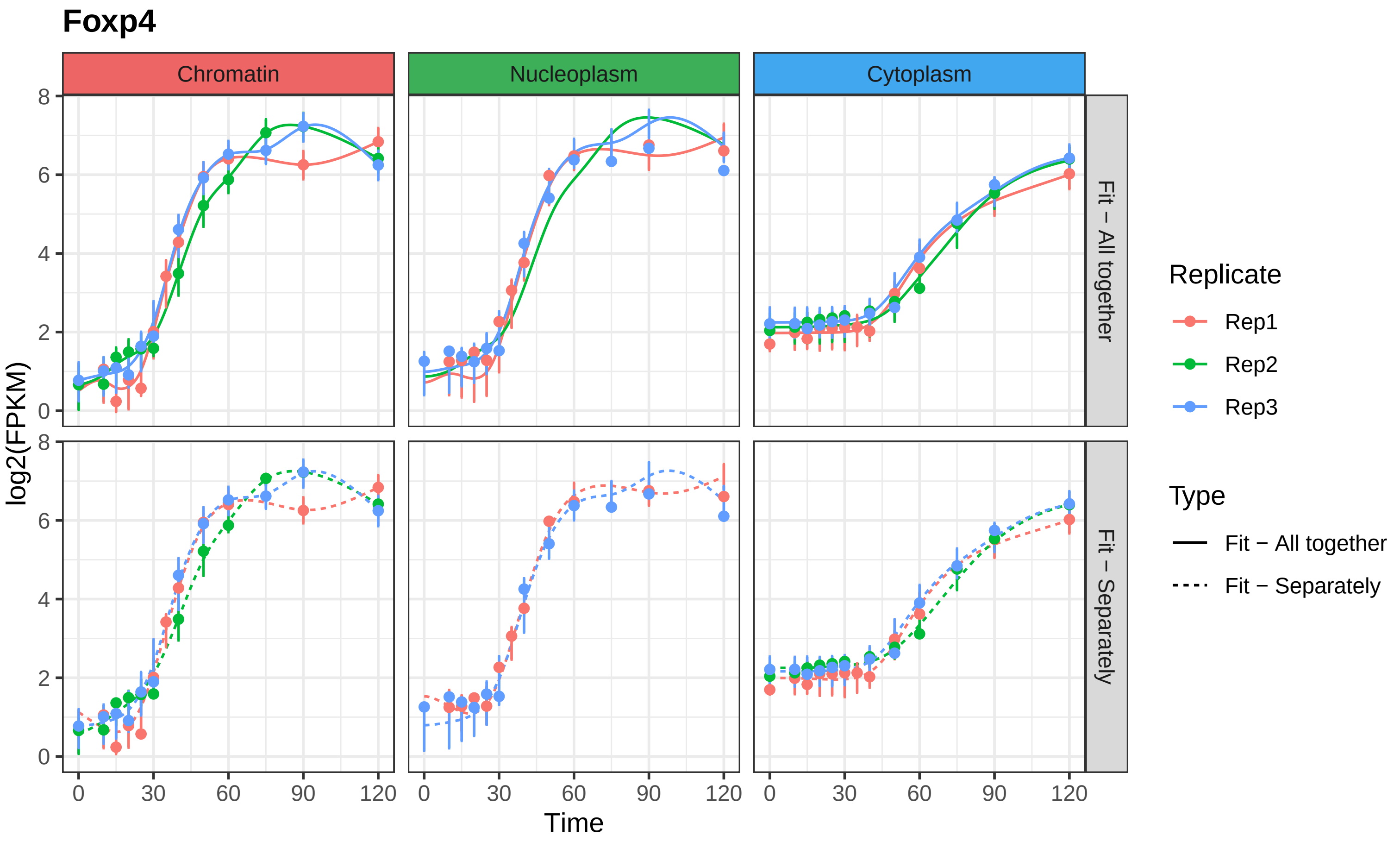

	Together	b1	b2	b3
**−NLL b1 ca**	−2.409	−1.01		
**−NLL b1 np**	7.059	1.407		
**−NLL b1 cyto**	−4.83	−5.207		
**−NLL b2 ca**	−5.147		−4.976	
**−NLL b2 np**				
**−NLL b2 cyto**	−4.411		−9.811	
**−NLL b3 ca**	−5.3			−2.969
**−NLL b3 np**	12.11			8.009
**−NLL b3 cyto**	−5.101			−4.901
Total	−8.031	−4.81	−14.79	0.1395
**Total with regul**	−4.917	−5.738	−16.38	0.4307

	Together	b1	b2	b3
spar	0.30760	0.4029	0.3919	0.35410
σ_b	0.12270	0.1125	1.149e−06	0.12090
σ_t	0.92990	0.4117	1.594	1.38000
ca_{0,b_1}	0.49140	1.1340		
$\log_{10}(k_1')$	−0.52810	−0.5333		−0.53200
$\log_{10}(k_2)$	−0.59330	−0.6545	−0.8009 or −2.42	−0.54430
$\log_{10}(k_2')$	−1.99200	−1.9990		−1.94200
$\log_{10}(k_{deg})$	−2.37000	−2.1400	−2.42 or −0.8009	−2.35400
$\log_{10}(k_1'k_2')$	−2.52000	−2.5330	−2.712	−2.47400
$\log_{10}(k_1'/k_2)$	0.06511	0.1213		0.01235
transport = $\log_{10}(k_1'k_2'/k_2)$	−1.92700	−1.8780	−1.912 or −0.292	−1.92900

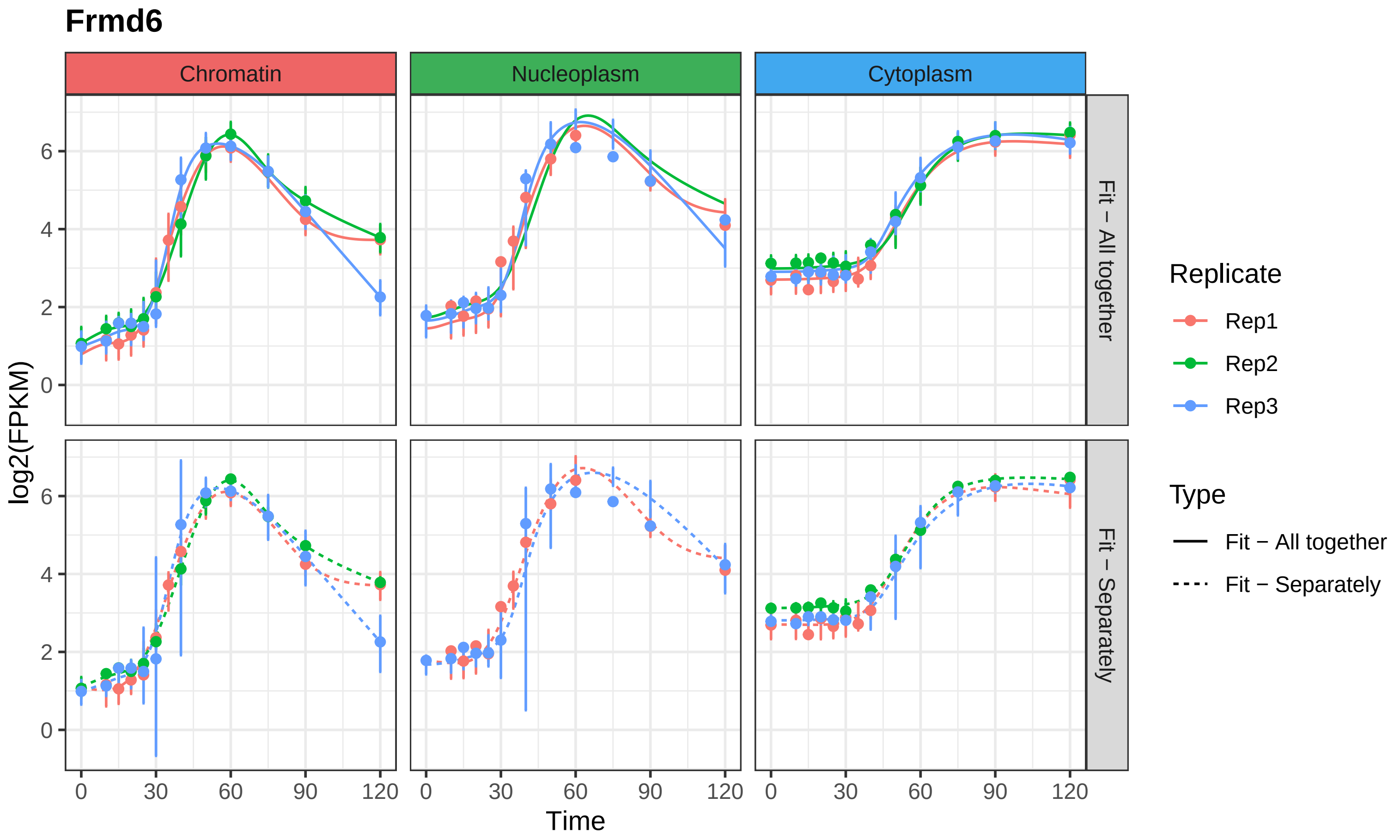

	Together	b1	b2	b3
**−NLL b1 ca**	−4.612	−4.046		
**−NLL b1 np**	6.076	1.652		
**−NLL b1 cyto**	−5.256	−3.994		
**−NLL b2 ca**	−6.511		−10.26	
**−NLL b2 np**				
**−NLL b2 cyto**	−5.196		−10.91	
**−NLL b3 ca**	−4.186			−0.07981
**−NLL b3 np**	14.3			6.739
**−NLL b3 cyto**	−6.008			−10.38
Total	−11.39	−6.388	−21.17	−3.725
**Total with regul**	−8.558	−7.567	−21.54	−2.651

	Together	b1	b2	b3
spar	0.3060	0.4255	0.3529	0.32930
σ_b	0.1172	0.1173	0.02714	0.02772
σ_t	1.7130	0.8565	1.068	4.68700
ca_{0,b_1}	0.7808	1.1170		
$\log_{10}(k_1')$	−0.6141	−0.4448		−0.88930
$\log_{10}(k_2)$	−0.8148	−0.6483	−0.6575 or −2.015	−1.09900
$\log_{10}(k_2')$	−1.5940	−1.5700		−1.61700
$\log_{10}(k_{deg})$	−1.9710	−1.8530	−2.015 or −0.6575	−1.95800
$\log_{10}(k_1' k_2')$	−2.2080	−2.0150	−2.063	−2.50600
$\log_{10}(k_1'/k_2)$	0.2006	0.2036		0.20970
$\text{transport} = \log_{10}(k_1' k_2'/k_2)$	−1.3940	−1.3670	−1.405 or −0.04735	−1.40700

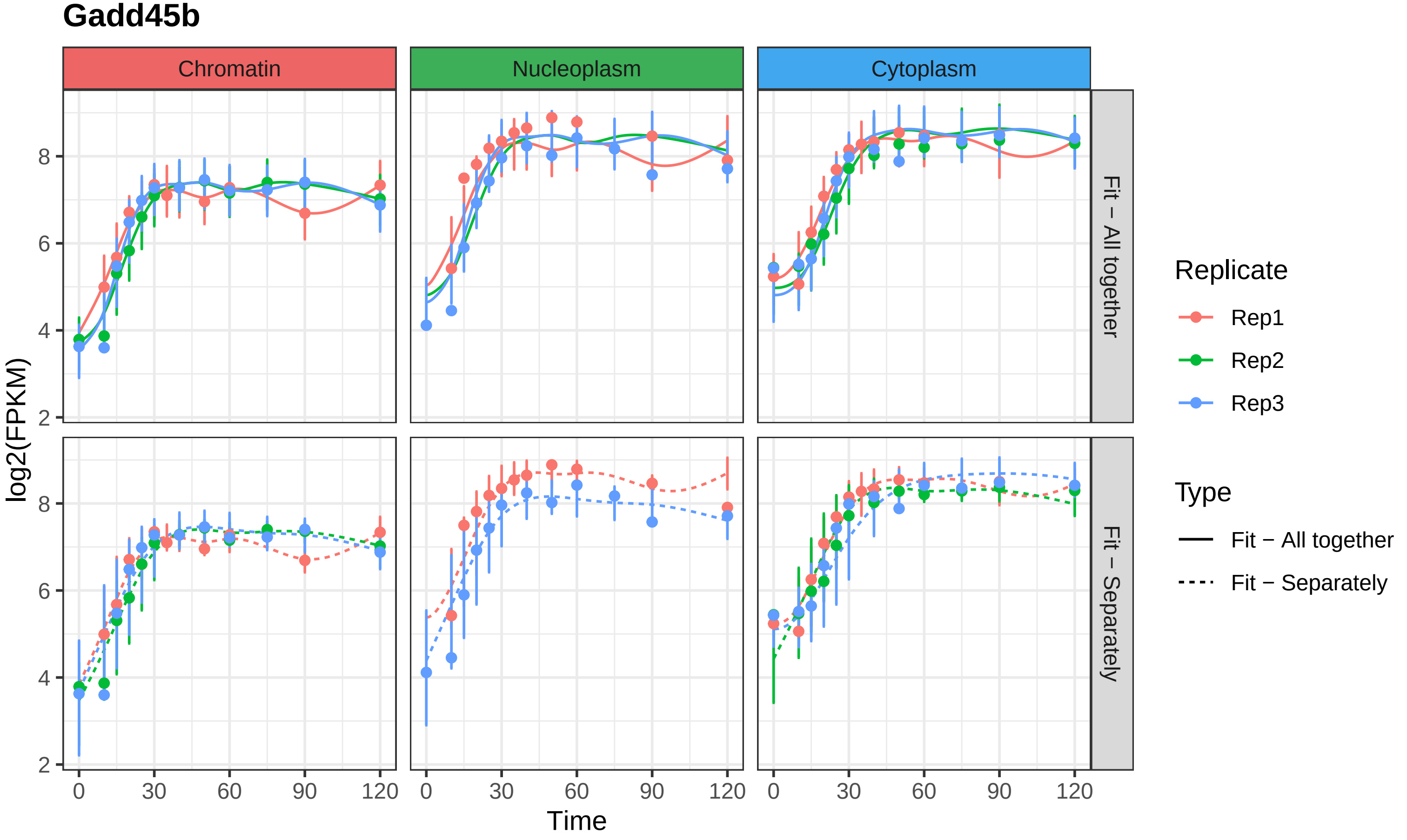

	Together	b1	b2	b3
**−NLL b1 ca**	−1.69	−3.514		
**−NLL b1 np**	12.78	6.853		
**−NLL b1 cyto**	−0.02353	−2.674		
**−NLL b2 ca**	−1.489		−2.388	
**−NLL b2 np**				
**−NLL b2 cyto**	2.238		4.331	
**−NLL b3 ca**	−0.09162			1.233
**−NLL b3 np**	10.24			3.895
**−NLL b3 cyto**	4.205			4.053
Total	26.17	0.664	1.943	9.181
**Total with regul**	31.1	2.447	0.3168	9.406

	Together	b1	b2	b3
spar	0.3280	0.3925	0.461	0.5295
σ_b	0.1994	0.1011	0.08553	0.1323
σ_t	1.2610	3.5570	3.889	4.4910
ca_{0,b_1}	3.9610	3.8610		
$\log_{10}(k_1')$	0.1083	−0.2944		5.1490
$\log_{10}(k_2)$	−0.2195	−0.7566	4.476 or 3.118	4.9400
$\log_{10}(k_2')$	−0.7311	−0.6787		−1.1350
$\log_{10}(k_{deg})$	−0.7809	−0.6380	3.118 or 4.476	−1.3530
$\log_{10}(k_1'k_2')$	−0.6228	−0.9731	7.881	4.0140
$\log_{10}(k_1'/k_2)$	0.3277	0.4622		0.2093
$\text{transport} = \log_{10}(k_1'k_2'/k_2)$	−0.4034	−0.2165	3.405 or 4.763	−0.9260

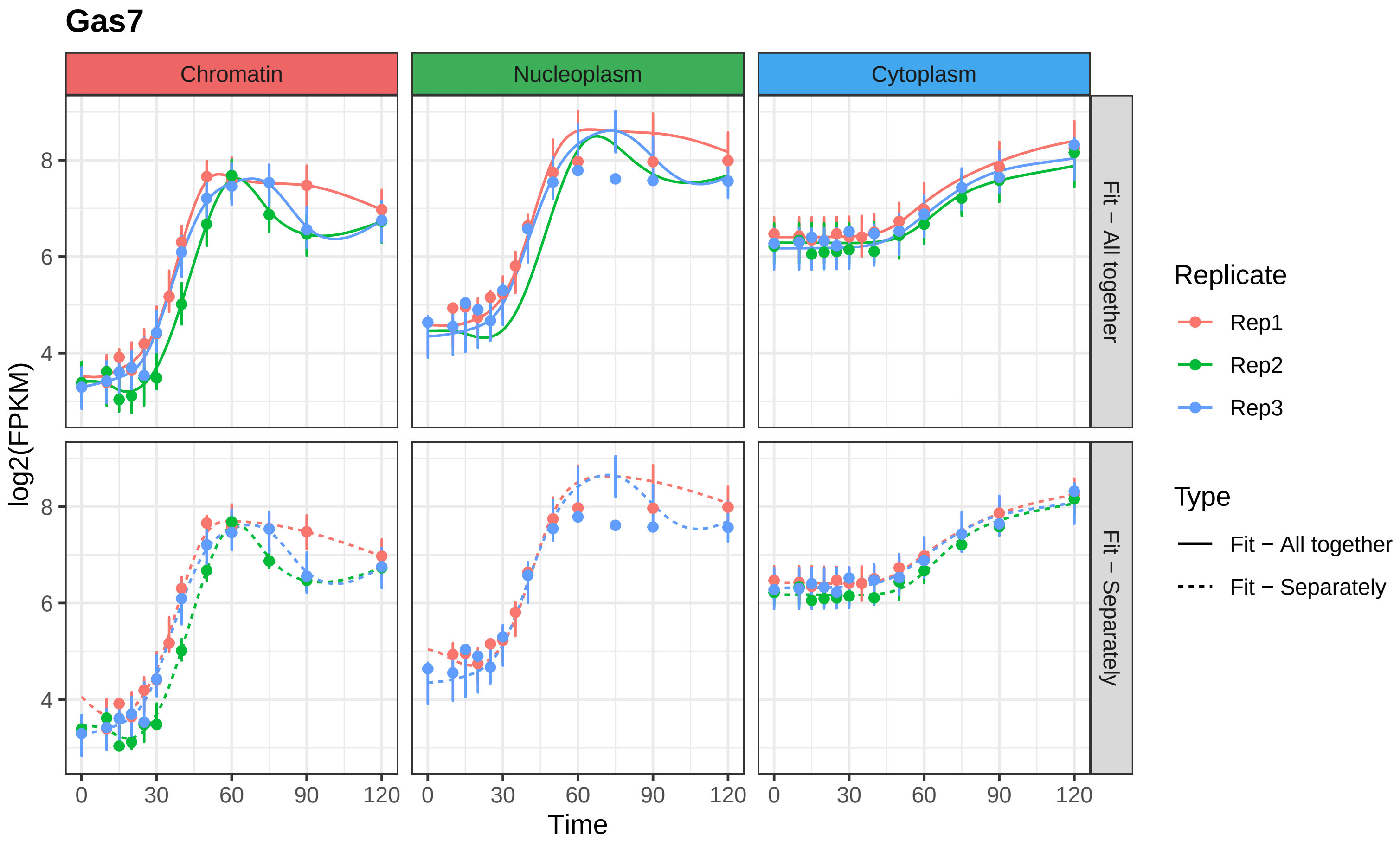

	Together	b1	b2	b3
**−NLL b1 ca**	−4.829	−3.838		
**−NLL b1 np**	5.751	3.719		
**−NLL b1 cyto**	−6.523	−9.187		
**−NLL b2 ca**	−5.275		−6.124	
**−NLL b2 np**				
**−NLL b2 cyto**	−4.255		−9.781	
**−NLL b3 ca**	−5.543			−3.819
**−NLL b3 np**	14.83			6.144
**−NLL b3 cyto**	−3.886			−5.216
Total	−9.732	−9.307	−15.91	−2.891
**Total with regul**	−8.473	−8.771	−15.62	−3.037

	Together	b1	b2	b3
spar	0.3484000	0.41290	0.3422	3.752e−01
σ_b	0.1472000	0.12280	0.07419	1.451e−01
σ_t	0.0001119	0.00421	1.101e−06	2.352e−06
ca_{0,b_1}	3.5360000	4.11200		
$\log_{10}(k_1')$	−0.4982000	−0.51670		−4.130e−01
$\log_{10}(k_2)$	−0.8167000	−0.81010	−1.034 or −2.308	−7.390e−01
$\log_{10}(k_2')$	−1.9500000	−1.96400		−1.971e+00
$\log_{10}(k_{deg})$	−2.4990000	−2.38300	−2.308 or −1.034	−2.561e+00
$\log_{10}(k_1' k_2')$	−2.4480000	−2.48100	−2.523	−2.384e+00
$\log_{10}(k_1'/k_2)$	0.3184000	0.29340		3.259e−01
$\text{transport} = \log_{10}(k_1' k_2'/k_2)$	−1.6310000	−1.67100	−1.489 or −0.2155	−1.645e+00

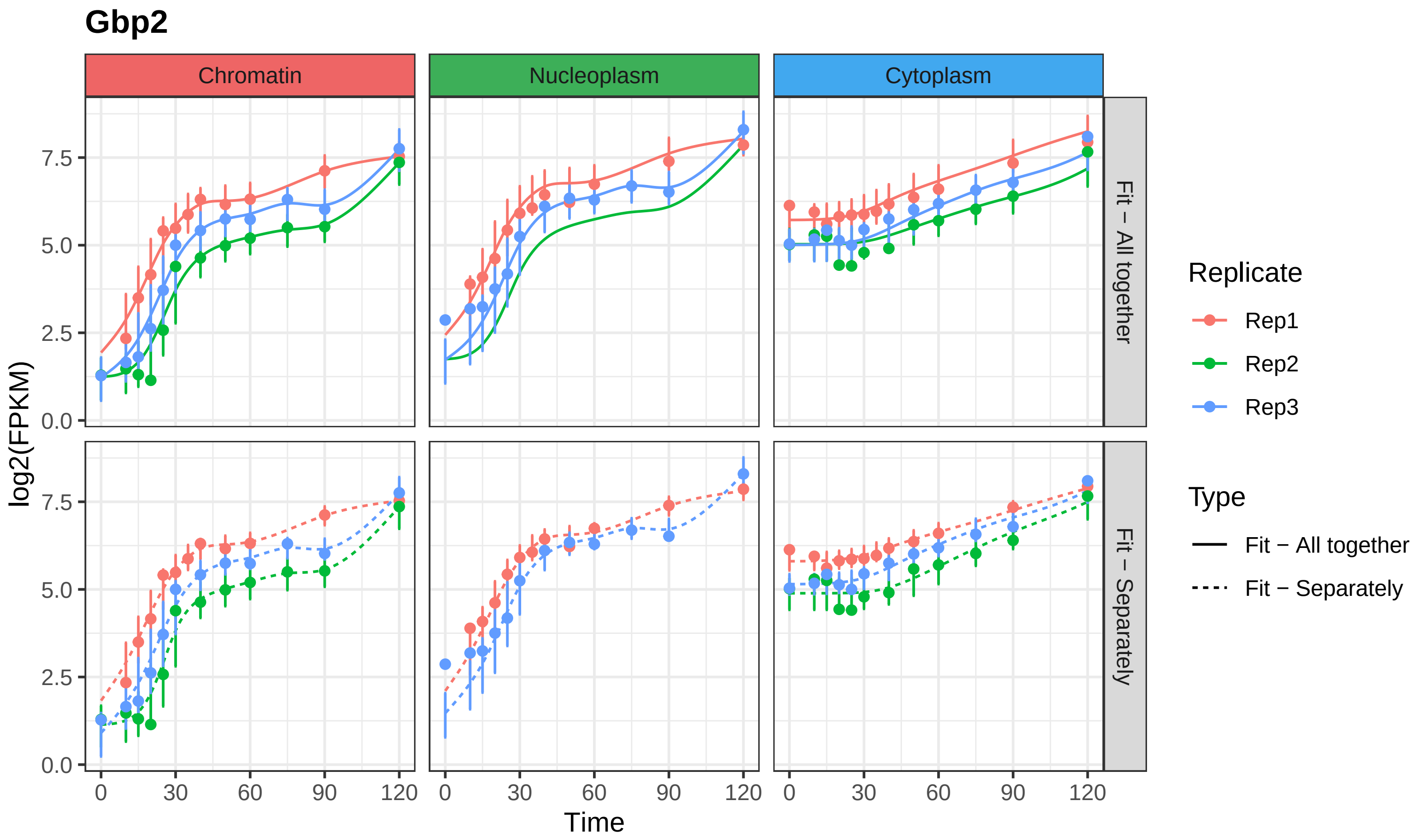

	Together	b1	b2	b3
**−NLL b1 ca**	−1.057	−3.735		
**−NLL b1 np**	3.393	−0.7474		
**−NLL b1 cyto**	−1.277	−6.642		
**−NLL b2 ca**	1.823		1.276	
**−NLL b2 np**				
**−NLL b2 cyto**	5.682		2.938	
**−NLL b3 ca**	−0.1755			−0.631
**−NLL b3 np**	10.41			2.343
**−NLL b3 cyto**	−1.164			−3.483
Total	17.64	−11.12	4.215	−1.771
**Total with regul**	17.07	−12.71	4.326	−3.386

	Together	b1	b2	b3
spar	0.4204	0.44720	0.3762	0.43070
σ_b	0.1562	0.08556	0.1562	0.09876
σ_t	2.5140	2.04500	2.445	2.64600
ca_{0,b_1}	1.9950	1.88900		
$\log_{10}(k_1')$	8.1530	6.46100		6.12200
$\log_{10}(k_2)$	8.0010	6.37800	−1.456 or −2.285	5.95200
$\log_{10}(k_2')$	−1.7350	−1.82300		−1.71000
$\log_{10}(k_{deg})$	−2.7220	−2.93600	−2.285 or −1.456	−2.81900
$\log_{10}(k_1'k_2')$	6.4180	4.63900	−2.611	4.41100
$\log_{10}(k_1'/k_2)$	0.1521	0.08339		0.16920
$transport = \log_{10}(k_1'k_2'/k_2)$	−1.5830	−1.73900	−1.156 or −0.3268	−1.54100

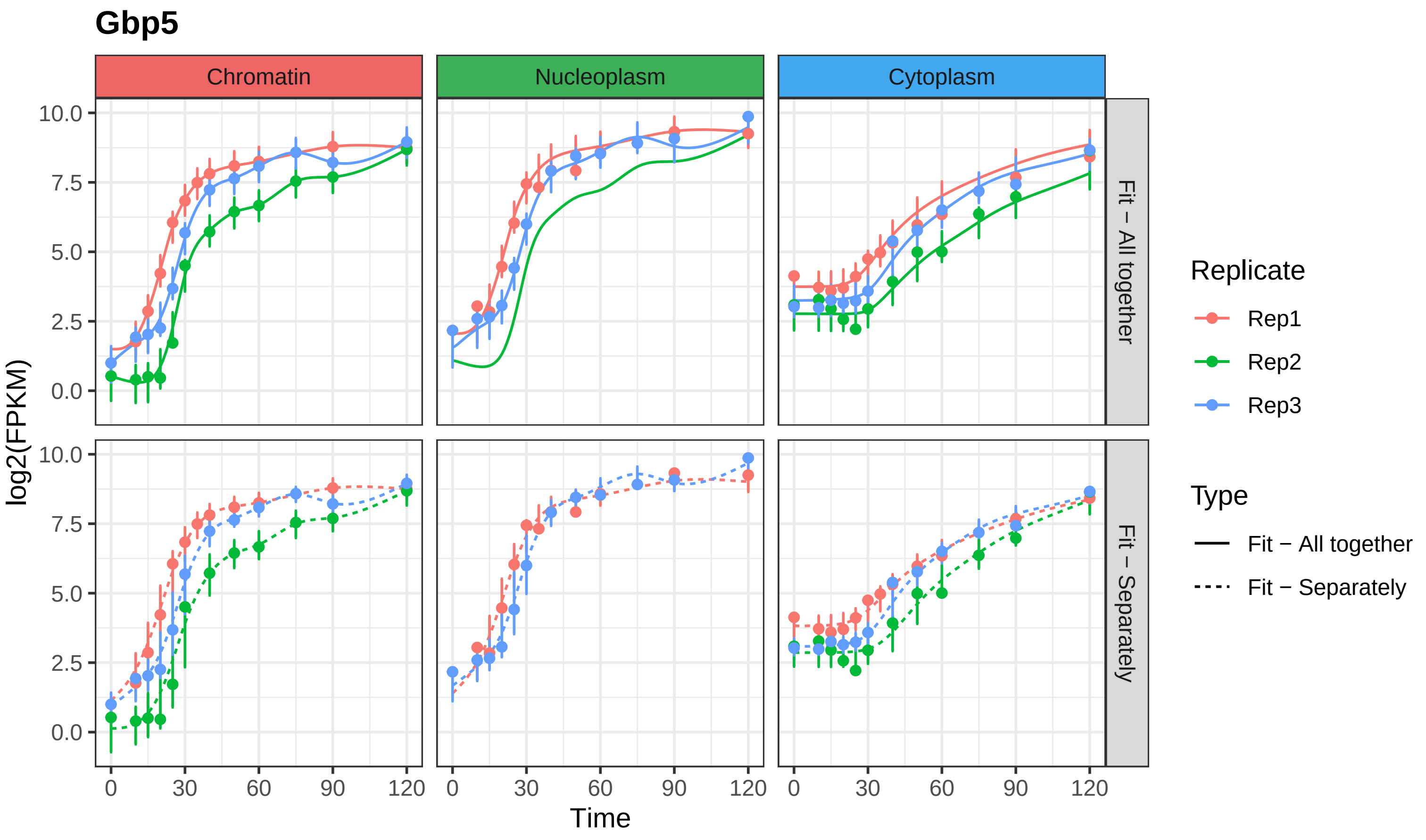

	Together	b1	b2	b3
**−NLL b1 ca**	−3.353	−2.702		
**−NLL b1 np**	7.948	5.995		
**−NLL b1 cyto**	4.873	−3.022		
**−NLL b2 ca**	−0.1549		2.219	
**−NLL b2 np**				
**−NLL b2 cyto**	7.968		5.249	
**−NLL b3 ca**	−2.412			−2.279
**−NLL b3 np**	1.086			−0.2482
**−NLL b3 cyto**	1.908			0.1763
Total	17.86	0.2716	7.468	−2.351
**Total with regul**	25.83	0.273	8.064	−0.5861

	Together	b1	b2	b3
spar	0.2472000	0.37150	0.3623	0.31620
σ_b	0.1890000	0.12270	0.1589	0.09326
σ_t	0.0008895	1.42300	2.382	1.87500
ca_{0,b_1}	1.5120000	1.18200		
$\log_{10}(k_1')$	0.2911000	1.51200		3.39800
$\log_{10}(k_2)$	0.1223000	1.43300	−0.8048 or −2.395	3.17800
$\log_{10}(k_2')$	−1.9630000	−2.06300		−2.02100
$\log_{10}(k_{deg})$	−2.4700000	−2.79300	−2.395 or −0.8048	−2.44400
$\log_{10}(k_1'k_2')$	−1.6720000	−0.55060	−2.377	1.37700
$\log_{10}(k_1'/k_2)$	0.1688000	0.07882		0.22010
transport = $\log_{10}(k_1'k_2'/k_2)$	−1.7940000	−1.98400	−1.572 or 0.01726	−1.80100

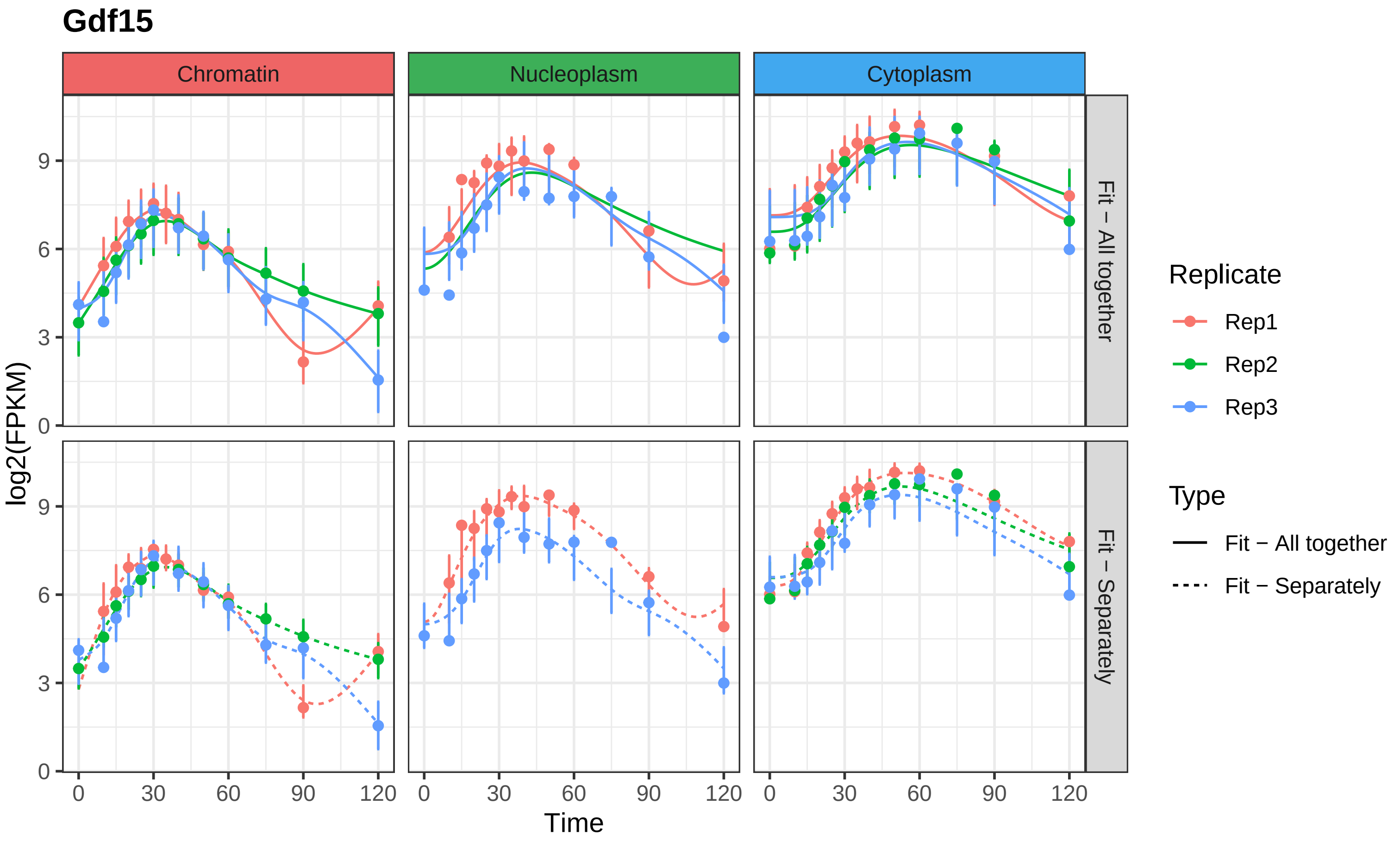

	Together	b1	b2	b3
**−NLL b1 ca**	3.275	−1.409		
**−NLL b1 np**	11.31	7.19		
**−NLL b1 cyto**	10.43	−0.5342		
**−NLL b2 ca**	2.812		−2.339	
**−NLL b2 np**				
**−NLL b2 cyto**	9.429		8.309	
**−NLL b3 ca**	4.95			2.901
**−NLL b3 np**	19.11			5.501
**−NLL b3 cyto**	11.47			8.764
Total	72.79	5.248	5.97	17.17
**Total with regul**	79.5	18.18	5.996	18.88

	Together	b1	b2	b3
spar	3.865e−01	0.3489	0.406	0.385500
σ_b	3.249e−01	0.1225	0.1979	0.252800
σ_t	3.572e−05	2.6160	0.0008936	0.000156
ca_{0,b_1}	4.024e+00	2.7300		
$\log_{10}(k_1')$	−4.298e−01	−0.3372		−0.404900
$\log_{10}(k_2)$	−9.890e−01	−1.0300	−0.9791 or −1.017	−0.769800
$\log_{10}(k_2')$	−8.345e−01	−0.9172		−0.755600
$\log_{10}(k_{deg})$	−1.212e+00	−1.2840	−1.017 or −0.9791	−1.237000
$\log_{10}(k_1'k_2')$	−1.264e+00	−1.2540	−1.059	−1.161000
$\log_{10}(k_1'/k_2)$	5.591e−01	0.6928		0.364900
$\text{transport} = \log_{10}(k_1'k_2'/k_2)$	−2.754e−01	−0.2243	−0.07957 or −0.04184	−0.390700

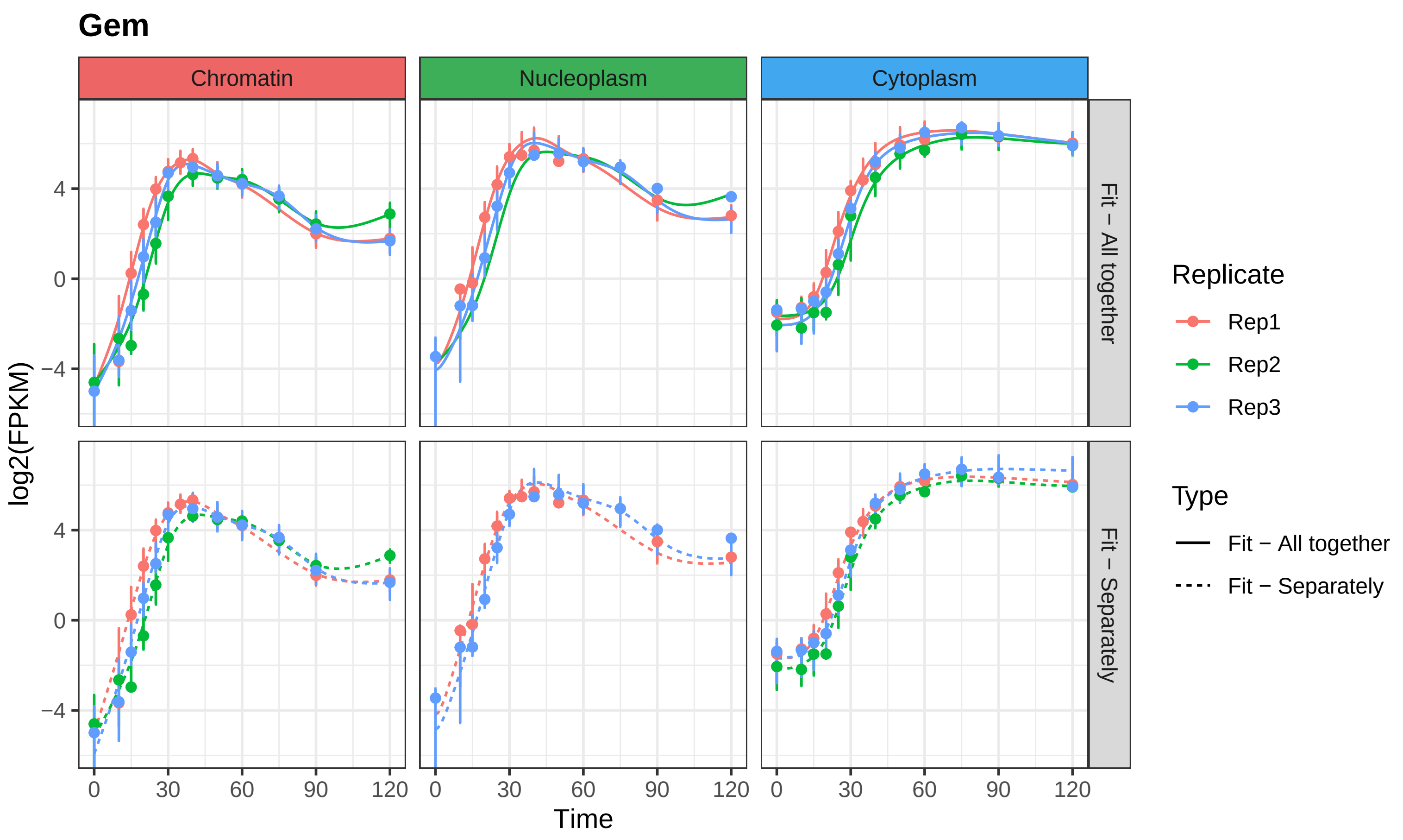

	Together	b1	b2	b3
**−NLL b1 ca**	1.748	1.696		
**−NLL b1 np**	8.113	8.748		
**−NLL b1 cyto**	2.388	−1.713		
**−NLL b2 ca**	2.521		−0.732	
**−NLL b2 np**				
**−NLL b2 cyto**	6.861		0.9033	
**−NLL b3 ca**	2.002			2.39
**−NLL b3 np**	13.96			10.42
**−NLL b3 cyto**	1.747			3.875
Total	39.34	8.731	0.1713	16.68
**Total with regul**	42.95	8.504	0.6625	18.84

	Together	b1	b2	b3
spar	0.31790	0.3888	0.337	0.3472000
σ_b	0.16980	0.1269	0.0679	0.2174000
σ_t	0.88450	1.2190	1.107	0.0003711
ca_{0,b_1}	−4.72300	−4.9630		
$\log_{10}(k_1')$	−0.08125	−0.1070		−0.0473200
$\log_{10}(k_2)$	−0.37860	−0.3508	4.341 or −1.883	−0.3760000
$\log_{10}(k_2')$	−1.23600	−1.3140		−1.3190000
$\log_{10}(k_{deg})$	−1.83500	−2.0660	−1.883 or 4.341	−2.2760000
$\log_{10}(k_1' k_2')$	−1.31800	−1.4210	3.36	−1.3660000
$\log_{10}(k_1'/k_2)$	0.29740	0.2438		0.3287000
$\text{transport} = \log_{10}(k_1' k_2'/k_2)$	−0.93900	−1.0700	−0.9808 or 5.243	−0.9904000

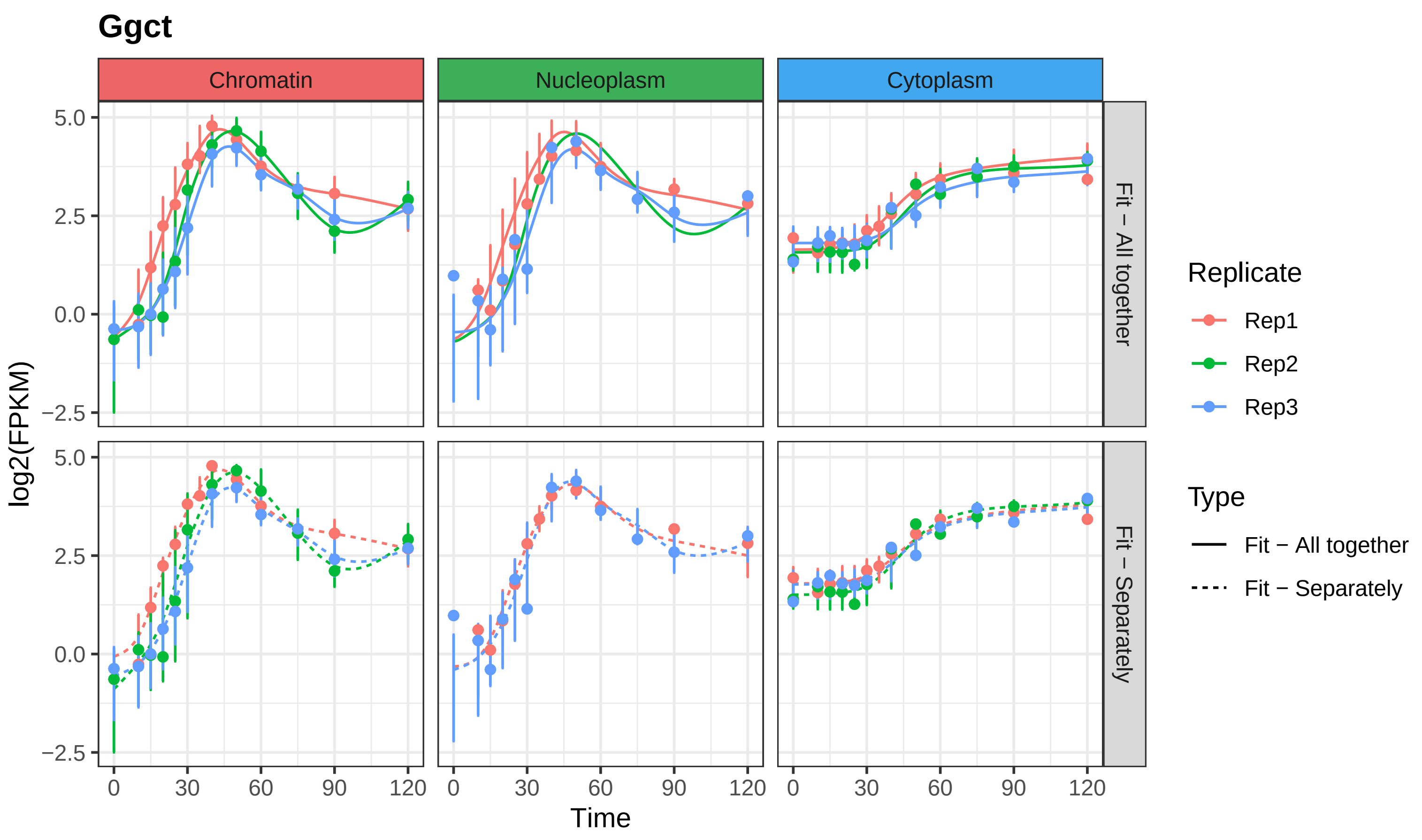

	Together	b1	b2	b3
**−NLL b1 ca**	−0.602	−3.104		
**−NLL b1 np**	8.65	−0.5368		
**−NLL b1 cyto**	−0.524	−4.038		
**−NLL b2 ca**	0.3788		1.796	
**−NLL b2 np**				
**−NLL b2 cyto**	−0.5249		−1.495	
**−NLL b3 ca**	−1.467			−1.125
**−NLL b3 np**	12.04			3.2
**−NLL b3 cyto**	1.553			0.1605
Total	19.5	−7.678	0.3014	2.236
**Total with regul**	19.99	−7.393	−1.55	1.094

	Together	b1	b2	b3
spar	0.35370	3.703e−01	0.4185	0.38290
σ_b	0.10700	5.868e−02	4.836e−06	0.05591
σ_t	2.31900	1.396e−06	3.663	2.44600
ca_{0,b_1}	−0.51930	1.695e−02		
$\log_{10}(k_1')$	−0.26890	−7.950e−01		4.66500
$\log_{10}(k_2)$	−0.25470	−7.197e−01	0.1675 or −2.594	4.61900
$\log_{10}(k_2')$	−1.87600	−1.880e+00		−1.89800
$\log_{10}(k_{deg})$	−2.55700	−2.514e+00	−2.594 or 0.1675	−2.55100
$\log_{10}(k_1'k_2')$	−2.14500	−2.675e+00	−1.705	2.76700
$\log_{10}(k_1'/k_2)$	−0.01411	−7.532e−02		0.04654
transport $= \log_{10}(k_1'k_2'/k_2)$	−1.89000	−1.955e+00	−1.872 or 0.8896	−1.85200

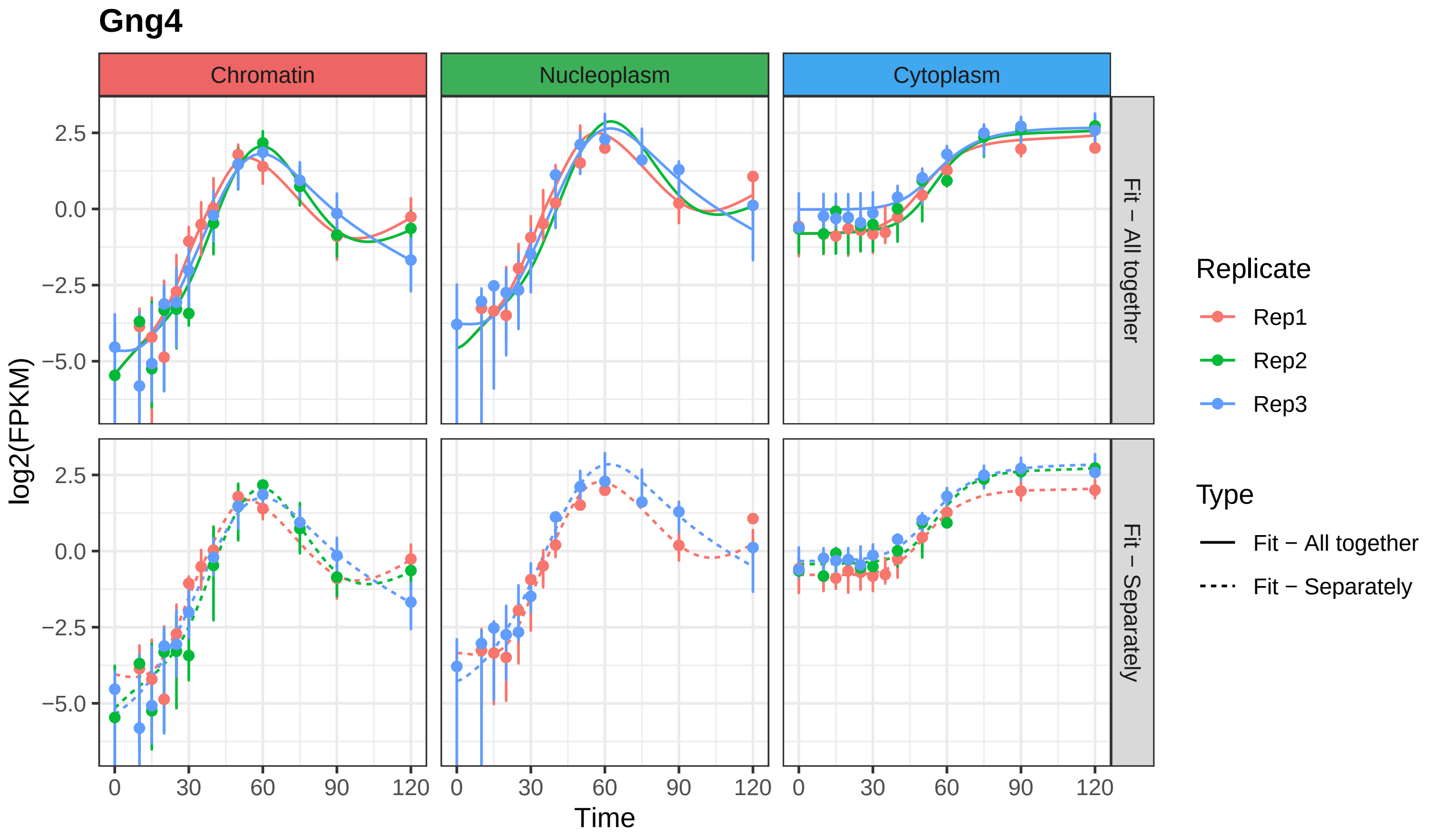

	Together	b1	b2	b3
**−NLL b1 ca**	5.144	3.63		
**−NLL b1 np**	8.994	10.28		
**−NLL b1 cyto**	0.5856	−6.514		
**−NLL b2 ca**	6.503		5.412	
**−NLL b2 np**				
**−NLL b2 cyto**	5.746		0.5306	
**−NLL b3 ca**	4.892			3.943
**−NLL b3 np**	11.01			9.466
**−NLL b3 cyto**	1.854			−3.091
Total	44.72	7.392	5.943	10.32
**Total with regul**	47.88	5.958	4.143	8.76

	Together	b1	b2	b3
spar	0.4136	0.41360	0.4096	0.4567
σ_b	0.1675	0.08467	8.929e−06	0.1219
σ_t	1.3370	0.80750	3.096	0.6277
ca_{0,b_1}	−5.4380	−4.00300		
$\log_{10}(k_1')$	−0.1951	−0.43520		0.1014
$\log_{10}(k_2)$	−0.4576	−0.64540	−0.5303 or −2.732	−0.2329
$\log_{10}(k_2')$	−1.6080	−1.61100		−1.6140
$\log_{10}(k_{deg})$	−2.7380	−2.38400	−2.732 or −0.5303	−2.7980
$\log_{10}(k_1'k_2')$	−1.8030	−2.04600	−1.837	−1.5130
$\log_{10}(k_1'/k_2)$	0.2624	0.21020		0.3343
transport $= \log_{10}(k_1'k_2'/k_2)$	−1.3460	−1.40000	−1.307 or 0.8945	−1.2800

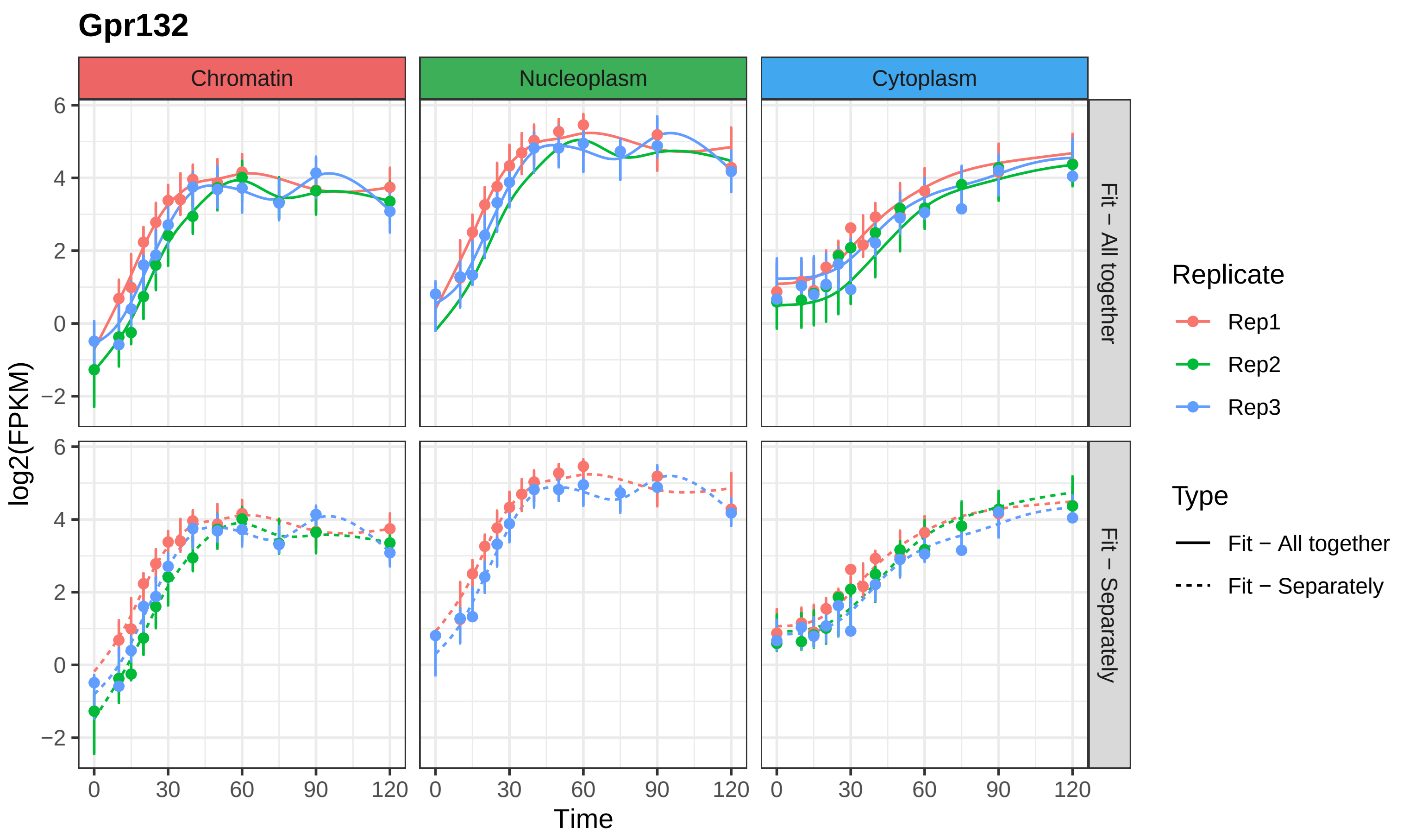

	Together	b1	b2	b3
**−NLL b1 ca**	−2.148	−3.718		
**−NLL b1 np**	1.142	1.984		
**−NLL b1 cyto**	1.894	1.482		
**−NLL b2 ca**	−1.834		−2.251	
**−NLL b2 np**				
**−NLL b2 cyto**	13.94		4.874	
**−NLL b3 ca**	−0.6818			−1.943
**−NLL b3 np**	−1.349			−2.997
**−NLL b3 cyto**	9.973			1.642
Total	20.94	−0.2516	2.623	−3.298
**Total with regul**	23.65	−0.3435	1.659	−4.315

	Together	b1	b2	b3
spar	0.3781000	0.397500	0.4296	0.3975
σ_b	0.1916000	0.148300	0.1595	0.1220
σ_t	0.0002747	0.004094	0.005327	0.5351
ca_{0,b_1}	−0.7023000	−0.136700		
$\log_{10}(k_1')$	6.6890000	0.740200		8.5940
$\log_{10}(k_2)$	6.3550000	0.402100	4.981 or −2.232	8.2610
$\log_{10}(k_2')$	−1.9340000	−1.913000		−2.0060
$\log_{10}(k_{deg})$	−2.1420000	−1.950000	−2.232 or 4.981	−2.1690
$\log_{10}(k_1' k_2')$	4.7550000	−1.173000	3.471	6.5880
$\log_{10}(k_1'/k_2)$	0.3339000	0.338100		0.3336
$\text{transport} = \log_{10}(k_1' k_2'/k_2)$	−1.6000000	−1.575000	−1.51 or 5.703	−1.6730

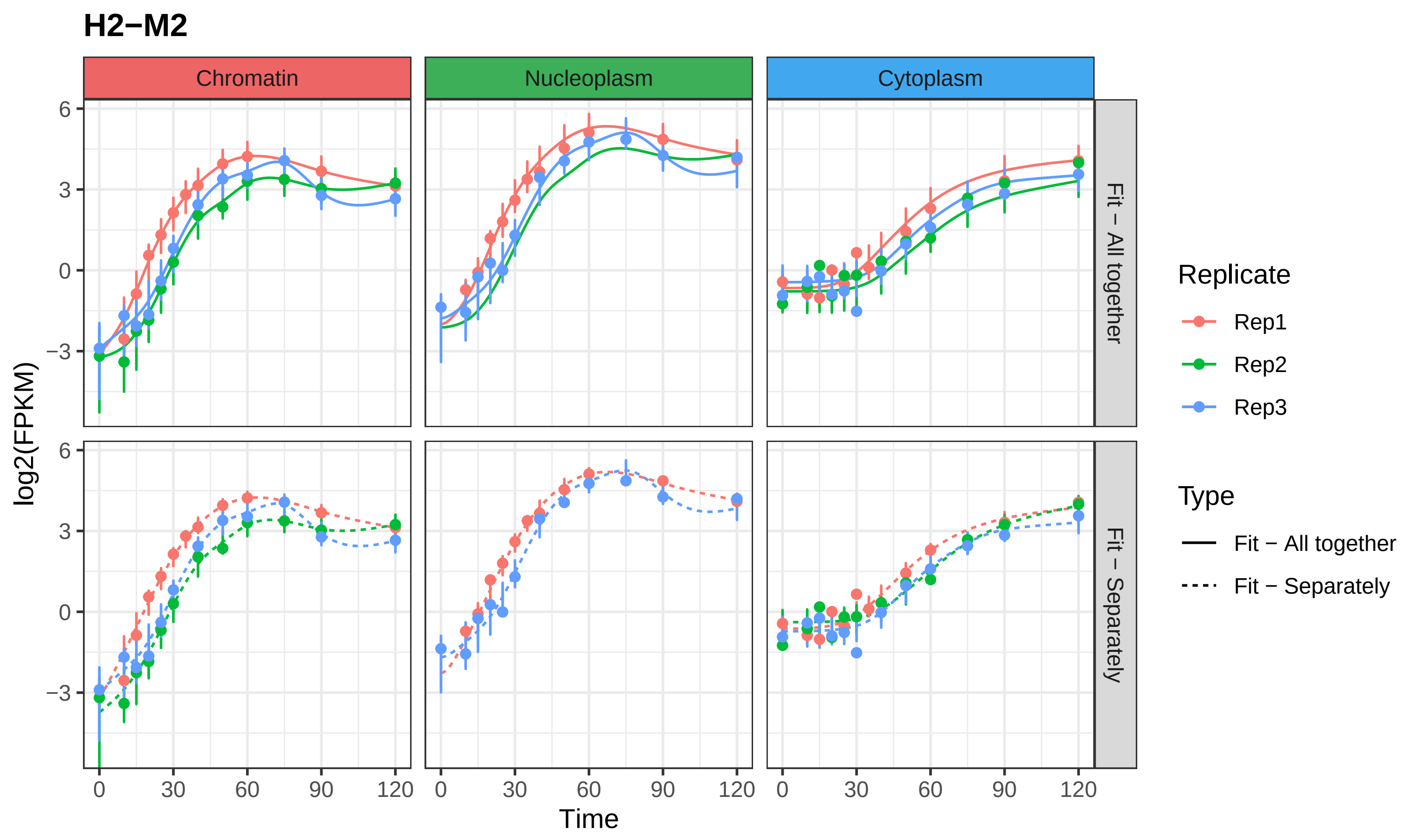

	Together	b1	b2	b3
**−NLL b1 ca**	−0.6555	−4.222		
**−NLL b1 np**	0.4402	−5.153		
**−NLL b1 cyto**	5.688	6.157		
**−NLL b2 ca**	0.7527		−0.6781	
**−NLL b2 np**				
**−NLL b2 cyto**	13.26		5.795	
**−NLL b3 ca**	0.6194			−0.624
**−NLL b3 np**	4.953			4.104
**−NLL b3 cyto**	8.894			3.099
Total	33.95	−3.218	5.117	6.58
**Total with regul**	35	−4.862	4.01	6.069

	Together	b1	b2	b3
spar	0.3684000	0.47810	0.4031	0.3809000
σ_b	0.1904000	0.07165	0.1243	0.1305000
σ_t	0.0001114	0.33800	1.883e−06	0.0001302
ca_{0,b_1}	−3.0670000	−3.17500		
$\log_{10}(k_1')$	−0.1888000	−0.23210		−0.1061000
$\log_{10}(k_2)$	−0.5252000	−0.52230	−1.078 or −2.576	−0.4843000
$\log_{10}(k_2')$	−2.1360000	−2.17600		−2.2410000
$\log_{10}(k_{deg})$	−2.5390000	−2.67200	−2.576 or −1.078	−2.5300000
$\log_{10}(k_1' k_2')$	−2.3240000	−2.40800	−2.647	−2.3470000
$\log_{10}(k_1'/k_2)$	0.3364000	0.29020		0.3782000
$\text{transport} = \log_{10}(k_1' k_2'/k_2)$	−1.7990000	−1.88600	−1.568 or −0.07076	−1.8630000

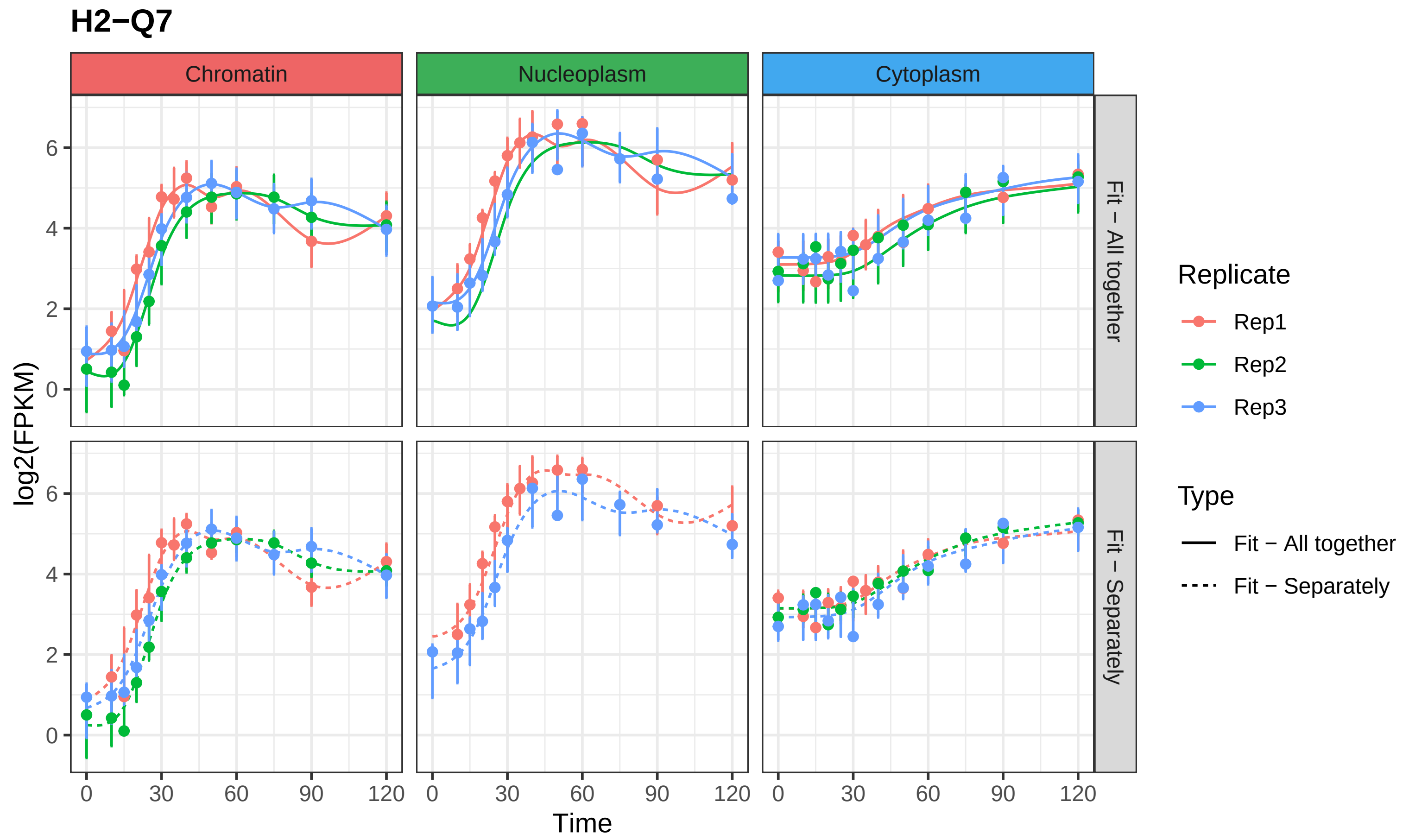

	Together	b1	b2	b3
**−NLL b1 ca**	2.249	1.938		
**−NLL b1 np**	4.717	1.737		
**−NLL b1 cyto**	2.016	1.997		
**−NLL b2 ca**	−0.3566		−3.033	
**−NLL b2 np**				
**−NLL b2 cyto**	5.333		−0.4037	
**−NLL b3 ca**	−1.442			−1.116
**−NLL b3 np**	6.544			3.888
**−NLL b3 cyto**	8.825			3.57
Total	27.89	5.672	−3.436	6.342
**Total with regul**	30.82	6	−3.669	6.252

	Together	b1	b2	b3
spar	3.536e−01	0.3926	0.364	0.3952000
σ_b	2.065e−01	0.1484	0.1096	0.1805000
σ_t	5.882e−05	2.1050	0.4321	0.0002589
ca_{0,b_1}	7.489e−01	0.9337		
$\log_{10}(k_1')$	5.926e−01	−0.1491		0.7793000
$\log_{10}(k_2)$	2.127e−01	−0.6256	3.988 or −2.633	0.4845000
$\log_{10}(k_2')$	−2.189e+00	−2.2880		−2.1280000
$\log_{10}(k_{deg})$	−2.525e+00	−2.4960	−2.633 or 3.988	−2.5120000
$\log_{10}(k_1'k_2')$	−1.596e+00	−2.4370	2.228	−1.3480000
$\log_{10}(k_1'/k_2)$	3.800e−01	0.4765		0.2948000
transport = $\log_{10}(k_1'k_2'/k_2)$	−1.809e+00	−1.8110	−1.76 or 4.861	−1.8330000

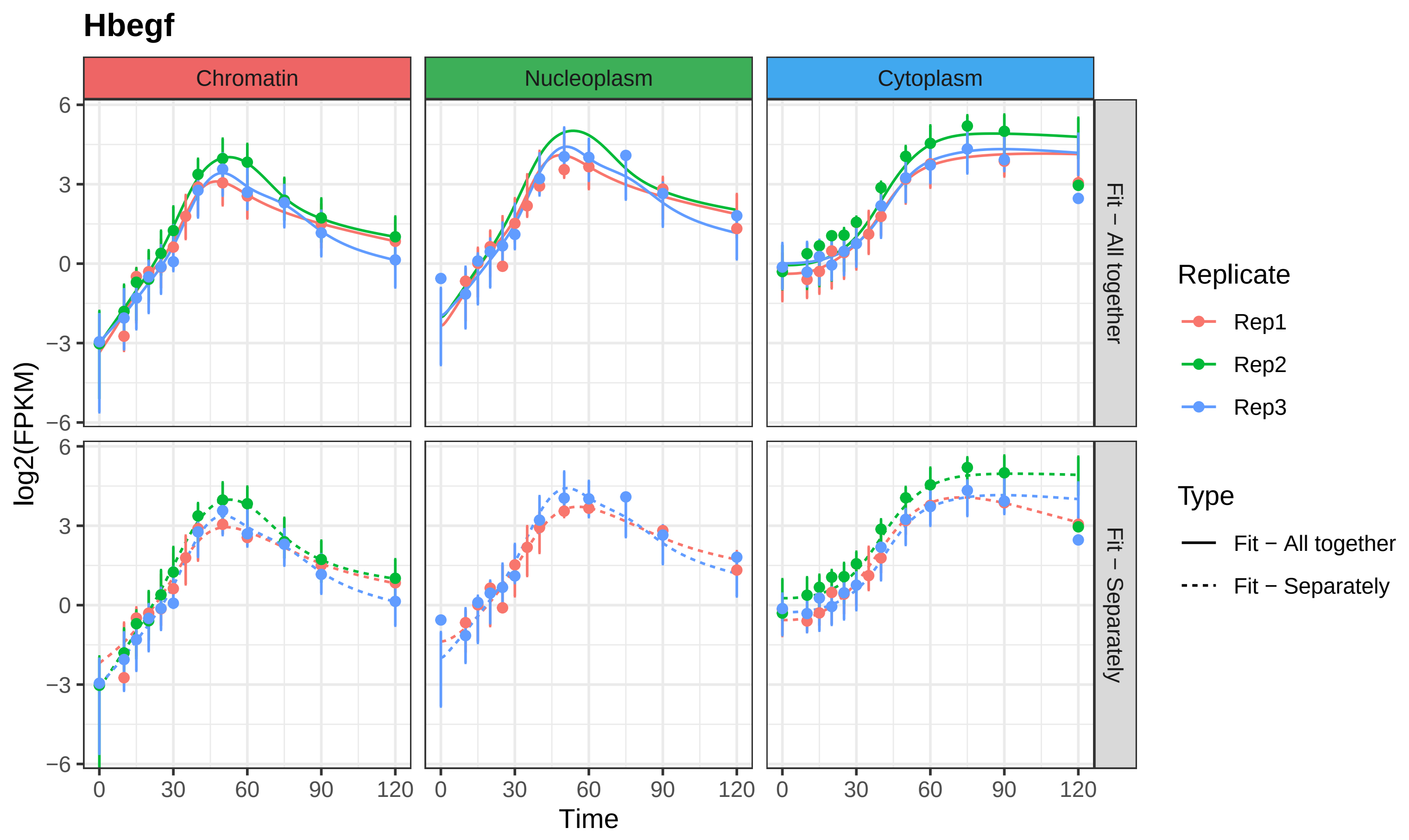

	Together	b1	b2	b3
**−NLL b1 ca**	3.645	4.642		
**−NLL b1 np**	7.857	3.108		
**−NLL b1 cyto**	7.089	−0.2149		
**−NLL b2 ca**	2.223		1.54	
**−NLL b2 np**				
**−NLL b2 cyto**	13.79		11.73	
**−NLL b3 ca**	3.167			2.493
**−NLL b3 np**	10.2			4.249
**−NLL b3 cyto**	9.436			9.107
Total	57.41	7.534	13.27	15.85
**Total with regul**	60.65	12.13	14.22	16.83

	Together	b1	b2	b3
spar	0.3655000	4.917e−01	0.4238	3.910e−01
σ_b	0.2634000	8.487e−07	0.2503	2.280e−01
σ_t	0.0002655	3.034e+00	1.298e−05	3.702e−05
ca_{0,b_1}	−3.3320000	−2.046e+00		
$\log_{10}(k_1')$	0.2689000	−3.414e−01		3.605e−01
$\log_{10}(k_2)$	−0.0324700	−5.876e−01	8.094 or −2.199	4.649e−02
$\log_{10}(k_2')$	−1.4540000	−1.067e+00		−1.511e+00
$\log_{10}(k_{deg})$	−2.0470000	−1.310e+00	−2.199 or 8.094	−2.031e+00
$\log_{10}(k_1'k_2')$	−1.1850000	−1.408e+00	6.919	−1.151e+00
$\log_{10}(k_1'/k_2)$	0.3014000	2.462e−01		3.140e−01
transport $= \log_{10}(k_1'k_2'/k_2)$	−1.1520000	−8.206e−01	−1.175 or 9.118	−1.197e+00

Hilpda

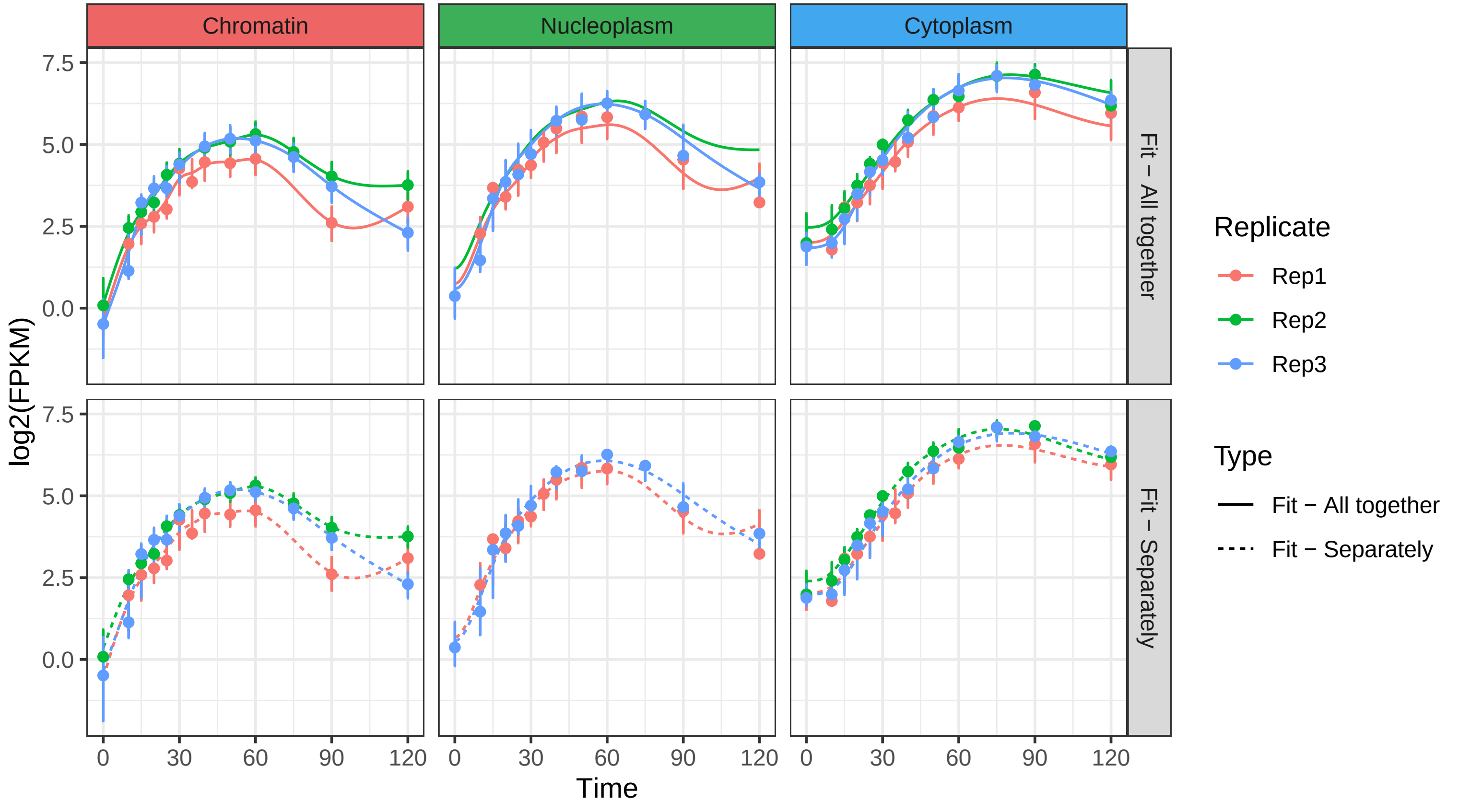

	Together	b1	b2	b3
**−NLL b1 ca**	−2.853	−1.549		
**−NLL b1 np**	7.966	6.002		
**−NLL b1 cyto**	1.168	−1.546		
**−NLL b2 ca**	−5.176		−7.23	
**−NLL b2 np**				
**−NLL b2 cyto**	0.244		−1.545	
**−NLL b3 ca**	−2.811			−3.272
**−NLL b3 np**	3.647			3.449
**−NLL b3 cyto**	−1.842			−3.541
Total	0.3429	2.907	−8.775	−3.363
**Total with regul**	4.434	4.276	−7.79	−4.458

	Together	b1	b2	b3
spar	0.2926	0.3549	0.3302	0.38410
σ_b	0.1398	0.1291	0.09295	0.07191
σ_t	1.0460	1.8020	0.0003494	2.19800
ca_{0,b_1}	−0.3703	−0.6482		
$\log_{10}(k_1')$	−0.4150	−0.4052		−0.47630
$\log_{10}(k_2)$	−0.7432	−0.7838	−0.7963 or −1.224	−0.75650
$\log_{10}(k_2')$	−1.0830	−1.1300		−1.12500
$\log_{10}(k_{deg})$	−1.4610	−1.5370	−1.224 or −0.7963	−1.55100
$\log_{10}(k_1'k_2')$	−1.4980	−1.5350	−1.405	−1.60200
$\log_{10}(k_1'/k_2)$	0.3282	0.3786		0.28030
transport = $\log_{10}(k_1'k_2'/k_2)$	−0.7545	−0.7511	−0.609 or −0.181	−0.84500

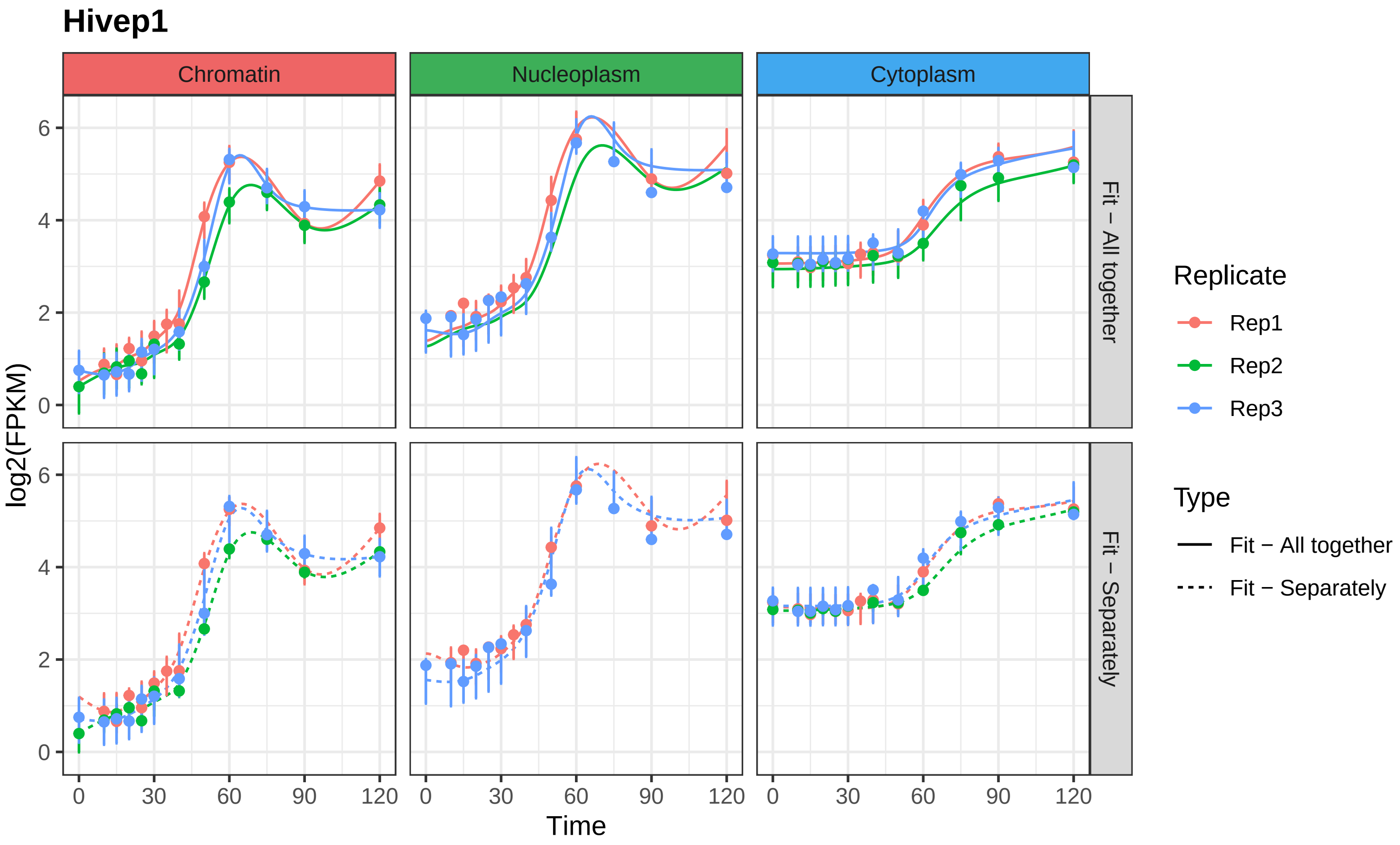

	Together	b1	b2	b3
**−NLL b1 ca**	−4.418	−3.864		
**−NLL b1 np**	3.227	1.442		
**−NLL b1 cyto**	−5.094	−7.614		
**−NLL b2 ca**	−5.859		−8.277	
**−NLL b2 np**				
**−NLL b2 cyto**	−5.312		−16.28	
**−NLL b3 ca**	−6.261			−2.725
**−NLL b3 np**	8.261			5.994
**−NLL b3 cyto**	−1.714			−3.334
Total	−17.17	−10.04	−24.56	−0.06533
**Total with regul**	−14.39	−9.937	−23.61	−0.06817

	Together	b1	b2	b3
spar	0.3098000	0.3709000	0.3224	0.3687
σ_b	0.1265000	0.1074000	0.01743	0.1378
σ_t	0.0001139	0.0001011	0.0005571	1.5040
ca_{0,b_1}	0.5167000	1.2100000		
$\log_{10}(k_1')$	0.0401800	−0.2968000		6.3490
$\log_{10}(k_2)$	−0.2219000	−0.5779000	−0.6076 or −2.308	6.0950
$\log_{10}(k_2')$	−1.8010000	−1.8030000		−1.8220
$\log_{10}(k_{deg})$	−2.3040000	−2.1050000	−2.308 or −0.6076	−2.3050
$\log_{10}(k_1'k_2')$	−1.7600000	−2.1000000	−2.118	4.5270
$\log_{10}(k_1'/k_2)$	0.2621000	0.2811000		0.2542
$\text{transport} = \log_{10}(k_1'k_2'/k_2)$	−1.5390000	−1.5220000	−1.511 or 0.19	−1.5680

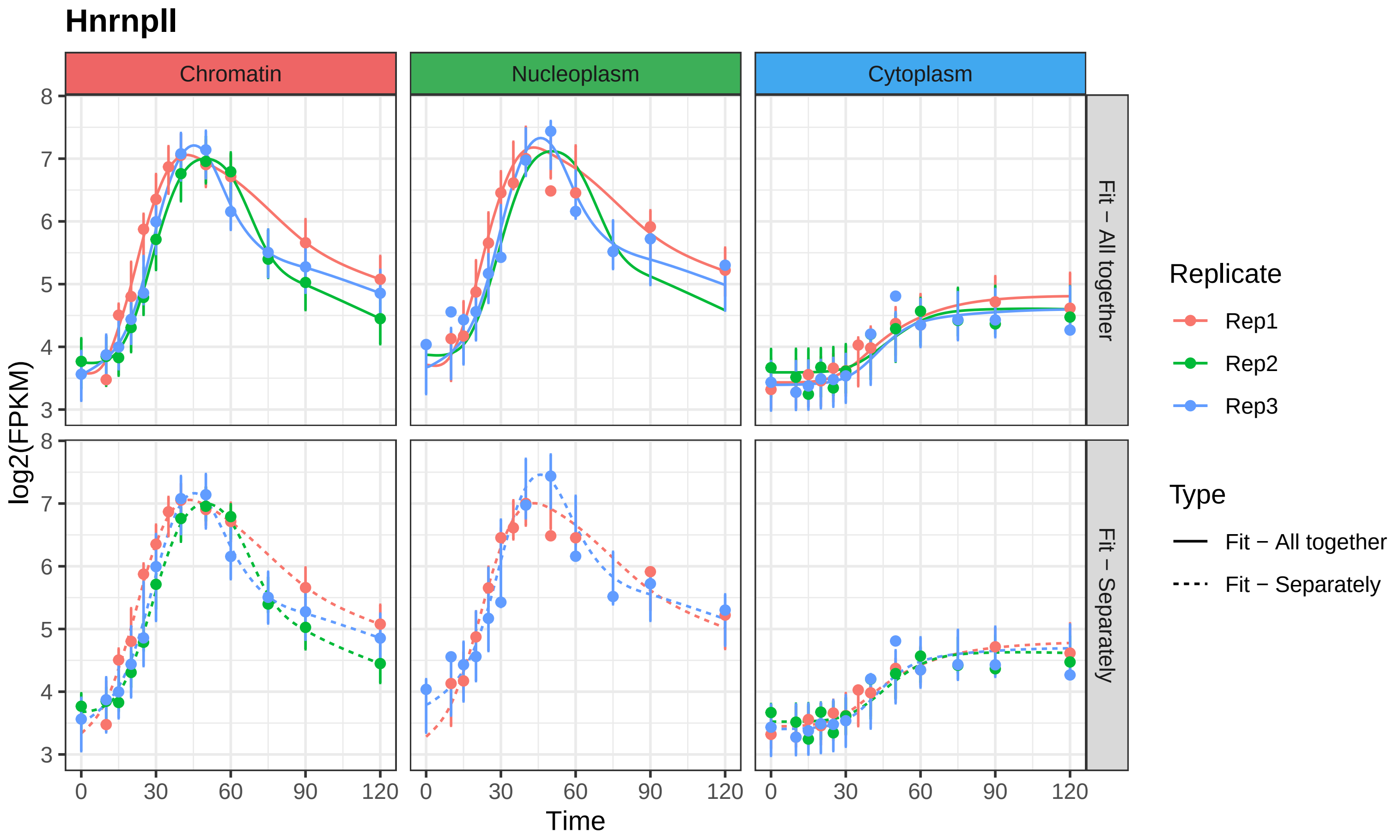

	Together	b1	b2	b3
**−NLL b1 ca**	−5.501	−5.722		
**−NLL b1 np**	1.865	0.216		
**−NLL b1 cyto**	−5.751	−7.108		
**−NLL b2 ca**	−7.705		−8.065	
**−NLL b2 np**				
**−NLL b2 cyto**	−2.451		−1.558	
**−NLL b3 ca**	−7.422			−3.702
**−NLL b3 np**	7.322			3.106
**−NLL b3 cyto**	1.485			1.052
Total	−18.16	−12.61	−9.623	0.4565
**Total with regul**	−16.44	−13.45	−10.35	0.3043

	Together	b1	b2	b3
spar	0.3340000	0.393300	0.3748	0.37370
σ_b	0.1310000	0.107700	0.09596	0.13650
σ_t	0.0003284	0.003977	0.0001869	1.88500
ca_{0,b_1}	3.6420000	3.412000		
$\log_{10}(k_1')$	0.1973000	4.806000		0.40350
$\log_{10}(k_2)$	0.1610000	4.822000	5.64 or −2.4	0.31420
$\log_{10}(k_2')$	−2.5140000	−2.522000		−2.51400
$\log_{10}(k_{deg})$	−2.4300000	−2.572000	−2.4 or 5.64	−2.39700
$\log_{10}(k_1' k_2')$	−2.3170000	2.284000	3.194	−2.11000
$\log_{10}(k_1'/k_2)$	0.0363600	−0.015950		0.08935
transport = $\log_{10}(k_1' k_2'/k_2)$	−2.4780000	−2.538000	−2.446 or 5.594	−2.42400

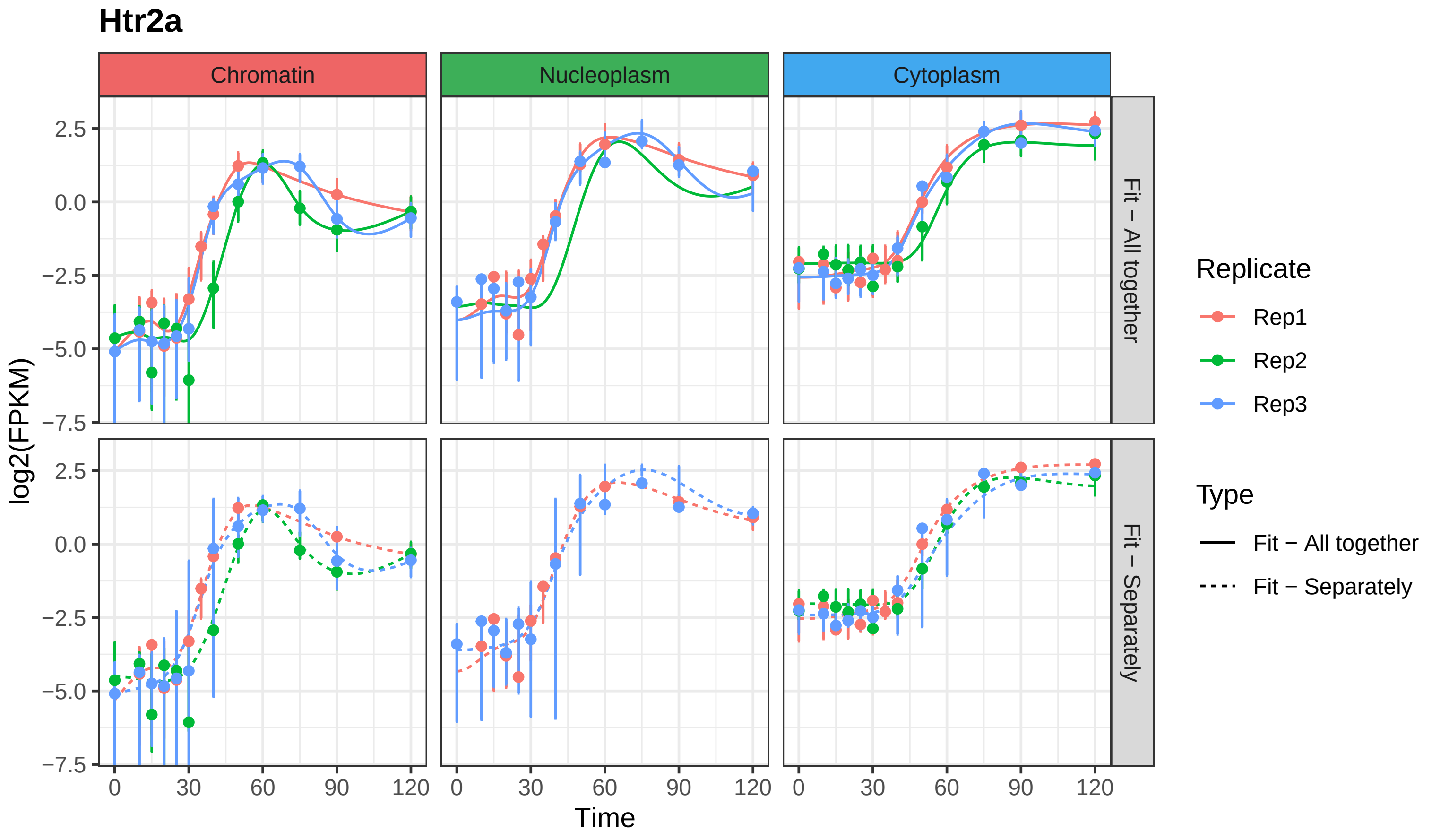

	Together	b1	b2	b3
**−NLL b1 ca**	2.149	0.6513		
**−NLL b1 np**	4.776	3.825		
**−NLL b1 cyto**	3.097	−1.296		
**−NLL b2 ca**	5.321		6.038	
**−NLL b2 np**				
**−NLL b2 cyto**	3.213		2.013	
**−NLL b3 ca**	2.924			7.167
**−NLL b3 np**	11.68			11.31
**−NLL b3 cyto**	5.086			2.172
Total	38.24	3.18	8.051	20.65
**Total with regul**	41.72	2.527	6.812	19.53

	Together	b1	b2	b3
spar	0.3014000	3.697e−01	0.3964	3.957e−01
σ_b	0.1518000	1.469e−06	0.1005	3.327e−06
σ_t	0.0001939	5.331e−07	1.173e−05	5.498e+00
ca_{0,b_1}	−5.0930000	−5.341e+00		
$\log_{10}(k_1')$	−0.5244000	−6.230e−01		−7.097e−01
$\log_{10}(k_2)$	−0.8450000	−9.201e−01	−0.8578 or −1.567	−1.155e+00
$\log_{10}(k_2')$	−1.2770000	−1.315e+00		−1.521e+00
$\log_{10}(k_{deg})$	−1.7160000	−1.855e+00	−1.567 or −0.8578	−1.881e+00
$\log_{10}(k_1'k_2')$	−1.8020000	−1.938e+00	−1.677	−2.230e+00
$\log_{10}(k_1'/k_2)$	0.3205000	2.971e−01		4.454e−01
transport = $\log_{10}(k_1'k_2'/k_2)$	−0.9567000	−1.018e+00	−0.8191 or −0.1101	−1.075e+00

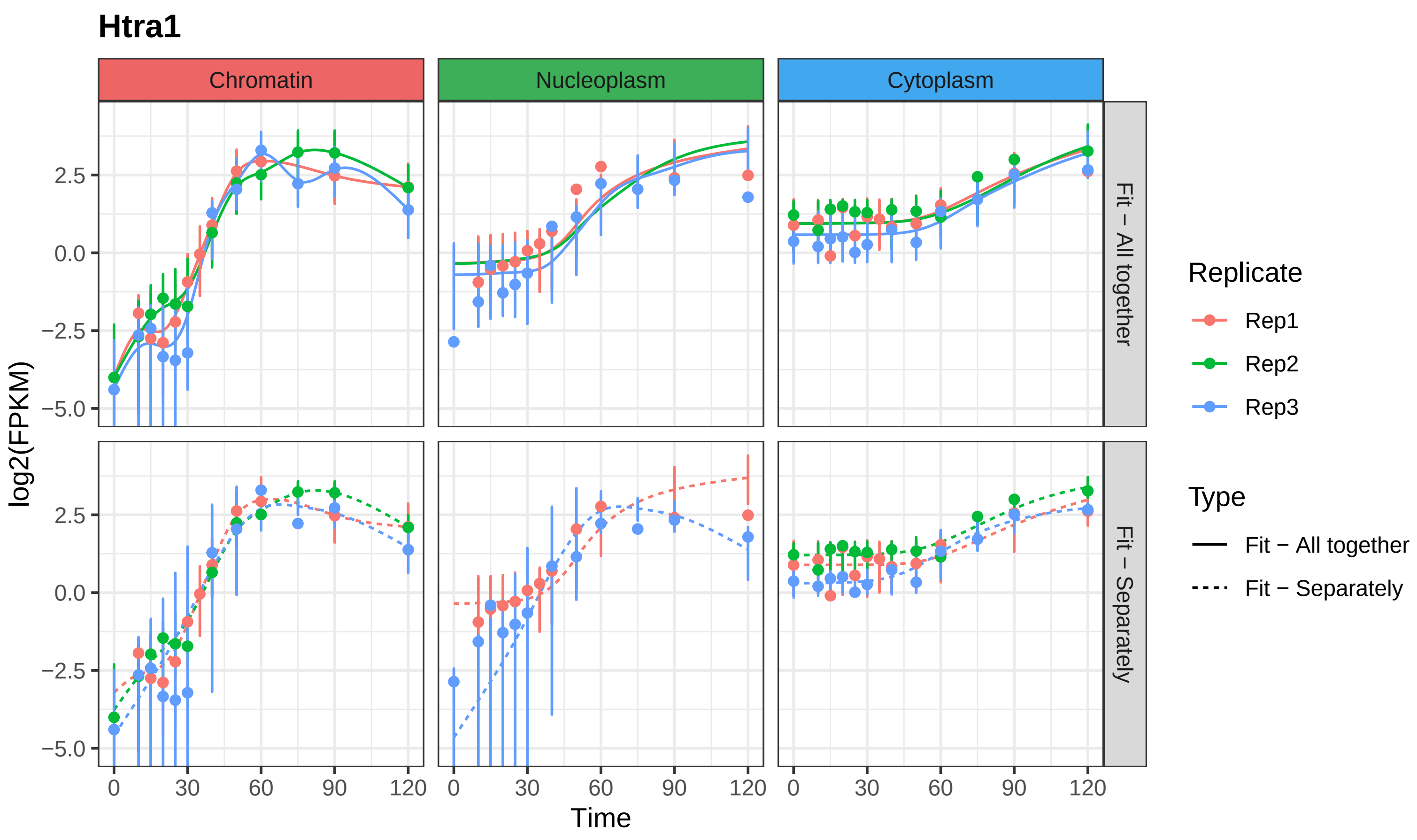

	Together	b1	b2	b3
−NLL b1 ca	4.254	4.696		
−NLL b1 np	14.51	13.76		
−NLL b1 cyto	6.49	5.721		
−NLL b2 ca	4.376		2.091	
−NLL b2 np				
−NLL b2 cyto	5.699		0.9971	
−NLL b3 ca	7.686			12.64
−NLL b3 np	20.03			13.79
−NLL b3 cyto	4.437			−1.738
Total	67.48	24.17	3.088	24.69
Total with regul	72.13	25.73	1.729	25.52

	Together	b1	b2	b3
spar	3.280e−01	4.051e−01	0.4059	5.517e−01
σ_b	2.510e−01	2.509e−01	0.1067	2.145e−05
σ_t	1.139e−05	1.355e−05	1.627e−06	7.334e+00
ca_{0,b_1}	−4.032e+00	−3.258e+00		
$\log_{10}(k_1')$	−1.667e+00	−1.509e+00		5.290e+00
$\log_{10}(k_2)$	−2.766e+00	−2.371e+00	−1.087 or −3.259	5.310e+00
$\log_{10}(k_2')$	−1.592e+00	−1.898e+00		−1.868e+00
$\log_{10}(k_{deg})$	−1.979e+00	−2.271e+00	−3.259 or −1.087	−3.363e+00
$\log_{10}(k_1' k_2')$	−3.259e+00	−3.407e+00	−2.847	3.421e+00
$\log_{10}(k_1'/k_2)$	1.099e+00	8.615e−01		−2.052e−02
transport = $\log_{10}(k_1' k_2'/k_2)$	−4.933e−01	−1.037e+00	−1.76 or 0.4124	−1.889e+00

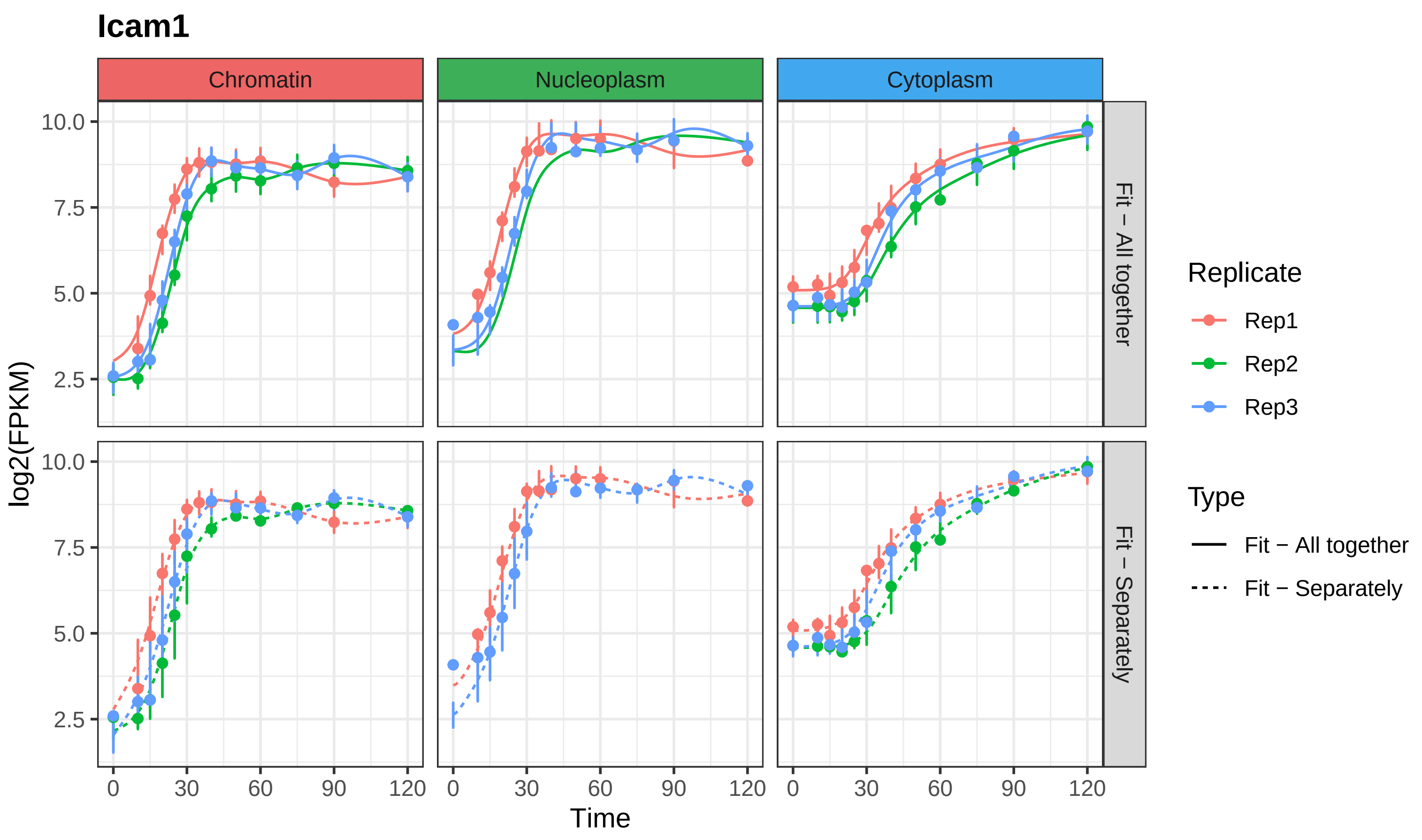

	Together	b1	b2	b3
**−NLL b1 ca**	−3.409	−2.482		
**−NLL b1 np**	3.372	3.814		
**−NLL b1 cyto**	−4.399	−5.638		
**−NLL b2 ca**	−5.234		−7.019	
**−NLL b2 np**				
**−NLL b2 cyto**	−4.575		−6.394	
**−NLL b3 ca**	−2.956			−0.8622
**−NLL b3 np**	9.131			1.819
**−NLL b3 cyto**	−3.179			−3.266
Total	−11.25	−4.306	−13.41	−2.309
**Total with regul**	−8.412	−4.622	−13.23	−3.174

	Together	b1	b2	b3
spar	0.310600	0.3820	0.3412	0.38120
σ_b	0.142300	0.1089	0.02746	0.09392
σ_t	0.002811	1.4260	2.223	1.81800
ca_{0,b_1}	3.082000	2.8700		
$\log_{10}(k_1')$	0.017840	−0.1620		0.22300
$\log_{10}(k_2)$	−0.222700	−0.3751	−0.8594 or −2.154	0.04136
$\log_{10}(k_2')$	−1.747000	−1.7340		−1.70100
$\log_{10}(k_{deg})$	−2.127000	−2.2130	−2.154 or −0.8594	−2.29700
$\log_{10}(k_1'k_2')$	−1.729000	−1.8960	−2.282	−1.47800
$\log_{10}(k_1'/k_2)$	0.240600	0.2130		0.18160
transport = $\log_{10}(k_1'k_2'/k_2)$	−1.507000	−1.5210	−1.423 or −0.1279	−1.52000

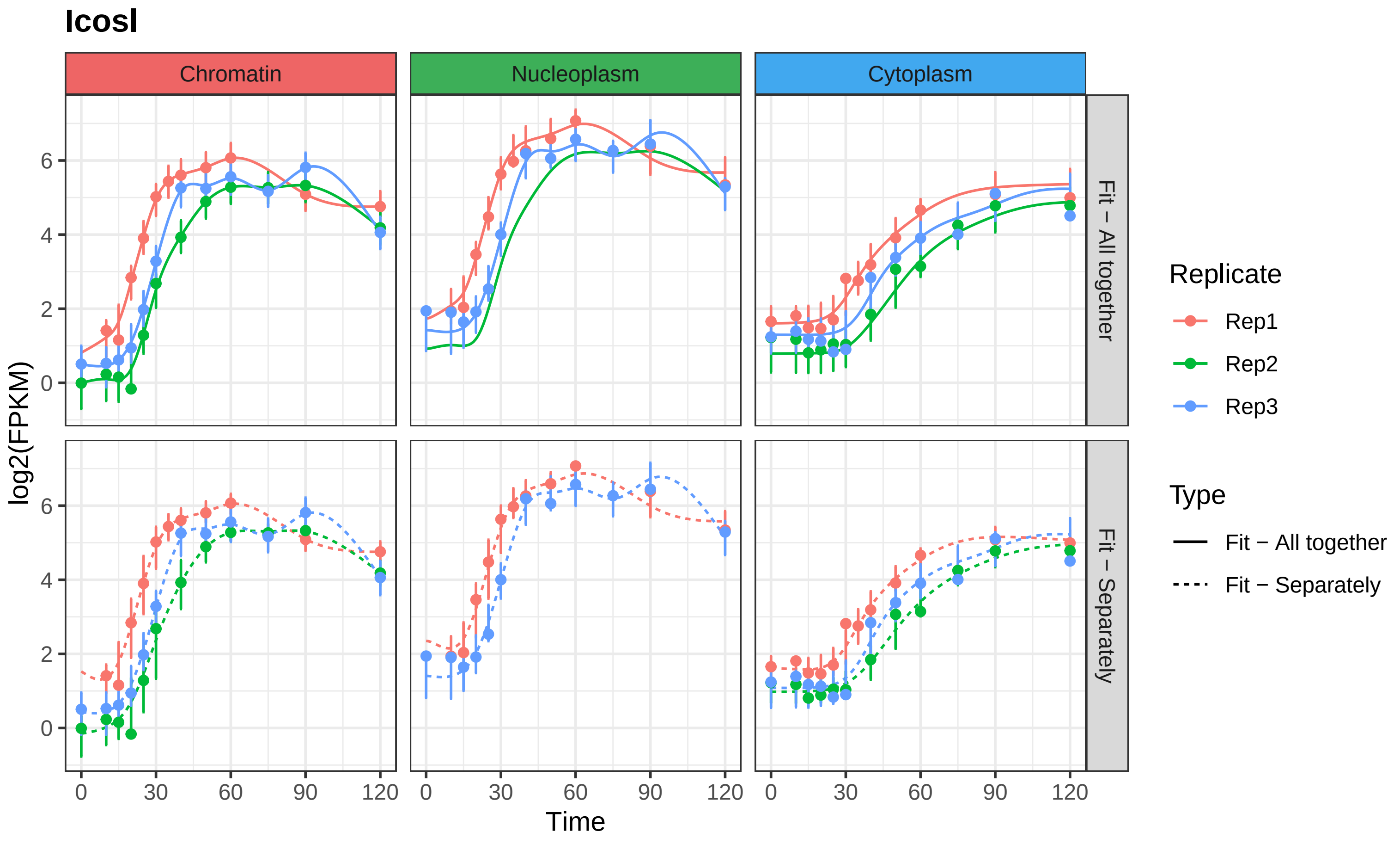

	Together	b1	b2	b3
**−NLL b1 ca**	−4.051	−4.881		
**−NLL b1 np**	−0.2519	1.943		
**−NLL b1 cyto**	−0.2052	−3.237		
**−NLL b2 ca**	−3.895		−1.641	
**−NLL b2 np**				
**−NLL b2 cyto**	2.098		−1.293	
**−NLL b3 ca**	−5.928			−3.667
**−NLL b3 np**	0.3109			0.2581
**−NLL b3 cyto**	9.327			5.189
Total	−2.594	−6.176	−2.933	1.78
**Total with regul**	3.948	−4.406	−3.775	4.366

	Together	b1	b2	b3
spar	0.2613000	0.32330	0.3754	3.144e−01
σ_b	0.1466000	0.09429	0.06992	1.565e−01
σ_t	0.0003046	1.56900	2.429	4.082e−05
ca_{0,b_1}	0.8140000	1.56300		
$\log_{10}(k_1')$	0.1829000	−0.04396		2.663e−01
$\log_{10}(k_2)$	−0.0944600	−0.29180	6.082 or −2.192	−2.650e−02
$\log_{10}(k_2')$	−2.1210000	−2.02400		−2.106e+00
$\log_{10}(k_{deg})$	−2.0830000	−1.80000	−2.192 or 6.082	−2.008e+00
$\log_{10}(k_1'k_2')$	−1.9380000	−2.06800	4.23	−1.839e+00
$\log_{10}(k_1'/k_2)$	0.2773000	0.24780		2.928e−01
$\text{transport} = \log_{10}(k_1'k_2'/k_2)$	−1.8440000	−1.77700	−1.852 or 6.421	−1.813e+00

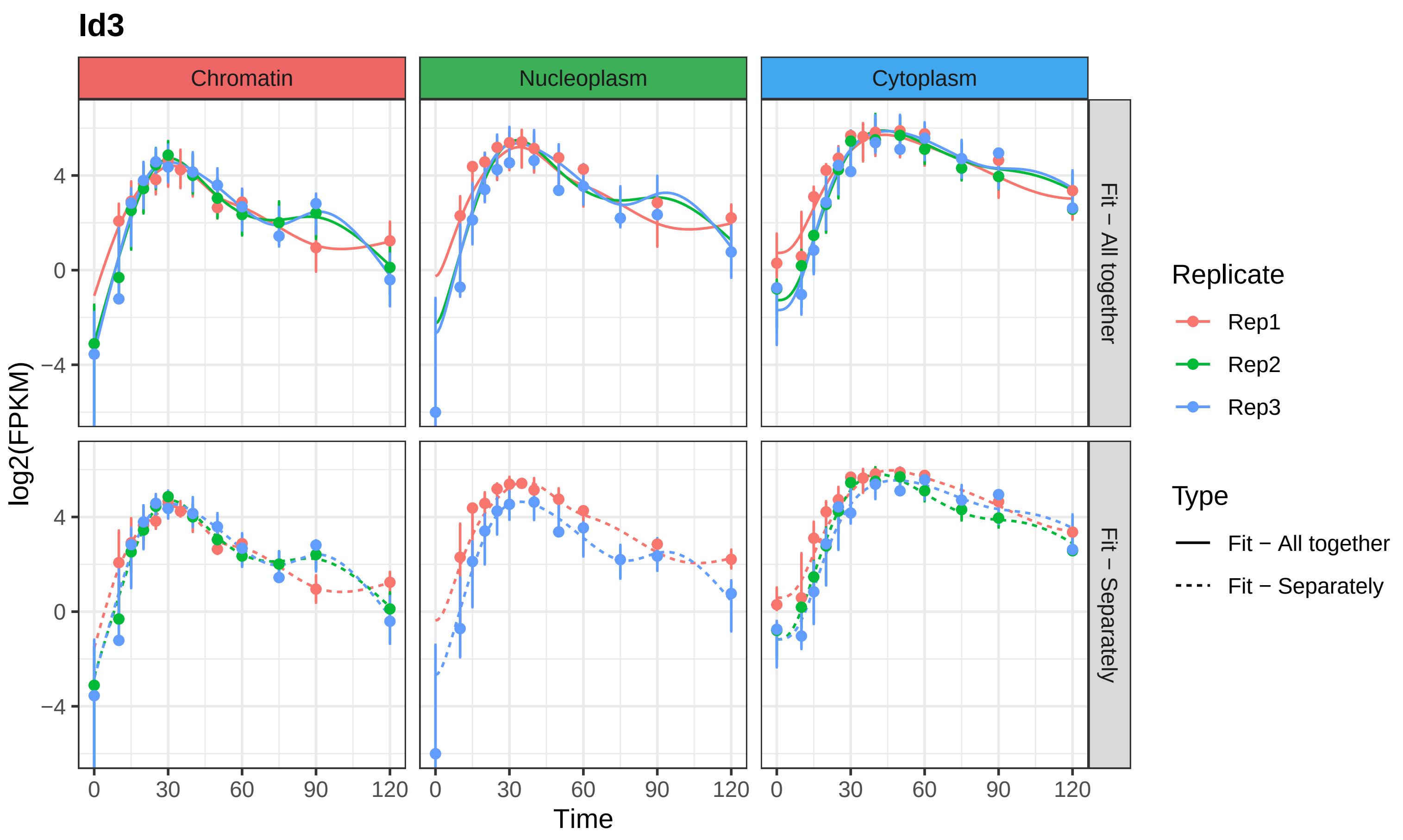

	Together	b1	b2	b3
**−NLL b1 ca**	2.542	0.6265		
**−NLL b1 np**	9.701	1.857		
**−NLL b1 cyto**	8.468	−0.4887		
**−NLL b2 ca**	4.317		−0.1361	
**−NLL b2 np**				
**−NLL b2 cyto**	5.987		−0.03806	
**−NLL b3 ca**	7.078			4.628
**−NLL b3 np**	19.42			3.828
**−NLL b3 cyto**	12.36			10.53
Total	69.88	1.995	−0.1742	18.98
**Total with regul**	73.01	4.697	−1.434	19.07

	Together	b1	b2	b3
spar	0.3908	3.480e−01	0.3988	0.40550
σ_b	0.2664	3.166e−05	0.1021	0.19760
σ_t	1.2780	3.094e+00	0.7928	1.72900
ca_{0,b_1}	−1.1810	−1.618e+00		
$\log_{10}(k_1')$	−0.1367	−3.733e−01		−0.48030
$\log_{10}(k_2)$	−0.3843	−7.247e−01	−0.3077 or −1.031	−0.52300
$\log_{10}(k_2')$	−0.8665	−8.878e−01		−0.82930
$\log_{10}(k_{deg})$	−1.1630	−1.178e+00	−1.031 or −0.3077	−1.28100
$\log_{10}(k_1'k_2')$	−1.0030	−1.261e+00	−0.8687	−1.31000
$\log_{10}(k_1'/k_2)$	0.2476	3.514e−01		0.04266
$\text{transport} = \log_{10}(k_1'k_2'/k_2)$	−0.6189	−5.364e−01	−0.5611 or 0.1622	−0.78670

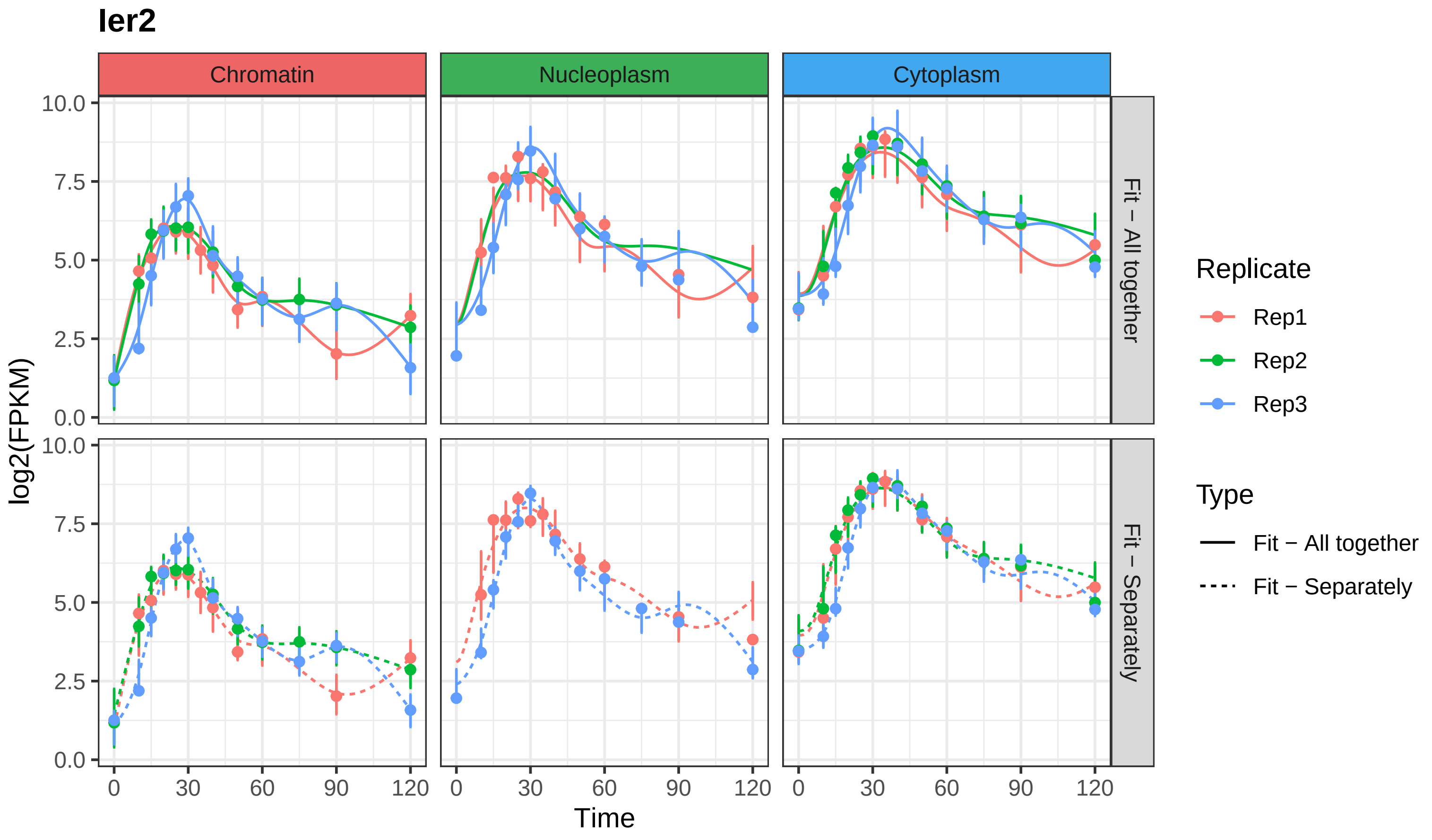

	Together	b1	b2	b3
−NLL b1 ca	0.03114	−0.4785		
−NLL b1 np	14.43	9.84		
−NLL b1 cyto	7.206	2.935		
−NLL b2 ca	−0.2501		−2.462	
−NLL b2 np				
−NLL b2 cyto	5.175		7.678	
−NLL b3 ca	1.296			−2.38
−NLL b3 np	12.39			3.477
−NLL b3 cyto	4.329			−1.539
Total	44.61	12.3	5.217	−0.4412
Total with regul	54.8	20.04	7.398	5.851

	Together	b1	b2	b3
spar	0.28940	0.34960	0.3295	2.570e−01
σ_b	0.24620	0.18990	0.1775	1.522e−01
σ_t	0.46820	1.53200	1.051	2.921e−05
ca_{0,b_1}	1.22300	0.92510		
$\log_{10}(k_1')$	0.03984	0.04760		2.048e−01
$\log_{10}(k_2)$	−0.48350	−0.58210	−0.6084 or −0.6488	−1.994e−01
$\log_{10}(k_2')$	−0.54170	−0.59610		−5.767e−01
$\log_{10}(k_{deg})$	−0.82340	−0.85220	−0.6488 or −0.6084	−9.111e−01
$\log_{10}(k_1'k_2')$	−0.50190	−0.54850	−0.4514	−3.719e−01
$\log_{10}(k_1'/k_2)$	0.52330	0.62970		4.042e−01
$\text{transport} = \log_{10}(k_1'k_2'/k_2)$	−0.01839	0.03361	0.157 or 0.1974	−1.725e−01

Ifnb1

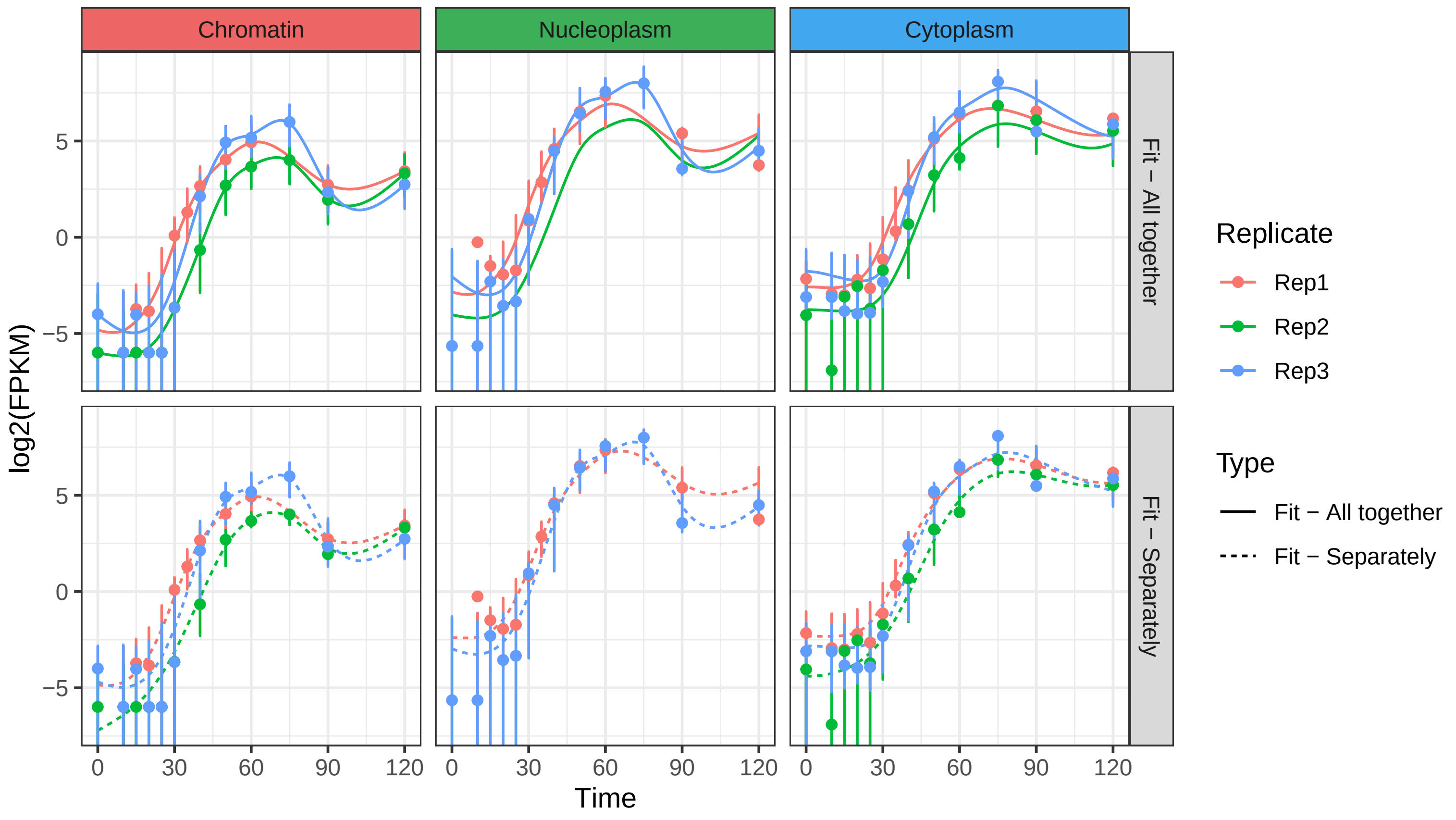

	Together	b1	b2	b3
**−NLL b1 ca**	12.32	11.52		
**−NLL b1 np**	22.38	20.26		
**−NLL b1 cyto**	12.35	9.589		
**−NLL b2 ca**	13.35		10.36	
**−NLL b2 np**				
**−NLL b2 cyto**	17.49		9.537	
**−NLL b3 ca**	14.36			14.07
**−NLL b3 np**	18.69			13.84
**−NLL b3 cyto**	21.44			17.39
Total	132.4	41.37	19.89	45.3
**Total with regul**	140	45.09	17.81	48.29

	Together	b1	b2	b3
spar	0.3160	3.602e−01	0.4222	0.3512
σ_b	0.3457	2.881e−01	1.127e−06	0.2552
σ_t	1.0560	6.866e−05	1.973	2.1680
ca_{0,b_1}	−4.8010	−4.825e+00		
$\log_{10}(k_1')$	3.9030	9.924e−02		7.4370
$\log_{10}(k_2)$	3.3090	−6.408e−01	−0.03362 or −1.338	6.9200
$\log_{10}(k_2')$	−1.1150	−1.175e+00		−1.2610
$\log_{10}(k_{deg})$	−1.1950	−1.197e+00	−1.338 or −0.03362	−1.3080
$\log_{10}(k_1'k_2')$	2.7880	−1.076e+00	−0.522	6.1760
$\log_{10}(k_1'/k_2)$	0.5938	7.400e−01		0.5169
transport $= \log_{10}(k_1'k_2'/k_2)$	−0.5213	−4.350e−01	−0.4884 or 0.8156	−0.7443

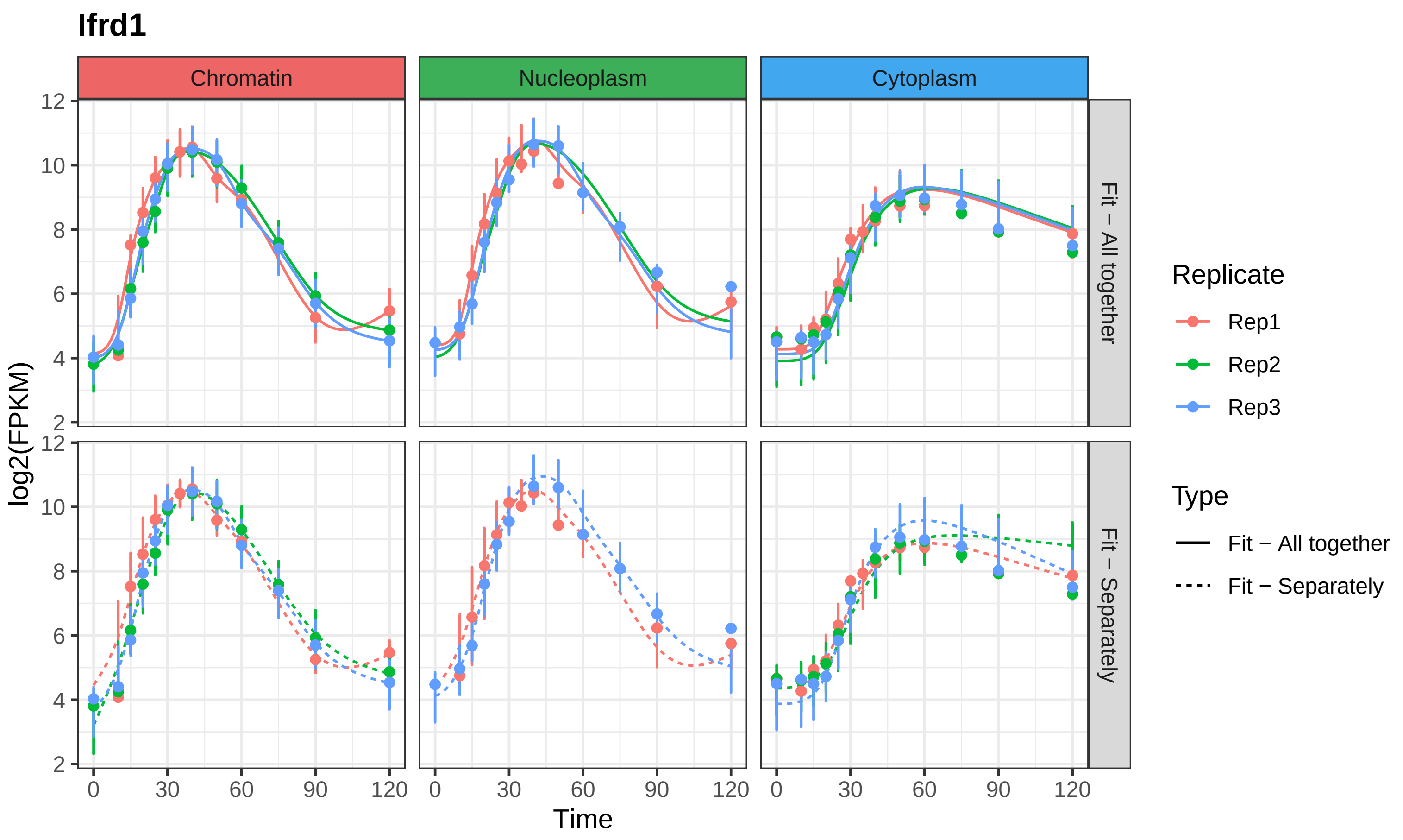

	Together	b1	b2	b3
**−NLL b1 ca**	3.877	2.809		
**−NLL b1 np**	5.112	6.829		
**−NLL b1 cyto**	5.103	2.598		
**−NLL b2 ca**	0.2976		2.358	
**−NLL b2 np**				
**−NLL b2 cyto**	14.18		11.4	
**−NLL b3 ca**	0.09398			1.307
**−NLL b3 np**	10.83			8.39
**−NLL b3 cyto**	5.084			7.885
Total	44.57	12.24	13.76	17.58
**Total with regul**	54.07	14.39	15.81	22.5

	Together	b1	b2	b3
spar	0.2508000	0.35790	0.3817	3.084e−01
σ_b	0.2497000	0.13350	0.2622	2.542e−01
σ_t	0.0003294	2.52700	0.01197	3.487e−07
ca_{0,b_1}	4.1890000	4.54100		
$\log_{10}(k_1')$	−0.2031000	−0.29020		−3.669e−01
$\log_{10}(k_2)$	−0.2796000	−0.30450	5.035 or −2.175	−5.055e−01
$\log_{10}(k_2')$	−1.7170000	−1.80100		−1.658e+00
$\log_{10}(k_{deg})$	−1.6780000	−1.75900	−2.175 or 5.035	−1.578e+00
$\log_{10}(k_1'k_2')$	−1.9200000	−2.09200	3.208	−2.025e+00
$\log_{10}(k_1'/k_2)$	0.0765000	0.01432		1.386e−01
transport $= \log_{10}(k_1'k_2'/k_2)$	−1.6410000	−1.78700	−1.828 or 5.382	−1.519e+00

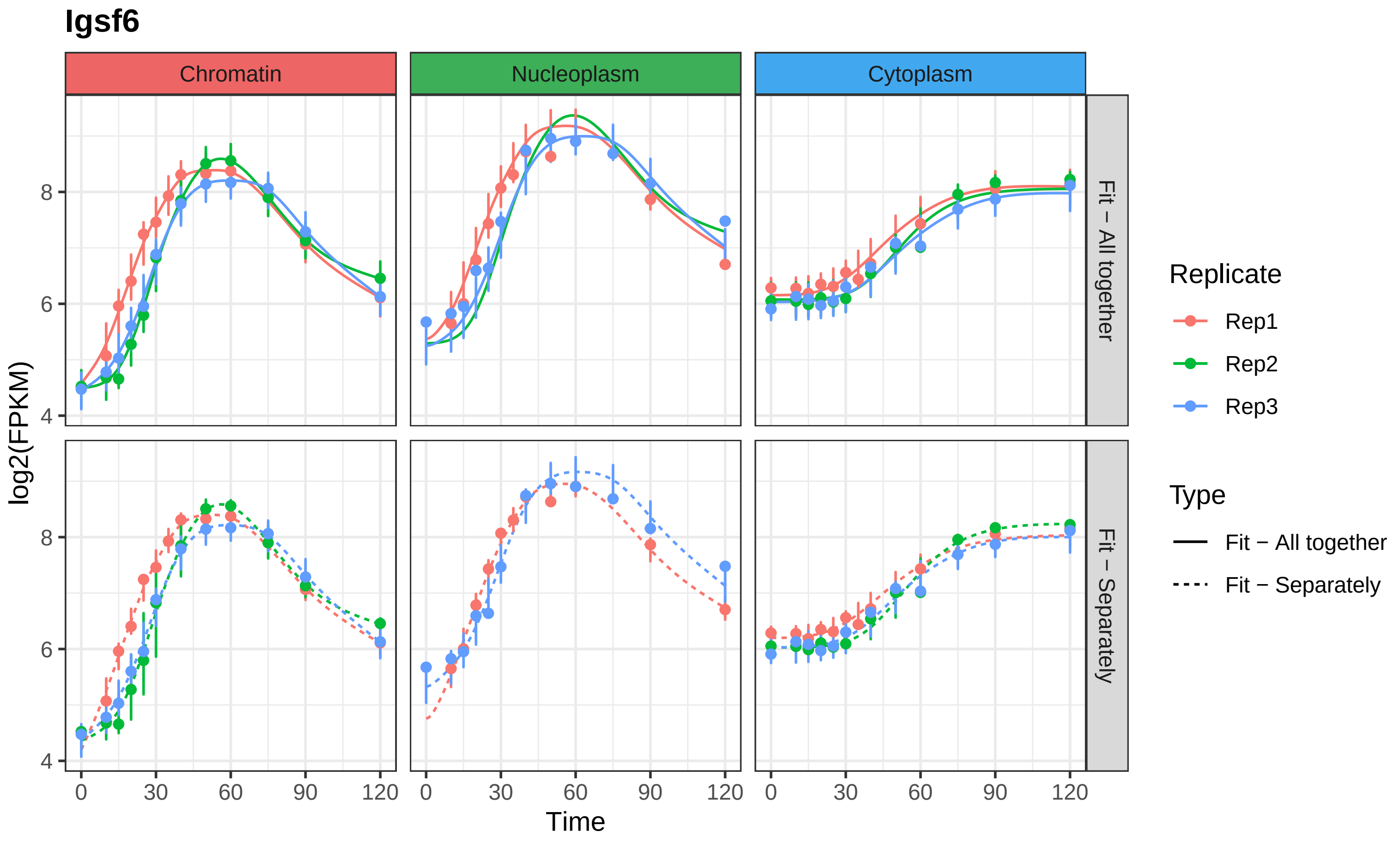

	Together	b1	b2	b3
**−NLL b1 ca**	−7.457	−10.54		
**−NLL b1 np**	4.13	−6.979		
**−NLL b1 cyto**	−7.55	−10.91		
**−NLL b2 ca**	−8.605		−8.158	
**−NLL b2 np**				
**−NLL b2 cyto**	−6.149		−13.41	
**−NLL b3 ca**	−9.21			−8.302
**−NLL b3 np**	9.013			0.0763
**−NLL b3 cyto**	−7.167			−6.95
Total	−33	−28.43	−21.57	−15.18
**Total with regul**	−32.52	−28.62	−23.03	−16.07

	Together	b1	b2	b3
spar	0.3535	0.38300	0.3908	0.38050
σ_b	0.1080	0.06829	0.01375	0.09396
σ_t	1.0000	0.38410	2.658	0.79400
ca_{0,b_1}	4.6140	4.22200		
$\log_{10}(k_1')$	−0.2161	−0.24770		0.10040
$\log_{10}(k_2)$	−0.4559	−0.41610	−0.8487 or −2.244	−0.18610
$\log_{10}(k_2')$	−2.0700	−2.09200		−2.11600
$\log_{10}(k_{deg})$	−2.3050	−2.52600	−2.244 or −0.8487	−2.32700
$\log_{10}(k_1'k_2')$	−2.2860	−2.33900	−2.592	−2.01600
$\log_{10}(k_1'/k_2)$	0.2398	0.16840		0.28660
$\text{transport} = \log_{10}(k_1'k_2'/k_2)$	−1.8300	−1.92300	−1.743 or −0.3476	−1.82900

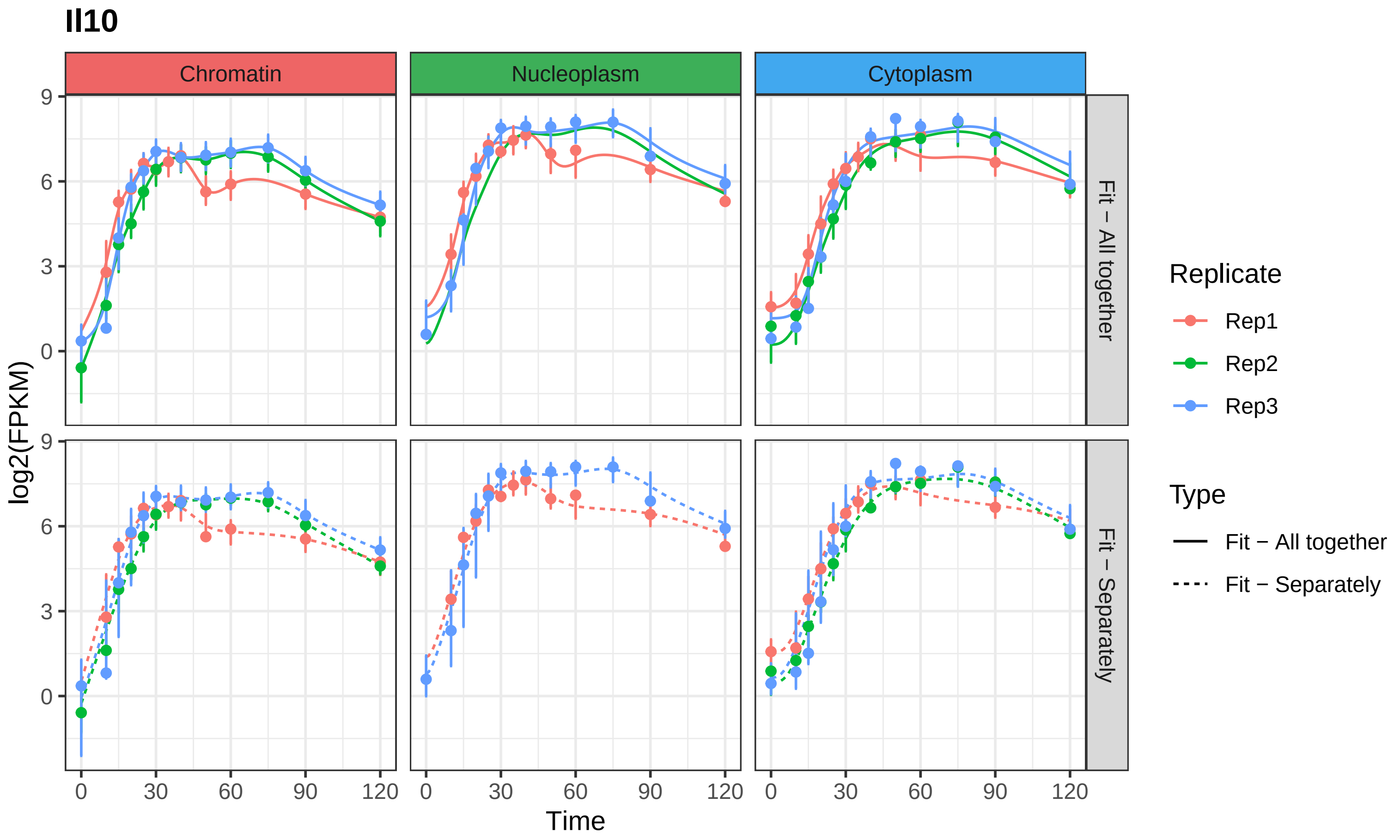

	Together	b1	b2	b3
**−NLL b1 ca**	−3.006	0.239		
**−NLL b1 np**	0.5466	1.859		
**−NLL b1 cyto**	3.842	1.403		
**−NLL b2 ca**	−3.003		−3.474	
**−NLL b2 np**				
**−NLL b2 cyto**	2.633		−1.372	
**−NLL b3 ca**	−0.9905			1.69
**−NLL b3 np**	2.757			3.129
**−NLL b3 cyto**	15.05			11.01
Total	17.83	3.501	−4.846	15.83
**Total with regul**	29.19	5.003	−6.716	15.79

	Together	b1	b2	b3
spar	0.21600	0.3907	0.428	0.37710
σ_b	0.16840	0.1437	0.08127	0.14800
σ_t	0.82720	1.2860	1.036	2.50600
ca_{0,b_1}	0.73020	0.5055		
$\log_{10}(k_1')$	−0.08029	−0.2558		−0.03058
$\log_{10}(k_2)$	−0.34180	−0.5131	−0.7264 or −0.7826	−0.28930
$\log_{10}(k_2')$	−1.07600	−1.0450		−0.92230
$\log_{10}(k_{deg})$	−1.06500	−1.0900	−0.7826 or −0.7264	−0.88070
$\log_{10}(k_1'k_2')$	−1.15600	−1.3010	−1.29	−0.95280
$\log_{10}(k_1'/k_2)$	0.26150	0.2574		0.25870
transport $= \log_{10}(k_1'k_2'/k_2)$	−0.81430	−0.7879	−0.5632 or −0.507	−0.66350

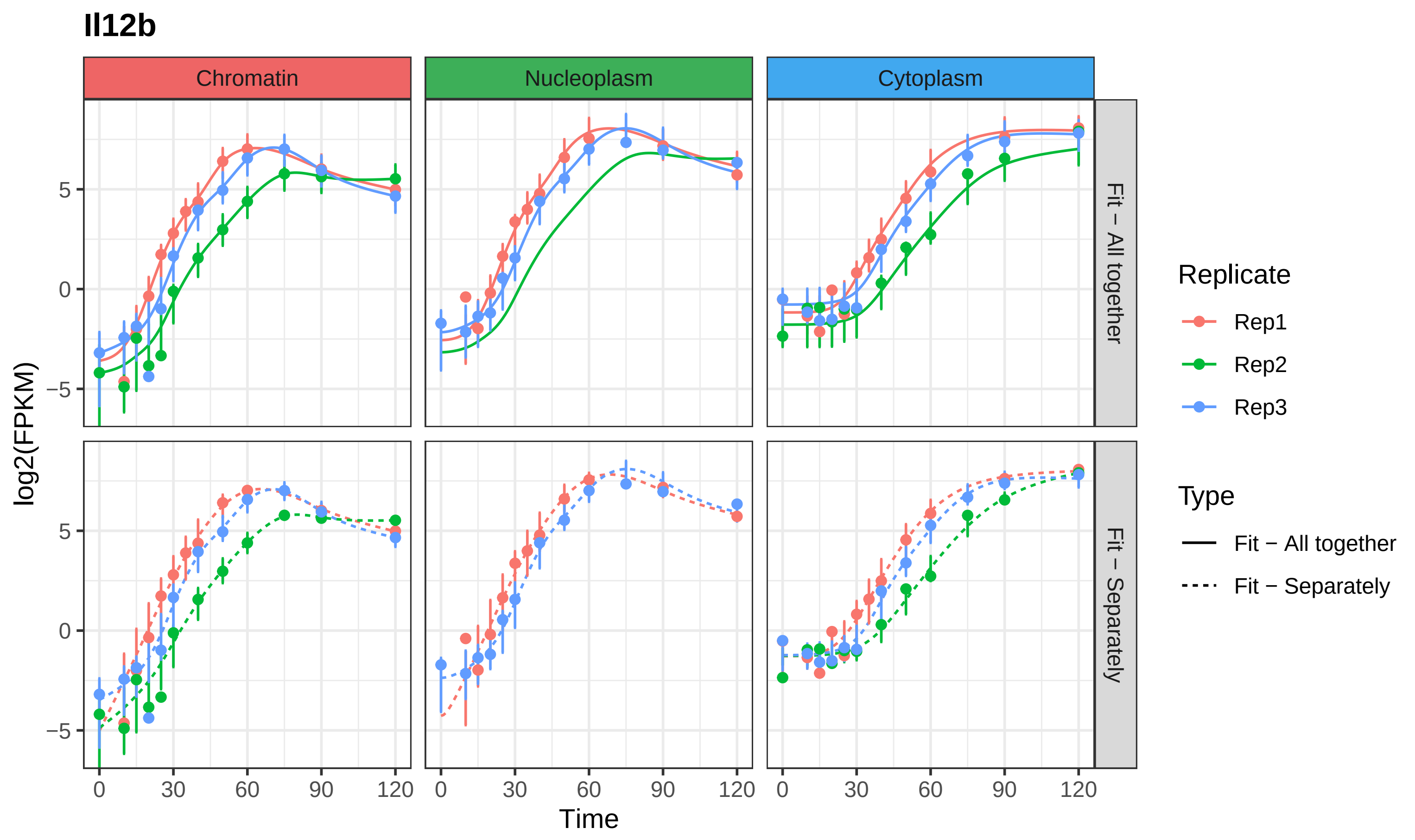

	Together	b1	b2	b3
**−NLL b1 ca**	4.344	4.064		
**−NLL b1 np**	11.96	7.322		
**−NLL b1 cyto**	9.391	13.19		
**−NLL b2 ca**	9.769		4.783	
**−NLL b2 np**				
**−NLL b2 cyto**	11.9		3.348	
**−NLL b3 ca**	10.8			9.567
**−NLL b3 np**	6.032			4.576
**−NLL b3 cyto**	8.43			4.492
Total	72.62	24.57	8.131	18.64
**Total with regul**	79.84	23.3	6.388	20.05

	Together	b1	b2	b3
spar	0.3178000	0.49180	0.4002	0.3360
σ_b	0.2612000	0.05307	7.066e−07	0.1472
σ_t	0.0006183	2.42200	1.985	1.6360
ca_{0,b_1}	−3.5690000	−4.99000		
$\log_{10}(k_1')$	−0.3364000	−0.20840		−0.3607
$\log_{10}(k_2)$	−0.6497000	−0.43430	−1.384 or −1.858	−0.6933
$\log_{10}(k_2')$	−1.4910000	−1.55700		−1.5420
$\log_{10}(k_{deg})$	−1.9080000	−2.45700	−1.858 or −1.384	−1.8840
$\log_{10}(k_1'k_2')$	−1.8270000	−1.76500	−2.155	−1.9020
$\log_{10}(k_1'/k_2)$	0.3133000	0.22590		0.3326
transport $= \log_{10}(k_1'k_2'/k_2)$	−1.1780000	−1.33100	−0.7712 or −0.2973	−1.2090

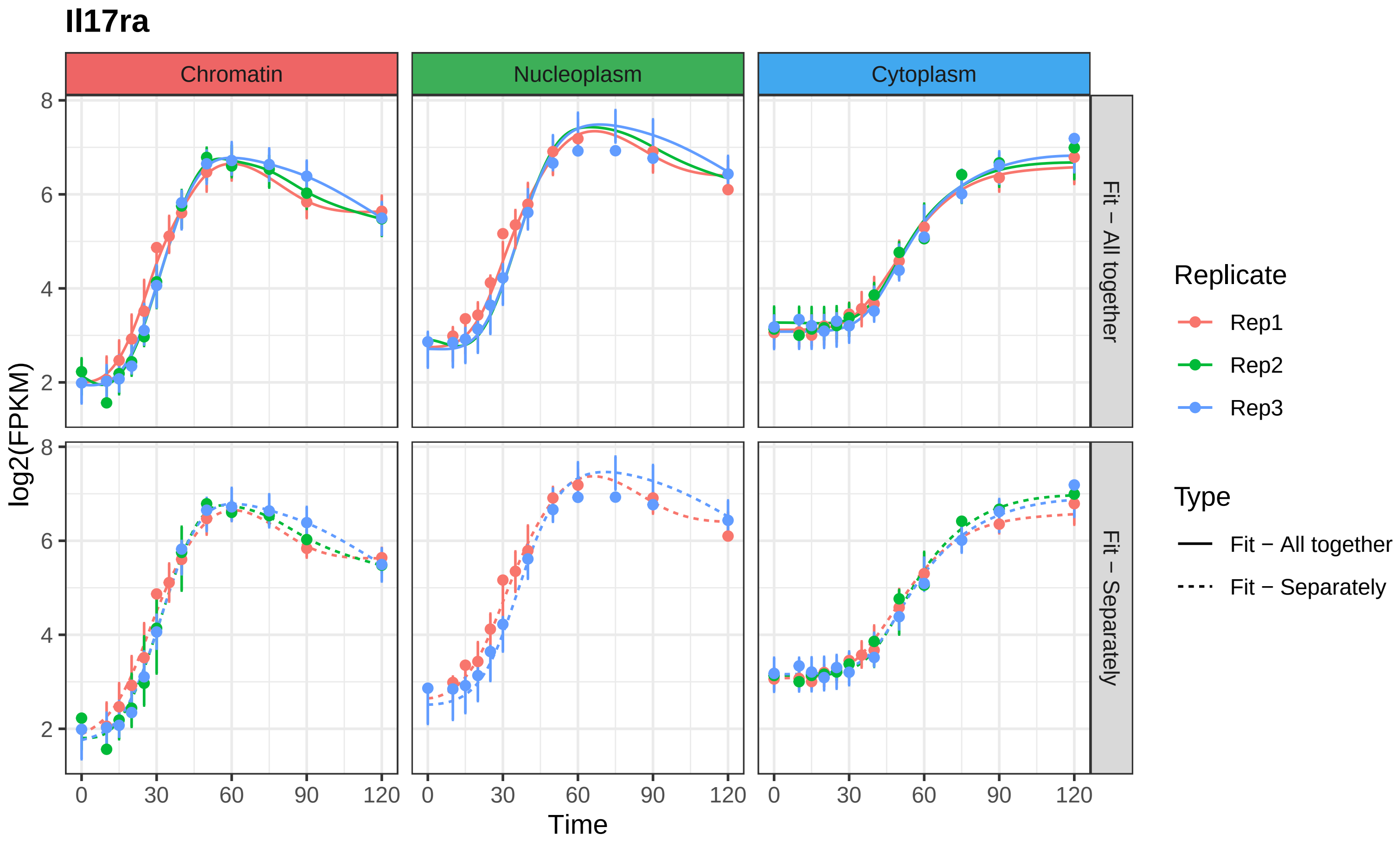

	Together	b1	b2	b3
**−NLL b1 ca**	−5.398	−5.501		
**−NLL b1 np**	0.9818	−1.153		
**−NLL b1 cyto**	−7.067	−9.028		
**−NLL b2 ca**	−4.935		−1.341	
**−NLL b2 np**				
**−NLL b2 cyto**	−2.033		−9.958	
**−NLL b3 ca**	−6.953			−4.844
**−NLL b3 np**	4.72			1.729
**−NLL b3 cyto**	−2.559			−4.692
Total	−23.24	−15.68	−11.3	−7.806
**Total with regul**	−23.69	−17.13	−13.19	−9.032

	Together	b1	b2	b3
spar	0.3913	0.44940	0.4167	4.086e−01
σ_b	0.1199	0.07617	0.04337	1.224e−01
σ_t	0.8012	1.45900	2.41	2.761e−05
ca_{0,b_1}	2.0350	1.97000		
$\log_{10}(k_1')$	−0.5743	−0.47880		−6.607e−01
$\log_{10}(k_2)$	−0.8038	−0.71040	−1.138 or −1.849	−8.879e−01
$\log_{10}(k_2')$	−1.8150	−1.84800		−1.843e+00
$\log_{10}(k_{deg})$	−1.9260	−1.97700	−1.849 or −1.138	−2.038e+00
$\log_{10}(k_1'k_2')$	−2.3890	−2.32700	−2.586	−2.504e+00
$\log_{10}(k_1'/k_2)$	0.2295	0.23160		2.272e−01
$\text{transport} = \log_{10}(k_1'k_2'/k_2)$	−1.5850	−1.61600	−1.448 or −0.7371	−1.616e+00

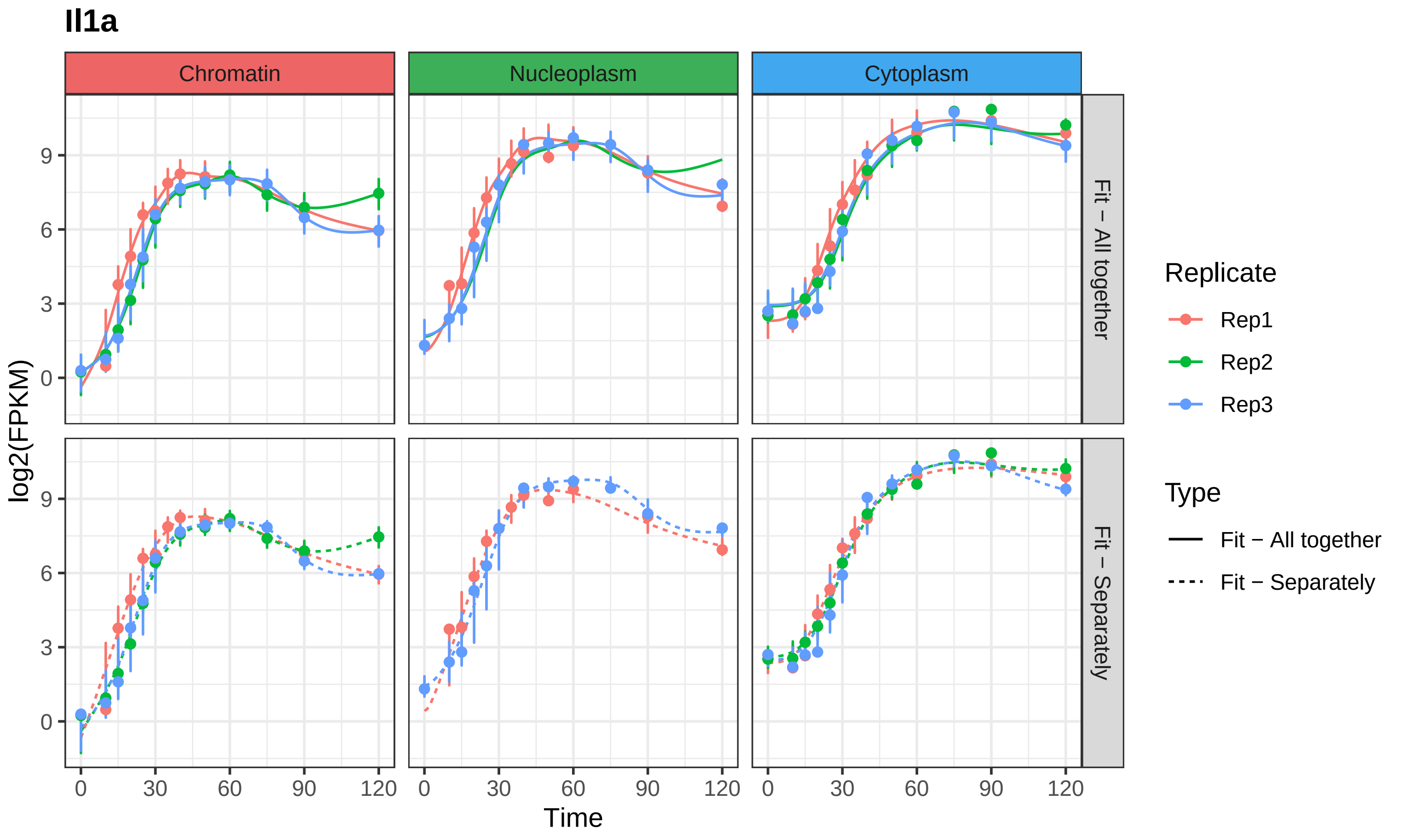

	Together	b1	b2	b3
**−NLL b1 ca**	2.726	1.169		
**−NLL b1 np**	8.535	4.273		
**−NLL b1 cyto**	7.324	1.453		
**−NLL b2 ca**	−0.2374		−2.022	
**−NLL b2 np**				
**−NLL b2 cyto**	7.31		1.329	
**−NLL b3 ca**	0.756			−2.431
**−NLL b3 np**	4.759			1.345
**−NLL b3 cyto**	11.12			5.086
Total	42.3	6.895	−0.6926	4
**Total with regul**	47.29	6.371	−0.6116	4.285

	Together	b1	b2	b3
spar	0.2950	0.412700	0.3657	0.34180
σ_b	0.2066	0.115000	0.1394	0.05728
σ_t	1.3600	1.759000	0.469	2.23700
ca_{0,b_1}	−0.3404	−0.637700		
$\log_{10}(k_1')$	0.0313	0.007322		0.02270
$\log_{10}(k_2)$	−0.4046	−0.323000	−0.2904 or −1.481	−0.49770
$\log_{10}(k_2')$	−1.0510	−1.164000		−1.01400
$\log_{10}(k_{deg})$	−1.4220	−1.750000	−1.481 or −0.2904	−1.32200
$\log_{10}(k_1'k_2')$	−1.0190	−1.157000	−0.8659	−0.99090
$\log_{10}(k_1'/k_2)$	0.4359	0.330400		0.52040
transport = $\log_{10}(k_1'k_2'/k_2)$	−0.6149	−0.833900	−0.5755 or 0.6152	−0.49320

Il1b

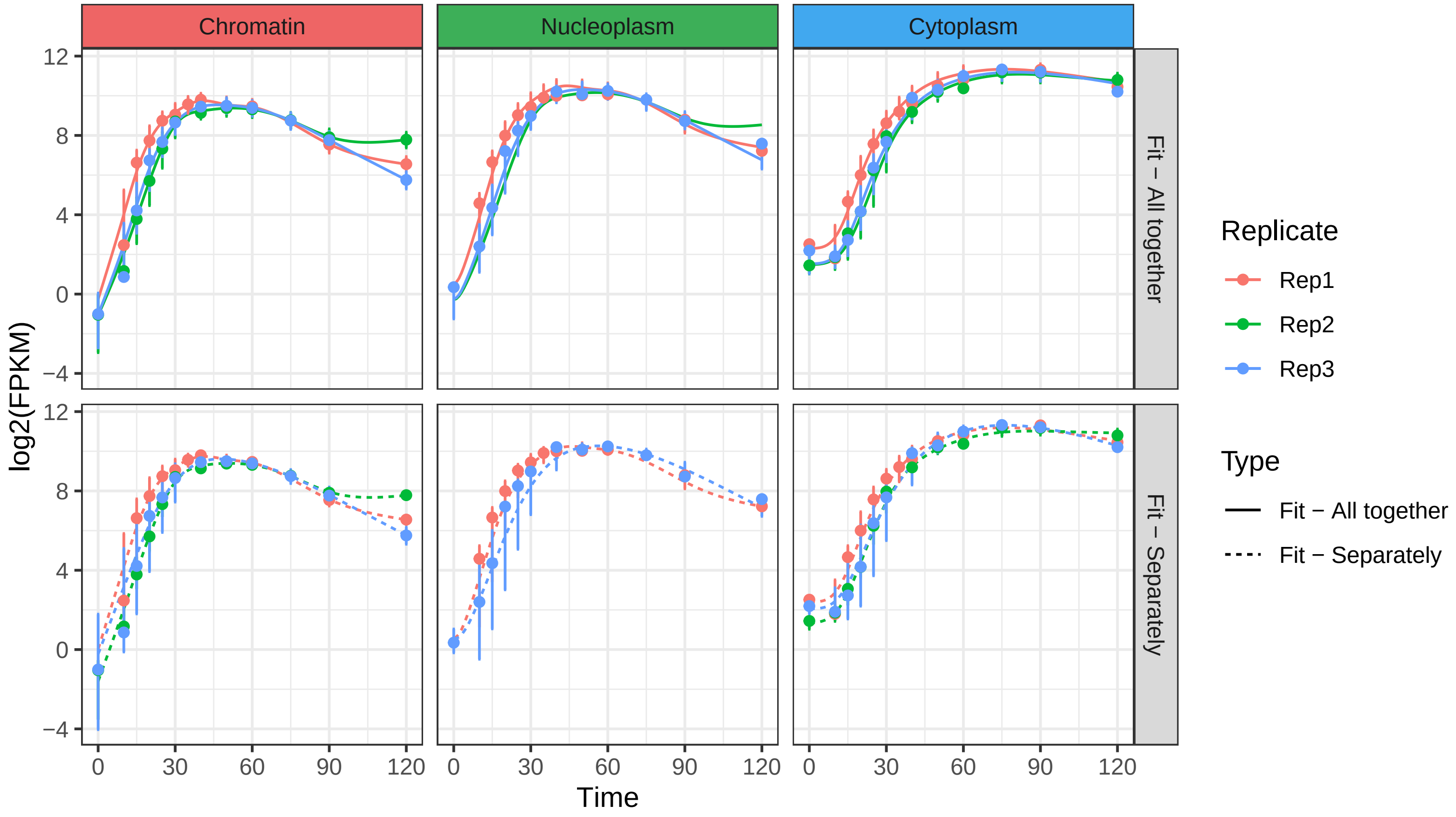

	Together	b1	b2	b3
**−NLL b1 ca**	−1.198	−3.109		
**−NLL b1 np**	3.653	1.255		
**−NLL b1 cyto**	5.645	3.717		
**−NLL b2 ca**	−0.9447		−3.923	
**−NLL b2 np**				
**−NLL b2 cyto**	0.8593		−1.561	
**−NLL b3 ca**	0.7685			1.907
**−NLL b3 np**	8.773			8.57
**−NLL b3 cyto**	5.005			1.15
Total	22.56	1.863	−5.484	11.63
**Total with regul**	25.9	2.074	−5.908	9.589

	Together	b1	b2	b3
spar	0.3170	0.36040	0.3598	0.44120
σ_b	0.1376	0.07278	0.07028	0.04105
σ_t	1.3800	2.07100	1.01	2.99900
ca_{0,b_1}	−0.2442	0.01638		
$\log_{10}(k_1')$	−0.2425	−0.46620		−0.64700
$\log_{10}(k_2)$	−0.4806	−0.63750	7.267 or −1.919	−0.87800
$\log_{10}(k_2')$	−1.1500	−1.11200		−0.94480
$\log_{10}(k_{deg})$	−1.6840	−1.65800	−1.919 or 7.267	−1.40300
$\log_{10}(k_1' k_2')$	−1.3930	−1.57900	6.236	−1.59200
$\log_{10}(k_1'/k_2)$	0.2380	0.17130		0.23090
$\text{transport} = \log_{10}(k_1' k_2'/k_2)$	−0.9125	−0.94110	−1.031 or 8.155	−0.71380

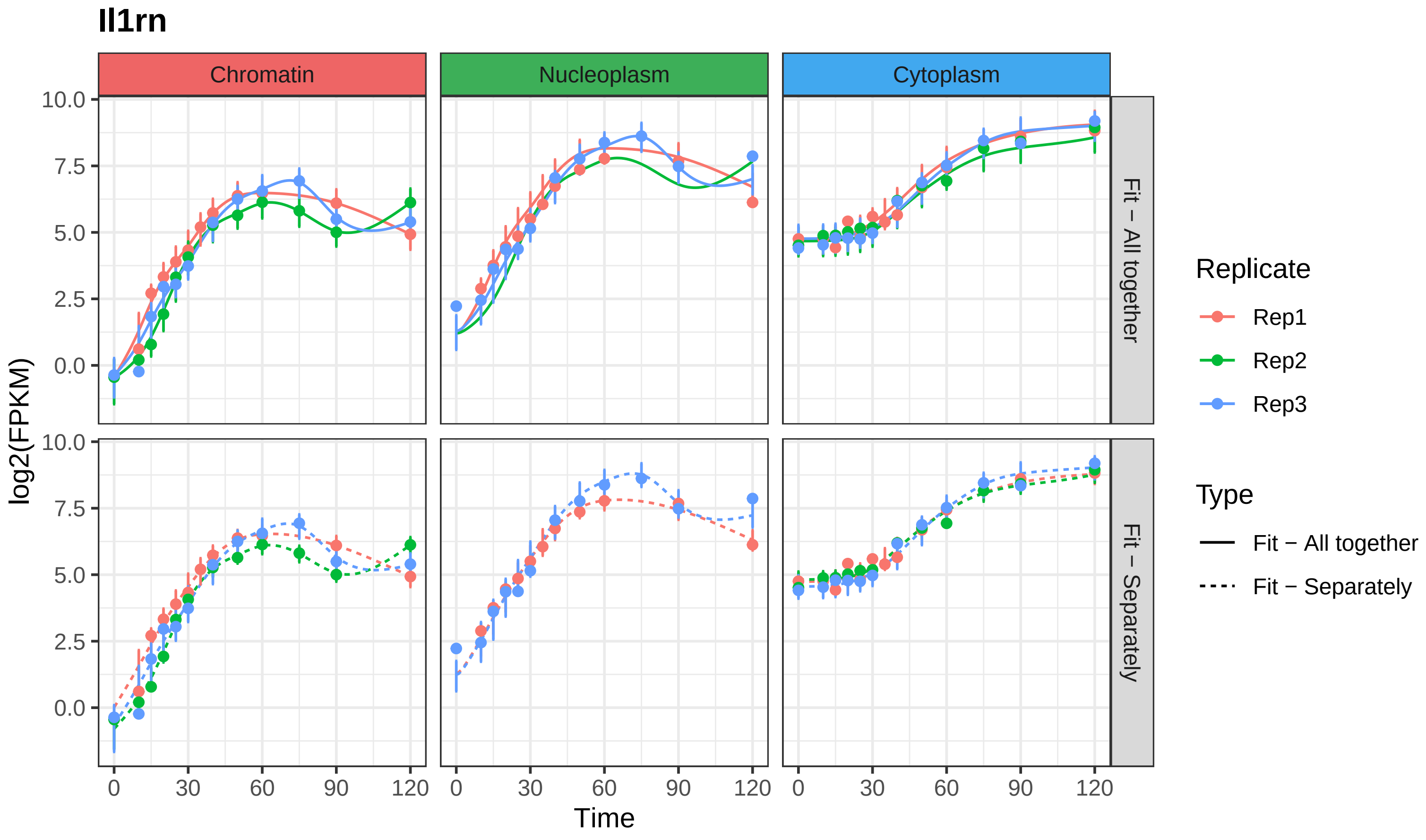

	Together	b1	b2	b3
**−NLL b1 ca**	−0.6805	−0.7546		
**−NLL b1 np**	7.88	−2.143		
**−NLL b1 cyto**	2.811	1.429		
**−NLL b2 ca**	−2.011		−5.433	
**−NLL b2 np**				
**−NLL b2 cyto**	0.8554		−1.913	
**−NLL b3 ca**	2.12			2.154
**−NLL b3 np**	10.91			5.23
**−NLL b3 cyto**	0.42			−1.367
Total	22.31	−1.469	−7.346	6.017
**Total with regul**	25.41	−2.699	−7.509	5.568

	Together	b1	b2	b3
spar	0.3365	0.45140	0.3607	0.3920
σ_b	0.1873	0.12320	0.1057	0.1508
σ_t	0.9449	1.45500	0.1513	1.7120
ca_{0,b_1}	−0.4294	0.04688		
$\log_{10}(k_1')$	0.1920	0.11850		0.3962
$\log_{10}(k_2)$	−0.3140	−0.26900	−0.05935 or −2.661	−0.1732
$\log_{10}(k_2')$	−1.5180	−1.48500		−1.5810
$\log_{10}(k_{deg})$	−2.5630	−2.52100	−2.661 or −0.05935	−2.5800
$\log_{10}(k_1' k_2')$	−1.3260	−1.36600	−1.03	−1.1850
$\log_{10}(k_1'/k_2)$	0.5060	0.38740		0.5694
transport $= \log_{10}(k_1' k_2'/k_2)$	−1.0120	−1.09700	−0.9708 or 1.631	−1.0110

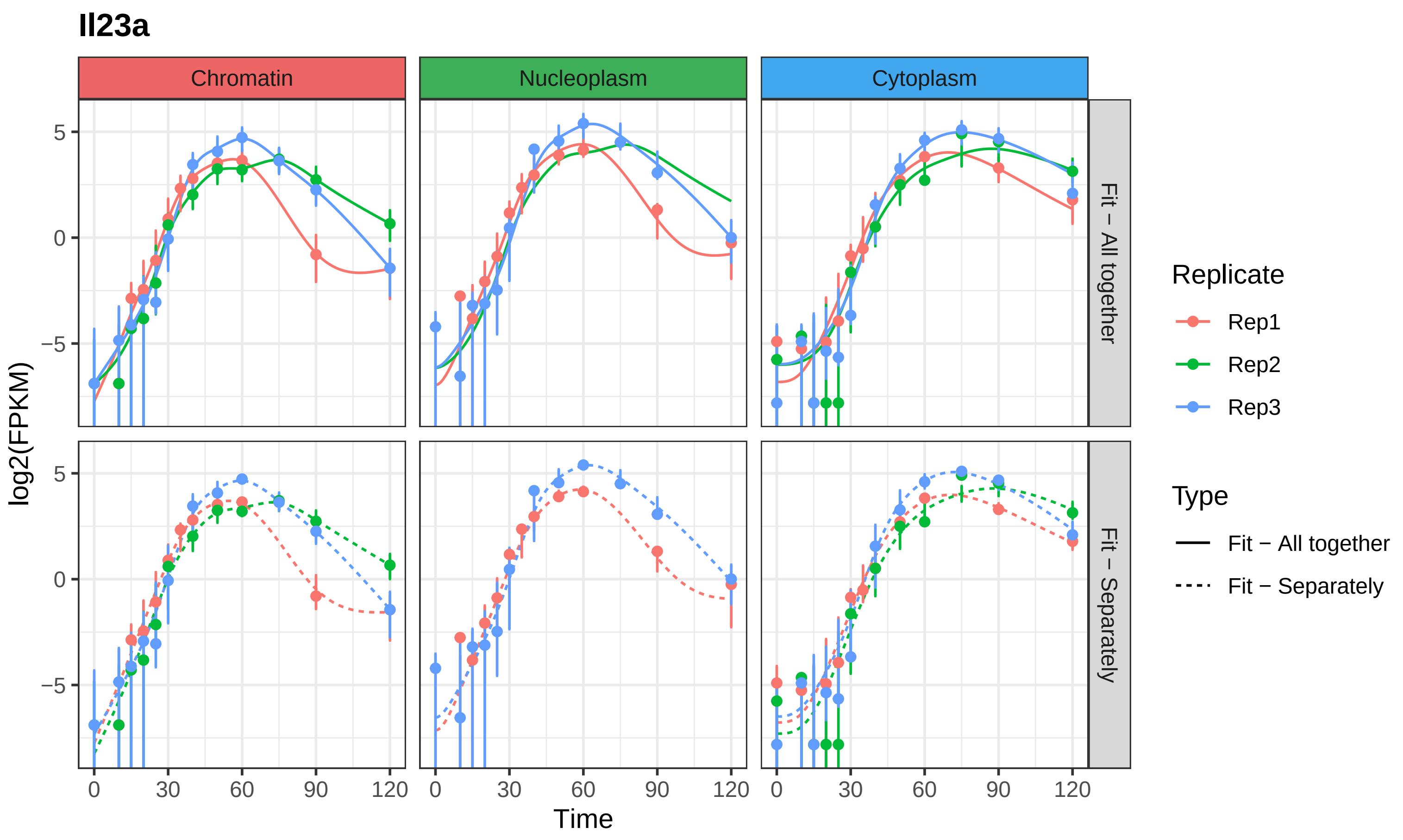

	Together	b1	b2	b3
**−NLL b1 ca**	5.329	3.904		
**−NLL b1 np**	7.895	7.191		
**−NLL b1 cyto**	12.93	9.062		
**−NLL b2 ca**	6.877		5.769	
**−NLL b2 np**				
**−NLL b2 cyto**	23.09		19.12	
**−NLL b3 ca**	6.715			5.678
**−NLL b3 np**	11.33			7.806
**−NLL b3 cyto**	17.89			15.7
Total	92.06	20.16	24.89	29.18
**Total with regul**	95.5	18.32	23.95	28.24

	Together	b1	b2	b3
spar	0.3145	4.220e−01	0.3927	3.701e−01
σ_b	0.1877	1.653e−05	0.1144	2.504e−06
σ_t	1.3200	1.405e+00	1.835	2.214e+00
ca_{0,b_1}	−7.7230	−7.737e+00		
$\log_{10}(k_1')$	−0.3792	−4.876e−01		−3.040e−01
$\log_{10}(k_2)$	−0.6113	−6.714e−01	−1.014 or −1.063	−5.390e−01
$\log_{10}(k_2')$	−1.2340	−1.223e+00		−1.124e+00
$\log_{10}(k_{deg})$	−1.2770	−1.334e+00	−1.063 or −1.014	−1.138e+00
$\log_{10}(k_1' k_2')$	−1.6130	−1.711e+00	−1.791	−1.428e+00
$\log_{10}(k_1'/k_2)$	0.2320	1.838e−01		2.350e−01
$transport = \log_{10}(k_1' k_2'/k_2)$	−1.0010	−1.039e+00	−0.7773 or −0.7283	−8.886e−01

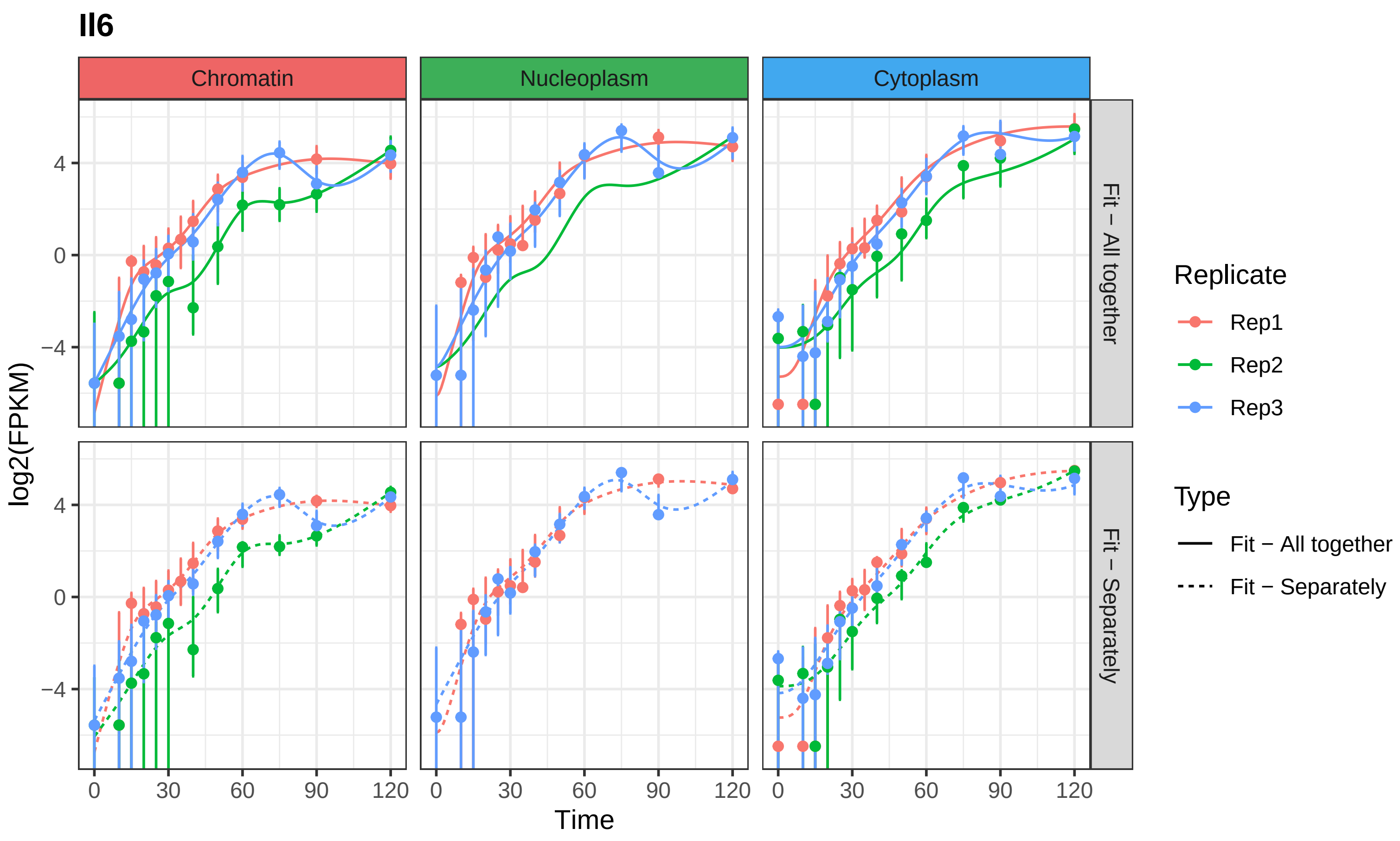

	Together	b1	b2	b3
**−NLL b1 ca**	6.041	3.274		
**−NLL b1 np**	9.588	9.175		
**−NLL b1 cyto**	15.29	9.329		
**−NLL b2 ca**	10.02		6.63	
**−NLL b2 np**				
**−NLL b2 cyto**	18.06		9.871	
**−NLL b3 ca**	5.748			2.983
**−NLL b3 np**	11.21			7.264
**−NLL b3 cyto**	11.76			9.492
Total	87.72	21.78	16.5	19.74
**Total with regul**	88.96	20.67	14.59	18.84

	Together	b1	b2	b3
spar	0.37080	3.894e−01	0.4068	0.3973000
σ_b	0.19180	3.924e−05	0.00041	0.1313000
σ_t	1.83000	2.886e+00	1.231	0.0009792
ca_{0,b_1}	−6.87800	−6.786e+00		
$\log_{10}(k_1')$	−0.04275	−3.471e−01		5.6550000
$\log_{10}(k_2)$	−0.26180	−6.038e−01	−1.053 or −1.091	5.4430000
$\log_{10}(k_2')$	−1.06300	−1.186e+00		−1.1740000
$\log_{10}(k_{deg})$	−1.31600	−1.383e+00	−1.091 or −1.053	−1.3220000
$\log_{10}(k_1'k_2')$	−1.10600	−1.533e+00	−1.486	4.4810000
$\log_{10}(k_1'/k_2)$	0.21900	2.567e−01		0.2114000
$\text{transport} = \log_{10}(k_1'k_2'/k_2)$	−0.84440	−9.293e−01	−0.4327 or −0.3952	−0.9627000

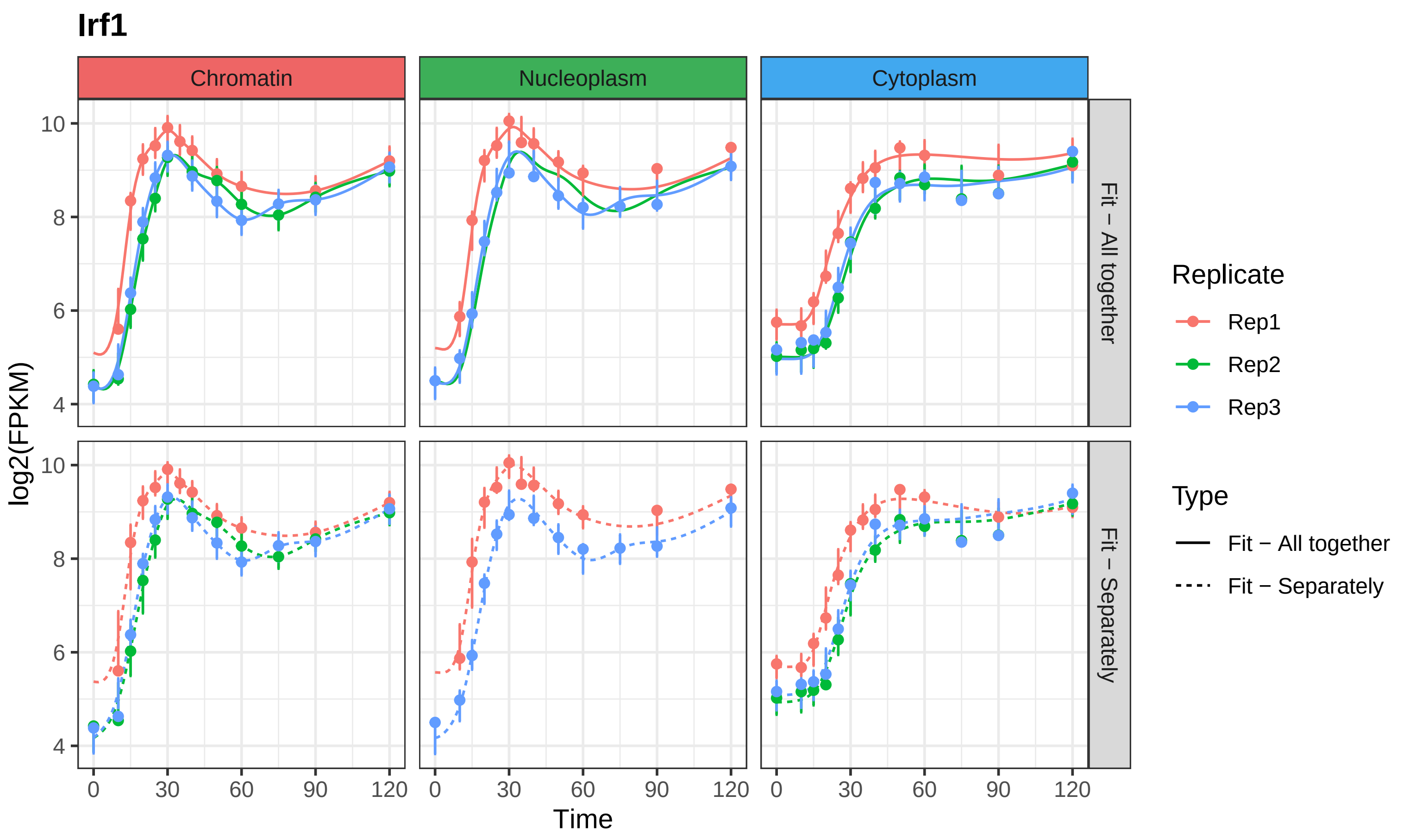

	Together	b1	b2	b3
**−NLL b1 ca**	−5.98	−7.602		
**−NLL b1 np**	−2.692	−0.5861		
**−NLL b1 cyto**	−4.136	−8.284		
**−NLL b2 ca**	−9.221		−5.65	
**−NLL b2 np**				
**−NLL b2 cyto**	−2.109		−2.552	
**−NLL b3 ca**	−8.898			−4.446
**−NLL b3 np**	−4.224			−6.099
**−NLL b3 cyto**	2.204			−1.679
Total	−35.06	−16.47	−8.202	−12.22
**Total with regul**	−25.27	−10.25	−4.775	−7.872

	Together	b1	b2	b3
spar	0.21440	0.25040	0.2868	2.756e−01
σ_b	0.10990	0.08021	0.08964	1.086e−01
σ_t	0.32460	0.96920	0.9346	2.386e−05
ca_{0,b_1}	5.12700	5.41600		
$\log_{10}(k_1')$	−0.15900	−0.20570		−2.778e−01
$\log_{10}(k_2)$	−0.18880	−0.26500	0.3775 or −1.709	−2.776e−01
$\log_{10}(k_2')$	−1.44200	−1.40700		−1.419e+00
$\log_{10}(k_{deg})$	−1.59500	−1.44200	−1.709 or 0.3775	−1.694e+00
$\log_{10}(k_1'k_2')$	−1.60100	−1.61300	−1.101	−1.697e+00
$\log_{10}(k_1'/k_2)$	0.02989	0.05931		−2.268e−04
transport $= \log_{10}(k_1'k_2'/k_2)$	−1.41200	−1.34800	−1.478 or 0.608	−1.420e+00

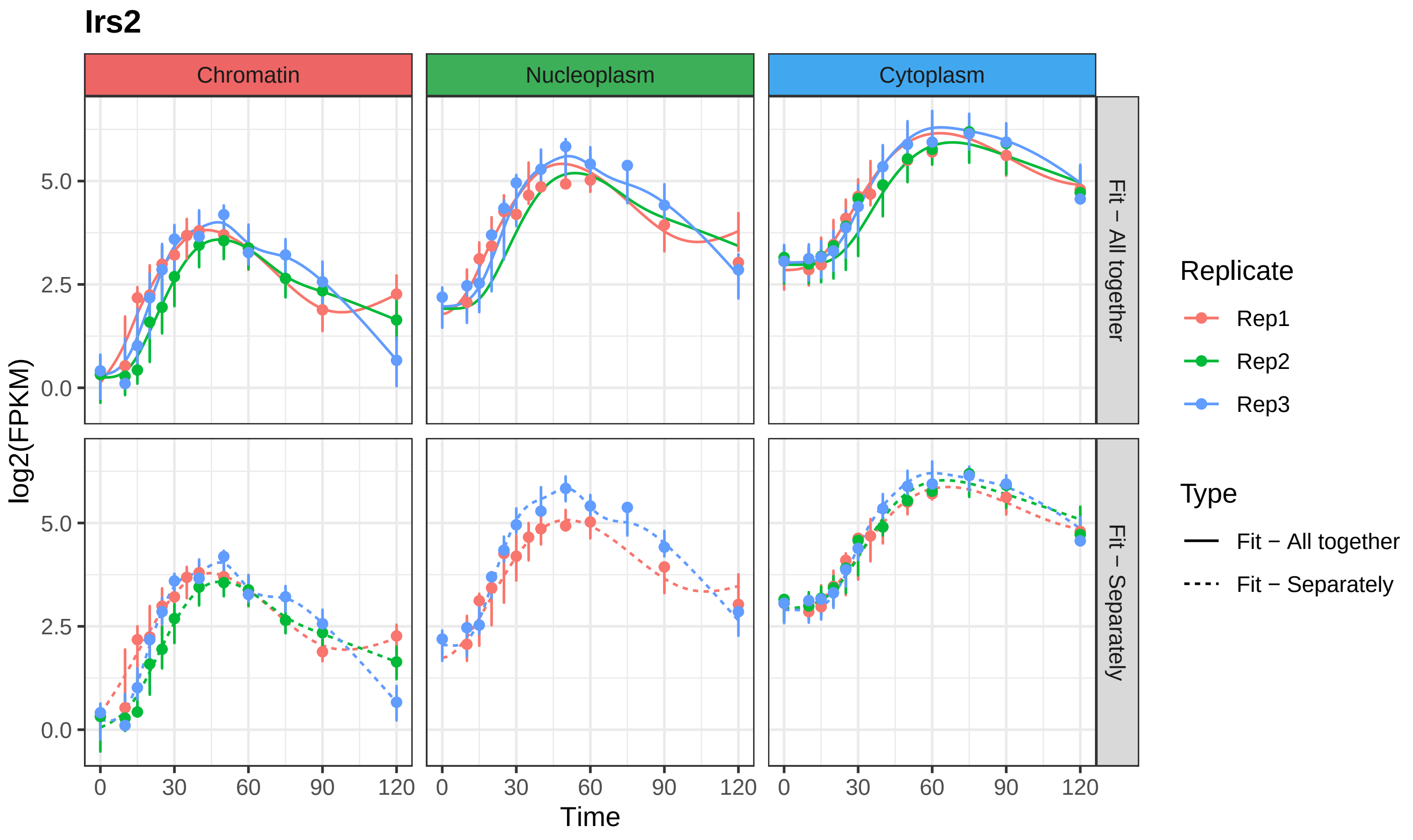

	Together	b1	b2	b3
**−NLL b1 ca**	−2.164	−2.647		
**−NLL b1 np**	6.993	2.404		
**−NLL b1 cyto**	1.469	−4.722		
**−NLL b2 ca**	−3.58		−4.195	
**−NLL b2 np**				
**−NLL b2 cyto**	5.235		−0.2114	
**−NLL b3 ca**	−0.9489			−4.266
**−NLL b3 np**	2.247			−3.404
**−NLL b3 cyto**	−3.08			−4.324
Total	6.171	−4.965	−4.406	−11.99
**Total with regul**	7.397	−6.347	−5.665	−10.17

	Together	b1	b2	b3
spar	0.3605	0.47420	0.4034	0.3150000
σ_b	0.1482	0.08195	0.108	0.0998300
σ_t	1.7840	2.78200	1.429	0.0002376
ca_{0,b_1}	0.1780	0.47830		
$\log_{10}(k_1')$	−0.2104	−0.38800		0.1957000
$\log_{10}(k_2)$	−0.7102	−0.79780	4.555 or −1.339	−0.3501000
$\log_{10}(k_2')$	−0.9305	−0.97200		−1.0580000
$\log_{10}(k_{deg})$	−1.2510	−1.32200	−1.339 or 4.555	−1.3130000
$\log_{10}(k_1'k_2')$	−1.1410	−1.36000	4.084	−0.8621000
$\log_{10}(k_1'/k_2)$	0.4997	0.40980		0.5458000
$\text{transport} = \log_{10}(k_1'k_2'/k_2)$	−0.4307	−0.56220	−0.4702 or 5.423	−0.5120000

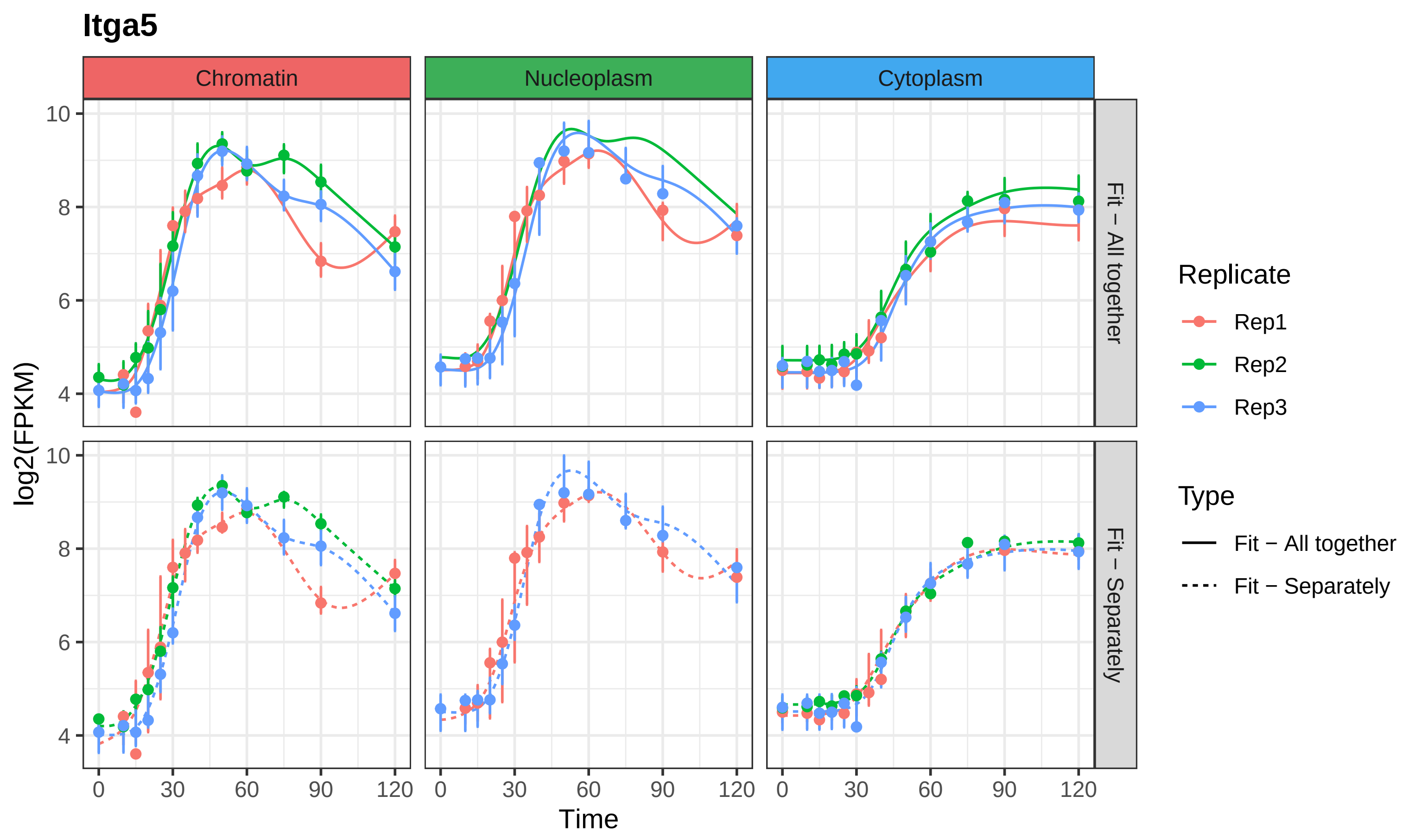

	Together	b1	b2	b3
**−NLL b1 ca**	0.7078	−0.0765		
**−NLL b1 np**	−0.627	−0.494		
**−NLL b1 cyto**	−1.352	−8.952		
**−NLL b2 ca**	−5.347		−7.553	
**−NLL b2 np**				
**−NLL b2 cyto**	−3.354		−10.55	
**−NLL b3 ca**	−5.667			−5.369
**−NLL b3 np**	3.492			2.37
**−NLL b3 cyto**	−3.534			−3.596
Total	−15.68	−9.523	−18.1	−6.595
**Total with regul**	−14.63	−9.664	−17.38	−5.288

	Together	b1	b2	b3
spar	0.3443	0.3776	0.3304	0.3317000
σ_b	0.1070	0.0398	0.05729	0.1285000
σ_t	1.8740	2.9170	0.6735	0.0002157
ca_{0,b_1}	4.0570	3.8480		
$\log_{10}(k_1')$	−0.5340	−0.6554		−0.1859000
$\log_{10}(k_2)$	−0.6746	−0.8119	−0.5296 or −2.1	−0.3302000
$\log_{10}(k_2')$	−1.9640	−1.8640		−2.0150000
$\log_{10}(k_{deg})$	−1.9450	−1.8900	−2.1 or −0.5296	−2.0180000
$\log_{10}(k_1'k_2')$	−2.4980	−2.5190	−2.492	−2.2010000
$\log_{10}(k_1'/k_2)$	0.1406	0.1565		0.1444000
$\text{transport} = \log_{10}(k_1'k_2'/k_2)$	−1.8230	−1.7070	−1.962 or −0.3916	−1.8710000

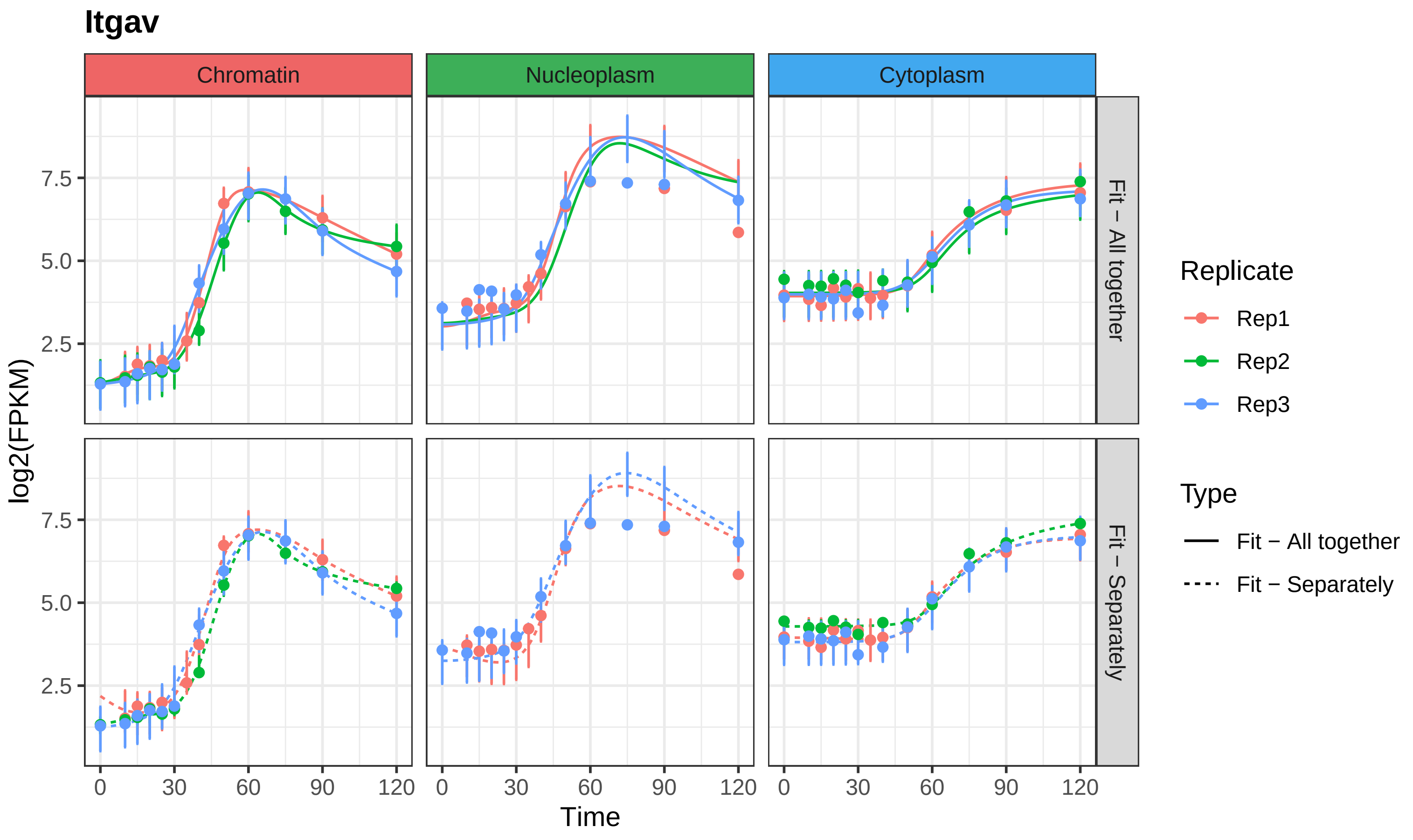

	Together	b1	b2	b3
**−NLL b1 ca**	−0.2795	0.4985		
**−NLL b1 np**	17.08	12.55		
**−NLL b1 cyto**	0.3102	−1.593		
**−NLL b2 ca**	−0.5194		−9.468	
**−NLL b2 np**				
**−NLL b2 cyto**	2.335		−8.448	
**−NLL b3 ca**	−0.08834			−4.178e−05
**−NLL b3 np**	19.09			12.42
**−NLL b3 cyto**	1.386			−0.2929
Total	39.31	11.46	−17.92	12.12
**Total with regul**	42.1	13.31	−16.59	13.21

	Together	b1	b2	b3
spar	3.509e−01	4.061e−01	0.3184	0.3814000
σ_b	2.390e−01	2.129e−01	0.06018	0.2219000
σ_t	1.349e−05	7.647e−05	0.4089	0.0000012
ca_{0,b_1}	1.226e+00	2.213e+00		
$\log_{10}(k_1')$	−4.283e−01	−4.276e−01		−0.3885000
$\log_{10}(k_2)$	−9.698e−01	−8.602e−01	−1.287 or −2.285	−0.9938000
$\log_{10}(k_2')$	−2.127e+00	−2.096e+00		−2.2180000
$\log_{10}(k_{deg})$	−2.400e+00	−2.194e+00	−2.285 or −1.287	−2.3870000
$\log_{10}(k_1'k_2')$	−2.555e+00	−2.524e+00	−2.68	−2.6060000
$\log_{10}(k_1'/k_2)$	5.414e−01	4.326e−01		0.6053000
transport = $\log_{10}(k_1'k_2'/k_2)$	−1.585e+00	−1.664e+00	−1.393 or −0.3947	−1.6120000

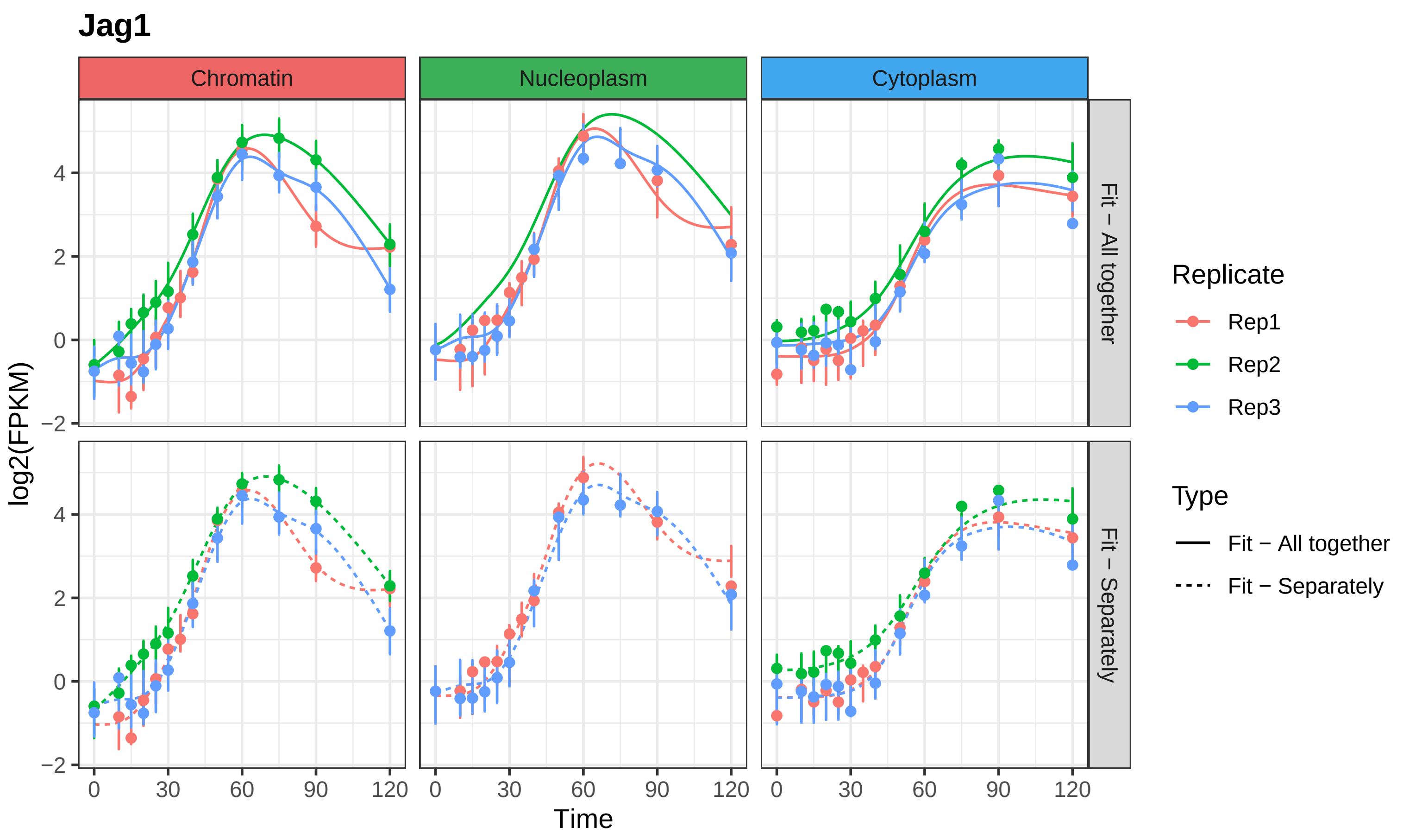

	Together	b1	b2	b3
**−NLL b1 ca**	−1.586	−2.223		
**−NLL b1 np**	4.386	3.183		
**−NLL b1 cyto**	−1.551	−3.235		
**−NLL b2 ca**	−4.25		−5.705	
**−NLL b2 np**				
**−NLL b2 cyto**	2.925		0.6447	
**−NLL b3 ca**	−1.487			−0.253
**−NLL b3 np**	2.881			1.047
**−NLL b3 cyto**	10.03			5.708
Total	11.35	−2.275	−5.06	6.502
**Total with regul**	11.9	−3.137	−6.413	6.237

	Together	b1	b2	b3
spar	0.382500	0.4159000	0.4077	3.970e−01
σ_b	0.161700	0.1146000	0.1102	1.727e−01
σ_t	0.000121	0.0002223	7.315e−06	3.679e−05
ca_{0,b_1}	−0.964100	−1.0110000		
$\log_{10}(k_1')$	−0.200300	−0.3090000		−2.638e−01
$\log_{10}(k_2)$	−0.351800	−0.5179000	−0.4265 or −1.988	−3.722e−01
$\log_{10}(k_2')$	−1.756000	−1.7780000		−1.633e+00
$\log_{10}(k_{deg})$	−1.781000	−1.7660000	−1.988 or −0.4265	−1.589e+00
$\log_{10}(k_1'k_2')$	−1.957000	−2.0870000	−2.126	−1.896e+00
$\log_{10}(k_1'/k_2)$	0.151500	0.2089000		1.085e−01
$\text{transport} = \log_{10}(k_1'k_2'/k_2)$	−1.605000	−1.5690000	−1.699 or −0.1376	−1.524e+00

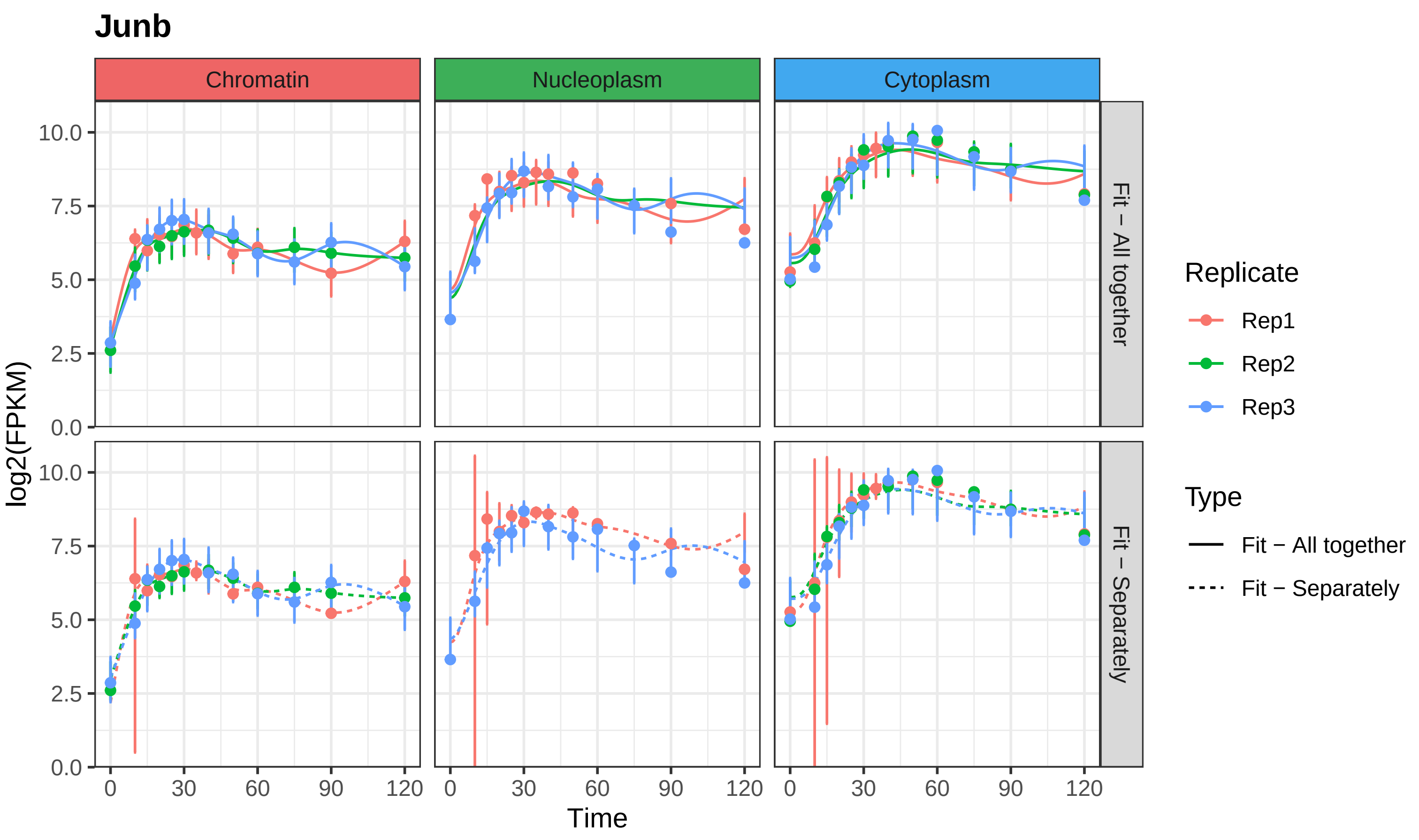

	Together	b1	b2	b3
**−NLL b1 ca**	0.8108	−3.466		
**−NLL b1 np**	9.603	4.422		
**−NLL b1 cyto**	5.452	6.391		
**−NLL b2 ca**	−0.002037		−2.011	
**−NLL b2 np**				
**−NLL b2 cyto**	7.201		8.999	
**−NLL b3 ca**	0.06684			0.3515
**−NLL b3 np**	12.77			6.607
**−NLL b3 cyto**	10.49			10.45
Total	46.39	7.347	6.988	17.4
**Total with regul**	52.88	16.37	9.572	19.32

	Together	b1	b2	b3
spar	0.3297000	0.33470	0.3319	3.812e−01
σ_b	0.2564000	0.03586	0.2053	2.566e−01
σ_t	0.0001499	6.68100	0.4186	7.494e−05
ca_{0,b_1}	2.8570000	2.01700		
$\log_{10}(k_1')$	−0.1905000	−0.31230		−1.508e−01
$\log_{10}(k_2)$	−0.6995000	−0.92800	−0.7001 or −0.7429	−5.521e−01
$\log_{10}(k_2')$	−0.6120000	−0.42650		−6.368e−01
$\log_{10}(k_{deg})$	−0.9673000	−0.74910	−0.7429 or −0.7001	−1.044e+00
$\log_{10}(k_1'k_2')$	−0.8024000	−0.73880	−0.6008	−7.877e−01
$\log_{10}(k_1'/k_2)$	0.5090000	0.61580		4.013e−01
$\text{transport} = \log_{10}(k_1'k_2'/k_2)$	−0.1029000	0.18920	0.09931 or 0.142	−2.356e−01

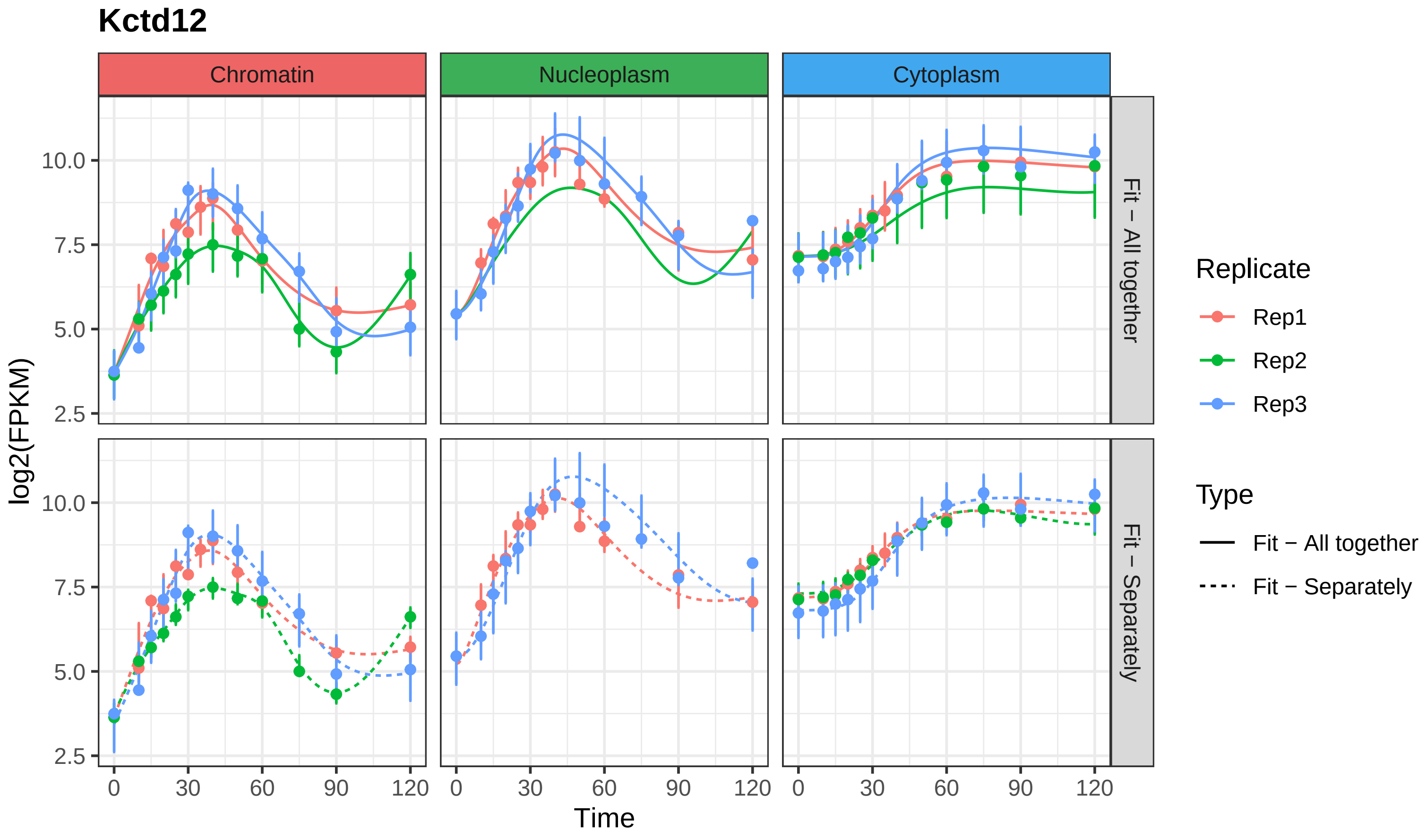

	Together	b1	b2	b3
**−NLL b1 ca**	2.851	1.722		
**−NLL b1 np**	5.574	5.33		
**−NLL b1 cyto**	0.1308	−6.001		
**−NLL b2 ca**	−0.2245		−7.686	
**−NLL b2 np**				
**−NLL b2 cyto**	7.725		−1.493	
**−NLL b3 ca**	3.182			4.145
**−NLL b3 np**	16.69			13.69
**−NLL b3 cyto**	5.358			0.5337
Total	41.29	1.05	−9.178	18.37
**Total with regul**	42.9	0.2337	−8.955	19.54

	Together	b1	b2	b3
spar	3.939e−01	0.47250	0.3502	4.311e−01
σ_b	2.441e−01	0.12980	0.105	2.614e−01
σ_t	2.549e−06	2.04100	0.0003608	1.645e−05
ca_{0,b_1}	3.697e+00	3.56600		
$\log_{10}(k_1')$	−1.739e−01	0.04641		−3.965e−01
$\log_{10}(k_2)$	−7.093e−01	−0.42890	−0.8027 or −1.851	−9.955e−01
$\log_{10}(k_2')$	−1.596e+00	−1.65100		−1.721e+00
$\log_{10}(k_{deg})$	−2.104e+00	−2.24600	−1.851 or −0.8027	−2.137e+00
$\log_{10}(k_1'k_2')$	−1.770e+00	−1.60500	−1.588	−2.117e+00
$\log_{10}(k_1'/k_2)$	5.354e−01	0.47530		5.990e−01
$\text{transport} = \log_{10}(k_1'k_2'/k_2)$	−1.061e+00	−1.17600	−0.7852 or 0.2628	−1.122e+00

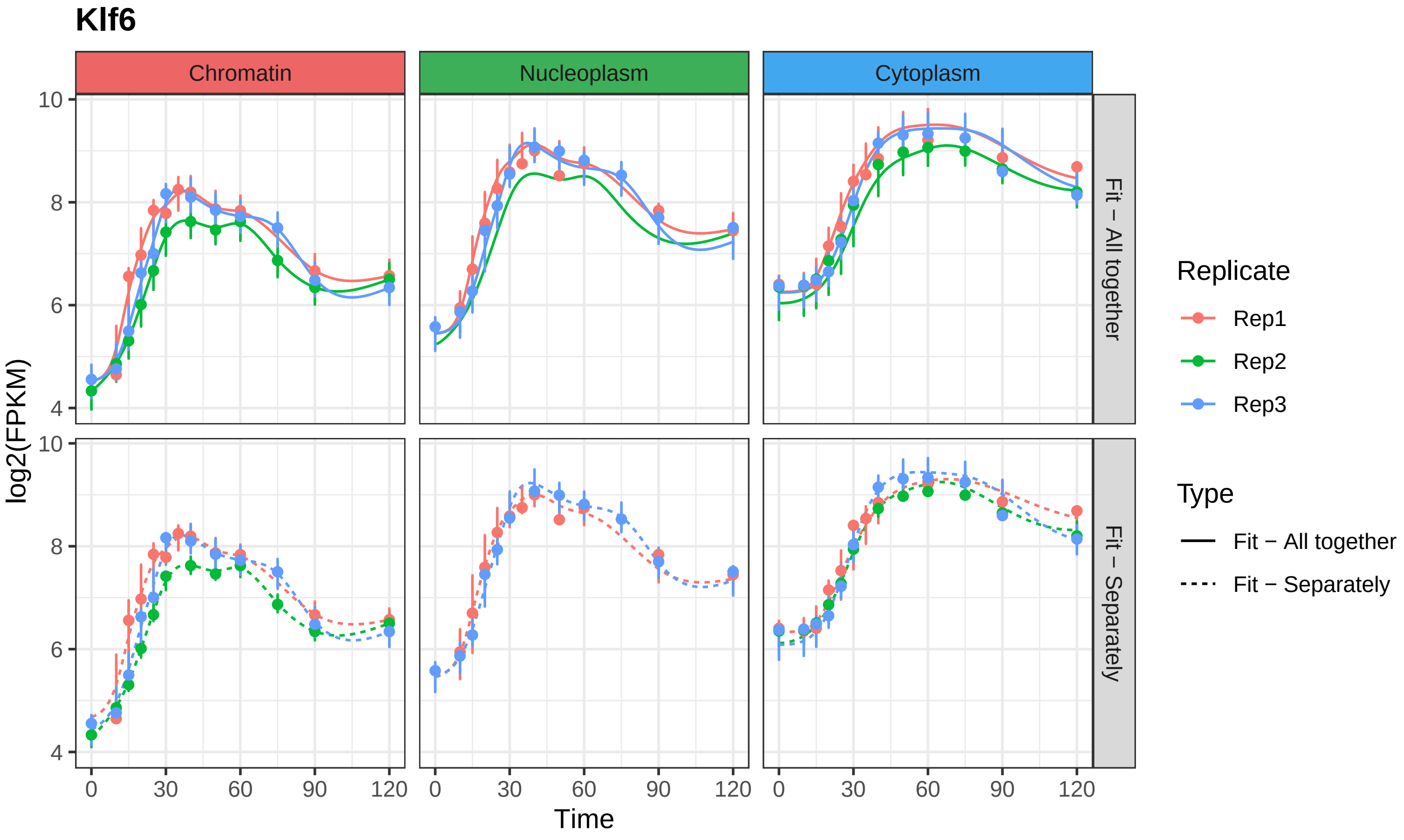

	Together	b1	b2	b3
**−NLL b1 ca**	−4.453	−5.528		
**−NLL b1 np**	−1.873	−2.793		
**−NLL b1 cyto**	1.743	−5.287		
**−NLL b2 ca**	−9.557		−13.81	
**−NLL b2 np**				
**−NLL b2 cyto**	0.3404		−7.888	
**−NLL b3 ca**	−7.573			−5.719
**−NLL b3 np**	−4.611			−5.794
**−NLL b3 cyto**	−3.268			−2.184
Total	−29.25	−13.61	−21.69	−13.7
**Total with regul**	−24.95	−11.65	−19.11	−11.77

	Together	b1	b2	b3
spar	0.28320	0.32240	0.2962	0.31310
σ_b	0.11130	0.07674	0.05824	0.09878
σ_t	0.87710	1.88000	0.0009613	0.57400
ca_{0,b_1}	4.58700	4.74300		
$\log_{10}(k_1')$	0.06897	−0.03789		0.02365
$\log_{10}(k_2)$	−0.20890	−0.28260	4.721 or −1.246	−0.28680
$\log_{10}(k_2')$	−1.04900	−1.19700		−1.01100
$\log_{10}(k_{deg})$	−1.28900	−1.45100	−1.246 or 4.721	−1.19800
$\log_{10}(k_1'k_2')$	−0.97980	−1.23500	4.027	−0.98700
$\log_{10}(k_1'/k_2)$	0.27780	0.24480		0.31050
$\text{transport} = \log_{10}(k_1'k_2'/k_2)$	−0.77090	−0.95230	−0.6947 or 5.273	−0.70020

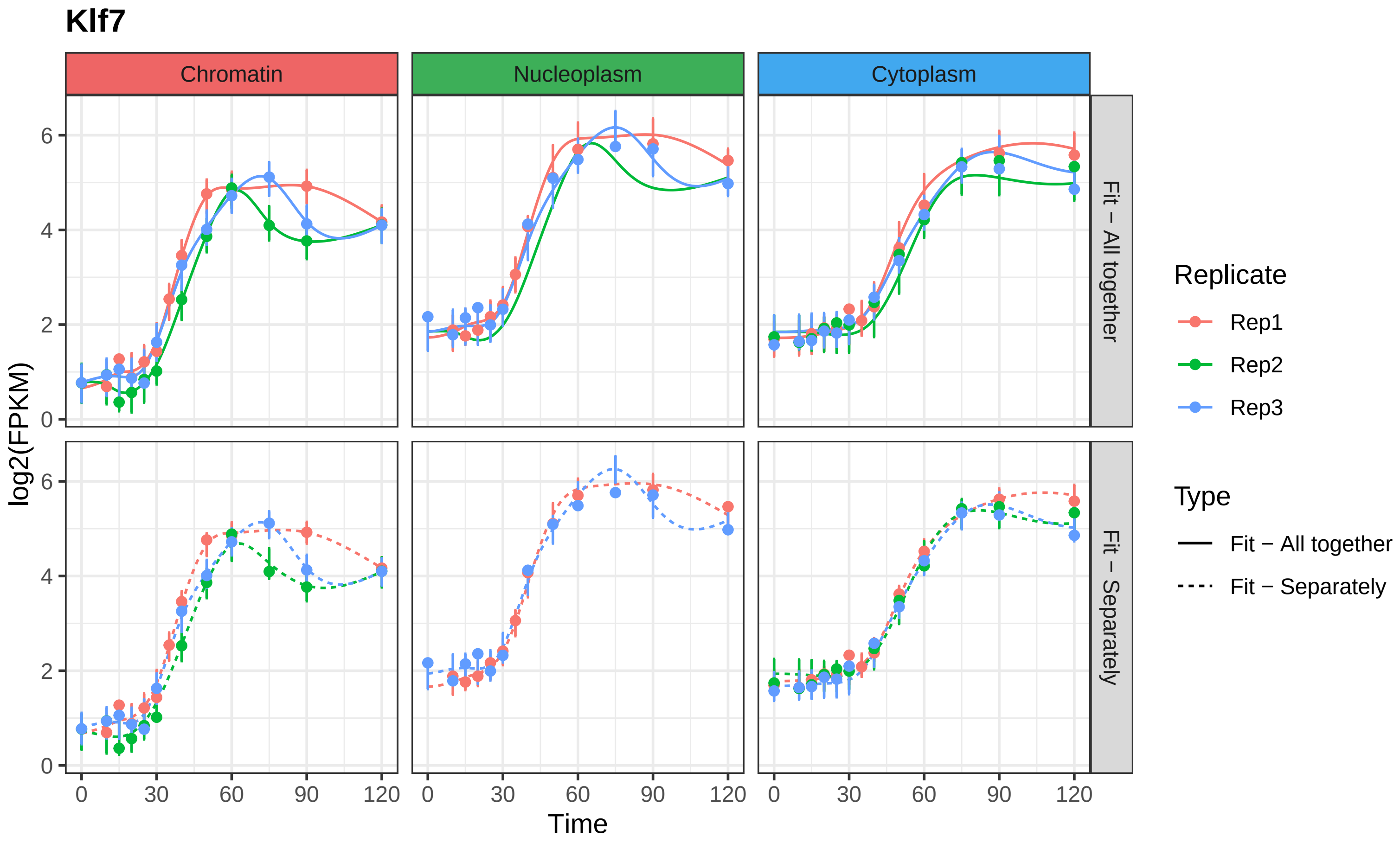

	Together	b1	b2	b3
**−NLL b1 ca**	−5.769	−5.799		
**−NLL b1 np**	−3.833	−6.4		
**−NLL b1 cyto**	−3.738	−7.768		
**−NLL b2 ca**	−6.968		−3.011	
**−NLL b2 np**				
**−NLL b2 cyto**	4.04		−3.436	
**−NLL b3 ca**	−6.866			−6.764
**−NLL b3 np**	1.634			−1.064
**−NLL b3 cyto**	−2.204			−5.652
Total	−23.7	−19.97	−6.447	−13.48
**Total with regul**	−21.74	−20.42	−8.135	−11.86

	Together	b1	b2	b3
spar	3.224e−01	0.36970	0.4365	0.3184000
σ_b	1.235e−01	0.07744	0.1091	0.0980000
σ_t	7.542e−05	0.43980	9.461e−06	0.0007084
ca_{0,b_1}	6.606e−01	0.67930		
$\log_{10}(k_1')$	−2.541e−01	−0.29420		−0.1243000
$\log_{10}(k_2)$	−5.776e−01	−0.59210	−0.6745 or −1.243	−0.4710000
$\log_{10}(k_2')$	−1.362e+00	−1.47000		−1.4100000
$\log_{10}(k_{deg})$	−1.359e+00	−1.50600	−1.243 or −0.6745	−1.3300000
$\log_{10}(k_1' k_2')$	−1.616e+00	−1.76400	−1.555	−1.5350000
$\log_{10}(k_1'/k_2)$	3.235e−01	0.29790		0.3467000
transport $= \log_{10}(k_1' k_2'/k_2)$	−1.039e+00	−1.17200	−0.8809 or −0.3127	−1.0640000

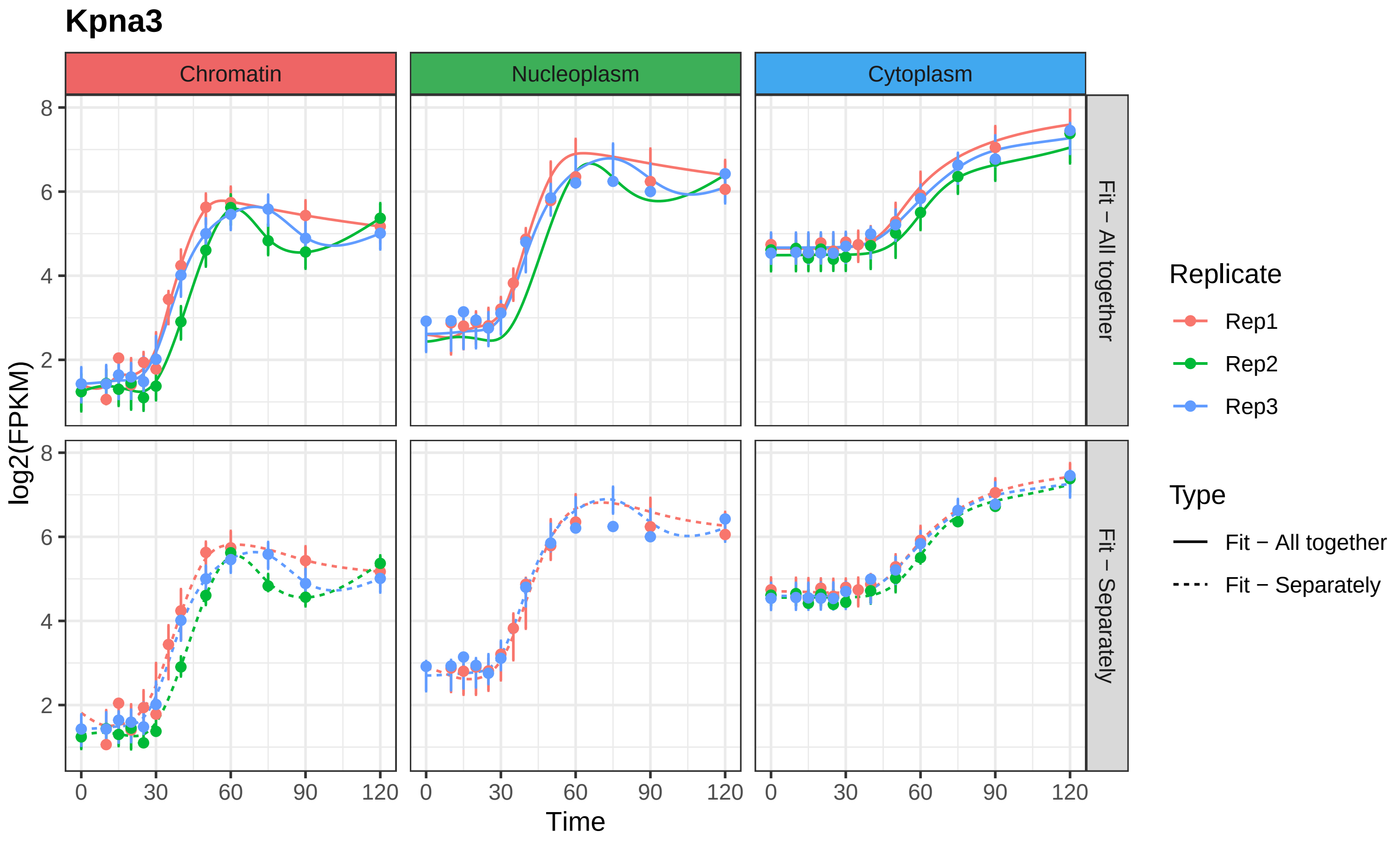

	Together	b1	b2	b3
**−NLL b1 ca**	−0.5327	3.132		
**−NLL b1 np**	6.538	0.7625		
**−NLL b1 cyto**	−6.513	−8.283		
**−NLL b2 ca**	−7.23		−8.279	
**−NLL b2 np**				
**−NLL b2 cyto**	−5.341		−7.93	
**−NLL b3 ca**	−7.098			−6.127
**−NLL b3 np**	6.382			2.27
**−NLL b3 cyto**	−6.091			−6.715
Total	−19.88	−4.388	−16.21	−10.57
**Total with regul**	−16.64	−3.776	−16.51	−9.254

	Together	b1	b2	b3
spar	0.307000	0.4080	0.3552	3.270e−01
σ_b	0.127600	0.1169	0.06551	1.104e−01
σ_t	0.001852	1.4600	2.802e−05	2.832e−05
ca_{0,b_1}	1.425000	1.8630		
$\log_{10}(k_1')$	−0.291100	−0.4720		−1.403e−01
$\log_{10}(k_2)$	−0.648400	−0.7887	−0.7951 or −2.04	−5.250e−01
$\log_{10}(k_2')$	−1.521000	−1.5080		−1.546e+00
$\log_{10}(k_{deg})$	−2.139000	−2.0600	−2.04 or −0.7951	−2.115e+00
$\log_{10}(k_1' k_2')$	−1.812000	−1.9800	−1.851	−1.686e+00
$\log_{10}(k_1'/k_2)$	0.357300	0.3167		3.847e−01
$\text{transport} = \log_{10}(k_1' k_2'/k_2)$	−1.163000	−1.1910	−1.056 or 0.1889	−1.161e+00

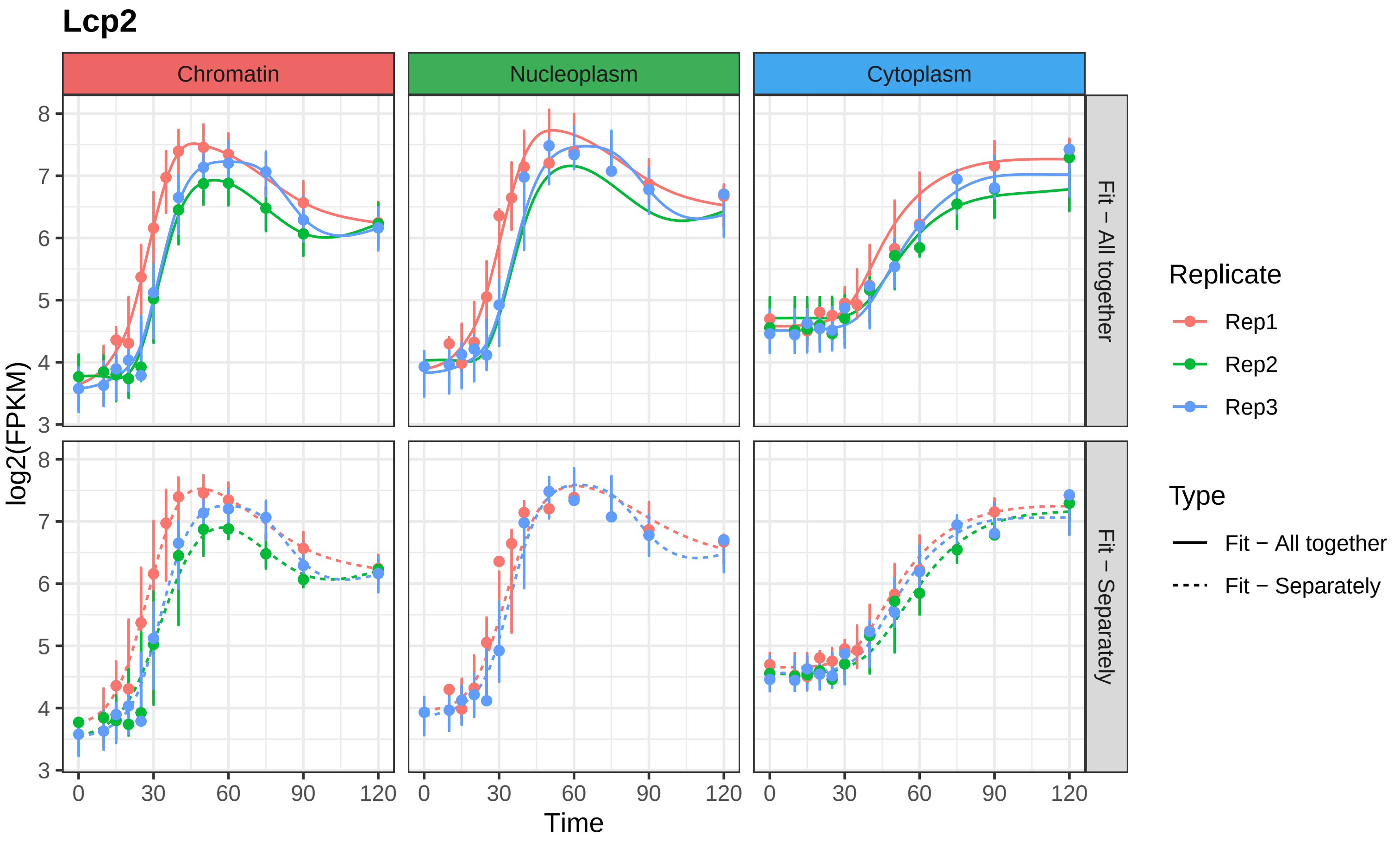

	Together	b1	b2	b3
**−NLL b1 ca**	−5.74	−4.879		
**−NLL b1 np**	2.909	3.581		
**−NLL b1 cyto**	−1.23	−7.779		
**−NLL b2 ca**	−7.491		−3.753	
**−NLL b2 np**				
**−NLL b2 cyto**	−0.4471		−6.593	
**−NLL b3 ca**	−5.864			−4.545
**−NLL b3 np**	0.09913			−1.814
**−NLL b3 cyto**	−2.806			−2.908
**Total**	−20.57	−9.077	−10.35	−9.268
**Total with regul**	−18.52	−10.54	−12.15	−9.648

	Together	b1	b2	b3
spar	0.32020	0.42200	0.4705	0.36670
σ_b	0.11890	0.07669	0.05248	0.09639
σ_t	1.43400	2.95300	3.853	2.06400
ca_{0,b_1}	3.65500	3.83200		
$\log_{10}(k_1')$	−0.51120	−0.94160		−0.26010
$\log_{10}(k_2)$	−0.58880	−1.00700	−1.441 or −1.488	−0.36360
$\log_{10}(k_2')$	−1.71700	−1.70300		−1.74700
$\log_{10}(k_{deg})$	−1.92100	−1.90800	−1.488 or −1.441	−1.95300
$\log_{10}(k_1'k_2')$	−2.22800	−2.64400	−2.627	−2.00800
$\log_{10}(k_1'/k_2)$	0.07763	0.06499		0.10350
transport $= \log_{10}(k_1'k_2'/k_2)$	−1.63900	−1.63800	−1.185 or −1.138	−1.64400

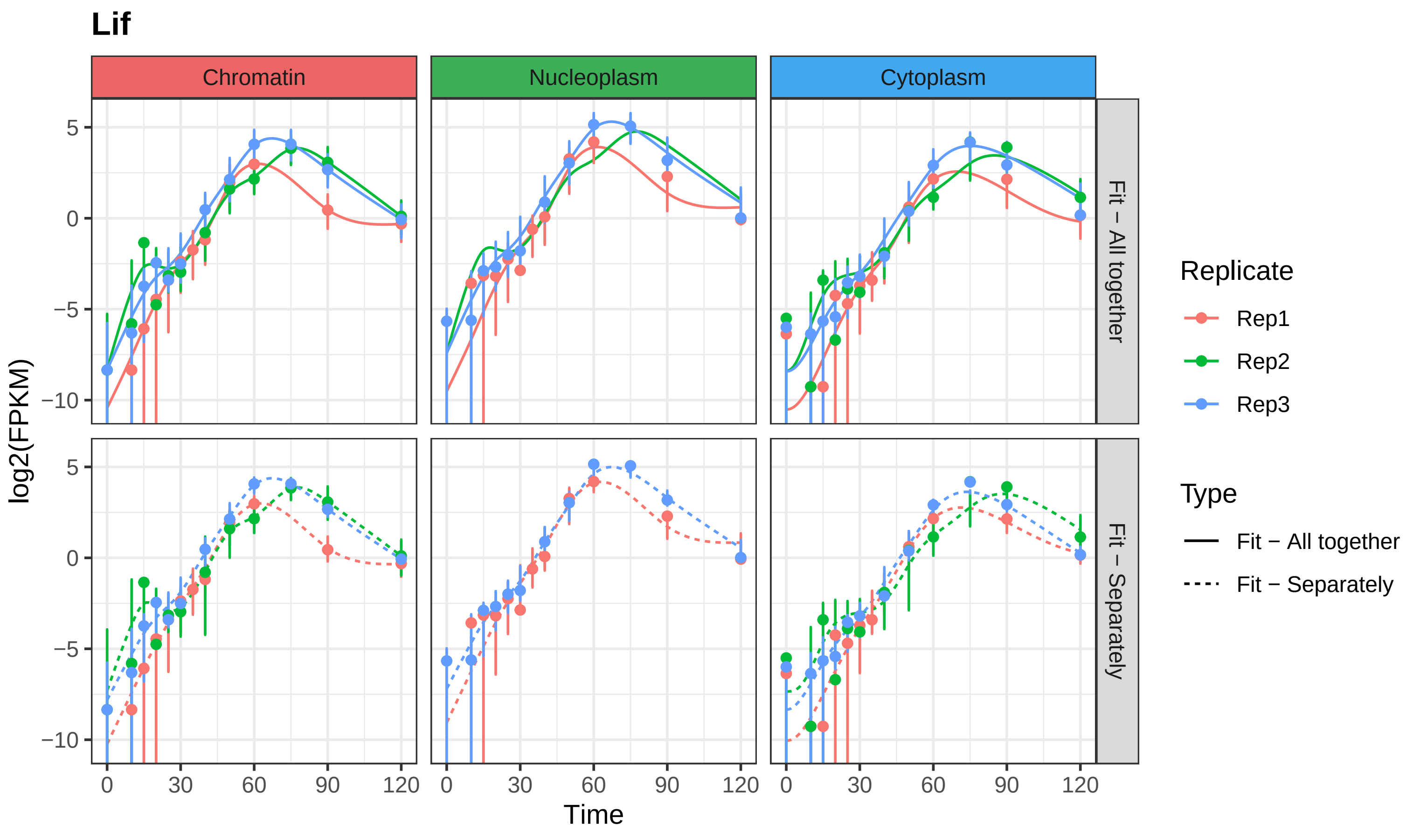

	Together	b1	b2	b3
**−NLL b1 ca**	7.399	6.259		
**−NLL b1 np**	18.38	18.99		
**−NLL b1 cyto**	15.13	12.81		
**−NLL b2 ca**	16.27		13.41	
**−NLL b2 np**				
**−NLL b2 cyto**	26.14		17.99	
**−NLL b3 ca**	9.101			5.169
**−NLL b3 np**	10.47			7.663
**−NLL b3 cyto**	12.7			6.837
Total	115.6	38.06	31.39	19.67
**Total with regul**	120.6	37.73	35.74	20.07

	Together	b1	b2	b3
spar	0.3192	0.4173	0.2918	0.3350000
σ_b	0.2665	0.1523	0.1655	0.0001952
σ_t	1.9880	2.1470	3.784	1.8970000
ca_{0,b_1}	−10.4300	−10.2500		
$\log_{10}(k_1')$	5.8870	4.1210		5.9260000
$\log_{10}(k_2)$	5.6100	3.7660	−0.7711 or −0.8039	5.7410000
$\log_{10}(k_2')$	−1.3020	−1.3790		−1.2120000
$\log_{10}(k_{deg})$	−0.9998	−1.0810	−0.8039 or −0.7711	−0.8673000
$\log_{10}(k_1'k_2')$	4.5850	2.7420	−1.588	4.7140000
$\log_{10}(k_1'/k_2)$	0.2768	0.3556		0.1853000
$\text{transport} = \log_{10}(k_1'k_2'/k_2)$	−1.0250	−1.0230	−0.817 or −0.7842	−1.0270000

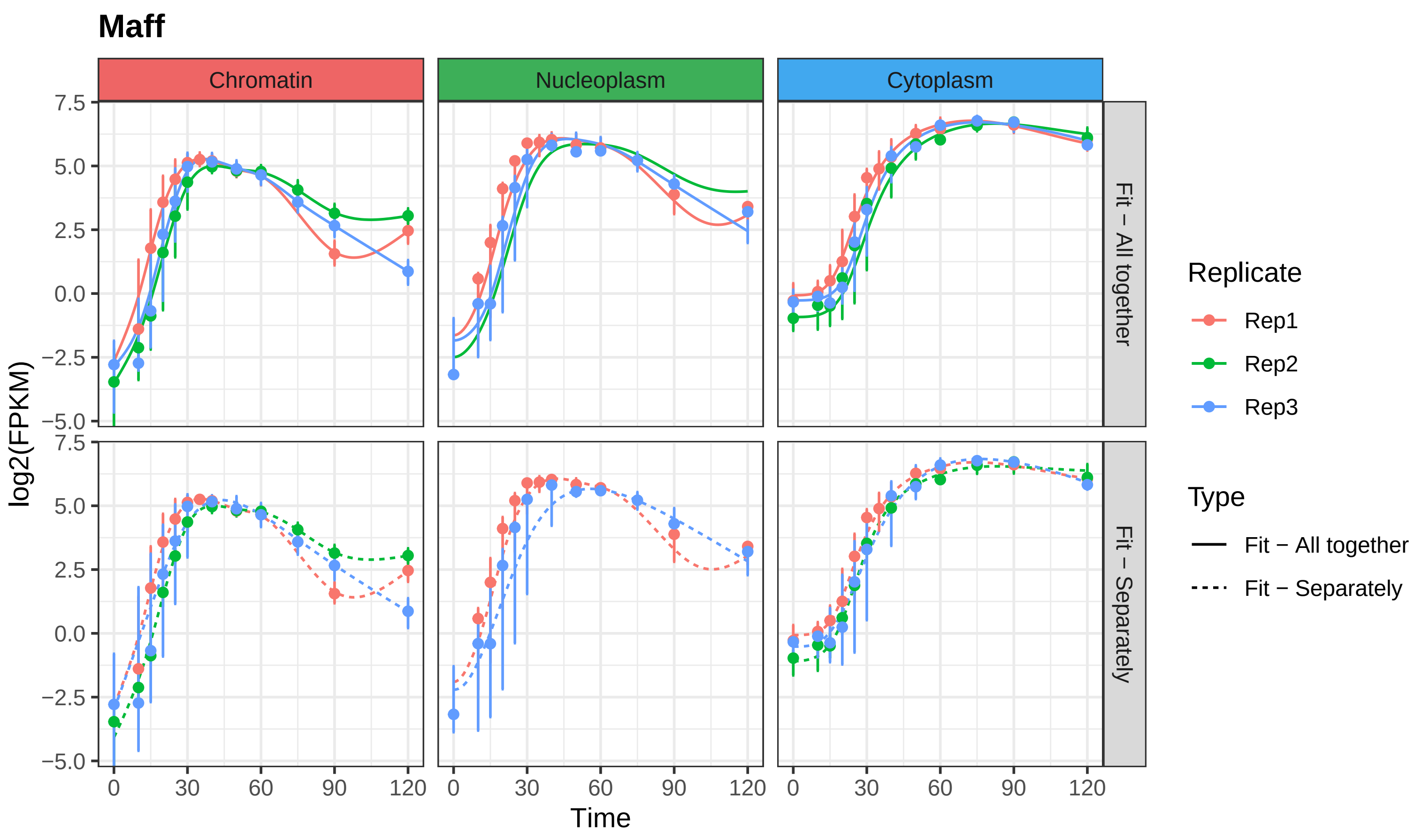

	Together	b1	b2	b3
**−NLL b1 ca**	−1.843	−4.377		
**−NLL b1 np**	5.332	5.19		
**−NLL b1 cyto**	−1.513	−5.2		
**−NLL b2 ca**	−1.114		−5.194	
**−NLL b2 np**				
**−NLL b2 cyto**	1.832		−1.351	
**−NLL b3 ca**	1.482			5.746
**−NLL b3 np**	17.78			8.714
**−NLL b3 cyto**	−1.322			0.8825
Total	20.63	−4.386	−6.545	15.34
**Total with regul**	22.9	−2.193	−4.707	13.67

	Together	b1	b2	b3
spar	0.32070	3.239e−01	0.313	4.905e−01
σ_b	0.08783	4.490e−06	0.08986	1.623e−06
σ_t	2.20800	2.385e+00	0.01135	4.004e+00
ca_{0,b_1}	−2.60600	−2.793e+00		
$\log_{10}(k_1')$	−0.58650	−5.058e−01		−9.107e−01
$\log_{10}(k_2)$	−0.89290	−7.874e−01	7.781 or −1.925	−1.149e+00
$\log_{10}(k_2')$	−1.16700	−1.230e+00		−9.128e−01
$\log_{10}(k_{deg})$	−1.64000	−1.783e+00	−1.925 or 7.781	−1.422e+00
$\log_{10}(k_1'k_2')$	−1.75400	−1.735e+00	6.756	−1.824e+00
$\log_{10}(k_1'/k_2)$	0.30640	2.816e−01		2.381e−01
$\text{transport} = \log_{10}(k_1'k_2'/k_2)$	−0.86070	−9.480e−01	−1.025 or 8.68	−6.747e−01

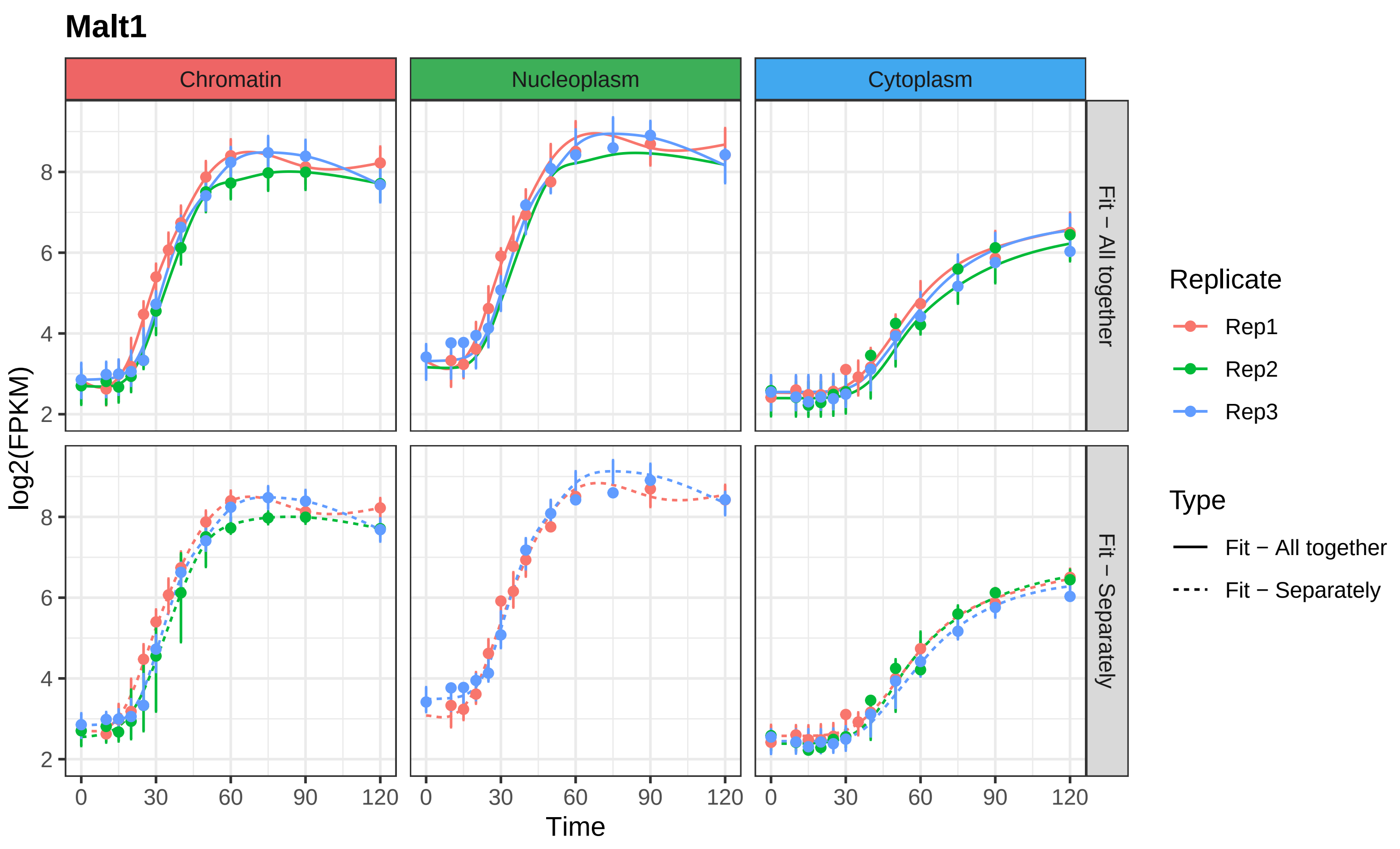

	Together	b1	b2	b3
**−NLL b1 ca**	−5.603	−6.516		
**−NLL b1 np**	2.053	0.2036		
**−NLL b1 cyto**	−3.858	−6.462		
**−NLL b2 ca**	−6.136		−3.915	
**−NLL b2 np**				
**−NLL b2 cyto**	7.758		−2.409	
**−NLL b3 ca**	−5.312			−5.83
**−NLL b3 np**	1.362			−1.024
**−NLL b3 cyto**	0.6016			−4.859
Total	−9.133	−12.78	−6.324	−11.71
**Total with regul**	−6.079	−13.64	−7.673	−10.05

	Together	b1	b2	b3
spar	0.3115000	0.39220	0.3949	0.31820
σ_b	0.1465000	0.08687	0.05353	0.09896
σ_t	0.0001823	1.15600	3.298	0.87590
ca_{0,b_1}	2.8490000	2.78300		
$\log_{10}(k_1')$	0.4700000	−0.01754		0.74280
$\log_{10}(k_2)$	0.3310000	−0.12030	4.844 or −2.142	0.54850
$\log_{10}(k_2')$	−2.4400000	−2.46000		−2.59400
$\log_{10}(k_{deg})$	−2.2090000	−2.30800	−2.142 or 4.844	−2.28000
$\log_{10}(k_1' k_2')$	−1.9700000	−2.47800	2.653	−1.85100
$\log_{10}(k_1'/k_2)$	0.1390000	0.10280		0.19430
transport $= \log_{10}(k_1' k_2'/k_2)$	−2.3010000	−2.35700	−2.191 or 4.795	−2.40000

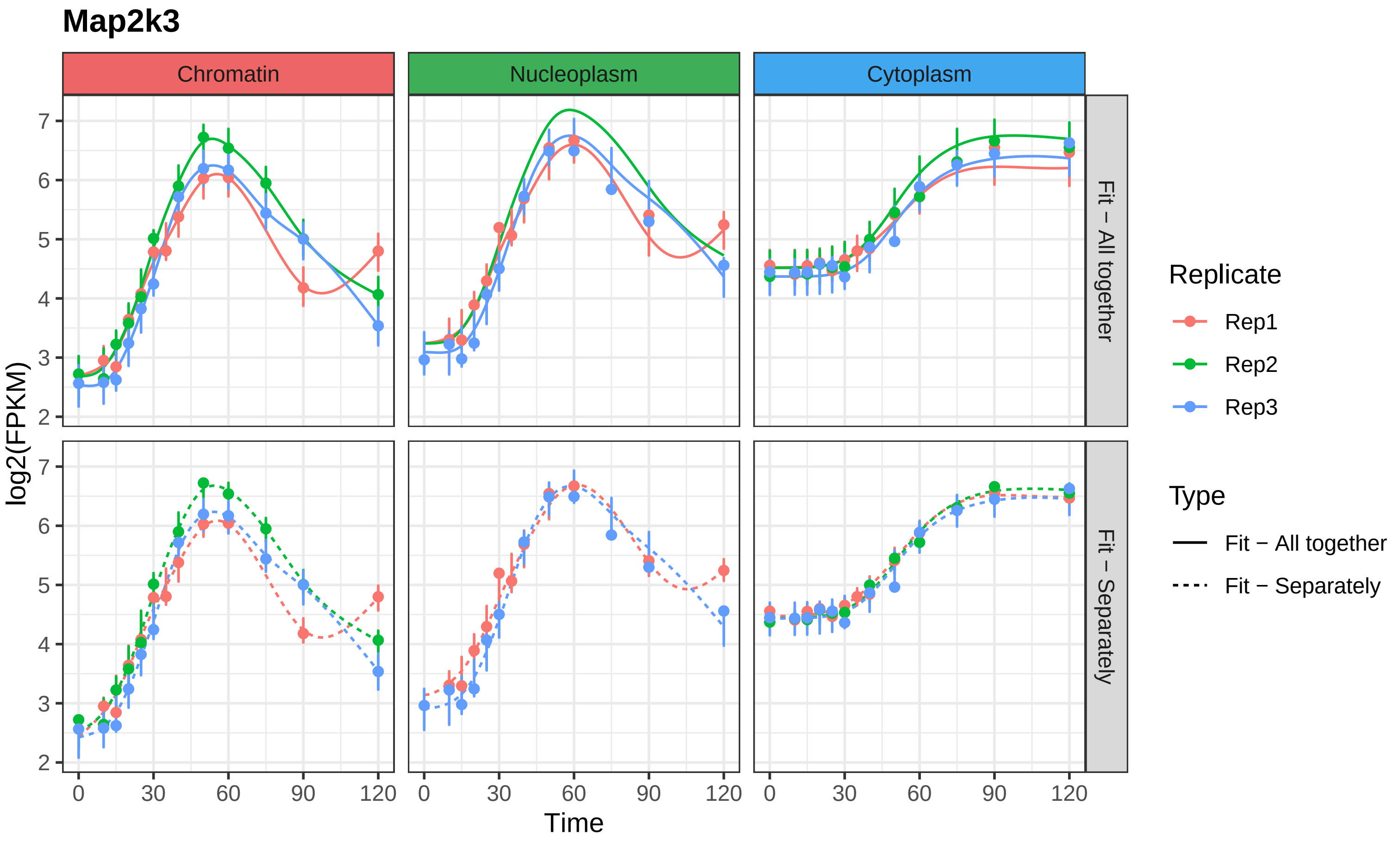

	Together	b1	b2	b3
**−NLL b1 ca**	−7.395	−8.497		
**−NLL b1 np**	−0.6671	−5.982		
**−NLL b1 cyto**	−6.048	−15.27		
**−NLL b2 ca**	−8.936		−7.863	
**−NLL b2 np**				
**−NLL b2 cyto**	−4.123		−12.04	
**−NLL b3 ca**	−9.691			−7.85
**−NLL b3 np**	0.622			−1.409
**−NLL b3 cyto**	−5.489			−5.97
Total	−41.73	−29.75	−19.91	−15.23
**Total with regul**	−40.99	−30.25	−21.4	−16.45

	Together	b1	b2	b3
spar	3.460e−01	0.38150	0.3898	3.918e−01
σ_b	1.003e−01	0.01492	0.03583	9.133e−02
σ_t	8.467e−09	1.84500	1.366	2.837e−07
ca_{0,b_1}	2.704e+00	2.44800		
$\log_{10}(k_1')$	−4.927e−01	−0.61880		−5.106e−01
$\log_{10}(k_2)$	−6.600e−01	−0.83210	−0.7741 or −2.204	−6.578e−01
$\log_{10}(k_2')$	−1.722e+00	−1.63400		−1.696e+00
$\log_{10}(k_{deg})$	−2.107e+00	−2.03500	−2.204 or −0.7741	−2.153e+00
$\log_{10}(k_1'k_2')$	−2.215e+00	−2.25300	−2.4	−2.206e+00
$\log_{10}(k_1'/k_2)$	1.673e−01	0.21340		1.471e−01
$\text{transport} = \log_{10}(k_1'k_2'/k_2)$	−1.555e+00	−1.42000	−1.626 or −0.1962	−1.548e+00

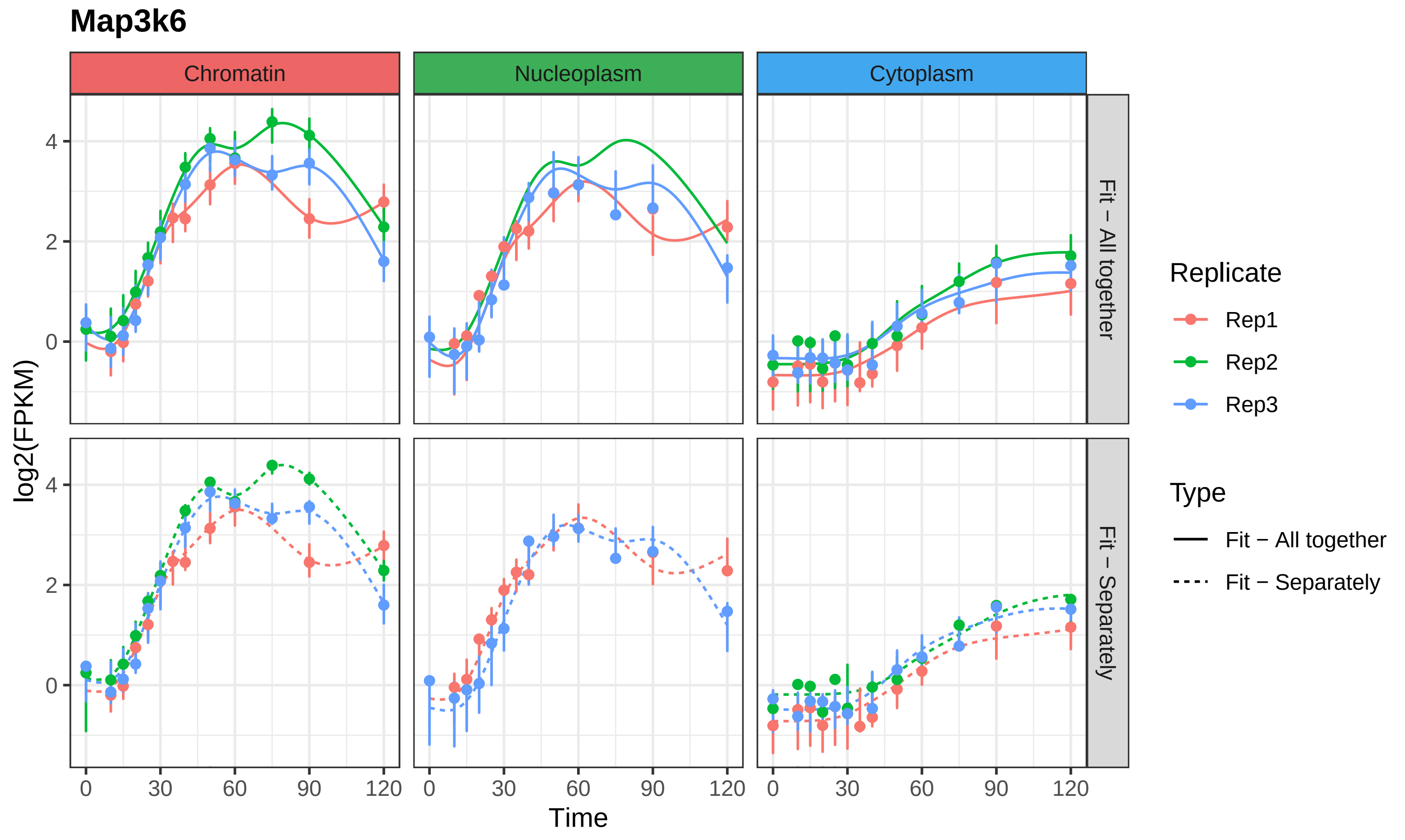

	Together	b1	b2	b3
**−NLL b1 ca**	−5.656	−5.55		
**−NLL b1 np**	4.718	−0.5781		
**−NLL b1 cyto**	−0.9068	−1.485		
**−NLL b2 ca**	−7.11		−10.46	
**−NLL b2 np**				
**−NLL b2 cyto**	0.6959		−2.184	
**−NLL b3 ca**	−6.487			−4.241
**−NLL b3 np**	7.399			0.06343
**−NLL b3 cyto**	−1.087			−3.217
Total	−8.433	−7.613	−12.64	−7.395
**Total with regul**	−8.417	−8.944	−11.75	−9.169

	Together	b1	b2	b3
spar	0.364000	0.407700	0.3232	0.40940
σ_b	0.109900	0.083570	1.954e−10	0.05773
σ_t	0.003715	0.002912	3.739e−07	1.55200
ca_{0,b_1}	0.022450	−0.032380		
$\log_{10}(k_1')$	0.780900	4.270000		−0.30320
$\log_{10}(k_2)$	0.883400	4.317000	−0.9895 or −2.457	−0.13360
$\log_{10}(k_2')$	−2.432000	−2.430000		−2.27400
$\log_{10}(k_{deg})$	−2.340000	−2.294000	−2.457 or −0.9895	−2.26500
$\log_{10}(k_1' k_2')$	−1.651000	1.839000	−3.544	−2.57700
$\log_{10}(k_1'/k_2)$	−0.102500	−0.047430		−0.16960
$\text{transport} = \log_{10}(k_1' k_2'/k_2)$	−2.535000	−2.478000	−2.555 or −1.088	−2.44400

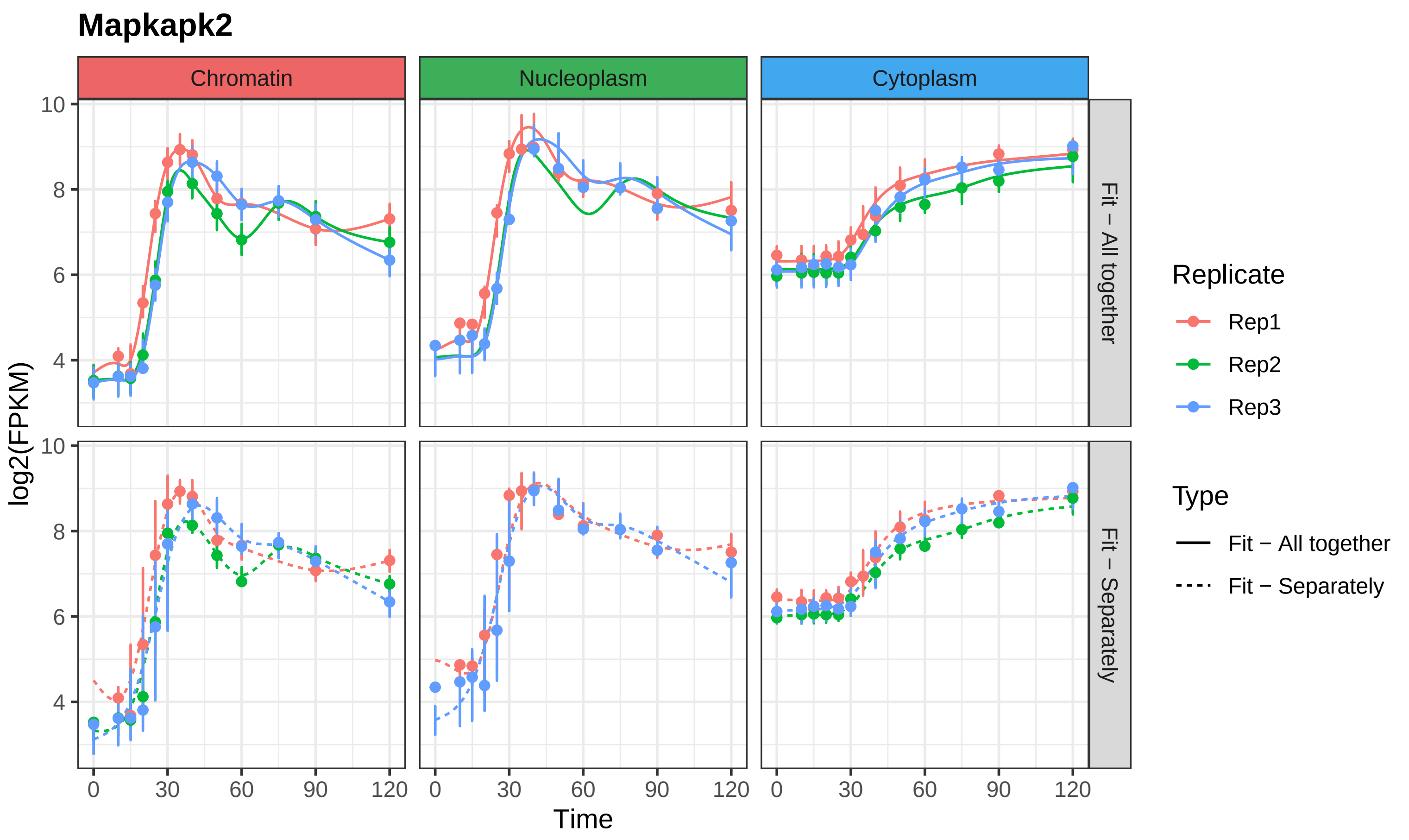

	Together	b1	b2	b3
**−NLL b1 ca**	−6.564	−2.624		
**−NLL b1 np**	5.552	4.86		
**−NLL b1 cyto**	−5.055	−7.432		
**−NLL b2 ca**	−8.558		−2.462	
**−NLL b2 np**				
**−NLL b2 cyto**	−6.255		−9.675	
**−NLL b3 ca**	−7.768			0.3181
**−NLL b3 np**	8.61			8.383
**−NLL b3 cyto**	−4.641			−6.129
Total	−24.68	−5.196	−12.14	2.572
**Total with regul**	−12.99	−4.032	−11.86	1.75

	Together	b1	b2	b3
spar	1.965e−01	0.34860	0.3417	0.38470
σ_b	1.252e−01	0.08395	0.06221	0.09748
σ_t	5.634e−05	2.50800	1.583	2.90400
ca_{0,b_1}	3.708e+00	4.57200		
$\log_{10}(k_1')$	−1.688e−01	−0.69150		5.14800
$\log_{10}(k_2)$	−3.338e−01	−0.83100	−0.4469 or −2.41	5.01000
$\log_{10}(k_2')$	−1.780e+00	−1.64000		−1.75300
$\log_{10}(k_{deg})$	−2.400e+00	−2.06800	−2.41 or −0.4469	−2.52400
$\log_{10}(k_1'k_2')$	−1.949e+00	−2.33200	−2.044	3.39500
$\log_{10}(k_1'/k_2)$	1.650e−01	0.13950		0.13780
transport $= \log_{10}(k_1'k_2'/k_2)$	−1.615e+00	−1.50000	−1.597 or 0.3663	−1.61500

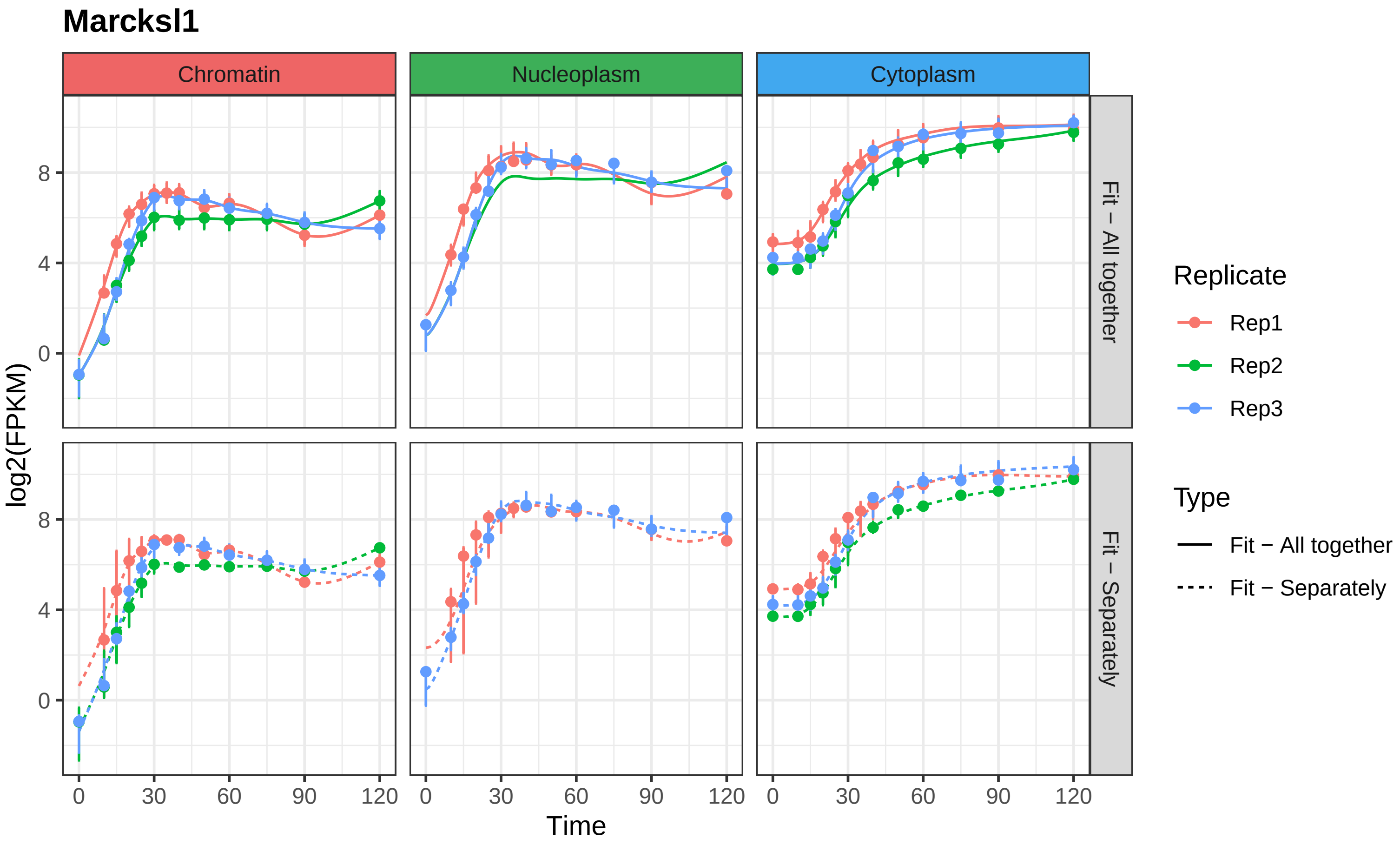

	Together	b1	b2	b3
**−NLL b1 ca**	−4.846	−6.086		
**−NLL b1 np**	7.766	2.24		
**−NLL b1 cyto**	−3.037	−11.48		
**−NLL b2 ca**	−2.422		−7.472	
**−NLL b2 np**				
**−NLL b2 cyto**	−1.629		−8.853	
**−NLL b3 ca**	−3.061			−1.066
**−NLL b3 np**	5.635			2.432
**−NLL b3 cyto**	−0.9685			−2.148
Total	−2.562	−15.33	−16.32	−0.7828
**Total with regul**	5.549	−11.55	−14.07	1.293

	Together	b1	b2	b3
spar	2.741e−01	2.875e−01	0.2997	3.220e−01
σ_b	1.536e−01	1.217e−07	7.725e−05	1.494e−01
σ_t	1.393e−06	3.062e+00	1.641	1.046e−05
ca_{0,b_1}	−8.647e−02	6.578e−01		
$\log_{10}(k_1')$	3.939e−01	−4.199e−01		2.914e−01
$\log_{10}(k_2)$	−1.422e−01	−9.298e−01	4.968 or −2.233	−2.749e−01
$\log_{10}(k_2')$	−1.228e+00	−1.131e+00		−1.228e+00
$\log_{10}(k_{deg})$	−2.179e+00	−1.909e+00	−2.233 or 4.968	−2.340e+00
$\log_{10}(k_1' k_2')$	−8.344e−01	−1.551e+00	4.228	−9.368e−01
$\log_{10}(k_1'/k_2)$	5.361e−01	5.099e−01		5.664e−01
$\text{transport} = \log_{10}(k_1' k_2'/k_2)$	−6.922e−01	−6.210e−01	−0.7404 or 6.461	−6.618e−01

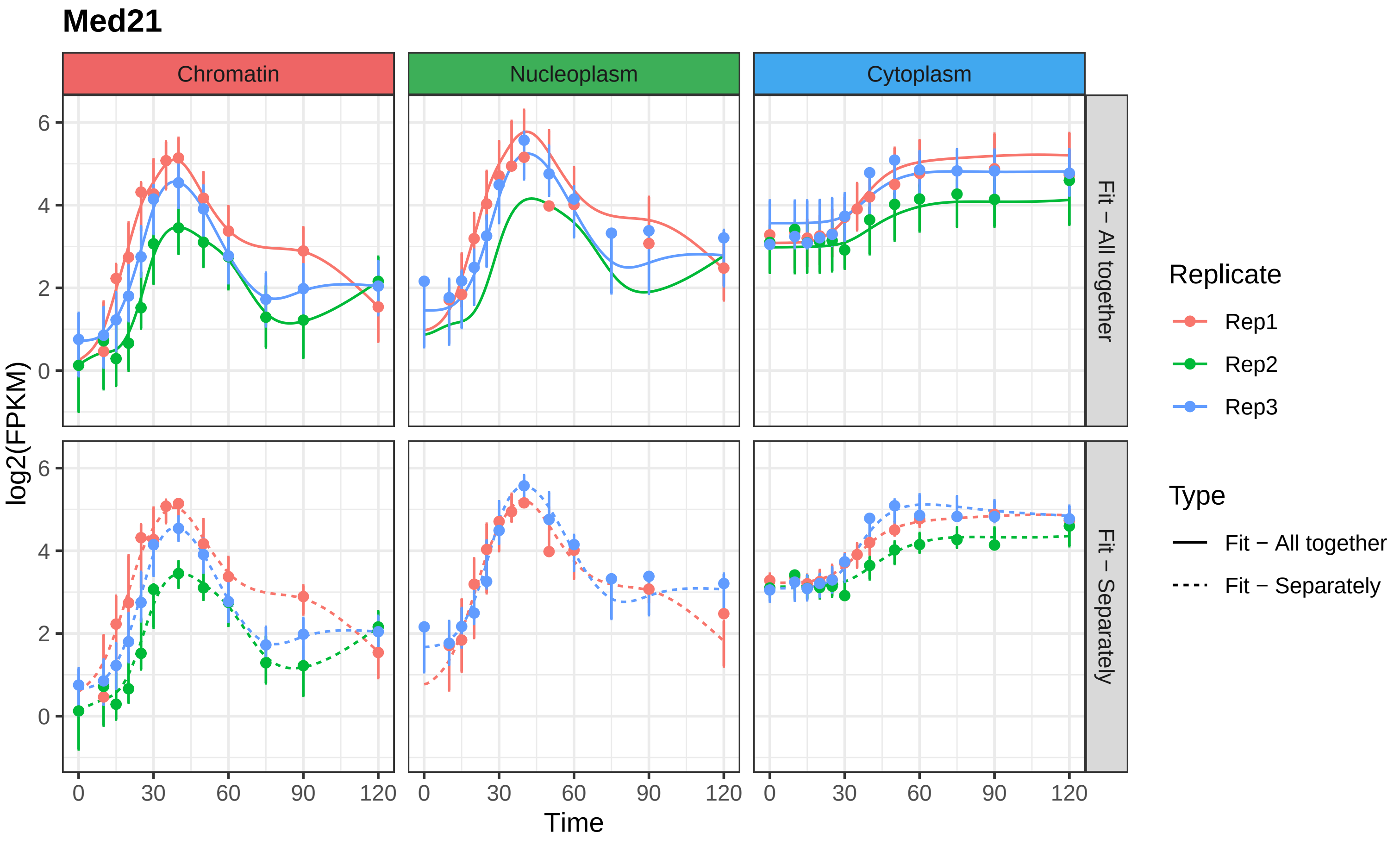

	Together	b1	b2	b3
**−NLL b1 ca**	0.5266	0.1899		
**−NLL b1 np**	13.12	3.479		
**−NLL b1 cyto**	0.4648	−11.82		
**−NLL b2 ca**	0.2476		−0.8398	
**−NLL b2 np**				
**−NLL b2 cyto**	−0.1207		−4.28	
**−NLL b3 ca**	−1.613			−3.712
**−NLL b3 np**	7.529			1.374
**−NLL b3 cyto**	4.932			−4.707
Total	25.09	−8.153	−5.12	−7.045
**Total with regul**	28.14	−8.741	−5.445	−6.348

	Together	b1	b2	b3
spar	0.3229000	0.39020	0.358	0.33530
σ_b	0.1925000	0.01836	0.07379	0.08462
σ_t	0.0006947	2.32300	1.211	1.15600
ca_{0,b_1}	0.2965000	0.69390		
$\log_{10}(k_1')$	−0.2348000	−0.09510		0.19560
$\log_{10}(k_2)$	−0.4547000	−0.14940	−0.5679 or −2.278	−0.11000
$\log_{10}(k_2')$	−1.7130000	−1.72000		−1.53000
$\log_{10}(k_{deg})$	−2.3480000	−2.45900	−2.278 or −0.5679	−1.95700
$\log_{10}(k_1' k_2')$	−1.9480000	−1.81500	−1.947	−1.33400
$\log_{10}(k_1'/k_2)$	0.2199000	0.05425		0.30560
$\text{transport} = \log_{10}(k_1' k_2'/k_2)$	−1.4930000	−1.66600	−1.379 or 0.3311	−1.22400

Mmp13

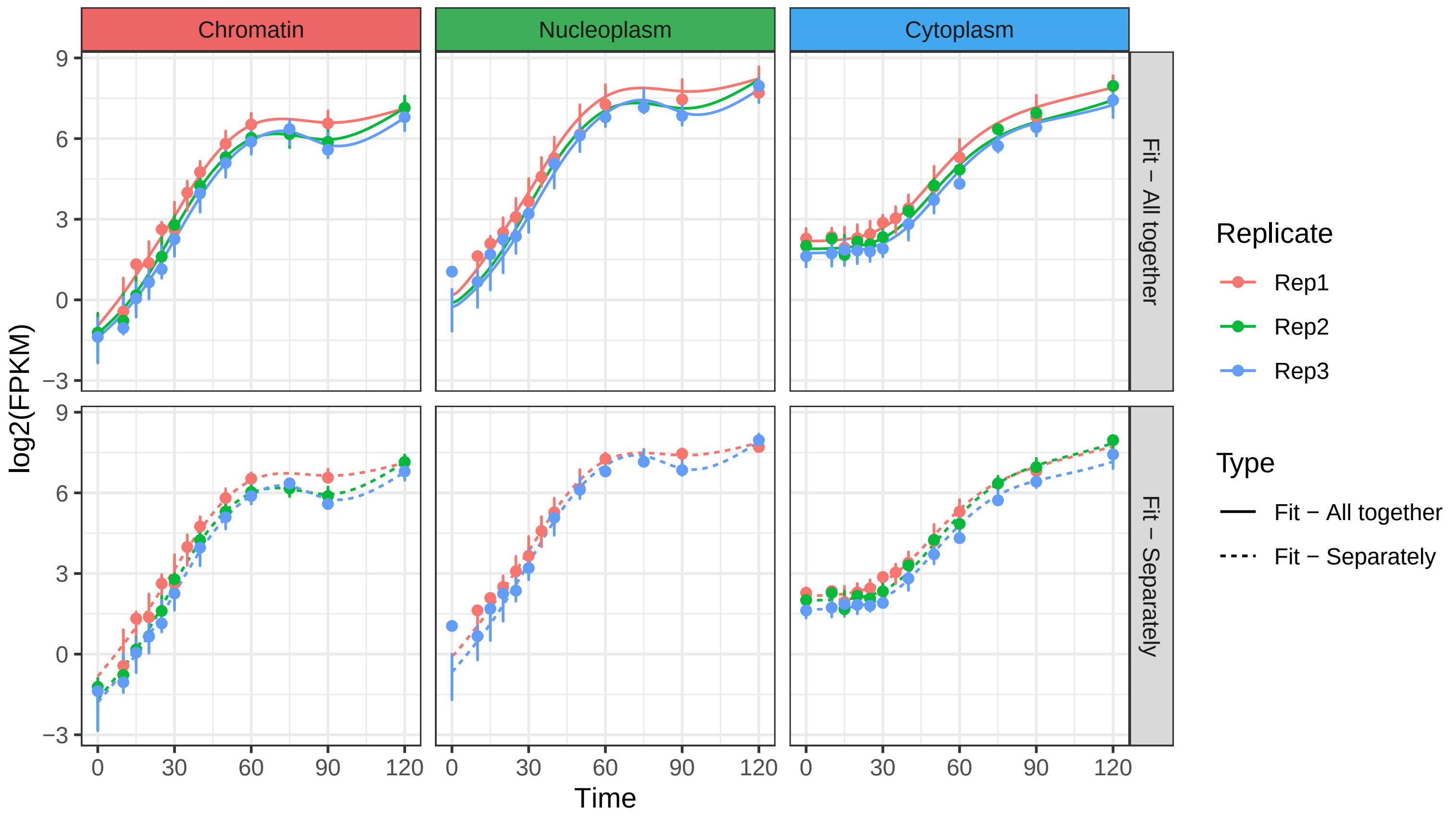

	Together	b1	b2	b3
**−NLL b1 ca**	0.9323	0.1685		
**−NLL b1 np**	6.352	−0.7271		
**−NLL b1 cyto**	−2.243	−4.078		
**−NLL b2 ca**	−2.131		−5.449	
**−NLL b2 np**				
**−NLL b2 cyto**	1.921		−1.437	
**−NLL b3 ca**	−1.827			−2.624
**−NLL b3 np**	12.63			−0.2631
**−NLL b3 cyto**	−2.089			−2.906
Total	13.55	−4.637	−6.886	−5.793
**Total with regul**	12.76	−5.785	−8.603	−7.724

	Together	b1	b2	b3
spar	0.43570	0.50230	0.4211	0.4430
σ_b	0.15600	0.08244	0.09251	0.0815
σ_t	0.86650	1.65900	0.5834	1.5020
ca_{0,b_1}	−0.94640	−0.78190		
$\log_{10}(k_1')$	0.06556	0.49600		3.6080
$\log_{10}(k_2)$	−0.28260	0.26690	−0.8598 or −2.374	3.2700
$\log_{10}(k_2')$	−1.78800	−1.74100		−1.8420
$\log_{10}(k_{deg})$	−2.39000	−2.41500	−2.374 or −0.8598	−2.5460
$\log_{10}(k_1' k_2')$	−1.72200	−1.24500	−2.126	1.7660
$\log_{10}(k_1'/k_2)$	0.34820	0.22910		0.3378
transport $= \log_{10}(k_1' k_2'/k_2)$	−1.44000	−1.51200	−1.266 or 0.2482	−1.5040

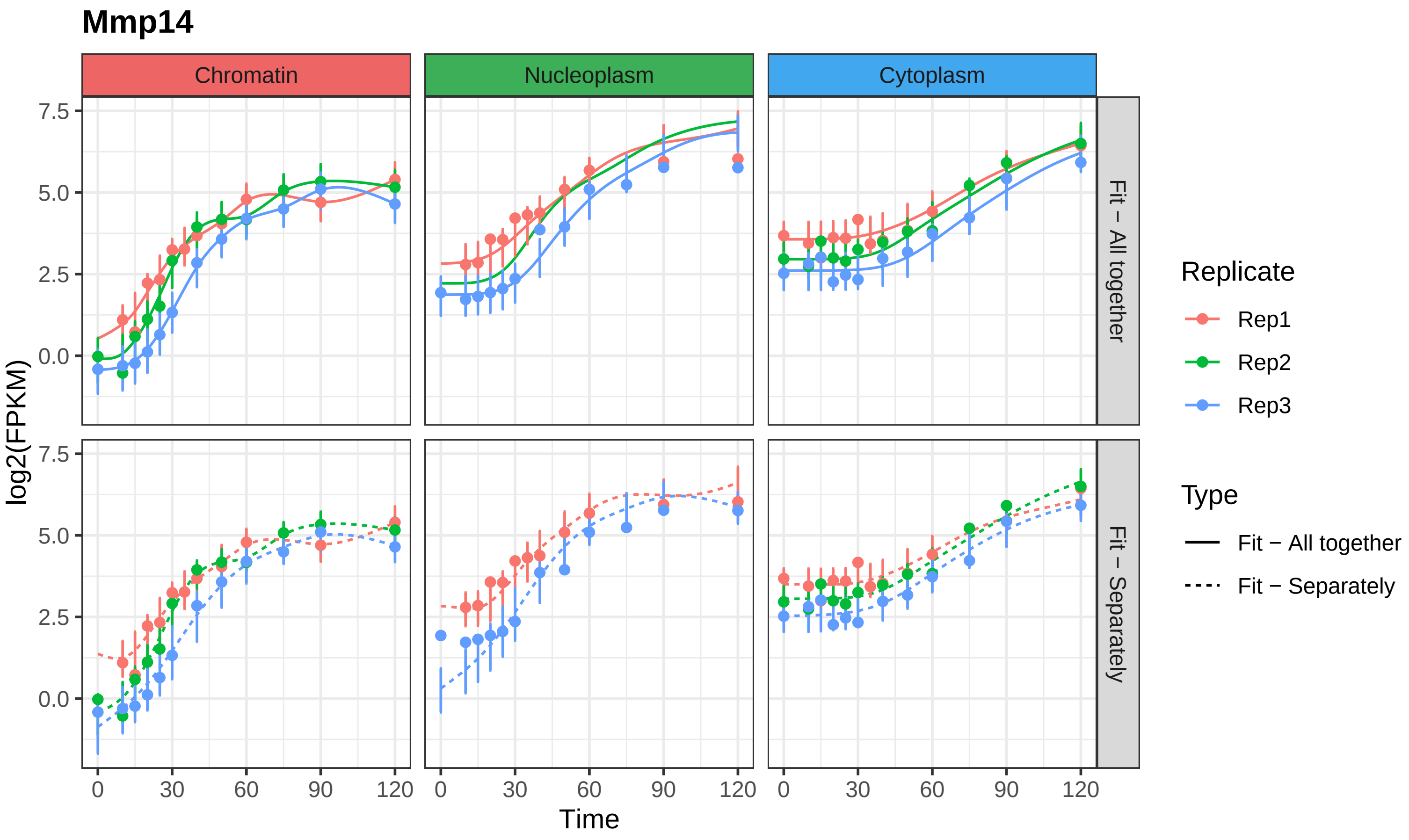

	Together	b1	b2	b3
**−NLL b1 ca**	−0.3263	0.4216		
**−NLL b1 np**	7.587	3.362		
**−NLL b1 cyto**	2.305	2.369		
**−NLL b2 ca**	−0.6741		−1.395	
**−NLL b2 np**				
**−NLL b2 cyto**	1.105		0.8016	
**−NLL b3 ca**	−2.91			−0.1711
**−NLL b3 np**	10.21			8.077
**−NLL b3 cyto**	0.9687			0.2938
Total	18.27	6.152	−0.5935	8.199
**Total with regul**	20.45	5.812	−1.24	8.009

	Together	b1	b2	b3
spar	0.3694000	0.4213	0.3898	0.5093
σ_b	0.1911000	0.1720	0.1366	0.1596
σ_t	0.0007469	1.8420	0.8827	2.7400
ca_{0,b_1}	0.5380000	1.4420		
$\log_{10}(k_1')$	−0.9645000	−0.5148		6.0830
$\log_{10}(k_2)$	−1.6580000	−0.9551	−1.652 or −2.088	5.7290
$\log_{10}(k_2')$	−1.6990000	−1.7860		−1.8450
$\log_{10}(k_{deg})$	−1.9220000	−1.9870	−2.088 or −1.652	−2.5150
$\log_{10}(k_1'k_2')$	−2.6630000	−2.3000	−2.696	4.2380
$\log_{10}(k_1'/k_2)$	0.6931000	0.4403		0.3541
$\text{transport} = \log_{10}(k_1'k_2'/k_2)$	−1.0060000	−1.3450	−1.044 or −0.608	−1.4910

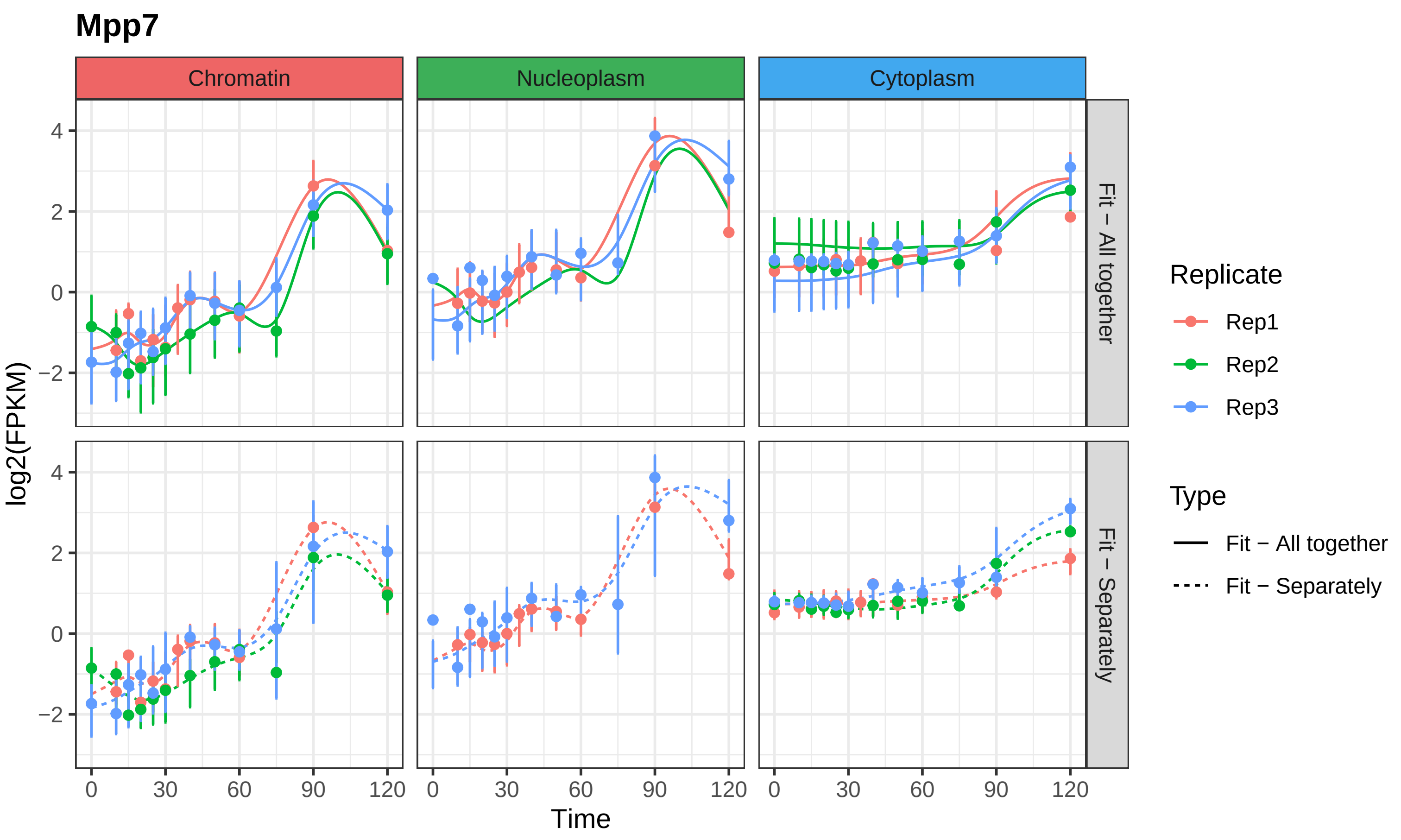

	Together	b1	b2	b3
**−NLL b1 ca**	2.228	1.192		
**−NLL b1 np**	3.37	−1.859		
**−NLL b1 cyto**	5.818	−3.682		
**−NLL b2 ca**	1.299		0.1989	
**−NLL b2 np**				
**−NLL b2 cyto**	7.78		−7.962	
**−NLL b3 ca**	0.8287			1.394
**−NLL b3 np**	12.17			7.824
**−NLL b3 cyto**	7.291			−6.426
Total	40.78	−4.349	−7.763	2.793
**Total with regul**	44.26	−4.635	−9.917	1.765

	Together	b1	b2	b3
spar	0.32760	0.37030	0.4561	4.332e−01
σ_b	0.22460	0.08399	9.457e−06	1.761e−05
σ_t	0.01175	1.69700	2.804	7.856e+00
ca_{0,b_1}	−1.40400	−1.49600		
$\log_{10}(k_1')$	7.94900	4.50200		6.453e+00
$\log_{10}(k_2)$	7.62500	4.25200	4.819 or −1.763	6.109e+00
$\log_{10}(k_2')$	−1.83500	−2.31000		−1.769e+00
$\log_{10}(k_{deg})$	−2.12300	−2.73200	−1.763 or 4.819	−2.201e+00
$\log_{10}(k_1'k_2')$	6.11400	2.19100	3.567	4.684e+00
$\log_{10}(k_1'/k_2)$	0.32410	0.25000		3.439e−01
transport $= \log_{10}(k_1'k_2'/k_2)$	−1.51100	−2.06000	−1.252 or 5.33	−1.425e+00

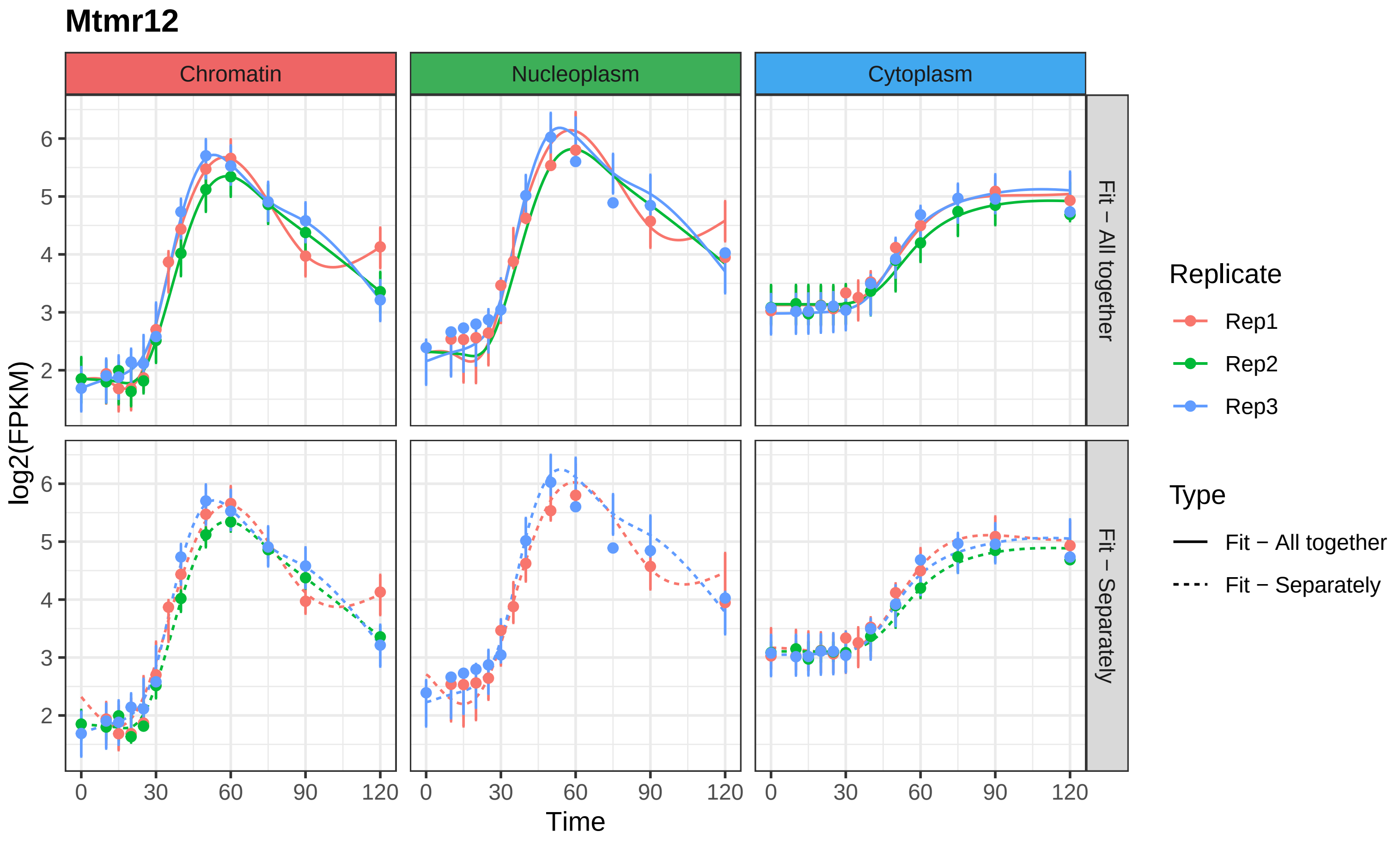

	Together	b1	b2	b3
**−NLL b1 ca**	−7.385	−2.667		
**−NLL b1 np**	9.679	1.078		
**−NLL b1 cyto**	−7.23	−7.11		
**−NLL b2 ca**	−8.03		−9.44	
**−NLL b2 np**				
**−NLL b2 cyto**	−7.725		−9.273	
**−NLL b3 ca**	−7.815			−5.732
**−NLL b3 np**	6.982			3.227
**−NLL b3 cyto**	−5.999			−5.572
Total	−27.52	−8.699	−18.71	−8.077
**Total with regul**	−25.81	−9.471	−18.57	−7.071

	Together	b1	b2	b3
spar	0.3248000	0.4631	0.3425	0.3356000
σ_b	0.1160000	0.1155	0.05338	0.1190000
σ_t	0.0001676	0.2240	0.001727	0.0007996
ca_{0,b_1}	1.8270000	2.4130		
$\log_{10}(k_1')$	0.5936000	0.4139		0.4467000
$\log_{10}(k_2)$	0.4527000	0.2972	5.343 or −2.161	0.2857000
$\log_{10}(k_2')$	−1.9130000	−1.7580		−1.9800000
$\log_{10}(k_{deg})$	−2.1600000	−1.8960	−2.161 or 5.343	−2.2260000
$\log_{10}(k_1'k_2')$	−1.3190000	−1.3440	3.559	−1.5330000
$\log_{10}(k_1'/k_2)$	0.1409000	0.1167		0.1609000
transport $= \log_{10}(k_1'k_2'/k_2)$	−1.7720000	−1.6410	−1.783 or 5.721	−1.8190000

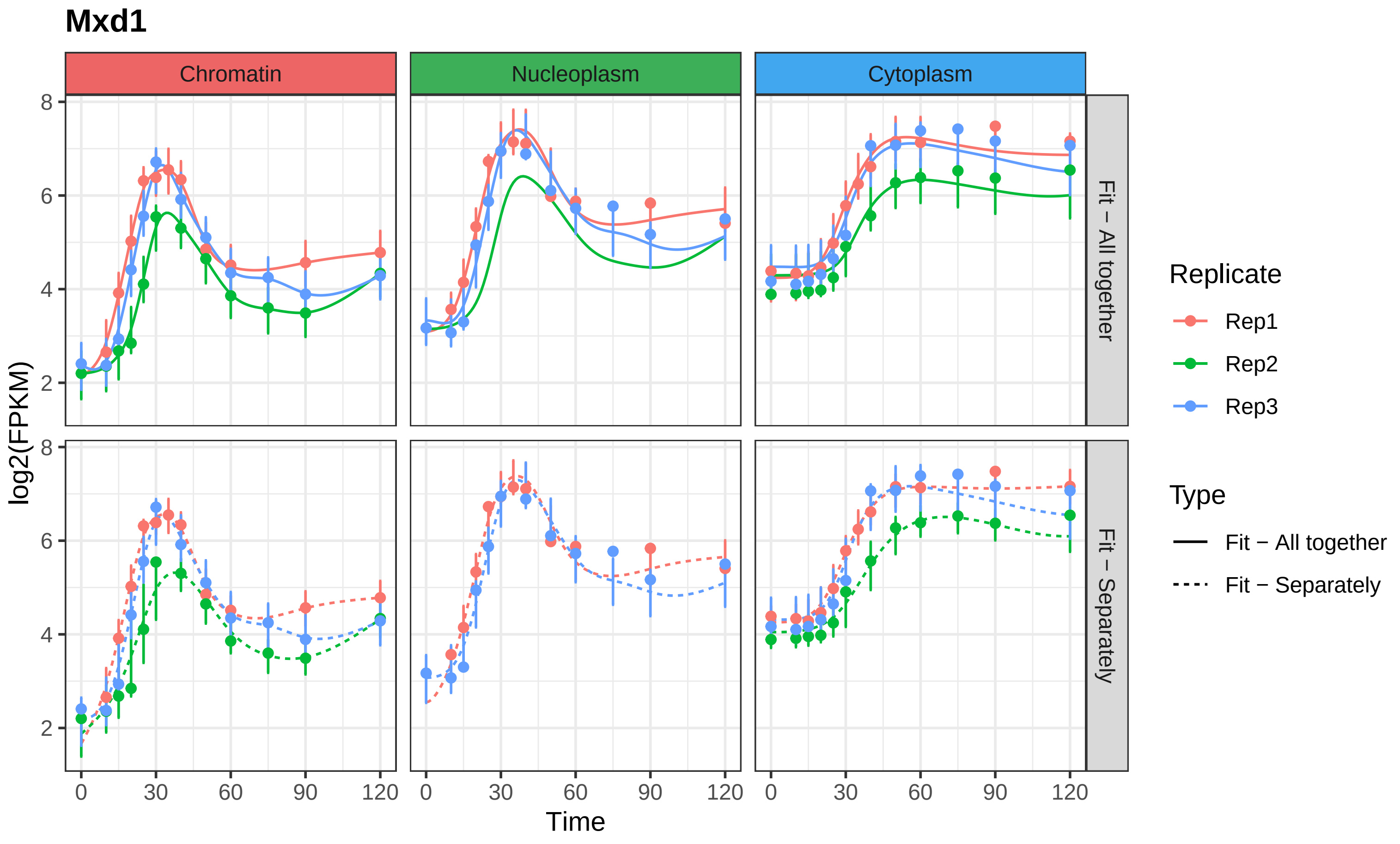

	Together	b1	b2	b3
**−NLL b1 ca**	−4.369	−5.633		
**−NLL b1 np**	1.667	3.14		
**−NLL b1 cyto**	−1.001	−5.637		
**−NLL b2 ca**	−4.268		0.2898	
**−NLL b2 np**				
**−NLL b2 cyto**	3.783		−1.219	
**−NLL b3 ca**	−4.791			−1.961
**−NLL b3 np**	3.977			1.621
**−NLL b3 cyto**	7.025			2.87
Total	2.024	−8.13	−0.9292	2.531
**Total with regul**	7.061	−5.011	−2.416	3.883

	Together	b1	b2	b3
spar	2.855e−01	0.3310000	0.4305	0.343800
σ_b	1.648e−01	0.1256000	0.1124	0.168300
σ_t	2.614e−05	0.0002714	2.486	0.001704
ca_{0,b_1}	2.170e+00	1.7020000		
$\log_{10}(k_1')$	−3.658e−01	−0.2762000		−0.331000
$\log_{10}(k_2)$	−6.532e−01	−0.5431000	−1.309 or −1.331	−0.604500
$\log_{10}(k_2')$	−1.322e+00	−1.4300000		−1.283000
$\log_{10}(k_{deg})$	−1.668e+00	−1.9440000	−1.331 or −1.309	−1.657000
$\log_{10}(k_1'k_2')$	−1.688e+00	−1.7060000	−1.986	−1.614000
$\log_{10}(k_1'/k_2)$	2.875e−01	0.2670000		0.273500
$\text{transport} = \log_{10}(k_1'k_2'/k_2)$	−1.035e+00	−1.1630000	−0.6771 or −0.6554	−1.010000

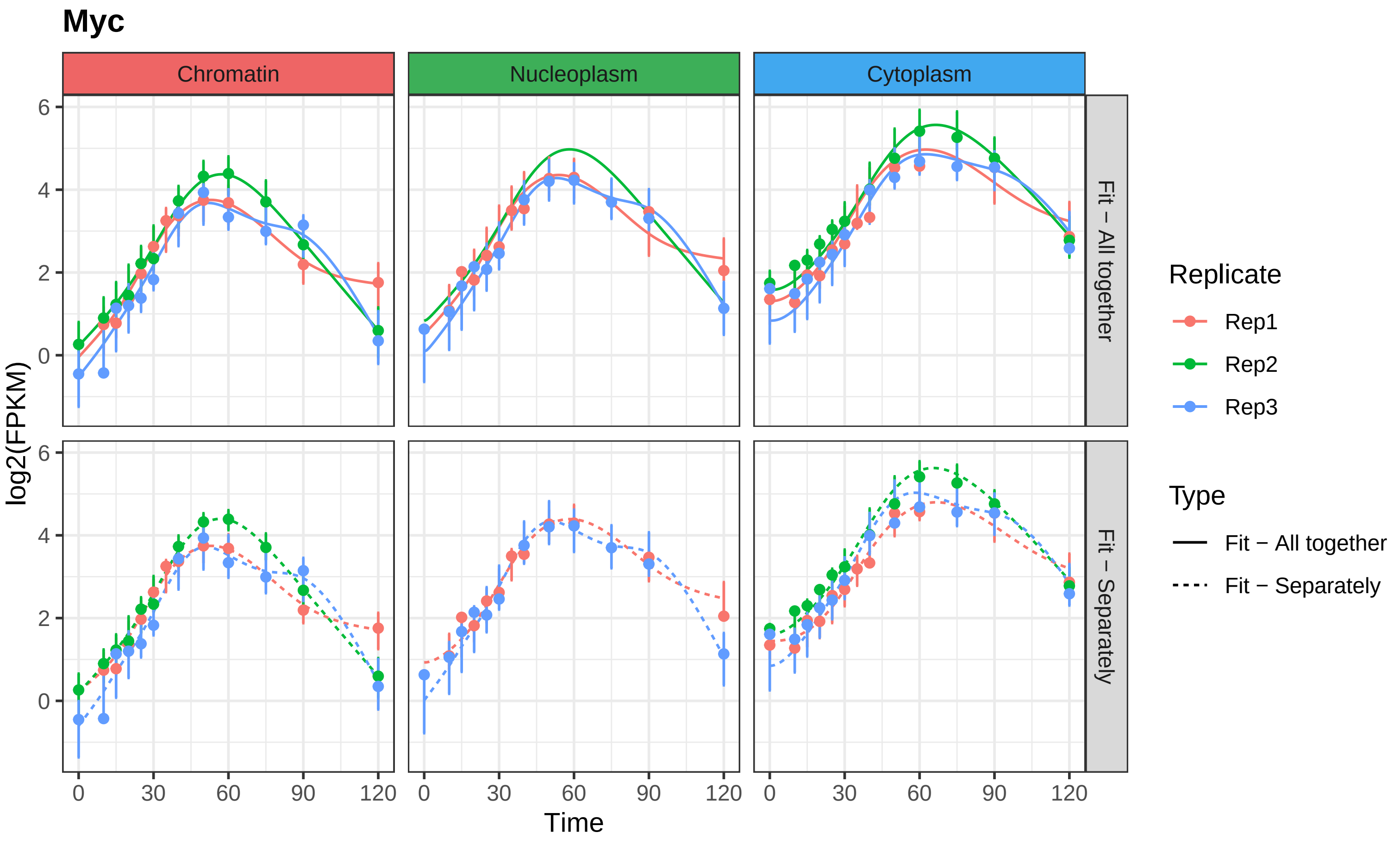

	Together	b1	b2	b3
**−NLL b1 ca**	−3.17	−4.799		
**−NLL b1 np**	3.323	0.08518		
**−NLL b1 cyto**	5.953	−0.8516		
**−NLL b2 ca**	−3.407		−5.572	
**−NLL b2 np**				
**−NLL b2 cyto**	−1.586		−0.9544	
**−NLL b3 ca**	2.606			2.049
**−NLL b3 np**	0.7053			−0.06679
**−NLL b3 cyto**	6.417			4.105
**Total**	10.84	−5.565	−6.527	6.088
**Total with regul**	10.43	−6.699	−8.416	5.293

	Together	b1	b2	b3
spar	0.462300	4.824e−01	0.428	0.4341000
σ_b	0.159900	1.213e−01	0.07427	0.1717000
σ_t	1.166000	9.388e−05	1.681	0.0003932
ca_{0,b_1}	−0.002191	3.308e−01		
$\log_{10}(k_1')$	0.202900	−5.110e−01		5.5750000
$\log_{10}(k_2)$	0.020800	−7.204e−01	4.749 or −1.003	5.3890000
$\log_{10}(k_2')$	−0.753700	−7.976e−01		−0.6239000
$\log_{10}(k_{deg})$	−0.978200	−9.538e−01	−1.003 or 4.749	−0.8748000
$\log_{10}(k_1'k_2')$	−0.550800	−1.309e+00	4.168	4.9510000
$\log_{10}(k_1'/k_2)$	0.182100	2.094e−01		0.1855000
transport $= \log_{10}(k_1'k_2'/k_2)$	−0.571600	−5.882e−01	−0.5808 or 5.17	−0.4384000

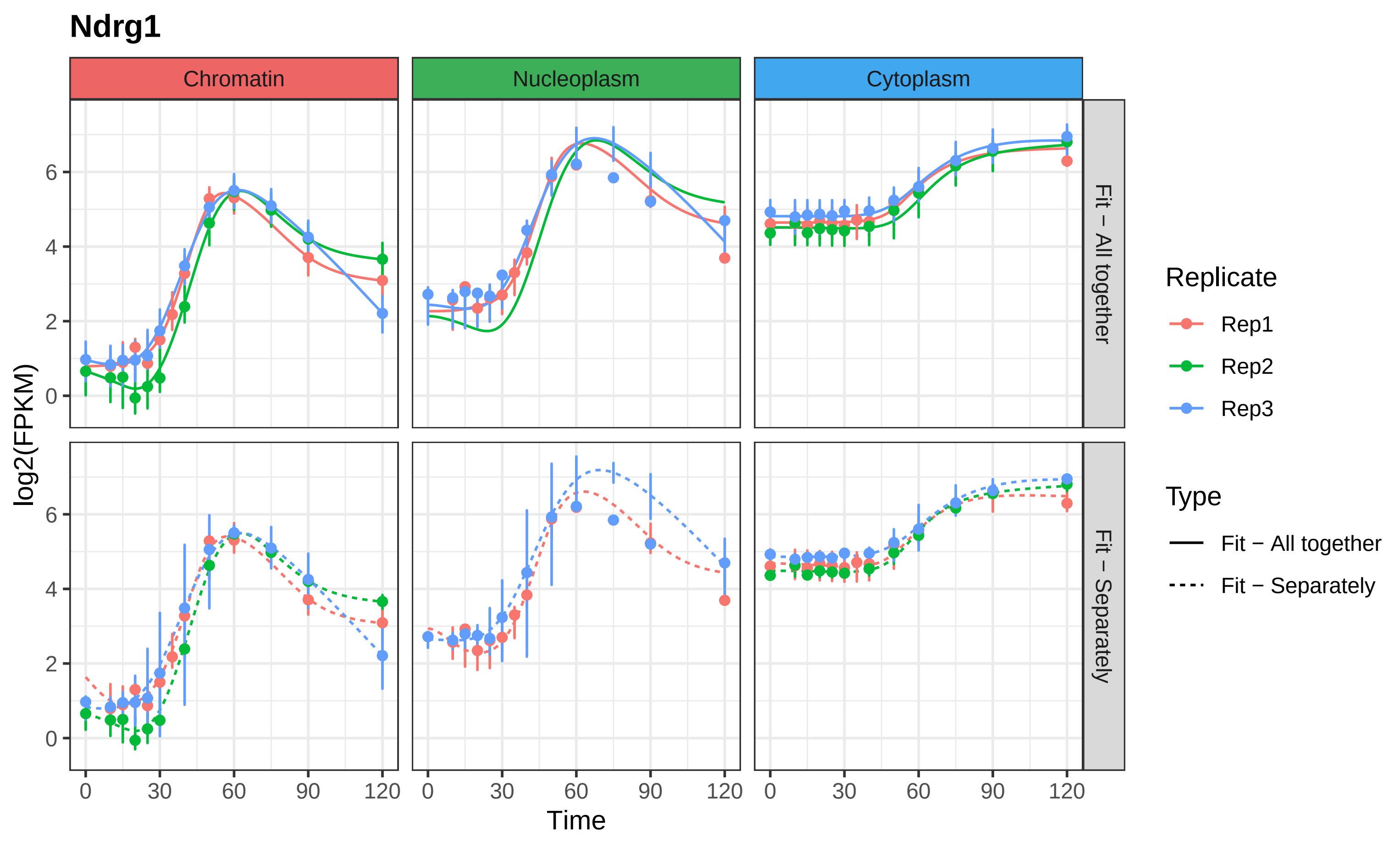

	Together	b1	b2	b3
**−NLL b1 ca**	−3.43	−2.833		
**−NLL b1 np**	9.345	5.969		
**−NLL b1 cyto**	−4.948	−6.647		
**−NLL b2 ca**	−3.727		−6.607	
**−NLL b2 np**				
**−NLL b2 cyto**	−4.976		−11.07	
**−NLL b3 ca**	−5.042			0.02676
**−NLL b3 np**	18.39			8.727
**−NLL b3 cyto**	−6.109			−14.58
Total	−0.5001	−3.511	−17.68	−5.823
**Total with regul**	0.3234	−3.184	−18.34	−7.384

	Together	b1	b2	b3
spar	0.3585000	0.4029000	0.362	0.42460
σ_b	0.1549000	0.1336000	0.04593	0.01551
σ_t	0.0002341	0.0003738	0.5567	5.55000
ca_{0,b_1}	0.7989000	1.6820000		
$\log_{10}(k_1')$	−0.2936000	−0.3031000		−0.29850
$\log_{10}(k_2)$	−0.7378000	−0.6948000	0.2368 or −2.38	−0.84500
$\log_{10}(k_2')$	−1.6860000	−1.5870000		−1.76100
$\log_{10}(k_{deg})$	−2.4020000	−2.1120000	−2.38 or 0.2368	−2.42800
$\log_{10}(k_1'k_2')$	−1.9800000	−1.8900000	−0.9942	−2.05900
$\log_{10}(k_1'/k_2)$	0.4442000	0.3918000		0.54650
transport = $\log_{10}(k_1'k_2'/k_2)$	−1.2420000	−1.1950000	−1.231 or 1.385	−1.21400

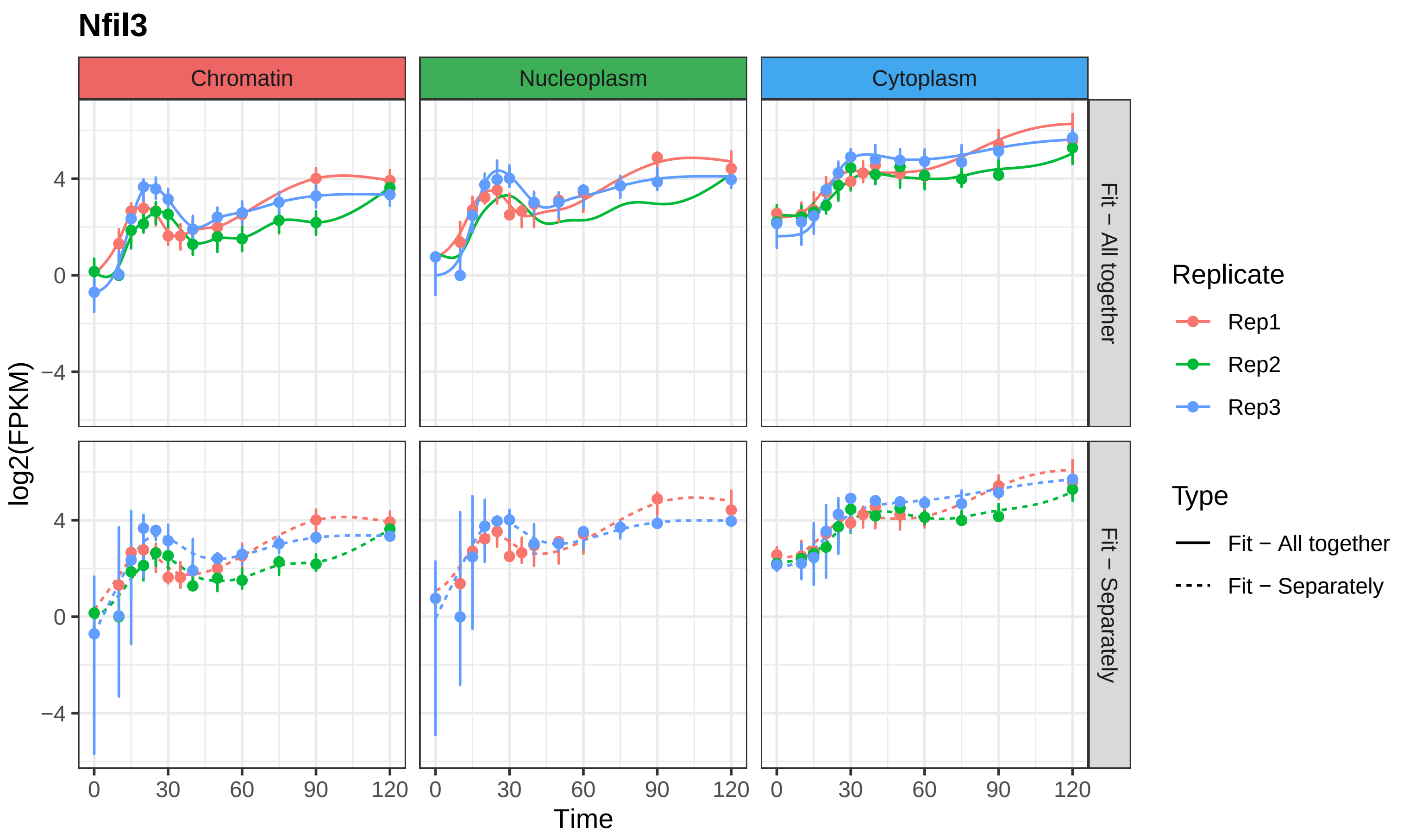

	Together	b1	b2	b3
**−NLL b1 ca**	−4.825	−1.738		
**−NLL b1 np**	3.391	4.368		
**−NLL b1 cyto**	3.468	1.185		
**−NLL b2 ca**	−3.69		0.1801	
**−NLL b2 np**				
**−NLL b2 cyto**	0.4162		−2.097	
**−NLL b3 ca**	−4.31			2.106
**−NLL b3 np**	2.756			−0.5793
**−NLL b3 cyto**	0.9565			−1.992
Total	−1.837	3.815	−1.916	−0.465
**Total with regul**	8.827	5.194	−2.865	−2.046

	Together	b1	b2	b3
spar	0.2284000	0.3697	0.3849	0.4296000
σ_b	0.1451000	0.1464	0.08657	0.0001086
σ_t	0.0007523	2.1440	2.718	5.7180000
ca_{0,b_1}	0.0491300	0.3069		
$\log_{10}(k_1')$	−0.2144000	−0.2828		6.9010000
$\log_{10}(k_2)$	−0.4379000	−0.5281	−0.9418 or −0.977	6.7120000
$\log_{10}(k_2')$	−0.8186000	−0.8656		−1.1180000
$\log_{10}(k_{deg})$	−1.3060000	−1.2680	−0.977 or −0.9418	−1.7650000
$\log_{10}(k_1'k_2')$	−1.0330000	−1.1480	−1.206	5.7830000
$\log_{10}(k_1'/k_2)$	0.2235000	0.2453		0.1889000
transport $= \log_{10}(k_1'k_2'/k_2)$	−0.5950000	−0.6203	−0.264 or −0.2288	−0.9288000

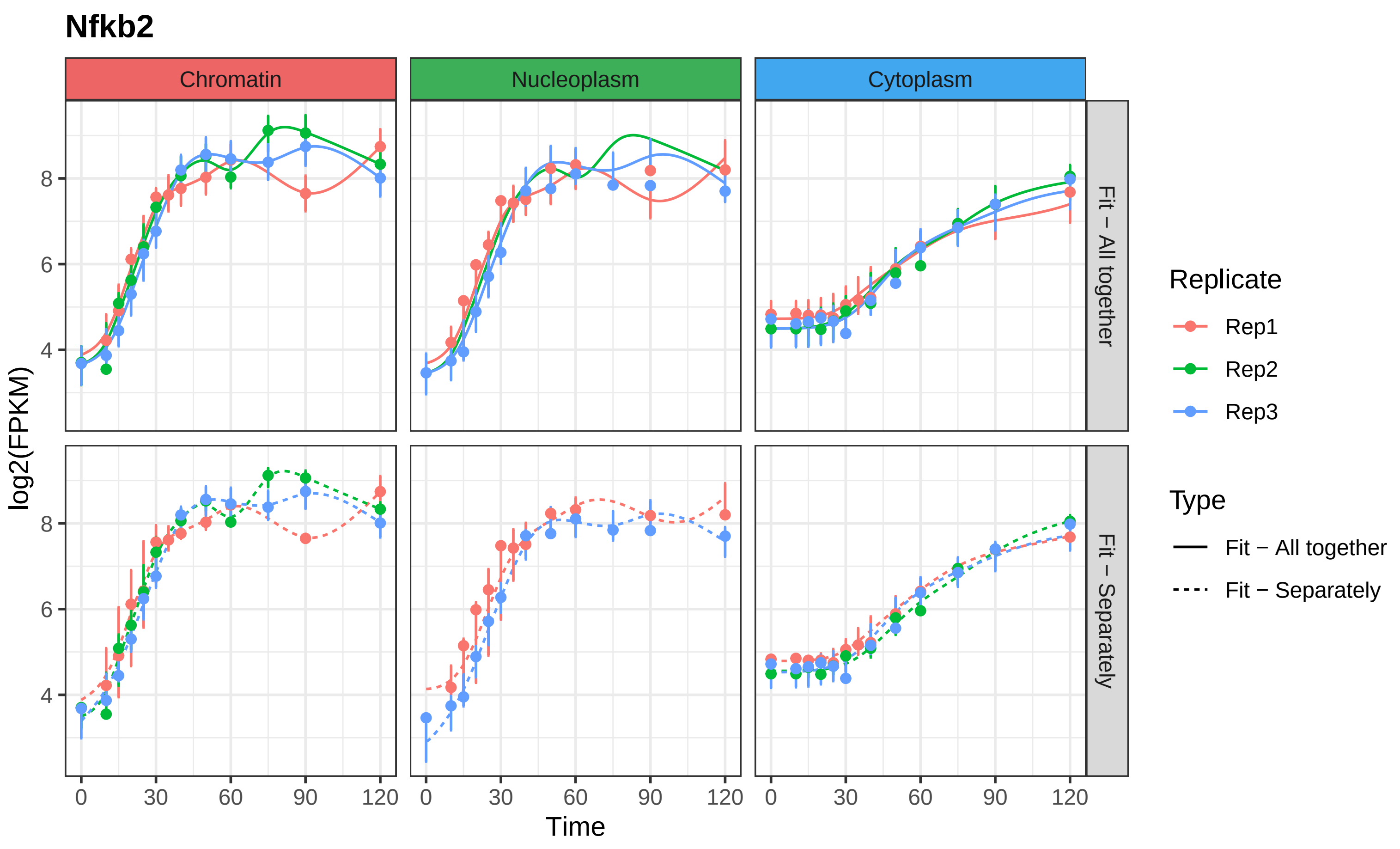

	Together	b1	b2	b3
**−NLL b1 ca**	−4.434	−6.583		
**−NLL b1 np**	8.39	2.316		
**−NLL b1 cyto**	−3.134	−12.71		
**−NLL b2 ca**	−2.385		−4.617	
**−NLL b2 np**				
**−NLL b2 cyto**	−3.77		−9.575	
**−NLL b3 ca**	−6.166			−5.405
**−NLL b3 np**	5.553			−2.234
**−NLL b3 cyto**	−2.113			−1.038
Total	−8.058	−16.98	−14.19	−8.677
**Total with regul**	−5.863	−16.96	−11.58	−9.813

	Together	b1	b2	b3
spar	0.32600	3.567e−01	0.2957	4.007e−01
σ_b	0.14350	2.392e−06	0.04675	1.180e−01
σ_t	0.69960	3.289e+00	1.712	2.107e−05
ca_{0,b_1}	3.90900	3.920e+00		
$\log_{10}(k_1')$	−0.17370	−8.574e−01		−4.165e−02
$\log_{10}(k_2)$	−0.11840	−9.352e−01	−1.165 or −2.344	1.011e−01
$\log_{10}(k_2')$	−2.07800	−2.034e+00		−2.005e+00
$\log_{10}(k_{deg})$	−2.38700	−2.230e+00	−2.344 or −1.165	−2.490e+00
$\log_{10}(k_1'k_2')$	−2.25100	−2.892e+00	−3.188	−2.046e+00
$\log_{10}(k_1'/k_2)$	−0.05528	7.784e−02		−1.427e−01
transport $= \log_{10}(k_1'k_2'/k_2)$	−2.13300	−1.957e+00	−2.024 or −0.844	−2.147e+00

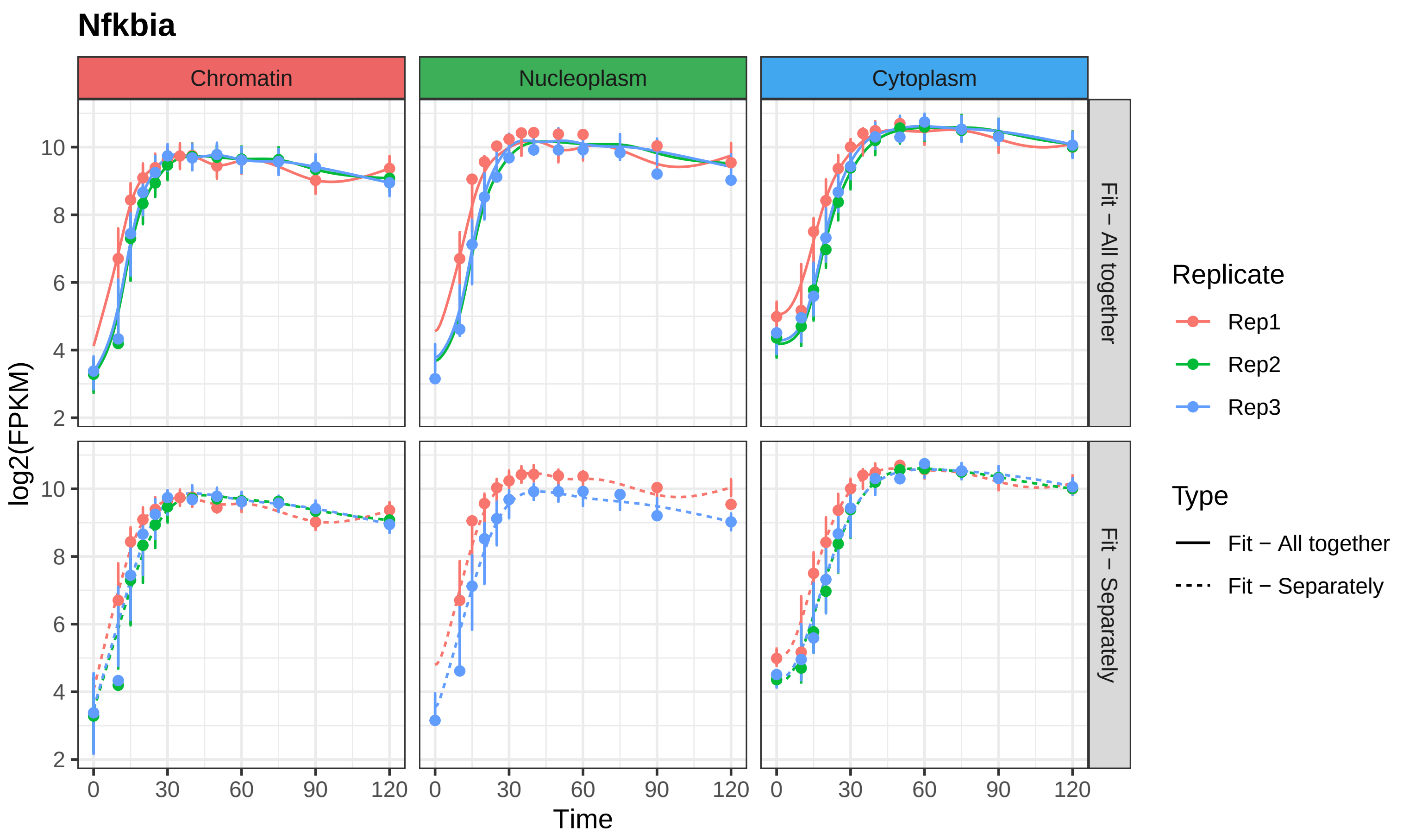

	Together	b1	b2	b3
**−NLL b1 ca**	−6.335	−8.71		
**−NLL b1 np**	9.913	2.501		
**−NLL b1 cyto**	−1.473	−4.764		
**−NLL b2 ca**	−3.925		−3.328	
**−NLL b2 np**				
**−NLL b2 cyto**	−4.963		−9.434	
**−NLL b3 ca**	−3.776			−0.9904
**−NLL b3 np**	11.98			0.009819
**−NLL b3 cyto**	−3.286			−1.192
Total	−1.864	−10.97	−12.76	−2.172
**Total with regul**	11.81	−5.092	−14.96	−4.003

	Together	b1	b2	b3
spar	0.2327	0.36420	0.4457	0.43280
σ_b	0.1322	0.08257	0.0219	0.08229
σ_t	1.1060	1.55000	2.131	2.11700
ca_{0,b_1}	4.1280	4.02700		
$\log_{10}(k_1')$	−0.2033	−0.27590		−0.12660
$\log_{10}(k_2)$	−0.3345	−0.49830	−0.6553 or −0.711	−0.14310
$\log_{10}(k_2')$	−0.8475	−0.81040		−0.86260
$\log_{10}(k_{deg})$	−0.9976	−0.88170	−0.711 or −0.6553	−1.11500
$\log_{10}(k_1'k_2')$	−1.0510	−1.08600	−1.111	−0.98920
$\log_{10}(k_1'/k_2)$	0.1312	0.22240		0.01655
$\text{transport} = \log_{10}(k_1'k_2'/k_2)$	−0.7163	−0.58800	−0.4556 or −0.3999	−0.84610

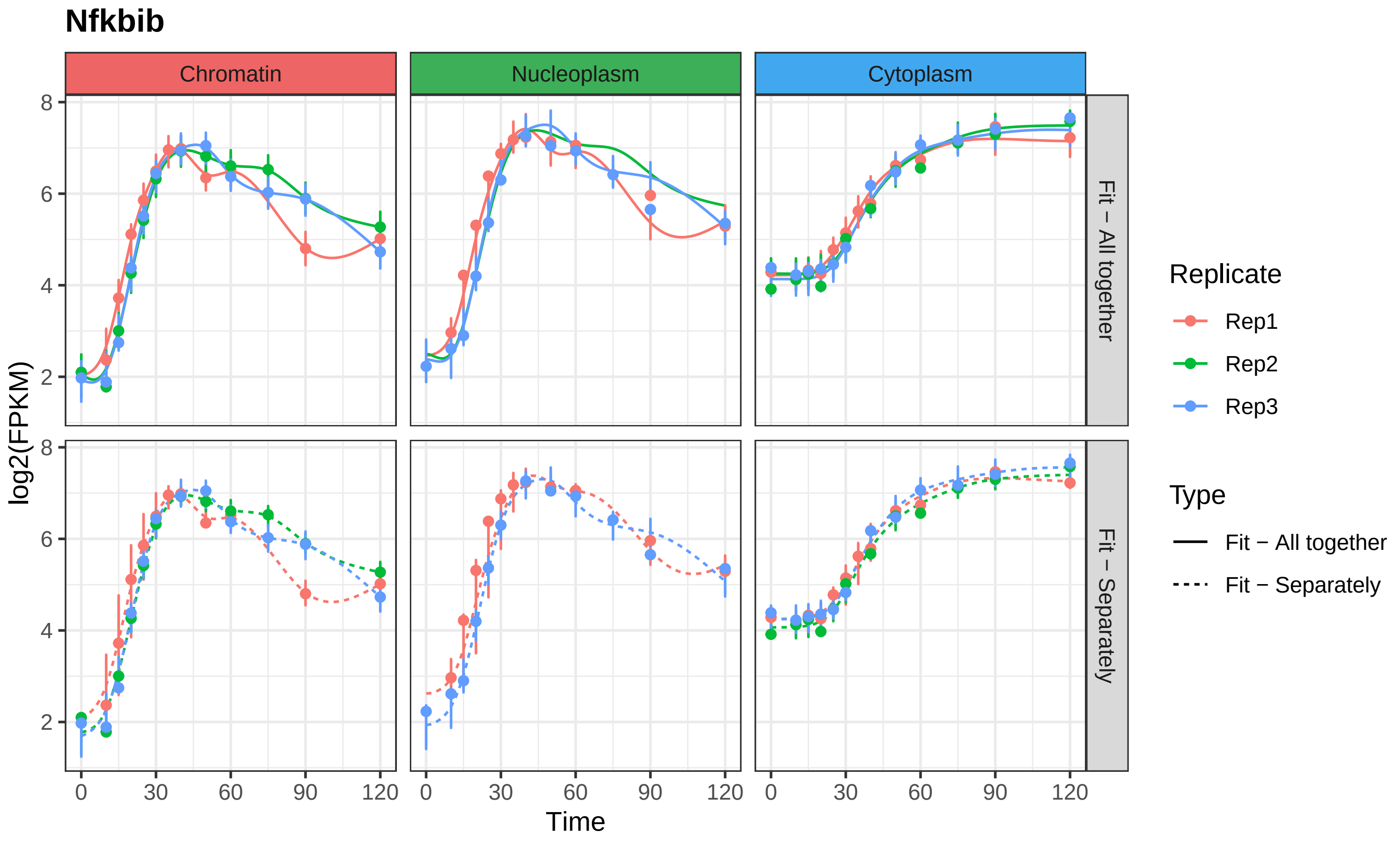

	Together	b1	b2	b3
**−NLL b1 ca**	−7.207	−5.899		
**−NLL b1 np**	4.776	−0.4381		
**−NLL b1 cyto**	−6.649	−8.24		
**−NLL b2 ca**	−7.431		−7.323	
**−NLL b2 np**				
**−NLL b2 cyto**	−2.795		−5.408	
**−NLL b3 ca**	−7.457			−4.805
**−NLL b3 np**	4.268			0.1968
**−NLL b3 cyto**	−4.833			−7.289
Total	−27.33	−14.58	−12.73	−11.9
**Total with regul**	−22.39	−12.63	−11.18	−10.47

	Together	b1	b2	b3
spar	0.2794	0.31910	0.3158	3.226e−01
σ_b	0.1157	0.04638	0.07321	9.972e−02
σ_t	0.1699	2.20000	0.000579	3.928e−05
ca_{0,b_1}	2.0510	2.12100		
$\log_{10}(k_1')$	−0.1439	−0.58050		−2.152e−01
$\log_{10}(k_2)$	−0.2784	−0.74710	0.5106 or −2.244	−2.889e−01
$\log_{10}(k_2')$	−1.6310	−1.59400		−1.551e+00
$\log_{10}(k_{deg})$	−2.1590	−2.08400	−2.244 or 0.5106	−2.246e+00
$\log_{10}(k_1'k_2')$	−1.7750	−2.17400	−1.043	−1.766e+00
$\log_{10}(k_1'/k_2)$	0.1345	0.16660		7.372e−02
transport $= \log_{10}(k_1'k_2'/k_2)$	−1.4960	−1.42700	−1.554 or 1.201	−1.477e+00

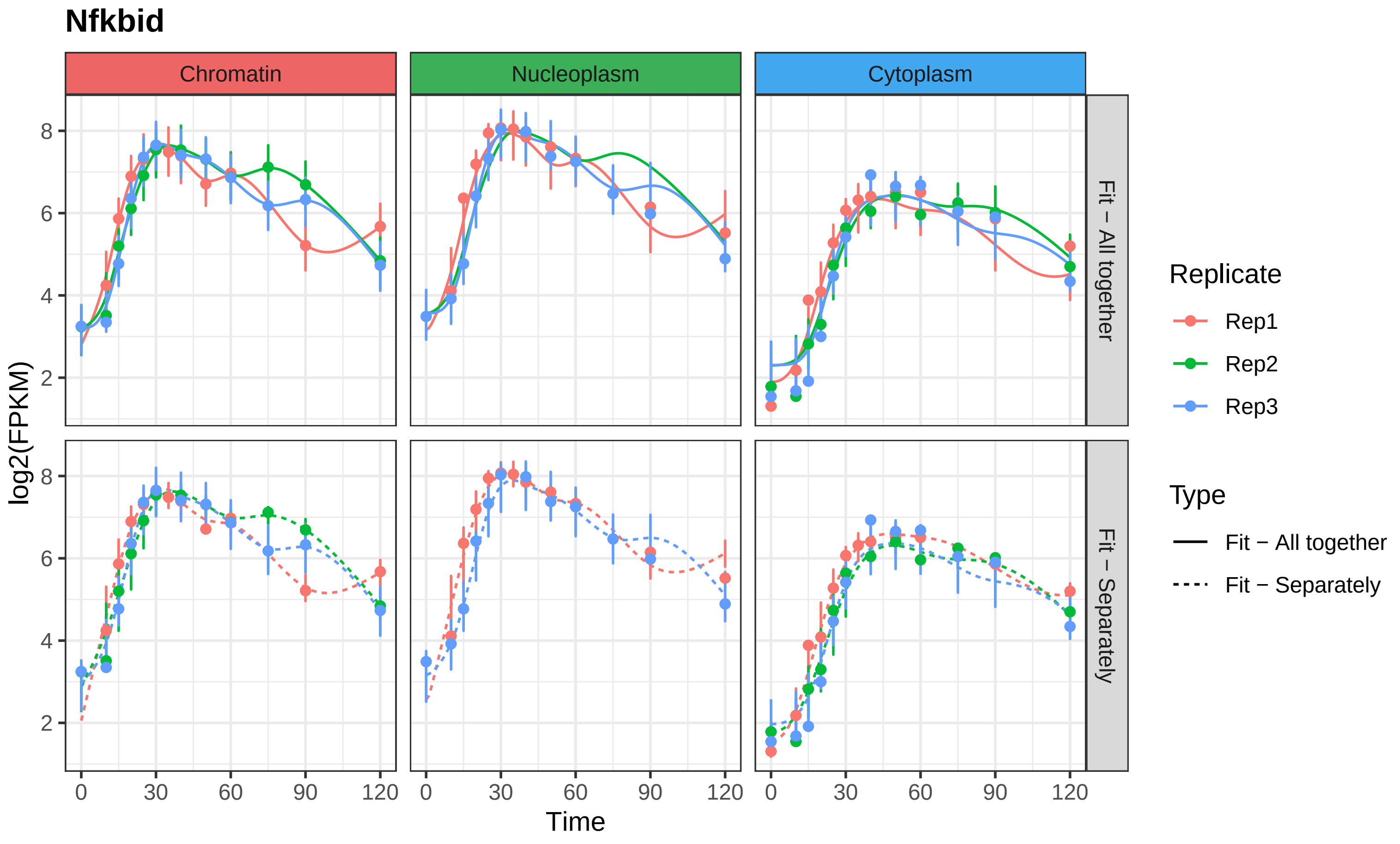

	Together	b1	b2	b3
**−NLL b1 ca**	−2.654	−5.475		
**−NLL b1 np**	3.948	3.534		
**−NLL b1 cyto**	9.036	−3.68		
**−NLL b2 ca**	−2.09		−3.807	
**−NLL b2 np**				
**−NLL b2 cyto**	4.6		0.2384	
**−NLL b3 ca**	−2.352			−0.4101
**−NLL b3 np**	0.8623			0.4383
**−NLL b3 cyto**	12.05			9.186
Total	23.41	−5.62	−3.568	9.215
**Total with regul**	32.6	1.853	−4.403	11.87

	Together	b1	b2	b3
spar	0.27450	0.38050	0.3737	0.3290000
σ_b	0.20140	0.10150	0.06841	0.2026000
σ_t	0.23780	1.41200	2.053	0.0001156
ca_{0,b_1}	2.81600	2.05500		
$\log_{10}(k_1')$	−0.03855	0.13080		−0.1759000
$\log_{10}(k_2)$	−0.14840	−0.02075	−0.2813 or −0.9755	−0.2425000
$\log_{10}(k_2')$	−1.45900	−1.54300		−1.4420000
$\log_{10}(k_{deg})$	−1.08000	−1.25100	−0.9755 or −0.2813	−1.0820000
$\log_{10}(k_1' k_2')$	−1.49700	−1.41200	−1.585	−1.6180000
$\log_{10}(k_1'/k_2)$	0.10990	0.15160		0.0665900
transport $= \log_{10}(k_1' k_2'/k_2)$	−1.34900	−1.39100	−1.304 or −0.6097	−1.3750000

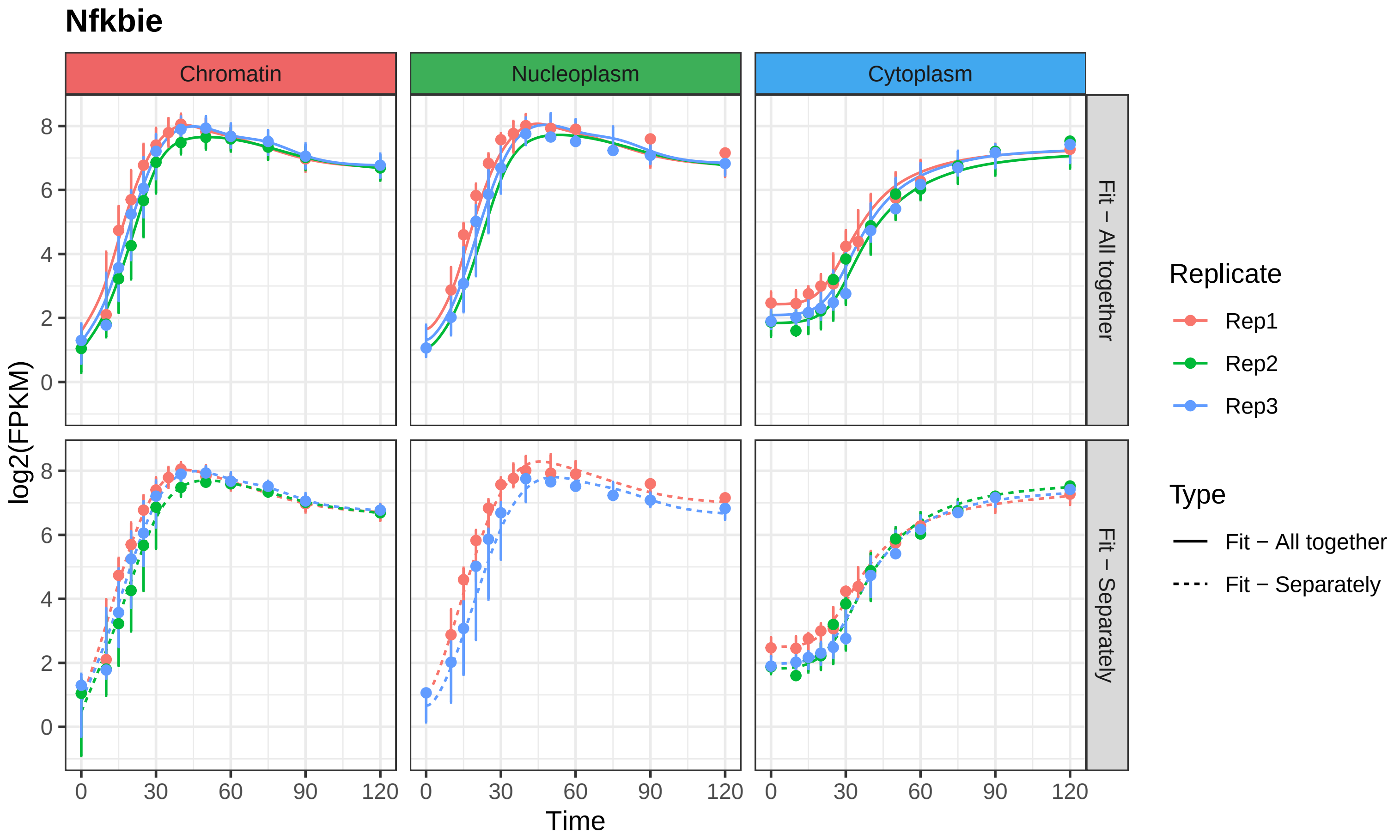

	Together	b1	b2	b3
**−NLL b1 ca**	−1.509	−3.827		
**−NLL b1 np**	4.483	1.875		
**−NLL b1 cyto**	0.3269	−4.519		
**−NLL b2 ca**	−2.885		−4.97	
**−NLL b2 np**				
**−NLL b2 cyto**	6.201		−0.2449	
**−NLL b3 ca**	−1.69			−3.118
**−NLL b3 np**	3.086			3.243
**−NLL b3 cyto**	2.657			−4.161
Total	10.67	−6.471	−5.215	−4.037
**Total with regul**	12.48	−6.757	−7.153	−5.315

	Together	b1	b2	b3
spar	0.32900	0.40120	0.4188	0.38970
σ_b	0.13060	0.09091	0.02186	0.06322
σ_t	1.93700	1.53900	2.848	2.38100
ca_{0,b_1}	1.63900	0.82450		
$\log_{10}(k_1')$	−0.40680	−0.38200		−0.73930
$\log_{10}(k_2)$	−0.42820	−0.47060	−0.6525 or −2.16	−0.70140
$\log_{10}(k_2')$	−1.89800	−2.06400		−1.85000
$\log_{10}(k_{deg})$	−2.13100	−2.49200	−2.16 or −0.6525	−2.24700
$\log_{10}(k_1'k_2')$	−2.30500	−2.44600	−2.404	−2.59000
$\log_{10}(k_1'/k_2)$	0.02132	0.08862		−0.03784
$transport = \log_{10}(k_1'k_2'/k_2)$	−1.87600	−1.97500	−1.752 or −0.2444	−1.88800

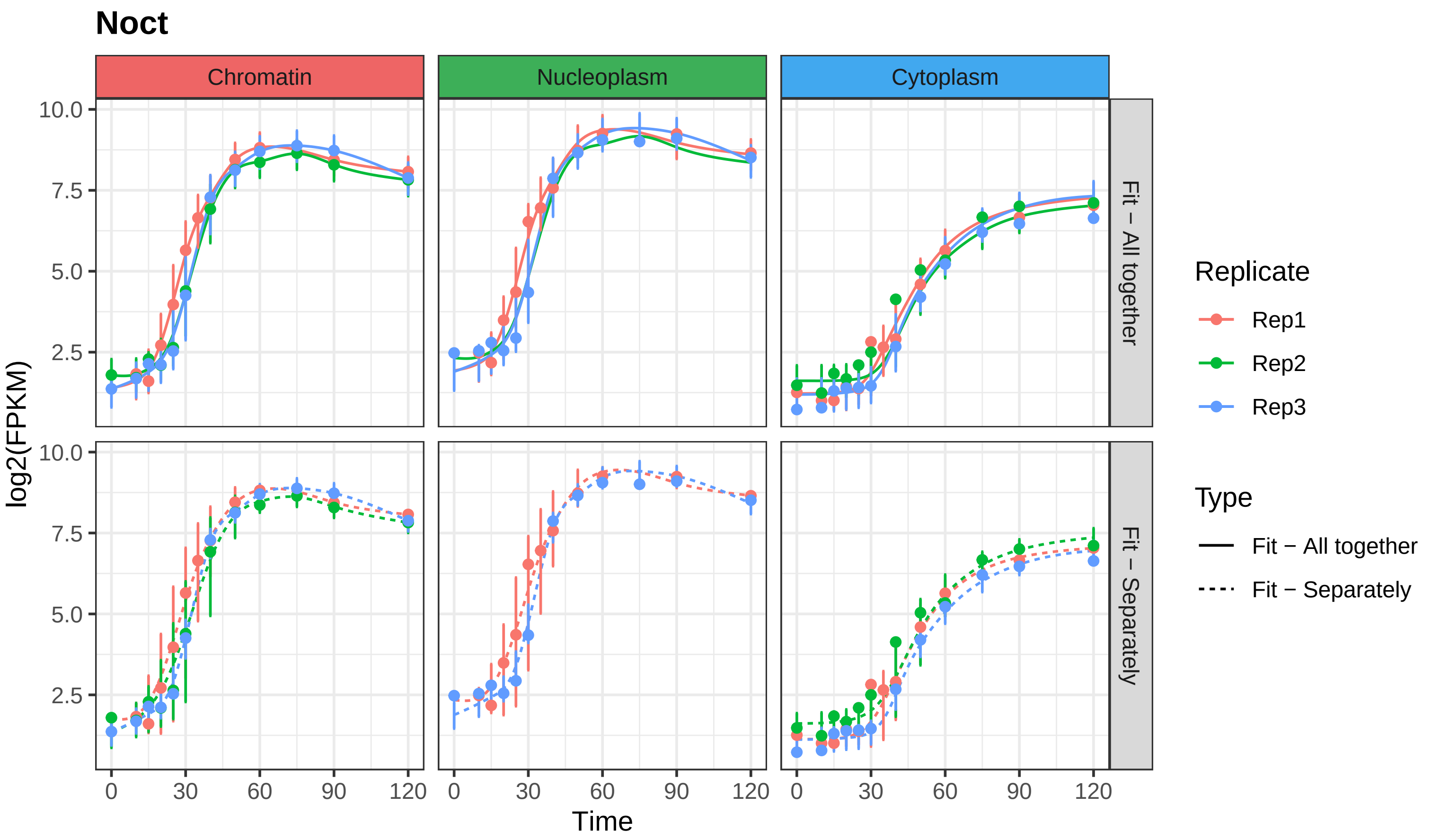

	Together	b1	b2	b3
**−NLL b1 ca**	−0.4862	−2.065		
**−NLL b1 np**	1.782	2.756		
**−NLL b1 cyto**	3.982	1.597		
**−NLL b2 ca**	−1.593		0.8082	
**−NLL b2 np**				
**−NLL b2 cyto**	12.95		4.263	
**−NLL b3 ca**	−1.684			−5.051
**−NLL b3 np**	4.777			0.683
**−NLL b3 cyto**	5.832			−0.5881
Total	25.56	2.288	5.072	−4.956
**Total with regul**	30.27	1.048	3.905	1.271

	Together	b1	b2	b3
spar	0.2895	3.963e−01	0.4069	0.2510
σ_b	0.1668	2.137e−06	0.1033	0.1107
σ_t	1.8260	3.552e+00	3.513	0.7782
ca_{0,b_1}	1.3980	1.816e+00		
$\log_{10}(k_1')$	3.4430	1.304e−01		0.9024
$\log_{10}(k_2)$	3.2820	−4.307e−02	5.803 or −2.138	0.7435
$\log_{10}(k_2')$	−2.2990	−2.346e+00		−2.4440
$\log_{10}(k_{deg})$	−2.0860	−1.984e+00	−2.138 or 5.803	−2.2130
$\log_{10}(k_1'k_2')$	1.1440	−2.215e+00	3.747	−1.5410
$\log_{10}(k_1'/k_2)$	0.1612	1.735e−01		0.1589
transport $= \log_{10}(k_1'k_2'/k_2)$	−2.1380	−2.172e+00	−2.057 or 5.885	−2.2850

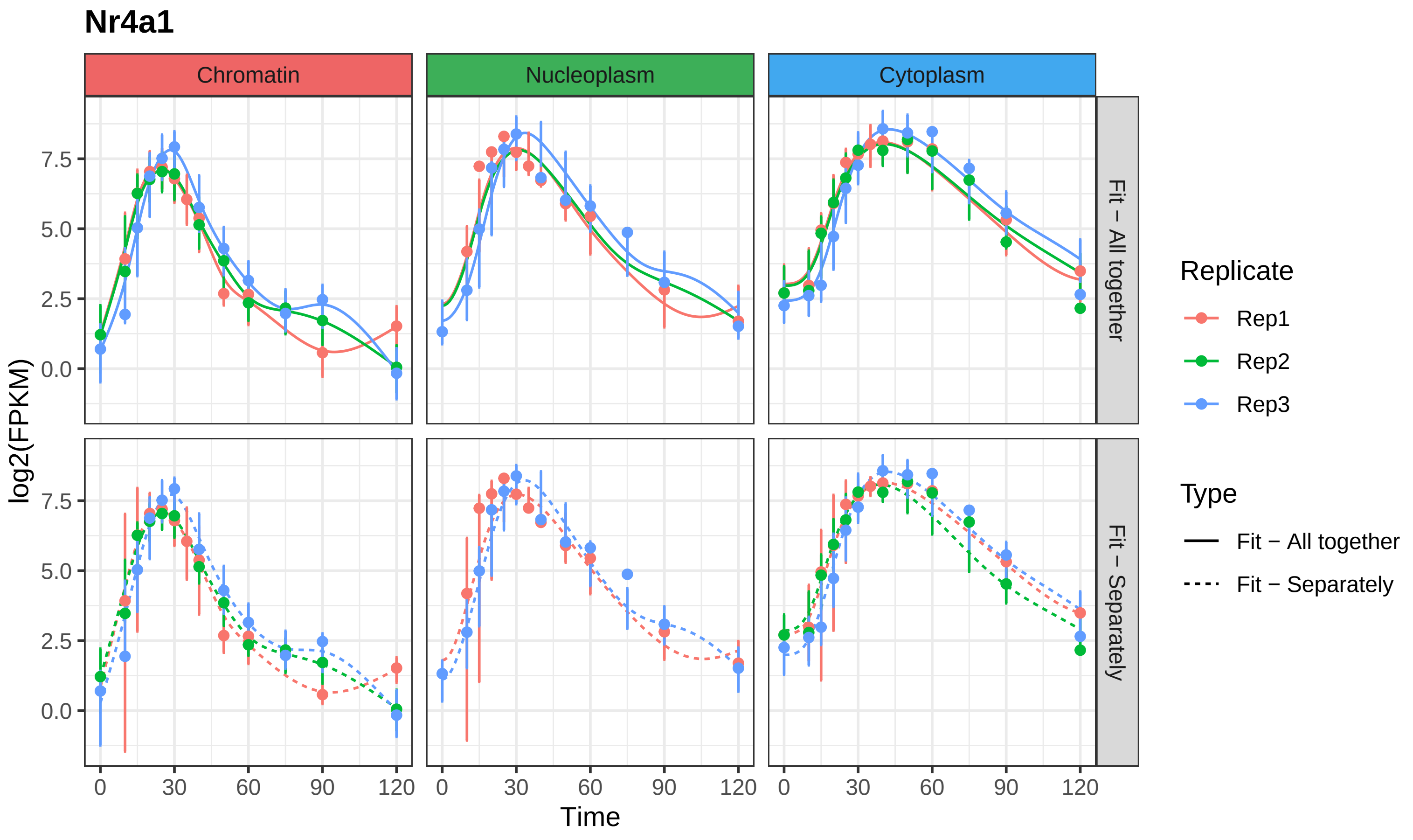

	Together	b1	b2	b3
**−NLL b1 ca**	2.8	2.911		
**−NLL b1 np**	12.57	11.99		
**−NLL b1 cyto**	4.729	−2.158		
**−NLL b2 ca**	2.791		1.657	
**−NLL b2 np**				
**−NLL b2 cyto**	10.89		8.897	
**−NLL b3 ca**	4.196			4.715
**−NLL b3 np**	13.12			8.453
**−NLL b3 cyto**	8.751			8.399
Total	59.85	12.74	10.55	21.57
**Total with regul**	66.45	16.18	11.79	22.89

	Together	b1	b2	b3
spar	0.3107	3.602e−01	0.3631	0.3762
σ_b	0.2497	4.209e−08	0.2005	0.2235
σ_t	1.4930	3.709e+00	1.4	1.6620
ca_{0,b_1}	1.2640	8.232e−01		
$\log_{10}(k_1')$	−0.5937	−6.671e−01		−0.5719
$\log_{10}(k_2)$	−0.9139	−9.583e−01	−0.9275 or −0.9635	−0.8262
$\log_{10}(k_2')$	−0.9112	−8.702e−01		−0.8552
$\log_{10}(k_{deg})$	−1.1270	−1.153e+00	−0.9635 or −0.9275	−1.1180
$\log_{10}(k_1'k_2')$	−1.5050	−1.537e+00	−1.384	−1.4270
$\log_{10}(k_1'/k_2)$	0.3202	2.912e−01		0.2543
$\text{transport} = \log_{10}(k_1'k_2'/k_2)$	−0.5911	−5.790e−01	−0.4569 or −0.4209	−0.6009

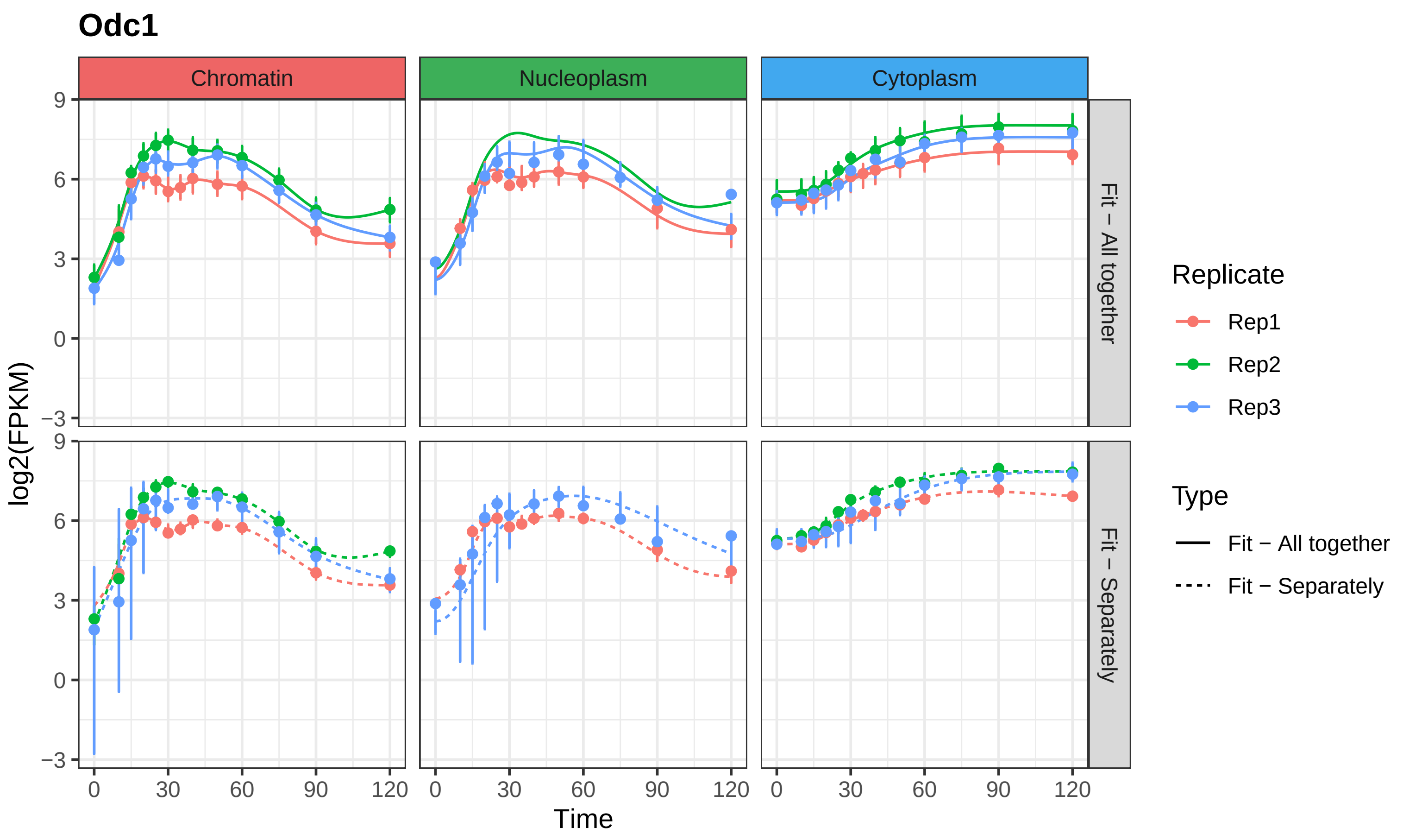

	Together	b1	b2	b3
**−NLL b1 ca**	−4.527	−9.877		
**−NLL b1 np**	−0.8995	−0.3058		
**−NLL b1 cyto**	−5.715	−14.13		
**−NLL b2 ca**	−3.352		−7.321	
**−NLL b2 np**				
**−NLL b2 cyto**	−2.677		−8.554	
**−NLL b3 ca**	−3.1			2.527
**−NLL b3 np**	24.3			11.44
**−NLL b3 cyto**	−3.136			−2.125
Total	0.8919	−24.32	−15.87	11.84
**Total with regul**	7.5	−20.69	−15.57	10.93

	Together	b1	b2	b3
spar	0.2731	0.28410	0.3395	0.42700
σ_b	0.1545	0.05580	0.05327	0.12180
σ_t	0.6635	1.54500	1.43	5.28900
ca_{0,b_1}	1.9410	2.81600		
$\log_{10}(k_1')$	−0.4478	−0.71580		−1.11100
$\log_{10}(k_2)$	−0.5582	−0.80180	4.797 or −2.534	−1.20000
$\log_{10}(k_2')$	−1.6180	−1.42200		−1.50200
$\log_{10}(k_{deg})$	−2.4930	−2.03700	−2.534 or 4.797	−2.44000
$\log_{10}(k_1'k_2')$	−2.0660	−2.13800	3.229	−2.61300
$\log_{10}(k_1'/k_2)$	0.1103	0.08595		0.08894
$\text{transport} = \log_{10}(k_1'k_2'/k_2)$	−1.5080	−1.33600	−1.568 or 5.763	−1.41300

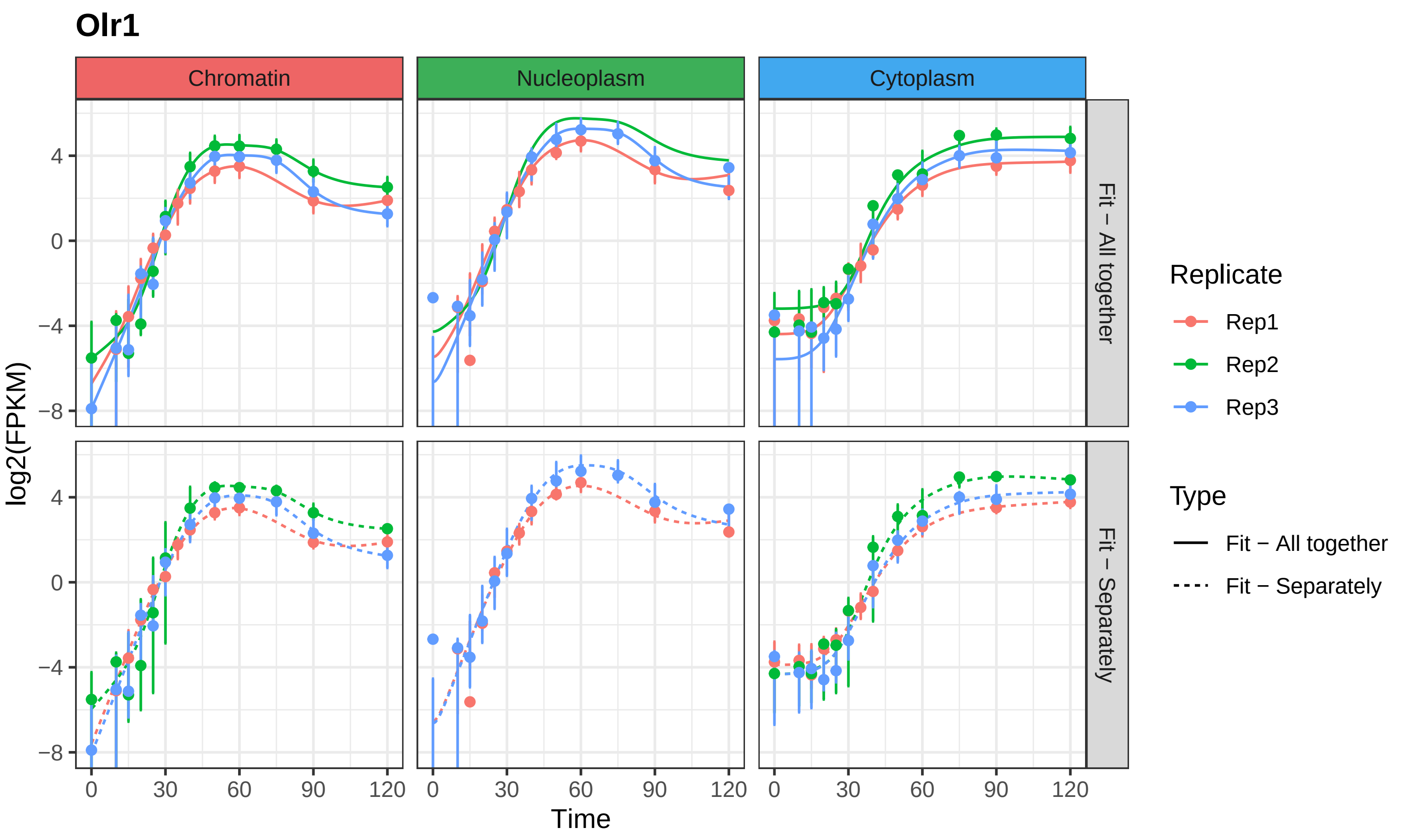

	Together	b1	b2	b3
**−NLL b1 ca**	1.33	−1.96		
**−NLL b1 np**	10.99	11.81		
**−NLL b1 cyto**	4.586	0.8661		
**−NLL b2 ca**	6.149		3.524	
**−NLL b2 np**				
**−NLL b2 cyto**	14.32		6.15	
**−NLL b3 ca**	6.831			5.526
**−NLL b3 np**	21.91			9.83
**−NLL b3 cyto**	11.99			6.813
Total	78.11	10.71	9.674	22.17
**Total with regul**	81.36	9.139	9.637	21.71

	Together	b1	b2	b3
spar	0.330100	0.45030	0.3478	0.40500
σ_b	0.168800	0.09232	2.556e−06	0.16700
σ_t	1.468000	0.91170	3.101	1.67400
ca_{0,b_1}	−6.704000	−7.60300		
$\log_{10}(k_1')$	0.009809	−0.03122		0.09751
$\log_{10}(k_2)$	−0.365900	−0.35130	−0.6602 or −1.819	−0.33230
$\log_{10}(k_2')$	−1.841000	−1.86800		−2.00100
$\log_{10}(k_{deg})$	−2.168000	−2.66600	−1.819 or −0.6602	−2.70100
$\log_{10}(k_1'k_2')$	−1.832000	−1.89900	−1.984	−1.90400
$\log_{10}(k_1'/k_2)$	0.375700	0.32010		0.42980
$\text{transport} = \log_{10}(k_1'k_2'/k_2)$	−1.466000	−1.54800	−1.324 or −0.1651	−1.57200

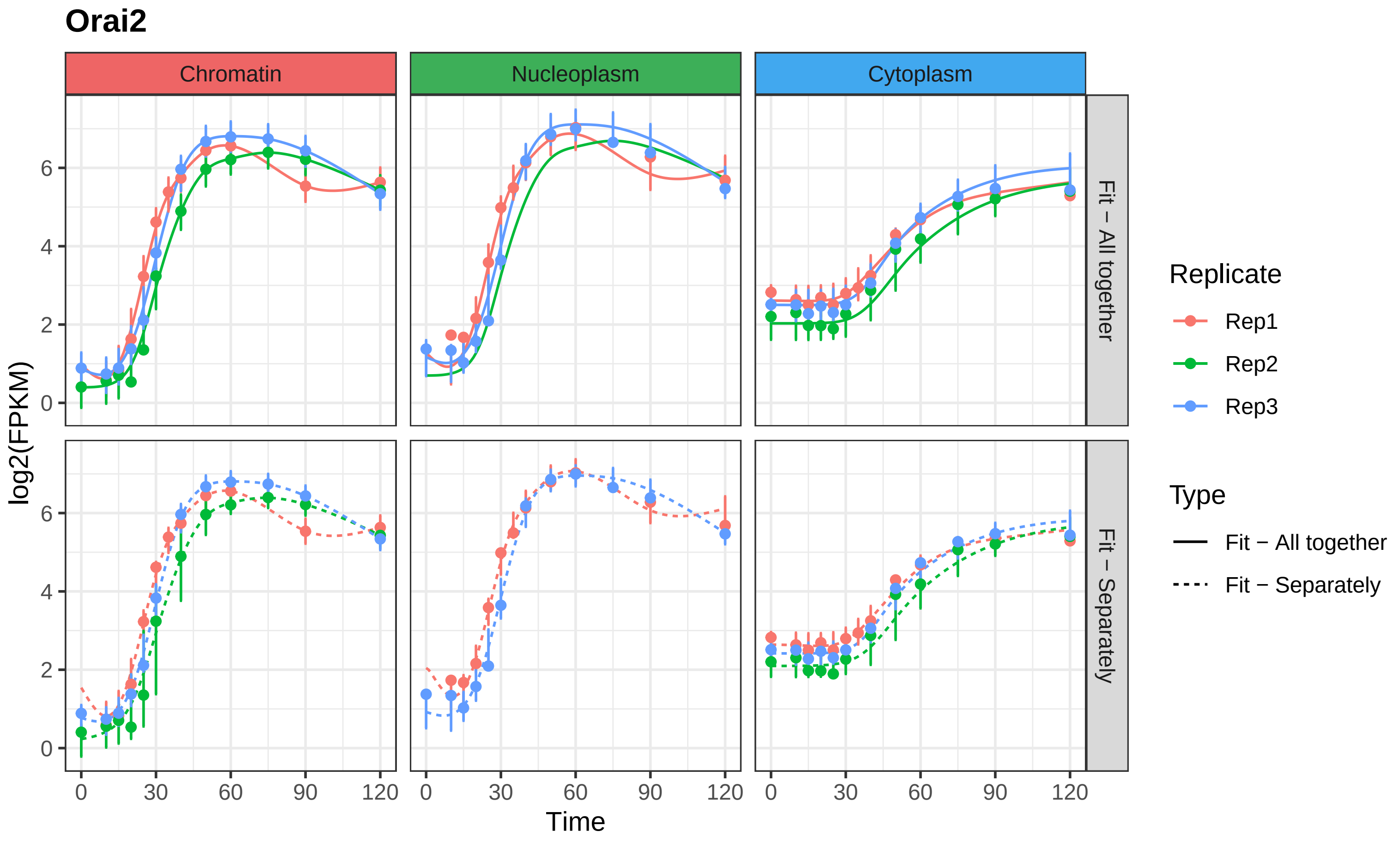

	Together	b1	b2	b3
**−NLL b1 ca**	−5.214	−5.38		
**−NLL b1 np**	6.659	0.189		
**−NLL b1 cyto**	−4.572	−6.062		
**−NLL b2 ca**	−3.257		−1.545	
**−NLL b2 np**				
**−NLL b2 cyto**	2.667		−0.5517	
**−NLL b3 ca**	−5.796			−6.782
**−NLL b3 np**	3.252			−0.04445
**−NLL b3 cyto**	−2.204			−3.996
Total	−8.464	−11.25	−2.096	−10.82
**Total with regul**	−5.787	−8.579	−2.604	−8.943

	Together	b1	b2	b3
spar	0.31800	0.347800	0.3708	0.31300
σ_b	0.13510	0.109900	0.08763	0.09273
σ_t	0.72190	0.004462	2.939	0.79860
ca_{0,b_1}	1.00800	1.603000		
$\log_{10}(k_1')$	8.17000	0.232900		1.24800
$\log_{10}(k_2)$	8.07900	0.082090	5.914 or −2.591	1.20300
$\log_{10}(k_2')$	−2.12100	−2.159000		−2.14600
$\log_{10}(k_{deg})$	−2.52200	−2.338000	−2.591 or 5.914	−2.59700
$\log_{10}(k_1'k_2')$	6.04900	−1.926000	3.886	−0.89810
$\log_{10}(k_1'/k_2)$	0.09059	0.150800		0.04465
transport $= \log_{10}(k_1'k_2'/k_2)$	−2.03100	−2.008000	−2.029 or 6.477	−2.10100

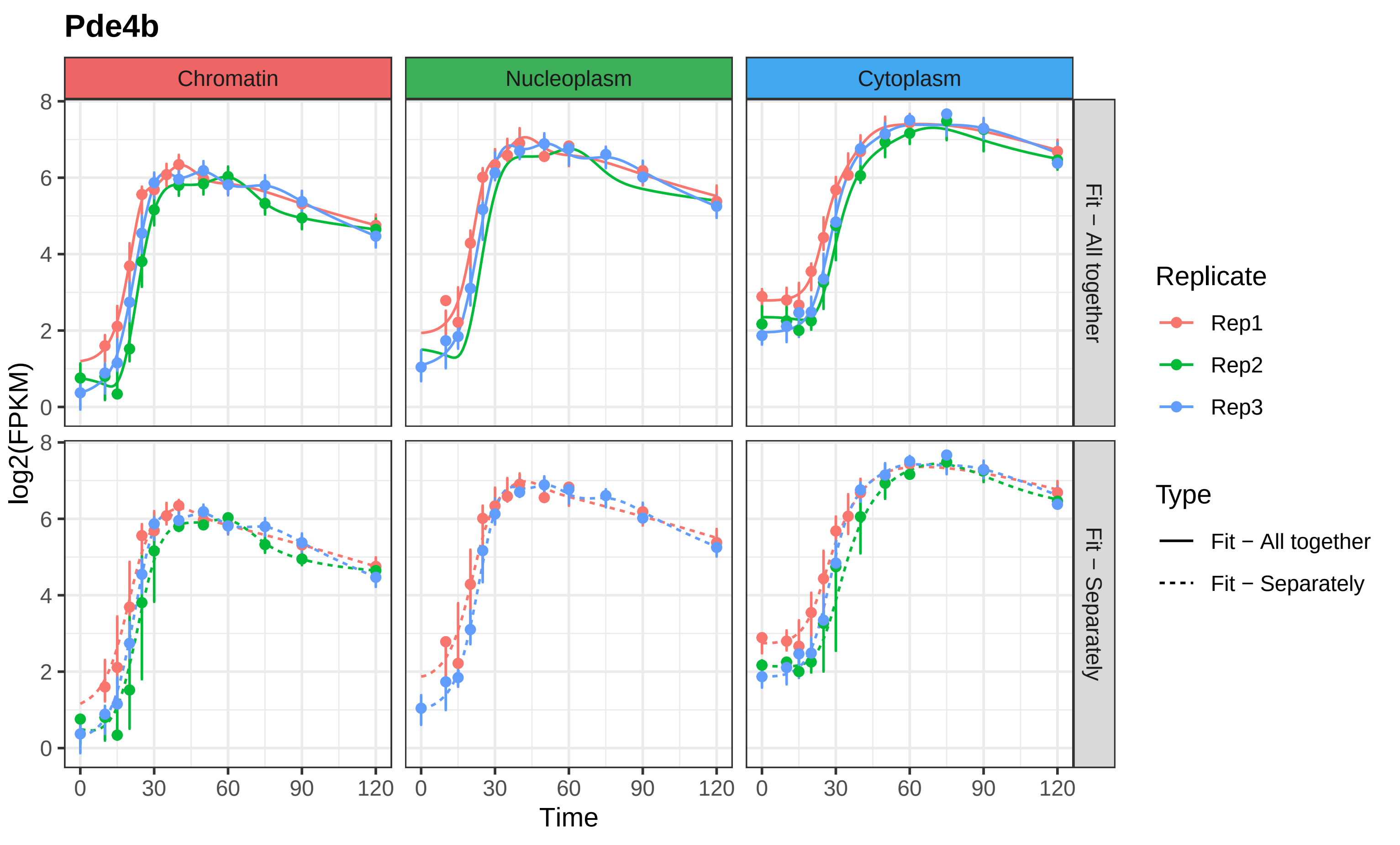

	Together	b1	b2	b3
**−NLL b1 ca**	−8.868	−5.243		
**−NLL b1 np**	6.277	3.305		
**−NLL b1 cyto**	−5.557	−5.322		
**−NLL b2 ca**	−7.437		−3.882	
**−NLL b2 np**				
**−NLL b2 cyto**	−1.141		−3.845	
**−NLL b3 ca**	−9.03			−7.163
**−NLL b3 np**	−6.083			−3.836
**−NLL b3 cyto**	−2.83			−2.73
Total	−34.67	−7.26	−7.726	−13.73
**Total with regul**	−24.46	−7.085	−6.939	−8.091

	Together	b1	b2	b3
spar	0.20700	0.352600	0.329	0.25460
σ_b	0.09546	0.075060	0.03444	0.07947
σ_t	0.59110	1.869000	2.416	0.59280
ca_{0,b_1}	1.20200	1.227000		
$\log_{10}(k_1')$	0.11650	0.009303		0.01966
$\log_{10}(k_2)$	−0.10670	−0.206200	−0.961 or −0.984	−0.20560
$\log_{10}(k_2')$	−1.00500	−1.056000		−0.96330
$\log_{10}(k_{deg})$	−1.26100	−1.319000	−0.984 or −0.961	−1.21600
$\log_{10}(k_1' k_2')$	−0.88890	−1.047000	−1.449	−0.94370
$\log_{10}(k_1'/k_2)$	0.22310	0.215500		0.22530
transport $= \log_{10}(k_1' k_2'/k_2)$	−0.78220	−0.840700	−0.4883 or −0.4653	−0.73810

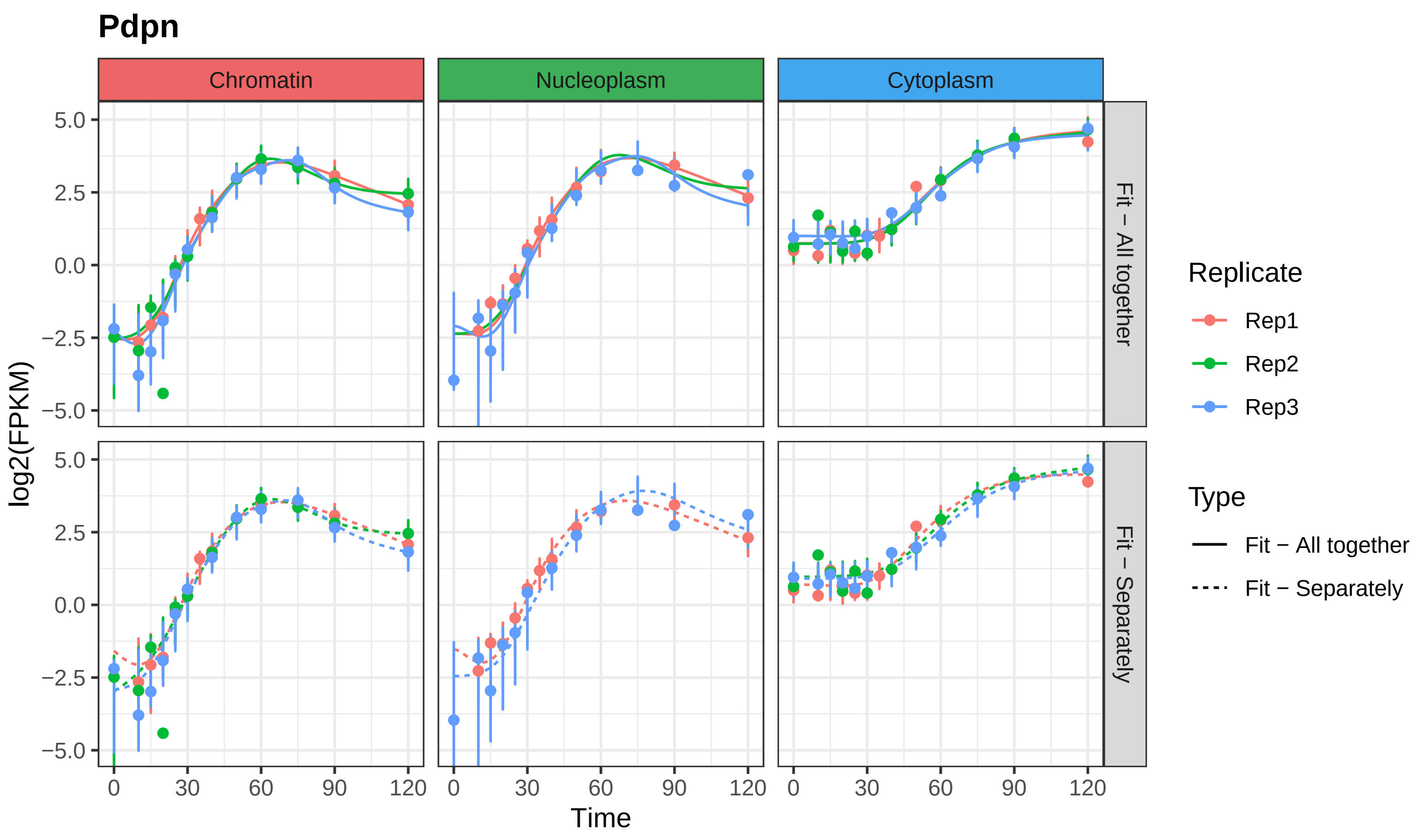

	Together	b1	b2	b3
**−NLL b1 ca**	0.2515	−0.3955		
**−NLL b1 np**	1.876	0.8171		
**−NLL b1 cyto**	3.748	3.023		
**−NLL b2 ca**	8.044		8.804	
**−NLL b2 np**				
**−NLL b2 cyto**	6.197		5.048	
**−NLL b3 ca**	2.42			3.69
**−NLL b3 np**	16.29			10.13
**−NLL b3 cyto**	0.8147			0.1623
Total	39.64	3.445	13.85	13.98
**Total with regul**	41.83	2.335	12.87	13.28

	Together	b1	b2	b3
spar	3.672e−01	4.141e−01	0.4141	4.201e−01
σ_b	1.698e−01	1.165e−01	0.1471	1.694e−01
σ_t	6.096e−05	3.868e−05	0.0002212	6.768e−05
ca_{0,b_1}	−2.481e+00	−1.470e+00		
$\log_{10}(k_1')$	−5.251e−01	−2.529e−01		−8.488e−01
$\log_{10}(k_2)$	−5.761e−01	−2.686e−01	−0.8498 or −2.544	−9.919e−01
$\log_{10}(k_2')$	−1.437e+00	−1.332e+00		−1.499e+00
$\log_{10}(k_{deg})$	−2.370e+00	−2.002e+00	−2.544 or −0.8498	−2.509e+00
$\log_{10}(k_1'k_2')$	−1.963e+00	−1.585e+00	−2.209	−2.348e+00
$\log_{10}(k_1'/k_2)$	5.104e−02	1.577e−02		1.431e−01
transport $= \log_{10}(k_1'k_2'/k_2)$	−1.386e+00	−1.316e+00	−1.359 or 0.3352	−1.356e+00

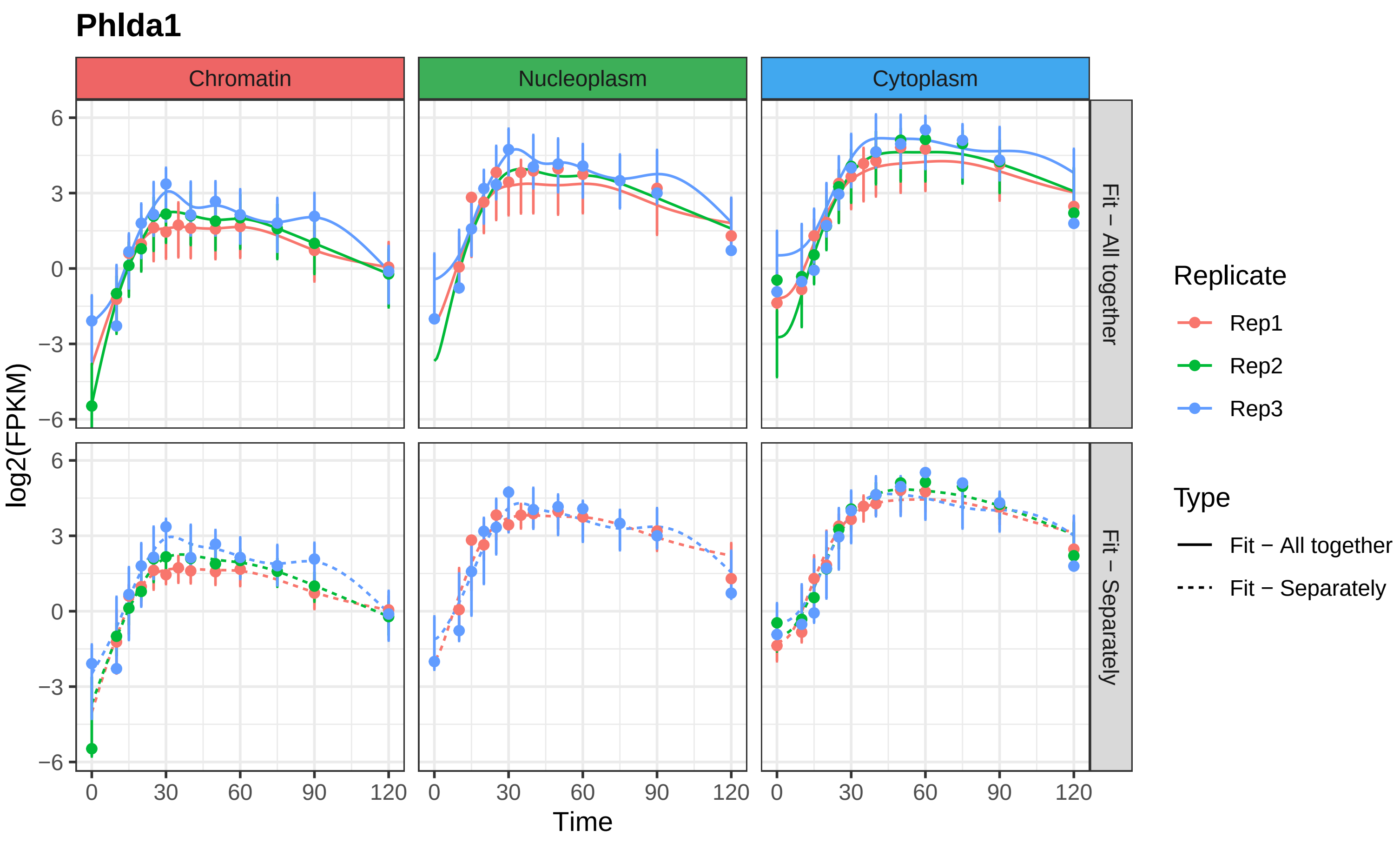

	Together	b1	b2	b3
**−NLL b1 ca**	4.004	−1.153		
**−NLL b1 np**	9.677	6.745		
**−NLL b1 cyto**	6.876	5.275		
**−NLL b2 ca**	5.145		−1.318	
**−NLL b2 np**				
**−NLL b2 cyto**	19.38		5.504	
**−NLL b3 ca**	7.216			6.401
**−NLL b3 np**	13.15			7.683
**−NLL b3 cyto**	20.93			12.86
Total	86.38	10.87	4.186	26.94
**Total with regul**	94.88	11.6	3.566	29.26

	Together	b1	b2	b3
spar	0.308800	0.4245	0.4258	0.38030
σ_b	0.350000	0.1648	0.1778	0.26690
σ_t	0.001134	1.8260	0.1006	2.23700
ca_{0,b_1}	−3.856000	−4.1600		
$\log_{10}(k_1')$	0.237100	0.3310		0.05184
$\log_{10}(k_2)$	−0.281800	−0.3085	−0.752 or −0.7789	−0.36390
$\log_{10}(k_2')$	−0.774100	−0.7535		−0.75610
$\log_{10}(k_{deg})$	−1.059000	−0.9656	−0.7789 or −0.752	−0.95370
$\log_{10}(k_1'k_2')$	−0.537000	−0.4225	−0.7081	−0.70430
$\log_{10}(k_1'/k_2)$	0.518900	0.6395		0.41580
transport = $\log_{10}(k_1'k_2'/k_2)$	−0.255200	−0.1140	0.04385 or 0.07072	−0.34040

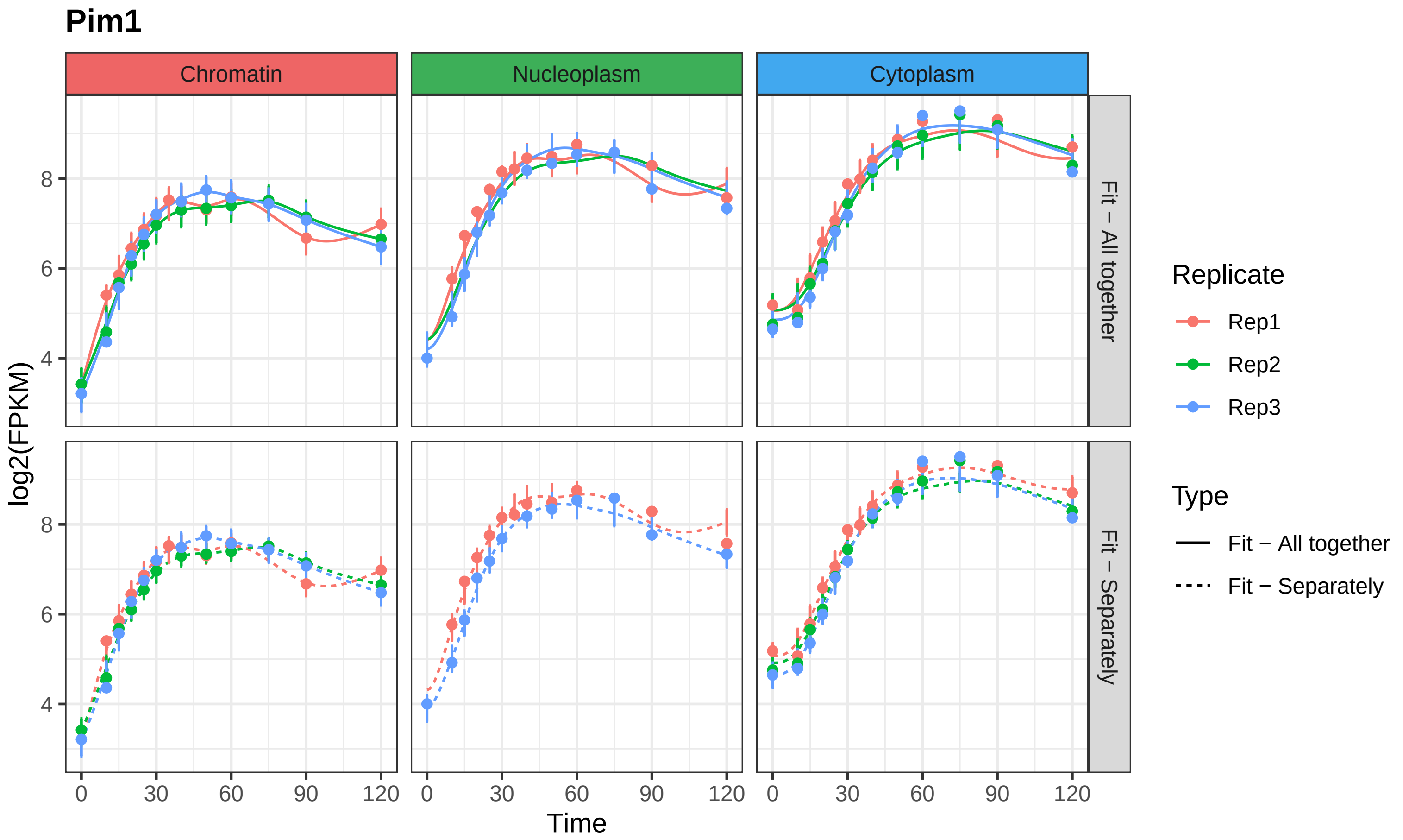

	Together	b1	b2	b3
**−NLL b1 ca**	−7.916	−9.312		
**−NLL b1 np**	0.9459	−1.385		
**−NLL b1 cyto**	−0.2267	−5.882		
**−NLL b2 ca**	−8.106		−9.953	
**−NLL b2 np**				
**−NLL b2 cyto**	−0.5038		−3.622	
**−NLL b3 ca**	−7.741			−7.486
**−NLL b3 np**	−1.018			−7.317
**−NLL b3 cyto**	0.01936			−1.695
Total	−24.55	−16.58	−13.58	−16.5
**Total with regul**	−21.45	−13.91	−13.82	−17.01

	Together	b1	b2	b3
spar	0.3313000	3.643e−01	0.3556	0.36770
σ_b	0.1264000	1.003e−01	0.07559	0.09544
σ_t	0.0001218	8.869e−05	0.5848	0.09303
ca_{0,b_1}	3.3630000	3.068e+00		
$\log_{10}(k_1')$	−0.3705000	−3.192e−01		−0.28130
$\log_{10}(k_2)$	−0.6755000	−6.738e−01	−0.8533 or −0.8714	−0.51310
$\log_{10}(k_2')$	−0.9507000	−1.057e+00		−0.94190
$\log_{10}(k_{deg})$	−1.1450000	−1.286e+00	−0.8714 or −0.8533	−1.16400
$\log_{10}(k_1' k_2')$	−1.3210000	−1.376e+00	−1.265	−1.22300
$\log_{10}(k_1'/k_2)$	0.3050000	3.546e−01		0.23180
$\text{transport} = \log_{10}(k_1' k_2'/k_2)$	−0.6457000	−7.027e−01	−0.4114 or −0.3933	−0.71000

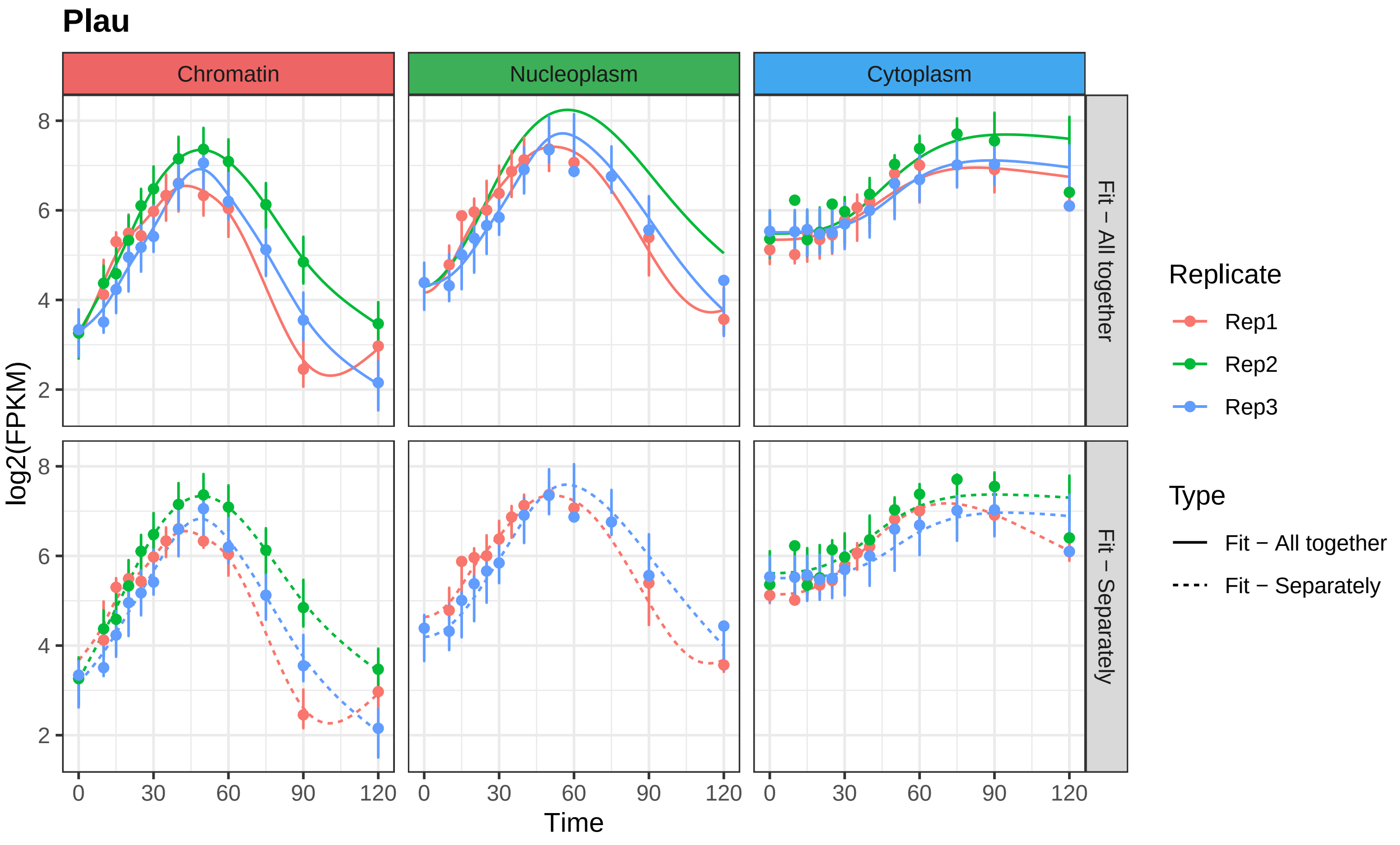

	Together	b1	b2	b3
**−NLL b1 ca**	−2.349	−5.521		
**−NLL b1 np**	0.536	−2.363		
**−NLL b1 cyto**	1.681	−8.625		
**−NLL b2 ca**	−4.43		−3.33	
**−NLL b2 np**				
**−NLL b2 cyto**	11.41		6.577	
**−NLL b3 ca**	−3.128			−1.539
**−NLL b3 np**	5.074			3.813
**−NLL b3 cyto**	0.4115			0.9912
Total	9.209	−16.51	3.247	3.266
**Total with regul**	10.04	−16.45	2.586	2.473

	Together	b1	b2	b3
spar	3.761e−01	0.35630	0.43	4.368e−01
σ_b	1.774e−01	0.06096	0.1776	1.727e−01
σ_t	2.526e−06	2.32300	2.958e−05	5.821e−05
ca_{0,b_1}	3.125e+00	3.70600		
$\log_{10}(k_1')$	−6.711e−01	−0.67970		−7.498e−01
$\log_{10}(k_2)$	−9.856e−01	−0.97170	5.374 or −2.411	−1.060e+00
$\log_{10}(k_2')$	−1.809e+00	−1.45500		−1.892e+00
$\log_{10}(k_{deg})$	−2.164e+00	−1.60700	−2.411 or 5.374	−2.287e+00
$\log_{10}(k_1'k_2')$	−2.480e+00	−2.13500	3.68	−2.642e+00
$\log_{10}(k_1'/k_2)$	3.146e−01	0.29200		3.104e−01
transport = $\log_{10}(k_1'k_2'/k_2)$	−1.494e+00	−1.16300	−1.693 or 6.092	−1.582e+00

Plaur

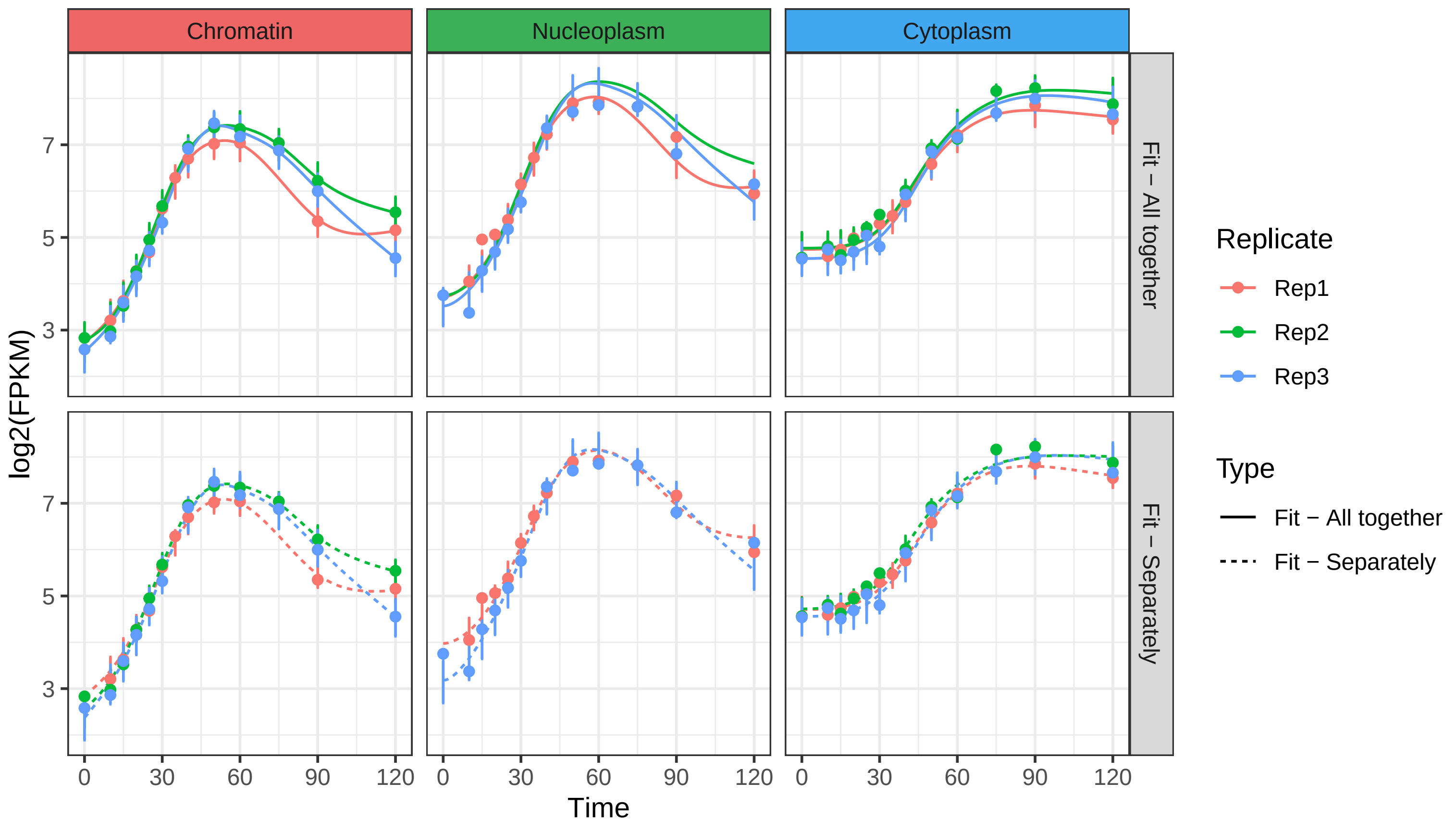

	Together	b1	b2	b3
**−NLL b1 ca**	−7.521	−7.523		
**−NLL b1 np**	3.417	−0.4477		
**−NLL b1 cyto**	−7.603	−10.12		
**−NLL b2 ca**	−7.73		−9.015	
**−NLL b2 np**				
**−NLL b2 cyto**	−2.769		−3.967	
**−NLL b3 ca**	−7.251			−4.991
**−NLL b3 np**	7.464			3.407
**−NLL b3 cyto**	−3.795			−3.343
Total	−25.79	−18.09	−12.98	−4.926
**Total with regul**	−26.38	−19.77	−14.4	−6.062

	Together	b1	b2	b3
spar	3.916e−01	0.4562000	0.3968	4.110e−01
σ_b	1.200e−01	0.0882700	0.08227	1.325e−01
σ_t	4.091e−05	0.0001295	0.0003627	7.022e−05
ca_{0,b_1}	2.768e+00	2.9320000		
$\log_{10}(k_1')$	−3.696e−01	−0.4543000		−3.507e−01
$\log_{10}(k_2)$	−6.654e−01	−0.7958000	7.967 or −2.063	−5.941e−01
$\log_{10}(k_2')$	−1.598e+00	−1.5740000		−1.592e+00
$\log_{10}(k_{deg})$	−1.906e+00	−1.7940000	−2.063 or 7.967	−2.008e+00
$\log_{10}(k_1'k_2')$	−1.967e+00	−2.0280000	6.557	−1.943e+00
$\log_{10}(k_1'/k_2)$	2.958e−01	0.3415000		2.434e−01
$\text{transport} = \log_{10}(k_1'k_2'/k_2)$	−1.302e+00	−1.2320000	−1.41 or 8.621	−1.349e+00

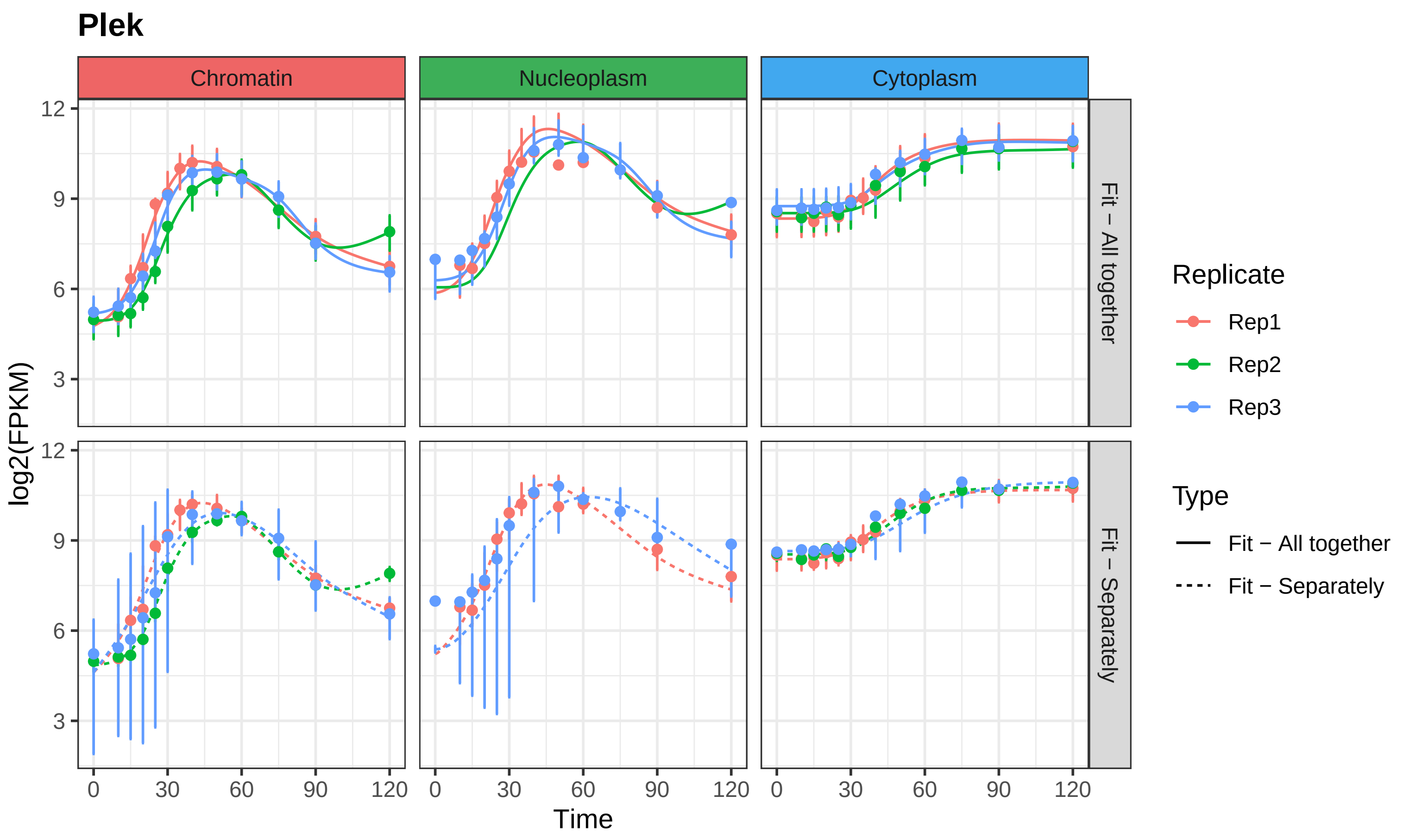

	Together	b1	b2	b3
**−NLL b1 ca**	0.2641	−0.5123		
**−NLL b1 np**	12.67	8.332		
**−NLL b1 cyto**	−1.073	−6.544		
**−NLL b2 ca**	−2.252		−7.022	
**−NLL b2 np**				
**−NLL b2 cyto**	−0.8755		−5.854	
**−NLL b3 ca**	−1.368			6.202
**−NLL b3 np**	16.3			12.19
**−NLL b3 cyto**	−1.874			−9.73
Total	21.79	1.276	−12.88	8.666
**Total with regul**	23.76	0.2798	−12.77	8.39

	Together	b1	b2	b3
spar	0.3498000	0.42370	0.3469	0.50740
σ_b	0.2020000	0.12830	0.07375	0.01633
σ_t	0.0002472	1.81200	1.154	8.27300
ca_{0,b_1}	4.8210000	4.71600		
$\log_{10}(k_1')$	−0.0926200	0.24450		−0.86610
$\log_{10}(k_2)$	−0.4255000	0.05939	5.017 or −2.454	−1.09900
$\log_{10}(k_2')$	−1.7280000	−1.71600		−1.61900
$\log_{10}(k_{deg})$	−2.4700000	−2.66400	−2.454 or 5.017	−2.60100
$\log_{10}(k_1'k_2')$	−1.8210000	−1.47200	3.673	−2.48600
$\log_{10}(k_1'/k_2)$	0.3329000	0.18510		0.23260
$\text{transport} = \log_{10}(k_1'k_2'/k_2)$	−1.3950000	−1.53100	−1.344 or 6.127	−1.38700

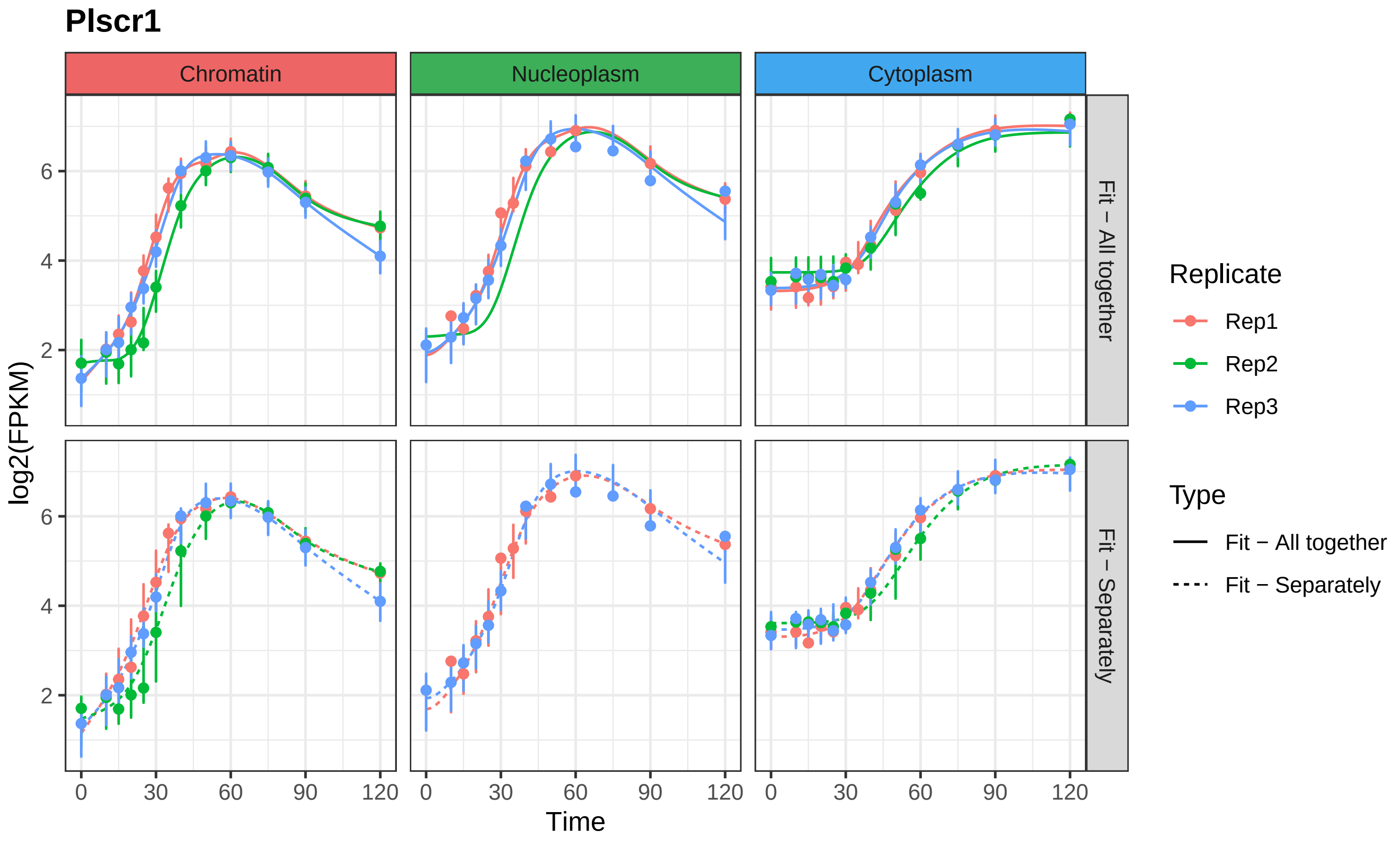

	Together	b1	b2	b3
**−NLL b1 ca**	−6.679	−4.716		
**−NLL b1 np**	−0.4249	−1.393		
**−NLL b1 cyto**	−4.477	−8.96		
**−NLL b2 ca**	−6.398		−2.608	
**−NLL b2 np**				
**−NLL b2 cyto**	−2.696		−7.953	
**−NLL b3 ca**	−7.473			−4.521
**−NLL b3 np**	7.625			5.137
**−NLL b3 cyto**	−5.806			−4.876
Total	−26.33	−15.07	−10.56	−4.26
**Total with regul**	−24.77	−16.77	−12.66	−4.667

	Together	b1	b2	b3
spar	0.3285	4.649e−01	0.4419	3.758e−01
σ_b	0.1037	5.613e−06	2.745e−06	1.274e−01
σ_t	0.5559	2.055e+00	3.197	4.719e−05
ca_{0,b_1}	1.2970	1.213e+00		
$\log_{10}(k_1')$	−0.4585	−5.676e−01		−4.979e−01
$\log_{10}(k_2)$	−0.6366	−7.302e−01	−1.072 or −1.913	−6.947e−01
$\log_{10}(k_2')$	−1.5890	−1.585e+00		−1.612e+00
$\log_{10}(k_{deg})$	−2.0210	−2.073e+00	−1.913 or −1.072	−2.074e+00
$\log_{10}(k_1'k_2')$	−2.0470	−2.153e+00	−2.342	−2.110e+00
$\log_{10}(k_1'/k_2)$	0.1781	1.626e−01		1.968e−01
$transport = \log_{10}(k_1'k_2'/k_2)$	−1.4110	−1.423e+00	−1.27 or −0.4289	−1.415e+00

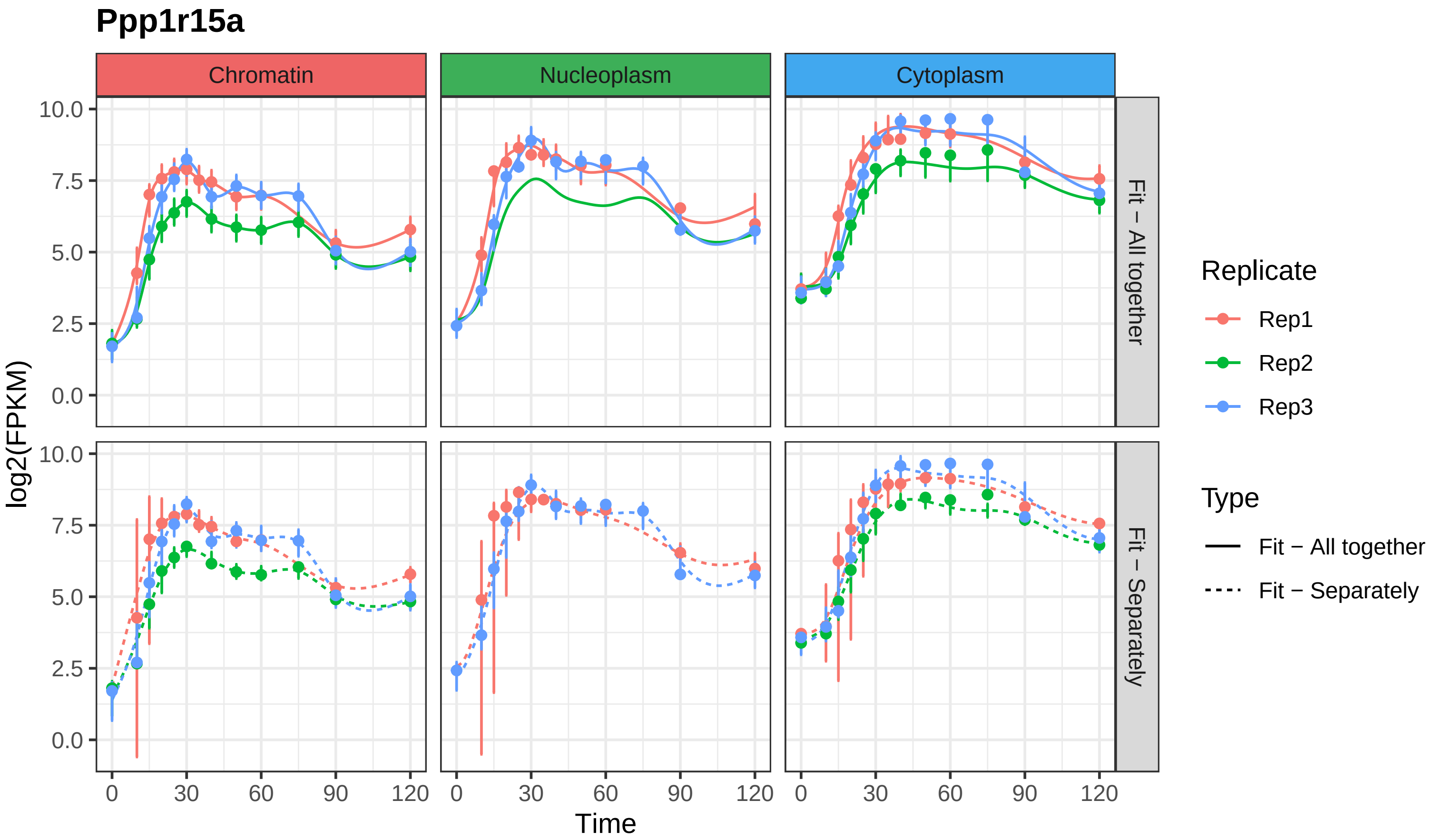

	Together	b1	b2	b3
**−NLL b1 ca**	−4.657	−3.674		
**−NLL b1 np**	2.839	4.041		
**−NLL b1 cyto**	2.767	−6.262		
**−NLL b2 ca**	−5.222		−4.029	
**−NLL b2 np**				
**−NLL b2 cyto**	4.345		−1.858	
**−NLL b3 ca**	−3.689			0.07798
**−NLL b3 np**	−1.648			1.121
**−NLL b3 cyto**	7.404			5.233
Total	2.138	−5.895	−5.886	6.432
**Total with regul**	19.32	−2.377	−6.126	10.86

	Together	b1	b2	b3
spar	0.1928	3.874e−01	0.3576	0.28300
σ_b	0.1591	3.647e−06	0.08166	0.15080
σ_t	0.4617	4.081e+00	1.384	1.20800
ca_{0,b_1}	1.7960	1.841e+00		
$\log_{10}(k_1')$	0.1267	−6.473e−01		−0.01289
$\log_{10}(k_2)$	−0.1298	−8.666e−01	−0.8152 or −0.8284	−0.27130
$\log_{10}(k_2')$	−0.8206	−8.480e−01		−0.70670
$\log_{10}(k_{deg})$	−1.1690	−1.191e+00	−0.8284 or −0.8152	−1.06400
$\log_{10}(k_1' k_2')$	−0.6939	−1.495e+00	−1.014	−0.71960
$\log_{10}(k_1'/k_2)$	0.2565	2.193e−01		0.25840
transport $= \log_{10}(k_1' k_2'/k_2)$	−0.5641	−6.287e−01	−0.1989 or −0.1857	−0.44820

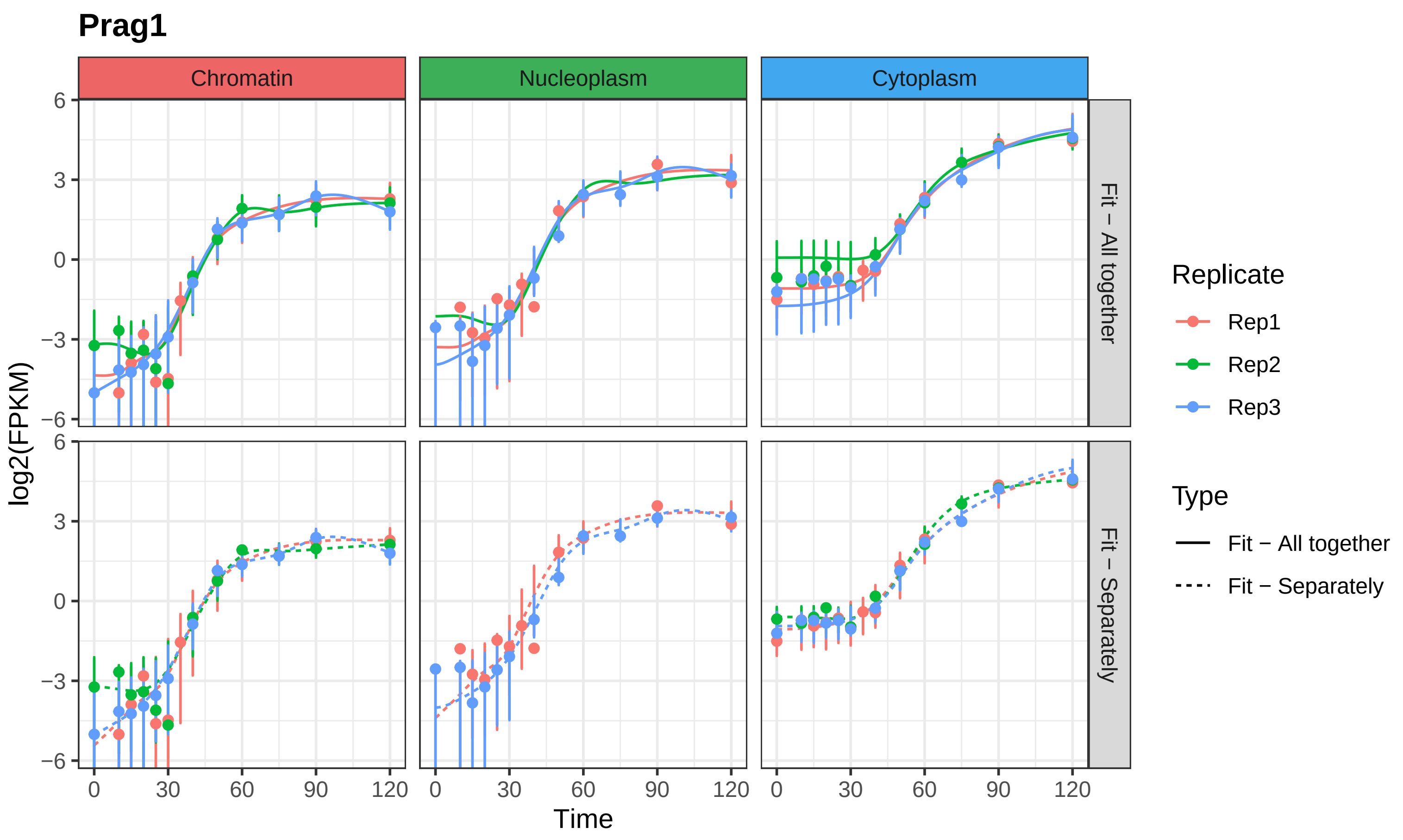

	Together	b1	b2	b3
**−NLL b1 ca**	7.97	8.098		
**−NLL b1 np**	13.01	13.13		
**−NLL b1 cyto**	2.878	3.029		
**−NLL b2 ca**	6.343		5.438	
**−NLL b2 np**				
**−NLL b2 cyto**	11.38		−3.318	
**−NLL b3 ca**	4.987			3.35
**−NLL b3 np**	7.948			4.682
**−NLL b3 cyto**	11.63			0.9147
Total	66.15	24.26	2.12	8.947
**Total with regul**	70.75	23.7	−0.1481	7.543

	Together	b1	b2	b3
spar	0.3870000	0.4153	0.4548	4.087e−01
σ_b	0.2002000	0.1388	0.0001323	1.071e−01
σ_t	0.0002433	2.7180	1.51	1.137e−05
ca_{0,b_1}	−4.3270000	−5.4230		
$\log_{10}(k_1')$	−0.2331000	5.2170		−3.765e−01
$\log_{10}(k_2)$	−0.5522000	4.9070	−1.047 or −1.09	−6.890e−01
$\log_{10}(k_2')$	−1.0690000	−1.2520		−1.127e+00
$\log_{10}(k_{deg})$	−1.7330000	−2.2560	−1.09 or −1.047	−2.050e+00
$\log_{10}(k_1'k_2')$	−1.3020000	3.9650	−1.357	−1.504e+00
$\log_{10}(k_1'/k_2)$	0.3191000	0.3104		3.125e−01
transport $= \log_{10}(k_1'k_2'/k_2)$	−0.7500000	−0.9420	−0.3094 or −0.267	−8.148e−01

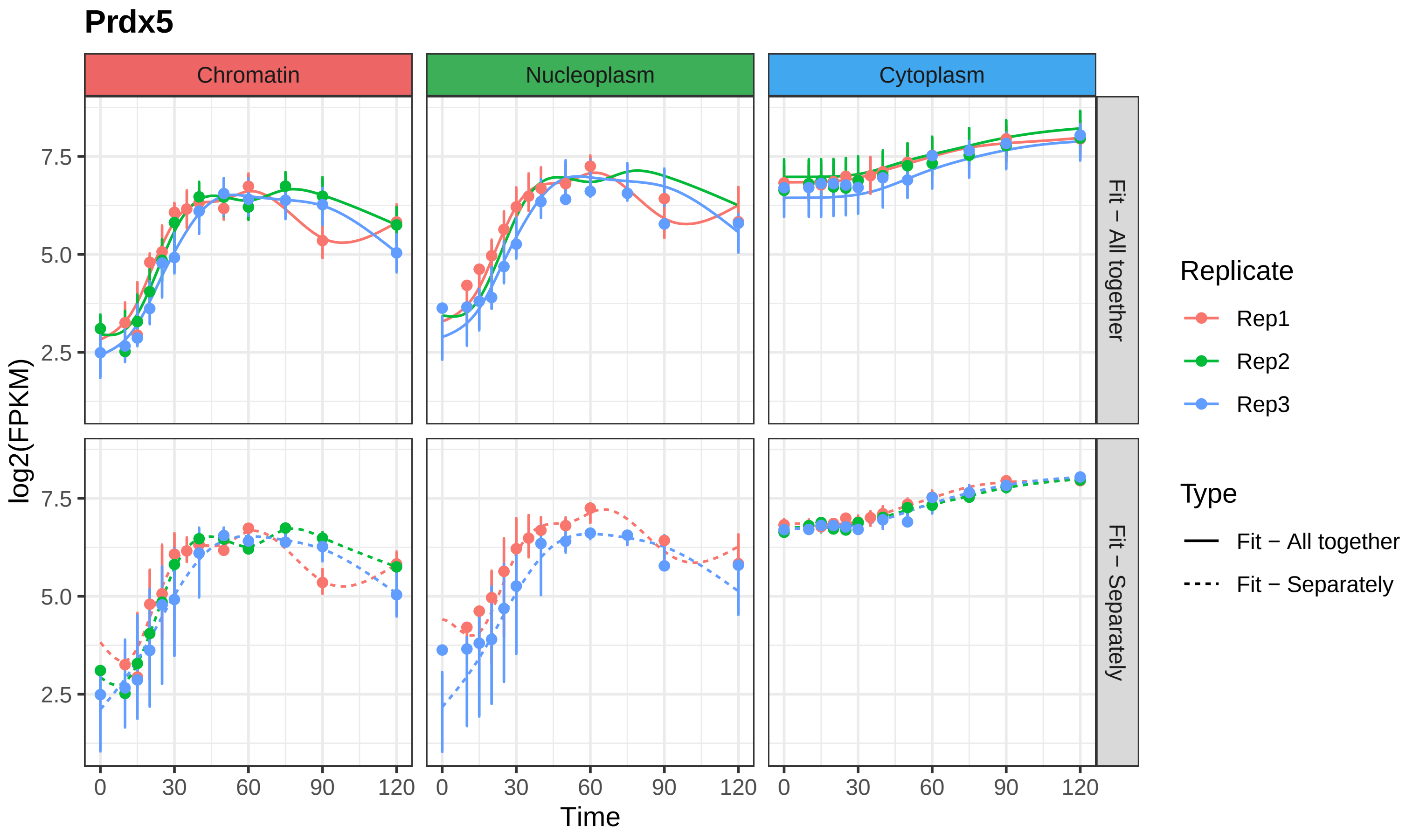

	Together	b1	b2	b3
**−NLL b1 ca**	0.8385	−3.997		
**−NLL b1 np**	2.825	3.134		
**−NLL b1 cyto**	−5.905	−16.37		
**−NLL b2 ca**	−2.079		−11.33	
**−NLL b2 np**				
**−NLL b2 cyto**	−0.1403		−13.78	
**−NLL b3 ca**	−2.983			1.785
**−NLL b3 np**	12.46			7.628
**−NLL b3 cyto**	0.2407			−13.76
Total	5.262	−17.24	−25.11	−4.352
**Total with regul**	5.697	−16.78	−23.43	−5.769

	Together	b1	b2	b3
spar	0.39640	0.35490	0.3099	0.47810
σ_b	0.16130	0.03358	0.03798	0.02671
σ_t	0.79830	3.83300	0.0003109	5.95000
ca_{0,b_1}	2.89600	3.90500		
$\log_{10}(k_1')$	0.23390	−0.37170		6.29300
$\log_{10}(k_2)$	0.09115	−0.54650	7.079 or −2.799	6.27500
$\log_{10}(k_2')$	−1.71700	−1.60700		−1.58600
$\log_{10}(k_{deg})$	−2.78300	−2.34400	−2.799 or 7.079	−2.95700
$\log_{10}(k_1' k_2')$	−1.48300	−1.97800	5.432	4.70800
$\log_{10}(k_1'/k_2)$	0.14280	0.17480		0.01792
transport $= \log_{10}(k_1' k_2'/k_2)$	−1.57500	−1.43200	−1.647 or 8.231	−1.56800

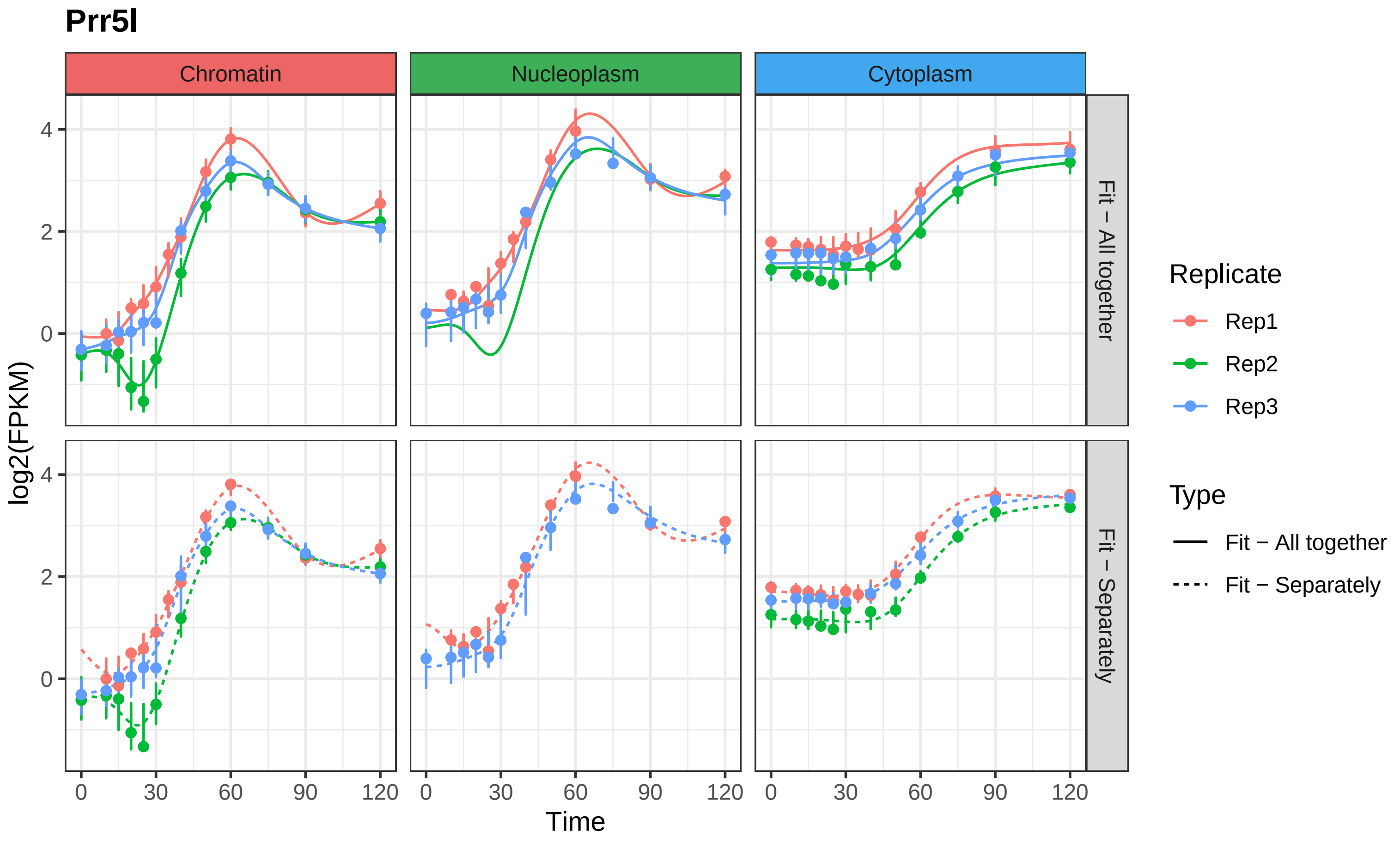

	Together	b1	b2	b3
**−NLL b1 ca**	−9.134	−9.134		
**−NLL b1 np**	−2.101	−4.366		
**−NLL b1 cyto**	−9.522	−15.37		
**−NLL b2 ca**	−8.423		−6.916	
**−NLL b2 np**				
**−NLL b2 cyto**	−5.023		−8.628	
**−NLL b3 ca**	−9.756			−7.361
**−NLL b3 np**	−1.15			−3.511
**−NLL b3 cyto**	−7.952			−12.94
Total	−53.06	−28.87	−15.54	−23.81
**Total with regul**	−50.81	−29.6	−15.92	−24.57

	Together	b1	b2	b3
spar	0.30650	0.4139000	0.3518	3.645e−01
σ_b	0.06512	0.0296700	0.01271	1.487e−05
σ_t	0.55730	0.0000813	4.055e−06	2.170e+00
ca_{0,b_1}	−0.05644	0.6384000		
$\log_{10}(k_1')$	−0.35890	−0.3080000		−5.391e−01
$\log_{10}(k_2)$	−0.51560	−0.4531000	−0.8045 or −1.935	−7.067e−01
$\log_{10}(k_2')$	−1.73700	−1.6550000		−1.719e+00
$\log_{10}(k_{deg})$	−2.09000	−1.8480000	−1.935 or −0.8045	−2.106e+00
$\log_{10}(k_1'k_2')$	−2.09600	−1.9630000	−2.284	−2.258e+00
$\log_{10}(k_1'/k_2)$	0.15670	0.1451000		1.676e−01
$\text{transport} = \log_{10}(k_1'k_2'/k_2)$	−1.58100	−1.5100000	−1.48 or −0.3492	−1.551e+00

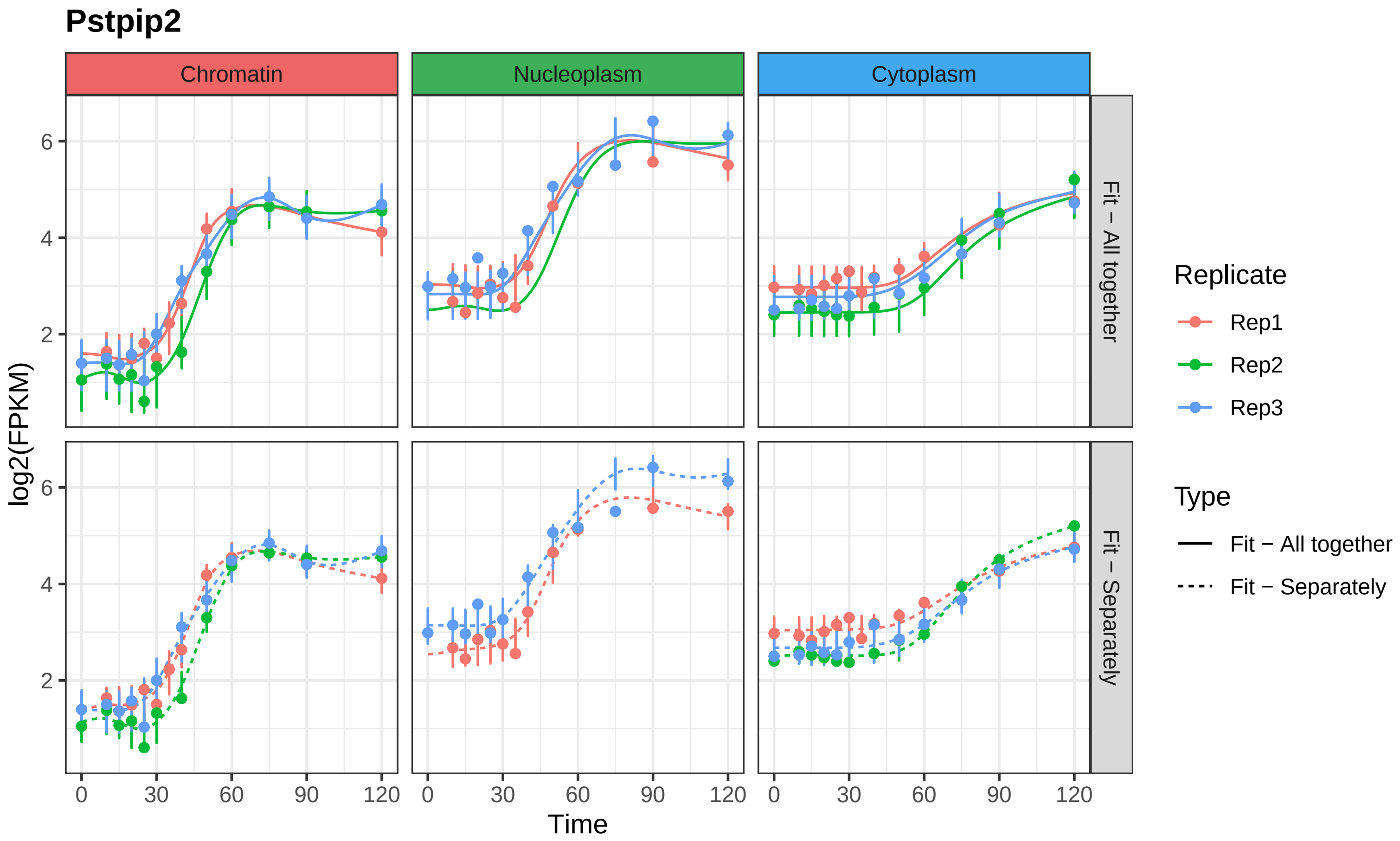

	Together	b1	b2	b3
**−NLL b1 ca**	−3.815	−6.119		
**−NLL b1 np**	5.476	−0.9977		
**−NLL b1 cyto**	−3.149	−6.495		
**−NLL b2 ca**	−3.43		−6.255	
**−NLL b2 np**				
**−NLL b2 cyto**	−2.498		−11.6	
**−NLL b3 ca**	−3.498			−2.838
**−NLL b3 np**	10.06			0.4868
**−NLL b3 cyto**	−0.7555			−4.062
Total	−1.604	−13.61	−17.85	−6.413
**Total with regul**	−1.038	−14.3	−19.1	−7.708

	Together	b1	b2	b3
spar	0.3632000	0.37820	0.3767	0.4036
σ_b	0.1477000	0.08152	2.378e−06	0.1027
σ_t	0.0001297	1.08600	6.449e−05	1.7990
ca_{0,b_1}	1.5970000	1.34400		
$\log_{10}(k_1')$	−0.5476000	−0.61110		−0.5371
$\log_{10}(k_2)$	−0.9776000	−0.96820	−1.18 or −1.796	−1.0640
$\log_{10}(k_2')$	−2.0420000	−2.11400		−2.2050
$\log_{10}(k_{deg})$	−2.0240000	−2.26300	−1.796 or −1.18	−2.0640
$\log_{10}(k_1'k_2')$	−2.5890000	−2.72500	−2.562	−2.7420
$\log_{10}(k_1'/k_2)$	0.4300000	0.35720		0.5264
transport = $\log_{10}(k_1'k_2'/k_2)$	−1.6120000	−1.75700	−1.382 or −0.7653	−1.6780

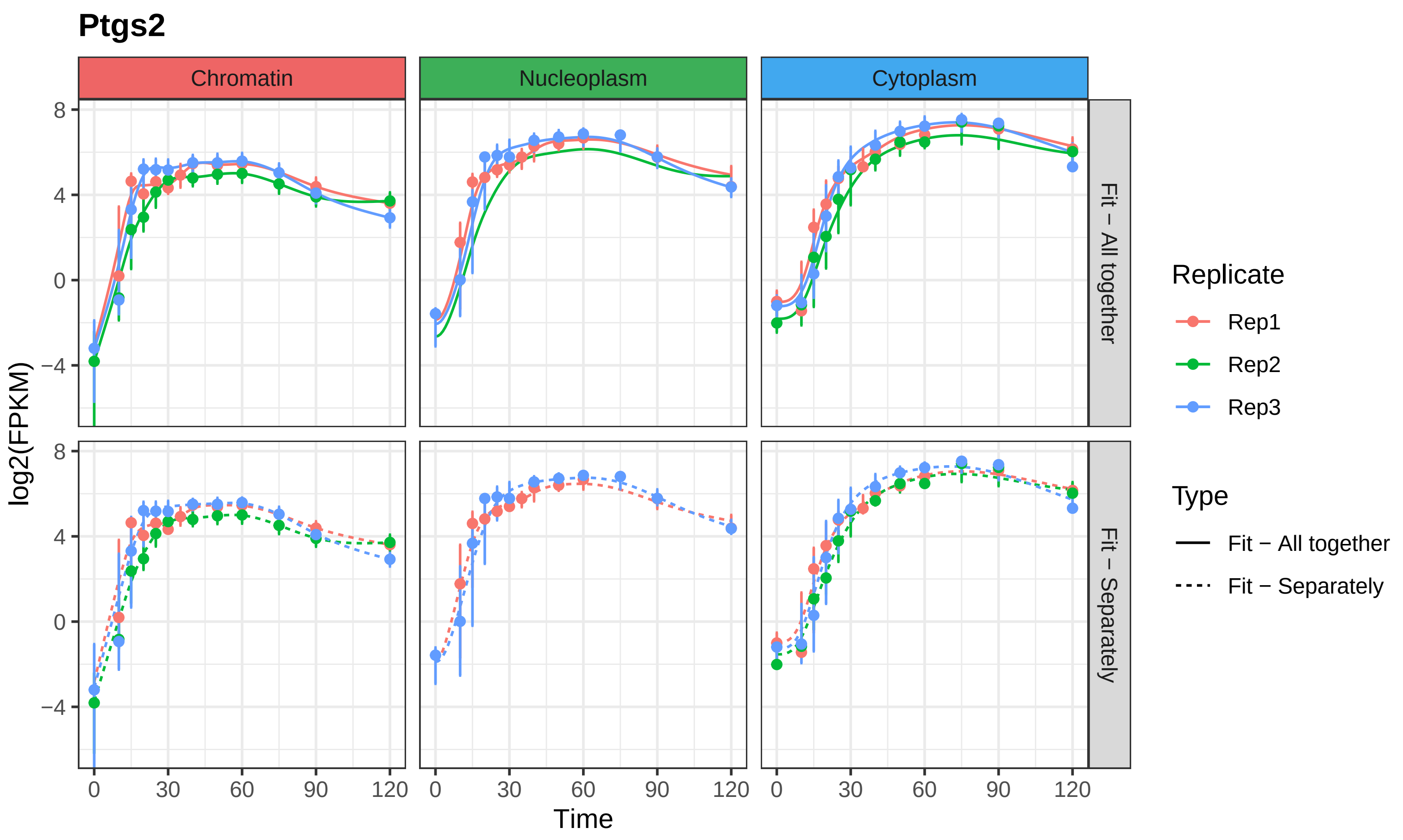

	Together	b1	b2	b3
**−NLL b1 ca**	0.004649	0.07523		
**−NLL b1 np**	1.825	0.8865		
**−NLL b1 cyto**	3.525	0.1802		
**−NLL b2 ca**	−1.253		−2.056	
**−NLL b2 np**				
**−NLL b2 cyto**	12.16		5.958	
**−NLL b3 ca**	−0.4443			−0.7867
**−NLL b3 np**	3.168			1.311
**−NLL b3 cyto**	5.221			6.737
Total	24.21	1.142	3.903	7.261
**Total with regul**	30.52	1.865	5.872	7.556

	Together	b1	b2	b3
spar	0.2676	0.3533	0.3192	0.3481
σ_b	0.1423	0.0902	0.1288	0.0920
σ_t	1.6830	2.2490	1.322	2.3730
ca_{0,b_1}	−3.0100	−2.8930		
$\log_{10}(k_1')$	−0.4948	−0.3668		−0.5392
$\log_{10}(k_2)$	−0.8479	−0.6721	−0.5542 or −1.325	−0.9050
$\log_{10}(k_2')$	−0.9493	−1.0660		−0.9095
$\log_{10}(k_{deg})$	−1.2000	−1.3250	−1.325 or −0.5542	−1.0970
$\log_{10}(k_1'k_2')$	−1.4440	−1.4330	−1.214	−1.4490
$\log_{10}(k_1'/k_2)$	0.3531	0.3054		0.3659
transport $= \log_{10}(k_1'k_2'/k_2)$	−0.5963	−0.7610	−0.6601 or 0.1107	−0.5437

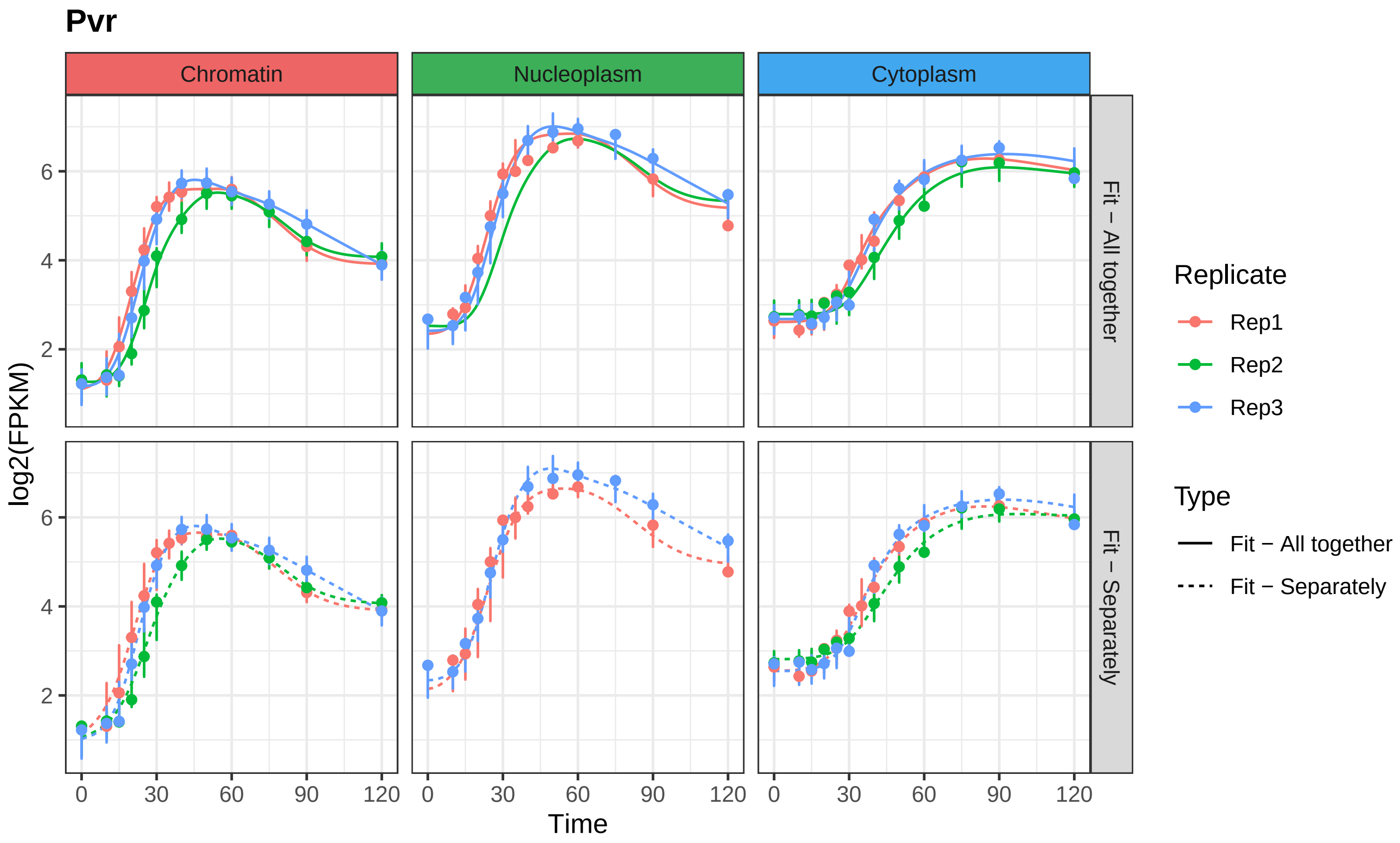

	Together	b1	b2	b3
**−NLL b1 ca**	−6.845	−5.259		
**−NLL b1 np**	4.407	0.4516		
**−NLL b1 cyto**	−4.348	−7.238		
**−NLL b2 ca**	−7.244		−5.842	
**−NLL b2 np**				
**−NLL b2 cyto**	−4.808		−7.276	
**−NLL b3 ca**	−6.303			−5.106
**−NLL b3 np**	−3.212			−5.028
**−NLL b3 cyto**	−1.593			−0.1179
Total	−29.95	−12.05	−13.12	−10.25
**Total with regul**	−29.22	−13.57	−14.78	−9.72

	Together	b1	b2	b3
spar	0.3430	0.40920	0.4011	0.34170
σ_b	0.1018	0.04976	0.05085	0.09928
σ_t	0.9371	2.20800	1.602	0.70170
ca_{0,b_1}	1.1630	1.25000		
$\log_{10}(k_1')$	−0.2391	−0.38630		−0.11700
$\log_{10}(k_2)$	−0.6140	−0.69330	−0.3177 or −1.889	−0.51410
$\log_{10}(k_2')$	−1.6550	−1.57900		−1.67600
$\log_{10}(k_{deg})$	−1.7340	−1.70100	−1.889 or −0.3177	−1.73900
$\log_{10}(k_1'k_2')$	−1.8940	−1.96500	−1.676	−1.79300
$\log_{10}(k_1'/k_2)$	0.3749	0.30700		0.39710
transport $= \log_{10}(k_1'k_2'/k_2)$	−1.2800	−1.27200	−1.358 or 0.2128	−1.27900

Rab20

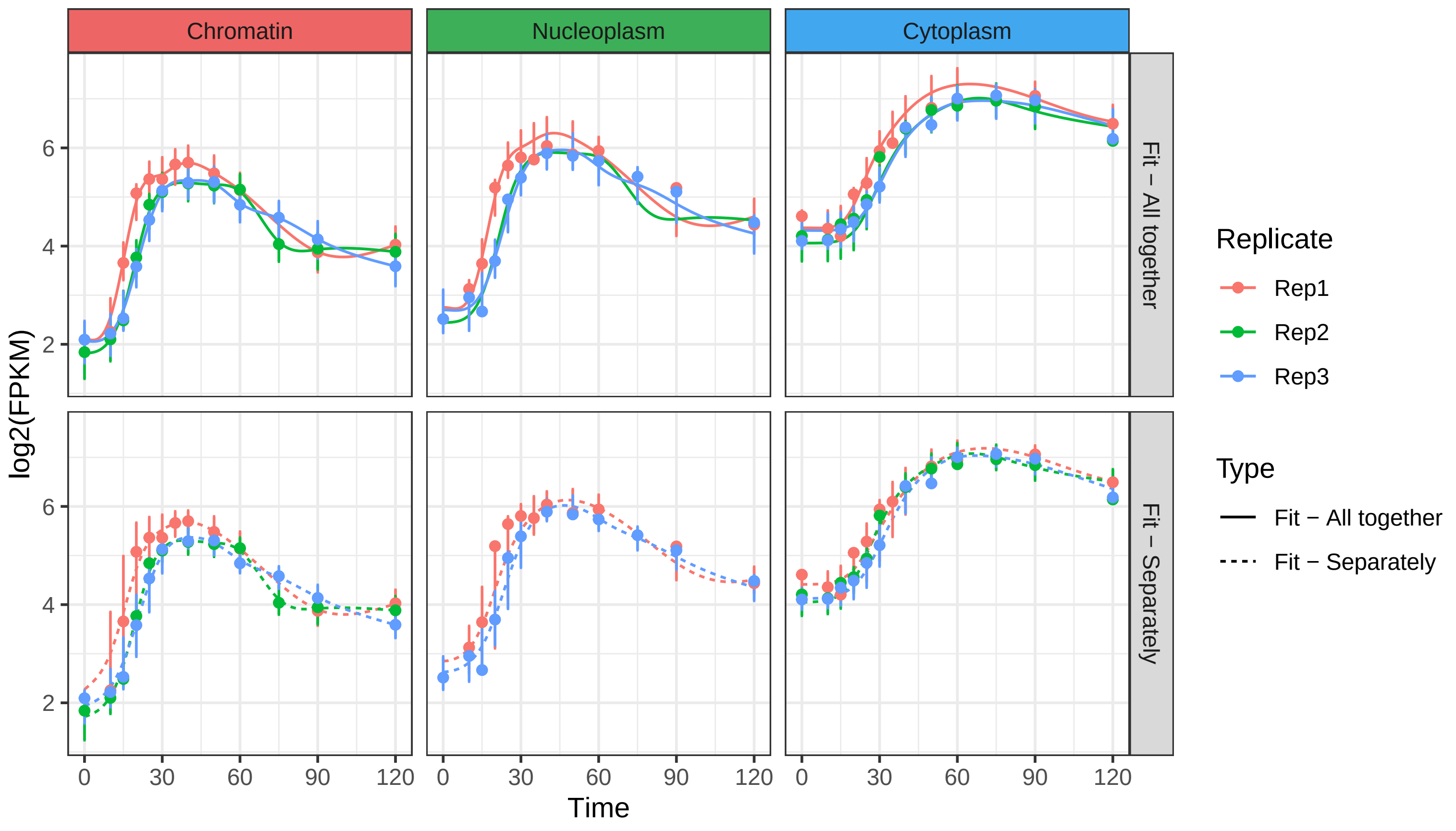

	Together	b1	b2	b3
**−NLL b1 ca**	−6.505	−4.079		
**−NLL b1 np**	4.65	1.201		
**−NLL b1 cyto**	−0.08735	−2.8		
**−NLL b2 ca**	−7.548		−7.302	
**−NLL b2 np**				
**−NLL b2 cyto**	0.7387		−3.687	
**−NLL b3 ca**	−8.162			−6.516
**−NLL b3 np**	−2.687			−2.439
**−NLL b3 cyto**	−4.837			−5.244
Total	−24.44	−5.677	−10.99	−14.2
**Total with regul**	−19.5	−6.049	−8.658	−14.23

	Together	b1	b2	b3
spar	0.2729	0.37880	0.3041	0.3496
σ_b	0.1209	0.07509	0.08889	0.0660
σ_t	0.0020	3.19400	0.003856	1.7020
ca_{0,b_1}	2.1700	2.39200		
$\log_{10}(k_1')$	−0.2779	−0.79120		−0.4543
$\log_{10}(k_2)$	−0.4666	−0.96500	5.198 or −1.541	−0.6625
$\log_{10}(k_2')$	−1.0290	−1.01500		−0.9462
$\log_{10}(k_{deg})$	−1.5160	−1.48500	−1.541 or 5.198	−1.3960
$\log_{10}(k_1'k_2')$	−1.3070	−1.80600	4.36	−1.4000
$\log_{10}(k_1'/k_2)$	0.1887	0.17380		0.2082
transport $= \log_{10}(k_1'k_2'/k_2)$	−0.8402	−0.84080	−0.8383 or 5.901	−0.7380

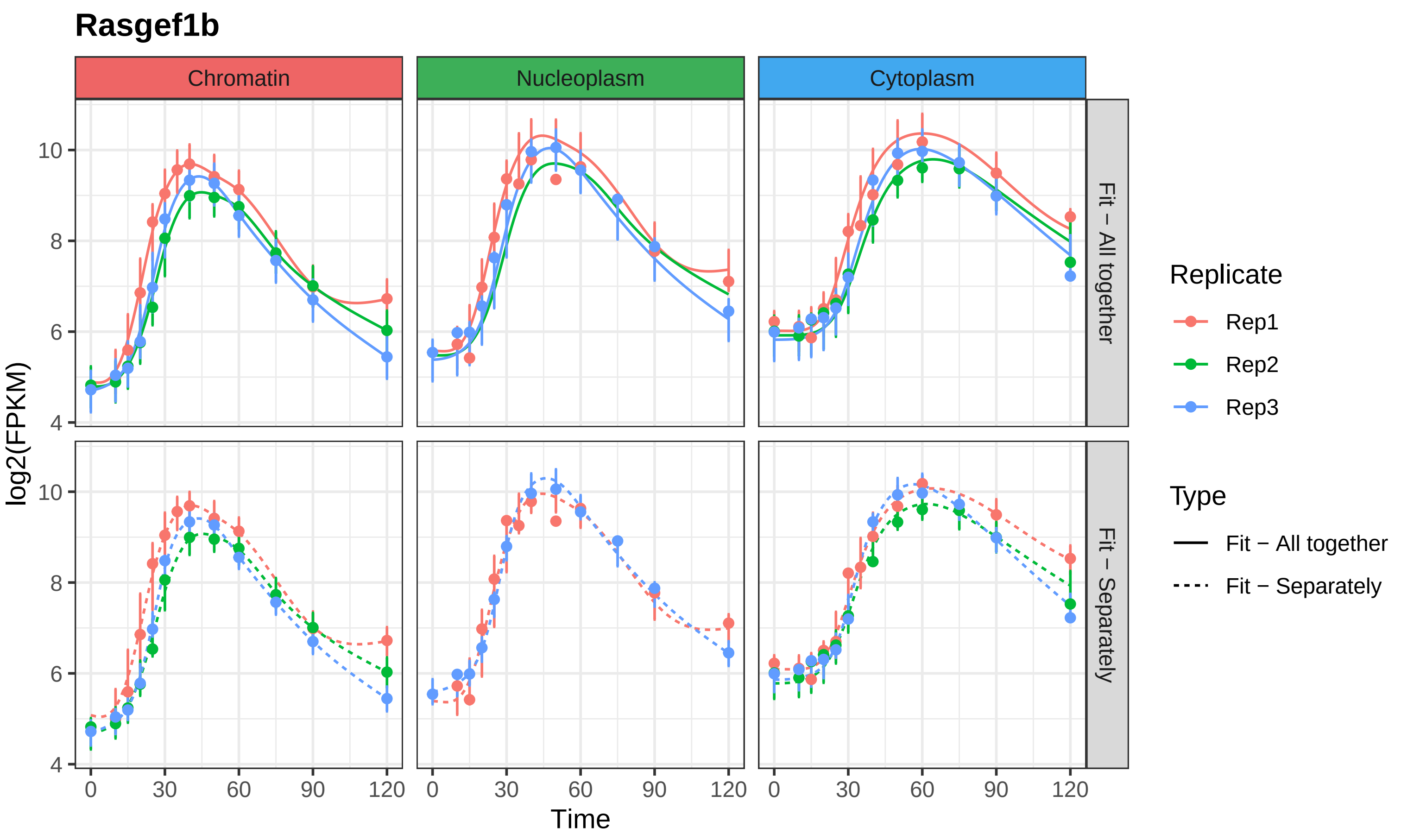

	Together	b1	b2	b3
**−NLL b1 ca**	−3.971	−4.591		
**−NLL b1 np**	11.61	4.518		
**−NLL b1 cyto**	4.129	−3.767		
**−NLL b2 ca**	−4.912		−5.797	
**−NLL b2 np**				
**−NLL b2 cyto**	−0.8384		0.1148	
**−NLL b3 ca**	−4.493			−8.689
**−NLL b3 np**	2.309			−6.765
**−NLL b3 cyto**	−0.07345			−2.061
Total	3.757	−3.84	−5.682	−17.51
**Total with regul**	6.248	−3.147	−5.046	−13.42

	Together	b1	b2	b3
spar	0.3214	0.35260	0.3432	0.276900
σ_b	0.1562	0.10860	0.1154	0.092450
σ_t	0.9709	1.53300	0.6338	0.000558
ca_{0,b_1}	4.9390	5.15100		
$\log_{10}(k_1')$	−0.3659	−0.44110		−0.102400
$\log_{10}(k_2)$	−0.5715	−0.53400	6.219 or −1.264	−0.375600
$\log_{10}(k_2')$	−1.0730	−1.16500		−1.061000
$\log_{10}(k_{deg})$	−1.2050	−1.37700	−1.264 or 6.219	−1.139000
$\log_{10}(k_1'k_2')$	−1.4390	−1.60600	5.286	−1.163000
$\log_{10}(k_1'/k_2)$	0.2057	0.09297		0.273200
$\text{transport} = \log_{10}(k_1'k_2'/k_2)$	−0.8670	−1.07200	−0.9337 or 6.55	−0.787700

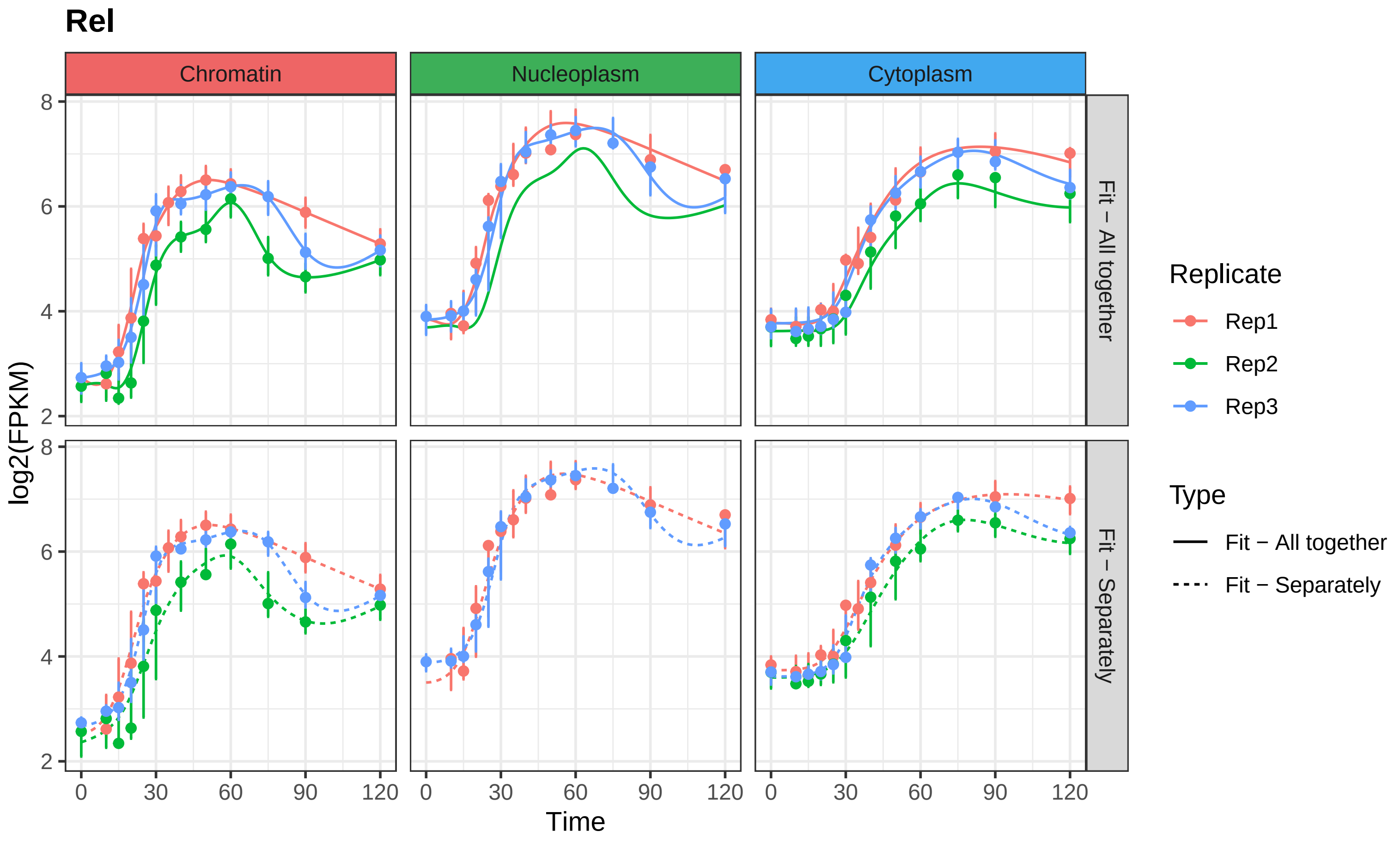

	Together	b1	b2	b3
**−NLL b1 ca**	−6.799	−5.2		
**−NLL b1 np**	4.24	3.833		
**−NLL b1 cyto**	−3.467	−6.774		
**−NLL b2 ca**	−6.545		1.404	
**−NLL b2 np**				
**−NLL b2 cyto**	−1.257		−6.987	
**−NLL b3 ca**	−8.072			−7.484
**−NLL b3 np**	−2.985			−2.509
**−NLL b3 cyto**	−5.834			−10.76
Total	−30.72	−8.141	−5.584	−20.75
**Total with regul**	−26.63	−8.799	−7.324	−20.01

	Together	b1	b2	b3
spar	0.28580	0.38510	0.4285	0.33010
σ_b	0.09546	0.09153	0.07005	0.04865
σ_t	1.66900	2.04100	3.468	1.83000
ca_{0,b_1}	2.75800	2.59300		
$\log_{10}(k_1')$	−0.29220	−0.23740		−0.30800
$\log_{10}(k_2)$	−0.62710	−0.53670	−0.7843 or −1.408	−0.67340
$\log_{10}(k_2')$	−1.46000	−1.58700		−1.47200
$\log_{10}(k_{deg})$	−1.43900	−1.65800	−1.408 or −0.7843	−1.39300
$\log_{10}(k_1'k_2')$	−1.75200	−1.82400	−1.821	−1.78000
$\log_{10}(k_1'/k_2)$	0.33490	0.29940		0.36540
transport $= \log_{10}(k_1'k_2'/k_2)$	−1.12500	−1.28800	−1.036 or −0.4123	−1.10700

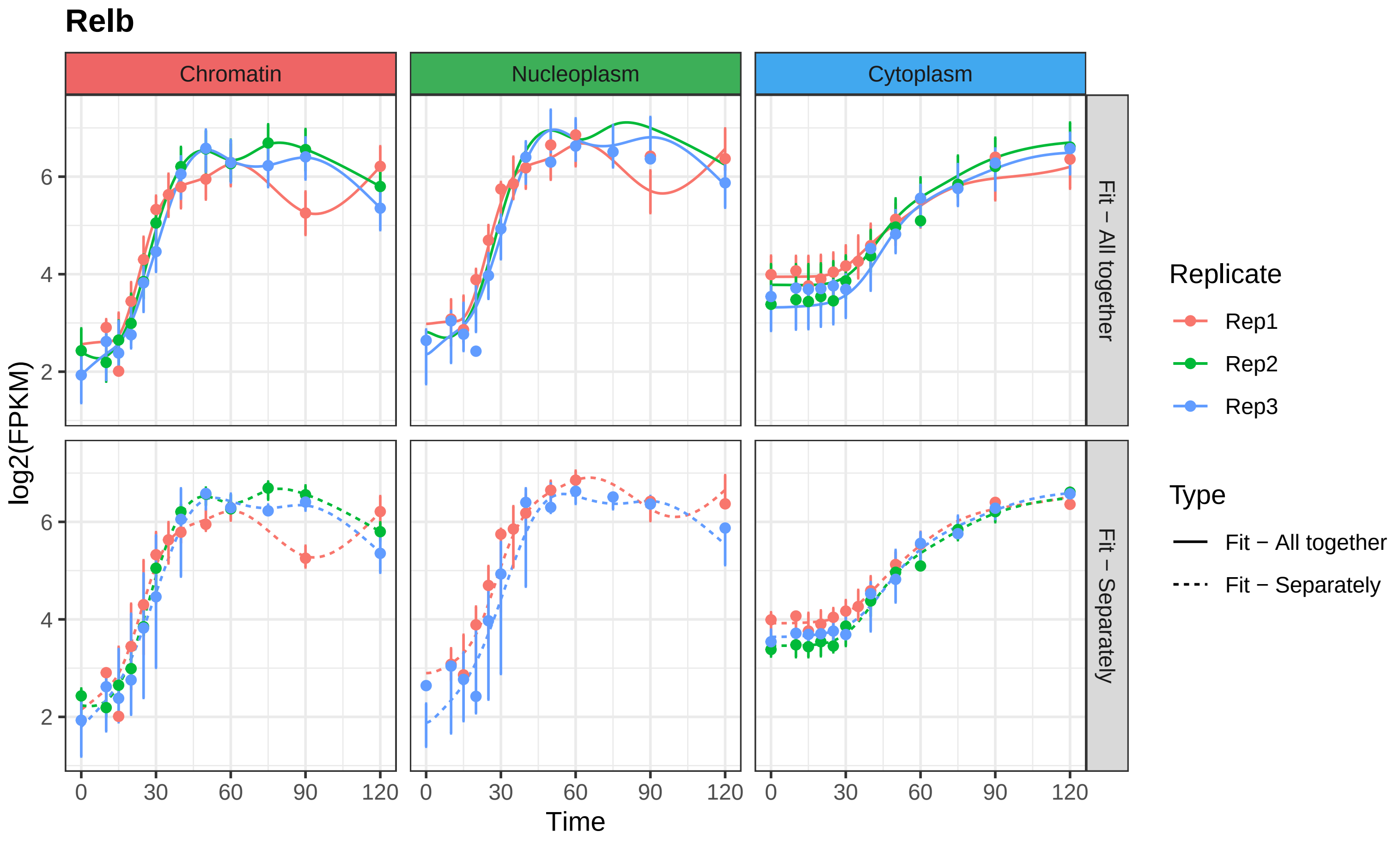

	Together	b1	b2	b3
**−NLL b1 ca**	−1.641	−1.029		
**−NLL b1 np**	3.203	−0.7313		
**−NLL b1 cyto**	−3.71	−8.698		
**−NLL b2 ca**	−5.691		−8.177	
**−NLL b2 np**				
**−NLL b2 cyto**	2.525		−7.494	
**−NLL b3 ca**	−4.924			−0.5261
**−NLL b3 np**	7.342			4.665
**−NLL b3 cyto**	0.08752			−11.31
Total	−2.809	−10.46	−15.67	−7.17
**Total with regul**	−0.3399	−10.85	−16	−8.974

	Together	b1	b2	b3
spar	3.189e−01	0.38450	0.3548	0.422900
σ_b	1.478e−01	0.05563	0.05801	0.009321
σ_t	5.276e−05	2.70700	0.6335	4.260000
ca_{0,b_1}	2.561e+00	2.16100		
$\log_{10}(k_1')$	1.470e−01	−0.61280		−0.180900
$\log_{10}(k_2)$	2.132e−02	−0.83810	5.613 or −2.202	−0.206900
$\log_{10}(k_2')$	−1.887e+00	−1.85200		−1.800000
$\log_{10}(k_{deg})$	−2.178e+00	−2.16000	−2.202 or 5.613	−2.329000
$\log_{10}(k_1'k_2')$	−1.740e+00	−2.46500	3.78	−1.981000
$\log_{10}(k_1'/k_2)$	1.257e−01	0.22530		0.026010
$\text{transport} = \log_{10}(k_1'k_2'/k_2)$	−1.762e+00	−1.62700	−1.833 or 5.982	−1.774000

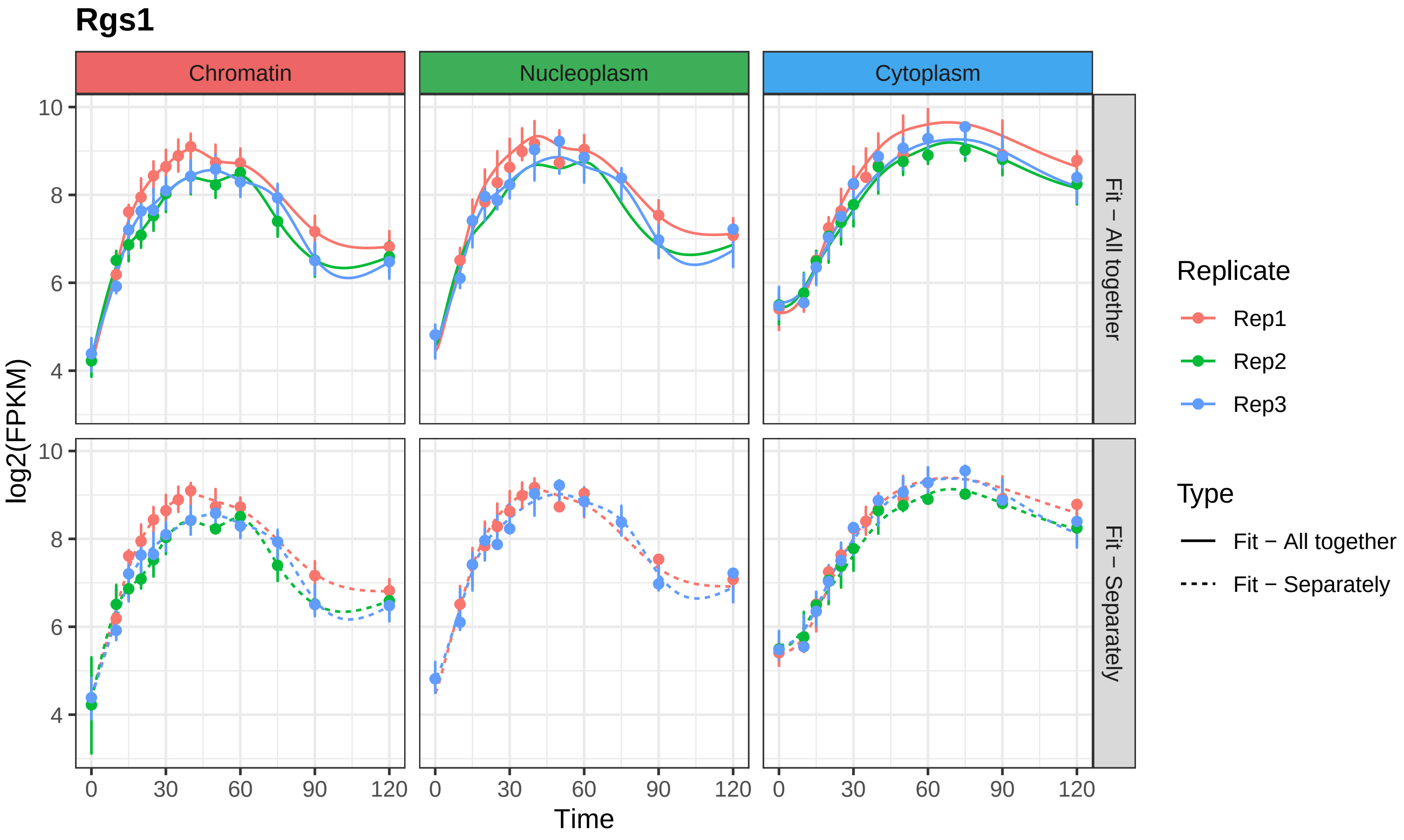

	Together	b1	b2	b3
−NLL b1 ca	−7.291	−7.663		
−NLL b1 np	0.2734	−2.879		
−NLL b1 cyto	3.969	−2.785		
−NLL b2 ca	−8.524		−13.34	
−NLL b2 np				
−NLL b2 cyto	−5.991		−8.503	
−NLL b3 ca	−7.596			−6.047
−NLL b3 np	1.007			0.04192
−NLL b3 cyto	−0.467			−2.8
Total	−24.62	−13.33	−21.84	−8.805
Total with regul	−20.19	−14.1	−15.27	−7.366

	Together	b1	b2	b3
spar	2.814e−01	0.38580	0.2403	0.32510
σ_b	1.263e−01	0.09659	0.01056	0.11150
σ_t	3.161e−05	1.03800	2.064	0.91710
ca_{0,b_1}	4.143e+00	4.35000		
$\log_{10}(k_1')$	8.641e−02	0.38800		−0.01553
$\log_{10}(k_2)$	−3.559e−03	0.35510	7.441 or −1.534	−0.15790
$\log_{10}(k_2')$	−1.201e+00	−1.28100		−1.16800
$\log_{10}(k_{deg})$	−1.464e+00	−1.55900	−1.534 or 7.441	−1.38700
$\log_{10}(k_1' k_2')$	−1.115e+00	−0.89260	6.266	−1.18400
$\log_{10}(k_1'/k_2)$	8.997e−02	0.03297		0.14230
transport $= \log_{10}(k_1' k_2'/k_2)$	−1.111e+00	−1.24800	−1.175 or 7.799	−1.02600

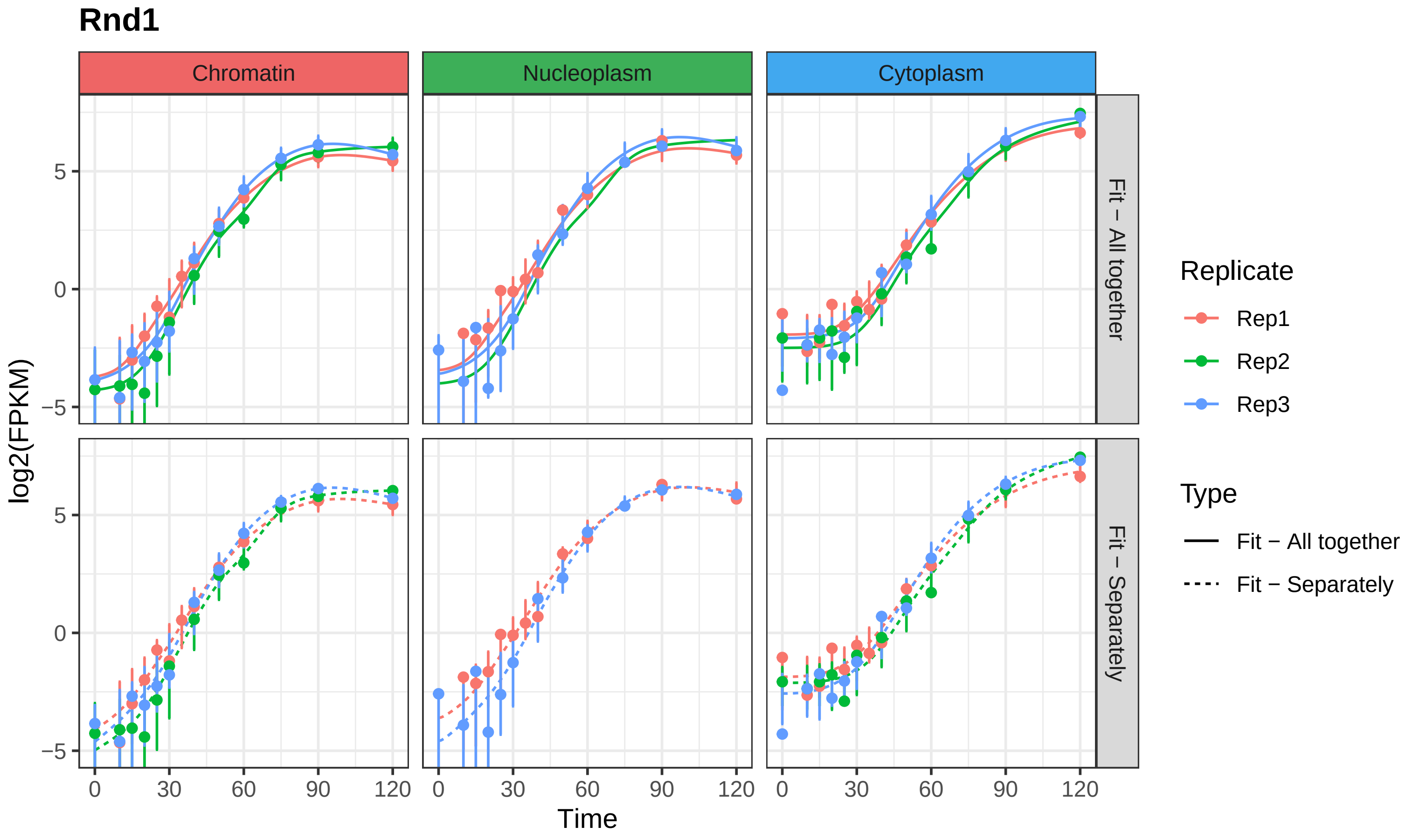

	Together	b1	b2	b3
**−NLL b1 ca**	4.08	3.92		
**−NLL b1 np**	8.78	6.99		
**−NLL b1 cyto**	10.82	10.61		
**−NLL b2 ca**	5.461		2.763	
**−NLL b2 np**				
**−NLL b2 cyto**	9.226		4.912	
**−NLL b3 ca**	4.633			1.893
**−NLL b3 np**	11.96			8.219
**−NLL b3 cyto**	11			6.129
Total	65.97	21.52	7.674	16.24
**Total with regul**	65.99	20.65	5.59	14

	Together	b1	b2	b3
spar	0.41380	0.45400	0.4242	4.474e−01
σ_b	0.13560	0.14270	3.31e−07	5.119e−08
σ_t	1.86700	1.51800	2.245	1.972e+00
ca_{0,b_1}	−3.69100	−4.06700		
$\log_{10}(k_1')$	−0.02946	0.01099		−1.054e−01
$\log_{10}(k_2)$	−0.11630	−0.13860	−0.6768 or −1.94	−1.154e−01
$\log_{10}(k_2')$	−1.20100	−1.34400		−1.151e+00
$\log_{10}(k_{deg})$	−1.65400	−1.86800	−1.94 or −0.6768	−1.756e+00
$\log_{10}(k_1' k_2')$	−1.23000	−1.33300	−1.756	−1.256e+00
$\log_{10}(k_1'/k_2)$	0.08681	0.14960		1.002e−02
transport $= \log_{10}(k_1' k_2'/k_2)$	−1.11400	−1.19500	−1.079 or 0.1838	−1.141e+00

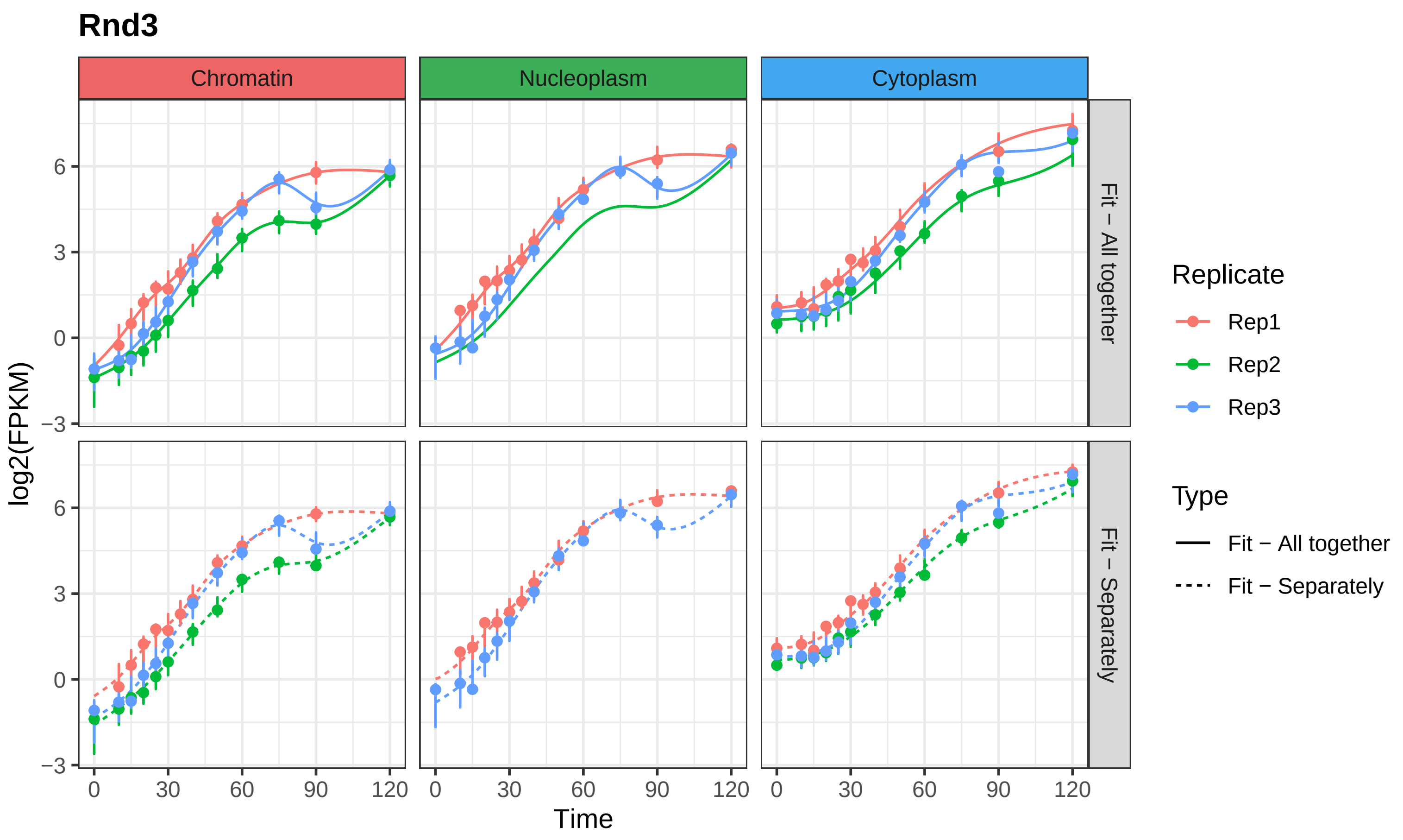

	Together	b1	b2	b3
**−NLL b1 ca**	−5.14	−5.534		
**−NLL b1 np**	−1.028	−1.941		
**−NLL b1 cyto**	−0.3747	−2.021		
**−NLL b2 ca**	−5.495		−4.946	
**−NLL b2 np**				
**−NLL b2 cyto**	2.287		−3.409	
**−NLL b3 ca**	−4.81			−3.548
**−NLL b3 np**	−2.444			−2.47
**−NLL b3 cyto**	1.86			1.015
Total	−15.14	−9.495	−8.355	−5.004
**Total with regul**	−15.54	−10.87	−10.15	−6.503

	Together	b1	b2	b3
spar	0.394600	0.40740	0.4765	0.4279000
σ_b	0.125300	0.07902	0.08742	0.1200000
σ_t	0.003939	1.38500	0.3669	0.0001349
ca_{0,b_1}	−0.965000	−0.56050		
$\log_{10}(k_1')$	5.461000	0.25200		5.5320000
$\log_{10}(k_2)$	5.298000	0.07104	4.907 or −1.598	5.3690000
$\log_{10}(k_2')$	−1.140000	−1.13700		−1.2010000
$\log_{10}(k_{deg})$	−1.590000	−1.46400	−1.598 or 4.907	−1.6830000
$\log_{10}(k_1'k_2')$	4.322000	−0.88450	3.996	4.3320000
$\log_{10}(k_1'/k_2)$	0.163400	0.18100		0.1637000
$\text{transport} = \log_{10}(k_1'k_2'/k_2)$	−0.976100	−0.95560	−0.9107 or 5.594	−1.0370000

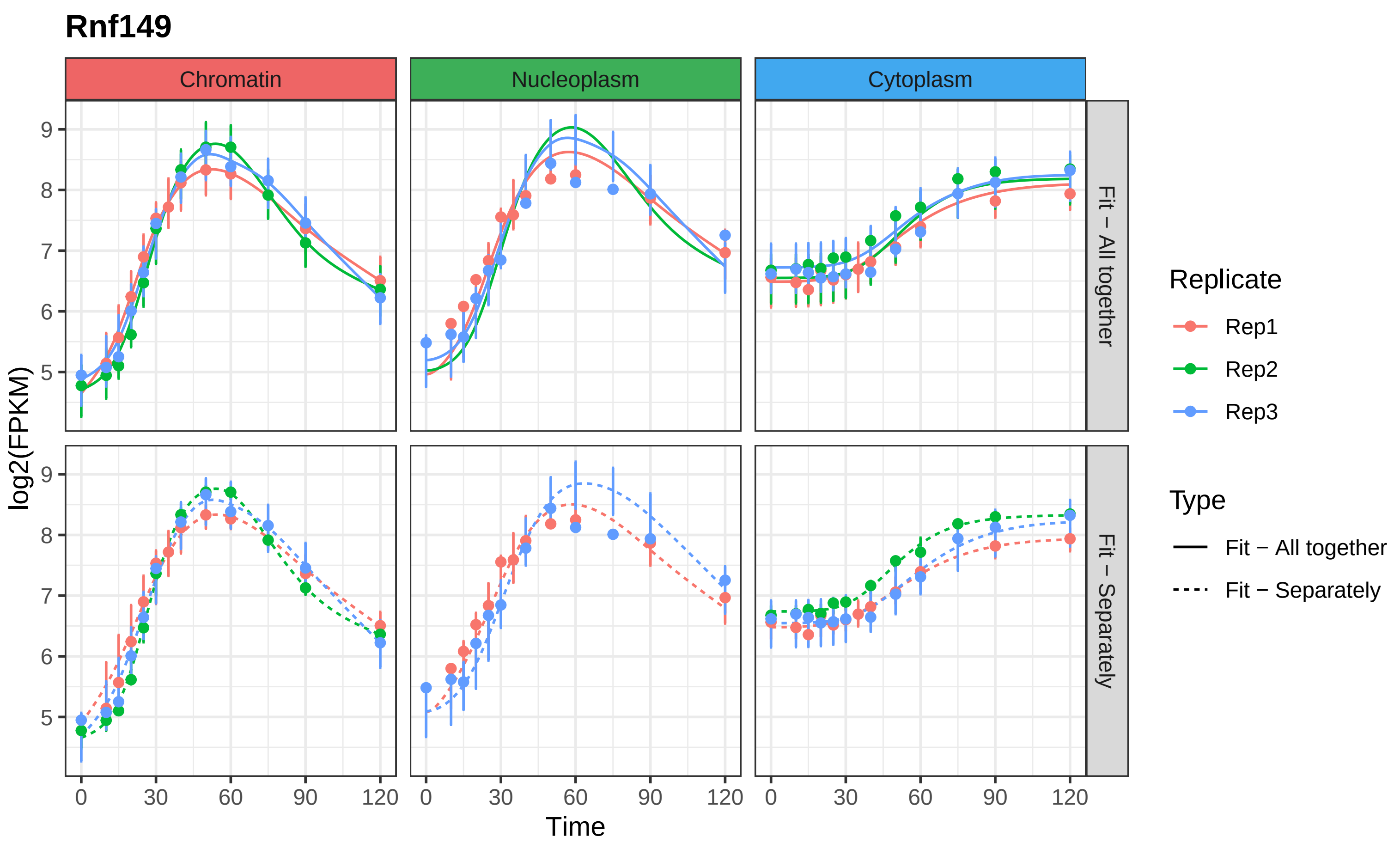

	Together	b1	b2	b3
**−NLL b1 ca**	−6.512	−5.368		
**−NLL b1 np**	4.355	0.8236		
**−NLL b1 cyto**	−6.258	−13.46		
**−NLL b2 ca**	−6.181		−13.1	
**−NLL b2 np**				
**−NLL b2 cyto**	−1.385		−14.77	
**−NLL b3 ca**	−6.125			−4.361
**−NLL b3 np**	10.87			5.651
**−NLL b3 cyto**	−2.037			−6.556
Total	−13.27	−18	−27.87	−5.265
**Total with regul**	−13.7	−18.91	−28.36	−6.646

	Together	b1	b2	b3
spar	0.3999000	0.51510	0.3558	4.353e−01
σ_b	0.1406000	0.06479	0.03155	1.338e−01
σ_t	0.0001929	2.33000	0.7603	1.783e−05
ca_{0,b_1}	4.6960000	5.07800		
$\log_{10}(k_1')$	−0.5667000	−0.50090		−8.696e−01
$\log_{10}(k_2)$	−0.6617000	−0.55690	0.01141 or −2.52	−9.932e−01
$\log_{10}(k_2')$	−2.0110000	−2.03900		−2.031e+00
$\log_{10}(k_{deg})$	−2.4700000	−2.45900	−2.52 or 0.01141	−2.468e+00
$\log_{10}(k_1'k_2')$	−2.5770000	−2.54000	−1.881	−2.901e+00
$\log_{10}(k_1'/k_2)$	0.0949100	0.05606		1.235e−01
transport $= \log_{10}(k_1'k_2'/k_2)$	−1.9160000	−1.98300	−1.893 or 0.6382	−1.907e+00

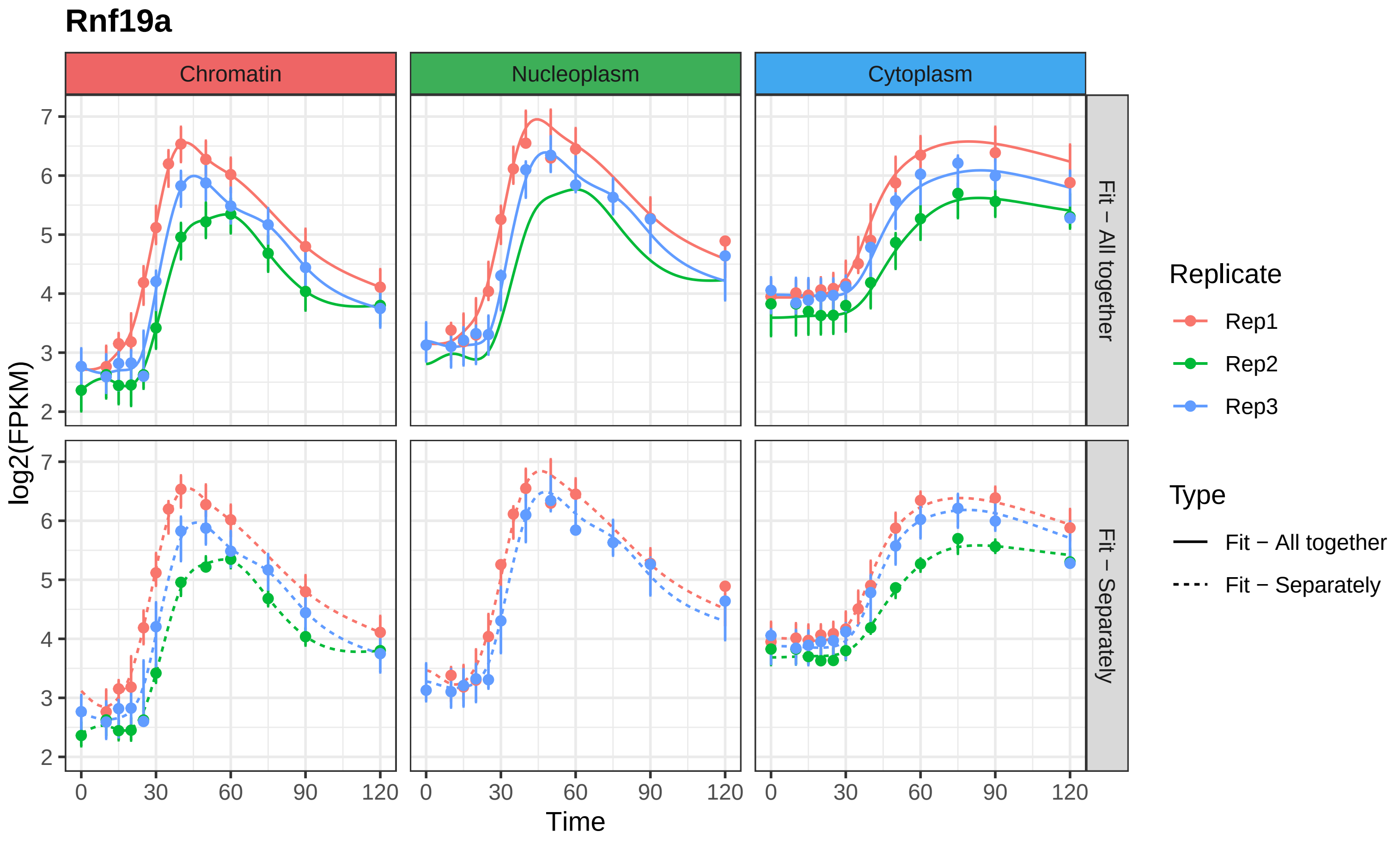

	Together	b1	b2	b3
**−NLL b1 ca**	−8.991	−8.527		
**−NLL b1 np**	2.952	1.917		
**−NLL b1 cyto**	−4.665	−10.88		
**−NLL b2 ca**	−10.38		−12.49	
**−NLL b2 np**				
**−NLL b2 cyto**	−7.73		−12.58	
**−NLL b3 ca**	−6.67			−4.076
**−NLL b3 np**	−2.794			−1.875
**−NLL b3 cyto**	−2.288			−4.424
Total	−40.56	−17.49	−25.07	−10.38
**Total with regul**	−35.03	−16.22	−22.78	−8.961

	Together	b1	b2	b3
spar	0.2616	0.336700	0.2993	0.32340
σ_b	0.1028	0.093740	0.03626	0.09942
σ_t	0.2658	0.002932	1.17e−05	1.25400
ca_{0,b_1}	2.7200	3.146000		
$\log_{10}(k_1')$	−0.2586	−0.372000		−0.01445
$\log_{10}(k_2)$	−0.3898	−0.475700	5.135 or −1.814	−0.17440
$\log_{10}(k_2')$	−1.5060	−1.498000		−1.42600
$\log_{10}(k_{deg})$	−1.7430	−1.663000	−1.814 or 5.135	−1.60700
$\log_{10}(k_1'k_2')$	−1.7650	−1.870000	3.709	−1.44100
$\log_{10}(k_1'/k_2)$	0.1312	0.103700		0.16000
transport $= \log_{10}(k_1'k_2'/k_2)$	−1.3750	−1.394000	−1.426 or 5.523	−1.26600

S100a10

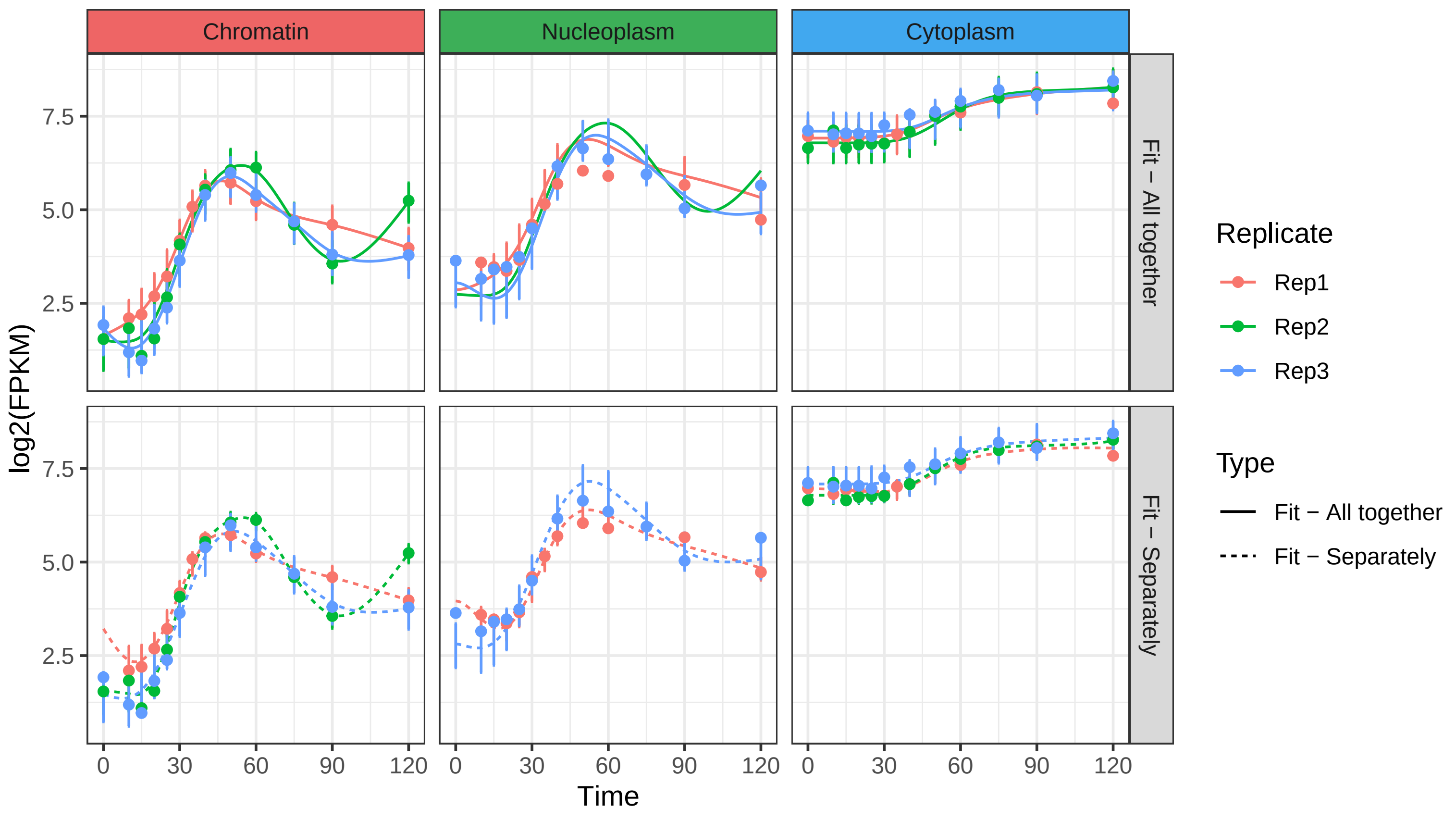

	Together	b1	b2	b3
**−NLL b1 ca**	−3.137	−6.548		
**−NLL b1 np**	14.11	−0.04578		
**−NLL b1 cyto**	−3.098	−9.429		
**−NLL b2 ca**	0.3393		−4.117	
**−NLL b2 np**				
**−NLL b2 cyto**	−3.351		−7.075	
**−NLL b3 ca**	−1.883			0.3189
**−NLL b3 np**	16.29			8.294
**−NLL b3 cyto**	−2.821			−4.123
Total	16.45	−16.02	−11.19	4.49
**Total with regul**	17.71	−15.27	−9.994	3.544

	Together	b1	b2	b3
spar	0.3830000	4.074e−01	0.3226	0.4361000
σ_b	0.1773000	8.724e−02	0.07355	0.1634000
σ_t	0.0004703	4.452e−05	0.0005972	0.0002638
ca_{0,b_1}	1.6560000	3.345e+00		
$\log_{10}(k_1')$	−0.3652000	−5.013e−01		0.2406000
$\log_{10}(k_2)$	−0.7330000	−7.252e−01	4.013 or −2.698	−0.1635000
$\log_{10}(k_2')$	−1.4520000	−1.172e+00		−1.4470000
$\log_{10}(k_{deg})$	−2.6720000	−2.075e+00	−2.698 or 4.013	−2.7350000
$\log_{10}(k_1' k_2')$	−1.8180000	−1.674e+00	2.888	−1.2070000
$\log_{10}(k_1'/k_2)$	0.3678000	2.240e−01		0.4040000
$\text{transport} = \log_{10}(k_1' k_2'/k_2)$	−1.0850000	−9.484e−01	−1.125 or 5.586	−1.0430000

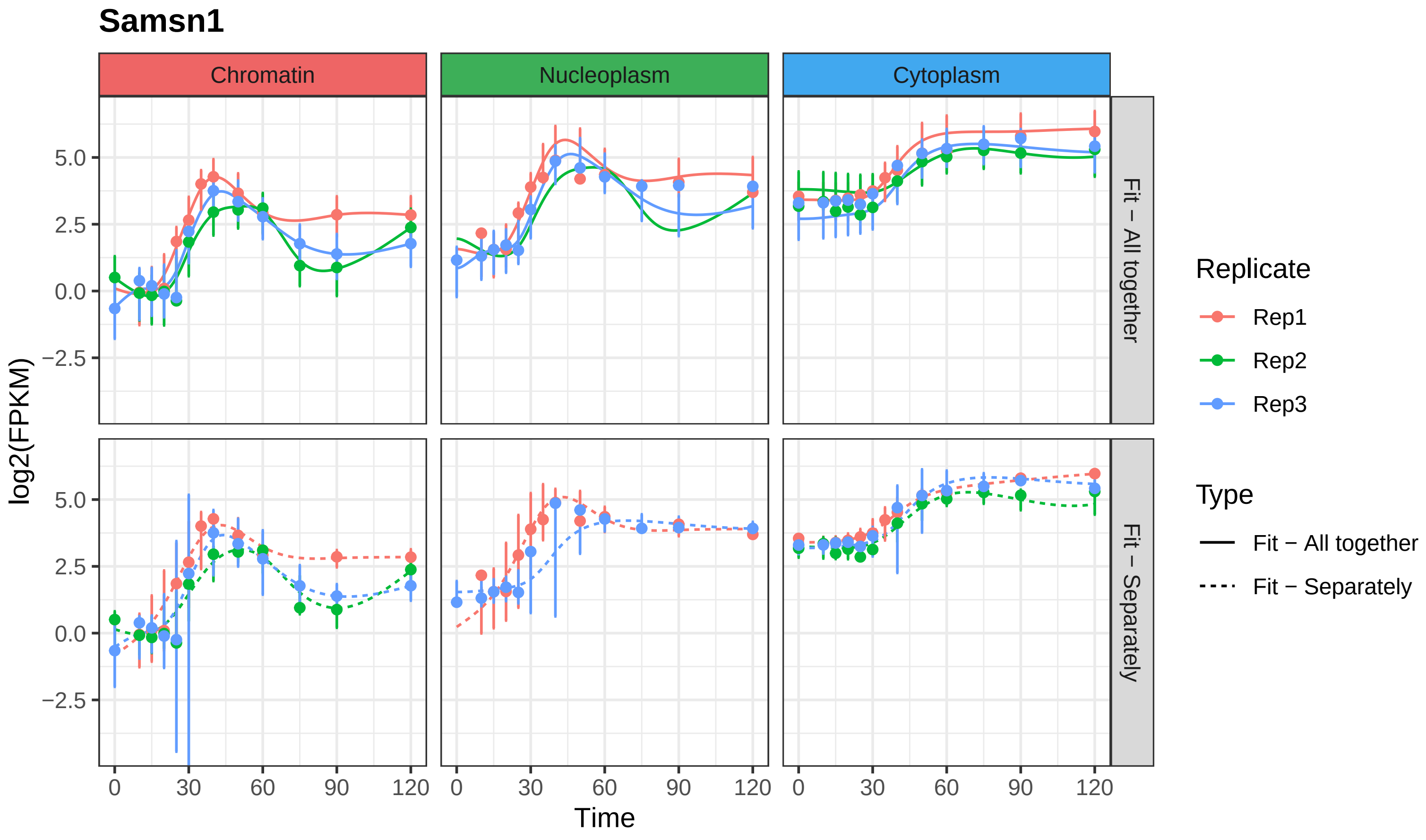

	Together	b1	b2	b3
**−NLL b1 ca**	1.183	3.129		
**−NLL b1 np**	11.4	11.61		
**−NLL b1 cyto**	2.095	−9.876		
**−NLL b2 ca**	3.078		3.037	
**−NLL b2 np**				
**−NLL b2 cyto**	8.451		0.1503	
**−NLL b3 ca**	3.728			5.494
**−NLL b3 np**	8.678			4.137
**−NLL b3 cyto**	7.338			−3.368
Total	45.95	4.867	3.187	6.263
**Total with regul**	49.97	3.628	1.818	6.379

	Together	b1	b2	b3
spar	0.3297000	0.46790	0.4562	0.37160
σ_b	0.2410000	0.03699	0.1273	0.05053
σ_t	0.0001528	4.13500	2.571	7.33800
ca_{0,b_1}	0.1154000	−0.71560		
$\log_{10}(k_1')$	−0.0393900	5.41300		−1.17600
$\log_{10}(k_2)$	−0.4828000	5.09700	0.1049 or −1.615	−1.79500
$\log_{10}(k_2')$	−1.2220000	−1.41800		−0.32530
$\log_{10}(k_{deg})$	−1.7780000	−2.37100	−1.615 or 0.1049	−0.82130
$\log_{10}(k_1'k_2')$	−1.2610000	3.99600	−0.5801	−1.50100
$\log_{10}(k_1'/k_2)$	0.4434000	0.31620		0.61960
transport = $\log_{10}(k_1'k_2'/k_2)$	−0.7783000	−1.10100	−0.685 or 1.035	0.29430

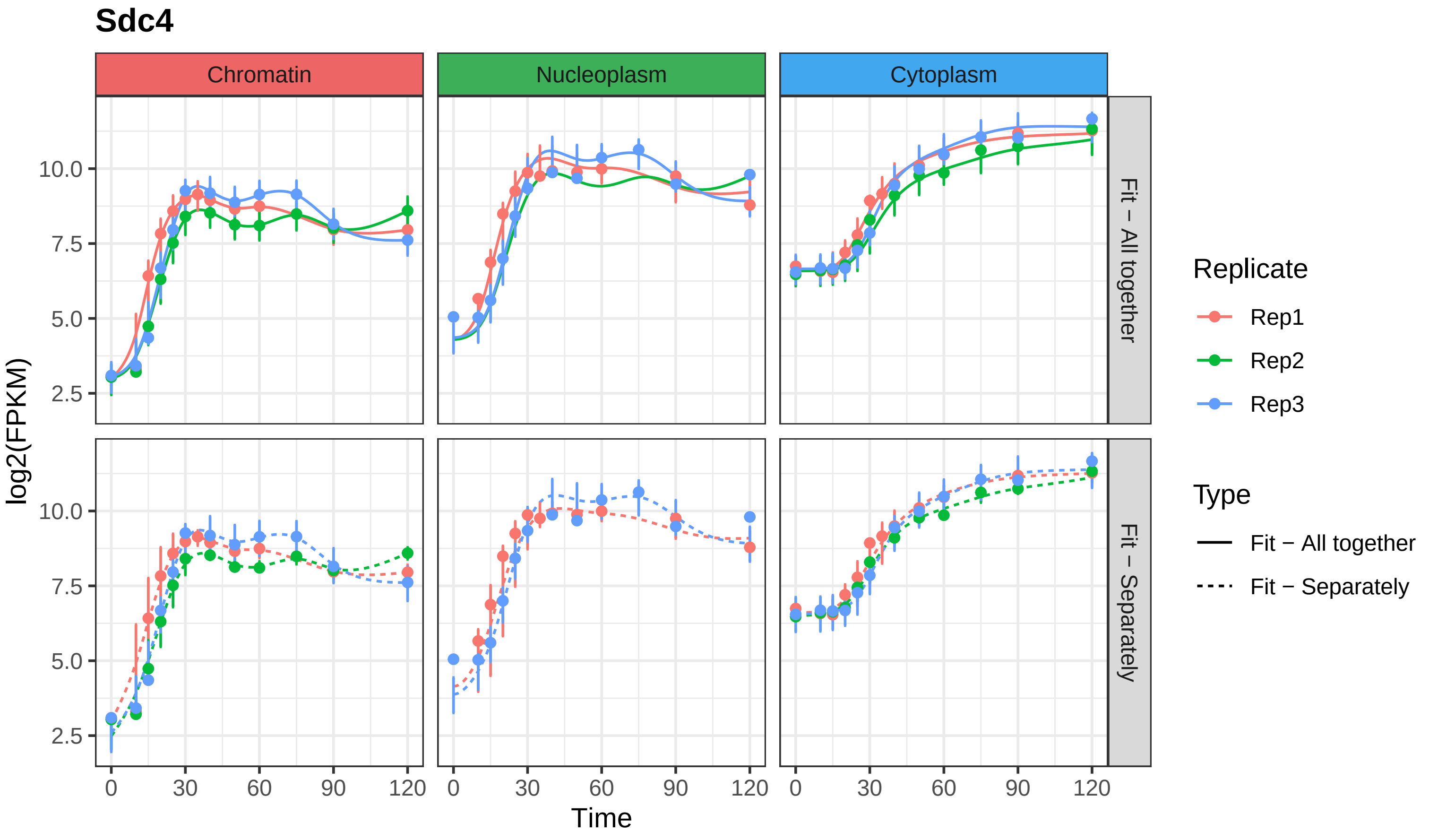

	Together	b1	b2	b3
**−NLL b1 ca**	0.8131	−2.859		
**−NLL b1 np**	4.601	6.191		
**−NLL b1 cyto**	−3.101	−4.577		
**−NLL b2 ca**	−2.864		−4.39	
**−NLL b2 np**				
**−NLL b2 cyto**	−0.6917		−6.74	
**−NLL b3 ca**	−1.931			1.713
**−NLL b3 np**	16.65			8.948
**−NLL b3 cyto**	−1.773			−2.245
Total	11.7	−1.245	−11.13	8.416
**Total with regul**	16.14	−1.405	−11.55	10.67

	Together	b1	b2	b3
spar	0.2905	0.36620	0.3585	0.3366000
σ_b	0.1693	0.08847	0.06086	0.2000000
σ_t	0.8397	2.62200	1.429	0.0005304
ca_{0,b_1}	3.0470	3.05400		
$\log_{10}(k_1')$	−0.1943	−0.54440		−0.2831000
$\log_{10}(k_2)$	−0.5877	−0.89850	0.04789 or −2.23	−0.6726000
$\log_{10}(k_2')$	−1.3960	−1.31400		−1.4610000
$\log_{10}(k_{deg})$	−2.0870	−2.05600	−2.23 or 0.04789	−2.2710000
$\log_{10}(k_1'k_2')$	−1.5910	−1.85800	−0.9696	−1.7440000
$\log_{10}(k_1'/k_2)$	0.3934	0.35420		0.3894000
transport = $\log_{10}(k_1'k_2'/k_2)$	−1.0030	−0.95950	−1.017 or 1.261	−1.0720000

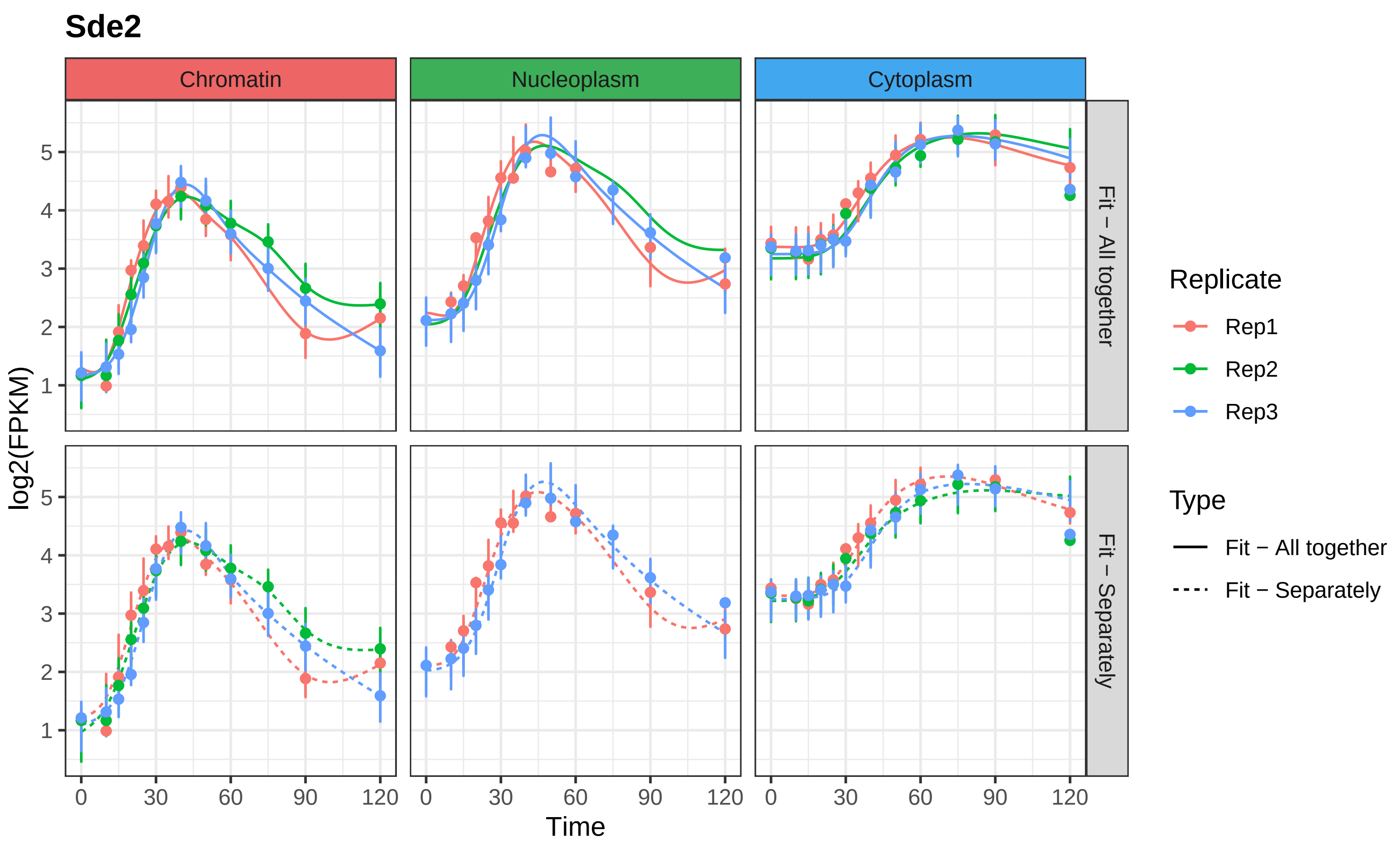

	Together	b1	b2	b3
**−NLL b1 ca**	−4.829	−3.082		
**−NLL b1 np**	1.067	0.2001		
**−NLL b1 cyto**	−6.89	−8.995		
**−NLL b2 ca**	−7.755		−6.139	
**−NLL b2 np**				
**−NLL b2 cyto**	3.423		0.6839	
**−NLL b3 ca**	−7.711			−5.578
**−NLL b3 np**	−0.8544			−0.4324
**−NLL b3 cyto**	−3.073			−1.621
Total	−26.62	−11.88	−5.455	−7.632
**Total with regul**	−25.77	−12.89	−5.741	−8.117

	Together	b1	b2	b3
spar	3.533e−01	0.40670	0.3682	3.745e−01
σ_b	1.168e−01	0.07539	0.1177	1.178e−01
σ_t	4.064e−05	1.93700	0.000291	5.774e−05
ca_{0,b_1}	1.382e+00	1.35400		
$\log_{10}(k_1')$	−3.926e−01	−0.48960		−4.174e−01
$\log_{10}(k_2)$	−6.760e−01	−0.75600	6.112 or −2.011	−6.991e−01
$\log_{10}(k_2')$	−1.430e+00	−1.34100		−1.483e+00
$\log_{10}(k_{deg})$	−1.771e+00	−1.70200	−2.011 or 6.112	−1.850e+00
$\log_{10}(k_1'k_2')$	−1.822e+00	−1.83000	4.777	−1.900e+00
$\log_{10}(k_1'/k_2)$	2.834e−01	0.26630		2.817e−01
$transport = \log_{10}(k_1'k_2'/k_2)$	−1.146e+00	−1.07400	−1.335 or 6.788	−1.201e+00

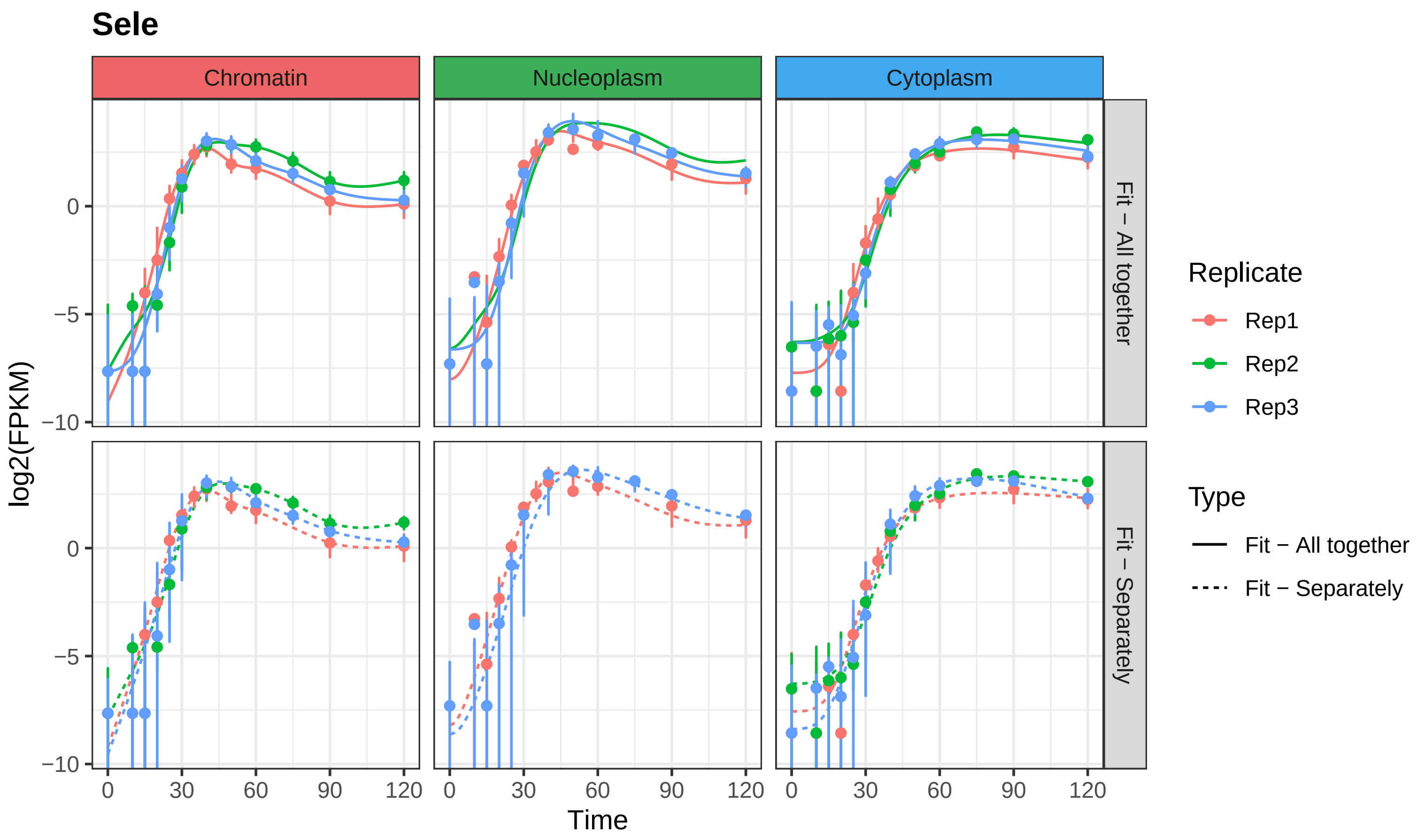

	Together	b1	b2	b3
**−NLL b1 ca**	0.7039	2.018		
**−NLL b1 np**	15.48	14		
**−NLL b1 cyto**	9.216	7.653		
**−NLL b2 ca**	5.184		5.317	
**−NLL b2 np**				
**−NLL b2 cyto**	8.24		7.421	
**−NLL b3 ca**	4.211			6.899
**−NLL b3 np**	14.2			13.44
**−NLL b3 cyto**	6.89			8.581
Total	64.12	23.67	12.74	28.92
**Total with regul**	68.51	23.9	12.19	27.27

	Together	b1	b2	b3
spar	0.2792	0.3679	0.3578	0.3988000
σ_b	0.1091	0.1394	8.13e−07	0.0004104
σ_t	0.9666	0.2102	1.878	2.6930000
ca_{0,b_1}	−9.0470	−9.1710		
$\log_{10}(k_1')$	−0.5056	−0.4223		−0.7412000
$\log_{10}(k_2)$	−0.8206	−0.7255	−0.8441 or −1.73	−1.0230000
$\log_{10}(k_2')$	−1.5410	−1.6550		−1.3190000
$\log_{10}(k_{deg})$	−1.6330	−1.8450	−1.73 or −0.8441	−1.3880000
$\log_{10}(k_1'k_2')$	−2.0470	−2.0770	−2.108	−2.0600000
$\log_{10}(k_1'/k_2)$	0.3150	0.3032		0.2820000
$\text{transport} = \log_{10}(k_1'k_2'/k_2)$	−1.2260	−1.3510	−1.263 or −0.3776	−1.0370000

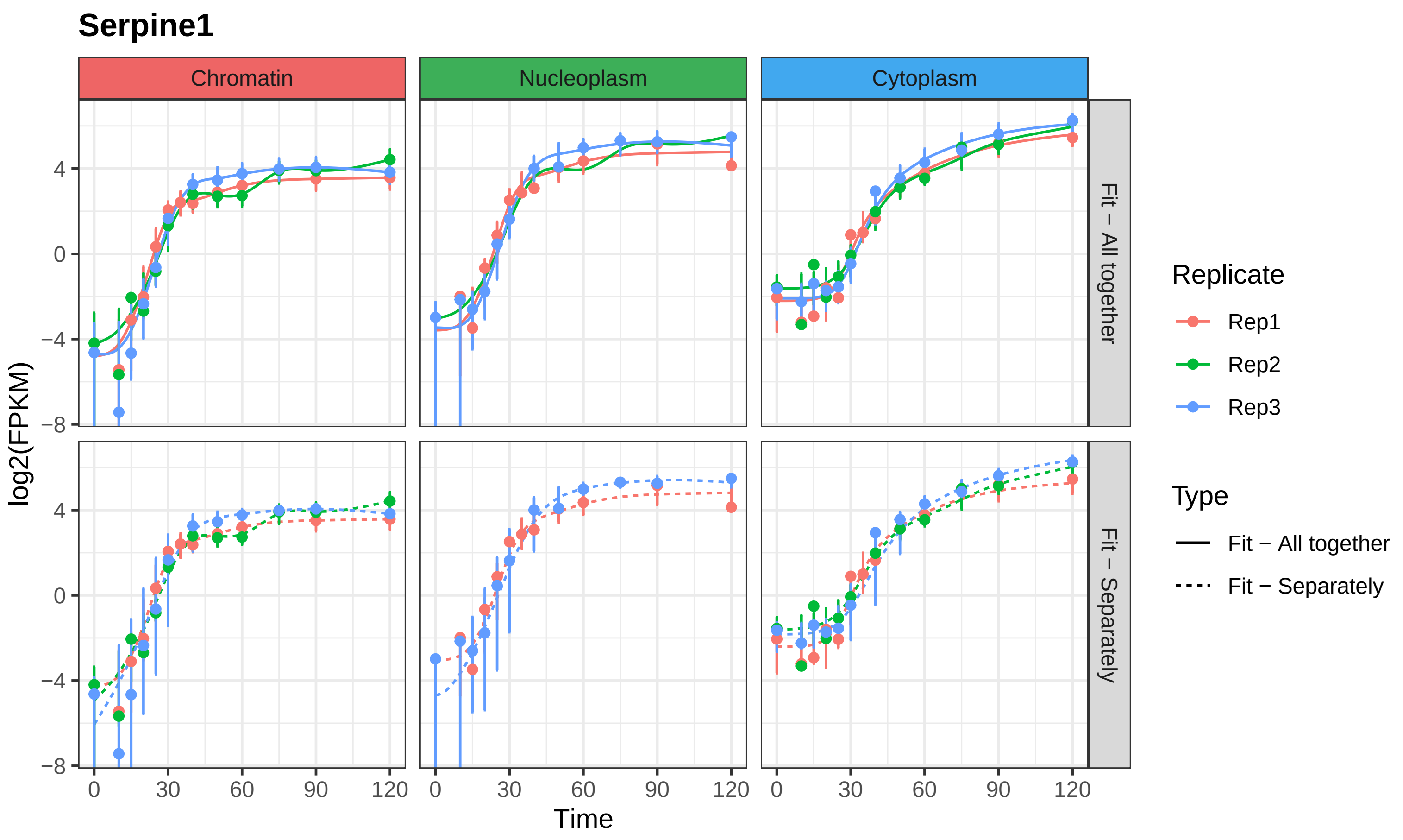

	Together	b1	b2	b3
**−NLL b1 ca**	1.725	3.408		
**−NLL b1 np**	10.61	9.886		
**−NLL b1 cyto**	9.278	7.404		
**−NLL b2 ca**	6.338		5.359	
**−NLL b2 np**				
**−NLL b2 cyto**	12.96		11.5	
**−NLL b3 ca**	5.696			6.366
**−NLL b3 np**	5.684			7.914
**−NLL b3 cyto**	4.225			3.981
Total	56.51	20.7	16.86	18.26
**Total with regul**	63.25	22.9	17.01	16.51

	Together	b1	b2	b3
spar	0.3266	0.3683	0.3679	0.42580
σ_b	0.1752	0.1600	0.1487	0.06395
σ_t	0.9779	1.6650	0.8805	3.49900
ca_{0,b_1}	−4.7580	−4.2060		
$\log_{10}(k_1')$	−0.1588	−0.3722		−0.46450
$\log_{10}(k_2)$	−0.5252	−0.7472	−0.1519 or −2.107	−0.88000
$\log_{10}(k_2')$	−1.3670	−1.2590		−1.48700
$\log_{10}(k_{deg})$	−1.7820	−1.4480	−2.107 or −0.1519	−2.34600
$\log_{10}(k_1' k_2')$	−1.5250	−1.6320	−1.254	−1.95200
$\log_{10}(k_1'/k_2)$	0.3665	0.3750		0.41550
transport $= \log_{10}(k_1' k_2'/k_2)$	−1.0000	−0.8844	−1.102 or 0.8523	−1.07200

Sh3bgrl2

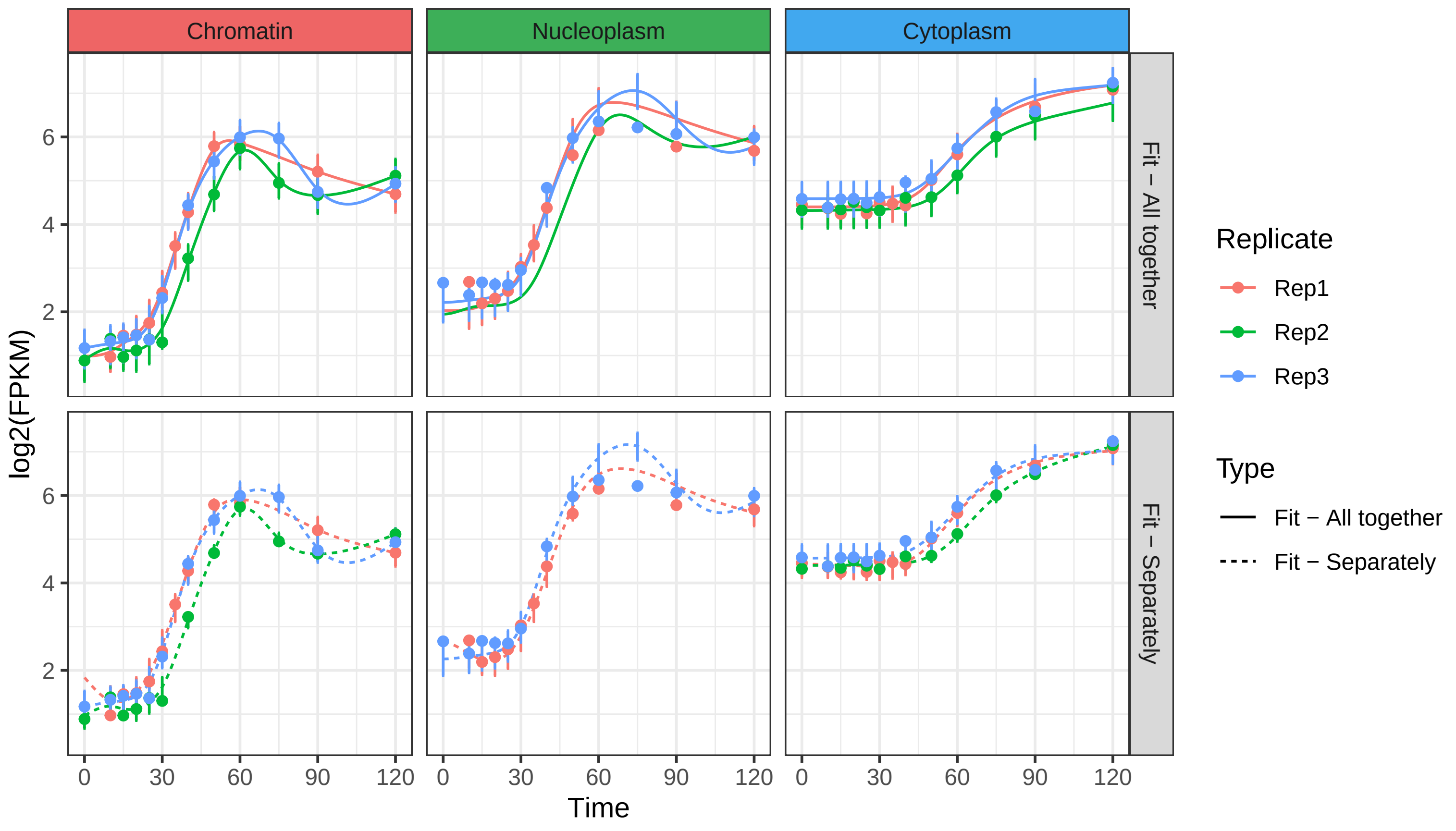

	Together	b1	b2	b3
**−NLL b1 ca**	−6.059	−5.749		
**−NLL b1 np**	9.079	0.8546		
**−NLL b1 cyto**	−6.355	−9.262		
**−NLL b2 ca**	−5.361		−7.663	
**−NLL b2 np**				
**−NLL b2 cyto**	−5.291		−13.1	
**−NLL b3 ca**	−5.785			−5.723
**−NLL b3 np**	10.69			0.438
**−NLL b3 cyto**	−5.021			−5.745
Total	−14.1	−14.16	−20.76	−11.03
**Total with regul**	−11.89	−12.28	−19.63	−9.682

	Together	b1	b2	b3
spar	3.251e−01	4.138e−01	0.3206	0.326100
σ_b	1.372e−01	1.014e−01	0.04308	0.109200
σ_t	5.823e−05	8.098e−05	0.0002459	0.000354
ca_{0,b_1}	9.951e−01	1.906e+00		
$\log_{10}(k_1')$	−5.739e−01	−6.193e−01		−0.359600
$\log_{10}(k_2)$	−8.879e−01	−8.616e−01	−1.391 or −2.081	−0.685700
$\log_{10}(k_2')$	−1.621e+00	−1.530e+00		−1.700000
$\log_{10}(k_{deg})$	−2.335e+00	−2.069e+00	−2.081 or −1.391	−2.395000
$\log_{10}(k_1'k_2')$	−2.195e+00	−2.150e+00	−2.437	−2.060000
$\log_{10}(k_1'/k_2)$	3.140e−01	2.423e−01		0.326100
$\text{transport} = \log_{10}(k_1'k_2'/k_2)$	−1.307e+00	−1.288e+00	−1.046 or −0.3564	−1.374000

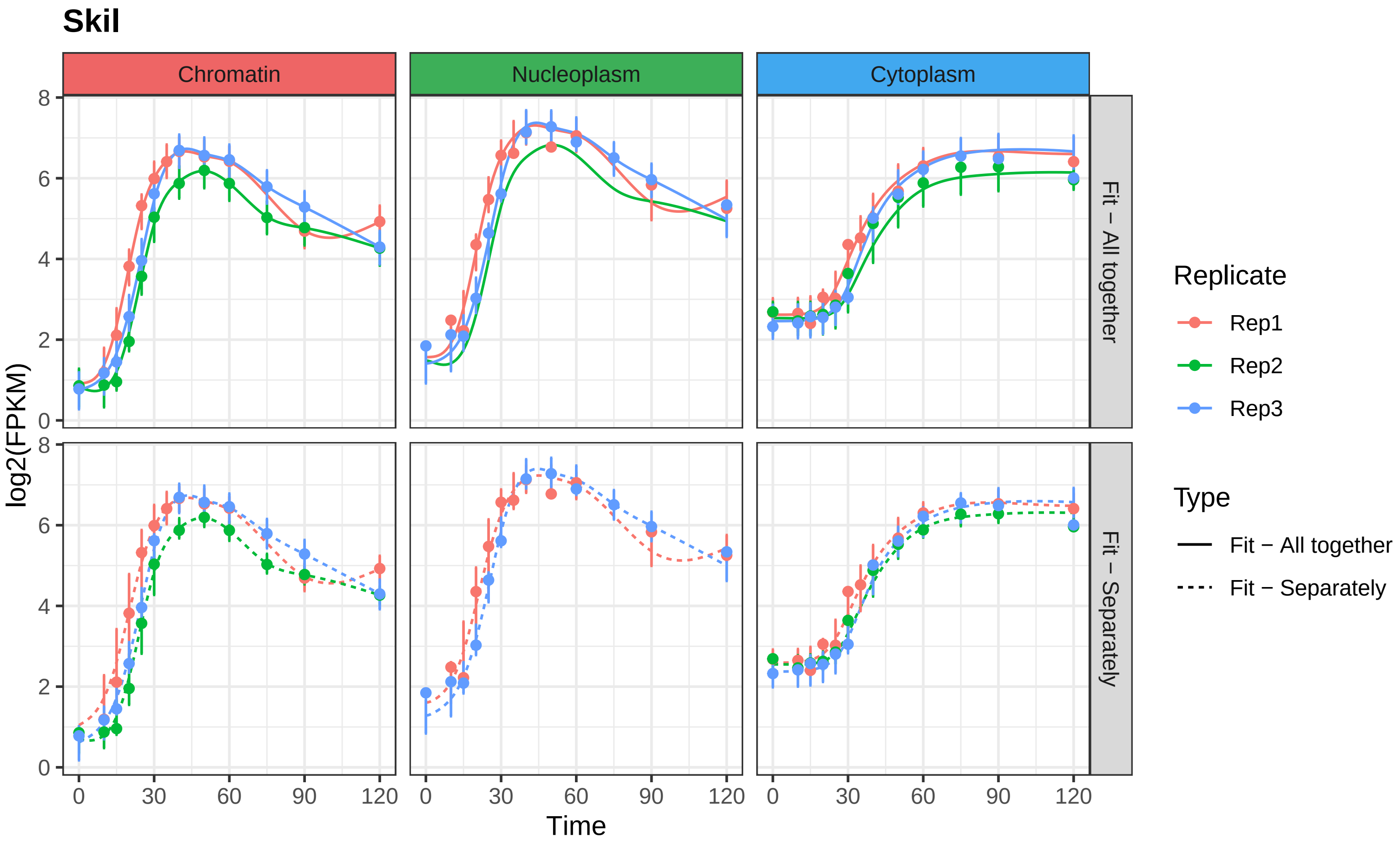

	Together	b1	b2	b3
**−NLL b1 ca**	−5.604	−2.459		
**−NLL b1 np**	7.696	5.797		
**−NLL b1 cyto**	−1.571	−3.65		
**−NLL b2 ca**	−5.68		−6.421	
**−NLL b2 np**				
**−NLL b2 cyto**	2.385		−3.524	
**−NLL b3 ca**	−6.178			−5.238
**−NLL b3 np**	−0.1804			−0.5219
**−NLL b3 cyto**	−0.5388			−1.037
Total	−9.671	−0.3129	−9.945	−6.797
**Total with regul**	−5.465	−0.2702	−7.733	−4.407

	Together	b1	b2	b3
spar	0.287700	0.36620	0.3048	0.3102000
σ_b	0.143300	0.11110	0.07546	0.1265000
σ_t	0.233000	1.80700	1.195	0.0003641
ca_{0,b_1}	0.952600	1.13500		
$\log_{10}(k_1')$	0.196400	−0.05808		0.1609000
$\log_{10}(k_2)$	0.001171	−0.22650	5.249 or −2.039	−0.0394000
$\log_{10}(k_2')$	−1.731000	−1.73200		−1.8000000
$\log_{10}(k_{deg})$	−2.048000	−2.02900	−2.039 or 5.249	−2.1290000
$\log_{10}(k_1'k_2')$	−1.535000	−1.79000	3.768	−1.6390000
$\log_{10}(k_1'/k_2)$	0.195300	0.16840		0.2003000
$\text{transport} = \log_{10}(k_1'k_2'/k_2)$	−1.536000	−1.56400	−1.481 or 5.807	−1.6000000

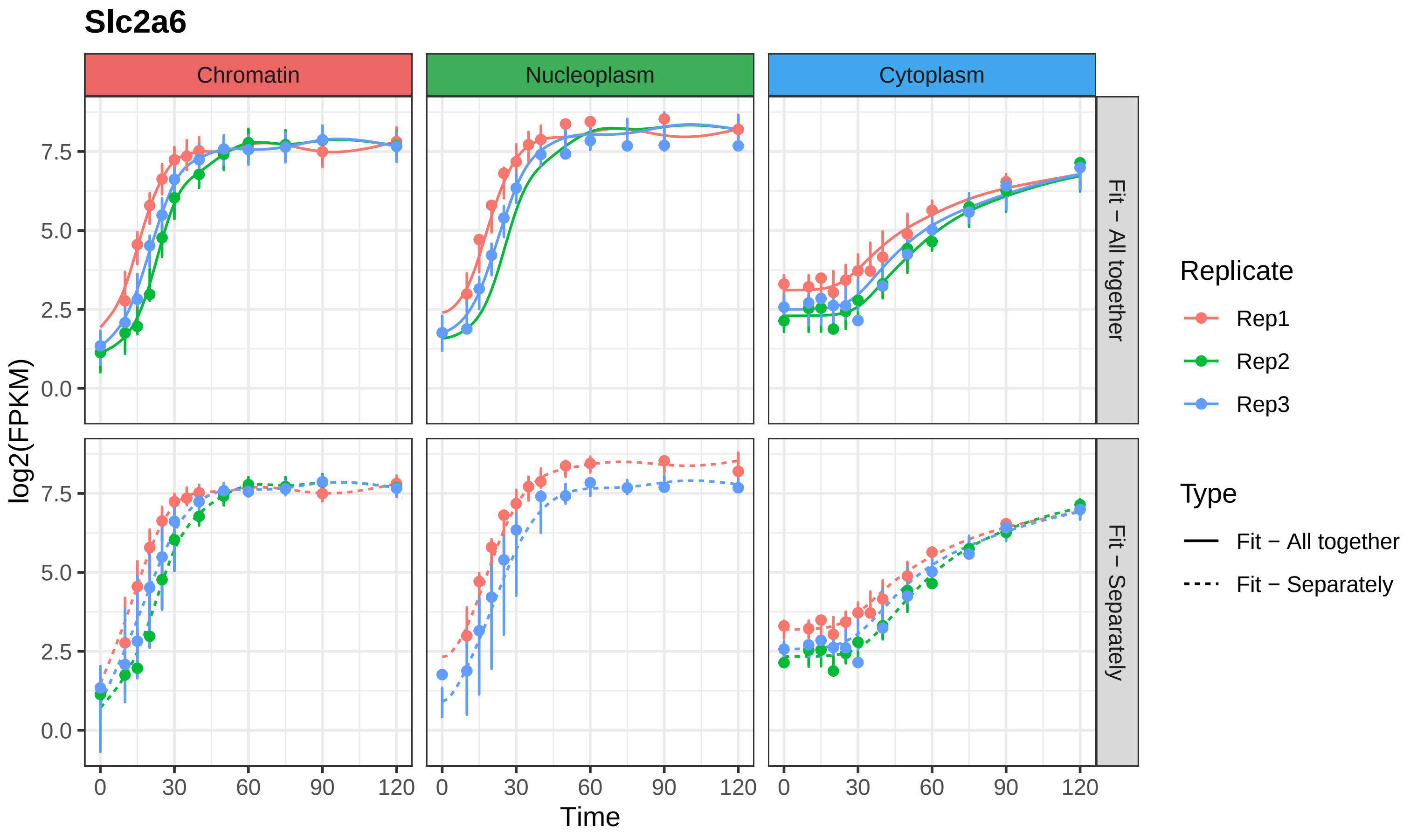

	Together	b1	b2	b3
**−NLL b1 ca**	−3.892	−5.679		
**−NLL b1 np**	3.852	0.04373		
**−NLL b1 cyto**	−0.3171	−3.793		
**−NLL b2 ca**	−3.869		−2.944	
**−NLL b2 np**				
**−NLL b2 cyto**	−0.02014		−0.1222	
**−NLL b3 ca**	−4.462			−1.415
**−NLL b3 np**	5.366			2.424
**−NLL b3 cyto**	5.418			2.889
Total	2.077	−9.428	−3.066	3.898
**Total with regul**	6.381	−9.968	−3.508	2.174

	Together	b1	b2	b3
spar	0.2909	0.40030	0.3665	0.43230
σ_b	0.1615	0.08526	0.09303	0.07376
σ_t	0.2773	1.66900	1.559	3.60700
ca_{0,b_1}	1.9660	1.52700		
$\log_{10}(k_1')$	−0.4554	−0.68160		−0.66720
$\log_{10}(k_2)$	−0.5966	−0.93560	−0.9694 or −2.534	−0.68500
$\log_{10}(k_2')$	−2.3160	−2.37500		−2.15500
$\log_{10}(k_{deg})$	−2.5300	−2.63700	−2.534 or −0.9694	−2.65100
$\log_{10}(k_1'k_2')$	−2.7720	−3.05700	−3.016	−2.82200
$\log_{10}(k_1'/k_2)$	0.1412	0.25400		0.01786
$\text{transport} = \log_{10}(k_1'k_2'/k_2)$	−2.1750	−2.12200	−2.046 or −0.4816	−2.13700

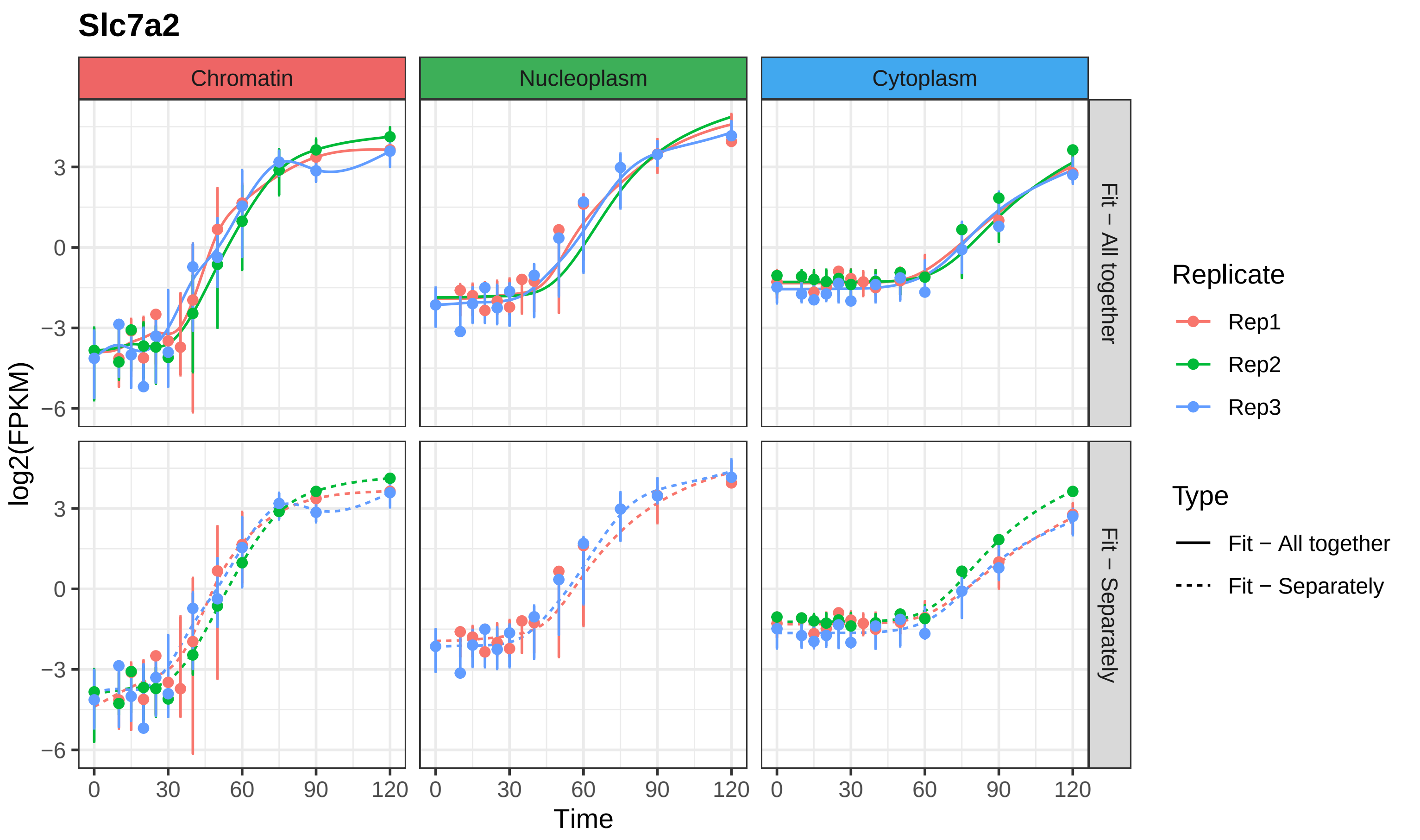

	Together	b1	b2	b3
**−NLL b1 ca**	6.484	7.688		
**−NLL b1 np**	9.436	8.555		
**−NLL b1 cyto**	0.2887	−0.5908		
**−NLL b2 ca**	4.469		−2.317	
**−NLL b2 np**				
**−NLL b2 cyto**	1.855		−6.951	
**−NLL b3 ca**	7.899			9.401
**−NLL b3 np**	7.981			8.52
**−NLL b3 cyto**	3.94			1.681
Total	42.35	15.65	−9.268	19.6
**Total with regul**	44.65	14.65	−11.17	18.98

	Together	b1	b2	b3
spar	0.3409	0.44410	0.4046	0.4015
σ_b	0.1198	0.08885	1.068e−08	0.1365
σ_t	4.3460	5.61500	4.762e−05	3.8290
ca_{0,b_1}	−3.8800	−4.39000		
$\log_{10}(k_1')$	−1.2350	−1.38200		−1.1360
$\log_{10}(k_2)$	−1.8310	−2.11900	−1.135 or −2.369	−1.6540
$\log_{10}(k_2')$	−1.8740	−1.92600		−2.0570
$\log_{10}(k_{deg})$	−2.0470	−2.11400	−2.369 or −1.135	−2.2060
$\log_{10}(k_1'k_2')$	−3.1090	−3.30800	−2.7	−3.1930
$\log_{10}(k_1'/k_2)$	0.5956	0.73770		0.5177
transport = $\log_{10}(k_1'k_2'/k_2)$	−1.2780	−1.18900	−1.565 or −0.3314	−1.5400

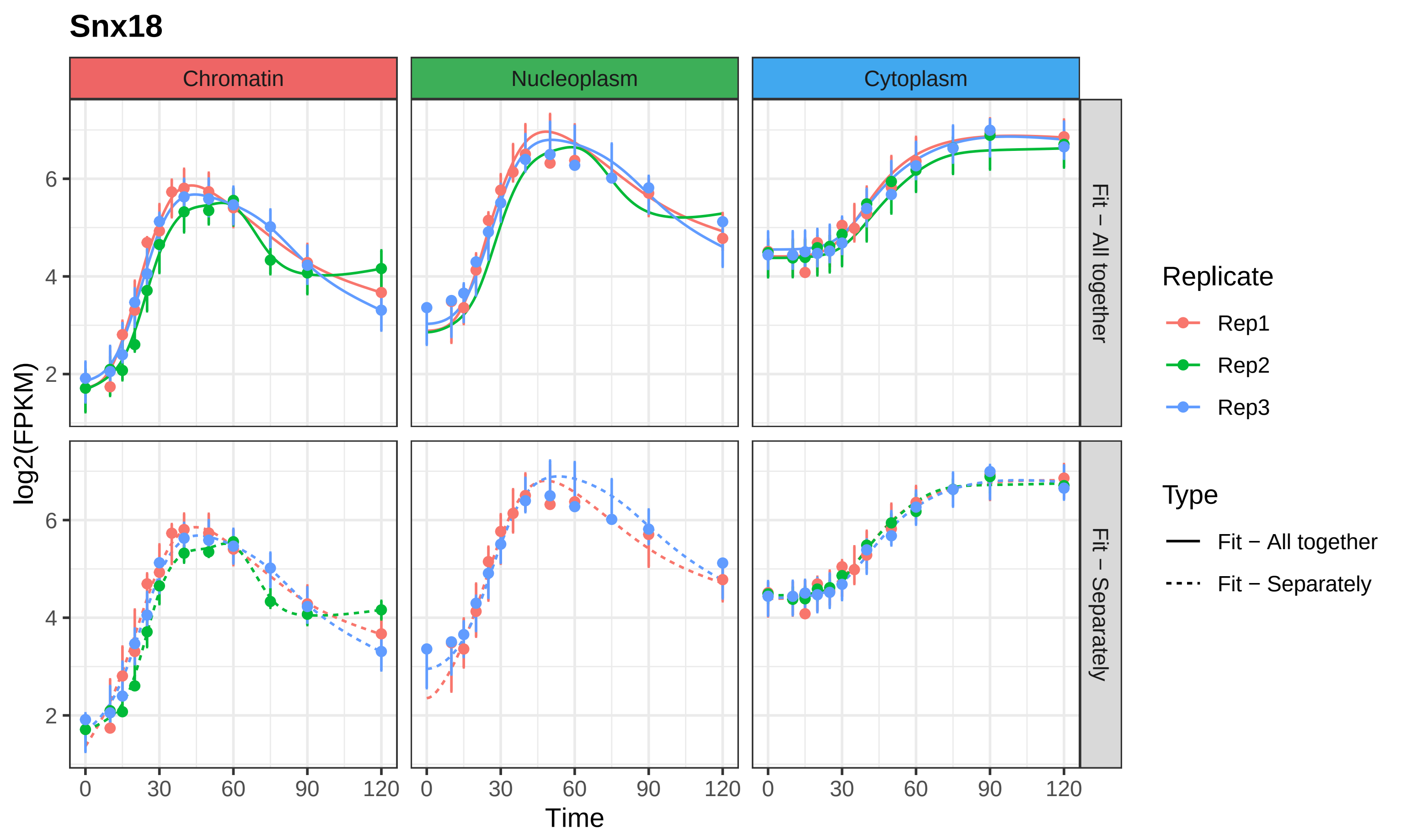

	Together	b1	b2	b3
**−NLL b1 ca**	−3.889	−2.298		
**−NLL b1 np**	3.769	2.364		
**−NLL b1 cyto**	−3.488	−4.628		
**−NLL b2 ca**	−5.609		−8.892	
**−NLL b2 np**				
**−NLL b2 cyto**	−2.865		−9.556	
**−NLL b3 ca**	−6.32			−4.156
**−NLL b3 np**	5			3.207
**−NLL b3 cyto**	−4.718			−7.046
Total	−18.12	−4.562	−18.45	−7.994
**Total with regul**	−17.19	−5.897	−16.79	−8.907

	Together	b1	b2	b3
spar	0.3509000	0.4489	0.3122	3.925e−01
σ_b	0.1326000	0.1197	0.05894	1.220e−01
σ_t	0.0008877	1.4210	0.5727	6.905e−07
ca_{0,b_1}	1.7750000	1.4840		
$\log_{10}(k_1')$	−0.3351000	−0.2477		−4.036e−01
$\log_{10}(k_2)$	−0.6876000	−0.5446	−0.2733 or −2.054	−7.902e−01
$\log_{10}(k_2')$	−1.6210000	−1.6620		−1.678e+00
$\log_{10}(k_{deg})$	−2.0790000	−2.2750	−2.054 or −0.2733	−2.116e+00
$\log_{10}(k_1'k_2')$	−1.9560000	−1.9100	−1.492	−2.082e+00
$\log_{10}(k_1'/k_2)$	0.3525000	0.2969		3.866e−01
$\text{transport} = \log_{10}(k_1'k_2'/k_2)$	−1.2680000	−1.3650	−1.218 or 0.5623	−1.292e+00

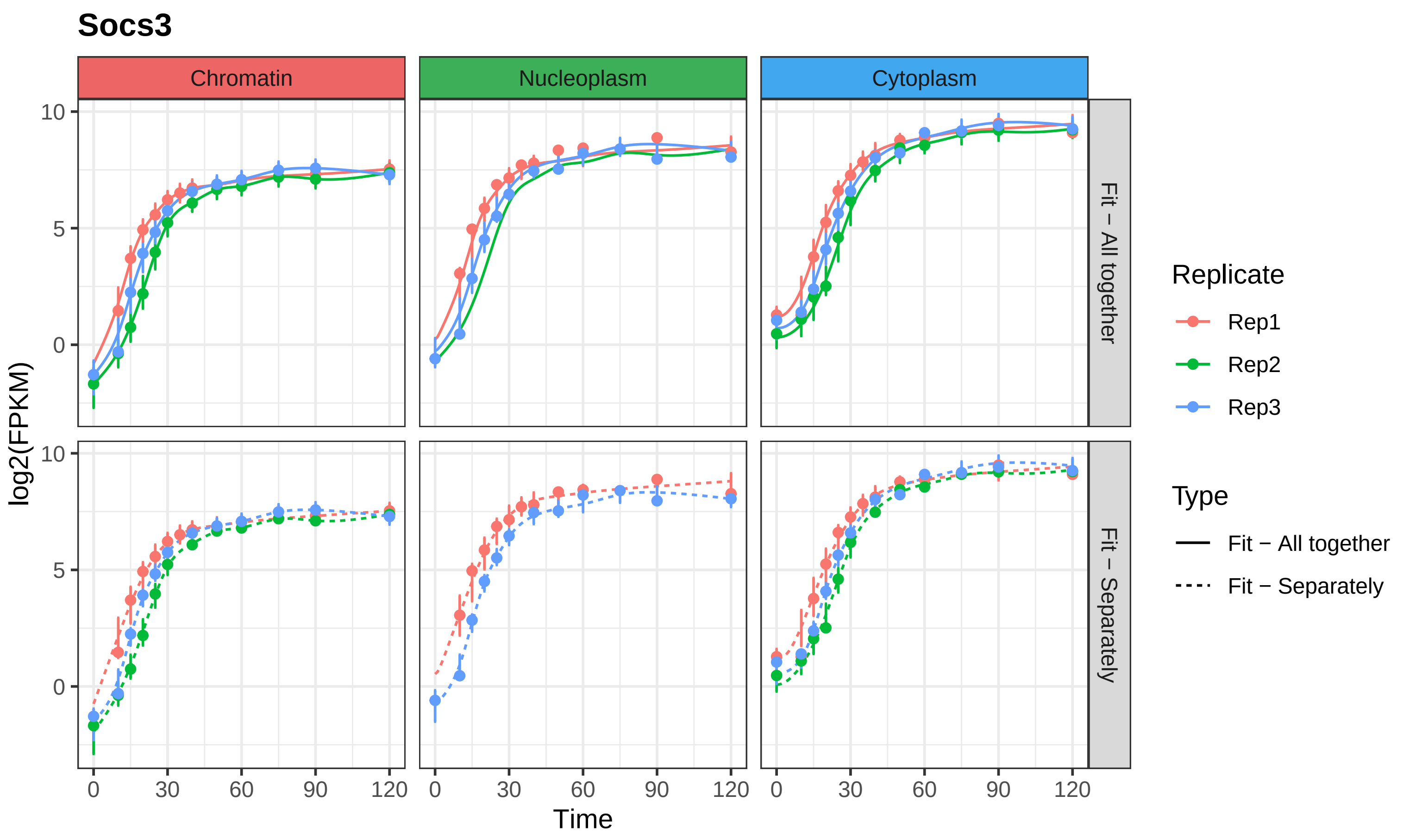

	Together	b1	b2	b3
**−NLL b1 ca**	−5.641	−4.284		
**−NLL b1 np**	7.131	2.169		
**−NLL b1 cyto**	2.558	2.535		
**−NLL b2 ca**	−5.313		−10.16	
**−NLL b2 np**				
**−NLL b2 cyto**	−1.905		−1.067	
**−NLL b3 ca**	−3.321			−4.549
**−NLL b3 np**	7.848			−0.2634
**−NLL b3 cyto**	−2.199			−1.53
Total	−0.8426	0.4208	−11.23	−6.343
**Total with regul**	9.844	1.718	−7.13	4.831

	Together	b1	b2	b3
spar	0.23340	0.4121	0.2724	1.970e−01
σ_b	0.13210	0.1215	0.04316	1.173e−01
σ_t	0.84290	1.3020	0.8242	1.066e−05
ca_{0,b_1}	−0.79780	−0.7577		
$\log_{10}(k_1')$	0.57140	0.2766		5.891e−01
$\log_{10}(k_2)$	0.26210	−0.1090	4.712 or −0.8709	3.636e−01
$\log_{10}(k_2')$	−0.62520	−0.6788		−5.768e−01
$\log_{10}(k_{deg})$	−0.92070	−0.8828	−0.8709 or 4.712	−9.750e−01
$\log_{10}(k_1'k_2')$	−0.05383	−0.4022	4.45	1.224e−02
$\log_{10}(k_1'/k_2)$	0.30930	0.3856		2.255e−01
$transport = \log_{10}(k_1'k_2'/k_2)$	−0.31590	−0.2932	−0.2619 or 5.321	−3.513e−01

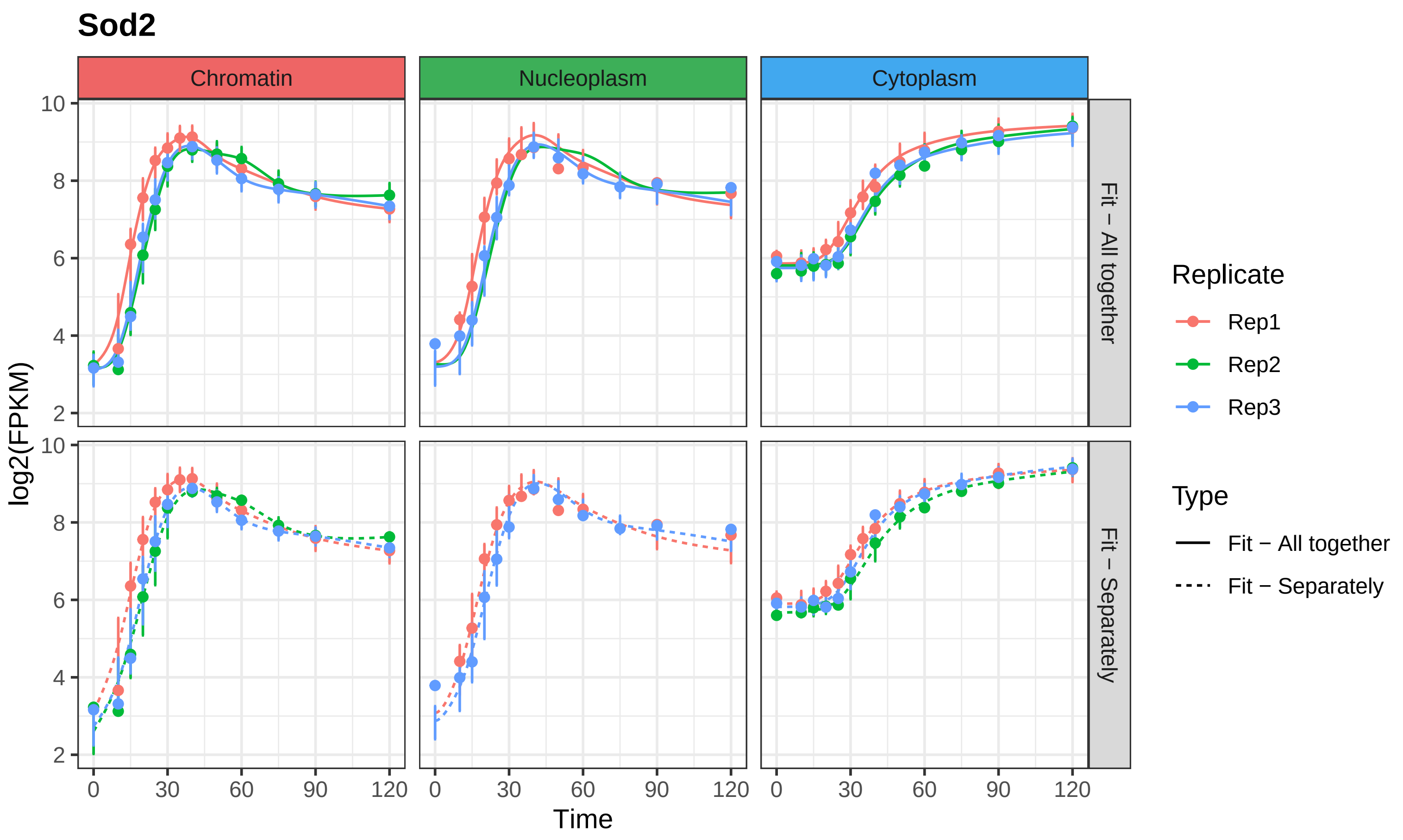

	Together	b1	b2	b3
**−NLL b1 ca**	−3.818	−2.303		
**−NLL b1 np**	6.566	5.229		
**−NLL b1 cyto**	−6.786	−8.161		
**−NLL b2 ca**	−5.771		−5.181	
**−NLL b2 np**				
**−NLL b2 cyto**	−5.94		−9.908	
**−NLL b3 ca**	−5.896			−3.723
**−NLL b3 np**	2.959			1.338
**−NLL b3 cyto**	−0.8759			−6.095
Total	−19.56	−5.235	−15.09	−8.479
**Total with regul**	−14.78	−4.234	−16.11	−8.137

	Together	b1	b2	b3
spar	0.27700	0.348600	0.3734	0.34240
σ_b	0.11100	0.107200	0.03548	0.07730
σ_t	0.89840	1.355000	1.797	1.55400
ca_{0,b_1}	3.28200	3.178000		
$\log_{10}(k_1')$	−0.43890	−0.579600		−0.32680
$\log_{10}(k_2)$	−0.46230	−0.572000	−0.4453 or −2.518	−0.36910
$\log_{10}(k_2')$	−1.57500	−1.586000		−1.55500
$\log_{10}(k_{deg})$	−2.34200	−2.434000	−2.518 or −0.4453	−2.44100
$\log_{10}(k_1'k_2')$	−2.01300	−2.165000	−2.04	−1.88200
$\log_{10}(k_1'/k_2)$	0.02344	−0.007523		0.04232
transport = $\log_{10}(k_1'k_2'/k_2)$	−1.55100	−1.593000	−1.594 or 0.4788	−1.51300

Sowahc

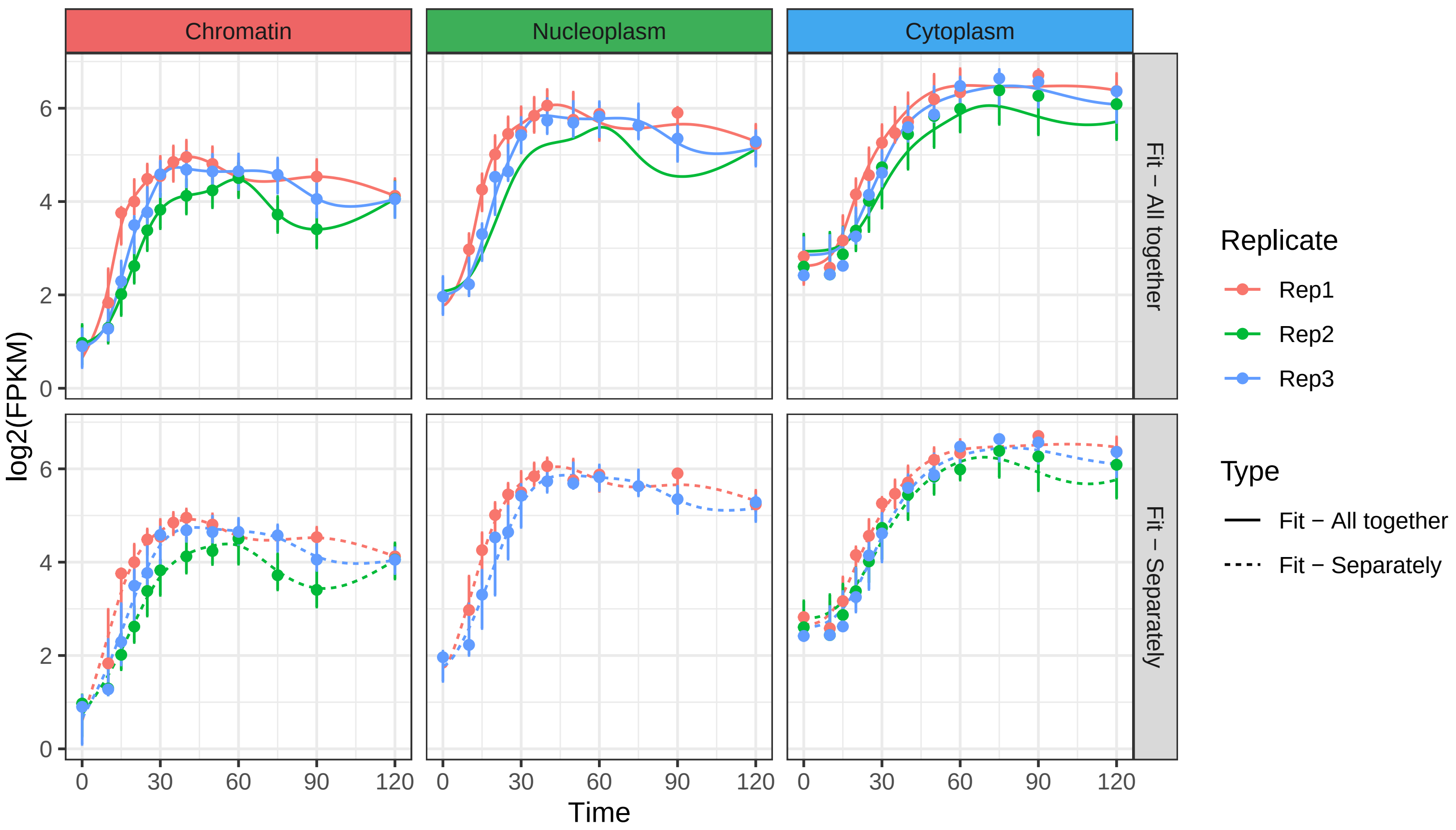

	Together	b1	b2	b3
**−NLL b1 ca**	−5.486	−7.151		
**−NLL b1 np**	−5.436	−3.746		
**−NLL b1 cyto**	−3.353	−5.223		
**−NLL b2 ca**	−8.192		−4.821	
**−NLL b2 np**				
**−NLL b2 cyto**	10.75		1.547	
**−NLL b3 ca**	−7.101			−3.93
**−NLL b3 np**	−4.241			−2.828
**−NLL b3 cyto**	3.176			0.4763
Total	−19.88	−16.12	−3.274	−6.281
**Total with regul**	−11.63	−15.39	−4.498	−7.636

	Together	b1	b2	b3
spar	0.24790	0.37910	0.4175	0.40270
σ_b	0.13050	0.07723	0.1329	0.09385
σ_t	0.24520	1.39400	6.913e−05	1.95000
ca_{0,b_1}	0.67230	0.62410		
$\log_{10}(k_1')$	0.05658	−0.05937		−0.15880
$\log_{10}(k_2)$	−0.28270	−0.40190	−0.5633 or −1.171	−0.50010
$\log_{10}(k_2')$	−1.10900	−1.20400		−1.11900
$\log_{10}(k_{deg})$	−1.36600	−1.48300	−1.171 or −0.5633	−1.36800
$\log_{10}(k_1'k_2')$	−1.05300	−1.26300	−1.115	−1.27700
$\log_{10}(k_1'/k_2)$	0.33930	0.34260		0.34130
$\text{transport} = \log_{10}(k_1'k_2'/k_2)$	−0.76990	−0.86120	−0.5514 or 0.05663	−0.77730

Sphk1

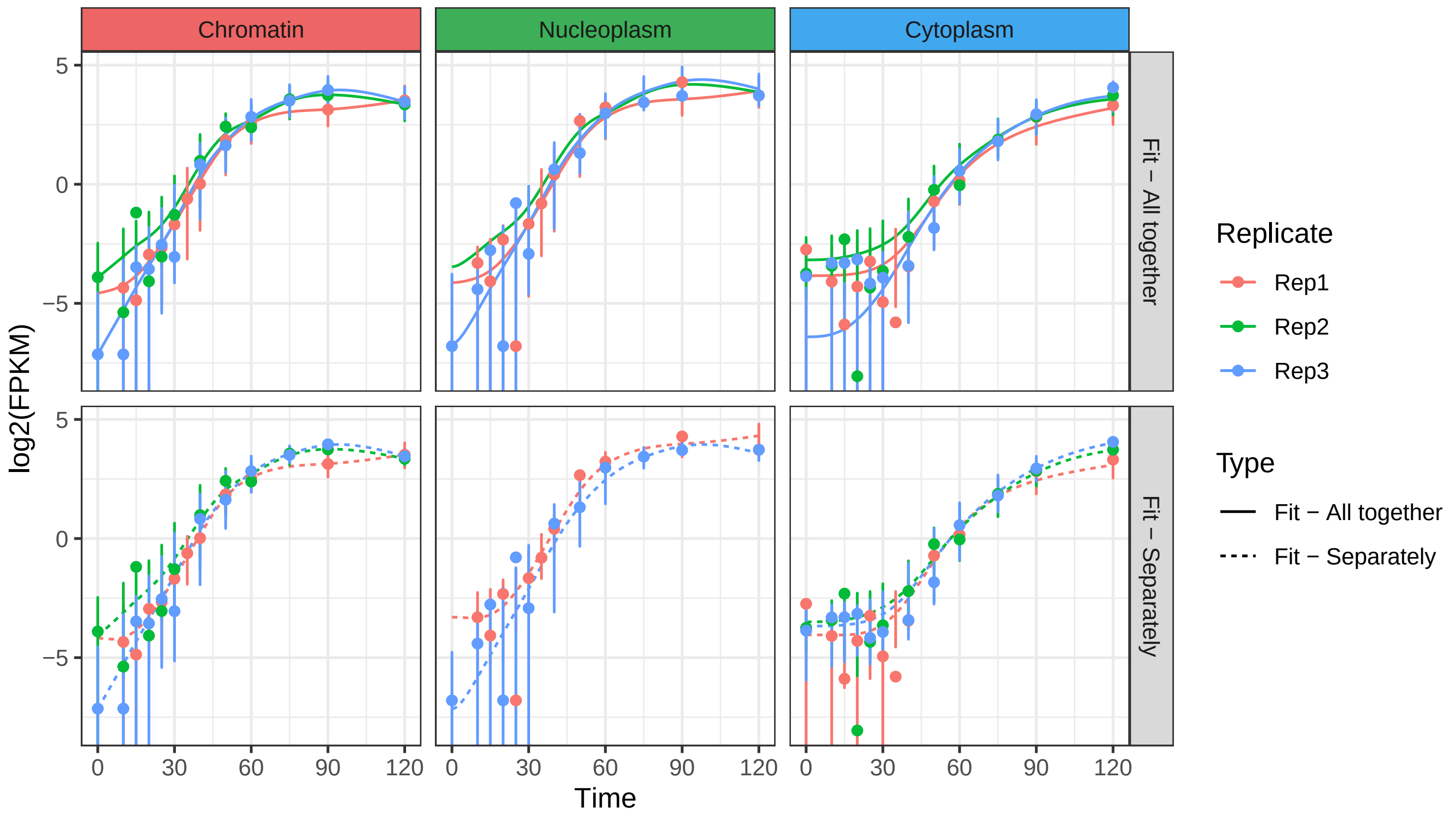

	Together	b1	b2	b3
**−NLL b1 ca**	6.726	3.395		
**−NLL b1 np**	15.25	15.11		
**−NLL b1 cyto**	18.33	17.7		
**−NLL b2 ca**	16.22		12.09	
**−NLL b2 np**				
**−NLL b2 cyto**	19.03		14.57	
**−NLL b3 ca**	10.3			6.949
**−NLL b3 np**	18.34			14.75
**−NLL b3 cyto**	32.23			7.978
Total	136.4	36.21	26.66	29.67
**Total with regul**	138.7	36.53	24.87	27.77

	Together	b1	b2	b3
spar	0.4255	0.4086	0.4646	4.528e−01
σ_b	0.2107	0.1718	0.003183	8.955e−08
σ_t	3.5250	1.1210	4.913	4.562e+00
ca_{0,b_1}	−4.5460	−4.1380		
$\log_{10}(k_1')$	−0.3715	−0.4488		−5.792e−01
$\log_{10}(k_2)$	−0.5062	−0.7108	−1.397 or −1.429	−5.835e−01
$\log_{10}(k_2')$	−1.7490	−1.7410		−1.676e+00
$\log_{10}(k_{deg})$	−1.8370	−1.5170	−1.429 or −1.397	−2.721e+00
$\log_{10}(k_1' k_2')$	−2.1210	−2.1900	−2.646	−2.255e+00
$\log_{10}(k_1'/k_2)$	0.1347	0.2620		4.310e−03
transport $= \log_{10}(k_1' k_2'/k_2)$	−1.6150	−1.4790	−1.249 or −1.217	−1.671e+00

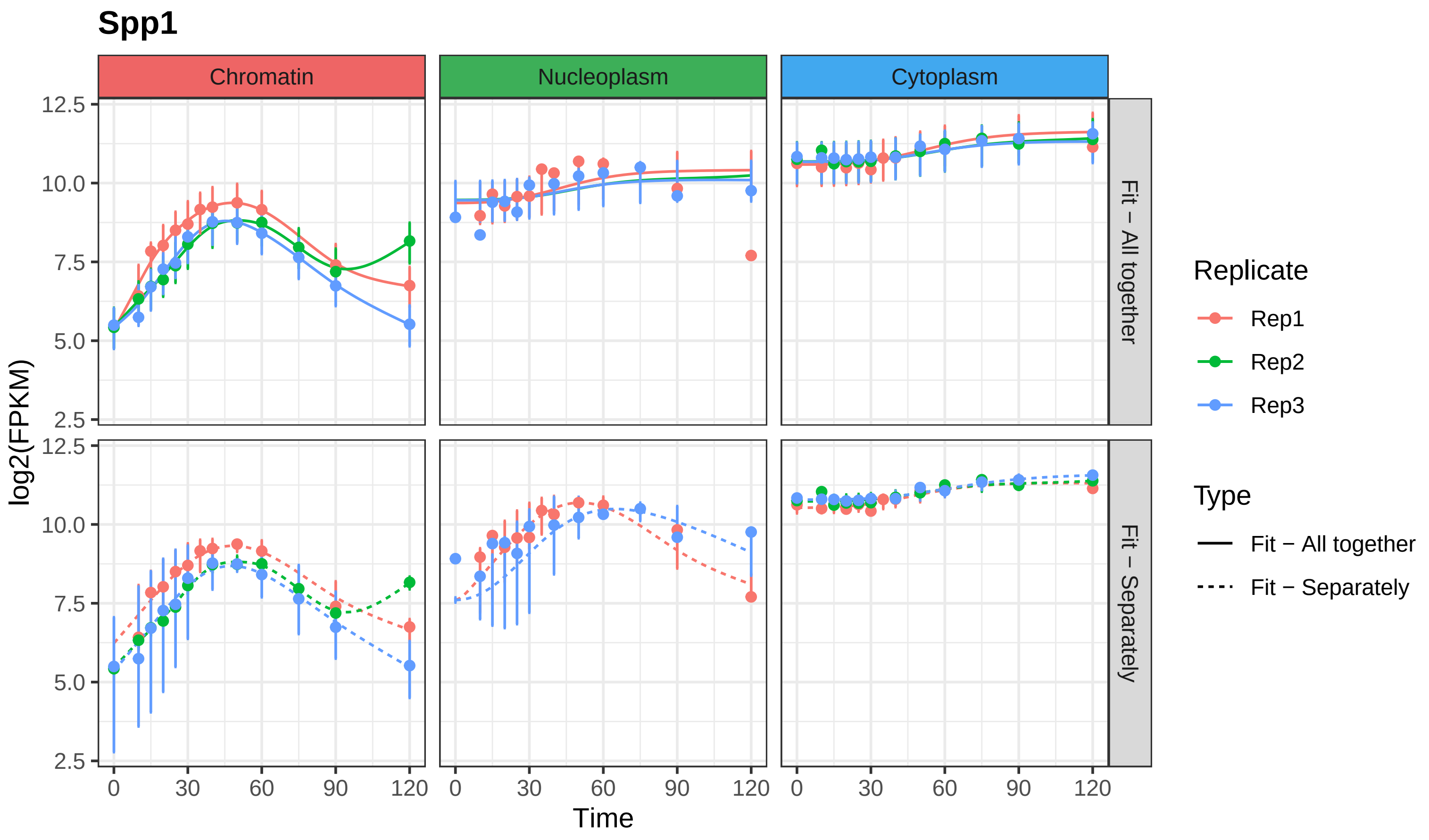

	Together	b1	b2	b3
**−NLL b1 ca**	−0.8478	−1.917		
**−NLL b1 np**	28.95	4.901		
**−NLL b1 cyto**	−0.4889	−9.674		
**−NLL b2 ca**	−1.934		−10.78	
**−NLL b2 np**				
**−NLL b2 cyto**	−1.443		−7.144	
**−NLL b3 ca**	−1.157			3.871
**−NLL b3 np**	9.659			11.28
**−NLL b3 cyto**	−1.732			−17.43
**Total**	31.01	−6.691	−17.92	−2.283
**Total with regul**	32.22	−6.104	−18.76	−1.482

	Together	b1	b2	b3
spar	4.084e−01	0.53730	0.3714	0.52930
σ_b	2.214e−01	0.06201	0.06996	0.02114
σ_t	3.351e−06	5.33700	7.347e−05	9.58800
ca_{0,b_1}	5.344e+00	6.28900		
$\log_{10}(k_1')$	−1.640e+00	0.05592		−0.72180
$\log_{10}(k_2)$	−2.853e+00	−0.35840	−0.3626 or −2.939	−1.39900
$\log_{10}(k_2')$	−6.545e−01	−1.81200		−1.76900
$\log_{10}(k_{deg})$	−1.022e+00	−2.69100	−2.939 or −0.3626	−2.72700
$\log_{10}(k_1'k_2')$	−2.294e+00	−1.75600	−1.713	−2.49100
$\log_{10}(k_1'/k_2)$	1.213e+00	0.41430		0.67680
$\text{transport} = \log_{10}(k_1'k_2'/k_2)$	5.586e−01	−1.39800	−1.351 or 1.226	−1.09300

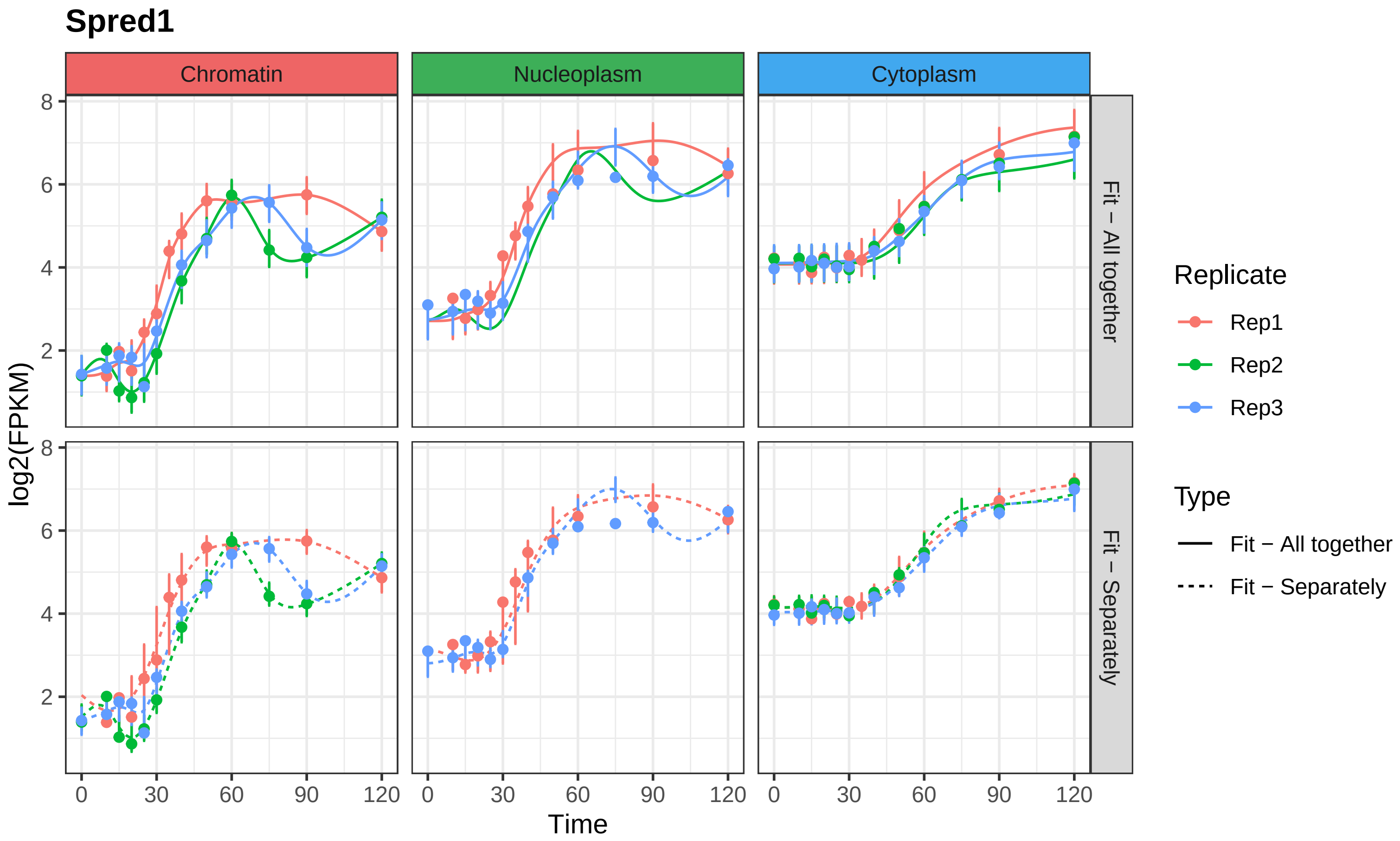

	Together	b1	b2	b3
**−NLL b1 ca**	−3.409	−0.3918		
**−NLL b1 np**	9.923	2.54		
**−NLL b1 cyto**	−2.108	−7.589		
**−NLL b2 ca**	−4.938		−5.962	
**−NLL b2 np**				
**−NLL b2 cyto**	0.04317		−4.65	
**−NLL b3 ca**	−3.157			−3.137
**−NLL b3 np**	3.481			−2.14
**−NLL b3 cyto**	−5.131			−7.538
Total	−5.296	−5.44	−10.61	−12.82
**Total with regul**	0.2583	−4.332	−6.775	−7.537

	Together	b1	b2	b3
spar	0.2744000	0.42840	0.2809	0.2619000
σ_b	0.1525000	0.09513	0.0934	0.1020000
σ_t	0.0001746	2.82300	8.65e−05	0.0004857
ca_{0,b_1}	1.3970000	2.11900		
$\log_{10}(k_1')$	−0.3907000	−0.61260		−0.3175000
$\log_{10}(k_2)$	−0.7881000	−0.94100	−0.618 or −1.846	−0.7338000
$\log_{10}(k_2')$	−1.6140000	−1.56700		−1.6220000
$\log_{10}(k_{deg})$	−2.0230000	−1.87400	−1.846 or −0.618	−1.9920000
$\log_{10}(k_1'k_2')$	−2.0050000	−2.18000	−1.67	−1.9390000
$\log_{10}(k_1'/k_2)$	0.3974000	0.32830		0.4163000
transport $= \log_{10}(k_1'k_2'/k_2)$	−1.2160000	−1.23900	−1.052 or 0.1768	−1.2060000

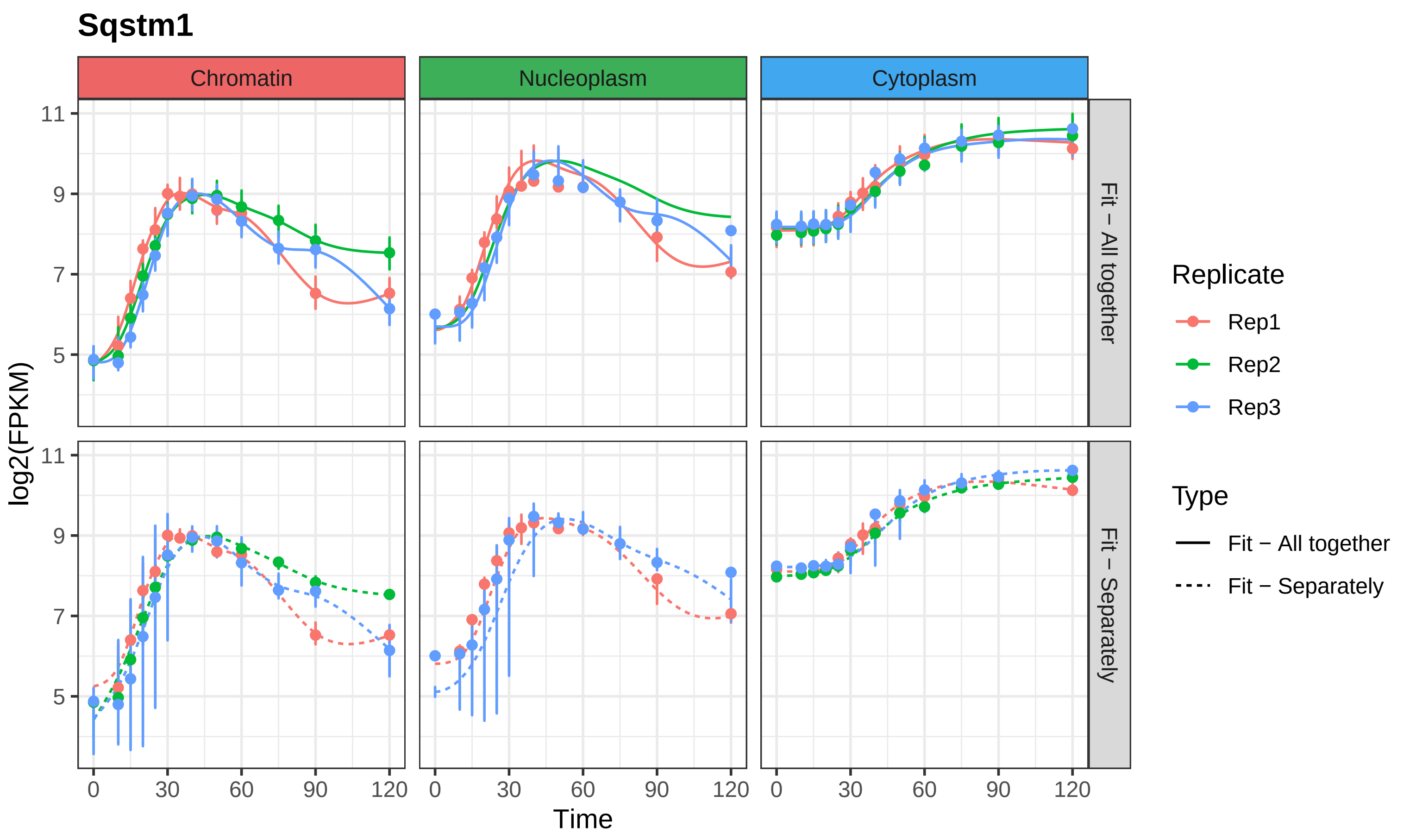

	Together	b1	b2	b3
**−NLL b1 ca**	−4.921	−5.79		
**−NLL b1 np**	5.151	−0.08883		
**−NLL b1 cyto**	−7.123	−12.79		
**−NLL b2 ca**	−6.585		−8.874	
**−NLL b2 np**				
**−NLL b2 cyto**	−4.398		−18.55	
**−NLL b3 ca**	−6.774			1.984
**−NLL b3 np**	10.45			7.015
**−NLL b3 cyto**	−2.442			−13.75
Total	−16.64	−18.67	−27.42	−4.755
**Total with regul**	−14.46	−18.78	−29.57	−6.299

	Together	b1	b2	b3
spar	0.327800	0.35740	0.429	4.256e−01
σ_b	0.136600	0.04402	0.003675	2.229e−07
σ_t	0.001129	2.55800	2.079	5.726e+00
ca_{0,b_1}	4.778000	5.32700		
$\log_{10}(k_1')$	−0.386100	−0.68120		−8.094e−01
$\log_{10}(k_2)$	−0.650800	−0.85090	−0.09567 or −2.405	−1.010e+00
$\log_{10}(k_2')$	−1.473000	−1.30400		−1.330e+00
$\log_{10}(k_{deg})$	−2.219000	−1.99500	−2.405 or −0.09567	−2.264e+00
$\log_{10}(k_1'k_2')$	−1.859000	−1.98600	−1.422	−2.140e+00
$\log_{10}(k_1'/k_2)$	0.264700	0.16970		2.008e−01
$\text{transport} = \log_{10}(k_1'k_2'/k_2)$	−1.209000	−1.13500	−1.326 or 0.9834	−1.129e+00

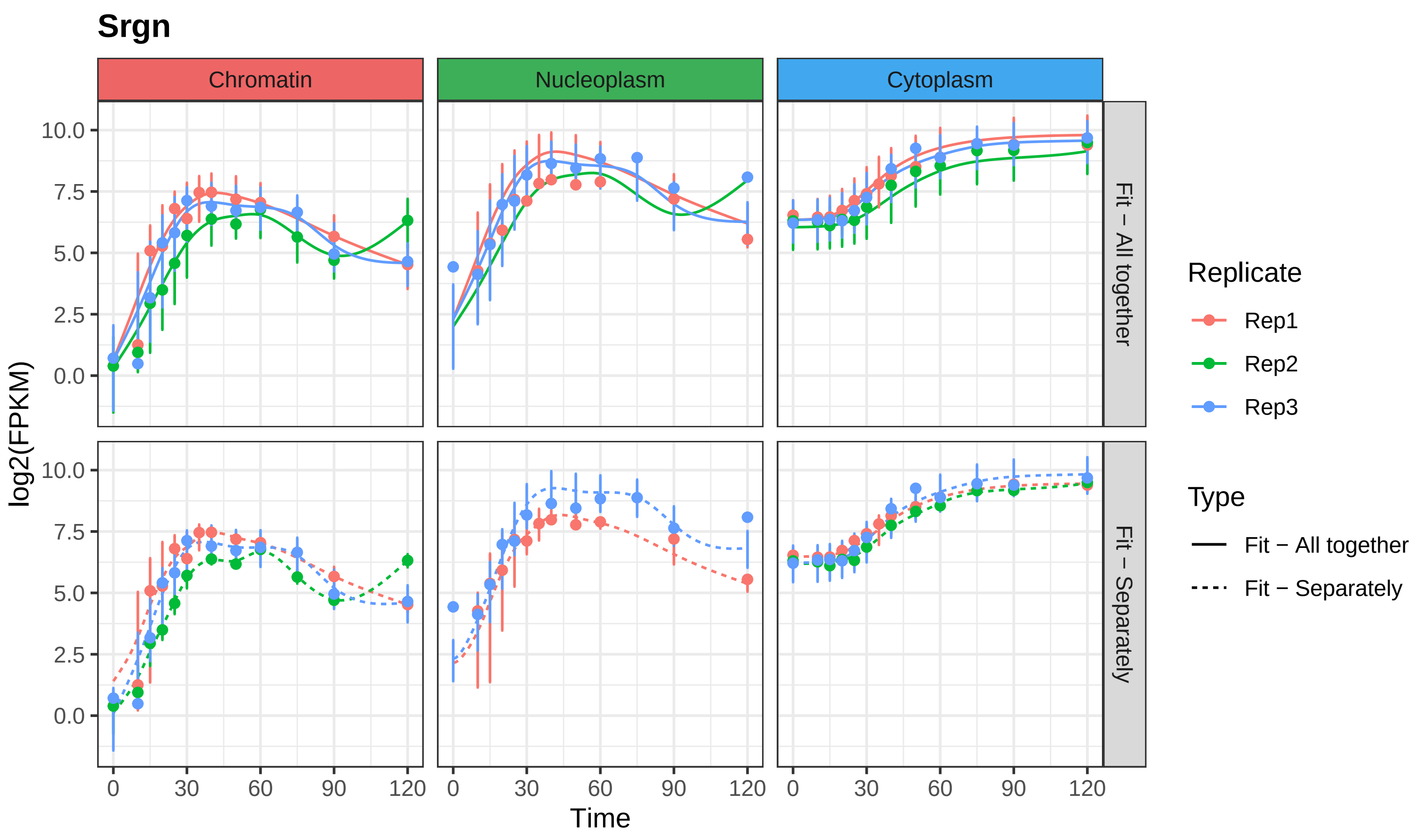

	Together	b1	b2	b3
**−NLL b1 ca**	6.605	0.03376		
**−NLL b1 np**	22.23	8.51		
**−NLL b1 cyto**	4.107	−10.4		
**−NLL b2 ca**	6.301		−4.254	
**−NLL b2 np**				
**−NLL b2 cyto**	3.22		−9.645	
**−NLL b3 ca**	8.62			6.356
**−NLL b3 np**	26.1			13.57
**−NLL b3 cyto**	3.249			1.785
**Total**	80.42	−1.859	−13.9	21.71
**Total with regul**	83.19	−1.123	−11.64	23.37

	Together	b1	b2	b3
spar	0.4255	0.34560	0.3016	0.3893
σ_b	0.2881	0.03377	0.05372	0.2523
σ_t	3.1060	3.91200	1.129	1.6660
ca_{0,b_1}	0.6412	1.46800		
$\log_{10}(k_1')$	7.5750	−0.24090		0.1452
$\log_{10}(k_2)$	7.0700	−0.46650	3.751 or −2.719	−0.5285
$\log_{10}(k_2')$	−1.5110	−1.33600		−1.5920
$\log_{10}(k_{deg})$	−2.7300	−2.63900	−2.719 or 3.751	−2.7690
$\log_{10}(k_1'k_2')$	6.0640	−1.57700	2.855	−1.4470
$\log_{10}(k_1'/k_2)$	0.5049	0.22560		0.6737
$\text{transport} = \log_{10}(k_1'k_2'/k_2)$	−1.0060	−1.11100	−0.8966 or 5.574	−0.9186

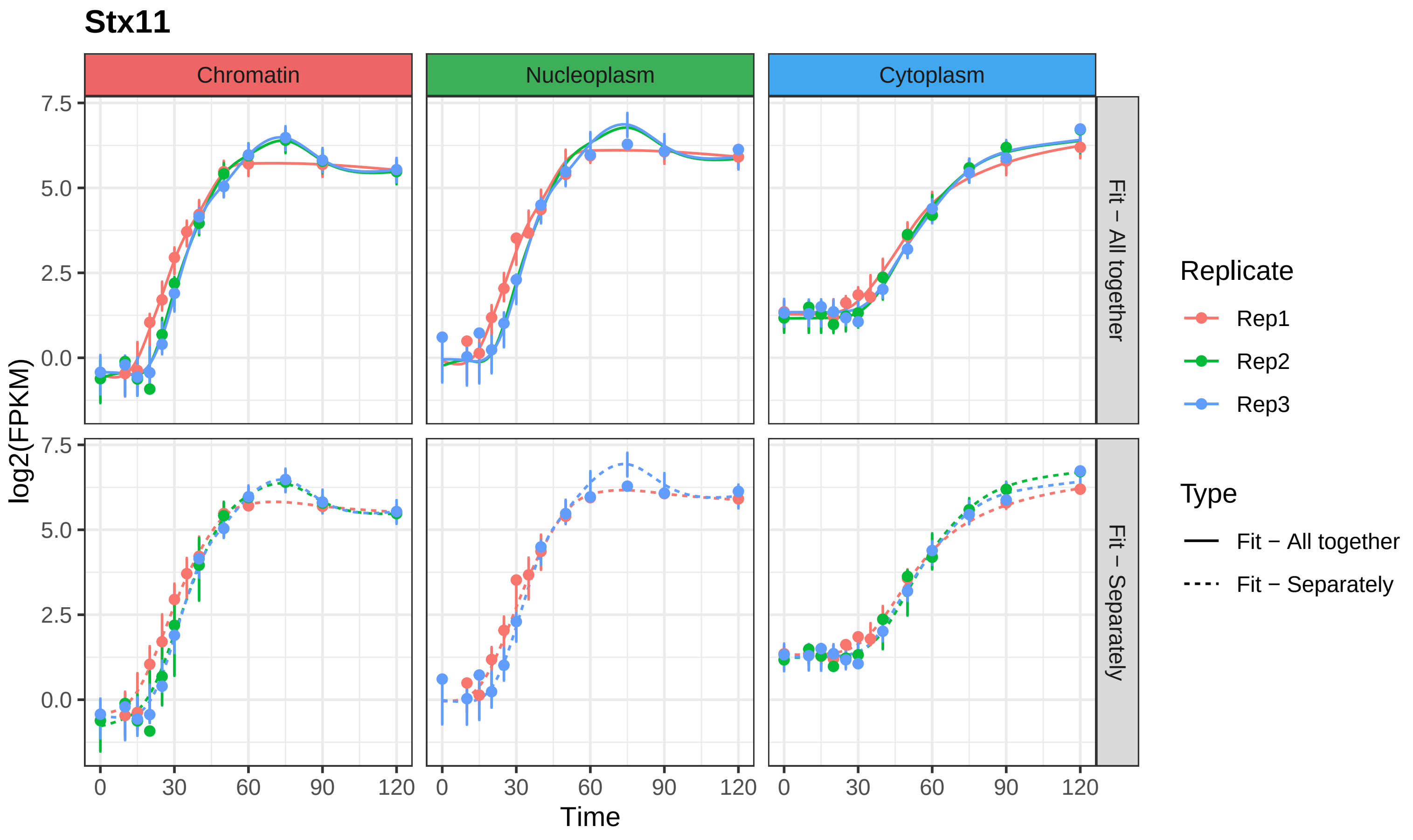

	Together	b1	b2	b3
**−NLL b1 ca**	−5.807	−5.889		
**−NLL b1 np**	2.421	−2.128		
**−NLL b1 cyto**	−4.178	−9.323		
**−NLL b2 ca**	−2.984		0.8034	
**−NLL b2 np**				
**−NLL b2 cyto**	−1.874		−4.266	
**−NLL b3 ca**	−5.732			−2.893
**−NLL b3 np**	9.536			2.64
**−NLL b3 cyto**	−3.089			−2.858
Total	−11.71	−17.34	−3.463	−3.112
**Total with regul**	−6.949	−18.54	−4.707	−1.923

	Together	b1	b2	b3
spar	2.786e−01	4.096e−01	0.3811	0.3317000
σ_b	1.209e−01	1.086e−06	3.222e−06	0.1188000
σ_t	7.294e−05	1.800e+00	2.494	0.0005264
ca_{0,b_1}	−4.750e−01	−3.262e−01		
$\log_{10}(k_1')$	3.265e−01	−2.939e−01		0.3877000
$\log_{10}(k_2)$	2.114e−01	−3.984e−01	−0.6864 or −2.108	0.2489000
$\log_{10}(k_2')$	−1.727e+00	−1.716e+00		−1.7470000
$\log_{10}(k_{deg})$	−2.144e+00	−2.130e+00	−2.108 or −0.6864	−2.1350000
$\log_{10}(k_1'k_2')$	−1.400e+00	−2.010e+00	−2.186	−1.3600000
$\log_{10}(k_1'/k_2)$	1.151e−01	1.045e−01		0.1388000
$\text{transport} = \log_{10}(k_1'k_2'/k_2)$	−1.612e+00	−1.611e+00	−1.499 or −0.0783	−1.6080000

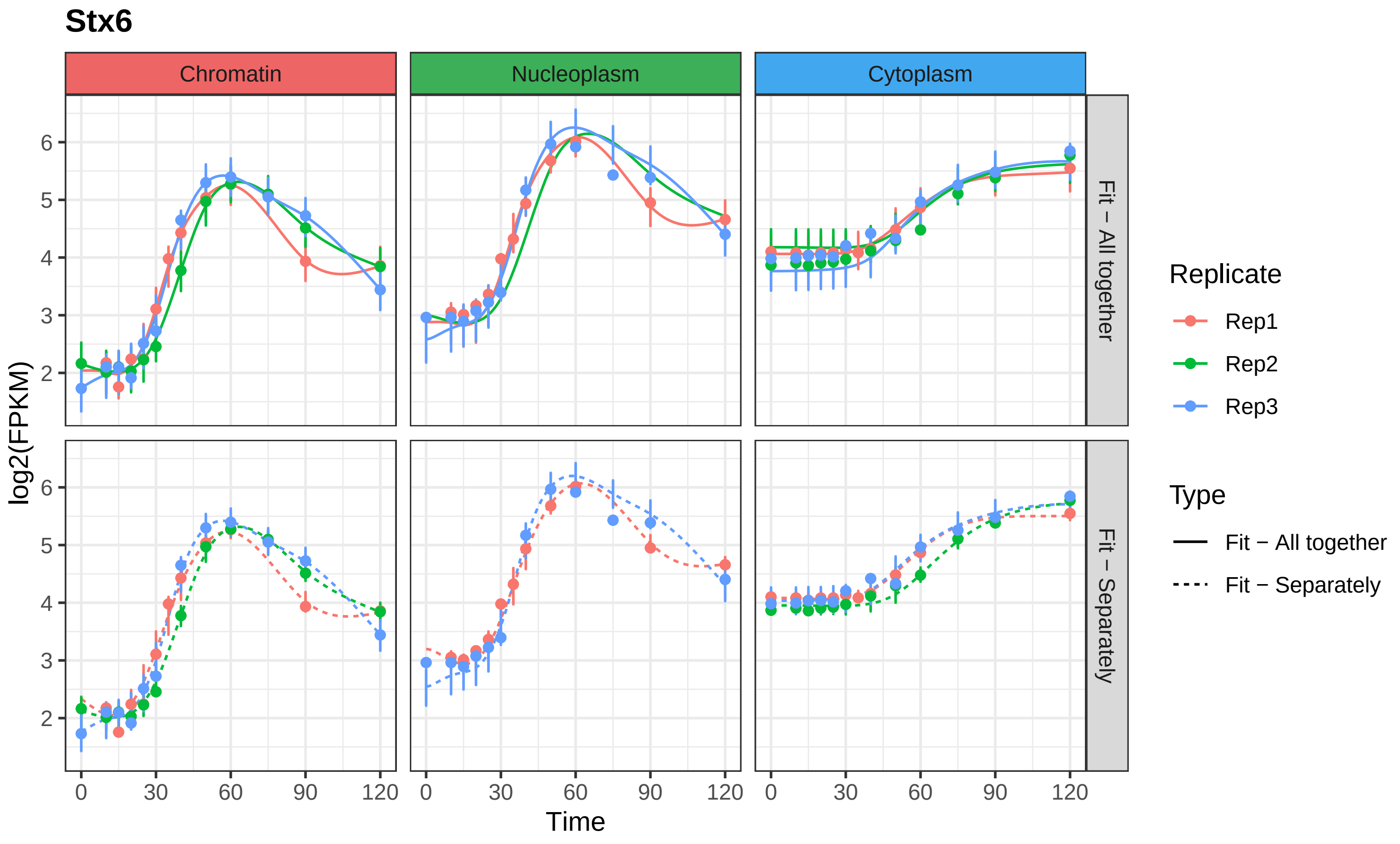

	Together	b1	b2	b3
**−NLL b1 ca**	−6.477	−5.937		
**−NLL b1 np**	−4.641	−10.89		
**−NLL b1 cyto**	−10.18	−22.34		
**−NLL b2 ca**	−9.117		−11.1	
**−NLL b2 np**				
**−NLL b2 cyto**	1.213		−12.18	
**−NLL b3 ca**	−7.087			−7.015
**−NLL b3 np**	1.892			−3.04
**−NLL b3 cyto**	1.603			−8.292
Total	−32.8	−39.16	−23.28	−18.35
**Total with regul**	−32.26	−40.99	−24.66	−18.01

	Together	b1	b2	b3
spar	0.3493000	4.495e−01	0.384	0.34140
σ_b	0.1090000	3.168e−07	0.03632	0.07614
σ_t	0.0001624	1.229e+00	9.686e−08	0.65900
ca_{0,b_1}	2.0320000	2.404e+00		
$\log_{10}(k_1')$	−0.0560700	−2.619e−01		0.04707
$\log_{10}(k_2)$	−0.3095000	−5.215e−01	−1.143 or −2.041	−0.18870
$\log_{10}(k_2')$	−1.9040000	−1.819e+00		−1.92700
$\log_{10}(k_{deg})$	−2.2590000	−2.086e+00	−2.041 or −1.143	−2.37500
$\log_{10}(k_1'k_2')$	−1.9600000	−2.081e+00	−2.634	−1.88000
$\log_{10}(k_1'/k_2)$	0.2534000	2.596e−01		0.23570
transport $= \log_{10}(k_1'k_2'/k_2)$	−1.6500000	−1.559e+00	−1.49 or −0.5931	−1.69200

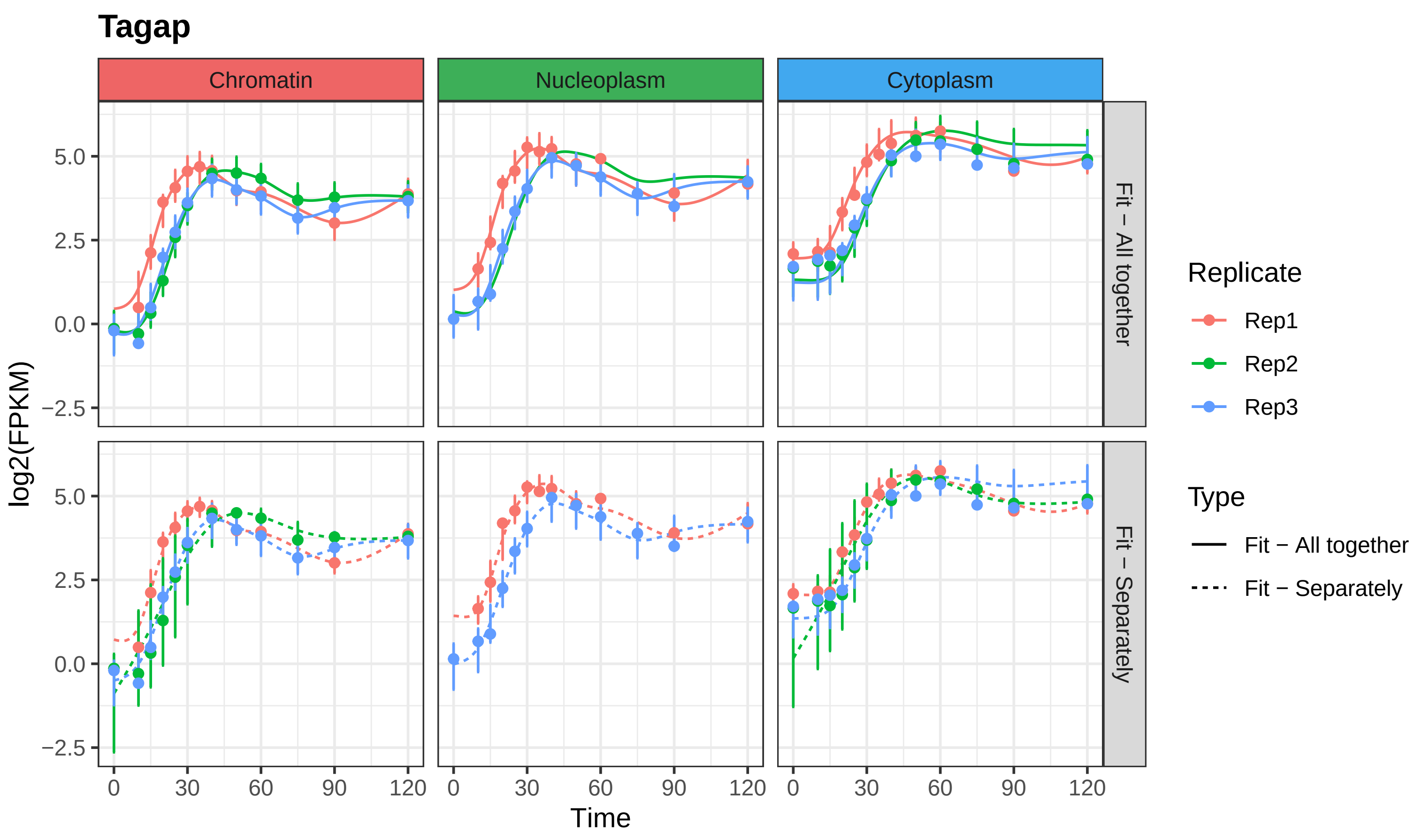

	Together	b1	b2	b3
**−NLL b1 ca**	−2.488	−5.07		
**−NLL b1 np**	−0.1411	−0.7739		
**−NLL b1 cyto**	−0.1393	−4.388		
**−NLL b2 ca**	−4.704		−1.318	
**−NLL b2 np**				
**−NLL b2 cyto**	7.314		4.132	
**−NLL b3 ca**	−3.107			−1.387
**−NLL b3 np**	−1.679			−1.292
**−NLL b3 cyto**	8.338			7.449
Total	3.393	−10.23	2.814	4.771
**Total with regul**	6.361	−8.15	1.818	6.466

	Together	b1	b2	b3
spar	3.148e−01	0.31380	0.5155	0.338000
σ_b	1.606e−01	0.09322	0.0142	0.173500
σ_t	9.736e−05	1.11400	4.767	0.002391
ca_{0,b_1}	5.191e−01	0.79350		
$\log_{10}(k_1')$	5.016e+00	−0.06186		5.285000
$\log_{10}(k_2)$	4.847e+00	−0.27500	2.348 or 2.319	5.139000
$\log_{10}(k_2')$	−9.004e−01	−0.90700		−0.940200
$\log_{10}(k_{deg})$	−1.184e+00	−1.09700	2.319 or 2.348	−1.351000
$\log_{10}(k_1' k_2')$	4.116e+00	−0.96890	4.984	4.345000
$\log_{10}(k_1'/k_2)$	1.696e−01	0.21310		0.146000
$\text{transport} = \log_{10}(k_1' k_2'/k_2)$	−7.308e−01	−0.69390	2.636 or 2.665	−0.794200

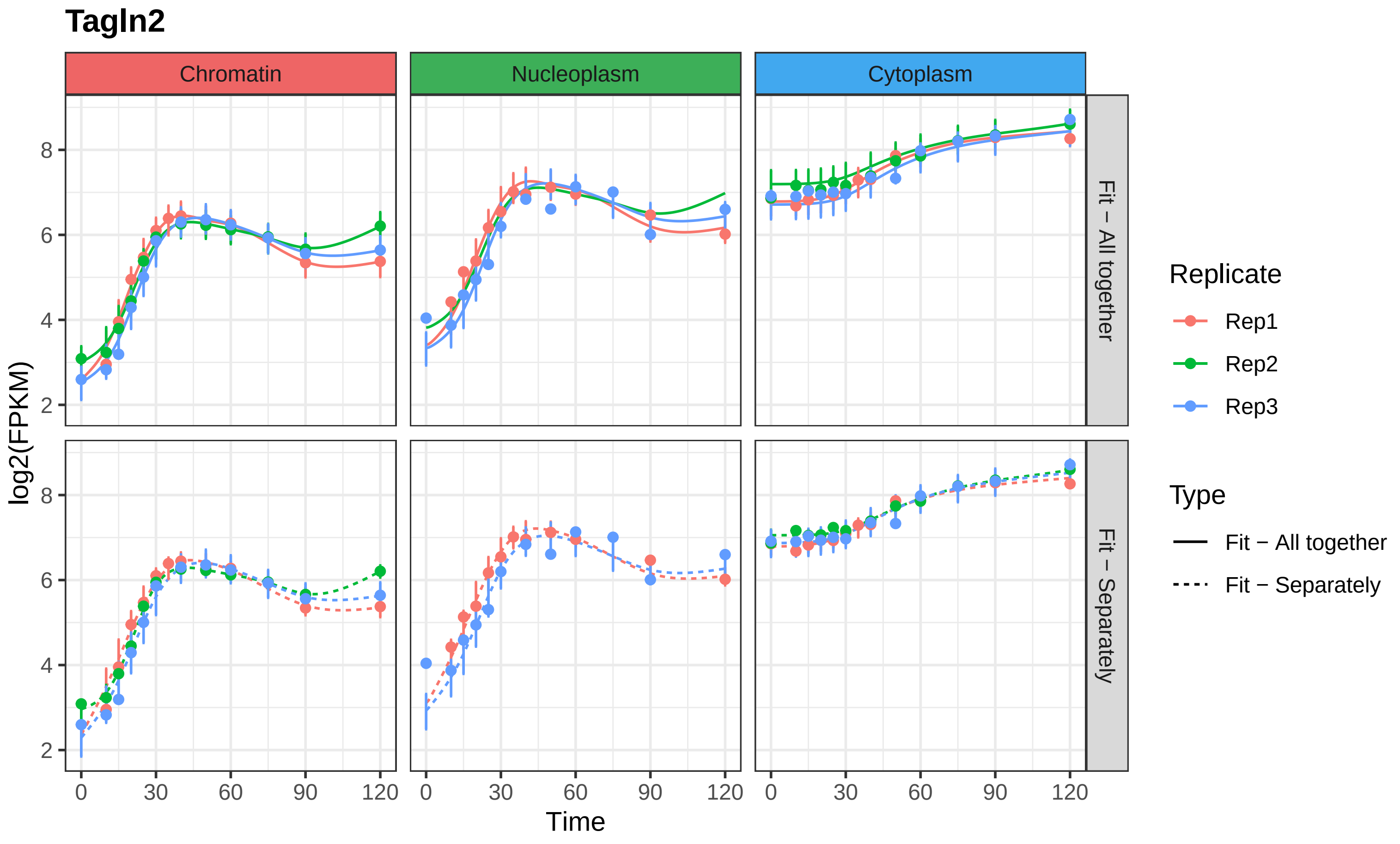

	Together	b1	b2	b3
**−NLL b1 ca**	−5.825	−5.568		
**−NLL b1 np**	−0.3074	−2.39		
**−NLL b1 cyto**	−8.49	−11.91		
**−NLL b2 ca**	−7.318		−13.17	
**−NLL b2 np**				
**−NLL b2 cyto**	−4.889		−12.07	
**−NLL b3 ca**	−6.343			−3.777
**−NLL b3 np**	12.41			2.331
**−NLL b3 cyto**	−3.034			−6.346
Total	−23.8	−19.87	−25.24	−7.792
**Total with regul**	−24.2	−21.43	−24.5	−9.34

	Together	b1	b2	b3
spar	0.3806	0.46710	0.3285	0.4250
σ_b	0.1173	0.06981	0.04482	0.1110
σ_t	0.8875	1.48100	0.1021	1.2100
ca_{0,b_1}	2.6700	2.45900		
$\log_{10}(k_1')$	0.2212	0.56600		1.1370
$\log_{10}(k_2)$	−0.0232	0.34180	−0.7986 or −2.469	0.9452
$\log_{10}(k_2')$	−1.5220	−1.54800		−1.4800
$\log_{10}(k_{deg})$	−2.5390	−2.65700	−2.469 or −0.7986	−2.6670
$\log_{10}(k_1' k_2')$	−1.3010	−0.98170	−2.035	−0.3430
$\log_{10}(k_1'/k_2)$	0.2444	0.22420		0.1921
transport = $\log_{10}(k_1' k_2'/k_2)$	−1.2780	−1.32400	−1.236 or 0.4344	−1.2880

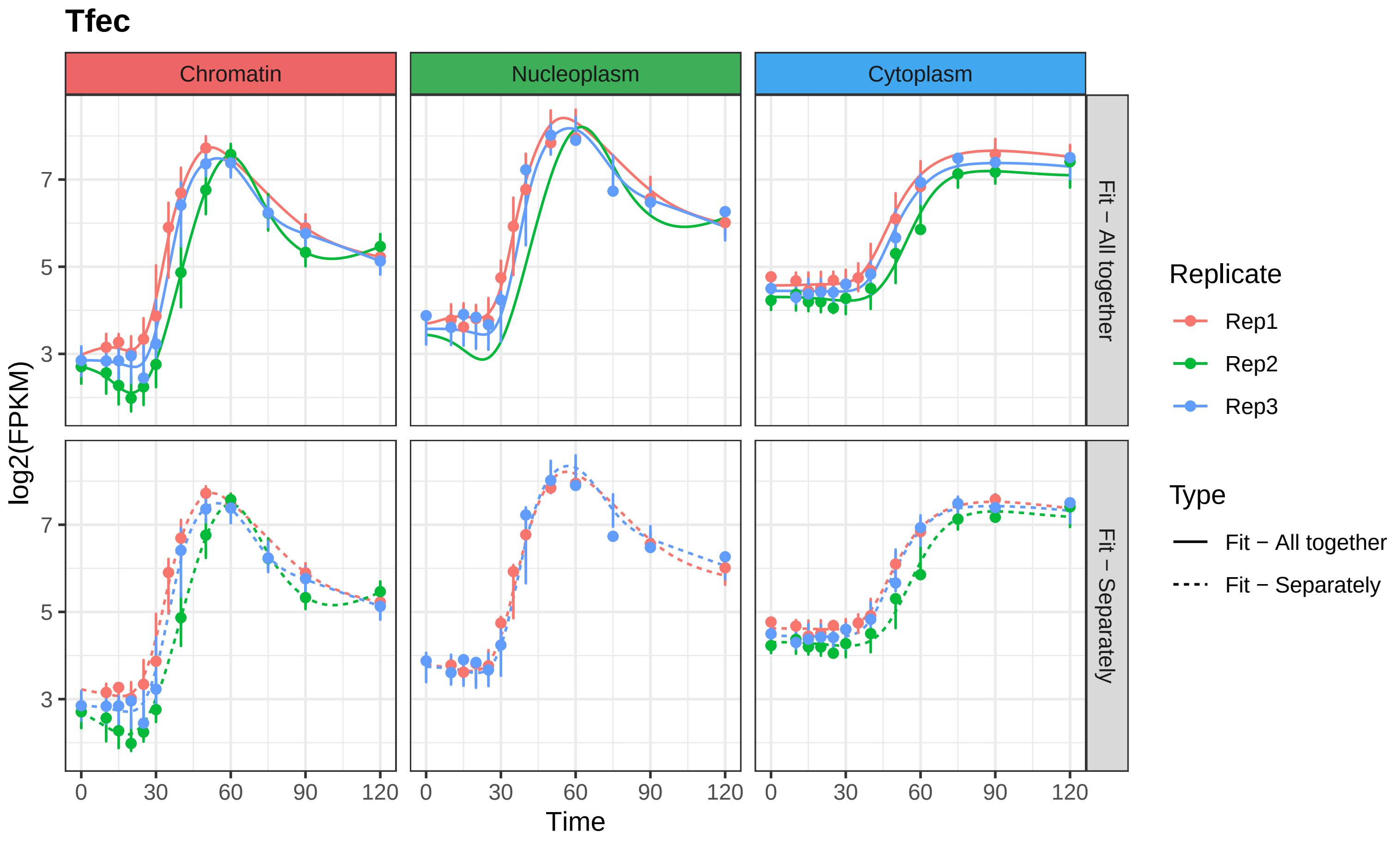

	Together	b1	b2	b3
**−NLL b1 ca**	−6.02	−6.497		
**−NLL b1 np**	1.201	−2.622		
**−NLL b1 cyto**	−6.383	−11.22		
**−NLL b2 ca**	−7.494		−4.888	
**−NLL b2 np**				
**−NLL b2 cyto**	−4.631		−4.175	
**−NLL b3 ca**	−5.75			−2.854
**−NLL b3 np**	9.02			1.894
**−NLL b3 cyto**	−7.247			−6.987
Total	−27.3	−20.34	−9.063	−7.947
**Total with regul**	−23.7	−20.4	−10.04	−6.768

	Together	b1	b2	b3
spar	0.29080	0.35430	0.3795	0.32780
σ_b	0.09542	0.05958	0.07897	0.09523
σ_t	1.48300	1.20900	1.332	1.67400
ca_{0,b_1}	2.97500	3.22300		
$\log_{10}(k_1')$	−0.23860	−0.40080		−0.10850
$\log_{10}(k_2)$	−0.45600	−0.56870	−0.723 or −1.855	−0.37340
$\log_{10}(k_2')$	−1.65300	−1.63900		−1.67300
$\log_{10}(k_{deg})$	−1.91500	−1.89200	−1.855 or −0.723	−1.88700
$\log_{10}(k_1' k_2')$	−1.89200	−2.04000	−2.095	−1.78200
$\log_{10}(k_1'/k_2)$	0.21740	0.16790		0.26480
transport $= \log_{10}(k_1' k_2'/k_2)$	−1.43600	−1.47100	−1.372 or −0.2406	−1.40800

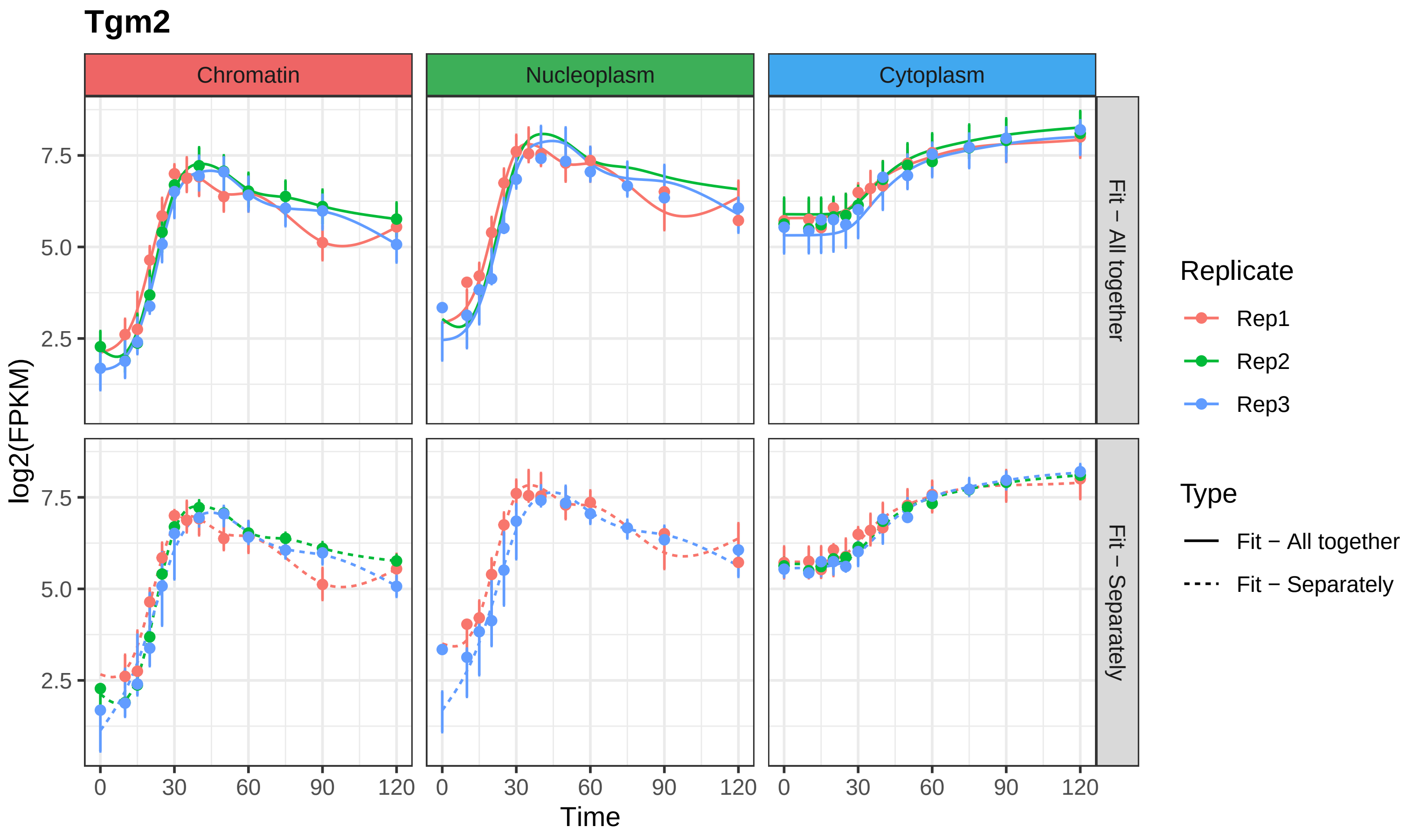

	Together	b1	b2	b3
**−NLL b1 ca**	−2.518	−0.9981		
**−NLL b1 np**	5.511	3.902		
**−NLL b1 cyto**	−3.812	−4.442		
**−NLL b2 ca**	−3.894		−11.49	
**−NLL b2 np**				
**−NLL b2 cyto**	−0.93		−9.256	
**−NLL b3 ca**	−4.004			−1.185
**−NLL b3 np**	12.65			3.337
**−NLL b3 cyto**	−0.05041			−7.198
Total	2.955	−1.538	−20.75	−5.046
**Total with regul**	6.491	−0.6202	−16.27	−6.625

	Together	b1	b2	b3
spar	3.147e−01	3.527e−01	0.2678	0.41100
σ_b	1.636e−01	1.493e−01	0.05604	0.08296
σ_t	5.847e−06	2.218e−07	0.0001124	2.13800
ca_{0,b_1}	2.143e+00	2.736e+00		
$\log_{10}(k_1')$	1.516e+00	1.441e+00		6.59100
$\log_{10}(k_2)$	1.271e+00	1.188e+00	6.386 or −2.571	6.42600
$\log_{10}(k_2')$	−1.698e+00	−1.642e+00		−1.60100
$\log_{10}(k_{deg})$	−2.560e+00	−2.315e+00	−2.571 or 6.386	−2.77100
$\log_{10}(k_1'k_2')$	−1.825e−01	−2.010e−01	4.885	4.99000
$\log_{10}(k_1'/k_2)$	2.450e−01	2.527e−01		0.16550
$\text{transport} = \log_{10}(k_1'k_2'/k_2)$	−1.453e+00	−1.389e+00	−1.501 or 7.456	−1.43600

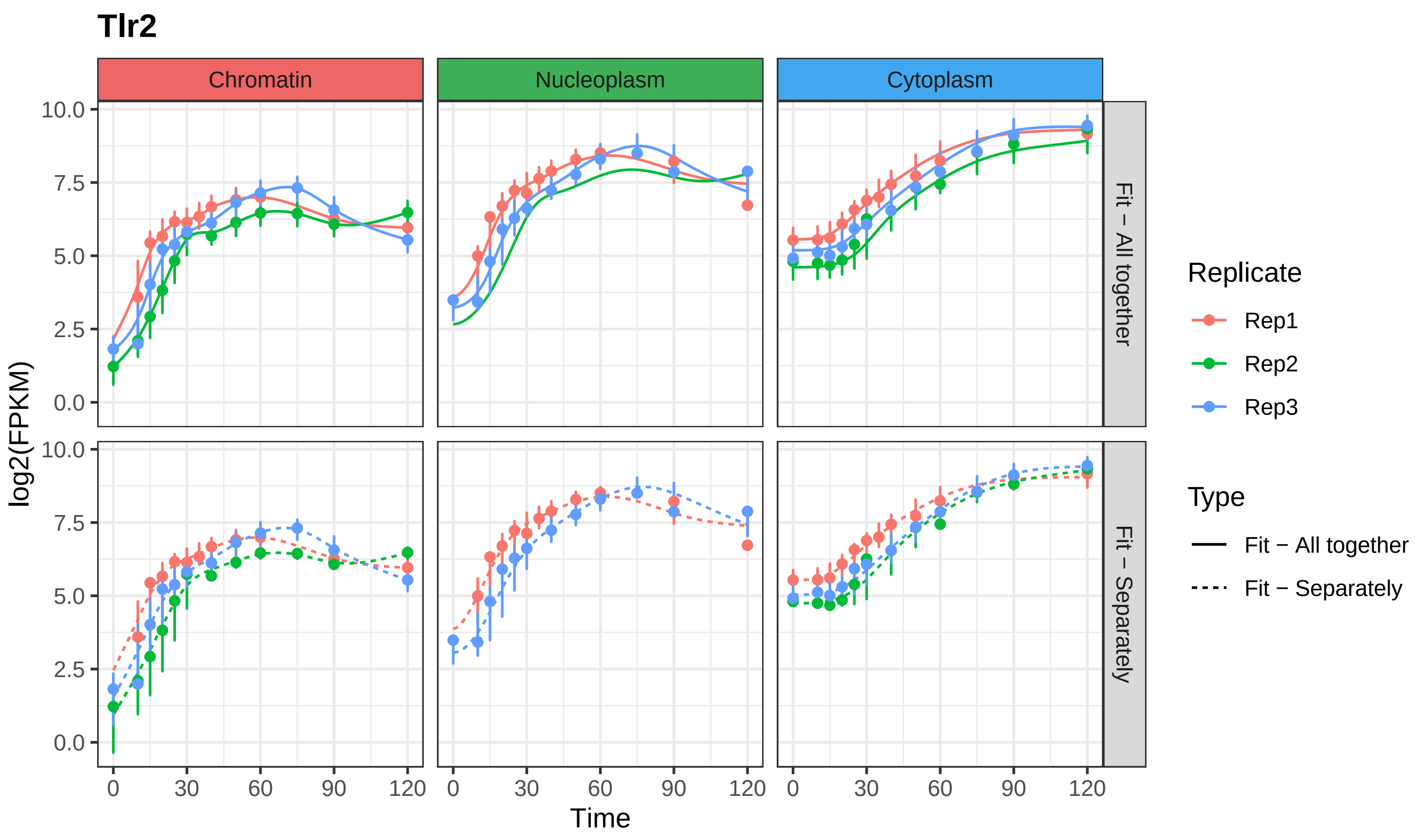

	Together	b1	b2	b3
**−NLL b1 ca**	−4.43	−4.281		
**−NLL b1 np**	4.428	5.179		
**−NLL b1 cyto**	−4.201	−6.591		
**−NLL b2 ca**	−4.554		−2.592	
**−NLL b2 np**				
**−NLL b2 cyto**	5.294		−4.031	
**−NLL b3 ca**	−2.358			−0.6401
**−NLL b3 np**	6.915			6.774
**−NLL b3 cyto**	−1.484			−5.046
Total	−0.3901	−5.693	−6.623	1.088
**Total with regul**	4.509	−5.789	−8.419	0.1705

	Together	b1	b2	b3
spar	0.3020	0.3980	0.4183	0.3941
σ_b	0.1396	0.1192	0.02393	0.1139
σ_t	1.6680	1.5290	3.83	2.5350
ca_{0,b_1}	2.1770	2.4750		
$\log_{10}(k_1')$	−0.3572	−0.2270		−0.5448
$\log_{10}(k_2)$	−0.7977	−0.6534	−1.008 or −1.971	−1.0020
$\log_{10}(k_2')$	−1.3880	−1.4280		−1.4150
$\log_{10}(k_{deg})$	−1.9730	−1.9280	−1.971 or −1.008	−2.0090
$\log_{10}(k_1'k_2')$	−1.7450	−1.6550	−1.834	−1.9600
$\log_{10}(k_1'/k_2)$	0.4405	0.4264		0.4569
transport $= \log_{10}(k_1'k_2'/k_2)$	−0.9476	−1.0020	−0.8253 or 0.1373	−0.9585

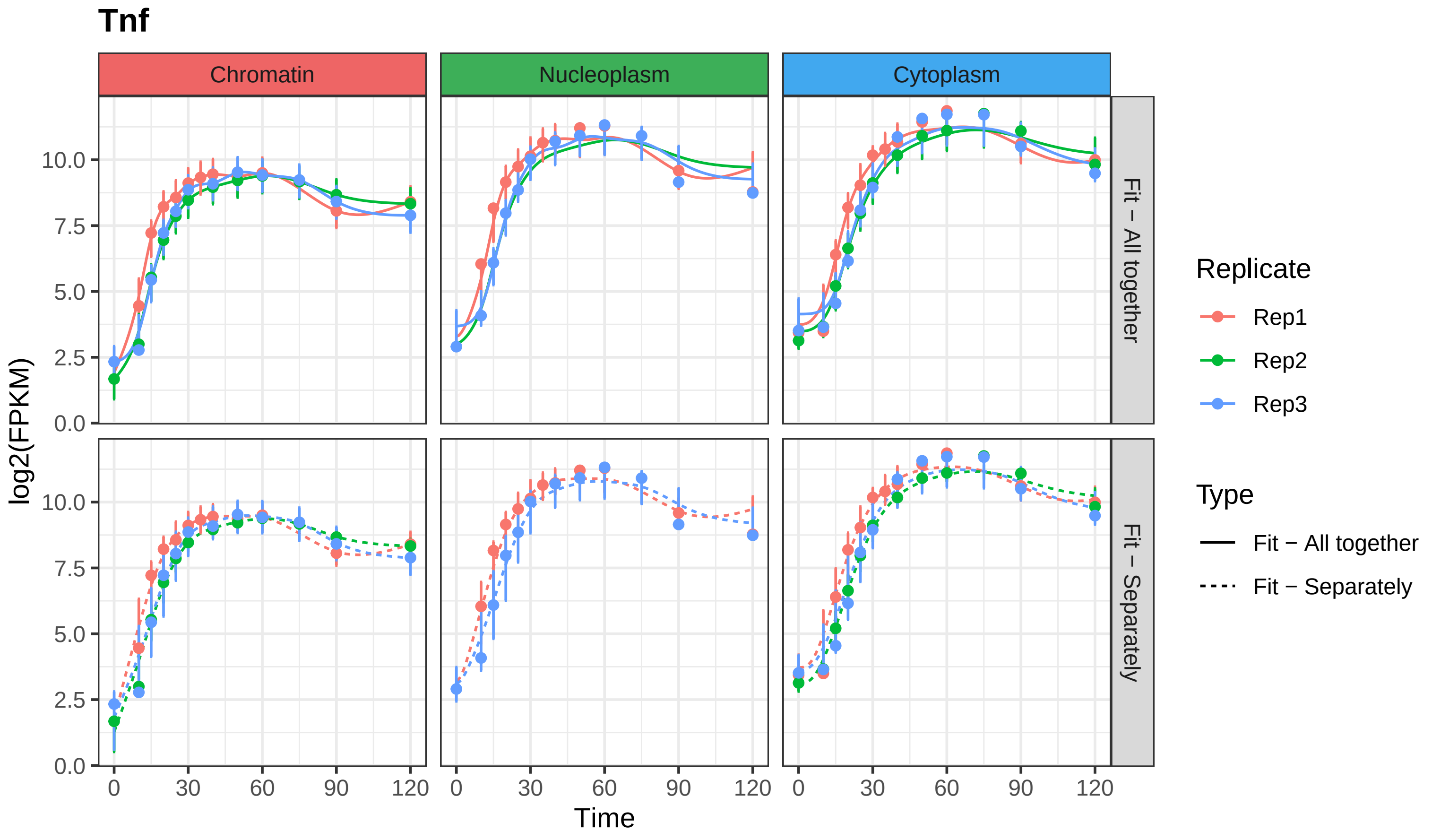

	Together	b1	b2	b3
**−NLL b1 ca**	−1.65	−1.133		
**−NLL b1 np**	5.843	6.81		
**−NLL b1 cyto**	6.422	5.268		
**−NLL b2 ca**	−1.309		−3.327	
**−NLL b2 np**				
**−NLL b2 cyto**	2.27		−0.1581	
**−NLL b3 ca**	−0.4534			2.884
**−NLL b3 np**	7.033			7.618
**−NLL b3 cyto**	11.74			8.982
Total	29.89	10.94	−3.485	19.48
**Total with regul**	47.82	17.54	−3.417	20.97

	Together	b1	b2	b3
spar	0.20150	0.39770	0.3525	0.36240
σ_b	0.21320	0.17170	0.09604	0.21000
σ_t	0.38840	1.47800	1.046	1.84700
ca_{0,b_1}	1.93100	1.75500		
$\log_{10}(k_1')$	0.02254	−0.06675		−0.07739
$\log_{10}(k_2)$	−0.39070	−0.49530	−0.7108 or −0.731	−0.47010
$\log_{10}(k_2')$	−0.81890	−0.79310		−0.69170
$\log_{10}(k_{deg})$	−0.95620	−0.94330	−0.731 or −0.7108	−0.83850
$\log_{10}(k_1'k_2')$	−0.79640	−0.85980	−0.8885	−0.76910
$\log_{10}(k_1'/k_2)$	0.41320	0.42860		0.39280
transport $= \log_{10}(k_1'k_2'/k_2)$	−0.40570	−0.36450	−0.1777 or −0.1575	−0.29890

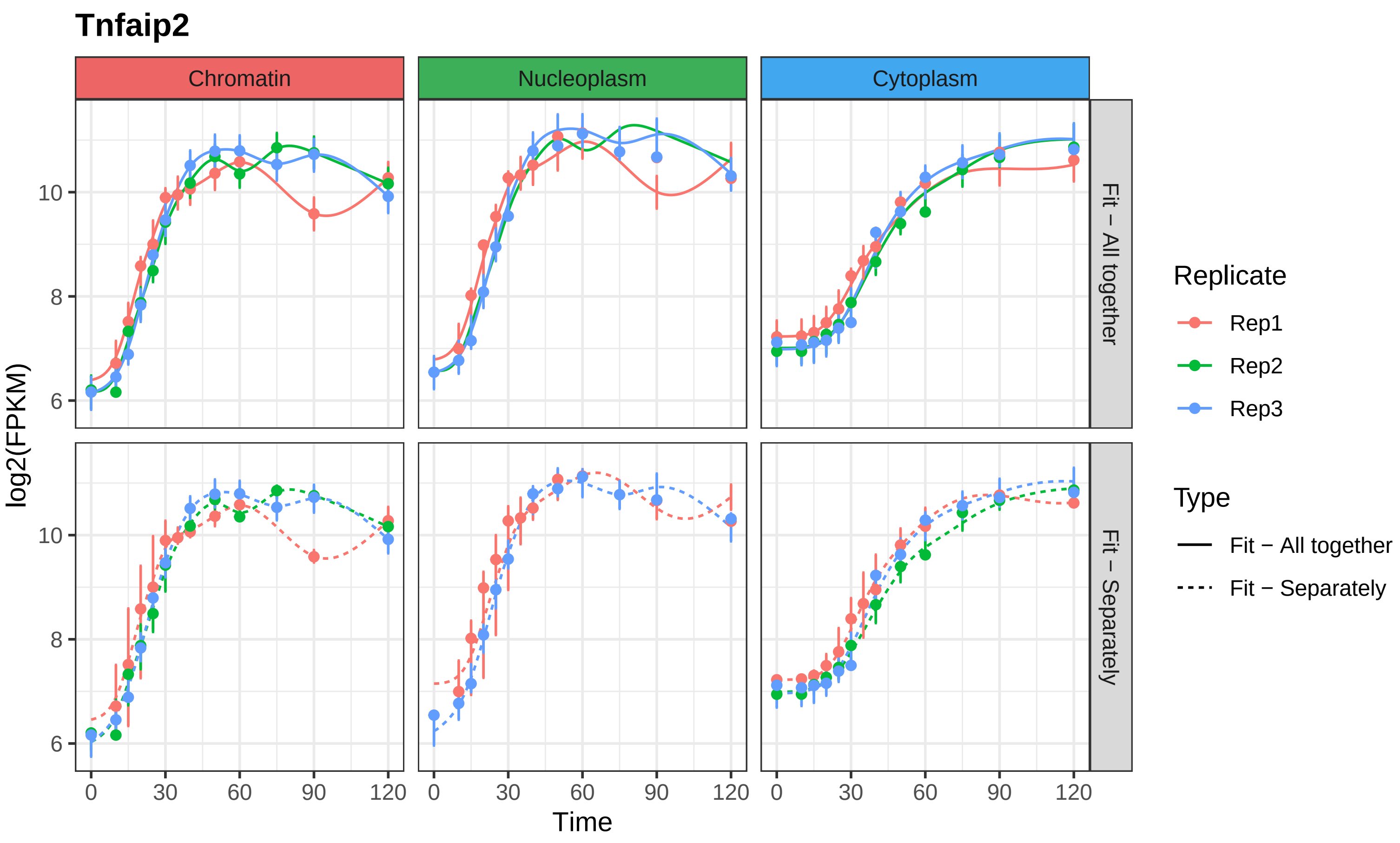

	Together	b1	b2	b3
**−NLL b1 ca**	−8.565	−9.496		
**−NLL b1 np**	8.436	5.547		
**−NLL b1 cyto**	−6.242	−17.28		
**−NLL b2 ca**	−7.725		−7.009	
**−NLL b2 np**				
**−NLL b2 cyto**	−6.723		−12.44	
**−NLL b3 ca**	−10.53			−9.208
**−NLL b3 np**	−3.02			−6.425
**−NLL b3 cyto**	−4.333			−1.728
Total	−38.71	−21.23	−19.45	−17.36
**Total with regul**	−34.16	−18.69	−17.7	−16.33

	Together	b1	b2	b3
spar	2.771e−01	0.30150	0.3094	0.3294000
σ_b	1.095e−01	0.00368	0.03907	0.0935900
σ_t	1.059e−05	3.20300	1.447	0.0005637
ca_{0,b_1}	6.414e+00	6.48800		
$\log_{10}(k_1')$	2.344e−01	−0.60560		0.5351000
$\log_{10}(k_2)$	1.147e−01	−0.81400	4.789 or −1.953	0.4699000
$\log_{10}(k_2')$	−1.666e+00	−1.49100		−1.6390000
$\log_{10}(k_{deg})$	−1.798e+00	−1.51300	−1.953 or 4.789	−1.8560000
$\log_{10}(k_1'k_2')$	−1.431e+00	−2.09600	3.125	−1.1030000
$\log_{10}(k_1'/k_2)$	1.197e−01	0.20850		0.0651900
$\text{transport} = \log_{10}(k_1'k_2'/k_2)$	−1.546e+00	−1.28200	−1.663 or 5.078	−1.5730000

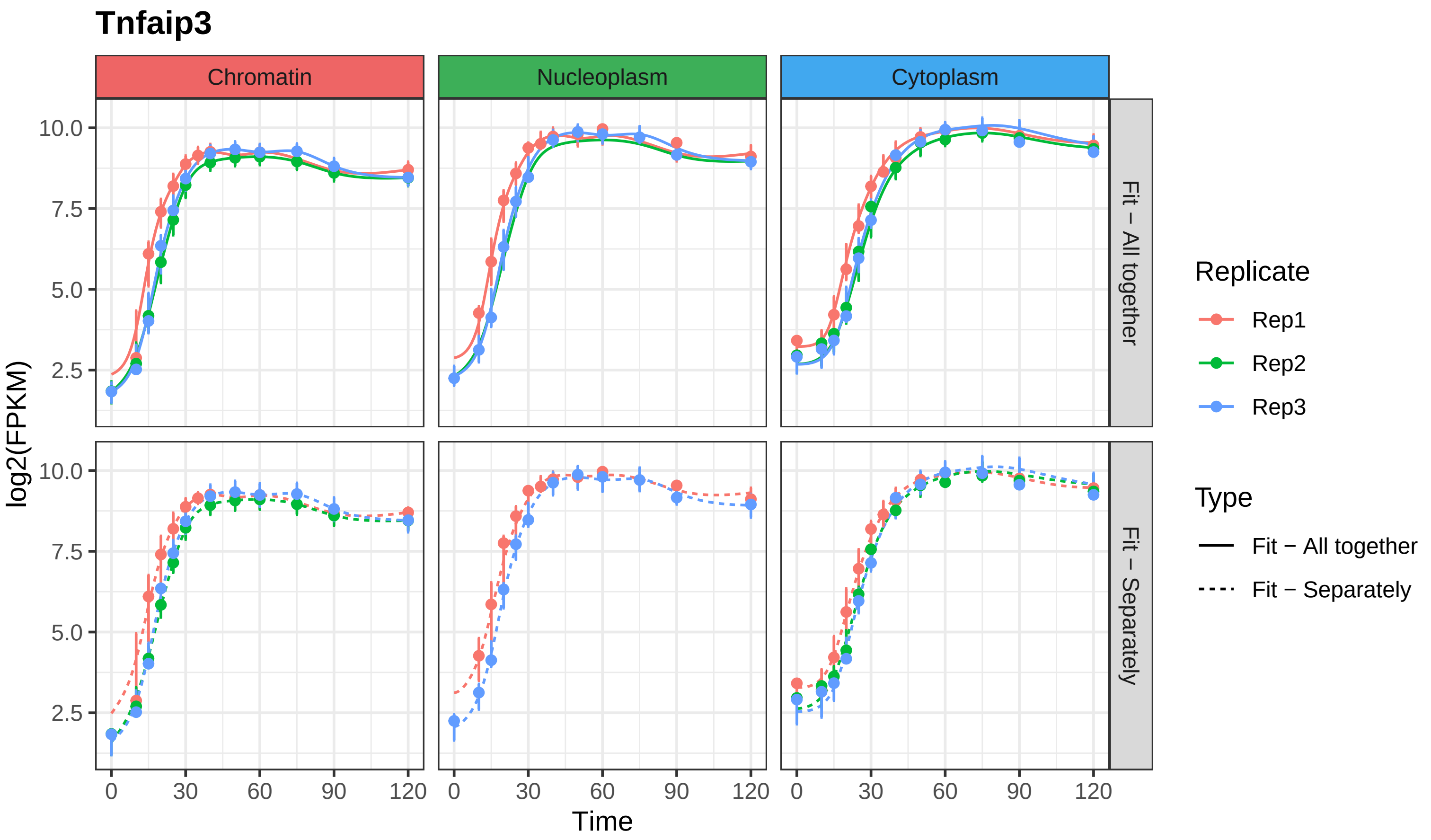

	Together	b1	b2	b3
**−NLL b1 ca**	−5.579	−7.075		
**−NLL b1 np**	−2.9	−1.566		
**−NLL b1 cyto**	−4.611	−6.401		
**−NLL b2 ca**	−9.521		−7.58	
**−NLL b2 np**				
**−NLL b2 cyto**	0.48		−0.1438	
**−NLL b3 ca**	−8.299			−4.731
**−NLL b3 np**	−6.396			−5.3
**−NLL b3 cyto**	2.904			3.472
Total	−33.92	−15.04	−7.723	−6.558
**Total with regul**	−25.74	−11.54	−1.75	1.984

	Together	b1	b2	b3
spar	0.22970	0.29100	0.2537	0.2257
σ_b	0.08853	0.05224	0.108	0.1262
σ_t	0.79220	1.44500	0.008286	0.1834
ca_{0,b_1}	2.39700	2.53100		
$\log_{10}(k_1')$	0.05811	−0.34730		−0.0342
$\log_{10}(k_2)$	−0.09831	−0.54030	4.658 or −1.249	−0.1721
$\log_{10}(k_2')$	−1.07100	−1.05300		−1.0800
$\log_{10}(k_{deg})$	−1.17400	−1.10200	−1.249 or 4.658	−1.2210
$\log_{10}(k_1'k_2')$	−1.01300	−1.40100	3.724	−1.1140
$\log_{10}(k_1'/k_2)$	0.15640	0.19310		0.1379
transport $= \log_{10}(k_1'k_2'/k_2)$	−0.91430	−0.86020	−0.9347 or 4.972	−0.9421

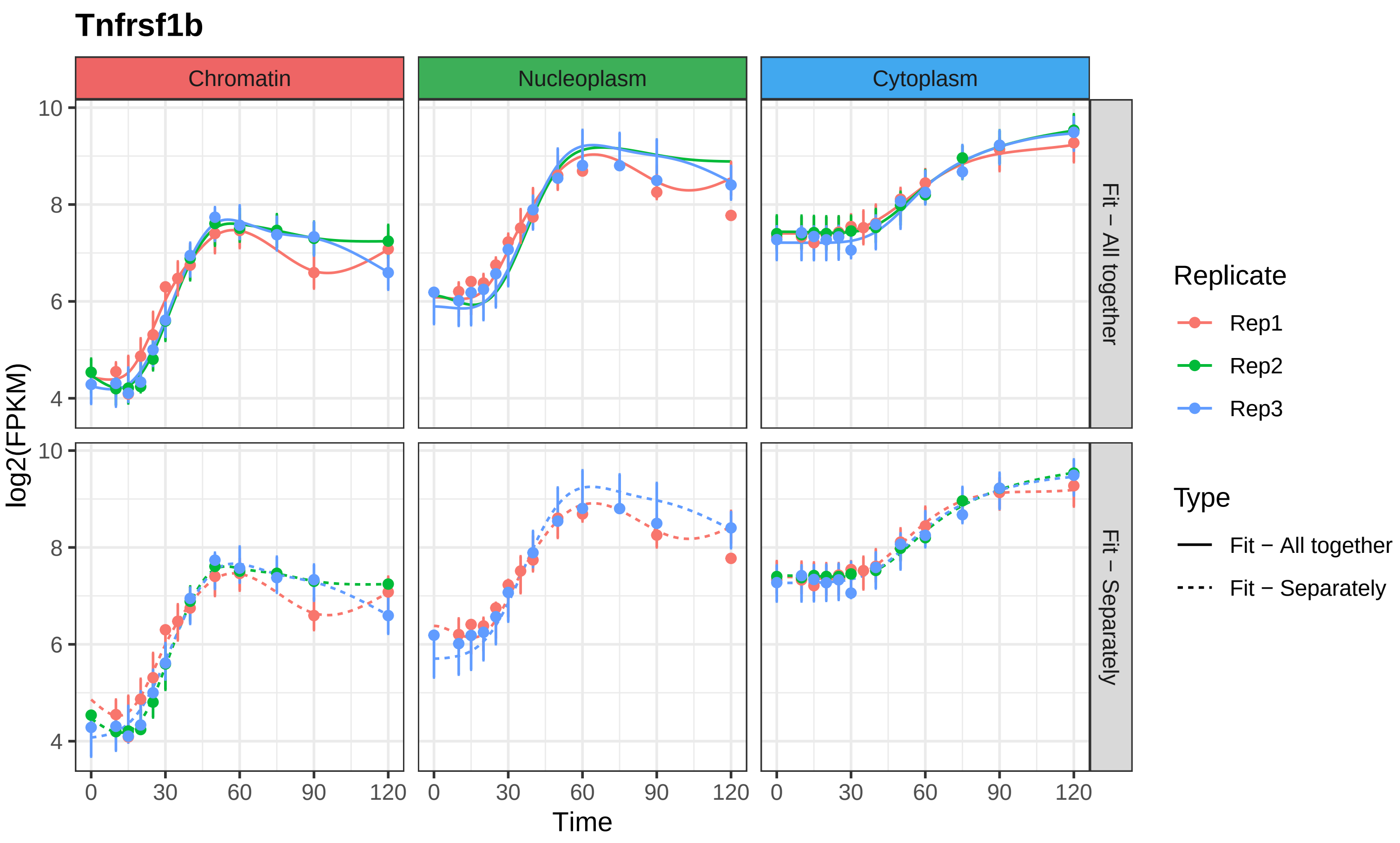

	Together	b1	b2	b3
**−NLL b1 ca**	−4.01	−3.157		
**−NLL b1 np**	6.205	2.347		
**−NLL b1 cyto**	−8.502	−8.826		
**−NLL b2 ca**	−7.862		−13.6	
**−NLL b2 np**				
**−NLL b2 cyto**	−8.984		−21.06	
**−NLL b3 ca**	−7.587			−3.648
**−NLL b3 np**	8.559			3.357
**−NLL b3 cyto**	−6.035			−6.17
Total	−28.22	−9.636	−34.66	−6.461
**Total with regul**	−28.94	−10.79	−35.01	−7.929

	Together	b1	b2	b3
spar	0.398900	0.4265	0.3522	0.4473000
σ_b	0.121300	0.1168	0.01314	0.1302000
σ_t	0.000041	0.9670	1.654	0.0001181
ca_{0,b_1}	4.474000	4.9570		
$\log_{10}(k_1')$	−0.366900	−0.3799		−0.1992000
$\log_{10}(k_2)$	−0.862100	−0.8377	−0.9923 or −2.009	−0.6897000
$\log_{10}(k_2')$	−1.642000	−1.4510		−1.6960000
$\log_{10}(k_{deg})$	−2.039000	−1.7560	−2.009 or −0.9923	−2.1660000
$\log_{10}(k_1' k_2')$	−2.009000	−1.8310	−2.112	−1.8950000
$\log_{10}(k_1'/k_2)$	0.495100	0.4577		0.4905000
$\text{transport} = \log_{10}(k_1' k_2'/k_2)$	−1.147000	−0.9934	−1.12 or −0.1037	−1.2050000

Tnfsf4

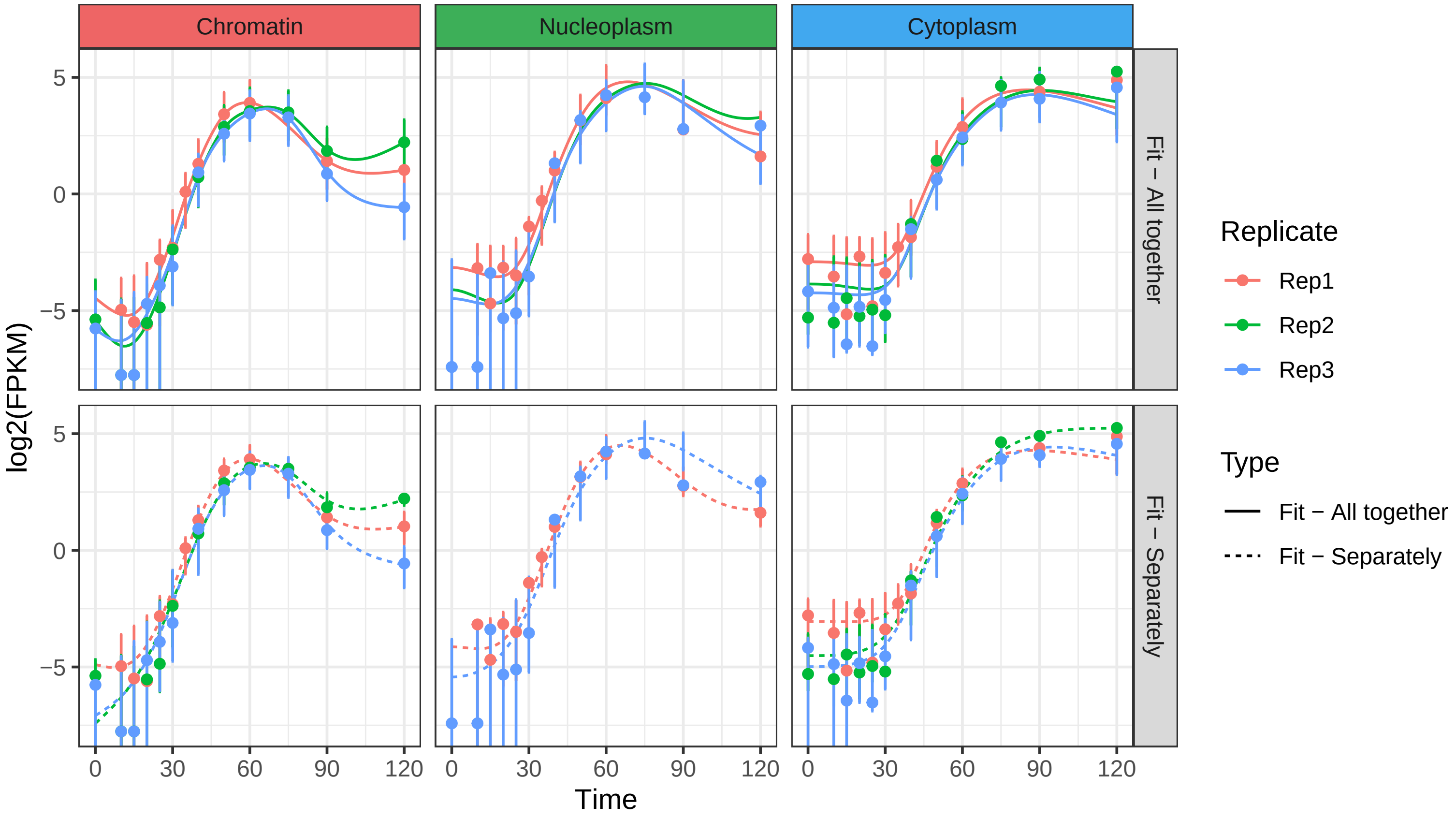

	Together	b1	b2	b3
**−NLL b1 ca**	7.834	5.797		
**−NLL b1 np**	11.43	5.42		
**−NLL b1 cyto**	16.41	18.66		
**−NLL b2 ca**	10.78		8.564	
**−NLL b2 np**				
**−NLL b2 cyto**	19.86		7.778	
**−NLL b3 ca**	11.06			10.29
**−NLL b3 np**	23.71			20.43
**−NLL b3 cyto**	16			11.87
Total	117.1	29.88	16.34	42.59
**Total with regul**	130.2	38.81	14.12	44.01

	Together	b1	b2	b3
spar	3.204e−01	0.4157000	0.4483	0.4084
σ_b	3.519e−01	0.2105000	0.0004049	0.2576
σ_t	3.551e−05	0.0001251	2.165	1.9490
ca_{0,b_1}	−4.451e+00	−4.8460000		
$\log_{10}(k_1')$	−7.111e−01	−0.6019000		−0.7082
$\log_{10}(k_2)$	−1.106e+00	−0.8307000	−1.493 or −1.513	−1.2050
$\log_{10}(k_2')$	−1.292e+00	−1.3850000		−1.4070
$\log_{10}(k_{deg})$	−1.366e+00	−1.7130000	−1.513 or −1.493	−1.5400
$\log_{10}(k_1'k_2')$	−2.003e+00	−1.9870000	−2.132	−2.1150
$\log_{10}(k_1'/k_2)$	3.951e−01	0.2288000		0.4967
$\text{transport} = \log_{10}(k_1'k_2'/k_2)$	−8.966e−01	−1.1570000	−0.6393 or −0.6189	−0.9101

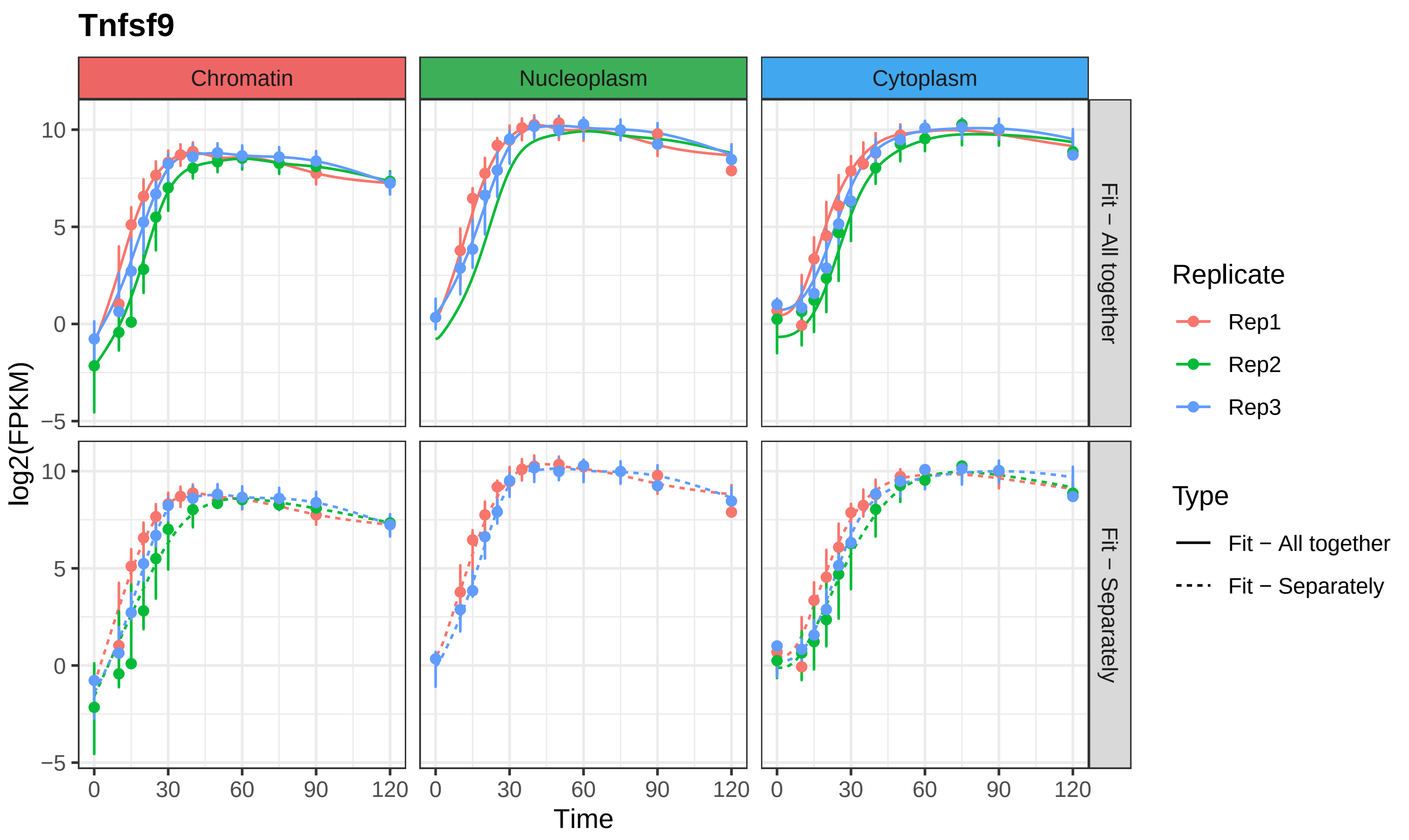

	Together	b1	b2	b3
**−NLL b1 ca**	1.211	1.634		
**−NLL b1 np**	6.993	7.36		
**−NLL b1 cyto**	8.765	6.365		
**−NLL b2 ca**	2.923		5.741	
**−NLL b2 np**				
**−NLL b2 cyto**	12.95		6.034	
**−NLL b3 ca**	1.909			0.3161
**−NLL b3 np**	3.656			1.982
**−NLL b3 cyto**	8.175			8.434
Total	46.58	15.36	11.78	10.73
**Total with regul**	50	15.34	10.85	15.36

	Together	b1	b2	b3
spar	0.3225	0.4240	0.514	0.2918
σ_b	0.1827	0.1690	0.09	0.1971
σ_t	1.4950	1.6300	2.874	0.3649
ca_{0,b_1}	−1.0660	−1.0190		
$\log_{10}(k_1')$	0.2953	0.1479		0.6078
$\log_{10}(k_2)$	−0.1300	−0.3143	−0.7981 or −0.8321	0.1984
$\log_{10}(k_2')$	−1.2410	−1.3310		−1.4230
$\log_{10}(k_{deg})$	−1.2760	−1.2980	−0.8321 or −0.7981	−1.4980
$\log_{10}(k_1' k_2')$	−0.9455	−1.1830	−1.188	−0.8152
$\log_{10}(k_1'/k_2)$	0.4253	0.4622		0.4094
$\text{transport} = \log_{10}(k_1' k_2'/k_2)$	−0.8154	−0.8692	−0.3902 or −0.3562	−1.0140

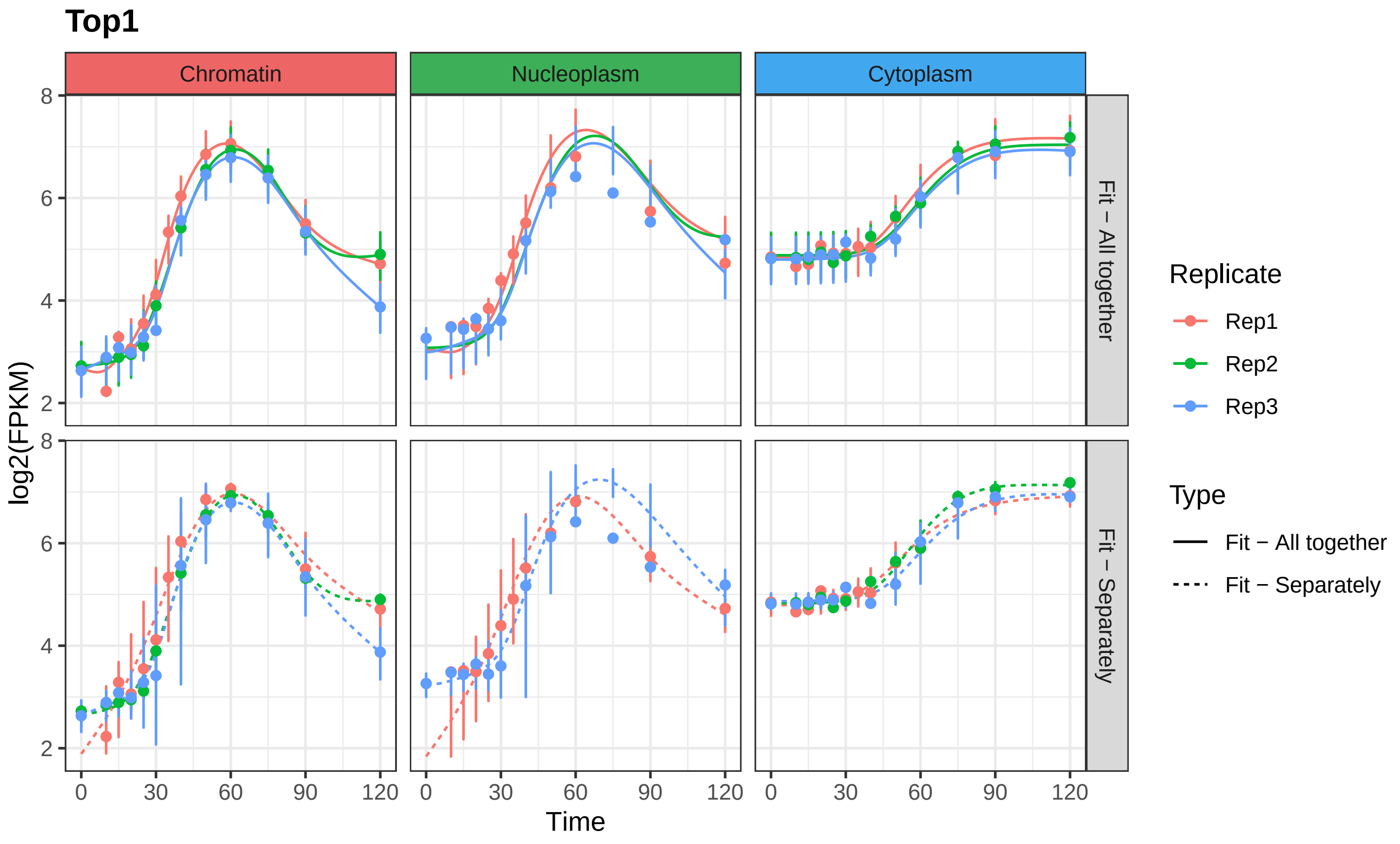

	Together	b1	b2	b3
**−NLL b1 ca**	−1.95	1.395		
**−NLL b1 np**	9.538	5.822		
**−NLL b1 cyto**	−3.132	−8.668		
**−NLL b2 ca**	−5.483		−7.719	
**−NLL b2 np**				
**−NLL b2 cyto**	−4.33		−10.81	
**−NLL b3 ca**	−4.103			−2.168
**−NLL b3 np**	13.65			6.714
**−NLL b3 cyto**	−4.658			−8.444
Total	−0.4623	−1.451	−18.53	−3.899
**Total with regul**	0.9909	−1.453	−20.35	−4.378

	Together	b1	b2	b3
spar	0.3677000	0.53880	0.4081	0.37450
σ_b	0.1581000	0.06428	0.03103	0.04821
σ_t	0.0001368	3.89800	2.264	5.40200
ca_{0,b_1}	2.7240000	2.01100		
$\log_{10}(k_1')$	−0.6874000	4.85800		−0.76900
$\log_{10}(k_2)$	−0.7950000	4.87300	−0.5383 or −2.166	−0.94860
$\log_{10}(k_2')$	−1.6690000	−1.75200		−1.74300
$\log_{10}(k_{deg})$	−2.2120000	−2.64100	−2.166 or −0.5383	−2.23400
$\log_{10}(k_1'k_2')$	−2.3560000	3.10600	−2.049	−2.51200
$\log_{10}(k_1'/k_2)$	0.1076000	−0.01497		0.17960
$\text{transport} = \log_{10}(k_1'k_2'/k_2)$	−1.5610000	−1.76700	−1.511 or 0.1164	−1.56300

Trib1

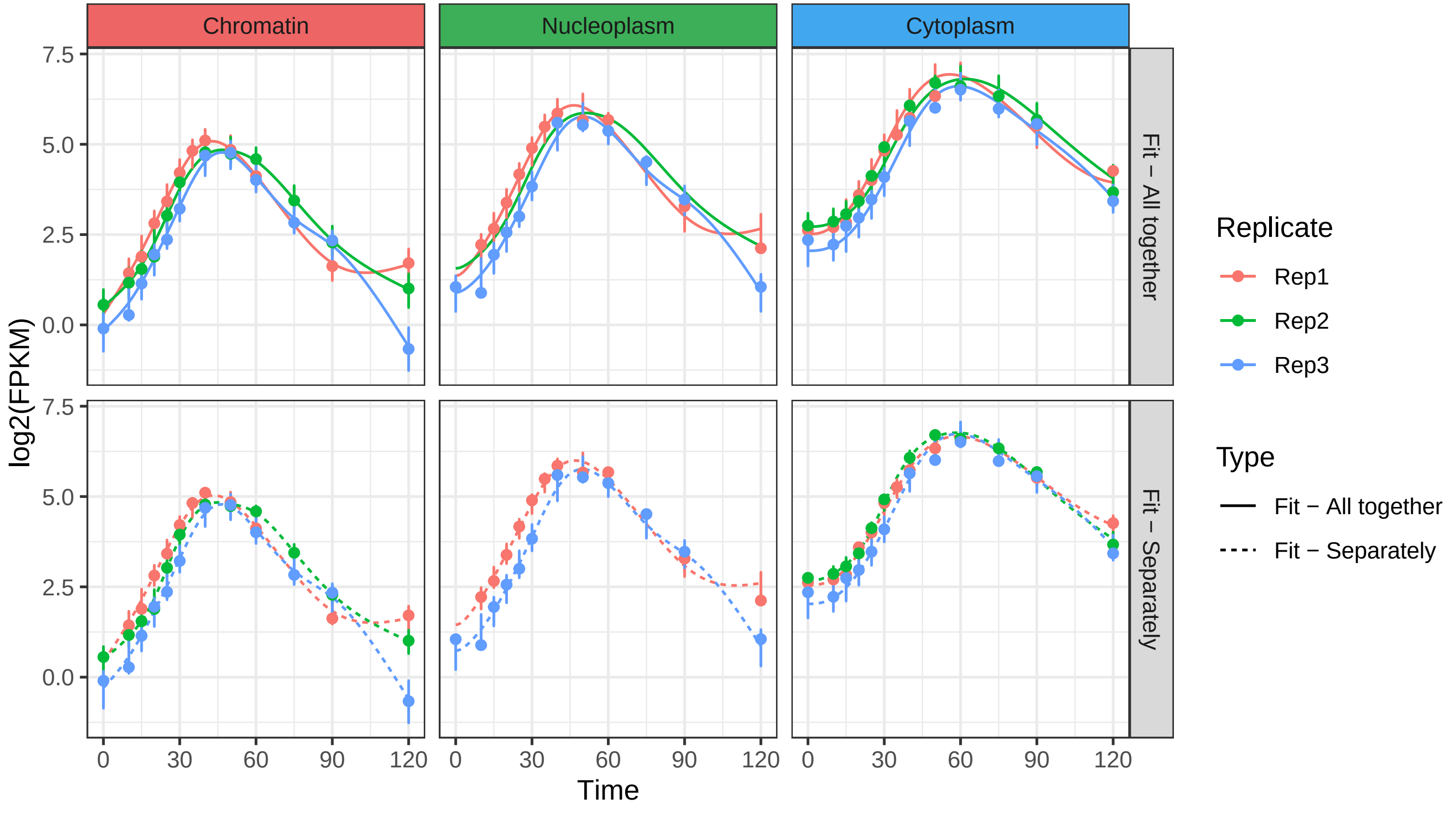

	Together	b1	b2	b3
**−NLL b1 ca**	−6.673	−7.398		
**−NLL b1 np**	−1.448	−1.7		
**−NLL b1 cyto**	3.746	−8.796		
**−NLL b2 ca**	−5.301		−8.501	
**−NLL b2 np**				
**−NLL b2 cyto**	0.3996		−9.043	
**−NLL b3 ca**	−4.77			−4.391
**−NLL b3 np**	−1.542			−1.497
**−NLL b3 cyto**	−2.113			−1.104
Total	−17.7	−17.89	−17.54	−6.992
**Total with regul**	−17.62	−19.2	−16.9	−7.882

	Together	b1	b2	b3
spar	3.966e−01	0.4534000	0.332	3.900e−01
σ_b	1.296e−01	0.0856600	0.0589	1.191e−01
σ_t	1.726e−05	0.0001393	0.2878	2.612e−05
ca_{0,b_1}	3.238e−01	0.4850000		
$\log_{10}(k_1')$	−2.541e−01	−0.2501000		−2.243e−01
$\log_{10}(k_2)$	−5.715e−01	−0.5561000	7.362 or −1.078	−5.344e−01
$\log_{10}(k_2')$	−7.167e−01	−0.8873000		−6.733e−01
$\log_{10}(k_{deg})$	−1.064e+00	−1.2190000	−1.078 or 7.362	−1.063e+00
$\log_{10}(k_1'k_2')$	−9.708e−01	−1.1370000	6.934	−8.977e−01
$\log_{10}(k_1'/k_2)$	3.174e−01	0.3060000		3.101e−01
$\text{transport} = \log_{10}(k_1'k_2'/k_2)$	−3.993e−01	−0.5813000	−0.4287 or 8.012	−3.632e−01

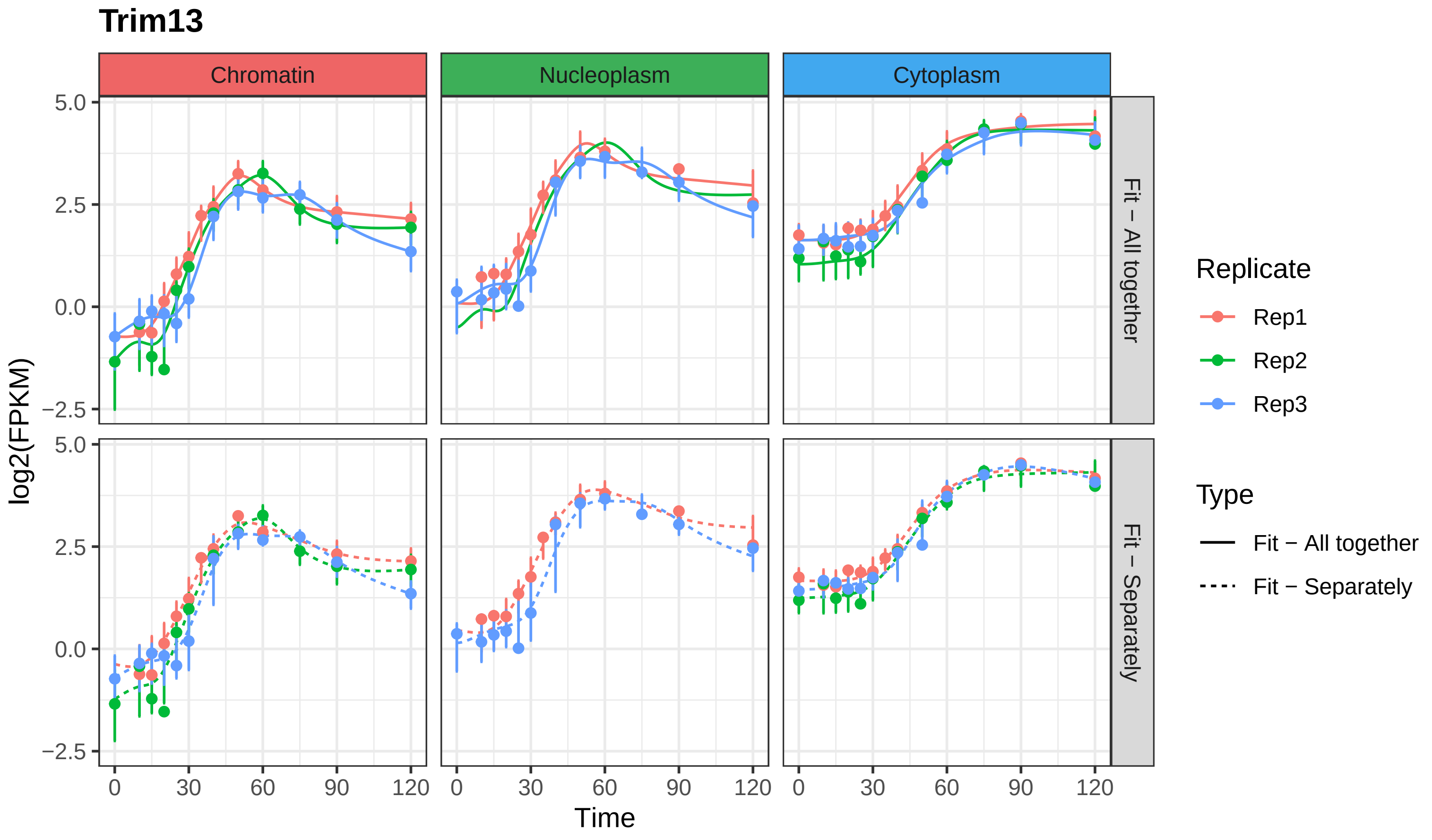

	Together	b1	b2	b3
**−NLL b1 ca**	−4.644	−3.463		
**−NLL b1 np**	3.305	−1.495		
**−NLL b1 cyto**	−4.906	−8.399		
**−NLL b2 ca**	−0.9264		1.027	
**−NLL b2 np**				
**−NLL b2 cyto**	0.7856		−1.91	
**−NLL b3 ca**	−4.765			−2.821
**−NLL b3 np**	0.8837			−0.2497
**−NLL b3 cyto**	−1.037			−6.246
Total	−11.3	−13.36	−0.8834	−9.316
**Total with regul**	−8.271	−13.06	−1.121	−10.1

	Together	b1	b2	b3
spar	0.310100	0.458100	0.361	3.674e−01
σ_b	0.108400	0.064140	0.09953	6.786e−06
σ_t	0.001057	0.000159	0.002477	2.929e+00
ca_{0,b_1}	−0.710500	−0.226600		
$\log_{10}(k_1')$	0.062590	−0.182000		−2.896e−01
$\log_{10}(k_2)$	−0.180600	−0.428900	5.87 or −1.89	−5.424e−01
$\log_{10}(k_2')$	−1.327000	−1.274000		−1.130e+00
$\log_{10}(k_{deg})$	−1.792000	−1.641000	−1.89 or 5.87	−1.523e+00
$\log_{10}(k_1'k_2')$	−1.264000	−1.456000	4.725	−1.420e+00
$\log_{10}(k_1'/k_2)$	0.243200	0.246900		2.527e−01
$\text{transport} = \log_{10}(k_1'k_2'/k_2)$	−1.084000	−1.027000	−1.145 or 6.615	−8.774e−01

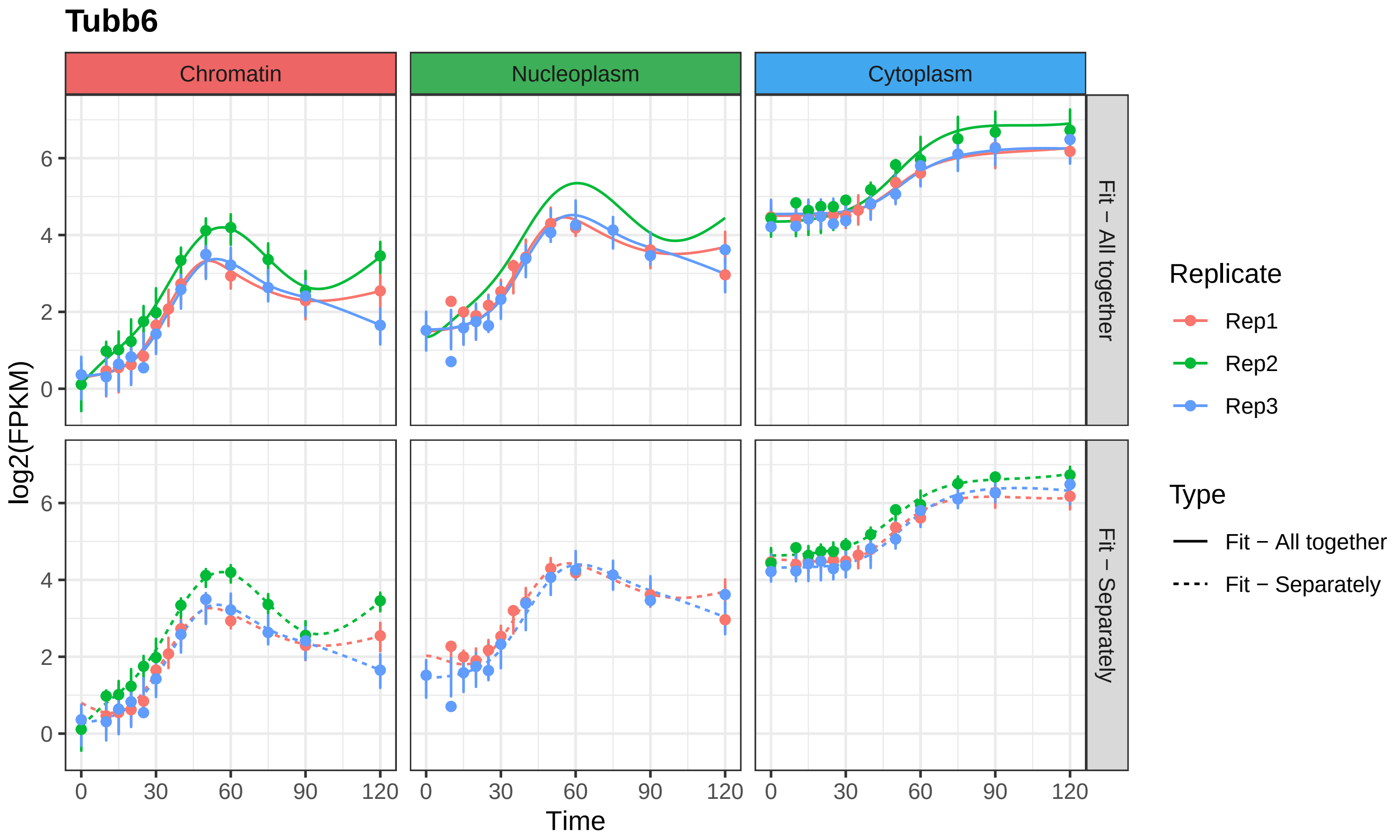

	Together	b1	b2	b3
**−NLL b1 ca**	−4.406	−4.692		
**−NLL b1 np**	7.047	4.666		
**−NLL b1 cyto**	−7.673	−10.52		
**−NLL b2 ca**	−5.642		−7.626	
**−NLL b2 np**				
**−NLL b2 cyto**	1.265		−8.135	
**−NLL b3 ca**	−3.941			−2.84
**−NLL b3 np**	5.621			6.727
**−NLL b3 cyto**	−2.252			−6.811
Total	−9.981	−10.55	−15.76	−2.924
**Total with regul**	−10.7	−10.8	−17.35	−4.378

	Together	b1	b2	b3
spar	0.4046000	4.496e−01	0.3988	4.213e−01
σ_b	0.1297000	9.537e−02	0.06377	1.183e−01
σ_t	0.0000998	3.258e−06	0.00308	9.161e−05
ca_{0,b_1}	0.2965000	8.889e−01		
$\log_{10}(k_1')$	−0.2812000	−2.758e−01		−5.077e−01
$\log_{10}(k_2)$	−0.6451000	−6.455e−01	4.138 or −2.228	−8.622e−01
$\log_{10}(k_2')$	−1.0780000	−9.067e−01		−8.874e−01
$\log_{10}(k_{deg})$	−1.9830000	−1.658e+00	−2.228 or 4.138	−1.750e+00
$\log_{10}(k_1'k_2')$	−1.3590000	−1.183e+00	3.25	−1.395e+00
$\log_{10}(k_1'/k_2)$	0.3639000	3.696e−01		3.545e−01
transport $= \log_{10}(k_1'k_2'/k_2)$	−0.7139000	−5.371e−01	−0.8887 or 5.478	−5.329e−01

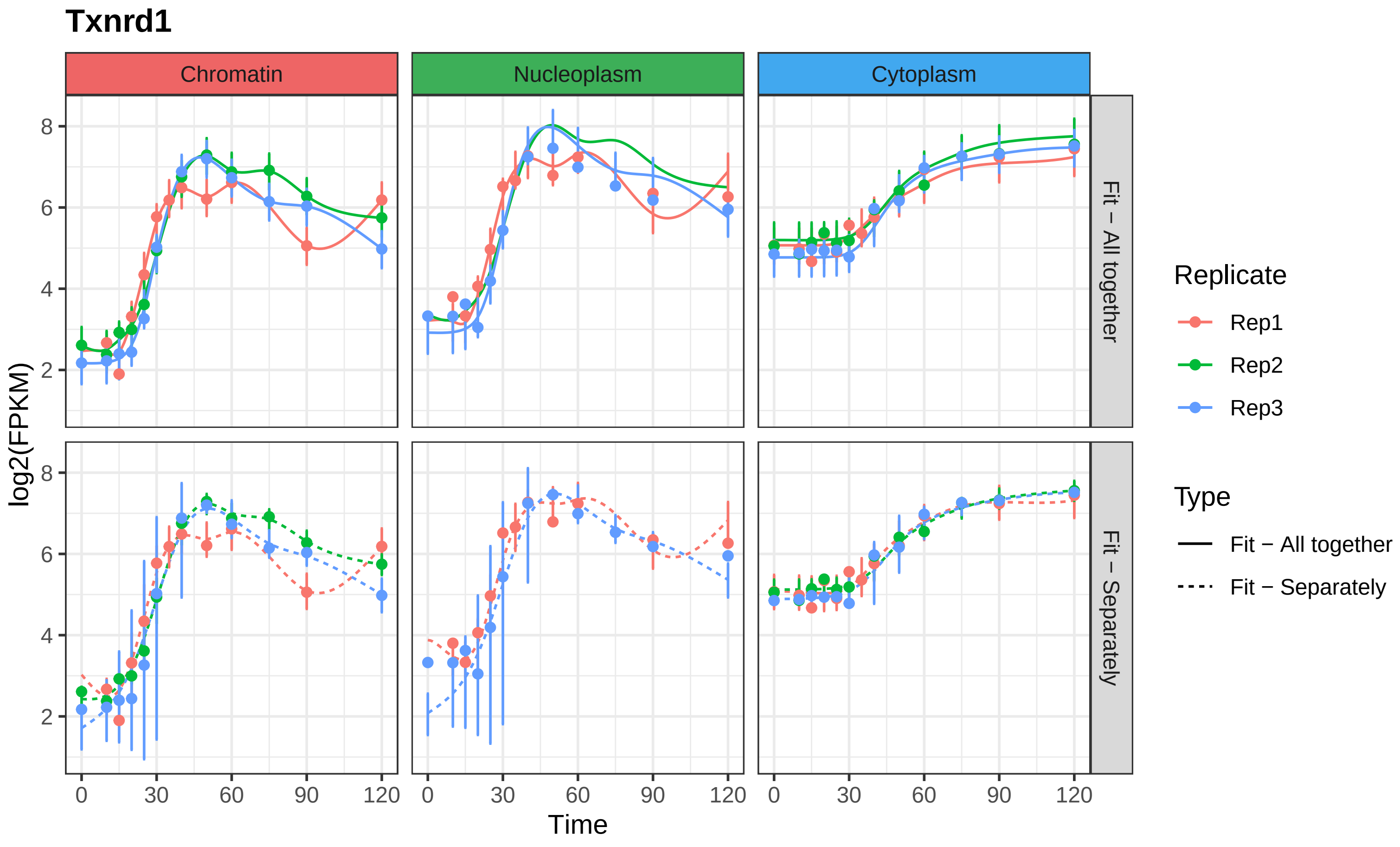

	Together	b1	b2	b3
**−NLL b1 ca**	−2.789	−0.07462		
**−NLL b1 np**	6.131	4.103		
**−NLL b1 cyto**	−1.025	−1.852		
**−NLL b2 ca**	−5.493		−5.263	
**−NLL b2 np**				
**−NLL b2 cyto**	−1.613		−4.255	
**−NLL b3 ca**	−5.186			2.476
**−NLL b3 np**	11.04			8.109
**−NLL b3 cyto**	−3.078			−12.09
Total	−2.016	2.176	−9.518	−1.509
**Total with regul**	4.198	3.371	−9.95	−3.223

	Together	b1	b2	b3
spar	0.2641000	0.3500	0.3637	4.455e−01
σ_b	0.1561000	0.1447	0.08546	9.820e−07
σ_t	0.0001784	1.5560	1.262	5.480e+00
ca_{0,b_1}	2.4620000	3.0690		
$\log_{10}(k_1')$	0.4333000	−0.2534		7.090e+00
$\log_{10}(k_2)$	0.2075000	−0.5101	5.244 or −2.462	6.979e+00
$\log_{10}(k_2')$	−1.7780000	−1.6270		−1.692e+00
$\log_{10}(k_{deg})$	−2.3360000	−1.9880	−2.462 or 5.244	−2.538e+00
$\log_{10}(k_1'k_2')$	−1.3450000	−1.8800	3.594	5.398e+00
$\log_{10}(k_1'/k_2)$	0.2259000	0.2566		1.111e−01
$\text{transport} = \log_{10}(k_1'k_2'/k_2)$	−1.5520000	−1.3700	−1.65 or 6.056	−1.581e+00

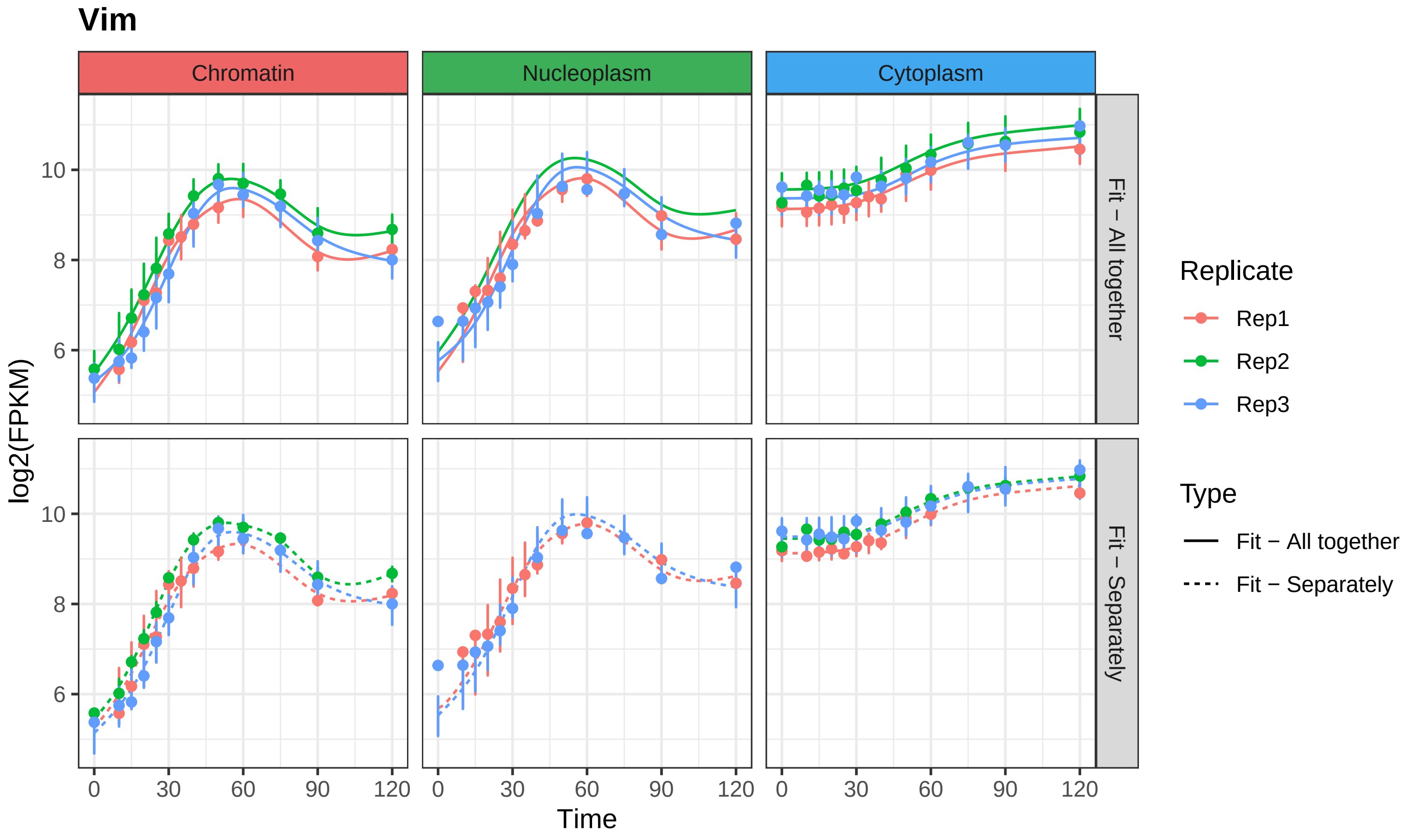

	Together	b1	b2	b3
**−NLL b1 ca**	−2.839	−2.622		
**−NLL b1 np**	4.709	1.391		
**−NLL b1 cyto**	−7.126	−11.31		
**−NLL b2 ca**	−4.402		−12.49	
**−NLL b2 np**				
**−NLL b2 cyto**	−4.57		−10.44	
**−NLL b3 ca**	−4.067			−3.834
**−NLL b3 np**	16.03			6.703
**−NLL b3 cyto**	−3.909			−4.88
Total	−6.178	−12.55	−22.94	−2.011
**Total with regul**	−7.335	−13.89	−23.69	−3.24

	Together	b1	b2	b3
spar	0.4524	0.48580	0.3659	0.4496
σ_b	0.1293	0.05805	0.05449	0.1473
σ_t	2.1950	3.44300	0.0001294	0.4679
ca_{0,b_1}	5.1430	5.37100		
$\log_{10}(k_1')$	8.2870	−0.15540		5.0110
$\log_{10}(k_2)$	8.1480	−0.29090	4.89 or −2.834	4.8930
$\log_{10}(k_2')$	−1.7090	−1.63600		−1.6930
$\log_{10}(k_{deg})$	−2.7950	−2.66700	−2.834 or 4.89	−2.8860
$\log_{10}(k_1'k_2')$	6.5780	−1.79100	3.262	3.3180
$\log_{10}(k_1'/k_2)$	0.1393	0.13550		0.1180
$\text{transport} = \log_{10}(k_1'k_2'/k_2)$	−1.5700	−1.50000	−1.628 or 6.096	−1.5750

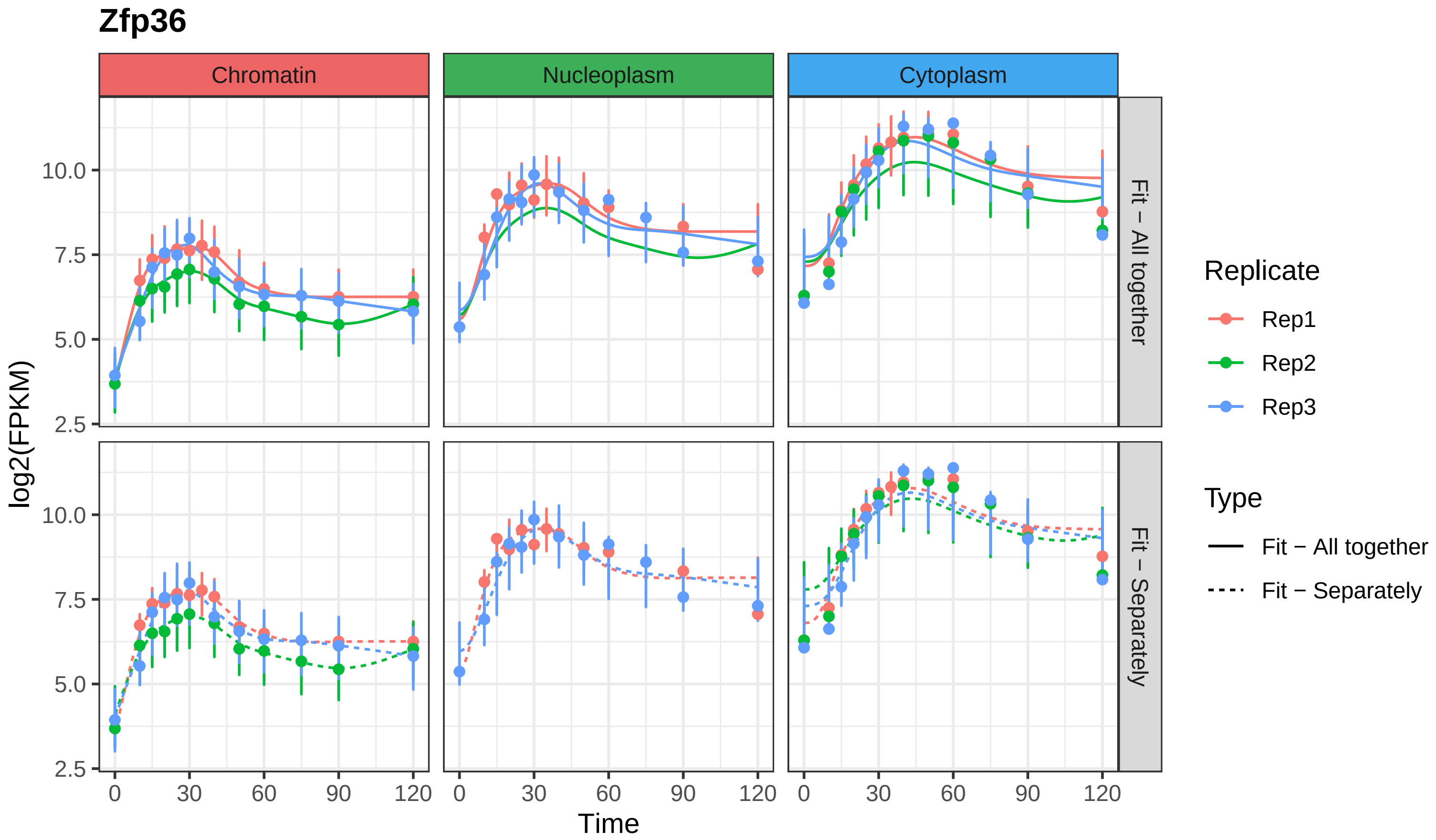

	Together	b1	b2	b3
**−NLL b1 ca**	1.317	−1.751		
**−NLL b1 np**	6.631	6.773		
**−NLL b1 cyto**	7.433	6.362		
**−NLL b2 ca**	1.55		1.334	
**−NLL b2 np**				
**−NLL b2 cyto**	16.55		15.08	
**−NLL b3 ca**	2.143			2.532
**−NLL b3 np**	6.56			5.565
**−NLL b3 cyto**	18.69			16.61
Total	60.87	11.38	16.42	24.7
**Total with regul**	67.82	16.49	19.62	27.87

	Together	b1	b2	b3
spar	3.475e−01	0.375300	0.3722	0.3872000
σ_b	2.935e−01	0.214600	0.2935	0.3066000
σ_t	1.723e−06	0.004546	0.4543	0.0001726
ca_{0,b_1}	3.563e+00	3.338000		
$\log_{10}(k_1')$	−1.326e−01	0.109700		−0.1900000
$\log_{10}(k_2)$	−7.132e−01	−0.457000	−0.8529 or −0.889	−0.7812000
$\log_{10}(k_2')$	−5.960e−01	−0.669500		−0.6347000
$\log_{10}(k_{deg})$	−1.069e+00	−1.097000	−0.889 or −0.8529	−1.0360000
$\log_{10}(k_1' k_2')$	−7.286e−01	−0.559800	−0.6316	−0.8246000
$\log_{10}(k_1'/k_2)$	5.807e−01	0.566700		0.5912000
transport $= \log_{10}(k_1' k_2'/k_2)$	−1.535e−02	−0.102800	0.2212 or 0.2574	−0.0434700

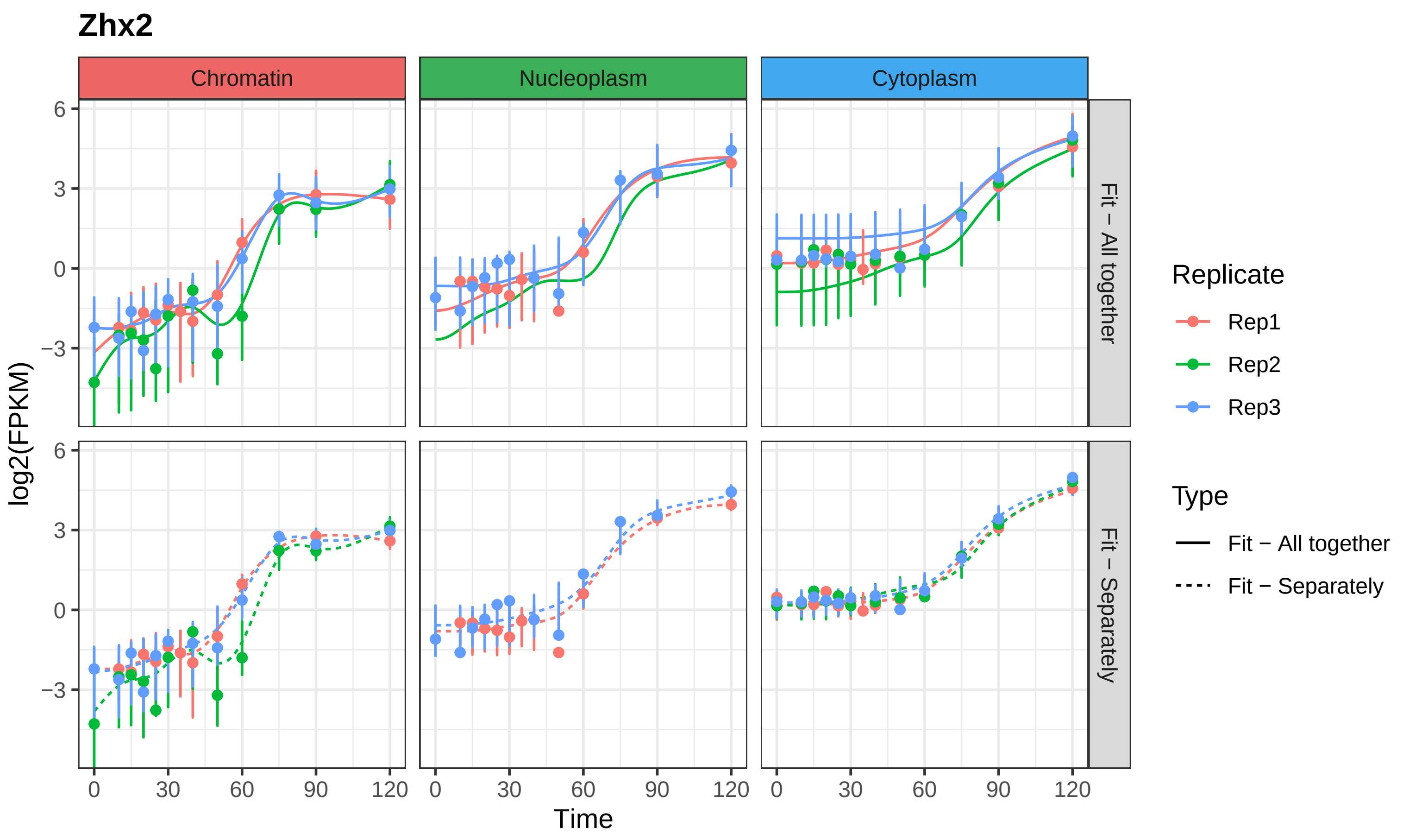

	Together	b1	b2	b3
**−NLL b1 ca**	6.01	1.063		
**−NLL b1 np**	9.248	2.334		
**−NLL b1 cyto**	6.974	−2.293		
**−NLL b2 ca**	10.27		6.738	
**−NLL b2 np**				
**−NLL b2 cyto**	20.37		1.72	
**−NLL b3 ca**	7.385			5.487
**−NLL b3 np**	10.05			10.25
**−NLL b3 cyto**	15.92			−0.1849
Total	86.22	1.104	8.458	15.55
**Total with regul**	90.49	0.4461	7.453	13.99

	Together	b1	b2	b3
spar	0.3714000	0.4053000	0.3882	0.4413
σ_b	0.3156000	0.0237100	0.1049	0.1196
σ_t	0.0001933	0.0000615	0.001917	1.0750
ca_{0,b_1}	−3.1710000	−2.1940000		
$\log_{10}(k_1')$	−0.8307000	−0.9529000		−0.8867
$\log_{10}(k_2)$	−1.3040000	−1.3760000	−1.019 or −2.073	−1.4150
$\log_{10}(k_2')$	−1.2220000	−1.1360000		−1.1020
$\log_{10}(k_{deg})$	−1.7600000	−1.4400000	−2.073 or −1.019	−1.3590
$\log_{10}(k_1'k_2')$	−2.0530000	−2.0890000	−1.891	−1.9890
$\log_{10}(k_1'/k_2)$	0.4734000	0.4231000		0.5282
transport $= \log_{10}(k_1'k_2'/k_2)$	−0.7489000	−0.7131000	−0.8717 or 0.1824	−0.5737

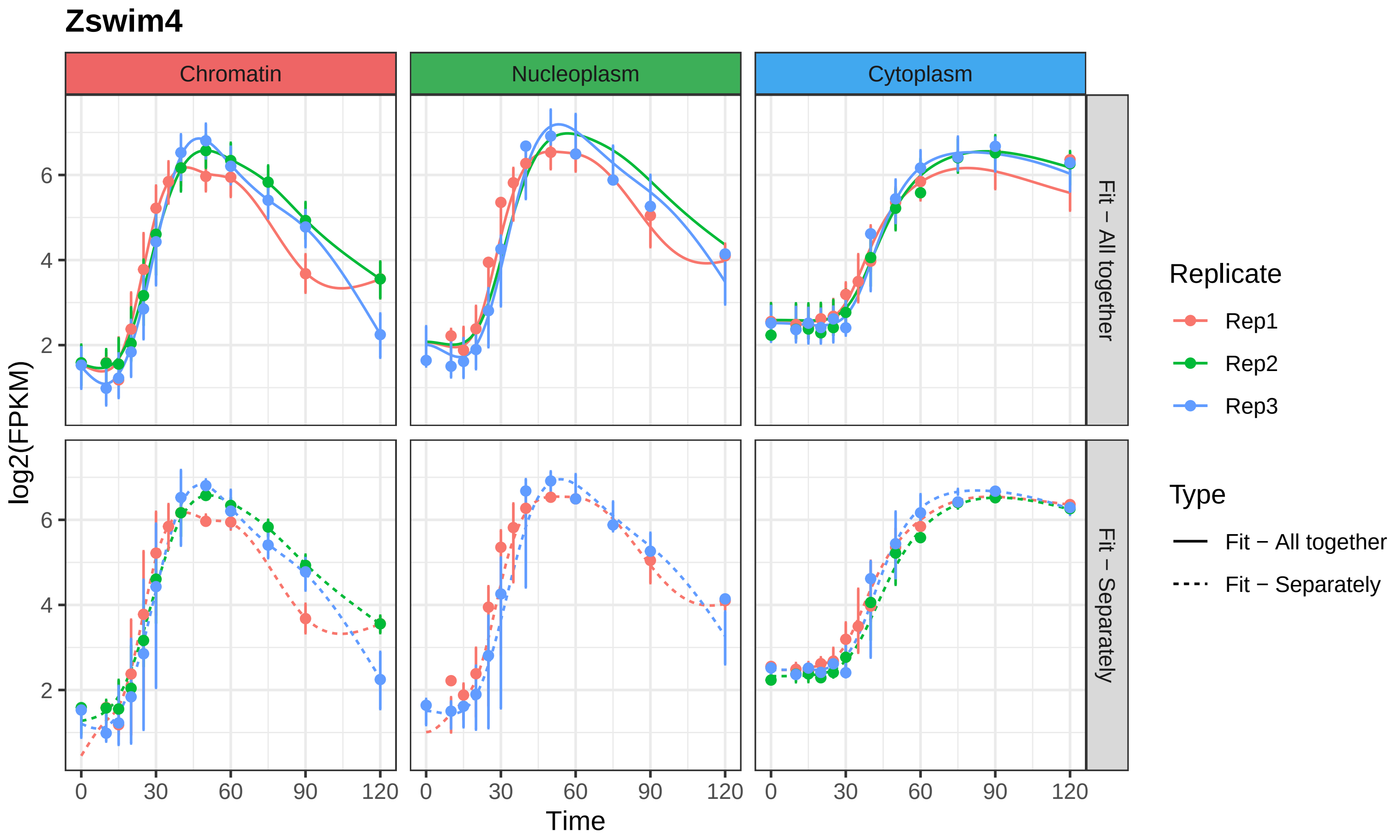

	Together	b1	b2	b3
−NLL b1 ca	−2.455	−4.6		
−NLL b1 np	0.3736	3.327		
−NLL b1 cyto	6.023	−11.87		
−NLL b2 ca	−4.603		−6.252	
−NLL b2 np				
−NLL b2 cyto	−0.8902		−11.16	
−NLL b3 ca	−4.118			−0.7176
−NLL b3 np	9.224			5.854
−NLL b3 cyto	−1.989			−9.241
Total	1.565	−13.14	−17.41	−4.104
Total with regul	4.022	−5.693	−18.88	−5.004

	Together	b1	b2	b3
spar	0.3198	2.862e−01	0.3876	3.727e−01
σ_b	0.1379	2.069e−06	6.849e−07	3.131e−06
σ_t	1.4010	2.684e+00	1.894	3.348e+00
ca_{0,b_1}	1.5730	4.443e−01		
$\log_{10}(k_1')$	−0.6407	−6.959e−01		−7.042e−01
$\log_{10}(k_2)$	−0.7955	−8.636e−01	−0.9809 or −1.694	−7.946e−01
$\log_{10}(k_2')$	−1.5270	−1.535e+00		−1.434e+00
$\log_{10}(k_{deg})$	−1.6810	−1.974e+00	−1.694 or −0.9809	−1.726e+00
$\log_{10}(k_1'k_2')$	−2.1680	−2.231e+00	−2.358	−2.138e+00
$\log_{10}(k_1'/k_2)$	0.1548	1.677e−01		9.039e−02
$transport = \log_{10}(k_1'k_2'/k_2)$	−1.3730	−1.367e+00	−1.377 or −0.6641	−1.344e+00
